# Performance of ChatGPT-4o and Four Open-Source Large Language Models in Generating Diagnoses Based on China’s Rare Disease Catalog: Comparative Study

**DOI:** 10.2196/69929

**Published:** 2025-06-18

**Authors:** Wei Zhong, YiFan Liu, Yan Liu, Kai Yang, HuiMin Gao, HuiHui Yan, WenJing Hao, YouSheng Yan, ChengHong Yin

**Affiliations:** 1 Department of Prenatal Diagnosis, Beijing Obstetrics and Gynecology Hospital, Capital Medical University, Beijing Maternal and Child Health Care Hospital, No. 251 Yaojiayuan Road, Chaoyang District, Beijing, China, 8618810963279

**Keywords:** large language models, ChatGPT, rare diseases, Llama, open-source LLMs, retrieval augmented generation, chain-of-thought, Deepseek

## Abstract

**Background:**

Diagnosing rare diseases remains challenging due to their inherent complexity and limited physician knowledge. Large language models (LLMs) offer new potential to enhance diagnostic workflows.

**Objective:**

This study aimed to evaluate the diagnostic accuracy of ChatGPT-4o and 4 open-source LLMs (qwen2.5:7b, Llama3.1:8b, qwen2.5:72b, and Llama3.1:70b) for rare diseases, assesses the language effect on diagnostic performance, and explore retrieval augmented generation (RAG) and chain-of-thought (CoT) reasoning.

**Methods:**

We extracted clinical manifestations of 121 rare diseases from China’s inaugural rare disease catalog. ChatGPT-4o generated a primary and 5 differential diagnoses, while 4 LLMs were assessed in both English and Chinese contexts. The lowest-performing model underwent RAG and CoT re-evaluation. Diagnostic accuracy was compared via the McNemar test. A survey evaluated 11 clinicians’ familiarity with rare diseases.

**Results:**

ChatGPT-4o demonstrated the highest diagnostic accuracy with 90.1%. Language effects varied across models: qwen2.5:7b showed comparable performance in Chinese (51.2%) and English (47.9%; *χ*²_1_=0.32, *P*=.57), whereas Llama3.1:8b exhibited significantly higher English accuracy (67.8% vs 31.4%; *χ*²_1_=40.20, *P*<.001). Among larger models, qwen2.5:72b maintained cross-lingual consistency considering the odds ratio (OR; Chinese: 82.6% vs English: 83.5%; OR 0.88, 95% CI 0.27-2.76,*P*=1.000), contrasting with Llama3.1:70b’s language-dependent variation (Chinese: 80.2% vs English: 90.1%; OR 0.29,95% CI 0.08-0.83, *P*=.02). Cross-model comparisons revealed Llama3.1:8b underperformed qwen2.5:7b in Chinese (*χ*²_1_=13.22,*P*<.001) but surpassed it in English (*χ*²_1_=13.92,*P*<.001). No significant differences were observed between qwen2.5:72b and Llama3.1:70b (English: OR 0.33, *P*=.08; Chinese: OR 1.5, 95% CI 0.48-5.12,*P*=.07); qwen2.5:72b matched ChatGPT-4o’s performance in both languages (English: OR 0.33, *P*=.08; Chinese: OR 0.44, *P*=.09); Llama3.1:70b mirrored ChatGPT-4o’s English accuracy (OR 1, *P*=1.000) but lagged in Chinese (OR 0.33; *P*=.02). RAG implementation enhanced qwen2.5:7b’s accuracy to 79.3% (*χ*²_1_=31.11, *P*<.001) with 85.9% retrieval precision. The distilled model Deepseek-R1:7b markedly underperformed (9.9% vs qwen2.5:7b; *χ*²_1_=42.19, *P*<.001). Clinician surveys revealed significant knowledge gaps in rare disease management.

**Conclusions:**

ChatGPT-4o demonstrated superior diagnostic performance for rare diseases. While Llama3.1:8b demonstrates viability for localized deployment in resource-constrained English diagnostic workflows, Chinese applications require larger models to achieve comparable diagnostic accuracy. This urgency is heightened by the release of open-source models like DeepSeek-R1, which may see rapid adoption without thorough validation. Successful clinical implementation of LLMs requires 3 core elements: model parameterization, user language, and pretraining data. The integration of RAG significantly enhanced open-source LLM accuracy for rare disease diagnosis, although caution remains warranted for low-parameter reasoning models showing substantial performance limitations. We recommend hospital IT departments and policymakers prioritize language relevance in model selection and consider integrating RAG with curated knowledge bases to enhance diagnostic utility in constrained settings, while exercising caution with low-parameter models.

## Introduction

Although the prevalence of individual rare diseases is low, their collective impact on the global population is considerable due to their vast diversity and number [1,2]. These conditions are primarily genetic in origin, characterized by limited treatment options and substantial financial burdens [3]. The diagnosis remains a complex and prolonged process [4], with patients often enduring a diagnostic odyssey averaging 5 years before receiving a conclusive diagnosis [1]. This prolonged uncertainty exacerbates patient distress and strain health care systems. While whole-exome sequencing has proven to be an efficacious diagnostic tool for most rare diseases in clinical practice, high cost and specialized expertise requirements limit its widespread use [5,6]. Alternatively, phenotype-driven diagnostic approaches present a more affordable and expedient solution, although their reliance on precise phenotypic terminology poses challenges for both patients and clinicians [6-8].

The recent advancements of large language models (LLMs) have expanded their applications in medical diagnostics [9,10]. With comprehensive medical knowledge, these models serve as diagnostic aids through natural language interactions [11], demonstrating promising capabilities in rare diseases diagnosis [12,13]. While clinicians are experienced in diagnosing common conditions, they often lack proficiency in managing the complex nature of rare diseases [8,14]. Consequently, integrating LLMs into the diagnostic workflow for rare diseases holds significant clinical value, especially for patients using these tools for self-diagnosis [12]. Existing studies have focused on specific rare diseases or limited disease groups [15,16]. However, assessing LLM performance across all rare diseases is challenging and impractical. Given geographic variations in rare disease prevalence [2], prioritizing regionally prevalent rare diseases for LLM development enhances diagnostic efficiency and improves local health care outcomes.

To advance rare disease management, China has established a national catalog of 207 rare diseases selected through established criteria including incidence, severity, and diagnostic feasibility. However, phenotype-based preliminary diagnosis remains challenging, with even specialists requiring guidance from senior clinicians [5,7]. Evaluating LLMs’ diagnostic capabilities within this framework holds critical value, enabling both assessment of clinical applicability in Chinese populations and generation of transferable insights for global health systems.

Furthermore, LLM performance variations stem from diverse architectures, parameter scales, and language configurations [11,12,17,18]. While commercial models excel in accuracy, open-source alternatives gain traction through local deployment advantages that safeguard patient privacy—a critical consideration given modern models’ ability to process sensitive data like medical images [12]. This necessitates rigorous evaluation of open-source models’ diagnostic suitability, particularly for rare diseases. Current research gaps persist, with limited studies on open-source LLMs for rare disease diagnosis and no systematic cross-linguistic evaluations addressing language, model parameters, and regional contexts. Such assessments are essential for optimizing LLM adoption in primary care settings and non-English health care systems, enabling evidence-based model selection aligned with local needs.

Open-source LLMs achieve domain competence through specialized fine-tuning, although their performance remains constrained by base architecture limitations [19]. Retrieval-augmented generation (RAG) enhances diagnostic accuracy by integrating domain-specific knowledge bases, effectively reducing hallucinations [20,21], yet its application in rare disease diagnostics remains underexplored, hindering clinical implementation.

The scalable DeepSeek-R1 architecture (1.5B-671B parameters) advances medical LLM development [22-25]. While its chain-of-thought (CoT) reasoning succeeds in general cognitive tasks, clinical validation for rare disease diagnosis , requiring specialized reasoning patterns, remains lacking.

This 3-phase investigation first evaluates ChatGPT-4o’s diagnostic accuracy using clinical manifestations from China’s rare disease catalog. We then assess 4 open-source LLMs (Chinese and United States–developed models with different parameters) in bilingual contexts (Chinese and English) to quantify language effects. Concurrently, we validate RAG and CoT for capability enhancement. Finally, clinician surveys across specialties (n=11) measure rare disease knowledge gaps, informing LLM implementation needs. This multilevel assessment aims to (1) determine clinical applicability of LLMs, (2) establish model selection criteria (parameters, language, and origin), and (3) guide development of region-specific diagnostic tools.

## Methods

### Diagnostic Trial Study Design

We evaluated LLM diagnostic performance through 3 sequential phases: (1) baseline establishment: ChatGPT-4o’s English performance as commercial reference standard. (2) Open-source model assessment: cross-linguistic accuracy (Chinese and English queries), parameter scaling effects (7b to 72b variants), architecture comparison (Chinese vs United States–developed models). (3) Capability enhancement: controlled RAG and CoT testing. This framework addresses 3 performance determinants (parametric scale, language alignment, and developmental origin), with the goal of resolving the diagnostic performance disparity between open-source and commercial LLMs in rare disease contexts.

### Data Source and Collection for China’s Rare Disease Catalog

The National Health Commission of China has published the Rare Disease Catalog, which currently includes 207 conditions that meet 4 mandatory criteria: (1) low prevalence (<1/500,000 or neonatal morbidity<1/10,000), (2) severe health impact, (3) established diagnostic protocols, and (4) actionable treatment pathways. The catalog can be updated only after a period of at least 2 years, with the inaugural version in 2018 listing 121 diseases and the 2023 update adding 86 new entries.

Clinical phenotypes were extracted from the National Rare Disease Registry System (NRDRS) [26], a centralized platform managed by Peking Union Medical College Hospital, integrating data from 107 collaborating institutions. As of 2025, the NRDRS contains containing 92,600 cases with demographic, diagnostic, therapeutic, and survival parameters from 107 institutions.

For LLM evaluation, we systematically curated deidentified symptom descriptions from NRDRS, removing disease-specific identifiers (nosology, pathogenic variants, and subtype classifications) to simulate patient narratives. Given genetic basis of most cataloged diseases, and their diagnostic criteria typically hinge on genetic testing rather than clinical symptoms or auxiliary diagnostic tests, we have designated genetic testing as a key criterion for classifying diseases as genetic within the catalog, in anticipation of subsequent analyses.

The clinical manifestations of all diseases were translated from Chinese to English using DeepL’s web interface [27], with each disease processed as an independent translation unit. Translated outputs underwent manual review by bilingual researchers to ensure completeness and preserve semantic accuracy, particularly for critical diagnostic descriptors. Verified translations preserved the source text’s original structure and were directly used as clinical case descriptions for LLM diagnostic evaluations in English.

### Diagnostic Flow

Using ChatGPT-4o’s application programming interface, we prompted the model: “As a doctor specializing in rare diseases, what kind of rare disease is most likely to be diagnosed based on the clinical manifestations of the disease I have provided you? In addition, 5 other possible diagnoses need to be listed.” To prevent conversational confounders, we used single-turn queries with history truncation, requiring models to output primary diagnosis and ranked differentials (top 5) solely from input data.

We clarify that LLMs cannot definitively diagnose rare diseases based solely on symptoms (clinical manifestations are insufficient for confirmation). Since most rare diseases require genetic testing or laboratory confirmation, absence of expected diagnoses in outputs does not imply model error but reflects symptom overlap among diseases. Our objective was to assess whether models can prioritize catalog diseases from clinical prompts, mimicking clinicians’ preliminary diagnose. Correct diagnosis required inclusion of exact disease names, accepted synonyms, or broader categories in primary and differential lists. Two clinical physicians reviewed the outcomes of the model’s outputs, and in cases of disagreement, a third physician was consulted to reach a consensus through thorough discussion.

### Selection of Open-Source LLMs

We evaluated 4 open-source LLMs: qwen2.5:7b/72b (Alibaba) and Llama3.1:8b/70b (Meta), representing diverse parameters and geographic origins. All models ran locally via the Ollama framework [28] with a custom interface “Chat” [29]. The models are configured with a temperature setting of 0.1 to ensure precision in outcomes, using prompt wording and diagnostic procedures analogous to ChatGPT-4o. Each model underwent sequential evaluation: English first, then Chinese.

### Statistical Analysis

ChatGPT-4o’s accuracy was assessed via Pearson chi-square (*χ*²) tests stratified by genetic status. In addition, 4 open-source models underwent bilingual testing (121 cases), generating 8 response sets. McNemar tests (*χ*² if discordant pairs ≥25; exact binomial otherwise) compared 36 model pairs plus 2 RAG and CoT interventions (38 comparisons). Total assessments (N=39) used Bonferroni-adjusted α=.001, and reported marginal findings (*P*<.05) to reduce type II errors. Analyses used R (R Core Team) version 4.3.2.

### Construction of Rare Disease Knowledge Base and Application of RAG

Leveraging the outputs from ChatGPT-4o, we used the open-source framework Maxkb (GitHub version 1.7.2) [30] to implement RAG for constructing a knowledge base specific to rare diseases. In addition, we did not correct the minority of diagnostic errors present in the ChatGPT-4o’s conversational content, as the primary objective of engaging in RAG was to explore the precision of its retrieval capabilities. It was also of interest to observe whether open-source LLMs would adhere to the principle of accurate retrieval despite the presence of incorrect conclusions within the knowledge base.

Text segmentation used “user” as delimiter, generating 121 text blocks (726 entries) representing complete diagnostic responses. The retrieval process used MaxKB’s proprietary embedding model (maxkb-embedding) for dense vector representations. For each query, we retrieved the top 1 most semantically relevant text block based on cosine similarity thresholding (>0.6). The system imposed a 2144-character constraint per retrieved segment to ensure clinical context preservation. When no qualifying segments were identified, a standardized null-response protocol (“There are no related contents.”) was triggered. The model with the lowest-performing in English was chosen for RAG to assess whether its diagnostic capabilities would improve post-RAG integration. It is defined as correct retrieval when the primary diagnosis output by the generative model is consistent with the primary diagnosis provided in the retrieved knowledge base (regardless of whether the diagnosis is correct).

### CoT Model Selection Rationale

The DeepSeek-R1 architecture (671B parameters) was distilled into 2 CoT variants: DeepSeek-R1:7B (from qwen2.5:7B) and DeepSeek-R1:8B (from Llama3.1:8B). While these distilled models were not directly evaluated, their base models underwent full diagnostic benchmarking in this study. This experimental design enables comparative analysis between a base model and its distilled counterpart to assess CoT-mediated performance. We selected the lowest-performing English base model (qwen2.5:7B) for CoT testing, ensuring performance changes reflected CoT architecture.

### Questionnaire for China’s Rare Disease Catalog

Initially aimed to compare physician diagnoses with LLM outputs. However, most surveyed diseases fell outside clinicians’ expertise. Moreover, in clinical practice, they rely heavily on whole-exome sequencing for diagnostics, suggesting that the accuracy of diagnoses for the diseases might be close to zero.

The revised survey assessed multidisciplinary specialists’ (prenatal diagnosticians, pediatricians, radiologists, etc) awareness of China’s Rare Disease Catalog to evaluate the need for LLM-assisted diagnostics. The selection of physicians was not random but rather aimed at maximizing the inclusion of professionals from relevant fields that our research team had access to, ensuring a diverse representation of perspectives.

### Ethical Considerations

This study used publicly available web-based textual data that did not contain any specific patient information. As a simulation of clinical diagnostic trials without intervention on real patients, this study complies with the institutional guidelines for ethics committee exemption.

## Results

### Diagnostic Accuracy of ChatGPT-4o

**Figure 1.**
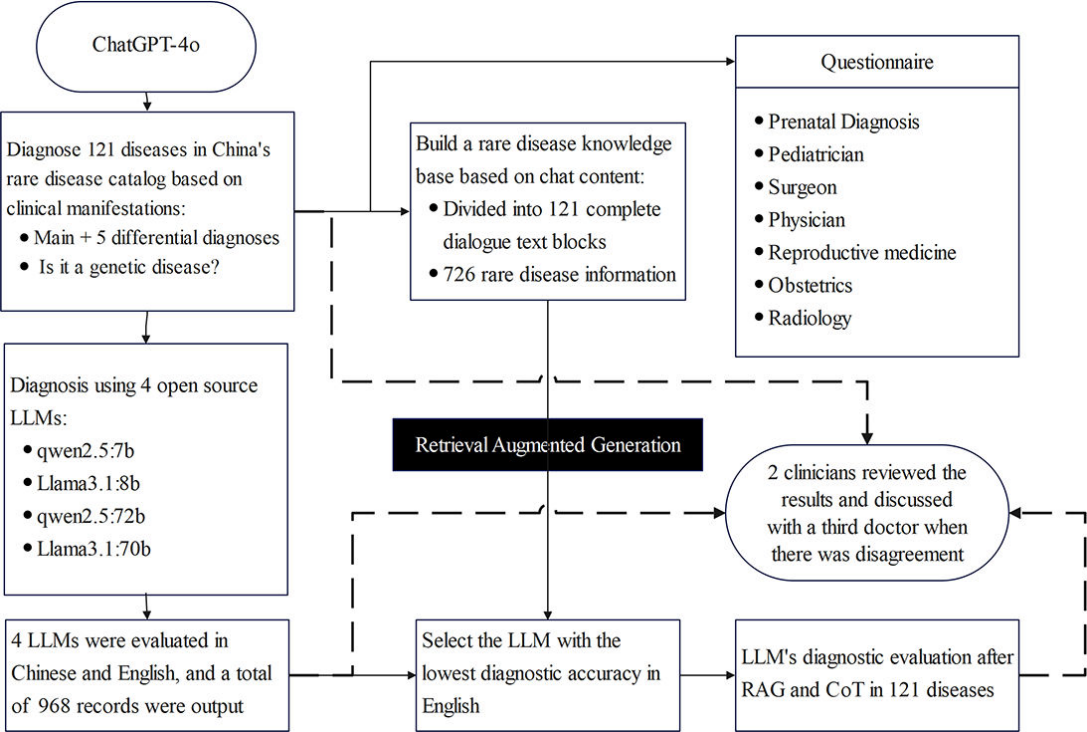
Comprehensive design flowchart of the study. ChatGPT-4o provided diagnostic assessments for 121 diseases in both languages. In parallel, 4 open-source large language models (LLMs) generated 8 response sets (4 models×2 languages); chain-of-thought integration with large language models outputs yielded 11 evaluation groups. CoT: chain-of-thought; LLM: large language model; RAG: retrieval augmented generation.

Figure 1 illustrates the methodological workflow encompassing all analytical phases. English-translated clinical cases (n=121) were evaluated (full outputs: MultimediaAppendices 1  2). The outputs comprised 86.78% genetic diseases and 12.22% nongenetic diseases, highlighting the genetic preponderance in China’s rare disease registry (Table 1). ChatGPT-4o achieved an overall accuracy of 90.1%, with no significant difference in performance between genetic and nongenetic diseases. Among correct cases, 13.8% (15/109) were listed as differentials.

**Table 1.** ChatGPT-4o diagnosis of 121 diseases in the China’s first catalog of rare diseases.

	Diagnosis, n (%)		Total, n (%)	95% CI
	Accuracy	Misdiagnosis		
Genetic^a^^,^^b^^,^^c^	94 (89.5)	11 (10.5)	105 (86.8)	0.83‐1.10
Nongenetic^a^^,^^b^^,^^c^	15 (93.8)	1 (6.3)	16 (13.2)	
Total	109 (90.1)	12 (9.9)	121 (100)	

a *χ*2: 0.28.

b *P*=.60.

c Relative risk (RR)=0.96.

### Diagnosis of Rare Diseases by 4 OpenSource LLMs in Chinese and English

#### Results of Diagnosis

**Figure 2.**
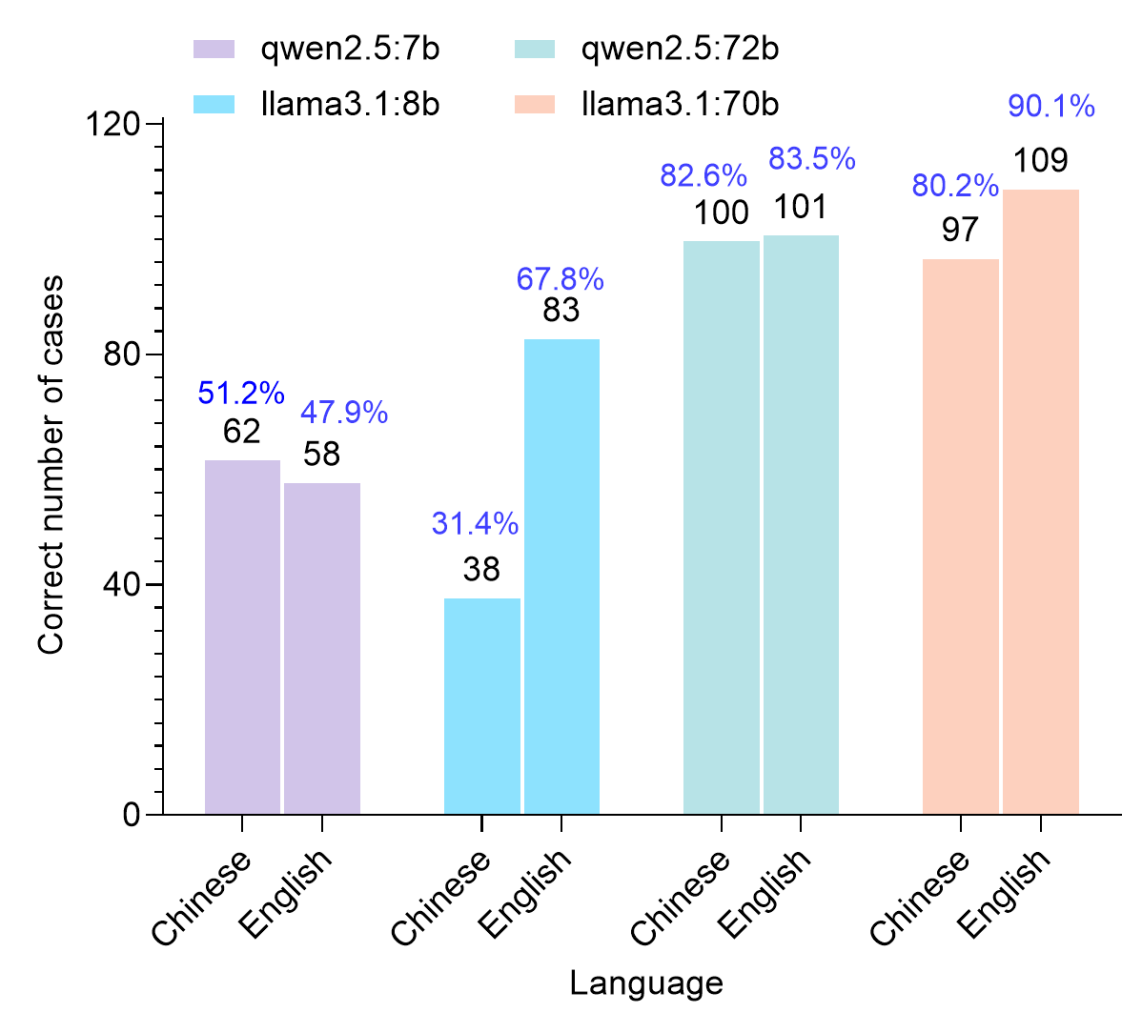
Correct number and proportion of cases by 4 open-source large language models in English and Chinese. Bilingual accuracy patterns reveal model-specific language dependencies, with Chinese qwen2.5 models demonstrating linguistic invariance contrasting with US Llama3.1 models’ English-dominant profiles.

Furthermore, 4 open-source LLMs diagnosed 121 rare diseases in both English and Chinese. Their performance, as illustrated in Figure 2, revealed notable variations across models and languages.

#### Performance in English

The LLMs exhibited a range of diagnostic capabilities, with Llama3.1:70b achieving the highest accuracy, followed by qwen2.5:72b, Llama3.1:8b, and qwen2.5:7b. Among the correct diagnoses, some were identified through differential diagnosis: qwen2.5:7b resolved 14 cases (24.1%), Llama3.1:8b resolved 15 cases (18.1%), qwen2.5:72b resolved 12 cases (11.9%), and Llama3.1:70b resolved 14 cases (12.8%). Notably, Llama3.1:8b initially refused to respond to one specific case (congenital hyperinsulinemic hypoglycemia) but provided a diagnosis upon re-engagement with identical settings.

#### Performance in Chinese

Qwen2.5:72b achieved the highest number of correct diagnoses, closely approaching Llama3.1:70b, followed by qwen2.5:7b, and finally Llama3.1:8b.

Moreover, qwen2.5 models (7b and 72b) demonstrated consistent performance across languages, maintaining strong diagnostic accuracy. In contrast, the Llama3.1 models (8b and 70b) showed reduced accuracy in Chinese compared with English, highlighting language-specific limitations. For differential diagnoses, qwen2.5:7b identified 11 cases (17.74%), Llama3.1:8b identified 6 cases (15.8%), qwen2.5:72b identified 10 cases (10%), and Llama3.1:70b identified 9 cases (9.3%). In addition, Llama3.1:70b frequently defaulted to English responses, requiring prompt adjustments to elicit Chinese outputs.

The analysis reveals significant language-mediated accuracy variations in LLM diagnostics. Chinese qwen2.5 models (7b/72b) showed cross-lingual consistency, whereas US Llama3.1 models (8b/70b) demonstrated language-specific performance, with English accuracy surpassing Chinese baselines.

### Multiple McNemar Tests for Diagnostic Accuracy of LLMs

ChatGPT-4o and 4 open-source LLMs generated 9 response sets. A total number of 36 McNemar test comparisons across the sets revealed distinct patterns (Multimedia Appendix 2). For language effects, qwen2.5:7b showed comparable Chinese (51.2%) and English accuracy (47.9%; *χ*²_1_=0.32, *P*=.57) and Llama3.1:8b exhibited English superiority (67.8% vs 31.4%; *χ*²_1_=40.20, *P*<.001). Larger models displayed architectural divergence where qwen2.5:72b maintained cross-lingual consistency (82.6% vs 83.5%; OR 0.88, *P*=1.000), while Llama3.1:70b showed language-dependent performance (80.2% vs 90.1%; OR 0.29, *P*=.02, marginally significant).

**Figure 3.**
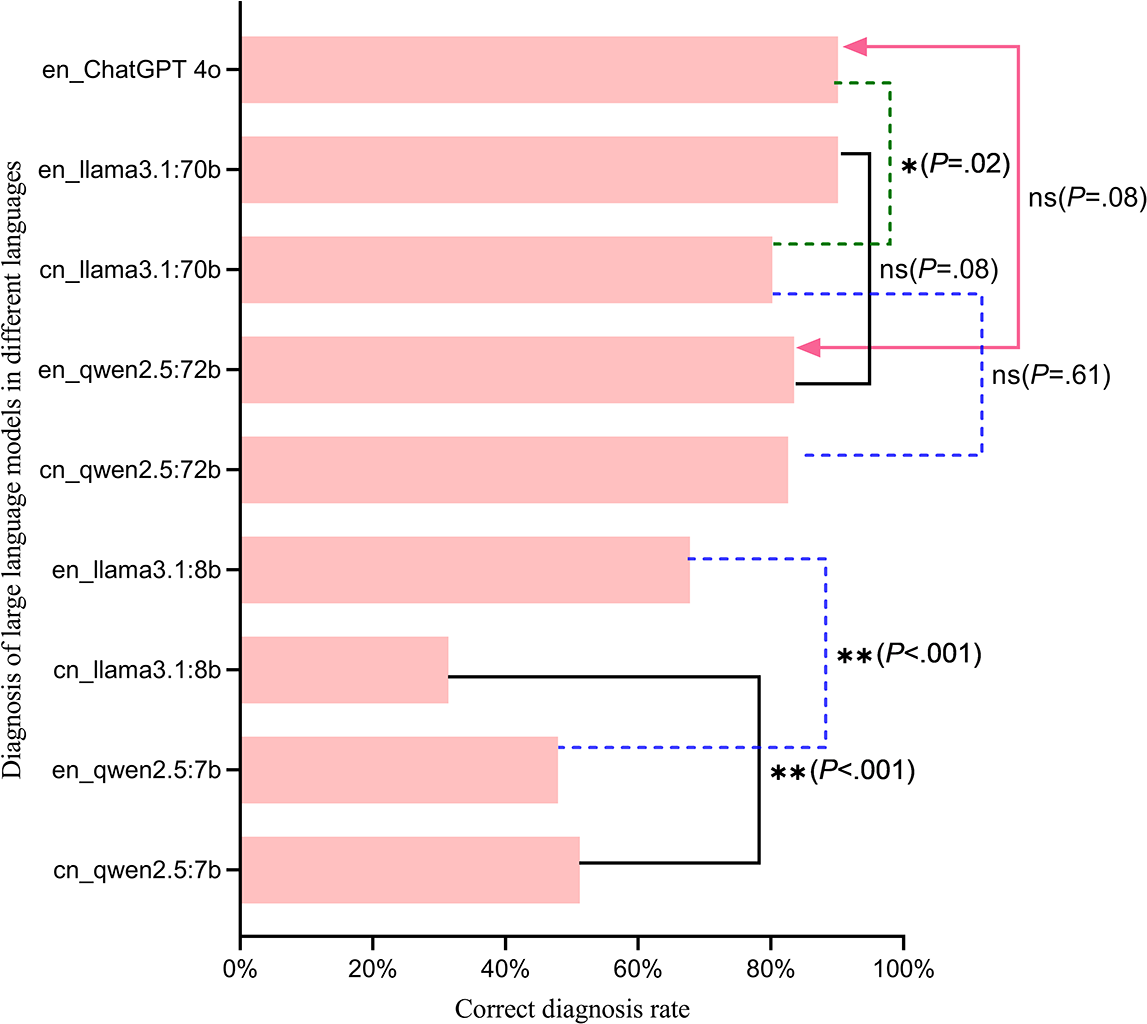
Cross-model diagnostic accuracy comparisons using McNemar tests. Performance evaluation based on rare disease, with models tested in Chinese and English. Significance notation: non-significant (*P*≥.05); *marginally significant (*P*<.05) ;**statistically significant (*P*<.001 post-Bonferroni correction).

Cross-model comparisons (Figure 3) revealed Llama3.1:8b underperformed qwen2.5:7b in Chinese (*χ*²_1_=13.22, *P*<.001) but excelled in English (*χ*²_1_=13.92, *P*<.001) No significant differences between qwen2.5:72b and Llama3.1:70b (English: OR 0.33, *P*=.08; Chinese: OR 1.5, *P*=.07) were found. qwen2.5:72b matched ChatGPT-4o’s performance in both languages (English: OR 0.33, *P*=.08; Chinese: OR 0.44, *P*=.09), and Llama3.1:70b mirrored ChatGPT-4o’s English accuracy (OR 1, *P*=1) but lagged in Chinese (OR 0.33; *P*=.02; marginally significant). Moreover, diagnostic accuracy scaled positively with parameter count across both languages (Figures3  4).

**Figure 4.**
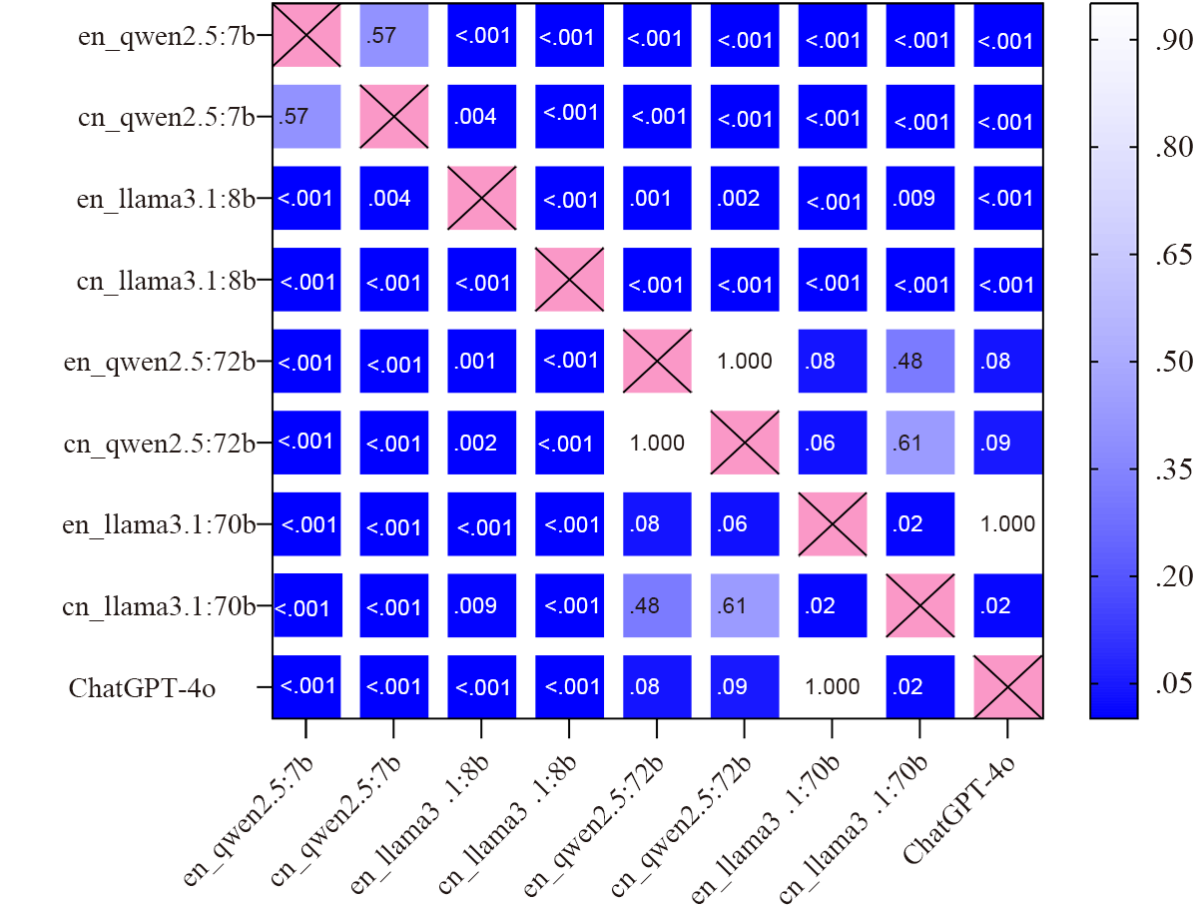
Diagnostic accuracy comparison matrix. Heat map visualization of cross-model *P *values (n=36 comparisons). Language identifiers: English and Chinese. Diagonal: identical dataset pairs (noncomparisons). Symmetric mirroring: Upper/lower triangle equivalence. Color encoding: darker hues indicate stronger significance.

### Enhancing Diagnostic Accuracy for Rare Diseases With RAG Technology

Among the evaluated models, qwen2.5:7b showed the lowest English diagnostic accuracy. Implementing RAG with a knowledge base built from ChatGPT-4o’s diagnostic outputs significantly improved its performance: accuracy increased to 79.3% (Δ+31.4% from baseline; Table 2), with qwen2.5:7b achieving 85.9% retrieval precision under imperfect knowledge bases. Theoretically revealed data suggests that RAG-enhanced accuracy could reach 85.9% with perfect retrieval. This is comparable to ChatGPT-4o’s benchmark of 90.1% when using identical clinical case inputs.

**Table 2.** Diagnostic performance of qwen2.5:7B with retrieval augmented generation intervention.

	Before RAG^a^, n (%)	After RAG, n (%)
Correct diagnosis^b^^,^^c^	58 (47.9)	96 (79.3)
Incorrect diagnosis^b^^,^^c^	63 (52.1)	25 (20.7)
Correct retrieval	—^d^	104 (85.9)
Incorrect retrieval	—	17 (14.1)

a RAG: retrieval augmented generation.

b *X*2_1_=31.11.

c *P* value <.001.

d Not applicable.

For disease 35 (glycogen storage disease), qwen2.5:7b incorrectly diagnosed the case and omitted differentials. Notably, the disease was correctly identified before RAG implementation with accurate knowledge base entries (primary diagnosis). Moreover, qwen2.5:7b consistently failed to diagnose cases with inaccurate knowledge base content.

### CoT Diagnostic Performance

The distilled DeepSeek-R1:7b demonstrated significant accuracy degradation when assessed on the same 121 rare diseases. Compared with its base model qwen2.5:7b (47.9% accuracy), DeepSeek-R1:7b achieved only 9.9% diagnostic precision (12/121 cases; Δ–38.0%, *χ*²_1_=42.19, *P*<.001).

### Survey on Awareness of China’s Rare Disease Catalog

We surveyed 11 clinical physicians from various departments in top-tier Chinese hospitals to assess their familiarity with diseases in China’s rare disease catalog. These physicians, especially 3 prenatal diagnosticians, are likely to encounter a wider range of rare diseases due to their practice settings. Departments like obstetrics, pediatrics, surgery, internal medicine, and radiology also commonly encounter rare diseases, while specialists in reproductive medicine may less frequently encounter them.

China’s rare disease catalog has been public since 2018. Data (Table 3) show that even experienced prenatal diagnosticians have limited awareness of the catalog’s diseases. Specialists in departments with a narrow disease focus, such as reproductive medicine, also tend to lack understanding of rare diseases. Our findings indicate that physicians’ knowledge of rare diseases is related to their department, experience, and training. Senior physicians generally know more about rare diseases but lack comprehensive knowledge, while junior attending physicians have more knowledge gaps.

**Table 3.** Clinical physicians’ familiarity with diseases from the first batch of China’s rare disease catalog.

Specialties and titles	Years of experience	Aware of catalog, n (%)	Able to diagnose, n (%)
Prenatal diagnosis			
Chief physician	21	112/121 (92.91)	71/121 (58.51)
Associate chief	29	103/121 (85.21)	98/121 (80.99)
Attending physician	3	85/121 (7.02)	23/121 (18.93)
Obstetrics, n (%)			
Attending physician	12	22/121 (18.2)	5/121 (4.13)
Attending physician	12	35/121 (28.93)	15/121 (12.40%)
Pediatrics			
Attending physician	7	65/121 (53.7)	10/121 (8.26)
Radiology			
Attending physician	5	37/121 (30.58)	15/121 (12.40)
Surgery			
Attending physician	4	30/121 (24.79)	16/121 (13.22)
Internal medicine			
Attending physician	4	17/121 (14.05)	8/121 (6.61)
Reproductive medicine			
Attending physician	5	21/121 (17.36%)	1/121 (0.83%)
Attending physician	4	40/121 (33.31)	13/121 (10.74)

## Discussion

### Principal Findings

Our cross-linguistic evaluation identified 3 critical factors governing LLM diagnostic performance. ChatGPT-4o achieved the highest diagnostic accuracy in rare disease assessment, highlighting LLMs’ potential in this domain. Open-source models showed strong dependence on parameter scale, input language, and developer origin. Implementation of RAG markedly improved qwen2.5:7b’s diagnostic accuracy, contrasting sharply with the performance degradation observed in CoT-enhanced models. Clinician surveys reinforced these technical findings, revealing substantial knowledge gaps among specialists—even senior practitioners demonstrated limited familiarity with China’s Rare Disease Catalog entries, underscoring the clinical necessity for LLM-assisted diagnostic systems.

### Limitations

First, our evaluation focused on China’s first rare disease catalog, potentially limiting generalizability to newly recognized or ultra-rare conditions. However, this focused approach prioritizes clinically impactful diseases over obscure textbook entries, enhancing practical relevance.

Second, the diagnostic evaluation was based solely on clinical manifestations, which may not fully reflect the complexity of real-world scenarios. This limitation could potentially reduce the diagnostic accuracy of the models. However, using clinical manifestations is still important for guiding clinicians to consider rare diseases, thereby reducing misdiagnoses and diagnostic delays. In addition, phenotype-driven strategies are valuable for minimizing the costs of genetic testing. Future work should integrate multimodal data (imaging and biomarkers) to better replicate real-world decision-making.

Third, Chinese-to-English translation via DeepL may introduce subtle inaccuracies. Although the translations were reviewed manually, some errors were inevitably, which potentially reduced the accuracy of the model in English. However, given the strong language comprehension ability of LLMs and the fact that the study results did not indicate higher accuracy for LLMs in Chinese, we believe that the translation process did not affect the statistical conclusions.

Finally, this study did not assess open-source models with parameters between 8 billion and 70 billion. This was due to the selection of two pairs of LLMs with similar parameters, which allowed for a more detailed comparison across different model aspects. Some models with fewer than 70b parameters may also perform well in diagnosing rare diseases, especially as technology keeps advancing.

### Comparison With Previous Work

This study’s clinical phenotypes derive from NRDRS, specifically targeting conditions in the nationally mandated catalog. The curated phenotypic profiles intentionally incorporate diagnostic uncertainty through multidisease symptom overlaps, rigorously simulating real-world differential diagnosis challenges for LLM evaluation. Catalog inclusion prioritizes diseases with elevated clinical urgency in China’s epidemiological context, characterized by high disease burden, actionable diagnostic criteria, and treatment-responsive outcomes. While global repositories (eg, OMIM [Online Mendelian Inheritance in Man] and Orphanet) provide extensive rare disease data, their geographic prevalence distributions introduce significant epidemiological mismatches for regional implementation. This justifies prioritizing region-specific frameworks for clinical LLM validation.

Recent studies show ChatGPT can help diagnose rare and complex diseases by analyzing medical histories and test results, offering potential diagnoses and treatment plans [31,32]. This aids health care professionals in faster, more accurate rare disease diagnosis [33]. However, ChatGPT-4 and Llama2 are more proficient in diagnosing common diseases than rare ones [12], which can be attributed to the limited training data in LLMs. Nevertheless, as LLMs improve, this gap should narrow [12,17]. In our investigation, the latest ChatGPT-4o achieved excellent accuracy diagnosing China’s initial rare disease catalog. While we recognize the transformative potential of ChatGPT-4o in rare diseases, we also concur that ChatGPT should be used as a supplementary tool, rather than a substitute for medical expertise [10,34,35].

Open-source LLMs enable hospital-specific diagnostic tools through local deployment, showing potential to surpass commercial models via targeted fine-tuning [36,37]. However, the proliferation of heterogeneous models (varying parameters or developers) complicates optimal model selection for rare disease applications.

This study involved deploying 4 open-source LLMs from different vendors with varying parameters to test their ability to diagnose rare diseases in Chinese and English. The goal was to compare diagnostic accuracy across models and assess their performance. Recent research shows ChatGPT performs unevenly across languages, with a big gap between its English skills and other languages [18,38]. For example, ChatGPT scores 82.67% in English sentence completion but only 35.85% in Arabic [39]. Its response accuracy drops from 71.49% in English to 42.74% in Arabic. In medical settings, ChatGPT works better with English prompts, even when analyzing Chinese medical reports [38]. These findings stress the need for better multilingual models and fixing language biases.

Our study did not assess the diagnostic prowess of ChatGPT-4o in Chinese, given its established English proficiency. Benchmarking against ChatGPT-4o’s English performance enabled systematic assessment of open-source models’ diagnostic parity with commercial counterparts.

Our findings confirm a strong positive correlation between model parameters and diagnostic accuracy. The qwen2.5:72b demonstrated a significant improvement in English accuracy over its smaller 7b-parameter counterpart, while Llama3.1:70b achieved accuracy comparable to ChatGPT-4o. Notably, language adaptation capabilities varied significantly by model origin: Chinese-developed qwen2.5 series maintained minimal accuracy variance between Chinese and English, attributable to balanced bilingual training data. In contrast, United States–developed Llama3.1 exhibited English-centric biases: the 8B-parameter version showed higher English versus Chinese accuracy, requiring 70b parameters to achieve Chinese diagnostic parity with qwen2.5:72b. In addition, despite significant marginally, Llama3.1:70b’s English accuracy surpassed its Chinese performance, highlighting persistent linguistic disparities in cross-regional model development. These results align with emerging evidence that model architecture and training corpus composition (not just parameter scaling) critically determine multilingual diagnostic capability [12,17].

The current data does not show that ChatGPT-4o’s diagnostic capabilities are significantly higher than those of open-source models with parameters around 70 billion, even when compared with the worst-performing diagnostic scenario (Chinese_Llama3.1:70b). Although this study established a strict significance level using Bonferroni adjustment, which prevented the demonstration of a significant difference between Chinese_Llama3.1:70b and ChatGPT-4o, an accuracy exceeding 80% is already sufficient for a foundational model. Domain-specific fine-tuning could further enhance performance for clinical deployment. In addition, in English-speaking countries, smaller models (Llama3.1:8b) with reinforcement strategies achieve acceptable accuracy at reduced computational costs.

Using the local language for diagnosing rare diseases may offer benefits, as models can access knowledge aligned with a country’s disease incidence rates. For example, in our review of generated outputs, Chinese_Llama3.1:70b correctly diagnosed non syndromic deafness (case 83), while English Llama3.1:70b and ChatGPT-4o did not. Chinese Llama3.1:70b identified the *GJB2* gene mutation–linked nonsyndromic hearing loss, a common cause in China due to widespread prenatal deafness gene screening and internet-based information. In contrast, English Llama3.1:70b suggested Pendred syndrome, which Chinese physicians usually call “Goiter-deafness syndrome” and is not in China’s rare disease catalog. This suggests LLMs may adapt responses to a user’s national context based on language, which could improve diagnostic relevance and accuracy. Instead of just translating to English, incorporating the user’s national background in LLM-driven diagnostics could be more effective, especially in models with large parameters.

Recent studies show RAG can boost LLM performance in medical tasks by integrating external databases, tackling issues like hallucination and outdated knowledge [21,40]. A case study on retrieving medical guidelines and treatment recommendations from curated medical resources demonstrated that RAG significantly improved model performance in terms of factual accuracy, completeness, user preference, and safety compared with standard LLMs [41]. Another study developed a customized LLM framework that combines RAG and prompt engineering to accurately interpret medical guidelines for the management of patients with chronic hepatitis C virus infection, thereby improving clinical decision support systems with promising results [42]. These studies highlight RAG’s potential in medical LLMs. Our study explored RAG to bridge the gap between open-source LLMs and ChatGPT-4o in the diagnosis of rare diseases. Results show that even small-parameter LLMs with RAG and a good knowledge base can match ChatGPT-4o’s accuracy. However, issues were identified with the qwen2.5:7b model using RAG, like not following prompts to generate differential diagnoses and incorrectly retrieving answers, even with matching queries. This suggests RAG might struggle with flexible clinical descriptions of rare diseases. RAG’s retrieval traceability remains its paramount strength, enabling clinicians to independently verify source validity rather than blindly trusting LLM outputs. Overall, RAG improves open-source models’ rare disease diagnostic performance, potentially surpassing commercial models, yet underscore the necessity for further optimization to ensure reliable retrievals.

DeepSeek’s recently released CoT-optimized models [22-25] present both opportunities and challenges for clinical AI integration. While CoT architectures excel in complex reasoning tasks, particularly arithmetic and commonsense inference, their computational intensity creates latency-performance tradeoffs. Emerging evidence suggests CoT frameworks may reduce diagnostic hallucinations and enhance decision interpretability in medicine [43-46], although effectiveness varies significantly across model scales, with larger architectures like GPT-4 showing superior adaptability [47]. Our findings reveal a paradoxical 38% accuracy decline in DeepSeek-R1:7b versus its base model, likely stemming from domain-specific knowledge loss during medical-focused distillation. This contrasts with code-oriented tasks where distillation typically improves efficacy, suggesting medical diagnosis requires preservation of specialized clinical reasoning patterns vulnerable to compression. To mitigate these limitations, we propose (1) postdistillation medical domain adaptation through targeted fine-tuning, or (2) deployment of larger CoT variants (eg, DeepSeek-R1:32b) to maintain diagnostic fidelity.

The physician survey confirmed the anticipated correlation between physicians’ knowledge of rare diseases and factors such as their specialty, professional experience, and training exposure. The scarcity of physicians proficient in diagnosing rare diseases from clinical phenotypes [6,48] contributes to the protracted time frames, often several years, for the confirmation of most rare diseases [1]. Phenotype-based tools and research aimed at prioritizing disease-causing genes in genetic disorders have long been pivotal in the diagnosis of rare diseases [7,49,50]. However, the conversion of natural language into standardized Human Phenotype Ontology terms present a persistent challenge that taxes the knowledge base of clinical physicians, and it is also a process that is both time-consuming and labor-intensive. Given that physicians are adept at medical record documentation, interfacing with LLMs using the medical terminology of clinical diagnostic tasks significantly boosts the efficiency of phenotype-based rare disease diagnosis.

Despite the current limitations of even the most sophisticated LLM in achieving comprehensive diagnostic accuracy across all rare diseases, the clinical integration of LLM diagnostic tools necessitates a strategic equilibrium between technological innovation and patient safety. To ensure privacy protection, LLMs should be implemented via hospital-based on-premises deployment, operating within isolated intranet environments and achieving seamless integration with existing electronic health record systems to eliminate risks of sensitive data exposure. Throughout diagnostic workflows, LLM outputs must be explicitly labeled as “auxiliary recommendations,” requiring mandatory human validation by attending physicians who must corroborate findings against comprehensive patient histories, laboratory evidence, and updated clinical guidelines. In rare disease scenarios, definitive diagnoses shall only be established following multidisciplinary consultations and evidence-based literature verification of LLM-generated hypotheses.

### Conclusions

In clinical practice where physicians frequently struggle to promptly diagnoses rare diseases, ChatGPT-4o demonstrated superior accuracy in China’s rare disease catalog. Moreover, optimizing parameters, language alignment, and pretraining data origins in open-source LLMs, combined with RAG augmentation, enhanced diagnostic precision to near-commercial performance. Caution remains warranted for low-parameter reasoning models showing substantial performance limitations. These findings establish hospital-specific LLM assistants as a feasible pathway for high-accuracy rare disease diagnosis.

## Supplementary material

10.2196/69929 Multimedia Appendix 1 This supplementary Material includes all the chat records of diagnoses for China's first batch of rare disease catalog by ChatGPT-4o and the four LLMs in this study. The document contains 11 collections of chat contents. The diagnostic sequence of the LLMs for the cases can be found in Multimedia Appendix 2.

10.2196/69929 Multimedia Appendix 2 This supplementary material encompasses the detailed diagnostic outcomes of ChatGPT-4o and the four LLMs for the cases in China's first batch of rare disease catalog. It includes a total of 12 sheets, presenting the specific diagnostic results and the comparative summary.

10.2196/69929 Multimedia Appendix 3 This file contains the R code used in this study.

## Abbreviations

CoT: chain-of-thought
LLM: large language model
NRDRS: National Rare Disease Registry System
OR: odds ratio
RAG: Retrieval Augmented Generation
