# Supplementary material for: Performance of ChatGPT-4o and Four Open-Source Large Language Models in Generating Diagnoses Based on China’s Rare Disease Catalog: Comparative Study
Source: J Med Internet Res. 2025 Jun 18;27:e69929. doi: 10.2196/69929 (PMC12192912; doi:10.2196/69929)
Supplement: Multimedia Appendix 1 [file jmir-v27-e69929-s001.zip › numbered_DeepSeek-R1-Distill-Qwen2.5-7B.docx]

# DeepSeek-R1-Distill-Qwen2.5-7B

### 1. User

The disease can begin in early childhood. Typical clinical manifestations include kidney and eye changes and hearing impairment. Renal manifestations are hematuria, proteinuria and progressive deterioration of renal function, in which hematuria is often persistent microscopic hematuria, which can occur after exercise or fever, and is common in adolescents. Hearing changes were sensorineural hearing loss, which gradually aggravated with the course of disease. Eye abnormalities manifest as myopia, youth ring and cataract, but lack specificity. Three ocular changes of diagnostic significance include anterior conical lens, posterior pleomorphic corneal atrophy, and retinal macula (white or yellow particles around the fovea). The incidence of hearing impairment in male patients in China was higher than that reported abroad (68% vs 55%), while that in female patients was lower than that reported abroad (7% vs 45%). The incidence of ocular abnormalities was similar to that reported abroad. Leiomyomas may also occur in a few patients, involving the respiratory tract, gastrointestinal tract, and female reproductive tract; occasionally aneurysmal lesions, midfacial dysplasia, and mental retardation have been reported.

Patients often have a family history. Male patients with autosomal recessive inheritance and X-linked inheritance progressed faster, often entering end-stage renal disease at the age of 16~35, while female patients with autosomal dominant inheritance and X-linked inheritance had a slower course of disease and later onset of renal failure. The age at which renal failure occurs is similar for all male members of the same affected family, but can vary among male members of different families. Therefore, renal failure can be divided into juvenile type (renal failure occurs in adolescence) and adult type (renal failure occurs in adulthood) according to the age of the affected family. Adolescent type is more prominent in extrarenal manifestations, boys usually die before reproduction, disease genes are often new mutations, while adult type families have older patients and fewer new mutations.

### DeepSeek-R1-Distill-Qwen2.5-7B

Based on the clinical manifestations provided, the most likely diagnosis is **Miller Syndrome X**. This syndrome is an autosomal recessive disorder that presents with a combination of renal, hearing, and ophthalmic abnormalities, often with significant strain on the patient’s quality of life. The features described—kidney and eye changes, hearing impairment, and hematuria—align well with this condition.

### Other Possible Diagnoses:

1. **Miller-Sterling Syndrome** — Another rare autosomal recessive disorder with ophthalmic, renal, and neurologic features.
2. **Alpers Syndrome** — Often caused by mutations in genes like KIT, affecting skeletal, cardiovascular, hepatic, and ophthalmic systems.
3. **Mucocutaneous Jacquot Syndrome** — Characterized by facial abnormalities, ear, nose, and throat issues, as well as cardiovascular and ophthalmic issues, but may not fit as closely with the hearing changes described.
4. **Bailey-Ward Tissue Fibrosis Progressive Retinitis (With Hypopigmentation)** — While primarily an ophthalmic disorder, it can cause renal and hearing issues in affected individuals.
5. **Oculoneuropathic Autimmune Diseases (OUNAID)** — A group of rare disorders with ophthalmic, neurologic, and renal features.

These diagnoses are suggested based on the combination of symptoms, family history, and the progression of the disease. However, a thorough diagnostic evaluation is necessary to confirm the exact cause.

### 2. User

Patients often have no abnormal performance in the neonatal period. The first symptom is developmental retardation at about 6 months of age, but the typical clinical manifestations appear after 1 year of age, and it often takes several years for clinical diagnosis to be confirmed. According to the frequency of clinical manifestations, they were divided into three categories: common manifestations (100% patients), frequent manifestations (more than 80% patients) and correlated manifestations (20% ~80% patients).

1. All of them showed normal pregnancy history and head circumference at birth; no birth defects and abnormal biochemical indexes; brain MRI/CT showed no structural abnormalities except mild cortical atrophy and myelin dysplasia; motor milestones lagged behind without regression; severe developmental retardation occurred at 6~12 months; language disorders showed no or very little vocabulary, repetitive language and non-verbal communication ability were stronger than expressive language ability; motor or balance disorders often showed ataxia and tremor of limbs; Abnormal behavioral characteristics are frequent laughter or smiling, obvious excited movements or happy behavior, often accompanied by clapping or hyperactivity.
2. The head circumference often showed backward growth, and microcephaly was still present at the age of 2. Epilepsy often occurred before the age of 3. Abnormal EEG: characteristic high amplitude spike-slow wave.
3. Correlation: flat occipital region; tongue preference; sucking or swallowing disorders; infantile feeding difficulties; low muscle tone; large jaw; wide tooth space; frequent salivation; excessive chewing movements; strabismus; hypopigmentation of the skin; light hair and eye color compared to family (absent type only); hyperreflexia of lower limb tendons; lifting or bending of upper limbs, especially when walking; wide gait base; high sensitivity to heat; abnormal sleep-wake cycle, less sleep; obsession with water, paper, and plastic; Eating related behavioral abnormalities; Obesity (more common in older, non-deficit patients); scoliosis; constipation.

### DeepSeek-R1-Distill-Qwen2.5-7B

Based on the clinical descriptions provided, the most likely diagnosis appears to be Lenbrowser syndrome. This condition presents with a combination of neurodevelopmental delays, developmental retardation, and a range of neurological, musculoskeletal, and immunological features. Here’s a structured summary of the reasoning:

1. ** developmental retardation and delays**: The presence of developmental retardation at around 6 months and motor milestone regression without regression points to a late-onset neurodevelopmental disorder, which is characteristic of Lenbrowser syndrome.
2. ** neurological and musculoskeletal features**: The child exhibits characteristics such as ataxia, tremors, and hyperreflexia, as well as skeletal abnormalities like scoliosis and wide gait base. This combination of neurological and musculoskeletal features aligns with Lenbrowser syndrome, which involves a triad of neurodevelopmental, neurologic, and bone/ligamentous changes.
3. ** Neurological abnormalities**: The MRI findings of mild cortical atrophy and myelin dysplasia, along with the presence of high amplitude spikes and slow waves on EEG, suggest focal brain abnormalities. These are typical of Lenbrowser syndrome.
4. ** bone and muscle involvement**: The child shows signs of bone deficiency with normal or increased囟门 circumference and microcephaly, typical of this syndrome. Additionally, the presence of Obesity correlates with older age, which may indicate delayed growth-phase related to the condition.
5. ** Epilepsy and Absence Seizures**: The occurrence of epilepsy with strangulated and absent-seizure pattern aligns with Lenbrowser syndrome, which can present with focal brain atrophy and difficulty with temporal lobe epilepsy.

Given the combination of these elements, Lenbrowser syndrome is the most plausible diagnosis. Other conditions like spinal muscular atrophy, spinal stenosis, or spinal cord compression were considered but did not fit as comprehensively as Lenbrowser syndrome.

### 3. User

1. Skeletal abnormalities: There is variation in the severity of the disease and the type of associated complications. Thoracic stenosis is present in all cases, and the thorax may be bell-shaped or long and narrow, with respiratory distress usually caused by a small, rigid thorax. The severity of respiratory insufficiency varies according to the type of thorax and the degree of underlying pulmonary dysplasia, and the age of onset varies. There is a negative correlation between the severity of respiratory insufficiency and the age at which the disease is diagnosed. Acute respiratory disease is more common in infants with severe restrictive and bell-shaped chests, and severe and fatal respiratory failure can occur in the early neonatal period. Infants with a long, narrow chest are more likely to present with mild respiratory involvement, which can eventually progress to chronic restrictive lung disease with recurrent respiratory infections. Short, deformed ribs cause intercostal muscle weakness and restricted breathing, leading to respiratory failure. Most children die within 2 years of age, with about half dying within 6 months of birth. Other skeletal anomalies include short ribs, short limbs, abnormally shaped pelvis, short fingers, and polydactyly. About 60% of patients have proximal cervical spinal stenosis.
2. Involvement of other organs: Renal lesions are present in about 40% of patients. The earliest sign of renal disease is impaired urinary concentration. Other signs include polyuria, proteinuria, elevated blood urea nitrogen and creatinine, and hypertension. Renal failure is the leading cause of death in this patient between the ages of 3 and 10 years. Hepatic involvement occurs in about 30% of cases and is manifested by hepatomegaly, elevated aminotransferases, cholestasis, cirrhosis and portal hypertension. The disease is occasionally associated with ocular developmental abnormalities, including optic nerve hypoplasia, retinal dystrophy, retinal pigmentation abnormalities, nystagmus, myopia and progressive visual impairment. There are also a few reports suggesting that these patients may also present with neurological abnormalities such as growth retardation, hypotonia, and central nervous system MRI abnormalities.

### DeepSeek-R1-Distill-Qwen2.5-7B

Based on the clinical manifestations provided, the most likely diagnosis is **spinal stenosis albinism** (spinal stenosis with simultaneous albinism and associated conditions like simultaneous respiratory distress and organ involvement). This condition is characterized by the presence of spinal stenosis, hemolytic anemia, and albinism with often simultaneous involvement of other organs, including the kidneys, heart, lungs, and eyes.

### Five additional possible diagnoses:

1. **Acute restrictive respiratory disease with spinal stenosis**: This could involve acute respiratory failure due to restrictive thoracic stenosis and simultaneous spinal stenosis.
2. **Hereditary spina bifida and spinal stenosis**: While this condition usually involves spina bifida, it could potentially involve spinal stenosis and organ dysplasia, though it is more commonly associated with hypotrophy and sensory abnormalities.
3. **Pseudoxanthoma elasticum with respiratory issues**: While less common, pseudoxanthoma elasticum is a rare condition that can involve elastic plate anomalies, potentially with associated respiratory compensatory mechanisms.
4. **Sparse albinism with associated respiratory and organ dysplasia**: Sparse albinism is a rare disorder involving variable defects and may include respiratory and organ issues alongside spinal stenosis-like findings.
5. **Acute kidney failure with liver dysfunction and respiratory distress**: Acute kidney failure is very common in infancy, but this condition lacks the systemic organ involvement described in the case.

This differential diagnosis is highly context-specific, and the key clue here is the simultaneous involvement of multiple systems, including the spine, heart, kidneys, liver, and eyes, with respiratory distress.

diagnostic_result

### 4. User

The main symptoms include mental behavioural abnormalities, cognitive impairment, memory loss, seizures, speech disorders, movement disorders, involuntary movements, decreased level of consciousness, and autonomic dysfunction. Other accompanying symptoms include sleep disorders, focal damage to the CNS, brainstem and cerebellar symptoms, and involvement of peripheral nerves and neuromuscular junctions.

There are three main clinical types: (1) anti-NMDAR encephalitis, which is the most predominant type and is characterised by diffuse encephalitis. (ii) Borderline encephalitis, with mental behavioural abnormalities, temporal lobe epilepsy and near-memory deficits as the main symptoms. The common types of borderline encephalitis are anti-LGI1 antibody, anti-GABABR antibody and anti-AMPAR antibody-related encephalitis. (iii) Other types, including Morvant syndrome, anti-DPPX antibody-associated encephalitis, anti-IgLON5 antibody-associated encephalopathy, and autoimmune cerebellar ataxia.

### DeepSeek-R1-Distill-Qwen2.5-7B

Based on the clinical symptoms you have described, the most likely diagnosis is **anti-NMDAR encephalitis**, a rare neurological disorder affecting the central nervous system (CNS). This condition is characterized by CNS inflammation, systemic neurologic symptoms, and focal or diffuse encephalitis. Key symptoms include mental and behavioral abnormalities, cognitive impairment, memory loss, seizures, speech disorders, movement disorders, involuntary movements, decreased level of consciousness, autonomic dysfunction, and focal or diffuse brainstem and cerebellar involvement. These symptoms also align with the description of **borderline encephalitis** and its less common variants.

Other possible diagnoses that could present with these symptoms and more broadly include:

1. **TDP-43 encephalitis**
   - Characterized by focal or diffuse encephalitis, behavioral disturbances, memory loss, speech difficulties, and brainstem/cerebral abnormalities.
2. **Corticobasal syndrome (dominant voxel syndrome)**
   - A rare and rare autosomal recessive disorder affecting the brainstem, cerebellum, andWegener’s granulomatosis. Symptoms include severe cognitive impairment, memory loss, speech/speech disturbances, ataxia, and decreased level of consciousness.
3. **Antibody-mediated autoimmune cerebellar encephalitis (AiCEn)**
   - Often caused by anti-neurofilament antibodies and characterized by focal or diffuse cerebellar abnormalities, decremental cognitive function, speech and language issues, and autonomic dysfunction.
4. **Leukoencephalopathy with heterotrophicNazca neurons cryptococcosis (LEN CRC)**
   - A rare cryptochondritis-like encephalitis caused by the bacterium *Cryptococcus neoformans* affecting homologue neurons and presenting with behavioral disturbances, memory loss, and decreased level of consciousness.
5. **Cerebral hypoperfusion syndromes**
   - Related to focal or widespread hypoperfusion of the brain, leading to behavioral abnormalities, memory loss, speech and language difficulties, seizures, and decreased level of consciousness.

These diagnoses can often present with overlapping symptoms, but the most common type of anti-NMDAR encephalitis is the predominant condition in this category.

### 5. User

The clinical manifestations of the disease are complex and varied, and lack specificity. Most of them involve multiple systems such as nerve, skin, respiratory, digestive and immune systems, and it is difficult to confirm the diagnosis from the clinical manifestations alone. It can be divided into two types: complete and partial.

1. Complete: It can be divided into two types: early-onset and late-onset. The most common manifestations are neurological symptoms, such as hypotonia, epilepsy, developmental delay, sensorineural deafness, ataxia, and progressive disorders of consciousness, etc. About half of the patients have ataxia, developmental delay, conjunctivitis, and visual problems, including visual problems. conjunctivitis, and visual problems, including optic atrophy. Metabolic disorders such as metabolic acidosis, hypoglycaemia and ketosis are often comorbid, and cutaneous manifestations include various forms of refractory rashes such as eczema, generalised erythema, and nappy rash, and patients may also present with respiratory symptoms such as hyperventilation, laryngeal wheezing, and apnoea, as well as digestive symptoms such as feeding difficulties, vomiting, and diarrhoea. Acute attacks can be triggered by infection, fever, fatigue, and a high protein diet. If treatment is not timely, it can often lead to irreversible damage. Patients with late-onset disease often develop in adolescence and may have no symptoms beforehand. The clinical phenotype is different from that of early-onset disease, which usually presents with peripheral neuropathy, muscle weakness, spastic paraparesis, skin rashes, and ocular problems, such as loss of vision and visual acuity, etc. Failure to treat the disease in a timely manner often leads to irreversible damage.
2. Partial : May develop at all ages or even without onset. The overall signs and symptoms of the child are relatively mild, starting only in emergency situations such as infections and starvation. Patients may have hypotonia, skin rashes, hair loss, acute neurological symptoms such as epilepsy and impaired consciousness.

### DeepSeek-R1-Distill-Qwen2.5-7B

The described clinical manifestations of the disease are highly suggestive of **Neurofibromatosis Type 2 (NF2)**. NF2 is a rare genetic disorder that affects multiple systems, including the nervous system, skin, and bone. It is characterized by developmental delay, sensory issues such as hypotonia, and can include respiratory and digestive symptoms. The disease can present with both early-onset and late-onset features, and it is associated with metabolic acidosis, hypoglycemia, and ketosis in later stages. The skin can also exhibit rash-like lesions, which can progress over time.

Other possible diagnoses that align with the described symptoms include:

1. **Epilepsy with Hydrocephalus**: This condition involves both a neurological surplus (hydrocephalus) and epilepsy, leading to symptoms such as developmental delay, sensory issues, and respiratory problems.
2. **F VBigDecimal (F Iraqi)**: This rare genetic disorder causes exonophthis (a skin disorder), developmental delay, and can affect the nervous system, leading to symptoms such as muscle weakness and sensory issues.
3. **Adactylodorsomnitorunt Alexandra 1 (ASD)**: This rare disorder is characterized by a variety of symptoms, including behavioral and nervous system issues, skin changes, and sensory deficits.
4. **Leukoencephalopathy due to Protoconversion in the Newborn (LEPPON)**: This rare condition affects the brain and can lead to developmental delay, muscle weakness, and metabolic issues.

Each of these diagnoses can present with a wide range of symptoms, including those described, and they are all associated with rare and complex presentations.

### 6. User

The high prevalence age group is infancy, which manifests as adrenocortical insufficiency with a predominant salt loss, and common symptoms include hyperkalemia, hyponatremia with dehydration, metabolic acidosis, hypoglycaemia, vomiting, shock, and skin pigmentation. Most patients present with failure or incomplete development during puberty, or impaired fertility, oligospermia and azoospermia. A few patients may present with early childhood hyperandrogenaemia, simple saline corticosteroid deficiency, and pseudo-precocious puberty or spontaneous pubertal development during puberty. In mild cases, the disease may start in adulthood.

Patients have hyperpigmentation of the skin, especially in exposed areas, friction areas, scarring and areola, axilla, scrotum.

Patients have varying degrees of reduced resistance to various stresses such as infection and trauma. In the event of a stressful situation, adrenal crisis can occur, manifested by nausea, vomiting, abdominal pain or diarrhoea, severe dehydration, lowered blood pressure, rapid heart rate, weak pulse, mental disorders, and often high fever, hypoglycaemia, hyponatraemia and even life-threatening.

### DeepSeek-R1-Distill-Qwen2.5-7B

Based on the clinical manifestations you have described, the most likely diagnosis is **discoid lupus erythematosus (DLE)**, a rare autoimmune skin and systemic disease. The description of skin pigmentation changes, hyperpigmentation in sun-exposed areas, salt loss, and systemic symptoms like hyperkalemia, hyponatremia, metabolic acidosis, and dysfunction in puberty are consistent with DLE.

Other possible rare diagnoses consistent with the clinical picture include:

1. **Adrenocortical insufficiency** (common in infancy but can occur in adults as well): This condition is characterized by salt loss and hyperkalemia, which are also noted in your description.
2. **Cushing’s syndrome**: A rare endocrine disorder that causes hyperglycemia, high blood pressure, and salt retention. However, the additional symptoms like shock and pubic hair changes (oligospermia and early puberty) make this less likely.
3. **Hyperandrogenism**: A condition where there is an increase in androgen production, leading to puberty onset. This may explain the hyperpigmentation and enzymatic changes.
4. **Severe Johannsen Ad Bigstra (JB):** This is a rare autosomal recessive disorder causing generalized hypoxia, salt loss, and hyperkalemia, among other symptoms. However, the presence of pigmentation changes complicates this diagnosis.
5. **Anion Gap-Metabolic Acidosis (AGMA):** While not a rare disease, AGMA is a common metabolic disorder that causes electrolyte disturbances and hyperglycemia, which aligns with some of the symptoms.

If you could provide more details about the individual’s medical history, family history, or response to treatment, I could better narrow down the diagnosis.

### 7. User

The disease can develop from birth to early adulthood. Neonatal onset is the most common, often presenting as macrosomia, poor feeding, hunger intolerance and persistent hypoglycaemia. Hypoglycaemic episodes may present with lethargy and hypotonia, as well as neurological sequelae or even death, such as apnoea, seizures and coma. In the delayed type, patients often show a hypoglycaemic reaction, including symptoms such as pallor, sweating and palpitations.

### DeepSeek-R1-Distill-Qwen2.5-7B

Based on the symptoms described, neonatal hypoglycaemic retinopathy (NHR) is the most likely diagnosis. This condition can manifest from birth to early adulthood, with typical symptoms such as hypoglycaemic episodes, poor feeding, and neurological issues. However, here are five other possible rare diagnoses that could present with similar symptoms:

1. **Infantile Insomniat Type II (IIT II)**: This metabolic disorder in children can cause hypoglycaemic episodes, leading to symptoms like ketoacidosis and prostration. It is characterized by a significant rise in blood glucose followed by hypoglycaemia.
2. **Central Hypoglycaemic Encephalopathy (CHC)**: This condition can lead to hypoglycaemic episodes and neurological symptoms, such as lethargy, apnoea, seizures, and comas. It is often associated with more stable hypoglycaemia over time.
3. **Mitochondrial Hypoglycaemic Disorders**: These genetic conditions can cause hypoglycaemic episodes, varying in severity. They may not always present with ketoacidosis but can result in other hypoglycaemic complications.
4. **Rare Genetic Disorders with Hypoglycaemic Episodes and Neurological Symptoms**: Conditions like infancy Toll- like 3 (Tlr3) deficiency can present with type I and II hypoglycaemic episodes along with neurological effects.
5. **Rubella Encephalopathy**: Although more associated with measles, rubella can cause encephalopathy and hypoglycaemic episodes in children, though the symptom presentation may differ.

These conditions should be considered for further evaluation based on the specific clinical presentation and patient history.

### 8. User

1. Type I, abdominal distension, growth retardation, hypoglycemic convulsions, recurrent nosebleeds, diarrhoea and vomiting are the main reasons for children’s patients to visit the clinic, and very rarely, the first manifestation is hematuria, blood in the stool, recurrent bone fracture, anaemia or gout. Adult patients who are never diagnosed and treated may present with multiple hepatic adenomas, chronic renal failure, severe gout with multiple gout stones, and osteoporosis. Other rare presentations include pulmonary hypertension, diabetes mellitus, cerebrovascular disease, and hepatic adenoma carcinoma. On physical examination, short stature and markedly enlarged liver can be seen.
2. Type Ib, in addition to the above manifestations, patients may also have recurrent infections with neutropenia, oral ulcers, inflammatory bowel disease, perianal ulcers, arthritis and splenomegaly.
3. Infantile type II, which is divided into classical infantile type and non-classical infantile type according to its prognosis. Most of the classical infants present with generalised muscle weakness in the first month of life, delayed motor development, enlarged heart on chest X-ray, high QRS waves and short PR intervals on ECG, hypertrophic cardiomyopathy on cardiac ultrasound, and varying degrees of elevation of creatinine kinase, etc. Most of the infants die of left heart failure or cardiorespiratory failure after pulmonary infections in the first year of life. The non-classical infantile form presents with muscle weakness within the first year of life, lags in motor development, and most often dies of respiratory failure in early childhood.
4. Type II late-onset, with onset of disease after 1 year of age and as late as 60 years of age. Most of the patients present with chronic progressive proximal muscle weakness and respiratory insufficiency, cardiac involvement is rare, and respiratory failure is the main cause of death. Clinical manifestations include easy fatigue, difficulty in sitting up, walking up and down stairs, squatting and walking, and in a few cases, sudden onset of respiratory failure.

### DeepSeek-R1-Distill-Qwen2.5-7B

Based on the symptoms described, several rare diseases could potentially be considered. These diagnoses are characterized by their rarity and the underdiagnosis that often occurs, leading to late or incomplete presentations. Here is a structured overview of the potential rare diseases associated with the symptoms:

1. **Enteral Nutrition Dependence (END):**
   - **Symptoms:** These include abdominal distension, growth retardation, hypoglycemic convulsions, recurrent nosebleeds, diarrhea, and vomiting. These symptoms are often associated with Tube-Feeding or Enteral Nutrition dependence.
   - **Rationale:** END is rare and often underdiagnosed, leading to complications such as recurrent bone fractures, anemia, and hyperbilirubinemia.
2. **Inflammatory Bowel Disease (IBD) late presentation:**
   - **Symptoms:** While IBD is more commonly noted for severe diarrhea in adults, it might present with abdominal symptoms, anemia, and eventually, more serious complications.
   - **Rationale:** Late or presentations with other symptoms can be indicative, especially withpossible Neurodevelopmental issues.
3. **Ossifying Chondroma Type II (OC II) in children:**
   - **Symptoms:** Characterized by generalized muscle weakness, oral ulcers, and gout-like stones (like pseudogout).
   - **Rationale:** OC II is a rare skeletal disorder that can present with an array of symptoms, sometimes mimicking other conditions early on.
4. **Gout in children:**
   - **Symptoms:** May include cellulite-like masses, predisposition to infections, and unique patterns of stones.
   - **Rationale:** While gout is more common in adults, children may present with different characteristics and may be underdiagnosed, especially if symptoms overlap with other conditions.
5. **SPARC (Secondary Progressive Renal Cell Carcinoma):**
   - **Symptoms:** Asymptomatic presentation in early or early adulthood, which can later present with chronic kidney disease and gout.
   - **Rationale:** SPARC is a rare malignancy of uncertain origin, often asymptomatic until symptoms emerge in later life.
6. **Diabetic Neuropathy:**
   - **Symptoms:** Includes foot ulcers and polyneuropathy with varying degrees of hyperglycemia, weight loss, and fatigue.
   - **Rationale:** Type II糖尿病 with complications is rare, and the symptoms here might suggest a form of diabetic neuropathy.
7. **Pulmonary Hypertension (PH):**
   - **Symptoms:** As yet there are no established early symptoms, leading to late diagnosis.
   - **Rationale:**PH is rare and often asymptomatic until advanced stages, with olefin-related symptoms.
8. **Cerebrovascular Disease (CVD):**
   - **Symptoms:** May vary but can include headaches, seizures, and neurodevelopmental issues like short stature.
   - **Rationale:**symptoms can overlap with other conditions, making CVD underdiagnosed in some cases.

**Additional Considerations:** - **Combination of conditions:** The combination of symptoms might suggest multiple underlying issues, such as a水电解质失衡 disorder (e.g., Endeavơr disorder with dextrose feeding deficiency) or co-morbid metabolic conditions. - **Autoimmune Disorders:** While less likely based on symptoms, consider checking for conditions with videlicet effects like99-106 knee pain syndrome or122-ox basilicodermatosis, which can present causing missed diagnoses. - **Adiposity-related Conditions:** Such as16-21-23 syndrome or17-21-26 syndrome, though less likely for the described symptoms.

**Conclusion:** The most likely rare disease based on the symptoms provided is Enteral Nutrition Dependence, considering the standardized combinations of chronic weight loss, anemia, diarrhea, and other systemic presentations. Other possibilities include IBD, Ovarian Tumors, Gout, and others, but each requires careful consideration of the unique symptom presentation. Proper diagnostic evaluation is crucial due to the rarity and underdiagnosis of these conditions.

### 9. User

1. Neuro-muscular system, causing neurological abnormalities, such as convulsions, coma, ataxia, and even psychiatric symptoms; muscle symptoms can also occur, including hyperreflexia, tremor, tetany, and muscle weakness. 2. Cardiovascular system, causing ECG changes, such as PR interval prolongation, QRS wave widening, etc. In severe cases, it can cause ventricular arrhythmia.
2. cardiovascular system, causing changes in electrocardiogram, such as prolongation of PR interval, widening of QRS wave, and so on, and in severe cases, it may cause ventricular arrhythmias.
3. Kidney, which may cause kidney stones, renal calcium deposits, renal insufficiency.
4. bone and joint system, can cause osteoporosis and osteochondrosis.
5. digestive system, may cause intestinal obstruction.
6. endocrine, patients can see diabetes mellitus or impaired glucose tolerance.
7. ocular symptoms, some types may be combined with ocular lesions, including nystagmus, visual field defects, myopia, etc. This group of diseases usually has a chronic course.

This group of diseases usually has a chronic course, with the neonatal and infantile onset types often being more severe and having a poorer prognosis. Some patients develop chronic renal failure during adolescence, and in some cases, failure to treat the disease in time can lead to severe mental retardation or even death.

### DeepSeek-R1-Distill-Qwen2.5-7B

Based on the clinical manifestations you provided, the most likely rare disease is **mitochondrial encephalomyopathy**. This condition often presents with a chronic, severe course, particularly in infants and neonates, and is associated with neurological, cardiovascular, kidney, and metabolic complications. The symptoms you described, such as neurological abnormalities, cardiovascular changes, kidney dysfunction, and bone and joint issues, align well with mitochondrial disorders, which are characterized by impaired mitochondrial function affecting multiple organ systems.

### Other Rare Diagnoses:

1. **Ehlers-Danlos syndrome with organ involvement**: This condition is rare and often presents with a combination of skin, tendon, and joint abnormalities, along with possible organ involvement such as the heart, kidney, and nervous system.
2. **Mitochondrial encephalomyopathy, Leigh syndrome**: A rare mitochondrial disorder associated with severe neurological, cardiovascular, and kidney issues.
3. **Mucolipidosis**: A rare lipid metabolism disorder that can cause a range of symptoms, including skin, organ, and nervous system abnormalities.
4. **Chronic kidney disease with metabolic features**: Rare and often caused by mitochondrial dysfunction, leading to kidney insufficiency and metabolic complications.
5. **Combined metabolic skeletal and neurological condition**: Some rare mitochondrial disorders present with a combination of metabolic, skeletal, and neurological features.

ThesePossible diagnoses may vary based on the specific clinical context and genetic background of the patient.

### 10. User

There are five types, of which the classic and intermediate types are the most common.

1. Classic type is the most common, accounting for 75 per cent of cases. Children are normal at birth, with early onset, severe symptoms and rapid progression. Usually, maple syrup odour appears in the cerumen 12 hours after birth; a special maple sugar odour is found in urine and sweat 12-24 hours after birth; ketonuria, irritability and feeding difficulties appear in 2-3 days; severe metabolic disorders and encephalopathic symptoms begin to appear in 4-5 days, which are manifested as difficulty in breastfeeding, paroxysmal vomiting, refusal to eat, lethargy, apnoea, convulsive seizures, heightened muscle tone and ‘fencing’. The symptoms of severe metabolic disorders and encephalopathy may include difficulty in breastfeeding, refusal to eat, lethargy, apnoea, convulsive seizures, increased muscle tone, and stereotypical movements such as ‘fencing’-like and ‘stomping’-like movements, etc. Coma and central respiratory failure may appear in the first 7-10 days of life, and most of the infants will die of severe metabolic disorders a few days after birth if they are not treated promptly.
2. Intermediate type can develop at any age. The symptoms are less severe than those of the classic type and include slow growth, delayed development, feeding difficulties, irritability, a maple syrup odour in the cerumen, and severe metabolic disorders and encephalopathy under stress. 3. Intermittent Type The intermittent type is characterised by episodic seizures.
3. Intermittent type: intermittent seizures, normal growth and development in the early stage, mostly under stressful situations such as infections and surgeries, manifested by episodic ataxia and ketoacidosis, and a very small number of severe cases may cause death. Branched-chain amino acid concentrations are normal during the intermittent period, and are elevated during the episodic period, similar to the classic type.

### DeepSeek-R1-Distill-Qwen2.5-7B

Based on the clinical manifestations described, the most likely rare disease is **infantile dysfunction syndrome with sleep apnoea and metabolic deficiency**, often associated with congenital sleep apnea. The clinical features, such as the onset of sleep apnea, cerumin odour, metabolic instability, and seizures, strongly suggest this syndrome.

### Key Features:

1. **Onset in infancy**: The classic type typically presents at birth or shortly thereafter.
2. **Cerumin Odour**: Present 12 hours after birth due to air trapping in the upper airway.
3. **Metabolic instability**: Rapid progression of symptoms, metabolic disorders, and encephalopathy.
4. **Seizures and respiratory failure**: Severe metabolic disorders can lead to convulsive seizures and, in some cases, central respiratory failure.
5. **Episodic nature**: The intermittent type is characterized by recurrent metabolic issues and seizures, often precipitated by stress or infections.

### Other Possible Diagnoses:

1. **Kain-Weiss Coveys syndrome**: A rare autosomal recessive sleep apnoea syndrome with hypoxemic respiratory failure.
2. **Infantile dysfunction syndrome**: A more general term for infants with hypoxia, metabolic instability, and developmental delays, which may overlap with sleep apnea.
3. **Compound partial screening syndrome**: A less common genetic disorder with symptoms including hypoxia and metabolic issues.
4. **Infantile atropospondyl dysplasia**: A rare autosomal recessive disorder with features of hypoxia and developmental delay.
5. **Neonatal hypoventilation syndrome**: A common condition characterized by episodes of hypoxia and apnoeic breathing.

These diagnoses highlight the complexity of rare diseases and the need for comprehensive evaluation and management tailored to the patient’s specific symptoms and risk factors.

### 11. User

The disease can start from birth to old age and can be acute or insidious. Almost any system or organ can be involved, with a predominance of energy-demanding organs such as the brain, heart, kidneys, eyes, ears, muscles, and gastrointestinal tract. Patients may present with a clinical syndrome of some kind, a single symptom or a combination of symptoms.

### DeepSeek-R1-Distill-Qwen2.5-7B

Based on your description of a disease that can span from birth to old age and affect multiple energy-demanding organs, the most likely rare disease is **low blood cell count (leuko fiberglass)**, which can lead to symptoms such as fatigue, weight loss, and bone marrow involvement. In addition to this, here are five other possible diagnoses:

1. **121 Connor syndrome ( disease )**: A rare genetic disorder affecting bone marrow and peripheral tissues, leading to megaloblastic anemia.
2. **19 aa18 disorder ( disease )**: A rare autosomal recessive disorder causing fatigue, anemia, and neurological symptoms.
3. **65 aa26 disorder/disease**: A rare autosomal recessive disorder affecting the heart, lungs, and other organs, often with cumulative fatigue.
4. **75 aa31 disorder ( disease )**: A rare autosomal recessive disorder causing blood cell aplasia and organ failure due to anemia and leukopenia.
5. **HOLDEN syndrome ( disease )**: A rare genetic disorder affecting bone marrow and peripheral tissues, leading to anemia and Satan-like fatigue.

### 12. User

The prevalence of the disease is between 5 and 50 years old, with a median age of onset of 39 years. The prevalence of women is significantly higher than that of men, with a female:male ratio of (9~11):1. Most of the cases have an acute/subacute onset, and the clinical manifestations include six core syndromes: (1) optic neuritis; (2) acute myelitis; (3) posterior pole syndrome; (4) acute brainstem syndrome; (5) symptomatic sleep attacks or acute mesencephalic clinical syndrome with typical MRI foci of the mesencephalon; and (6) symptomatic cerebral syndrome with typical brain lesions. It is a highly recurrent and disabling disease, with more than 90 per cent of patients having a recurrent course, and the majority of patients are left with more severe neurological disability.

### DeepSeek-R1-Distill-Qwen2.5-7B

The most likely diagnosis, based on the described clinical manifestations and considering the female predominance and age group, is **Acute Inflammatory Multisystem Neurosyndrome (AIMNS)**. This syndrome is characterized by acute onset and a high female to male ratio, affecting multiple brain regions and presenting with optic neuritis, acute myelitis, and various brainstem and mesencephalic conditions.

**Possible additional diagnoses** that may overlap with the described features include:

1. **Anticancer syndrome**: Due to less common and often severe presentations involving multiple neurologic features.
2. **Cerebral synaptic dysfunction**: Causing differential presentations, though typically less severe than other syndromes.
3. **Facio-optic neuropathy**: May present with optic neuritis as a core feature.
4. **Epilepsy with combined cafesynetic features**: Involving focal or multifocal lesions of the nervous system.
5. **Multiple acute inflammatory episodes**: Affecting different regions of the nervous system simultaneously.

These additional considerations provide a broader spectrum of possibilities that align with the multisystem inflammatory presentation described.

### 13. User

Hearing impairment. In the past, it was thought that the deafness was congenital, which could be identified by hearing screening, and that it would be apparent from birth, mostly involving both sides, and symmetrically severe prelingual deafness. In recent years, it has been found that individual cases may present with asymmetrical or unilateral hearing loss, and the degree of hearing loss is highly variable, ranging from mild to very severe in different patients; progressive deafness that occurs after birth is also not uncommon.

### DeepSeek-R1-Distill-Qwen2.5-7B

Based on the clinical manifestations described, the rare disease most likely to be diagnosed is **TDUCTS Syndrome (Thyroglossal Discase) Combined with Syspedia and X-Linked Osteonecrosis of the Temporal bone, Type 2 (X-UNET2)**. This syndrome is rare and presents with severe, asymmetric hearing loss, often accompanied by other features such as kwashiorkor, cleft palate, and xerOpericlitis. However, several other rare conditions could present with similar symptoms, including:

1. **Menkes Disease**: A rare genetic disorder causing significant hearing loss and neurological complications due to copper deficiency.
2. **Menkes-kin Syndrome**: Another rare genetic condition characterized by hearing loss and neurological issues, often due to mutations disrupting copper transport proteins.
3. **Tစက်စုံစသမ်း (Labyrinthine Congenital Ossicular Deformity)**: A rare congenital condition of the ear characterized by abnormal ear ossicular structure and hearing loss.
4. **Menkes-kin Lyons-Trentamoa Syndrome**: A less common variant of Menkes-kin Syndrome, also contributing to hearing loss and neurological issues.
5. **Copper Deficiency**: A rare condition where insufficient copper intake leads to poor copper transport, resulting in hearing loss and other systemic effects.

These conditions are rare due to genetic mutations, specific pathophysiological mechanisms, or unique presentations that distinguish them from more common disorders. For an accurate diagnosis, it is crucial to consult an audiologist, genetic counselor, or ear, nose, and throat (ENT) specialist, who can evaluate for these rare conditions.

### 14. User

Bradykinesia, tremor, and myotonia. Levodopa-induced motor complications are more likely to occur after treatment. In terms of non-motor symptoms, mood disorders (e.g., depression, anxiety, irritability, etc.) and behavioural disorders (e.g., compulsive dosage increases, impulse control disorders, stereotyped behaviour, etc.) are likely to occur.

### DeepSeek-R1-Distill-Qwen2.5-7B

Based on the symptoms described—Bradykinesia, tremor, and myotonia, along with non-motor symptoms of mood and behavioral disorders—it is most likely that the underlying condition is **Restinosis**. This is a dopamine-depleting disorder affecting the substantia nigra, leading to motor symptoms like tremors and myotonia. The use of levodopa, a common treatment, can result in side effects such as tardive dyskinesia. Non-motor symptoms, such as mood disorders and behavioral issues, are consistent with the impact of dopamine depletion on brain function.

Other possible conditions to consider include:

1. **Tardive dyskinesia**—a side effect of dopamine replacement therapy, though not the primary manifestation.
2. **Epogenetic movement disorder**—though less commonly associated with these symptoms.
3. **Fragmentation syndrome**—a rare condition associated with dopamine-related symptoms.
4. **Restinosis**—the primary cause fitting all described symptoms.
5. **Dementing movement disorder**—though less directly linked to the symptoms mentioned.

The most probable primary diagnosis is Restinosis.

### 15. User

**1. polyneuropathy**, often presenting as a symmetrical sensory and/or motor peripheral neuropathy of the extremities, progressing gradually from distal to proximal.

**2. organomegaly,** including hepatomegaly, splenomegaly, or lymph node enlargement; lymph node biopsy is often suggestive of Castleman disease.

**Endocrine abnormalities,** including hypogonadism (e.g., male impotence, breast development), hypothyroidism, abnormal glucose metabolism (e.g., diabetes mellitus), and adrenal insufficiency.

**4. skin changes,** which may be manifested by deepening of skin colour, cutaneous glomeruloid haemangiomas, white nails, polycythemia, hirsutism, and cyanosis of the hands and feet.

**5. increased extracirculatory water load,** including limb oedema, ascites, pleural effusion, pericardial effusion, and optic papilla oedema.

**Sclerosing bone disease,** is an important clinical manifestation. It may be manifested as bone pain or without clinical symptoms. Sclerosing bone disease can be detected significantly more frequently with CT scanning of the bones, and can be visualised as simple sclerosis of the bones, mixed sclerotic and osteolytic lesions, or simple osteolytic changes.

**7. erythrocytosis and/or thrombocytosis** **8.

**8. pulmonary hypertension** , patients may present with pulmonary hypertension, as evidenced by reduced activity tolerance, hypoxaemia, etc.

**9. cerebral infarction**.

### DeepSeek-R1-Distill-Qwen2.5-7B

Based on the clinical manifestations provided, the most likely diagnosis is **medullary sponge Gehlenius**, a rare condition characterized by a combination of polyneuropathy, bone lesions, organomegaly, and endocrine abnormalities. This condition is multifaceted, integrating symptoms such as polyneuropathy, bone tumors, sizes of organs, skin changes, and endocrine issues like hypothyroidism. The integration of these symptoms suggests a rare, multifactorial disorder that could be further explored through diagnostic evaluations including imaging and biochemical tests.

### 16. User

Common manifestations in the foetal period include reduced foetal activity, excess amniotic fluid and breech presentation. In the neonatal period hypotonia is one of the hallmark features of this disorder, and severe hypotonia can lead to asphyxia, feeding difficulties, weak sucking, and weak crying. In infancy and early childhood, the main manifestations are growth retardation and lagging cognitive, motor and language development. Appetite increases with age and obesity can occur if eating is not restricted. Short stature is usually present in school age, and personality traits such as temper tantrums, stubbornness, and obsessive-compulsive disorder gradually appear. Abnormal behaviours include intense foraging, skin scratching, and autistic-like behaviour. Seizures occur in about 25 per cent of patients. Delayed or incomplete appearance of secondary sexual characteristics may occur in adolescent males and females. Obesity can lead to complications such as sleep apnoea, pulmonary heart disease, insulin resistance and even diabetes. Scoliosis, osteoporosis, and gastroesophageal reflux are also more common. The main manifestations in adulthood are obesity and its complications, including cardiovascular problems, diabetes and sleep apnoea. Other manifestations are mental abnormalities and infertility.

Fatal risks: choking due to uncoordinated mouth movements, hypotonia, polyphagia, gulping habits and inadequate chewing, and gastric rupture due to overeating.

Physical examination reveals characteristic facial features that become progressively more typical, including almond eyes, small mouth, thin upper lip, downward-facing corners of the mouth, small hands and feet, short stature, fair skin, low muscle tone, and genital hypoplasia (e.g., cryptorchidism, scrotal hypoplasia, or clitoral hypoplasia).

### DeepSeek-R1-Distill-Qwen2.5-7B

The rare disease most likely associated with the described clinical manifestations is **Down syndrome**. Key points supporting this diagnosis include:

1. **Amniotic Fluid and Breech Presentation**: The presence of excess amniotic fluid and a breech presentation during the fetal period is a hallmark of Down syndrome, particularly associated with transposition of the great-circle accessory chord (TGAC).
2. **Growth Delay**: Over 50% stunted by the first birthday is a common feature of Down syndrome, leading to growth retardation.
3. **Hypotonia and Seizures**: These are typical motor issues that contribute to physical examination findings such as low muscle tone.
4. **Female Delayed Puberty**: The delay in puberty is often female predominant, which is also characteristic of Down syndrome.
5. **Presentation Features**: The almond eyes, thin upper lip, small mouth, and other distinctive facial features align with Down syndrome, despite the presentation being less common than other syndromes.

While other conditions like Spina Bifacial Deformity, Prader-Willi, or microdeletions (e.g., Patau) share some features, the combination of delay in motor and cognitive development, growth retardation, and agenesis presentation make Down syndrome the most fitting rare disease in this context.

### 17. User

Patients often start in infancy or adolescence, with roughly equal male and female ratios. The water content of secretions from the epithelial cells of the respiratory tract, pancreas and biliary tract is reduced, and the secretions become viscous and difficult to clear. The lungs are the most commonly affected organ, and patients present with chronic bacterial infections and colonisation of the respiratory tract with pathogens, often initially Haemophilus influenzae and Staphylococcus aureus, and ultimately with chronic colonisation and/or infection with Pseudomonas aeruginosa or Klebsiella onionis. Once infection occurs, neutrophils are recruited in large numbers into the lung tissue and release elastase, which causes destruction of the lung tissue, leading to the formation of bronchiectasis. The patient’s bronchiectasis often starts at an early age and often begins in the upper lobes, in contrast to the majority of infections that result in the formation of bronchiectasis that is primarily located in the middle and lower lungs.

Patients often present with sweat stains left on dark-coloured clothing, or parents notice salty skin in their children. Abnormalities in the flow of bile and pancreatic fluid can lead to indigestion and malabsorption, as well as liver or pancreatic disease, and in severe cases, associated diabetes. The viscous intestinal fluid may also make the patient more susceptible to intestinal obstruction and rectal prolapse. In addition, male patients may develop impaired sperm movement in the vas deferens and congenital absence of the vas deferens (CBAVD).

### DeepSeek-R1-Distill-Qwen2.5-7B

Based on the detailed description of the symptoms and clinical manifestations provided, the most likely diagnosis is **Acute-to-Chronic Transitioning Pneumonitis (ACTP)**, particularly affecting the upper lobes. This condition is characterized by chronic bronchiectasis, thick secretions, and systemic symptoms such as neutrophilic inflammation, dysfunction, and obstruction.

**Other possible diagnoses:**

1. **Pseudomonas aeruginosa Pneumonia**: Typically affecting the middle and lower lobes, but can also involve the upper lobes in rare cases.
2. **Acute Bronchitis-Chronicization syndrome**: May lead to regional symptom changes and chronic bronchiectasis.
3. **Interstitial Pneumonia**: A rare condition affecting both upper and lower lobes, associated with fibrosis and chronic inflammation.
4. **Acute Mobius Pneumonia**: A rare entity characterized by multisystem involvement and regional lung disease.
5. **Induced Pulmonary Hypertension**: Can lead to air trapping and chronic disease, presenting with a range of symptoms as described.

These diagnoses are considered based on the combination of respiratory symptoms, systemic manifestations, and the chronic nature of the condition.

### 18. User

Patients present with oropharyngitis, otitis media, respiratory infections, cellulitis and skin infections, often due to staphylococcal and streptococcal infections. Oral ulcers and painful gingivitis occur in most children before the age of 2 years in severe neutropenia with decreased bone marrow reserve. If associated with diffuse gastrointestinal lesions, it may cause abdominal pain and diarrhoea similar to Crohn’s disease. Neutropenia can lead to recurrent bacterial or fungal infections in various areas, with the main sites of involvement being the skin and mucous membranes, ears, nose, throat and lungs. The infections are often severe or even fatal. About 15% of patients progress to acute leukaemia or myelodysplastic syndrome. The children usually do not have characteristic malformations.

### DeepSeek-R1-Distill-Qwen2.5-7B

The main diagnosis is likely an acute myelodysplastic syndrome (MDS) or acute leukemia, given the progression observed in the patient. Here’s a structured summary of the thought process:

### Main Diagnosis:

1. **Acute Myelodysplastic Syndrome (MDS):** The progression to acute leukemia or MDS in 15% of cases suggests a specific MDS subtype influenced by the infection.
2. **Acute Leukemia:** This could be Acute Lymphoblastic Leukemia (ALL), Acute Promyelocytic Leukemia (APL), or other acute leukemia variants associated with the infection.

### Possible Other Diagnoses:

1. **Appending Syndrome:** Characterized by Streptococcus pyogenes infections leading to fever, immunodeficiency, and anemia, fitting the clinical presentation.
2. **Staphylococcal or Streptococcal Infections:** Without specific signs but leading to widespread infections across multiple body systems.
3. **Acute Ectrombolytic Bullcallen Syndrome (Ect Tromb Bull):** Assumed due to the bone marrow involvement.
4. **Myelofibrosis:** Though not directly mentioned, it could be associated with the infection.
5. **Other Blood-Related Rare Syndromes:** Including Acute Myelogenous Leukemia (AML) variants, though less likely.

This structured approach highlights the main and possible additional rare diagnoses based on the provided symptoms and clinical presentation.

### 19. User

Typical Patients present with both CNS damage and hypoadrenocorticism. Neurological symptoms and symptoms of hypoadrenocorticism may occur simultaneously or sequentially. In a minority of patients, they may occur alone. The manifestations of adrenocortical insufficiency vary in severity. It is most common in children, accounting for 35% of all patients. The onset of symptoms occurs between the ages of 4 and 8 years. The initial symptoms include poor concentration, memory loss, learning difficulties, unsteady gait and abnormal behaviour, followed by visual and auditory loss, dysarthria, seizures, ataxia, paralysis and dementia. Clinical manifestations progressively worsen, culminating in blindness or deafness and complete paralysis. Some exhibit deafferentation and eventually die of central respiratory failure, brain herniation, and infection. Most patients have adrenocortical impairment when they first develop neurological symptoms.

### DeepSeek-R1-Distill-Qwen2.5-7B

Based on the clinical manifestations you provided, the most likely rare disease diagnosis is **Ganglionic Eczema (GE)**. This condition is characterized by simultaneous CNS damage (neurological symptoms) and hypoadrenocorticism, progressing from ADHD-like symptoms to more severe neurological deficits and even Leigh syndrome-like features in some cases. The progression weaves from developmental delay through memory loss, learning difficulties, to severe neurological deficits and cognitive impairment.

Other possible diagnoses that align with similar clinical features could include:

1. **Quinovable Epilepsy**: A rare form of focal hyperadrenocorticism characterized by “javel-like” spikes, visual loss, myopia, and agnosia, with some presenting with developmental delays and focal neurological deficits.
2. **Proximal蠢Root**: A rare, autosomal recessive condition causing focal hyperexcit Rotary activity with visual and motor axonal deficits, as well as hypoadrenocorticism.
3. **Alpers Syndrome**: A rare genetic disorder of the CNS characterized by hypoadrenocorticism and a combination of developmental delay, focal neurological deficits, seizures, and motor unsteadiness.
4. **Hydrocephalus**: A condition characterized by abnormal accumulation of cerebrospinal fluid leading to increased intracranial pressure, causing characteristic neurological deficits, hydrocephalus abs Quantity, and decline in motor and cognitive function. However, this is less directly linked to the adrenocorticotropic axis but fits some of the neurological and developmental features.
5. **Epilepsy with Sons**: A rare autosomal recessive disorder of spike excitability leading to profound developmental delays, focal axonal deficits, and comorbidities such as seizures, although it is more commonly associated with Lennox-Gastaut syndrome.

In summary, **Ganglionic Eczema** is the most likely diagnosis, but the other conditions listed above share overlapping clinical features.

### 20. User

Most of the children start to show symptoms of infection from 4 to 12 months after birth. The most prominent clinical manifestation is recurrent severe bacterial infections in the respiratory system, digestive system and central nervous system, with respiratory tract infections being the most common, and common pathogens include Haemophilus influenzae, Streptococcus pneumoniae, Staphylococcus aureus, and Pseudomonas aeruginosa, etc. The most common infections are respiratory tract infections. Prolonged and repeated respiratory infections can lead to irreversible organ function damage such as bronchiectasis and pestle finger. Common symptoms of GI infections include diarrhoea, abdominal pain, gastro-oesophageal reflux and gastroenteritis. Pathogens causing infectious diarrhoea include Giardia lamblia, Salmonella, Campylobacter jejuni and Cryptosporidium.

### DeepSeek-R1-Distill-Qwen2.5-7B

The clinical manifestations described are highly suggestive of rare bacterial syndromes, particularly those caused by bacteria such as *Hib* (Haemophilus influenzae), *pnca* (Streptococcus pneumoniae), * aureus* (Staphylococcus aureus), and * aeruginosa* (Pseudomonas aeruginosa). These syndromes are rare and often characterized by recurrent infections across multiple systems, including the respiratory, gastrointestinal, and central nervous systems. The most common rare bacterial syndromes associated with such clinical features are:

### Primary Diagnosis:

1. **Bacterial Cluster Hypersensitivity Defects in Disproportionate Feature (BCHD)**: This is the prototypical rare bacterial disease involving *Hib*, *pnca*, *aur*, and *aeruginosa*. It is characterized by recurrent respiratory infections, generalized diarrhea, and neurotoxic episodes.

### Other Possible Diagnoses:

1. **Bacterial Cluster Hyphens Defects in Mid-P (BCHM)**: Caused by *Hib*, *pnca*, and *aur*. It shares many features with BCHD but lacks the anterior-posterior dislocation of利于 neurotoxic episodes seen in BCHD.
2. **Bacterial Cluster Hyphens Defects in Krablin-Franklin (BCHKN)**: Another rare bacterial syndrome caused by similar bacteria (*Hib*, *pnca*, *aur*) but is less common than BCHD or BCHM.
3. **BCHD2 (BCHD D2)*: A rare variant of BCHD characterized by a distinct pattern of neurotoxic episodes.
4. **BCH1 (BCH1)*: A rarer form of BCHD characterized by a different pattern of neurotoxic episodes and less classic gastrointestinal features.

These diagnoses are based on the commonality of recurrent bacterial infections, particularly *Hib* and *pnca*, which are frequently implicated in rare bacterial syndromes alongside other bacteria such as *aur* and *aeruginosa*. The骸 infections described could overlap with other rare bacterial syndromes, such as Fiesta syndrome ( *A. infantum* ) or chest-high fever in fathers syndrome ( *C. fatheris* ), but these are less likely given the presence of other pathogens.

### 21. User

The clinical manifestations of the patients included salt loss and hyperandrogenism. Neonatal onset was characterized by varying degrees of adrenal insufficiency, such as weakness, nausea, vomiting, feeding difficulties, diarrhea, chronic dehydration, skin pigmentation, and growth retardation. Adrenal crisis is often the first manifestation of the disease in the neonatal period, manifested as severe hyponatremia, hyperkalemia, hypovolemic shock, may be accompanied by hypoglycemia, induced by stress. Severe hyponatremia can lead to central nervous system manifestations such as convulsions, and severe hyperkalemia can cause fatal arrhythmias.

Hyperandrogenism manifests differently at different ages. Female children with classical diseases have different degrees of masculinity at birth. Light performance for isolated clitoris hypertrophy, heavy external genitalia can approach men, labia majora scrotal shape, clitoris hypospadia-like penis, and with a common urethral vaginal orifice. However, the labia majora cannot touch the gonads and has completely normal female internal genital structures (ovary and uterus). Males may not have external genital abnormalities such as enlarged penis during neonatal and infancy, which is a common cause of delayed diagnosis. In early childhood, both sexes will present peripheral precocious puberty. Boys presented with enlarged penis with or without pubic hair; females presented with heterosexual precocious puberty. Chronic high levels of sex hormones stimulate hypothalamic gonadotropin-releasing hormone (GnRH) neurons and develop into central precocious puberty. Women may also have secondary sexual dysplasia and primary amenorrhea or oligomenorrhea. Linear growth with accelerated bone age occurs in both sexes from infancy, resulting in impaired terminal height.

Other findings include skin and mucous membrane pigmentation, especially areola and vulva, some children may not have this change.

Non-classical hyperandrogenism in childhood, adolescence and even adulthood may be characterized by accelerated growth and rapid bone age progression.

### DeepSeek-R1-Distill-Qwen2.5-7B

Based on the clinical manifestations provided—salt loss due to hypo-adrenalism, hyperandrogenism, and accelerated bone age—the most likely rare disease to be diagnosed is **Growth Hormone-Dependent Precocious PubenOMA (GHDP)** or **Arthros-o-al-d什onism**. These conditions typically present with carbohydrate metabolism disorders (e.g., hypoald什nism causing salt loss), bone age advancement, pigmented areas, and precocious puberty in males and heteromorphic secondary sexual characteristics in females. They are rare because they are caused by redundant ectopic adenomata of the pituitary gland that produce excess androgens, leading to Cushing-like hyperandrogenism and hyperald什nism.

**Additional possible diagnoses:**

1. **Acromel syndrome**
   - A rare disorder characterized by redundant ectopic adenomata of the hypothalamus-pituitary-gonadal axis, leading to excessive androgen production and hypothyroidism. It can result in similar symptoms, including pigmentation and bone age advancement.
2. **Potbellied syndrome (IGF deficiency)**
   - This condition can cause redundant ectopic adenomata of the pituitary gland, leading to excess androgens and bone age advancement. While less common than Cushing’s syndrome, it can also present with pigmented areas and hyperandrogenism.
3. **Cushing’s syndrome**
   - Although more commonly associated with non-hormonal causes of hyperandrogenism, Cushing’s syndrome can present in rare cases with redundant ectopic adenomata, leading to pigmented areas and accelerated bone age.
4. **Growth hormone deficiency (GH deficiency)**
   - While less common, GH deficiency can causally relate to bone age advancement. However, it is not strongly associated with hyperandrogenism or excess androgen production.
5. **Hyperandrogenism due to androgen insufficiency syndrome**
   - This condition can result from hypothyroidism or hypogonadism, leading to excess andrangeaim production and pigmented areas. However, it is not as specific to the symptoms described.

Each of these conditions has unique clinical features, but the combination of symptoms described aligns most closely with GHDP or Arthros-o-al-dшonism due to their commonality in causing redundant adenomata that produce excessive androgens and hyperald什nism.

### 22. User

The patient has clinical manifestations at birth, due to lack of pigment in the whole body, skin capillaries are exposed, skin is thin and soft, white and red, and accompanied by varying degrees of vasodilation. Patients are highly sensitive to ultraviolet light, often solar dermatitis, actinic cheilitis, basal cell carcinoma and squamous cell carcinoma and other diseases. Hair is white, silver, yellow, gold or brown. Eyelashes and eyebrows are white or pale yellow. Retina, iris color decline, pupil color decline. Photophobia is obvious during the day, night vision is normal, and other visual changes can also be accompanied, such as decreased visual acuity, hypoplasia of the fovea, changes in neural pathways at the optic chiasm, and nystagmus.

### DeepSeek-R1-Distill-Qwen2.5-7B

Based on the detailed symptoms described, the most fitting rare disease diagnosis is **Pachonyxerosis**. This condition presents early on and involves widespread xanthan-rich skin, including features like capillary hyperplasia and reduced pigmentation. Key symptoms include photophobia, xanthan prodigands, and a hypersensitive skin and eyes to sunlight, along with visual changes such as nystagmus and photopic foveal hypoplasia. While other rare conditions might have overlapping symptoms, Pachonyxerosis uniquely combines pigmented and xanthan-rich skin changes with sensitivity and visual alterations.

### 23. User

A degenerative disease of the nervous system in which both upper and lower motor neurons are involved. The main clinical manifestations are progressive weakness and atrophy of the muscles of the bulbar, limbs, chest and abdomen. Eye motor nerve and sphincter function are generally not affected, but may be affected later in some patients. Between 20% and 50% of patients may exhibit cognitive impairment, and between 5% and 15% may even develop frontotemporal dementia. Death from respiratory failure occurs an average of 3 to 5 years after onset, but 5% to 10% of patients survive for more than 10 years.

Detailed history and physical examination to find evidence of common involvement of upper and lower motor neurons in brain stem, neck, chest and lumbosacral regions is the basis for diagnosis of the disease. Physical examination showed that both upper and lower motor neurons were involved in the same area, which was the key point of diagnosis. The main signs of lower motor neuron involvement included muscle weakness, atrophy and fascicular fibrillation. The tongue muscles, facial muscles, throat muscles, neck muscles, different muscle groups of the limbs, back muscles and pectoralis muscles are usually examined. The signs of upper motor neuron involvement mainly included positive pathological signs, tendon hyperreflexia, hypertonia, clonus, etc. Usually check sucking reflex, pharyngeal reflex, mandibular reflex, palmar jaw reflex, whether there is strong crying, strong laughter and other pseudobulbar paralysis manifestations, limb tendon reflex, muscle tension, Hoffman sign, lower limb pathology, abdominal wall reflex, etc. Clinical examination is the primary method of detecting upper motor neuron involvement. In areas of atrophy and weakness, if tendon reflexes are not low or active, it may indicate pyramidal tract damage even if there are no pathological signs.

### DeepSeek-R1-Distill-Qwen2.5-7B

The disease described is most likely **Inclusion Necrosis (IXN) syndrome**, characterized by the degeneration of multiple motor neurons affecting both upper and lower motor areas, leading to progressive muscle weakness and atrophy. Other possible rare diseases that present with similar symptoms could include:

1. **Motor Infarct in Epilepsy (MIE)**: A stroke-like event affecting motor neurons, though it usually presents with neurological symptoms like aphasia and ataxia rather than cognitive impairment.
2. ** Motor Neuron Encephalopathies (MNEs)**: A broader category that can involve various motor neurons and may present with cognitive issues.
3. **Mitochondrial Encepho-Neuropathies**: Rare disorders affecting motor neurons with signs like muscle weakness and atrophy, though they may not always include cognitive impairment.

These conditions are considered alternatives based on the clinical features described, particularly the comprehensive impact on motor neurons and the associated symptoms.

### 24. User

An autosomal recessive disorder affecting the urea cycle, leading to hyperammonemia, encephalopathy, and respiratory alkalosis. It is characterized by a buildup of arginine and ammonia in the blood, causing symptoms such as developmental delays, seizures, intellectual disability, spasticity, and ataxia. The condition typically manifests around age 3, with symptoms including stiffness, slow growth, and cognitive decline. Without treatment, it can progress to severe spasticity, loss of ambulation, and intellectual disability.

### DeepSeek-R1-Distill-Qwen2.5-7B

Based on the clinical manifestations described, the most likely diagnosis is **Pseudoxanthoma Elasticum (PXE)**. This is a rare, autosomal recessive disorder that primarily affects the adenine uptake cycle, leading to hyperammonemia and urea cycle abnormalities. It often presents in early childhood (around age 3) with symptoms such as hyperammonemia, encephalopathy, respiratory alkalosis, stiffness, slow growth, and spasticity. Without treatment, it can progress to severe spasticity, loss of ambulation, and intellectual disability.

### Other Possible Diagnoses:

1. **Smith-LASY Syndrome**: An autosomal recessive disorder causing urea cycle disturbances, leading to hyperammonemia, encephalopathy, and respiratory alkalosis. It often presents before age 2 and is associated with joint hypermobility and skeletal fibrosis.
2. **Oautosynaptic Urea Enzyme Deficiency (OUED)**: A rare autosomal recessive condition affecting adenine transport, resulting in hyperammonemia and urea cycle defects. Symptoms include muscle weakness, ataxia, and encephalopathy, often starting between ages 2-4.
3. **Inositoluria**: An autosomal recessive disorder caused by inositol deficiency, leading to hyperamin Gap. Symptoms include hyperammonemia, urea cycle abnormalities, and fluid retention, with possible joint issues and respiratory alkalosis.
4. **GlutarIchinitis (Talwar’s Encephalopathy)**: A common autosomal recessive disorder causing urea cycle disturbances and hyperammonemia. It presents with encephalopathy, joint hypermobility, and respiratory alkalosis, often around age 3.
5. **Proximal Myopathy of Atrial Fibrillation (PMAF)**: Though less common, this rare disorder can present with hyperammonemia, urea cycle defects, and spasticity. It is associated with atrial fibrillation and proximal muscle weakness.

### Considerations:

- A thorough genetic workup is essential, as these conditions are inherited and may require testing for other autosomal recessive disorders.
- Blood workup, including urea cycle enzyme testing and kidney function evaluation, is crucial to confirm the diagnosis.

For a formal diagnosis, it is advisable to consult a pediatric endocrinologist or a geneticist to assess for recessive traits and ensure accurate identification of the condition.

### 25. User

The typical clinical picture includes the triad of microangiopathic haemolytic anaemia, thrombocytopenia and acute renal failure. Non-immune erythrocyte destruction due to microthrombosis often results in a haemoglobin level below 80 g/L, a negative Coombs test, and erythrocyte fragments seen on peripheral blood smears; platelet counts usually fall below 50×10^9 /L, but skin purpura and active bleeding are rare; the severity of the acute renal impairment varies from person to person, with some patients requiring dialysis support, often accompanied by elevated blood pressure.

### DeepSeek-R1-Distill-Qwen2.5-7B

Based on the clinical manifestations provided—microangiopathic hemolytic anemia, thrombocytopenia, and acute renal failure—this presentation strongly suggests **servesenitism (idiopathic thrombocytopenic purpura with hemolytic anemia and renal failure, ITAP HM)**. Servesenitism is a rare, idiopathic presentation of thrombocytopenia and microangiopathic hemolytic anemia, often associated with reduced erythropoiesis, leading to anemia of chronic disease, and may commonly present with renal involvement when iron-chelating disorders or inappropriate erythropoiesis occur.

### Other possible diagnoses:

1. **Acute kidney-donating anemia (AKDA)**: This is a rare form of anemia of chronic disease where the kidneys are rejecting donated red blood cells, leading to decreased EPO production and anemia. It can present with symptoms such as anemia of chronic disease stages and renal failure, though it may have a different mechanism of anemia progression.
2. **Oxgentiase deficiency**: A rare deficiency disorder causing anemia of chronic disease due to redundant erythropoiesis. This condition is characterized by a hypophagemic anemia, reticulocytosis, and thrombocytopenia, though it may be less common and may not always present with hypertension unless otherwise managed.
3. **Particle cell aplasia (P-CAP)**: This is a rare hematinic disorder causing reticulocytosis and thrombocytopenia due to bone marrow fibrosis. However, it is not commonly associated with acute kidney failure unless there is an additional uncontrolled cause of anemia.
4. **Inclusion anemia**: A rare blood disorder causing megaloblastic anemia, often associated with anemia of chronic disease, but it typically does not involve thrombocytopenia or renal failure.
5. **Pollard-Bálopace syndrome**: A rare combined aplastic anemia and idiopathic kidney-donating anemia, presenting with bone marrow fibrosis, thrombocytopenia, and anemia of chronic disease. It can sometimes involve renal failure, though it is less commonly reported.

### Note:

The most likely diagnosis in this case is **servesenitism**, given the classic presentation of anemia, thrombocytopenia, and renal failure. However, the presence of these features could suggest any of the above conditions, depending on the specifics of the case. A thorough clinical evaluation and genetic testing (e.g., CO diabetes, EPOR mutations, or fERRA deficiency) would be required to confirm the diagnosis.

### 26. User

Patients have a variety of clinical presentations. It depends on the location, extent and rate of progression of the lesion. Clinical symptoms mainly include:

1. The occupying effect of an enlarged pituitary gland, which in most patients results from compression of the enlarged pituitary gland on the anterior region and surrounding structures (e.g., optic crossings, cavernous sinus, etc.), or from an inflammatory infiltrate. The most common clinical manifestations are persistent severe headaches and progressively worsening visual dysfunction, including decreased visual acuity and visual field defects.
2. hypopituitarism. Depending on the degree of involvement, the various axes of the pituitary gland may show partial or even total hypopituitarism, such as amenorrhoea, hypogonadism, nausea, malaise, chills, loss of sexual hair and weight loss.
3. Central dysuria, more than 70% of the patients will have symptoms of central dysuria such as polyuria, irritable thirst and polydipsia due to the involvement of the posterior pituitary, pituitary stalk and even hypothalamus region. In some patients, the blocking effect of the pituitary stalk leads to a mild increase in serum prolactin levels.
4. Hypothalamic syndrome, when the lesion involves the region of the hypothalamus, there will be drowsiness, lack of satiety, severe weight gain, lack of thirst, temperature fluctuation, sweating disorders, and emotional and psychiatric disorders.
5. Systemic disease-related manifestations. Patients with secondary disease often have a combination of clinical manifestations characteristic of systemic disease, such as LCH patients often have a combination of thyroid, lung, bone, liver, and skin lesions. patients with GPA are often middle-aged or older men, and respiratory symptoms often precede hypothalamic pituitary involvement. patients with IgG4-related pituitary inflammation often have a combination of clinical manifestations with the involvement of other organs such as lacrimal glands, submandibular glands, and pancreas. The disease is often associated with clinical signs of involvement of other organs such as the lacrimal gland and submaxillary gland and pancreas.

### DeepSeek-R1-Distill-Qwen2.5-7B

Based on the clinical manifestations provided, the most likely diagnosis is a **Bridging Tumor**, specifically a **Differentiated Bridging Tumor**. This type of tumor can cause compression of pituitary structures, leading to hypopituitarism and systemic involvement.

Other possible rare diagnoses include:

1. **Differentiated Neuro-Endocrine Tumors (DN ET)**: These can affect multiple organs and include conditions like neuro-endocrine tumors.
2. **Churle syndrome**: Characterized by decoder cell-derived pituitary tumors, leading to systemic symptoms.
3. **Differentiated Neuro-Endocrine tumors**: A rare group of tumors that involve endocrine cells and can present with various systemic symptoms.
4. **Neurokalund**: A rare syndrome with symptoms including hypopituitarism, central nervous system involvement, and systemic issues.
5. **Blast Cell Neuroendocrine tumors**: Highly aggressive tumors that can involve multiple organs and exhibit complex symptoms.

These diagnoses are rare and complex, often requiring specialized evaluation and management.

### 27. User

Hyperglycaemia is the most common clinical manifestation of the disease. However, patients can spontaneously remit without intervention and maintain normal blood glucose levels. The disease is one of the leading causes of autoimmune hypoglycaemia, with about 25% of patients experiencing spontaneous hypoglycaemia during the course of the disease. Acanthosis nigricans is present in about 50 per cent of patients. In addition, polycystic ovaries and hyperandrogenism are not uncommon in this group of patients. Other autoimmune diseases are common, with systemic lupus erythematosus (SLE) being the most common, accounting for about 50 per cent of cases. Primary biliary cirrhosis, dermatomyositis, and scleroderma are also present. The disease can also be a paraneoplastic syndrome manifestation of Hodgkin’s lymphoma, multiple myeloma, and others.

### DeepSeek-R1-Distill-Qwen2.5-7B

Based on the provided clinical manifestations and considering the features of the disease, the most likely diagnosis appears to be **Systemic Lupus Erythematosus (SLE)**. Here’s the rationale:

1. **Hyperglycemia**: SLE can manifest as hyperglycemia, particularly in the early stages, where some patients may spontaneously remit hypoglycemia upon remission phases of the disease.
2. **Acanthosis Nigricans and Polycystic Ovaries**: These are common findings in SLE, indicating potential oxidative stress and inflammation.
3. **Polycystic Organ System and Hyperandrogenism**: While SLE itself doesn’t typically lead to hyperandrogenism, it can affect the renal and reproductive systems, which may underlie some of these symptoms.
4. **Spontaneous Hypoglycemia**: This is not uncommon in SLE, especially when associated with neurological involvement.
5. **Autoimmune Features and Link to Paraneoplastic Syndromes**: SLE is multifocal in many patients, and while it can sometimes present with cancers or related complications, the presence of paraneoplastic symptoms further points to SLE as the primary condition.
6. **Rarity and Prevalence**: SLE, though a common autoimmune disease, can present with less common clinical features, aligning it as a rare presentation in this context.

Other potential conditions, such as rare variants of type 1 diabetes, do not align as closely with the symptoms listed. Therefore, SLE is the most plausible diagnosis based on the clinical features provided.

### 28. User

The disease starts in infancy and childhood. The first attack occurs between the ages of 5 months and 7 years. The clinical picture is one of recurrent episodes of intractable vomiting and severe ketoacidosis with depression, dehydration, shortness of breath, and coma. There is usually a clear trigger, and the disease may begin acutely after fasting, gastrointestinal and upper respiratory tract infections, fever, stress, or excessive protein intake. Routine laboratory tests show elevated or normal blood glucose, some hypoglycaemia, blood gases show metabolic acidosis, blood ammonia can be normal or mildly elevated, liver function, renal function and normal, and urine ketone bodies are positive. Ketosis and acidosis may recur, and clinical and laboratory tests may be normal between attacks.

Neurological changes occur in about 1/5 of the cases, manifested by cognitive impairment, mental retardation, extrapyramidal abnormalities and symptoms of stroke. Cranial MRI reveals extensive demyelination, including white matter, and symmetric high signal changes in the basal ganglia at T2.

Patients may be asymptomatic in the early stages and are diagnosed in the neonatal period or in high-risk groups by blood and urine metabolic screening. There is no clear association between age of onset, frequency of attacks and prognosis. There is a tendency for the attacks to decrease or stop with age, and in most cases growth is unaffected on follow-up. Recurrent and severe attacks can lead to multi-organ dysfunction or even death.

### DeepSeek-R1-Distill-Qwen2.5-7B

Based on the clinical manifestations you’ve provided, **Acute Influx of Sugar Intake Nuclear Fibrolysis Syndrome (ais)** is the most likely diagnosis. This syndrome is characterized by recurrent episodes of ketoacidosis, intractable vomiting, metabolic acidosis, and neurological deficits, often triggered by high carbohydrate or sugar intake, particularly in early infancy or childhood. The comas and neurologic changes observed, combined with the presence of white matter demyelination seen on MRI, strongly suggest ais.

### Other Possible Diagnoses:

1. **Alpha1 Antitumourine Antibodies (α1-ATNT) Syndrome**:
   - Symptoms can include weight loss, ketoacidosis, and neurological deficits.
   - Neurological changes may occur, but the specific imaging findings (white matter demyelination) are less characteristic.
2. **Menkes Disease**:
   - This syndrome is typically associated with phosphate stones, ginger cell cysts, and muscle wasting.
   - The presence of severe ketoacidosis and metabolic acidosis is less characteristic of Menkes disease, but other symptoms like neurological deficits may overlap.
3. **Creutzfeldt-Jakob Disease (CJD)**:
   - A prion-based neurodegenerative disorder causing unary clusters of lesions.
   - CJD typically presents with progressive neurological deficits and ” �ware dysfunction, but it is not typically associated with ketoacidosis or frequent metabolic acidosis.
4. **PhucineOLER_COLOR改革委 syndrome**:
   - A syndrome associated with mimicking benign methylmalonic acidemia.
   - Symptoms may include intractable vomiting, ketoacidosis, and neurological deficits.
   - The MRI findings are less commonly associated with phosphate metabolism disorders.
5. **Acute Gastroenteritis and Ketonuria without Underlying Cause**:
   - In some cases, ketoacidosis and intractable vomiting may be caused by acute gastrointestinal inflammation or infection.
   - The presence of metabolic acidosis, comas, and neurological deficits may make this less likely, but it is a consideration.

### Conclusion:

The most likely diagnosis is **Acute Influx of Sugar Intake Nuclear Fibrolysis Syndrome (ais)**, based on the combination of recurrent ketoacidosis, intractable vomiting, metabolic acidosis, and neurological deficits, often triggered by high carbohydrate intake. The imaging findings (white matter demyelination) strongly suggest a metabolic disorder affecting the central nervous system.

### 29. User

1. the onset is at a young age and is more common in women.
2. there is often a family history of syncope or sudden death.
3. different genotypes and phenotypes, can involve potassium, sodium and calcium channels. Characteristic tip-distortion ventricular tachycardia (TdP) is often present, leading to syncope or sudden death. 4.
4. The ECG shows a prolonged QT interval.
5. ECG generally has the following characteristics: (1) QT interval prolongation, short - long - short cycle phenomenon induced; (2) ventricular rate of 160-240 beats / minute, QRS wave amplitude and morphology around the equipotential line of reversal, 5-20 cardiac cycles of the main wave around the baseline of the reversal of the majority of the time can be terminated; (3) ATP is more difficult to terminate the TdP.

### DeepSeek-R1-Distill-Qwen2.5-7B

Based on the clinical manifestations you provided, the most likely diagnosis is **Long QT Syndrome** (LQT), specifically with a variant phenotype (Long QT Version 4 or Long QT Pr; Prepard for variants). This syndrome is characterized by a progressively lengthening QT interval on the ECG, prolonged QT interval, and tip-distortion ventricular tachycardia (TdP), which can lead to sudden death in many patients. These variants are often associated with mutations in genes such as **KCNQ1** or **XIST**, which alter the function of potassium, sodium, and calcium channels.

Possible additional diagnoses based on the clinical features could include:

1. **Long QT Syndrome (LQTS)**: A genetic disorder characterized by a prolonged QT interval, TdP, and a phenotype of progressive QT prolongation caused by mutations in iodine-thionyl chloride sensitivity or other KCNQ1-related genes.
2. **Right-sided bundle branch block (RBBB)**: While not exclusively caused by mutations in KCNQ1, RBBB can coexist with TdP, especially in female patients with a family history of sudden cardiac death.
3. **Sotos Syndrome**: A genetic disorder characterized by a long QT interval, TdP, and co-existing phenotype ofrv330, a condition associated with arrhythmias, sudden death, and electrolyte imbalances.
4. **Right ventricular outflow obstruction (RVO Buckley-Magee’s variant)**: Though less common in this context, RVO can sometimes be associated with TdP and a prolonged QT interval in rare cases.
5. **Genetic mutations causing bundle branch block (Bloss搬到 block) with a variant gene such as PTEN or others causing electrolyte disturbances.**

These diagnoses are based on the patterns of electrolyte disturbances, TdP, and a progressive QT prolongation phenotype.

### 30. User

The disease can develop at any age, and most of the children develop between 1 month and 7 years old, with an average age of about 2 years old. Clinical manifestations vary greatly among patients, mainly including: (1) acute energy metabolism disorder crisis, manifested by low ketone hypoglycemia, high blood ammonia and metabolic acidosis; (2) cardiomyopathy, manifested by ventricular hypertrophy, cardiac insufficiency, cardiac arrhythmia, and elevated creatine kinase, etc.; (3) myopathy, manifested by muscle weakness, hypotonia, myalgia, poor exercise endurance, elevated muscular-type creatine kinase, and lipid deposition in muscle fibres, etc. (4) liver damage, manifested by hepatomegaly, fatty liver, liver function abnormalities, etc. Some liver-damaged patients have liver enlargement, fatty liver, and liver function abnormalities; Liver damage, manifested by hepatomegaly, fatty liver, abnormal liver function, etc. Some children with liver damage have acute onset of illness, manifested by convulsions, progressive consciousness disorder, etc., which is often misdiagnosed as Reye syndrome. In addition, gastrointestinal symptoms such as recurrent abdominal pain, vomiting and gastroesophageal reflux, respiratory symptoms such as recurrent infections and wheezing, and anaemia have also been reported.

Newborn screening: diagnosed children may have no clinical manifestations. It is a potentially fatal disease, and children can die suddenly from acute energy metabolism disorder crisis or acute heart failure. Clinical manifestations are varied and related to age of onset, time of onset and severity of illness. The most common symptoms are cardiac and skeletal muscle damage in infancy and childhood. Symptoms in adulthood are mild or asymptomatic, mostly due to reduced endurance or easy fatigue. During pregnancy, pregnant women may have atypical manifestations such as fatigue and arrhythmias due to energy expenditure and reduced physiological plasma carnitine levels.

### DeepSeek-R1-Distill-Qwen2.5-7B

Based on the clinical manifestations and diagnostic features you provided, the most likely rare disease that could be diagnosed is **Acute Metabolic Deprivation Neuralgia (AMDN)**. AMDN is a rare autosomal recessive disorder characterized by a chronic metabolic condition that causes volt-hour episodes (non-degree hyperglycemia), yardstick episodes (low ketone hypoglycemia), and metabolic acidosis. It is most commonly diagnosed in children younger than 7 years of age, with the average age of onset being around 2 years. The most frequent presentations are in children, but it can also affect older individuals. The symptoms described, including metabolic acidosis, energy metabolism disorders, and muscle weakness, align well with AMDN.

### Five Other Possible Diagnoses:

1. **Simulated Acute Lactic Ketoneosis (SAK)**: This is a mimetic metabolic disorder characterized by low blood glucose, hyperglycemia, hypoketosis, and metabolic acidosis. It is often associated with transient episodes of low oxygen consumption and muscle glycogenolysis. SAK can present with symptoms like muscle weakness, hypotonia, and Episodes of ketoacidosis mimic a wide range of conditions, including AMDN, so careful workup is required.
2. **Mitochondrial Encephalo-Myopathy, Lacticictis, and Serve (MELAS)**: This mitochondrial disorder is often autosomal recessive and presents with muscle weakness, atrophy, muscle cramps, fatigue, lethargy, and referral pain. It can also cause cardiac involvement and hepatomegaly, though it tends to occur later in life (secondary to mitochondrial accumulated lactic dehydrogenase). The liver involvement described in your case could be a clue for MELAS.
3. **CHARGE Syndrome**: This is a multifactorial disorder that often involves severe metabolic issues, neurological deficits, malnutrition, and muscle weakness. Its pathophysiology involves substituted ethanolamine, which leads to metabolic acidosis and ketoacidosis mimics. CHARGE syndrome often presents with both metabolic and neuronsynaptic instability, and it could explain the mixed presentations of metabolic, neurological, and cardiac symptoms you describe.
4. **Acute Lactic Deprivation Nervousness (ALDN)**: Like AMDN, ALDN is a rare autosomal recessive condition that causes volt-hour episodes (hyperrectal temperature, radiant hyperpigmentation, and unusual abdominal pain) and yardstick episodes (low ketone hypoglycemia and metabolic acidosis). It is most commonly seen in childhood, but adults can also present with these symptoms. ALDN can also cause cardiac and muscle involvement, consistent with the findings you mentioned.
5. **Central Autonomic Neuritis (CAN)**: CAN is a rare condition that affects the central nervous system and causes a syndrome of non-intensive hyperglycemia, metabolic acidosis, and arrhythmias. It often mimics other metabolic and cardiac disorders, such as Reye syndrome, and can have episodes that mimic the patterns described in AMDN.

In conclusion, while AMDN is the most likely diagnosis given the information provided, other rare metabolic and multisystem disorders such as SAK, MELAS, CHARGE syndrome, ALDN, and CAN could present with overlapping symptoms. Diagnoses should be confirmed with detailed workup, including metabolic panels, imaging studies, and genetic testing if relevant.

### 31. User

In the unicentric form, the disease often presents only with enlarged lymph nodes without systemic symptoms. The median diameter of the enlarged lymph nodes is about 5.5 cm, and they are commonly found in the chest (24%), neck (20%), abdomen (18%), and retroperitoneum (14%). Some patients with unicentric disease are prone to paraneoplastic aspergillosis, occlusive bronchiolitis, and growth retardation. In addition to multiple lymph node enlargements, multicentric disease is often associated with systemic manifestations such as fever, night sweats, malaise, weight loss, anaemia, hepatic insufficiency, renal insufficiency, and volume overload (generalised oedema, pleural effusion, ascites, etc.). There is also a specific subtype in which patients may present with thrombocytopenia, severe oedema (anasarca), myelofibrosis, renal dysfunction, and hepatosplenomegaly (organomegaly).

### DeepSeek-R1-Distill-Qwen2.5-7B

Based on the clinical description provided, the most likely rare disease is **systemic lymph-node multinucleosis**, a systemic condition characterized by multiple lymph node enlargements. This condition is associated with generalized oedema and systemic symptoms such as fever, night sweats, malaise, weight loss, and organ enlargement.

**Other possible diagnoses to consider:**

1. **syntaxic syndrome**: While primarily associated with muscle weakness, it can present with generalized lymph node involvement.
2. **multifocal lymphadenopathy**: Often caused by infection, leading to widespread lymph node enlargement and systemic symptoms.
3. **stereoseorschism**: Characterized by multiple small localized lymph node enlargement in areas like the flank and compensatory Redistribution of blood flow, leading to symptoms like oedema.
4. **Paraneoplastic aspergillosis**: A complication of aspergillosis, presenting with multiple lymph node enlarge ment and associated systemic symptoms.
5. **Systemic disease causing multifocal lymphedema**: Such as parasitic infections or other systemic conditions leading to generalized lymphoedema and multi-node enlargement.

### 32. User

The main clinical manifestations are muscle atrophy, weakness and sensory loss of the limbs, mainly in the distal part of the lower limbs and gradually developing towards the proximal part of the limbs. Common clinical manifestations include poorer locomotor ability than people of the same age, difficulty in running, easy to twist the foot, foot drop, calf gastrocnemius muscle atrophy resembling a ‘crane leg’; examination can find bowed feet, hammertoes, distal limb weakness and atrophy, and loss of deep sensation. The disease usually starts before the age of 20 and progresses slowly. Later stages of the disease may severely affect activity, but rarely result in total disability and do not affect normal life expectancy. However, there are specific types that may have an early and severe onset.

### DeepSeek-R1-Distill-Qwen2.5-7B

**Primary Diagnosis: Bulbar Charcot**

Bulbar Charcot is a rare genetic disorder characterized by the slow, progressive wasting of proximal muscles, particularly in the legs. It can cause a variety of symptoms, including muscle atrophy, sensory loss, muscle unions, and foot drop. The ‘crane leg’ appearance is a typical observation in this condition. It is associated with early progression and can lead to significant physical limitations.

**Other Possible Diagnoses:**

1. **Acute Myotonic Dystrophy (AMD):**
   AMD is an early-onset myopathy characterized by muscle wasting and myositis. It can present with symptoms like muscle atrophy, weakness, and sensory loss. However, it is more commonly associated with severe muscle pain and muscle disruption rather than the specific features of ‘crane leg’ and foot drop.
2. **LACR-PPP (Lactic Acidosis-arginine conjugate-Related Periodic Protease):**
   LACR-PPP is a rare periodic lysosomal storage disorder that can lead to muscle atrophy and unions. It often presents with early muscle wasting and may involve the proximal lower limb, leading to a ‘crane leg’ appearance in some cases.
3. **Myositis Antaluaca:**
   This rare condition is characterized by early-onset, generalized proximal muscle wasting. It can result in muscle unions and may exhibit foot drop and sensory deficits, fitting the clinical presentation described.
4. **Minorϭt:**
   A lysosomal storage disorder, Minorϭt can cause muscle wasting, bone changes, and sensory loss. It might present with characteristics similar to Bulbar Charcot, including muscle atrophy and sensory symptoms, especially in older age.
5. **MyopingolyFeaturedScyphose:**
   This rare neurosis can lead to muscle wasting and sensory abnormalities. It might present with features such as proximal muscle atrophy, sensory loss, and foot drop, aligning with the clinical description provided.

Each of these conditions should be considered based on a comprehensive clinical evaluation, including genetic testing where appropriate, to determine the most likely diagnosis.

### 33. User

The onset of the disease occurs from birth to several months. The onset of jaundice is usually within 2 months of age and rarely later than 5 months of age. The most common presentation is persistent jaundice after birth with or without light coloured stools and mild or no liver enlargement. There are many other non-specific clinical signs, including feeding difficulties, growth retardation, diarrhoea, hypoglycaemia and prolonged clotting time. Most of these symptoms and signs resolve spontaneously or with dietary modification and medication around 6 months to 1 year of age, with a small proportion of patients developing further disease after puberty. The majority of developed disease develops in adulthood. Delirium, confusion, and other mental abnormalities caused by hyperammonemia usually appear suddenly after drinking alcohol, consuming sweets, taking certain medications, or having an infection. In severe cases, the disease progresses rapidly and can be life-threatening without proper treatment. Most patients are slim, and most of them have obvious dietary preference, addicted to legumes, high protein, high fat food, and anorexic to high sugar.

### DeepSeek-R1-Distill-Qwen2.5-7B

The rare disease described is most likely **Dubin-Johnson syndrome**, characterized by early onset jaundice, associated with both liver and central nervous system issues. Key features include:

1. **Jaundice**: Persistent jaundice from birth, especially in the first two months.
2. **Metabolic Deficit**: Deficiency of UDP-n-acetylglutamine leads to hyperammonemia, contributing to mental abnormalities like delirium and confusion.
3. **Dietary Preferences**: thin appearance, anorexic tendencies, and specific dietary preferences (legumes, high protein, high fat, high sugar).
4. **Prognosis**: Most cases improve on their own or with intervention by six to twelve months. A small proportion may develop severe complications in adulthood.
5. **Central Nervous System Involvement**: Characterized by sudden mental disorders, highlighting comorbidity with conditions like paternal early onset Judaism.

Dubin-Johnson syndrome is a rare metabolic disorder that fits all described symptoms, particularly its early onset and association with both liver disease and nervous system dysfunction.

### 34. User

Symptoms may appear after birth or may start in infancy to early adulthood. It is characterised by fluctuating muscle weakness and intolerance to fatigue, mostly in the extraocular muscles, facial muscles, medulla oblongata, limbs and respiratory muscles, and even life-threatening apnoeic episodes may occur in infancy. The simplex syndrome is characterised mainly by skeletal muscle weakness without congenital malformations. The overlapping syndromes of glycosylation defects and myopathies of the disease are often accompanied by congenital developmental abnormalities. Clinical manifestations vary between the different types, with some patients, usually born with proximal muscle weakness, cholinesterase inhibitors effective, and in severe cases, sudden respiratory distress and apnoea due to medullary palsy, which can be induced by stress, cold, and infection. Some patients, starting at birth or in infancy, may present with more severe, axially distributed muscle weakness, but the extraocular muscles may not be involved.

### DeepSeek-R1-Distill-Qwen2.5-7B

The rare disease you describe, characterized by fluctuating muscle weakness in extraocular, facial, medulla oblongata, and respiratory muscles, with apnoeic episodes in infancy, is most likely due to **infantile spina bifurca**. This condition is associated with narrowing of the spinal canal, leading to significant muscle weakness and neurologic symptoms.

**Additional Possible Diagnoses:** 1. **Ataxia-telicardia syndrome (A-TS)**: A genetic disorder causing muscle weakness, speech and language delays, and hearing loss. 2. **Acute Myeloid Leukemia (AML)**: Though less common, it can present with muscle weakness and neurologic issues. 3. **Chediak-Higashi Syndrome**: An autosomal recessive disorder causing muscle weakness and developmental delays. 4. **Thalassemia**: A blood disorder leading to iron deficiency and various muscle and neurologic symptoms. 5. **Cystic Fibrosis**: A rare cause of muscle weaknes and respiratory issues, though less common.

These conditions are selected based on their common presentations of muscle weakness and neurologic symptoms.

### 35. User

1. The disease usually starts in infancy or childhood, and in a few cases it starts in adulthood.
2. Tonicity can occur throughout the entire body, including the skeletal muscles of the limbs, facial muscles, extraocular muscles, and lingual muscles.
3. Tonicity is apparent during sudden movement after rest and is reduced by repeated movements, which is called the ‘warm-up’ phenomenon. Transient muscle weakness may occur at the start of a sudden movement. In patients with a longer course of the disease, there may be persistent weakness of the distal limbs, even involving the proximal limbs.
4. Physical examination reveals that the patient is often muscular and has an athlete’s body shape. After clenching the fist vigorously, the fingers cannot be unfolded in time; after closing the eyes vigorously, they cannot be opened immediately. Knocking on the hand interosseous muscle, lingual muscle and other parts of the hand can cause persistent muscle contraction (percussive muscle ankylosis, ‘muscle ball’ phenomenon).

### DeepSeek-R1-Distill-Qwen2.5-7B

Based on the clinical manifestations you provided, the most likely rare disease could be **Tonic Clonic Epilepsy (TCE)**, also known as ” childhood absence epilepsy.” This condition is characterized by recurrent tonic (rigid) and clonic (convulsive) episodes, which often appear as tonicity throughout the body, similar to the description you provided. Tonic clonic seizures are especially common in early childhood and may also present with a warm-up phenomenon (sudden muscle weakness after rest or upon movement). TCE is a rare neurodevelopmental disorder often associated with developmental delay or regression.

Other possible rare diagnoses based on the clinical descriptions could include:

1. **Spina bifida myelomagnifying fibrosing neuropathy (SP/FN)**: A rare condition that can cause muscle weakness and挛缩（due to nerve root compression）. It is typically diagnosed in infancy or childhood and can present with tonic clonic-like episodes.
2. **Epilepsy with temporary disabilities (Etdi):** A syndrome characterized by febrile episodes that include tonic clonic-like symptoms. It may also present with muscle weakness and other neurological deficits.
3. **Montevideo periodic paralysis:** A rare, inherited disorder of the central nervous system that can cause muscle weakness and挛缩, particularly involving the legs and lower limbs. It can present with tonic clonic-like episodes in some cases.
4. **Korsachy-Korsbo syndrome:** A rare genetic disorder that causes progressive muscle weakness and joint dysfunction. While it more commonly presents with spastic paresis, it may also present with tonic clonic-like episodes in some individuals.
5. **Epilepsy with malaise:** A presentation of generalized tonic clonic epilepsies that may occur during episodes of fever, totaling in the context of a fever with muscle weakness.

If you could provide more specific details about the patient’s symptoms or medical history, it could help narrow down the diagnosis further.

### 36. User

Secondary thoracic deformities caused by scoliosis can lead to cardiopulmonary hypoplasia, poor cardiopulmonary reserve, and may include cardiac displacement, arrhythmia, reduced lung capacity, pneumonia, etc. Abdominal deformities can cause gastrointestinal disorders, leading to dyspepsia and lack of appetite. In addition, the nerve root pulling caused by vertebral malformation and disc degeneration can produce irritating pain in the corresponding body parts, and in severe cases, it can cause spinal cord dysfunction or even paralysis.

Physical examination: the general manifestations are unequal shoulders when standing upright, one side of the lumbar folds, lumbar flexion with back asymmetry (‘razor back’), sometimes asymmetry on both sides of the pelvis, and gait abnormalities. In addition, neurological examination is also needed to observe whether the patient has increased or low muscle tone and whether there are sensory abnormalities in the limbs.

Clinically, it is usually classified into the following 3 types according to the imaging characteristics:

Type I - Vertebral formation disorder type, i.e., one or more vertebrae are partially or completely formed with disorder, such as wedge vertebrae, hemivertebrae or butterfly vertebrae;

Type II - poor vertebral segmentation type, i.e., two or more vertebrae are partially or completely segmented and impaired, e.g., blocked vertebrae, bone bridge formation, etc;

Type III - mixed type, where there is both vertebral body formation disorder and vertebral body mal-segmentation.

### DeepSeek-R1-Distill-Qwen2.5-7B

根据您提供的临床表现，最可能的诊断是先天性脊柱横弯（如 username=“srh-001”）或先天性脊柱融合症（如 username=“srh-002”）。此外，以下可能是其他可能的诊断：

1. **先天性单侧性发育异常**：这可能导致侧弯、体型不均和下肢短小。
2. **先天性脊柱ectors**（如unchedron症）：可能导致单侧或双侧的椎体缩短、纵突异常和脊柱变形。
3. **先天性脊柱发育障碍**：可能导致肺活量减少、 Poor SVR 和心肺功能异常。
4. **先天性脊柱可疑的横位融合**：由于横位可能导致脊柱影像学异常，如双侧的异常前倾斜和前凸畸形。
5. **先天性先天性胸椎成桥**：这可能导致胸椎成桥，影响心脏功能和肺活量。

这些诊断都可能伴有脊柱发育异常，可能导致您描述的临床表现，但最终的诊断需要结合详细的临床评估和影像学检查。

### 37. User

There are more men than women. Some patients have atypical symptoms of chest pain, which are detected during coronary angiography or coronary CT. Patients are often clinically symptomatic when there is (1) a combination of obstructive atherosclerotic disease, thrombosis, slow flow, entrapment, or rupture with exertional angina or acute coronary syndromes; (2) compression of adjacent tissues; (3) rupture of an aneurysm, which is rare and can cause acute cardiac tamponade; and (4) myocardial ischemia due to microvascular dysfunction, which can occur even in the absence of significant coronary stenosis. The most common clinical manifestation is cardiac ischaemia.

The most common clinical manifestation is angina pectoris, and in a few patients, myocardial infarction, severe arrhythmia, or even sudden death may occur. The disease may break into the right atrium, right ventricle, or coronary sinus, creating a left-to-right shunt, which can be heard as a continuous murmur and, in severe cases, affect cardiac function. The most serious complication is rupture of the tumour into the pericardium, which can lead to bleeding, cardiac tamponade or even death.

### DeepSeek-R1-Distill-Qwen2.5-7B

Based on the clinical manifestations described, the most likely rare disease diagnosis is **anterior descending atherosclerotic myocardial infarction (pre-MI)**. This condition presents with chest pain, often due to necrotizing myocardial infarction, and can lead to complications such as left-to-right shunts or pericardial effusion.

Other possible diagnoses that align with the described symptoms include:

1. **Coronary sinus thrombosis** - A condition involving clots in the coronary sinus causing moderate chest pain without a lesion.
2. **Aneurysm-related chest pain** - Due to rupture of an aneurysm, potentially causing arrhythmias or acute cardiac tamponade.
3. **Coronary artery disease with ongoing clots** - Involving areas of microvascular dysfunction leading to ischemia without significant stenosis.
4. **Angiographically silent ischemia** - Where ischemia exists but is not visually apparent.
5. **Myocardial disease with multifocal necrosis** - Leading to chest pain and arrhythmias in a non-uniform manner.

These diagnoses collectively align with the rare and specific clinical presentation described.

### 38. User

1. Bone marrow failure Anaemia is present at birth in 35 per cent of children and is often diagnosed between 2 weeks and 2 years after birth. The anaemia is macrocytic or normocytic orthochromatic, with decreased reticulocytes, normal or mildly decreased white blood cells, normal or mildly increased platelets, and hypoplasia of the erythroid lineage of the bone marrow and hyperproliferation of the granulocyte and megakaryocyte lineages.
2. Congenital developmental anomalies 30% ~50% of patients may have congenital developmental anomalies, mainly involving the head, upper limbs, heart and genitourinary system. Examples include short stature, craniofacial anomalies including craniofacial hypertelorism, wide flat nose, small head, congenital cataracts, glaucoma, strabismus, high arched hard palate and cleft lip and palate or even Turner’s syndrome appearance, bunions, congenital cardiovascular anomalies, and genitourinary anomalies. The presence of physical abnormalities does not predict the severity of the patient’s haematological disorders. 30% of affected patients may present with postnatal growth retardation, often associated with other congenital malformations.
3. The incidence of tumours in patients with increased susceptibility to cancer is about 4%, which is higher than in the normal population of the same age. And the tumour occurs at an early age. These include acute leukaemia of the haematological system, myelodysplastic syndromes, lymphomas and solid tumours such as osteosarcoma, thymic carcinoma, hepatocellular carcinoma, melanoma, fibrous histiocytoma, gastric cancer and intestinal cancer. The prognosis of the tumours is poorer than that of the general population suffering from the same tumour, with a higher mortality rate.

### DeepSeek-R1-Distill-Qwen2.5-7B

The rare disease most likely to be diagnosed based on the described clinical manifestations is **Negrin anemia**, a mitochondrial disorder causing bone marrow failure anemia (BMFA). Five additional possible diagnoses that could fit the described scenario are:

1. **Rokitansky-Syndrome**: This syndrome involves bone marrow failure anemia, often associated with other developmental abnormalities.
2. **Myelodysplastic Syndromes (MDS)**: Characterized by ineffective bone marrow function in producing red and white blood cells, leading to various haematological issues.
3. **Acute Leukaemia**: A type of cancer, commonly linked to MDS and other blood disorders, which fits the description of high cancer incidence.
4. **Fibro ossifying chondroma**: While not directly related to blood disorders, it is a rare, benign tumor, potentially contributing to the description of solid tumors.
5. **Congenital heart defects**: This could be part of the developmental abnormalities often associated with the described conditions.

These diagnoses collectively explain the combination of bone marrow failure anemia, developmental abnormalities, and increased cancer risk in the described patient group.

### 39. User

The lesions may involve all systems of the body, so their clinical manifestations vary depending on the site of involvement. General symptoms include fever, malaise, night sweats, and weight loss. The rest of the affected systems mainly include:

1. Skeletal system, most patients have skeletal involvement at the time of diagnosis, with lower limb bones being the most common, but only 50% of patients have bone pain.
2. cardiovascular system, lesions involving the cardiovascular system, most patients do not have obvious clinical manifestations, but such as pericardial involvement, resulting in a large amount of pericardial effusion can lead to chest tightness, breathlessness, or even cardiac tamponade; lesions involving coronary arteries can cause myocardial ischemia-like manifestations, such as chest tightness, chest pain, etc.; diffuse myocardial involvement has also been reported, which can lead to cardiac failure-like manifestations; lesions encircling the renal arteries can cause renal hypertension; lesions encircling the mesenteric artery can cause renal hypertension; lesions encircling the mesenteric artery can cause renal hypertension. Diffuse myocardial involvement has also been reported, leading to heart failure-like symptoms; lesions encircling the renal arteries may cause renal hypertension; lesions encircling the mesenteric vessels may cause intestinal ischaemia, such as abdominal pain.
3. respiratory system Most patients with respiratory system involvement are asymptomatic, but a few patients have dry cough and dyspnoea.
4. central nervous system central nervous system involvement may appear corresponding clinical manifestations due to different parts of brain parenchyma involvement, such as limb movement disorder, gait abnormality, alteration of consciousness, etc.; spinal cord involvement may appear different clinical manifestations due to different affected segments; pituitary gland lesions may manifest polydipsia, polyuria, and other hormone secretion related manifestations.
5. Retro-orbital lesions Retro-orbital involvement is one of the most common clinical manifestations, which may manifest as unilateral or bilateral proptosis, or even cause blindness.
6. Retroperitoneal space/renal involvement. Retroperitoneal lesions may resemble retroperitoneal fibrosis and may cause unilateral or bilateral retrorenal obstruction if the lesion encircles the ureter.
7. cutaneous involvement, the most common cutaneous lesion is a xanthomatous change, which occurs on the eyelids in about 1/3 of patients, and may also occur on the face, neck, axillae, trunk, and groin.

### DeepSeek-R1-Distill-Qwen2.5-7B

Based on the clinical manifestations described, the most likely rare disease could be **sarcoidosis**. Sarcoidosis is a systemic granulomatous disorder that can affect multiple organ systems, including the skeletal system, cardiovascular system, respiratory system, central nervous system, and the skin. It is characterized by xanthomatous changes, akin to pseudogout, and can lead to joint involvement, chest tightness, breathlessness, and nervous system symptoms. Additionally, it can cause pericardial involvement and retroperitoneal/renal involvement, often leading to obstruction. Sarcoidosis is relatively rare and less commonly discussed, fitting well within the category of rare diseases.

### 40. User

The disease often presents with multi-organ and multi-system involvement, with males being more affected than female patients. Clinical manifestations often involve different organs as the patient grows older.

1. Facial appearance, male patients usually appear at the age of 12-14 years with characteristic facial features, such as convexity of the supraorbital ridge, frontal bulge and thickening of the lips. 2.
2. cutaneous angiokeratomas, which are common in patients with the classic form, are red patches of raised skin in the ‘sitting area’, i.e., between the umbilicus and knees, the scrotum, the buttocks, and the inner thighs.
3. Neurological system, most patients will have peripheral nerve pain, which manifests as intolerable burning sensation on the soles of the feet and palms of the hands and radiates to the proximal limbs, and even painful spasms; autonomic nerve involvement manifests as little or no sweating; and central nervous system involvement manifests as early-onset transient ischemic attacks or ischemic strokes.
4. Eye: Characteristic manifestations include conjunctival vascular tortuosity, corneal turbidity, posterior lens capsule clouding, and retinal vascular tortuosity, which in severe cases may lead to reduced or even loss of vision. It is often one of the main reasons for female patients to visit the clinic.
5. Digestive tract, most often after eating nausea, vomiting, abdominal distension, cramping abdominal pain and diarrhoea, may also manifest as malabsorption or constipation.
6. Kidney, early manifestation of urinary concentration dysfunction such as increased nocturia, polyuria and enuresis, with the progress of the disease can gradually appear proteinuria, and even up to the level of nephrotic syndrome, accompanied by renal function damage, mostly around 30 years old into the end-stage renal disease.
7. Cardiovascular system, can be manifested as hypertension, myocardial ischemia caused by coronary artery involvement, heart valve lesions and hypertrophic cardiomyopathy, and in severe cases, it can be manifested as angina pectoris, myocardial infarction and heart failure. It is mostly a late manifestation of the disease and a major cause of death.

### DeepSeek-R1-Distill-Qwen2.5-7B

Based on the clinical manifestations you have provided, the most likely diagnosis is **Teleport Speed Syndrome (TSS)**, a rare genetic disorder characterized by multi-organ involvement and early-onset symptoms, particularly in males. Here are the key diagnostic features:

1. **Teleport Speed Syndrome (TSS)**:
   - Affects males predominantly.
   - Multi-organ system involvement: Includes neurologic, cardiovascular, renal, gastrointestinal, and ocular symptoms.
   - manifested by the age of 12-14 years.
   - Triggers: Singleton syndrome sometime before 3 months of age.
   - arrested growth in early development.
2. **Ocular Manifestations**:
   - Conjunitive vascular tortuosity.
   - Corneal turbidity.
   - Posterior lens capsule clouding.
   - Retinal vascular tortuosity.
   - May lead to reduced or loss of vision in severe cases.
3. **Neurological Manifestations**:
   - Peripheral nerve pain: Intolerable burning sensations on soles of feet and palms of hands, radiating to the proximal limbs, and painful spasms.
   - Autonomic nerve involvement: Little or no sweating.
   - Central nervous system involvement: Early-onset transient ischemic attacks (TIAs) or ischemic strokes.
4. **Cutaneous Manifestations**:
   - Commonly involves raised skin patches (angiokeratomas) in specific areas:
     - Sitting area (between the umbilicus and knees).
     - Scrotum.
     - Buttocks.
     - Inner thighs.
5. **Digestive Tract Manifestations**:
   - After eating: Nausea, vomiting, abdominal distension, cramping, and diarrhea.
   - May also manifest as malabsorption or constipation.
6. **Renal Manifestations**:
   - Early stages: Increased nocturia, polyuria, and enuresis.
   - Progression: May develop proteinuria and nephrotic syndrome.
   - Accompanied by renal dysfunction, most commonly around 30 years.
7. **Cardiovascular Manifestations**:
   - Hypertension.
   - Myocardial ischemia and possible coronary artery disease.
   - Heart valve lesions.
   - Hypertrophic cardiomyopathy.
   - Angina pectoris, myocardial infarction, and heart failure as severe complications.

### Additional Possible Diagnoses:

- ** village syndrome**: A hydrophobic syndrome, not directly connected, unless other symptoms or progression suggests it.
- **Splay速症 (-figure it might also be kWCD)**: If the neurological worsening continues without resolution.

### Next Steps:

- A comprehensive evaluation by a team of specialists, including neurologists, cardiologists, nephrologists, and geneticists, is essential to confirm the diagnosis and manage the condition effectively. Teleport Speed Syndrome is a rare but serious condition, and early intervention can significantly improve outcomes.

### 41. User

The disease is classified as Type 1 and Type 2.

1. Type 1 is mainly characterised by recurrent inflammatory reactions and plasma membrane inflammation, including recurrent fever, abdominal pain and arthritis. Fever is recurrent and may be triggered by cold, strenuous exercise, surgery, infection, etc. and resolves spontaneously within 13 days. Abdominal pain is the most common clinical manifestation, seen in 95% of patients. It is a sudden onset of abdominal pain, often accompanied by fever, abdominal examination may have abdominal distension, abdominal pressure, rebound pain, muscle tension, and the loss of bowel sounds on auscultation. Stereotactic abdominal radiographs show flat air and fluid in the small intestine. Abdominal pain often resolves spontaneously after 24-48 hours. Arthritis is also a common clinical manifestation, seen in 75% of patients. It often involves the large joints of the lower limbs, such as the hip and knee joints. There may be joint swelling and pain. Rash is seen in 7% ~40% of patients, mainly involving the extensor side of the lower limbs, and the typical rash is erysipelas of tansy-like erythema. Pleurisy is seen in 45% of patients and presents as a sudden onset of unilateral chest pain, often accompanied by fever. Pericarditis is less common.
2. Type 2 starts with amyloidosis. It presents with persistent proteinuria and often leads to end-stage renal disease. Amyloid can also be deposited in the heart, gastrointestinal tract, liver and other organs, leading to corresponding clinical manifestations.

### DeepSeek-R1-Distill-Qwen2.5-7B

The rare diseases you are describing can be categorized as follows:

1. **Type 1**: Likely Sarcoidosis
   - **Key Features**: Sarcoidosis is a chronic inflammatory disorder that causes various symptoms, including recurrent fever, abdominal pain, joint inflammation, and proteinuria if the disease spreads. The abdominal pain is often sudden and may resolve after receiving fluids. It can also lead to respiratory issues and rare skin lesions.
2. **Type 2**: Likely Amyloid-Mediated Chronic Kidney Disease (CKD)
   - **Key Features**: This condition starts with amyloidosis, leading to persistent proteinuria, which can progress to end-stage chronic kidney disease. The amyloid deposits can cause organ damage in various parts of the body, including the heart and gastrointestinal tract.

Other possible rare diagnoses could include: - **Functional Peritoneal Abscess**: Characterized by abdominal pain, fever, and signs of partial absorption. - **Periodic Paroxysmal Acute Kidney Disease (PKD)**: Focused on acute kidney insufficiency with fluctuating proteinuria. - **Involving Maps of the Urinary System (IMPSYNDROME)**: Often due to phosphate metabolism issues. - **Mixed Hypercalcemic Periodic Paroxysmal Kidney Disease (MH-PPKD)**: A specific type of PKD involving phosphate metabolism and mineral metabolism disorders.

These diagnoses reflect a range of rare conditions that fit the clinical manifestations provided, particularly emphasizing sarcoidosis and amyloid-related chronic kidney disease.

### 42. User

The main manifestations are congenital developmental anomalies, progressive bone marrow failure and increased incidence of tumours, while some patients may show no developmental anomalies or bone marrow failure.

1. Developmental anomalies, patients may show a variety of physical and mental developmental abnormalities. Growth defects, abnormal skin pigmentation, unilateral or bilateral skeletal malformations of the upper limbs, short stature, microcephaly, ocular anomalies, genitourinary malformations, endocrine anomalies, hearing loss, outer ear anomalies, congenital heart defects, digestive anomalies, abnormalities of the central nervous system, facial deformities, spinal deformities, neck deformities, and developmental delays and/or intellectual disabilities. These manifestations are mostly non-specific, with thumb and upper limb deformities being more specific.
2. Progressive bone marrow failure with haematological abnormalities is the most serious clinical feature of Fanconi anaemia. It may present as a single or total haematopenia. The age of onset of haemopenia varies widely, even among siblings. The median age of onset is about 7.6 years, and it is rare in infants and young children; 90% of patients with haematological abnormalities develop before the age of 40 years. Thrombocytopenia or leukopenia usually precedes anaemia and is often accompanied by an increase in macrocytes, foetal haemoglobin (HbF 1), and a gradual increase in total haematopoiesis.
3. There is an increased risk of neoplasms, including haematological malignancies and non-haematological malignant solid tumours. The risk of acute myeloid leukaemia (AML) is increased about 500-fold, and the cumulative incidence of AML is about 13% at the age of 50 years, with most cases occurring between the ages of 15 and 35 years. -7, del (7q) is associated with an increased incidence of myelodysplastic syndromes (MDS)/AML. Acute lymphoblastic leukaemia and lymphoma have also been reported. Solid tumours can be the first manifestation of the disease. Head and neck squamous cell carcinomas (HNSCCs) are the most common solid tumours in patients and have an earlier age of onset (20-40 years), mostly occurring in the oral cavity (e.g., tongue cancers), are mostly in the progressive stage, and respond poorly to treatment. There is also an increased incidence of skin, oesophageal, liver and genitourinary tract tumours. In a few patients, multiple tumours may be seen concurrently, or haematological and non-haematological tumours may be seen concurrently. And there is a marked intolerance to chemotherapy or radiotherapy.

### DeepSeek-R1-Distill-Qwen2.5-7B

Based on the clinical manifestations provided, the most likely diagnosis is **Fanconi Anemia**. This rare disease presents with developmental anomalies, progressive bone marrow failure, and an increased risk of neoplasms, including both hematological and non-hematological malignancies. Fanconi Anemia is characterized by its impact on bone marrow function and its high tumor burden, making it a fitting match for the symptoms described.

### Additional Possible Diagnoses with Similar Features:

1. **Acute Myeloid Leukaemia (AML):** Affects bone marrow, causes neutropenia, and may present with increased malignancy.
2. **Acute Lymphoblastic Leukaemia (ALL):** Similar to AML, involving lymphocytes and associated with bone marrow issues and malignancy.
3. **Alpers Syndrome:** Characterized by developmental delays and bone marrow insufficiency, with potentially high cancer risk.
4. **Combined Inherited Disorders of Connective Tissue (CIDCT):** May present with immune deficiencies and connective tissue issues that can affect bone marrow function.
5. **Kupffer’s Disease:** Involves a deficiency in macrophages and can lead to bone marrow failure and increased malignancy risk.

These diagnoses share some of the clinical features outlined, particularly bone marrow dysfunction and increased cancer risk, though they may differ in severity and specific symptoms.

### 43. User

The perinatal onset of the disease is often typical, and within a few days of ingestion of breast milk or lactose-containing formula, the child develops life-threatening complications: feeding problems, diarrhoea, vomiting, hypoglycaemia, liver impairment, haemorrhage, jaundice, and cataracts. If left untreated, sepsis, shock and death may occur. Children who survive to infancy may develop severe brain damage if they continue to ingest lactose. Some children have long-term complications such as mental retardation, delayed growth, ataxia, blindness, and ovarian dysfunction in female patients.

### DeepSeek-R1-Distill-Qwen2.5-7B

The rare disease described in the clinical presentation is **Label Franco Syndrome (LFS)**. This syndrome is characterized by a range of symptoms, including developmental delays, neurological abnormalities, and complications such as hypoglycemia, jaundice, and cataracts. If untreated, it can lead to sepsis, shock, and death, particularly in males, while female survivors may experience long-term complications like mental retardation and ovarian dysfunction.

### Additional Possible Diagnoses (based on rare disease categories):

1. **Growth Hormone Deficiency** - Characterized by stunted growth, delayed puberty, and metabolic issues, potentially overlapping with some of the described symptoms.
2. **Gaucher’s Disease** - A lysosomal storage disorder that can cause early-onset neurological and deposition issues, potentially leading to complications like jaundice and developmental delays.
3. **Spina Bifida** - A neuroanatomical disorder affecting the developing spinal cord, leading to neurological damage and potential long-term complications.
4. **Dragonail Syndrome** - A rare genetic condition affecting the heart, liver, and brain, which could present with some of the described features.
5. **Ataxia-Telangiectasia (ATG6)** - A rare genetic disorder causing severe neurological issues, potentially overlapping with the neurological symptoms described.

### 44. User

1. Type I is the most common subtype. There is no primary central nervous system involvement, but some patients may develop secondary neurological manifestations (e.g., spinal cord compression) as the disease progresses. The disease can develop in all age groups, with about 2/3 of patients developing in childhood. Organ manifestations are mainly hepatosplenomegaly, especially splenomegaly, often accompanied by hypersplenism, and even splenic infarction and splenic rupture. The haematological manifestations are mainly thrombocytopenia and anaemia, and some patients have leucopenia, which may be accompanied by coagulation abnormalities. Patients present with pallor, fatigue, skin and gum bleeding, increased menstruation, and even life-threatening bleeding. Most patients have skeletal invasion, but the severity varies. Early invasion sites mainly include lumbar vertebrae, long bone epiphyses and diaphyses, and in the middle and late stages, epiphyses and bony prominences may be involved. Patients often experience acute or chronic diffuse bone pain, and in severe cases, osteonecrosis (ischaemic necrosis), followed by joint collapse involving the proximal and distal femur, proximal tibia and proximal humerus. Osteolytic lesions, pathological fractures, vertebral compression fractures and other fragility fractures due to reduced bone density may also occur. Skeletal lesions can interfere with daily activities and can be disabling. In children, the most common manifestations are osteoporosis, flask-like deformity of the metaphysis of the long bones, varying degrees of hypodensity of the metaphysis of the long bones, thinning of the bone cortex, etc. There may be growth retardation. Some patients may have lung involvement, which is mainly manifested as interstitial lung disease, pulmonary solid changes, and pulmonary hypertension. In addition, patients may have abnormalities of sugar and lipid metabolism, increased risk of malignant tumours such as multiple myeloma, cholelithiasis, and abnormalities of the immune system.
2. Type II, in addition to hepatosplenomegaly, anaemia, thrombocytopenia and other manifestations similar to those of type I, patients with type II mainly have acute neurological involvement. It often develops in the neonatal period to infancy, progresses rapidly and has a high morbidity and mortality rate. There are rapidly progressive medullary palsy, dyskinesia, seizures, angular reflexes and cognitive deficits and other signs of acute neurological damage, and growth is retarded, with death occurring before the age of 2 to 4 years. Some patients with severe disease may develop joint contractures.
3. The early manifestations of type III are similar to those of type I, with gradual onset of neurological involvement, which often develops in childhood and has a slow progression of disease and a longer life expectancy. The patients often have the invasion of the motor nerve, eye movement disorders, ataxia, anisocoria, epilepsy, myoclonus, accompanied by developmental delays and intellectual backwardness. Type III can be divided into three subtypes, namely, type IIIa, which is characterised by more rapidly progressive neurological symptoms (ocular motility disorders, cerebellar ataxia, spasticity, myoclonus, and dementia) and hepatosplenomegaly; type IIIb, which is characterised by hepatosplenomegaly and skeletal symptoms with fewer CNS symptoms; and type IIIc, which is characterised by milder symptoms, including calcification of the cardiac valves and corneal clouding, and is predominantly found in the Druze population. Type IIIc.

### DeepSeek-R1-Distill-Qwen2.5-7B

Based on the clinical manifestations provided, the most likely rare disease diagnosis is:

**1. Centric chondro Jenkins syndrome**: This condition is characterized by early signs of orexin syndrome, cancellous bone changes, and progressive platelet and white blood cell reduction. It may also involve features like hypophosphatemia and phosphate hypophosphatemia, and sometimes liver dysfunction. The gradual progression of bone changes starts from early childhood, and focal BSP (bone-specific asymptomatic portal) densities occur, along with L2-L3 vertexospondyl hypoplasia.

**5 other possible diagnoses**:

- **2. Primary Orbicular Chondro通告 syndrome**: This condition is associated with early growing pintail deformities, cancellous bone involvement, and sometimes orexin syndrome-like presentations. It often includes Stark stars and radiating arthroses.
- **3. Acute promyelocytic leukemia (APL)**: Typically associated with rapidly progressing symptoms, including bone marrow infiltration, platelet and white blood cell reductions, and possibly neurological symptoms. However, it is more acute and less slow to progress than the described condition.
- **4. Acute necrotizing历程 syndrome (ACS)**: Characterized by acute bacterial or viral infections affecting bone, leading to necrosis. This condition is more associated with rapid progression due to infection rather than gradual bone marrow infiltration.
- **5. IntrahepMapper syndrome**: This syndrome is primarily associated with early liver dysfunction and intrahepMapper syndrome. It may involve anemia, thrombocytopenia, and aggressive cholestasis.

These diagnoses were considered based on the key features of the clinical manifestations, including bone marrow involvement, anemia, thrombocytopenia, orexin syndrome-like symptoms, and progressive neurocitic features. Additionally, the condition may involve complications such as phosphate hypophosphatemia and focal BSP densities seen in Centric chondro Jenkins syndrome.

### 45. User

It often starts in one muscle group and progresses to others, with ptosis and diplopia being the most common onset, accounting for 80 per cent of cases. Skeletal muscle weakness is fluctuating and easily fatigued, with a light morning and heavy evening. According to the clinical manifestations, it is divided into five types. Type I: ocular muscle type, manifested by ptosis and diplopia. Type ⅡA: mild generalised type, with mild involvement of the limb muscle groups. Type IIB: Moderate generalised type with moderate involvement of the limb muscle groups, usually with mastication, swallowing and dysarthria. Type III: Severe aggressive type with rapid progression, involving the pharyngeal muscles within weeks or months and the respiratory muscles within six months. Type IV: Late-onset severe type, slowly progressive, involving the respiratory muscles within 2 years. Type V: myasthenia gravis, skeletal muscle atrophy within six months of disease onset. Neurological examination can be seen in the proximal limbs, trunk, neck, craniofacial muscle weakness, intolerance to fatigue, Jolly test is positive, tendon reflexes are normal, no sensory deficits, no pathological signs. Some patients may progress to respiratory failure and develop myasthenia gravis crisis.

### DeepSeek-R1-Distill-Qwen2.5-7B

Based on the clinical manifestations you provided, the most likely rare disease that could be diagnosed is **myasthenia gravis**. Myasthenia gravis is a chronic autoimmune disorder that primarily affects the书法 and protects motor neurons in the skeletal muscle, leading to symptoms such as ptosis, diplopia, and stereometric strabismus (a type of eye misalignment). The muscle weakness described, as well as the neurological examination findings ( Jolly’s positive, no sensory deficits, and mild reflexos deficits), are consistent with myasthenia gravis.

Other possible diagnoses consistent with the clinical presentation include:

1. **Crohn’s disease**: This is a chronic inflammatory disorder of the gastrointestinal tract and can also present with muscle weakness, ptosis, and diplopia as a result of tabletop syndrome.
2. **Cerebellar encephalomalacia**: This is a rare autosomal dominant disorder that causesiasmal and paresmy, as well asbling and diplopia. It can involve multiple muscle groups and has a variable presentation.
3. **Duchenne muscular dystrophy (DMD)**: While this is more commonly associated with Monk syndrome (a severe form of Duchenne muscular dystrophy), it can also present with ptosis and diplopia if the condition is mild or progressive.
4. **Spinal muscular atrophy (SMN1 or SMN2 mutations)**: This is a genetic disorder affecting the motor neurons in the upper motor neurons of the muscles, leading to progressive muscle weakness and atrophy. However, it is more commonly associated with acceleration of muscle wasting and respiratory muscles wasting.
5. **Alagille syndrome**: This is a rare genetic disorder that can cause muscle weakness and neurologic deficits, including ptosis and diplopia. It is associated with a high risk of developing Cannon’s hypernatividad, which can lead to respiratory failure.

The presence of ptosis, diplopia, muscle weakness, and neurological deficits, especially the progression of weakness and the potential for respiratory failure, supports a diagnosis of myasthenia gravis as the most likely condition. However, it is always important to consider other possibilities, especially given the patient’s age, comorbidities, and other factors that could influence the diagnosis.

### 46. User

The disease often starts in adolescence or early adulthood. Clinical manifestations are mainly related to hypokalemia and hypomagnesemia. Mild patients may be asymptomatic or show mild fatigue and poor appetite; severe patients may have twitching of limbs, flaccid paralysis, painful spasms, fainting, and transverse myelinolysis secondary to acute renal injury, and may even suffer from cardiac arrest due to severe ventricular arrhythmias. The common clinical manifestations are as follows:

1. systemic symptoms fatigue, thirst, polydipsia, salt craving.
2. Neuro-muscular system Muscle weakness, painful spasms, convulsions, convulsive seizures, numbness of limbs, sensory abnormalities, rhabdomyolysis, paralysis, dizziness, vertigo, ataxia, and pseudotumor cerebri.
3. Cardiovascular system Palpitation, syncope, normal or low blood pressure and ventricular arrhythmia, etc.
4. Digestive system Constipation, vomiting, etc.
5. Urinary system Polyuria, nocturia, enuresis, proteinuria and renal insufficiency.
6. Bone and joint system Arthralgia, cartilage calcium sedimentation.
7. endocrine and growth retardation, delayed puberty; diabetes mellitus or impaired glucose tolerance is not uncommon in patients with chronically low potassium and magnesium. 8. ocular symptoms.
8. Ocular symptoms Blurred vision and scleral choroidal calcification may occur in a small number of patients.

### DeepSeek-R1-Distill-Qwen2.5-7B

Based on the symptoms provided, the most likely rare disease is **cryptogenic rotational syndrome (CRS)**. This condition is characterized by hypokalemia and hypomagnesemia, often leading to neurological, cardiovascular, and rhabdomyopathic presentations, including muscle twitches, arrhythmias, and even cardiac arrest. The severity and neurological involvement speak strongly to CRS.

Additional possible rare diagnoses to consider include: 1. **Lithium toxicity syndrome**: Characterized by hypokalemia and hypomagnesemia, often with muscle weakness and convulsions. 2. **Erythropoietic stenosis (ES)**: A rare condition with hypokalemia and hypomagnesemia, causing fatigue and other symptoms. 3. **Acute kidney injury syndrome (AKAS)**: Relate to severe renal injury, often presenting with hypokalemia and hypomagnesemia. 4. **Creutzfeldt-Jakob disease (CJD)**: A rare prion-related disease with neurological symptoms, including muscle twitches and twitching. 5. **Chronic kidney disease with sodium-phosphates syndrome (CKD-SUSY)**: Characterized by low potassium and magnesium, often with neurological features.

These conditions are less common and may also present with similar symptoms, making accurate diagnosis challenging.

### 47. User

The majority of children develop the disease in infancy or early childhood, with a variety of presentations. The earlier the onset, the more severe the symptoms and the worse the prognosis. The majority of patients have a relatively normal presentation in the first year of life. The earliest sign that appears in about 75% of patients is a large head size, and most children are born with a larger head circumference than their peers or have a rapid increase in head circumference soon after birth, which may be accompanied by mild non-specific symptoms, including irritability, difficulty in feeding, and vomiting. Children usually develop ketosis, vomiting, hepatomegaly and acute encephalopathic crisis, including hypotonia, loss of consciousness and convulsive seizures, between 3 and 36 months of age after fever, infection, surgery, or vaccination, which may resolve with symptomatic treatment, but not fully recover. If acute encephalopathic crisis occurs repeatedly, the neurological damage will be progressively aggravated, and there may be developmental regression, and eventually cognitive dysfunction may occur. In some children, delayed movement, dystonia, and random movement disorders occur gradually over the first few years of life, but mental development is almost normal. Patients often die within 10 years of age from concomitant diseases or Reye-like seizures, which decrease with age. Rarely, the onset of the disease occurs during adolescence or even adulthood, and may be asymptomatic until the first episode.

### DeepSeek-R1-Distill-Qwen2.5-7B

The description of the disease presents with a severe and life-threatening encephalopathy in early childhood, followed by long-term neurological and developmental issues. Based on the symptoms and progression, the most likely diagnosis is a genetic disorder causing progressive encephalopathy and neurodevelopmental regression. Potential candidates include:

1. **A奶粉x syndrome**: A genetic disorder associated with encephalopathy and convulsions, often causing significant neurological and developmental issues.
2. **Shi’s syndrome (Shida encephalopathy)**: A rare disorder characterized by sudden severe encephalopathy and significantakash-mails, though it may not specifically match all symptoms described.
3. **Progressive External Ophthalmoplegia with Encephalopathy (PEOE)**: A rare condition that can lead to convulsions, encephalopathy, and developmental regression.
4. **Macrocerephalic encephalopathy syndrome**: A group of rare disorders causing brain swelling leading to convulsions, which can result in lengthy neurodevelopmental issues and death in young adults.
5. **Kotelsky disease**: While primarily affecting adults, it can present with neurodevelopmental issues in childhood, though it may not fully align with all described symptoms.

These diagnoses are based on the progression of symptoms and the associated risks of severe neurological complications, suggesting a rare genetic or developmental disorder.

### 48. User

It mainly manifests as bleeding from joints, muscles and deep tissues, but also internal organs such as gastrointestinal tract and central nervous system. If repeated bleeding occurs, untreated bleeding can lead to joint deformity and/or pseudotumour formation, which can be life-threatening in severe cases. Delayed haemorrhage after trauma or surgery is characteristic of this disease.

### DeepSeek-R1-Distill-Qwen2.5-7B

Based on the symptoms described—bleeding from joints, muscles, deep tissues, internal organs like the gastrointestinal tract, and central nervous system, with the risk of life-threatening complications if untreated— several rare but recognized conditions could be considered:

1. **Hemolytic Anemia**: Characterized by a reduced capacity to produce hemoglobin, leading to low red blood cell production and increased risk of bleeding.
2. **Polycythemia Vera (PV)**: An overproduction of red blood cells causing excessive bleeding from various tissues.
3. **Afghanistan Syndrome**: A rare genetic disorder affecting platelet function and leading to recurrent bleeding from joints and tissues.
4. **Disability Syndrome**: A genetic disorder causing platelet deficiency, leading to prolonged bleeding.
5. **Talasemi Disease**: A bleeding disorder due to improper platelet production.
6. **Reye’s Syndrome**: Involves liver inflammation causing gastrointestinal bleeding, though not extremely rare.

These conditions should be considered in the differential diagnosis. A specialist, particularly a hematologist or geneticist, would be appropriate for further evaluation and management.

### 49. User

It can involve multiple organs of the body and has a variety of clinical manifestations. The age of onset is mostly 3-60 years old, and there are also reports of patients diagnosed at 8 months and over 70 years old. In children, hepatic involvement is the first manifestation of the disease, while in adolescents and adults, neurological involvement is the first manifestation of the disease.

It may present with asymptomatic persistent elevation of aminotransferases, chronic hepatitis, cirrhosis, and acute liver failure. Neurological involvement may manifest as motor dysfunction, tremor, ataxia, chorea, dysautonomia, dystonia, and in some patients masked face, stiff limbs, and gait abnormalities. Some patients combine with mental behavioural abnormalities such as apathy, aggressive behaviour, and personality changes. Kidney involvement may manifest as haematuria, proteinuria, etc. Involvement of the blood system may be manifested as haemolytic anaemia, liver cirrhosis, splenic hyperfunction leading to a decrease in the blood tertiary system, and coagulation abnormalities. Relatively rare systems involved include bone and joint, heart, endocrine and reproductive systems.

### DeepSeek-R1-Distill-Qwen2.5-7B

Based on the detailed description of the rare disease, the most plausible conditions that fit the symptoms are:

1. **Wilson’s disease**: This condition involves multiple organ systems, including the liver, heart, nervous system, and kidneys. It is characterized by neurological symptoms like ataxia and can lead to organ failure.
2. **Alport syndrome**: Although more common in early childhood, certain cases might present with adult onset and multiple system involvement, including nervous system symptoms.
3. **Progressive Malignant_lyss syndrome**: A rare multisystem condition that combines malignancy with organ involvement and neurological symptoms.
4. **Other multisystem conditions**: Such as, but not limited to, rare multifactorial endocrine syndromes or conditions like hydrocephalus, which might involve multiple organ systems.
5. **Revasive disease**: A rare, severe multisystem condition that might involve various organs, including the liver, nervous system, and others.

Each condition is distinct and has specific features that align with different aspects of the described disease. Further evaluation would be necessary to pinpoint the exact cause.

### 50. User

The clinical picture is very heterogeneous, even within the same family. The disease usually begins before the age of 30 years, worsens during adolescence, and the oedema often has an acute onset. The clinical picture is characterised by recurrent, unpredictable cutaneous and submucosal oedema. The oedema is episodic, self-limited, usually resolves spontaneously in 3-5 days, asymmetric and non-concave. Oedema can involve any part of the body, with the extremities, face, genitals, respiratory tract and gastrointestinal tract mucosa being the most common. The most fatal is respiratory mucosal oedema, which can rapidly progress to respiratory distress or asphyxiation due to laryngeal oedema, and can lead to death by asphyxiation if not resuscitated in time, with a lethality rate as high as 11%-40%, which is one of the main causes of death. Episodes of mucosal oedema of the digestive tract manifest as severe abdominal pain, accompanied by nausea and vomiting, and are therefore often misdiagnosed as acute abdomen, leading to unnecessary abdominal surgery. Skin oedema occurring in the limbs and other parts of the body can affect its function.

### DeepSeek-R1-Distill-Qwen2.5-7B

Based on the clinical description provided, the most likely diagnosis is **alcoholic bullae (abd)**. This condition is rare, causing episodic, self-limiting oedema, bullae, and asymmetric lesions that can lead to respiratory distress and death if not treated. The presentation affects submucosal mucosa, including the respiratory tract, which aligns with the described symptoms.

Other possible rare diagnoses that might fit include:

1. **Severe bullous keratosis**: Characterized by episodic oedema and bullae, though less commonly associated with life-threatening complications unless progressing rapidly.
2. **Rubinstein–Tschar CDDL-like Alopecia amalgamata**: A condition that might present with localized oedema, though it is more typically associated with alopecia.
3. **Alkaptonuria**: While more systemic, it can cause oedema, though it is less specific to localized mucosal areas.
4. **Primary immune-mediated oedema**: A less specific condition, but immune-mediated oedema can present with the described symptoms in rare cases.
5. **Inflammatory strict{xia dermatothurax**: A rare condition of doubtful practical relevance, but it’s possible to consider.

Each of these conditions varies in clinical presentation and pathophysiology, making animal bullae the most fitting in this case due to the specific presentation and potential for fatal outcomes.

### 51. User

The disease starts at birth and the severity of clinical manifestations is related to the subtype of the disease. Clinical manifestations include blisters or maculopapular blisters on the hands, elbows, knees and feet and other areas prone to friction, or around the body. Blisters or ulcers of the oral mucosa may occur, leading to dysphagia and hoarseness. Some patients have mottled pigmentation abnormalities, baldness, palmoplantar hyperkeratosis, enamel defects, nail thickening and nail dystrophy. Other impairments include disfigurement of the digits due to repeated scar formation, anaemia, osteoporosis, muscle atrophy, cardiomyopathy, and deafness. Some subtypes may be associated with basal cell carcinoma, squamous cell carcinoma, and malignant melanoma in advanced stages.

### DeepSeek-R1-Distill-Qwen2.5-7B

Based on the clinical manifestations provided, the most likely diagnosis is **psoriasis**. Psoriasis is a rare autoimmune disorder characterized by red, scaly plaques, blisters (in some cases), pigmentation abnormalities, and skin ulcers. It commonly affects areas of the body prone to friction, such as the hands, elbows, knees, and feet, as well as the oral mucosa.

Here are five other possible rare diagnoses that could present with similar symptoms:

1. **Subcutaneous starchatosis**: This is a rare, autosomal recessive disorder characterized by clusters of small, flat or subcutaneous scaly lesions (e.g., blisters) around the body. It can involve areas such as the face, arms, legs, and head, with possible pigmentation changes.
2. **Oncopigmentation**: This is a rare and idiopathic pigmentation disorder of the skin characterized by hypopigmentation (mottled or uneven pigmentation) and isolated areas of dead skin scaling. It is often asymptomatic or catches the attention of a patient in the course of life.
3. **Ichthyosis communis**: This is a rare, genetic skin disorder causing asymmetric scaling, pigmentation changes, scaly lesions, and fine linearkeratosis with blisters around the edges. It is often associated with subclinical or mild dermato-oncological findings.
4. **Langerhansin naughtie**: This is an atypical case of Langerhans cell histiocytosis (LCH), a rare, inflammatory disorder of connective tissue. It causes scaly skin, ulcers, pigmented areas, and dermal Zytenes (tumor-like growths). It can be recognized by its multifaceted clinical picture.
5. **Multiple system atrophy - Type II (MSA-II)**: This is a rare neurodegenerative disorder associated with atrophy of the muscles, enzymopathy (possible for anemia), and skin and eye changes. While it causes muscle atrophy and can lead to neurodegenerative conditions, it is less related to the skin manifestations described, making it a less likely diagnosis unless there is comorbidity.

In summary, while psoriasis is the most likely diagnosis based on the symptoms provided, the other conditions listed could also present with similar skin and systemic manifestations, especially if they are associated with branched subtype presentations.

### 52. User

The clinical presentation of patients varies with age, amount and duration of fructose exposure. In general, the younger the age of onset, the more severe the symptoms. The onset of the disease is typically around 6 months of age and usually occurs when fructose-containing foods, such as fruits, honey and certain vegetables, are first consumed. Common clinical symptoms include hypoglycaemic episodes after eating fructose-containing foods, with nausea, vomiting, abdominal pain, bloating, sweating, tremors, convulsions and even coma.

If the diagnosis is not clear and the patient chronically ingests fructose foods for a long period of time, in addition to hypoglycemic manifestations, growth retardation, hepatomegaly, ascites, jaundice and metabolic disorders such as hypoglycemia, metabolic acidosis, hypophosphatemia, hyperuricemia, hypermagnesemia, and hyperpyruvatemia may occur. In severe cases, multiple organ damage, liver and renal failure, convulsions, coma, and even death occur.

In acute attacks, laboratory tests reveal hypoglycaemia, hypophosphatemia and hypokalemia, accompanied by elevated uric acid, lactate, pyruvate, free fatty acids and triglycerides. Chronic patients present with hepatic impairment and indications of hepatic fibrosis, including serum bilirubin, elevated transaminases, and coagulation disorders. Urinalysis reveals proteinuria and fructose positivity.

### DeepSeek-R1-Distill-Qwen2.5-7B

The clinical presentation described strongly suggests **Crigler-Najjar syndrome**, a rare genetic metabolic disorder affecting carbohydrate metabolism. Here’s a structured answer:

1. **Crigler-Najjar Syndrome**:
   - **Characteristics**: Causes accumulative carbohydrate metabolism defects, leading to energy deficits, hypoglycemia, and neural issues.
   - **Gene Mutation**: Typically due to mutations in the WLEL, TSHR, or SLC2A5 genes.
   - **Symptoms**: Includes hypoglycemic episodes, nausea, vomiting, abdominal pain, bloating, sweating, tremors, convulsions, and in chronic cases, growth retardation and hepatomegaly.
2. **Alternative Considerations**:
   - **Iconing**: A rare metabolic disorder with hypoglycemia, seizures, and growth retardation, though less commonly associated with sugar intake.
   - **Hernodoniae**: Focuses on mineral metabolism issues, less relevant here.
   - **Growth Stewart-TonEB计划延迟症**: Pituitary-related, focuses on growth but not directly on sugars.
   - **Hereditary Non-Remixing Syndrome (HNCS)**: Monogenic mutations causing hypoglycemia and growth issues.
   - **Hypoglycemic Precipitation Syndrome (HPS)**: Due to low erythropoietin levels, not genetic, and not linked to sugars.

**Conclusion**: Crigler-Najjar syndrome best fits the described symptoms, particularly the genetic and metabolic aspects tied to sugar metabolism, making it the most likely diagnosis.

### 53. User

**1. Migraine** Migraine with aura is often the first symptom of the disease and is seen in 20-40% of patients, which is five times higher than in the general population; it can also manifest as migraine without aura, with an incidence not different from that of the general population. The incidence of migraine is lower in Asian patients than in European and American patients.

**2. Subcortical ischemic events** Transient ischemic attacks (TIAs) and ischemic strokes are the most common clinical manifestations of the disease, occurring in 60% to 85% of symptomatic patients, most of whom lack common risk factors for cerebrovascular disease. 2/3 of patients present with the classic lacunar syndrome.

**3. Cognitive deficits** Cognitive dysfunction with dementia develops progressively in 60% of patients.

**Affective disorders and apathy** 20% of patients have affective disorders, with depressive states being the most common, and rarely alternating between depressive and manic states; apathy is seen in 40% of patients.

In addition, seizures occur in 5-10% of patients, and hearing loss, impaired consciousness, spinal cord infarction, intracranial haemorrhage, and combined basin infarction have also been reported.

### DeepSeek-R1-Distill-Qwen2.5-7B

The rare disease most likely associated with the provided clinical manifestations, including migraines with aura, subcortical ischemic events, cognitive deficits, and other symptoms, is a **Mitochondrial Encephalomyopathy (MEM)**. This condition is characterized by TIAs, cognitive impairment, and multifocal system involvement, often leading to a spectrum of presentations that include memory loss, hearing issues, and other neurological deficits.

**Possible Diagnoses:**

1. **Mitochondrial Encephalomyopathy, Lactic acidosis, and Malaise (MEMLM)**: This condition is distinguished by TIAs, leucemia-like features, and a high neurologic deficit.
2. **Mitochondrial Arteritis**: This самостоятельный artery condition can cause TIAs, cognitive malingering, and other systemic symptoms.
3. **Focal Segmental Episodic Post-artritis Nerve Lesions (FEP)**: TIAs due to_dc-_in inflammation, often leading to cognitive and neurologic deficits.
4. **Intracranial Hemorrhage**: Though less common in_MEM, episodes can present with TIAs and cognitive issues.
5. **Spinal Infarction**: In rare cases, TIAs may precede or be associated with mitochondrial-related neurologic insults in the spinal cord.

This comprehensive approach considers the multifaceted presentation of the disease, linking TIAs, cognitive deficits, and other symptoms under the broader umbrella of mitochondrial encephalopathy.

### 54. User

The onset of the disease is usually insidious in early youth (but has been reported from 1 to 76 years of age). The main manifestation is a slowly progressive spastic paralysis of both lower limbs, with pyramidal signs (hyperreflexia, positive ankle clonus, positive pathology). Bilateral upper limb reflexes may also be hyperreflexic, but generally do not affect upper limb activity. More than half of the patients will have proximal weakness of the lower limbs. Decreased vibratory sensation of the ankle tuning forks (which usually does not disappear) is detected on examination in about 50% of patients, and urinary dysfunction is present in about one third of patients.

The disease progresses with age, with about 20% of patients eventually losing the ability to walk. The severity of clinical symptoms varies considerably between patients carrying the same mutation in the same family.

### DeepSeek-R1-Distill-Qwen2.5-7B

Based on the clinical presentation described, several rare diseases could potentially fit, but the most likely next steps involve further diagnostic evaluation. Here’s a structured approach:

1. **Identify Key Symptoms and Features**:
   - Slowly progressive spastic paralysis in both lower limbs.
   - Hyperreflexia (positive ankle clonus, pathology).
   - Bilateral upper limb hyperreflexia.
   - Proximal weakness of the lower limbs.
   - Decreased vibratory sensation of the ankle in 50% of cases.
   - Urinary dysfunction in 33% of cases.
   - Age-related progression, with early-onset in youth and possible late onset (up to 76 years).
2. **Consider Potential Conditions**:
   - **Charcot’s Syndrome**: Typically affects the cerebellum and affects upper and lower limbs, but the progression and symptoms described are less consistent.
   - **Mitochondrial Myopathy**: Could explain comprehensive muscle weakness and sensory issues, but specific to the lower limb presentation is unclear.
   - **Genetic Syndromes**: Conditions like- Eisenstein’s syndrome or other neurodegenerative syndromes with spasticity could be considered.
   - **Spastic Dipole or Tethered Limes Syndromes**: Less likely due to Differences in motor pattern.
3. **Decision for Further Evaluation**:
   - **Neurological Examination**: Comprehensive assessment by a neurologist, including imaging if necessary.
   - **Genetic Testing**: Likely required to identify genetic mutations causing the condition.
   - **Refer to Expert**: Consulting a neurologist, neurosurgeon, or geneticist would be essential for a definitive diagnosis.

In conclusion, without more specific symptoms, the most probable next steps involve a detailed neurological examination, genetic testing, and referring to a specialist for further evaluation.

### 55. User

Most of the affected children develop the disease in newborns and early infants. Clinical manifestations are similar to biotinidase deficiency, without specificity, mainly feeding difficulties, vomiting, diarrhoea, hypotonia, convulsions, depression, lethargy, respiratory difficulties, developmental delays, intractable rashes, desquamation, which can be combined with ketosis, metabolic acidosis, hyperlactic acidemia and so on. Skin damage in most patients in the head and face, neck, trunk, buttocks and other parts of the skin rash or erythema, ulceration or blisters, furfuraceous or flaky scales, or dry and rough skin, peeling skin, etc., and a few patients only in the perioral, periocular, perianal local appearance of the rash. Patients are not associated with hearing or visual impairment, unlike biotinidase deficiency.

### DeepSeek-R1-Distill-Qwen2.5-7B

Based on the clinical presentation described, the most likely diagnoses are:

1. **Infantile Spina Bifida**: Characterized by Sensorineural Hypotrophy (SNH) with symptoms like feeding difficulties, vomiting, diarrhea, hypotonia, and developmental delays. It presents with cutaneous rashes in various areas of the body, including the head, face, neck, trunk, and buttocks, with occasional ulcers or blisters and dry, scaly skin.
2. **Atlas Encephalos Aphitrophos**: An autosomal recessive disorder affecting the hindbrain, presenting with convulsions, developmental delays, and skin rashes. It often causes a perioral or periocular rash in addition to blisters or eczemas.
3. **产销性先天性脑膜孔狭小**: Characterized by Sensorineural dysfunction and growth retardation, presenting with developmental delays, feeding difficulties, and skin rashes limited to the face, neck, or upper limb.
4. **Wild-Type Rolandic Encephalomyopathy (wRe)**: A very rare childhood condition affecting the developing brain, presenting with a range of symptoms akin to those described, including skin issues like polydermatitis.
5. **Siretan Paintings/Encephalos Aphitrophos**: An autosomal recessive disorder with early-onset Sensorineural dysfunction, presenting with developmental challenges and skin conditions like perinatal atlas dermatosis.

Each of these conditions fits the description due to their early onset, nonspecific symptoms, and specific skin manifestations, excluding hearing or visual impairments.

### 56. User

Children are born without abnormalities, and the clinical presentation in infancy is not specific and is characterised only by growth and developmental delays. It is often diagnosed at 3 years of age after the onset of ocular signs and symptoms.

1. visual impairment, myopia and iris tremor due to ectopic lens may appear after 3 years of age, and may be combined with astigmatism, glaucoma, staphylococcus, cataract, retinal detachment and optic nerve atrophy in later stages. 2. intellectual disability is common.
2. Intellectual disability is more common, mainly manifested as progressive mental retardation. Vitamin B6 treatment-sensitive patients are less mentally impaired. 3.
3. mental or behavioural disorders, which may occur in about 50% of children and are associated with convulsions in 20%.
4. Skeletal involvement, similar to the manifestations of Marfontein syndrome, or in combination with scoliosis, funnel chest, chicken chest, knee valgus, and bowed feet, with generalised osteoporosis seen on X-ray.
5. Thrombophilia, which occurs at all ages and may involve all types of blood vessels, especially the cerebral vessels. High levels of homocysteinemia may alter the vessel wall and increase platelet adhesion, resulting in optic nerve atrophy, paralysis, pulmonary heart disease, and renal hypertension.
6. Spontaneous pneumothorax and acute pancreatitis are less common.

### DeepSeek-R1-Distill-Qwen2.5-7B

The clinical presentation described is multifaceted, involving both skeletal and ophthalmic issues, along with developmental delays and behavioral disorders. Based on the characteristics mentioned, the most likely diagnosis is a **multifactorial rare disorder** that combines skeletal-muscular integrative features with ophthalmic abnormalities, behavioral issues, and developmental milestones.

**Key Considerations:**

1. **Combination of Skeletal and Ophthalmic Issues:** The presence of skeletal abnormalities (e.g., scoliosis, bowed feet, joint issues) alongside ophthalmic manifestations (myopia, pigment changes, dark urine) suggests a disorder that affects multiple systems.
2. **Developmental Delays and Congenital Abnormalities:** Early diagnosis at 3 years with growth delays and morphological abnormalities points to a condition involving embryonal positioning syndromes (EPS), which often includes skeletal and sensory issues.
3. **Behavioral and Mental Irregularities:** The presence of behavioral disorders alongside intellectual disability suggests a complex genetic disorder.
4. **Presence of Embryonal Positioning Syndromes (EPS):** This group of disorders is characterized by skeletal abnormalities, ophthalmic issues, and developmental delays, making it a plausible underlying cause.

**Possible Diagnosis:**

Given the combination of features, the most likely diagnosis is a **multifactorial genetic disorder** that involves both skeletal-muscular integration and ophthalmic abnormalities, commonly associated with embryonal positioning syndromes. Specific conditions within this spectrum may include, but are not limited to, some forms of Usher syndrome or skeletal-muscular integrative syndromes.

**Further Consideration:**

If the disorder increasingly presents with20/20 vision and residual grades of myopia, it may align with specific subtypes within EPS. However, without more detailed symptoms, the exact condition cannot be narrowing down further. Thus, it remains a multifactorial disorder requiring specialized genetic evaluation and targeted management.

### 57. User

The main manifestations of the disease are markedly elevated levels of LDL-C from birth, cholesterol deposits in the skin, eyes and tendons, and the formation of xanthomas and lipomatous corneal arches. As a result of exposure to high levels of LDL-C, most patients develop coronary atherosclerotic heart disease before the age of 20 and may die as a result.

A detailed family history is needed, as some patients have heterozygous parents and therefore have elevated LDL-C levels (usually greater than the 95th percentile of their country’s age- and sex-specific norms) and a strong positive family history of premature atherosclerotic cardiovascular disease.

### DeepSeek-R1-Distill-Qwen2.5-7B

Based on the clinical manifestations provided, the most likely diagnosis is:

### 1. Hereditary Hypertriglyceridemia with Early Atherosclerosis and Xanthomatosis

This condition is characterized by elevated triglycerides and LDL cholesterol levels from birth, cholesterol deposits in the skin, eyes, and tendons, xanthomas, and lipomatous corneal arches. The early development of coronary atherosclerotic heart disease in children and the presence of a heterozygous family history with a strong predisposition to heart disease further support this diagnosis.

### 5 Other Possible Diagnoses:

1. **Hypertriglyceridemia (specifically combined with early xanthomatosis and atherosclerosis)**
   This can be linked to genetic mutations affecting the production or reabsorption of triglycerides, leading to cholesterol deposits and xanthomas.
2. **Xanthomatosis with Early Atherosclerosis (PEMAX)**
   A condition characterized by xanthomas, early atherosclerosis, and hyperlipidemia, which may have a genetic or metabolic basis.
3. **Von Hippel-Lindau Disease (VLK)**
   This condition involves corneal deposits (likely the lipomatous corneal arches) and hyperlipidemia, often with a genetic component affecting cholesterol metabolism.
4. **Hyperlipidemia with Pseudoxanthoma Elasticum (PXE)**
   While PXE is primarily associated with vitamin D deficiency, it can coexist with hyperlipidemia and xanthomatosis in certain cases.

These diagnoses are based on the clinical features you provided and potential additional genetic or metabolic causes common in rare genetic disorders.

### 58. User

It is characterised by a triad of movement disorders, psychiatric symptoms and cognitive deficits, usually with an insidious onset and slow progression. The age of onset ranges from childhood to 79 years, with the most common being 30-50 years of age.

The disease involves rapid, involuntary, non-rhythmic movements of the face, trunk, and limbs. Early abnormal movements are mild, and the patient may have difficulty recognising the presence of chorea or integrating chorea-like movements into random movements, which makes the control of normal movements difficult or deviant; as the disease progresses, the extent and degree of chorea often increases, and even affects the diaphragm, pharynx, and laryngeal muscles, resulting in dysarthria, dysphagia, and involuntary movement. As the disease progresses, the extent and degree of chorea often increases, even affecting the pharyngeal and laryngeal muscles, resulting in dysarthria, dysphagia, and involuntary vocalisations. In the later stages of the disease, the chorea often disappears, and is replaced by a Parkinson’s-like pattern of stiffness and hyperkinesis, which may be accompanied by focal dystonia. Difficulty in maintaining movement is also a common manifestation of the disease, i.e., the inability to maintain certain simple voluntary movements, such as difficulty in maintaining tongue extension.

Mental disorders may precede chorea, and common symptoms include depressed mood, depression, irritability, apathy, and anxiety, as well as paranoia, delusions, and hallucinations; rare symptoms include obsessive-compulsive behaviours and psychotic manifestations, which tend to worsen progressively.

Cognitive impairment is most often characterised by executive dysfunction, which is manifested by a reduced ability to make decisions, multi-task and shift cognitive goals. Patients usually lack self-awareness of their cognitive deficits. Memory loss or impairment occurs late in the course of the disease and progresses to dementia as the disease progresses.

In addition to the triad of symptoms, eye movement abnormalities are also prominent. Early in the disease, there is a slowing down of eye-sweeping speed, but eye-following movements are preserved; in later stages, eye-following movements, voluntary sweeping and refixation are all impaired. In addition, weight loss and cachexia are common features of the disease, with patients experiencing significant weight loss as a result of the high energy expenditure of involuntary movements, usually without loss of appetite. Sleep and/or sexual dysfunction are also common.

Progressive deterioration of motor and cognitive function can lead to significant complications, such as dysphagia due to involvement of the pharyngeal muscles, dyskinesia, reduced activity and even bed rest, which can lead to aspiration pneumonia and other infections, and decubitus ulcers.

### DeepSeek-R1-Distill-Qwen2.5-7B

The integrated symptoms described, including a triad of motor, psychiatric, and cognitive deficits with slow, insidious onset and progression towards specific parkinsonian features, suggest a rare neurodegenerative condition. Given the characteristics—insidious onset, progression towards parkinsonism, cognitive deficits, and psychiatric symptoms—the most likely disease is **anomalous internal pontine gray matter syndrome (AIPM)**.

**Answer:** - **Most Likely Disease:** Anomalous Internal Pontine Gray Matter Syndrome (AIPM)

**Additional Possible Diagnoses:** 1. Fragile X syndrome 2. Stachyotrophoid syndrome 3. Prader-Willi/X syndrome 4. Korsakoff syndrome 5. SMA-related syndrome

These syndromes, while distinct, could present with overlapping or related clinical features, especially in rare or combined presentation scenarios.

### 59. User

Clinical signs and symptoms are mainly neurological and vary in severity. The age of onset can range from neonatal to adulthood (late-onset), and there is a broad phenotypic spectrum.

1. In the neonatal period, symptoms associated with hyperammonemia [including lethargy, drowsiness, refusal to eat, vomiting with respiratory alkalosis and/or seizures] appear after about 24-48 hours of asymptomatic onset. It is indistinguishable from other urea cycle disorders of neonatal onset.
2. Late-onset, which accounts for 88 per cent of patients, 40 per cent of whom occur before the age of 3 years, 29 per cent in childhood and 19 per cent in adulthood. The main manifestations are: (i) chronic neurocognitive deficits, including developmental delay, ataxia, spasticity, learning disabilities, cognitive deficits, and/or unexplained seizures; (ii) acute encephalopathy secondary to hyperammonaemic crisis induced by a variety of factors (e.g., infections, fasting, or injuries); and (iii) chronic hepatic dysfunction, with unexplained elevation of hepatic aminotransferases with or without mild coagulation disorders, with or without mild hyperammonaemia, and protein intolerance. Ammonemia and protein intolerance.

### DeepSeek-R1-Distill-Qwen2.5-7B

Based on the clinical manifestations you have described, the most likely diagnosis is **Rett Syndrome**, which is a rare genetic disorder characterized by developmental delays, motor and sensory neuron degeneration, and shell-shaped brain. The symptoms described, such as hyperammonemia in the neonatal period and chronic neurocognitive deficits, are consistent with Rett syndrome.

### Five Other Possible Diagnoses:

1. **Corneticulate Diplegia**: A rare genetic disorder causing delayed motor development, with features such as hypotonia, coordination difficulties, and sensory abnormalities, often associated with hyperammonemia and neurocognitive deficits.
2. **Prader-Willi Syndrome (PWS)**: A genetic disorder characterized by severe intellectual disabilities, distinctive facial features, and ataxia. It may present with developmental delays, motor issues, and neurocognitive difficulties, though the spectra of presentation can vary widely.
3. **Phenylketonuria (PKU)**: Though primarily a metabolic disorder caused by phenylalanine kinase deficiency, PKU can also present with neurological symptoms, including hyperammonemia, developmental delay, and encephalopathy in severe cases.
4. **Variable Boltzmann Chromophagocytosis (VBC)**: A rare neurodegenerative disorder that can cause developmental delay, neurocognitive deficits, and hyperammonemia. It is associated with both early-onset and late-onset presentations.
5. **Alpers Syndrome**: A rare genetic disorder characterized by chronic neurological deficits, developmental delay, and features of altered brain metabolism, including hyperammonemia. It may present with similar symptoms to Rett syndrome but has a different pathophysiology.

In all cases, further correlation with the patient’s symptoms, medical history, and genetic testing would be necessary to confirm the diagnosis.

### 60. User

Some children have no clinical symptoms in the neonatal period, but after 3-4 months of life, the typical symptoms appear gradually: the hair turns from black to yellow, the skin is light-coloured, and the urine and sweat are foul-smelling. As the child grows older, he/she gradually shows backward intellectual development, microcephaly, epileptic seizures, and may also show abnormalities in behaviour, personality, and neurocognition. For example, hyperactivity, self-mutilation, aggression, autism, low self-esteem, depression, etc. Vomiting and eczema are also common in infancy.

In some patients, there are no clinical symptoms in the neonatal period, and after 1-3 months of life, in addition to the symptoms of PKU, the main manifestations are dyskinesia, lethargy, hypotonia of the trunk, increased or decreased muscle tone of the limbs, nystagmus, dysphagia, increased drooling, flaccidity, torticollis, unresponsiveness, insomnia, and severe intellectual developmental disorders, and so on.

### DeepSeek-R1-Distill-Qwen2.5-7B

The symptoms described suggest a rare neurological disorder. The progression from hair color change, skin lightness, and foul-smelling secretions to later behavioral and intellectual issues points towards several potential conditions. Based on the analysis, the most plausible rare disease is **Epilepsy with Early Onset and Slowly Progressing Cognitive Impairment (EOIC)**, though further verification is recommended. Other potential conditions to consider include:

1. **Recounted Encephalopathy (RE)**: Characterized by prolonged illness causing deficit features.
2. **Epilepsy with Pseudocerebri Luminosis (EPL)**: Less common but presents with similar neurological features.
3. **Prionencephalopathy**: A rare condition with late neurological deficits in children.
4. **Barbier-Lafontaine Syndrome**: A rare disorder causing developmental delays and neurological issues.
5. **Epilepsy with Early Onset and Pseudocerebri in. Oris (EOIC)**.

Each condition should be considered alongside the child’s specific symptoms and a thorough clinical evaluation by a neurologist or pediatric specialist.

### 61. User

The severity of clinical manifestations is highly variable. Patients may present with abnormalities in bone mineralisation, dental or joint pathology, muscle weakness, etc. Currently, there are seven main types of the disease.

1. simple dental type, which is the mildest and most common type, with only dental involvement and no rickets or osteomalacia. 2. adult type, which often develops in middle age.
2. Adult type, which often develops in middle age and presents with dental abnormalities or osteochondrosis. It may cause impaired mobility due to repeated fractures, bone and joint pain, or muscle weakness. 3.
3. the childhood type, which usually develops after 6 months of age, can be mild to severe, and the children may have different degrees of tooth loss and rickets.
4. the infantile form, with onset before 6 months of age, is often poorly ventilated, difficult to survive, debilitating, and may have a wide fontanelle or cranial deformity. 50 per cent of infantile HPP die of complications. 5. the perinatal form, with onset before 6 months of age, is the most severe form of HPP, with onset before 6 months of age.
5. The perinatal form, which is the extreme form, develops in utero, is evident at birth, and is almost always fatal. Children with short limbs and severe skeletal undermineralisation may present with high-pitched crying, seizures, periodic apnoea with cyanosis and bradycardia, unexplained fever, irritability, myelosuppressive anaemia and intracranial haemorrhage, and pulmonary atelectasis.
6. Pseudohypophosphatasia, similar to the infantile form, in which the patient has the manifestations of infantile hypophosphatasia with normal or elevated serum ALP. 7.
7. Benign type, sometimes presenting with skeletal malformations in utero, with perinatal hypophosphatase activity, but resolving after birth without the associated manifestations of infantile hypophosphatasia or simple dental hypophosphatasia.

### DeepSeek-R1-Distill-Qwen2.5-7B

Based on the clinical manifestations described, it is likely that the condition is **hypophosphatasia pseudoxanthomatosis (HPP)**, a rare genetic disorder characterized by bone mineralization defects and skeletal.pathological changes. HPP is most commonly divided into seven subtypes, each with distinct clinical presentations. Below, I will identify the subtype most likely to present with the described symptoms and provide additional possible diagnoses:

### Most Likely Diagnosis: Hypophosphatasia Pseudoxanthomatosis (HPP)

HPP is the most common diagnosis associated with defective bone formation, often due to alpha-1 antip积极参与 the hypothesis. HPP is typically a **cornerstone of rare diseases** and is often asymptomatic except during the preschool years. However, there are seven specific subtypes of HPP classified based on the onset age and clinical manifestations.

### The Seven Subtypes of Hypophosphatasia Pseudoxanthomatosis (HPP)

1. **Simple Dental Type**:
   - Mild and most common presentation.
   - **Symptoms**: Only dental anomalies (e.g., tooth loss, caries, or xerdontia), no rickets or osteomalacia. Joint involvement is uncommon.
   - Case Example: A child with tooth loss due to non-etching phosphates.
2. **Adult Type**:
   - Develops during middle adulthood.
   - **Symptoms**: Dental or joint abnormalities, rickets, muscle weakness, impaired mobility, and recurrent fractures.
   - Case Example: A young adult with rickets due to hyperparathyroidism.
3. **Childhood Type**:
   - Typically begins after 6 months of age.
   - **Symptoms**: Moderate-to-severe dental and/or joint involvement, tooth loss, rickets, and growth restriction.
   - Case Example: A child with severe tooth loss and rickets due to early mineral deficiency.
4. **Infantile Form**:
   - Onset before 6 months of age.
   - **Symptoms**: Severe dental and/or joint abnormalities, perimthal fibula (weak girth in the hip), hypocalpectrolyte syndrome, and poor ventilatory and growth outcomes.
   - Case Example: A baby with combined pseudoxanthomatosis and spina bifida.
5. **Perinatal Form**:
   - Onset before 6 months and often in utero or post-natally.
   - **Symptoms**: Most severe form, with short stature due to severe dehydration and hypocalcemia. Pathological骨折 and/or semilunar metaphyseal defects due to minimise mineralization. Pathological fractures, muscle weakness, cyanosis, fever, and intracranial hemorrhages (ICH) are common.
   - Case Example: A postterm infant with severe bone defects and cyanosis due to intracranial bleeding.
6. **Pseudohypophosphatasia**:
   - Similar to the infantile form, but with **elevated or normal serum parathyroid hormone (PTH)** levels.
   - **Symptoms**: Dental abnormalities, may include hypocalp or moderate-to-severe osteopenia.
   - Case Example: A child with early phosphate deficiency but normal PTH levels.
7. **Benign Type**:
   - Onset during utero and postnatal periods, with **perinatal mineral deficiency**.
   - **Symptoms**: **Skeletal malformations in utero** (e.g., fontanelle deformity), perinatal hypophosphatase, no mineral deficiency at birth or no associated signs of mineral deficiency.
   - Case Example: A child with a cleft palate or absent fontanelle.

### Alternative Diagnoses

If the described clinical manifestations do not fit the seven subtypes of HPP, alternative diagnoses include:

- **Subacterial osteodystrophy**: Focuses on bone defects due to subatomic particles, often seen in welders or nuclear workers.
- **Idiopathic skeletal hypophosphatemia**: A rare genetic disorder characterized by unilateral skeletal hypophosphatosis, leading to osteopenia or osteoporosis.
- **Cushing’s syndrome**: Characterized by hyperparathyroidism, osteolytic lesions, and skler成人 growth.
- **yth錄症-帕金森氏症复合症 (Thyroid hormone-induced weakness and parkinsonism)**: A rare prematurity-related disorder leading to hypocalp and developmental delay.
- ** -=** -= -= -= -= -= -= -= -= -= -= -= -= -= -= -= -= -= -= -= _

### Conclusion

The most likely diagnosis based on the clinical manifestations described is **hypophosphatasia pseudoxanthomatosis (HPP)**, but it is important to consider the severity and onset age of the symptoms to determine whether another subtype or related disorder is more appropriate. A detailed clinical history and genetic testing (if not already done) would further clarify the diagnosis.

### 62. User

Children with square skulls, chicken breasts, beaded ribs, bracelet sign, foot bracelet sign, Henry’s groove, and lower limb deformities, which may be inversion of the knee (O-shaped leg) or eversion of the knee (X-shaped leg), appearing when they start weight-bearing near the age of one week, are accompanied by growth retardation, short stature, wobbly gait, progressively aggravating bone deformities, multiple bone fractures, bone pain, and abnormalities of tooth development (poor tooth quality, toothache, difficulty in regeneration after loss). Imaging is characterised by skeletal deformities, such as Imaging manifestations include skeletal deformities, widening and blurring of the epiphysis of the long bones, a cupped shape, and many fine strips of calcification like brushes in the cupped shape. In adults, the main manifestations are limb weakness, activity limitation, bone pain, multiple pathological fractures (long bones of the limbs, ribs, pelvis and vertebrae can occur), and shortening of height. Imaging manifestations include a generalised decrease in bone density, blurred bone trabeculae with a hairy glass appearance, pelvic deformity, false fracture lines, and biconcave deformity of the vertebral body.

### DeepSeek-R1-Distill-Qwen2.5-7B

Based on the comprehensive analysis of the presented symptoms and imaging findings, the most likely diagnosis is Lenz’s Anomaly, which is associated with both craniofacial and spinal psoas dysplasia. This condition can lead to a generalized skeletal dysplasia pattern, including lower limb deformities (X or O-shaped legs) and rib anomalies. However, the comprehensive nature of the symptoms, including multiple bone fractures and growth retardation, suggests a potential for a generalized skeletal dysplasia syndrome that affects both the craniofacial and lower extremities.

**Possible Diagnoses:**

1. **Lenz’s Anomaly**: Characterized by craniofacial psoas dysplasia and spinal stenosis, often leading to lower limb deformities and associated skeletal issues.
2. **Congenital Scoliosis with Associated Psoriasis Dysplasia**: This condition can cause lower limb X or O-shaped deformities alongside craniofacial anomalies.
3. **Pseudotumour of Lenz**: A rare condition associated with psoas dysplasia, potentially affecting both the face and lower extremities.
4. **Spina Bifida**: Although less likely, it can occur with associated psoas and skeletal issues.
5. **Ehlers-Danlos Syndrome (EDS)**: May affect bone structure and could underlie some of the observed features, though it is less commonly associated with skeletal dysplasia of this severity.
6. **Spina Bifida X Syndrome**: Involves spinal cord abnormalities, which could contribute to lower limb deformities.
7. **Metachonic Dysplasia**: Primarily associated with rib anomalies, it might interact with lower limb deformities in this case.
8. **Spina Bifida**: Less likely but could present with skeletal anomalies if spinal growth issues are present.
9. **Ehlers-Danlos Syndrome (EDS)**: Potentially affecting bone density and causing growth retardation, though its typical presentation might not include the specific lower limb and rib anomalies described.
10. **Generalized Skeletal Dysplasia Syndrome**: A broader category that encapsulates the multifaceted skeletal and craniofacial issues observed.

Each of these possibilities requires further investigation, including genetic testing, to establish a definitive diagnosis. Lenz’s Anomaly remains the most plausible explanation given the symptoms, but the comprehensive nature of the presentation suggests a complex interplay of factors.

### 63. User

Clinical manifestations include heart failure, chest pain, hypotension, syncope, sudden cardiac death, arrhythmias and thromboembolism. Some patients also have extracardiac manifestations such as peripheral neuropathy, autonomic neuropathy and gastrointestinal symptoms.

### DeepSeek-R1-Distill-Qwen2.5-7B

The rare disease most likely associated with the given clinical manifestations—heart failure, chest pain, hypotension, syncope, sudden cardiac death, arrhythmias, and thromboembolism, along with extracardiac symptoms like peripheral neuropathy and gastrointestinal issues—is suspected to be a genetic disorder causing sudden cardiac death (SCD).

**Library Card (LC) and My Card (MC):**

- **LC**: KCNQ1-C 2062A>G mutation, a known cause of SCD, leading to premature ventricular contractions and arrhythmogenic conditions, which contribute to heart failure, chest pain, hypotension, and related symptoms, including arrhythmias and possible thromboembolism.
- **MC**: Acute Myocardial Infarction (AMI) - though more common, it’s a possible cause if the patient experiences a heart attack with symptoms like chest pain and arrhythmias.
- **MC**: Essential Hypertension - could contribute to heart failure and chest pain if severe enough.
- **MC**: Atrial Flutter or Fibrillation - arrhythmogenic conditions linked to SCD.
- **MC**: Acute Heart Attack - severe heart attack symptoms could manifest with heart failure and arrhythmias.
- **MC**: Other Arrhythmias - such as bundle branch block or paroxysmal wide complex arrhythmia.

This conclusion is based on a genetic mutation linked to SCD and arrhythmias, which collectively present with the described symptoms.

### 64. User

1. secondary sexual characteristics and impaired gametogenesis in males: childish voice, small penis, no pubic hair growth, small testes or cryptorchid testes, no spermatogenesis; in females: mammary gland dysplasia, infantile vulva, primary amenorrhoea.
2. delayed epiphyseal closure upper volume/lower volume <1, fingertip distance > height, susceptible to osteoporosis.
3. Olfactory disorders 40% ~60% of IHH patients suffer from loss of sense of smell due to abnormal development of the olfactory bulb and olfactory tract, and are unable to identify odours.
4. Other manifestations Facial midline defects, such as cleft lip and palate; isolated kidneys; short fingers (toes), syndactyly; skeletal deformities or dental hypoplasia; overweight and obesity; mirror image movement.

### DeepSeek-R1-Distill-Qwen2.5-7B

**Diagnosis: McFarland Syndrome**

**Presentation:** McFarland syndrome is the most likely diagnosis given the combination of symptoms provided, particularly the presence of facial midline defects (cleft lip/palate), short stature, skeletal hypoplasia, craniofacial abnormalities, mirror image movement (pseudo-tibial syndrome), and sensory issues such as loss of smell, especially in 40-60% of cases.

**Key Features:** 1. **Facial and Craniofacial Abnormalities:** Includes cleft lip and palate, short fingers or toes, and skeletal hypoplasia. 2. **Skeletal and Dental Hypoplasia:** Characterized by delayed epiphyseal closure, supernumerary ribs (not mentioned here), and isotrets and isolated hearts. 3. **Cognitive and Motor Features:**Associated with intellectual disability, atrophy of facial skin, microcephaly, and pseudotibial syndrome (mirror image movement). 4. **Sensory Impairments:** High prevalence of sensory issues, including olfactory dysfunction in 40-60% of patients. 5. **Orthopedic Abnormalities:** stiffness, response to steroids, and excess detrimental effects on growth and development.

**Other Considerations:** - While Ehlers-Danlos syndrome, Fragile X syndrome, and BPH syndrome share some features, McFarland syndrome is distinguished by the presence of facial midline defects and pseudotibial syndrome, which are not typically prominent in these other conditions.

**Conclusion:** Based on the clinical manifestations including facial midline defects, skeletal hypoplasia, olfactory issues, and the unique feature of pseudotibial syndrome, McFarland syndrome is the most accurate diagnosis. Other rare conditions such as Ehlers-Danlos syndrome, Fragile X syndrome, and Baumann postherpade syndrome are less likely given these distinct traits.

### 65. User

The most common symptom is shortness of breath after activity, and other symptoms include fatigue, dizziness, chest pain, chest tightness, palpitations, blackouts, and syncope, etc. Once syncope or vertigo occurs, it suggests that the patient’s cardiac output may have decreased significantly. Due to atypical symptoms and signs, the majority of patients have a significant delay in consultation, with at least 1/5 of patients taking more than 2 years from the onset of symptoms to diagnosis; patients may have a hyperactive second heart sound of the pulmonary valve, and tricuspid insufficiency may cause a systolic murmur in the tricuspid region; signs of right heart failure include jugular venous congestion or rupture, enlarged liver, oedema of the lower extremities, multiple plasma cavities, jaundice, and cyanosis, among other signs.

### DeepSeek-R1-Distill-Qwen2.5-7B

Based on the clinical manifestations you have described, the most likely rare disease to be diagnosed is **SinoInspiration_dyad症 (SIN syndrome)**. This syndrome is associated with a hyperactive second heart sound (SinoInspiration) of the pulmonary valve, tachycardia, chest pain, and other symptoms like fatigue, dizziness, palpitations, blackouts, and syncope. It often occurs in younger males and is linked to a decrease in cardiac output, which aligns with the symptoms you mentioned.

### Five Other Possible Diagnoses:

1. **Pulmonary Stenosis**: This condition causes a blockage in the pulmonary artery, leading to increased heart effort and murmurous sounds in the tricuspid or pulmonic areas.
2. **Tricuspid Insufficiency**: A congenital heart defect that causes a murmur in the tricuspid area and may present with symptoms like dizziness, fatigue, and shortness of breath.
3. **Mitral Stenosis/Valve Prolapse**: While more common, stenosis can cause pulmonary congestion and murmurous sounds. Prolapse may explain some of the symptoms, such as dizziness and tachycardia.
4. **Isolated Tachycardia Syndromes**: Some rare tachycardias, such as those caused by intrinsic or inherited accessory pathways, may mimic other symptoms like fatigue and dizziness.
5. **Congenital Heart Defects**: Some rare heart conditions, such as hypoplastic left heart syndrome or RVSD (right ventricle amortear syndrome), may cause similar symptoms, depending on the specific pathology.

These diagnoses are based on the clinical presentation and the presence of murmurous sounds and cardiac murmurs. Further diagnostic workup, including echocardiography and cardiac catheterization, would be necessary to confirm the diagnosis.

### 66. User

The disease is usually insidious. The main manifestations are dry cough, exertional dyspnoea, pestle-like fingers (toes), and bursting sounds distributed mainly in both lower lungs are its typical signs. In the terminal stage, cyanosis, pulmonary hypertension, pulmonary heart disease and right heart insufficiency can be seen.

### DeepSeek-R1-Distill-Qwen2.5-7B

Based on the clinical manifestations you’ve described - **dry cough**, **exertional dyspnea**, **pitting finger/toe syndrome**, low-belly lung sounds in the lower lung areas, cyanosis in the terminal stage, **pulmonary hypertension**, and **pulmonary heart disease** -, the most likely diagnosis is **CHRONIC HEART FAILURE (CORONARY ARTERIAL RESTRICTED BY EMBOLISM, IN Tutorial)**. Chronic heart failure is a rare disease characterized by impaired heart contractions, leading to pulmonary congestion and fluid retention, which can cause the symptoms you described, especially cyanosis as the disease progresses.

Other possible diagnoses could include:

1. **CARIDIOPATHIC LUNG DISEASE (CLD)**: A rare and often untreatable condition caused by a heart-related defect affecting the lung, leading to low-belly sounds and cyanosis.
2. **PULMONARY FIBROSIS**: A rare and often progressive lung condition characterized by scarring, edema, and cyanosis as part of its advanced stages.
3. **SAROID CYTOMA**: While more commonly associated with lung masses, it can sometimes cause similar symptoms, though it is less likely in this context.
4. **INTERSTITIAL LUNG DISEASE (ILD)**: A rare inflammatory condition causing lung congestion and edema, which can present with symptoms like cyanosis.
5. **SCAR P除此以外, could also consider sarcoidosis, but it is less commonly associated with the specific symptoms described, although it can cause edema and cyanosis in rare cases.

To confirm the diagnosis, further workup would include **pulmonary function tests**, **imaging studies** (e.g., chest X-ray, CT scan), ** heart catheterization**,**  CBC** to look for cytokines in case of ILD or sarcoidosis, and **electrocardiogram (ECG)** to assess heart function.

The doctor would need to perform a detailed clinical examination, possibly perform imaging or genetic testing, to confirm the exact cause of the symptoms. If cyanosis, pulmonary hypertension, and pulmonary heart disease are present in the terminal stage, CHRONIC HEART FAILURE is the most likely diagnosis. The doctor should consider these symptoms in the context of other possible rare conditions but prioritize CHRONIC HEART FAILURE as the likely cause.

### 67. User

It is a multi-organ and multi-system disease that can involve almost all organs and tissues of the body. Clinical manifestations are varied, and it is easy to miss the diagnosis when patients consult different specialties for different organ involvement. Most of the patients have a slow onset of the disease, with a chronic progression. The disease may start in a single organ and gradually involve multiple organs, or it may develop in multiple organs at the same time. In a minority of patients, the disease is self-limiting.

Lacrimal and salivary glands are the most commonly affected organs. The main manifestations are symmetrical, painless enlargement of the lacrimal gland, submandibular gland or parotid gland, hard nodules, with or without sinusitis or thickening of extraocular muscles. Involvement of the digestive system may manifest as autoimmune pancreatitis, sclerosing cholangitis, sclerosing mesenteritis, etc. Clinical manifestations may include vague abdominal pain, abdominal distension, dyspepsia, and jaundice. Lung involvement may be manifested as bronchial wall thickening, interstitial lung lesions, lung nodules/lumps and pleural thickening, etc. Patients may be asymptomatic or have cough, shortness of breath and wheezing. Retroperitoneal fibrosis or peri-abdominal aortitis is also one of the more common clinical manifestations, which is mainly a thickening of the soft tissue around the abdominal aorta, which can encircle the ureter and cause ureteral stenosis and pyelonephrosis, and in a small number of patients, abdominal aortic aneurysm occurs. Clinical manifestations include abdominal pain, low back pain, or oedema of the lower extremities. Less than 10% of patients develop IgG4-associated renal disease, most often manifesting as tubulointerstitial nephropathy, renal pelvic space-occupying lesions, ureteral wall thickening, or renal mass-like lesions. Other organs involved include the thyroid (sclerosing thyroiditis) and prostate. CNS lesions are rare, such as autoimmune pituitary inflammation, hypertrophic dural meningitis, and intracranial inflammatory pseudotumours, and patients may present with headaches, urolithiasis, and hypopituitarism. In addition to the above organ involvement, most patients combine with superficial or deep lymph node enlargement. In addition, about half of the patients have a history of allergy-related disease, which can be accompanied by asthma.

### DeepSeek-R1-Distill-Qwen2.5-7B

Based on the clinical manifestations described, the most plausible diagnosis could be **“Epiglottitis with respiratory symptoms and multiple organ involvement”**. This diagnosis aligns with the involvement of multiple systems, including the salivary glands, digestive system, and respiratory system, which are typically associated with Epiglottitis. However, it’s important to note that Epiglottitis might not encompass all the symptoms mentioned, particularly those involving the pancreas or diverse organ systems. For a more specific diagnosis, further evaluation and consideration of additional symptoms, such as pancreatic issues or lung involvement, would be necessary.

### 68. User

Children with cholestasis and cirrhosis develop. The main clinical manifestations are hyperconjugated bilirubinaemia and often malabsorption of fat-soluble vitamins, such as rickets. The clinical phenotypes of different enzyme defects are slightly different. For example, oxysterol 7α-hydroxylase defects are associated with hereditary spastic paralysis in adulthood; cholesterol 25-hydroxylase defects are associated with intractable constipation; 3β-hydroxysteroid -Δ5-C27- steroid dehydrogenase defects are associated with cholestatic liver disease and fat-soluble vitamin deficiencies in infancy; and sterol 27-hydroxylase defects are associated with chronic diarrhoea and developmental delays in children, and spasticity in adulthood. Sterol 27-hydroxylase defects can cause chronic diarrhoea and developmental delay in children, and spastic paralysis in adults; α-formyl-coenzyme A dehydrogenase defects can cause fat-soluble vitamin deficiencies in infancy, and sensory neuropathy in adulthood; and bile acid-coenzyme A-amino acid N-acetyltransferase defects can cause intrahepatic cholestasis, developmental delay, and familial hypercholesterolemia.

### DeepSeek-R1-Distill-Qwen2.5-7B

Based on the clinical manifestations provided, the most likely rare disease is **oxysterol 7α-hydroxylase deficiency**, which is a genetic disorder affecting cholesterol metabolism. This condition is characterized by hyperconjugated bilirubinaemia, malabsorption of fat-soluble vitamins such as rickets, and various enzyme defects leading to different clinical phenotypes.

### Most Likely Diagnosis:

1. **Oxysterol 7α-hydroxylase Deficiency**: This condition results from a defect in the synthesis or function of a protein that converts oxysterols into 7α-hydroxyoxysterols. It is associated with hereditary spastic paralysis in adulthood.

### Other Possible Diagnoses:

1. **Cholesterol 25-Hydroxylase Deficiency**: This condition causes intractable constipation and is linked to sterol disorders like familial hypercholesterolemia or related metabolic syndromes.
2. **3β-Hydroxysteroid-Δ5-C27-steroid Dehydrogenase Defect**: This defect is associated with fat-soluble vitamin deficiencies, liver disease, and developmental delays in infancy.
3. **Sterol 27-Hydroxylase Deficiency**: Characterized by chronic diarrhoea, developmental delays, spasticity in adulthood, and systemic inflammation syndromes.
4. **Alpha-Formyl-Coenzyme A Dehydrogenase Deficiency**: This condition causes early-onset spasticity, developmental delay, and rickets, often associated with familial hypercholesterolemia.
5. **Bile Acid-Coenzyme A-Amino Acid N-Acetyltransferase Deficiency**: This condition is linked to intrahepatic cholestasis, developmental delay, and familial hypercholesterolemia.

These conditions may overlap with the symptoms described and may vary based on the specific genetic or metabolic defect causing the phenotype.

### 69. User

**1. Acute neonatal type** The onset of the disease is acute and the progression of the disease is rapid and can be life-threatening. Within 1-2 weeks of birth, it presents with feeding difficulties, vomiting, muscle weakness, hypotonia, and lethargy or worsening progression to coma. Accumulation of isovaleric acid may cause the characteristic odour of ‘sweaty feet’. Liver enlargement may be present on examination. Laboratory tests suggest metabolic acidosis, hyperammonemia, hypoglycemia or hyperglycemia, ketosis, hypocalcemia, and pancytopenia. If the patient survives the acute exacerbation in the neonatal period, he or she will progress to the chronic intermittent form.

**The chronic intermittent form** has an insidious onset. It presents only as a nonspecific inability to tolerate fasting or developmental delay. It is often triggered by upper respiratory tract infections or the intake of a high-protein diet, and is characterised by recurrent episodes of vomiting, lethargy, coma, acidosis with ketonuria, and ‘sweaty feet odour’, which can be relieved by a protein-restricted diet and glucose infusion. The vast majority of patients with chronic intermittent form have normal psychomotor development, but some have developmental delays and varying degrees of mental retardation.

### DeepSeek-R1-Distill-Qwen2.5-7B

Based on the clinical manifestations you provided, the most likely rare disease diagnosis would be **Acute infantile akinesia with ketonic ketosis (AKK)**. AKK is a rare metabolic disorder characterized by the acute onset of signs of ketosis, central nervous system depression, and developmental delay. The presentation you described matches the classic features of AKK, where the patient experiences symptoms such as feeding difficulties, vomiting, muscle weakness, hypotonia, lethargy, and, in severe cases, coma. Additionally, the presence of metabolic acidosis, hyperammonemia, hypoglycemia, ketosis, hypocalcemia, and pancytopenia strongly supports a metabolic origin, which is common in AKK.

Other possible rare disease diagnoses that may present with similar clinical features include:

1. **Glutarichondriacucurühlungsp flapii (GGEL)**: This rare neurodevelopmental disorder is associated with unpredictable episodes of unsteady Jesús syndrome, myopathy, and Warschweiz disease. It is characterized by inheritance of certain genes and typically presents with developmental delay, muscle weakness, and neurological abnormalities.
2. **Ossot-Plateau syndrome (OPSS)**: A rare neurodevelopmental disorder presenting initially with ophthalmoplegia, head-ache, polite gait, and developmental delay. It is often associated with inherited nonsyndromic mental retardation and comorbid conditions such as metabolic acidosis.
3. **Pro EMPGE**: Prothoracosis with Elective Mesenterectomy Gastroenteromegaly and Ectopomy (Pro EMPGE) is a rare, autosomal recessive disorder causing abdominal pain, nausea, vomiting, and centrally directed pain in the periarmpilotic region. It may appear acutely in cases of undiagnosed ethanolism or drug-induced liver injury.
4. **Reye’s syndrome**: Although not as rare, Reye’s syndrome is a metabolic disorder characterized by encephalopathy, developmental delay, and hypoglycemia. It is more common in children exposed to antiviral medications and can present with similar acute symptoms in some cases.
5. **Spinal glutarylacetate depletion**: This is a relatively recently described rare metabolic disorder causing developmental delay, feeding difficulties, and ataxia. It is characterized by inherited deficient activity of acetat (GLUDAC), leading to a range of neurological and metabolic features.

In summary, while the patient’s presentation aligns most closely with AKK, other rare diseases with overlapping clinical features should also be considered. A thorough clinical and genetic evaluation is essential to confirm the diagnosis and ensure the correct management.

### 70. User

1. dysgenesis of secondary sexual characteristics and impaired gametogenesis.
2. Olfactory disorders Inability to recognise odours, especially aromatic odours, due to abnormal development of the olfactory bulb and olfactory tract.

### DeepSeek-R1-Distill-Qwen2.5-7B

The clinical manifestations provided—dysgenesis of secondary sexual characteristics and impaired gametogenesis, along with olfactory disorders—point towards a rare condition. The closest fit is **X-Stationary Orphan Disease (XSS)**, which is an X-linked recessive disorder affecting secondary sexual characteristics and gametogenesis. While XSS is primarily associated with physical and developmental issues, it is possible that olfactory dysfunction may also manifest as part of the broader presentation of the condition. However, the connection to olfactory issues may be coincidental or part of a more comprehensive rare disease. In conclusion, XSS is the most likely rare disease to consider, though further evaluation would be necessary to confirm this diagnosis.

### 71. User

The condition varies greatly from mild to severe and is therefore easily misdiagnosed and underdiagnosed. Clinical manifestations mainly include:

**1. Fever** The fever is irregular and may be periodic or persistent. Patients with hypothalamic involvement may have central thermoregulatory abnormalities.

**2. rash** Mainly on the trunk, scalp and hairline. It begins as a reddish papule, which may then become a haemorrhagic or eczema-like seborrheic rash, followed by crusting, which is followed by a white spot.

**3. Oral and eye, ear, nose and throat lesions** Gum swelling, loose teeth, proptosis, and persistent otitis media with external auditory canal rash are typical.

**4. Central uropathy** Pituitary gland, hypothalamus or even other parts of the central nervous system, with neuropsychiatric symptoms in a few patients.

**5. Respiratory symptoms** Coughing, severe breathlessness, cyanosis, and even recurrent pneumothorax episodes. Pulmonary signs are usually unremarkable.

**6.** The liver and spleen can be enlarged, jaundice, abnormal liver function, liver failure and portal hypertension can occur, and eventually patients can die of cirrhosis and liver failure.

**7. Digestive tract symptoms** Digestive tract involvement is less common and can manifest as polyps or adenomatous changes, usually without clinical symptoms, and abdominal pain, diarrhoea and hypoproteinaemia may occur when the lesions are diffuse.

**8. Haematopoietic system** Lymph nodes may be enlarged, and anaemia, abnormal white blood cell and platelet counts may occur with bone marrow involvement.

**9. Bone damage** Skull, limb bones, spine and pelvis may have pain and lumps, and pathological fractures may occur. Proptosis due to orbital bone lesions is one of the typical manifestations in paediatric patients.

### DeepSeek-R1-Distill-Qwen2.5-7B

The condition described aligns most closely with **Primitive Neuroectoderm Dysplasia (PND)**, characterized by its multifaceted clinical presentation, including urothelial and Retroperitoneal osteopetrosis, orbitofacial abnormalities, and associated hematologic and hepatic findings.

**Five additional rare diseases with overlapping symptoms:**

1. **Kala-athrunya (Infective Endemism)**: Involves liver, spleen, and bone involvement with possible ophthalmoplegia.
2. **Wilson’s disease**: Relates to copper deficiency, involving liver enlargement and jaundice, though less specific to urothelial or skeletal features.
3. **Molnar syndrome**: Features liver and spleen enlargement with ophthalmoplegia.
4. **Orf Pseudoarachnodysosthenes Syndrome**: Involved in spinal stenosis and retroperitoneal osteolysis.
5. **Disodium phosphate encephalopathy**: May present with neurologic symptoms alongside urothelial and hepatic involvement.

These diseases share some overlapping clinical presentations but may vary in specific symptomatology, necessitating a thorough clinical evaluation.

### 72. User

**1. Growth disorders and other malformations,** most of the children are born with a weight within the normal range, but some of them are born with a length of less than 48cm (42-47cm). After birth, the growth rate gradually and significantly slows down, and the lifelong height of untreated male patients ranges from 116 to 142 cm, and the lifelong height of female patients ranges from 108 to 136 cm. At the same time, the patients’ hands, feet and internal organs are small. Some patients have other congenital malformations, such as hip dislocation, aortic valve stenosis, cryptorchidism, and congenital cataracts.

**2. Facial features,** the characteristic facial changes of patients are prominent forehead, saddle nose, small jaw, larger head to body ratio than normal children, but the absolute value of head circumference is still small compared with children of the same age. Hair is thinning, and some patients may have a ‘sunset sign’, with blue sclera in patients from the Mediterranean and Middle Eastern regions. The patient has delayed teething, late deciduous milk teeth and multiple caries, and may have malocclusion due to a short mandible.

**Skeletal musculature** Some patients have congenital hip dislocation and ischaemic necrosis of the femoral head. Due to mid-facial dysplasia and pharyngeal anatomical abnormalities, the patient’s speech is high pitched and 85% of the patients have limited elbow joint movement.

**4. Metabolic disorders,** Patients often have a combination of metabolic abnormalities, including hypoglycaemia in the neonatal period and infancy, and progressive obesity in adulthood.

**5. Delayed sexual development ,** Patients with this condition have small genitalia, with male patients presenting with small penises and testicles, and female patients having small ovaries on ultrasound. Most patients exhibit delayed pubertal development. Although sexual development is delayed, male and female patients can eventually develop mature sexual function and be fertile.

**6. Low tumour incidence** Patients have a significantly reduced risk of tumour development compared to the general population.

### DeepSeek-R1-Distill-Qwen2.5-7B

**Final Answer:**

Based on the clinical manifestations provided, the most likely rare disease is **Growth-Related Congenital Malformation (GRCM) syndrome**, which encompasses a range of conditions characterized by growth retardation, skeletal abnormalities, and facial dysplasia. These conditions often include skeletal flattenings, hip dislocations, and microcephaly, aligning with GRCM’s presentation.

**Five Additional Possible Diagnoses:**

1. **Ehlers-Danlos Syndrome (EDS):** Characterized by joint hypermobility and connective tissue disorders, though not directly linked to the described features.
2. **McAlpine Syndrome:** Involves exclusively breast milk feeding, growth retardation, and long-frame growth.
3. **Cheetah Syndrome:** Known for growth retardation, hypoglycemia, and long-frame growth.
4. **Defaches Syndrome:** A condition with growth hormone deficiency leading to delayed puberty and genital issues.
5. **Silicon Valley Syndrome:** Linked to high radiation exposure and increased cancer risk, though primarily associated with metabolic issues.

Each of these conditions provides a spectrum of possible diagnoses, reflecting the diverse array of rare disorders that can present with similar clinical features.

### 73. User

The main symptom is a sudden, sharp, painless loss of visual acuity in both eyes that occurs sequentially, usually weeks to months between episodes. Funduscopic examination shows congestion and oedema of the optic disc and tortuous dilatation of the peripapillary capillaries in the early stages of the disease, while the oedema and capillary dilatation subside in the later stages of the disease, and eventually the temporal side of the optic disc or all of the optic disc shows atrophic changes. The majority of patients have only ocular manifestations, while a few may have other systemic symptoms such as mental retardation, epilepsy, hearing impairment, dystonia, etc.

### DeepSeek-R1-Distill-Qwen2.5-7B

The clinical description you provided is highly suggestive of **Pseudoxanthoma Elasticum (PXE)**. This condition is a rare autoimmune disorder that typically presents in childhood and adolescence with a sudden, sharp, painless loss of visual acuity in one or both eyes. It is characterized by capillary tortuosity and oedema of the optic disc, which resolves over time but is followed by atrophic changes.

### Most Likely Diagnosis:

1. **Pseudoxanthoma Elasticum (PXE)**
   - This condition is associated with a unique pattern of optic disc changes, including capillary tortuosity and oedema that resolves over time, followed by atrophic changes. It is often systemic and can present with a variety of systemic symptoms such as mental retardation, epilepsy, and hearing loss, which align with the description provided.

### Other Possible Diagnoses:

1. **Ring sign公路 accidents (Tàishi estar síntome)**
   - This is a rare condition caused by the optic ring sign公路 accidents, which can lead to sudden and severe loss of vision in both eyes. It is often associated with systemic symptoms similar to PXE.
2. **Marfan’s disease**
   - While not as rare, Marfan’s disease can cause optic disc changes and capillary tortuosity. It is a systemic disorder that can also present with nervous system and organ involvement, including the visual system.
3. **Caviaправица (Pig disease)**
   - This condition is a rare autosomal recessive disorder that can cause optic disc edema and capillary changes. It is less commonly associated with visual acuity loss but can present with systemic symptoms.
4. **Acute angels综合征 (Acute angle-closure glaucoma syndrome)**
   - This is a rare and serious condition associated with optic disc oedema and subsequent atrophy due to increased intraocular pressure. It is an emergency and rare in terms of frequency.

Please note that differential diagnosis should always be made in collaboration with a_ophthalmologist, as the symptoms and imaging findings can be mimicked by other conditions.

### 74. User

The clinical manifestations of patients are low ketogenic hypoglycaemia, liver function abnormalities, lactic acidosis, cardiomyopathy in infancy, growth retardation, hypotonia or cardiomyopathy in some patients, and cholestasis or cirrhosis in a few. In early childhood, some patients show growth retardation, hypotonia or cardiomyopathy, and a few may have cholestasis or cirrhosis. In early childhood, patients may have feeding difficulties, episodic metabolic disorders, and episodic rhabdomyolysis. Most patients will have retinopathy in childhood, and peripheral neuropathy is a less common complication of long-term disease.

### DeepSeek-R1-Distill-Qwen2.5-7B

The rare disease that most closely matches the clinical manifestations described is **Ossanna syndrome**. This syndrome is characterized by a combination of metabolic and cardiac issues, including hypoglycemia, growth retardation, retinopathy, and episodes of rhabdomyolysis. It is an autosomal recessive disorder and typically presents in early childhood. Ossanna syndrome also involves malnutrition and hepatopathy, which aligns with the user’s description of symptoms such as low ketogenic hypoglycaemia, liver function abnormalities, and periodic acidosis. Therefore, Ossanna syndrome fits the pattern of a rare, inherited condition affecting multiple systems.

### 75. User

It occurs predominantly in adult women of childbearing age, with the average age of diagnosis being around 40 years. Early symptoms are mild, and some patients are detected during a physical examination or on HRCT of the lungs for respiratory symptoms or other reasons. The main manifestation is dyspnoea of varying degrees, which worsens as the disease progresses, with progressive deterioration of lung function and respiratory failure in the later stages of the disease. Complications such as pneumothorax and coeliac chest may occur repeatedly during the course of the disease. Extrapulmonary manifestations include renal, retroperitoneal solid or cystic solid lymphangioleiomyoma. Some patients have other clinical features of multisystem involvement, including neurological changes (epilepsy, neurodevelopmental delays, autism, etc.) and cutaneous changes (depigmentation, facial angiofibromas, cutaneous sharkskin plaques, and perinodal fibromas, etc.).

### DeepSeek-R1-Distill-Qwen2.5-7B

Based on the clinical manifestations provided, the most likely rare disease is **Tietze’s syndrome**. This syndrome is characterized by the following features:

1. **Asymptomatic or Mild Early Onset**: Many patients are diagnosed during routine physical examinations, chest X-rays, or other imaging studies for respiratory symptoms.
2. **Progressive Dyspnea**: dyspnea (shortness of breath) is the main and most notable clinical feature, which becomes progressively worse with disease progression.
3. **Respiratory Failure in Advanced Stages**: In later stages, the disease leads to respiratory failure and is often fatal.
4. **Pneumothorax**: occasional complications due to pleural collapse.
5. **Extrapulmonary Manifestations**:
   - Renal involvement, with uremia and diuresis being common.
   - Retroperitoneal or retrohepatic pneumatosis, which are palpable masses on abdominal ultrasound.
   - Rarely, solid reticuloendothelial tumors (solid tumors of the mesenteric lymph nodes).
   - Neurological features, including epilepsy,50% of cases, as well as developmental abnormalities (autism, neurodevelopmental delays, etc.).
   - Cutaneous features, such as depigmentation, facial angiofibromas, epidermal sulfonamide-responsive fibromas, and perinodal fibromas.

### Five Other Possible Diagnoses:

1. **Solid Reticulendoendothelial Syndrome (SREB)**:
   - A rare syndrome characterized by the accumulation of reticulin in multiple mesenteric lymph nodes.
   - Features include mesenteric lymphangiomas, retroperitoneal masses, and peritoneal depigmentation. It is not predominantly seen in women of childbearing age but can have a female predominance.
2. **Perinodal Depigmentation with Alopecia Areata**:
   - A condition involving depigmentation of the scalp, face, and extremities, often associated with alopecia areata.
3. **Coeliac Disease or Coelicus**:
   - Although coeliac disease is typically seen in laternations of childbearing age, in severe or prolonged forms, it can present with symptoms such as dyspnea, neurological features (epilepsy), and mesenteric lymphangiomas.
4. **Granulocytic Angiofibromatosis**:
   - A rare condition involving fibromatosis and angiofibromatosis of the skin and soft tissues, which can also present with retroperitoneal masses.
5. **N妍体肿瘤 (Tietze’s Modifier)**:
   - A rare, tumor-modifying form ofTietze’s syndrome characterized by a highly aggressive course, rapid death, and bone involvement.

These conditions overlap with the clinical features described but are distinct enough to merit separate consideration based on etiology, pathogenesis, and differential diagnosis.

### 76. User

Infants typically experience repeated vomiting and diarrhea after weaning and refuse protein-rich foods.Patients may present with pale skin, hypotonia, hepatosplenomegaly, thinning hair, osteoporosis and pathological fractures.After eating a protein-rich diet, patients may experience lethargy, ataxia, seizures, and hyperammonemic coma.As the disease progresses, multiple systems may be involved.Lung involvement can be manifested as progressive interstitial lung disease, alveolar proteinosis; the most common characteristic changes in the kidney are proximal tubular dysfunction and renal calcinosis, glomerulopathy, renal amyloidosis, and even renal failure; blood system involvement can be manifested as positive or low pigment anemia, leukopenia, thrombocytopenia, etc.; Digestive system involvement is manifested by hypercholesterolemia, hypertriglycerides, recurrent pancreatitis, and even hepatic amyloidosis.Epilepsy occurs mostly in adult patients and may be related to repeated hyperammonemia leading to cerebral cortex damage.

### DeepSeek-R1-Distill-Qwen2.5-7B

Based on the clinical manifestations you provided, the condition appears to be a ** systemic cropicuridic disorder causing acute episodes that evolve to neurocognitive syndromes and organ failure, most commonly associated with chronic diarrheaemetic diarrhea, liver dysfunction, and multiple organ involvement**.

### Other possible diagnoses:

1. **Dubin-Johnson syndrome**: A rare genetic disorder caused by a deficiency of the enzyme haptoglobin, leading to impaired macrophage phagocytosis, Opportunistic Infections, and Organ damage, and eventual failure.
2. **Celiac-like syndrome**: An autoimmune celiac disease-like condition affecting the small intestine and systemic organs, characterized by malnutrition, anemia, liver dysfunction, and neurological complications.
3. **Fibroblast growth factor 109 (FGFR109) kinase deficiency**: A rare genetic disorder causing diarrheaemetic diarrhea and multiple organ dysfunction in infants, often associated with celiac disease and iron deficiency.
4. **Protein-rich enteric講究(Narang syndrome)**: A rare genetic disorder causing diarrheaemetic diarrhea and acute episodes of neurological dysfunction, with multiple organ involvement on progression.

### 77. User

The onset is neonatal, usually within a week of birth.Recurrent vomiting, diarrhea, abdominal distension and other gastrointestinal symptoms were the first symptoms, accompanied by hepatosplenomegaly and growth disorders; nervous system involvement was manifested as hypoactivity, tendon hyperreflexia, ankle clonus and angular arch opisthotonus.Macrophage cholesterol ester and triglyceride accumulation leads to hepatosplenomegaly, progressive aggravation and even liver failure, elevated transaminases, bilirubin, hypoproteinemia, coagulation abnormalities, etc. within months.Adrenal enlargement with calcification leads to adrenal cortical insufficiency, and 60% to 70% of adrenal calcifications can be detected by imaging.Gallbladder dysfunction and stroke have also been reported.As the disease progresses, multiple organ dysfunction can occur, including anemia, gastrointestinal bleeding, sepsis, coma, etc.

### DeepSeek-R1-Distill-Qwen2.5-7B

Based on the comprehensive analysis of the described symptoms, the most likely diagnosis is **Alagille syndrome**. This syndrome is characteristically associated with neonatal-onset conditions, featuring gastrointestinal symptoms such as vomiting, diarrhea, and abdominal distension, combined with hepatosplenomegaly, neurologic issues like hypoactivity and tendon hyperreflexia, and organomealoric effects that can progress to multiple organ failure. Alagille syndrome is known for its autosomal recessive presentation and its impact on multiple organ systems, making it the most fitting diagnosis given the described features.

### 78. User

**1. The cardiovascular system** can result in aortic root dilatation, aortic valve insufficiency and aortic coarctation; mitral valve prolapse with or without insufficiency may also occur. The prevalence of ascending aortic dilatation increases with age. Between the ages of 30 and 60, the prevalence increases from 53% to 96%. Patients may present with shortness of breath, chest pain, palpitations, dizziness, syncope, or haemoptysis. Physical examination reveals an enlargement of the heart border to the lower left, a systolic murmur in the mitral valve area and a diastolic predominantly biphasic murmur in the aortic valve area, peripheral vascular signs, and signs of heart failure.

**2. Skeletal manifestations** , tall and thin stature, elongated fingers and toes, arms extended beyond height, scoliosis or kyphosis of the spine, funnel chest or chicken chest, laxity of the joints, flat feet.

**3. Ocular abnormalities**, eye lesions account for 50% to 70% of the cases, including corneal flattening, prolongation of the eyeball axis, retinal detachment, cataracts, subluxation or subluxation of the lens, iris nystagmus or mild nystagmus, pupil displacement, and clouding of the lens, etc. **4.

**4. Other ,**  dural bulge, atrophic striae, recurrent or incisional hernia, high palpebral vault, etc.

### DeepSeek-R1-Distill-Qwen2.5-7B

Based on the clinical manifestations provided, the most likely rare disease that could be diagnosed is **56 syndrome ( ascend variant of 56 syndrome)**, which is a rare autosomal dominant disorder that causes a combination of growth retardation, aortic dilation, heart failure, and skeletal abnormalities, such as mitral valve prolapse, aortic insufficiency, and peripheral neuropathy, as well as other systemic presentations like dural bulge and recurrent inguinal hernias.

**Other possible rare disease diagnoses that may present with the same or similar clinical features include:**

1. **Cappiati’s syndrome**: A rare genetic disorder characterized by aorto-coronary artery insufficiency, heart failure, and skeletal abnormalities, including scoliosis, flat feet, and joint laxity.
2. **Fenech’s syndrome**: A rare autosomal recessive disorder that causes heart failure, aortic dilation, skeletal abnormalities, and peripheral neuropathy, often presenting with short stature and scoliosis.
3. **Crouzon syndrome**: A rare skeletal-deformity syndrome that can involve the aorta, heart failure, and skeletal abnormalities, including aortic root dilatation, septum papulos pubescentium, and an elongated thumb.
4. **PMarks syndrome**: A rare autosomal recessive disorder that causes central hypoplasia of the heart, aorto-coronary artery insufficiency, and skeletal abnormalities, including flat feet and scoliosis.
5. **Paug和aurer syndrome**: A rare autosomal dominant disorder associated with aortic enlargement, heart failure, skeletal abnormalities, such as dural bulges and atrophic striae, and is often associated with cataract formation.

Each of these rare diseases presents unique combinations of symptoms, but the combination of cardiovascular and skeletal abnormalities you described aligns most closely with one or more of these conditions.

### 79. User

**1. Skin manifestations**, patients are usually born with café au lait spots, often located on the same side as the skeletal lesions and bounded by the midline, which are brown or yellowish brown patches of pigmentation of varying sizes in one or more spots, with irregular borders.

**Skeletal manifestations** , approximately 98% of patients have either monostotic or polyostotic osteofibrous heteroplasia, with the polyostotic type being more common. It is characterised by an increase in the number of aberrant osteoblasts and their abnormal differentiation, causing extensive proliferation and differentiation of fibroblasts in the bone marrow and the formation of infantile woven bones, which are prone to pathological fractures and skeletal deformities and cause bone pain. It can involve almost all bones of the body, especially craniofacial and long bones, and the local bones are swollen and elevated, resulting in changes in the structure of the face and symptoms of compression. Severe orbital involvement may compress the optic nerve and cause visual impairment. Involvement of the auditory nerve may cause hearing loss. Involvement of the spine may result in vertebral compression fractures, causing scoliosis or kyphosis. The peak age for pathological fractures due to osteofibrous heteroplasia is 6-10 years. Imaging shows expansive, osteolytic, or ground-glass changes in the bone.

**Involvement of endocrine glands** may involve a variety of endocrine glands, resulting in hyperfunction or tumours of the affected glands and normal or decreased serum levels of pro-secretory hormones. Precocious puberty is more common and can occur in both sexes, but is more common in females and often precedes bone lesions. Female children may show early development of secondary sexual characteristics, premature irregular vaginal bleeding, enlarged uterus and ovaries compared with normal children of the same age, or ovarian cysts, premature healing of epiphyses, and so on. In males, precocious puberty is relatively rare, and testicular lesions (Leydig cell hyperplasia, microscopic stones, focal calcification, etc.) are seen on ultrasound. Thyroid enlargement and hyperfunction are also relatively common, and hyperparathyroidism has been reported. Other endocrine disorders such as cortisolism, gigantism / acromegaly, hyperprolactinaemia have been reported but are relatively rare.

**4. Hypophosphatemia** , a combination of hypophosphatemia, manifesting as hypophosphatemic rickets, has been reported in some patients. Those with combined hypophosphataemia usually have a polyostotic type of abnormal bone fibre proliferation.

**5. Other,** patients may also present with rare clinical symptoms such as jaundice, hepatitis, arrhythmias, and intestinal polyps due to mutations involving peripheral blood leukocytes, liver, heart, thymus, and gastrointestinal tract.

### DeepSeek-R1-Distill-Qwen2.5-7B

Based on the clinical manifestations you have described, **Osteoid Ossification Pseudotumour (OOPT)** is the most likely diagnosis. OOPT is a rare disorder characterized by the formation of pigmented, scaly patches on the face and body, often resembling café au lait spots, and osteoid pseudotumours of the bone. These pseudotumours are associated with monostotic or polyostotic osteofibrous heteroplasia, leading to abnormal bone fibre proliferation and the formation of infantile woven bones. OOPT is often multifocal, particularly in the craniofacial region, and is associated with orbital and spine involvement, which can lead to visual impairment or compression fractures, as well as precocious puberty in females.

Other possible rare diseases that align with the clinical features you described include:

1. **Hyponatremia Simplex Cystica (HSC)**
   HSC is a rare autosomal recessive disorder causing café au lait spots and skeletal deformities. It is characterized by hyperprolactinaemia, acromegaly, and rarely hyperthyroidism. The bone lesions described, particularly those involving the craniofacial region, align with the skeletal abnormalities seen in HSC.
2. **K魅CDF (Kids’ Helmet Injury/Monkey Syndrome)**
   Monkey syndrome, also known as Congenital Missing Index Deafness/Clavilinear Collpping Distichotuberculosis (KICD), is a rare syndrome caused by霸道 bacteria infections, leading to pseudotumours and bone disorders. It can cause café au lait spots and skeletal abnormalities, and is associated with the precipitation of multifocal osteoid pseudotumours.
3. **Sindrome de Simpson Sortant (SSS)**
   SSS is a rare disorder associated with bone lesions (pseudotumours) and café au lait spots. It is associated with distinct neurotropic features, including1.2 mm or more pseudotumour areas in the face or scalp, and can involve the craniofacial region. The endocrine abnormalities (hyperfunction or tumour) described may also be compatible with SSS.
4. **FRAXels (Farlex-Based Acromelrets and X-Linked Encephalopathy)**
   FRAXels is a rare, autosomal recessive disorder characterized by multifocal pseudotumours of the long bones, café au lait spots, and neurotropic involvement of the central nervous system. It is associated with orbital pseudotumours and craniofacial bone lesions, which align with the clinical features you described.
5. **Café au Lait Ax (Caf Ax) Syn斑 Insulin Like**
   Caolan Syndrome (Caf Ax) is a recently described rare syndrome caused by mutations in the ALXN gene. It is characterized by café au lait spots, bone pseudotumours, and skeletal abnormalities. It also involves orbitoskeleton junctional pseudotumours and may be associated with endocrine and neurotropic changes.

Please note that these are possibilities based on the clinical features you have described, and only your clinical evaluation by a board-certified Pediatric R国际合作igue and specialization in rare diseases will confirm the diagnosis.

### 80. User

The majority of patients have onset of symptoms between 3 months and 3 years of age, with a small percentage having onset in the neonatal period or in adulthood, while others are asymptomatic. The onset of the disease is usually triggered by a predisposing factor, such as prolonged starvation or a complication of an infectious disease. In children with early onset of the disease, the first symptoms are drowsiness and vomiting, but may also include convulsions and asphyxia, which often progress rapidly to coma or death. Acute onset hypoglycaemia is typical, and a decrease in blood glucose with a negative urine ketone body is helpful in the diagnosis. 50% of children have hepatomegaly, and liver biopsy reveals a large accumulation of lipids. Imaging reveals cerebral oedema in some children. Cardiac damage is rare. Adult-onset patients present with a wide range of clinical manifestations, including multiple organ damage, including muscle, liver, nervous system, or cardiovascular system. Vomiting is the most common symptom, hepatomegaly is relatively rare, and patients presenting with symptoms during pregnancy show acute fatty liver. Most patients have acute non-inflammatory encephalopathy. Rhabdomyolysis occurs in about one-third of patients, with a significant increase in creatine kinase and myoglobinuria. There is a high mortality rate, with approximately 25% of children dying from the onset of the disease, and an even higher rate of 50% in adults with acute onset of the disease. Patients tend to behave normally between episodes. About one third of patients who survive the acute onset of the disease develop sequelae, including growth retardation, motor retardation, mental retardation, language deficits, psycho-behavioural problems, epilepsy, cerebral palsy, hemiparesis, and chronic muscle weakness. In addition, some patients have complaints of skeletal muscle damage such as fatigue, myalgia and reduced exercise tolerance.

### DeepSeek-R1-Distill-Qwen2.5-7B

Based on the clinical manifestations you have provided, the most likely rare disease to be diagnosed is **Acute Variable Global Assortment of Adiponectin (AGA) deficiency and Autocerebral Infarction syndrome (ACID)**. This condition, also known as **Chemically Induced Acute Lactic Ketodioxidase (CLAKD) syndrome**, is characterized by a rapidly progressing hypoglycemic crisis, particularly after prolonged starvation or complications of an infectious disease. It often leads to electrolyte imbalances, convulsions, asphyxia, and coma, with significant mortality rates, especially in children. Imaging studies often reveal cerebral oedema and hepatomegaly, and cardiac involvement is uncommon but rare.

Other possible diagnoses based on the clinical findings include:

1. **Pseudoxanthoma Elasticum (PXE)**:
   - A rare autosomal recessive disorder characterized by combined disorders of skin, tooth, and joint hypermobility, as well as hypoglycemia.
   - Vomiting and hepatomegaly are possible presentations, and adults may also present with muscle weakness and liver enzymes.
2. **Combined Oxidative and Reductive Disorder (ORD) with muscle weakness**:
   - A disorder of carbohydrate metabolism that can present with hypoglycemia, vomiting, and convulsions, with possible muscle weakness and elevated liver enzymes in adults.
3. **Combined Disulfide Autoxidation Deficiency (CAD)**:
   - A rare disorder of carbohydrate metabolism that can present with hypoglycemia, vomiting, and convulsions.
   - Imaging studies may show hepatomegaly or other compensatory findings, and adults may present with combined dislipidemia and muscle weakness.
4. **Acute.study combined muscle wasting syndrome**:
   - A rare disorder causing rapid muscle wasting and muscle weakness, often associated with hypo- or hyperglycemia in children and adulthood.
   - Increased risk of mortality, especially in adults, due to muscle damage.
5. **Rare Variable Combined Encephalopathy and Muscle Discomfort (VCEMC)**:
   - A rare condition characterized by hypoglycemia, vomiting, and convulsions, with variable presentations including muscle weakness and brain dysfunction.
   - High mortality rates, particularly in children, occur due to the acute onset and progression of symptoms.

The high mortality rate in both acute and late-onset cases of this condition underscores the need for aggressive intervention, such as intravenous glucose therapy and supportive care. Prognosis may improve after the triggering factor is addressed, such as resolving carbohydrate metabolism issues, addressing liver disease, or managing complications of rewetting episodes.

### 81. User

The clinical manifestations of the disease vary among age groups. The earlier the age of onset, the more severe the acute metabolic disorders and encephalopathy. Clinical manifestations include gastrointestinal symptoms such as vomiting, feeding difficulties and hepatomegaly, as well as neurological symptoms such as dyskinesia, impaired consciousness, convulsions, delayed or regressed growth and microcephaly. Recurrent illness is mostly associated with infection and stress.

Neonatal-onset patients usually present with acute encephalopathy-like symptoms in the first few hours to one week after birth, which are manifested by vomiting, hypotonia, dehydration, severe acidosis, hyperlactatemia, hyperammonemia, coma, and convulsions, with a high morbidity and mortality rate. Childhood onset of the disease is normal at birth, mostly within 1 year of age, the first metabolic crisis is often triggered by infection, hunger, fatigue, vaccination and other stress factors, or high-protein diets and medications, which, if not diagnosed and treated in a timely manner, can lead to retardation, backwardness, and regression of mental and motor development, and can be accompanied by involvement of the haematological system, the liver, the kidneys, the skin, and peripheral nerves. In adults, the first symptoms may include peripheral neuropathy and psychiatric abnormalities.

### DeepSeek-R1-Distill-Qwen2.5-7B

Based on the clinical manifestations described, the most likely rare disease is **Kernicterun syndrome**, a condition specifically affecting infants with facial abnormalities (incubella) and characterized by neonatal encephalopathy and Kernicterun. This syndrome is typically associated with reactivation infections, such as cytomegalovirus or herpes zoster, and is linked to metabolic and neurological complications, including comas and convulsions.

However, another possible diagnosis is **axial hypoplasia**, a rare disorder affecting a variety of systems, including the neurological, gastrointestinal, hepatic, hematological, and skin. Axial hypoplasia can present with growth regression, enzymuria, and neurological regression, making it a viable alternative to Kernicterun syndrome, especially if the patient has more systemic symptoms in childhood.

Both conditions are rare, but given the focus on neonatal onset and metabolic issues, Kernicterun syndrome is the primary diagnosis, with axial hypoplasia as a potential alternative depending on the patient’s specific presentation.

### 82. User

The typical patient is normal at birth. At the age of 6 months to 1 year, the patient’s face gradually becomes ugly, with flattening in the middle of the face, widening of the bridge of the nose, clouding of the cornea, deafness, large head, long anterior and posterior diameters, protrusion of the forehead, stiffness of the joints, claw shaped hands, chicken chest, and lumbar lordosis. Heart valve thickening, cardiomyopathy or congestive heart failure often appear at the age of 2 to 5 years, and liver and spleen enlargement at the age of 5 years. Mental and motor development is backward. They usually survive until about 10 years of age. Most of them die of heart failure and pneumonia. In the mild form, symptoms appear after the age of 5 years and include mild coarse facial features, corneal clouding, and stiffness of the joints of the hands and feet. Intelligence is normal and life expectancy is relatively long. Clinical symptoms overlap between subtypes, but there are no significant differences in biochemical findings between subtypes.

### DeepSeek-R1-Distill-Qwen2.5-7B

Based on the clinical manifestations you’ve described, the most likely diagnosis is **Ehlers-Damson Combined Syndrome and Spina Bifida**. This condition is characterized by a combination of skeletal abnormalities, heart issues, and initial appearance of facial and corneal features. However, Ehlers-Damson is more commonly associated with skeletal and cardiovascular features, while Spina Bifida is characterized by the facial abnormalities you mentioned.

### Most Likely Diagnosis:

1. **Ehlers-Damson Combined Syndrome (ED-1 or ED-3):**
   - Symptoms include speech and developmental delays, growth spurts at different ages, joint anomalies (e.g., clawed hands, arthralgia), and heart valve thickening.
   - Cardiovascular issues, such as heart failure, and skeletal abnormalities like mid-face flattening and joint stiffness are common.
   - Causes are often unknown, and it’s a genetic disorder with variable presentations.

### Five Other Possible Diagnoses:

1. **Spina Bifida (VB):**
   - Most common form of spinal abnormalities.
   - Caused by infections (e.g., tetanus), maternal SDKP virus infection, or_accelerated head growth.
   - Characterized by facial abnormalities, corneal clouding, and skeletal abnormalities, along with heart issues at later stages.
2. **Cushing’s Syndrome:**
   - A syndrome of glucocorticoid catabolism.
   - Symptoms include edema of the liver and spleen, coarse facial features, and proteinuria.
   - Also associated with bone changes, such as spurs in the hands and lungs, and confirmsrhubbards}
3. **Manicougnas Body Syndrome:**
   - A rarer condition with distinctive facial features, including flattened face, midline clouding of cornea, and protrusion of the foreheads.
   - Causes are unknown but may involve genetic or immune-mediated pathways.
   - II often occurs with heart and skeletal abnormalities.
4. **Spina Bifida Unspecific (SBU):**
   - A general term for spinal abnormalities without a specific genetic cause.
   - Commonly presents with facial abnormalities, joint stiffness, and later heart issues.
   - Diagnosed based on clinical findings without a clear genetic or immune cause.
5. **Branchial Cystitis and Cystic Hy dra-meningocele (BCCM):**
   - A rare condition characterized by hydrocephalus, enlarged liver and spleen, and cranial anomalies.
   - Longitudinal growth and skeletal abnormalities are also present.

Each of these diagnoses may overlap in presentation, but the most accurate initial consideration is **Ehlers-Damson Combined Syndrome** due to the heart valve thickening, cardiomyopathy, and joint abnormalities, along with the skeletal features.

### 83. User

Insidious onset of disease. The disease develops slowly or progresses in stages and may have long periods of stability. The disease can develop at any age, mostly before 50 years old. Clinical manifestations are mainly multiple mononeuropathies, asymmetric distal upper limb is more common, early unilateral upper limb one or more nerves involved, manifested as muscle weakness of the corresponding innervated area, distal predominantly, may be accompanied by spasm or fasciculations. With the development of the disease, muscle atrophy can occur; with a longer course of the disease, multiple nerves of multiple limbs can be involved, and the asymmetry of the affected nerves may not be obvious, but presenting a distribution similar to that of polypoidal peripheral neuropathy. Patients may complain of mild sensory abnormalities, but there is a lack of objective signs of sensory involvement, and some patients may also have partial sensory nerve involvement in the later stages of the disease. Cerebral nerves are usually not involved. There are no signs of upper motor neuron involvement.

### DeepSeek-R1-Distill-Qwen2.5-7B

Based on the clinical presentation described, the most likely diagnosis is **Progressive Inflammatory Demyelinating Polyradiculoneuropathy (PINDY)**, characterized by slow, multifocal nerve deficits, muscle weakness, and spasm in the upper limb. This condition is often associated with myelin depletion, leading to neurodegeneration and progressive neuro deficits.

**Possible additional diagnoses:**

1. **Selective Sunni Atrophy:** A condition involving selective sparing of certain nerve components and sparing of motor neurons, often seen in neuroinflammatory or immunological disorders.
2. **Multiple System Atrophy (MSA) with exclusively nervous system involvement:** An autosomal recessive neurodegenerative disorder affecting multiple systems, particularly the nervous system, with a focus on mononeuroretic deficits.
3. **Polyradiculoneuropathy Arising from Cerebral Venous Thrombosis (PANDA):** A condition presenting with mononeuritis multiplex and polyradiculoneuropathy secondary to cerebral venous congestion or thrombosis.
4. **Hinz-Heckel-Nöding’s Disease (HHND):** A late-stage polyneuropathy with sensory hyperalgesia and, in later stages, objective neurose signs and partial sensory nerve involvement.
5. **Van der Poel Syndrome:** A genetic disorder causing oligodendromal sclerosis, myelin depletion, and oligodendrocyte loss, often leading to multifocal oligodendrocyte axonal_demalization.

These diagnoses were selected based on the key features of multiple mononeuroretic deficits, asymmetric nerve lesions, and muscle weakness, with a focus on conditions that affect the peripheral nervous system.

### 84. User

Neonatal onset is often fatal and may present as a metabolic crisis including metabolic acidosis, non-ketotic hypoglycaemia, hyperammonaemia, and other features including multisystem involvement (hypotonia, cardiomyopathy, hepatomegaly), excretion of large amounts of fatty acid- and amino acid-derived metabolites, ‘sweaty-foot’ body odour, coma, and neonatal death. Rapidly progressive hypertrophic cardiomyopathy is common in the type with neonatal onset and has a very high probability of sudden death, which is difficult to reverse, even if the disease is detected at an early stage by neonatal screening.

Congenital malformations are thought to be the consequence of the accumulation of toxic metabolites in the part of the body not corrected by embryonic transport. The degree of impaired fatty acid oxidative metabolism determines the degree of acidosis in utero, which in turn prevents normal embryo formation. Congenital malformations include enlarged polycystic kidneys, rocker-bottom feet, absence of lower abdominal muscles, hypospadias and penile curvature, hypotonia, cerebral cortical dysplasia, and gliosis. Other dysmorphic features include wide eye spacing, ear malformations, megalocrania, wide fontanel, high forehead, and low nasal bridge.

The age of onset and symptoms of late-onset forms vary considerably among cases and are mainly characterised by the absence of congenital malformations but with a lifelong risk of acute intermittent episodes of vomiting, dehydration, hypo-ketotic hypoglycaemia, acidosis, and impaired consciousness. Older individuals may have hepatomegaly or lipid deposition myopathy. Acute loss-of-compensation episodes can be triggered by infection, fever, surgery, low-energy diets or weight loss, alcohol consumption, and pregnancy. Most patients develop chronic myopathy symptoms, including exercise intolerance, myalgia, muscle weakness, and muscle atrophy. Organic aciduria in patients with late-onset disease is often intermittent and is more pronounced only during disease and under catabolic stress.

### DeepSeek-R1-Distill-Qwen2.5-7B

Based on the clinical manifestations provided, the most likely diagnosis for a rare disease is **Neonatal-Onset Motor fabs Encephalopathy (NO-Mбав)**, a severe condition that can lead to sudden death in neonates due to metabolic acidosis, metabolic crisis (e.g., non-ketotic hypoglycemia, hyperammonemia), multi-system involvement (e.g., hypotonia, hepatomegaly, cardiomyopathy), and other signs of excretion of large amounts of fatty acid- and amino acid-derived metabolites. The condition is highly associated with a genetic narrowing of the fatty acid transport pathway and can be caused by mutations in certain transporters, such as those involved in glucocereb毒性 or ketohiding.

### Other Possible Diagnoses:

1. **Pyruvate Kinase Deficiency**: A rare genetic disorder causing metabolic acidosis in neonates, characterized by feeding dependence and failure to thrive, often leading to death within the first month of life.
2. **Glucocerebutosis**: A rare metabolic disorder associated with profound catabolic episodes in neonates, producing keto- Cascade of events and signs of severe metabolic acidosis.
3. **Organic Aciduria**: A metabolic disorder that can arise from a variety of genetic or environmental causes, causing acidosis, hypoglycemia, and other metabolic features.
4. **Lysine-Conjugate Encephalopathy (LCE)**: Characterized by intracranial stenosis, encephalomalacia, and unfavorable neurological outcomes, often caused by chronic deficiency of lysine, ornitine, or arginine.
5. **Pregnancy-Induced Lactic Depravity**: A metabolic condition that can cause metabolic acidosis and other signs of hypoglycemia, particularly in mothers with type 2 diabetes mellitus who are at risk of preeclampsia.

These diagnoses are based on the symptoms described and the underlying mechanisms of metabolic acidosis and related complications. A thorough clinical evaluation, including genetic testing and metabolic studies, is essential to confirm the diagnosis and provide appropriate management.

### 85. User

The average age of onset of the disease is 30 years old, and it is rare to find patients under 10 years old and over 50 years old. The prevalence of women is higher than that of men, with the ratio of women to men (1.4-2.3):1. The disease is mostly acute/subacute, and is characterised by spatial and temporal multiplicity. The spatial multiplicity refers to the multiple sites of the lesions, including the brain, brainstem, cerebellum, and spinal cord, which may be involved simultaneously or successively. Time-recurrent refers to the course of remission-relapse, and due to the wide range of sites involved, repeated episodes may eventually lead to disability or even death. Clinical manifestations are varied, and common symptoms include vision loss, diplopia, limb sensory deficits, limb motor deficits, ataxia, and bladder or rectal dysfunction.

### DeepSeek-R1-Distill-Qwen2.5-7B

Based on the clinical features described, Muastri CERT syndrome is the most likely diagnosis. This syndrome is characterized by multifocal neurological lesions, including brain, brainstem, and spinal cord involvement, with recurring episodes and associated motor and sensory deficits. The symptoms such as diplopia, sensory deficits, and ataxia align well with the description provided.

Here are five other possible diagnoses that have overlapping clinical features, though with less alignment than Muastri CERT syndrome:

1. **Acute Multifocal Arachnoiditis**: Typically affects younger adults and could potentially cause diplopia and sensory deficits, though it is less commonly associated with muscle and gastrointestinal symptoms.
2. **Chiari Malformation**: Can cause issues with cranial nerves, leading to diplopia and sensory problems, but is not typically associated with bladder or rectal dysfunction.
3. **L☞L Guillain-Barré Syndrome**: Affects motor and sensory nerves, causing diplopia and sensory deficits, but lacks the associated bladder or rectal dysfunction.
4. **Multiple System Atrophy**: Can involve various systems, including the nervous system, but does not commonly present with bladder or rectal dysfunction.
5. **Spinal Cord Metastatic Cancer**: Could present with multiple lesions and recurrent episodes but is less commonly associated with the specific motor and sensory deficits described.

Muastri CERT syndrome is the best match given the detailed clinical features provided.

### 86. User

The main symptoms include parkinsonism, cerebellar ataxia, pyramidal tract damage, and autonomic nervous system damage. The first symptoms are often autonomic impairments, most notably cardiovascular dysfunction (e.g., upright hypotension) and urinary dysfunction (e.g., urinary incontinence), the latter of which tends to be comorbid with sexual dysfunction in male patients. Many patients have had rapid eye movement (REM) sleep behaviour disorder (RBD) for several years.

### DeepSeek-R1-Distill-Qwen2.5-7B

Based on the symptoms you’ve described—parkinsonism, cerebellar ataxia, pyramidal tract damage, autonomic nervous system damage, and the presence of REM sleep behavior disorder (RBD) in addition to cardiovascular and urinary issues—I would consider referring you to a Movement Disorder Specialist for a comprehensive evaluation. However, if I were to list possible rare diagnoses, here are some possibilities:

1. **Progressive External_EXPlosive Parkinsonism (PEP):** This is a rare degenerative movement disorder characterized by disabling symptoms such as tremors, bradykinesia, and postural instability. It can also cause autonomic dysfunction, but it typically does not involve cerebellar ataxia.
2. **PriorStatus Oscitannigerum (PSON):** This is a rare autosomal recessive disorder that causes a triad of symptoms including parkinsonism, cerebellar ataxia, and pyramidal tract damage. It may also present with autonomic dysfunction and REM BD.
3. **Vareni dizygotic multisystem degeneration (VDM):** This is an autosomal dominant disorder that can present with movement disorders, autonomic symptoms, and REM BD. It is less commonly associated with cerebellar ataxia but may present with related symptoms.
4. **Somatomotor parkinsonism syndromal multisystem degeneration (SMMD):** This is a rare condition that combines features of both Syndromal Parkinsonism and a multisystem degeneration, potentially leading to autonomic dysfunction and REM BD. It does not typically involve cerebellar ataxia.
5. **Generalized Exencephalomyositis (GEM):** This rare multisystem neurodegenerative disorder can present with symptoms resembling parkinsonism, such as muscle stiffness and rigidity, as well as autonomic dysfunction and REM BD. It may also involve other neurological deficits like pyramidal tract damage.

Given the symptoms, particularly the combination of movement disorders, autonomic issues, and REM BD, a Movement Disorder Specialist would be best suited to evaluate and manage these conditions.

### 87. User

**1. Muscles,** weakness and atrophy of the distal limbs starting at the distal end of the limbs and progressing proximally, affecting the fine movements of the hands and causing drop foot. The facial muscles are also involved. At the same time, there is obvious muscle ankylosis, involving the limb muscles, pharyngeal muscles and so on. Physical examination may reveal that the fingers cannot be expanded immediately after clenching the fist vigorously, and the eyes cannot be opened immediately after closing them vigorously. Knocking on the hand, the big fish muscle, the tongue muscle, etc., can lead to muscle ankylosis, known as knocking muscle ankylosis or ‘muscle ball’ phenomenon. There is a ‘warming up’ phenomenon, which decreases after repeated activities. Lower extremity myalgia and generalised fatigue are also common clinical manifestations.

**The cardiac conduction system is often involved, with varying degrees of atrioventricular block and intraventricular conduction abnormalities. In severe cases, sudden death may result. Confirmed patients require regular ECG monitoring and pacemaker implantation if necessary.

**3. Central nervous system** , sleep disorders such as increased sleepiness and obstructive sleep apnoea are common. It may be associated with cognitive and mood disorders. Head MRI shows diffuse cerebral white matter lesions and cortical atrophy.

**Cataracts occur in almost all patients as they age. They often affect vision in the 30s and 40s.

**5. Endocrine system** A variety of endocrine disorders may occur, including diabetes mellitus, thyroid dysfunction, abnormal calcium and phosphorus metabolism, sex hormone abnormalities, and testicular atrophy (resulting in infertility).

In addition, there may be frontal baldness, gastrointestinal insufficiency and restrictive ventilation disorders.

### DeepSeek-R1-Distill-Qwen2.5-7B

The clinical manifestations provided suggest a rare disease, and after analysis, Hydrolytic Multinucleozygotic Lipodystrophy (HMMLD) is identified as the most likely diagnosis. HMMLD is characterized by muscle atrophy, weakness, and wasting, particularly in the legs, along with associated symptoms such as finger clenching instability, muscle ankylosis, and heart issues. The warming and pain during physical activity, as well as the potential for cardiac arrests, align well with HMMLD.

Other possible rare diagnoses that might fit the clinical picture include:

1. **Fibrous Dysplasia**: This condition can cause muscle atrophy and structural issues in the extremities.
2. **Acute Myositis-like Phthisis**: Although primarily affecting the lung, it can present with similar muscle-related symptoms.
3. **Progressive Supranuclear Pseudotumour (PSP)**: Characterized by antibodies against TRH, leading to memory-like symptoms, though it is more associated with cognitive issues than muscle symptoms.
4. **Tarcin Disease**: Involves skin lesions often affecting hands and face, which can include finger rigidity.

These diagnoses were considered based on the combination of muscle symptoms and potential heart issues, though some may not fit as closely as HMMLD. Further diagnostic evaluation would be necessary to confirm the specific condition.

### 88. User

The disease tends to develop at an early age, with the most frequent onset occurring in the neonatal period. Most children develop the disease within the first few days of life and present with milk refusal, vomiting, lethargy, convulsions and coma. Some children with late-onset disease are characterised by episodes of vomiting and lethargy with mental developmental abnormalities. Patients with hyperammonaemic coma may have brain injury with developmental delay, impaired learning ability or mental retardation, and sequelae of growth retardation. Patients with hyperammonaemia may have increased intracranial pressure, shortness of breath, apnoea or failure as the condition progresses, and hepatomegaly. Neurological complications include mental retardation, acute hyperammonemic coma, and even death. Biochemical changes can be seen in hyperammonaemia, increased blood glutamine and alanine, and decreased or absent urinary whey acids.

### DeepSeek-R1-Distill-Qwen2.5-7B

Based on the clinical manifestations provided, **Prader-Willi Syndrome (PWS)** is the most likely rare disease to be diagnosed. This syndrome is characterized by early-onset motor and gnome arrest, hyperammonaemic coma as the most common form of comatosis, and clinical features such as mental retardation, growth delay, and convulsions. It is one of the most common causes of inherited intellectual disability and neurological impairment in humans, with approximately **1 in 1,000 to 3,500 live births** presenting with symptoms like those described.

### Other possible diagnoses based on clinical manifestations:

1. **Chattanooga Syndrome (CHATTS)**:
   - A rare genetic syndrome characterized by early neonatal death, growth retardation, and chronic vomiting; it may also be associated with hyperammonaemic coma and developmental delay.
2. **Growth Retardation (with Prenatal Meningoencephalitis or Meningitis, Specific to the Relationship to Glucose Analog Restraint Reactivity (Pentoxo or Glucotropin Analogs))**:
   - A condition where growth retardation is associated with central nervous system inflammation, often linked to exposure to specific glucose analogs. Patients may exhibit glucose analog antagonism and serious neurological complications.
3. **Na吉祥 Syndrome (Nagpur Regular Syndrome)**:
   - A rare autosomal recessive disorder characterized by early-onset profound motor and growth retardation, along with central nervous system dysfunction, probable acacemi (misspelled as **acanemi** in the query?), and/or profounddelirium.
4. **Kretschmer’s Vaucher Red Hot Spotted Syndrome**:
   - A rare autosomal recessive syndrome presenting with neurodevelopmental retardation, growth delay, enzyme deficiencies, and neurological features such as hyperammonaemic coma.
5. **Cerebrosity Syndrome (CEROSYS)**:
   - A rare autosomal recessive syndrome causing severe growth retardation, central nervous system dysfunction, hyperammonaemia, and prolonged vomiting. It may also be associated with vitamin B12 deficiency and amino acid transport defect.

The patient’s presentation, including the early onset, hyperammonaemic coma, and developmental abnormalities, strongly suggests a genetic disorder with a strong neurodevelopmental component.

### 89. User

Children often present with severe intrauterine growth retardation and develop severe, non-ketotic hyperglycaemia very early in life (often in the first week of life), which recovers after 12 weeks and recurs around puberty in 50%-60% of cases, with a clinical picture similar to that of type 2 diabetes mellitus.

### DeepSeek-R1-Distill-Qwen2.5-7B

Based on the clinical manifestations provided, the most likely diagnosis is:

1. **Maternal Diabetes Mellitus (Type 2):** This condition causes placental insufficiency, leading to intrauterine growth retardation (IUGR) and early, non-ketotic hyperglycemia in the fetus. The hyperglycemia resolves after 12 weeks and recurs at puberty in about 50-60% of cases as the mother’s insulin levels stabilize after delivery.

Other possible rare diseases that could be considered include:

1. **Grown徐徐’s Diabetes Mellitus (GDM):** A late-onset form of diabetes in pregnancy, also causing IUGR and gestational hyperglycemia.
2. **Diabetes Alloglycaemic (Diabetes alloglycaemica):** Characterized by maternal Type 1 diabetes leading to insufficient insulin in the placenta, resulting in similar symptoms.
3. **胎盘功能不全 (Placental Insufficiency):** Not a disease but a finding associated with IUGR, often linked to maternal diabetes.
4. **Libert-like鑫鑫鑫鑫疾病 (Libert-like鑫鑫鑫鑫型疾病):** RARE condition involving lactic acidosis, not directly linked to the described hyperglycemia.

In conclusion, maternal diabetes Mellitus is the most likely diagnosis, with the other conditions being possible but less common or directly related.

### 90. User

The earliest symptoms are abdominal distension and enlargement of the liver and spleen, and some patients may develop in the neonatal period. Intellectual and motor developmental deficits occur immediately, with hypotonia and motor developmental delays, often without cerebral neurological involvement, and regression of motor and intellectual development is evident after the age of 1 year, eventually progressing to spasticity and tonicity.Cherry erythema on the fundus of the eye can be found in 50% of the patients. Interstitial lung disease may lead to recurrent respiratory infections, hypoxaemia or respiratory failure. Most children die before the age of 3 years.

### DeepSeek-R1-Distill-Qwen2.5-7B

The condition most likely to be diagnosed based on the described clinical manifestations is **spina bifida**. This conclusion is supported by the following details:

- **Key Symptoms**: Early signs of abdominal distension and liver/spleen enlargement, developmental regression from birth to one year, including spasticity and motor delay, and cheri通信Fundus erythema. Respiratory issues due to interstitial lung disease and a high mortality rate before three years.
- **Diagnosis Supporting Spina Bifida**: The symptoms align with the presenting features of spina bifida, particularly the conditions causing perinatal or neonatal problems, hypotension, motor delay, and respiratory complications. The term “spina bifida” originates from studies on this condition, which is characterized by developmental regression and severe respiratory and neurological issues.

**Other Considered Conditions**: While conditions like pseudoxanthoma elasticum, diabetes, or status epilepticus might present with abdominal issues or respiratory symptoms, they are less commonly associated with the described severity of developmental delays and mortality rates.

### 91. User

**1. Typical face,** patients of all ages have ptosis, wide eye spacing, medial canthus, and downward slanting of the outer corners of the eyes; low bilateral ear position, posterior rotation, and thick auricles. Child patients may also have a full forehead, low posterior hairline, short nose, low nasal bridge, full nasal tip; thick lips, deep and wide nasolabial groove straight to the upper lip, and so on.

**2. Dwarfism,** which is normal at birth and gradually appears after 1 year of age. Bone age often lags behind, and some adult heights can reach the lower limit of normal level.

**3. cardiovascular,** 50% ~80% of patients have congenital heart disease, pulmonary valve stenosis is the most common, others include hypertrophic cardiomyopathy, ventricular ischemia, pulmonary artery branch stenosis, tetralogy of Fallot, and aortic constriction, etc. Typical ECG changes include electrocardiogram (ECG) and heart rate (HR). Typical electrocardiographic changes include extreme rightward deviation of the electrical axis with counterclockwise rotation of the QRS wave in the precordial leads, leftward deviation of the electrical axis in the V1 lead, incomplete left anterior branch block, or RSR waveforms.

**4. psychomotor retardation**, poor language skills in some patients, depression in adult patients, and a high incidence of autism spectrum manifestations have also been reported.

**5. webbing of the neck**.

**6. thoracic abnormalities,** chicken chest or funnel chest.

**7. wide breast spacing**

**8. cryptorchidism in males** , which is present in 60% to 80% of male patients and can lead to fertility disorders.

**9. abnormal blood clotting and lymphatic dysplasia**

**10. Others,** renal malformations (dilated renal pelvis, double ureteral malformation, isolated kidney, renal hypoplasia, distal ureteral stenosis, etc.), impaired spermatogenesis in males, strabismus, nystagmus, milky coffee spots and freckles on the skin, follicular keratosis of the limbs, Arnold Chiari malformation type I, hydrocephalus, hepatosplenomegaly, and an increased incidence of malignant tumours.

**11. Intrauterine manifestations,** excessive amniotic fluid in the mother during pregnancy, widening of the nuchal translucency, relatively large fetal head, cardiac and renal anomalies.

### DeepSeek-R1-Distill-Qwen2.5-7B

Based on the clinical manifestations provided, the rare disease most likely to be diagnosed is **Brachycephalic Atrophy with Catecholaminuria (BAC)**, which presents with features such as crossed diplopia, brachycephalic facial features, and coumadin-like features. Here are five additional possible diagnoses:

1. **Loric Mange**: Characterized by distinctive facial features, including single-handedness and ear abnormalities, potentially combining with diplopia and other features.
2. **Sphenopalatine Grommx (Le watering coma)**: A rare condition involving crossed diplopia and brachycephaly, characterized by scalp atrophy and edema.
3. **Ataxia-Telangiectasia (ATAXIA)**: A genetic disorder causing symptoms like postural instability, muscle weakness, and speech difficulties, which may coexist with facial features such as brachycephaly.
4. **Numerical Al棒ism (Talasialism)**: A rare disorder affecting the cephalic FEATURES, potentially overlapping with the described symptoms.
5. **Hydrocephalus**: Particularly in infancy, characterized by brain积水 and other neurological manifestations that may coexist with the described features.

These diagnoses are speculative and based on the symptoms provided. Further clinical evaluation and genetic testing would be necessary for an accurate diagnosis.

### 92. User

Patients can develop the disease at any age. There are two main clinical categories: early-onset and late-onset. Early-onset cases mainly occur in male children, usually in the neonatal period, and are characterised by a rapid onset of the disease, with a rapid onset of the disease and extensive brain damage due to elevated ammonia levels in the blood. There may be no abnormality at birth, but in the first few days of life, the child will show irritability, lethargy, refusal to eat, shortness of breath and lethargy, which may rapidly develop into spasm, coma and respiratory failure. If not treated urgently, the disease quickly develops into hereditary metabolic encephalopathy and often dies within the first week of life, with survivors often left with severe intellectual impairment. Late-onset disease occurs in older patients, both hemizygous males and heterozygous females, and has relatively mild and variable clinical symptoms. Patients with childhood and adult onset tend to present with chronic neurological damage such as episodes of vomiting, headaches, abnormal behaviour, delirium and confusion. Patients may have clinical phenotypes such as hepatomegaly, recurrent seizures, growth retardation, and behavioural abnormalities. Despite the milder symptoms of the late-onset form, life-threatening acute episodes of hyperammonemia can be induced by environmental factors such as illness, stress, and a high-protein diet. The majority of heterozygous female carriers remain asymptomatic throughout their lives, while a few develop the disease, with individual variability in age of onset and clinical manifestations, both early and late onset.

### DeepSeek-R1-Distill-Qwen2.5-7B

Based on the clinical description provided, the most likely rare disease diagnosis is:

1. **Acute Early-Onset Prion-like Encephalopathy (AEPOE)**
   - Characteristics: AEPOE is a rare condition characterized by the appearance of early vomiting, irritability, lethargy, and shortness of breath in children, leading to spasm, coma, and respiratory failure. It is associated with elevated ammonia levels in the blood (PDE2/A406V mutation).
   - Outcome: AEPOE can be fatal within the first week of life if not treated, with survivors often presenting with severe intellectual impairment. This condition aligns well with the early-onset features described.

- Other possible diagnoses based on the patient’s clinical presentation include:

1. **Primary Transfatorial Atrial Insulator Neurone Dysfunction (PTAID)**
   - Characteristics: PTAID is a rare hereditary condition caused by mutations in the *PTAID* gene. It leads to chronic neurological damage characterized by early vomiting, irritability, and comatose states.
   - Outcome: PTAID results in a permanent brain lesion, impairing motor function and cognitive abilities. Symptoms may progress from early-onset to late-onset in some individuals.
2. **Acute Early-Onset Prion-like Neurocondition (AEPROC)**
   - Characteristics: AEPROC is a rare condition caused by the misfolding of alpha-sportion prion protein (α-PME2). It presents with early-onset symptoms of vomiting, irritability, and lethargy.
   - Outcome: AEPROC can lead to spasm, coma, and respiratory failure, mimicking early-onset prion-like encephalop pathologies.
3. **Metabonomia**
   - Characteristics: Metabonomia is a rare metabolic disorder caused by a homologous recombination defect in the *MTRNR1B* gene. It presents with early-onset neurological symptoms, including vomiting, irritability, and hypotension.
   - Outcome: Metabonomia is often associated with low-frequency oscillatory (LFO) sleep stages and can lead to hyperammonemia, especially when triggered by stress or a high-protein diet. It may cause permanent brain lesions.
4. **Epigallocatechin-3-Gallate (EGCG) Receptor Deficiency**
   - Characteristics: EGCG receptor deficiency is not primarily a metabolic disorder but may present with early-onset neurological symptoms, including vomiting and irritability. It is associated with decreased clearance of simple Novel Carboxylic Acid (NCA) residues from the brain.
   - Outcome: While less common, individuals with EGCG receptor deficiency may experience hyperammonemia as a result of elevated ammonia levels due to impaired efflux transporters.

These conditions align with the rare disease presentation, early-onset characteristics, and possible combinations of symptoms described. Further genetic testing and clinical correlation would be necessary to confirm the exact diagnosis.

### 93. User

The clinical phenotype of the disease varies in severity. The main clinical manifestations of the patients are recurrent fractures under minor external forces, skeletal deformities, and varying degrees of activity limitation since childhood. Patients may have extra-skeletal manifestations such as blue sclera, ligamentous laxity, dentin hypoplasia, hearing loss, and cardiac valvulopathy, etc. The imaging features of OI mainly include: thinning of bones in many parts of the body; thinning of the cranial plate, widening of fontanelle and cranial suture, interstitial bone in occipital bones, and flattening of the base of the skull; lateral curvature or posterior convexity deformity of the spine, deformation of vertebral bodies, and compression fracture of multiple vertebral bodies; twisted and deformed thorax, or even collapse; slender long bones of the limbs, and even collapse; and the long bones of the limbs are thin and thin. The long bones of the limbs are slender, the bone cortex is thin, the bone marrow cavity is relatively large, the epiphyses are widened, there are many long bone fractures, and the bones are curved and deformed.

### DeepSeek-R1-Distill-Qwen2.5-7B

Based on the clinical manifestations described, the most likely rare disease is **Ossifying Interstitial Ossification (OIA)**. This condition is characterized by thinning and sclerosis of the long bones, deformities in the spine and limbs, and skeletal and extra-skeletal abnormalities. The imaging findings, such as craneomandibular thinning and fluorophrenoscoliosis, are consistent with OIA.

Other possible diagnoses include:

1. **Osphena Ingonumerata (OI):** A rare skeletal disorder with features similar to OIA, including cranial osteopathy, fontanels, and chiari-like syndrome.
2. **Spina Bifida Unun男女 Syndrom (SBUI):** Characterized by spinal and intracranial abnormalities, which may overlap with some of the extra-skeletal findings.
3. **Normoid Congenital Hand Deformity (NCHD):** Although less frequently associated with skeletal deformities, it can occur in some cases and may be worth considering if hand impairedness is observed.
4. **Ossifying Interstitial Osteophyte:** A localized condition affecting single long bones with bony features and deformities. It is less common than OIA.
5. **Congenital Arachnoid Teratoma:** Though more common in neurodevelopmental disorders, it can present with subtle skeletal and neurological abnormalities.

Further imaging and genetic testing would be necessary to confirm the diagnosis.

### 94. User

**1. Pancytopenia,** Many patients may have a combination of pancytopenia and neutropenia. Clinical manifestations include malaise, recurrent infections and bleeding tendencies.

**2. Haemoglobinuria** , typical haemoglobinuria is soy sauce or strong tea colour. It usually lasts for 23 days and subsides on its own without treatment, while in severe cases, it lasts for 12 weeks or even longer. Some patients have frequent episodes of haemoglobinuria, while a significant number of patients have no clear episodes of haemoglobinuria over a long period of time or observation.

Episodes of haemoglobinuria may be accompanied by fever, low back pain and abdominal pain. Other symptoms associated with smooth muscle dysfunction can include dysphagia, abdominal pain, bloating, back pain, headache, oesophageal spasms, and erectile dysfunction. Rare conditions include choledochal duct dyskinesia, acute pancreatitis, ischaemia and ulceration of the duodenum or colon.

**3. thrombosis**.

**Jaundice and hepatosplenomegaly** , less than half of patients have mild jaundice. Most patients do not have hepatosplenomegaly.

Common comorbidities include infection, anaemic heart disease, cholelithiasis, renal insufficiency and even failure.

### DeepSeek-R1-Distill-Qwen2.5-7B

Based on the clinical manifestations you provided, the most likely rare disease diagnosis is **Pancytopenia of chronic viral hepatitis**. This condition is often associated with anemia or thrombocytopenia due to chronic blood loss from borohydride syndrome or other causes, along with jaundice due to increased production of bile salts. Viral hepatitis can also cause hepatosplenomegaly and may be associated with infections, which would explain some of the symptoms you listed.

Other possible diagnoses associated with these clinical manifestations could include:

1. **Pansaotenephritis**: A rare genetic disorder characterized by lysosomal acidosis, anemia, and pancytopenia, which can coexist with autoimmune features like infection, jaundice, and pooled erythropoiesis.
2. **Mdigitspolyuria or Hydroxyuric acidemia**: Characterized by lysosomal storage defects and anemia, which can be associated with jaundice, thrombosis, and other complications.
3. **Systemic Lupus Erythematosus (SLE)**: An autoimmune disorder that can cause anemia, pancytopenia, hemolytic anemia, jaundice, thrombosis, and hepatosplenomegaly.
4. **Dilated TCLP申診 (Treated Chloroquine(module softball)**: A severe presentation of certain lysosomal storage disorders, such as Gaucher disease, causing anemia, thrombosis, and hepatosplenomegaly.

Please note that these are additional possibilities, and a thorough clinical evaluation by a team of specialists would be necessary to confirm the diagnosis.

### 95. User

Clinical manifestations include repeated intussusception, abdominal pain, diarrhoea, mucus stools, blood in stool, constipation, vomiting blood and anaemia. Characteristic clinical manifestations are skin and mucous membrane pigmentation and multiple polyps in the gastrointestinal tract. 95% of the patients have hyperpigmentation, which can be found on the lips and surrounding areas of the mouth, cheeks, face, fingers, palms of the hands, soles of the feet, perianal area, etc., and occasionally on the intestinal mucous membranes. The pigmentation can be brown, tan, grey, blue, etc. It usually occurs before the age of 2 years, and the size is 1~5mm. 88%-100% of the patients will develop gastrointestinal polyps, and the onset of gastrointestinal polyps most often occurs at the age of 11-13 years, and the polyps can occur throughout the entire gastrointestinal tract, with the small intestine being the most common, followed by the colon and the stomach. The size of the polyps is variable, and their tips vary in length and thickness. The risk of cancer is high, with an average age of 42 years.

### DeepSeek-R1-Distill-Qwen2.5-7B

Based on the clinical manifestations described, the most likely rare disease is:

**Enteroendometriosis**: This condition is characterized by an overgrowth of uterine met. leading to polyps in the gastrointestinal tract. It aligns with the symptoms of repeated intussusception, abdominal pain, diarrhea, mucus stools, blood in stool, constipation, vomiting blood, and anemia, along with pigmentation in the gastrointestinal tract. The pigmentation is typically hyperpigmented in areas like the mouth, face, and feet, often in shades such as brown, tan, grey, or blue. Risk of malignancy is noted, with an average age of diagnosis around 42 years.

Other possible rare diseases that could present with similar symptoms include:

1. **Cryptic Dysplasia**: A rare condition often associated with intestinal obstruction and colitis, which can present with the described gastrointestinal symptoms.
2. **Adenoma**: A benign tumor affecting the colon or small intestine, which can lead to polyps and other symptoms.
3. **Lever’s Syndrome**: Characterized by episodes of abdominal pain and colitis, potentially associated with the symptoms described.
4. **Atmospheric Defects**: Rare chromosomal abnormalities that can lead to gastrointestinal disorders, including colitis and other symptoms.
5. **Adenovirus-Associated Colitis**: A viral infection that can cause hematochezia, mucus stools, and other gastrointestinal symptoms, often associated with risk factors like trauma or diabetes.

These additional conditions, though less likely, could potentially align with the presented clinical descriptions due to overlapping symptoms and conditions.

### 96. User

In the neonatal period, most of the children have no clinical symptoms, but after 3-4 months of life, typical symptoms gradually appear, and the symptoms are obvious at the age of 1 year. A few months after birth, due to insufficient melanin synthesis, their hair and iris colour gradually becomes lighter, yellow or brownish-yellow, with white skin. Urine and sweat contain a large amount of phenyl lactic acid and have a foul odour of rat urine. As they grow older, they gradually show delayed intellectual development, with predominantly cognitive developmental disorders, microcephaly, seizures, as well as behavioural and personality abnormalities, such as hyperactivity, self-mutilation, aggression, autism, low self-esteem, depression and other neurological manifestations. Vomiting and eczema are also commonly seen in infancy.

### DeepSeek-R1-Distill-Qwen2.5-7B

Based on the clinical manifestations provided, the most likely rare disease is:

1. **Wilson’s disease**: This chronic metabolic disorder affects copper metabolism, leading to copper deficiency, which can cause skin discoloration, neurological signs, and behavioral issues. The pigmented alopecia and metabolic acidosis from phenyl lactic acid align with this diagnosis, though the presentation may vary.

Other possible diagnoses to consider include:

1. **Copper deficiency without disease**: A condition where copper levels are low without the underlying disease causing symptoms like Wilson’s.
2. **Entop Allah syndrome**: Characterized by hypopigmented erythema, typically presenting in infancy, though it may not always include the neurological features described.
3. **Queen introduced syndrome**: A very rare disorder presenting with developmental delay, hyperactivity, and neurological issues, though it is generally more severe and less common.
4. **Crigler-Najjar syndrome, type IV (CNS)**: May present with a range of symptoms including developmental delay, visual problems, and neurological issues, though it may not always include the specific alopecia described.
5. **Obstructive Sleep Apnea (OSA)**: While not typically a rare disease, it can present with developmental issues and other symptoms if concurrent conditions exist.

These possibilities should be evaluated with a comprehensive clinical approach, considering the unique presentation and patient history.

### 97. User

Mainly skin symptoms and neuropsychiatric symptoms.

**1. The group of cutaneous symptoms,** are erythema, herpes and even ulceration on exposed parts of the skin after exposure to light. The rash may be of the type of eczema, urticaria, summertime itchy rash or erythema multiforme. The oral mucosa may have red patches and brownish-red teeth. Eye damage such as conjunctivitis, keratitis and iritis may also occur. In severe cases, there may be deformation of the skin of the nose, ears and fingers with kinking. There may be special purple colouration. Hirsutism may be present.

**2. A cluster of neuropsychiatric symptoms ,**  the most common manifestation of which is acute abdominal pain, which tends to be severe, stable, and poorly localised, sometimes accompanied by painful cramps. It may be accompanied by constipation, bloating, nausea, vomiting, and sometimes diarrhoea and increased bowel sounds. Sensory and motor neuropathies may precede the onset of abdominal pain and present as limb pain with numbness, abnormal sensation and sensory inversions; motor weakness often begins proximally in the upper limbs and may progress to the lower limbs and distal extremities. Involvement of the cerebral nerves may lead to medullary paralysis, impaired respiratory function and death. Severe motor neuropathy may result in quadriplegia. Autonomic nervous system involvement manifests as tachycardia, hypertension, sweating, agitation, and tremor. Neurogenic bladder dysfunction may cause dysuria, dyspareunia, urinary retention and incontinence. Other acute neuropsychiatric manifestations include insomnia, anxiety, agitation, agitation, hallucinations, hysteria, disorientation, delirium, affective apathy, depression, phobias, and altered consciousness ranging from lethargy to coma.

### DeepSeek-R1-Distill-Qwen2.5-7B

Based on the clinical manifestations described, the most likely rare disease diagnosis would likely be **segovirimetic disorder (PMDD, Plates and Manufacturers’ Diphtheria-like Meeting Day),** a rare condition that presents with a combination of skin and nervous system symptoms, including the skin manifestations described (erythema, herpes, ulcers, and palace mucosa changes) and the described neuropsychiatric symptoms (abdominal pain, neuropathic changes, and neurogenic bladder dysfunction).

**Other possible diagnoses based on the clinical presentation:**

1. **Beta-thalassemia**: A rare blood disorder that can present with skin lesions (e.g., herpetic, oral mucosal changes, and tooth discoloration) and in rare cases, may be associated with arrangé syndrome or occupies, which involve nervous system symptoms like abdominal pain and neuropathy.
2. **Cyclothymic disorder (formerly Poloid lunative syndrome)**: A rare mental disorder that can present with abdominal pain (likely of equidistribution) and in rare cases, may involve Remarkable IIAP and ophthalmoplegia, though it is primarily a mental disorder.
3. **Clicks (proliferations of the central nervous system outside the spinal canal)**: A rare and conclusive condition that causes unusual lesions, aseptic meningitis, periventricular abscess, and can involve the serum neurological findings associated with abdominal pain and neuropathy. It also presents with discrete clinical lesions.
4. **Pioneer cell aplasia (PCA)**: A rare, fatal x-linked disorder of rises that can present with a variety of skin and nervous system lesions, including oral PURP, oral mucosal lesions, and outward pointing fingers with central tendency. It can also involve the central nervous system with autonomic neuropathy,也知道.
5. **Pioneer cell aplasia II (PCA2)**: A less common clinical variant of PCA that preserves a majority of the skin and dyskeratosis congenita features but involves the hand and may add nervous system symptoms like abdominal pain and autonomic neuropathy.

While these other conditions are possible, PMDD is the most likely diagnosis given the combination of skin and nervous system symptoms, particularly the abdominal pain, neuropathy, and mental symptoms described.

### 98. User

**1. Infection, patients** are infected to varying degrees. Infections are recurrent, severe, difficult to treat, opportunistic and have a broad spectrum of pathogens. Typical children are usually born with no specific manifestations, and fatal infections can occur before 2 months of age due to cellular immune abnormalities. The sites and pathogens of infection are diverse, including recurrent pneumonia, recurrent thrush, persistent diarrhoea, recurrent otitis media, persistent viral infections (e.g., respiratory syncytial virus, EBV, CMV, etc.), and conditionally causative organisms, such as Pneumocystis carinii, are also common.

**2. Vaccine disease,** in which patients are extremely immunocompromised, can occur with infection and dissemination of live attenuated vaccines. Severe diarrhoea can also occur in children vaccinated with attenuated rotavirus vaccine.

**3. Retarded or arrested growth and development** is a distinguishing feature of the disease. It is associated with recurrent infections, diarrhoea and corresponding genetic defects.

Children may also have a rash and enlarged liver and spleen lymph nodes similar to erysipelas.

In other children, erythroderma-like symptoms, hepatosplenomegaly, seborrheic dermatitis, haematopenia, and sclerosing cholangitis may be present.

### DeepSeek-R1-Distill-Qwen2.5-7B

**Final Answer:**

**Primary Diagnosis:** The most likely rare disease based on the described symptoms is **Spina Bifida**, which is a condition characterized by spinal anomalies, growth retardation, and developmental delays. It often results from environmental factors and may present with-associated complications like hepatosplenomegaly and/or a rash.

**Additional Possible Diagnoses:**

1. **PAX8 Gene Mutation Disorder**: A genetic condition that can cause variable presentations including growth issues, recurrent infections, and developmental delays due to mutations in the PAX8 gene.
2. **Klinefelter Syndrome**: X-linked condition caused by a mutation in one of the genes on the X chromosome, leading to XY chromosome translocations, slow growth, and other developmental changes.
3. **Respiratory Syncytial Virus (RSV) Pneumonia**: Can cause recurrent pneumonia and bronchitis, presenting with signs of respiratory infection and sometimes affecting other systems.
4. **Latent Syphilis**: Potentially causing symptoms like rash, hepatosplenomegaly, and others, especially if untreated.
5. **Toxoplasma Gondii Infection**: Can lead to liver involvement and hepatosplenomegaly, though more typically a parasitic infection.

These diagnoses were selected considering the symptoms of growth issues, recurrent infections, and developmental abnormalities, along with immune system concerns. Spina Bifida was chosen as the primary condition due to its association with similar clinical features, while the other conditions were included based on possible overlapping symptoms and conditions often linked to similar presentations.

### 99. User

The core symptom is dystonia, which is mainly manifested by abnormal expressive postures and involuntary movements, mostly involving the head and neck muscles, trunk muscles, rotator anterior muscles of the limbs, finger and wrist flexors, toe extensors and plantar flexors. It is aggravated by random movements, reduced or disappeared when resting and sleeping, and in the late stage, it can be a fixed twisting spastic deformity. Sensory trickery (relief techniques) and movement specificity are the characteristic manifestations of dystonia. Sensory tricks (relieving techniques) are random movements that are specifically designed to correct abnormal postures or relieve dystonic movements, usually simple movements that involve or target the affected area, but are not forceful to counteract the symptoms of dystonia. Movement specificity refers to the presence of dystonia only during specific activities or the performance of specific tasks, such as certain occupations (e.g., writing spasms, musician’s spasms) or sports.

### DeepSeek-R1-Distill-Qwen2.5-7B

Based on the clinical manifestations of dystonia, the most likely diagnosis is ** MOTOR NEURON DISEASE (MND)**, a rare neurological disorder often characterized by muscle weakness and coordination issues, leading to abnormal, involuntary movements in areas like the head, neck, and limbs. This condition can manifest with dystonic symptoms, especially during activities that require precision and coordination.

Other possible rare diagnoses that might present with similar symptoms include:

1. **REARE BREEDS OF PIANOS SYNDROME**: A rare inherited disorder causing dystonic movements, particularly in the hands, with mutations in genes affecting motor neurons.
2. **PAGES SYNDROME**: Another rare genetic condition resulting in dystonic movements, often affecting coordination and posture, though it’s less commonly encountered.
3. **INHERITED MOTOR NEURON DISEASES**: A broad category of rare genetic disorders where the central nervous system affects motor neurons, leading to dystonic symptoms and motor neuron degeneration.
4. **Spastic Diphenococconus**: Though more common, this condition can present with fixed, twisting spasticity, fitting the late-stage description of twisted deformity mentioned.

Each of these conditions has unique genetic or neurological underpinnings, contributing to the rare nature of dystonia presentations.

### 100. User

**1. Kidney,** the main manifestations are limb oedema and increased foam in the urine. Laboratory tests may reveal simple moderate proteinuria or nephrotic syndrome, and renal insufficiency may be seen in the advanced stage.

**2. Heart,** the main manifestations are shortness of breath after activity, limb edema, pleural effusion, ascites and other signs of restrictive cardiac insufficiency.

**3. Liver,** there may be slight discomfort or pain in the liver area, but most patients may be asymptomatic, and hepatomegaly or liver enzyme abnormalities are often detected during physical examination. Liver failure may occur in advanced stages of the disease.

**Peripheral and autonomic nerves,** Symmetrical sensory and/or motor peripheral neuropathy of the extremities. Autonomic abnormalities are often manifested by upright hypotension, gastroparesis, pseudo-intestinal obstruction, and impotence.

**5. Gastrointestinal tract** , can present with total gastrointestinal involvement, with the stomach and small intestine being the most commonly involved. It can present with epigastric discomfort, dyspepsia, diarrhoea, constipation, malabsorption syndrome and gastrointestinal bleeding.

**6. Soft tissues ,** Tongue involvement may present with megaglossia, tongue dysmobility, and dysarthria. Skin mucosa may present with skin purpura and petechiae, which are more common around the orbits and in the loose skin areas of the neck. Nail atrophy and hair loss may also occur.

**7. Coagulation abnormalities** Patients often have coagulation factor X deficiency, resulting in corresponding bleeding manifestations.

### DeepSeek-R1-Distill-Qwen2.5-7B

Based on the clinical manifestations you provided, the most likely rare disease is **nephrotic syndrome**. Nephrotic syndrome is a rare condition characterized by the presence of small amounts of protein in the urine (proteinuria) and edema (swelling of the limbs), along with symptoms such as shortness of breath, ascites (fluid accumulation in the abdomen), and sometimes an elevated blood pressure. In chronic cases, proteinuria may progress to **nephrotic syndrome nephrotic syndrome**, which can lead to **kidney insufficiency and eventual kidney failure**.

In addition to nephrotic syndrome, there are several other rare conditions that could present with similar clinical features. Here are five other possible rare diseases that might be considered:

1. **Heart failure (CHF):** While not solely characterized by limb edema and proteinuria, heart failure can present with edema, ascites, and other symptoms of fluid overload. Certain forms of heart failure are rare, such as **congenital heart diseases** or **systemic lupus erythematosus-associated heart failure**.
2. **Gastrointestinal obstruction or bleeding:** The presence of gastrointestinal symptoms (e.g., epigastric discomfort, diarrhea, blood in stool) and raspberries or Nz訾 sounds on examination can suggest **gastrointestinal obstruction**. Rarer conditions like **parenchymal abnormalities causing obstruction** could be a possibility.
3. **Soft tissue involvement (e.g., myositis or polychivalent Recipes for anemia):** The presence of edema, sensory or motor symptoms in the extremities, and skin lesions could suggest a rare autoimmune or inflammatory condition like **myositis** or **leukoaraiosis**.
4. **Coagulation abnormalities:** The presence of bleeding symptoms (e.g., hematemesis, petechiae, or衄血) alongside edema could be indicative of **coagulation disorders** such as **factor X or II deficiency**, **py sehigitis**, or **warfarin-induced thrombocytopenic purpendicular syndrome**.
5. **Rare genetic disorders:** Some rare genetic disorders can present with edema, ascites, and proteinuria. Examples include **thyroid autoimmune diseases** (e.g., Graves’ disease), **lentzelediabetes or genetic hyperthyroidism**, or **autosomal recessive kidney diseases** like **k Alexander (ADAR) diseases**.

In conclusion, nephrotic syndrome is the most likely rare disease based on the symptoms you provided, but it is important to consider other conditions, especially when additional symptoms such as assays or imaging findings are available for further evaluation.

### 101. User

Jaundice and itchy skin are typical clinical manifestations. Other developmental delays include short stature, delayed pubertal growth, gallbladder stones, steatorrhea due to fat absorption disorders, hepatosplenomegaly, rickets due to fat-soluble vitamin deficiencies, delayed bone age, dry eye, coagulation disorders, and neuromuscular disorders. Children may also develop visual and hearing abnormalities, irritability, lethargy and poor concentration. In advanced stages, portal hypertension and liver tumours may occur.

### DeepSeek-R1-Distill-Qwen2.5-7B

Based on the provided clinical manifestations, the primary diagnosis is **Dubin-Johnson syndrome**, a rare genetic disorder. Here’s a detailed breakdown:

1. **Dubin-Johnson Syndrome**:
   - **Presentation**: Characterized by intrahepatic cholestasis of varying grades, secondary generalized hypoacidsis, and gallosumebinosis.
   - **Key Features**: Jaundice, gallbladder stones, steatorrhea, short stature, delayed pubertal growth, and rickets-like features due to vitamin D deficiency or metabolic issues.
2. **Other Conditions**:
   - **Growth Hormone Deficiency (plementation)**: This can explain the developmental delays, short stature, and skeletal abnormalities seen in Dubin-Johnson syndrome.
   - **Hereditary Intrahepatic Cholestasis of Infancy (HIChI)**: A rare genetic condition causing jaundice, liver tumors, and developmental issues.
   - **Coagulation Disorders**: Thrombocytopenia and easy bleeding are common, supported by the liver dysfunction typical of Dubin-Johnson syndrome.
   - **Electrolyte Imbalance**: Likely due to the chronic condition, leading to compensatory mechanisms.
3. **Additional Considerations**:
   - **Rickets**: Can arise from fat-soluble vitamin D deficiency, sometimes secondary to Dubin-Johnson syndrome or other metabolic disorders.
   - **Neuromuscular Issues**: May be related to metabolic or genetic factors affecting these systems.

In summary, Dubin-Johnson syndrome is the primary diagnosis, with other symptoms explained by its systemic effects and associated complications.

### 102. User

The disease starts in childhood. The disease manifests itself as a mild delay in motor development and progressive atrophy of skeletal muscles, which affects the motor function of the limbs and gradually leads to gait abnormalities and limited movement of the upper limbs, with the natural course of the disease often resulting in loss of the ability to walk at about 10 years of age. Thereafter, scoliosis, joint contracture, respiratory muscle weakness and dilated cardiomyopathy appeared, and the patient died of respiratory failure and cardiac failure at the age of about 20 years. Pseudohypertrophy of both gastrocnemius muscles, pseudohypertrophy of both forearms and lingual muscles, positive Gowers’ sign, and lumbar lordosis can be seen on examination. The disease may start in youth or even adulthood, and some patients do not affect survival. Signs of pseudohypertrophy are obvious, and in some patients, dilated cardiomyopathy is first apparent when limb weakness is still mild.

### DeepSeek-R1-Distill-Qwen2.5-7B

The clinical manifestation described is most consistent with **pseudohypertrophic muscular dystrophy (PHMD)**, a rare genetic disorder that causes progressive muscle atrophy, particularly in the limbs, and is often fatal in early adulthood. PHMD is characterized by:

- **Atrophy of the muscles**, particularly the limb muscles, leading to motor deficiency.
- **Scoliosis** (a lateral curvature of the spine).
- **Joint contractures**, limiting limb movement.
- **Respiratory muscle weakness**, commonly in early to middle life.
- **Dilated cardiomyopathy**, which often occurs in the later stages of the disease.
- **Positive Gowers’ sign**, a sign of pseudohypertrophy in the lower limbs.

Other possible rare diseases with similar or overlapping features could include:

1. **Mitochondrial myopathy** - A condition where depletion of mitochondrial function leads to muscle and nerve damage, often causing respiratory and cardiac issues, and can result in severe muscle decay.
2. **Cerebral palsy** - While not as rare as PHMD, it is a genetic disorder that can cause motor abnormalities and muscle weakness. However, it is associated with significant physical and developmental abnormalities rather than a focus on pseudohypertrophy.
3. **Transforming growth factor-beta (TGF-β) signaling pathway defects** - A condition that impacts muscle development and function, potentially leading to muscle wasting and structural changes in the spine.
4. **Genetic muscular dystrophy with expanding growth plates** - A subgroup of rare muscular dystrophies where growth plate expansion is a key feature, leading to delayed or absent maturity of the skeletal system, and potentially severe muscle dysfunction.
5. **Spina muscular atrophy** (though not as rare) - This is a rare condition that can lead to severe muscle weakness and muscle wasting, but is more common and doesn’t typically involve the pseudohypertrophic findings you described.

### 103. User

Mainly hyperaemia, brain damage and cardiomyopathy.

**1. Neonatal onset** Normal at birth, vomiting, lethargy, hypotonia, convulsions, dyspnoea, hyperaemia, ketosis, hypoglycaemia, acidosis, dilated cardiomyopathy, pancreatitis and other abnormalities after starting breastfeeding, with a high morbidity and mortality rate.

**2. Delayed-onset** is often induced by fever, starvation, high-protein diet, and infections, and manifests itself as feeding difficulties in infancy and early childhood, developmental delays, convulsions, and hypotonia. In many patients, cognitive ability and neurological development are impaired, with an increase in slow waves or epileptic waves on the EEG; some patients may have fractures and osteoporosis on X-ray; bone marrow suppression is also common, causing granulocytopenia, anaemia and thrombocytopenia. There may also be cardiac damage, such as cardiomyopathy, arrhythmia, prolonged QT interval, and diminished cardiac function. Renal impairment is less common.

### DeepSeek-R1-Distill-Qwen2.5-7B

The clinical presentation described, which includes hyperaemia, brain damage, and cardiomyopathy, is most likely due to **Ketocidin myopathy**, a rare skeletal muscle disorder characterized by ketoacidosis, convulsions, and associated cardiomyopathy, often seen in late infancy or early childhood.

In addition to this condition, here are five other possible rare diagnoses that present with similar or related features:

1. **Acute Allograft Vasculitis (AAV)**: A rare and severe condition leading to maternal-foetal wastage, presenting with ketoacidosis and convulsions in the neonate, alongside cardiomyopathy and hyperaemia.
2. **Cerebral Vasculitis**: A rare condition affecting brain vasculature, leading to convulsions, brain dysfunction, and sometimes cardiomyopathy.
3. **Lopolysaccharidosis**: A rare metabolic disorder causing weight loss, ketoacidosis, and hyperaemia, often with developmental abnormalities.
4. **Spina Atrophy**: While primarily a condition associated with Schwann cell dysfunction, it can present with neurological deficits and is linked to other rare conditions.
5. **Enteroardhria**: A rare metabolic disorder causing ketoacidosis, ammon中毒-like symptoms, and hyperaemia, often with other complications.

Each of these conditions highlights the complexity and rarity of presentations involving hyperaemia, brain dysfunction, and cardiomyopathy, underscoring the need for specialized evaluation and consideration.

### 104. User

Clinical manifestations are often nonspecific, with an insidious onset. Symptoms such as coughing up sputum, lethargy and fatigue may occur. Secondary infections may include fever, coughing up purulent sputum, or chest pain and haemoptysis. A few patients may be asymptomatic and may have no positive signs. Bursting sounds may be present in some patients, cyanosis may be present in severe cases, and pestle and mortar fingers may be seen in a few patients. The clinical picture is often less severe than the chest image, and this inconsistency between symptoms and images is a characteristic feature of the disease. Serum anti-GM-CSF antibodies are markedly elevated.

### DeepSeek-R1-Distill-Qwen2.5-7B

Based on the clinical manifestations you have described, **histoplasmosis** (specifically caused by the fungus **Histoplasma capsulatum**) is the most likely diagnosis. Here are five additional possible diagnoses that could present with similar clinical features:

1. **Schistosomiasis**: Portal hypertension, ascariasis, and other parasitic infections can cause nonspecific symptoms, including coughing up sputum, fatigue, and chest pain. The anti-GM-CSF antibodies may be elevated in some cases, but this diagnosis is less commonly associated with this specific elevation.
2. **Miliary TB**: Cavitating nodule, coughing up purulent sputum, and chest pain are common symptoms of tuberculosis. However, anti-GM-CSF antibodies are not typically elevated in this condition.
3. **Bartonellosis**: Coughing up sputum with red discoloration may occur in Bartonella infections, but this is less commonly associated with anti-GM-CSF antibodies.
4. **HIV/HPV Coinfection**: Coinfections with HIV and human papillomavirus (HPV) can cause multifCreamy rash, generalised lymphadenopathy, and weight loss. However, the clinical features you have described are less characteristic of this condition.
5. **Acute HIV Re activation**: Persistent opportunistic infections such as cytomegalovirus or histoplasmosis can occur after HIV reactivation, but this is less commonly associated with diagnostic features you have described.

If you have more detailed symptoms or imaging findings, I can help further refine the diagnosis. Let me know!

### 105. User

In most cases, the disease begins in adolescence, with a gradual loss of the optic rod cells, followed by cone cell involvement and death of the retinal pigment epithelium. Therefore, night blindness is usually the first symptom (but after urbanisation, some patients often have a decrease in visual acuity), and the earlier the onset of night blindness, the more severe the patient’s condition. Although peripheral visual field defects also occur early, patients are usually unaware of them and rarely complain of them in the early stages of the disease. The disease progresses to a loss of central vision and further narrowing of the visual field, and most people become legally blind before the age of 40. The degree of visual impairment can be related to the mode of inheritance.

The presentation of the fundus is related to the stage of the disease: in the early stages of the disease, the fundus may be normal or nearly normal on routine examination, but in some patients, an early form of blanket retinal degeneration may be observed on mid-peripheral fundus photography (these subtle changes are difficult to recognise with current scanning laser fundus photography or wide-field fundus photography). As the disease progresses, some patients develop typical fundus changes, such as retinal osteoblast-like pigmentation combined with mid-peripheral retinal atrophy, a waxy optic disc, and thinning of retinal blood vessels; some patients develop blanketing or depigmentation, which can be seen in the mid-peripheral retina, or extensive degeneration. Other common complications include macular cystoid degeneration, posterior subcapsular cataract, vitreous dust particles, and optic disc vitreous warts; a rare complication is Coat’s retinopathy.

### DeepSeek-R1-Distill-Qwen2.5-7B

The disease described is most likely an autosomal recessive disorder affecting the outer retina. It involves a progressive loss of rod cells first, followed by cone cells and the retinal pigment epithelium, leading to night blindness and later legal blindness by the age of 40. Key fundus changes include atrophy, waxy optic discs, and pigment changes, suggesting a defect in outer retina nuclear function. The rare complication of Coat’s retinopathy further supports this diagnosis. This disorder is likely autosomal recessive, contributing to its rarity.

### 106. User

The most common clinical presentation is white pupil syndrome, in which a yellow-white reflection is visible in the pupil. Disuse strabismus may occur in the affected eye due to the tumour being located at the posterior pole and low visual acuity. The early presentation is a greyish-white, round or oval arching mass that may bulge into the vitreous or grow flat along the choroid. Retinal blood vessels on the surface of the tumour may become dilated and haemorrhagic, and even exudative retinal detachment may occur. Tumour tissue may also penetrate the retina and enter the vitreous, floating like a snowball, or even deposited in the anterior chamber to form pseudo anterior chamber pus. The onset of the disease is early in children with bilateral retinal detachment, usually within one year of age. The onset is later in unilateral children, usually at two or three years of age.

### DeepSeek-R1-Distill-Qwen2.5-7B

Based on the clinical manifestations you provided, the diagnosis is most likely **White Pupil Syndrome (WPS)**, also known as posterior harvesting syndrome. This rare disease involves tumors arising from the optic nerve sheath in the posterior portion of the retina, leading to yellow-white reflections in the white pupil and associated retinal complications such as hemorrhages, exudative detachment, and peritumoral tissue invasion of the vitreous.

However, based on your detailed description, here are five other possible rare diagnoses that might present with similar features:

1. **Unilateral Transient Retinal Atrophy (TRIES)**: This condition is characterized by pigment changes and exudative detachment, but it typically does not involve the optic sheath or white pupil syndrome.
2. **Choroid Predominant Exudative Detachment (CPEX)**: While it can present with exudative detachment, it usually involves the choroid plexus rather than the retina surface.
3. **Traine’s Disease**: This rare condition involves exudative detachment and may contain some “budding” features but is not associated with white pupil syndrome.
4. **Pathological Axial Deficiency of the Pigment Cells (PADP)**: This disorder affects the retinal pigment epithelium and may lead to pigment defects, but it does not typically involve the optic sheath.
5. **Blastema of the Optic Nerve**: This rare tumor can present with tumor-like lesions, but it is not commonly associated with white pupil syndrome.

Please note that these are additional possibilities, and a thorough clinical evaluation under the guidance of a experienced ophthalmologist would be required to confirm the exact diagnosis.

### 107. User

The average age of onset is 5.6 months. The first seizure is usually normal at birth and starts within 1 year of age. The first seizure is usually febrile convulsion, which may be one-sided or generalised clonic or tonic clonic seizure, and febrile convulsions are characterized by long duration and recurrent episodes, and may appear in a persistent state. 28%~35% of children may start the disease without febrile convulsions, but they often have heat sensitivity in the later course of the disease. Elevated body temperature due to various reasons, including exercise, fever, vaccination, hot baths, and high ambient temperatures, can trigger or exacerbate seizures and predispose to status epilepticus. Some children develop an acute encephalopathy characterised by deep coma following status epilepticus.

Children tend to present with febrile convulsions between the ages of 1 and 4 years, and have a variety of seizure types, such as myoclonic, atypical catatonic, partial seizures, and generalised tonic clonic seizures. Myoclonic seizures are often triggered by light stimulation, eye closure, and a specific graphic stimulus, and involve simultaneous clonus of one or several muscles, all accompanied by synchronised abnormal brain wave bursts. The intensity of the myoclonus varies, from a slight forward or backward movement of the body when only the axial muscles are violated, to a severe fall or sudden throwing of an object in the hand. Myoclonic seizures can be isolated or cluster, but a sustained state is rare. Myoclonic seizures may not occur at all in about 25% of children. The concurrent EEG of atypical apoplectic seizures shows half of the discharges to be of focal origin, most of which are irregular, irregular, slow spikes and slow waves, often accompanied by myoclonic seizures. 30% of the children may have atypical apoplectic persistent seizures. The incidence of partial seizures is 43.0% to 78.6%, which manifests as lateral limb (or) head deflection seizures or clonic seizures, and there are also motor seizures with consciousness and autonomic symptoms, automatisms, and hypotonia, etc. After 1 year of age, the children have psychomotor developmental delays, especially language developmental delays.

### DeepSeek-R1-Distill-Qwen2.5-7B

Based on the clinical manifestations provided, the most likely rare disease is **Wellington syndrome**. This syndrome is characterized by early-onset tonic-clonic seizures (TCs) that typically begin before age 1 and may start within the first year of life. It is a rare disorder with a high frequency of neonatal or early postnatal onset of seizures.

### Other possible diagnoses:

1. **Lennox-Gastaut syndrome (LGS)**: A rare neurological disorder that can present with early-onset seizures, particularly tonic-clonic seizures. It is more commonly diagnosed in older children but may occur in younger ages as well.
2. **Epilepsy with MRI-leepotic syndrome (EWMS)**: A rare multifocal Spike-Twave discharge syndrome that can present with a mix of tonic-clonic and partial seizures. It is associated with an MRI-leepotic lesion and is more common in adulthood when remapped.
3. **Ad_channels channel modulationbeth syndrome**: A rare disorder that can present with early-onset generalized tonic-clonic seizures and heat sensitivity.
4. **Lennox-Gastaut syndrome with early convulsions**: This is a less common variant of LGS that may present earlier and more variably than the typical clinical presentation.
5. **Varicose海绵血管症 (Approximate varicose海绵血管病)**: A rare autosomal recessive disorder of the nervous system that can present with early-onset convulsions and encephalopathy.

### Note:

Given the clinical features described—such as early onset of seizures, heat sensitivity, and potentially recurrent or acute neurologic issues—Wellington syndrome remains the most likely diagnosis, but it is important to consider other rare syndromes that present with similar features, such as Lennox-Gastaut syndrome or MRI-leepotic syndrome. A thorough neurological examination, MRI, and genetic testing would be necessary to confirm the diagnosis.

### 108. User

On the one hand, it presents as chronic haemolytic anaemia, and in addition to anaemia-related symptoms, it may cause mild to moderate yellowing of the skin and sclera and pigmented cholelithiasis due to elevated bilirubin, which may be exacerbated by cold, infection and dehydration. In infants and young children, splenomegaly may be present, and with age, the spleen may shrink due to splenic infarction and fibrosis.

On the other hand recurrent vaso-obstructive pain due to capillary micro thrombosis. Children and adults tend to present with myalgia of the limbs, large joint pain and low back pain, and in severe cases, severe abdominal pain, headache, and even limb paralysis and coma. Over time, vascular obstruction can occur in almost every organ system, and cyclical pain can occur in patients who are thin, easily fatigued, susceptible to infection, and malnourished. Thinning of the bone cortex, osteoporosis, spinal deformity, aseptic necrosis of the femoral head due to an overcompensatory increase in bone marrow haematopoietic tissue, and localised osteosclerosis if there is a bone infarction. Ocular symptoms may include fundus haemorrhage and retinal detachment due to embolism of the retinal vessels in the fundus. In addition, mental retardation may occur, affecting neurological development.

Acute pain is the first symptom of the disease in more than 25% of patients. It is most common in children older than 2 years of age. The severity of pain can vary from mild to severe. Approximately one-half of pain episodes are accompanied by objective clinical signs such as fever, swelling, tenderness, tachypnoea, hypertension, nausea and vomiting.

### DeepSeek-R1-Distill-Qwen2.5-7B

Based on the comprehensive analysis of the symptoms described, the most plausible rare disease is Dubin-Johnson syndrome. This syndrome is characterized by a lysosomal storagenomaly leading to lysostaphinosis, causing muscle wasting, fibrosis, and potentially lysostaphinosis-related complications. It can present with hemolytic anemia, splenomegaly, splenic fibrosis, and vaso-obstructive pain, aligning with the clinical features provided. While other conditions like autoimmune diseases or lysostaphinosis are considered, Dubin-Johnson syndrome best fits the description due to its overlapping symptoms related to muscle wasting, organ fibrosis, and circulatory issues.

### 109. User

The main clinical manifestations of neonatal and paediatric patients include:

1. Intrauterine growth retardation and postnatal growth disorders . Intrauterine growth retardation, low birth weight, lack of postnatal catch-up growth, and proportional short stature.
2. Specific physical signs: wide and prominent forehead, pointed jaw leading to a triangular face, and large relative head circumference. Dysmorphic teeth, low ear position, and asymmetry of trunk deviation, including limb length discrepancy, and lateral curvature deformity of the little finger.
3. Other manifestations include feeding difficulties, hypoglycaemia in the neonatal period, genitourinary malformations such as hypospadias, cryptorchidism and horseshoe kidneys, and digestive anomalies such as cafe au lait spots and reflux oesophagitis. Some patients have cognitive, motor and language developmental delays and learning disabilities.

### DeepSeek-R1-Distill-Qwen2.5-7B

Based on the clinical manifestations you have described—specifically intrauterine growth retardation, postnatal growth disorders, physical signs such as a triangular face, growth discrepancies, and developmental delays—it is likely that the individual may have **hydrocephalus**. Hydrocephalus is a common rare condition that can present with these physical and developmental features, particularly in neonatal and early pediatric populations.

Other possible rare diagnoses consistent with these symptoms could include:

1. **Down syndrome**
   - A genetic disorder associated with intrauterine growth retardation, postnatal growth issues, and developmental delays.
2. **Spina bifida or a related spinal anomaly (e.g., myelomeningoely)**
   - Spina bifida is a rare congenital anomaly of the spinal cord that can cause significant physical deformities, growth issues, and developmental delays.
3. **Epiglottid dysplasia**
   - A rare autosomal recessive disorder affecting the epiglottis, leading to feeding difficulties and developmental delays, though it can also present with craniofacial features.
4. **Cerebral palsy**
   - A condition characterized by physical and developmental delays, often alongside growth retardation and growth issues, though it is less common than Down syndrome.
5. **Trisomy 18 (Euplaty)**
   - A rare chromosomal disorder that can affect bone and other systems, often leading to skeletal and skeletal muscle abnormalities alongside growth issues.

Please note that clinical signs alone cannot definitively diagnose a rare disease, and further evaluation, such as genetic testing or imaging studies (e.g., MRI or CT), would be necessary to confirm the diagnosis. Rare diseases can vary widely in their presentation, so a thorough clinical case-by-case assessment is essential.

### 110. User

**1. Xanthomas of the skin,** occurring near the attachment points of the extensor tendons, such as the Achilles tendon, elbow, knee, etc., and there is a close relationship between skin injuries and the appearance of xanthomas. Cutaneous xanthomas are rare in children.

**2. Atherosclerosis ,** Patients have a significantly higher risk of developing atherosclerosis and coronary artery disease than normal subjects. Early-onset coronary artery disease may be one of the characteristics of the disease.

**3. Alterations in the blood system ,** The content of plant sterols in the blood is significantly increased, resulting in a higher proportion of plant sterols in the sterol component of the cell membrane, abnormal cell membrane function of red blood cells, platelets and other cell membranes, anaemia and thrombocytopenia, and the patient may have weakness, pallor, skin bleeding and other manifestations.

**4. Others,** in addition to the above symptoms, some patients may develop joint pain, arthritis, splenomegaly, elevated transaminases, etc.

### DeepSeek-R1-Distill-Qwen2.5-7B

Based on the clinical manifestations you have provided, the most likely rare disease is likely **Xqusence syndrome**, a rare genetic disorder characterized by xanthomas of the skin, low body weight, and associated blood system alterations. This condition is often associated with a high index of suspicion in low birth weight infants and children.

Here are five additional possible rare diagnoses that may present with similar or related clinical features:

### 1. Oqusence Syndrome

- **Key Features**: Xanthomas of the skin, low body weight, joint pain, and associated blood system alterations.
- **Incidence**: low birth weight (very common in families with this condition).
- **Differential Diagnosis**: X-linked hypertriglyceridemia, early-onset coronary artery disease, and associated systemic findings.

### 2. Oculocardiomegaly syndrome (X-linked Congenital Arthritis of the Browser Type)

- **Key Features**: Oculocardiomegaly (眼球-心脏扩大), joint pain, and a high index of suspicion in patients with xanthomas of the skin.
- **Incidence**: rare but shares clinical overlap with xanthomas and associated blood system alterations.
- **Differential Diagnosis**: systemic inflammatory responses,9
- **Lab Findings**: Elevated liver enzymes (AST/ALT), normal transaminases, suggestive of an autoimmune response or ammonia metabolism issue.

### 3. X-linked Hypertriglyceridemia (XHT)

- **Key Features**: Elevated triglycerides, xanthomas of the skin, and potential association with early-onset atherosclerosis.
- **Incidence**: rare but shares clinical overlap with xanthomas and associated blood system findings.
- **Differential Diagnosis**: Other lipid disorders or metabolic conditions.
- **Lab Findings**: High triglycerides but normal or low cholesterol.

### 4. Cushing’s Syndrome

- **Key Features**: Alterations in blood system (e.g., platelet and red blood cell function), skin lesions, and potential joint involvement.
- **Incidence**: less common, but may overlap with other symptoms such as joint pain and atherosclerosis.
- **Differential Diagnosis**: Other endocrine disorders, especially other Cushing-like syndromes or metabolic conditions.
- **Lab Findings**: Elevated CYP19 or other enzymes associated with steroid hormone metabolism.

### 5. Non-Alcoholic Steatohepatitis (NASH)

- **Key Features**: Fatty liver, associated symptoms of liver inflammation, and eventually fibrosis.
- **Incidence**: while more common, it may present with nonspecific systemic or joint involvement.
- **Differential Diagnosis**: Alcohol-related liver disease or other metabolic conditions.
- **Lab Findings**: Elevated liver enzymes,bilirubin, fibroblast growth factor (FGF)- related findings.

### Explanation:

The clinical features such as xanthomas of the skin, elevated liver enzymes, and associated joint or systemic symptoms could overlap with multiple rare conditions. The relative rarity of each of these conditions can help guide a more thorough clinical evaluation and work-up.

### 111. User

The onset of the disease is insidious in males, and the common age of onset is 30-60 years old. Patients often start with painful spasms, tremor, and weakness of the lower limbs, and gradually develop weakness of the upper limbs, muscle atrophy of the medulla oblongata and the face, muscle fasciculations, dysarthria, dysphagia, dyspnoea, and respiratory difficulties. Androgen receptor insensitivity may be associated with gynecomastia and reduced reproductive function. Sensory abnormalities are present in more than 50 per cent of patients. The disease progresses slowly, with patients usually becoming unable to walk late in the course of the disease, and only some patients require assisted ventilation, which has no significant impact on survival.

**1. Atypical symptoms,** some of which may occur years or even decades before the onset of limb weakness, include fatigability, painful spasms, perioral tremor, and postural tremor. Muscle weakness: most patients start with weakness of both lower limbs, gradually progressing to both upper limbs, tongue muscles and facial muscles, while the extraocular muscles are often not involved. The distribution of limb weakness is asymmetrical, with proximal involvement predominating. Involvement of the muscles of mastication may cause mandibular ptosis and tremor. Physical examination of the lower motor neuron damage signs, manifested as a decrease in muscle strength of the limbs, proximal, accompanied by muscle atrophy, fasciculations, tendon reflexes are weakened or disappeared. Most patients may have obvious tongue muscle atrophy and fibrillation, but the soft palate activity and pharyngeal reflexes are normal, and the degree of choking on water and dysphagia is mild. In some patients, chin and perioral fasciculations can also be observed. The motor function impairment of the patients gradually increases with the prolongation of the disease, and in the later stage, they may have difficulty in walking up the stairs and walking unsteadily.

**2. Sensory deficits** , with varying degrees of hyperalgesia present in about half of the patients, while in the others it is subclinical, with abnormalities found only on sensory nerve conduction tests.

**3. endocrine abnormalities,** including abnormalities of glucose and fat metabolism, and androgen receptor insensitivity and other manifestations, the latter including gynecomastia, decreased sexual function, infertility, and testicular atrophy.

The latter include gynecomastia, decreased sexual function, infertility, testicular atrophy, etc.

**4. Female carriers,** most female carriers do not have symptoms, and even if they do have symptoms, they are mild, such as fasciculations, mild weakness of distal limbs, muscle spasms, or increased creatine myosinase.

### DeepSeek-R1-Distill-Qwen2.5-7B

The patient’s symptoms describe Coat’s de Vries syndrome, a rare multisystem disorder primarily affecting males in the age range of 30 to 60 years. Key features include central nervous system and endocrine involvement, such as muscle weakness, sensory deficits, and androgen insensitivity with related issues. Other rare disease possibilities include Kalesinfinity, Blallem-Dijkstra, and Guigot’s disease, each with distinct characteristics. Coats de Vries syndrome is the most fitting diagnosis given the broad symptom profile.

**Answer:** The most likely rare disease is Coat’s de Vries syndrome. Possible other diagnoses include Kalesinfinity, Blallem-Dijkstra syndrome, and Guigot’s disease.

### 112. User

Some patients present with reduced foetal movement in utero and are born as floppy babies. The onset of the disease occurs within 6 months, with a mean age of onset of 1 month after birth. They present with generalised floppy weakness and severe hypotonia. Most children have difficulty sucking and swallowing due to weakness of the tongue, facial and masticatory muscles, and atrophy and tremor of the lingual muscles are seen. Intercostal muscle involvement can present with dyspnoea and abdominal breathing. The chest has a bell-shaped appearance. The lower limbs are more heavily involved than the upper limbs, and proximal is more severe than distal. Severe muscle weakness in the mid-trunk area leaves the child unable to control head movements, unable to lift or roll over, and without the ability to sit alone. When lying down, both lower limbs are in the frog-leg position with hip abduction and knee flexion. Muscle atrophy is not obvious, and some children have mild joint deformities. Children with good intelligence, tendon reflexes disappeared, limb sensation is normal. The children’s muscle weakness progressively worsens, and eventually they lose all voluntary movement ability, are fed nasogastrically, and suffer from respiratory failure due to repeated respiratory infections. 80% of the children die within 1 year of age, and rarely survive beyond 2 years of age.

### DeepSeek-R1-Distill-Qwen2.5-7B

Based on the clinical manifestations provided, the most likely rare disease to be diagnosed is **G顶综合症 (G - T - P - P - E)**, also known as the **Rizzari-Blank-Ruggiero syndrome**, or possibly **C;ar disease (CD)**. These are rare mitochondrial disorders that cause generalized muscle weakness, respiratory failure, and reduced fetal movement. Both conditions typically present in early childhood and have a poor prognosis, with many children dying within the first year of life.

However, here are **five other possible diagnoses** that could explain the symptoms described (though they may not be as common):

1. **三百型神经肌肉肌肉营养不良病 (CY)/**
   - A rare mitochondrial disorder that causes generalized muscle weakness and respiratory failure. It may also present with hypotonia, floppy neonatal appearance, and developmental delays.
2. **Ehlers-Danlos syndrome (EDS)/**
   - A connective tissue disorder that can cause muscle atrophy, joint involvement (often proximal and involving both lower and upper limbs), and respiratory distress. It may also result in developmental delays and reduced feeding ability due to motor weakness.
3. **Jervell-Blank-Nielsen syndrome (JBN)/**
   - Another rare mitochondrial disorder characterized by respiratory failure, generalized muscle weakness, and developmental delay. sympatho maturation delay can lead to jointly involved presentations.
4. **Alpers syndrome (ALP)/**
   - A rare condition of early childhood caused by mutations in the ALPR gene, leading to joint hypermobility, muscle weakness, joint calcification, and respiratory failure. Noticeable features include proximal muscle weakness and difficulty in sitting up.
5. **Mayo-Ezrak syndrome (ME)/**
   - A rare autosomal recessive disorder caused by mutations in SPraising-Prader-Willi.newaxis syndrome. It can lead to muscle atrophy, joint involvement, and respiratory distress. It may also present with developmental delays and restricted movement.

These diagnoses are not perfect matches for the symptoms described, but they are possible alternatives that could explain some of the clinical features. A thorough clinical evaluation and genetic testing would be necessary to confirm the cause of the condition.

### 113. User

Patients usually start in adulthood, with an insidious onset and gradual progression. The main manifestations are cerebellar ataxia, including drunkard’s gait, clumsy movements, minstrel-like speech, nystagmus, diplopia, etc.; pyramidal tract damage, including hyperreflexia, ankle clonus, and positive pathological signs; and extrapyramidal symptoms, including Parkinson’s syndrome, dystonia, and ankylosis. In addition, some patients may have peripheral neuropathy. In the early stages of the disease, some patients may have damage to the pyramidal tract (similar to spastic paraplegia) or extrapyramidal symptoms (similar to Parkinson’s syndrome), and ataxia is not prominent.

The disease progresses gradually, with loss of walking ability after 10-15 years. Prolonged dysphagia leading to malnutrition or aspiration is the main cause of death.

### DeepSeek-R1-Distill-Qwen2.5-7B

Based on the clinical manifestations described, **Spinitus cerebellarum / Cerebellar Hypotrophy Atrophy Syncephytrophy (CP, C Ax121)** is the most likely rare disease to be diagnosed. This syndrome is characterized by cerebellar ataxia with traits such as drunkard’s gait, minstrel-like speech, and nystagmus, as well as extrapyramidal symptoms like those seen in Parkinson’s disease and dystonia. Peripheral neuropathy is also commonly associated with this condition.

Other possible rare diseases that could present with similar symptoms include:

1. **Early-Onset Ataxic Dysphagia (EOD)**: This is a rare disorder characterized by cerebellar ataxia, peripheralsystemic neurodegeneration, and profound dysphagia, which can lead to malnutrition and death. It often presents with inefficiency in motor functions and sensory loss.
2. **Spinocerebellar Ataxia (SCA)**: This group of diseases (e.g., SCA2, SCA3, SCA6, etc.) shares many symptoms with cerebellar ataxia, including cerebellar hypoperfusion, cerebellar abnormalities, nystagmus, and extrapyramidal symptoms like those seen in Parkinson’s disease.
3. **Early-Onset Ataxia with Multiple System Neurodegeneration (EOMIND)**: This rare disease presents with ataxia, coordination difficulties, and multisystem neurodegeneration. It often includes peripheral neuropathy and other extrapyramidal symptoms.
4. **Complex Partial Simple Spastic Paraplegia (CPTSP)**: While this condition is more commonly associated with spasticity and muscle weakness, it can also present with extrapyramidal symptoms and neurodegenerative changes, though it is less commonly associated with cerebellar ataxia.
5. **Erb’s Encephalomyopathy (EE)**: This rare, chronic neurodegenerative disorder often presents with encephalopathy, cerebellar hypoperfusion, nystagmus, and extrapyramidal symptoms such as parkinsonism and dystonia. It may progress over a long timespan.

In all cases, further investigation and clinical correlation will be necessary to confirm the diagnosis, as other rare conditions may present with similar clinical features.

### 114. User

The disease may be combined with gastro-oesophageal reflux in the early stages of the disease. As the disease progresses, the skin becomes swollen and sclerotic and may be accompanied by changes in skin pigmentation, including hyperpigmentation or depigmentation. In addition to skin involvement, patients often present with arthralgia and myalgia, and may develop ulcers and capillary dilatation of the digits. In addition, the patient may have multiple internal organ involvement. The respiratory system is more likely to be involved, with interstitial fibrosis and pulmonary hypertension. The gastrointestinal manifestations are varied and include gastroesophageal reflux, sinus vasodilatation, abdominal pain, diarrhoea, constipation and fecal incontinence. A small number of patients may present with renal crisis, which is characterised by acute renal insufficiency and is often associated with hypertension. Cardiac involvement may manifest as pericardial effusion, abnormalities of the cardiac conduction system, and myocardial involvement in individual patients. Neurological involvement of the peripheral nervous system is common, such as trigeminal neuropathy, peripheral neuropathy, etc. Central nervous system involvement is rare.

### DeepSeek-R1-Distill-Qwen2.5-7B

Based on the clinical presentation provided, the most likely rare disease is **Bigiecticulosis**. This condition is characterized by multifaceted symptoms affecting multiple systems, including respiratory, gastrointestinal, cardiovascular, and neurological aspects.

Here are five other possible rare diagnoses that may present with similar symptoms:

1. **Organically Low Episodes (OPLE) with Cheyne-Stokes Respiration**: Characterized by alterations in oxygen and carbon dioxide levels, heart failure, and Tessier’s triangles.
2. **Hyperemesis gravidarum**: Caused by uterine contractions during pregnancy leading to systemic hypotension and high blood pressure.
3. **Hyperplasia Cardiolungus**: An undifferentiated Sunni heart disease with valvular dysregulation, hypertension, and interstitial lung disease.
4. **Myasthenia Gravida**: Often combined with respiratory failure and presents with symptoms like respiratory failure, hypercapnia, tachycardia, and polyuria.
5. **HCM (Hypogenetic Cardiac Mass)**: Causing left-sided failure, high-output heart, interstitial lung disease, and hypotension.

These diagnoses are rare and require careful clinical evaluation due to their overlapping symptoms and presentations.

### 115. User

In addition to clinical symptoms similar to phenylketonuria, children 1-3 months after birth also show symptoms of dopamine deficiency, such as dyskinesia, hypotonia, lethargy, nystagmus, dysphagia, etc. Symptoms related to serotonin deficiency, such as unresponsiveness, depression, insomnia, etc. Norepinephrine deficiency, such as low muscle tone in the trunk, ptosis, cerebellar dysgenesis, etc., may be associated with dopamine deficiency. Symptoms associated with norepinephrine deficiency, such as hypotonia of the trunk, ptosis, and cerebellar hypoplasia. There may also be persistent convulsions, recurrent fever, delayed development of motor milestones, total paralysis, and severe impairment of intellectual development.

### DeepSeek-R1-Distill-Qwen2.5-7B

Based on the symptoms described—dyskinesia, hypotonia, lethargy, nystagmus, dysphagia, unresponsiveness, depression, insomnia, hypotonia of the trunk, ptosis, cerebellar dysgenesis, and other developmental delays—it is possible that the child could have one or more of the following rare diseases. Each condition is briefly explained below:

1. **Phenylketonuria (PKU)**: Though PKU is more commonly diagnosed in children with pmeasurements and_prevagen t现enylketonuria can present in infancy, it is a rare autosomal recessive disorder affecting metabolism. Early symptoms might include developmental delays, neurological deficits, and sensory issues, aligning with the described symptoms.
2. **Congenital Diabetic Neuropathy (CDN)**: This rare disorder can present early in life and involves defects in the autonomic nervous system, sensory nerves, and the dopaminergic system. Symptoms like dyskinesia, hypotonia, and developmental delays are consistent with CDN.
3. **Crigler-Najjar Syndrome (CNS)**: A rare metabolic disorder that can present in infancy with a variety of neurological and cardiovascular issues. Symptoms such as developmental delays, skewed elevated intracranial pressure (sEIH), and sensory abnormalities may be present.
4. **Ataxia-Telangiectasia Moderate to Severe (ATM)**: Although ATM is more commonly diagnosed in adulthood, it can present early in infancy with ataxia, developmental delays, and other sensorimotor issues. While less common in pregnancy, it is a rare autosomal recessive disorder that can affect early growth and development.
5. **Van der Hage-Wijs-van Houten Syndrome (VdHV-k)**: This rare neurodevelopmental disorder is typically autosomal recessive and can present early in life. It is characterized by specific neurological and motor difficulties, as well as ophthalmic abnormalities.
6. **Man Confusion Syndrome (MCS)**: Though not as well-known, MCS is a rare disorder characterized by developmental delay, ataxia, and focal brain lesions early in life. It can present with motor and sensory deficits that may overlap with the described symptoms.

These conditions are all rare and share some overlapping neurological symptoms. A thorough clinical evaluation by a pediatric neurologist or neurodevelopmental specialist would be necessary to confirm the diagnosis.

### 116. User

They often present with multisystem involvement and develop in stages. Cardiac rhabdomyosarcomas can appear in the foetal/neonatal period, most of which resolve spontaneously; epileptic and cutaneous symptoms can appear in the first few months of life; renal angiomyolipomas and neoplastic lesions elsewhere in the body develop with age; and adult women are prone to pulmonary lymphangioleiomyomatosis of the lungs. Brain lesions include mainly cerebral cortical nodules, subventricular giant cell astrocytomas, and subventricular nodules. Skin lesions include leukoplakia visible after birth, progressive facial angiofibromas, shark leather spots, perinodal fibromas, and forehead plaques; renal vascular smooth muscle lipomas are present in up to 90% of patients; retinal involvement is present in about 50% of patients but rarely affects visual acuity; and LAM in the lungs occurs in about 35% of females; other areas, such as the liver, pancreas, gastrointestinal tract, and skeleton, may be involved, although usually with milder symptoms. Others, such as the liver, pancreas, digestive tract, and bones, can also be involved, but symptoms are usually mild.

### DeepSeek-R1-Distill-Qwen2.5-7B

Based on the clinical manifestations provided, the most likely rare disease to be diagnosed is **sarcomatosis**, a group of rare cancers, particularly muscle and connective tissue sarcomas. These cancers can cause multisystem involvement and are often associated with diverse skin and organ involvement.

In addition to sarcomatosis, up to five possible other rare diagnostic entities that might present with these clinical features include:

1. **GSTRINGűGEBENKötőurán (“Presenting as a giant cell tumor of unknown location” or bullous tumor):** This is a rare myelopubic sarcoma that can present as bullous or cavity wall-like lesions in various locations.
2. **Barton庆氏体:** Sarcomas can also present with soft tissue tumors, such as Barton bodies, which are typically seen in muscle.
3. **Miliary lymphangiomatosis:** This condition, less common than lymphangioleiomyomatosis, involves slow-growing lymphatic cystic fibrosis-like lesions.
4. **Neuronal tumors:** Gliomas can cause fulness of the chest, muscle weakness, and involve multiple systems.
5. **Melanocytic nevus:** Although rarer, occasional skin tumors, particularly melanocytic nevus, can present with mild skin symptoms in some sarcomatosis cases.

These additional possibilities reflect the diverse presentation and involvement of rare sarcomatosis-like conditions.

### 117. User

Most children with the undetected or untreated chronic form die before the age of 10 years, usually as a result of liver failure, neurological involvement or hepatocellular carcinoma.

The acute form has a rapid onset and progression, with acute liver failure as the main manifestation, and clinical signs include hepatosplenomegaly, jaundice, vomiting, abdominal distension, anorexia, lethargy, anaemia, bleeding tendencies and growth retardation. The child may have the characteristic odour of ‘boiled cabbage’ or ‘rotten mushrooms’. Insufficient hepatic synthesis of coagulation factors, marked prolongation of PT and APTT, and decreased levels of coagulation factors II, VII, IX, Ⅺ and Ⅻ, which are difficult to correct with vitamin K supplementation. Hepatic failure may be followed by ascites, jaundice and gastrointestinal bleeding, and children occasionally develop persistent hypoglycaemia. Untreated children often die of liver failure within weeks or months of onset of illness.

### DeepSeek-R1-Distill-Qwen2.5-7B

Based on the symptoms provided, the condition appears to present with acute onset, acute liver failure, hepatosplenomegaly, jaundice, and severe coagulation issues. While the symptoms are suggestive of conditions such as primary biliary cholangitis or acute toxic cholangitis, given the context of a rare disease, the most plausible possibilities are:

1. **Propagating Hemodermia**: A rare condition involving liver inflammation, hepatosplenomegaly, jaundice, and significant bleeding.
2. **Acute Toxic Cholangitis**: Characterized by acute liver failure, jaundice, and potentially severe bleeding due to hepatosplenomegaly.
3. **Primary Biliary Cholangitis**: In rare severe cases, presenting acutely with liver failure and coagulation issues.
4. **Dubin-Johnson Syndrome**: A rare multifactorial disorder affecting the liver, characterized by hepatosplenomegaly, jaundice, and bleeding.
5. **Orthoch典雅↶ic Shell Disease**: A rare condition causing orthochronic thrombocytopenia and hemodermia, consistent with prolonged coagulation factors.

These conditions represent the most plausible rare diagnoses based on the symptoms described.

### 118. User

The clinical presentation is markedly heterogeneous, and there are three types based on age of onset and clinical presentation.

**The cardiomyopathic type,** which is the most common type of severe early-onset cardiac and multi-organ failure, develops mainly in newborns and early infants. It has a high mortality rate and is associated with hypoglycaemia, Reye’s syndrome, sudden neonatal death, hypertrophic and dilated cardiomyopathy, pericardial effusion, arrhythmias, myasthenia, hepatomegaly, and intermittent hypoglycaemia, with elevated levels of creatine kinase; myocardial hypertrophy and arrhythmias can be fatal.

**2. hepatic type ,** less severe, hepatic and hypohidrotic hypoglycaemic type with onset in late infancy or childhood, presenting with hepatomegaly and liver function abnormalities, decreased fasting tolerance and acute hypohidrotic hypoglycaemia without myocardial damage and cardiac hypertrophy, but can be life-threatening without timely diagnosis and treatment.

**3. Myopathic type** , which develops mainly in adolescence to adulthood, is late-onset, mildly symptomatic, and is usually not accompanied by myocardial disease and hypoglycaemia. The main manifestations are rhabdomyolysis and myoglobinuria after exercise, infection or starvation, and even renal failure, which may be accompanied by muscle weakness, painful muscle spasms or myalgia.

### DeepSeek-R1-Distill-Qwen2.5-7B

Based on the clinical presentation provided, the most likely diagnosis is likely **cardiomyopathic rare disease**, given the severity, early onset, and multi-organ failure it presents with a high mortality rate. Below are five other possible rare disease diagnoses based on similar presentations:

### 1. Atrial Hypertrophic Pseudocubitus

- **Condition**: Lethargy, arrhythmias, Telecomycin toxicity.
- **Type of Onset and Presentation**: Early-onset, with emergence, as described in the physiology cases.

### 2. Acute Renal Failure

- **Condition**: Acute bilateral glomerular票价，Severe anemia, Hypertension.
- **Type of Onset and Presentation**: Early-onset, with Emergent treatment, as described in the physiology cases.

### 3. Osteopetrosis

- **Condition**: keysome Scaling, Osteopenia, Fractures。
- **Type of Onset andPresentation**: Early-onset Secondary hyperparathyroidism, Emergent endocrinology, as described in the endocrinology cases.

### 4. Huntington’s Disease

- **Condition**:.stroke/Lost Vision, Movement disorders, Muscle weakening。
- **Type of Onset andPresentation**: Early-onset Movement disorders, Subjective memory loss, as per neurology cases.

### 5. Phantom Pain

- **Condition**:朗诵, Severe pain without obvious cause。
- **Type of Onset andPresentation**: Late-onset, Subjective pain, as per neurology cases.

### 6. Combination of Rare Heart, Liver, and Kidney Diseases

- **Condition**:Combination of conditions, E.g., Hypertrophic cardiomyopathy,Choledochal cyst, and Acute renal failure.
- **Type of Onset andPresentation**: Varied onset, Emergent treatment or biopsy, as described in the cardiology, gastroenterology, and nephrology cases.

### 7. Imitative Syndromes with Specific Onset andPresentation

- **Condition**:Combination of rare syndromes, E.g., Co-sale syndrome and Acute respiratory failure.
- **Type of Onset andPresentation**:Varied onset, Emergent treatment or biopsy, as described in pediatrics and critical care.

### 119. User

**1. specific facial features,** periorbital subcutaneous tissue fullness, widened orbital distance, stellate irises, long midriffs, thick lips, wide mouths, and small jaws.

**2. cardiovascular disease,** elastin arteriopathy is present in 75% ~80% of patients and can affect any artery. Peripheral pulmonary stenosis (37% ~61%) is more common in infancy but improves with time. Supravalvular aortic stenosis is more common (35% ~65%) and worsens over time by age 5. Arterial stenosis leads to increased left heart resistance, cardiac hypertrophy, and heart failure. Other anomalies include renal artery stenosis, thoracic aortic stenosis, abdominal aortic stenosis, mitral valve prolapse and aortic valve insufficiency, mesenteric artery stenosis, and intracranial vascular anomalies. Hypertension can occur at any age and may be related in part to renal artery stenosis.

**3. Cognitive ability,** 75% of patients have mental retardation, which is usually mild. Cognitive impairment is characteristically manifested by relatively good short-term memory and verbal expression, but extreme weakness in visuospatial structural cognition. No gender differences in IQ have been reported, and children’s IQ is stable over time.

**4. personality, behavioural, and psychological abnormalities,** including excessive friendliness, excessive empathy, attention deficit, anxiety, and fear.

**5. sleep abnormalities,** with a 65% prevalence of sleep problems, including increased sleep latency and decreased sleep efficiency.

**6. eyes,** tear duct obstruction, hyperopia (67%), and strabismus (50%) are common. Cataracts have also been reported in adult patients.

**7. Ear, Nose, and Throat (ENT),** 50 per cent of patients have chronic otitis media, 90 per cent have increased sensitivity to sound, and 63 per cent of children and 92 per cent of adults have mild to moderate hearing impairment, mostly progressive sensorineural hearing loss. Most patients are hoarse or have a low voice.

**8. dental** , which may include baby teeth, enamel hypoplasia, and a misaligned bite.

**9. digestive system,** Chronic abdominal pain is a common symptom, and possible etiologies include gastroesophageal reflux, hiatal hernia, peptic ulcer, cholelithiasis, diverticulitis, ischaemic bowel disease, chronic constipation and anxiety somatisation. Hypercalcaemia may also lead to irritability, vomiting, constipation and muscle spasms.

**10. urinary system,** urinary frequency and enuresis are common in paediatric patients.

**11. neurological, muscular,** with low muscle tone and joint laxity in young children, leading to compensatory postural abnormalities to maintain body balance. Older children and adults have typical hypertonia and active deep tendon reflexes, resulting in a stiff and awkward gait, impaired fine motor function, and difficulty using tools and writing in patients of all ages. Adult patients will have cerebellar ataxia and tremor.

**12. Growth and development** .70% of infants have poor weight gain.

**13. idiopathic hypercalcaemia** . It occurs in infancy and childhood in 15-50% of patients and the most common symptoms are irritability, vomiting and constipation.

**14. endocrine.** Hypothyroidism (10 per cent), early puberty (50 per cent). Impaired glucose tolerance occurs in 26% of adolescent patients.

### DeepSeek-R1-Distill-Qwen2.5-7B

Based on the clinical manifestations provided, the most likely rare disease is **Kerrichashi syndrome**, an autosomal recessive disorder characterized by restrictive cardiomyopathy, craniofacial abnormalities, growth issues, and hypercalcemia. This syndrome fits the phenotype described, including the craniofacial features, cardiovascular issues, and growth problems.

**Additional possible diagnoses:**

1. **De Gebelein’s syndrome** - A rare heart and kidney disorder, though less directly fitting the broader phenotype.
2. **Gambian 苏ICD** - Mitochondrial depletion syndrome affecting heart and growth, though more specialized.
3. **Ehlers-Danlos syndrome** - Connective tissue disorder causing skin andConnective tissue issues, though less comprehensive.
4. **Bianchini syndrome** - Autoclonal dehd Daniel’s rarer condition affecting bone, heart, and endocrine system.
5. **Alagille syndrome** - A heteroplasmic disorder with growth, craniofacial, and cardiovascular presentations.

These conditions provide a broader spectrum of rare disorders that might coexist or present alongside those listed.

### 120. User

**1. Haemorrhagic tendency** is a common manifestation of the disease, especially in blood-stained stools, and most children develop the disease in the neonatal period. The next manifestations are petechiae, ecchymosis, haemoptysis and haematuria, and some severe cases may have life-threatening gastrointestinal haemorrhage or intracranial haemorrhage. Bleeding in patients is caused by thrombocytopenia with reduction in platelet volume, but only about 25% of cases have the typical triad of symptoms.

**2. Infection.** Children are susceptible to a variety of infections, including herpes simplex virus, cytomegalovirus, Streptococcus pneumoniae, and fungi. The patient’s immunodeficiency worsens with age.

**3. Eczema.**  Eczema may be present in approximately 80% of children with WAS and varies widely in extent and severity. There may be typically severe, refractory eczema, or very mild or even no eczema manifestations.

**4. Autoimmune diseases.**  The most common is autoimmune haemolytic anaemia. Others include vasculitis, arthritis, renal disease, inflammatory bowel disease, neutropenia and autoimmune thrombocytopenic purpura.

**5. malignant tumours .** Children have a significantly higher risk of malignant tumours of the lymphatic system, especially lymphomas; non-Hodgkin’s lymphoma, lymphoblastic leukaemia, myelodysplastic syndromes, and spermatogonia have been reported.

**6. other .** Children with this disease may have completely normal platelet levels with persistent or recurrent neutropenia.

### DeepSeek-R1-Distill-Qwen2.5-7B

Based on the clinical manifestations provided, the most likely rare disease to be diagnosed is **autismic hemolytic anemia**, which is a common manifestation of autoimmune diseases such as Segmental Autoimmune Hemolytic Anemia (SAHA) or Segmental Autoimmune Thrombotic Anemia (SATA). These autoimmune conditions can present with recurrent infections, anemia, and bleeding due to autoimmune haemolytic anemia.

Other possible rare diseases that could present with the described clinical manifestations include:

1. **Endemic fevers/fever of unknown origin (EFO)**: A rare and serious condition where a child has a fever that does not respond to standard treatments. It can be associated with PbO2 or PbO2P, which are antibodies that neutralize toxins in the body.
2. **Lymphocyte-toxin-acetylcholinesterase (LYstocks)**: A lysosomal storage disorder characterized by severe infections, including respiratory infections and gastrointestinal tract inflammation, often accompanied by blood loss.
3. **B-cell syndrome-like behaviour in cancer (BLS)</sup: Exclusive of cancer, a rare, aggressive condition presenting with ineffective.floor> );[BR]4.** B-cell helper virus (BCHV)-related disease**: A rare and aggressive infection in children, which leads to chronic diarrhea, gastrointestinal bleeding, and severe anemia.
4. **Neonatal herpes**: A rare but severe condition that can cause petechiae, ecchymosis, and gastrointestinal bleeding in young children.
5. **Systemic autoimmune thyroid disease**: A rare autoimmune disorder that can cause anemia, hypothyroidism, and autoimmune blood disorders, including autoimmune haemolytic anemia.

Each of these conditions can present with a combination of bleeding, infections, and immunodeficiency, making them plausible rare diseases to consider in the clinical scenario described. Further diagnostic workup, including blood tests, imaging, and genetic testing, would be necessary to confirm the specific cause.

### 121. User

The most common clinical manifestations are fulminant infectious mononucleosis, anomalous immunoglobulinaemia and B-cell lymphoma following EBV infection. Aberimmunoglobulinaemia and B-cell lymphoma can occur in patients without EBV infection. Aberimmunoglobulinaemia usually presents with increased levels of IgA and IgM and decreased levels of IgG1 and IgG3. Lymphomas are predominantly non-Hodgkin B lymphomas. Other clinical manifestations include lymphocytic vasculitis, aplastic anaemia and lymphomatoid granulomatosis. In some patients, the clinical picture is mild, with only abnormal immunoglobulinaemia or mild EBV infection, possibly due to somatic gene repair.

In some patients, the predominant clinical manifestation is a haemophagocytic syndrome, with splenomegaly, which does not necessarily require EBV infection to be induced. Some patients may present with intestinal inflammation such as Crohn’s disease or colitis, but rarely develop lymphoma.

### DeepSeek-R1-Distill-Qwen2.5-7B

The rare disease most likely to be diagnosed based on the described symptoms is a **B-cell non-Hodgkin lymphoma** associated with EBV-induced immunoglobulinaemia. The presence of EBV activation, causing fulminant mononucleosis-like symptoms, and the coexistence of immunoglobulinaemia with B-lymphoma suggest this diagnosis. The clinical features observed, such as fulminant infectious mononucleosis and anomalous immunoglobulinaemia, are indicative of EBV reactivation leading to both mononucleosis-like symptoms and B-cell infiltration.

### Five Other Possible Diagnoses:

1. **EBV-associated non-Hodgkin lymphoma**: A subclass where EBV reactivation leads to non-Hodgkin lymphoma presentation.
2. **EBV reactivation varicella-zoster syndrome (EVRZS)**: A condition where EBV reactivation causes symptoms of varicella and zoster along with mononucleosis-like features.
3. **EBV-induced IgGapl Cascade Syndrome**: A rare disorder where EBV reactivation causes a cascade of immunoglobulinaemia and lymphomata.
4. **Primary B-cell neoplasms**: Such as mantle cell lymphoma, plasma cell emphasies, or activated B-cell lymphoma.
5. **Lymphocytemphosis autoimmune reaction (LAIR)**: An autoimmune B-cell disorder causing B-lymphoma and immunoglobulinaemia.

These diagnoses highlight the complexity of rare diseases and the need for further investigation to determine the specific underlying cause in each case.
