# Supplementary material for: Performance of ChatGPT-4o and Four Open-Source Large Language Models in Generating Diagnoses Based on China’s Rare Disease Catalog: Comparative Study
Source: J Med Internet Res. 2025 Jun 18;27:e69929. doi: 10.2196/69929 (PMC12192912; doi:10.2196/69929)
Supplement: Multimedia Appendix 1 [file jmir-v27-e69929-s001.zip › numbered_llama3.1_8b_cn.docx]

# numbered_llama3.1_8b_cn

**1.user** 2024-11-30T22:44:02Z:

该疾病可于儿童期早期起病。典型的临床表现包括肾脏、眼部改变及听力受损。肾脏表现为血尿、蛋白尿和肾功能进行性恶化，其中血尿常为持续性镜下血尿，可在运动后或发热时出现肉眼血尿，常见于青少年型。听力改变为感音神经性听力损失， 并随病程逐渐加重。眼部异常表现为近视、青年环和白内障，但缺乏特异性。三种具有诊断意义的眼部改变包括：前圆锥形晶状体、后多形性角膜萎缩和视网膜斑（视网膜中心凹区域周围的白色或黄色颗粒）。我国男性患者听力受损发生率高于国外报道（68％比 55％），女性则低于国外报道（7％比 45％），眼异常发生率与国外报道相近。少数患者还可出现平滑肌瘤，可累及呼吸道、胃肠道及女性生殖道；偶有动脉瘤病变、面中部发育异常和精神发育迟滞的报道。

患者常有家族史。常染色体隐性遗传患者和 X 连锁遗传的男性患者病情进展较快，常于 16~35 岁进入终末期肾病；而常染色体显性遗传和 X 连锁遗传的女性患者多病程较为缓慢，肾功能衰竭出现较晚。在同一个患病家系中，所有男性成员发生肾功能衰竭的年龄相似，但不同家系中的男性成员发生肾功能衰竭的年龄可以不同。因此，可根据患病家系发生肾功能衰竭的年龄分为：青少年型（肾衰竭发生在青少年时期） 与成人型（肾衰竭发生在成年以后）。青少年型肾外表现更为突出，男孩通常在繁衍后代前即死亡，致病基因常为新发突变；而成人型家系中的患者较年长，少为新发突变。

**llama3.1_8b** 2024-11-30T22:44:12Z:

根据您提供的临床表现，这种罕见疾病最可能诊断出的是**阿尔伯特氏症（Alport syndrome）**。

阿尔伯特氏症是一种遗传性肾脏疾病，其典型临床表现包括：

1. 肾脏改变：血尿、蛋白尿和肾功能进行性恶化。
2. 听力受损：感音神经性听力损失，随病程逐渐加重。
3. 眼部异常：近视、青年环和白内障，以及特异性的前圆锥形晶状体、后多形性角膜萎缩和视网膜斑。

除此之外，还有五种可能的诊断：

1. **轻链型肾病（Light chain nephropathy）**：是一种因为免疫球蛋白异常轻链产生过多而导致的肾脏疾病。
2. **亨利-特伦伯格综合征（Henoch-Schönlein purpura, HSP）**：是一种常见的血管炎，表现为皮肤、关节和肾脏等多个系统受累。
3. **Goodpasture病症（Goodpasture disease）**：是一种罕见的自身免疫性疾病，表现为肾脏和神经肌肉受累。
4. **肾绒毛膜癌综合征（Focal segmental glomerulosclerosis, FSGS）**：是一种常见的肾脏疾病，表现为肾功能进行性恶化。
5. **X-linked polycystic kidney disease (PKD)**：是一种遗传性的肾脏疾病，其特征是肾脏内形成许多囊肿。

请注意，这些可能的诊断都需要根据具体临床表现和实验室检查结果进行综合评估。

**2.user** 2024-11-30T22:44:36Z:

患者在新生儿期常无异常表现。首发症状是 6 月龄左右出现发育迟滞表现，但典型临床表现多在 1 岁后出现，临床确定诊断往往需要数年时间。按照临床表现出现频率不同分为：均出现的表现（100％患者）、经常性表现（80％以上患者）、相关性表现（20％ ~80％ 患者）3 类。

**1. 均出现的表现** 正常孕产史和出生头围；无出生缺陷和生化指标异常；颅脑 MRI/CT 除轻微皮质萎缩、髓鞘发育不良外，多无结构异常；运动里程碑落后而无倒退；6~12 个月出现严重发育迟缓；语言障碍显示为无或极少量词汇，重复性语言和非语言交往能力强于表达性语言能力；运动或平衡障碍常表现为共济失调以及四肢震颤；异常的行为特征表现为频繁大笑或微笑、明显的兴奋动作或快乐举止、常伴拍手或多动。

**2. 经常性表现** 头围增长落后，随访至 2 岁仍表现为小头畸形；3 岁前常出现癫痫；异常脑电图：特征性高波幅棘 - 慢波。

**3. 相关性表现** 枕部扁平；喜吐舌；吸吮或吞咽障碍；婴儿期喂养困难；肌张力低；巨大下颌；牙间隙宽；频繁流涎；过度咀嚼动作；斜视；皮肤色素减退，与家人相比头发和眼睛颜色浅（仅见于缺失型）；下肢腱反射亢进；上举或弯曲上肢，尤其是行走时；步基宽；对热敏感性高；异常睡眠 - 觉醒周期、睡眠少；迷恋水、纸、塑料；进食相关行为异常；肥胖（多见于年长、非缺失型患者）；脊柱侧凸；便秘。

**llama3.1_8b** 2024-11-30T22:44:42Z:

根据您提供的信息，这个病症最有可能是伯克特-谢德尔综合征（BBS）。这个罕见疾病影响到生殖系统和中枢神经系统，表现为发育迟滞、运动困难、语言障碍等。

其他五种可能的诊断包括：

1. 甲状腺功能减退症（Hypothyroidism）：这种疾病会导致新生儿期或婴儿期的发育迟缓，后来发展为运动和认知功能的障碍。
2. 精神发育迟滞伴有运动问题的唐氏综合征（Down syndrome）：虽然通常与智力低下有关，但也会出现运动困难、语言障碍等特点。
3. 遗传性肌营养不良症（Friedreich’s ataxia）：这种疾病影响到神经系统，导致运动功能的衰退和平衡问题。
4. 脑瘫（Cerebral palsy）：这是一种由于脑部发育障碍引起的运动功能损伤，其特点包括肌肉强直、震颤、平衡困难等。
5. 克汀综合征（Klinefelter syndrome）：这种疾病影响到性腺系统和神经系统，表现为智力低下、运动功能障碍以及语言问题。

**3.user** 2024-11-30T22:45:20Z:

1. 骨骼异常表现：疾病严重程度和相关并发症的类型存在差异。所有 病例均存在胸腔狭窄，胸廓可呈钟形或长而窄，呼吸窘迫通常由小而僵硬的胸腔引起。根据胸廓类型和潜在的肺发育不良的程度，呼吸功能不全严重程度不同，发病年龄不同。呼吸功能不全严重程度与 该疾病诊断年龄呈负相关。存在严重的限制性胸廓和钟形胸廓的婴儿更多见急性呼吸系统疾病，在新生儿早期可出现严重和致命的呼吸衰竭。胸腔长而窄的婴儿，更多表现为轻度呼吸系统受累，最终可逐渐发展为慢性限制性肺病伴反复呼吸道感染。肋骨短小、畸形引起肋间肌无力、呼吸受限，进而出现呼吸衰竭。多数患儿于 2 岁内死亡，其中约有半数患儿于生后 6 个月内死亡。患者其他骨骼异常包括肋骨短、四肢短小、骨盆形状异常、短指、多指（趾） 畸形等。约有 60％患者伴有近端颈椎狭窄。
2. 其他脏器受累表现： 约 40％的患者会出现肾脏病变。患者肾脏病变的最早表现是尿液浓缩功能受损，其他表现包括多尿、蛋白尿、血尿素氮和肌酐升高以及高血压。肾功能衰竭是 该患者在 3~10 岁死亡的主要原因。约 30％的病例出现肝脏受累，表现为肝肿大、转氨酶升高、胆汁淤积、肝硬化和门静脉高压。该疾病偶与眼睛发育异常相关，包括视神经发育不全、视网膜营养不良、视网膜色素异常、眼球震颤、近视和进行性视力障碍。亦有少数报道提示这些患者也可出现神经系统异常如发育落后、肌张力低下、中枢神经系统MRI 异常等。

**llama3.1_8b** 2024-11-30T22:45:28Z:

根据您的描述，我认为最可能的诊断是：

1. **胸廓管状狭窄症（Jeune Syndrome）**：这是一种罕见的遗传性疾病，表现为胸廓管状畸形、肺发育不良和其他多系统受累。

其他五种可能的诊断是：

1. **马尔曼-史塔福综合征（Marfan Syndrome）**：这是一种影响身體各个系统的遗传性疾病，表现为长瘦体型、长腿、大头等。
2. **埃德沃兹综合征（Edwards Syndrome）**：这是一种染色体异常引起的遗传性疾病，表现为脑发育不全、心脏问题和肺部受累等。
3. **斯托帕-比洛综合征（Stickler Syndrome）**：这是一种罕见的遗传性疾病，影响面部、关节和眼部等系统，且常伴有听力丧失和视力障碍。
4. **魏尔-布鲁纳综合征（Weil-Brodel syndrome）**：这是一种罕见的遗传性疾病，表现为胸廓管状畸形、肺发育不良和其他多系统受累，与胸廓管状狭窄症相似，但有所不同。
5. **维尔-布罗德综合征（Vell-Brodel syndrome）**：这是一种罕见的遗传性疾病，影响面部、关节和眼部等系统，且常伴有听力丧失和视力障碍。

请注意，这些诊断仅基于您提供的信息，实际上可能需要进行更详细的检查和评估才能确切地确定。

**4.user** 2024-11-30T22:45:51Z:

主要症状包括精神行为异常、认知障碍、记忆力下降、癫痫发作、言语障碍、运动障碍、不自主运动，意识水平下降、自主神经功能障碍等。其他伴随症状包括睡眠障碍、CNS 局灶性损害、脑干、小脑症状以及周围神经和神经肌肉接头受累等。

临床可分为 3 种主要类型：①抗 NMDAR 脑炎，是 最主要类型，其特征性临床表现符合弥漫性脑炎。②边缘性脑炎，以精神行为异常、颞叶癫痫和近记忆力障碍为主要症状，常见的边缘性脑炎有抗 LGI1 抗体、抗 GABABR 抗体与抗 AMPAR 抗体相关的脑炎。③其他类型，包括莫旺综合征、抗 DPPX 抗体相关脑炎、抗 IgLON5 抗体相关脑病、自身免疫性小脑性共济失调等。

**llama3.1_8b** 2024-11-30T22:45:55Z:

根据提供的信息，我认为最有可能的诊断是：

1. 弥漫性脑炎（Encephalitis）特别是由于抗NMDAR抗体引起的脑炎。 其他五种可能的诊断包括：
2. 边缘性脑炎（Limbic Encephalitis）
3. 莫旺综合征（Morvan Syndrome）
4. 自身免疫性小脑性共济失调（Autoimmune Cerebellar Ataxia）
5. 抗DPPX抗体相关脑炎（Anti-DPPX Antibody-Related Encephalitis）
6. 抗IgLON5抗体相关脑病（Anti-IgLON5 Antibody-Related Encephalopathy）

请注意，这些诊断都是基于提供的临床表现和症状，并且需要进一步的检查和确认来确定最终的诊断。

**5.user** 2024-11-30T22:46:13Z:

疾病临床表现复杂多样，缺乏特异性。多累及神经、皮肤、呼吸、消化和免疫等多系统，单从临床表现难以确诊。可以分为完全性及部分性两种类型。

1. 完全性： 又可分为早发型及晚发型两种。①早发型患者多在新生儿或婴儿早期起病，通常症状出现在生后早期起病，通常症状出现在生后 1 周至 10 岁期间，平均发病年龄为 3 个月，最常见的表现为神经系统症状，如肌张力低下、癫痫、发育迟缓、感音性耳聋、共济失调、进行性意识障碍等，大约一半的患者出现共济失调，发育迟缓，结膜炎和视觉问题，包括视神经萎缩。常合并代谢性酸中毒、低血糖、酮症等代谢紊乱，皮肤表现包括各种形态的难治性皮疹如湿疹、全身性红斑、尿布疹，患者也可出现过度通气、喉部喘鸣和呼吸暂停等呼吸系统症状，此外还有消化系统的症状如喂养困难、呕吐、腹泻等。感染、发热、疲劳、高蛋白饮食等可诱发急性发作。若治疗不及时，常可导致不可逆损害。②晚发型患者常于青春期发病，之前可无任何症状，临床表型较早发型不同，通常表现为周围神经病，肌无力、痉挛性轻瘫，皮疹和眼部问题，如视力丧失和视力下降等，如不能及时治疗，常导致不可逆性损害。
2. 部分性 ： 可在各个年龄段发病，甚至不发病。患儿整体症状及体征相对较轻，仅在感染、饥饿等应急状态下起病。患者可能有肌张力减退、皮疹、脱发，可出现急性神经系统症状癫痫、意识障碍等。

**llama3.1_8b** 2024-11-30T22:46:18Z:

根据您的描述，我认为最有可能的诊断是克拉格-尼尔森综合征（Krabbe disease）。这是一种遗传性的神经退行性疾病，影响新生儿或婴幼儿，表现为肌张力低下、癫痫、发育迟缓、共济失调等神经系统症状，以及皮肤和呼吸系统的并发症。

五种可能的诊断：

1. 克拉格-尼尔森综合征（Krabbe disease）
2. 遗传性肌无力病（Friedreich’s ataxia）
3. 迁延性白质失养症（Leukodystrophy）
4. 小细胞脑病变综合征（Canavan disease）
5. 皮肤和神经系统的遗传性疾病，如多动症（Tay-Sachs syndrome）等。

请注意，这些诊断需要通过进一步的检查和确认，例如实验室检测、影像学检查等，以确定最准确的诊断。

**6.user** 2024-11-30T22:46:44Z:

高发年龄段为婴儿期，表现为以失盐为主的肾上腺皮质功能不全，常见症状包括高血钾、低血钠与脱水、代谢性酸中毒、低血糖、呕吐、休克、皮肤色素沉着等。多数患者在青春期表现为不发育或发育不完全，或生育能力受损，少精和无精症等。少数患者可以表现为儿童早期高雄激素血症、单纯性盐皮质激素缺乏，青春期可出现假性性早熟或自发性青春发育等。轻者可成年期起病。

患者皮肤有色素沉着，尤其是在暴露处、摩擦处、瘢痕及乳晕、腋窝、阴囊处。

患者对感染、外伤等各种应激的抵抗力有不同程度的减弱。在发生应激情况时，可出现肾上腺危象，表现为恶心、呕吐、腹痛或腹泻、严重脱水、血压降低、心率快、脉细弱、精神失常，常有高热、低血糖症、低钠血症，甚至危及生命。

**llama3.1_8b** 2024-11-30T22:46:50Z:

基于您提供的信息，我认为可能的诊断是 Congenital Adrenal Hyperplasia（CAH）。这是一种罕见疾病，它影响肾上腺皮质的功能，从而导致激素水平的异常变化。

其他五种可能的诊断包括：

1. **Cushing综合征**：由长期接触糖皮质激素引起的一系列症状，包括皮肤色素沉着、体重增加、血压升高等。
2. **Addison病**：一种因肾上腺皮质功能不全导致的疾病，表现为皮肤色素减少、低血糖、脱水等症状。
3. **肾上腺增生症**：一种由肾上腺皮质功能亢进引起的一系列症状，包括高血压、体重增加、皮肤色素沉着等。
4. **Pernicious anemia**：是一种因为缺乏维生素B12导致的贫血和神经系统问题的疾病。
5. **Turner综合征**：一种因性染色体异常（45,X）导致的疾病，表现为生长迟缓、性早熟等症状。

**7.user** 2024-11-30T22:47:07Z:

从出生到成年早期均可发病。其中新生儿起病最为常见，常表现为巨大儿、摄食不良、不耐受饥饿和持续性低血糖等。低血糖发作时可表现为嗜睡、肌张力低下等，也可表现为神经系统后遗症甚至死亡，如呼吸暂停、癫痫发作和昏迷等。在迟发性类型中，患者常表现为低血糖反应，包括面色苍白、出汗和心悸等症状。

**llama3.1_8b** 2024-11-30T22:47:13Z:

根据你提供的临床表现，这种疾病最可能是糖尿病病毒感染（Cytomegalovirus，CMV）所致的巨大儿综合征，尤其是在新生儿中较为常见。

其他五种可能的诊断：

1. **克汀症**：一种由于胸腺功能不全导致的免疫缺陷疾病，表现为巨大儿、摄食不良和低血糖等。
2. **肾上腺素能性肿瘤**：一种罕见的肿瘤，可以引起肾上腺素过量分泌，导致低血糖、嗜睡和肌张力低下等症状。
3. **甲状腺功能亢进**：一种内分泌疾病，可以引起代谢率增高，导致摄食不良、出汗和心悸等症状。
4. **多囊卵巢综合征**：一种内分泌疾病，可以引起体重增加、摄食不良和代谢率增高等症状。
5. **维生素D缺乏性佝偻病**：一种营养相关的疾病，可以导致低血糖、肌张力低下和骨骼发育迟缓等症状。

**8.user** 2024-11-30T22:47:36Z:

1. Ⅰ型 ，腹部膨隆、生长迟缓、低血糖抽搐、反复鼻出血、腹泻和呕吐为儿童患者主要就诊原因，极少数以肉眼血尿、便血、反复骨折、贫血或痛风等为首发表现。从未确诊及治疗的成年患者可以多发肝腺瘤、慢性肾衰、严重痛风伴多发痛风石、骨质疏松等就诊。其他少见表现包括肺动脉高压、糖尿病、脑血管病和肝腺瘤癌变等。查体可见身材矮小和肝脏明显增大。
2. Ⅰb 型 ， 患者除以上表现外，还可有反复感染伴中性粒细胞减少、口腔溃疡、炎症性肠病、肛周溃疡、关节炎和脾大等。
3. Ⅱ型婴儿型， 根据预后分为经典婴儿型和非经典婴儿型。经典婴儿型大部分在生后第 1 个月即出现全身性肌肉无力，运动发育迟缓，胸部 X 线片示心脏增大，心电图见高 QRS 波和短 PR 间期，心脏彩超见肥厚型心肌病改变，血肌酸激酶不同程度升高等， 多于生后 1 年之内死于左心衰竭或肺部感染后心肺功能衰竭。非经典婴儿型在生后 1 年内出现肌肉无力，运动发育落后，多于幼儿期死于呼吸衰竭。
4. Ⅱ型晚发型 ， 患者于 1 岁后起病，可晚至 60 岁发病。多表现为慢性进行性近端肌力下降和呼吸功能不全，心脏受累少见，呼吸功能衰竭是主要的致死原因。临床表现为易疲劳、仰卧起坐、上下楼梯、蹲起困难和行走无力，少数以突发呼吸衰竭起病。

**llama3.1_8b** 2024-11-30T22:47:43Z:

根据你的描述，我认为最可能的诊断是：

1. 巨球蛋白血症（Alpha-Galactosidase A deficiency）或称为 Hunter综合征。

巨球蛋白血症是一种X连锁遗传性疾病，影响约6人中的一人。这种疾病涉及体内一种名为α-Gal A的酶缺乏，这种酶对处理糖分子非常重要。如果这个酶缺乏，就会导致身体积累巨球蛋白，从而引起一系列临床表现，如：

- 生长迟缓
- 低血糖抽搐
- 反复鼻出血、腹泻和呕吐
- 肝脏增大
- 骨骼问题（如骨折或骨质疏松）
- 肾脏疾病
- 神经系统问题

巨球蛋白血症的诊断通常是基于临床表现、实验室检查和遗传学分析。治疗主要集中在控制肝脏负担和减少肌肉损伤。

请注意，以上提到的其他可能性可能与患者的具体情况有关，但并非总能准确诊断巨球蛋白血症。因此，最正确的诊断应基于详尽的临床评估、实验室检查和遗传学分析。

**9.user** 2024-11-30T22:48:13Z:

1. 神经 - 肌肉系统， 引起神经系统的异常，如惊厥、昏迷、共济失调、甚至精神症状；也可出现肌肉症状，包括腱反射亢进、震颤、手足搐搦、肌无力等。
2. 心血管系统， 引起心电图的改变，可见 PR 间期延长、QRS 波增宽等，严重者可引起室性心律失常。
3. 肾脏 ，可引起肾结石、肾钙质沉着、肾功能不全。
4. 骨关节系统， 可引起骨质疏松、骨软化。
5. 消化系统， 可引起肠梗阻表现。
6. 内分泌， 患者可见糖尿病或者糖耐量减低。
7. 眼部症状， 部分类型可合并眼部病变，包括眼球震颤、视野缺损、近视等。

该组疾病通常为慢性病程，其中新生儿期、婴儿期起病的类型往往病情重、预后差。部分患者在青春期就发展为慢性肾衰竭，部分患者若未能及时治疗，会导致患儿严重精神发育迟滞甚至死亡。

**llama3.1_8b** 2024-11-30T22:48:21Z:

根据您的描述，我认为最可能的诊断是**加里病**(Pseudohypoparathyroidism type 1a)。

此外，以下五种疾病也可能与这些症状和体征有关：

1. **维生素D依赖性氯化钙减少症**（Vitamin D-dependent hypophosphatemia）：这是一个罕见的遗传性疾病，特点是血液中磷酸盐水平降低，可能导致肾脏问题、骨骼异常和肌肉无力等。
2. **皮肤系统的铅中毒**（Lead intoxication）：长期接触铅会损害神经系统，并引起一系列身体症状，如头晕、恶心、周围神经病变等。
3. **内分泌系统的克汀病**（Hypopituitarism）：这种疾病是由于下丘脑或腺体的一部分功能受损，导致激素水平降低，引起一系列身体问题，如生长迟缓、性发育不全等。
4. **代谢系统的酮症酸中毒**（Diabetic ketoacidosis）：这是一个罕见的糖尿病并发症，会导致血液中的酮体水平升高，并引起一系列身体问题，如呼吸急促、恶心、头晕等。
5. **遗传系统的肌营养不良**（Muscular dystrophy）：这种疾病是由于肌肉细胞无法正常分泌蛋白质，从而导致肌肉萎缩和无力等症状。

这些都是罕见的疾病，可能与您的描述相吻合。然而，最终诊断将取决于进一步的检查和医学评估。

**10.user** 2024-11-30T22:48:46Z:

可分为 5 种类型，其中以经典型和中间型最常见。

**1. 经典型** 最常见，占 75％。患儿出生时正常，发病早、症状重并发展迅速。通常患儿出生 12 小时后，在耵聍中即出现枫糖浆气味；生后 12~24 小时在尿液和汗液中有特殊的枫糖味；2~3 天出现酮尿、易激惹和喂养困难；4~5 天开始出现严重的代谢紊乱和脑病症状，表现为哺乳困难、阵发性呕吐、拒食、嗜睡、呼吸暂停、惊厥发作、肌张力增高以及“击剑”样和“蹬车”样刻板动作等；生后 7~10 天可出现昏迷和中枢性呼吸衰竭，若不及时治疗， 大多数的患儿在出生后数天死于严重的代谢紊乱。

**2. 中间型** 在任何年龄阶段均可发病。患者症状较经典型轻，表现为生长缓慢、发育落后、喂养困难、易激惹、在耵聍中有枫糖浆气味，应激情况下可以出现严重的代谢紊乱和脑病。

**3. 间歇型** 呈间歇发作，早期生长发育均正常，多在感染和手术等应激情况下发作，表现为发作性共济失调和酮症酸中毒，极少数的严重者可引起死亡。间歇期支链氨基酸浓度正常，发作期与经典型类似有支链氨基酸浓度升高。

**llama3.1_8b** 2024-11-30T22:48:52Z:

根据您提供的信息，我认为最可能的诊断是 Maple Syrup Urine Disease（MSUD），也称为白糖尿病。这是一种遗传性疾病，导致体内没有足够的酶来分解某些氨基酸，从而引起严重的代谢紊乱。

其他五种可能的诊断：

1. **丙氯前臭症**：一种影响肌肉和神经的罕见疾病，表现为肌肉无力、麻木和瘫痪。
2. **戈尔德氏症候群**：一种遗传性疾病，导致体内钙水平升高，引起肌肉无力、骨折和其他问题。
3. **白细胞减少症**：一种影响白细胞数量的罕见疾病，可能导致感染和免疫功能不全。
4. **酮症酸中毒**：一种代谢紊乱，表现为体内酮体积聚，从而引起头痛、恶心和其他问题。
5. **帕金森病**：一种影响运动系统的神经退行性疾病，可能导致肌肉僵硬、抖动和其他运动功能障碍。

**11.user** 2024-11-30T22:49:06Z:

出生后至老年均可起病，可急性或隐袭起病。几乎任何系统或器官均可受累，以能量需求旺盛的器官，如脑、心、肾、眼、耳、肌肉、胃肠道等多见。患者可以表现为某种临床综合征，也可仅表现单个症状或者多种症状组合。

**llama3.1_8b** 2024-11-30T22:49:12Z:

根据你的描述，我认为你可能是在描述一种名为”病毒诱发综合征”(Viral Triggered Syndrome)的罕见疾病。这种疾病可能由各种不同的病毒引起，包括重复感染、潜伏病毒重新激活等。

下面是五种其他可能的诊断：

1. **自体免疫性疾病**：例如系统性红斑狼疮（SLE）、类风湿关节炎（RA）等，这些疾病会影响身体多个系统和器官，表现为各种不同的临床症状。
2. **遗传性肌肉病**：例如 Duchenne 综合征、Becker 综合征等，这些疾病会导致肌肉力量下降，从而引起运动功能障碍。
3. **神经系统疾病**：例如多发性硬化症（MS）、 Guillain-Barré 综合征等，这些疾病会影响神经系统，导致各种神经功能障碍。
4. **内分泌疾病**：例如 Grave 病、甲状腺危象等，这些疾病会影响身体的内分泌系统，从而引起多种不同的临床症状。
5. **免疫缺陷疾病**：例如 acquired 免疫缺陷综合征（AIDS）、再生障碍性贫血（Coombs 综合征）等，这些疾病会导致身体的免疫功能受损，从而增加感染风险和体内炎症反应。

**12.user** 2024-11-30T22:49:28Z:

好发年龄 5~50 岁，中位发病年龄 39 岁。女性患病率明显高于男性，女：男比例为(9~11) ：1。多急性 / 亚急性起病，临床表现包括 6 组核心症候群：①视神经炎；②急性脊髓炎；③极后区综合征；④急性脑干综合征；⑤症状性睡眠发作或急性间脑临床综合征伴典型的间脑 MRI 病灶；⑥症状性大脑综合征伴典型的脑部病变。 为高复发、高致残性疾病，90％以上的患者为复发性病程，多数患者遗留较为严重的神经功能残疾。

**llama3.1_8b** 2024-11-30T22:49:35Z:

根据你的描述，我认为可能诊断出的罕见病是多发症（ Guillain-Barré Syndrome, GBS）。以下是一些其他可能的诊断：

1. **脊髓炎**：急性脊髓炎 (Transverse Myelitis) 或脊髓脏瘤可能导致相似的临床表现，包括视神经炎、极后区综合征和症状性睡眠发作。
2. **脑部肿瘤或腫瘤**：某些类型的脑部肿瘤，如脑膜瘤或颅内甲状腺瘤，可以导致相似的临床表现，包括视神经炎、急性脊髓炎和症状性睡眠发作。
3. **多系统退行性疾病**：某些类型的多系统退行性疾病（如多系统萎缩症）可能导致神经功能的广泛衰竭，包括视神经炎、脊髓损伤和脑部功能障碍。
4. **帕金森综合征**：虽然主要特点是运动迟缓，但是某些类型的帕金森综合征也可能导致睡眠困难、视觉问题和认知功能下降等症状。
5. **克罗恩病**：一些研究表明，克罗恩病可能与神经系统症状有关，包括视神经炎、脊髓炎和脑部炎症。

**13user** 2024-11-30T22:50:01Z:

听力损伤。以往多认为该耳聋为听力筛查即可明确的先天性， 初生即外显，多累及双侧，对称的重度语前聋。近年来逐渐发现个别病例可表现为不对称或单侧听力受损，听力损失程度变异较大，不同病人的表现可从轻度至极重度不等；渐进性、出生后才发生的耳聋也并不少见。

**llama3.1_8b** 2024-11-30T22:50:09Z:

根据您提供的信息，我推测罕见病可能是遗传性的听力损伤或先天性听力障碍（Congenital hearing loss）。这是因为您的描述提到了“先天性”、“重度语前聋”，这些术语都与遗传和听觉系统的早期发育有关。

然而，遗传性听力损伤或先天性听力障碍是多种原因综合的，可以由多种不同的基因或染色体异常引起，而不是一个具体的疾病名称。因此，下面列出的几种可能的诊断更具体一些：

1. **Usher 综合征**（Usher syndrome）：是一种遗传性的听力损伤和视觉障碍综合征。
2. **遗传性耳聋**（Genetic hearing loss）：这是一个较为广泛的术语，指的是由遗传因素引起的听力损失，可以由于多种不同的基因或染色体异常导致。
3. **Pendred 综合征**：是一种罕见的遗传性疾病，主要表现为耳聋和甲状腺功能减退。
4. **遗传性脑瘫与听力损伤综合征**（Friedreich’s ataxia）：虽然主要影响运动功能，但也可能伴有听力损失或其他听觉问题。
5. **遗传性中枢性听觉障碍**（Genetic central auditory processing disorder）：这是一个较为少见的状况，涉及到听觉系统在处理和理解声音方面的问题。

请注意，这些诊断只是可能的选项，而不是具体的答案。罕见病的诊断需要根据个体情况、家族史、遗传分析以及其他相关检查来综合考虑。

**14.user** 2024-11-30T22:51:49Z:

运动迟缓、震颤、肌强直。治疗后更容易出现左旋多巴诱导的运动并发症。在非运动症状方面，容易发生情绪障碍(如抑郁、焦虑、易激惹等) 及行为障碍(如强迫性增加药量、冲动控制障碍、刻板行为等)。

**llama3.1_8b** 2024-11-30T22:51:59Z:

根据您提供的临床表现，这名患者最有可能患有的罕见病是帕金森病（Parkinson’s Disease，PD）。帕金森病是一种神经退行性疾病，主要影响运动功能，症状包括运动迟缓、震颤、肌强直等。左旋多巴（Levodopa）是一种常用药物来治疗帕金森病，但也可能诱发一些并发症。

以下是五种其他可能的诊断：

1. **亨廷顿病（Huntington’s Disease）**：这是一种遗传性疾病，主要影响运动、认知和情绪。患者可能表现出类似的运动迟缓、震颤等症状，以及严重的情绪障碍和行为问题。
2. **多系统阿尔茨海默病（Multi-System Atrophy, MSA）**：这是一种罕见的神经退行性疾病，影响运动、自主神经系统和认知功能。患者可能表现出类似的运动迟缓、震颤等症状，以及自主神经系统障碍。
3. **运动神经元病变（Motor Neuron Disease, MND）**：这是一种罕见的疾病，影响运动神经元，导致肌肉萎缩和无力。患者可能表现出类似的运动迟缓、震颤等症状，以及肌肉萎缩。
4. **创伤后脑损伤（Traumatic Brain Injury, TBI）**：这是一种因外伤导致的大脑损伤，可能影响运动、认知和情绪功能。患者可能表现出类似的运动迟缓、震颤等症状，以及严重的情绪障碍和行为问题。
5. **精神病性疾病（Psychotic Disorder）**：这是一种罕见的精神健康状况，影响认知、情绪和行为功能。患者可能表现出类似的运动迟缓、震颤等症状，以及严重的情绪障碍和行为问题。

需要注意的是，这些诊断都是临床上可能的，但不是唯一的解释。如果您有进一步的信息或细节，可能可以更准确地进行诊断。

**15.user** 2024-11-30T22:52:12Z:

**1. 多发神经病** ，往往表现为对称性的四肢感觉和(或) 运动性周围神经病，逐步由远端向近端进展。

**2. 器官肿大，** 包括肝大、脾大或淋巴结肿大；淋巴结活检常提示为 Castleman 病。

**3. 内分泌异常 ，**常见的包括性功能减退(如男性阳痿、乳房发育)、甲状腺功能减退、糖代谢异常(如糖尿病)、肾上腺功能不全。

**4. 皮肤改变，** 可表现为皮肤颜色加深、皮肤肾小球样血管瘤、白甲、多血质、多毛症和手足发绀等。

**5. 循环外水负荷增加，** 包括肢体水肿、腹水、胸腔积液、心包积液、视乳头水肿等。

**6. 硬化性骨病 ，**是重要临床表现。可表现为骨痛，亦可无临床症状。骨骼 CT 检查可以显著提高硬化性骨病的检出率，影像学上可以表现为单纯骨骼硬化、或硬化和溶骨混合病灶、或者单纯溶骨性改变。

**7. 红细胞增多和(或) 血小板增多**

**8. 肺动脉高压** ，患者可出现肺动脉高压，表现为活动耐量减低、低氧血症等。

**9. 脑梗死**

**llama3.1_8b** 2024-11-30T22:52:18Z:

根据您提供的临床表现，我推测可能诊断的是 Waldenström氏淋巴瘤（一种罕见的白细胞相关肿瘤）。

以下是其他五种可能的诊断：

1. **Castleman病**：这是一个罕见的疾病，表现为淋巴结肿大、组织内淋巴细胞增多和炎症反应。
2. **血管肉瘤**：这是一种罕见的血管壁恶性肿瘤，可能影响任何年龄段的人，表现为皮肤颜色加深、血管瘤、淋巴结肿大等。
3. **多发神经病**（如 Guillain-Barré综合征）：这是一个罕见的疾病，表现为周围神经系统症状，如感觉和运动功能障碍。
4. **皮肌炎**：这是一个罕见的自身免疫性疾病，表现为肌肉疼痛、肿胀和炎症反应，以及可能的皮肤和内脏器官受累。
5. **血小板相关综合征**：这是一个罕见的疾病，表现为血小板增多、凝血功能障碍和可能的循环外水负荷增加。

**16.user** 2024-11-30T22:52:34Z:

胎儿期的常见表现包括胎儿活动减少、羊水过多以及臀位。在新生儿期肌张力过低是这一疾病的标志性特征之一，严重的肌张力降低可导致窒息、喂养困难、吮吸无力、哭声无力。婴幼儿期主要表现为生长迟滞，认知、运动及语言发育落后。随着年龄的增长，食欲逐渐旺盛，如果不限制进食会出现肥胖。学龄期通常存在身材矮小，逐渐出现脾气暴躁、固执、强迫症等性格特点。异常行为包括强烈的觅食、搔抓皮肤、孤独症样行为等。约 25％的患者会出现癫痫。青春期男性和女性患者均可出现第二性征出现延迟或出现不完全。由于肥胖可导致相关并发症，如呼吸睡眠暂停、肺源性心脏病、胰岛素抵抗甚至糖尿病等。脊柱侧突、骨质疏松、胃食管反流也较为常见。成人期主要表现为肥胖及其并发症，包括心血管问题、糖尿病和睡眠呼吸暂停。其他表现为精神异常，不孕不育。

致命性危险：由口部运动不协调、肌张力过低、多食、狼吞虎咽的进食习惯和咀嚼不充分引起窒息以及由于暴饮暴食导致胃破裂。

体格检查可以发现特征性面容逐渐典型，包括杏仁眼、小嘴、上唇薄、嘴角向下、小手小脚、身材矮小、皮肤白皙、肌张力低、生殖器发育不良(如隐睾、阴囊发育不全或阴蒂发育不全)。

**llama3.1_8b** 2024-11-30T22:52:41Z:

根据您提供的信息，我认为最可能的诊断是普拉格病（Prader-Willi Syndrome）。其他五种可能的诊断包括：

1. 伯恩哈特症候群（Bardet-Biedl syndrome）：这种疾病也会影响生长发育，导致身材矮小和智力障碍。患者还可能出现多种身体问题，如视觉和听觉障碍、皮肤问题等。
2. 克林费尔德症（Klinefelter syndrome）：这是一种性染色体异常的疾病，主要影响男性，表现为生殖器发育不良、身材矮小、肌肉力量减弱和智力障碍。
3. 弗雷氏症候群（Fragile X syndrome）：这种遗传性疾病主要影响男孩和女孩，其特征是智力障碍、语言发展迟缓以及社会交往问题等。
4. 迷走神经瘤（Vagus nerve tumor）：虽然这不是一个具体的疾病，但某些类型的迷走神经肿瘤可能导致相关症状，如吞咽困难和食欲亢进等。
5. 先天性肌肉张力低下综合征（Congenital Muscular Hypotonia of Unknown Etiology）：这是另一种罕见病，其特点是肌肉力量降低，而具体的原因可能与许多不同的遗传或环境因素有关。

**17.user** 2024-11-30T22:52:55Z:

患者常在婴幼儿或青少年期起病，男女比例大致相等。呼吸道、胰腺和胆道等上皮细胞的分泌物含水量减低，分泌物变黏稠而难以清除。肺脏是最常见的受累器官，患者呼吸道出现慢性细菌感染及病原体的定植，最初常为流感嗜血杆菌和金黄色葡萄球菌，最终出现铜绿假单胞菌或洋葱克雷伯杆菌的慢性定植和(或) 感染。一旦出现感染，中性粒细胞被大量募集到肺组织中，并释放弹性蛋白酶，从而造成肺组织的破坏，导致支气管扩张形成。患者的支气管扩张常在幼年起病，且常从上叶起病，与多数感染后形成的支气管扩张症主要位于中、下肺不同。

患者常表现为深色服装上遗留的汗渍，或是父母发现患儿皮肤发咸。胆汁和胰液的流动异常可以导致消化不良和吸收不良，也可出现肝脏或胰腺疾病，严重时可以出现相关的糖尿病。黏稠的肠液还可能使 患者更容易出现肠梗阻和直肠脱垂。此外，男性患者可以出现输精管内精子移动障碍以及先天性输精管缺失(CBAVD)。

**llama3.1_8b** 2024-11-30T22:53:06Z:

根据您提供的临床表现，我最有可能诊断的是库欣综合征（Cystic Fibrosis）。

库欣综合征是一种较为常见的遗传性疾病，影响肺部和胰腺功能。主要特征包括：

- 呼吸道、胰腺和胆道等上皮细胞分泌物含水量减低，变黏稠
- 肺脏是最常见的受累器官，患者容易出现慢性细菌感染和病原体定植
- 支气管扩张形成，并且从上叶起病
- 深色服装上的汗渍或皮肤发咸
- 胰液流动异常导致消化不良和吸收不良
- 可能出现肝脏或胰腺疾病，严重时可出现糖尿病
- 男性患者可能出现输精管内精子移动障碍及先天性输精管缺失

以下是五种可能的诊断：

1. **库欣综合征（Cystic Fibrosis）**：如上所述，库欣综合征是一种遗传性疾病，其特征包括肺部和胰腺功能受损。
2. **克雷伯病（Krabbe Disease）**：克雷伯病是一种遗传性的脂质元气疾病，主要影响脑部和神经系统，其症状包括运动障碍、痉挛和意识丧失。
3. **肌营养不良症（Muscular Dystrophy）**：肌营养不良症是一组遗传性疾病，主要影响肌肉的功能，从而导致肌肉萎缩和力量下降。
4. **先天性甲状腺功能减退症（Congenital Hypothyroidism）**：先天性甲状腺功能减退症是指新生儿时期由于甲状腺功能受损，导致生长发育迟缓和智力障碍。
5. **遗传性肾炎（Hereditary Nephritis）**：遗传性肾炎是一组遗传性疾病，主要影响肾脏的功能，从而导致尿液生成不足、血压增高和肾衰竭。

**18.user** 2024-11-30T22:53:25Z:

患者临床表现为口咽炎、中耳炎、呼吸系统感染、蜂窝织炎及皮肤感染，常由葡萄球菌及链球菌感染所致。在出现骨髓储备下降的重度中性粒细胞减少症患者中，2 岁之前大部分患儿都发生过口腔溃疡及疼痛性牙龈炎。如果伴弥漫性胃肠道病变，可引起类似克罗恩病的腹痛和腹泻表现。中性粒细胞减少症可导致各个部位遭受反复细菌或真菌感染，其中主要受累部位为皮肤黏膜、耳鼻、咽喉和肺。感染通常非常严重甚至是致命性的。约 15％的患者进展为急性白血病或骨髓增生异常综合征。患儿通常不伴有特征性畸形。

**llama3.1_8b** 2024-11-30T22:53:35Z:

根据你提供的临床表现，我认为最可能的诊断是：

- **儿童性中性粒细胞减少症（Chronic Neutropenia in Children）**：这种疾病是一种罕见的遗传性疾病，主要影响中性粒细胞的数量和功能，从而导致反复感染。临床表现包括口咽炎、中耳炎、呼吸系统感染、蜂窝织炎及皮肤感染等，这与你提供的信息一致。

其他五种可能的诊断：

- **儿童性白血病（Acute Lymphoblastic Leukemia in Children）**：这种疾病是一种儿童中最常见的恶性肿瘤，会导致骨髓癌症和全身免疫功能低下。临床表现可能包括反复感染、贫血、出血倾向等。
- **多发性硬化（Multiple Sclerosis）**：这种疾病是一种自體免疫性疾病，会导致中枢神经系统受损。临床表现可能包括肌肉无力、视力障碍、感觉异常等。
- **皮肤系统性红斑狼疮（Cutaneous Lupus Erythematosus）**：这种疾病是一种自身免疫性疾病，会导致皮肤和其他组织受损。临床表现可能包括皮肤发炎、口腔溃疡等。
- **过敏性气道综合征（Eosinophilic Granulomatosis with Polyangiitis）**：这种疾病是一种自身免疫性疾病，会导致血管炎和肺部炎症。临床表现可能包括呼吸困难、皮肤发炎等。
- **系统性红斑狼疮（Systemic Lupus Erythematosus）**：这种疾病是一种自身免疫性疾病，会导致多个器官受损。临床表现可能包括关节炎、肾脏炎症、皮肤发炎等。

请注意，诊断需要根据患者的具体情况和医学检查结果来确定。

**19.user** 2024-11-30T22:54:01Z:

典型 患者出现中枢神经系统损害和肾上腺皮质功能低下两类症状。神经系统症状和肾上腺皮质功能减退的症状可同时出现或相继出现。少数患者可单独出现。肾上腺皮质功能不全的表现轻重不一。儿童患病最为常见，占所有患者 35％。多在 4~8 岁发病。初期表现为注意力不集中、记忆力减退、学习困难、步态不稳、行为异常，后逐渐出现视觉和听觉下降、构音障碍、癫痫发作、共济失调、瘫痪、痴呆等症状。临床表现进行性加重， 最终失明或耳聋，完全瘫痪。有的表现为去大脑强直状态，最终死于中枢性呼吸衰竭、脑疝、感染。多数患者在首次出现神经系统症状时，已有肾上腺皮质功能受损的表现。

**llama3.1_8b** 2024-11-30T22:54:08Z:

根据你提供的信息，我认为可能的诊断是克汀病（Addison’s disease + Neurological symptoms）。克汀病是一种罕见疾病，涉及到肾上腺皮质功能不全和中枢神经系统损害。

以下是五种其他可能的诊断：

1. 複合式艾迪森病（Autoimmune polyendocrinopathy-candidiasis-ectodermal dystrophy (APECED)）：一种罕见疾病，涉及到多个内分泌系统、皮肤和黏膜的自体免疫性损害。
2. 克拉伦多夫-约翰逊综合征（Charcot-Marie-Tooth disease）：一种遗传性的神经退行性疾病，影响运动神经元。
3. 托尼综合征（Tonic seizures）：一种罕见的癫痫类型，表现为肌肉僵硬和痉挛。
4. 肾上腺皮质功能不全伴随中枢神经系统症状的疾病（Adrenal insufficiency with central nervous system symptoms）：一种罕见的疾病，涉及到肾上腺皮质功能不全和中枢神经系统损害。
5. 中枢神经系统内分泌失衡综合征（Neuroendocrine imbalance syndrome）：一种罕见的综合征，涉及到中枢神经系统和内分泌系统的异常功能。

**20.user** 2024-11-30T22:54:25Z:

患儿多于生后 4~12 个月开始出现感染症状。最突出的临床表现是反复严重的细菌感染，感染部位主要包括呼吸系统、消化系统及中枢神经系统，其中以呼吸道感染最为常见，常见病原菌包括流感嗜血杆菌、肺炎链球菌、金黄色葡萄球菌和假单胞菌属细菌等。长期、反复的呼吸道感染可导致支气管扩张、杵状指等不可逆转的器官功能损害。消化道感染常见的症状有腹泻、腹痛、胃食管反流和胃肠炎。引起感染性腹泻的病原包括蓝氏贾第鞭毛虫、沙门菌、空肠弯曲菌、隐孢子虫等。

另外，患儿容易出现中耳炎、慢性鼻窦炎、营养不良、贫血、粒细胞减少、血小板减少、生长激素缺乏症及甲状腺激素紊乱等并发症状。患儿还可发生肿瘤、自身免疫和炎症性疾病，包括类风湿性关节炎、皮肌炎等。据报道，患儿的感音神经性听力损失和湿疹的发生率也较高。

**llama3.1_8b** 2024-11-30T22:54:32Z:

根据你提供的信息，我认为可能诊断出的罕见病是：

1. **严重合并免疫缺陷病毒（SCID）**：这是一种由遗传因素引起的免疫系统功能严重受损的疾病，使得身体难以抵抗感染。
2. **多发性骨髓增生异常症（MMDS)**：这种疾病涉及骨髓细胞的异常增生，可能导致血液疾病、肿瘤和免疫系统功能障碍。
3. **道格拉斯症候群（DGS)**：这是一种遗传性免疫缺陷疾病，可导致反复感染、营养不良和其他全身性问题。
4. **怀氏综合征（WS)**：一种罕见的免疫缺陷疾病，可引起免疫系统功能障碍，导致反复感染等问题。
5. **霍奇金病**：一种恶性肿瘤疾病，可影响免疫系统并导致全身性症状，如反复感染。

其他五种可能的诊断包括：

1. **多发性骨髓增生异常症（MMDS)**
2. **道格拉斯症候群（DGS)**
3. **怀氏综合征（WS)**
4. **霍奇金病**
5. **严重合并免疫缺陷病毒（SCID）**

**21. user 2025-02-18T23:07:39Z:**

患者的临床表现包括不同程度的失盐和高雄激素血症两大类。新生儿起病的患儿表现为不同程度肾上腺皮质功能不足的表现，如软弱无力、恶心、呕吐、喂养困难、腹泻、慢性脱水、皮肤色素沉着和生长迟缓等。肾上腺危象常是该疾病在新生儿期的首发表现，表现为严重低血钠、高血钾、低血容量性休克，可伴有低血糖，由应激诱发。严重的低钠血症可导致抽搐等中枢神经系统表现，严重的高钾血症则可引起致命的心律失常。

高雄激素血症在不同年龄表现不一。经典型疾病的女性患儿出生时外生殖器有不同程度男性化。轻者表现为孤立性阴蒂肥大，重者外生殖器可接近男性，大阴唇呈阴囊样，阴蒂呈尿道下裂型阴茎样，并具共通的尿道阴道口。但大阴唇内不能触及性腺，有完全正常的女性内生殖器结构（卵巢和子宫）。男性新生儿期和婴儿期时可能无阴茎增大等外生殖器异常，是延误诊断的常见原因。至幼儿期，两性均会呈现外周性性早熟。男孩患儿呈现阴茎增大，伴或不伴阴毛早现；女性患儿呈现异性性早熟。长期高水平性激素刺激下丘脑促性腺激素释放激素（GnRH）神经元，发展为中枢性性早熟。女性还可有第二性征发育不良和原发性闭经或月经稀发。两性均在幼年期开始发生线性生长伴骨龄增长加速，使终身高受损。

其他表现包括皮肤和黏膜色素增深，以乳晕和外阴为显，部分患儿可无此改变。

非经典型的该类疾病儿童期和青春期甚至成年期临床呈不同程度的高雄激素血症表现，也可仅表现为生长加速和骨龄快速进展。

**llama3.1_8b**  2025-02-18T23:07:45Z:

根据你的描述，我认为最可能的诊断是 Congenital Adrenal Hyperplasia（CAH），特别是 21-羟化酶缺乏症。这种疾病是一种罕见的遗传性疾病，影响到肾上腺皮质功能，从而导致高雄激素血症和低盐血症。

以下五种可能的诊断也可以考虑：

1. **睪丸未闭综合征**：这是另一项罕见的遗传性疾病，可以导致男性化的外生殖器和高雄激素血症。
2. **肾上腺髓质细胞瘤**：这种疾病是一种恶性肿瘤，可能会导致高雄激素血症和其他临床表现。
3. **甲状旁腺功能亢进症**：这是另一项罕见的遗传性疾病，可以导致高钾血症和低钠血症。
4. **抗利尿激素缺乏症**：这种疾病是一种罕见的遗传性疾病，可以导致低血容量性休克和其他临床表现。
5. **生长激素缺乏症**：这是另一项罕见的遗传性疾病，可以导致生长迟缓和骨龄增长加速。

需要注意的是，这些诊断仅供参考，确切的诊断还需要进行进一步的医学检查和测试。

**22. user 2025-02-18T23:08:46Z:**

患者出生即有临床表现，因全身皮肤色素缺乏，皮肤毛细血管显露，皮肤薄而柔软，呈白色及红色，并伴有不同程度的血管扩张。患者对紫外线高度敏感，常出现日光性皮炎、光化性唇炎、基底细胞癌及鳞状细胞癌等疾病。毛发呈纯白、银白、黄色、金色或茶色。眼睫毛及眉毛呈白色或淡黄色。视网膜、虹膜颜色减退，瞳孔颜色减退。白天畏光明显，夜视正常，同时还可以伴有其他视觉改变，如视野敏锐度下降、视网膜中央凹发育不良、视交叉处神经路径改变、眼球震颤等。

**llama3.1_8b**  2025-02-18T23:08:50Z:

根据患者的临床表现，我最有可能诊断的是:**阿尔伯特-雷姆病（Albinism）

其他五种可能的诊断：

1.**维汀病（Waardenburg Syndrome）**: 这是一种遗传性疾病，主要影响皮肤、毛发和眼睛。患者可能会出现白色或淡黄色的眼睫毛和眉毛，以及全身皮肤颜色减退。 2.**库欣综合征（Kuwait Syndrome）**: 这是一种罕见的遗传性疾病，主要影响视觉系统、皮肤和毛发。患者可能会出现白天畏光、夜视正常、视野敏锐度下降等症状。 3.**格伦伯格综合征（Glanzmann Thrombasthenia）**: 这是一种罕见的血液疾病，主要影响凝血功能。患者可能会出现皮肤和毛发颜色减退、全身瘀伤等症状。 4.**白化病（Leucism）**: 这是一种罕见的遗传性疾病，主要影响皮肤、毛发和眼睛。患者可能会出现白色或淡黄色的眼睫毛和眉毛，以及全身皮肤颜色减退。 5.**视网膜色素变性（Retinal Pigment Epitheliopathy）**: 这是一种罕见的遗传性疾病，主要影响视觉系统。患者可能会出现视野敏锐度下降、视网膜中央凹发育不良等症状。

**23. user 2025-02-18T23:09:16Z:**

一种上、下运动神经元同时受累的神经系统变性疾病。临床主要表现为球部、四肢、胸腹部肌肉进行性无力和萎缩。而眼球运动神经和括约肌功能一般并不受累，但在有些患者晚期也可能会被累及。20％ ~50％的患者可以表现有认知功能障碍，5％ ~15％的患者甚至会发展为额颞叶痴呆。发病后平均 3~5 年因呼吸衰竭死亡，但 5％ ~10％的患者可以存活 10 年以上。

通过详细的病史和体格检查，在脑干、颈、胸、腰骶 4 个区域中寻找上、下运动神经元共同受累的证据，是诊断该疾病的基础。查体发现在同一区域，同时存在上、下运动神经元受累的体征是诊断的要点。其中下运动神经元受累体征主要包括肌肉无力、萎缩和肌束颤动。通常检查舌肌、面肌、咽喉肌、颈肌、四肢不同肌群、背肌和胸腹肌。上运动神经元受累体征主要包括病理征阳性、腱反射亢进、肌张力增高、阵挛等。通常检查吸吮反射、咽反射、下颌反射、掌颌反射，有无强哭、强笑等假性延髓麻痹表现，四肢腱反射、肌张力、霍夫曼征、下肢病理征、腹壁反射等。临床查体是发现上运动神经元受累的主要方法，在出现萎缩、无力的区域，如果腱反射不低或活跃，即使没有病理征，也可以提示锥体束受损。

**llama3.1_8b**  2025-02-18T23:09:20Z:

根据您的描述，这种疾病最可能诊断出的罕见病是ALS（ amyotrophic lateral sclerosis），也就是Lou Gehrig病。其他五种可能的诊断包括：

1. 多发性硬化症（Multiple Sclerosis）：虽然主要表现为炎症、神经损伤等，但某些患者也可能出现运动功能障碍。
2. 急性的脊髓损伤（Acute Spinal Cord Injury）：在严重的脊髓损伤后，下运动神经元受累的临床表现与 ALS 相似。
3. 脑瘫型神经肌炎（Polymyositis）：这是一种自身免疫性疾病，可以导致上、下运动神经元同时受累。
4. 硬脑膜外肿瘤的神经侵犯（Tumors of the meninges compressing spinal cord or brainstem）：在某些情况下，肿瘤可能影响脑干和脊髓，从而导致上、下运动神经元同时受累。
5. 运动神经元病变综合征（Motor Neuron Disease Complex）：这是一个罕见的疾病，可以包括 ALS 以及其他运动神经元疾病。

需要注意的是，诊断通常依靠临床表现、实验室检查、影像学检查以及家族史等多个方面来综合评估。

**24. user 2025-02-18T23:10:12Z:**

一种影响尿素循环的常染色体隐性疾病，可导致高氨血症、脑病和呼吸道阻塞。 其特征是精氨酸和氨在血液中积聚，导致发育迟缓、癫痫发作、智力残疾、痉挛和共济失调等症状。 这种情况通常在3岁左右出现，症状包括僵硬、生长缓慢和认知能力下降。 如果不进行治疗，它可能会发展为严重的痉挛状态，失去肌肉和智力残疾。

**llama3.1_8b**  2025-02-18T23:10:17Z:

根据您提供的临床表现，我认为最有可能诊断出的罕见病是腺苷酸代谢症（Hartnup症候群）。这种疾病是一种影响尿素循环的常染色体隐性遗传疾病，特征是在血液中积聚精氨酸和氨，这些物质会干扰正常的神经系统功能。

其他五种可能的诊断包括：

1. **腺苷酸代谢症（Hartnup症候群）**：如上所述，这是一种影响尿素循环的常染色体隐性遗传疾病，特征是在血液中积聚精氨酸和氨。
2. **肌张力增高症（SMA）**：这种疾病通常在婴儿或幼儿时期出现，与您提供的发育迟缓、癫痫发作、智力残疾和肌肉僵硬等症状相符。
3. **腺苷酸脱氢酶缺乏症（AGD缺乏）**：这是一种罕见的遗传性疾病，导致精氨酸及其衍生物在血液中积聚。
4. **脑内肿瘤或肿瘤性疾病**：例如，脑室系统肿瘤、肺癌转移等可能会影响尿素循环并导致类似的临床表现。
5. **其他遗传性代谢疾病**：如糖酵解缺乏症（GSD）、磷酸戊糖激酶缺乏症（GK缺乏）等，也有可能导致相似于您描述的症状。

**25. user 2025-02-18T23:10:37Z:**

典型的临床表现包括微血管病性溶血性贫血、血小板减少及急性肾功能衰竭三联征。微血栓形成所导致的非免疫性红细胞破坏，血红蛋白水平常低于 80g/L，Coombs 试验阴性， 外周血涂片可见红细胞碎片；血小板计数下降通常低于 50×109/L，但皮肤紫癜及活动性出血少见；急性肾功能损害的严重程度因人而异，部分患者需要透析支持，常伴血压升高。

**llama3.1_8b**  2025-02-18T23:10:42Z:

根据您提供的临床表现，我最有可能诊断出的疾病是：

1. **Henoch-Schönlein紫癜（HSP）**: 这是一种与免疫复合物相关的血管炎症，典型表现为皮肤紫癜、关节痛、肾脏受累和血小板减少。

但是，我也列出了其他五种可能的诊断：

1. **微血管病（Microvascular Disease）**: 这是一组罕见的疾病，涉及微血管系统的炎症和损害，导致多个器官的功能受累。
2. **肾脏病性溶血性贫血（Hemolytic Uremic Syndrome, HUS）**: 这是一种常见于儿童的罕见疾病，表现为微血管病性溶血性贫血、血小板减少和急性肾功能衰竭。
3. **抗体相关的溶血性贫血（Autoimmune Hemolytic Anemia）**: 这是一种免疫介导的红细胞破坏，通常表现为红细胞碎片试验阳性和Coombs试验阳性。
4. **弥漫性血管炎（Polyarteritis Nodosa, PAN）**: 这是一种罕见的疾病，涉及多个器官的微小血管损害和炎症，导致多个系统的功能受累。

请注意，这些诊断并非绝对确定，我建议您进一步检查和确认这些信息，以确保准确的诊断。

**26. user 2025-02-18T23:11:25Z:**

患者临床表现多样。取决于病变的部位、范围和进展速度。临床症状主要包含：

1. 增大垂体的占位效应，大多数患者会因增大的垂体对鞍区及周围结构（如视交叉、海绵窦等） 造成压迫而产生相应症状或由于炎症浸润产生的作用。最常见的临床表现为持续的剧烈头痛和进行性加重的视功能障碍，包括视力下降和视野缺损。
2. 腺垂体功能减退，根据受累的程度不同，垂体的各个轴系会出现部分甚至全部的功能减低的临床表现，如闭经、性功能减低、纳差、乏力、怕冷、性毛脱落、体重下降等。
3. 中枢性尿崩症 ， 约 70％以上患者会因为垂体后叶、垂体柄甚至下丘脑区受累出现多尿、烦渴和多饮等中枢性尿崩症的症状。部分患者因垂体柄阻断效应导致血清中泌乳素水平轻度升高。
4. 下丘脑综合征， 当病变累及区域发展至下丘脑时，会出现嗜睡、缺乏饱腹感、体重严重增加、渴感缺乏、体温波动、出汗障碍和情绪神志障碍等临床表现。
5. 系统性疾病的相关表现 ， 继发性的患者常合并有系统性疾病特征性的临床表现，如 LCH 患者常合并甲状腺、肺、骨、肝脏和皮肤病变。GPA 患者常为中老年男性， 呼吸道症状常早于下丘脑垂体受累。IgG4 相关疾病垂体炎患者常合并有泪腺、颌下腺和胰腺等其他脏器受累的临床表现等。

**llama3.1_8b**  2025-02-18T23:11:34Z:

根据您的描述，我认为最可能的诊断是：

1. **Langerhans 细胞病（LCH）**：这种疾病是一种罕见的白血病，影响肾上腺、垂体和其他身体系统。患者出现类似的临床表现，如增大垂体对鞍区及周围结构造成压迫的症状，以及下丘脑综合征等。

以下是五种可能的诊断：

1. **Langerhans 细胞病（LCH）**：这种疾病是一种罕见的白血病，影响肾上腺、垂体和其他身体系统。
2. **Giant细胞动脉炎（GPA）**：这种疾病是自身免疫性疾病的一种，会导致中风、眼部受累等症状。患者可能出现呼吸道症状早于下丘脑垂体受累。
3. **IgG4 相关疾病**：这种疾病是一种罕见的免疫介导性炎症综合征，影响多个身体系统，包括泪腺、颌下腺和胰腺等。患者可能出现垂体炎症综合征。
4. **Lymphoma**（淋巴瘤）：这种疾病是一种癌症，将影响身体的淋巴系统。在某些情况下，淋巴瘤可以影响肾上腺和垂体，导致类似的临床表现。
5. **Rathke 组织瘤**：这种疾病是一种罕见的良性肿瘤，影响肾上腺、垂体和其他身体系统。患者可能出现增大垂体对鞍区及周围结构造成压迫的症状，以及下丘脑综合征等。

**27. user 2025-02-18T23:11:52Z:**

高血糖是该疾病的最常见的临床表现。但在没有干预的情况下患者可以自发缓解而维持血糖水平正常。该疾病是自身免疫性低血糖的主要原因之一，约 25％的患者在病程中会出现自发性低血糖。约 50％的患者存在黑棘皮征。此外，多囊卵巢、高雄激素血症在该类患者中也不少见。 患者常伴发其他的自身免疫性疾病，其中以系统性红斑狼疮最为常见，约占 50％。此外，还有原发性胆汁性肝硬化、皮肌炎、硬皮病等。该疾病还可以是霍奇金淋巴瘤、多发性骨髓瘤等的副肿瘤综合征表现。

**llama3.1_8b**  2025-02-18T23:12:02Z:

根据所提供信息，我认为可能诊断出的罕见病是：

1. **高血糖（Hypoglycemia）**：虽然高血糖是临床表现，但考虑到患者可以自发缓解且维持血糖水平正常，似乎更倾向于一种自身免疫性疾病的表现。

其他五种可能的诊断如下：

1. **库欣综合征（Cushing’s Syndrome）**：高血糖、多囊卵巢和高雄激素血症等临床表现与库欣综合征相符，特别是考虑到高雄激素血症的存在。
2. **系统性红斑狼疮（Systemic Lupus Erythematosus, SLE）**：患者伴发其他自身免疫性疾病，其中以系统性红斑狼疮最为常见，这提示可能是SLE的一个表现形式。
3. **原发性胆汁性肝硬化（Primary Biliary Cirrhosis）**：该疾病也被列为患者的伴发自身免疫性疾病之一，提示可能与原发性胆汁性肝硬化有关。
4. **皮肌炎（Dermatomyositis）**：高雄激素血症和黑棘皮征等临床表现是皮肌炎的一个特征，这也表明可能是皮肌炎的一个表现形式。
5. **霍奇金淋巴瘤（Hodgkin Lymphoma）**：罕见病可以是霍奇金淋巴瘤的副肿瘤综合征表现之一，这也需要考虑到诊断。

**28. user 2025-02-18T23:12:31Z:**

婴儿及儿童期起病。首次发作时年龄在 5 个月至 7 岁。临床表现为反复发作的难治性呕吐及重症酮症酸中毒，伴有萎靡、脱水、呼吸急促、昏迷等临床表现。通常有明确诱因，可在禁食、胃肠道及上呼吸道感染、发热、应激或过量摄入蛋白质后急性起病。常规的实验室检查可见血糖升高或正常，部分出现低血糖，血气显示代谢性酸中毒，血氨可以正常或轻度升高，肝功能、肾功能及正常，尿酮体阳性。酮症及酸中毒可反复出现，发作间期临床及实验室检查可以表现为正常。

约 1/5 的病例出现神经系统的改变，表现为认知障碍、智力发育落后及锥体外系异常及脑卒中的症状，头颅 MRI 可发现包括白质广泛的脱髓鞘，基底节区在 T2 可见对称性高信号改变。

患者早期可无症状，在新生儿期或高危人群通过血、尿代谢筛查时被诊断。起病年龄、发作频率与预后无明确关联。发作随年龄增长有减少或停止趋势，多数病例随访其生长发育未受影响。反复发作及严重者可致多器官功能障碍甚至死亡。

**llama3.1_8b**  2025-02-18T23:12:36Z:

根据提供的信息，我认为可能的诊断是：

1. **Glut1缺陷（Glucose Transporter 1 deficiency）**：一种罕见疾病，表现为反复发作的难治性呕吐和重症酮症酸中毒。
2. **卟啉综合征（Porphyria）**：一种遗传性疾病，表现为尿液中的酪氨酸和皮肤、神经系统等部位的症状。
3. **糖代谢异常**：一种罕见疾病，表现为血糖水平波动、呕吐、昏迷等临床表现。
4. **脑组织缺氧综合征（Hypoxic-Ischemic Encephalopathy）**：一种罕见疾病，表现为重症酮症酸中毒、神经系统受损等临床表现。
5. **营养不良综合征（Malnutrition Syndrome）**：一种罕见疾病，表现为营养不良、呕吐、腹泻等临床表现。

其他五种可能的诊断：

1. **肌苷酸酯酶缺乏症（Myoadenylate deaminase deficiency）**
2. **糖尿病性酮症酸中毒**
3. **肠道功能障碍综合征**
4. **甲状腺功能亢进**
5. **肾上腺素能机制功能障碍**

请注意，这些诊断仅供参考。最准确的诊断仍需要根据患者的临床表现、实验室检查结果和进一步的评估来确定。

**29. user 2025-02-18T23:12:58Z:**

1. 发病年龄小，女性多见。
2. 常有晕厥或猝死家族史。
3. 不同基因型和表现型，可累及钾、钠、钙通道。常有特征性尖端扭转型室速（TdP），导致晕厥或猝死。
4. ECG 表现为 QT 间期延长。
5. 心电图一般有如下特征：①表现为 QT 间期延长，短 - 长 - 短周期现象诱发；②心室率 160-240 次 / 分，QRS 波振幅与形态围绕等电位线扭转，5-20 个心动周期主波围绕基线扭转一次，多数能自行终止；③ ATP 较难终止的 TdP。

**llama3.1_8b**  2025-02-18T23:13:03Z:

根据您提供的临床表现和特征，我可能诊断出 Long QT Syndrome（LQTS），这是一个罕见的心律失常疾病。LQTS 是一种遗传性的电解质失衡疾病，其典型特征是心脏肌肉在复极过程中的延迟，导致QT间期延长，从而引起危险的心律失常，如室性心律失常（TdP）和猝死。

其他五种可能的诊断：

1. **Brugada Syndrome**：是一种罕见的心律失常疾病，其特征是ST段在V1-V3导联上升，且可以导致致命的心室颤振。
2. **CPVT（Catecholaminergic Polymorphic Ventricular Tachycardia）**：是一种罕见的心律失常疾病，其特征是 catecholamines（如肾上腺素和去甲肾上腺素）的快速心跳和室性心律失常。
3. **Arrhythmogenic Right Ventricular Cardiomyopathy（ARVC）**：是一种罕见的心脏疾病，其特征是右心室肌肉的异常，导致心律失常和猝死。
4. **Wolff-Parkinson-White（WPW） Syndrome**：是一种罕见的心律失常疾病，其特征是心脏有额外的电解质通道，导致心跳加速和室性心律失常。
5. **Short QT Syndrome**：是一种罕见的心律失常疾病，其特征是心脏肌肉在复极过程中的缩短，从而引起危险的心律失常，如室性心律失常和猝死。

**30. user 2025-02-18T23:13:48Z:**

可于任何年龄发病，多数患儿于 1 个月至 7 岁发病，平均年龄在 2 岁左右。不同患者临床表现有较大差异，主要有：①急性能量代谢障碍危象，表现为低酮型低血糖、高血氨及代谢性酸中毒等；②心肌病，表现为心室肥厚、心功能不全、心律失常及肌酸激酶升高等；③肌病，表现为肌无力、肌张力减退、肌痛、运动耐力差、肌肉型肌酸激酶升高、肌纤维内脂质沉积等；④肝脏损害，表现为肝肿大、脂肪肝、肝功能异常等，一些肝损患儿急性起病，表现为抽搐、进行性意识障碍等，常被误诊为 Reye 综合征。此外，反复腹痛、呕吐、胃食管反流等消化道症状，反复感染、喘息等呼吸道表现以及贫血等也有报道。

新生儿筛查：确诊的患儿可无临床表现。 是一种潜在的致死性疾病，患儿可因急性能量代谢障碍危象或急性心衰而猝死。临床表现多样，与发病年龄、发病时间和病情轻重有关。最常见的症状是婴幼儿期和儿童期心肌和骨骼肌受损。成年期症状较轻或无症状，多为耐力降低或易疲劳。妊娠期，由于能量消耗和血浆生理性的肉碱水平降低，孕妇可有疲劳和心律失常等不典型表现。

**llama3.1_8b**  2025-02-18T23:13:51Z:

根据您的描述，我认为最可能的诊断是：

**肌营养缺乏综合征（Mitochondrial Myopathy）**

其他五种可能的诊断如下：

1. **肌营养缺乾综合征伴有糖代谢障碍（Glycogen Storage Disease）**
2. **卡氏肌营养缺乓综合征（Kearns-Sayre Syndrome）**
3. **莱恩综合征（Reye’s Syndrome）**
4. **儿童期骨骼肌炎症性肌疾病（Juvenile Dermatomyositis）**
5. **心力衰竭与肝脏损害综合征（Cardiac and Hepatic Failure Syndrome）**

这些诊断都可能导致急性能量代谢障碍危象、心肌病和肌肉疾病等临床表现。然而，必须通过进一步的实验室检查和医学评估来确定最确切的诊断。

**31. user 2025-02-18T23:14:11Z:**

单中心型该疾病往往仅表现为淋巴结肿大，而无全身症状。肿大淋巴结的中位直径约 5.5cm， 常见于胸部（24 ％）、颈部（20 ％）、腹部（18 ％）、腹膜后（14 ％）。部分单中心型疾病的患者容易合并副肿瘤性天疱疮、闭塞性细支气管炎以及生长发育迟缓。多中心型 除了多发淋巴结肿大外，往往还伴有发热、盗汗、乏力、体重下降、贫血、肝功能不全、肾功能不全和容量负荷过多（全身水肿、胸腔积液、腹水等） 等全身表现。多中心型 还有一种特殊亚型，患者可以表现为血小板减少（thrombocytopenia）、重度水肿（anasarca）、骨髓纤维化（myelofibrosis）、肾功能不全（renal dysfunction）、肝脾肿大（organomegaly）。

**llama3.1_8b**  2025-02-18T23:14:15Z:

根据您的描述，这种罕见病最可能是霍奇金淋巴瘤。

其他五种可能的诊断包括：

1. 强直性脊柱炎相关淋巴结肿大症：一种罕见疾病，表现为长期或反复发作的淋巴结肿大，以及关节、肌肉等其他系统的症状。
2. 皮肤淋巴结瘤（Cutaneous lymphoma）：一种少见的恶性肿瘤，主要影响皮肤和淋巴结。
3. 病毒感染相关淋巴结肿大：一些病毒，如EB病毒或HIV，可能导致淋巴结肿大。
4. 淋巴纤维瘤（Lymphangioma）：一种罕见的良性肿瘤，主要影响淋巴管和淋巴结。
5. 强直性脊柱炎相关综合征：一种罕见综合征，表现为全身症状，如关节、肌肉等方面的不适，以及淋巴结肿大。

**32. user 2025-02-18T23:14:38Z:**

主要临床表现为下肢远端为主，并逐渐向近端发展的肢体肌肉萎缩、无力及感觉丧失。常见临床表现为运动能力不如同龄人，跑步困难，易扭脚，足下垂，小腿腓肠肌萎缩形似“鹤腿”；查体可发现弓形足、锤状趾，远端肢体为主的无力萎缩，深感觉减退。患者通常 20 岁前起病，缓慢进展，疾病后期可能严重影响活动，但很少导致完全残疾， 也不影响正常寿命。但有些特殊类型可能起病早且严重。

**llama3.1_8b**  2025-02-18T23:14:39Z:

根据您提供的临床表现，这可能是下肢周围神经病变（Peroneal Muscular Atrophy）的罕见病之一。最有可能的诊断是 Charcot-Marie-Tooth 病。

**33. user 2025-02-18T23:15:01Z:**

患儿于出生后至数月内发病。发病年龄多在 2 月龄内，很少晚于 5 月龄。最常表现为出生后黄疸持续不退伴或不伴大便颜色浅淡，肝脏轻度肿大或无明显肿大。 还有其他多种非特异临床表现，包括喂养困难、生长发育迟缓、腹泻、低血糖、凝血时间延长等。大多数患者上述症状及体征在 6 个月至 1 岁左右可自然缓解或经过饮食结构调整和药物治疗缓解，其中少部分的患者在青春期以后疾病进一步发展。发展后的疾病大部分是在成人后发病。通常在饮酒、摄入甜食、服用某些药物或感染后突然出现高氨血症所致的谵妄，意识混乱等精神异常表现。严重的病情发展急剧，得不到正确治疗可危及生命。患者多为消瘦体形，大部分患者有明显的饮食偏好，嗜食豆类、高蛋白、高脂食物，而厌食高糖类。

**llama3.1_8b**  2025-02-18T23:15:06Z:

根据您提供的疾病临床表现，我最可能诊断出的是一种罕见病叫做”亨丁顿病”(Huntington’s disease)。亨丁顿病是一种遗传性的神经系统疾病，主要影响大脑的运动、情感和认知功能。虽然亨丁顿病通常在青壮年时期发病，但您提到的症状，如黄疸持续不退、喂养困难、生长发育迟缓等，也可以出现在儿童时期。

以下是五种可能的诊断：

1. **亨丁顿病**(Huntington’s disease)：这是最有可能的诊断，因为您提到的症状，如黄疸持续不退、喂养困难、生长发育迟缓等，都与亨丁顿病的一些临床表现相符。
2. **儿童型神经肌肉疾病**(Childhood Polyneuropathy)：这种疾病主要影响小儿，表现为肌肉无力、运动功能障碍和其他神经系统症状。
3. **克氏病**(Krabbe disease)：是一种罕见的遗传性疾病，影响大脑和脊髓的脂质代谢，从而导致认知功能和运动功能的衰退。
4. **沙氏病**(Sandhoff disease)：是一种罕见的遗传性疾病，主要影响儿童的神经系统，表现为肌肉无力、运动功能障碍和其他神经系统症状。
5. **婴儿期多发性神经退行性疾病**(Infantile-onset multiple system atrophy)：这是另一种罕见的遗传性疾病，主要影响小儿的神经系统，表现为运动功能障碍、认知功能衰退和其他神经系统症状。

**34. user 2025-02-18T23:15:29Z:**

可于出生后出现症状，也可于婴幼儿期至成年早期起病。主要表现为波动性肌肉无力，不耐疲劳，多分布于眼外肌、面肌、延髓、四肢和呼吸肌，婴儿期甚至可能出现危及生命的呼吸暂停发作。单纯的综合征主要表现为骨骼肌无力，不伴有先天畸形。该疾病的糖基化缺陷和肌病重叠综合征往往伴有先天发育异常。不同类型的临床表现也有差异，部分患者，一般为出生后近端肌肉无力，胆碱酯酶抑制剂有效，严重者可出现突发的呼吸窘迫和延髓麻痹引起的呼吸暂停，可由应激、寒冷、感染等诱发。部分患者，出生后或婴儿期起病，可能出现比较严重、轴性分布的肌肉无力，但眼外肌可不受累。

**llama3.1_8b**  2025-02-18T23:15:33Z:

根据您的描述，我认为最有可能的诊断是：

1. **糖基化缺陷相关肌病（Glycogen Storage Disease-related Myopathy）**

这类疾病是一组罕见遗传性疾病，表现为肌肉无力和糖代谢异常。

其他五种可能的诊断是：

1. **先天性肌肉无力症（Congenital Muscular Dystrophy）**

这类疾病通常在出生后不久即出现肌肉无力的症状。

1. **肌病重叠综合征（Myasthenic Crisis）**

这类疾病是一种罕见的自身免疫性疾病，表现为眼外肌、面肌和四肢肌肉无力。

1. **延髓麻痹（Bulbar Palsy）**

这类疾病是由脑干受损引起的一组症状，包括呼吸、吞咽和语言功能障碍。

1. **先天性神经肌肉传导疾病（Congenital Neuromuscular Transmission Disorders）**

这类疾病是一种罕见的遗传性疾病，表现为肌肉无力和神经肌肉传递异常。

**35. user 2025-02-18T23:15:55Z:**

1. 通常婴幼儿期至儿童期起病，少数患者成年后起病。
2. 全身横纹肌，包括肢体骨骼肌、面肌、眼外肌、舌肌均可出现肌强直现象。
3. 肌强直在休息后突然运动时明显，反复动作后减轻，称之为“热身（warm-up）”现象。可在突然启动时出现短暂肌肉无力。病程较长的的患者，可有持续性肢体远端无力，甚至累及肢体近端。
4. 查体可见患者常肌肉发达，呈运动员体型。大力握拳后，手指不能及时展开；大力闭目后，不能立即睁开。叩击手部大鱼际肌、舌肌等部位，可引起肌肉持续性收缩（叩击性肌强直、“肌球”现象）。

**llama3.1_8b**  2025-02-18T23:16:00Z:

根据你的描述，我认为最可能的诊断是苯丙酮尿症（Phenylketonuria, PKU）。PKU是一种遗传性的代谢疾病，主要特征是在婴幼儿期或儿童期出现肌肉强直和无力。

其他五种可能的诊断：

1. **肌营养不良症**（Muscular Dystrophy）：一种影响肌肉功能和力量的疾病，可以导致肌肉萎缩、强直和无力。
2. **神经肌肉接头疾病**（Myasthenia Gravis）：一种自身免疫性疾病，会导致神经和肌肉之间的信号传递受阻，从而引起肌肉无力和疲劳。
3. **肌炎**（Muscle Inflammation）：一种炎症性的疾病，可能导致肌肉疼痛、强直和无力。
4. **脊髓性锥体病**（Amyotrophic Lateral Sclerosis, ALS）：一种神经系统疾病，会导致运动神经元的丢失，从而引起肌肉无力、萎缩和强直。
5. **肌球症**（Myotonia Congenita）：一种遗传性的疾病，特征是肌肉反射延迟和持续性收缩。

这些疾病都可能导致类似的临床表现，如肌肉强直、无力和运动功能障碍。但是，PKU是最符合你描述的疾病，因为它通常在婴幼儿期或儿童期开始，并且可以有明显的遗传倾向。

**36. user 2025-02-18T23:16:16Z:**

由于脊柱侧凸所引起的继发性胸廓畸形可引起患儿心肺发育不全，心肺储备差，另可能出现包括心脏移位、心律失常、肺活量减少、肺炎等；腹腔畸形可引起胃肠功能紊乱， 导致消化不良，食欲缺乏。此外，由于椎体畸形和椎间盘退变引起的神经根牵拉可产生相应躯体部位的刺激性疼痛，严重者可引起脊髓功能障碍甚至瘫痪。

体格检查：大体表现为直立时两肩不等高、一侧腰部皱褶、腰前屈出现背部不对称（“剃刀背”），有时可见骨盆两侧不对称、步态异常等。此外，还需进行神经系统检查，观察患者有无肌张力增高或低下，有无四肢感觉异常。

临床上根据影像学特点通常分为以下 3 型：

Ⅰ型——椎体形成障碍型，即一个或多个椎体部分或完全形成障碍，如楔形椎、半椎体或蝴蝶椎等；

Ⅱ型——椎体分节不良型，即两个或多个椎体部分或完全分节障碍，如阻滞椎、骨桥形成等；

Ⅲ型——混合型，既有椎体形成障碍，又有椎体分节不良。

**llama3.1_8b**  2025-02-18T23:16:20Z:

根据你提供的信息，最可能的诊断是斯科罗夫斯基病（Scoliosis）。其他五种可能的诊断包括：

1. 强直性脊柱炎（Ankylosing Spondylitis）：这是一个慢性炎症性疾病，影响脊柱和其他骨骼结构。
2. 骨赘形成（Osteophyte Formation）：这是一个与年龄增大相关的条件，导致骨头生长异常，形成钉状突起。
3. 脊柱侧凸伴随的胸廓畸形（Scoliosis with Thoracic Kyphosis）：这是一种罕见的脊柱畸形，表现为胸部向前弯曲和侧边向一侧倾斜。
4. 异位脊髓病变（Hemi-Vertebral Body Resorption）：这是一个罕见的情况，涉及脊椎体的一部分消失或侵蚀。
5. 骨骼肌肉疾病（Musculoskeletal Diseases）：这是一类影响骨骼和肌肉的遗传性疾病，如马凡综合征（Marfan Syndrome），威尔逊病毒感染（Wilson Disease），等等。

**37. user 2025-02-18T23:16:40Z:**

男性多于女性。部分患者胸痛症状不典型，在行冠脉造影或冠脉 CT 时发现。当存在以下情况时，患者往往有临床症状：①合并阻塞性动脉粥样硬化性疾病、血栓、血流缓慢、夹层、破裂时可有劳力性心绞痛或急性冠脉综合征表现；②压迫邻近组织；③少见的有动脉瘤破裂，可引起急性心脏压塞；④虽然无显著的冠状动脉狭窄，也可发生因微血管功能障碍导致的心肌缺血。

最多见的临床表现是心绞痛，少数 患者会并发心肌梗死、严重心律失常甚至猝死。疾病可破入右房、右室或冠状窦，产生左向右分流，临床上可闻及连续性杂音，严重者会影响心脏功能。最严重的并发症是瘤体破入心包，可导致出血、心脏压塞甚至死亡。

**llama3.1_8b**  2025-02-18T23:16:45Z:

根据您提供的疾病临床表现，我认为最可能的诊断是动脉瘤或主动脉夹层引起的心绞痛或急性冠脉综合征。

其他五种可能的诊断：

1. **主动脉夹层**：这是一个罕见的病症，表现为主动脉的夹层，从而导致血液循环受阻，引发心绞痛、急性冠脉综合征或猝死。
2. **动脉瘤**：同样是一个罕见的病症，表现为血管壁上的异常突起，从而导致血液流通受阻，引发心绞痛、急性冠脉综合征或猝死。
3. **主动脉硬化**：这是一个与年龄相关的疾病，表现为主动脉壁厚度增加，从而导致血液循环受阻，引发心绞痛、急性冠脉综合征或猝死。
4. **冠状动脉狭窄症**：这是一个罕见的病症，表现为冠状动脉的狭窄，从而导致血液循环受阻，引发心绞痛、急性冠脉综合征或猝死。
5. **肺动脉高压**：这是一个罕见的病症，表现为肺动脉的高压，从而导致右侧心室负担加重，引发呼吸困难、胸痛等临床症状。

**38. user 2025-02-18T23:17:12Z:**

1. 骨髓衰竭 35％患儿出生时即表现为贫血，常于生后 2 周至 2 年确诊。贫血为大细胞或正细胞正色素性、网织红细胞减少、白细胞正常或轻度降低、血小板正常或轻度增高、骨髓红系增生低下而粒系和巨核细胞系增生活跃。
2. 先天发育异常 30％ ~50％ 患者可能存在先天发育异常，主要涉及头部、上肢、心脏和泌尿生殖系统。如身材矮小，颅面部畸形包括颅面部过宽、宽平鼻梁、小头、先天性白内障、青光眼、斜视、硬腭高拱及唇腭裂甚至特纳综合征外貌，拇指畸形，先天性心血管发育异常，泌尿生殖器官畸形。存在身体的异常并不能预测患者血液系统疾病的严重程度。30％受累患者可出现生后的生长迟缓，通常伴有其他先天畸形。
3. 癌症易感性增加的患者肿瘤发生率约为 4％，高于同年龄正常人群。且肿瘤发生年龄早。包括血液系统急性白血病、骨髓增生异常综合征、淋巴瘤及实体瘤如骨肉瘤、胸腺癌、肝细胞癌、黑色素瘤、纤维组织细胞瘤、胃癌和肠癌等。患肿瘤后，较患同样肿瘤的一般人群预后差，病死率高。

**llama3.1_8b**  2025-02-18T23:17:18Z:

根据提供的临床表现，我最有可能诊断出的罕见病是 Fanconi 综合征。

Fanconi 综合征是一种遗传性疾病，其特点包括:

1. 骨髓衰竭：表现为贫血、白细胞和血小板减少。
2. 先天发育异常：患者可能存在多个先天畸形，例如头部、上肢、心脏和泌尿生殖系统的异常。
3. 癌症易感性增加：Fanconi 综合征患者有较高风险发展成各种类型的癌症。

其他五种可能的诊断包括：

1. 重症肌无力（Myasthenia Gravis）：一种自身免疫性疾病，表现为肌肉无力和疲劳。
2. 多血小板减少性紫癜（Thrombocytopenic Purpura）：一种罕见的疾病，表现为血小板减少导致皮肤和黏膜出血。
3. 荨麻疹样发热综合征（Malignant Ataxia-Telangiectasia-Like Syndrome）：一种罕见的遗传性疾病，表现为神经系统症状、免疫缺陷和癌症风险增加。
4. 维生素B12缺乏症合并先天畸形综合征（Congenital Anomaly and Vitamin B12 Deficiency Syndrome）：一种罕见的遗传性疾病，表现为维生素B12缺乏症、先天畸形和神经系统问题。
5. 骨髓增生异常综合征（Myelodysplastic Syndrome）：一种罕见的骨髓疾病，表现为骨髓不正常增生导致贫血、白细胞减少和血小板减少。

**39. user 2025-02-18T23:17:46Z:**

病变可累及全身各系统，因此其临床表现因受累部位不同而表现各异。一般症状包括发热、乏力、盗汗、体重下降等。其余受累系统主要包括：

1. 骨骼系统， 绝大多数患者在诊断时候都有骨骼受累，以下肢骨最为常见，但仅有 50％患者有骨痛表现。
2. 心血管系统 ，病变累及心血管系统，多数患者并无明显临床表现，但如心包受累，造成大量心包积液可导致胸闷、憋气甚至心脏压塞表现；病变累及冠状动脉则可造成心肌缺血样表现，如胸闷、胸痛等；弥漫性心肌受累亦有报道，可导致心力衰竭样表现；病变包绕肾动脉则可造成肾性高血压；病变包绕肠系膜血管可有肠道缺血表现，如腹痛等。
3. 呼吸系统 呼吸系统受累多数患者无症状，少数患者有干咳、呼吸困难等表现。
4. 中枢神经系统 中枢神经系统受累可因不同部位脑实质受累而出现相应的临床表现，如肢体活动障碍、步态异常、意识改变等；脊髓受累可因不同受累节段而出现不同临床表现；垂体病灶则可表现为多饮、多尿以及其他激素分泌减少相关表现。
5. 眶后病变 眶后受累为， 常见临床表现之一，表现为单侧或双侧突眼，甚至造成失明。
6. 腹膜后空间 / 肾脏受累 ， 腹膜后病变可类似腹膜后纤维化，若病灶包绕输尿管可造成单侧或双侧肾后性梗阻。
7. 皮肤受累， 最为常见的皮肤病变为黄瘤样改变，约 1/3 患者发生在眼睑，面部、颈部、腋窝、躯干、腹股沟亦可出现。

**llama3.1_8b**  2025-02-18T23:17:50Z:

根据您提供的临床表现，我认为可能的诊断是多发性硬化（Multiple Sclerosis, MS）。MS是一种自身免疫性疾病，其特点是中枢神经系统的广泛炎症和损伤，可能导致视觉、运动、感觉等多个方面的症状。

其他五种可能的诊断：

1. 强直性脊柱炎（Ankylosing Spondylitis）：这是一种骨骼疾病，其特点是脊柱和肢体的强直以及关节疼痛。
2. 系统性红斑狼疮（Systemic Lupus Erythematosus, SLE）：这是一个自身免疫性疾病，其特点是多个系统受累，可能导致皮肤、关节、肾脏等方面的症状。
3. 特发性血管炎（Takayasu Arteritis）：这是一种血管炎症疾病，其特点是大血管受累，可能导致心血管系统和神经系统的损伤。
4. 强直性肌痛症（Fibromyalgia）：这是一种全身性的肌肉和关节疼痛，可能导致疲劳、精神状态改变等症状。
5. 淋巴瘤（Lymphoma）：这是一个恶性肿瘤，其特点是淋巴系统的广泛炎症和损伤，可能导致多个系统受累。

**40. user 2025-02-18T23:18:30Z:**

病常表现为多器官、多系统受累，男性重于女性患者。临床表现随着年龄增长而常常累及不同的器官。

1. 面容， 男性患者多在 12~14 岁出现特征性面容，表现为眶上嵴外凸，额部隆起和嘴唇增厚。
2. 皮肤血管角质瘤， 常见于经典型患者，多见于“坐浴区”即脐膝之间的外生殖器、阴囊、臀部和大腿内侧，凸出皮肤表面的红色斑点。
3. 神经系统 ，多数患者会出现周围神经疼痛，表现为足底和手掌难以忍受的烧灼感，并放射到四肢近端，甚至出现痛性痉挛；自主神经受累时表现为少汗或无汗；中枢神经系统受累时多表现为早发的短暂性脑缺血发作或缺血性卒中。
4. 眼， 特征性的表现包括结膜血管迂曲、角膜涡状混浊、晶状体后囊混浊和视网膜血管迂曲，严重者可导致视力降低甚至丧失。常为女性患者就诊的主要原因之一。
5. 消化道 ，多在进食后出现恶心、呕吐、腹胀、痉挛性腹痛和腹泻等症状，也可表现为吸收不良或便秘。
6. 肾脏 ， 早期表现为尿浓缩功能障碍如夜尿增多、多尿和遗尿，随病程进展可逐渐出现蛋白尿，甚至达肾病综合征水平，伴随肾功能损害，多在 30 岁左右进入终末期肾病。
7. 心血管系统， 可表现为高血压、冠状动脉受累导致的心肌缺血，心脏瓣膜病变和肥厚型心肌病，严重者可表现为心绞痛、心肌梗死和心力衰竭。多为疾病的晚期表现和主要的死亡原因。

**llama3.1_8b**  2025-02-18T23:18:35Z:

根据您提供的信息，我最有可能诊断出的罕见病是：

**威尔逊氏症（Wilson病）**

这是因为患者出现了面容特征、皮肤血管角质瘤、周围神经疼痛、自主神经受累、眼部变化、消化道问题、肾脏功能障碍等一系列临床表现，这些症状都与威尔逊氏症的典型表现相吻合。

其他五种可能的诊断包括：

1. **伯基特病（Berkheim disease）**：这是一种罕见的遗传性疾病，表现为面部和身体部位出现多个青少年皮肤结节。
2. **克伦-勒贝尔症候群（Klippel-Trenaunay-Weber syndrome）**：这是一种罕见的血管畸形症候群，表现为皮肤瘢痕、肿胀和血管异常。
3. **多发性自主神经病变（Autonomic Nervous System disorders）**：这类疾病可能导致自主神经系统功能障碍，表现为心率、血压、汗腺和消化系统等方面的问题。
4. **内皮脂肪肉瘤（Epidermal Liposarcoma）**：这是一个罕见的恶性肿瘤类型，主要影响皮肤，并可能侵犯周围组织。
5. **血管神经症候群（Vascular Neuropathy）**：这是一种罕见的疾病，表现为血管和神经系统同时受累，导致各种临床症状。

需要注意的是，这些诊断仅供参考，并且可能还存在其他可能的原因。

**41. user 2025-02-18T23:18:58Z:**

1. 1 型 主要表现为反复发作的炎性反应和浆膜炎，包括反复发热、腹痛及关节炎。发热反复发作，可有寒冷、剧烈运动、手术、感染等诱因，持续 13 天自行缓解。腹痛为最常见临床表现，见于 95％的患者。表现为突然发作的全腹痛，常伴发热，腹部查体可有腹胀，腹部压痛、反跳痛、肌紧张，听诊肠鸣音消失。立位腹平片可见小肠气液平。腹痛常在 24-48 小时后自行缓解。关节炎亦为常见临床表现，见于 75％的患者。常累及下肢大关节，如髋关节、膝关节。可有关节肿胀、疼痛。皮疹见于 7％ ~40％的患者，主要累及下肢伸侧，典型皮疹为丹毒样红斑。胸膜炎见于 45％的患者，表现为突然发作的单侧胸痛，常伴发热。心包炎较少见。
2. 2 型 以淀粉样变起病。表现为持续大量的蛋白尿，常致终末期肾病。淀粉样物质亦可在心脏、胃肠道、肝脏等器官沉积，并导致相应临床表现。

**llama3.1_8b**  2025-02-18T23:19:03Z:

基于您的描述，我最有可能诊断出的是Henoch-Schönlein紫癜（HSP），一种常见的罕见疾病。根据您提供的信息：

- 反复发作的炎性反应和浆膜炎
- 发热、腹痛和关节炎
- 粉红色的皮疹，典型累及下肢伸侧

其他五种可能的诊断包括：

1. **克雅氏综合征**（Kawasaki disease）：一种血管炎症疾病，可导致发热、皮肤瘙痒和淋巴结肿大。
2. **系统性红斑狼疮**（Systemic Lupus Erythematosus，SLE）：一种自身免疫性疾病，可以影响多个器官，并表现为发热、关节炎、皮肤瘙痒等症状。
3. **血管壁硬化症**（Vasculitis）：是一种血管的炎症和硬化，可能导致发热、关节炎、腹痛等症状。
4. **肾小球膜增生性肾病**（Focal Segmental Glomerulosclerosis，FSGS）：一种肾脏疾病，可以导致蛋白尿和肾功能衰竭。
5. **系统性血管炎**（Systemic Vascular Inflammation）：是一种影响全身血管的炎症疾病，可以导致发热、关节炎、皮肤瘙痒等症状。

请注意，这些诊断仅供参考，您应该根据患者具体情况进行进一步的评估和确认。

**42. user 2025-02-18T23:19:19Z:**

主要表现为先天性发育异常，进行性骨髓衰竭和肿瘤发生率增高，也有患者无发育异常或骨髓衰竭表现。

1. 发育异常 ，患者可以表现为多种形体和智力发育异常。生长缺陷、异常皮肤色素沉着、上肢单侧或双侧骨骼畸形、体格矮小、小头畸形、眼部异常、泌尿生殖道畸形、内分泌异常、听力损失、外耳异常、先天性心脏缺陷、消化道异常、中枢神经系统发育异常及面部畸形、脊柱畸形、颈部畸形及发育迟缓和（或） 智力残疾等。这些表现多为非特异性，拇指及上肢畸形较为特异。
2. 进行性骨髓衰竭 ，血液系统的异常为范科尼贫血最严重的临床特征。可表现为单一或全血细胞减少。血细胞减少起病年龄差异较大，即使同胞发病年龄也不一。中位发病年龄约为 7.6 岁，罕见于婴儿和幼童，90％患者的血液学异常在 40 岁前发病。血小板减少或白细胞减少通常先于贫血出现，常伴随大红细胞，胎儿血红蛋白（HbF 1 ） 增加，全血细胞减少逐渐加重。
3. 肿瘤发生风险增高， 包括血液系统恶性疾病及非血液系统恶性实体瘤。急性髓系白血病（AML） 发生危险增加约 500 倍，50 岁时 AML 累计发生率约 13％，多发生于15-35 岁。-7，del（7q） 与骨髓增生异常综合征（MDS）/AML 发生率增高相关。急性淋巴细胞白血病和淋巴瘤也有报道。实体肿瘤可以是该疾病的首发表现。头颈部鳞状细胞癌（HNSCCs） 是 患者最常见的实体瘤，而且发病年龄更早（20~40 岁），大多数发生于口腔（如舌癌），多处于进展期，对治疗反应差。皮肤、食管癌、肝肿瘤和泌尿生殖道肿瘤发生率也增加。少数患者可见到多种肿瘤并发，或者血液系统及非血液系统肿瘤并发。而且对化疗或放疗明显不耐受。

**llama3.1_8b**  2025-02-18T23:19:23Z:

根据您提供的信息，可能诊断出的是 Fanconi 综合征（Fanconi Anemia）。其他五种可能的诊断包括：

1. 伯基特综合征（Buerger’s disease）：这是一个罕见的血管疾病，可以导致手臂和脚部血管狭窄，从而引起局部症状。
2. 迁延性肌营养不良症（Carnitine Palmitoyltransferase II deficiency）：这是一种遗传性的代谢疾病，可以影响肌肉功能，导致肌肉无力和骨骼畸形。
3. 肾上腺素毒性综合征（Cathecholamine toxicosis）：这是一种罕见的代谢疾病，可以导致血液系统异常、心脏问题和其他多器官系统的问题。
4. 特定类型的心脏瓣膜病：某些类型的心脏瓣膜病，如复杂瓣膜病（Complex valve disease），可以与先天性发育异常、骨髓衰竭和肿瘤发生率增高有关。
5. 迁延性肌营养不良症（Myopathy, Carnitine Palmitoyltransferase II deficiency）：这是一种遗传性的代谢疾病，可以影响肌肉功能，导致肌肉无力和骨骼畸形。

请注意，这些诊断都与您提供的信息有关，但最可能的诊断仍然是 Fanconi 综合征。

**43. user 2025-02-18T23:19:41Z:**

经典型患儿常在围生期发病，在摄取母乳或含乳糖配方奶粉数天内，患儿会出现危及生命的并发症：喂养问题、腹泻、呕吐、低血糖、肝功能损伤、出血、黄疸、 白内障。如果未及时治疗，可能会发生败血症、休克和死亡。存活至婴儿期的患儿，如果继续摄取乳糖，可能会出现严重的脑损伤。一些患儿有智力落后、生长发育延迟、共济失调、失明以及女性患者的卵巢功能障碍等远期并发症。

**llama3.1_8b**  2025-02-18T23:19:43Z:

基于你提供的临床表现，我认为最可能的诊断是乳糖不耐受（Lactose intolerance）。但是，这个疾病通常在几个月大后出现，而不是围生期。

更可能的答案是乳糖血症（Galactosemia），这是一个罕见的遗传性疾病，主要影响新生儿。在摄取含有牛奶或乳糖的食物后，患儿会出现严重的健康问题，如喂养困难、腹泻、呕吐、低血糖、肝功能损伤等。

**44. user 2025-02-18T23:20:29Z:**

1. Ⅰ型最常见亚型。无原发性中枢神经系统受累表现，一些患者随着疾病进展可能出现继发神经系统临床表现（如脊髓受压等）。各年龄段均可发病，约 2/3 患者在儿童期发病。脏器表现主要为肝脾肿大，尤以脾肿大显著，常伴脾功能亢进，甚至出现脾梗死、脾破裂等。血液学主要表现为血小板减少和贫血，部分患者白细胞减少，可伴有凝血功能异常。患者表现为面色苍白、疲乏无力、皮肤及牙龈出血、月经增多，甚至出现危及生命的出血现象。多数患者有骨骼受侵，但轻重不一。早期受侵犯部位主要包括腰椎、长骨干骺端和骨干，中后期可累及骨骺和骨突。患者常有急性或慢性弥漫性骨痛，严重者可出现骨坏死（缺血性坏死），随后可出现关节塌陷累及股骨近端和远端、胫骨近端和肱骨近端。还可发生溶骨性病变、病理性骨折、椎体压缩骨折及其他因骨密度降低导致的脆性骨折。骨骼病变可影响日常活动，并可致残。儿童患者常见的表现依次是骨质疏松、长骨干骺端烧瓶样畸形、长骨干骺端密度不同程度的减低、骨皮质变薄等，可有生长发育迟缓。部分患者可有肺部受累，主要表现为间质性肺病、肺实变、肺动脉高压等。此外，患者还会出现糖和脂类代谢异常、多发性骨髓瘤等恶性肿瘤发病风险增高、胆石症、免疫系统异常等表现。
2. Ⅱ型，Ⅱ型患者除有与Ⅰ型相似的肝脾肿大、贫血、血小板减少等表现外，主要为急性神经系统受累表现。常发病于新生儿期至婴儿期，进展较快，病死率高。有迅速进展的延髓麻痹、动眼障碍、癫痫发作、角弓反张及认知障碍等急性神经系统受损表现，生长发育落后，2~4 岁前死亡。一些重度患者会出现关节挛缩。
3. Ⅲ型早期表现与Ⅰ型相似，逐渐出现神经系统受累表现，常发病于儿童期，病情进展缓慢，寿命可较长。患者常有动眼神经受侵、眼球运动障碍，并有共济失调、角弓反张、癫痫、肌阵挛，伴发育迟缓、智力落后。Ⅲ型可分为 3 种亚型，即以较快进展的神经系统症状（眼球运动障碍、小脑共济失调、痉挛、肌阵挛及痴呆） 及肝脾肿大为主要表现的Ⅲa 型；以肝脾肿大及骨骼症状为主要表现而中枢神经系统症状较少的Ⅲb 型；其他症状较轻，以心脏瓣膜钙化及角膜混浊为特殊表现， 主要出现在德鲁兹人群的Ⅲc型。

**llama3.1_8b**  2025-02-18T23:20:33Z:

根据您的描述，我认为最有可能的诊断是：

1. Ⅰ型的病情表现，即一种罕见的骨髓疾病，与骨骼、肝脾、血液学以及神经系统受累有关。这种疾病也可以导致多种并发症，如骨折、关节塌陷等。

其他五种可能的诊断是：

1. 霍奇金淋巴瘤：一种罕见的癌症，可以影响骨髓和其他器官。
2. 贝尔病：一种罕见的神经系统疾病，主要影响儿童和青少年。
3. 蕾因-霍夫 曲（RhD）免疫反应障碍：一种罕见的血液学疾病，可以导致溶血性贫血等症状。
4. 苯丙酮症：一种罕见的遗传性疾病，可以影响多个器官系统，包括神经系统、骨骼和其他身体部分。
5. 组织细胞淋巴瘤（T-ALL）：一种罕见的癌症，可以影响骨髓和其他器官。

**45. user 2025-02-18T23:21:13Z:**

经常从一组肌群开始，逐渐累及其他肌群，以上睑下垂和复视为最常见的发病症状， 占 80％。骨骼肌无力表现为波动性和易疲劳性，晨轻暮重。根据临床表现，分成五型。Ⅰ型：眼肌型，表现为上睑下垂、复视等。ⅡA 型：轻度全身型，四肢肌群轻度受累。ⅡB 型：中度全身型，四肢肌群中度受累，通常有咀嚼、吞咽和构音障碍。Ⅲ型： 重度激进型，进展快，数周或数月内累及咽喉肌，半年内累及呼吸肌。Ⅳ型：迟发重度型，缓慢进展，2 年内累及呼吸肌。Ⅴ型：肌萎缩型，起病半年内出现骨骼肌萎缩。神经科查体可见四肢近端、躯干、颈部、颅面部肌肉无力，不耐疲劳，Jolly 试验阳性，腱反射正常，无感觉障碍，无病理征。部分病人可进展至呼吸衰竭，出现重症肌无力危象。

**llama3.1_8b**  2025-02-18T23:21:16Z:

根据你提供的临床表现，似乎是诊断为重症肌无力的可能性较大。其他五种可能的诊断包括：

1. 维生素D缺乏性佝偻病（Rickets）：虽然主要见于儿童，但成年人也可以出现类似的骨骼和肌肉问题。
2. 神经系统肌炎（Neuromuscular junction disorders）：这种疾病影响了神经和肌肉之间的连接，导致肌肉无力和麻痹。
3. 遗传性肌营养不良症（Familial Muscular Dystrophy）：这是一种遗传性的肌肉疾病，会导致肌肉无力和萎缩。
4. 肌肉炎（Myositis）：这是一个影响肌肉的炎症过程，可以导致肌肉无力、疼痛和肿胀。
5. 自身免疫性肌肉疾病（Autoimmune myopathies）：这是一类由于自身免疫反应引起的肌肉疾病，会导致肌肉无力、萎缩和破坏。

需要注意的是，这些疾病都有不同的临床表现，所以需要根据具体情况进行综合诊断。

**46. user 2025-02-18T23:21:42Z:**

常于青少年或成年早期起病。临床表现主要与低血钾和低血镁相关，轻型患者可无症状或表现为轻度乏力和纳差；严重患者会出现四肢抽搐、软瘫、痛性痉挛、晕厥和横纹肌溶解继发急性肾损伤，甚至因为严重室性心律失常导致心搏骤停。常见临床表现如下：

1. 全身症状 疲乏、口渴、多饮、嗜盐。
2. 神经 - 肌肉系统 肌无力、痛性痉挛、抽搐、惊厥发作、肢体麻木、感觉异常、横纹肌溶解、瘫痪、头晕、眩晕、共济失调和假性脑瘤等。
3. 心血管系统 心悸、晕厥、血压正常或偏低和室性心律失常等。
4. 消化系统 便秘、呕吐等。
5. 泌尿系统 多尿、夜尿增多、遗尿、蛋白尿和肾功能不全。
6. 骨关节系统 关节痛、软骨钙质沉着症。
7. 内分泌和生长发育 生长迟缓、青春期延迟；长期低钾和低镁的患者糖尿病或者糖耐量减低的比例并不少见。
8. 眼部症状 少数患者会出现视物模糊和巩膜脉络膜钙化。

**llama3.1_8b**  2025-02-18T23:21:46Z:

根据您的描述，我认为最可能的诊断是镁缺乏性低钾综合征（Hypokalemic Periodic Paralysis, HPP）。这是一个罕见的遗传疾病，表现为周期性的低血钾和低血镁，并导致肌肉无力、抽搐等症状。

其他五种可能的诊断包括：

1. 维生素D缺乏性佝偻病：虽然主要影响儿童，但成年人也可能出现类似的临床表现，如骨骼钙质沉着和低血镁。
2. 肾脏疾病（如肾病综合征）：可能导致电解质紊乱，包括低血镁和低血钾，并影响多个身体系统。
3. 迟发性肌无力（Duchenne 运动力）或贝克特-谢氏综合征：这两种疾病都可以导致慢性低血镁，但临床表现更为复杂，包括肌肉无力、萎缩等。
4. 崩溃酸症：一种罕见的代谢疾病，可以导致电解质紊乱和肌肉无力等症状。
5. 神经肌肉接头疾病（如 Guillain-Barré 综合征）：虽然主要影响神经系统，但也可能导致肌肉无力和低血镁。

**47. user 2025-02-18T23:22:11Z:**

大多数患儿于婴幼儿期发病，表现多样。发病越早，症状越重，预后越差。大部分患者生后 1 年内表现相对正常，约 75％的患者最早出现的体征是头大，多数患儿出生时头围较同龄儿大或生后不久头围迅速增大，可伴轻微的非特异性症状，包括易激惹、喂养困难和呕吐等。患儿通常生后 3~36 月龄间，在发热、感染、手术或预防接种等诱因后出现酮症、呕吐、肝大和急性脑病危象表现，包括肌张力低下、意识丧失和惊厥发作等，对症治疗后症状可缓解，但不能完全恢复。如果急性脑病危象反复发生，神经系统损伤将进行性加重，可有发育倒退现象，最终可出现认知功能障碍。部分患儿在生后数年逐渐出现运动延缓、肌张力异常和随意运动障碍，但智力发育基本正常。患者常在 10 岁内死于伴发疾病或 Reye 样发作，随年龄增长发作减少。极少数患者于青春期甚至成年时期发病，首次发病之前可无症状。

**llama3.1_8b**  2025-02-18T23:22:15Z:

根据你的描述，我认为可能的诊断是苯丙酮尿症（Phenylketonuria, PKU）。PKU是一种遗传性疾病，由于体内缺乏某一酶而导致苯丙氨酸在身体中积累，进而引起一系列神经系统的损害。这种疾病如果未及时诊断和治疗，将会导致严重的神经发育障碍、智力下降甚至死亡。

其他五种可能的诊断是：

1. 甲状腺功能减退症：这是一种内分泌系统疾病，表现为生长迟缓、智力低下和其他多个身体方面的问题。
2. 肝脏疾病（如肝炎）：虽然你的描述中没有明确提到肝脏问题，但某些罕见的肝脏疾病也可能导致类似的临床表现，包括发热、肌肉无力和神经系统损害。
3. 神经元退行性疾病（如 Huntington）：这些疾病通常在青少年或成年时期开始表现，但某些罕见的形式也可能在婴幼儿期开始。
4. 脑白质疾病（如多发性硬化症）：这类疾病通常会导致肌肉无力、感知异常和运动功能障碍等临床表现，尽管它们也可能以不同的方式表现出来。
5. 遗传代谢病（如 Krebs-Enzypathie）：这些疾病通常会导致脑部、神经系统和其他身体系统的损害，表现为发热、肌肉无力、智力下降等问题。

**48. user 2025-02-18T23:22:36Z:**

主要表现为关节、肌肉和深部组织出血，也可表现为胃肠道、中枢神经系统等内部脏器出血等。若反复出血，不及时治疗可导致关节畸形和(或) 假肿瘤形成，严重者可危及生命。外伤或手术后延迟性出血是本病的特点。

**llama3.1_8b**  2025-02-18T23:22:40Z:

根据你的描述，我最有可能诊断出的罕见病是:

1. **血友病**：这是一个遗传性的凝血障碍，表现为关节、肌肉和深部组织的出血，并且外伤或手术后延迟性出血。

以下是其他五种可能的诊断：

1. **亨奇-法利症候群**：是一种罕见的遗传性疾病，表现为凝血功能障碍和多系统受累。
2. **维生素K依赖性凝血因子缺乏症**：是一种罕见的凝血障碍，表现为外伤或手术后延迟性出血。
3. **Factor XIII缺乏症**：是一种罕见的凝血障碍，表现为重度出血和假肿瘤形成。
4. **血小板凝集素缺乏症**：是一种罕见的凝血障碍，表现为重度出血和关节、肌肉等组织受累。

请注意，这些诊断并不是绝对的，并且需要通过进一步的医疗检查和实验室测试来确认。

**49. user 2025-02-18T23:22:59Z:**

可累及全身多个脏器，临床表现多样。发病年龄多为 3~60 岁，也有8 个月及 70 多岁诊断的患者报道。儿童患者多以肝脏受累为首发表现，青少年及成人以神经系统受累为首发症状的比例增高。

可表现为无症状的转氨酶持续升高、慢性肝炎、肝硬化和急性肝功能衰竭。神经系统受累可表现为运动功能障碍、震颤、共济失调、舞蹈症、自主运动障碍、肌张力障碍，一些患者表现为面具脸、四肢僵硬、步态异常等。一些患者合并精神行为异常，如淡漠、攻击行为、性格改变等。肾脏受累可表现为血尿、蛋白尿等。血液系统受累可表现为溶血性贫血、肝硬化、脾功能亢进致血液三系下降、凝血功能异常等。相对少见的受累系统还包括骨关节、心脏、内分泌和生殖系统等。

**llama3.1_8b**  2025-02-18T23:23:04Z:

根据您的描述，我认为可能诊断出的是威尔逊病（Wilson’s disease），是一种遗传性疾病，由于铜离子在体内的异常积累，导致肝脏、神经系统、血液系统和其他多个脏器受累。患者可能表现为肝炎、肝硬化、神经系统症状，如舞蹈症、震颤等。

以下是五种可能的诊断：

1. **威尔逊病（Wilson’s disease）**：由铜离子在体内的异常积累，导致肝脏、神经系统、血液系统和其他多个脏器受累。
2. **亨廷顿病（Huntington’s disease）**：一种遗传性疾病，由于CAG-repeat的扩增，导致基因编码蛋白质的异常积累，从而影响大脑的功能，最终导致运动功能障碍、认知功能障碍和精神行为异常。
3. **帕金森病（Parkinson’s disease）**：一种神经系统疾病，由于多巴胺能神经元损伤，导致运动功能障碍、震颤等症状。
4. **苯丙酮尿症（Phenylketonuria, PKU）**：一种遗传性疾病，由于苯丙氨酸在体内的异常积累，导致肾脏受累，并可能影响神经系统和其他多个脏器。
5. **高铁血红蛋白病（Hemochromatosis）**：一种遗传性疾病，由于铁离子在体内的异常积累，导致肝脏、肾脏和其他多个脏器受累，并可能影响血液系统。

**50. user 2025-02-18T23:23:26Z:**

临床表现具有很大的异质性，甚至同一家系中的患者也有很大差异。 通常在 30 岁前起病，青春期加重，水肿常呈急性发作。临床上以反复发作、难以预测的皮肤和黏膜下水肿为特征。水肿的特点呈发作性，自限性，一般 3~5 天自然缓解，非对称性，非可凹性。水肿可累及身体任何部位，以四肢、颜面、生殖器、呼吸道和胃肠道黏膜较为常见。其中最致命的是呼吸道黏膜水肿，可因喉水肿迅速进展导致呼吸困难或窒息， 如抢救不及时可窒息死亡，致死率高达 11％ ~40％，是 主要死因之一。消化道黏膜水肿发作表现为剧烈腹痛，伴恶心、呕吐，因此常被误诊为急腹症，导致不必要的腹部手术。发生于肢体等部位的皮肤水肿则会影响其功能。

**llama3.1_8b**  2025-02-18T23:23:30Z:

根据您提供的临床表现，我最有可能诊断出的疾病是：

1. 血管性紫癜（Henoch-Schönlein Purpura，HSP）：是一种自身免疫性疾病，其特征是在皮肤、黏膜和内脏器官出现水肿，常见于儿童和青少年，水肿的特点是发作性、自限性，并且常伴有血管炎。

以下五种疾病也可能与临床表现相吻合：

1. 荨麻疹（Urticaria）：是一种皮肤过敏反应，其特征是在皮肤出现水肿，通常呈现为荨麻疹样皮疹。
2. 皮肌炎（Dermatomyositis）：是一种自身免疫性疾病，其特征是在皮肤和肌肉出现水肿和炎症，常见于儿童和成人。
3. 肾脏综合征（Nephrotic Syndrome）：是一种肾脏疾病，其特征是大量蛋白尿、水肿、脂肪肝等症状。
4. 维持细胞增多症（Hereditary Angioedema，HAE）：是一种遗传性疾病，其特征是在皮肤和黏膜出现水肿，并常伴有血管炎。

请注意，这些诊断并不是绝对的，最准确的诊断需要结合临床表现、实验室检查结果以及其他相关信息。

**51. user 2025-02-18T23:23:47Z:**

出生即起病，临床表现的轻重与疾病亚型相关。临床表现为手部、肘部、膝及足部和其他容易摩擦的部位或周身出现水疱或大疱。可以出现口腔黏膜水疱或溃疡，导致吞咽困难和声音嘶哑。部分患者伴有斑驳性色素异常、秃发、掌跖角化过度、牙釉质缺陷、甲增厚和甲营养不良。其他损害包括反复瘢痕形成导致的指端残毁、贫血、骨质疏松、肌肉萎缩、心肌病、耳聋。部分亚型晚期可伴发基底细胞癌、鳞状细胞癌及恶性黑色素瘤。

**llama3.1_8b**  2025-02-18T23:23:52Z:

根据您的描述，我认为可能的诊断是：

1. **皮肤粘液样肉芽疹病（Epidermolysis Bullosa, EB）**：这是一个罕见的遗传性疾病，表现为皮肤和黏膜出现水疱或大疱，尤其是在易摩擦的部位。一些EB亚型还会引起其他系统性的问题，如口腔黏膜损伤、色素异常、甲增厚等。
2. **库欣病（Kushner Syndrome）**：这是一个罕见的综合征，表现为皮肤和黏膜出现水疱或大疱，伴有其他症状如口腔黏膜损伤、色素异常、甲增厚等。
3. **白癜风（Lupus）**：虽然不是典型的罕见病，但是某些白癜风患者可能出现皮肤和黏膜水疱或大疱，尤其是在易摩擦的部位。
4. **真菌感染导致的皮肤病变（Fungal Infections）**：某些真菌感染，如环形斑型红斑（Ringworm），可以出现皮肤和黏膜水疱或大疱，尤其是在易摩擦的部位。
5. **特发性高铁血红蛋白病（Erythropoietic Protoporphyria, EPP）**：这是一个罕见的遗传性疾病，表现为皮肤和黏膜出现水疱或大疱，尤其是在易摩擦的部位。
6. **基底细胞癌（Basal Cell Carcinoma）**：虽然不是一种罕见病，但是某些基底细胞癌患者可能出现皮肤水疱或大疱。

请注意，上述诊断仅为推测，并不排除其他可能性。最准确的诊断需要根据具体情况进行临床检查和实验室检查。

**52. user 2025-02-18T23:24:08Z:**

患者的临床表现随年龄、接触果糖的量和时间而存在差异。一般而言，发病年龄越小，症状越重。经典型患儿于 6 个月左右起病，通常在首次食用了含有果糖的食物，如水果、蜂蜜和某些蔬菜时发病。常见临床症状包括进食含果糖食物后出现低血糖发作，表现为恶心、呕吐、腹痛、腹胀、出汗、震颤、抽搐甚至昏迷。

如果诊断未明，患者长期慢性摄入果糖食物，除了低血糖表现外，会出现生长发育迟缓，肝肿大、腹水、黄疸及代谢紊乱，如低血糖、代谢性酸中毒、低磷酸盐血症、高尿酸血症、高镁血症、高丙酮酸血症。病情严重者出现多脏器损害，肝、肾功能衰竭，抽搐、昏迷，甚至死亡。

在急性发作时，实验室检查会发现低血糖、低血磷和低血钾，同时伴有尿酸、乳酸、丙酮酸、游离脂肪酸和甘油三酯升高。慢性患者出现肝功能损害及肝纤维化的指征，包括血清胆红素，转氨酶升高，凝血障碍。尿液检查可见蛋白尿及果糖阳性。

**llama3.1_8b**  2025-02-18T23:24:11Z:

根据您的描述，最可能的诊断是**果糖血症（Hereditary Fructose Intolerance）**。

此外，还有以下五种罕见病可能与这些临床表现相关：

1. **葡萄糖-6-磷酸脱氢酶缺乏症（Gluconolactone Dehydrogenase Deficiency）**
2. **乳酸血症（Lactic Acidemia）**
3. **低磷酸盐血症（Hypophosphatemia）**：可能是由于果糖血症引起的代谢紊乱
4. **肝脏衰竭（Liver Failure）**
5. **遗传性内分泌缺陷（Inherited Endocrine Deficiency）**

请注意，这些都是罕见疾病，不一定会出现所有这些临床表现。最准确的诊断需要进一步的实验室检查和专家的评估。

**53. user 2025-02-18T23:24:36Z:**

**1. 偏头痛** 先兆偏头痛常为该疾病的首发症状，见于 20％ ~40％的患者，是普通人群的 5倍；也可表现为无先兆偏头痛，发病率与普通人群无差异。亚裔患者偏头痛发生率较欧美患者低。

**2. 皮层下缺血性事件** 短暂性脑缺血发作（TIA） 和缺血性卒中是该疾病最常见的临床表现，见于 60％ ~85％的症状性患者，且大多数患者缺乏常见的脑血管病危险因素。2/3 的患者表现为经典的腔隙综合征。

**3. 认知缺陷** 60％的患者逐渐出现认知功能障碍与痴呆。

**4. 情感障碍与淡漠** 20％的患者具有情感障碍，抑郁状态最常见，极少数可表现为抑郁与躁狂状态交替出现；淡漠见于 40％的患者。

此外，5％ ~10％患者有癫痫发作，也有听力下降、意识障碍、脊髓梗死、颅内出血以及合并流域性梗死的报道。

**llama3.1_8b**  2025-02-18T23:24:38Z:

根据你的描述，我最可能诊断出的罕见病是**遗传性肌营养不全（MMD）**。以下五种疾病也是可能的诊断：

1. **沃森病**：一种遗传性的神经退行性疾病，影响运动和认知功能。
2. **克罗恩病相关性脑炎**：一种罕见的自身免疫性疾病，影响大脑和身体其他部位。
3. **韦尼克-卡加斯病**：一种罕见的神经退行性疾病，影响运动、感觉和认知功能。
4. **多动症**：一种神经系统疾病，其特征是注意力缺陷、过度活动和其他行为问题。
5. **马登综合征**：一种罕见的遗传性疾病，导致认知障碍、情感问题和运动功能障碍。

这些疾病与你的描述中提到的临床表现有相似之处，但需要进一步检查和诊断才能确认。

**54. user 2025-02-18T23:24:58Z:**

通常在青年早期（但 1 岁至 76 岁起病均有报道） 隐匿起病。主要表现为缓慢进展的双下肢痉挛性瘫痪，查体可见锥体束征（腱反射亢进，踝阵挛阳性、病理征阳性）。双上肢反射也可亢进，但一般不影响上肢活动。超过一半的患者会有下肢近端力弱表现。约 50％的患者查体中可发现双踝音叉震动觉减低（一般不会消失），另约 1/3 的患者有排尿障碍。

随着年龄增长，疾病逐渐进展，约 20％的患者最终会丧失行走能力。在同一家系中， 携带同一突变的不同患者，临床症状轻重可有较大差异。

**llama3.1_8b**  2025-02-18T23:24:59Z:

根据您提供的信息，这可能是罕见病遗传性肌营养不良（Spinal Muscular Atrophy, SMA）的特征。SMA是一种影响神经系统和肌肉的遗传性疾病，主要表现为肌肉萎缩和无力。

**55. user 2025-02-18T23:25:26Z:**

患儿多在新生儿、婴儿早期发病。临床表现与生物素酶缺乏相似，无特异性，主要有喂养困难、呕吐、腹泻、肌张力低下、惊厥、精神萎靡、嗜睡、呼吸困难、发育落后、顽固皮疹、脱皮，可合并酮症、代谢性酸中毒、高乳酸血症等。皮肤损害中大部分患者在头面部、颈部、躯干、臀部等部位皮肤红疹或红斑、溃烂或水疱、糠状或片状鳞屑，或皮肤干糙、脱皮等，少数仅在口周、眼周、肛周局部出现皮疹。患者不伴听力或视力障碍，不同于生物素酶缺乏症。

**llama3.1_8b**  2025-02-18T23:25:31Z:

基于您提供的临床表现，我认为最有可能的诊断是：

1. **原发性草酸尿症（Hartnup病）**：这种罕见疾病主要影响皮肤和神经系统，表现为皮肤红斑、溃烂或水疱、糠状或片状鳞屑，以及肌张力低下、惊厥等神经症状。

其它五种可能的诊断：

1. **生物素酶缺乏**：这种疾病也会导致皮肤问题，但与原发性草酸尿症相比，其临床表现更加全面，包括更严重的营养困难和神经系统的问题。
2. **牛奶四联症（Lactose intolerance）**：虽然可能有喂养困难和呕吐，但这通常是由于乳糖不耐受引起的，与其他临床表现相比，这个诊断似乎不太可能。
3. **新生儿溶血病（Hemolytic Disease of the Newborn）**：这种疾病主要影响红细胞，并不会导致皮肤红斑或溃烂，主要症状是黄疸、贫血等，与原发性草酸尿症不太相似。
4. **新生儿低钙血症（Hypocalcemia）**：这个诊断可能会引起肌张力低下和惊厥，但并不典型地与皮肤红斑或溃烂相关联。
5. **婴幼儿急性化脓性坏死性脑炎（Meningococcemia）**：这种疾病主要影响中枢神经系统，并可能伴有全身性感染，临床表现与原发性草酸尿症不同。

**56. user 2025-02-18T23:25:48Z:**

该病患儿出生时多无异常，婴儿期临床表现并无特异性，仅表现为生长和发育迟缓。常于 3 岁有眼部症状和体征后得以诊断。

1. 视力受损， 异位晶状体所致的近视和虹膜震颤等症状可在 3 岁后出现，晚期可合并散光、青光眼、葡萄肿、白内障、视网膜剥离及视神经萎缩。
2. 智力障碍较常见，主要表现为进展性智力障碍。维生素 B6 治疗敏感型患者智力障碍较轻。
3. 精神或行为障碍， 大约 50％患儿可出现，20％患儿合并惊厥。
4. 骨骼受累， 类似马方综合征表现，或合并脊柱侧凸，漏斗胸，鸡胸，膝外翻及弓形足，X 线可见全身性骨质疏松。
5. 血栓性疾病， 发生于各个年龄段，可累及各类血管，尤其是脑血管。高水平同型半胱氨酸血症可改变血管壁及增加血小板黏附性，出现视神经萎缩、瘫痪、肺心病及肾性高血压。
6. 自发性气胸和急性胰腺炎 较少见。

**llama3.1_8b**  2025-02-18T23:25:54Z:

基于提供的临床表现和症状，我认为最可能的诊断是：

1. **亨丁顿病（Huntington’s disease）**：这是一个遗传性的神经退行性疾病，主要影响大脑。它的典型特征包括运动功能障碍、认知问题和行为变化。这与提供的临床表现相符，如智力障碍、精神或行为障碍以及骨骼受累等。

其他可能的诊断为：

1. **纤维体病（Fibrocytic disease）**：一种罕见的遗传性疾病，影响肌肉和神经系统。它的症状包括运动功能障碍、智力障碍和骨骼受累等。
2. **巴尔德施密特症候群（Bardet-Biedl syndrome）**：一种罕见的遗传性疾病，影响多个身体系统，包括视觉、认知和运动功能。它的典型特征包括视力受损、智力障碍和骨骼受累等。
3. **纽伦堡综合症（Nuerenbergs syndrome）**：一种罕见的遗传性疾病，影响大脑和神经系统。它的典型特征包括智力障碍、运动功能障碍和行为问题等。
4. **克莱因-弗勒斯彻综合症（Klein-Fleischer syndrome）**：一种罕见的遗传性疾病，影响大脑和神经系统。它的典型特征包括智力障碍、运动功能障碍和骨骼受累等。
5. **卡德综合症（Card syndrome）**：一种罕见的遗传性疾病，影响多个身体系统，包括视觉、认知和运动功能。它的典型特征包括视力受损、智力障碍和骨骼受累等。

需要注意的是，这些可能的诊断都与提供的临床表现相符，但最终的诊断还需要根据进一步的检查和评估进行确认。

**57. user 2025-02-18T23:26:10Z:**

患者的主要表现有：出生后即发现 LDL-C 水平明显升高，胆固醇沉积在皮肤、眼睛以及肌腱形成黄色瘤和脂性角膜弓。由于机体暴露于高水平的 LDL-C 中， 患者多数会在 20 岁前就出现冠状动脉粥样硬化性心脏病并可能因此死亡。

需要详细询问患者的家族史，部分患者父母均为杂合子并因此出现 LDL-C 水平升高（通常大于本国特定年龄和性别标准的 95百分位数） 以及早发动脉粥样硬化性心血管疾病强阳性家族史。

**llama3.1_8b**  2025-02-18T23:26:15Z:

根据您提供的信息，我认为可能诊断出的罕见病是：

1.**弗里德雷希-蒂恩症候群（FHDD，Familial Hypercholesterolemia）**：这是一种遗传性的疾病，表现为 LDL-C 水平显著升高，并导致胆固醇沉积在皮肤、眼睛和肌腱。患者往往在年轻时即出现冠状动脉粥样硬化性心脏病。

其他五种可能的诊断：

2.**家族性高胆固醇血症（FHLS，Familial Hypercholesterolemia）**：这也是一种遗传性的疾病，与 FHDD 相似，但 LDL-C 水平升高不如前者那么显著。

3.**塔尔特综合征（TS，Tangier Disease）**：这是另一项罕见的脂质代谢障碍症状，表现为 LDL-C 水平降低，并伴有过量的肝脏脂肪酸（VLDL）。

4.**家族性高剂量脂蛋白血症（FHC，Familial Hyperalphalipoproteinemia）**：这是一种罕见的疾病，其特征是极高水平的 HDL-C 和相对较低的 LDL-C。

5.**卡拉赫综合征（CKCS，Cerebrotendinous Xanthomatosis）**：这是另一种罕见的脂质代谢疾病，其特点是存在黄色瘤和肌腱炎等症状，但与您的描述相比，这种疾病主要影响的是中枢神经系统。

6.**家族性高剂量脂蛋白血症综合征（FHC，Familial Hyperalphalipoproteinemia）**：这是一种罕见的疾病，其特点是极高水平的 HDL-C 和相对较低的 LDL-C。

**58. user 2025-02-18T23:26:37Z:**

临床特征表现为运动障碍、精神症状和认知障碍三联征，通常隐匿起病，缓慢进展。发病年龄从儿童期至 79 岁不等，最常见于 30~50 岁。

疾病累及面部、躯干和肢体的快速，不自主，无节律运动；早期异常动作轻微，患者可能难以意识到舞蹈症状的存在，或将舞蹈样动作整合到随意运动中，使得正常动作的控制出现困难或偏差；随着疾病的进展，舞蹈症的范围和程度常逐渐加重，甚至影响膈肌、咽和喉部肌肉，从而产生构音障碍、吞咽困难和不自主发声；疾病晚期舞蹈症常消失，代之以僵直、少动为主的帕金森症样表现，可伴有局灶性肌张力障碍；最后常导致卧床。运动保持困难也是该病常见表现，即不能保持某些简单的自主动作，如保持伸舌动作困难等。

精神障碍可先于舞蹈症出现，常见症状包括情绪低落、抑郁、易激惹、淡漠和焦虑， 也可出现偏执、妄想和幻觉；少见症状包括强迫行为和精神病表现，以上症状多呈进行性加重。

认知障碍最主要的特征是执行功能障碍，表现为做决定、执行多重任务和转换认知目标的能力下降。患者通常对其认知缺陷缺乏自知力。记忆减退或丧失多出现在病程晚期， 随疾病进展，最终可发展为痴呆。

除以上三联征外，眼球运动异常也是一个突出表现。早期表现为眼球扫视速度减慢，但眼球追随运动保留；晚期则眼球追随运动、自主扫视和再固定全部受损。此外，体重减轻和恶病质也是该病的常见特征，患者的不自主运动大量消耗能量可使体重明显下降，而通常无食欲减退。睡眠和(或) 性功能障碍也比较常见。

运动和认知功能的逐渐恶化会导致显著的并发症，如咽喉部肌肉受累导致吞咽困难， 运动障碍、活动减少甚至卧床可导致吸入性肺炎和其他感染、褥疮等。

**llama3.1_8b**  2025-02-18T23:26:39Z:

根据你提供的临床表现，我认为最可能的诊断是亨廷顿病（Huntington’s disease）。亨廷τον病是一种遗传性的神经退行性疾病，特征是运动障碍、精神症状和认知障碍三联征。其他五种可能的诊断包括：

1. 强直性痉挛性球形细胞瘤（Huntington-like disorder）
2. 扑翼舞舞蹈病（Chorea-acanthocytosis）
3. 病理性舞蹈症（Syndromes with chorea）
4. 舞蹈样动作障碍（Dyskinesias）
5. 複合运动障碍（Complex movement disorders）

**59. user 2025-02-18T23:26:58Z:**

临床症状、体征主要以神经系统症状为主，临床表现严重程度亦不同。发病年龄可以从新生儿期到成年期（晚发型），并且具有广泛的表型谱。

1. 新生儿期, 约24~48 小时内无明显症状，随后出现与高氨血症相关的症状［包括嗜睡、困倦、拒食、呕吐，伴呼吸性碱中毒和（或） 癫痫发作］。与新生儿发病的其他尿素循环障碍疾病难以区分。
2. 晚发型 , 该型患者占 88％，其中 40％发生在 3 岁前，29％在儿童期，19％在成人期。主要表现为：①慢性神经认知缺陷，包括发育迟缓、共济失调、痉挛、学习障碍、认知缺陷和（或） 无法解释的癫痫发作；②由多种因素（如感染，禁食或损伤） 诱发的继发于高氨血症危象的急性脑病；③慢性肝功能障碍，肝转氨酶不明原因升高伴有或不伴有轻度凝血功能障碍，伴或不伴轻度高氨血症和蛋白质不耐受。

**llama3.1_8b**  2025-02-18T23:27:01Z:

根据你的描述，我可能会诊断出的是克汀综合征（Citrullinemia），它是一种遗传性的尿素循环障碍疾病。其他五种可能的诊断包括：

1. **尿素循环障碍**：一组与氨清除和尿素合成有关的代谢异常。
2. **高氨血症**：一种由于肝脏无法正常处理氨而导致的血液中氨含量升高的状况。
3. **肝性脑病**：由肝功能障碍引起的脑部疾病，可能包括嗜睡、困倦、意识丧失等症状。
4. **新生儿代谢疾病**：一组由于新生儿在出生时就存在的遗传性代谢异常所致的疾病，可能影响到新生儿的身体各个系统。
5. **脑炎或神经系统感染**：某些感染（如脑炎）或其他神经系统疾病可能导致类似的临床表现。

然而，需要注意的是，每种疾病都有其特定的症状和体征，所以只有通过综合考虑临床表现、实验室检查结果和家族史等信息才能确切地诊断。

**60. user 2025-02-18T23:27:25Z:**

部分患儿在新生儿期多无临床症状，出生 3-4 个月后逐渐出现典型症状：头发由黑变黄，皮肤颜色浅淡，尿液、汗液鼠臭味。随着年龄增长，逐渐表现出智力发育落后、小头畸形、癫痫发作，也可出现行为、性格、神经认知等异常。如多动、自残、攻击、自闭症、自卑、忧郁等，婴儿期还常出现呕吐、湿疹等。 部分 患者在新生儿期也多无临床症状，出生 1-3 个月后除表现 PKU 症状外，主要表现为运动障碍、嗜睡、躯干肌张力低下、四肢肌张力增高或低下、眼震颤、吞咽困难、口水增多、松软、角弓反张，反应迟钝、失眠、智力发育严重障碍等。

**llama3.1_8b**  2025-02-18T23:27:29Z:

根据提供的临床表现，我认为可能的诊断是：

1. 苯丙酮尿症（PKU）：这是一个遗传性疾病，由于体内缺乏苯丙氨酸脱氢酶，导致苯丙氨酸积累，引起一系列临床表现。
2. 克氏肌张力增高症：这是一个罕见的神经系统疾病，表现为肌张力增高、四肢僵硬和姿势异常等。
3. 强直-痉挛型脑瘫：这是一种常见于儿童的神经系统疾病，表现为运动障碍、吞咽困难、口水增多等。
4. 癫痫：这是一个由于神经系统异常导致的临床综合征，表现为意识改变、肌肉抽搐和癫痫发作等。
5. 智力发育迟缓症：这是一种罕见的遗传性疾病，表现为智力发育迟缓、行为异常和神经认知功能障碍等。

其他五种可能的诊断包括：

1. 白细胞缺陷症（SCID）
2. 过敏性休克综合征
3. 肾上腺髓样体减退症
4. 褪色素失落症
5. 皮肤病变综合征

需要注意的是，以上所有疾病都是罕见的，诊断和治疗需要根据具体临床表现进行调整。

**61. user 2025-02-18T23:28:09Z:**

临床表现的严重程度具有很大的差异。患者可以表现为骨骼矿化异常、牙齿或关节病变、肌肉乏力等。目前，疾病主要分为 7 种类型。

1. 单纯牙型， 是最轻、也是最常见的类型，仅有牙齿受累表现，无佝偻病或骨软化症的表现。
2. 成人型，常中年发病，表现为牙齿异常或骨软化症。由于反复骨折、骨骼和关节疼痛或肌肉无力，可引起活动障碍。
3. 儿童型，常在 6 月龄后发病，可轻度到重度，患儿可有不同程度的牙齿脱落、佝偻病的表现。
4. 婴儿型 ，于 6 月龄前发病，常表现为纳差、难以存活、衰弱，可有囟门宽阔或头颅畸形。50％的婴儿型 HPP 会死于各种并发症。
5. 围生期型，是极端类型，在宫内即发病，出生时症状明显，几乎均是致死型。患儿四肢短小、严重骨骼矿化不足，可表现为高调哭喊、癫痫发作、周期性呼吸暂停、伴有发绀和心动过缓、不明原因发热、易激惹、骨髓抑制性贫血和颅内出血、肺不张。
6. 假性低磷酸酯酶症， 类似于婴儿型，患者具有婴儿型低磷酸酯酶症的表现，但血清 ALP 正常或升高。
7. 良性型， 有时在宫内出现骨骼畸形，有围生期低磷酸酶活性，但出生后病情缓解，没有婴儿型低磷酸酯酶症或单纯牙型低磷酸酯酶症的相关表现。

**llama3.1_8b**  2025-02-18T23:28:10Z:

根据您提供的信息，可能的诊断是：

1. 低磷酸酯酶症（HPP）
2. 骨骼矿化异常
3. 牙齿或关节病变
4. 肌肉乏力
5. 佝偻病
6. 骨软化症
7. 围生期低磷酸酯酶症

**62. user 2025-02-18T23:28:42Z:**

患儿主要表现为方颅、鸡胸、串珠肋、手镯征、足镯征、亨利氏沟，多在将近周岁开始负重时出现下肢畸形，可表现为膝内翻（O 形腿） 或膝外翻（X 形腿），并伴有生长迟缓、身材矮小、步态摇摆、进行性加重的骨畸形、多发性骨折、骨骼疼痛以及牙齿发育异常（牙质差、牙痛、脱落后不易再生） 等。影像学表现为骨骼畸形、长骨干骺端增宽和模糊，呈杯口样，杯口内可见许多细条状钙化影如毛刷状。在成人期主要表现为肢体乏力、活动受限、骨痛、多发病理性骨折（四肢长骨、肋骨、骨盆和椎体均可发生）、身高变矮。影像学表现为骨密度普遍减低，骨小梁模糊，呈毛玻璃状，骨盆畸形、假骨折线、椎体呈双凹变形等。

**llama3.1_8b**  2025-02-18T23:28:45Z:

根据你的描述，这名专门研究罕见病的医生最有可能诊断出的是：

**奥斯特里彻-劳格斯病（Osteogenesis imperfecta，OI）**

其他五种可能的诊断是：

1. **骨肉瘤（Osteosarcoma）**
2. **骨骼肌炎症性疾病（Inflammatory bone disease）**
3. **多关节强直性风湿热（Polyarticular Juvenile Idiopathic Arthritis, JIA）**
4. **神经管畸形症（Neurofibromatosis）**
5. **骨性纤维化症（Fibrous dysplasia of bone）**

请注意，这些诊断都是基于你的描述，并不一定准确。实际上，正确的诊断可能需要更多信息和检查结果。

**63. user 2025-02-18T23:30:18Z:**

临床表现有心力衰竭、胸痛、低血压、晕厥、心源性猝死、心律失常和血栓栓塞等。部分患者还会有心脏外的表现，如周围神经病、自主神经病和胃肠道症状等。

**llama3.1_8b**  2025-02-18T23:31:29Z:

根据你提供的临床表现，似乎这个疾病与心血管系统有关。作为一名专门研究罕见病的医生，我认为最有可能诊断出的疾病是：

1. **卡特-赫尔伯特综合征**（Carpenter’s syndrome）：这是一个罕见的先天性心脏疾病，表现为心力衰竭、胸痛、低血压等心血管症状，以及周围神经病和自主神经病。

其他可能的诊断包括：

1. **多发性血管瘤**（Multiple Arterial Aneurysms）：这是一个罕见的血管疾病，表现为胸痛、低血压等心血管症状，以及血栓栓塞和心律失常。
2. **先天性心脏病**（Congenital Heart Disease）：这是一个广泛的范畴，包括许多不同类型的心脏问题，表现为心力衰竭、胸痛等心血管症状，以及周围神经病和自主神经病。
3. **威尔逊病**（Wilson’s disease）：这是一个罕见的遗传性疾病，影响到肝脏和大脑，表现为周围神经病、自主神经病等非心血管症状，以及心律失常和血栓栓塞。
4. **库欣综合征**（Kushner syndrome）：这是一个罕见的免疫系统疾病，表现为周围神经病、自主神经病等非心血管症状，以及心律失常和血栓栓塞。
5. **多发性肌炎**（Multiple Myositis）：这是一个罕见的肌肉疾病，表现为周围神经病、自主神经病等非心血管症状，以及心律失常和血栓栓塞。

这些诊断需要通过进一步的检查和测试来确认。

**64. user 2025-02-18T23:32:42Z:**

**1. 第二性征不发育和配子生成障碍** 男性表现为童声、小阴茎、无阴毛生长、小睾丸或隐睾、无精子生成；女性表现为乳腺不发育、幼稚外阴和原发闭经。

**2. 骨骺闭合延迟** 上部量 / 下部量<1，指尖距 > 身高，易患骨质疏松症。

**3. 嗅觉障碍** 因嗅球和嗅束发育异常，40％ ~60％ IHH 患者合并嗅觉丧失，不能识别气味。

**4. 其他表现** 面中线缺陷，如唇腭裂；孤立肾；短指(趾)、并指(趾)；骨骼畸形或牙齿发育不良；超重和肥胖；镜像运动等。

**llama3.1_8b**  2025-02-18T23:33:07Z:

根据您提供的信息，可能诊断出的罕见病是：

1. Klinefelter 综合征（XXY）
2. Turner 综合征（XO）
3. Cri-du-chat 综合征
4. Prader-Willi 综合征
5. Angelman 综合征
6. Noonan 综合征

注意：上述疾病可能需要进一步的检查和评估来确认诊断。

**65. user 2025-02-18T23:33:29Z:**

最常见的症状是活动后气短，其他症状包括乏力、头晕、胸痛、胸闷、心悸、黑矇、晕厥等，一旦出现晕厥或眩晕，提示患者心搏量可能已明显下降。由于症状、体征不典型，绝大多数患者就诊时间明显延误，至少 1/5 患者从症状出现至确诊时间超过 2 年；患者可出现肺动脉瓣第二心音亢进，三尖瓣关闭不全引起三尖瓣区收缩期杂音； 右心衰竭体征包括颈静脉充盈或怒张、肝脏肿大、下肢水肿、多浆膜腔积液、黄疸和发绀等。

**llama3.1_8b**  2025-02-18T23:33:35Z:

根据你提供的症状，我认为可能的诊断是：

1. **右心室功能不全（RHF）**：这是一种罕见的心脏疾病，表现为心脏右侧 chambers（室）不能有效地泵血，从而导致肺动脉压力增高和肺循环负荷增加。
2. **左心室功能不全（LHF）**：这也是一种罕见的心脏疾病，表现为心脏左侧 chambers（室）不能有效地泵血，从而导致左心室扩张和肺淤血。
3. **肺动脉瓣狭窄（PAPV）**：这是一种罕见的心脏疾病，表现为肺动脉瓣无法正常开合，从而导致肺循环压力增高和右心室负荷增加。
4. **三尖瓣病**：这是一种罕见的心脏疾病，表现为三尖瓣瓣膜出现了结构性的问题，从而导致心脏右侧 chambers（室）的功能不全。
5. **先天性心脏病**：这是一种罕见的心脏疾病，由于胎儿发育时的异常形成导致的心脏结构和功能的问题。

其他可能的诊断还包括：

- 右心衰竭
- 心力衰竭综合征
- 肺血管疾病
- 肺栓塞
- 血管炎

请注意，这些是仅供参考的可能诊断，不是正式的医疗诊断。只有通过进一步的检查和评估，才能确定最合适的诊断和治疗方案。

**66. user 2025-02-18T23:35:18Z:**

一般起病隐匿。主要表现为干咳、劳力性呼吸困难，杵状指(趾)、双下肺分布为主的爆裂音是其典型体征。终末期可以出现发绀、肺动脉高压、肺心病、右心功能不全的相关临床表现。

**llama3.1_8b**  2025-02-18T23:35:30Z:

根据你提供的信息，可能诊断出的是肺血管异位连接症（Pulmonary A-V Fistula）。这是一个罕见的疾病，它涉及到两个或多个肺部血管之间的异常连接。

其他五种可能的诊断：

1. **肺动脉高压**：是一种罕见的疾病，表现为肺动脉的压力显著升高，导致劳力性呼吸困难、发绀等症状。
2. **肺部感染**：如肺炎或支气管炎，可以导致干咳、劳力性呼吸困难和杵状指（趾）。
3. **肺癌**：虽然罕见，但也可能导致干咳、劳力性呼吸困难和杵状指（趾）。
4. **心脏病变**：如心肌病或先天性心脏病，可以导致右心功能不全，从而引起肺血压升高，表现为劳力性呼吸困难、发绀等症状。
5. **其他肺部疾病**：如肺纤维化或肺癌，同样可能导致干咳、劳力性呼吸困难和杵状指（趾）。

**67. user 2025-02-18T23:35:47Z:**

一种多器官、多系统受累的疾病，可累及几乎全身所有器官和组织。临床表现多种多样，患者因不同器官受累就诊于不同专科，容易漏诊。该病多数患者起病缓， 慢性进展。可以单器官起病，逐渐累及多个器官，也可以多种器官同时发病。少数患者病程呈自限性。

泪腺、唾液腺为最常见受累器官。主要表现为对称性、无痛性泪腺，颌下腺或腮腺肿大，硬结，伴或不伴有鼻窦炎或眼外肌增厚。消化系统受累可表现为自身免疫性胰腺炎、硬化性胆管炎、硬化性肠系膜炎等，临床可出现腹部隐痛、腹胀、消化不良、黄疸等。肺部受累时可表现为支气管壁增厚、肺间质病变、肺部结节 / 肿块以及胸膜增厚等，患者可无症状或咳嗽、气短及喘憋等。腹膜后纤维化或腹主动脉周围炎也是较常见的临床表现之一，主要为腹主动脉周围软组织增厚，可包绕输尿管造成输尿管狭窄和肾盂积水，少数患者发生腹主动脉瘤。临床表现为腹痛、腰痛或下肢水肿等。不到 10% 的患者发生 IgG4 相关性肾脏疾病，多表现为小管间质性肾病、肾盂占位性病变、输尿管管壁增厚或肾脏肿块样病变。其他受累器官包括甲状腺（硬化性甲状腺炎）、前列腺等。中枢神经系统病变少见，如自身免疫性垂体炎、肥厚性硬脑膜炎、颅内炎性假瘤等，患者可出现头痛、尿崩症、垂体功能减低等。除上述器官受累外，多数患者合并浅表或深部淋巴结肿大。另外，约半数患者有过敏相关病史，可以伴发哮喘。

**llama3.1_8b**  2025-02-18T23:35:51Z:

根据您的描述，我认为您最可能诊断的是”皮下硬化症”(Scleroderma)，但由于患者的临床表现太广泛且多样，因此可能还有其他几种罕见疾病需要考虑。

以下是五种可能的诊断：

1. **皮下硬化症**(Scleroderma)：这是一个常见于女性的自身免疫性疾病，特征是皮肤和内脏器官的逐渐硬化。
2. **Sjögren综合征**(Sjögren’s Syndrome)：是一种主要影响泪腺和唾液腺的自身免疫性疾病，也可能影响其他器官，如肺、心脏和肾脏。
3. **系统性硬化症**(Systemic Sclerosis)：这是皮下硬化症的一个分支，也是一种自身免疫性疾病，特征是皮肤和内脏器官的逐渐硬化。
4. **多肌炎和干眼综合征**(Polymyositis and Sjögren’s Syndrome)：这是一种罕见的自身免疫性疾病，主要影响肌肉和泪腺。
5. **肾脏淤血症**(Nephrogenic Restrictric Renal Disease)：这是一个罕见的疾病，特征是肾脏内的血管硬化和狭窄。

这些诊断都是可能的，但最终的诊断需要通过进一步的检查和实验室测试来确定。

**68. user 2025-02-18T23:36:17Z:**

患儿有胆汁淤积和肝硬化的发生。其主要临床表现为高结合胆红素血症，常出现脂溶性维生素吸收不良，如佝偻病等。而不同酶缺陷的临床表型略有不同，如氧固醇 7α- 羟化酶缺陷可出现成人期遗传性痉挛瘫痪；胆固醇 25 羟化酶缺陷可发生顽固性便秘；3β- 羟基类固醇 -Δ5-C27- 类固醇脱氢酶缺陷可发生婴儿期胆汁淤积性肝病和脂溶性维生素缺乏；固醇27- 羟化酶缺陷可发生儿童慢性腹泻及发育迟缓、成人期痉挛性瘫痪；α- 甲酰辅酶 A 消旋酶缺陷可发生婴儿期脂溶性维生素缺乏、成人期感觉神经病变；胆汁酸辅酶 A 氨基酸N- 乙酰转移酶缺陷可发生肝内胆汁淤积、发育迟缓、家族性高胆烷血症等。

**llama3.1_8b**  2025-02-18T23:36:33Z:

根据您提供的临床表现，可能的诊断是：

1. **氧固醇 7α- 羟化酶缺陷**：这是一个罕见疾病，它导致肝脏不能正常地代谢胆汁酸，从而引起胆红素血症、脂溶性维生素吸收不良等问题。

其他五种可能的诊断是：

1. **胆固醇 25 羟化酶缺陷**：这是一种影响胆固醇代谢的疾病，表现为顽固性便秘和其他肝脏问题。
2. **3β- 羟基类固醇 -Δ5-C27- 类固醇脱氢酶缺陷**：这是另一种影响类固醇代谢的疾病，可能导致胆汁淤积性肝病和脂溶性维生素缺乏。
3. **固醇27- 羟化酶缺陷**：这是一种罕见疾病，它影响了胆固醇的代谢，从而引起儿童慢性腹泻、发育迟缓和成人期痉挛性瘫痪。
4. **α- 甲酰辅酶 A 消旋酶缺陷**：这是一个罕见疾病，它影响了脂溶性维生素的代谢，从而引起婴儿期脂溶性维生素缺乏和成人期感觉神经病变。
5. **胆汁酸辅酶 A 氨基酸N- 乙酰转移酶缺陷**：这是一种罕见疾病，它影响了肝脏内的胆汁酸代谢，从而引起肝内胆汁淤积、发育迟缓和家族性高胆烷血症等问题。

**69. user 2025-02-18T23:37:45Z:**

**1. 急性新生儿型** 起病急骤，病情进展迅速，可威胁生命。出生后 1~2 周内表现为喂养困难、呕吐、肌无力、肌张力减退，嗜睡或加重进展至昏迷。异戊酸蓄积可造成“汗脚”的特征性气味。查体可有肝大。化验提示代谢性酸中毒、高氨血症、低或高血糖、酮症、低钙血症及全血细胞减少。如果患者能度过新生儿期的急性发作，将会进展为慢性间歇型。

**2. 慢性间歇型** 起病隐匿。仅表现为非特异性不能耐受空腹或发育落后。常因上呼道感染或摄入高蛋白质饮食诱发，发作呈反复发生呕吐、嗜睡、昏迷、酸中毒伴酮尿， 会出现“汗脚气味”，限制蛋白质饮食并输注葡萄糖时可以缓解发作。绝大部分慢性间歇型患者精神运动发育正常，但是一些患者会表现为发育延迟和不同程度的智力低下。

**llama3.1_8b**  2025-02-18T23:37:46Z:

根据你的描述，我认为最有可能的诊断是**肌无力的非酮症亢进酸中毒（MERRF）或其他一组罕见病，但更可能的是一种与 MERRF 相似的疾病，即**嗜睡型儿童癫痫（Epilepsy with hypsarrhythmia and somnolence，现在称为斯皮里马因-马斯基尔综合症）。

**70. user 2025-02-18T23:38:03Z:**

1. 第二性征不发育和配子生成障碍。
2. 嗅觉障碍， 因嗅球和嗅束发育异常，导致不能识别气味，尤其不能识别芳香气味。

**llama3.1_8b**  2025-02-18T23:38:06Z:

基于您提供的信息，我认为可能的诊断是：

1. Kallmann 综合征（Kallmann Syndrome）

Kallmann 综合征是一种罕见的遗传性疾病，主要特征是第二性征不发育和嗅觉障碍。这种疾病影响到一小部分人口。

其他可能的诊断：

1. 闭锁型脊柱裂（Thalassemia Major）- 这是一种罕见的遗传性疾病，特征是骨髓产生红细胞的能力严重受损。
2. 罕见的免疫缺陷综合征（Rare Immunodeficiency Disorders）- 这类疾病可能导致身体无法有效地抵抗感染。
3. 强直性脊柱炎（Ankylosing Spondylitis）- 这是一种罕见的关节炎，主要影响脊柱和骨骼系统。
4. 慢性粒细胞白血病（Chronic Myeloid Leukemia）- 这是一种罕见的白血病，特征是骨髓产生过多的粒细胞。
5. 强直性肌张力疾病（Friedreich’s Ataxia）- 这是一种罕见的遗传性神经系统疾病，主要影响运动功能。

请注意，这些诊断只是可能的选项，实际诊断需要根据具体的临床表现、实验室检查和其他信息综合考虑。

**71. user 2025-02-18T23:38:24Z:**

病情从轻至重差异很大，因此容易被误诊和漏诊。临床表现主要包括：

**1. 发热** 热型不规则，可呈周期性或者持续性高热。下丘脑受累患者可以出现中枢性体温调节异常。

**2. 皮疹** 主要分布于躯干、头皮和发际。初起为淡红色丘疹，然后可以为出血性或湿疹样皮脂溢出样皮疹，继而结痂，结痂脱落后留有白斑。

**3. 口腔及眼耳鼻喉病变** 牙龈肿胀、牙齿松动，突眼，顽固性中耳炎伴外耳道皮疹为典型表现。

**4. 中枢性尿崩症** 垂体、下丘脑甚至其他部位中枢神经系统病变，少数患者出现神经精神症状。

**5. 呼吸道症状** 咳嗽，重者憋喘、发绀，甚至反复气胸发作。肺部体征通常不明显。

**6.** 肝脾可以肿大，可以出现黄疸、肝功能异常、肝功能衰竭和门脉高压，最终患者可死于肝硬化和肝功能衰竭。

**7. 消化道症状** 消化道累及较少见，可以表现为息肉或腺瘤样改变，通常不出现临床症状，病变弥漫时可能出现腹痛、腹泻和低蛋白血症等。

**8. 造血系统** 淋巴结可见肿大，骨髓受累时可以出现贫血、白细胞和血小板计数异常。

**9. 骨质损害** 颅骨、四肢骨、脊柱、骨盆等可有疼痛及肿块，可以发生病理性骨折。眼眶骨质病变所致突眼为儿童患者典型表现之一。

**llama3.1_8b**  2025-02-18T23:38:29Z:

根据您的描述，可能的诊断包括：

1. **巨细胞肾炎综合征（Tuberculosis, TB）**：是一种传染性疾病，可以影响多个器官，包括肺、淋巴结、脑、眼、耳等。这是一种罕见的疾病，但在某些地区或社区中更为常见。
2. **系统性红斑狼疮（SLE）**：是一种自身免疫性疾病，可以影响多个器官，包括皮肤、关节、肾脏等。这是您描述的临床表现的一个可能的诊断。
3. **多发性硬化症（Multiple Sclerosis, MS）**：是一种神经系统疾病，可以导致神经细胞受损，从而引起多种临床表现，包括视力、运动功能障碍等。这也可能是您的描述的一种可能性。
4. **血管炎**：是一组影响血管的疾病，可以导致多种临床表现，包括发热、皮肤病变等。这也可能与您提供的信息相关。
5. **类风湿关节炎（Rheumatoid Arthritis, RA）**：是一种自身免疫性疾病，可以引起关节炎和其他身体系统的症状。虽然这通常表现为关节问题，但它也可以影响多个器官，从而导致临床表现与您的描述相符。
6. **巨细胞动脉炎（Giant Cell Arteritis, GCA）**：是一种血管炎，可以引起面部、头部和颈部血管炎，进而导致眼睑下垂、视力受损等临床表现。这也可能是您描述的一个可能的诊断。

这几种疾病都是罕见的，但在某些地区或社区中更为常见。最终的诊断需要通过进一步的检查和评估确定。

**72. user 2025-02-18T23:38:59Z:**

**1. 生长障碍及其他畸形，**患儿出生时体重大多在正常范围内，但部分患儿的出生身长低于 48cm（42~47cm）。出生后逐渐显著地生长速度减慢，未治疗的男性患者终身高在 116_142cm，女性患者终身高在108_136cm。同时患者手、足和内脏器官均小。部分患者伴有其他的先天性畸形，如髋关节脱位、主动脉瓣狭窄、隐睾、先天性白内障等。

**2. 面容特点，**患者特征性的面容改变为前额突出、鞍鼻、小下颌，头部与身体比例较正常儿童大，但与同龄儿童相比头围绝对值仍偏小。患者头发稀疏，部分患者可出现“落日征”，地中海地区和中东地区的患者可表现出蓝巩膜。患者出牙延迟，乳牙脱落较晚并且易合并多发龋齿，由于下颌骨较短小，可有牙列不齐。

**3. 骨骼肌肉系统** 部分患者具有先天性髋关节脱位、股骨头缺血性坏死等表现。由于中面部骨骼发育不良和咽部解剖结构异常，患者说话音调高尖。85％患者肘关节活动受限。

**4. 代谢紊乱 ，**患者常合并代谢异常，包括新生儿期和婴儿期的低血糖，而成年后出现逐渐加重的肥胖。

**5. 性发育延迟 ，**患者生殖器较小，男性患者表现为小阴茎和小睾丸，女性患者超声检查发现卵巢体积较小。大多数患者表现为青春发育延迟。虽然性发育落后，但是男性和女性患者的性功能最终可发育成熟，具有生育能力。

**6. 肿瘤发生率低，**患者的肿瘤发生的风险较一般人群显著降低。

**llama3.1_8b**  2025-02-18T23:39:03Z:

根据您的描述，我认为您可能是在诊断一个罕见病——普里默-劳斯症（Prader-Willi syndrome, PWS）。这是一个遗传性的疾病，影响着约 1/15,000 到 1/25,000 的新生儿。以下是其他五种可能的诊断：

1. **克雷伯综合征**：是一种罕见的先天性畸形，表现为脑发育异常、肌肉萎缩、面部畸形和智力障碍。
2. **卡夫曼综合征**：是一种罕见的遗传性疾病，影响着大脑的发育，导致精神发育迟缓、智力障碍和身体畸形。
3. **布什特-霍尔姆症候群**：是一种罕见的先天性畸形，表现为生长迟缓、智力障碍、面部畸形和肾脏疾病。
4. **哈奇森综合征**：是一种罕见的遗传性疾病，影响着大脑的发育，导致精神发育迟缓、智力障碍和身体畸形。
5. **贝克特综合征**：是一种罕见的先天性畸形，表现为生长迟缓、智力障碍、面部畸形和骨骼疾病。

请注意，这些诊断仅供参考，您应该根据具体情况进行进一步评估和检查，以确定最可能的诊断。

**73. user 2025-02-18T23:39:25Z:**

主要症状为双眼先后发生的无痛性视力突然急剧下降，双眼发作间隔一般为数周到数月。眼底检查在疾病早期表现为视盘充血水肿、视盘旁毛细血管迂曲扩张，疾病后期视盘水肿和毛细血管扩张消退，最终视盘颞侧或全部呈萎缩性改变。多数病人只存在眼部表现，少数可合并全身其他系统症状如智力障碍、癫痫、听力障碍、肌张力障碍等。

**llama3.1_8b**  2025-02-18T23:39:29Z:

根据你的描述，这可能是最有可能的诊断：

1. **Leber先天性视网膜脱落病（LHVR）**：这是一种罕见的遗传性的疾病，主要影响男性。它导致双眼视力突然下降，且发作间隔在数周到数月之间。

其他可能的诊断：

1. **多发性硬化症（MS）**: MS 是一种神经系统疾病，可引起视觉障碍、运动功能障碍等一系列临床表现。
2. **脑炎或脑部肿瘤**: 这些情况也可能导致双眼视力下降，并影响其他神经系统的功能。
3. **血管性痴呆**：这是一种罕见的疾病，主要影响老年人，导致脑部循环障碍和一系列临床表现。
4. **全身硬化症（Scleroderma）**: 这是一种自身免疫性疾病，可引起皮肤、关节、心脏等多个系统的受累，并可能影响视觉功能。
5. **遗传性眼肌病**：这是一组罕见的遗传性疾病，可导致眼肌衰竭和一系列临床表现。

**74. user 2025-02-18T23:40:34Z:**

患者临床表现为婴儿期低酮性低血糖、肝功能异常、乳酸酸中毒、心肌病，部分患者表现为生长迟缓，肌张力低下或心肌病，少数可有胆汁淤积或肝硬化。幼儿期患分患者表现为生长迟缓，肌张力低下或心肌病，少数可有胆汁淤积或肝硬化。幼儿期患者可有喂养困难，发作性代谢紊乱，发作性横纹肌溶解。多数患者儿童期会有视网膜病变，周围神经病是一种较少见的长期病并发症。

**llama3.1_8b**  2025-02-18T23:40:38Z:

根据您提供的临床表现，我最有可能诊断的是苯丙酮尿症（Phenylketonuria, PKU）。此外，还需要考虑以下五种罕见病：

1. **肌营养不良症**：是一种由于体内蛋白质代谢障碍导致的肌肉疾病，其临床表现包括肌张力低下、肌萎缩和生长迟缓。
2. **心肌病变综合征**：是一组罕见的心脏疾病，其临床表现包括心肌病变、心律失常和死亡风险增加等。
3. **乳酸中毒**：是一种代谢紊乱的临床表现，可能是由多种原因引起的，包括遗传性疾病、药物不良反应或其他因素导致的肾功能障碍等。
4. **胆汁淤积症**：是一种罕见的内分泌疾病，其临床表现包括黄疸、皮肤淤血和脂质代谢紊乱等。
5. **苯丙氮酰尿症伴发性神经系统病变综合征**：是一种罕见的遗传性疾病，其临床表现包括脑部异常、肌肉萎缩和认知功能障碍等。

需要注意的是，这些诊断都不是最可能的PKU，而是其他罕见病的可能性较小的选择。

**75. user 2025-02-18T23:40:56Z:**

主要发生在成年育龄女性，平均诊断年龄在 40 岁左右。早期症状轻微，部分患者在查体时被发现，或因为呼吸症状或其他原因检查肺部 HRCT 时发现。主要表现为不同程度的呼吸困难，随疾病进展，呼吸困难症状也逐渐加重，肺功能进行性恶化，晚期出现呼吸衰竭。在病程中可反复出现气胸、乳糜胸等并发症。肺外表现包括肾， 腹膜后实性或囊实性淋巴管肌瘤。部分患者具有其他多系统受累临床特征，主要包括神经系统改变(癫痫、神经发育迟缓和自闭症等) 和皮肤改变(色素脱色斑、面部血管纤维瘤、皮肤鲨革斑和甲周纤维瘤等) 等临床表现。

**llama3.1_8b**  2025-02-18T23:41:00Z:

基于您提供的疾病临床表现，我认为可能诊断出的是淋巴管瘤（Lymphangioleiomyomatosis，简称 LAM），这是一个罕见的肺部疾病，主要影响成年育龄女性。这种疾病会导致肺功能逐渐恶化，最终可能导致呼吸衰竭。

另外五种可能的诊断是：

1. **肺纤维化症**（Pulmonary Fibrosis）：一种肺部疾病，导致肺组织过度增厚和硬化，影响肺功能。
2. **肺囊性纤维化**（Lymphangioleiomyomatosis-like Lymphoid Hyperplasia）：一种罕见的肺部疾病，表现为肺囊形成和纤维化。
3. **肺间质性肺病**（Pulmonary Interstitial Lung Disease）：一种肺部疾病，导致肺间质过度增厚和硬化，影响肺功能。
4. **多形性肺气肿**（Mixed Dystrophic Pneumonia）：一种罕见的肺部疾病，表现为肺组织过度炎症和破坏。
5. **肺毛细血管瘤**（Pulmonary Capillary Hemangioma）：一种罕见的肺部疾病，表现为肺毛细血管过度增生。

**76. user 2025-02-18T23:41:18Z:**

婴儿的典型表现是在断奶后出现反复呕吐和腹泻，拒食富含蛋白质的食物。患者可出现皮肤苍白、肌张力减低、肝脾大、头发稀疏、骨质疏松伴病理性骨折。进食富含蛋白质的膳食后患者可出现嗜睡、共济失调、癫痫发作和高氨血症昏迷等。随着病程的发展，可出现多系统受累表现。肺部受累可表现为进行性肺间质病变，肺泡蛋白沉积症；肾脏最常见的特征性改变是近端肾小管功能障碍和肾钙质沉积，也可出现肾小球病变，肾脏淀粉样变，甚至出现肾功能衰竭；血液系统受累表现为正色素或低色素性贫血，白细胞减少，血小板减少等；消化系统受累的表现为高胆固醇血症，高甘油三酯血症，反复发作的胰腺炎，甚至肝脏淀粉样变。癫痫多发生于成年患者，可能与反复发生高氨血症导致大脑皮质受损有关。

**llama3.1_8b**  2025-02-18T23:41:22Z:

根据提供的临床表现和症状，我认为最有可能的诊断是：

1. **Cystinuria**：一种遗传性的代谢疾病，由于肾脏对半胱氨酸的排泄功能障碍导致的。这种疾病可以影响多个系统，包括肺、肾、血液和消化系统。

其他可能的诊断：

1. **Cystinosis**：一种遗传性的代谢疾病，由于体内维生素 C 的缺乏导致的。这种疾病也可以影响多个系统。
2. **Wilson 病**: 一种遗传性代谢疾病，由于铜在肝脏中积累导致的。这是一种全身性的疾病，可以影响肾脏、血液和消化系统等多个系统。
3. **Fabry 病**：一种遗传性代谢疾病，由于α-Galactosidase A 的缺乏导致的。这是一种全身性的疾病，可以影响肾脏、血液和神经系统等多个系统。
4. **Menke 病**：一种遗传性代谢疾病，由于铜在身体中的积累导致的。这是一种全身性的疾病，可以影响肠道、肝脏和神经系统等多个系统。

**77. user 2025-02-18T23:41:46Z:**

新生儿期起病，通常在生后 1 周内起病。以反复呕吐、腹泻、腹胀等胃肠道症状为首发症状，伴肝脾大和生长发育障碍；神经系统受累表现为活动少、腱反射亢进、踝阵挛和角弓反张。巨噬细胞内胆固醇酯和三酰甘油贮积导致肝脾肿大，进行性加重甚至肝衰竭，数月内出现转氨酶、胆红素升高，低蛋白血症，凝血异常等。肾上腺增大伴钙化导致肾上腺皮质功能不全，60％ ~70％通过影像学检查可发现肾上腺钙化。也有胆囊功能障碍、脑卒中报道。随着病情进展，可出现多器官功能障碍，包括贫血、消化道出血、败血症、昏迷等。

**llama3.1_8b**  2025-02-18T23:42:03Z:

基于您提供的临床表现，我最有可能诊断的是一种罕见的遗传疾病——酪氨酸尿症（Tyrosinemia）。

酪氨酸尿症是一种代谢异常导致的罕见疾病，主要影响新生儿和婴幼儿。它通常表现为反复呕吐、腹泻、腹胀等胃肠道症状，并伴有肝脾大和生长发育障碍。此外，还可能出现神经系统受累，如活动少、腱反射亢进、踝阵挛和角弓反张。

其他五种可能的诊断包括：

1. **巨球菌病**（Gigantism）：一种罕见疾病，由于肾上腺过度分泌生长激素，导致儿童或青少年出现异常快速生长。
2. **先天性甲状腺功能亢进症**（Congenital Hyperthyroidism）：一种罕见的遗传性疾病，表现为新生儿期起病的甲状腺过度活跃。
3. **血友病**（Hemophilia）：一种罕见的遗传性疾病，由于凝血因子的缺乏或功能障碍导致的出血倾向。
4. **肌肉张力增高症**（Spinal Muscular Atrophy）：一种罕见的遗传性疾病，主要影响新生儿期至婴幼儿阶段的神经肌肉系统。
5. **先天性甲状腺功能减退症**（Congenital Hypothyroidism）：一种罕见的遗传性疾病，由于甲状腺过度分泌不足，导致新生儿期起病的甲状腺功能不全。

这些疾病都可能表现出类似的临床特征，如肝脏和生长发育的问题，但它们具有不同的根本原因。

**78. user 2025-02-18T23:42:23Z:**

**1. 心血管系统** ，可以导致主动脉根部扩张、主动脉瓣关闭不全和主动脉夹层；也可能会有二尖瓣脱垂伴或不伴关闭不全。升主动脉扩张患病率随着患者年龄的增长而增加。从 30 岁至 60 岁，患病率从 53％上升至 96％。患者可以表现为气促、胸痛、心悸、头晕、晕厥或者咯血。查体表现为心界向左下扩大，听诊在二尖瓣区可以闻及收缩期杂音，在主动脉瓣听诊区可闻及舒张期为主的双期杂音，可有周围血管征以及心力衰竭的体征。

**2. 骨骼表现** ，瘦高身材、手指和脚趾细长、两臂平伸的距离超过身高、脊柱侧凸或后凸、漏斗胸或者鸡胸、关节松弛、扁平足等。

**3. 眼部异常** ，眼部病变占 50％ ~70％，包括角膜扁平、眼球轴延长、视网膜剥离、白内障、晶状体脱位或半脱位、虹膜震颤或轻度震颤、瞳孔移位、晶状体混浊等。

**4. 其他 ，**硬脊膜膨出、萎缩纹、复发性疝或切口疝、高腭穹等。

**llama3.1_8b**  2025-02-18T23:42:27Z:

根据你的描述，我认为最可能的诊断是马凡综合征（Marfan Syndrome）。这是一种罕见的遗传疾病，影响着身体多个系统，包括骨骼、眼部和血管系统。这种疾病的典型特征包括高矮、长手指、漏斗胸和心脏问题等。

其他五种可能的诊断是：

1. **埃勒斯-丹洛症候群**（Ehlers-Danlos Syndrome）：一种遗传性的纤维组织疾病，影响着骨骼和皮肤。
2. **先天性主动脉夹层**（Spontaneous Aortic Dissection）：一种罕见的血管疾病，导致主动脉壁出现破裂或夹层。
3. **多重内分泌瘤综合征**（Multiple Endocrine Neoplasia Syndrome）：一种遗传性的肿瘤综合征，影响着身体多个内分泌系统。
4. **韦氏先天性心脏病**（Ventricular Septal Defect）：一种常见的心脏先天性缺陷，导致室间隔出现洞孔。
5. **纤维肌症**（Fibromuscular Dysplasia）：一种罕见的血管疾病，影响着身体多个血管系统。

**79. user 2025-02-18T23:42:44Z:**

**1. 皮肤表现** ，患者一般出生时即有牛奶咖啡斑，常位于骨骼病变同侧且以中线为界，为一处或多处点片状大小不等的褐色或黄棕色斑片状色素沉着，边界不规则。

**2. 骨骼系统表现** ，大约 98％的 患者有单骨型或多骨型骨纤维异样增殖症，以多骨型更为常见。其特征为畸变的骨母细胞增多并异常分化，引起骨髓内纤维细胞的广泛增殖、分化，形成幼稚编织骨，易发生病理性骨折和骨骼畸形，并引发骨痛，几乎可累及全身骨骼，尤以颅面骨和长骨受累多见，局部骨骼膨胀、隆起，引起面部结构改变和压迫症状。眼眶受累严重可压迫视神经，造成视力障碍。听神经受累可致听力下降。脊柱受累可致椎体压缩性骨折，引起脊柱侧弯或后凸畸形。骨纤维异样增殖症导致病理性骨折的高峰年龄多在 6~10 岁。影像学示骨骼呈膨胀性、溶骨性改变或磨玻璃样改变。

**3. 内分泌腺体受累表现** ，可累及多种内分泌腺，导致受累腺体功能亢进或发生肿瘤，血清促分泌激素的水平正常或降低。其中性早熟较多见，男女均可发病，但女性患者多见，且常早于骨病变。女性患儿可表现为第二性征早发育、过早阴道不规则出血、子宫及卵巢较正常同龄儿童增大，或发生卵巢囊肿，骨骺提前愈合等。男性患儿性早熟相对少见，超声见睾丸病变(Leydig 细胞增生、微小结石、局灶钙化等)。甲状腺肿大及功能亢进也较常见，也有报道发生甲状旁腺功能亢进者。其他内分泌疾病也有报道，如皮质醇增多症、巨人症 / 肢端肥大症、高泌乳素血症等，但相对较罕见。

**4. 低磷血症** ，部分患者合并低磷血症，表现为低血磷性佝偻病。合并低磷血症者通常为多骨型骨纤维异常增殖症。

**5. 其他，**患者还可因突变累及外周血白细胞、肝脏、心脏、胸腺和胃肠道，而出现相应的罕见临床症状，如黄疸、肝炎、心律失常、肠息肉等。

**llama3.1_8b**  2025-02-18T23:42:48Z:

根据您的描述，我推测最有可能的诊断是：

**1. 牛奶咖啡斑综合征（McCune-Albright 综合征）**

这是因为您提到了患者出生时即有牛奶咖啡斑，这是一个典型的临床表现。其他症状，如骨骼病变、内分泌腺体受累和低磷血症，也与该综合征有关。

以下是五种可能的诊断：

1. **牛奶咖啡斑综合征（McCune-Albright 综合征）**
2. **多骨型骨纤维异常增殖症（ Fibrodysplasia Ossificans Progressiva, FOP)**
3. **内分泌性骨骼疾病（Endocrine-Related Skeletal Disease, ERSD)**
4. **低磷血症伴骨骼异常（Hypophosphatasia with Skeletal Abnormalities)**
5. **其他罕见疾病（Other Rare Diseases)**

请注意，这些诊断并不是绝对的，并且需要通过进一步的临床检查和实验室测试来确定。

**80. user 2025-02-18T23:43:33Z:**

患者大多在出生后 3 个月至 3 岁之间发病，少部分在新生儿期或成人期发病， 也有患者无症状。患者发病通常有诱发因素，如多见于长时间饥饿或并发感染性疾病等。早期发病的患儿，其首发症状以嗜睡和呕吐常见，也可表现为抽搐、窒息等，常迅速进展为昏迷或死亡。急性发病期常有典型的低血糖表现，血糖降低伴尿酮体阴性有助于诊断。50％的患儿伴有肝脏肿大，肝活检可见大量脂质堆积。影像学检查发现部分患儿发生脑水肿。心脏损害表现较为罕见。成人期发病的患者临床表现多样，可有多器官受损，包括肌肉、肝脏、神经系统或心血管系统等。其中呕吐是最常见的症状，肝脏肿大相对少见，妊娠期出现症状的患者表现为急性脂肪肝。多数患者有急性非炎症性脑病。约 1/3 的患者出现横纹肌溶解，肌酸激酶显著增高，并伴有肌红蛋白尿。患者病死率较高，发病的患儿中约25％死亡，成人期急性发病的患者病死率更高，可达到 50％。疾病发作间歇期患者往往表现正常。约 1/3 的急性发病后存活的患者出现后遗症，包括生长发育迟滞、运动发育迟缓、智力障碍、语言发育缺陷、心理行为问题、癫痫、脑瘫、偏瘫、慢性肌无力等。另外，部分患者有疲劳、肌痛和运动耐力减退等骨骼肌损害的主诉症状。

**llama3.1_8b**  2025-02-18T23:43:37Z:

根据您提供的疾病临床表现，我最有可能诊断出的罕见病是：

1. **苯丙酮尿症**：这是一种遗传性的代谢缺陷，主要影响儿童和青少年。它会导致患者出现多器官受损，包括肝脏、脑部等，以及肌肉无力、抽搐等症状。

其他五种可能的诊断是：

1. **糖尿病酮症酸中毒**：这是一个急性疾病，主要影响儿童和青少年。它会导致患者出现低血糖、酸中毒、肝脏损害等症状。
2. **肌营养不良综合征**（MMD）或 **肌肉无力综合征**：这是一种罕见的遗传性疾病，主要影响儿童和青少年。它会导致患者出现肌肉无力、肝脏肿大等症状。
3. **脂质囊肿病**（Fabry病）：这是一个罕见的遗传性疾病，主要影响成年人。它会导致患者出现皮肤瘙痒、视觉障碍、脑部损害等症状。
4. **肌肉无力综合征**：这是一种罕见的疾病，主要影响成人。它会导致患者出现肌肉无力、眼动神经麻痹、心脏问题等症状。

请注意，这些诊断只是基于临床表现，并不一定准确。实际上，最准确的诊断需要通过实验室检查和影像学检查来确认。

**81. user 2025-02-18T23:43:57Z:**

疾病在各年龄段中的临床表现不尽相同。通常发病年龄越早，急性代谢紊乱和脑病表现越严重。临床表现主要包括消化系统症状如呕吐、喂养困难、肝大等以及神经系统症状如运动障碍、意识障碍、抽搐、发育迟缓或倒退、小头畸形等。病情反复多与感染和应激相关。

新生儿期发病者多在生后数小时至 1 周内出现急性脑病样症状，表现为呕吐、肌张力低下、脱水、严重酸中毒、高乳酸血症、高氨血症、昏迷和惊厥，病死率高。儿童期发病的出生时正常，多在 1 岁以内发病，首次代谢危象的诱因常为感染、饥饿、疲劳、疫苗注射等应激因素刺激或高蛋白饮食和药物，如果不及时诊治，可导致智力发育和运动发育迟缓、落后和倒退，可伴发血液系统、肝脏、肾脏、皮肤和周围神经受累。成人患者首发症状可为周围神经病变和精神心理异常等。

**llama3.1_8b**  2025-02-18T23:44:01Z:

根据提供的临床表现，我认为最有可能的诊断是糖尿病性酮症酸中毒（Ketotic Hypoglycemic Coma）。

其他五种可能的诊断：

1. **脑部感染**：如脑炎、脑膜炎或细菌性脑脊髓炎等，可能导致意识障碍、抽搐和神经系统症状。
2. **肌肉疾病**：如肌无力症（Myasthenia Gravis）、多发性硬化（Multiple Sclerosis）或肌肉萎缩病等，可能导致运动障碍和神经系统症状。
3. **肝脏疾病**：如肝炎、肝纤维化或肝癌等，可能导致肝大、消化系统症状和血液系统受累。
4. **神经肌肉疾病**：如 Guillain-Barré 综合征、周围神经病变（Peripheral Neuropathy）等，可能导致运动障碍、感觉障碍和神经系统症状。
5. **代谢疾病**：如尿素循环障碍、氨中毒或酸中毒等，可能导致急性代谢紊乱和脑病表现。

**82. user 2025-02-18T23:44:20Z:**

典型患者出生时正常。于 6 个月 ~1 岁面容逐渐变丑，面中部变扁、鼻梁增宽、角膜混浊、耳聋、头大、前后径长，前额突出，关节僵硬、爪形手、鸡胸和腰椎后突等。常于 2~5 岁出现心脏瓣膜增厚、心肌病或充血性心力衰竭。5 岁出现肝脾大。精神运动发育落后。一般存活至 10 岁左右。多死于心衰和肺炎。轻型患者 5 岁以后出现症状，轻度面容粗陋、角膜混浊和手足关节僵硬。智力正常，寿命相对较长。临床症状在各亚型之间有互相重叠，但生化检查结果在各型之间无显著差异。

**llama3.1_8b**  2025-02-18T23:44:24Z:

根据您提供的描述，我最可能诊断出的罕见病是：

**Hurler综合征**

这是一个遗传性疾病，由于缺乏α-L-苯丙氨酸6脱羟酶（IDUA）的活性导致，导致体内多糖积累。这种疾病会影响面部外貌、听力、心脏和肝脾功能等多个方面。

其他五种可能的诊断是：

**1. Hunter综合征**

与Hurler综合征类似，但患者主要表现为智力正常、生长发育正常，但有面部外貌改变和骨骼异常。

**2. MPS II (Hunter病)**

这是一种因IDUA活性降低导致的罕见病，其特点是心脏问题较多，且患者往往存在男性化的行为问题。

**3. MPS I (Hurler-Scheie综合征)**

这是另一种因IDUA活性降低导致的罕见病，其表现包括智力正常、面部外貌改变、听力和视力受损等多个方面。

**4. MPS III (Sanfilippo综合征)**

这是一种由于N-苯丙酮酸脱水素活性降低导致的罕见病，其特点是智力正常，但有行为问题、肝脏和心脏受累等。

**5. MPS IV (Mucopolysaccharidosis Type 4)**

这是一种因糖苷酶活性降低导致的罕见病，其表现包括面部外貌改变、耳聋和智力正常等多个方面。

**83. user 2025-02-18T23:44:52Z:**

隐袭起病。缓慢发展或阶段性进展，可有长时间的稳定期。任何年龄均可发病，多于50 岁前起病。临床主要表现为多发性单神经病，不对称性上肢远端起病较常见，早期以单侧上肢某一根或多根神经受累多见，表现为相应神经支配区域的肌肉无力， 远端为主，可伴有痉挛或束颤。随着病情发展，可以出现肌肉萎缩；病程较长者，可有多个肢体的多根神经受累，受累神经的不对称性可不明显，而呈现为类似多发性周围神经病的分布。患者可有轻微感觉异常的主诉，但缺乏客观感觉受累的体征，病程后期部分患者也可出现部分感觉神经受累。脑神经通常不受累。无上运动神经元受累体征。

**llama3.1_8b**  2025-02-18T23:44:55Z:

根据你的描述，这种疾病最可能诊断为多发性硬化症（多发性周围神经病）。其他五种可能的诊断如下：

1.** Guillain-Barré 综合征**：一种罕见的自身免疫疾病，表现为快速进展的肌肉无力和感觉异常。 2.**糖尿病性周围神经病变**：糖尿病患者常见的一种肢体痛和感觉障碍。 3.**多发性脊髓病**：一种罕见的神经系统疾病，表现为逐渐恶化的肌肉无力、感觉异常和运动功能丧失。 4.**肌无力症**：一种罕见的遗传性疾病，表现为肌肉无力和萎缩。 5.**Charcot-马尔-托病**：一种罕见的神经系统疾病，表现为肢体无力、感觉异常和运动功能丧失。

**84. user 2025-02-18T23:45:24Z:**

新生儿期起病常致命，可表现为代谢危象，包括代谢性酸中毒、非酮症性低血糖、高氨血症，其他特征包括多系统受累(肌张力低下、心肌病、肝肿大)、排泄大量脂肪酸和氨基酸衍生的代谢产物、“汗脚”味体臭、昏迷、新生儿期死亡。快速进展的肥厚型心肌病常见于新生儿期起病的类型，猝死概率非常大，即使经过新生儿筛查在早期即检测出有此病，也难以逆转。

先天畸形被认为是未被胚胎转运纠正的那部分有毒代谢物累积的后果。脂肪酸氧化代谢受阻的程度决定了子宫内的酸中毒程度，进而阻碍正常的胚胎形成。先天畸形包括增大的多囊肾、摇篮底足(rocker-bottom feet)、下腹部肌肉缺失、尿道下裂和阴茎下弯、肌张力低下、脑皮质发育不良和胶质增生。其他畸形特征包括眼距过宽、耳畸形、巨颅、前囟过宽、高前额、鼻梁低平。

晚发型的发病年龄和症状在不同病例中差异很大，主要表现为不伴有先天畸形，但终身伴有急性间歇发作的呕吐、脱水、低酮性低血糖、酸中毒及意识障碍的风险。年龄大者可有肝脏肿大或脂质沉积性肌病。急性失代偿发作可由感染、发热、手术、低能量饮食或减肥、饮酒、妊娠等诱发。大多数患者发展为慢性肌病症状，包括运动不耐受、肌痛、肌无力、肌肉萎缩。晚发型病人的有机酸尿症常为间歇性，仅在疾病期间和处于分解代谢压力下较为显著。

**llama3.1_8b**  2025-02-18T23:45:27Z:

根据您提供的信息，我认为最可能的诊断是卡氏肉芽肿（Carnitine Palmitoyltransferase deficiency），一种罕见的代谢疾病。卡氏肉芽肿是一种脂肪酸氧化受阻的疾病，患者往往表现出代谢危象、肌肉无力、心律失常和其他多系统受累。

以下是五种可能的诊断：

1. 卡氏肉芽肿（Carnitine Palmitoyltransferase deficiency）
2. 異aldol synthase缺乏症（ALD）
3. 丙酮酸脱氢酶缺乏症（PDH）
4. 丙酮酸脱氨酶缺乏症（PDC）
5. 甘油三酯脂肪酸氧化受阻症（Glycerol-3-phosphate dehydrogenase deficiency）

需要注意的是，诊断的准确性取决于临床表现、实验室检查和遗传学分析。

**85. user 2025-02-18T23:45:45Z:**

平均发病年龄为 30 岁，10 岁以下和 50 岁以上患者少见。女性患病率高于男性，女：男比例为（1.4~2.3） ：1。多急性 / 亚急性起病，具有空间多发和时间多发特点。空间多发指病变部位多发，大脑、脑干、小脑、脊髓可同时或相继受累。时间多发指缓解—复发的病程，由于累及部位广泛，反复发作后可最终致残，甚至致死。临床表现多样，常见症状包括视力下降、复视、肢体感觉障碍、肢体运动障碍、共济失调、膀胱或直肠功能障碍等。

**llama3.1_8b**  2025-02-18T23:45:47Z:

根据您提供的信息，我认为可能的诊断是：

1. **多发性硬化病（Multiple Sclerosis, MS）**

其他五种可能的诊断包括：

1. **Guillain-Barré综合征（GBS）**
2. **系统性红斑狼疮（Systemic Lupus Erythematosus, SLE）**
3. **多发性脊髓病变（Tritrichomoniasis）**
4. **脑炎/脑部感染（Encephalitis/Meningitis）**

这些疾病都可能表现为神经系统的多种症状，包括视力障碍、肢体感觉和运动障碍、共济失调等。然而，请注意，每个疾病的特点和患者群都有所不同。

再次强调一下，一定要根据您的专业知识和进一步的诊断信息来确定最终的诊断。

**86. user 2025-02-18T23:46:06Z:**

主要包括帕金森症、小脑性共济失调、锥体束损害以及自主神经系统损害。首发症状常为自主神经损害，最主要的是心血管功能障碍(如直立性低血压) 和排尿障碍(如尿失禁)，后者在男性患者还容易合并性功能障碍。很多患者病前有长达数年的快速眼动期睡眠行为障碍(RBD)。

**llama3.1_8b**  2025-02-18T23:46:29Z:

基于您提供的信息，我认为可能诊断出的罕见病是：

1. **多系统萎缩症（Machado-Joseph Disease，MJD）**：这是一个遗传性的神经退行性疾病，其特征是神经细胞逐渐丢失导致运动功能、自主功能和认知功能受损。它可以引起帕金森综合征、小脑性共济失调、锥体束损害以及心血管系统和排尿系统的症状。

其他可能的诊断如下：

1. **罕见遗传性疾病—** **多肌肽瘤病（Multiple Endocrine Neoplasia，MEN）**

- 这是一种由遗传决定的多发性内分泌腺体良性或恶性肿瘤综合征。它可能导致各种内分泌系统的功能障碍，从而引起一系列症状，如心血管功能不全和排尿功能障碍。

1. **遗传性肌肉疾病—** **施瓦茨-雅金斯病（Schwartz-Jampel Syndrome）**

- 这是一种罕见的遗传性疾病，其特点是体重减轻、肌张力增高、腺体大小变大和心脏问题等。患者可能出现自主神经功能障碍，例如心血管不稳定。

1. **遗传性神经退行性疾病—** **斯宾格尔-塞林综合征（Spastic Paraplegia, Type 7）**

- 这是一种罕见的遗传性疾病，其特征是逐渐恶化的运动功能障碍，主要影响下肢肌肉。同时，也可能出现心脏问题。

1. **其他遗传性疾病—** **家族性乙型主动脉瘤（Familial Aortic Dissection）**

- 这是指由于遗传因素导致的主动脉瘤综合征，其特点是在长期内随着年龄增长，主动脉壁逐渐剥离引起严重后果。

1. **生化学变化导致的疾病—** **酪氨酸氨基转移酶缺陷**

- 这是一种罕见的遗传性疾病，其特点是体内酪氨酸和其他氨基酸代谢异常。可能引起一系列全身性症状，如神经系统、心血管系统和肌肉功能等。

以上列表中提到的这些罕见疾病可能与您提供的症状相符，但最终诊断必须根据患者的临床表现、实验室检查结果、遗传检测以及其他相关信息综合考虑确定。

**87. user 2025-02-18T23:47:01Z:**

**1. 肌肉，** 四肢远端起始并逐渐向近端进展的无力萎缩，影响手部精细动作，造成垂足。面肌亦可受累。同时有明显肌强直现象，累及肢体肌、咽喉肌等。查体可发现大力握拳后手指不能立即展开，用力闭目后不能立即睁开。叩击手部大鱼际肌、舌肌等，可引出肌强直，称为叩击性肌强直或“肌球”现象。肌强直存在“热身”现象，反复活动后，肌强直减轻。下肢肌痛和全身疲乏感也是常见临床表现。

**2. 心脏，** 心脏传导系统常有受累，出现不同程度的房室传导阻滞和室内传导异常。严重时可造成猝死。确诊患者需定期监测心电图，必要时植入起搏器。

**3. 中枢神经系统** ，睡眠增多、阻塞性睡眠呼吸暂停等睡眠障碍多见。可伴有认知及情绪障碍。头 MRI 可见弥漫性脑白质病变及皮层萎缩。

**4. 眼** ，随着年龄增长，几乎所有患者均会出现白内障。常在 30~40 岁影响视力。

**5. 内分泌系统** ，可出现多种内分泌疾病，包括糖尿病、甲状腺功能异常、钙磷代谢异常、性激素异常、睾丸萎缩(造成不育) 等。

此外，还可有额秃、胃肠动力不足、限制性通气障碍等。

**llama3.1_8b**  2025-02-18T23:47:06Z:

根据提供的临床表现，可能的诊断是肌营养缺乏症（Muscular Dystrophy）的一个类型，即 Duchenne肌营养缺乏症（Duchenne Muscular Dystrophy, DMD）。以下是一些其他可能的诊断：

1. **肌营养缺乏症（Muscular Dystrophy）**：这是一个更广泛的医疗条件，涵盖多种类型的肌肉疾病，其中一些类型与DMD类似，如 Becker肌营养缺乏症、肱膊肌营养缺乑等。
2. **强直性肌痉挛（Stiff Person Syndrome）**：这种罕见疾病特征是肌肉强直，但主要影响腹部和四肢近端区域。
3. **威尔逊氏病（Wilson Disease）**：虽然主要涉及肝脏和脑部的金属积累，偶尔也会表现出肌肉症状如无力和肌痛，这取决于病情的具体情况。
4. **肌肉强直性神经炎（Myotonic Dystrophy）**：这一罕见疾病主要特征是肌肉强直，而不是DMD典型的肌肉萎缩和无力。但它也可能引起眼部问题、内分泌失调等。
5. **克雅氏病（Kabuki Syndrome）**：这是一个罕见综合症，其特点包括面貌特殊、肌肉强直、智力障碍以及一些其他身体上的变化。

请注意，这些可能的诊断都是基于给出的临床表现，并且需要通过进一步的检查和医生评估来确认确切的疾病类型。

**88. user 2025-02-18T23:47:45Z:**

该病发病年龄往往较早，最常发病的年龄为新生儿时期。大多患儿生后数日内发病，表现为拒奶、呕吐、嗜睡、惊厥、昏迷。部分晚发型患儿以发作性呕吐、嗜睡，伴精神发育异常为特点。高氨血症昏迷的患者可能有脑部伤害与发展迟缓、学习能力受损或智力障碍以及生长发育落后的后遗症。高氨血症患者可能造成颅内压增高、呼吸急促，病情进展会出现呼吸暂停或衰竭，肝肿大。神经系统并发症包括智力障碍、急性高血氨昏迷，甚至死亡。生化改变可见高氨血症、血谷氨酰胺和丙氨酸增高，尿中乳清酸降低或缺如。

**llama3.1_8b**  2025-02-18T23:47:49Z:

根据你的描述，我认为最可能的诊断是”新生儿颅内压增高综合征（NECPS）“，也称为”乳清酸减少症”。这种罕见病通常在新生儿时期发作，表现为高氨血症、嗜睡、惊厥、昏迷和呼吸暂停或衰竭等临床表现。

其他五种可能的诊断包括：

1. **肝脏疾病**：如先天性肝脏疾病、肝炎或肝硬化。
2. **代谢疾病**：如乳清酸减少症、丙酮酸脱氢酶缺乏症等。
3. **神经系统疾病**：如脑部感染、脑损伤或脑瘫。
4. **呼吸系统疾病**：如先天性气管支气管狭窄、肺动脉高压等。
5. **免疫系统疾病**：如先天性免疫缺陷综合征（SCID）。

需要注意的是，这些诊断都有可能与新生儿颅内压增高综合征（NECPS）共享一些临床表现，因此进一步的实验室检查和影像学检查是必要的，以帮助确定准确的诊断。

**89. user 2025-02-18T23:48:08Z:**

患儿常表现为严重的宫内发育迟缓，出生后很早(常在出生后 1 周) 即出现严重的、非酮症性高血糖，12 周后可恢复，50％ ~60％在青春期前后复发，复发后临床表现类似于2 型糖尿病。

**llama3.1_8b**  2025-02-18T23:48:30Z:

根据你的描述，我最有可能诊断出的是 Congenital Insulin Resistance 或 McIntosh 症候群（一种罕见的代谢疾病）。

其他五种可能的诊断包括：

1. **MODY3**：一种先天性糖尿病，表现为儿童时期高血糖。
2. **Cushing 症候群**：由于肾上腺激素过量导致的临床综合征，可能引起高血糖和其他代谢异常。
3. **Gestational Diabetes Mellitus（GDM）相关的遗传因素**：某些个体可能由于基因变异而易感于 GDM，这种情况在儿童中也可能出现。
4. **先天性肾上腺功能减退综合征（Congenital Adrenal Hypoplasia Syndrome，CAH）相关的代谢异常**：这种疾病可能导致严重的代谢紊乱，包括高血糖。
5. **其他罕见的代谢疾病，如 McCune-Albright 综合征或 Carpenter 综合征等**：这些疾病也可能表现为高血糖和代谢紊乱。

需要注意的是，这些诊断都需要根据详细的临床表现、实验室检查结果和遗传学分析来确定。

**90. user 2025-02-18T23:48:52Z:**

患者最早出现的症状是腹部膨隆，肝脾增大，部分患者可在新生儿期发病。智力和运动发育落后随即出现，肌张力低下，运动发育迟缓，而脑神经功能常不受累，1 岁后运动智力发育倒退明显，最后进展为痉挛强直状态。50％患者可以发现眼底樱桃红斑。间质性肺部病变可导致反复呼吸道感染、低氧血症或呼吸功能衰竭。多数患儿 3 岁前死亡。

**llama3.1_8b**  2025-02-18T23:48:56Z:

基于提供的临床表现，我认为可能的罕见病诊断是：

1. **苯丙酮尿症（PKU）**: PKU是一种遗传性疾病，特征是在新生儿期发病，随后出现智力和运动发育落后、肌张力低下等症状。眼底樱桃红斑是该病的一种常见表现。

其他五种可能的诊断：

1. **巨细胞毒症（GMHS）**: GMHS是一种罕见的免疫系统疾病，特征是在新生儿期发病，导致肝脾增大、智力和运动发育落后等症状。
2. **肌苷酸激酶缺乏症（McArdle病）**: McArdle病是一种遗传性疾病，特征是在运动发育迟缓、肌张力低下等方面出现问题，尤其是在运动时表现明显。
3. **戈克兰综合征（GCK deficiency）**: GCK是磷酸甘油激酶的一种酶，戈克兰综合征是一种罕见的疾病，其特征包括新生儿期发病、智力和运动发育落后等症状。
4. **肌肉内神经系统疾病（Muscular Dystrophy）**: 肌肉内神经系统疾病是一组遗传性疾病，特征包括肌张力低下、运动发育迟缓等症状。某些类型的肌肉内神经系统疾病也可能导致智力和运动发育落后。

请注意，这些诊断只是基于所提供的临床表现，并不一定是最终确定的结果。进一步的检查和评估是必要的，以确保准确的诊断。

**91. user 2025-02-18T23:49:19Z:**

**1. 典型面容，** 所有年龄患者均有上睑下垂，眼距宽，内眦赘皮，双眼外角下斜；双耳位低，后旋，耳廓厚。儿童患者还可有前额饱满，后发际低，鼻短，鼻梁低，鼻尖饱满；唇厚，鼻唇沟深而宽直达上唇等。

**2. 矮小，，** 出生时正常，1 岁后渐出现矮小。骨龄常落后，部分成年身高可达正常下限水平。

**3. 心血管 ，**50％ ~80％患者有先心病，肺动脉瓣狭窄最常见，其他包括肥厚型心肌病，室缺，肺动脉分支狭窄，法洛四联症和主动脉缩窄等。典型心电图改变包括心电轴极度右偏伴胸前导联 QRS 波逆时针旋转，V1 导联心电轴左偏、左前支不全传导阻滞或 RSR 波形。

**4. 精神运动发育落后** ，部分患者语言能力差，成年患者易有抑郁情绪，也有报道此病患者自闭症谱系表现发生率较高。

**5. 颈蹼，**

**6. 胸廓异常 ，**鸡胸或漏斗胸。

**7. 乳距宽**

**8. 男性隐睾** ，60％ ~80％男性患者合并隐睾，可致生育障碍。

**9. 凝血功能异常和淋巴管发育不良**

**10. 其他，** 肾脏畸形(肾盂扩张、双输尿管畸形、孤立肾、肾发育不良、远端输尿管狭窄等)，男性精子生成障碍，斜视，眼颤，皮肤牛奶咖啡斑和雀斑，四肢毛囊角化症， Ⅰ型阿诺德 - 基亚里畸形，脑积水，肝脾大，恶性肿瘤发生率增加等。

**11. 宫内表现，** 母亲孕期羊水过多，胎儿颈部透明带增宽，胎头相对较大，心脏和肾脏异常。

**llama3.1_8b**  2025-02-18T23:49:26Z:

根据您提供的信息，我认为最有可能的诊断是“ Noonan 综合征”。Noonan 综合征是一种罕见的先天性疾病，其临床表现包括：

- 上睑下垂、眼距宽和内眦赘皮
- 矮小（体格发育落后）
- 先心病（如肺动脉瓣狭窄、肥厚型心肌病等）
- 精神运动发育落后
- 颈蹼
- 胸廓异常（鸡胸或漏斗胸）
- 乳距宽
- 男性隐睾

其他可能的诊断包括：

1. **Down 综合征**：也是一种常见的先天性疾病，其临床表现包括上睑下垂、眼距宽、内眦赘皮、矮小、心血管异常和精神运动发育落后。
2. **Turner 综合征**：又称为“45,X”综合征，是一种女性单纯性遗传疾病，其临床表现包括上睑下垂、眼距宽、内眦赘皮、矮小、心血管异常和精神运动发育落后。
3. **Lenz-Majewski 综合征**：是一种罕见的先天性疾病，其临床表现包括上睑下垂、眼距宽、内眦赘皮、矮小、心血管异常和精神运动发育落后，以及特有的“鸡胸”或漏斗胸。
4. **Cornelia 代谢综合征**：是一种罕见的先天性疾病，其临床表现包括上睑下垂、眼距宽、内眦赘皮、矮小、心血管异常和精神运动发育落后，以及特有的“四肢毛囊角化症”。
5. **Prader-Willi 综合征**：是一种罕见的先天性疾病，其临床表现包括上睑下垂、眼距宽、内眦赘皮、矮小、心血管异常和精神运动发育落后，以及特有的“肥胖”和“男性隐睾”。

请注意，这些都是可能的诊断，但最终的诊断需要根据病人的具体临床表现和检查结果进行综合评估。

**92. user 2025-02-18T23:49:40Z:**

患者可以在任何年龄阶段发病。临床主要分为早发型和晚发型两种。早发型主要发生在男性患儿，一般在新生儿期发病，临床表现为起病急，病情凶险，因血氨升高，使得大脑广泛性损害。出生时可无异常，生后数天即表现出易激惹、嗜睡、拒食、呼吸急促和昏睡等，可迅速发展为痉挛、昏迷和呼吸衰竭。如果不给予紧急处理，很快发展成遗传代谢性脑病，并常在刚出生的 1 周内死亡，幸存者多遗留严重的智力损害。晚发型多发生在较大年龄的患者中，可以是半合子的男性和杂合子的女性，临床症状相对轻，且表现多样。儿童期和成人期发病的患者多表现为慢性神经系统损伤，如发作性呕吐、头痛、行为异常、谵妄、精神错乱等。患者可有肝大、反复癫痫发作、生长发育迟缓、行为异常等临床表型。尽管晚发型症状较轻，但是在疾病、应激、高蛋白饮食等环境因素应激下会诱发高氨血症的急性发作而威胁生命。杂合子女性携带者多数终身无症状，少数发病，发病年龄及临床表现有个体差异性，既有早发型，也可表现为晚发型。

**llama3.1_8b**  2025-02-18T23:49:44Z:

根据您提供的临床表现，我认为最可能的诊断是:

1. 崩坏性角膜营养障碍（Batten disease），一种罕见遗传病。
2. 苯丙氨酸尿症（Phenylketonuria，PKU），一种遗传性代谢疾病。
3. 异酰胺症（Isovaleric acidemia），一种罕见的遗传性代谢疾病。
4. 丙酮酸血症（Ketotic hypoglycemia），一种罕见的代谢疾病。
5. 褪色素失养（Albinism），一种遗传性疾病。

这些疾病都可能导致神经系统、肝脏和其他身体系统的功能障碍。特别是崩坏性角膜营养障碍（Batten disease）和苯丙氨酸尿症（PKU）这两种疾病，早发型确实会导致严重的神经系统损害和高氨血症。

其他可能的诊断包括：

- 苯丙酮酸血症（Phenylalaninemia）
- 丝氨酸缺乏症（Silent mutation）
- 乳糖不耐受症（Lactose intolerance）
- 糖尿病相关性神经损害（Diabetic neuropathy）

这些疾病都可能导致神经系统、肝脏和其他身体系统的功能障碍。然而，我仍然认为崩坏性角膜营养障碍（Batten disease）和苯丙氨酸尿症（PKU）是最有可能的诊断。

**93. user 2025-02-18T23:50:04Z:**

疾病的临床表型轻重不一。患者的主要临床表现是自幼起病的轻微外力下反复骨折、骨骼畸形，不同程度活动受限。患者可有蓝巩膜、韧带松弛、牙本质发育不全、听力下降、心脏瓣膜病变等骨骼外表现。OI 的影像学特征主要包括：全身多部位骨质稀疏；颅板变薄，囟门和颅缝增宽，枕骨可有缝间骨， 颅底扁平；脊柱可有侧弯或后凸畸形，椎体呈变形，可有多椎体压缩性骨折；胸廓可扭曲、变形，甚至塌陷；四肢长骨纤细、骨皮质菲薄，骨髓腔相对较大，干骺端增宽，多发长骨骨折，骨骼弯曲畸形等。

**llama3.1_8b**  2025-02-18T23:50:10Z:

根据你提供的信息，这位患者最有可能患有天生骨骼变性（Osteogenesis Imperfecta, OI）。此外，还有五种其他可能的诊断：

1. 佝僂病：虽然佝僂病主要影响肋骨，但它也可以导致脊柱侧弯和长骨畸形。患者的听力下降和心脏瓣膜病变也可以在佝僂病中看到。
2. 强直性肌痛（Duchenne Muscular Dystrophy）：这个疾病主要影响肌肉，但它也可以导致骨骼问题，例如脊柱侧弯和长骨畸形。患者的听力下降和心脏瓣膜病变也可以在强直性肌痛中看到。
3. 多发性骨折症：这个疾病主要影响骨骼，但它也可以导致肌肉无力和其他问题，例如蓝巩膜、牙本质发育不全。患者的听力下降和心脏瓣膜病变在多发性骨折症中较少见。
4. 扭转综合征（Torticollis Sequence）：这个疾病主要影响颈部，但它也可以导致脊柱侧弯和长骨畸形。患者的听力下降和心脏瓣膜病变在扭转综合征中较少见。
5. 多系统畸形（Multiple Congenital Anomalies）：这个疾病主要影响身体多个部位，但它也可以导致骨骼、肌肉和其他问题，例如蓝巩膜、牙本质发育不全。患者的听力下降和心脏瓣膜病变在多系统畸形中较少见。

请注意，这些疾病之间可能有重叠的临床表现，因此要确保准确诊断，需要进行进一步的检查和评估。

**94. user 2025-02-18T23:50:34Z:**

**1. 全血细胞减少，** 许多患者可以合并全血细胞减少、中性粒细胞降低。临床上表现为乏力、反复感染和出血倾向。

**2. 血红蛋白尿** ，典型的血红蛋白尿呈酱油或浓茶色。一般持续 2~3 天，不加处理自行消退，重者 1~2 周，甚至持续更长时间。有些患者的血红蛋白尿频繁发作，也有相当多的患者在很长的病程或观察期内始终无明确血红蛋白尿发作。

血红蛋白尿发作时可伴有发热、腰痛、腹痛等症状。其他与平滑肌功能障碍相关的症状可以有吞咽困难、腹痛、胃胀、背痛、头痛、食管痉挛、勃起障碍等。少见的情况包括：胆囊管运动障碍、急性胰腺炎、十二指肠或结肠的缺血和溃疡。

**3. 血栓形成**

**4. 黄疸与肝脾肿大** ，不到一半 患者有轻度黄疸。多数患者没有肝脾肿大。

常见的合并症包括感染、贫血性心脏病、胆石症、肾功能不全甚至衰竭等。

**llama3.1_8b**  2025-02-18T23:50:37Z:

根据提供的临床表现，可能的诊断是：

**1. 血红蛋白尿综合征（Hemolytic-Uremic Syndrome, HUS)**：这是一个罕见的疾病，其特点是血红蛋白尿、全血细胞减少和肾功能不全。

其他五种可能的诊断包括：

**2. 血液性溶血性贫血（Hemolytic Anemia）**：这是一个因为红细胞破坏速度超过生成速度而导致的贫血。

**3. 自身免疫性溶血性贫血（Autoimmune Hemolytic Anemia, AIHA)**：是一种由于自身免疫反应导致的溶血性贫血。

**4. 複合系统炎症综合征（Cryoglobulinemic Vasculitis）**：这是一个罕见的疾病，其特点是血栓形成、全血细胞减少和黄疸。

**5. 血管炎综合征（Vasculitic Syndrome）**：这是一个涉及血管炎症的疾病，其临床表现包括全血细胞减少、血红蛋白尿和血栓形成。

**95. user 2025-02-18T23:50:55Z:**

临床表现为反复肠套叠、腹痛、腹泻、黏液便、便血、便秘、呕血、贫血等。特征性临床表现为皮肤黏膜色素斑和胃肠道多发息肉。95% 以上患者出现色素沉着，色素沉着多见于口唇及其四周、颊部、面部、手指、手掌、足底、肛周等，偶见于肠黏膜。色素可呈褐色、棕褐色、灰色和蓝色等，通常发生 2 岁前，大小 1_5mm。约88%_100% 患者会发生胃肠道息肉，胃肠道息肉发病多发生于 11~13 岁，息肉可发生在整个胃肠道，以小肠多见，其次是结肠和胃。息肉大小不定，蒂长短粗细不一。该类疾病患癌风险较高，平均患癌年龄为 42 岁。

**llama3.1_8b**  2025-02-18T23:50:57Z:

根据您的描述，这种罕见病最有可能的诊断是：

**Peutz-Jeghers综合征（PJS）**

其他五种可能的诊断是：

1. **amiloidosis**
2. **克利氏症**
3. **家族性非息肉性腺瘤病变**
4. **家族性胃肠道息肉病**
5. **多发性腺瘤病**

需要注意的是，这些疾病的临床表现可能相似，但它们都是不同的罕见病。为了确诊，需要进行进一步的医学检查和实验室检测，如遗传学分析、内镜检查等。

**96. user 2025-02-18T23:51:13Z:**

患儿在新生儿期多无临床症状，出生 3~4 个月后逐渐出现典型症状，1 岁时症状明显。出生数月后因黑色素合成不足，其毛发和虹膜色泽逐渐变浅，为黄色或棕黄色，皮肤白。由于尿液、汗液含有大量苯乳酸而有鼠尿臭味。随着年龄增长，逐渐表现出智能发育迟缓，以认知发育障碍为主、小头畸形、癫痫发作，也可出现行为、性格等异常，如多动、自残、攻击、自闭症、自卑、忧郁等神经系统表现。婴儿期还常出现呕吐、湿疹等。

**llama3.1_8b**  2025-02-18T23:51:19Z:

根据您提供的临床表现，我最可能诊断出的罕见病是”苯丙酮尿症”(Phenylketonuria, PKU)。

PKU是一种遗传性的代谢异常，导致身体无法正确地分解苯丙氨酸，从而引起脑部损伤和其他临床表现。

以下是五种可能的诊断：

1. **苯丙酮尿症**(Phenylketonuria, PKU)：如上所述，这是一种遗传性的代谢异常，导致身体无法正确地分解苯丙氨酸，从而引起脑部损伤和其他临床表现。
2. **多糖利尿症**(Mucopolysaccharidosis, MPS)：这是另一种遗传性疾病，涉及到体内的糖原代谢异常，导致身体积累了大量的多糖物质，从而引起神经系统、骨骼和皮肤等系统的损害。
3. **苯丙酮尿症似综合征**(Phenylketonuria-like syndrome)：这是另一种罕见病，表现为类似的临床表现，如黑色素合成不足、智力发育迟缓和神经系统异常等，但其遗传学原因可能与PKU不同。
4. **儿童肌营养不良症**(Childhood Muscular Dystrophy)：这是一个群体名称，包括多种不同的肌肉疾病，如 Duchenne 肌肉萎缩症、Becker 肌肉萎缩症等，这些疾病都可能导致肌肉萎缩和智力发育迟缓等临床表现。
5. **遗传性神经系统缺陷**(Hereditary Neuropathy)：这是另一种罕见病，涉及到体内的神经系统开发异常，从而引起智力发育迟缓、运动功能障碍和其他临床表现。

需要注意的是，这些诊断仅供参考，您应该根据具体情况对疾病进行更加详细和准确的诊断。

**97. user 2025-02-18T23:51:34Z:**

主要为皮肤症状和神经精神症状。

**1. 皮肤症状群，** 为光照后在皮肤暴露部位出现红斑、疱疹甚至溃烂。皮疹可为湿疹、荨麻疹、夏令痒疹或多形性红斑等类型。口腔黏膜可有红色斑点，牙呈棕红色。同时可并发眼损害如结膜炎、角膜炎及虹膜炎等。严重者可有鼻、耳、手指皮肤结瘢变形。可有特殊紫色面容。可有多毛症。

**2. 神经精神症状群 ，**最常见的表现为急性腹痛，腹痛往往为重度、稳定、定位不明，有时会伴有痛性痉挛。可以伴随便秘、腹胀、恶心、呕吐，有时出现腹泻和肠鸣音增加。感觉和运动神经病可能先于腹痛出现，表现为肢体痛，伴麻木感、感觉异常和感觉倒错； 运动无力往往始于上肢近端，可向下肢和远端进展。累及脑神经可能导致延髓麻痹、呼吸功能受损和死亡。严重运动神经病可导致四肢瘫痪。自主神经系统受累表现为心动过速、高血压、出汗、躁动和震颤等。神经源性膀胱功能障碍可能引起尿痛、排尿困难、尿潴留和尿失禁。其他急性神经精神表现包括失眠、焦虑、躁动、激越、幻觉、癔症、定向障碍、谵妄、情感淡漠、抑郁、恐惧症和意识改变，从嗜睡到昏迷程度不一。

**llama3.1_8b**  2025-02-18T23:51:39Z:

根据您提供的疾病临床表现，我最有可能诊断出的是**多系统萎缩症（MPS）**。这是因为该疾病的临床表现包括皮肤、神经精神等多个系统的受累，例如：

- 皮肤症状：光照后出现红斑、疱疹、溃烂，口腔黏膜和牙齿改变，以及结膜炎、角膜炎、虹膜炎等眼损害。
- 神经精神症状：急性腹痛、感觉和运动神经病变、脑神经累及导致呼吸功能受损等。

另外五种可能的诊断：

1. **牛皮癣**（Psoriasis）：是一种慢性自身免疫疾病，表现为皮肤鳞状细胞增生，从而导致红斑、鳞屑、疱疹等症状。
2. **急性血管炎**（Henoch-Schönlein Purpura）：是一种血管炎症，主要影响小血管，表现为皮肤紫癜、关节炎、腹痛等。
3. **多重系统神经退行性疾病**（MND）：是一种渐进性的神经细胞损伤和死亡，导致肌肉无力、瘫痪等症状。
4. **过敏性尿路综合征**（Interstitial Cystitis/Bladder Pain Syndrome）：是一种慢性炎症性疾病，主要影响膀胱，表现为尿痛、排尿困难、尿潴留等。
5. **自主神经系统障碍**（Autonomic Nervous System Disorder）：是一种影响自主神经系统的疾病，导致心率、血压、出汗等功能失常。

**98. user 2025-02-18T23:52:04Z:**

**1. 感染，患者**均有不同程度感染。 感染具有反复、严重、难治、机会致病、病原谱广等特点。典型患儿出生时一般没有特别表现，由于患儿存在细胞免疫异常，2 月龄前也可发生致命感染。感染部位及病原多种多样，包括反复肺炎，反复鹅口疮，持续腹泻，反复中耳炎，持续病毒感染(如呼吸道合胞病毒、EBV、CMV 等)，卡氏肺囊虫等条件致病菌感染也较常见。

**2. 疫苗病，** 患者免疫功能极度低下，可发生减毒活疫苗感染和播散。 患儿接种减毒轮状病毒疫苗也可发生严重腹泻。

**3. 生长发育落后或停滞** 是 疾病的显著特征。与患儿反复感染、腹泻和相应基因缺陷均有关。

患儿还可伴有皮疹、肝脾淋巴结肿大类似红皮病表现

另一部分患儿可出现类似红皮病、肝脾肿大、脂溢性皮炎、血细胞减少症，硬化性胆管炎。

**llama3.1_8b**  2025-02-18T23:52:07Z:

根据你提供的临床表现，我最有可能诊断出的是:**严重结合免疫缺陷病（SCID）。**

其他五种可能的诊断：

1. **红皮病**：一种罕见的、遗传性的皮肤疾病，导致患者出现红色的皮疹。
2. **骨髓增殖性疾病**：是一类罕见的、遗传性的骨髓疾病，可能影响到患者的免疫系统和血细胞生成。
3. **卡氏肺囊虫感染**：一种罕见的、细菌性的感染，可以导致肺部炎症和其他问题。
4. **免疫缺陷综合征（AIDS）**：一种较为常见的、由病毒引起的免疫系统疾病，可能影响到患者的免疫系统功能。
5. **遗传性溶血性贫血**：是一种罕见的、遗传性的疾病，可以导致患者的红细胞生成不足，从而出现贫血。

**99. user 2025-02-18T23:52:21Z:**

核心症状是肌张力不全，主要表现为异常的表情姿势和不自主的动作，多累及头颈部肌肉、躯干肌、肢体的旋前肌、指腕屈肌、趾伸肌和跖屈肌等。随意运动时加重，休息睡眠时减轻或消失，晚期可呈固定扭曲痉挛畸形。感觉诡计(缓解技巧) 和动作特异性是肌张力不全的特征性表现。感觉诡计(缓解技巧) 是指专门用于纠正异常姿势或缓解肌张力不全性运动的随意动作，通常是涉及或针对受累部位的简单运动，但不是用力对抗肌张力不全症状。动作特异性是指仅在特定活动或执行特定任务时出现肌张力不全，例如某些职业(如书写痉挛、音乐家痉挛) 或运动。

**llama3.1_8b**  2025-02-18T23:52:25Z:

根据你的描述，我认为最有可能的诊断是：

1. 肌张力不全症（Dystonia）：这是一个涉及肌肉张力异常增加和扭曲的罕见神经系统疾病。该症状可以影响身体任何部位，包括面部、头颈部、躯干、肢体等。

其他五种可能的诊断是：

1. 展开性脊柱侧弯（Scoliosis）：这是一个涉及脊柱异常侧弯和扭曲的疾病，可以导致身体不对称和姿势异常。
2. 肌肉假性麻痹（Pseudoparalysis）：这是一个罕见的神经系统疾病，特点是肌肉张力降低和运动功能丧失，通常影响肢体和躯干部位。
3. 神经管瘤综合征（Neurofibromatosis）：这是一个涉及神经管异常增生和扭曲的遗传性疾病，可以导致多个神经系统症状，如肌张力不全、运动功能丧失等。
4. 腰椎动脉综合征（Cervicogenic Dystonia）：这是一个罕见的神经系统疾病，特点是腰椎异常增生和扭曲导致的肌肉张力异常增加和扭曲。

请注意，这些诊断都是基于你的描述，但最终确定确切的诊断需要进行进一步的临床检查、影像学检查和实验室检查。

**100. user 2025-02-18T23:52:48Z:**

**1. 肾脏 ，**主要表现为肢体水肿和尿中泡沫增多，实验室检查可以发现单纯的中等量蛋白尿或肾病综合征，晚期可出现肾功能不全。

**2. 心脏 ，**主要表现为活动后气短、肢体水肿、胸腔积液、腹水等限制性心功能不全表现。

**3. 肝脏 ，**可有轻微肝区不适或疼痛，但多数患者可无症状，往往为体检时发现肝大或者肝酶异常。疾病晚期可出现肝功能衰竭。

**4. 周围神经和自主神经 ，**为对称性的四肢感觉和(或) 运动性周围神经病。自主神经异常多表现为直立性低血压、胃轻瘫、假性肠梗阻和阳痿等。

**5. 胃肠道** ，可以出现全胃肠道受累，以胃部和小肠受累多见。可以表现为上腹不适、消化不良、腹泻、便秘、吸收不良综合征和消化道出血等。

**6. 软组织 ，**舌体受累可出现巨舌、舌体活动障碍和构音异常等。皮肤黏膜可以出现皮肤紫癜和瘀斑，以眼眶周围和颈部皮肤松弛部位较为常见。也可以出现指甲萎缩脱落和毛发脱落等。

**7. 凝血功能异常** 患者常会伴发凝血因子Ⅹ缺乏，造成相应的出血表现。

**llama3.1_8b**  2025-02-18T23:52:54Z:

根据您提供的疾病临床表现，我认为最可能的诊断是肾脏相关疾病。但是需要注意的是，这些症状也可以出现在其他一些罕见病中。因此，以下五种可能性也是非常重要的：

1. **弥漫性系统性硬化症（DSS）**：这是一个罕见的自身免疫性疾病，可以影响多个器官和系统，包括肾脏、心脏、肝脏、周围神经和自主神经以及胃肠道。这个疾病的临床表现与您提供的症状非常相似。
2. **皮肌症**：这是另一个罕见的自身免疫性疾病，主要影响皮肤和肌肉系统，可以导致肌肉无力、关节痛、皮肤紫癜等症状。这个疾病也可能影响肾脏和其他器官。
3. **过敏性血管炎（Henoch-Schönleinpurpura, HSP）**：这是一个罕见的自身免疫性疾病，主要影响皮肤、关节、肾脏和胃肠道系统。这个疾病可以导致紫癜、关节痛、尿中蛋白质异常等症状。
4. **威尔逊病（Wilson’s disease）**：这是一个罕见的遗传性代谢疾病，主要影响肝脏和脑部，可以导致肝功能衰竭、神经系统问题以及其他器官受累的症状。
5. **库欣综合征（Kushner syndrome）**：这是一个罕见的自身免疫性疾病，主要影响肾脏、心脏和胃肠道系统，可以导致肾功能不全、限制性心功能不全以及消化问题等症状。

因此，我认为最可能的诊断是弥漫性系统性硬化症（DSS），但是也需要考虑其他五种可能性。

**101. user 2025-02-18T23:53:08Z:**

黄疸和皮肤瘙痒是典型临床表现。其他有身材矮小、青春期发育落后等发育迟缓表现，胆囊结石，脂肪吸收障碍所致的脂肪泻，肝脾肿大以及脂溶性维生素缺乏所致佝偻病、骨龄延迟、干眼症、凝血障碍和神经肌肉病变等症状。患儿亦可以出现视觉及听力异常，出现烦躁、嗜睡及注意力不集中等改变。晚期可出现门静脉高压症和肝脏肿瘤等。

**llama3.1_8b**  2025-02-18T23:53:11Z:

根据您的描述，这个罕见病最有可能是：

苯丙酮尿症（Phenylketonuria, PKU）

其他五种可能的诊断为：

1. 法林酸血症（Familial Hypercholesterolemia）
2. 异质性甲状腺功能减退症（Congenital Hypothyroidism）
3. 锥体肌萎缩症（Huntington’s Disease）
4. 疱疹样变性病（Porphyrias）
5. 维生素D抵抗性佝偻病（Vitamin D-Resistant Rickets）

这些疾病都可能表现出与您的描述相似的临床特征，如黄疸、皮肤瘙痒、发育迟缓等。然而，PKU是最可能的诊断，因为它可以导致脂溶性维生素缺乏、凝血障碍和神经肌肉病变等症状，与您的描述相符。

**102. user 2025-02-18T23:53:28Z:**

在儿童期起病。表现为运动发育轻度迟滞，骨骼肌进行性无力萎缩，影响肢体运动功能，逐渐出现步态异常、上肢活动受限，自然病程常在 10 岁左右丧失行走能力。此后出现脊柱侧弯、关节挛缩、呼吸肌无力、扩张型心肌病，20 岁左右因呼吸衰竭、心功能衰竭死亡。查体可见双腓肠肌假肥大，同时可有双前臂及舌肌假肥大，Gowers 征阳性，腰椎前凸等。可青年甚至成年起病， 部分患者不影响生存期。假肥大体征明显，部分患者在肢体无力尚轻时，先出现明显的扩张型心肌病。

**llama3.1_8b**  2025-02-18T23:53:31Z:

根据你提供的信息，我认为最有可能的诊断是 Duchenne 肌营养不良症（DMD）。

五种可能的诊断：

1. Becker 肌营养不良症（BMD）：与 DMD 类似，但进展较慢，影响较少。
2. Facioscapulohumeral 肌营养不良症（FSHD）：主要影响面部肌肉、肩blade 和胸肌，但也可能影响下肢肌肉。
3. Myotonic 变性：主要表现为肌肉张力增高和持续性痉挛，不同于 DMD 的无力和萎缩特征。
4. Limb-Girdle 肌营养不良症（LGMD）：影响肩膀和下肢区域的肌肉，进展较慢且变异较大。
5. Congenital 肌营养不良症：一种罕见的遗传性疾病，从出生时就表现出肌肉无力和萎缩。

**103. user 2025-02-18T23:54:03Z:**

主要为高血氨、脑损伤和心肌病等。

**1. 新生儿起病型** 出生时正常，开始哺乳后出现呕吐、嗜睡、肌张力低下、惊厥、呼吸困难、高血氨、酮症、低血糖、酸中毒、扩张型心肌病、胰腺炎等异常，病死率高。

**2. 迟发型** 常因发热、饥饿、高蛋白饮食和感染等诱发，表现为婴幼儿期喂养困难、发育落后、惊厥、肌张力低下等。许多患者的认知能力及神经系统发育受到损害，脑电图慢波增多或见癫痫波；一些患者可有骨折，X 线见骨质疏松；还常造成骨髓抑制，引起粒细胞减少、贫血、血小板减少。也可有心脏损害，如心肌病、心律失常、QT 间期延长、心功能减弱等。肾功能损害较为少见。

**llama3.1_8b**  2025-02-18T23:54:07Z:

根据你提供的信息，我认为最可能的诊断是甲状腺激素代谢疾病——原发性高血氨症（也称为危及生命的高氨症）。这种罕见病表现为高血氨、脑损伤和心肌病等。

以下是五种其他可能的诊断：

1. **巨细胞病毒感染**: 这是一种严重的感染，主要影响新生儿，可以导致肺炎、心脏问题和肾衰竭。
2. **先天性代谢疾病（如酮症酸中毒）**: 这类疾病会导致体内积累异常物质，引起严重的代谢紊乱，可能影响脑功能和心血管系统。
3. **新生儿肺炎综合征**: 这是一种较为常见的新生儿疾病，表现为呼吸困难、皮肤发绀等，并且可能导致严重的心脏问题。
4. **先天性甲状腺功能减退症**: 这类疾病会影响甲状腺激素的产生，从而导致高血氨、肌张力低下和心脏问题等。
5. **新生儿代谢综合征（如伯格氏病）**: 这是一种罕见的疾病，表现为肌张力低下、心脏问题以及代谢紊乱等。

**104. user 2025-02-18T23:54:35Z:**

临床表现常没有特异性，隐匿起病。可出现咳痰、消瘦、乏力等症状。继发感染时可有发热、咳脓性痰，也可出现胸痛、咯血。少数患者可无症状，可以没有阳性体征。部分患者可以出现爆裂音，重症患者可有发绀，少数患者可见杵状指。临床表现常比胸部影像受累的程度轻，这种症状和影像不一致的表现也是该病 的一个特征。血清抗 GM-CSF 抗体测定明显增高。

**llama3.1_8b**  2025-02-18T23:54:45Z:

根据您提供的信息，我认为可能诊断的是Good的硬化症（也称为好型白血病性干骺状细胞综合征）。这种罕见疾病主要影响儿童和青少年，表现为肺部感染、发热、咯血等症状，以及某些患者出现杵状指等特异性的体征。然而，请注意，这仅是一种可能的诊断，并且需要进一步的医疗检查来确认。

五种其他可能的诊断包括：

1. 急性髓样白血病（AML）：这种疾病是儿童和成人的一种罕见白血病，表现为骨髓增殖异常、肺部感染等。
2. 淋巴瘤：某些类型的淋巴瘤可能会导致肺部受累、咯血等症状。
3. 纤维化性疾病：这是一种罕见的全身疾病，表现为肺部纤维化等。
4. 慢性骨髓增殖异常综合征（CMML）：这种罕见疾病主要影响成年人，表现为骨髓增殖异常、肺部感染等。
5. 急进性粒细胞白血病（APL）：这种罕见疾病是儿童和成人的一种白血病，表现为骨髓增殖异常、肺部感染等。

**105. user 2025-02-18T23:55:27Z:**

大多患者青少年时期起病，首先是视杆细胞逐渐丧失，随后出现视锥细胞受累， 视网膜色素上皮细胞死亡。因此通常以夜盲为首发症状(但城市化后部分患者常以视力下降为首发症状)，而且夜盲出现得越早，患者的病情往往越严重。虽然周边视野缺损也发生较早，但患者通常意识不到，很少以此作为疾病早期的主诉。病情逐渐发展为中心视力下降，视野进一步缩窄，多数在 40 岁之前就成为法定盲人。患者的视功能损伤程度可与遗传方式相关。

患者的眼底表现与疾病所处的阶段相关：疾病早期患者眼底在常规检查下可表现正常或接近正常，但部分患者在中周部眼底照相下可以观察到早期毯层样视网膜变性(这类细微改变在目前的扫描激光眼底照相或广域眼底照相下很难识别)。随着病情的进展，部分 患者会出现典型的眼底改变，表现为视网膜骨细胞样色素沉积合并中周部视网膜萎缩，视盘蜡黄，视网膜血管变细等；部分病人会出现毯层样视网膜变性或脱色素改变，见于中周部视网膜或广泛变性。其他常见的并发症还包括黄斑囊样变性、后囊下白内障、玻璃体尘样颗粒、视盘玻璃膜疣等并发症；少见的并发症有 Coat’s 样视网膜病变。

**llama3.1_8b**  2025-02-18T23:55:31Z:

根据您提供的临床表现，这名患者最可能患有”Leber’s 绝对性夜盲症”（即 Leber congenital amaurosis）。这个疾病是由视网膜色素上皮细胞死亡引起的一种罕见遗传性眼疾。它通常在青少年时期出现，最初表现为夜盲，随着时间的推移会发展成中心视力下降和视野缩窄，最终导致 blindness。

以下是五种可能的诊断：

1. **Leber’s 绝对性夜盲症**（Leber congenital amaurosis）：这是患者最可能患有的疾病。
2. **红斑性夜盲症**（Retinitis pigmentosa）：这种疾病也会导致视杆细胞和锥细胞受损，但通常首先表现为周边视野缺损。
3. **Batten 绝对性夜盲症**（Batten disease）：这是一个遗传性的神经退行性疾病，常见于儿童，可能导致视力下降以及其他神经系统问题。
4. **Stargardt 绝对性夜盲症**（Stargardt disease）：这是另一种罕见的遗传性眼疾，通常首先表现为黄斑区域色素沉积。
5. **Leber’s 细胞色素上皮变性**（Leber’s hereditary retinal degeneration）：这种疾病是由视网膜色素上皮细胞死亡引起的一种罕见的遗传性眼疾，可能与患者提供的临床表现相吻合。

**106. user 2025-02-18T23:56:15Z:**

最常见的临床表现是白瞳症，即瞳孔可见黄白色反光。患眼可因肿瘤位于后极部，视力低下，而发生失用性斜视。早期表现为灰白色，圆形或椭圆形拱状肿物，可向玻璃体隆起，或沿脉络膜扁平生长。肿瘤表面的视网膜血管可出现扩张、出血，甚至发生渗出性视网膜脱离。瘤组织也可穿破视网膜进入玻璃体，如雪球状漂浮，甚至沉积于前房形成假性前房积脓。双侧性患儿发病较早，通常一岁以内发病。单侧性患儿发病较迟，通常在两岁或三岁时发生。

**llama3.1_8b**  2025-02-18T23:56:18Z:

根据您提供的临床表现，我认为最可能诊断出的罕见病是retinoblastoma（视网膜母细胞瘤）。这是一个常见于儿童的恶性肿瘤，其特征是在后极部形成白色或黄白色的肿物，可能引起瞳孔反光、失用性斜视和视力下降。

其他五种可能的诊断包括：

1. **Hemangioma of the retina**：这是一种良性的血管瘤，可表现为圆形或椭圆形拱状肿物。
2. **Coats disease**：这是一种罕见的视网膜疾病，可能导致玻璃体内出现血管扩张和渗出性视网膜脱离。
3. **Persistent fetal vasculature (PFV)**：这是一种较为少见的视网膜疾病，表现为异常的血管形成和扩张。
4. **Retinal dysplasia**：这是一种罕见的视网膜疾病，可能导致视网膜组织异常生长和发展。
5. **Optic nerve glioma**：这是一种神经胶质瘤，可影响视神经，并导致各种视觉问题。

**107. user 2025-02-18T23:56:37Z:**

患儿出生时多正常，在 1 岁以内起病，平均发病年龄为 5.6 个月。首次发作常为热性惊厥，可为一侧性或全面性阵挛或强直阵挛发作，热性惊厥具有发作持续时间长和反复发作特点，可出现持续状态。28％ ~35％患儿可能以无热惊厥起病，但在以后病程中常有热敏感特点。各种原因引起的体温升高，包括运动、发热、疫苗接种、洗热水澡和环境温度高等，均容易诱发或使发作加重，并易发生癫痫持续状态。有些患儿会出现以癫痫持续状态之后深昏迷为特征的急性脑病。

患儿多于 1~4 岁期间出现无热惊厥，具有多种发作类型，如肌阵挛、不典型失神、部分性发作、全面强直阵挛发作等。肌阵挛发作常由光刺激、闭眼和某种特定的图形刺激诱发，一块或数块肌肉同时阵挛，均伴有同步异常脑电波阵发性发放。肌阵挛强度不一，仅侵犯轴性肌肉时，出现身体轻微向前或向后的动作，严重者跌倒或突然抛掷手中物体。肌阵挛发作可以是孤立的，也可群发，但持续状态比较罕见。约 25％的 儿可以始终不出现肌阵挛发作。不典型失神发作同期脑电图显示半数为局灶起源放电，多数呈不规则不节律的慢棘慢波发放，经常伴随肌阵挛发作。30％的患儿可发生不典型失神持续状态。部分性发作发生率为 43.0％ ~78.6％，表现为偏侧肢体(或) 头部偏转性发作或阵挛性发作，亦有运动性发作伴有意识障碍和自主神经症状、自动症、肌张力降低等。1 岁以后患儿出现精神运动发育迟滞，尤其是语言发育迟缓。

**llama3.1_8b**  2025-02-18T23:56:44Z:

根据您提供的临床表现，我认为最可能诊断的是西尼尔综合征（West Syndrome）。西尼尔综合征是一种罕见的先天性脑发育障碍，其典型特征包括：

1. 一岁以内起病：患者通常在1岁以内出现症状。
2. 热性惊厥：热性惊厥是西尼尔综合征最常见的首发症状，表现为阵挛或强直阵挛发作，可能伴有体温升高。
3. 肌阵挛：肌阵挛发作是西尼尔综合征另一个特征性的症状，通常由光刺激、闭眼和某种特定的图形刺激诱发。
4. 依赖于体温变化的癫痫：西尼尔综合征患者往往对体温变化敏感，这可能导致发作加重或诱发。
5. 精神运动发育迟滞：1岁以后，西尼尔综合征患者可能会出现精神运动发育迟滞，尤其是语言发育迟缓。

其他五种可能的诊断包括：

1. **Lennox-贡戈病**（Lennox-Gastaut Syndrome）：一种罕见的儿童癫痫疾病，其特征性症状包括多种类型的发作，包括肌阵挛、不典型失神和全面强直阵挛。
2. **多发性脱髓鞘病**（Multiple Sclerosis）：一种自身免疫性疾病，可能导致癫痫、视觉障碍、运动功能障碍等症状。
3. **脑炎**：一种由病毒或细菌引起的脑部炎症，可能导致热性惊厥、肌阵挛和其他神经系统症状。
4. **癫痫发作综合征**（Epileptic Encephalopathy）：一种罕见的先天性脑发育障碍，其特征是多种类型的癫痫发作，包括热性惊厥、肌阵挛和不典型失神。
5. **儿童全身性硬化症**（Juvenile Idiopathic Arthritis）：一种罕见的自身免疫性疾病，可能导致身体各部位的炎症和组织损伤。

**108. user 2025-02-18T23:57:02Z:**

一方面表现为慢性溶血性贫血，除贫血相关症状，因胆红素升高可引起皮肤、巩膜轻中度黄染、色素性胆石症，当寒冷、感染、脱水时症状可能加重。婴幼儿可出现脾大，随年龄增长，脾脏可因脾梗死、纤维化而缩小。

另一方面由于毛细血管微血栓而引起反复发作的血管阻塞性疼痛。儿童和成人多表现为四肢肌痛、大关节疼痛和腰背疼痛，严重者可出现剧烈腹痛、头痛，甚至肢体瘫痪、昏迷。随着时间的推移，血管阻塞几乎可发生于每个器官系统，可以出现周期性疼痛，患者瘦弱、易疲劳、易感染、营养不良。因骨髓造血组织过度代偿性增加使骨皮质变薄、骨质疏松、脊柱变形、股骨头无菌坏死，如有骨梗死则可导致局部骨质硬化。眼部症状可能出现眼底视网膜血管栓塞引起眼底出血、视网膜脱离。另外，尚可影响神经系统的发育而出现智力低下。

在超过 25％的患者中急性疼痛为疾病的首发症状。在大于 2 岁的患儿中是最常见的症状。疼痛的严重程度可从轻微到剧烈不等。大约 1/2 的疼痛发作伴随客观的临床体征，如发热、肿胀、压痛、呼吸过速、高血压、恶心和呕吐。

**llama3.1_8b**  2025-02-18T23:57:05Z:

根据您提供的症状，这可能是一种罕见的疾病：球形细胞减少症（Sickle Cell Disease，SCD）。其他五种可能的诊断是：

1. 库欣综合征（Kawasaki Syndrome）：一种儿童急性感染性血管炎，表现为高热、皮疹、淋巴结肿大等。
2. 血小板减少症（Thrombocytopenia）：由于血小板数量过低导致的出血和易血栓风险。
3. 细胞毒性贫血（Hemolytic Anemia）：一种由于红细胞破坏过多导致的贫血，表现为黄疸、脾大等症状。
4. 血管炎（Vasculitis）：一种血管炎症和损伤导致的各类器官功能障碍。
5. 遗传性血栓性凝固倾向疾病（Hereditary Hemorrhagic Telangiectasia，HHT）：一种家族性遗传性的疾病，表现为出血和易血栓风险。

但是，请注意这些诊断都需要根据具体的临床表现、实验室检查结果和其他相关因素来进行综合评估。

**109. user 2025-02-18T23:57:22Z:**

新生儿和儿童患者的主要临床表现包括：

**1. 宫内发育迟缓及出生后生长障碍** 宫内发育迟缓，低出生体重，出生后缺乏追赶生长，成比例的身材矮小。

**2. 特殊体征** 前额宽而突出、尖下颌导致三角形脸，相对头围偏大。齿列不齐、耳位低，躯体偏身不对称，包括四肢长度差异，小指侧弯畸形等。

**3. 其他表现** 喂养困难、新生儿期可出现低血糖，尿道下裂、隐睾和马蹄肾等泌尿生殖系统畸形，牛奶咖啡斑，反流性食管炎等消化系统异常。部分患者出现认知、运动和语言发育迟缓，学习障碍等。

**llama3.1_8b**  2025-02-18T23:57:24Z:

基于您提供的临床表现，我最有可能诊断出的罕见病是：

**1. 普里斯特林综合征（Prader-Willi Syndrome）**

其他五种可能的诊断：

**2. 强直性脊柱炎相关综合征（Stickler Syndrome）** **3. 海尔氏症候群（Hallervorden-Spatz Disease）** **4. 皮埃特罗综合征（Pitt-Hopkins Syndrome）** **5. 贝克综合征（Beckwith-Wiedemann Syndrome）** **6. 克伦伯格-伦纳德综合征（Klinefelter Syndrome)**

请注意，这些诊断仅基于您提供的临床表现，需要结合其他测试和检查来确认。

**110. user 2025-02-18T23:57:43Z:**

**1. 皮肤黄瘤，** 好发于伸肌肌腱附着点附近，例如跟腱、肘关节、膝关节等，皮肤损伤与黄瘤出现关系密切。皮肤黄瘤少见于儿童患者。

**2. 动脉粥样硬化 ，**患者出现动脉粥样硬化、冠心病的风险显著高于正常人。早发性冠心病可以作为本病的特点之一。

**3. 血液系统改变 ，**血液中植物固醇含量明显增加，导致细胞膜中固醇成分中植物固醇比例增高，红细胞、血小板等细胞膜功能异常，出现贫血和血小板减少，患者可出现乏力、面色苍白、皮肤出血等表现。

**4. 其他，**除上述症状，部分患者会出现关节痛、关节炎、脾大、转氨酶升高等。

**llama3.1_8b**  2025-02-18T23:57:46Z:

根据您提供的临床表现，可能的罕见病诊断是：

1. **皮肤黄瘤**

其他五种可能的诊断是：

1. **多动脉粥样硬化症**：这种疾病会导致动脉粘液壁的损伤和炎症，从而引起多个动脉的粥样硬化。
2. **特发性血小板减少性紫癜（ITP）**：这是一个罕见的疾病，主要表现为血小板数量显著降低，并可能导致皮肤和黏膜出血。
3. **红斑狼疮（SLE）**：这是一种自身免疫性疾病，可以影响多个器官系统，包括皮肤、关节、肾脏等，表现为皮肤黄瘤、关节痛、贫血等。
4. **全身性硬化症（Scleroderma）**：这种疾病会导致身体各部位的皮肤和软组织逐渐硬化，可能引起皮肤黄瘤、关节炎、肾脏受累等。

**111. user 2025-02-18T23:58:11Z:**

男性发病，起病隐匿，常见发病年龄为 30~60 岁。患者常以痛性痉挛、震颤、双下肢无力等症状起病，逐渐出现双上肢无力、延髓部和面部的肌肉萎缩、肌束颤动、构音障碍、吞咽困难、呼吸困难等。可伴有男性乳房发育、生殖功能降低等雄激素受体不敏感表现。超过 50％的患者存在感觉异常。该病进展缓慢，患者通常在病程晚期才出现行走不能，仅有部分患者需要辅助通气，对生存期无显著影响。

**1. 不典型症状 ，**某些不典型症状可在出现肢体力弱前数年甚至数十年出现，包括易疲劳、痛性痉挛、口周震颤及姿势性震颤。肌无力：多数患者以双下肢无力起病，逐渐进展至双上肢、舌肌以及面肌，而眼外肌往往不受累。肢体无力的分布多不对称，以近端受累为主。咀嚼肌受累时可引起下颌下垂及震颤。体检以下运动神经元损害体征为主，表现为肢体肌力下降，近端为著，伴肌肉萎缩、束颤，腱反射多减弱或消失。多数患者可出现明显舌肌萎缩、纤颤，但软腭活动、咽反射多正常，患者饮水呛咳、吞咽困难的程度也较轻。某些患者还可观察到颏部以及口周束颤。患者运动功能损害随病程延长逐渐加重，后期可出现上楼费力、行走不稳等。

**2. 感觉障碍** ，约半数患者存在不同程度的感觉减退，而在其他患者为亚临床性，仅在感觉神经传导检查中发现异常。

**3. 内分泌异常，** 包括葡萄糖及脂肪代谢的异常，雄激素受体不敏感等表现，后者包括男性乳房发育、性功能下降、不孕不育、睾丸萎缩等。

**4. 女性携带者，** 多数女性携带者并不出现症状，即使出现症状程度也较轻，可仅表现为束颤、轻度远端肢体无力、肌肉痉挛或肌酸肌酶增高等。

**llama3.1_8b**  2025-02-18T23:58:13Z:

根据提供的临床表现，可能的诊断是:

1. 肌营养病（Amyotrophic Lateral Sclerosis, ALS）

其他五种可能的诊断是：

1. 多系统萎缩症（Multi-System Atrophy, MSA）
2. 进行性核上空间皮质性麻痹症（Progressive Supranuclear Palsy, PSP）
3. 桑地亚综合征（Sandhoff Disease）
4. 褪色素变性病（Huntington’s Disease）
5. 肾性神经退行性疾病（Spinal and Bulbar Muscular Atrophy, SBMA）

**112. user 2025-02-18T23:58:33Z:**

部分患者在宫内表现为胎动减少，出生时为松软儿。患者在 6 个月内发病，平均发病年龄在生后 1 个月。表现为全身松软无力，严重肌张力低下。由于舌、面和咀嚼肌无力，大多数患儿出现吸吮和吞咽困难，可见舌肌萎缩和震颤。肋间肌受累可以出现呼吸困难，腹式呼吸。胸部呈钟型外观。下肢较上肢受累重，近端较远端严重。严重躯体中轴部位肌无力使患儿不能控制头部运动，不会抬头或翻身，没有独坐能力。卧位时，双下肢呈髋外展、膝屈曲的蛙腿体位。肌肉萎缩多不明显， 部分患儿轻度关节畸形。患儿智力好，腱反射消失，四肢感觉正常。患儿肌无力进行性加重，最终失去所有自主运动能力，鼻饲喂养，反复呼吸道感染而致呼吸衰竭。80％患儿 1岁内死亡，很少活过 2 岁。

**llama3.1_8b**  2025-02-18T23:58:39Z:

根据您的描述，我认为可能的诊断是：

1. **肌营养不良症（Spinal Muscular Atrophy, SMA）**：这是一个遗传性疾病，由于肌肉中的肌动蛋白基因缺失或变异导致。SMA表现为肌无力、萎缩和瘫痪，尤其是在早产儿和新生儿中较常见。

其他可能的诊断：

1. **先天性肌无力症（Congenital Muscular Dystrophy）**：这是一个群体疾病，表现为出生时或婴儿期出现全身性肌肉无力、萎缩和瘫痪。不同的形式可能有不同的遗传因素和基因变异。
2. **先天性锥体系统疾病（Congenital Motor Neuron Disease）**：这是一个群体疾病，表现为新生儿或婴儿期出现肌无力、萎缩和瘫痪。不同的形式可能有不同的遗传因素和基因变异。
3. **肌肉病毒感染（Muscular Dystrophy Viral Infection）**：这是一个罕见的疾病，表现为新生儿或婴儿期出现全身性肌无力、萎缩和瘫痪。不同的形式可能有不同的基因变异。
4. **神经肌肉接头障碍（Neuromuscular Junction Disorder）**：这是一个群体疾病，表现为新生儿或婴儿期出现肌无力、萎缩和瘫痪。不同的形式可能有不同的遗传因素和基因变异。
5. **先天性脑部脏器受累症（Congenital Cerebral and Visceral Abnormalities）**：这是一个群体疾病，表现为新生儿或婴儿期出现全身性肌无力、萎缩和瘫痪。不同的形式可能有不同的遗传因素和基因变异。

请注意，以上这些诊断仅供参考，而真正的诊断应根据更多的临床信息、实验室检查和遗传学分析等综合考虑得出。

**113. user 2025-02-18T23:58:52Z:**

患者通常成年起病，隐匿起始，逐渐进展。主要表现为小脑性共济失调，包括醉汉步态、动作笨拙、吟诗样语言、眼震、复视等；锥体束损害，包括腱反射亢进、踝阵挛、病理征阳性；锥体外系症状，包括帕金森综合征、肌张力障碍、强直等。此外，部分患者可有周围神经病。部分患者在疾病早期可以锥体束损害(类似痉挛性截瘫) 或锥体外系症状(类似帕金森综合征) 为主，共济失调症状不突出。

疾病逐渐进展，起病 10~15 年后丧失行走能力。长期吞咽困难致营养不良或误吸是导致死亡的主要原因。

**llama3.1_8b**  2025-02-18T23:58:58Z:

根据您的描述，我最有可能诊断出的罕见病是**斯奇综合征（Spinocerebellar ataxia, SCA）**。

其他五种可能的诊断：

1. **多系统萎缩症（Multisystem atrophy, MSA）**：这是一个罕见的神经退行性疾病，其临床表现包括小脑性共济失调、锥体束损害和锥体外系症状。
2. **富利-阿尔德综合征（Friedreich’s ataxia）**：这是一种遗传性的神经退行性疾病，主要影响小脑功能，导致共济失调等症状。
3. **帕金森综合征伴同行性共济失调（Parkinson-plus syndromes）**：这一类疾病包括一些罕见的神经退行性疾病，其临床表现与斯奇综合征相似，但主要特点是锥体外系症状。
4. **脊髓小细胞瘤（Spinal cord oligodendroglioma）**：这是一种罕见的脑肿瘤，可能导致共济失调、腱反射亢进和周围神经病等临床表现。
5. **克罗恩氏病相关的神经系统疾病（Crohn’s disease-associated neurologic disorders）**：有些患者在克罗恩氏病中可能出现神经系统症状，如共济失调、锥体束损害和周围神经病。

需要注意的是，这些诊断都是基于临床表现的初步分析，进一步的检查和确认是必要的。

**114. user 2025-02-18T23:59:16Z:**

疾病早期可能会合并胃食管反流。随着病程的进展，患者逐渐出现皮肤肿胀及硬化，并可伴皮肤色素改变，包括色素沉着或色素脱失。除皮肤受累之外，患者常会伴有关节痛及肌痛等症状，并可出现指端溃疡和毛细血管扩张。此外，患者可能会出现多个内脏器官受累。其中呼吸系统较易受累，主要表现为肺间质纤维化和肺动脉高压。患者的消化系统表现多样， 包括胃食管反流、胃窦血管扩张症、腹痛、腹泻、便秘、大便失禁等。少数患者可能会出现肾危象，主要表现为急性肾功能不全，并且常合并高血压。心脏受累可表现为心包积液、心脏传导系统异常，个别患者可出现心肌受累。神经系统方面，外周神经系统受累多见，例如三叉神经病变、周围神经病变等，罕见中枢神经受累。

**llama3.1_8b**  2025-02-18T23:59:20Z:

根据您提供的疾病临床表现，我最有可能诊断出的罕见病是**威尔逊氏症候群**（Werdnig-Hoffmann病），一种罕见的先天性肌肉退行性疾病。然而，其他五种可能的诊断如下：

1. **多发性肌炎**(Polymyositis)：是一种自身免疫性疾病，影响肌肉，导致肌肉疼痛和无力。
2. **系统性红斑狼疮**(Systemic Lupus Erythematosus, SLE)：是一种免疫系统紊乱引起的多器官疾病，可以影响皮肤、关节、肾脏等多个系统。
3. **全身硬化症**(Scleroderma)：是一种自身免疫性疾病，导致身体组织和器官逐渐硬化和纤维化。
4. **皮肌炎**(Dermatomyositis)：是全身肌肉疼痛的症状伴随着皮肤炎症，如色素沉积等。
5. **系统性红斑狼疮样疾病**(Systemic Lupus Erythematosus-like Disease, SLELD)：是一种较为罕见的自身免疫性疾病，类似于SLE，但与SLE有所区别。

**115. user 2025-02-18T23:59:41Z:**

患儿在出生 1~3 个月后除了出现类似苯丙酮尿症的临床症状外，还表现为多巴胺缺乏症状，如运动障碍、肌张力低下、嗜睡、眼震颤、吞咽困难等。5- 羟色胺缺乏相关症状，如反应迟钝、抑郁、失眠等。去甲肾上腺素缺乏相关症状，如躯干肌张力低下、眼睑下垂、小脑发育障碍等。还会有顽固性抽搐、反复发热，运动里程碑发育迟滞， 全身瘫痪，智能发育严重障碍等。

**llama3.1_8b**  2025-02-18T23:59:44Z:

根据您提供的信息，我认为最可能的诊断是:

**苯丙酮尿症和多巴胺缺乏综合征（Tyrosinemia and Dopa Deficiency Syndrome）**

但是，由于苯丙酮尿症是一种遗传性疾病，而多巴胺缺乏综合征则是一种较为罕见的神经递质代谢异常，我怀疑这种诊断可能还不足以解释所有的临床表现。

因此，我列出的其他五种可能的诊断是：

1. **苯丙酮尿症和多巴胺缺乏综合征**
2. **多巴胺能神经元缺失综合征（Dopa-Responsive Dystonia）**
3. **苯丙酮尿症伴发的运动障碍综合征**
4. **神经递质代谢异常综合征**（如5-羟色胺、去甲肾上腺素等相关疾病）
5. **智力障碍与运动功能障碍综合征**（如Down综合征等）

**116. user 2025-02-19T00:00:04Z:**

常表现为多系统受累，其发生发展有一定的阶段性。在胎儿 / 新生儿期可以出现心脏横纹肌瘤，大部分可自发消退；出生后数月就可出现癫痫症状和皮肤症状；随着年龄的增大，出现肾血管平滑肌脂肪瘤和全身其他部位的肿瘤性病变；成年女性肺部容易出现肺部淋巴管肌瘤病。脑部病变主要包括脑皮质结节、室管膜下巨细胞星形细胞瘤和室管膜下结节等。皮肤病变包括出生后可见的白斑，逐渐生长的面部血管纤维瘤、鲨革斑、甲周纤维瘤及前额斑块等；高达 90％的患者有肾脏血管平滑肌脂肪瘤；约 50％患者病变累及视网膜，但很少影响视力；约 35％女性患者肺部出现 LAM；其他如肝脏、胰腺、消化道、骨骼也可以受累，但一般症状较轻。

**llama3.1_8b**  2025-02-19T00:00:07Z:

根据您提供的信息，我认为最有可能的诊断是： Tuberous Sclerosis Complex（结节性硬化症）

其他五种可能的诊断包括：

1. Neurofibromatosis Type 1（神经纤维瘤病型1）
2. Sturge-Weber Syndrome（史图尔格-韦伯综合征）
3. von Hippel-Lindau Disease（威泼-林道综合征）
4. Li-Fraumeni Syndrome（李-弗拉明综合征）
5. Gorlin Syndrome（戈林综合征）

这些疾病都与多系统受累和肿瘤形成有关，但是每种疾病的特定临床表现和遗传特征可能有所不同。

**117. user 2025-02-19T00:00:28Z:**

未被发现或未治疗的慢性型儿童大多在 10 岁以前死亡，死因通常是肝功能衰竭、神经系统受累或肝细胞癌。

急性型起病急骤、进展迅速，以急性肝功能衰竭为主要表现，临床表现为肝脾肿大、黄疸、呕吐、腹胀、厌食、嗜睡、贫血、出血倾向及生长迟缓。患儿可能具有“煮白菜” 或“烂蘑菇”的特征性气味。肝脏合成凝血因子不足，PT、APTT 明显延长，凝血因子Ⅱ、Ⅶ、Ⅸ、Ⅺ和Ⅻ水平降低，补充维生素 K 后难以纠正。肝功能衰竭可继发腹水、黄疸和消化道出血，患儿偶尔会出现持续低血糖。未治疗的患儿多于起病数周或数月内因肝功能衰竭死亡。

**llama3.1_8b**  2025-02-19T00:00:33Z:

根据您提供的临床表现，我最有可能诊断的是威廉斯综合征（Williams syndrome）。这是一个罕见的遗传性疾病，其特征包括：

- 智力发育迟缓或正常
- 心脏异常，尤其是肺动脉狭窄和四联瓣膜病变
- 面部、颈部和胸部皮肤黯色
- 高血压和血管疾病
- 吃进食物后出现“烂蘑菇”的特殊气味

但是，根据您提供的其他症状，如肝脏问题和凝血功能障碍，我认为另一个可能的诊断是：

维生素K依赖性凝血因子缺陷（VKCFD）。这是一个罕见的遗传病，其特征包括：

- 肝功能衰竭
- 血液凝固问题，表现为PT和APTT延长
- 补充维生素K后难以纠正凝血因子水平

其他五种可能的诊断包括：

1. 罕见肝脏疾病，如原发性肝硬化或肝脏转移瘤。
2. 神经系统疾病，如遗传性的肌肉营养不良（GME）或苯丙氨酸血症。
3. 代谢疾病，如甘油脂质储存病或脂肪酸氧化障碍。
4. 迟发性胰腺炎，表现为黄疸、腹水和消化道出血。
5. 肝脏肿瘤，如肝细胞癌。

请注意，这些是仅有的可能性，并且我建议您进一步的检查和评估才能得到最准确的诊断。

**118. user 2025-02-19T00:00:52Z:**

临床表现具有明显异质性，根据起病年龄和临床表现分为 3 个类型。

**1. 心肌病型，** 是最常见的一种严重早发心脏和多脏器衰竭型，主要在新生儿和婴儿早期发病。此型发病凶险，患儿病死率高，表现为低血糖、瑞氏综合征、新生儿猝死、肥厚型和扩张型心肌病、心包积液、心律失常、肌无力、肝肿大和间歇性低血糖，肌酸激酶水平升高；心肌肥厚和心律失常可致死。

**2. 肝型 ，**较轻，为肝脏和低酮性低血糖型，婴儿后期或儿童期发病，表现为肝大和肝功能异常，空腹耐力下降和急性低酮性低血糖，不伴心肌损害和心肌肥厚，但未经及时诊断和治疗也会有生命危险。

**3. 肌病型** ，主要在青少年至成年期发病，为迟发型，症状轻，一般不伴有心肌疾病和低血糖。主要表现为运动、感染或饥饿后的横纹肌溶解和肌红蛋白尿，甚至可发生肾功能衰竭，可伴有肌无力、肌肉痛性痉挛或肌痛。

**llama3.1_8b**  2025-02-19T00:00:56Z:

根据您的描述，我认为可能的诊断是：

1. **Miyoshi体肌营养不良症**：这是一种罕见的遗传性肌病，主要影响儿童和青少年。它表现为肌肉溶解、横纹肌溶解和肌红蛋白尿。

其他五种可能的诊断：

1. **Denny-Brown综合征**：一种罕见的神经肌肉疾病，表现为肌肉无力、肌肉痛性痉挛或肌痛，并伴有肾功能衰竭。
2. **Muscular dystrophy（MD）-Becker型**：一种罕见的遗传性肌病，主要影响青少年和成年人。它表现为运动能力下降、肌肉无力和肌肉溶解。
3. **Myositis**：一种罕见的自身免疫性肌炎疾病，表现为肌肉痛性痉挛或肌痛，并伴有肾功能衰竭。
4. **Glycogen storage disease（GSD）-type III**：一种罕见的代谢性肌病，主要影响儿童和青少年。它表现为低血糖、肌肉溶解和横纹肌溶解。
5. **Kearns-Sayre综合征**：一种罕见的遗传性肌病，主要影响儿童和青少年。它表现为肌肉无力、肌肉痛性痉挛或肌痛，并伴有肾功能衰竭。

请注意，这些诊断仅是可能的选择，并且需要进一步的临床检查和实验室测试来确认。

**119. user 2025-02-19T00:01:13Z:**

**1. 特殊面容，** 眶周皮下组织饱满、眶距增宽、星状虹膜、人中长、嘴唇厚、宽嘴以及小下颌。

**2. 心血管疾病，** 75％ ~80％的患者存在弹性蛋白动脉病，可影响任何动脉。周围型肺动脉狭窄(37％ ~61％) 在婴儿期较常见，但随着时间的推移可有改善。瓣上型主动脉狭窄较常见(35％ ~65％)，并且随着时间的推移在 5 岁前恶化。动脉狭窄导致左心阻力增高、心肌肥厚和心力衰竭。其他异常包括肾动脉狭窄、胸主动脉狭窄、腹主动脉狭窄、二尖瓣脱垂和主动脉瓣关闭不全、肠系膜动脉狭窄、颅内血管异常等。高血压可出现在任何年龄，部分可能和肾动脉狭窄有关。

**3. 认知能力，** 75％的患者有智力障碍，通常较为轻微。认知障碍具有特征性，表现为短期记忆和语言表达能力相对较好，但在视觉空间结构性认知方面却极其薄弱。在智商方面没有性别差异的报道，且随着时间的推移，儿童的智商是稳定的。

**4. 性格、行为、心理异常，** 包括过度友好、过度移情、注意力缺陷、焦虑、恐惧。

**5. 睡眠异常 ，**睡眠问题的发生率为 65％，包括睡眠潜伏期增加和睡眠效率降低。

**6. 眼睛，** 泪道阻塞、远视(67％) 和斜视(50％) 常见。成人患者也有白内障的报道。

**7. 耳鼻喉，** 50％的患者有慢性中耳炎，90％对声音的敏感性增加，63％的儿童和92％的成人有轻度至中度听力损害，多为进行性感音神经性听力损失。大多数患者声音嘶哑或声音低沉。

**8. 牙齿** ，可有小牙、牙釉质发育不良和错位咬合。

**9. 消化系统，** 慢性腹痛是常见症状，可能的病因包括胃食管反流、裂孔疝、消化性溃疡、胆石症、憩室炎、缺血性肠病、慢性便秘和焦虑躯体化。高钙血症也可能导致易怒、呕吐、便秘和肌肉痉挛。

**10. 泌尿系统，** 尿频和遗尿症在儿童患者中很常见。

**11. 神经、肌肉** ，幼童的肌张力偏低，关节松弛，导致代偿性姿势异常，以保持身体平衡。年龄较大的儿童和成人有典型的肌张力增高和深肌腱反射活跃，导致步态僵硬而笨拙，精细运动功能受损，所有年龄段患者都有使用工具和书写困难。成人患者会有小脑共济失调和震颤。

**12. 生长发育** 。70％的婴儿体重增长不佳。

**13. 特发性高钙血症** 。多在婴幼儿期出现，见于 15％ ~50％的患者，最常见的症状为易激惹、呕吐和便秘。

**14. 内分泌。**甲状腺功能减退(10％)、青春期提前(50％)。糖耐量受损在青少年患者中的发生率为 26％。

**llama3.1_8b**  2025-02-19T00:01:19Z:

根据您的描述，这些临床表现最有可能诊断的是马尔曼-斯图皮特病（Marden-Walker Syndrome）。该疾病是一种罕见的遗传性综合征，其特点包括特殊的面部外观、心血管疾病、认知障碍、行为问题、睡眠异常、眼睛问题、耳鼻喉问题、牙齿问题、消化系统问题、泌尿系统问题、神经肌肉问题和生长发育迟缓等。

其他可能的诊断包括：

1. **威尔逊病（Wilson’s Disease）**：一种遗传性疾病，特征是肝脏损伤、精神健康问题、运动功能障碍和神经系统问题。
2. **巴斯德-阿勒综合症（Bassen-Kornzweig Syndrome）**：一种罕见的脂质代谢疾病，其特点包括肝脏损伤、视觉问题、肌肉无力等。
3. **克拉夫-亨丁顿病（Krabbe Disease）**：一种遗传性神经系统疾病，其特征是早产儿死亡、高热、发育迟缓和运动功能障碍。
4. **法比-格雷厄姆症候群（Fabry Disease）**：一种罕见的遗传性脂质代谢疾病，其特点包括肾脏损伤、神经系统问题和肌肉无力等。
5. **加氏综合征（Gaucher’s Disease）**：一种罕见的脂质代谢疾病，其特点包括肝脏损伤、骨骼变性和运动功能障碍等。

**120. user 2025-02-19T00:02:11Z:**

**1. 出血倾向** ，为患者常见表现，尤其血丝便多见，大部分患儿在新生儿期即可发病。其次表现为瘀斑、瘀点、咯血和血尿等出血倾向，部分严重者可出现威胁生命的消化道大出血或颅内出血等。患者出血由血小板减少引起，同时伴有血小板体积减小，但仅约 25％的病例同时具有典型三联征表现。

**2. 感染 。**患儿易患各种感染，包括单纯疱疹病毒、巨细胞病毒、肺炎链球菌、真菌等。患者免疫缺陷随年龄增长而加重。

**3. 湿疹。** 约 80％的 WAS 患儿可出现湿疹，范围和严重程度差异很大。可有典型严重、难治性湿疹，也可非常轻微，甚至无湿疹表现。

**4. 自身免疫性疾病。** 最常见为自身免疫性溶血性贫血。其他包括血管炎、关节炎、肾脏疾病、炎症性肠病、中性粒细胞减少症和自身免疫性血小板减少性紫癜等。

**5. 恶性肿瘤 。**患儿发生淋巴系统恶性肿瘤的风险明显增高，尤其是淋巴瘤，非霍奇金淋巴瘤、淋巴细胞白血病、骨髓增生异常综合征、精原细胞瘤等均有报道。

**6. 其他 。**该病患儿可具有完全正常的血小板水平，但中性粒细胞持续或反复减少。

**llama3.1_8b**  2025-02-19T00:02:17Z:

根据您提供的临床表现，我最可能诊断出的是”威尔逊氏病（WAS）“。但是，需要注意的是，这个列表并非是排他性的，也可能存在其他罕见疾病与这些症状相关联。在临床实践中，鉴于患者的具体情况和检查结果，才可以做出准确的诊断。

以下五种可能的诊断：

1. **血小板减少性紫癜**：这种疾病表现为血小板减少、出血倾向等症状，与您提供的症状相似，但与威尔逊氏病区别在于，后者通常伴有其他免疫缺陷和自身免疫性疾病。
2. **儿童期多发性细胞异常综合征**：这种综合征可能表现为血小板减少、感染等症状，并且可能与威尔逊氏病相似，但具有不同的遗传特征和临床表现。
3. **白血病或淋巴瘤**：这些恶性肿瘤可能表现出类似的症状，如出血倾向、感染等，但是主要特点是有异常的细胞增殖和累积。
4. **自体免疫性疾病**，如自身免疫性溶血性贫血或自身免疫性血小板减少性紫癜，可能表现为出血倾向、感染等症状，但不一定伴有遗传特征。
5. **其他骨髓疾病**，例如骨髓增生异常综合征，也可能导致类似的临床表现，但这需要更多的详细信息来区分。

请注意，这些诊断仅基于您提供的初步症状，而不是完整的病例描述或实验室结果。任何确切的诊断都应该在与医生的讨论和进一步检查后确定。

**121. user 2025-02-19T00:03:54Z:**

最常见的临床表现是 EB 病毒感染后的暴发性传染性单核细胞增多症、异常免疫球蛋白血症及 B 细胞淋巴瘤。异常免疫球蛋白血症及 B 细胞来源的淋巴瘤可发生于没有 EB 病毒感染的患者。异常免疫球蛋白血症通常表现为 IgA 及 IgM水平升高，而 IgG1 和 IgG3 减少。淋巴瘤以非霍奇金 B 淋巴细胞瘤为主。其他临床表现包括淋巴细胞性脉管炎、再生障碍性贫血和淋巴瘤样肉芽肿病等。某些患者临床表现轻微， 仅有异常免疫球蛋白血症或轻度 EB 病毒感染，可能是由于发生了体细胞基因修复。

部分患者主要临床表现为噬血细胞综合征，以脾肿大多见，且不一定需要 EBV 感染诱发。部分患者可出现肠道炎症如克罗恩病或结肠炎，但很少发展为淋巴瘤。

**llama3.1_8b**  2025-02-19T00:04:25Z:

根据您的描述，最可能的诊断是 Waldenström病（一种罕见的B细胞淋巴瘤），以及EB病毒相关的综合征。其他五种可能的诊断包括：

1. Burkitt淋巴瘤：一种快速进展的非霍奇金淋巴瘤，常见于儿童和青少年。
2. 强直性脊柱炎：是一种慢性炎症性疾病，影响脊柱和其他身体部位。
3. 先天性免疫缺陷综合征（PID）：是一组罕见的遗传性疾病，影响个体的免疫系统功能。
4. 迟发性硬化症：一种罕见的神经系统疾病，表现为运动功能障碍和感觉问题。
5. 异常免疫球蛋白血症（IgM多黏度血症）：是一种罕见的免疫系统疾病，特征是 IgM水平升高。
