# Supplementary material for: Performance of ChatGPT-4o and Four Open-Source Large Language Models in Generating Diagnoses Based on China’s Rare Disease Catalog: Comparative Study
Source: J Med Internet Res. 2025 Jun 18;27:e69929. doi: 10.2196/69929 (PMC12192912; doi:10.2196/69929)
Supplement: Multimedia Appendix 1 [file jmir-v27-e69929-s001.zip › numbered_Llama3.1-70B-cn.docx]

numbered_llama3.1_70b_cn

**1. user 2024-11-30T22:09:29Z:**

该疾病可于儿童期早期起病。典型的临床表现包括肾脏、眼部改变及听力受损。肾脏表现为血尿、蛋白尿和肾功能进行性恶化，其中血尿常为持续性镜下血尿，可在运动后或发热时出现肉眼血尿，常见于青少年型。听力改变为感音神经性听力损失， 并随病程逐渐加重。眼部异常表现为近视、青年环和白内障，但缺乏特异性。三种具有诊断意义的眼部改变包括：前圆锥形晶状体、后多形性角膜萎缩和视网膜斑（视网膜中心凹区域周围的白色或黄色颗粒）。我国男性患者听力受损发生率高于国外报道（68％比 55％），女性则低于国外报道（7％比 45％），眼异常发生率与国外报道相近。少数患者还可出现平滑肌瘤，可累及呼吸道、胃肠道及女性生殖道；偶有动脉瘤病变、面中部发育异常和精神发育迟滞的报道。

患者常有家族史。常染色体隐性遗传患者和 X 连锁遗传的男性患者病情进展较快，常于 16~35 岁进入终末期肾病；而常染色体显性遗传和 X 连锁遗传的女性患者多病程较为缓慢，肾功能衰竭出现较晚。在同一个患病家系中，所有男性成员发生肾功能衰竭的年龄相似，但不同家系中的男性成员发生肾功能衰竭的年龄可以不同。因此，可根据患病家系发生肾功能衰竭的年龄分为：青少年型（肾衰竭发生在青少年时期） 与成人型（肾衰竭发生在成年以后）。青少年型肾外表现更为突出，男孩通常在繁衍后代前即死亡，致病基因常为新发突变；而成人型家系中的患者较年长，少为新发突变。

**llama3.1:70b** 2024-11-30T22:09:56Z:

根据您提供的临床表现，我最可能诊断出的罕见病是：

**Alport 综合征**

其他五种可能的诊断包括：

1. **Fabry 病**: 一种遗传性疾病，由于α-Gal A 酶缺乏导致的糖脂质代谢异常，临床表现包括肾脏受损、眼部改变和听力损失。
2. **Bardet-Biedl 综合征**: 一种罕见的遗传性疾病，临床表现包括肥胖、学习障碍、夜盲症、肾脏囊肿以及生殖系统异常。
3. **Polycystic 肾病（PKD）**: 一种常染色体显性遗传病，特征是双侧肾脏出现多个囊肿，最终导致慢性肾脏疾病和肾衰竭。
4. **Nail-Patella 综合征**: 一种罕见的遗传性疾病，临床表现包括指甲畸形、膝盖骨异常以及肾脏受损。
5. **Focal Segmental Glomerulosclerosis（FSGS）**: 一种常见的肾脏疾病，特征是肾小球部分纤维化和硬化，最终导致慢性肾脏疾病和肾衰竭。

请注意，这些诊断都是可能的，但需要进一步的检查和测试来确认确切的诊断。

**2. user 2024-11-30T22:10:00Z:**

患者在新生儿期常无异常表现。首发症状是 6 月龄左右出现发育迟滞表现，但典型临床表现多在 1 岁后出现，临床确定诊断往往需要数年时间。按照临床表现出现频率不同分为：均出现的表现（100％患者）、经常性表现（80％以上患者）、相关性表现（20％ ~80％ 患者）3 类。

**1. 均出现的表现** 正常孕产史和出生头围；无出生缺陷和生化指标异常；颅脑 MRI/CT 除轻微皮质萎缩、髓鞘发育不良外，多无结构异常；运动里程碑落后而无倒退；6~12 个月出现严重发育迟缓；语言障碍显示为无或极少量词汇，重复性语言和非语言交往能力强于表达性语言能力；运动或平衡障碍常表现为共济失调以及四肢震颤；异常的行为特征表现为频繁大笑或微笑、明显的兴奋动作或快乐举止、常伴拍手或多动。

**2. 经常性表现** 头围增长落后，随访至 2 岁仍表现为小头畸形；3 岁前常出现癫痫；异常脑电图：特征性高波幅棘 - 慢波。

**3. 相关性表现** 枕部扁平；喜吐舌；吸吮或吞咽障碍；婴儿期喂养困难；肌张力低；巨大下颌；牙间隙宽；频繁流涎；过度咀嚼动作；斜视；皮肤色素减退，与家人相比头发和眼睛颜色浅（仅见于缺失型）；下肢腱反射亢进；上举或弯曲上肢，尤其是行走时；步基宽；对热敏感性高；异常睡眠 - 觉醒周期、睡眠少；迷恋水、纸、塑料；进食相关行为异常；肥胖（多见于年长、非缺失型患者）；脊柱侧凸；便秘。

**llama3.1:70b** 2024-11-30T22:10:25Z:

根据您提供的临床表现，我最可能诊断出的罕见病是：

**Angelman 综合征**

Angelman 综合征是一种罕见的遗传性疾病，主要影响神经系统和行为。它通常在婴儿期无明显异常，但在6个月左右开始出现发育迟滞，随着时间推移，典型临床表现逐渐出现。

其他可能的诊断包括：

1. **Prader-Willi 综合征**: 一种罕见的遗传性疾病，主要影响食欲调节、生长和发展，以及行为。
2. **Smith-Magenis 综合征**: 一种罕见的遗传性疾病，主要影响神经系统、行为和身体发育。
3. **Wolf-Hirschhorn 综合征**: 一种罕见的遗传性疾病，主要影响生长和发展，以及神经系统。
4. **Cri-du-chat 综合征**: 一种罕见的遗传性疾病，主要影响神经系统、行为和身体发育。
5. **Phelan-McDermid 综合征**: 一种罕见的遗传性疾病，主要影响神经系统、行为和身体发育。

请注意，这些诊断都是可能的，但需要进一步的检查和测试来确认确切的诊断。

**3. user 2024-11-30T22:10:28Z:**

**1.骨骼异常表现**：疾病严重程度和相关并发症的类型存在差异。所有 病例均存在胸腔狭窄，胸廓可呈钟形或长而窄，呼吸窘迫通常由小而僵硬的胸腔引起。根据胸廓类型和潜在的肺发育不良的程度，呼吸功能不全严重程度不同，发病年龄不同。呼吸功能不全严重程度与 该疾病诊断年龄呈负相关。存在严重的限制性胸廓和钟形胸廓的婴儿更多见急性呼吸系统疾病，在新生儿早期可出现严重和致命的呼吸衰竭。胸腔长而窄的婴儿，更多表现为轻度呼吸系统受累，最终可逐渐发展为慢性限制性肺病伴反复呼吸道感染。肋骨短小、畸形引起肋间肌无力、呼吸受限，进而出现呼吸衰竭。多数患儿于 2 岁内死亡，其中约有半数患儿于生后 6 个月内死亡。患者其他骨骼异常包括肋骨短、四肢短小、骨盆形状异常、短指、多指（趾） 畸形等。约有 60％患者伴有近端颈椎狭窄。

**2.其他脏器受累表现：** 约 40％的患者会出现肾脏病变。患者肾脏病变的最早表现是尿液浓缩功能受损，其他表现包括多尿、蛋白尿、血尿素氮和肌酐升高以及高血压。肾功能衰竭是 该患者在 3~10 岁死亡的主要原因。约 30％的病例出现肝脏受累，表现为肝肿大、转氨酶升高、胆汁淤积、肝硬化和门静脉高压。该疾病偶与眼睛发育异常相关，包括视神经发育不全、视网膜营养不良、视网膜色素异常、眼球震颤、近视和进行性视力障碍。亦有少数报道提示这些患者也可出现神经系统异常如发育落后、肌张力低下、中枢神经系统MRI 异常等。

**llama3.1:70b** 2024-11-30T22:11:06Z:

根据您提供的临床表现，我最可能诊断出的罕见病是：

**Jeune 综合征**

Jeune 综合征是一种罕见的遗传性疾病，主要影响骨骼、呼吸和肾脏系统。患者通常在婴儿期出现胸腔狭窄和呼吸功能不全，严重程度不同。其他常见表现包括肋骨短小、四肢短小、骨盆形状异常、近端颈椎狭窄等。

根据您的描述，Jeune 综合征的特点包括：

- 胸腔狭窄和呼吸功能不全是主要特征
- 肋骨短小、畸形引起肋间肌无力、呼吸受限
- 多数患儿于 2 岁内死亡，其中约有半数患儿于生后 6 个月内死亡
- 约 40％的患者会出现肾脏病变，包括尿液浓缩功能受损、多尿、蛋白尿等
- 约 30％的病例出现肝脏受累，包括肝肿大、转氨酶升高、胆汁淤积等

其他可能的诊断包括：

**1.Spondyloepiphyseal dysplasia congenita**: 一种罕见的遗传性疾病，主要影响骨骼系统。

**2.Caffey 综合征**: 一种罕见的遗传性疾病，主要影响骨骼和神经系统。

**3.Osteogenesis imperfecta**: 一种罕见的遗传性疾病，主要影响骨骼系统。

**4.Campomelic dysplasia**: 一种罕见的遗传性疾病，主要影响骨骼系统。

请注意，这些诊断都是可能的，但需要进一步的检查和测试来确认确切的诊断。

**4. user 2024-11-30T22:11:08Z:**

主要症状包括精神行为异常、认知障碍、记忆力下降、癫痫发作、言语障碍、运动障碍、不自主运动，意识水平下降、自主神经功能障碍等。其他伴随症状包括睡眠障碍、CNS 局灶性损害、脑干、小脑症状以及周围神经和神经肌肉接头受累等。

临床可分为 3 种主要类型：①抗 NMDAR 脑炎，是 最主要类型，其特征性临床表现符合弥漫性脑炎。②边缘性脑炎，以精神行为异常、颞叶癫痫和近记忆力障碍为主要症状，常见的边缘性脑炎有抗 LGI1 抗体、抗 GABABR 抗体与抗 AMPAR 抗体相关的脑炎。③其他类型，包括莫旺综合征、抗 DPPX 抗体相关脑炎、抗 IgLON5 抗体相关脑病、自身免疫性小脑性共济失调等。

**llama3.1:70b** 2024-11-30T22:11:42Z:

根据您提供的临床表现，我最可能诊断出的罕见病是：

**抗 NMDAR 脑炎**

抗 NMDA 受体（N-Methyl-D-Aspartate）脑炎是一种自身免疫性疾病，主要影响中枢神经系统。患者通常出现精神行为异常、认知障碍、记忆力下降、癫痫发作等症状。

根据您的描述，抗 NMDAR 脑炎的特点包括：

- 精神行为异常，如幻觉、妄想、情绪波动
- 认知障碍，如注意力不集中、记忆力下降
- 癫痫发作，如全身强直性癫痫或局部性癫痫
- 言语障碍，如词汇贫乏、语句结构混乱
- 运动障碍，如肌肉无力、不自主运动

其他可能的诊断包括：

1. **抗 LGI1 脑炎**: 一种自身免疫性疾病，主要影响中枢神经系统。
2. **抗 GABABR 脑炎**: 一种自身免疫性疾病，主要影响中枢神经系统。
3. **抗 AMPAR 脑炎**: 一种自身免疫性疾病，主要影响中枢神经系统。
4. **莫旺综合征**: 一种罕见的遗传性疾病，主要影响神经肌肉接头和自主神经功能。
5. **自身免疫性小脑性共济失调**: 一种自身免疫性疾病，主要影响小脑和运动系统。

请注意，这些诊断都是可能的，但需要进一步的检查和测试来确认确切的诊断。

**5. user 2024-11-30T22:11:46Z:**

疾病临床表现复杂多样，缺乏特异性。多累及神经、皮肤、呼吸、消化和免疫等多系统，单从临床表现难以确诊。可以分为完全性及部分性两种类型。

1. 完全性： 又可分为早发型及晚发型两种。①早发型患者多在新生儿或婴儿早期起病，通常症状出现在生后早期起病，通常症状出现在生后 1 周至 10 岁期间，平均发病年龄为 3 个月，最常见的表现为神经系统症状，如肌张力低下、癫痫、发育迟缓、感音性耳聋、共济失调、进行性意识障碍等，大约一半的患者出现共济失调，发育迟缓，结膜炎和视觉问题，包括视神经萎缩。常合并代谢性酸中毒、低血糖、酮症等代谢紊乱，皮肤表现包括各种形态的难治性皮疹如湿疹、全身性红斑、尿布疹，患者也可出现过度通气、喉部喘鸣和呼吸暂停等呼吸系统症状，此外还有消化系统的症状如喂养困难、呕吐、腹泻等。感染、发热、疲劳、高蛋白饮食等可诱发急性发作。若治疗不及时，常可导致不可逆损害。②晚发型患者常于青春期发病，之前可无任何症状，临床表型较早发型不同，通常表现为周围神经病，肌无力、痉挛性轻瘫，皮疹和眼部问题，如视力丧失和视力下降等，如不能及时治疗，常导致不可逆性损害。
2. 部分性 ： 可在各个年龄段发病，甚至不发病。患儿整体症状及体征相对较轻，仅在感染、饥饿等应急状态下起病。患者可能有肌张力减退、皮疹、脱发，可出现急性神经系统症状癫痫、意识障碍等。

**llama3.1:70b** 2024-11-30T22:12:21Z:

根据您提供的临床表现，我最可能诊断出的罕见病是：

** Maple 糖尿病**

Maple 糖尿病是一种遗传性疾病，主要影响多个系统，包括神经、皮肤、呼吸、消化和免疫等。患者通常出现复杂的临床表现，缺乏特异性。

根据您的描述，Maple 糖尿病的特点包括：

- 完全性类型：早发型和晚发型两种
  - 早发型：新生儿或婴儿早期起病，神经系统症状如肌张力低下、癫痫、发育迟缓等
  - 晚发型：青春期发病，周围神经病、肌无力、痉挛性轻瘫、皮疹和眼部问题等
- 部分性类型：可在各个年龄段发病，症状及体征相对较轻，只在感染、饥饿等应急状态下起病

其他可能的诊断包括：

1. **Propionic 酸尿病**: 一种遗传性疾病，主要影响多个系统。
2. **Methylmalonic 酸尿病**: 一种遗传性疾病，主要影响多个系统。
3. **Isovaleric 酸尿病**: 一种遗传性疾病，主要影响多个系统。
4. **Glutaric 酸尿病**: 一种遗传性疾病，主要影响多个系统。
5. **Organic 酸尿病**: 一种遗传性疾病，主要影响多个系统。

请注意，这些诊断都是可能的，但需要进一步的检查和测试来确认确切的诊断。

**6. user 2024-11-30T22:12:36Z:**

高发年龄段为婴儿期，表现为以失盐为主的肾上腺皮质功能不全，常见症状包括高血钾、低血钠与脱水、代谢性酸中毒、低血糖、呕吐、休克、皮肤色素沉着等。多数患者在青春期表现为不发育或发育不完全，或生育能力受损，少精和无精症等。少数患者可以表现为儿童早期高雄激素血症、单纯性盐皮质激素缺乏，青春期可出现假性性早熟或自发性青春发育等。轻者可成年期起病。

患者皮肤有色素沉着，尤其是在暴露处、摩擦处、瘢痕及乳晕、腋窝、阴囊处。

患者对感染、外伤等各种应激的抵抗力有不同程度的减弱。在发生应激情况时，可出现肾上腺危象，表现为恶心、呕吐、腹痛或腹泻、严重脱水、血压降低、心率快、脉细弱、精神失常，常有高热、低血糖症、低钠血症，甚至危及生命。

**llama3.1:70b** 2024-11-30T22:13:14Z:

根据您提供的临床表现，我最可能诊断出的罕见病是：

**肾上腺皮质功能不全（ Congenital Adrenal Hyperplasia, CAH）**

特别是 21-羟化酶缺乏型 CAH，这是一种遗传性疾病，主要影响肾上腺皮质的功能。

根据您的描述，CAH 的特点包括：

*婴儿期：以失盐为主的肾上腺皮质功能不全，常见症状包括高血钾、低血钠与脱水、代谢性酸中毒、低血糖、呕吐、休克等。* 青春期：不发育或发育不完全，或生育能力受损，少精和无精症等。 *皮肤色素沉着：尤其是在暴露处、摩擦处、瘢痕及乳晕、腋窝、阴囊处。* 对感染、外伤等各种应激的抵抗力有不同程度的减弱，在发生应激情况时，可出现肾上腺危象，表现为恶心、呕吐、腹痛或腹泻、严重脱水、血压降低、心率快、脉细弱、精神失常等。

其他可能的诊断包括：

1. **CYP21A2 基因突变**: 这是一种遗传性疾病，主要影响肾上腺皮质的功能。
2. **17α-羟化酶缺乏症**: 这是一种遗传性疾病，主要影响肾上腺皮质的功能。
3. **11β-羟化酶缺乏症**: 这是一种遗传性疾病，主要影响肾上腺皮质的功能。

请注意，这些诊断都是可能的，但需要进一步的检查和测试来确认确切的诊断。

**7. user 2024-11-30T22:13:17Z:**

从出生到成年早期均可发病。其中新生儿起病最为常见，常表现为巨大儿、摄食不良、不耐受饥饿和持续性低血糖等。低血糖发作时可表现为嗜睡、肌张力低下等，也可表现为神经系统后遗症甚至死亡，如呼吸暂停、癫痫发作和昏迷等。在迟发性类型中，患者常表现为低血糖反应，包括面色苍白、出汗和心悸等症状。

**llama3.1:70b** 2024-11-30T22:13:45Z:

根据您提供的临床表现，我最可能诊断出的罕见病是：

**先天性高胰岛素症（ Congenital Hyperinsulinism, CHI）**

特别是持续性高胰岛素分泌型 CHI，这是一种遗传性疾病，主要影响胰岛功能。

根据您的描述，CHI 的特点包括：

*新生儿期：巨大儿、摄食不良、不耐受饥饿和持续性低血糖等。* 低血糖发作时：嗜睡、肌张力低下、呼吸暂停、癫痫发作和昏迷等神经系统后遗症。 *迟发性类型中：患者常表现为低血糖反应，包括面色苍白、出汗和心悸等症状。

其他可能的诊断包括：

1. **胰岛素受体基因突变**: 这是一种遗传性疾病，主要影响胰岛功能。
2. **葡萄糖激酶缺乏症**: 这是一种遗传性疾病，主要影响胰岛功能。
3. **先天性多囊肾病**: 这是一种遗传性疾病，主要影响胰岛功能。

请注意，这些诊断都是可能的，但需要进一步的检查和测试来确认确切的诊断。

**8. user 2024-11-30T22:13:57Z:**

1. Ⅰ型 ，腹部膨隆、生长迟缓、低血糖抽搐、反复鼻出血、腹泻和呕吐为儿童患者主要就诊原因，极少数以肉眼血尿、便血、反复骨折、贫血或痛风等为首发表现。从未确诊及治疗的成年患者可以多发肝腺瘤、慢性肾衰、严重痛风伴多发痛风石、骨质疏松等就诊。其他少见表现包括肺动脉高压、糖尿病、脑血管病和肝腺瘤癌变等。查体可见身材矮小和肝脏明显增大。
2. Ⅰb 型 ， 患者除以上表现外，还可有反复感染伴中性粒细胞减少、口腔溃疡、炎症性肠病、肛周溃疡、关节炎和脾大等。
3. Ⅱ型婴儿型， 根据预后分为经典婴儿型和非经典婴儿型。经典婴儿型大部分在生后第 1 个月即出现全身性肌肉无力，运动发育迟缓，胸部 X 线片示心脏增大，心电图见高 QRS 波和短 PR 间期，心脏彩超见肥厚型心肌病改变，血肌酸激酶不同程度升高等， 多于生后 1 年之内死于左心衰竭或肺部感染后心肺功能衰竭。非经典婴儿型在生后 1 年内出现肌肉无力，运动发育落后，多于幼儿期死于呼吸衰竭。
4. Ⅱ型晚发型 ， 患者于 1 岁后起病，可晚至 60 岁发病。多表现为慢性进行性近端肌力下降和呼吸功能不全，心脏受累少见，呼吸功能衰竭是主要的致死原因。临床表现为易疲劳、仰卧起坐、上下楼梯、蹲起困难和行走无力，少数以突发呼吸衰竭起病。

**llama3.1:70b** 2024-11-30T22:14:32Z:

根据您提供的临床表现，我最可能诊断出的罕见病是：

**糖原病（ Glycogen Storage Disease, GSD）**

特别是GSD Ⅰ型、GSD Ib 型和GSD Ⅱ型婴儿型、非经典婴儿型和晚发型。

根据您的描述，GSD 的特点包括：

- GSD Ⅰ型：腹部膨隆、生长迟缓、低血糖抽搐、反复鼻出血、腹泻和呕吐等。
- GSD Ib 型：除以上表现外，还可有反复感染伴中性粒细胞减少、口腔溃疡、炎症性肠病、肛周溃疡、关节炎和脾大等。
- GSD Ⅱ型婴儿型：全身性肌肉无力，运动发育迟缓，胸部 X 线片示心脏增大，心电图见高 QRS 波和短 PR 间期，心脏彩超见肥厚型心肌病改变等。
- GSD Ⅱ型晚发型：慢性进行性近端肌力下降和呼吸功能不全，心脏受累少见，呼吸功能衰竭是主要的致死原因。

其他可能的诊断包括：

1. **磷酸化酶缺乏症**: 这是一种遗传性疾病，主要影响糖原代谢。
2. **丙酮酸脱氢酶复合体缺乏症**: 这是一种遗传性疾病，主要影响能量代谢。

请注意，这些诊断都是可能的，但需要进一步的检查和测试来确认确切的诊断。

**9. user 2024-11-30T22:14:34Z:**

1. 神经 - 肌肉系统， 引起神经系统的异常，如惊厥、昏迷、共济失调、甚至精神症状；也可出现肌肉症状，包括腱反射亢进、震颤、手足搐搦、肌无力等。
2. 心血管系统， 引起心电图的改变，可见 PR 间期延长、QRS 波增宽等，严重者可引起室性心律失常。
3. 肾脏 ，可引起肾结石、肾钙质沉着、肾功能不全。
4. 骨关节系统， 可引起骨质疏松、骨软化。
5. 消化系统， 可引起肠梗阻表现。
6. 内分泌， 患者可见糖尿病或者糖耐量减低。
7. 眼部症状， 部分类型可合并眼部病变，包括眼球震颤、视野缺损、近视等。

该组疾病通常为慢性病程，其中新生儿期、婴儿期起病的类型往往病情重、预后差。部分患者在青春期就发展为慢性肾衰竭，部分患者若未能及时治疗，会导致患儿严重精神发育迟滞甚至死亡。

**llama3.1:70b** 2024-11-30T22:15:02Z:

根据您提供的临床表现，我最可能诊断出的罕见病是：

**尿素循环疾病（ Urea Cycle Disorder, UCD）**

特别是一组影响氮质代谢和尿素循环的遗传性疾病。

根据您的描述，UCD 的特点包括：

- 神经-肌肉系统：惊厥、昏迷、共济失调、精神症状等。
- 心血管系统：心电图改变，如 PR 间期延长、QRS 波增宽等，严重者可引起室性心律失常。
- 肾脏：肾结石、肾钙质沉着、肾功能不全。
- 骨关节系统：骨质疏松、骨软化。
- 消化系统：肠梗阻表现。
- 内分泌：糖尿病或者糖耐量减低。
- 眼部症状：眼球震颤、视野缺损、近视等。

其他可能的诊断包括：

1. **氨基酸代谢疾病**: 一组影响氨基酸代谢的遗传性疾病。
2. **有机酸代谢疾病**: 一组影响有机酸代谢的遗传性疾病。

请注意，这些诊断都是可能的，但需要进一步的检查和测试来确认确切的诊断。

**10. user 2024-11-30T22:15:14Z:**

可分为 5 种类型，其中以经典型和中间型最常见。

**1. 经典型** 最常见，占 75％。患儿出生时正常，发病早、症状重并发展迅速。通常患儿出生 12 小时后，在耵聍中即出现枫糖浆气味；生后 12~24 小时在尿液和汗液中有特殊的枫糖味；2~3 天出现酮尿、易激惹和喂养困难；4~5 天开始出现严重的代谢紊乱和脑病症状，表现为哺乳困难、阵发性呕吐、拒食、嗜睡、呼吸暂停、惊厥发作、肌张力增高以及“击剑”样和“蹬车”样刻板动作等；生后 7~10 天可出现昏迷和中枢性呼吸衰竭，若不及时治疗， 大多数的患儿在出生后数天死于严重的代谢紊乱。

**2. 中间型** 在任何年龄阶段均可发病。患者症状较经典型轻，表现为生长缓慢、发育落后、喂养困难、易激惹、在耵聍中有枫糖浆气味，应激情况下可以出现严重的代谢紊乱和脑病。

**3. 间歇型** 呈间歇发作，早期生长发育均正常，多在感染和手术等应激情况下发作，表现为发作性共济失调和酮症酸中毒，极少数的严重者可引起死亡。间歇期支链氨基酸浓度正常，发作期与经典型类似有支链氨基酸浓度升高。

**llama3.1:70b** 2024-11-30T22:15:37Z:

根据您的描述，我确认了这些疾病是：

**枫糖尿症（Maple Syrup Urine Disease, MSUD）**

这是一个遗传性疾病，影响支链氨基酸（BCAAs）的代谢。

根据您的描述，这些类型的MSUD特点包括：

- **经典型**: 最常见，占75％。患儿出生时正常，但发病早、症状重并发展迅速。通常在耵聍中出现枫糖浆气味，并伴有酮尿、易激惹和喂养困难等。
- **中间型**: 在任何年龄阶段均可发病，患者症状较经典型轻，表现为生长缓慢、发育落后、喂养困难、易激惹等。
- **间歇型**: 呈间歇发作，早期生长发育均正常，但在感染和手术等应激情况下可出现严重的代谢紊乱和脑病。

其他类型还有：

1. **轻度型**
2. **不典型**

这些类型的MSUD都可能导致不同程度的症状和并发症。

**11. user 2024-11-30T22:15:47Z:**

出生后至老年均可起病，可急性或隐袭起病。几乎任何系统或器官均可受累，以能量需求旺盛的器官，如脑、心、肾、眼、耳、肌肉、胃肠道等多见。患者可以表现为某种临床综合征，也可仅表现单个症状或者多种症状组合。

**llama3.1:70b** 2024-11-30T22:16:09Z:

根据您的描述，我认为这些疾病可能是：

**线粒体病（Mitochondrial Disease）**

这是一个影响线粒体功能的遗传性疾病，导致细胞内能量生产异常。

根据您的描述，这些疾病的特点包括：

- 出生后至老年均可起病，可急性或隐袭起病。
- 几乎任何系统或器官均可受累，以能量需求旺盛的器官，如脑、心、肾、眼、耳、肌肉、胃肠道等多见。
- 患者可以表现为某种临床综合征，也可仅表现单个症状或者多种症状组合。

线粒体病的常见类型包括：

- **Kearns-Sayre综合征（KSS）**
- **MELAS综合征（Mitochondrial Encephalomyopathy, Lactic Acidosis, and Stroke-like episodes）**
- **MERRF综合征（Myoclonus Epilepsy with Ragged-Red Fibers）**
- **NARP综合征（Neuropathy, Ataxia, and Retinitis Pigmentosa）**

这些疾病都可能导致不同程度的症状和并发症。

**12. user 2024-11-30T22:16:21Z:**

好发年龄 5~50 岁，中位发病年龄 39 岁。女性患病率明显高于男性，女：男比例为(9~11) ：1。多急性 / 亚急性起病，临床表现包括 6 组核心症候群：①视神经炎；②急性脊髓炎；③极后区综合征；④急性脑干综合征；⑤症状性睡眠发作或急性间脑临床综合征伴典型的间脑 MRI 病灶；⑥症状性大脑综合征伴典型的脑部病变。 为高复发、高致残性疾病，90％以上的患者为复发性病程，多数患者遗留较为严重的神经功能残疾。

**llama3.1:70b** 2024-11-30T22:16:49Z:

根据您的描述，我认为这些疾病可能是：

**系统性多发性脑脊髓炎（Neuromyelitis Optica, NMO）**

这是一个自身免疫性疾病，影响中枢神经系统，特别是视神经和脊髓。

根据您的描述，这些疾病的特点包括：

- 好发年龄 5~50 岁，中位发病年龄 39 岁。
- 女性患病率明显高于男性，女：男比例为(9~11) ：1。
- 多急性 / 亚急性起病，临床表现包括 6 组核心症候群：
  - 视神经炎
  - 急性脊髓炎
  - 极后区综合征
  - 急性脑干综合征
  - 症状性睡眠发作或急性间脑临床综合征伴典型的间脑 MRI 病灶
  - 症状性大脑综合征伴典型的脑部病变。
- 为高复发、高致残性疾病，90％以上的患者为复发性病程，多数患者遗留较为严重的神经功能残疾。

NMO是一种罕见但严重的自身免疫性疾病，需要早期诊断和治疗以避免长期并发症。

**13. user 2024-11-30T22:16:56Z:**

听力损伤。以往多认为该耳聋为听力筛查即可明确的先天性， 初生即外显，多累及双侧，对称的重度语前聋。近年来逐渐发现个别病例可表现为不对称或单侧听力受损，听力损失程度变异较大，不同病人的表现可从轻度至极重度不等；渐进性、出生后才发生的耳聋也并不少见。

**llama3.1:70b** 2024-11-30T22:17:18Z:

根据您的描述，我认为这些疾病可能是：

**先天性听觉障碍（Congenital Hearing Loss）**

特别是由于 **GJB2 基因突变引起的非综合征性遗传性听力损伤（DFNB1）**

这种情况下，患者可能表现为：

- 听力损伤
- 以往多认为该耳聋为听力筛查即可明确的先天性，初生即外显，多累及双侧，对称的重度语前聋。
- 但是近年来逐渐发现个别病例可表现为不对称或单侧听力受损，听力损失程度变异较大，不同病人的表现可从轻度至极重度不等。
- 渐进性、出生后才发生的耳聋也并不少见。

GJB2 基因突变是最常见的非综合征性遗传性听力损伤的原因，占所有先天性听觉障碍病例的约20-50%。

**14. user 2024-11-30T22:20:02Z:**

运动迟缓、震颤、肌强直。治疗后更容易出现左旋多巴诱导的运动并发症。在非运动症状方面，容易发生情绪障碍(如抑郁、焦虑、易激惹等) 及行为障碍(如强迫性增加药量、冲动控制障碍、刻板行为等)。列出主要诊断并且给出5个鉴别。

**llama3.1:70b** 2024-11-30T22:20:34Z:

根据您的描述，我认为这些疾病可能是：

**多巴依赖综合征（Dopamine Dysregulation Syndrome, DDS）**

特别是在帕金森病患者中。

这种情况下，患者可能表现为：

- 运动迟缓、震颤、肌强直。
- 治疗后更容易出现左旋多巴诱导的运动并发症。
- 在非运动症状方面，容易发生情绪障碍（如抑郁、焦虑、易激惹等）及行为障碍（如强迫性增加药量、冲动控制障碍、刻板行为等）。

鉴别诊断：

1. **帕金森病（Parkinson’s Disease）**：虽然DDS经常发生在帕金森病患者中，但两者是不同的临床实体。
2. **多巴激增症（Dopamine Agonist-Induced Psychosis）**：与DDS相似，但主要是由多巴激增剂引起的精神症状。
3. **强迫症（Obsessive-Compulsive Disorder, OCD）**：虽然DDS患者可能表现出强迫性行为，但两者是不同的临床实体。
4. **注意力缺陷多动障碍（Attention Deficit Hyperactivity Disorder, ADHD）**：虽然DDS患者可能表现出注意力不集中和冲动行为，但两者是不同的临床实体。
5. **药物诱导的精神病（Substance-Induced Psychosis）**：与DDS相似，但主要是由其他药物引起的精神症状。

**15. user 2024-11-30T22:22:16Z:**

**1. 多发神经病** ，往往表现为对称性的四肢感觉和(或) 运动性周围神经病，逐步由远端向近端进展。

**2. 器官肿大，** 包括肝大、脾大或淋巴结肿大；淋巴结活检常提示为 Castleman 病。

**3. 内分泌异常 ，**常见的包括性功能减退(如男性阳痿、乳房发育)、甲状腺功能减退、糖代谢异常(如糖尿病)、肾上腺功能不全。

**4. 皮肤改变，** 可表现为皮肤颜色加深、皮肤肾小球样血管瘤、白甲、多血质、多毛症和手足发绀等。

**5. 循环外水负荷增加，** 包括肢体水肿、腹水、胸腔积液、心包积液、视乳头水肿等。

**6. 硬化性骨病 ，**是重要临床表现。可表现为骨痛，亦可无临床症状。骨骼 CT 检查可以显著提高硬化性骨病的检出率，影像学上可以表现为单纯骨骼硬化、或硬化和溶骨混合病灶、或者单纯溶骨性改变。

**7. 红细胞增多和(或) 血小板增多**

**8. 肺动脉高压** ，患者可出现肺动脉高压，表现为活动耐量减低、低氧血症等。

**9. 脑梗死**

**llama3.1:70b** 2024-11-30T22:24:17Z:

根据您的描述，我认为这些疾病可能是：

**POEMS 综合征**

POEMS 综合征是一种罕见的综合征，其名称来源于以下几个方面：

- **P**: Polyneuropathy（多发神经病）
- **O**: Organomegaly（器官肿大）
- **E**: Endocrinopathy（内分泌异常）
- **M**: Monoclonal gammopathy（单克隆性γ-球蛋白血症）
- **S**: Skin changes（皮肤改变）

POEMS 综合征是一种自身免疫性疾病，其临床表现复杂多样，可以包括上述的 9 个方面。该综合征通常影响中年男性，治疗方法包括激素、放疗和化疗等。

鉴别诊断：

1. **多发神经病**
2. **Castleman 病**
3. **内分泌疾病**（如糖尿病、甲状腺功能减退）
4. **皮肤疾病**（如白癜风、多毛症）
5. **骨骼疾病**（如骨软化症、Paget 病）
6. **血液疾病**（如单克隆性γ-球蛋白血症、红细胞增多症）
7. **肺动脉高压**
8. **脑血管疾病**（如脑梗死）

**16. user 2024-11-30T22:24:41Z:**

胎儿期的常见表现包括胎儿活动减少、羊水过多以及臀位。在新生儿期肌张力过低是这一疾病的标志性特征之一，严重的肌张力降低可导致窒息、喂养困难、吮吸无力、哭声无力。婴幼儿期主要表现为生长迟滞，认知、运动及语言发育落后。随着年龄的增长，食欲逐渐旺盛，如果不限制进食会出现肥胖。学龄期通常存在身材矮小，逐渐出现脾气暴躁、固执、强迫症等性格特点。异常行为包括强烈的觅食、搔抓皮肤、孤独症样行为等。约 25％的患者会出现癫痫。青春期男性和女性患者均可出现第二性征出现延迟或出现不完全。由于肥胖可导致相关并发症，如呼吸睡眠暂停、肺源性心脏病、胰岛素抵抗甚至糖尿病等。脊柱侧突、骨质疏松、胃食管反流也较为常见。成人期主要表现为肥胖及其并发症，包括心血管问题、糖尿病和睡眠呼吸暂停。其他表现为精神异常，不孕不育。

致命性危险：由口部运动不协调、肌张力过低、多食、狼吞虎咽的进食习惯和咀嚼不充分引起窒息以及由于暴饮暴食导致胃破裂。

体格检查可以发现特征性面容逐渐典型，包括杏仁眼、小嘴、上唇薄、嘴角向下、小手小脚、身材矮小、皮肤白皙、肌张力低、生殖器发育不良(如隐睾、阴囊发育不全或阴蒂发育不全)。

**llama3.1:70b** 2024-11-30T22:25:12Z:

根据您的描述，我认为这些疾病可能是：

**普瑞德威利综合征（Prader-Willi 综合征）**

普瑞德威利综合征是一种罕见的遗传性疾病，其特点包括：

- 胎胎期：胎儿活动减少、羊水过多、臀位
- 新生儿期：肌张力过低，窒息、喂养困难、吮吸无力、哭声无力
- 婴幼儿期：生长迟滞，认知、运动及语言发育落后
- 学龄期：身材矮小，脾气暴躁、固执、强迫症等性格特点
- 青春期：第二性征出现延迟或不完全
- 成人期：肥胖及其并发症，如心血管问题、糖尿病和睡眠呼吸暂停

普瑞德威利综合征的致命性危险包括：

- 窒息
- 胃破裂

体格检查可以发现特征性面容，包括：

- 杏仁眼
- 小嘴
- 上唇薄
- 嘴角向下
- 小手小脚
- 身材矮小
- 皮肤白皙
- 肌张力低
- 生殖器发育不良

普瑞德威利综合征是一种非常罕见的疾病，据估计每年出生约 1/15,000 到 1/30,000 的婴儿。

**17. user 2024-11-30T22:25:18Z:**

患者常在婴幼儿或青少年期起病，男女比例大致相等。呼吸道、胰腺和胆道等上皮细胞的分泌物含水量减低，分泌物变黏稠而难以清除。肺脏是最常见的受累器官，患者呼吸道出现慢性细菌感染及病原体的定植，最初常为流感嗜血杆菌和金黄色葡萄球菌，最终出现铜绿假单胞菌或洋葱克雷伯杆菌的慢性定植和(或) 感染。一旦出现感染，中性粒细胞被大量募集到肺组织中，并释放弹性蛋白酶，从而造成肺组织的破坏，导致支气管扩张形成。患者的支气管扩张常在幼年起病，且常从上叶起病，与多数感染后形成的支气管扩张症主要位于中、下肺不同。

患者常表现为深色服装上遗留的汗渍，或是父母发现患儿皮肤发咸。胆汁和胰液的流动异常可以导致消化不良和吸收不良，也可出现肝脏或胰腺疾病，严重时可以出现相关的糖尿病。黏稠的肠液还可能使 患者更容易出现肠梗阻和直肠脱垂。此外，男性患者可以出现输精管内精子移动障碍以及先天性输精管缺失(CBAVD)。

**llama3.1:70b** 2024-11-30T22:25:45Z:

根据您的描述，我认为这些疾病可能是：

**囊性纤维化症（Cystic Fibrosis, CF）**

囊性纤维化症是一种遗传性疾病，影响多个器官系统，包括呼吸道、胰腺和胆道等。患者的分泌物含水量减低，变得黏稠而难以清除，导致慢性感染和炎症。

囊性纤维化症的特点包括：

- 呼吸道：慢性细菌感染、病原体定植、肺组织破坏、支气管扩张形成
- 胰腺：消化不良、吸收不良、糖尿病
- 胆道：胆汁和胰液流动异常、肝脏疾病
- 男性患者：输精管内精子移动障碍、先天性输精管缺失（CBAVD）

囊性纤维化症是一种常见的遗传性疾病，影响约每 2,500 名新生儿中的 1 人。其发病机制是由于CFTR基因突变导致的细胞内钠离子浓度升高，从而影响分泌物的水含量和黏稠度。

**18. user 2024-11-30T22:25:48Z:**

患者临床表现为口咽炎、中耳炎、呼吸系统感染、蜂窝织炎及皮肤感染，常由葡萄球菌及链球菌感染所致。在出现骨髓储备下降的重度中性粒细胞减少症患者中，2 岁之前大部分患儿都发生过口腔溃疡及疼痛性牙龈炎。如果伴弥漫性胃肠道病变，可引起类似克罗恩病的腹痛和腹泻表现。中性粒细胞减少症可导致各个部位遭受反复细菌或真菌感染，其中主要受累部位为皮肤黏膜、耳鼻、咽喉和肺。感染通常非常严重甚至是致命性的。约 15％的患者进展为急性白血病或骨髓增生异常综合征。患儿通常不伴有特征性畸形。

**llama3.1:70b** 2024-11-30T22:26:13Z:

根据您的描述，我认为这些疾病可能是：

**先天性中性粒细胞减少症（Severe Congenital Neutropenia, SCN）**

先天性中性粒细胞减少症是一种罕见的遗传性疾病，表现为重度中性粒细胞减少和反复感染。患者常由葡萄球菌及链球菌感染所致。

其临床特点包括：

- 口咽炎、中耳炎、呼吸系统感染
- 皮肤感染和蜂窝织炎
- 骨髓储备下降的重度中性粒细胞减少症患者常出现口腔溃疡及疼痛性牙龈炎 *弥漫性胃肠道病变可引起类似克罗恩病的腹痛和腹泻表现

感染通常非常严重甚至是致命性的。约 15％的患者进展为急性白血病或骨髓增生异常综合征。

先天性中性粒细胞减少症是一种极为罕见的疾病，预后较差。如果不及时治疗，患者可能面临严重的感染风险甚至死亡。

**19. user 2024-11-30T22:26:58Z:**

典型 患者出现中枢神经系统损害和肾上腺皮质功能低下两类症状。神经系统症状和肾上腺皮质功能减退的症状可同时出现或相继出现。少数患者可单独出现。肾上腺皮质功能不全的表现轻重不一。儿童患病最为常见，占所有患者 35％。多在 4~8 岁发病。初期表现为注意力不集中、记忆力减退、学习困难、步态不稳、行为异常，后逐渐出现视觉和听觉下降、构音障碍、癫痫发作、共济失调、瘫痪、痴呆等症状。临床表现进行性加重， 最终失明或耳聋，完全瘫痪。有的表现为去大脑强直状态，最终死于中枢性呼吸衰竭、脑疝、感染。多数患者在首次出现神经系统症状时，已有肾上腺皮质功能受损的表现。给出主要诊断和5个鉴别诊断。

**llama3.1:70b** 2024-11-30T22:27:46Z:

根据您的描述，我认为这些疾病可能是：

**阿德森-比尔默病（Adrenoleukodystrophy, ALD）**

阿德森-比尔默病是一种罕见的遗传性疾病，表现为中枢神经系统损害和肾上腺皮质功能低下。患者常出现注意力不集中、记忆力减退、学习困难等症状，并逐渐恶化至失明、耳聋、完全瘫痪。

其临床特点包括：

- 中枢神经系统损害：注意力不集中、记忆力减退、学习困难、步态不稳、行为异常
- 肾上腺皮质功能低下：肾上腺皮质功能不全的表现轻重不一

鉴别诊断：

1. **克罗恩病**：克罗恩病是一种慢性炎症性肠病，主要影响消化系统。虽然两者都可能出现神经系统症状，但克罗恩病通常不伴有肾上腺皮质功能低下。
2. **多发性硬化症**：多发性硬化症是一种自身免疫性疾病，影响中枢神经系统。虽然两者都可能出现神经系统症状，但多发性硬化症通常不伴有肾上腺皮质功能低下。
3. **雷耶综合征**：雷耶综合征是一种罕见的遗传性疾病，主要影响中枢神经系统和肝脏。虽然两者都可能出现神经系统症状，但雷耶综合征通常不伴有肾上腺皮质功能低下。
4. **加兰德-格雷戈里综合征**：加兰德-格雷戈里综合征是一种罕见的遗传性疾病，主要影响中枢神经系统和骨骼。虽然两者都可能出现神经系统症状，但加兰德-格雷戈里综合征通常不伴有肾上腺皮质功能低下。
5. **马尔切斯科综合征**：马尔切斯科综合征是一种罕见的遗传性疾病，主要影响中枢神经系统和骨骼。虽然两者都可能出现神经系统症状，但马尔切斯科综合征通常不伴有肾上腺皮质功能低下。

**20. user 2024-11-30T22:27:47Z:**

患儿多于生后 4~12 个月开始出现感染症状。最突出的临床表现是反复严重的细菌感染，感染部位主要包括呼吸系统、消化系统及中枢神经系统，其中以呼吸道感染最为常见，常见病原菌包括流感嗜血杆菌、肺炎链球菌、金黄色葡萄球菌和假单胞菌属细菌等。长期、反复的呼吸道感染可导致支气管扩张、杵状指等不可逆转的器官功能损害。消化道感染常见的症状有腹泻、腹痛、胃食管反流和胃肠炎。引起感染性腹泻的病原包括蓝氏贾第鞭毛虫、沙门菌、空肠弯曲菌、隐孢子虫等。

另外，患儿容易出现中耳炎、慢性鼻窦炎、营养不良、贫血、粒细胞减少、血小板减少、生长激素缺乏症及甲状腺激素紊乱等并发症状。患儿还可发生肿瘤、自身免疫和炎症性疾病，包括类风湿性关节炎、皮肌炎等。据报道，患儿的感音神经性听力损失和湿疹的发生率也较高。

**llama3.1:70b** 2024-11-30T22:28:38Z:

根据您的描述，我认为这些疾病可能是：

**Wiskott-Aldrich 综合征（WAS）**

Wiskott-Aldrich 综合征是一种罕见的遗传性疾病，表现为反复严重的细菌感染、血小板减少和生长激素缺乏症。患者常出现呼吸道感染、消化道感染和中枢神经系统感染等症状。

其临床特点包括：

- 反复严重的细菌感染，主要影响呼吸系统、消化系统及中枢神经系统
- 血小板减少和生长激素缺乏症
- 中耳炎、慢性鼻窦炎、营养不良、贫血等并发症状

鉴别诊断：

1. **先天性免疫缺陷病**：先天性免疫缺陷病是一组遗传性疾病，影响机体的免疫系统。虽然两者都可能出现感染症状，但先天性免疫缺陷病通常不伴有血小板减少和生长激素缺乏症。
2. **慢性肉芽肿病**：慢性肉芽肿病是一种遗传性疾病，影响机体的炎症反应。虽然两者都可能出现感染症状，但慢性肉芽肿病通常不伴有血小板减少和生长激素缺乏症。
3. **常见变异型免疫缺陷病**：常见变异型免疫缺陷病是一种遗传性疾病，影响机体的免疫系统。虽然两者都可能出现感染症状，但常见变异型免疫缺陷病通常不伴有血小板减少和生长激素缺乏症。
4. **X连锁结合免疫缺陷病**：X连锁结合免疫缺陷病是一种遗传性疾病，影响机体的免疫系统。虽然两者都可能出现感染症状，但X连锁结合免疫缺陷病通常不伴有血小板减少和生长激素缺乏症。
5. **选择性IgA缺乏症**：选择性IgA缺乏症是一种遗传性疾病，影响机体的免疫系统。虽然两者都可能出现感染症状，但选择性IgA缺乏症通常不伴有血小板减少和生长激素缺乏症。

注意：上述鉴别诊断仅供参考，实际诊断应根据患者的具体临床表现和实验室检查结果进行。

### **21. User**

患者的临床表现包括不同程度的失盐和高雄激素血症两大类。新生儿起病的患儿表现为不同程度肾上腺皮质功能不足的表现，如软弱无力、恶心、呕吐、喂养困难、腹泻、慢性脱水、皮肤色素沉着和生长迟缓等。肾上腺危象常是该疾病在新生儿期的首发表现，表现为严重低血钠、高血钾、低血容量性休克，可伴有低血糖，由应激诱发。严重的低钠血症可导致抽搐等中枢神经系统表现，严重的高钾血症则可引起致命的心律失常。

高雄激素血症在不同年龄表现不一。经典型疾病的女性患儿出生时外生殖器有不同程度男性化。轻者表现为孤立性阴蒂肥大，重者外生殖器可接近男性，大阴唇呈阴囊样，阴蒂呈尿道下裂型阴茎样，并具共通的尿道阴道口。但大阴唇内不能触及性腺，有完全正常的女性内生殖器结构（卵巢和子宫）。男性新生儿期和婴儿期时可能无阴茎增大等外生殖器异常，是延误诊断的常见原因。至幼儿期，两性均会呈现外周性性早熟。男孩患儿呈现阴茎增大，伴或不伴阴毛早现；女性患儿呈现异性性早熟。长期高水平性激素刺激下丘脑促性腺激素释放激素（GnRH）神经元，发展为中枢性性早熟。女性还可有第二性征发育不良和原发性闭经或月经稀发。两性均在幼年期开始发生线性生长伴骨龄增长加速，使终身高受损。

其他表现包括皮肤和黏膜色素增深，以乳晕和外阴为显，部分患儿可无此改变。

非经典型的该类疾病儿童期和青春期甚至成年期临床呈不同程度的高雄激素血症表现，也可仅表现为生长加速和骨龄快速进展。

### Llama3.1:70b

根据您提供的临床表现，我认为最可能的诊断是先天性肾上腺增生症（Congenital Adrenal Hyperplasia，CAH）。这是一个由于肾上腺皮质激素合成酶缺陷引起的疾病，导致肾上腺皮质激素合成减少，雄激素合成增加。

其他五种可能的诊断包括：

1. **先天性肾上腺皮质功能减退症**（Congenital Adrenal Insufficiency）：这是一种由于肾上腺皮质激素合成减少或缺陷引起的疾病，导致肾上腺皮质功能不足。
2. **21-羟化酶缺陷症**（21-Hydroxylase Deficiency）：这是CAH的一种常见类型，导致肾上腺皮质激素合成减少，雄激素合成增加。
3. **17-羟化酶缺陷症**（17-Hydroxylase Deficiency）：这是一种较为罕见的CAH类型，导致肾上腺皮质激素合成减少，雄激素合成增加。
4. **肾上腺皮质功能减退症**（Adrenal Insufficiency）：这是一种由于肾上腺皮质激素合成减少或缺陷引起的疾病，导致肾上腺皮质功能不足。
5. **Cushing综合征**（Cushing’s Syndrome）：这是一种由于肾上腺皮质激素过量引起的疾病，导致一系列临床表现，包括高血压、糖尿病、骨质疏松等。

需要注意的是，以上诊断需要通过进一步的检查和实验室测试来确认。

### **22. User**

患者出生即有临床表现，因全身皮肤色素缺乏，皮肤毛细血管显露，皮肤薄而柔软，呈白色及红色，并伴有不同程度的血管扩张。患者对紫外线高度敏感，常出现日光性皮炎、光化性唇炎、基底细胞癌及鳞状细胞癌等疾病。毛发呈纯白、银白、黄色、金色或茶色。眼睫毛及眉毛呈白色或淡黄色。视网膜、虹膜颜色减退，瞳孔颜色减退。白天畏光明显，夜视正常，同时还可以伴有其他视觉改变，如视野敏锐度下降、视网膜中央凹发育不良、视交叉处神经路径改变、眼球震颤等。

### Llama3.1:70b

根据您提供的临床表现，医生可能诊断出的是**阿尔宾症（Albinism）**。阿尔宾症是一种罕见的遗传性疾病，主要影响皮肤、毛发和眼睛的色素形成。患者通常表现为皮肤、毛发和眼睛的色素严重缺乏，容易受到紫外线的伤害，并伴有视觉障碍。

其他五种可能的诊断包括：

1. **皮肤白化病（Vitiligo）**：一种自身免疫性疾病，导致皮肤色素细胞破坏，出现白色斑块。
2. **皮肤苷酶缺乏症（Chédiak-Higashi病）**：一种罕见的遗传性疾病，影响皮肤、毛发和眼睛的色素形成，并伴有免疫系统功能障碍。
3. **格里森综合征（Griscelli综合征）**：一种罕见的遗传性疾病，影响皮肤、毛发和眼睛的色素形成，并伴有免疫系统功能障碍和神经系统问题。
4. **赫尔曼斯基-普德拉克综合征（Hermansky-Pudlak综合征）**：一种罕见的遗传性疾病，影响皮肤、毛发和眼睛的色素形成，并伴有血小板功能障碍和肺部问题。
5. **Waardenburg综合征**：一种罕见的遗传性疾病，影响皮肤、毛发和眼睛的色素形成，并伴有听力丧失和其他神经系统问题。

### **23. User**

一种上、下运动神经元同时受累的神经系统变性疾病。临床主要表现为球部、四肢、胸腹部肌肉进行性无力和萎缩。而眼球运动神经和括约肌功能一般并不受累，但在有些患者晚期也可能会被累及。20％ ~50％的患者可以表现有认知功能障碍，5％ ~15％的患者甚至会发展为额颞叶痴呆。发病后平均 3~5 年因呼吸衰竭死亡，但 5％ ~10％的患者可以存活 10 年以上。

通过详细的病史和体格检查，在脑干、颈、胸、腰骶 4 个区域中寻找上、下运动神经元共同受累的证据，是诊断该疾病的基础。查体发现在同一区域，同时存在上、下运动神经元受累的体征是诊断的要点。其中下运动神经元受累体征主要包括肌肉无力、萎缩和肌束颤动。通常检查舌肌、面肌、咽喉肌、颈肌、四肢不同肌群、背肌和胸腹肌。上运动神经元受累体征主要包括病理征阳性、腱反射亢进、肌张力增高、阵挛等。通常检查吸吮反射、咽反射、下颌反射、掌颌反射，有无强哭、强笑等假性延髓麻痹表现，四肢腱反射、肌张力、霍夫曼征、下肢病理征、腹壁反射等。临床查体是发现上运动神经元受累的主要方法，在出现萎缩、无力的区域，如果腱反射不低或活跃，即使没有病理征，也可以提示锥体束受损。

### Llama3.1:70b

根据您提供的临床表现，这种疾病最有可能是**肌萎缩侧索硬化症（Amyotrophic Lateral Sclerosis, ALS）**。ALS是一种进行性的神经退行性疾病，影响上运动神经元和下运动神经元，导致肌肉无力和萎缩，最终影响呼吸功能。

其他五种可能的诊断包括：

1. **多灶性运动神经病（Multifocal Motor Neuropathy, MMN）：**一种主要影响上肢的周围神经病，通常表现为不对称的肌肉无力和萎缩，但不伴有上运动神经元的体征。
2. **脊髓性肌萎缩症（Spinal Muscular Atrophy, SMA）：**一种遗传性神经肌肉疾病，主要影响下运动神经元，导致肌肉无力和萎缩，但通常不伴有上运动神经元的体征。
3. **进行性延髓麻痹（Progressive Bulbar Palsy, PBP）：**一种主要影响脑干的运动神经元疾病，表现为球部肌肉无力和萎缩，但可能不伴有明显的四肢肌肉受累。
4. **进行性脊肌萎缩症（Progressive Spinal Muscular Atrophy）：**一种主要影响脊髓前角细胞的疾病，表现为四肢肌肉无力和萎缩，但通常不伴有上运动神经元的体征。
5. **肯尼迪病（Kennedy's Disease）：**一种X连锁显性遗传病，主要影响下运动神经元，表现为肌肉无力和萎缩，但通常不伴有上运动神经元的体征。

这些疾病的诊断需要综合病史、体格检查、电生理检查（如神经传导速度和肌电图）以及可能的基因检测等多方面的信息。

### **24. User**

一种影响尿素循环的常染色体隐性疾病，可导致高氨血症、脑病和呼吸道阻塞。 其特征是精氨酸和氨在血液中积聚，导致发育迟缓、癫痫发作、智力残疾、痉挛和共济失调等症状。 这种情况通常在3岁左右出现，症状包括僵硬、生长缓慢和认知能力下降。 如果不进行治疗，它可能会发展为严重的痉挛状态，失去肌肉和智力残疾。

### Llama3.1:70b

基于您提供的症状，我最可能的诊断是:

1. **尿素循环缺陷（Urea Cycle Disorder，UCD）**：这是一组影响尿素循环的遗传性疾病，导致体内无法正常清除氨，导致高氨血症。这种情况可能导致神经系统受损，引起一系列症状，包括发育迟缓、癫痫、智力障碍等。

其他可能的诊断包括：

1. ** Ornithine Transcarbamylase (OTC) 缺陷**：这是一种影响尿素循环的X连锁隐性遗传病，主要影响男性，导致高氨血症和其他神经系统症状。
2. **氨基甲酰磷酸合成酶（CPS）缺陷**：这是一种影响尿素循环的常染色体隐性遗传病，导致高氨血症和神经系统症状。
3. **精氨酸血症**：这是一种影响尿素循环的常染色体隐性遗传病，导致精氨酸在血液中积累，引起神经系统症状。
4. **氨基甲酰磷酸合成酶（CPS）和氨基甲酰磷酸合成酶（OTC）缺陷**：这是一种影响尿素循环的复合遗传病，导致高氨血症和神经系统症状。
5. **尿素循环缺陷综合征**：这是一种影响尿素循环的综合征，包括多种遗传病，导致高氨血症和神经系统症状。

需要注意的是，以上诊断需要通过遗传学检测和临床表现进行确认。

### **25. User**

典型的临床表现包括微血管病性溶血性贫血、血小板减少及急性肾功能衰竭三联征。微血栓形成所导致的非免疫性红细胞破坏，血红蛋白水平常低于 80g/L，Coombs 试验阴性， 外周血涂片可见红细胞碎片；血小板计数下降通常低于 50×109/L，但皮肤紫癜及活动性出血少见；急性肾功能损害的严重程度因人而异，部分患者需要透析支持，常伴血压升高。

### Llama3.1:70b

根据您提供的临床表现，医生可能诊断出的是血栓性血小板减少性紫癜（Thrombotic Thrombocytopenic Purpura，TTP）。此外，其他五种可能的诊断包括：

1. 血栓性血小板减少性紫癜（Hemolytic Uremic Syndrome，HUS）：主要表现为溶血性贫血、血小板减少和急性肾功能衰竭，常见于儿童。
2. 血栓性血小板减少性紫癜（Disseminated Intravascular Coagulation，DIC）：一种全身性血栓形成和出血的综合征，常见于感染、创伤、癌症等情况。
3. 血栓性血小板减少性紫癜（Antiphospholipid Syndrome，APS）：一种自身免疫性疾病，表现为血栓形成、血小板减少和反复流产等。
4. 血栓性血小板减少性紫癜（Thrombotic Microangiopathy，TMA）：一种微血管病变，表现为溶血性贫血、血小板减少和急性肾功能衰竭。
5. 血栓性血小板减少性紫癜（Atypical Hemolytic Uremic Syndrome，aHUS）：一种罕见的遗传性疾病，表现为溶血性贫血、血小板减少和急性肾功能衰竭。

### **26. User**

患者临床表现多样。取决于病变的部位、范围和进展速度。临床症状主要包含：

1. 增大垂体的占位效应，大多数患者会因增大的垂体对鞍区及周围结构（如视交叉、海绵窦等） 造成压迫而产生相应症状或由于炎症浸润产生的作用。最常见的临床表现为持续的剧烈头痛和进行性加重的视功能障碍，包括视力下降和视野缺损。
2. 腺垂体功能减退，根据受累的程度不同，垂体的各个轴系会出现部分甚至全部的功能减低的临床表现，如闭经、性功能减低、纳差、乏力、怕冷、性毛脱落、体重下降等。
3. 中枢性尿崩症 ， 约 70％以上患者会因为垂体后叶、垂体柄甚至下丘脑区受累出现多尿、烦渴和多饮等中枢性尿崩症的症状。部分患者因垂体柄阻断效应导致血清中泌乳素水平轻度升高。
4. 下丘脑综合征， 当病变累及区域发展至下丘脑时，会出现嗜睡、缺乏饱腹感、体重严重增加、渴感缺乏、体温波动、出汗障碍和情绪神志障碍等临床表现。
5. 系统性疾病的相关表现 ， 继发性的患者常合并有系统性疾病特征性的临床表现，如 LCH 患者常合并甲状腺、肺、骨、肝脏和皮肤病变。GPA 患者常为中老年男性， 呼吸道症状常早于下丘脑垂体受累。IgG4 相关疾病垂体炎患者常合并有泪腺、颌下腺和胰腺等其他脏器受累的临床表现等。

### Llama3.1:70b

根据您提供的临床表现，我最有可能诊断出的罕见病是：

1. **垂体炎**：垂体炎是一种罕见的炎症性疾病，主要影响垂体和下丘脑。患者可能会出现垂体功能减退、视功能障碍、尿崩症和下丘脑综合征等症状。
2. **Langerhans 细胞组织增生症（LCH）**：LCH是一种罕见的疾病，主要影响儿童和青少年。患者可能会出现多器官受累，包括骨骼、皮肤、肺、肝脏和中枢神经系统等。
3. **Granulomatosis 与多血管炎（GPA）**：GPA是一种罕见的自身免疫性疾病，主要影响呼吸道和肾脏。患者可能会出现呼吸道症状、肾脏损害和下丘脑垂体受累等症状。
4. **IgG4 相关疾病**：IgG4 相关疾病是一种罕见的自身免疫性疾病，主要影响多个器官，包括泪腺、颌下腺、胰腺和下丘脑垂体等。患者可能会出现多器官受累和下丘脑垂体受累等症状。
5. **垂体瘤**：垂体瘤是一种罕见的肿瘤，主要影响垂体。患者可能会出现垂体功能减退、视功能障碍和尿崩症等症状。

### **27. User**

高血糖是该疾病的最常见的临床表现。但在没有干预的情况下患者可以自发缓解而维持血糖水平正常。该疾病是自身免疫性低血糖的主要原因之一，约 25％的患者在病程中会出现自发性低血糖。约 50％的患者存在黑棘皮征。此外，多囊卵巢、高雄激素血症在该类患者中也不少见。 患者常伴发其他的自身免疫性疾病，其中以系统性红斑狼疮最为常见，约占 50％。此外，还有原发性胆汁性肝硬化、皮肌炎、硬皮病等。该疾病还可以是霍奇金淋巴瘤、多发性骨髓瘤等的副肿瘤综合征表现。

### Llama3.1:70b

根据您提供的临床表现，可能的罕见病诊断是：

1. **多发性内分泌腺体功能亢进综合征（MEN）**：该综合征涉及多个内分泌腺体的功能亢进，可能导致高血糖、黑棘皮征、多囊卵巢和高雄激素血症等症状。
2. **自身免疫性多腺体综合征（APECED）**：该综合征是一种罕见的自身免疫性疾病，可能导致多个内分泌腺体的功能障碍，包括高血糖、低血糖和黑棘皮征等症状。
3. **系统性红斑狼疮（SLE）**：该疾病是一种自身免疫性疾病，可能导致多个器官的炎症，包括皮肤、关节、肾脏等，高血糖和低血糖是SLE的常见表现。
4. **原发性胆汁性肝硬化（PBC）**：该疾病是一种自身免疫性肝脏疾病，可能导致肝脏纤维化和肝硬化，高血糖和低血糖是PBC的常见表现。
5. **皮肌炎/皮肌炎样皮肤病（DM/PM）**：该疾病是一种自身免疫性肌肉疾病，可能导致肌肉无力和皮肤病变，高血糖和低血糖是DM/PM的常见表现。
6. **硬皮病（SSc）**：该疾病是一种自身免疫性疾病，可能导致皮肤和内脏器官的纤维化，高血糖和低血糖是SSc的常见表现。

### **28. User**

婴儿及儿童期起病。首次发作时年龄在 5 个月至 7 岁。临床表现为反复发作的难治性呕吐及重症酮症酸中毒，伴有萎靡、脱水、呼吸急促、昏迷等临床表现。通常有明确诱因，可在禁食、胃肠道及上呼吸道感染、发热、应激或过量摄入蛋白质后急性起病。常规的实验室检查可见血糖升高或正常，部分出现低血糖，血气显示代谢性酸中毒，血氨可以正常或轻度升高，肝功能、肾功能及正常，尿酮体阳性。酮症及酸中毒可反复出现，发作间期临床及实验室检查可以表现为正常。

约 1/5 的病例出现神经系统的改变，表现为认知障碍、智力发育落后及锥体外系异常及脑卒中的症状，头颅 MRI 可发现包括白质广泛的脱髓鞘，基底节区在 T2 可见对称性高信号改变。

患者早期可无症状，在新生儿期或高危人群通过血、尿代谢筛查时被诊断。起病年龄、发作频率与预后无明确关联。发作随年龄增长有减少或停止趋势，多数病例随访其生长发育未受影响。反复发作及严重者可致多器官功能障碍甚至死亡。

### Llama3.1:70b

根据您提供的临床表现，我最有可能诊断出的是**丙酮酸脱氢酶复合体缺陷症**（Pyruvate Dehydrogenase Complex Deficiency，PDCD）。这种疾病是一种罕见的遗传性代谢疾病，主要影响能量代谢，导致丙酮酸不能正常转化为乙酰-CoA，从而引起一系列临床表现。

其他五种可能的诊断包括：

1. ** maple 糖尿病**（Maple Syrup Urine Disease，MSUD）：一种遗传性代谢疾病，主要影响分支链氨基酸（亮氨酸、异亮氨酸和缬氨酸）的代谢，导致这些氨基酸及其酮体在体内积累，引起呕吐、昏迷等症状。
2. **丙氨酸血症**（Propionic Acidemia，PA）：一种遗传性代谢疾病，主要影响丙氨酸的代谢，导致丙氨酸及其相关化合物在体内积累，引起呕吐、昏迷等症状。
3. **异亮氨酸血症**（Isovaleric Acidemia，IVA）：一种遗传性代谢疾病，主要影响异亮氨酸的代谢，导致异亮氨酸及其相关化合物在体内积累，引起呕吐、昏迷等症状。
4. **3-甲基戊二酸血症**（3-Methylcrotonyl-CoA Carboxylase Deficiency，3-MCC）：一种遗传性代谢疾病，主要影响戊二酸的代谢，导致戊二酸及其相关化合物在体内积累，引起呕吐、昏迷等症状。
5. **长链 3-羟酰-CoA 脱氢酶缺陷症**（Long-Chain 3-Hydroxyacyl-CoA Dehydrogenase Deficiency，LCHADD）：一种遗传性代谢疾病，主要影响脂肪酸的代谢，导致脂肪酸及其相关化合物在体内积累，引起呕吐、昏迷等症状。

这些疾病都属于有机酸代谢疾病，主要影响体内的能量代谢，导致一系列临床表现。

### **29. User**

1. 发病年龄小，女性多见。
2. 常有晕厥或猝死家族史。
3. 不同基因型和表现型，可累及钾、钠、钙通道。常有特征性尖端扭转型室速（TdP），导致晕厥或猝死。
4. ECG 表现为 QT 间期延长。
5. 心电图一般有如下特征：①表现为 QT 间期延长，短 - 长 - 短周期现象诱发；②心室率 160-240 次 / 分，QRS 波振幅与形态围绕等电位线扭转，5-20 个心动周期主波围绕基线扭转一次，多数能自行终止；③ ATP 较难终止的 TdP。

### Llama3.1:70b

根据您提供的临床表现，我最有可能诊断出的是**长 QT 间期综合征（Long QT Syndrome，LQTS）**。

其他五种可能的诊断是：

1. **布鲁加达综合征（Brugada Syndrome）**：表现为特征性的ST段抬高和QRS波形态改变，常见于东南亚男性，家族史阳性。
2. ** catecholaminergic 多形性室性心动过速（CPVT）**：表现为儿童或青少年发病，运动或情绪激动时诱发室性心动过速，常有家族史。
3. **心肌病（Hypertrophic Cardiomyopathy，HCM）**：表现为左心室壁厚度增加，常见于年轻人，家族史阳性。
4. **阿米达隆心律失常（Amiodarone-Induced Torsades de Pointes）**：表现为长期使用阿米达隆后出现TdP，常见于老年人。
5. **低血钾心律失常（Hypokalemia-Induced Torsades de Pointes）**：表现为低血钾状态下出现TdP，常见于老年人或慢性疾病患者。

### **30. User**

可于任何年龄发病，多数患儿于 1 个月至 7 岁发病，平均年龄在 2 岁左右。不同患者临床表现有较大差异，主要有：①急性能量代谢障碍危象，表现为低酮型低血糖、高血氨及代谢性酸中毒等；②心肌病，表现为心室肥厚、心功能不全、心律失常及肌酸激酶升高等；③肌病，表现为肌无力、肌张力减退、肌痛、运动耐力差、肌肉型肌酸激酶升高、肌纤维内脂质沉积等；④肝脏损害，表现为肝肿大、脂肪肝、肝功能异常等，一些肝损患儿急性起病，表现为抽搐、进行性意识障碍等，常被误诊为 Reye 综合征。此外，反复腹痛、呕吐、胃食管反流等消化道症状，反复感染、喘息等呼吸道表现以及贫血等也有报道。

新生儿筛查：确诊的患儿可无临床表现。 是一种潜在的致死性疾病，患儿可因急性能量代谢障碍危象或急性心衰而猝死。临床表现多样，与发病年龄、发病时间和病情轻重有关。最常见的症状是婴幼儿期和儿童期心肌和骨骼肌受损。成年期症状较轻或无症状，多为耐力降低或易疲劳。妊娠期，由于能量消耗和血浆生理性的肉碱水平降低，孕妇可有疲劳和心律失常等不典型表现。

### Llama3.1:70b

根据您提供的临床表现，我最有可能诊断出的是**卡尔尼틴缺乏症**（Carnitine Deficiency）。此病是一种遗传性疾病，影响卡尔尼틴的合成或运输，卡尔尼틴是一种对脂肪酸代谢和能量生产至关重要的物质。

其他五种可能的诊断包括：

1. **脂肪酸氧化疾病**（Fatty Acid Oxidation Disorders）：这是一组遗传性疾病，影响脂肪酸的分解和能量生产，导致类似的临床表现。
2. **肌营养不良症**（Muscular Dystrophy）：这是一组遗传性疾病，影响肌肉的结构和功能，导致肌肉无力和萎缩。
3. **心肌病**（Cardiomyopathy）：这是一组影响心脏肌肉的疾病，导致心脏功能不全和心律失常。
4. **Reye 综合征**（Reye’s Syndrome）：这是一种罕见的疾病，主要影响儿童，导致肝脏和脑部损害。
5. **线粒体病**（Mitochondrial Disease）：这是一组影响线粒体功能的疾病，导致能量生产不良和多器官损害。

需要注意的是，以上诊断需要通过进一步的检查和测试来确认，包括遗传分析、血液检查和组织活检等。

### **31. User**

单中心型该疾病往往仅表现为淋巴结肿大，而无全身症状。肿大淋巴结的中位直径约 5.5cm， 常见于胸部（24 ％）、颈部（20 ％）、腹部（18 ％）、腹膜后（14 ％）。部分单中心型疾病的患者容易合并副肿瘤性天疱疮、闭塞性细支气管炎以及生长发育迟缓。多中心型 除了多发淋巴结肿大外，往往还伴有发热、盗汗、乏力、体重下降、贫血、肝功能不全、肾功能不全和容量负荷过多（全身水肿、胸腔积液、腹水等） 等全身表现。多中心型 还有一种特殊亚型，患者可以表现为血小板减少（thrombocytopenia）、重度水肿（anasarca）、骨髓纤维化（myelofibrosis）、肾功能不全（renal dysfunction）、肝脾肿大（organomegaly）。

### Llama3.1:70b

根据您提供的临床表现，以下是一些可能的罕见病诊断：

1. **Langerhans 细胞组织增生症（Langerhans Cell Histiocytosis，LCH）**：该疾病可能导致淋巴结肿大、全身症状以及多器官受累，包括骨骼、皮肤、肺、肝、脾和中枢神经系统等。
2. **Castleman病**：该疾病是一种淋巴增生性疾病，可能导致淋巴结肿大、全身症状以及多器官受累，包括骨髓、肝、脾和肾等。
3. **Rosai-Dorfman病**：该疾病是一种罕见的淋巴增生性疾病，可能导致淋巴结肿大、全身症状以及多器官受累，包括骨髓、肝、脾和肾等。
4. **Kikuchi-Fujimoto病**：该疾病是一种罕见的淋巴增生性疾病，可能导致淋巴结肿大、全身症状以及多器官受累，包括骨髓、肝、脾和肾等。
5. **Sjögren 综合征**：该疾病是一种自身免疫性疾病，可能导致淋巴结肿大、全身症状以及多器官受累，包括唾液腺、泪腺、肾和肺等。
6. **淋巴瘤**：该疾病是一种恶性淋巴增生性疾病，可能导致淋巴结肿大、全身症状以及多器官受累，包括骨髓、肝、脾和肾等。

### **32. User**

主要临床表现为下肢远端为主，并逐渐向近端发展的肢体肌肉萎缩、无力及感觉丧失。常见临床表现为运动能力不如同龄人，跑步困难，易扭脚，足下垂，小腿腓肠肌萎缩形似“鹤腿”；查体可发现弓形足、锤状趾，远端肢体为主的无力萎缩，深感觉减退。患者通常 20 岁前起病，缓慢进展，疾病后期可能严重影响活动，但很少导致完全残疾， 也不影响正常寿命。但有些特殊类型可能起病早且严重。

### Llama3.1:70b

根据您提供的临床表现，主要诊断为 **Charcot-Marie-Tooth病（CMT）**。CMT是一组遗传性神经疾病，主要影响周围神经，导致远端肢体（尤其是下肢）的肌肉萎缩、无力和感觉丧失。这种疾病通常在20岁前开始，进展缓慢，严重程度因人而异。

其他可能的诊断包括：

1. **肌营养不良症（Duchenne/Becker肌营养不良症）**：这是一种遗传性肌肉疾病，主要影响男孩，导致肌肉无力和萎缩，尤其是下肢。
2. **脊髓性肌肉萎缩症（SMA）**：这是一组遗传性神经疾病，影响脊髓运动神经元，导致肌肉无力和萎缩。
3. **周围神经病变（CIDP）**：这是一种自身免疫性疾病，影响周围神经，导致肌肉无力、萎缩和感觉丧失。
4. **肌无力症（Myasthenia Gravis）**：这是一种自身免疫性疾病，影响神经与肌肉之间的信号传递，导致肌肉无力和疲劳。
5. **肌萎缩侧索硬化（ALS）**：这是一种神经退行性疾病，影响运动神经元，导致肌肉无力和萎缩，尤其是下肢。

请注意，这些疾病的临床表现可能相似，但有不同的病因和进展特点。确诊需要进一步的检查和测试，包括遗传分析、神经电生理检查和影像学检查等。

### **33. User**

患儿于出生后至数月内发病。发病年龄多在 2 月龄内，很少晚于 5 月龄。最常表现为出生后黄疸持续不退伴或不伴大便颜色浅淡，肝脏轻度肿大或无明显肿大。 还有其他多种非特异临床表现，包括喂养困难、生长发育迟缓、腹泻、低血糖、凝血时间延长等。大多数患者上述症状及体征在 6 个月至 1 岁左右可自然缓解或经过饮食结构调整和药物治疗缓解，其中少部分的患者在青春期以后疾病进一步发展。发展后的疾病大部分是在成人后发病。通常在饮酒、摄入甜食、服用某些药物或感染后突然出现高氨血症所致的谵妄，意识混乱等精神异常表现。严重的病情发展急剧，得不到正确治疗可危及生命。患者多为消瘦体形，大部分患者有明显的饮食偏好，嗜食豆类、高蛋白、高脂食物，而厌食高糖类。

### Llama3.1:70b

根据您提供的临床表现，我最有可能诊断出的罕见病是**先天性肝脏酮症（Congenital Hepatic Ketosis）**，也称为**先天性肝脏酮症病（Congenital Hepatic Ketosis Disease）**。

其他五种可能的诊断包括：

1. **先天性肝脏酶缺陷病（Congenital Hepatic Enzyme Deficiency Disease）**：这种疾病是由于肝脏酶缺陷导致的，表现为黄疸、肝脏肿大、生长发育迟缓等。
2. **肝脏先天性纤维化（Congenital Hepatic Fibrosis）**：这种疾病是由于肝脏纤维化导致的，表现为黄疸、肝脏肿大、门脉高压等。
3. **先天性胆汁淤积症（Congenital Biliary Atresia）**：这种疾病是由于胆汁淤积导致的，表现为黄疸、肝脏肿大、生长发育迟缓等。
4. **肝脏先天性囊肿（Congenital Hepatic Cyst）**：这种疾病是由于肝脏囊肿导致的，表现为黄疸、肝脏肿大、腹痛等。
5. **先天性肝脏癌（Congenital Hepatic Cancer）**：这种疾病是由于肝脏癌导致的，表现为黄疸、肝脏肿大、体重减轻等。

### **34. User**

可于出生后出现症状，也可于婴幼儿期至成年早期起病。主要表现为波动性肌肉无力，不耐疲劳，多分布于眼外肌、面肌、延髓、四肢和呼吸肌，婴儿期甚至可能出现危及生命的呼吸暂停发作。单纯的综合征主要表现为骨骼肌无力，不伴有先天畸形。该疾病的糖基化缺陷和肌病重叠综合征往往伴有先天发育异常。不同类型的临床表现也有差异，部分患者，一般为出生后近端肌肉无力，胆碱酯酶抑制剂有效，严重者可出现突发的呼吸窘迫和延髓麻痹引起的呼吸暂停，可由应激、寒冷、感染等诱发。部分患者，出生后或婴儿期起病，可能出现比较严重、轴性分布的肌肉无力，但眼外肌可不受累。

### Llama3.1:70b

根据您提供的临床表现，医生可能诊断出的是**肌无力症（Myasthenia Gravis）**。肌无力症是一种自身免疫性疾病，主要影响神经与肌肉之间的信号传递，导致肌肉无力和疲劳。其特征性症状包括眼睑下垂、双视、吞咽困难、呼吸肌无力等。

其他五种可能的诊断包括：

1. **肌营养不良症（Muscular Dystrophy）**：一组遗传性疾病，导致肌肉逐渐萎缩和无力。
2. **肌炎（Myositis）**：肌肉发炎，可能导致肌肉无力和疼痛。
3. **肌无力性侧索硬化症（Amyotrophic Lateral Sclerosis, ALS）**：一种神经退行性疾病，影响运动神经元，导致肌肉无力和萎缩。
4. **重复发作性肌无力症（Periodic Paralysis）**：一组遗传性疾病，导致肌肉周期性无力，通常与血液中的钾离子水平波动有关。
5. **肌无力性肌病（Myopathic Myasthenia）**：一种罕见的肌肉疾病，表现为肌肉无力和萎缩，可能与肌肉的结构或功能异常有关。

### **35. User**

1. 通常婴幼儿期至儿童期起病，少数患者成年后起病。
2. 全身横纹肌，包括肢体骨骼肌、面肌、眼外肌、舌肌均可出现肌强直现象。
3. 肌强直在休息后突然运动时明显，反复动作后减轻，称之为“热身（warm-up）”现象。可在突然启动时出现短暂肌肉无力。病程较长的的患者，可有持续性肢体远端无力，甚至累及肢体近端。
4. 查体可见患者常肌肉发达，呈运动员体型。大力握拳后，手指不能及时展开；大力闭目后，不能立即睁开。叩击手部大鱼际肌、舌肌等部位，可引起肌肉持续性收缩（叩击性肌强直、“肌球”现象）。

### Llama3.1:70b

根据您提供的临床表现，我最有可能诊断出的是**肌强直症（Myotonia Congenita）**。

肌强直症是一种罕见的遗传性疾病，主要特征是肌肉强直和运动迟缓。患者在休息后突然运动时会出现肌肉强直，反复动作后会减轻，这被称为“热身（warm-up）”现象。患者还可能出现肌肉无力，特别是在突然启动时。随着病程的进展，患者可能会出现持续性肢体远端无力，甚至累及肢体近端。

其他五种可能的诊断包括：

1. **肌营养不良症（Muscular Dystrophy）**：一种遗传性疾病，主要特征是肌肉无力和萎缩。
2. **肌强直性肌营养不良症（Myotonic Muscular Dystrophy）**：一种遗传性疾病，主要特征是肌肉强直和肌肉无力。
3. **肌无力症（Myasthenia Gravis）**：一种自身免疫性疾病，主要特征是肌肉无力和疲劳。
4. **肌强直性肌病（Myotonia Congenita）**：一种遗传性疾病，主要特征是肌肉强直和运动迟缓。
5. **帕金森病（Parkinson’s Disease）**：一种神经退行性疾病，主要特征是运动迟缓、僵硬和震颤。

### **36. User**

由于脊柱侧凸所引起的继发性胸廓畸形可引起患儿心肺发育不全，心肺储备差，另可能出现包括心脏移位、心律失常、肺活量减少、肺炎等；腹腔畸形可引起胃肠功能紊乱， 导致消化不良，食欲缺乏。此外，由于椎体畸形和椎间盘退变引起的神经根牵拉可产生相应躯体部位的刺激性疼痛，严重者可引起脊髓功能障碍甚至瘫痪。

体格检查：大体表现为直立时两肩不等高、一侧腰部皱褶、腰前屈出现背部不对称（“剃刀背”），有时可见骨盆两侧不对称、步态异常等。此外，还需进行神经系统检查，观察患者有无肌张力增高或低下，有无四肢感觉异常。

临床上根据影像学特点通常分为以下 3 型：

Ⅰ型——椎体形成障碍型，即一个或多个椎体部分或完全形成障碍，如楔形椎、半椎体或蝴蝶椎等；

Ⅱ型——椎体分节不良型，即两个或多个椎体部分或完全分节障碍，如阻滞椎、骨桥形成等；

Ⅲ型——混合型，既有椎体形成障碍，又有椎体分节不良。

### Llama3.1:70b

根据您提供的疾病临床表现，我最有可能诊断出的是**先天性脊柱侧凸**（Congenital Scoliosis）。这种疾病是由于胎儿发育过程中脊柱异常形成所致，表现为脊柱侧凸，常伴有其他畸形，如胸廓畸形、腹腔畸形等。

其他五种可能的诊断包括：

1. **马凡综合征**（Marfan Syndrome）：一种遗传性疾病，表现为脊柱侧凸、胸廓畸形、心脏问题等。
2. **埃勒斯-丹洛斯综合征**（Ehlers-Danlos Syndrome）：一种遗传性疾病，表现为皮肤超弹性、关节过度活动、脊柱侧凸等。
3. **脊柱裂**（Spina Bifida）：一种先天性疾病，表现为脊柱未完全闭合，常伴有神经系统问题。
4. **脊柱侧凸伴有神经纤维瘤病**（Neurofibromatosis Type 1 with Scoliosis）：一种遗传性疾病，表现为神经纤维瘤、脊柱侧凸等。
5. **脊柱畸形伴有肌营养不良**（Muscular Dystrophy with Scoliosis）：一种遗传性疾病，表现为肌肉无力、脊柱畸形等。

### **37. User**

男性多于女性。部分患者胸痛症状不典型，在行冠脉造影或冠脉 CT 时发现。当存在以下情况时，患者往往有临床症状：①合并阻塞性动脉粥样硬化性疾病、血栓、血流缓慢、夹层、破裂时可有劳力性心绞痛或急性冠脉综合征表现；②压迫邻近组织；③少见的有动脉瘤破裂，可引起急性心脏压塞；④虽然无显著的冠状动脉狭窄，也可发生因微血管功能障碍导致的心肌缺血。

最多见的临床表现是心绞痛，少数 患者会并发心肌梗死、严重心律失常甚至猝死。疾病可破入右房、右室或冠状窦，产生左向右分流，临床上可闻及连续性杂音，严重者会影响心脏功能。最严重的并发症是瘤体破入心包，可导致出血、心脏压塞甚至死亡。

### Llama3.1:70b

根据您提供的临床表现，我最有可能诊断出的是冠状动脉畸形（Coronary Artery Anomaly）。这种罕见病是指冠状动脉的异常解剖结构，可能导致冠状动脉狭窄、血栓形成、心肌缺血等症状。

其他五种可能的诊断包括：

1. 冠状动脉瘤（Coronary Artery Aneurysm）：冠状动脉壁局部扩张，可能导致血栓形成、心肌缺血等症状。
2. 冠状动脉夹层（Coronary Artery Dissection）：冠状动脉壁内层与中层分离，可能导致冠状动脉狭窄、血栓形成等症状。
3. 冠状动脉桥（Coronary Artery Bridge）：冠状动脉在心肌表面形成桥梁样结构，可能导致冠状动脉狭窄、心肌缺血等症状。
4. 冠状动脉 Fistula（Coronary Artery Fistula）：冠状动脉与心房或心室之间形成异常通道，可能导致左向右分流、心脏功能受损等症状。
5. 冠状动脉动脉瘤样病变（Coronary Artery Aneurysmal Disease）：冠状动脉壁局部扩张，可能导致血核形成、心肌缺血等症状。

### **38. User**

1. 骨髓衰竭 35％患儿出生时即表现为贫血，常于生后 2 周至 2 年确诊。贫血为大细胞或正细胞正色素性、网织红细胞减少、白细胞正常或轻度降低、血小板正常或轻度增高、骨髓红系增生低下而粒系和巨核细胞系增生活跃。
2. 先天发育异常 30％ ~50％ 患者可能存在先天发育异常，主要涉及头部、上肢、心脏和泌尿生殖系统。如身材矮小，颅面部畸形包括颅面部过宽、宽平鼻梁、小头、先天性白内障、青光眼、斜视、硬腭高拱及唇腭裂甚至特纳综合征外貌，拇指畸形，先天性心血管发育异常，泌尿生殖器官畸形。存在身体的异常并不能预测患者血液系统疾病的严重程度。30％受累患者可出现生后的生长迟缓，通常伴有其他先天畸形。
3. 癌症易感性增加的患者肿瘤发生率约为 4％，高于同年龄正常人群。且肿瘤发生年龄早。包括血液系统急性白血病、骨髓增生异常综合征、淋巴瘤及实体瘤如骨肉瘤、胸腺癌、肝细胞癌、黑色素瘤、纤维组织细胞瘤、胃癌和肠癌等。患肿瘤后，较患同样肿瘤的一般人群预后差，病死率高。

### Llama3.1:70b

根据您提供的临床表现，我最有可能诊断出的是法布瑞氏贫血（Fanconi贫血）。此病是一种罕见的遗传性疾病，主要特征包括骨髓衰竭、先天发育异常和癌症易感性增加。

其他五种可能的诊断包括：

1. **骨髓衰竭综合征（Aplastic Anemia）**：虽然骨髓衰竭是法布瑞氏贫血的特征，但也可能是其他疾病的表现，如骨髓衰竭综合征。
2. **先天性巨细胞贫血（Congenital Dyserythropoietic Anemia）**：这种疾病也表现为贫血和骨髓异常，但通常不伴有先天发育异常或癌症易感性。
3. **骨髓增生异常综合征（Myelodysplastic Syndromes）**：这种疾病也可能导致骨髓功能障碍和贫血，但通常发生在成人中。
4. **Shwachman-Diamond 综合征**：这种疾病也表现为骨髓衰竭和先天发育异常，但通常伴有胰腺功能不全和其他特征。
5. **Diamond-Blackfan 贫血（Diamond-Blackfan Anemia）**：这种疾病主要表现为红细胞生成障碍和贫血，但通常不伴有先天发育异常或癌症易感性。

需要注意的是，确诊需要进一步的实验室检查和遗传分析。

### **39. User**

病变可累及全身各系统，因此其临床表现因受累部位不同而表现各异。一般症状包括发热、乏力、盗汗、体重下降等。其余受累系统主要包括：

1. 骨骼系统， 绝大多数患者在诊断时候都有骨骼受累，以下肢骨最为常见，但仅有 50％患者有骨痛表现。
2. 心血管系统 ，病变累及心血管系统，多数患者并无明显临床表现，但如心包受累，造成大量心包积液可导致胸闷、憋气甚至心脏压塞表现；病变累及冠状动脉则可造成心肌缺血样表现，如胸闷、胸痛等；弥漫性心肌受累亦有报道，可导致心力衰竭样表现；病变包绕肾动脉则可造成肾性高血压；病变包绕肠系膜血管可有肠道缺血表现，如腹痛等。
3. 呼吸系统 呼吸系统受累多数患者无症状，少数患者有干咳、呼吸困难等表现。
4. 中枢神经系统 中枢神经系统受累可因不同部位脑实质受累而出现相应的临床表现，如肢体活动障碍、步态异常、意识改变等；脊髓受累可因不同受累节段而出现不同临床表现；垂体病灶则可表现为多饮、多尿以及其他激素分泌减少相关表现。
5. 眶后病变 眶后受累为， 常见临床表现之一，表现为单侧或双侧突眼，甚至造成失明。
6. 腹膜后空间 / 肾脏受累 ， 腹膜后病变可类似腹膜后纤维化，若病灶包绕输尿管可造成单侧或双侧肾后性梗阻。
7. 皮肤受累， 最为常见的皮肤病变为黄瘤样改变，约 1/3 患者发生在眼睑，面部、颈部、腋窝、躯干、腹股沟亦可出现。

### Llama3.1:70b

根据您提供的临床表现，我最有可能诊断出的是**多发性骨髓瘤（Multiple Myeloma）**。

多发性骨髓瘤是一种骨髓癌，表现为骨骼系统受累，常见于老年人。其临床表现包括骨痛、骨折、贫血、肾功能不全、感染等。根据您的描述，患者的骨骼系统受累，且有骨痛表现，这与多发性骨髓瘤的典型表现相符。

其他五种可能的诊断包括：

1. **淋巴瘤（Lymphoma）**：淋巴瘤是一种免疫系统癌症，表现为淋巴结肿大、发热、盗汗、体重下降等。根据您的描述，患者的全身系统受累，包括骨骼系统、心血管系统、呼吸系统等，这与淋巴瘤的表现相似。
2. **骨肉瘤（Osteosarcoma）**：骨肉瘤是一种骨骼癌，表现为骨痛、骨折、肿块等。根据您的描述，患者的骨骼系统受累，且有骨痛表现，这与骨肉瘤的典型表现相符。
3. **系统性红斑狼疮（Systemic Lupus Erythematosus，SLE）**：SLE是一种自身免疫疾病，表现为全身系统受累，包括骨骼系统、心血管系统、呼吸系统等。根据您的描述，患者的全身系统受累，这与SLE的表现相似。
4. **结节病（Sarcoidosis）**：结节病是一种自身免疫疾病，表现为全身系统受累，包括骨骼系统、心血管系统、呼吸系统等。根据您的描述，患者的全身系统受累，这与结节病的表现相似。
5. **骨髓纤维化（Myelofibrosis）**：骨髓纤维化是一种骨髓疾病，表现为骨骼系统受累、贫血、肾功能不全等。根据您的描述，患者的骨骼系统受累，这与骨髓纤维化的典型表现相符。

需要注意的是，这些诊断都是基于您的描述的临床表现，实际诊断需要通过进一步的检查和实验室测试来确认。

### **40. User**

病常表现为多器官、多系统受累，男性重于女性患者。临床表现随着年龄增长而常常累及不同的器官。

1. 面容， 男性患者多在 12~14 岁出现特征性面容，表现为眶上嵴外凸，额部隆起和嘴唇增厚。
2. 皮肤血管角质瘤， 常见于经典型患者，多见于“坐浴区”即脐膝之间的外生殖器、阴囊、臀部和大腿内侧，凸出皮肤表面的红色斑点。
3. 神经系统 ，多数患者会出现周围神经疼痛，表现为足底和手掌难以忍受的烧灼感，并放射到四肢近端，甚至出现痛性痉挛；自主神经受累时表现为少汗或无汗；中枢神经系统受累时多表现为早发的短暂性脑缺血发作或缺血性卒中。
4. 眼， 特征性的表现包括结膜血管迂曲、角膜涡状混浊、晶状体后囊混浊和视网膜血管迂曲，严重者可导致视力降低甚至丧失。常为女性患者就诊的主要原因之一。
5. 消化道 ，多在进食后出现恶心、呕吐、腹胀、痉挛性腹痛和腹泻等症状，也可表现为吸收不良或便秘。
6. 肾脏 ， 早期表现为尿浓缩功能障碍如夜尿增多、多尿和遗尿，随病程进展可逐渐出现蛋白尿，甚至达肾病综合征水平，伴随肾功能损害，多在 30 岁左右进入终末期肾病。
7. 心血管系统， 可表现为高血压、冠状动脉受累导致的心肌缺血，心脏瓣膜病变和肥厚型心肌病，严重者可表现为心绞痛、心肌梗死和心力衰竭。多为疾病的晚期表现和主要的死亡原因。

### Llama3.1:70b

根据您提供的临床表现，我最有可能诊断出的是 Fabry 病。Fabry 病是一种由α-半乳糖苷酶 A（α-Gal A）缺乏引起的罕见遗传性疾病，主要影响男性患者。该病的临床表现包括面容改变、皮肤血管角质瘤、神经系统受累、眼部改变、消化道症状、肾脏损害和心血管系统受累等。

其他五种可能的诊断包括：

1. Hunter 病：一种由苷酶缺乏引起的罕见遗传性疾病，主要影响男性患者，临床表现包括面容改变、皮肤血管角质瘤、神经系统受累和肾脏损害等。
2. Hurler 病：一种由α-L-偶氮糖苷酶缺乏引起的罕见遗传性疾病，主要影响儿童，临床表现包括面容改变、皮肤血管角质瘤、神经系统受累和肾脏损害等。
3. Sanfilippo 病：一种由葡萄糖苷酶缺乏引起的罕见遗传性疾病，主要影响儿童，临床表现包括面容改变、皮肤血管角质瘤、神经系统受累和肾脏损害等。
4. Pompe 病：一种由酸α-葡萄糖苷酶缺乏引起的罕见遗传性疾病，主要影响儿童，临床表现包括肌肉无力、心脏增大和肾脏损害等。
5. Gaucher 病：一种由葡萄糖苷酶缺乏引起的罕见遗传性疾病，主要影响儿童，临床表现包括肝脏增大、脾脏增大、骨骼损害和肾脏损害等。

### **41. User**

疾病分为 1 型和 2 型。

1. 1 型 主要表现为反复发作的炎性反应和浆膜炎，包括反复发热、腹痛及关节炎。发热反复发作，可有寒冷、剧烈运动、手术、感染等诱因，持续 13 天自行缓解。腹痛为最常见临床表现，见于 95％的患者。表现为突然发作的全腹痛，常伴发热，腹部查体可有腹胀，腹部压痛、反跳痛、肌紧张，听诊肠鸣音消失。立位腹平片可见小肠气液平。腹痛常在 24-48 小时后自行缓解。关节炎亦为常见临床表现，见于 75％的患者。常累及下肢大关节，如髋关节、膝关节。可有关节肿胀、疼痛。皮疹见于 7％ ~40％的患者，主要累及下肢伸侧，典型皮疹为丹毒样红斑。胸膜炎见于 45％的患者，表现为突然发作的单侧胸痛，常伴发热。心包炎较少见。
2. 2 型 以淀粉样变起病。表现为持续大量的蛋白尿，常致终末期肾病。淀粉样物质亦可在心脏、胃肠道、肝脏等器官沉积，并导致相应临床表现。

### Llama3.1:70b

根据您提供的疾病临床表现，我最有可能诊断出的是家族性地中海热（Familial Mediterranean Fever，FMF）。FMF是一种常见于地中海沿岸地区的罕见遗传性疾病，主要特征包括反复发作的炎症反应和浆膜炎，包括反复发热、腹痛、关节炎和胸膜炎等。这些症状与您提供的临床表现相符。

其他五种可能的诊断包括：

1. **成人史蒂文斯-约翰逊综合征（Adult-onset Still’s disease，AOSD）**：这是一种自身炎症性疾病，表现为反复发热、关节炎和皮疹等。
2. **系统性红斑狼疮（Systemic Lupus Erythematosus，SLE）**：这是一种自身免疫性疾病，表现为多器官炎症，包括关节炎、皮疹、肾炎等。
3. **血管炎（Vasculitis）**：这是一组疾病的总称，表现为血管壁炎症，导致血管狭窄或阻塞，引起多器官损害。
4. **淀粉样变性（Amyloidosis）**：这是一种因淀粉样蛋白沉积于器官而导致的疾病，表现为肾脏、心脏、肝脏等器官功能障碍。
5. **多器官炎症性疾病（Multisystem Inflammatory Disease）**：这是一种自身炎症性疾病，表现为多器官炎症，包括关节炎、皮疹、肾炎等。

### **42. User**

主要表现为先天性发育异常，进行性骨髓衰竭和肿瘤发生率增高，也有患者无发育异常或骨髓衰竭表现。

1. 发育异常 ，患者可以表现为多种形体和智力发育异常。生长缺陷、异常皮肤色素沉着、上肢单侧或双侧骨骼畸形、体格矮小、小头畸形、眼部异常、泌尿生殖道畸形、内分泌异常、听力损失、外耳异常、先天性心脏缺陷、消化道异常、中枢神经系统发育异常及面部畸形、脊柱畸形、颈部畸形及发育迟缓和（或） 智力残疾等。这些表现多为非特异性，拇指及上肢畸形较为特异。
2. 进行性骨髓衰竭 ，血液系统的异常为范科尼贫血最严重的临床特征。可表现为单一或全血细胞减少。血细胞减少起病年龄差异较大，即使同胞发病年龄也不一。中位发病年龄约为 7.6 岁，罕见于婴儿和幼童，90％患者的血液学异常在 40 岁前发病。血小板减少或白细胞减少通常先于贫血出现，常伴随大红细胞，胎儿血红蛋白（HbF 1 ） 增加，全血细胞减少逐渐加重。
3. 肿瘤发生风险增高， 包括血液系统恶性疾病及非血液系统恶性实体瘤。急性髓系白血病（AML） 发生危险增加约 500 倍，50 岁时 AML 累计发生率约 13％，多发生于15-35 岁。-7，del（7q） 与骨髓增生异常综合征（MDS）/AML 发生率增高相关。急性淋巴细胞白血病和淋巴瘤也有报道。实体肿瘤可以是该疾病的首发表现。头颈部鳞状细胞癌（HNSCCs） 是 患者最常见的实体瘤，而且发病年龄更早（20~40 岁），大多数发生于口腔（如舌癌），多处于进展期，对治疗反应差。皮肤、食管癌、肝肿瘤和泌尿生殖道肿瘤发生率也增加。少数患者可见到多种肿瘤并发，或者血液系统及非血液系统肿瘤并发。而且对化疗或放疗明显不耐受。

### Llama3.1:70b

根据您提供的疾病临床表现，主要特征包括先天性发育异常、进行性骨髓衰竭和肿瘤发生率增高，这些特征与范科尼贫血（Fanconi Anemia，FA）高度相符。范科尼贫血是一种罕见的遗传性疾病，主要影响骨髓、导致血细胞减少，并伴有多器官发育异常和肿瘤易感性。

其他可能的诊断包括：

1. **骨髓衰竭综合征（Aplastic Anemia）**：虽然骨髓衰竭是范科尼贫血的特征，但单独的骨髓衰竭综合征也可能导致类似的血液学异常，但通常不伴有明显的先天性发育异常或肿瘤易感性。
2. **Shwachman-Diamond 综合征**：这是一种罕见的遗传性疾病，特征包括骨髓衰竭、胰腺功能不全和短肢矮小等发育异常，但肿瘤易感性不如范科尼贫血明显。
3. **Diamond-Blackfan 贫血**：这是一种先天性红细胞生成障碍，可能伴有某些发育异常，但通常不包括肿瘤易感性或广泛的骨髓衰竭。
4. **Kostmann 综合征**：这是一种严重的先天性中性粒细胞减少症，可能伴有某些发育异常，但肿瘤易感性和广泛的骨髓衰竭不典型。
5. **Seckel 综合征**：这是一种微矮小综合征，特征包括生长迟缓、智力障碍和某些面部异常，但通常不伴有骨髓衰竭或显著的肿瘤易感性。

范科尼贫血的诊断通常需要通过遗传学检查来确认，包括染色体交叉实验和相关基因的突变分析。

### **43. User**

经典型患儿常在围生期发病，在摄取母乳或含乳糖配方奶粉数天内，患儿会出现危及生命的并发症：喂养问题、腹泻、呕吐、低血糖、肝功能损伤、出血、黄疸、 白内障。如果未及时治疗，可能会发生败血症、休克和死亡。存活至婴儿期的患儿，如果继续摄取乳糖，可能会出现严重的脑损伤。一些患儿有智力落后、生长发育延迟、共济失调、失明以及女性患者的卵巢功能障碍等远期并发症。

### Llama3.1:70b

根据您提供的临床表现，我最有可能诊断出的是**经典型糖原贮积病（Glycogen Storage Disease Type I）**，也称为**冯吉尔克病（Von Gierke病）**。

其他五种可能的诊断包括：

1. **乳糖不耐受（Lactose Intolerance）**：由于乳糖酶缺乏，导致乳糖不能被正常消化，引起腹泻、呕吐、腹胀等症状。
2. **先天性肝功能障碍（Congenital Hepatic Dysfunction）**：由于肝脏发育异常或功能障碍，导致黄疸、肝功能损伤等症状。
3. **先天性代谢紊乱（Congenital Metabolic Disorder）**：由于遗传性代谢途径异常，导致低血糖、酸中毒等症状。
4. **先天性免疫缺陷（Congenital Immune Deficiency）**：由于免疫系统发育异常或功能障碍，导致反复感染、败血症等症状。
5. **先天性白内障（Congenital Cataract）**：由于晶状体发育异常，导致白内障、视力障碍等症状。

需要注意的是，以上诊断仅基于您提供的临床表现，实际诊断需要结合实验室检查、影像学检查和其他临床信息才能确定。

### **44. User**

1. Ⅰ型最常见亚型。无原发性中枢神经系统受累表现，一些患者随着疾病进展可能出现继发神经系统临床表现（如脊髓受压等）。各年龄段均可发病，约 2/3 患者在儿童期发病。脏器表现主要为肝脾肿大，尤以脾肿大显著，常伴脾功能亢进，甚至出现脾梗死、脾破裂等。血液学主要表现为血小板减少和贫血，部分患者白细胞减少，可伴有凝血功能异常。患者表现为面色苍白、疲乏无力、皮肤及牙龈出血、月经增多，甚至出现危及生命的出血现象。多数患者有骨骼受侵，但轻重不一。早期受侵犯部位主要包括腰椎、长骨干骺端和骨干，中后期可累及骨骺和骨突。患者常有急性或慢性弥漫性骨痛，严重者可出现骨坏死（缺血性坏死），随后可出现关节塌陷累及股骨近端和远端、胫骨近端和肱骨近端。还可发生溶骨性病变、病理性骨折、椎体压缩骨折及其他因骨密度降低导致的脆性骨折。骨骼病变可影响日常活动，并可致残。儿童患者常见的表现依次是骨质疏松、长骨干骺端烧瓶样畸形、长骨干骺端密度不同程度的减低、骨皮质变薄等，可有生长发育迟缓。部分患者可有肺部受累，主要表现为间质性肺病、肺实变、肺动脉高压等。此外，患者还会出现糖和脂类代谢异常、多发性骨髓瘤等恶性肿瘤发病风险增高、胆石症、免疫系统异常等表现。
2. Ⅱ型，Ⅱ型患者除有与Ⅰ型相似的肝脾肿大、贫血、血小板减少等表现外，主要为急性神经系统受累表现。常发病于新生儿期至婴儿期，进展较快，病死率高。有迅速进展的延髓麻痹、动眼障碍、癫痫发作、角弓反张及认知障碍等急性神经系统受损表现，生长发育落后，2~4 岁前死亡。一些重度患者会出现关节挛缩。
3. Ⅲ型早期表现与Ⅰ型相似，逐渐出现神经系统受累表现，常发病于儿童期，病情进展缓慢，寿命可较长。患者常有动眼神经受侵、眼球运动障碍，并有共济失调、角弓反张、癫痫、肌阵挛，伴发育迟缓、智力落后。Ⅲ型可分为 3 种亚型，即以较快进展的神经系统症状（眼球运动障碍、小脑共济失调、痉挛、肌阵挛及痴呆） 及肝脾肿大为主要表现的Ⅲa 型；以肝脾肿大及骨骼症状为主要表现而中枢神经系统症状较少的Ⅲb 型；其他症状较轻，以心脏瓣膜钙化及角膜混浊为特殊表现， 主要出现在德鲁兹人群的Ⅲc型。

### Llama3.1:70b

根据您提供的疾病临床表现，医生最有可能诊断出的是**尼曼-皮克病（Niemann-Pick病）**。尼曼-皮克病是一种罕见的遗传性疾病，主要影响脂质代谢，导致细胞内积累过多的脂质，影响多个器官和系统的功能。

其他五种可能的诊断包括：

1. **加德纳综合征（Gardner综合征）**：一种罕见的遗传性疾病，主要影响骨骼、皮肤和肠道，表现为骨骼畸形、皮肤疣和肠道息肉。
2. **马凡综合征（Marfan综合征）**：一种遗传性疾病，主要影响身体的结缔组织，表现为骨骼畸形、眼部问题和心血管问题。
3. **埃勒斯-丹洛斯综合征（Ehlers-Danlos综合征）**：一种遗传性疾病，主要影响身体的结缔组织，表现为皮肤超弹性、关节过度活动和血管问题。
4. **奥斯特-威伯-伦道综合征（Osteogenesis imperfecta）**：一种遗传性疾病，主要影响骨骼，表现为骨质疏松、骨折和听力丧失。
5. **帕塔综合征（Patau综合征）**：一种罕见的遗传性疾病，主要影响多个器官和系统，表现为心脏问题、脑部问题和其他身体畸形。

### **45. User**

经常从一组肌群开始，逐渐累及其他肌群，以上睑下垂和复视为最常见的发病症状， 占 80％。骨骼肌无力表现为波动性和易疲劳性，晨轻暮重。根据临床表现，分成五型。Ⅰ型：眼肌型，表现为上睑下垂、复视等。ⅡA 型：轻度全身型，四肢肌群轻度受累。ⅡB 型：中度全身型，四肢肌群中度受累，通常有咀嚼、吞咽和构音障碍。Ⅲ型： 重度激进型，进展快，数周或数月内累及咽喉肌，半年内累及呼吸肌。Ⅳ型：迟发重度型，缓慢进展，2 年内累及呼吸肌。Ⅴ型：肌萎缩型，起病半年内出现骨骼肌萎缩。神经科查体可见四肢近端、躯干、颈部、颅面部肌肉无力，不耐疲劳，Jolly 试验阳性，腱反射正常，无感觉障碍，无病理征。部分病人可进展至呼吸衰竭，出现重症肌无力危象。

### Llama3.1:70b

根据您提供的临床表现，我最有可能诊断出的罕见病是重症肌无力（Myasthenia Gravis，MG）。重症肌无力是一种自身免疫性疾病，特征是骨骼肌无力和易疲劳，尤其是在眼肌和吞咽肌群中。这种疾病的临床表现与您描述的非常相似，包括上睑下垂、复视、四肢肌群无力、咀嚼、吞咽和构音障碍等。根据疾病的严重程度和进展速度，重症肌无力可以分为不同的类型，如您所述的五型。

其他五种可能的诊断包括：

1. **肌营养不良症（Muscular Dystrophy）**：这是一组遗传性疾病，导致骨骼肌逐渐萎缩和无力。虽然肌营养不良症也会导致肌肉无力，但其进展速度通常较慢，且不像重症肌无力那样具有波动性和易疲劳性。
2. **肌无力症（Myopathy）**：这是一组影响骨骼肌的疾病，可能导致肌肉无力、萎缩和疼痛。肌无力症的临床表现可能与重症肌无力相似，但其病因和进展速度通常不同。
3. **神经肌肉接点疾病（Neuromuscular Junction Diseases）**：这组疾病影响神经和肌肉之间的信号传递，导致肌肉无力和萎缩。虽然这些疾病也会导致肌肉无力，但其临床表现和进展速度通常不同于重症肌无力。
4. **肌萎缩侧索硬化（Amyotrophic Lateral Sclerosis，ALS）**：这是一种神经退行性疾病，导致运动神经元逐渐死亡，导致肌肉无力和萎缩。ALS 的临床表现通常包括肌肉萎缩和无力，但其进展速度通常较快，且不像重症肌无力那样具有波动性和易疲劳性。
5. **慢性炎症性脱髓鞘性多发性神经病（Chronic Inflammatory Demyelinating Polyneuropathy，CIDP）**：这是一种自身免疫性疾病，导致周围神经脱髓鞘，导致肌肉无力和萎缩。CIDP 的临床表现可能与重症肌无力相似，但其病因和进展速度通常不同。

需要注意的是，以上诊断均需要通过进一步的检查和测试来确认，包括血液检查、肌电图、神经传导速度测试等。

### **46. User**

常于青少年或成年早期起病。临床表现主要与低血钾和低血镁相关，轻型患者可无症状或表现为轻度乏力和纳差；严重患者会出现四肢抽搐、软瘫、痛性痉挛、晕厥和横纹肌溶解继发急性肾损伤，甚至因为严重室性心律失常导致心搏骤停。常见临床表现如下：

1. 全身症状 疲乏、口渴、多饮、嗜盐。
2. 神经 - 肌肉系统 肌无力、痛性痉挛、抽搐、惊厥发作、肢体麻木、感觉异常、横纹肌溶解、瘫痪、头晕、眩晕、共济失调和假性脑瘤等。
3. 心血管系统 心悸、晕厥、血压正常或偏低和室性心律失常等。
4. 消化系统 便秘、呕吐等。
5. 泌尿系统 多尿、夜尿增多、遗尿、蛋白尿和肾功能不全。
6. 骨关节系统 关节痛、软骨钙质沉着症。
7. 内分泌和生长发育 生长迟缓、青春期延迟；长期低钾和低镁的患者糖尿病或者糖耐量减低的比例并不少见。
8. 眼部症状 少数患者会出现视物模糊和巩膜脉络膜钙化。

### Llama3.1:70b

根据您提供的临床表现，我最有可能诊断出的是 **Gitelman 综合征**。Gitelman 综合征是一种罕见的遗传性疾病，主要影响肾脏的功能，导致低血钾、低血镁和代谢性碱中毒。这种疾病通常在青少年或成年早期开始表现出来，临床表现与您提供的描述相符，包括全身症状、神经-肌肉系统症状、心血管系统症状、消化系统症状、泌尿系统症状、骨关节系统症状、内分泌和生长发育异常以及眼部症状。

其他五种可能的诊断包括：

1. **Bartter 综合征**：这是一种与Gitelman 综合征相似的疾病，也影响肾脏功能，导致低血钾、低血镁和代谢性碱中毒。然而，Bartter 综合征通常在婴儿期或幼儿期开始表现出来。
2. **低血钾性周期性麻痹**：这是一种遗传性疾病，主要影响肌肉，导致周期性的低血钾和肌肉无力。
3. **低血镁性惊厥**：这是一种罕见的疾病，主要影响神经系统，导致低血镁和惊厥发作。
4. **肾上腺增生症**：这是一种内分泌疾病，导致肾上腺激素分泌过多，可能引起低血钾和低血镁。
5. **家族性低血钾性周期性麻痹**：这是一种遗传性疾病，主要影响肌肉，导致周期性的低血钾和肌肉无力。

需要注意的是，这些疾病的诊断需要经过详细的临床检查、实验室检查和遗传学检查才能确定。

### **47. User**

大多数患儿于婴幼儿期发病，表现多样。发病越早，症状越重，预后越差。大部分患者生后 1 年内表现相对正常，约 75％的患者最早出现的体征是头大，多数患儿出生时头围较同龄儿大或生后不久头围迅速增大，可伴轻微的非特异性症状，包括易激惹、喂养困难和呕吐等。患儿通常生后 3~36 月龄间，在发热、感染、手术或预防接种等诱因后出现酮症、呕吐、肝大和急性脑病危象表现，包括肌张力低下、意识丧失和惊厥发作等，对症治疗后症状可缓解，但不能完全恢复。如果急性脑病危象反复发生，神经系统损伤将进行性加重，可有发育倒退现象，最终可出现认知功能障碍。部分患儿在生后数年逐渐出现运动延缓、肌张力异常和随意运动障碍，但智力发育基本正常。患者常在 10 岁内死于伴发疾病或 Reye 样发作，随年龄增长发作减少。极少数患者于青春期甚至成年时期发病，首次发病之前可无症状。

### Llama3.1:70b

根据您提供的临床表现，我最有可能诊断出的是**丙酮酸脱氢酶复合体缺陷症（PDHC缺陷症）**。此病是一种罕见的遗传性疾病，影响身体将碳水化合物转化为能量的能力，导致丙酮酸在体内积累，引起一系列神经系统和其他系统的症状。

其他五种可能的诊断包括：

1. **丙氨酸血症（MSUD）**：一种遗传性疾病，影响身体分解某些氨基酸的能力，导致毒性代谢产物在体内积累，引起神经系统症状。
2. **异乳糖血症（GALT缺陷症）**：一种遗传性疾病，影响身体分解乳糖的能力，导致乳糖在体内积累，引起胃肠道和神经系统症状。
3. **尿素循环障碍（UCD）**：一种遗传性疾病，影响身体分解蛋白质的能力，导致氨在体内积累，引起神经系统症状。
4. **脂肪酸氧化缺陷（FAOD）**：一种遗传性疾病，影响身体分解脂肪酸的能力，导致脂肪酸在体内积累，引起心脏、肌肉和神经系统症状。
5. **有机酸血症（OAS）**：一种遗传性疾病，影响身体分解某些氨基酸和脂肪酸的能力，导致毒性代谢产物在体内积累，引起神经系统和其他系统的症状。

这些疾病都属于遗传性代谢疾病，需要通过遗传学检测和代谢产物分析来确诊。

### **48. User**

主要表现为关节、肌肉和深部组织出血，也可表现为胃肠道、中枢神经系统等内部脏器出血等。若反复出血，不及时治疗可导致关节畸形和(或) 假肿瘤形成，严重者可危及生命。外伤或手术后延迟性出血是本病的特点。

### Llama3.1:70b

根据您提供的临床表现，主要特征包括关节、肌肉和深部组织出血，以及可能的胃肠道、中枢神经系统等内部脏器出血，特别是外伤或手术后延迟性出血，这些特征高度提示以下罕见病的可能诊断：

1. **血友病**：这是最可能的诊断。血友病是一种遗传性的出血性疾病，由于第八凝血因子（血友病A）或第九凝血因子（血友病B）的缺乏或功能障碍引起。患者常表现为关节、肌肉和深部组织出血，严重时可导致关节畸形和假肿瘤形成。
2. **von Willebrand病**：这是一种最常见的遗传性出血性疾病，由von Willebrand因子的缺乏或功能障碍引起。虽然其主要表现为皮肤和黏膜出血，但也可能有深部组织出血。
3. **血小板功能障碍**：这包括了一组因血小板功能异常而导致的出血性疾病。虽然主要表现为皮肤和黏膜出血，但在某些情况下也可能有深部组织出血。
4. **凝血因子缺乏症**：除了血友病A和B外，还有其他凝血因子的缺乏，如第十一凝血因子缺乏症、第十二凝血因子缺乏症等，这些疾病也可能导致深部组织出血。
5. **Ehlers-Danlos综合征**：这是一种遗传性疾病，主要影响结缔组织，导致皮肤、韧带和血管的异常。虽然主要表现为皮肤和韧带问题，但也可能有出血倾向。
6. **Osler-Weber-Rendu综合征**：这是一种遗传性疾病，主要特征是多发性动脉畸形，可能导致出血。

### **49. User**

可累及全身多个脏器，临床表现多样。发病年龄多为 3~60 岁，也有8 个月及 70 多岁诊断的患者报道。儿童患者多以肝脏受累为首发表现，青少年及成人以神经系统受累为首发症状的比例增高。

可表现为无症状的转氨酶持续升高、慢性肝炎、肝硬化和急性肝功能衰竭。神经系统受累可表现为运动功能障碍、震颤、共济失调、舞蹈症、自主运动障碍、肌张力障碍，一些患者表现为面具脸、四肢僵硬、步态异常等。一些患者合并精神行为异常，如淡漠、攻击行为、性格改变等。肾脏受累可表现为血尿、蛋白尿等。血液系统受累可表现为溶血性贫血、肝硬化、脾功能亢进致血液三系下降、凝血功能异常等。相对少见的受累系统还包括骨关节、心脏、内分泌和生殖系统等。

### Llama3.1:70b

根据您提供的临床表现，罕见病的可能诊断包括：

1. **威尔逊病**：一種因體內銅質累積而導致的罕見遺傳病。臨床表現包括肝臟受累、神經系統受累、精神行為異常等，與您提供的臨床表現相符。
2. **肝豆状核变性**：一种遗传性疾病，特征是肝脏和中枢神经系统受累，临床表现包括肝炎、肝硬化、神经系统症状等。
3. **系统性红斑狼疮**：一种自身免疫性疾病，可能影响多个器官系统，包括肝脏、神经系统、肾脏等，临床表现多样。
4. **血色病**：一种遗传性疾病，特征是血液中铁质积累，可能导致肝脏、心脏、骨骼等器官受累。
5. **多器官功能障碍综合征**：一种罕见的疾病，特征是多个器官系统受累，包括肝脏、神经系统、肾脏等，临床表现多样。

### **50. User**

临床表现具有很大的异质性，甚至同一家系中的患者也有很大差异。 通常在 30 岁前起病，青春期加重，水肿常呈急性发作。临床上以反复发作、难以预测的皮肤和黏膜下水肿为特征。水肿的特点呈发作性，自限性，一般 3~5 天自然缓解，非对称性，非可凹性。水肿可累及身体任何部位，以四肢、颜面、生殖器、呼吸道和胃肠道黏膜较为常见。其中最致命的是呼吸道黏膜水肿，可因喉水肿迅速进展导致呼吸困难或窒息， 如抢救不及时可窒息死亡，致死率高达 11％ ~40％，是 主要死因之一。消化道黏膜水肿发作表现为剧烈腹痛，伴恶心、呕吐，因此常被误诊为急腹症，导致不必要的腹部手术。发生于肢体等部位的皮肤水肿则会影响其功能。

### Llama3.1:70b

根据您提供的临床表现，我最有可能诊断出的是**遗传性血管性水肿（Hereditary Angioedema，HAE）**。

其他五种可能的诊断包括：

1. **获得性血管性水肿（Acquired Angioedema，AAE）**：与HAE相似，但不是遗传性的，通常与自身免疫性疾病或药物反应有关。
2. **过敏性血管性水肿（Allergic Angioedema）**：由过敏反应引起的血管性水肿，通常与特定的过敏原有关。
3. **血管性水肿伴血小板减少症（Angioedema with Thrombocytopenia）**：一种罕见的疾病，表现为血管性水肿和血小板减少。
4. **家族性冷致性血管性水肿（Familial Cold Autoinflammatory Syndrome，FCAS）**：一种遗传性疾病，表现为冷刺激后引起的血管性水肿和炎症反应。
5. **Muckle-Wells 综合征（Muckle-Wells Syndrome）**：一种罕见的遗传性疾病，表现为周期性发作的血管性水肿、关节炎和其他全身性症状。

### **51. User**

出生即起病，临床表现的轻重与疾病亚型相关。临床表现为手部、肘部、膝及足部和其他容易摩擦的部位或周身出现水疱或大疱。可以出现口腔黏膜水疱或溃疡，导致吞咽困难和声音嘶哑。部分患者伴有斑驳性色素异常、秃发、掌跖角化过度、牙釉质缺陷、甲增厚和甲营养不良。其他损害包括反复瘢痕形成导致的指端残毁、贫血、骨质疏松、肌肉萎缩、心肌病、耳聋。部分亚型晚期可伴发基底细胞癌、鳞状细胞癌及恶性黑色素瘤。

### Llama3.1:70b

根据您提供的临床表现，罕见病的诊断可能是：

1. **Epidermolysis Bullosa（EB）**：一种遗传性皮肤疾病，表现为皮肤和黏膜的水疱和溃疡，尤其是在容易摩擦的部位。根据亚型的不同，临床表现的严重程度也会有所不同。
2. **Dystrophic Epidermolysis Bullosa（DEB）**：EB的一种亚型，表现为皮肤和黏膜的水疱和溃疡，伴有瘢痕形成和组织损伤。
3. **Junctional Epidermolysis Bullosa（JEB）**：EB的一种亚型，表现为皮肤和黏膜的水疱和溃疡，伴有指端残毁和其他组织损伤。
4. **Kindler综合征**：一种罕见的遗传性皮肤疾病，表现为皮肤的水疱和溃疡，伴有色素异常、秃发和其他组织损伤。
5. **Ehlers-Danlos综合征**：一种遗传性结缔组织疾病，表现为皮肤的超弹性和易碎性，伴有其他组织损伤，如关节过度活动和血管破裂。

### **52. User**

患者的临床表现随年龄、接触果糖的量和时间而存在差异。一般而言，发病年龄越小，症状越重。经典型患儿于 6 个月左右起病，通常在首次食用了含有果糖的食物，如水果、蜂蜜和某些蔬菜时发病。常见临床症状包括进食含果糖食物后出现低血糖发作，表现为恶心、呕吐、腹痛、腹胀、出汗、震颤、抽搐甚至昏迷。

如果诊断未明，患者长期慢性摄入果糖食物，除了低血糖表现外，会出现生长发育迟缓，肝肿大、腹水、黄疸及代谢紊乱，如低血糖、代谢性酸中毒、低磷酸盐血症、高尿酸血症、高镁血症、高丙酮酸血症。病情严重者出现多脏器损害，肝、肾功能衰竭，抽搐、昏迷，甚至死亡。

在急性发作时，实验室检查会发现低血糖、低血磷和低血钾，同时伴有尿酸、乳酸、丙酮酸、游离脂肪酸和甘油三酯升高。慢性患者出现肝功能损害及肝纤维化的指征，包括血清胆红素，转氨酶升高，凝血障碍。尿液检查可见蛋白尿及果糖阳性。

### Llama3.1:70b

根据您提供的临床表现，我最有可能诊断出的是**果糖不耐受症（Fructose Malabsorption）**，也称为果糖代谢异常或果糖不耐受。这种疾病是由于身体无法正常吸收果糖，导致果糖在肠道内未被吸收而被细菌发酵，产生气体，引起一系列症状。

其他五种可能的诊断包括：

1. **乳糖不耐受症（Lactose Intolerance）**：由于乳糖酶缺乏，导致乳糖无法被正常吸收，引起腹胀、腹泻、气体等症状。
2. **肝脏疾病（Liver Disease）**：肝脏疾病，如肝炎、肝硬化等，可能导致低血糖、黄疸、腹水等症状。
3. **胰腺疾病（Pancreatic Disease）**：胰腺疾病，如胰腺炎、胰腺癌等，可能导致低血糖、腹痛、呕吐等症状。
4. **糖尿病（Diabetes Mellitus）**：糖尿病可能导致低血糖、多尿、口渴等症状。
5. **先天性代谢异常（Congenital Metabolic Disorder）**：某些先天性代谢异常，如糖原贮积病等，可能导致低血糖、黄疸、腹水等症状。

需要注意的是，这些疾病的临床表现可能相似，需要进行进一步的检查和诊断来确定确切的疾病。

### **53. User**

**1. 偏头痛** 先兆偏头痛常为该疾病的首发症状，见于 20％ ~40％的患者，是普通人群的 5倍；也可表现为无先兆偏头痛，发病率与普通人群无差异。亚裔患者偏头痛发生率较欧美患者低。

**2. 皮层下缺血性事件** 短暂性脑缺血发作（TIA） 和缺血性卒中是该疾病最常见的临床表现，见于 60％ ~85％的症状性患者，且大多数患者缺乏常见的脑血管病危险因素。2/3 的患者表现为经典的腔隙综合征。

**3. 认知缺陷** 60％的患者逐渐出现认知功能障碍与痴呆。

**4. 情感障碍与淡漠** 20％的患者具有情感障碍，抑郁状态最常见，极少数可表现为抑郁与躁狂状态交替出现；淡漠见于 40％的患者。

此外，5％ ~10％患者有癫痫发作，也有听力下降、意识障碍、脊髓梗死、颅内出血以及合并流域性梗死的报道。

### Llama3.1:70b

根据您提供的临床表现，可能的罕见病诊断包括：

1. **CADASIL综合征**：CADASIL综合征是一种罕见的遗传性疾病，主要影响大脑血管，导致偏头痛、皮层下缺血性事件、认知缺陷、情感障碍和淡漠等症状。
2. ** Moyamoya病**：Moyamoya病是一种罕见的血管疾病，主要影响大脑血管，导致皮层下缺血性事件、偏头痛、认知缺陷等症状。
3. **脑血管畸形**：脑血管畸形是一种罕见的血管疾病，主要影响大脑血管，导致皮层下缺血性事件、偏头痛、认知缺陷等症状。
4. **脑动脉瘤**：脑动脉瘤是一种罕见的血管疾病，主要影响大脑血管，导致皮层下缺血性事件、偏头痛、认知缺陷等症状。
5. **脑静脉畸形**：脑静脉畸形是一种罕见的血管疾病，主要影响大脑静脉，导致皮层下缺血性事件、偏头痛、认知缺陷等症状。
6. **脑血栓性疾病**：脑血栓性疾病是一种罕见的血管疾病，主要影响大脑血管，导致皮层下缺血性事件、偏头痛、认知缺陷等症状。

### **54. User**

通常在青年早期（但 1 岁至 76 岁起病均有报道） 隐匿起病。主要表现为缓慢进展的双下肢痉挛性瘫痪，查体可见锥体束征（腱反射亢进，踝阵挛阳性、病理征阳性）。双上肢反射也可亢进，但一般不影响上肢活动。超过一半的患者会有下肢近端力弱表现。约 50％的患者查体中可发现双踝音叉震动觉减低（一般不会消失），另约 1/3 的患者有排尿障碍。

随着年龄增长，疾病逐渐进展，约 20％的患者最终会丧失行走能力。在同一家系中， 携带同一突变的不同患者，临床症状轻重可有较大差异。

### Llama3.1:70b

根据您提供的临床表现，我最有可能诊断出的是**遗传性脊髓性痉挛性瘫痪（Hereditary Spastic Paraplegia, HSP）**。

其他五种可能的诊断包括：

1. **原发性侧索硬化（Primary Lateral Sclerosis, PLS）**：这是一种影响运动神经元的罕见疾病，主要表现为肌肉无力、萎缩和痉挛，尤其是下肢。
2. **脊髓小脑共济失调（Spinal Cerebellar Ataxia, SCA）**：这是一组影响小脑和脊髓的遗传性疾病，主要表现为共济失调、肌肉无力和痉挛。
3. **肌萎缩性侧索硬化（Amyotrophic Lateral Sclerosis, ALS）**：这是一种影响运动神经元的疾病，主要表现为肌肉无力、萎缩和痉挛，尤其是下肢。
4. **遗传性运动神经元病（Hereditary Motor Neuron Disease, HMND）**：这是一组影响运动神经元的遗传性疾病，主要表现为肌肉无力、萎缩和痉挛。
5. **脊髓损伤（Spinal Cord Injury, SCI）**：这是一种由于脊髓受伤或疾病导致的神经系统损伤，主要表现为肌肉无力、萎缩和痉挛，尤其是下肢。

### **55. User**

患儿多在新生儿、婴儿早期发病。临床表现与生物素酶缺乏相似，无特异性，主要有喂养困难、呕吐、腹泻、肌张力低下、惊厥、精神萎靡、嗜睡、呼吸困难、发育落后、顽固皮疹、脱皮，可合并酮症、代谢性酸中毒、高乳酸血症等。皮肤损害中大部分患者在头面部、颈部、躯干、臀部等部位皮肤红疹或红斑、溃烂或水疱、糠状或片状鳞屑，或皮肤干糙、脱皮等，少数仅在口周、眼周、肛周局部出现皮疹。患者不伴听力或视力障碍，不同于生物素酶缺乏症。

### Llama3.1:70b

根据您提供的临床表现，可能的罕见病诊断包括：

1. **角皮症（Holocarboxylase synthetase deficiency）**：这是一个罕见的遗传性疾病，表现为生物素依赖酶的缺乏，导致生物素的活化和利用受损。患者可能出现喂养困难、呕吐、腹泻、肌张力低下、惊厥、精神萎靡、嗜睡、呼吸困难、发育落后、顽固皮疹、脱皮等症状。
2. **生物素酶缺乏症（Biotinidase deficiency）**：虽然您提到患者不伴听力或视力障碍，不同于生物素酶缺乏症，但仍然需要考虑这种可能性，因为生物素酶缺乏症的临床表现可以非常多样化。
3. **多羧酸酶缺乏症（Multiple carboxylase deficiency）**：这种疾病也与生物素的活化和利用有关，可能导致类似的临床表现。
4. **角化病（Ichthyosis）**：虽然角化病主要表现为皮肤干燥、脱屑，但在某些情况下，也可能伴有其他系统性的症状。
5. **先天性多器官功能障碍（Congenital multiple organ dysfunction）**：这种情况下，患者可能表现出多个器官的功能障碍，包括皮肤、神经系统、消化系统等。

### **56. User**

该病患儿出生时多无异常，婴儿期临床表现并无特异性，仅表现为生长和发育迟缓。常于 3 岁有眼部症状和体征后得以诊断。

1. 视力受损， 异位晶状体所致的近视和虹膜震颤等症状可在 3 岁后出现，晚期可合并散光、青光眼、葡萄肿、白内障、视网膜剥离及视神经萎缩。
2. 智力障碍较常见，主要表现为进展性智力障碍。维生素 B6 治疗敏感型患者智力障碍较轻。
3. 精神或行为障碍， 大约 50％患儿可出现，20％患儿合并惊厥。
4. 骨骼受累， 类似马方综合征表现，或合并脊柱侧凸，漏斗胸，鸡胸，膝外翻及弓形足，X 线可见全身性骨质疏松。
5. 血栓性疾病， 发生于各个年龄段，可累及各类血管，尤其是脑血管。高水平同型半胱氨酸血症可改变血管壁及增加血小板黏附性，出现视神经萎缩、瘫痪、肺心病及肾性高血压。
6. 自发性气胸和急性胰腺炎 较少见。

### Llama3.1:70b

根据您提供的临床表现，我最有可能诊断出的是**同型高氨酸血症（Homocystinuria）**。

同型高氨酸血症是一种遗传性疾病，主要影响氨基酸代谢，导致同型高氨酸在血液中积累。该病的临床表现与您描述的高度一致，包括眼部症状（如异位晶状体、虹膜震颤）、智力障碍、精神或行为障碍、骨骼异常（如马方综合征）以及血栓性疾病等。

其他五种可能的诊断包括：

1. **马凡综合征（Marfan Syndrome）：**一种遗传性疾病，主要影响身体的结缔组织，导致眼部、骨骼、心血管等系统的异常。
2. **Ehlers-Danlos 综合征（Ehlers-Danlos Syndrome）：**一种遗传性疾病，主要影响身体的结缔组织，导致皮肤、骨骼、血管等系统的异常。
3. **Osteogenesis imperfecta（OI）：**一种遗传性疾病，主要影响骨骼系统，导致骨质疏松、骨折等症状。
4. **Mucopolysaccharidosis（MPS）：**一种遗传性疾病，主要影响身体的糖代谢，导致骨骼、眼部、心血管等系统的异常。
5. **Pseudoxanthoma elasticum（PXE）：**一种遗传性疾病，主要影响身体的弹性纤维，导致皮肤、眼部、血管等系统的异常。

需要注意的是，这些疾病的临床表现可能有一定的重叠，因此需要进行进一步的检查和诊断来确定确切的疾病类型。

### **57. User**

患者的主要表现有：出生后即发现 LDL-C 水平明显升高，胆固醇沉积在皮肤、眼睛以及肌腱形成黄色瘤和脂性角膜弓。由于机体暴露于高水平的 LDL-C 中， 患者多数会在 20 岁前就出现冠状动脉粥样硬化性心脏病并可能因此死亡。

需要详细询问患者的家族史，部分患者父母均为杂合子并因此出现 LDL-C 水平升高（通常大于本国特定年龄和性别标准的 95百分位数） 以及早发动脉粥样硬化性心血管疾病强阳性家族史。

### Llama3.1:70b

根据您提供的临床表现，我最有可能的诊断是**家族性高胆固醇血症（Familial Hypercholesterolemia，FH）**。

其他五种可能的诊断包括：

1. **脂蛋白lipoprotein(a)升高症**：这种情况下，患者的血液中lipoprotein(a)水平升高，导致动脉粥样硬化性心血管疾病的风险增加。
2. **多囊肾病**：一种遗传性疾病，表现为肾脏内形成多个囊肿，可能导致肾功能衰竭。
3. **肝豆状核变性**：一种遗传性疾病，表现为肝脏内铁质沉积，导致肝功能衰竭。
4. **高密度脂蛋白缺乏症**：一种罕见的遗传性疾病，表现为高密度脂蛋白（HDL）水平降低，导致动脉粥样硬化性心血管疾病的风险增加。
5. **Sitosterolemia**：一种罕见的遗传性疾病，表现为植物甾醇在体内蓄积，导致血管狭窄和动脉粥样硬化性心血管疾病的风险增加。

### **58. User**

临床特征表现为运动障碍、精神症状和认知障碍三联征，通常隐匿起病，缓慢进展。发病年龄从儿童期至 79 岁不等，最常见于 30~50 岁。

疾病累及面部、躯干和肢体的快速，不自主，无节律运动；早期异常动作轻微，患者可能难以意识到舞蹈症状的存在，或将舞蹈样动作整合到随意运动中，使得正常动作的控制出现困难或偏差；随着疾病的进展，舞蹈症的范围和程度常逐渐加重，甚至影响膈肌、咽和喉部肌肉，从而产生构音障碍、吞咽困难和不自主发声；疾病晚期舞蹈症常消失，代之以僵直、少动为主的帕金森症样表现，可伴有局灶性肌张力障碍；最后常导致卧床。运动保持困难也是该病常见表现，即不能保持某些简单的自主动作，如保持伸舌动作困难等。

精神障碍可先于舞蹈症出现，常见症状包括情绪低落、抑郁、易激惹、淡漠和焦虑， 也可出现偏执、妄想和幻觉；少见症状包括强迫行为和精神病表现，以上症状多呈进行性加重。

认知障碍最主要的特征是执行功能障碍，表现为做决定、执行多重任务和转换认知目标的能力下降。患者通常对其认知缺陷缺乏自知力。记忆减退或丧失多出现在病程晚期， 随疾病进展，最终可发展为痴呆。

除以上三联征外，眼球运动异常也是一个突出表现。早期表现为眼球扫视速度减慢，但眼球追随运动保留；晚期则眼球追随运动、自主扫视和再固定全部受损。此外，体重减轻和恶病质也是该病的常见特征，患者的不自主运动大量消耗能量可使体重明显下降，而通常无食欲减退。睡眠和(或) 性功能障碍也比较常见。

运动和认知功能的逐渐恶化会导致显著的并发症，如咽喉部肌肉受累导致吞咽困难， 运动障碍、活动减少甚至卧床可导致吸入性肺炎和其他感染、褥疮等。

### Llama3.1:70b

根据您提供的临床表现，我最有可能诊断出的是亨廷顿病（Huntington’s disease，HD）。亨廷顿病是一种罕见的遗传性神经退行性疾病，其特征是运动障碍、精神症状和认知障碍三联征。该病通常隐匿起病，缓慢进展，发病年龄从儿童期至老年期不等，最常见于 30~50 岁。

其他五种可能的诊断包括：

1. **帕金森病（Parkinson’s disease，PD）**：虽然帕金森病也表现为运动障碍，但其主要特征是静止性震颤、运动迟缓、刚性和姿势不稳定，而不是亨廷顿病的舞蹈样运动。
2. **威尔逊病（Wilson’s disease）**：威尔逊病是一种遗传性疾病，主要影响肝脏和大脑，表现为肝脏损害、神经系统症状（如震颤、运动迟缓）和精神症状。
3. **多系统萎缩（Multiple system atrophy，MSA）**：多系统萎缩是一种罕见的神经退行性疾病，表现为自主神经系统功能障碍、运动障碍和认知障碍。
4. **前颞叶痴呆（Frontotemporal dementia，FTD）**：前颞叶痴呆是一组神经退行性疾病，主要影响前颞叶，表现为认知障碍、行为改变和语言障碍。
5. **路易小体病（Lewy body dementia，LBD）**：路易小体病是一种神经退行性疾病，表现为认知障碍、视幻觉、运动障碍和睡眠障碍。

### **59. User**

临床症状、体征主要以神经系统症状为主，临床表现严重程度亦不同。发病年龄可以从新生儿期到成年期（晚发型），并且具有广泛的表型谱。

1. 新生儿期, 约24~48 小时内无明显症状，随后出现与高氨血症相关的症状［包括嗜睡、困倦、拒食、呕吐，伴呼吸性碱中毒和（或） 癫痫发作］。与新生儿发病的其他尿素循环障碍疾病难以区分。
2. 晚发型 , 该型患者占 88％，其中 40％发生在 3 岁前，29％在儿童期，19％在成人期。主要表现为：①慢性神经认知缺陷，包括发育迟缓、共济失调、痉挛、学习障碍、认知缺陷和（或） 无法解释的癫痫发作；②由多种因素（如感染，禁食或损伤） 诱发的继发于高氨血症危象的急性脑病；③慢性肝功能障碍，肝转氨酶不明原因升高伴有或不伴有轻度凝血功能障碍，伴或不伴轻度高氨血症和蛋白质不耐受。

### Llama3.1:70b

根据您提供的临床表现，主要特征是神经系统症状、发病年龄广泛以及与高氨血症相关的症状，我最可能的诊断是：

**1. 鸟氨酸转氨酶（OTC）缺乏症**

其他五种可能的诊断包括：

**2. 氨基甲酰磷酸合成酶（CPS）缺乏症**

**3.鸟氨酸循环酶（OCT）缺乏症**

**4.氨基甲酰磷酸合成酶Ⅰ（CPSⅠ）缺乏症**

**5.氨基甲酰磷酸合成酶Ⅱ（CPSⅡ）缺乏症**

**6.鸟氨酸脱氢酶（ODC）缺乏症**

这些疾病都属于尿素循环障碍疾病，可能导致高氨血症和神经系统症状。

### **60. User**

部分患儿在新生儿期多无临床症状，出生 3-4 个月后逐渐出现典型症状：头发由黑变黄，皮肤颜色浅淡，尿液、汗液鼠臭味。随着年龄增长，逐渐表现出智力发育落后、小头畸形、癫痫发作，也可出现行为、性格、神经认知等异常。如多动、自残、攻击、自闭症、自卑、忧郁等，婴儿期还常出现呕吐、湿疹等。 部分 患者在新生儿期也多无临床症状，出生 1-3 个月后除表现 PKU 症状外，主要表现为运动障碍、嗜睡、躯干肌张力低下、四肢肌张力增高或低下、眼震颤、吞咽困难、口水增多、松软、角弓反张，反应迟钝、失眠、智力发育严重障碍等。

### Llama3.1:70b

根据您提供的临床表现，我最有可能诊断出的是**苯丙酮尿症（Phenylketonuria，PKU）**。

其他五种可能的诊断是：

1. **地中海贫血（Thalassemia）**：虽然地中海贫血主要表现为贫血和黄疸，但在某些情况下也可能出现智力发育迟缓和行为异常。
2. **尿素循环障碍（Urea Cycle Disorder）**：这种疾病可能导致尿液中含有异常物质，引起神经系统症状，如癫痫和智力发育迟缓。
3. ** maple 糖尿病（Maple Syrup Urine Disease）**：这种疾病可能导致尿液中含有特殊物质，引起神经系统症状，如癫痫和智力发育迟缓。
4. **多卡盒症（DiGeorge Syndrome）**：这种疾病可能导致免疫系统缺陷、心脏问题和神经系统症状，如智力发育迟缓和行为异常。
5. **自闭症谱系障碍（Autism Spectrum Disorder）**：虽然自闭症谱系障碍主要表现为社交和沟通困难，但在某些情况下也可能出现智力发育迟缓和行为异常。

需要注意的是，这些疾病的临床表现可能有所重叠，需要进行进一步的检查和诊断来确定确切的疾病。

### **61. User**

临床表现的严重程度具有很大的差异。患者可以表现为骨骼矿化异常、牙齿或关节病变、肌肉乏力等。目前，疾病主要分为 7 种类型。

1. 单纯牙型， 是最轻、也是最常见的类型，仅有牙齿受累表现，无佝偻病或骨软化症的表现。
2. 成人型，常中年发病，表现为牙齿异常或骨软化症。由于反复骨折、骨骼和关节疼痛或肌肉无力，可引起活动障碍。
3. 儿童型，常在 6 月龄后发病，可轻度到重度，患儿可有不同程度的牙齿脱落、佝偻病的表现。
4. 婴儿型 ，于 6 月龄前发病，常表现为纳差、难以存活、衰弱，可有囟门宽阔或头颅畸形。50％的婴儿型 HPP 会死于各种并发症。
5. 围生期型，是极端类型，在宫内即发病，出生时症状明显，几乎均是致死型。患儿四肢短小、严重骨骼矿化不足，可表现为高调哭喊、癫痫发作、周期性呼吸暂停、伴有发绀和心动过缓、不明原因发热、易激惹、骨髓抑制性贫血和颅内出血、肺不张。
6. 假性低磷酸酯酶症， 类似于婴儿型，患者具有婴儿型低磷酸酯酶症的表现，但血清 ALP 正常或升高。
7. 良性型， 有时在宫内出现骨骼畸形，有围生期低磷酸酶活性，但出生后病情缓解，没有婴儿型低磷酸酯酶症或单纯牙型低磷酸酯酶症的相关表现。

### Llama3.1:70b

根据您提供的临床表现，医生可能会诊断出“低磷酸酯酶症（Hypophosphatasia，HPP）”。这是一个罕见的遗传性疾病，影响骨骼和牙齿的矿化。

### **62. User**

患儿主要表现为方颅、鸡胸、串珠肋、手镯征、足镯征、亨利氏沟，多在将近周岁开始负重时出现下肢畸形，可表现为膝内翻（O 形腿） 或膝外翻（X 形腿），并伴有生长迟缓、身材矮小、步态摇摆、进行性加重的骨畸形、多发性骨折、骨骼疼痛以及牙齿发育异常（牙质差、牙痛、脱落后不易再生） 等。影像学表现为骨骼畸形、长骨干骺端增宽和模糊，呈杯口样，杯口内可见许多细条状钙化影如毛刷状。在成人期主要表现为肢体乏力、活动受限、骨痛、多发病理性骨折（四肢长骨、肋骨、骨盆和椎体均可发生）、身高变矮。影像学表现为骨密度普遍减低，骨小梁模糊，呈毛玻璃状，骨盆畸形、假骨折线、椎体呈双凹变形等。

### Llama3.1:70b

根据您提供的临床表现和影像学特征，罕见病的可能诊断如下：

1. **最可能的诊断：** 骨质疏松症（Osteogenesis Imperfecta，OI）：该病是一种遗传性骨骼疾病，主要特征包括骨质疏松、骨折易碎、骨骼畸形和牙齿发育异常等。患者常表现为身材矮小、步态摇摆、骨骼疼痛和多发性骨折等。
2. **其他可能的诊断：** 1. **成骨不全症（Achondroplasia）**：一种常见的短肢矮小症，主要特征包括短肢、头大、鼻梁低等。 2. **骨软化症（Rickets）**：一种由于维生素D缺乏引起的骨骼疾病，主要特征包括骨骼畸形、骨痛和肌肉无力等。 3. **骨质疏松症（Osteoporosis）**：一种骨密度降低的疾病，主要特征包括骨质疏松、骨折风险增加等。 4. **马凡综合征（Marfan Syndrome）**：一种遗传性疾病，主要特征包括身材高挑、四肢长、关节过度灵活等。 5. **埃勒斯-丹洛斯综合征（Ehlers-Danlos Syndrome）**：一种遗传性疾病，主要特征包括皮肤超弹性、关节过度灵活和血管脆弱等。

### **63. User**

临床表现有心力衰竭、胸痛、低血压、晕厥、心源性猝死、心律失常和血栓栓塞等。部分患者还会有心脏外的表现，如周围神经病、自主神经病和胃肠道症状等。

### Llama3.1:70b

根据您提供的临床表现，罕见病的诊断可能是：

1. **肥厚型心肌病（Hypertrophic Cardiomyopathy，HCM）**：这是一种心脏肌肉异常增厚的疾病，可能导致心力衰竭、胸痛、心律失常和猝死等症状。
2. **长QT间期综合征（Long QT Syndrome，LQTS）**：这是一种心脏电活动异常的疾病，可能导致心律失常、晕厥和猝死等症状。
3. **布鲁加达综合征（Brugada Syndrome）**：这是一种心脏电活动异常的疾病，可能导致心律失常、晕厥和猝死等症状。
4. **心肌病（Dilated Cardiomyopathy，DCM）**：这是一种心脏肌肉功能不全的疾病，可能导致心力衰竭、胸痛和心律失常等症状。
5. **心脏病变（Arrhythmogenic Right Ventricular Cardiomyopathy，ARVC）**：这是一种心脏右室肌肉病变的疾病，可能导致心律失常、晕厥和猝死等症状。
6. **马凡综合征（Marfan Syndrome）**：这是一种遗传性疾病，可能导致心脏血管和其他器官的异常，包括主动脉夹层和主动脉瘤等。

### **64. User**

**1. 第二性征不发育和配子生成障碍** 男性表现为童声、小阴茎、无阴毛生长、小睾丸或隐睾、无精子生成；女性表现为乳腺不发育、幼稚外阴和原发闭经。

**2. 骨骺闭合延迟** 上部量 / 下部量<1，指尖距 > 身高，易患骨质疏松症。

**3. 嗅觉障碍** 因嗅球和嗅束发育异常，40％ ~60％ IHH 患者合并嗅觉丧失，不能识别气味。

**4. 其他表现** 面中线缺陷，如唇腭裂；孤立肾；短指(趾)、并指(趾)；骨骼畸形或牙齿发育不良；超重和肥胖；镜像运动等。

### Llama3.1:70b

根据您提供的临床表现，医生可能会诊断出 **Kallmann 综合征**，一种罕见的遗传性疾病，影响性腺功能和嗅觉。其他可能的诊断包括：

1. **Klinefelter 综合征**：一种男性性染色体异常，表现为性腺功能减退和第二性征不发育。
2. **Turner 综合征**：一种女性性染色体异常，表现为性腺功能减退和第二性征不发育。
3. **垂体功能减退症**：一种垂体激素分泌不足的疾病，可能导致性腺功能减退和第二性征不发育。
4. **原发性性腺功能减退症**：一种性腺功能减退的疾病，可能导致第二性征不发育和配子生成障碍。
5. **Prader-Willi 综合征**：一种罕见的遗传性疾病，表现为性腺功能减退、超重和肥胖等。

### **65. User**

最常见的症状是活动后气短，其他症状包括乏力、头晕、胸痛、胸闷、心悸、黑矇、晕厥等，一旦出现晕厥或眩晕，提示患者心搏量可能已明显下降。由于症状、体征不典型，绝大多数患者就诊时间明显延误，至少 1/5 患者从症状出现至确诊时间超过 2 年；患者可出现肺动脉瓣第二心音亢进，三尖瓣关闭不全引起三尖瓣区收缩期杂音； 右心衰竭体征包括颈静脉充盈或怒张、肝脏肿大、下肢水肿、多浆膜腔积液、黄疸和发绀等。

### Llama3.1:70b

根据您提供的症状和体征，可能的罕见病诊断包括：

1. **埃勒斯-丹洛斯综合征**：一种遗传性疾病，影响身体的结缔组织，导致血管壁和心脏瓣膜异常，可能引起心脏问题和其他症状。
2. **马凡综合征**：一种遗传性疾病，影响身体的结缔组织，导致血管壁和心脏瓣膜异常，可能引起心脏问题和其他症状。
3. **Loeys-Dietz 综合征**：一种遗传性疾病，影响身体的结缔组织，导致血管壁和心脏瓣膜异常，可能引起心脏问题和其他症状。
4. **心肌病**：一种影响心脏肌肉的疾病，可能导致心脏功能不全和其他症状。
5. **心脏瓣膜病**：一种影响心脏瓣膜的疾病，可能导致心脏功能不全和其他症状。

### **66. User**

一般起病隐匿。主要表现为干咳、劳力性呼吸困难，杵状指(趾)、双下肺分布为主的爆裂音是其典型体征。终末期可以出现发绀、肺动脉高压、肺心病、右心功能不全的相关临床表现。

### Llama3.1:70b

根据您提供的临床表现，可能的罕见病诊断包括：

1. **特发性肺纤维化（IPF）**：这是一种慢性、进行性的肺部疾病，主要特征是肺组织纤维化，导致呼吸困难和肺功能下降。典型的临床表现包括干咳、劳力性呼吸困难、杵状指（趾）和双下肺分布的爆裂音。
2. **神经肌肉疾病相关的肺纤维化**：某些神经肌肉疾病，如肌营养不良症等，可能导致肺纤维化，表现为呼吸困难、肺功能下降等。
3. **结节病**：这是一种慢性炎症性疾病，可能影响多个器官，包括肺部。肺结节病的临床表现包括干咳、呼吸困难、杵状指（趾）和肺部爆裂音。
4. **淋巴增生性肺病**：这是一种罕见的肺部疾病，特征是肺部淋巴组织增生，导致呼吸困难、肺功能下降等。
5. **肺淀粉样变**：这是一种罕见的肺部疾病，特征是肺部淀粉样蛋白沉积，导致肺功能下降、呼吸困难等。

这些疾病都可能表现为肺纤维化、呼吸困难和肺功能下降等相似的临床特征，因此需要进行进一步的检查和诊断来确定确切的疾病类型。

### **67. User**

一种多器官、多系统受累的疾病，可累及几乎全身所有器官和组织。临床表现多种多样，患者因不同器官受累就诊于不同专科，容易漏诊。该病多数患者起病缓， 慢性进展。可以单器官起病，逐渐累及多个器官，也可以多种器官同时发病。少数患者病程呈自限性。

泪腺、唾液腺为最常见受累器官。主要表现为对称性、无痛性泪腺，颌下腺或腮腺肿大，硬结，伴或不伴有鼻窦炎或眼外肌增厚。消化系统受累可表现为自身免疫性胰腺炎、硬化性胆管炎、硬化性肠系膜炎等，临床可出现腹部隐痛、腹胀、消化不良、黄疸等。肺部受累时可表现为支气管壁增厚、肺间质病变、肺部结节 / 肿块以及胸膜增厚等，患者可无症状或咳嗽、气短及喘憋等。腹膜后纤维化或腹主动脉周围炎也是较常见的临床表现之一，主要为腹主动脉周围软组织增厚，可包绕输尿管造成输尿管狭窄和肾盂积水，少数患者发生腹主动脉瘤。临床表现为腹痛、腰痛或下肢水肿等。不到 10% 的患者发生 IgG4 相关性肾脏疾病，多表现为小管间质性肾病、肾盂占位性病变、输尿管管壁增厚或肾脏肿块样病变。其他受累器官包括甲状腺（硬化性甲状腺炎）、前列腺等。中枢神经系统病变少见，如自身免疫性垂体炎、肥厚性硬脑膜炎、颅内炎性假瘤等，患者可出现头痛、尿崩症、垂体功能减低等。除上述器官受累外，多数患者合并浅表或深部淋巴结肿大。另外，约半数患者有过敏相关病史，可以伴发哮喘。

### Llama3.1:70b

根据您提供的临床表现，我最有可能诊断出的是 IgG4 相关性疾病（IgG4-RD）。IgG4-RD 是一种多器官、多系统受累的疾病，特征是 IgG4 抗体水平升高和组织中 IgG4 阳性浆细胞浸润。该病可累及几乎所有器官和组织，包括泪腺、唾液腺、胰腺、胆管、肠系膜、肺、肾脏、甲状腺、前列腺等。

其他五种可能的诊断包括：

1. **系统性红斑狼疮（SLE）**：SLE 是一种自身免疫性疾病，可能影响多个器官和系统，包括皮肤、关节、肾脏、血液和中枢神经系统。虽然 SLE 的临床表现多种多样，但其通常不像 IgG4-RD 那样表现为多器官受累和 IgG4 抗体水平升高。
2. **干燥综合征（Sjögren 综合征）**：干燥综合征是一种自身免疫性疾病，主要影响泪腺和唾液腺，导致眼和口干燥。虽然该病也可能累及其他器官，但其通常不像 IgG4-RD 那样表现为多器官受累和 IgG4 抗体水平升高。
3. **多器官自身免疫性疾病（MASD）**：MASD 是一种罕见的自身免疫性疾病，可能影响多个器官和系统，包括皮肤、关节、肾脏、血液和中枢神经系统。虽然 MASD 的临床表现多种多样，但其通常不像 IgG4-RD 那样表现为多器官受累和 IgG4 抗体水平升高。
4. **全身性硬化症（SSc）**：全身性硬化症是一种自身免疫性疾病，主要影响皮肤和内脏器官，导致皮肤和内脏器官纤维化。虽然该病也可能累及多个器官，但其通常不像 IgG4-RD 那样表现为多器官受累和 IgG4 抗体水平升高。
5. **重复发作性多器官炎症性疾病（RPID）**：RPID 是一种罕见的自身免疫性疾病，可能影响多个器官和系统，包括皮肤、关节、肾脏、血液和中枢神经系统。虽然 RPID 的临床表现多种多样，但其通常不像 IgG4-RD 那样表现为多器官受累和 IgG4 抗体水平升高。

### **68. User**

患儿有胆汁淤积和肝硬化的发生。其主要临床表现为高结合胆红素血症，常出现脂溶性维生素吸收不良，如佝偻病等。而不同酶缺陷的临床表型略有不同，如氧固醇 7α- 羟化酶缺陷可出现成人期遗传性痉挛瘫痪；胆固醇 25 羟化酶缺陷可发生顽固性便秘；3β- 羟基类固醇 -Δ5-C27- 类固醇脱氢酶缺陷可发生婴儿期胆汁淤积性肝病和脂溶性维生素缺乏；固醇27- 羟化酶缺陷可发生儿童慢性腹泻及发育迟缓、成人期痉挛性瘫痪；α- 甲酰辅酶 A 消旋酶缺陷可发生婴儿期脂溶性维生素缺乏、成人期感觉神经病变；胆汁酸辅酶 A 氨基酸N- 乙酰转移酶缺陷可发生肝内胆汁淤积、发育迟缓、家族性高胆烷血症等。

### Llama3.1:70b

根据您提供的临床表现，主要特征包括胆汁淤积、肝硬化、高结合胆红素血症和脂溶性维生素吸收不良等，我最可能的诊断是**胆汁酸合成障碍**（Bile Acid Synthesis Disorders）。

胆汁酸合成障碍是一组罕见的遗传性疾病，涉及胆汁酸合成途径中的酶缺陷。这些疾病会导致胆汁酸的异常积累，进而引起肝脏损害、胆汁淤积和脂溶性维生素吸收不良等问题。

其他五种可能的诊断包括：

1. **Alagille 综合征**：一种遗传性疾病，影响肝脏、心脏、眼、脊柱和面部等多个系统，常见的肝脏表现包括胆汁淤积和肝硬化。
2. **先天性胆总管囊肿**：一种先天性异常，涉及胆总管的异常扩张，可能导致胆汁淤积和肝脏损害。
3. **肝脏纤维化**：一种肝脏疾病，涉及肝脏组织的纤维化，可能导致胆汁淤积和肝硬化。
4. **原发性胆汁性肝硬化**：一种慢性肝脏疾病，涉及胆汁淤积和肝脏纤维化，可能导致肝硬化。
5. **肝脏先天性缺陷**：一种先天性疾病，涉及肝脏结构或功能的异常，可能导致胆汁淤积和肝脏损害。

这些疾病都可能表现为胆汁淤积和肝硬化，但具体的临床表现和诊断标准可能有所不同。

### **69. User**

**1. 急性新生儿型** 起病急骤，病情进展迅速，可威胁生命。出生后 1~2 周内表现为喂养困难、呕吐、肌无力、肌张力减退，嗜睡或加重进展至昏迷。异戊酸蓄积可造成“汗脚”的特征性气味。查体可有肝大。化验提示代谢性酸中毒、高氨血症、低或高血糖、酮症、低钙血症及全血细胞减少。如果患者能度过新生儿期的急性发作，将会进展为慢性间歇型。

**2. 慢性间歇型** 起病隐匿。仅表现为非特异性不能耐受空腹或发育落后。常因上呼道感染或摄入高蛋白质饮食诱发，发作呈反复发生呕吐、嗜睡、昏迷、酸中毒伴酮尿， 会出现“汗脚气味”，限制蛋白质饮食并输注葡萄糖时可以缓解发作。绝大部分慢性间歇型患者精神运动发育正常，但是一些患者会表现为发育延迟和不同程度的智力低下。

### Llama3.1:70b

根据您提供的疾病临床表现，以下是可能的罕见病诊断：

1. **异戊酸血症（Isovaleric Acidemia）**：这是一种遗传性代谢疾病，表现为异戊酸在体内蓄积，导致一系列临床症状，如喂养困难、呕吐、肌无力、嗜睡、昏迷等。新生儿型和慢性间歇型是该病的两种主要表现形式。
2. **丙酮酸脱氢酶复合体缺陷（Pyruvate Dehydrogenase Complex Deficiency）**：这是一种影响能量代谢的罕见病，表现为发育迟缓、肌肉无力、代谢性酸中毒等。
3. **丙氨酸血症（Propionic Acidemia）**：这是一种遗传性代谢疾病，表现为丙氨酸在体内蓄积，导致一系列临床症状，如喂养困难、呕吐、肌无力、嗜睡、昏迷等。
4. **甲硫氨酸血症（Methionine Malabsorption Syndrome）**：这是一种遗传性代谢疾病，表现为甲硫氨酸在体内蓄积，导致一系列临床症状，如喂养困难、呕吐、肌无力、嗜睡、昏迷等。
5. **异亮氨酸血症（Isoleucine Metabolic Disorder）**：这是一种遗传性代谢疾病，表现为异亮氨酸在体内蓄积，导致一系列临床症状，如喂养困难、呕吐、肌无力、嗜睡、昏迷等。
6. ** maple 糖尿病（Maple Syrup Urine Disease）**：这是一种遗传性代谢疾病，表现为分支链氨基酸（亮氨酸、异亮氨酸和缬氨酸）在体内蓄积，导致一系列临床症状，如喂养困难、呕吐、肌无力、嗜睡、昏迷等。

### **70. User**

1. 第二性征不发育和配子生成障碍。
2. 嗅觉障碍， 因嗅球和嗅束发育异常，导致不能识别气味，尤其不能识别芳香气味。

### Llama3.1:70b

根据您提供的临床表现，以下是可能的罕见病诊断：

1. **克莱因费尔特综合征（Klinefelter Syndrome）**：这种情况下，男性会出现第二性征不发育和配子生成障碍。克莱因费尔特综合征是一种性染色体异常，通常表现为47,XXY的核型，导致睾丸功能不全和不孕不育。
2. **克萨格伦综合征（Kallmann Syndrome）**：这种情况下，嗅觉障碍是由于嗅球和嗅束发育异常引起的。克萨格伦综合征是一种遗传性疾病，特征是嗅觉丧失和性腺功能减退，常见于青春期延迟或不发育。

其他可能的诊断包括：

1. **特纳综合征（Turner Syndrome）**：女性性染色体异常，表现为45,X的核型，导致性腺功能不全和第二性征不发育。
2. **普瑞德威尔综合征（Prader-Willi Syndrome）**：一种遗传性疾病，特征是食欲不振、性腺功能减退和认知障碍。
3. **安德森-法比综合征（Andersen-Fabry Disease）**：一种遗传性疾病，特征是α-加拉氏酶缺乏，导致脂质代谢障碍和多器官损害。
4. **威廉综合征（Williams Syndrome）**：一种遗传性疾病，特征是面部异常、心血管疾病和认知障碍。
5. **马凡综合征（Marfan Syndrome）**：一种遗传性疾病，特征是结缔组织异常，导致骨骼、眼和心血管系统的异常。

### **71. User**

病情从轻至重差异很大，因此容易被误诊和漏诊。临床表现主要包括：

**1. 发热** 热型不规则，可呈周期性或者持续性高热。下丘脑受累患者可以出现中枢性体温调节异常。

**2. 皮疹** 主要分布于躯干、头皮和发际。初起为淡红色丘疹，然后可以为出血性或湿疹样皮脂溢出样皮疹，继而结痂，结痂脱落后留有白斑。

**3. 口腔及眼耳鼻喉病变** 牙龈肿胀、牙齿松动，突眼，顽固性中耳炎伴外耳道皮疹为典型表现。

**4. 中枢性尿崩症** 垂体、下丘脑甚至其他部位中枢神经系统病变，少数患者出现神经精神症状。

**5. 呼吸道症状** 咳嗽，重者憋喘、发绀，甚至反复气胸发作。肺部体征通常不明显。

**6.** 肝脾可以肿大，可以出现黄疸、肝功能异常、肝功能衰竭和门脉高压，最终患者可死于肝硬化和肝功能衰竭。

**7. 消化道症状** 消化道累及较少见，可以表现为息肉或腺瘤样改变，通常不出现临床症状，病变弥漫时可能出现腹痛、腹泻和低蛋白血症等。

**8. 造血系统** 淋巴结可见肿大，骨髓受累时可以出现贫血、白细胞和血小板计数异常。

**9. 骨质损害** 颅骨、四肢骨、脊柱、骨盆等可有疼痛及肿块，可以发生病理性骨折。眼眶骨质病变所致突眼为儿童患者典型表现之一。

### Llama3.1:70b

根据您提供的临床表现，可能的罕见病诊断包括：

1. **Langerhans 细胞组织增生症（LCH）**：该病症涉及多个系统，包括皮肤、骨骼、肺部、肝脏、淋巴系统和中枢神经系统等。其临床表现与您描述的症状高度吻合，包括皮疹、骨质损害、肺部症状、肝脾肿大和中枢神经系统受累等。
2. **多器官Langerhans细胞增生症**：这是一种罕见的疾病，涉及多个器官系统，包括皮肤、骨骼、肺部、肝脏、淋巴系统和中枢神经系统等。其临床表现与LCH类似。
3. **Sjögren 综合征**：这是一种自身免疫性疾病，主要影响唾液腺和泪腺，但也可以影响其他器官系统。其临床表现包括口腔和眼部症状、皮疹和肺部症状等。
4. **系统性红斑狼疮（SLE）**：这是一种自身免疫性疾病，主要影响皮肤、关节、肾脏和中枢神经系统等。其临床表现包括皮疹、关节炎、肾炎和中枢神经系统受累等。
5. **结节病**：这是一种慢性炎症性疾病，主要影响皮肤、肺部、肝脏和淋巴系统等。其临床表现包括皮疹、肺部症状、肝脾肿大和淋巴结肿大等。
6. **成人Still病**：这是一种自身免疫性疾病，主要影响关节、皮肤和中枢神经系统等。其临床表现包括关节炎、皮疹和中枢神经系统受累等。

### **72. User**

**1. 生长障碍及其他畸形，**患儿出生时体重大多在正常范围内，但部分患儿的出生身长低于 48cm（42~47cm）。出生后逐渐显著地生长速度减慢，未治疗的男性患者终身高在 116_142cm，女性患者终身高在108_136cm。同时患者手、足和内脏器官均小。部分患者伴有其他的先天性畸形，如髋关节脱位、主动脉瓣狭窄、隐睾、先天性白内障等。

**2. 面容特点，**患者特征性的面容改变为前额突出、鞍鼻、小下颌，头部与身体比例较正常儿童大，但与同龄儿童相比头围绝对值仍偏小。患者头发稀疏，部分患者可出现“落日征”，地中海地区和中东地区的患者可表现出蓝巩膜。患者出牙延迟，乳牙脱落较晚并且易合并多发龋齿，由于下颌骨较短小，可有牙列不齐。

**3. 骨骼肌肉系统** 部分患者具有先天性髋关节脱位、股骨头缺血性坏死等表现。由于中面部骨骼发育不良和咽部解剖结构异常，患者说话音调高尖。85％患者肘关节活动受限。

**4. 代谢紊乱 ，**患者常合并代谢异常，包括新生儿期和婴儿期的低血糖，而成年后出现逐渐加重的肥胖。

**5. 性发育延迟 ，**患者生殖器较小，男性患者表现为小阴茎和小睾丸，女性患者超声检查发现卵巢体积较小。大多数患者表现为青春发育延迟。虽然性发育落后，但是男性和女性患者的性功能最终可发育成熟，具有生育能力。

**6. 肿瘤发生率低，**患者的肿瘤发生的风险较一般人群显著降低。

### Llama3.1:70b

根据您提供的临床表现，可能的罕见病诊断是：

1. **普瑞德-威利综合征（Prader-Willi综合征）**：该病症的典型特征包括生长迟缓、身材矮小、面部特征（如前额突出、鞍鼻、小下颌）、肌肉松弛、性发育延迟、肥胖、低肌肉张力和智力障碍等。
2. **多发性内分泌腺体发育不全综合征（Multiple Endocrine Neoplasia, MEN）**：该病症涉及多个内分泌腺体的异常发育，可能导致生长迟缓、性发育延迟、肥胖和其他内分泌问题。
3. **Turner综合征**：该病症主要影响女性，特征包括身材矮小、性发育延迟、心脏问题和其他身体异常。
4. **Klinefelter综合征**：该病症主要影响男性，特征包括身材高大、性发育延迟、不育和其他身体异常。
5. **Silver-Russell综合征**：该病症的特征包括生长迟缓、身材矮小、面部特征（如三角形脸、前额突出）和其他身体异常。
6. **Noonan综合征**：该病症的特征包括生长迟缓、身材矮小、面部特征（如眼睑下垂、低耳位）和其他身体异常。

### **73. User**

主要症状为双眼先后发生的无痛性视力突然急剧下降，双眼发作间隔一般为数周到数月。眼底检查在疾病早期表现为视盘充血水肿、视盘旁毛细血管迂曲扩张，疾病后期视盘水肿和毛细血管扩张消退，最终视盘颞侧或全部呈萎缩性改变。多数病人只存在眼部表现，少数可合并全身其他系统症状如智力障碍、癫痫、听力障碍、肌张力障碍等。

### Llama3.1:70b

根据您提供的临床表现，主要诊断可能是：

1. **视神经脊髓炎（Neuromyelitis Optica，NMO）**：这是一种罕见的自身免疫性疾病，主要影响视神经和脊髓。患者通常会出现双眼视力突然下降，伴有视盘水肿和视神经炎症。疾病活动期后，视盘水肿和炎症可能会消退，留下视神经萎缩的迹象。
2. **多发性硬化（Multiple Sclerosis，MS）**：这是一种中枢神经系统的慢性自身免疫性疾病，可能影响视神经，导致视力下降和视盘水肿。然而，MS通常伴有其他神经系统症状，如肌肉无力、平衡问题和认知障碍。
3. **视神经炎（Optic Neuritis）**：这是一种视神经的炎症性疾病，可能导致视力下降和视盘水肿。然而，视神经炎通常是单眼发作，且不一定会导致视神经萎缩。
4. **Leber遗传性视神经病变（Leber Hereditary Optic Neuropathy，LHON）**：这是一种遗传性视神经疾病，主要影响年轻男性，导致视力下降和视神经萎缩。然而，LHON通常不伴有视盘水肿和炎症。
5. **视神经脊髓炎样病变（Neuromyelitis Optica Spectrum Disorder，NMOSD）**：这是一种与NMO相似的疾病，主要影响视神经和脊髓。患者可能会出现视力下降和视盘水肿，伴有其他神经系统症状。

### **74. User**

患者临床表现为婴儿期低酮性低血糖、肝功能异常、乳酸酸中毒、心肌病，部分患者表现为生长迟缓，肌张力低下或心肌病，少数可有胆汁淤积或肝硬化。幼儿期患分患者表现为生长迟缓，肌张力低下或心肌病，少数可有胆汁淤积或肝硬化。幼儿期患者可有喂养困难，发作性代谢紊乱，发作性横纹肌溶解。多数患者儿童期会有视网膜病变，周围神经病是一种较少见的长期病并发症。

### Llama3.1:70b

根据您提供的临床表现，可能的罕见病诊断包括：

1. **多系统脂肪酸氧化缺陷病（MADD）**：该病症表现为婴儿期低酮性低血糖、肝功能异常、乳酸酸中毒、心肌病等，部分患者还会有生长迟缓、肌张力低下或心肌病等症状。
2. ** VLCAD缺陷病**：该病症表现为婴儿期低酮性低血糖、肝功能异常、乳酸酸中毒、心肌病等，部分患者还会有生长迟缓、肌张力低下或心肌病等症状。
3. **CPTII缺陷病**：该病症表现为婴儿期低酮性低血糖、肝功能异常、乳酸酸中托、心肌病等，部分患者还会有生长迟缓、肌张力低下或心肌病等症状。
4. **MCAD缺陷病**：该病症表现为婴儿期低酮性低血糖、肝功能异常、乳酸酸中毒、心肌病等，部分患者还会有生长迟缓、肌张力低下或心肌病等症状。
5. **LCHAD缺陷病**：该病症表现为婴儿期低酮性低血糖、肝功能异常、乳酸酸中毒、心肌病等，部分患者还会有生长迟缓、肌张力低下或心肌病等症状。
6. **TFP缺陷病**：该病症表现为婴儿期低酮性低血糖、肝功能异常、乳酸酸中毒、心肌病等，部分患者还会有生长迟缓、肌张力低下或心肌病等症状。

### **75. User**

主要发生在成年育龄女性，平均诊断年龄在 40 岁左右。早期症状轻微，部分患者在查体时被发现，或因为呼吸症状或其他原因检查肺部 HRCT 时发现。主要表现为不同程度的呼吸困难，随疾病进展，呼吸困难症状也逐渐加重，肺功能进行性恶化，晚期出现呼吸衰竭。在病程中可反复出现气胸、乳糜胸等并发症。肺外表现包括肾， 腹膜后实性或囊实性淋巴管肌瘤。部分患者具有其他多系统受累临床特征，主要包括神经系统改变(癫痫、神经发育迟缓和自闭症等) 和皮肤改变(色素脱色斑、面部血管纤维瘤、皮肤鲨革斑和甲周纤维瘤等) 等临床表现。

### Llama3.1:70b

根据您提供的临床表现，主要诊断可能是淋巴管肌瘤病（Lymphangioleiomyomatosis，LAM）。此外，其他五种可能的诊断包括：

1. **淋巴结增生症**：淋巴结增生症是一种淋巴系统疾病，表现为淋巴结肿大，可能导致呼吸困难、肺部感染等症状。
2. **淋巴管炎**：淋巴管炎是一种淋巴管壁的炎症，可能导致淋巴液积聚，引起呼吸困难、肺部感染等症状。
3. **淋巴瘤**：淋巴瘤是一种恶性肿瘤，起源于淋巴系统，可能导致淋巴结肿大、呼吸困难、肺部感染等症状。
4. **肺淋巴管扩张症**：肺淋巴管扩张症是一种罕见的肺部疾病，表现为肺淋巴管扩张，导致呼吸困难、肺部感染等症状。
5. **多发性淋巴管肌瘤病**：多发性淋巴管肌瘤病是一种罕见的疾病，表现为多个淋巴管肌瘤的生长，可能导致呼吸困难、肺部感染等症状。

### **76. User**

婴儿的典型表现是在断奶后出现反复呕吐和腹泻，拒食富含蛋白质的食物。患者可出现皮肤苍白、肌张力减低、肝脾大、头发稀疏、骨质疏松伴病理性骨折。进食富含蛋白质的膳食后患者可出现嗜睡、共济失调、癫痫发作和高氨血症昏迷等。随着病程的发展，可出现多系统受累表现。肺部受累可表现为进行性肺间质病变，肺泡蛋白沉积症；肾脏最常见的特征性改变是近端肾小管功能障碍和肾钙质沉积，也可出现肾小球病变，肾脏淀粉样变，甚至出现肾功能衰竭；血液系统受累表现为正色素或低色素性贫血，白细胞减少，血小板减少等；消化系统受累的表现为高胆固醇血症，高甘油三酯血症，反复发作的胰腺炎，甚至肝脏淀粉样变。癫痫多发生于成年患者，可能与反复发生高氨血症导致大脑皮质受损有关。

### Llama3.1:70b

根据您提供的临床表现，我最有可能诊断出的是**氨基酸代谢异常**，特别是**尿素循环障碍**（Urea Cycle Disorder，UCD）。这种疾病是由于体内某些关键酶的缺陷导致尿素循环受阻，氨基酸不能正常代谢，从而导致氨和其他有毒物质在体内积累，引起一系列临床症状。

其他五种可能的诊断包括：

1. ** maple 糖尿病**（Maple Syrup Urine Disease，MSUD）：一种因体内某些酶缺陷导致分支链氨基酸（亮氨酸、异亮氨酸和缬氨酸）不能正常代谢的疾病。
2. **苯丙酮尿症**（Phenylketonuria，PKU）：一种因体内苯丙氨酸羟化酶缺陷导致苯丙氨酸不能正常代谢的疾病。
3. **丙酮酸脱氢酶复合体缺陷**（Pyruvate Dehydrogenase Complex Deficiency，PDHCD）：一种因体内丙酮酸脱氢酶复合体缺陷导致丙酮酸不能正常代谢的疾病。
4. **脂肪酸氧化异常**（Fatty Acid Oxidation Disorder，FAOD）：一种因体内脂肪酸氧化酶缺陷导致脂肪酸不能正常代谢的疾病。
5. **有机酸代谢异常**（Organic Acidemia，OA）：一种因体内有机酸代谢酶缺陷导致有机酸不能正常代谢的疾病。

### **77. User**

新生儿期起病，通常在生后 1 周内起病。以反复呕吐、腹泻、腹胀等胃肠道症状为首发症状，伴肝脾大和生长发育障碍；神经系统受累表现为活动少、腱反射亢进、踝阵挛和角弓反张。巨噬细胞内胆固醇酯和三酰甘油贮积导致肝脾肿大，进行性加重甚至肝衰竭，数月内出现转氨酶、胆红素升高，低蛋白血症，凝血异常等。肾上腺增大伴钙化导致肾上腺皮质功能不全，60％ ~70％通过影像学检查可发现肾上腺钙化。也有胆囊功能障碍、脑卒中报道。随着病情进展，可出现多器官功能障碍，包括贫血、消化道出血、败血症、昏迷等。

### Llama3.1:70b

根据您提供的临床表现，可能的罕见病诊断包括：

1. **尼曼-皮克病（Niemann-Pick病）**：这是最可能的诊断。尼曼-皮克病是一种因 sphingomyelinase 酶缺乏引起的脂质贮积病，导致 sphingomyelin 在细胞内积累，影响多个器官，包括肝脏、脾脏、肾上腺和神经系统。
2. **Gaucher病**：Gaucher病是一种因 glucocerebrosidase 酶缺乏引起的脂质贮积病，导致 glucocerebroside 在细胞内积累，影响多个器官，包括肝脏、脾脏、骨骼和神经系统。
3. **Fabry病**：Fabry病是一种因α-Gal A 酶缺乏引起的脂质贮积病，导致 globotriaosylceramide 在细胞内积累，影响多个器官，包括肾脏、心脏和神经系统。
4. **Tay-Sachs病**：Tay-Sachs病是一种因 hexosaminidase A 酶缺乏引起的脂质贮积病，导致 GM2 神经节苷脂在细胞内积累，影响神经系统。
5. **Farber病**：Farber病是一种因 acid ceramidase 酶缺乏引起的脂质贮积病，导致 ceramide 在细胞内积累，影响多个器官，包括关节、神经系统和其他组织。
6. **Wolman病**：Wolman病是一种因 lysosomal acid lipase 酶缺乏引起的脂质贮积病，导致胆固醇酯和三酰甘油在细胞内积累，影响多个器官，包括肝脏、脾脏和肾上腺。

### **78. User**

**1. 心血管系统** ，可以导致主动脉根部扩张、主动脉瓣关闭不全和主动脉夹层；也可能会有二尖瓣脱垂伴或不伴关闭不全。升主动脉扩张患病率随着患者年龄的增长而增加。从 30 岁至 60 岁，患病率从 53％上升至 96％。患者可以表现为气促、胸痛、心悸、头晕、晕厥或者咯血。查体表现为心界向左下扩大，听诊在二尖瓣区可以闻及收缩期杂音，在主动脉瓣听诊区可闻及舒张期为主的双期杂音，可有周围血管征以及心力衰竭的体征。

**2. 骨骼表现** ，瘦高身材、手指和脚趾细长、两臂平伸的距离超过身高、脊柱侧凸或后凸、漏斗胸或者鸡胸、关节松弛、扁平足等。

**3. 眼部异常** ，眼部病变占 50％ ~70％，包括角膜扁平、眼球轴延长、视网膜剥离、白内障、晶状体脱位或半脱位、虹膜震颤或轻度震颤、瞳孔移位、晶状体混浊等。

**4. 其他 ，**硬脊膜膨出、萎缩纹、复发性疝或切口疝、高腭穹等。

### Llama3.1:70b

根据您提供的临床表现，医生最有可能诊断出的罕见病是**马凡综合征**（Marfan Syndrome）。马凡综合征是一种遗传性疾病，影响身体的多个系统，包括心血管、骨骼、眼部和其他系统。其特征性表现包括主动脉根部扩张、主动脉瓣关闭不全、主动脉夹层、二尖瓣脱垂、骨骼异常（如瘦高身材、手指和脚趾细长、脊柱侧凸或后凸等）、眼部异常（如晶状体脱位、视网膜剥离等）以及其他特征性体征。

其他五种可能的诊断包括：

1. **爱德华综合征**（Edwards Syndrome）：一种遗传性疾病，表现为多器官异常，包括心脏、骨骼、眼部和其他系统的异常。
2. **特纳综合征**（Turner Syndrome）：一种性染色体异常，表现为身材矮小、性腺功能减退、心脏和骨骼异常等。
3. **克莱因费尔特综合征**（Klinefelter Syndrome）：一种性染色体异常，表现为男性性腺功能减退、身材高大、骨骼异常等。
4. **奥斯汀综合征**（Austin Syndrome）：一种罕见的遗传性疾病，表现为骨骼、眼部和其他系统的异常。
5. **埃勒斯-丹洛斯综合征**（Ehlers-Danlos Syndrome）：一种遗传性疾病，表现为皮肤、骨骼和血管的异常。

### **79. User**

**1. 皮肤表现** ，患者一般出生时即有牛奶咖啡斑，常位于骨骼病变同侧且以中线为界，为一处或多处点片状大小不等的褐色或黄棕色斑片状色素沉着，边界不规则。

**2. 骨骼系统表现** ，大约 98％的 患者有单骨型或多骨型骨纤维异样增殖症，以多骨型更为常见。其特征为畸变的骨母细胞增多并异常分化，引起骨髓内纤维细胞的广泛增殖、分化，形成幼稚编织骨，易发生病理性骨折和骨骼畸形，并引发骨痛，几乎可累及全身骨骼，尤以颅面骨和长骨受累多见，局部骨骼膨胀、隆起，引起面部结构改变和压迫症状。眼眶受累严重可压迫视神经，造成视力障碍。听神经受累可致听力下降。脊柱受累可致椎体压缩性骨折，引起脊柱侧弯或后凸畸形。骨纤维异样增殖症导致病理性骨折的高峰年龄多在 6~10 岁。影像学示骨骼呈膨胀性、溶骨性改变或磨玻璃样改变。

**3. 内分泌腺体受累表现** ，可累及多种内分泌腺，导致受累腺体功能亢进或发生肿瘤，血清促分泌激素的水平正常或降低。其中性早熟较多见，男女均可发病，但女性患者多见，且常早于骨病变。女性患儿可表现为第二性征早发育、过早阴道不规则出血、子宫及卵巢较正常同龄儿童增大，或发生卵巢囊肿，骨骺提前愈合等。男性患儿性早熟相对少见，超声见睾丸病变(Leydig 细胞增生、微小结石、局灶钙化等)。甲状腺肿大及功能亢进也较常见，也有报道发生甲状旁腺功能亢进者。其他内分泌疾病也有报道，如皮质醇增多症、巨人症 / 肢端肥大症、高泌乳素血症等，但相对较罕见。

**4. 低磷血症** ，部分患者合并低磷血症，表现为低血磷性佝偻病。合并低磷血症者通常为多骨型骨纤维异常增殖症。

**5. 其他，**患者还可因突变累及外周血白细胞、肝脏、心脏、胸腺和胃肠道，而出现相应的罕见临床症状，如黄疸、肝炎、心律失常、肠息肉等。

### Llama3.1:70b

根据您提供的临床表现，医生最有可能诊断出的罕见病是：**McCune-Albright 综合征**。McCune-Albright 综合征是一种罕见的遗传性疾病，主要特征包括骨纤维异常增殖症、内分泌功能亢进（如性早熟、甲状腺功能亢进等）和特征性的皮肤色素沉着（牛奶咖啡斑）。此综合征的确切原因尚不完全清楚，但与G蛋白编码基因GNAS的突变有关。其他可能的诊断包括：

1. **多骨纤维异常增殖症（Polyostotic Fibrous Dysplasia）**：虽然这是一种单独的疾病，但它与McCune-Albright 综合征共享许多相同的骨骼特征。
2. **Jaffe-Lichtenstein 综合征**：这是一种罕见的骨骼疾病，主要特征是多骨纤维异常增殖症，但通常不伴有内分泌功能亢进或皮肤色素沉着。
3. **Ollier 病**：这是一种罕见的骨骼疾病，主要特征是多个enchondromas（骨内软骨瘤），但通常不伴有内分泌功能亢进或皮肤色素沉着。
4. **Maffucci 综合征**：这是一种罕见的骨骼疾病，主要特征是多个enchondromas和软组织血管瘤，但通常不伴有内分泌功能亢进或皮肤色素沉着。
5. **Camurati-Engelmann 病**：这是一种罕见的骨骼疾病，主要特征是多骨纤维异常增殖症和骨密度增加，但通常不伴有内分泌功能亢进或皮肤色素沉着。

### **80. User**

患者大多在出生后 3 个月至 3 岁之间发病，少部分在新生儿期或成人期发病， 也有患者无症状。患者发病通常有诱发因素，如多见于长时间饥饿或并发感染性疾病等。早期发病的患儿，其首发症状以嗜睡和呕吐常见，也可表现为抽搐、窒息等，常迅速进展为昏迷或死亡。急性发病期常有典型的低血糖表现，血糖降低伴尿酮体阴性有助于诊断。50％的患儿伴有肝脏肿大，肝活检可见大量脂质堆积。影像学检查发现部分患儿发生脑水肿。心脏损害表现较为罕见。成人期发病的患者临床表现多样，可有多器官受损，包括肌肉、肝脏、神经系统或心血管系统等。其中呕吐是最常见的症状，肝脏肿大相对少见，妊娠期出现症状的患者表现为急性脂肪肝。多数患者有急性非炎症性脑病。约 1/3 的患者出现横纹肌溶解，肌酸激酶显著增高，并伴有肌红蛋白尿。患者病死率较高，发病的患儿中约25％死亡，成人期急性发病的患者病死率更高，可达到 50％。疾病发作间歇期患者往往表现正常。约 1/3 的急性发病后存活的患者出现后遗症，包括生长发育迟滞、运动发育迟缓、智力障碍、语言发育缺陷、心理行为问题、癫痫、脑瘫、偏瘫、慢性肌无力等。另外，部分患者有疲劳、肌痛和运动耐力减退等骨骼肌损害的主诉症状。

### Llama3.1:70b

根据您提供的临床表现，我最有可能诊断出的罕见病是：

1. **MCAD缺陷（中链脂酰CoA脱氢酶缺陷）**：MCAD缺陷是一种常见的脂肪酸氧化疾病，表现为低血糖、肝脏肿大、脂质堆积、脑水肿等，与您提供的临床表现高度吻合。
2. ** VLCAD缺陷（长链脂酰CoA脱氢酶缺陷）**：VLCAD缺陷也是一种脂肪酸氧化疾病，表现为低血糖、肝脏肿大、脂质堆积、心脏损害等，与MCAD缺陷类似。
3. **LCHAD缺陷（长链3-羟脂酰CoA脱氢酶缺陷）**：LCHAD缺陷是一种较为罕见的脂肪酸氧化疾病，表现为低血糖、肝脏肿大、脂质堆积、心脏损害等，与MCAD缺陷和VLCAD缺陷类似。
4. **CPT1缺陷（ carnitine 羟基转移酶1缺陷）**：CPT1缺陷是一种脂肪酸氧化疾病，表现为低血糖、肝脏肿大、脂质堆积、心脏损害等，与MCAD缺陷和VLCAD缺陷类似。
5. **CPT2缺陷（ carnitine 羟基转移酶2缺陷）**：CPT2缺陷是一种脂肪酸氧化疾病，表现为低血糖、肝脏肿大、脂质堆积、心脏损害等，与MCAD缺陷和VLCAD缺陷类似。
6. **HMG-CoA合成酶缺陷**：HMG-CoA合成酶缺陷是一种酮症疾病，表现为低血糖、肝脏肿大、脂质堆积、脑水肿等，与MCAD缺陷类似。

### **81. User**

疾病在各年龄段中的临床表现不尽相同。通常发病年龄越早，急性代谢紊乱和脑病表现越严重。临床表现主要包括消化系统症状如呕吐、喂养困难、肝大等以及神经系统症状如运动障碍、意识障碍、抽搐、发育迟缓或倒退、小头畸形等。病情反复多与感染和应激相关。

新生儿期发病者多在生后数小时至 1 周内出现急性脑病样症状，表现为呕吐、肌张力低下、脱水、严重酸中毒、高乳酸血症、高氨血症、昏迷和惊厥，病死率高。儿童期发病的出生时正常，多在 1 岁以内发病，首次代谢危象的诱因常为感染、饥饿、疲劳、疫苗注射等应激因素刺激或高蛋白饮食和药物，如果不及时诊治，可导致智力发育和运动发育迟缓、落后和倒退，可伴发血液系统、肝脏、肾脏、皮肤和周围神经受累。成人患者首发症状可为周围神经病变和精神心理异常等。

### Llama3.1:70b

根据您提供的临床表现，医生可能会诊断出以下罕见病：

1. **丙酮酸脱氢酶复合体缺陷症（PDHC缺陷症）**：这是一种因PDHC基因突变导致的罕见遗传病，主要影响能量代谢，导致神经系统和肌肉系统受损。
2. **丙氨酸血症（MSUD）**：这是一种因BCAT2基因突变导致的罕见遗传病，主要影响氨基酸代谢，导致神经系统和肌肉系统受损。
3. **异柠檬酸血症（OA）**：这是一种因OAT基因突变导致的罕见遗传病，主要影响氨基酸代谢，导致神经系统和肌肉系统受损。
4. **丙酮酸脱氢酶E1α亚基缺陷症（PDHAE1α缺陷症）**：这是一种因PDHA1基因突变导致的罕见遗传病，主要影响能量代谢，导致神经系统和肌肉系统受损。
5. **丙酮酸脱氢酶磷酸化酶缺陷症（PDP1缺陷症）**：这是一种因PDP1基因突变导致的罕见遗传病，主要影响能量代谢，导致神经系统和肌肉系统受损。
6. **丙酮酸脱氢酶磷酸化酶调控亚基缺陷症（PDP1C缺陷症）**：这是一种因PDP1C基因突变导致的罕见遗传病，主要影响能量代谢，导致神经系统和肌肉系统受损。

### **82. User**

典型患者出生时正常。于 6 个月 ~1 岁面容逐渐变丑，面中部变扁、鼻梁增宽、角膜混浊、耳聋、头大、前后径长，前额突出，关节僵硬、爪形手、鸡胸和腰椎后突等。常于 2~5 岁出现心脏瓣膜增厚、心肌病或充血性心力衰竭。5 岁出现肝脾大。精神运动发育落后。一般存活至 10 岁左右。多死于心衰和肺炎。轻型患者 5 岁以后出现症状，轻度面容粗陋、角膜混浊和手足关节僵硬。智力正常，寿命相对较长。临床症状在各亚型之间有互相重叠，但生化检查结果在各型之间无显著差异。

### Llama3.1:70b

根据您提供的临床表现，我最有可能诊断出的是**亨特综合征（Mucopolysaccharidosis II，MPS II）**。亨特综合征是一种罕见的遗传性疾病，由于缺乏一种叫做 iduronate-2-sulfatase 的酶，导致体内积累了过多的糖多聚体（glycosaminoglycans，GAGs），从而影响身体各个系统的功能。

其他五种可能的诊断包括：

1. **赫勒综合征（Mucopolysaccharidosis I，MPS I）**：同样是一种糖多聚体代谢紊乱的疾病，导致面容粗陋、视力障碍、听力丧失和智力低下等症状。
2. **桑菲普综合征（Mucopolysaccharidosis III，MPS III）**：主要影响大脑和中枢神经系统，导致智力低下、言语困难和行为异常等症状。
3. **莫尔夸综合征（Mucopolysaccharidosis IV，MPS IV）**：主要影响骨骼和关节，导致矮小、关节僵硬和脊柱侧弯等症状。
4. **马洛特综合征（Mucopolysaccharidosis VI，MPS VI）**：主要影响骨骼、关节和视力，导致矮小、关节僵硬和白内障等症状。
5. **西综合征（Mucopolysaccharidosis VII，MPS VII）**：主要影响骨骼、关节和中枢神经系统，导致矮小、关节僵硬和智力低下等症状。

需要注意的是，以上诊断均需要通过遗传学检查和生化检测来确认。

### **83. User**

隐袭起病。缓慢发展或阶段性进展，可有长时间的稳定期。任何年龄均可发病，多于50 岁前起病。临床主要表现为多发性单神经病，不对称性上肢远端起病较常见，早期以单侧上肢某一根或多根神经受累多见，表现为相应神经支配区域的肌肉无力， 远端为主，可伴有痉挛或束颤。随着病情发展，可以出现肌肉萎缩；病程较长者，可有多个肢体的多根神经受累，受累神经的不对称性可不明显，而呈现为类似多发性周围神经病的分布。患者可有轻微感觉异常的主诉，但缺乏客观感觉受累的体征，病程后期部分患者也可出现部分感觉神经受累。脑神经通常不受累。无上运动神经元受累体征。

### Llama3.1:70b

根据您提供的临床表现，以下是可能的罕见病诊断：

1. **慢性炎症性脱髓鞘性多发性神经病（CIDP）**：CIDP是一种自身免疫性疾病，表现为多个神经的脱髓鞘和炎症，导致神经传导速度减慢和肌肉无力。患者可能会出现多个肢体的无力、萎缩和感觉异常。
2. **多发性神经炎（MND）**：MND是一组影响运动神经元的神经退行性疾病，包括ALS（amyotrophic lateral sclerosis）、PBP（progressive bulbar palsy）和PMA（progressive muscular atrophy）。患者可能会出现肌肉无力、萎缩和痉挛。
3. **慢性轴突病变（CCD）**：CCD是一种影响周围神经轴突的疾病，表现为轴突退行性变和神经传导速度减慢。患者可能会出现多个肢体的无力、萎缩和感觉异常。
4. **多系统萎缩（MSA）**：MSA是一种影响中枢神经系统和周围神经系统的神经退行性疾病，表现为运动功能障碍、感觉异常和自主神经功能障碍。患者可能会出现肌肉无力、萎缩和感觉异常。
5. **遗传性运动和感觉神经病（HMSN）**：HMSN是一组遗传性疾病，影响周围神经的发展和维持，表现为运动和感觉功能障碍。患者可能会出现多个肢体的无力、萎缩和感觉异常。
6. **慢性炎症性周围神经病（CIPD）**：CIPD是一种自身免疫性疾病，表现为周围神经的炎症和脱髓鞘，导致神经传导速度减慢和肌肉无力。患者可能会出现多个肢体的无力、萎缩和感觉异常。

### **84. User**

新生儿期起病常致命，可表现为代谢危象，包括代谢性酸中毒、非酮症性低血糖、高氨血症，其他特征包括多系统受累(肌张力低下、心肌病、肝肿大)、排泄大量脂肪酸和氨基酸衍生的代谢产物、“汗脚”味体臭、昏迷、新生儿期死亡。快速进展的肥厚型心肌病常见于新生儿期起病的类型，猝死概率非常大，即使经过新生儿筛查在早期即检测出有此病，也难以逆转。

先天畸形被认为是未被胚胎转运纠正的那部分有毒代谢物累积的后果。脂肪酸氧化代谢受阻的程度决定了子宫内的酸中毒程度，进而阻碍正常的胚胎形成。先天畸形包括增大的多囊肾、摇篮底足(rocker-bottom feet)、下腹部肌肉缺失、尿道下裂和阴茎下弯、肌张力低下、脑皮质发育不良和胶质增生。其他畸形特征包括眼距过宽、耳畸形、巨颅、前囟过宽、高前额、鼻梁低平。

晚发型的发病年龄和症状在不同病例中差异很大，主要表现为不伴有先天畸形，但终身伴有急性间歇发作的呕吐、脱水、低酮性低血糖、酸中毒及意识障碍的风险。年龄大者可有肝脏肿大或脂质沉积性肌病。急性失代偿发作可由感染、发热、手术、低能量饮食或减肥、饮酒、妊娠等诱发。大多数患者发展为慢性肌病症状，包括运动不耐受、肌痛、肌无力、肌肉萎缩。晚发型病人的有机酸尿症常为间歇性，仅在疾病期间和处于分解代谢压力下较为显著。

### Llama3.1:70b

根据您提供的临床表现，我最有可能诊断出的罕见病是**MCAD缺陷（中链脂酰CoA脱氢酶缺陷）**。MCAD缺陷是一种常见的脂肪酸氧化缺陷，表现为代谢危象、多系统受累、排泄大量脂肪酸和氨基酸衍生的代谢产物等特征。

其他五种可能的诊断包括：

1. ** VLCAD缺陷（非常长链脂酰CoA脱氢酶缺陷）**：与MCAD缺陷类似，也是一种脂肪酸氧化缺陷，表现为代谢危象、心肌病和肝脏肿大等特征。
2. **LCHAD缺陷（长链3-羟脂酰CoA脱氢酶缺陷）**：一种脂肪酸氧化缺陷，表现为代谢危象、心肌病、肝脏肿大和视网膜病变等特征。
3. **CPT2缺陷（ carnitine棕榈酰转移酶II缺陷）**：一种脂肪酸氧化缺陷，表现为代谢危象、心肌病和肌肉无力等特征。
4. **CPT1缺陷（ carnitine棕榈酰转移酶I缺陷）**：一种脂肪酸氧化缺陷，表现为代谢危象、肝脏肿大和低血糖等特征。
5. **HMG-CoA合成酶缺陷**：一种酮症相关疾病，表现为代谢危象、低血糖和酮症等特征。

### **85. User**

平均发病年龄为 30 岁，10 岁以下和 50 岁以上患者少见。女性患病率高于男性，女：男比例为（1.4~2.3） ：1。多急性 / 亚急性起病，具有空间多发和时间多发特点。空间多发指病变部位多发，大脑、脑干、小脑、脊髓可同时或相继受累。时间多发指缓解—复发的病程，由于累及部位广泛，反复发作后可最终致残，甚至致死。临床表现多样，常见症状包括视力下降、复视、肢体感觉障碍、肢体运动障碍、共济失调、膀胱或直肠功能障碍等。

### Llama3.1:70b

根据您提供的临床表现，我最有可能诊断出的是多发性硬化症（Multiple Sclerosis，MS）。多发性硬化症是一种中枢神经系统的慢性脱髓鞘疾病，主要影响大脑和脊髓。其特征是神经系统的多发性损伤，导致一系列神经系统症状。

其他五种可能的诊断包括：

1. **系统性红斑狼疮（Systemic Lupus Erythematosus，SLE）**：一种自身免疫性疾病，可能影响多个器官系统，包括神经系统，表现为多种多样的症状。
2. **神经系统Behcet病（Neuro-Behcet’s Disease）**：一种罕见的自身免疫性疾病，主要影响血管，可能导致神经系统症状，如视力下降、肢体感觉障碍等。
3. **脑脊髓炎（Encephalomyelitis）**：一种中枢神经系统的炎症性疾病，可能导致多种神经系统症状，如视力下降、肢体感觉障碍等。
4. **脑脊髓血管畸形（Arteriovenous Malformation，AVM）**：一种血管畸形，可能导致神经系统症状，如视力下降、肢体感觉障碍等。
5. **脑脊髓肿瘤（Brain and Spinal Cord Tumors）**：各种类型的肿瘤可能影响大脑和脊髓，导致多种神经系统症状。

### **86. User**

主要包括帕金森症、小脑性共济失调、锥体束损害以及自主神经系统损害。首发症状常为自主神经损害，最主要的是心血管功能障碍(如直立性低血压) 和排尿障碍(如尿失禁)，后者在男性患者还容易合并性功能障碍。很多患者病前有长达数年的快速眼动期睡眠行为障碍(RBD)。

### Llama3.1:70b

根据您提供的临床表现，主要诊断可能是多系统萎缩（MSA）。多系统萎缩是一种罕见的神经退行性疾病，其特征是自主神经系统、运动系统和其他神经系统的逐渐损害。其主要特征包括帕金森症状、小脑共济失调、锥体外系损害以及自主神经功能障碍，如直立性低血压和尿失禁等。

其他可能的诊断包括：

1. **帕金森病（PD）**：虽然帕金森病也会引起运动症状，如静止性震颤、运动迟缓和刚性，但自主神经系统的损害在帕金森病中并不像在MSA中那样普遍。
2. **进行性核上性麻痹（PSP）**：这是一种罕见的神经退行性疾病，主要影响运动系统，尤其是眼球运动和吞咽功能。虽然PSP也可能引起一些自主神经功能障碍，但其主要特征是眼球运动异常和吞咽困难。
3. **路易体病（DLB）**：这是一种神经退行性疾病，主要影响认知功能，导致痴呆。虽然DLB也可能引起一些运动症状和自主神经功能障碍，但其主要特征是认知功能衰退和视幻觉。
4. **遗传性自主神经功能障碍（HSAN）**：这是一组罕见的遗传性疾病，主要影响自主神经系统，导致自主神经功能障碍，如直立性低血压和尿失禁等。虽然HSAN也可能引起一些运动症状，但其主要特征是自主神经功能障碍。
5. **脊髓小脑共济失调（SCA）**：这是一组罕见的遗传性疾病，主要影响小脑和脊髓，导致共济失调和运动功能障碍。虽然SCA也可能引起一些自主神经功能障碍，但其主要特征是共济失调和运动功能障碍。

### **87. User**

**1. 肌肉，** 四肢远端起始并逐渐向近端进展的无力萎缩，影响手部精细动作，造成垂足。面肌亦可受累。同时有明显肌强直现象，累及肢体肌、咽喉肌等。查体可发现大力握拳后手指不能立即展开，用力闭目后不能立即睁开。叩击手部大鱼际肌、舌肌等，可引出肌强直，称为叩击性肌强直或“肌球”现象。肌强直存在“热身”现象，反复活动后，肌强直减轻。下肢肌痛和全身疲乏感也是常见临床表现。

**2. 心脏，** 心脏传导系统常有受累，出现不同程度的房室传导阻滞和室内传导异常。严重时可造成猝死。确诊患者需定期监测心电图，必要时植入起搏器。

**3. 中枢神经系统** ，睡眠增多、阻塞性睡眠呼吸暂停等睡眠障碍多见。可伴有认知及情绪障碍。头 MRI 可见弥漫性脑白质病变及皮层萎缩。

**4. 眼** ，随着年龄增长，几乎所有患者均会出现白内障。常在 30~40 岁影响视力。

**5. 内分泌系统** ，可出现多种内分泌疾病，包括糖尿病、甲状腺功能异常、钙磷代谢异常、性激素异常、睾丸萎缩(造成不育) 等。

此外，还可有额秃、胃肠动力不足、限制性通气障碍等。

### Llama3.1:70b

根据您提供的临床表现，医生可能诊断出的是肌强直性肌营养不良症（Myotonic Dystrophy，简称DM）。这种罕见病是一种遗传性疾病，主要影响肌肉、心脏、神经系统、眼和内分泌系统等多个系统。

其他五种可能的诊断包括：

1. **肌营养不良症（Duchenne Muscular Dystrophy，DMD）**：一种遗传性疾病，主要影响肌肉，导致肌肉无力和萎缩。
2. ** facioscapulohumeral 肌营养不良症（FSHD）**：一种遗传性疾病，主要影响面部、肩膀和上肢肌肉，导致肌肉无力和萎缩。
3. **肌强直性肌病（Myotonia Congenita）**：一种遗传性疾病，主要影响肌肉，导致肌肉强直和无力。
4. **肌萎缩侧索硬化（Amyotrophic Lateral Sclerosis，ALS）**：一种神经退行性疾病，主要影响运动神经元，导致肌肉无力和萎缩。
5. **多系统萎缩（Multiple System Atrophy，MSA）**：一种神经退行性疾病，主要影响自主神经系统、运动系统和认知功能，导致多个系统受累。

### **88. User**

该病发病年龄往往较早，最常发病的年龄为新生儿时期。大多患儿生后数日内发病，表现为拒奶、呕吐、嗜睡、惊厥、昏迷。部分晚发型患儿以发作性呕吐、嗜睡，伴精神发育异常为特点。高氨血症昏迷的患者可能有脑部伤害与发展迟缓、学习能力受损或智力障碍以及生长发育落后的后遗症。高氨血症患者可能造成颅内压增高、呼吸急促，病情进展会出现呼吸暂停或衰竭，肝肿大。神经系统并发症包括智力障碍、急性高血氨昏迷，甚至死亡。生化改变可见高氨血症、血谷氨酰胺和丙氨酸增高，尿中乳清酸降低或缺如。

### Llama3.1:70b

根据您提供的临床表现，可能的罕见病诊断包括：

1. **尿素循环障碍（Urea Cycle Disorder，UCD）**：这是一组遗传性疾病，影响体内氮质代谢，导致血液中氨的积累。尿素循环障碍的典型症状包括高氨血症、呕吐、嗜睡、惊厥、昏迷等，与您描述的临床表现相符。
2. **丙酮酸脱氢酶复合体缺陷（Pyruvate Dehydrogenase Complex Deficiency，PDHC）**：这是一种影响能量代谢的遗传性疾病，导致丙酮酸不能正常转化为乙酰CoA，进而影响能量生成。患者可能表现为高氨血症、发作性呕吐、嗜睡、精神发育异常等。
3. **乳清酸血症（Lactic Acidemia）**：这是一组影响能量代谢的疾病，导致乳清酸在体内积累。患者可能表现为高氨血症、发作性呕吐、嗜睡、精神发育异常等。
4. **高氨血症伴发性疾病（Hyperammonemia Syndromes）**：这是一组因各种原因导致高氨血症的疾病，可能包括遗传性疾病、感染、药物不良反应等。患者可能表现为高氨血症、呕吐、嗜睡、惊厥、昏迷等。
5. **先天性代谢疾病（Inborn Errors of Metabolism，IEM）**：这是一组遗传性疾病，影响体内代谢过程，导致各种临床表现。患者可能表现为高氨血症、发作性呕吐、嗜睡、精神发育异常等。

### **89. User**

患儿常表现为严重的宫内发育迟缓，出生后很早(常在出生后 1 周) 即出现严重的、非酮症性高血糖，12 周后可恢复，50％ ~60％在青春期前后复发，复发后临床表现类似于2 型糖尿病。

### Llama3.1:70b

根据您提供的临床表现，可能的罕见病诊断是：

1. **先天性高胰岛素血症（Congenital Hyperinsulinism）**：这种疾病通常表现为严重的宫内发育迟缓和出生后早期的非酮症性高血糖。然而，先天性高胰岛素血症通常表现为低血糖，而不是高血糖。
2. **永久性先天性糖尿病（Permanent Neonatal Diabetes Mellitus, PNDM）**：这种疾病表现为出生后 6 个月内出现的糖尿病，通常伴有宫内发育迟缓和其他异常。然而，PNDM 通常不会在青春期前后复发。
3. **暂时性先天性糖尿病（Transient Neonatal Diabetes Mellitus, TNDM）**：这种疾病表现为出生后 1 周内出现的糖尿病，通常在 12 周后恢复。然而，TNDM 通常不会在青春期前后复发。
4. **Wolfram 综合征（Wolfram Syndrome）**：这种疾病表现为糖尿病、视神经萎缩、听力丧失和其他神经系统异常。然而，Wolfram 综合征通常不会在出生后 1 周内出现糖尿病。
5. **Kabuki 综合征（Kabuki Syndrome）**：这种疾病表现为多器官异常，包括糖尿病、心脏病和神经系统异常。然而，Kabuki 综合征通常不会在出生后 1 周内出现糖尿病。

最可能的诊断是 **暂时性先天性糖尿病（Transient Neonatal Diabetes Mellitus, TNDM）**，因为它符合您提供的临床表现，包括出生后 1 周内出现的糖尿病和 12 周后恢复。然而，需要进一步的检查和诊断来确认最终的诊断。

### **90. User**

患者最早出现的症状是腹部膨隆，肝脾增大，部分患者可在新生儿期发病。智力和运动发育落后随即出现，肌张力低下，运动发育迟缓，而脑神经功能常不受累，1 岁后运动智力发育倒退明显，最后进展为痉挛强直状态。50％患者可以发现眼底樱桃红斑。间质性肺部病变可导致反复呼吸道感染、低氧血症或呼吸功能衰竭。多数患儿 3 岁前死亡。

### Llama3.1:70b

根据您提供的临床表现，以下是可能的罕见病诊断：

1. **Niemann-Pick病**：该病是一种因缺乏酸性鞘脂酶而导致的脂质代谢紊乱疾病。患者常表现为肝脾增大、智力和运动发育迟缓、眼底樱桃红斑等特征。
2. **Tay-Sachs病**：该病是一种因缺乏六己酰苷脂酶A而导致的GM2神经节苷脂贮积症。患者常表现为智力和运动发育迟缓、眼底樱桃红斑等特征。
3. **克山病**：该病是一种因缺乏葡萄糖-6-磷酸脱氢酶而导致的葡萄糖代谢紊乱疾病。患者常表现为肝脾增大、智力和运动发育迟缓等特征。
4. **Hurler综合征**：该病是一种因缺乏α-L-伊多酮酸酶而导致的黏多糖代谢紊乱疾病。患者常表现为肝脾增大、智力和运动发育迟缓、眼底樱桃红斑等特征。
5. **Sanfilippo综合征**：该病是一种因缺乏α-N-乙酰葡萄糖氨酸-6-磺酸酶而导致的黏多糖代谢紊乱疾病。患者常表现为肝脾增大、智力和运动发育迟缓、眼底樱桃红斑等特征。
6. **Morquio综合征**：该病是一种因缺乏N-乙酰葡萄糖氨酸-6-磺酸酶而导致的黏多糖代谢紊乱疾病。患者常表现为肝脾增大、智力和运动发育迟缓、眼底樱桃红斑等特征。

### **91. User**

**1. 典型面容，** 所有年龄患者均有上睑下垂，眼距宽，内眦赘皮，双眼外角下斜；双耳位低，后旋，耳廓厚。儿童患者还可有前额饱满，后发际低，鼻短，鼻梁低，鼻尖饱满；唇厚，鼻唇沟深而宽直达上唇等。

**2. 矮小，，** 出生时正常，1 岁后渐出现矮小。骨龄常落后，部分成年身高可达正常下限水平。

**3. 心血管 ，**50％ ~80％患者有先心病，肺动脉瓣狭窄最常见，其他包括肥厚型心肌病，室缺，肺动脉分支狭窄，法洛四联症和主动脉缩窄等。典型心电图改变包括心电轴极度右偏伴胸前导联 QRS 波逆时针旋转，V1 导联心电轴左偏、左前支不全传导阻滞或 RSR 波形。

**4. 精神运动发育落后** ，部分患者语言能力差，成年患者易有抑郁情绪，也有报道此病患者自闭症谱系表现发生率较高。

**5. 颈蹼，**

**6. 胸廓异常 ，**鸡胸或漏斗胸。

**7. 乳距宽**

**8. 男性隐睾** ，60％ ~80％男性患者合并隐睾，可致生育障碍。

**9. 凝血功能异常和淋巴管发育不良**

**10. 其他，** 肾脏畸形(肾盂扩张、双输尿管畸形、孤立肾、肾发育不良、远端输尿管狭窄等)，男性精子生成障碍，斜视，眼颤，皮肤牛奶咖啡斑和雀斑，四肢毛囊角化症， Ⅰ型阿诺德 - 基亚里畸形，脑积水，肝脾大，恶性肿瘤发生率增加等。

**11. 宫内表现，** 母亲孕期羊水过多，胎儿颈部透明带增宽，胎头相对较大，心脏和肾脏异常。

### Llama3.1:70b

根据您提供的临床表现，罕见病的诊断可能是 **诺恩综合征（Noonan Syndrome）**。

诺恩综合征是一种常见的遗传性疾病，表现为多器官异常，包括心血管、骨骼、面部、生殖系统等方面的异常。其典型特征包括上睑下垂、眼距宽、内眦赘皮、双眼外角下斜、前额饱满、后发际低、鼻短、鼻梁低、鼻尖饱满、唇厚、鼻唇沟深等。

其他可能的诊断包括：

1. **威廉综合征（Williams Syndrome）**：表现为心血管疾病、面部异常、生长迟缓、智力障碍等。
2. **阿尔波特综合征（Alport Syndrome）**：表现为肾脏疾病、听力丧失、眼部异常等。
3. **马凡综合征（Marfan Syndrome）**：表现为骨骼、心血管、眼部异常等。
4. **埃勒斯-丹洛斯综合征（Ehlers-Danlos Syndrome）**：表现为皮肤、骨骼、血管异常等。
5. **普瑞德威尔综合征（Prader-Willi Syndrome）**：表现为生长迟缓、智力障碍、肥胖、性腺功能减退等。

### **92. User**

患者可以在任何年龄阶段发病。临床主要分为早发型和晚发型两种。早发型主要发生在男性患儿，一般在新生儿期发病，临床表现为起病急，病情凶险，因血氨升高，使得大脑广泛性损害。出生时可无异常，生后数天即表现出易激惹、嗜睡、拒食、呼吸急促和昏睡等，可迅速发展为痉挛、昏迷和呼吸衰竭。如果不给予紧急处理，很快发展成遗传代谢性脑病，并常在刚出生的 1 周内死亡，幸存者多遗留严重的智力损害。晚发型多发生在较大年龄的患者中，可以是半合子的男性和杂合子的女性，临床症状相对轻，且表现多样。儿童期和成人期发病的患者多表现为慢性神经系统损伤，如发作性呕吐、头痛、行为异常、谵妄、精神错乱等。患者可有肝大、反复癫痫发作、生长发育迟缓、行为异常等临床表型。尽管晚发型症状较轻，但是在疾病、应激、高蛋白饮食等环境因素应激下会诱发高氨血症的急性发作而威胁生命。杂合子女性携带者多数终身无症状，少数发病，发病年龄及临床表现有个体差异性，既有早发型，也可表现为晚发型。

### Llama3.1:70b

根据您提供的临床表现，我最有可能诊断出的是**尿素循环障碍（Urea Cycle Disorder，UCD）**，特别是**鸟氨酸转氨酶缺乏症（Ornithine Transcarbamylase Deficiency，OTCD）**。这种疾病是由于鸟氨酸转氨酶基因突变引起的，导致尿素循环受阻，氨在体内蓄积，引起神经系统损害。

其他五种可能的诊断包括：

1. **氨基甲酰磷酸合成酶缺乏症（Carbamoyl Phosphate Synthetase Deficiency，CPSD）**：同样是尿素循环障碍的一种，导致氨在体内蓄积。
2. **氨基甲酰磷酸合成酶Ⅰ缺乏症（Carbamoyl Phosphate Synthetase I Deficiency，CPSID）**：也是一种尿素循环障碍，导致氨在体内蓄积。
3. **鸟氨酸转氨酶缺乏症（Ornithine Transcarbamylase Deficiency，OTCD）**：如前所述，这是尿素循环障碍的一种，导致氨在体内蓄积。
4. **丙酮酸脱氢酶复合体缺乏症（Pyruvate Dehydrogenase Complex Deficiency，PDHD）**：一种导致丙酮酸不能正常转化为乙酰-CoA 的疾病，导致神经系统损害。
5. **异柠檬酸脱氢酶缺乏症（Isocitrate Dehydrogenase Deficiency，IDHD）**：一种柠檬酸循环障碍，导致神经系统损害。

需要注意的是，这些疾病的诊断需要通过遗传检测、血液检查和其他辅助检查来确认。

### **93. User**

疾病的临床表型轻重不一。患者的主要临床表现是自幼起病的轻微外力下反复骨折、骨骼畸形，不同程度活动受限。患者可有蓝巩膜、韧带松弛、牙本质发育不全、听力下降、心脏瓣膜病变等骨骼外表现。OI 的影像学特征主要包括：全身多部位骨质稀疏；颅板变薄，囟门和颅缝增宽，枕骨可有缝间骨， 颅底扁平；脊柱可有侧弯或后凸畸形，椎体呈变形，可有多椎体压缩性骨折；胸廓可扭曲、变形，甚至塌陷；四肢长骨纤细、骨皮质菲薄，骨髓腔相对较大，干骺端增宽，多发长骨骨折，骨骼弯曲畸形等。

### Llama3.1:70b

根据您提供的临床表现和影像学特征，OI（骨质脆弱症）是最可能的诊断。其他五种可能的诊断包括：

1. **骨软化症**：一种由于维生素D缺乏或钙磷代谢异常引起的骨骼疾病，表现为骨质软化、畸形和骨折。
2. **成骨不全症**：一种遗传性骨骼疾病，表现为骨骼发育不全、畸形和骨折。
3. **骨巨细胞瘤**：一种骨骼肿瘤，表现为骨质破坏、畸形和骨折。
4. **Paget病**：一种骨骼疾病，表现为骨质增生、畸形和骨折。
5. **骨质疏松症**：一种由于激素变化、营养不良或其他因素引起的骨骼疾病，表现为骨质疏松、畸形和骨折。

### **94. User**

**1. 全血细胞减少，** 许多患者可以合并全血细胞减少、中性粒细胞降低。临床上表现为乏力、反复感染和出血倾向。

**2. 血红蛋白尿** ，典型的血红蛋白尿呈酱油或浓茶色。一般持续 2~3 天，不加处理自行消退，重者 1~2 周，甚至持续更长时间。有些患者的血红蛋白尿频繁发作，也有相当多的患者在很长的病程或观察期内始终无明确血红蛋白尿发作。

血红蛋白尿发作时可伴有发热、腰痛、腹痛等症状。其他与平滑肌功能障碍相关的症状可以有吞咽困难、腹痛、胃胀、背痛、头痛、食管痉挛、勃起障碍等。少见的情况包括：胆囊管运动障碍、急性胰腺炎、十二指肠或结肠的缺血和溃疡。

**3. 血栓形成**

**4. 黄疸与肝脾肿大** ，不到一半 患者有轻度黄疸。多数患者没有肝脾肿大。

常见的合并症包括感染、贫血性心脏病、胆石症、肾功能不全甚至衰竭等。

### Llama3.1:70b

根据您提供的临床表现，以下是一些罕见病的可能诊断：

1. **帕金森病**：帕金森病是一种影响运动系统的神经退行性疾病。其典型症状包括静止性震颤、运动迟缓、刚性以及姿势和平衡问题。虽然帕金森病主要影响运动系统，但也可能出现非运动症状，如认知障碍、情绪变化和自主神经功能障碍。
2. **多发性硬化症**：多发性硬化症是一种影响中枢神经系统（CNS）的慢性自身免疫性疾病。其症状因人而异，可能包括视力问题、肌肉无力、平衡和协调问题、认知困难以及感觉异常。
3. **肌营养不良症**：肌营养不良症是一组影响骨骼肌的遗传性疾病。其特征是肌肉无力和萎缩，通常从婴儿期或幼儿期开始。肌营养不良症的严重程度和进展速度因人而异。
4. **亨廷顿病**：亨廷顿病是一种遗传性神经退行性疾病，主要影响大脑的基底节。其典型症状包括不自主的舞蹈样运动（舞蹈病）、认知障碍和情绪变化。
5. **肌强直症**：肌强直症是一种影响肌肉的神经疾病，表现为肌肉僵硬和痉挛。其严重程度和影响的肌肉群因人而异。

### **95. User**

临床表现为反复肠套叠、腹痛、腹泻、黏液便、便血、便秘、呕血、贫血等。特征性临床表现为皮肤黏膜色素斑和胃肠道多发息肉。95% 以上患者出现色素沉着，色素沉着多见于口唇及其四周、颊部、面部、手指、手掌、足底、肛周等，偶见于肠黏膜。色素可呈褐色、棕褐色、灰色和蓝色等，通常发生 2 岁前，大小 1_5mm。约88%_100% 患者会发生胃肠道息肉，胃肠道息肉发病多发生于 11~13 岁，息肉可发生在整个胃肠道，以小肠多见，其次是结肠和胃。息肉大小不定，蒂长短粗细不一。该类疾病患癌风险较高，平均患癌年龄为 42 岁。

### Llama3.1:70b

根据您提供的临床表现，特别是皮肤黏膜色素斑和胃肠道多发息肉，我最有可能诊断出的是**佩茨-耶格尔综合征（Peutz-Jeghers Syndrome, PJS）**。

佩茨-耶格尔综合征是一种罕见的遗传性疾病，其特征是出现特征性的皮肤和黏膜色素沉着，以及胃肠道多发性息肉。这种疾病确实会增加患癌风险，尤其是胃肠道癌。

其他五种可能的诊断包括：

1. **家族性腺瘤性息肉症（Familial Adenomatous Polyposis, FAP）**：这种疾病也会导致胃肠道多发性息肉，但主要特征是家族性遗传和高风险发展为结肠癌。
2. ** Lynch 综合征**：又称为遗传性非息肉性结肠癌综合征，主要特征是家族性遗传和高风险发展为结肠癌、子宫内膜癌等。
3. **Cowden 综合征**：一种罕见的遗传性疾病，特征是多器官良性肿瘤和高风险发展为乳腺癌、甲状腺癌等。
4. **Birt-Hogg-Dubé 综合征**：一种罕见的遗传性疾病，特征是皮肤良性肿瘤和高风险发展为肾癌。
5. **Li-Fraumeni 综合征**：一种罕见的遗传性疾病，特征是家族性遗传和高风险发展为多种类型的癌症，包括乳腺癌、脑癌、骨肉瘤等。

这些疾病都有不同的特征和遗传模式，需要通过进一步的检查和家族史调查来确诊。

### **96. User**

患儿在新生儿期多无临床症状，出生 3~4 个月后逐渐出现典型症状，1 岁时症状明显。出生数月后因黑色素合成不足，其毛发和虹膜色泽逐渐变浅，为黄色或棕黄色，皮肤白。由于尿液、汗液含有大量苯乳酸而有鼠尿臭味。随着年龄增长，逐渐表现出智能发育迟缓，以认知发育障碍为主、小头畸形、癫痫发作，也可出现行为、性格等异常，如多动、自残、攻击、自闭症、自卑、忧郁等神经系统表现。婴儿期还常出现呕吐、湿疹等。

### Llama3.1:70b

根据您提供的临床表现，医生可能诊断出的是**苯丙酮尿症（Phenylketonuria，PKU）**。这是一个遗传性疾病，由于体内缺乏苯丙氨酸羟化酶，导致苯丙氨酸及其代谢产物在体内积累，引起一系列神经系统和身体的症状。

其他五种可能的诊断包括：

1. **地中海贫血**：一种遗传性贫血，主要影响地中海沿岸地区的人群，表现为贫血、黄疸、脾脏增大等。
2. **白化病**：一种遗传性疾病，影响黑色素的合成，表现为皮肤、毛发、虹膜色泽减退或消失。
3. ** maple 糖尿病**：一种遗传性疾病，由于体内缺乏支链氨基酸氨基转移酶，导致支链氨基酸及其代谢产物在体内积累，引起一系列神经系统和身体的症状。
4. **肝豆状核变性**：一种遗传性疾病，由于体内缺乏铜蛋白，导致铜在体内积累，引起肝脏、脑部等器官损害。
5. **肌营养不良症**：一种遗传性疾病，由于体内缺乏肌肉蛋白，导致肌肉萎缩、无力等症状。

### **97. User**

主要为皮肤症状和神经精神症状。

**1. 皮肤症状群，** 为光照后在皮肤暴露部位出现红斑、疱疹甚至溃烂。皮疹可为湿疹、荨麻疹、夏令痒疹或多形性红斑等类型。口腔黏膜可有红色斑点，牙呈棕红色。同时可并发眼损害如结膜炎、角膜炎及虹膜炎等。严重者可有鼻、耳、手指皮肤结瘢变形。可有特殊紫色面容。可有多毛症。

**2. 神经精神症状群 ，**最常见的表现为急性腹痛，腹痛往往为重度、稳定、定位不明，有时会伴有痛性痉挛。可以伴随便秘、腹胀、恶心、呕吐，有时出现腹泻和肠鸣音增加。感觉和运动神经病可能先于腹痛出现，表现为肢体痛，伴麻木感、感觉异常和感觉倒错； 运动无力往往始于上肢近端，可向下肢和远端进展。累及脑神经可能导致延髓麻痹、呼吸功能受损和死亡。严重运动神经病可导致四肢瘫痪。自主神经系统受累表现为心动过速、高血压、出汗、躁动和震颤等。神经源性膀胱功能障碍可能引起尿痛、排尿困难、尿潴留和尿失禁。其他急性神经精神表现包括失眠、焦虑、躁动、激越、幻觉、癔症、定向障碍、谵妄、情感淡漠、抑郁、恐惧症和意识改变，从嗜睡到昏迷程度不一。

### Llama3.1:70b

根据您提供的临床表现，主要为皮肤症状和神经精神症状，可能的罕见病诊断包括：

1. **维生素B12缺乏症**：维生素B12缺乏症可能导致神经系统和皮肤症状。神经系统症状包括神经炎、肌肉无力、感觉异常和运动功能障碍等。皮肤症状包括皮肤苍白、黄疸、口腔溃疡和皮肤瘙痒等。
2. **维生素B6缺乏症**：维生素B6缺乏症可能导致神经系统和皮肤症状。神经系统症状包括神经炎、肌肉无力、感觉异常和运动功能障碍等。皮肤症状包括皮肤炎、口腔溃疡和皮肤瘙痒等。
3. **维生素B1缺乏症**：维生素B1缺乏症可能导致神经系统和皮肤症状。神经系统症状包括神经炎、肌肉无力、感觉异常和运动功能障碍等。皮肤症状包括皮肤炎、口腔溃疡和皮肤瘙痒等。
4. **维生素B3缺乏症**：维生素B3缺乏症可能导致皮肤症状。皮肤症状包括皮肤炎、口腔溃疡和皮肤瘙痒等。
5. **维生素B7缺乏症**：维生素B7缺乏症可能导致皮肤症状。皮肤症状包括皮肤炎、口腔溃疡和皮肤瘙痒等。

### **98. User**

**1. 感染，患者**均有不同程度感染。 感染具有反复、严重、难治、机会致病、病原谱广等特点。典型患儿出生时一般没有特别表现，由于患儿存在细胞免疫异常，2 月龄前也可发生致命感染。感染部位及病原多种多样，包括反复肺炎，反复鹅口疮，持续腹泻，反复中耳炎，持续病毒感染(如呼吸道合胞病毒、EBV、CMV 等)，卡氏肺囊虫等条件致病菌感染也较常见。

**2. 疫苗病，** 患者免疫功能极度低下，可发生减毒活疫苗感染和播散。 患儿接种减毒轮状病毒疫苗也可发生严重腹泻。

**3. 生长发育落后或停滞** 是 疾病的显著特征。与患儿反复感染、腹泻和相应基因缺陷均有关。

患儿还可伴有皮疹、肝脾淋巴结肿大类似红皮病表现

另一部分患儿可出现类似红皮病、肝脾肿大、脂溢性皮炎、血细胞减少症，硬化性胆管炎。

### Llama3.1:70b

根据您提供的临床表现，以下是一些可能的罕见病诊断：

1. **重症联合免疫缺陷病（Severe Combined Immunodeficiency，SCID）**：该病症表现为严重的感染、疫苗病、生长发育落后等，与您描述的临床表现相符。
2. **Wiskott-Aldrich 综合征**：该病症表现为反复感染、血细胞减少症、皮疹和淋巴结肿大等，与您描述的临床表现有一些相似之处。
3. **DiGeorge 综合征**：该病症表现为免疫功能低下、反复感染、生长发育落后等，与您描述的临床表现有一些相似之处。
4. **X-连锁重症联合免疫缺陷病（X-linked SCID）**：该病症表现为严重的感染、疫苗病、生长发育落后等，与您描述的临床表现相符。
5. **Omenn 综合征**：该病症表现为重度免疫缺陷、反复感染、生长发育落后等，与您描述的临床表现有一些相似之处。
6. **ZAP70缺陷**：该病症表现为重度免疫缺陷、反复感染、生长发育落后等，与您描述的临床表现有一些相似之处。

### **99. User**

核心症状是肌张力不全，主要表现为异常的表情姿势和不自主的动作，多累及头颈部肌肉、躯干肌、肢体的旋前肌、指腕屈肌、趾伸肌和跖屈肌等。随意运动时加重，休息睡眠时减轻或消失，晚期可呈固定扭曲痉挛畸形。感觉诡计(缓解技巧) 和动作特异性是肌张力不全的特征性表现。感觉诡计(缓解技巧) 是指专门用于纠正异常姿势或缓解肌张力不全性运动的随意动作，通常是涉及或针对受累部位的简单运动，但不是用力对抗肌张力不全症状。动作特异性是指仅在特定活动或执行特定任务时出现肌张力不全，例如某些职业(如书写痉挛、音乐家痉挛) 或运动。

### Llama3.1:70b

根据您提供的症状描述，主要特征包括肌张力不全、异常表情姿势、不自主的动作、随意运动时加重、休息睡眠时减轻或消失等，这些症状与**肌张力不全症（Dystonia）**高度相符。肌张力不全症是一组运动障碍，其特点是肌肉张力异常，导致不自主的肌肉收缩，引起异常姿势和不自主的动作。感觉诡计（缓解技巧）和动作特异性是肌张力不全症的特征性表现。

其他可能的诊断包括：

1. **帕金森病（Parkinson’s Disease）**：虽然帕金森病也会导致运动障碍，但其主要特征是静止性震颤、运动迟缓、刚性以及姿势不稳定等，与肌张力不全症的临床表现有所不同。
2. **肌强直症（Stiff Person Syndrome）**：这是一种罕见的神经疾病，主要表现为肌肉强直和疼痛，通常影响躯干和下肢，与肌张力不全症的临床表现有一些相似之处，但强直和疼痛是其主要特征。
3. **Wilson病（Wilson’s Disease）**：这是一种遗传性疾病，主要影响肝脏和大脑，临床表现包括肝脏损害、神经系统症状（如肌张力不全、震颤等）和精神症状等。
4. **Huntington病（Huntington’s Disease）**：这是一种遗传性神经退行性疾病，主要表现为舞蹈样运动、认知功能障碍和精神症状等，与肌张力不全症的临床表现有一些相似之处，但舞蹈样运动是其主要特征。
5. **肌营养不良症（Myotonic Dystrophy）**：这是一种遗传性肌肉疾病，主要表现为肌肉萎缩、肌肉强直和运动迟缓等，与肌张力不全症的临床表现有一些相似之处，但肌肉萎缩和肌肉强直是其主要特征。

### **100. User**

**1. 肾脏 ，**主要表现为肢体水肿和尿中泡沫增多，实验室检查可以发现单纯的中等量蛋白尿或肾病综合征，晚期可出现肾功能不全。

**2. 心脏 ，**主要表现为活动后气短、肢体水肿、胸腔积液、腹水等限制性心功能不全表现。

**3. 肝脏 ，**可有轻微肝区不适或疼痛，但多数患者可无症状，往往为体检时发现肝大或者肝酶异常。疾病晚期可出现肝功能衰竭。

**4. 周围神经和自主神经 ，**为对称性的四肢感觉和(或) 运动性周围神经病。自主神经异常多表现为直立性低血压、胃轻瘫、假性肠梗阻和阳痿等。

**5. 胃肠道** ，可以出现全胃肠道受累，以胃部和小肠受累多见。可以表现为上腹不适、消化不良、腹泻、便秘、吸收不良综合征和消化道出血等。

**6. 软组织 ，**舌体受累可出现巨舌、舌体活动障碍和构音异常等。皮肤黏膜可以出现皮肤紫癜和瘀斑，以眼眶周围和颈部皮肤松弛部位较为常见。也可以出现指甲萎缩脱落和毛发脱落等。

**7. 凝血功能异常** 患者常会伴发凝血因子Ⅹ缺乏，造成相应的出血表现。

### Llama3.1:70b

根据您提供的临床表现，可能的罕见病诊断包括：

1. **Fabry病**：一种由α-半乳糖苷酶A缺乏引起的遗传性疾病，主要影响肾脏、心脏、周围神经和皮肤等系统。
2. **家族性 Transthyretin 淀粉样蛋白沉积症（ATTR）**：一种由变异的 Transthyretin 蛋白引起的遗传性疾病，主要影响周围神经、自主神经和心脏等系统。
3. **轻链病**：一种由异常的轻链蛋白引起的淀粉样蛋白沉积症，主要影响肾脏、心脏和周围神经等系统。
4. **系统性淀粉样蛋白沉积症（AL淀粉样蛋白病）**：一种由异常的轻链蛋白引起的淀粉样蛋白沉积症，主要影响肾脏、心脏、周围神经和皮肤等系统。
5. **家族性淀粉样蛋白多发性神经病变（FAP）**：一种由变异的 Transthyretin 蛋白引起的遗传性疾病，主要影响周围神经和自主神经等系统。
6. **淀粉样蛋白沉积症（AA淀粉样蛋白病）**：一种由异常的轻链蛋白引起的淀粉样蛋白沉积症，主要影响肾脏、心脏、周围神经和皮肤等系统。

### **101. User**

黄疸和皮肤瘙痒是典型临床表现。其他有身材矮小、青春期发育落后等发育迟缓表现，胆囊结石，脂肪吸收障碍所致的脂肪泻，肝脾肿大以及脂溶性维生素缺乏所致佝偻病、骨龄延迟、干眼症、凝血障碍和神经肌肉病变等症状。患儿亦可以出现视觉及听力异常，出现烦躁、嗜睡及注意力不集中等改变。晚期可出现门静脉高压症和肝脏肿瘤等。

### Llama3.1:70b

根据您提供的临床表现，可能的罕见病诊断包括：

1. **原发性胆汁性肝硬化（PBC）**：这是一种慢性肝脏疾病，主要影响胆管，导致胆汁淤积，引起黄疸、皮肤瘙痒等症状。
2. **Alagille 综合征**：这是一种罕见的遗传性疾病，影响肝脏、心脏和其他器官，表现为胆汁淤积、黄疸、皮肤瘙痒等。
3. **肝脏先天性胆管扩张症**：这是一种罕见的先天性疾病，表现为胆管扩张，导致胆汁淤积、黄疸、皮肤瘙痒等。
4. **肝脏脂质沉积症**：这是一种罕见的遗传性疾病，表现为肝脏脂质沉积，导致脂肪吸收障碍、脂肪泻等。
5. **肝脏 Wilson 病**：这是一种罕见的遗传性疾病，表现为肝脏铜离子沉积，导致肝脏损害、黄疸、皮肤瘙痒等。
6. **肝脏先天性纤维化**：这是一种罕见的先天性疾病，表现为肝脏纤维化，导致肝脏损害、黄疸、皮肤瘙痒等。

### **102. User**

在儿童期起病。表现为运动发育轻度迟滞，骨骼肌进行性无力萎缩，影响肢体运动功能，逐渐出现步态异常、上肢活动受限，自然病程常在 10 岁左右丧失行走能力。此后出现脊柱侧弯、关节挛缩、呼吸肌无力、扩张型心肌病，20 岁左右因呼吸衰竭、心功能衰竭死亡。查体可见双腓肠肌假肥大，同时可有双前臂及舌肌假肥大，Gowers 征阳性，腰椎前凸等。可青年甚至成年起病， 部分患者不影响生存期。假肥大体征明显，部分患者在肢体无力尚轻时，先出现明显的扩张型心肌病。

### Llama3.1:70b

根据您提供的临床表现，医生最有可能诊断出的罕见病是**杜兴肌营养不良症（Duchenne Muscular Dystrophy，DMD）**。

其他五种可能的诊断包括：

1. **贝克尔肌营养不良症（Becker Muscular Dystrophy，BMD）**：与DMD相似，但进展较慢，患者可能在成年后才出现明显症状。
2. **肢带肌营养不良症（Limb-Girdle Muscular Dystrophy，LGMD）**：影响肢体和骨盆区域的肌肉，进展速度因人而异。
3. **面肩肱型肌营养不良症（Facioscapulohumeral Muscular Dystrophy，FSHD）**：主要影响面部、肩膀和上肢肌肉，进展速度较慢。
4. **肌营养不良症的其他类型**：如Emery-Dreifuss肌营养不良症、米托康德里肌营养不良症等。
5. **其他神经肌肉疾病**：如脊髓性肌萎缩症、慢性炎症性脱髓鞘性多发性神经病等。

### **103. User**

主要为高血氨、脑损伤和心肌病等。

**1. 新生儿起病型** 出生时正常，开始哺乳后出现呕吐、嗜睡、肌张力低下、惊厥、呼吸困难、高血氨、酮症、低血糖、酸中毒、扩张型心肌病、胰腺炎等异常，病死率高。

**2. 迟发型** 常因发热、饥饿、高蛋白饮食和感染等诱发，表现为婴幼儿期喂养困难、发育落后、惊厥、肌张力低下等。许多患者的认知能力及神经系统发育受到损害，脑电图慢波增多或见癫痫波；一些患者可有骨折，X 线见骨质疏松；还常造成骨髓抑制，引起粒细胞减少、贫血、血小板减少。也可有心脏损害，如心肌病、心律失常、QT 间期延长、心功能减弱等。肾功能损害较为少见。

### Llama3.1:70b

根据您提供的临床表现，主要特征包括高血氨、脑损伤和心肌病等，我最有可能诊断出的罕见病是**丙氨酸血症**（Propionic Acidemia）。

丙氨酸血症是一种遗传性代谢疾病，由于体内丙氨酸脱氢酶的缺陷，导致丙氨酸及其相关化合物在体内积累，引起一系列临床问题。新生儿起病型和迟发型是两种常见的临床表现形式，分别对应于不同的发病时间和症状严重程度。

其他五种可能的诊断包括：

1. **异亮氨酸血症**（Isovaleric Acidemia）：一种遗传性代谢疾病，由于体内异亮氨酸脱氢酶的缺陷，导致异亮氨酸及其相关化合物在体内积累，引起高血氨、酸中毒、惊厥等症状。
2. **甲硫氨酸血症**（Methioninemia）：一种遗传性代谢疾病，由于体内甲硫氨酸脱氢酶的缺陷，导致甲硫氨酸及其相关化合物在体内积累，引起高血氨、酸中毒、惊厥等症状。
3. **酮症性高血氨症**（Ketotic Hyperammonemia）：一种临床综合征，由于体内酮体生成增多，导致高血氨、酸中毒、惊厥等症状。
4. **尿素循环障碍**（Urea Cycle Disorder）：一种遗传性代谢疾病，由于体内尿素循环酶的缺陷，导致尿素及其相关化合物在体内积累，引起高血氨、酸中毒、惊厥等症状。
5. **有机酸血症**（Organic Acidemia）：一种临床综合征，由于体内有机酸生成增多，导致高血氨、酸中毒、惊厥等症状。

这些疾病都可能导致高血氨、脑损伤和心肌病等临床表现，因此需要进行进一步的诊断和鉴别。

### **104. User**

临床表现常没有特异性，隐匿起病。可出现咳痰、消瘦、乏力等症状。继发感染时可有发热、咳脓性痰，也可出现胸痛、咯血。少数患者可无症状，可以没有阳性体征。部分患者可以出现爆裂音，重症患者可有发绀，少数患者可见杵状指。临床表现常比胸部影像受累的程度轻，这种症状和影像不一致的表现也是该病 的一个特征。血清抗 GM-CSF 抗体测定明显增高。

### Llama3.1:70b

根据您提供的临床表现，医生可能诊断出的是**肺部淤积病（Pulmonary Alveolar Proteinosis，PAP）**。这种罕见病是由于肺泡中淤积了过多的蛋白质和脂质所致，导致呼吸困难和其他呼吸系统症状。

其他五种可能的诊断包括：

1. **慢性阻塞性肺疾病（COPD）**：这是一种常见的肺部疾病，表现为呼吸困难、咳嗽和咳痰等症状。
2. **肺结核**：这是一种由细菌引起的肺部感染，表现为咳嗽、咳痰、发热和体重减轻等症状。
3. **肺纤维化**：这是一种肺部疾病，表现为肺组织纤维化，导致呼吸困难和其他呼吸系统症状。
4. **肺炎**：这是一种肺部感染，表现为发热、咳嗽、咳痰和呼吸困难等症状。
5. **囊性纤维化**：这是一种遗传性疾病，表现为肺部囊性病变，导致呼吸困难和其他呼吸系统症状。

需要注意的是，以上诊断仅为可能的诊断，需要进一步的检查和测试来确认确切的诊断。

### **105. User**

大多患者青少年时期起病，首先是视杆细胞逐渐丧失，随后出现视锥细胞受累， 视网膜色素上皮细胞死亡。因此通常以夜盲为首发症状(但城市化后部分患者常以视力下降为首发症状)，而且夜盲出现得越早，患者的病情往往越严重。虽然周边视野缺损也发生较早，但患者通常意识不到，很少以此作为疾病早期的主诉。病情逐渐发展为中心视力下降，视野进一步缩窄，多数在 40 岁之前就成为法定盲人。患者的视功能损伤程度可与遗传方式相关。

患者的眼底表现与疾病所处的阶段相关：疾病早期患者眼底在常规检查下可表现正常或接近正常，但部分患者在中周部眼底照相下可以观察到早期毯层样视网膜变性(这类细微改变在目前的扫描激光眼底照相或广域眼底照相下很难识别)。随着病情的进展，部分 患者会出现典型的眼底改变，表现为视网膜骨细胞样色素沉积合并中周部视网膜萎缩，视盘蜡黄，视网膜血管变细等；部分病人会出现毯层样视网膜变性或脱色素改变，见于中周部视网膜或广泛变性。其他常见的并发症还包括黄斑囊样变性、后囊下白内障、玻璃体尘样颗粒、视盘玻璃膜疣等并发症；少见的并发症有 Coat’s 样视网膜病变。

### Llama3.1:70b

根据您提供的临床表现，可能的罕见病诊断是：

1. **视网膜色素变性病（Retinitis Pigmentosa, RP）**：这是一种常见的遗传性眼病，主要影响视网膜，导致视力逐渐下降，尤其是在夜间或低光照条件下。患者通常在青少年时期开始出现症状，包括夜盲、视野缩窄和视力下降。
2. **Leber 先天性黑夜盲（Leber Congenital Amaurosis, LCA）**：这是一种罕见的遗传性眼病，主要影响视网膜，导致严重的视力障碍，尤其是在夜间或低光照条件下。患者通常在婴儿期或幼儿期开始出现症状。
3. **视网膜色素上皮病变（Retinal Pigment Epithelium Disease, RPED）**：这是一种罕见的遗传性眼病，主要影响视网膜色素上皮细胞，导致视力逐渐下降，尤其是在夜间或低光照条件下。患者通常在青少年时期开始出现症状。
4. **Usher 综合征（Usher Syndrome）**：这是一种罕见的遗传性疾病，主要影响视网膜和听力，导致视力逐渐下降和听力丧失。患者通常在青少年时期开始出现症状。
5. **Bardet-Biedl 综合征（Bardet-Biedl Syndrome）**：这是一种罕见的遗传性疾病，主要影响视网膜、听力、肾脏和其他器官，导致视力逐渐下降和听力丧失。患者通常在青少年时期开始出现症状。
6. **视网膜色素变性病伴黄斑变性（Retinitis Pigmentosa with Macular Dystrophy）**：这是一种罕见的遗传性眼病，主要影响视网膜和黄斑，导致视力逐渐下降，尤其是在夜间或低光照条件下。患者通常在青少年时期开始出现症状。

### **106. User**

最常见的临床表现是白瞳症，即瞳孔可见黄白色反光。患眼可因肿瘤位于后极部，视力低下，而发生失用性斜视。早期表现为灰白色，圆形或椭圆形拱状肿物，可向玻璃体隆起，或沿脉络膜扁平生长。肿瘤表面的视网膜血管可出现扩张、出血，甚至发生渗出性视网膜脱离。瘤组织也可穿破视网膜进入玻璃体，如雪球状漂浮，甚至沉积于前房形成假性前房积脓。双侧性患儿发病较早，通常一岁以内发病。单侧性患儿发病较迟，通常在两岁或三岁时发生。

### Llama3.1:70b

根据您提供的临床表现，医生最有可能诊断出的是视网膜母细胞瘤（Retinoblastoma）。其他五种可能的诊断包括：

1. 视网膜母细胞瘤（Retinoblastoma）：一种起源于视网膜的恶性肿瘤，主要影响儿童。
2. 视网膜血管瘤（Retinal Hemangioblastoma）：一种良性肿瘤，起源于视网膜的血管。
3. 视网膜母细胞瘤样病变（Retinoblastoma-like Lesions）：一种非肿瘤性病变，表现为视网膜异常增生。
4. 视网膜脉络膜母细胞瘤（Retinocytoma）：一种良性肿瘤，起源于视网膜的神经上皮细胞。
5. 视网膜母细胞瘤样病变（Retinoblastoma-like Lesions）：一种非肿瘤性病变，表现为视网膜异常增生。

### **107. User**

患儿出生时多正常，在 1 岁以内起病，平均发病年龄为 5.6 个月。首次发作常为热性惊厥，可为一侧性或全面性阵挛或强直阵挛发作，热性惊厥具有发作持续时间长和反复发作特点，可出现持续状态。28％ ~35％患儿可能以无热惊厥起病，但在以后病程中常有热敏感特点。各种原因引起的体温升高，包括运动、发热、疫苗接种、洗热水澡和环境温度高等，均容易诱发或使发作加重，并易发生癫痫持续状态。有些患儿会出现以癫痫持续状态之后深昏迷为特征的急性脑病。

患儿多于 1~4 岁期间出现无热惊厥，具有多种发作类型，如肌阵挛、不典型失神、部分性发作、全面强直阵挛发作等。肌阵挛发作常由光刺激、闭眼和某种特定的图形刺激诱发，一块或数块肌肉同时阵挛，均伴有同步异常脑电波阵发性发放。肌阵挛强度不一，仅侵犯轴性肌肉时，出现身体轻微向前或向后的动作，严重者跌倒或突然抛掷手中物体。肌阵挛发作可以是孤立的，也可群发，但持续状态比较罕见。约 25％的 儿可以始终不出现肌阵挛发作。不典型失神发作同期脑电图显示半数为局灶起源放电，多数呈不规则不节律的慢棘慢波发放，经常伴随肌阵挛发作。30％的患儿可发生不典型失神持续状态。部分性发作发生率为 43.0％ ~78.6％，表现为偏侧肢体(或) 头部偏转性发作或阵挛性发作，亦有运动性发作伴有意识障碍和自主神经症状、自动症、肌张力降低等。1 岁以后患儿出现精神运动发育迟滞，尤其是语言发育迟缓。

### Llama3.1:70b

根据您提供的临床表现，可能的罕见病诊断包括：

1. **Dravet 综合征**：一种罕见的遗传性癫痫综合征，特征是婴儿期开始的热性惊厥和后续的多种类型的癫痫发作，包括肌阵挛、不典型失神和部分性发作等。
2. **Lennox-Gastaut 综合征**：一种严重的癫痫综合征，特征是多种类型的癫痫发作，包括肌阵挛、不典型失神和强直-阵挛发作等，常伴有智力障碍和行为问题。
3. **Doose 综合征**：一种罕见的遗传性癫痫综合征，特征是婴儿期开始的肌阵挛和其他类型的癫痫发作，常伴有智力障碍和行为问题。
4. **West 综合征**：一种罕见的遗传性癫痫综合征，特征是婴儿期开始的肌阵挛和其他类型的癫痫发作，常伴有智力障碍和行为问题。
5. **Landau-Kleffner 综合征**：一种罕见的癫痫综合征，特征是儿童期开始的语言发育迟缓和癫痫发作，常伴有智力障碍和行为问题。

### **108. User**

一方面表现为慢性溶血性贫血，除贫血相关症状，因胆红素升高可引起皮肤、巩膜轻中度黄染、色素性胆石症，当寒冷、感染、脱水时症状可能加重。婴幼儿可出现脾大，随年龄增长，脾脏可因脾梗死、纤维化而缩小。

另一方面由于毛细血管微血栓而引起反复发作的血管阻塞性疼痛。儿童和成人多表现为四肢肌痛、大关节疼痛和腰背疼痛，严重者可出现剧烈腹痛、头痛，甚至肢体瘫痪、昏迷。随着时间的推移，血管阻塞几乎可发生于每个器官系统，可以出现周期性疼痛，患者瘦弱、易疲劳、易感染、营养不良。因骨髓造血组织过度代偿性增加使骨皮质变薄、骨质疏松、脊柱变形、股骨头无菌坏死，如有骨梗死则可导致局部骨质硬化。眼部症状可能出现眼底视网膜血管栓塞引起眼底出血、视网膜脱离。另外，尚可影响神经系统的发育而出现智力低下。

在超过 25％的患者中急性疼痛为疾病的首发症状。在大于 2 岁的患儿中是最常见的症状。疼痛的严重程度可从轻微到剧烈不等。大约 1/2 的疼痛发作伴随客观的临床体征，如发热、肿胀、压痛、呼吸过速、高血压、恶心和呕吐。

### Llama3.1:70b

根据您提供的临床表现，医生可能诊断出的是**镰状细胞病**（Sickle Cell Disease，SCD）。镰状细胞病是一种遗传性疾病，由于基因突变导致血红蛋白异常，形成镰状红细胞，导致慢性溶血性贫血和血管阻塞。

其他可能的诊断包括：

1. **地中海贫血**：一种遗传性贫血，主要影响地中海沿岸地区的人群，表现为慢性溶血性贫血和脾大。
2. **溶血性贫血**：一种因红细胞破坏过快导致的贫血，可能由多种原因引起，包括遗传性疾病、自身免疫性疾病等。
3. **血管炎**：一种血管壁炎症，可能导致血管阻塞和器官损害。
4. **系统性红斑狼疮**：一种自身免疫性疾病，可能导致多器官损害，包括血管炎和溶血性贫血。
5. **骨髓纤维化**：一种骨髓疾病，导致骨髓纤维化和造血功能障碍，可能引起贫血和骨质疏松。

### **109. User**

新生儿和儿童患者的主要临床表现包括：

**1. 宫内发育迟缓及出生后生长障碍** 宫内发育迟缓，低出生体重，出生后缺乏追赶生长，成比例的身材矮小。

**2. 特殊体征** 前额宽而突出、尖下颌导致三角形脸，相对头围偏大。齿列不齐、耳位低，躯体偏身不对称，包括四肢长度差异，小指侧弯畸形等。

**3. 其他表现** 喂养困难、新生儿期可出现低血糖，尿道下裂、隐睾和马蹄肾等泌尿生殖系统畸形，牛奶咖啡斑，反流性食管炎等消化系统异常。部分患者出现认知、运动和语言发育迟缓，学习障碍等。

### Llama3.1:70b

根据您提供的临床表现，以下是可能的罕见病诊断：

1. **Silver-Russell 综合征（SRS）**：这是一种罕见的遗传性疾病，主要特征包括宫内发育迟缓、出生后生长障碍、特殊面部特征（如前额宽而突出、尖下颌）和躯体不对称等。
2. **Prader-Willi 综合征（PWS）**：这是一种因父源性15号染色体长臂缺失或不活跃引起的罕见遗传性疾病。主要特征包括出生后生长迟缓、食欲不振、特殊面部特征（如前额宽而突出、上颌突出）和智力障碍等。
3. **Turner 综合征**：这是一种女性性染色体异常，主要特征包括宫内发育迟缓、出生后生长障碍、特殊面部特征（如低耳位、鱼尾纹）和躯体不对称等。
4. **Noonan 综合征**：这是一种遗传性疾病，主要特征包括特殊面部特征（如眼睑下垂、低耳位）、心脏畸形和生长迟缓等。
5. **Williams 综合征**：这是一种因7号染色体长臂缺失引起的罕见遗传性疾病。主要特征包括特殊面部特征（如眼睑下垂、鼻梁低）、心脏畸形和智力障碍等。
6. ** Floating-Harbor 综合征**：这是一种罕见的遗传性疾病，主要特征包括特殊面部特征（如前额宽而突出、尖下颌）、生长迟缓和智力障碍等。

### **110. User**

**1. 皮肤黄瘤，** 好发于伸肌肌腱附着点附近，例如跟腱、肘关节、膝关节等，皮肤损伤与黄瘤出现关系密切。皮肤黄瘤少见于儿童患者。

**2. 动脉粥样硬化 ，**患者出现动脉粥样硬化、冠心病的风险显著高于正常人。早发性冠心病可以作为本病的特点之一。

**3. 血液系统改变 ，**血液中植物固醇含量明显增加，导致细胞膜中固醇成分中植物固醇比例增高，红细胞、血小板等细胞膜功能异常，出现贫血和血小板减少，患者可出现乏力、面色苍白、皮肤出血等表现。

**4. 其他，**除上述症状，部分患者会出现关节痛、关节炎、脾大、转氨酶升高等。

### Llama3.1:70b

根据您提供的临床表现，可能的罕见病诊断包括：

1. **西蒂尔病（Sitosterolemia）**：一种因植物固醇代谢异常导致的罕见遗传病，表现为皮肤黄瘤、动脉粥样硬化、血液系统改变等。
2. **家族性高胆固醇血症（Familial Hypercholesterolemia）**：一种遗传性疾病，表现为胆固醇水平升高，导致早发性动脉粥样硬化和冠心病。
3. **皮质醇增多症（Cushing’s Syndrome）**：一种因皮质醇水平升高导致的内分泌疾病，表现为皮肤黄瘤、动脉粥样硬化、血液系统改变等。
4. **脂质代谢异常（Lipid Metabolism Disorder）**：一种因脂质代谢异常导致的疾病，表现为皮肤黄瘤、动脉粥样硬化、血液系统改变等。
5. **遗传性血管疾病（Hereditary Vascular Disease）**：一种因遗传因素导致的血管疾病，表现为动脉粥样硬化、冠心病等。

### **111. User**

男性发病，起病隐匿，常见发病年龄为 30~60 岁。患者常以痛性痉挛、震颤、双下肢无力等症状起病，逐渐出现双上肢无力、延髓部和面部的肌肉萎缩、肌束颤动、构音障碍、吞咽困难、呼吸困难等。可伴有男性乳房发育、生殖功能降低等雄激素受体不敏感表现。超过 50％的患者存在感觉异常。该病进展缓慢，患者通常在病程晚期才出现行走不能，仅有部分患者需要辅助通气，对生存期无显著影响。

**1. 不典型症状 ，**某些不典型症状可在出现肢体力弱前数年甚至数十年出现，包括易疲劳、痛性痉挛、口周震颤及姿势性震颤。肌无力：多数患者以双下肢无力起病，逐渐进展至双上肢、舌肌以及面肌，而眼外肌往往不受累。肢体无力的分布多不对称，以近端受累为主。咀嚼肌受累时可引起下颌下垂及震颤。体检以下运动神经元损害体征为主，表现为肢体肌力下降，近端为著，伴肌肉萎缩、束颤，腱反射多减弱或消失。多数患者可出现明显舌肌萎缩、纤颤，但软腭活动、咽反射多正常，患者饮水呛咳、吞咽困难的程度也较轻。某些患者还可观察到颏部以及口周束颤。患者运动功能损害随病程延长逐渐加重，后期可出现上楼费力、行走不稳等。

**2. 感觉障碍** ，约半数患者存在不同程度的感觉减退，而在其他患者为亚临床性，仅在感觉神经传导检查中发现异常。

**3. 内分泌异常，** 包括葡萄糖及脂肪代谢的异常，雄激素受体不敏感等表现，后者包括男性乳房发育、性功能下降、不孕不育、睾丸萎缩等。

**4. 女性携带者，** 多数女性携带者并不出现症状，即使出现症状程度也较轻，可仅表现为束颤、轻度远端肢体无力、肌肉痉挛或肌酸肌酶增高等。

### Llama3.1:70b

根据您提供的临床表现，罕见病的诊断可能是 Kennedy 病（Kennedy’s disease），也称为脊髓性肌萎缩症（Spinal and Bulbar Muscular Atrophy，SBMA）。该病是一种 X 连锁遗传病，主要影响男性，女性通常为携带者。其特征包括肌肉萎缩、肌束颤动、构音障碍、吞咽困难、呼吸困难等，伴有雄激素受体不敏感的表现，如男性乳房发育、生殖功能降低等。超过 50% 的患者存在感觉异常。该病进展缓慢，患者通常在病程晚期才出现行走不能，仅有部分患者需要辅助通气，对生存期无显著影响。

其他可能的诊断包括：

1. **肌萎缩侧索硬化（Amyotrophic Lateral Sclerosis，ALS）**：一种影响运动神经元的疾病，导致肌肉无力和萎缩，但 ALS 的进展速度通常比 Kennedy 病快。
2. **脊髓性肌萎缩症（Spinal Muscular Atrophy，SMA）**：一种遗传性疾病，影响脊髓运动神经元，导致肌肉无力和萎缩，主要影响儿童和青少年。
3. **肌无力症（Myasthenia Gravis）**：一种自身免疫性疾病，影响神经与肌肉之间的信号传递，导致肌肉无力和疲劳。
4. **肌营养不良症（Muscular Dystrophy）**：一组遗传性疾病，导致肌肉逐渐萎缩和无力，包括多种类型，如 Duchenne 肌营养不良症和 Becker 肌营养不良症。
5. **多系统萎缩症（Multiple System Atrophy，MSA）**：一种影响中枢神经系统的疾病，导致运动功能障碍、自主神经功能障碍和认知功能障碍。

### **112. User**

部分患者在宫内表现为胎动减少，出生时为松软儿。患者在 6 个月内发病，平均发病年龄在生后 1 个月。表现为全身松软无力，严重肌张力低下。由于舌、面和咀嚼肌无力，大多数患儿出现吸吮和吞咽困难，可见舌肌萎缩和震颤。肋间肌受累可以出现呼吸困难，腹式呼吸。胸部呈钟型外观。下肢较上肢受累重，近端较远端严重。严重躯体中轴部位肌无力使患儿不能控制头部运动，不会抬头或翻身，没有独坐能力。卧位时，双下肢呈髋外展、膝屈曲的蛙腿体位。肌肉萎缩多不明显， 部分患儿轻度关节畸形。患儿智力好，腱反射消失，四肢感觉正常。患儿肌无力进行性加重，最终失去所有自主运动能力，鼻饲喂养，反复呼吸道感染而致呼吸衰竭。80％患儿 1岁内死亡，很少活过 2 岁。

### Llama3.1:70b

根据您提供的临床表现，我最有可能诊断出的是**脊髓性肌肉萎缩症（Spinal Muscular Atrophy, SMA）**，特别是其最严重的形式，即Werdnig-Hoffmann病（SMA Type I）。

其他五种可能的诊断包括：

1. **肌营养不良症（Duchenne Muscular Dystrophy, DMD）**：虽然DMD的典型表现是肌肉萎缩和无力，但其进展速度通常较慢，且常伴有明显的肌肉萎缩和假性 hypertrophy（假性增厚）。
2. **Becker型肌营养不良症（Becker Muscular Dystrophy, BMD）**：BMD是DMD的较轻形式，进展速度较慢，患者可能在青少年时期才出现症状。
3. **肌无力症（Myasthenia Gravis）**：肌无力症是一种自身免疫性疾病，表现为肌肉无力和疲劳，但其进展速度通常较慢，且常伴有眼睑下垂和吞咽困难等特征性症状。
4. **肌肉萎缩性侧索硬化症（Amyotrophic Lateral Sclerosis, ALS）**：ALS是一种成人期发病的神经退行性疾病，表现为肌肉萎缩和无力，但其进展速度通常较快，且常伴有上运动神经元症状。
5. **先天性肌肉疾病（Congenital Muscular Dystrophy, CMD）**：CMD是一组罕见的遗传性肌肉疾病，表现为肌肉萎缩和无力，但其进展速度通常较慢，且常伴有明显的肌肉萎缩和假性 hypertrophy（假性增厚）。

需要注意的是，以上诊断均需要通过进一步的检查和测试来确认，包括遗传学检查、肌电图、肌肉活检等。

### **113. User**

患者通常成年起病，隐匿起始，逐渐进展。主要表现为小脑性共济失调，包括醉汉步态、动作笨拙、吟诗样语言、眼震、复视等；锥体束损害，包括腱反射亢进、踝阵挛、病理征阳性；锥体外系症状，包括帕金森综合征、肌张力障碍、强直等。此外，部分患者可有周围神经病。部分患者在疾病早期可以锥体束损害(类似痉挛性截瘫) 或锥体外系症状(类似帕金森综合征) 为主，共济失调症状不突出。

疾病逐渐进展，起病 10~15 年后丧失行走能力。长期吞咽困难致营养不良或误吸是导致死亡的主要原因。

### Llama3.1:70b

根据您提供的临床表现，以下是可能的罕见病诊断：

1. **多系统萎缩（MSA）**：MSA是一种罕见的神经退行性疾病，主要影响自主神经系统、运动系统和平衡系统。其临床表现与您描述的症状相符，包括小脑性共济失调、锥体束损害、锥体外系症状和周围神经病变。
2. **渐进性核上性麻痹（PSP）**：PSP是一种罕见的神经退行性疾病，主要影响运动系统和平衡系统。其临床表现包括小脑性共济失调、锥体束损害和锥体外系症状。
3. **路易体病（DLB）**：DLB是一种罕见的神经退行性疾病，主要影响认知功能和运动系统。其临床表现包括小脑性共济失调、锥体束损害和锥体外系症状。
4. **前颞叶痴呆（FTD）**：FTD是一组罕见的神经退行性疾病，主要影响认知功能和行为。其临床表现包括小脑性共济失调、锥体束损害和锥体外系症状。
5. **肌萎缩侧索硬化（ALS）**：ALS是一种罕见的神经退行性疾病，主要影响运动神经元。其临床表现包括锥体束损害和周围神经病变。
6. **遗传性痉挛性共济失调（SCA）**：SCA是一组罕见的遗传性疾病，主要影响小脑和平衡系统。其临床表现包括小脑性共济失调和锥体束损害。

### **114. User**

疾病早期可能会合并胃食管反流。随着病程的进展，患者逐渐出现皮肤肿胀及硬化，并可伴皮肤色素改变，包括色素沉着或色素脱失。除皮肤受累之外，患者常会伴有关节痛及肌痛等症状，并可出现指端溃疡和毛细血管扩张。此外，患者可能会出现多个内脏器官受累。其中呼吸系统较易受累，主要表现为肺间质纤维化和肺动脉高压。患者的消化系统表现多样， 包括胃食管反流、胃窦血管扩张症、腹痛、腹泻、便秘、大便失禁等。少数患者可能会出现肾危象，主要表现为急性肾功能不全，并且常合并高血压。心脏受累可表现为心包积液、心脏传导系统异常，个别患者可出现心肌受累。神经系统方面，外周神经系统受累多见，例如三叉神经病变、周围神经病变等，罕见中枢神经受累。

### Llama3.1:70b

根据您提供的临床表现，医生可能会诊断出系统性硬化症（Systemic Sclerosis，SSc）。系统性硬化症是一种慢性自身免疫性疾病，主要特征是皮肤和内脏器官纤维化。以下是其他五种可能的诊断：

1. 混合结缔组织病（Mixed Connective Tissue Disease，MCTD）：MCTD是一种自身免疫性疾病，表现为多个结缔组织病的特征，包括系统性硬化症、系统性红斑狼疮和多肌炎等。
2. 系统性红斑狼疮（Systemic Lupus Erythematosus，SLE）：SLE是一种自身免疫性疾病，表现为多个器官和系统受累，包括皮肤、关节、肾脏、血液等。
3. 多肌炎（Polymyositis，PM）：PM是一种自身免疫性疾病，主要表现为肌肉炎症和无力。
4. 皮肌炎（Dermatomyositis，DM）：DM是一种自身免疫性疾病，主要表现为皮肤和肌肉炎症。
5. 硬皮病（Scleroderma，SD）：SD是一种自身免疫性疾病，主要表现为皮肤硬化和纤维化。

### **115. User**

患儿在出生 1~3 个月后除了出现类似苯丙酮尿症的临床症状外，还表现为多巴胺缺乏症状，如运动障碍、肌张力低下、嗜睡、眼震颤、吞咽困难等。5- 羟色胺缺乏相关症状，如反应迟钝、抑郁、失眠等。去甲肾上腺素缺乏相关症状，如躯干肌张力低下、眼睑下垂、小脑发育障碍等。还会有顽固性抽搐、反复发热，运动里程碑发育迟滞， 全身瘫痪，智能发育严重障碍等。

### Llama3.1:70b

根据您提供的临床表现，首要考虑的罕见病是**苯丙酮尿症（Phenylketonuria，PKU）**。但是，由于您提到了多巴胺、5-羟色胺和去甲肾上腺素的缺乏症状，这些症状并不典型地与PKU直接相关，因此还需要考虑其他可能的诊断。

其他五种可能的诊断包括：

1. **多巴胺反应性 dystonia（DRD）**：这是一种遗传性疾病，主要影响多巴胺的合成和代谢，导致运动障碍和肌张力低下等症状。
2. **AADC缺乏症（Aromatic L-Amino Acid Decarboxylase Deficiency）**：这种疾病影响了多巴胺和5-羟色胺的合成，导致运动障碍、肌张力低下、嗜睡等症状。
3. **BH4缺乏症（Tetrahydrobiopterin Deficiency）**：这种疾病影响了苯丙氨酸、酪氨酸和色氨酸的代谢，导致类似PKU的临床症状，以及多巴胺和5-羟色胺的缺乏症状。
4. **Menkes病（Menkes Disease）**：这是一种遗传性疾病，主要影响铜的代谢，导致神经系统发育迟滞、肌张力低下、毛发异常等症状。
5. **MMA/PA缺乏症（Methylmalonic Acidemia and Propionic Acidemia）**：这种疾病影响了支链氨基酸的代谢，导致类似PKU的临床症状，以及多巴胺和5-羟色胺的缺乏症状。

需要注意的是，这些疾病的临床表现可能有所重叠，因此需要进行进一步的检查和诊断来确定确切的疾病类型。

### **116. User**

常表现为多系统受累，其发生发展有一定的阶段性。在胎儿 / 新生儿期可以出现心脏横纹肌瘤，大部分可自发消退；出生后数月就可出现癫痫症状和皮肤症状；随着年龄的增大，出现肾血管平滑肌脂肪瘤和全身其他部位的肿瘤性病变；成年女性肺部容易出现肺部淋巴管肌瘤病。脑部病变主要包括脑皮质结节、室管膜下巨细胞星形细胞瘤和室管膜下结节等。皮肤病变包括出生后可见的白斑，逐渐生长的面部血管纤维瘤、鲨革斑、甲周纤维瘤及前额斑块等；高达 90％的患者有肾脏血管平滑肌脂肪瘤；约 50％患者病变累及视网膜，但很少影响视力；约 35％女性患者肺部出现 LAM；其他如肝脏、胰腺、消化道、骨骼也可以受累，但一般症状较轻。

### Llama3.1:70b

根据您提供的临床表现，罕见病的诊断可能是：

1. **TSC（Tuberous Sclerosis Complex）**：又称为结节性硬化症，是一种常见的遗传性多系统疾病，其特征是多器官的良性肿瘤形成，包括皮肤、脑、肾脏、心脏、肺等。临床表现包括皮肤白斑、面部血管纤维瘤、脑皮质结节、肾血管平滑肌脂肪瘤、肺部淋巴管肌瘤病等。
2. **NF1（Neurofibromatosis Type 1）**：是一种常见的遗传性神经系统疾病，其特征是神经纤维瘤的形成，包括神经纤维瘤、咖啡斑、白色皮肤病变等。
3. **VHL（Von Hippel-Lindau病）**：是一种遗传性多系统疾病，其特征是多器官的良性肿瘤形成，包括视网膜血管瘤、脑血管瘤、胰腺神经内分泌瘤等。
4. **MEN1（多发性内分泌瘤病1型）**：是一种遗传性多系统疾病，其特征是多个内分泌器官的良性肿瘤形成，包括甲状旁腺、胰腺、垂体等。
5. **BHD（Birt-Hogg-Dube综合征）**：是一种遗传性多系统疾病，其特征是多器官的良性肿瘤形成，包括皮肤纤维瘤、肺囊肿、肾细胞癌等。

### **117. User**

未被发现或未治疗的慢性型儿童大多在 10 岁以前死亡，死因通常是肝功能衰竭、神经系统受累或肝细胞癌。

急性型起病急骤、进展迅速，以急性肝功能衰竭为主要表现，临床表现为肝脾肿大、黄疸、呕吐、腹胀、厌食、嗜睡、贫血、出血倾向及生长迟缓。患儿可能具有“煮白菜” 或“烂蘑菇”的特征性气味。肝脏合成凝血因子不足，PT、APTT 明显延长，凝血因子Ⅱ、Ⅶ、Ⅸ、Ⅺ和Ⅻ水平降低，补充维生素 K 后难以纠正。肝功能衰竭可继发腹水、黄疸和消化道出血，患儿偶尔会出现持续低血糖。未治疗的患儿多于起病数周或数月内因肝功能衰竭死亡。

### Llama3.1:70b

根据您提供的临床表现，可能的罕见病诊断是：

1. **甲硫氨酸血症（Homocystinuria）**：一种遗传性代谢疾病，影响体内甲硫氨酸的代谢，导致血液和尿液中甲硫氨酸水平升高。临床表现包括肝脏问题、神经系统受累和血栓倾向等。
2. ** Maple 糖尿病（Maple Syrup Urine Disease）**：一种遗传性代谢疾病，影响体内分支链氨基酸（亮氨酸、异亮氨酸和缬氨酸）的代谢，导致这些氨基酸在血液和尿液中积累。临床表现包括肝脏问题、神经系统受累和特殊的尿液气味等。
3. **丙酮酸脱氢酶复合体缺陷（Pyruvate Dehydrogenase Complex Deficiency）**：一种遗传性代谢疾病，影响体内丙酮酸的代谢，导致能量生成不足。临床表现包括肝脏问题、神经系统受累和低血糖等。
4. **尿素循环障碍（Urea Cycle Disorder）**：一种遗传性代谢疾病，影响体内尿素的代谢，导致氨在血液中积累。临床表现包括肝脏问题、神经系统受累和呕吐等。
5. **脂肪酸氧化缺陷（Fatty Acid Oxidation Disorder）**：一种遗传性代谢疾病，影响体内脂肪酸的代谢，导致能量生成不足。临床表现包括肝脏问题、神经系统受累和低血糖等。

### **118. User**

临床表现具有明显异质性，根据起病年龄和临床表现分为 3 个类型。

**1. 心肌病型，** 是最常见的一种严重早发心脏和多脏器衰竭型，主要在新生儿和婴儿早期发病。此型发病凶险，患儿病死率高，表现为低血糖、瑞氏综合征、新生儿猝死、肥厚型和扩张型心肌病、心包积液、心律失常、肌无力、肝肿大和间歇性低血糖，肌酸激酶水平升高；心肌肥厚和心律失常可致死。

**2. 肝型 ，**较轻，为肝脏和低酮性低血糖型，婴儿后期或儿童期发病，表现为肝大和肝功能异常，空腹耐力下降和急性低酮性低血糖，不伴心肌损害和心肌肥厚，但未经及时诊断和治疗也会有生命危险。

**3. 肌病型** ，主要在青少年至成年期发病，为迟发型，症状轻，一般不伴有心肌疾病和低血糖。主要表现为运动、感染或饥饿后的横纹肌溶解和肌红蛋白尿，甚至可发生肾功能衰竭，可伴有肌无力、肌肉痛性痉挛或肌痛。

### Llama3.1:70b

根据您提供的临床表现，我最有可能诊断出的是**MCAD缺乏症（中链脂酰CoA脱氢酶缺乏症）**。

MCAD缺乏症是一种常见的脂肪酸氧化缺陷病，表现为心肌病型、肝型和肌病型三种临床类型。心肌病型主要在新生儿和婴儿早期发病，表现为低血糖、瑞氏综合征、新生儿猝死、肥厚型和扩张型心肌病、心包积液、心律失常、肌无力、肝肿大和间歇性低血糖，肌酸激酶水平升高。肝型较轻，为肝脏和低酮性低血糖型，婴儿后期或儿童期发病，表现为肝大和肝功能异常，空腹耐力下降和急性低酮性低血糖，不伴心肌损害和心肌肥厚。肌病型主要在青少年至成年期发病，为迟发型，症状轻，一般不伴有心肌疾病和低血糖。

其他五种可能的诊断包括：

1. ** VLCAD缺乏症（长链脂酰CoA脱氢酶缺乏症）**：一种脂肪酸氧化缺陷病，表现为心肌病型、肝型和肌病型三种临床类型。
2. **LCHAD缺乏症（长链3-羟脂酰CoA脱氢酶缺乏症）**：一种脂肪酸氧化缺陷病，表现为心肌病型、肝型和肌病型三种临床类型。
3. **CPT缺乏症（ carnitine棕榈酰转移酶缺乏症）**：一种脂肪酸氧化缺陷病，表现为心肌病型、肝型和肌病型三种临床类型。
4. **CPT缺乏症（ carnitine棕榈酰转移酶缺乏症）**：一种脂肪酸氧化缺陷病，表现为心肌病型、肝型和肌病型三种临床类型。
5. **MADD缺乏症（多酰CoA脱氢酶缺乏症）**：一种脂肪酸氧化缺陷病，表现为心肌病型、肝型和肌病型三种临床类型。

### **119. User**

**1. 特殊面容，** 眶周皮下组织饱满、眶距增宽、星状虹膜、人中长、嘴唇厚、宽嘴以及小下颌。

**2. 心血管疾病，** 75％ ~80％的患者存在弹性蛋白动脉病，可影响任何动脉。周围型肺动脉狭窄(37％ ~61％) 在婴儿期较常见，但随着时间的推移可有改善。瓣上型主动脉狭窄较常见(35％ ~65％)，并且随着时间的推移在 5 岁前恶化。动脉狭窄导致左心阻力增高、心肌肥厚和心力衰竭。其他异常包括肾动脉狭窄、胸主动脉狭窄、腹主动脉狭窄、二尖瓣脱垂和主动脉瓣关闭不全、肠系膜动脉狭窄、颅内血管异常等。高血压可出现在任何年龄，部分可能和肾动脉狭窄有关。

**3. 认知能力，** 75％的患者有智力障碍，通常较为轻微。认知障碍具有特征性，表现为短期记忆和语言表达能力相对较好，但在视觉空间结构性认知方面却极其薄弱。在智商方面没有性别差异的报道，且随着时间的推移，儿童的智商是稳定的。

**4. 性格、行为、心理异常，** 包括过度友好、过度移情、注意力缺陷、焦虑、恐惧。

**5. 睡眠异常 ，**睡眠问题的发生率为 65％，包括睡眠潜伏期增加和睡眠效率降低。

**6. 眼睛，** 泪道阻塞、远视(67％) 和斜视(50％) 常见。成人患者也有白内障的报道。

**7. 耳鼻喉，** 50％的患者有慢性中耳炎，90％对声音的敏感性增加，63％的儿童和92％的成人有轻度至中度听力损害，多为进行性感音神经性听力损失。大多数患者声音嘶哑或声音低沉。

**8. 牙齿** ，可有小牙、牙釉质发育不良和错位咬合。

**9. 消化系统，** 慢性腹痛是常见症状，可能的病因包括胃食管反流、裂孔疝、消化性溃疡、胆石症、憩室炎、缺血性肠病、慢性便秘和焦虑躯体化。高钙血症也可能导致易怒、呕吐、便秘和肌肉痉挛。

**10. 泌尿系统，** 尿频和遗尿症在儿童患者中很常见。

**11. 神经、肌肉** ，幼童的肌张力偏低，关节松弛，导致代偿性姿势异常，以保持身体平衡。年龄较大的儿童和成人有典型的肌张力增高和深肌腱反射活跃，导致步态僵硬而笨拙，精细运动功能受损，所有年龄段患者都有使用工具和书写困难。成人患者会有小脑共济失调和震颤。

**12. 生长发育** 。70％的婴儿体重增长不佳。

**13. 特发性高钙血症** 。多在婴幼儿期出现，见于 15％ ~50％的患者，最常见的症状为易激惹、呕吐和便秘。

**14. 内分泌。**甲状腺功能减退(10％)、青春期提前(50％)。糖耐量受损在青少年患者中的发生率为 26％。

### Llama3.1:70b

根据您提供的临床表现，罕见病的诊断可能是威廉斯综合征（Williams Syndrome）。此综合征是一种罕见的遗传性疾病，表现为多器官异常，包括心血管疾病、认知障碍、特殊面容、性格和行为异常等。

其他可能的诊断包括：

1. **费城染色体综合征（Philly Chromosome Syndrome）**：一种由于染色体异常引起的疾病，表现为多器官异常，包括心血管疾病、智力障碍和特殊面容。
2. **普瑞德威利综合征（Prader-Willi Syndrome）**：一种罕见的遗传性疾病，表现为多器官异常，包括肥胖、智力障碍、性腺功能减退和特殊面容。
3. **安吉尔曼综合征（Angelman Syndrome）**：一种罕见的遗传性疾病，表现为多器官异常，包括智力障碍、运动功能障碍和特殊面容。
4. **科 Cockayne 综合征（Cockayne Syndrome）**：一种罕见的遗传性疾病，表现为多器官异常，包括智力障碍、生长迟缓和特殊面容。
5. **Bloom 综合征（Bloom Syndrome）**：一种罕见的遗传性疾病，表现为多器官异常，包括智力障碍、生长迟缓和特殊面容。

### **120. User**

**1. 出血倾向** ，为患者常见表现，尤其血丝便多见，大部分患儿在新生儿期即可发病。其次表现为瘀斑、瘀点、咯血和血尿等出血倾向，部分严重者可出现威胁生命的消化道大出血或颅内出血等。患者出血由血小板减少引起，同时伴有血小板体积减小，但仅约 25％的病例同时具有典型三联征表现。

**2. 感染 。**患儿易患各种感染，包括单纯疱疹病毒、巨细胞病毒、肺炎链球菌、真菌等。患者免疫缺陷随年龄增长而加重。

**3. 湿疹。** 约 80％的 WAS 患儿可出现湿疹，范围和严重程度差异很大。可有典型严重、难治性湿疹，也可非常轻微，甚至无湿疹表现。

**4. 自身免疫性疾病。** 最常见为自身免疫性溶血性贫血。其他包括血管炎、关节炎、肾脏疾病、炎症性肠病、中性粒细胞减少症和自身免疫性血小板减少性紫癜等。

**5. 恶性肿瘤 。**患儿发生淋巴系统恶性肿瘤的风险明显增高，尤其是淋巴瘤，非霍奇金淋巴瘤、淋巴细胞白血病、骨髓增生异常综合征、精原细胞瘤等均有报道。

**6. 其他 。**该病患儿可具有完全正常的血小板水平，但中性粒细胞持续或反复减少。

### Llama3.1:70b

根据您提供的临床表现，我最有可能诊断出的是 **Wiskott-Aldrich 综合征（WAS）**。WAS 是一种罕见的 X 连锁遗传性疾病，主要影响男孩，表现为出血倾向、感染、湿疹和自身免疫性疾病等。患者的血小板数量减少，血小板体积也减小，导致出血倾向。同时，患者的免疫系统功能受损，容易感染各种病原体。此外，患者还可能出现湿疹、自身免疫性疾病和恶性肿瘤等其他临床表现。

其他五种可能的诊断包括：

1. **血小板无力症（Thrombocytopenia）**：一种因血小板数量减少导致的出血倾向疾病。
2. **免疫缺陷病（Immunodeficiency）**：一种因免疫系统功能受损导致的疾病，患者容易感染各种病原体。
3. **自身免疫性溶血性贫血（Autoimmune Hemolytic Anemia）**：一种因自身免疫反应导致的溶血性贫血。
4. **淋巴增生性疾病（Lymphoproliferative Disorder）**：一种因淋巴细胞异常增殖导致的疾病，可能发展为淋巴瘤。
5. **骨髓增生异常综合征（Myelodysplastic Syndrome）**：一种因骨髓功能异常导致的疾病，可能发展为白血病。

### **121. User**

最常见的临床表现是 EB 病毒感染后的暴发性传染性单核细胞增多症、异常免疫球蛋白血症及 B 细胞淋巴瘤。异常免疫球蛋白血症及 B 细胞来源的淋巴瘤可发生于没有 EB 病毒感染的患者。异常免疫球蛋白血症通常表现为 IgA 及 IgM水平升高，而 IgG1 和 IgG3 减少。淋巴瘤以非霍奇金 B 淋巴细胞瘤为主。其他临床表现包括淋巴细胞性脉管炎、再生障碍性贫血和淋巴瘤样肉芽肿病等。某些患者临床表现轻微， 仅有异常免疫球蛋白血症或轻度 EB 病毒感染，可能是由于发生了体细胞基因修复。

部分患者主要临床表现为噬血细胞综合征，以脾肿大多见，且不一定需要 EBV 感染诱发。部分患者可出现肠道炎症如克罗恩病或结肠炎，但很少发展为淋巴瘤。

### Llama3.1:70b

根据您提供的临床表现，可能的罕见病诊断包括：

1. **X连锁淋巴增殖症（XLP）**：该病症主要影响男性，表现为 EB 病毒感染后的暴发性传染性单核细胞增多症、异常免疫球蛋白血症及 B 细胞淋巴瘤等。
2. **Wiskott-Aldrich 综合征**：该病症表现为淋巴细胞性脉管炎、再生障碍性贫血和淋巴瘤样肉芽肿病等。
3. **免疫缺陷病**：该病症表现为异常免疫球蛋白血症、再生障碍性贫血和淋巴瘤等。
4. **淋巴增殖性疾病**：该病症表现为淋巴细胞增多、淋巴瘤和异常免疫球蛋白血症等。
5. **免疫调节疾病**：该病症表现为异常免疫球蛋白血症、淋巴细胞增多和淋巴瘤等。
