# Supplementary material for: Performance of ChatGPT-4o and Four Open-Source Large Language Models in Generating Diagnoses Based on China’s Rare Disease Catalog: Comparative Study
Source: J Med Internet Res. 2025 Jun 18;27:e69929. doi: 10.2196/69929 (PMC12192912; doi:10.2196/69929)
Supplement: Multimedia Appendix 1 [file jmir-v27-e69929-s001.zip › numbered_Llama3.1-70B-en.docx]

numbered_llama3.1_70b_en

**1. user 2024-11-30T14:07:09Z:**

“1.Skeletal abnormalities: There is variation in the severity of the disease and the type of associated complications. Thoracic stenosis is present in all cases, and the thorax may be bell-shaped or long and narrow, with respiratory distress usually caused by a small, rigid thorax. The severity of respiratory insufficiency varies according to the type of thorax and the degree of underlying pulmonary dysplasia, and the age of onset varies. There is a negative correlation between the severity of respiratory insufficiency and the age at which the disease is diagnosed. Acute respiratory disease is more common in infants with severe restrictive and bell-shaped chests, and severe and fatal respiratory failure can occur in the early neonatal period. Infants with a long, narrow chest are more likely to present with mild respiratory involvement, which can eventually progress to chronic restrictive lung disease with recurrent respiratory infections. Short, deformed ribs cause intercostal muscle weakness and restricted breathing, leading to respiratory failure. Most children die within 2 years of age, with about half dying within 6 months of birth. Other skeletal anomalies include short ribs, short limbs, abnormally shaped pelvis, short fingers, and polydactyly. About 60% of patients have proximal cervical spinal stenosis. 2. Involvement of other organs: Renal lesions are present in about 40% of patients. The earliest sign of renal disease is impaired urinary concentration. Other signs include polyuria, proteinuria, elevated blood urea nitrogen and creatinine, and hypertension. Renal failure is the leading cause of death in this patient between the ages of 3 and 10 years. Hepatic involvement occurs in about 30% of cases and is manifested by hepatomegaly, elevated aminotransferases, cholestasis, cirrhosis and portal hypertension. The disease is occasionally associated with ocular developmental abnormalities, including optic nerve hypoplasia, retinal dystrophy, retinal pigmentation abnormalities, nystagmus, myopia and progressive visual impairment. There are also a few reports suggesting that these patients may also present with neurological abnormalities such as growth retardation, hypotonia, and central nervous system MRI abnormalities.

”

**llama3.1:70b** 2024-11-30T14:07:36Z:

What a fascinating case!

Based on the clinical manifestations you provided, I would propose the following diagnosis:

**Primary Diagnosis:** Asphyxiating Thoracic Dystrophy (ATD), also known as Jeune syndrome.

This rare genetic disorder is characterized by a small thorax, short ribs, and respiratory distress, which aligns with the skeletal abnormalities described in your case. The involvement of other organs, such as renal lesions and hepatic dysfunction, also matches the presentation of ATD.

**Five Other Possible Diagnoses:**

1. **Spondyloepiphyseal dysplasia congenita (SEDC)**: This rare genetic disorder affects bone growth and development, leading to skeletal abnormalities, including thoracic stenosis and short limbs.
2. **Cerebrocostomandibular syndrome**: This rare condition is characterized by a small thorax, rib anomalies, and other skeletal abnormalities, as well as potential neurological involvement.
3. **Ellis-van Creveld syndrome**: This genetic disorder affects bone growth and development, leading to short limbs, polydactyly, and other skeletal abnormalities, including thoracic stenosis.
4. **Thoracic-pelvic-phalangeal dystrophy**: This rare condition is characterized by a small thorax, rib anomalies, and other skeletal abnormalities, as well as potential involvement of the pelvis and phalanges.
5. **Short-rib polydactyly syndrome (SRPS)**: This genetic disorder affects bone growth and development, leading to short ribs, polydactyly, and other skeletal abnormalities, including thoracic stenosis.

Please note that these alternative diagnoses are based on the clinical manifestations you provided, but a definitive diagnosis can only be made after a comprehensive evaluation, including imaging studies, laboratory tests, and genetic analysis.

**2. user 2024-11-30T14:07:55Z:**

The disease can start from birth to old age and can be acute or insidious. Almost any system or organ can be involved, with a predominance of energy-demanding organs such as the brain, heart, kidneys, eyes, ears, muscles, and gastrointestinal tract. Patients may present with a clinical syndrome of some kind, a single symptom or a combination of symptoms.

**llama3.1:70b** 2024-11-30T14:08:21Z:

What a fascinating case!

Based on your description, I’m going to take an educated guess that the rare disease most likely to be diagnosed is:

**Mitochondrial Myopathy**

This condition affects the mitochondria, which are the energy-producing structures within cells. Mitochondrial myopathies can present with a wide range of symptoms affecting various organs and systems, including those you mentioned (brain, heart, kidneys, eyes, ears, muscles, and gastrointestinal tract). The age of onset can vary from birth to old age, and the disease can be acute or insidious.

Now, here are five other possible diagnoses that could be considered:

- **Ehlers-Danlos Syndrome**: A group of genetic disorders affecting connective tissue, which can lead to a wide range of symptoms involving multiple organ systems.
- **Familial Amyloid Polyneuropathy (FAP)**: A rare inherited disorder caused by mutations in the transthyretin gene, leading to amyloid deposits in various organs and tissues, resulting in diverse clinical manifestations.
- **MELAS Syndrome**: A mitochondrial encephalomyopathy, lactic acidosis, and stroke-like episodes syndrome, which can present with a range of symptoms including seizures, muscle weakness, and gastrointestinal issues.
- **Cystic Fibrosis-related Disorders (CFRD)**: While cystic fibrosis is typically associated with respiratory symptoms, CFRD can involve other organs such as the pancreas, liver, and intestines, leading to diverse clinical presentations.
- **Fabry Disease**: A genetic disorder caused by deficiency of the enzyme alpha-Galactosidase A, which leads to accumulation of globotriaosylceramide in various cells and tissues, resulting in a range of symptoms including pain, gastrointestinal issues, and renal problems.

These diagnoses are all relatively rare and can present with complex, multi-systemic symptoms.

**3. user 2024-11-30T14:08:42Z:**

The prevalence of the disease is between 5 and 50 years old, with a median age of onset of 39 years. The prevalence of women is significantly higher than that of men, with a female:male ratio of (9~11):1. Most of the cases have an acute/subacute onset, and the clinical manifestations include six core syndromes: (1) optic neuritis; (2) acute myelitis; (3) posterior pole syndrome; (4) acute brainstem syndrome; (5) symptomatic sleep attacks or acute mesencephalic clinical syndrome with typical MRI foci of the mesencephalon; and (6) symptomatic cerebral syndrome with typical brain lesions. It is a highly recurrent and disabling disease, with more than 90 per cent of patients having a recurrent course, and the majority of patients are left with more severe neurological disability.

**llama3.1:70b** 2024-11-30T14:09:15Z:

A fascinating case!

Based on the clinical manifestations you provided, I would propose the following diagnosis:

**Primary Diagnosis:** Neuromyelitis Optica Spectrum Disorder (NMOSD)

Reasoning:

- Demographics: NMOSD predominantly affects women, with a female:male ratio of 9-11:1, which matches your description.
- Age of onset: The median age of onset is around 39 years, consistent with your information.
- Clinical manifestations: All six core syndromes you mentioned are characteristic of NMOSD:
  - Optic neuritis (syndrome 1)
  - Acute myelitis (syndrome 2)
  - Posterior pole syndrome (syndrome 3), also known as posterior reversible encephalopathy syndrome
  - Acute brainstem syndrome (syndrome 4)
  - Symptomatic sleep attacks or acute mesencephalic clinical syndrome with typical MRI foci of the mesencephalon (syndrome 5)
  - Symptomatic cerebral syndrome with typical brain lesions (syndrome 6)

**Alternative Diagnoses:**

- **Multiple Sclerosis (MS)**: While MS is also a demyelinating disease, its clinical manifestations and demographics differ from NMOSD. However, some cases of MS may present similarly.
- **Aquaporin-4 Negative Neuromyelitis Optica**: A subtype of NMOSD with similar clinical features but lacking the presence of aquaporin-4 antibodies.
- **Relapsing-remitting Multiple Sclerosis with Aquaporin-4 Antibodies**: Some cases of relapsing-remitting MS may present with aquaporin-4 antibodies, making them difficult to distinguish from NMOSD.
- **Systemic Lupus Erythematosus (SLE)**: Although SLE is an autoimmune disease that can cause neurological symptoms, its clinical manifestations and demographics are different from those of NMOSD.
- **CNS Vasculitis**: A rare condition characterized by inflammation of blood vessels in the central nervous system, which may present with a range of neurological symptoms.

Please note that these alternative diagnoses should be considered based on further diagnostic testing and evaluation to rule out other possibilities.

What do you think? Do these suggestions align with your thinking?

**4. user 2024-11-30T14:09:25Z:**

Hearing impairment. In the past, it was thought that the deafness was congenital, which could be identified by hearing screening, and that it would be apparent from birth, mostly involving both sides, and symmetrically severe prelingual deafness. In recent years, it has been found that individual cases may present with asymmetrical or unilateral hearing loss, and the degree of hearing loss is highly variable, ranging from mild to very severe in different patients; progressive deafness that occurs after birth is also not uncommon.

**llama3.1:70b** 2024-11-30T14:09:54Z:

A fascinating case! Based on the clinical manifestations you provided, I’m going to take a educated guess:

**Most likely diagnosis:** Pendred Syndrome (also known as Pendrin Syndrome or Bilateral Sensorineural Hearing Loss with Euthyroid Goiter)

Pendred syndrome is a rare genetic disorder that affects approximately 1 in 15,000 individuals. It is characterized by bilateral sensorineural hearing loss, which can be congenital or progressive, and often associated with thyroid abnormalities (euthyroid goiter). The degree of hearing loss can vary greatly among affected individuals.

**Five other possible diagnoses:**

- **Usher Syndrome**: A rare genetic disorder that affects approximately 1 in 30,000 individuals, characterized by sensorineural hearing loss, progressive vision loss, and balance problems.
- **Stickler Syndrome**: A rare genetic disorder that affects approximately 1 in 75,000 individuals, characterized by sensorineural hearing loss, eye abnormalities (e.g., vitreoretinal degeneration), and joint hypermobility.
- **Branchio-Oto-Renal (BOR) Syndrome**: A rare genetic disorder that affects approximately 1 in 40,000 individuals, characterized by hearing loss, branchial clefts or cysts, and renal anomalies.
- **CHARGE Syndrome**: A rare genetic disorder that affects approximately 1 in 10,000 individuals, characterized by Coloboma (eye abnormalities), Heart defects, Atresia choanae (nasal passage blockage), Restricted growth and development, Genital hypoplasia, and Ear anomalies (hearing loss).
- **Klippel-Feil Syndrome**: A rare genetic disorder that affects approximately 1 in 42,000 individuals, characterized by sensorineural hearing loss, fusion of two or more cervical vertebrae, and other skeletal abnormalities.

Please note that a definitive diagnosis can only be made through a comprehensive evaluation, including medical history, physical examination, laboratory tests (e.g., genetic testing), and imaging studies.

**5. user 2024-11-30T14:10:45Z:**

“**1. polyneuropathy**, often presenting as a symmetrical sensory and/or motor peripheral neuropathy of the extremities, progressing gradually from distal to proximal.

**2. organomegaly,** including hepatomegaly, splenomegaly, or lymph node enlargement; lymph node biopsy is often suggestive of Castleman disease.

**Endocrine abnormalities,** including hypogonadism (e.g., male impotence, breast development), hypothyroidism, abnormal glucose metabolism (e.g., diabetes mellitus), and adrenal insufficiency.

**4. skin changes,** which may be manifested by deepening of skin colour, cutaneous glomeruloid haemangiomas, white nails, polycythemia, hirsutism, and cyanosis of the hands and feet.

**5. increased extracirculatory water load,** including limb oedema, ascites, pleural effusion, pericardial effusion, and optic papilla oedema.

**Sclerosing bone disease,** is an important clinical manifestation. It may be manifested as bone pain or without clinical symptoms. Sclerosing bone disease can be detected significantly more frequently with CT scanning of the bones, and can be visualised as simple sclerosis of the bones, mixed sclerotic and osteolytic lesions, or simple osteolytic changes.

**7. erythrocytosis and/or thrombocytosis** **8.

**8. pulmonary hypertension** , patients may present with pulmonary hypertension, as evidenced by reduced activity tolerance, hypoxaemia, etc.

**9. cerebral infarction**.

”

**llama3.1:70b** 2024-11-30T14:11:10Z:

Based on the clinical manifestations you provided, I would say that the most likely rare disease to be diagnosed is **POEMS syndrome**, a paraneoplastic disorder associated with plasma cell dyscrasias, such as multiple myeloma or Waldenström’s macroglobulinemia.

Here are five other possible diagnoses that could be considered based on these clinical manifestations:

- **Multicentric Castleman disease**: This is a rare lymphoproliferative disorder characterized by the presence of human herpesvirus 8 (HHV-8) and can present with similar systemic symptoms, including polyneuropathy, organomegaly, and skin changes.
- **Amyloidosis**: Amyloid light chain (AL) amyloidosis or transthyretin-related hereditary amyloidosis could be considered given the presence of neuropathy, organomegaly, and skin changes.
- **Familial Mediterranean fever**: This is an autosomal recessive disorder characterized by recurring episodes of fever, polyserositis, and erysipelas-like rash, which can also present with systemic symptoms similar to POEMS syndrome.
- **Sjögren’s syndrome**: Although more commonly associated with autoimmune disorders, Sjögren’s syndrome can also present with systemic manifestations such as neuropathy, organomegaly, and skin changes.
- **Mastocytosis**: Systemic mastocytosis is a rare disorder characterized by the accumulation of mast cells in various organs, including bone marrow, lymph nodes, liver, spleen, and skin, leading to similar systemic symptoms.

Keep in mind that these diagnoses are speculative based on the provided clinical manifestations and would require further evaluation and testing for confirmation.

**6. user 2024-11-30T21:07:05Z:**

The disease can begin in early childhood. Typical clinical manifestations include kidney and eye changes and hearing impairment. Renal manifestations are hematuria, proteinuria and progressive deterioration of renal function, in which hematuria is often persistent microscopic hematuria, which can occur after exercise or fever, and is common in adolescents. Hearing changes were sensorineural hearing loss, which gradually aggravated with the course of disease. Eye abnormalities manifest as myopia, youth ring and cataract, but lack specificity. Three ocular changes of diagnostic significance include anterior conical lens, posterior pleomorphic corneal atrophy, and retinal macula (white or yellow particles around the fovea). The incidence of hearing impairment in male patients in China was higher than that reported abroad (68% vs 55%), while that in female patients was lower than that reported abroad (7% vs 45%). The incidence of ocular abnormalities was similar to that reported abroad. Leiomyomas may also occur in a few patients, involving the respiratory tract, gastrointestinal tract, and female reproductive tract; occasionally aneurysmal lesions, midfacial dysplasia, and mental retardation have been reported.

Patients often have a family history. Male patients with autosomal recessive inheritance and X-linked inheritance progressed faster, often entering end-stage renal disease at the age of 16~35, while female patients with autosomal dominant inheritance and X-linked inheritance had a slower course of disease and later onset of renal failure. The age at which renal failure occurs is similar for all male members of the same affected family, but can vary among male members of different families. Therefore, renal failure can be divided into juvenile type (renal failure occurs in adolescence) and adult type (renal failure occurs in adulthood) according to the age of the affected family. Adolescent type is more prominent in extrarenal manifestations, boys usually die before reproduction, disease genes are often new mutations, while adult type families have older patients and fewer new mutations.

**llama3.1:70b** 2024-11-30T21:07:35Z:

What a fascinating case!

Based on the clinical manifestations you provided, I’m going to take a educated guess that the most likely rare disease diagnosis is:

**Alport Syndrome**

This genetic disorder affects approximately 1 in 50,000 births and is characterized by kidney disease (nephritis), hearing loss, and eye abnormalities. The disease can begin in early childhood, and the clinical manifestations you described, including hematuria, proteinuria, progressive renal deterioration, sensorineural hearing loss, and specific ocular changes (anterior conical lens, posterior pleomorphic corneal atrophy, and retinal macula changes), are all consistent with Alport Syndrome.

Here are five other possible diagnoses to consider:

- **Fabry Disease**: A genetic disorder caused by a deficiency of the enzyme alpha-Gal A, leading to the accumulation of globotriaosylsphingosine in various organs, including the kidneys, heart, and nervous system.
- **Bartter Syndrome**: A rare genetic disorder characterized by impaired function of the Na-K-Cl cotransporter in the kidney, leading to hypokalemia, alkalosis, and hypercalciuria.
- **Nail-Patella Syndrome**: A rare genetic disorder affecting the development of nails, kneecaps, elbows, and kidneys, often associated with renal disease and hearing loss.
- **Townes-Brockes Syndrome**: A rare genetic disorder characterized by a combination of anomalies, including kidney disease, hearing loss, and eye abnormalities.
- **Branchio-Oto-Renal (BOR) Syndrome**: A rare genetic disorder affecting the development of the branchial arches, ears, and kidneys, often associated with renal disease, hearing loss, and eye abnormalities.

Please note that a definitive diagnosis can only be made through a combination of clinical evaluation, laboratory tests, and genetic analysis.

**7. user 2024-11-30T21:07:45Z:**

Patients often have no abnormal performance in the neonatal period. The first symptom is developmental retardation at about 6 months of age, but the typical clinical manifestations appear after 1 year of age, and it often takes several years for clinical diagnosis to be confirmed. According to the frequency of clinical manifestations, they were divided into three categories: common manifestations (100% patients), frequent manifestations (more than 80% patients) and correlated manifestations (20% ~80% patients).

- All of them showed normal pregnancy history and head circumference at birth; no birth defects and abnormal biochemical indexes; brain MRI/CT showed no structural abnormalities except mild cortical atrophy and myelin dysplasia; motor milestones lagged behind without regression; severe developmental retardation occurred at 6~12 months; language disorders showed no or very little vocabulary, repetitive language and non-verbal communication ability were stronger than expressive language ability; motor or balance disorders often showed ataxia and tremor of limbs; Abnormal behavioral characteristics are frequent laughter or smiling, obvious excited movements or happy behavior, often accompanied by clapping or hyperactivity.
- The head circumference often showed backward growth, and microcephaly was still present at the age of 2. Epilepsy often occurred before the age of 3. Abnormal EEG: characteristic high amplitude spike-slow wave.
- Correlation: flat occipital region; tongue preference; sucking or swallowing disorders; infantile feeding difficulties; low muscle tone; large jaw; wide tooth space; frequent salivation; excessive chewing movements; strabismus; hypopigmentation of the skin; light hair and eye color compared to family (absent type only); hyperreflexia of lower limb tendons; lifting or bending of upper limbs, especially when walking; wide gait base; high sensitivity to heat; abnormal sleep-wake cycle, less sleep; obsession with water, paper, and plastic; Eating related behavioral abnormalities; Obesity (more common in older, non-deficit patients); scoliosis; constipation.

**llama3.1:70b** 2024-11-30T21:08:16Z:

A fascinating case! Based on the clinical manifestations you provided, I would propose that the most likely rare disease diagnosis is:

**Angelman Syndrome**

This neurogenetic disorder was first described by Dr. Harry Angelman in 1965. The characteristic features of Angelman syndrome include developmental delay, intellectual disability, severe speech impairment, ataxia, and unusual behaviors such as frequent laughter and hyperactivity.

The clinical manifestations you provided match many of the diagnostic criteria for Angelman syndrome:

- Normal prenatal and perinatal history
- Developmental delay and intellectual disability
- Severe speech impairment with minimal or no expressive language
- Ataxia and tremors
- Epilepsy (common in AS patients)
- Characteristic EEG pattern (high-amplitude spike-slow wave complexes)
- Microcephaly (common in AS patients)
- Unusual behaviors such as frequent laughter, hyperactivity, and hand clapping

Now, here are five other possible diagnoses that could be considered based on some of the clinical features you provided:

- **Rett Syndrome**: a genetic disorder characterized by developmental delay, intellectual disability, seizures, and stereotypical hand movements.
- **Prader-Willi Syndrome**: a genetic disorder characterized by developmental delay, intellectual disability, obesity, short stature, and behavioral problems such as hyperphagia and skin picking.
- **Williams Syndrome**: a genetic disorder characterized by developmental delay, intellectual disability, distinctive facial features, and cardiovascular abnormalities.
- **Smith-Magenis Syndrome**: a rare genetic disorder characterized by developmental delay, intellectual disability, seizures, and self-injurious behaviors.
- **15q13.3 Microdeletion Syndrome**: a rare genetic disorder characterized by developmental delay, intellectual disability, seizures, and behavioral problems such as anxiety and attention deficit hyperactivity disorder (ADHD).

Please note that each of these alternative diagnoses would require further evaluation and diagnostic testing to confirm or rule out.

**8. user 2024-11-30T21:08:21Z:**

The main symptoms include mental behavioural abnormalities, cognitive impairment, memory loss, seizures, speech disorders, movement disorders, involuntary movements, decreased level of consciousness, and autonomic dysfunction. Other accompanying symptoms include sleep disorders, focal damage to the CNS, brainstem and cerebellar symptoms, and involvement of peripheral nerves and neuromuscular junctions.

There are three main clinical types: (1) anti-NMDAR encephalitis, which is the most predominant type and is characterised by diffuse encephalitis. (ii) Borderline encephalitis, with mental behavioural abnormalities, temporal lobe epilepsy and near-memory deficits as the main symptoms. The common types of borderline encephalitis are anti-LGI1 antibody, anti-GABABR antibody and anti-AMPAR antibody-related encephalitis. (iii) Other types, including Morvant syndrome, anti-DPPX antibody-associated encephalitis, anti-IgLON5 antibody-associated encephalopathy, and autoimmune cerebellar ataxia.

**llama3.1:70b** 2024-11-30T21:08:54Z:

Based on the clinical manifestations you provided, the rare disease most likely to be diagnosed is:

**Anti-NMDAR Encephalitis**

This condition is characterized by diffuse encephalitis and is known for its association with mental behavioral abnormalities, cognitive impairment, memory loss, seizures, speech disorders, movement disorders, and decreased level of consciousness. Anti-NMDAR encephalitis is an autoimmune disorder caused by the presence of antibodies against N-methyl-D-aspartate receptors (NMDARs), which are critical components of the nervous system.

Here are five other possible diagnoses that may be considered based on the symptoms:

- **Hashimoto Encephalopathy**: This rare autoimmune condition involves inflammation of the brain and is associated with Hashimoto’s thyroiditis, an autoimmune disorder affecting the thyroid gland. Symptoms include seizures, cognitive impairment, memory loss, and behavioral changes.
- **Rasmussen Encephalitis**: A rare inflammatory neurological disorder that primarily affects children, characterized by focal damage to the CNS, leading to hemispheric atrophy and resulting in mental behavioral abnormalities, seizures, and motor dysfunction.
- **Progressive Multifocal Leukoencephalopathy (PML)**: A rare viral disease of the CNS caused by the JC virus. Symptoms include cognitive impairment, memory loss, speech disorders, movement disorders, and decreased level of consciousness, often with a subacute progression.
- **Creutzfeldt-Jakob Disease**: A rare neurodegenerative disorder caused by an abnormal form of prion protein. Symptoms include rapid cognitive decline, memory loss, personality changes, difficulty with coordination and balance, and vision problems.
- **Bickerstaff Encephalitis**: An autoimmune condition that affects the brainstem, characterized by symptoms such as sudden onset of mental status changes, confusion, seizures, ataxia, ophthalmoplegia (paralysis or weakness of eye muscles), and areflexia.

These conditions should be considered in the differential diagnosis, but anti-NMDAR encephalitis is the most likely diagnosis based on the provided clinical manifestations.

**9. user 2024-11-30T21:09:43Z:**

The clinical manifestations of the disease are complex and varied, and lack specificity. Most of them involve multiple systems such as nerve, skin, respiratory, digestive and immune systems, and it is difficult to confirm the diagnosis from the clinical manifestations alone. It can be divided into two types: complete and partial.

- Complete: It can be divided into two types: early-onset and late-onset. The most common manifestations are neurological symptoms, such as hypotonia, epilepsy, developmental delay, sensorineural deafness, ataxia, and progressive disorders of consciousness, etc. About half of the patients have ataxia, developmental delay, conjunctivitis, and visual problems, including visual problems. conjunctivitis, and visual problems, including optic atrophy. Metabolic disorders such as metabolic acidosis, hypoglycaemia and ketosis are often comorbid, and cutaneous manifestations include various forms of refractory rashes such as eczema, generalised erythema, and nappy rash, and patients may also present with respiratory symptoms such as hyperventilation, laryngeal wheezing, and apnoea, as well as digestive symptoms such as feeding difficulties, vomiting, and diarrhoea. Acute attacks can be triggered by infection, fever, fatigue, and a high protein diet. If treatment is not timely, it can often lead to irreversible damage. Patients with late-onset disease often develop in adolescence and may have no symptoms beforehand. The clinical phenotype is different from that of early-onset disease, which usually presents with peripheral neuropathy, muscle weakness, spastic paraparesis, skin rashes, and ocular problems, such as loss of vision and visual acuity, etc. Failure to treat the disease in a timely manner often leads to irreversible damage.
- Partial : May develop at all ages or even without onset. The overall signs and symptoms of the child are relatively mild, starting only in emergency situations such as infections and starvation. Patients may have hypotonia, skin rashes, hair loss, acute neurological symptoms such as epilepsy and impaired consciousness.

**llama3.1:70b** 2024-11-30T21:10:14Z:

Based on the clinical manifestations you provided, I would consider the following possible diagnoses:

**Most likely diagnosis:**

- **Mitochondrial Neurogastrointestinal Encephalomyopathy (MNGIE)**: This rare genetic disorder affects multiple systems, including the nervous system, skin, respiratory, and digestive systems. It is characterized by complex and varied manifestations, including neurological symptoms, metabolic disorders, cutaneous manifestations, and digestive problems.

**Other possible diagnoses:**

1. **Ehlers-Danlos Syndrome (EDS)**: A group of genetic disorders that affect the body’s connective tissue, leading to skin hyperextensibility, joint hypermobility, and tissue fragility. Some patients with EDS may present with neurological symptoms, such as developmental delay and seizures.
2. **Familial Hemiplegic Migraine (FHM)**: A rare genetic disorder characterized by recurrent episodes of migraine headaches, often accompanied by neurological symptoms, such as hemiparesis, seizures, and ataxia.
3. **Leigh Syndrome**: A rare neurometabolic disorder that affects the nervous system, causing progressive degeneration of the brain and spinal cord. It is characterized by developmental delay, seizures, and metabolic disorders.
4. **Alpers-Huttenlocher Syndrome (AHS)**: A rare genetic disorder that affects the liver and nervous system, leading to progressive liver disease and neurological symptoms, such as seizures, ataxia, and developmental delay.

**Additional considerations:**

Given the variability of clinical manifestations and the lack of specificity, other diagnoses may need to be considered. A thorough diagnostic workup, including genetic testing, imaging studies, and laboratory tests (e.g., metabolic panels), will likely be necessary to confirm a diagnosis.

It is essential to note that these conditions are extremely rare, and an accurate diagnosis can only be made by a multidisciplinary team of specialists with expertise in the relevant fields.

**10. user 2024-11-30T21:11:18Z:**

The high prevalence age group is infancy, which manifests as adrenocortical insufficiency with a predominant salt loss, and common symptoms include hyperkalemia, hyponatremia with dehydration, metabolic acidosis, hypoglycaemia, vomiting, shock, and skin pigmentation. Most patients present with failure or incomplete development during puberty, or impaired fertility, oligospermia and azoospermia. A few patients may present with early childhood hyperandrogenaemia, simple saline corticosteroid deficiency, and pseudo-precocious puberty or spontaneous pubertal development during puberty. In mild cases, the disease may start in adulthood.

Patients have hyperpigmentation of the skin, especially in exposed areas, friction areas, scarring and areola, axilla, scrotum.

Patients have varying degrees of reduced resistance to various stresses such as infection and trauma. In the event of a stressful situation, adrenal crisis can occur, manifested by nausea, vomiting, abdominal pain or diarrhoea, severe dehydration, lowered blood pressure, rapid heart rate, weak pulse, mental disorders, and often high fever, hypoglycaemia, hyponatraemia and even life-threatening.

**llama3.1:70b** 2024-11-30T21:11:45Z:

Based on the clinical manifestations you provided, I would say that the most likely diagnosis is Congenital Adrenal Hyperplasia (CAH) due to 21-Hydroxylase Deficiency.

Here are five other possible diagnoses:

- **Familial Glucocorticoid Deficiency**: This rare genetic disorder affects the production of cortisol and, in some cases, aldosterone, leading to adrenal insufficiency, hyperpigmentation, and increased susceptibility to infections.
- **Triple A Syndrome (Allgrove Syndrome)**: This is a rare autosomal recessive disorder characterized by achalasia, alacrima, and adrenal insufficiency due to ACTH resistance, which may present with similar symptoms of adrenal crisis and skin hyperpigmentation.
- **X-Linked Adrenoleukodystrophy**: Although primarily a neurological disorder, X-linked adrenoleukodystrophy can also affect the adrenal glands, leading to Addison’s disease-like symptoms, including hyperpigmentation and increased susceptibility to infections.
- **Adrenal Hypoplasia Congenita**: This rare genetic disorder affects the development of the adrenal glands, leading to primary adrenal insufficiency, which may present with similar symptoms of salt loss, dehydration, and skin hyperpigmentation.
- **Triple H Syndrome (Hereditary Adrenal Insufficiency)**: This is a rare autosomal recessive disorder characterized by hereditary adrenal insufficiency due to impaired ACTH stimulation, which may present with similar symptoms of adrenal crisis and skin hyperpigmentation.

Please note that these diagnoses are speculative and would require further investigation, including genetic testing and hormonal evaluations, to confirm the diagnosis.

**11. user 2024-11-30T21:11:52Z:**

The disease can develop from birth to early adulthood. Neonatal onset is the most common, often presenting as macrosomia, poor feeding, hunger intolerance and persistent hypoglycaemia. Hypoglycaemic episodes may present with lethargy and hypotonia, as well as neurological sequelae or even death, such as apnoea, seizures and coma. In the delayed type, patients often show a hypoglycaemic reaction, including symptoms such as pallor, sweating and palpitations.

**llama3.1:70b** 2024-11-30T21:12:25Z:

Based on the clinical manifestations you provided, I would suspect that the rare disease most likely to be diagnosed is:

**Congenital Hyperinsulinism (CHI)**: This is a group of rare genetic disorders characterized by excessive insulin secretion, leading to hypoglycemia. The symptoms you described, such as macrosomia, poor feeding, hunger intolerance, and persistent hypoglycemia in neonates, are consistent with CHI. Additionally, the delayed type presentation with hypoglycaemic reactions, including pallor, sweating, and palpitations, is also typical of CHI.

Here are five other possible diagnoses to consider:

- **Kabuki Syndrome**: A rare genetic disorder characterized by developmental delay, intellectual disability, and various physical anomalies. Some patients may present with hypoglycemia, macrosomia, and seizures.
- **Beckwith-Wiedemann Syndrome (BWS)**: A rare genetic overgrowth disorder that can present with macrosomia, hypoglycemia, and omphalocele (a congenital abdominal wall defect). Patients may also experience apnea, seizures, or coma due to hypoglycemia.
- **Pearson Syndrome**: A rare mitochondrial disorder characterized by sideroblastic anemia, pancreatic insufficiency, and various other systemic manifestations. Hypoglycemia and macrosomia can be present in some cases.
- **Congenital Adrenal Hyperplasia (CAH)**: A group of genetic disorders affecting adrenal steroidogenesis. Some forms of CAH, such as 21-hydroxylase deficiency, may present with hypoglycemia, macrosomia, and seizures due to cortisol and aldosterone deficiencies.
- **Pancreatic Agenesis or Hypoplasia**: Rare congenital anomalies characterized by partial or complete absence of pancreatic tissue. Patients may present with neonatal diabetes, hypoglycemia, and macrosomia.

Please note that these alternative diagnoses are less likely based on the provided clinical manifestations, but should still be considered in a differential diagnosis approach.

**12. user 2024-11-30T21:12:35Z:**

1. Type I, abdominal distension, growth retardation, hypoglycemic convulsions, recurrent nosebleeds, diarrhoea and vomiting are the main reasons for children’s patients to visit the clinic, and very rarely, the first manifestation is hematuria, blood in the stool, recurrent bone fracture, anaemia or gout. Adult patients who are never diagnosed and treated may present with multiple hepatic adenomas, chronic renal failure, severe gout with multiple gout stones, and osteoporosis. Other rare presentations include pulmonary hypertension, diabetes mellitus, cerebrovascular disease, and hepatic adenoma carcinoma. On physical examination, short stature and markedly enlarged liver can be seen.

1. Type Ib, in addition to the above manifestations, patients may also have recurrent infections with neutropenia, oral ulcers, inflammatory bowel disease, perianal ulcers, arthritis and splenomegaly.
2. Infantile type II, which is divided into classical infantile type and non-classical infantile type according to its prognosis. Most of the classical infants present with generalised muscle weakness in the first month of life, delayed motor development, enlarged heart on chest X-ray, high QRS waves and short PR intervals on ECG, hypertrophic cardiomyopathy on cardiac ultrasound, and varying degrees of elevation of creatinine kinase, etc. Most of the infants die of left heart failure or cardiorespiratory failure after pulmonary infections in the first year of life. The non-classical infantile form presents with muscle weakness within the first year of life, lags in motor development, and most often dies of respiratory failure in early childhood.
3. Type II late-onset, with onset of disease after 1 year of age and as late as 60 years of age. Most of the patients present with chronic progressive proximal muscle weakness and respiratory insufficiency, cardiac involvement is rare, and respiratory failure is the main cause of death. Clinical manifestations include easy fatigue, difficulty in sitting up, walking up and down stairs, squatting and walking, and in a few cases, sudden onset of respiratory failure.

**llama3.1:70b** 2024-11-30T21:13:02Z:

Based on the clinical manifestations you provided, the most likely diagnosis is:

**Glycogen Storage Disease (GSD) Type I**

Also known as von Gierke disease, this rare genetic disorder affects the body’s ability to convert glycogen into glucose, leading to severe hypoglycemia and other systemic complications.

Five other possible diagnoses that could be considered based on some of the clinical manifestations include:

- **Pompe Disease (GSD Type II)**: This diagnosis is likely for patients presenting with muscle weakness, hypertrophic cardiomyopathy, and elevated creatinine kinase levels.
- **McArdle’s Disease (GSD Type V)**: Patients who experience exercise-induced muscle cramps, weakness, and fatigue might be considered for this diagnosis.
- **Debranching Enzyme Deficiency (GSD Type III)**: Clinical manifestations such as growth retardation, hepatomegaly, and progressive muscle weakness could lead to consideration of this diagnosis.
- **Hepatic Phosphorylase Deficiency (GSD Type VI)**: Patients presenting with mild hypoglycemia, hepatomegaly, and growth retardation might be considered for this diagnosis.
- **Forbe’s Disease (GSD Type IIIb)**: Clinical manifestations such as failure to thrive, hepatosplenomegaly, and progressive muscle weakness could lead to consideration of this diagnosis.

It is essential to note that a definitive diagnosis can only be made through genetic testing, enzyme assays, or other specialized tests. These possible diagnoses should be further investigated through diagnostic workup and consultation with a specialist in rare genetic disorders.

**13. user 2024-11-30T21:13:07Z:**

1. Neuro-muscular system, causing neurological abnormalities, such as convulsions, coma, ataxia, and even psychiatric symptoms; muscle symptoms can also occur, including hyperreflexia, tremor, tetany, and muscle weakness. 2. Cardiovascular system, causing ECG changes, such as PR interval prolongation, QRS wave widening, etc. In severe cases, it can cause ventricular arrhythmia.

1. cardiovascular system, causing changes in electrocardiogram, such as prolongation of PR interval, widening of QRS wave, and so on, and in severe cases, it may cause ventricular arrhythmias.
2. Kidney, which may cause kidney stones, renal calcium deposits, renal insufficiency.
3. bone and joint system, can cause osteoporosis and osteochondrosis.
4. digestive system, may cause intestinal obstruction.
5. endocrine, patients can see diabetes mellitus or impaired glucose tolerance.
6. ocular symptoms, some types may be combined with ocular lesions, including nystagmus, visual field defects, myopia, etc. This group of diseases usually has a chronic course.

This group of diseases usually has a chronic course, with the neonatal and infantile onset types often being more severe and having a poorer prognosis. Some patients develop chronic renal failure during adolescence, and in some cases, failure to treat the disease in time can lead to severe mental retardation or even death.

**llama3.1:70b** 2024-11-30T21:13:47Z:

What a fascinating set of symptoms!

Based on the clinical manifestations you’ve provided, I’m going to take a stab at diagnosing a rare disease that might fit this profile.

**Most likely diagnosis:** Hypoparathyroidism (a rare genetic disorder affecting calcium and phosphorus metabolism)

Here’s why:

- **Neuro-muscular symptoms**: The presence of convulsions, coma, ataxia, psychiatric symptoms, muscle weakness, hyperreflexia, tremor, and tetany are consistent with hypocalcemia (low calcium levels), which is a hallmark of hypoparathyroidism.
- **Cardiovascular system involvement**: Prolongation of the PR interval, widening of the QRS wave, and ventricular arrhythmias can occur due to hypocalcemia-induced changes in cardiac ion channels.
- **Kidney manifestations**: Kidney stones, renal calcium deposits, and renal insufficiency are consistent with hyperphosphatemia (elevated phosphorus levels) and abnormal calcium metabolism.
- **Bone and joint symptoms**: Osteoporosis and osteochondrosis can occur due to the abnormal mineralization of bone tissue secondary to hypoparathyroidism.
- **Other systems involvement**: The presence of impaired glucose tolerance or diabetes mellitus, intestinal obstruction, and ocular lesions (nystagmus, visual field defects) are less common but possible manifestations of hypoparathyroidism.

Now, here are five alternative diagnoses that might also be considered based on the provided symptoms:

**Alternative Diagnoses:**

- **Pseudohypoparathyroidism**: A rare genetic disorder affecting calcium and phosphorus metabolism, with similar symptoms to hypoparathyroidism.
- **Bartter syndrome**: A rare genetic disorder affecting electrolyte balance, which can cause neuromuscular symptoms, cardiovascular abnormalities, and kidney manifestations.
- **Ehlers-Danlos syndrome**: A group of disorders that affect connective tissue, leading to joint hypermobility, skin hyperextensibility, and potential cardiovascular and gastrointestinal complications.
- **Mitochondrial myopathies**: A group of disorders caused by mutations in mitochondrial DNA, which can lead to muscle weakness, neurological symptoms, and other systemic manifestations.
- **Cockayne syndrome**: A rare genetic disorder affecting the repair of damaged DNA, leading to a range of systemic symptoms including neuromuscular abnormalities, cardiovascular issues, and renal insufficiency.

Please note that these diagnoses are speculative and would require further investigation and laboratory testing to confirm.

**14. user 2024-11-30T21:14:34Z:**

There are five types, of which the classic and intermediate types are the most common.

- Classic type is the most common, accounting for 75 per cent of cases. Children are normal at birth, with early onset, severe symptoms and rapid progression. Usually, maple syrup odour appears in the cerumen 12 hours after birth; a special maple sugar odour is found in urine and sweat 12-24 hours after birth; ketonuria, irritability and feeding difficulties appear in 2-3 days; severe metabolic disorders and encephalopathic symptoms begin to appear in 4-5 days, which are manifested as difficulty in breastfeeding, paroxysmal vomiting, refusal to eat, lethargy, apnoea, convulsive seizures, heightened muscle tone and ‘fencing’. The symptoms of severe metabolic disorders and encephalopathy may include difficulty in breastfeeding, refusal to eat, lethargy, apnoea, convulsive seizures, increased muscle tone, and stereotypical movements such as ‘fencing’-like and ‘stomping’-like movements, etc. Coma and central respiratory failure may appear in the first 7-10 days of life, and most of the infants will die of severe metabolic disorders a few days after birth if they are not treated promptly.
- Intermediate type can develop at any age. The symptoms are less severe than those of the classic type and include slow growth, delayed development, feeding difficulties, irritability, a maple syrup odour in the cerumen, and severe metabolic disorders and encephalopathy under stress. 3. Intermittent Type The intermittent type is characterised by episodic seizures.
- Intermittent type: intermittent seizures, normal growth and development in the early stage, mostly under stressful situations such as infections and surgeries, manifested by episodic ataxia and ketoacidosis, and a very small number of severe cases may cause death. Branched-chain amino acid concentrations are normal during the intermittent period, and are elevated during the episodic period, similar to the classic type.

**llama3.1:70b** 2024-11-30T21:15:07Z:

Based on the clinical manifestations you provided, I’m going to take a educated guess that the rare disease most likely to be diagnosed is:

**Maple Syrup Urine Disease (MSUD)**

This is a metabolic disorder caused by a deficiency of the branched-chain alpha-keto acid dehydrogenase complex (BCKDC), leading to the accumulation of branched-chain amino acids and their toxic metabolites in the body.

The symptoms you described, such as:

- Maple syrup odor in cerumen, urine, and sweat
- Ketonuria
- Feeding difficulties
- Irritability
- Lethargy
- Apnoea
- Convulsive seizures
- Heightened muscle tone
- Stereotypical movements (e.g., “fencing” and “stomping”)

are all consistent with MSUD.

Here are five other possible diagnoses that could be considered:

- **Phenylketonuria (PKU)**: Another metabolic disorder caused by a deficiency of phenylalanine hydroxylase, leading to the accumulation of phenylalanine in the body.
- **Propionic acidemia**: A metabolic disorder caused by a deficiency of propionyl-CoA carboxylase, leading to the accumulation of propionate and its toxic metabolites in the body.
- **Methylmalonic acidemia**: A metabolic disorder caused by a deficiency of methylmalonyl-CoA mutase or other enzymes involved in the metabolism of branched-chain amino acids.
- **Isovaleric acidemia**: A metabolic disorder caused by a deficiency of isovaleryl-CoA dehydrogenase, leading to the accumulation of isovalerate and its toxic metabolites in the body.
- **Glutaric aciduria type 1**: A metabolic disorder caused by a deficiency of glutaryl-CoA dehydrogenase, leading to the accumulation of glutarate and its toxic metabolites in the body.

Please note that these alternative diagnoses are less likely based on the specific symptoms you provided, but they should still be considered in the differential diagnosis.

**15. user 2024-11-30T21:15:10Z:**

Bradykinesia, tremor, and myotonia. Levodopa-induced motor complications are more likely to occur after treatment. In terms of non-motor symptoms, mood disorders (e.g., depression, anxiety, irritability, etc.) and behavioural disorders (e.g., compulsive dosage increases, impulse control disorders, stereotyped behaviour, etc.) are likely to occur.

**llama3.1:70b** 2024-11-30T21:15:37Z:

What a fascinating set of clinical manifestations!

Based on the symptoms you provided, I would like to propose a primary diagnosis:

**Dopa-Responsive Dystonia (DRD) or more specifically, GCH1-related Dopa-Responsive Dystonia**

This rare neurological disorder is characterized by dystonic movements (e.g., tremor, myotonia), bradykinesia, and marked responsiveness to levodopa treatment. The development of levodopa-induced motor complications is consistent with this diagnosis.

And here are five other possible diagnoses that may need to be considered:

- **Parkinson’s Disease**: While Parkinson’s disease typically presents with rigidity, tremors, bradykinesia, and postural instability, the presence of myotonia and levodopa-induced motor complications might suggest a different underlying condition.
- **Dystonic Parkinsonism**: This rare disorder shares some features with DRD but often has a more parkinsonian phenotype.
- **Wilson’s Disease**: A genetic disorder characterized by abnormal copper metabolism, leading to neurological symptoms like tremors, bradykinesia, and dystonia, as well as psychiatric manifestations (e.g., mood disorders).
- **Fahr’s Syndrome**: A rare condition marked by idiopathic calcification of the basal ganglia, which can lead to a range of motor symptoms including dystonia, parkinsonism, and tremors.
- **Pantothenate Kinase-Associated Neurodegeneration (PKAN)**: A rare genetic disorder characterized by progressive dystonia, parkinsonism, and neuropsychiatric symptoms.

Please note that these differential diagnoses require thorough evaluation, including imaging studies, laboratory tests, and detailed clinical assessments to confirm or rule out each condition.

**16. user 2024-11-30T21:15:58Z:**

Common manifestations in the foetal period include reduced foetal activity, excess amniotic fluid and breech presentation. In the neonatal period hypotonia is one of the hallmark features of this disorder, and severe hypotonia can lead to asphyxia, feeding difficulties, weak sucking, and weak crying. In infancy and early childhood, the main manifestations are growth retardation and lagging cognitive, motor and language development. Appetite increases with age and obesity can occur if eating is not restricted. Short stature is usually present in school age, and personality traits such as temper tantrums, stubbornness, and obsessive-compulsive disorder gradually appear. Abnormal behaviours include intense foraging, skin scratching, and autistic-like behaviour. Seizures occur in about 25 per cent of patients. Delayed or incomplete appearance of secondary sexual characteristics may occur in adolescent males and females. Obesity can lead to complications such as sleep apnoea, pulmonary heart disease, insulin resistance and even diabetes. Scoliosis, osteoporosis, and gastroesophageal reflux are also more common. The main manifestations in adulthood are obesity and its complications, including cardiovascular problems, diabetes and sleep apnoea. Other manifestations are mental abnormalities and infertility.

Fatal risks: choking due to uncoordinated mouth movements, hypotonia, polyphagia, gulping habits and inadequate chewing, and gastric rupture due to overeating.

Physical examination reveals characteristic facial features that become progressively more typical, including almond eyes, small mouth, thin upper lip, downward-facing corners of the mouth, small hands and feet, short stature, fair skin, low muscle tone, and genital hypoplasia (e.g., cryptorchidism, scrotal hypoplasia, or clitoral hypoplasia).

**llama3.1:70b** 2024-11-30T21:16:34Z:

What a fascinating set of clinical manifestations!

Based on the symptoms you’ve described, I’m going to take a educated guess that the rare disease most likely to be diagnosed is **Prader-Willi Syndrome (PWS)**.

Here’s why:

- The characteristic facial features, such as almond eyes, small mouth, thin upper lip, and downward-facing corners of the mouth, are highly suggestive of PWS.
- The hypotonia, growth retardation, lagging cognitive and motor development, and short stature in childhood are all consistent with PWS.
- The increased appetite and obesity, which can lead to complications like sleep apnea, pulmonary heart disease, insulin resistance, and diabetes, are also hallmarks of the condition.
- The behavioral features, such as temper tantrums, stubbornness, obsessive-compulsive disorder, intense foraging, skin scratching, and autistic-like behavior, are commonly seen in individuals with PWS.
- The delayed or incomplete appearance of secondary sexual characteristics in adolescents is another characteristic feature of the syndrome.

Now, here are five other possible diagnoses that could be considered:

- **Down Syndrome**: While some features overlap, Down syndrome typically presents with distinct facial features, such as a flat face, small ears, and protruding tongue.
- **Angelman Syndrome**: This condition shares some similarities with PWS, including developmental delays and behavioral abnormalities, but it often presents with microcephaly, seizures, and a happy demeanor.
- **Smith-Magenis Syndrome**: This rare genetic disorder can present with similar facial features, developmental delays, and behavioral problems, but it is typically associated with a deletion on chromosome 17p11.2.
- **Cohen Syndrome**: This rare condition shares some similarities with PWS, including obesity, intellectual disability, and distinctive facial features, but it often presents with a more significant degree of intellectual disability and microcephaly.
- **Myotonic Dystrophy**: While not as likely, myotonic dystrophy can present with hypotonia, growth retardation, and cognitive impairment, but it is typically associated with muscle stiffness, wasting, and myotonia (involuntary muscle contractions).

Please note that a definitive diagnosis requires genetic testing and/or other diagnostic evaluations.

**17. user 2024-11-30T21:17:08Z:**

Patients often start in infancy or adolescence, with roughly equal male and female ratios. The water content of secretions from the epithelial cells of the respiratory tract, pancreas and biliary tract is reduced, and the secretions become viscous and difficult to clear. The lungs are the most commonly affected organ, and patients present with chronic bacterial infections and colonisation of the respiratory tract with pathogens, often initially Haemophilus influenzae and Staphylococcus aureus, and ultimately with chronic colonisation and/or infection with Pseudomonas aeruginosa or Klebsiella onionis. Once infection occurs, neutrophils are recruited in large numbers into the lung tissue and release elastase, which causes destruction of the lung tissue, leading to the formation of bronchiectasis. The patient’s bronchiectasis often starts at an early age and often begins in the upper lobes, in contrast to the majority of infections that result in the formation of bronchiectasis that is primarily located in the middle and lower lungs.

Patients often present with sweat stains left on dark-coloured clothing, or parents notice salty skin in their children. Abnormalities in the flow of bile and pancreatic fluid can lead to indigestion and malabsorption, as well as liver or pancreatic disease, and in severe cases, associated diabetes. The viscous intestinal fluid may also make the patient more susceptible to intestinal obstruction and rectal prolapse. In addition, male patients may develop impaired sperm movement in the vas deferens and congenital absence of the vas deferens (CBAVD).

**llama3.1:70b** 2024-11-30T21:17:40Z:

Based on the clinical manifestations you provided, I strongly suspect that the rare disease is:

**1. Cystic Fibrosis (CF)**: The combination of recurrent respiratory infections with Pseudomonas aeruginosa or Klebsiella pneumoniae, bronchiectasis, pancreatic insufficiency leading to malabsorption, liver disease, and salty skin all point towards CF as the most likely diagnosis.

Here are five other possible diagnoses that could be considered:

**2. Primary Ciliary Dyskinesia (PCD)**: Although less common than CF, PCD also presents with chronic respiratory infections, bronchiectasis, and situs inversus in some cases. However, the symptoms you described do not specifically suggest PCD.

**3. Young’s Syndrome**: This rare condition is characterized by obstructive azoospermia (blockage of sperm) and chronic sinopulmonary disease. While it shares some features with CF, such as respiratory infections, it does not typically present with pancreatic insufficiency or salty skin.

**4. Kartagener Syndrome**: This is a subset of Primary Ciliary Dyskinesia characterized by situs inversus (reversal of internal organs), chronic sinusitis, and bronchiectasis. However, the symptoms you described do not specifically suggest this condition.

**5. Shwachman-Diamond Syndrome**: This rare disorder presents with pancreatic insufficiency, bone marrow dysfunction, and skeletal abnormalities. While it shares some features with CF, such as pancreatic insufficiency, it does not typically present with chronic respiratory infections or salty skin.

**6. Congenital Bilateral Absence of the Vas Deferens (CBAVD)**: This condition is often associated with CF and presents with infertility due to absence or blockage of the vas deferens. However, CBAVD alone would not account for the other symptoms you described.

Please note that a definitive diagnosis can only be made through genetic testing, sweat chloride testing, or other specific diagnostic tests.

**18. user 2024-11-30T21:17:42Z:**

Patients present with oropharyngitis, otitis media, respiratory infections, cellulitis and skin infections, often due to staphylococcal and streptococcal infections. Oral ulcers and painful gingivitis occur in most children before the age of 2 years in severe neutropenia with decreased bone marrow reserve. If associated with diffuse gastrointestinal lesions, it may cause abdominal pain and diarrhoea similar to Crohn’s disease. Neutropenia can lead to recurrent bacterial or fungal infections in various areas, with the main sites of involvement being the skin and mucous membranes, ears, nose, throat and lungs. The infections are often severe or even fatal. About 15% of patients progress to acute leukaemia or myelodysplastic syndrome. The children usually do not have characteristic malformations.

**llama3.1:70b** 2024-11-30T21:18:12Z:

What a fascinating set of clinical manifestations!

Based on the symptoms you’ve described, I’m going to take a educated guess that the rare disease most likely to be diagnosed is:

**Kostmann Syndrome (Severe Congenital Neutropenia)**

This condition is characterized by severe neutropenia present at birth or within the first few months of life, leading to recurrent bacterial and fungal infections. The symptoms you’ve described, including oropharyngitis, otitis media, respiratory infections, cellulitis, skin infections, oral ulcers, painful gingivitis, and gastrointestinal involvement, are all consistent with Kostmann Syndrome.

Here are five other possible diagnoses that might be considered:

- **Shwachman-Diamond Syndrome**: Another rare congenital disorder characterized by neutropenia, pancreatic insufficiency, skeletal abnormalities, and a risk of developing leukemia or myelodysplastic syndrome.
- **Leukocyte Adhesion Deficiency (LAD)**: A rare immunodeficiency disorder that affects the ability of white blood cells to adhere to and migrate through the endothelial lining of blood vessels, leading to recurrent infections.
- **Chronic Granulomatous Disease (CGD)**: A genetic disorder characterized by impaired phagocytic function, leading to chronic granulomatous inflammation and recurrent infections with catalase-positive bacteria and fungi.
- **Glycogen Storage Disease type Ib**: A rare genetic disorder that affects glycogen metabolism, leading to neutropenia, Crohn-like gastrointestinal symptoms, and an increased risk of infections and leukemia.
- **Bare Lymphocyte Syndrome (BLS)**: A rare immunodeficiency disorder characterized by impaired T-cell activation and a decreased ability to fight off viral and bacterial infections.

These conditions all share some similarities with the clinical manifestations you’ve described, but Kostmann Syndrome is the one that seems most likely based on the specific combination of symptoms.

**19. user 2024-11-30T21:18:25Z:**

Typical Patients present with both CNS damage and hypoadrenocorticism. Neurological symptoms and symptoms of hypoadrenocorticism may occur simultaneously or sequentially. In a minority of patients, they may occur alone. The manifestations of adrenocortical insufficiency vary in severity. It is most common in children, accounting for 35% of all patients. The onset of symptoms occurs between the ages of 4 and 8 years. The initial symptoms include poor concentration, memory loss, learning difficulties, unsteady gait and abnormal behaviour, followed by visual and auditory loss, dysarthria, seizures, ataxia, paralysis and dementia. Clinical manifestations progressively worsen, culminating in blindness or deafness and complete paralysis. Some exhibit deafferentation and eventually die of central respiratory failure, brain herniation, and infection. Most patients have adrenocortical impairment when they first develop neurological symptoms.

**llama3.1:70b** 2024-11-30T21:18:49Z:

What a fascinating and complex clinical presentation! Based on the combination of CNS damage and hypoadrenocorticism, I’m going to take a educated guess that the rare disease most likely to be diagnosed is:

**Adrenoleukodystrophy (ALD)**: A genetic disorder characterized by the accumulation of very long-chain fatty acids in the body, leading to demyelination and inflammation in the central nervous system, as well as adrenal insufficiency.

And here are five other possible diagnoses that come to mind:

- **Adrenomyeloneuropathy (AMN)**: A variant of ALD with a milder course, primarily affecting the spinal cord and peripheral nerves.
- **Schilder’s disease**: A rare demyelinating disorder of the central nervous system, which can also involve adrenal insufficiency in some cases.
- **Krabbe disease**: A lysosomal storage disorder that affects the myelin sheath, leading to CNS damage and potentially adrenal insufficiency.
- **Leukoencephalopathy with vanishing white matter (LEUVWM)**: A rare genetic disorder affecting the white matter of the brain, which can also involve endocrine dysfunction, including adrenal insufficiency.
- **Mitochondrial neurogastrointestinal encephalomyopathy (MNGIE)**: A mitochondrial disorder that affects multiple systems, including the nervous system and adrenal glands.

Please note that these diagnoses are speculative, and a definitive diagnosis would require further evaluation, imaging studies, genetic testing, and potentially other specialized investigations.

**20. user 2024-11-30T21:19:44Z:**

Most of the children start to show symptoms of infection from 4 to 12 months after birth. The most prominent clinical manifestation is recurrent severe bacterial infections in the respiratory system, digestive system and central nervous system, with respiratory tract infections being the most common, and common pathogens include Haemophilus influenzae, Streptococcus pneumoniae, Staphylococcus aureus, and Pseudomonas aeruginosa, etc. The most common infections are respiratory tract infections. Prolonged and repeated respiratory infections can lead to irreversible organ function damage such as bronchiectasis and pestle finger. Common symptoms of GI infections include diarrhoea, abdominal pain, gastro-oesophageal reflux and gastroenteritis. Pathogens causing infectious diarrhoea include Giardia lamblia, Salmonella, Campylobacter jejuni and Cryptosporidium.

In addition, children are prone to otitis media, chronic sinusitis, malnutrition, anaemia, granulocytopenia, thrombocytopenia, growth hormone deficiency and thyroid hormone disorders. Children may also develop tumours, autoimmune and inflammatory diseases, including rheumatoid arthritis and dermatomyositis. A higher incidence of sensorineural hearing loss and eczema has also been reported in children.

**llama3.1:70b** 2024-11-30T21:20:10Z:

What a complex set of clinical manifestations!

Based on the symptoms you’ve described, I would propose the following:

**Most likely diagnosis:**

- **Severe Combined Immunodeficiency (SCID)**: SCID is a rare primary immunodeficiency disorder characterized by impaired development and function of T cells and/or B cells, leading to severe recurrent infections, particularly in the respiratory, gastrointestinal, and central nervous systems.

**Other possible diagnoses:**

1. **X-linked Agammaglobulinemia (XLA)**: XLA is another primary immunodeficiency disorder that affects males, characterized by low or absent levels of all classes of immunoglobulins, leading to recurrent bacterial infections.
2. **Common Variable Immunodeficiency (CVID)**: CVID is a heterogeneous group of disorders characterized by impaired antibody production, leading to recurrent infections and autoimmune phenomena.
3. **Chronic Granulomatous Disease (CGD)**: CGD is a rare primary immunodeficiency disorder caused by mutations in genes encoding subunits of the NADPH oxidase complex, leading to impaired killing of certain bacteria and fungi.
4. **Wiskott-Aldrich Syndrome (WAS)**: WAS is a rare X-linked recessive disorder characterized by eczema, thrombocytopenia, immune deficiency, and an increased risk of autoimmune disorders and malignancies.

Please note that these diagnoses are not exhaustive, and other conditions might also be considered based on the clinical presentation. A comprehensive diagnostic workup, including laboratory tests (e.g., immunoglobulin levels, flow cytometry) and genetic analysis, would be necessary to establish a definitive diagnosis.

### **21. User**

The clinical manifestations of the patients included salt loss and hyperandrogenism. Neonatal onset was characterized by varying degrees of adrenal insufficiency, such as weakness, nausea, vomiting, feeding difficulties, diarrhea, chronic dehydration, skin pigmentation, and growth retardation. Adrenal crisis is often the first manifestation of the disease in the neonatal period, manifested as severe hyponatremia, hyperkalemia, hypovolemic shock, may be accompanied by hypoglycemia, induced by stress. Severe hyponatremia can lead to central nervous system manifestations such as convulsions, and severe hyperkalemia can cause fatal arrhythmias.

Hyperandrogenism manifests differently at different ages. Female children with classical diseases have different degrees of masculinity at birth. Light performance for isolated clitoris hypertrophy, heavy external genitalia can approach men, labia majora scrotal shape, clitoris hypospadia-like penis, and with a common urethral vaginal orifice. However, the labia majora cannot touch the gonads and has completely normal female internal genital structures (ovary and uterus). Males may not have external genital abnormalities such as enlarged penis during neonatal and infancy, which is a common cause of delayed diagnosis. In early childhood, both sexes will present peripheral precocious puberty. Boys presented with enlarged penis with or without pubic hair; females presented with heterosexual precocious puberty. Chronic high levels of sex hormones stimulate hypothalamic gonadotropin-releasing hormone (GnRH) neurons and develop into central precocious puberty. Women may also have secondary sexual dysplasia and primary amenorrhea or oligomenorrhea. Linear growth with accelerated bone age occurs in both sexes from infancy, resulting in impaired terminal height.

Other findings include skin and mucous membrane pigmentation, especially areola and vulva, some children may not have this change.

Non-classical hyperandrogenism in childhood, adolescence and even adulthood may be characterized by accelerated growth and rapid bone age progression.

### Llama3.1:70b

Based on the clinical manifestations you provided, the most likely diagnosis is:

**1. Congenital Adrenal Hyperplasia (CAH) due to 21-Hydroxylase Deficiency**

This is a rare genetic disorder that affects the adrenal glands, leading to impaired production of cortisol and aldosterone, and overproduction of androgens. The symptoms you described, such as salt loss, hyperandrogenism, adrenal insufficiency, and precocious puberty, are all consistent with CAH due to 21-hydroxylase deficiency.

Here are five other possible diagnoses that may be considered:

**2. Congenital Adrenal Hyperplasia (CAH) due to 11-Hydroxylase Deficiency**: This is another form of CAH that can present with similar symptoms, including hyperandrogenism and adrenal insufficiency.

**3. Congenital Adrenal Hyperplasia (CAH) due to 3-Beta-Hydroxysteroid Dehydrogenase Deficiency**: This is a rare form of CAH that can present with salt loss, hyperandrogenism, and adrenal insufficiency.

**4. Adrenal Insufficiency due to Adrenal Hypoplasia Congenita**: This is a rare disorder that affects the development of the adrenal glands, leading to adrenal insufficiency and potentially hyperandrogenism.

**5. P450 Oxidoreductase Deficiency**: This is a rare genetic disorder that affects the production of steroid hormones, including cortisol and androgens, leading to symptoms such as adrenal insufficiency and hyperandrogenism.

**6. Denys-Drash Syndrome**: This is a rare genetic disorder that affects the development of the adrenal glands and kidneys, leading to symptoms such as adrenal insufficiency, hyperandrogenism, and renal abnormalities.

It’s worth noting that these diagnoses are all relatively rare and may require specialized testing and evaluation to confirm. A diagnosis of CAH due to 21-hydroxylase deficiency is the most likely based on the symptoms you described, but further evaluation and testing would be necessary to confirm the diagnosis.

### **22. User**

The patient has clinical manifestations at birth, due to lack of pigment in the whole body, skin capillaries are exposed, skin is thin and soft, white and red, and accompanied by varying degrees of vasodilation. Patients are highly sensitive to ultraviolet light, often solar dermatitis, actinic cheilitis, basal cell carcinoma and squamous cell carcinoma and other diseases. Hair is white, silver, yellow, gold or brown. Eyelashes and eyebrows are white or pale yellow. Retina, iris color decline, pupil color decline. Photophobia is obvious during the day, night vision is normal, and other visual changes can also be accompanied, such as decreased visual acuity, hypoplasia of the fovea, changes in neural pathways at the optic chiasm, and nystagmus.

### Llama3.1:70b

Based on the clinical manifestations you provided, the most likely diagnosis is:

**Albinism**

Specifically, the symptoms suggest Oculocutaneous Albinism (OCA), a group of rare genetic disorders characterized by the reduction or complete lack of melanin production in the skin, hair, and eyes.

Here are five other possible diagnoses that may be considered:

- **Hermansky-Pudlak Syndrome (HPS)**: A rare genetic disorder characterized by albinism, platelet dysfunction, and other systemic complications.
- **Chédiak-Higashi Syndrome**: A rare genetic disorder characterized by albinism, immune system dysfunction, and other systemic complications.
- **Griscelli Syndrome**: A rare genetic disorder characterized by albinism, immune system dysfunction, and other systemic complications.
- **Waardenburg Syndrome**: A rare genetic disorder characterized by deafness, albinism, and other developmental abnormalities.
- **Vitiligo**: A condition characterized by the loss of melanin-producing cells in the skin, leading to white patches, but it typically does not affect the hair or eyes.

Please note that a definitive diagnosis can only be made through a comprehensive medical evaluation, including genetic testing, and a thorough review of the patient’s medical history and physical examination.

### **23. User**

A degenerative disease of the nervous system in which both upper and lower motor neurons are involved. The main clinical manifestations are progressive weakness and atrophy of the muscles of the bulbar, limbs, chest and abdomen. Eye motor nerve and sphincter function are generally not affected, but may be affected later in some patients. Between 20% and 50% of patients may exhibit cognitive impairment, and between 5% and 15% may even develop frontotemporal dementia. Death from respiratory failure occurs an average of 3 to 5 years after onset, but 5% to 10% of patients survive for more than 10 years.

Detailed history and physical examination to find evidence of common involvement of upper and lower motor neurons in brain stem, neck, chest and lumbosacral regions is the basis for diagnosis of the disease. Physical examination showed that both upper and lower motor neurons were involved in the same area, which was the key point of diagnosis. The main signs of lower motor neuron involvement included muscle weakness, atrophy and fascicular fibrillation. The tongue muscles, facial muscles, throat muscles, neck muscles, different muscle groups of the limbs, back muscles and pectoralis muscles are usually examined. The signs of upper motor neuron involvement mainly included positive pathological signs, tendon hyperreflexia, hypertonia, clonus, etc. Usually check sucking reflex, pharyngeal reflex, mandibular reflex, palmar jaw reflex, whether there is strong crying, strong laughter and other pseudobulbar paralysis manifestations, limb tendon reflex, muscle tension, Hoffman sign, lower limb pathology, abdominal wall reflex, etc. Clinical examination is the primary method of detecting upper motor neuron involvement. In areas of atrophy and weakness, if tendon reflexes are not low or active, it may indicate pyramidal tract damage even if there are no pathological signs.

### Llama3.1:70b

Based on the clinical manifestations you provided, the most likely diagnosis is:

**Amyotrophic Lateral Sclerosis (ALS)**

ALS is a progressive neurodegenerative disease that affects both upper and lower motor neurons, leading to muscle weakness, atrophy, and eventually paralysis. The disease typically affects the bulbar, limb, chest, and abdominal muscles, while sparing the eye motor nerves and sphincter function, at least initially. Cognitive impairment and frontotemporal dementia are also common comorbidities in ALS patients.

Here are five other possible diagnoses that may be considered:

1. **Progressive Muscular Atrophy (PMA)**: A rare motor neuron disease that primarily affects lower motor neurons, leading to muscle weakness and atrophy. While PMA can present similarly to ALS, it typically lacks upper motor neuron involvement.
2. **Primary Lateral Sclerosis (PLS)**: A rare motor neuron disease that primarily affects upper motor neurons, leading to progressive weakness, stiffness, and spasticity. PLS can be distinguished from ALS by the absence of lower motor neuron involvement.
3. **Kennedy’s Disease**: A rare genetic disorder that affects motor neurons, leading to progressive muscle weakness, atrophy, and tremors. Kennedy’s disease typically affects males and is characterized by a slower progression than ALS.
4. **Spinal Muscular Atrophy (SMA)**: A genetic disorder that affects motor neurons, leading to progressive muscle weakness and atrophy. SMA can present in various forms, including a severe infantile form (Werdnig-Hoffmann disease) and a milder adult-onset form.
5. **Multifocal Motor Neuropathy (MMN)**: A rare autoimmune disorder that affects motor neurons, leading to progressive muscle weakness and atrophy. MMN can present with a more patchy distribution of weakness and is often responsive to immunotherapy.

It’s essential to note that a definitive diagnosis of ALS or other motor neuron diseases requires a comprehensive evaluation, including electromyography (EMG), nerve conduction studies (NCS), and imaging studies, in addition to a thorough clinical examination.

### **24. User**

An autosomal recessive disorder affecting the urea cycle, leading to hyperammonemia, encephalopathy, and respiratory alkalosis. It is characterized by a buildup of arginine and ammonia in the blood, causing symptoms such as developmental delays, seizures, intellectual disability, spasticity, and ataxia. The condition typically manifests around age 3, with symptoms including stiffness, slow growth, and cognitive decline. Without treatment, it can progress to severe spasticity, loss of ambulation, and intellectual disability.

### Llama3.1:70b

Based on the clinical manifestations you provided, the rare disease that is most likely to be diagnosed is:

**Argininosuccinic Aciduria (ASA)**

This is a rare genetic disorder that affects the urea cycle, leading to the accumulation of arginine and ammonia in the blood. The symptoms you described, such as developmental delays, seizures, intellectual disability, spasticity, and ataxia, are consistent with ASA.

Here are five other possible diagnoses that may be considered:

1. **Citrullinemia**: Another urea cycle disorder that can cause hyperammonemia, encephalopathy, and respiratory alkalosis. However, citrullinemia typically presents with more severe symptoms in infancy.
2. **Arginemia**: A rare disorder that affects the breakdown of arginine, leading to its accumulation in the blood. While it shares some similarities with ASA, arginemia typically presents with more mild symptoms.
3. **Hyperornithinemia-Hyperammonemia-Homocitrullinuria (HHH) syndrome**: A rare disorder that affects the urea cycle and can cause hyperammonemia, encephalopathy, and respiratory alkalosis. However, HHH syndrome typically presents with more episodic symptoms and is often associated with liver dysfunction.
4. **Carbamoyl phosphate synthetase I (CPS I) deficiency**: A rare disorder that affects the urea cycle and can cause hyperammonemia, encephalopathy, and respiratory alkalosis. However, CPS I deficiency typically presents with more severe symptoms in infancy and is often associated with liver dysfunction.
5. **Ornithine transcarbamylase (OTC) deficiency**: A rare disorder that affects the urea cycle and can cause hyperammonemia, encephalopathy, and respiratory alkalosis. However, OTC deficiency typically presents with more severe symptoms in infancy and is often associated with liver dysfunction.

It’s worth noting that a definitive diagnosis of ASA or any of these other conditions would require genetic testing and/or biochemical analysis to confirm the presence of specific enzyme deficiencies or genetic mutations.

### **25. User**

The typical clinical picture includes the triad of microangiopathic haemolytic anaemia, thrombocytopenia and acute renal failure. Non-immune erythrocyte destruction due to microthrombosis often results in a haemoglobin level below 80 g/L, a negative Coombs test, and erythrocyte fragments seen on peripheral blood smears; platelet counts usually fall below 50×10^9 /L, but skin purpura and active bleeding are rare; the severity of the acute renal impairment varies from person to person, with some patients requiring dialysis support, often accompanied by elevated blood pressure.

### Llama3.1:70b

Based on the clinical manifestations you provided, the rare disease that is most likely to be diagnosed is:

**Thrombotic Thrombocytopenic Purpura (TTP)**

TTP is a rare blood disorder characterized by the formation of blood clots in small blood vessels throughout the body, leading to microangiopathic hemolytic anemia, thrombocytopenia, and acute renal failure. The clinical presentation you described, including the triad of microangiopathic hemolytic anemia, thrombocytopenia, and acute renal failure, is consistent with TTP.

Here are five other possible diagnoses that may be considered:

1. **Hemolytic Uremic Syndrome (HUS)**: HUS is a rare disorder characterized by the triad of microangiopathic hemolytic anemia, thrombocytopenia, and acute renal failure, similar to TTP. However, HUS is often associated with a more prominent renal involvement and a higher risk of chronic kidney disease.
2. **Disseminated Intravascular Coagulation (DIC)**: DIC is a rare condition characterized by the widespread activation of the coagulation cascade, leading to the formation of blood clots in small blood vessels throughout the body. While DIC can present with similar laboratory findings, it is often associated with a more severe coagulopathy and a higher risk of bleeding.
3. **Atypical Hemolytic Uremic Syndrome (aHUS)**: aHUS is a rare disorder characterized by the triad of microangiopathic hemolytic anemia, thrombocytopenia, and acute renal failure, similar to TTP and HUS. However, aHUS is often associated with a more prominent complement system dysregulation and a higher risk of chronic kidney disease.
4. **Thrombotic Microangiopathy (TMA)**: TMA is a rare condition characterized by the formation of blood clots in small blood vessels throughout the body, leading to microangiopathic hemolytic anemia and thrombocytopenia. While TMA can present with similar laboratory findings, it is often associated with a more variable clinical presentation and a higher risk of bleeding.
5. **Catastrophic Antiphospholipid Syndrome (CAPS)**: CAPS is a rare autoimmune disorder characterized by the formation of blood clots in small blood vessels throughout the body, leading to microangiopathic hemolytic anemia, thrombocytopenia, and acute renal failure. While CAPS can present with similar laboratory findings, it is often associated with a more prominent autoimmune component and a higher risk of thrombotic events.

It’s worth noting that these conditions can have overlapping clinical and laboratory features, and a definitive diagnosis often requires a combination of clinical evaluation, laboratory testing, and imaging studies.

### **26. User**

Patients have a variety of clinical presentations. It depends on the location, extent and rate of progression of the lesion. Clinical symptoms mainly include:

1. The occupying effect of an enlarged pituitary gland, which in most patients results from compression of the enlarged pituitary gland on the anterior region and surrounding structures (e.g., optic crossings, cavernous sinus, etc.), or from an inflammatory infiltrate. The most common clinical manifestations are persistent severe headaches and progressively worsening visual dysfunction, including decreased visual acuity and visual field defects.
2. hypopituitarism. Depending on the degree of involvement, the various axes of the pituitary gland may show partial or even total hypopituitarism, such as amenorrhoea, hypogonadism, nausea, malaise, chills, loss of sexual hair and weight loss.
3. Central dysuria, more than 70% of the patients will have symptoms of central dysuria such as polyuria, irritable thirst and polydipsia due to the involvement of the posterior pituitary, pituitary stalk and even hypothalamus region. In some patients, the blocking effect of the pituitary stalk leads to a mild increase in serum prolactin levels.
4. Hypothalamic syndrome, when the lesion involves the region of the hypothalamus, there will be drowsiness, lack of satiety, severe weight gain, lack of thirst, temperature fluctuation, sweating disorders, and emotional and psychiatric disorders.
5. Systemic disease-related manifestations. Patients with secondary disease often have a combination of clinical manifestations characteristic of systemic disease, such as LCH patients often have a combination of thyroid, lung, bone, liver, and skin lesions. patients with GPA are often middle-aged or older men, and respiratory symptoms often precede hypothalamic pituitary involvement. patients with IgG4-related pituitary inflammation often have a combination of clinical manifestations with the involvement of other organs such as lacrimal glands, submandibular glands, and pancreas. The disease is often associated with clinical signs of involvement of other organs such as the lacrimal gland and submaxillary gland and pancreas.

### Llama3.1:70b

Based on the clinical manifestations you provided, the most likely diagnosis is:

**Lymphocytic Hypophysitis (LH)**

Lymphocytic hypophysitis is a rare inflammatory condition of the pituitary gland, which can cause a range of symptoms including headaches, visual disturbances, hypopituitarism, and central diabetes insipidus. The involvement of the hypothalamus can also lead to hypothalamic syndrome.

Here are five other possible diagnoses:

1. **Langerhans Cell Histiocytosis (LCH)**: LCH is a rare disorder characterized by the proliferation of Langerhans cells, which can infiltrate various organs, including the pituitary gland, hypothalamus, and other parts of the brain. Clinical manifestations can include diabetes insipidus, hypopituitarism, and hypothalamic syndrome.
2. **Granulomatosis with Polyangiitis (GPA)**: GPA is a rare autoimmune disorder that can cause inflammation in various organs, including the pituitary gland and hypothalamus. Clinical manifestations can include respiratory symptoms, headaches, visual disturbances, and hypopituitarism.
3. **IgG4-related Hypophysitis**: IgG4-related hypophysitis is a rare inflammatory condition of the pituitary gland, which can cause a range of symptoms including headaches, visual disturbances, hypopituitarism, and central diabetes insipidus. The disease is often associated with involvement of other organs such as the lacrimal gland, submandibular gland, and pancreas.
4. **Sarcoidosis**: Sarcoidosis is a rare autoimmune disorder that can cause inflammation in various organs, including the pituitary gland and hypothalamus. Clinical manifestations can include headaches, visual disturbances, hypopituitarism, and hypothalamic syndrome.
5. **Tolosa-Hunt Syndrome**: Tolosa-Hunt syndrome is a rare disorder characterized by inflammation of the cavernous sinus and superior orbital fissure, which can cause a range of symptoms including headaches, visual disturbances, and hypopituitarism. The disease can also involve the hypothalamus and pituitary gland.

It’s worth noting that these diagnoses are rare and often require a comprehensive diagnostic workup, including imaging studies, laboratory tests, and histopathological examination, to confirm the diagnosis.

### **27. User**

Hyperglycaemia is the most common clinical manifestation of the disease. However, patients can spontaneously remit without intervention and maintain normal blood glucose levels. The disease is one of the leading causes of autoimmune hypoglycaemia, with about 25% of patients experiencing spontaneous hypoglycaemia during the course of the disease. Acanthosis nigricans is present in about 50 per cent of patients. In addition, polycystic ovaries and hyperandrogenism are not uncommon in this group of patients. Other autoimmune diseases are common, with systemic lupus erythematosus (SLE) being the most common, accounting for about 50 per cent of cases. Primary biliary cirrhosis, dermatomyositis, and scleroderma are also present. The disease can also be a paraneoplastic syndrome manifestation of Hodgkin’s lymphoma, multiple myeloma, and others.

### Llama3.1:70b

What a fascinating case!

Based on the clinical manifestations you provided, I’m going to take a stab at the most likely diagnosis:

**Primary diagnosis:** Autoimmune Polyendocrine Syndrome Type 2 (APS-2), also known as Schmidt’s syndrome or PAS II.

APS-2 is a rare autoimmune disorder characterized by the presence of multiple autoimmune diseases, including autoimmune hypoglycemia, hyperglycemia, and other endocrine disorders. The syndrome is often associated with other autoimmune diseases, such as SLE, primary biliary cirrhosis, and dermatomyositis, which aligns with the clinical manifestations you described.

The presence of acanthosis nigricans, polycystic ovaries, and hyperandrogenism in some patients also supports this diagnosis, as these features are commonly seen in APS-2.

Now, here are five other possible diagnoses that might be considered:

1. **Multiple Endocrine Neoplasia Type 1 (MEN1)**: This rare genetic disorder is characterized by the development of multiple endocrine tumors, which can lead to hyperglycemia, hypoglycemia, and other endocrine disorders.
2. **Polyglandular Autoimmune Syndrome Type 1 (APS-1)**: Also known as APECED syndrome, this rare autoimmune disorder is characterized by the presence of multiple autoimmune diseases, including autoimmune hypoparathyroidism, Addison’s disease, and mucocutaneous candidiasis.
3. **Insulin Autoimmune Syndrome (IAS)**: This rare condition is characterized by the presence of autoantibodies against insulin, leading to hypoglycemia and hyperglycemia.
4. **POEMS Syndrome**: This rare paraneoplastic syndrome is characterized by the presence of polyneuropathy, organomegaly, endocrinopathy, monoclonal gammopathy, and skin changes, which can include acanthosis nigricans.
5. **Stiff Person Syndrome (SPS)**: This rare autoimmune disorder is characterized by progressive stiffness and rigidity of the muscles, which can be associated with autoimmune hypoglycemia and other endocrine disorders.

Please note that these alternative diagnoses are less likely, and APS-2 remains the most probable diagnosis based on the clinical manifestations you provided.

### **28. User**

The disease starts in infancy and childhood. The first attack occurs between the ages of 5 months and 7 years. The clinical picture is one of recurrent episodes of intractable vomiting and severe ketoacidosis with depression, dehydration, shortness of breath, and coma. There is usually a clear trigger, and the disease may begin acutely after fasting, gastrointestinal and upper respiratory tract infections, fever, stress, or excessive protein intake. Routine laboratory tests show elevated or normal blood glucose, some hypoglycaemia, blood gases show metabolic acidosis, blood ammonia can be normal or mildly elevated, liver function, renal function and normal, and urine ketone bodies are positive. Ketosis and acidosis may recur, and clinical and laboratory tests may be normal between attacks.

Neurological changes occur in about 1/5 of the cases, manifested by cognitive impairment, mental retardation, extrapyramidal abnormalities and symptoms of stroke. Cranial MRI reveals extensive demyelination, including white matter, and symmetric high signal changes in the basal ganglia at T2.

Patients may be asymptomatic in the early stages and are diagnosed in the neonatal period or in high-risk groups by blood and urine metabolic screening. There is no clear association between age of onset, frequency of attacks and prognosis. There is a tendency for the attacks to decrease or stop with age, and in most cases growth is unaffected on follow-up. Recurrent and severe attacks can lead to multi-organ dysfunction or even death.

### Llama3.1:70b

Based on the clinical manifestations you provided, the most likely diagnosis is:

**Glutaric Acidemia Type 1 (GA-1)**

GA-1 is a rare genetic disorder caused by a deficiency of the enzyme glutaryl-CoA dehydrogenase, leading to the accumulation of glutaric acid and 3-hydroxyglutaric acid in the body. The clinical presentation, including recurrent episodes of intractable vomiting, ketoacidosis, and neurological changes, is consistent with GA-1. The disease often presents in infancy and childhood, and the triggers you mentioned, such as fasting, infections, and stress, are common precipitants of acute episodes.

Here are five other possible diagnoses that should be considered:

1. **Medium-Chain Acyl-CoA Dehydrogenase (MCAD) Deficiency**: Another rare genetic disorder that affects the metabolism of fatty acids, leading to episodes of ketoacidosis and hypoglycemia.
2. **Very-Long-Chain Acyl-CoA Dehydrogenase (VLCAD) Deficiency**: A similar disorder to MCAD deficiency, affecting the metabolism of very-long-chain fatty acids.
3. **Carnitine Palmitoyltransferase (CPT) Deficiency**: A genetic disorder that affects the transport of fatty acids into the mitochondria, leading to episodes of ketoacidosis and hypoglycemia.
4. **Ketothiolase Deficiency**: A rare genetic disorder that affects the metabolism of ketone bodies, leading to episodes of ketoacidosis and neurological changes.
5. **Mitochondrial Trifunctional Protein (MTP) Deficiency**: A genetic disorder that affects the metabolism of fatty acids and ketone bodies, leading to episodes of ketoacidosis and neurological changes.

These disorders all have similar clinical presentations and should be considered in the differential diagnosis. However, GA-1 remains the most likely diagnosis based on the specific combination of clinical features and laboratory findings you provided.

### **29. User**

1. the onset is at a young age and is more common in women.
2. there is often a family history of syncope or sudden death.
3. different genotypes and phenotypes, can involve potassium, sodium and calcium channels. Characteristic tip-distortion ventricular tachycardia (TdP) is often present, leading to syncope or sudden death. 4.
4. The ECG shows a prolonged QT interval.
5. ECG generally has the following characteristics: (1) QT interval prolongation, short - long - short cycle phenomenon induced; (2) ventricular rate of 160-240 beats / minute, QRS wave amplitude and morphology around the equipotential line of reversal, 5-20 cardiac cycles of the main wave around the baseline of the reversal of the majority of the time can be terminated; (3) ATP is more difficult to terminate the TdP.

### Llama3.1:70b

Based on the clinical manifestations you provided, the most likely diagnosis is:

**Long QT Syndrome (LQTS)**

This is a rare genetic disorder characterized by a prolonged QT interval on the ECG, which can lead to life-threatening arrhythmias, such as Torsades de Pointes (TdP) and sudden cardiac death. The condition often presents at a young age, with a higher incidence in women, and may have a family history of syncope or sudden death.

Here are five other possible diagnoses that may be considered:

1. **Catecholaminergic Polymorphic Ventricular Tachycardia (CPVT)**: This is another rare genetic disorder that can cause abnormal heart rhythms, including TdP, in response to emotional stress or physical activity.
2. **Brugada Syndrome**: This is a genetic disorder that affects the sodium channels in the heart, leading to abnormal ECG patterns and an increased risk of sudden cardiac death.
3. **Arrhythmogenic Right Ventricular Cardiomyopathy (ARVC)**: This is a rare genetic disorder that affects the muscle in the right ventricle, leading to abnormal heart rhythms and an increased risk of sudden cardiac death.
4. **Hypokalemic Periodic Paralysis**: This is a rare genetic disorder that affects the potassium channels in the body, leading to episodes of muscle weakness and paralysis, as well as cardiac arrhythmias.
5. **Andersen-Tawil Syndrome**: This is a rare genetic disorder that affects the potassium channels in the body, leading to episodes of muscle weakness and paralysis, as well as cardiac arrhythmias, including TdP.

It’s worth noting that these conditions can have overlapping clinical features, and a definitive diagnosis often requires genetic testing and other specialized evaluations.

### **30. User**

The disease can develop at any age, and most of the children develop between 1 month and 7 years old, with an average age of about 2 years old. Clinical manifestations vary greatly among patients, mainly including: (1) acute energy metabolism disorder crisis, manifested by low ketone hypoglycemia, high blood ammonia and metabolic acidosis; (2) cardiomyopathy, manifested by ventricular hypertrophy, cardiac insufficiency, cardiac arrhythmia, and elevated creatine kinase, etc.; (3) myopathy, manifested by muscle weakness, hypotonia, myalgia, poor exercise endurance, elevated muscular-type creatine kinase, and lipid deposition in muscle fibres, etc. (4) liver damage, manifested by hepatomegaly, fatty liver, liver function abnormalities, etc. Some liver-damaged patients have liver enlargement, fatty liver, and liver function abnormalities; Liver damage, manifested by hepatomegaly, fatty liver, abnormal liver function, etc. Some children with liver damage have acute onset of illness, manifested by convulsions, progressive consciousness disorder, etc., which is often misdiagnosed as Reye syndrome. In addition, gastrointestinal symptoms such as recurrent abdominal pain, vomiting and gastroesophageal reflux, respiratory symptoms such as recurrent infections and wheezing, and anaemia have also been reported.

Newborn screening: diagnosed children may have no clinical manifestations. It is a potentially fatal disease, and children can die suddenly from acute energy metabolism disorder crisis or acute heart failure. Clinical manifestations are varied and related to age of onset, time of onset and severity of illness. The most common symptoms are cardiac and skeletal muscle damage in infancy and childhood. Symptoms in adulthood are mild or asymptomatic, mostly due to reduced endurance or easy fatigue. During pregnancy, pregnant women may have atypical manifestations such as fatigue and arrhythmias due to energy expenditure and reduced physiological plasma carnitine levels.

### Llama3.1:70b

Based on the clinical manifestations you provided, the most likely diagnosis is:

**Carnitine Palmitoyltransferase II (CPT II) Deficiency**

CPT II deficiency is a rare genetic disorder that affects the metabolism of fatty acids, leading to a range of clinical manifestations, including acute energy metabolism disorder crises, cardiomyopathy, myopathy, liver damage, and other symptoms you described.

Here are five other possible diagnoses that may be considered based on the clinical manifestations:

1. **Very Long-Chain Acyl-CoA Dehydrogenase (VLCAD) Deficiency**: This is another rare genetic disorder that affects the metabolism of fatty acids, leading to similar clinical manifestations, including acute energy metabolism disorder crises, cardiomyopathy, and myopathy.
2. **Medium-Chain Acyl-CoA Dehydrogenase (MCAD) Deficiency**: This is a rare genetic disorder that affects the metabolism of medium-chain fatty acids, leading to clinical manifestations such as acute energy metabolism disorder crises, cardiomyopathy, and myopathy.
3. **Long-Chain 3-Hydroxyacyl-CoA Dehydrogenase (LCHAD) Deficiency**: This is a rare genetic disorder that affects the metabolism of long-chain fatty acids, leading to clinical manifestations such as cardiomyopathy, myopathy, and liver damage.
4. **Carnitine Transporter Deficiency**: This is a rare genetic disorder that affects the transport of carnitine, a molecule essential for fatty acid metabolism, leading to clinical manifestations such as cardiomyopathy, myopathy, and liver damage.
5. **Mitochondrial Trifunctional Protein (MTP) Deficiency**: This is a rare genetic disorder that affects the metabolism of fatty acids and other molecules within the mitochondria, leading to clinical manifestations such as cardiomyopathy, myopathy, and liver damage.

It’s worth noting that these diagnoses are all related to disorders of fatty acid metabolism and may have overlapping clinical manifestations, making diagnosis challenging. A definitive diagnosis typically requires genetic testing and/or biochemical analysis of fatty acid metabolism.

### **31. User**

In the unicentric form, the disease often presents only with enlarged lymph nodes without systemic symptoms. The median diameter of the enlarged lymph nodes is about 5.5 cm, and they are commonly found in the chest (24%), neck (20%), abdomen (18%), and retroperitoneum (14%). Some patients with unicentric disease are prone to paraneoplastic aspergillosis, occlusive bronchiolitis, and growth retardation. In addition to multiple lymph node enlargements, multicentric disease is often associated with systemic manifestations such as fever, night sweats, malaise, weight loss, anaemia, hepatic insufficiency, renal insufficiency, and volume overload (generalised oedema, pleural effusion, ascites, etc.). There is also a specific subtype in which patients may present with thrombocytopenia, severe oedema (anasarca), myelofibrosis, renal dysfunction, and hepatosplenomegaly (organomegaly).

### Llama3.1:70b

Based on the clinical manifestations you provided, the rare disease that is most likely to be diagnosed is:

**Castleman Disease**

Castleman disease is a rare disorder characterized by the abnormal growth of cells in the body’s lymph nodes, which can lead to unicentric or multicentric disease. The clinical manifestations you described, such as enlarged lymph nodes, systemic symptoms, and paraneoplastic syndromes, are consistent with Castleman disease.

Here are five other possible diagnoses that could be considered:

1. **Lymphoma**: Lymphoma is a type of cancer that can cause enlarged lymph nodes and systemic symptoms, similar to Castleman disease.
2. **Sarcoidosis**: Sarcoidosis is a rare inflammatory disease that can cause enlarged lymph nodes and systemic symptoms, including fever, weight loss, and organ dysfunction.
3. **Histiocytosis**: Histiocytosis is a group of rare disorders characterized by the abnormal growth of histiocytes, which can lead to enlarged lymph nodes and systemic symptoms.
4. **Multicentric Reticulohistiocytosis**: This is a rare disorder characterized by the abnormal growth of histiocytes, which can lead to enlarged lymph nodes, systemic symptoms, and joint involvement.
5. **Rosai-Dorfman Disease**: This is a rare disorder characterized by the abnormal growth of histiocytes, which can lead to enlarged lymph nodes, systemic symptoms, and paraneoplastic syndromes.

It’s worth noting that a definitive diagnosis can only be made with a combination of clinical evaluation, imaging studies, and histopathological examination of affected tissues.

### **32. User**

The main clinical manifestations are muscle atrophy, weakness and sensory loss of the limbs, mainly in the distal part of the lower limbs and gradually developing towards the proximal part of the limbs. Common clinical manifestations include poorer locomotor ability than people of the same age, difficulty in running, easy to twist the foot, foot drop, calf gastrocnemius muscle atrophy resembling a ‘crane leg’; examination can find bowed feet, hammertoes, distal limb weakness and atrophy, and loss of deep sensation. The disease usually starts before the age of 20 and progresses slowly. Later stages of the disease may severely affect activity, but rarely result in total disability and do not affect normal life expectancy. However, there are specific types that may have an early and severe onset.

### Llama3.1:70b

Based on the clinical manifestations you provided, the most likely diagnosis is:

**Charcot-Marie-Tooth disease (CMT)**

Charcot-Marie-Tooth disease is a group of inherited disorders that affect the peripheral nerves, leading to muscle atrophy, weakness, and sensory loss in the limbs, particularly in the distal parts of the lower limbs. The disease typically starts before the age of 20 and progresses slowly, with symptoms worsening over time. The characteristic “crane leg” appearance due to calf muscle atrophy is a classic feature of CMT.

Here are five other possible diagnoses that may be considered:

1. **Hereditary motor and sensory neuropathy (HMSN)**: This is a broader category of inherited disorders that affect the peripheral nerves, leading to muscle weakness, atrophy, and sensory loss. While CMT is a specific type of HMSN, other subtypes may present with similar symptoms.
2. **Friedreich’s ataxia**: This is an autosomal recessive disorder that affects the peripheral nerves and spinal cord, leading to progressive damage to the nervous system. While the primary symptoms are ataxia and loss of coordination, muscle weakness and atrophy can also occur.
3. **Spinal muscular atrophy (SMA)**: This is a group of inherited disorders that affect the motor neurons in the spinal cord, leading to muscle weakness and atrophy. While SMA typically presents in infancy or early childhood, some forms of the disease may have a later onset and milder symptoms.
4. **Distal spinal muscular atrophy (DSMA)**: This is a rare inherited disorder that affects the motor neurons in the spinal cord, leading to muscle weakness and atrophy primarily in the distal parts of the limbs.
5. **Refsum disease**: This is a rare autosomal recessive disorder that affects the metabolism of fatty acids, leading to peripheral neuropathy, muscle weakness, and atrophy. While the primary symptoms are related to vision and hearing loss, muscle weakness and atrophy can also occur.

It’s essential to note that a definitive diagnosis can only be made through a combination of clinical evaluation, genetic testing, and other diagnostic tests, such as electromyography (EMG) and nerve conduction studies (NCS).

### **33. User**

The onset of the disease occurs from birth to several months. The onset of jaundice is usually within 2 months of age and rarely later than 5 months of age. The most common presentation is persistent jaundice after birth with or without light coloured stools and mild or no liver enlargement. There are many other non-specific clinical signs, including feeding difficulties, growth retardation, diarrhoea, hypoglycaemia and prolonged clotting time. Most of these symptoms and signs resolve spontaneously or with dietary modification and medication around 6 months to 1 year of age, with a small proportion of patients developing further disease after puberty. The majority of developed disease develops in adulthood. Delirium, confusion, and other mental abnormalities caused by hyperammonemia usually appear suddenly after drinking alcohol, consuming sweets, taking certain medications, or having an infection. In severe cases, the disease progresses rapidly and can be life-threatening without proper treatment. Most patients are slim, and most of them have obvious dietary preference, addicted to legumes, high protein, high fat food, and anorexic to high sugar.

### Llama3.1:70b

Based on the clinical manifestations you provided, the rare disease that is most likely to be diagnosed is:

**1. Ornithine Transcarbamylase (OTC) Deficiency**

OTC deficiency is a rare genetic disorder that affects the urea cycle, leading to the accumulation of ammonia in the blood. The symptoms you described, such as jaundice, feeding difficulties, growth retardation, and mental abnormalities caused by hyperammonemia, are consistent with OTC deficiency. The disease often presents in infancy, but some patients may not develop symptoms until later in life, triggered by factors such as alcohol consumption or certain medications.

Here are five other possible diagnoses that could be considered based on the clinical manifestations:

**2. Citrullinemia Type I**: Another urea cycle disorder that can present with similar symptoms, including jaundice, feeding difficulties, and hyperammonemia.

**3. Argininosuccinic Aciduria**: A rare genetic disorder that affects the urea cycle, leading to the accumulation of argininosuccinic acid in the blood. Symptoms can include jaundice, feeding difficulties, and growth retardation.

**4. Maple Syrup Urine Disease (MSUD)**: A metabolic disorder that affects the breakdown of certain amino acids, leading to the accumulation of toxic compounds in the blood. Symptoms can include feeding difficulties, growth retardation, and mental abnormalities.

**5. Glycogen Storage Disease Type I (GSD I)**: A rare genetic disorder that affects the breakdown of glycogen, leading to the accumulation of glycogen in the liver and kidneys. Symptoms can include jaundice, feeding difficulties, and growth retardation.

**6. Carnitine Palmitoyltransferase (CPT) Deficiency**: A rare genetic disorder that affects the breakdown of fatty acids, leading to the accumulation of toxic compounds in the blood. Symptoms can include feeding difficulties, growth retardation, and mental abnormalities.

It’s worth noting that these diagnoses are speculative and would require further testing and evaluation to confirm. A comprehensive diagnostic workup, including genetic testing, biochemical analysis, and imaging studies, would be necessary to determine the underlying cause of the patient’s symptoms.

### **34. User**

Symptoms may appear after birth or may start in infancy to early adulthood. It is characterised by fluctuating muscle weakness and intolerance to fatigue, mostly in the extraocular muscles, facial muscles, medulla oblongata, limbs and respiratory muscles, and even life-threatening apnoeic episodes may occur in infancy. The simplex syndrome is characterised mainly by skeletal muscle weakness without congenital malformations. The overlapping syndromes of glycosylation defects and myopathies of the disease are often accompanied by congenital developmental abnormalities. Clinical manifestations vary between the different types, with some patients, usually born with proximal muscle weakness, cholinesterase inhibitors effective, and in severe cases, sudden respiratory distress and apnoea due to medullary palsy, which can be induced by stress, cold, and infection. Some patients, starting at birth or in infancy, may present with more severe, axially distributed muscle weakness, but the extraocular muscles may not be involved.

### Llama3.1:70b

Based on the clinical manifestations you provided, the rare disease that is most likely to be diagnosed is:

**Congenital Myasthenic Syndrome (CMS)**

CMS is a group of rare genetic disorders that affect the transmission of nerve impulses to muscles, leading to fluctuating muscle weakness and fatigue. The symptoms you described, such as muscle weakness in the extraocular muscles, facial muscles, limbs, and respiratory muscles, as well as life-threatening apnoeic episodes in infancy, are consistent with CMS.

Here are five other possible diagnoses that may be considered:

1. **Myotonic Dystrophy**: a genetic disorder characterized by muscle stiffness, weakness, and wasting, particularly in the face, neck, and distal limbs.
2. **Glycogen Storage Disease Type II (Pompe Disease)**: a genetic disorder that affects the breakdown of glycogen in muscles, leading to muscle weakness, cardiomegaly, and respiratory failure.
3. **Mitochondrial Myopathies**: a group of disorders caused by mutations in mitochondrial DNA, leading to muscle weakness, fatigue, and other systemic symptoms.
4. **Congenital Fiber-Type Disproportion (CFTD)**: a rare muscle disorder characterized by muscle weakness, hypotonia, and delayed motor development, often with a distinctive pattern of muscle fiber distribution on biopsy.
5. **Centronuclear Myopathy**: a rare genetic disorder characterized by muscle weakness, particularly in the face, neck, and distal limbs, with a distinctive “centronuclear” pattern of muscle fibers on biopsy.

Please note that a definitive diagnosis can only be made through a combination of clinical evaluation, laboratory tests (e.g., genetic testing, electromyography, muscle biopsy), and imaging studies.

### **35. User**

1. The disease usually starts in infancy or childhood, and in a few cases it starts in adulthood.
2. Tonicity can occur throughout the entire body, including the skeletal muscles of the limbs, facial muscles, extraocular muscles, and lingual muscles.
3. Tonicity is apparent during sudden movement after rest and is reduced by repeated movements, which is called the ‘warm-up’ phenomenon. Transient muscle weakness may occur at the start of a sudden movement. In patients with a longer course of the disease, there may be persistent weakness of the distal limbs, even involving the proximal limbs.
4. Physical examination reveals that the patient is often muscular and has an athlete’s body shape. After clenching the fist vigorously, the fingers cannot be unfolded in time; after closing the eyes vigorously, they cannot be opened immediately. Knocking on the hand interosseous muscle, lingual muscle and other parts of the hand can cause persistent muscle contraction (percussive muscle ankylosis, ‘muscle ball’ phenomenon).

### Llama3.1:70b

Based on the clinical manifestations you provided, the most likely diagnosis is:

**Myotonia Congenita**

Myotonia Congenita is a rare genetic disorder characterized by delayed relaxation of skeletal muscles after voluntary contraction, leading to stiffness and difficulty moving. The “warm-up” phenomenon, where repeated movements reduce muscle stiffness, is a hallmark of this condition. The physical examination findings you described, such as difficulty unfolding the fingers after clenching the fist and persistent muscle contraction after percussion (percussive muscle ankylosis or “muscle ball” phenomenon), are also consistent with Myotonia Congenita.

Here are five other possible diagnoses that may be considered:

1. **Paramyotonia Congenita**: A rare genetic disorder that also affects muscle relaxation, but is often associated with cold-induced stiffness and weakness.
2. **Dystrophia Myotonica**: A genetic disorder that causes progressive muscle wasting and weakness, as well as myotonia (delayed muscle relaxation).
3. **Brody Disease**: A rare genetic disorder that affects muscle relaxation, causing stiffness and weakness, particularly in the limbs.
4. **Stiff Person Syndrome**: A rare autoimmune disorder that causes progressive stiffness and rigidity of the muscles, often accompanied by spasms and weakness.
5. **Schwartz-Jampel Syndrome**: A rare genetic disorder that causes muscle stiffness, weakness, and wasting, often accompanied by skeletal abnormalities and other systemic features.

Please note that a definitive diagnosis can only be made after a comprehensive evaluation, including medical history, physical examination, laboratory tests, and genetic analysis.

### **36. User**

Secondary thoracic deformities caused by scoliosis can lead to cardiopulmonary hypoplasia, poor cardiopulmonary reserve, and may include cardiac displacement, arrhythmia, reduced lung capacity, pneumonia, etc. Abdominal deformities can cause gastrointestinal disorders, leading to dyspepsia and lack of appetite. In addition, the nerve root pulling caused by vertebral malformation and disc degeneration can produce irritating pain in the corresponding body parts, and in severe cases, it can cause spinal cord dysfunction or even paralysis.

Physical examination: the general manifestations are unequal shoulders when standing upright, one side of the lumbar folds, lumbar flexion with back asymmetry (‘razor back’), sometimes asymmetry on both sides of the pelvis, and gait abnormalities. In addition, neurological examination is also needed to observe whether the patient has increased or low muscle tone and whether there are sensory abnormalities in the limbs.

Clinically, it is usually classified into the following 3 types according to the imaging characteristics:

Type I - Vertebral formation disorder type, i.e., one or more vertebrae are partially or completely formed with disorder, such as wedge vertebrae, hemivertebrae or butterfly vertebrae;

Type II - poor vertebral segmentation type, i.e., two or more vertebrae are partially or completely segmented and impaired, e.g., blocked vertebrae, bone bridge formation, etc;

Type III - mixed type, where there is both vertebral body formation disorder and vertebral body mal-segmentation.

### Llama3.1:70b

Based on the clinical manifestations you provided, the rare disease that is most likely to be diagnosed is:

**Congenital Scoliosis**

This condition is characterized by a lateral curvature of the spine that is present at birth, often due to vertebral malformations or abnormalities in the formation or segmentation of the vertebrae. The clinical manifestations you described, such as unequal shoulders, lumbar folds, and back asymmetry, are consistent with congenital scoliosis. Additionally, the classification into three types based on imaging characteristics (Type I: vertebral formation disorder, Type II: poor vertebral segmentation, and Type III: mixed type) is also consistent with the classification of congenital scoliosis.

Here are five other possible diagnoses that may be considered:

1. **Neurofibromatosis Type 1 (NF1)**: This genetic disorder can cause scoliosis, as well as other skeletal abnormalities, skin changes, and neurological problems.
2. **Marfan Syndrome**: This genetic disorder affects the body’s connective tissue and can cause scoliosis, as well as other skeletal, cardiovascular, and ocular abnormalities.
3. **Ehlers-Danlos Syndrome**: This group of genetic disorders affects the body’s connective tissue and can cause scoliosis, as well as other skeletal, skin, and joint abnormalities.
4. **Osteogenesis Imperfecta**: This genetic disorder affects the production of collagen, leading to brittle bones and scoliosis, as well as other skeletal abnormalities.
5. **Klippel-Feil Syndrome**: This rare genetic disorder is characterized by the congenital fusion of two or more cervical vertebrae, which can cause scoliosis, as well as other skeletal and neurological abnormalities.

It’s worth noting that a definitive diagnosis can only be made after a thorough medical evaluation, including imaging studies and genetic testing, if necessary.

### **37. User**

There are more men than women. Some patients have atypical symptoms of chest pain, which are detected during coronary angiography or coronary CT. Patients are often clinically symptomatic when there is (1) a combination of obstructive atherosclerotic disease, thrombosis, slow flow, entrapment, or rupture with exertional angina or acute coronary syndromes; (2) compression of adjacent tissues; (3) rupture of an aneurysm, which is rare and can cause acute cardiac tamponade; and (4) myocardial ischemia due to microvascular dysfunction, which can occur even in the absence of significant coronary stenosis. The most common clinical manifestation is cardiac ischaemia.

The most common clinical manifestation is angina pectoris, and in a few patients, myocardial infarction, severe arrhythmia, or even sudden death may occur. The disease may break into the right atrium, right ventricle, or coronary sinus, creating a left-to-right shunt, which can be heard as a continuous murmur and, in severe cases, affect cardiac function. The most serious complication is rupture of the tumour into the pericardium, which can lead to bleeding, cardiac tamponade or even death.

### Llama3.1:70b

Based on the clinical manifestations you provided, the rare disease that is most likely to be diagnosed is:

**Coronary Artery Anomaly, specifically Coronary Artery Fistula or Coronary Artery Aneurysm**

This condition is characterized by an abnormal connection between the coronary arteries and other cardiac chambers or vessels, leading to symptoms such as angina, myocardial infarction, and arrhythmias. The presence of a continuous murmur and the risk of rupture into the pericardium, leading to cardiac tamponade, are also consistent with this diagnosis.

Here are five other possible diagnoses that could be considered:

1. **Spontaneous Coronary Artery Dissection (SCAD)**: This condition involves a tear in the inner lining of the coronary artery, leading to symptoms such as chest pain, myocardial infarction, and arrhythmias.
2. **Myocardial Bridging**: This condition involves a portion of the coronary artery being compressed by the myocardium, leading to symptoms such as angina and myocardial infarction.
3. **Coronary Artery Vasospasm**: This condition involves a sudden constriction of the coronary artery, leading to symptoms such as chest pain and myocardial infarction.
4. **Cardiac Sarcoidosis**: This condition involves inflammation of the heart tissue, leading to symptoms such as arrhythmias, heart failure, and sudden death.
5. **Fibromuscular Dysplasia (FMD) of the Coronary Arteries**: This condition involves a non-atherosclerotic, non-inflammatory vascular disease that can lead to symptoms such as angina, myocardial infarction, and arrhythmias.

It’s worth noting that these conditions are relatively rare and may require further testing and evaluation to confirm the diagnosis.

### **38. User**

1. Bone marrow failure Anaemia is present at birth in 35 per cent of children and is often diagnosed between 2 weeks and 2 years after birth. The anaemia is macrocytic or normocytic orthochromatic, with decreased reticulocytes, normal or mildly decreased white blood cells, normal or mildly increased platelets, and hypoplasia of the erythroid lineage of the bone marrow and hyperproliferation of the granulocyte and megakaryocyte lineages.
2. Congenital developmental anomalies 30% ~50% of patients may have congenital developmental anomalies, mainly involving the head, upper limbs, heart and genitourinary system. Examples include short stature, craniofacial anomalies including craniofacial hypertelorism, wide flat nose, small head, congenital cataracts, glaucoma, strabismus, high arched hard palate and cleft lip and palate or even Turner’s syndrome appearance, bunions, congenital cardiovascular anomalies, and genitourinary anomalies. The presence of physical abnormalities does not predict the severity of the patient’s haematological disorders. 30% of affected patients may present with postnatal growth retardation, often associated with other congenital malformations.
3. The incidence of tumours in patients with increased susceptibility to cancer is about 4%, which is higher than in the normal population of the same age. And the tumour occurs at an early age. These include acute leukaemia of the haematological system, myelodysplastic syndromes, lymphomas and solid tumours such as osteosarcoma, thymic carcinoma, hepatocellular carcinoma, melanoma, fibrous histiocytoma, gastric cancer and intestinal cancer. The prognosis of the tumours is poorer than that of the general population suffering from the same tumour, with a higher mortality rate.

### Llama3.1:70b

Based on the clinical manifestations you provided, the rare disease that is most likely to be diagnosed is:

**Fanconi Anemia (FA)**

Fanconi Anemia is a rare genetic disorder characterized by bone marrow failure, congenital developmental anomalies, and an increased susceptibility to cancer. The clinical manifestations you described, such as bone marrow failure with macrocytic or normocytic anemia, decreased reticulocytes, and hypoplasia of the erythroid lineage, are consistent with FA. Additionally, the presence of congenital developmental anomalies, such as craniofacial abnormalities, heart defects, and genitourinary anomalies, is also typical of FA. The increased risk of cancer, particularly at a young age, is another hallmark of the disease.

Here are five other possible diagnoses that may be considered:

1. **Dyskeratosis Congenita (DC)**: A rare genetic disorder characterized by bone marrow failure, skin hyperpigmentation, and an increased risk of cancer. While DC shares some similarities with FA, it typically presents with more pronounced skin and nail abnormalities.
2. **Shwachman-Diamond Syndrome (SDS)**: A rare genetic disorder characterized by bone marrow failure, pancreatic insufficiency, and skeletal abnormalities. While SDS shares some similarities with FA, it typically presents with more pronounced pancreatic and skeletal abnormalities.
3. **Diamond-Blackfan Anemia (DBA)**: A rare genetic disorder characterized by bone marrow failure, particularly affecting the erythroid lineage. While DBA shares some similarities with FA, it typically presents with more pronounced erythroid hypoplasia and fewer congenital developmental anomalies.
4. **Seckel Syndrome**: A rare genetic disorder characterized by primordial dwarfism, microcephaly, and an increased risk of cancer. While Seckel Syndrome shares some similarities with FA, it typically presents with more pronounced growth retardation and microcephaly.
5. **Bloom Syndrome (BS)**: A rare genetic disorder characterized by an increased risk of cancer, particularly at a young age, and a variety of congenital developmental anomalies. While BS shares some similarities with FA, it typically presents with more pronounced telangiectatic erythema and fewer hematological abnormalities.

It is essential to note that a definitive diagnosis of Fanconi Anemia or any of these alternative diagnoses requires genetic testing and a comprehensive evaluation by a multidisciplinary team of specialists.

### **39. User**

The lesions may involve all systems of the body, so their clinical manifestations vary depending on the site of involvement. General symptoms include fever, malaise, night sweats, and weight loss. The rest of the affected systems mainly include:

1. Skeletal system, most patients have skeletal involvement at the time of diagnosis, with lower limb bones being the most common, but only 50% of patients have bone pain.
2. cardiovascular system, lesions involving the cardiovascular system, most patients do not have obvious clinical manifestations, but such as pericardial involvement, resulting in a large amount of pericardial effusion can lead to chest tightness, breathlessness, or even cardiac tamponade; lesions involving coronary arteries can cause myocardial ischemia-like manifestations, such as chest tightness, chest pain, etc.; diffuse myocardial involvement has also been reported, which can lead to cardiac failure-like manifestations; lesions encircling the renal arteries can cause renal hypertension; lesions encircling the mesenteric artery can cause renal hypertension; lesions encircling the mesenteric artery can cause renal hypertension. Diffuse myocardial involvement has also been reported, leading to heart failure-like symptoms; lesions encircling the renal arteries may cause renal hypertension; lesions encircling the mesenteric vessels may cause intestinal ischaemia, such as abdominal pain.
3. respiratory system Most patients with respiratory system involvement are asymptomatic, but a few patients have dry cough and dyspnoea.
4. central nervous system central nervous system involvement may appear corresponding clinical manifestations due to different parts of brain parenchyma involvement, such as limb movement disorder, gait abnormality, alteration of consciousness, etc.; spinal cord involvement may appear different clinical manifestations due to different affected segments; pituitary gland lesions may manifest polydipsia, polyuria, and other hormone secretion related manifestations.
5. Retro-orbital lesions Retro-orbital involvement is one of the most common clinical manifestations, which may manifest as unilateral or bilateral proptosis, or even cause blindness.
6. Retroperitoneal space/renal involvement. Retroperitoneal lesions may resemble retroperitoneal fibrosis and may cause unilateral or bilateral retrorenal obstruction if the lesion encircles the ureter.
7. cutaneous involvement, the most common cutaneous lesion is a xanthomatous change, which occurs on the eyelids in about 1/3 of patients, and may also occur on the face, neck, axillae, trunk, and groin.

### Llama3.1:70b

Based on the clinical manifestations you provided, the rare disease that is most likely to be diagnosed is:

**Erdheim-Chester disease (ECD)**

Erdheim-Chester disease is a rare, non-Langerhans cell histiocytosis characterized by the proliferation of histiocytes in various organs, leading to a wide range of clinical manifestations. The disease can affect multiple systems, including the skeletal, cardiovascular, respiratory, central nervous, and cutaneous systems, among others. The clinical features you described, such as skeletal involvement, cardiovascular lesions, respiratory system involvement, central nervous system manifestations, retro-orbital lesions, retroperitoneal space/renal involvement, and cutaneous xanthomatous changes, are all consistent with ECD.

Here are five other possible diagnoses that may be considered based on the clinical manifestations:

1. **Langerhans cell histiocytosis (LCH)**: While LCH is a different entity from ECD, it can also present with similar clinical manifestations, such as skeletal lesions, skin involvement, and central nervous system manifestations.
2. **Sarcoidosis**: Sarcoidosis is a systemic granulomatous disease that can affect multiple organs, including the lungs, skin, eyes, and central nervous system. While the clinical features you described are not typical of sarcoidosis, some patients may present with similar manifestations.
3. **Wegener’s granulomatosis (Granulomatosis with polyangiitis)**: This is a rare autoimmune disorder that can affect multiple organs, including the respiratory tract, kidneys, and skin. While the clinical features you described are not typical of Wegener’s granulomatosis, some patients may present with similar manifestations.
4. **Castleman disease**: Castleman disease is a rare lymphoproliferative disorder that can present with systemic symptoms, such as fever, weight loss, and night sweats, as well as lymphadenopathy and organ involvement.
5. **Multicentric reticulohistiocytosis**: This is a rare disease characterized by the proliferation of histiocytes in multiple organs, leading to a wide range of clinical manifestations, including skin lesions, joint involvement, and systemic symptoms.

It is essential to note that these alternative diagnoses are less likely than ECD, and a definitive diagnosis can only be made through a combination of clinical evaluation, imaging studies, and histopathological examination.

### **40. User**

The disease often presents with multi-organ and multi-system involvement, with males being more affected than female patients. Clinical manifestations often involve different organs as the patient grows older.

1. Facial appearance, male patients usually appear at the age of 12-14 years with characteristic facial features, such as convexity of the supraorbital ridge, frontal bulge and thickening of the lips. 2.
2. cutaneous angiokeratomas, which are common in patients with the classic form, are red patches of raised skin in the ‘sitting area’, i.e., between the umbilicus and knees, the scrotum, the buttocks, and the inner thighs.
3. Neurological system, most patients will have peripheral nerve pain, which manifests as intolerable burning sensation on the soles of the feet and palms of the hands and radiates to the proximal limbs, and even painful spasms; autonomic nerve involvement manifests as little or no sweating; and central nervous system involvement manifests as early-onset transient ischemic attacks or ischemic strokes.
4. Eye: Characteristic manifestations include conjunctival vascular tortuosity, corneal turbidity, posterior lens capsule clouding, and retinal vascular tortuosity, which in severe cases may lead to reduced or even loss of vision. It is often one of the main reasons for female patients to visit the clinic.
5. Digestive tract, most often after eating nausea, vomiting, abdominal distension, cramping abdominal pain and diarrhoea, may also manifest as malabsorption or constipation.
6. Kidney, early manifestation of urinary concentration dysfunction such as increased nocturia, polyuria and enuresis, with the progress of the disease can gradually appear proteinuria, and even up to the level of nephrotic syndrome, accompanied by renal function damage, mostly around 30 years old into the end-stage renal disease.
7. Cardiovascular system, can be manifested as hypertension, myocardial ischemia caused by coronary artery involvement, heart valve lesions and hypertrophic cardiomyopathy, and in severe cases, it can be manifested as angina pectoris, myocardial infarction and heart failure. It is mostly a late manifestation of the disease and a major cause of death.

### Llama3.1:70b

What a fascinating case!

Based on the clinical manifestations you provided, I’m going to take a educated guess that the rare disease most likely to be diagnosed is:

**Fabry disease**

Fabry disease is a genetic disorder caused by a deficiency of the enzyme alpha-Gal A, leading to the accumulation of globotriaosylceramide (Gb3 or GL-3) in various organs and tissues. The disease is X-linked, which explains the higher prevalence in males. The clinical manifestations you described, including the characteristic facial features, cutaneous angiokeratomas, neurological symptoms, eye involvement, gastrointestinal issues, kidney problems, and cardiovascular complications, are all consistent with Fabry disease.

Here are five other possible diagnoses that might be considered:

1. **Mucopolysaccharidosis type I (MPS I)**: This is a genetic disorder caused by a deficiency of the enzyme alpha-L-iduronidase, leading to the accumulation of glycosaminoglycans in various organs and tissues. While the clinical manifestations are similar to Fabry disease, MPS I typically presents with more pronounced skeletal and joint abnormalities.
2. **Gaucher disease**: This is a genetic disorder caused by a deficiency of the enzyme glucocerebrosidase, leading to the accumulation of glucocerebroside in various organs and tissues. While Gaucher disease can present with similar systemic involvement, it typically lacks the characteristic facial features and cutaneous angiokeratomas seen in Fabry disease.
3. **Niemann-Pick disease**: This is a group of genetic disorders caused by deficiencies in various enzymes involved in lipid metabolism, leading to the accumulation of lipids in various organs and tissues. While Niemann-Pick disease can present with similar systemic involvement, it typically lacks the characteristic facial features and cutaneous angiokeratomas seen in Fabry disease.
4. **Mitochondrial myopathy, encephalopathy, lactic acidosis, and stroke-like episodes (MELAS) syndrome**: This is a mitochondrial disorder that can present with similar neurological and systemic manifestations, including stroke-like episodes and gastrointestinal issues. However, MELAS syndrome typically lacks the characteristic facial features and cutaneous angiokeratomas seen in Fabry disease.
5. **Anderson-Fabry disease variant: Late-onset Fabry disease**: This is a variant of Fabry disease that presents later in life, often with milder symptoms. While the clinical manifestations are similar to classic Fabry disease, late-onset Fabry disease typically lacks the characteristic facial features and cutaneous angiokeratomas seen in classic Fabry disease.

Please note that these alternative diagnoses are speculative and would require further evaluation and testing to confirm or rule out.

### **41. User**

The disease is classified as Type 1 and Type 2.

1. Type 1 is mainly characterised by recurrent inflammatory reactions and plasma membrane inflammation, including recurrent fever, abdominal pain and arthritis. Fever is recurrent and may be triggered by cold, strenuous exercise, surgery, infection, etc. and resolves spontaneously within 13 days. Abdominal pain is the most common clinical manifestation, seen in 95% of patients. It is a sudden onset of abdominal pain, often accompanied by fever, abdominal examination may have abdominal distension, abdominal pressure, rebound pain, muscle tension, and the loss of bowel sounds on auscultation. Stereotactic abdominal radiographs show flat air and fluid in the small intestine. Abdominal pain often resolves spontaneously after 24-48 hours. Arthritis is also a common clinical manifestation, seen in 75% of patients. It often involves the large joints of the lower limbs, such as the hip and knee joints. There may be joint swelling and pain. Rash is seen in 7% ~40% of patients, mainly involving the extensor side of the lower limbs, and the typical rash is erysipelas of tansy-like erythema. Pleurisy is seen in 45% of patients and presents as a sudden onset of unilateral chest pain, often accompanied by fever. Pericarditis is less common.
2. Type 2 starts with amyloidosis. It presents with persistent proteinuria and often leads to end-stage renal disease. Amyloid can also be deposited in the heart, gastrointestinal tract, liver and other organs, leading to corresponding clinical manifestations.

### Llama3.1:70b

Based on the clinical manifestations you provided, the rare disease that is most likely to be diagnosed is Familial Mediterranean Fever (FMF).

FMF is a hereditary autoimmune disorder characterized by recurrent episodes of fever, abdominal pain, and arthritis, which are similar to the symptoms described in Type 1. The disease is most commonly found in populations from the Mediterranean region, such as Armenians, Arabs, Greeks, and Turks.

Here are five other possible diagnoses that may be considered:

1. **Tumor Necrosis Factor Receptor-Associated Periodic Syndrome (TRAPS)**: This is another hereditary autoimmune disorder that presents with recurrent episodes of fever, abdominal pain, and arthritis, similar to FMF.
2. **Hyper-IgD Syndrome (HIDS)**: This is a rare genetic disorder characterized by recurrent episodes of fever, abdominal pain, and arthritis, often triggered by minor infections or vaccinations.
3. **Cryopyrin-Associated Periodic Syndrome (CAPS)**: This is a group of rare genetic disorders that present with recurrent episodes of fever, abdominal pain, and arthritis, often accompanied by skin rash and conjunctivitis.
4. **Systemic Juvenile Idiopathic Arthritis (sJIA)**: This is a subtype of juvenile idiopathic arthritis characterized by high fever, rash, and arthritis, often accompanied by abdominal pain and serositis.
5. **Autoinflammatory Syndrome with Lipodystrophy (Majeed Syndrome)**: This is a rare genetic disorder characterized by recurrent episodes of fever, abdominal pain, and arthritis, often accompanied by lipodystrophy (loss of body fat) and amyloidosis.

Type 2, which presents with amyloidosis, may be a secondary complication of FMF or another autoinflammatory disorder, or it may be a separate entity, such as **Hereditary Amyloidosis**, which is a group of rare genetic disorders characterized by the deposition of amyloid fibrils in various organs.

### **42. User**

The main manifestations are congenital developmental anomalies, progressive bone marrow failure and increased incidence of tumours, while some patients may show no developmental anomalies or bone marrow failure.

1. Developmental anomalies, patients may show a variety of physical and mental developmental abnormalities. Growth defects, abnormal skin pigmentation, unilateral or bilateral skeletal malformations of the upper limbs, short stature, microcephaly, ocular anomalies, genitourinary malformations, endocrine anomalies, hearing loss, outer ear anomalies, congenital heart defects, digestive anomalies, abnormalities of the central nervous system, facial deformities, spinal deformities, neck deformities, and developmental delays and/or intellectual disabilities. These manifestations are mostly non-specific, with thumb and upper limb deformities being more specific.
2. Progressive bone marrow failure with haematological abnormalities is the most serious clinical feature of Fanconi anaemia. It may present as a single or total haematopenia. The age of onset of haemopenia varies widely, even among siblings. The median age of onset is about 7.6 years, and it is rare in infants and young children; 90% of patients with haematological abnormalities develop before the age of 40 years. Thrombocytopenia or leukopenia usually precedes anaemia and is often accompanied by an increase in macrocytes, foetal haemoglobin (HbF 1), and a gradual increase in total haematopoiesis.
3. There is an increased risk of neoplasms, including haematological malignancies and non-haematological malignant solid tumours. The risk of acute myeloid leukaemia (AML) is increased about 500-fold, and the cumulative incidence of AML is about 13% at the age of 50 years, with most cases occurring between the ages of 15 and 35 years. -7, del (7q) is associated with an increased incidence of myelodysplastic syndromes (MDS)/AML. Acute lymphoblastic leukaemia and lymphoma have also been reported. Solid tumours can be the first manifestation of the disease. Head and neck squamous cell carcinomas (HNSCCs) are the most common solid tumours in patients and have an earlier age of onset (20-40 years), mostly occurring in the oral cavity (e.g., tongue cancers), are mostly in the progressive stage, and respond poorly to treatment. There is also an increased incidence of skin, oesophageal, liver and genitourinary tract tumours. In a few patients, multiple tumours may be seen concurrently, or haematological and non-haematological tumours may be seen concurrently. And there is a marked intolerance to chemotherapy or radiotherapy.

### Llama3.1:70b

Based on the clinical manifestations you provided, the most likely diagnosis is:

**Fanconi Anemia (FA)**

Fanconi anemia is a rare genetic disorder characterized by congenital developmental anomalies, progressive bone marrow failure, and an increased risk of tumors, including hematological malignancies and non-hematological solid tumors. The clinical features you described, such as thumb and upper limb deformities, growth defects, and increased risk of acute myeloid leukemia (AML) and head and neck squamous cell carcinomas (HNSCCs), are consistent with Fanconi anemia.

Here are five other possible diagnoses that may be considered:

1. **Dyskeratosis Congenita (DC)**: A rare genetic disorder characterized by skin hyperpigmentation, nail dystrophy, and mucosal leukoplakia, with an increased risk of bone marrow failure and cancer.
2. **Shwachman-Diamond Syndrome (SDS)**: A rare genetic disorder characterized by exocrine pancreatic insufficiency, bone marrow failure, and skeletal abnormalities, with an increased risk of leukemia and other cancers.
3. **Seckel Syndrome**: A rare genetic disorder characterized by primordial dwarfism, microcephaly, and intellectual disability, with an increased risk of cancer and bone marrow failure.
4. **Bloom Syndrome (BS)**: A rare genetic disorder characterized by short stature, facial anomalies, and an increased risk of cancer, including leukemia and lymphoma.
5. **Ataxia-Telangiectasia (AT)**: A rare genetic disorder characterized by progressive ataxia, telangiectasias, and immunodeficiency, with an increased risk of cancer, including leukemia and lymphoma.

These disorders share some similarities with Fanconi anemia, but each has distinct clinical features that can help differentiate them from FA.

### **43. User**

The perinatal onset of the disease is often typical, and within a few days of ingestion of breast milk or lactose-containing formula, the child develops life-threatening complications: feeding problems, diarrhoea, vomiting, hypoglycaemia, liver impairment, haemorrhage, jaundice, and cataracts. If left untreated, sepsis, shock and death may occur. Children who survive to infancy may develop severe brain damage if they continue to ingest lactose. Some children have long-term complications such as mental retardation, delayed growth, ataxia, blindness, and ovarian dysfunction in female patients.

### Llama3.1:70b

What a fascinating and challenging case!

Based on the clinical manifestations you provided, I’m going to take a educated guess that the most likely diagnosis is:

**Galactosemia**

Galactosemia is a rare genetic disorder that affects the body’s ability to metabolize galactose, a sugar found in milk and other dairy products. The symptoms you described, such as feeding problems, diarrhea, vomiting, hypoglycemia, liver impairment, and cataracts, are all consistent with galactosemia. The disease is typically diagnosed in the neonatal period, and if left untreated, can lead to severe complications, including sepsis, shock, and death.

Here are five other possible diagnoses that could be considered:

1. **Hereditary Fructose Intolerance**: This is a rare genetic disorder that affects the body’s ability to metabolize fructose, a sugar found in many fruits and vegetables. While the symptoms are similar to galactosemia, the trigger is different (fructose vs. galactose).
2. **Glycogen Storage Disease Type I**: This is a rare genetic disorder that affects the body’s ability to metabolize glycogen, a complex carbohydrate. The symptoms can include hypoglycemia, liver impairment, and growth retardation.
3. **Maple Syrup Urine Disease**: This is a rare genetic disorder that affects the body’s ability to metabolize certain amino acids. The symptoms can include feeding problems, vomiting, and neurological impairment.
4. **Urea Cycle Disorder**: This is a group of rare genetic disorders that affect the body’s ability to remove ammonia from the blood. The symptoms can include feeding problems, vomiting, and neurological impairment.
5. **Pyruvate Dehydrogenase Complex Deficiency**: This is a rare genetic disorder that affects the body’s ability to metabolize pyruvate, a key energy-producing molecule. The symptoms can include feeding problems, vomiting, and neurological impairment.

Please note that these alternative diagnoses are less likely, and galactosemia remains the most probable diagnosis based on the clinical manifestations you provided.

### **44. User**

1. Type I is the most common subtype. There is no primary central nervous system involvement, but some patients may develop secondary neurological manifestations (e.g., spinal cord compression) as the disease progresses. The disease can develop in all age groups, with about 2/3 of patients developing in childhood. Organ manifestations are mainly hepatosplenomegaly, especially splenomegaly, often accompanied by hypersplenism, and even splenic infarction and splenic rupture. The haematological manifestations are mainly thrombocytopenia and anaemia, and some patients have leucopenia, which may be accompanied by coagulation abnormalities. Patients present with pallor, fatigue, skin and gum bleeding, increased menstruation, and even life-threatening bleeding. Most patients have skeletal invasion, but the severity varies. Early invasion sites mainly include lumbar vertebrae, long bone epiphyses and diaphyses, and in the middle and late stages, epiphyses and bony prominences may be involved. Patients often experience acute or chronic diffuse bone pain, and in severe cases, osteonecrosis (ischaemic necrosis), followed by joint collapse involving the proximal and distal femur, proximal tibia and proximal humerus. Osteolytic lesions, pathological fractures, vertebral compression fractures and other fragility fractures due to reduced bone density may also occur. Skeletal lesions can interfere with daily activities and can be disabling. In children, the most common manifestations are osteoporosis, flask-like deformity of the metaphysis of the long bones, varying degrees of hypodensity of the metaphysis of the long bones, thinning of the bone cortex, etc. There may be growth retardation. Some patients may have lung involvement, which is mainly manifested as interstitial lung disease, pulmonary solid changes, and pulmonary hypertension. In addition, patients may have abnormalities of sugar and lipid metabolism, increased risk of malignant tumours such as multiple myeloma, cholelithiasis, and abnormalities of the immune system.
2. Type II, in addition to hepatosplenomegaly, anaemia, thrombocytopenia and other manifestations similar to those of type I, patients with type II mainly have acute neurological involvement. It often develops in the neonatal period to infancy, progresses rapidly and has a high morbidity and mortality rate. There are rapidly progressive medullary palsy, dyskinesia, seizures, angular reflexes and cognitive deficits and other signs of acute neurological damage, and growth is retarded, with death occurring before the age of 2 to 4 years. Some patients with severe disease may develop joint contractures.
3. The early manifestations of type III are similar to those of type I, with gradual onset of neurological involvement, which often develops in childhood and has a slow progression of disease and a longer life expectancy. The patients often have the invasion of the motor nerve, eye movement disorders, ataxia, anisocoria, epilepsy, myoclonus, accompanied by developmental delays and intellectual backwardness. Type III can be divided into three subtypes, namely, type IIIa, which is characterised by more rapidly progressive neurological symptoms (ocular motility disorders, cerebellar ataxia, spasticity, myoclonus, and dementia) and hepatosplenomegaly; type IIIb, which is characterised by hepatosplenomegaly and skeletal symptoms with fewer CNS symptoms; and type IIIc, which is characterised by milder symptoms, including calcification of the cardiac valves and corneal clouding, and is predominantly found in the Druze population. Type IIIc.

### Llama3.1:70b

Based on the clinical manifestations you provided, the most likely diagnosis is:

**Gaucher’s disease**

Gaucher’s disease is a rare genetic disorder caused by a deficiency of the enzyme glucocerebrosidase, leading to the accumulation of glucocerebroside in various organs, including the spleen, liver, bones, and central nervous system. The disease has three main subtypes, which match the descriptions you provided:

- Type I: Non-neuronopathic Gaucher’s disease, characterized by hepatosplenomegaly, thrombocytopenia, anemia, and skeletal involvement, without primary central nervous system involvement.
- Type II: Acute neuronopathic Gaucher’s disease, characterized by severe neurological involvement, including seizures, dyskinesia, and cognitive deficits, with a poor prognosis.
- Type III: Subacute neuronopathic Gaucher’s disease, characterized by a gradual onset of neurological symptoms, including eye movement disorders, ataxia, and developmental delays, with a slower progression than Type II.

Other possible diagnoses that may be considered based on the clinical manifestations include:

1. **Niemann-Pick disease**: A group of genetic disorders caused by a deficiency of the enzyme sphingomyelinase, leading to the accumulation of sphingomyelin in various organs.
2. **Fabry disease**: A genetic disorder caused by a deficiency of the enzyme alpha-Gal A, leading to the accumulation of globotriaosylceramide in various organs, including the kidneys, heart, and nervous system.
3. **Mucopolysaccharidosis**: A group of genetic disorders caused by a deficiency of enzymes involved in the breakdown of glycosaminoglycans, leading to their accumulation in various organs.
4. **Osteopetrosis**: A rare genetic disorder characterized by an increase in bone density, leading to skeletal abnormalities, including osteonecrosis and fractures.
5. **Lysosomal storage disorders**: A group of genetic disorders caused by a deficiency of enzymes involved in the breakdown of cellular waste products, leading to their accumulation in various organs.

Note that these alternative diagnoses may not perfectly match the clinical manifestations you provided, and further testing and evaluation would be necessary to confirm a diagnosis.

### **45. User**

It often starts in one muscle group and progresses to others, with ptosis and diplopia being the most common onset, accounting for 80 per cent of cases. Skeletal muscle weakness is fluctuating and easily fatigued, with a light morning and heavy evening. According to the clinical manifestations, it is divided into five types. Type I: ocular muscle type, manifested by ptosis and diplopia. Type ⅡA: mild generalised type, with mild involvement of the limb muscle groups. Type IIB: Moderate generalised type with moderate involvement of the limb muscle groups, usually with mastication, swallowing and dysarthria. Type III: Severe aggressive type with rapid progression, involving the pharyngeal muscles within weeks or months and the respiratory muscles within six months. Type IV: Late-onset severe type, slowly progressive, involving the respiratory muscles within 2 years. Type V: myasthenia gravis, skeletal muscle atrophy within six months of disease onset. Neurological examination can be seen in the proximal limbs, trunk, neck, craniofacial muscle weakness, intolerance to fatigue, Jolly test is positive, tendon reflexes are normal, no sensory deficits, no pathological signs. Some patients may progress to respiratory failure and develop myasthenia gravis crisis.

### Llama3.1:70b

Based on the clinical manifestations you provided, the most likely diagnosis is:

**Myasthenia Gravis (MG)**

Myasthenia Gravis is a rare autoimmune disease characterized by fluctuating muscle weakness and fatigue, particularly affecting the ocular, bulbar, and proximal limb muscles. The disease often starts in one muscle group and progresses to others, with ptosis and diplopia being common initial symptoms. The five types of MG you described match the typical classification of the disease.

Here are five other possible diagnoses that may be considered, although they are less likely:

1. **Lambert-Eaton Myasthenic Syndrome (LEMS)**: An autoimmune disorder that affects the neuromuscular junction, causing muscle weakness, fatigue, and autonomic dysfunction. While LEMS shares some similarities with MG, it typically presents with more proximal muscle weakness and autonomic symptoms.
2. **Congenital Myasthenic Syndrome (CMS)**: A group of rare genetic disorders that affect the neuromuscular junction, causing muscle weakness and fatigue. CMS typically presents at birth or in early childhood, and its symptoms are often more severe and progressive than those of MG.
3. **Mitochondrial Myopathies**: A group of rare genetic disorders that affect the mitochondria, leading to muscle weakness, fatigue, and other systemic symptoms. While some mitochondrial myopathies may present with similar symptoms to MG, they often have distinct clinical and laboratory features.
4. **Inflammatory Myopathies**: A group of autoimmune disorders that cause muscle inflammation and weakness, such as polymyositis and dermatomyositis. While these conditions may present with similar symptoms to MG, they often have distinct clinical and laboratory features, such as muscle inflammation and skin rash.
5. **Botulism**: A rare neurological disorder caused by the toxin produced by the bacterium Clostridium botulinum. Botulism can cause muscle weakness, fatigue, and cranial nerve dysfunction, but its symptoms are often more acute and severe than those of MG.

It’s essential to note that a definitive diagnosis of Myasthenia Gravis or any other condition requires a comprehensive evaluation, including laboratory tests (e.g., acetylcholine receptor antibodies), electromyography, and imaging studies.

### **46. User**

The disease often starts in adolescence or early adulthood. Clinical manifestations are mainly related to hypokalemia and hypomagnesemia. Mild patients may be asymptomatic or show mild fatigue and poor appetite; severe patients may have twitching of limbs, flaccid paralysis, painful spasms, fainting, and transverse myelinolysis secondary to acute renal injury, and may even suffer from cardiac arrest due to severe ventricular arrhythmias. The common clinical manifestations are as follows:

1. systemic symptoms fatigue, thirst, polydipsia, salt craving.
2. Neuro-muscular system Muscle weakness, painful spasms, convulsions, convulsive seizures, numbness of limbs, sensory abnormalities, rhabdomyolysis, paralysis, dizziness, vertigo, ataxia, and pseudotumor cerebri.
3. Cardiovascular system Palpitation, syncope, normal or low blood pressure and ventricular arrhythmia, etc.
4. Digestive system Constipation, vomiting, etc.
5. Urinary system Polyuria, nocturia, enuresis, proteinuria and renal insufficiency.
6. Bone and joint system Arthralgia, cartilage calcium sedimentation.
7. endocrine and growth retardation, delayed puberty; diabetes mellitus or impaired glucose tolerance is not uncommon in patients with chronically low potassium and magnesium. 8. ocular symptoms.
8. Ocular symptoms Blurred vision and scleral choroidal calcification may occur in a small number of patients.

### Llama3.1:70b

Based on the clinical manifestations you provided, the most likely diagnosis is:

**Bartter syndrome**

Bartter syndrome is a rare genetic disorder characterized by hypokalemia, hypomagnesemia, and metabolic alkalosis. The symptoms you described, such as muscle weakness, painful spasms, convulsions, and cardiac arrhythmias, are consistent with the clinical presentation of Bartter syndrome. Additionally, the presence of systemic symptoms like fatigue, thirst, and polydipsia, as well as the involvement of multiple organ systems, including the neuromuscular, cardiovascular, and urinary systems, are also characteristic of Bartter syndrome.

Here are five other possible diagnoses that may be considered based on the clinical manifestations:

1. **Gitelman syndrome**: Another rare genetic disorder that affects the kidneys and is characterized by hypokalemia, hypomagnesemia, and metabolic alkalosis. While the symptoms are similar to Bartter syndrome, Gitelman syndrome tends to have a milder course.
2. **Liddle syndrome**: A rare genetic disorder that affects the kidneys and is characterized by hypokalemia, hypertension, and metabolic alkalosis. While the symptoms are similar to Bartter syndrome, Liddle syndrome tends to have a more pronounced effect on blood pressure.
3. **East syndrome**: A rare genetic disorder that affects the kidneys and is characterized by hypokalemia, hypomagnesemia, and metabolic alkalosis, similar to Bartter syndrome. However, East syndrome tends to have a more pronounced effect on the eyes, with symptoms like blurred vision and scleral choroidal calcification.
4. **Familial hypokalemic periodic paralysis**: A rare genetic disorder that affects the muscles and is characterized by episodes of muscle weakness, paralysis, and hypokalemia. While the symptoms are similar to Bartter syndrome, this disorder tends to have a more episodic course.
5. **Renal tubular acidosis (RTA) type 1**: A rare genetic disorder that affects the kidneys and is characterized by hypokalemia, metabolic acidosis, and kidney stones. While the symptoms are similar to Bartter syndrome, RTA type 1 tends to have a more pronounced effect on the kidneys and urinary system.

It’s worth noting that these diagnoses are not mutually exclusive, and some patients may have overlapping features of multiple disorders. A thorough diagnostic evaluation, including genetic testing and imaging studies, would be necessary to confirm the diagnosis.

### **47. User**

The majority of children develop the disease in infancy or early childhood, with a variety of presentations. The earlier the onset, the more severe the symptoms and the worse the prognosis. The majority of patients have a relatively normal presentation in the first year of life. The earliest sign that appears in about 75% of patients is a large head size, and most children are born with a larger head circumference than their peers or have a rapid increase in head circumference soon after birth, which may be accompanied by mild non-specific symptoms, including irritability, difficulty in feeding, and vomiting. Children usually develop ketosis, vomiting, hepatomegaly and acute encephalopathic crisis, including hypotonia, loss of consciousness and convulsive seizures, between 3 and 36 months of age after fever, infection, surgery, or vaccination, which may resolve with symptomatic treatment, but not fully recover. If acute encephalopathic crisis occurs repeatedly, the neurological damage will be progressively aggravated, and there may be developmental regression, and eventually cognitive dysfunction may occur. In some children, delayed movement, dystonia, and random movement disorders occur gradually over the first few years of life, but mental development is almost normal. Patients often die within 10 years of age from concomitant diseases or Reye-like seizures, which decrease with age. Rarely, the onset of the disease occurs during adolescence or even adulthood, and may be asymptomatic until the first episode.

### Llama3.1:70b

Based on the clinical manifestations you provided, the most likely diagnosis is:

**MCAD Deficiency (Medium-Chain Acyl-CoA Dehydrogenase Deficiency)**

MCAD deficiency is a rare genetic disorder that affects the body’s ability to convert certain fats to energy. The symptoms you described, such as large head size, ketosis, vomiting, hepatomegaly, and acute encephalopathic crisis, are consistent with MCAD deficiency. The disease often presents in infancy or early childhood, and the severity of symptoms can vary depending on the age of onset.

Here are five other possible diagnoses that may be considered based on the clinical manifestations:

1. **Glutaric Acidemia Type 1 (GA1)**: This is a rare genetic disorder that affects the body’s ability to break down certain amino acids. Symptoms can include large head size, developmental delay, and acute encephalopathic crisis.
2. **Carnitine Palmitoyltransferase 1 (CPT1) Deficiency**: This is a rare genetic disorder that affects the body’s ability to transport fatty acids into the mitochondria for energy production. Symptoms can include ketosis, vomiting, hepatomegaly, and acute encephalopathic crisis.
3. **Very Long-Chain Acyl-CoA Dehydrogenase (VLCAD) Deficiency**: This is a rare genetic disorder that affects the body’s ability to break down certain fatty acids. Symptoms can include large head size, developmental delay, and acute encephalopathic crisis.
4. **Reye’s Syndrome**: This is a rare but serious condition that causes swelling in the liver and brain. Symptoms can include vomiting, seizures, and altered mental status, often triggered by a viral infection or vaccination.
5. **Mitochondrial Encephalomyopathy, Lactic Acidosis, and Stroke-like Episodes (MELAS)**: This is a rare genetic disorder that affects the mitochondria, leading to symptoms such as developmental delay, seizures, and stroke-like episodes.

It’s essential to note that these diagnoses are speculative and may require further testing and evaluation to confirm. A comprehensive diagnostic workup, including genetic testing, imaging studies, and laboratory tests, would be necessary to determine the underlying cause of the symptoms.

### **48. User**

It mainly manifests as bleeding from joints, muscles and deep tissues, but also internal organs such as gastrointestinal tract and central nervous system. If repeated bleeding occurs, untreated bleeding can lead to joint deformity and/or pseudotumour formation, which can be life-threatening in severe cases. Delayed haemorrhage after trauma or surgery is characteristic of this disease.

### Llama3.1:70b

Based on the clinical manifestations you provided, the rare disease that is most likely to be diagnosed is:

**Hemophilia A**

Hemophilia A is a genetic disorder caused by a deficiency of clotting factor VIII, leading to recurrent bleeding episodes, particularly into joints, muscles, and internal organs. The disease is characterized by delayed bleeding after trauma or surgery, and repeated bleeding can lead to joint deformity and pseudotumor formation.

Here are five other possible diagnoses that may be considered:

1. **Hemophilia B**: Similar to Hemophilia A, but caused by a deficiency of clotting factor IX.
2. **Von Willebrand Disease**: A bleeding disorder caused by a deficiency or dysfunction of von Willebrand factor, a protein that helps platelets adhere to injured blood vessels.
3. **Factor XI Deficiency**: A rare bleeding disorder caused by a deficiency of clotting factor XI, which can lead to bleeding episodes, particularly after trauma or surgery.
4. **Glanzmann Thrombasthenia**: A rare bleeding disorder caused by a deficiency or dysfunction of platelet glycoproteins, leading to impaired platelet aggregation and bleeding episodes.
5. **Afibrinogenemia**: A rare bleeding disorder caused by a deficiency of fibrinogen, a protein essential for blood clotting, leading to bleeding episodes, particularly after trauma or surgery.

These diagnoses should be considered in the differential diagnosis, but Hemophilia A is the most likely diagnosis based on the clinical manifestations you provided.

### **49. User**

It can involve multiple organs of the body and has a variety of clinical manifestations. The age of onset is mostly 3-60 years old, and there are also reports of patients diagnosed at 8 months and over 70 years old. In children, hepatic involvement is the first manifestation of the disease, while in adolescents and adults, neurological involvement is the first manifestation of the disease.

It may present with asymptomatic persistent elevation of aminotransferases, chronic hepatitis, cirrhosis, and acute liver failure. Neurological involvement may manifest as motor dysfunction, tremor, ataxia, chorea, dysautonomia, dystonia, and in some patients masked face, stiff limbs, and gait abnormalities. Some patients combine with mental behavioural abnormalities such as apathy, aggressive behaviour, and personality changes. Kidney involvement may manifest as haematuria, proteinuria, etc. Involvement of the blood system may be manifested as haemolytic anaemia, liver cirrhosis, splenic hyperfunction leading to a decrease in the blood tertiary system, and coagulation abnormalities. Relatively rare systems involved include bone and joint, heart, endocrine and reproductive systems.

### Llama3.1:70b

What a fascinating case!

Based on the clinical manifestations you provided, I’m going to take a educated guess that the rare disease most likely to be diagnosed is:

**Wilson’s Disease**

Wilson’s Disease is a rare genetic disorder characterized by excessive accumulation of copper in the body, particularly in the liver, brain, and other organs. The clinical manifestations you described, such as liver involvement (elevated aminotransferases, chronic hepatitis, cirrhosis, and acute liver failure), neurological involvement (motor dysfunction, tremor, ataxia, chorea, dysautonomia, dystonia), and psychiatric symptoms (apathy, aggressive behavior, personality changes), are all consistent with Wilson’s Disease.

Additionally, the disease can also affect the kidneys (hematuria, proteinuria), blood system (hemolytic anemia, coagulation abnormalities), and other systems (bone and joint, heart, endocrine, and reproductive systems).

Here are five other possible diagnoses that may be considered:

1. **Mitochondrial Neurogastrointestinal Encephalomyopathy (MNGIE)**: a rare genetic disorder characterized by mitochondrial dysfunction, leading to gastrointestinal, neurological, and other systemic symptoms.
2. **Friedreich’s Ataxia**: a rare genetic disorder causing progressive damage to the nervous system, leading to ataxia, dysarthria, and other neurological symptoms.
3. **Hallervorden-Spatz Syndrome (Pantothenate Kinase-Associated Neurodegeneration)**: a rare genetic disorder characterized by iron accumulation in the brain, leading to neurological symptoms such as dystonia, parkinsonism, and cognitive decline.
4. **Ceroid Lipofuscinosis**: a group of rare genetic disorders characterized by accumulation of abnormal lipids in cells, leading to neurological, psychiatric, and other systemic symptoms.
5. **Lysosomal Storage Disorders (e.g., Niemann-Pick Disease)**: a group of rare genetic disorders characterized by accumulation of abnormal lipids in cells, leading to systemic symptoms such as liver dysfunction, neurological symptoms, and other organ involvement.

Please note that these diagnoses are speculative and may require further investigation, including genetic testing, imaging studies, and other diagnostic tests to confirm the diagnosis.

### **50. User**

The clinical picture is very heterogeneous, even within the same family. The disease usually begins before the age of 30 years, worsens during adolescence, and the oedema often has an acute onset. The clinical picture is characterised by recurrent, unpredictable cutaneous and submucosal oedema. The oedema is episodic, self-limited, usually resolves spontaneously in 3-5 days, asymmetric and non-concave. Oedema can involve any part of the body, with the extremities, face, genitals, respiratory tract and gastrointestinal tract mucosa being the most common. The most fatal is respiratory mucosal oedema, which can rapidly progress to respiratory distress or asphyxiation due to laryngeal oedema, and can lead to death by asphyxiation if not resuscitated in time, with a lethality rate as high as 11%-40%, which is one of the main causes of death. Episodes of mucosal oedema of the digestive tract manifest as severe abdominal pain, accompanied by nausea and vomiting, and are therefore often misdiagnosed as acute abdomen, leading to unnecessary abdominal surgery. Skin oedema occurring in the limbs and other parts of the body can affect its function.

### Llama3.1:70b

Based on the clinical manifestations you provided, the rare disease that is most likely to be diagnosed is:

**Hereditary Angioedema (HAE)**

HAE is a rare genetic disorder characterized by recurrent episodes of severe, unpredictable, and potentially life-threatening angioedema, which is a rapid swelling of the skin and mucous membranes. The symptoms you described, such as recurrent cutaneous and submucosal oedema, episodic and self-limited oedema, and the potential for respiratory and gastrointestinal tract involvement, are all consistent with HAE.

Here are five other possible diagnoses that may be considered:

1. **Acquired Angioedema (AAE)**: AAE is a non-hereditary form of angioedema that can be caused by various factors, such as autoimmune disorders, infections, or medications. While the symptoms are similar to HAE, AAE typically has a later onset and may not have a clear family history.
2. **Mast Cell Activation Syndrome (MCAS)**: MCAS is a condition characterized by excessive mast cell activation, leading to symptoms such as angioedema, abdominal pain, and respiratory distress. While MCAS can present with similar symptoms to HAE, it is often associated with other mast cell-related symptoms, such as flushing and itching.
3. **Erythema Multiforme (EM)**: EM is an immune-mediated disorder that can cause skin and mucous membrane lesions, including angioedema. However, EM typically presents with a more widespread rash and is often associated with infections or medications.
4. **Systemic Capillary Leak Syndrome (SCLS)**: SCLS is a rare disorder characterized by recurrent episodes of capillary leak, leading to angioedema, hypotension, and hemoconcentration. While SCLS can present with similar symptoms to HAE, it is often associated with more severe hypotension and hemoconcentration.
5. **Gleich’s Syndrome**: Gleich’s Syndrome is a rare condition characterized by episodic angioedema, weight gain, and elevated eosinophil counts. While it can present with similar symptoms to HAE, Gleich’s Syndrome is often associated with a more pronounced eosinophilic response and weight gain.

It’s essential to note that a definitive diagnosis can only be made through a comprehensive medical evaluation, including laboratory tests, medical history, and family history.

### **51. User**

The disease starts at birth and the severity of clinical manifestations is related to the subtype of the disease. Clinical manifestations include blisters or maculopapular blisters on the hands, elbows, knees and feet and other areas prone to friction, or around the body. Blisters or ulcers of the oral mucosa may occur, leading to dysphagia and hoarseness. Some patients have mottled pigmentation abnormalities, baldness, palmoplantar hyperkeratosis, enamel defects, nail thickening and nail dystrophy. Other impairments include disfigurement of the digits due to repeated scar formation, anaemia, osteoporosis, muscle atrophy, cardiomyopathy, and deafness. Some subtypes may be associated with basal cell carcinoma, squamous cell carcinoma, and malignant melanoma in advanced stages.

### Llama3.1:70b

Based on the clinical manifestations you provided, the rare disease that is most likely to be diagnosed is:

**Epidermolysis Bullosa (EB)**

Epidermolysis Bullosa is a group of rare genetic disorders characterized by blistering of the skin and mucous membranes, often due to minor trauma or friction. The severity of the disease varies depending on the subtype, and the clinical manifestations you described are consistent with the more severe forms of EB, such as dystrophic EB or junctional EB.

Here are five other possible diagnoses that may be considered:

1. **Kindler syndrome**: A rare genetic disorder characterized by congenital blistering, photosensitivity, and skin atrophy, often associated with gastrointestinal and genitourinary complications.
2. **Pachyonychia congenita**: A rare genetic disorder characterized by thickening of the nails, palmoplantar keratoderma, and blistering of the skin, often associated with other ectodermal abnormalities.
3. **Erythropoietic protoporphyria**: A rare genetic disorder characterized by photosensitivity, blistering of the skin, and anemia, often associated with liver disease and other systemic complications.
4. **Xeroderma pigmentosum**: A rare genetic disorder characterized by photosensitivity, blistering of the skin, and an increased risk of skin cancers, including basal cell carcinoma, squamous cell carcinoma, and malignant melanoma.
5. **Dyskeratosis congenita**: A rare genetic disorder characterized by blistering of the skin, nail dystrophy, and mucosal lesions, often associated with bone marrow failure, pulmonary fibrosis, and an increased risk of cancer.

These diagnoses may be considered based on the clinical manifestations you provided, but Epidermolysis Bullosa remains the most likely diagnosis.

### **52. User**

The clinical presentation of patients varies with age, amount and duration of fructose exposure. In general, the younger the age of onset, the more severe the symptoms. The onset of the disease is typically around 6 months of age and usually occurs when fructose-containing foods, such as fruits, honey and certain vegetables, are first consumed. Common clinical symptoms include hypoglycaemic episodes after eating fructose-containing foods, with nausea, vomiting, abdominal pain, bloating, sweating, tremors, convulsions and even coma.

If the diagnosis is not clear and the patient chronically ingests fructose foods for a long period of time, in addition to hypoglycemic manifestations, growth retardation, hepatomegaly, ascites, jaundice and metabolic disorders such as hypoglycemia, metabolic acidosis, hypophosphatemia, hyperuricemia, hypermagnesemia, and hyperpyruvatemia may occur. In severe cases, multiple organ damage, liver and renal failure, convulsions, coma, and even death occur.

In acute attacks, laboratory tests reveal hypoglycaemia, hypophosphatemia and hypokalemia, accompanied by elevated uric acid, lactate, pyruvate, free fatty acids and triglycerides. Chronic patients present with hepatic impairment and indications of hepatic fibrosis, including serum bilirubin, elevated transaminases, and coagulation disorders. Urinalysis reveals proteinuria and fructose positivity.

### Llama3.1:70b

Based on the clinical manifestations you provided, the most likely diagnosis is:

**Hereditary Fructose Intolerance (HFI)**

HFI is a rare genetic disorder caused by a deficiency of the enzyme aldolase B, which is necessary for the breakdown of fructose in the liver. The symptoms and laboratory findings you described are consistent with HFI, including hypoglycemic episodes, nausea, vomiting, abdominal pain, and liver dysfunction after consuming fructose-containing foods.

Here are five other possible diagnoses that may be considered:

1. **Essential Fructosuria**: A benign condition characterized by the inability to absorb fructose, leading to fructose in the urine. However, it does not typically cause hypoglycemic episodes or liver dysfunction.
2. **Fructose Malabsorption**: A condition where the body is unable to absorb fructose, leading to gastrointestinal symptoms such as bloating, abdominal pain, and diarrhea. However, it does not typically cause hypoglycemic episodes or liver dysfunction.
3. **Glycogen Storage Disease Type I (GSD I)**: A genetic disorder caused by a deficiency of the enzyme glucose-6-phosphatase, leading to hypoglycemia, lactic acidosis, and hepatomegaly. However, it is not typically associated with fructose intolerance.
4. **Pyruvate Kinase Deficiency**: A genetic disorder caused by a deficiency of the enzyme pyruvate kinase, leading to hemolytic anemia, jaundice, and liver dysfunction. However, it is not typically associated with fructose intolerance.
5. **Galactosemia**: A genetic disorder caused by a deficiency of the enzyme galactose-1-phosphate uridyltransferase, leading to galactose accumulation in the body. It can cause similar symptoms to HFI, including hypoglycemia, liver dysfunction, and jaundice, but is typically associated with galactose-containing foods rather than fructose.

It’s worth noting that a definitive diagnosis of HFI typically requires a combination of clinical evaluation, laboratory tests, and genetic analysis to confirm the presence of a mutation in the aldolase B gene.

### **53. User**

**1. Migraine** Migraine with aura is often the first symptom of the disease and is seen in 20-40% of patients, which is five times higher than in the general population; it can also manifest as migraine without aura, with an incidence not different from that of the general population. The incidence of migraine is lower in Asian patients than in European and American patients.

**2. Subcortical ischemic events** Transient ischemic attacks (TIAs) and ischemic strokes are the most common clinical manifestations of the disease, occurring in 60% to 85% of symptomatic patients, most of whom lack common risk factors for cerebrovascular disease. 2/3 of patients present with the classic lacunar syndrome.

**3. Cognitive deficits** Cognitive dysfunction with dementia develops progressively in 60% of patients.

**Affective disorders and apathy** 20% of patients have affective disorders, with depressive states being the most common, and rarely alternating between depressive and manic states; apathy is seen in 40% of patients.

In addition, seizures occur in 5-10% of patients, and hearing loss, impaired consciousness, spinal cord infarction, intracranial haemorrhage, and combined basin infarction have also been reported.

### Llama3.1:70b

Based on the clinical manifestations you provided, the rare disease that is most likely to be diagnosed is:

**1. Cerebral Autosomal Dominant Arteriopathy with Subcortical Infarcts and Leukoencephalopathy (CADASIL)**

CADASIL is a rare, hereditary condition characterized by migraine with aura, subcortical ischemic events, cognitive deficits, and psychiatric symptoms, which matches the clinical manifestations you described.

Here are five other possible diagnoses that may be considered:

**2. Moyamoya Disease**: A rare condition caused by narrowing or blockage of the blood vessels in the brain, leading to ischemic strokes, cognitive deficits, and psychiatric symptoms.

**3. Fabry Disease**: A genetic disorder that can cause stroke, cognitive decline, and psychiatric symptoms, as well as other systemic manifestations such as kidney disease and cardiac problems.

**4. Mitochondrial Encephalomyopathy, Lactic Acidosis, and Stroke-like episodes (MELAS)**: A rare mitochondrial disorder that can cause stroke-like episodes, cognitive decline, and psychiatric symptoms, as well as other systemic manifestations such as muscle weakness and hearing loss.

**5. Susac Syndrome**: A rare autoimmune disorder that can cause subcortical ischemic events, cognitive deficits, and psychiatric symptoms, as well as other systemic manifestations such as hearing loss and vision problems.

**6. Hereditary Endotheliopathy with Retinopathy, Nephropathy, and Stroke (HERNS)**: A rare genetic disorder that can cause stroke, cognitive decline, and psychiatric symptoms, as well as other systemic manifestations such as kidney disease and retinopathy.

Note that these alternative diagnoses may not perfectly match the clinical manifestations you provided, but they share some similarities and may be considered in the differential diagnosis.

### **54. User**

The onset of the disease is usually insidious in early youth (but has been reported from 1 to 76 years of age). The main manifestation is a slowly progressive spastic paralysis of both lower limbs, with pyramidal signs (hyperreflexia, positive ankle clonus, positive pathology). Bilateral upper limb reflexes may also be hyperreflexic, but generally do not affect upper limb activity. More than half of the patients will have proximal weakness of the lower limbs. Decreased vibratory sensation of the ankle tuning forks (which usually does not disappear) is detected on examination in about 50% of patients, and urinary dysfunction is present in about one third of patients.

The disease progresses with age, with about 20% of patients eventually losing the ability to walk. The severity of clinical symptoms varies considerably between patients carrying the same mutation in the same family.

### Llama3.1:70b

Based on the clinical manifestations you provided, the rare disease that is most likely to be diagnosed is:

**Hereditary Spastic Paraplegia (HSP)**

Hereditary Spastic Paraplegia is a group of rare genetic disorders characterized by progressive spastic paralysis of the lower limbs, often with pyramidal signs, proximal weakness, and decreased vibratory sensation. The disease typically presents in early youth, but can occur at any age, and progresses slowly over time, with significant variability in severity between individuals carrying the same mutation.

Here are five other possible diagnoses that could be considered based on the clinical manifestations:

1. **Primary Lateral Sclerosis (PLS)**: A rare motor neuron disease characterized by progressive spastic paralysis of the limbs, often with pyramidal signs, but typically without sensory involvement.
2. **Spinal Muscular Atrophy (SMA)**: A genetic disorder characterized by progressive muscle weakness and wasting, often with proximal weakness and decreased reflexes, but typically without significant sensory involvement.
3. **Adrenomyeloneuropathy (AMN)**: A rare genetic disorder characterized by progressive spastic paralysis of the lower limbs, often with sensory involvement, and adrenal insufficiency.
4. **Leukodystrophy**: A group of rare genetic disorders characterized by progressive damage to the white matter of the brain and spinal cord, often with spastic paralysis, sensory involvement, and cognitive decline.
5. **Spastic Ataxia**: A rare genetic disorder characterized by progressive spastic paralysis of the limbs, often with ataxia, dysarthria, and sensory involvement.

It’s worth noting that a definitive diagnosis would require a comprehensive evaluation, including genetic testing, imaging studies, and electrophysiological tests, to rule out other possible causes of the patient’s symptoms.

### **55. User**

Most of the affected children develop the disease in newborns and early infants. Clinical manifestations are similar to biotinidase deficiency, without specificity, mainly feeding difficulties, vomiting, diarrhoea, hypotonia, convulsions, depression, lethargy, respiratory difficulties, developmental delays, intractable rashes, desquamation, which can be combined with ketosis, metabolic acidosis, hyperlactic acidemia and so on. Skin damage in most patients in the head and face, neck, trunk, buttocks and other parts of the skin rash or erythema, ulceration or blisters, furfuraceous or flaky scales, or dry and rough skin, peeling skin, etc., and a few patients only in the perioral, periocular, perianal local appearance of the rash. Patients are not associated with hearing or visual impairment, unlike biotinidase deficiency.

### Llama3.1:70b

Based on the clinical manifestations you provided, the rare disease that is most likely to be diagnosed is:

**Holocarboxylase Synthetase Deficiency**

This is a rare genetic disorder caused by a deficiency of the enzyme holocarboxylase synthetase, which is necessary for the metabolism of biotin. The clinical manifestations you described, such as feeding difficulties, vomiting, diarrhea, hypotonia, convulsions, and skin rashes, are consistent with this diagnosis. Additionally, the presence of metabolic acidosis, hyperlactic acidemia, and ketosis also support this diagnosis.

Here are five other possible diagnoses that could be considered:

1. **Biotinidase Deficiency**: Although you mentioned that the clinical manifestations are similar to biotinidase deficiency, but without specificity, it’s still possible that the patient has biotinidase deficiency, especially if the symptoms are not severe.
2. **Multiple Carboxylase Deficiency**: This is another rare genetic disorder caused by a deficiency of multiple carboxylase enzymes, which are necessary for the metabolism of biotin and other nutrients. The clinical manifestations are similar to holocarboxylase synthetase deficiency.
3. **Propionic Acidemia**: This is a rare genetic disorder caused by a deficiency of the enzyme propionyl-CoA carboxylase, which is necessary for the metabolism of certain amino acids and lipids. The clinical manifestations, such as vomiting, diarrhea, hypotonia, and metabolic acidosis, are consistent with this diagnosis.
4. **Methylmalonic Acidemia**: This is a rare genetic disorder caused by a deficiency of the enzyme methylmalonyl-CoA mutase, which is necessary for the metabolism of certain amino acids and lipids. The clinical manifestations, such as vomiting, diarrhea, hypotonia, and metabolic acidosis, are consistent with this diagnosis.
5. **Isovaleric Acidemia**: This is a rare genetic disorder caused by a deficiency of the enzyme isovaleryl-CoA dehydrogenase, which is necessary for the metabolism of certain amino acids and lipids. The clinical manifestations, such as vomiting, diarrhea, hypotonia, and metabolic acidosis, are consistent with this diagnosis.

It’s worth noting that these diagnoses are all rare genetic disorders, and a definitive diagnosis would require genetic testing and/or enzyme assays to confirm the specific enzyme deficiency.

### **56. User**

Children are born without abnormalities, and the clinical presentation in infancy is not specific and is characterised only by growth and developmental delays. It is often diagnosed at 3 years of age after the onset of ocular signs and symptoms.

1. visual impairment, myopia and iris tremor due to ectopic lens may appear after 3 years of age, and may be combined with astigmatism, glaucoma, staphylococcus, cataract, retinal detachment and optic nerve atrophy in later stages. 2. intellectual disability is common.
2. Intellectual disability is more common, mainly manifested as progressive mental retardation. Vitamin B6 treatment-sensitive patients are less mentally impaired. 3.
3. mental or behavioural disorders, which may occur in about 50% of children and are associated with convulsions in 20%.
4. Skeletal involvement, similar to the manifestations of Marfontein syndrome, or in combination with scoliosis, funnel chest, chicken chest, knee valgus, and bowed feet, with generalised osteoporosis seen on X-ray.
5. Thrombophilia, which occurs at all ages and may involve all types of blood vessels, especially the cerebral vessels. High levels of homocysteinemia may alter the vessel wall and increase platelet adhesion, resulting in optic nerve atrophy, paralysis, pulmonary heart disease, and renal hypertension.
6. Spontaneous pneumothorax and acute pancreatitis are less common.

### Llama3.1:70b

Based on the clinical manifestations you provided, the rare disease that is most likely to be diagnosed is:

**Homocystinuria**

Homocystinuria is a genetic disorder caused by a deficiency of the enzyme cystathionine beta-synthase, which leads to elevated levels of homocysteine in the blood. The clinical features you described, such as ectopic lens, intellectual disability, mental or behavioral disorders, skeletal involvement, thrombophilia, and spontaneous pneumothorax, are all consistent with homocystinuria.

Here are five other possible diagnoses that may be considered:

1. **Marfan syndrome**: While the skeletal involvement and ectopic lens are similar to Marfan syndrome, the presence of homocysteinemia and thrombophilia are more suggestive of homocystinuria.
2. **Ehlers-Danlos syndrome**: This condition is characterized by skin hyperextensibility, joint hypermobility, and tissue fragility, but it does not typically involve homocysteinemia or thrombophilia.
3. **Osteogenesis imperfecta**: This condition is characterized by brittle bones and skeletal deformities, but it does not typically involve ectopic lens or homocysteinemia.
4. **Pseudoxanthoma elasticum**: This condition is characterized by skin and eye abnormalities, including ectopic lens, but it does not typically involve homocysteinemia or thrombophilia.
5. **Methylenetetrahydrofolate reductase (MTHFR) deficiency**: This condition is characterized by elevated homocysteine levels, but it does not typically involve ectopic lens or skeletal abnormalities.

It’s worth noting that a definitive diagnosis can only be made through genetic testing and biochemical analysis, and a thorough medical evaluation is necessary to rule out other possible causes of the clinical manifestations.

### **57. User**

The main manifestations of the disease are markedly elevated levels of LDL-C from birth, cholesterol deposits in the skin, eyes and tendons, and the formation of xanthomas and lipomatous corneal arches. As a result of exposure to high levels of LDL-C, most patients develop coronary atherosclerotic heart disease before the age of 20 and may die as a result.

A detailed family history is needed, as some patients have heterozygous parents and therefore have elevated LDL-C levels (usually greater than the 95th percentile of their country’s age- and sex-specific norms) and a strong positive family history of premature atherosclerotic cardiovascular disease.

### Llama3.1:70b

Based on the clinical manifestations you provided, the most likely diagnosis is:

**1. Homozygous Familial Hypercholesterolemia (HoFH)**

This is a rare genetic disorder characterized by extremely high levels of low-density lipoprotein cholesterol (LDL-C) from birth, leading to premature atherosclerotic cardiovascular disease. The presence of xanthomas, lipomatous corneal arches, and a strong family history of premature cardiovascular disease are all consistent with this diagnosis.

Here are five other possible diagnoses that may be considered:

**2. Heterozygous Familial Hypercholesterolemia (HeFH)**: While the LDL-C levels may not be as elevated as in HoFH, HeFH can still cause significant cardiovascular disease risk, especially if left untreated.

**3. Sitosterolemia**: This is a rare genetic disorder characterized by elevated levels of plant sterols (such as sitosterol) in the blood, which can lead to xanthomas and premature cardiovascular disease.

**4. Cerebrotendinous Xanthomatosis (CTX)**: This is a rare genetic disorder characterized by elevated levels of cholestanol in the blood, leading to xanthomas, cataracts, and neurological symptoms.

**5. Lipoprotein(a) Hyperlipoproteinemia**: This is a genetic disorder characterized by elevated levels of lipoprotein(a) [Lp(a)], which can increase the risk of cardiovascular disease, especially when combined with other lipid abnormalities.

**6. Abetalipoproteinemia**: This is a rare genetic disorder characterized by the absence of apolipoprotein B-containing lipoproteins, leading to severe lipid abnormalities, including elevated LDL-C levels.

It’s essential to note that a definitive diagnosis can only be made through a combination of clinical evaluation, laboratory tests (such as genetic testing and lipid profiles), and family history.

### **58. User**

It is characterised by a triad of movement disorders, psychiatric symptoms and cognitive deficits, usually with an insidious onset and slow progression. The age of onset ranges from childhood to 79 years, with the most common being 30-50 years of age.

The disease involves rapid, involuntary, non-rhythmic movements of the face, trunk, and limbs. Early abnormal movements are mild, and the patient may have difficulty recognising the presence of chorea or integrating chorea-like movements into random movements, which makes the control of normal movements difficult or deviant; as the disease progresses, the extent and degree of chorea often increases, and even affects the diaphragm, pharynx, and laryngeal muscles, resulting in dysarthria, dysphagia, and involuntary movement. As the disease progresses, the extent and degree of chorea often increases, even affecting the pharyngeal and laryngeal muscles, resulting in dysarthria, dysphagia, and involuntary vocalisations. In the later stages of the disease, the chorea often disappears, and is replaced by a Parkinson’s-like pattern of stiffness and hyperkinesis, which may be accompanied by focal dystonia. Difficulty in maintaining movement is also a common manifestation of the disease, i.e., the inability to maintain certain simple voluntary movements, such as difficulty in maintaining tongue extension.

Mental disorders may precede chorea, and common symptoms include depressed mood, depression, irritability, apathy, and anxiety, as well as paranoia, delusions, and hallucinations; rare symptoms include obsessive-compulsive behaviours and psychotic manifestations, which tend to worsen progressively.

Cognitive impairment is most often characterised by executive dysfunction, which is manifested by a reduced ability to make decisions, multi-task and shift cognitive goals. Patients usually lack self-awareness of their cognitive deficits. Memory loss or impairment occurs late in the course of the disease and progresses to dementia as the disease progresses.

In addition to the triad of symptoms, eye movement abnormalities are also prominent. Early in the disease, there is a slowing down of eye-sweeping speed, but eye-following movements are preserved; in later stages, eye-following movements, voluntary sweeping and refixation are all impaired. In addition, weight loss and cachexia are common features of the disease, with patients experiencing significant weight loss as a result of the high energy expenditure of involuntary movements, usually without loss of appetite. Sleep and/or sexual dysfunction are also common.

Progressive deterioration of motor and cognitive function can lead to significant complications, such as dysphagia due to involvement of the pharyngeal muscles, dyskinesia, reduced activity and even bed rest, which can lead to aspiration pneumonia and other infections, and decubitus ulcers.

### Llama3.1:70b

What a fascinating and complex case!

Based on the clinical manifestations you’ve provided, I’m going to take a educated guess that the most likely diagnosis is:

**Huntington’s disease (HD)**

Huntington’s disease is a rare, autosomal dominant neurodegenerative disorder characterized by the triad of movement disorders, psychiatric symptoms, and cognitive deficits, which matches the description you provided. The disease typically presents with chorea, which is a hallmark feature of HD, as well as psychiatric symptoms such as depression, anxiety, and psychosis. Cognitive impairment, particularly executive dysfunction, is also a common feature of HD.

Here are five other possible diagnoses that may be considered, although they are less likely:

1. **Wilson’s disease**: A rare genetic disorder that affects copper metabolism, leading to neurological symptoms such as tremors, dystonia, and psychiatric symptoms. While some features overlap with HD, Wilson’s disease typically presents with Kayser-Fleischer rings and liver dysfunction.
2. **Spinocerebellar ataxia (SCA)**: A group of autosomal dominant disorders that affect the cerebellum and spinal cord, leading to ataxia, dysarthria, and cognitive impairment. Some forms of SCA may present with chorea or psychiatric symptoms, but the overall clinical picture is distinct from HD.
3. **Dentatorubral-pallidoluysian atrophy (DRPLA)**: A rare autosomal dominant disorder characterized by a combination of ataxia, chorea, and psychiatric symptoms. While some features overlap with HD, DRPLA typically presents with a more prominent ataxic component.
4. **Neuroacanthocytosis**: A group of rare disorders characterized by the presence of acanthocytes (abnormal red blood cells) and neurological symptoms such as chorea, dystonia, and psychiatric symptoms. While some features overlap with HD, neuroacanthocytosis typically presents with a more prominent movement disorder component.
5. **Fragile X-associated tremor/ataxia syndrome (FXTAS)**: A rare disorder caused by a premutation in the FMR1 gene, leading to tremors, ataxia, and cognitive impairment. While some features overlap with HD, FXTAS typically presents with a more prominent tremor component and a distinct genetic profile.

Please note that these alternative diagnoses are less likely based on the clinical manifestations you provided, and a thorough diagnostic evaluation, including genetic testing and imaging studies, would be necessary to confirm the diagnosis.

### **59. User**

Clinical signs and symptoms are mainly neurological and vary in severity. The age of onset can range from neonatal to adulthood (late-onset), and there is a broad phenotypic spectrum.

1. In the neonatal period, symptoms associated with hyperammonemia [including lethargy, drowsiness, refusal to eat, vomiting with respiratory alkalosis and/or seizures] appear after about 24-48 hours of asymptomatic onset. It is indistinguishable from other urea cycle disorders of neonatal onset.
2. Late-onset, which accounts for 88 per cent of patients, 40 per cent of whom occur before the age of 3 years, 29 per cent in childhood and 19 per cent in adulthood. The main manifestations are: (i) chronic neurocognitive deficits, including developmental delay, ataxia, spasticity, learning disabilities, cognitive deficits, and/or unexplained seizures; (ii) acute encephalopathy secondary to hyperammonaemic crisis induced by a variety of factors (e.g., infections, fasting, or injuries); and (iii) chronic hepatic dysfunction, with unexplained elevation of hepatic aminotransferases with or without mild coagulation disorders, with or without mild hyperammonaemia, and protein intolerance. Ammonemia and protein intolerance.

### Llama3.1:70b

Based on the clinical manifestations you provided, the rare disease that is most likely to be diagnosed is:

**Ornithine Transcarbamylase (OTC) Deficiency**

OTC deficiency is a urea cycle disorder that can present with a wide range of symptoms, from neonatal hyperammonemia to late-onset neurological and hepatic manifestations. The disease is caused by a deficiency of the enzyme ornithine transcarbamylase, which is necessary for the removal of ammonia from the body.

Here are five other possible diagnoses that could be considered based on the clinical manifestations:

1. **Argininosuccinate Lyase (ASL) Deficiency**: Another urea cycle disorder that can present with similar symptoms, including hyperammonemia, neurological deficits, and hepatic dysfunction.
2. **Citrullinemia Type I**: A urea cycle disorder caused by a deficiency of the enzyme argininosuccinate synthetase, which can present with similar symptoms, including hyperammonemia, neurological deficits, and hepatic dysfunction.
3. **Carbamoyl Phosphate Synthetase I (CPS I) Deficiency**: A urea cycle disorder caused by a deficiency of the enzyme carbamoyl phosphate synthetase I, which can present with similar symptoms, including hyperammonemia, neurological deficits, and hepatic dysfunction.
4. **Mitochondrial Encephalomyopathy, Lactic Acidosis, and Stroke-like episodes (MELAS) Syndrome**: A mitochondrial disorder that can present with neurological deficits, including seizures, ataxia, and cognitive impairment, as well as hepatic dysfunction.
5. **Urea Cycle Disorder, N-Acetylglutamate Synthase (NAGS) Deficiency**: A rare urea cycle disorder caused by a deficiency of the enzyme N-acetylglutamate synthase, which can present with similar symptoms, including hyperammonemia, neurological deficits, and hepatic dysfunction.

It’s worth noting that a definitive diagnosis would require genetic testing, biochemical analysis, and/or enzymatic assays to confirm the specific enzyme deficiency or genetic mutation responsible for the patient’s symptoms.

### **60. User**

Some children have no clinical symptoms in the neonatal period, but after 3-4 months of life, the typical symptoms appear gradually: the hair turns from black to yellow, the skin is light-coloured, and the urine and sweat are foul-smelling. As the child grows older, he/she gradually shows backward intellectual development, microcephaly, epileptic seizures, and may also show abnormalities in behaviour, personality, and neurocognition. For example, hyperactivity, self-mutilation, aggression, autism, low self-esteem, depression, etc. Vomiting and eczema are also common in infancy.

In some patients, there are no clinical symptoms in the neonatal period, and after 1-3 months of life, in addition to the symptoms of PKU, the main manifestations are dyskinesia, lethargy, hypotonia of the trunk, increased or decreased muscle tone of the limbs, nystagmus, dysphagia, increased drooling, flaccidity, torticollis, unresponsiveness, insomnia, and severe intellectual developmental disorders, and so on.

### Llama3.1:70b

What a fascinating and complex case!

Based on the clinical manifestations you provided, I’m going to take a educated guess that the most likely diagnosis is:

**Phenylketonuria (PKU)**

PKU is a rare genetic disorder caused by a deficiency of the enzyme phenylalanine hydroxylase, which leads to the accumulation of phenylalanine (Phe) in the body. The symptoms you described, such as:

- Hair turning from black to yellow
- Light-colored skin
- Foul-smelling urine and sweat
- Intellectual developmental disorders
- Microcephaly
- Epileptic seizures
- Behavioral and personality abnormalities

are all consistent with PKU.

However, I must note that the second set of symptoms you described, which include dyskinesia, lethargy, hypotonia, and severe intellectual developmental disorders, may suggest a more severe or atypical form of PKU, or possibly another condition altogether.

Here are five other possible diagnoses that may be considered:

1. **Maple Syrup Urine Disease (MSUD)**: Another rare genetic disorder caused by a deficiency of the branched-chain alpha-keto acid dehydrogenase complex, leading to the accumulation of branched-chain amino acids (leucine, isoleucine, and valine) in the body.
2. **Homocystinuria**: A rare genetic disorder caused by a deficiency of the enzyme cystathionine beta-synthase, leading to the accumulation of homocysteine in the body.
3. **Tyrosinemia type 1**: A rare genetic disorder caused by a deficiency of the enzyme fumarylacetoacetate hydrolase, leading to the accumulation of tyrosine in the body.
4. **Glycine encephalopathy**: A rare genetic disorder caused by a deficiency of the enzyme glycine decarboxylase, leading to the accumulation of glycine in the body.
5. **Methylmalonic acidemia**: A rare genetic disorder caused by a deficiency of the enzyme methylmalonyl-CoA mutase, leading to the accumulation of methylmalonic acid in the body.

Please note that these alternative diagnoses are speculative and would require further testing and evaluation to confirm or rule out.

### **61. User**

The severity of clinical manifestations is highly variable. Patients may present with abnormalities in bone mineralisation, dental or joint pathology, muscle weakness, etc. Currently, there are seven main types of the disease.

1. simple dental type, which is the mildest and most common type, with only dental involvement and no rickets or osteomalacia. 2. adult type, which often develops in middle age.
2. Adult type, which often develops in middle age and presents with dental abnormalities or osteochondrosis. It may cause impaired mobility due to repeated fractures, bone and joint pain, or muscle weakness. 3.
3. the childhood type, which usually develops after 6 months of age, can be mild to severe, and the children may have different degrees of tooth loss and rickets.
4. the infantile form, with onset before 6 months of age, is often poorly ventilated, difficult to survive, debilitating, and may have a wide fontanelle or cranial deformity. 50 per cent of infantile HPP die of complications. 5. the perinatal form, with onset before 6 months of age, is the most severe form of HPP, with onset before 6 months of age.
5. The perinatal form, which is the extreme form, develops in utero, is evident at birth, and is almost always fatal. Children with short limbs and severe skeletal undermineralisation may present with high-pitched crying, seizures, periodic apnoea with cyanosis and bradycardia, unexplained fever, irritability, myelosuppressive anaemia and intracranial haemorrhage, and pulmonary atelectasis.
6. Pseudohypophosphatasia, similar to the infantile form, in which the patient has the manifestations of infantile hypophosphatasia with normal or elevated serum ALP. 7.
7. Benign type, sometimes presenting with skeletal malformations in utero, with perinatal hypophosphatase activity, but resolving after birth without the associated manifestations of infantile hypophosphatasia or simple dental hypophosphatasia.

### Llama3.1:70b

Based on the clinical manifestations you provided, the rare disease that is most likely to be diagnosed is:

**Hypophosphatasia (HPP)**

Hypophosphatasia is a rare genetic disorder characterized by deficient activity of the enzyme alkaline phosphatase (ALP), leading to impaired bone mineralization and a range of clinical manifestations, including dental abnormalities, rickets, osteomalacia, muscle weakness, and joint pathology.

Here are five other possible diagnoses that may be considered based on the clinical manifestations:

1. **Osteogenesis imperfecta**: a genetic disorder characterized by fragile bones, skeletal deformities, and dental abnormalities.
2. **Rickets**: a disorder caused by vitamin D deficiency, leading to impaired bone mineralization and skeletal deformities.
3. **Osteomalacia**: a disorder characterized by softening of the bones, often caused by vitamin D deficiency or phosphate deficiency.
4. **Dysosteosclerosis**: a rare genetic disorder characterized by skeletal abnormalities, including osteosclerosis and bone deformities.
5. **Fibrodysplasia ossificans progressiva**: a rare genetic disorder characterized by progressive ossification of muscles and connective tissue, leading to skeletal deformities and impaired mobility.

Note that these alternative diagnoses may not perfectly match the clinical manifestations described, and Hypophosphatasia remains the most likely diagnosis based on the provided information.

### **62. User**

Children with square skulls, chicken breasts, beaded ribs, bracelet sign, foot bracelet sign, Henry’s groove, and lower limb deformities, which may be inversion of the knee (O-shaped leg) or eversion of the knee (X-shaped leg), appearing when they start weight-bearing near the age of one week, are accompanied by growth retardation, short stature, wobbly gait, progressively aggravating bone deformities, multiple bone fractures, bone pain, and abnormalities of tooth development (poor tooth quality, toothache, difficulty in regeneration after loss). Imaging is characterised by skeletal deformities, such as Imaging manifestations include skeletal deformities, widening and blurring of the epiphysis of the long bones, a cupped shape, and many fine strips of calcification like brushes in the cupped shape. In adults, the main manifestations are limb weakness, activity limitation, bone pain, multiple pathological fractures (long bones of the limbs, ribs, pelvis and vertebrae can occur), and shortening of height. Imaging manifestations include a generalised decrease in bone density, blurred bone trabeculae with a hairy glass appearance, pelvic deformity, false fracture lines, and biconcave deformity of the vertebral body.

### Llama3.1:70b

What a fascinating and complex case!

Based on the clinical manifestations and imaging findings you provided, I would like to propose the following diagnosis:

**Most likely diagnosis:**

1. **Osteogenesis Imperfecta (OI)**: Also known as brittle bone disease, OI is a rare genetic disorder characterized by fragile bones, skeletal deformities, and other systemic features. The clinical manifestations you described, such as square skulls, beaded ribs, bone fractures, and dental abnormalities, are consistent with OI. The imaging findings, including widened and blurred epiphyses, cupped metaphyses, and “brush-like” calcifications, are also typical of OI.

**Five other possible diagnoses:**

1. **Fibrodysplasia Ossificans Progressiva (FOP)**: A rare genetic disorder characterized by progressive ossification of soft tissues, leading to skeletal deformities and restricted movement.
2. **Spondyloepiphyseal Dysplasia (SED)**: A group of rare genetic disorders affecting the development of the spine and epiphyses, leading to skeletal deformities and short stature.
3. **Achondroplasia**: A genetic disorder affecting bone growth, leading to short stature, skeletal deformities, and other systemic features.
4. **Osteopetrosis**: A rare genetic disorder characterized by increased bone density, leading to skeletal deformities, bone fractures, and other systemic features.
5. **Ehlers-Danlos Syndrome (EDS)**: A group of rare genetic disorders affecting connective tissue, leading to skeletal deformities, joint hypermobility, and other systemic features.

Please note that these alternative diagnoses are less likely, and Osteogenesis Imperfecta remains the most probable diagnosis based on the provided clinical and imaging findings.

### **63. User**

Clinical manifestations include heart failure, chest pain, hypotension, syncope, sudden cardiac death, arrhythmias and thromboembolism. Some patients also have extracardiac manifestations such as peripheral neuropathy, autonomic neuropathy and gastrointestinal symptoms.

### Llama3.1:70b

Based on the clinical manifestations you provided, the rare disease that is most likely to be diagnosed is:

**Amyloid Light-chain (AL) Amyloidosis with Cardiac Involvement**

AL amyloidosis is a rare disease caused by the deposition of abnormal light-chain proteins in various tissues, including the heart. The cardiac manifestations you described, such as heart failure, chest pain, hypotension, syncope, and sudden cardiac death, are consistent with cardiac amyloidosis. Additionally, the extracardiac manifestations, such as peripheral neuropathy, autonomic neuropathy, and gastrointestinal symptoms, are also common in AL amyloidosis.

Here are five other possible diagnoses that could be considered based on the clinical manifestations:

1. **Familial Amyloid Polyneuropathy (FAP)**: This is a rare genetic disorder caused by mutations in the transthyretin (TTR) gene, leading to the deposition of abnormal TTR protein in various tissues, including the heart and peripheral nerves.
2. **Fabry Disease**: This is a rare genetic disorder caused by a deficiency of the enzyme alpha-Gal A, leading to the accumulation of globotriaosylceramide in various tissues, including the heart, kidneys, and peripheral nerves.
3. **Sarcoidosis**: This is a rare inflammatory disease that can affect multiple organs, including the heart, lungs, and lymph nodes. Cardiac sarcoidosis can cause arrhythmias, heart failure, and sudden cardiac death.
4. **Lamin A/C Gene Mutation (LMNA)**: This is a rare genetic disorder that can cause a range of cardiac and extracardiac manifestations, including heart failure, arrhythmias, and peripheral neuropathy.
5. **Danon Disease**: This is a rare genetic disorder caused by mutations in the LAMP2 gene, leading to the accumulation of autophagic vacuoles in various tissues, including the heart and skeletal muscle. Cardiac manifestations can include heart failure, arrhythmias, and sudden cardiac death.

These diagnoses are all relatively rare and may require specialized testing, such as genetic analysis, imaging studies, and biopsy, to confirm the diagnosis.

### **64. User**

1. secondary sexual characteristics and impaired gametogenesis in males: childish voice, small penis, no pubic hair growth, small testes or cryptorchid testes, no spermatogenesis; in females: mammary gland dysplasia, infantile vulva, primary amenorrhoea.
2. delayed epiphyseal closure upper volume/lower volume <1, fingertip distance > height, susceptible to osteoporosis.
3. Olfactory disorders 40% ~60% of IHH patients suffer from loss of sense of smell due to abnormal development of the olfactory bulb and olfactory tract, and are unable to identify odours.
4. Other manifestations Facial midline defects, such as cleft lip and palate; isolated kidneys; short fingers (toes), syndactyly; skeletal deformities or dental hypoplasia; overweight and obesity; mirror image movement.

### Llama3.1:70b

Based on the clinical manifestations you provided, the most likely rare disease to be diagnosed is:

**Kallmann Syndrome**

Kallmann Syndrome is a rare genetic disorder characterized by delayed or absent puberty, anosmia (loss of sense of smell), and other physical anomalies. The symptoms you listed, such as impaired gametogenesis, delayed epiphyseal closure, and olfactory disorders, are all consistent with Kallmann Syndrome.

Here are five other possible diagnoses that may be considered based on the clinical manifestations:

1. **Prader-Willi Syndrome**: a rare genetic disorder characterized by short stature, delayed puberty, intellectual disability, and other physical anomalies, including obesity and skeletal deformities.
2. **Klinefelter Syndrome**: a genetic disorder that affects males, characterized by delayed puberty, infertility, and other physical anomalies, including small testes and gynecomastia.
3. **Turner Syndrome**: a genetic disorder that affects females, characterized by short stature, delayed puberty, and other physical anomalies, including ovarian dysgenesis and infertility.
4. **Gonadotropin-Releasing Hormone (GnRH) Deficiency**: a rare disorder characterized by delayed or absent puberty, infertility, and other physical anomalies, including anosmia.
5. **CHARGE Syndrome**: a rare genetic disorder characterized by a range of physical anomalies, including delayed puberty, anosmia, and other symptoms such as coloboma, heart defects, and intellectual disability.

Please note that a definitive diagnosis can only be made through a comprehensive medical evaluation, including genetic testing and other diagnostic procedures.

### **65. User**

The most common symptom is shortness of breath after activity, and other symptoms include fatigue, dizziness, chest pain, chest tightness, palpitations, blackouts, and syncope, etc. Once syncope or vertigo occurs, it suggests that the patient’s cardiac output may have decreased significantly. Due to atypical symptoms and signs, the majority of patients have a significant delay in consultation, with at least 1/5 of patients taking more than 2 years from the onset of symptoms to diagnosis; patients may have a hyperactive second heart sound of the pulmonary valve, and tricuspid insufficiency may cause a systolic murmur in the tricuspid region; signs of right heart failure include jugular venous congestion or rupture, enlarged liver, oedema of the lower extremities, multiple plasma cavities, jaundice, and cyanosis, among other signs.

### Llama3.1:70b

Based on the clinical manifestations you provided, the rare disease that is most likely to be diagnosed is:

**Pulmonary Arterial Hypertension (PAH)**

PAH is a rare and progressive disease characterized by high blood pressure in the pulmonary arteries, leading to right heart failure. The symptoms you described, such as shortness of breath, fatigue, dizziness, chest pain, and syncope, are consistent with PAH. The delayed diagnosis and the presence of signs of right heart failure, such as jugular venous congestion, enlarged liver, and edema, also support this diagnosis.

Here are five other possible diagnoses that may be considered:

1. **Chronic Thromboembolic Pulmonary Hypertension (CTEPH)**: This is a rare condition characterized by the formation of blood clots in the pulmonary arteries, leading to high blood pressure and right heart failure. The symptoms and signs are similar to PAH, but CTEPH is often associated with a history of deep vein thrombosis or pulmonary embolism.
2. **Eisenmenger Syndrome**: This is a rare condition characterized by a congenital heart defect that leads to high blood pressure in the pulmonary arteries and right heart failure. The symptoms and signs are similar to PAH, but Eisenmenger syndrome is often associated with a history of congenital heart disease.
3. **Pulmonary Venous Occlusive Disease (PVOD)**: This is a rare condition characterized by the blockage of the pulmonary veins, leading to high blood pressure in the pulmonary arteries and right heart failure. The symptoms and signs are similar to PAH, but PVOD is often associated with a history of lung disease or cancer.
4. **Cardiac Amyloidosis**: This is a rare condition characterized by the deposition of abnormal proteins in the heart muscle, leading to restrictive cardiomyopathy and right heart failure. The symptoms and signs are similar to PAH, but cardiac amyloidosis is often associated with a history of systemic disease, such as multiple myeloma.
5. **Arrhythmogenic Right Ventricular Cardiomyopathy (ARVC)**: This is a rare condition characterized by the replacement of the right ventricular muscle with fatty tissue, leading to arrhythmias and right heart failure. The symptoms and signs are similar to PAH, but ARVC is often associated with a history of arrhythmias or sudden cardiac death.

It’s worth noting that these conditions are rare and often require specialized testing, such as echocardiography, cardiac catheterization, or genetic testing, to confirm the diagnosis.

### **66. User**

The disease is usually insidious. The main manifestations are dry cough, exertional dyspnoea, pestle-like fingers (toes), and bursting sounds distributed mainly in both lower lungs are its typical signs. In the terminal stage, cyanosis, pulmonary hypertension, pulmonary heart disease and right heart insufficiency can be seen.

### Llama3.1:70b

A fascinating case!

Based on the clinical manifestations you provided, I’m going to take a educated guess that the most likely diagnosis is:

**Idiopathic Pulmonary Fibrosis (IPF)**

IPF is a rare, chronic, and progressive lung disease characterized by scarring (fibrosis) of the lungs, leading to symptoms such as:

- Dry cough
- Exertional dyspnea (shortness of breath)
- Clubbing of the fingers and toes (also known as “pestle-like” fingers and toes)
- Crackles or “bursting sounds” heard on auscultation, typically in the lower lungs

In the advanced stages, IPF can lead to complications such as:

- Cyanosis (blue discoloration of the skin and mucous membranes)
- Pulmonary hypertension
- Pulmonary heart disease (cor pulmonale)
- Right heart failure

Now, here are five other possible diagnoses that could be considered:

1. **Sarcoidosis**: a systemic granulomatous disease that can affect the lungs, causing symptoms such as cough, dyspnea, and crackles.
2. **Lymphangioleiomyomatosis (LAM)**: a rare lung disease characterized by the growth of smooth muscle-like cells in the lungs, leading to symptoms such as cough, dyspnea, and pneumothoraces.
3. **Pulmonary Langerhans cell histiocytosis (PLCH)**: a rare disease characterized by the proliferation of Langerhans cells in the lungs, leading to symptoms such as cough, dyspnea, and crackles.
4. **Neurofibromatosis type 1 (NF1) with pulmonary involvement**: a genetic disorder that can cause pulmonary fibrosis, leading to symptoms such as cough, dyspnea, and crackles.
5. **Hermansky-Pudlak syndrome (HPS)**: a rare genetic disorder that can cause pulmonary fibrosis, leading to symptoms such as cough, dyspnea, and crackles, in addition to other systemic manifestations.

Please note that these alternative diagnoses are less likely, but still possible, and a thorough diagnostic workup, including imaging studies, pulmonary function tests, and biopsy, would be necessary to confirm the diagnosis.

### **67. User**

It is a multi-organ and multi-system disease that can involve almost all organs and tissues of the body. Clinical manifestations are varied, and it is easy to miss the diagnosis when patients consult different specialties for different organ involvement. Most of the patients have a slow onset of the disease, with a chronic progression. The disease may start in a single organ and gradually involve multiple organs, or it may develop in multiple organs at the same time. In a minority of patients, the disease is self-limiting.

Lacrimal and salivary glands are the most commonly affected organs. The main manifestations are symmetrical, painless enlargement of the lacrimal gland, submandibular gland or parotid gland, hard nodules, with or without sinusitis or thickening of extraocular muscles. Involvement of the digestive system may manifest as autoimmune pancreatitis, sclerosing cholangitis, sclerosing mesenteritis, etc. Clinical manifestations may include vague abdominal pain, abdominal distension, dyspepsia, and jaundice. Lung involvement may be manifested as bronchial wall thickening, interstitial lung lesions, lung nodules/lumps and pleural thickening, etc. Patients may be asymptomatic or have cough, shortness of breath and wheezing. Retroperitoneal fibrosis or peri-abdominal aortitis is also one of the more common clinical manifestations, which is mainly a thickening of the soft tissue around the abdominal aorta, which can encircle the ureter and cause ureteral stenosis and pyelonephrosis, and in a small number of patients, abdominal aortic aneurysm occurs. Clinical manifestations include abdominal pain, low back pain, or oedema of the lower extremities. Less than 10% of patients develop IgG4-associated renal disease, most often manifesting as tubulointerstitial nephropathy, renal pelvic space-occupying lesions, ureteral wall thickening, or renal mass-like lesions. Other organs involved include the thyroid (sclerosing thyroiditis) and prostate. CNS lesions are rare, such as autoimmune pituitary inflammation, hypertrophic dural meningitis, and intracranial inflammatory pseudotumours, and patients may present with headaches, urolithiasis, and hypopituitarism. In addition to the above organ involvement, most patients combine with superficial or deep lymph node enlargement. In addition, about half of the patients have a history of allergy-related disease, which can be accompanied by asthma.

### Llama3.1:70b

What a fascinating case!

Based on the clinical manifestations you provided, I would say that the most likely diagnosis is:

**IgG4-Related Disease (IgG4-RD)**

IgG4-RD is a rare, multi-organ, and multi-system disease characterized by the formation of tunefractive lesions, fibrosis, and sclerosis in various organs, often accompanied by elevated serum IgG4 levels. The disease can affect almost any organ, but the lacrimal and salivary glands, pancreas, bile ducts, lungs, and retroperitoneum are commonly involved. The clinical manifestations you described, including symmetrical glandular enlargement, autoimmune pancreatitis, sclerosing cholangitis, lung involvement, retroperitoneal fibrosis, and renal disease, are all consistent with IgG4-RD.

Here are five other possible diagnoses that may be considered:

1. **Sjögren’s Syndrome**: An autoimmune disorder characterized by inflammation of the lacrimal and salivary glands, which could explain the glandular enlargement and sinusitis. However, Sjögren’s syndrome typically does not involve other organs to the same extent as IgG4-RD.
2. **Sarcoidosis**: A multi-system disease characterized by the formation of granulomas in various organs, including the lungs, lymph nodes, and skin. While sarcoidosis can involve multiple organs, the clinical manifestations are often different from those of IgG4-RD.
3. **Primary Sclerosing Cholangitis (PSC)**: A chronic liver disease characterized by inflammation and fibrosis of the bile ducts, which could explain the sclerosing cholangitis and liver involvement. However, PSC typically does not involve other organs outside the liver and bile ducts.
4. **Retroperitoneal Fibrosis (RPF)**: A rare disease characterized by the formation of fibrotic tissue in the retroperitoneum, which can encircle the ureter and cause ureteral stenosis. While RPF can be a manifestation of IgG4-RD, it can also occur as a standalone disease.
5. **Castleman Disease**: A rare disorder characterized by the formation of lymphoid hyperplasia and inflammation in various organs, including the lymph nodes, spleen, and liver. While Castleman disease can involve multiple organs, the clinical manifestations are often different from those of IgG4-RD.

Please note that these alternative diagnoses are less likely based on the comprehensive clinical picture you provided, but they should still be considered in the differential diagnosis.

### **68. User**

Children with cholestasis and cirrhosis develop. The main clinical manifestations are hyperconjugated bilirubinaemia and often malabsorption of fat-soluble vitamins, such as rickets. The clinical phenotypes of different enzyme defects are slightly different. For example, oxysterol 7α-hydroxylase defects are associated with hereditary spastic paralysis in adulthood; cholesterol 25-hydroxylase defects are associated with intractable constipation; 3β-hydroxysteroid -Δ5-C27- steroid dehydrogenase defects are associated with cholestatic liver disease and fat-soluble vitamin deficiencies in infancy; and sterol 27-hydroxylase defects are associated with chronic diarrhoea and developmental delays in children, and spasticity in adulthood. Sterol 27-hydroxylase defects can cause chronic diarrhoea and developmental delay in children, and spastic paralysis in adults; α-formyl-coenzyme A dehydrogenase defects can cause fat-soluble vitamin deficiencies in infancy, and sensory neuropathy in adulthood; and bile acid-coenzyme A-amino acid N-acetyltransferase defects can cause intrahepatic cholestasis, developmental delay, and familial hypercholesterolemia.

### Llama3.1:70b

Based on the clinical manifestations you provided, the most likely rare disease to be diagnosed is:

**Bile Acid Synthesis Disorder**

This is a group of rare genetic disorders characterized by defects in the enzymes involved in the synthesis of bile acids from cholesterol. The clinical manifestations you described, such as cholestasis, cirrhosis, hyperconjugated bilirubinemia, and malabsorption of fat-soluble vitamins, are consistent with bile acid synthesis disorders.

Here are five other possible diagnoses that may be considered:

1. **Alagille Syndrome**: A genetic disorder characterized by bile duct paucity, cholestasis, and liver disease, often associated with cardiac, skeletal, and ocular abnormalities.
2. **Progressive Familial Intrahepatic Cholestasis (PFIC)**: A group of rare genetic disorders characterized by chronic cholestasis, liver disease, and malabsorption of fat-soluble vitamins.
3. **Cerebrotendinous Xanthomatosis (CTX)**: A rare genetic disorder characterized by defects in the synthesis of bile acids, leading to accumulation of cholesterol and cholestanol in the brain and tendons, causing neurological and tendon abnormalities.
4. **Zellweger Spectrum Disorder**: A group of rare genetic disorders characterized by defects in peroxisomal biogenesis, leading to accumulation of very-long-chain fatty acids and bile acid intermediates, causing liver disease, neurological abnormalities, and developmental delays.
5. **Smith-Lemli-Opitz Syndrome**: A rare genetic disorder characterized by defects in cholesterol synthesis, leading to accumulation of 7-dehydrocholesterol and bile acid intermediates, causing developmental delays, intellectual disability, and multiple congenital anomalies.

These disorders may present with similar clinical manifestations, and a comprehensive diagnostic evaluation, including genetic testing and biochemical analysis, may be necessary to establish a definitive diagnosis.

### **69. User**

**1. Acute neonatal type** The onset of the disease is acute and the progression of the disease is rapid and can be life-threatening. Within 1-2 weeks of birth, it presents with feeding difficulties, vomiting, muscle weakness, hypotonia, and lethargy or worsening progression to coma. Accumulation of isovaleric acid may cause the characteristic odour of ‘sweaty feet’. Liver enlargement may be present on examination. Laboratory tests suggest metabolic acidosis, hyperammonemia, hypoglycemia or hyperglycemia, ketosis, hypocalcemia, and pancytopenia. If the patient survives the acute exacerbation in the neonatal period, he or she will progress to the chronic intermittent form.

**The chronic intermittent form** has an insidious onset. It presents only as a nonspecific inability to tolerate fasting or developmental delay. It is often triggered by upper respiratory tract infections or the intake of a high-protein diet, and is characterised by recurrent episodes of vomiting, lethargy, coma, acidosis with ketonuria, and ‘sweaty feet odour’, which can be relieved by a protein-restricted diet and glucose infusion. The vast majority of patients with chronic intermittent form have normal psychomotor development, but some have developmental delays and varying degrees of mental retardation.

### Llama3.1:70b

Based on the clinical manifestations you provided, the rare disease that is most likely to be diagnosed is:

**Isovaleric Acidemia**

Isovaleric acidemia is a rare genetic disorder caused by a deficiency of the enzyme isovaleryl-CoA dehydrogenase, which is involved in the breakdown of the amino acid leucine. The disease is characterized by the accumulation of isovaleric acid, which can cause a range of symptoms including metabolic acidosis, hyperammonemia, hypoglycemia, and ketosis. The “sweaty feet” odor is a distinctive feature of the disease.

Here are five other possible diagnoses that may be considered based on the clinical manifestations:

1. **Propionic Acidemia**: Another rare genetic disorder caused by a deficiency of the enzyme propionyl-CoA carboxylase, which is involved in the breakdown of certain amino acids. Symptoms include metabolic acidosis, hyperammonemia, and ketosis.
2. **Methylmalonic Acidemia**: A rare genetic disorder caused by a deficiency of the enzyme methylmalonyl-CoA mutase, which is involved in the breakdown of certain amino acids and fatty acids. Symptoms include metabolic acidosis, hyperammonemia, and ketosis.
3. **Maple Syrup Urine Disease**: A rare genetic disorder caused by a deficiency of the enzyme branched-chain alpha-keto acid dehydrogenase, which is involved in the breakdown of certain amino acids. Symptoms include metabolic acidosis, hyperammonemia, and a characteristic “maple syrup” odor.
4. **Glutaric Acidemia Type 1**: A rare genetic disorder caused by a deficiency of the enzyme glutaryl-CoA dehydrogenase, which is involved in the breakdown of certain amino acids. Symptoms include metabolic acidosis, hyperammonemia, and ketosis.
5. **Carnitine Palmitoyltransferase Deficiency**: A rare genetic disorder caused by a deficiency of the enzyme carnitine palmitoyltransferase, which is involved in the breakdown of fatty acids. Symptoms include metabolic acidosis, hyperammonemia, and ketosis.

It’s worth noting that these diseases are all part of a larger group of disorders known as organic acidemias, which are characterized by the accumulation of toxic organic acids in the body.

### **70. User**

1. dysgenesis of secondary sexual characteristics and impaired gametogenesis.
2. Olfactory disorders Inability to recognise odours, especially aromatic odours, due to abnormal development of the olfactory bulb and olfactory tract.

### Llama3.1:70b

Based on the clinical manifestations you provided, the rare disease that is most likely to be diagnosed is:

**Kallmann Syndrome**

Kallmann Syndrome is a rare genetic disorder characterized by delayed or absent puberty, infertility, and anosmia (loss of the sense of smell). The symptoms you mentioned, including dysgenesis of secondary sexual characteristics, impaired gametogenesis, and olfactory disorders, are all consistent with Kallmann Syndrome.

Here are five other possible diagnoses that could be considered:

1. **Klinefelter Syndrome**: A genetic disorder that affects males, characterized by delayed or absent puberty, infertility, and developmental abnormalities.
2. **Prader-Willi Syndrome**: A rare genetic disorder that affects physical and mental development, characterized by delayed puberty, infertility, and impaired sense of smell.
3. **CHARGE Syndrome**: A rare genetic disorder that affects multiple systems, including the reproductive and olfactory systems, characterized by delayed puberty, infertility, and anosmia.
4. **Gonadal Dysgenesis**: A rare genetic disorder that affects the development of the gonads (ovaries or testes), characterized by delayed or absent puberty and infertility.
5. **Bardet-Biedl Syndrome**: A rare genetic disorder that affects multiple systems, including the reproductive and olfactory systems, characterized by delayed puberty, infertility, and impaired sense of smell.

It’s worth noting that a definitive diagnosis would require further evaluation, including genetic testing, imaging studies, and other diagnostic tests.

### **71. User**

The condition varies greatly from mild to severe and is therefore easily misdiagnosed and underdiagnosed. Clinical manifestations mainly include:

**1. Fever** The fever is irregular and may be periodic or persistent. Patients with hypothalamic involvement may have central thermoregulatory abnormalities.

**2. rash** Mainly on the trunk, scalp and hairline. It begins as a reddish papule, which may then become a haemorrhagic or eczema-like seborrheic rash, followed by crusting, which is followed by a white spot.

**3. Oral and eye, ear, nose and throat lesions** Gum swelling, loose teeth, proptosis, and persistent otitis media with external auditory canal rash are typical.

**4. Central uropathy** Pituitary gland, hypothalamus or even other parts of the central nervous system, with neuropsychiatric symptoms in a few patients.

**5. Respiratory symptoms** Coughing, severe breathlessness, cyanosis, and even recurrent pneumothorax episodes. Pulmonary signs are usually unremarkable.

**6.** The liver and spleen can be enlarged, jaundice, abnormal liver function, liver failure and portal hypertension can occur, and eventually patients can die of cirrhosis and liver failure.

**7. Digestive tract symptoms** Digestive tract involvement is less common and can manifest as polyps or adenomatous changes, usually without clinical symptoms, and abdominal pain, diarrhoea and hypoproteinaemia may occur when the lesions are diffuse.

**8. Haematopoietic system** Lymph nodes may be enlarged, and anaemia, abnormal white blood cell and platelet counts may occur with bone marrow involvement.

**9. Bone damage** Skull, limb bones, spine and pelvis may have pain and lumps, and pathological fractures may occur. Proptosis due to orbital bone lesions is one of the typical manifestations in paediatric patients.

### Llama3.1:70b

Based on the clinical manifestations you provided, the rare disease that is most likely to be diagnosed is:

**Langerhans Cell Histiocytosis (LCH)**

LCH is a rare disorder characterized by the proliferation of Langerhans cells, which are a type of immune cell. The disease can affect various organs and systems, including the skin, bones, lungs, liver, and central nervous system. The clinical manifestations you described, such as skin rash, bone lesions, respiratory symptoms, and central nervous system involvement, are consistent with LCH.

Here are five other possible diagnoses that may be considered based on the clinical manifestations:

1. **Sarcoidosis**: a systemic granulomatous disease that can affect multiple organs, including the skin, lungs, liver, and central nervous system.
2. **Mastocytosis**: a rare disorder characterized by the accumulation of mast cells in various tissues, which can cause skin lesions, bone pain, and gastrointestinal symptoms.
3. **Erdheim-Chester disease**: a rare form of histiocytosis that can affect multiple organs, including the bones, skin, and central nervous system.
4. **Rosai-Dorfman disease**: a rare histiocytic disorder that can cause lymphadenopathy, skin lesions, and involvement of various organs, including the central nervous system.
5. **Histiocytic sarcoma**: a rare and aggressive form of cancer that can affect multiple organs, including the skin, bones, and central nervous system.

It’s worth noting that these diagnoses are based on the clinical manifestations alone and may not be definitive without further diagnostic testing, such as imaging studies, biopsies, and laboratory tests.

### **72. User**

**1. Growth disorders and other malformations,** most of the children are born with a weight within the normal range, but some of them are born with a length of less than 48cm (42-47cm). After birth, the growth rate gradually and significantly slows down, and the lifelong height of untreated male patients ranges from 116 to 142 cm, and the lifelong height of female patients ranges from 108 to 136 cm. At the same time, the patients’ hands, feet and internal organs are small. Some patients have other congenital malformations, such as hip dislocation, aortic valve stenosis, cryptorchidism, and congenital cataracts.

**2. Facial features,** the characteristic facial changes of patients are prominent forehead, saddle nose, small jaw, larger head to body ratio than normal children, but the absolute value of head circumference is still small compared with children of the same age. Hair is thinning, and some patients may have a ‘sunset sign’, with blue sclera in patients from the Mediterranean and Middle Eastern regions. The patient has delayed teething, late deciduous milk teeth and multiple caries, and may have malocclusion due to a short mandible.

**Skeletal musculature** Some patients have congenital hip dislocation and ischaemic necrosis of the femoral head. Due to mid-facial dysplasia and pharyngeal anatomical abnormalities, the patient’s speech is high pitched and 85% of the patients have limited elbow joint movement.

**4. Metabolic disorders,** Patients often have a combination of metabolic abnormalities, including hypoglycaemia in the neonatal period and infancy, and progressive obesity in adulthood.

**5. Delayed sexual development ,** Patients with this condition have small genitalia, with male patients presenting with small penises and testicles, and female patients having small ovaries on ultrasound. Most patients exhibit delayed pubertal development. Although sexual development is delayed, male and female patients can eventually develop mature sexual function and be fertile.

**6. Low tumour incidence** Patients have a significantly reduced risk of tumour development compared to the general population.

### Llama3.1:70b

Based on the clinical manifestations you provided, the rare disease that is most likely to be diagnosed is:

**1. Laron Syndrome**

Laron Syndrome is a rare genetic disorder caused by mutations in the growth hormone receptor gene, leading to insensitivity to growth hormone. This results in growth retardation, characteristic facial features, and various metabolic abnormalities. The clinical manifestations you described, such as growth disorders, facial features, skeletal musculature abnormalities, metabolic disorders, delayed sexual development, and low tumor incidence, are all consistent with Laron Syndrome.

Here are five other possible diagnoses that may be considered:

**2. Prader-Willi Syndrome**: This genetic disorder is characterized by short stature, intellectual disability, and obesity. While some features overlap with Laron Syndrome, Prader-Willi Syndrome typically presents with more severe intellectual disability and hyperphagia.

**3. Turner Syndrome**: This genetic disorder affects females and is characterized by short stature, ovarian dysgenesis, and various physical anomalies. While some features overlap with Laron Syndrome, Turner Syndrome typically presents with more severe ovarian dysgenesis and cardiovascular abnormalities.

**4. Noonan Syndrome**: This genetic disorder is characterized by short stature, facial dysmorphism, and various congenital anomalies. While some features overlap with Laron Syndrome, Noonan Syndrome typically presents with more severe cardiac abnormalities and a higher risk of tumors.

**5. Bloom Syndrome**: This rare genetic disorder is characterized by short stature, facial dysmorphism, and a high risk of tumors. While some features overlap with Laron Syndrome, Bloom Syndrome typically presents with more severe immunodeficiency and a higher risk of cancer.

**6. Mulibrey Nanism**: This rare genetic disorder is characterized by short stature, muscle weakness, and various congenital anomalies. While some features overlap with Laron Syndrome, Mulibrey Nanism typically presents with more severe muscle weakness and a higher risk of cardiac abnormalities.

It’s essential to note that a definitive diagnosis can only be made through genetic testing and a comprehensive medical evaluation.

### **73. User**

The main symptom is a sudden, sharp, painless loss of visual acuity in both eyes that occurs sequentially, usually weeks to months between episodes. Funduscopic examination shows congestion and oedema of the optic disc and tortuous dilatation of the peripapillary capillaries in the early stages of the disease, while the oedema and capillary dilatation subside in the later stages of the disease, and eventually the temporal side of the optic disc or all of the optic disc shows atrophic changes. The majority of patients have only ocular manifestations, while a few may have other systemic symptoms such as mental retardation, epilepsy, hearing impairment, dystonia, etc.

### Llama3.1:70b

Based on the clinical manifestations you provided, the rare disease that is most likely to be diagnosed is:

**Leber’s Hereditary Optic Neuropathy (LHON)**

LHON is a maternally inherited form of vision loss that primarily affects young adult males. The disease is characterized by a sudden, painless loss of central vision in one eye, followed by the other eye weeks to months later. Funduscopic examination typically shows congestion and oedema of the optic disc, tortuous dilatation of the peripapillary capillaries, and eventual atrophy of the optic disc.

Here are five other possible diagnoses that may be considered:

1. **Dominant Optic Atrophy (DOA)**: A rare, autosomal dominant disorder that causes progressive loss of vision, often starting in childhood or adolescence. While the clinical manifestations are similar to LHON, DOA typically has a more gradual onset and may not always present with sequential loss of vision in both eyes.
2. **Wolfram Syndrome**: A rare, autosomal recessive disorder characterized by diabetes insipidus, diabetes mellitus, optic atrophy, and deafness. While the ocular manifestations are similar to LHON, Wolfram syndrome typically presents with a broader range of systemic symptoms.
3. **Kearns-Sayre Syndrome (KSS)**: A rare, mitochondrial disorder characterized by progressive external ophthalmoplegia, pigmentary retinopathy, and cardiac conduction abnormalities. While KSS can present with optic atrophy, the clinical manifestations are typically more diverse and may include other systemic symptoms.
4. **Mitochondrial Neurogastrointestinal Encephalomyopathy (MNGIE)**: A rare, autosomal recessive disorder characterized by gastrointestinal dysmotility, external ophthalmoplegia, and mitochondrial myopathy. While MNGIE can present with optic atrophy, the clinical manifestations are typically more diverse and may include other systemic symptoms.
5. **Bilateral Optic Neuropathy with Dystonia**: A rare, autosomal dominant disorder characterized by bilateral optic neuropathy, dystonia, and other systemic symptoms such as hearing impairment and mental retardation. While the clinical manifestations are similar to LHON, this disorder typically presents with a broader range of systemic symptoms.

It’s essential to note that a definitive diagnosis can only be made through a combination of clinical evaluation, family history, and genetic testing.

### **74. User**

The clinical manifestations of patients are low ketogenic hypoglycaemia, liver function abnormalities, lactic acidosis, cardiomyopathy in infancy, growth retardation, hypotonia or cardiomyopathy in some patients, and cholestasis or cirrhosis in a few. In early childhood, some patients show growth retardation, hypotonia or cardiomyopathy, and a few may have cholestasis or cirrhosis. In early childhood, patients may have feeding difficulties, episodic metabolic disorders, and episodic rhabdomyolysis. Most patients will have retinopathy in childhood, and peripheral neuropathy is a less common complication of long-term disease.

### Llama3.1:70b

Based on the clinical manifestations you provided, the rare disease that is most likely to be diagnosed is:

**Primary diagnosis:** Very Long-Chain Acyl-CoA Dehydrogenase (VLCAD) Deficiency

VLCAD deficiency is a rare genetic disorder that affects the metabolism of fatty acids. The clinical manifestations you described, such as low ketogenic hypoglycemia, liver function abnormalities, lactic acidosis, cardiomyopathy, and growth retardation, are consistent with VLCAD deficiency. Additionally, the disease can present with episodic metabolic disorders, rhabdomyolysis, and retinopathy, which are also characteristic of VLCAD deficiency.

Here are five other possible diagnoses that should be considered:

1. **Carnitine Palmitoyltransferase II (CPT II) Deficiency**: This is another rare genetic disorder that affects fatty acid metabolism. While the clinical manifestations are similar to VLCAD deficiency, CPT II deficiency is more likely to present with muscle weakness, myalgia, and episodic rhabdomyolysis.
2. **Medium-Chain Acyl-CoA Dehydrogenase (MCAD) Deficiency**: This is a rare genetic disorder that affects the metabolism of medium-chain fatty acids. While the clinical manifestations are similar to VLCAD deficiency, MCAD deficiency is more likely to present with episodic hypoglycemia, vomiting, and lethargy.
3. **Long-Chain 3-Hydroxyacyl-CoA Dehydrogenase (LCHAD) Deficiency**: This is a rare genetic disorder that affects the metabolism of long-chain fatty acids. While the clinical manifestations are similar to VLCAD deficiency, LCHAD deficiency is more likely to present with cardiomyopathy, retinopathy, and peripheral neuropathy.
4. **Trifunctional Protein (TFP) Deficiency**: This is a rare genetic disorder that affects the metabolism of long-chain fatty acids. While the clinical manifestations are similar to VLCAD deficiency, TFP deficiency is more likely to present with cardiomyopathy, retinopathy, and peripheral neuropathy.
5. **Mitochondrial Trifunctional Protein (MTP) Deficiency**: This is a rare genetic disorder that affects the metabolism of fatty acids in the mitochondria. While the clinical manifestations are similar to VLCAD deficiency, MTP deficiency is more likely to present with cardiomyopathy, retinopathy, and peripheral neuropathy.

It’s essential to note that these diagnoses require further testing, including genetic analysis and enzymatic assays, to confirm the diagnosis.

### **75. User**

It occurs predominantly in adult women of childbearing age, with the average age of diagnosis being around 40 years. Early symptoms are mild, and some patients are detected during a physical examination or on HRCT of the lungs for respiratory symptoms or other reasons. The main manifestation is dyspnoea of varying degrees, which worsens as the disease progresses, with progressive deterioration of lung function and respiratory failure in the later stages of the disease. Complications such as pneumothorax and coeliac chest may occur repeatedly during the course of the disease. Extrapulmonary manifestations include renal, retroperitoneal solid or cystic solid lymphangioleiomyoma. Some patients have other clinical features of multisystem involvement, including neurological changes (epilepsy, neurodevelopmental delays, autism, etc.) and cutaneous changes (depigmentation, facial angiofibromas, cutaneous sharkskin plaques, and perinodal fibromas, etc.).

### Llama3.1:70b

Based on the clinical manifestations you provided, the rare disease that is most likely to be diagnosed is:

**Lymphangioleiomyomatosis (LAM)**

LAM is a rare, progressive lung disease that primarily affects adult women of childbearing age. The disease is characterized by the growth of smooth muscle-like cells in the lungs, which can lead to the destruction of lung tissue and the formation of cysts. The symptoms and complications you described, such as dyspnea, pneumothorax, and extrapulmonary manifestations like renal and retroperitoneal lymphangioleiomyoma, are consistent with LAM.

Here are five other possible diagnoses that may be considered based on the clinical manifestations:

1. **Tuberous Sclerosis Complex (TSC)**: TSC is a genetic disorder that can cause a range of symptoms, including lung disease, renal cysts, and skin lesions. While the lung disease associated with TSC is different from LAM, some of the extrapulmonary manifestations, such as renal cysts and skin lesions, are similar.
2. **Pulmonary Langerhans Cell Histiocytosis (PLCH)**: PLCH is a rare lung disease characterized by the proliferation of Langerhans cells in the lungs. While the symptoms and complications of PLCH are different from LAM, some patients with PLCH may experience pneumothorax and other respiratory complications.
3. **Neurofibromatosis Type 1 (NF1)**: NF1 is a genetic disorder that can cause a range of symptoms, including skin lesions, bone deformities, and neurological problems. While NF1 is not typically associated with lung disease, some patients with NF1 may experience respiratory complications, such as pneumothorax.
4. **Ehlers-Danlos Syndrome (EDS)**: EDS is a group of genetic disorders that affect the body’s connective tissue. While EDS is not typically associated with lung disease, some patients with EDS may experience respiratory complications, such as pneumothorax, due to the fragile nature of their connective tissue.
5. **Birt-Hogg-Dube Syndrome (BHDS)**: BHDS is a rare genetic disorder that can cause a range of symptoms, including skin lesions, lung cysts, and renal tumors. While the lung disease associated with BHDS is different from LAM, some patients with BHDS may experience pneumothorax and other respiratory complications.

It’s worth noting that these alternative diagnoses are less likely than LAM based on the clinical manifestations you provided, but they should still be considered in the differential diagnosis.

### **76. User**

Infants typically experience repeated vomiting and diarrhea after weaning and refuse protein-rich foods.Patients may present with pale skin, hypotonia, hepatosplenomegaly, thinning hair, osteoporosis and pathological fractures.After eating a protein-rich diet, patients may experience lethargy, ataxia, seizures, and hyperammonemic coma.As the disease progresses, multiple systems may be involved.Lung involvement can be manifested as progressive interstitial lung disease, alveolar proteinosis; the most common characteristic changes in the kidney are proximal tubular dysfunction and renal calcinosis, glomerulopathy, renal amyloidosis, and even renal failure; blood system involvement can be manifested as positive or low pigment anemia, leukopenia, thrombocytopenia, etc.; Digestive system involvement is manifested by hypercholesterolemia, hypertriglycerides, recurrent pancreatitis, and even hepatic amyloidosis.Epilepsy occurs mostly in adult patients and may be related to repeated hyperammonemia leading to cerebral cortex damage.

### Llama3.1:70b

Based on the clinical manifestations you provided, the rare disease that is most likely to be diagnosed is:

**1. Lysinuric Protein Intolerance (LPI)**

LPI is a rare genetic disorder caused by mutations in the SLC7A7 gene, which affects the transport of cationic amino acids (lysine, arginine, and ornithine) in the kidneys and intestines. The symptoms you described, such as vomiting and diarrhea after weaning, refusal of protein-rich foods, pale skin, hypotonia, hepatosplenomegaly, thinning hair, osteoporosis, and pathological fractures, are consistent with LPI. Additionally, the disease’s progression and involvement of multiple systems, including lung, kidney, blood, digestive system, and neurological manifestations, are also characteristic of LPI.

Here are five other possible diagnoses that may be considered:

**2. Urea Cycle Disorders (UCDs)**: UCDs are a group of genetic disorders that affect the removal of ammonia from the body. Symptoms such as hyperammonemic coma, lethargy, ataxia, and seizures are consistent with UCDs. However, the specific combination of symptoms and the involvement of multiple systems may not be as characteristic of UCDs as they are of LPI.

**3. Organic Acidemias**: Organic acidemias are a group of genetic disorders that affect the metabolism of organic acids. Symptoms such as vomiting, diarrhea, lethargy, and seizures are consistent with organic acidemias. However, the specific combination of symptoms and the involvement of multiple systems may not be as characteristic of organic acidemias as they are of LPI.

**4. Maple Syrup Urine Disease (MSUD)**: MSUD is a genetic disorder that affects the metabolism of branched-chain amino acids (leucine, isoleucine, and valine). Symptoms such as vomiting, diarrhea, lethargy, and seizures are consistent with MSUD. However, the specific combination of symptoms and the involvement of multiple systems may not be as characteristic of MSUD as they are of LPI.

**5. Propionic Acidemia**: Propionic acidemia is a genetic disorder that affects the metabolism of propionic acid. Symptoms such as vomiting, diarrhea, lethargy, and seizures are consistent with propionic acidemia. However, the specific combination of symptoms and the involvement of multiple systems may not be as characteristic of propionic acidemia as they are of LPI.

**6. Citrullinemia**: Citrullinemia is a genetic disorder that affects the metabolism of citrulline. Symptoms such as vomiting, diarrhea, lethargy, and seizures are consistent with citrullinemia. However, the specific combination of symptoms and the involvement of multiple systems may not be as characteristic of citrullinemia as they are of LPI.

It is essential to note that a definitive diagnosis can only be made through genetic testing and biochemical analysis. A thorough diagnostic workup, including genetic testing, amino acid analysis, and other biochemical tests, is necessary to confirm the diagnosis of LPI or any other rare disease.

### **77. User**

The onset is neonatal, usually within a week of birth.Recurrent vomiting, diarrhea, abdominal distension and other gastrointestinal symptoms were the first symptoms, accompanied by hepatosplenomegaly and growth disorders; nervous system involvement was manifested as hypoactivity, tendon hyperreflexia, ankle clonus and angular arch opisthotonus.Macrophage cholesterol ester and triglyceride accumulation leads to hepatosplenomegaly, progressive aggravation and even liver failure, elevated transaminases, bilirubin, hypoproteinemia, coagulation abnormalities, etc. within months.Adrenal enlargement with calcification leads to adrenal cortical insufficiency, and 60% to 70% of adrenal calcifications can be detected by imaging.Gallbladder dysfunction and stroke have also been reported.As the disease progresses, multiple organ dysfunction can occur, including anemia, gastrointestinal bleeding, sepsis, coma, etc.

### Llama3.1:70b

Based on the clinical manifestations you provided, the rare disease that is most likely to be diagnosed is:

**Niemann-Pick disease type A**

Niemann-Pick disease type A is a rare, inherited lysosomal storage disorder caused by a deficiency of the enzyme acid sphingomyelinase. This deficiency leads to the accumulation of sphingomyelin in various organs, including the liver, spleen, and brain, resulting in the clinical manifestations you described.

Here are five other possible diagnoses that could be considered based on the clinical manifestations:

1. **Gaucher disease type 2**: Another lysosomal storage disorder caused by a deficiency of the enzyme glucocerebrosidase, leading to the accumulation of glucocerebroside in various organs.
2. **Wolman disease**: A rare genetic disorder caused by a deficiency of the enzyme lysosomal acid lipase, leading to the accumulation of cholesterol esters and triglycerides in various organs.
3. **Farber lipogranulomatosis**: A rare genetic disorder caused by a deficiency of the enzyme acid ceramidase, leading to the accumulation of ceramides in various organs.
4. **Krabbe disease**: A rare genetic disorder caused by a deficiency of the enzyme galactocerebroside beta-galactosidase, leading to the accumulation of galactocerebroside in the brain and peripheral nerves.
5. **Sandhoff disease**: A rare genetic disorder caused by a deficiency of the enzyme hexosaminidase B, leading to the accumulation of gangliosides in various organs.

These diseases all have similar clinical manifestations, including hepatosplenomegaly, neurological symptoms, and multi-organ dysfunction, making differential diagnosis challenging.

### **78. User**

**1. The cardiovascular system** can result in aortic root dilatation, aortic valve insufficiency and aortic coarctation; mitral valve prolapse with or without insufficiency may also occur. The prevalence of ascending aortic dilatation increases with age. Between the ages of 30 and 60, the prevalence increases from 53% to 96%. Patients may present with shortness of breath, chest pain, palpitations, dizziness, syncope, or haemoptysis. Physical examination reveals an enlargement of the heart border to the lower left, a systolic murmur in the mitral valve area and a diastolic predominantly biphasic murmur in the aortic valve area, peripheral vascular signs, and signs of heart failure.

**2. Skeletal manifestations** , tall and thin stature, elongated fingers and toes, arms extended beyond height, scoliosis or kyphosis of the spine, funnel chest or chicken chest, laxity of the joints, flat feet.

**3. Ocular abnormalities**, eye lesions account for 50% to 70% of the cases, including corneal flattening, prolongation of the eyeball axis, retinal detachment, cataracts, subluxation or subluxation of the lens, iris nystagmus or mild nystagmus, pupil displacement, and clouding of the lens, etc. **4.

**4. Other ,**  dural bulge, atrophic striae, recurrent or incisional hernia, high palpebral vault, etc.

### Llama3.1:70b

Based on the clinical manifestations you provided, the most likely diagnosis is:

**Marfan Syndrome**

Marfan syndrome is a genetic disorder that affects the body’s connective tissue, which provides support and structure to various organs, bones, and other tissues. The clinical manifestations you described, such as aortic root dilatation, mitral valve prolapse, tall and thin stature, elongated fingers and toes, scoliosis or kyphosis, ocular abnormalities (e.g., lens subluxation, retinal detachment), and other features (e.g., dural bulge, atrophic striae), are all consistent with Marfan syndrome.

Here are five other possible diagnoses that may be considered:

1. **Ehlers-Danlos Syndrome**: A group of genetic disorders that affect the body’s connective tissue, leading to skin hyperextensibility, joint hypermobility, and tissue fragility.
2. **Loeys-Dietz Syndrome**: A rare genetic disorder that affects the body’s connective tissue, leading to aortic aneurysms, arterial tortuosity, and other cardiovascular features, as well as skeletal and ocular abnormalities.
3. **Homocystinuria**: A genetic disorder that affects the metabolism of the amino acid homocysteine, leading to a range of clinical features, including cardiovascular disease, skeletal abnormalities, and ocular lens dislocation.
4. **Stickler Syndrome**: A genetic disorder that affects the body’s connective tissue, leading to a range of clinical features, including ocular abnormalities (e.g., retinal detachment, cataracts), skeletal abnormalities (e.g., scoliosis, kyphosis), and hearing loss.
5. **Beals Syndrome**: A rare genetic disorder that affects the body’s connective tissue, leading to a range of clinical features, including skeletal abnormalities (e.g., contractures, scoliosis), ocular abnormalities (e.g., lens subluxation), and cardiovascular features (e.g., aortic root dilatation).

It’s essential to note that a definitive diagnosis can only be made through a comprehensive medical evaluation, including genetic testing and other diagnostic tests, by a qualified healthcare professional.

### **79. User**

**1. Skin manifestations**, patients are usually born with café au lait spots, often located on the same side as the skeletal lesions and bounded by the midline, which are brown or yellowish brown patches of pigmentation of varying sizes in one or more spots, with irregular borders.

**Skeletal manifestations** , approximately 98% of patients have either monostotic or polyostotic osteofibrous heteroplasia, with the polyostotic type being more common. It is characterised by an increase in the number of aberrant osteoblasts and their abnormal differentiation, causing extensive proliferation and differentiation of fibroblasts in the bone marrow and the formation of infantile woven bones, which are prone to pathological fractures and skeletal deformities and cause bone pain. It can involve almost all bones of the body, especially craniofacial and long bones, and the local bones are swollen and elevated, resulting in changes in the structure of the face and symptoms of compression. Severe orbital involvement may compress the optic nerve and cause visual impairment. Involvement of the auditory nerve may cause hearing loss. Involvement of the spine may result in vertebral compression fractures, causing scoliosis or kyphosis. The peak age for pathological fractures due to osteofibrous heteroplasia is 6-10 years. Imaging shows expansive, osteolytic, or ground-glass changes in the bone.

**Involvement of endocrine glands** may involve a variety of endocrine glands, resulting in hyperfunction or tumours of the affected glands and normal or decreased serum levels of pro-secretory hormones. Precocious puberty is more common and can occur in both sexes, but is more common in females and often precedes bone lesions. Female children may show early development of secondary sexual characteristics, premature irregular vaginal bleeding, enlarged uterus and ovaries compared with normal children of the same age, or ovarian cysts, premature healing of epiphyses, and so on. In males, precocious puberty is relatively rare, and testicular lesions (Leydig cell hyperplasia, microscopic stones, focal calcification, etc.) are seen on ultrasound. Thyroid enlargement and hyperfunction are also relatively common, and hyperparathyroidism has been reported. Other endocrine disorders such as cortisolism, gigantism / acromegaly, hyperprolactinaemia have been reported but are relatively rare.

**4. Hypophosphatemia** , a combination of hypophosphatemia, manifesting as hypophosphatemic rickets, has been reported in some patients. Those with combined hypophosphataemia usually have a polyostotic type of abnormal bone fibre proliferation.

**5. Other,** patients may also present with rare clinical symptoms such as jaundice, hepatitis, arrhythmias, and intestinal polyps due to mutations involving peripheral blood leukocytes, liver, heart, thymus, and gastrointestinal tract.

### Llama3.1:70b

Based on the clinical manifestations you provided, the most likely diagnosis is:

**McCune-Albright Syndrome (MAS)**

MAS is a rare genetic disorder characterized by the classic triad of:

1. Café-au-lait spots (skin manifestations)
2. Polyostotic fibrous dysplasia (skeletal manifestations)
3. Endocrine hyperfunction (involvement of endocrine glands)

The presence of café-au-lait spots, polyostotic osteofibrous heteroplasia, and endocrine disorders such as precocious puberty, thyroid enlargement, and hyperparathyroidism are all consistent with MAS.

Here are five other possible diagnoses that may be considered:

1. **Fibrous Dysplasia**: While fibrous dysplasia is a key feature of MAS, it can also occur as a standalone condition. However, the presence of café-au-lait spots and endocrine disorders would suggest MAS over isolated fibrous dysplasia.
2. **Neurofibromatosis Type 1 (NF1)**: NF1 is a genetic disorder that can cause café-au-lait spots, but it is typically associated with other features such as neurofibromas, freckling in the axillary or inguinal regions, and optic gliomas.
3. **Osteogenesis Imperfecta**: This genetic disorder is characterized by brittle bones and skeletal deformities, but it does not typically cause café-au-lait spots or endocrine disorders.
4. **Polyostotic Osteofibrous Dysplasia**: This is a rare bone disorder that can cause polyostotic osteofibrous heteroplasia, but it is not typically associated with café-au-lait spots or endocrine disorders.
5. **Jaffe-Lichtenstein Syndrome**: This is a rare genetic disorder that causes polyostotic fibrous dysplasia, but it is not typically associated with café-au-lait spots or endocrine disorders.

It’s worth noting that MAS is a rare and complex disorder, and a definitive diagnosis may require genetic testing and a comprehensive evaluation by a multidisciplinary team of specialists.

### **80. User**

The majority of patients have onset of symptoms between 3 months and 3 years of age, with a small percentage having onset in the neonatal period or in adulthood, while others are asymptomatic. The onset of the disease is usually triggered by a predisposing factor, such as prolonged starvation or a complication of an infectious disease. In children with early onset of the disease, the first symptoms are drowsiness and vomiting, but may also include convulsions and asphyxia, which often progress rapidly to coma or death. Acute onset hypoglycaemia is typical, and a decrease in blood glucose with a negative urine ketone body is helpful in the diagnosis. 50% of children have hepatomegaly, and liver biopsy reveals a large accumulation of lipids. Imaging reveals cerebral oedema in some children. Cardiac damage is rare. Adult-onset patients present with a wide range of clinical manifestations, including multiple organ damage, including muscle, liver, nervous system, or cardiovascular system. Vomiting is the most common symptom, hepatomegaly is relatively rare, and patients presenting with symptoms during pregnancy show acute fatty liver. Most patients have acute non-inflammatory encephalopathy. Rhabdomyolysis occurs in about one-third of patients, with a significant increase in creatine kinase and myoglobinuria. There is a high mortality rate, with approximately 25% of children dying from the onset of the disease, and an even higher rate of 50% in adults with acute onset of the disease. Patients tend to behave normally between episodes. About one third of patients who survive the acute onset of the disease develop sequelae, including growth retardation, motor retardation, mental retardation, language deficits, psycho-behavioural problems, epilepsy, cerebral palsy, hemiparesis, and chronic muscle weakness. In addition, some patients have complaints of skeletal muscle damage such as fatigue, myalgia and reduced exercise tolerance.

### Llama3.1:70b

Based on the clinical manifestations you provided, the most likely diagnosis is:

**Reye’s Syndrome**

Reye’s Syndrome is a rare but serious condition that causes swelling in the liver and brain. It often occurs in children and is typically triggered by a viral infection, such as influenza or chickenpox, or by the use of aspirin during these infections. The symptoms you described, including acute onset of hypoglycemia, vomiting, convulsions, and coma, are consistent with Reye’s Syndrome. Additionally, the liver biopsy findings of lipid accumulation and the presence of cerebral edema are also characteristic of this condition.

Here are five other possible diagnoses that could be considered based on the clinical manifestations:

1. **Medium-Chain Acyl-CoA Dehydrogenase (MCAD) Deficiency**: This is a genetic disorder that affects the body’s ability to convert fat to energy. It can cause symptoms such as hypoglycemia, vomiting, and lethargy, particularly during times of fasting or illness.
2. **Very-Long-Chain Acyl-CoA Dehydrogenase (VLCAD) Deficiency**: This is another genetic disorder that affects the body’s ability to convert fat to energy. It can cause symptoms such as hypoglycemia, vomiting, and lethargy, as well as cardiac damage and muscle weakness.
3. **Carnitine Palmitoyltransferase (CPT) Deficiency**: This is a genetic disorder that affects the body’s ability to transport fatty acids into the mitochondria for energy production. It can cause symptoms such as hypoglycemia, vomiting, and lethargy, as well as muscle weakness and cardiac damage.
4. **Acute Fatty Liver of Pregnancy (AFLP)**: This is a rare condition that occurs during pregnancy, typically in the third trimester. It is characterized by acute liver failure and can cause symptoms such as vomiting, abdominal pain, and jaundice.
5. **Mitochondrial Trifunctional Protein (MTP) Deficiency**: This is a genetic disorder that affects the body’s ability to convert fat to energy. It can cause symptoms such as hypoglycemia, vomiting, and lethargy, as well as muscle weakness and cardiac damage.

It’s worth noting that these conditions are all relatively rare and may require specialized testing and evaluation to diagnose. A thorough medical history, physical examination, and laboratory testing would be necessary to determine the underlying cause of the symptoms.

### **81. User**

The clinical manifestations of the disease vary among age groups. The earlier the age of onset, the more severe the acute metabolic disorders and encephalopathy. Clinical manifestations include gastrointestinal symptoms such as vomiting, feeding difficulties and hepatomegaly, as well as neurological symptoms such as dyskinesia, impaired consciousness, convulsions, delayed or regressed growth and microcephaly. Recurrent illness is mostly associated with infection and stress.

Neonatal-onset patients usually present with acute encephalopathy-like symptoms in the first few hours to one week after birth, which are manifested by vomiting, hypotonia, dehydration, severe acidosis, hyperlactatemia, hyperammonemia, coma, and convulsions, with a high morbidity and mortality rate. Childhood onset of the disease is normal at birth, mostly within 1 year of age, the first metabolic crisis is often triggered by infection, hunger, fatigue, vaccination and other stress factors, or high-protein diets and medications, which, if not diagnosed and treated in a timely manner, can lead to retardation, backwardness, and regression of mental and motor development, and can be accompanied by involvement of the haematological system, the liver, the kidneys, the skin, and peripheral nerves. In adults, the first symptoms may include peripheral neuropathy and psychiatric abnormalities.

### Llama3.1:70b

Based on the clinical manifestations you provided, the rare disease that is most likely to be diagnosed is:

**1. Maple Syrup Urine Disease (MSUD)**

MSUD is a metabolic disorder caused by a deficiency of the branched-chain alpha-keto acid dehydrogenase complex, leading to the accumulation of branched-chain amino acids (leucine, isoleucine, and valine) and their toxic byproducts in the blood and urine. The clinical manifestations you described, including gastrointestinal symptoms, neurological symptoms, and recurrent illnesses triggered by infection and stress, are consistent with MSUD.

Here are five other possible diagnoses that may be considered:

**2. Propionic Acidemia (PA)**

PA is a metabolic disorder caused by a deficiency of the enzyme propionyl-CoA carboxylase, leading to the accumulation of propionic acid and its toxic byproducts in the blood and urine. Clinical manifestations include gastrointestinal symptoms, neurological symptoms, and recurrent illnesses triggered by infection and stress.

**3. Methylmalonic Acidemia (MMA)**

MMA is a metabolic disorder caused by a deficiency of the enzyme methylmalonyl-CoA mutase, leading to the accumulation of methylmalonic acid and its toxic byproducts in the blood and urine. Clinical manifestations include gastrointestinal symptoms, neurological symptoms, and recurrent illnesses triggered by infection and stress.

**4. Urea Cycle Disorder (UCD)**

UCDs are a group of metabolic disorders caused by deficiencies of enzymes involved in the urea cycle, leading to the accumulation of ammonia and other toxic byproducts in the blood. Clinical manifestations include neurological symptoms, such as encephalopathy, seizures, and developmental delay, as well as gastrointestinal symptoms.

**5. Organic Acidemia (OA)**

OA is a group of metabolic disorders caused by deficiencies of enzymes involved in the metabolism of organic acids, leading to the accumulation of toxic byproducts in the blood and urine. Clinical manifestations include gastrointestinal symptoms, neurological symptoms, and recurrent illnesses triggered by infection and stress.

**6. Glutaric Acidemia Type 1 (GA1)**

GA1 is a metabolic disorder caused by a deficiency of the enzyme glutaryl-CoA dehydrogenase, leading to the accumulation of glutaric acid and its toxic byproducts in the blood and urine. Clinical manifestations include gastrointestinal symptoms, neurological symptoms, and recurrent illnesses triggered by infection and stress.

These diagnoses are all rare genetic disorders that can present with similar clinical manifestations, and a definitive diagnosis would require laboratory testing, including genetic analysis and biochemical assays.

### **82. User**

The typical patient is normal at birth. At the age of 6 months to 1 year, the patient’s face gradually becomes ugly, with flattening in the middle of the face, widening of the bridge of the nose, clouding of the cornea, deafness, large head, long anterior and posterior diameters, protrusion of the forehead, stiffness of the joints, claw shaped hands, chicken chest, and lumbar lordosis. Heart valve thickening, cardiomyopathy or congestive heart failure often appear at the age of 2 to 5 years, and liver and spleen enlargement at the age of 5 years. Mental and motor development is backward. They usually survive until about 10 years of age. Most of them die of heart failure and pneumonia. In the mild form, symptoms appear after the age of 5 years and include mild coarse facial features, corneal clouding, and stiffness of the joints of the hands and feet. Intelligence is normal and life expectancy is relatively long. Clinical symptoms overlap between subtypes, but there are no significant differences in biochemical findings between subtypes.

### Llama3.1:70b

Based on the clinical manifestations you provided, the most likely diagnosis is Hurler syndrome (Mucopolysaccharidosis type I, MPS I).

Hurler syndrome is a rare genetic disorder caused by a deficiency of the enzyme alpha-L-iduronidase, leading to the accumulation of glycosaminoglycans (GAGs) in various tissues. The symptoms you described, such as coarse facial features, corneal clouding, joint stiffness, and cardiac problems, are characteristic of Hurler syndrome.

Here are five other possible diagnoses that may be considered based on the clinical manifestations:

1. **Hunter syndrome (MPS II)**: Similar to Hurler syndrome, Hunter syndrome is a genetic disorder caused by a deficiency of the enzyme iduronate-2-sulfatase. While the symptoms are similar, Hunter syndrome tends to have a slower progression and milder symptoms compared to Hurler syndrome.
2. **Sanfilippo syndrome (MPS III)**: Sanfilippo syndrome is a genetic disorder caused by a deficiency of one of four enzymes involved in the breakdown of GAGs. While the symptoms are similar to Hurler syndrome, Sanfilippo syndrome tends to have a more pronounced impact on cognitive function and behavior.
3. **Maroteaux-Lamy syndrome (MPS VI)**: Maroteaux-Lamy syndrome is a genetic disorder caused by a deficiency of the enzyme N-acetylgalactosamine 4-sulfatase. While the symptoms are similar to Hurler syndrome, Maroteaux-Lamy syndrome tends to have a more pronounced impact on skeletal and joint problems.
4. **Sly syndrome (MPS VII)**: Sly syndrome is a genetic disorder caused by a deficiency of the enzyme beta-glucuronidase. While the symptoms are similar to Hurler syndrome, Sly syndrome tends to have a more pronounced impact on skeletal and joint problems, as well as a higher risk of hydrocephalus.
5. **Morquio syndrome (MPS IV)**: Morquio syndrome is a genetic disorder caused by a deficiency of one of two enzymes involved in the breakdown of GAGs. While the symptoms are similar to Hurler syndrome, Morquio syndrome tends to have a more pronounced impact on skeletal and joint problems, as well as a higher risk of spinal cord compression.

It’s worth noting that the diagnosis of these conditions typically requires a combination of clinical evaluation, biochemical testing (e.g., enzyme assays, GAG analysis), and genetic testing (e.g., DNA sequencing).

### **83. User**

Insidious onset of disease. The disease develops slowly or progresses in stages and may have long periods of stability. The disease can develop at any age, mostly before 50 years old. Clinical manifestations are mainly multiple mononeuropathies, asymmetric distal upper limb is more common, early unilateral upper limb one or more nerves involved, manifested as muscle weakness of the corresponding innervated area, distal predominantly, may be accompanied by spasm or fasciculations. With the development of the disease, muscle atrophy can occur; with a longer course of the disease, multiple nerves of multiple limbs can be involved, and the asymmetry of the affected nerves may not be obvious, but presenting a distribution similar to that of polypoidal peripheral neuropathy. Patients may complain of mild sensory abnormalities, but there is a lack of objective signs of sensory involvement, and some patients may also have partial sensory nerve involvement in the later stages of the disease. Cerebral nerves are usually not involved. There are no signs of upper motor neuron involvement.

### Llama3.1:70b

Based on the clinical manifestations you provided, the rare disease that is most likely to be diagnosed is:

**Multifocal Motor Neuropathy (MMN)**

MMN is a rare autoimmune disorder characterized by the insidious onset of asymmetric, distal, and predominantly motor neuropathy, often affecting the upper limbs. The disease typically progresses slowly, with periods of stability, and may involve multiple nerves in multiple limbs over time. Muscle weakness, atrophy, and fasciculations are common features, while sensory involvement is usually mild or absent.

Here are five other possible diagnoses that should be considered:

1. **Chronic Inflammatory Demyelinating Polyradiculoneuropathy (CIDP)**: While CIDP typically presents with more widespread and symmetric involvement, some cases can have an asymmetric or multifocal presentation, making it a differential diagnosis.
2. **Amyotrophic Lateral Sclerosis (ALS)**: Although ALS typically involves both upper and lower motor neurons, some cases can present with a predominantly lower motor neuron syndrome, which might be considered in the differential diagnosis.
3. **Lewis-Sumner Syndrome**: This is a rare variant of CIDP characterized by a multifocal, asymmetric presentation, often with a predilection for the upper limbs.
4. **Neuropathy with Anti-GM1 Antibodies**: This is a rare autoimmune neuropathy characterized by the presence of anti-GM1 antibodies, which can present with a multifocal, motor-predominant neuropathy.
5. **Distal Spinal Muscular Atrophy**: This is a rare genetic disorder characterized by a slowly progressive, distal muscle weakness and atrophy, often affecting the upper limbs.

It is essential to note that a definitive diagnosis can only be made after a comprehensive evaluation, including electromyography, nerve conduction studies, laboratory tests, and, in some cases, nerve biopsy.

### **84. User**

Neonatal onset is often fatal and may present as a metabolic crisis including metabolic acidosis, non-ketotic hypoglycaemia, hyperammonaemia, and other features including multisystem involvement (hypotonia, cardiomyopathy, hepatomegaly), excretion of large amounts of fatty acid- and amino acid-derived metabolites, ‘sweaty-foot’ body odour, coma, and neonatal death. Rapidly progressive hypertrophic cardiomyopathy is common in the type with neonatal onset and has a very high probability of sudden death, which is difficult to reverse, even if the disease is detected at an early stage by neonatal screening.

Congenital malformations are thought to be the consequence of the accumulation of toxic metabolites in the part of the body not corrected by embryonic transport. The degree of impaired fatty acid oxidative metabolism determines the degree of acidosis in utero, which in turn prevents normal embryo formation. Congenital malformations include enlarged polycystic kidneys, rocker-bottom feet, absence of lower abdominal muscles, hypospadias and penile curvature, hypotonia, cerebral cortical dysplasia, and gliosis. Other dysmorphic features include wide eye spacing, ear malformations, megalocrania, wide fontanel, high forehead, and low nasal bridge.

The age of onset and symptoms of late-onset forms vary considerably among cases and are mainly characterised by the absence of congenital malformations but with a lifelong risk of acute intermittent episodes of vomiting, dehydration, hypo-ketotic hypoglycaemia, acidosis, and impaired consciousness. Older individuals may have hepatomegaly or lipid deposition myopathy. Acute loss-of-compensation episodes can be triggered by infection, fever, surgery, low-energy diets or weight loss, alcohol consumption, and pregnancy. Most patients develop chronic myopathy symptoms, including exercise intolerance, myalgia, muscle weakness, and muscle atrophy. Organic aciduria in patients with late-onset disease is often intermittent and is more pronounced only during disease and under catabolic stress.

### Llama3.1:70b

Based on the clinical manifestations you provided, the most likely diagnosis is:

**MCAD Deficiency (Medium-Chain Acyl-CoA Dehydrogenase Deficiency)**

MCAD deficiency is a rare genetic disorder that affects the body’s ability to convert certain fats to energy. The symptoms you described, such as metabolic crisis, non-ketotic hypoglycemia, hyperammonemia, hypertrophic cardiomyopathy, and “sweaty-foot” body odor, are consistent with MCAD deficiency. The disease can present in the neonatal period with severe symptoms, including metabolic crisis and sudden death, or later in life with intermittent episodes of illness triggered by various factors.

Here are five other possible diagnoses that may be considered based on the clinical manifestations:

1. **Very Long-Chain Acyl-CoA Dehydrogenase (VLCAD) Deficiency**: Similar to MCAD deficiency, VLCAD deficiency is a rare genetic disorder that affects the body’s ability to convert certain fats to energy. The symptoms are similar, including metabolic crisis, cardiomyopathy, and myopathy.
2. **Carnitine Palmitoyltransferase (CPT) Deficiency**: CPT deficiency is a rare genetic disorder that affects the body’s ability to transport fatty acids into the mitochondria for energy production. The symptoms are similar to MCAD deficiency, including metabolic crisis, cardiomyopathy, and myopathy.
3. **Long-Chain 3-Hydroxyacyl-CoA Dehydrogenase (LCHAD) Deficiency**: LCHAD deficiency is a rare genetic disorder that affects the body’s ability to convert certain fats to energy. The symptoms are similar to MCAD deficiency, including metabolic crisis, cardiomyopathy, and myopathy.
4. **Glutaric Acidemia Type II (GA2)**: GA2 is a rare genetic disorder that affects the body’s ability to convert certain amino acids and fatty acids to energy. The symptoms are similar to MCAD deficiency, including metabolic crisis, cardiomyopathy, and myopathy.
5. **Multiple Acyl-CoA Dehydrogenase Deficiency (MADD)**: MADD is a rare genetic disorder that affects the body’s ability to convert certain fats and amino acids to energy. The symptoms are similar to MCAD deficiency, including metabolic crisis, cardiomyopathy, and myopathy.

These diagnoses are all part of a group of disorders known as fatty acid oxidation disorders, which are characterized by impaired energy production from fatty acids.

### **85. User**

The average age of onset of the disease is 30 years old, and it is rare to find patients under 10 years old and over 50 years old. The prevalence of women is higher than that of men, with the ratio of women to men (1.4-2.3):1. The disease is mostly acute/subacute, and is characterised by spatial and temporal multiplicity. The spatial multiplicity refers to the multiple sites of the lesions, including the brain, brainstem, cerebellum, and spinal cord, which may be involved simultaneously or successively. Time-recurrent refers to the course of remission-relapse, and due to the wide range of sites involved, repeated episodes may eventually lead to disability or even death. Clinical manifestations are varied, and common symptoms include vision loss, diplopia, limb sensory deficits, limb motor deficits, ataxia, and bladder or rectal dysfunction.

### Llama3.1:70b

Based on the clinical manifestations you provided, the rare disease that is most likely to be diagnosed is:

**Multiple Sclerosis (MS)**

Multiple Sclerosis is a chronic and often disabling autoimmune disease that affects the central nervous system (CNS), which includes the brain, spinal cord, and optic nerves. The disease is characterized by spatial and temporal multiplicity, with multiple sites of lesions in the CNS, and a course of remission-relapse. The symptoms you listed, such as vision loss, diplopia, limb sensory and motor deficits, ataxia, and bladder or rectal dysfunction, are all common manifestations of MS.

Here are five other possible diagnoses that may be considered:

1. **Neuromyelitis Optica (NMO)**: Also known as Devic’s disease, NMO is a rare autoimmune disorder that affects the optic nerves and spinal cord, leading to vision loss, pain, and weakness.
2. **Acute Disseminated Encephalomyelitis (ADEM)**: ADEM is a rare autoimmune disease that affects the brain and spinal cord, causing inflammation and damage to the myelin sheath. Symptoms can include vision loss, weakness, and cognitive impairment.
3. **Transverse Myelitis**: This is a rare inflammatory condition that affects the spinal cord, leading to symptoms such as weakness, numbness, and bladder or bowel dysfunction.
4. **Sarcoidosis**: This is a rare autoimmune disorder that can affect multiple organs, including the CNS, leading to symptoms such as vision loss, weakness, and cognitive impairment.
5. **Behçet’s Disease**: This is a rare autoimmune disorder that can cause inflammation in multiple parts of the body, including the CNS, leading to symptoms such as vision loss, weakness, and cognitive impairment.

It’s worth noting that these alternative diagnoses may have some overlapping symptoms with MS, but they have distinct clinical features and diagnostic criteria. A thorough diagnostic evaluation, including imaging studies, laboratory tests, and clinical examination, would be necessary to confirm the diagnosis.

### **86. User**

The main symptoms include parkinsonism, cerebellar ataxia, pyramidal tract damage, and autonomic nervous system damage. The first symptoms are often autonomic impairments, most notably cardiovascular dysfunction (e.g., upright hypotension) and urinary dysfunction (e.g., urinary incontinence), the latter of which tends to be comorbid with sexual dysfunction in male patients. Many patients have had rapid eye movement (REM) sleep behaviour disorder (RBD) for several years.

### Llama3.1:70b

Based on the clinical manifestations you provided, the rare disease that is most likely to be diagnosed is:

**Multiple System Atrophy (MSA)**

MSA is a rare, progressive neurodegenerative disorder characterized by a combination of parkinsonism, cerebellar ataxia, pyramidal tract damage, and autonomic nervous system damage. The disease often presents with autonomic dysfunction, including orthostatic hypotension and urinary incontinence, which are consistent with the symptoms you described. Additionally, RBD is a common feature of MSA, and many patients experience significant autonomic dysfunction, including cardiovascular and urinary symptoms.

Here are five other possible diagnoses that may be considered:

1. **Progressive Supranuclear Palsy (PSP)**: While PSP is primarily characterized by parkinsonism and supranuclear gaze palsy, some patients may exhibit cerebellar ataxia and autonomic dysfunction, which could overlap with the symptoms you described.
2. **Corticobasal Degeneration (CBD)**: CBD is a rare neurodegenerative disorder that can present with a combination of parkinsonism, cerebellar ataxia, and cognitive decline. Autonomic dysfunction is not a primary feature of CBD, but it can occur in some patients.
3. **Lewy Body Dementia (LBD)**: LBD is a neurodegenerative disorder characterized by cognitive decline, parkinsonism, and visual hallucinations. Autonomic dysfunction, including RBD, can occur in LBD, but it is not a primary feature of the disease.
4. **Autonomic Dysfunction with Parkinsonism (ADP)**: ADP is a rare disorder characterized by autonomic dysfunction, including orthostatic hypotension and urinary incontinence, in combination with parkinsonism. However, cerebellar ataxia is not a primary feature of ADP.
5. **Spinocerebellar Ataxia (SCA)**: SCA is a group of rare genetic disorders characterized by cerebellar ataxia and, in some cases, parkinsonism and autonomic dysfunction. However, the combination of symptoms you described is not typical of most SCA subtypes.

It is essential to note that these diagnoses are speculative and may require further evaluation, including imaging studies, laboratory tests, and clinical assessments, to confirm the diagnosis.

### **87. User**

**1. Muscles,** weakness and atrophy of the distal limbs starting at the distal end of the limbs and progressing proximally, affecting the fine movements of the hands and causing drop foot. The facial muscles are also involved. At the same time, there is obvious muscle ankylosis, involving the limb muscles, pharyngeal muscles and so on. Physical examination may reveal that the fingers cannot be expanded immediately after clenching the fist vigorously, and the eyes cannot be opened immediately after closing them vigorously. Knocking on the hand, the big fish muscle, the tongue muscle, etc., can lead to muscle ankylosis, known as knocking muscle ankylosis or ‘muscle ball’ phenomenon. There is a ‘warming up’ phenomenon, which decreases after repeated activities. Lower extremity myalgia and generalised fatigue are also common clinical manifestations.

**The cardiac conduction system is often involved, with varying degrees of atrioventricular block and intraventricular conduction abnormalities. In severe cases, sudden death may result. Confirmed patients require regular ECG monitoring and pacemaker implantation if necessary.

**3. Central nervous system** , sleep disorders such as increased sleepiness and obstructive sleep apnoea are common. It may be associated with cognitive and mood disorders. Head MRI shows diffuse cerebral white matter lesions and cortical atrophy.

**Cataracts occur in almost all patients as they age. They often affect vision in the 30s and 40s.

**5. Endocrine system** A variety of endocrine disorders may occur, including diabetes mellitus, thyroid dysfunction, abnormal calcium and phosphorus metabolism, sex hormone abnormalities, and testicular atrophy (resulting in infertility).

In addition, there may be frontal baldness, gastrointestinal insufficiency and restrictive ventilation disorders.

### Llama3.1:70b

Based on the clinical manifestations you provided, the rare disease that is most likely to be diagnosed is:

**Myotonic Dystrophy Type 1 (DM1)**

Myotonic Dystrophy Type 1 is a genetic disorder characterized by muscle weakness and atrophy, myotonia (muscle stiffness), and other systemic features. The clinical manifestations you described, such as muscle weakness and atrophy starting in the distal limbs, myotonia, facial muscle involvement, and cardiac conduction abnormalities, are all consistent with DM1.

Here are five other possible diagnoses that may be considered:

1. **Myotonic Dystrophy Type 2 (DM2)**: Similar to DM1, but with a milder course and fewer systemic features.
2. **Proximal Myotonic Myopathy (PROMM)**: A rare genetic disorder characterized by proximal muscle weakness and myotonia, without the systemic features of DM1.
3. **Limb-Girdle Muscular Dystrophy (LGMD)**: A group of genetic disorders characterized by muscle weakness and atrophy, primarily affecting the proximal muscles.
4. **Facioscapulohumeral Muscular Dystrophy (FSHD)**: A genetic disorder characterized by muscle weakness and atrophy, primarily affecting the facial and shoulder muscles.
5. **Mitochondrial Myopathy, Encephalopathy, Lactic Acidosis, and Stroke-like episodes (MELAS)**: A rare genetic disorder characterized by muscle weakness, encephalopathy, and other systemic features, including lactic acidosis and stroke-like episodes.

It’s worth noting that a definitive diagnosis can only be made through a combination of clinical evaluation, genetic testing, and other diagnostic tests, such as electromyography (EMG) and muscle biopsy.

### **88. User**

The disease tends to develop at an early age, with the most frequent onset occurring in the neonatal period. Most children develop the disease within the first few days of life and present with milk refusal, vomiting, lethargy, convulsions and coma. Some children with late-onset disease are characterised by episodes of vomiting and lethargy with mental developmental abnormalities. Patients with hyperammonaemic coma may have brain injury with developmental delay, impaired learning ability or mental retardation, and sequelae of growth retardation. Patients with hyperammonaemia may have increased intracranial pressure, shortness of breath, apnoea or failure as the condition progresses, and hepatomegaly. Neurological complications include mental retardation, acute hyperammonemic coma, and even death. Biochemical changes can be seen in hyperammonaemia, increased blood glutamine and alanine, and decreased or absent urinary whey acids.

### Llama3.1:70b

Based on the clinical manifestations you provided, the most likely diagnosis is:

**Urea Cycle Disorder (UCD)**, specifically **Ornithine Transcarbamylase (OTC) Deficiency**.

The symptoms you described, such as hyperammonemia, vomiting, lethargy, convulsions, and coma, are consistent with a urea cycle disorder. The urea cycle is a metabolic pathway that removes excess ammonia from the body. Defects in this pathway can lead to the accumulation of ammonia, which is toxic to the brain and other organs.

Here are five other possible diagnoses that could be considered:

1. **Propionic Acidemia**: a rare genetic disorder caused by a deficiency of the enzyme propionyl-CoA carboxylase, leading to the accumulation of propionic acid and its metabolites, which can cause hyperammonemia, vomiting, lethargy, and developmental delay.
2. **Methylmalonic Acidemia**: a rare genetic disorder caused by a deficiency of the enzyme methylmalonyl-CoA mutase, leading to the accumulation of methylmalonic acid and its metabolites, which can cause hyperammonemia, vomiting, lethargy, and developmental delay.
3. **Citrullinemia**: a rare genetic disorder caused by a deficiency of the enzyme argininosuccinate synthetase, leading to the accumulation of citrulline and ammonia, which can cause hyperammonemia, vomiting, lethargy, and developmental delay.
4. **Argininosuccinic Aciduria**: a rare genetic disorder caused by a deficiency of the enzyme argininosuccinate lyase, leading to the accumulation of argininosuccinic acid and ammonia, which can cause hyperammonemia, vomiting, lethargy, and developmental delay.
5. **Carbamoyl Phosphate Synthetase I (CPS I) Deficiency**: a rare genetic disorder caused by a deficiency of the enzyme CPS I, leading to the accumulation of ammonia and other metabolites, which can cause hyperammonemia, vomiting, lethargy, and developmental delay.

These disorders are all rare and can present with similar symptoms, so a thorough diagnostic evaluation, including genetic testing and biochemical analysis, is necessary to confirm the diagnosis.

### **89. User**

Children often present with severe intrauterine growth retardation and develop severe, non-ketotic hyperglycaemia very early in life (often in the first week of life), which recovers after 12 weeks and recurs around puberty in 50%-60% of cases, with a clinical picture similar to that of type 2 diabetes mellitus.

### Llama3.1:70b

Based on the clinical manifestations you provided, the rare disease that is most likely to be diagnosed is:

**Transient Neonatal Diabetes Mellitus (TNDM)**

TNDM is a rare form of diabetes that typically presents in the first few weeks of life, often with severe intrauterine growth retardation. The disease is characterized by non-ketotic hyperglycemia, which usually resolves by 12 weeks of age. However, in approximately 50-60% of cases, the disease recurs around puberty, often with a clinical picture similar to type 2 diabetes mellitus.

Here are five other possible diagnoses that could be considered:

1. **Permanent Neonatal Diabetes Mellitus (PNDM)**: Similar to TNDM, but the diabetes does not resolve and persists throughout life.
2. **Wolfram Syndrome**: A rare genetic disorder characterized by early-onset diabetes mellitus, optic atrophy, and other systemic abnormalities.
3. **Kabuki Syndrome**: A rare genetic disorder characterized by developmental delay, intellectual disability, and a range of systemic abnormalities, including diabetes mellitus.
4. **Pearson Syndrome**: A rare mitochondrial disorder characterized by early-onset diabetes mellitus, pancreatic exocrine dysfunction, and other systemic abnormalities.
5. **Thiamine-responsive megaloblastic anemia syndrome (TRMA)**: A rare genetic disorder characterized by megaloblastic anemia, diabetes mellitus, and sensorineural deafness, which responds to thiamine supplementation.

Please note that these alternative diagnoses are less likely based on the provided clinical manifestations, but they should still be considered in the differential diagnosis.

### **90. User**

The earliest symptoms are abdominal distension and enlargement of the liver and spleen, and some patients may develop in the neonatal period. Intellectual and motor developmental deficits occur immediately, with hypotonia and motor developmental delays, often without cerebral neurological involvement, and regression of motor and intellectual development is evident after the age of 1 year, eventually progressing to spasticity and tonicity.Cherry erythema on the fundus of the eye can be found in 50% of the patients. Interstitial lung disease may lead to recurrent respiratory infections, hypoxaemia or respiratory failure. Most children die before the age of 3 years.

### Llama3.1:70b

Based on the clinical manifestations you provided, the rare disease that is most likely to be diagnosed is:

**Niemann-Pick disease type A**

Niemann-Pick disease type A is a rare, inherited lysosomal storage disorder caused by a deficiency of the enzyme acid sphingomyelinase. The disease is characterized by the accumulation of sphingomyelin in various organs, including the liver, spleen, and brain. The clinical manifestations you described, such as abdominal distension, hepatosplenomegaly, intellectual and motor developmental deficits, hypotonia, and cherry-red spots on the fundus of the eye, are consistent with Niemann-Pick disease type A.

Here are five other possible diagnoses that may be considered based on the clinical manifestations:

1. **Gaucher disease type II**: Another lysosomal storage disorder caused by a deficiency of the enzyme glucocerebrosidase, which can present with similar symptoms, including hepatosplenomegaly, intellectual and motor developmental deficits, and cherry-red spots on the fundus of the eye.
2. **Farber lipogranulomatosis**: A rare lysosomal storage disorder caused by a deficiency of the enzyme acid ceramidase, which can present with similar symptoms, including hepatosplenomegaly, intellectual and motor developmental deficits, and joint deformities.
3. **Mucopolysaccharidosis type I (Hurler syndrome)**: A rare genetic disorder caused by a deficiency of the enzyme alpha-L-iduronidase, which can present with similar symptoms, including hepatosplenomegaly, intellectual and motor developmental deficits, and clouded corneas.
4. **Krabbe disease**: A rare genetic disorder caused by a deficiency of the enzyme galactosylceramidase, which can present with similar symptoms, including intellectual and motor developmental deficits, hypotonia, and seizures.
5. **Sandhoff disease**: A rare genetic disorder caused by a deficiency of the enzyme hexosaminidase B, which can present with similar symptoms, including intellectual and motor developmental deficits, hypotonia, and cherry-red spots on the fundus of the eye.

It’s worth noting that these diagnoses are based on the clinical manifestations you provided, and a definitive diagnosis would require further testing, including genetic analysis and enzyme assays.

### **91. User**

**1. Typical face,** patients of all ages have ptosis, wide eye spacing, medial canthus, and downward slanting of the outer corners of the eyes; low bilateral ear position, posterior rotation, and thick auricles. Child patients may also have a full forehead, low posterior hairline, short nose, low nasal bridge, full nasal tip; thick lips, deep and wide nasolabial groove straight to the upper lip, and so on.

**2. Dwarfism,** which is normal at birth and gradually appears after 1 year of age. Bone age often lags behind, and some adult heights can reach the lower limit of normal level.

**3. cardiovascular,** 50% ~80% of patients have congenital heart disease, pulmonary valve stenosis is the most common, others include hypertrophic cardiomyopathy, ventricular ischemia, pulmonary artery branch stenosis, tetralogy of Fallot, and aortic constriction, etc. Typical ECG changes include electrocardiogram (ECG) and heart rate (HR). Typical electrocardiographic changes include extreme rightward deviation of the electrical axis with counterclockwise rotation of the QRS wave in the precordial leads, leftward deviation of the electrical axis in the V1 lead, incomplete left anterior branch block, or RSR waveforms.

**4. psychomotor retardation**, poor language skills in some patients, depression in adult patients, and a high incidence of autism spectrum manifestations have also been reported.

**5. webbing of the neck**.

**6. thoracic abnormalities,** chicken chest or funnel chest.

**7. wide breast spacing**

**8. cryptorchidism in males** , which is present in 60% to 80% of male patients and can lead to fertility disorders.

**9. abnormal blood clotting and lymphatic dysplasia**

**10. Others,** renal malformations (dilated renal pelvis, double ureteral malformation, isolated kidney, renal hypoplasia, distal ureteral stenosis, etc.), impaired spermatogenesis in males, strabismus, nystagmus, milky coffee spots and freckles on the skin, follicular keratosis of the limbs, Arnold Chiari malformation type I, hydrocephalus, hepatosplenomegaly, and an increased incidence of malignant tumours.

**11. Intrauterine manifestations,** excessive amniotic fluid in the mother during pregnancy, widening of the nuchal translucency, relatively large fetal head, cardiac and renal anomalies.

### Llama3.1:70b

Based on the clinical manifestations you provided, the most likely diagnosis is:

**Noonan Syndrome**

Noonan Syndrome is a rare genetic disorder characterized by distinctive facial features, short stature, congenital heart defects, and developmental delays. The clinical manifestations you listed, such as the typical face, dwarfism, cardiovascular abnormalities, psychomotor retardation, webbing of the neck, thoracic abnormalities, and cryptorchidism in males, are all consistent with Noonan Syndrome.

Here are five other possible diagnoses that may be considered:

1. **Turner Syndrome**: While Turner Syndrome typically affects females, some of the clinical manifestations, such as short stature, webbing of the neck, and cardiovascular abnormalities, may overlap with Noonan Syndrome.
2. **Williams Syndrome**: Williams Syndrome is a rare genetic disorder characterized by distinctive facial features, short stature, and congenital heart defects. While the facial features and cardiovascular abnormalities may be similar to Noonan Syndrome, Williams Syndrome typically presents with a more friendly and outgoing personality.
3. **Kabuki Syndrome**: Kabuki Syndrome is a rare genetic disorder characterized by distinctive facial features, short stature, and developmental delays. While the facial features and developmental delays may be similar to Noonan Syndrome, Kabuki Syndrome typically presents with more pronounced intellectual disability and seizures.
4. **Cardiofaciocutaneous Syndrome**: Cardiofaciocutaneous Syndrome is a rare genetic disorder characterized by congenital heart defects, distinctive facial features, and skin abnormalities. While the cardiovascular abnormalities and facial features may be similar to Noonan Syndrome, Cardiofaciocutaneous Syndrome typically presents with more pronounced skin abnormalities and intellectual disability.
5. **Costello Syndrome**: Costello Syndrome is a rare genetic disorder characterized by distinctive facial features, short stature, and congenital heart defects. While the facial features and cardiovascular abnormalities may be similar to Noonan Syndrome, Costello Syndrome typically presents with more pronounced skin abnormalities and an increased risk of malignant tumors.

It’s essential to note that a definitive diagnosis can only be made through genetic testing and a comprehensive medical evaluation.

### **92. User**

Patients can develop the disease at any age. There are two main clinical categories: early-onset and late-onset. Early-onset cases mainly occur in male children, usually in the neonatal period, and are characterised by a rapid onset of the disease, with a rapid onset of the disease and extensive brain damage due to elevated ammonia levels in the blood. There may be no abnormality at birth, but in the first few days of life, the child will show irritability, lethargy, refusal to eat, shortness of breath and lethargy, which may rapidly develop into spasm, coma and respiratory failure. If not treated urgently, the disease quickly develops into hereditary metabolic encephalopathy and often dies within the first week of life, with survivors often left with severe intellectual impairment. Late-onset disease occurs in older patients, both hemizygous males and heterozygous females, and has relatively mild and variable clinical symptoms. Patients with childhood and adult onset tend to present with chronic neurological damage such as episodes of vomiting, headaches, abnormal behaviour, delirium and confusion. Patients may have clinical phenotypes such as hepatomegaly, recurrent seizures, growth retardation, and behavioural abnormalities. Despite the milder symptoms of the late-onset form, life-threatening acute episodes of hyperammonemia can be induced by environmental factors such as illness, stress, and a high-protein diet. The majority of heterozygous female carriers remain asymptomatic throughout their lives, while a few develop the disease, with individual variability in age of onset and clinical manifestations, both early and late onset.

### Llama3.1:70b

Based on the clinical manifestations you provided, the rare disease that is most likely to be diagnosed is:

**Ornithine Transcarbamylase (OTC) Deficiency**

OTC deficiency is a genetic disorder that affects the urea cycle, leading to elevated ammonia levels in the blood. The clinical manifestations you described, including the rapid onset of symptoms in male children, extensive brain damage, and the potential for life-threatening acute episodes of hyperammonemia in late-onset cases, are consistent with OTC deficiency.

Here are five other possible diagnoses that may be considered:

1. **Citrullinemia**: Another urea cycle disorder that can present with similar symptoms, including elevated ammonia levels and neurological damage.
2. **Argininosuccinic Aciduria**: A rare genetic disorder that affects the urea cycle, leading to elevated ammonia levels and potential neurological damage.
3. **Carbamoyl Phosphate Synthetase I (CPS I) Deficiency**: A genetic disorder that affects the urea cycle, leading to elevated ammonia levels and potential neurological damage.
4. **Hyperornithinemia-Hyperammonemia-Homocitrullinuria (HHH) Syndrome**: A rare genetic disorder that affects the urea cycle, leading to elevated ammonia levels and potential neurological damage.
5. **Urea Cycle Disorder (UCD) due to N-Acetylglutamate Synthase (NAGS) Deficiency**: A rare genetic disorder that affects the urea cycle, leading to elevated ammonia levels and potential neurological damage.

It’s worth noting that these disorders are all relatively rare and may require specialized testing and expertise to diagnose. A definitive diagnosis would typically require a combination of clinical evaluation, laboratory testing (including genetic testing), and imaging studies.

### **93. User**

The clinical phenotype of the disease varies in severity. The main clinical manifestations of the patients are recurrent fractures under minor external forces, skeletal deformities, and varying degrees of activity limitation since childhood. Patients may have extra-skeletal manifestations such as blue sclera, ligamentous laxity, dentin hypoplasia, hearing loss, and cardiac valvulopathy, etc. The imaging features of OI mainly include: thinning of bones in many parts of the body; thinning of the cranial plate, widening of fontanelle and cranial suture, interstitial bone in occipital bones, and flattening of the base of the skull; lateral curvature or posterior convexity deformity of the spine, deformation of vertebral bodies, and compression fracture of multiple vertebral bodies; twisted and deformed thorax, or even collapse; slender long bones of the limbs, and even collapse; and the long bones of the limbs are thin and thin. The long bones of the limbs are slender, the bone cortex is thin, the bone marrow cavity is relatively large, the epiphyses are widened, there are many long bone fractures, and the bones are curved and deformed.

### Llama3.1:70b

Based on the clinical manifestations and imaging features you provided, the most likely diagnosis is:

**Osteogenesis Imperfecta (OI)**

OI is a rare genetic disorder characterized by fragile bones, skeletal deformities, and other extra-skeletal manifestations. The clinical features you described, such as recurrent fractures, skeletal deformities, blue sclera, ligamentous laxity, dentin hypoplasia, hearing loss, and cardiac valvulopathy, are all consistent with OI.

Here are five other possible diagnoses that may be considered:

1. **Ehlers-Danlos Syndrome (EDS)**: While EDS is primarily characterized by skin hyperextensibility, joint hypermobility, and tissue fragility, some patients may also experience skeletal deformities and fractures.
2. **Fibrodysplasia Ossificans Progressiva (FOP)**: FOP is a rare genetic disorder characterized by progressive heterotopic ossification, leading to skeletal deformities and limited mobility. However, the presence of blue sclera and other extra-skeletal manifestations is not typical of FOP.
3. **Brittle Bone Disease (BBD)**: BBD is a rare genetic disorder characterized by fragile bones and skeletal deformities, but it does not typically involve extra-skeletal manifestations.
4. **Spondyloepiphyseal Dysplasia (SED)**: SED is a group of rare genetic disorders characterized by skeletal deformities, particularly affecting the spine and epiphyses. While some patients may experience fractures, the extra-skeletal manifestations you described are not typical of SED.
5. **Marfan Syndrome**: Marfan syndrome is a genetic disorder characterized by tall stature, skeletal deformities, and cardiovascular manifestations. While some patients may experience fractures, the presence of blue sclera and other extra-skeletal manifestations is not typical of Marfan syndrome.

It’s essential to note that a definitive diagnosis can only be made through a combination of clinical evaluation, imaging studies, and genetic testing.

### **94. User**

**1. Pancytopenia,** Many patients may have a combination of pancytopenia and neutropenia. Clinical manifestations include malaise, recurrent infections and bleeding tendencies.

**2. Haemoglobinuria** , typical haemoglobinuria is soy sauce or strong tea colour. It usually lasts for 23 days and subsides on its own without treatment, while in severe cases, it lasts for 12 weeks or even longer. Some patients have frequent episodes of haemoglobinuria, while a significant number of patients have no clear episodes of haemoglobinuria over a long period of time or observation.

Episodes of haemoglobinuria may be accompanied by fever, low back pain and abdominal pain. Other symptoms associated with smooth muscle dysfunction can include dysphagia, abdominal pain, bloating, back pain, headache, oesophageal spasms, and erectile dysfunction. Rare conditions include choledochal duct dyskinesia, acute pancreatitis, ischaemia and ulceration of the duodenum or colon.

**3. thrombosis**.

**Jaundice and hepatosplenomegaly** , less than half of patients have mild jaundice. Most patients do not have hepatosplenomegaly.

Common comorbidities include infection, anaemic heart disease, cholelithiasis, renal insufficiency and even failure.

### Llama3.1:70b

Based on the clinical manifestations you provided, the most likely rare disease diagnosis is:

**Paroxysmal Nocturnal Hemoglobinuria (PNH)**

PNH is a rare, acquired, life-threatening disease of the blood characterized by the destruction of red blood cells (hemolysis), bone marrow failure, and the presence of blood clots (thrombosis). The clinical manifestations you described, such as pancytopenia, hemoglobinuria, thrombosis, and jaundice, are consistent with PNH.

Here are five other possible diagnoses that may be considered:

1. **Thrombotic Thrombocytopenic Purpura (TTP)**: a rare blood disorder characterized by the formation of blood clots in small blood vessels, leading to thrombocytopenia, microangiopathic hemolytic anemia, and organ dysfunction.
2. **Hemophagocytic Lymphohistiocytosis (HLH)**: a rare disorder of the immune system characterized by the overproduction of inflammatory cytokines, leading to hemophagocytosis, pancytopenia, and organ dysfunction.
3. **Myelodysplastic Syndrome (MDS)**: a group of rare blood disorders characterized by the abnormal production of blood cells, leading to pancytopenia, anemia, and increased risk of infection and bleeding.
4. **Sickle Cell Disease**: a genetic disorder that affects hemoglobin production, leading to anemia, jaundice, and increased risk of infection and thrombosis.
5. **Autoimmune Hemolytic Anemia (AIHA)**: a rare disorder characterized by the immune system’s attack on red blood cells, leading to hemolysis, anemia, and jaundice.

Please note that these diagnoses are speculative and may require further testing and evaluation to confirm.

### **95. User**

Clinical manifestations include repeated intussusception, abdominal pain, diarrhoea, mucus stools, blood in stool, constipation, vomiting blood and anaemia. Characteristic clinical manifestations are skin and mucous membrane pigmentation and multiple polyps in the gastrointestinal tract. 95% of the patients have hyperpigmentation, which can be found on the lips and surrounding areas of the mouth, cheeks, face, fingers, palms of the hands, soles of the feet, perianal area, etc., and occasionally on the intestinal mucous membranes. The pigmentation can be brown, tan, grey, blue, etc. It usually occurs before the age of 2 years, and the size is 1~5mm. 88%-100% of the patients will develop gastrointestinal polyps, and the onset of gastrointestinal polyps most often occurs at the age of 11-13 years, and the polyps can occur throughout the entire gastrointestinal tract, with the small intestine being the most common, followed by the colon and the stomach. The size of the polyps is variable, and their tips vary in length and thickness. The risk of cancer is high, with an average age of 42 years.

### Llama3.1:70b

Based on the clinical manifestations you provided, the most likely diagnosis is:

**Peutz-Jeghers Syndrome (PJS)**

Peutz-Jeghers Syndrome is a rare autosomal dominant genetic disorder characterized by the development of benign hamartomatous polyps in the gastrointestinal tract and an increased risk of certain cancers. The syndrome is also associated with distinctive mucocutaneous melanin deposits, which cause skin and mucous membrane pigmentation.

The clinical manifestations you described, including repeated intussusception, abdominal pain, diarrhea, mucus stools, blood in stool, constipation, vomiting blood, and anemia, are consistent with the gastrointestinal polyps and potential complications associated with PJS. The characteristic skin and mucous membrane pigmentation, particularly the presence of brown, tan, grey, or blue spots on the lips, mouth, face, fingers, palms, soles, and perianal area, is a hallmark feature of PJS.

Here are five other possible diagnoses that may be considered, although they are less likely:

1. **Familial Adenomatous Polyposis (FAP)**: While FAP is also characterized by the development of gastrointestinal polyps, it typically presents with hundreds to thousands of adenomatous polyps, which have a higher malignant potential than the hamartomatous polyps seen in PJS. Skin and mucous membrane pigmentation is not a characteristic feature of FAP.
2. **Juvenile Polyposis Syndrome (JPS)**: JPS is a rare genetic disorder characterized by the development of multiple juvenile polyps in the gastrointestinal tract. While JPS can present with similar gastrointestinal symptoms, it is not typically associated with skin and mucous membrane pigmentation.
3. **Cowden Syndrome**: Cowden syndrome is a rare genetic disorder characterized by the development of multiple hamartomatous lesions, including gastrointestinal polyps. While it can present with similar gastrointestinal symptoms, it is not typically associated with the characteristic skin and mucous membrane pigmentation seen in PJS.
4. **Bannayan-Riley-Ruvalcaba Syndrome**: This is a rare genetic disorder characterized by the development of multiple hamartomatous lesions, including gastrointestinal polyps. While it can present with similar gastrointestinal symptoms, it is not typically associated with the characteristic skin and mucous membrane pigmentation seen in PJS.
5. **Lynch Syndrome**: Lynch syndrome is a rare genetic disorder characterized by an increased risk of certain cancers, including colorectal cancer. While it can present with similar gastrointestinal symptoms, it is not typically associated with the characteristic skin and mucous membrane pigmentation seen in PJS.

It is essential to note that a definitive diagnosis of Peutz-Jeghers Syndrome requires a combination of clinical evaluation, genetic testing, and histopathological examination of the polyps.

### **96. User**

In the neonatal period, most of the children have no clinical symptoms, but after 3-4 months of life, typical symptoms gradually appear, and the symptoms are obvious at the age of 1 year. A few months after birth, due to insufficient melanin synthesis, their hair and iris colour gradually becomes lighter, yellow or brownish-yellow, with white skin. Urine and sweat contain a large amount of phenyl lactic acid and have a foul odour of rat urine. As they grow older, they gradually show delayed intellectual development, with predominantly cognitive developmental disorders, microcephaly, seizures, as well as behavioural and personality abnormalities, such as hyperactivity, self-mutilation, aggression, autism, low self-esteem, depression and other neurological manifestations. Vomiting and eczema are also commonly seen in infancy.

### Llama3.1:70b

Based on the clinical manifestations you provided, the most likely diagnosis is:

**Phenylketonuria (PKU)**

PKU is a rare genetic disorder caused by a deficiency of the enzyme phenylalanine hydroxylase, which leads to the accumulation of phenylalanine and its metabolites, such as phenyl lactic acid, in the body. The symptoms you described, including the lightening of hair and iris color, musty odor of urine and sweat, delayed intellectual development, and behavioral abnormalities, are all consistent with PKU.

Here are five other possible diagnoses that may be considered:

1. **Tyrosinemia type 1**: A rare genetic disorder caused by a deficiency of the enzyme fumarylacetoacetate hydrolase, leading to the accumulation of toxic metabolites, including succinylacetone, which can cause similar symptoms to PKU.
2. **Homocystinuria**: A rare genetic disorder caused by a deficiency of the enzyme cystathionine beta-synthase, leading to the accumulation of homocysteine and its metabolites, which can cause similar symptoms to PKU, including intellectual disability and behavioral abnormalities.
3. **Maple syrup urine disease**: A rare genetic disorder caused by a deficiency of the enzyme branched-chain alpha-keto acid dehydrogenase, leading to the accumulation of branched-chain amino acids and their metabolites, which can cause similar symptoms to PKU, including intellectual disability and behavioral abnormalities.
4. **Glycine encephalopathy**: A rare genetic disorder caused by a deficiency of the enzyme glycine decarboxylase, leading to the accumulation of glycine and its metabolites, which can cause similar symptoms to PKU, including intellectual disability and behavioral abnormalities.
5. **Histidinemia**: A rare genetic disorder caused by a deficiency of the enzyme histidase, leading to the accumulation of histidine and its metabolites, which can cause similar symptoms to PKU, including intellectual disability and behavioral abnormalities.

It’s worth noting that these alternative diagnoses are less likely than PKU, given the specific combination of symptoms you described. However, a comprehensive diagnostic evaluation, including genetic testing and biochemical analysis, would be necessary to confirm the diagnosis.

### **97. User**

Mainly skin symptoms and neuropsychiatric symptoms.

**1. The group of cutaneous symptoms,** are erythema, herpes and even ulceration on exposed parts of the skin after exposure to light. The rash may be of the type of eczema, urticaria, summertime itchy rash or erythema multiforme. The oral mucosa may have red patches and brownish-red teeth. Eye damage such as conjunctivitis, keratitis and iritis may also occur. In severe cases, there may be deformation of the skin of the nose, ears and fingers with kinking. There may be special purple colouration. Hirsutism may be present.

**2. A cluster of neuropsychiatric symptoms ,**  the most common manifestation of which is acute abdominal pain, which tends to be severe, stable, and poorly localised, sometimes accompanied by painful cramps. It may be accompanied by constipation, bloating, nausea, vomiting, and sometimes diarrhoea and increased bowel sounds. Sensory and motor neuropathies may precede the onset of abdominal pain and present as limb pain with numbness, abnormal sensation and sensory inversions; motor weakness often begins proximally in the upper limbs and may progress to the lower limbs and distal extremities. Involvement of the cerebral nerves may lead to medullary paralysis, impaired respiratory function and death. Severe motor neuropathy may result in quadriplegia. Autonomic nervous system involvement manifests as tachycardia, hypertension, sweating, agitation, and tremor. Neurogenic bladder dysfunction may cause dysuria, dyspareunia, urinary retention and incontinence. Other acute neuropsychiatric manifestations include insomnia, anxiety, agitation, agitation, hallucinations, hysteria, disorientation, delirium, affective apathy, depression, phobias, and altered consciousness ranging from lethargy to coma.

### Llama3.1:70b

What a fascinating and complex case!

Based on the clinical manifestations you provided, I’m going to take a educated guess that the most likely diagnosis is:

**Porphyria**

Specifically, I suspect **Acute Intermittent Porphyria (AIP)** or **Variegate Porphyria (VP)**, which are both types of acute porphyrias that can present with a combination of skin and neuropsychiatric symptoms.

Here’s why:

1. **Skin symptoms**: The skin manifestations you described, such as erythema, blistering, and ulceration on sun-exposed areas, are consistent with porphyria cutanea tarda (PCT), a subtype of porphyria. The presence of hirsutism and purple discoloration also supports this diagnosis.
2. **Neuropsychiatric symptoms**: The acute abdominal pain, neuropathies, and autonomic nervous system involvement are all characteristic of acute porphyrias, particularly AIP and VP. The neuropsychiatric symptoms, such as anxiety, agitation, hallucinations, and altered consciousness, are also consistent with these conditions.

Now, here are five other possible diagnoses that might be considered:

1. **Erythropoietic Protoporphyria (EPP)**: Another type of porphyria that can cause skin symptoms, although the neuropsychiatric manifestations are less common.
2. **Systemic Lupus Erythematosus (SLE)**: An autoimmune disease that can cause a wide range of symptoms, including skin rashes, neuropsychiatric manifestations, and abdominal pain.
3. **Sjögren’s Syndrome**: An autoimmune disorder that can cause skin symptoms, neuropathies, and neuropsychiatric manifestations, although the abdominal pain is less typical.
4. **Fabry Disease**: A genetic disorder that can cause skin symptoms, neuropathies, and autonomic nervous system involvement, although the abdominal pain and neuropsychiatric manifestations are less common.
5. **Mitochondrial Neurogastrointestinal Encephalomyopathy (MNGIE)**: A rare genetic disorder that can cause a combination of gastrointestinal, neurological, and neuropsychiatric symptoms, although the skin manifestations are less typical.

Please note that these alternative diagnoses are less likely, and a thorough diagnostic workup, including laboratory tests and genetic analysis, would be necessary to confirm or rule out these conditions.

### **98. User**

**1. Infection, patients** are infected to varying degrees. Infections are recurrent, severe, difficult to treat, opportunistic and have a broad spectrum of pathogens. Typical children are usually born with no specific manifestations, and fatal infections can occur before 2 months of age due to cellular immune abnormalities. The sites and pathogens of infection are diverse, including recurrent pneumonia, recurrent thrush, persistent diarrhoea, recurrent otitis media, persistent viral infections (e.g., respiratory syncytial virus, EBV, CMV, etc.), and conditionally causative organisms, such as Pneumocystis carinii, are also common.

**2. Vaccine disease,** in which patients are extremely immunocompromised, can occur with infection and dissemination of live attenuated vaccines. Severe diarrhoea can also occur in children vaccinated with attenuated rotavirus vaccine.

**3. Retarded or arrested growth and development** is a distinguishing feature of the disease. It is associated with recurrent infections, diarrhoea and corresponding genetic defects.

Children may also have a rash and enlarged liver and spleen lymph nodes similar to erysipelas.

In other children, erythroderma-like symptoms, hepatosplenomegaly, seborrheic dermatitis, haematopenia, and sclerosing cholangitis may be present.

### Llama3.1:70b

Based on the clinical manifestations you provided, the most likely rare disease diagnosis is:

**Severe Combined Immunodeficiency (SCID)**

SCID is a group of rare, potentially life-threatening disorders caused by mutations in genes involved in the development and function of the immune system. The symptoms you described, such as recurrent and severe infections, opportunistic infections, and vaccine-related illnesses, are consistent with SCID. Additionally, the presence of growth and developmental delays, rash, hepatosplenomegaly, and other systemic symptoms are also characteristic of SCID.

Here are five other possible diagnoses that may be considered based on the clinical manifestations:

1. **Wiskott-Aldrich Syndrome (WAS)**: a rare X-linked recessive disorder characterized by eczema, thrombocytopenia, and recurrent infections.
2. **Hyper-IgE Syndrome (HIES)**: a rare disorder characterized by recurrent skin and lung infections, high levels of IgE, and eczema.
3. **Chronic Granulomatous Disease (CGD)**: a rare disorder caused by mutations in genes involved in the production of reactive oxygen species, leading to recurrent infections and granulomatous disease.
4. **DiGeorge Syndrome (22q11.2 deletion syndrome)**: a rare disorder characterized by thymic hypoplasia, congenital heart defects, and immunodeficiency, leading to recurrent infections and other systemic symptoms.
5. **Omenn Syndrome**: a rare form of SCID characterized by erythroderma, hepatosplenomegaly, and lymphadenopathy, often associated with a specific genetic mutation.

These diagnoses should be considered in the differential diagnosis, but SCID remains the most likely diagnosis based on the clinical manifestations you provided.

### **99. User**

The core symptom is dystonia, which is mainly manifested by abnormal expressive postures and involuntary movements, mostly involving the head and neck muscles, trunk muscles, rotator anterior muscles of the limbs, finger and wrist flexors, toe extensors and plantar flexors. It is aggravated by random movements, reduced or disappeared when resting and sleeping, and in the late stage, it can be a fixed twisting spastic deformity. Sensory trickery (relief techniques) and movement specificity are the characteristic manifestations of dystonia. Sensory tricks (relieving techniques) are random movements that are specifically designed to correct abnormal postures or relieve dystonic movements, usually simple movements that involve or target the affected area, but are not forceful to counteract the symptoms of dystonia. Movement specificity refers to the presence of dystonia only during specific activities or the performance of specific tasks, such as certain occupations (e.g., writing spasms, musician’s spasms) or sports.

### Llama3.1:70b

Based on the clinical manifestations you provided, the most likely diagnosis is:

**Primary Dystonia (also known as Idiopathic Torsion Dystonia)**

This is a rare neurological disorder characterized by involuntary muscle contractions, leading to abnormal postures and movements, often affecting the head, neck, trunk, and limbs. The presence of sensory tricks and movement specificity are hallmark features of primary dystonia.

Here are five other possible diagnoses to consider:

1. **Dopa-Responsive Dystonia (DRD)**: A rare genetic disorder that responds well to levodopa treatment. Patients with DRD often exhibit diurnal fluctuations in symptoms, with worsening of dystonia in the afternoon and evening.
2. **Wilson’s Disease**: A rare genetic disorder characterized by excessive copper accumulation in the body, leading to neurological symptoms, including dystonia, tremors, and parkinsonism.
3. **Huntington’s Disease**: A rare genetic disorder that causes progressive damage to the brain, leading to motor symptoms, including dystonia, chorea, and rigidity, as well as cognitive decline and psychiatric symptoms.
4. **Tardive Dyskinesia**: A rare side effect of long-term antipsychotic medication use, characterized by involuntary movements, including dystonia, chorea, and athetosis.
5. **Myoclonus-Dystonia Syndrome**: A rare genetic disorder characterized by a combination of myoclonic jerks and dystonic movements, often affecting the neck, trunk, and limbs.

These alternative diagnoses should be considered in the differential diagnosis, and further evaluation, including genetic testing, imaging studies, and laboratory tests, may be necessary to confirm the diagnosis.

### **100. User**

**1. Kidney,** the main manifestations are limb oedema and increased foam in the urine. Laboratory tests may reveal simple moderate proteinuria or nephrotic syndrome, and renal insufficiency may be seen in the advanced stage.

**2. Heart,** the main manifestations are shortness of breath after activity, limb edema, pleural effusion, ascites and other signs of restrictive cardiac insufficiency.

**3. Liver,** there may be slight discomfort or pain in the liver area, but most patients may be asymptomatic, and hepatomegaly or liver enzyme abnormalities are often detected during physical examination. Liver failure may occur in advanced stages of the disease.

**Peripheral and autonomic nerves,** Symmetrical sensory and/or motor peripheral neuropathy of the extremities. Autonomic abnormalities are often manifested by upright hypotension, gastroparesis, pseudo-intestinal obstruction, and impotence.

**5. Gastrointestinal tract** , can present with total gastrointestinal involvement, with the stomach and small intestine being the most commonly involved. It can present with epigastric discomfort, dyspepsia, diarrhoea, constipation, malabsorption syndrome and gastrointestinal bleeding.

**6. Soft tissues ,** Tongue involvement may present with megaglossia, tongue dysmobility, and dysarthria. Skin mucosa may present with skin purpura and petechiae, which are more common around the orbits and in the loose skin areas of the neck. Nail atrophy and hair loss may also occur.

**7. Coagulation abnormalities** Patients often have coagulation factor X deficiency, resulting in corresponding bleeding manifestations.

### Llama3.1:70b

Based on the clinical manifestations you provided, the rare disease that is most likely to be diagnosed is:

**Amyloidosis**

Amyloidosis is a group of diseases characterized by the deposition of abnormal proteins (amyloid) in various tissues and organs, leading to their dysfunction. The clinical manifestations you described, including kidney involvement (proteinuria, nephrotic syndrome), heart involvement (restrictive cardiomyopathy), liver involvement (hepatomegaly, liver enzyme abnormalities), peripheral and autonomic neuropathy, gastrointestinal involvement (malabsorption, gastrointestinal bleeding), soft tissue involvement (tongue, skin, nail, and hair abnormalities), and coagulation abnormalities (factor X deficiency), are all consistent with amyloidosis.

Here are five other possible diagnoses that may be considered:

1. **Multiple Myeloma**: a plasma cell dyscrasia that can cause kidney damage, anemia, bone lesions, and hypercalcemia, and may also involve the heart, liver, and nervous system.
2. **Sarcoidosis**: a systemic granulomatous disease that can affect multiple organs, including the lungs, skin, eyes, liver, and nervous system, and may cause symptoms similar to those described.
3. **Systemic Lupus Erythematosus (SLE)**: a autoimmune disease that can affect multiple organs, including the kidneys, heart, liver, and nervous system, and may cause symptoms similar to those described.
4. **Waldenström’s Macroglobulinemia**: a lymphoproliferative disorder that can cause anemia, bleeding, and neurological symptoms, and may also involve the kidneys, heart, and liver.
5. **Primary Sjögren’s Syndrome**: an autoimmune disease that can cause dry eyes, dry mouth, and systemic symptoms, including kidney, heart, and liver involvement, and may also cause neurological symptoms.

Note that these alternative diagnoses are less likely than amyloidosis, based on the specific combination of clinical manifestations you provided.

### **101. User**

Jaundice and itchy skin are typical clinical manifestations. Other developmental delays include short stature, delayed pubertal growth, gallbladder stones, steatorrhea due to fat absorption disorders, hepatosplenomegaly, rickets due to fat-soluble vitamin deficiencies, delayed bone age, dry eye, coagulation disorders, and neuromuscular disorders. Children may also develop visual and hearing abnormalities, irritability, lethargy and poor concentration. In advanced stages, portal hypertension and liver tumours may occur.

### Llama3.1:70b

What a fascinating case!

Based on the clinical manifestations you’ve provided, I’m going to take a educated guess that the rare disease most likely to be diagnosed is:

**Alagille Syndrome**

Alagille Syndrome is a rare genetic disorder characterized by bile duct paucity, leading to chronic cholestasis, jaundice, and pruritus (itchy skin). The disease often presents with a range of systemic manifestations, including:

- Growth retardation and short stature
- Delayed puberty
- Gallstones and cholelithiasis
- Steatorrhea and fat-soluble vitamin deficiencies
- Hepatosplenomegaly
- Rickets and osteoporosis
- Ocular and auditory abnormalities
- Neuromuscular disorders

The disease is caused by mutations in the JAG1 gene, which codes for a protein involved in cell signaling and development.

Now, here are five other possible diagnoses that could be considered based on the clinical manifestations:

1. **Progressive Familial Intrahepatic Cholestasis (PFIC)**: A group of rare genetic disorders characterized by chronic cholestasis, jaundice, and pruritus, often leading to liver failure.
2. **Bile Acid Synthesis Disorders**: A group of rare genetic disorders affecting the synthesis of bile acids, leading to chronic cholestasis, jaundice, and pruritus.
3. **Cystic Fibrosis**: A genetic disorder affecting the respiratory, digestive, and reproductive systems, often presenting with steatorrhea, malabsorption, and liver disease.
4. **Abetalipoproteinemia**: A rare genetic disorder affecting the absorption of fat-soluble vitamins, leading to steatorrhea, malabsorption, and liver disease.
5. **Byler Disease**: A rare genetic disorder characterized by chronic cholestasis, jaundice, and pruritus, often leading to liver failure and other systemic complications.

Please note that these diagnoses are speculative and would require further evaluation, including genetic testing, imaging studies, and laboratory tests, to confirm the diagnosis.

### **102. User**

The disease starts in childhood. The disease manifests itself as a mild delay in motor development and progressive atrophy of skeletal muscles, which affects the motor function of the limbs and gradually leads to gait abnormalities and limited movement of the upper limbs, with the natural course of the disease often resulting in loss of the ability to walk at about 10 years of age. Thereafter, scoliosis, joint contracture, respiratory muscle weakness and dilated cardiomyopathy appeared, and the patient died of respiratory failure and cardiac failure at the age of about 20 years. Pseudohypertrophy of both gastrocnemius muscles, pseudohypertrophy of both forearms and lingual muscles, positive Gowers’ sign, and lumbar lordosis can be seen on examination. The disease may start in youth or even adulthood, and some patients do not affect survival. Signs of pseudohypertrophy are obvious, and in some patients, dilated cardiomyopathy is first apparent when limb weakness is still mild.

### Llama3.1:70b

Based on the clinical manifestations you provided, the most likely diagnosis is:

**Duchenne Muscular Dystrophy (DMD)**

DMD is a genetic disorder caused by a mutation in the dystrophin gene, leading to progressive muscle degeneration and weakness. The disease typically presents in childhood, with symptoms such as delayed motor development, muscle atrophy, and pseudohypertrophy of certain muscles (e.g., calf muscles). The disease progresses to loss of ambulation, scoliosis, joint contractures, respiratory muscle weakness, and dilated cardiomyopathy, ultimately leading to respiratory and cardiac failure.

Here are five other possible diagnoses that may be considered:

1. **Becker Muscular Dystrophy (BMD)**: Similar to DMD, but with a milder course and later onset. Patients with BMD may remain ambulatory into adulthood.
2. **Limb-Girdle Muscular Dystrophy (LGMD)**: A group of disorders characterized by muscle weakness and wasting, primarily affecting the proximal muscles of the limbs. Some forms of LGMD may present with similar symptoms to DMD.
3. **Spinal Muscular Atrophy (SMA)**: A genetic disorder caused by mutations in the SMN1 gene, leading to progressive muscle weakness and wasting. While SMA typically presents in infancy, some forms may have a later onset and milder course.
4. **Friedreich’s Ataxia**: A genetic disorder caused by a mutation in the frataxin gene, leading to progressive damage to the nervous system and muscle weakness. While the primary symptoms are ataxia and sensory deficits, some patients may develop muscle weakness and wasting.
5. **Emery-Dreifuss Muscular Dystrophy**: A genetic disorder characterized by muscle weakness, wasting, and contractures, primarily affecting the shoulders, upper arms, and lower legs. Some patients may develop cardiac conduction abnormalities and dilated cardiomyopathy.

These alternative diagnoses may be considered based on the presence of specific symptoms, such as ataxia or sensory deficits, or the absence of certain features, such as pseudohypertrophy of the calf muscles. However, DMD remains the most likely diagnosis based on the clinical manifestations you provided.

### **103. User**

Mainly hyperaemia, brain damage and cardiomyopathy.

**1. Neonatal onset** Normal at birth, vomiting, lethargy, hypotonia, convulsions, dyspnoea, hyperaemia, ketosis, hypoglycaemia, acidosis, dilated cardiomyopathy, pancreatitis and other abnormalities after starting breastfeeding, with a high morbidity and mortality rate.

**2. Delayed-onset** is often induced by fever, starvation, high-protein diet, and infections, and manifests itself as feeding difficulties in infancy and early childhood, developmental delays, convulsions, and hypotonia. In many patients, cognitive ability and neurological development are impaired, with an increase in slow waves or epileptic waves on the EEG; some patients may have fractures and osteoporosis on X-ray; bone marrow suppression is also common, causing granulocytopenia, anaemia and thrombocytopenia. There may also be cardiac damage, such as cardiomyopathy, arrhythmia, prolonged QT interval, and diminished cardiac function. Renal impairment is less common.

### Llama3.1:70b

Based on the clinical manifestations you provided, the rare disease that is most likely to be diagnosed is:

**1. Carnitine Palmitoyltransferase II (CPT II) Deficiency**

CPT II deficiency is a rare genetic disorder that affects the metabolism of fatty acids. The clinical manifestations you described, such as hyperaemia, brain damage, cardiomyopathy, and other abnormalities, are consistent with CPT II deficiency. The disease can present in the neonatal period or later in childhood, and the symptoms can be triggered by fasting, high-protein diet, or infections.

Here are five other possible diagnoses that may be considered based on the clinical manifestations:

**2. Medium-Chain Acyl-CoA Dehydrogenase (MCAD) Deficiency**: This is another rare genetic disorder that affects the metabolism of fatty acids. The symptoms are similar to CPT II deficiency, including hyperaemia, brain damage, cardiomyopathy, and other abnormalities.

**3. Very-Long-Chain Acyl-CoA Dehydrogenase (VLCAD) Deficiency**: This is a rare genetic disorder that affects the metabolism of fatty acids. The symptoms are similar to CPT II deficiency, including hyperaemia, brain damage, cardiomyopathy, and other abnormalities.

**4. Barth Syndrome**: This is a rare genetic disorder that affects the metabolism of fatty acids and the function of mitochondria. The symptoms include cardiomyopathy, skeletal muscle weakness, and other abnormalities.

**5. Kearns-Sayre Syndrome (KSS)**: This is a rare mitochondrial disorder that affects the function of mitochondria. The symptoms include cardiomyopathy, skeletal muscle weakness, and other abnormalities, including developmental delays and cognitive impairment.

**6. MELAS Syndrome (Mitochondrial Encephalomyopathy, Lactic Acidosis, and Stroke-like episodes)**: This is a rare mitochondrial disorder that affects the function of mitochondria. The symptoms include stroke-like episodes, lactic acidosis, and other abnormalities, including developmental delays and cognitive impairment.

Note that these diagnoses are based on the clinical manifestations you provided, and a definitive diagnosis would require further testing, including genetic testing and other diagnostic evaluations.

### **104. User**

Clinical manifestations are often nonspecific, with an insidious onset. Symptoms such as coughing up sputum, lethargy and fatigue may occur. Secondary infections may include fever, coughing up purulent sputum, or chest pain and haemoptysis. A few patients may be asymptomatic and may have no positive signs. Bursting sounds may be present in some patients, cyanosis may be present in severe cases, and pestle and mortar fingers may be seen in a few patients. The clinical picture is often less severe than the chest image, and this inconsistency between symptoms and images is a characteristic feature of the disease. Serum anti-GM-CSF antibodies are markedly elevated.

### Llama3.1:70b

Based on the clinical manifestations you provided, the rare disease that is most likely to be diagnosed is:

**Pulmonary Alveolar Proteinosis (PAP)**

PAP is a rare lung disease characterized by the accumulation of surfactant-like protein and phospholipids in the alveoli, leading to impaired gas exchange. The clinical manifestations you described, such as coughing up sputum, lethargy, fatigue, and secondary infections, are consistent with PAP. The presence of elevated serum anti-GM-CSF antibodies is also a hallmark of autoimmune PAP, which is the most common form of the disease.

Here are five other possible diagnoses that may be considered based on the clinical manifestations:

1. **Idiopathic Pulmonary Fibrosis (IPF)**: A chronic and progressive lung disease characterized by scarring of the lung tissue, leading to symptoms such as coughing, fatigue, and shortness of breath.
2. **Sarcoidosis**: A systemic granulomatous disease that can affect the lungs, leading to symptoms such as coughing, fatigue, and shortness of breath, as well as skin and eye manifestations.
3. **Lymphangioleiomyomatosis (LAM)**: A rare lung disease characterized by the growth of smooth muscle-like cells in the lungs, leading to symptoms such as shortness of breath, coughing, and chest pain.
4. **Pulmonary Langerhans Cell Histiocytosis (PLCH)**: A rare lung disease characterized by the proliferation of Langerhans cells in the lungs, leading to symptoms such as coughing, shortness of breath, and chest pain.
5. **Cryptogenic Organizing Pneumonia (COP)**: A rare lung disease characterized by the presence of organizing pneumonia in the lungs, leading to symptoms such as coughing, shortness of breath, and fever.

It’s worth noting that these alternative diagnoses may not fully explain the presence of elevated serum anti-GM-CSF antibodies, which is a distinctive feature of autoimmune PAP.

### **105. User**

In most cases, the disease begins in adolescence, with a gradual loss of the optic rod cells, followed by cone cell involvement and death of the retinal pigment epithelium. Therefore, night blindness is usually the first symptom (but after urbanisation, some patients often have a decrease in visual acuity), and the earlier the onset of night blindness, the more severe the patient’s condition. Although peripheral visual field defects also occur early, patients are usually unaware of them and rarely complain of them in the early stages of the disease. The disease progresses to a loss of central vision and further narrowing of the visual field, and most people become legally blind before the age of 40. The degree of visual impairment can be related to the mode of inheritance.

The presentation of the fundus is related to the stage of the disease: in the early stages of the disease, the fundus may be normal or nearly normal on routine examination, but in some patients, an early form of blanket retinal degeneration may be observed on mid-peripheral fundus photography (these subtle changes are difficult to recognise with current scanning laser fundus photography or wide-field fundus photography). As the disease progresses, some patients develop typical fundus changes, such as retinal osteoblast-like pigmentation combined with mid-peripheral retinal atrophy, a waxy optic disc, and thinning of retinal blood vessels; some patients develop blanketing or depigmentation, which can be seen in the mid-peripheral retina, or extensive degeneration. Other common complications include macular cystoid degeneration, posterior subcapsular cataract, vitreous dust particles, and optic disc vitreous warts; a rare complication is Coat’s retinopathy.

### Llama3.1:70b

Based on the clinical manifestations you provided, the most likely diagnosis is:

**Retinitis Pigmentosa (RP)**

Retinitis Pigmentosa is a group of rare, genetic disorders that involve a breakdown and loss of cells in the retina, which is the light-sensitive tissue that lines the back of the eye. The symptoms and progression of the disease you described, including night blindness, peripheral visual field defects, loss of central vision, and characteristic fundus changes, are all consistent with RP.

Here are five other possible diagnoses that could be considered, although they are less likely:

1. **Leber Congenital Amaurosis (LCA)**: A rare, inherited eye disease that affects the retina and causes severe visual impairment or blindness at birth or in early childhood.
2. **Choroideremia**: A rare, inherited disorder that causes progressive loss of vision due to degeneration of the choroid, a layer of blood vessels between the sclera and retina.
3. **Batten Disease**: A rare, inherited disorder that affects the nervous system and can cause progressive loss of vision, as well as other symptoms such as seizures and cognitive decline.
4. **Usher Syndrome**: A rare, inherited disorder that affects the retina and inner ear, causing progressive loss of vision and hearing.
5. **Cone-Rod Dystrophy**: A rare, inherited disorder that affects the retina and causes progressive loss of central vision and color vision, often with a later onset than RP.

It’s worth noting that a definitive diagnosis would require a comprehensive ophthalmological examination, including electroretinography (ERG) and genetic testing, to confirm the presence of RP or one of these other conditions.

### **106. User**

The most common clinical presentation is white pupil syndrome, in which a yellow-white reflection is visible in the pupil. Disuse strabismus may occur in the affected eye due to the tumour being located at the posterior pole and low visual acuity. The early presentation is a greyish-white, round or oval arching mass that may bulge into the vitreous or grow flat along the choroid. Retinal blood vessels on the surface of the tumour may become dilated and haemorrhagic, and even exudative retinal detachment may occur. Tumour tissue may also penetrate the retina and enter the vitreous, floating like a snowball, or even deposited in the anterior chamber to form pseudo anterior chamber pus. The onset of the disease is early in children with bilateral retinal detachment, usually within one year of age. The onset is later in unilateral children, usually at two or three years of age.

### Llama3.1:70b

Based on the clinical manifestations you provided, the most likely diagnosis is:

**Retinoblastoma**

Retinoblastoma is a rare, malignant tumor of the retina that typically affects young children. The clinical presentation you described, including white pupil syndrome (leukocoria), disuse strabismus, low visual acuity, and a greyish-white, round or oval mass in the posterior pole of the eye, is consistent with retinoblastoma.

Here are five other possible diagnoses that may be considered:

1. **Coats disease**: A rare eye disorder characterized by abnormal development of blood vessels in the retina, leading to retinal detachment and vision loss. While Coats disease can present with similar clinical features, it is typically unilateral and affects older children.
2. **Medulloepithelioma**: A rare, malignant tumor of the ciliary body or retina that can present with similar clinical features, including a white pupillary reflex and retinal detachment.
3. **Retinal astrocytoma**: A rare, benign tumor of the retina that can present with a similar clinical appearance, including a greyish-white mass in the posterior pole of the eye.
4. **Congenital retinal fold**: A rare, non-neoplastic condition characterized by a fold in the retina that can present with similar clinical features, including a white pupillary reflex and retinal detachment.
5. **Persistent fetal vasculature (PFV)**: A rare, congenital condition characterized by the persistence of fetal blood vessels in the eye, which can present with similar clinical features, including a white pupillary reflex and retinal detachment.

It’s worth noting that a definitive diagnosis of retinoblastoma or any of these alternative diagnoses would require further evaluation, including imaging studies (e.g., ultrasound, CT, or MRI) and potentially a biopsy or other diagnostic procedures.

### **107. User**

The average age of onset is 5.6 months. The first seizure is usually normal at birth and starts within 1 year of age. The first seizure is usually febrile convulsion, which may be one-sided or generalised clonic or tonic clonic seizure, and febrile convulsions are characterized by long duration and recurrent episodes, and may appear in a persistent state. 28%~35% of children may start the disease without febrile convulsions, but they often have heat sensitivity in the later course of the disease. Elevated body temperature due to various reasons, including exercise, fever, vaccination, hot baths, and high ambient temperatures, can trigger or exacerbate seizures and predispose to status epilepticus. Some children develop an acute encephalopathy characterised by deep coma following status epilepticus.

Children tend to present with febrile convulsions between the ages of 1 and 4 years, and have a variety of seizure types, such as myoclonic, atypical catatonic, partial seizures, and generalised tonic clonic seizures. Myoclonic seizures are often triggered by light stimulation, eye closure, and a specific graphic stimulus, and involve simultaneous clonus of one or several muscles, all accompanied by synchronised abnormal brain wave bursts. The intensity of the myoclonus varies, from a slight forward or backward movement of the body when only the axial muscles are violated, to a severe fall or sudden throwing of an object in the hand. Myoclonic seizures can be isolated or cluster, but a sustained state is rare. Myoclonic seizures may not occur at all in about 25% of children. The concurrent EEG of atypical apoplectic seizures shows half of the discharges to be of focal origin, most of which are irregular, irregular, slow spikes and slow waves, often accompanied by myoclonic seizures. 30% of the children may have atypical apoplectic persistent seizures. The incidence of partial seizures is 43.0% to 78.6%, which manifests as lateral limb (or) head deflection seizures or clonic seizures, and there are also motor seizures with consciousness and autonomic symptoms, automatisms, and hypotonia, etc. After 1 year of age, the children have psychomotor developmental delays, especially language developmental delays.

### Llama3.1:70b

Based on the clinical manifestations you provided, the most likely diagnosis is:

**Dravet Syndrome**

Dravet Syndrome is a rare genetic disorder that affects approximately 1 in 40,000 births. It is characterized by severe, uncontrolled seizures that begin in infancy, often triggered by fever or hot temperatures. The seizures can be prolonged and recurrent, and may lead to status epilepticus. Children with Dravet Syndrome often experience developmental delays, particularly in language and cognitive skills.

Here are five other possible diagnoses that may be considered based on the clinical manifestations:

1. **Lennox-Gastaut Syndrome**: A rare and severe form of epilepsy characterized by multiple seizure types, including atonic, tonic, and atypical absence seizures. Children with Lennox-Gastaut Syndrome often experience developmental delays and intellectual disability.
2. **Myoclonic-Astatic Epilepsy (Doose Syndrome)**: A rare form of epilepsy characterized by myoclonic seizures, often triggered by visual stimuli. Children with Doose Syndrome may experience developmental delays and intellectual disability.
3. **West Syndrome**: A rare and severe form of epilepsy characterized by infantile spasms, often accompanied by developmental regression. Children with West Syndrome may experience a range of seizure types, including myoclonic and tonic seizures.
4. **Epilepsy with Myoclonic-Atonic Seizures (EMA)**: A rare form of epilepsy characterized by myoclonic and atonic seizures, often triggered by visual stimuli. Children with EMA may experience developmental delays and intellectual disability.
5. **Generalized Epilepsy with Febrile Seizures Plus (GEFS+)**: A rare genetic disorder characterized by febrile seizures and a range of other seizure types, including myoclonic and tonic-clonic seizures. Children with GEFS+ may experience developmental delays and intellectual disability.

It’s essential to note that a definitive diagnosis can only be made by a qualified healthcare professional, such as a pediatric neurologist, after a comprehensive evaluation, including a thorough medical history, physical examination, EEG, and genetic testing.

### **108. User**

On the one hand, it presents as chronic haemolytic anaemia, and in addition to anaemia-related symptoms, it may cause mild to moderate yellowing of the skin and sclera and pigmented cholelithiasis due to elevated bilirubin, which may be exacerbated by cold, infection and dehydration. In infants and young children, splenomegaly may be present, and with age, the spleen may shrink due to splenic infarction and fibrosis.

On the other hand recurrent vaso-obstructive pain due to capillary micro thrombosis. Children and adults tend to present with myalgia of the limbs, large joint pain and low back pain, and in severe cases, severe abdominal pain, headache, and even limb paralysis and coma. Over time, vascular obstruction can occur in almost every organ system, and cyclical pain can occur in patients who are thin, easily fatigued, susceptible to infection, and malnourished. Thinning of the bone cortex, osteoporosis, spinal deformity, aseptic necrosis of the femoral head due to an overcompensatory increase in bone marrow haematopoietic tissue, and localised osteosclerosis if there is a bone infarction. Ocular symptoms may include fundus haemorrhage and retinal detachment due to embolism of the retinal vessels in the fundus. In addition, mental retardation may occur, affecting neurological development.

Acute pain is the first symptom of the disease in more than 25% of patients. It is most common in children older than 2 years of age. The severity of pain can vary from mild to severe. Approximately one-half of pain episodes are accompanied by objective clinical signs such as fever, swelling, tenderness, tachypnoea, hypertension, nausea and vomiting.

### Llama3.1:70b

Based on the clinical manifestations you provided, the most likely diagnosis is:

**Sickle Cell Disease (SCD)**

Sickle Cell Disease is a genetic disorder that affects hemoglobin production, leading to chronic hemolytic anemia, vaso-occlusive crises, and various systemic complications. The symptoms you described, such as chronic anemia, jaundice, splenomegaly, vaso-occlusive pain, and increased risk of infections, are all consistent with SCD.

Here are five other possible diagnoses that may be considered:

1. **Beta-Thalassemia Major**: This is a genetic disorder that affects hemoglobin production, leading to severe anemia, jaundice, and splenomegaly. While it shares some similarities with SCD, the clinical presentation is generally more severe, and vaso-occlusive crises are less common.
2. **Hereditary Spherocytosis**: This is a genetic disorder that affects the production of red blood cells, leading to chronic hemolytic anemia, jaundice, and splenomegaly. While it shares some similarities with SCD, the clinical presentation is generally milder, and vaso-occlusive crises are less common.
3. **Paroxysmal Nocturnal Hemoglobinuria (PNH)**: This is a rare acquired disorder that affects the production of red blood cells, leading to chronic hemolytic anemia, jaundice, and thrombosis. While it shares some similarities with SCD, the clinical presentation is generally more variable, and vaso-occlusive crises are less common.
4. **Thalassemia Intermedia**: This is a genetic disorder that affects hemoglobin production, leading to chronic anemia, jaundice, and splenomegaly. While it shares some similarities with SCD, the clinical presentation is generally milder, and vaso-occlusive crises are less common.
5. **Hemoglobin SC Disease**: This is a genetic disorder that affects hemoglobin production, leading to chronic hemolytic anemia, jaundice, and splenomegaly. While it shares some similarities with SCD, the clinical presentation is generally milder, and vaso-occlusive crises are less common.

It’s worth noting that a definitive diagnosis of SCD or any of these alternative diagnoses would require laboratory tests, such as hemoglobin electrophoresis, genetic testing, and other diagnostic evaluations.

### **109. User**

The main clinical manifestations of neonatal and paediatric patients include:

1. Intrauterine growth retardation and postnatal growth disorders . Intrauterine growth retardation, low birth weight, lack of postnatal catch-up growth, and proportional short stature.
2. Specific physical signs: wide and prominent forehead, pointed jaw leading to a triangular face, and large relative head circumference. Dysmorphic teeth, low ear position, and asymmetry of trunk deviation, including limb length discrepancy, and lateral curvature deformity of the little finger.
3. Other manifestations include feeding difficulties, hypoglycaemia in the neonatal period, genitourinary malformations such as hypospadias, cryptorchidism and horseshoe kidneys, and digestive anomalies such as cafe au lait spots and reflux oesophagitis. Some patients have cognitive, motor and language developmental delays and learning disabilities.

### Llama3.1:70b

Based on the clinical manifestations you provided, the most likely diagnosis is Silver-Russell Syndrome (SRS). SRS is a rare genetic disorder characterized by intrauterine growth restriction, postnatal growth failure, and distinct physical features, including a triangular face, prominent forehead, and limb length discrepancy.

Here are five other possible diagnoses that may be considered based on the clinical manifestations:

1. **Prader-Willi Syndrome**: This rare genetic disorder is characterized by growth restriction, feeding difficulties, and distinct physical features, including a narrow forehead, almond-shaped eyes, and a prominent jaw. While some features overlap with SRS, Prader-Willi Syndrome typically presents with more pronounced hypotonia and hyperphagia.
2. **Noonan Syndrome**: This genetic disorder is characterized by growth restriction, distinctive facial features (including a broad forehead and low-set ears), and various congenital anomalies, including heart defects and genitourinary malformations. While some features overlap with SRS, Noonan Syndrome typically presents with more pronounced cardiac abnormalities.
3. **Beckwith-Wiedemann Syndrome**: This rare genetic disorder is characterized by overgrowth, rather than growth restriction, and is often associated with distinctive physical features, including a large tongue and abdominal wall defects. However, some patients with Beckwith-Wiedemann Syndrome may present with growth restriction and other features that overlap with SRS.
4. **Kabuki Syndrome**: This rare genetic disorder is characterized by growth restriction, distinctive facial features (including a broad forehead and arched eyebrows), and various congenital anomalies, including heart defects and genitourinary malformations. While some features overlap with SRS, Kabuki Syndrome typically presents with more pronounced intellectual disability and behavioral abnormalities.
5. **3-M Syndrome**: This rare genetic disorder is characterized by growth restriction, distinctive facial features (including a broad forehead and prominent jaw), and various congenital anomalies, including heart defects and genitourinary malformations. While some features overlap with SRS, 3-M Syndrome typically presents with more pronounced skeletal abnormalities and a higher risk of cancer.

It is essential to note that a definitive diagnosis can only be made through a comprehensive evaluation, including genetic testing, imaging studies, and a thorough physical examination.

### **110. User**

**1. Xanthomas of the skin,** occurring near the attachment points of the extensor tendons, such as the Achilles tendon, elbow, knee, etc., and there is a close relationship between skin injuries and the appearance of xanthomas. Cutaneous xanthomas are rare in children.

**2. Atherosclerosis ,** Patients have a significantly higher risk of developing atherosclerosis and coronary artery disease than normal subjects. Early-onset coronary artery disease may be one of the characteristics of the disease.

**3. Alterations in the blood system ,** The content of plant sterols in the blood is significantly increased, resulting in a higher proportion of plant sterols in the sterol component of the cell membrane, abnormal cell membrane function of red blood cells, platelets and other cell membranes, anaemia and thrombocytopenia, and the patient may have weakness, pallor, skin bleeding and other manifestations.

**4. Others,** in addition to the above symptoms, some patients may develop joint pain, arthritis, splenomegaly, elevated transaminases, etc.

### Llama3.1:70b

Based on the clinical manifestations you provided, the rare disease that is most likely to be diagnosed is:

**1. Sitosterolemia**

Sitosterolemia is a rare genetic disorder characterized by the accumulation of plant sterols, such as sitosterol, in the body. The disease is caused by mutations in the ABCG5 and ABCG8 genes, which are responsible for the transport of plant sterols out of the body. The clinical manifestations of sitosterolemia include xanthomas of the skin, particularly near the attachment points of the extensor tendons, atherosclerosis, and alterations in the blood system, including anemia and thrombocytopenia.

Here are five other possible diagnoses that may be considered:

**2. Cerebrotendinous xanthomatosis (CTX)**: CTX is a rare genetic disorder caused by mutations in the CYP27A1 gene, leading to the accumulation of cholestanol in the body. Clinical manifestations include xanthomas of the skin, atherosclerosis, and neurological symptoms.

**3. Familial hypercholesterolemia (FH)**: FH is a genetic disorder caused by mutations in the LDLR gene, leading to high levels of low-density lipoprotein (LDL) cholesterol in the blood. Clinical manifestations include xanthomas of the skin, atherosclerosis, and an increased risk of coronary artery disease.

**4. Phytosterolemia**: Phytosterolemia is a rare genetic disorder caused by mutations in the ABCG5 and ABCG8 genes, similar to sitosterolemia. However, phytosterolemia is characterized by the accumulation of plant sterols, such as campesterol and stigmasterol, in the body.

**5. Lysosomal acid lipase deficiency (LAL-D)**: LAL-D is a rare genetic disorder caused by mutations in the LIPA gene, leading to the accumulation of lipids in the lysosomes of cells. Clinical manifestations include liver dysfunction, splenomegaly, and an increased risk of atherosclerosis.

**6. Niemann-Pick disease type B**: Niemann-Pick disease type B is a rare genetic disorder caused by mutations in the SMPD1 gene, leading to the accumulation of sphingomyelin in the lysosomes of cells. Clinical manifestations include liver dysfunction, splenomegaly, and an increased risk of atherosclerosis.

Note that these diagnoses are based on the clinical manifestations provided and may not be exhaustive. A comprehensive diagnostic evaluation, including genetic testing and laboratory studies, would be necessary to confirm the diagnosis.

### **111. User**

The onset of the disease is insidious in males, and the common age of onset is 30-60 years old. Patients often start with painful spasms, tremor, and weakness of the lower limbs, and gradually develop weakness of the upper limbs, muscle atrophy of the medulla oblongata and the face, muscle fasciculations, dysarthria, dysphagia, dyspnoea, and respiratory difficulties. Androgen receptor insensitivity may be associated with gynecomastia and reduced reproductive function. Sensory abnormalities are present in more than 50 per cent of patients. The disease progresses slowly, with patients usually becoming unable to walk late in the course of the disease, and only some patients require assisted ventilation, which has no significant impact on survival.

**1. Atypical symptoms,** some of which may occur years or even decades before the onset of limb weakness, include fatigability, painful spasms, perioral tremor, and postural tremor. Muscle weakness: most patients start with weakness of both lower limbs, gradually progressing to both upper limbs, tongue muscles and facial muscles, while the extraocular muscles are often not involved. The distribution of limb weakness is asymmetrical, with proximal involvement predominating. Involvement of the muscles of mastication may cause mandibular ptosis and tremor. Physical examination of the lower motor neuron damage signs, manifested as a decrease in muscle strength of the limbs, proximal, accompanied by muscle atrophy, fasciculations, tendon reflexes are weakened or disappeared. Most patients may have obvious tongue muscle atrophy and fibrillation, but the soft palate activity and pharyngeal reflexes are normal, and the degree of choking on water and dysphagia is mild. In some patients, chin and perioral fasciculations can also be observed. The motor function impairment of the patients gradually increases with the prolongation of the disease, and in the later stage, they may have difficulty in walking up the stairs and walking unsteadily.

**2. Sensory deficits** , with varying degrees of hyperalgesia present in about half of the patients, while in the others it is subclinical, with abnormalities found only on sensory nerve conduction tests.

**3. endocrine abnormalities,** including abnormalities of glucose and fat metabolism, and androgen receptor insensitivity and other manifestations, the latter including gynecomastia, decreased sexual function, infertility, and testicular atrophy.

The latter include gynecomastia, decreased sexual function, infertility, testicular atrophy, etc.

**4. Female carriers,** most female carriers do not have symptoms, and even if they do have symptoms, they are mild, such as fasciculations, mild weakness of distal limbs, muscle spasms, or increased creatine myosinase.

### Llama3.1:70b

Based on the clinical manifestations you provided, the most likely diagnosis is:

**Kennedy’s Disease (Spinal and Bulbar Muscular Atrophy, SBMA)**

This is a rare, X-linked recessive disorder caused by an expansion of CAG repeats in the androgen receptor gene. The disease primarily affects males, with an onset age of 30-60 years, and is characterized by progressive muscle weakness, atrophy, and fasciculations, particularly in the lower limbs, as well as androgen receptor insensitivity leading to endocrine abnormalities.

Other possible diagnoses to consider:

1. **Amyotrophic Lateral Sclerosis (ALS)**: While ALS is a more common motor neuron disease, some patients with Kennedy’s Disease may be misdiagnosed with ALS due to similar clinical features. However, ALS typically has a more rapid progression and often involves the upper motor neurons.
2. **Spinal Muscular Atrophy (SMA)**: SMA is a genetic disorder that affects the motor neurons, leading to muscle weakness and atrophy. However, SMA typically presents in infancy or early childhood, and the clinical features are more severe than those seen in Kennedy’s Disease.
3. **Myotonic Dystrophy**: This is a genetic disorder characterized by muscle weakness, wasting, and myotonia (delayed muscle relaxation). While some patients with Kennedy’s Disease may exhibit myotonia, the clinical features are distinct, and myotonic dystrophy typically involves other systems, such as the heart and eyes.
4. **Hereditary Spastic Paraplegia (HSP)**: HSP is a group of genetic disorders characterized by progressive lower limb weakness and spasticity. While some patients with Kennedy’s Disease may exhibit spasticity, the clinical features are distinct, and HSP typically does not involve the upper motor neurons or endocrine abnormalities.
5. **Friedreich’s Ataxia**: This is a genetic disorder characterized by progressive ataxia, dysarthria, and sensory deficits. While some patients with Kennedy’s Disease may exhibit sensory deficits, the clinical features are distinct, and Friedreich’s Ataxia typically involves the cerebellum and peripheral nerves.

It is essential to note that a definitive diagnosis of Kennedy’s Disease requires genetic testing to identify the expansion of CAG repeats in the androgen receptor gene.

### **112. User**

Some patients present with reduced foetal movement in utero and are born as floppy babies. The onset of the disease occurs within 6 months, with a mean age of onset of 1 month after birth. They present with generalised floppy weakness and severe hypotonia. Most children have difficulty sucking and swallowing due to weakness of the tongue, facial and masticatory muscles, and atrophy and tremor of the lingual muscles are seen. Intercostal muscle involvement can present with dyspnoea and abdominal breathing. The chest has a bell-shaped appearance. The lower limbs are more heavily involved than the upper limbs, and proximal is more severe than distal. Severe muscle weakness in the mid-trunk area leaves the child unable to control head movements, unable to lift or roll over, and without the ability to sit alone. When lying down, both lower limbs are in the frog-leg position with hip abduction and knee flexion. Muscle atrophy is not obvious, and some children have mild joint deformities. Children with good intelligence, tendon reflexes disappeared, limb sensation is normal. The children’s muscle weakness progressively worsens, and eventually they lose all voluntary movement ability, are fed nasogastrically, and suffer from respiratory failure due to repeated respiratory infections. 80% of the children die within 1 year of age, and rarely survive beyond 2 years of age.

### Llama3.1:70b

Based on the clinical manifestations you provided, the most likely diagnosis is:

**Spinal Muscular Atrophy (SMA) Type I, also known as Werdnig-Hoffmann disease**

This is a severe and rare genetic disorder that affects the nerve cells responsible for controlling voluntary muscle movement. The symptoms you described, such as generalized floppy weakness, severe hypotonia, difficulty sucking and swallowing, and progressive muscle weakness, are all consistent with SMA Type I.

Here are five other possible diagnoses that may be considered:

1. **Congenital Myotonic Dystrophy**: This is a rare genetic disorder that affects muscle tone and relaxation. While it shares some similarities with SMA, it typically presents with myotonia (difficulty relaxing muscles after contraction) and other distinct features.
2. **Congenital Muscular Dystrophy**: This is a group of rare genetic disorders that affect muscle strength and tone. While it shares some similarities with SMA, it typically presents with muscle weakness and wasting, as well as joint contractures and other skeletal abnormalities.
3. **Pompe Disease**: This is a rare genetic disorder that affects the breakdown of glycogen in muscles. It can present with muscle weakness, hypotonia, and respiratory difficulties, but typically also involves cardiac and other systemic symptoms.
4. **Myasthenia Gravis**: This is a rare autoimmune disorder that affects the nerve-muscle junction, leading to muscle weakness and fatigue. While it can present with similar symptoms to SMA, it typically involves fluctuating weakness and responsiveness to treatment with acetylcholinesterase inhibitors.
5. **Congenital Fiber-Type Disproportion**: This is a rare genetic disorder that affects the development of muscle fibers. It can present with muscle weakness, hypotonia, and other symptoms similar to SMA, but typically involves distinct histopathological features on muscle biopsy.

It’s worth noting that a definitive diagnosis of SMA Type I is typically made through a combination of clinical evaluation, genetic testing (e.g., SMN1 gene deletion), and electromyography (EMG) and muscle biopsy findings.

### **113. User**

Patients usually start in adulthood, with an insidious onset and gradual progression. The main manifestations are cerebellar ataxia, including drunkard’s gait, clumsy movements, minstrel-like speech, nystagmus, diplopia, etc.; pyramidal tract damage, including hyperreflexia, ankle clonus, and positive pathological signs; and extrapyramidal symptoms, including Parkinson’s syndrome, dystonia, and ankylosis. In addition, some patients may have peripheral neuropathy. In the early stages of the disease, some patients may have damage to the pyramidal tract (similar to spastic paraplegia) or extrapyramidal symptoms (similar to Parkinson’s syndrome), and ataxia is not prominent.

The disease progresses gradually, with loss of walking ability after 10-15 years. Prolonged dysphagia leading to malnutrition or aspiration is the main cause of death.

### Llama3.1:70b

Based on the clinical manifestations you provided, the most likely diagnosis is:

**Multiple System Atrophy (MSA)**

MSA is a rare, progressive neurodegenerative disorder characterized by a combination of cerebellar ataxia, pyramidal tract damage, and extrapyramidal symptoms, which matches the clinical presentation you described. The disease typically starts in adulthood and progresses gradually, leading to loss of walking ability and eventually death due to complications such as dysphagia and aspiration.

Here are five other possible diagnoses that may be considered:

1. **Friedreich’s Ataxia**: A rare genetic disorder that causes progressive damage to the nervous system, leading to cerebellar ataxia, pyramidal tract damage, and peripheral neuropathy. However, Friedreich’s Ataxia typically starts in childhood or adolescence, which may not match the adult onset described.
2. **Spinocerebellar Ataxia (SCA)**: A group of rare genetic disorders that cause progressive cerebellar ataxia, which may be accompanied by pyramidal tract damage and extrapyramidal symptoms. However, SCA typically has a more prominent ataxic component and may not always include extrapyramidal symptoms.
3. **Amyotrophic Lateral Sclerosis (ALS)**: A rare neurodegenerative disorder that affects motor neurons, leading to progressive muscle weakness and atrophy. While ALS can cause pyramidal tract damage and some patients may have cerebellar ataxia, the primary clinical feature is muscle weakness, which is not emphasized in the description.
4. **Progressive Supranuclear Palsy (PSP)**: A rare neurodegenerative disorder characterized by progressive damage to the brainstem and basal ganglia, leading to extrapyramidal symptoms, including Parkinson’s syndrome and dystonia. While PSP can cause some cerebellar ataxia, the primary clinical feature is supranuclear gaze palsy, which is not mentioned in the description.
5. **Ataxia-Telangiectasia**: A rare genetic disorder that causes progressive cerebellar ataxia, peripheral neuropathy, and immunodeficiency. While Ataxia-Telangiectasia can cause some extrapyramidal symptoms, the primary clinical feature is cerebellar ataxia, and the disease typically starts in childhood, which may not match the adult onset described.

Please note that these alternative diagnoses are speculative and may not be as likely as Multiple System Atrophy based on the clinical manifestations provided. A thorough diagnostic evaluation, including imaging studies, laboratory tests, and genetic analysis, would be necessary to confirm the diagnosis.

### **114. User**

The disease may be combined with gastro-oesophageal reflux in the early stages of the disease. As the disease progresses, the skin becomes swollen and sclerotic and may be accompanied by changes in skin pigmentation, including hyperpigmentation or depigmentation. In addition to skin involvement, patients often present with arthralgia and myalgia, and may develop ulcers and capillary dilatation of the digits. In addition, the patient may have multiple internal organ involvement. The respiratory system is more likely to be involved, with interstitial fibrosis and pulmonary hypertension. The gastrointestinal manifestations are varied and include gastroesophageal reflux, sinus vasodilatation, abdominal pain, diarrhoea, constipation and fecal incontinence. A small number of patients may present with renal crisis, which is characterised by acute renal insufficiency and is often associated with hypertension. Cardiac involvement may manifest as pericardial effusion, abnormalities of the cardiac conduction system, and myocardial involvement in individual patients. Neurological involvement of the peripheral nervous system is common, such as trigeminal neuropathy, peripheral neuropathy, etc. Central nervous system involvement is rare.

### Llama3.1:70b

Based on the clinical manifestations you provided, the rare disease that is most likely to be diagnosed is:

**Systemic Sclerosis (Scleroderma)**

Systemic sclerosis is a chronic autoimmune disease characterized by thickening and hardening of the skin (sclerosis) and internal organs, including the lungs, gastrointestinal tract, kidneys, and heart. The disease often presents with gastroesophageal reflux, skin thickening and sclerosis, arthralgia, myalgia, and digital ulcers, which are all consistent with the clinical manifestations you described.

Here are five other possible diagnoses that may be considered:

1. **Mixed Connective Tissue Disease (MCTD)**: MCTD is an autoimmune disease that combines features of systemic sclerosis, systemic lupus erythematosus, and rheumatoid arthritis. While it shares some similarities with systemic sclerosis, MCTD tends to have more prominent musculoskeletal and hematologic manifestations.
2. **Eosinophilic Fasciitis**: This is a rare inflammatory disease characterized by thickening and inflammation of the fascia, which can lead to skin sclerosis and internal organ involvement. However, eosinophilic fasciitis tends to have more prominent eosinophilia and fascial involvement.
3. **Sjögren’s Syndrome**: This is an autoimmune disease that primarily affects the exocrine glands, leading to dry eyes and dry mouth. However, Sjögren’s syndrome can also involve the skin, lungs, and other internal organs, and may present with similar manifestations to systemic sclerosis.
4. **Limited Systemic Sclerosis (CREST Syndrome)**: CREST syndrome is a subtype of systemic sclerosis characterized by Calcinosis, Raynaud’s phenomenon, Esophageal dysmotility, Sclerodactyly, and Telangiectasia. While it shares some similarities with systemic sclerosis, CREST syndrome tends to have more limited skin involvement and fewer internal organ manifestations.
5. **Undifferentiated Connective Tissue Disease (UCTD)**: UCTD is a condition characterized by the presence of autoimmune antibodies and clinical manifestations that do not meet the full criteria for a specific connective tissue disease, such as systemic sclerosis or lupus. UCTD can present with a wide range of symptoms, including skin, joint, and internal organ involvement.

It’s worth noting that these diagnoses are not mutually exclusive, and some patients may have overlapping features of multiple conditions. A thorough diagnostic evaluation, including laboratory tests, imaging studies, and clinical assessment, is necessary to determine the underlying diagnosis.

### **115. User**

In addition to clinical symptoms similar to phenylketonuria, children 1-3 months after birth also show symptoms of dopamine deficiency, such as dyskinesia, hypotonia, lethargy, nystagmus, dysphagia, etc. Symptoms related to serotonin deficiency, such as unresponsiveness, depression, insomnia, etc. Norepinephrine deficiency, such as low muscle tone in the trunk, ptosis, cerebellar dysgenesis, etc., may be associated with dopamine deficiency. Symptoms associated with norepinephrine deficiency, such as hypotonia of the trunk, ptosis, and cerebellar hypoplasia. There may also be persistent convulsions, recurrent fever, delayed development of motor milestones, total paralysis, and severe impairment of intellectual development.

### Llama3.1:70b

Based on the clinical manifestations you provided, the rare disease that is most likely to be diagnosed is:

**Aromatic L-Amino Acid Decarboxylase (AADC) Deficiency**

AADC deficiency is a rare genetic disorder that affects the production of neurotransmitters such as dopamine, serotonin, and norepinephrine. The symptoms you described, including dopamine deficiency (dyskinesia, hypotonia, lethargy, nystagmus, dysphagia), serotonin deficiency (unresponsiveness, depression, insomnia), and norepinephrine deficiency (low muscle tone in the trunk, ptosis, cerebellar dysgenesis), are consistent with AADC deficiency. Additionally, the presence of persistent convulsions, recurrent fever, delayed development of motor milestones, total paralysis, and severe impairment of intellectual development are also characteristic of this disorder.

Here are five other possible diagnoses that may be considered:

1. **Tyrosine Hydroxylase Deficiency**: This is another rare genetic disorder that affects the production of dopamine and norepinephrine. While the symptoms may overlap with AADC deficiency, tyrosine hydroxylase deficiency typically presents with more severe parkinsonian features.
2. **Dopa-Responsive Dystonia (DRD)**: This is a rare genetic disorder that affects the production of dopamine. While the symptoms may overlap with AADC deficiency, DRD typically presents with more prominent dystonic features and a better response to levodopa treatment.
3. **Monoamine Oxidase A (MAOA) Deficiency**: This is a rare genetic disorder that affects the breakdown of neurotransmitters such as serotonin, norepinephrine, and dopamine. While the symptoms may overlap with AADC deficiency, MAOA deficiency typically presents with more prominent behavioral and psychiatric features.
4. **Tetrahydrobiopterin (BH4) Deficiency**: This is a rare genetic disorder that affects the production of neurotransmitters such as dopamine, serotonin, and norepinephrine. While the symptoms may overlap with AADC deficiency, BH4 deficiency typically presents with more prominent hyperphenylalaninemia and a better response to BH4 supplementation.
5. **Pterin-4a-Carbinolamine Dehydratase (PCD) Deficiency**: This is a rare genetic disorder that affects the production of neurotransmitters such as dopamine, serotonin, and norepinephrine. While the symptoms may overlap with AADC deficiency, PCD deficiency typically presents with more prominent hyperphenylalaninemia and a better response to BH4 supplementation.

It’s essential to note that these diagnoses are based on the clinical manifestations provided and may not be definitive without further diagnostic testing, such as genetic analysis and biochemical assays.

### **116. User**

They often present with multisystem involvement and develop in stages. Cardiac rhabdomyosarcomas can appear in the foetal/neonatal period, most of which resolve spontaneously; epileptic and cutaneous symptoms can appear in the first few months of life; renal angiomyolipomas and neoplastic lesions elsewhere in the body develop with age; and adult women are prone to pulmonary lymphangioleiomyomatosis of the lungs. Brain lesions include mainly cerebral cortical nodules, subventricular giant cell astrocytomas, and subventricular nodules. Skin lesions include leukoplakia visible after birth, progressive facial angiofibromas, shark leather spots, perinodal fibromas, and forehead plaques; renal vascular smooth muscle lipomas are present in up to 90% of patients; retinal involvement is present in about 50% of patients but rarely affects visual acuity; and LAM in the lungs occurs in about 35% of females; other areas, such as the liver, pancreas, gastrointestinal tract, and skeleton, may be involved, although usually with milder symptoms. Others, such as the liver, pancreas, digestive tract, and bones, can also be involved, but symptoms are usually mild.

### Llama3.1:70b

Based on the clinical manifestations you provided, the most likely diagnosis is:

**Tuberous Sclerosis Complex (TSC)**

TSC is a rare genetic disorder characterized by the growth of non-cancerous tumors in various parts of the body, including the skin, brain, kidneys, and lungs. The clinical features you described, such as cardiac rhabdomyosarcomas, epileptic and cutaneous symptoms, renal angiomyolipomas, and pulmonary lymphangioleiomyomatosis, are all consistent with TSC.

Here are five other possible diagnoses that may be considered:

1. **Neurofibromatosis type 1 (NF1)**: While NF1 is a distinct condition, some of the skin lesions you described, such as café-au-lait spots and neurofibromas, can be similar to those seen in TSC.
2. **Sturge-Weber syndrome**: This rare disorder is characterized by a port-wine stain on the face, glaucoma, and seizures, which may be similar to some of the symptoms described.
3. **Von Hippel-Lindau disease**: This genetic disorder is characterized by the development of tumors in multiple organs, including the kidneys, pancreas, and adrenal glands, which may be similar to some of the symptoms described.
4. **Multiple endocrine neoplasia type 1 (MEN1)**: This rare genetic disorder is characterized by the development of tumors in multiple endocrine glands, which may be similar to some of the symptoms described.
5. **Birt-Hogg-Dubé syndrome**: This rare genetic disorder is characterized by the development of skin lesions, lung cysts, and renal tumors, which may be similar to some of the symptoms described.

It’s essential to note that a definitive diagnosis can only be made through a combination of clinical evaluation, imaging studies, and genetic testing.

### **117. User**

Most children with the undetected or untreated chronic form die before the age of 10 years, usually as a result of liver failure, neurological involvement or hepatocellular carcinoma.

The acute form has a rapid onset and progression, with acute liver failure as the main manifestation, and clinical signs include hepatosplenomegaly, jaundice, vomiting, abdominal distension, anorexia, lethargy, anaemia, bleeding tendencies and growth retardation. The child may have the characteristic odour of ‘boiled cabbage’ or ‘rotten mushrooms’. Insufficient hepatic synthesis of coagulation factors, marked prolongation of PT and APTT, and decreased levels of coagulation factors II, VII, IX, Ⅺ and Ⅻ, which are difficult to correct with vitamin K supplementation. Hepatic failure may be followed by ascites, jaundice and gastrointestinal bleeding, and children occasionally develop persistent hypoglycaemia. Untreated children often die of liver failure within weeks or months of onset of illness.

### Llama3.1:70b

Based on the clinical manifestations you provided, the rare disease that is most likely to be diagnosed is:

**Tyrosinemia Type 1**

Tyrosinemia Type 1 is a rare genetic disorder caused by a deficiency of the enzyme fumarylacetoacetate hydrolase (FAH), which leads to the accumulation of toxic metabolites in the liver. The disease is characterized by acute liver failure, hepatosplenomegaly, jaundice, vomiting, abdominal distension, anorexia, lethargy, anemia, bleeding tendencies, and growth retardation. The “boiled cabbage” or “rotten mushroom” odor is a distinctive feature of the disease. The disease can lead to liver failure, hepatocellular carcinoma, and death if left untreated.

Here are five other possible diagnoses that may be considered based on the clinical manifestations:

1. **Wilson’s Disease**: A rare genetic disorder caused by a defect in the ATP7B gene, leading to copper accumulation in the liver and brain. Clinical manifestations include liver failure, jaundice, vomiting, abdominal distension, anorexia, lethargy, and neurological symptoms.
2. **Glycogen Storage Disease Type IV**: A rare genetic disorder caused by a deficiency of the enzyme branching enzyme, leading to the accumulation of abnormal glycogen in the liver. Clinical manifestations include liver failure, hepatosplenomegaly, jaundice, vomiting, abdominal distension, anorexia, and lethargy.
3. **Hereditary Fructose Intolerance**: A rare genetic disorder caused by a deficiency of the enzyme aldolase B, leading to the accumulation of fructose-1-phosphate in the liver. Clinical manifestations include liver failure, jaundice, vomiting, abdominal distension, anorexia, lethargy, and hypoglycemia.
4. **Alpha-1 Antitrypsin Deficiency**: A rare genetic disorder caused by a deficiency of the enzyme alpha-1 antitrypsin, leading to liver damage and lung disease. Clinical manifestations include liver failure, jaundice, vomiting, abdominal distension, anorexia, lethargy, and respiratory symptoms.
5. **Citrin Deficiency**: A rare genetic disorder caused by a deficiency of the enzyme citrin, leading to the accumulation of citrulline and other amino acids in the liver. Clinical manifestations include liver failure, jaundice, vomiting, abdominal distension, anorexia, lethargy, and hypoglycemia.

Note that these diagnoses are based on the clinical manifestations provided and may not be exhaustive. A definitive diagnosis can only be made through genetic testing, biochemical analysis, and other diagnostic tests.

### **118. User**

The clinical presentation is markedly heterogeneous, and there are three types based on age of onset and clinical presentation.

**The cardiomyopathic type,** which is the most common type of severe early-onset cardiac and multi-organ failure, develops mainly in newborns and early infants. It has a high mortality rate and is associated with hypoglycaemia, Reye’s syndrome, sudden neonatal death, hypertrophic and dilated cardiomyopathy, pericardial effusion, arrhythmias, myasthenia, hepatomegaly, and intermittent hypoglycaemia, with elevated levels of creatine kinase; myocardial hypertrophy and arrhythmias can be fatal.

**2. hepatic type ,** less severe, hepatic and hypohidrotic hypoglycaemic type with onset in late infancy or childhood, presenting with hepatomegaly and liver function abnormalities, decreased fasting tolerance and acute hypohidrotic hypoglycaemia without myocardial damage and cardiac hypertrophy, but can be life-threatening without timely diagnosis and treatment.

**3. Myopathic type** , which develops mainly in adolescence to adulthood, is late-onset, mildly symptomatic, and is usually not accompanied by myocardial disease and hypoglycaemia. The main manifestations are rhabdomyolysis and myoglobinuria after exercise, infection or starvation, and even renal failure, which may be accompanied by muscle weakness, painful muscle spasms or myalgia.

### Llama3.1:70b

Based on the clinical manifestations you provided, the rare disease that is most likely to be diagnosed is:

**Very Long-Chain Acyl-CoA Dehydrogenase (VLCAD) Deficiency**

VLCAD deficiency is a rare genetic disorder that affects the body’s ability to convert certain fats to energy. The clinical presentation of VLCAD deficiency matches the three types you described:

1. **Cardiomyopathic type**: VLCAD deficiency can cause severe cardiac and multi-organ failure in newborns and early infants, leading to high mortality rates.
2. **Hepatic type**: The hepatic type of VLCAD deficiency presents with hepatomegaly, liver function abnormalities, and hypoglycemia, which is consistent with the clinical manifestations you described.
3. **Myopathic type**: The myopathic type of VLCAD deficiency typically presents in adolescence to adulthood with rhabdomyolysis, myoglobinuria, and muscle weakness after exercise, infection, or starvation.

Other possible diagnoses that may be considered based on the clinical manifestations include:

1. **Medium-Chain Acyl-CoA Dehydrogenase (MCAD) Deficiency**: Another rare genetic disorder that affects the body’s ability to convert certain fats to energy, which can present with similar clinical manifestations to VLCAD deficiency.
2. **Carnitine Palmitoyltransferase (CPT) Deficiency**: A rare genetic disorder that affects the body’s ability to transport fatty acids into the mitochondria for energy production, which can present with similar clinical manifestations to VLCAD deficiency.
3. **Long-Chain 3-Hydroxyacyl-CoA Dehydrogenase (LCHAD) Deficiency**: A rare genetic disorder that affects the body’s ability to convert certain fats to energy, which can present with similar clinical manifestations to VLCAD deficiency.
4. **Trifunctional Protein (TFP) Deficiency**: A rare genetic disorder that affects the body’s ability to convert certain fats to energy, which can present with similar clinical manifestations to VLCAD deficiency.
5. **Carnitine-Acylcarnitine Translocase (CACT) Deficiency**: A rare genetic disorder that affects the body’s ability to transport fatty acids into the mitochondria for energy production, which can present with similar clinical manifestations to VLCAD deficiency.

It’s essential to note that a definitive diagnosis can only be made through genetic testing and enzyme assays.

### **119. User**

**1. specific facial features,** periorbital subcutaneous tissue fullness, widened orbital distance, stellate irises, long midriffs, thick lips, wide mouths, and small jaws.

**2. cardiovascular disease,** elastin arteriopathy is present in 75% ~80% of patients and can affect any artery. Peripheral pulmonary stenosis (37% ~61%) is more common in infancy but improves with time. Supravalvular aortic stenosis is more common (35% ~65%) and worsens over time by age 5. Arterial stenosis leads to increased left heart resistance, cardiac hypertrophy, and heart failure. Other anomalies include renal artery stenosis, thoracic aortic stenosis, abdominal aortic stenosis, mitral valve prolapse and aortic valve insufficiency, mesenteric artery stenosis, and intracranial vascular anomalies. Hypertension can occur at any age and may be related in part to renal artery stenosis.

**3. Cognitive ability,** 75% of patients have mental retardation, which is usually mild. Cognitive impairment is characteristically manifested by relatively good short-term memory and verbal expression, but extreme weakness in visuospatial structural cognition. No gender differences in IQ have been reported, and children’s IQ is stable over time.

**4. personality, behavioural, and psychological abnormalities,** including excessive friendliness, excessive empathy, attention deficit, anxiety, and fear.

**5. sleep abnormalities,** with a 65% prevalence of sleep problems, including increased sleep latency and decreased sleep efficiency.

**6. eyes,** tear duct obstruction, hyperopia (67%), and strabismus (50%) are common. Cataracts have also been reported in adult patients.

**7. Ear, Nose, and Throat (ENT),** 50 per cent of patients have chronic otitis media, 90 per cent have increased sensitivity to sound, and 63 per cent of children and 92 per cent of adults have mild to moderate hearing impairment, mostly progressive sensorineural hearing loss. Most patients are hoarse or have a low voice.

**8. dental** , which may include baby teeth, enamel hypoplasia, and a misaligned bite.

**9. digestive system,** Chronic abdominal pain is a common symptom, and possible etiologies include gastroesophageal reflux, hiatal hernia, peptic ulcer, cholelithiasis, diverticulitis, ischaemic bowel disease, chronic constipation and anxiety somatisation. Hypercalcaemia may also lead to irritability, vomiting, constipation and muscle spasms.

**10. urinary system,** urinary frequency and enuresis are common in paediatric patients.

**11. neurological, muscular,** with low muscle tone and joint laxity in young children, leading to compensatory postural abnormalities to maintain body balance. Older children and adults have typical hypertonia and active deep tendon reflexes, resulting in a stiff and awkward gait, impaired fine motor function, and difficulty using tools and writing in patients of all ages. Adult patients will have cerebellar ataxia and tremor.

**12. Growth and development** .70% of infants have poor weight gain.

**13. idiopathic hypercalcaemia** . It occurs in infancy and childhood in 15-50% of patients and the most common symptoms are irritability, vomiting and constipation.

**14. endocrine.** Hypothyroidism (10 per cent), early puberty (50 per cent). Impaired glucose tolerance occurs in 26% of adolescent patients.

### Llama3.1:70b

Based on the clinical manifestations you provided, the most likely diagnosis is:

**Williams Syndrome**

Williams syndrome is a rare genetic disorder characterized by a distinctive set of facial features, cardiovascular disease, cognitive impairment, and other systemic abnormalities. The clinical manifestations you listed, such as the specific facial features, elastin arteriopathy, cardiovascular disease, cognitive impairment, and behavioral abnormalities, are all consistent with Williams syndrome.

Here are five other possible diagnoses that may be considered:

1. **Noonan Syndrome**: This is a genetic disorder that shares some similarities with Williams syndrome, including cardiovascular disease, cognitive impairment, and distinctive facial features. However, Noonan syndrome typically has a different set of facial features and is often associated with coagulopathy.
2. **Fragile X Syndrome**: This is a genetic disorder that is the most common cause of inherited intellectual disability. While it shares some similarities with Williams syndrome, such as cognitive impairment and behavioral abnormalities, it typically has a different set of physical features and is often associated with a long face and large ears.
3. **Prader-Willi Syndrome**: This is a genetic disorder that is characterized by short stature, intellectual disability, and a range of systemic abnormalities, including cardiovascular disease and sleep apnea. While it shares some similarities with Williams syndrome, it typically has a different set of physical features and is often associated with hyperphagia and obesity.
4. **Smith-Magenis Syndrome**: This is a genetic disorder that is characterized by a range of systemic abnormalities, including cardiovascular disease, cognitive impairment, and behavioral abnormalities. While it shares some similarities with Williams syndrome, it typically has a different set of physical features and is often associated with a deletion on chromosome 17.
5. **Kabuki Syndrome**: This is a rare genetic disorder that is characterized by a range of systemic abnormalities, including cardiovascular disease, cognitive impairment, and distinctive facial features. While it shares some similarities with Williams syndrome, it typically has a different set of physical features and is often associated with a range of other systemic abnormalities, including skeletal and gastrointestinal anomalies.

It’s worth noting that these diagnoses are all relatively rare and may require genetic testing and other diagnostic evaluations to confirm. A comprehensive medical evaluation and genetic testing would be necessary to determine the underlying diagnosis.

### **120. User**

**1. Haemorrhagic tendency** is a common manifestation of the disease, especially in blood-stained stools, and most children develop the disease in the neonatal period. The next manifestations are petechiae, ecchymosis, haemoptysis and haematuria, and some severe cases may have life-threatening gastrointestinal haemorrhage or intracranial haemorrhage. Bleeding in patients is caused by thrombocytopenia with reduction in platelet volume, but only about 25% of cases have the typical triad of symptoms.

**2. Infection.** Children are susceptible to a variety of infections, including herpes simplex virus, cytomegalovirus, Streptococcus pneumoniae, and fungi. The patient’s immunodeficiency worsens with age.

**3. Eczema.**  Eczema may be present in approximately 80% of children with WAS and varies widely in extent and severity. There may be typically severe, refractory eczema, or very mild or even no eczema manifestations.

**4. Autoimmune diseases.**  The most common is autoimmune haemolytic anaemia. Others include vasculitis, arthritis, renal disease, inflammatory bowel disease, neutropenia and autoimmune thrombocytopenic purpura.

**5. malignant tumours .** Children have a significantly higher risk of malignant tumours of the lymphatic system, especially lymphomas; non-Hodgkin’s lymphoma, lymphoblastic leukaemia, myelodysplastic syndromes, and spermatogonia have been reported.

**6. other .** Children with this disease may have completely normal platelet levels with persistent or recurrent neutropenia.

### Llama3.1:70b

Based on the clinical manifestations you provided, the most likely diagnosis is:

**Wiskott-Aldrich Syndrome (WAS)**

WAS is a rare X-linked recessive disorder characterized by eczema, thrombocytopenia, immune deficiency, and a predisposition to autoimmune diseases and lymphoid malignancies. The clinical features you described, including hemorrhagic tendency, infections, eczema, autoimmune diseases, and increased risk of malignant tumors, are all consistent with WAS.

Here are five other possible diagnoses that may be considered:

1. **X-linked thrombocytopenia**: This is a milder form of WAS, characterized by thrombocytopenia and bleeding tendency, but without the other features of WAS.
2. **Immune thrombocytopenic purpura (ITP)**: ITP is an autoimmune disease characterized by thrombocytopenia and bleeding tendency, but it does not typically present with eczema, infections, or autoimmune diseases.
3. **Severe combined immunodeficiency (SCID)**: SCID is a group of disorders characterized by severe immune deficiency, which can present with recurrent infections and autoimmune diseases, but it does not typically present with thrombocytopenia or eczema.
4. **Bleeding disorder due to platelet dysfunction**: This is a group of disorders characterized by platelet dysfunction, which can present with bleeding tendency, but it does not typically present with eczema, infections, or autoimmune diseases.
5. **Autoimmune lymphoproliferative syndrome (ALPS)**: ALPS is a disorder characterized by autoimmune diseases, lymphadenopathy, and splenomegaly, which can present with thrombocytopenia and bleeding tendency, but it does not typically present with eczema or infections.

Note that these alternative diagnoses may not fully explain all the clinical features you described, and WAS remains the most likely diagnosis based on the combination of symptoms and signs presented.

### **121. User**

The most common clinical manifestations are fulminant infectious mononucleosis, anomalous immunoglobulinaemia and B-cell lymphoma following EBV infection. Aberimmunoglobulinaemia and B-cell lymphoma can occur in patients without EBV infection. Aberimmunoglobulinaemia usually presents with increased levels of IgA and IgM and decreased levels of IgG1 and IgG3. Lymphomas are predominantly non-Hodgkin B lymphomas. Other clinical manifestations include lymphocytic vasculitis, aplastic anaemia and lymphomatoid granulomatosis. In some patients, the clinical picture is mild, with only abnormal immunoglobulinaemia or mild EBV infection, possibly due to somatic gene repair.

In some patients, the predominant clinical manifestation is a haemophagocytic syndrome, with splenomegaly, which does not necessarily require EBV infection to be induced. Some patients may present with intestinal inflammation such as Crohn’s disease or colitis, but rarely develop lymphoma.

### Llama3.1:70b

Based on the clinical manifestations you provided, the rare disease that is most likely to be diagnosed is:

**X-linked Lymphoproliferative Disease (XLP)**

XLP is a rare genetic disorder caused by mutations in the SH2D1A or XIAP genes, which are involved in the regulation of immune responses. The clinical manifestations you described, such as fulminant infectious mononucleosis, anomalous immunoglobulinaemia, and B-cell lymphoma following EBV infection, are consistent with XLP. Additionally, the presence of lymphocytic vasculitis, aplastic anaemia, and lymphomatoid granulomatosis in some patients also supports this diagnosis.

Here are five other possible diagnoses that could be considered based on the clinical manifestations:

1. **Autoimmune Lymphoproliferative Syndrome (ALPS)**: This rare disorder is characterized by abnormal lymphocyte proliferation, autoimmune cytopenias, and increased risk of lymphoma. While the clinical manifestations are similar to XLP, ALPS is typically associated with mutations in the FAS or FASLG genes.
2. **Common Variable Immunodeficiency (CVID)**: CVID is a heterogeneous group of disorders characterized by impaired antibody production and increased susceptibility to infections. Some patients with CVID may present with lymphoma or autoimmune manifestations, but the clinical picture is often milder than XLP.
3. **Wiskott-Aldrich Syndrome (WAS)**: WAS is a rare X-linked disorder characterized by eczema, thrombocytopenia, and immunodeficiency. While the clinical manifestations are distinct from XLP, some patients with WAS may develop lymphoma or autoimmune disorders.
4. **Chronic Active EBV (CAEBV) Disease**: CAEBV is a rare disorder characterized by persistent EBV infection and chronic inflammation. While the clinical manifestations are similar to XLP, CAEBV is not typically associated with genetic mutations or familial inheritance.
5. **Multicentric Castleman Disease (MCD)**: MCD is a rare disorder characterized by lymphoid hyperplasia and autoimmune manifestations. While the clinical picture is distinct from XLP, some patients with MCD may develop lymphoma or autoimmune disorders.

It is essential to note that a definitive diagnosis can only be made through genetic testing and careful evaluation of the patient’s clinical manifestations and family history.
