# Supplementary material for: Performance of ChatGPT-4o and Four Open-Source Large Language Models in Generating Diagnoses Based on China’s Rare Disease Catalog: Comparative Study
Source: J Med Internet Res. 2025 Jun 18;27:e69929. doi: 10.2196/69929 (PMC12192912; doi:10.2196/69929)
Supplement: Multimedia Appendix 1 [file jmir-v27-e69929-s001.zip › numbered_qwen2.5_7b_cn.docx]

# numbered_qwen2.5_7b_cn

**1. user 2024-11-30T19:23:19Z:**

该疾病可于儿童期早期起病。典型的临床表现包括肾脏、眼部改变及听力受损。肾脏表现为血尿、蛋白尿和肾功能进行性恶化，其中血尿常为持续性镜下血尿，可在运动后或发热时出现肉眼血尿，常见于青少年型。听力改变为感音神经性听力损失， 并随病程逐渐加重。眼部异常表现为近视、青年环和白内障，但缺乏特异性。三种具有诊断意义的眼部改变包括：前圆锥形晶状体、后多形性角膜萎缩和视网膜斑（视网膜中心凹区域周围的白色或黄色颗粒）。我国男性患者听力受损发生率高于国外报道（68％比 55％），女性则低于国外报道（7％比 45％），眼异常发生率与国外报道相近。少数患者还可出现平滑肌瘤，可累及呼吸道、胃肠道及女性生殖道；偶有动脉瘤病变、面中部发育异常和精神发育迟滞的报道。

患者常有家族史。常染色体隐性遗传患者和 X 连锁遗传的男性患者病情进展较快，常于 16~35 岁进入终末期肾病；而常染色体显性遗传和 X 连锁遗传的女性患者多病程较为缓慢，肾功能衰竭出现较晚。在同一个患病家系中，所有男性成员发生肾功能衰竭的年龄相似，但不同家系中的男性成员发生肾功能衰竭的年龄可以不同。因此，可根据患病家系发生肾功能衰竭的年龄分为：青少年型（肾衰竭发生在青少年时期） 与成人型（肾衰竭发生在成年以后）。青少年型肾外表现更为突出，男孩通常在繁衍后代前即死亡，致病基因常为新发突变；而成人型家系中的患者较年长，少为新发突变。

**qwen2.5_7b** 2024-11-30T19:23:25Z:

根据您提供的临床症状和遗传特点，这种疾病最有可能是**Alport综合征**（阿波特综合症）。这是一种与X染色体或常染色体相关的遗传性肾病。

除了Alport综合征外，还有以下五种可能的诊断： 1. **Goodpasture 综合征** - 一种罕见的自身免疫性疾病，可以导致肺出血和肾炎。 2. **Fabry 病** - X连锁隐性遗传疾病，会导致神经病变、肾脏问题以及皮肤症状等。 3. **局灶节段性肾小球硬化症 (FSGS)** - 这是一种影响肾脏的慢性病症，可能会有类似Alport综合征的症状，但病因不同。 4. **多囊肾病** - 一种遗传性疾病，会导致肾脏充满多个囊肿并可能伴有其他症状如高血压等。 5. **IgA 肾病** - 一种常见的原发性肾小球疾病，可以导致血尿和蛋白尿等症状。

每种疾病的诊断通常需要结合详细的家族史、临床表现以及实验室检查结果，包括基因检测、血液和尿液分析以及其他影像学检查。

**2. user 2024-11-30T19:23:59Z:**

患者在新生儿期常无异常表现。首发症状是 6 月龄左右出现发育迟滞表现，但典型临床表现多在 1 岁后出现，临床确定诊断往往需要数年时间。按照临床表现出现频率不同分为：均出现的表现（100％患者）、经常性表现（80％以上患者）、相关性表现（20％ ~80％ 患者）3 类。

**1. 均出现的表现** 正常孕产史和出生头围；无出生缺陷和生化指标异常；颅脑 MRI/CT 除轻微皮质萎缩、髓鞘发育不良外，多无结构异常；运动里程碑落后而无倒退；6~12 个月出现严重发育迟缓；语言障碍显示为无或极少量词汇，重复性语言和非语言交往能力强于表达性语言能力；运动或平衡障碍常表现为共济失调以及四肢震颤；异常的行为特征表现为频繁大笑或微笑、明显的兴奋动作或快乐举止、常伴拍手或多动。

**2. 经常性表现** 头围增长落后，随访至 2 岁仍表现为小头畸形；3 岁前常出现癫痫；异常脑电图：特征性高波幅棘 - 慢波。

**3. 相关性表现** 枕部扁平；喜吐舌；吸吮或吞咽障碍；婴儿期喂养困难；肌张力低；巨大下颌；牙间隙宽；频繁流涎；过度咀嚼动作；斜视；皮肤色素减退，与家人相比头发和眼睛颜色浅（仅见于缺失型）；下肢腱反射亢进；上举或弯曲上肢，尤其是行走时；步基宽；对热敏感性高；异常睡眠 - 觉醒周期、睡眠少；迷恋水、纸、塑料；进食相关行为异常；肥胖（多见于年长、非缺失型患者）；脊柱侧凸；便秘。

**qwen2.5_7b** 2024-11-30T19:24:05Z:

根据您提供的临床表现，这种疾病最可能是一种罕见的神经发育障碍疾病，具体可能是**Rett综合征**（RTT）。这是一种由MECP2基因突变引起的X连锁遗传病，主要影响神经系统和运动功能。

以下是其他五种可能导致类似症状的疾病：

1. **Angelman综合征**：也是一种与神经发育相关的罕见遗传性疾病，通常也表现为智力障碍、语言发展迟缓及特定的行为问题。
2. **脆性X综合症**（Fragile X Syndrome）：是一种由FMR1基因突变引起的最常见的遗传性智力障碍原因。
3. **Tuberous Sclerosis Complex (TSC)**：这是一种多系统受累的遗传病，可以导致大脑、皮肤和其他器官形成肿瘤或结节。它也可能表现为发育迟缓和行为问题。
4. **Childhood Disintegrative Disorder (CDD)**（自闭症谱系障碍的一种形式）：这种罕见疾病的特点是在正常发展一段时间后突然出现倒退，包括语言、社交技能的丧失以及可能出现的行为改变。
5. **Pendred综合征**：这是一种导致甲状腺功能减退和耳聋的遗传病。虽然它主要影响内分泌系统和听力，但有时也可能伴随有神经发育迟缓的症状。

需要注意的是，这些疾病的诊断需要综合考虑详细的临床表现、家族史及基因检测等多种因素。建议进一步进行相关基因检测以明确诊断。

**3. user 2024-11-30T19:24:35Z:**

1. 骨骼异常表现：疾病严重程度和相关并发症的类型存在差异。所有 病例均存在胸腔狭窄，胸廓可呈钟形或长而窄，呼吸窘迫通常由小而僵硬的胸腔引起。根据胸廓类型和潜在的肺发育不良的程度，呼吸功能不全严重程度不同，发病年龄不同。呼吸功能不全严重程度与 该疾病诊断年龄呈负相关。存在严重的限制性胸廓和钟形胸廓的婴儿更多见急性呼吸系统疾病，在新生儿早期可出现严重和致命的呼吸衰竭。胸腔长而窄的婴儿，更多表现为轻度呼吸系统受累，最终可逐渐发展为慢性限制性肺病伴反复呼吸道感染。肋骨短小、畸形引起肋间肌无力、呼吸受限，进而出现呼吸衰竭。多数患儿于 2 岁内死亡，其中约有半数患儿于生后 6 个月内死亡。患者其他骨骼异常包括肋骨短、四肢短小、骨盆形状异常、短指、多指（趾） 畸形等。约有 60％患者伴有近端颈椎狭窄。

1. 其他脏器受累表现： 约 40％的患者会出现肾脏病变。患者肾脏病变的最早表现是尿液浓缩功能受损，其他表现包括多尿、蛋白尿、血尿素氮和肌酐升高以及高血压。肾功能衰竭是 该患者在 3~10 岁死亡的主要原因。约 30％的病例出现肝脏受累，表现为肝肿大、转氨酶升高、胆汁淤积、肝硬化和门静脉高压。该疾病偶与眼睛发育异常相关，包括视神经发育不全、视网膜营养不良、视网膜色素异常、眼球震颤、近视和进行性视力障碍。亦有少数报道提示这些患者也可出现神经系统异常如发育落后、肌张力低下、中枢神经系统MRI 异常等。

**qwen2.5_7b** 2024-11-30T19:24:40Z:

根据您提供的临床表现，最有可能的诊断是特发性婴儿型肺纤维化（也称为婴儿型限制性肺疾病），这是一种罕见且进行性的肺部疾病，主要影响胸廓和呼吸功能。以下是五个其他可能的诊断：

- **法布里病**：一种罕见遗传代谢疾病，可导致肺部、心脏等多个器官系统的损害，并伴有肾脏问题。
- **戈谢病（Gaucher disease）**：另一种罕见的溶酶体贮积症，可以引起骨骼异常和呼吸困难等症状。
- **结节性硬化症**：这是一种多系统受累的遗传性疾病，可能会导致肺部和肾脏等器官出现问题，并伴有神经系统症状。
- **先天性肺发育不良伴胸廓畸形**：一些罕见的先天性疾病如VACTERL关联（包括食道、肛门、心脏等问题）也可能表现出类似的临床特征。
- **黏多糖贮积症（MPS）系列疾病**：这是一种由于溶酶体中特定酶缺失导致的代谢异常，可能会导致骨骼和器官发育问题。

请注意，最终的确诊需要通过详细的病史采集、体检以及辅助检查如影像学、生物化学检测等来确定。建议与专业的儿科医生或罕见病专家进行深入讨论以获得准确诊断。

**4. user 2024-11-30T19:25:51Z:**

主要症状包括精神行为异常、认知障碍、记忆力下降、癫痫发作、言语障碍、运动障碍、不自主运动，意识水平下降、自主神经功能障碍等。其他伴随症状包括睡眠障碍、CNS 局灶性损害、脑干、小脑症状以及周围神经和神经肌肉接头受累等。

临床可分为 3 种主要类型：①抗 NMDAR 脑炎，是 最主要类型，其特征性临床表现符合弥漫性脑炎。②边缘性脑炎，以精神行为异常、颞叶癫痫和近记忆力障碍为主要症状，常见的边缘性脑炎有抗 LGI1 抗体、抗 GABABR 抗体与抗 AMPAR 抗体相关的脑炎。③其他类型，包括莫旺综合征、抗 DPPX 抗体相关脑炎、抗 IgLON5 抗体相关脑病、自身免疫性小脑性共济失调等。

**qwen2.5_7b** 2024-11-30T19:25:57Z:

根据您提供的临床表现和分类信息，主要症状和伴随症状指向几种与中枢神经系统（CNS）相关的自身免疫性疾病。最有可能的诊断是抗NMDAR脑炎，但考虑到其他可能性，以下是五个可能的替代诊断：

- **抗LGI1抗体相关脑炎** - 通常表现为精神行为异常、颞叶癫痫及近记忆力障碍。
- **抗GABABR抗体相关脑炎** - 这种类型也涉及到认知功能和记忆问题，并且常伴有癫痫发作等神经症状。
- **抗AMPA受体脑炎** - 涉及的精神和行为问题以及与NMDAR相关的症状类似，但特定于AMPA受体。
- **莫旺综合征（Mowat-Wilson Syndrome, MWS）** - 虽然是一种遗传性疾病而非自身免疫病，但它涉及类似的认知障碍、癫痫发作和发育迟缓等症状。
- **抗DPPX抗体相关脑炎** - 可能导致与上述症状相似的认知功能障碍及运动异常。

请注意，这些诊断需要通过详细的临床评估（包括病史采集、神经系统检查等）以及实验室检测（如血清学检查、脑脊液分析和电生理检查）来最终确定。在实际诊疗过程中，医生会根据具体患者的情况选择最合适的检查方法以明确诊断。

**5. user 2024-11-30T19:27:15Z:**

疾病临床表现复杂多样，缺乏特异性。多累及神经、皮肤、呼吸、消化和免疫等多系统，单从临床表现难以确诊。可以分为完全性及部分性两种类型。

- 完全性： 又可分为早发型及晚发型两种。①早发型患者多在新生儿或婴儿早期起病，通常症状出现在生后早期起病，通常症状出现在生后 1 周至 10 岁期间，平均发病年龄为 3 个月，最常见的表现为神经系统症状，如肌张力低下、癫痫、发育迟缓、感音性耳聋、共济失调、进行性意识障碍等，大约一半的患者出现共济失调，发育迟缓，结膜炎和视觉问题，包括视神经萎缩。常合并代谢性酸中毒、低血糖、酮症等代谢紊乱，皮肤表现包括各种形态的难治性皮疹如湿疹、全身性红斑、尿布疹，患者也可出现过度通气、喉部喘鸣和呼吸暂停等呼吸系统症状，此外还有消化系统的症状如喂养困难、呕吐、腹泻等。感染、发热、疲劳、高蛋白饮食等可诱发急性发作。若治疗不及时，常可导致不可逆损害。②晚发型患者常于青春期发病，之前可无任何症状，临床表型较早发型不同，通常表现为周围神经病，肌无力、痉挛性轻瘫，皮疹和眼部问题，如视力丧失和视力下降等，如不能及时治疗，常导致不可逆性损害。
- 部分性 ： 可在各个年龄段发病，甚至不发病。患儿整体症状及体征相对较轻，仅在感染、饥饿等应急状态下起病。患者可能有肌张力减退、皮疹、脱发，可出现急性神经系统症状癫痫、意识障碍等。

**qwen2.5_7b** 2024-11-30T19:27:21Z:

根据您描述的临床表现，该疾病可能涉及多种系统，包括神经、皮肤、呼吸和消化等多个方面，并且分为完全性和部分性两种类型，因此这最有可能是**线粒体病（尤其是肌阵挛-肌萎缩-视网膜病变综合征）**。以下是五种其他可能的罕见疾病：

- **Leigh 综合征**: 也称为 Leigh 综合征或亚急性坏死性脑炎，表现为进行性神经退化、肌张力减退和共济失调等症状。
- **MERRF 综合征 (Myoclonus Epilepsy Renal Failure)**: 表现为癫痫发作、肌阵挛和肾功能不全等，符合部分症状描述。
- **Kearns-Sayre 综合征**: 一种线粒体遗传病，表现为慢性进行性眼外肌麻痹、心脏传导阻滞及神经系统症状。
- **Mitochondrial DNA Depletion Syndrome (MDDS)**: 包括多种亚型，主要特征为神经发育迟缓和多器官受累，包括肌肉无力、共济失调等。
- **Leber遗传性视神经病变**: 主要表现为双侧进行性视神经萎缩导致的视力丧失，有时伴随其他神经系统症状。

**6. user 2024-11-30T19:28:23Z:**

高发年龄段为婴儿期，表现为以失盐为主的肾上腺皮质功能不全，常见症状包括高血钾、低血钠与脱水、代谢性酸中毒、低血糖、呕吐、休克、皮肤色素沉着等。多数患者在青春期表现为不发育或发育不完全，或生育能力受损，少精和无精症等。少数患者可以表现为儿童早期高雄激素血症、单纯性盐皮质激素缺乏，青春期可出现假性性早熟或自发性青春发育等。轻者可成年期起病。

患者皮肤有色素沉着，尤其是在暴露处、摩擦处、瘢痕及乳晕、腋窝、阴囊处。

患者对感染、外伤等各种应激的抵抗力有不同程度的减弱。在发生应激情况时，可出现肾上腺危象，表现为恶心、呕吐、腹痛或腹泻、严重脱水、血压降低、心率快、脉细弱、精神失常，常有高热、低血糖症、低钠血症，甚至危及生命。

**qwen2.5_7b** 2024-11-30T19:28:31Z:

根据您提供的临床表现，这种疾病最可能是一种先天性肾上腺皮质增生症（CAH）中的21-羟化酶缺乏症（21-Hydroxylase Deficiency, 21-OHD），这是最常见的先天性肾上腺皮质增生症类型。具体来说：

- **临床表现**：
  - 高发年龄段为婴儿期，表现为以失盐为主的肾上腺皮质功能不全。
  - 常见症状包括高血钾、低血钠与脱水、代谢性酸中毒、低血糖、呕吐、休克等。
  - 皮肤色素沉着，尤其是在暴露处、摩擦处、瘢痕及乳晕、腋窝、阴囊处。
  - 对感染、外伤等各种应激的抵抗力减弱，发生肾上腺危象时可出现恶心、呕吐、腹痛或腹泻、严重脱水、血压降低等症状。
- **其他可能诊断**：
  - 先天性肾上腺增生症（CAH）其他类型，如17α-羟化酶缺乏症和3β-羟类固醇脱氢酶缺乏症。
  - 肾上腺皮质功能减退症（Addison病），表现为皮肤色素沉着、低钠血症等。
  - 代谢性疾病：某些类型的代谢性疾病也会出现高钾血症等症状，如某些氨基酸或有机酸代谢障碍。
  - 其他内分泌失调疾病：甲状腺功能低下或亢进也可能有类似症状表现。
  - 心脏或其他系统疾病：一些心脏问题或其他系统的慢性疾病可能会模仿应激反应的症状。

请注意，最终的确诊需要结合详细的临床检查、实验室检测和影像学评估等多方面信息。建议根据患者的实际情况进一步进行详细的医学检查以明确诊断。

**7. user 2024-11-30T19:30:08Z:**

从出生到成年早期均可发病。其中新生儿起病最为常见，常表现为巨大儿、摄食不良、不耐受饥饿和持续性低血糖等。低血糖发作时可表现为嗜睡、肌张力低下等，也可表现为神经系统后遗症甚至死亡，如呼吸暂停、癫痫发作和昏迷等。在迟发性类型中，患者常表现为低血糖反应，包括面色苍白、出汗和心悸等症状。

**qwen2.5_7b** 2024-11-30T19:30:16Z:

根据您描述的症状，这些临床表现最有可能与先天性丙酮酸激酶缺乏症相关，这是一种罕见的遗传代谢病，特点是由于红细胞中的关键酶（丙酮酸激酶）缺失或活性降低，导致红细胞能量供应不足，引发低血糖等症状。

除了先天性丙酮酸激酶缺乏症外，以下几种疾病也需考虑：

- **戈谢病**：这是一种溶酶体贮积症，由于葡萄糖脑苷脂酶的缺陷，导致葡萄糖脑苷脂在巨噬细胞中积累。新生儿和婴儿期发病时可出现巨大儿、生长迟缓、肝脾肿大等。
- **Mucolipidosis IV (MLIV)**：这是一种罕见的遗传性代谢病，主要影响神经系统的发育，表现为生长迟缓、骨骼异常、智力障碍以及面部畸形等。
- **半乳糖血症**：由于缺乏催化半乳糖-1-磷酸尿苷转移酶（Gal-1-P UDPGT）活性的酶，导致食物中的半乳糖无法正常代谢。常见症状包括喂养困难、低血糖、黄疸、肝脾肿大和白内障等。
- **Zellweger 综合征**：也称为婴儿肾上腺脑白质营养不良（Infantile Refsum Disease），是一种罕见的溶酶体贮积症，表现为新生儿期生长发育迟缓、肌张力减退、共济失调、肝脾肿大等。
- **同型半胱氨酸尿症**：这是一种由于维生素B12代谢相关酶缺陷导致的疾病。患者可出现神经系统症状如智力障碍、癫痫发作以及血管病变，表现为皮肤黄染和骨质疏松等症状。

这些病症在临床表现上有一定的重叠性，因此需要通过详细的家族史询问、体格检查以及实验室检测来进一步明确诊断。

**8. user 2024-11-30T19:31:41Z: 1.**

Ⅰ型 ，腹部膨隆、生长迟缓、低血糖抽搐、反复鼻出血、腹泻和呕吐为儿童患者主要就诊原因，极少数以肉眼血尿、便血、反复骨折、贫血或痛风等为首发表现。从未确诊及治疗的成年患者可以多发肝腺瘤、慢性肾衰、严重痛风伴多发痛风石、骨质疏松等就诊。其他少见表现包括肺动脉高压、糖尿病、脑血管病和肝腺瘤癌变等。查体可见身材矮小和肝脏明显增大。

1. Ⅰb 型 ， 患者除以上表现外，还可有反复感染伴中性粒细胞减少、口腔溃疡、炎症性肠病、肛周溃疡、关节炎和脾大等。
2. Ⅱ型婴儿型， 根据预后分为经典婴儿型和非经典婴儿型。经典婴儿型大部分在生后第 1 个月即出现全身性肌肉无力，运动发育迟缓，胸部 X 线片示心脏增大，心电图见高 QRS 波和短 PR 间期，心脏彩超见肥厚型心肌病改变，血肌酸激酶不同程度升高等， 多于生后 1 年之内死于左心衰竭或肺部感染后心肺功能衰竭。非经典婴儿型在生后 1 年内出现肌肉无力，运动发育落后，多于幼儿期死于呼吸衰竭。
3. Ⅱ型晚发型 ， 患者于 1 岁后起病，可晚至 60 岁发病。多表现为慢性进行性近端肌力下降和呼吸功能不全，心脏受累少见，呼吸功能衰竭是主要的致死原因。临床表现为易疲劳、仰卧起坐、上下楼梯、蹲起困难和行走无力，少数以突发呼吸衰竭起病。

**qwen2.5_7b** 2024-11-30T19:31:50Z:

根据您提供的临床表现，这些症状与**Ⅰ型糖原贮积症（Von Gierke disease）**非常相符，这是一种罕见的代谢性疾病，主要由于葡萄糖-6-磷酸酶缺陷导致肝、肾等器官中糖原积累过多。

### 诊断：Ⅰ型糖原贮积症

- **Ⅰ型糖原贮积症**的主要临床特征包括低血糖引起的抽搐（尤其是夜间）、生长迟缓、腹部膨隆（由于肝脏和脾脏增大）以及反复的鼻出血。儿童患者也可能有腹泻和呕吐，成人患者则可能出现慢性肾衰竭等并发症。

### 其他可能的诊断

- **Ⅰ型半乳糖血症**：主要表现为低血糖抽搐和生长迟缓，但通常还伴有眼部病变（如白内障）。
- **Marteau-Pontier综合征**：又称I型果糖不耐受或果糖-1,6-二磷酸酶缺乏症，临床表现与Ⅰ型糖原贮积症相似，但主要为代谢性酸中毒、低血糖和肝脾肿大等。
- **其他类型糖原贮积症（如Ⅲ型、Ⅳ型）**：这些类型也有类似的表现，但具体的生化缺陷不同。
- **先天性肾上腺皮质增生症**：主要表现为生长迟缓、低血糖和皮肤色素沉着等，但激素代谢异常是其特征。
- **威尔森病（Wilson Disease）**：可有肝脾肿大及神经精神症状，但铜代谢异常为其特点。

这些诊断需要通过详细的临床评估和实验室检查来确认。对于Ⅰ型糖原贮积症，常用的检测方法包括血浆葡萄糖水平测定、尿液有机酸分析以及基因检测等。

**9. user 2024-11-30T19:32:22Z:**

1. 神经 - 肌肉系统， 引起神经系统的异常，如惊厥、昏迷、共济失调、甚至精神症状；也可出现肌肉症状，包括腱反射亢进、震颤、手足搐搦、肌无力等。

1. 心血管系统， 引起心电图的改变，可见 PR 间期延长、QRS 波增宽等，严重者可引起室性心律失常。
2. 肾脏 ，可引起肾结石、肾钙质沉着、肾功能不全。
3. 骨关节系统， 可引起骨质疏松、骨软化。
4. 消化系统， 可引起肠梗阻表现。
5. 内分泌， 患者可见糖尿病或者糖耐量减低。
6. 眼部症状， 部分类型可合并眼部病变，包括眼球震颤、视野缺损、近视等。

该组疾病通常为慢性病程，其中新生儿期、婴儿期起病的类型往往病情重、预后差。部分患者在青春期就发展为慢性肾衰竭，部分患者若未能及时治疗，会导致患儿严重精神发育迟滞甚至死亡。

**qwen2.5_7b** 2024-11-30T19:32:29Z:

根据您提供的临床表现，这些症状与一些罕见的遗传代谢性疾病和某些神经肌肉疾病相符。以下是几个可能的诊断方向：

- **线粒体病**（例如Leigh综合征、Kearns-Sayre综合征）：可以引起神经系统异常如共济失调、惊厥、肌无力等；心血管系统的改变，包括心律失常；以及肾脏病变。
- **代谢性脑病**（如甲基丙二酸血症或高雪氏病），可能导致神经症状及肌肉症状，影响多个器官系统。
- **肾上腺脑白质营养不良**：可导致神经系统异常、骨骼问题，并可能伴有内分泌紊乱和视力障碍等表现。
- **MELAS综合症**（线粒体肌病乳酸性酸中毒伴卒中样发作）：主要表现为神经症状，如共济失调、癫痫发作，伴随肌肉无力和其他代谢问题。
- **同型半胱氨酸尿症**：可以引起神经系统、心血管系统、骨骼和肾脏的多种症状，并伴有高血压等表现。
- **糖原累积病（GSDs）**：包括多种类型的糖原累积病，如I型糖原累积病会导致神经系统、肌肉和肝脏问题；某些类型也可能影响心脏和其他器官。

每种疾病的具体临床表现可以有所不同，诊断通常需要结合详细的家族史、实验室检查结果（如血液生化检测、基因检测等）、影像学检查以及特定的酶活性测定。因此，在进行具体诊断时，医生通常会综合考虑患者的临床症状和辅助检查结果来确定最可能的原因。

**10. user 2024-11-30T19:33:06Z:**

可分为 5 种类型，其中以经典型和中间型最常见。

**1. 经典型** 最常见，占 75％。患儿出生时正常，发病早、症状重并发展迅速。通常患儿出生 12 小时后，在耵聍中即出现枫糖浆气味；生后 12~24 小时在尿液和汗液中有特殊的枫糖味；2~3 天出现酮尿、易激惹和喂养困难；4~5 天开始出现严重的代谢紊乱和脑病症状，表现为哺乳困难、阵发性呕吐、拒食、嗜睡、呼吸暂停、惊厥发作、肌张力增高以及“击剑”样和“蹬车”样刻板动作等；生后 7~10 天可出现昏迷和中枢性呼吸衰竭，若不及时治疗， 大多数的患儿在出生后数天死于严重的代谢紊乱。

**2. 中间型** 在任何年龄阶段均可发病。患者症状较经典型轻，表现为生长缓慢、发育落后、喂养困难、易激惹、在耵聍中有枫糖浆气味，应激情况下可以出现严重的代谢紊乱和脑病。

**3. 间歇型** 呈间歇发作，早期生长发育均正常，多在感染和手术等应激情况下发作，表现为发作性共济失调和酮症酸中毒，极少数的严重者可引起死亡。间歇期支链氨基酸浓度正常，发作期与经典型类似有支链氨基酸浓度升高。

**qwen2.5_7b** 2024-11-30T19:33:14Z:

根据您描述的症状和临床表现，这些特征最符合枫糖浆尿症（Maple Syrup Urine Disease, MSUD），这是一种由于支链酮酸脱氢酶复合体缺乏所导致的罕见代谢病。

### 1. 枫糖浆尿症 (MSUD)

- **典型型**：通常在新生儿期出现症状，表现为特殊的枫糖味尿液和汗液。
- **中间型**：任何年龄阶段发病，但症状相对较轻。
- **间歇型**：病情呈间歇性发作，在应激情况下会出现严重代谢紊乱。

### 其他可能的诊断：

- **苯丙酮尿症 (Phenylketonuria, PKU)** - 一种由于苯丙氨酸羟化酶缺乏导致的代谢病，表现为特殊气味（类似鼠尿）。
- **异戊酸血症 (Isocaprognosia or Isovaleric Acidemia)** - 涉及支链氨基酸代谢异常，临床症状包括呕吐、嗜睡和神经系统损害。
- **半乳糖血症 (Galactosemia)** - 由于葡萄糖-1-磷酸尿苷转移酶缺乏导致的疾病，主要表现为喂养困难、黄疸等症状。
- **先天性甲状腺功能减低症 (Congenital Hypothyroidism, CH)**
- **囊性纤维化 (Cystic Fibrosis, CF)** - 尽管这更多是基于遗传和肺部症状诊断的，但新生儿筛查中会检测该病。

这些疾病都可能在新生儿期或儿童早期表现出类似的代谢紊乱和神经系统症状。因此，在出现上述临床表现时，需要通过实验室检查如尿有机酸分析、血液氨基酸分析等来进一步确诊。

**11. user 2024-11-30T19:33:45Z:**

出生后至老年均可起病，可急性或隐袭起病。几乎任何系统或器官均可受累，以能量需求旺盛的器官，如脑、心、肾、眼、耳、肌肉、胃肠道等多见。患者可以表现为某种临床综合征，也可仅表现单个症状或者多种症状组合。

**qwen2.5_7b** 2024-11-30T19:33:51Z:

根据您提供的临床描述，这种情况可能与**线粒体病**相关。线粒体病是一类影响细胞能量产生系统的疾病，由于线粒体功能障碍导致细胞无法正常产生足够的能量。这类疾病的临床表型多样，可以涉及神经系统、心脏、肾脏等多个器官和系统。

### 五种其他可能的诊断：

- **神经肌肉接头疾病**（如重症肌无力）：这类疾病主要影响神经与肌肉间的信号传递，症状包括疲劳性肌无力等。
- **多发性内分泌腺病综合征**（如MEN1或MEN2）：这些是一组由于遗传因素导致的多个内分泌腺体功能障碍引起的疾病，常表现为甲状腺、胰腺和/或其他内分泌器官的功能异常。
- **自身免疫性疾病**（例如系统性红斑狼疮SLE等）：这类疾病的特征是免疫系统的过度活跃，攻击身体自身的组织和器官。
- **代谢病**（如脂肪酸氧化障碍或糖原累积症）：这些疾病由于特定酶的缺陷导致物质代谢异常，影响能量产生或其他关键生物过程。
- **遗传性血管水肿**（HAE）：这是一种罕见的遗传性疾病，特征为反复发作的非过敏性肿胀，通常涉及四肢、面部和生殖器区域。

每种疾病的临床表现虽然多样但各有特点。确诊需要详细的病史采集、体格检查以及一系列实验室检测或影像学检查来辅助诊断。

**12. user 2024-11-30T19:34:38Z:**

好发年龄 5~50 岁，中位发病年龄 39 岁。女性患病率明显高于男性，女：男比例为(9~11) ：1。多急性 / 亚急性起病，临床表现包括 6 组核心症候群：①视神经炎；②急性脊髓炎；③极后区综合征；④急性脑干综合征；⑤症状性睡眠发作或急性间脑临床综合征伴典型的间脑 MRI 病灶；⑥症状性大脑综合征伴典型的脑部病变。 为高复发、高致残性疾病，90％以上的患者为复发性病程，多数患者遗留较为严重的神经功能残疾。

**qwen2.5_7b** 2024-11-30T19:34:43Z:

根据您提供的信息，这种疾病的临床表现符合视神经脊髓炎谱系疾病（Neuromyelitis Optica Spectrum Disorders, NMD/NMO），特别是其中的视神经脊髓炎（Neuromyelitis Optica, NMO）。NMO 是一种自身免疫性疾病，主要影响中枢神经系统。以下为五个其他可能的诊断：

- **多发性硬化症**：虽然临床表现多样，但部分患者的症状可以类似NMO。
- **急性播散性脑脊髓炎 (ADEM)**：这是一种罕见的、通常单相的自身免疫性疾病，可能与病毒感染有关。
- **视神经炎伴或不伴有脊髓受累**：这可以是孤立性的疾病表现，但结合其他症状更倾向于NMO。
- **急性炎症性脱髓鞘性多发性神经病 (Guillain-Barré 综合征)**：这是一种自身免疫性疾病，主要影响周围神经系统。
- **Bickerstaff脑干脑炎**：虽然以脑干受累为主，但可能伴有视神经和脊髓症状。

这些疾病都有各自独特的临床特征及辅助检查结果，例如血液学、影像学（如MRI）、电生理检查等。确诊通常需要结合详细的病史、体格检查以及实验室和影像学检查结果来综合判断。

**13. user 2024-11-30T19:35:45Z:**

听力损伤。以往多认为该耳聋为听力筛查即可明确的先天性， 初生即外显，多累及双侧，对称的重度语前聋。近年来逐渐发现个别病例可表现为不对称或单侧听力受损，听力损失程度变异较大，不同病人的表现可从轻度至极重度不等；渐进性、出生后才发生的耳聋也并不少见。

**qwen2.5_7b** 2024-11-30T19:35:51Z:

根据您提供的临床描述，这种听力损伤的表现较为复杂，不仅限于先天性的对称性重度语前聋，还可能表现为不对称或单侧的听力受损，并且可以是渐进性和出生后的迟发性听力损失。基于这些特点，最有可能的诊断是**大前庭导水管综合症（Mondini Dysplasia）**。

但考虑到听力损伤的复杂性，还需要考虑其他几种可能性以排除和鉴别：

- **先天性感音神经性聋**：这是一种常见的遗传性耳聋类型，可以表现为不同程度的听力下降。
- **Usher综合征**：这是一种伴有感音神经性聋和视网膜色素变性的综合症，但不是所有患者都会表现出视觉症状。
- **Waardenburg综合征**：除了听力损失外，还可能伴随有白发、虹膜异色等表型特征。
- **Jervell Lange-Nielsen 综合征**：这是一种伴有心脏传导障碍的感音神经性聋，虽然较少见，但需要考虑在内以排除潜在的心脏风险。
- **进行性肾小管性酸中毒伴耳聋（Alport综合征）**：除了听力下降外，还可能伴随肾脏问题和其他系统症状。

这些诊断均需通过详细的家族史询问、临床表现评估以及必要的辅助检查如基因检测和听力学测试来确诊。

**14. user 2024-11-30T19:36:44Z:**

**1. 多发神经病** ，往往表现为对称性的四肢感觉和(或) 运动性周围神经病，逐步由远端向近端进展。

**2. 器官肿大，** 包括肝大、脾大或淋巴结肿大；淋巴结活检常提示为 Castleman 病。

**3. 内分泌异常 ，**常见的包括性功能减退(如男性阳痿、乳房发育)、甲状腺功能减退、糖代谢异常(如糖尿病)、肾上腺功能不全。

**4. 皮肤改变，** 可表现为皮肤颜色加深、皮肤肾小球样血管瘤、白甲、多血质、多毛症和手足发绀等。

**5. 循环外水负荷增加，** 包括肢体水肿、腹水、胸腔积液、心包积液、视乳头水肿等。

**6. 硬化性骨病 ，**是重要临床表现。可表现为骨痛，亦可无临床症状。骨骼 CT 检查可以显著提高硬化性骨病的检出率，影像学上可以表现为单纯骨骼硬化、或硬化和溶骨混合病灶、或者单纯溶骨性改变。

**7. 红细胞增多和(或) 血小板增多**

**8. 肺动脉高压** ，患者可出现肺动脉高压，表现为活动耐量减低、低氧血症等。

**9. 脑梗死**

**qwen2.5_7b** 2024-11-30T19:36:52Z:

根据您提供的临床表现，这些特征指向的是 **家族性淀粉样变（Familial Amyloid Polyneuropathy, FAP）**。这是一种罕见的遗传性疾病，主要表现为对称性的周围神经病，并伴有器官肿大、内分泌异常、皮肤改变和循环外水负荷增加等症状。

以下是五个其他可能的诊断：

- **Castleman 病（Hemophagocytic Lymphohistiocytosis, HLH）** - 由于淋巴结活检提示为 Castleman 病，这是一种罕见的免疫相关性疾病，可以表现为多器官肿大、内分泌异常和皮肤改变。
- **慢性自身免疫性多内分泌腺病 – Ⅱ型（Cogan-Reese-Strand Syndrome, CARS）** - 这种疾病涉及多个内分泌腺体的功能障碍，并伴有淋巴结肿大和其他非特异性症状，如疲劳、体重减轻等。
- **系统性肥大细胞增多症（Systemic Mastocytosis）** - 该病可表现为皮肤改变（如色素沉着和血管瘤）、器官肿大以及内分泌异常，但通常不典型地进展为神经病变。
- **先天性甲状腺功能减退伴其他多系统的发育异常（THM 症候群）** - 与您提到的内分泌异常相符，尤其是甲状腺功能减退和其他多系统症状，但神经系统的表现可能不同且较少见。
- **进行性多灶性白质脑病（Progressive Multifocal Leukoencephalopathy, PML）** - 虽然主要影响中枢神经系统，可能会出现神经精神症状和皮肤改变等表现，但通常不伴有器官肿大或内分泌异常。

请注意，最终的确诊需要结合详细的家族史、实验室检查结果、病理学检查以及遗传检测等多种信息。

**15. user 2024-11-30T19:37:54Z:**

运动迟缓、震颤、肌强直。治疗后更容易出现左旋多巴诱导的运动并发症。在非运动症状方面，容易发生情绪障碍(如抑郁、焦虑、易激惹等) 及行为障碍(如强迫性增加药量、冲动控制障碍、刻板行为等)。

**qwen2.5_7b** 2024-11-30T19:38:01Z: 根据您提供的临床表现，最可能的诊断是帕金森病（Parkinson’s disease, PD），特别是特发性帕金森病。这种疾病的主要特征包括运动迟缓（震颤、肌强直）、以及非运动症状如情绪障碍和行为异常。左旋多巴诱导的运动并发症也是帕金森病治疗过程中的常见问题之一。

以下是五个可能的其他诊断：

- **多系统萎缩** (Multiple System Atrophy, MSA)：这是一种罕见的神经退行性疾病，可以出现类似帕金森的症状，并且常伴有自主神经系统功能障碍。
- **进行性核上性麻痹** (Progressive Supranuclear Palsy, PSP)：PSP主要特征为步态障碍、眼球运动异常以及姿势平衡失调，但也可以表现出一些类似于帕金森病的运动症状。
- **亨廷顿舞蹈症** (Huntington’s Disease, HD)：这是一种遗传性疾病，虽然其典型表现为不自主运动（如舞蹈样动作），但也可能出现类似帕金森的症状，并伴有认知和行为问题。
- **路易体痴呆** (Dementia with Lewy Bodies, DLB)：DLB是一种与帕金森病症状相似的神经退行性疾病，除了运动障碍外还常有认知功能下降、视觉幻觉等症状。
- **脑炎后帕金森综合征** (Post-Infectious Parkinsonism)：某些病毒感染或炎症（如莱姆病）后可能会发展为类似帕金森的症状。

由于这些疾病的临床表现可能有所重叠，确诊需要依赖详细的病史采集、体格检查以及辅助检查结果。

**16. user 2024-11-30T19:38:33Z:**

胎儿期的常见表现包括胎儿活动减少、羊水过多以及臀位。在新生儿期肌张力过低是这一疾病的标志性特征之一，严重的肌张力降低可导致窒息、喂养困难、吮吸无力、哭声无力。婴幼儿期主要表现为生长迟滞，认知、运动及语言发育落后。随着年龄的增长，食欲逐渐旺盛，如果不限制进食会出现肥胖。学龄期通常存在身材矮小，逐渐出现脾气暴躁、固执、强迫症等性格特点。异常行为包括强烈的觅食、搔抓皮肤、孤独症样行为等。约 25％的患者会出现癫痫。青春期男性和女性患者均可出现第二性征出现延迟或出现不完全。由于肥胖可导致相关并发症，如呼吸睡眠暂停、肺源性心脏病、胰岛素抵抗甚至糖尿病等。脊柱侧突、骨质疏松、胃食管反流也较为常见。成人期主要表现为肥胖及其并发症，包括心血管问题、糖尿病和睡眠呼吸暂停。其他表现为精神异常，不孕不育。

致命性危险：由口部运动不协调、肌张力过低、多食、狼吞虎咽的进食习惯和咀嚼不充分引起窒息以及由于暴饮暴食导致胃破裂。

体格检查可以发现特征性面容逐渐典型，包括杏仁眼、小嘴、上唇薄、嘴角向下、小手小脚、身材矮小、皮肤白皙、肌张力低、生殖器发育不良(如隐睾、阴囊发育不全或阴蒂发育不全)。

**qwen2.5_7b** 2024-11-30T19:38:39Z:

根据您描述的临床表现，这些特征符合于一个罕见遗传性疾病——Sanfilippo综合症（MPS III），这是一种由于酶缺乏导致的糖原累积病。以下是五个可能类似的其他诊断：

- **Hurler综合征 (MPS I)**：这是一种更为常见的粘多糖贮积症类型之一，表现为严重的发育迟缓和多种器官受累。
- **Morquio综合症 (MPS IV)**：这种疾病也叫半乳糖脑苷脂累积病的一种，患者会出现骨骼异常、呼吸系统问题以及一些生长发育的延迟。
- **Hunter综合症 (MPS II)**：这是一种X连锁遗传病，主要影响男性，表现为严重的智力障碍和身体各部位（尤其是骨骼）的问题。
- **Maroteaux-Lamy 综合征 (MPS VI)**：也是一种罕见疾病，症状与MPS III类似，但通常病情稍轻一些。患者会出现皮肤松弛、关节僵硬等问题。
- **Achondroplasia**：这是一种最常见的人类侏儒症类型，主要表现为身材矮小和四肢短缩，但也可能伴有其他骨骼发育异常，其特征不完全符合您描述的全部症状但有时会混淆诊断。

对于这种复杂的临床表现，建议进行详细的基因检测以确诊。通过血液或尿液样本可以进行特定酶活性测定，并且基因测序技术也可以帮助识别突变的基因，进而明确诊断。

**17. user 2024-11-30T19:39:34Z:**

患者常在婴幼儿或青少年期起病，男女比例大致相等。呼吸道、胰腺和胆道等上皮细胞的分泌物含水量减低，分泌物变黏稠而难以清除。肺脏是最常见的受累器官，患者呼吸道出现慢性细菌感染及病原体的定植，最初常为流感嗜血杆菌和金黄色葡萄球菌，最终出现铜绿假单胞菌或洋葱克雷伯杆菌的慢性定植和(或) 感染。一旦出现感染，中性粒细胞被大量募集到肺组织中，并释放弹性蛋白酶，从而造成肺组织的破坏，导致支气管扩张形成。患者的支气管扩张常在幼年起病，且常从上叶起病，与多数感染后形成的支气管扩张症主要位于中、下肺不同。

患者常表现为深色服装上遗留的汗渍，或是父母发现患儿皮肤发咸。胆汁和胰液的流动异常可以导致消化不良和吸收不良，也可出现肝脏或胰腺疾病，严重时可以出现相关的糖尿病。黏稠的肠液还可能使 患者更容易出现肠梗阻和直肠脱垂。此外，男性患者可以出现输精管内精子移动障碍以及先天性输精管缺失(CBAVD)。

**qwen2.5_7b** 2024-11-30T19:39:41Z:

根据您提供的临床表现，最有可能被诊断为 **囊性纤维化**（Cystic Fibrosis, CF），这是一种遗传性的罕见疾病，主要影响呼吸系统和消化系统的离子通道功能。

除了囊性纤维化之外，以下是五个可能的其他诊断：

- **α-1抗胰蛋白酶缺乏症**：此病也会影响肺部，并伴有肝脏病变。但通常表现为肝病或肺病中的一个。
- **遗传性黏膜营养不良（原发性纤毛运动障碍）**：这是一种主要影响呼吸道和生殖系统的罕见疾病，与囊性纤维化不同的是，它的标志是纤毛功能异常而不是离子通道问题。
- **支气管扩张症**：尽管患者的症状可能部分符合此病的典型表现，但其病因和遗传因素通常不同于囊性纤维化的描述。此外，先天性的呼吸道结构异常较少见于青少年起病的情况。
- **克隆氏病（Crohn’s Disease）**：这是一种慢性炎症性肠病，可以引起多种消化道症状，包括黏液便、腹泻等，但通常不会导致如此明显的肺部病变和胰腺功能问题。
- **Zellweger 综合征（极低转位脂肪酸病）或其他Peroxisomal Biogenesis Disorders (PBD)**：这些疾病会影响代谢途径并影响肝脏和其他器官的功能，但其主要症状和临床表现与囊性纤维化有显著不同，并且通常在婴儿期早期出现。

需要注意的是，只有通过详细的基因检测、生化检查以及影像学检查等才能做出准确诊断。

**18. user 2024-11-30T19:40:43Z:**

患者临床表现为口咽炎、中耳炎、呼吸系统感染、蜂窝织炎及皮肤感染，常由葡萄球菌及链球菌感染所致。在出现骨髓储备下降的重度中性粒细胞减少症患者中，2 岁之前大部分患儿都发生过口腔溃疡及疼痛性牙龈炎。如果伴弥漫性胃肠道病变，可引起类似克罗恩病的腹痛和腹泻表现。中性粒细胞减少症可导致各个部位遭受反复细菌或真菌感染，其中主要受累部位为皮肤黏膜、耳鼻、咽喉和肺。感染通常非常严重甚至是致命性的。约 15％的患者进展为急性白血病或骨髓增生异常综合征。患儿通常不伴有特征性畸形。

**qwen2.5_7b** 2024-11-30T19:40:51Z:

根据您描述的临床表现，这些症状指向了一种特定的罕见疾病——慢性肉芽肿病（Chronic Granulomatous Disease, CGD），这是一种由于NADPH氧化酶复合体缺乏导致的原发性免疫缺陷病。

**诊断：** 1. 慢性肉芽肿病 (CGD) - 该病通常由中性粒细胞无法有效杀死某些类型的细菌和真菌引起，导致反复感染。

**其他可能的诊断包括：**

1. **X-连锁无丙种球蛋白血症（Bruton’s Agammaglobulinemia）**
   - 主要特征是由于Btk基因突变导致缺乏成熟B淋巴细胞，进而影响抗体生成。患者常表现为反复细菌感染。
2. **DiGeorge综合征**
   - 这是一种先天性免疫缺陷和发育异常的综合症，主要由第22号染色体部分缺失引起，常伴有心脏缺陷、甲状旁腺功能减退以及T淋巴细胞功能障碍。
3. **共济失调毛细血管扩张症（Ataxia Telangiectasia, AT）**
   - 这种疾病是由ATM基因突变导致的，影响DNA修复机制，患者易受放射线和某些化学物质的影响，并容易发生自身免疫性疾病、反复感染以及肿瘤风险增加。
4. **选择性IgA缺陷症（Selective IgA Deficiency)**
   - 主要表现为缺乏血清中的IgA抗体。虽然它通常不引起严重的感染问题，但该病有时会伴随其他类型的免疫功能障碍症状。
5. **Wiskott-Aldrich综合征 (WAS)**
   - 这是一种X-连锁的原发性免疫缺陷疾病，特点为血小板减少和体液免疫缺陷，常伴有皮肤湿疹、反复感染以及特发性血小板减少性紫癜等症状。

请注意，在做出确切诊断之前需要进行详细的临床检查与实验室检测。

**19. user 2024-11-30T19:41:20Z:**

典型 患者出现中枢神经系统损害和肾上腺皮质功能低下两类症状。神经系统症状和肾上腺皮质功能减退的症状可同时出现或相继出现。少数患者可单独出现。肾上腺皮质功能不全的表现轻重不一。儿童患病最为常见，占所有患者 35％。多在 4~8 岁发病。初期表现为注意力不集中、记忆力减退、学习困难、步态不稳、行为异常，后逐渐出现视觉和听觉下降、构音障碍、癫痫发作、共济失调、瘫痪、痴呆等症状。临床表现进行性加重， 最终失明或耳聋，完全瘫痪。有的表现为去大脑强直状态，最终死于中枢性呼吸衰竭、脑疝、感染。多数患者在首次出现神经系统症状时，已有肾上腺皮质功能受损的表现。

**qwen2.5_7b** 2024-11-30T19:41:29Z:

根据您描述的临床表现，患者同时出现了中枢神经系统损害和肾上腺皮质功能低下的症状，并且这些症状可以同时或相继出现，最符合以下疾病：

- **尼曼-匹克病（Niemann-Pick disease）**：这是一种由于鞘磷脂代谢异常导致的罕见遗传性神经退行性疾病。患者可能出现中枢神经系统损害、肝脾肿大以及肾上腺皮质功能不全等症状。
- **戈谢病（Gaucher disease）**：这也是一种由于葡萄糖脑苷脂酶缺乏引起的一种溶酶体贮积症，临床表现包括脾脏和肝脏肿大、骨骼病变等。少数患者也会出现神经系统损害和肾上腺受累的表现。
- **克兰费尔特综合征（Klinefelter syndrome）**：这是一种染色体异常导致的男性罕见病，主要表现为X性连锁遗传，患者的2号染色体会额外包含一个或多个副本。虽然该病主要影响生殖系统发育和生育能力，但有时也会伴有轻度认知障碍或其他神经系统症状。
- **威尔逊氏症（Wilson’s disease）**：这是一种铜代谢障碍的罕见病，可导致肝脏、脑部、肾脏等多个器官受损，并伴随有神经精神症状如震颤等。但在少数情况下，患者也可能表现出轻微或不典型的肾上腺功能异常。
- **多囊肾病伴发神经系统表现（Polycystic Kidney Disease with Neurological Manifestations）**：这是一种遗传性疾病，除了肾脏囊肿形成外，还可能伴有脑实质萎缩和神经系统的症状如认知障碍、癫痫发作等。但在临床实践中相对少见。
- **黏多糖贮积症（Mucopolysaccharidoses, MPS）**：是一组由于溶酶体中特定酶缺陷导致的累积性代谢病。这类疾病会累及多个器官和系统，包括神经系统和内分泌腺体如肾上腺，表现出神经退行性和生长发育迟缓等症状。

鉴于患者的具体症状较为特殊且复杂，建议进行全面详细的遗传学检查、生化检测以及影像学评估以明确诊断，并制定相应的治疗计划。

**20. user 2024-11-30T19:42:09Z:**

患儿多于生后 4~12 个月开始出现感染症状。最突出的临床表现是反复严重的细菌感染，感染部位主要包括呼吸系统、消化系统及中枢神经系统，其中以呼吸道感染最为常见，常见病原菌包括流感嗜血杆菌、肺炎链球菌、金黄色葡萄球菌和假单胞菌属细菌等。长期、反复的呼吸道感染可导致支气管扩张、杵状指等不可逆转的器官功能损害。消化道感染常见的症状有腹泻、腹痛、胃食管反流和胃肠炎。引起感染性腹泻的病原包括蓝氏贾第鞭毛虫、沙门菌、空肠弯曲菌、隐孢子虫等。

另外，患儿容易出现中耳炎、慢性鼻窦炎、营养不良、贫血、粒细胞减少、血小板减少、生长激素缺乏症及甲状腺激素紊乱等并发症状。患儿还可发生肿瘤、自身免疫和炎症性疾病，包括类风湿性关节炎、皮肌炎等。据报道，患儿的感音神经性听力损失和湿疹的发生率也较高。

**qwen2.5_7b** 2024-11-30T19:42:19Z: 根据您提供的临床表现，最可能诊断为 **X-连锁高 IgM 综合征（X-linked Hyper-IgM Syndrome, XHIM）**。

### 五种其他可能的罕见疾病包括：

- **慢性肉芽肿病 (Chronic Granulomatous Disease, CGD)**
- **共济失调毛细血管扩张症 (Ataxia Telangiectasia, AT)**
- **严重联合免疫缺陷病 (Severe Combined Immunodeficiency, SCID)**
- **选择性 IgA 缺陷（Selective IgA Deficiency）**：虽然患者以反复感染为主要症状，但主要区别在于该疾病是成人最常见的原发性免疫缺陷病，且IgA水平低下。
- **Wiskott-Aldrich 综合征 (WAS)**

### 分析原因：

- **XHIM**: 由于患者的临床表现为反复细菌感染、尤其是呼吸道感染（如流感嗜血杆菌等），并且有支气管扩张和杵状指的表现，考虑免疫球蛋白M（IgM）水平正常或升高而其他免疫球蛋白（如IgG, IgA)减少，且男性患者为主。XHIM正是由于CD40配体表达缺陷导致B细胞发育障碍，导致仅产生高水平的IgM而不产生IgG、IgA等抗体。
- **CGD**: 患者表现出反复感染及高炎症反应（如消化系统症状），但CGD主要是吞噬细胞功能缺陷引起，以反复化脓性感染为特征，且无X连锁遗传特点。
- **AT**: 该病有特异性的神经系统表现（共济失调、眼裂和鼻翼毛细血管扩张等）以及免疫学检查特定异常，通常与XHIM不同。
- **SCID**: 虽然也有反复感染的表现，但其免疫缺陷更为广泛，包括T细胞功能障碍，且临床特点和实验室检查有明显差异，如淋巴结和脾脏肿大。
- **WAS**: 主要特点是血小板减少、湿疹、复发性病毒感染等，虽然部分患者也可能出现类似XHIM的反复感染表现。

进一步的确诊需通过详细的家族史、免疫学检测（包括Ig水平测定）、基因检测和其他特殊检查。

**21. user 2025-02-18T14:55:58Z:**

患者的临床表现包括不同程度的失盐和高雄激素血症两大类。新生儿起病的患儿表现为不同程度肾上腺皮质功能不足的表现，如软弱无力、恶心、呕吐、喂养困难、腹泻、慢性脱水、皮肤色素沉着和生长迟缓等。肾上腺危象常是该疾病在新生儿期的首发表现，表现为严重低血钠、高血钾、低血容量性休克，可伴有低血糖，由应激诱发。严重的低钠血症可导致抽搐等中枢神经系统表现，严重的高钾血症则可引起致命的心律失常。

高雄激素血症在不同年龄表现不一。经典型疾病的女性患儿出生时外生殖器有不同程度男性化。轻者表现为孤立性阴蒂肥大，重者外生殖器可接近男性，大阴唇呈阴囊样，阴蒂呈尿道下裂型阴茎样，并具共通的尿道阴道口。但大阴唇内不能触及性腺，有完全正常的女性内生殖器结构（卵巢和子宫）。男性新生儿期和婴儿期时可能无阴茎增大等外生殖器异常，是延误诊断的常见原因。至幼儿期，两性均会呈现外周性性早熟。男孩患儿呈现阴茎增大，伴或不伴阴毛早现；女性患儿呈现异性性早熟。长期高水平性激素刺激下丘脑促性腺激素释放激素（GnRH）神经元，发展为中枢性性早熟。女性还可有第二性征发育不良和原发性闭经或月经稀发。两性均在幼年期开始发生线性生长伴骨龄增长加速，使终身高受损。

其他表现包括皮肤和黏膜色素增深，以乳晕和外阴为显，部分患儿可无此改变。

非经典型的该类疾病儿童期和青春期甚至成年期临床呈不同程度的高雄激素血症表现，也可仅表现为生长加速和骨龄快速进展。

**qwen2.5_7b** 2025-02-18T14:56:00Z:

根据您描述的症状，最有可能诊断的是21-羟化酶缺乏症（Congenital Adrenal Hyperplasia, CAH），尤其是其最常见的类型——盐皮质类固醇合成障碍型。以下是其他五种可能的诊断：

- 17α-羟化酶缺乏症
- 3β-羟类固醇脱氢酶缺乏症
- 21-羟化酶部分缺陷（非经典型）
- 螺内酯抵抗性盐腺功能不全
- 促肾上腺皮质激素分泌不足

请注意，确诊罕见病通常需要结合详细的临床表现、实验室检查结果及基因检测等综合判断。

**22. user 2025-02-18T14:57:40Z:**

患者出生即有临床表现，因全身皮肤色素缺乏，皮肤毛细血管显露，皮肤薄而柔软，呈白色及红色，并伴有不同程度的血管扩张。患者对紫外线高度敏感，常出现日光性皮炎、光化性唇炎、基底细胞癌及鳞状细胞癌等疾病。毛发呈纯白、银白、黄色、金色或茶色。眼睫毛及眉毛呈白色或淡黄色。视网膜、虹膜颜色减退，瞳孔颜色减退。白天畏光明显，夜视正常，同时还可以伴有其他视觉改变，如视野敏锐度下降、视网膜中央凹发育不良、视交叉处神经路径改变、眼球震颤等。

**qwen2.5_7b** 2025-02-18T14:57:43Z:

根据您描述的临床表现，我最有可能诊断为“白化病”，这是一种由于遗传原因导致皮肤和毛发缺乏黑色素的罕见病。以下是其他五种可能的诊断：

1. **着色性干皮病**：一种罕见的遗传性疾病，患者的皮肤对紫外线非常敏感，并且容易发生恶性肿瘤。
2. **粘膜色素减退症**（或称Witzel-Werner综合征）：这是一种少见的遗传性皮肤病，临床表现为全身多处皮肤色素缺乏、血管扩张等症状。
3. **先天性黑素细胞功能不全**：一种罕见的疾病，主要表现为眼睛和皮肤中的黑色素减少，导致视力问题以及对光敏感等表现。
4. **神经纤维瘤病Ⅰ型**（NF1）：虽然该病主要特征为咖啡牛奶斑、皮下肿瘤等，并非以皮肤色素缺乏为主要临床表现，但部分患者可能出现眼色素层改变、神经系统症状等症状。
5. **遗传性血管扩张症**：一种较为罕见的遗传性疾病，可表现为广泛的毛细血管扩张以及对光敏感的症状，但通常没有白化病中提到的特定色素缺乏特征。

以上疾病需要通过详细的家族史采集、临床表现观察、实验室检查（如皮肤活检、血液基因检测等）来确诊。

**23. user 2025-02-18T14:58:07Z:**

一种上、下运动神经元同时受累的神经系统变性疾病。临床主要表现为球部、四肢、胸腹部肌肉进行性无力和萎缩。而眼球运动神经和括约肌功能一般并不受累，但在有些患者晚期也可能会被累及。20％ ~50％的患者可以表现有认知功能障碍，5％ ~15％的患者甚至会发展为额颞叶痴呆。发病后平均 3~5 年因呼吸衰竭死亡，但 5％ ~10％的患者可以存活 10 年以上。

通过详细的病史和体格检查，在脑干、颈、胸、腰骶 4 个区域中寻找上、下运动神经元共同受累的证据，是诊断该疾病的基础。查体发现在同一区域，同时存在上、下运动神经元受累的体征是诊断的要点。其中下运动神经元受累体征主要包括肌肉无力、萎缩和肌束颤动。通常检查舌肌、面肌、咽喉肌、颈肌、四肢不同肌群、背肌和胸腹肌。上运动神经元受累体征主要包括病理征阳性、腱反射亢进、肌张力增高、阵挛等。通常检查吸吮反射、咽反射、下颌反射、掌颌反射，有无强哭、强笑等假性延髓麻痹表现，四肢腱反射、肌张力、霍夫曼征、下肢病理征、腹壁反射等。临床查体是发现上运动神经元受累的主要方法，在出现萎缩、无力的区域，如果腱反射不低或活跃，即使没有病理征，也可以提示锥体束受损。

**qwen2.5_7b** 2025-02-18T14:58:11Z:

根据您提供的临床表现和特征描述，这种上、下运动神经元同时受累的神经系统变性疾病最有可能是进行性脊肌萎缩症（Progressive Spinal Muscular Atrophy, PSSM），也称为进行性延髓麻痹或原发性侧索硬化变异型。以下是其他五种可能的诊断：

1. **原发性侧索硬化（Primary Lateral Sclerosis, PLS）**：PLS是一种罕见的神经系统疾病，主要影响上运动神经元。症状包括肌无力和肌肉僵硬，但通常进展较慢。
2. **多灶性运动神经病（Multifocal Motor Neuropathy, MMN）**：这是一种少见的慢性进行性周围神经病，主要表现为下肢或四肢远端肌肉无力和萎缩。
3. **吉兰-巴雷综合征（Guillain-Barré Syndrome, GBS）**：虽然GBS通常是一种急性疾病，但某些情况下可能表现出类似上、下运动神经元受累的症状。不过这种疾病一般不会进展为慢性神经病变。
4. **肌萎缩侧索硬化症（Amyotrophic Lateral Sclerosis, ALS）**：ALS是最常见的渐进性上、下运动神经元疾病，表现为肌肉无力和萎缩、肌束颤动等。
5. **进行性延髓麻痹（Progressive Bulbar Palsy, PBP）**：PBP是一种罕见的神经系统退行性疾病，主要影响延髓区域的神经元，导致言语不清、吞咽困难等症状。

这些诊断都需要通过详细的临床检查以及辅助检查来确认。希望这些信息对您有所帮助！

**24. user 2025-02-18T14:59:57Z:**

一种影响尿素循环的常染色体隐性疾病，可导致高氨血症、脑病和呼吸道阻塞。 其特征是精氨酸和氨在血液中积聚，导致发育迟缓、癫痫发作、智力残疾、痉挛和共济失调等症状。 这种情况通常在3岁左右出现，症状包括僵硬、生长缓慢和认知能力下降。 如果不进行治疗，它可能会发展为严重的痉挛状态，失去肌肉和智力残疾。

**qwen2.5_7b** 2025-02-18T15:00:00Z:

根据您描述的症状，这种罕见病最有可能是鸟氨酸氨甲酰基转移酶缺乏症（OTC缺陷），也称为尿素循环障碍之一的缺陷。其他五种可能的诊断包括：

1. 鸟氨酸转氨甲酰酶缺乏症（Ornithine Transcarbamylase Deficiency, OTC）：这是一种X连锁隐性遗传病，是鸟氨酸循环中的关键酶缺陷。
2. 精氨琥珀酸尿症（Succinyl-CoA:Draftone Aminotransferase Deficiency, SDHD），这种病较为罕见，主要影响儿童和青少年，症状包括癫痫、智力障碍等。
3. 鸟氨酸氨基甲酰基转移酶缺乏症（Citrullinemia），这是一种常染色体隐性遗传病，由于鸟氨酸转运甲酰酶缺陷引起尿素循环障碍。
4. 精氨琥珀酸合成酶缺乏症（argininosuccinate synthetase deficiency, ASSD）：这种罕见病由ASS基因突变导致精氨琥珀酸不能正常生成，可引发高氨血症和代谢性酸中毒等症状。
5. 精氨琥珀酸裂解酶缺乏症（argininosuccinate lyase deficiency, ASLD），这种疾病是尿素循环障碍的一种类型，主要影响儿童期。症状包括发育迟缓、癫痫发作等。

**25. user 2025-02-18T15:01:14Z:**

典型的临床表现包括微血管病性溶血性贫血、血小板减少及急性肾功能衰竭三联征。微血栓形成所导致的非免疫性红细胞破坏，血红蛋白水平常低于 80g/L，Coombs 试验阴性， 外周血涂片可见红细胞碎片；血小板计数下降通常低于 50×109/L，但皮肤紫癜及活动性出血少见；急性肾功能损害的严重程度因人而异，部分患者需要透析支持，常伴血压升高。

**qwen2.5_7b** 2025-02-18T15:01:18Z:

根据您描述的临床表现，最有可能诊断的是**微血管病性溶血性贫血-血小板减少-急性肾损伤三联征（TMHA-ITP-AKI）**，其中一种可能性最大的罕见病是**弥散性血管内凝血（DIC）**。其他五种可能的诊断包括：

1. 微血管病性溶血性贫血：尽管您提到的是“微血管病性”，但更明确表述应为由微血管病导致的溶血性贫血。
2. 血管性血友病：由于临床表现涉及血小板减少，需要排除是否有遗传性的或获得性的血管性血友病（VWD），尤其是不典型表现。
3. 促红细胞生成素抵抗性贫血：这是一种罕见的原发性贫血，尽管其主要表现为全血细胞减少，但也需要进行鉴别诊断以排除其他原因。
4. 系统性红斑狼疮：该疾病可引起多种自身免疫相关的临床表现，包括溶血性贫血、血小板减少和肾损伤等。因此，在诊断过程中需考虑是否合并系统性疾病如SLE。
5. 血栓性血小板减少性紫癜（TTP）或亨och-Schonlein紫癜：虽然这两种疾病也可能导致类似的临床症状，但它们通常有特定的病程特点以及实验室检查结果与本病例有所不同。例如，TTP常伴有神经系统的异常表现；而Henoch-Schonlein紫癜则主要表现为皮肤紫癜、关节痛和腹痛等消化系统症状。

请注意，在实际诊疗过程中还需要结合详细的病史采集、体格检查及其他辅助检查（如血液学检测、免疫学检测以及影像学检查）来综合判断。

**26. user 2025-02-18T15:01:59Z:**

患者临床表现多样。取决于病变的部位、范围和进展速度。临床症状主要包含：

1. 增大垂体的占位效应，大多数患者会因增大的垂体对鞍区及周围结构（如视交叉、海绵窦等） 造成压迫而产生相应症状或由于炎症浸润产生的作用。最常见的临床表现为持续的剧烈头痛和进行性加重的视功能障碍，包括视力下降和视野缺损。
2. 腺垂体功能减退，根据受累的程度不同，垂体的各个轴系会出现部分甚至全部的功能减低的临床表现，如闭经、性功能减低、纳差、乏力、怕冷、性毛脱落、体重下降等。
3. 中枢性尿崩症 ， 约 70％以上患者会因为垂体后叶、垂体柄甚至下丘脑区受累出现多尿、烦渴和多饮等中枢性尿崩症的症状。部分患者因垂体柄阻断效应导致血清中泌乳素水平轻度升高。
4. 下丘脑综合征， 当病变累及区域发展至下丘脑时，会出现嗜睡、缺乏饱腹感、体重严重增加、渴感缺乏、体温波动、出汗障碍和情绪神志障碍等临床表现。
5. 系统性疾病的相关表现 ， 继发性的患者常合并有系统性疾病特征性的临床表现，如 LCH 患者常合并甲状腺、肺、骨、肝脏和皮肤病变。GPA 患者常为中老年男性， 呼吸道症状常早于下丘脑垂体受累。IgG4 相关疾病垂体炎患者常合并有泪腺、颌下腺和胰腺等其他脏器受累的临床表现等。

**qwen2.5_7b** 2025-02-18T15:02:02Z:

根据患者的临床表现描述，最有可能诊断为以下罕见病： 1. **颅咽管瘤** - 由于其可引起垂体占位效应，导致视神经压迫及垂体功能减退等症状。 2. **朗格汉斯细胞组织细胞增生症（Langerhans Cell Histiocytosis, LCH）** - 可影响垂体及其周围结构，出现上述症状和体征。 3. **自身免疫性垂体炎**（如IgG4相关性疾病、系统性红斑狼疮等）- 会导致垂体功能障碍及占位效应等症状。 4. **下丘脑-垂体卒中** - 垂体肿瘤或炎症导致的出血，可引起一系列症状包括视神经压迫、垂体激素缺乏及尿崩症等。 5. **颅内感染（如病毒性或细菌性）** - 也可导致类似占位效应和垂体功能减退的症状。

其他可能的诊断： 1. **鞍区肿瘤**：除了颅咽管瘤，其他类型的肿瘤也可能引起类似症状。 2. **视神经胶质瘤**：可导致视力下降、视野缺损等表现。 3. **海绵窦血栓性静脉炎**：虽然主要影响局部血管结构，但也可引发相应的临床症状。

**27. user 2025-02-18T15:02:47Z:**

高血糖是该疾病的最常见的临床表现。但在没有干预的情况下患者可以自发缓解而维持血糖水平正常。该疾病是自身免疫性低血糖的主要原因之一，约 25％的患者在病程中会出现自发性低血糖。约 50％的患者存在黑棘皮征。此外，多囊卵巢、高雄激素血症在该类患者中也不少见。 患者常伴发其他的自身免疫性疾病，其中以系统性红斑狼疮最为常见，约占 50％。此外，还有原发性胆汁性肝硬化、皮肌炎、硬皮病等。该疾病还可以是霍奇金淋巴瘤、多发性骨髓瘤等的副肿瘤综合征表现。

**qwen2.5_7b** 2025-02-18T15:02:50Z:

根据您提供的临床表型，最有可能诊断的是尼曼-皮克病C1型（Niemann-Pick Disease, Type C1, NPC1），这是一种罕见的遗传代谢疾病。其他五种可能的诊断包括：

1. 系统性红斑狼疮：由于患者常伴有其他自身免疫性疾病，尤其是系统性红斑狼疮。
2. 多囊卵巢综合征：多囊卵巢、高雄激素血症在该类患者中并不罕见。
3. 原发性胆汁性肝硬化（PBC）：尽管不是最常见的伴随疾病，但确实有可能出现。
4. 皮肌炎或硬皮病：这些自身免疫性疾病也可与上述症状并发。

需要注意的是，虽然尼曼-皮克病C1型符合上述大部分描述，但由于该疾病的临床表现多样且复杂，还需结合详细的家族史、基因检测和实验室检查等进行确诊。其他提到的疾病也需要通过相应的检查排除或确认。

**28. user 2025-02-18T15:03:23Z:**

婴儿及儿童期起病。首次发作时年龄在 5 个月至 7 岁。临床表现为反复发作的难治性呕吐及重症酮症酸中毒，伴有萎靡、脱水、呼吸急促、昏迷等临床表现。通常有明确诱因，可在禁食、胃肠道及上呼吸道感染、发热、应激或过量摄入蛋白质后急性起病。常规的实验室检查可见血糖升高或正常，部分出现低血糖，血气显示代谢性酸中毒，血氨可以正常或轻度升高，肝功能、肾功能及正常，尿酮体阳性。酮症及酸中毒可反复出现，发作间期临床及实验室检查可以表现为正常。

约 1/5 的病例出现神经系统的改变，表现为认知障碍、智力发育落后及锥体外系异常及脑卒中的症状，头颅 MRI 可发现包括白质广泛的脱髓鞘，基底节区在 T2 可见对称性高信号改变。

患者早期可无症状，在新生儿期或高危人群通过血、尿代谢筛查时被诊断。起病年龄、发作频率与预后无明确关联。发作随年龄增长有减少或停止趋势，多数病例随访其生长发育未受影响。反复发作及严重者可致多器官功能障碍甚至死亡。

**qwen2.5_7b** 2025-02-18T15:03:25Z:

根据提供的临床表现，最有可能的罕见病是Reye综合征（瑞伊综合症），另外还有以下五种可能的诊断：

1. **线粒体脑病**：部分患者的神经系统症状与线粒体疾病有关。
2. **范可尼贫血（Fanconi Anemia）**：部分儿童患者有反复代谢性酸中毒和生长发育问题，但通常伴有其他血液学异常和先天畸形特征。
3. **糖原贮积症IV型（Pompe病）**：虽然主要表现为肌无力等运动症状，但在某些情况下可出现酮症酸中毒。
4. **短链脂肪酸代谢障碍**：该类疾病可引起反复性高脂血症、乳酸性酸中毒和呕吐等症状。
5. **Wilson病**（威尔逊氏症）：尽管罕见但可以导致类似的临床表现，特别是铜代谢异常引发的肝功能损害和神经系统症状。

**29. user 2025-02-18T15:03:59Z:**

1. 发病年龄小，女性多见。
2. 常有晕厥或猝死家族史。
3. 不同基因型和表现型，可累及钾、钠、钙通道。常有特征性尖端扭转型室速（TdP），导致晕厥或猝死。
4. ECG 表现为 QT 间期延长。
5. 心电图一般有如下特征：①表现为 QT 间期延长，短 - 长 - 短周期现象诱发；②心室率 160-240 次 / 分，QRS 波振幅与形态围绕等电位线扭转，5-20 个心动周期主波围绕基线扭转一次，多数能自行终止；③ ATP 较难终止的 TdP。

**qwen2.5_7b** 2025-02-18T15:04:04Z:

根据您提供的临床表现和心电图特征，我最有可能诊断出的是长QT综合征（Long QT Syndrome, LQTS）。这是一种由于心脏复极异常导致QT间期延长的心律失常疾病。LQTS是罕见病的一种，可因基因突变引起，通常与钾、钠、钙通道的缺陷有关，女性发病率略高于男性。

除了长QT综合征以外，根据您提供的信息还应考虑以下几种可能诊断：

1. **Brugada 综合征**：一种以特异性心电图改变和猝死为特征的心脏病。尽管心电图上可能会有类似LQTS的表现（如ST段抬高），但Brugada综合征通常与钠通道的突变有关。
2. **儿茶酚胺敏感性多形性室速 (CPVT)**：这是一种以运动或情绪激动诱发的多形性室速为特征的心脏病。虽然心电图表现可能与LQTS相似，但其病理基础是钙释放调控蛋白的缺陷。
3. **短QT综合征**（Short QT Syndrome, SQTS）：尽管名字相反，SQTS也是一种罕见遗传性心脏病，但由于心脏复极异常导致QT间期缩短。它也会表现为心律失常和晕厥，但在心电图上与LQTS正好相反。
4. **左束支传导阻滞 (Left Bundle Branch Block, LBBB)**：虽然不是一种罕见病，但LBBB会导致心电图上出现类似长QT综合征的表现（如QRS波增宽、QT间期延长等），需要鉴别诊断。
5. **心脏致心律失常右室发育不良 (Arrhythmogenic Right Ventricular Cardiomyopathy, ARVC)**：这是一种以心室肌纤维化和脂肪组织替代为特征的心脏病，可能会导致晕厥或猝死。虽然其主要病理基础与LQTS不同，但在临床上有时也会伴有心律失常的表现。

请注意，以上诊断是基于您提供的信息进行推测，实际诊断需要综合考虑详细的临床资料、家族史及基因检测结果等多方面因素。

**30. user 2025-02-18T15:04:42Z:**

可于任何年龄发病，多数患儿于 1 个月至 7 岁发病，平均年龄在 2 岁左右。不同患者临床表现有较大差异，主要有：①急性能量代谢障碍危象，表现为低酮型低血糖、高血氨及代谢性酸中毒等；②心肌病，表现为心室肥厚、心功能不全、心律失常及肌酸激酶升高等；③肌病，表现为肌无力、肌张力减退、肌痛、运动耐力差、肌肉型肌酸激酶升高、肌纤维内脂质沉积等；④肝脏损害，表现为肝肿大、脂肪肝、肝功能异常等，一些肝损患儿急性起病，表现为抽搐、进行性意识障碍等，常被误诊为 Reye 综合征。此外，反复腹痛、呕吐、胃食管反流等消化道症状，反复感染、喘息等呼吸道表现以及贫血等也有报道。

新生儿筛查：确诊的患儿可无临床表现。 是一种潜在的致死性疾病，患儿可因急性能量代谢障碍危象或急性心衰而猝死。临床表现多样，与发病年龄、发病时间和病情轻重有关。最常见的症状是婴幼儿期和儿童期心肌和骨骼肌受损。成年期症状较轻或无症状，多为耐力降低或易疲劳。妊娠期，由于能量消耗和血浆生理性的肉碱水平降低，孕妇可有疲劳和心律失常等不典型表现。

**qwen2.5_7b** 2025-02-18T15:04:45Z:

根据您提供的临床表现，最有可能的诊断是贝克威思-德莱恩综合征（Beckwith-Wiedemann Syndrome, BWS）。然而，考虑到多种可能，以下是其他五种可能导致类似症状的罕见病：

1. 线粒体肌病：这是一种遗传性线粒体功能障碍导致的能量产生问题。患者可能出现急性能量代谢障碍危象以及心肌和骨骼肌受损等症状。
2. 甲状腺相关性眼病（Graves’眼病）：虽然主要表现为眼部症状，但部分重症病例也可能出现心脏受累、肌肉无力等多系统表现。
3. 糖原累积症（Glycogen Storage Disease, GSD）：这是一种糖代谢异常导致的遗传性疾病。不同类型的GSD可表现出低血糖、心肌病以及肝损伤等症状。
4. 肾上腺脑白质营养不良（Adrenoleukodystrophy, ALD）: 这是一种X连锁遗传疾病，主要表现为急性肾上腺功能不全和进行性神经系统症状。但在某些病例中也可能出现肌肉无力等临床表现。
5. 代谢性疾病：如丙酸血症、肉碱缺乏症等，它们均能导致类似的能量代谢障碍危象，伴有低血糖、高氨血症及代谢性酸中毒等症状。

请注意，这些诊断需要通过详细的病史采集、体格检查以及相应的实验室检测和基因检测来确诊。

**31. user 2025-02-18T15:05:17Z:**

单中心型该疾病往往仅表现为淋巴结肿大，而无全身症状。肿大淋巴结的中位直径约 5.5cm， 常见于胸部（24 ％）、颈部（20 ％）、腹部（18 ％）、腹膜后（14 ％）。部分单中心型疾病的患者容易合并副肿瘤性天疱疮、闭塞性细支气管炎以及生长发育迟缓。多中心型 除了多发淋巴结肿大外，往往还伴有发热、盗汗、乏力、体重下降、贫血、肝功能不全、肾功能不全和容量负荷过多（全身水肿、胸腔积液、腹水等） 等全身表现。多中心型 还有一种特殊亚型，患者可以表现为血小板减少（thrombocytopenia）、重度水肿（anasarca）、骨髓纤维化（myelofibrosis）、肾功能不全（renal dysfunction）、肝脾肿大（organomegaly）。

**qwen2.5_7b** 2025-02-18T15:05:20Z:

根据您描述的临床表现，最有可能诊断为Castleman病。其他五种可能的诊断包括：

1. 淋巴瘤：淋巴结肿大伴有全身症状和器官损害是淋巴瘤的常见表现，但需要通过病理学检查来明确。
2. EB病毒感染相关的淋巴增生性疾病（如传染性单核细胞增多症）：EB病毒可以引起淋巴结肿大，并且常伴有发热、乏力等症状，但是通常伴随有血清学证据支持该诊断。
3. 结缔组织病：某些类型的结缔组织疾病也可能导致淋巴结肿大和全身症状，如系统性红斑狼疮或硬皮症等，但这些疾病的临床特征与Castleman病有所不同。
4. 艾滋病相关淋巴增生性疾病：HIV感染可引起多种淋巴组织异常，包括Castleman病。因此在患者具有高风险因素的情况下，需要排除艾滋病的可能。
5. 淋巴结反应性增生或炎症（如慢性感染）：某些细菌、真菌或寄生虫感染可以导致局部或全身淋巴结肿大和相应症状，需通过实验室检查和影像学评估来鉴别。

**32. user 2025-02-18T15:05:56Z:**

主要临床表现为下肢远端为主，并逐渐向近端发展的肢体肌肉萎缩、无力及感觉丧失。常见临床表现为运动能力不如同龄人，跑步困难，易扭脚，足下垂，小腿腓肠肌萎缩形似“鹤腿”；查体可发现弓形足、锤状趾，远端肢体为主的无力萎缩，深感觉减退。患者通常 20 岁前起病，缓慢进展，疾病后期可能严重影响活动，但很少导致完全残疾， 也不影响正常寿命。但有些特殊类型可能起病早且严重。

**qwen2.5_7b** 2025-02-18T15:06:00Z:

根据您提供的临床表现，这种罕见病最有可能是脊肌萎缩症（Spinal Muscular Atrophy, SMA），这是一种由于运动神经元存活基因1（SMN1）突变导致的遗传性神经肌肉疾病。

除此之外，其他五种可能的诊断包括：

1. **进行性肌营养不良**：特别是假肥大型肌营养不良（Duchenne型和 Becker 型），这类疾病多见于男孩，通常从儿童早期开始，表现为肢体无力、步态异常等症状。
2. **线粒体病**：例如MELAS综合征或Kearns-Sayre 综合征等，这类疾病是由于线粒体DNA突变导致的能量代谢障碍引起的一系列症状，包括肌肉无力和感觉丧失。
3. **Charcot-Marie-Tooth 病（CMT）**：这是一种常见的遗传性周围神经病，表现为远端肢体的萎缩、无力和感觉减退。与您描述的症状相似，患者通常也有多足畸形如弓形足等。
4. **先天性肌营养不良**：包括各种类型的先天性肌肉发育不全，症状可有早发性的肌无力和肌肉萎缩。
5. **神经源性疾病**：例如多灶性运动神经病（Multifocal Motor Neuropathy, MMN），是一种以反复发作的肢体远端肌群无力为特征的周围神经病。

对于上述疾病的确诊通常需要结合详细的家族史、临床表现及辅助检查如电生理检测、基因检测等。

**33. user 2025-02-18T15:06:42Z:**

患儿于出生后至数月内发病。发病年龄多在 2 月龄内，很少晚于 5 月龄。最常表现为出生后黄疸持续不退伴或不伴大便颜色浅淡，肝脏轻度肿大或无明显肿大。 还有其他多种非特异临床表现，包括喂养困难、生长发育迟缓、腹泻、低血糖、凝血时间延长等。大多数患者上述症状及体征在 6 个月至 1 岁左右可自然缓解或经过饮食结构调整和药物治疗缓解，其中少部分的患者在青春期以后疾病进一步发展。发展后的疾病大部分是在成人后发病。通常在饮酒、摄入甜食、服用某些药物或感染后突然出现高氨血症所致的谵妄，意识混乱等精神异常表现。严重的病情发展急剧，得不到正确治疗可危及生命。患者多为消瘦体形，大部分患者有明显的饮食偏好，嗜食豆类、高蛋白、高脂食物，而厌食高糖类。

**qwen2.5_7b** 2025-02-18T15:06:43Z:

根据您描述的症状和临床表现，最有可能诊断的罕见病是“经典型有机酸血症—— maple syrup urine disease（MSUD）”，其他五种可能的诊断如下： 1. 先天性肾上腺皮质增生症（CAH） 2. 低血糖症 3. 高氨血症 4. 丙酸血症 5. 苯丙酮尿症

以上病症均有一定的临床表现和症状与您描述的情况相符。但请注意，最终的确诊需要通过实验室检查、基因检测等手段进行确认。建议及时就医并进行全面检查以明确诊断。

**34. user 2025-02-18T15:07:20Z:**

可于出生后出现症状，也可于婴幼儿期至成年早期起病。主要表现为波动性肌肉无力，不耐疲劳，多分布于眼外肌、面肌、延髓、四肢和呼吸肌，婴儿期甚至可能出现危及生命的呼吸暂停发作。单纯的综合征主要表现为骨骼肌无力，不伴有先天畸形。该疾病的糖基化缺陷和肌病重叠综合征往往伴有先天发育异常。不同类型的临床表现也有差异，部分患者，一般为出生后近端肌肉无力，胆碱酯酶抑制剂有效，严重者可出现突发的呼吸窘迫和延髓麻痹引起的呼吸暂停，可由应激、寒冷、感染等诱发。部分患者，出生后或婴儿期起病，可能出现比较严重、轴性分布的肌肉无力，但眼外肌可不受累。

**qwen2.5_7b** 2025-02-18T15:07:23Z:

根据您描述的症状，这可能是肢带型肌营养不良症（Limb Girdle Muscular Dystrophy, LGMD），这是一种罕见遗传性疾病。它主要影响肩胛骨和髋部附近的骨骼肌，并且有多种亚型。

以下是其他五种可能的诊断：

1. **先天性肌营养不良**：包括眼肌型、面肩肱型等，部分类型也可能伴有呼吸系统问题。
2. **进行性肌营养不良（Duchenne或Becker型）**：主要影响下肢肌肉，并可累及呼吸和心脏功能。尽管该疾病通常表现为严重的症状，但某些亚型可能症状较轻。
3. **眼咽型肌营养不良**：以眼外肌受累为主，也可能导致吞咽困难等其他症状。
4. **先天性线粒体肌病**：由能量产生障碍引起，可能导致广泛的肌肉无力和其他器官系统的损害。
5. **重症肌无力**：虽然不是遗传性疾病，但这种自身免疫疾病也可以表现为波动性的肌肉无力和疲劳。

这些诊断都需要通过详细的临床检查、基因检测以及可能的肌肉活检来确定。请咨询专业的罕见病医生以获得准确的诊断和治疗建议。

**35. user 2025-02-18T15:08:39Z:**

1. 通常婴幼儿期至儿童期起病，少数患者成年后起病。
2. 全身横纹肌，包括肢体骨骼肌、面肌、眼外肌、舌肌均可出现肌强直现象。
3. 肌强直在休息后突然运动时明显，反复动作后减轻，称之为“热身（warm-up）”现象。可在突然启动时出现短暂肌肉无力。病程较长的的患者，可有持续性肢体远端无力，甚至累及肢体近端。
4. 查体可见患者常肌肉发达，呈运动员体型。大力握拳后，手指不能及时展开；大力闭目后，不能立即睁开。叩击手部大鱼际肌、舌肌等部位，可引起肌肉持续性收缩（叩击性肌强直、“肌球”现象）。

**qwen2.5_7b** 2025-02-18T15:08:42Z:

根据您描述的临床表现特征，这些症状与罕见病中的一种称为“眼咽型肌营养不良症”(Ophtalmopharyngeal muscular dystrophy, OPMD)非常契合。OPMD属于Duchenne/Becker型肌营养不良（D/BMD）谱系疾病之一。

其他五种可能的诊断包括： 1. 先天性肌强直(Congenital myotonia): 一种以肌肉僵硬和运动困难为特征的遗传性疾病，通常在儿童期出现。 2. 肌强直性肌病(Myotonic dystrophy, DM): 包括DM1和DM2两种类型。DM是一种多系统受累的遗传性疾病，临床表现多样，可伴有心律失常、糖尿病等非肌肉症状。 3. 肉毒碱缺乏症（Carnitine deficiency）: 以代谢障碍为特征的一组疾病，可能表现为肌无力等症状。 4. 班达明氏病(Bandnamian syndrome): 一种罕见的线粒体遗传性疾病，主要影响骨骼肌和神经系统。 5. 勒-雪氏症（Lysosomal storage diseases, LSDs）: 是一类因溶酶体中酶缺陷导致代谢产物在细胞内积累而引发的一组遗传性代谢病。

**36. user 2025-02-18T15:09:28Z:**

由于脊柱侧凸所引起的继发性胸廓畸形可引起患儿心肺发育不全，心肺储备差，另可能出现包括心脏移位、心律失常、肺活量减少、肺炎等；腹腔畸形可引起胃肠功能紊乱， 导致消化不良，食欲缺乏。此外，由于椎体畸形和椎间盘退变引起的神经根牵拉可产生相应躯体部位的刺激性疼痛，严重者可引起脊髓功能障碍甚至瘫痪。

体格检查：大体表现为直立时两肩不等高、一侧腰部皱褶、腰前屈出现背部不对称（“剃刀背”），有时可见骨盆两侧不对称、步态异常等。此外，还需进行神经系统检查，观察患者有无肌张力增高或低下，有无四肢感觉异常。

临床上根据影像学特点通常分为以下 3 型：

Ⅰ型——椎体形成障碍型，即一个或多个椎体部分或完全形成障碍，如楔形椎、半椎体或蝴蝶椎等；

Ⅱ型——椎体分节不良型，即两个或多个椎体部分或完全分节障碍，如阻滞椎、骨桥形成等；

Ⅲ型——混合型，既有椎体形成障碍，又有椎体分节不良。

**qwen2.5_7b** 2025-02-18T15:09:31Z:

根据您描述的临床表现和特征，最有可能诊断为脊柱侧凸（Scoliosis），这是一种常见的脊柱畸形疾病。

其他五种可能的诊断包括：

1. 先天性脊柱侧凸：与脊柱发育过程中存在异常有关。
2. 神经肌肉型脊柱侧凸：常见于神经肌肉性疾病，如肌营养不良、脑瘫等。
3. 姿势性脊柱侧凸：多由长期姿势不良引起，常无结构性问题。
4. 胸廓畸形合并脊柱侧凸：伴有胸骨、肋骨或其他胸廓结构的异常。
5. 先天性肺发育不全伴脊柱侧凸：肺部发育不全导致心脏和呼吸系统受到影响。

**37. user 2025-02-18T15:09:58Z:**

男性多于女性。部分患者胸痛症状不典型，在行冠脉造影或冠脉 CT 时发现。当存在以下情况时，患者往往有临床症状：①合并阻塞性动脉粥样硬化性疾病、血栓、血流缓慢、夹层、破裂时可有劳力性心绞痛或急性冠脉综合征表现；②压迫邻近组织；③少见的有动脉瘤破裂，可引起急性心脏压塞；④虽然无显著的冠状动脉狭窄，也可发生因微血管功能障碍导致的心肌缺血。

最多见的临床表现是心绞痛，少数 患者会并发心肌梗死、严重心律失常甚至猝死。疾病可破入右房、右室或冠状窦，产生左向右分流，临床上可闻及连续性杂音，严重者会影响心脏功能。最严重的并发症是瘤体破入心包，可导致出血、心脏压塞甚至死亡。

**qwen2.5_7b** 2025-02-18T15:10:02Z:

根据您描述的症状和临床表现，这种罕见病可能是动脉夹层，特别是冠状动脉的主动脉夹层。

1. 冠状动脉粥样硬化性心脏病：尽管您提到患者多见于男性，并且症状可能不典型，但这是更为常见的疾病之一。
2. 冠状动脉微血管功能障碍：该病可导致心肌缺血的症状，但是通常无显著的冠状动脉狭窄。
3. 大动脉炎：这是一种罕见的血管炎症，可以影响到主动脉及其分支，包括冠状动脉。
4. 动脉瘤：尤其是心脏或大血管的动脉瘤，可能会压迫邻近组织或者破裂引起心包积血甚至心脏压塞。但是您所描述的症状更加符合动静脉畸形（AVM）的表现。
5. 冠状动脉瘘：这是一种罕见情况，其中冠状动脉与右房、右室或冠状窦之间存在异常连接，导致左向右分流和连续性杂音。

值得注意的是，上述所有诊断都需通过详细的病史采集、体格检查以及一系列辅助检查（如心脏超声、CT血管成像等）来确认。

**38. user 2025-02-18T15:10:48Z:**

1. 骨髓衰竭 35％患儿出生时即表现为贫血，常于生后 2 周至 2 年确诊。贫血为大细胞或正细胞正色素性、网织红细胞减少、白细胞正常或轻度降低、血小板正常或轻度增高、骨髓红系增生低下而粒系和巨核细胞系增生活跃。
2. 先天发育异常 30％ ~50％ 患者可能存在先天发育异常，主要涉及头部、上肢、心脏和泌尿生殖系统。如身材矮小，颅面部畸形包括颅面部过宽、宽平鼻梁、小头、先天性白内障、青光眼、斜视、硬腭高拱及唇腭裂甚至特纳综合征外貌，拇指畸形，先天性心血管发育异常，泌尿生殖器官畸形。存在身体的异常并不能预测患者血液系统疾病的严重程度。30％受累患者可出现生后的生长迟缓，通常伴有其他先天畸形。
3. 癌症易感性增加的患者肿瘤发生率约为 4％，高于同年龄正常人群。且肿瘤发生年龄早。包括血液系统急性白血病、骨髓增生异常综合征、淋巴瘤及实体瘤如骨肉瘤、胸腺癌、肝细胞癌、黑色素瘤、纤维组织细胞瘤、胃癌和肠癌等。患肿瘤后，较患同样肿瘤的一般人群预后差，病死率高。

**qwen2.5_7b** 2025-02-18T15:10:51Z:

根据您提供的临床表现，这种罕见病最有可能是范可尼贫血（Fanconi Anemia, FA），这是一种遗传性血液病。其他五种可能的诊断包括：

1. **先天性角化不良（Dyskeratosis Congenita）**：这是一种与端粒缩短相关的罕见遗传性疾病，可能会导致多种器官和组织的问题。
2. **骨髓增生异常综合征/骨髓增殖性肿瘤（Myelodysplastic Syndromes/Malignant Myeloproliferative Neoplasms, MDS/MPN）**：这类疾病可以影响血液干细胞，导致血细胞生成异常，并可能发展为急性白血病。
3. **免疫缺陷综合症（Immunodeficiency Syndrome）**：一些罕见的免疫系统缺陷病症也可能表现出类似的症状或并发症。
4. **先天性造血系统疾病（Congenital Hematopoietic Disorders）**：这包括某些特定类型的血液疾病，如纯红细胞再生障碍、Fanconi贫血等，都可能伴有发育异常及癌症易感性的特征。
5. **遗传性恶性肿瘤综合症（Hereditary Cancer Predisposition Syndromes, HCPs）**：这类综合征涉及多个基因突变，使得患者更易患某些类型的癌症。

需要注意的是，以上诊断需要通过详细的临床检查、血液学检测以及可能的基因检测来进一步确认。

**39. user 2025-02-18T15:11:26Z:**

病变可累及全身各系统，因此其临床表现因受累部位不同而表现各异。一般症状包括发热、乏力、盗汗、体重下降等。其余受累系统主要包括：

1. 骨骼系统， 绝大多数患者在诊断时候都有骨骼受累，以下肢骨最为常见，但仅有 50％患者有骨痛表现。
2. 心血管系统 ，病变累及心血管系统，多数患者并无明显临床表现，但如心包受累，造成大量心包积液可导致胸闷、憋气甚至心脏压塞表现；病变累及冠状动脉则可造成心肌缺血样表现，如胸闷、胸痛等；弥漫性心肌受累亦有报道，可导致心力衰竭样表现；病变包绕肾动脉则可造成肾性高血压；病变包绕肠系膜血管可有肠道缺血表现，如腹痛等。
3. 呼吸系统 呼吸系统受累多数患者无症状，少数患者有干咳、呼吸困难等表现。
4. 中枢神经系统 中枢神经系统受累可因不同部位脑实质受累而出现相应的临床表现，如肢体活动障碍、步态异常、意识改变等；脊髓受累可因不同受累节段而出现不同临床表现；垂体病灶则可表现为多饮、多尿以及其他激素分泌减少相关表现。
5. 眶后病变 眶后受累为， 常见临床表现之一，表现为单侧或双侧突眼，甚至造成失明。
6. 腹膜后空间 / 肾脏受累 ， 腹膜后病变可类似腹膜后纤维化，若病灶包绕输尿管可造成单侧或双侧肾后性梗阻。
7. 皮肤受累， 最为常见的皮肤病变为黄瘤样改变，约 1/3 患者发生在眼睑，面部、颈部、腋窝、躯干、腹股沟亦可出现。

**qwen2.5_7b** 2025-02-18T15:11:30Z:

根据您提供的临床表现和系统累及情况，最有可能的诊断是多发性硬化症（尽管它通常不是罕见病），但基于您的描述更可能的是朗格汉斯组织细胞增生症中的Hand-Schüller-Christian病型或Letterer-Siwe病型。然而，考虑到您的具体描述，以下五种其他可能性也可能需要考虑：

1. **朗汉斯细胞组织细胞增生症**：包括了不典型类肉瘤、巨细胞肉芽肿（Hand-Schüller-Christian病）以及全体型LCH（Letterer-Siwe病），这些疾病可以影响多个系统，且有类似的表现。
2. **结节病**：这是一种原因不明的多系统炎症性肉芽肿性疾病，可累及全身多个器官和组织。
3. **嗜酸粒细胞性胃肠炎**：伴随有嗜酸粒细胞增多的胃肠系统炎症反应，可能伴有皮肤、血液学以及肺部受累。
4. **淀粉样变性病**：包括多种类型的蛋白质在组织中异常沉积导致的症状群，可影响多个器官和系统。
5. **肉芽肿性多血管炎（GPA）/ 显微镜下多血管炎 (MPA)**：这些自身免疫性疾病可以引起全身多系统的炎症反应。

**40. user 2025-02-18T15:12:18Z:**

病常表现为多器官、多系统受累，男性重于女性患者。临床表现随着年龄增长而常常累及不同的器官。

1. 面容， 男性患者多在 12~14 岁出现特征性面容，表现为眶上嵴外凸，额部隆起和嘴唇增厚。
2. 皮肤血管角质瘤， 常见于经典型患者，多见于“坐浴区”即脐膝之间的外生殖器、阴囊、臀部和大腿内侧，凸出皮肤表面的红色斑点。
3. 神经系统 ，多数患者会出现周围神经疼痛，表现为足底和手掌难以忍受的烧灼感，并放射到四肢近端，甚至出现痛性痉挛；自主神经受累时表现为少汗或无汗；中枢神经系统受累时多表现为早发的短暂性脑缺血发作或缺血性卒中。
4. 眼， 特征性的表现包括结膜血管迂曲、角膜涡状混浊、晶状体后囊混浊和视网膜血管迂曲，严重者可导致视力降低甚至丧失。常为女性患者就诊的主要原因之一。
5. 消化道 ，多在进食后出现恶心、呕吐、腹胀、痉挛性腹痛和腹泻等症状，也可表现为吸收不良或便秘。
6. 肾脏 ， 早期表现为尿浓缩功能障碍如夜尿增多、多尿和遗尿，随病程进展可逐渐出现蛋白尿，甚至达肾病综合征水平，伴随肾功能损害，多在 30 岁左右进入终末期肾病。
7. 心血管系统， 可表现为高血压、冠状动脉受累导致的心肌缺血，心脏瓣膜病变和肥厚型心肌病，严重者可表现为心绞痛、心肌梗死和心力衰竭。多为疾病的晚期表现和主要的死亡原因。

**qwen2.5_7b** 2025-02-18T15:12:20Z:

根据您描述的症状，最有可能诊断出的是Fabry病（法布里病）。其他五种可能的诊断包括：

1. 卡斯特利综合征：与Fabry病类似，也是一种罕见的遗传性代谢疾病，会导致多系统受累。
2. 艾迪生病：一种由于肾上腺皮质功能减退引起的内分泌疾病，可能会出现疲劳、色素沉着等症状。
3. 家族性良性型糖尿（Mucolipidosis IV）：一种溶酶体贮积症，会导致多器官受累的症状，包括神经系统和消化系统等。
4. 遗传性神经纤维瘤病Ⅰ型（HNP1）：这是一种常染色体显性遗传的疾病，会导致神经系统、皮肤和其他组织的异常增生。
5. 罕见的慢性嗜酸粒细胞性白血病（CEL）：一种罕见的骨髓增殖性疾病，可导致多器官受累和神经系统症状。

**41. user 2025-02-18T15:12:47Z:**

疾病分为 1 型和 2 型。

1. 1 型 主要表现为反复发作的炎性反应和浆膜炎，包括反复发热、腹痛及关节炎。发热反复发作，可有寒冷、剧烈运动、手术、感染等诱因，持续 13 天自行缓解。腹痛为最常见临床表现，见于 95％的患者。表现为突然发作的全腹痛，常伴发热，腹部查体可有腹胀，腹部压痛、反跳痛、肌紧张，听诊肠鸣音消失。立位腹平片可见小肠气液平。腹痛常在 24-48 小时后自行缓解。关节炎亦为常见临床表现，见于 75％的患者。常累及下肢大关节，如髋关节、膝关节。可有关节肿胀、疼痛。皮疹见于 7％ ~40％的患者，主要累及下肢伸侧，典型皮疹为丹毒样红斑。胸膜炎见于 45％的患者，表现为突然发作的单侧胸痛，常伴发热。心包炎较少见。
2. 2 型 以淀粉样变起病。表现为持续大量的蛋白尿，常致终末期肾病。淀粉样物质亦可在心脏、胃肠道、肝脏等器官沉积，并导致相应临床表现。

**qwen2.5_7b** 2025-02-18T15:12:50Z:

根据您提供的临床表现描述，最有可能的诊断是：

1. 家族性地中海热 (Familial Mediterranean Fever, FMF) - 该疾病是一种遗传性的自身炎症性疾病，符合您的描述。
2. 白细胞功能障碍症候群（如慢性中性粒细胞白血病伴发热和血管炎）- 与反复出现的炎性反应、浆膜炎及关节炎表现一致。
3. 巨球蛋白血症 - 某些类型的巨球蛋白血症也可能有类似腹痛的表现，但较少见。
4. 血栓性疾病（如抗磷脂综合征或高凝状态）- 该类疾病可能导致反复的炎症和血管问题，包括腹痛、关节炎等，但通常伴有其他血液检查异常。
5. 系统性淀粉样变 - 其临床表现与2型描述的一致，但由于1型也有部分患者的淀粉样物质沉积，需要鉴别。

请注意，最终的确诊需结合详细的病史采集、体格检查及辅助检查结果（如血常规、C反应蛋白测定、血沉等急性期反应物、自身抗体检测、遗传学分析以及必要时的组织活检等）。

**42. user 2025-02-18T15:13:17Z:**

主要表现为先天性发育异常，进行性骨髓衰竭和肿瘤发生率增高，也有患者无发育异常或骨髓衰竭表现。

1. 发育异常 ，患者可以表现为多种形体和智力发育异常。生长缺陷、异常皮肤色素沉着、上肢单侧或双侧骨骼畸形、体格矮小、小头畸形、眼部异常、泌尿生殖道畸形、内分泌异常、听力损失、外耳异常、先天性心脏缺陷、消化道异常、中枢神经系统发育异常及面部畸形、脊柱畸形、颈部畸形及发育迟缓和（或） 智力残疾等。这些表现多为非特异性，拇指及上肢畸形较为特异。
2. 进行性骨髓衰竭 ，血液系统的异常为范科尼贫血最严重的临床特征。可表现为单一或全血细胞减少。血细胞减少起病年龄差异较大，即使同胞发病年龄也不一。中位发病年龄约为 7.6 岁，罕见于婴儿和幼童，90％患者的血液学异常在 40 岁前发病。血小板减少或白细胞减少通常先于贫血出现，常伴随大红细胞，胎儿血红蛋白（HbF 1 ） 增加，全血细胞减少逐渐加重。
3. 肿瘤发生风险增高， 包括血液系统恶性疾病及非血液系统恶性实体瘤。急性髓系白血病（AML） 发生危险增加约 500 倍，50 岁时 AML 累计发生率约 13％，多发生于15-35 岁。-7，del（7q） 与骨髓增生异常综合征（MDS）/AML 发生率增高相关。急性淋巴细胞白血病和淋巴瘤也有报道。实体肿瘤可以是该疾病的首发表现。头颈部鳞状细胞癌（HNSCCs） 是 患者最常见的实体瘤，而且发病年龄更早（20~40 岁），大多数发生于口腔（如舌癌），多处于进展期，对治疗反应差。皮肤、食管癌、肝肿瘤和泌尿生殖道肿瘤发生率也增加。少数患者可见到多种肿瘤并发，或者血液系统及非血液系统肿瘤并发。而且对化疗或放疗明显不耐受。

**qwen2.5_7b** 2025-02-18T15:13:20Z:

根据您描述的临床表现，最有可能诊断出的是范可尼贫血（Fanconi Anemia, FA），这是一种罕见遗传性疾病。

此外，还有以下五种可能的诊断： 1. 遗传性小头畸形伴发育迟缓 (Hutchinson-Gilford Progeria Syndrome)：尽管症状与FA有所不同，但部分患者可能会表现出类似的生长缺陷。 2. 乳腺癌易感基因突变综合症 (Li-Fraumeni syndrome, LFS): 患者会表现出肿瘤发生风险增高，尤其在儿童时期出现恶性肉瘤和脑瘤。 3. 贝克威思-威德纳综合征（Beckwith-Wiedemann Syndrome）：主要表现为器官过大、先天性肿瘤易发等特征。 4. 神经纤维瘤病1型 (Neurofibromatosis Type 1, NF1) ：患者可能会表现出骨骼异常和其他非血液系统恶性肿瘤的高风险，但一般不包括范科尼贫血特有的骨髓衰竭症状。 5. 贝吉尔综合症（Bloom Syndrome）：虽然此病通常表现为免疫缺陷和光敏感性皮肤问题，但它也会导致细胞不稳定和肿瘤发生率增加。

**43. user 2025-02-18T15:13:38Z:**

经典型患儿常在围生期发病，在摄取母乳或含乳糖配方奶粉数天内，患儿会出现危及生命的并发症：喂养问题、腹泻、呕吐、低血糖、肝功能损伤、出血、黄疸、 白内障。如果未及时治疗，可能会发生败血症、休克和死亡。存活至婴儿期的患儿，如果继续摄取乳糖，可能会出现严重的脑损伤。一些患儿有智力落后、生长发育延迟、共济失调、失明以及女性患者的卵巢功能障碍等远期并发症。

**qwen2.5_7b** 2025-02-18T15:13:42Z:

根据描述的经典型患儿临床表现，最有可能诊断为“经典型半乳糖血症”，该病主要由于半乳糖-1-磷酸尿苷转移酶（GALT）缺乏导致代谢异常，引发临床症状。

其他五种可能的诊断包括： 1. 先天性代谢缺陷：与经典型半乳糖血症相似的症状可由其他先天性代谢缺陷引起，如果糖不耐受、葡萄糖-6-磷酸脱氢酶缺乏症等。 2. 乳糜泻（非乳糖相关）：这是一种免疫介导的小肠疾病，可能会出现类似的消化道症状和营养吸收不良，但主要是对麸质过敏引起的。不过这种情况通常没有肝功能损伤或出血等症状。 3. 先天性感染性疾病：某些先天性感染（如巨细胞病毒、风疹等），也可以在新生儿期表现出与经典型半乳糖血症相似的临床症状，特别是低血糖和黄疸。 4. 代谢性酸中毒：一些罕见的代谢异常也可能导致类似的婴儿期症状，比如丙酮酸尿症或3-磷酸甘油醛脱氢酶缺乏症等。 5. 先天性肝病：某些先天性的肝脏疾病可能也会出现类似于该病例中的肝功能损伤和黄疸表现。

**44. user 2025-02-18T15:14:31Z:**

1. Ⅰ型最常见亚型。无原发性中枢神经系统受累表现，一些患者随着疾病进展可能出现继发神经系统临床表现（如脊髓受压等）。各年龄段均可发病，约 2/3 患者在儿童期发病。脏器表现主要为肝脾肿大，尤以脾肿大显著，常伴脾功能亢进，甚至出现脾梗死、脾破裂等。血液学主要表现为血小板减少和贫血，部分患者白细胞减少，可伴有凝血功能异常。患者表现为面色苍白、疲乏无力、皮肤及牙龈出血、月经增多，甚至出现危及生命的出血现象。多数患者有骨骼受侵，但轻重不一。早期受侵犯部位主要包括腰椎、长骨干骺端和骨干，中后期可累及骨骺和骨突。患者常有急性或慢性弥漫性骨痛，严重者可出现骨坏死（缺血性坏死），随后可出现关节塌陷累及股骨近端和远端、胫骨近端和肱骨近端。还可发生溶骨性病变、病理性骨折、椎体压缩骨折及其他因骨密度降低导致的脆性骨折。骨骼病变可影响日常活动，并可致残。儿童患者常见的表现依次是骨质疏松、长骨干骺端烧瓶样畸形、长骨干骺端密度不同程度的减低、骨皮质变薄等，可有生长发育迟缓。部分患者可有肺部受累，主要表现为间质性肺病、肺实变、肺动脉高压等。此外，患者还会出现糖和脂类代谢异常、多发性骨髓瘤等恶性肿瘤发病风险增高、胆石症、免疫系统异常等表现。
2. Ⅱ型，Ⅱ型患者除有与Ⅰ型相似的肝脾肿大、贫血、血小板减少等表现外，主要为急性神经系统受累表现。常发病于新生儿期至婴儿期，进展较快，病死率高。有迅速进展的延髓麻痹、动眼障碍、癫痫发作、角弓反张及认知障碍等急性神经系统受损表现，生长发育落后，2~4 岁前死亡。一些重度患者会出现关节挛缩。
3. Ⅲ型早期表现与Ⅰ型相似，逐渐出现神经系统受累表现，常发病于儿童期，病情进展缓慢，寿命可较长。患者常有动眼神经受侵、眼球运动障碍，并有共济失调、角弓反张、癫痫、肌阵挛，伴发育迟缓、智力落后。Ⅲ型可分为 3 种亚型，即以较快进展的神经系统症状（眼球运动障碍、小脑共济失调、痉挛、肌阵挛及痴呆） 及肝脾肿大为主要表现的Ⅲa 型；以肝脾肿大及骨骼症状为主要表现而中枢神经系统症状较少的Ⅲb 型；其他症状较轻，以心脏瓣膜钙化及角膜混浊为特殊表现， 主要出现在德鲁兹人群的Ⅲc型。

**qwen2.5_7b** 2025-02-18T15:14:34Z:

根据您提供的临床表现，最有可能诊断出的是**进行性骨化性纤维发育不良（FOP）**，尤其是其最常见的类型：I型。

另外五种可能的诊断包括：

1. **先天性肌强直** - 一种遗传性肌肉疾病，表现为僵硬和运动障碍。
2. **成骨不全症（脆骨病）** - 一种以骨骼脆弱和易骨折为主要特征的遗传性疾病。
3. **戈谢病** - 一种由于葡萄糖脑苷脂酶缺乏导致神经鞘脂积累而引发的溶酶体贮积病，可能表现出肝脾肿大、贫血等。
4. **法布里病** - 另一种溶酶体贮积症，常表现为疼痛、神经系统症状和器官功能损害。
5. **多发性骨髓瘤** - 一种浆细胞恶性肿瘤，通常会导致骨骼破坏、贫血以及凝血功能异常等症状。

请注意，虽然这些罕见病的临床表现可能与您描述的情况有相似之处，但最终的确诊仍然需要通过详细的医学检查、实验室检测和影像学等手段来确定。

**45. user 2025-02-18T15:15:00Z:**

经常从一组肌群开始，逐渐累及其他肌群，以上睑下垂和复视为最常见的发病症状， 占 80％。骨骼肌无力表现为波动性和易疲劳性，晨轻暮重。根据临床表现，分成五型。Ⅰ型：眼肌型，表现为上睑下垂、复视等。ⅡA 型：轻度全身型，四肢肌群轻度受累。ⅡB 型：中度全身型，四肢肌群中度受累，通常有咀嚼、吞咽和构音障碍。Ⅲ型： 重度激进型，进展快，数周或数月内累及咽喉肌，半年内累及呼吸肌。Ⅳ型：迟发重度型，缓慢进展，2 年内累及呼吸肌。Ⅴ型：肌萎缩型，起病半年内出现骨骼肌萎缩。神经科查体可见四肢近端、躯干、颈部、颅面部肌肉无力，不耐疲劳，Jolly 试验阳性，腱反射正常，无感觉障碍，无病理征。部分病人可进展至呼吸衰竭，出现重症肌无力危象。

**qwen2.5_7b** 2025-02-18T15:15:03Z:

根据您提供的临床表现描述，这类疾病最有可能是重症肌无力（Myasthenia Gravis, MG），这是一种获得性自身免疫性疾病，主要表现为肌肉易疲劳和波动性的无力。具体临床特征如下：

1. **重症肌无力**：最为符合所述的症状。
2. **周期性麻痹**：一种以反复发作的骨骼肌弛缓性瘫痪为特点的疾病。
3. **多发性肌炎/皮肌炎**：表现为肌肉炎症和相关皮肤病变，但通常没有波动性和易疲劳性的特点。
4. **先天性肌无力综合症**：这是一种遗传性疾病，但根据您的描述，患者一般在中年后发病，且病程较为缓慢，因此不符合此诊断标准。
5. **慢性肉芽肿性肌炎**：表现为进行性肌肉萎缩和炎症反应，通常伴有明显的局部压痛。

请注意，以上诊断需结合详细的临床检查、实验室检测（如抗体检测）、电生理检查以及影像学等综合评估后才能确定。如果需要进一步明确诊断，请咨询专业的神经科医生或罕见病专家。

**46. user 2025-02-18T15:15:28Z:**

常于青少年或成年早期起病。临床表现主要与低血钾和低血镁相关，轻型患者可无症状或表现为轻度乏力和纳差；严重患者会出现四肢抽搐、软瘫、痛性痉挛、晕厥和横纹肌溶解继发急性肾损伤，甚至因为严重室性心律失常导致心搏骤停。常见临床表现如下：

1. 全身症状 疲乏、口渴、多饮、嗜盐。
2. 神经 - 肌肉系统 肌无力、痛性痉挛、抽搐、惊厥发作、肢体麻木、感觉异常、横纹肌溶解、瘫痪、头晕、眩晕、共济失调和假性脑瘤等。
3. 心血管系统 心悸、晕厥、血压正常或偏低和室性心律失常等。
4. 消化系统 便秘、呕吐等。
5. 泌尿系统 多尿、夜尿增多、遗尿、蛋白尿和肾功能不全。
6. 骨关节系统 关节痛、软骨钙质沉着症。
7. 内分泌和生长发育 生长迟缓、青春期延迟；长期低钾和低镁的患者糖尿病或者糖耐量减低的比例并不少见。
8. 眼部症状 少数患者会出现视物模糊和巩膜脉络膜钙化。

**qwen2.5_7b** 2025-02-18T15:15:34Z:

根据您描述的症状，最有可能诊断为遗传性肾上腺皮质功能不全（Addison病）合并低钾血症或原发性醛固酮增多症（Conn综合征），但是由于症状多样且复杂，在未明确检查前还需要排除以下其他罕见疾病：

1. 低钾型周期性瘫痪：患者常表现为发作性对称性四肢弛缓性瘫痪，通常伴有肌痛、乏力等症状。部分病例可见眼睑下垂等眼部症状。
2. 肾小管酸中毒：该病以肾功能异常为特点，可出现代谢性酸中毒以及电解质紊乱（如低钾血症）的表现；
3. 先天性溶酶体贮积症（如Mucolipidosis type IV、Gaucher病等）：该类疾病常表现为发育迟缓、肌无力等症状；
4. 甲状腺功能减退症：可能引起疲劳、嗜盐增多、便秘等非特异性症状，且有神经肌肉症状如肌肉疼痛、痉挛等；
5. 特发性低钾血症或低镁血症。

**47. user 2025-02-18T15:16:07Z:**

大多数患儿于婴幼儿期发病，表现多样。发病越早，症状越重，预后越差。大部分患者生后 1 年内表现相对正常，约 75％的患者最早出现的体征是头大，多数患儿出生时头围较同龄儿大或生后不久头围迅速增大，可伴轻微的非特异性症状，包括易激惹、喂养困难和呕吐等。患儿通常生后 3~36 月龄间，在发热、感染、手术或预防接种等诱因后出现酮症、呕吐、肝大和急性脑病危象表现，包括肌张力低下、意识丧失和惊厥发作等，对症治疗后症状可缓解，但不能完全恢复。如果急性脑病危象反复发生，神经系统损伤将进行性加重，可有发育倒退现象，最终可出现认知功能障碍。部分患儿在生后数年逐渐出现运动延缓、肌张力异常和随意运动障碍，但智力发育基本正常。患者常在 10 岁内死于伴发疾病或 Reye 样发作，随年龄增长发作减少。极少数患者于青春期甚至成年时期发病，首次发病之前可无症状。

**qwen2.5_7b** 2025-02-18T15:16:08Z:

根据提供的临床表现，最有可能的诊断是MIM 251950 - 中链酰基辅酶A脱氢酶缺乏症（MCADD），这是一种罕见的遗传性代谢病。

其他可能的诊断包括： 1. 葡萄糖-6-磷酸脱氢酶缺乏症 2. 苯丙酮尿症 3. 线粒体脑肌病 4. 有机酸血症 5. 先天性肾上腺皮质增生

**48. user 2025-02-18T15:16:32Z:**

主要表现为关节、肌肉和深部组织出血，也可表现为胃肠道、中枢神经系统等内部脏器出血等。若反复出血，不及时治疗可导致关节畸形和(或) 假肿瘤形成，严重者可危及生命。外伤或手术后延迟性出血是本病的特点。

**qwen2.5_7b** 2025-02-18T15:16:35Z:

根据您提供的临床表现，最有可能诊断出的是血管性血友病（Von Willebrand Disease, VWD）。VWD是一种常见的遗传性出血性疾病，主要由于von Willebrand因子的缺乏或功能异常导致。

此外还有以下五种可能的诊断：

1. 血友病：血友病A和B是男性患者较多见的遗传性凝血障碍疾病，表现为关节、肌肉等部位反复出血。
2. 白血病或其他血液系统恶性肿瘤：某些类型的白血病或淋巴瘤也会导致不明原因的自发性或轻微创伤后出血倾向。
3. 血小板功能障碍性疾病（如特发性血小板减少性紫癜）：部分患者可以出现皮肤黏膜出血等症状，但通常不会引起关节畸形等严重后果。
4. 遗传性毛细血管扩张症：以反复鼻衄、牙龈出血及皮肤瘀斑为主要特征，有时会累及其他器官如消化道或泌尿系统。
5. 凝血因子缺乏症（如凝血因子XI缺乏症）：该病临床表现与VWD类似，但患者体内存在其他类型的凝血因子缺陷。

**49. user 2025-02-18T15:17:22Z:**

可累及全身多个脏器，临床表现多样。发病年龄多为 3~60 岁，也有8 个月及 70 多岁诊断的患者报道。儿童患者多以肝脏受累为首发表现，青少年及成人以神经系统受累为首发症状的比例增高。

可表现为无症状的转氨酶持续升高、慢性肝炎、肝硬化和急性肝功能衰竭。神经系统受累可表现为运动功能障碍、震颤、共济失调、舞蹈症、自主运动障碍、肌张力障碍，一些患者表现为面具脸、四肢僵硬、步态异常等。一些患者合并精神行为异常，如淡漠、攻击行为、性格改变等。肾脏受累可表现为血尿、蛋白尿等。血液系统受累可表现为溶血性贫血、肝硬化、脾功能亢进致血液三系下降、凝血功能异常等。相对少见的受累系统还包括骨关节、心脏、内分泌和生殖系统等。

**qwen2.5_7b** 2025-02-18T15:17:25Z:

根据您描述的症状，最有可能诊断为威尔森病（Wilson’s disease）。此外，其他五种可能的诊断包括：

1. 肝豆状核变性：是一种铜代谢障碍性疾病，临床表现与威尔森病类似。
2. 遗传性共济失调：如弗里德赖希共济失调、脊髓小脑共济失调等，这些疾病常表现出运动功能障碍和自主神经功能障碍等症状。
3. 肝脏疾病：如自身免疫性肝炎、病毒性肝炎（如乙型、丙型肝炎）、药物性肝损伤等。由于肝脏受累的表现多样且常见，所以不能排除其他肝脏疾病的可能。
4. 神经系统变性疾病：如多发性硬化症、帕金森病等，这些疾病可引起神经系统功能障碍。
5. 血管炎或血管性疾病：如巨细胞动脉炎、大动脉炎等。由于这些疾病也可导致肝脾肿大和神经系统症状，因此也不能排除其可能性。

**50. user 2025-02-18T15:18:00Z:**

临床表现具有很大的异质性，甚至同一家系中的患者也有很大差异。 通常在 30 岁前起病，青春期加重，水肿常呈急性发作。临床上以反复发作、难以预测的皮肤和黏膜下水肿为特征。水肿的特点呈发作性，自限性，一般 3~5 天自然缓解，非对称性，非可凹性。水肿可累及身体任何部位，以四肢、颜面、生殖器、呼吸道和胃肠道黏膜较为常见。其中最致命的是呼吸道黏膜水肿，可因喉水肿迅速进展导致呼吸困难或窒息， 如抢救不及时可窒息死亡，致死率高达 11％ ~40％，是 主要死因之一。消化道黏膜水肿发作表现为剧烈腹痛，伴恶心、呕吐，因此常被误诊为急腹症，导致不必要的腹部手术。发生于肢体等部位的皮肤水肿则会影响其功能。

**qwen2.5_7b** 2025-02-18T15:18:03Z:

根据您描述的症状，最有可能诊断的是遗传性血管水肿（HAE），这是一种罕见的遗传性疾病，由C1酯酶抑制剂（C1-INH）缺陷或功能障碍引起。

其他五种可能的诊断包括： 1. 药物诱导的血管神经性水肿：某些药物可能会导致血管神经性水肿，但这种疾病通常有明确用药史。 2. 特发性水肿：这是一种病因不明的水肿，可能与多种因素有关，如激素、饮食等。 3. 心源性水肿：心脏疾病引起的全身性或局部性水肿，常伴有心力衰竭的症状和体征。临床表现与HAE有一定相似之处，但是通常有心脏病史。 4. 肾源性水肿：肾脏疾病导致的水肿，一般会伴随尿检异常、高血压等症状。 5. 血管炎相关水肿：血管炎症性疾病引起的水肿，可能伴有皮疹、关节痛等其他症状。

**51. user 2025-02-18T15:18:23Z:**

出生即起病，临床表现的轻重与疾病亚型相关。临床表现为手部、肘部、膝及足部和其他容易摩擦的部位或周身出现水疱或大疱。可以出现口腔黏膜水疱或溃疡，导致吞咽困难和声音嘶哑。部分患者伴有斑驳性色素异常、秃发、掌跖角化过度、牙釉质缺陷、甲增厚和甲营养不良。其他损害包括反复瘢痕形成导致的指端残毁、贫血、骨质疏松、肌肉萎缩、心肌病、耳聋。部分亚型晚期可伴发基底细胞癌、鳞状细胞癌及恶性黑色素瘤。

**qwen2.5_7b** 2025-02-18T15:18:26Z:

根据您提供的症状，这种罕见病最有可能是大疱性表皮松解症（Epidermolysis Bullosa, EB），这是一种遗传性皮肤脆弱性疾病，会导致身体受到轻微摩擦或损伤时出现水泡或大疱。

以下是另外五种可能的诊断：

1. **先天性鱼鳞病样红皮病**：表现为婴儿期即起病的全身性红斑、丘疹和鳞屑，有时伴有水疱。
2. **掌跖角化过度症**：主要特征为手掌和足底过度角化，通常没有水疱症状，但有明显的皮肤增厚和角质层堆积。
3. **先天性大疱性表皮松解症-肢端型**（Epidermolysis Bullosa Dystrophica）：此类型EB亚型主要影响肢体末端，可伴有其他全身症状如口腔溃疡、色素异常等。
4. **遗传性牙釉质发育不全**：表现为牙齿表面出现多个小凹陷或缺损，但通常无皮肤症状。
5. **先天性角化不良症（Congenital Ichthyosiform Erythroderma, CIE）**：为一种罕见的遗传性皮肤病，其特点是出生时即存在的红皮病和鱼鳞状脱屑。

请注意，以上诊断只是基于您提供的临床表现做出的推测，最终的确诊仍需通过专业的医学检查和化验。

**52. user 2025-02-18T15:18:43Z:**

患者的临床表现随年龄、接触果糖的量和时间而存在差异。一般而言，发病年龄越小，症状越重。经典型患儿于 6 个月左右起病，通常在首次食用了含有果糖的食物，如水果、蜂蜜和某些蔬菜时发病。常见临床症状包括进食含果糖食物后出现低血糖发作，表现为恶心、呕吐、腹痛、腹胀、出汗、震颤、抽搐甚至昏迷。

如果诊断未明，患者长期慢性摄入果糖食物，除了低血糖表现外，会出现生长发育迟缓，肝肿大、腹水、黄疸及代谢紊乱，如低血糖、代谢性酸中毒、低磷酸盐血症、高尿酸血症、高镁血症、高丙酮酸血症。病情严重者出现多脏器损害，肝、肾功能衰竭，抽搐、昏迷，甚至死亡。

在急性发作时，实验室检查会发现低血糖、低血磷和低血钾，同时伴有尿酸、乳酸、丙酮酸、游离脂肪酸和甘油三酯升高。慢性患者出现肝功能损害及肝纤维化的指征，包括血清胆红素，转氨酶升高，凝血障碍。尿液检查可见蛋白尿及果糖阳性。

**qwen2.5_7b** 2025-02-18T15:18:47Z:

根据您提供的临床表现，这种罕见病最有可能是果糖不耐症或果糖-1-磷酸醛缩酶缺乏症（简称F1PAL）。其他五种可能的诊断包括：

1. 磷酸甘油酸变位酶缺乏症：这是一种以低血糖和丙酮酸升高为主要临床表现的罕见病，但患者不会出现果糖代谢问题。
2. 葡萄糖-6-磷酸脱氢酶缺乏症：此病通常会导致溶血性贫血及高铁血红蛋白血症等，并不是主要因为摄入果糖引发症状。
3. 糖原累积病（尤其是I型）：这是一种代谢障碍，但临床表现以肝脾肿大、低血糖为主，并伴有其他器官受累症状，如发育迟缓和肝功能异常等。然而与患者描述的症状不完全相符。
4. 脂肪酸氧化障碍疾病：这是一组罕见的遗传性代谢病，会导致脂质积累在肝脏和其他组织中，但通常不会出现果糖摄入后的低血糖发作。
5. 苯丙酮尿症（PKU）：这是一种先天性的氨基酸代谢异常，导致苯丙氨酸无法正常代谢。临床表现为生长发育迟缓、智力障碍等，且患者需要避免食用含苯丙氨酸的食物。然而，与果糖不耐症不同的是，其并不会出现摄入果糖后低血糖发作的症状。

综上所述，在您描述的罕见病症状中，果糖-1-磷酸醛缩酶缺乏症（F1PAL）是最有可能的诊断。其他五种可能的诊断需要通过详细的基因检测和生化检查进一步排除。

**53. user 2025-02-18T15:19:18Z:**

**1. 偏头痛** 先兆偏头痛常为该疾病的首发症状，见于 20％ ~40％的患者，是普通人群的 5倍；也可表现为无先兆偏头痛，发病率与普通人群无差异。亚裔患者偏头痛发生率较欧美患者低。

**2. 皮层下缺血性事件** 短暂性脑缺血发作（TIA） 和缺血性卒中是该疾病最常见的临床表现，见于 60％ ~85％的症状性患者，且大多数患者缺乏常见的脑血管病危险因素。2/3 的患者表现为经典的腔隙综合征。

**3. 认知缺陷** 60％的患者逐渐出现认知功能障碍与痴呆。

**4. 情感障碍与淡漠** 20％的患者具有情感障碍，抑郁状态最常见，极少数可表现为抑郁与躁狂状态交替出现；淡漠见于 40％的患者。

此外，5％ ~10％患者有癫痫发作，也有听力下降、意识障碍、脊髓梗死、颅内出血以及合并流域性梗死的报道。

**qwen2.5_7b** 2025-02-18T15:19:19Z:

根据您提供的临床表现，最有可能诊断为**腔隙性脑梗塞（小血管闭塞性脑病）**。

其他五种可能的诊断包括：

1. **进展型多灶性白质脑病伴常染色体显性遗传倾向（PMLD）**
2. **进行性核上性麻痹（PSP）**
3. **亨廷顿舞蹈症**
4. **慢性进展性脑炎（如自身免疫性脑炎）**
5. **额颞叶痴呆**

**54. user 2025-02-18T15:19:44Z:**

通常在青年早期（但 1 岁至 76 岁起病均有报道） 隐匿起病。主要表现为缓慢进展的双下肢痉挛性瘫痪，查体可见锥体束征（腱反射亢进，踝阵挛阳性、病理征阳性）。双上肢反射也可亢进，但一般不影响上肢活动。超过一半的患者会有下肢近端力弱表现。约 50％的患者查体中可发现双踝音叉震动觉减低（一般不会消失），另约 1/3 的患者有排尿障碍。

随着年龄增长，疾病逐渐进展，约 20％的患者最终会丧失行走能力。在同一家系中， 携带同一突变的不同患者，临床症状轻重可有较大差异。

**qwen2.5_7b** 2025-02-18T15:19:46Z:

根据您提供的临床表现，我最有可能诊断出的罕见病为：进行性脊肌萎缩症（Progressive Spinal Muscular Atrophy, PSMA）。

此外，其他五种可能的诊断包括： 1. 进行性延髓麻痹 (PMD) 2. 肌阵挛-癫痫伴锥体束征 (MELAS) 3. 先天性肌营养不良 4. 重症肌无力 5. 多系统萎缩 (MSA) 中的帕金森型或小脑型

**55. user 2025-02-18T15:20:37Z:**

患儿多在新生儿、婴儿早期发病。临床表现与生物素酶缺乏相似，无特异性，主要有喂养困难、呕吐、腹泻、肌张力低下、惊厥、精神萎靡、嗜睡、呼吸困难、发育落后、顽固皮疹、脱皮，可合并酮症、代谢性酸中毒、高乳酸血症等。皮肤损害中大部分患者在头面部、颈部、躯干、臀部等部位皮肤红疹或红斑、溃烂或水疱、糠状或片状鳞屑，或皮肤干糙、脱皮等，少数仅在口周、眼周、肛周局部出现皮疹。患者不伴听力或视力障碍，不同于生物素酶缺乏症。

**qwen2.5_7b** 2025-02-18T15:20:40Z:

根据提供的症状信息，我最有可能的诊断是BCKDHA（乙酰辅酶A羧化酶α亚基）基因突变引起的丙酸血症。这种罕见病可导致类似的临床表现。

其他五种可能的诊断包括：

1. 2,4-二硝基苯酚中毒：类似于代谢性酸中毒和高乳酸血症的症状。
2. 先天性肾上腺皮质增生：与发育落后、皮肤损害相似，但通常伴有电解质紊乱等其他症状。
3. 半乳糖血症1型（GALT基因突变）：表现为喂养困难、呕吐等症状，但通常是由于摄入含半乳糖食物导致的代谢障碍。
4. 长链脂肪酸氧化障碍：可能会出现类似的皮疹、发育落后和代谢性酸中毒等症状。
5. 有机酸血症-其他类型（如丙酮酸羧化酶缺乏）：症状与乙酰辅酶A羧化酶α亚基相关缺陷相似，但具体基因或酶有所不同。

**56. user 2025-02-18T15:21:09Z:**

该病患儿出生时多无异常，婴儿期临床表现并无特异性，仅表现为生长和发育迟缓。常于 3 岁有眼部症状和体征后得以诊断。

1. 视力受损， 异位晶状体所致的近视和虹膜震颤等症状可在 3 岁后出现，晚期可合并散光、青光眼、葡萄肿、白内障、视网膜剥离及视神经萎缩。
2. 智力障碍较常见，主要表现为进展性智力障碍。维生素 B6 治疗敏感型患者智力障碍较轻。
3. 精神或行为障碍， 大约 50％患儿可出现，20％患儿合并惊厥。
4. 骨骼受累， 类似马方综合征表现，或合并脊柱侧凸，漏斗胸，鸡胸，膝外翻及弓形足，X 线可见全身性骨质疏松。
5. 血栓性疾病， 发生于各个年龄段，可累及各类血管，尤其是脑血管。高水平同型半胱氨酸血症可改变血管壁及增加血小板黏附性，出现视神经萎缩、瘫痪、肺心病及肾性高血压。
6. 自发性气胸和急性胰腺炎 较少见。

**qwen2.5_7b** 2025-02-18T15:21:12Z:

根据提供的临床表现，最有可能的罕见病诊断为**同型半胱氨酸尿症（Homocystinuria, HCY）**。这是一种由于代谢缺陷导致同型半胱氨酸代谢异常增加的遗传性疾病。

此外，以下是其他五种可能的诊断：

1. **马方综合征（Marfan Syndrome）**：骨骼和眼部症状与提供的临床表现有些许相似之处。
2. **成骨不全症（Osteogenesis Imperfecta, OI）**：患者常表现出生长发育迟缓、易骨折以及全身性骨质疏松等症状，但其骨骼异常模式不同于马方综合征。
3. **肾上腺脑白质营养不良（Adrenoleukodystrophy, ALD）**：这是一种以神经系统和肾上腺功能障碍为特征的遗传性疾病，可能表现出视力受损、智力下降等临床表现。
4. **黏多糖贮积症（Mucopolysaccharidoses, MPSs）**：这类罕见病可引起生长发育迟缓、骨骼异常及眼部症状。
5. **半乳糖血症（Galactosemia）**：这是一种由于代谢途径缺陷导致的遗传性疾病，虽然不常见上述所有表现，但可以表现出生长和智力障碍等症状。

**57. user 2025-02-18T16:01:16Z:**

患者的主要表现有：出生后即发现 LDL-C 水平明显升高，胆固醇沉积在皮肤、眼睛以及肌腱形成黄色瘤和脂性角膜弓。由于机体暴露于高水平的 LDL-C 中， 患者多数会在 20 岁前就出现冠状动脉粥样硬化性心脏病并可能因此死亡。

需要详细询问患者的家族史，部分患者父母均为杂合子并因此出现 LDL-C 水平升高（通常大于本国特定年龄和性别标准的 95百分位数） 以及早发动脉粥样硬化性心血管疾病强阳性家族史。

**qwen2.5_7b** 2025-02-18T16:01:26Z:

根据您提供的临床表现，最有可能诊断的是以下几种罕见病：

1. **家族性高胆固醇血症**：这是一种常见的常染色体显性遗传性脂质代谢障碍疾病，由于LDL受体基因突变导致血液中低密度脂蛋白（LDL-C）水平显著升高，且患者通常在青少年期就会出现动脉粥样硬化性心血管疾病的症状。
2. **家族性混合型高脂血症**：这种病同样具有遗传背景，表现为高胆固醇和高三酸甘油酯的特征。但相较于家族性高胆固醇血症，其临床表现更为广泛一些。
3. **家族性低密度脂蛋白受体功能障碍综合征**：这是由于LDLR基因突变导致LDL受体无法有效去除血液中的LDL颗粒而引起的一种罕见病，临床症状与家族性高胆固醇血症类似。
4. **Birt-Hogg-Dubé 综合征（BHD）**：虽然主要特征是皮肤和肾脏的良性肿瘤、肺部气囊样改变等，但部分患者也可能伴有血脂代谢异常的表现。不过这种病相对少见。
5. **Smith-Lemli-Opitz综合征（SLOS）**：这是一种常染色体隐性遗传疾病，由于7α-羟化酶缺陷导致胆甾醇合成障碍，可能引起多种临床表现包括高胆固醇血症。然而该病症主要表现为发育迟缓、智力障碍等症状。
6. **Hypobetalipoproteinemia（低β脂蛋白血症）**：这是一种少见的遗传性代谢病，特征是血液中β-脂蛋白水平降低。尽管患者可能表现出与家族性高胆固醇血症类似的血脂异常，但其病因及临床表现有所不同。

以上五种疾病都需通过详细的家族史询问、实验室检查如基因检测等来明确诊断。在实际诊疗过程中，医生还需要结合患者的实际情况综合判断最有可能的诊断。

**58. user 2025-02-18T16:01:57Z:**

临床特征表现为运动障碍、精神症状和认知障碍三联征，通常隐匿起病，缓慢进展。发病年龄从儿童期至 79 岁不等，最常见于 30~50 岁。

疾病累及面部、躯干和肢体的快速，不自主，无节律运动；早期异常动作轻微，患者可能难以意识到舞蹈症状的存在，或将舞蹈样动作整合到随意运动中，使得正常动作的控制出现困难或偏差；随着疾病的进展，舞蹈症的范围和程度常逐渐加重，甚至影响膈肌、咽和喉部肌肉，从而产生构音障碍、吞咽困难和不自主发声；疾病晚期舞蹈症常消失，代之以僵直、少动为主的帕金森症样表现，可伴有局灶性肌张力障碍；最后常导致卧床。运动保持困难也是该病常见表现，即不能保持某些简单的自主动作，如保持伸舌动作困难等。

精神障碍可先于舞蹈症出现，常见症状包括情绪低落、抑郁、易激惹、淡漠和焦虑， 也可出现偏执、妄想和幻觉；少见症状包括强迫行为和精神病表现，以上症状多呈进行性加重。

认知障碍最主要的特征是执行功能障碍，表现为做决定、执行多重任务和转换认知目标的能力下降。患者通常对其认知缺陷缺乏自知力。记忆减退或丧失多出现在病程晚期， 随疾病进展，最终可发展为痴呆。

除以上三联征外，眼球运动异常也是一个突出表现。早期表现为眼球扫视速度减慢，但眼球追随运动保留；晚期则眼球追随运动、自主扫视和再固定全部受损。此外，体重减轻和恶病质也是该病的常见特征，患者的不自主运动大量消耗能量可使体重明显下降，而通常无食欲减退。睡眠和(或) 性功能障碍也比较常见。

运动和认知功能的逐渐恶化会导致显著的并发症，如咽喉部肌肉受累导致吞咽困难， 运动障碍、活动减少甚至卧床可导致吸入性肺炎和其他感染、褥疮等。

**qwen2.5_7b** 2025-02-18T16:01:59Z:

根据您描述的症状三联征（运动障碍、精神症状和认知障碍），该罕见病最有可能是亨廷顿舞蹈症。其他五种可能的诊断包括：

1. 亨廷顿舞蹈症：一种遗传性的神经退行性疾病，通常表现为不自主的运动、精神症状以及认知障碍。
2. 额颞叶痴呆（FTD）：以额叶和/或颞叶功能障碍为特征的一类进行性神经退行性疾病，常伴有行为、情绪和社会功能障碍。
3. 路易体痴呆：一种以帕金森病样运动障碍和认知功能障碍为主要临床表现的疾病，可出现精神症状及睡眠障碍等非运动症状。
4. 皮克病（Pick’s Disease）：一种罕见的额颞叶退化性疾病，常表现为行为、语言、情感和社会性衰退等症状。
5. 克雅氏症（CJD）：由朊病毒引起的一种致命性脑部疾病，早期可表现出类似亨廷顿舞蹈症的症状。

**59. user 2025-02-18T16:02:18Z:**

临床症状、体征主要以神经系统症状为主，临床表现严重程度亦不同。发病年龄可以从新生儿期到成年期（晚发型），并且具有广泛的表型谱。

1. 新生儿期, 约24~48 小时内无明显症状，随后出现与高氨血症相关的症状［包括嗜睡、困倦、拒食、呕吐，伴呼吸性碱中毒和（或） 癫痫发作］。与新生儿发病的其他尿素循环障碍疾病难以区分。
2. 晚发型 , 该型患者占 88％，其中 40％发生在 3 岁前，29％在儿童期，19％在成人期。主要表现为：①慢性神经认知缺陷，包括发育迟缓、共济失调、痉挛、学习障碍、认知缺陷和（或） 无法解释的癫痫发作；②由多种因素（如感染，禁食或损伤） 诱发的继发于高氨血症危象的急性脑病；③慢性肝功能障碍，肝转氨酶不明原因升高伴有或不伴有轻度凝血功能障碍，伴或不伴轻度高氨血症和蛋白质不耐受。

**qwen2.5_7b** 2025-02-18T16:02:20Z:

根据您提供的临床表现，最有可能诊断为尿素循环障碍中的精氨琥珀酸尿症（Argininosuccinic Aciduria, ASA）。

此外，其他五种可能的诊断包括：

1. 尿素循环障碍中其他的疾病：如高血氨症、瓜氨酸血症等；
2. 代谢性脑病：如线粒体脑肌病、乳酸酸中毒伴卒中样发作（MELAS）等；
3. 慢性神经系统发育障碍：如自闭症谱系障碍、神经纤维瘤病等；
4. 肝脏疾病：如肝豆状核变性、威尔逊氏症等；
5. 代谢性高氨血症相关疾病：如先天性甲硫氨酸酶缺乏症等。

**60. user 2025-02-18T16:02:55Z:**

部分患儿在新生儿期多无临床症状，出生 3-4 个月后逐渐出现典型症状：头发由黑变黄，皮肤颜色浅淡，尿液、汗液鼠臭味。随着年龄增长，逐渐表现出智力发育落后、小头畸形、癫痫发作，也可出现行为、性格、神经认知等异常。如多动、自残、攻击、自闭症、自卑、忧郁等，婴儿期还常出现呕吐、湿疹等。 部分 患者在新生儿期也多无临床症状，出生 1-3 个月后除表现 PKU 症状外，主要表现为运动障碍、嗜睡、躯干肌张力低下、四肢肌张力增高或低下、眼震颤、吞咽困难、口水增多、松软、角弓反张，反应迟钝、失眠、智力发育严重障碍等。

**qwen2.5_7b** 2025-02-18T16:02:57Z:

根据您提供的临床表现，最有可能诊断为苯丙酮尿症（Phenylketonuria, PKU）。以下是其他五种可能的诊断：

1. 脑白质营养不良：表现为小头畸形、智力发育落后等症状。
2. 假性PKU: 该病与PKU类似，但并非由于苯丙氨酸代谢异常引起的。症状包括智力发育迟缓和运动障碍等。
3. 先天性甲状腺功能减退症（先天性甲低）：可出现智力发育落后、肌张力低下等症状，并且有时会有皮肤颜色浅淡的表现。
4. 苯环氨基酸尿症：表现为头发变黄、鼠臭味及神经系统症状等，但不涉及苯丙氨酸代谢异常。
5. 神经系统代谢病：如线粒体疾病或神经酰胺沉积病等，可表现出多动、自残等症状。

**61. user 2025-02-18T16:03:39Z:**

临床表现的严重程度具有很大的差异。患者可以表现为骨骼矿化异常、牙齿或关节病变、肌肉乏力等。目前，疾病主要分为 7 种类型。

1. 单纯牙型， 是最轻、也是最常见的类型，仅有牙齿受累表现，无佝偻病或骨软化症的表现。
2. 成人型，常中年发病，表现为牙齿异常或骨软化症。由于反复骨折、骨骼和关节疼痛或肌肉无力，可引起活动障碍。
3. 儿童型，常在 6 月龄后发病，可轻度到重度，患儿可有不同程度的牙齿脱落、佝偻病的表现。
4. 婴儿型 ，于 6 月龄前发病，常表现为纳差、难以存活、衰弱，可有囟门宽阔或头颅畸形。50％的婴儿型 HPP 会死于各种并发症。
5. 围生期型，是极端类型，在宫内即发病，出生时症状明显，几乎均是致死型。患儿四肢短小、严重骨骼矿化不足，可表现为高调哭喊、癫痫发作、周期性呼吸暂停、伴有发绀和心动过缓、不明原因发热、易激惹、骨髓抑制性贫血和颅内出血、肺不张。
6. 假性低磷酸酯酶症， 类似于婴儿型，患者具有婴儿型低磷酸酯酶症的表现，但血清 ALP 正常或升高。
7. 良性型， 有时在宫内出现骨骼畸形，有围生期低磷酸酶活性，但出生后病情缓解，没有婴儿型低磷酸酯酶症或单纯牙型低磷酸酯酶症的相关表现。

**qwen2.5_7b** 2025-02-18T16:03:42Z:

根据您描述的临床表现在7种类型中，最有可能诊断出的是婴儿型低磷酸酯酶症（Hypophosphatasia, HPP）。其他五种可能的诊断包括：

1. 单纯牙型：只有牙齿受累的表现，没有其他骨骼或肌肉症状。
2. 成人型：表现为成人期发病，通常在中年时期出现牙齿异常或骨软化症相关症状。
3. 儿童型：儿童期发病，从6月龄开始到较重的不同程度的临床表现，包括牙齿脱落、佝偻病等。
4. 围生期型：这是极罕见的一种类型，在宫内即发病，并在出生时出现明显症状。通常表现为四肢短小、严重骨骼矿化不足等症状。
5. 假性低磷酸酯酶症：这是一种类似婴儿型HPP的表现，但血清碱性磷酸酶(ALP)水平正常或升高。

以上这7种类型是由不同的基因突变引起的，其中I型（婴儿型）最为常见且病情最严重。其他类型在临床表现上有所不同，因此需要通过详细的遗传学检测和生化指标来明确诊断。

**62. user 2025-02-18T16:04:07Z:**

患儿主要表现为方颅、鸡胸、串珠肋、手镯征、足镯征、亨利氏沟，多在将近周岁开始负重时出现下肢畸形，可表现为膝内翻（O 形腿） 或膝外翻（X 形腿），并伴有生长迟缓、身材矮小、步态摇摆、进行性加重的骨畸形、多发性骨折、骨骼疼痛以及牙齿发育异常（牙质差、牙痛、脱落后不易再生） 等。影像学表现为骨骼畸形、长骨干骺端增宽和模糊，呈杯口样，杯口内可见许多细条状钙化影如毛刷状。在成人期主要表现为肢体乏力、活动受限、骨痛、多发病理性骨折（四肢长骨、肋骨、骨盆和椎体均可发生）、身高变矮。影像学表现为骨密度普遍减低，骨小梁模糊，呈毛玻璃状，骨盆畸形、假骨折线、椎体呈双凹变形等。

**qwen2.5_7b** 2025-02-18T16:04:10Z:

根据描述的临床表现，最有可能诊断为佝偻病（维生素D缺乏性佝偻病），其他五种可能的诊断包括：

1. 成人型佝偻病（也称为假性佝偻病）：这是一种罕见的遗传性疾病，与维生素D代谢相关基因突变有关。
2. 骨质疏松症：表现为骨密度减低、骨折风险增加等症状。但该病症通常在老年人中较为常见，且症状特点可能有所不同。
3. 软骨营养不良：一种常染色体隐性遗传病，主要影响骨骼发育和成熟过程，导致骨骼畸形和生长迟缓等临床表现。
4. 成骨不全症（脆骨病）：这是一种罕见的遗传性疾病，以骨质脆弱、易骨折为特征。然而，在该病症中，佝偻病特有的X形腿或O形腿并不常见，且多表现为先天性症状，而非后天获得性的骨骼变化。
5. 石骨症：一种极为罕见的骨骼发育障碍疾病，导致全身骨骼异常钙化和硬化。临床表现包括广泛性骨痛、骨折倾向及生长迟缓等症状。

**63. user 2025-02-18T16:05:16Z:**

临床表现有心力衰竭、胸痛、低血压、晕厥、心源性猝死、心律失常和血栓栓塞等。部分患者还会有心脏外的表现，如周围神经病、自主神经病和胃肠道症状等。

**qwen2.5_7b** 2025-02-18T16:05:20Z:

根据您提供的临床表现，我将尝试列出几种可能性较高的罕见疾病：

1. **遗传性心肌病**：包括肥厚型心肌病或扩张型心肌病，这类疾病可能导致心力衰竭、心律失常及晕厥等症状。
2. **长QT综合征**：这是一种遗传性心脏电生理异常导致的心脏节律问题，可能会引起晕厥甚至猝死等严重症状，同时伴有心律失常的临床表现。
3. **法布里病**：这是一种罕见的X连锁隐性遗传代谢疾病，主要由于α-半乳糖苷酶A缺乏导致脂质在体内蓄积。除了心脏受累外，患者还可能出现神经病变、肾功能衰竭等非心脏症状。
4. **特发性心肌炎**：虽然病因不明，但本病可引起类似心血管疾病的多种临床表现，包括心力衰竭、低血压和晕厥。
5. **家族性高胆固醇血症**：这是一种由LDL受体基因突变引起的遗传性疾病，患者往往在年轻时就表现出严重的冠状动脉疾病症状（如胸痛），但部分病例也可能伴有心脏外的表现。
6. **肺栓塞或心包炎**：这两种疾病也可引起类似的临床表现。肺栓塞表现为突发性呼吸困难、胸痛等；而心包炎则可导致剧烈的胸部疼痛，且疼痛在深呼吸时加剧。

以上列举的六种罕见病都与您提供的症状有一定关联，但具体诊断需要结合详细的病史询问、体格检查及必要的辅助检查来确定。

**64. user 2025-02-18T16:05:55Z:**

**1. 第二性征不发育和配子生成障碍** 男性表现为童声、小阴茎、无阴毛生长、小睾丸或隐睾、无精子生成；女性表现为乳腺不发育、幼稚外阴和原发闭经。

**2. 骨骺闭合延迟** 上部量 / 下部量<1，指尖距 > 身高，易患骨质疏松症。

**3. 嗅觉障碍** 因嗅球和嗅束发育异常，40％ ~60％ IHH 患者合并嗅觉丧失，不能识别气味。

**4. 其他表现** 面中线缺陷，如唇腭裂；孤立肾；短指(趾)、并指(趾)；骨骼畸形或牙齿发育不良；超重和肥胖；镜像运动等。

**qwen2.5_7b** 2025-02-18T16:05:59Z:

根据您提供的临床表现，最有可能诊断为特发性嗅觉缺失（ISSH）/嗅球无癥候群，其他可能的诊断包括：

1. 特纳综合症：第二性征不发育、原发闭经是特纳综合症患者常见的症状之一。此外，患者的骨骺闭合可能会延迟。
2. 苯丙酮尿症：这是一种代谢障碍疾病，可能导致智力低下、生长迟缓和第二性征不发育等症状，部分患儿还可能出现嗅觉减退或丧失。
3. 高促性腺激素性闭经-原发性睾丸功能减退症（HPO）：表现为闭经、无生育能力及第二性征发育不良等典型临床表现，骨骺可能闭合延迟，但与IHH的遗传模式不同。此外，部分患者可出现嗅觉障碍。
4. 软骨发育不全：该病是一种常染色体显性遗传疾病，主要表现为身材矮小、特殊面容（短头畸形、鼻梁塌陷等）、骨骼畸形等症状，也可能伴有第二性征发育不良等问题。
5. 先天性肾上腺皮质增生症：由于酶缺陷导致激素合成异常，可能引起第二性征不发育或幼稚表现，并且部分患者可合并嗅觉障碍。

**65. user 2025-02-18T16:06:32Z:**

最常见的症状是活动后气短，其他症状包括乏力、头晕、胸痛、胸闷、心悸、黑矇、晕厥等，一旦出现晕厥或眩晕，提示患者心搏量可能已明显下降。由于症状、体征不典型，绝大多数患者就诊时间明显延误，至少 1/5 患者从症状出现至确诊时间超过 2 年；患者可出现肺动脉瓣第二心音亢进，三尖瓣关闭不全引起三尖瓣区收缩期杂音； 右心衰竭体征包括颈静脉充盈或怒张、肝脏肿大、下肢水肿、多浆膜腔积液、黄疸和发绀等。

**qwen2.5_7b** 2025-02-18T16:06:35Z:

根据您描述的症状表现，这种罕见病最有可能是右室流出道梗阻型肺动脉高压（Right Ventricular Outflow Tract Obstruction, RVOOTO），也称为特发性肺动脉瓣下狭窄。其他可能的诊断包括：

1. 限制型心肌病：表现为心脏扩大、充血性心力衰竭和静脉压升高，可引起乏力、头晕、胸痛等症状。
2. 肺动脉闭锁不全：由于瓣膜或瓣环发育异常导致肺动脉瓣关闭不全，主要症状为呼吸困难和乏力。
3. 非对称性肥厚型心肌病（非梗阻型）：表现为心脏杂音、乏力、胸痛等症状，可能伴有黑矇、晕厥等。
4. 限制型心包炎：由于心包增厚导致心脏舒张功能受限，主要症状为呼吸困难和乏力。
5. 慢性肺源性心脏病（肺心病）：由于慢性肺部疾病引起右心室肥大，临床表现为肺动脉高压、右心衰竭等症状。

**66. user 2025-02-18T16:07:48Z:**

一般起病隐匿。主要表现为干咳、劳力性呼吸困难，杵状指(趾)、双下肺分布为主的爆裂音是其典型体征。终末期可以出现发绀、肺动脉高压、肺心病、右心功能不全的相关临床表现。

**qwen2.5_7b** 2025-02-18T16:07:51Z:

根据您描述的临床表现，最有可能诊断出的是特发性肺纤维化。

1. 特发性肺纤维化：主要表现为干咳、劳力性呼吸困难和杵状指(趾)，是肺间质性疾病的一种。
2. 肺泡蛋白沉积症：除了干咳、呼吸困难外，该病患者常有肺部爆裂音，并且部分患者也会有杵状指（趾）的表现。但肺泡蛋白沉积症比较罕见。
3. 矽肺/尘肺：这类疾病主要由于长期吸入粉尘引起，也可出现干咳、呼吸困难等症状，但其特征性表现为胸部X线或CT上可见到典型的结节和纤维化改变。
4. 慢性支气管炎：可表现出类似的症状，如干咳、劳力性呼吸困难，但常伴有慢性咳嗽史，并且肺部听诊爆裂音较少见。
5. 纤维性肺泡炎：是一种病因不明的肺间质疾病，主要表现为进行性的呼吸困难和干咳。但该病较少出现杵状指(趾)的表现。

请注意以上诊断并非最终结论，需要根据患者的具体情况进行综合判断并结合实验室检查及影像学检查结果来明确诊断。

**67. user 2025-02-18T16:08:13Z:**

一种多器官、多系统受累的疾病，可累及几乎全身所有器官和组织。临床表现多种多样，患者因不同器官受累就诊于不同专科，容易漏诊。该病多数患者起病缓， 慢性进展。可以单器官起病，逐渐累及多个器官，也可以多种器官同时发病。少数患者病程呈自限性。

泪腺、唾液腺为最常见受累器官。主要表现为对称性、无痛性泪腺，颌下腺或腮腺肿大，硬结，伴或不伴有鼻窦炎或眼外肌增厚。消化系统受累可表现为自身免疫性胰腺炎、硬化性胆管炎、硬化性肠系膜炎等，临床可出现腹部隐痛、腹胀、消化不良、黄疸等。肺部受累时可表现为支气管壁增厚、肺间质病变、肺部结节 / 肿块以及胸膜增厚等，患者可无症状或咳嗽、气短及喘憋等。腹膜后纤维化或腹主动脉周围炎也是较常见的临床表现之一，主要为腹主动脉周围软组织增厚，可包绕输尿管造成输尿管狭窄和肾盂积水，少数患者发生腹主动脉瘤。临床表现为腹痛、腰痛或下肢水肿等。不到 10% 的患者发生 IgG4 相关性肾脏疾病，多表现为小管间质性肾病、肾盂占位性病变、输尿管管壁增厚或肾脏肿块样病变。其他受累器官包括甲状腺（硬化性甲状腺炎）、前列腺等。中枢神经系统病变少见，如自身免疫性垂体炎、肥厚性硬脑膜炎、颅内炎性假瘤等，患者可出现头痛、尿崩症、垂体功能减低等。除上述器官受累外，多数患者合并浅表或深部淋巴结肿大。另外，约半数患者有过敏相关病史，可以伴发哮喘。

**qwen2.5_7b** 2025-02-18T16:08:19Z:

根据您提供的临床表现，这种多系统受累的罕见疾病最有可能是IgG4相关性疾病（IgG4-related disease, IgG4-RD），其特点是多个器官和组织被IgG4阳性的浆细胞浸润，并伴有进行性纤维化。此外，还需要考虑以下五种可能的诊断：

1. **多发性硬化症**：神经系统症状相似，但通常伴随视神经病变、共济失调等特征。
2. **肉芽肿性疾病**（如结节病）：肺部受累较为常见，可有非干酪样坏死性肉芽肿形成。
3. **纤维肌痛综合征**：主要表现为慢性全身多处肌肉疼痛，但无器官特异性病变。
4. **淋巴瘤或恶性组织细胞增生症**：需要排除肿瘤性疾病导致的相似临床表现。
5. **自身免疫性肝病（如原发性硬化性胆管炎）**：消化系统受累症状类似，但通常以肝脏和胆道系统为主。

请注意，以上诊断仅根据提供的信息进行推断，并不代表最终确诊。建议患者进行全面检查及专业评估后由医生做出最终判断。

**68. user 2025-02-18T16:08:32Z:**

患儿有胆汁淤积和肝硬化的发生。其主要临床表现为高结合胆红素血症，常出现脂溶性维生素吸收不良，如佝偻病等。而不同酶缺陷的临床表型略有不同，如氧固醇 7α- 羟化酶缺陷可出现成人期遗传性痉挛瘫痪；胆固醇 25 羟化酶缺陷可发生顽固性便秘；3β- 羟基类固醇 -Δ5-C27- 类固醇脱氢酶缺陷可发生婴儿期胆汁淤积性肝病和脂溶性维生素缺乏；固醇27- 羟化酶缺陷可发生儿童慢性腹泻及发育迟缓、成人期痉挛性瘫痪；α- 甲酰辅酶 A 消旋酶缺陷可发生婴儿期脂溶性维生素缺乏、成人期感觉神经病变；胆汁酸辅酶 A 氨基酸N- 乙酰转移酶缺陷可发生肝内胆汁淤积、发育迟缓、家族性高胆烷血症等。

**qwen2.5_7b** 2025-02-18T16:08:37Z:

根据您描述的临床表现，我首先考虑诊断为原发性胆汁淤积症中的遗传性类固醇生成障碍，特别是与胆固醇代谢相关的酶缺陷（如氧固醇 7α- 羟化酶、胆固醇25羟化酶和3β-羟基类固醇 -Δ5-C27- 类固醇脱氢酶）的罕见病。以下为其他可能的诊断：

1. 继发性胆汁淤积症：虽然主要与原发性因素相关，但也需要考虑肝内外胆道梗阻、自身免疫性疾病（如原发性硬化性胆管炎）、药物或毒素等导致的继发性胆汁淤积。
2. 肝内胆汁淤积症：包括暂时性的胆汁淤积，例如新生儿高胆红素血症；以及肝细胞功能异常所引起的长期胆汁淤积（如Alagille综合征、Budd-Chiari 综合征等）。
3. 血色病或其他代谢性肝病：某些罕见的遗传性疾病或获得性疾病可能会导致肝脏功能障碍和胆汁淤积，比如血色素沉着症、威尔森氏症（铜沉积症）、α1-抗胰蛋白酶缺乏症等。
4. 脂肪酸氧化障碍：一些罕见的脂代谢疾病也可引起肝损伤及胆汁异常，例如肉碱棕榈酰转移酶I缺陷症、长链脂肪酸氧化缺陷等。
5. 胆道闭锁或先天性胆管发育不全：这是一种较为常见的导致婴儿期出现严重黄疸和胆汁淤积的肝脏疾病，但通常与其他酶缺陷引起的遗传病有所区别。

**69. user 2025-02-18T16:09:29Z:**

**1. 急性新生儿型** 起病急骤，病情进展迅速，可威胁生命。出生后 1~2 周内表现为喂养困难、呕吐、肌无力、肌张力减退，嗜睡或加重进展至昏迷。异戊酸蓄积可造成“汗脚”的特征性气味。查体可有肝大。化验提示代谢性酸中毒、高氨血症、低或高血糖、酮症、低钙血症及全血细胞减少。如果患者能度过新生儿期的急性发作，将会进展为慢性间歇型。

**2. 慢性间歇型** 起病隐匿。仅表现为非特异性不能耐受空腹或发育落后。常因上呼道感染或摄入高蛋白质饮食诱发，发作呈反复发生呕吐、嗜睡、昏迷、酸中毒伴酮尿， 会出现“汗脚气味”，限制蛋白质饮食并输注葡萄糖时可以缓解发作。绝大部分慢性间歇型患者精神运动发育正常，但是一些患者会表现为发育延迟和不同程度的智力低下。

**qwen2.5_7b** 2025-02-18T16:09:53Z:

根据您描述的临床表现，最有可能诊断的是 **异戊酸血症**（Isovaleric Acidemia, IVA）。

其他五种可能的诊断包括： 1. 长链脂肪酸氧化障碍 (LCAO)：如长链羟脂酰CoA脱氢酶缺乏症。 2. 丙酸血症 (Propionic Acidemia, PA)：这是一种遗传性有机酸代谢障碍，与IV型异戊酸血症类似但有不同的代谢途径和临床表现。 3. 草酰乙酸-琥珀酰CoA转硫酶缺乏症 (Oxalosuccinic Aciduria, OSA)。 4. 甲基丙二酸尿症 (Methylmalonic Acidemia, MMA)，特别是其中的辅酶B12依赖型亚型。 5. 肉碱缺乏症：如肉碱棕榈酰转移酶I缺乏症。

**70. user 2025-02-18T16:10:22Z:**

1. 第二性征不发育和配子生成障碍。
2. 嗅觉障碍， 因嗅球和嗅束发育异常，导致不能识别气味，尤其不能识别芳香气味。

**qwen2.5_7b** 2025-02-18T16:10:29Z:

根据您提供的临床表现，我会考虑以下几种罕见病诊断：

1. Kallmann综合征 - 该疾病表现为性腺功能减退、第二性征不发育及嗅觉障碍。
2. 脑颜面血管瘤病（Sturge-Weber综合症） - 可引起面部血管痣和/或大脑半球血管畸形，但本病例中主要表现更符合Kallmann综合征。
3. XY纯合性睾丸缺失症（又称为Swyer综合征） - 该疾病表现为男性伴染色体组成而没有睾丸，导致第二性征不发育和配子生成障碍。
4. 性腺功能减退-特纳综合症（Turner Syndrome） - 尽管更常见于女性，但也可在极少数情况下影响男性；表现为生殖系统异常、身材矮小以及第二性征不发育。但是该病一般不会单独引起嗅觉障碍。
5. Klinefelter综合征 - 通常表现为X连锁的智力障碍和睾丸功能减退，可能伴随轻微的性别认同问题。

请注意，这些诊断是基于您提供的信息进行初步推断。要确诊疾病，还需要进一步检查、实验室测试和其他临床评估。

**71. user 2025-02-18T16:10:51Z:**

病情从轻至重差异很大，因此容易被误诊和漏诊。临床表现主要包括：

**1. 发热** 热型不规则，可呈周期性或者持续性高热。下丘脑受累患者可以出现中枢性体温调节异常。

**2. 皮疹** 主要分布于躯干、头皮和发际。初起为淡红色丘疹，然后可以为出血性或湿疹样皮脂溢出样皮疹，继而结痂，结痂脱落后留有白斑。

**3. 口腔及眼耳鼻喉病变** 牙龈肿胀、牙齿松动，突眼，顽固性中耳炎伴外耳道皮疹为典型表现。

**4. 中枢性尿崩症** 垂体、下丘脑甚至其他部位中枢神经系统病变，少数患者出现神经精神症状。

**5. 呼吸道症状** 咳嗽，重者憋喘、发绀，甚至反复气胸发作。肺部体征通常不明显。

**6.** 肝脾可以肿大，可以出现黄疸、肝功能异常、肝功能衰竭和门脉高压，最终患者可死于肝硬化和肝功能衰竭。

**7. 消化道症状** 消化道累及较少见，可以表现为息肉或腺瘤样改变，通常不出现临床症状，病变弥漫时可能出现腹痛、腹泻和低蛋白血症等。

**8. 造血系统** 淋巴结可见肿大，骨髓受累时可以出现贫血、白细胞和血小板计数异常。

**9. 骨质损害** 颅骨、四肢骨、脊柱、骨盆等可有疼痛及肿块，可以发生病理性骨折。眼眶骨质病变所致突眼为儿童患者典型表现之一。

**qwen2.5_7b** 2025-02-18T16:11:02Z:

根据您提供的临床症状，该疾病最有可能是Sturge-Weber综合征（SWS），也称为面部血管瘤伴脑血管畸形综合症。

1. Sturge-Weber 综合征：是一种罕见的神经皮肤综合征，主要特征为面部血管瘤和脑血管畸形。符合发热、皮疹、中枢性尿崩症、呼吸道症状及消化道症状的表现。
2. 肝豆状核变性（Wilson病）：该病可以导致肝脾肿大，黄疸，肝功能异常等，但通常不会出现中枢神经系统病变，所以排除了这个可能性。
3. 朗格汉斯细胞组织细胞增生症（Langerhans cell histiocytosis, LCH）: 可能会出现发热、皮疹、呼吸道症状和肝脾肿大等症状。但是其病因及临床表现与Sturge-Weber综合征不同，且神经系统受累相对较少。
4. 遗传性出血性毛细血管扩张症（Hereditary Hemorrhagic Telangiectasia, HHT）：该病可以表现为反复鼻衄、皮肤黏膜的毛细血管扩张和肺部动静脉畸形等。但是HHT主要以血管病变为主，中枢神经系统受累的表现不明显。
5. 颅内肿瘤：某些颅内肿瘤可能会导致类似的症状，如发热、皮疹、中枢性尿崩症以及呼吸系统症状。但根据临床表现，Sturge-Weber综合征具有更明显的面部血管瘤和脑血管畸形的特点，因此与颅内肿瘤的诊断相比可能性较低。
6. 神经纤维瘤病（Neurofibromatosis, NF）：是一种遗传性疾病，主要表现为皮肤神经纤维瘤、咖啡牛奶斑以及神经系统异常等。虽然NF可以导致中枢神经系统受累，但其临床表现和Sturge-Weber综合征有所不同，且面部血管病变较轻。 综上所述，根据所提供的临床症状及排除其他疾病后，最有可能的诊断是Sturge-Weber综合征。建议进一步进行相关检查以确诊并制定合适的治疗方案。

**72. user 2025-02-18T16:28:06Z:**

**1. 生长障碍及其他畸形，**患儿出生时体重大多在正常范围内，但部分患儿的出生身长低于 48cm（42~47cm）。出生后逐渐显著地生长速度减慢，未治疗的男性患者终身高在 116_142cm，女性患者终身高在108_136cm。同时患者手、足和内脏器官均小。部分患者伴有其他的先天性畸形，如髋关节脱位、主动脉瓣狭窄、隐睾、先天性白内障等。

**2. 面容特点，**患者特征性的面容改变为前额突出、鞍鼻、小下颌，头部与身体比例较正常儿童大，但与同龄儿童相比头围绝对值仍偏小。患者头发稀疏，部分患者可出现“落日征”，地中海地区和中东地区的患者可表现出蓝巩膜。患者出牙延迟，乳牙脱落较晚并且易合并多发龋齿，由于下颌骨较短小，可有牙列不齐。

**3. 骨骼肌肉系统** 部分患者具有先天性髋关节脱位、股骨头缺血性坏死等表现。由于中面部骨骼发育不良和咽部解剖结构异常，患者说话音调高尖。85％患者肘关节活动受限。

**4. 代谢紊乱 ，**患者常合并代谢异常，包括新生儿期和婴儿期的低血糖，而成年后出现逐渐加重的肥胖。

**5. 性发育延迟 ，**患者生殖器较小，男性患者表现为小阴茎和小睾丸，女性患者超声检查发现卵巢体积较小。大多数患者表现为青春发育延迟。虽然性发育落后，但是男性和女性患者的性功能最终可发育成熟，具有生育能力。

**6. 肿瘤发生率低，**患者的肿瘤发生的风险较一般人群显著降低。

**qwen2.5_7b** 2025-02-18T16:28:42Z:

根据您提供的临床表现，最有可能的诊断是萨尔蒙-布兰顿综合征（Salmon-Brown Syndrome），这是一种罕见遗传病，主要影响生长、骨骼和面部发育。

此外，其他五种可能的诊断包括： 1. 马凡综合症：一种以心血管系统异常为主的心脏血管结缔组织疾病。 2. 艾尔沃德氏症候群（Elwes Syndrome）：罕见遗传性神经系统疾病，影响生长、面部特征和牙齿。 3. 雷诺氏病（Renpenning Syndrome）：一种非常罕见的遗传性疾病，表现为身材矮小、面部畸形以及特定的身体特征等。 4. 小胖威利综合症（Prader-Willi 综合征）：以过度饥饿、生长障碍和发育迟缓为主要特征，但没有提到蓝巩膜等症状，因此可能性较低。 5. 肥大性骨关节病（Digeorge Syndrome 或 22q11.2 缺失综合症）：一种由于染色体 22 上的基因缺失而导致的症状复杂多样疾病。

**73. user 2025-02-18T16:29:29Z:**

主要症状为双眼先后发生的无痛性视力突然急剧下降，双眼发作间隔一般为数周到数月。眼底检查在疾病早期表现为视盘充血水肿、视盘旁毛细血管迂曲扩张，疾病后期视盘水肿和毛细血管扩张消退，最终视盘颞侧或全部呈萎缩性改变。多数病人只存在眼部表现，少数可合并全身其他系统症状如智力障碍、癫痫、听力障碍、肌张力障碍等。

**qwen2.5_7b** 2025-02-18T16:29:34Z:

根据您提供的临床表现，这种罕见病可能为视神经脊髓炎谱系疾病（Neuromyelitis Optica Spectrum Disorders, NMOSD）或Leber先天性黑蒙1型（LCA1），此外还有以下几种可能性：

1. 视神经脊髓炎谱系疾病：此病是由于自身免疫反应攻击中枢神经系统导致的一种罕见病。临床表现为视神经炎和脊髓炎，可伴有其他症状如脑干受累、小脑症状等。
2. Leber先天性黑蒙（LCA）是一种遗传性的视网膜色素变性疾病，常在儿童早期出现视力下降的症状。这种疾病通常由RPE65、CNGA3、CNGB3等多个基因突变引起，而您所描述的症状与Leber先天性黑蒙1型（LCA1，由RLBP1基因突变导致）较为符合。
3. 视神经炎：视神经炎是指视神经的炎症反应，主要症状为视力急剧下降。在一些情况下，视盘充血水肿是早期的表现之一。后期可能会出现萎缩性改变，但通常不会像NMOSD那样持续时间更长或伴有其他神经系统症状。
4. 脱髓鞘疾病：这类罕见病包括多发性硬化症和自身免疫性脱髓鞘脑白质病等。它们会导致大脑、脊髓和其他神经组织的损伤，并可能引起视力下降等症状，但通常不会出现视盘水肿和萎缩性改变。
5. 视网膜血管炎：这是一种影响视网膜血管的炎症反应，可能会导致无痛性的视力突然急剧下降。然而，它的症状一般不包括视盘充血、水肿或最终的萎缩性改变。 以上仅为参考建议，具体诊断需要结合患者详细的病史资料以及临床检查结果（如血液检测、脑脊液分析、眼底荧光素血管造影等），并由专业医生进行综合判断。

**74. user 2025-02-18T16:30:10Z:**

患者临床表现为婴儿期低酮性低血糖、肝功能异常、乳酸酸中毒、心肌病，部分患者表现为生长迟缓，肌张力低下或心肌病，少数可有胆汁淤积或肝硬化。幼儿期患分患者表现为生长迟缓，肌张力低下或心肌病，少数可有胆汁淤积或肝硬化。幼儿期患者可有喂养困难，发作性代谢紊乱，发作性横纹肌溶解。多数患者儿童期会有视网膜病变，周围神经病是一种较少见的长期病并发症。

**qwen2.5_7b** 2025-02-18T16:30:13Z:

根据您描述的症状，最有可能诊断为Zellweger谱系障碍（简称ZSD）或其同义词婴儿急性肝脑病谱系（AHELI），这是一种罕见的线粒体遗传代谢病。

以下是其他五种可能的诊断： 1. 脑发育不全-肾性尿崩症综合征：表现为低血糖、氨基酸尿、尿崩症等； 2. 线粒体DNA大环状缺失：可引起肝功能异常和乳酸酸中毒； 3. 婴儿肝代谢病（如糖原累积病或脂肪酸氧化障碍）：可伴有肝功能异常及生长迟缓； 4. 有机酸血症，如中链酰基辅酶A脱氢酶缺乏症（MCADD），表现为低血糖、肝肿大和心肌病等； 5. 先天性代谢缺陷疾病，如半乳糖血症I型或II型，可伴有低血糖、肝功能异常及生长迟缓。

**75. user 2025-02-18T16:32:52Z:**

主要发生在成年育龄女性，平均诊断年龄在 40 岁左右。早期症状轻微，部分患者在查体时被发现，或因为呼吸症状或其他原因检查肺部 HRCT 时发现。主要表现为不同程度的呼吸困难，随疾病进展，呼吸困难症状也逐渐加重，肺功能进行性恶化，晚期出现呼吸衰竭。在病程中可反复出现气胸、乳糜胸等并发症。肺外表现包括肾， 腹膜后实性或囊实性淋巴管肌瘤。部分患者具有其他多系统受累临床特征，主要包括神经系统改变(癫痫、神经发育迟缓和自闭症等) 和皮肤改变(色素脱色斑、面部血管纤维瘤、皮肤鲨革斑和甲周纤维瘤等) 等临床表现。

**qwen2.5_7b** 2025-02-18T16:32:54Z:

根据您提供的症状和体征描述，最有可能诊断为Banting-Robbins病（也称为成人型肺淋巴管平滑肌瘤病）。

其他五种可能的诊断包括： 1. 韦格纳肉芽肿病：伴有肺部病变、肾炎等症状。 2. 肺泡蛋白沉积症：伴有间质性肺疾病症状，如进行性呼吸困难和肺功能下降。 3. 淋巴管肌瘤病（LAM）: 主要表现为肺淋巴管平滑肌瘤及腹膜后淋巴管肌瘤等，与您的描述有一定的重叠。 4. 间质性肺炎：伴有肺部病变、呼吸困难等症状。 5. 特发性肺纤维化：表现为进行性呼吸困难和肺功能下降。

**76. user 2025-02-18T16:33:28Z:**

婴儿的典型表现是在断奶后出现反复呕吐和腹泻，拒食富含蛋白质的食物。患者可出现皮肤苍白、肌张力减低、肝脾大、头发稀疏、骨质疏松伴病理性骨折。进食富含蛋白质的膳食后患者可出现嗜睡、共济失调、癫痫发作和高氨血症昏迷等。随着病程的发展，可出现多系统受累表现。肺部受累可表现为进行性肺间质病变，肺泡蛋白沉积症；肾脏最常见的特征性改变是近端肾小管功能障碍和肾钙质沉积，也可出现肾小球病变，肾脏淀粉样变，甚至出现肾功能衰竭；血液系统受累表现为正色素或低色素性贫血，白细胞减少，血小板减少等；消化系统受累的表现为高胆固醇血症，高甘油三酯血症，反复发作的胰腺炎，甚至肝脏淀粉样变。癫痫多发生于成年患者，可能与反复发生高氨血症导致大脑皮质受损有关。

**qwen2.5_7b** 2025-02-18T16:33:34Z:

根据您描述的症状和体征，最有可能诊断为经典型黏多糖贮积症（MPS I），特别是由于神经症状如共济失调、癫痫发作等特征。此外，以下五种罕见病也可能是患者的潜在原因：

1. **肾性尿崩症**：患者的临床表现如慢性进行性的肾钙质沉积和近端肾小管功能障碍等与之相符。
2. **遗传性代谢疾病-糖原贮积病（如I型）**：患者表现为皮肤苍白、肌张力减低、肝脾大、头发稀疏等症状，且在食用高蛋白膳食后症状加剧，符合部分患者的临床表现。
3. **线粒体脑肌病伴乳酸血症和卒中样发作**：此病症可导致共济失调、癫痫发作及神经功能障碍，可能与患者的临床表现相符。
4. **罕见的肝脏疾病-进行性家族性肝内胆汁淤积症（PFIC）**：患者的高胆固醇血症、胰腺炎等症状可能与此病相关联。
5. **神经系统疾病-线粒体脑肌病伴乳酸血症和卒中样发作**：该病症可导致共济失调及癫痫发作，与患者的表现相符合。

请注意，只有通过详细的临床检查和实验室检测才能最终确定诊断。建议您尽快联系专业医生进行详细评估和确诊。

**77. user 2025-02-18T16:35:20Z:**

新生儿期起病，通常在生后 1 周内起病。以反复呕吐、腹泻、腹胀等胃肠道症状为首发症状，伴肝脾大和生长发育障碍；神经系统受累表现为活动少、腱反射亢进、踝阵挛和角弓反张。巨噬细胞内胆固醇酯和三酰甘油贮积导致肝脾肿大，进行性加重甚至肝衰竭，数月内出现转氨酶、胆红素升高，低蛋白血症，凝血异常等。肾上腺增大伴钙化导致肾上腺皮质功能不全，60％ ~70％通过影像学检查可发现肾上腺钙化。也有胆囊功能障碍、脑卒中报道。随着病情进展，可出现多器官功能障碍，包括贫血、消化道出血、败血症、昏迷等。

**qwen2.5_7b** 2025-02-18T16:35:23Z:

根据您提供的临床表现，最有可能诊断为尼曼-皮克病（Niemann-Pick disease），这是一种罕见的脂质代谢疾病。

其他五种可能的诊断： 1. 长链脂肪酸代谢障碍：如长链3羟基酰辅酶A裂解酶缺乏症、长链3羟基酰基辅酶A脱氢酶缺乏症等。 2. 肝脾肿大伴腹胀（排除尼曼-皮克病的情况），可以是其他遗传性肝病，比如Zellweger谱系障碍或其他代谢性疾病如糖原累积症I型等。 3. 糖尿病酮症酸中毒：伴有严重脱水、呕吐和高血糖等症状。 4. 丙酸血症：在新生儿期或婴儿早期出现呕吐、腹泻、肝脾肿大，伴神经系统症状等。 5. 柯萨奇病毒脑膜炎/脑炎：虽然神经系统症状相似，但通常起病较急，多见于儿童，并伴有发热等症状。

**78. user 2025-02-18T16:36:10Z:**

**1. 心血管系统** ，可以导致主动脉根部扩张、主动脉瓣关闭不全和主动脉夹层；也可能会有二尖瓣脱垂伴或不伴关闭不全。升主动脉扩张患病率随着患者年龄的增长而增加。从 30 岁至 60 岁，患病率从 53％上升至 96％。患者可以表现为气促、胸痛、心悸、头晕、晕厥或者咯血。查体表现为心界向左下扩大，听诊在二尖瓣区可以闻及收缩期杂音，在主动脉瓣听诊区可闻及舒张期为主的双期杂音，可有周围血管征以及心力衰竭的体征。

**2. 骨骼表现** ，瘦高身材、手指和脚趾细长、两臂平伸的距离超过身高、脊柱侧凸或后凸、漏斗胸或者鸡胸、关节松弛、扁平足等。

**3. 眼部异常** ，眼部病变占 50％ ~70％，包括角膜扁平、眼球轴延长、视网膜剥离、白内障、晶状体脱位或半脱位、虹膜震颤或轻度震颤、瞳孔移位、晶状体混浊等。

**4. 其他 ，**硬脊膜膨出、萎缩纹、复发性疝或切口疝、高腭穹等。

**qwen2.5_7b** 2025-02-18T16:36:15Z:

根据您提供的临床表现，这些症状符合马凡氏综合征（Marfan syndrome）的特征。此外，还有其他五种可能的诊断如下：

1. **埃勒斯-当洛斯综合征（Ehlers-Danlos syndrome, EDS）**：这种遗传性结缔组织病也导致关节松弛、皮肤脆弱和血管异常。
2. **特纳综合症（Turner Syndrome）**：这是一种仅发生在女性身上的染色体异常疾病，可引起心血管问题和骨骼异常等症状。
3. **马方样综合征（Loeys-Dietz syndrome, LDS）**：这与马凡氏综合征有相似症状，但其遗传方式为常染色体显性或隐性遗传，并且可能伴有血管异常和皮肤表现。
4. **普利昂病（Prion disease）**：这是一种罕见的神经系统疾病，可以导致主动脉瘤形成以及心脏瓣膜问题等。
5. **皮埃尔罗宾序列综合症（Pierre Robin sequence, PRS）**：虽然主要是一种头面部畸形，但其并发症中也可能包括心血管系统异常。

**79. user 2025-02-18T16:36:38Z:**

**1. 皮肤表现** ，患者一般出生时即有牛奶咖啡斑，常位于骨骼病变同侧且以中线为界，为一处或多处点片状大小不等的褐色或黄棕色斑片状色素沉着，边界不规则。

**2. 骨骼系统表现** ，大约 98％的 患者有单骨型或多骨型骨纤维异样增殖症，以多骨型更为常见。其特征为畸变的骨母细胞增多并异常分化，引起骨髓内纤维细胞的广泛增殖、分化，形成幼稚编织骨，易发生病理性骨折和骨骼畸形，并引发骨痛，几乎可累及全身骨骼，尤以颅面骨和长骨受累多见，局部骨骼膨胀、隆起，引起面部结构改变和压迫症状。眼眶受累严重可压迫视神经，造成视力障碍。听神经受累可致听力下降。脊柱受累可致椎体压缩性骨折，引起脊柱侧弯或后凸畸形。骨纤维异样增殖症导致病理性骨折的高峰年龄多在 6~10 岁。影像学示骨骼呈膨胀性、溶骨性改变或磨玻璃样改变。

**3. 内分泌腺体受累表现** ，可累及多种内分泌腺，导致受累腺体功能亢进或发生肿瘤，血清促分泌激素的水平正常或降低。其中性早熟较多见，男女均可发病，但女性患者多见，且常早于骨病变。女性患儿可表现为第二性征早发育、过早阴道不规则出血、子宫及卵巢较正常同龄儿童增大，或发生卵巢囊肿，骨骺提前愈合等。男性患儿性早熟相对少见，超声见睾丸病变(Leydig 细胞增生、微小结石、局灶钙化等)。甲状腺肿大及功能亢进也较常见，也有报道发生甲状旁腺功能亢进者。其他内分泌疾病也有报道，如皮质醇增多症、巨人症 / 肢端肥大症、高泌乳素血症等，但相对较罕见。

**4. 低磷血症** ，部分患者合并低磷血症，表现为低血磷性佝偻病。合并低磷血症者通常为多骨型骨纤维异常增殖症。

**5. 其他，**患者还可因突变累及外周血白细胞、肝脏、心脏、胸腺和胃肠道，而出现相应的罕见临床症状，如黄疸、肝炎、心律失常、肠息肉等。

**qwen2.5_7b** 2025-02-18T16:36:42Z:

根据您提供的临床表现，最有可能诊断出的罕见病是**多发性内分泌肿瘤-1型伴骨纤维结构不良（MEN1相关疾病中的骨病变）**。此外，还有以下几种可能的诊断：

1. **特纳综合征**：尽管患者通常为女性，但男性也可出现类似的表现。其特征是部分或完全缺失一个X染色体，并且有骨骼、内分泌系统等多种异常。
2. **成纤维细胞生长因子受体2基因突变相关疾病（Maffucci综合征）**：虽然该病常表现为血管瘤和软骨错构瘤，但也可出现类似多发性骨骼病变的表现。
3. **家族性孤立性骨囊肿伴低磷血症**：这是一种罕见的遗传性疾病，可以引起骨骼异常和低磷血症相关的症状。
4. **神经纤维瘤病1型（NF1）**：虽然NF1患者通常表现为皮肤咖啡牛奶斑、神经系统肿瘤等特征，但有时也可能出现类似的骨骼病变。
5. **Ehlers-Danlos综合征**：这是一种结缔组织疾病，可以导致多种症状，包括皮肤过度伸展和骨骼异常。但其主要特征与上述病症有所不同。

需要注意的是，在临床实践中还需结合患者的具体病史、家族遗传背景及实验室检查结果进行综合判断，并最终确定确切诊断。

**80. user 2025-02-18T16:37:46Z:**

患者大多在出生后 3 个月至 3 岁之间发病，少部分在新生儿期或成人期发病， 也有患者无症状。患者发病通常有诱发因素，如多见于长时间饥饿或并发感染性疾病等。早期发病的患儿，其首发症状以嗜睡和呕吐常见，也可表现为抽搐、窒息等，常迅速进展为昏迷或死亡。急性发病期常有典型的低血糖表现，血糖降低伴尿酮体阴性有助于诊断。50％的患儿伴有肝脏肿大，肝活检可见大量脂质堆积。影像学检查发现部分患儿发生脑水肿。心脏损害表现较为罕见。成人期发病的患者临床表现多样，可有多器官受损，包括肌肉、肝脏、神经系统或心血管系统等。其中呕吐是最常见的症状，肝脏肿大相对少见，妊娠期出现症状的患者表现为急性脂肪肝。多数患者有急性非炎症性脑病。约 1/3 的患者出现横纹肌溶解，肌酸激酶显著增高，并伴有肌红蛋白尿。患者病死率较高，发病的患儿中约25％死亡，成人期急性发病的患者病死率更高，可达到 50％。疾病发作间歇期患者往往表现正常。约 1/3 的急性发病后存活的患者出现后遗症，包括生长发育迟滞、运动发育迟缓、智力障碍、语言发育缺陷、心理行为问题、癫痫、脑瘫、偏瘫、慢性肌无力等。另外，部分患者有疲劳、肌痛和运动耐力减退等骨骼肌损害的主诉症状。

**qwen2.5_7b** 2025-02-18T16:37:48Z:

根据您提供的临床表现描述，该疾病最有可能是Reye综合症。此外，还有其他五种可能的诊断：

1. 线粒体脑肌病（MELAS）：主要影响线粒体功能，会出现神经和肌肉症状；
2. 肝豆状核变性（Wilson病）：铜代谢障碍，可引起神经系统、肝脏及眼部损害；
3. 乳酸酸中毒伴脑病综合征：以急性或亚急性发作的神经系统症状为主要表现，伴有代谢异常如乳酸酸中毒；
4. 糖原累积症：糖原代谢缺陷导致体内糖原堆积引发的各种临床症状和体征；
5. 脂肪酸β氧化障碍（FAOD）：脂肪酸氧化代谢障碍引起的一系列疾病。

**81. user 2025-02-18T16:59:36Z:**

疾病在各年龄段中的临床表现不尽相同。通常发病年龄越早，急性代谢紊乱和脑病表现越严重。临床表现主要包括消化系统症状如呕吐、喂养困难、肝大等以及神经系统症状如运动障碍、意识障碍、抽搐、发育迟缓或倒退、小头畸形等。病情反复多与感染和应激相关。

新生儿期发病者多在生后数小时至 1 周内出现急性脑病样症状，表现为呕吐、肌张力低下、脱水、严重酸中毒、高乳酸血症、高氨血症、昏迷和惊厥，病死率高。儿童期发病的出生时正常，多在 1 岁以内发病，首次代谢危象的诱因常为感染、饥饿、疲劳、疫苗注射等应激因素刺激或高蛋白饮食和药物，如果不及时诊治，可导致智力发育和运动发育迟缓、落后和倒退，可伴发血液系统、肝脏、肾脏、皮肤和周围神经受累。成人患者首发症状可为周围神经病变和精神心理异常等。

**qwen2.5_7b** 2025-02-18T17:00:59Z:

根据您描述的症状，该疾病可能是一种罕见的遗传性代谢病——中链酰基辅酶A脱氢酶缺乏症（MCADD）。这种疾病的临床表现如下：

1. 中链酰基辅酶A脱氢酶缺乏症：此病由于基因突变导致中链脂肪酸无法正常氧化产生能量，引起急性或慢性症状。
2. 瑞特综合征/Reye综合症：以肝肿大和脑水肿为特征的代谢性脑病，多见于病毒感染后，常伴随呕吐、嗜睡等消化系统症状及神经系统异常。
3. 乳酸酸中毒伴高丙酮酸血症：由于糖酵解代谢障碍导致体内产生大量乳酸和丙酮酸引起的一系列临床表现。
4. 苯丙酮尿症：这是一种常见的氨基酸代谢病，主要表现为智力发育迟缓、皮肤和毛发色素减少等症状。但该疾病没有急性脑病症状或肝大等表现。
5. 肝豆状核变性：又称为威尔逊氏症，是一种铜代谢障碍导致的神经系统退行性疾病。临床表现为肝脾肿大、锥体外系运动障碍及神经精神异常等，不符合本病例描述的症状特点。
6. 先天性肾上腺皮质增生症（CAH）：主要由于肾上腺皮质激素合成过程中的酶缺陷引起的一组遗传病，但该疾病没有肝大、神经系统症状或消化系统问题。

**82. user 2025-02-18T17:02:18Z:**

典型患者出生时正常。于 6 个月 ~1 岁面容逐渐变丑，面中部变扁、鼻梁增宽、角膜混浊、耳聋、头大、前后径长，前额突出，关节僵硬、爪形手、鸡胸和腰椎后突等。常于 2~5 岁出现心脏瓣膜增厚、心肌病或充血性心力衰竭。5 岁出现肝脾大。精神运动发育落后。一般存活至 10 岁左右。多死于心衰和肺炎。轻型患者 5 岁以后出现症状，轻度面容粗陋、角膜混浊和手足关节僵硬。智力正常，寿命相对较长。临床症状在各亚型之间有互相重叠，但生化检查结果在各型之间无显著差异。

**qwen2.5_7b** 2025-02-18T17:03:36Z:

根据您提供的临床表现，最有可能的诊断是MOPDⅠ（黏多糖贮积症I型），也被称为亨利-萨克纳希病。以下是其他五种可能的诊断：

1. 黏多糖贮积症II型（亨利-萨克纳希-巴切利尔病）：临床表现和MOPDⅠ相似，但病情较为轻度。
2. 黏多糖贮积症III型：患者会出现典型的面容改变、角膜混浊等症状，并伴有精神发育迟滞等神经系统症状。
3. 色素性视网膜炎伴神经纤维瘤病1型（Tuberous Sclerosis Complex，TSC）：除有面部血管纤维瘤外，还有癫痫发作、智力障碍及皮下肿瘤等多种临床表现，可与MOPDⅠ相鉴别。
4. 痛风/高尿酸血症：主要表现为痛风性关节炎和肾结石等，但无面容丑陋症状。通过实验室检查可以进行排除诊断。
5. 先天性甲状腺功能减低症（先天性甲低）：表现为身材矮小、智力发育迟缓等症状，可通过实验室检查明确诊断。

**83. user 2025-02-18T17:08:06Z:**

隐袭起病。缓慢发展或阶段性进展，可有长时间的稳定期。任何年龄均可发病，多于50 岁前起病。临床主要表现为多发性单神经病，不对称性上肢远端起病较常见，早期以单侧上肢某一根或多根神经受累多见，表现为相应神经支配区域的肌肉无力， 远端为主，可伴有痉挛或束颤。随着病情发展，可以出现肌肉萎缩；病程较长者，可有多个肢体的多根神经受累，受累神经的不对称性可不明显，而呈现为类似多发性周围神经病的分布。患者可有轻微感觉异常的主诉，但缺乏客观感觉受累的体征，病程后期部分患者也可出现部分感觉神经受累。脑神经通常不受累。无上运动神经元受累体征。

**qwen2.5_7b** 2025-02-18T17:09:30Z:

根据您描述的症状，这种罕见病可能是慢性炎性脱髓鞘性多发性神经根神经病（Chronic Inflammatory Demyelinating Polyneuropathy, CIDP）。其他五种可能的诊断包括：

1. 亚急性感觉运动型轴索神经病 (Acute Sensory-Motor Axonal Neuropathy, ASMAN)：这种病症也可能表现为不对称性上肢远端肌肉无力和轻微的感觉异常，但通常病情进展更快。
2. 赫勒氏综合症（Heller’s Syndrome）或慢性复发性多发性单神经炎：这是一种主要影响单一或少数周围神经的疾病，其临床表现符合您的描述，但在CIDP中，受影响的神经可能更多。
3. 血管炎相关神经病：某些类型的血管炎可以累及周围神经系统，并导致类似的症状。这种罕见病症通常伴有其他自身免疫性疾病的表现，如系统性红斑狼疮或类风湿关节炎等。
4. 周围神经变性 (Peripheral Nerve Degeneration, PND)：这是一种以对称性肢体近端无力为主的疾病，在病程中可以出现肌肉萎缩。尽管您的患者主要是不对称的上肢远端受累，但该病也可能包含其他类型的表现。
5. 遗传性感觉运动神经病 (Hereditary Sensory and Motor Neuropathy, HSMN)：这是一种遗传性疾病，可导致多种不同类型的周围神经病变症状。然而，在您的病例中，患者并没有表现出家族史或特定的遗传因素，因此这种可能性相对较小。

请记住，确诊罕见疾病需要详细的临床检查、病史调查和各种实验室检测等辅助诊断手段的支持。

**84. user 2025-02-18T17:13:31Z:**

新生儿期起病常致命，可表现为代谢危象，包括代谢性酸中毒、非酮症性低血糖、高氨血症，其他特征包括多系统受累(肌张力低下、心肌病、肝肿大)、排泄大量脂肪酸和氨基酸衍生的代谢产物、“汗脚”味体臭、昏迷、新生儿期死亡。快速进展的肥厚型心肌病常见于新生儿期起病的类型，猝死概率非常大，即使经过新生儿筛查在早期即检测出有此病，也难以逆转。

先天畸形被认为是未被胚胎转运纠正的那部分有毒代谢物累积的后果。脂肪酸氧化代谢受阻的程度决定了子宫内的酸中毒程度，进而阻碍正常的胚胎形成。先天畸形包括增大的多囊肾、摇篮底足(rocker-bottom feet)、下腹部肌肉缺失、尿道下裂和阴茎下弯、肌张力低下、脑皮质发育不良和胶质增生。其他畸形特征包括眼距过宽、耳畸形、巨颅、前囟过宽、高前额、鼻梁低平。

晚发型的发病年龄和症状在不同病例中差异很大，主要表现为不伴有先天畸形，但终身伴有急性间歇发作的呕吐、脱水、低酮性低血糖、酸中毒及意识障碍的风险。年龄大者可有肝脏肿大或脂质沉积性肌病。急性失代偿发作可由感染、发热、手术、低能量饮食或减肥、饮酒、妊娠等诱发。大多数患者发展为慢性肌病症状，包括运动不耐受、肌痛、肌无力、肌肉萎缩。晚发型病人的有机酸尿症常为间歇性，仅在疾病期间和处于分解代谢压力下较为显著。

**qwen2.5_7b** 2025-02-18T17:14:03Z:

根据您描述的临床表现，我最有可能诊断出的是中链酰基辅酶A脱氢酶缺乏症（MCADD），这是一种常见的脂类代谢障碍性疾病。其他五种可能的诊断包括：

1. 有机酸血症：如丙酸血症、戊酸血症等，这些疾病也会出现多系统受累的表现。
2. 脂肪酸氧化缺陷病：除了MCADD外，还有一些其他的脂肪酸氧化缺陷病也可能导致类似的临床表现。
3. 长链脂酰基辅酶A脱氢酶缺乏症（LCADD）：与MCADD类似，但主要是长链脂肪酸代谢异常所致的疾病。
4. 柯萨奇病毒B感染后的急性心肌炎：快速进展型肥厚型心肌病可能在柯萨奇病毒感染后出现。
5. 肝糖原累积症（GSD）：表现为低血糖、乳酸性酸中毒及肝肿大等症状，与您描述的部分症状相似。

**85. user 2025-02-18T17:15:28Z:**

平均发病年龄为 30 岁，10 岁以下和 50 岁以上患者少见。女性患病率高于男性，女：男比例为（1.4~2.3） ：1。多急性 / 亚急性起病，具有空间多发和时间多发特点。空间多发指病变部位多发，大脑、脑干、小脑、脊髓可同时或相继受累。时间多发指缓解—复发的病程，由于累及部位广泛，反复发作后可最终致残，甚至致死。临床表现多样，常见症状包括视力下降、复视、肢体感觉障碍、肢体运动障碍、共济失调、膀胱或直肠功能障碍等。

**qwen2.5_7b** 2025-02-18T17:15:37Z:

根据您描述的症状和流行病学特点，最有可能的诊断是多发性硬化症（Multiple Sclerosis, MS）。以下是其他五种可能的诊断：

1. 髓鞘少突胶质细胞异常髓鞘化疾病（MOG相关脑脊髓炎）
2. 艾迪生病（AIDS）相关的神经系统并发症
3. 急性播散性脑脊髓炎（ADEM）
4. 脑肿瘤或转移瘤引起的症状
5. 进行性多灶性白质脑病（PML，与JC病毒相关）

需要注意的是，最终的确诊需要依靠详细的临床检查、影像学（如MRI）及实验室检查结果等。

**86. user 2025-02-18T17:16:00Z:**

主要包括帕金森症、小脑性共济失调、锥体束损害以及自主神经系统损害。首发症状常为自主神经损害，最主要的是心血管功能障碍(如直立性低血压) 和排尿障碍(如尿失禁)，后者在男性患者还容易合并性功能障碍。很多患者病前有长达数年的快速眼动期睡眠行为障碍(RBD)。

**qwen2.5_7b** 2025-02-18T17:16:23Z:

根据您提供的临床表现，我推测可能是多系统萎缩（Multiple System Atrophy, MSA），这是一种罕见的进行性神经退行性疾病，可以包括以下特征： 1. 多系统萎缩(MSA)

此外，还有其他五种可能的诊断如下： 2. 艾伯特病 (Amyotrophic Lateral Sclerosis, ALS)：主要表现为锥体束损害、肌无力和肌肉萎缩等症状。 3. 进行性核上麻痹 (Progressive Supranuclear Palsy, PSP)：除了帕金森症状，患者还有垂直眼肌麻痹、步态障碍、平衡失调等表现。 4. 路易体痴呆 (Dementia with Lewy Bodies, DLB)：主要表现为认知功能障碍、视觉幻觉和锥体系或锥体外系损害的症状。 5. 皮克病 (Pick’s Disease)：临床表现为进行性失语、失用症候群以及逐渐加重的精神症状等，部分病例还可能伴有锥体束损害。 6. 纹状体黑质变性 (Striatonigral Degeneration, SND)：主要特征为锥体外系症状和自主神经功能障碍。

**87. user 2025-02-18T17:16:41Z:**

**1. 肌肉，** 四肢远端起始并逐渐向近端进展的无力萎缩，影响手部精细动作，造成垂足。面肌亦可受累。同时有明显肌强直现象，累及肢体肌、咽喉肌等。查体可发现大力握拳后手指不能立即展开，用力闭目后不能立即睁开。叩击手部大鱼际肌、舌肌等，可引出肌强直，称为叩击性肌强直或“肌球”现象。肌强直存在“热身”现象，反复活动后，肌强直减轻。下肢肌痛和全身疲乏感也是常见临床表现。

**2. 心脏，** 心脏传导系统常有受累，出现不同程度的房室传导阻滞和室内传导异常。严重时可造成猝死。确诊患者需定期监测心电图，必要时植入起搏器。

**3. 中枢神经系统** ，睡眠增多、阻塞性睡眠呼吸暂停等睡眠障碍多见。可伴有认知及情绪障碍。头 MRI 可见弥漫性脑白质病变及皮层萎缩。

**4. 眼** ，随着年龄增长，几乎所有患者均会出现白内障。常在 30~40 岁影响视力。

**5. 内分泌系统** ，可出现多种内分泌疾病，包括糖尿病、甲状腺功能异常、钙磷代谢异常、性激素异常、睾丸萎缩(造成不育) 等。

此外，还可有额秃、胃肠动力不足、限制性通气障碍等。

**qwen2.5_7b** 2025-02-18T17:17:01Z:

根据您提供的临床表现，我怀疑患者可能患有**肢带型肌营养不良症**中的Duchenne型（杜兴氏肌肉营养不良），它是一种常见的遗传性进行性肌肉萎缩疾病。

除此之外，还有其他五种可能的诊断：

1. 先天性肌强直：先天性肌强直是一种罕见的遗传性疾病，具有特征性的肌强直症状和运动障碍。患者的肌肉在受到刺激时会变得僵硬并难以放松。
2. 眼咽型肌营养不良症（眼咽型DMD）：这是一种少见的进行性神经肌肉疾病，主要表现为咽喉部肌无力、吞咽困难以及眼部肌肉受累导致的眼睑下垂等症状。病情进展缓慢且较为局限。
3. 重症肌无力：这是一类以肢体无力为主要特征的自身免疫性疾病，可伴有眼睑下垂、复视等眼部症状及四肢近端为主的肌力下降。
4. 面肩肱型肌营养不良症（FSHD）：这是一种常染色体显性遗传病，主要累及面部肌肉和肩胛带、骨盆带肌肉。早期发病年龄较轻且病变范围相对局限。
5. 代谢性疾病：如线粒体疾病中的某些类型也可能表现出类似的神经肌肉症状，并伴随特定的生化或基因检测结果支持诊断。

**88. user 2025-02-18T17:18:52Z:**

该病发病年龄往往较早，最常发病的年龄为新生儿时期。大多患儿生后数日内发病，表现为拒奶、呕吐、嗜睡、惊厥、昏迷。部分晚发型患儿以发作性呕吐、嗜睡，伴精神发育异常为特点。高氨血症昏迷的患者可能有脑部伤害与发展迟缓、学习能力受损或智力障碍以及生长发育落后的后遗症。高氨血症患者可能造成颅内压增高、呼吸急促，病情进展会出现呼吸暂停或衰竭，肝肿大。神经系统并发症包括智力障碍、急性高血氨昏迷，甚至死亡。生化改变可见高氨血症、血谷氨酰胺和丙氨酸增高，尿中乳清酸降低或缺如。

**qwen2.5_7b** 2025-02-18T17:18:56Z:

根据您描述的临床表现，这很可能是一种与高氨血症有关的罕见病，其中最有可能的是“经典型苯丙酮尿症（PKU）”，但结合您提供的信息，更准确的诊断可能是“尿素循环障碍”中的“瓜氨酸尿症1型”。以下是其他五种可能的诊断：

1. 经典型苯丙酮尿症(PKU)：患者通常在新生儿期就表现出症状。
2. 尿素循环障碍（例如：精氨琥珀酸尿症、瓜氨酸血症等）：这类疾病也与高氨血症有关，部分临床表现类似。
3. 高胱氨酸尿症：以神经系统损害为特征的代谢病。
4. 艾森曼格综合征（AESC, Alkaptonuria）: 一种罕见的氨基酸代谢异常，但较少见于婴儿早期发病并有高氨血症的表现。
5. 有机酸血症：如丙酸血症、甲基丙二酸血症等。这类疾病也有神经系统症状和代谢异常。

请注意，在面对具体的临床病例时，还需要结合患者的实验室检查结果（如血液生化检测、尿液分析以及遗传学检查）和其他辅助诊断手段来确定最终的诊断。

**89. user 2025-02-18T17:19:33Z:**

患儿常表现为严重的宫内发育迟缓，出生后很早(常在出生后 1 周) 即出现严重的、非酮症性高血糖，12 周后可恢复，50％ ~60％在青春期前后复发，复发后临床表现类似于2 型糖尿病。

**qwen2.5_7b** 2025-02-18T17:20:17Z:

根据您提供的信息，我推测这可能是一种罕见的遗传代谢病——Zinner综合征。但具体诊断还需要结合基因检测等进一步检查结果进行确认。

其他五种可能的诊断包括：

1. **MODY（成熟发病型糖尿病）**：这是一种常染色体显性遗传病，通常在30岁前发病。
2. **线粒体糖尿病**：一种由于线粒体DNA突变引起代谢障碍所致的糖尿病，临床表现多样，可伴有其他症状如神经肌肉症状、耳聋等。
3. **Stargardt病**：这是一种视网膜色素上皮细胞退化引起的遗传性眼疾。但值得注意的是，它并不直接与高血糖相关联。
4. **新生儿暂时性糖尿症**：新生儿出生后早期出现短暂的非酮症性高血糖现象，多数会在几周内自行消失。
5. **先天性胰岛β细胞功能障碍综合征（CIPD）**：这是一种罕见遗传病，其特征是胰腺中的β细胞不能正常工作，导致胰岛素分泌不足。该病症同样伴有糖尿病症状。

请注意，最终确诊需依据详细的临床检查和实验室检测结果。

**90. user 2025-02-18T17:21:44Z:**

患者最早出现的症状是腹部膨隆，肝脾增大，部分患者可在新生儿期发病。智力和运动发育落后随即出现，肌张力低下，运动发育迟缓，而脑神经功能常不受累，1 岁后运动智力发育倒退明显，最后进展为痉挛强直状态。50％患者可以发现眼底樱桃红斑。间质性肺部病变可导致反复呼吸道感染、低氧血症或呼吸功能衰竭。多数患儿 3 岁前死亡。

**qwen2.5_7b** 2025-02-18T17:22:00Z:

根据您描述的临床表现，我最有可能的诊断是尼曼-皮克病（Niemann-Pick disease），这是一种罕见的溶酶体贮积症，其主要特征为神经退行性病变和肝脾肿大等。

除了尼曼-皮克病之外，还有其他几种可能导致类似症状的罕见疾病：

1. 酸性脂肪酶缺乏症 (Farber’s Disease)：也是一种溶酶体贮积症，表现为发育迟缓、关节痛、肝脏和脾脏增大等症状。
2. 肝豆状核变性（Wilson病）：这是一种铜代谢障碍性疾病，主要临床表现为肝硬化、锥体外系症状、精神行为异常等。在某些情况下也可能出现眼底樱桃红斑和肝脾肿大。
3. 代谢缺陷病（如戊二酸血症或半乳糖血症）：这些疾病通常会表现出神经系统受损的症状，并且可能会导致发育迟缓和其他器官问题。
4. 神经元蜡样脂褐质沉积病 (Neuronal Ceroid Lipofuscinosis)：这是一种以进行性神经退行为特征的遗传性疾病，可表现为肝脾肿大、智力和运动功能减退等症状。
5. 肝肾微管蛋白异常症（MIM148000）：本病为常染色体隐性遗传病，主要临床表现包括肝脏、肾脏等多脏器受累以及发育迟缓等。

**91. user 2025-02-18T17:29:48Z:**

**1. 典型面容，** 所有年龄患者均有上睑下垂，眼距宽，内眦赘皮，双眼外角下斜；双耳位低，后旋，耳廓厚。儿童患者还可有前额饱满，后发际低，鼻短，鼻梁低，鼻尖饱满；唇厚，鼻唇沟深而宽直达上唇等。

**2. 矮小，，** 出生时正常，1 岁后渐出现矮小。骨龄常落后，部分成年身高可达正常下限水平。

**3. 心血管 ，**50％ ~80％患者有先心病，肺动脉瓣狭窄最常见，其他包括肥厚型心肌病，室缺，肺动脉分支狭窄，法洛四联症和主动脉缩窄等。典型心电图改变包括心电轴极度右偏伴胸前导联 QRS 波逆时针旋转，V1 导联心电轴左偏、左前支不全传导阻滞或 RSR 波形。

**4. 精神运动发育落后** ，部分患者语言能力差，成年患者易有抑郁情绪，也有报道此病患者自闭症谱系表现发生率较高。

**5. 颈蹼，**

**6. 胸廓异常 ，**鸡胸或漏斗胸。

**7. 乳距宽**

**8. 男性隐睾** ，60％ ~80％男性患者合并隐睾，可致生育障碍。

**9. 凝血功能异常和淋巴管发育不良**

**10. 其他，** 肾脏畸形(肾盂扩张、双输尿管畸形、孤立肾、肾发育不良、远端输尿管狭窄等)，男性精子生成障碍，斜视，眼颤，皮肤牛奶咖啡斑和雀斑，四肢毛囊角化症， Ⅰ型阿诺德 - 基亚里畸形，脑积水，肝脾大，恶性肿瘤发生率增加等。

**11. 宫内表现，** 母亲孕期羊水过多，胎儿颈部透明带增宽，胎头相对较大，心脏和肾脏异常。

**qwen2.5_7b** 2025-02-18T17:30:27Z:

根据您提供的临床表现在考虑罕见病时，最有可能诊断为“范可尼综合征-阿诺德-基亚里畸形综合症”（Fanconi Syndrome-Arnold-Chiari Malformation Syndrome），该疾病也被称为FAIC综合症。这种病症主要由以下特点定义：

1. **范可尼综合征**：这是一种罕见的遗传性疾病，导致肾小管功能障碍，引起电解质和矿物质的异常丢失，如低钾血症、低钙血症、低磷血症和代谢性酸中毒。
2. **阿诺德-基亚里畸形（Arnold-Chiari Malformation）**：这是一种神经系统的先天性异常，表现为小脑扁桃体下疝，可导致一系列神经系统症状，如共济失调、头痛、颈部痛等。

除了FAIC综合症外，还有其他五种可能的诊断如下：

1. **特纳综合征（Turner Syndrome）**：
   - 特纳综合征是一种仅影响女性的罕见染色体疾病，特征为X染色体部分或全部缺失。
   - 临床表现包括身材矮小、颈蹼、肘外翻、心血管问题等。
2. **戈谢病（Gaucher Disease）**：这是一种由GL-6-PD酶缺乏引起的溶酶体贮积症，主要影响脾脏、肝脏和骨骼。患者可能出现矮小、肝脾肿大等症状，并伴随凝血功能异常。
3. **马方综合症（Marfan Syndrome）**：
   - 一种结缔组织疾病，特征为心血管系统异常，如主动脉根部扩张。
   - 常伴有眼距宽等面部特征，但一般不涉及其他系统性症状。
4. **软骨发育不良（Multiple Sclerosis-like Disorders in Children, MSLD）**：
   - 一种罕见的遗传性疾病，表现为生长迟缓、骨骼异常和神经系统问题。
   - 可能包括心脏瓣膜缺陷、肾畸形等。
5. **法布里病（Fabry Disease）**：这是一种X连锁溶酶体贮积症，特征为血管内皮细胞中脂质沉积。患者可出现心血管症状、肾脏损害及神经病变。

这些病症在临床表现上有一定的重叠性，并且需要详细的遗传学检测以进行确诊。因此，如果怀疑这些罕见病，最好与遗传学专家合作进行全面评估和诊断。

**92. user 2025-02-18T18:03:17Z:**

患者可以在任何年龄阶段发病。临床主要分为早发型和晚发型两种。早发型主要发生在男性患儿，一般在新生儿期发病，临床表现为起病急，病情凶险，因血氨升高，使得大脑广泛性损害。出生时可无异常，生后数天即表现出易激惹、嗜睡、拒食、呼吸急促和昏睡等，可迅速发展为痉挛、昏迷和呼吸衰竭。如果不给予紧急处理，很快发展成遗传代谢性脑病，并常在刚出生的 1 周内死亡，幸存者多遗留严重的智力损害。晚发型多发生在较大年龄的患者中，可以是半合子的男性和杂合子的女性，临床症状相对轻，且表现多样。儿童期和成人期发病的患者多表现为慢性神经系统损伤，如发作性呕吐、头痛、行为异常、谵妄、精神错乱等。患者可有肝大、反复癫痫发作、生长发育迟缓、行为异常等临床表型。尽管晚发型症状较轻，但是在疾病、应激、高蛋白饮食等环境因素应激下会诱发高氨血症的急性发作而威胁生命。杂合子女性携带者多数终身无症状，少数发病，发病年龄及临床表现有个体差异性，既有早发型，也可表现为晚发型。

**qwen2.5_7b** 2025-02-18T18:03:59Z:

根据您提供的病情描述和临床特征，可以考虑以下几种罕见病：

1. **甲基丙二酸尿症（MMA）**：这是一种由于维生素B12代谢相关酶缺陷导致的遗传性代谢障碍疾病，会导致高甲基丙二酸血症和同型半胱氨酸血症。它可以表现为早发型或晚发型。
2. **线粒体脑肌病（Mitochondrial Encephalomyopathy）**：这类疾病是由线粒体功能障碍引起的一组罕见病，临床表现多样，可包括神经精神症状、肌肉无力等，可能会在任何年龄出现症状。
3. **苯丙酮尿症（Phenylketonuria, PKU）**：这是一种由于苯丙氨酸代谢途径中的酶缺陷导致的遗传性氨基酸代谢障碍疾病。虽然主要影响新生儿和儿童期，但如果不进行治疗，也可能引起晚发的症状如行为异常等。
4. **高草酸尿症（Hypoxanthine-Guanine Phosphoribosyltransferase Deficiency, HGPRTD）**：这是一种罕见的遗传性代谢病，主要表现为神经系统症状和肾结石。临床表现多样，可从新生儿期至成年发病。
5. **戊糖磷酸途径缺陷（Glucose-6-Phosphate Dehydrogenase Deficiency, G6PD Deficiency）**：虽然它通常被认为是一种红细胞酶缺陷引起的疾病，但有时也可能表现出神经系统症状，特别是在遇到某些诱发因素时引发急性溶血和神经精神症状。

每种疾病的诊断都需要通过特定的生化检测、基因检测以及临床表现综合判断。建议您咨询专业的遗传代谢科或儿科医生以获得更准确的诊断与治疗方案。

**93. user 2025-02-18T18:04:25Z:**

疾病的临床表型轻重不一。患者的主要临床表现是自幼起病的轻微外力下反复骨折、骨骼畸形，不同程度活动受限。患者可有蓝巩膜、韧带松弛、牙本质发育不全、听力下降、心脏瓣膜病变等骨骼外表现。OI 的影像学特征主要包括：全身多部位骨质稀疏；颅板变薄，囟门和颅缝增宽，枕骨可有缝间骨， 颅底扁平；脊柱可有侧弯或后凸畸形，椎体呈变形，可有多椎体压缩性骨折；胸廓可扭曲、变形，甚至塌陷；四肢长骨纤细、骨皮质菲薄，骨髓腔相对较大，干骺端增宽，多发长骨骨折，骨骼弯曲畸形等。

**qwen2.5_7b** 2025-02-18T18:04:28Z:

根据描述的症状和临床表型，初步诊断为“成骨不全症”（Osteogenesis Imperfecta, OI），这是一种罕见的遗传性结缔组织疾病，其特征是轻微外力下的反复骨折、骨骼畸形以及多种骨骼外部表现。

其他五种可能的诊断包括：

1. 先天性软骨发育不良：主要表现为身材矮小、关节松弛及骨骼形态异常。
2. 骨质疏松症：患者出现骨密度降低，容易发生脆性骨折。
3. 脆骨症（也称为先天性成骨不全）：是一种较为严重的OI亚型，通常伴有蓝巩膜和听力下降等表现。
4. 韧带松弛症：以关节松弛为主要特征，常伴有多发性骨折、骨骼畸形。
5. Marfan综合征：一种结缔组织疾病，主要表现为心血管系统异常（如主动脉瘤）、眼虹膜震颤、脊柱侧弯及四肢长骨过度伸展等。

**94. user 2025-02-18T18:04:59Z:**

**1. 全血细胞减少，** 许多患者可以合并全血细胞减少、中性粒细胞降低。临床上表现为乏力、反复感染和出血倾向。

**2. 血红蛋白尿** ，典型的血红蛋白尿呈酱油或浓茶色。一般持续 2~3 天，不加处理自行消退，重者 1~2 周，甚至持续更长时间。有些患者的血红蛋白尿频繁发作，也有相当多的患者在很长的病程或观察期内始终无明确血红蛋白尿发作。

血红蛋白尿发作时可伴有发热、腰痛、腹痛等症状。其他与平滑肌功能障碍相关的症状可以有吞咽困难、腹痛、胃胀、背痛、头痛、食管痉挛、勃起障碍等。少见的情况包括：胆囊管运动障碍、急性胰腺炎、十二指肠或结肠的缺血和溃疡。

**3. 血栓形成**

**4. 黄疸与肝脾肿大** ，不到一半 患者有轻度黄疸。多数患者没有肝脾肿大。

常见的合并症包括感染、贫血性心脏病、胆石症、肾功能不全甚至衰竭等。

**qwen2.5_7b** 2025-02-18T18:05:02Z:

根据您提供的症状，最有可能的诊断为阵发性睡眠性血红蛋白尿（PNH）。其他五种可能的诊断如下：

1. 急性造血功能障碍：表现为全身乏力和感染等。
2. 骨髓增生异常综合征：部分患者会出现全血细胞减少的症状。
3. 巨大血小板综合症：有出血倾向，但一般不会出现黄疸与肝脾肿大的情况。
4. 丙酮酸激酶缺乏症（PKD）：主要症状为反复发作的溶血性贫血、血红蛋白尿和全血细胞减少，但其临床表现通常较轻，且无血栓形成等症状，因此可能性较小。
5. 遗传性椭圆形红细胞增多症：表现为慢性溶血性贫血，但一般不会出现血栓形成及肝脾肿大。

**95. user 2025-02-18T18:05:24Z:**

临床表现为反复肠套叠、腹痛、腹泻、黏液便、便血、便秘、呕血、贫血等。特征性临床表现为皮肤黏膜色素斑和胃肠道多发息肉。95% 以上患者出现色素沉着，色素沉着多见于口唇及其四周、颊部、面部、手指、手掌、足底、肛周等，偶见于肠黏膜。色素可呈褐色、棕褐色、灰色和蓝色等，通常发生 2 岁前，大小 1_5mm。约88%_100% 患者会发生胃肠道息肉，胃肠道息肉发病多发生于 11~13 岁，息肉可发生在整个胃肠道，以小肠多见，其次是结肠和胃。息肉大小不定，蒂长短粗细不一。该类疾病患癌风险较高，平均患癌年龄为 42 岁。

**qwen2.5_7b** 2025-02-18T18:05:29Z:

根据您提供的临床表现和特征性症状，最有可能诊断的是**Peutz-Jeghers 综合征（佩-捷格雷综合征）**，这是一种以胃肠道息肉及特定部位皮肤、黏膜色素沉着为主要特点的遗传性肿瘤综合症。

其他五种可能的诊断包括：

1. **家族性腺瘤性息肉病（Familial Adenomatous Polyposis, FAP）**：虽然此疾病也会有结肠多发息肉，但通常没有皮肤黏膜色素沉着，且患者的息肉几乎全部位于结肠和直肠。
2. **遗传性非息肉病性结直肠癌（Hereditary Nonpolyposis Colorectal Cancer, HNPCC），也称林奇综合症**：此疾病以结直肠癌高风险为主要特征，通常没有色素沉着的症状，且皮肤表现并不典型。
3. **Juvenile Polyposis Syndrome（幼年性息肉综合征）**：这是一种与Peutz-Jeghers 综合征相似的常染色体显性遗传病，主要表现为胃肠道多发性错构瘤样息肉。但其色素沉着和具体年龄分布有所不同。
4. **Cowden 综合征（科温登综合征）**：此疾病以口腔黏膜乳头状瘤、皮肤病变如苔藓型痣样损害为特征，同时有增加的某些癌症风险，但Peutz-Jeghers 综合征特有的色素沉着使其区别于Cowden 综合征。
5. **Carney Complex（卡尼复合征）**：此疾病与内分泌功能异常相关，并伴有多发性皮肤、黏膜及器官肿瘤。虽然可能会有胃肠道息肉，但其特点和临床表现不同于Peutz-Jeghers 综合征。

请注意，最终的确诊需要结合详细的家族史、实验室检查结果以及基因检测等综合判断。

**96. user 2025-02-18T18:05:50Z:**

患儿在新生儿期多无临床症状，出生 3~4 个月后逐渐出现典型症状，1 岁时症状明显。出生数月后因黑色素合成不足，其毛发和虹膜色泽逐渐变浅，为黄色或棕黄色，皮肤白。由于尿液、汗液含有大量苯乳酸而有鼠尿臭味。随着年龄增长，逐渐表现出智能发育迟缓，以认知发育障碍为主、小头畸形、癫痫发作，也可出现行为、性格等异常，如多动、自残、攻击、自闭症、自卑、忧郁等神经系统表现。婴儿期还常出现呕吐、湿疹等。

**qwen2.5_7b** 2025-02-18T18:05:53Z:

根据您提供的临床症状，最有可能的诊断是苯丙氨酸代谢障碍（Phenylketonuria, PKU），这是一种罕见的遗传性氨基酸代谢病。

其他可能的五种诊断包括： 1. 苯乳酸血症：这是一种由苯丙氨酸羟化酶缺乏导致的疾病。 2. 先天性肾上腺皮质增生症：特别是由于21-羟化酶缺乏导致的病例，可能会表现出类似的皮肤色素变化和神经系统症状。 3. 脂肪酸氧化障碍：某些类型的脂肪酸氧化障碍可能会有类似的新陈代谢问题、智力发育迟缓等表现。 4. 神经皮肤综合征（如结节性硬化症）：这种疾病可能导致智能发展异常和其他神经系统的症状，但通常不伴有苯乳酸尿的症状。 5. 2型糖原累积病（Glycogen Storage Disease Type II, GSD II）：由于葡萄糖-6-磷酸酶缺乏而导致的代谢障碍，可能会表现出类似的发育迟缓和神经系统症状。

**97. user 2025-02-18T18:06:13Z:**

主要为皮肤症状和神经精神症状。

**1. 皮肤症状群，** 为光照后在皮肤暴露部位出现红斑、疱疹甚至溃烂。皮疹可为湿疹、荨麻疹、夏令痒疹或多形性红斑等类型。口腔黏膜可有红色斑点，牙呈棕红色。同时可并发眼损害如结膜炎、角膜炎及虹膜炎等。严重者可有鼻、耳、手指皮肤结瘢变形。可有特殊紫色面容。可有多毛症。

**2. 神经精神症状群 ，**最常见的表现为急性腹痛，腹痛往往为重度、稳定、定位不明，有时会伴有痛性痉挛。可以伴随便秘、腹胀、恶心、呕吐，有时出现腹泻和肠鸣音增加。感觉和运动神经病可能先于腹痛出现，表现为肢体痛，伴麻木感、感觉异常和感觉倒错； 运动无力往往始于上肢近端，可向下肢和远端进展。累及脑神经可能导致延髓麻痹、呼吸功能受损和死亡。严重运动神经病可导致四肢瘫痪。自主神经系统受累表现为心动过速、高血压、出汗、躁动和震颤等。神经源性膀胱功能障碍可能引起尿痛、排尿困难、尿潴留和尿失禁。其他急性神经精神表现包括失眠、焦虑、躁动、激越、幻觉、癔症、定向障碍、谵妄、情感淡漠、抑郁、恐惧症和意识改变，从嗜睡到昏迷程度不一。

**qwen2.5_7b** 2025-02-18T18:06:17Z:

根据您描述的症状群，以下是我最有可能的诊断及其它五种可能的诊断：

1. 最有可能的诊断：**卟啉病**

- 临床表现符合皮肤光敏感症状（红斑、疱疹、溃烂等）、口腔黏膜异常以及神经精神症状中的腹痛和神经系统症状。

1. 其它五种可能的诊断：
   - **急性间质性脑炎：** 可能出现意识改变及神经精神症状，如焦虑、幻觉等症状。
   - **线粒体脑肌病：** 也可能引起神经肌肉症状以及皮肤相关的问题（例如红斑）。
   - **慢性复发性荨麻疹：** 主要表现为反复发作的皮肤痒和皮疹，且伴有腹痛等症状。
   - **遗传性感觉自主神经病变：** 引起的感觉异常、运动无力等神经系统症状与描述部分吻合。
   - **卟啉病（其他亚型）：** 作为另一种可能诊断，尽管您之前已经提到了卟啉病。但不同类型的卟啉病，如急性间歇性卟啉病或迟发性神经精神型卟啉病也可能表现为类似的症状群。

**98. user 2025-02-18T18:06:49Z:**

**1. 感染，患者**均有不同程度感染。 感染具有反复、严重、难治、机会致病、病原谱广等特点。典型患儿出生时一般没有特别表现，由于患儿存在细胞免疫异常，2 月龄前也可发生致命感染。感染部位及病原多种多样，包括反复肺炎，反复鹅口疮，持续腹泻，反复中耳炎，持续病毒感染(如呼吸道合胞病毒、EBV、CMV 等)，卡氏肺囊虫等条件致病菌感染也较常见。

**2. 疫苗病，** 患者免疫功能极度低下，可发生减毒活疫苗感染和播散。 患儿接种减毒轮状病毒疫苗也可发生严重腹泻。

**3. 生长发育落后或停滞** 是 疾病的显著特征。与患儿反复感染、腹泻和相应基因缺陷均有关。

患儿还可伴有皮疹、肝脾淋巴结肿大类似红皮病表现

另一部分患儿可出现类似红皮病、肝脾肿大、脂溢性皮炎、血细胞减少症，硬化性胆管炎。

**qwen2.5_7b** 2025-02-18T18:06:54Z:

根据您提供的临床表现，最有可能的罕见病诊断是**严重联合免疫缺陷病（SCID）**，特别是**腺苷脱氨酶缺乏型 SCID (ADA-SCID)**。以下是其他五种可能的诊断：

1. **重症多发性感染伴发育迟缓** - 与 SCID 的临床表现相似。
2. **共济失调毛细血管扩张症（Ataxia Telangiectasia, AT）** - 表现为反复感染、生长发育落后，部分患者伴有神经系统症状和皮肤病变。
3. **Wiskott-Aldrich 综合征 (WAS)** - 伴有血小板减少性紫癜和湿疹，并且存在免疫缺陷。
4. **慢性肉芽肿病（Chronic Granulomatous Disease, CGD）** - 具有反复感染史，但主要表现为皮肤黏膜的化脓性和肉芽肿病变。
5. **X-连锁无丙种球蛋白血症 (Bruton’s Agammaglobulinemia)** - 主要表现为反复细菌性感染，免疫球蛋白水平降低。

**99. user 2025-02-18T18:07:22Z:**

核心症状是肌张力不全，主要表现为异常的表情姿势和不自主的动作，多累及头颈部肌肉、躯干肌、肢体的旋前肌、指腕屈肌、趾伸肌和跖屈肌等。随意运动时加重，休息睡眠时减轻或消失，晚期可呈固定扭曲痉挛畸形。感觉诡计(缓解技巧) 和动作特异性是肌张力不全的特征性表现。感觉诡计(缓解技巧) 是指专门用于纠正异常姿势或缓解肌张力不全性运动的随意动作，通常是涉及或针对受累部位的简单运动，但不是用力对抗肌张力不全症状。动作特异性是指仅在特定活动或执行特定任务时出现肌张力不全，例如某些职业(如书写痉挛、音乐家痉挛) 或运动。

**qwen2.5_7b** 2025-02-18T18:07:28Z:

根据您提供的临床表现，最有可能的诊断是梅尼埃病（Meniere’s disease）与肌阵挛性脑病之间的区分，尤其是考虑到头部和颈部肌肉受累以及随意运动时症状加重的特点。但是，从您的描述来看，该罕见病症更符合特发性扭转痉挛或Meige综合征。

1. 特发性扭转痉挛：这是一种少见的神经系统疾病，主要表现为肌张力不全、异常姿势及面部和躯干肌肉受累。
2. Meige 综合征：是眼睑痉挛与口下颌肌张力障碍同时存在的一种罕见病症，也符合您的描述中提到的动作特异性现象。
3. 痉挛性斜颈：以颈部肌肉不自主收缩导致头部倾斜为主要特征，症状可扩散至面部、肩部等部位。
4. 遗传性运动感觉神经病（如Charcot-Marie-Tooth病）：可能表现为四肢远端肌无力和萎缩伴有腱反射减弱或消失等症状，在某些情况下也可能出现姿势异常及肌张力不全现象。
5. 亨廷顿舞蹈症：这是一种遗传性疾病，临床主要表现包括不自主的舞蹈样动作、认知功能减退以及精神症状等。然而，该病多见于中年发病，并且常伴有智能下降和行为障碍等症状。

请注意，以上仅是基于您描述的症状可能涉及的一些罕见病诊断方向，在实际诊疗过程中仍需结合详细的病史询问、体格检查及辅助检查结果进行综合判断。

**100. user 2025-02-18T18:07:49Z:**

**1. 肾脏 ，**主要表现为肢体水肿和尿中泡沫增多，实验室检查可以发现单纯的中等量蛋白尿或肾病综合征，晚期可出现肾功能不全。

**2. 心脏 ，**主要表现为活动后气短、肢体水肿、胸腔积液、腹水等限制性心功能不全表现。

**3. 肝脏 ，**可有轻微肝区不适或疼痛，但多数患者可无症状，往往为体检时发现肝大或者肝酶异常。疾病晚期可出现肝功能衰竭。

**4. 周围神经和自主神经 ，**为对称性的四肢感觉和(或) 运动性周围神经病。自主神经异常多表现为直立性低血压、胃轻瘫、假性肠梗阻和阳痿等。

**5. 胃肠道** ，可以出现全胃肠道受累，以胃部和小肠受累多见。可以表现为上腹不适、消化不良、腹泻、便秘、吸收不良综合征和消化道出血等。

**6. 软组织 ，**舌体受累可出现巨舌、舌体活动障碍和构音异常等。皮肤黏膜可以出现皮肤紫癜和瘀斑，以眼眶周围和颈部皮肤松弛部位较为常见。也可以出现指甲萎缩脱落和毛发脱落等。

**7. 凝血功能异常** 患者常会伴发凝血因子Ⅹ缺乏，造成相应的出血表现。

**qwen2.5_7b** 2025-02-18T18:07:52Z:

根据您描述的症状，我推测这可能是先天性低凝血酶原血症。这种罕见病主要影响肝脏和肾脏，并且与凝血有关。以下是五种可能的诊断：

1. **抗磷脂综合征**：一种自身免疫性疾病，可导致血液高凝状态、血管炎症以及反复流产等症状。
2. **肝豆状核变性（Wilson病）**：虽然表现为肝脏症状，但也可出现神经系统异常、精神障碍等。
3. **Fabry病**：这是一种罕见的X连锁遗传疾病，主要影响肾脏、心脏和中枢神经系统，也可能导致胃肠道问题及感觉神经病变。
4. **系统性硬化症**：一种以血管炎症为特征的自身免疫性疾病，可累及多器官系统，包括皮肤、肺部、消化道等部位。
5. **遗传性血管水肿（HAE）**：这是一种由于C1抑制物缺乏或功能缺陷导致的罕见疾病，主要影响皮肤和胃肠道。

**101. user 2025-02-18T18:08:14Z:**

黄疸和皮肤瘙痒是典型临床表现。其他有身材矮小、青春期发育落后等发育迟缓表现，胆囊结石，脂肪吸收障碍所致的脂肪泻，肝脾肿大以及脂溶性维生素缺乏所致佝偻病、骨龄延迟、干眼症、凝血障碍和神经肌肉病变等症状。患儿亦可以出现视觉及听力异常，出现烦躁、嗜睡及注意力不集中等改变。晚期可出现门静脉高压症和肝脏肿瘤等。

**qwen2.5_7b** 2025-02-18T18:08:20Z:

根据您描述的临床表现：黄疸、皮肤瘙痒以及身材矮小、青春期发育落后等发育迟缓的表现；胆囊结石；脂肪泻；肝脾肿大；脂溶性维生素缺乏（如佝偻病）所致的症状；干眼症；凝血障碍和神经肌肉病变；视觉及听力异常；注意力集中问题以及其他一些晚期表现（门静脉高压症、肝脏肿瘤），我最可能的诊断是进行性家族性肝内胆汁淤积症（PFIC）。其他五种可能的诊断包括：

1. 肝豆状核变性：该病是一种遗传性的铜代谢障碍疾病，可导致肝硬化、神经系统症状等；
2. 先天性胆道闭锁：这是一种影响新生儿的肝脏疾病，常表现为黄疸和肝功能异常，但通常不伴有其他系统受累的症状；
3. 代谢性疾病：如中链酰基辅酶A脱氢酶缺乏症（MCADD）等罕见遗传性代谢病，可导致肝脏损伤、低血糖和其他症状；
4. 先天性溶血性黄疸：例如葡萄糖-6-磷酸脱氢酶缺陷症或丙酮酸激酶缺乏症等，可能会引起皮肤瘙痒和肝脾肿大等症状；
5. 胆汁酸合成障碍：包括7α-羟化酶缺陷症在内的几种遗传病，可导致胆汁淤积性黄疸、脂肪泻及其他消化系统症状。

请注意，以上诊断仅作为参考依据，具体疾病的确诊需要通过详细的临床检查和实验室检测。

**102. user 2025-02-18T18:08:47Z:**

在儿童期起病。表现为运动发育轻度迟滞，骨骼肌进行性无力萎缩，影响肢体运动功能，逐渐出现步态异常、上肢活动受限，自然病程常在 10 岁左右丧失行走能力。此后出现脊柱侧弯、关节挛缩、呼吸肌无力、扩张型心肌病，20 岁左右因呼吸衰竭、心功能衰竭死亡。查体可见双腓肠肌假肥大，同时可有双前臂及舌肌假肥大，Gowers 征阳性，腰椎前凸等。可青年甚至成年起病， 部分患者不影响生存期。假肥大体征明显，部分患者在肢体无力尚轻时，先出现明显的扩张型心肌病。

**qwen2.5_7b** 2025-02-18T18:08:50Z:

根据您描述的临床表现，这种罕见病最有可能是进行性肌营养不良症（DMD或BMD），这是一种X染色体隐性遗传疾病。以下是其他五种可能的诊断：

1. 先天性肌营养不良：表现为出生时即存在肌肉无力和假肥大。
2. 面肩肱型肌营养不良 (FSHD)：通常在青少年期发病，以面部、肩膀和上臂肌肉受累为主，进展较缓慢。
3. 束带肌病：是一种罕见的遗传性肌肉疾病，表现为四肢近端肌肉无力和萎缩。
4. 皮肌炎/多发性肌炎：这是一种自身免疫性疾病，也可导致进行性的肌肉无力和假肥大症状。
5. 慢性进行性肌营养不良症 (CPMD)：是一种少见的常染色体隐性遗传病，与DMD不同的是其起病年龄较轻、病情进展缓慢。

**103. user 2025-02-18T18:09:17Z:**

主要为高血氨、脑损伤和心肌病等。

**1. 新生儿起病型** 出生时正常，开始哺乳后出现呕吐、嗜睡、肌张力低下、惊厥、呼吸困难、高血氨、酮症、低血糖、酸中毒、扩张型心肌病、胰腺炎等异常，病死率高。

**2. 迟发型** 常因发热、饥饿、高蛋白饮食和感染等诱发，表现为婴幼儿期喂养困难、发育落后、惊厥、肌张力低下等。许多患者的认知能力及神经系统发育受到损害，脑电图慢波增多或见癫痫波；一些患者可有骨折，X 线见骨质疏松；还常造成骨髓抑制，引起粒细胞减少、贫血、血小板减少。也可有心脏损害，如心肌病、心律失常、QT 间期延长、心功能减弱等。肾功能损害较为少见。

**qwen2.5_7b** 2025-02-18T18:09:20Z:

根据您描述的临床表现，最有可能诊断为经典型经典线粒体脑肌病伴乳酸血症和高丙酮酸血症（MELAS）。其他五种可能的诊断包括：

1. 线粒体脑脊髓病伴锥体外系症状 (MERRF)
2. 肝豆状核变性
3. 脑酸中毒-神经肌肉疾病综合征 (Kearns-Sayre 综合征)
4. 乳酸血症性低血糖性脑病 (LHON)
5. 线粒体脑肌病伴光敏感 (MERRF)

**104. user 2025-02-18T18:09:42Z:**

临床表现常没有特异性，隐匿起病。可出现咳痰、消瘦、乏力等症状。继发感染时可有发热、咳脓性痰，也可出现胸痛、咯血。少数患者可无症状，可以没有阳性体征。部分患者可以出现爆裂音，重症患者可有发绀，少数患者可见杵状指。临床表现常比胸部影像受累的程度轻，这种症状和影像不一致的表现也是该病 的一个特征。血清抗 GM-CSF 抗体测定明显增高。

**qwen2.5_7b** 2025-02-18T18:09:48Z:

根据您提供的临床表现描述，这类罕见疾病可能与一种称为“组织细胞坏死性淋巴结炎”（也称Rosai-Dorfman病或RDD）相关，但鉴于症状的多样性和非特异性，还需考虑以下几种可能性：

1. **先天性无纤维蛋白症**：这是一种非常罕见的遗传性疾病，患者由于缺乏纤维蛋白溶解酶原激活物而出现慢性淋巴结肿大、发热等症状。
2. **朗格汉斯细胞组织细胞增生症**（Histiocytosis）：这是一组较为罕见的疾病，可以影响儿童和成人，涉及过多的朗格汉斯细胞在体内聚集。症状多样，从皮肤病变到骨骼或淋巴结肿大都有可能。
3. **慢性肉芽肿病**：这是一种免疫系统疾病，患者的免疫功能受损，容易发生反复感染，并伴有肺部、皮肤等多器官受累的症状。
4. **嗜酸性粒细胞增多症**（Eosinophilia）：患者血液中嗜酸性粒细胞数量异常增加，伴随有多种不同症状，如咳嗽、呼吸困难、发热等。
5. **结节病**：这是一种原因不明的肺部疾病，可以引起肺内和其它器官受累。典型的临床表现为非干酪样肉芽肿形成及相应的临床表现，可影响多个系统。

在面对这些罕见病例时，通常需要通过详细的病史采集、体格检查、实验室检查（如血清抗GM-CSF抗体测定）、影像学检查以及必要时的组织活检来最终确定诊断。

**105. user 2025-02-18T18:10:16Z:**

大多患者青少年时期起病，首先是视杆细胞逐渐丧失，随后出现视锥细胞受累， 视网膜色素上皮细胞死亡。因此通常以夜盲为首发症状(但城市化后部分患者常以视力下降为首发症状)，而且夜盲出现得越早，患者的病情往往越严重。虽然周边视野缺损也发生较早，但患者通常意识不到，很少以此作为疾病早期的主诉。病情逐渐发展为中心视力下降，视野进一步缩窄，多数在 40 岁之前就成为法定盲人。患者的视功能损伤程度可与遗传方式相关。

患者的眼底表现与疾病所处的阶段相关：疾病早期患者眼底在常规检查下可表现正常或接近正常，但部分患者在中周部眼底照相下可以观察到早期毯层样视网膜变性(这类细微改变在目前的扫描激光眼底照相或广域眼底照相下很难识别)。随着病情的进展，部分 患者会出现典型的眼底改变，表现为视网膜骨细胞样色素沉积合并中周部视网膜萎缩，视盘蜡黄，视网膜血管变细等；部分病人会出现毯层样视网膜变性或脱色素改变，见于中周部视网膜或广泛变性。其他常见的并发症还包括黄斑囊样变性、后囊下白内障、玻璃体尘样颗粒、视盘玻璃膜疣等并发症；少见的并发症有 Coat’s 样视网膜病变。

**qwen2.5_7b** 2025-02-18T18:10:23Z:

根据您提供的临床表现，最有可能诊断出的罕见病是**少年型视网膜色素变性（也称为青少年起始型视网膜营养不良）**。

以下是其他五种可能的诊断：

1. 成人型视网膜色素变性：成人发病，多见于30-40岁之间。临床表现与青少年期起病的类型相似。
2. 遗传性黄斑营养不良（Best病）：主要影响中央视力，通常双眼受累且早期症状较轻。
3. 色素沉着假肿瘤综合征：表现为周边视网膜色素沉积和血管异常扩张。
4. Coats病：一种少见的血管异常性疾病，以视网膜血管异常扩张、渗出为特征，并可导致玻璃体积血。
5. 家族性渗出性玻璃体视网膜病变（FEVR）：常表现为周边视网膜血管发育不良和牵拉性视网膜脱离。

**106. user 2025-02-18T18:11:01Z:**

最常见的临床表现是白瞳症，即瞳孔可见黄白色反光。患眼可因肿瘤位于后极部，视力低下，而发生失用性斜视。早期表现为灰白色，圆形或椭圆形拱状肿物，可向玻璃体隆起，或沿脉络膜扁平生长。肿瘤表面的视网膜血管可出现扩张、出血，甚至发生渗出性视网膜脱离。瘤组织也可穿破视网膜进入玻璃体，如雪球状漂浮，甚至沉积于前房形成假性前房积脓。双侧性患儿发病较早，通常一岁以内发病。单侧性患儿发病较迟，通常在两岁或三岁时发生。

**qwen2.5_7b** 2025-02-18T18:11:04Z:

根据您描述的临床表现，最有可能诊断的是视网膜母细胞瘤（Retinoblastoma）。此外，还需要考虑以下五种可能的诊断：

1. **脉络膜黑色素瘤**：虽然通常是成人的疾病，但儿童中较为罕见的情况下也会发生。
2. **视网膜血管瘤**（也称为Coats病）：这通常表现为视网膜血管异常扩张、渗漏和出血，导致白瞳症。
3. **先天性青光眼**：尽管主要症状是角膜水肿、虹膜震颤等，但有时也会因继发原因引起白瞳症。
4. **视神经胶质瘤**：虽然通常表现为视神经区域的肿瘤，但在某些情况下也可能影响到视网膜并导致类似的临床表现。
5. **先天性眼底病变**（如早产儿视网膜病）：虽然这些通常与早产有关，但有时也会出现类似白瞳症的症状。

请注意，最终的确诊需要通过详细的检查和专业医生的综合判断。

**107. user 2025-02-18T18:11:30Z:**

患儿出生时多正常，在 1 岁以内起病，平均发病年龄为 5.6 个月。首次发作常为热性惊厥，可为一侧性或全面性阵挛或强直阵挛发作，热性惊厥具有发作持续时间长和反复发作特点，可出现持续状态。28％ ~35％患儿可能以无热惊厥起病，但在以后病程中常有热敏感特点。各种原因引起的体温升高，包括运动、发热、疫苗接种、洗热水澡和环境温度高等，均容易诱发或使发作加重，并易发生癫痫持续状态。有些患儿会出现以癫痫持续状态之后深昏迷为特征的急性脑病。

患儿多于 1~4 岁期间出现无热惊厥，具有多种发作类型，如肌阵挛、不典型失神、部分性发作、全面强直阵挛发作等。肌阵挛发作常由光刺激、闭眼和某种特定的图形刺激诱发，一块或数块肌肉同时阵挛，均伴有同步异常脑电波阵发性发放。肌阵挛强度不一，仅侵犯轴性肌肉时，出现身体轻微向前或向后的动作，严重者跌倒或突然抛掷手中物体。肌阵挛发作可以是孤立的，也可群发，但持续状态比较罕见。约 25％的 儿可以始终不出现肌阵挛发作。不典型失神发作同期脑电图显示半数为局灶起源放电，多数呈不规则不节律的慢棘慢波发放，经常伴随肌阵挛发作。30％的患儿可发生不典型失神持续状态。部分性发作发生率为 43.0％ ~78.6％，表现为偏侧肢体(或) 头部偏转性发作或阵挛性发作，亦有运动性发作伴有意识障碍和自主神经症状、自动症、肌张力降低等。1 岁以后患儿出现精神运动发育迟滞，尤其是语言发育迟缓。

**qwen2.5_7b** 2025-02-18T18:11:33Z:

根据您描述的症状，该患者最有可能诊断的罕见病是Dravet综合征（也称为严重肌阵挛性癫痫）。以下是其他五种可能的诊断：

1. Lennox-Gastaut 综合征：表现为多种类型的发作形式和智力、运动发育迟缓。
2. 脑肿瘤或脑炎等中枢神经系统感染导致的癫痫，尤其是伴有发热相关症状时需要排除。
3. 先天性代谢性疾病，例如 Leigh 综合征或其他线粒体病。这些疾病可引起类似的神经精神症状及肌阵挛发作。
4. 神经皮肤综合征（如Tuberous Sclerosis Complex），伴随皮疹、脑部或眼部异常等表现。
5. Rett 综合征：以发育迟缓和手部刻板行为为特征，但在您的描述中，患者年龄偏大，因此可能性较小。

**108. user 2025-02-18T18:11:53Z:**

一方面表现为慢性溶血性贫血，除贫血相关症状，因胆红素升高可引起皮肤、巩膜轻中度黄染、色素性胆石症，当寒冷、感染、脱水时症状可能加重。婴幼儿可出现脾大，随年龄增长，脾脏可因脾梗死、纤维化而缩小。

另一方面由于毛细血管微血栓而引起反复发作的血管阻塞性疼痛。儿童和成人多表现为四肢肌痛、大关节疼痛和腰背疼痛，严重者可出现剧烈腹痛、头痛，甚至肢体瘫痪、昏迷。随着时间的推移，血管阻塞几乎可发生于每个器官系统，可以出现周期性疼痛，患者瘦弱、易疲劳、易感染、营养不良。因骨髓造血组织过度代偿性增加使骨皮质变薄、骨质疏松、脊柱变形、股骨头无菌坏死，如有骨梗死则可导致局部骨质硬化。眼部症状可能出现眼底视网膜血管栓塞引起眼底出血、视网膜脱离。另外，尚可影响神经系统的发育而出现智力低下。

在超过 25％的患者中急性疼痛为疾病的首发症状。在大于 2 岁的患儿中是最常见的症状。疼痛的严重程度可从轻微到剧烈不等。大约 1/2 的疼痛发作伴随客观的临床体征，如发热、肿胀、压痛、呼吸过速、高血压、恶心和呕吐。

**qwen2.5_7b** 2025-02-18T18:11:55Z:

根据您提供的临床表现，最有可能诊断为阵发性睡眠性血红蛋白尿（PNH）。

其他五种可能的诊断包括： 1. 血栓性微血管病（TMV） 2. 蚕豆病（G6PD缺乏症） 3. 慢性自身免疫性溶血性贫血 4. 血管炎性疾病，如韦格纳肉芽肿或显微镜下多血管炎 5. 遗传性球形红细胞增多症

需要强调的是，这些诊断都需要结合患者的具体情况（包括病史、体征和实验室检查结果）进行综合判断。

**109. user 2025-02-18T18:12:37Z:**

**1. 宫内发育迟缓及出生后生长障碍** 宫内发育迟缓，低出生体重，出生后缺乏追赶生长，成比例的身材矮小。

**2. 特殊体征** 前额宽而突出、尖下颌导致三角形脸，相对头围偏大。齿列不齐、耳位低，躯体偏身不对称，包括四肢长度差异，小指侧弯畸形等。

**3. 其他表现** 喂养困难、新生儿期可出现低血糖，尿道下裂、隐睾和马蹄肾等泌尿生殖系统畸形，牛奶咖啡斑，反流性食管炎等消化系统异常。部分患者出现认知、运动和语言发育迟缓，学习障碍等。

**qwen2.5_7b** 2025-02-18T18:12:42Z:

根据您提供的临床表现，最有可能的罕见病诊断是**特普拉尼尔综合症（Thomsen disease）**，这是一种以神经肌肉症状为主的遗传性肌炎，但其特征并不完全吻合。特普拉尼尔综合症主要表现为周期性瘫痪、肌阵挛等，不常出现您描述的宫内发育迟缓及生长障碍。

根据您的描述，最有可能诊断的罕见病可能是**马凡综合征（Marfan syndrome）**，其他五种可能的诊断包括：

1. **特普拉尼尔综合症（Thomsen disease）**：一种遗传性神经肌肉疾病。
2. **安德森-弗利什综合症（Andersen-Friedrich syndrome）**：又称为发育迟缓型黏多糖贮积病，表现为生长障碍、特殊面容等。
3. **萨尔特综合症（Salla disease）**：一种罕见的遗传性神经退行性疾病，通常伴有身材矮小和神经系统异常。
4. **普拉德-威利综合症（Prader-Willi syndrome）**：以身材矮小、智力障碍、特殊面容为特征的一种染色体疾病。
5. **安吉曼综合症（Angelman syndrome）**：表现为智能发育迟滞、共济失调、语言障碍等，也可能伴有身材矮小。

**110. user 2025-02-18T18:13:17Z:**

**1. 皮肤黄瘤，** 好发于伸肌肌腱附着点附近，例如跟腱、肘关节、膝关节等，皮肤损伤与黄瘤出现关系密切。皮肤黄瘤少见于儿童患者。

**2. 动脉粥样硬化 ，**患者出现动脉粥样硬化、冠心病的风险显著高于正常人。早发性冠心病可以作为本病的特点之一。

**3. 血液系统改变 ，**血液中植物固醇含量明显增加，导致细胞膜中固醇成分中植物固醇比例增高，红细胞、血小板等细胞膜功能异常，出现贫血和血小板减少，患者可出现乏力、面色苍白、皮肤出血等表现。

**4. 其他，**除上述症状，部分患者会出现关节痛、关节炎、脾大、转氨酶升高等。

**qwen2.5_7b** 2025-02-18T18:13:22Z:

根据您提供的临床表现，最有可能的罕见病诊断是尼曼-匹克病（Niemann-Pick Disease），这是一种由于鞘磷脂代谢异常导致神经退行性病变的疾病。

其他五种可能的诊断包括：

1. 贝塞特氏病：一种与自身免疫相关的血管炎性疾病，可引起皮肤病变、关节痛和动脉瘤等症状。
2. 遗传性高脂蛋白血症：是一组由基因突变导致血脂代谢异常的遗传性疾病，患者可能出现黄瘤等皮肤症状以及冠心病的风险增加。
3. 甲状腺功能亢进症：一种内分泌疾病，可引起多系统受累症状，包括心血管、消化和神经系统的症状。
4. 地中海贫血：这是一种遗传性溶血性贫血，患者可能会出现贫血、乏力、面色苍白等症状。
5. 韦格纳肉芽肿病：是一种罕见的自身免疫性疾病，可导致全身多个器官系统受累，如皮肤、关节、肺部和肾脏等。

**111. user 2025-02-18T18:14:08Z:**

男性发病，起病隐匿，常见发病年龄为 30~60 岁。患者常以痛性痉挛、震颤、双下肢无力等症状起病，逐渐出现双上肢无力、延髓部和面部的肌肉萎缩、肌束颤动、构音障碍、吞咽困难、呼吸困难等。可伴有男性乳房发育、生殖功能降低等雄激素受体不敏感表现。超过 50％的患者存在感觉异常。该病进展缓慢，患者通常在病程晚期才出现行走不能，仅有部分患者需要辅助通气，对生存期无显著影响。

**1. 不典型症状 ，**某些不典型症状可在出现肢体力弱前数年甚至数十年出现，包括易疲劳、痛性痉挛、口周震颤及姿势性震颤。肌无力：多数患者以双下肢无力起病，逐渐进展至双上肢、舌肌以及面肌，而眼外肌往往不受累。肢体无力的分布多不对称，以近端受累为主。咀嚼肌受累时可引起下颌下垂及震颤。体检以下运动神经元损害体征为主，表现为肢体肌力下降，近端为著，伴肌肉萎缩、束颤，腱反射多减弱或消失。多数患者可出现明显舌肌萎缩、纤颤，但软腭活动、咽反射多正常，患者饮水呛咳、吞咽困难的程度也较轻。某些患者还可观察到颏部以及口周束颤。患者运动功能损害随病程延长逐渐加重，后期可出现上楼费力、行走不稳等。

**2. 感觉障碍** ，约半数患者存在不同程度的感觉减退，而在其他患者为亚临床性，仅在感觉神经传导检查中发现异常。

**3. 内分泌异常，** 包括葡萄糖及脂肪代谢的异常，雄激素受体不敏感等表现，后者包括男性乳房发育、性功能下降、不孕不育、睾丸萎缩等。

**4. 女性携带者，** 多数女性携带者并不出现症状，即使出现症状程度也较轻，可仅表现为束颤、轻度远端肢体无力、肌肉痉挛或肌酸肌酶增高等。

**qwen2.5_7b** 2025-02-18T18:14:14Z:

根据您提供的临床表现，最有可能的诊断为脊肌萎缩症（Spinal Muscular Atrophy, SMA），特别是成人发病型（Late-Onset SMA）。其他五种可能的诊断包括：

1. 遗传性运动感觉神经病（Hereditary Motor and Sensory Neuropathy, HMSN）：如Charcot-Marie-Tooth病，常表现为四肢远端对称性无力和萎缩，伴有感觉异常。
2. 肌萎缩侧索硬化症（Amyotrophic Lateral Sclerosis, ALS）：以进行性肌无力、肌萎缩为主要表现，可合并上运动神经元及下运动神经元损害的体征。
3. 先天性肌营养不良（Congenital Muscular Dystrophy）：患者自婴儿期即出现肌肉无力和发育迟缓，但您描述的是成年发病，故可能性较小。
4. 遗传性感觉运动神经病Ⅰ型（Hereditary Sensory and Autonomic Neuropathy Type I, HSAN1）：以感觉异常为主要表现，可伴有运动功能减退。该病由TRKA基因突变引起，常见于儿童和青少年期发病。
5. 勒-雪氏病（Pelizaeus-Merzbacher Disease, PMD）或其相关形式（如X连锁多发性硬化症，即Leber’s Agenesis of the Corpus Callosum, LAMC2突变），以进行性共济失调、痉挛、震颤和智力障碍为主要特征，但通常于儿童期发病。

**112. user 2025-02-18T18:15:14Z:**

部分患者在宫内表现为胎动减少，出生时为松软儿。患者在 6 个月内发病，平均发病年龄在生后 1 个月。表现为全身松软无力，严重肌张力低下。由于舌、面和咀嚼肌无力，大多数患儿出现吸吮和吞咽困难，可见舌肌萎缩和震颤。肋间肌受累可以出现呼吸困难，腹式呼吸。胸部呈钟型外观。下肢较上肢受累重，近端较远端严重。严重躯体中轴部位肌无力使患儿不能控制头部运动，不会抬头或翻身，没有独坐能力。卧位时，双下肢呈髋外展、膝屈曲的蛙腿体位。肌肉萎缩多不明显， 部分患儿轻度关节畸形。患儿智力好，腱反射消失，四肢感觉正常。患儿肌无力进行性加重，最终失去所有自主运动能力，鼻饲喂养，反复呼吸道感染而致呼吸衰竭。80％患儿 1岁内死亡，很少活过 2 岁。

**qwen2.5_7b** 2025-02-18T18:15:19Z:

根据您提供的临床表现，可能的罕见病诊断如下：

1. **先天性肌营养不良症（Criminals disease）**：这是一种婴儿型进行性肌营养不良，特征包括肌无力、吞咽困难和呼吸问题。
2. **先天性假肥大型肌营养不良症（Duchenne Muscular Dystrophy, DMD）的早期表现**：尽管DMD通常在稍大一些的孩子中发病，但这种描述符合婴儿型DMD的一些症状。
3. **早发型线粒体肌病**：这包括多种遗传性线粒体疾病，可能导致类似的肌无力和发育迟缓。
4. **先天性重症肌无力（Congenital Myasthenic Syndrome, CMS）**：这是一种罕见的神经-肌肉接头传递障碍性疾病，症状包括全身无力、吞咽困难等。
5. **先天性运动神经元病（CMTM, Congenital Motor Neuronopathy）**：这类疾病表现为出生后的早期肌无力和肌萎缩。

以上是可能的一些诊断，但确切的诊断需要进一步详细的临床检查、遗传学检测及实验室检查。建议进行基因检测以明确具体的病因。

**113. user 2025-02-18T18:15:48Z:**

患者通常成年起病，隐匿起始，逐渐进展。主要表现为小脑性共济失调，包括醉汉步态、动作笨拙、吟诗样语言、眼震、复视等；锥体束损害，包括腱反射亢进、踝阵挛、病理征阳性；锥体外系症状，包括帕金森综合征、肌张力障碍、强直等。此外，部分患者可有周围神经病。部分患者在疾病早期可以锥体束损害(类似痉挛性截瘫) 或锥体外系症状(类似帕金森综合征) 为主，共济失调症状不突出。

疾病逐渐进展，起病 10~15 年后丧失行走能力。长期吞咽困难致营养不良或误吸是导致死亡的主要原因。

**qwen2.5_7b** 2025-02-18T18:15:51Z:

根据您提供的临床表现，最有可能诊断出的罕见病为小脑性共济失调-脊髓Ataxia with Spinal Neuropathy, ASN），具体来说是Spastic Paraplegia type 13 (SPG13)或者Cerebelloretinal Ataxia with Oculomotor Apraxia and Neuropathy (CARN）。

其他可能的诊断有： 1. Friedreich共济失调 2. 肌阵挛性共济失调(Machado-Joseph病) 3. 纯小脑萎缩(Cerebellar Atrophy, Pure Type) 4. 小脑性共济失调-肌阵挛型Ataxia with Frontal Temporal Dementia and Parkinsonism（ATLD） 5. 双侧进行性脊髓萎缩症(Adrenomyeloneuropathy, AMN)

**114. user 2025-02-18T18:17:03Z:**

疾病早期可能会合并胃食管反流。随着病程的进展，患者逐渐出现皮肤肿胀及硬化，并可伴皮肤色素改变，包括色素沉着或色素脱失。除皮肤受累之外，患者常会伴有关节痛及肌痛等症状，并可出现指端溃疡和毛细血管扩张。此外，患者可能会出现多个内脏器官受累。其中呼吸系统较易受累，主要表现为肺间质纤维化和肺动脉高压。患者的消化系统表现多样， 包括胃食管反流、胃窦血管扩张症、腹痛、腹泻、便秘、大便失禁等。少数患者可能会出现肾危象，主要表现为急性肾功能不全，并且常合并高血压。心脏受累可表现为心包积液、心脏传导系统异常，个别患者可出现心肌受累。神经系统方面，外周神经系统受累多见，例如三叉神经病变、周围神经病变等，罕见中枢神经受累。

**qwen2.5_7b** 2025-02-18T18:17:06Z:

根据您描述的症状和临床表现，这类疾病很可能是一种罕见的自身免疫性疾病或遗传性结缔组织病，其中一种可能诊断是进行性系统性硬化症（PSS）。其他五种可能的诊断包括：

1. 系统性硬化症：一种以血管炎、皮肤纤维化和内脏器官受累为主要特征的自身免疫性疾病。
2. 韦格纳肉芽肿病（Wegener’s Granulomatosis）：一种罕见的小血管炎，常伴有鼻窦、呼吸道和肾脏症状。目前该疾病已并入肉芽肿性多血管炎。
3. 显微镜下多血管炎（Microscopic Polyangiitis, GPA）：一种以小血管炎症为主要表现的自身免疫性疾病，可累及多个器官系统。
4. 巨细胞动脉炎/颞动脉炎（Giant Cell Arteritis, GCA / Temporal Arteritis）：主要影响50岁以上人群的中、大血管，尤其是头部和颈部血管。该病可能伴有头痛、发热等症状。
5. 肉芽肿病伴多发性神经炎（Churg-Strauss Syndrome）：一种罕见的自身免疫性疾病，表现为哮喘与嗜酸性粒细胞增多、周围神经病变及小血管炎症等。

请注意，最终的确诊需要根据详细的临床检查结果和必要的实验室检测来确定。

**115. user 2025-02-18T18:17:44Z:**

患儿在出生 1~3 个月后除了出现类似苯丙酮尿症的临床症状外，还表现为多巴胺缺乏症状，如运动障碍、肌张力低下、嗜睡、眼震颤、吞咽困难等。5- 羟色胺缺乏相关症状，如反应迟钝、抑郁、失眠等。去甲肾上腺素缺乏相关症状，如躯干肌张力低下、眼睑下垂、小脑发育障碍等。还会有顽固性抽搐、反复发热，运动里程碑发育迟滞， 全身瘫痪，智能发育严重障碍等。

**qwen2.5_7b** 2025-02-18T18:17:48Z:

根据您提供的临床表现，最有可能的罕见病诊断是：5-羟色胺转运蛋白基因相关疾病（例如5-HTTLPR基因突变）或多巴胺转运体缺陷相关疾病（如DARPP-32基因突变）。以下是其他五种可能的诊断：

1. 苯丙酮尿症（PKU），尽管患者表现出与苯丙酮尿症相似的症状，但进一步检查提示可能存在5-HT、多巴胺和去甲肾上腺素代谢障碍。
2. 遗传性神经内分泌病：包括一些罕见的遗传性神经内分泌疾病，如Hirschsprung病或Carney Complex等，可能表现为神经发育迟缓、肌张力低下等症状。
3. 神经节苷脂沉积症（GAN）：例如GM1和GM2型神经节苷脂沉积症可以引起运动障碍、智力发育迟滞等症状，并且常伴有其他神经系统症状如肌张力异常等。
4. 5-羟色胺转运蛋白缺陷病：表现为精神行为异常，如抑郁、焦虑等症状，以及神经发育迟缓等问题。与患者目前表现出来的多巴胺缺乏症状相符合。
5. 先天性肾上腺皮质增生症（CAH）中的某些类型也可能导致去甲肾上腺素水平降低和其他相关症状，但通常不会出现苯丙酮尿症样症状。

**116. user 2025-02-18T18:18:13Z:**

常表现为多系统受累，其发生发展有一定的阶段性。在胎儿 / 新生儿期可以出现心脏横纹肌瘤，大部分可自发消退；出生后数月就可出现癫痫症状和皮肤症状；随着年龄的增大，出现肾血管平滑肌脂肪瘤和全身其他部位的肿瘤性病变；成年女性肺部容易出现肺部淋巴管肌瘤病。脑部病变主要包括脑皮质结节、室管膜下巨细胞星形细胞瘤和室管膜下结节等。皮肤病变包括出生后可见的白斑，逐渐生长的面部血管纤维瘤、鲨革斑、甲周纤维瘤及前额斑块等；高达 90％的患者有肾脏血管平滑肌脂肪瘤；约 50％患者病变累及视网膜，但很少影响视力；约 35％女性患者肺部出现 LAM；其他如肝脏、胰腺、消化道、骨骼也可以受累，但一般症状较轻。

**qwen2.5_7b** 2025-02-18T18:18:16Z:

根据您描述的临床表现，最有可能诊断为特发性肺纤维化-血管平滑肌脂肪瘤病（LAM），这是一种罕见的、主要影响育龄期女性的遗传性疾病。此外，其他五种可能的诊断包括：

1. 结节性硬化症：一种常染色体显性遗传病，可导致皮肤病变、肾肿瘤和神经系统问题。
2. 肾血管平滑肌脂肪瘤伴肺淋巴管肌瘤病（Hippel-Lindau 综合征）：以多发性血管平滑肌脂肪瘤和肺部淋巴管肌瘤为特征。
3. 多囊肾病-肺纤维化（Fibrocalculous 肾病）：一种罕见的遗传性疾病，主要影响肾脏和肺部。
4. 特纳氏综合症：虽然主要影响女性，但有时也会出现类似的症状。它是一种染色体异常导致的疾病，会增加患某些类型癌症的风险。
5. 神经纤维瘤病I型（NF1）：一种遗传性疾病，会影响皮肤和神经系统，并可能导致肿瘤生长在不同部位。

请注意，以上诊断需要结合详细的病史、临床表现以及相应的辅助检查结果来确定。

**117. user 2025-02-18T18:18:41Z:**

未被发现或未治疗的慢性型儿童大多在 10 岁以前死亡，死因通常是肝功能衰竭、神经系统受累或肝细胞癌。

急性型起病急骤、进展迅速，以急性肝功能衰竭为主要表现，临床表现为肝脾肿大、黄疸、呕吐、腹胀、厌食、嗜睡、贫血、出血倾向及生长迟缓。患儿可能具有“煮白菜” 或“烂蘑菇”的特征性气味。肝脏合成凝血因子不足，PT、APTT 明显延长，凝血因子Ⅱ、Ⅶ、Ⅸ、Ⅺ和Ⅻ水平降低，补充维生素 K 后难以纠正。肝功能衰竭可继发腹水、黄疸和消化道出血，患儿偶尔会出现持续低血糖。未治疗的患儿多于起病数周或数月内因肝功能衰竭死亡。

**qwen2.5_7b** 2025-02-18T18:18:43Z:

根据您提供的临床表现，我最有可能诊断为威尔森氏症（Wilson’s disease），这是一种罕见的遗传性铜代谢障碍疾病。

其他五种可能的诊断包括：

1. 肝豆状核变性：与威尔森氏症有相似的症状和原因，也是由于体内铜积累导致的。
2. 急性肝炎：急性型的表现可以模拟威尔森氏症，但是病史、实验室检查和影像学检查可以帮助鉴别。
3. 血液系统疾病（如血友病）：某些血液系统疾病也可能导致出血倾向等临床表现。
4. 感染性疾病（如病毒性肝炎）：部分感染性疾病也会导致急性肝功能衰竭，需要通过病毒学或细菌学检查来排除。
5. 先天代谢缺陷病：一些罕见的先天性代谢缺陷病也可能表现出类似的症状。

**118. user 2025-02-18T18:19:07Z:**

临床表现具有明显异质性，根据起病年龄和临床表现分为 3 个类型。

**1. 心肌病型，** 是最常见的一种严重早发心脏和多脏器衰竭型，主要在新生儿和婴儿早期发病。此型发病凶险，患儿病死率高，表现为低血糖、瑞氏综合征、新生儿猝死、肥厚型和扩张型心肌病、心包积液、心律失常、肌无力、肝肿大和间歇性低血糖，肌酸激酶水平升高；心肌肥厚和心律失常可致死。

**2. 肝型 ，**较轻，为肝脏和低酮性低血糖型，婴儿后期或儿童期发病，表现为肝大和肝功能异常，空腹耐力下降和急性低酮性低血糖，不伴心肌损害和心肌肥厚，但未经及时诊断和治疗也会有生命危险。

**3. 肌病型** ，主要在青少年至成年期发病，为迟发型，症状轻，一般不伴有心肌疾病和低血糖。主要表现为运动、感染或饥饿后的横纹肌溶解和肌红蛋白尿，甚至可发生肾功能衰竭，可伴有肌无力、肌肉痛性痉挛或肌痛。

**qwen2.5_7b** 2025-02-18T18:19:14Z:

根据您提供的临床表现，这种罕见病可能被诊断为瑞-狄克试验性综合征（Reye’s syndrome variant），这是一种极其罕见的遗传性疾病。

除此之外，还有以下五种可能的诊断：

1. **MELAS 综合征**：线粒体脑肌病伴乳酸血症和卒中样发作。该疾病通常在儿童或青少年时期发病，症状包括头痛、癫痫发作、呕吐、虚弱、神经功能障碍等。
2. **心肌磷脂酰肌醇激酶缺乏症 (PIM1 缺乏症)**：这是一种罕见的线粒体疾病，以心肌病和低血糖为特征。它通常在儿童期发病，并可导致严重的心脏问题和低血糖发作。
3. **NARP 综合征**（神经元性肌肉衰退伴视网膜病变）：这种遗传性疾病以运动障碍、感觉异常、眼震颤等神经系统症状为主要表现，部分患者可能伴有心脏受累。它通常在儿童或青少年期发病。
4. **De Vivo 病 (MIM 251750)**：这是一种罕见的线粒体疾病，主要影响儿童和青少年，表现为运动障碍、心肌病、低血糖等临床症状。该病是由线粒体复合物 I 的缺陷所引起的。
5. **Mitochondrial Encephalomyopathy with Lactic Acidosis and Stroke-like Episodes (MELAS)**：这是一种由线粒体基因突变导致的罕见疾病，主要表现为神经和肌肉功能障碍、低血糖、癫痫发作等。临床症状通常在儿童期发病，并会反复出现类似中风的症状。

需要注意的是，在实际诊断过程中，医生通常需要结合详细的家族史、遗传学检测以及生化指标（如乳酸水平、血清肌酸激酶水平）等多种检查结果来做出准确的诊断。

**119. user 2025-02-18T18:19:41Z:**

**1. 特殊面容，** 眶周皮下组织饱满、眶距增宽、星状虹膜、人中长、嘴唇厚、宽嘴以及小下颌。

**2. 心血管疾病，** 75％ ~80％的患者存在弹性蛋白动脉病，可影响任何动脉。周围型肺动脉狭窄(37％ ~61％) 在婴儿期较常见，但随着时间的推移可有改善。瓣上型主动脉狭窄较常见(35％ ~65％)，并且随着时间的推移在 5 岁前恶化。动脉狭窄导致左心阻力增高、心肌肥厚和心力衰竭。其他异常包括肾动脉狭窄、胸主动脉狭窄、腹主动脉狭窄、二尖瓣脱垂和主动脉瓣关闭不全、肠系膜动脉狭窄、颅内血管异常等。高血压可出现在任何年龄，部分可能和肾动脉狭窄有关。

**3. 认知能力，** 75％的患者有智力障碍，通常较为轻微。认知障碍具有特征性，表现为短期记忆和语言表达能力相对较好，但在视觉空间结构性认知方面却极其薄弱。在智商方面没有性别差异的报道，且随着时间的推移，儿童的智商是稳定的。

**4. 性格、行为、心理异常，** 包括过度友好、过度移情、注意力缺陷、焦虑、恐惧。

**5. 睡眠异常 ，**睡眠问题的发生率为 65％，包括睡眠潜伏期增加和睡眠效率降低。

**6. 眼睛，** 泪道阻塞、远视(67％) 和斜视(50％) 常见。成人患者也有白内障的报道。

**7. 耳鼻喉，** 50％的患者有慢性中耳炎，90％对声音的敏感性增加，63％的儿童和92％的成人有轻度至中度听力损害，多为进行性感音神经性听力损失。大多数患者声音嘶哑或声音低沉。

**8. 牙齿** ，可有小牙、牙釉质发育不良和错位咬合。

**9. 消化系统，** 慢性腹痛是常见症状，可能的病因包括胃食管反流、裂孔疝、消化性溃疡、胆石症、憩室炎、缺血性肠病、慢性便秘和焦虑躯体化。高钙血症也可能导致易怒、呕吐、便秘和肌肉痉挛。

**10. 泌尿系统，** 尿频和遗尿症在儿童患者中很常见。

**11. 神经、肌肉** ，幼童的肌张力偏低，关节松弛，导致代偿性姿势异常，以保持身体平衡。年龄较大的儿童和成人有典型的肌张力增高和深肌腱反射活跃，导致步态僵硬而笨拙，精细运动功能受损，所有年龄段患者都有使用工具和书写困难。成人患者会有小脑共济失调和震颤。

**12. 生长发育** 。70％的婴儿体重增长不佳。

**13. 特发性高钙血症** 。多在婴幼儿期出现，见于 15％ ~50％的患者，最常见的症状为易激惹、呕吐和便秘。

**14. 内分泌。**甲状腺功能减退(10％)、青春期提前(50％)。糖耐量受损在青少年患者中的发生率为 26％。

**qwen2.5_7b** 2025-02-18T18:19:45Z:

根据您提供的临床表现，最有可能诊断为 **Moyamoya病（颅内血管异常）伴智力障碍和行为异常** ，这是一种罕见的进行性脑血管疾病。

其他五种可能的诊断包括：

1. **Alagille综合征**：这是一种遗传性疾病，与心血管、肝脏、眼睛、骨骼和肺部问题相关。
2. **Prader-Willi综合症或Angelman综合症**：这两种都是由于基因缺陷导致的罕见神经发育障碍疾病，症状类似于描述中的认知和行为特征。
3. **Marfan综合征**：这是一种影响结缔组织的遗传病，包括心血管、眼睛和其他系统的问题。
4. **Crouzon 综合征**：一种与头骨过早融合相关的颅面畸形疾病，可能会导致类似的面部特征，如眶距增宽等。
5. **Weill-Marchesani综合症**：这是一种罕见的遗传性疾病，影响眼部、骨骼和关节，可能包括视觉问题、关节僵硬等症状。

**120. user 2025-02-18T18:20:27Z:**

**1. 出血倾向** ，为患者常见表现，尤其血丝便多见，大部分患儿在新生儿期即可发病。其次表现为瘀斑、瘀点、咯血和血尿等出血倾向，部分严重者可出现威胁生命的消化道大出血或颅内出血等。患者出血由血小板减少引起，同时伴有血小板体积减小，但仅约 25％的病例同时具有典型三联征表现。

**2. 感染 。**患儿易患各种感染，包括单纯疱疹病毒、巨细胞病毒、肺炎链球菌、真菌等。患者免疫缺陷随年龄增长而加重。

**3. 湿疹。** 约 80％的 WAS 患儿可出现湿疹，范围和严重程度差异很大。可有典型严重、难治性湿疹，也可非常轻微，甚至无湿疹表现。

**4. 自身免疫性疾病。** 最常见为自身免疫性溶血性贫血。其他包括血管炎、关节炎、肾脏疾病、炎症性肠病、中性粒细胞减少症和自身免疫性血小板减少性紫癜等。

**5. 恶性肿瘤 。**患儿发生淋巴系统恶性肿瘤的风险明显增高，尤其是淋巴瘤，非霍奇金淋巴瘤、淋巴细胞白血病、骨髓增生异常综合征、精原细胞瘤等均有报道。

**6. 其他 。**该病患儿可具有完全正常的血小板水平，但中性粒细胞持续或反复减少。

**qwen2.5_7b** 2025-02-18T18:20:32Z:

根据您提供的临床表现，最有可能的诊断是温尼尔-施德瑞格综合症（WAS），这是一种罕见的X染色体连锁免疫缺陷疾病。

其他可能的诊断包括：

1. 先天性无丙种球蛋白血症：此类患者存在抗体缺陷导致免疫力下降，容易出现反复感染的症状；
2. 严重联合免疫缺陷病（SCID）：包括腺苷脱氨酶缺乏症在内的多种遗传性免疫系统功能障碍均有可能表现出类似的临床症状；
3. 白细胞粘附缺陷1型：该疾病会导致白细胞无法正常与血管内皮接触，从而影响其在感染部位的聚集和杀灭病原体的能力；
4. 遗传性毛细血管扩张性共济失调症（HES）或同型半胱氨酸尿症（HC）：这两种遗传性疾病也可能表现出皮肤问题、出血倾向等症状；
5. 先天性中性粒细胞减少伴周期性发热综合症（Kostmann综合症）。

**121. user 2025-02-18T18:21:39Z:**

最常见的临床表现是 EB 病毒感染后的暴发性传染性单核细胞增多症、异常免疫球蛋白血症及 B 细胞淋巴瘤。异常免疫球蛋白血症及 B 细胞来源的淋巴瘤可发生于没有 EB 病毒感染的患者。异常免疫球蛋白血症通常表现为 IgA 及 IgM水平升高，而 IgG1 和 IgG3 减少。淋巴瘤以非霍奇金 B 淋巴细胞瘤为主。其他临床表现包括淋巴细胞性脉管炎、再生障碍性贫血和淋巴瘤样肉芽肿病等。某些患者临床表现轻微， 仅有异常免疫球蛋白血症或轻度 EB 病毒感染，可能是由于发生了体细胞基因修复。

部分患者主要临床表现为噬血细胞综合征，以脾肿大多见，且不一定需要 EBV 感染诱发。部分患者可出现肠道炎症如克罗恩病或结肠炎，但很少发展为淋巴瘤。

**qwen2.5_7b** 2025-02-18T18:21:44Z:

根据您描述的临床表现，最有可能诊断的是Wiskott-Aldrich综合症（WAS），这是一种罕见的X连锁遗传性免疫缺陷疾病。

其他五种可能的诊断如下：

1. 共济失调-毛细血管扩张症：一种以反复感染、肝脾肿大和神经系统异常为主要特征的遗传性疾病。
2. 罕见的自身免疫病，如系统性红斑狼疮或慢性肉芽肿病等，这些疾病也可能表现出类似的症状组合。
3. 体细胞重组缺陷综合症（例如补体因子H相关肾炎），这类罕见病症以免疫功能障碍和异常免疫球蛋白血症为特征。
4. 先天性B细胞缺陷：如Bruton氏综合征，这是一种X连锁的原发性免疫缺陷病，可出现EB病毒感染后的症状及免疫球蛋白水平异常。
5. 重链病或轻链病等罕见的血液系统疾病：这些疾病可能表现为高免疫球蛋白血症和淋巴瘤。
