# Supplementary material for: Performance of ChatGPT-4o and Four Open-Source Large Language Models in Generating Diagnoses Based on China’s Rare Disease Catalog: Comparative Study
Source: J Med Internet Res. 2025 Jun 18;27:e69929. doi: 10.2196/69929 (PMC12192912; doi:10.2196/69929)
Supplement: Multimedia Appendix 1 [file jmir-v27-e69929-s001.zip › numbered_qwen2.5_7b_en.docx]

# numbered_qwen2.5_7b_en

**1. user 2024-11-30T14:17:20Z:**

“1.Skeletal abnormalities: There is variation in the severity of the disease and the type of associated complications. Thoracic stenosis is present in all cases, and the thorax may be bell-shaped or long and narrow, with respiratory distress usually caused by a small, rigid thorax. The severity of respiratory insufficiency varies according to the type of thorax and the degree of underlying pulmonary dysplasia, and the age of onset varies. There is a negative correlation between the severity of respiratory insufficiency and the age at which the disease is diagnosed. Acute respiratory disease is more common in infants with severe restrictive and bell-shaped chests, and severe and fatal respiratory failure can occur in the early neonatal period. Infants with a long, narrow chest are more likely to present with mild respiratory involvement, which can eventually progress to chronic restrictive lung disease with recurrent respiratory infections. Short, deformed ribs cause intercostal muscle weakness and restricted breathing, leading to respiratory failure. Most children die within 2 years of age, with about half dying within 6 months of birth. Other skeletal anomalies include short ribs, short limbs, abnormally shaped pelvis, short fingers, and polydactyly. About 60% of patients have proximal cervical spinal stenosis. 2. Involvement of other organs: Renal lesions are present in about 40% of patients. The earliest sign of renal disease is impaired urinary concentration. Other signs include polyuria, proteinuria, elevated blood urea nitrogen and creatinine, and hypertension. Renal failure is the leading cause of death in this patient between the ages of 3 and 10 years. Hepatic involvement occurs in about 30% of cases and is manifested by hepatomegaly, elevated aminotransferases, cholestasis, cirrhosis and portal hypertension. The disease is occasionally associated with ocular developmental abnormalities, including optic nerve hypoplasia, retinal dystrophy, retinal pigmentation abnormalities, nystagmus, myopia and progressive visual impairment. There are also a few reports suggesting that these patients may also present with neurological abnormalities such as growth retardation, hypotonia, and central nervous system MRI abnormalities.

**qwen2.5_7b** 2024-11-30T14:17:26Z:

Based on the clinical manifestations you’ve provided, the most likely rare disease is **Osteogenesis Imperfecta Type II (OI Type II)**, often referred to as “Periostosis-Bell-Shaped Thorax Syndrome.” This condition has several characteristic features that align with the description:

- **Skeletal Abnormalities**: OI Type II involves multiple skeletal abnormalities including short ribs, thoracic stenosis leading to a bell-shaped or long and narrow chest, and severe respiratory distress.
- **Respiratory Involvement**: The presentation of acute respiratory issues in infants is common, as well as the progression to chronic restrictive lung disease with recurrent infections.
- **Other Organ Involvement**: Renal and hepatic involvement are seen, including renal failure which can be a significant cause of morbidity and mortality.

However, given the complexity and the presence of other organ systems involved, here are five additional possible diagnoses that might also fit some of the clinical features described:

- **Marfan Syndrome**: This condition often involves thoracic abnormalities (like a long, narrow chest) as well as ocular issues like retinal detachment, but it is typically associated with more cardiovascular symptoms.
- **Mucopolysaccharidosis (MPS)**: Depending on the specific subtype, MPS can involve skeletal and respiratory issues along with organ involvement such as kidneys and liver, though the severity and pattern might differ from OI Type II.
- **Nephronophthisis**: This is a renal disease that often presents in children and can be associated with other developmental abnormalities, but it usually does not include severe thoracic or skeletal deformities.
- **Ehlers-Danlos Syndrome (EDS)**: Some subtypes of EDS can present with musculoskeletal issues including short ribs and joint hypermobility, but respiratory involvement is less common in the classic types.
- **Hypophosphatasia**: This metabolic disorder affects bone mineralization and can cause various skeletal abnormalities, but typically does not include renal or hepatic complications as described.

Each of these conditions should be evaluated based on a comprehensive clinical assessment, imaging studies, biochemical tests, and genetic testing where appropriate to confirm the diagnosis.

**2. user 2024-11-30T14:18:17Z:**

The disease can start from birth to old age and can be acute or insidious. Almost any system or organ can be involved, with a predominance of energy-demanding organs such as the brain, heart, kidneys, eyes, ears, muscles, and gastrointestinal tract. Patients may present with a clinical syndrome of some kind, a single symptom or a combination of symptoms.

**qwen2.5_7b** 2024-11-30T14:18:21Z:

Based on the broad clinical manifestations you’ve described—starting from birth to old age, involving almost any system or organ, with a focus on energy-demanding organs such as the brain, heart, kidneys, eyes, ears, muscles, and gastrointestinal tract—the disease most likely to be considered is **Systemic Lupus Erythematosus (SLE)**. SLE can affect multiple organ systems and its clinical presentation can vary widely.

However, given the wide range of potential diagnoses, here are five other possible conditions that could also fit these broad criteria:

- **Multisystem Inflammatory Syndrome in Children (MIS-C)** - This condition primarily affects children who have or recently had COVID-19 and can involve multiple organ systems including the heart, kidneys, brain, eyes, skin, lips, and tongue.
- **Chronic Autoimmune Polyglandular Syndrome (APSA Types 1 & 2)** - These syndromes involve autoimmune destruction of endocrine glands such as the thyroid, adrenal, and parathyroid glands, and can also affect other organs like the kidneys or lungs.
- **Cogan’s Syndrome** - This condition primarily affects the eyes and ears but can also include involvement of the cardiovascular system, skin, and mucous membranes.
- **Sjögren’s Syndrome** - While often associated with dryness in the eyes and mouth, it can affect multiple organs including the lungs, kidneys, and occasionally the nervous system.
- **Autoimmune Hepatitis (AIH)** - This is an autoimmune disease that causes inflammation of the liver. It can present with a variety of symptoms affecting other organ systems as well.

Each of these conditions would require specific diagnostic criteria to confirm, such as blood tests, imaging studies, and sometimes biopsies, depending on the clinical presentation.

**3. user 2024-11-30T14:19:03Z:**

The prevalence of the disease is between 5 and 50 years old, with a median age of onset of 39 years. The prevalence of women is significantly higher than that of men, with a female:male ratio of (9~11):1. Most of the cases have an acute/subacute onset, and the clinical manifestations include six core syndromes: (1) optic neuritis; (2) acute myelitis; (3) posterior pole syndrome; (4) acute brainstem syndrome; (5) symptomatic sleep attacks or acute mesencephalic clinical syndrome with typical MRI foci of the mesencephalon; and (6) symptomatic cerebral syndrome with typical brain lesions. It is a highly recurrent and disabling disease, with more than 90 per cent of patients having a recurrent course, and the majority of patients are left with more severe neurological disability.

**qwen2.5_7b** 2024-11-30T14:19:08Z:

Based on the clinical manifestations provided, this description aligns closely with Multiple Sclerosis (MS), which is one of the most common rare diseases that present in young adults. Here’s why:

- **Age Range**: The typical age range for MS onset is 20 to 50 years old.
- **Sex Ratio**: Females are significantly more affected than males, with a female-to-male ratio often around (9:1) or higher.
- **Symptom Patterns**:
  - **Optic Neuritis**: A common early symptom of MS where inflammation causes pain and blurred vision in one or both eyes.
  - **Acute Myelitis**: Inflammation in the spinal cord leading to sudden weakness, numbness, and other neurological symptoms.
  - **Posterior Pole Syndrome**: This can include macular edema, retinal lesions, and visual field deficits, often seen with optic neuritis.
  - **Brainstem Syndromes**: Symptoms such as dizziness, vertigo, difficulty swallowing, or facial weakness due to brainstem involvement.
  - **Cerebral Lesions**: MRI showing characteristic demyelinating plaques in the brain.

Given these details, here are five other possible diagnoses that could be considered but are less likely based on the provided information:

- **Neuromyelitis Optica Spectrum Disorder (NMOSD)**: Similar to MS, NMOSD can present with optic neuritis and myelitis, but it has a different antibody profile, often involving AQP4 antibodies.
- **Primary Progressive Multiple Sclerosis**: This form of MS is less common and tends to have a more gradual progression without the relapsing-remitting pattern.
- **Relapsing-Remitting Multiple Sclerosis (RRMS)**: The most common type of MS, characterized by distinct relapses followed by partial recovery periods.
- **Acute Disseminated Encephalomyelitis (ADEM)**: An inflammatory condition affecting the brain and spinal cord that often follows an infection or vaccination, with a more acute onset than typical MS.
- **Transverse Myelitis**: This can occur independently or as part of a wider neurological syndrome like MS or NMOSD, but typically presents more acutely without the optic neuritis component.

Each of these diagnoses has unique features that could be considered in differential diagnosis, but based on the provided clinical picture, Multiple Sclerosis is highly likely to be the most probable condition.

**4. user 2024-11-30T14:19:32Z:**

Hearing impairment. In the past, it was thought that the deafness was congenital, which could be identified by hearing screening, and that it would be apparent from birth, mostly involving both sides, and symmetrically severe prelingual deafness. In recent years, it has been found that individual cases may present with asymmetrical or unilateral hearing loss, and the degree of hearing loss is highly variable, ranging from mild to very severe in different patients; progressive deafness that occurs after birth is also not uncommon.

**qwen2.5_7b** 2024-11-30T14:19:37Z:

Based on the clinical manifestations you’ve provided for congenital bilateral symmetric prelingual severe sensorineural hearing impairment with recent findings of asymmetrical or unilateral cases and progressive loss, the most likely rare disease could be:

- **Jervell and Lange-Nielsen Syndrome (JLNS)**: This is a rare autosomal recessive disorder characterized by congenital sensorineural deafness associated with episodic attacks of syncope due to prolonged QT interval in the heart. However, it does not typically present with progressive hearing loss.
- **Usher Syndrome Type I**: This condition involves early-onset severe to profound sensorineural hearing loss and is often accompanied by retinitis pigmentosa leading to vision impairment. There can be variable degrees of deafness and the progression can vary from patient to patient.
- **Gilles de la Tourette Syndrome (GT):** While primarily characterized by motor and vocal tics, some patients with GT may also experience a form of sensory disturbance that includes hearing issues, though this is not common or typical.
- **Alport Syndrome**: This is an inherited disorder affecting the kidneys, ears, and eyes. It can present with progressive sensorineural hearing loss in addition to other symptoms such as hematuria (blood in urine) and eye abnormalities.
- **VACTERL Association**: This acronym stands for a collection of congenital anomalies that may include vertebral malformations, anal atresia, cardiac defects, tracheoesophageal fistula with esophageal atresia, renal anomalies, and limb defects. Hearing impairment is one of the less common features but can occur.
- **Waardenburg Syndrome**: This syndrome includes hearing loss (sensorineural or mixed), white forelock, heterochromia iridis, and occasionally cleft palate. It comes in different types, with Type I being the most common and involving all these symptoms.

Given your description, Usher Syndrome Type I seems to be the closest match due to its presentation of severe prelingual hearing loss along with retinitis pigmentosa, though it’s important to note that progressive hearing loss can occur. Each of these conditions requires specific genetic testing for a definitive diagnosis.

**5. user 2024-11-30T14:20:38Z:**

“**1. polyneuropathy**, often presenting as a symmetrical sensory and/or motor peripheral neuropathy of the extremities, progressing gradually from distal to proximal.

**2. organomegaly,** including hepatomegaly, splenomegaly, or lymph node enlargement; lymph node biopsy is often suggestive of Castleman disease.

**Endocrine abnormalities,** including hypogonadism (e.g., male impotence, breast development), hypothyroidism, abnormal glucose metabolism (e.g., diabetes mellitus), and adrenal insufficiency.

**4. skin changes,** which may be manifested by deepening of skin colour, cutaneous glomeruloid haemangiomas, white nails, polycythemia, hirsutism, and cyanosis of the hands and feet.

**5. increased extracirculatory water load,** including limb oedema, ascites, pleural effusion, pericardial effusion, and optic papilla oedema.

**Sclerosing bone disease,** is an important clinical manifestation. It may be manifested as bone pain or without clinical symptoms. Sclerosing bone disease can be detected significantly more frequently with CT scanning of the bones, and can be visualised as simple sclerosis of the bones, mixed sclerotic and osteolytic lesions, or simple osteolytic changes.

**7. erythrocytosis and/or thrombocytosis** **8.

**8. pulmonary hypertension** , patients may present with pulmonary hypertension, as evidenced by reduced activity tolerance, hypoxaemia, etc.

**9. cerebral infarction**.

”

**qwen2.5_7b** 2024-11-30T14:20:44Z:

Based on the clinical manifestations provided, one rare disease that is highly likely to be diagnosed is **Castleman Disease**, given the presence of organomegaly (specifically hepatomegaly and splenomegaly), lymphadenopathy with a suggestive biopsy result for Castleman disease, and possibly other associated systemic symptoms like polyneuropathy, endocrine abnormalities, skin changes, and increased extracirculatory water load.

However, due to the diverse clinical manifestations, several other rare diseases could also fit these symptoms. Here are five other possible diagnoses:

- **Primary Sjögren’s Syndrome** - This autoimmune disease can present with a polyneuropathy (though usually less common), organomegaly (specifically lymphadenopathy and hepatosplenomegaly), endocrine abnormalities, and various skin manifestations.
- **Multiple Endocrine Neoplasia Type 1 (MEN1)** - This condition often involves multiple endocrine glands and can present with polyneuropathy, organomegaly (especially splenomegaly), endocrine abnormalities (hypogonadism, hypothyroidism, abnormal glucose metabolism), and skin changes.
- **Lyme Disease** - Chronic or disseminated Lyme disease can manifest as a wide range of symptoms including polyneuropathy, hepatosplenomegaly, skin changes, and potentially other systemic manifestations depending on the stage of infection.
- **Maffucci Syndrome** - This rare congenital disorder is characterized by multiple hemangiomas and osteochondromas (bone tumors). It can present with polyneuropathy, organomegaly, and skin changes but generally does not include the other symptoms like endocrine abnormalities or pulmonary hypertension.
- **Hereditary Hemochromatosis** - This condition leads to iron overload in the body and can cause various manifestations including hepatosplenomegaly, bone pain (sclerosing bone disease), polyneuropathy, endocrine abnormalities due to secondary hypogonadism, and skin changes.

Each of these conditions requires careful clinical evaluation, specific diagnostic tests, and often a thorough history to determine the correct diagnosis.

**6. user 2024-11-30T17:05:52Z:**

The disease can begin in early childhood. Typical clinical manifestations include kidney and eye changes and hearing impairment. Renal manifestations are hematuria, proteinuria and progressive deterioration of renal function, in which hematuria is often persistent microscopic hematuria, which can occur after exercise or fever, and is common in adolescents. Hearing changes were sensorineural hearing loss, which gradually aggravated with the course of disease. Eye abnormalities manifest as myopia, youth ring and cataract, but lack specificity. Three ocular changes of diagnostic significance include anterior conical lens, posterior pleomorphic corneal atrophy, and retinal macula (white or yellow particles around the fovea). The incidence of hearing impairment in male patients in China was higher than that reported abroad (68% vs 55%), while that in female patients was lower than that reported abroad (7% vs 45%). The incidence of ocular abnormalities was similar to that reported abroad. Leiomyomas may also occur in a few patients, involving the respiratory tract, gastrointestinal tract, and female reproductive tract; occasionally aneurysmal lesions, midfacial dysplasia, and mental retardation have been reported.

Patients often have a family history. Male patients with autosomal recessive inheritance and X-linked inheritance progressed faster, often entering end-stage renal disease at the age of 16~35, while female patients with autosomal dominant inheritance and X-linked inheritance had a slower course of disease and later onset of renal failure. The age at which renal failure occurs is similar for all male members of the same affected family, but can vary among male members of different families. Therefore, renal failure can be divided into juvenile type (renal failure occurs in adolescence) and adult type (renal failure occurs in adulthood) according to the age of the affected family. Adolescent type is more prominent in extrarenal manifestations, boys usually die before reproduction, disease genes are often new mutations, while adult type families have older patients and fewer new mutations.

**qwen2.5_7b** 2024-11-30T17:06:08Z:

Based on the clinical manifestations you’ve described—particularly kidney and eye changes along with hearing impairment—the rare disease most likely to be diagnosed is **Fabry Disease**.

### Other Possible Diagnoses:

- **Alport Syndrome**: This genetic disorder affects the kidneys, ears, and eyes. It can also present with similar renal manifestations like hematuria, proteinuria, and progressive kidney function decline.
- **Nephrotic Syndrome due to Primary Glomerular Disease**: Certain forms of glomerular disease such as focal segmental glomerulosclerosis (FSGS) or minimal change nephropathy might show similar clinical profiles in terms of proteinuria and hematuria but without the specific ocular manifestations.
- **Diabetic Nephropathy**: While typically associated with diabetes, diabetic nephropathy can present similarly to Fabry Disease in terms of progressive kidney dysfunction, especially if not well managed.
- **Gordon Syndrome (OMIM 237500)**: This condition also presents with ocular changes like cataracts and retinal changes along with hearing loss and renal impairment, though the age of onset and severity can vary more widely than in Fabry Disease.
- **Chronic Kidney Diseases of Genetic Origin**: Other genetic causes of chronic kidney disease could mimic some aspects of Fabry Disease but would not be expected to have the specific ocular and hearing abnormalities described.

Each of these conditions shares clinical features with Fabry Disease, particularly affecting multiple organ systems, which makes Fabry Disease the most likely diagnosis based on your description.

**7. user 2024-11-30T17:09:44Z:**

Patients often have no abnormal performance in the neonatal period. The first symptom is developmental retardation at about 6 months of age, but the typical clinical manifestations appear after 1 year of age, and it often takes several years for clinical diagnosis to be confirmed. According to the frequency of clinical manifestations, they were divided into three categories: common manifestations (100% patients), frequent manifestations (more than 80% patients) and correlated manifestations (20% ~80% patients).

- All of them showed normal pregnancy history and head circumference at birth; no birth defects and abnormal biochemical indexes; brain MRI/CT showed no structural abnormalities except mild cortical atrophy and myelin dysplasia; motor milestones lagged behind without regression; severe developmental retardation occurred at 6~12 months; language disorders showed no or very little vocabulary, repetitive language and non-verbal communication ability were stronger than expressive language ability; motor or balance disorders often showed ataxia and tremor of limbs; Abnormal behavioral characteristics are frequent laughter or smiling, obvious excited movements or happy behavior, often accompanied by clapping or hyperactivity.
- The head circumference often showed backward growth, and microcephaly was still present at the age of 2. Epilepsy often occurred before the age of 3. Abnormal EEG: characteristic high amplitude spike-slow wave.
- Correlation: flat occipital region; tongue preference; sucking or swallowing disorders; infantile feeding difficulties; low muscle tone; large jaw; wide tooth space; frequent salivation; excessive chewing movements; strabismus; hypopigmentation of the skin; light hair and eye color compared to family (absent type only); hyperreflexia of lower limb tendons; lifting or bending of upper limbs, especially when walking; wide gait base; high sensitivity to heat; abnormal sleep-wake cycle, less sleep; obsession with water, paper, and plastic; Eating related behavioral abnormalities; Obesity (more common in older, non-deficit patients); scoliosis; constipation.

**qwen2.5_7b** 2024-11-30T17:09:50Z:

Based on the clinical manifestations provided, the most likely rare disease is **Rett Syndrome**. Here are five other possible diagnoses that could present similar symptoms:

- **Rett Syndrome**: This condition typically affects females and involves developmental delays, motor skills regression, intellectual disability, and various behavioral issues.
- **Childhood Disintegrative Disorder (CDD)**: Also known as Heller’s syndrome, this condition involves normal development followed by significant loss of social, communication, and other cognitive functions after the age of 2 years.
- **Sotos Syndrome**: This is a genetic disorder characterized by overgrowth in infancy, developmental delays, and often some behavioral issues, but typically does not involve the specific neurodevelopmental regression seen in Rett Syndrome.
- **Angelman Syndrome**: While it involves severe intellectual disability, seizures, and sometimes ataxia, Angelman syndrome has distinct features such as happy demeanor, frequent laughter, and a distinctive movement disorder different from Rett Syndrome.
- **Tourette Syndrome**: This neurological disorder is characterized by multiple motor tics and one or more vocal tics, often with the onset in childhood but typically does not include the specific pattern of developmental regression described here.

Each of these conditions has unique characteristics that differentiate them from each other, making a thorough differential diagnosis essential for accurate treatment planning.

**8. user 2024-11-30T17:11:19Z:**

The main symptoms include mental behavioural abnormalities, cognitive impairment, memory loss, seizures, speech disorders, movement disorders, involuntary movements, decreased level of consciousness, and autonomic dysfunction. Other accompanying symptoms include sleep disorders, focal damage to the CNS, brainstem and cerebellar symptoms, and involvement of peripheral nerves and neuromuscular junctions.

There are three main clinical types: (1) anti-NMDAR encephalitis, which is the most predominant type and is characterised by diffuse encephalitis. (ii) Borderline encephalitis, with mental behavioural abnormalities, temporal lobe epilepsy and near-memory deficits as the main symptoms. The common types of borderline encephalitis are anti-LGI1 antibody, anti-GABABR antibody and anti-AMPAR antibody-related encephalitis. (iii) Other types, including Morvant syndrome, anti-DPPX antibody-associated encephalitis, anti-IgLON5 antibody-associated encephalopathy, and autoimmune cerebellar ataxia.

**qwen2.5_7b** 2024-11-30T17:11:27Z:

Based on the clinical manifestations provided, the most likely rare disease to be diagnosed is **Encephalitis Lethargica**, which can present with a wide array of neurological symptoms including mental behavioral abnormalities, cognitive impairment, memory loss, seizures, speech disorders, movement disorders, involuntary movements, and decreased level of consciousness. However, given the specific antibodies mentioned in your clinical types, it suggests an autoimmune encephalitis, particularly related to anti-NMDAR (N-Methyl-D-Aspartate Receptor) encephalitis.

Here are five other possible diagnoses that could also fit the described symptoms:

- **Anti-NMDAR Encephalitis**: This is a well-known autoimmune condition where antibodies target the NMDA receptor, leading to severe neurological and psychiatric symptoms as you’ve detailed.
- **Autoimmune Cerebellar Ataxias (e.g., Anti-IgLON5 Antibody-Associated Encephalopathy)**: These conditions can present with cerebellar ataxia combined with other neurological symptoms.
- **Anti-LGI1 Encephalitis**: LGI1 antibodies are associated with limbic encephalitis and often present with cognitive decline, seizures, and memory problems.
- **Anti-GABABR Encephalitis**: This condition involves the presence of GABAB receptor antibodies, leading to a wide range of neurological symptoms including those you have mentioned.
- **Morvan Syndrome (Painful Arcuate Nucleus Disease)**: While not as common, this rare disorder can present with sleep disorders, autonomic dysfunction, and peripheral nerve involvement.

Each of these conditions can overlap in their clinical presentations, making a precise diagnosis challenging without further specific tests such as antibody profiling or imaging studies.

**9. user 2024-11-30T17:13:15Z:**

The clinical manifestations of the disease are complex and varied, and lack specificity. Most of them involve multiple systems such as nerve, skin, respiratory, digestive and immune systems, and it is difficult to confirm the diagnosis from the clinical manifestations alone. It can be divided into two types: complete and partial.

- Complete: It can be divided into two types: early-onset and late-onset. The most common manifestations are neurological symptoms, such as hypotonia, epilepsy, developmental delay, sensorineural deafness, ataxia, and progressive disorders of consciousness, etc. About half of the patients have ataxia, developmental delay, conjunctivitis, and visual problems, including visual problems. conjunctivitis, and visual problems, including optic atrophy. Metabolic disorders such as metabolic acidosis, hypoglycaemia and ketosis are often comorbid, and cutaneous manifestations include various forms of refractory rashes such as eczema, generalised erythema, and nappy rash, and patients may also present with respiratory symptoms such as hyperventilation, laryngeal wheezing, and apnoea, as well as digestive symptoms such as feeding difficulties, vomiting, and diarrhoea. Acute attacks can be triggered by infection, fever, fatigue, and a high protein diet. If treatment is not timely, it can often lead to irreversible damage. Patients with late-onset disease often develop in adolescence and may have no symptoms beforehand. The clinical phenotype is different from that of early-onset disease, which usually presents with peripheral neuropathy, muscle weakness, spastic paraparesis, skin rashes, and ocular problems, such as loss of vision and visual acuity, etc. Failure to treat the disease in a timely manner often leads to irreversible damage.
- Partial : May develop at all ages or even without onset. The overall signs and symptoms of the child are relatively mild, starting only in emergency situations such as infections and starvation. Patients may have hypotonia, skin rashes, hair loss, acute neurological symptoms such as epilepsy and impaired consciousness.

**qwen2.5_7b** 2024-11-30T17:13:25Z:

Based on the clinical manifestations described, this patient’s case strongly suggests a diagnosis related to Zellweger spectrum disorders (ZSD), which include Zellweger syndrome, Neonatal Adrenoleukodystrophy (NALD), and Infantile Refeeding Syndrome (IRF). These are all part of a spectrum of peroxisome biogenesis disorders characterized by severe neurological impairment, multisystem involvement, and metabolic abnormalities.

### Primary Diagnosis:

- **Zellweger Spectrum Disorders**

### Five Possible Diagnoses:

1. **Hypohidrotic Ectodermal Dysplasia (HED)**
   - Although the symptoms of HED are more specific to dental, hair, and sweat gland issues, there can be some overlapping features in skin rashes and neurological impairment.
2. **Metachromatic Leukodystrophy (MLD)**
   - MLD involves similar neurological symptoms like developmental delay and ataxia, but the primary diagnostic marker is the deficiency of arylsulfatase A leading to accumulation of sulfatides in the central nervous system.
3. **Hypomagnesemia with Secondary Hypocalcemia (HSH)**
   - This condition can present with neurological symptoms such as seizures and ataxia, but typically involves electrolyte disturbances which are not a primary feature in Zellweger spectrum disorders.
4. **Cerebrotendineous Xanthomatosis (CTX)**
   - CTX presents with similar skin rashes and some neurological issues, but it is more specific to the accumulation of cholestanol and xanthomas affecting tendons, bones, and brain.
5. **Fragile X Syndrome**
   - While primarily a genetic condition affecting intellectual disability and autism spectrum disorders, Fragile X can also present with sensory processing abnormalities and some neurological symptoms, which might overlap in diagnosis without thorough testing.

Given the complexity and severity of the clinical manifestations, a comprehensive genetic evaluation including targeted next-generation sequencing (NGS) panels for peroxisome biogenesis disorders is crucial to confirm the specific subtype within Zellweger spectrum disorders. Additionally, metabolic workup and imaging studies such as MRI brain can aid in diagnosis and management.

**10. user 2024-11-30T17:14:29Z:**

The high prevalence age group is infancy, which manifests as adrenocortical insufficiency with a predominant salt loss, and common symptoms include hyperkalemia, hyponatremia with dehydration, metabolic acidosis, hypoglycaemia, vomiting, shock, and skin pigmentation. Most patients present with failure or incomplete development during puberty, or impaired fertility, oligospermia and azoospermia. A few patients may present with early childhood hyperandrogenaemia, simple saline corticosteroid deficiency, and pseudo-precocious puberty or spontaneous pubertal development during puberty. In mild cases, the disease may start in adulthood.

Patients have hyperpigmentation of the skin, especially in exposed areas, friction areas, scarring and areola, axilla, scrotum.

Patients have varying degrees of reduced resistance to various stresses such as infection and trauma. In the event of a stressful situation, adrenal crisis can occur, manifested by nausea, vomiting, abdominal pain or diarrhoea, severe dehydration, lowered blood pressure, rapid heart rate, weak pulse, mental disorders, and often high fever, hypoglycaemia, hyponatraemia and even life-threatening.

**qwen2.5_7b** 2024-11-30T17:14:37Z:

Based on the clinical manifestations you have described, the most likely diagnosis is **Congenital Adrenal Hyperplasia (CAH), specifically 21-hydroxylase deficiency**. This condition is one of the most common inherited endocrine disorders affecting the adrenal glands and occurs due to a deficiency in the enzyme 21-hydroxylase.

Here are five other possible diagnoses that could be considered based on similar clinical features:

- **Congenital Adrenal Hyperplasia (CAH) due to 11β-Hydroxylase Deficiency**: This is another form of CAH, but it is less common than the 21-hydroxylase deficiency.
- **Primary Aldosteronism**: Although not as common in infancy, this condition can present with salt loss and hypertension, which might overlap some symptoms seen in CAH.
- **Congenital Adrenal Hyperplasia (CAH) due to 17α-Hydroxylase Deficiency**: This is a rare form of CAH but may share similar clinical features with the other forms mentioned.
- **Primary Hypothyroidism**: While less likely given the adrenal gland focus, hypothyroidism can present with growth and puberty issues and may need to be ruled out in the differential diagnosis.
- **Adrenal Tuberculosis or Adrenal Cancer**: These are very rare but should be considered if there is any suspicion of a mass or tumor, which could explain some of the symptoms observed, especially if they appear later in life.

Given the specific combination of clinical manifestations such as adrenocortical insufficiency, salt loss, hyperpigmentation, and issues with puberty, 21-hydroxylase deficiency (CAH) is the most probable diagnosis. However, thorough diagnostic workup including biochemical tests for adrenal hormones, genetic testing, and imaging studies should be performed to confirm the specific subtype of CAH or rule out other conditions.

**11. user 2024-11-30T17:15:07Z:**

The disease can develop from birth to early adulthood. Neonatal onset is the most common, often presenting as macrosomia, poor feeding, hunger intolerance and persistent hypoglycaemia. Hypoglycaemic episodes may present with lethargy and hypotonia, as well as neurological sequelae or even death, such as apnoea, seizures and coma. In the delayed type, patients often show a hypoglycaemic reaction, including symptoms such as pallor, sweating and palpitations.

**qwen2.5_7b** 2024-11-30T17:15:18Z:

Based on the clinical manifestations provided, the most likely diagnosis is **congenital diabetes insipidus (nephrogenic diabetes insipidus)**, but considering the symptoms of persistent hypoglycemia, the primary condition that fits this description more accurately is **congenital hyperinsulinism**.

### Primary Diagnosis: Congenital Hyperinsulinism

- **Characteristics**: This disorder involves excessive production and release of insulin by the beta cells in the pancreas. Symptoms often include recurrent hypoglycemia, especially in neonates.
- **Clinical Manifestations**:
  - Macrosomia due to high insulin levels leading to increased nutrient storage as fat.
  - Poor feeding and hunger intolerance.
  - Persistent hypoglycaemia with symptoms such as lethargy, hypotonia, apnoea, seizures, and coma in severe cases.
- **Management**: Treatment options include dietary management, drugs like octreotide or diazoxide, and sometimes surgical intervention.

### Five Other Possible Diagnoses

- **Reye’s Syndrome**
  - **Characteristics**: A rare disorder usually occurring in children recovering from a viral infection (like chickenpox or flu), presenting with vomiting, altered mental status, and changes in consciousness.
- **Mitochondrial Disorders** (e.g., MELAS)
  - **Characteristics**: These can present with a variety of symptoms including hypoglycemia, seizures, developmental delays, and other neurological deficits.
- **Prader-Willi Syndrome**
  - **Characteristics**: A genetic disorder that affects multiple body systems. It often presents in infancy with poor muscle tone, feeding difficulties, and later with excessive eating leading to obesity.
- **Mucopolysaccharidosis (MPS) Syndromes**
  - **Characteristics**: A group of rare lysosomal storage diseases affecting various organs and tissues, causing progressive physical symptoms including joint stiffness, coarse facial features, and growth abnormalities.
- **Panhypopituitarism**
  - **Characteristics**: A condition where the pituitary gland does not produce enough of one or more hormones, often leading to hypoglycemia due to insufficient production of antidiuretic hormone (ADH) among others.

These conditions are listed based on their potential for presenting with symptoms such as hypoglycemia and other overlapping features, but the primary diagnosis from the given clinical manifestations is congenital hyperinsulinism.

**12. user 2024-11-30T18:15:20Z:**

1. Type I, abdominal distension, growth retardation, hypoglycemic convulsions, recurrent nosebleeds, diarrhoea and vomiting are the main reasons for children’s patients to visit the clinic, and very rarely, the first manifestation is hematuria, blood in the stool, recurrent bone fracture, anaemia or gout. Adult patients who are never diagnosed and treated may present with multiple hepatic adenomas, chronic renal failure, severe gout with multiple gout stones, and osteoporosis. Other rare presentations include pulmonary hypertension, diabetes mellitus, cerebrovascular disease, and hepatic adenoma carcinoma. On physical examination, short stature and markedly enlarged liver can be seen.

1. Type Ib, in addition to the above manifestations, patients may also have recurrent infections with neutropenia, oral ulcers, inflammatory bowel disease, perianal ulcers, arthritis and splenomegaly.
2. Infantile type II, which is divided into classical infantile type and non-classical infantile type according to its prognosis. Most of the classical infants present with generalised muscle weakness in the first month of life, delayed motor development, enlarged heart on chest X-ray, high QRS waves and short PR intervals on ECG, hypertrophic cardiomyopathy on cardiac ultrasound, and varying degrees of elevation of creatinine kinase, etc. Most of the infants die of left heart failure or cardiorespiratory failure after pulmonary infections in the first year of life. The non-classical infantile form presents with muscle weakness within the first year of life, lags in motor development, and most often dies of respiratory failure in early childhood.
3. Type II late-onset, with onset of disease after 1 year of age and as late as 60 years of age. Most of the patients present with chronic progressive proximal muscle weakness and respiratory insufficiency, cardiac involvement is rare, and respiratory failure is the main cause of death. Clinical manifestations include easy fatigue, difficulty in sitting up, walking up and down stairs, squatting and walking, and in a few cases, sudden onset of respiratory failure.

**qwen2.5_7b** 2024-11-30T18:15:31Z:

Based on the clinical presentations you have described, it seems most likely that these patients are suffering from **Fabry Disease** (or more specifically, variants of Fabry Disease).

### Diagnoses Based on Clinical Manifestations:

- **Fabry Disease (Type I, Type Ib)**
- **Mucopolysaccharidosis VI (Maroteaux-Lamy Syndrome)**
- **Gaucher Disease**
- **Zellweger Spectrum Disorders (Peroxisomal Biogenesis Disorders)**
- **Fragile X Syndrome**

### Explanation:

- **Fabry Disease** is a rare genetic disorder caused by mutations in the *GLA* gene, leading to the accumulation of glycosphingolipids in various tissues, including the kidneys, heart, and nervous system. The clinical manifestations you described, such as abdominal distension, growth retardation, hypoglycemic convulsions, recurrent nosebleeds, diarrhea, and vomiting; and later complications like hepatic adenomas, chronic renal failure, severe gout, osteoporosis, pulmonary hypertension, diabetes mellitus, cerebrovascular disease, and hepatic adenoma carcinoma are all consistent with this condition.
- **Mucopolysaccharidosis VI (Maroteaux-Lamy Syndrome)**: This is a lysosomal storage disorder that can present with symptoms like skeletal abnormalities, progressive joint stiffness, coarse facial features, and in some cases, hematological manifestations such as neutropenia. However, the wide range of presentations you listed for Fabry Disease makes this less likely.
- **Gaucher Disease**: This is also a lysosomal storage disorder caused by mutations in the *GBA* gene. It can present with symptoms like hepatosplenomegaly, bone pain, and anemia, but the specific combination of clinical manifestations does not fit as closely as Fabry Disease.
- **Zellweger Spectrum Disorders**: These are peroxisomal biogenesis disorders that involve multiple organ systems including the liver, brain, and kidneys. However, they do not typically present with the gastrointestinal symptoms described for Fabry Disease.
- **Fragile X Syndrome**: This is a genetic disorder affecting the X chromosome leading to intellectual disability and various physical characteristics. While it can affect multiple organs, its primary manifestations do not align well with the clinical picture you provided.

Given the detailed and specific nature of your descriptions, particularly in Type I Fabry Disease, this condition seems to be the most likely diagnosis among those listed.

**13. user 2024-11-30T18:16:45Z:**

1. Neuro-muscular system, causing neurological abnormalities, such as convulsions, coma, ataxia, and even psychiatric symptoms; muscle symptoms can also occur, including hyperreflexia, tremor, tetany, and muscle weakness. 2. Cardiovascular system, causing ECG changes, such as PR interval prolongation, QRS wave widening, etc. In severe cases, it can cause ventricular arrhythmia.

1. cardiovascular system, causing changes in electrocardiogram, such as prolongation of PR interval, widening of QRS wave, and so on, and in severe cases, it may cause ventricular arrhythmias.
2. Kidney, which may cause kidney stones, renal calcium deposits, renal insufficiency.
3. bone and joint system, can cause osteoporosis and osteochondrosis.
4. digestive system, may cause intestinal obstruction.
5. endocrine, patients can see diabetes mellitus or impaired glucose tolerance.
6. ocular symptoms, some types may be combined with ocular lesions, including nystagmus, visual field defects, myopia, etc. This group of diseases usually has a chronic course.

This group of diseases usually has a chronic course, with the neonatal and infantile onset types often being more severe and having a poorer prognosis. Some patients develop chronic renal failure during adolescence, and in some cases, failure to treat the disease in time can lead to severe mental retardation or even death.

**qwen2.5_7b** 2024-11-30T18:16:58Z:

Based on the clinical manifestations provided, one rare disease that fits these symptoms is **Kearns-Sayre Syndrome (KSS)**, which is a type of mitochondrial disorder. Here are the key points and five other possible diagnoses:

### 1. Kearns-Sayre Syndrome (KSS)

- **Neurological Manifestations:** Can cause neurological abnormalities such as ataxia, myopathy, seizures, and ophthalmoplegia.
- **Cardiovascular System:** May show ECG changes like PR interval prolongation and QRS wave widening.
- **Ocular Symptoms:** Often includes ocular movement disorders and visual disturbances.

### 5 Other Possible Diagnoses:

- **Mitochondrial Myopathy, Encephalopathy, Lactic Acidosis, and Stroke-like Episodes (MELAS)**
  - **Neurological Manifestations:** Seizures, stroke-like episodes, headaches.
  - **Cardiovascular System:** May cause cardiac conduction defects.
  - **Ocular Symptoms:** Vision loss.
- **Leigh Syndrome (Subacute Myelencephalopathy)**:
  - **Neurological Manifestations:** Progressive neurological decline, ataxia, seizures, and developmental delay.
  - **Cardiovascular System:** May show ECG abnormalities.
  - **Ocular Symptoms:** None typically, but can be part of the broader mitochondrial disease spectrum.
- **MERRF (Myoclonic Epilepsy with Ragged Red Fibers) Syndrome**:
  - **Neurological Manifestations:** Myoclonus, epilepsy, ataxia.
  - **Cardiovascular System:** Mild cardiac involvement.
  - **Ocular Symptoms:** Nystagmus.
- **Mitochondrial DNA Deletion Syndromes (Dejerine-Sottas Neuropathy, Kearns-Sayre Syndrome)**:
  - **Neurological Manifestations:** Severe sensory and motor neuropathy.
  - **Cardiovascular System:** ECG abnormalities.
  - **Ocular Symptoms:** Visual field defects.
- **Chronic Progressive External Ophthalmoplegia (CPEO)**
  - **Neurological Manifestations:** Weakness of extraocular muscles leading to ptosis, ophthalmoplegia.
  - **Cardiovascular System:** ECG changes can occur.
  - **Ocular Symptoms:** Ptosis, external ophthalmoplegia.
- **Muscle-Renal Syndrome (MRS)**
  - **Neurological Manifestations:** None typically, but patients can have myopathy and renal involvement.
  - **Cardiovascular System:** Rare ECG changes.
  - **Ocular Symptoms:** None typical, but part of a broader syndrome.

These diagnoses are based on the wide range of symptoms and their chronic nature, often with onset in childhood or adolescence. Each has its specific clinical features and diagnostic criteria that can be further investigated through genetic testing, muscle biopsy, and other specialized tests.

**14. user 2024-11-30T18:18:14Z:**

There are five types, of which the classic and intermediate types are the most common.

- Classic type is the most common, accounting for 75 per cent of cases. Children are normal at birth, with early onset, severe symptoms and rapid progression. Usually, maple syrup odour appears in the cerumen 12 hours after birth; a special maple sugar odour is found in urine and sweat 12-24 hours after birth; ketonuria, irritability and feeding difficulties appear in 2-3 days; severe metabolic disorders and encephalopathic symptoms begin to appear in 4-5 days, which are manifested as difficulty in breastfeeding, paroxysmal vomiting, refusal to eat, lethargy, apnoea, convulsive seizures, heightened muscle tone and ‘fencing’. The symptoms of severe metabolic disorders and encephalopathy may include difficulty in breastfeeding, refusal to eat, lethargy, apnoea, convulsive seizures, increased muscle tone, and stereotypical movements such as ‘fencing’-like and ‘stomping’-like movements, etc. Coma and central respiratory failure may appear in the first 7-10 days of life, and most of the infants will die of severe metabolic disorders a few days after birth if they are not treated promptly.
- Intermediate type can develop at any age. The symptoms are less severe than those of the classic type and include slow growth, delayed development, feeding difficulties, irritability, a maple syrup odour in the cerumen, and severe metabolic disorders and encephalopathy under stress. 3. Intermittent Type The intermittent type is characterised by episodic seizures.
- Intermittent type: intermittent seizures, normal growth and development in the early stage, mostly under stressful situations such as infections and surgeries, manifested by episodic ataxia and ketoacidosis, and a very small number of severe cases may cause death. Branched-chain amino acid concentrations are normal during the intermittent period, and are elevated during the episodic period, similar to the classic type.

**qwen2.5_7b** 2024-11-30T18:18:19Z:

Based on the clinical manifestations provided, the most likely diagnosis is **Maple Syrup Urine Disease (MSUD)**.

### Other Possible Diagnoses:

- **Congenital Hypothyroidism**: This condition can cause symptoms of lethargy, feeding difficulties, and developmental delays similar to those seen in MSUD.
- **Reye’s Syndrome**: Although less common today due to the use of aspirin in children being restricted, it still presents with severe metabolic disturbances and encephalopathy.
- **Biotinidase Deficiency**: This condition can cause symptoms like lethargy, poor feeding, and developmental delay as well.
- **Medium-Chain Acyl-CoA Dehydrogenase Deficiency (MCADD)**: MCADD can present with similar metabolic crises, especially after fasting or illness.
- **Sepsis/Infection**: Particularly in newborns, infections can lead to symptoms of lethargy, poor feeding, and metabolic disturbances that might be confused with MSUD.

These conditions need to be ruled out through appropriate biochemical tests and genetic evaluations.

**15. user 2024-11-30T18:19:57Z: Bradykinesia, tremor, and myotonia. Levodopa-induced motor complications are more likely to occur after treatment. In terms of non-motor symptoms, mood disorders (e.g., depression, anxiety, irritability, etc.) and behavioural disorders (e.g., compulsive dosage increases, impulse control disorders, stereotyped behaviour, etc.) are likely to occur.**

**qwen2.5_7b** 2024-11-30T18:20:03Z:

Based on the clinical manifestations you’ve provided—bradykinesia, tremor, myotonia, levodopa-induced motor complications, mood disorders, and behavioral disorders—the most likely rare disease is **Dopa-Responsive Dystonia (DRD)**, also known as Segawa disease.

### Five Other Possible Diagnoses:

- **Myoclonus-Dystonia Syndrome**: This syndrome involves both myoclonic jerks and dystonic postures or movements.
- **Corticobasal Degeneration (CBD)**: Characterized by a combination of motor symptoms (like dystonia, tremor, rigidity) and cognitive decline.
- **Wilson’s Disease**: An inherited disorder that causes copper to accumulate in the liver, brain, kidneys, corneas, and other organs, leading to neurological and psychiatric symptoms.
- **Progressive Supranuclear Palsy (PSP)**: A progressive neurodegenerative disease affecting movement and gait, with common symptoms including balance difficulties, stiffness, and postural instability.
- **Parkinson’s Disease**: While Parkinson’s is more common, some patients can develop levodopa-induced dyskinesias after long-term treatment.

Each of these conditions should be considered based on the full clinical picture and further diagnostic tests.

**16. user 2024-11-30T18:20:43Z:**

Common manifestations in the foetal period include reduced foetal activity, excess amniotic fluid and breech presentation. In the neonatal period hypotonia is one of the hallmark features of this disorder, and severe hypotonia can lead to asphyxia, feeding difficulties, weak sucking, and weak crying. In infancy and early childhood, the main manifestations are growth retardation and lagging cognitive, motor and language development. Appetite increases with age and obesity can occur if eating is not restricted. Short stature is usually present in school age, and personality traits such as temper tantrums, stubbornness, and obsessive-compulsive disorder gradually appear. Abnormal behaviours include intense foraging, skin scratching, and autistic-like behaviour. Seizures occur in about 25 per cent of patients. Delayed or incomplete appearance of secondary sexual characteristics may occur in adolescent males and females. Obesity can lead to complications such as sleep apnoea, pulmonary heart disease, insulin resistance and even diabetes. Scoliosis, osteoporosis, and gastroesophageal reflux are also more common. The main manifestations in adulthood are obesity and its complications, including cardiovascular problems, diabetes and sleep apnoea. Other manifestations are mental abnormalities and infertility.

Fatal risks: choking due to uncoordinated mouth movements, hypotonia, polyphagia, gulping habits and inadequate chewing, and gastric rupture due to overeating.

Physical examination reveals characteristic facial features that become progressively more typical, including almond eyes, small mouth, thin upper lip, downward-facing corners of the mouth, small hands and feet, short stature, fair skin, low muscle tone, and genital hypoplasia (e.g., cryptorchidism, scrotal hypoplasia, or clitoral hypoplasia).

**qwen2.5_7b** 2024-11-30T18:20:49Z:

Based on the clinical manifestations described, the rare disease most likely to be diagnosed is Prader-Willi Syndrome (PWS), a genetic disorder caused by the loss of function of genes on chromosome 15.

### Other Possible Diagnoses:

- **Congenital Hypothyroidism**: While it does not fully match the described clinical manifestations, it can present with some similar symptoms like hypotonia and growth retardation.
- **Cerebral Palsy**: This condition primarily affects movement and muscle tone but may have some overlapping features in early infancy such as hypotonia.
- **Tay-Sachs Disease**: Although more common in Ashkenazi Jewish populations, it can present with developmental delays, hypotonia, and other neurological symptoms.
- **Mucopolysaccharidosis (MPS) Types**: A group of lysosomal storage disorders that can cause a range of symptoms including growth retardation, cognitive issues, and skeletal abnormalities.
- **Smith-Lemli-Opitz Syndrome**: This genetic disorder affects cholesterol metabolism and can present with a variety of developmental delays, facial anomalies, and other physical characteristics.

These diagnoses are based on the broad clinical picture provided, but only Prader-Willi Syndrome has a comprehensive set of overlapping features that closely match the described condition.

**17. user 2024-11-30T18:21:31Z:**

Patients often start in infancy or adolescence, with roughly equal male and female ratios. The water content of secretions from the epithelial cells of the respiratory tract, pancreas and biliary tract is reduced, and the secretions become viscous and difficult to clear. The lungs are the most commonly affected organ, and patients present with chronic bacterial infections and colonisation of the respiratory tract with pathogens, often initially Haemophilus influenzae and Staphylococcus aureus, and ultimately with chronic colonisation and/or infection with Pseudomonas aeruginosa or Klebsiella onionis. Once infection occurs, neutrophils are recruited in large numbers into the lung tissue and release elastase, which causes destruction of the lung tissue, leading to the formation of bronchiectasis. The patient’s bronchiectasis often starts at an early age and often begins in the upper lobes, in contrast to the majority of infections that result in the formation of bronchiectasis that is primarily located in the middle and lower lungs.

Patients often present with sweat stains left on dark-coloured clothing, or parents notice salty skin in their children. Abnormalities in the flow of bile and pancreatic fluid can lead to indigestion and malabsorption, as well as liver or pancreatic disease, and in severe cases, associated diabetes. The viscous intestinal fluid may also make the patient more susceptible to intestinal obstruction and rectal prolapse. In addition, male patients may develop impaired sperm movement in the vas deferens and congenital absence of the vas deferens (CBAVD).

**qwen2.5_7b** 2024-11-30T18:21:39Z:

Based on the clinical manifestations provided, the most likely diagnosis is cystic fibrosis (CF), a common genetic disorder that affects multiple organ systems.

### Other Possible Diagnoses:

- **Primary Ciliary Dyskinesia (PCD):**
  - PCD can also cause chronic respiratory infections and bronchiectasis due to impaired mucociliary clearance, but the pattern of lung involvement is often different from CF.
- **Alpha-1 Antitrypsin Deficiency:**
  - This condition can lead to lung disease (emphysema) and liver disease similar to CF, but typically presents later in life and with a specific deficiency in alpha-1 antitrypsin.
- **Bronchiolitis Obliterans Organizing Pneumonia (BOOP):**
  - BOOP is an inflammatory condition that can cause symptoms of chronic cough and respiratory infections, but it does not typically present with the viscous secretions or the characteristic saltiness noted in CF.
- **Immunodeficiency Syndromes:**
  - Certain primary immunodeficiencies like Severe Combined Immunodeficiency (SCID) or Chronic Granulomatous Disease (CGD) can cause recurrent infections but do not typically present with the specific lung pathology described for CF.
- **Mucoviscidosis Variants:**
  - There are rare variants of cystic fibrosis that may have different clinical presentations, although they would still primarily affect the respiratory and digestive systems similarly to classic CF.

These diagnoses should be considered in the differential based on the patient’s specific symptoms and family history, and further diagnostic tests such as sweat chloride testing, genetic testing for CFTR mutations, and imaging studies are typically required to confirm a diagnosis of cystic fibrosis.

**18. user 2024-11-30T18:22:07Z:**

Patients present with oropharyngitis, otitis media, respiratory infections, cellulitis and skin infections, often due to staphylococcal and streptococcal infections. Oral ulcers and painful gingivitis occur in most children before the age of 2 years in severe neutropenia with decreased bone marrow reserve. If associated with diffuse gastrointestinal lesions, it may cause abdominal pain and diarrhoea similar to Crohn’s disease. Neutropenia can lead to recurrent bacterial or fungal infections in various areas, with the main sites of involvement being the skin and mucous membranes, ears, nose, throat and lungs. The infections are often severe or even fatal. About 15% of patients progress to acute leukaemia or myelodysplastic syndrome. The children usually do not have characteristic malformations.

**qwen2.5_7b** 2024-11-30T18:22:18Z:

Based on the clinical manifestations provided, one rare disease that could be considered is **Chronic Benign Neutropenia with Granulomatous Inflammation (CBNGI)**, also known as **Chediak-Higashi Syndrome** in its more severe form or occasionally with neutropenia. However, let’s focus on CBNGI for this scenario:

- **Chronic Benign Neutropenia with Granulomatous Inflammation (CBNGI)**
  - This is a rare disorder characterized by recurrent bacterial infections, neutropenia, and granulomas in the skin, mucous membranes, and other organs.
- **Shwachman-Diamond Syndrome (SDS)**
  - SDS is an autosomal-recessive disease affecting bone marrow function leading to severe neutropenia, exocrine pancreatic insufficiency, short stature, and hematologic abnormalities.
- **Chronic Granulomatous Disease (CGD)**
  - A primary immunodeficiency characterized by recurrent infections due to defects in the NADPH oxidase component of phagocytes, leading to chronic granulomas.
- **Wiskott-Aldrich Syndrome (WAS)**
  - This X-linked recessive disorder is characterized by thrombocytopenia, eczema, and immunodeficiency including recurrent infections due to defects in the WASP protein.
- **Severe Chronic Granulomatous Neutropenia with Febrile Episodes (SCGNFE)**
  - A rare neutrophil disorder characterized by chronic granulomatous disease-like symptoms, severe neutropenia, and febrile episodes.
- **Chronic Myeloid Leukemia in Blast Crisis (CML-BC)**
  - Although not as common for this age group, CML can progress to a blast crisis stage where patients may present with similar features including recurrent infections, fever, and neutropenia.

Given the clinical presentation of recurrent infections, severe neutropenia, oral ulcers, painful gingivitis, and the possibility of progressing to acute leukemia or myelodysplastic syndrome, chronic benign neutropenia with granulomatous inflammation is a likely diagnosis. However, it’s crucial to conduct further diagnostic tests such as blood work, bone marrow biopsy, genetic testing, and possibly imaging studies for an accurate diagnosis.

**19. user 2024-11-30T18:23:05Z:**

Typical Patients present with both CNS damage and hypoadrenocorticism. Neurological symptoms and symptoms of hypoadrenocorticism may occur simultaneously or sequentially. In a minority of patients, they may occur alone. The manifestations of adrenocortical insufficiency vary in severity. It is most common in children, accounting for 35% of all patients. The onset of symptoms occurs between the ages of 4 and 8 years. The initial symptoms include poor concentration, memory loss, learning difficulties, unsteady gait and abnormal behaviour, followed by visual and auditory loss, dysarthria, seizures, ataxia, paralysis and dementia. Clinical manifestations progressively worsen, culminating in blindness or deafness and complete paralysis. Some exhibit deafferentation and eventually die of central respiratory failure, brain herniation, and infection. Most patients have adrenocortical impairment when they first develop neurological symptoms.

**qwen2.5_7b** 2024-11-30T18:23:13Z:

Based on the clinical manifestations provided, the most likely diagnosis is **neurocristopathy**, specifically related to a condition such as **Doose syndrome (Myoclonic-Astatic Epilepsy)** or **Rasmussen’s encephalitis** with concurrent hypoadrenocorticism. However, given the specific combination of CNS damage and adrenocortical insufficiency in this presentation, the following rare diseases should be considered:

- **Neurocristopathy (Hypothalamic Hamartoma-Adrenal Insufficiency Syndrome)** - This condition involves a hamartoma in the hypothalamus that can lead to both neurological issues and adrenal insufficiency.
- **Cushing-Turner syndrome** - This is an extremely rare autosomal recessive condition characterized by pituitary underdevelopment, adrenocortical insufficiency, and neurological deficits.
- **Albright hereditary osteodystrophy (AHO)** - This condition can present with a variety of symptoms including hypoparathyroidism, skeletal abnormalities, and sometimes adrenal dysfunction leading to secondary hypoadrenocorticism.
- **Liddle syndrome with neurocristopathy** - A rare genetic disorder that includes neurological deficits alongside primary hyperaldosteronism, which can be associated with adrenal insufficiency due to secondary causes.
- **Alport syndrome** - While primarily a renal disease, it can also present with sensorineural hearing loss and other neurological symptoms. In some cases, there may be concurrent adrenal involvement leading to hypoadrenocorticism.

Given the specific combination of neurological damage and adrenocortical insufficiency described, Neurocristopathy or conditions similar in presentation would likely be the primary focus for diagnosis.

**20. user 2024-11-30T18:24:02Z:**

Most of the children start to show symptoms of infection from 4 to 12 months after birth. The most prominent clinical manifestation is recurrent severe bacterial infections in the respiratory system, digestive system and central nervous system, with respiratory tract infections being the most common, and common pathogens include Haemophilus influenzae, Streptococcus pneumoniae, Staphylococcus aureus, and Pseudomonas aeruginosa, etc. The most common infections are respiratory tract infections. Prolonged and repeated respiratory infections can lead to irreversible organ function damage such as bronchiectasis and pestle finger. Common symptoms of GI infections include diarrhoea, abdominal pain, gastro-oesophageal reflux and gastroenteritis. Pathogens causing infectious diarrhoea include Giardia lamblia, Salmonella, Campylobacter jejuni and Cryptosporidium.

In addition, children are prone to otitis media, chronic sinusitis, malnutrition, anaemia, granulocytopenia, thrombocytopenia, growth hormone deficiency and thyroid hormone disorders. Children may also develop tumours, autoimmune and inflammatory diseases, including rheumatoid arthritis and dermatomyositis. A higher incidence of sensorineural hearing loss and eczema has also been reported in children.

**qwen2.5_7b** 2024-11-30T18:24:08Z:

Based on the clinical manifestations provided, the most likely rare disease is **Severe Combined Immunodeficiency (SCID)**, specifically a form known as SCID with enteropathy or sometimes referred to as Omenn syndrome, which is one of several subtypes of SCID.

### Five Other Possible Diagnoses:

- **X-linked Chronic Granulomatous Disease (CGD)** - This condition involves recurrent infections and neutrophil function defects.
- **Wiskott-Aldrich Syndrome** - Characterized by eczema, thrombocytopenia, and immunodeficiency leading to repeated infections.
- **Adenosine Deaminase Deficiency (ADA-SCID)** - A form of SCID that can present with severe infections and poor growth.
- **Selective IgA Deficiency** - While primarily a defect in IgA production, it can be associated with recurrent infections and other immune dysfunctions.
- **Common Variable Immunodeficiency (CVID)** - Characterized by hypogammaglobulinemia leading to frequent bacterial infections.

Each of these conditions can present with a variety of symptoms overlapping with the ones described, making accurate diagnosis crucial through detailed clinical assessment, genetic testing, and specific immunological tests.

**21. user 2025-02-18T09:37:27Z:**

The clinical manifestations of the patients included salt loss and hyperandrogenism. Neonatal onset was characterized by varying degrees of adrenal insufficiency, such as weakness, nausea, vomiting, feeding difficulties, diarrhea, chronic dehydration, skin pigmentation, and growth retardation. Adrenal crisis is often the first manifestation of the disease in the neonatal period, manifested as severe hyponatremia, hyperkalemia, hypovolemic shock, may be accompanied by hypoglycemia, induced by stress. Severe hyponatremia can lead to central nervous system manifestations such as convulsions, and severe hyperkalemia can cause fatal arrhythmias.

Hyperandrogenism manifests differently at different ages. Female children with classical diseases have different degrees of masculinity at birth. Light performance for isolated clitoris hypertrophy, heavy external genitalia can approach men, labia majora scrotal shape, clitoris hypospadia-like penis, and with a common urethral vaginal orifice. However, the labia majora cannot touch the gonads and has completely normal female internal genital structures (ovary and uterus). Males may not have external genital abnormalities such as enlarged penis during neonatal and infancy, which is a common cause of delayed diagnosis. In early childhood, both sexes will present peripheral precocious puberty. Boys presented with enlarged penis with or without pubic hair; females presented with heterosexual precocious puberty. Chronic high levels of sex hormones stimulate hypothalamic gonadotropin-releasing hormone (GnRH) neurons and develop into central precocious puberty. Women may also have secondary sexual dysplasia and primary amenorrhea or oligomenorrhea. Linear growth with accelerated bone age occurs in both sexes from infancy, resulting in impaired terminal height.

Other findings include skin and mucous membrane pigmentation, especially areola and vulva, some children may not have this change.

Non-classical hyperandrogenism in childhood, adolescence and even adulthood may be characterized by accelerated growth and rapid bone age progression.

**qwen2.5_7b** 2025-02-18T09:37:33Z:

Based on the clinical manifestations provided, the most likely diagnosis is **Congenital Adrenal Hyperplasia (CAH)**, specifically the type 21-hydroxylase deficiency, which is the most common form of CAH. This condition leads to a severe lack of cortisol and aldosterone production, resulting in salt loss and adrenal insufficiency. The hyperandrogenism observed, especially in females with ambiguous genitalia, supports this diagnosis.

Here are five other possible diagnoses that might be considered:

- **McCune-Albright Syndrome (MAS)** - This is an uncommon condition characterized by skin pigmentation (cafe-au-lait spots), bone lesions, and endocrine disorders such as hyperandrogenism and precocious puberty. However, it typically does not present with the severe salt loss seen in CAH.
- **Ovarian Proliferation or Hyperplasia** - This can cause excessive production of sex hormones leading to hyperandrogenism and precocious puberty but usually lacks the systemic manifestations such as salt wasting and adrenal crisis seen in CAH.
- **Nephroblastomatosis** - While rare, this condition involves kidney tumors and may present with features related to hormonal imbalances, but it typically does not include the severe salt loss or adrenal insufficiency observed in CAH.
- **Congenital Adrenal Hyperplasia (CAH) due to 11β-Hydroxylase Deficiency** - This is a less common form of CAH that can also present with hyperandrogenism, but it does not typically involve the severe salt loss seen in type 21-hydroxylase deficiency.
- **Congenital Adrenal Hyperplasia (CAH) due to 3β-Hydroxysteroid Dehydrogenase Deficiency** - This is a very rare form of CAH that can lead to hyperandrogenism and adrenal insufficiency but usually does not present with the severe salt loss seen in type 21-hydroxylase deficiency.

Given the clinical findings, CAH due to 21-hydroxylase deficiency remains the most probable diagnosis.

**22. user 2025-02-18T09:39:08Z:**

The patient has clinical manifestations at birth, due to lack of pigment in the whole body, skin capillaries are exposed, skin is thin and soft, white and red, and accompanied by varying degrees of vasodilation. Patients are highly sensitive to ultraviolet light, often solar dermatitis, actinic cheilitis, basal cell carcinoma and squamous cell carcinoma and other diseases. Hair is white, silver, yellow, gold or brown. Eyelashes and eyebrows are white or pale yellow. Retina, iris color decline, pupil color decline. Photophobia is obvious during the day, night vision is normal, and other visual changes can also be accompanied, such as decreased visual acuity, hypoplasia of the fovea, changes in neural pathways at the optic chiasm, and nystagmus.

**qwen2.5_7b** 2025-02-18T09:39:13Z:

Based on the clinical manifestations you’ve described, the patient likely has a diagnosis of **Albinism**. Albinism is a group of genetic conditions characterized by reduced or absent pigmentation in the skin, hair, and eyes due to a deficiency in melanin production. The specific type can be further determined through additional tests such as DNA analysis.

Here are five other possible diagnoses that could have similar clinical manifestations but are less likely given the primary features described:

- **Vitiligo**: This is an autoimmune disorder leading to depigmentation of the skin, but it typically develops later in life and does not usually involve the other systemic symptoms such as photosensitivity and ocular changes.
- **Hypoplastic Anemia with Albinism (Griscelli Syndrome)**: This rare genetic condition can cause albinism along with immunodeficiency issues, but it generally involves more specific hematological abnormalities that are less common in your case.
- **Waardenburg Syndrome**: This syndrome includes pigmentary changes and hearing loss, but the primary focus is on pigmentation of the skin and hair rather than systemic photosensitivity or ocular manifestations as prominent.
- **Neurocutaneous Syndromes** (e.g., Neurofibromatosis Type 1): While these can present with various skin changes, they typically do not include the specific pattern of albinism noted in this case without additional neurological features.
- **Pigmentary Mosaicism**: This condition involves variable pigment distribution but is usually associated with more localized patterns and does not typically present with the systemic findings described here.

Given the detailed clinical picture you’ve provided, Albinism remains the most likely diagnosis, particularly Oculocutaneous Albinism (OCA), which encompasses all the symptoms mentioned.

**23. user 2025-02-18T09:40:25Z:**

A degenerative disease of the nervous system in which both upper and lower motor neurons are involved. The main clinical manifestations are progressive weakness and atrophy of the muscles of the bulbar, limbs, chest and abdomen. Eye motor nerve and sphincter function are generally not affected, but may be affected later in some patients. Between 20% and 50% of patients may exhibit cognitive impairment, and between 5% and 15% may even develop frontotemporal dementia. Death from respiratory failure occurs an average of 3 to 5 years after onset, but 5% to 10% of patients survive for more than 10 years.

Detailed history and physical examination to find evidence of common involvement of upper and lower motor neurons in brain stem, neck, chest and lumbosacral regions is the basis for diagnosis of the disease. Physical examination showed that both upper and lower motor neurons were involved in the same area, which was the key point of diagnosis. The main signs of lower motor neuron involvement included muscle weakness, atrophy and fascicular fibrillation. The tongue muscles, facial muscles, throat muscles, neck muscles, different muscle groups of the limbs, back muscles and pectoralis muscles are usually examined. The signs of upper motor neuron involvement mainly included positive pathological signs, tendon hyperreflexia, hypertonia, clonus, etc. Usually check sucking reflex, pharyngeal reflex, mandibular reflex, palmar jaw reflex, whether there is strong crying, strong laughter and other pseudobulbar paralysis manifestations, limb tendon reflex, muscle tension, Hoffman sign, lower limb pathology, abdominal wall reflex, etc. Clinical examination is the primary method of detecting upper motor neuron involvement. In areas of atrophy and weakness, if tendon reflexes are not low or active, it may indicate pyramidal tract damage even if there are no pathological signs.

**qwen2.5_7b** 2025-02-18T09:40:30Z:

Based on the clinical manifestations described, the most likely diagnosis is **Progressive Bulbar Palsy (PBP)**, which is often a manifestation of Amyotrophic Lateral Sclerosis (ALS). ALS is a neurodegenerative disease characterized by degeneration of both upper and lower motor neurons. The progressive weakness and atrophy in various muscle groups, including those affecting speech and swallowing (bulbar), along with cognitive impairment and potential frontotemporal dementia align well with the clinical profile of ALS.

Here are five other possible diagnoses to consider:

- **Primary Lateral Sclerosis (PLS)**: This is a less common form of ALS where the initial symptoms are primarily upper motor neuron signs without evidence of lower motor neuron degeneration for several years, though it can progress to involve both upper and lower motor neurons over time.
- **Cervical Myelopathy**: While typically not progressive in nature like ALS or PLS, cervical myelopathy can present with a similar pattern of weakness and atrophy due to spinal cord compression from conditions such as herniated discs or spondylosis.
- **Metachromatic Leukodystrophy (MLD)**: This is an inherited disorder characterized by the accumulation of sulfatide in the brain and other tissues, leading to progressive neurological deterioration, which can include muscle weakness and atrophy.
- **Vasculitis**: Inflammatory processes affecting blood vessels within the nervous system can lead to similar symptoms due to ischemia or direct inflammation of motor neurons.
- **Chronic Progressive Multifocal Leukoencephalopathy (PML)**: Caused by reactivation of the JC virus, this condition leads to progressive demyelination and can present with a variety of neurological deficits including muscle weakness and atrophy in multiple areas of the body.

Each of these conditions has its specific diagnostic criteria, which would be further evaluated through clinical history, physical examination, and possibly additional tests such as EMG/NCS, MRI, CSF analysis, genetic testing, or nerve biopsy.

**24. user 2025-02-18T09:42:18Z:**

An autosomal recessive disorder affecting the urea cycle, leading to hyperammonemia, encephalopathy, and respiratory alkalosis. It is characterized by a buildup of arginine and ammonia in the blood, causing symptoms such as developmental delays, seizures, intellectual disability, spasticity, and ataxia. The condition typically manifests around age 3, with symptoms including stiffness, slow growth, and cognitive decline. Without treatment, it can progress to severe spasticity, loss of ambulation, and intellectual disability.

**qwen2.5_7b** 2025-02-18T09:42:22Z:

Based on the clinical manifestations provided, the most likely rare disease diagnosis is **argininemia**. This condition arises from a deficiency in the enzyme arginase, which is necessary for the urea cycle.

Five other possible diagnoses that could present with similar symptoms include:

- **Ornithine Transcarbamylase Deficiency (OTCD)**: A disorder of the urea cycle caused by a deficiency in ornithine transcarbamylase enzyme.
- **Citrullinemia**: A rare genetic disorder resulting from a deficiency of the argininosuccinate synthetase enzyme, which is part of the urea cycle.
- **Hartnup Disease**: Although primarily affecting the amino acid transport system in the gut and kidney, it can present with neurological symptoms including ataxia and seizures due to tryptophan deficiency.
- **MAP (Methylmalonic Acidemia) Type C**: A disorder caused by a defect in cobalamin metabolism leading to accumulation of methylmalonic acid in the blood, which can also cause hyperammonemia and similar neurological manifestations.
- **Glutaric Aciduria Type 1**: A metabolic disorder characterized by a deficiency in glutaryl-CoA dehydrogenase, leading to accumulation of glutaric acid, causing symptoms such as developmental delay and seizures.

Each of these conditions shares some clinical overlap with argininemia, particularly in the areas of hyperammonemia, neurological manifestations, and developmental issues. However, specific biochemical testing is essential for accurate diagnosis.

**25. user 2025-02-18T09:43:47Z:**

The typical clinical picture includes the triad of microangiopathic haemolytic anaemia, thrombocytopenia and acute renal failure. Non-immune erythrocyte destruction due to microthrombosis often results in a haemoglobin level below 80 g/L, a negative Coombs test, and erythrocyte fragments seen on peripheral blood smears; platelet counts usually fall below 50×10^9 /L, but skin purpura and active bleeding are rare; the severity of the acute renal impairment varies from person to person, with some patients requiring dialysis support, often accompanied by elevated blood pressure.

**qwen2.5_7b** 2025-02-18T09:43:52Z:

Based on the clinical manifestations you’ve described—microangiopathic hemolytic anemia (MAHA), thrombocytopenia, and acute renal failure—the most likely diagnosis is **Thrombotic Thrombocytopenic Purpura (TTP)**.

Here are five other possible diagnoses:

- **Hemolytic-Uremic Syndrome (HUS)**
  - This syndrome typically presents with MAHA, thrombocytopenia, and acute kidney injury.
  - It is often associated with Shiga toxin-producing Escherichia coli (STEC) infection or can be related to other factors like antibiotic use or certain drugs.
- **Disseminated Intravascular Coagulation (DIC)**
  - DIC can cause microangiopathic hemolysis, thrombocytopenia, and multi-organ failure.
  - It is typically a complication of severe sepsis, trauma, cancer, or obstetric complications.
- **Evans Syndrome**
  - This syndrome involves both autoimmune hemolytic anemia (HA) and immune thrombocytopenic purpura (ITP).
  - Symptoms can include MAHA, thrombocytopenia, and sometimes acute kidney injury if there is significant blood loss or vasculitis.
- **Severe Preeclampsia with Eclampsia**
  - This condition during pregnancy can lead to similar symptoms including hemolysis, thrombocytopenia, and renal dysfunction.
  - It is related to endothelial dysfunction and can be life-threatening for both the mother and fetus.
- **Malignancy-Related Microangiopathic Hemolytic Anemia**
  - Certain malignancies, particularly those involving the hematopoietic system or metastatic tumors affecting small blood vessels, can present with MAHA.
  - This is often accompanied by systemic symptoms related to the underlying cancer.

Each of these conditions requires a specific diagnostic approach and may involve different treatment strategies.

**26. user 2025-02-18T09:44:46Z:**

Patients have a variety of clinical presentations. It depends on the location, extent and rate of progression of the lesion. Clinical symptoms mainly include:

- The occupying effect of an enlarged pituitary gland, which in most patients results from compression of the enlarged pituitary gland on the anterior region and surrounding structures (e.g., optic crossings, cavernous sinus, etc.), or from an inflammatory infiltrate. The most common clinical manifestations are persistent severe headaches and progressively worsening visual dysfunction, including decreased visual acuity and visual field defects.
- hypopituitarism. Depending on the degree of involvement, the various axes of the pituitary gland may show partial or even total hypopituitarism, such as amenorrhoea, hypogonadism, nausea, malaise, chills, loss of sexual hair and weight loss.
- Central dysuria, more than 70% of the patients will have symptoms of central dysuria such as polyuria, irritable thirst and polydipsia due to the involvement of the posterior pituitary, pituitary stalk and even hypothalamus region. In some patients, the blocking effect of the pituitary stalk leads to a mild increase in serum prolactin levels.
- Hypothalamic syndrome, when the lesion involves the region of the hypothalamus, there will be drowsiness, lack of satiety, severe weight gain, lack of thirst, temperature fluctuation, sweating disorders, and emotional and psychiatric disorders.
- Systemic disease-related manifestations. Patients with secondary disease often have a combination of clinical manifestations characteristic of systemic disease, such as LCH patients often have a combination of thyroid, lung, bone, liver, and skin lesions. patients with GPA are often middle-aged or older men, and respiratory symptoms often precede hypothalamic pituitary involvement. patients with IgG4-related pituitary inflammation often have a combination of clinical manifestations with the involvement of other organs such as lacrimal glands, submandibular glands, and pancreas. The disease is often associated with clinical signs of involvement of other organs such as the lacrimal gland and submaxillary gland and pancreas.

**qwen2.5_7b** 2025-02-18T09:44:51Z:

Based on the clinical manifestations you’ve provided, the most likely rare disease to be diagnosed would be **Sheehan Syndrome**, which results from pituitary infarction following hemorrhagic shock or severe postpartum bleeding. However, given the additional symptoms such as optic nerve compression and hypopituitarism, several other conditions could also fit these clinical manifestations:

- **Sheehan Syndrome**: This condition occurs due to ischemic necrosis of the anterior pituitary gland secondary to hemorrhagic shock or massive postpartum hemorrhage.
- **Infectious Pituitary Hypophyseal Disease (e.g., Meningoencephalitis, Tuberculosis)**: Infections can cause inflammation and compression of the pituitary leading to similar symptoms as Sheehan’s syndrome but with additional signs of systemic infection.
- **Hypothalamic Pituitary Adenomas**: These tumors can compress surrounding structures including the optic nerves, causing visual disturbances and may also result in hormonal deficiencies due to their proximity to the pituitary gland.
- **Granulomatous Hypophysitis (e.g., Sarcoidosis or Tuberculosis)**: Granulomatous inflammation of the pituitary gland can lead to compression symptoms as well as hypopituitarism and other systemic manifestations depending on the extent of involvement.
- **Autoimmune Pituitary Disease**: Conditions like Lymphocytic Hypophysitis (e.g., Autoimmune Hypophysitis) can cause similar symptoms due to infiltration and inflammation of pituitary tissue leading to hormonal deficiencies and ocular compression signs.
- **Vasculitides (e.g., Granulomatosis with Polyangiitis, Wegener’s Granulomatosis)**: These conditions can involve the hypothalamic-pituitary region causing a combination of neurological symptoms including visual disturbances and endocrine manifestations due to vasculitis affecting the blood supply to the pituitary.

Each of these diagnoses would require specific diagnostic tools such as MRI of the brain, hormonal assessments (ACTH, TSH, GH, PRL), and in some cases, further imaging or biopsy for confirmation.

**27. user 2025-02-18T09:46:07Z:**

Hyperglycaemia is the most common clinical manifestation of the disease. However, patients can spontaneously remit without intervention and maintain normal blood glucose levels. The disease is one of the leading causes of autoimmune hypoglycaemia, with about 25% of patients experiencing spontaneous hypoglycaemia during the course of the disease. Acanthosis nigricans is present in about 50 per cent of patients. In addition, polycystic ovaries and hyperandrogenism are not uncommon in this group of patients. Other autoimmune diseases are common, with systemic lupus erythematosus (SLE) being the most common, accounting for about 50 per cent of cases. Primary biliary cirrhosis, dermatomyositis, and scleroderma are also present. The disease can also be a paraneoplastic syndrome manifestation of Hodgkin’s lymphoma, multiple myeloma, and others.

**qwen2.5_7b** 2025-02-18T09:46:12Z:

Based on the clinical manifestations you’ve described, the most likely rare disease is **Reactive Hypoglycemia** or more specifically, **Autoimmune Polyendocrine Syndrome Type 2 (APS-2)**, also known as Schmidt’s syndrome.

Here are five other possible diagnoses:

- **Type 1 Diabetes Mellitus**: While it can present with hyperglycaemia, the spontaneous remission and subsequent hypoglycaemic episodes could suggest an autoimmune component not typically seen in type 1 diabetes alone.
- **Hypothalamic Pituitary Adrenal Axis Dysfunction (HPA Axis Dysfunction)**: This condition can result from various causes including autoimmune disorders and can manifest with hyperglycaemia, acanthosis nigricans, and other endocrine abnormalities.
- **Gestational Diabetes Mellitus**: If the patient has a history of pregnancy or if the symptoms started during pregnancy, gestational diabetes needs to be considered, although it is less likely given the ongoing nature of the symptoms post-pregnancy.
- **Hereditary Hemochromatosis**: This condition can present with hyperglycaemia and endocrine abnormalities but usually does not cause spontaneous hypoglycaemia or acanthosis nigricans without concurrent liver disease.
- **Primary Focal Adenomatous Hyperplasia (PFACH)**: This is a rare condition that can affect the pancreas, leading to insulin resistance and other metabolic issues, though it is less likely given the wide range of symptoms you’ve described.

Given the complexity and specific set of clinical findings, APS-2 is the most fitting diagnosis due to its characteristic endocrine manifestations, including diabetes insipidus or adrenal insufficiency (which can present with hypoglycaemia), along with acanthosis nigricans and other autoimmune features.

**28. user 2025-02-18T09:47:35Z:**

The disease starts in infancy and childhood. The first attack occurs between the ages of 5 months and 7 years. The clinical picture is one of recurrent episodes of intractable vomiting and severe ketoacidosis with depression, dehydration, shortness of breath, and coma. There is usually a clear trigger, and the disease may begin acutely after fasting, gastrointestinal and upper respiratory tract infections, fever, stress, or excessive protein intake. Routine laboratory tests show elevated or normal blood glucose, some hypoglycaemia, blood gases show metabolic acidosis, blood ammonia can be normal or mildly elevated, liver function, renal function and normal, and urine ketone bodies are positive. Ketosis and acidosis may recur, and clinical and laboratory tests may be normal between attacks.

Neurological changes occur in about 1/5 of the cases, manifested by cognitive impairment, mental retardation, extrapyramidal abnormalities and symptoms of stroke. Cranial MRI reveals extensive demyelination, including white matter, and symmetric high signal changes in the basal ganglia at T2.

Patients may be asymptomatic in the early stages and are diagnosed in the neonatal period or in high-risk groups by blood and urine metabolic screening. There is no clear association between age of onset, frequency of attacks and prognosis. There is a tendency for the attacks to decrease or stop with age, and in most cases growth is unaffected on follow-up. Recurrent and severe attacks can lead to multi-organ dysfunction or even death.

**qwen2.5_7b** 2025-02-18T09:47:40Z:

Based on the clinical manifestations provided, the disease most likely diagnosed is **Maple Syrup Urine Disease (MSUD)**, a metabolic disorder caused by the deficiency of branched-chain alpha-keto acid dehydrogenase complex. The key features that point to this diagnosis include:

- **Maple Syrup Urine Disease (MSUD)** - This rare genetic condition affects the metabolism of certain amino acids, particularly leucine, isoleucine, and valine. It leads to the accumulation of these branched-chain amino acids and their metabolites in the blood and urine, causing characteristic symptoms like recurrent ketoacidosis and hyperammonemia.
- **Classic MSUD** - This form usually presents early in life with symptoms starting between 5 months and 7 years as described.
- **Intermediate MSUD** - Similar to Classic MSUD but milder and often diagnosed later due to less severe initial symptoms.
- **Weak Phenylketonuria (PKU)** - Although PKU primarily affects phenylalanine metabolism, it can sometimes present with neurological complications, making it a potential differential diagnosis.
- **Congenital Hypothyroidism** - This is another condition that can cause lethargy, poor feeding, and growth retardation in infants but typically does not involve the specific metabolic features of MSUD.
- **Reye’s Syndrome** - While this is a rare condition associated with aspirin use during viral infections causing liver failure and brain swelling, it often presents later than 7 years and has different etiological factors.

These differential diagnoses account for the recurrent episodes of ketoacidosis, the specific triggers such as fasting or infection, and the neurological complications seen in some cases. The characteristic urine odor (reminiscent of maple syrup) is also a hallmark of MSUD, though it may not always be present depending on the severity and age at presentation.

**29. user 2025-02-18T09:48:38Z:**

- the onset is at a young age and is more common in women.
- there is often a family history of syncope or sudden death.
- different genotypes and phenotypes, can involve potassium, sodium and calcium channels. Characteristic tip-distortion ventricular tachycardia (TdP) is often present, leading to syncope or sudden death. 4.
- The ECG shows a prolonged QT interval.
- ECG generally has the following characteristics: (1) QT interval prolongation, short - long - short cycle phenomenon induced; (2) ventricular rate of 160-240 beats / minute, QRS wave amplitude and morphology around the equipotential line of reversal, 5-20 cardiac cycles of the main wave around the baseline of the reversal of the majority of the time can be terminated; (3) ATP is more difficult to terminate the TdP.

**qwen2.5_7b** 2025-02-18T09:48:42Z:

Based on the clinical manifestations provided, the rare disease most likely to be diagnosed is Long QT Syndrome (LQTS). This syndrome is characterized by a prolonged QT interval on the electrocardiogram (ECG), syncope, and an increased risk of torsades de pointes (TdP) ventricular tachycardia leading to sudden cardiac death.

Here are five other possible diagnoses that may present similar symptoms or overlapping characteristics:

- **Brugada Syndrome**: This is a genetic heart rhythm disorder characterized by right bundle branch block-like ECG changes and an increased risk of sudden cardiac arrest, often associated with syncope and sudden unexplained nocturnal death syndrome (SUNDS).
- **Catecholaminergic Polymorphic Ventricular Tachycardia (CPVT)**: This condition is characterized by polymorphic ventricular tachycardia that can be triggered by exercise or emotional stress, often in the context of a family history.
- **Early Repolarization Syndrome**: While generally not as life-threatening, it can present with an ECG showing J-point elevation and ST-segment elevation, mimicking LQTS.
- **Hypertrophic Cardiomyopathy (HCM)**: This condition involves left ventricular hypertrophy and can sometimes present with syncope or sudden death, though the ECG typically shows a pattern of left ventricular hypertrophy without a prolonged QT interval.
- **Arrhythmogenic Right Ventricular Dysplasia/Cardiomyopathy (ARVD/C)**: This is a genetic disorder affecting the right ventricle, leading to progressive myocardial fibrosis and fat infiltration, which can cause arrhythmias including sudden cardiac death.

Each of these conditions needs careful evaluation with appropriate diagnostic testing such as electrophysiology studies, family history assessment, and possibly genetic testing.

**30. user 2025-02-18T09:50:36Z:**

The disease can develop at any age, and most of the children develop between 1 month and 7 years old, with an average age of about 2 years old. Clinical manifestations vary greatly among patients, mainly including: (1) acute energy metabolism disorder crisis, manifested by low ketone hypoglycemia, high blood ammonia and metabolic acidosis; (2) cardiomyopathy, manifested by ventricular hypertrophy, cardiac insufficiency, cardiac arrhythmia, and elevated creatine kinase, etc.; (3) myopathy, manifested by muscle weakness, hypotonia, myalgia, poor exercise endurance, elevated muscular-type creatine kinase, and lipid deposition in muscle fibres, etc. (4) liver damage, manifested by hepatomegaly, fatty liver, liver function abnormalities, etc. Some liver-damaged patients have liver enlargement, fatty liver, and liver function abnormalities; Liver damage, manifested by hepatomegaly, fatty liver, abnormal liver function, etc. Some children with liver damage have acute onset of illness, manifested by convulsions, progressive consciousness disorder, etc., which is often misdiagnosed as Reye syndrome. In addition, gastrointestinal symptoms such as recurrent abdominal pain, vomiting and gastroesophageal reflux, respiratory symptoms such as recurrent infections and wheezing, and anaemia have also been reported.

Newborn screening: diagnosed children may have no clinical manifestations. It is a potentially fatal disease, and children can die suddenly from acute energy metabolism disorder crisis or acute heart failure. Clinical manifestations are varied and related to age of onset, time of onset and severity of illness. The most common symptoms are cardiac and skeletal muscle damage in infancy and childhood. Symptoms in adulthood are mild or asymptomatic, mostly due to reduced endurance or easy fatigue. During pregnancy, pregnant women may have atypical manifestations such as fatigue and arrhythmias due to energy expenditure and reduced physiological plasma carnitine levels.

**qwen2.5_7b** 2025-02-18T09:50:41Z:

Based on the clinical manifestations provided, the disease most likely diagnosed is **Carnitine Palmitoyltransferase II (CPT-II) Deficiency**. This rare genetic disorder fits well with the symptoms described:

- **Acute Energy Metabolism Disorder Crisis**: Patients often experience episodes of hypoglycemia, ketosis, and metabolic acidosis.
- **Cardiomyopathy**: Ventricular hypertrophy, cardiac insufficiency, arrhythmias, and elevated creatine kinase levels.
- **Myopathy**: Muscle weakness, hypotonia, myalgia, poor exercise endurance, elevated muscular-type creatine kinase, lipid deposition in muscle fibers.
- **Liver Damage**: Hepatomegaly, fatty liver, abnormal liver function.

### Five Other Possible Diagnoses

- **Methylmalonic Aciduria (C2 Variant)**: This is a different type of metabolic disorder that can also present with similar symptoms involving the metabolism of fatty acids and amino acids.
- **Long-Chain 3-Hydroxyacyl-CoA Dehydrogenase Deficiency (LCHAD Deficiency)**: This condition affects fat metabolism and can lead to cardiomyopathy, liver problems, and acute metabolic crises.
- **Carnitine Uptake Disorder**: Impaired ability to transport carnitine into cells can also cause similar symptoms as CPT-II deficiency due to impaired fatty acid oxidation.
- **Glycogen Storage Disease Type V (McArdle’s Disease)**: While primarily affecting muscle, it can present with myopathy and may have overlapping features of fatigue and exercise intolerance.
- **Cystinosis**: A rare genetic disorder that affects the kidneys and eyes, but also has muscular symptoms which can be similar to those seen in CPT-II deficiency.

These diagnoses are based on the clinical profile provided and are among the conditions that could potentially present with similar symptoms involving metabolic crises, cardiac issues, muscle weakness, and liver involvement.

**31. user 2025-02-18T09:52:38Z:**

In the unicentric form, the disease often presents only with enlarged lymph nodes without systemic symptoms. The median diameter of the enlarged lymph nodes is about 5.5 cm, and they are commonly found in the chest (24%), neck (20%), abdomen (18%), and retroperitoneum (14%). Some patients with unicentric disease are prone to paraneoplastic aspergillosis, occlusive bronchiolitis, and growth retardation. In addition to multiple lymph node enlargements, multicentric disease is often associated with systemic manifestations such as fever, night sweats, malaise, weight loss, anaemia, hepatic insufficiency, renal insufficiency, and volume overload (generalised oedema, pleural effusion, ascites, etc.). There is also a specific subtype in which patients may present with thrombocytopenia, severe oedema (anasarca), myelofibrosis, renal dysfunction, and hepatosplenomegaly (organomegaly).

**qwen2.5_7b** 2025-02-18T09:52:42Z:

Based on the clinical manifestations you’ve described, the most likely diagnosis is **Primary Cutaneous Anaplastic Large Cell Lymphoma (PC-ALCL)** in its unicentric form. This disease typically presents with a solitary or multiple nodal enlargements and can occasionally be associated with systemic symptoms as well.

Here are five other possible diagnoses that should be considered:

- **Langerhans Cell Histiocytosis (LCH)**: This condition, especially if the lymph node involvement is unicentric, can present similarly to PC-ALCL but involves Langerhans cells rather than T-cell mediated anaplastic large cell lymphoma.
- **Solitary Fibrous Tumor**: This tumor can also cause localized lymphadenopathy and may be mistaken for a malignant lymphoma, especially if not biopsied properly. It is more common in the pleura but can present as a solitary mass elsewhere.
- **Cystic Lymphangioma or Cavernous Hemangioma**: These benign conditions can sometimes present with isolated enlarged lymph nodes that could mimic malignancy.
- **Metastatic Tumor**: A patient may have metastatic disease from another primary cancer, which can present with lymphadenopathy and possibly systemic symptoms depending on the source of the metastasis.
- ** Reactive Hyperplasia or Infection (e.g., Tuberculosis, Mycobacterium avium complex)**: These infections can cause significant enlargement of lymph nodes and may require investigation to rule out infectious causes before considering a malignant diagnosis.

Given the complexity and potential for systemic involvement, thorough diagnostic workup including biopsies, imaging studies like PET-CT, and appropriate blood tests are essential in confirming the exact nature of the disease.

**32. user 2025-02-18T09:53:51Z:**

The main clinical manifestations are muscle atrophy, weakness and sensory loss of the limbs, mainly in the distal part of the lower limbs and gradually developing towards the proximal part of the limbs. Common clinical manifestations include poorer locomotor ability than people of the same age, difficulty in running, easy to twist the foot, foot drop, calf gastrocnemius muscle atrophy resembling a ‘crane leg’; examination can find bowed feet, hammertoes, distal limb weakness and atrophy, and loss of deep sensation. The disease usually starts before the age of 20 and progresses slowly. Later stages of the disease may severely affect activity, but rarely result in total disability and do not affect normal life expectancy. However, there are specific types that may have an early and severe onset

**qwen2.5_7b** 2025-02-18T09:53:55Z:

Based on the clinical manifestations provided, the most likely rare disease is **Charcot-Marie-Tooth Disease (CMT)**, specifically CMT type 1 or 2.

### Five Other Possible Diagnoses:

- **Friedreich’s Ataxia**: This is a neurodegenerative disorder characterized by progressive ataxia, scoliosis, and cardiomyopathy. While it primarily affects the cerebellum and spinal cord, peripheral neuropathy can also occur.
- **Hereditary Motor and Sensory Neuropathies (HMSN) - Type 3**: Also known as Dejerine-Sottas disease, this is a more severe form of CMT that typically presents with early-onset and rapid progression of symptoms.
- **Acquired Immunodeficiency Syndrome (AIDS)-Related Peripheral Neuropathy**: This can present with similar symptoms but often in conjunction with other signs of immunodeficiency such as recurrent infections or opportunistic illnesses.
- **Leber Hereditary Optic Neuropathy (LHON)**: Although primarily affecting vision, it can sometimes be associated with peripheral neuropathy and muscle weakness due to mitochondrial dysfunction.
- **Guillain-Barré Syndrome**: This is an acute inflammatory polyneuropathy that can present with sudden onset of limb weakness and sensory loss but usually has a more acute course compared to CMT.

These diagnoses are based on the clinical presentation described, which includes progressive muscle atrophy, weakness, and sensory loss in the limbs. The specific types of CMT (such as 1A or 2B) can vary widely in their genetic basis and severity, but Charcot-Marie-Tooth Disease is a common and well-recognized diagnosis for these symptoms.

**33. user 2025-02-18T09:54:48Z:**

The onset of the disease occurs from birth to several months. The onset of jaundice is usually within 2 months of age and rarely later than 5 months of age. The most common presentation is persistent jaundice after birth with or without light coloured stools and mild or no liver enlargement. There are many other non-specific clinical signs, including feeding difficulties, growth retardation, diarrhoea, hypoglycaemia and prolonged clotting time. Most of these symptoms and signs resolve spontaneously or with dietary modification and medication around 6 months to 1 year of age, with a small proportion of patients developing further disease after puberty. The majority of developed disease develops in adulthood. Delirium, confusion, and other mental abnormalities caused by hyperammonemia usually appear suddenly after drinking alcohol, consuming sweets, taking certain medications, or having an infection. In severe cases, the disease progresses rapidly and can be life-threatening without proper treatment. Most patients are slim, and most of them have obvious dietary preference, addicted to legumes, high protein, high fat food, and anorexic to high sugar.

**qwen2.5_7b** 2025-02-18T09:54:54Z:

Based on the clinical manifestations provided, the rare disease that is most likely to be diagnosed is **Classic Phenylketonuria (PKU)**.

Here are five other possible diagnoses: 1. **Glycogen Storage Disease Type I (von Gierke Disease)** 2. **Maple Syrup Urine Disease (MSUD)** 3. **Zellweger Spectrum Disorder (Peroxisomal Disorders)** 4. **Cystic Fibrosis (CF)** 5. **Homocystinuria**

### Detailed Explanation:

- **Classic Phenylketonuria (PKU)**: The key features of PKU include persistent jaundice, feeding difficulties, growth retardation, and the resolution of symptoms with dietary modification. Hyperammonemia can cause delirium or confusion, especially after consuming protein-rich foods like legumes.
- **Glycogen Storage Disease Type I (von Gierke Disease)**: This disease typically presents with hypoglycemia, hepatomegaly (liver enlargement), and growth failure. However, the description notes mild or no liver enlargement in PKU, which is not typical of von Gierke Disease.
- **Maple Syrup Urine Disease (MSUD)**: MSUD causes a characteristic odor in the urine similar to maple syrup due to branched-chain amino acid accumulation. Symptoms can include feeding difficulties, vomiting, and neurological symptoms like delirium or confusion. However, the timeline of symptom resolution does not match PKU.
- **Zellweger Spectrum Disorder (Peroxisomal Disorders)**: These are a group of metabolic disorders that affect the breakdown of certain fats and lead to progressive liver disease and neurological problems. The presentation with jaundice, growth retardation, and liver involvement is seen but the spontaneous resolution is not typical.
- **Cystic Fibrosis (CF)**: CF typically presents with respiratory symptoms, digestive issues like chronic diarrhea, and malnutrition. While there can be liver involvement in severe cases, it does not fit the pattern of rapid symptom resolution noted in PKU.
- **Homocystinuria**: This is a metabolic disorder where the body cannot properly process certain amino acids. It can lead to growth failure, cognitive issues, and other symptoms but does not typically present with the characteristic resolution timeline described for PKU.

Given these details, Classic Phenylketonuria (PKU) remains the most likely diagnosis due to the specific pattern of persistent jaundice, dietary preferences, and spontaneous symptom resolution.

**34. user 2025-02-18T09:55:57Z:**

Symptoms may appear after birth or may start in infancy to early adulthood. It is characterised by fluctuating muscle weakness and intolerance to fatigue, mostly in the extraocular muscles, facial muscles, medulla oblongata, limbs and respiratory muscles, and even life-threatening apnoeic episodes may occur in infancy. The simplex syndrome is characterised mainly by skeletal muscle weakness without congenital malformations. The overlapping syndromes of glycosylation defects and myopathies of the disease are often accompanied by congenital developmental abnormalities. Clinical manifestations vary between the different types, with some patients, usually born with proximal muscle weakness, cholinesterase inhibitors effective, and in severe cases, sudden respiratory distress and apnoea due to medullary palsy, which can be induced by stress, cold, and infection. Some patients, starting at birth or in infancy, may present with more severe, axially distributed muscle weakness, but the extraocular muscles may not be involved.

**qwen2.5_7b** 2025-02-18T09:56:01Z:

Based on the clinical manifestations you provided, this description is most consistent with **Glycogen Storage Disease Type 9 (GSD IX), also known as Andersen Disease**. GSD IX is a rare genetic disorder characterized by a deficiency of debranching enzyme, which leads to glycogen accumulation in muscle and liver tissues.

Here are five other possible diagnoses that could be considered based on the symptoms described:

- **Congenital Myasthenic Syndrome (CMS)**: This group of disorders involves neuromuscular junction defects leading to fluctuating muscle weakness, fatigue, and potentially respiratory involvement.
- **Myotonic Dystrophy**: Although primarily a type 2 presentation is more common with later onset, a variant can present in infancy or early childhood with muscle weakness and other symptoms.
- **Congenital Muscular Dystrophy (CMD)**: A group of diseases that include various genetic defects leading to muscle weakness, often with additional congenital anomalies like joint contractures.
- **Inherently Weak Syndrome (HWS)**: Also known as Central Core Disease, this condition involves muscle weakness and periodic paralysis due to abnormalities in the sarcoplasmic reticulum.
- **Lambert-Eaton Myasthenic Syndrome (LEMS)**: While typically an adult-onset disorder, some patients can present with early symptoms involving fluctuating muscle weakness, particularly affecting the eyes and face.

Each of these conditions presents unique clinical features, but given the specific mention of glycosylation defects, GSD IX remains a strong candidate.

**35. user 2025-02-18T09:56:46Z:**

- The disease usually starts in infancy or childhood, and in a few cases it starts in adulthood.
- Tonicity can occur throughout the entire body, including the skeletal muscles of the limbs, facial muscles, extraocular muscles, and lingual muscles.
- Tonicity is apparent during sudden movement after rest and is reduced by repeated movements, which is called the ‘warm-up’ phenomenon. Transient muscle weakness may occur at the start of a sudden movement. In patients with a longer course of the disease, there may be persistent weakness of the distal limbs, even involving the proximal limbs.
- Physical examination reveals that the patient is often muscular and has an athlete’s body shape. After clenching the fist vigorously, the fingers cannot be unfolded in time; after closing the eyes vigorously, they cannot be opened immediately. Knocking on the hand interosseous muscle, lingual muscle and other parts of the hand can cause persistent muscle contraction (percussive muscle ankylosis, ‘muscle ball’ phenomenon).

**qwen2.5_7b** 2025-02-18T09:56:51Z:

Based on the clinical manifestations you’ve provided, the disease most likely to be diagnosed is **Benign Exercise-Induced Muscular Hypercontraction (BEIMH)**, which is a rare form of periodic paralysis. The key features that point to this diagnosis include:

- **Age of Onset**: Starting in infancy or childhood but occasionally beginning in adulthood.
- **Tonicity and Weakness**: Tonic muscle contractions and weakness that can affect the skeletal muscles, including those responsible for facial expression, eye movement, and speech (extraocular and lingual muscles).
- **Warm-Up Phenomenon**: Reduced muscle tone with repeated movements and persistent distal muscle weakness.
- **Muscle Ankylosis**: The “muscle ball” phenomenon observed during physical examination, where voluntary movement of the fingers or eyes is delayed due to sustained muscle contraction.

### Five Other Possible Diagnoses:

- **Thomsen Disease (Periodic Paralysis)**: Another form of periodic paralysis that can present with similar symptoms, including muscle weakness and episodes of hyperkalemia-induced muscle stiffness.
- **Paramyotonia Congenita**: This is a channelopathy associated with cold-induced myotonia where muscles are stiff when first moved after rest, improving with activity or warmth.
- **Myasthenia Gravis**: A neuromuscular disorder characterized by fluctuating muscle weakness and fatigability that can involve ocular, bulbar, and limb muscles.
- **Lambert-Eaton Myasthenic Syndrome (LEMS)**: Often associated with tumors, especially small cell lung cancer, and presents with muscle weakness that improves with sustained activity.
- **Hyperthyroidism**: Can cause generalized weakness and myopathy, particularly in the proximal muscles of the limbs.

These diagnoses are based on similar clinical presentations to BEIMH but each has distinct characteristics and underlying mechanisms. A thorough diagnostic workup including genetic testing, electromyography (EMG), and blood tests would be necessary for a definitive diagnosis.

**36. user 2025-02-18T09:58:02Z:**

Secondary thoracic deformities caused by scoliosis can lead to cardiopulmonary hypoplasia, poor cardiopulmonary reserve, and may include cardiac displacement, arrhythmia, reduced lung capacity, pneumonia, etc. Abdominal deformities can cause gastrointestinal disorders, leading to dyspepsia and lack of appetite. In addition, the nerve root pulling caused by vertebral malformation and disc degeneration can produce irritating pain in the corresponding body parts, and in severe cases, it can cause spinal cord dysfunction or even paralysis.

Physical examination: the general manifestations are unequal shoulders when standing upright, one side of the lumbar folds, lumbar flexion with back asymmetry (‘razor back’), sometimes asymmetry on both sides of the pelvis, and gait abnormalities. In addition, neurological examination is also needed to observe whether the patient has increased or low muscle tone and whether there are sensory abnormalities in the limbs.

Clinically, it is usually classified into the following 3 types according to the imaging characteristics:

Type I - Vertebral formation disorder type, i.e., one or more vertebrae are partially or completely formed with disorder, such as wedge vertebrae, hemivertebrae or butterfly vertebrae;

Type II - poor vertebral segmentation type, i.e., two or more vertebrae are partially or completely segmented and impaired, e.g., blocked vertebrae, bone bridge formation, etc;

Type III - mixed type, where there is both vertebral body formation disorder and vertebral body mal-segmentation.

**qwen2.5_7b** 2025-02-18T09:58:06Z:

Based on the clinical manifestations provided, the most likely rare disease diagnosis is **Scoliosis**, specifically a severe case that leads to secondary complications such as cardiopulmonary hypoplasia and gastrointestinal disorders. Scoliosis can be further categorized based on the imaging characteristics into three types: Type I (vertebral formation disorder), Type II (poor vertebral segmentation), or Type III (mixed type).

### Five Other Possible Diagnoses:

- **Kyphoscoliosis**: This is a combination of scoliosis and kyphosis, which can lead to similar complications as described.
- **Congenital Scoliosis**: A form of scoliosis that develops in infancy or early childhood due to abnormalities in the development of the vertebrae.
- **Paraspinous Tumor**: Growth near the spine that can cause deformities and compress surrounding nerves, leading to pain and other symptoms similar to those described.
- **Tetralogy of Fallot with Pulmonary Hypertension**: While primarily a cardiac condition, it can lead to cardiopulmonary issues and may present some thoracic deformities.
- **Risser Sign Abnormality**: This can indicate skeletal maturity in adolescents but is not a primary disease; however, it could be relevant if the patient is undergoing evaluation for scoliosis treatment or monitoring.

Each of these conditions would require specific diagnostic tests and clinical evaluations to confirm the diagnosis. Imaging studies such as X-rays, MRI, and possibly CT scans are essential to determine the type of scoliosis and its severity, which will guide further management decisions.

**37. user 2025-02-18T09:58:43Z:**

There are more men than women. Some patients have atypical symptoms of chest pain, which are detected during coronary angiography or coronary CT. Patients are often clinically symptomatic when there is (1) a combination of obstructive atherosclerotic disease, thrombosis, slow flow, entrapment, or rupture with exertional angina or acute coronary syndromes; (2) compression of adjacent tissues; (3) rupture of an aneurysm, which is rare and can cause acute cardiac tamponade; and (4) myocardial ischemia due to microvascular dysfunction, which can occur even in the absence of significant coronary stenosis. The most common clinical manifestation is cardiac ischaemia.

The most common clinical manifestation is angina pectoris, and in a few patients, myocardial infarction, severe arrhythmia, or even sudden death may occur. The disease may break into the right atrium, right ventricle, or coronary sinus, creating a left-to-right shunt, which can be heard as a continuous murmur and, in severe cases, affect cardiac function. The most serious complication is rupture of the tumour into the pericardium, which can lead to bleeding, cardiac tamponade or even death.

**qwen2.5_7b** 2025-02-18T09:58:47Z:

Based on the clinical manifestations you provided, the rare disease that fits best appears to be **Familial Mediterranean Fever (FMF)**, but it seems there might be a mix-up with the symptoms described. The description of angina pectoris and chest pain in combination with atherosclerotic diseases, thrombosis, slow flow, entrapment, rupture, and myocardial ischemia due to microvascular dysfunction is more characteristic of **Coronary Microvascular Dysfunction (CMD)** or possibly **Prinzmetal’s Angina**. However, the mention of a continuous murmur and shunt suggests a condition involving cardiac tumors or aneurysms.

Here are five possible diagnoses based on the provided symptoms:

- **Coronary Microvascular Dysfunction (CMD)** - CMD can cause angina pectoris in patients without traditional coronary artery disease.
- **Prinzmetal’s Angina** - This is a type of variant angina where vasospasm causes chest pain, often occurring at rest and associated with microvascular dysfunction.
- **Coronary Aneurysm or Coronary Artery Fistula** - These can lead to symptoms similar to those described, including myocardial ischemia and the possibility of cardiac shunts.
- **Cardiac Tumors (e.g., Rhabdomyoma or Leiomyoma)** - These can cause a variety of symptoms, including angina, arrhythmias, and the potential for shunts that could explain the continuous murmur.
- **Chagas Disease** - This condition from Latin America can lead to cardiac dysfunction and may present with chest pain due to myocardial involvement.

Given the complexity and severity of some of the symptoms mentioned (such as sudden death, myocardial infarction, severe arrhythmias), a thorough clinical evaluation, including angiography, echocardiography, and possibly an MRI or CT scan, would be necessary to confirm the diagnosis.

**38. user 2025-02-18T10:01:04Z:**

- Bone marrow failure Anaemia is present at birth in 35 per cent of children and is often diagnosed between 2 weeks and 2 years after birth. The anaemia is macrocytic or normocytic orthochromatic, with decreased reticulocytes, normal or mildly decreased white blood cells, normal or mildly increased platelets, and hypoplasia of the erythroid lineage of the bone marrow and hyperproliferation of the granulocyte and megakaryocyte lineages.
- Congenital developmental anomalies 30% ~50% of patients may have congenital developmental anomalies, mainly involving the head, upper limbs, heart and genitourinary system. Examples include short stature, craniofacial anomalies including craniofacial hypertelorism, wide flat nose, small head, congenital cataracts, glaucoma, strabismus, high arched hard palate and cleft lip and palate or even Turner’s syndrome appearance, bunions, congenital cardiovascular anomalies, and genitourinary anomalies. The presence of physical abnormalities does not predict the severity of the patient’s haematological disorders. 30% of affected patients may present with postnatal growth retardation, often associated with other congenital malformations.
- The incidence of tumours in patients with increased susceptibility to cancer is about 4%, which is higher than in the normal population of the same age. And the tumour occurs at an early age. These include acute leukaemia of the haematological system, myelodysplastic syndromes, lymphomas and solid tumours such as osteosarcoma, thymic carcinoma, hepatocellular carcinoma, melanoma, fibrous histiocytoma, gastric cancer and intestinal cancer. The prognosis of the tumours is poorer than that of the general population suffering from the same tumour, with a higher mortality rate.

**qwen2.5_7b** 2025-02-18T10:01:10Z:

Based on the clinical manifestations provided, the rare disease most likely to be diagnosed appears to be **Dyskeratosis Congenita (DC)**, particularly the Hoyeraal-Hreidarsson variant. This condition is characterized by bone marrow failure, congenital anomalies, and increased susceptibility to cancer.

### Possible Diagnoses:

- **Dyskeratosis Congenita** - Specifically the Hoyeraal-Hreidarsson variant
  - Clinical Manifestations: Bone marrow failure with macrocytic or normocytic anemia, congenital developmental anomalies (e.g., short stature, craniofacial abnormalities), and increased susceptibility to cancer.
- **Shwachman-Diamond Syndrome** - While it primarily affects bone marrow function, it also includes digestive issues and skeletal abnormalities.
  - Clinical Manifestations: Bone marrow failure with pancytopenia, pancreatic insufficiency, short stature, and skeletal dysplasia.
- **Fanconi Anemia** - Characterized by progressive bone marrow failure, congenital anomalies, and increased risk of developing leukemia or other cancers.
  - Clinical Manifestations: Bone marrow failure leading to anemia, thrombocytopenia, pancytopenia, congenital malformations (e.g., limb defects), and predisposition to leukemia.
- **Ataxia Telangiectasia** - A rare genetic disorder that affects the nervous system, immune system, and other organs.
  - Clinical Manifestations: Progressive ataxia, telangiectasias, immunodeficiency, increased risk of cancer (especially lymphoma), and possible hematological abnormalities.
- **Chronic Benign Neutral Leukocytosis** - A rare condition characterized by persistent neutrophilia without symptoms or associated signs.
  - Clinical Manifestations: Elevated white blood cell count but with no clinical evidence of infection or other underlying disease, which does not fit well the presented profile but is listed for completeness.
- **Wiskott-Aldrich Syndrome** - An X-linked recessive disorder that affects the immune system and also has hematological manifestations.
  - Clinical Manifestations: Immune dysregulation (eczema, thrombocytopenia), recurrent infections, and increased risk of autoimmunity or certain cancers.

These conditions are all rare but align with some aspects of the clinical picture described. Dyskeratosis Congenita is particularly well-matched due to its overlapping features of bone marrow failure, congenital anomalies, and early onset cancer susceptibility.

**39. user 2025-02-18T10:01:39Z:**

The lesions may involve all systems of the body, so their clinical manifestations vary depending on the site of involvement. General symptoms include fever, malaise, night sweats, and weight loss. The rest of the affected systems mainly include:

- Skeletal system, most patients have skeletal involvement at the time of diagnosis, with lower limb bones being the most common, but only 50% of patients have bone pain.
- cardiovascular system, lesions involving the cardiovascular system, most patients do not have obvious clinical manifestations, but such as pericardial involvement, resulting in a large amount of pericardial effusion can lead to chest tightness, breathlessness, or even cardiac tamponade; lesions involving coronary arteries can cause myocardial ischemia-like manifestations, such as chest tightness, chest pain, etc.; diffuse myocardial involvement has also been reported, which can lead to cardiac failure-like manifestations; lesions encircling the renal arteries can cause renal hypertension; lesions encircling the mesenteric artery can cause renal hypertension; lesions encircling the mesenteric artery can cause renal hypertension. Diffuse myocardial involvement has also been reported, leading to heart failure-like symptoms; lesions encircling the renal arteries may cause renal hypertension; lesions encircling the mesenteric vessels may cause intestinal ischaemia, such as abdominal pain.
- respiratory system Most patients with respiratory system involvement are asymptomatic, but a few patients have dry cough and dyspnoea.
- central nervous system central nervous system involvement may appear corresponding clinical manifestations due to different parts of brain parenchyma involvement, such as limb movement disorder, gait abnormality, alteration of consciousness, etc.; spinal cord involvement may appear different clinical manifestations due to different affected segments; pituitary gland lesions may manifest polydipsia, polyuria, and other hormone secretion related manifestations.
- Retro-orbital lesions Retro-orbital involvement is one of the most common clinical manifestations, which may manifest as unilateral or bilateral proptosis, or even cause blindness.
- Retroperitoneal space/renal involvement. Retroperitoneal lesions may resemble retroperitoneal fibrosis and may cause unilateral or bilateral retrorenal obstruction if the lesion encircles the ureter.
- cutaneous involvement, the most common cutaneous lesion is a xanthomatous change, which occurs on the eyelids in about 1/3 of patients, and may also occur on the face, neck, axillae, trunk, and groin.

**qwen2.5_7b** 2025-02-18T10:01:45Z:

Based on the clinical manifestations you have provided, the rare disease that most closely matches these symptoms is **Multisystem Lymphoproliferative Disorder**, specifically a form resembling **Waldenström Macroglobulinemia** or potentially another lymphoproliferative disorder. However, without more specific blood work and imaging findings, this can only be an educated guess based on the symptomatology:

### Diagnosis: Potential Multisystem Lymphoproliferative Disorder (Waldenström Macroglobulinemia)

- **Multisystem Involvement**: The lesions involve multiple systems including skeletal, cardiovascular, respiratory, central nervous system, retro-orbital, cutaneous, and retroperitoneal spaces.
- **Common Symptoms**: Fever, malaise, night sweats, weight loss.
- **Skeletal Involvement**: Lower limb bone involvement with variable pain.
- **Cardiovascular Involvement**: Pericardial effusion, myocardial ischemia, renal hypertension.
- **Respiratory Involvement**: Asymptomatic or minor respiratory symptoms like dry cough and dyspnea.
- **Nervous System Involvement**: Central nervous system manifestations such as limb movement disorders, gait abnormalities, alteration of consciousness. Spinal cord involvement can lead to varied neurological deficits. Pituitary gland lesions causing endocrine changes.
- **Ocular Involvement**: Retro-orbital lesions leading to proptosis and potential blindness.
- **Renal and Cutaneous Involvement**: Retroperitoneal involvement mimicking retroperitoneal fibrosis, xanthomatous skin changes.

### Five Other Possible Diagnoses:

- **Hodgkin Lymphoma**: Given the multisystem involvement and systemic symptoms, this can be a potential consideration.
- **Systemic Vasculitis (e.g., Granulomatosis with Polyangiitis)**: This condition can involve multiple organ systems similarly as described.
- **Primary Amyloidosis**: Can present with widespread organ involvement and similar clinical signs including cardiovascular and renal manifestations.
- **Lymphoma of Unknown Primary Origin**: A diagnosis where the primary site is not initially identified but involves multiple organs.
- **Sarcoidosis**: Although typically granulomatous in nature, it can also involve a wide range of organs as described.

Each of these diagnoses would require further investigation including detailed clinical history, physical examination, imaging studies (such as MRI, CT scans), laboratory tests (including blood work and possibly biopsies), and sometimes specific antibody testing or genetic analysis to confirm.

**40. user 2025-02-18T10:02:18Z:**

The disease often presents with multi-organ and multi-system involvement, with males being more affected than female patients. Clinical manifestations often involve different organs as the patient grows older.

- Facial appearance, male patients usually appear at the age of 12-14 years with characteristic facial features, such as convexity of the supraorbital ridge, frontal bulge and thickening of the lips. 2.
- cutaneous angiokeratomas, which are common in patients with the classic form, are red patches of raised skin in the ‘sitting area’, i.e., between the umbilicus and knees, the scrotum, the buttocks, and the inner thighs.
- Neurological system, most patients will have peripheral nerve pain, which manifests as intolerable burning sensation on the soles of the feet and palms of the hands and radiates to the proximal limbs, and even painful spasms; autonomic nerve involvement manifests as little or no sweating; and central nervous system involvement manifests as early-onset transient ischemic attacks or ischemic strokes.
- Eye: Characteristic manifestations include conjunctival vascular tortuosity, corneal turbidity, posterior lens capsule clouding, and retinal vascular tortuosity, which in severe cases may lead to reduced or even loss of vision. It is often one of the main reasons for female patients to visit the clinic.
- Digestive tract, most often after eating nausea, vomiting, abdominal distension, cramping abdominal pain and diarrhoea, may also manifest as malabsorption or constipation.
- Kidney, early manifestation of urinary concentration dysfunction such as increased nocturia, polyuria and enuresis, with the progress of the disease can gradually appear proteinuria, and even up to the level of nephrotic syndrome, accompanied by renal function damage, mostly around 30 years old into the end-stage renal disease.
- Cardiovascular system, can be manifested as hypertension, myocardial ischemia caused by coronary artery involvement, heart valve lesions and hypertrophic cardiomyopathy, and in severe cases, it can be manifested as angina pectoris, myocardial infarction and heart failure. It is mostly a late manifestation of the disease and a major cause of death.

**qwen2.5_7b** 2025-02-18T10:02:24Z:

Based on the clinical manifestations you’ve described, this patient appears to have Fabry Disease, which is a rare X-linked genetic disorder that affects multiple organ systems due to the accumulation of glycolipids in cells.

### Most Likely Diagnosis: Fabry Disease

**Key Characteristics of Fabry Disease:** 1. Multi-organ and multi-system involvement. 2. More common in males than females. 3. Progressive nature as patients age, with diverse symptoms appearing at different ages. 4. Characteristic facial appearance (e.g., “angiookeratic facies”) often seen in older boys and young men. 5. Neurological issues like neuropathic pain, especially in the extremities. 6. Renal involvement leading to proteinuria, nephrotic syndrome, and renal failure. 7. Cardiovascular symptoms including hypertension and heart disease. 8. Gastrointestinal complaints such as abdominal pain, nausea, and vomiting.

### Other Possible Diagnoses:

- **Chronic Kidney Disease (CKD) with Genetic Underlying Causes**: Some cases of CKD may have a genetic basis that could present with similar renal manifestations to Fabry Disease but without the characteristic facial changes or neurological symptoms.
- **Fabry Disease Mimics**:
  - **Mucopolysaccharidosis Type I (Hurler Syndrome)**: Presents with skeletal abnormalities, cardiac issues, and developmental delays, but typically affects both males and females.
- **Chronic Progressive Inflammatory Demyelinating Polyneuropathy (CPIDP)**: Can present with painful neuropathies similar to those seen in Fabry Disease, though it is not linked to the X chromosome.
- **Alagille Syndrome**: A multisystem disorder affecting liver, heart, bones, and eyes, often presenting with distinctive facies but without the typical skin angiokeratomas or characteristic eye changes of Fabry Disease.
- **Nephrotic Syndrome with Underlying Genetic Causes**: Can present with proteinuria, edema, and renal failure similar to late-stage Fabry Disease but may have different initial clinical presentations.

Each of these conditions needs a specific diagnostic approach, including genetic testing for confirmation in cases where the clinical presentation is not definitive.

**41. user 2025-02-18T10:02:59Z:**

The disease is classified as Type 1 and Type 2.

- Type 1 is mainly characterised by recurrent inflammatory reactions and plasma membrane inflammation, including recurrent fever, abdominal pain and arthritis. Fever is recurrent and may be triggered by cold, strenuous exercise, surgery, infection, etc. and resolves spontaneously within 13 days. Abdominal pain is the most common clinical manifestation, seen in 95% of patients. It is a sudden onset of abdominal pain, often accompanied by fever, abdominal examination may have abdominal distension, abdominal pressure, rebound pain, muscle tension, and the loss of bowel sounds on auscultation. Stereotactic abdominal radiographs show flat air and fluid in the small intestine. Abdominal pain often resolves spontaneously after 24-48 hours. Arthritis is also a common clinical manifestation, seen in 75% of patients. It often involves the large joints of the lower limbs, such as the hip and knee joints. There may be joint swelling and pain. Rash is seen in 7% ~40% of patients, mainly involving the extensor side of the lower limbs, and the typical rash is erysipelas of tansy-like erythema. Pleurisy is seen in 45% of patients and presents as a sudden onset of unilateral chest pain, often accompanied by fever. Pericarditis is less common.
- Type 2 starts with amyloidosis. It presents with persistent proteinuria and often leads to end-stage renal disease. Amyloid can also be deposited in the heart, gastrointestinal tract, liver and other organs, leading to corresponding clinical manifestations.

**qwen2.5_7b** 2025-02-18T10:03:06Z:

Based on the detailed clinical manifestations provided, it sounds like you are describing a rare inflammatory disorder potentially related to **Primary Amyloidosis (Type 1) or Familial Mediterranean Fever (FMF)**. Here’s why:

### Type 1: Familial Mediterranean Fever (FMF)

- **Clinical Manifestations**:
  - Recurrent fever
  - Abdominal pain, seen in almost all patients
  - Joint inflammation, particularly affecting large joints like the hips and knees
  - Erythema nodosum (a skin manifestation) in some patients
  - Other symptoms include pleurisy and pericarditis

### Type 2: Primary Amyloidosis

- **Clinical Manifestations**:
  - Persistent proteinuria, often leading to end-stage renal disease
  - Organ involvement due to amyloid deposition (heart, liver, gastrointestinal tract)

Given the detailed descriptions:

- **Primary Amyloidosis**: Characterized by chronic inflammation and progressive organ damage due to amyloid deposits.
- **Familial Mediterranean Fever (FMF)**: A genetic disorder causing periodic inflammatory attacks, particularly affecting the abdomen and joints.

### Other Possible Diagnoses:

1. **Relapsing Polychondritis**: This condition involves recurrent inflammation of cartilaginous structures, leading to symptoms like arthritis, ear pain, eye problems, and nasal issues.
2. **Systemic Vasculitis (e.g., Giant Cell Arteritis, Polyarteritis Nodosa)**: Involves inflammation in blood vessels, causing various symptoms including fever, joint pain, and organ involvement.
3. **Cogan Syndrome (Neurolalicular Uveitis)**: A rare condition affecting the eyes and inner ear, presenting with uveitis and hearing loss, though it may not fit all the described features perfectly but can sometimes involve recurrent inflammation.
4. **Reactive Arthritis**: Often triggered by an infection, this condition leads to arthritis, conjunctivitis, urethritis, and skin lesions.

### Summary:

- **Primary Amyloidosis (Type 2)**
- **Familial Mediterranean Fever (FMF) (Type 1)**

**Other Potential Diagnoses:** 3. Relapsing Polychondritis 4. Systemic Vasculitis 5. Cogan Syndrome 6. Reactive Arthritis

Given the specific clinical features, Familial Mediterranean Fever is highly likely to be considered as Type 1, while Primary Amyloidosis fits well with the proteinuria and organ involvement described in Type 2.

**42. user 2025-02-18T10:04:03Z:**

The main manifestations are congenital developmental anomalies, progressive bone marrow failure and increased incidence of tumours, while some patients may show no developmental anomalies or bone marrow failure.

- Developmental anomalies, patients may show a variety of physical and mental developmental abnormalities. Growth defects, abnormal skin pigmentation, unilateral or bilateral skeletal malformations of the upper limbs, short stature, microcephaly, ocular anomalies, genitourinary malformations, endocrine anomalies, hearing loss, outer ear anomalies, congenital heart defects, digestive anomalies, abnormalities of the central nervous system, facial deformities, spinal deformities, neck deformities, and developmental delays and/or intellectual disabilities. These manifestations are mostly non-specific, with thumb and upper limb deformities being more specific.
- Progressive bone marrow failure with haematological abnormalities is the most serious clinical feature of Fanconi anaemia. It may present as a single or total haematopenia. The age of onset of haemopenia varies widely, even among siblings. The median age of onset is about 7.6 years, and it is rare in infants and young children; 90% of patients with haematological abnormalities develop before the age of 40 years. Thrombocytopenia or leukopenia usually precedes anaemia and is often accompanied by an increase in macrocytes, foetal haemoglobin (HbF 1), and a gradual increase in total haematopoiesis.
- There is an increased risk of neoplasms, including haematological malignancies and non-haematological malignant solid tumours. The risk of acute myeloid leukaemia (AML) is increased about 500-fold, and the cumulative incidence of AML is about 13% at the age of 50 years, with most cases occurring between the ages of 15 and 35 years. -7, del (7q) is associated with an increased incidence of myelodysplastic syndromes (MDS)/AML. Acute lymphoblastic leukaemia and lymphoma have also been reported. Solid tumours can be the first manifestation of the disease. Head and neck squamous cell carcinomas (HNSCCs) are the most common solid tumours in patients and have an earlier age of onset (20-40 years), mostly occurring in the oral cavity (e.g., tongue cancers), are mostly in the progressive stage, and respond poorly to treatment. There is also an increased incidence of skin, oesophageal, liver and genitourinary tract tumours. In a few patients, multiple tumours may be seen concurrently, or haematological and non-haematological tumours may be seen concurrently. And there is a marked intolerance to chemotherapy or radiotherapy.

**qwen2.5_7b** 2025-02-18T10:04:08Z:

Based on the clinical manifestations you’ve described, the most likely rare disease diagnosis is **Fanconi Anemia** (FA). The combination of congenital developmental anomalies, progressive bone marrow failure with hemopoenia, and an increased risk of tumors strongly points to this condition. Here are five other possible diagnoses that might present similarly:

- **Ataxia Telangiectasia (AT)**: Patients with AT often have a combination of neurologic symptoms like ataxia and cerebellar dysfunction, immunodeficiency, progressive failure of bone marrow function leading to anemia and/or immunodeficiency, and an increased risk of cancer.
- **Dyskeratosis Congenita**: This condition involves multiple organ systems, including the skin (telangiectasias), mucous membranes, and blood cells. Patients can have a variety of hematologic manifestations and an increased risk of cancers, especially those affecting the head and neck region.
- **Shwachman-Diamond Syndrome (SDS)**: This is characterized by exocrine pancreatic insufficiency, skeletal abnormalities, and growth failure in childhood. There’s also an increased incidence of hematological complications like neutropenia and thrombocytopenia, as well as a predisposition to certain cancers.
- **Hematopoietic Stem Cell Deficiencies (HSCL)**: This is a group of rare diseases characterized by variable clinical features including bone marrow failure, immunodeficiency, and developmental abnormalities. Some forms can present with similar hematological and neoplastic features.
- **Retinoblastoma Syndrome**: While primarily known for its association with retinal tumors, this condition can also have systemic manifestations including skeletal abnormalities, an increased risk of leukemia, and other solid tumors, making it a possible consideration in cases with overlapping symptoms.

Each of these conditions has distinct characteristics that need to be considered along with the patient’s history, physical examination, and diagnostic testing. Comprehensive genetic testing is often crucial for definitive diagnosis.

**43. user 2025-02-18T10:04:34Z:**

The perinatal onset of the disease is often typical, and within a few days of ingestion of breast milk or lactose-containing formula, the child develops life-threatening complications: feeding problems, diarrhoea, vomiting, hypoglycaemia, liver impairment, haemorrhage, jaundice, and cataracts. If left untreated, sepsis, shock and death may occur. Children who survive to infancy may develop severe brain damage if they continue to ingest lactose. Some children have long-term complications such as mental retardation, delayed growth, ataxia, blindness, and ovarian dysfunction in female patients.

**qwen2.5_7b** 2025-02-18T10:04:39Z:

Based on the clinical manifestations you’ve provided, the disease is most likely **Classic Galactosemia (Galactose-1-Phosphate Uridyltransferase Deficiency)**. Here are five other possible diagnoses to consider:

- **Zellweger Syndrome (Peroxisome Biogenesis Disorder Type 0)**: This is a severe form of peroxisomal biogenesis disorder that often presents with similar neurological and liver issues, but it usually has a more variable presentation and does not typically have the specific lactose intolerance and galactose-specific symptoms.
- **Lysinuric Protein Intolerance (LPI)**: This rare genetic disorder can present with symptoms like vomiting, diarrhea, and liver dysfunction following the ingestion of protein, particularly those containing lysine-rich proteins. However, LPI does not specifically target galactose or lactose as a trigger for all the symptoms.
- **Congenital Hypertyrosinemia (CHT)**: While it is rare, CHT can present with severe liver and kidney problems, vomiting, and other systemic issues, but it is more commonly associated with tyrosine metabolism issues rather than galactose or lactose.
- **Severe Combined Immunodeficiency (SCID) due to Adenosine Deaminase Deficiency**: SCID can present with life-threatening infections early in infancy, but the specific combination of symptoms including liver impairment and cataracts is less common without the characteristic immunological defects.
- **Crigler-Najjar Syndrome Type 1 (CN1)**: This condition involves severe unconjugated hyperbilirubinemia leading to kernicterus and brain damage, but it does not typically include the gastrointestinal issues or specific lactose intolerance seen in galactosemia.

Each of these conditions can present with significant complications early in life, but the combination of symptoms, particularly those related to galactose metabolism and the specific pattern of organ involvement, points strongly towards classic galactosemia.

**44. user 2025-02-18T10:05:32Z:**

- Type I is the most common subtype. There is no primary central nervous system involvement, but some patients may develop secondary neurological manifestations (e.g., spinal cord compression) as the disease progresses. The disease can develop in all age groups, with about 2/3 of patients developing in childhood. Organ manifestations are mainly hepatosplenomegaly, especially splenomegaly, often accompanied by hypersplenism, and even splenic infarction and splenic rupture. The haematological manifestations are mainly thrombocytopenia and anaemia, and some patients have leucopenia, which may be accompanied by coagulation abnormalities. Patients present with pallor, fatigue, skin and gum bleeding, increased menstruation, and even life-threatening bleeding. Most patients have skeletal invasion, but the severity varies. Early invasion sites mainly include lumbar vertebrae, long bone epiphyses and diaphyses, and in the middle and late stages, epiphyses and bony prominences may be involved. Patients often experience acute or chronic diffuse bone pain, and in severe cases, osteonecrosis (ischaemic necrosis), followed by joint collapse involving the proximal and distal femur, proximal tibia and proximal humerus. Osteolytic lesions, pathological fractures, vertebral compression fractures and other fragility fractures due to reduced bone density may also occur. Skeletal lesions can interfere with daily activities and can be disabling. In children, the most common manifestations are osteoporosis, flask-like deformity of the metaphysis of the long bones, varying degrees of hypodensity of the metaphysis of the long bones, thinning of the bone cortex, etc. There may be growth retardation. Some patients may have lung involvement, which is mainly manifested as interstitial lung disease, pulmonary solid changes, and pulmonary hypertension. In addition, patients may have abnormalities of sugar and lipid metabolism, increased risk of malignant tumours such as multiple myeloma, cholelithiasis, and abnormalities of the immune system.
- Type II, in addition to hepatosplenomegaly, anaemia, thrombocytopenia and other manifestations similar to those of type I, patients with type II mainly have acute neurological involvement. It often develops in the neonatal period to infancy, progresses rapidly and has a high morbidity and mortality rate. There are rapidly progressive medullary palsy, dyskinesia, seizures, angular reflexes and cognitive deficits and other signs of acute neurological damage, and growth is retarded, with death occurring before the age of 2 to 4 years. Some patients with severe disease may develop joint contractures.
- The early manifestations of type III are similar to those of type I, with gradual onset of neurological involvement, which often develops in childhood and has a slow progression of disease and a longer life expectancy. The patients often have the invasion of the motor nerve, eye movement disorders, ataxia, anisocoria, epilepsy, myoclonus, accompanied by developmental delays and intellectual backwardness. Type III can be divided into three subtypes, namely, type IIIa, which is characterised by more rapidly progressive neurological symptoms (ocular motility disorders, cerebellar ataxia, spasticity, myoclonus, and dementia) and hepatosplenomegaly; type IIIb, which is characterised by hepatosplenomegaly and skeletal symptoms with fewer CNS symptoms; and type IIIc, which is characterised by milder symptoms, including calcification of the cardiac valves and corneal clouding, and is predominantly found in the Druze population. Type IIIc.

**qwen2.5_7b** 2025-02-18T10:05:37Z:

Based on the clinical manifestations provided, the rare disease that matches these descriptions most closely is **Alpha 1-Antitrypsin Deficiency (AATD)**. This condition can present with a variety of symptoms ranging from liver and lung diseases to skeletal abnormalities and neurological issues depending on the specific subtype.

### Five Other Possible Diagnoses:

- **Gaucher Disease**
  - Gaucher disease is an inherited disorder characterized by the accumulation of glucocerebroside in macrophages, leading to hepatosplenomegaly, bone complications (osteopenia, osteoporosis), and occasionally neurological involvement. The type 3 variant can present with more severe neurological manifestations.
- **Fabry Disease**
  - This is a lysosomal storage disease caused by the deficiency of alpha-galactosidase A. It typically presents with renal, cardiac, and cerebrovascular complications as well as peripheral neuropathy and gastrointestinal symptoms.
- **Hypophosphatasia**
  - Hypophosphatasia is a rare metabolic disorder characterized by defective activity of alkaline phosphatase in various tissues leading to bone disease (osteomalacia, osteopetrosis) and soft tissue calcification. It can cause progressive skeletal deformities and may have neurological involvement.
- **Cystinosis**
  - Cystinosis is an inherited metabolic disorder caused by a deficiency of the cystinosin protein in lysosomes leading to excessive accumulation of cystine crystals, primarily affecting the kidneys but also involving the eyes, liver, muscles, and brain.
- **Scheie Syndrome (Mild Hurler Syndrome)**
  - This is one of the milder forms of Mucopolysaccharidosis type I (MPS I). Patients with Scheie syndrome typically have mild to moderate skeletal abnormalities and may develop corneal clouding, but they usually do not have severe cognitive or neurological impairment.

These conditions can present with overlapping symptoms, especially in terms of hematological, skeletal, and sometimes neurological manifestations. However, the specific combination described for each type aligns most closely with Alpha 1-Antitrypsin Deficiency (AATD).

**45. user 2025-02-18T10:06:54Z:**

It often starts in one muscle group and progresses to others, with ptosis and diplopia being the most common onset, accounting for 80 per cent of cases. Skeletal muscle weakness is fluctuating and easily fatigued, with a light morning and heavy evening. According to the clinical manifestations, it is divided into five types. Type I: ocular muscle type, manifested by ptosis and diplopia. Type ⅡA: mild generalised type, with mild involvement of the limb muscle groups. Type IIB: Moderate generalised type with moderate involvement of the limb muscle groups, usually with mastication, swallowing and dysarthria. Type III: Severe aggressive type with rapid progression, involving the pharyngeal muscles within weeks or months and the respiratory muscles within six months. Type IV: Late-onset severe type, slowly progressive, involving the respiratory muscles within 2 years. Type V: myasthenia gravis, skeletal muscle atrophy within six months of disease onset. Neurological examination can be seen in the proximal limbs, trunk, neck, craniofacial muscle weakness, intolerance to fatigue, Jolly test is positive, tendon reflexes are normal, no sensory deficits, no pathological signs. Some patients may progress to respiratory failure and develop myasthenia gravis crisis.

**qwen2.5_7b** 2025-02-18T10:06:59Z:

Based on the clinical manifestations provided, the most likely diagnosis is **Myasthenia Gravis (MG)**. The characteristics of fluctuating muscle weakness, particularly affecting ocular muscles initially, followed by progressive involvement of other skeletal muscles, with a positive Jolly test, are highly indicative of MG.

### Five Other Possible Diagnoses:

- **Lambert-Eaton Myasthenic Syndrome (LEMS):**
  - This condition typically presents with muscle weakness and fatigue that worsens with activity and improves with rest.
  - Unlike MG, LEMS often affects distal muscles more than proximal ones.
- **Chronic Inflammatory Demyelinating Polyneuropathy (CIDP):**
  - CIDP is characterized by progressive or relapsing weakness of the peripheral nerves, which can also show muscle atrophy.
  - It usually presents with symmetric limb weakness and sensory disturbances.
- **Congenital Myasthenic Syndromes:**
  - These are genetic disorders that affect the neuromuscular junction similar to MG but occur from birth or early childhood.
  - They often present with severe weakness, especially in the neonatal period, and can have distinctive inheritance patterns.
- **Inclusion Body Myositis (IBM):**
  - IBM is a chronic inflammatory myopathy affecting adults, particularly males over 50 years old.
  - It typically presents with progressive muscle weakness of the quadriceps, hand grip, and fingers without preceding muscle fatigability.
- **Botulism:**
  - This rare condition results from toxins produced by Clostridium botulinum affecting the neuromuscular junction.
  - Symptoms include generalized muscle weakness, diplopia, dysphagia, and respiratory muscle involvement leading to respiratory failure.
  - Diagnosis is based on a history of food or wound contamination.

Each of these conditions has distinct clinical features that differentiate them from MG. The final diagnosis would be confirmed through various tests including serum autoantibody testing for acetylcholine receptor antibodies (common in MG), electromyography, and nerve conduction studies.

**46. user 2025-02-18T10:07:27Z:**

The disease often starts in adolescence or early adulthood. Clinical manifestations are mainly related to hypokalemia and hypomagnesemia. Mild patients may be asymptomatic or show mild fatigue and poor appetite; severe patients may have twitching of limbs, flaccid paralysis, painful spasms, fainting, and transverse myelinolysis secondary to acute renal injury, and may even suffer from cardiac arrest due to severe ventricular arrhythmias. The common clinical manifestations are as follows:

- systemic symptoms fatigue, thirst, polydipsia, salt craving.
- Neuro-muscular system Muscle weakness, painful spasms, convulsions, convulsive seizures, numbness of limbs, sensory abnormalities, rhabdomyolysis, paralysis, dizziness, vertigo, ataxia, and pseudotumor cerebri.
- Cardiovascular system Palpitation, syncope, normal or low blood pressure and ventricular arrhythmia, etc.
- Digestive system Constipation, vomiting, etc.
- Urinary system Polyuria, nocturia, enuresis, proteinuria and renal insufficiency.
- Bone and joint system Arthralgia, cartilage calcium sedimentation.
- endocrine and growth retardation, delayed puberty; diabetes mellitus or impaired glucose tolerance is not uncommon in patients with chronically low potassium and magnesium. 8. ocular symptoms.
- Ocular symptoms Blurred vision and scleral choroidal calcification may occur in a small number of patients.

**qwen2.5_7b** 2025-02-18T10:07:32Z:

Based on the clinical manifestations you have provided, the disease most likely to be diagnosed is **Adolescent Benign Myoclonic Epilepsy with Chvostek’s Sign and Periodic Paralysis (AIMEC)**, also known as benign myoclonus-renal syndrome. This condition involves periodic paralysis, hypokalemia, and hypomagnesemia, which align closely with the symptoms described.

However, given the wide range of clinical manifestations you’ve provided, here are five other possible diagnoses to consider:

- **Mg-K-Associated Periodic Paralysis (MKPP)**: This condition is characterized by episodes of weakness and paralysis associated with fluctuations in serum magnesium and potassium levels.
- **Thonney Syndrome**: A rare condition that includes periodic paralysis, neuromuscular symptoms, and other systemic manifestations such as renal dysfunction, which fits the clinical profile provided.
- **Pseudohypoparathyroidism (Type 1A, 1B)**: This endocrine disorder can present with muscle weakness, growth retardation, cognitive impairment, and other symptoms like those described, though it typically includes specific resistance to parathyroid hormone.
- **Familial Hypokalemic Periodic Paralysis**: This is a genetic condition characterized by recurrent episodes of weakness, low potassium levels (hypokalemia), and can include neurological symptoms such as twitching and paralysis.
- **Gitelman Syndrome**: An inherited disorder that primarily affects the kidneys and involves hypokalemia, hypomagnesemia, and hypocalciuria with normal to slightly elevated renin activity, which could present with some of the symptoms you mentioned, especially in a chronic context.

Each of these conditions has unique characteristics but can overlap with the clinical picture described. Diagnostic confirmation would typically involve detailed genetic testing, blood electrolyte levels, muscle biopsy, and other specialized tests to distinguish between them.

**47. user 2025-02-18T10:08:01Z:**

The majority of children develop the disease in infancy or early childhood, with a variety of presentations. The earlier the onset, the more severe the symptoms and the worse the prognosis. The majority of patients have a relatively normal presentation in the first year of life. The earliest sign that appears in about 75% of patients is a large head size, and most children are born with a larger head circumference than their peers or have a rapid increase in head circumference soon after birth, which may be accompanied by mild non-specific symptoms, including irritability, difficulty in feeding, and vomiting. Children usually develop ketosis, vomiting, hepatomegaly and acute encephalopathic crisis, including hypotonia, loss of consciousness and convulsive seizures, between 3 and 36 months of age after fever, infection, surgery, or vaccination, which may resolve with symptomatic treatment, but not fully recover. If acute encephalopathic crisis occurs repeatedly, the neurological damage will be progressively aggravated, and there may be developmental regression, and eventually cognitive dysfunction may occur. In some children, delayed movement, dystonia, and random movement disorders occur gradually over the first few years of life, but mental development is almost normal. Patients often die within 10 years of age from concomitant diseases or Reye-like seizures, which decrease with age. Rarely, the onset of the disease occurs during adolescence or even adulthood, and may be asymptomatic until the first episode.

**qwen2.5_7b** 2025-02-18T10:08:05Z:

Based on the clinical manifestations provided, the most likely diagnosis is **Hyponatremic Encephalopathy Due to Syndrome of Inappropriate Antidiuretic Hormone Secretion (SIADH) with Head Growth Abnormalities**, which often manifests as a condition known in the medical literature as **Cerebral Salt-Wasting Syndrome** (CSS) or similar conditions that present with abnormalities in head growth and recurrent encephalopathic crises. However, given the specific details of the clinical course and prognosis, the disease most likely fits the description of **Mucopolysaccharidosis Type I (MPS I)**, specifically the severe form.

Here are five other possible diagnoses:

- **Mucopolysaccharidosis Type I (MPS I)**: As mentioned above, this is a severe lysosomal storage disease characterized by progressive neurological deterioration and developmental regression with head growth abnormalities.
- **Cerebral Edema Due to Reye’s Syndrome**: This condition can present similarly, especially in the acute encephalopathic crisis phase, but typically follows specific risk factors like aspirin use during viral infections.
- **Severe Congenital Hyperammonemia (e.g., Argininosuccinic Aciduria or Citrullinemia)**: These conditions can cause neurological dysfunction and episodes of encephalopathy due to metabolic disturbances.
- **Metachromatic Leukodystrophy (MLD)**: This is a lysosomal storage disorder that causes progressive neurodegeneration, often with early symptoms including developmental delay and enlarged head circumference.
- **Sanfilippo Syndrome**: A form of mucopolysaccharidosis characterized by progressive cognitive decline and behavioral issues, which might present with similar neurological manifestations over time.

Each of these conditions has distinct biochemical and clinical features that can help in differential diagnosis once specific tests are performed.

**48. user 2025-02-18T10:09:02Z:**

It mainly manifests as bleeding from joints, muscles and deep tissues, but also internal organs such as gastrointestinal tract and central nervous system. If repeated bleeding occurs, untreated bleeding can lead to joint deformity and/or pseudotumour formation, which can be life-threatening in severe cases. Delayed haemorrhage after trauma or surgery is characteristic of this disease.

**qwen2.5_7b** 2025-02-18T10:09:06Z:

Based on the clinical manifestations provided, the rare disease that most likely fits this description is **Hemophilia**. Hemophilia is a genetic disorder characterized by a deficiency in clotting factors, primarily factor VIII (hemophilia A) and less commonly factor IX (hemophilia B), which leads to excessive bleeding.

Here are five other possible diagnoses that could also present with similar symptoms:

- **von Willebrand Disease (vWD)**: This is the most common hereditary bleeding disorder. It involves a defect in von Willebrand factor, which can lead to prolonged bleeding episodes and can affect multiple sites including joints, muscles, and internal organs.
- **Disseminated Intravascular Coagulation (DIC)**: While typically associated with underlying diseases such as sepsis or cancer, DIC can present with spontaneous bleeding due to the consumption of clotting factors and platelets leading to microthrombi formation throughout the body.
- **Lupus Erythematosus (SLE) - Hemarthrosis Variant**: In some severe cases of SLE, particularly those involving vasculitis, patients may experience recurrent joint hemorrhage and bleeding into deep tissues.
- **Hereditary Telangiectasia with Epistaxis (Osler-Weber-Rendu Syndrome)**: This condition is primarily characterized by telangiectasias but can also involve spontaneous bleeding from various sites, including the joints, muscles, and internal organs.
- **Mucopolysaccharidosis (MPS) Types**: These are a group of lysosomal storage diseases that can present with musculoskeletal abnormalities, joint involvement, and bleeding due to tissue damage and inflammation.

Given the severity and specific nature of the symptoms you’ve described, hemophilia is highly likely. However, a thorough diagnostic evaluation including genetic testing, laboratory assessments (such as clotting factor levels), imaging studies, and other clinical evaluations would be necessary for an accurate diagnosis.

**49. user 2025-02-18T10:09:36Z:**

It can involve multiple organs of the body and has a variety of clinical manifestations. The age of onset is mostly 3-60 years old, and there are also reports of patients diagnosed at 8 months and over 70 years old. In children, hepatic involvement is the first manifestation of the disease, while in adolescents and adults, neurological involvement is the first manifestation of the disease.

It may present with asymptomatic persistent elevation of aminotransferases, chronic hepatitis, cirrhosis, and acute liver failure. Neurological involvement may manifest as motor dysfunction, tremor, ataxia, chorea, dysautonomia, dystonia, and in some patients masked face, stiff limbs, and gait abnormalities. Some patients combine with mental behavioural abnormalities such as apathy, aggressive behaviour, and personality changes. Kidney involvement may manifest as haematuria, proteinuria, etc. Involvement of the blood system may be manifested as haemolytic anaemia, liver cirrhosis, splenic hyperfunction leading to a decrease in the blood tertiary system, and coagulation abnormalities. Relatively rare systems involved include bone and joint, heart, endocrine and reproductive systems.

**qwen2.5_7b** 2025-02-18T10:09:41Z:

Based on the clinical manifestations you have described, this condition is most likely to be Wilson’s Disease (also known as Hepatolenticular Degeneration), a rare genetic disorder that primarily affects the liver and brain. Here are five other possible diagnoses:

- **Wilson’s Disease** - As previously mentioned, it is characterized by copper accumulation in various organs such as the liver, brain, kidneys, and cornea leading to neurological, hepatic, and renal symptoms.
- **Neonatal Hemochromatosis** - A rare genetic disorder that affects infants, causing excessive iron absorption, leading to organ damage including liver, heart, and pancreas issues.
- **Alpha-1 Antitrypsin Deficiency** - A hereditary condition where the body does not produce enough of a protein (alpha-1 antitrypsin) which helps protect the lungs and liver from damage.
- **Copper Toxicity or Wilson’s Disease Mimickers** - Conditions that may mimic Wilson’s disease but are due to excess copper in the diet, genetic disorders affecting copper metabolism other than Wilson’s, or secondary copper accumulation due to conditions like chronic hepatitis or cirrhosis.
- **Hepatitis C Virus (HCV) Infection** - Chronic HCV can lead to a wide range of liver diseases and may present with symptoms such as fatigue, jaundice, hepatic dysfunction, and in some cases, neurological manifestations secondary to liver failure.
- **Autoimmune Hepatitis** - An autoimmune condition that can cause inflammation of the liver leading to various clinical features including elevated liver enzymes, jaundice, and sometimes neurological symptoms due to liver dysfunction.

These conditions need to be ruled out or considered based on a detailed medical history, physical examination, laboratory tests (such as liver function tests, serum copper levels, ceruloplasmin levels, genetic testing for Wilson’s disease), and specific imaging or other diagnostic tools.

**50. user 2025-02-18T10:10:07Z:**

The clinical picture is very heterogeneous, even within the same family. The disease usually begins before the age of 30 years, worsens during adolescence, and the oedema often has an acute onset. The clinical picture is characterised by recurrent, unpredictable cutaneous and submucosal oedema. The oedema is episodic, self-limited, usually resolves spontaneously in 3-5 days, asymmetric and non-concave. Oedema can involve any part of the body, with the extremities, face, genitals, respiratory tract and gastrointestinal tract mucosa being the most common. The most fatal is respiratory mucosal oedema, which can rapidly progress to respiratory distress or asphyxiation due to laryngeal oedema, and can lead to death by asphyxiation if not resuscitated in time, with a lethality rate as high as 11%-40%, which is one of the main causes of death. Episodes of mucosal oedema of the digestive tract manifest as severe abdominal pain, accompanied by nausea and vomiting, and are therefore often misdiagnosed as acute abdomen, leading to unnecessary abdominal surgery. Skin oedema occurring in the limbs and other parts of the body can affect its function.

**qwen2.5_7b** 2025-02-18T10:10:11Z:

Based on the clinical manifestations you’ve provided, the most likely diagnosis is Hereditary Angioedema (HAE). HAE is a rare genetic disorder that causes recurrent episodes of swelling, primarily affecting the skin but also involving mucosal layers such as the respiratory and gastrointestinal tracts. The heterogeneity of the disease within families and its typical onset before age 30 align with known characteristics of HAE.

Here are five other possible diagnoses to consider:

- **Acute Angioedema**: This can occur due to various triggers including foods, medications, or infections, but it typically does not have a familial pattern seen in HAE.
- **Angioedema Due to C1-Inhibitor Deficiency (Hereditary Angioedema)**: This is the primary diagnosis based on your description, given its episodic nature and involvement of both cutaneous and mucosal oedema.
- **Idiopathic Angioedema**: This can occur without a known genetic cause and often presents with similar symptoms but does not typically have a familial history.
- **Allergic Angioedema**: While this can present similarly, it is usually triggered by an allergic reaction and tends to be less severe in frequency and intensity than HAE episodes.
- **Traumatic Angioedema**: This can occur due to trauma or physical injury, but the recurrent nature and lack of a clear history of trauma make this less likely.

Given the severity and potential lethality associated with respiratory oedema, it is crucial to confirm the diagnosis through genetic testing for C1 inhibitor deficiency, which would be specific for HAE.

**51. user 2025-02-18T10:11:10Z:**

The disease starts at birth and the severity of clinical manifestations is related to the subtype of the disease. Clinical manifestations include blisters or maculopapular blisters on the hands, elbows, knees and feet and other areas prone to friction, or around the body. Blisters or ulcers of the oral mucosa may occur, leading to dysphagia and hoarseness. Some patients have mottled pigmentation abnormalities, baldness, palmoplantar hyperkeratosis, enamel defects, nail thickening and nail dystrophy. Other impairments include disfigurement of the digits due to repeated scar formation, anaemia, osteoporosis, muscle atrophy, cardiomyopathy, and deafness. Some subtypes may be associated with basal cell carcinoma, squamous cell carcinoma, and malignant melanoma in advanced stages.

**qwen2.5_7b** 2025-02-18T10:11:15Z:

Based on the clinical manifestations you’ve described, this patient’s symptoms align closely with **Epidermolysis Bullosa (EB)**, specifically a form of Junctional Epidermolysis Bullosa (JEB). Here are five other possible diagnoses to consider:

- **Epidermolysis Bullosa (EB):**
  - Subtypes like Dystrophic EB or Junctional EB could fit the clinical picture given the presence of blisters, mucosal involvement, and systemic complications.
- **Linear IgA Bullous Dermatosis:**
  - This condition involves blistering on areas that are prone to friction, similar to what is seen in EB but typically does not involve oral mucosa or systemic symptoms as severely.
- **Pemphigus:**
  - A group of autoimmune bullous diseases where blisters form due to autoantibodies targeting desmoglein proteins leading to blister formation. It can affect mucous membranes and skin, though it usually does not present with the severe musculoskeletal and systemic involvement seen here.
- **Stevens-Johnson Syndrome (SJS) or Toxic Epidermal Necrolysis (TEN):**
  - These are acute dermatological conditions resulting from hypersensitivity reactions that can affect mucous membranes, skin, and may have some systemic manifestations but typically do not present with the chronic and multifaceted nature of EB.
- **Cockayne Syndrome:**
  - This rare genetic disorder can cause a wide array of symptoms including neurological problems, growth retardation, and photosensitivity leading to skin issues like blisters, though it does not usually involve mucosal involvement or the systemic complications described here as extensively.

Given the detailed clinical manifestations, EB is most likely the primary diagnosis, with these other conditions being less probable based on the comprehensive nature of the symptoms. A biopsy for direct evidence of blistering, immunopathological studies, and genetic testing would be crucial in confirming the exact subtype of EB.

**52. user 2025-02-18T10:12:29Z:**

The clinical presentation of patients varies with age, amount and duration of fructose exposure. In general, the younger the age of onset, the more severe the symptoms. The onset of the disease is typically around 6 months of age and usually occurs when fructose-containing foods, such as fruits, honey and certain vegetables, are first consumed. Common clinical symptoms include hypoglycaemic episodes after eating fructose-containing foods, with nausea, vomiting, abdominal pain, bloating, sweating, tremors, convulsions and even coma.

If the diagnosis is not clear and the patient chronically ingests fructose foods for a long period of time, in addition to hypoglycemic manifestations, growth retardation, hepatomegaly, ascites, jaundice and metabolic disorders such as hypoglycemia, metabolic acidosis, hypophosphatemia, hyperuricemia, hypermagnesemia, and hyperpyruvatemia may occur. In severe cases, multiple organ damage, liver and renal failure, convulsions, coma, and even death occur.

In acute attacks, laboratory tests reveal hypoglycaemia, hypophosphatemia and hypokalemia, accompanied by elevated uric acid, lactate, pyruvate, free fatty acids and triglycerides. Chronic patients present with hepatic impairment and indications of hepatic fibrosis, including serum bilirubin, elevated transaminases, and coagulation disorders. Urinalysis reveals proteinuria and fructose positivity.

**qwen2.5_7b** 2025-02-18T10:12:35Z:

Based on the clinical manifestations described, the most likely rare disease is **Hereditary Fructose Intolerance (HFI)**, which is caused by a deficiency of the enzyme aldolase B, leading to the accumulation of fructose-1-phosphate. Here are five other possible diagnoses that might need to be considered:

- **Glycogen Storage Diseases (GSDs)**: Depending on the specific subtype, symptoms can include hypoglycemia, fatigue, and growth retardation similar to those seen in HFI.
- **Phosphofructokinase Deficiency**: This is a rare inherited metabolic disorder that affects red blood cells but can sometimes present with metabolic acidosis and other systemic signs due to altered metabolism of fructose and glucose.
- **Hereditary Glucose-6-Phosphate Dehydrogenase (G6PD) Deficiency**: While primarily affecting hemolysis under oxidative stress, severe cases might present with more general metabolic derangements including those seen in HFI, especially if there is an interaction between G6PD and fructose metabolism.
- **Fructose Malabsorption or Fructose Intolerance (Non-Enzymatic)**: This condition can mimic some symptoms of HFI but typically presents without the severe systemic signs and does not require a genetic defect; rather, it involves poor absorption of fructose.
- **Diabetic Ketoacidosis (DKA) with Severe Hyperglycemia**: Although this is more commonly associated with diabetes, in rare cases where glucose metabolism is severely impaired and there are other metabolic abnormalities, symptoms might be misattributed to a different condition like HFI if not properly differentiated based on history of dietary fructose exposure.

In each case, the specific clinical presentation would guide further diagnostic testing such as enzyme assays (e.g., aldolase B for HFI), genetic testing, and metabolic panel analysis.

**53. user 2025-02-18T10:13:29Z:**

**1. Migraine** Migraine with aura is often the first symptom of the disease and is seen in 20-40% of patients, which is five times higher than in the general population; it can also manifest as migraine without aura, with an incidence not different from that of the general population. The incidence of migraine is lower in Asian patients than in European and American patients.

**2. Subcortical ischemic events** Transient ischemic attacks (TIAs) and ischemic strokes are the most common clinical manifestations of the disease, occurring in 60% to 85% of symptomatic patients, most of whom lack common risk factors for cerebrovascular disease. 2/3 of patients present with the classic lacunar syndrome.

**3. Cognitive deficits** Cognitive dysfunction with dementia develops progressively in 60% of patients.

**Affective disorders and apathy** 20% of patients have affective disorders, with depressive states being the most common, and rarely alternating between depressive and manic states; apathy is seen in 40% of patients.

In addition, seizures occur in 5-10% of patients, and hearing loss, impaired consciousness, spinal cord infarction, intracranial haemorrhage, and combined basin infarction have also been reported.

**qwen2.5_7b** 2025-02-18T10:13:36Z:

Based on the clinical manifestations provided, it seems most likely that the disease being described is **Lewy Body Dementia (LBD)**. LBD is a type of dementia characterized by cognitive decline, visual hallucinations, Parkinsonism, and fluctuations in alertness or attention. The symptoms you’ve listed are highly consistent with LBD:

- **Migraine**: While migraines can occur in the general population, their frequency and specific association with LBD are not prominent enough to be the primary focus here.
- **Subcortical ischemic events** (including TIAs and strokes): These are more commonly associated with vascular dementias such as **Vascular Dementia**, but the prominence of these symptoms in your list is less characteristic compared to other diagnoses.
- **Cognitive deficits**: The progressive cognitive decline seen in LBD aligns well, especially when combined with affective disorders.

### Five Other Possible Diagnoses

Given the clinical manifestations provided, here are five additional rare diseases that should be considered:

- **Parkinson’s Disease Dementia (PDD)**: Parkinson’s disease can progress to a dementia phase, which would explain the cognitive deficits and parkinsonian symptoms.
- **Progressive Supranuclear Palsy (PSP)**: PSP is associated with difficulties with balance, gait, eye movements, and progressive cognitive decline. The presence of lacunar infarctions could also be relevant.
- **Corticobasal Syndrome (CBS)**: CBS involves a combination of movement disorders, cognitive decline, and various other neurological symptoms that might fit the described clinical picture.
- **Frontotemporal Dementia (FTD)**: FTD is characterized by changes in behavior and personality early on, along with language or motor difficulties. The mention of affective disorders and apathy could be relevant here.
- **Chronic Autoimmune Encephalitis**: This condition can present with a wide range of neurological symptoms including cognitive decline, seizures, and mood disturbances, which might overlap with the described symptoms.

Each of these diagnoses requires further investigation through detailed history taking, clinical examination, and possibly specific neuroimaging or laboratory tests to differentiate them accurately.

**54. user 2025-02-18T10:14:27Z:**

The onset of the disease is usually insidious in early youth (but has been reported from 1 to 76 years of age). The main manifestation is a slowly progressive spastic paralysis of both lower limbs, with pyramidal signs (hyperreflexia, positive ankle clonus, positive pathology). Bilateral upper limb reflexes may also be hyperreflexic, but generally do not affect upper limb activity. More than half of the patients will have proximal weakness of the lower limbs. Decreased vibratory sensation of the ankle tuning forks (which usually does not disappear) is detected on examination in about 50% of patients, and urinary dysfunction is present in about one third of patients.

The disease progresses with age, with about 20% of patients eventually losing the ability to walk. The severity of clinical symptoms varies considerably between patients carrying the same mutation in the same family.

**qwen2.5_7b** 2025-02-18T10:14:36Z:

Based on the provided clinical manifestations, the most likely diagnosis is **Primary Lateral Sclerosis (PLS)**, a form of motor neuron disease characterized by upper motor neuron signs and symptoms without lower motor neuron involvement. Here are five other possible diagnoses to consider:

- **Amyotrophic Lateral Sclerosis (ALS)** - While ALS typically presents with both upper and lower motor neuron signs, it can sometimes start primarily as an upper motor neuron disorder.
- **Progressive Spastic Paraplegia (PSP)** - This is a group of rare hereditary disorders characterized by progressive spasticity and weakness in the legs, which may or may not include upper limb involvement. PSP typically progresses more quickly than PLS.
- **Corticospinal Tract Infiltration/Inflammation (e.g., due to Multiple Sclerosis)** - MS can sometimes present with a spastic paraparesis as an initial symptom, although it usually involves other symptoms like sensory disturbances and visual problems.
- **Chronic Inflammatory Demyelinating Polyneuropathy (CIDP)** - Although CIDP primarily affects the peripheral nervous system, some patients may initially present with central nervous system signs that could mimic PLS, especially if the spinal cord is involved early in the disease course.
- **Metachromatic Leukodystrophy (MLD)** - This is a rare lysosomal storage disorder that can sometimes present with spastic paraplegia and other neurological symptoms, though it typically presents earlier in life and may involve cognitive decline and other symptoms not mentioned here.

Each of these conditions needs to be evaluated by considering the patient’s full history, additional clinical findings, and possibly neuroimaging or genetic testing.

**55. user 2025-02-18T10:16:37Z:**

Most of the affected children develop the disease in newborns and early infants. Clinical manifestations are similar to biotinidase deficiency, without specificity, mainly feeding difficulties, vomiting, diarrhoea, hypotonia, convulsions, depression, lethargy, respiratory difficulties, developmental delays, intractable rashes, desquamation, which can be combined with ketosis, metabolic acidosis, hyperlactic acidemia and so on. Skin damage in most patients in the head and face, neck, trunk, buttocks and other parts of the skin rash or erythema, ulceration or blisters, furfuraceous or flaky scales, or dry and rough skin, peeling skin, etc., and a few patients only in the perioral, periocular, perianal local appearance of the rash. Patients are not associated with hearing or visual impairment, unlike biotinidase deficiency.

**qwen2.5_7b** 2025-02-18T10:17:03Z:

Based on the clinical manifestations you’ve provided, one rare disease that fits these symptoms is **Maple Syrup Urine Disease (MSUD)**. MSUD is an inborn error of metabolism characterized by a buildup of branched-chain amino acids and their derivatives due to a defect in branched-chain alpha-ketoacid dehydrogenase (BCKD) complex, which leads to the production of toxic metabolites.

Here are five other possible diagnoses:

- **Propionic Acidemia** - Another inborn error of metabolism that affects the breakdown of certain amino acids and can cause similar symptoms including feeding difficulties, vomiting, developmental delays, and skin lesions.
- **Glutaric Aciduria Type 1 (GA1)** - This is a rare genetic disorder affecting the metabolism of amino acids. It can present with similar neurological symptoms and dermatological issues.
- **Carnitine Urea Cycle Disorder** - A group of disorders related to the urea cycle, which might present with metabolic acidosis, hyperlactic acidemia, and skin rashes.
- **Homocystinuria** - Characterized by elevated levels of homocysteine due to a deficiency in cystathionine beta-synthase (CBS) or other related enzymes, leading to symptoms like developmental delays, dermatological issues, and eye problems.
- **Mucopolysaccharidosis Types I, II, IIIA** - These are lysosomal storage diseases that can present with multiple systemic symptoms including developmental delay, skin changes, and neurological impairment.

Each of these conditions involves metabolic abnormalities and can have overlapping clinical presentations, making differential diagnosis challenging without specific biochemical tests.

**56. user 2025-02-18T10:17:33Z:**

Children are born without abnormalities, and the clinical presentation in infancy is not specific and is characterised only by growth and developmental delays. It is often diagnosed at 3 years of age after the onset of ocular signs and symptoms.

- visual impairment, myopia and iris tremor due to ectopic lens may appear after 3 years of age, and may be combined with astigmatism, glaucoma, staphylococcus, cataract, retinal detachment and optic nerve atrophy in later stages. 2. intellectual disability is common.
- Intellectual disability is more common, mainly manifested as progressive mental retardation. Vitamin B6 treatment-sensitive patients are less mentally impaired. 3.
- mental or behavioural disorders, which may occur in about 50% of children and are associated with convulsions in 20%.
- Skeletal involvement, similar to the manifestations of Marfontein syndrome, or in combination with scoliosis, funnel chest, chicken chest, knee valgus, and bowed feet, with generalised osteoporosis seen on X-ray.
- Thrombophilia, which occurs at all ages and may involve all types of blood vessels, especially the cerebral vessels. High levels of homocysteinemia may alter the vessel wall and increase platelet adhesion, resulting in optic nerve atrophy, paralysis, pulmonary heart disease, and renal hypertension.
- Spontaneous pneumothorax and acute pancreatitis are less common.

**qwen2.5_7b** 2025-02-18T10:17:54Z:

Based on the clinical manifestations you have provided, the most likely diagnosis is **Sotos Syndrome** (also known as cerebral gigantism). Sotos Syndrome is a genetic disorder characterized by distinctive facial features, overgrowth in infancy, and developmental delays. The ocular signs and symptoms such as visual impairment, myopia, iris tremor due to ectopic lens, and later complications like glaucoma, cataract, retinal detachment, optic nerve atrophy, along with intellectual disability, are consistent with Sotos Syndrome.

Here are five other possible diagnoses that could present with similar clinical features:

- **Neurofibromatosis Type 1 (NF1)**: This genetic disorder can cause learning disabilities and may be associated with visual impairment, ocular signs like optic nerve anomalies, and a variety of other neurological symptoms.
- **Tuberous Sclerosis Complex (TSC)**: This condition often includes intellectual disability, developmental delays, and various manifestations that could involve the eyes, such as retinal hamartomas and optic nerve atrophy.
- **Cornelia de Lange Syndrome (CdLS)**: This genetic disorder can cause similar growth issues, developmental delays, and ocular abnormalities including visual impairment and eye movements disorders like iris tremor.
- **Mucopolysaccharidosis (MPS) Types**: These are a group of lysosomal storage diseases that can present with progressive neurological symptoms, growth and development delay, and ocular complications such as glaucoma and corneal clouding.
- **Smith-Magenis Syndrome (SMS)**: This genetic disorder often involves developmental delays, behavioral problems, and sleep disturbances. While less likely to have the specific ocular findings like ectopic lens, it can still present with growth and developmental issues.

Each of these conditions should be considered in the differential diagnosis based on the patient’s clinical presentation and additional diagnostic tests such as genetic testing may help confirm or rule out each possibility.

**57. user 2025-02-18T10:18:42Z:**

The main manifestations of the disease are markedly elevated levels of LDL-C from birth, cholesterol deposits in the skin, eyes and tendons, and the formation of xanthomas and lipomatous corneal arches. As a result of exposure to high levels of LDL-C, most patients develop coronary atherosclerotic heart disease before the age of 20 and may die as a result.

A detailed family history is needed, as some patients have heterozygous parents and therefore have elevated LDL-C levels (usually greater than the 95th percentile of their country’s age- and sex-specific norms) and a strong positive family history of premature atherosclerotic cardiovascular disease.

**qwen2.5_7b** 2025-02-18T10:19:01Z:

Based on the clinical manifestations you’ve provided, the most likely diagnosis is Familial Hypercholesterolemia (FH). FH is a rare genetic disorder characterized by markedly elevated levels of LDL cholesterol from birth, which leads to early-onset coronary artery disease and other clinical manifestations such as tendon xanthomas and cholesterol deposits in various tissues.

### Possible Alternatives:

- **Mönckern Syndrome** - This condition involves familial hypercholesterolemia but can also include additional features like nail changes (subungual keratosis) which might be less prominent in FH.
- **Apolipoprotein B100 Deficiency** - A rare genetic disorder where the body does not produce functional ApoB, leading to low LDL cholesterol levels, although this is quite distinct from elevated levels seen in FH.
- **Familial Combined Hyperlipidemia (FCHL)** - This condition involves multiple lipoprotein abnormalities including elevated LDL and triglycerides but typically without the same severity or early onset of cardiovascular disease as seen in FH.
- **Hypothyroidism** - While not a genetic disorder, hypothyroidism can lead to elevated cholesterol levels; however, it generally does not present with tendon xanthomas or the specific cutaneous and ocular manifestations mentioned.
- **Lipoprotein(a) Deficiency** - A rare condition where Lp(a) is severely reduced or absent, which might cause slightly lower LDL levels but typically without the clinical manifestations of FH.

Each of these conditions can be ruled out through detailed family history, genetic testing, and specific laboratory tests for lipoproteins.

**58. user 2025-02-18T10:20:24Z:**

It is characterised by a triad of movement disorders, psychiatric symptoms and cognitive deficits, usually with an insidious onset and slow progression. The age of onset ranges from childhood to 79 years, with the most common being 30-50 years of age.

The disease involves rapid, involuntary, non-rhythmic movements of the face, trunk, and limbs. Early abnormal movements are mild, and the patient may have difficulty recognising the presence of chorea or integrating chorea-like movements into random movements, which makes the control of normal movements difficult or deviant; as the disease progresses, the extent and degree of chorea often increases, and even affects the diaphragm, pharynx, and laryngeal muscles, resulting in dysarthria, dysphagia, and involuntary movement. As the disease progresses, the extent and degree of chorea often increases, even affecting the pharyngeal and laryngeal muscles, resulting in dysarthria, dysphagia, and involuntary vocalisations. In the later stages of the disease, the chorea often disappears, and is replaced by a Parkinson’s-like pattern of stiffness and hyperkinesis, which may be accompanied by focal dystonia. Difficulty in maintaining movement is also a common manifestation of the disease, i.e., the inability to maintain certain simple voluntary movements, such as difficulty in maintaining tongue extension.

Mental disorders may precede chorea, and common symptoms include depressed mood, depression, irritability, apathy, and anxiety, as well as paranoia, delusions, and hallucinations; rare symptoms include obsessive-compulsive behaviours and psychotic manifestations, which tend to worsen progressively.

Cognitive impairment is most often characterised by executive dysfunction, which is manifested by a reduced ability to make decisions, multi-task and shift cognitive goals. Patients usually lack self-awareness of their cognitive deficits. Memory loss or impairment occurs late in the course of the disease and progresses to dementia as the disease progresses.

In addition to the triad of symptoms, eye movement abnormalities are also prominent. Early in the disease, there is a slowing down of eye-sweeping speed, but eye-following movements are preserved; in later stages, eye-following movements, voluntary sweeping and refixation are all impaired. In addition, weight loss and cachexia are common features of the disease, with patients experiencing significant weight loss as a result of the high energy expenditure of involuntary movements, usually without loss of appetite. Sleep and/or sexual dysfunction are also common.

Progressive deterioration of motor and cognitive function can lead to significant complications, such as dysphagia due to involvement of the pharyngeal muscles, dyskinesia, reduced activity and even bed rest, which can lead to aspiration pneumonia and other infections, and decubitus ulcers.

**qwen2.5_7b** 2025-02-18T10:20:38Z:

Based on the clinical manifestations provided, the most likely diagnosis is **Chorea Huntington’s disease (Huntington’s Disease)**. The key features that point towards this diagnosis include:

- **Choreiform Movements**: Rapid, involuntary, non-rhythmic movements affecting the face, trunk, and limbs.
- **Psychiatric Symptoms**: Depressed mood, irritability, apathy, anxiety, paranoia, delusions, hallucinations.
- **Cognitive Deficits**: Executive dysfunction leading to difficulty in decision-making, multitasking, and shifting cognitive goals.
- **Triad of Movement Disorders, Psychiatric Symptoms, and Cognitive Deficits**.

Here are five other possible diagnoses that could present with similar symptoms:

- **Wilson’s Disease**
  - **Symptoms**: Tremors, dysarthria, ataxia, psychiatric symptoms like depression, anxiety, and personality changes.
- **Ménière’s Disease (with Neuropsychiatric Manifestations)**
  - **Symptoms**: Vertigo, tinnitus, hearing loss, mood swings, cognitive decline.
- **Progressive Supranuclear Palsy (PSP)**
  - **Symptoms**: Gait disturbance, postural instability, falls, cognitive impairment with executive dysfunction, and parkinsonian features.
- **Neuropsychiatric Systemic Lupus Erythematosus (NPSLE)**
  - **Symptoms**: Psychosis, depression, anxiety, cognitive decline, movement disorders.
- **Corticobasal Degeneration (CBD)**
  - **Symptoms**: Progressive motor impairment with rigidity and bradykinesia, apraxia, cognitive decline, and psychiatric symptoms like depression and apathy.

These other diagnoses can present with overlapping symptoms but generally have distinct clinical features that help differentiate them from Huntington’s Disease.

**59. user 2025-02-18T10:21:33Z:**

Clinical signs and symptoms are mainly neurological and vary in severity. The age of onset can range from neonatal to adulthood (late-onset), and there is a broad phenotypic spectrum.

- In the neonatal period, symptoms associated with hyperammonemia [including lethargy, drowsiness, refusal to eat, vomiting with respiratory alkalosis and/or seizures] appear after about 24-48 hours of asymptomatic onset. It is indistinguishable from other urea cycle disorders of neonatal onset.
- Late-onset, which accounts for 88 per cent of patients, 40 per cent of whom occur before the age of 3 years, 29 per cent in childhood and 19 per cent in adulthood. The main manifestations are: (i) chronic neurocognitive deficits, including developmental delay, ataxia, spasticity, learning disabilities, cognitive deficits, and/or unexplained seizures; (ii) acute encephalopathy secondary to hyperammonaemic crisis induced by a variety of factors (e.g., infections, fasting, or injuries); and (iii) chronic hepatic dysfunction, with unexplained elevation of hepatic aminotransferases with or without mild coagulation disorders, with or without mild hyperammonaemia, and protein intolerance. Ammonemia and protein intolerance.

**qwen2.5_7b** 2025-02-18T10:21:38Z:

Based on the clinical manifestations you have provided, the most likely rare disease is **Argininosuccinate Synthase 1 (ASNS) Deficiency** (also known as Argininosuccinic Aciduria Type I). This condition affects the urea cycle, which is responsible for detoxifying ammonia in the body. Here are five other possible diagnoses that could be considered:

- **Citrullinemia (Argininosuccinate Lyase Deficiency)** - Another urea cycle disorder with similar neurological symptoms and hyperammonemic crises.
- **Ornithine Transcarbamylase Deficiency** - This is the most common urea cycle disorder, often presenting similarly but usually in males due to its X-linked inheritance pattern.
- **Argininosuccinate Lyase Deficiency (ASL Deficiency)** - Another urea cycle enzyme deficiency that can present with neurological symptoms and hyperammonemia.
- **N-Acetylglutamate Synthetase Deficiency** - An error in the first step of ammonia metabolism, leading to similar symptoms as other urea cycle disorders.
- **Methylmalonic Acidemia (CblB Type)** - While primarily a disorder affecting the cobalamin pathway, it can present with neurological symptoms and metabolic acidosis due to elevated levels of methylmalonic acid in the blood.

Each of these conditions should be considered based on the clinical findings and biochemical testing for specific enzyme deficiencies.

**60. user 2025-02-18T10:22:03Z:**

Some children have no clinical symptoms in the neonatal period, but after 3-4 months of life, the typical symptoms appear gradually: the hair turns from black to yellow, the skin is light-coloured, and the urine and sweat are foul-smelling. As the child grows older, he/she gradually shows backward intellectual development, microcephaly, epileptic seizures, and may also show abnormalities in behaviour, personality, and neurocognition. For example, hyperactivity, self-mutilation, aggression, autism, low self-esteem, depression, etc. Vomiting and eczema are also common in infancy.

In some patients, there are no clinical symptoms in the neonatal period, and after 1-3 months of life, in addition to the symptoms of PKU, the main manifestations are dyskinesia, lethargy, hypotonia of the trunk, increased or decreased muscle tone of the limbs, nystagmus, dysphagia, increased drooling, flaccidity, torticollis, unresponsiveness, insomnia, and severe intellectual developmental disorders, and so on.

**qwen2.5_7b** 2025-02-18T10:23:22Z:

Based on the clinical manifestations you have provided, these symptoms are highly indicative of **Mucopolysaccharidosis (MPS)**, specifically MPS I (Hurler Syndrome), which can present with some features of Phenylketonuria (PKU) as well. However, the specific combination of symptoms points more towards a mucopolysaccharidosis subtype. Here are the detailed diagnoses:

- **Mucopolysaccharidosis I (MPS I)** - Given the progressive physical and neurological signs such as intellectual decline, backward development, microcephaly, epileptic seizures, dyskinesia, and developmental disorders.
- **Phenylketonuria (PKU)** - The initial symptom of hair turning yellowish and foul-smelling sweat can indicate PKU, though this is usually associated with a more pronounced cognitive impairment if not treated early.
- **Mucopolysaccharidosis IIIA (Sanfilippo Syndrome A)** - Characterized by progressive neurodegeneration with symptoms such as behavioral abnormalities, intellectual decline, and sometimes epileptic seizures.
- **Hunter Syndrome (MPS II)** - This condition presents with similar neurological symptoms but often includes more pronounced skeletal issues and a broader range of physical abnormalities.
- **Hurler Syndrome (MPS I H)** - The combination of neurodevelopmental delay, respiratory issues, cardiac problems, and the specific clinical signs you mentioned are highly suggestive of this condition.

Each of these diagnoses has its characteristic symptoms and biochemical tests that can be used for definitive diagnosis. It is crucial to conduct a thorough genetic evaluation including enzyme assays and molecular testing to confirm the exact subtype of mucopolysaccharidosis or other conditions.

**61. user 2025-02-18T10:24:23Z:**

The severity of clinical manifestations is highly variable. Patients may present with abnormalities in bone mineralisation, dental or joint pathology, muscle weakness, etc. Currently, there are seven main types of the disease.

- simple dental type, which is the mildest and most common type, with only dental involvement and no rickets or osteomalacia. 2. adult type, which often develops in middle age.
- Adult type, which often develops in middle age and presents with dental abnormalities or osteochondrosis. It may cause impaired mobility due to repeated fractures, bone and joint pain, or muscle weakness. 3.
- the childhood type, which usually develops after 6 months of age, can be mild to severe, and the children may have different degrees of tooth loss and rickets.
- the infantile form, with onset before 6 months of age, is often poorly ventilated, difficult to survive, debilitating, and may have a wide fontanelle or cranial deformity. 50 per cent of infantile HPP die of complications. 5. the perinatal form, with onset before 6 months of age, is the most severe form of HPP, with onset before 6 months of age.
- The perinatal form, which is the extreme form, develops in utero, is evident at birth, and is almost always fatal. Children with short limbs and severe skeletal undermineralisation may present with high-pitched crying, seizures, periodic apnoea with cyanosis and bradycardia, unexplained fever, irritability, myelosuppressive anaemia and intracranial haemorrhage, and pulmonary atelectasis.
- Pseudohypophosphatasia, similar to the infantile form, in which the patient has the manifestations of infantile hypophosphatasia with normal or elevated serum ALP. 7.
- Benign type, sometimes presenting with skeletal malformations in utero, with perinatal hypophosphatase activity, but resolving after birth without the associated manifestations of infantile hypophosphatasia or simple dental hypophosphatasia.

**qwen2.5_7b** 2025-02-18T10:24:37Z:

Based on the clinical manifestations provided, the most likely rare disease is **Infantile Hypophosphatasia (IHPP)**. This condition fits the detailed descriptions given for the severe forms, particularly the infantile and perinatal types.

Here are five other possible diagnoses:

- **Hypophosphatasia** - The general term for a group of rare genetic disorders affecting the mineralization of bone and teeth due to deficiencies in an enzyme called alkaline phosphatase (ALP). The severity can vary, with infantile hypophosphatasia being one of its severe forms.
- **Vitamin D-Resistant Rickets** - A form of rickets resistant to treatment with vitamin D, which might present with similar bone mineralization abnormalities as hypophosphatasia but is caused by a different genetic mutation affecting vitamin D metabolism.
- **Metaphyseal Chondrodysplasia** - A group of rare skeletal dysplasias characterized by short stature and abnormal development of the epiphyses, which might also involve bone and joint pathology as described.
- **Hypophosphatemic Rickets (X-linked or Autosomal)** - A condition where there is a chronic low level of phosphate in the blood due to various genetic causes, leading to poor mineralization of bones and teeth.
- **Osteogenesis Imperfecta** - Also known as “brittle bone disease,” this rare genetic disorder affects the bones, causing them to break easily without significant trauma. While it primarily involves skeletal issues, the variability in clinical manifestations can sometimes overlap with hypophosphatasia or other conditions involving mineralization.

These diagnoses are based on the specific clinical presentations and differential characteristics of the diseases that fit the clinical descriptions provided.

**62. user 2025-02-18T10:25:50Z:**

Children with square skulls, chicken breasts, beaded ribs, bracelet sign, foot bracelet sign, Henry’s groove, and lower limb deformities, which may be inversion of the knee (O-shaped leg) or eversion of the knee (X-shaped leg), appearing when they start weight-bearing near the age of one week, are accompanied by growth retardation, short stature, wobbly gait, progressively aggravating bone deformities, multiple bone fractures, bone pain, and abnormalities of tooth development (poor tooth quality, toothache, difficulty in regeneration after loss). Imaging is characterised by skeletal deformities, such as Imaging manifestations include skeletal deformities, widening and blurring of the epiphysis of the long bones, a cupped shape, and many fine strips of calcification like brushes in the cupped shape. In adults, the main manifestations are limb weakness, activity limitation, bone pain, multiple pathological fractures (long bones of the limbs, ribs, pelvis and vertebrae can occur), and shortening of height. Imaging manifestations include a generalised decrease in bone density, blurred bone trabeculae with a hairy glass appearance, pelvic deformity, false fracture lines, and biconcave deformity of the vertebral body.

**qwen2.5_7b** 2025-02-18T10:26:52Z:

Based on the clinical manifestations provided, the most likely rare disease is **Osteogenesis Imperfecta (OI)**. OI is a genetic disorder that affects the bones, making them fragile and prone to breaking easily due to thin, brittle, defective collagen.

### Other Possible Diagnoses:

- **Rickets** - A childhood condition caused by vitamin D deficiency leading to softening and weakening of the bones.
- **Marfan Syndrome** - An inherited disorder affecting connective tissue that can affect bone structure among other systems.
- **Mucopolysaccharidosis (MPS)** - A group of rare genetic disorders where the body is unable to break down large sugar molecules, leading to progressive damage throughout the body including the skeletal system.
- **Crouzon Syndrome** - An autosomal dominant disorder that causes premature fusion of certain skull bones (craniosynostosis), preventing normal growth and affecting the shape of the head and face.
- **Multiple Hereditary Exostoses (MHE)** - A condition involving multiple benign bone tumors, which can cause skeletal abnormalities.

Each of these conditions has specific clinical features and imaging findings that could be similar or different from OI, but given the characteristic presentation with square skull, various bone deformities, and progressive skeletal changes, OI remains the most likely diagnosis.

**63. user 2025-02-18T10:34:11Z:**

Clinical manifestations include heart failure, chest pain, hypotension, syncope, sudden cardiac death, arrhythmias and thromboembolism. Some patients also have extracardiac manifestations such as peripheral neuropathy, autonomic neuropathy and gastrointestinal symptoms.

**qwen2.5_7b** 2025-02-18T10:34:21Z:

Based on the clinical manifestations you’ve described—heart failure, chest pain, hypotension, syncope, sudden cardiac death, arrhythmias, and thromboembolism, along with extracardiac manifestations like peripheral neuropathy, autonomic neuropathy, and gastrointestinal symptoms—the most likely rare disease is **Fabry Disease**. This condition is caused by a deficiency of the enzyme α-galactosidase A, leading to the accumulation of globotriaosylceramide in various organs.

Here are five other possible diagnoses that could present with similar clinical manifestations:

- **Lysosomal Storage Diseases (e.g., Niemann-Pick Disease Type C)**: These conditions can cause cardiomyopathy and neurologic symptoms such as peripheral neuropathy, cognitive decline, and ataxia.
- **Carney Complex**: This rare genetic condition can lead to heart disease including arrhythmias and cardiomyopathies, along with skin pigmentation abnormalities and endocrine tumors.
- **Brugada Syndrome**: This is an inherited cardiac disorder characterized by electrocardiographic (ECG) changes that increase the risk of sudden cardiac death in young people.
- **Hypertrophic Cardiomyopathy (HCM)**: A genetic heart condition where the walls of the heart become abnormally thick and can lead to various symptoms including chest pain, arrhythmias, and even syncope.
- **Leukoencephalopathy with Demyelination and Sulfatidosis**: This rare neurological disorder can present with both cardiac issues like cardiomyopathy and extracardiac manifestations such as peripheral neuropathy.

Each of these conditions would require specific diagnostic testing to confirm the diagnosis, including biochemical tests for enzyme deficiencies, genetic testing, ECGs, echocardiograms, and other specialized examinations.

**64. user 2025-02-18T10:35:25Z:**

- secondary sexual characteristics and impaired gametogenesis in males: childish voice, small penis, no pubic hair growth, small testes or cryptorchid testes, no spermatogenesis; in females: mammary gland dysplasia, infantile vulva, primary amenorrhoea.
- delayed epiphyseal closure upper volume/lower volume <1, fingertip distance > height, susceptible to osteoporosis.
- Olfactory disorders 40% ~60% of IHH patients suffer from loss of sense of smell due to abnormal development of the olfactory bulb and olfactory tract, and are unable to identify odours.
- Other manifestations Facial midline defects, such as cleft lip and palate; isolated kidneys; short fingers (toes), syndactyly; skeletal deformities or dental hypoplasia; overweight and obesity; mirror image movement.

**qwen2.5_7b** 2025-02-18T10:35:30Z:

Based on the clinical manifestations you’ve provided, the most likely rare disease is **Kallmann Syndrome**. This condition typically involves both hypogonadotropic hypogonadism (affecting sexual development) and anosmia or hyposmia (reduced sense of smell). Here are five other possible diagnoses to consider:

- **Kallmann Syndrome**: This is the most likely diagnosis given the combination of impaired gametogenesis, olfactory disorders, and other associated manifestations such as midline facial defects.
- **Hypothalamic Hamartoma**: Can cause anosmia, hypogonadism, and sometimes other neurodevelopmental issues due to its location near the hypothalamus.
- **McCune-Albright Syndrome (MAS)**: Characterized by polyostotic fibrous dysplasia of bones, café-au-lait skin lesions, and endocrine abnormalities including precocious puberty or hypogonadism. However, it does not typically present with anosmia as a primary feature.
- **Prader-Willi Syndrome**: Known for its combination of intellectual disability, behavioral problems, short stature, and obesity. While it can sometimes have some skeletal features, it is less likely to be the main diagnosis given the specific combination of symptoms provided.
- **Apert Syndrome**: A type of acrocephalosyndactyl dysplasia with craniofacial abnormalities, syndactyly, and other bone deformities. While the skeletal findings are present, the absence of a consistent olfactory disorder makes this less likely compared to Kallmann Syndrome.
- **Klinefelter Syndrome**: Primarily affects males, causing small testes, reduced facial and body hair, and infertility due to extra X chromosome. However, it does not typically include the olfactory disorders or craniofacial defects mentioned.

Given these symptoms, Kallmann Syndrome is the most likely diagnosis, but a thorough genetic and endocrine evaluation would be necessary for confirmation.

**65. user 2025-02-18T10:36:12Z:**

The most common symptom is shortness of breath after activity, and other symptoms include fatigue, dizziness, chest pain, chest tightness, palpitations, blackouts, and syncope, etc. Once syncope or vertigo occurs, it suggests that the patient’s cardiac output may have decreased significantly. Due to atypical symptoms and signs, the majority of patients have a significant delay in consultation, with at least 1/5 of patients taking more than 2 years from the onset of symptoms to diagnosis; patients may have a hyperactive second heart sound of the pulmonary valve, and tricuspid insufficiency may cause a systolic murmur in the tricuspid region; signs of right heart failure include jugular venous congestion or rupture, enlarged liver, oedema of the lower extremities, multiple plasma cavities, jaundice, and cyanosis, among other signs.

**qwen2.5_7b** 2025-02-18T10:36:18Z:

Based on the clinical manifestations you’ve provided, the most likely rare disease is **Hemochromatosis**, given that it can present with symptoms like fatigue, shortness of breath, chest pain, and heart murmurs due to iron overload. However, since hemochromatosis is not typically associated with the specific cardiac and respiratory issues detailed here, let’s consider another possibility: **Primary Pulmonary Hypertension (PPH)** or more broadly **Group 1 Pulmonary Arterial Hypertension (PAH)**.

### Most Likely Diagnosis:

- **Pulmonary Arterial Hypertension (Group 1 PAH)**

### Five Other Possible Diagnoses:

- **Congenital Heart Disease (Specifically, Eisenmenger Syndrome)**
  - This can present with similar symptoms including right-to-left shunting which can lead to pulmonary hypertension and the signs of cor pulmonale.
- **Fabry Disease**
  - This rare genetic disorder affects the lysosomes in cells throughout the body, leading to a variety of symptoms including cardiovascular issues like angina, myocardial infarction, and arrhythmias.
- **Lupus Erythematosus (SLE) Cardiac Complications**
  - SLE can affect multiple organs, including the heart, and can present with pericarditis or myocarditis leading to symptoms of shortness of breath, chest pain, and syncope.
- **Chronic Thromboembolic Pulmonary Hypertension (CTEPH)**
  - This is a rare form of pulmonary hypertension where blood clots that travel from the legs are trapped in the lungs over time, causing progressive narrowing and scarring of the pulmonary arteries.
- **Arrhythmogenic Right Ventricular Dysplasia Cardiomyopathy (ARVD/C)**
  - This condition causes the right ventricle to deteriorate, leading to cardiac arrhythmias which can result in symptoms like palpitations, syncope, chest pain, and even sudden death.

Each of these conditions has specific diagnostic criteria and treatments that differ from one another. A thorough clinical evaluation including a detailed medical history, physical examination, and additional diagnostic tests such as echocardiograms, right heart catheterization, genetic testing for hemochromatosis or Fabry disease, and others would be essential in making an accurate diagnosis.

**66. user 2025-02-18T10:37:30Z:**

The disease is usually insidious. The main manifestations are dry cough, exertional dyspnoea, pestle-like fingers (toes), and bursting sounds distributed mainly in both lower lungs are its typical signs. In the terminal stage, cyanosis, pulmonary hypertension, pulmonary heart disease and right heart insufficiency can be seen.

**qwen2.5_7b** 2025-02-18T10:37:34Z:

Based on the clinical manifestations you provided, the most likely rare disease is **Sarcoidosis**. Sarcoidosis is a multisystem inflammatory disorder that causes granulomatous inflammation in various organs but commonly affects the lungs and lymph nodes. The other possible diagnoses to consider are:

- **Lymphangioleiomyomatosis (LAM)** - This is a rare lung disease primarily affecting women of childbearing age, characterized by abnormal cell growth in the lungs that can lead to cyst formation and respiratory symptoms.
- **Idiopathic Pulmonary Fibrosis (IPF)** - A chronic condition characterized by scarring of the lung tissue leading to progressive lung stiffness and impaired gas exchange.
- **Churg-Strauss Syndrome (CSS)** - An autoimmune disease where patients initially have asthma followed by the development of vasculitis, often affecting the respiratory system with eosinophilia as a key feature.
- **Hamman-Rosenthal Syndrome** - A rare condition characterized by pulmonary fibrosis and diaphragmatic hernia, though it is less likely given the insidious onset and specific symptoms you mentioned.
- **Berylliosis** - An occupational lung disease caused by exposure to beryllium, leading to granulomatous inflammation of the lungs similar to sarcoidosis but with a known environmental cause.

Each of these conditions can present with some overlapping symptoms, so further diagnostic testing such as biopsy, imaging studies (CT scans), and laboratory tests would be necessary to confirm the diagnosis.

**67. user 2025-02-18T10:38:36Z:**

It is a multi-organ and multi-system disease that can involve almost all organs and tissues of the body. Clinical manifestations are varied, and it is easy to miss the diagnosis when patients consult different specialties for different organ involvement. Most of the patients have a slow onset of the disease, with a chronic progression. The disease may start in a single organ and gradually involve multiple organs, or it may develop in multiple organs at the same time. In a minority of patients, the disease is self-limiting.

Lacrimal and salivary glands are the most commonly affected organs. The main manifestations are symmetrical, painless enlargement of the lacrimal gland, submandibular gland or parotid gland, hard nodules, with or without sinusitis or thickening of extraocular muscles. Involvement of the digestive system may manifest as autoimmune pancreatitis, sclerosing cholangitis, sclerosing mesenteritis, etc. Clinical manifestations may include vague abdominal pain, abdominal distension, dyspepsia, and jaundice. Lung involvement may be manifested as bronchial wall thickening, interstitial lung lesions, lung nodules/lumps and pleural thickening, etc. Patients may be asymptomatic or have cough, shortness of breath and wheezing. Retroperitoneal fibrosis or peri-abdominal aortitis is also one of the more common clinical manifestations, which is mainly a thickening of the soft tissue around the abdominal aorta, which can encircle the ureter and cause ureteral stenosis and pyelonephrosis, and in a small number of patients, abdominal aortic aneurysm occurs. Clinical manifestations include abdominal pain, low back pain, or oedema of the lower extremities. Less than 10% of patients develop IgG4-associated renal disease, most often manifesting as tubulointerstitial nephropathy, renal pelvic space-occupying lesions, ureteral wall thickening, or renal mass-like lesions. Other organs involved include the thyroid (sclerosing thyroiditis) and prostate. CNS lesions are rare, such as autoimmune pituitary inflammation, hypertrophic dural meningitis, and intracranial inflammatory pseudotumours, and patients may present with headaches, urolithiasis, and hypopituitarism. In addition to the above organ involvement, most patients combine with superficial or deep lymph node enlargement. In addition, about half of the patients have a history of allergy-related disease, which can be accompanied by asthma.

**qwen2.5_7b** 2025-02-18T10:38:41Z:

Based on the clinical manifestations provided, this patient likely has **IgG4-Related Disease (IgG4-RD)**, also known as IgG4-Associated Diseases. This condition is characterized by chronic inflammation and fibrosis involving multiple organs with increased levels of the immunoglobulin IgG4.

Here are five other possible diagnoses that may present similarly but differ in key aspects:

- **Sjögren’s Syndrome**: While primarily affecting the salivary and lacrimal glands, it can also involve other organs such as the lungs (interstitial lung disease) and kidneys. However, Sjögren’s is typically associated with specific serological markers like anti-SSA/Ro and anti-SSB/La antibodies.
- **Autoimmune Hepatitis**: This condition can cause chronic liver inflammation and may present with symptoms involving the digestive system, such as abdominal pain and jaundice. However, it primarily affects the liver and often has distinct laboratory findings including elevated transaminases and aminotransferase levels.
- **Multifocal Organ System Involvement due to Sarcoidosis**: This condition can also present with multi-organ involvement affecting the lungs, skin, eyes, and other organs. However, it is characterized by non-caseating granulomas which are seen in biopsy samples, and there may be specific radiographic findings like bilateral hilar lymphadenopathy.
- **Hashimoto’s Thyroiditis**: This autoimmune condition can affect the thyroid gland leading to hypothyroidism. While it can cause gland enlargement (goiter), it is typically limited to the thyroid and has specific laboratory markers such as elevated anti-thyroid peroxidase antibodies (TPOAb) or anti-thyroglobulin antibodies.
- **Pseudotumoral Lesions of Various Organs**: These lesions can occur in multiple organs but are more likely to present without the characteristic IgG4 positivity and multi-organ involvement typical of IgG4-RD. They may be due to other inflammatory processes or neoplasms that require further diagnostic workup.

Each of these conditions would need specific tests and clinical evaluations for a definitive diagnosis, particularly given the broad range of possible organ involvements described in IgG4-RD.

**68. user 2025-02-18T10:39:06Z:**

Children with cholestasis and cirrhosis develop. The main clinical manifestations are hyperconjugated bilirubinaemia and often malabsorption of fat-soluble vitamins, such as rickets. The clinical phenotypes of different enzyme defects are slightly different. For example, oxysterol 7α-hydroxylase defects are associated with hereditary spastic paralysis in adulthood; cholesterol 25-hydroxylase defects are associated with intractable constipation; 3β-hydroxysteroid -Δ5-C27- steroid dehydrogenase defects are associated with cholestatic liver disease and fat-soluble vitamin deficiencies in infancy; and sterol 27-hydroxylase defects are associated with chronic diarrhoea and developmental delays in children, and spasticity in adulthood. Sterol 27-hydroxylase defects can cause chronic diarrhoea and developmental delay in children, and spastic paralysis in adults; α-formyl-coenzyme A dehydrogenase defects can cause fat-soluble vitamin deficiencies in infancy, and sensory neuropathy in adulthood; and bile acid-coenzyme A-amino acid N-acetyltransferase defects can cause intrahepatic cholestasis, developmental delay, and familial hypercholesterolemia.

**qwen2.5_7b** 2025-02-18T10:39:11Z:

Based on the clinical manifestations provided, the most likely rare disease is **Smith-Lemli-Opitz Syndrome (SLOS)**. This condition results from a defect in the enzyme 3β-hydroxysteroid -Δ5-C27-sterol dehydrogenase, which leads to a deficiency of cholesterol and associated problems such as cholestasis, rickets, developmental delays, and malabsorption.

Here are five other possible diagnoses that could also present with similar symptoms:

- **Smith-Lemli-Opitz Syndrome (SLOS)**: As mentioned earlier, this is caused by a defect in 3β-hydroxysteroid -Δ5-C27-sterol dehydrogenase, leading to cholesterol deficiency and associated issues.
- **Sterol 27-Hydroxylase Deficiency**: This condition causes chronic diarrhea, developmental delays, and spasticity due to defects in sterol 27-hydroxylase.
- **Cholesterol 25-Hydroxylase Deficiency**: Characterized by intractable constipation due to a defect in this enzyme, leading to impaired vitamin D metabolism.
- **Oxysterol 7α-Hydroxylase Deficiency**: Associated with hereditary spastic paralysis in adulthood but can be suspected early if the pattern of cholestasis and developmental delay is observed.
- **Bile Acid-Coenzyme A-Amino Acid N-Acetyltransferase (BAAT) Deficiency**: This rare disorder causes intrahepatic cholestasis, developmental delays, and familial hypercholesterolemia due to a defect in this enzyme.

Each of these conditions can present with some overlapping symptoms like cholestasis, cirrhosis, and fat-soluble vitamin deficiencies, but the specific clinical features and age of onset help differentiate between them.

**69. user 2025-02-18T10:39:43Z:**

**1. Acute neonatal type** The onset of the disease is acute and the progression of the disease is rapid and can be life-threatening. Within 1-2 weeks of birth, it presents with feeding difficulties, vomiting, muscle weakness, hypotonia, and lethargy or worsening progression to coma. Accumulation of isovaleric acid may cause the characteristic odour of ‘sweaty feet’. Liver enlargement may be present on examination. Laboratory tests suggest metabolic acidosis, hyperammonemia, hypoglycemia or hyperglycemia, ketosis, hypocalcemia, and pancytopenia. If the patient survives the acute exacerbation in the neonatal period, he or she will progress to the chronic intermittent form.

**The chronic intermittent form** has an insidious onset. It presents only as a nonspecific inability to tolerate fasting or developmental delay. It is often triggered by upper respiratory tract infections or the intake of a high-protein diet, and is characterised by recurrent episodes of vomiting, lethargy, coma, acidosis with ketonuria, and ‘sweaty feet odour’, which can be relieved by a protein-restricted diet and glucose infusion. The vast majority of patients with chronic intermittent form have normal psychomotor development, but some have developmental delays and varying degrees of mental retardation.

**qwen2.5_7b** 2025-02-18T10:39:47Z:

Based on the clinical manifestations provided, the rare disease most likely to be diagnosed is **Isolated Isovaleric Acidemia (IVA)** or **IVD (Isovaleryl-CoA Dehydrogenase Deficiency)**. This condition is characterized by the acute and chronic forms as described:

- **Isolated Isovaleric Acidemia (IVA) / IVD** - This is a rare genetic metabolic disorder caused by a deficiency in the enzyme isoamylase or isoamylene dehydrogenase, which leads to the accumulation of isovaleryl-CoA and its metabolites.
- **Possible Diagnoses:**
  - **Medium-Chain Acyl-CoA Dehydrogenase Deficiency (MCADD)**: Another metabolic disorder that causes high levels of medium-chain fatty acids in the blood.
  - **Propionic Acidemia**: A disorder caused by a deficiency in propionyl-CoA carboxylase, leading to accumulation of propionic acid and other metabolites.
  - **Methylmalonic Acidemia (CblAdeficiency)**: An inherited metabolic disorder where there is a defect in the conversion of certain amino acids. It can present with similar symptoms but is due to different biochemical pathways.
  - **Glutaric Aciduria Type 1**: A rare metabolic disorder that affects the breakdown of some amino acids and leads to accumulation of toxic substances like glutaric acid.
  - **Homozygous Pyruvate Dehydrogenase Deficiency (PDHD)**: A severe form of a metabolic condition where there is an absence of the pyruvate dehydrogenase complex, leading to lactic acidosis and other metabolic derangements.

Each of these disorders can present with similar symptoms such as feeding difficulties, vomiting, lethargy, and hypotonia, making differential diagnosis crucial through biochemical tests and genetic confirmation.

**70. user 2025-02-18T10:40:33Z:**

- dysgenesis of secondary sexual characteristics and impaired gametogenesis.
- Olfactory disorders Inability to recognise odours, especially aromatic odours, due to abnormal development of the olfactory bulb and olfactory tract.

**qwen2.5_7b** 2025-02-18T10:40:37Z:

Based on the clinical manifestations you’ve provided, it appears that the most likely rare disease is Kallmann syndrome. This is a condition characterized by hypogonadotropic hypogonadism (impaired gametogenesis) and anosmia (inability to recognize odors due to olfactory dysfunction). Here are five other possible diagnoses:

- **Kallmann Syndrome**: Characterized by secondary sexual characteristics dysgenesis, impaired gametogenesis, and anosmia.
- **Isolated Hypogonadotropic Hypogonadism (IHH)**: This condition involves only the absence of pubertal development and hypogonadism without anosmia or other sensory deficits.
- **McCune-Albright Syndrome**: Features include polyostotic fibrous dysplasia, skin pigmentation abnormalities, and often endocrine disorders such as hyperthyroidism and precocious puberty, but it is less likely given the specific olfactory dysfunction.
- **Craniofacial Disorders with Hypogonadotropic Hypogonadism**: Some rare craniofacial syndromes can present with hypogonadotropic hypogonadism but do not typically include anosmia as a primary symptom.
- **Hypothalamic Hamartoma**: This is a benign tumor in the hypothalamus that can cause anosmia and other symptoms related to hormonal dysfunction, though it’s less common than Kallmann syndrome.

Each of these conditions has unique clinical features, so further diagnostic testing would be necessary to confirm the exact diagnosis.

**71. user 2025-02-18T10:41:03Z:**

The condition varies greatly from mild to severe and is therefore easily misdiagnosed and underdiagnosed. Clinical manifestations mainly include:

**1. Fever** The fever is irregular and may be periodic or persistent. Patients with hypothalamic involvement may have central thermoregulatory abnormalities.

**2. rash** Mainly on the trunk, scalp and hairline. It begins as a reddish papule, which may then become a haemorrhagic or eczema-like seborrheic rash, followed by crusting, which is followed by a white spot.

**3. Oral and eye, ear, nose and throat lesions** Gum swelling, loose teeth, proptosis, and persistent otitis media with external auditory canal rash are typical.

**4. Central uropathy** Pituitary gland, hypothalamus or even other parts of the central nervous system, with neuropsychiatric symptoms in a few patients.

**5. Respiratory symptoms** Coughing, severe breathlessness, cyanosis, and even recurrent pneumothorax episodes. Pulmonary signs are usually unremarkable.

**6.** The liver and spleen can be enlarged, jaundice, abnormal liver function, liver failure and portal hypertension can occur, and eventually patients can die of cirrhosis and liver failure.

**7. Digestive tract symptoms** Digestive tract involvement is less common and can manifest as polyps or adenomatous changes, usually without clinical symptoms, and abdominal pain, diarrhoea and hypoproteinaemia may occur when the lesions are diffuse.

**8. Haematopoietic system** Lymph nodes may be enlarged, and anaemia, abnormal white blood cell and platelet counts may occur with bone marrow involvement.

**9. Bone damage** Skull, limb bones, spine and pelvis may have pain and lumps, and pathological fractures may occur. Proptosis due to orbital bone lesions is one of the typical manifestations in paediatric patients.

**qwen2.5_7b** 2025-02-18T10:41:09Z:

Based on the clinical manifestations you’ve provided, the condition most likely points towards **Bloom Syndrome (BS)**. Bloom syndrome is a rare genetic disorder characterized by multiple system involvement and specific physical features. The symptoms align well with BS, which includes:

- **Frequent Infections and Fever**: Irregular and periodic fevers are common in BS patients due to immunodeficiency.
- **Rash**: Characteristic photosensitive rash that appears on the trunk, scalp, and hairline.
- **Oral and Eye, Ear, Nose, Throat Lesions**: Gum swelling and loose teeth are common; proptosis and otitis media can occur in some patients.
- **Central Nervous System Involvement**: Neuropsychiatric symptoms and hypothalamic dysfunction leading to irregular fevers.
- **Respiratory Symptoms**: Coughing, breathlessness, and cyanosis may be present due to pulmonary involvement; recurrent pneumothorax is rare but possible.
- **Liver and Spleen Enlargement**: Hepatosplenomegaly with potential for cirrhosis and liver failure in severe cases.
- **Digestive Tract Involvement**: Gastrointestinal polyps or adenomatous changes are common, though often asymptomatic.
- **Haematopoietic System**: Lymphadenopathy and blood dyscrasias such as anaemia can occur due to bone marrow involvement.
- **Bone Damage**: Bone pain, fractures, and proptosis from orbital bone lesions.

### Five Other Possible Diagnoses

- **Ataxia-Telangiectasia (AT)**: A rare genetic disorder characterized by progressive ataxia, telangiectasias, immunodeficiency, and an increased risk of cancer.
- **X-linked Agammaglobulinemia**: An X-linked primary immunodeficiency where the patient lacks B cells and antibodies, leading to frequent infections.
- **Severe Combined Immunodeficiency (SCID)**: A group of rare genetic disorders that affect the immune system, causing severe, life-threatening infections from a young age.
- **Chronic Granulomatous Disease (CGD)**: An inherited disorder where certain white blood cells cannot kill bacteria and fungi, leading to recurrent infections.
- **Pleomorphic Adenoma**: A benign tumor of the salivary glands that can cause swelling in the face or neck, though this is less likely given the systemic nature of the described symptoms.

These other conditions share some similar symptoms but are distinct from Bloom Syndrome, and their prevalence or specific clinical features may help narrow down the diagnosis further.

**72. user 2025-02-18T11:41:01Z:**

**1. Growth disorders and other malformations,** most of the children are born with a weight within the normal range, but some of them are born with a length of less than 48cm (42-47cm). After birth, the growth rate gradually and significantly slows down, and the lifelong height of untreated male patients ranges from 116 to 142 cm, and the lifelong height of female patients ranges from 108 to 136 cm. At the same time, the patients’ hands, feet and internal organs are small. Some patients have other congenital malformations, such as hip dislocation, aortic valve stenosis, cryptorchidism, and congenital cataracts.

**2. Facial features,** the characteristic facial changes of patients are prominent forehead, saddle nose, small jaw, larger head to body ratio than normal children, but the absolute value of head circumference is still small compared with children of the same age. Hair is thinning, and some patients may have a ‘sunset sign’, with blue sclera in patients from the Mediterranean and Middle Eastern regions. The patient has delayed teething, late deciduous milk teeth and multiple caries, and may have malocclusion due to a short mandible.

**Skeletal musculature** Some patients have congenital hip dislocation and ischaemic necrosis of the femoral head. Due to mid-facial dysplasia and pharyngeal anatomical abnormalities, the patient’s speech is high pitched and 85% of the patients have limited elbow joint movement.

**4. Metabolic disorders,** Patients often have a combination of metabolic abnormalities, including hypoglycaemia in the neonatal period and infancy, and progressive obesity in adulthood.

**5. Delayed sexual development ,** Patients with this condition have small genitalia, with male patients presenting with small penises and testicles, and female patients having small ovaries on ultrasound. Most patients exhibit delayed pubertal development. Although sexual development is delayed, male and female patients can eventually develop mature sexual function and be fertile.

**6. Low tumour incidence** Patients have a significantly reduced risk of tumour development compared to the general population.

**qwen2.5_7b** 2025-02-18T11:41:11Z:

Based on the clinical manifestations you’ve provided, the most likely rare disease diagnosis is **Spondylsotomeleia-Rachitica (Silver-Russell Syndrome, SRS)**. This condition is characterized by growth restriction, facial features, skeletal abnormalities, and other associated symptoms that match your description.

### Five Other Possible Diagnoses:

- **Turner Syndrome** - Characterized by short stature, delayed puberty, gonadal dysgenesis, and a variety of physical anomalies.
- **Prader-Willi Syndrome (PWS)** - Known for growth failure in infancy, followed by excessive eating and obesity in childhood, along with other features like hypotonia and cognitive issues.
- **Hypohidrotic Ectodermal Dysplasia** - This condition involves abnormalities of the skin, hair, nails, and sweat glands, which can present with a distinctive facial appearance but is less likely to match all your described symptoms.
- **Bloom Syndrome (BS)** - A rare genetic disorder characterized by growth retardation, sun-sensitive skin, and an increased risk of cancer. It does not fit the overall profile as well due to its more prominent features related to immunodeficiency and cancer predisposition.
- **Hypothalamic Hypopituitarism** - This condition involves deficiencies in pituitary hormones that can affect growth, metabolism, and other bodily functions, but typically presents with more specific hormonal signs rather than the unique combination of symptoms described.

Given the detailed clinical picture, Silver-Russell Syndrome (SRS) is the most fitting diagnosis.

**73. user 2025-02-18T11:41:51Z:**

The main symptom is a sudden, sharp, painless loss of visual acuity in both eyes that occurs sequentially, usually weeks to months between episodes. Funduscopic examination shows congestion and oedema of the optic disc and tortuous dilatation of the peripapillary capillaries in the early stages of the disease, while the oedema and capillary dilatation subside in the later stages of the disease, and eventually the temporal side of the optic disc or all of the optic disc shows atrophic changes. The majority of patients have only ocular manifestations, while a few may have other systemic symptoms such as mental retardation, epilepsy, hearing impairment, dystonia, etc.

**qwen2.5_7b** 2025-02-18T11:42:00Z:

Based on the clinical description provided, the most likely rare disease is Leber’s Hereditary Optic Neuropathy (LHON), which affects the optic nerve and can lead to sudden visual loss. However, given the additional systemic symptoms mentioned, it is important to consider other conditions that might present with similar ocular findings but also have associated systemic manifestations.

- **Leber’s Hereditary Optic Neuropathy (LHON)**: This mitochondrial disorder primarily affects young adults and causes rapid and painless loss of vision due to damage to the optic nerves. The ophthalmological signs you described are characteristic, including disc swelling in the early stages and later atrophy.
- **Optic Neuritis**: While typically associated with multiple sclerosis (MS), optic neuritis can also occur as a standalone condition or in association with other systemic diseases like sarcoidosis or systemic lupus erythematosus. It presents with acute visual loss and optic disc swelling, which may subside over time.
- **Cogan’s Syndrome**: This rare autoimmune disorder affects the eyes (uveitis) and inner ear (vestibular neuritis), causing a combination of ocular inflammation and vestibular symptoms like dizziness or vertigo. It can sometimes present with visual disturbances, although not typically as acute as in LHON.
- **Oguchi Disease**: A rare condition characterized by intermittent painless visual blurring that worsens at dusk and improves overnight. Fundus examination shows characteristic dark red spots (hemorrhages) on the optic disc which resolve over time. This disease is not associated with systemic symptoms but fits the pattern of episodic vision loss.
- **Eales’ Disease**: Primarily affecting young males, this condition involves non-inflammatory neovascularization in the retina and can cause recurrent episodes of painless vision loss. It is often unilateral at presentation but may become bilateral over time. Systemic manifestations are rare, but there might be an association with other vascular diseases.
- **MELAS Syndrome (Mitochondrial Encephalomyopathy, Lactic Acidosis, and Stroke-like Episodes)**: This mitochondrial disorder can present with a variety of symptoms including visual loss due to optic neuropathy, stroke-like episodes in the brain, hearing impairment, and muscle weakness or dystonia. The visual symptoms are often associated with systemic involvement.

Given the clinical presentation, Leber’s Hereditary Optic Neuropathy (LHON) is the most likely diagnosis, but it is crucial to consider other conditions that might present similarly for a thorough differential diagnosis.

**74. user 2025-02-18T11:42:35Z:**

The clinical manifestations of patients are low ketogenic hypoglycaemia, liver function abnormalities, lactic acidosis, cardiomyopathy in infancy, growth retardation, hypotonia or cardiomyopathy in some patients, and cholestasis or cirrhosis in a few. In early childhood, some patients show growth retardation, hypotonia or cardiomyopathy, and a few may have cholestasis or cirrhosis. In early childhood, patients may have feeding difficulties, episodic metabolic disorders, and episodic rhabdomyolysis. Most patients will have retinopathy in childhood, and peripheral neuropathy is a less common complication of long-term disease.

**qwen2.5_7b** 2025-02-18T11:42:42Z:

Based on the clinical manifestations you’ve described—low ketogenic hypoglycaemia, liver function abnormalities, lactic acidosis, cardiomyopathy in infancy, growth retardation, hypotonia or cardiomyopathy in some patients, cholestasis or cirrhosis in a few, and retinopathy in childhood—the most likely diagnosis is ** VLCAD deficiency (Very Long Chain Acyl-CoA Dehydrogenase Deficiency)**. This rare disorder primarily affects fatty acid oxidation.

Here are five other possible diagnoses that could present with similar clinical manifestations:

- **Medium-Chain Acyl-CoA Dehydrogenase Deficiency (MCADD)**: MCADD can also cause hypoglycemia, liver dysfunction, and lactic acidosis, but it is typically less severe than VLCAD deficiency.
- **Isobutyryl-CoA Dehydrogenase Deficiency**: This condition involves metabolic issues similar to those seen in VLCAD deficiency, including hypoketotic hypoglycemia, hepatomegaly, and growth failure.
- **Primary Carnitine Deficiency**: Patients can exhibit similar symptoms such as cardiomyopathy, hepatic dysfunction, and hypoketotic hypoglycemia, but the primary issue here is with carnitine transport or metabolism.
- **Mitochondrial Encephalomyopathies (e.g., MELAS Syndrome)**: While typically more focused on neurological manifestations, some mitochondrial disorders can present with cardiomyopathy, liver dysfunction, and growth failure.
- **Zellweger Spectrum Disorders (Peroxisomal Biogenesis Disorders)**: These conditions often include symptoms like hepatomegaly, retinopathy, and neurodevelopmental delay, though they are less common and more typically associated with peroxisomal dysfunction rather than fatty acid oxidation defects.

Each of these diagnoses would need to be evaluated based on a thorough clinical assessment, biochemical tests (like acylcarnitine profiles), genetic testing, and possibly imaging studies.

**75. user 2025-02-18T11:44:50Z:**

It occurs predominantly in adult women of childbearing age, with the average age of diagnosis being around 40 years. Early symptoms are mild, and some patients are detected during a physical examination or on HRCT of the lungs for respiratory symptoms or other reasons. The main manifestation is dyspnoea of varying degrees, which worsens as the disease progresses, with progressive deterioration of lung function and respiratory failure in the later stages of the disease. Complications such as pneumothorax and coeliac chest may occur repeatedly during the course of the disease. Extrapulmonary manifestations include renal, retroperitoneal solid or cystic solid lymphangioleiomyoma. Some patients have other clinical features of multisystem involvement, including neurological changes (epilepsy, neurodevelopmental delays, autism, etc.) and cutaneous changes (depigmentation, facial angiofibromas, cutaneous sharkskin plaques, and perinodal fibromas, etc.).

**qwen2.5_7b** 2025-02-18T11:44:55Z:

Based on the clinical manifestations provided, the rare disease most likely to be diagnosed is Lymphangioleiomyomatosis (LAM). This condition predominantly affects adult women of childbearing age, often presenting with respiratory symptoms like dyspnea that worsen over time, and can lead to complications such as pneumothorax. The presence of renal involvement and the characteristic cutaneous and neurological manifestations further support this diagnosis.

Here are five other possible diagnoses:

- **Pulmonary Lymphangioleiomyomatosis (LAM)**: This is the primary condition based on the clinical features described, but for diagnostic alternatives, it’s important to consider similar conditions.
- **Bronchiolitis Obliterans**: This condition involves inflammation and scarring of the small airways leading to progressive lung damage, often seen in patients with certain occupational exposures or autoimmune disorders.
- **Cystic Fibrosis (CF)**: CF is a genetic disorder that primarily affects the lungs but can also cause various other health issues, including digestive problems and diabetes. It typically presents earlier than LAM, usually during childhood.
- **Interstitial Lung Disease (ILD) with Pulmonary Hypertension**: Various types of ILD can present similarly to LAM in terms of progressive lung function decline and respiratory symptoms but may have different underlying causes like autoimmune diseases or environmental exposures.
- **Pulmonary Alveolar Proteinosis (PAP)**: This rare condition involves the accumulation of surfactant protein A in the alveoli leading to respiratory symptoms, but it is typically diagnosed through specific blood tests and bronchoalveolar lavage.

Each of these conditions would need further investigation including imaging studies like HRCT, pulmonary function tests, genetic testing for LAM, and possibly other specialized tests depending on the clinical context.

**76. user 2025-02-18T11:45:20Z:**

Infants typically experience repeated vomiting and diarrhea after weaning and refuse protein-rich foods.Patients may present with pale skin, hypotonia, hepatosplenomegaly, thinning hair, osteoporosis and pathological fractures.After eating a protein-rich diet, patients may experience lethargy, ataxia, seizures, and hyperammonemic coma.As the disease progresses, multiple systems may be involved.Lung involvement can be manifested as progressive interstitial lung disease, alveolar proteinosis; the most common characteristic changes in the kidney are proximal tubular dysfunction and renal calcinosis, glomerulopathy, renal amyloidosis, and even renal failure; blood system involvement can be manifested as positive or low pigment anemia, leukopenia, thrombocytopenia, etc.; Digestive system involvement is manifested by hypercholesterolemia, hypertriglycerides, recurrent pancreatitis, and even hepatic amyloidosis.Epilepsy occurs mostly in adult patients and may be related to repeated hyperammonemia leading to cerebral cortex damage.

**qwen2.5_7b** 2025-02-18T11:45:28Z:

Based on the clinical manifestations you have described, the most likely diagnosis is **Maple Syrup Urine Disease (MSUD)**, which is a rare genetic disorder that leads to the accumulation of branched-chain amino acids and their corresponding ketoacids due to a deficiency in branched-chain alpha-ketoacid dehydrogenase (BCKD) activity.

**Five Other Possible Diagnoses:**

- **Cystinosis**: A rare metabolic disease caused by cystinosis, characterized by lysosomal storage of the amino acid cystine. It can present with renal tubular dysfunction, hypothyroidism, and retinal degeneration, but does not typically include severe hyperammonemia or specific dietary response like MSUD.
- **Fabry Disease**: An X-linked lysosomal storage disease resulting from a deficiency in alpha-galactosidase A. It can present with intermittent abdominal pain, gastrointestinal issues, renal dysfunction, and neuropathy, but does not usually have the characteristic acute episodes of hyperammonemia or dietary response seen in MSUD.
- **Wilson’s Disease**: An inherited disorder that leads to copper accumulation in various organs. It typically presents with neurologic symptoms such as tremors, hepatic involvement, and Kayser-Fleischer rings, but does not include the specific dietary sensitivity and metabolic issues seen in MSUD.
- **Mucopolysaccharidosis (MPS) Types**: A group of lysosomal storage diseases caused by deficiencies of enzymes necessary for normal metabolism of mucopolysaccharides. These conditions can present with hepatosplenomegaly, skeletal abnormalities, and progressive neurological decline but do not have the acute metabolic crisis seen in MSUD.
- **Reye Syndrome (RS)**: A rare but serious condition that affects the brain and liver caused by aspirin use during viral infections. Although it can cause vomiting, lethargy, and seizures, its progression and dietary triggers are distinct from those of MSUD.

Each of these conditions has specific clinical features and biochemical tests that differentiate them from Maple Syrup Urine Disease. A definitive diagnosis in such cases would likely require genetic testing for BCKD or other specific enzyme deficiencies.

**77. user 2025-02-18T11:46:32Z:**

The onset is neonatal, usually within a week of birth.Recurrent vomiting, diarrhea, abdominal distension and other gastrointestinal symptoms were the first symptoms, accompanied by hepatosplenomegaly and growth disorders; nervous system involvement was manifested as hypoactivity, tendon hyperreflexia, ankle clonus and angular arch opisthotonus.Macrophage cholesterol ester and triglyceride accumulation leads to hepatosplenomegaly, progressive aggravation and even liver failure, elevated transaminases, bilirubin, hypoproteinemia, coagulation abnormalities, etc. within months.Adrenal enlargement with calcification leads to adrenal cortical insufficiency, and 60% to 70% of adrenal calcifications can be detected by imaging.Gallbladder dysfunction and stroke have also been reported.As the disease progresses, multiple organ dysfunction can occur, including anemia, gastrointestinal bleeding, sepsis, coma, etc.

**qwen2.5_7b** 2025-02-18T11:46:38Z:

Based on the clinical manifestations provided, the most likely rare disease is Niemann-Pick Disease Type C (NPC), which primarily affects the brain and liver. Here are five other possible diagnoses to consider:

- **Niemann-Pick Disease Types A and B**: These types involve significant neurological involvement and organ dysfunction but typically present earlier in life with more pronounced neurologic symptoms.
- **Gaucher Disease**: This is another lysosomal storage disorder that can affect the liver, spleen, and bone marrow, leading to hepatosplenomegaly and other systemic issues. However, it usually does not involve such severe neurological manifestations early on.
- **Fabry Disease**: A rare X-linked genetic disorder characterized by pain in the hands and feet, renal impairment, cardiovascular complications, and gastrointestinal symptoms. It can also present with growth disorders but typically without hepatosplenomegaly as severe or calcified adrenal involvement.
- **Zellweger Spectrum Disorder (Peroxisomal Biogenesis Disorders)**: This is a group of peroxisomal disorders that can cause liver dysfunction, neurological issues, and other systemic problems. However, it usually presents earlier with more pronounced signs in the neonatal period.
- **Sideroblastic Anemias**: These are a group of anemias where there is defective iron utilization by the erythroid precursors leading to macrocytic anemia, often associated with growth retardation and organomegaly but without the distinctive features like hepatosplenomegaly with calcified adrenal glands.

The characteristic features of Niemann-Pick Disease Type C, including the late-onset nature (neonatal onset is more common for types A and B), progressive neurological decline, liver dysfunction, and adrenal calcification, are highly suggestive of NPC.

**78. user 2025-02-18T11:47:03Z:**

**1. The cardiovascular system** can result in aortic root dilatation, aortic valve insufficiency and aortic coarctation; mitral valve prolapse with or without insufficiency may also occur. The prevalence of ascending aortic dilatation increases with age. Between the ages of 30 and 60, the prevalence increases from 53% to 96%. Patients may present with shortness of breath, chest pain, palpitations, dizziness, syncope, or haemoptysis. Physical examination reveals an enlargement of the heart border to the lower left, a systolic murmur in the mitral valve area and a diastolic predominantly biphasic murmur in the aortic valve area, peripheral vascular signs, and signs of heart failure.

**2. Skeletal manifestations** , tall and thin stature, elongated fingers and toes, arms extended beyond height, scoliosis or kyphosis of the spine, funnel chest or chicken chest, laxity of the joints, flat feet.

**3. Ocular abnormalities**, eye lesions account for 50% to 70% of the cases, including corneal flattening, prolongation of the eyeball axis, retinal detachment, cataracts, subluxation or subluxation of the lens, iris nystagmus or mild nystagmus, pupil displacement, and clouding of the lens, etc. **4.

**4. Other ,**  dural bulge, atrophic striae, recurrent or incisional hernia, high palpebral vault, etc.

**qwen2.5_7b** 2025-02-18T11:47:08Z:

Based on the clinical manifestations you have provided, it appears that these symptoms align closely with Marfan syndrome, a connective tissue disorder characterized by cardiovascular abnormalities, skeletal features, ocular issues, and other systemic manifestations. Here are five other possible diagnoses to consider:

- **Marfan Syndrome**:
  - Cardiovascular: Aortic root dilatation, aortic valve insufficiency, coarctation of the aorta.
  - Skeletal: Tall stature, long slender fingers (arachnodactyly), pectus deformities.
  - Ocular: Lens dislocation, ectopia lentis, retinal detachments.
- **Ehlers-Danlos Syndrome (EDS)**:
  - Connective tissue disorder with joint hypermobility, skin hyperelasticity, and vascular fragility.
  - Skeletal: Hypermobility of joints, scoliosis, rib notches.
  - Skin: Hyperextensible skin, easy bruising.
- **Loeys-Dietz Syndrome**:
  - Cardiovascular: Aortic aneurysm, aortic dissection, pulmonary hypertension.
  - Skeletal: Arachnodactyly, dural ectasia (related to the bulging of the dura mater in the cranium).
  - Ocular: Lens subluxation.
- **Vasovagal Syncope Syndromes**:
  - Cardiovascular: Episodes of syncope due to vasovagal reflex.
  - Not typically associated with skeletal or ocular abnormalities but can present with cardiovascular manifestations like palpitations and dizziness.
- **Homocystinuria**:
  - Metabolic disorder affecting the metabolism of homocysteine, leading to various symptoms including osteoporosis, lens subluxation, and intellectual disability.
  - Cardiovascular: Aortic root dilatation but less common than in Marfan syndrome.
  - Skeletal: Bone fragility.

Each of these conditions should be evaluated with appropriate clinical assessment, genetic testing where applicable, and imaging studies to confirm the diagnosis.

**79. user 2025-02-18T11:47:38Z:**

**1. Skin manifestations**, patients are usually born with café au lait spots, often located on the same side as the skeletal lesions and bounded by the midline, which are brown or yellowish brown patches of pigmentation of varying sizes in one or more spots, with irregular borders.

**Skeletal manifestations** , approximately 98% of patients have either monostotic or polyostotic osteofibrous heteroplasia, with the polyostotic type being more common. It is characterised by an increase in the number of aberrant osteoblasts and their abnormal differentiation, causing extensive proliferation and differentiation of fibroblasts in the bone marrow and the formation of infantile woven bones, which are prone to pathological fractures and skeletal deformities and cause bone pain. It can involve almost all bones of the body, especially craniofacial and long bones, and the local bones are swollen and elevated, resulting in changes in the structure of the face and symptoms of compression. Severe orbital involvement may compress the optic nerve and cause visual impairment. Involvement of the auditory nerve may cause hearing loss. Involvement of the spine may result in vertebral compression fractures, causing scoliosis or kyphosis. The peak age for pathological fractures due to osteofibrous heteroplasia is 6-10 years. Imaging shows expansive, osteolytic, or ground-glass changes in the bone.

**Involvement of endocrine glands** may involve a variety of endocrine glands, resulting in hyperfunction or tumours of the affected glands and normal or decreased serum levels of pro-secretory hormones. Precocious puberty is more common and can occur in both sexes, but is more common in females and often precedes bone lesions. Female children may show early development of secondary sexual characteristics, premature irregular vaginal bleeding, enlarged uterus and ovaries compared with normal children of the same age, or ovarian cysts, premature healing of epiphyses, and so on. In males, precocious puberty is relatively rare, and testicular lesions (Leydig cell hyperplasia, microscopic stones, focal calcification, etc.) are seen on ultrasound. Thyroid enlargement and hyperfunction are also relatively common, and hyperparathyroidism has been reported. Other endocrine disorders such as cortisolism, gigantism / acromegaly, hyperprolactinaemia have been reported but are relatively rare.

**4. Hypophosphatemia** , a combination of hypophosphatemia, manifesting as hypophosphatemic rickets, has been reported in some patients. Those with combined hypophosphataemia usually have a polyostotic type of abnormal bone fibre proliferation.

**5. Other,** patients may also present with rare clinical symptoms such as jaundice, hepatitis, arrhythmias, and intestinal polyps due to mutations involving peripheral blood leukocytes, liver, heart, thymus, and gastrointestinal tract.

**qwen2.5_7b** 2025-02-18T11:47:44Z:

Based on the provided clinical manifestations, the most likely diagnosis is **McCune-Albright Syndrome (MAS)**. This rare disorder often presents with a combination of bone lesions, skin pigmentation, and endocrine abnormalities which closely match the symptoms described:

- **McCune-Albright Syndrome (MAS)**
- **Multiple Endocrine Neoplasia Type 1 (MEN1)**
- **Gardner Syndrome** - Characterized by polyostotic fibrous dysplasia, gastrointestinal polyps, and bone deformities, but often with more specific colonic symptoms.
- **McCune-Lampron Disease** - Similar to MAS but typically associated with a later age of onset and less frequent endocrine manifestations.
- **Fibrous Dysplasia (FD)**
- **Osteitis Fibrosa Cystica**

### Detailed Breakdown:

- **McCune-Albright Syndrome (MAS):**
  - Characterized by polyostotic fibrous dysplasia of the bones, café-au-lait spots on the skin, and endocrine abnormalities.
- **Multiple Endocrine Neoplasia Type 1 (MEN1):**
  - Involves tumors in multiple endocrine glands like the parathyroid, pituitary, and pancreas, which can present with similar symptoms to MAS but without the bone lesions or café-au-lait spots.
- **Gardner Syndrome:**
  - Features include polyposis coli (intestinal polyps) along with fibrous dysplasia of the bones, often presenting with gastrointestinal symptoms like blood in stools and skin manifestations, though less frequent than in MAS.
- **McCune-Lampron Disease:**
  - Similar to MAS but typically presents later in life and with a different pattern of endocrine involvement.
- **Fibrous Dysplasia (FD):**
  - Primarily affects the bones, leading to characteristic bone deformities and pain due to fibrous tissue replacing normal bone. However, it does not usually include skin pigmentation or significant endocrine abnormalities like those seen in MAS.
- **Osteitis Fibrosa Cystica:**
  - Characterized by severe hypophosphatemia leading to rickets-like symptoms, but typically without the café-au-lait spots and endocrine issues observed in MAS.

Each of these diagnoses would be considered based on their specific clinical presentations and the unique combination of symptoms you have outlined. Further diagnostic testing such as genetic analysis or imaging studies might be necessary for a definitive diagnosis.

**80. user 2025-02-18T11:48:20Z:**

The majority of patients have onset of symptoms between 3 months and 3 years of age, with a small percentage having onset in the neonatal period or in adulthood, while others are asymptomatic. The onset of the disease is usually triggered by a predisposing factor, such as prolonged starvation or a complication of an infectious disease. In children with early onset of the disease, the first symptoms are drowsiness and vomiting, but may also include convulsions and asphyxia, which often progress rapidly to coma or death. Acute onset hypoglycaemia is typical, and a decrease in blood glucose with a negative urine ketone body is helpful in the diagnosis. 50% of children have hepatomegaly, and liver biopsy reveals a large accumulation of lipids. Imaging reveals cerebral oedema in some children. Cardiac damage is rare. Adult-onset patients present with a wide range of clinical manifestations, including multiple organ damage, including muscle, liver, nervous system, or cardiovascular system. Vomiting is the most common symptom, hepatomegaly is relatively rare, and patients presenting with symptoms during pregnancy show acute fatty liver. Most patients have acute non-inflammatory encephalopathy. Rhabdomyolysis occurs in about one-third of patients, with a significant increase in creatine kinase and myoglobinuria. There is a high mortality rate, with approximately 25% of children dying from the onset of the disease, and an even higher rate of 50% in adults with acute onset of the disease. Patients tend to behave normally between episodes. About one third of patients who survive the acute onset of the disease develop sequelae, including growth retardation, motor retardation, mental retardation, language deficits, psycho-behavioural problems, epilepsy, cerebral palsy, hemiparesis, and chronic muscle weakness. In addition, some patients have complaints of skeletal muscle damage such as fatigue, myalgia and reduced exercise tolerance.

**qwen2.5_7b** 2025-02-18T11:48:28Z:

Based on the clinical manifestations you’ve provided, the most likely rare disease is **Reye’s Syndrome** (also known as Reye Syndrome or Viral Illness-Induced Hepatic Encephalopathy). This condition primarily affects children who are recovering from viral infections, such as influenza or chickenpox. Key features include:

- **Reye’s Syndrome**
  - **Clinical Manifestations:**
    - Onset between 3 months and 3 years of age (although cases in neonates and adults can occur).
    - Often triggered by a predisposing factor like viral infections.
    - Early onset includes drowsiness, vomiting, convulsions, asphyxia, rapid progression to coma or death.
    - Acute hypoglycemia, hepatomegaly, cerebral edema observed on imaging.
    - Cardiac damage is rare.
    - Adult-onset presents with diverse clinical manifestations, including acute fatty liver and non-inflammatory encephalopathy.
- **Other Possible Diagnoses:**
  - **Lipid Storage Diseases:** Such as Niemann-Pick disease or Gaucher’s disease can present with hepatomegaly, lipid accumulation in the brain, and neurodevelopmental issues.
    - **Clinical Manifestations:**
      - Early onset of symptoms (can vary).
      - Neurological involvement (drowsiness, encephalopathy) and organ involvement (hepatic and/or neurologic).
      - Lipid storage within cells leading to various clinical presentations.
  - **Acute Interstitial Nephritis:** Can present with acute kidney injury, vomiting, and hepatomegaly.
    - **Clinical Manifestations:**
      - Rapid onset of symptoms including nausea, vomiting, and abdominal pain.
      - Hepatomegaly can occur due to drug reactions or other causes.
      - Acute renal failure and altered mental status in severe cases.
  - **Autoimmune Hepatitis:** Can present with hepatomegaly, jaundice, and encephalopathy.
    - **Clinical Manifestations:**
      - Autoimmune-mediated liver injury leading to elevated transaminases and bilirubin levels.
      - Encephalopathy due to hepatic dysfunction.
      - Possible muscle symptoms such as myalgia.
  - **Metabolic Disorders:** Such as Maple Syrup Urine Disease (MSUD) or Urea Cycle Disorders can present with acute encephalopathy, metabolic acidosis, and hepatomegaly.
    - **Clinical Manifestations:**
      - Onset during infancy or early childhood.
      - Metabolic crisis triggered by high-protein diet leading to ketoacidosis and encephalopathy.
      - Unique odor of urine (MSUD) and elevated blood ammonia levels.
  - **Toxic Encephalopathy:** Caused by various toxins, including methanol or ethylene glycol poisoning.
    - **Clinical Manifestations:**
      - Rapid onset of neurological symptoms such as confusion, ataxia, and seizures.
      - Hepatic dysfunction with acute liver failure.
      - Vomiting and altered mental status.

These diagnoses are based on the clinical features described, including age of onset, triggers, organ involvement, and characteristic symptoms. Each condition requires specific diagnostic criteria and tests to confirm the diagnosis.

**81. user 2025-02-18T11:48:58Z:**

The clinical manifestations of the disease vary among age groups. The earlier the age of onset, the more severe the acute metabolic disorders and encephalopathy. Clinical manifestations include gastrointestinal symptoms such as vomiting, feeding difficulties and hepatomegaly, as well as neurological symptoms such as dyskinesia, impaired consciousness, convulsions, delayed or regressed growth and microcephaly. Recurrent illness is mostly associated with infection and stress.

Neonatal-onset patients usually present with acute encephalopathy-like symptoms in the first few hours to one week after birth, which are manifested by vomiting, hypotonia, dehydration, severe acidosis, hyperlactatemia, hyperammonemia, coma, and convulsions, with a high morbidity and mortality rate. Childhood onset of the disease is normal at birth, mostly within 1 year of age, the first metabolic crisis is often triggered by infection, hunger, fatigue, vaccination and other stress factors, or high-protein diets and medications, which, if not diagnosed and treated in a timely manner, can lead to retardation, backwardness, and regression of mental and motor development, and can be accompanied by involvement of the haematological system, the liver, the kidneys, the skin, and peripheral nerves. In adults, the first symptoms may include peripheral neuropathy and psychiatric abnormalities.

**qwen2.5_7b** 2025-02-18T11:49:19Z:

Based on the clinical manifestations described, the most likely diagnosis is **Classic Onset Medium-Chain Acyl-CoA Dehydrogenase Deficiency (MCADD)**. This condition is one of the more common fatty acid oxidation disorders that can present with a wide range of symptoms depending on age at onset.

### Five Possible Diagnoses:

- **Very Long Chain Acyl-CoA Dehydrogenase Deficiency (VLCADD)**
- **Carnitine Palmitoyltransferase I Deficiency (CPTI deficiency)**
- **Long-Chain 3-Hydroxyacyl-CoA Dehydrogenase Deficiency (LCHAD deficiency)**
- **Short-Chain Lysine Oxidase Deficiency (SCLOD)**
- **Very Long Chain Acyl-CoA Dehydrogenase Deficiency with Hepatopathy (VLCADD-Hep)**

### Explanation:

- **Classic Onset MCADD:**
  - This condition is characterized by the accumulation of medium-chain fatty acids, which can lead to metabolic crises in response to fasting or illness.
  - It typically presents with symptoms like vomiting, hypoglycemia, acidosis, and encephalopathy, especially in neonates.
- **VLCADD:**
  - Involves the deficiency of very long-chain acyl-CoA dehydrogenase (VLCDH), leading to accumulation of very-long-chain fatty acids.
  - Symptoms can be similar but might include more severe liver involvement and neurological symptoms in adults.
- **CPTI Deficiency:**
  - Affects carnitine palmitoyltransferase I, which is involved in the transport of fatty acids into mitochondria for beta-oxidation.
  - Presents with acute metabolic decompensations like hypoglycemia and liver dysfunction, especially after fasting.
- **LCHAD Deficiency:**
  - Involves long-chain 3-hydroxyacyl-CoA dehydrogenase, leading to the accumulation of very-long-chain fatty acids.
  - Symptoms include hepatomegaly, encephalopathy, and hypoglycemia, particularly in children.
- **SCLOD:**
  - Short-Chain Lysine Oxidase Deficiency is a rare condition that can present with symptoms like lethargy, vomiting, and metabolic acidosis.
  - It often has a late-onset form associated with neurological complications.
- **VLCADD-Hep (Very Long Chain Acyl-CoA Dehydrogenase Deficiency with Hepatopathy):**
  - This variant of VLCADD involves more significant liver involvement compared to classical VLCADD, leading to chronic liver disease and other systemic manifestations.

Each of these conditions has distinct biochemical abnormalities that can be diagnosed through specialized laboratory tests such as acylcarnitine profiles, genetic testing for mutations in the relevant genes, and sometimes specific enzyme assays. Early diagnosis and management are crucial to prevent severe metabolic crises and improve outcomes.

**82. user 2025-02-18T11:50:44Z:**

The typical patient is normal at birth. At the age of 6 months to 1 year, the patient’s face gradually becomes ugly, with flattening in the middle of the face, widening of the bridge of the nose, clouding of the cornea, deafness, large head, long anterior and posterior diameters, protrusion of the forehead, stiffness of the joints, claw shaped hands, chicken chest, and lumbar lordosis. Heart valve thickening, cardiomyopathy or congestive heart failure often appear at the age of 2 to 5 years, and liver and spleen enlargement at the age of 5 years. Mental and motor development is backward. They usually survive until about 10 years of age. Most of them die of heart failure and pneumonia. In the mild form, symptoms appear after the age of 5 years and include mild coarse facial features, corneal clouding, and stiffness of the joints of the hands and feet. Intelligence is normal and life expectancy is relatively long. Clinical symptoms overlap between subtypes, but there are no significant differences in biochemical findings between subtypes.

**qwen2.5_7b** 2025-02-18T11:50:56Z:

Based on the clinical manifestations you’ve described, the most likely rare disease is **Mandibulofacial Dysostosis (MFD) with or without deafness**, also known as Pfeiffer syndrome. This condition is characterized by craniofacial dysostosis, skeletal abnormalities, and often hearing loss.

Here are five other possible diagnoses that could present similar clinical features:

- **Apert Syndrome**: Characterized by a distinctive facies (including broad, short head), syndactyly of the hands and feet, and midface hypoplasia. Hearing loss can also be present.
- **Crouzon Syndrome**: Involves premature fusion of certain skull bones (craniosynostosis), causing the head to misshape. It can also lead to facial abnormalities including a beaked nose and eyes that bulge outward.
- **Saethre-Chop端正脸综合征**（Saethre-Chop状脸综合症）：这是一种罕见的遗传性颅骨发育异常，表现为面部不对称、前额低平或分裂等。患者通常还会出现手指或脚趾部分融合。
- **Kabuki Syndrome**: A genetic disorder that can cause a wide range of symptoms, including craniofacial abnormalities such as an asymmetric face and large ears. It also often includes developmental delays, learning difficulties, and other physical features.
- **Treacher Collins Syndrome (TCS)**: This syndrome affects the development of bones in the face and jaw and can lead to hearing loss, cleft palate, and ear malformations. Patients may also exhibit a distinctive facial appearance with downslanted eyes and zygomatic bone hypoplasia.

These conditions often overlap in their clinical features, making differential diagnosis complex. Detailed genetic testing and further clinical evaluation are typically required for accurate diagnosis.

**83. user 2025-02-18T11:51:41Z:**

Insidious onset of disease. The disease develops slowly or progresses in stages and may have long periods of stability. The disease can develop at any age, mostly before 50 years old. Clinical manifestations are mainly multiple mononeuropathies, asymmetric distal upper limb is more common, early unilateral upper limb one or more nerves involved, manifested as muscle weakness of the corresponding innervated area, distal predominantly, may be accompanied by spasm or fasciculations. With the development of the disease, muscle atrophy can occur; with a longer course of the disease, multiple nerves of multiple limbs can be involved, and the asymmetry of the affected nerves may not be obvious, but presenting a distribution similar to that of polypoidal peripheral neuropathy. Patients may complain of mild sensory abnormalities, but there is a lack of objective signs of sensory involvement, and some patients may also have partial sensory nerve involvement in the later stages of the disease. Cerebral nerves are usually not involved. There are no signs of upper motor neuron involvement.

**qwen2.5_7b** 2025-02-18T11:52:04Z:

Based on the clinical manifestations provided, the most likely rare disease to be diagnosed is **Chronic Inflammatory Demyelinating Polyneuropathy (CIDP)**. CIDP is a chronic disorder characterized by symmetric or asymmetric limb weakness and peripheral neuropathy that usually has an insidious onset.

Here are five other possible diagnoses:

- **Multifocal Motor Neuropathy (MMN)**
- **Acute Inflammatory Demyelinating Polyneuropathy (AIDP, Guillain-Barré Syndrome)**
- **Hereditary Motor and Sensory Neuropathies (HMSN or Charcot-Marie-Tooth disease)**
- **Complex Regional Pain Syndrome (CRPS) with Neurogenic Component**
- **Vitamin B12 Deficiency with Associated Neuropathy**

These diagnoses are based on the clinical features you’ve outlined, such as insidious onset, asymmetric distal muscle weakness, and the absence of upper motor neuron involvement or significant sensory abnormalities early in the disease course.

**84. user 2025-02-18T11:52:49Z:**

Neonatal onset is often fatal and may present as a metabolic crisis including metabolic acidosis, non-ketotic hypoglycaemia, hyperammonaemia, and other features including multisystem involvement (hypotonia, cardiomyopathy, hepatomegaly), excretion of large amounts of fatty acid- and amino acid-derived metabolites, ‘sweaty-foot’ body odour, coma, and neonatal death. Rapidly progressive hypertrophic cardiomyopathy is common in the type with neonatal onset and has a very high probability of sudden death, which is difficult to reverse, even if the disease is detected at an early stage by neonatal screening.

Congenital malformations are thought to be the consequence of the accumulation of toxic metabolites in the part of the body not corrected by embryonic transport. The degree of impaired fatty acid oxidative metabolism determines the degree of acidosis in utero, which in turn prevents normal embryo formation. Congenital malformations include enlarged polycystic kidneys, rocker-bottom feet, absence of lower abdominal muscles, hypospadias and penile curvature, hypotonia, cerebral cortical dysplasia, and gliosis. Other dysmorphic features include wide eye spacing, ear malformations, megalocrania, wide fontanel, high forehead, and low nasal bridge.

The age of onset and symptoms of late-onset forms vary considerably among cases and are mainly characterised by the absence of congenital malformations but with a lifelong risk of acute intermittent episodes of vomiting, dehydration, hypo-ketotic hypoglycaemia, acidosis, and impaired consciousness. Older individuals may have hepatomegaly or lipid deposition myopathy. Acute loss-of-compensation episodes can be triggered by infection, fever, surgery, low-energy diets or weight loss, alcohol consumption, and pregnancy. Most patients develop chronic myopathy symptoms, including exercise intolerance, myalgia, muscle weakness, and muscle atrophy. Organic aciduria in patients with late-onset disease is often intermittent and is more pronounced only during disease and under catabolic stress.

**qwen2.5_7b** 2025-02-18T11:52:54Z:

Based on the clinical manifestations provided, the most likely rare disease is Medium-Chain Acyl-CoA Dehydrogenase Deficiency (MCADD). This condition fits many of the described symptoms, including:

- **Medium-Chain Acyl-CoA Dehydrogenase Deficiency (MCADD)** - This is a disorder that affects the metabolism of fatty acids and can present with metabolic crises in neonates and later in life due to fasting or illness.
- **Long-Chain 3-Hydroxyacyl-CoA Dehydrogenase Deficiency (LCHAD Deficiency)** - Similar to MCADD, this condition involves issues with fatty acid oxidation but affects longer chain fatty acids and can present similarly.
- **Very Long Chain Acyl-CoA Dehydrogenase Deficiency (VLCADD/ALD)** - This is another form of fatty acid oxidation disorder that can cause metabolic crises in infants and young children.
- **Fatty Acid Oxidation Disorders (FAODs) Group** - This includes a variety of disorders such as Medium-Chain Acyl-CoA Dehydrogenase Deficiency, Long-Chain 3-Hydroxyacyl-CoA Dehydrogenase Deficiency, and Very Long Chain Acyl-CoA Dehydrogenase Deficiency.
- **Organic Acidemias** - This group includes various inborn errors of metabolism that can present with organic aciduria and metabolic crises. Examples include Methylmalonic Acidemia (MMA) and Propionic Acidemia (PA).
- **Congenital Lactic Acidosis Syndromes** - These conditions often present with lactic acidosis, hypoglycemia, and other symptoms like those described. They are less specific to fatty acid oxidation but can overlap in clinical presentation.

Given the detailed nature of the provided clinical manifestations, MCADD is the most likely diagnosis due to its wide range of symptoms from neonatal lethality to lifelong episodic metabolic crises triggered by various stressors.

**85. user 2025-02-18T11:54:11Z:**

The average age of onset of the disease is 30 years old, and it is rare to find patients under 10 years old and over 50 years old. The prevalence of women is higher than that of men, with the ratio of women to men (1.4-2.3):1. The disease is mostly acute/subacute, and is characterised by spatial and temporal multiplicity. The spatial multiplicity refers to the multiple sites of the lesions, including the brain, brainstem, cerebellum, and spinal cord, which may be involved simultaneously or successively. Time-recurrent refers to the course of remission-relapse, and due to the wide range of sites involved, repeated episodes may eventually lead to disability or even death. Clinical manifestations are varied, and common symptoms include vision loss, diplopia, limb sensory deficits, limb motor deficits, ataxia, and bladder or rectal dysfunction.

**qwen2.5_7b** 2025-02-18T11:54:15Z:

Based on the clinical manifestations provided, the disease that most likely fits this description is **Acute Disseminated Encephalomyelitis (ADEM)**. ADEM typically presents with acute/subacute onset and can affect multiple regions of the central nervous system including the brain, brainstem, cerebellum, and spinal cord. The spatial multiplicity and time-recurrent nature of symptoms align well with this condition.

### Five Other Possible Diagnoses:

- **Multiple Sclerosis (MS)** - Although MS is more chronic in its progression, it can present acutely or subacutely and can involve multiple areas of the CNS, causing a wide range of neurological deficits.
- **Neuromyelitis Optica Spectrum Disorder (NMOSD)** - This condition often involves vision loss and can cause acute spinal cord inflammation leading to motor and sensory deficits.
- **Acute Cerebellar Ataxia with Encephalopathy** - While less common, this condition could present with ataxia, which is a common symptom mentioned, alongside other neurological symptoms.
- **Hereditary Inclusion Body Myopathies (HIBM)** - Although primarily affecting the muscles, some cases can have central nervous system involvement leading to neurological deficits that may match those described.
- **Paraneoplastic Neurological Syndromes** - These can present with a variety of neurological signs and symptoms, often associated with underlying malignancies but can sometimes be seen in the absence of an obvious tumor.

Each of these conditions presents with a range of clinical manifestations similar to ADEM, making differential diagnosis important for appropriate treatment.

**86. user 2025-02-18T11:54:41Z:**

The main symptoms include parkinsonism, cerebellar ataxia, pyramidal tract damage, and autonomic nervous system damage. The first symptoms are often autonomic impairments, most notably cardiovascular dysfunction (e.g., upright hypotension) and urinary dysfunction (e.g., urinary incontinence), the latter of which tends to be comorbid with sexual dysfunction in male patients. Many patients have had rapid eye movement (REM) sleep behaviour disorder (RBD) for several years.

**qwen2.5_7b** 2025-02-18T11:54:45Z:

Based on the clinical manifestations you’ve provided, the most likely diagnosis is Multiple System Atrophy (MSA), specifically the autonomic-predominant or cerebellar subtype. MSA is a rare, progressive disorder that affects the autonomic nervous system, movement control centers in the brain, and balance.

Here are five other possible diagnoses:

- **Shy-Drager Syndrome** - This is an older term for the autonomic symptoms of MSA, which includes cardiovascular and urinary dysfunction.
- **Progressive Supranuclear Palsy (PSP)** - PSP can present with parkinsonism and ataxia but often has distinct features like vertical gaze palsy and a more consistent cognitive decline.
- **Corticobasal Degeneration (CBD)** - This condition may include parkinsonian symptoms, ataxia, and other signs such as limb rigidity and apraxia.
- **Pick Disease** - A form of frontotemporal dementia that can present with behavioral changes, language problems, and motor decline, though it typically does not cause autonomic dysfunction.
- **Dementia with Lewy Bodies (DLB)** - DLB may include parkinsonian features but is characterized by visual hallucinations, fluctuating cognition, and sleep disturbances.

Given the specific combination of symptoms, MSA remains a strong candidate for this presentation.

**87. user 2025-02-18T11:55:11Z:**

**1. Muscles,** weakness and atrophy of the distal limbs starting at the distal end of the limbs and progressing proximally, affecting the fine movements of the hands and causing drop foot. The facial muscles are also involved. At the same time, there is obvious muscle ankylosis, involving the limb muscles, pharyngeal muscles and so on. Physical examination may reveal that the fingers cannot be expanded immediately after clenching the fist vigorously, and the eyes cannot be opened immediately after closing them vigorously. Knocking on the hand, the big fish muscle, the tongue muscle, etc., can lead to muscle ankylosis, known as knocking muscle ankylosis or ‘muscle ball’ phenomenon. There is a ‘warming up’ phenomenon, which decreases after repeated activities. Lower extremity myalgia and generalised fatigue are also common clinical manifestations.

**The cardiac conduction system is often involved, with varying degrees of atrioventricular block and intraventricular conduction abnormalities. In severe cases, sudden death may result. Confirmed patients require regular ECG monitoring and pacemaker implantation if necessary.

**3. Central nervous system** , sleep disorders such as increased sleepiness and obstructive sleep apnoea are common. It may be associated with cognitive and mood disorders. Head MRI shows diffuse cerebral white matter lesions and cortical atrophy.

**Cataracts occur in almost all patients as they age. They often affect vision in the 30s and 40s.

**5. Endocrine system** A variety of endocrine disorders may occur, including diabetes mellitus, thyroid dysfunction, abnormal calcium and phosphorus metabolism, sex hormone abnormalities, and testicular atrophy (resulting in infertility).

In addition, there may be frontal baldness, gastrointestinal insufficiency and restrictive ventilation disorders.

**qwen2.5_7b** 2025-02-18T11:55:17Z:

Based on the clinical manifestations you’ve provided, the most likely diagnosis is **Charcot-Marie-Tooth Disease (CMT)**, specifically CMT type 1A. This condition is one of the most common hereditary peripheral neuropathies and fits well with several aspects of your description:

- **Muscle Weakness and Atrophy**: Distal muscle weakness and atrophy starting from the distal limbs and progressing proximally.
- **Facial Muscle Involvement**: Facial muscle involvement leading to expressions that are difficult to make, as seen in cases of “muscle ball” phenomena (knock-on ankylosis).
- **Cardiac Conduction System Involvement**: Heart rhythm disturbances such as atrioventricular block.
- **Central Nervous System Manifestations**: Cognitive and mood disorders along with diffuse cerebral white matter lesions on MRI.
- **Cataracts**: Common in older patients, often developing in the 30s or 40s.
- **Endocrine System Involvement**: Various endocrine abnormalities including thyroid dysfunction, abnormal calcium metabolism, and testicular atrophy leading to infertility.

### Other Possible Diagnoses:

- **Hypokalemic Periodic Paralysis (HPP)**: This condition can present with muscle weakness and cramps but does not typically involve the wide range of central nervous system symptoms seen in CMT.
- **Mitochondrial Myopathy, Encephalopathy, Lactic Acidosis, and Stroke-like Episodes (MELAS)**: This mitochondrial disorder could explain some of the neurological features such as headaches, stroke-like episodes, and cognitive decline but does not typically have the characteristic CMT pattern of muscle weakness and atrophy.
- **Familial Hypertrophic Cardiomyopathy (FHCM)**: While this condition can present with cardiac conduction abnormalities, it is less likely to explain the peripheral neuropathy and other systemic symptoms in your case.
- **Dermatomyositis**: This inflammatory myopathy could cause muscle weakness and atrophy but does not typically affect the central nervous system or lead to cataracts or endocrine disorders.
- **Axonal Hereditary Sensory and Autonomic Neuropathy (HSAN)**: This condition can present with similar peripheral neuropathy symptoms but is less likely to involve such a wide range of systemic manifestations.

These other conditions may have overlapping features, but the combination of peripheral nerve involvement, muscle atrophy, cardiac conduction issues, central nervous system manifestations, and endocrine disorders strongly points towards CMT.

**88. user 2025-02-18T11:55:43Z:**

The disease tends to develop at an early age, with the most frequent onset occurring in the neonatal period. Most children develop the disease within the first few days of life and present with milk refusal, vomiting, lethargy, convulsions and coma. Some children with late-onset disease are characterised by episodes of vomiting and lethargy with mental developmental abnormalities. Patients with hyperammonaemic coma may have brain injury with developmental delay, impaired learning ability or mental retardation, and sequelae of growth retardation. Patients with hyperammonaemia may have increased intracranial pressure, shortness of breath, apnoea or failure as the condition progresses, and hepatomegaly. Neurological complications include mental retardation, acute hyperammonemic coma, and even death. Biochemical changes can be seen in hyperammonaemia, increased blood glutamine and alanine, and decreased or absent urinary whey acids.

**qwen2.5_7b** 2025-02-18T11:55:47Z:

Based on the clinical manifestations provided, the most likely diagnosis is **Classic Phenylketonuria (PKU)**. PKU is a rare genetic disorder that leads to the accumulation of phenylalanine in the body due to a deficiency in the enzyme phenylalanine hydroxylase.

### Five Other Possible Diagnoses:

- **Hereditary Ammonia Disease (HAMD)**
  - This includes conditions like argininosuccinic aciduria and citrullinemia, which can present with hyperammonemia similar to PKU but are due to different enzyme deficiencies.
- **Methylenetetrahydrofolate Reductase Deficiency (MTHFR)**
  - While primarily affecting folate metabolism, it can also lead to neurodevelopmental issues and may present similarly in some cases.
- **Carnitine Palmitoyltransferase II (CPT-II) Deficiency**
  - This disorder affects fatty acid oxidation and can cause metabolic crises with symptoms including vomiting, lethargy, and encephalopathy.
- **Propionic Acidemia (PA)**
  - PA is a catabolic defect in the metabolism of branched-chain amino acids, leading to hyperammonemia and metabolic acidosis, which can present similarly to PKU but with distinct biochemical features.
- **Cystinuria**
  - While primarily a disorder of renal tubular transport proteins for neutral amino acids, it can lead to hyperammonemia in cases where there is significant kidney stone formation leading to obstruction and subsequent hyperammonemia.

Each of these conditions shares some clinical similarities with PKU but have distinct biochemical pathways and genetic origins. Proper diagnosis would require detailed metabolic testing and genetic analysis.

**89. user 2025-02-18T11:56:10Z:**

Children often present with severe intrauterine growth retardation and develop severe, non-ketotic hyperglycaemia very early in life (often in the first week of life), which recovers after 12 weeks and recurs around puberty in 50%-60% of cases, with a clinical picture similar to that of type 2 diabetes mellitus.

**qwen2.5_7b** 2025-02-18T11:56:15Z:

Based on the clinical manifestations you provided—severe intrauterine growth retardation followed by severe non-ketotic hyperglycemia early in life that resolves after 12 weeks but recurs around puberty, mimicking type 2 diabetes mellitus—the most likely diagnosis is **MODY (Maturity-Onset Diabetes of the Young)**. Specifically, this presentation aligns closely with **MODY3**, which is caused by mutations in the HNF1A gene.

### Possible Diagnoses:

- **MODY3 (HNF1A-Related Maturity-Onset Diabetes)**
- **Permanent Neonatal Diabetes Mellitus (PNDM)**: This can be due to a variety of genetic defects, including mutations in the KCNJ11 or ABCC8 genes.
- **Type 1 Diabetes Mellitus** (T1DM): While T1DM typically presents with ketoacidosis early on, some cases may present similarly and need careful differentiation through autoantibody testing.
- **Monogenic Diabetes Syndromes**: Other monogenic forms of diabetes can also fit this profile, such as those caused by mutations in the INS (Insulin) gene or GCK (Glucokinase) gene.
- **Congenital Hyperinsulinism** (CHI): This condition involves persistent hypoglycemia and may present with hyperglycemia in neonates due to compensatory mechanisms, but would typically not follow a pattern of resolution followed by recurrence.

### Diagnostic Approach:

- **Genetic Testing**: Testing for mutations in the HNF1A gene is highly recommended given the clinical presentation.
- **Metabolic Panel**: To assess electrolyte levels and other metabolic markers.
- **Autoantibody Testing**: For T1DM to rule it out or confirm if MODY3 is the cause.
- **Urine Analysis**: To check for ketones, which would be absent in non-ketotic hyperglycemia.

Given the specific pattern of symptoms and age-related recurrence, genetic testing will likely provide a definitive diagnosis.

**90. user 2025-02-18T12:01:26Z:**

The earliest symptoms are abdominal distension and enlargement of the liver and spleen, and some patients may develop in the neonatal period. Intellectual and motor developmental deficits occur immediately, with hypotonia and motor developmental delays, often without cerebral neurological involvement, and regression of motor and intellectual development is evident after the age of 1 year, eventually progressing to spasticity and tonicity.Cherry erythema on the fundus of the eye can be found in 50% of the patients. Interstitial lung disease may lead to recurrent respiratory infections, hypoxaemia or respiratory failure. Most children die before the age of 3 years.

**qwen2.5_7b** 2025-02-18T12:01:35Z:

Based on the clinical manifestations provided, the most likely rare disease is **Zellweger Spectrum Disorder (ZSD)**, which includes Zellweger syndrome and Neonatal Adrenoleukodystrophy (NALD) and Infantile Refsum Disease (IRD). These conditions are collectively known as Peroxisome Biogenesis Disorders (PBD).

Here are five other possible diagnoses that could present with similar features:

- **Hepatorenal Syndrome**: While primarily associated with liver failure, it can sometimes be seen in peroxisomal disorders and may include symptoms of liver dysfunction.
- **Fatty Acid Oxidation Disorders**: Such as Medium-Chain Acyl-CoA Dehydrogenase Deficiency (MCADD) or Long-Chain 3-Hydroxyacyl-CoA Dehydrogenase Deficiency, which can present with similar metabolic issues and liver/spleen enlargement.
- **Sjögren-Larsson Syndrome**: A rare genetic disorder that primarily affects the skin, hair, and nervous system but can also include hepatosplenomegaly and intellectual disabilities.
- **Dyserythropoietic Anemia (Pernicious Erythroblastosis)**: This condition involves abnormal red blood cell formation and can sometimes be associated with liver and spleen enlargement, though it is less commonly linked to the neurological symptoms described here.
- **Congenital Adrenoleukodystrophy (CALD)**: Although typically more focused on adrenal gland dysfunction leading to adrenal crisis early in life, some cases may show overlapping features such as liver/spleen involvement and progressive neurodegeneration.

Given the specific combination of hepatosplenomegaly, neurological symptoms, cherry erythema, and respiratory issues, Zellweger Spectrum Disorder remains a highly probable diagnosis. However, definitive diagnosis would require further biochemical tests (such as peroxisomal function assays) and genetic testing for known mutations associated with these disorders.

**91. user 2025-02-18T12:02:14Z:**

**1. Typical face,** patients of all ages have ptosis, wide eye spacing, medial canthus, and downward slanting of the outer corners of the eyes; low bilateral ear position, posterior rotation, and thick auricles. Child patients may also have a full forehead, low posterior hairline, short nose, low nasal bridge, full nasal tip; thick lips, deep and wide nasolabial groove straight to the upper lip, and so on.

**2. Dwarfism,** which is normal at birth and gradually appears after 1 year of age. Bone age often lags behind, and some adult heights can reach the lower limit of normal level.

**3. cardiovascular,** 50% ~80% of patients have congenital heart disease, pulmonary valve stenosis is the most common, others include hypertrophic cardiomyopathy, ventricular ischemia, pulmonary artery branch stenosis, tetralogy of Fallot, and aortic constriction, etc. Typical ECG changes include electrocardiogram (ECG) and heart rate (HR). Typical electrocardiographic changes include extreme rightward deviation of the electrical axis with counterclockwise rotation of the QRS wave in the precordial leads, leftward deviation of the electrical axis in the V1 lead, incomplete left anterior branch block, or RSR waveforms.

**4. psychomotor retardation**, poor language skills in some patients, depression in adult patients, and a high incidence of autism spectrum manifestations have also been reported.

**5. webbing of the neck**.

**6. thoracic abnormalities,** chicken chest or funnel chest.

**7. wide breast spacing**

**8. cryptorchidism in males** , which is present in 60% to 80% of male patients and can lead to fertility disorders.

**9. abnormal blood clotting and lymphatic dysplasia**

**10. Others,** renal malformations (dilated renal pelvis, double ureteral malformation, isolated kidney, renal hypoplasia, distal ureteral stenosis, etc.), impaired spermatogenesis in males, strabismus, nystagmus, milky coffee spots and freckles on the skin, follicular keratosis of the limbs, Arnold Chiari malformation type I, hydrocephalus, hepatosplenomegaly, and an increased incidence of malignant tumours.

**11. Intrauterine manifestations,** excessive amniotic fluid in the mother during pregnancy, widening of the nuchal translucency, relatively large fetal head, cardiac and renal anomalies.

**qwen2.5_7b** 2025-02-18T12:02:19Z:

Based on the clinical manifestations you’ve provided, these symptoms are highly indicative of **Treacher Collins Syndrome (TCS)**. Treacher Collins Syndrome is a rare genetic disorder that affects the development of bones and facial muscles before birth, leading to a characteristic facial appearance and other associated health issues.

Here are five additional possible diagnoses that could also fit some aspects of the clinical picture:

- **Nager Syndrome**: A rare genetic disorder characterized by craniofacial abnormalities similar to those in Treacher Collins syndrome but often with additional limb malformations.
- **Bardet-Biedl Syndrome (BBS)**: This is a complex genetic disorder that can affect multiple organs and systems, including the eyes, kidneys, liver, pancreas, and brain. It also causes retinitis pigmentosa, cognitive impairment, and obesity.
- **Kabuki Syndrome**: A rare genetic condition characterized by distinctive facial features, growth retardation, and developmental delays. It often includes additional symptoms like joint hypermobility, learning disabilities, and certain types of cancer.
- **Goldenhar Syndrome (Oculo-Auriculo-Vertebral Spectrum)**: This is a group of congenital abnormalities that involve the face, ears, neck, and spine. Symptoms include a spectrum from mild to severe facial defects, ear problems, eye issues, and skeletal malformations.
- **Pfeiffer Syndrome**: A rare genetic disorder characterized by craniofacial deformities (including a wide forehead, beaked nose, and widely spaced eyes), fusion of the fingers or toes, and other abnormalities like hearing loss, vision impairment, and heart defects.

These conditions can overlap in some symptoms but generally present with distinct differences. Given the comprehensive nature of your list, Treacher Collins Syndrome is the most likely primary diagnosis based on the wide range of systemic issues you’ve described.

**92. user 2025-02-18T12:02:53Z:**

Patients can develop the disease at any age. There are two main clinical categories: early-onset and late-onset. Early-onset cases mainly occur in male children, usually in the neonatal period, and are characterised by a rapid onset of the disease, with a rapid onset of the disease and extensive brain damage due to elevated ammonia levels in the blood. There may be no abnormality at birth, but in the first few days of life, the child will show irritability, lethargy, refusal to eat, shortness of breath and lethargy, which may rapidly develop into spasm, coma and respiratory failure. If not treated urgently, the disease quickly develops into hereditary metabolic encephalopathy and often dies within the first week of life, with survivors often left with severe intellectual impairment. Late-onset disease occurs in older patients, both hemizygous males and heterozygous females, and has relatively mild and variable clinical symptoms. Patients with childhood and adult onset tend to present with chronic neurological damage such as episodes of vomiting, headaches, abnormal behaviour, delirium and confusion. Patients may have clinical phenotypes such as hepatomegaly, recurrent seizures, growth retardation, and behavioural abnormalities. Despite the milder symptoms of the late-onset form, life-threatening acute episodes of hyperammonemia can be induced by environmental factors such as illness, stress, and a high-protein diet. The majority of heterozygous female carriers remain asymptomatic throughout their lives, while a few develop the disease, with individual variability in age of onset and clinical manifestations, both early and late onset.

**qwen2.5_7b** 2025-02-18T12:02:57Z:

Based on the clinical manifestations provided, the most likely rare disease is **Classic Canavan Disease** (Canavan disease). This condition is an inherited disorder that leads to severe brain damage. Here are five other possible diagnoses:

- **Classic Canavan Disease**: Characterized by rapid deterioration in neonates due to high ammonia levels leading to extensive brain damage and often death within the first year of life.
- **Methylmalonic Acidemia (c-Met Form)**: This is a metabolic disorder that leads to accumulation of methylmalonic acid, causing neurological symptoms similar to those seen in Canavan disease but with different biochemical markers.
- **Niemann-Pick Disease Type C**: A lysosomal storage disorder leading to progressive neurological deterioration. It can present with similar neurological symptoms including tremors, ataxia, and cognitive decline, though the onset is generally later than in Canavan disease.
- **Metachromatic Leukodystrophy (MLD)**: An autosomal recessive disorder due to arylsulfatase A deficiency leading to demyelination of the brain. Symptoms include progressive neurological deterioration with cognitive and motor dysfunction.
- **Glycogen Storage Disease Type IV (Mellitoleptic Glycogenosis)**: This is a rare inherited metabolic disorder characterized by defects in liver glycogen metabolism, presenting with acute encephalopathy, hypoglycemia, and recurrent hyperammonemia.

Each of these diseases has distinct biochemical pathways involved but can present with overlapping clinical features related to brain dysfunction, particularly in the context of hyperammonemia. Diagnostic confirmation would typically involve a combination of genetic testing, biochemical analysis, and imaging studies.

**93. user 2025-02-18T12:03:24Z:**

The clinical phenotype of the disease varies in severity. The main clinical manifestations of the patients are recurrent fractures under minor external forces, skeletal deformities, and varying degrees of activity limitation since childhood. Patients may have extra-skeletal manifestations such as blue sclera, ligamentous laxity, dentin hypoplasia, hearing loss, and cardiac valvulopathy, etc. The imaging features of OI mainly include: thinning of bones in many parts of the body; thinning of the cranial plate, widening of fontanelle and cranial suture, interstitial bone in occipital bones, and flattening of the base of the skull; lateral curvature or posterior convexity deformity of the spine, deformation of vertebral bodies, and compression fracture of multiple vertebral bodies; twisted and deformed thorax, or even collapse; slender long bones of the limbs, and even collapse; and the long bones of the limbs are thin and thin. The long bones of the limbs are slender, the bone cortex is thin, the bone marrow cavity is relatively large, the epiphyses are widened, there are many long bone fractures, and the bones are curved and deformed.

**qwen2.5_7b** 2025-02-18T12:03:28Z:

Based on the clinical manifestations you’ve described, the most likely rare disease diagnosis is **Osteogenesis Imperfecta (OI)**. This condition, also known as brittle bone disease, involves fragile bones that can break easily from minor stress or even spontaneously without any obvious trauma.

Five other possible diagnoses to consider are:

- **Mucopolysaccharidosis (MPS)**: A group of metabolic disorders where the body is unable to break down large sugar molecules (mucopolysaccharides), leading to skeletal abnormalities and various physical symptoms such as blue sclera, joint stiffness, heart problems, and respiratory issues.
- **Marfan Syndrome**: An autosomal dominant connective tissue disorder that can affect multiple organ systems, including the skeleton, cardiovascular system, eyes, and lungs. Patients often have tall stature, long limbs, and a curved spine (scoliosis). Cardiac valvulopathy is common, which could explain some of the symptoms you mentioned.
- **Spondylochalasis**: A rare hereditary disorder characterized by softening of the vertebral bone cortex and can lead to deformities such as kyphosis or scoliosis. It typically affects the spine but may also involve other skeletal changes.
- **Pseudohypoparathyroidism (PHP)**: An endocrine disorder that can present with various skeletal abnormalities, including short stature and brittle bones, though it is less likely to have the range of symptoms described compared to OI or similar conditions.
- **Hypophosphatasia**: A rare metabolic bone disease caused by deficiency in the enzyme alkaline phosphatase, leading to defective bone mineralization and resulting in weak and fragile bones, fractures, and skeletal deformities.

Each of these conditions should be considered based on a thorough clinical evaluation, imaging studies, biochemical tests, and genetic testing when necessary.

**94. user 2025-02-18T12:04:15Z:**

**1. Pancytopenia,** Many patients may have a combination of pancytopenia and neutropenia. Clinical manifestations include malaise, recurrent infections and bleeding tendencies.

**2. Haemoglobinuria** , typical haemoglobinuria is soy sauce or strong tea colour. It usually lasts for 23 days and subsides on its own without treatment, while in severe cases, it lasts for 12 weeks or even longer. Some patients have frequent episodes of haemoglobinuria, while a significant number of patients have no clear episodes of haemoglobinuria over a long period of time or observation.

Episodes of haemoglobinuria may be accompanied by fever, low back pain and abdominal pain. Other symptoms associated with smooth muscle dysfunction can include dysphagia, abdominal pain, bloating, back pain, headache, oesophageal spasms, and erectile dysfunction. Rare conditions include choledochal duct dyskinesia, acute pancreatitis, ischaemia and ulceration of the duodenum or colon.

**3. thrombosis**.

**Jaundice and hepatosplenomegaly** , less than half of patients have mild jaundice. Most patients do not have hepatosplenomegaly.

Common comorbidities include infection, anaemic heart disease, cholelithiasis, renal insufficiency and even failure.

**qwen2.5_7b** 2025-02-18T12:04:19Z:

Based on the clinical manifestations provided, the most likely rare disease is **Shwachman-Diamond Syndrome (SDS)**. SDS is a genetic disorder characterized by bone marrow failure leading to pancytopenia (reduction in all blood cell types) and neutropenia, as well as issues with exocrine pancreatic insufficiency.

Here are five other possible diagnoses that could be considered:

- **Thrombocytopenia-Absence of Pancreas Syndrome (TAPS)**: This is a rare genetic disorder causing pancytopenia along with the absence or underdevelopment of the pancreas, which can lead to issues similar to SDS but often associated with pancreatic insufficiency and diabetes.
- **Fanconi Anemia**: A rare genetic condition that leads to bone marrow failure (pancytopenia), malformation syndrome, and a predisposition to leukemia and solid tumors.
- **Hereditary Hemorrhagic Telangiectasia (HHT)**: While not primarily characterized by pancytopenia, HHT can present with bleeding tendencies and may be associated with other symptoms like those mentioned in the second case, such as haemoglobinuria and smooth muscle dysfunction.
- **Pernicious Anemia**: Although less likely given the absence of vitamin B12 deficiency symptoms typically seen, pernicious anemia can cause pancytopenia due to its effect on red blood cell production in the bone marrow.
- **Aplastic Anemia**: This condition involves a failure of the bone marrow to produce new blood cells, leading to pancytopenia and other associated symptoms like recurrent infections and bleeding tendencies.

Each of these conditions has specific diagnostic criteria that would help differentiate them from SDS or support another diagnosis if the clinical picture fits better.

**95. user 2025-02-18T12:04:50Z:**

Clinical manifestations include repeated intussusception, abdominal pain, diarrhoea, mucus stools, blood in stool, constipation, vomiting blood and anaemia. Characteristic clinical manifestations are skin and mucous membrane pigmentation and multiple polyps in the gastrointestinal tract. 95% of the patients have hyperpigmentation, which can be found on the lips and surrounding areas of the mouth, cheeks, face, fingers, palms of the hands, soles of the feet, perianal area, etc., and occasionally on the intestinal mucous membranes. The pigmentation can be brown, tan, grey, blue, etc. It usually occurs before the age of 2 years, and the size is 1~5mm. 88%-100% of the patients will develop gastrointestinal polyps, and the onset of gastrointestinal polyps most often occurs at the age of 11-13 years, and the polyps can occur throughout the entire gastrointestinal tract, with the small intestine being the most common, followed by the colon and the stomach. The size of the polyps is variable, and their tips vary in length and thickness. The risk of cancer is high, with an average age of 42 years.

**qwen2.5_7b** 2025-02-18T12:04:55Z:

Based on the clinical manifestations provided, the disease most likely to be diagnosed is Peutz-Jeghers Syndrome (PJS), a hereditary gastrointestinal hamartomatous polyposis disorder.

### Five Other Possible Diagnoses:

- **Juvenile Polyposis Syndrome (JPS)**: This condition involves multiple juvenile polyps in the gastrointestinal tract and can sometimes present with similar symptoms, but typically does not have the characteristic mucocutaneous pigmentation.
- **Lynch Syndrome (Hereditary Nonpolyposis Colorectal Cancer, HNPCC)**: While it is associated with colorectal cancer, Lynch syndrome does not usually cause the specific gastrointestinal polyps or skin/mucosal pigmentation seen in PJS.
- **Gardner Syndrome**: This condition involves multiple adenomatous colon polyps and extracolonic manifestations like osteomas, but typically does not include the characteristic mucocutaneous pigmentation of PJS.
- **Turcot Syndrome**: Similar to Gardner syndrome, it includes both colorectal cancer and central nervous system tumors, but usually without the skin/mucosal pigmentation seen in PJS.
- **Cowden Syndrome (PTEN Hamartoma Tumor Syndrome)**: This condition involves multiple hamartomatous polyps of the gastrointestinal tract along with other manifestations like mucocutaneous papules and an increased risk of breast cancer, but does not typically cause the specific gastrointestinal symptoms or skin/mucosal pigmentation as seen in PJS.

### Summary

- **Primary Diagnosis**: Peutz-Jeghers Syndrome (PJS)
- **Other Possible Diagnoses**:
  1. Juvenile Polyposis Syndrome (JPS)
  2. Lynch Syndrome (Hereditary Nonpolyposis Colorectal Cancer, HNPCC)
  3. Gardner Syndrome
  4. Turcot Syndrome
  5. Cowden Syndrome (PTEN Hamartoma Tumor Syndrome)

**96. user 2025-02-18T12:05:22Z:**

In the neonatal period, most of the children have no clinical symptoms, but after 3-4 months of life, typical symptoms gradually appear, and the symptoms are obvious at the age of 1 year. A few months after birth, due to insufficient melanin synthesis, their hair and iris colour gradually becomes lighter, yellow or brownish-yellow, with white skin. Urine and sweat contain a large amount of phenyl lactic acid and have a foul odour of rat urine. As they grow older, they gradually show delayed intellectual development, with predominantly cognitive developmental disorders, microcephaly, seizures, as well as behavioural and personality abnormalities, such as hyperactivity, self-mutilation, aggression, autism, low self-esteem, depression and other neurological manifestations. Vomiting and eczema are also commonly seen in infancy.

**qwen2.5_7b** 2025-02-18T12:05:26Z:

Based on the clinical manifestations provided, the most likely diagnosis is **Phenylketonuria (PKU)**. PKU is a genetic disorder that leads to an accumulation of phenylalanine due to a deficiency of the enzyme phenylalanine hydroxylase, which is necessary for converting phenylalanine into tyrosine.

Here are five other possible diagnoses that could present similar or overlapping symptoms:

- **Mucopolysaccharidosis (MPS) I** - Also known as Hurler syndrome, this is a group of rare genetic disorders caused by the deficiency of specific enzymes involved in breaking down complex carbohydrates called glycosaminoglycans.
- **Tay-Sachs Disease** - A fatal genetic disorder characterized by progressive damage to the nervous system that leads to mental retardation and death due to nerve cell deterioration.
- **Sickle Cell Disease** - While primarily associated with hemolytic anemia, it can present with neurological symptoms, delayed development, and other complications affecting multiple organ systems.
- **Wilson’s Disease** - An inherited disorder of copper metabolism that leads to copper toxicity in the liver, brain, and other organs. It is characterized by neurologic and psychiatric features along with liver dysfunction.
- **Homocystinuria** - A genetic metabolic disorder where the body cannot properly process certain proteins due to a deficiency of an enzyme called cystathionine beta-synthase (CBS). This can lead to intellectual disability, skeletal abnormalities, and cardiovascular problems.

These diagnoses are based on the specific signs and symptoms described such as delayed cognitive development, behavioral issues, neurological manifestations, and biochemical markers like the presence of phenyl lactic acid in urine and sweat.

**97. user 2025-02-18T12:06:20Z:**

Mainly skin symptoms and neuropsychiatric symptoms.

**1. The group of cutaneous symptoms,** are erythema, herpes and even ulceration on exposed parts of the skin after exposure to light. The rash may be of the type of eczema, urticaria, summertime itchy rash or erythema multiforme. The oral mucosa may have red patches and brownish-red teeth. Eye damage such as conjunctivitis, keratitis and iritis may also occur. In severe cases, there may be deformation of the skin of the nose, ears and fingers with kinking. There may be special purple colouration. Hirsutism may be present.

**2. A cluster of neuropsychiatric symptoms ,**  the most common manifestation of which is acute abdominal pain, which tends to be severe, stable, and poorly localised, sometimes accompanied by painful cramps. It may be accompanied by constipation, bloating, nausea, vomiting, and sometimes diarrhoea and increased bowel sounds. Sensory and motor neuropathies may precede the onset of abdominal pain and present as limb pain with numbness, abnormal sensation and sensory inversions; motor weakness often begins proximally in the upper limbs and may progress to the lower limbs and distal extremities. Involvement of the cerebral nerves may lead to medullary paralysis, impaired respiratory function and death. Severe motor neuropathy may result in quadriplegia. Autonomic nervous system involvement manifests as tachycardia, hypertension, sweating, agitation, and tremor. Neurogenic bladder dysfunction may cause dysuria, dyspareunia, urinary retention and incontinence. Other acute neuropsychiatric manifestations include insomnia, anxiety, agitation, agitation, hallucinations, hysteria, disorientation, delirium, affective apathy, depression, phobias, and altered consciousness ranging from lethargy to coma.

**qwen2.5_7b** 2025-02-18T12:06:24Z:

Based on the clinical manifestations you have provided, the rare disease most likely to be diagnosed is **Porphyria Cutanea Tarda (PCT)** with an acute intermittent variant or another form of Acute Intermittent Porphyria (AIP), as both conditions can present with a combination of severe photosensitivity leading to skin lesions and neuropsychiatric symptoms.

### Five Other Possible Diagnoses:

- **Cutaneous Lupus Erythematosus (CLE)** - Can present with light-sensitive rashes, particularly on the face and extremities, but typically does not include neurological symptoms.
- **Systemic Lupus Erythematosus (SLE)** - Often involves skin lesions and can have neuropsychiatric manifestations like cognitive impairment or psychosis, but usually presents with more systemic involvement such as joint pain, kidney issues, and blood abnormalities.
- **Vasculitis** - Especially if it is cutaneous vasculitis, which can cause skin ulcers and rashes. Neuropsychiatric symptoms may occur due to inflammation in the central nervous system.
- **Neuromyelitis Optica (NMO)** - While primarily affecting the optic nerves and spinal cord, it can present with diverse neuropsychiatric manifestations including pain, depression, and cognitive impairment. Skin findings are less common but can occur secondary to vasculitic changes.
- **Paraneoplastic Neurological Syndromes** - These conditions arise from a tumor producing antibodies that target nerve cells. They can cause various neurological symptoms and skin changes, particularly if the underlying cancer is in the liver or lymph nodes.

Each of these diagnoses would require specific diagnostic tests such as blood work, genetic testing for porphyrias, ANA titers for lupus, imaging studies, and sometimes biopsies to rule out or confirm a diagnosis.

**98. user 2025-02-18T12:07:25Z:**

**1. Infection, patients** are infected to varying degrees. Infections are recurrent, severe, difficult to treat, opportunistic and have a broad spectrum of pathogens. Typical children are usually born with no specific manifestations, and fatal infections can occur before 2 months of age due to cellular immune abnormalities. The sites and pathogens of infection are diverse, including recurrent pneumonia, recurrent thrush, persistent diarrhoea, recurrent otitis media, persistent viral infections (e.g., respiratory syncytial virus, EBV, CMV, etc.), and conditionally causative organisms, such as Pneumocystis carinii, are also common.

**2. Vaccine disease,** in which patients are extremely immunocompromised, can occur with infection and dissemination of live attenuated vaccines. Severe diarrhoea can also occur in children vaccinated with attenuated rotavirus vaccine.

**3. Retarded or arrested growth and development** is a distinguishing feature of the disease. It is associated with recurrent infections, diarrhoea and corresponding genetic defects.

Children may also have a rash and enlarged liver and spleen lymph nodes similar to erysipelas.

In other children, erythroderma-like symptoms, hepatosplenomegaly, seborrheic dermatitis, haematopenia, and sclerosing cholangitis may be present.

**qwen2.5_7b** 2025-02-18T12:07:32Z:

Based on the clinical manifestations provided, the most likely diagnosis is **Severe Combined Immunodeficiency (SCID)**, specifically a variant known as **X-linked SCID** or **Reticular Dysgenesis** due to its association with severe and recurrent infections, growth retardation, and other systemic symptoms.

Here are five other possible diagnoses that could also present with similar symptoms:

- **Wiskott-Aldrich Syndrome (WAS)**: This is a rare primary immunodeficiency characterized by eczema, thrombocytopenia, and increased susceptibility to infections. The recurrent infections can be severe and the immune system issues often lead to growth retardation.
- **DiGeorge Syndrome (DGS)**: Also known as 22q11.2 deletion syndrome, this condition results from a microdeletion in chromosome 22 that affects thymus development, leading to T-cell immunodeficiency. It can present with similar symptoms including recurrent infections, growth delays, and developmental issues.
- **Adenosine Deaminase Deficiency (ADA-SCID)**: This is one of the more well-known types of SCID where ADA enzyme deficiency leads to severe immune system failure. Patients often have recurrent infections and immunodeficiencies like those described, but may also have specific metabolic concerns related to adenosine metabolism.
- **Severe Chronic Granulomatous Disease (SCGD)**: This condition is due to NADPH oxidase defects in phagocytes and can lead to recurrent, severe infections despite normal T-cell function. Patients often have growth issues and immune-related symptoms that may overlap with those described for SCID variants.
- **Chronic Granulomatous Disease (CGD)**: Similar to SCGD but less common, this condition results from NADPH oxidase defects leading to recurrent, severe infections particularly involving the skin and lungs. The systemic manifestations can also include growth issues and other immune-related symptoms.

Each of these conditions has unique features, but they all present with significant immunodeficiency leading to recurrent infections, and many have associated developmental or growth issues that could explain the clinical picture described.

**99. user 2025-02-18T12:08:20Z:**

The core symptom is dystonia, which is mainly manifested by abnormal expressive postures and involuntary movements, mostly involving the head and neck muscles, trunk muscles, rotator anterior muscles of the limbs, finger and wrist flexors, toe extensors and plantar flexors. It is aggravated by random movements, reduced or disappeared when resting and sleeping, and in the late stage, it can be a fixed twisting spastic deformity. Sensory trickery (relief techniques) and movement specificity are the characteristic manifestations of dystonia. Sensory tricks (relieving techniques) are random movements that are specifically designed to correct abnormal postures or relieve dystonic movements, usually simple movements that involve or target the affected area, but are not forceful to counteract the symptoms of dystonia. Movement specificity refers to the presence of dystonia only during specific activities or the performance of specific tasks, such as certain occupations (e.g., writing spasms, musician’s spasms) or sports.

**qwen2.5_7b** 2025-02-18T12:08:24Z:

Based on the clinical manifestations you’ve provided, the most likely diagnosis is **Dystonias**. More specifically, this pattern suggests that the patient might be experiencing a form of **Task-Specific Dystonia**, also known as **Occupational Dystonia** (e.g., writer’s cramp, musician’s dystonia).

Here are five other possible diagnoses to consider:

- **Cervical Dystonia**: Often manifests with spasms and twisting in the neck area due to involuntary contractions of muscles in the head, neck, and shoulders.
- **Task-Specific Dystonia (Muscle Contraction Disorders)**: This includes conditions like writer’s cramp or musician’s dystonia, which are characterized by dystonic movements occurring only during specific tasks or activities.
- **Habit-Related Dystonia**: Sometimes referred to as “psychogenic” dystonias, these can occur due to repetitive habits or behaviors that have become involuntary.
- **Dopa-Responsive Dystonia (DRD)**: A form of dystonia that responds well to levodopa therapy and often has its onset in childhood or early adolescence.
- **Primary Dystonia**: This is a genetic condition characterized by sustained, twisting movements and abnormal postures due to abnormalities in the basal ganglia region of the brain.

Given the specific nature of the dystonia described (task-specific and responsive to sensory tricks), cervical dystonia and task-specific dystonia are particularly relevant. However, further diagnostic tests such as MRI scans or genetic testing might be necessary to confirm the exact type of dystonia and rule out other conditions.

**100. user 2025-02-18T12:09:22Z:**

**1. Kidney,** the main manifestations are limb oedema and increased foam in the urine. Laboratory tests may reveal simple moderate proteinuria or nephrotic syndrome, and renal insufficiency may be seen in the advanced stage.

**2. Heart,** the main manifestations are shortness of breath after activity, limb edema, pleural effusion, ascites and other signs of restrictive cardiac insufficiency.

**3. Liver,** there may be slight discomfort or pain in the liver area, but most patients may be asymptomatic, and hepatomegaly or liver enzyme abnormalities are often detected during physical examination. Liver failure may occur in advanced stages of the disease.

**Peripheral and autonomic nerves,** Symmetrical sensory and/or motor peripheral neuropathy of the extremities. Autonomic abnormalities are often manifested by upright hypotension, gastroparesis, pseudo-intestinal obstruction, and impotence.

**5. Gastrointestinal tract** , can present with total gastrointestinal involvement, with the stomach and small intestine being the most commonly involved. It can present with epigastric discomfort, dyspepsia, diarrhoea, constipation, malabsorption syndrome and gastrointestinal bleeding.

**6. Soft tissues ,** Tongue involvement may present with megaglossia, tongue dysmobility, and dysarthria. Skin mucosa may present with skin purpura and petechiae, which are more common around the orbits and in the loose skin areas of the neck. Nail atrophy and hair loss may also occur.

**7. Coagulation abnormalities** Patients often have coagulation factor X deficiency, resulting in corresponding bleeding manifestations.

**qwen2.5_7b** 2025-02-18T12:09:27Z:

Based on the clinical manifestations provided, it appears that you might be referring to **Fabry Disease**, a rare genetic disorder caused by the deficiency of the enzyme alpha-galactosidase A. Fabry Disease primarily affects the kidneys and the cardiovascular system, causing symptoms like limb oedema, proteinuria (leading to nephrotic syndrome in severe cases), and renal insufficiency.

Here are five other possible diagnoses based on the provided manifestations:

- **Nephrotic Syndrome** - This is a clinical condition characterized by heavy protein leakage from the kidneys into the urine, which can lead to swelling, particularly in the lower limbs (oedema) and an increase in foam in the urine. It often results from various kidney diseases.
- **Cardiac Amyloidosis** - A rare type of heart disease where abnormal proteins accumulate in the heart tissue, leading to restrictive cardiac insufficiency symptoms such as shortness of breath after activity, pleural effusion, and ascites.
- **Alpha-1 Antitrypsin Deficiency** - This is a genetic disorder that can lead to liver disease (causing hepatomegaly or liver enzyme abnormalities) and lung disease due to the deficiency of alpha-1 antitrypsin, which protects the lungs from damage by enzymes in the airways.
- **Guillain-Barré Syndrome** - This is an autoimmune disorder where the body’s immune system attacks part of its peripheral nervous system. Symptoms include symmetrical sensory and motor peripheral neuropathy (which can affect the extremities) along with possible autonomic abnormalities such as hypotension upon standing, which can mimic some of the symptoms listed.
- **Scleroderma** - An autoimmune disease characterized by hardening and tightening of the skin due to excessive scar tissue formation. It can involve various organs including the gastrointestinal tract, causing symptoms like dyspepsia, diarrhea, constipation, and nail atrophy, along with possible skin purpura and petechiae.

Each of these conditions could present with overlapping symptoms, which is why a thorough diagnostic approach involving clinical history, physical examination, laboratory tests, and possibly specialized imaging or biopsy would be necessary to determine the exact cause.

**101. user 2025-02-18T12:09:43Z:**

Jaundice and itchy skin are typical clinical manifestations. Other developmental delays include short stature, delayed pubertal growth, gallbladder stones, steatorrhea due to fat absorption disorders, hepatosplenomegaly, rickets due to fat-soluble vitamin deficiencies, delayed bone age, dry eye, coagulation disorders, and neuromuscular disorders. Children may also develop visual and hearing abnormalities, irritability, lethargy and poor concentration. In advanced stages, portal hypertension and liver tumours may occur.

**qwen2.5_7b** 2025-02-18T12:09:50Z:

Based on the clinical manifestations you’ve provided, these symptoms strongly suggest **Alagille Syndrome**. Alagille syndrome is a genetic disorder affecting multiple organ systems and can include:

- **Jaundice and Itchy Skin (Pruritus):** Due to bile duct abnormalities.
- **Short Stature:** Often due to poor nutritional status from fat malabsorption.
- **Delayed Pubertal Growth:** May be related to liver dysfunction or other systemic effects.
- **Gallbladder Stones:** Bile duct abnormalities can lead to gallstones formation.
- **Steatorrhea:** Fat malabsorption is common due to bile deficiency.
- **Hepatosplenomegaly:** Enlargement of the liver and spleen.
- **Rickets Due to Vitamin D Deficiency:** Fat-soluble vitamin deficiencies can lead to rickets.
- **Delayed Bone Age:** Growth retardation seen in skeletal development.
- **Dry Eye:** May be associated with meibomian gland dysfunction or other systemic issues.
- **Coagulation Disorders:** Reduced synthesis of clotting factors due to liver insufficiency.

**Five Other Possible Diagnoses:**

1. **Cystic Fibrosis (CF):**
   - **Steatorrhea and Poor Nutritional Status:** Characteristic of CF due to pancreatic insufficiency.
   - **Hepatomegaly:** Can occur secondary to fat malabsorption or other metabolic issues.
   - **Rickets:** Common in CF, related to vitamin D deficiency.
2. **Primary Sclerosing Cholangitis (PSC):**
   - **Jaundice and Itchy Skin:** Due to bile duct obstruction.
   - **Gallbladder Stones:** Associated with PSC.
   - **Steatorrhea:** Can occur due to secondary effects of bile duct damage.
3. **Metachromatic Leukodystrophy (MLD):**
   - **Neuromuscular Disorders and Developmental Delays:** Characteristic progressive neurological decline.
   - **Visual and Hearing Abnormalities:** Common in advanced stages of MLD.
   - **Irritability, Lethargy, and Poor Concentration:** Early signs of neurodegeneration.
4. **Wilson’s Disease:**
   - **Hepatomegaly and Jaundice:** Liver damage due to copper accumulation.
   - **Coagulation Disorders:** Due to liver dysfunction.
   - **Neuromuscular Symptoms (in advanced cases):** Tremors, dysarthria, dystonia.
5. **Biliary Atresia:**
   - **Jaundice and Itchy Skin:** Bile duct obstruction in the extrahepatic biliary tree.
   - **Gallbladder Stones:** Possible secondary to bile duct blockage.
   - **Hepatomegaly:** Enlarged liver due to obstruction.

These diagnoses cover a range of conditions that could present with similar clinical manifestations, and thorough genetic testing or other specific diagnostic tests would be necessary for a definitive diagnosis.

**102. user 2025-02-18T12:10:28Z:**

The disease starts in childhood. The disease manifests itself as a mild delay in motor development and progressive atrophy of skeletal muscles, which affects the motor function of the limbs and gradually leads to gait abnormalities and limited movement of the upper limbs, with the natural course of the disease often resulting in loss of the ability to walk at about 10 years of age. Thereafter, scoliosis, joint contracture, respiratory muscle weakness and dilated cardiomyopathy appeared, and the patient died of respiratory failure and cardiac failure at the age of about 20 years. Pseudohypertrophy of both gastrocnemius muscles, pseudohypertrophy of both forearms and lingual muscles, positive Gowers’ sign, and lumbar lordosis can be seen on examination. The disease may start in youth or even adulthood, and some patients do not affect survival. Signs of pseudohypertrophy are obvious, and in some patients, dilated cardiomyopathy is first apparent when limb weakness is still mild.

**qwen2.5_7b** 2025-02-18T12:10:32Z:

Based on the clinical manifestations provided, the most likely diagnosis for this rare disease is **Duchenne Muscular Dystrophy (DMD)**. Duchenne Muscular Dystrophy is a type of muscular dystrophy that primarily affects boys and is characterized by progressive muscle degeneration and weakness. The symptoms you described align well with DMD:

- **Duchenne Muscular Dystrophy (DMD)** - This is the primary diagnosis given the clinical features such as delay in motor development, progressive muscle atrophy, gait abnormalities, respiratory muscle weakness, cardiac issues, pseudohypertrophy of muscles, and positive Gowers’ sign.

### Five Other Possible Diagnoses

- **Becker Muscular Dystrophy (BMD)** - Similar to DMD but with a later onset and slower progression.
- **Facioscapulohumeral Muscular Dystrophy (FSHD)** - Characterized by progressive weakness of facial, scapular, and upper arm muscles.
- **Limb-Girdle Muscular Dystrophy (LGMD) Types 1 and 2** - A group of muscle diseases that can present with various combinations of limb-girdle muscle weakness.
- **Congenital Muscular Dystrophies (CMDs)** - These include disorders like Bethlem Myopathy, which may show some overlapping features but typically presents earlier in life.
- **Myotonic Dystrophy Type 1 (DM1)** - While less common for the described pattern of muscle weakness and respiratory issues, it can present with a range of symptoms including myotonia and cardiac involvement.

Each of these conditions has its specific clinical profile that may need to be differentiated from DMD based on additional diagnostic tests such as genetic testing, electromyography (EMG), and muscle biopsy.

**103. user 2025-02-18T12:11:46Z:**

Mainly hyperaemia, brain damage and cardiomyopathy.

**1. Neonatal onset** Normal at birth, vomiting, lethargy, hypotonia, convulsions, dyspnoea, hyperaemia, ketosis, hypoglycaemia, acidosis, dilated cardiomyopathy, pancreatitis and other abnormalities after starting breastfeeding, with a high morbidity and mortality rate.

**2. Delayed-onset** is often induced by fever, starvation, high-protein diet, and infections, and manifests itself as feeding difficulties in infancy and early childhood, developmental delays, convulsions, and hypotonia. In many patients, cognitive ability and neurological development are impaired, with an increase in slow waves or epileptic waves on the EEG; some patients may have fractures and osteoporosis on X-ray; bone marrow suppression is also common, causing granulocytopenia, anaemia and thrombocytopenia. There may also be cardiac damage, such as cardiomyopathy, arrhythmia, prolonged QT interval, and diminished cardiac function. Renal impairment is less common.

**qwen2.5_7b** 2025-02-18T12:11:51Z:

Based on the clinical manifestations provided, the most likely rare disease appears to be **Reye’s Syndrome**, a condition that primarily affects children and young adults who are recovering from viral infections such as influenza or chickenpox. The symptoms of Reye’s syndrome include vomiting, altered mental status (lethargy), seizures, convulsions, and in some cases, hyperaemia and cardiomyopathy.

However, the clinical picture described also suggests several other rare conditions that could be considered:

- **Reye’s Syndrome**: As mentioned, this condition is characterized by acute encephalopathy, vomiting, and abnormal liver function tests. Cardiomyopathy can occur but is less common.
- **MELAS Syndrome (Mitochondrial Encephalomyopathy, Lactic Acidosis, and Stroke-like Episodes)**: This is a mitochondrial disorder that presents with stroke-like episodes, seizures, headache, developmental delay, muscle weakness, lactic acidosis, and cardiomyopathy.
- **Fabry Disease**: This genetic lysosomal storage disease affects males more commonly than females. Symptoms include pain in the hands and feet, kidney dysfunction, cardiac problems (such as cardiomyopathy), and hyperaemia due to vasculature issues.
- **Kearns-Sayre Syndrome**: A rare mitochondrial disorder that can present with progressive external ophthalmoplegia, pigmentary retinopathy, cardiomyopathy, and myopathy. It can also have features of Leigh syndrome (a condition that causes neurological problems).
- **Cerebrotendineous Xanthomatosis (CTX)**: This is a rare autosomal recessive disorder characterized by tendon xanthomas, cerebrovascular disease (such as stroke-like episodes), diabetes insipidus, and often cardiomyopathy.
- **Friedreich’s Ataxia**: Although not typically associated with hyperaemia or significant brain damage, this hereditary condition can present with cardiomyopathy, ataxia, and other neurological symptoms, making it another possibility to consider in the differential diagnosis.

Each of these conditions requires specific diagnostic tests for confirmation, such as genetic testing, MRI, EEG, liver function tests, muscle biopsies, or mitochondrial DNA analysis.

**104. user 2025-02-18T12:12:11Z:**

Clinical manifestations are often nonspecific, with an insidious onset. Symptoms such as coughing up sputum, lethargy and fatigue may occur. Secondary infections may include fever, coughing up purulent sputum, or chest pain and haemoptysis. A few patients may be asymptomatic and may have no positive signs. Bursting sounds may be present in some patients, cyanosis may be present in severe cases, and pestle and mortar fingers may be seen in a few patients. The clinical picture is often less severe than the chest image, and this inconsistency between symptoms and images is a characteristic feature of the disease. Serum anti-GM-CSF antibodies are markedly elevated.

**qwen2.5_7b** 2025-02-18T12:12:16Z:

Based on the provided clinical manifestations, the most likely diagnosis is **hypereosinophilic syndrome (HES)**, given that HES can present with nonspecific symptoms like coughing up sputum, lethargy, and fatigue. Secondary infections, such as fever, purulent sputum, chest pain, haemoptysis, and the presence of bursting sounds could indicate a secondary infection common in chronic lung conditions seen in HES patients. Severe cases might show cyanosis due to potential underlying respiratory compromise.

Here are five other possible diagnoses that should be considered:

- **Churg-Strauss Syndrome (CSS)**: This is an inflammatory disorder involving the vasculitis of small and medium-sized blood vessels, leading to a similar presentation with respiratory symptoms including coughing up sputum, wheezing, and chest pain.
- **Idiopathic Pulmonary Fibrosis (IPF)**: IPF typically presents with progressive dyspnea and a dry, hacking cough. While it does not usually present with secondary infections or fever as prominently, the presence of “pestle and mortar” fingers could suggest underlying fibrotic changes.
- **Bronchiectasis**: This condition often leads to chronic productive cough, fatigue, and can result in bacterial superinfections leading to fever and purulent sputum.
- **Lymphangioleiomyomatosis (LAM)**: While typically associated with females of reproductive age, LAM can present with dyspnea, coughing up sputum, and chest pain due to the proliferation of smooth muscle cells in the lungs.
- **Nontuberculous Mycobacterial Infection**: This condition can cause nonspecific symptoms like chronic cough, fatigue, and lethargy, often complicated by secondary bacterial infections leading to fever, purulent sputum, and chest pain.

Given the characteristic serum anti-GM-CSF antibodies being markedly elevated, this further supports a diagnosis of hypereosinophilic syndrome (HES), as eosinophils play a significant role in the pathogenesis through GM-CSF-mediated mechanisms.

**105. user 2025-02-18T12:13:04Z:**

In most cases, the disease begins in adolescence, with a gradual loss of the optic rod cells, followed by cone cell involvement and death of the retinal pigment epithelium. Therefore, night blindness is usually the first symptom (but after urbanisation, some patients often have a decrease in visual acuity), and the earlier the onset of night blindness, the more severe the patient’s condition. Although peripheral visual field defects also occur early, patients are usually unaware of them and rarely complain of them in the early stages of the disease. The disease progresses to a loss of central vision and further narrowing of the visual field, and most people become legally blind before the age of 40. The degree of visual impairment can be related to the mode of inheritance.

The presentation of the fundus is related to the stage of the disease: in the early stages of the disease, the fundus may be normal or nearly normal on routine examination, but in some patients, an early form of blanket retinal degeneration may be observed on mid-peripheral fundus photography (these subtle changes are difficult to recognise with current scanning laser fundus photography or wide-field fundus photography). As the disease progresses, some patients develop typical fundus changes, such as retinal osteoblast-like pigmentation combined with mid-peripheral retinal atrophy, a waxy optic disc, and thinning of retinal blood vessels; some patients develop blanketing or depigmentation, which can be seen in the mid-peripheral retina, or extensive degeneration. Other common complications include macular cystoid degeneration, posterior subcapsular cataract, vitreous dust particles, and optic disc vitreous warts; a rare complication is Coat’s retinopathy.

**qwen2.5_7b** 2025-02-18T12:13:08Z:

Based on the clinical manifestations provided, the most likely diagnosis for this patient would be **Retinitis Pigmentosa (RP)**. Retinitis Pigmentosa is a group of inherited degenerative eye diseases that primarily affect the retina and can lead to significant visual impairment over time due to progressive loss of rod and cone cells.

Here are five other possible diagnoses that could also present with similar symptoms:

- **Usher Syndrome**: This syndrome combines retinal dystrophy (similar to RP) with hearing impairment, making it a distinct possibility if there is any family history or additional sensorineural deafness in the patient.
- **Leber Congenital Amaurosis (LCA)**: Although typically diagnosed earlier and often in infancy, LCA can also present similarly to RP, especially later on when symptoms such as night blindness and loss of peripheral vision become more pronounced.
- **Best Disease**: This is a relatively rare genetic disorder that affects the retina and can lead to progressive visual impairment, though it usually presents with more distinct macular changes compared to classic RP.
- **Joubert Syndrome**: A neurodevelopmental disorder characterized by distinctive cerebellar malformations that can be associated with retinal dystrophy; however, this would also present with additional neurological symptoms like ataxia and breathing abnormalities.
- **Cone-rod Dystrophy (CRD)**: This condition involves the progressive degeneration of both cone and rod cells in the retina but often presents later in adolescence or early adulthood, making it a relevant consideration based on your description of the disease beginning during this period.

Each of these conditions can affect retinal function, leading to similar visual symptoms as Retinitis Pigmentosa. Accurate diagnosis typically requires detailed genetic testing, clinical examination, and specialized imaging techniques like fundus photography and optical coherence tomography (OCT).

**106. user 2025-02-18T12:13:26Z:**

The most common clinical presentation is white pupil syndrome, in which a yellow-white reflection is visible in the pupil. Disuse strabismus may occur in the affected eye due to the tumour being located at the posterior pole and low visual acuity. The early presentation is a greyish-white, round or oval arching mass that may bulge into the vitreous or grow flat along the choroid. Retinal blood vessels on the surface of the tumour may become dilated and haemorrhagic, and even exudative retinal detachment may occur. Tumour tissue may also penetrate the retina and enter the vitreous, floating like a snowball, or even deposited in the anterior chamber to form pseudo anterior chamber pus. The onset of the disease is early in children with bilateral retinal detachment, usually within one year of age. The onset is later in unilateral children, usually at two or three years of age.

**qwen2.5_7b** 2025-02-18T12:13:30Z:

Based on the clinical manifestations you’ve described—white pupil syndrome (leukocoria), disuse strabismus, a posteriorly located mass with possible growth into the vitreous, haemorrhage and exudation in retinal blood vessels, retinal detachment, and pseudo anterior chamber pus—the most likely diagnosis is Retinoblastoma.

### Five Other Possible Diagnoses:

- **Coats’ Disease**: A rare disease that affects the retina, leading to leakage of fluid into the outer layers of the retina, causing retinal detachment.
- **Retinal Hemangioma (Retinopathy of Prematurity)**: An abnormal vascular proliferation in the retina commonly seen in premature infants.
- **Retinoblastoma Variant (Type II)**: A less common variant that may present with different clinical features but shares some characteristics with classic retinoblastoma.
- **Malignant Retinopapilloma**: A benign tumor that can mimic retinoblastoma and may have similar symptoms, including leukocoria.
- **Juvenile Xanthogranuloma (JXG)**: An uncommon disorder characterized by the presence of lipid-laden macrophages in various tissues, sometimes presenting with a white reflex due to localized inflammation or tumor-like lesions.

Each of these conditions can present with some overlapping symptoms but have distinct differences in pathology and clinical course. Proper imaging studies like ultrasound of the eye, MRI, and detailed ophthalmoscopic examination will help confirm the diagnosis.

**107. user 2025-02-18T12:14:44Z:**

The average age of onset is 5.6 months. The first seizure is usually normal at birth and starts within 1 year of age. The first seizure is usually febrile convulsion, which may be one-sided or generalised clonic or tonic clonic seizure, and febrile convulsions are characterized by long duration and recurrent episodes, and may appear in a persistent state. 28%~35% of children may start the disease without febrile convulsions, but they often have heat sensitivity in the later course of the disease. Elevated body temperature due to various reasons, including exercise, fever, vaccination, hot baths, and high ambient temperatures, can trigger or exacerbate seizures and predispose to status epilepticus. Some children develop an acute encephalopathy characterised by deep coma following status epilepticus.

Children tend to present with febrile convulsions between the ages of 1 and 4 years, and have a variety of seizure types, such as myoclonic, atypical catatonic, partial seizures, and generalised tonic clonic seizures. Myoclonic seizures are often triggered by light stimulation, eye closure, and a specific graphic stimulus, and involve simultaneous clonus of one or several muscles, all accompanied by synchronised abnormal brain wave bursts. The intensity of the myoclonus varies, from a slight forward or backward movement of the body when only the axial muscles are violated, to a severe fall or sudden throwing of an object in the hand. Myoclonic seizures can be isolated or cluster, but a sustained state is rare. Myoclonic seizures may not occur at all in about 25% of children. The concurrent EEG of atypical apoplectic seizures shows half of the discharges to be of focal origin, most of which are irregular, irregular, slow spikes and slow waves, often accompanied by myoclonic seizures. 30% of the children may have atypical apoplectic persistent seizures. The incidence of partial seizures is 43.0% to 78.6%, which manifests as lateral limb (or) head deflection seizures or clonic seizures, and there are also motor seizures with consciousness and autonomic symptoms, automatisms, and hypotonia, etc. After 1 year of age, the children have psychomotor developmental delays, especially language developmental delays.

**qwen2.5_7b** 2025-02-18T12:14:49Z:

Based on the clinical manifestations you provided, the most likely diagnosis is Dravet syndrome (DS), a type of severe myoclonic epilepsy in infancy. Here are five other possible diagnoses that could be considered:

- **Dravet Syndrome (Severe Myoclonic Epilepsy of Infancy)**
  - This condition typically presents with early onset febrile seizures, followed by non-febrile seizures characterized by multiple seizure types including myoclonus and atypical absence-like seizures.
- **Lennox-Gastaut Syndrome (LGS)**
  - LGS usually has a later onset than Dravet syndrome, around 1-3 years of age, with a variety of seizure types such as drop attacks and typical absence seizures, often accompanied by developmental delay.
- **West Syndrome (Infantile Spasms)**
  - This condition is characterized by sudden, brief, and synchronous muscle contractions, usually in clusters, which may be triggered by stimuli or changes in position.
- **Rett Syndrome**
  - While primarily a neurodevelopmental disorder with significant cognitive and motor delays, Rett syndrome can present with seizures that are often multifocal or generalized.
- **Ohtahara Syndrome (Early Infantile Epileptic Encephalopathy)**
  - This is an early-onset, severe form of epilepsy characterized by multiple seizure types, including focal seizures, myoclonic jerks, and status epilepticus, often with a poor prognosis.
- **Crigler-Najjar Syndrome (Type I)**
  - Though this condition primarily affects liver function leading to bilirubinuria and kernicterus, in rare cases, it can also present with seizures due to hyperbilirubinemia which can affect the central nervous system.

Given the specific clinical manifestations you’ve described, Dravet syndrome is highly likely, but considering other conditions such as West Syndrome or Rett Syndrome may be necessary for a comprehensive differential diagnosis.

**108. user 2025-02-18T12:15:11Z:**

On the one hand, it presents as chronic haemolytic anaemia, and in addition to anaemia-related symptoms, it may cause mild to moderate yellowing of the skin and sclera and pigmented cholelithiasis due to elevated bilirubin, which may be exacerbated by cold, infection and dehydration. In infants and young children, splenomegaly may be present, and with age, the spleen may shrink due to splenic infarction and fibrosis.

On the other hand recurrent vaso-obstructive pain due to capillary micro thrombosis. Children and adults tend to present with myalgia of the limbs, large joint pain and low back pain, and in severe cases, severe abdominal pain, headache, and even limb paralysis and coma. Over time, vascular obstruction can occur in almost every organ system, and cyclical pain can occur in patients who are thin, easily fatigued, susceptible to infection, and malnourished. Thinning of the bone cortex, osteoporosis, spinal deformity, aseptic necrosis of the femoral head due to an overcompensatory increase in bone marrow haematopoietic tissue, and localised osteosclerosis if there is a bone infarction. Ocular symptoms may include fundus haemorrhage and retinal detachment due to embolism of the retinal vessels in the fundus. In addition, mental retardation may occur, affecting neurological development.

Acute pain is the first symptom of the disease in more than 25% of patients. It is most common in children older than 2 years of age. The severity of pain can vary from mild to severe. Approximately one-half of pain episodes are accompanied by objective clinical signs such as fever, swelling, tenderness, tachypnoea, hypertension, nausea and vomiting.

**qwen2.5_7b** 2025-02-18T12:15:15Z:

Based on the clinical manifestations provided, this patient most likely has **Hereditary Spherocytosis** (HS), which is a common hereditary hemolytic anemia characterized by red blood cells that are misshapen and prone to breakage. The symptoms such as chronic hemolytic anemia, jaundice due to elevated bilirubin, pigmented gallstones, and splenomegaly followed by splenic infarction, along with the recurrent vaso-occlusive pain episodes, are consistent with HS.

Here are five other possible diagnoses that could be considered:

- **Thalassemia** - This genetic disorder involves a defect in hemoglobin synthesis, leading to chronic anemia and potential splenomegaly.
- **Glucose-6-phosphate Dehydrogenase (G6PD) Deficiency** - A common enzyme deficiency that can lead to hemolytic crises triggered by certain drugs, foods, or infections.
- **Paroxysmal Nocturnal Hemoglobinuria (PNH)** - An acquired disorder involving the bone marrow and blood, often presenting with intermittent hemolysis, anemia, and thrombosis.
- **Autoimmune Hemolytic Anemia** - Can cause chronic hemolysis due to immune-mediated destruction of red blood cells; symptoms can include jaundice and splenomegaly but typically do not present with vaso-occlusive pain as described.
- **Sickle Cell Disease (SCD)** - While more commonly known for its acute painful crises, SCD also presents with chronic hemolytic anemia, though the pattern of symptoms does not entirely match HS.

Each of these conditions has specific criteria and tests that can help confirm or rule out their diagnosis in further diagnostic evaluations.

**109. user 2025-02-18T12:15:55Z:**

The main clinical manifestations of neonatal and paediatric patients include:

- Intrauterine growth retardation and postnatal growth disorders . Intrauterine growth retardation, low birth weight, lack of postnatal catch-up growth, and proportional short stature.
- Specific physical signs: wide and prominent forehead, pointed jaw leading to a triangular face, and large relative head circumference. Dysmorphic teeth, low ear position, and asymmetry of trunk deviation, including limb length discrepancy, and lateral curvature deformity of the little finger.
- Other manifestations include feeding difficulties, hypoglycaemia in the neonatal period, genitourinary malformations such as hypospadias, cryptorchidism and horseshoe kidneys, and digestive anomalies such as cafe au lait spots and reflux oesophagitis. Some patients have cognitive, motor and language developmental delays and learning disabilities.

**qwen2.5_7b** 2025-02-18T12:16:02Z:

Based on the clinical manifestations provided, the most likely rare disease to be diagnosed is **Klippel-Feil Syndrome** (KFS). This condition is characterized by a range of physical anomalies that often include:

- **Intrauterine Growth Retardation and Postnatal Growth Disorders**: Common in KFS due to various underlying genetic factors.
- **Dysmorphic Physical Signs**: The wide and prominent forehead, pointed jaw leading to a triangular face, and large relative head circumference are typical features of KFS.
- **Genitourinary Malformations**: Hypospadias, cryptorchidism, and horseshoe kidneys can be seen in some cases.
- **Feeding Difficulties and Hypoglycemia**: These may occur due to associated digestive anomalies or other underlying conditions.
- **Developmental Delays**: Cognitive, motor, and language developmental delays are often reported.

### Other Possible Diagnoses

- **VACTERL Association**:
  - **V**: Vertebral anomalies
  - **A**: Anorectal malformations
  - **C**: Cardiac defects
  - **T**: Tracheoesophageal fistula and esophageal atresia
  - **R**: Renal abnormalities
  - **L**: Limb deficiencies
- **DiGeorge Syndrome (22q11.2 Deletion Syndrome)**:
  - Characterized by thymic hypoplasia, resulting in immunodeficiency.
  - Cardiac defects such as tetralogy of Fallot or septal defects.
  - Other symptoms include low-set ears, cleft palate, and developmental delays.
- **Treacher Collins Syndrome**:
  - Ocular and facial anomalies including downward slanting palpebral fissures, colobomas, microtia (small external ear).
  - Skeletal dysplasia and hearing loss.
  - Often presents with dental issues similar to the described patient.
- **Spondyloepiphyseal Dysplasia Congenita**:
  - Short stature with disproportionate skeletal abnormalities.
  - Craniofacial features can vary but often include a high-arched palate, short neck, and a small lower jaw (micrognathia).
  - Can have joint hypermobility and dislocations.
- **Marfan Syndrome**:
  - While primarily associated with cardiovascular issues like aortic aneurysms, it can also present with skeletal abnormalities including scoliosis and limb deformities.
  - Patients may exhibit facial features such as a long face, small lower jaw, and widely spaced teeth (diastemata).

These conditions are less likely to be the primary diagnosis but should still be considered given their overlapping clinical presentations. The final diagnosis would typically require a thorough genetic evaluation and imaging studies.

**110. user 2025-02-18T12:16:26Z:**

**1. Xanthomas of the skin,** occurring near the attachment points of the extensor tendons, such as the Achilles tendon, elbow, knee, etc., and there is a close relationship between skin injuries and the appearance of xanthomas. Cutaneous xanthomas are rare in children.

**2. Atherosclerosis ,** Patients have a significantly higher risk of developing atherosclerosis and coronary artery disease than normal subjects. Early-onset coronary artery disease may be one of the characteristics of the disease.

**3. Alterations in the blood system ,** The content of plant sterols in the blood is significantly increased, resulting in a higher proportion of plant sterols in the sterol component of the cell membrane, abnormal cell membrane function of red blood cells, platelets and other cell membranes, anaemia and thrombocytopenia, and the patient may have weakness, pallor, skin bleeding and other manifestations.

**4. Others,** in addition to the above symptoms, some patients may develop joint pain, arthritis, splenomegaly, elevated transaminases, etc.

**qwen2.5_7b** 2025-02-18T12:16:30Z:

Based on the clinical manifestations you’ve provided, the most likely rare disease is ** Familial Hypercholesterolemia (FH)**. This condition is characterized by high levels of low-density lipoprotein cholesterol in the blood and can lead to atherosclerosis at an early age, with symptoms such as xanthomas and cardiovascular issues.

Here are five other possible diagnoses that may also fit these clinical manifestations:

- **Niemann-Pick Disease Type A/B** - This is a lysosomal storage disorder characterized by the accumulation of cholesterol in cells, leading to xanthomas and potentially affecting multiple organ systems including the liver and spleen.
- **Abetalipoproteinemia (Bassen-Kornzweig Syndrome)** - This is a rare genetic disorder that affects lipid metabolism, leading to elevated levels of plant sterols in the blood and causing skin symptoms similar to those described.
- **Lysinuric Protein Intolerance** - A rare genetic disorder affecting protein digestion, which can present with various clinical manifestations including neurological issues, metabolic abnormalities, and hematological disorders like anemia.
- **Cerebrotendineous Xanthomatosis (CTX)** - This is a very rare genetic condition characterized by xanthomas, progressive mental deterioration, and cataracts due to the accumulation of cholesterol and other lipids in tissues.
- **Familial Dysbetalipoproteinemia** - Another form of inherited hyperlipidemia that can lead to early-onset atherosclerosis, xanthomas, and other cardiovascular complications similar to familial hypercholesterolemia.

Each of these conditions has distinct features but might present with some overlapping symptoms, making differential diagnosis important. Genetic testing is crucial for confirming the exact condition in such cases.

**111. user 2025-02-18T12:16:53Z:**

The onset of the disease is insidious in males, and the common age of onset is 30-60 years old. Patients often start with painful spasms, tremor, and weakness of the lower limbs, and gradually develop weakness of the upper limbs, muscle atrophy of the medulla oblongata and the face, muscle fasciculations, dysarthria, dysphagia, dyspnoea, and respiratory difficulties. Androgen receptor insensitivity may be associated with gynecomastia and reduced reproductive function. Sensory abnormalities are present in more than 50 per cent of patients. The disease progresses slowly, with patients usually becoming unable to walk late in the course of the disease, and only some patients require assisted ventilation, which has no significant impact on survival.

**1. Atypical symptoms,** some of which may occur years or even decades before the onset of limb weakness, include fatigability, painful spasms, perioral tremor, and postural tremor. Muscle weakness: most patients start with weakness of both lower limbs, gradually progressing to both upper limbs, tongue muscles and facial muscles, while the extraocular muscles are often not involved. The distribution of limb weakness is asymmetrical, with proximal involvement predominating. Involvement of the muscles of mastication may cause mandibular ptosis and tremor. Physical examination of the lower motor neuron damage signs, manifested as a decrease in muscle strength of the limbs, proximal, accompanied by muscle atrophy, fasciculations, tendon reflexes are weakened or disappeared. Most patients may have obvious tongue muscle atrophy and fibrillation, but the soft palate activity and pharyngeal reflexes are normal, and the degree of choking on water and dysphagia is mild. In some patients, chin and perioral fasciculations can also be observed. The motor function impairment of the patients gradually increases with the prolongation of the disease, and in the later stage, they may have difficulty in walking up the stairs and walking unsteadily.

**2. Sensory deficits** , with varying degrees of hyperalgesia present in about half of the patients, while in the others it is subclinical, with abnormalities found only on sensory nerve conduction tests.

**3. endocrine abnormalities,** including abnormalities of glucose and fat metabolism, and androgen receptor insensitivity and other manifestations, the latter including gynecomastia, decreased sexual function, infertility, and testicular atrophy.

The latter include gynecomastia, decreased sexual function, infertility, testicular atrophy, etc.

**4. Female carriers,** most female carriers do not have symptoms, and even if they do have symptoms, they are mild, such as fasciculations, mild weakness of distal limbs, muscle spasms, or increased creatine myosinase.

**qwen2.5_7b** 2025-02-18T12:16:59Z:

Based on the clinical manifestations provided, the rare disease that best matches these symptoms is **Charcot-Marie-Tooth Disease (CMT)** type 2D. This is a hereditary peripheral neuropathy characterized by distal muscle weakness and atrophy in the lower limbs progressing to the upper limbs, with sensory loss often present.

### Five Other Possible Diagnoses

- **Multiple Sclerosis (MS)**
  - MS can present with progressive neurological symptoms including weakness, spasms, and sensory abnormalities. However, it is usually associated with a demyelinating process leading to relapsing-remitting episodes rather than the slow, insidious progression seen in CMT.
- **Motor Neuron Disease (MND)**
  - MND includes conditions like Amyotrophic Lateral Sclerosis (ALS), which can cause progressive weakness and atrophy of muscles. However, ALS typically progresses more rapidly and often involves respiratory muscle involvement earlier than described here.
- **Lambert-Eaton Myasthenic Syndrome (LEMS)**
  - LEMS is an autoimmune neuromuscular disorder characterized by muscle weakness that improves with sustained activity, fatigability, and sometimes sensory abnormalities. However, it usually does not cause muscle atrophy or the specific pattern of lower motor neuron signs described.
- **Familial Hypertrophic Pseudomyositis (FHPM)**
  - FHPM is a rare neuromuscular disorder characterized by progressive muscle weakness, muscle pain, and atrophy, often with sensory abnormalities. However, it typically presents more acutely and does not usually involve the medulla oblongata or the respiratory muscles as extensively as described.
- **Hereditary Sensory and Autonomic Neuropathy (HSAN)**
  - HSAN types can present with a range of symptoms including pain, temperature and touch insensitivity, autonomic dysfunction, and muscle weakness. However, they do not typically present with the specific combination of lower motor neuron signs and respiratory involvement described here.

Each of these conditions has distinct features that set them apart from CMT2D, particularly in their clinical presentation, progression, and associated symptoms. The detailed history and physical examination findings strongly suggest a diagnosis of Charcot-Marie-Tooth Disease type 2D.

**112. user 2025-02-18T12:17:51Z:**

Some patients present with reduced foetal movement in utero and are born as floppy babies. The onset of the disease occurs within 6 months, with a mean age of onset of 1 month after birth. They present with generalised floppy weakness and severe hypotonia. Most children have difficulty sucking and swallowing due to weakness of the tongue, facial and masticatory muscles, and atrophy and tremor of the lingual muscles are seen. Intercostal muscle involvement can present with dyspnoea and abdominal breathing. The chest has a bell-shaped appearance. The lower limbs are more heavily involved than the upper limbs, and proximal is more severe than distal. Severe muscle weakness in the mid-trunk area leaves the child unable to control head movements, unable to lift or roll over, and without the ability to sit alone. When lying down, both lower limbs are in the frog-leg position with hip abduction and knee flexion. Muscle atrophy is not obvious, and some children have mild joint deformities. Children with good intelligence, tendon reflexes disappeared, limb sensation is normal. The children’s muscle weakness progressively worsens, and eventually they lose all voluntary movement ability, are fed nasogastrically, and suffer from respiratory failure due to repeated respiratory infections. 80% of the children die within 1 year of age, and rarely survive beyond 2 years of age.

**qwen2.5_7b** 2025-02-18T12:17:56Z:

Based on the clinical manifestations provided, the most likely diagnosis is **Kearns-Sayre Syndrome (KSS)**, which is a rare mitochondrial disorder characterized by progressive ophthalmoplegia, pigmentary retinopathy, and cardiac conduction defects. However, given the specific presentation of severe hypotonia, generalized weakness, feeding difficulties, respiratory issues, and lack of obvious muscle atrophy in your description, other potential diagnoses should be considered as well.

Here are five other possible diagnoses:

- **Congenital Myasthenic Syndromes (CMS)** - A group of inherited disorders characterized by chronic, fluctuating muscle weakness due to abnormalities in the transmission of nerve impulses to muscles. CMS can present with symptoms like those described, including neonatal-onset hypotonia and feeding difficulties.
- **Mitochondrial Encephalomyopathy, Lactic Acidosis, and Stroke-like Events (MELAS)** - While MELAS typically presents later in childhood or adulthood, some cases may have early onset similar to KSS, with additional symptoms such as seizures and stroke-like episodes.
- **Metachromatic Leukodystrophy (MLD)** - An autosomal recessive disorder caused by a deficiency of the arylsulfatase A enzyme, leading to the accumulation of sulfatides in the central nervous system. MLD can present with progressive neurological symptoms including hypotonia and feeding difficulties.
- **Dejerine-Sottas Disease (DSD)** - This is a severe form of peripheral neuropathy caused by PMP22 duplication or deletion. Symptoms include severe hypotonia, developmental delay, and motor impairments similar to those described in your patient.
- **Congenital Muscular Dystrophy (CMD)** - CMD is a group of rare genetic muscle disorders characterized by weakness that begins in infancy or early childhood. While CMD typically presents with more obvious muscle atrophy and dystrophic changes, some forms can have less pronounced muscle atrophy and present similarly to the described condition.

Each of these conditions should be evaluated based on comprehensive clinical assessment, biochemical testing, and genetic analysis for accurate diagnosis.

**113. user 2025-02-18T12:18:22Z:**

Patients usually start in adulthood, with an insidious onset and gradual progression. The main manifestations are cerebellar ataxia, including drunkard’s gait, clumsy movements, minstrel-like speech, nystagmus, diplopia, etc.; pyramidal tract damage, including hyperreflexia, ankle clonus, and positive pathological signs; and extrapyramidal symptoms, including Parkinson’s syndrome, dystonia, and ankylosis. In addition, some patients may have peripheral neuropathy. In the early stages of the disease, some patients may have damage to the pyramidal tract (similar to spastic paraplegia) or extrapyramidal symptoms (similar to Parkinson’s syndrome), and ataxia is not prominent.

The disease progresses gradually, with loss of walking ability after 10-15 years. Prolonged dysphagia leading to malnutrition or aspiration is the main cause of death.

**qwen2.5_7b** 2025-02-18T12:18:26Z:

Based on the clinical manifestations you’ve provided, this patient most likely has **Spinocerebellar Ataxia Type 3 (SCA3)**, also known as Machado-Joseph Disease. This disease is characterized by a combination of cerebellar ataxia, pyramidal tract signs, and extrapyramidal symptoms, which aligns with the patient’s presentation.

### Five Other Possible Diagnoses:

- **Spinocerebellar Ataxia Type 7 (SCA7)**: This type is characterized by retinal dystrophy in addition to ataxia, but it can present similarly in terms of cerebellar and pyramidal tract involvement.
- **Vermian Cerebellar Ataxia**: While less common, this type of ataxia involves the vermis of the cerebellum and often includes prominent gait disturbances and speech abnormalities.
- **Hereditary Spastic Paraplegia (HSP)**: Some forms of HSP can present with both spasticity and ataxia, especially in the early stages as described.
- **Cerebral Autosomal Dominant Arteriopathy with Subcortical Infarcts and Leukoencephalopathy (CADASIL)**: This condition primarily affects the cerebral blood vessels but can lead to progressive ataxia due to chronic ischemic changes.
- **Wilson’s Disease**: Although it typically presents earlier in life, Wilson’s disease can cause neurological symptoms including ataxia and dystonia, along with other manifestations like liver dysfunction.

These diagnoses are based on the clinical features you’ve outlined, but definitive diagnosis would require further genetic testing and possibly neuroimaging or nerve conduction studies.

**114. user 2025-02-18T12:18:58Z:**

The disease may be combined with gastro-oesophageal reflux in the early stages of the disease. As the disease progresses, the skin becomes swollen and sclerotic and may be accompanied by changes in skin pigmentation, including hyperpigmentation or depigmentation. In addition to skin involvement, patients often present with arthralgia and myalgia, and may develop ulcers and capillary dilatation of the digits. In addition, the patient may have multiple internal organ involvement. The respiratory system is more likely to be involved, with interstitial fibrosis and pulmonary hypertension. The gastrointestinal manifestations are varied and include gastroesophageal reflux, sinus vasodilatation, abdominal pain, diarrhoea, constipation and fecal incontinence. A small number of patients may present with renal crisis, which is characterised by acute renal insufficiency and is often associated with hypertension. Cardiac involvement may manifest as pericardial effusion, abnormalities of the cardiac conduction system, and myocardial involvement in individual patients. Neurological involvement of the peripheral nervous system is common, such as trigeminal neuropathy, peripheral neuropathy, etc. Central nervous system involvement is rare.

**qwen2.5_7b** 2025-02-18T12:19:04Z:

Based on the clinical manifestations you’ve described, the disease appears to be **Bloom Syndrome** (BS), a rare genetic disorder characterized by its specific combination of features:

- **Bloom Syndrome**: This condition is caused by mutations in the BLM gene and results from a defect in the DNA repair mechanism. The symptoms often include skin sensitivity to sunlight leading to redness, rashes, and swelling; short stature; immune system deficiencies; increased susceptibility to infections; and an elevated risk of cancer.
- **Ehlers-Danlos Syndrome (EDS) - Hypermobility Type**: This type of EDS features hypermobile joints, skin that is highly elastic and stretchy, as well as pain in the joints and muscles. It can also involve gastrointestinal issues such as dyspepsia or irritable bowel syndrome.
- **Mucopolysaccharidosis (MPS) Types**: These are a group of lysosomal storage diseases characterized by the accumulation of glycosaminoglycans due to enzyme deficiencies, leading to progressive organ damage and other symptoms like skeletal deformities and skin changes.
- **Rothmund-Thomson Syndrome (RTS)**: This is another rare genetic condition that involves short stature, rashes, bone abnormalities, and a high risk of developing certain cancers such as osteosarcoma or squamous cell carcinoma. Skin issues include rashes that appear after sun exposure.
- **Cockayne Syndrome**: This is a rare autosomal recessive disorder characterized by severe growth retardation, progressive neurological degeneration leading to loss of motor skills and mental development, and increased sensitivity to sunlight causing skin lesions and eye problems.
- **Ataxia-Telangiectasia (AT)**: This condition involves ataxia (difficulty walking), telangiectasias (small blood vessels near the surface of the skin becoming enlarged and visible), and a predisposition to cancer due to immunodeficiency, cerebellar degeneration, and other symptoms like eye problems.

Each of these conditions presents with unique combinations of symptoms that can overlap but are distinct in their primary underlying causes. Given the specific details provided about skin changes, gastrointestinal issues, pulmonary involvement, and neurological manifestations, Bloom Syndrome remains a strong candidate for diagnosis due to its characteristic features including photosensitivity, short stature, and immunodeficiency, along with other systemic manifestations.

**115. user 2025-02-18T12:19:20Z:**

In addition to clinical symptoms similar to phenylketonuria, children 1-3 months after birth also show symptoms of dopamine deficiency, such as dyskinesia, hypotonia, lethargy, nystagmus, dysphagia, etc. Symptoms related to serotonin deficiency, such as unresponsiveness, depression, insomnia, etc. Norepinephrine deficiency, such as low muscle tone in the trunk, ptosis, cerebellar dysgenesis, etc., may be associated with dopamine deficiency. Symptoms associated with norepinephrine deficiency, such as hypotonia of the trunk, ptosis, and cerebellar hypoplasia. There may also be persistent convulsions, recurrent fever, delayed development of motor milestones, total paralysis, and severe impairment of intellectual development.

**qwen2.5_7b** 2025-02-18T12:19:25Z:

Based on the clinical manifestations you’ve described, the most likely diagnosis is **dopa-responsive dystonia (DRD)**, which is a form of hereditary dystonia that responds to levodopa therapy. DRD can also present with additional symptoms related to neurotransmitter deficiencies such as those involving dopamine, serotonin, and norepinephrine.

Here are five other possible diagnoses:

- **Mucopolysaccharidoses (e.g., Hurler Syndrome)** - These conditions involve the accumulation of mucopolysaccharides due to lysosomal enzyme deficiencies and can present with neurological symptoms such as dystonia, hypotonia, developmental delay, and dysmorphic features.
- **Aromatic L-Amino Acid Decarboxylase Deficiency (AAAD)** - This condition affects neurotransmitter metabolism, leading to a deficiency in dopamine and serotonin. Patients may exhibit symptoms similar to those of DRD, including dystonia, hypotonia, developmental delay, and neurological signs.
- **Mitochondrial Disorders** - Certain mitochondrial disorders can affect multiple organ systems, including the nervous system, and may present with muscle weakness, dystonia, hypotonia, and other neuropsychiatric symptoms. These conditions often involve complex inheritance patterns.
- **Neurodegeneration with Brain Iron Accumulation (NBIA)** - This group of diseases includes disorders like Hallervorden-Spatz disease that can manifest with progressive neurological deterioration including dystonia, dyskinesia, and other movement disorders. Some forms also exhibit cognitive decline and ataxia.
- **Zellweger Spectrum Disorder** - A peroxisomal biogenesis disorder characterized by severe developmental delay, liver dysfunction, and neurological abnormalities such as dystonia and hypotonia. This can be associated with additional systemic manifestations like renal issues or characteristic facial features.

Given the complex nature of these symptoms and their potential overlap, a thorough diagnostic workup including biochemical testing for neurotransmitters, genetic testing, and imaging studies would be crucial to confirm the diagnosis.

**116. user 2025-02-18T12:20:00Z:**

They often present with multisystem involvement and develop in stages. Cardiac rhabdomyosarcomas can appear in the foetal/neonatal period, most of which resolve spontaneously; epileptic and cutaneous symptoms can appear in the first few months of life; renal angiomyolipomas and neoplastic lesions elsewhere in the body develop with age; and adult women are prone to pulmonary lymphangioleiomyomatosis of the lungs. Brain lesions include mainly cerebral cortical nodules, subventricular giant cell astrocytomas, and subventricular nodules. Skin lesions include leukoplakia visible after birth, progressive facial angiofibromas, shark leather spots, perinodal fibromas, and forehead plaques; renal vascular smooth muscle lipomas are present in up to 90% of patients; retinal involvement is present in about 50% of patients but rarely affects visual acuity; and LAM in the lungs occurs in about 35% of females; other areas, such as the liver, pancreas, gastrointestinal tract, and skeleton, may be involved, although usually with milder symptoms. Others, such as the liver, pancreas, digestive tract, and bones, can also be involved, but symptoms are usually mild.

**qwen2.5_7b** 2025-02-18T12:20:04Z:

Based on the clinical manifestations you have provided, this patient most likely has **LAM (Lymphangioleiomyomatosis)**, specifically a variant known as LAMB2 or Tuberous Sclerosis Complex (TSC) related LAM. However, given the multisystem involvement and the presence of both rhabdomyosarcomas and LAM, this description more closely aligns with **Tuberous Sclerosis Complex (TSC)**.

### Primary Diagnosis:

- **Tuberous Sclerosis Complex (TSC)**

### Five Other Possible Diagnoses:

1. **LEOPARD Syndrome** - A rare genetic disorder characterized by distinctive skin pigmentation, cardiac abnormalities, and other features.
2. **Carney Complex** - An inherited condition that can lead to various benign tumors in the skin and endocrine glands, as well as other health issues.
3. **Neurofibromatosis Type 1 (NF1)** - A genetic disorder characterized by skin changes, neurofibromas, and a variety of other symptoms.
4. **McCune-Albright Syndrome** - A rare disorder involving bones, skin, and the endocrine system, leading to various symptoms such as café-au-lait spots and precocious puberty.
5. **Renal Cell Carcinoma with LAM-like Lesions (RCC)** - In some cases, renal cell carcinoma can present with features similar to TSC-related LAM.

Given the specific multisystem involvement described, TSC is most likely, but it’s important to consider these other conditions for a thorough diagnostic evaluation.

**117. user 2025-02-18T12:20:32Z:**

Most children with the undetected or untreated chronic form die before the age of 10 years, usually as a result of liver failure, neurological involvement or hepatocellular carcinoma.

The acute form has a rapid onset and progression, with acute liver failure as the main manifestation, and clinical signs include hepatosplenomegaly, jaundice, vomiting, abdominal distension, anorexia, lethargy, anaemia, bleeding tendencies and growth retardation. The child may have the characteristic odour of ‘boiled cabbage’ or ‘rotten mushrooms’. Insufficient hepatic synthesis of coagulation factors, marked prolongation of PT and APTT, and decreased levels of coagulation factors II, VII, IX, Ⅺ and Ⅻ, which are difficult to correct with vitamin K supplementation. Hepatic failure may be followed by ascites, jaundice and gastrointestinal bleeding, and children occasionally develop persistent hypoglycaemia. Untreated children often die of liver failure within weeks or months of onset of illness.

**qwen2.5_7b** 2025-02-18T12:20:38Z:

Based on the clinical manifestations described, the most likely rare disease is **Metachromatic Leukodystrophy (MLD)**, although it’s important to note that this presentation more closely aligns with the acute form of a lysosomal storage disorder called **Mucopolysaccharidosis I (MPS I), specifically the severe ‘Hurler’ or ‘Hurler-Scheie’ subtypes**. However, given the specific mention of liver failure and characteristic odour, it’s more likely MLD.

- **Metachromatic Leukodystrophy (MLD)**: This is an autosomal recessive disorder caused by a deficiency in arylsulfatase A leading to accumulation of sulfatides in the brain and other tissues. It can have acute and chronic forms, with neurological involvement being significant. The characteristic “musty” or “boiled cabbage” odor is due to released arylsulfate.
- **Mucopolysaccharidosis I (MPS I) - Hurler Syndrome**: This condition involves a deficiency of the enzyme alpha-L-iduronidase, leading to the accumulation of glycosaminoglycans in various tissues. The chronic form can lead to liver dysfunction and growth retardation, while the acute form presents rapidly with hepatosplenomegaly, hypoglycaemia, and coagulopathy.
- **Fabry Disease**: This is an X-linked lysosomal storage disorder due to a deficiency of alpha-galactosidase A leading to accumulation of globotriaosylceramide in the blood vessels and organs, including the liver. It can present with hepatomegaly, renal impairment, and cardiovascular complications.
- **Hemimethylmalonic Acidemia (HMM)**: This condition is caused by a deficiency of methylmalonyl-CoA mutase leading to elevated levels of methylmalonic acid in blood, which can cause metabolic acidosis, liver dysfunction, and neurological symptoms.
- **Sialidosis**: This is an autosomal recessive disorder due to a deficiency of neuraminidase enzyme leading to the accumulation of sialylated glycans in tissues. It can present with progressive neurodegeneration, hepatomegaly, and respiratory difficulties.

Given the severity and specific symptoms such as liver failure, coagulopathy, and the characteristic odour, the most likely diagnosis is **Mucopolysaccharidosis I (Hurler Syndrome)**, but Metachromatic Leukodystrophy should also be considered.

**118. user 2025-02-18T12:21:21Z:**

The clinical presentation is markedly heterogeneous, and there are three types based on age of onset and clinical presentation.

**The cardiomyopathic type,** which is the most common type of severe early-onset cardiac and multi-organ failure, develops mainly in newborns and early infants. It has a high mortality rate and is associated with hypoglycaemia, Reye’s syndrome, sudden neonatal death, hypertrophic and dilated cardiomyopathy, pericardial effusion, arrhythmias, myasthenia, hepatomegaly, and intermittent hypoglycaemia, with elevated levels of creatine kinase; myocardial hypertrophy and arrhythmias can be fatal.

**2. hepatic type ,** less severe, hepatic and hypohidrotic hypoglycaemic type with onset in late infancy or childhood, presenting with hepatomegaly and liver function abnormalities, decreased fasting tolerance and acute hypohidrotic hypoglycaemia without myocardial damage and cardiac hypertrophy, but can be life-threatening without timely diagnosis and treatment.

**3. Myopathic type** , which develops mainly in adolescence to adulthood, is late-onset, mildly symptomatic, and is usually not accompanied by myocardial disease and hypoglycaemia. The main manifestations are rhabdomyolysis and myoglobinuria after exercise, infection or starvation, and even renal failure, which may be accompanied by muscle weakness, painful muscle spasms or myalgia.

**qwen2.5_7b** 2025-02-18T12:21:25Z:

Based on the clinical presentations you have described, the most likely rare disease to fit this profile is **Zellweger Syndrome spectrum (Peroxisomal Biogenesis Disorders)**, specifically Zellweger Spectrum Disorder. This condition affects peroxisome biogenesis and results in a range of neurological and organ system dysfunction.

### Other Possible Diagnoses:

- **Neonatal Adrenoleukodystrophy (NALD):**
  - A milder form of adrenoleukodystrophy that can present with some similar neurological symptoms, including myelin loss in the brain, but typically does not affect cardiac function as severely.
- **Cerebrotendineous X-Lleanorhydrolase Deficiency (CTLD):**
  - A rare disorder characterized by progressive neurological deterioration and calcification of tendons and ligaments, though it does not usually present with severe cardiomyopathy.
- **Fatty Acid Oxidation Disorders:**
  - Such as Medium-Chain Acyl-CoA Dehydrogenase Deficiency (MCADD) or Very Long-Chain Acyl-CoA Dehydrogenase Deficiency (VLCADD), which can cause episodes of hypoglycemia, liver dysfunction, and myopathy but typically do not present with severe cardiomyopathy.
- **Dystrophic Epidermolysis Bullosa (DEB):**
  - Primarily affects the skin, causing blisters and erosions, but some subtypes can also present with muscle weakness or hypoglycemia, though usually without the cardiac manifestations described.
- **Metachromatic Leukodystrophy (MLD):**
  - A lysosomal storage disorder that primarily affects the nervous system but does not typically cause cardiomyopathy in its earlier stages.

These diagnoses cover a range of conditions from peroxisomal disorders to metabolic and neurological disorders, which can present with varying degrees of similarity based on their clinical manifestations.

**119. user 2025-02-18T12:21:57Z:**

**1. specific facial features,** periorbital subcutaneous tissue fullness, widened orbital distance, stellate irises, long midriffs, thick lips, wide mouths, and small jaws.

**2. cardiovascular disease,** elastin arteriopathy is present in 75% ~80% of patients and can affect any artery. Peripheral pulmonary stenosis (37% ~61%) is more common in infancy but improves with time. Supravalvular aortic stenosis is more common (35% ~65%) and worsens over time by age 5. Arterial stenosis leads to increased left heart resistance, cardiac hypertrophy, and heart failure. Other anomalies include renal artery stenosis, thoracic aortic stenosis, abdominal aortic stenosis, mitral valve prolapse and aortic valve insufficiency, mesenteric artery stenosis, and intracranial vascular anomalies. Hypertension can occur at any age and may be related in part to renal artery stenosis.

**3. Cognitive ability,** 75% of patients have mental retardation, which is usually mild. Cognitive impairment is characteristically manifested by relatively good short-term memory and verbal expression, but extreme weakness in visuospatial structural cognition. No gender differences in IQ have been reported, and children’s IQ is stable over time.

**4. personality, behavioural, and psychological abnormalities,** including excessive friendliness, excessive empathy, attention deficit, anxiety, and fear.

**5. sleep abnormalities,** with a 65% prevalence of sleep problems, including increased sleep latency and decreased sleep efficiency.

**6. eyes,** tear duct obstruction, hyperopia (67%), and strabismus (50%) are common. Cataracts have also been reported in adult patients.

**7. Ear, Nose, and Throat (ENT),** 50 per cent of patients have chronic otitis media, 90 per cent have increased sensitivity to sound, and 63 per cent of children and 92 per cent of adults have mild to moderate hearing impairment, mostly progressive sensorineural hearing loss. Most patients are hoarse or have a low voice.

**8. dental** , which may include baby teeth, enamel hypoplasia, and a misaligned bite.

**9. digestive system,** Chronic abdominal pain is a common symptom, and possible etiologies include gastroesophageal reflux, hiatal hernia, peptic ulcer, cholelithiasis, diverticulitis, ischaemic bowel disease, chronic constipation and anxiety somatisation. Hypercalcaemia may also lead to irritability, vomiting, constipation and muscle spasms.

**10. urinary system,** urinary frequency and enuresis are common in paediatric patients.

**11. neurological, muscular,** with low muscle tone and joint laxity in young children, leading to compensatory postural abnormalities to maintain body balance. Older children and adults have typical hypertonia and active deep tendon reflexes, resulting in a stiff and awkward gait, impaired fine motor function, and difficulty using tools and writing in patients of all ages. Adult patients will have cerebellar ataxia and tremor.

**12. Growth and development** .70% of infants have poor weight gain.

**13. idiopathic hypercalcaemia** . It occurs in infancy and childhood in 15-50% of patients and the most common symptoms are irritability, vomiting and constipation.

**14. endocrine.** Hypothyroidism (10 per cent), early puberty (50 per cent). Impaired glucose tolerance occurs in 26% of adolescent patients.

**qwen2.5_7b** 2025-02-18T12:22:01Z:

Based on the clinical manifestations provided, the most likely diagnosis is **Ehlers-Danlos Syndrome, Hypermobility Type with Cardiac Manifestations** (EDS-HCM), also known as EDS-VC (Vasculopathy and Cardiovascular Disease). This rare connective tissue disorder often includes multiple systems involvement, which aligns well with the symptoms described.

### Other Possible Diagnoses:

- **Marfan Syndrome**: Given the cardiovascular manifestations including arterial stenosis, supravalvular aortic stenosis, and other related cardiac issues, Marfan syndrome is another possibility.
- **Loeys-Dietz Syndrome**: This condition also involves significant vascular anomalies similar to EDS-VC, such as aortopathy and pulmonary artery stenosis, but can present with more severe systemic involvement.
- **VACTERL Association (Vertebral Abnormalities, Anal Atresia, Cardiac Defects, Tracheo-esophageal Fistula, Renal Anomalies, Limb Deformities)**: This condition often presents with a combination of congenital anomalies including cardiovascular defects and other organ malformations.
- **Hypertrophic Cardiomyopathy (HCM)**: Given the presence of heart failure, cardiac hypertrophy, and valve abnormalities, HCM is another plausible diagnosis to consider.
- **Chronic Kidney Disease**: The combination of renal artery stenosis and urinary symptoms could point towards chronic kidney disease as a contributing factor.

These conditions often present with overlapping clinical features that can be challenging to differentiate without further diagnostic testing such as genetic testing, imaging studies, or specialized evaluations.

**120. user 2025-02-18T12:22:37Z:**

**1. Haemorrhagic tendency** is a common manifestation of the disease, especially in blood-stained stools, and most children develop the disease in the neonatal period. The next manifestations are petechiae, ecchymosis, haemoptysis and haematuria, and some severe cases may have life-threatening gastrointestinal haemorrhage or intracranial haemorrhage. Bleeding in patients is caused by thrombocytopenia with reduction in platelet volume, but only about 25% of cases have the typical triad of symptoms.

**2. Infection.** Children are susceptible to a variety of infections, including herpes simplex virus, cytomegalovirus, Streptococcus pneumoniae, and fungi. The patient’s immunodeficiency worsens with age.

**3. Eczema.**  Eczema may be present in approximately 80% of children with WAS and varies widely in extent and severity. There may be typically severe, refractory eczema, or very mild or even no eczema manifestations.

**4. Autoimmune diseases.**  The most common is autoimmune haemolytic anaemia. Others include vasculitis, arthritis, renal disease, inflammatory bowel disease, neutropenia and autoimmune thrombocytopenic purpura.

**5. malignant tumours .** Children have a significantly higher risk of malignant tumours of the lymphatic system, especially lymphomas; non-Hodgkin’s lymphoma, lymphoblastic leukaemia, myelodysplastic syndromes, and spermatogonia have been reported.

**6. other .** Children with this disease may have completely normal platelet levels with persistent or recurrent neutropenia.

**qwen2.5_7b** 2025-02-18T12:22:42Z:

Based on the clinical manifestations provided, the most likely rare disease is **Wiskott-Aldrich Syndrome (WAS)**. This condition fits the described symptoms of bleeding diathesis due to thrombocytopenia and its complications, eczema, immune deficiencies leading to a wide range of infections, autoimmune diseases, and an increased risk of malignancies.

### Five Other Possible Diagnoses:

- **Severe Combined Immunodeficiency (SCID):**
  - SCID is characterized by severe immunodeficiency that leads to recurrent, life-threatening infections.
  - The clinical presentation includes poor growth, failure to thrive, chronic diarrhea, and persistent or fatal infections due to a variety of pathogens.
- **X-linked Lymphoproliferative Syndrome (XLP):**
  - XLP is also an X-linked disorder characterized by severe immunodeficiency with increased susceptibility to Epstein-Barr virus (EBV) infection.
  - The condition can manifest as fulminant and often fatal infectious mononucleosis, hemophagocytic lymphohistiocytosis, or lymphoproliferative disorders.
- **Chronic Granulomatous Disease (CGD):**
  - CGD is a primary immunodeficiency characterized by recurrent infections due to defective oxidative phosphorylation in phagocytes.
  - Clinical features include severe, recurrent bacterial and fungal infections, particularly of the skin, lungs, and bones.
- **X-linked Agammaglobulinemia (Bruton’s Disease):**
  - This condition results from mutations in the BTK gene, leading to a lack of mature B cells and a deficiency in immunoglobulins.
  - The primary symptom is recurrent pyogenic infections due to hypogammaglobulinemia.
- **Ataxia-Telangiectasia (AT):**
  - AT is an autosomal recessive disorder characterized by cerebellar ataxia, telangiectasias, and immunodeficiency.
  - Additional clinical manifestations include severe combined immunodeficiency with recurrent infections, progressive neurological deterioration, and a predisposition to malignancies.

These conditions, while distinct from WAS, share some overlapping symptoms such as infections, immune deficiencies, and other associated complications.

**121. user 2025-02-18T12:23:21Z:**

The most common clinical manifestations are fulminant infectious mononucleosis, anomalous immunoglobulinaemia and B-cell lymphoma following EBV infection. Aberimmunoglobulinaemia and B-cell lymphoma can occur in patients without EBV infection. Aberimmunoglobulinaemia usually presents with increased levels of IgA and IgM and decreased levels of IgG1 and IgG3. Lymphomas are predominantly non-Hodgkin B lymphomas. Other clinical manifestations include lymphocytic vasculitis, aplastic anaemia and lymphomatoid granulomatosis. In some patients, the clinical picture is mild, with only abnormal immunoglobulinaemia or mild EBV infection, possibly due to somatic gene repair.

In some patients, the predominant clinical manifestation is a haemophagocytic syndrome, with splenomegaly, which does not necessarily require EBV infection to be induced. Some patients may present with intestinal inflammation such as Crohn’s disease or colitis, but rarely develop lymphoma.

**qwen2.5_7b** 2025-02-18T12:23:26Z:

Based on the provided clinical manifestations and additional details, the most likely diagnosis is **X-linked Agammaglobulinemia (XLA)**, which is a rare primary immunodeficiency. The presence of abnormally high levels of IgA and IgM while having low levels of IgG1 and IgG3 is characteristic of XLA. Additionally, the occurrence of B-cell lymphoma following EBV infection or in patients without EBV infection suggests a condition that could be associated with XLA, such as **Common Variable Immunodeficiency (CVID)**.

Here are five other possible diagnoses to consider:

- **X-linked Agammaglobulinemia (XLA)**
- **Common Variable Immunodeficiency (CVID)**
- **Wiskott-Aldrich Syndrome (WAS)**
- **Hyper-IgM Syndrome** (can present with increased IgA and decreased IgG levels)
- **Severe Combined Immunodeficiency (SCID)**

Each of these conditions can present with immunoglobulin abnormalities, lymphoproliferative disorders, and various clinical manifestations as described in your case:

- **XLA**: Primarily affects males due to the X-linked inheritance, characterized by agammaglobulinemia.
- **CVID**: A heterogeneous group of primary immunodeficiencies with variable presentations including hypogammaglobulinemia.
- **WAS**: Characterized by eczema, thrombocytopenia, and recurrent infections.
- **Hyper-IgM Syndrome**: Caused by defects in the switching from IgM to other isotypes; can present with high IgM levels and low levels of other immunoglobulins.
- **SCID**: A severe primary immunodeficiency that can have a variety of causes including defects in T-cell function, which might not always show EBV-related manifestations.

Further diagnostic tests such as serology for specific antibodies, lymphocyte subset analysis, and genetic testing would be crucial to confirm the exact diagnosis.
