# Supplementary material for: Performance of ChatGPT-4o and Four Open-Source Large Language Models in Generating Diagnoses Based on China’s Rare Disease Catalog: Comparative Study
Source: J Med Internet Res. 2025 Jun 18;27:e69929. doi: 10.2196/69929 (PMC12192912; doi:10.2196/69929)
Supplement: Multimedia Appendix 1 [file jmir-v27-e69929-s001.zip › numbered_qwen2.5_7b_rag.docx]

# numbered_qwen2.5_7b_rag

**1. user:**

The disease can begin in early childhood. Typical clinical manifestations include kidney and eye changes and hearing impairment. Renal manifestations are hematuria, proteinuria and progressive deterioration of renal function, in which hematuria is often persistent microscopic hematuria, which can occur after exercise or fever, and is common in adolescents. Hearing changes were sensorineural hearing loss, which gradually aggravated with the course of disease. Eye abnormalities manifest as myopia, youth ring and cataract, but lack specificity. Three ocular changes of diagnostic significance include anterior conical lens, posterior pleomorphic corneal atrophy, and retinal macula (white or yellow particles around the fovea). The incidence of hearing impairment in male patients in China was higher than that reported abroad (68% vs 55%), while that in female patients was lower than that reported abroad (7% vs 45%). The incidence of ocular abnormalities was similar to that reported abroad. Leiomyomas may also occur in a few patients, involving the respiratory tract, gastrointestinal tract, and female reproductive tract; occasionally aneurysmal lesions, midfacial dysplasia, and mental retardation have been reported.

Patients often have a family history. Male patients with autosomal recessive inheritance and X-linked inheritance progressed faster, often entering end-stage renal disease at the age of 16~35, while female patients with autosomal dominant inheritance and X-linked inheritance had a slower course of disease and later onset of renal failure. The age at which renal failure occurs is similar for all male members of the same affected family, but can vary among male members of different families. Therefore, renal failure can be divided into juvenile type (renal failure occurs in adolescence) and adult type (renal failure occurs in adulthood) according to the age of the affected family. Adolescent type is more prominent in extrarenal manifestations, boys usually die before reproduction, disease genes are often new mutations, while adult type families have older patients and fewer new mutations.

**RAG-qwen2.5_7b**:

Based on the information provided, this description aligns with a rare genetic disorder known as Alport Syndrome. This condition typically begins in early childhood and is characterized by three primary clinical manifestations: kidney disease, hearing loss, and eye abnormalities.

### Renal Manifestations:

- **Hematuria**: Often persistent microscopic hematuria that can occur after exercise or fever, more common in adolescents.

- **Proteinuria**: Presence of protein in the urine.

- **Progressive Deterioration of Renal Function**: Leading to end-stage renal disease (ESRD) over time.

### Hearing Loss:

- **Sensorineural Hearing Loss**: Gradually worsening with the course of the disease, more prevalent among male patients in China compared to abroad (68% vs 55%).

### Eye Abnormalities:

- **Myopia**, **Youth Ring** and **Cataract**: These are common but lack specificity.

- **Diagnostic Significant Ocular Changes** include:

- Anterior conical lens

- Posterior pleomorphic corneal atrophy

- Retinal macula (white or yellow particles around the fovea)

### Genetic Inheritance Patterns and Prognosis:

- **Autosomal Recessive Inheritance**: Faster progression, often leading to ESRD between ages 16-35.

- **X-linked Inheritance**: Also rapid, with similar outcomes as autosomal recessive inheritance.

- **Autosomal Dominant Inheritance**: Slower disease course and later onset of renal failure.

- **Family History**: Often present; the age at which renal failure occurs is consistent within families but varies between different families.

### Additional Manifestations:

- **Leiomyomas** (benign smooth muscle tumors) may occur in respiratory tract, gastrointestinal tract, or female reproductive tract.

- **Aneurysmal Lesions**, **Midfacial Dysplasia**, and **Mental Retardation**: Occasional findings.

In summary, Alport Syndrome is a genetic disorder with these characteristic features. The clinical presentation can vary based on the mode of inheritance, with male patients generally experiencing more severe extrarenal manifestations compared to female patients.

**2. user:**

Patients often have no abnormal performance in the neonatal period. The first symptom is developmental retardation at about 6 months of age, but the typical clinical manifestations appear after 1 year of age, and it often takes several years for clinical diagnosis to be confirmed. According to the frequency of clinical manifestations, they were divided into three categories: common manifestations (100% patients), frequent manifestations (more than 80% patients) and correlated manifestations (20% ~80% patients).

1. All of them showed normal pregnancy history and head circumference at birth; no birth defects and abnormal biochemical indexes; brain MRI/CT showed no structural abnormalities except mild cortical atrophy and myelin dysplasia; motor milestones lagged behind without regression; severe developmental retardation occurred at 6~12 months; language disorders showed no or very little vocabulary, repetitive language and non-verbal communication ability were stronger than expressive language ability; motor or balance disorders often showed ataxia and tremor of limbs; Abnormal behavioral characteristics are frequent laughter or smiling, obvious excited movements or happy behavior, often accompanied by clapping or hyperactivity.
2. The head circumference often showed backward growth, and microcephaly was still present at the age of 2. Epilepsy often occurred before the age of 3. Abnormal EEG: characteristic high amplitude spike-slow wave.
3. Correlation: flat occipital region; tongue preference; sucking or swallowing disorders; infantile feeding difficulties; low muscle tone; large jaw; wide tooth space; frequent salivation; excessive chewing movements; strabismus; hypopigmentation of the skin; light hair and eye color compared to family (absent type only); hyperreflexia of lower limb tendons; lifting or bending of upper limbs, especially when walking; wide gait base; high sensitivity to heat; abnormal sleep-wake cycle, less sleep; obsession with water, paper, and plastic; Eating related behavioral abnormalities; Obesity (more common in older, non-deficit patients); scoliosis; constipation.

**RAG-qwen2.5_7b**:

Based on the provided information, this description aligns with a rare genetic disorder known as **Rett Syndrome**. Rett Syndrome is characterized by specific clinical manifestations that typically appear after the neonatal period and are often confirmed clinically over several years.

### Clinical Manifestations:

1. **Neonatal Period:**

- No abnormal performance.

- Normal pregnancy history and head circumference at birth.

- No birth defects or abnormal biochemical indexes.

- Brain MRI/CT shows no structural abnormalities, except mild cortical atrophy and myelin dysplasia.

- Motor milestones lag behind without regression.

- Severe developmental retardation occurs between 6 to 12 months.

2. **Language Disorders:**

- No or very little vocabulary.

- Repetitive language.

- Stronger non-verbal communication ability than expressive language ability.

- Ataxia and tremor of limbs due to motor or balance disorders.

3. **Behavioral Characteristics:**

- Frequent laughter or smiling.

- Obvious excited movements or happy behavior, often accompanied by clapping or hyperactivity.

4. **Head Circumference:**

- Backward growth in head circumference.

- Microcephaly present at age 2.

5. **Seizures and EEG:**

- Epilepsy often occurs before the age of 3.

- Abnormal EEG with characteristic high amplitude spike-slow wave patterns.

6. **Physical Features:**

- Flat occipital region.

- Tongue preference.

- Sucking or swallowing disorders.

- Infantile feeding difficulties.

- Low muscle tone.

- Large jaw and wide tooth space.

- Frequent salivation.

- Excessive chewing movements.

- Strabismus (crossed eyes).

- Hypopigmentation of the skin, light hair, and eye color compared to family.

- Hyperreflexia of lower limb tendons.

- Lifting or bending of upper limbs, especially when walking.

- Wide gait base.

7. **Sensory and Behavioral Issues:**

- High sensitivity to heat.

- Abnormal sleep-wake cycle with less sleep.

- Obsession with water, paper, and plastic.

- Eating-related behavioral abnormalities.

- Obesity more common in older, non-deficit patients.

- Scoliosis (spinal curvature).

- Constipation.

### Summary:

The clinical presentation described is consistent with Rett Syndrome, a rare genetic disorder affecting primarily girls. The key features include normal early development followed by severe regression in motor and language skills, characteristic behavioral patterns, and specific physical and neurological findings. Early diagnosis can be challenging but is crucial for appropriate management and support.

For further evaluation and confirmation, genetic testing for MECP2 mutations is recommended. If you need more detailed information or assistance with a specific aspect of Rett Syndrome, please let me know!

**3. user:**

1. Skeletal abnormalities: There is variation in the severity of the disease and the type of associated complications. Thoracic stenosis is present in all cases, and the thorax may be bell-shaped or long and narrow, with respiratory distress usually caused by a small, rigid thorax. The severity of respiratory insufficiency varies according to the type of thorax and the degree of underlying pulmonary dysplasia, and the age of onset varies. There is a negative correlation between the severity of respiratory insufficiency and the age at which the disease is diagnosed. Acute respiratory disease is more common in infants with severe restrictive and bell-shaped chests, and severe and fatal respiratory failure can occur in the early neonatal period. Infants with a long, narrow chest are more likely to present with mild respiratory involvement, which can eventually progress to chronic restrictive lung disease with recurrent respiratory infections. Short, deformed ribs cause intercostal muscle weakness and restricted breathing, leading to respiratory failure. Most children die within 2 years of age, with about half dying within 6 months of birth. Other skeletal anomalies include short ribs, short limbs, abnormally shaped pelvis, short fingers, and polydactyly. About 60% of patients have proximal cervical spinal stenosis.
2. Involvement of other organs: Renal lesions are present in about 40% of patients. The earliest sign of renal disease is impaired urinary concentration. Other signs include polyuria, proteinuria, elevated blood urea nitrogen and creatinine, and hypertension. Renal failure is the leading cause of death in this patient between the ages of 3 and 10 years. Hepatic involvement occurs in about 30% of cases and is manifested by hepatomegaly, elevated aminotransferases, cholestasis, cirrhosis and portal hypertension. The disease is occasionally associated with ocular developmental abnormalities, including optic nerve hypoplasia, retinal dystrophy, retinal pigmentation abnormalities, nystagmus, myopia and progressive visual impairment. There are also a few reports suggesting that these patients may also present with neurological abnormalities such as growth retardation, hypotonia, and central nervous system MRI abnormalities.

**RAG-qwen2.5_7b**:

Based on the provided information, it appears that the patient is likely suffering from **Jeune Syndrome (Asphyxiating Thoracic Dysplasia)**. Here’s a detailed breakdown of why this diagnosis fits:

1. **Skeletal Abnormalities:**

- **Thoracic Stenosis:** Present in all cases, with the thorax often being bell-shaped or long and narrow.

- **Respiratory Distress:** Caused by a small, rigid thorax, leading to varying degrees of respiratory insufficiency. The severity correlates negatively with the age at diagnosis, with more severe cases presenting earlier.

- **Acute Respiratory Disease:** More common in infants with severe restrictive and bell-shaped chests, potentially leading to fatal respiratory failure in early neonatal periods.

- **Short Ribs and Limbs:** Common skeletal anomalies include short ribs, short limbs, abnormally shaped pelvis, short fingers, and polydactyly.

- **Proximal Cervical Spinal Stenosis:** About 60% of patients have this condition.

2. **Involvement of Other Organs:**

- **Renal Lesions:** Present in about 40% of patients, with the earliest sign being impaired urinary concentration. Other signs include polyuria, proteinuria, elevated blood urea nitrogen and creatinine, hypertension, and renal failure.

- **Hepatic Involvement:** Occurs in about 30% of cases, manifested by hepatomegaly, elevated aminotransferases, cholestasis, cirrhosis, and portal hypertension.

- **Ocular Abnormalities:** Including optic nerve hypoplasia, retinal dystrophy, retinal pigmentation abnormalities, nystagmus, myopia, and progressive visual impairment.

- **Neurological Abnormalities:** Growth retardation, hypotonia, and central nervous system MRI abnormalities have been reported.

Given the clinical presentation of severe respiratory issues, skeletal anomalies, renal and hepatic involvement, and other systemic manifestations, Jeune Syndrome (Asphyxiating Thoracic Dysplasia) is a strong candidate for this patient's condition.

For further confirmation and management, it would be advisable to consult with specialists in pediatric pulmonology, nephrology, hepatology, ophthalmology, and neurology. Comprehensive multidisciplinary care is crucial for managing the complex clinical picture associated with Jeune Syndrome.

**4. user:**

The main symptoms include mental behavioural abnormalities, cognitive impairment, memory loss, seizures, speech disorders, movement disorders, involuntary movements, decreased level of consciousness, and autonomic dysfunction. Other accompanying symptoms include sleep disorders, focal damage to the CNS, brainstem and cerebellar symptoms, and involvement of peripheral nerves and neuromuscular junctions.

There are three main clinical types: (1) anti-NMDAR encephalitis, which is the most predominant type and is characterised by diffuse encephalitis. (ii) Borderline encephalitis, with mental behavioural abnormalities, temporal lobe epilepsy and near-memory deficits as the main symptoms. The common types of borderline encephalitis are anti-LGI1 antibody, anti-GABABR antibody and anti-AMPAR antibody-related encephalitis. (iii) Other types, including Morvant syndrome, anti-DPPX antibody-associated encephalitis, anti-IgLON5 antibody-associated encephalopathy, and autoimmune cerebellar ataxia.

**RAG-qwen2.5_7b**:

Based on the provided information, it appears that the patient is likely suffering from a form of **Anti-NMDAR Encephalitis** or one of its variants. Here’s a detailed breakdown of why this diagnosis fits and the key symptoms associated with each type:

### Main Symptoms:

- **Mental Behavioral Abnormalities:** Characterized by psychiatric symptoms such as depressed mood, irritability, apathy, anxiety, paranoia, delusions, and hallucinations.

- **Cognitive Impairment:** Manifested primarily through executive dysfunction, difficulty in decision-making, multitasking, and shifting cognitive goals. Memory loss may occur later in the disease course and can progress to dementia.

- **Speech Disorders:** Involving dysarthria (speech difficulties) and dysphagia (swallowing problems).

- **Movement Disorders:** Including chorea-like movements that affect the face, trunk, and limbs, leading to involuntary movements. As the disease progresses, these may be replaced by a Parkinson's-like pattern of stiffness and hyperkinesis.

- **Involuntary Movements:** Early mild, non-rhythmic movements that worsen over time, affecting diaphragm, pharynx, and laryngeal muscles.

- **Decreased Level of Consciousness:** Manifested as difficulty in maintaining certain voluntary movements, such as tongue extension.

- **Autonomic Dysfunction:** Including sleep disorders and other autonomic symptoms.

### Clinical Types:

1. **Anti-NMDAR Encephalitis (Most Predominant Type):**

- **Diffuse Encephalitis:** Characterized by widespread neurological involvement leading to a triad of movement disorders, psychiatric symptoms, and cognitive deficits.

2. **Borderline Encephalitis:**

- **Mental Behavioral Abnormalities:** Similar to Anti-NMDAR Encephalitis but with less severe or different patterns.

- **Temporal Lobe Epilepsy:** May present as seizures or epilepsy-like symptoms.

- **Near-Memory Deficits:** Memory impairments that are more localized compared to the diffuse nature seen in Anti-NMDAR Encephalitis.

3. **Other Types:**

- **Morvan Syndrome:** Characterized by severe autonomic dysfunction, including excessive sweating, salivation, and muscle weakness.

- **Anti-LGI1 Antibody-Associated Encephalitis:** Often involves limbic encephalitis with psychiatric symptoms and seizures.

- **Anti-GABABR Antibody-Related Encephalitis:** May present with ataxia (lack of coordination) and other neurological deficits.

- **Anti-AMPAR Antibody-Related Encephalitis:** Can cause cognitive impairment, memory loss, and psychiatric symptoms.

- **Autoimmune Cerebellar Ataxia:** Primarily affects the cerebellum leading to ataxic movements.

### Additional Symptoms:

- **Sleep Disorders:** Including insomnia or excessive sleepiness.

- **Focal Damage to CNS, Brainstem, and Cerebellar Symptoms:** May involve specific neurological deficits such as motor or sensory abnormalities.

- **Involvement of Peripheral Nerves and Neuromuscular Junctions:** Can lead to muscle weakness, fatigue, and other peripheral nerve symptoms.

### Summary:

Given the comprehensive set of symptoms including mental behavioral abnormalities, cognitive impairment, speech disorders, movement disorders, and autonomic dysfunction, the patient is most likely suffering from **Anti-NMDAR Encephalitis** or one of its variants. The specific type can be determined through detailed clinical assessment and antibody testing.

For accurate diagnosis and treatment, it would be advisable to consult a neurologist who specializes in autoimmune encephalitides for further evaluation and management. Antibody testing and other diagnostic tools will help confirm the exact subtype and guide appropriate therapy.

**5. user:**

The clinical manifestations of the disease are complex and varied, and lack specificity. Most of them involve multiple systems such as nerve, skin, respiratory, digestive and immune systems, and it is difficult to confirm the diagnosis from the clinical manifestations alone. It can be divided into two types: complete and partial.

1. Complete: It can be divided into two types: early-onset and late-onset. The most common manifestations are neurological symptoms, such as hypotonia, epilepsy, developmental delay, sensorineural deafness, ataxia, and progressive disorders of consciousness, etc. About half of the patients have ataxia, developmental delay, conjunctivitis, and visual problems, including visual problems. conjunctivitis, and visual problems, including optic atrophy. Metabolic disorders such as metabolic acidosis, hypoglycaemia and ketosis are often comorbid, and cutaneous manifestations include various forms of refractory rashes such as eczema, generalised erythema, and nappy rash, and patients may also present with respiratory symptoms such as hyperventilation, laryngeal wheezing, and apnoea, as well as digestive symptoms such as feeding difficulties, vomiting, and diarrhoea. Acute attacks can be triggered by infection, fever, fatigue, and a high protein diet. If treatment is not timely, it can often lead to irreversible damage. Patients with late-onset disease often develop in adolescence and may have no symptoms beforehand. The clinical phenotype is different from that of early-onset disease, which usually presents with peripheral neuropathy, muscle weakness, spastic paraparesis, skin rashes, and ocular problems, such as loss of vision and visual acuity, etc. Failure to treat the disease in a timely manner often leads to irreversible damage.
2. Partial : May develop at all ages or even without onset. The overall signs and symptoms of the child are relatively mild, starting only in emergency situations such as infections and starvation. Patients may have hypotonia, skin rashes, hair loss, acute neurological symptoms such as epilepsy and impaired consciousness.

**RAG-qwen2.5_7b**:

Based on the clinical manifestations provided, it seems that the disease you are describing is more consistent with Familial Mediterranean Fever (FMF) rather than the previously discussed conditions. Here’s a detailed analysis:

### Type 1 and Type 2 of FMF:

- **Type 1:**

- **Recurrent Inflammatory Episodes:** Characterized by recurrent fever, abdominal pain, and arthritis.

- **Fever:** Recurrent, triggered by various factors such as cold, strenuous exercise, surgery, infection, etc., resolving within 13 days.

- **Abdominal Pain:** Most common clinical manifestation (95% of patients), sudden onset with associated fever. Abdominal examination may show abdominal distension, tenderness, rebound pain, and loss of bowel sounds.

- **Arthritis:** Involves large joints of the lower limbs (75% of patients), presenting as joint swelling and pain.

- **Rash:** Seen in 7-40% of patients, typically involving the extensor side of the lower limbs with a rash resembling erysipelas.

- **Pleurisy:** Occurs in 45% of patients, presenting as sudden onset unilateral chest pain often accompanied by fever.

- **Type 2:**

- **Amyloidosis:** Starts with persistent proteinuria and often leads to end-stage renal disease. Amyloid can also deposit in the heart, gastrointestinal tract, liver, etc., leading to corresponding clinical manifestations.

### Comparison with the Described Condition:

The described condition involves multiple systems including nerve, skin, respiratory, digestive, and immune systems, which aligns well with FMF. However, there are some key differences:

- **Neurological Symptoms:** The described condition includes hypotonia, epilepsy, developmental delay, sensorineural deafness, ataxia, and progressive disorders of consciousness (early-onset), peripheral neuropathy, muscle weakness, spastic paraparesis, skin rashes, and ocular problems (late-onset). These symptoms are more comprehensive and involve a wider range of neurological and systemic issues.

- **Metabolic Disorders:** The described condition mentions metabolic acidosis, hypoglycemia, and ketosis, which are not typically associated with FMF but could be present in other conditions like familial Mediterranean fever-related amyloidosis (Type 2).

### Possible Diagnoses:

Given the complexity and specificity of the clinical manifestations, it is important to consider other hereditary inflammatory disorders such as:

- **Muckle-Wells Syndrome:** A related disorder that includes recurrent fevers, skin rashes, and sensorineural deafness.

- **TNF Receptor-Associated Periodic Syndrome (TRAPS):** Characterized by periodic fever, rash, and arthritis without the typical abdominal pain seen in FMF.

### Conclusion:

Based on the detailed clinical manifestations provided, the most likely diagnosis is Familial Mediterranean Fever (FMF). However, given the complexity of the described condition, it might be beneficial to consider other hereditary inflammatory disorders such as Muckle-Wells Syndrome or TRAPS for a more comprehensive evaluation. A thorough diagnostic workup including genetic testing and specific biomarkers would be essential for accurate diagnosis.

If you need further clarification or additional information, please let me know!

**6. user:**

The high prevalence age group is infancy, which manifests as adrenocortical insufficiency with a predominant salt loss, and common symptoms include hyperkalemia, hyponatremia with dehydration, metabolic acidosis, hypoglycaemia, vomiting, shock, and skin pigmentation. Most patients present with failure or incomplete development during puberty, or impaired fertility, oligospermia and azoospermia. A few patients may present with early childhood hyperandrogenaemia, simple saline corticosteroid deficiency, and pseudo-precocious puberty or spontaneous pubertal development during puberty. In mild cases, the disease may start in adulthood.

Patients have hyperpigmentation of the skin, especially in exposed areas, friction areas, scarring and areola, axilla, scrotum.

Patients have varying degrees of reduced resistance to various stresses such as infection and trauma. In the event of a stressful situation, adrenal crisis can occur, manifested by nausea, vomiting, abdominal pain or diarrhoea, severe dehydration, lowered blood pressure, rapid heart rate, weak pulse, mental disorders, and often high fever, hypoglycaemia, hyponatraemia and even life-threatening.

**RAG-qwen2.5_7b**:

Based on the clinical manifestations you've provided, the most likely rare disease diagnosis is **Congenital Adrenal Hyperplasia (CAH)**, specifically due to 21-hydroxylase deficiency. This condition is characterized by adrenocortical insufficiency with a predominant salt loss, leading to symptoms such as hyperkalemia, hyponatremia, dehydration, metabolic acidosis, hypoglycaemia, vomiting, and shock in infancy.

### Key Clinical Manifestations:

1. **Infancy:**

- Adrenocortical insufficiency with salt-wasting.

- Common symptoms include:

- Hyperkalemia

- Hyponatremia with dehydration

- Metabolic acidosis

- Hypoglycaemia

- Vomiting

- Shock

- Skin pigmentation

2. **Puberty and Adulthood:**

- Failure or incomplete development during puberty.

- Impaired fertility, oligospermia, and azoospermia.

- Early childhood hyperandrogenaemia.

- Simple saline corticosteroid deficiency.

- Pseudo-precocious puberty or spontaneous pubertal development.

3. **Adult Presentation:**

- Mild cases may start in adulthood with similar symptoms but less severe.

### Additional Clinical Manifestations:

1. **Skin Pigmentation:**

- Hyperpigmentation of the skin, especially in exposed areas, friction areas, scarring, areola, axilla, and scrotum.

2. **Adrenal Crisis:**

- Reduced resistance to various stresses such as infection and trauma.

- Adrenal crisis can occur during stressful situations, manifested by:

- Nausea

- Vomiting

- Abdominal pain or diarrhoea

- Severe dehydration

- Lowered blood pressure

- Rapid heart rate

- Weak pulse

- Mental disorders

- High fever

- Hyponatraemia

### Other Possible Diagnoses:

1. **Addison's Disease (Primary Adrenal Insufficiency):**

- Features adrenal insufficiency, hyperpigmentation, and electrolyte imbalances.

2. **X-linked Adrenal Hypoplasia Congenita:**

- Involves adrenal insufficiency and salt-wasting with issues related to puberty and fertility.

3. **Familial Glucocorticoid-Steering Enzyme Deficiency (FGSED):**

- A rare genetic disorder affecting cortisol production.

Given the detailed clinical manifestations, CAH due to 21-hydroxylase deficiency is the most likely diagnosis. For a definitive diagnosis, further testing such as adrenal function tests, electrolyte levels, and genetic testing for 21-hydroxylase deficiency would be necessary.

If you need more information or specific diagnostic steps, please let me know!

**7. user:**

The disease can develop from birth to early adulthood. Neonatal onset is the most common, often presenting as macrosomia, poor feeding, hunger intolerance and persistent hypoglycaemia. Hypoglycaemic episodes may present with lethargy and hypotonia, as well as neurological sequelae or even death, such as apnoea, seizures and coma. In the delayed type, patients often show a hypoglycaemic reaction, including symptoms such as pallor, sweating and palpitations.

**RAG-qwen2.5_7b**:

Based on the clinical manifestations you've provided, the most likely diagnosis is **Congenital Hyperinsulinism (CHI)**. This condition is characterized by excessive insulin secretion leading to persistent hypoglycemia, often presenting from birth or early childhood with symptoms such as poor feeding, hunger intolerance, and hypoglycemic episodes that can manifest with lethargy, hypotonia, and neurological sequelae including apnoea, seizures, and coma.

### Other Possible Diagnoses:

1. **Glycogen Storage Disease Type 1 (Von Gierke Disease):**

- This condition can lead to hypoglycemia during fasting, along with hepatomegaly, hyperlipidemia, and lactic acidosis.

2. **Congenital Adrenal Hyperplasia (CAH):**

- While CAH primarily affects the adrenal glands, it can cause hypoglycemia due to adrenal insufficiency, electrolyte imbalances, and poor feeding.

3. **Beckwith-Wiedemann Syndrome:**

- Characterized by neonatal hypoglycemia, macrosomia, and other physical anomalies.

4. **Mitochondrial Disorders:**

- These can cause energy production issues leading to hypoglycemia, hypotonia, and neurological symptoms.

5. **Insulinoma (in cases of very young onset):**

- Although extremely rare in neonates and young children, an insulin-producing tumor can cause persistent hypoglycemia.

### Diagnostic Steps:

To confirm the diagnosis, specific tests such as:

- Blood glucose monitoring

- Insulin levels

- C-peptide levels

- Genetic testing for CHI

For further evaluation of other potential diagnoses, additional tests may include:

- Liver function tests and glycogen storage studies for Glycogen Storage Disease Type 1

- Adrenal function tests for CAH

- Ultrasound or MRI for Beckwith-Wiedemann Syndrome

- Metabolic panel and genetic testing for mitochondrial disorders

If you need more detailed information on any of these conditions or specific diagnostic procedures, please let me know!

**8. user:**

1. Type I, abdominal distension, growth retardation, hypoglycemic convulsions, recurrent nosebleeds, diarrhoea and vomiting are the main reasons for children’s patients to visit the clinic, and very rarely, the first manifestation is hematuria, blood in the stool, recurrent bone fracture, anaemia or gout. Adult patients who are never diagnosed and treated may present with multiple hepatic adenomas, chronic renal failure, severe gout with multiple gout stones, and osteoporosis. Other rare presentations include pulmonary hypertension, diabetes mellitus, cerebrovascular disease, and hepatic adenoma carcinoma. On physical examination, short stature and markedly enlarged liver can be seen.
2. Type Ib, in addition to the above manifestations, patients may also have recurrent infections with neutropenia, oral ulcers, inflammatory bowel disease, perianal ulcers, arthritis and splenomegaly.
3. Infantile type II, which is divided into classical infantile type and non-classical infantile type according to its prognosis. Most of the classical infants present with generalised muscle weakness in the first month of life, delayed motor development, enlarged heart on chest X-ray, high QRS waves and short PR intervals on ECG, hypertrophic cardiomyopathy on cardiac ultrasound, and varying degrees of elevation of creatinine kinase, etc. Most of the infants die of left heart failure or cardiorespiratory failure after pulmonary infections in the first year of life. The non-classical infantile form presents with muscle weakness within the first year of life, lags in motor development, and most often dies of respiratory failure in early childhood.
4. Type II late-onset, with onset of disease after 1 year of age and as late as 60 years of age. Most of the patients present with chronic progressive proximal muscle weakness and respiratory insufficiency, cardiac involvement is rare, and respiratory failure is the main cause of death. Clinical manifestations include easy fatigue, difficulty in sitting up, walking up and down stairs, squatting and walking, and in a few cases, sudden onset of respiratory failure.

**RAG-qwen2.5_7b**:

Based on the provided information, it appears that these symptoms and presentations are characteristic of **Congenital Hyperinsulinism (CHI)**, particularly Type I. However, given the detailed descriptions, let's break down each type and their associated clinical features:

### Type I:

- **Main Symptoms in Children:**

- Abdominal distension

- Growth retardation

- Hypoglycemic convulsions

- Recurrent nosebleeds

- Diarrhea and vomiting

- **Rare First Manifestations:**

- Hematuria

- Blood in the stool

- Recurrent bone fractures

- Anemia or gout

- **Adult Presentations (if undiagnosed and untreated):**

- Multiple hepatic adenomas

- Chronic renal failure

- Severe gout with multiple gout stones

- Osteoporosis

- Pulmonary hypertension

- Diabetes mellitus

- Cerebrovascular disease

- Hepatic adenoma carcinoma

- **Physical Examination:**

- Short stature

- Markedly enlarged liver

### Type Ib:

- **Additional Symptoms:**

- Recurrent infections with neutropenia

- Oral ulcers

- Inflammatory bowel disease

- Perianal ulcers

- Arthritis and splenomegaly

### Infantile Type II:

- **Classical Infantile Type:**

- Generalized muscle weakness in the first month of life

- Delayed motor development

- Enlarged heart on chest X-ray

- High QRS waves and short PR intervals on ECG

- Hypertrophic cardiomyopathy on cardiac ultrasound

- Varying degrees of elevation of creatinine kinase

- **Prognosis:**

- Most infants die from left heart failure or cardiorespiratory failure after pulmonary infections in the first year of life.

- **Non-Classical Infantile Type:**

- Muscle weakness within the first year of life

- Lag in motor development

- Often dies from respiratory failure in early childhood

### Late-Onset Type II:

- **Onset After 1 Year of Age (up to 60 years):**

- Chronic progressive proximal muscle weakness

- Respiratory insufficiency

- Rare cardiac involvement

- Main cause of death: Respiratory failure

- **Clinical Manifestations:**

- Easy fatigue

- Difficulty sitting up, walking up and down stairs, squatting, and walking

- Sudden onset of respiratory failure in some cases

### Conclusion:

The symptoms described are consistent with CHI, particularly Type I. However, the detailed clinical features suggest a broader spectrum of presentations that could include other types or conditions within the broader category of metabolic disorders affecting insulin regulation.

For accurate diagnosis and management, it is crucial to consult a healthcare provider who can perform appropriate diagnostic tests such as blood glucose monitoring, genetic testing, and imaging studies. If you have specific cases or further details, please provide them for more targeted advice.

Would you like more information on any particular aspect of CHI or its management?

**9. user:**

1. Neuro-muscular system, causing neurological abnormalities, such as convulsions, coma, ataxia, and even psychiatric symptoms; muscle symptoms can also occur, including hyperreflexia, tremor, tetany, and muscle weakness. 2. Cardiovascular system, causing ECG changes, such as PR interval prolongation, QRS wave widening, etc. In severe cases, it can cause ventricular arrhythmia.
2. cardiovascular system, causing changes in electrocardiogram, such as prolongation of PR interval, widening of QRS wave, and so on, and in severe cases, it may cause ventricular arrhythmias.
3. Kidney, which may cause kidney stones, renal calcium deposits, renal insufficiency.
4. bone and joint system, can cause osteoporosis and osteochondrosis.
5. digestive system, may cause intestinal obstruction.
6. endocrine, patients can see diabetes mellitus or impaired glucose tolerance.
7. ocular symptoms, some types may be combined with ocular lesions, including nystagmus, visual field defects, myopia, etc. This group of diseases usually has a chronic course.

This group of diseases usually has a chronic course, with the neonatal and infantile onset types often being more severe and having a poorer prognosis. Some patients develop chronic renal failure during adolescence, and in some cases, failure to treat the disease in time can lead to severe mental retardation or even death.

**RAG-qwen2.5_7b**:

Based on the provided clinical manifestations, the most likely rare diseases that could fit this profile are **Bartter Syndrome** and **Gitelman Syndrome**. These renal tubular disorders affect electrolyte handling and can lead to a variety of systemic symptoms.

### Bartter Syndrome:

- **Neuro-muscular System:**

- Neurological abnormalities such as convulsions, coma, ataxia, and psychiatric symptoms.

- Muscular symptoms including hyperreflexia, tremor, tetany, and muscle weakness.

- **Cardiovascular System:**

- ECG changes such as PR interval prolongation and QRS wave widening.

- Severe cases can cause ventricular arrhythmias.

- **Kidney:**

- Kidney stones, renal calcium deposits, and renal insufficiency.

- **Bone and Joint System:**

- Osteoporosis and osteochondrosis.

- **Digestive System:**

- Intestinal obstruction.

- **Endocrine:**

- Diabetes mellitus or impaired glucose tolerance.

- **Ocular Symptoms:**

- Some types may be associated with ocular lesions such as nystagmus, visual field defects, and myopia.

### Gitelman Syndrome:

- **Neuro-muscular System:**

- Neurological abnormalities such as convulsions, coma, ataxia, and psychiatric symptoms.

- Muscular symptoms including hyperreflexia, tremor, tetany, and muscle weakness.

- **Cardiovascular System:**

- ECG changes such as PR interval prolongation and QRS wave widening.

- Severe cases can cause ventricular arrhythmias.

- **Kidney:**

- Kidney stones, renal calcium deposits, and renal insufficiency.

- **Bone and Joint System:**

- Osteoporosis and osteochondrosis.

- **Digestive System:**

- Intestinal obstruction.

- **Endocrine:**

- Diabetes mellitus or impaired glucose tolerance.

- **Ocular Symptoms:**

- Some types may be associated with ocular lesions such as nystagmus, visual field defects, and myopia.

### Other Possible Diagnoses:

1. **Hypoparathyroidism:**

- Can cause neuromuscular symptoms such as tetany and seizures due to hypocalcemia.

- Cardiovascular changes like prolonged QT interval.

2. **Familial Hypocalciuric Hypercalcemia (FHH):**

- Typically does not present with the wide range of systemic symptoms described, but can cause hypercalciuria and hypercalcemia.

3. **Lowe Syndrome:**

- A rare X-linked disorder affecting the kidneys, eyes, and brain.

- Can cause cataracts, renal tubular acidosis, and neurological abnormalities such as ataxia and intellectual disability.

4. **Cystinuria:**

- Characterized by kidney stones due to cystine accumulation in urine.

- May also present with neuromuscular symptoms and gastrointestinal issues.

5. **Fanconi Syndrome:**

- A renal tubular disorder causing malabsorption of various substances, including glucose and amino acids.

- Can lead to a variety of systemic symptoms such as muscle weakness, bone abnormalities, and renal failure.

Given the chronic course and severe neurological and cardiovascular manifestations, Bartter Syndrome or Gitelman Syndrome are highly likely diagnoses. However, further diagnostic tests such as electrolyte levels, urine analysis, and genetic testing would be necessary for definitive diagnosis.

Would you like more detailed information on any of these conditions or additional assistance with diagnosis?

**10. user:**

There are five types, of which the classic and intermediate types are the most common.

1. Classic type is the most common, accounting for 75 per cent of cases. Children are normal at birth, with early onset, severe symptoms and rapid progression. Usually, maple syrup odour appears in the cerumen 12 hours after birth; a special maple sugar odour is found in urine and sweat 12-24 hours after birth; ketonuria, irritability and feeding difficulties appear in 2-3 days; severe metabolic disorders and encephalopathic symptoms begin to appear in 4-5 days, which are manifested as difficulty in breastfeeding, paroxysmal vomiting, refusal to eat, lethargy, apnoea, convulsive seizures, heightened muscle tone and ‘fencing’. The symptoms of severe metabolic disorders and encephalopathy may include difficulty in breastfeeding, refusal to eat, lethargy, apnoea, convulsive seizures, increased muscle tone, and stereotypical movements such as ‘fencing’-like and ‘stomping’-like movements, etc. Coma and central respiratory failure may appear in the first 7-10 days of life, and most of the infants will die of severe metabolic disorders a few days after birth if they are not treated promptly.
2. Intermediate type can develop at any age. The symptoms are less severe than those of the classic type and include slow growth, delayed development, feeding difficulties, irritability, a maple syrup odour in the cerumen, and severe metabolic disorders and encephalopathy under stress. 3. Intermittent Type The intermittent type is characterised by episodic seizures.
3. Intermittent type: intermittent seizures, normal growth and development in the early stage, mostly under stressful situations such as infections and surgeries, manifested by episodic ataxia and ketoacidosis, and a very small number of severe cases may cause death. Branched-chain amino acid concentrations are normal during the intermittent period, and are elevated during the episodic period, similar to the classic type.

**RAG-qwen2.5_7b**:

Based on the clinical manifestations you've described, the most likely diagnosis is **Maple Syrup Urine Disease (MSUD)**. Specifically, this condition can manifest in different types based on the severity and age of onset:

1. **Classic Type**:

- This type accounts for approximately 75% of MSUD cases.

- Children are typically normal at birth but show early onset severe symptoms with rapid progression.

- Symptoms include:

- Appearance of maple syrup odor in cerumen (earwax) within 12 hours after birth.

- Special maple sugar odor in urine and sweat within 12-24 hours after birth.

- Ketonuria, irritability, and feeding difficulties appear around 2-3 days after birth.

- Severe metabolic disorders and encephalopathic symptoms begin to manifest around 4-5 days after birth. These symptoms include difficulty in breastfeeding, paroxysmal vomiting, refusal to eat, lethargy, apnea, convulsive seizures, heightened muscle tone, and stereotypical movements such as "fencing" or "stomping."

- Coma and central respiratory failure may occur within the first 7-10 days of life.

- Without prompt treatment, most infants will die due to severe metabolic disorders a few days after birth.

2. **Intermediate Type**:

- This type can develop at any age and has less severe symptoms compared to the classic type.

- Symptoms include slow growth, delayed development, feeding difficulties, irritability, and a maple syrup odor in the cerumen.

- Severe metabolic disorders and encephalopathy may occur under stress.

3. **Intermittent Type**:

- This type is characterized by episodic seizures.

- During normal periods, there are no significant symptoms, but during episodes, there can be intermittent ataxia (loss of coordination) and ketoacidosis.

- Branched-chain amino acid concentrations are normal during the interepisode period but elevated during the episode, similar to the classic type.

Given these clinical features, it is crucial to perform diagnostic tests such as blood and urine analysis for branched-chain amino acids, organic acid testing, and genetic testing for mutations in the BCKDHA or BCKDK genes, which are responsible for MSUD. Early diagnosis and prompt treatment with a low-protein diet can significantly improve outcomes.

Would you like more detailed information on any specific aspect of Maple Syrup Urine Disease?

**11. user:**

The disease can start from birth to old age and can be acute or insidious. Almost any system or organ can be involved, with a predominance of energy-demanding organs such as the brain, heart, kidneys, eyes, ears, muscles, and gastrointestinal tract. Patients may present with a clinical syndrome of some kind, a single symptom or a combination of symptoms.

**RAG-qwen2.5_7b**:

Based on the provided information, the broad and variable symptom presentation suggests a wide range of potential diagnoses. Given that the disease can start from birth to old age and affect almost any system or organ with a predominance in energy-demanding organs such as the brain, heart, kidneys, eyes, ears, muscles, and gastrointestinal tract, several conditions could fit this description:

1. **Mitochondrial Diseases**:

- These disorders arise due to dysfunctional mitochondria, which are crucial for cellular energy production.

- Symptoms can include neurological issues (e.g., seizures, developmental delays), cardiomyopathy, liver dysfunction, muscle weakness, and gastrointestinal problems.

2. **Lysosomal Storage Disorders**:

- These conditions result from defects in lysosomal function, leading to the accumulation of undigested substances within cells.

- Common symptoms can include neurological issues (e.g., cognitive decline, movement disorders), organomegaly, skeletal abnormalities, and skin changes.

3. **Peroxisomal Disorders**:

- Dysfunctions in peroxisomes can lead to a variety of symptoms affecting the nervous system, liver, eyes, and other organs.

- Symptoms might include neurological issues (e.g., ataxia, vision problems), liver dysfunction, hearing loss, and developmental delays.

4. **Congenital Disorders of Glycosylation (CDG)**:

- These are inherited metabolic disorders that affect glycosylation processes in the body.

- Symptoms can include neurological issues (e.g., intellectual disability, seizures), liver involvement, muscle weakness, and skin changes.

5. **Metabolic Myopathies**:

- Conditions like glycogen storage diseases and fatty acid oxidation disorders primarily affect muscles but can also impact other organs.

- Symptoms might include muscle pain, weakness, cardiomyopathy, and gastrointestinal issues.

6. **Multi-Systemic Atrophy Disorders**:

- These conditions can present with various neurological symptoms affecting multiple systems.

- Common symptoms include autonomic dysfunction (e.g., orthostatic hypotension), movement disorders, and cognitive decline.

Given the broad spectrum of potential diagnoses, it is crucial to perform comprehensive diagnostic tests such as genetic testing, biochemical assays, and imaging studies. Early diagnosis and appropriate management can significantly improve patient outcomes.

Would you like more detailed information on any specific condition or further assistance in understanding these diagnoses?

**12. user:**

The prevalence of the disease is between 5 and 50 years old, with a median age of onset of 39 years. The prevalence of women is significantly higher than that of men, with a female:male ratio of (9~11):1. Most of the cases have an acute/subacute onset, and the clinical manifestations include six core syndromes: (1) optic neuritis; (2) acute myelitis; (3) posterior pole syndrome; (4) acute brainstem syndrome; (5) symptomatic sleep attacks or acute mesencephalic clinical syndrome with typical MRI foci of the mesencephalon; and (6) symptomatic cerebral syndrome with typical brain lesions. It is a highly recurrent and disabling disease, with more than 90 per cent of patients having a recurrent course, and the majority of patients are left with more severe neurological disability.

**RAG-qwen2.5_7b**:

Based on the provided clinical manifestations and demographic information, the most likely diagnosis is **Neuromyelitis Optica Spectrum Disorder (NMOSD)**. Here’s a detailed explanation:

### Clinical Manifestations:

1. **Optic Neuritis**: Inflammation of the optic nerve leading to vision loss.

2. **Acute Myelitis**: Inflammation of the spinal cord causing symptoms such as paralysis, pain, and sensory disturbances.

3. **Posterior Pole Syndrome**: A specific type of optic neuritis affecting the posterior part of the retina.

4. **Acute Brainstem Syndrome**: Symptoms related to brainstem involvement, including cranial nerve palsies and respiratory issues.

5. **Symptomatic Sleep Attacks or Acute Mesencephalic Clinical Syndrome with Typical MRI Foci of the Mesencephalon**: Characterized by sudden sleep attacks and specific MRI findings in the midbrain.

6. **Symptomatic Cerebral Syndrome with Typical Brain Lesions**: Neurological symptoms due to brain lesions, often seen on imaging.

### Demographic Information:

- **Age of Onset**: Between 5 and 50 years old, with a median age of onset at 39 years.

- **Gender Prevalence**: Significantly higher in women, with a female:male ratio of (9~11):1.

### Recurrence and Disability:

- Highly recurrent disease: More than 90% of patients have a relapsing course.

- Significant neurological disability: Most patients are left with severe neurological impairment.

### Other Possible Diagnoses to Consider:

1. **Multiple Sclerosis (MS)**: While it can present with optic neuritis and myelitis, the demographic profile and MRI findings differ from NMOSD.

2. **Sarcoidosis (Neurosarcoidosis)**: Can cause similar neurological symptoms but is less common than NMOSD.

3. **Systemic Lupus Erythematosus (SLE) with CNS Involvement**: More common in females, can present with a variety of neurological symptoms including optic neuritis and myelitis.

4. **Antiphospholipid Syndrome (APS) with Central Nervous System Manifestations**: May cause recurrent neurological symptoms but is less specific for NMOSD.

5. **Behçet's Disease with Neurological Involvement (Neuro-Behçet's)**: Can lead to recurrent neurological symptoms, including brainstem and cerebral involvement.

### Conclusion:

Given the acute/subacute onset, the presence of multiple core syndromes, high female prevalence, and significant recurrence leading to severe disability, NMOSD is the most likely diagnosis. However, further diagnostic tests such as serum autoantibody testing (specifically NMO-IgG), MRI findings, and clinical evaluation are essential for confirmation.

Would you like more detailed information on any specific aspect of NMOSD or further assistance in understanding these diagnoses?

**13. user:**

Hearing impairment. In the past, it was thought that the deafness was congenital, which could be identified by hearing screening, and that it would be apparent from birth, mostly involving both sides, and symmetrically severe prelingual deafness. In recent years, it has been found that individual cases may present with asymmetrical or unilateral hearing loss, and the degree of hearing loss is highly variable, ranging from mild to very severe in different patients; progressive deafness that occurs after birth is also not uncommon.

**RAG-qwen2.5_7b**:

Based on the clinical manifestations you have described, the most likely rare disease to consider is **Alport Syndrome**. Here’s why:

1. **Hearing Loss**: Alport Syndrome can cause progressive hearing loss, which can be either prelingual (occurring before the acquisition of speech) or postlingual (occurring after the acquisition of speech). The degree of hearing loss can vary from mild to severe and is often asymmetrical or unilateral.

2. **Genetic Basis**: Alport Syndrome is a genetic disorder caused by mutations in the COL4A5, COL4A6, or COL4A3 genes, which encode for components of the glomerular basement membrane. This genetic basis explains the variability in the degree and pattern of hearing loss among patients.

3. **Other Symptoms**: In addition to hearing loss, Alport Syndrome can also cause kidney disease (often leading to renal failure) and eye abnormalities such as a thin lens or retinal detachment.

Given these characteristics, Alport Syndrome fits well with the clinical presentation you described, where hearing loss is variable in degree and pattern, and can occur both prenatally and postnatally.

Other possible diagnoses include:

1. **Usher Syndrome**: While it does involve hearing loss, it also includes a progressive vision disorder called retinitis pigmentosa.

2. **Jervell and Lange-Nielsen Syndrome**: This condition involves congenital deafness and long QT syndrome affecting the heart's electrical system.

3. **Wolfram Syndrome**: This rare genetic disorder causes diabetes insipidus, diabetes mellitus, optic atrophy, and deafness.

4. **Branchio-Oto-Renal (BOR) Syndrome**: This genetic disorder can lead to hearing loss, as well as kidney issues and branchial cysts or fistulas.

For a definitive diagnosis, genetic testing for Alport Syndrome would be recommended, along with comprehensive evaluations of renal function and ocular health if necessary.

Would you like more detailed information on any specific aspect of Alport Syndrome or further assistance in understanding these diagnoses?

**14. user:**

Bradykinesia, tremor, and myotonia. Levodopa-induced motor complications are more likely to occur after treatment. In terms of non-motor symptoms, mood disorders (e.g., depression, anxiety, irritability, etc.) and behavioural disorders (e.g., compulsive dosage increases, impulse control disorders, stereotyped behaviour, etc.) are likely to occur.

**RAG-qwen2.5_7b**:

Based on the clinical manifestations you've described—bradykinesia, tremor, and myotonia—with levodopa-induced motor complications and non-motor symptoms such as mood disorders and behavioral disorders—the most likely rare disease is **Dopa-Responsive Dystonia (DRD)**, also known as Segawa Syndrome.

### Key Characteristics of Dopa-Responsive Dystonia (DRD):

1. **Motor Symptoms**:

- **Bradykinesia**: Slowness of movement.

- **Tremor**: Often present and can be action or rest tremor.

- **Myotonia**: Delayed relaxation of muscles after contraction.

2. **Levodopa-Induced Complications**:

- Levodopa treatment is often effective initially but can lead to complications such as dyskinesias (involuntary movements) over time.

3. **Non-Motor Symptoms**:

- **Mood Disorders**: Depression, anxiety, irritability.

- **Behavioral Disorders**: Compulsive dosage increases, impulse control disorders, stereotyped behavior.

### Other Possible Diagnoses:

1. **Juvenile Parkinsonism**: Similar to Parkinson's disease but occurs in younger individuals. It can also present with bradykinesia and tremor, and may respond to levodopa treatment.

2. **Myotonic Dystrophy**: Primarily a muscular disorder characterized by myotonia, which can have neurological complications including mood and behavioral disorders.

3. **Wilson's Disease**: A genetic disorder affecting copper metabolism that can cause neurological symptoms like tremor and bradykinesia, as well as mood disturbances.

4. **Parkinson's Plus Syndromes**:

- **Multiple System Atrophy (MSA)**: Can present with Parkinson-like symptoms and mood/behavioral disorders but is generally less responsive to levodopa.

- **Progressive Supranuclear Palsy (PSP)**: Also presents with Parkinson-like symptoms and can have mood and behavioral disturbances, though it is not typically responsive to levodopa.

### Diagnostic Considerations:

- **Detailed Clinical History**: Reviewing the patient's history for any familial patterns or specific triggers.

- **Genetic Testing**: To rule out conditions like Wilson's disease or myotonic dystrophy.

- **Neuroimaging**: MRI or CT scans can help rule out other neurological disorders.

For an accurate diagnosis, a comprehensive evaluation including genetic testing and possibly neuroimaging would be necessary. If you need further assistance with any specific aspect of these diagnoses or additional tests, feel free to ask!

**15. user:**

**1. polyneuropathy**, often presenting as a symmetrical sensory and/or motor peripheral neuropathy of the extremities, progressing gradually from distal to proximal.

**2. organomegaly,** including hepatomegaly, splenomegaly, or lymph node enlargement; lymph node biopsy is often suggestive of Castleman disease.

**Endocrine abnormalities,** including hypogonadism (e.g., male impotence, breast development), hypothyroidism, abnormal glucose metabolism (e.g., diabetes mellitus), and adrenal insufficiency.

**4. skin changes,** which may be manifested by deepening of skin colour, cutaneous glomeruloid haemangiomas, white nails, polycythemia, hirsutism, and cyanosis of the hands and feet.

**5. increased extracirculatory water load,** including limb oedema, ascites, pleural effusion, pericardial effusion, and optic papilla oedema.

**Sclerosing bone disease,** is an important clinical manifestation. It may be manifested as bone pain or without clinical symptoms. Sclerosing bone disease can be detected significantly more frequently with CT scanning of the bones, and can be visualised as simple sclerosis of the bones, mixed sclerotic and osteolytic lesions, or simple osteolytic changes.

**7. erythrocytosis and/or thrombocytosis** **8.

**8. pulmonary hypertension** , patients may present with pulmonary hypertension, as evidenced by reduced activity tolerance, hypoxaemia, etc.

**9. cerebral infarction**.

**RAG-qwen2.5_7b**:

Based on the clinical manifestations you have described, the most likely rare disease diagnosis is **POEMS Syndrome** (Polyneuropathy, Organomegaly, Endocrinopathy, M-protein, and Skin changes). Here are the key features of POEMS syndrome that align with your description:

1. **Polyneuropathy**: Often presenting as a symmetrical sensory and/or motor peripheral neuropathy of the extremities, progressing gradually from distal to proximal.

2. **Organomegaly**: Including hepatomegaly, splenomegaly, or lymph node enlargement; lymph node biopsy is often suggestive of Castleman disease.

3. **Endocrine Abnormalities**: Including hypogonadism (e.g., male impotence, breast development), hypothyroidism, abnormal glucose metabolism (e.g., diabetes mellitus), and adrenal insufficiency.

4. **Skin Changes**: Manifested by deepening of skin color, cutaneous glomeruloid hemangiomas, white nails, polycythemia, hirsutism, and cyanosis of the hands and feet.

5. **Increased Extracirculatory Water Load**: Including limb edema, ascites, pleural effusion, pericardial effusion, and optic papilla edema.

6. **Sclerosing Bone Disease**: An important clinical manifestation that can be detected with CT scanning of the bones, often visualized as simple sclerosis or mixed sclerotic and osteolytic lesions.

7. **Erythrocytosis and/or Thrombocytosis**: Increased red blood cell count and/or platelet count.

8. **Pulmonary Hypertension**: Patients may present with reduced activity tolerance and hypoxemia.

### Other Possible Diagnoses:

1. **Castleman Disease**: This condition can be associated with organomegaly, endocrinopathy, and polyneuropathy, as well as inflammatory symptoms. It sometimes overlaps with POEMS syndrome.

2. **Amyloidosis**: Can cause peripheral neuropathy, organomegaly, and other systemic symptoms.

Given the comprehensive nature of your description, POEMS syndrome is a strong candidate for the diagnosis. However, further diagnostic tests such as blood work, imaging studies (e.g., CT scans), and specific biomarker testing would be necessary to confirm the diagnosis.

If you need more detailed information on any aspect of these conditions or require additional diagnostic steps, feel free to ask!

**16. user:**

Common manifestations in the foetal period include reduced foetal activity, excess amniotic fluid and breech presentation. In the neonatal period hypotonia is one of the hallmark features of this disorder, and severe hypotonia can lead to asphyxia, feeding difficulties, weak sucking, and weak crying. In infancy and early childhood, the main manifestations are growth retardation and lagging cognitive, motor and language development. Appetite increases with age and obesity can occur if eating is not restricted. Short stature is usually present in school age, and personality traits such as temper tantrums, stubbornness, and obsessive-compulsive disorder gradually appear. Abnormal behaviours include intense foraging, skin scratching, and autistic-like behaviour. Seizures occur in about 25 per cent of patients. Delayed or incomplete appearance of secondary sexual characteristics may occur in adolescent males and females. Obesity can lead to complications such as sleep apnoea, pulmonary heart disease, insulin resistance and even diabetes. Scoliosis, osteoporosis, and gastroesophageal reflux are also more common. The main manifestations in adulthood are obesity and its complications, including cardiovascular problems, diabetes and sleep apnoea. Other manifestations are mental abnormalities and infertility.

Fatal risks: choking due to uncoordinated mouth movements, hypotonia, polyphagia, gulping habits and inadequate chewing, and gastric rupture due to overeating.

Physical examination reveals characteristic facial features that become progressively more typical, including almond eyes, small mouth, thin upper lip, downward-facing corners of the mouth, small hands and feet, short stature, fair skin, low muscle tone, and genital hypoplasia (e.g., cryptorchidism, scrotal hypoplasia, or clitoral hypoplasia).

**RAG-qwen2.5_7b**:

Based on the clinical manifestations and features provided, the most likely diagnosis is **Prader-Willi Syndrome (PWS)**. Here is a detailed breakdown of the key characteristics that align with PWS:

1. **Foetal Period**:

- Reduced foetal activity.

- Excess amniotic fluid.

- Breech presentation.

2. **Neonatal Period**:

- Hypotonia, which is one of the hallmark features.

- Severe hypotonia can lead to asphyxia, feeding difficulties, weak sucking, and weak crying.

3. **Infancy and Early Childhood**:

- Growth retardation.

- Lagging cognitive, motor, and language development.

- Increased appetite with age, leading to obesity if not restricted.

- Short stature in school age.

- Personality traits such as temper tantrums, stubbornness, and obsessive-compulsive disorder.

4. **Abnormal Behaviors**:

- Intense foraging.

- Skin scratching.

- Autistic-like behavior.

- Seizures occur in about 25% of patients.

5. **Adolescence and Adulthood**:

- Delayed or incomplete appearance of secondary sexual characteristics.

- Obesity leading to complications such as sleep apnoea, pulmonary heart disease, insulin resistance, and diabetes.

- Scoliosis, osteoporosis, and gastroesophageal reflux are more common.

- Main manifestations include obesity and its complications (cardiovascular problems, diabetes, sleep apnoea).

- Mental abnormalities and infertility.

6. **Physical Examination**:

- Characteristic facial features: almond-shaped eyes, small mouth, thin upper lip, downward-facing corners of the mouth, small hands and feet.

- Short stature and fair skin.

- Low muscle tone.

- Genital hypoplasia (e.g., cryptorchidism, scrotal hypoplasia, or clitoral hypoplasia).

7. **Fatal Risks**:

- Choking due to uncoordinated mouth movements.

- Hypotonia.

- Polyphagia and gulping habits leading to inadequate chewing.

- Gastric rupture due to overeating.

Given these clinical features, Prader-Willi Syndrome is the most likely diagnosis. If further confirmation is needed, genetic testing can be performed to identify the specific genetic abnormalities associated with PWS.

**17. user:**

Patients often start in infancy or adolescence, with roughly equal male and female ratios. The water content of secretions from the epithelial cells of the respiratory tract, pancreas and biliary tract is reduced, and the secretions become viscous and difficult to clear. The lungs are the most commonly affected organ, and patients present with chronic bacterial infections and colonisation of the respiratory tract with pathogens, often initially Haemophilus influenzae and Staphylococcus aureus, and ultimately with chronic colonisation and/or infection with Pseudomonas aeruginosa or Klebsiella onionis. Once infection occurs, neutrophils are recruited in large numbers into the lung tissue and release elastase, which causes destruction of the lung tissue, leading to the formation of bronchiectasis. The patient’s bronchiectasis often starts at an early age and often begins in the upper lobes, in contrast to the majority of infections that result in the formation of bronchiectasis that is primarily located in the middle and lower lungs.

Patients often present with sweat stains left on dark-coloured clothing, or parents notice salty skin in their children. Abnormalities in the flow of bile and pancreatic fluid can lead to indigestion and malabsorption, as well as liver or pancreatic disease, and in severe cases, associated diabetes. The viscous intestinal fluid may also make the patient more susceptible to intestinal obstruction and rectal prolapse. In addition, male patients may develop impaired sperm movement in the vas deferens and congenital absence of the vas deferens (CBAVD).

**RAG-qwen2.5_7b**:

Based on the clinical manifestations you have described, the most likely diagnosis is **Cystic Fibrosis (CF)**. Here is a detailed breakdown of why this condition fits the provided symptoms:

1. **Age of Onset and Gender Ratio**:

- Patients often start in infancy or adolescence with roughly equal male and female ratios.

2. **Viscous Secretions**:

- The water content of secretions from the epithelial cells of the respiratory tract, pancreas, and biliary tract is reduced, leading to viscous and difficult-to-clear secretions.

- This condition affects multiple organs but primarily impacts the lungs, pancreas, and biliary system.

3. **Lung Infections**:

- The lungs are the most commonly affected organ.

- Patients present with chronic bacterial infections and colonization of the respiratory tract with pathogens such as Haemophilus influenzae and Staphylococcus aureus.

- Over time, these infections can lead to chronic colonization and/or infection by Pseudomonas aeruginosa or Klebsiella pneumoniae.

- Neutrophils are recruited into the lung tissue, release elastase, which causes destruction of the lung tissue, leading to bronchiectasis.

4. **Bronchiectasis**:

- Bronchiectasis often starts at an early age and typically begins in the upper lobes, unlike other infections that primarily affect the middle and lower lungs.

5. **Sweat Stains and Saltiness**:

- Patients may present with sweat stains on dark-colored clothing or parents noticing salty skin.

- This is due to the presence of high levels of sodium chloride in the sweat, a hallmark of CF.

6. **Digestive Issues**:

- Abnormalities in the flow of bile and pancreatic fluid can lead to indigestion and malabsorption.

- These issues may result in liver or pancreatic disease, and severe cases can be associated with diabetes.

- The viscous intestinal fluid increases susceptibility to intestinal obstruction and rectal prolapse.

7. **Male Infertility**:

- Male patients may develop impaired sperm movement in the vas deferens and congenital absence of the vas deferens (CBAVD).

Given these clinical features, Cystic Fibrosis is indeed the most likely diagnosis based on the provided information. This condition affects multiple organ systems, particularly the lungs, pancreas, and biliary tract, with characteristic symptoms such as thick secretions, recurrent infections, and digestive issues.

For further confirmation, diagnostic tests such as sweat chloride testing, genetic testing for CFTR mutations, and imaging studies like chest X-rays or CT scans would be recommended.

**18. user:**

Patients present with oropharyngitis, otitis media, respiratory infections, cellulitis and skin infections, often due to staphylococcal and streptococcal infections. Oral ulcers and painful gingivitis occur in most children before the age of 2 years in severe neutropenia with decreased bone marrow reserve. If associated with diffuse gastrointestinal lesions, it may cause abdominal pain and diarrhoea similar to Crohn’s disease. Neutropenia can lead to recurrent bacterial or fungal infections in various areas, with the main sites of involvement being the skin and mucous membranes, ears, nose, throat and lungs. The infections are often severe or even fatal. About 15% of patients progress to acute leukaemia or myelodysplastic syndrome. The children usually do not have characteristic malformations.

**RAG-qwen2.5_7b**:

Based on the clinical manifestations you described, the most likely diagnosis is **Severe Congenital Neutropenia (SCN), also known as Kostmann syndrome**. This condition is characterized by severe neutropenia leading to recurrent bacterial infections in various areas such as the skin, mucous membranes, ears, nose, throat, and lungs. The infections can be severe or even fatal, and about 15% of patients may progress to acute leukemia or myelodysplastic syndrome.

Here are some key points from your description that align with SCN:

1. **Recurrent Infections**:

- Oropharyngitis, otitis media, respiratory infections, cellulitis, and skin infections often due to staphylococcal and streptococcal infections.

- Oral ulcers and painful gingivitis in most children before the age of 2 years.

2. **Severe Neutropenia**:

- Severe neutropenia with decreased bone marrow reserve, leading to recurrent bacterial or fungal infections.

3. **Gastrointestinal Involvement**:

- Diffuse gastrointestinal lesions causing abdominal pain and diarrhea similar to Crohn's disease.

4. **Progression to Hematological Malignancies**:

- About 15% of patients may progress to acute leukemia or myelodysplastic syndrome.

5. **No Characteristic Malformations**:

- Children usually do not have characteristic malformations, which differentiates this condition from others with more pronounced physical abnormalities.

Given these clinical features, Severe Congenital Neutropenia (SCN) is the most appropriate diagnosis based on the provided information. For further confirmation, diagnostic tests such as absolute neutrophil count (ANC), bone marrow biopsy, and genetic testing for mutations in the ELANE gene would be recommended.

**19. user:**

Typical Patients present with both CNS damage and hypoadrenocorticism. Neurological symptoms and symptoms of hypoadrenocorticism may occur simultaneously or sequentially. In a minority of patients, they may occur alone. The manifestations of adrenocortical insufficiency vary in severity. It is most common in children, accounting for 35% of all patients. The onset of symptoms occurs between the ages of 4 and 8 years. The initial symptoms include poor concentration, memory loss, learning difficulties, unsteady gait and abnormal behaviour, followed by visual and auditory loss, dysarthria, seizures, ataxia, paralysis and dementia. Clinical manifestations progressively worsen, culminating in blindness or deafness and complete paralysis. Some exhibit deafferentation and eventually die of central respiratory failure, brain herniation, and infection. Most patients have adrenocortical impairment when they first develop neurological symptoms.

**RAG-qwen2.5_7b**

Based on the clinical manifestations provided, the rare disease most likely to be diagnosed is **Adrenoleukodystrophy (ALD)**. ALD is a genetic disorder that affects both the central nervous system (CNS) and the adrenal glands, leading to adrenocortical insufficiency (hypoadrenocorticism). Here are the key points that align with this diagnosis:

1. **Clinical Presentation**:

- Patients typically present with both CNS damage and hypoadrenocorticism.

- Neurological symptoms and symptoms of hypoadrenocorticism may occur simultaneously or sequentially.

- In a minority of patients, one set of symptoms may occur alone.

2. **Age of Onset**:

- The most common form (cerebral ALD) typically begins between the ages of 4 and 8 years.

3. **Initial Symptoms**:

- Poor concentration, memory loss, learning difficulties.

- Unsteady gait and abnormal behavior.

- Visual and auditory loss.

- Dysarthria (speech difficulties).

- Seizures.

- Ataxia (lack of muscle coordination).

- Paralysis.

- Dementia.

4. **Progression**:

- Clinical manifestations progressively worsen, culminating in blindness or deafness and complete paralysis.

- Some patients may exhibit deafferentation and eventually die from central respiratory failure, brain herniation, or infection.

5. **Prevalence**:

- ALD is most common in children, accounting for approximately 35% of all cases.

Given these clinical features, Adrenoleukodystrophy (ALD) is the most appropriate diagnosis based on the provided information. For further confirmation, diagnostic tests such as very long-chain fatty acid (VLCFA) levels, MRI of the brain, and genetic testing for mutations in the ABCD1 gene would be recommended.

Other possible diagnoses that could present with similar symptoms include:

1. **Refsum Disease**: A peroxisomal disorder leading to neurological symptoms, ataxia, and peripheral neuropathy, but typically includes retinitis pigmentosa and anosmia.

2. **Zellweger Spectrum Disorders**: A group of peroxisomal biogenesis disorders that can lead to neurological symptoms, including hypotonia, seizures, and hearing loss, as well as adrenal insufficiency.

3. **Metachromatic Leukodystrophy (MLD)**: Another leukodystrophy affecting the central nervous system with progressive neurodegeneration but typically does not include significant adrenocortical impairment.

These other conditions should be considered in the differential diagnosis, but ALD remains the most likely based on the described symptoms.

**20. user:**

Most of the children start to show symptoms of infection from 4 to 12 months after birth. The most prominent clinical manifestation is recurrent severe bacterial infections in the respiratory system, digestive system and central nervous system, with respiratory tract infections being the most common, and common pathogens include Haemophilus influenzae, Streptococcus pneumoniae, Staphylococcus aureus, and Pseudomonas aeruginosa, etc. The most common infections are respiratory tract infections. Prolonged and repeated respiratory infections can lead to irreversible organ function damage such as bronchiectasis and pestle finger. Common symptoms of GI infections include diarrhoea, abdominal pain, gastro-oesophageal reflux and gastroenteritis. Pathogens causing infectious diarrhoea include Giardia lamblia, Salmonella, Campylobacter jejuni and Cryptosporidium.

In addition, children are prone to otitis media, chronic sinusitis, malnutrition, anaemia, granulocytopenia, thrombocytopenia, growth hormone deficiency and thyroid hormone disorders. Children may also develop tumours, autoimmune and inflammatory diseases, including rheumatoid arthritis and dermatomyositis. A higher incidence of sensorineural hearing loss and eczema has also been reported in children.

**RAG-qwen2.5_7b**:

Based on the clinical manifestations provided, the most likely rare disease diagnosis is **Chronic Granulomatous Disease (CGD)**. CGD is a primary immunodeficiency disorder characterized by recurrent bacterial and fungal infections due to the inability of phagocytes to produce reactive oxygen species necessary for killing certain bacteria and fungi.

Here are the key points that support this diagnosis:

1. **Recurrent Severe Bacterial Infections**:

- The most common infections are respiratory tract infections, with pathogens including Haemophilus influenzae, Streptococcus pneumoniae, Staphylococcus aureus, and Pseudomonas aeruginosa.

- These recurrent infections can lead to irreversible organ damage such as bronchiectasis.

2. **Gastrointestinal Infections**:

- Common symptoms include diarrhea, abdominal pain, gastro-oesophageal reflux, and gastroenteritis.

- Pathogens causing infectious diarrhoea include Giardia lamblia, Salmonella, Campylobacter jejuni, and Cryptosporidium.

3. **Other Clinical Manifestations**:

- Children are prone to otitis media, chronic sinusitis, malnutrition, anaemia, granulocytopenia (low white blood cell count), thrombocytopenia (low platelet count), growth hormone deficiency, and thyroid hormone disorders.

- They may also develop tumors, autoimmune and inflammatory diseases such as rheumatoid arthritis and dermatomyositis.

- Higher incidences of sensorineural hearing loss and eczema have been reported.

Given these clinical features, CGD is the most appropriate diagnosis. For further confirmation, diagnostic tests such as nitroblue tetrazolium (NBT) test or diaphorase assay to assess phagocyte function would be recommended.

Other possible diagnoses that could present with similar symptoms include:

1. **Severe Combined Immunodeficiency (SCID)**: Characterized by the absence of functional T-lymphocytes and B-lymphocytes, leading to severe recurrent infections.

2. **Common Variable Immunodeficiency (CVID)**: A disorder characterized by low levels of serum immunoglobulins and an impaired ability to produce antibodies.

These other conditions should be considered in the differential diagnosis, but CGD remains the most likely based on the described symptoms.

# 21. User

The clinical manifestations of the patients included salt loss and hyperandrogenism. Neonatal onset was characterized by varying degrees of adrenal insufficiency, such as weakness, nausea, vomiting, feeding difficulties, diarrhea, chronic dehydration, skin pigmentation, and growth retardation. Adrenal crisis is often the first manifestation of the disease in the neonatal period, manifested as severe hyponatremia, hyperkalemia, hypovolemic shock, may be accompanied by hypoglycemia, induced by stress. Severe hyponatremia can lead to central nervous system manifestations such as convulsions, and severe hyperkalemia can cause fatal arrhythmias.

Hyperandrogenism manifests differently at different ages. Female children with classical diseases have different degrees of masculinity at birth. Light performance for isolated clitoris hypertrophy, heavy external genitalia can approach men, labia majora scrotal shape, clitoris hypospadia-like penis, and with a common urethral vaginal orifice. However, the labia majora cannot touch the gonads and has completely normal female internal genital structures (ovary and uterus). Males may not have external genital abnormalities such as enlarged penis during neonatal and infancy, which is a common cause of delayed diagnosis. In early childhood, both sexes will present peripheral precocious puberty. Boys presented with enlarged penis with or without pubic hair; females presented with heterosexual precocious puberty. Chronic high levels of sex hormones stimulate hypothalamic gonadotropin-releasing hormone (GnRH) neurons and develop into central precocious puberty. Women may also have secondary sexual dysplasia and primary amenorrhea or oligomenorrhea. Linear growth with accelerated bone age occurs in both sexes from infancy, resulting in impaired terminal height.

Other findings include skin and mucous membrane pigmentation, especially areola and vulva, some children may not have this change.

Non-classical hyperandrogenism in childhood, adolescence and even adulthood may be characterized by accelerated growth and rapid bone age progression.

Based on the clinical manifestations described, it appears that the patient is likely suffering from Congenital Adrenal Hyperplasia (CAH), specifically 21-hydroxylase deficiency. This condition is one of the most common forms of CAH and involves a defect in the enzyme 21-hydroxylase, which is crucial for cortisol and aldosterone synthesis.

### Neonatal Onset

- **Adrenal Insufficiency**: The neonatal presentation includes varying degrees of adrenal insufficiency characterized by symptoms such as weakness, nausea, vomiting, feeding difficulties, diarrhea, chronic dehydration, skin pigmentation, and growth retardation. Adrenal crisis is often the first manifestation in the neonatal period.
  - **Adrenal Crisis Symptoms**:
    - Severe hyponatremia (low sodium levels)
    - Hyperkalemia (high potassium levels)
    - Hypovolemic shock
    - May be accompanied by hypoglycemia (low blood sugar)

### Hyperandrogenism

- **Female Children**: Those with classical CAH often present at birth with varying degrees of masculinization. This can manifest as isolated clitoral hypertrophy, heavy external genitalia resembling those in males, labia majora with a scrotum-like appearance, and a clitoris that resembles a penis (hypospadic-like). The labia majora do not touch the gonads, and there are normal female internal genital structures (ovary and uterus).
- **Male Children**: Often, external genital abnormalities such as an enlarged penis may not be present during neonatal or infancy stages, leading to delayed diagnosis.
- **Early Childhood**:
  - Both sexes can experience peripheral precocious puberty. Boys might have an enlarged penis with or without pubic hair; girls might show heterosexual precocious puberty.
  - Chronic high levels of sex hormones stimulate the hypothalamic gonadotropin-releasing hormone (GnRH) neurons, leading to central precocious puberty in both sexes.

### Other Findings

- **Skin and Mucous Membrane Pigmentation**: Especially noted around the areola and vulva. Some children may not exhibit this change.
- **Non-Classical Hyperandrogenism**: In childhood, adolescence, and even adulthood, non-classical cases can be characterized by accelerated growth and rapid bone age progression.

### Management

Management of CAH typically involves: 1. **Salt Supplementation**: To correct hyponatremia. 2. **Steroid Replacement Therapy**: To maintain adequate levels of cortisol and aldosterone. 3. **Monitoring for Complications**: Regular follow-ups to monitor for adrenal crises, growth patterns, and sexual development.

If you suspect CAH in a patient based on these clinical manifestations, it is important to consult with an endocrinologist or a specialist in rare diseases for appropriate diagnostic testing (such as measurement of 17-hydroxyprogesterone levels) and management.

# 22. User

The patient has clinical manifestations at birth, due to lack of pigment in the whole body, skin capillaries are exposed, skin is thin and soft, white and red, and accompanied by varying degrees of vasodilation. Patients are highly sensitive to ultraviolet light, often solar dermatitis, actinic cheilitis, basal cell carcinoma and squamous cell carcinoma and other diseases. Hair is white, silver, yellow, gold or brown. Eyelashes and eyebrows are white or pale yellow. Retina, iris color decline, pupil color decline. Photophobia is obvious during the day, night vision is normal, and other visual changes can also be accompanied, such as decreased visual acuity, hypoplasia of the fovea, changes in neural pathways at the optic chiasm, and nystagmus.

Based on the clinical manifestations described, the most likely diagnosis is **Oculocutaneous Albinism (OCA)**. This condition involves a significant reduction or complete absence of melanin pigment in the skin, hair, and eyes, leading to many of the symptoms you’ve observed.

Here are additional details and considerations:

### Oculocutaneous Albinism (OCA)

- **Skin**: The patient exhibits a lack of pigmentation, with the skin appearing thin, soft, white, and red due to exposed capillaries. There is also varying degrees of vasodilation.
- **Hair**: Hair can be white, silver, yellow, gold, or brown, depending on the specific type of OCA (Type I to Type IV).
- **Eyes**: Retina, iris, and pupil color decline, photophobia during the day with normal night vision. Other visual changes include decreased visual acuity, foveal hypoplasia, optic chiasm pathway changes, and nystagmus.

### Additional Considerations

1. **Hermansky-Pudlak Syndrome (HPS)**: This condition involves albinism along with bleeding disorders and can affect the lungs, kidneys, or bowel. It is a rare but important differential diagnosis.
2. **Chediak-Higashi Syndrome**: Characterized by albinism, immunodeficiency, recurrent infections, and neurological issues due to large granules in white blood cells.
3. **Griscelli Syndrome**: Presents with pigmentary abnormalities of the skin and hair, along with potential immunodeficiency and neurological issues.
4. **Waardenburg Syndrome**: Primarily known for causing hearing loss but can also present with pigmentary abnormalities including heterochromia (different colored eyes) and patches of depigmentation.

Given the comprehensive nature of these symptoms, a thorough diagnostic evaluation should include: - Genetic testing for OCA to confirm the specific type. - Blood tests to rule out bleeding disorders in HPS. - Neurological assessments to evaluate potential complications from Chediak-Higashi Syndrome or Waardenburg Syndrome.

For accurate diagnosis and appropriate management, it is recommended to consult a dermatologist, ophthalmologist, and geneticist.

# 23. User

A degenerative disease of the nervous system in which both upper and lower motor neurons are involved. The main clinical manifestations are progressive weakness and atrophy of the muscles of the bulbar, limbs, chest and abdomen. Eye motor nerve and sphincter function are generally not affected, but may be affected later in some patients. Between 20% and 50% of patients may exhibit cognitive impairment, and between 5% and 15% may even develop frontotemporal dementia. Death from respiratory failure occurs an average of 3 to 5 years after onset, but 5% to 10% of patients survive for more than 10 years.

Detailed history and physical examination to find evidence of common involvement of upper and lower motor neurons in brain stem, neck, chest and lumbosacral regions is the basis for diagnosis of the disease. Physical examination showed that both upper and lower motor neurons were involved in the same area, which was the key point of diagnosis. The main signs of lower motor neuron involvement included muscle weakness, atrophy and fascicular fibrillation. The tongue muscles, facial muscles, throat muscles, neck muscles, different muscle groups of the limbs, back muscles and pectoralis muscles are usually examined. The signs of upper motor neuron involvement mainly included positive pathological signs, tendon hyperreflexia, hypertonia, clonus, etc. Usually check sucking reflex, pharyngeal reflex, mandibular reflex, palmar jaw reflex, whether there is strong crying, strong laughter and other pseudobulbar paralysis manifestations, limb tendon reflex, muscle tension, Hoffman sign, lower limb pathology, abdominal wall reflex, etc. Clinical examination is the primary method of detecting upper motor neuron involvement. In areas of atrophy and weakness, if tendon reflexes are not low or active, it may indicate pyramidal tract damage even if there are no pathological signs.

Based on the clinical manifestations provided, the most likely diagnosis is **Amyotrophic Lateral Sclerosis (ALS)**. Here is a detailed breakdown of the reasoning and key points:

### Clinical Manifestations

- **Progressive Weakness and Atrophy**: The patient experiences progressive weakness and atrophy in muscles of the bulbar region, limbs, chest, and abdomen.
- **Eye Motor Nerve and Sphincter Function**: Generally not affected initially but may be involved later. This is a key distinguishing feature from other conditions that might affect these areas.
- **Cognitive Impairment**: Up to 50% of patients may exhibit cognitive impairment, with about 15% developing frontotemporal dementia.
- **Respiratory Failure**: Death typically occurs within 3 to 5 years after onset due to respiratory failure. However, 5% to 10% of patients can survive for more than 10 years.

### Diagnostic Criteria

- **Involvement of Both Upper and Lower Motor Neurons**: The physical examination shows involvement of both upper and lower motor neurons in the brainstem, neck, chest, and lumbosacral regions.
- **Lower Motor Neuron Involvement**:
  - Muscle weakness
  - Atrophy
  - Fascicular fibrillation
  - Examination of specific muscle groups such as tongue muscles, facial muscles, throat muscles, neck muscles, limb muscles, back muscles, and pectoralis muscles.
- **Upper Motor Neuron Involvement**:
  - Positive pathological signs (e.g., Babinski sign)
  - Tendon hyperreflexia
  - Hypertonia
  - Clonus
  - Checking for pseudobulbar paralysis manifestations such as strong crying, strong laughter
  - Limb tendon reflexes, muscle tension, Hoffman sign, lower limb pathology, and abdominal wall reflex.

### Differential Diagnoses

- **Primary Lateral Sclerosis (PLS)**: This condition primarily affects upper motor neurons and may not progress to involve lower motor neurons. The course is generally slower compared to ALS.
- **Progressive Muscular Atrophy (PMA)**: A rare form of ALS that involves only lower motor neurons, with no involvement of upper motor neurons.
- **Primary Lateral Sclerosis (PLS) vs. Progressive Spastic Paraplegia**: PLS typically affects the lower limbs first and progresses slowly without significant atrophy or respiratory issues.
- **Corticobulbar Tract Lesions**: Can present with similar symptoms but usually involve more specific neurological deficits.

### Conclusion

Given the comprehensive clinical picture, including progressive muscle weakness, atrophy, involvement of both upper and lower motor neurons, and potential cognitive impairment, the most likely diagnosis is Amyotrophic Lateral Sclerosis (ALS).

If further confirmation is needed, additional diagnostic tools such as electromyography (EMG), nerve conduction studies, and possibly a lumbar puncture may be considered. Genetic testing for ALS-related genes might also provide valuable information in some cases.

For more detailed evaluation and management, consulting with a neurologist specializing in motor neuron diseases would be recommended.

# 24. User

An autosomal recessive disorder affecting the urea cycle, leading to hyperammonemia, encephalopathy, and respiratory alkalosis. It is characterized by a buildup of arginine and ammonia in the blood, causing symptoms such as developmental delays, seizures, intellectual disability, spasticity, and ataxia. The condition typically manifests around age 3, with symptoms including stiffness, slow growth, and cognitive decline. Without treatment, it can progress to severe spasticity, loss of ambulation, and intellectual disability.

Based on the clinical manifestations you’ve described, the most likely diagnosis is **Arginase Deficiency**, a rare autosomal recessive disorder affecting the urea cycle. Here are the key points and additional details:

### Clinical Manifestations:

- **Age of Onset**: Typically manifests around age 3.
- **Symptoms**:
  - Developmental delays
  - Seizures
  - Intellectual disability
  - Spasticity
  - Ataxia
  - Stiffness
  - Slow growth
  - Cognitive decline

### Biochemical Characteristics:

- **Buildup of Arginine and Ammonia**: Due to the deficiency in arginase, there is a buildup of these substances in the blood.
- **Hyperammonemia**: Elevated levels of ammonia in the blood.
- **Encephalopathy**: Brain dysfunction leading to neurological symptoms.
- **Respiratory Alkalosis**: Hyperventilation causing an increase in blood pH.

### Diagnosis and Management:

- **Diagnosis**:
  - **Blood Tests**: Measurement of plasma arginine, citrulline, and ammonia levels.
  - **Genetic Testing**: Identification of mutations in the *ARG1* gene.
  - **Urea Cycle Panel**: Comprehensive evaluation of urea cycle enzymes.
- **Management**:
  - **Dietary Management**: Low-protein diet to reduce ammonia production.
  - **Ornithine Transcarbamylase (OTC) Deaminase Supplementation**: For some cases, OTC supplementation might be beneficial.
  - **Pharmacological Interventions**: Use of medications like sodium benzoate and phenylbutyrate to help manage hyperammonemia.

### Differential Diagnoses:

- **Ornithine Transcarbamylase Deficiency (OTC Deficiency)**: Involves a different enzyme in the urea cycle but can present with similar symptoms.
- **Carbamoyl Phosphate Synthetase I Deficiency**: Another urea cycle disorder with distinct biochemical markers.
- **Citrullinemia Type I**: Characterized by citrulline accumulation rather than arginine and ammonia.
- **Argininosuccinic Aciduria**: Involves a different enzyme in the urea cycle pathway.
- **Hyperornithinemia-Hyperammonemia-Homocitrullinuria Syndrome (HHH Syndrome)**: A rare condition with distinct biochemical characteristics.

Given these details, Arginase Deficiency is the most likely diagnosis based on the clinical presentation and biochemical findings. For further confirmation and management, consulting a geneticist or a specialist in metabolic disorders would be recommended.

# 25. User

The typical clinical picture includes the triad of microangiopathic haemolytic anaemia, thrombocytopenia and acute renal failure. Non-immune erythrocyte destruction due to microthrombosis often results in a haemoglobin level below 80 g/L, a negative Coombs test, and erythrocyte fragments seen on peripheral blood smears; platelet counts usually fall below 50×10^9 /L, but skin purpura and active bleeding are rare; the severity of the acute renal impairment varies from person to person, with some patients requiring dialysis support, often accompanied by elevated blood pressure.

Based on the clinical picture you described, the most likely diagnosis is **Hemolytic Uremic Syndrome (HUS)**. HUS is characterized by a triad of microangiopathic hemolytic anemia, thrombocytopenia, and acute renal failure. Here are the key points that support this diagnosis:

1. **Microangiopathic Hemolytic Anemia**:
   - Non-immune erythrocyte destruction due to microthrombosis.
   - Hemoglobin levels often below 80 g/L.
   - Negative Coombs test (indicating non-immune hemolysis).
   - Erythrocyte fragments seen on peripheral blood smears.
2. **Thrombocytopenia**:
   - Platelet counts usually fall below 50×10^9 /L.
   - Skin purpura and active bleeding are rare, distinguishing it from other conditions like Disseminated Intravascular Coagulation (DIC).
3. **Acute Renal Failure**:
   - Severity varies from person to person.
   - Some patients may require dialysis support.
   - Often accompanied by elevated blood pressure.

### Differential Diagnoses:

1. **Thrombotic Thrombocytopenic Purpura (TTP)**:
   - Shares similarities with HUS, including microangiopathic hemolytic anemia and thrombocytopenia.
   - Often presents with neurological symptoms and fever, distinguishing it from typical HUS.
2. **Atypical Hemolytic Uremic Syndrome (aHUS)**:
   - A variant of HUS not associated with Shiga toxin-producing E. coli infection.
   - Involves genetic or acquired complement dysregulation.
3. **Systemic Lupus Erythematosus (SLE)**:
   - With renal involvement leading to lupus nephritis, it can present with anemia, thrombocytopenia, and renal failure.
   - Autoimmune features would be present.
4. **Disseminated Intravascular Coagulation (DIC)**:
   - Can present with thrombocytopenia, microangiopathic hemolytic anemia, and renal impairment.
   - Typically associated with systemic bleeding and clotting issues.
5. **Malignant Hypertension**:
   - Severe hypertension can lead to microangiopathic hemolytic anemia and renal failure.
   - Elevated blood pressure is a key component here, consistent with the provided symptoms.

### Diagnostic Tests and Considerations:

- **Complete Blood Count (CBC)**: To assess hemoglobin levels and platelet counts.
- **Peripheral Blood Smear**: To look for erythrocyte fragments.
- **Coombs Test**: To confirm non-immune hemolysis.
- **Renal Function Tests**: Including serum creatinine and blood urea nitrogen (BUN) to evaluate renal function.
- **Complement Levels**: For aHUS.
- **Autoantibody Testing**: For SLE.

Given the clinical presentation, HUS is the most likely diagnosis. However, further diagnostic tests will be necessary to confirm the exact cause and rule out other potential conditions.

# 26. User

Patients have a variety of clinical presentations. It depends on the location, extent and rate of progression of the lesion. Clinical symptoms mainly include:

1. The occupying effect of an enlarged pituitary gland, which in most patients results from compression of the enlarged pituitary gland on the anterior region and surrounding structures (e.g., optic crossings, cavernous sinus, etc.), or from an inflammatory infiltrate. The most common clinical manifestations are persistent severe headaches and progressively worsening visual dysfunction, including decreased visual acuity and visual field defects.
2. hypopituitarism. Depending on the degree of involvement, the various axes of the pituitary gland may show partial or even total hypopituitarism, such as amenorrhoea, hypogonadism, nausea, malaise, chills, loss of sexual hair and weight loss.
3. Central dysuria, more than 70% of the patients will have symptoms of central dysuria such as polyuria, irritable thirst and polydipsia due to the involvement of the posterior pituitary, pituitary stalk and even hypothalamus region. In some patients, the blocking effect of the pituitary stalk leads to a mild increase in serum prolactin levels.
4. Hypothalamic syndrome, when the lesion involves the region of the hypothalamus, there will be drowsiness, lack of satiety, severe weight gain, lack of thirst, temperature fluctuation, sweating disorders, and emotional and psychiatric disorders.
5. Systemic disease-related manifestations. Patients with secondary disease often have a combination of clinical manifestations characteristic of systemic disease, such as LCH patients often have a combination of thyroid, lung, bone, liver, and skin lesions. patients with GPA are often middle-aged or older men, and respiratory symptoms often precede hypothalamic pituitary involvement. patients with IgG4-related pituitary inflammation often have a combination of clinical manifestations with the involvement of other organs such as lacrimal glands, submandibular glands, and pancreas. The disease is often associated with clinical signs of involvement of other organs such as the lacrimal gland and submaxillary gland and pancreas.

Based on the provided information, the clinical presentations of patients with lesions affecting the pituitary gland can vary significantly depending on the location, extent, and rate of progression of the lesion. Here are the key clinical symptoms:

1. **Occupying Effect of an Enlarged Pituitary Gland**:
   - **Common Manifestations**: Persistent severe headaches and progressively worsening visual dysfunction.
     - **Visual Symptoms**: Decreased visual acuity and visual field defects due to compression by the enlarged pituitary gland on the optic crossings or cavernous sinus.
2. **Hypopituitarism**:
   - **Degree of Involvement**: Depending on the extent, various axes of the pituitary gland may show partial or total hypopituitarism.
     - **Symptoms**: Amenorrhoea, hypogonadism, nausea, malaise, chills, loss of sexual hair, and weight loss.
3. **Central Dysuria**:
   - **Prevalence**: More than 70% of patients experience symptoms such as polyuria, irritability, and increased thirst (polydipsia).
     - **Mechanism**: Involvement of the posterior pituitary, pituitary stalk, or hypothalamus region.
     - **Prolactin Levels**: Some patients may show a mild increase in serum prolactin levels due to the blocking effect on the pituitary stalk.
4. **Hypothalamic Syndrome**:
   - **Lesion Involvement**: When the lesion affects the hypothalamus, symptoms include drowsiness, lack of satiety, severe weight gain, loss of thirst, temperature fluctuations, sweating disorders, and emotional and psychiatric disorders.
5. **Systemic Disease-Related Manifestations**:
   - **Secondary Diseases**: Patients with secondary diseases often exhibit a combination of clinical manifestations characteristic of systemic disease.
     - **Examples**:
       - **Langerhans Cell Histiocytosis (LCH)**: Thyroid, lung, bone, liver, and skin lesions.
       - **Granulomatosis with Polyangiitis (GPA)**: Often affects middle-aged or older men; respiratory symptoms often precede hypothalamic pituitary involvement.
       - **IgG4-Related Pituitary Inflammation**: Clinical manifestations involving other organs such as lacrimal glands, submandibular glands, and pancreas.

These clinical presentations highlight the complexity of pituitary disorders and their potential to affect multiple systems within the body. The symptoms can vary widely depending on the specific location and extent of the lesion, making accurate diagnosis and treatment crucial for patient care.

Would you like more detailed information on any specific aspect or need further clarification?

# 27. User

Patients have a variety of clinical presentations. It depends on the location, extent and rate of progression of the lesion. Clinical symptoms mainly include:

1. The occupying effect of an enlarged pituitary gland, which in most patients results from compression of the enlarged pituitary gland on the anterior region and surrounding structures (e.g., optic crossings, cavernous sinus, etc.), or from an inflammatory infiltrate. The most common clinical manifestations are persistent severe headaches and progressively worsening visual dysfunction, including decreased visual acuity and visual field defects.
2. hypopituitarism. Depending on the degree of involvement, the various axes of the pituitary gland may show partial or even total hypopituitarism, such as amenorrhoea, hypogonadism, nausea, malaise, chills, loss of sexual hair and weight loss.
3. Central dysuria, more than 70% of the patients will have symptoms of central dysuria such as polyuria, irritable thirst and polydipsia due to the involvement of the posterior pituitary, pituitary stalk and even hypothalamus region. In some patients, the blocking effect of the pituitary stalk leads to a mild increase in serum prolactin levels.
4. Hypothalamic syndrome, when the lesion involves the region of the hypothalamus, there will be drowsiness, lack of satiety, severe weight gain, lack of thirst, temperature fluctuation, sweating disorders, and emotional and psychiatric disorders.
5. Systemic disease-related manifestations. Patients with secondary disease often have a combination of clinical manifestations characteristic of systemic disease, such as LCH patients often have a combination of thyroid, lung, bone, liver, and skin lesions. patients with GPA are often middle-aged or older men, and respiratory symptoms often precede hypothalamic pituitary involvement. patients with IgG4-related pituitary inflammation often have a combination of clinical manifestations with the involvement of other organs such as lacrimal glands, submandibular glands, and pancreas. The disease is often associated with clinical signs of involvement of other organs such as the lacrimal gland and submaxillary gland and pancreas.

Based on the provided information, patients with conditions affecting the pituitary gland or hypothalamus can present with a variety of clinical symptoms depending on the location, extent, and rate of progression of the lesion. Here are the main clinical manifestations:

1. **Occupying Effect of Enlarged Pituitary Gland:**
   - **Common Manifestations:** Persistent severe headaches and progressively worsening visual dysfunction.
     - **Visual Symptoms:** Decreased visual acuity and visual field defects.
   - **Causes:** Compression from an enlarged pituitary gland or inflammatory infiltrate on the anterior region and surrounding structures (e.g., optic crossings, cavernous sinus).
2. **Hypopituitarism:**
   - **Symptoms Vary by Degree of Involvement:**
     - **Partial Hypopituitarism:** Amenorrhea, hypogonadism, nausea, malaise, chills, loss of sexual hair, and weight loss.
     - **Total Hypopituitarism:** Severe cases with more extensive hormonal deficiencies.
3. **Central Dysuria:**
   - **Prevalence:** More than 70% of patients experience central dysuria.
     - **Symptoms:** Polyuria, irritability, increased thirst (polydipsia).
   - **Causes:** Involvement of the posterior pituitary, pituitary stalk, and hypothalamus region.
4. **Hypothalamic Syndrome:**
   - **Symptoms:** Drowsiness, lack of satiety, severe weight gain, lack of thirst, temperature fluctuations, sweating disorders, emotional and psychiatric disturbances.
   - **Causes:** Lesions involving the hypothalamus region.
5. **Systemic Disease-Related Manifestations:**
   - **Secondary Diseases:**
     - **LCH (Letterer-Siwe Disease):** Combination of thyroid, lung, bone, liver, and skin lesions.
     - **GPA (Granulomatosis with Polyangiitis):** Often affects middle-aged or older men; respiratory symptoms often precede hypothalamic-pituitary involvement.
     - **IgG4-Related Pituitary Inflammation:** Clinical manifestations involving other organs such as lacrimal glands, submandibular glands, and pancreas. The disease is associated with clinical signs of involvement in these organs.

These symptoms highlight the diverse and complex nature of conditions affecting the pituitary gland and hypothalamus, emphasizing the importance of comprehensive diagnostic approaches to identify and manage these conditions effectively.

# 28. User

Hyperglycaemia is the most common clinical manifestation of the disease. However, patients can spontaneously remit without intervention and maintain normal blood glucose levels. The disease is one of the leading causes of autoimmune hypoglycaemia, with about 25% of patients experiencing spontaneous hypoglycaemia during the course of the disease. Acanthosis nigricans is present in about 50 per cent of patients. In addition, polycystic ovaries and hyperandrogenism are not uncommon in this group of patients. Other autoimmune diseases are common, with systemic lupus erythematosus (SLE) being the most common, accounting for about 50 per cent of cases. Primary biliary cirrhosis, dermatomyositis, and scleroderma are also present. The disease can also be a paraneoplastic syndrome manifestation of Hodgkin’s lymphoma, multiple myeloma, and others.

Based on the clinical manifestations you’ve described, the most likely diagnosis is **Type B Insulin Resistance Syndrome**. This condition is characterized by severe insulin resistance often caused by autoantibodies against the insulin receptor, leading to hyperglycemia. Patients can experience spontaneous remissions and episodes of hypoglycemia due to fluctuating autoantibody levels. Acanthosis nigricans is commonly observed in about 50% of patients. Additionally, polycystic ovaries and hyperandrogenism are not uncommon.

Other autoimmune diseases are frequently associated with this condition: - **Systemic Lupus Erythematosus (SLE)**: Present in about 50% of cases. - **Primary Biliary Cirrhosis** - **Dermatomyositis** - **Scleroderma**

The disease can also manifest as a paraneoplastic syndrome associated with conditions such as: - **Hodgkin’s Lymphoma** - **Multiple Myeloma**

Given these clinical features, Type B Insulin Resistance Syndrome appears to be the most fitting diagnosis. However, it is important to consider other potential diagnoses such as:

1. **Polyendocrine Autoimmune Syndrome**: This encompasses a cluster of autoimmune diseases that might include diabetes, thyroiditis, and others, often with accompanying skin manifestations and polycystic ovarian disease.
2. **Polycystic Ovary Syndrome (PCOS) with Insulin Resistance and Autoimmunity**: Although primarily a reproductive disorder, PCOS can present with insulin resistance and has been associated with autoimmune conditions.
3. **Autoimmune Polyglandular Syndrome Type 1 or 2**: These involve multiple autoimmune conditions.

Further diagnostic testing and clinical evaluation will be necessary to confirm the diagnosis and rule out other potential causes.

# 29. User

The disease starts in infancy and childhood. The first attack occurs between the ages of 5 months and 7 years. The clinical picture is one of recurrent episodes of intractable vomiting and severe ketoacidosis with depression, dehydration, shortness of breath, and coma. There is usually a clear trigger, and the disease may begin acutely after fasting, gastrointestinal and upper respiratory tract infections, fever, stress, or excessive protein intake. Routine laboratory tests show elevated or normal blood glucose, some hypoglycaemia, blood gases show metabolic acidosis, blood ammonia can be normal or mildly elevated, liver function, renal function and normal, and urine ketone bodies are positive. Ketosis and acidosis may recur, and clinical and laboratory tests may be normal between attacks.

Neurological changes occur in about 1/5 of the cases, manifested by cognitive impairment, mental retardation, extrapyramidal abnormalities and symptoms of stroke. Cranial MRI reveals extensive demyelination, including white matter, and symmetric high signal changes in the basal ganglia at T2.

Patients may be asymptomatic in the early stages and are diagnosed in the neonatal period or in high-risk groups by blood and urine metabolic screening. There is no clear association between age of onset, frequency of attacks and prognosis. There is a tendency for the attacks to decrease or stop with age, and in most cases growth is unaffected on follow-up. Recurrent and severe attacks can lead to multi-organ dysfunction or even death.

Based on the clinical manifestations you’ve described, the most likely diagnosis is **Glutaric Aciduria Type I (GA-I)**. This rare metabolic disorder fits the profile of recurrent episodes of intractable vomiting, severe ketoacidosis with depression, dehydration, shortness of breath, and coma, often triggered by factors such as fasting, gastrointestinal infections, fever, stress, or excessive protein intake.

Key features include: - **Clinical Picture**: Recurrent episodes of intractable vomiting and severe ketoacidosis. - **Laboratory Findings**: - Elevated or normal blood glucose levels. - Some hypoglycemia. - Blood gases showing metabolic acidosis. - Normal liver function, renal function, and blood ammonia (can be normal or mildly elevated). - Positive urine ketone bodies. - **Recurrent Episodes**: Ketosis and acidosis may recur between attacks when clinical and laboratory tests are normal.

**Neurological Changes**: About 1 in 5 cases show neurological changes such as cognitive impairment, mental retardation, extrapyramidal abnormalities, and symptoms of stroke. Cranial MRI reveals extensive demyelination, including white matter, and symmetric high signal changes in the basal ganglia at T2.

Additional Considerations: - **Diagnosis**: Patients may be asymptomatic in early stages and are often diagnosed through blood and urine metabolic screening. - **Prognosis**: There is no clear association between age of onset, frequency of attacks, and prognosis. Attacks tend to decrease or stop with age, and growth is generally unaffected on follow-up. - **Severe Recurrent Attacks**: Can lead to multi-organ dysfunction or even death.

Given these clinical features, GA-I is the most likely diagnosis. Further diagnostic testing such as organic acid analysis, plasma amino acids, and genetic testing for GLDC mutations can confirm the diagnosis.

# 30. User

1. the onset is at a young age and is more common in women.
2. there is often a family history of syncope or sudden death.
3. different genotypes and phenotypes, can involve potassium, sodium and calcium channels. Characteristic tip-distortion ventricular tachycardia (TdP) is often present, leading to syncope or sudden death. 4.
4. The ECG shows a prolonged QT interval.
5. ECG generally has the following characteristics: (1) QT interval prolongation, short - long - short cycle phenomenon induced; (2) ventricular rate of 160-240 beats / minute, QRS wave amplitude and morphology around the equipotential line of reversal, 5-20 cardiac cycles of the main wave around the baseline of the reversal of the majority of the time can be terminated; (3) ATP is more difficult to terminate the TdP.

Based on the provided clinical manifestations, the most likely diagnosis is **Long QT Syndrome (LQTS)**. Here’s a detailed explanation:

1. **Onset at Young Age and More Common in Women**:
   - LQTS can onset from infancy to adulthood but often presents earlier in life. It is more common in women, which aligns with the given information.
2. **Family History of Syncope or Sudden Death**:
   - LQTS has a genetic basis and often runs in families. Family history of syncope or sudden death is a common feature.
3. **Different Genotypes and Phenotypes Involving Ion Channels**:
   - LQTS involves mutations in genes encoding cardiac ion channels, including potassium (KCNQ1, KCNH2), sodium (SCN5A), and calcium (CACNA1C) channels. These genetic variations can lead to characteristic symptoms such as torsades de pointes (TdP).
4. **Prolonged QT Interval on ECG**:
   - The ECG shows a prolonged QT interval, which is the hallmark of LQTS.
5. **ECG Characteristics**:
   - **QT Interval Prolongation**: This is consistent with LQTS.
   - **Short-Long-Short Cycle Phenomenon**: This phenomenon can be observed in some forms of LQTS, where the QT interval shortens during episodes of TdP and then returns to a prolonged state.
   - **Ventricular Rate (160-240 bpm)**: This rate is typical for TdP.
   - **QRS Wave Amplitude and Morphology**: The QRS waves often show a characteristic pattern with the main wave around the equipotential line of reversal, which can be terminated in 5-20 cardiac cycles.

Given these clinical features, LQTS fits the profile described. Other conditions such as Brugada Syndrome, Catecholaminergic Polymorphic Ventricular Tachycardia (CPVT), Short QT Syndrome, and Arrhythmogenic Right Ventricular Cardiomyopathy (ARVC) can be considered but are less likely based on the provided information.

**Other Possible Diagnoses to Consider**: 1. **Brugada Syndrome**: Characterized by a distinct ECG pattern (ST-segment elevation in leads V1-V3) and often associated with sudden cardiac death, though it typically does not present with prolonged QT intervals. 2. **Catecholaminergic Polymorphic Ventricular Tachycardia (CPVT)**: Triggered by physical activity or stress, leading to syncope or sudden death, but usually shows normal resting ECGs. 3. **Short QT Syndrome**: Characterized by a short QT interval and increased risk of arrhythmias, which is the opposite of LQTS. 4. **Arrhythmogenic Right Ventricular Cardiomyopathy (ARVC)**: A genetic condition leading to ventricular arrhythmias and syncope, often with specific ECG patterns.

In conclusion, based on the provided clinical features, Long QT Syndrome (LQTS) is the most likely diagnosis. Further diagnostic tests such as genetic testing for ion channel mutations and detailed ECG analysis would be beneficial for confirmation.

# 31. User

The disease can develop at any age, and most of the children develop between 1 month and 7 years old, with an average age of about 2 years old. Clinical manifestations vary greatly among patients, mainly including: (1) acute energy metabolism disorder crisis, manifested by low ketone hypoglycemia, high blood ammonia and metabolic acidosis; (2) cardiomyopathy, manifested by ventricular hypertrophy, cardiac insufficiency, cardiac arrhythmia, and elevated creatine kinase, etc.; (3) myopathy, manifested by muscle weakness, hypotonia, myalgia, poor exercise endurance, elevated muscular-type creatine kinase, and lipid deposition in muscle fibres, etc. (4) liver damage, manifested by hepatomegaly, fatty liver, liver function abnormalities, etc. Some liver-damaged patients have liver enlargement, fatty liver, and liver function abnormalities; Liver damage, manifested by hepatomegaly, fatty liver, abnormal liver function, etc. Some children with liver damage have acute onset of illness, manifested by convulsions, progressive consciousness disorder, etc., which is often misdiagnosed as Reye syndrome. In addition, gastrointestinal symptoms such as recurrent abdominal pain, vomiting and gastroesophageal reflux, respiratory symptoms such as recurrent infections and wheezing, and anaemia have also been reported.

Newborn screening: diagnosed children may have no clinical manifestations. It is a potentially fatal disease, and children can die suddenly from acute energy metabolism disorder crisis or acute heart failure. Clinical manifestations are varied and related to age of onset, time of onset and severity of illness. The most common symptoms are cardiac and skeletal muscle damage in infancy and childhood. Symptoms in adulthood are mild or asymptomatic, mostly due to reduced endurance or easy fatigue. During pregnancy, pregnant women may have atypical manifestations such as fatigue and arrhythmias due to energy expenditure and reduced physiological plasma carnitine levels.

Based on the provided clinical manifestations, the most likely diagnosis is **Fatty Acid Oxidation Disorder (FAOD)**. Here’s a detailed explanation:

1. **Age of Onset and Development**:
   - The disease can develop at any age, but most children develop between 1 month and 7 years old, with an average age of about 2 years old.
2. **Clinical Manifestations**:
   - **Acute Energy Metabolism Disorder Crisis**: Characterized by low ketone levels, hypoglycemia, high blood ammonia, and metabolic acidosis.
   - **Cardiomyopathy**: Manifested by ventricular hypertrophy, cardiac insufficiency, cardiac arrhythmia, and elevated creatine kinase.
   - **Myopathy**: Manifested by muscle weakness, hypotonia, myalgia, poor exercise endurance, elevated muscular-type creatine kinase, and lipid deposition in muscle fibers.
   - **Liver Damage**: Manifested by hepatomegaly, fatty liver, liver function abnormalities. Some children have acute onset of illness with convulsions and progressive consciousness disorder, often misdiagnosed as Reye syndrome.
3. **Gastrointestinal Symptoms**:
   - Recurrent abdominal pain, vomiting, and gastroesophageal reflux.
4. **Respiratory Symptoms**:
   - Recurrent infections and wheezing.
5. **Anemia**: Reported in some cases.
6. **Newborn Screening**:
   - Diagnosed children may have no clinical manifestations initially but are at risk of sudden death from acute energy metabolism disorder crisis or acute heart failure.
7. **Adult Symptoms**:
   - Mild or asymptomatic, mostly due to reduced endurance or easy fatigue.
8. **Pregnancy Manifestations**:
   - Atypical symptoms such as fatigue and arrhythmias due to increased energy expenditure and reduced physiological plasma carnitine levels.

Given the wide range of clinical manifestations and the potential for acute life-threatening events, FAOD is a strong candidate based on the described symptoms. Fatty Acid Oxidation Disorders are a group of metabolic disorders that affect how the body processes fatty acids, leading to various systemic issues as outlined above.

For further confirmation, genetic testing would be necessary to identify specific mutations in genes involved in fatty acid oxidation pathways. If you have any additional clinical data or specific symptoms to consider, please provide more details for a more precise diagnosis and management plan.

# 32. User

In the unicentric form, the disease often presents only with enlarged lymph nodes without systemic symptoms. The median diameter of the enlarged lymph nodes is about 5.5 cm, and they are commonly found in the chest (24%), neck (20%), abdomen (18%), and retroperitoneum (14%). Some patients with unicentric disease are prone to paraneoplastic aspergillosis, occlusive bronchiolitis, and growth retardation. In addition to multiple lymph node enlargements, multicentric disease is often associated with systemic manifestations such as fever, night sweats, malaise, weight loss, anaemia, hepatic insufficiency, renal insufficiency, and volume overload (generalised oedema, pleural effusion, ascites, etc.). There is also a specific subtype in which patients may present with thrombocytopenia, severe oedema (anasarca), myelofibrosis, renal dysfunction, and hepatosplenomegaly (organomegaly).

Based on the provided clinical manifestations, the most likely rare disease to be diagnosed is **Castleman disease**. Here’s a detailed explanation:

1. **Unicentric Castleman Disease (UCD)**:
   - **Clinical Presentation**: The disease often presents with enlarged lymph nodes without systemic symptoms.
   - **Lymph Node Enlargement**: The median diameter of the enlarged lymph nodes is about 5.5 cm, and they are commonly found in the chest (24%), neck (20%), abdomen (18%), and retroperitoneum (14%).
   - **Associated Conditions**: Some patients with UCD may be prone to paraneoplastic aspergillosis, occlusive bronchiolitis, and growth retardation.
2. **Multicentric Castleman Disease (MCD)**:
   - **Clinical Presentation**: This form is associated with multiple lymph node enlargements and systemic manifestations.
   - **Systemic Symptoms**: Fever, night sweats, malaise, weight loss, anaemia, hepatic insufficiency, renal insufficiency, and volume overload (generalized oedema, pleural effusion, ascites).
   - **Specific Subtype (TAFRO Syndrome)**: This subtype presents with additional symptoms such as thrombocytopenia, severe edema (anasarca), myelofibrosis, renal dysfunction, and hepatosplenomegaly.
3. **Other Possible Diagnoses**:
   - **Lymphoma**: Both Hodgkin and non-Hodgkin lymphomas can present with lymphadenopathy and systemic symptoms such as fever, night sweats, and weight loss.
   - **Sarcoidosis**: This condition can present with lymph node enlargement and systemic symptoms, although it typically has specific pulmonary involvement and non-caseating granulomas on biopsy.
   - **Systemic Lupus Erythematosus (SLE)**: SLE can present with systemic symptoms and lymphadenopathy, along with organ involvement such as renal and hepatic dysfunction.

Given the detailed clinical picture provided, Castleman disease appears to be the most fitting diagnosis, especially considering both unicentric and multicentric forms. The specific subtype mentioned aligns well with TAFRO syndrome, a subset of MCD characterized by thrombocytopenia, severe edema (anasarca), myelofibrosis, renal dysfunction, and hepatosplenomegaly.

If further diagnostic tests are available, such as biopsies or imaging studies, they would help confirm the specific subtype and guide appropriate treatment.

# 33. User

The main clinical manifestations are muscle atrophy, weakness and sensory loss of the limbs, mainly in the distal part of the lower limbs and gradually developing towards the proximal part of the limbs. Common clinical manifestations include poorer locomotor ability than people of the same age, difficulty in running, easy to twist the foot, foot drop, calf gastrocnemius muscle atrophy resembling a ‘crane leg’; examination can find bowed feet, hammertoes, distal limb weakness and atrophy, and loss of deep sensation. The disease usually starts before the age of 20 and progresses slowly. Later stages of the disease may severely affect activity, but rarely result in total disability and do not affect normal life expectancy. However, there are specific types that may have an early and severe onset.

Based on the clinical manifestations you provided, the most likely diagnosis is **Charcot-Marie-Tooth disease (CMT)**. Here’s a detailed explanation:

1. **Clinical Manifestations**:
   - **Muscle Atrophy and Weakness**: The symptoms include muscle atrophy and weakness in the limbs, particularly in the distal parts of the lower limbs and gradually progressing towards the proximal parts.
   - **Sensory Loss**: There is sensory loss, especially in the distal parts of the lower limbs.
   - **Locomotor Ability**: Patients often have poorer locomotor ability than people of the same age, with difficulties such as running and a tendency to twist their feet, leading to foot drop.
   - **Calf Gastrocnemius Muscle Atrophy**: The gastrocnemius muscles in the calf can atrophy, giving a “crane leg” appearance.
   - **Examination Findings**: During examination, you may find bowed feet, hammertoes, distal limb weakness and atrophy, and loss of deep sensation.
2. **Disease Progression**:
   - The disease typically starts before the age of 20 and progresses slowly.
   - Later stages can severely affect activity but rarely result in total disability or significantly impact life expectancy.
3. **Specific Types**:
   - There are specific types of CMT that may have an early and severe onset, which aligns with your description of certain cases having a more aggressive presentation.
4. **Other Possible Diagnoses**:
   - **Hereditary Neuropathy with Liability to Pressure Palsies (HNPP)**: This condition can cause similar symptoms but often involves episodic nerve compression.
   - **Friedreich’s Ataxia**: While primarily affecting coordination and balance, it can also present with distal muscle weakness and atrophy in the lower limbs.
   - **Spinal Muscular Atrophy (SMA)**: Some forms can present with gradual muscle weakness and atrophy but typically do not involve significant sensory loss.
   - **Distal Hereditary Motor Neuropathy (dHMN)**: This condition primarily affects motor nerves, leading to muscle weakness and atrophy without significant sensory involvement.
   - **Refsum Disease**: A rare metabolic disorder that can present with peripheral neuropathy among other systemic symptoms but often includes additional features like retinitis pigmentosa and ichthyosis.

Given the detailed clinical picture provided, Charcot-Marie-Tooth disease (CMT) is the most fitting diagnosis. If further diagnostic tests such as nerve conduction studies, electromyography (EMG), or genetic testing are available, they would help confirm the specific type of CMT.

# 34. User

The onset of the disease occurs from birth to several months. The onset of jaundice is usually within 2 months of age and rarely later than 5 months of age. The most common presentation is persistent jaundice after birth with or without light coloured stools and mild or no liver enlargement. There are many other non-specific clinical signs, including feeding difficulties, growth retardation, diarrhoea, hypoglycaemia and prolonged clotting time. Most of these symptoms and signs resolve spontaneously or with dietary modification and medication around 6 months to 1 year of age, with a small proportion of patients developing further disease after puberty. The majority of developed disease develops in adulthood. Delirium, confusion, and other mental abnormalities caused by hyperammonemia usually appear suddenly after drinking alcohol, consuming sweets, taking certain medications, or having an infection. In severe cases, the disease progresses rapidly and can be life-threatening without proper treatment. Most patients are slim, and most of them have obvious dietary preference, addicted to legumes, high protein, high fat food, and anorexic to high sugar.

Based on the clinical manifestations you’ve described, the most likely diagnosis is **Hereditary Fructose Intolerance (HFI)**. Here’s a detailed explanation:

1. **Clinical Manifestations**:
   - **Onset and Jaundice**: The disease typically starts from birth to several months of age, with jaundice usually appearing within 2 months and rarely later than 5 months.
   - **Persistent Jaundice**: Persistent jaundice is a common presentation, often accompanied by light-colored stools. Liver enlargement may be mild or absent.
   - **Other Non-Specific Signs**: The patient may experience feeding difficulties, growth retardation, diarrhea, hypoglycemia, and prolonged clotting time.
   - **Resolution of Symptoms**: Most symptoms resolve spontaneously or with dietary modifications and medication around 6 to 12 months of age. However, a small proportion of patients may develop further disease after puberty.
2. **Adult-Onset Disease**:
   - The majority of the developed disease typically manifests in adulthood. Delirium, confusion, and other mental abnormalities caused by hyperammonemia usually appear suddenly after consuming alcohol, sweets, certain medications, or having an infection.
   - In severe cases, these symptoms can progress rapidly and be life-threatening without proper treatment.
3. **Dietary Habits**:
   - Most patients are slim and have a clear dietary preference, often favoring legumes, high-protein, and high-fat foods while avoiding high-sugar items.
4. **Other Possible Diagnoses**:
   - **Galactosemia**: Presents with similar symptoms in infancy but primarily affects galactose metabolism.
   - **Tyrosinemia Type I**: A rare metabolic condition that can present with liver dysfunction and other systemic symptoms, but the specific pattern of jaundice and dietary preferences do not align as closely.

Given these clinical features, HFI is the most fitting diagnosis. Further diagnostic tests such as a fructose tolerance test or genetic testing for hereditary fructose intolerance would help confirm this diagnosis.

# 35. User

Symptoms may appear after birth or may start in infancy to early adulthood. It is characterised by fluctuating muscle weakness and intolerance to fatigue, mostly in the extraocular muscles, facial muscles, medulla oblongata, limbs and respiratory muscles, and even life-threatening apnoeic episodes may occur in infancy. The simplex syndrome is characterised mainly by skeletal muscle weakness without congenital malformations. The overlapping syndromes of glycosylation defects and myopathies of the disease are often accompanied by congenital developmental abnormalities. Clinical manifestations vary between the different types, with some patients, usually born with proximal muscle weakness, cholinesterase inhibitors effective, and in severe cases, sudden respiratory distress and apnoea due to medullary palsy, which can be induced by stress, cold, and infection. Some patients, starting at birth or in infancy, may present with more severe, axially distributed muscle weakness, but the extraocular muscles may not be involved.

Based on the clinical symptoms you’ve provided, the most likely diagnosis is **Congenital Myasthenic Syndromes (CMS)**. Here’s a detailed explanation:

1. **Clinical Manifestations**:
   - Symptoms may appear after birth or start in infancy to early adulthood.
   - Characterized by fluctuating muscle weakness and intolerance to fatigue, primarily affecting extraocular muscles, facial muscles, medulla oblongata, limbs, and respiratory muscles.
   - Life-threatening apnoeic episodes can occur in infancy due to involvement of the medulla oblongata.
2. **Subtypes**:
   - **Simplex Syndrome**: Characterized mainly by skeletal muscle weakness without congenital malformations.
   - **Overlapping Syndromes**: Often accompanied by congenital developmental abnormalities and involve glycosylation defects or myopathies.
3. **Clinical Variability**:
   - Some patients are born with proximal muscle weakness, and cholinesterase inhibitors can be effective in managing symptoms.
   - Severe cases may experience sudden respiratory distress and apnoea due to medullary palsy, which can be triggered by stress, cold, or infection.
4. **Other Possible Diagnoses**:
   - **Myasthenia Gravis (Juvenile or Congenital)**: While presenting similar symptoms, it typically starts later than infancy and is more often acquired.
   - **Congenital Muscular Dystrophy (CMD)**: This includes a variety of disorders that can present with muscle weakness and developmental abnormalities but may not exhibit the specific responsiveness to cholinesterase inhibitors.

Given these clinical features, CMS aligns well with the described symptoms. Further diagnostic tests such as electrophysiological studies (e.g., repetitive nerve stimulation), blood tests for autoantibodies, and genetic testing can help confirm this diagnosis.

# 36. User

1. The disease usually starts in infancy or childhood, and in a few cases it starts in adulthood.
2. Tonicity can occur throughout the entire body, including the skeletal muscles of the limbs, facial muscles, extraocular muscles, and lingual muscles.
3. Tonicity is apparent during sudden movement after rest and is reduced by repeated movements, which is called the ‘warm-up’ phenomenon. Transient muscle weakness may occur at the start of a sudden movement. In patients with a longer course of the disease, there may be persistent weakness of the distal limbs, even involving the proximal limbs.
4. Physical examination reveals that the patient is often muscular and has an athlete’s body shape. After clenching the fist vigorously, the fingers cannot be unfolded in time; after closing the eyes vigorously, they cannot be opened immediately. Knocking on the hand interosseous muscle, lingual muscle and other parts of the hand can cause persistent muscle contraction (percussive muscle ankylosis, ‘muscle ball’ phenomenon).

Based on the clinical manifestations you’ve provided, the most likely rare disease diagnosis is **Myotonia Congenita**. This condition is characterized by muscle stiffness (myotonia) that typically improves with repeated activity (the “warm-up” phenomenon), often starting in childhood or infancy. The description of the patient having a muscular build and experiencing difficulty relaxing muscles after contraction, such as not being able to quickly open the hand or eyes, aligns well with this condition.

Here are five other possible diagnoses that share some overlapping features:

1. **Paramyotonia Congenita**: This condition is similar to Myotonia Congenita but worsens with cold and exercise, and does not exhibit the “warm-up” phenomenon.
2. **Myotonic Dystrophy (Type 1 or Type 2)**: A multi-system disorder that includes progressive muscle wasting and weakness, myotonia, cataracts, and other systemic features. Unlike Myotonia Congenita, it often involves progressive weakness and atrophy.
3. **Hyperkalemic Periodic Paralysis**: Episodes of muscle weakness that can be triggered by hyperkalemia (high potassium levels) or certain medications, but does not typically involve the “warm-up” phenomenon.
4. **Thyrotoxic Myopathy**: Muscle weakness associated with thyroid dysfunction, often presenting with myotonia and other neuromuscular symptoms, but typically involving more systemic manifestations.
5. **Congenital Myotonic Dystrophy (Myotonia Congenita Type 1)**: A specific form of congenital myotonia that can present similarly to Myotonia Congenita but may have additional features.

To confirm the diagnosis, further diagnostic tests such as electromyography (EMG), genetic testing for known mutations associated with these conditions, and possibly muscle biopsy or other specialized tests would be necessary.

# 37. User

Secondary thoracic deformities caused by scoliosis can lead to cardiopulmonary hypoplasia, poor cardiopulmonary reserve, and may include cardiac displacement, arrhythmia, reduced lung capacity, pneumonia, etc. Abdominal deformities can cause gastrointestinal disorders, leading to dyspepsia and lack of appetite. In addition, the nerve root pulling caused by vertebral malformation and disc degeneration can produce irritating pain in the corresponding body parts, and in severe cases, it can cause spinal cord dysfunction or even paralysis.

Physical examination: the general manifestations are unequal shoulders when standing upright, one side of the lumbar folds, lumbar flexion with back asymmetry (‘razor back’), sometimes asymmetry on both sides of the pelvis, and gait abnormalities. In addition, neurological examination is also needed to observe whether the patient has increased or low muscle tone and whether there are sensory abnormalities in the limbs.

Clinically, it is usually classified into the following 3 types according to the imaging characteristics:

Type I - Vertebral formation disorder type, i.e., one or more vertebrae are partially or completely formed with disorder, such as wedge vertebrae, hemivertebrae or butterfly vertebrae;

Type II - poor vertebral segmentation type, i.e., two or more vertebrae are partially or completely segmented and impaired, e.g., blocked vertebrae, bone bridge formation, etc;

Type III - mixed type, where there is both vertebral body formation disorder and vertebral body mal-segmentation.

Based on the clinical manifestations and imaging characteristics you have described, the most likely rare disease diagnosis is **Congenital Scoliosis**. Congenital scoliosis involves spinal deformities due to malformations of the vertebrae, which can lead to secondary complications such as thoracic and abdominal deformities, impacting cardiopulmonary and gastrointestinal functions.

### Clinical Manifestations:

- **Cardiopulmonary Complications**: Secondary thoracic deformities caused by scoliosis can lead to cardiopulmonary hypoplasia, poor cardiopulmonary reserve, cardiac displacement, arrhythmias, reduced lung capacity, and pneumonia.
- **Gastrointestinal Disorders**: Abdominal deformities can cause gastrointestinal issues such as dyspepsia and lack of appetite.

### Physical Examination:

- **General Manifestations**: Unequal shoulders when standing upright, one side of the lumbar folds, lumbar flexion with back asymmetry (‘razor back’), sometimes asymmetry on both sides of the pelvis, and gait abnormalities.
- **Neurological Examination**: Increased or low muscle tone, and sensory abnormalities in the limbs.

### Imaging Characteristics:

- **Type I - Vertebral Formation Disorder Type**: One or more vertebrae are partially or completely formed with disorder, such as wedge vertebrae, hemivertebrae, or butterfly vertebrae.
- **Type II - Poor Vertebral Segmentation Type**: Two or more vertebrae are partially or completely segmented and impaired, e.g., blocked vertebrae, bone bridge formation.
- **Type III - Mixed Type**: Both vertebral body formation disorder and vertebral body mal-segmentation.

To confirm the diagnosis, further diagnostic tests such as X-rays, MRI, and CT scans would be necessary to visualize the spinal deformities and their specific types. Additionally, a thorough neurological examination can help assess any associated nerve root compression or spinal cord dysfunction.

If you need more detailed information on these conditions or have specific questions about the diagnostic process, feel free to ask!

# 38. User

There are more men than women. Some patients have atypical symptoms of chest pain, which are detected during coronary angiography or coronary CT. Patients are often clinically symptomatic when there is (1) a combination of obstructive atherosclerotic disease, thrombosis, slow flow, entrapment, or rupture with exertional angina or acute coronary syndromes; (2) compression of adjacent tissues; (3) rupture of an aneurysm, which is rare and can cause acute cardiac tamponade; and (4) myocardial ischemia due to microvascular dysfunction, which can occur even in the absence of significant coronary stenosis. The most common clinical manifestation is cardiac ischaemia.

The most common clinical manifestation is angina pectoris, and in a few patients, myocardial infarction, severe arrhythmia, or even sudden death may occur. The disease may break into the right atrium, right ventricle, or coronary sinus, creating a left-to-right shunt, which can be heard as a continuous murmur and, in severe cases, affect cardiac function. The most serious complication is rupture of the tumour into the pericardium, which can lead to bleeding, cardiac tamponade or even death.

Based on the clinical manifestations and symptoms you’ve described, the rare disease most likely involves **coronary artery fistula**. Here is a detailed explanation:

### Diagnosis: Coronary Artery Fistula

1. **Clinical Manifestations**:
   - **Chest Pain**: Atypical chest pain detected during coronary angiography or coronary CT.
   - **Cardiac Ischemia**: Common manifestation, often due to combinations of obstructive atherosclerotic disease, thrombosis, slow flow, entrapment, or rupture with exertional angina or acute coronary syndromes.
   - **Compression of Adjacent Tissues**: Compression leading to ischemic symptoms.
   - **Rupture of Aneurysm**: Rare but serious complication causing acute cardiac tamponade.
   - **Myocardial Ischemia Due to Microvascular Dysfunction**: Occurs even in the absence of significant coronary stenosis.
2. **Common Clinical Manifestations**:
   - **Angina Pectoris**: Most common symptom.
   - **Myocardial Infarction, Severe Arrhythmias, or Sudden Death**: In some cases.
   - **Left-to-Right Shunt**: Can occur when the fistula breaks into the right atrium, right ventricle, or coronary sinus, leading to a continuous murmur and potential cardiac dysfunction.
3. **Serious Complications**:
   - **Rupture of Tumor into Pericardium**: Leads to bleeding, cardiac tamponade, or even death.

### Supporting Evidence from the Description:

- The presence of more men than women is not specific to this condition but can be seen in various cardiovascular diseases.
- Atypical chest pain detected during coronary angiography or CT supports the involvement of a structural abnormality like a fistula.
- The combination of obstructive atherosclerotic disease, thrombosis, slow flow, entrapment, or rupture with exertional angina aligns with the clinical presentation of coronary artery fistulas.
- Rupture of an aneurysm causing acute cardiac tamponade is a rare but serious complication that can occur in such cases.
- Myocardial ischemia due to microvascular dysfunction without significant stenosis further supports the diagnosis.

### Additional Considerations:

1. **Coronary Artery Anomalies (CAAs)**:
   - While CAAs can lead to atypical coronary circulation issues, they typically do not present with the specific combination of symptoms and complications described.
2. **Coronary Artery Aneurysm**:
   - Although aneurysms can cause ischemic symptoms and risk of rupture leading to tamponade, they are less common than fistulas in this context.
3. **Kawasaki Disease (Later Stage)**:
   - Kawasaki disease typically involves vasculitis affecting multiple arteries but is not primarily associated with coronary artery anomalies or specific chest pain patterns as described here.

Given the detailed clinical manifestations and complications, **coronary artery fistula** remains the most likely diagnosis based on your description. Further diagnostic imaging such as angiography would be crucial to confirm this diagnosis.

If you need more information or further assistance, please let me know!

# 39. User

1. Bone marrow failure Anaemia is present at birth in 35 per cent of children and is often diagnosed between 2 weeks and 2 years after birth. The anaemia is macrocytic or normocytic orthochromatic, with decreased reticulocytes, normal or mildly decreased white blood cells, normal or mildly increased platelets, and hypoplasia of the erythroid lineage of the bone marrow and hyperproliferation of the granulocyte and megakaryocyte lineages.
2. Congenital developmental anomalies 30% ~50% of patients may have congenital developmental anomalies, mainly involving the head, upper limbs, heart and genitourinary system. Examples include short stature, craniofacial anomalies including craniofacial hypertelorism, wide flat nose, small head, congenital cataracts, glaucoma, strabismus, high arched hard palate and cleft lip and palate or even Turner’s syndrome appearance, bunions, congenital cardiovascular anomalies, and genitourinary anomalies. The presence of physical abnormalities does not predict the severity of the patient’s haematological disorders. 30% of affected patients may present with postnatal growth retardation, often associated with other congenital malformations.
3. The incidence of tumours in patients with increased susceptibility to cancer is about 4%, which is higher than in the normal population of the same age. And the tumour occurs at an early age. These include acute leukaemia of the haematological system, myelodysplastic syndromes, lymphomas and solid tumours such as osteosarcoma, thymic carcinoma, hepatocellular carcinoma, melanoma, fibrous histiocytoma, gastric cancer and intestinal cancer. The prognosis of the tumours is poorer than that of the general population suffering from the same tumour, with a higher mortality rate.

Based on the clinical manifestations you have provided, the most likely diagnosis is **Diamond-Blackfan Anemia (DBA)**. Here is a detailed explanation:

1. **Bone Marrow Failure and Anaemia**:
   - Anaemia is present at birth in 35% of children and often diagnosed between 2 weeks and 2 years after birth.
   - The anaemia is typically macrocytic or normocytic orthochromatic, with decreased reticulocytes, normal or mildly decreased white blood cells, normal or mildly increased platelets, and hypoplasia of the erythroid lineage of the bone marrow and hyperproliferation of the granulocyte and megakaryocyte lineages.
2. **Congenital Developmental Anomalies**:
   - 30% to 50% of patients may have congenital developmental anomalies, mainly involving the head, upper limbs, heart, and genitourinary system.
   - Examples include short stature, craniofacial anomalies such as hypertelorism, wide flat nose, small head, congenital cataracts, glaucoma, strabismus, high arched hard palate, and cleft lip and palate. Some patients may even exhibit a Turner’s syndrome appearance or genitourinary anomalies.
   - The presence of physical abnormalities does not predict the severity of the patient’s haematological disorders. 30% of affected patients may present with postnatal growth retardation, often associated with other congenital malformations.
3. **Increased Susceptibility to Cancer**:
   - The incidence of tumours in DBA is about 4%, which is higher than in the normal population of the same age.
   - Tumors occur at an early age and include acute leukaemia, myelodysplastic syndromes, lymphomas, and solid tumors such as osteosarcoma, thymic carcinoma, hepatocellular carcinoma, melanoma, fibrous histiocytoma, gastric cancer, and intestinal cancer.
   - The prognosis of these tumours is poorer than that of the general population suffering from the same tumour, with a higher mortality rate.

Given these clinical features, Diamond-Blackfan Anemia (DBA) fits the description provided. If further confirmation is needed, genetic testing for DBA can be performed to identify mutations in the RPS19 gene or other related genes involved in ribosome biogenesis.

Would you like more information on any specific aspect of DBA?

# 40. User

The lesions may involve all systems of the body, so their clinical manifestations vary depending on the site of involvement. General symptoms include fever, malaise, night sweats, and weight loss. The rest of the affected systems mainly include:

1. Skeletal system, most patients have skeletal involvement at the time of diagnosis, with lower limb bones being the most common, but only 50% of patients have bone pain.
2. cardiovascular system, lesions involving the cardiovascular system, most patients do not have obvious clinical manifestations, but such as pericardial involvement, resulting in a large amount of pericardial effusion can lead to chest tightness, breathlessness, or even cardiac tamponade; lesions involving coronary arteries can cause myocardial ischemia-like manifestations, such as chest tightness, chest pain, etc.; diffuse myocardial involvement has also been reported, which can lead to cardiac failure-like manifestations; lesions encircling the renal arteries can cause renal hypertension; lesions encircling the mesenteric artery can cause renal hypertension; lesions encircling the mesenteric artery can cause renal hypertension. Diffuse myocardial involvement has also been reported, leading to heart failure-like symptoms; lesions encircling the renal arteries may cause renal hypertension; lesions encircling the mesenteric vessels may cause intestinal ischaemia, such as abdominal pain.
3. respiratory system Most patients with respiratory system involvement are asymptomatic, but a few patients have dry cough and dyspnoea.
4. central nervous system central nervous system involvement may appear corresponding clinical manifestations due to different parts of brain parenchyma involvement, such as limb movement disorder, gait abnormality, alteration of consciousness, etc.; spinal cord involvement may appear different clinical manifestations due to different affected segments; pituitary gland lesions may manifest polydipsia, polyuria, and other hormone secretion related manifestations.
5. Retro-orbital lesions Retro-orbital involvement is one of the most common clinical manifestations, which may manifest as unilateral or bilateral proptosis, or even cause blindness.
6. Retroperitoneal space/renal involvement. Retroperitoneal lesions may resemble retroperitoneal fibrosis and may cause unilateral or bilateral retrorenal obstruction if the lesion encircles the ureter.
7. cutaneous involvement, the most common cutaneous lesion is a xanthomatous change, which occurs on the eyelids in about 1/3 of patients, and may also occur on the face, neck, axillae, trunk, and groin.

Based on the provided clinical manifestations, the patient’s symptoms suggest a condition that involves multiple systems of the body. Given the detailed information about various systemic involvements, one potential diagnosis to consider is **Lupus Erythematosus (Systemic Lupus Erythematosus, SLE)**. Here’s an analysis based on the given clinical features:

1. **General Symptoms**:
   - Fever
   - Malaise
   - Night sweats
   - Weight loss
2. **Skeletal System Involvement**:
   - Most patients have skeletal involvement at diagnosis.
   - Lower limb bones are the most common, but only 50% of patients experience bone pain.
3. **Cardiovascular System Involvement**:
   - Pericardial involvement can lead to large amounts of pericardial effusion, causing chest tightness and breathlessness (potentially cardiac tamponade).
   - Coronary artery lesions may cause myocardial ischemia-like symptoms such as chest tightness and chest pain.
   - Diffuse myocardial involvement can result in heart failure-like manifestations.
   - Lesions encircling the renal arteries or mesenteric vessels can lead to hypertension and intestinal ischemia, respectively.
4. **Respiratory System Involvement**:
   - Asymptomatic in most patients, but a few may experience dry cough and dyspnea.
5. **Central Nervous System (CNS) Involvement**:
   - Limb movement disorders
   - Gait abnormalities
   - Altered consciousness
   - Spinal cord involvement with varied clinical manifestations based on the affected segment.
   - Pituitary gland lesions may manifest as polydipsia and polyuria.
6. **Retro-orbital Lesions**:
   - Unilateral or bilateral proptosis, potentially leading to blindness.
7. **Retroperitoneal Space/Renal Involvement**:
   - Retroperitoneal fibrosis-like symptoms with unilateral or bilateral retrorenal obstruction if the lesion encircles the ureter.
8. **Cutaneous Involvement**:
   - Xanthomatous changes, commonly seen on the eyelids in about 1/3 of patients, and may also occur on the face, neck, axillae, trunk, and groin.

Given these clinical features, SLE is a systemic autoimmune disease that can affect multiple organs and systems. The presence of multi-system involvement, including skeletal, cardiovascular, respiratory, neurological, ocular, retroperitoneal, and cutaneous manifestations, aligns well with the diagnostic criteria for SLE.

To confirm this diagnosis, further evaluation would be necessary, including: - Comprehensive clinical history and physical examination. - Laboratory tests such as antinuclear antibodies (ANA), anti-double-stranded DNA (dsDNA) antibodies, complement levels (C3, C4). - Imaging studies like echocardiography for cardiac involvement, MRI or CT scans for neurological and retroperitoneal involvement.

It is crucial to consult with a rheumatologist or an immunologist for further diagnostic workup and management.

# 41. User

The disease often presents with multi-organ and multi-system involvement, with males being more affected than female patients. Clinical manifestations often involve different organs as the patient grows older.

1. Facial appearance, male patients usually appear at the age of 12-14 years with characteristic facial features, such as convexity of the supraorbital ridge, frontal bulge and thickening of the lips. 2.
2. cutaneous angiokeratomas, which are common in patients with the classic form, are red patches of raised skin in the ‘sitting area’, i.e., between the umbilicus and knees, the scrotum, the buttocks, and the inner thighs.
3. Neurological system, most patients will have peripheral nerve pain, which manifests as intolerable burning sensation on the soles of the feet and palms of the hands and radiates to the proximal limbs, and even painful spasms; autonomic nerve involvement manifests as little or no sweating; and central nervous system involvement manifests as early-onset transient ischemic attacks or ischemic strokes.
4. Eye: Characteristic manifestations include conjunctival vascular tortuosity, corneal turbidity, posterior lens capsule clouding, and retinal vascular tortuosity, which in severe cases may lead to reduced or even loss of vision. It is often one of the main reasons for female patients to visit the clinic.
5. Digestive tract, most often after eating nausea, vomiting, abdominal distension, cramping abdominal pain and diarrhoea, may also manifest as malabsorption or constipation.
6. Kidney, early manifestation of urinary concentration dysfunction such as increased nocturia, polyuria and enuresis, with the progress of the disease can gradually appear proteinuria, and even up to the level of nephrotic syndrome, accompanied by renal function damage, mostly around 30 years old into the end-stage renal disease.
7. Cardiovascular system, can be manifested as hypertension, myocardial ischemia caused by coronary artery involvement, heart valve lesions and hypertrophic cardiomyopathy, and in severe cases, it can be manifested as angina pectoris, myocardial infarction and heart failure. It is mostly a late manifestation of the disease and a major cause of death.

Based on the provided clinical manifestations and the characteristics described, the disease in question appears to be **Fabry Disease**. This rare genetic disorder primarily affects males due to X-linked inheritance, which aligns with the observation that males are more affected than females.

### Key Clinical Manifestations of Fabry Disease:

1. **Facial Appearance:**
   - Male patients often exhibit characteristic facial features at around 12-14 years of age, including:
     - Convexity of the supraorbital ridge
     - Frontal bulge
     - Thickening of the lips
2. **Cutaneous Angiokeratomas:**
   - Common in patients with the classic form, these are red patches of raised skin found in the “sitting area,” specifically between the umbilicus and knees, scrotum, buttocks, and inner thighs.
3. **Neurological System:**
   - Most patients experience peripheral nerve pain, characterized by:
     - Intolerable burning sensation on the soles of the feet and palms of the hands
     - Painful spasms that can radiate to proximal limbs
   - Autonomic nerve involvement manifests as little or no sweating.
   - Central nervous system involvement includes early-onset transient ischemic attacks or ischemic strokes.
4. **Eye:**
   - Characteristic manifestations include:
     - Conjunctival vascular tortuosity
     - Corneal turbidity
     - Posterior lens capsule clouding
     - Retinal vascular tortuosity, which can lead to reduced or even loss of vision.
   - These ocular symptoms are often a main reason for female patients to seek medical attention.
5. **Digestive Tract:**
   - Symptoms include:
     - Nausea and vomiting after eating
     - Abdominal distension
     - Cramping abdominal pain
     - Diarrhea, which may also manifest as malabsorption or constipation.
6. **Kidney:**
   - Early manifestations of urinary concentration dysfunction include:
     - Increased nocturia
     - Polyuria
     - Enuresis
   - With disease progression, proteinuria can develop and eventually lead to nephrotic syndrome.
   - Renal function damage typically occurs around 30 years old and may progress to end-stage renal disease.
7. **Cardiovascular System:**
   - Symptoms include:
     - Hypertension
     - Myocardial ischemia due to coronary artery involvement
     - Heart valve lesions
     - Hypertrophic cardiomyopathy.
   - Severe cases can manifest as angina pectoris, myocardial infarction, and heart failure.
   - These cardiovascular symptoms are often late manifestations of the disease and a major cause of death.

### Summary:

Fabry Disease is a rare genetic disorder characterized by multi-organ involvement, primarily affecting males due to X-linked inheritance. The clinical manifestations described align well with Fabry Disease, including characteristic facial features, cutaneous angiokeratomas, peripheral neuropathy, ocular symptoms, gastrointestinal issues, renal dysfunction, and late-onset cardiovascular complications.

For a definitive diagnosis, genetic testing for the GLA gene is recommended. If you suspect someone has Fabry Disease based on these symptoms, consulting with a specialist in rare diseases or a geneticist would be advisable.

# 42. User

The disease is classified as Type 1 and Type 2.

1. Type 1 is mainly characterised by recurrent inflammatory reactions and plasma membrane inflammation, including recurrent fever, abdominal pain and arthritis. Fever is recurrent and may be triggered by cold, strenuous exercise, surgery, infection, etc. and resolves spontaneously within 13 days. Abdominal pain is the most common clinical manifestation, seen in 95% of patients. It is a sudden onset of abdominal pain, often accompanied by fever, abdominal examination may have abdominal distension, abdominal pressure, rebound pain, muscle tension, and the loss of bowel sounds on auscultation. Stereotactic abdominal radiographs show flat air and fluid in the small intestine. Abdominal pain often resolves spontaneously after 24-48 hours. Arthritis is also a common clinical manifestation, seen in 75% of patients. It often involves the large joints of the lower limbs, such as the hip and knee joints. There may be joint swelling and pain. Rash is seen in 7% ~40% of patients, mainly involving the extensor side of the lower limbs, and the typical rash is erysipelas of tansy-like erythema. Pleurisy is seen in 45% of patients and presents as a sudden onset of unilateral chest pain, often accompanied by fever. Pericarditis is less common.
2. Type 2 starts with amyloidosis. It presents with persistent proteinuria and often leads to end-stage renal disease. Amyloid can also be deposited in the heart, gastrointestinal tract, liver and other organs, leading to corresponding clinical manifestations.

Based on the provided information, the disease described appears to be **Familial Mediterranean Fever (FMF)**. FMF is a hereditary inflammatory disorder characterized by recurrent episodes of fever and inflammation affecting various organs and tissues.

### Type 1:

- **Clinical Manifestations:**
  - **Recurrent Inflammatory Reactions:** The disease is marked by recurrent episodes of fever, abdominal pain, and arthritis.
  - **Fever:** Recurrent fever can be triggered by factors such as cold exposure, strenuous exercise, surgery, or infection. It typically resolves spontaneously within 13 days.
  - **Abdominal Pain:** This is the most common clinical manifestation, seen in 95% of patients. It is a sudden onset of abdominal pain often accompanied by fever. On physical examination, there may be signs such as abdominal distension, abdominal pressure, rebound tenderness, muscle tension, and loss of bowel sounds.
  - **Arthritis:** Arthritis affects the large joints of the lower limbs (hip and knee joints) in about 75% of patients, causing joint swelling and pain.
  - **Rash:** A rash is observed in 7% to 40% of patients, typically involving the extensor side of the lower limbs. The typical rash resembles erysipelas with a tansy-like erythema.
  - **Pleurisy:** Pleurisy occurs in about 45% of patients and presents as sudden onset unilateral chest pain often accompanied by fever.
  - **Pericarditis:** This is less common.

### Type 2:

- **Clinical Manifestations:**
  - **Amyloidosis:** Type 2 FMF starts with amyloidosis, which leads to persistent proteinuria.
  - **Renal Involvement:** Amyloid deposition in the kidneys often results in end-stage renal disease.
  - **Other Organ Involvement:** Amyloid can also be deposited in other organs such as the heart, gastrointestinal tract, and liver, leading to corresponding clinical manifestations.

### Summary:

The recurrent inflammatory reactions and specific clinical features described for both Type 1 and Type 2 align well with Familial Mediterranean Fever (FMF). The diagnostic criteria include the presence of recurrent fever, abdominal pain, arthritis, rash, pleurisy, and the progression to amyloidosis in Type 2.

For a definitive diagnosis, genetic testing for mutations in the MEFV gene is recommended. If you suspect FMF based on these symptoms, consulting with a specialist in hereditary inflammatory disorders would be advisable.

# 43. User

The main manifestations are congenital developmental anomalies, progressive bone marrow failure and increased incidence of tumours, while some patients may show no developmental anomalies or bone marrow failure.

1. Developmental anomalies, patients may show a variety of physical and mental developmental abnormalities. Growth defects, abnormal skin pigmentation, unilateral or bilateral skeletal malformations of the upper limbs, short stature, microcephaly, ocular anomalies, genitourinary malformations, endocrine anomalies, hearing loss, outer ear anomalies, congenital heart defects, digestive anomalies, abnormalities of the central nervous system, facial deformities, spinal deformities, neck deformities, and developmental delays and/or intellectual disabilities. These manifestations are mostly non-specific, with thumb and upper limb deformities being more specific.
2. Progressive bone marrow failure with haematological abnormalities is the most serious clinical feature of Fanconi anaemia. It may present as a single or total haematopenia. The age of onset of haemopenia varies widely, even among siblings. The median age of onset is about 7.6 years, and it is rare in infants and young children; 90% of patients with haematological abnormalities develop before the age of 40 years. Thrombocytopenia or leukopenia usually precedes anaemia and is often accompanied by an increase in macrocytes, foetal haemoglobin (HbF 1), and a gradual increase in total haematopoiesis.
3. There is an increased risk of neoplasms, including haematological malignancies and non-haematological malignant solid tumours. The risk of acute myeloid leukaemia (AML) is increased about 500-fold, and the cumulative incidence of AML is about 13% at the age of 50 years, with most cases occurring between the ages of 15 and 35 years. -7, del (7q) is associated with an increased incidence of myelodysplastic syndromes (MDS)/AML. Acute lymphoblastic leukaemia and lymphoma have also been reported. Solid tumours can be the first manifestation of the disease. Head and neck squamous cell carcinomas (HNSCCs) are the most common solid tumours in patients and have an earlier age of onset (20-40 years), mostly occurring in the oral cavity (e.g., tongue cancers), are mostly in the progressive stage, and respond poorly to treatment. There is also an increased incidence of skin, oesophageal, liver and genitourinary tract tumours. In a few patients, multiple tumours may be seen concurrently, or haematological and non-haematological tumours may be seen concurrently. And there is a marked intolerance to chemotherapy or radiotherapy.

Based on the provided information, the condition described appears to be **Fanconi Anemia (FA)**. Fanconi anemia is a rare genetic disorder characterized by congenital developmental anomalies, progressive bone marrow failure, and an increased risk of tumors.

### Main Manifestations:

1. **Developmental Anomalies:**
   - Growth defects
   - Abnormal skin pigmentation
   - Unilateral or bilateral skeletal malformations of the upper limbs
   - Short stature
   - Microcephaly
   - Ocular anomalies
   - Genitourinary malformations
   - Endocrine anomalies
   - Hearing loss
   - Outer ear anomalies
   - Congenital heart defects
   - Digestive anomalies
   - Abnormalities of the central nervous system
   - Facial deformities
   - Spinal deformities
   - Neck deformities
   - Developmental delays and/or intellectual disabilities
2. **Progressive Bone Marrow Failure:**
   - The most serious clinical feature is progressive bone marrow failure, which may present as a single or total haematopenia.
   - The age of onset varies widely; the median age of onset is about 7.6 years, with rare cases in infants and young children.
   - By the age of 40, 90% of patients develop haematological abnormalities.
   - Thrombocytopenia or leukopenia usually precedes anaemia and is often accompanied by an increase in macrocytes, foetal haemoglobin (HbF), and a gradual increase in total haematopoiesis.
3. **Increased Risk of Neoplasms:**
   - There is a significantly increased risk of both haematological malignancies and non-haematological malignant solid tumours.
   - The risk of acute myeloid leukaemia (AML) is about 500 times higher, with a cumulative incidence of AML at 13% by the age of 50 years. Most cases occur between ages 15 to 35 years.
   - Del(7q) is associated with an increased risk of myelodysplastic syndromes (MDS)/AML.
   - Acute lymphoblastic leukaemia and lymphoma have also been reported.
   - Solid tumours, particularly head and neck squamous cell carcinomas (HNSCCs), are common. These tumors often occur in the oral cavity, such as on the tongue, and tend to be progressive with poor response to treatment.
   - There is an increased incidence of skin, oesophageal, liver, and genitourinary tract tumours.
   - In some cases, multiple tumours may co-occur, or both haematological and non-haematological tumors may be present simultaneously.
   - Patients often show a marked intolerance to chemotherapy or radiotherapy.

This comprehensive overview highlights the complex nature of Fanconi Anemia, emphasizing its multi-faceted clinical manifestations. If you need further details or specific information on any aspect, please let me know!

# 44. User

The perinatal onset of the disease is often typical, and within a few days of ingestion of breast milk or lactose-containing formula, the child develops life-threatening complications: feeding problems, diarrhoea, vomiting, hypoglycaemia, liver impairment, haemorrhage, jaundice, and cataracts. If left untreated, sepsis, shock and death may occur. Children who survive to infancy may develop severe brain damage if they continue to ingest lactose. Some children have long-term complications such as mental retardation, delayed growth, ataxia, blindness, and ovarian dysfunction in female patients.

Based on the clinical manifestations you’ve described, the most likely diagnosis is **Classic Galactosemia**. This condition involves an individual’s inability to properly metabolize galactose, leading to severe complications shortly after ingestion of breast milk or lactose-containing formula.

### Key Features:

- **Perinatal Onset:** The disease often presents within a few days of feeding the child with breast milk or lactose-containing formula.
- **Immediate Complications:**
  - Feeding problems
  - Diarrhea
  - Vomiting
  - Hypoglycemia
  - Liver impairment
  - Hemorrhage
  - Jaundice
  - Cataracts

If left untreated, the complications can be life-threatening: - Sepsis - Shock - Death

### Long-Term Complications:

- Severe brain damage in infants who continue to ingest lactose.
- Mental retardation
- Delayed growth
- Ataxia
- Blindness
- Ovarian dysfunction in female patients

### Differential Diagnoses:

1. **Hereditary Fructose Intolerance (HFI):** This condition involves an inability to break down fructose, leading to liver and kidney damage after ingestion of fructose-containing foods. However, it typically presents later when fructose is introduced into the diet.
2. **Tyrosinemia Type I:** A metabolic disorder resulting from a deficiency in fumarylacetoacetate hydrolase, causing liver dysfunction, renal tubular acidosis, and a risk of bleeding. Cataracts are not typically associated with this condition.
3. **Glycogen Storage Disease Type I (Von Gierke Disease):** Characterized by hypoglycemia, lactic acidosis, hyperuricemia, and hepatomegaly. This disease involves glycogen metabolism rather than immediate post-ingestion reactions.
4. **Neonatal Hemochromatosis:** A disorder resulting in congenital liver disease with iron overload, presenting with liver failure but not typically involving cataracts or direct dietary triggers.

Given the specific symptoms and complications described, Classic Galactosemia is the most likely diagnosis. Early detection and prompt treatment are crucial to prevent severe long-term complications.

# 45. User

1. Type I is the most common subtype. There is no primary central nervous system involvement, but some patients may develop secondary neurological manifestations (e.g., spinal cord compression) as the disease progresses. The disease can develop in all age groups, with about 2/3 of patients developing in childhood. Organ manifestations are mainly hepatosplenomegaly, especially splenomegaly, often accompanied by hypersplenism, and even splenic infarction and splenic rupture. The haematological manifestations are mainly thrombocytopenia and anaemia, and some patients have leucopenia, which may be accompanied by coagulation abnormalities. Patients present with pallor, fatigue, skin and gum bleeding, increased menstruation, and even life-threatening bleeding. Most patients have skeletal invasion, but the severity varies. Early invasion sites mainly include lumbar vertebrae, long bone epiphyses and diaphyses, and in the middle and late stages, epiphyses and bony prominences may be involved. Patients often experience acute or chronic diffuse bone pain, and in severe cases, osteonecrosis (ischaemic necrosis), followed by joint collapse involving the proximal and distal femur, proximal tibia and proximal humerus. Osteolytic lesions, pathological fractures, vertebral compression fractures and other fragility fractures due to reduced bone density may also occur. Skeletal lesions can interfere with daily activities and can be disabling. In children, the most common manifestations are osteoporosis, flask-like deformity of the metaphysis of the long bones, varying degrees of hypodensity of the metaphysis of the long bones, thinning of the bone cortex, etc. There may be growth retardation. Some patients may have lung involvement, which is mainly manifested as interstitial lung disease, pulmonary solid changes, and pulmonary hypertension. In addition, patients may have abnormalities of sugar and lipid metabolism, increased risk of malignant tumours such as multiple myeloma, cholelithiasis, and abnormalities of the immune system.
2. Type II, in addition to hepatosplenomegaly, anaemia, thrombocytopenia and other manifestations similar to those of type I, patients with type II mainly have acute neurological involvement. It often develops in the neonatal period to infancy, progresses rapidly and has a high morbidity and mortality rate. There are rapidly progressive medullary palsy, dyskinesia, seizures, angular reflexes and cognitive deficits and other signs of acute neurological damage, and growth is retarded, with death occurring before the age of 2 to 4 years. Some patients with severe disease may develop joint contractures.
3. The early manifestations of type III are similar to those of type I, with gradual onset of neurological involvement, which often develops in childhood and has a slow progression of disease and a longer life expectancy. The patients often have the invasion of the motor nerve, eye movement disorders, ataxia, anisocoria, epilepsy, myoclonus, accompanied by developmental delays and intellectual backwardness. Type III can be divided into three subtypes, namely, type IIIa, which is characterised by more rapidly progressive neurological symptoms (ocular motility disorders, cerebellar ataxia, spasticity, myoclonus, and dementia) and hepatosplenomegaly; type IIIb, which is characterised by hepatosplenomegaly and skeletal symptoms with fewer CNS symptoms; and type IIIc, which is characterised by milder symptoms, including calcification of the cardiac valves and corneal clouding, and is predominantly found in the Druze population. Type IIIc.

Based on the information provided, it appears you are describing a rare disease with multiple subtypes, likely related to a metabolic disorder such as **Gaucher Disease**. Gaucher Disease is characterized by varying clinical manifestations depending on the subtype and age of onset. Let’s break down the key points for each subtype:

### Type I (Non-neuronopathic Gaucher Disease)

- **General Characteristics:**
  - No primary central nervous system involvement.
  - Some patients may develop secondary neurological manifestations such as spinal cord compression.
  - Can occur in any age group, with about 2/3 of cases developing in childhood.
- **Organ Manifestations:**
  - Hepatosplenomegaly, especially splenomegaly, often accompanied by hypersplenism, splenic infarction, and splenic rupture.
  - Haematological manifestations include thrombocytopenia, anaemia, and leucopenia with potential coagulation abnormalities.
- **Skeletal Involvement:**
  - Early skeletal involvement includes lumbar vertebrae, long bone epiphyses and diaphyses.
  - Chronic or acute diffuse bone pain, osteonecrosis (ischaemic necrosis), pathological fractures, vertebral compression fractures, and other fragility fractures due to reduced bone density.
- **Other Manifestations:**
  - Osteoporosis, flask-like deformity of the metaphysis of long bones, varying degrees of hypodensity, thinning of the bone cortex, growth retardation.
  - Pulmonary involvement includes interstitial lung disease, pulmonary solid changes, and pulmonary hypertension.
  - Metabolic abnormalities include sugar and lipid metabolism issues, increased risk of malignant tumours such as multiple myeloma, cholelithiasis, and immune system abnormalities.

### Type II (Neuronopathic Gaucher Disease)

- **General Characteristics:**
  - Rapidly progressive neurological involvement often developing in the neonatal period to infancy.
  - High morbidity and mortality rate.
- **Neurological Manifestations:**
  - Acute neurological involvement including medullary palsy, dyskinesia, seizures, angular reflexes, cognitive deficits.
  - Growth retardation with death occurring before age 2 to 4 years.
  - Joint contractures in severe cases.

### Type III (Subacute Neuronopathic Gaucher Disease)

- **General Characteristics:**
  - Gradual onset of neurological involvement developing in childhood.
  - Slow progression and longer life expectancy compared to Type II.
- **Neurological Manifestations:**
  - Involvement of motor nerves, eye movement disorders, ataxia, anisocoria, epilepsy, myoclonus.
  - Developmental delays and intellectual backwardness.
- **Subtypes:**
  - **Type IIIa:** Rapidly progressive neurological symptoms including ocular motility disorders, cerebellar ataxia, spasticity, myoclonus, and dementia; hepatosplenomegaly.
  - **Type IIIb:** Hepatosplenomegaly and skeletal symptoms with fewer central nervous system (CNS) symptoms.
  - **Type IIIc:** Milder symptoms including calcification of the cardiac valves and corneal clouding, predominantly found in the Druze population.

### Summary

- **Type I** is characterized by non-neurological manifestations such as hepatosplenomegaly, haematological abnormalities, skeletal involvement, and other systemic issues.
- **Type II** involves rapid neurological deterioration with a high mortality rate.
- **Type III** has a slower progression with gradual neurological involvement but longer life expectancy.

These subtypes highlight the complexity and variability of Gaucher Disease, which can affect multiple organ systems and have different clinical courses depending on the subtype. For accurate diagnosis and management, it is crucial to consult with specialists in metabolic disorders or genetic diseases.

Would you like more detailed information on any specific aspect of these subtypes?

# 46. User

It often starts in one muscle group and progresses to others, with ptosis and diplopia being the most common onset, accounting for 80 per cent of cases. Skeletal muscle weakness is fluctuating and easily fatigued, with a light morning and heavy evening. According to the clinical manifestations, it is divided into five types. Type I: ocular muscle type, manifested by ptosis and diplopia. Type ⅡA: mild generalised type, with mild involvement of the limb muscle groups. Type IIB: Moderate generalised type with moderate involvement of the limb muscle groups, usually with mastication, swallowing and dysarthria. Type III: Severe aggressive type with rapid progression, involving the pharyngeal muscles within weeks or months and the respiratory muscles within six months. Type IV: Late-onset severe type, slowly progressive, involving the respiratory muscles within 2 years. Type V: myasthenia gravis, skeletal muscle atrophy within six months of disease onset. Neurological examination can be seen in the proximal limbs, trunk, neck, craniofacial muscle weakness, intolerance to fatigue, Jolly test is positive, tendon reflexes are normal, no sensory deficits, no pathological signs. Some patients may progress to respiratory failure and develop myasthenia gravis crisis.

Based on the clinical manifestations provided, the most likely diagnosis is **Myasthenia Gravis** (MG). This condition is characterized by fluctuating muscle weakness and fatigue, often starting with ocular symptoms like ptosis and diplopia. The progression and types described align well with the classification of Myasthenia Gravis.

Here are the five types based on your description:

1. **Type I: Ocular Muscle Type**
   - Manifested by ptosis and diplopia.
   - Accounts for 80% of cases starting with ocular symptoms.
2. **Type ⅡA: Mild Generalized Type**
   - Mild involvement of limb muscle groups.
   - Proximal limb weakness is common, but generally mild.
3. **Type IIB: Moderate Generalized Type**
   - Moderate involvement of limb muscle groups.
   - Often includes mastication, swallowing, and dysarthria.
4. **Type III: Severe Aggressive Type**
   - Rapid progression with severe symptoms.
   - Involves pharyngeal muscles within weeks or months and respiratory muscles within six months.
5. **Type IV: Late-Onset Severe Type**
   - Slowly progressive.
   - Involves respiratory muscles within two years.
6. **Type V: Myasthenia Gravis with Skeletal Muscle Atrophy**
   - Skeletal muscle atrophy within six months of disease onset.

### Clinical Features

- **Fluctuating and Fatigable Weakness:** Symptoms worsen with activity and improve with rest.
- **Morning Light, Evening Heavy Pattern:** Muscle weakness is often more pronounced in the evening compared to the morning.
- **Proximal Limb, Trunk, Neck, Craniofacial Muscle Weakness:** Neurological examination reveals these areas as affected.
- **Intolerance to Fatigue:** Patients experience difficulty maintaining muscle strength during prolonged activity.
- **Jolly Test Positive:** This test is positive in patients with Myasthenia Gravis due to the presence of fatigable weakness.
- **Normal Tendon Reflexes:** No sensory deficits or pathological signs are observed.

### Possible Differential Diagnoses

1. **Lambert-Eaton Myasthenic Syndrome (LEMS):**
   - Typically starts with proximal limb muscle weakness rather than ocular symptoms.
   - Improvement with exertion is a key feature.
2. **Mitochondrial Myopathy:**
   - Progressive muscle weakness, often involving multiple muscle groups.
   - May present with similar fatigue and weakness but typically does not have the characteristic fluctuation seen in MG.

### Conclusion

Given the clinical presentation and progression described, Myasthenia Gravis is the most likely diagnosis. However, further diagnostic tests such as serum antibody testing (anti-AChR antibodies), nerve conduction studies, and electromyography (EMG) can help confirm the diagnosis and rule out other conditions. If respiratory failure or a myasthenia gravis crisis develops, prompt medical intervention will be necessary.

# 47. User

The disease often starts in adolescence or early adulthood. Clinical manifestations are mainly related to hypokalemia and hypomagnesemia. Mild patients may be asymptomatic or show mild fatigue and poor appetite; severe patients may have twitching of limbs, flaccid paralysis, painful spasms, fainting, and transverse myelinolysis secondary to acute renal injury, and may even suffer from cardiac arrest due to severe ventricular arrhythmias. The common clinical manifestations are as follows:

1. systemic symptoms fatigue, thirst, polydipsia, salt craving.
2. Neuro-muscular system Muscle weakness, painful spasms, convulsions, convulsive seizures, numbness of limbs, sensory abnormalities, rhabdomyolysis, paralysis, dizziness, vertigo, ataxia, and pseudotumor cerebri.
3. Cardiovascular system Palpitation, syncope, normal or low blood pressure and ventricular arrhythmia, etc.
4. Digestive system Constipation, vomiting, etc.
5. Urinary system Polyuria, nocturia, enuresis, proteinuria and renal insufficiency.
6. Bone and joint system Arthralgia, cartilage calcium sedimentation.
7. endocrine and growth retardation, delayed puberty; diabetes mellitus or impaired glucose tolerance is not uncommon in patients with chronically low potassium and magnesium. 8. ocular symptoms.
8. Ocular symptoms Blurred vision and scleral choroidal calcification may occur in a small number of patients.

Based on the clinical manifestations you provided, the disease most likely to be diagnosed is **Gitelman syndrome**. Gitelman syndrome is a rare inherited disorder characterized by hypokalemia (low potassium) and hypomagnesemia (low magnesium), which aligns with the symptoms described. Here are the key points that support this diagnosis:

1. **Systemic Symptoms:**
   - Fatigue, thirst, polydipsia, salt craving.
2. **Neuromuscular System:**
   - Muscle weakness, painful spasms, convulsions, convulsive seizures, numbness of limbs, sensory abnormalities, rhabdomyolysis, paralysis, dizziness, vertigo, ataxia, and pseudotumor cerebri.
3. **Cardiovascular System:**
   - Palpitation, syncope, normal or low blood pressure, ventricular arrhythmia.
4. **Digestive System:**
   - Constipation, vomiting.
5. **Urinary System:**
   - Polyuria, nocturia, enuresis, proteinuria, and renal insufficiency.
6. **Bone and Joint System:**
   - Arthralgia, cartilage calcium sedimentation.
7. **Endocrine and Growth Retardation:**
   - Delayed puberty, endocrine disturbances such as diabetes mellitus or impaired glucose tolerance in chronically low potassium and magnesium patients.
8. **Ocular Symptoms:**
   - Blurred vision and scleral choroidal calcification may occur in a small number of patients.

Gitelman syndrome is caused by mutations in the gene encoding the thiazide-sensitive sodium-chloride cotransporter (NCCT) in the distal convoluted tubule of the kidney. This leads to impaired reabsorption of sodium and chloride, resulting in hypokalemia and hypomagnesemia.

Other possible diagnoses to consider include: - **Bartter syndrome:** Another rare inherited disorder affecting kidney function, leading to hypokalemia and metabolic alkalosis. - **Liddle syndrome:** A rare genetic disorder characterized by hypertension and hypokalemia due to aldosterone receptor overactivity. - **Renal tubular acidosis (RTA):** Various types of RTA can cause electrolyte imbalances similar to Gitelman syndrome.

Given the comprehensive nature of the symptoms and the specific electrolyte abnormalities, Gitelman syndrome is the most likely diagnosis based on the information provided. Further diagnostic tests such as blood electrolytes, renal function tests, and genetic testing may be necessary for confirmation.

# 48. User

The majority of children develop the disease in infancy or early childhood, with a variety of presentations. The earlier the onset, the more severe the symptoms and the worse the prognosis. The majority of patients have a relatively normal presentation in the first year of life. The earliest sign that appears in about 75% of patients is a large head size, and most children are born with a larger head circumference than their peers or have a rapid increase in head circumference soon after birth, which may be accompanied by mild non-specific symptoms, including irritability, difficulty in feeding, and vomiting. Children usually develop ketosis, vomiting, hepatomegaly and acute encephalopathic crisis, including hypotonia, loss of consciousness and convulsive seizures, between 3 and 36 months of age after fever, infection, surgery, or vaccination, which may resolve with symptomatic treatment, but not fully recover. If acute encephalopathic crisis occurs repeatedly, the neurological damage will be progressively aggravated, and there may be developmental regression, and eventually cognitive dysfunction may occur. In some children, delayed movement, dystonia, and random movement disorders occur gradually over the first few years of life, but mental development is almost normal. Patients often die within 10 years of age from concomitant diseases or Reye-like seizures, which decrease with age. Rarely, the onset of the disease occurs during adolescence or even adulthood, and may be asymptomatic until the first episode.

Based on the clinical manifestations provided, the most likely diagnosis for this condition is **Medium-Chain Acyl-CoA Dehydrogenase Deficiency (MCAD Deficiency)**. This inherited disorder affects the body’s ability to break down medium-chain fatty acids into energy, especially during periods of fasting or illness, leading to the symptoms described.

### Key Clinical Manifestations:

1. **Early Onset and Severity:**
   - The majority of children develop the disease in infancy or early childhood.
   - Earlier onset is associated with more severe symptoms and a worse prognosis.
2. **Initial Presentation (First Year of Life):**
   - Relatively normal presentation for most patients during the first year of life.
   - About 75% of patients exhibit a large head size, often due to rapid increase in head circumference after birth, which may be accompanied by mild non-specific symptoms such as irritability, difficulty in feeding, and vomiting.
3. **Acute Episodes (3-36 Months):**
   - Ketosis, vomiting, hepatomegaly, and acute encephalopathic crisis.
   - Acute encephalopathic crises often occur after fever, infection, surgery, or vaccination.
   - Symptoms include hypotonia, loss of consciousness, and convulsive seizures.
   - These episodes may resolve with symptomatic treatment but do not fully recover.
4. **Progressive Neurological Damage:**
   - Repeated acute encephalopathic crises lead to progressive neurological damage.
   - Developmental regression and cognitive dysfunction may occur over time.
   - Some children may experience delayed movement, dystonia, and random movement disorders gradually over the first few years of life.
5. **Prognosis:**
   - Patients often die within 10 years of age due to concomitant diseases or Reye-like seizures.
   - The risk of Reye-like seizures decreases with age.
6. **Rare Onset in Adolescence or Adulthood:**
   - Rare cases may present asymptomatic until the first episode, which can occur during adolescence or even adulthood.

### Other Possible Diagnoses to Consider:

1. **Ornithine Transcarbamylase Deficiency (OTC Deficiency):** An X-linked genetic disorder affecting the urea cycle, leading to ammonia accumulation.
2. **Very Long-Chain Acyl-CoA Dehydrogenase Deficiency (VLCAD Deficiency):** Affects the breakdown of very long-chain fatty acids.
3. **Short-Chain Lysophosphatidic Acidacyltransferase 1 (SCALD1) Deficiency:** Involves defects in lipid metabolism.
4. **Fatty Acid Oxidation Disorders:** Other types such as Long-Chain Acyl-CoA Dehydrogenase Deficiency (LCAD Deficiency).
5. **Mitochondrial Disorders:** Such as Mitochondrial Encephalomyopathy, Lactic Acidosis, and Stroke-like Episodes (MELAS).

Given the detailed clinical picture provided, MCAD deficiency is the most likely diagnosis for this condition. Further diagnostic testing such as genetic testing, biochemical assays, and metabolic evaluations would be necessary to confirm the diagnosis.

# 49. User

It mainly manifests as bleeding from joints, muscles and deep tissues, but also internal organs such as gastrointestinal tract and central nervous system. If repeated bleeding occurs, untreated bleeding can lead to joint deformity and/or pseudotumour formation, which can be life-threatening in severe cases. Delayed haemorrhage after trauma or surgery is characteristic of this disease.

Based on the clinical manifestations you have provided, the most likely diagnosis is **Hemophilia**. Hemophilia is a rare genetic disorder characterized by a deficiency in specific clotting factors, leading to prolonged bleeding episodes. The symptoms of joint and muscle bleeding, as well as delayed hemorrhage after trauma or surgery, align with the typical presentation of hemophilia.

Here are five other possible diagnoses to consider: 1. **Von Willebrand Disease**: Although more common than hemophilia, severe forms can present with joint and tissue bleeding, particularly when von Willebrand factor is significantly decreased. 2. **Acquired Hemophilia**: This is an autoimmune disorder where the body produces antibodies against its own clotting factors, leading to similar bleeding symptoms as congenital hemophilia. 3. **Factor XIII Deficiency**: This is a rare bleeding disorder that can cause delayed bleeding, poor wound healing, and spontaneous bleeding similar to hemophilia. 4. **Factor VII Deficiency**: Another rare bleeding disorder where deficiency of factor VII leads to bleeding symptoms, though joint bleeding is less common than in hemophilia. 5. **Platelet Function Disorders**: While classically these disorders result in mucocutaneous bleeding, severe cases can sometimes present with bleeding symptoms that mimic those of hemophilia, especially after surgery or trauma.

Given the severity and specific nature of the symptoms you described, including joint deformity and pseudotumour formation from repeated untreated bleeding, Hemophilia remains the most probable diagnosis. Further diagnostic testing such as clotting factor assays and genetic testing would be necessary to confirm the exact type of hemophilia (A or B) and rule out other potential causes.

# 50. User

It can involve multiple organs of the body and has a variety of clinical manifestations. The age of onset is mostly 3-60 years old, and there are also reports of patients diagnosed at 8 months and over 70 years old. In children, hepatic involvement is the first manifestation of the disease, while in adolescents and adults, neurological involvement is the first manifestation of the disease.

It may present with asymptomatic persistent elevation of aminotransferases, chronic hepatitis, cirrhosis, and acute liver failure. Neurological involvement may manifest as motor dysfunction, tremor, ataxia, chorea, dysautonomia, dystonia, and in some patients masked face, stiff limbs, and gait abnormalities. Some patients combine with mental behavioural abnormalities such as apathy, aggressive behaviour, and personality changes. Kidney involvement may manifest as haematuria, proteinuria, etc. Involvement of the blood system may be manifested as haemolytic anaemia, liver cirrhosis, splenic hyperfunction leading to a decrease in the blood tertiary system, and coagulation abnormalities. Relatively rare systems involved include bone and joint, heart, endocrine and reproductive systems.

Based on the clinical manifestations provided, the disease most likely to be diagnosed is **Wilson’s Disease**. Wilson’s Disease is a rare genetic disorder characterized by excessive copper accumulation in the body, primarily affecting the liver and brain but potentially involving other organs.

Here are the key points that support this diagnosis: 1. **Age of Onset**: The age range of 3-60 years old with occasional reports from as young as 8 months to over 70 years old is consistent with Wilson’s Disease. 2. **Clinical Manifestations**: - **Hepatic Involvement**: Early in childhood, hepatic involvement is common and can present as asymptomatic persistent elevation of aminotransferases, chronic hepatitis, cirrhosis, or acute liver failure. - **Neurological Involvement**: In adolescents and adults, neurological symptoms are more prominent and can include motor dysfunction (tremor, ataxia, chorea), dysautonomia, dystonia, masked face, stiff limbs, and gait abnormalities. Mental behavioral changes such as apathy, aggressive behavior, and personality changes may also be observed. - **Renal Involvement**: Kidney involvement can manifest as hematuria and proteinuria. - **Blood System Involvement**: This includes hemolytic anemia, liver cirrhosis leading to splenic hyperfunction (decreased blood cell counts), coagulation abnormalities, and other systemic manifestations. - **Other Systems**: Relatively rare systems involved include bone and joint issues, heart problems, endocrine disorders, and reproductive system involvement.

Given these comprehensive clinical features, Wilson’s Disease is the most likely diagnosis. Further diagnostic tests such as serum ceruloplasmin levels, 24-hour urinary copper excretion, and liver biopsy can confirm the presence of excessive copper accumulation characteristic of this condition.

# 51. User

The clinical picture is very heterogeneous, even within the same family. The disease usually begins before the age of 30 years, worsens during adolescence, and the oedema often has an acute onset. The clinical picture is characterised by recurrent, unpredictable cutaneous and submucosal oedema. The oedema is episodic, self-limited, usually resolves spontaneously in 3-5 days, asymmetric and non-concave. Oedema can involve any part of the body, with the extremities, face, genitals, respiratory tract and gastrointestinal tract mucosa being the most common. The most fatal is respiratory mucosal oedema, which can rapidly progress to respiratory distress or asphyxiation due to laryngeal oedema, and can lead to death by asphyxiation if not resuscitated in time, with a lethality rate as high as 11%-40%, which is one of the main causes of death. Episodes of mucosal oedema of the digestive tract manifest as severe abdominal pain, accompanied by nausea and vomiting, and are therefore often misdiagnosed as acute abdomen, leading to unnecessary abdominal surgery. Skin oedema occurring in the limbs and other parts of the body can affect its function.

Based on the clinical picture you’ve described, the most likely diagnosis is **Hereditary Angioedema (HAE)**. This condition is characterized by recurrent episodes of severe swelling (angioedema) in various parts of the body, including the extremities, face, airway, and gastrointestinal tract, which matches the symptoms you’ve outlined.

Key features that support this diagnosis include: 1. **Heterogeneous Clinical Picture**: HAE can present with a wide range of symptoms within the same family. 2. **Age of Onset and Worsening During Adolescence**: The disease typically begins before the age of 30 years and worsens during adolescence, which aligns with your description. 3. **Acute Onset and Episodic Nature**: Episodes often have an acute onset and are episodic, self-limiting, resolving spontaneously in 3-5 days. 4. **Asymmetric and Non-Concave Oedema**: The oedema is typically asymmetric and non-concave, affecting various parts of the body including extremities, face, genitals, respiratory tract, and gastrointestinal tract mucosa. 5. **Fatal Respiratory Involvement**: Respiratory mucosal oedema can lead to severe respiratory distress or asphyxiation due to laryngeal oedema, with a high lethality rate (11%-40%), making it one of the main causes of death in HAE patients. 6. **Gastrointestinal Involvement**: Episodes manifesting as severe abdominal pain, nausea, and vomiting can be misdiagnosed as acute abdomen, leading to unnecessary surgical interventions.

Other possible diagnoses to consider include: 1. **Acquired Angioedema (AAE)**: Similar clinical features but occurs later in life and is often associated with another condition affecting the immune system. 2. **Idiopathic Angioedema**: Episodes of swelling without a known cause, typically without a hereditary component. 3. **Angioedema Due to ACE Inhibitors**: Drug-induced angioedema that can present similarly but is related to medication use.

Given these clinical features and the potential severity, it is crucial to confirm the diagnosis through appropriate tests such as C1 inhibitor levels, C4 binding protein (C4BP) activity, and genetic testing for HAE.

# 52. User

The disease starts at birth and the severity of clinical manifestations is related to the subtype of the disease. Clinical manifestations include blisters or maculopapular blisters on the hands, elbows, knees and feet and other areas prone to friction, or around the body. Blisters or ulcers of the oral mucosa may occur, leading to dysphagia and hoarseness. Some patients have mottled pigmentation abnormalities, baldness, palmoplantar hyperkeratosis, enamel defects, nail thickening and nail dystrophy. Other impairments include disfigurement of the digits due to repeated scar formation, anaemia, osteoporosis, muscle atrophy, cardiomyopathy, and deafness. Some subtypes may be associated with basal cell carcinoma, squamous cell carcinoma, and malignant melanoma in advanced stages.

Based on the clinical manifestations you’ve described, the most likely diagnosis is **Epidermolysis Bullosa (EB)**. This condition typically starts at birth and can have varying severities depending on the subtype. Here’s a detailed breakdown:

### Key Features of Epidermolysis Bullosa:

1. **Blisters or Maculopapular Blisters**: Commonly observed on areas prone to friction such as hands, elbows, knees, feet, and other body parts.
2. **Oral Mucosa Involvement**: Blisters or ulcers in the oral mucosa leading to dysphagia (difficulty swallowing) and hoarseness.
3. **Skin Pigmentation Abnormalities**: Mottled pigmentation abnormalities can be present.
4. **Hair Loss (Alopecia)**: Baldness may occur.
5. **Palmoplantar Hyperkeratosis**: Thickening of the skin on the palms and soles.
6. **Enamel Defects**: Dental issues such as enamel defects.
7. **Nail Changes**: Nail thickening and dystrophy.
8. **Disfigurement Due to Scar Formation**: Repeated scarring can lead to disfigurement, particularly in digits.
9. **Systemic Impairments**:
   - **Anemia**
   - **Osteoporosis**
   - **Muscle Atrophy**
   - **Cardiomyopathy**
   - **Deafness**

### Subtypes of Epidermolysis Bullosa:

- **Dystrophic EB (DEB)**: The most common subtype, characterized by severe blistering and scarring.
- **Junctional EB (JEB)**: Involves the junction between the epidermis and dermis, leading to more widespread and severe blisters.
- **Kindler Syndrome**: A rare form with similar symptoms but distinct genetic causes.

### Differential Diagnoses:

1. **Kindler Syndrome**: Characterized by skin blistering, photosensitivity, and progressive poikiloderma (skin changes).
2. **Porphyria**: A group of disorders affecting the skin and/or nervous system, some types can cause blistering on sun-exposed areas.
3. **Pemphigus Vulgaris**: An autoimmune disorder causing painful blistering of the skin and mucous membranes.
4. **Bullous Pemphigoid**: Another autoimmune blistering disorder more common in older adults.
5. **Dyskeratosis Congenita**: A rare genetic disorder with nail dystrophy, skin pigmentation changes, and oral leukoplakia.

### Conclusion:

Given the clinical manifestations you’ve described, Epidermolysis Bullosa (specifically Dystrophic EB or Junctional EB) is the most likely diagnosis. However, a definitive diagnosis would require further medical evaluation including genetic testing and possibly biopsies to confirm the subtype.

For accurate diagnosis and appropriate management, consultation with a dermatologist specializing in rare skin conditions and a geneticist is highly recommended.

# 53. User

The clinical presentation of patients varies with age, amount and duration of fructose exposure. In general, the younger the age of onset, the more severe the symptoms. The onset of the disease is typically around 6 months of age and usually occurs when fructose-containing foods, such as fruits, honey and certain vegetables, are first consumed. Common clinical symptoms include hypoglycaemic episodes after eating fructose-containing foods, with nausea, vomiting, abdominal pain, bloating, sweating, tremors, convulsions and even coma.

If the diagnosis is not clear and the patient chronically ingests fructose foods for a long period of time, in addition to hypoglycemic manifestations, growth retardation, hepatomegaly, ascites, jaundice and metabolic disorders such as hypoglycemia, metabolic acidosis, hypophosphatemia, hyperuricemia, hypermagnesemia, and hyperpyruvatemia may occur. In severe cases, multiple organ damage, liver and renal failure, convulsions, coma, and even death occur.

In acute attacks, laboratory tests reveal hypoglycaemia, hypophosphatemia and hypokalemia, accompanied by elevated uric acid, lactate, pyruvate, free fatty acids and triglycerides. Chronic patients present with hepatic impairment and indications of hepatic fibrosis, including serum bilirubin, elevated transaminases, and coagulation disorders. Urinalysis reveals proteinuria and fructose positivity.

Based on the clinical presentation you’ve described, the most likely diagnosis is **Hereditary Fructose Intolerance (HFI)**. This condition results from a deficiency in the enzyme aldolase B, leading to an inability to properly metabolize fructose and causing severe symptoms upon ingestion of fructose-containing foods.

### Key Features:

1. **Age of Onset**: Typically around 6 months of age, often occurring when the patient first consumes fructose-containing foods such as fruits, honey, or certain vegetables.
2. **Acute Symptoms**:
   - Hypoglycemic episodes
   - Nausea, vomiting
   - Abdominal pain, bloating
   - Sweating, tremors
   - Convulsions and even coma
3. **Chronic Symptoms**:
   - Growth retardation
   - Hepatomegaly (enlarged liver)
   - Ascites (accumulation of fluid in the abdomen)
   - Jaundice
   - Metabolic disorders such as hypoglycemia, metabolic acidosis, hypophosphatemia, hyperuricemia, hypermagnesemia, and hyperpyruvatemia
   - Severe cases may lead to multiple organ damage, liver and renal failure, convulsions, coma, and even death

### Laboratory Findings:

- **Acute Attacks**: Hypoglycemia, hypophosphatemia, and hypokalemia; elevated uric acid, lactate, pyruvate, free fatty acids, and triglycerides.
- **Chronic Patients**: Hepatic impairment with indications of hepatic fibrosis (elevated serum bilirubin, transaminases, coagulation disorders); proteinuria and fructose positivity on urinalysis.

### Differential Diagnoses:

1. **Fructose-1,6-bisphosphatase deficiency**: A disorder affecting gluconeogenesis with hypoglycemia, metabolic acidosis, and liver dysfunction.
2. **Glycogen storage disease type I (Von Gierke disease)**: This condition involves glycogen accumulation in the liver leading to hypoglycemia, lactic acidosis, and renal problems.

Given these clinical features and laboratory findings, HFI is the most likely diagnosis for this patient. It is crucial to confirm the diagnosis through specific enzyme assays or genetic testing to ensure appropriate management and dietary restrictions.

For further confirmation, consider conducting: - **Enzyme Assays**: Measurement of aldolase B activity in red blood cells. - **Genetic Testing**: Identification of mutations in the ALDOB gene.

If you need more detailed information on diagnosis and management, please let me know!

# 54. User

**1. Migraine** Migraine with aura is often the first symptom of the disease and is seen in 20-40% of patients, which is five times higher than in the general population; it can also manifest as migraine without aura, with an incidence not different from that of the general population. The incidence of migraine is lower in Asian patients than in European and American patients.

**2. Subcortical ischemic events** Transient ischemic attacks (TIAs) and ischemic strokes are the most common clinical manifestations of the disease, occurring in 60% to 85% of symptomatic patients, most of whom lack common risk factors for cerebrovascular disease. 2/3 of patients present with the classic lacunar syndrome.

**3. Cognitive deficits** Cognitive dysfunction with dementia develops progressively in 60% of patients.

**Affective disorders and apathy** 20% of patients have affective disorders, with depressive states being the most common, and rarely alternating between depressive and manic states; apathy is seen in 40% of patients.

In addition, seizures occur in 5-10% of patients, and hearing loss, impaired consciousness, spinal cord infarction, intracranial haemorrhage, and combined basin infarction have also been reported.

Based on the clinical manifestations you’ve provided, the disease most likely to be diagnosed is **CADASIL (Cerebral Autosomal Dominant Arteriopathy with Subcortical Infarcts and Leukoencephalopathy)**. CADASIL is a genetic disorder characterized by:

1. **Migraine**: Migraines are common in CADASIL, particularly those with aura, affecting 20-40% of patients, which is five times higher than in the general population. The incidence of migraine without aura is similar to that in the general population and is lower in Asian patients compared to European and American patients.
2. **Subcortical Ischemic Events**: Transient ischemic attacks (TIAs) and ischemic strokes are frequent, occurring in 60-85% of symptomatic patients. Most patients lack common risk factors for cerebrovascular disease, with about 2/3 presenting with the classic lacunar syndrome.
3. **Cognitive Deficits**: Cognitive dysfunction leading to dementia develops progressively in 60% of patients.
4. **Affective Disorders and Apathy**: Affective disorders are seen in 20% of patients, primarily depressive states, while apathy is observed in 40% of cases.
5. **Seizures**: Seizures occur in 5-10% of patients.
6. **Other Symptoms**: Additional symptoms include hearing loss, impaired consciousness, spinal cord infarction, intracranial hemorrhage, and combined basin infarctions.

CADASIL is a genetic condition caused by mutations in the NOTCH3 gene on chromosome 19, leading to progressive neurological decline characterized by these diverse clinical manifestations.

Here are five other possible diagnoses that could present with similar symptoms:

1. **Fabry Disease**: A genetic disorder affecting lysosomal storage, which can lead to strokes and cognitive impairment. It also includes neuropathic pain and angiokeratomas, though these specific symptoms were not mentioned.
2. **MELAS Syndrome (Mitochondrial Encephalomyopathy, Lactic Acidosis, and Stroke-like Episodes)**: This condition involves recurrent stroke-like episodes, encephalopathy, lactic acidosis, and progressive neurological decline, but it is less likely given the prevalence of migraines with aura in CADASIL.
3. **Hypoperfusion-Related Disorders**: Conditions such as cerebral small vessel disease or moyamoya syndrome can present with similar symptoms, including ischemic events and cognitive deficits.
4. **Vasculitis**: Certain types of vasculitis affecting small blood vessels can cause subcortical ischemic events, migraines, and cognitive decline.
5. **Hereditary Cerebrovascular Disorders**: Other genetic disorders such as CADASIL variants or related conditions might present similarly but are less common than CADASIL.

Given the detailed clinical picture provided, CADASIL is the most likely diagnosis based on the symptoms described. However, a thorough genetic and clinical evaluation would be necessary for definitive diagnosis.

# 55. User

The onset of the disease is usually insidious in early youth (but has been reported from 1 to 76 years of age). The main manifestation is a slowly progressive spastic paralysis of both lower limbs, with pyramidal signs (hyperreflexia, positive ankle clonus, positive pathology). Bilateral upper limb reflexes may also be hyperreflexic, but generally do not affect upper limb activity. More than half of the patients will have proximal weakness of the lower limbs. Decreased vibratory sensation of the ankle tuning forks (which usually does not disappear) is detected on examination in about 50% of patients, and urinary dysfunction is present in about one third of patients.

The disease progresses with age, with about 20% of patients eventually losing the ability to walk. The severity of clinical symptoms varies considerably between patients carrying the same mutation in the same family.

Based on the clinical manifestations you’ve described, the most likely diagnosis is **Hereditary Spastic Paraplegia (HSP)**. HSP is a group of inherited disorders characterized by progressive weakness and spasticity in the lower limbs. Here are the key points that support this diagnosis:

1. **Onset**: The disease typically begins insidiously during early youth but can be reported from 1 to 76 years of age.
2. **Main Manifestation**:
   - Slowly progressive spastic paralysis of both lower limbs with pyramidal signs (hyperreflexia, positive ankle clonus, positive Babinski sign).
   - Bilateral upper limb reflexes may also be hyperreflexic but generally do not affect upper limb activity.
3. **Proximal Weakness**: More than half of the patients will have proximal weakness in the lower limbs.
4. **Sensory Deficits**: Decreased vibratory sensation detected on examination in about 50% of patients using ankle tuning forks, which usually does not disappear.
5. **Urinary Dysfunction**: Present in approximately one-third of patients.
6. **Progression**: The disease progresses with age, and about 20% of patients eventually lose the ability to walk.
7. **Variability**: The severity of clinical symptoms can vary considerably between patients carrying the same mutation within the same family.

Given these characteristics, HSP is a strong candidate for this patient’s condition. However, it is important to consider other potential diagnoses that could present similarly:

1. **Primary Lateral Sclerosis (PLS)**: A rare motor neuron disease causing progressive weakness and stiffness in the legs.
2. **Multiple Sclerosis (MS)**: An autoimmune disease affecting the central nervous system, potentially leading to similar spasticity and gait disturbances.
3. **Adrenomyeloneuropathy (AMN)**: A form of adrenoleukodystrophy primarily affecting the spinal cord and peripheral nerves, leading to spastic paraparesis.
4. **Spinal Cord Tumor**: Though less likely given the genetic implications, a tumor could cause similar progressive spastic paraplegia.
5. **Tropical Spastic Paraparesis (TSP)/HTLV-1 associated myelopathy (HAM)**: A disease caused by the human T-lymphotropic virus type 1, leading to spasticity in the legs.

To definitively diagnose HSP or any of these other conditions, further diagnostic tests such as genetic testing, nerve conduction studies, and MRI scans would be necessary. These tests can help rule out other potential causes and confirm the specific diagnosis.

Would you like more information on any of these points or need assistance with further diagnostic steps?

# 56. User

Most of the affected children develop the disease in newborns and early infants. Clinical manifestations are similar to biotinidase deficiency, without specificity, mainly feeding difficulties, vomiting, diarrhoea, hypotonia, convulsions, depression, lethargy, respiratory difficulties, developmental delays, intractable rashes, desquamation, which can be combined with ketosis, metabolic acidosis, hyperlactic acidemia and so on. Skin damage in most patients in the head and face, neck, trunk, buttocks and other parts of the skin rash or erythema, ulceration or blisters, furfuraceous or flaky scales, or dry and rough skin, peeling skin, etc., and a few patients only in the perioral, periocular, perianal local appearance of the rash. Patients are not associated with hearing or visual impairment, unlike biotinidase deficiency.

Based on the clinical manifestations you’ve described, the most likely diagnosis is **holocarboxylase synthetase deficiency**. This condition is part of the biotin-thiamine-responsive basal ganglia disease spectrum and presents similarly to biotinidase deficiency but with some differences in symptoms and severity.

### Key Clinical Manifestations:

- **Neonatal Onset**: Most affected children develop the disease in newborns or early infants.
- **Clinical Symptoms**:
  - Feeding difficulties
  - Vomiting
  - Diarrhea
  - Hypotonia
  - Convulsions
  - Depression
  - Lethargy
  - Respiratory difficulties
  - Developmental delays
  - Intractable rashes, desquamation (peeling skin)
  - Ketosis
  - Metabolic acidosis
  - Hyperlactic acidemia
- **Skin Damage**:
  - Rash or erythema in the head and face, neck, trunk, buttocks, etc.
  - Ulceration or blisters
  - Furfuraceous (scaly) or flaky scales
  - Dry and rough skin with peeling

### Differential Diagnoses to Consider:

1. **Multiple Carboxylase Deficiency**: This includes both biotinidase deficiency and holocarboxylase synthetase deficiency. Symptoms overlap, and genetic testing is required to differentiate them.
2. **Propionic Acidemia**: A metabolic disorder that can cause similar symptoms, including metabolic acidosis, vomiting, hypotonia, and developmental delays.
3. **Methylmalonic Acidemia**: Another metabolic disorder presenting with similar metabolic derangements, such as metabolic acidosis and lethargy.
4. **Organic Acidemias**: A broader category that includes disorders like isovaleric acidemia and glutaric acidemia, which can present with metabolic crises and dermatologic manifestations.
5. **Maple Syrup Urine Disease (MSUD)**: Although not typically characterized by skin manifestations, it can cause metabolic acidosis and neurological symptoms, including lethargy and hypotonia.

Given the specific combination of feeding difficulties, vomiting, diarrhea, hypotonia, convulsions, rashes, and metabolic derangements, holocarboxylase synthetase deficiency is a strong candidate. Genetic testing will be crucial to confirm the diagnosis and differentiate it from other similar conditions.

Would you like more information on any specific aspect of this condition or need further diagnostic guidance?

# 57. User

Children are born without abnormalities, and the clinical presentation in infancy is not specific and is characterised only by growth and developmental delays. It is often diagnosed at 3 years of age after the onset of ocular signs and symptoms.

1. visual impairment, myopia and iris tremor due to ectopic lens may appear after 3 years of age, and may be combined with astigmatism, glaucoma, staphylococcus, cataract, retinal detachment and optic nerve atrophy in later stages. 2. intellectual disability is common.
2. Intellectual disability is more common, mainly manifested as progressive mental retardation. Vitamin B6 treatment-sensitive patients are less mentally impaired. 3.
3. mental or behavioural disorders, which may occur in about 50% of children and are associated with convulsions in 20%.
4. Skeletal involvement, similar to the manifestations of Marfontein syndrome, or in combination with scoliosis, funnel chest, chicken chest, knee valgus, and bowed feet, with generalised osteoporosis seen on X-ray.
5. Thrombophilia, which occurs at all ages and may involve all types of blood vessels, especially the cerebral vessels. High levels of homocysteinemia may alter the vessel wall and increase platelet adhesion, resulting in optic nerve atrophy, paralysis, pulmonary heart disease, and renal hypertension.
6. Spontaneous pneumothorax and acute pancreatitis are less common.

Based on the clinical manifestations you’ve described, the most likely diagnosis is **Homocystinuria**. This condition fits well with the ocular findings, skeletal abnormalities, intellectual disability, and thrombotic events mentioned.

### Key Clinical Manifestations:

1. **Ocular Symptoms**:
   - Visual impairment, myopia, and iris tremor due to ectopic lens may appear after 3 years of age.
   - Other ocular issues include astigmatism, glaucoma, staphylococcus, cataract, retinal detachment, and optic nerve atrophy in later stages.
2. **Intellectual Disability**:
   - Common, often表现为进行性智力减退。
   - Vitamin B6 treatment can help reduce the severity of intellectual disability.
3. **Mental or Behavioral Disorders**:
   - Occur in about 50% of children and are associated with convulsions in 20%.
4. **Skeletal Involvement**:
   - Similar to Marfan syndrome, but may include scoliosis, funnel chest, chicken breast, knee valgus, and bowed feet.
   - Generalized osteoporosis seen on X-ray.
5. **Thrombophilia**:
   - Occurs at all ages and can involve various blood vessels, especially cerebral vessels.
   - High levels of homocysteinemia may lead to optic nerve atrophy, paralysis, pulmonary heart disease, and renal hypertension.
6. **Other Less Common Manifestations**:
   - Spontaneous pneumothorax and acute pancreatitis are less common but still possible.

### Differential Diagnoses to Consider:

1. **Marfan Syndrome**: Connective tissue disorder with ocular, skeletal, and cardiovascular abnormalities.
2. **Ehlers-Danlos Syndrome**: A group of connective tissue disorders that can present with similar symptoms.
3. **Homocystinuria**: The most likely diagnosis given the specific combination of clinical features.
4. **Methylmalonic Acidemia**: Metabolic disorder causing developmental delays and neurological issues.
5. **Propionic Acidemia**: Another metabolic disorder presenting with similar symptoms.

### Diagnostic Testing:

- **Biochemical Tests**: Measurement of homocysteine levels in blood and urine.
- **Genetic Testing**: To confirm the diagnosis by identifying mutations in the genes responsible for cystathionine beta-synthase (CBS) deficiency.

Given these clinical findings, a thorough biochemical and genetic evaluation is recommended to confirm the diagnosis. If Homocystinuria is suspected, early intervention with vitamin B6 supplementation can help manage some of the symptoms and improve outcomes.

Would you like more information on specific diagnostic tests or treatment options?

# 58. User

The main manifestations of the disease are markedly elevated levels of LDL-C from birth, cholesterol deposits in the skin, eyes and tendons, and the formation of xanthomas and lipomatous corneal arches. As a result of exposure to high levels of LDL-C, most patients develop coronary atherosclerotic heart disease before the age of 20 and may die as a result.

A detailed family history is needed, as some patients have heterozygous parents and therefore have elevated LDL-C levels (usually greater than the 95th percentile of their country’s age- and sex-specific norms) and a strong positive family history of premature atherosclerotic cardiovascular disease.

Based on the clinical manifestations you provided, the most likely diagnosis is **Homozygous Familial Hypercholesterolemia (HoFH)**. This rare genetic disorder is characterized by extremely high levels of low-density lipoprotein cholesterol (LDL-C) from birth, leading to early-onset atherosclerotic cardiovascular disease, xanthomas, and other cholesterol deposits.

### Key Clinical Manifestations:

1. **Elevated LDL-C Levels**: Markedly elevated LDL-C from birth.
2. **Cholesterol Deposits**:
   - Skin: Xanthomas (fatty deposits under the skin).
   - Eyes: Cholesterol deposits in the cornea, forming lipomatous corneal arches.
   - Tendons: Tendon xanthomas.
3. **Cardiovascular Disease**: Most patients develop coronary atherosclerotic heart disease before the age of 20 and may die as a result.

### Diagnostic Considerations:

- **Detailed Family History**: Some patients have heterozygous parents, leading to elevated LDL-C levels (usually greater than the 95th percentile of their country’s age- and sex-specific norms) and a strong positive family history of premature atherosclerotic cardiovascular disease.
- **Laboratory Tests**:
  - Measurement of LDL-C levels in blood.
  - Genetic testing for mutations in the LDLR, APOB, or PCSK9 genes.

### Differential Diagnoses to Consider (Less Likely Given Specific Findings):

1. **Heterozygous Familial Hypercholesterolemia (HeFH)**: Although less severe than HoFH, it can still lead to elevated LDL-C levels and an increased risk of cardiovascular disease.
2. **Sitosterolemia**: A rare metabolic disorder leading to increased absorption and decreased excretion of plant sterols and cholesterol, causing xanthomas and early atherosclerosis.
3. **Polygenic Hypercholesterolemia**: A condition where multiple genetic factors contribute to high cholesterol levels, though it is typically less severe than familial hypercholesterolemia.
4. **Type III Hyperlipoproteinemia (Dysbetalipoproteinemia)**: Characterized by elevated cholesterol and triglycerides, along with xanthomas, although the lipid profile is different from that of HoFH.
5. **Lecithin-Cholesterol Acyltransferase (LCAT) Deficiency**: A rare disorder affecting cholesterol metabolism.

Given the specific clinical manifestations and family history, HoFH remains the most likely diagnosis. Further diagnostic testing, including genetic analysis, would be recommended to confirm the diagnosis.

Would you like more information on diagnostic procedures or treatment options for HoFH?

# 59. User

It is characterised by a triad of movement disorders, psychiatric symptoms and cognitive deficits, usually with an insidious onset and slow progression. The age of onset ranges from childhood to 79 years, with the most common being 30-50 years of age.

The disease involves rapid, involuntary, non-rhythmic movements of the face, trunk, and limbs. Early abnormal movements are mild, and the patient may have difficulty recognising the presence of chorea or integrating chorea-like movements into random movements, which makes the control of normal movements difficult or deviant; as the disease progresses, the extent and degree of chorea often increases, and even affects the diaphragm, pharynx, and laryngeal muscles, resulting in dysarthria, dysphagia, and involuntary movement. As the disease progresses, the extent and degree of chorea often increases, even affecting the pharyngeal and laryngeal muscles, resulting in dysarthria, dysphagia, and involuntary vocalisations. In the later stages of the disease, the chorea often disappears, and is replaced by a Parkinson’s-like pattern of stiffness and hyperkinesis, which may be accompanied by focal dystonia. Difficulty in maintaining movement is also a common manifestation of the disease, i.e., the inability to maintain certain simple voluntary movements, such as difficulty in maintaining tongue extension.

Mental disorders may precede chorea, and common symptoms include depressed mood, depression, irritability, apathy, and anxiety, as well as paranoia, delusions, and hallucinations; rare symptoms include obsessive-compulsive behaviours and psychotic manifestations, which tend to worsen progressively.

Cognitive impairment is most often characterised by executive dysfunction, which is manifested by a reduced ability to make decisions, multi-task and shift cognitive goals. Patients usually lack self-awareness of their cognitive deficits. Memory loss or impairment occurs late in the course of the disease and progresses to dementia as the disease progresses.

In addition to the triad of symptoms, eye movement abnormalities are also prominent. Early in the disease, there is a slowing down of eye-sweeping speed, but eye-following movements are preserved; in later stages, eye-following movements, voluntary sweeping and refixation are all impaired. In addition, weight loss and cachexia are common features of the disease, with patients experiencing significant weight loss as a result of the high energy expenditure of involuntary movements, usually without loss of appetite. Sleep and/or sexual dysfunction are also common.

Progressive deterioration of motor and cognitive function can lead to significant complications, such as dysphagia due to involvement of the pharyngeal muscles, dyskinesia, reduced activity and even bed rest, which can lead to aspiration pneumonia and other infections, and decubitus ulcers.

Based on the provided description, the disease you are describing aligns with **Huntington’s Disease (HD)**. Huntington’s Disease is a rare genetic disorder characterized by a triad of movement disorders, psychiatric symptoms, and cognitive deficits, typically with an insidious onset and slow progression.

### Key Clinical Manifestations:

1. **Movement Disorders**:
   - **Chorea**: Rapid, involuntary, non-rhythmic movements involving the face, trunk, and limbs.
     - Early on, chorea is mild and may be difficult for the patient to recognize or integrate into normal movements.
     - As the disease progresses, chorea becomes more pronounced and can affect muscles involved in breathing (diaphragm), speech (pharynx and laryngeal muscles), leading to dysarthria, dysphagia, and involuntary vocalizations.
     - In later stages, chorea may diminish and be replaced by a Parkinson’s-like pattern of stiffness and hyperkinesis, which can include focal dystonia.
   - **Difficulty in Maintaining Movement**: Patients struggle with maintaining simple voluntary movements, such as tongue extension.
2. **Psychiatric Symptoms**:
   - Early symptoms often include mood disorders like depression, irritability, apathy, anxiety, paranoia, delusions, and hallucinations.
   - Rare symptoms may include obsessive-compulsive behaviors and psychotic manifestations that worsen over time.
3. **Cognitive Impairment**:
   - Executive dysfunction: Reduced ability to make decisions, multitask, and shift cognitive goals.
   - Patients often lack self-awareness of their cognitive deficits.
   - Memory loss or impairment occurs later in the disease course and progresses to dementia.
4. **Eye Movement Abnormalities**:
   - Early in the disease, there is a slowing down of eye-sweeping speed.
   - In later stages, eye-following movements, voluntary sweeping, and refixation are all impaired.
5. **Additional Features**:
   - Weight loss and cachexia: High energy expenditure from involuntary movements without loss of appetite.
   - Sleep and/or sexual dysfunction: Common in patients with HD.
   - Complications due to progressive deterioration:
     - Dysphagia leading to aspiration pneumonia and other infections.
     - Reduced activity and bed rest, which can result in decubitus ulcers.

### Diagnosis and Management:

- **Genetic Testing**: Confirmatory diagnosis through genetic testing for the Huntington’s disease mutation (HTT gene).
- **Management**:
  - Medications: Antipsychotics, antidepressants, and anticonvulsants to manage psychiatric symptoms.
  - Speech Therapy: To address dysarthria and dysphagia.
  - Physical Therapy: To manage movement disorders and maintain muscle strength.
  - Cognitive Behavioral Therapy (CBT): To help with mood and cognitive issues.

### Prognosis:

- Progressive deterioration of motor and cognitive function, leading to significant complications such as aspiration pneumonia and decubitus ulcers.

Huntington’s Disease is a complex condition that requires multidisciplinary management involving neurologists, psychiatrists, physical therapists, and other healthcare professionals. Early diagnosis and intervention can help manage symptoms and improve quality of life for patients and their families.

# 60. User

Clinical signs and symptoms are mainly neurological and vary in severity. The age of onset can range from neonatal to adulthood (late-onset), and there is a broad phenotypic spectrum.

1. In the neonatal period, symptoms associated with hyperammonemia [including lethargy, drowsiness, refusal to eat, vomiting with respiratory alkalosis and/or seizures] appear after about 24-48 hours of asymptomatic onset. It is indistinguishable from other urea cycle disorders of neonatal onset.
2. Late-onset, which accounts for 88 per cent of patients, 40 per cent of whom occur before the age of 3 years, 29 per cent in childhood and 19 per cent in adulthood. The main manifestations are: (i) chronic neurocognitive deficits, including developmental delay, ataxia, spasticity, learning disabilities, cognitive deficits, and/or unexplained seizures; (ii) acute encephalopathy secondary to hyperammonaemic crisis induced by a variety of factors (e.g., infections, fasting, or injuries); and (iii) chronic hepatic dysfunction, with unexplained elevation of hepatic aminotransferases with or without mild coagulation disorders, with or without mild hyperammonaemia, and protein intolerance. Ammonemia and protein intolerance.

Based on the provided clinical signs and symptoms, the condition described is consistent with **Ornithine Transcarbamylase (OTC) Deficiency**, a rare X-linked urea cycle disorder. OTC deficiency is characterized by varying severity and age of onset, ranging from neonatal to adulthood.

### Key Clinical Manifestations:

1. **Neonatal Onset**:
   - Symptoms associated with hyperammonemia appear after about 24-48 hours of asymptomatic onset.
   - Common symptoms include lethargy, drowsiness, refusal to eat, vomiting, and respiratory alkalosis, which may be accompanied by seizures.
   - These symptoms are indistinguishable from other urea cycle disorders that present in the neonatal period.
2. **Late-Onset (88% of Patients)**:
   - 40% occur before the age of 3 years.
   - 29% occur during childhood.
   - 19% occur in adulthood.
   - Main manifestations include:
     - **Chronic Neurocognitive Deficits**: Developmental delay, ataxia, spasticity, learning disabilities, cognitive deficits, and/or unexplained seizures.
     - **Acute Encephalopathy**: Secondary to hyperammonaemic crises triggered by various factors such as infections, fasting, or injuries.
     - **Chronic Hepatic Dysfunction**: Unexplained elevation of hepatic aminotransferases with or without mild coagulation disorders, and protein intolerance.

### Additional Considerations:

- **Other Urea Cycle Disorders**:
  - **Carbamoyl Phosphate Synthetase I (CPS1) Deficiency**: Another urea cycle disorder that can present with severe neonatal hyperammonemia.
  - **Argininosuccinate Synthetase Deficiency (Citrullinemia Type I)**: This condition also leads to hyperammonemia and can cause similar neurological and hepatic symptoms.

### Diagnosis and Management:

- **Genetic Testing**: Confirmatory diagnosis through genetic testing for the OTC gene.
- **Management**:
  - **Acute Hyperammonemic Crises**: Treatment with intravenous arginine, sodium benzoate, or other medications to manage hyperammonemia.
  - **Long-Term Management**: Dietary restrictions and periodic monitoring of liver function tests.

### Prognosis:

- The prognosis varies depending on the severity and management of the condition. Early diagnosis and intervention can help manage symptoms and improve quality of life.

Given the broad phenotypic spectrum and the specific clinical features, OTC Deficiency is a strong candidate for this presentation. However, further diagnostic testing may be necessary to rule out other urea cycle disorders.

Would you like more detailed information on any aspect of OTC Deficiency or its management?

# 61. User

Some children have no clinical symptoms in the neonatal period, but after 3-4 months of life, the typical symptoms appear gradually: the hair turns from black to yellow, the skin is light-coloured, and the urine and sweat are foul-smelling. As the child grows older, he/she gradually shows backward intellectual development, microcephaly, epileptic seizures, and may also show abnormalities in behaviour, personality, and neurocognition. For example, hyperactivity, self-mutilation, aggression, autism, low self-esteem, depression, etc. Vomiting and eczema are also common in infancy.

In some patients, there are no clinical symptoms in the neonatal period, and after 1-3 months of life, in addition to the symptoms of PKU, the main manifestations are dyskinesia, lethargy, hypotonia of the trunk, increased or decreased muscle tone of the limbs, nystagmus, dysphagia, increased drooling, flaccidity, torticollis, unresponsiveness, insomnia, and severe intellectual developmental disorders, and so on.

Based on the clinical manifestations described, the most likely diagnosis is **Phenylketonuria (PKU)**. PKU is a rare genetic disorder caused by a deficiency of the enzyme phenylalanine hydroxylase, leading to the accumulation of phenylalanine in the body. The symptoms you mentioned, such as changes in hair and skin pigmentation, intellectual disability, neurological issues, and a musty odor in the urine and sweat, are classic signs of untreated PKU.

### Key Symptoms of PKU:

1. **Hair and Skin Changes**:
   - Hair turns from black to yellow.
   - Skin becomes lighter in color.
   - Urine and sweat have a foul-smelling, musty odor (due to phenylacetic acid).
2. **Intellectual Development and Neurological Issues**:
   - Gradual backward intellectual development.
   - Microcephaly (small head size).
   - Epileptic seizures.
   - Behavioral abnormalities such as hyperactivity, self-mutilation, aggression, autism, low self-esteem, depression.
3. **Infancy Symptoms**:
   - Vomiting and eczema are common in infancy.

### Other Possible Diagnoses to Consider:

1. **Hyperphenylalaninemia**: This refers to a group of conditions with elevated levels of phenylalanine, including variants of PKU with milder symptoms or other enzyme deficiencies affecting phenylalanine metabolism.
2. **Tyrosinemia Type I**: This is another metabolic disorder that can present with neurological symptoms, failure to thrive, and liver problems, although it typically has more severe hepatic manifestations.
3. **Maple Syrup Urine Disease (MSUD)**: This is a disorder of branched-chain amino acid metabolism. Symptoms include a characteristic maple syrup smell in urine and sweat, as well as neurological issues.

### Diagnostic Steps:

- **Genetic Testing**: Confirmatory diagnosis through genetic testing for the PKU gene.
- **Biochemical Tests**: Measurement of phenylalanine levels in blood or urine.
- **Clinical Evaluation**: Comprehensive evaluation by a pediatrician or metabolic specialist to assess developmental and neurological status.

Given the symptoms described, it is crucial to perform appropriate diagnostic tests to confirm the diagnosis and initiate treatment as early as possible. Early intervention with a low-phenylalanine diet can significantly improve outcomes in PKU patients.

If you need further assistance or have specific questions about these conditions, feel free to ask!

# 62. User

The severity of clinical manifestations is highly variable. Patients may present with abnormalities in bone mineralisation, dental or joint pathology, muscle weakness, etc. Currently, there are seven main types of the disease.

1. simple dental type, which is the mildest and most common type, with only dental involvement and no rickets or osteomalacia. 2. adult type, which often develops in middle age.
2. Adult type, which often develops in middle age and presents with dental abnormalities or osteochondrosis. It may cause impaired mobility due to repeated fractures, bone and joint pain, or muscle weakness. 3.
3. the childhood type, which usually develops after 6 months of age, can be mild to severe, and the children may have different degrees of tooth loss and rickets.
4. the infantile form, with onset before 6 months of age, is often poorly ventilated, difficult to survive, debilitating, and may have a wide fontanelle or cranial deformity. 50 per cent of infantile HPP die of complications. 5. the perinatal form, with onset before 6 months of age, is the most severe form of HPP, with onset before 6 months of age.
5. The perinatal form, which is the extreme form, develops in utero, is evident at birth, and is almost always fatal. Children with short limbs and severe skeletal undermineralisation may present with high-pitched crying, seizures, periodic apnoea with cyanosis and bradycardia, unexplained fever, irritability, myelosuppressive anaemia and intracranial haemorrhage, and pulmonary atelectasis.
6. Pseudohypophosphatasia, similar to the infantile form, in which the patient has the manifestations of infantile hypophosphatasia with normal or elevated serum ALP. 7.
7. Benign type, sometimes presenting with skeletal malformations in utero, with perinatal hypophosphatase activity, but resolving after birth without the associated manifestations of infantile hypophosphatasia or simple dental hypophosphatasia.

Based on the clinical manifestations described, the disease you are referring to is most likely **Hypophosphatasia (HPP)**. HPP is a rare genetic disorder characterized by varying degrees of severity and can manifest in several types:

1. **Simple Dental Type**: This is the mildest and most common type, involving only dental abnormalities with no rickets or osteomalacia.
2. **Adult Type**: Often develops in middle age and presents with dental abnormalities or osteochondrosis. It may cause impaired mobility due to repeated fractures, bone and joint pain, or muscle weakness.
3. **Childhood Type**: Usually develops after 6 months of age, can be mild to severe, and children may have different degrees of tooth loss and rickets.
4. **Infantile Form**: With onset before 6 months of age, this form is often poorly ventilated, difficult to survive, debilitating, and may present with a wide fontanelle or cranial deformity. Approximately 50% of infantile HPP cases result in death due to complications.
5. **Perinatal Form**: The most severe form, developing in utero and evident at birth, is almost always fatal. Children with short limbs and severe skeletal undermineralization may present with high-pitched crying, seizures, periodic apnea with cyanosis and bradycardia, unexplained fever, irritability, myelosuppressive anemia, intracranial hemorrhage, and pulmonary atelectasis.
6. **Pseudohypophosphatasia**: Similar to the infantile form but with normal or elevated serum ALP levels.
7. **Benign Type**: Sometimes presenting with skeletal malformations in utero, with perinatal hypophosphatase activity that resolves after birth without associated manifestations of infantile hypophosphatasia or simple dental hypophosphatasia.

Given the described symptoms and the varying degrees of severity, HPP fits the clinical profile provided.

# 63. User

Children with square skulls, chicken breasts, beaded ribs, bracelet sign, foot bracelet sign, Henry’s groove, and lower limb deformities, which may be inversion of the knee (O-shaped leg) or eversion of the knee (X-shaped leg), appearing when they start weight-bearing near the age of one week, are accompanied by growth retardation, short stature, wobbly gait, progressively aggravating bone deformities, multiple bone fractures, bone pain, and abnormalities of tooth development (poor tooth quality, toothache, difficulty in regeneration after loss). Imaging is characterised by skeletal deformities, such as Imaging manifestations include skeletal deformities, widening and blurring of the epiphysis of the long bones, a cupped shape, and many fine strips of calcification like brushes in the cupped shape. In adults, the main manifestations are limb weakness, activity limitation, bone pain, multiple pathological fractures (long bones of the limbs, ribs, pelvis and vertebrae can occur), and shortening of height. Imaging manifestations include a generalised decrease in bone density, blurred bone trabeculae with a hairy glass appearance, pelvic deformity, false fracture lines, and biconcave deformity of the vertebral body.

Based on the clinical manifestations and imaging findings you have described, the condition most likely corresponds to **X-linked Hypophosphatemic Rickets (XLH)**. Here is a detailed explanation:

### Clinical Manifestations:

- **Bony Deformities**:
  - Square skull (craniotabes)
  - Pectus carinatum (chicken breast)
  - Costochondral beading (beaded ribs)
  - Bracelet sign and foot bracelet sign
  - Henry’s groove
  - Lower limb deformities, including O-shaped legs (genu valgum) or X-shaped legs (genu varum)
- **Growth Retardation**:
  - Short stature
  - Wobbly gait
- **Bone Pain and Fractures**:
  - Multiple bone fractures
  - Progressive bone deformities
  - Bone pain
- **Tooth Development Abnormalities**:
  - Poor tooth quality
  - Toothache
  - Difficulty in regeneration after loss

### Imaging Findings:

- **Long Bones**:
  - Widening and blurring of the epiphyses
  - Cupped shape with fine strips of calcification resembling brushes
- **Generalized Decrease in Bone Density**:
  - Blurred trabeculae with a “hairy glass” appearance
  - Pelvic deformities
  - False fracture lines
  - Biconcave deformity of the vertebral body

### Differential Diagnoses to Consider:

1. **Osteogenesis Imperfecta**: Characterized by brittle bones, fractures, and growth issues.
2. **Hypophosphatasia**: A rare metabolic bone disease leading to rickets-like symptoms, bone pain, and fractures.
3. **Metaphyseal Dysplasia (Pyle’s Disease)**: Rare bone disorder causing widened metaphyses.

### Conclusion:

The combination of clinical signs such as square skull, bony deformities, growth retardation, bone pain, and imaging findings like widening epiphyses and blurred trabeculae strongly suggests **X-linked Hypophosphatemic Rickets (XLH)**. This condition is characterized by impaired mineralization due to a defect in the PHEX gene, leading to phosphate wasting and subsequent rickets-like symptoms.

For further confirmation and management, genetic testing for PHEX mutations may be recommended. Treatment typically involves phosphorus supplementation and active vitamin D analogs to manage the symptoms and prevent complications.

If you need more specific diagnostic or treatment recommendations, please consult a specialist in metabolic bone diseases.

# 64. User

Clinical manifestations include heart failure, chest pain, hypotension, syncope, sudden cardiac death, arrhythmias and thromboembolism. Some patients also have extracardiac manifestations such as peripheral neuropathy, autonomic neuropathy and gastrointestinal symptoms.

Based on the clinical manifestations you’ve described—heart failure, chest pain, hypotension, syncope, sudden cardiac death, arrhythmias, and thromboembolism, along with extracardiac manifestations such as peripheral neuropathy, autonomic neuropathy, and gastrointestinal symptoms—the rare disease most likely to be diagnosed is **Cardiac Amyloidosis**.

### Key Features of Cardiac Amyloidosis:

- **Cardiac Symptoms:**
  - Heart failure
  - Chest pain
  - Hypotension
  - Syncope
  - Sudden cardiac death
  - Arrhythmias
  - Thromboembolism
- **Extracardiac Manifestations:**
  - Peripheral neuropathy
  - Autonomic neuropathy
  - Gastrointestinal symptoms

### Other Possible Diagnoses:

1. **Hypertrophic Cardiomyopathy (HCM):**
   - Primarily affects the heart, causing thickening of the myocardium.
   - Can lead to heart failure, arrhythmias, and syncope.
2. **Arrhythmogenic Right Ventricular Cardiomyopathy (ARVC):**
   - Characterized by progressive replacement of cardiac muscle with fatty and fibrous tissue in the right ventricle.
   - Often results in arrhythmias and sudden cardiac death.
3. **Fabry Disease:**
   - A lysosomal storage disorder affecting multiple organ systems, including the heart.
   - Can cause hypertrophic cardiomyopathy, arrhythmias, and conduction blocks.
4. **Sarcoidosis:**
   - An inflammatory disease that can affect various organs, including the heart.
   - Cardiac involvement can lead to heart failure, arrhythmias, and sudden cardiac death.
5. **Chagas Disease:**
   - A parasitic infection caused by Trypanosoma cruzi.
   - Can cause chronic heart issues like heart failure and arrhythmias.
   - Often presents with peripheral and autonomic neuropathy and gastrointestinal symptoms.

### Diagnostic Considerations:

- **Cardiac Amyloidosis:** Confirmation typically requires a biopsy of the affected tissue to identify amyloid deposits. Additional tests may include echocardiography, MRI, and genetic testing for specific mutations.
- **HCM and ARVC:** Diagnosed through echocardiography, genetic testing, and sometimes endomyocardial biopsy.
- **Fabry Disease:** Confirmed by enzyme assays and genetic testing.
- **Sarcoidosis:** Diagnosed via imaging (CT, MRI), biopsy, and blood tests for inflammation markers.
- **Chagas Disease:** Diagnosed through serology tests, echocardiography, and sometimes cardiac MRI.

For a precise diagnosis, it is crucial to perform a thorough clinical evaluation and appropriate diagnostic tests. Consulting with specialists such as cardiologists, neurologists, and infectious disease experts would be beneficial.

# 65. User

1. secondary sexual characteristics and impaired gametogenesis in males: childish voice, small penis, no pubic hair growth, small testes or cryptorchid testes, no spermatogenesis; in females: mammary gland dysplasia, infantile vulva, primary amenorrhoea.
2. delayed epiphyseal closure upper volume/lower volume <1, fingertip distance > height, susceptible to osteoporosis.
3. Olfactory disorders 40% ~60% of IHH patients suffer from loss of sense of smell due to abnormal development of the olfactory bulb and olfactory tract, and are unable to identify odours.
4. Other manifestations Facial midline defects, such as cleft lip and palate; isolated kidneys; short fingers (toes), syndactyly; skeletal deformities or dental hypoplasia; overweight and obesity; mirror image movement.

Based on the clinical manifestations you’ve provided, the most likely rare disease to be diagnosed is **Isolated Hypogonadotropic Hypogonadism (IHH)**. This condition is characterized by a combination of hypogonadotropic hypogonadism and other associated features such as delayed epiphyseal closure, skeletal abnormalities, and olfactory disorders.

### Key Features of Isolated Hypogonadotropic Hypogonadism (IHH):

1. **Secondary Sexual Characteristics and Impaired Gametogenesis:**
   - In males: Childish voice, small penis, no pubic hair growth, small testes or cryptorchid testes, no spermatogenesis.
   - In females: Mammary gland dysplasia, infantile vulva, primary amenorrhoea.
2. **Delayed Epiphyseal Closure and Skeletal Abnormalities:**
   - Delayed epiphyseal closure (upper volume/lower volume <1).
   - Fingertip distance > height.
   - Susceptibility to osteoporosis.
3. **Olfactory Disorders:**
   - 40% to 60% of IHH patients suffer from loss of sense of smell due to abnormal development of the olfactory bulb and olfactory tract, and are unable to identify odours.
4. **Other Manifestations:**
   - Facial midline defects such as cleft lip and palate.
   - Isolated kidneys.
   - Short fingers (toes), syndactyly.
   - Skeletal deformities or dental hypoplasia.
   - Overweight and obesity.
   - Mirror image movement.

### Other Possible Diagnoses:

1. **Kallmann Syndrome**: Characterized by a combination of hypogonadotropic hypogonadism and anosmia, along with midline defects and skeletal abnormalities.
2. **Congenital Hypogonadotropic Hypogonadism (CHH) without anosmia**: Similar to IHH but typically without the loss of smell.
3. **Prader-Willi Syndrome**: Presents with hypogonadism, obesity, and characteristic behavioral features, though not usually associated with anosmia.
4. **CHARGE Syndrome**: A genetic condition that includes coloboma, heart defects, atresia of the choanae, growth retardation, genital abnormalities, and ear abnormalities.
5. **Turner Syndrome (in females)**: Primarily a chromosomal condition that can present with some overlapping features.

For accurate diagnosis, it is important to perform comprehensive evaluations including hormonal assessments, genetic testing, imaging studies, and detailed clinical examinations. Consulting with specialists such as endocrinologists, geneticists, and orthopedic surgeons would be beneficial.

# 66. User

The most common symptom is shortness of breath after activity, and other symptoms include fatigue, dizziness, chest pain, chest tightness, palpitations, blackouts, and syncope, etc. Once syncope or vertigo occurs, it suggests that the patient’s cardiac output may have decreased significantly. Due to atypical symptoms and signs, the majority of patients have a significant delay in consultation, with at least 1/5 of patients taking more than 2 years from the onset of symptoms to diagnosis; patients may have a hyperactive second heart sound of the pulmonary valve, and tricuspid insufficiency may cause a systolic murmur in the tricuspid region; signs of right heart failure include jugular venous congestion or rupture, enlarged liver, oedema of the lower extremities, multiple plasma cavities, jaundice, and cyanosis, among other signs.

Based on the clinical manifestations provided, the most likely rare disease that fits the described symptomatology is **Pulmonary Arterial Hypertension (PAH)**. This condition is characterized by increased pressure within the pulmonary arteries, leading to right heart strain and failure, which aligns with the symptoms of shortness of breath after activity, fatigue, syncope, and signs of right heart failure described.

### Key Features of Pulmonary Arterial Hypertension (PAH):

1. **Symptoms:**
   - Shortness of breath after activity.
   - Fatigue.
   - Dizziness.
   - Chest pain or tightness.
   - Palpitations.
   - Blackouts and syncope.
2. **Signs Suggestive of PAH:**
   - Hyperactive second heart sound (S2) of the pulmonary valve.
   - Tricuspid insufficiency causing a systolic murmur in the tricuspid region.
   - Signs of right heart failure:
     - Jugular venous congestion or rupture.
     - Enlarged liver.
     - Edema of the lower extremities.
     - Multiple pleural effusions (cavities).
     - Jaundice and cyanosis.

### Other Possible Diagnoses:

1. **Chronic Thromboembolic Pulmonary Hypertension (CTEPH):**
   - This condition involves unresolved blood clots in the lungs, leading to similar symptoms of PAH.
2. **Eisenmenger Syndrome:**
   - A type of congenital heart defect resulting in pulmonary hypertension, causing symptoms such as cyanosis and syncope.
3. **Constrictive Pericarditis:**
   - This condition can lead to reduced cardiac output due to the thickened pericardium constraining the heart.
4. **Cardiomyopathy:**
   - Various forms of cardiomyopathy, particularly those affecting the right side of the heart, can cause these symptoms.
5. **Cor Pulmonale:**
   - Right heart failure caused by chronic lung disease.

Given the atypical nature of the symptoms and the significant delay in diagnosis (with 1/5 of patients taking more than 2 years from symptom onset to diagnosis), it is crucial for patients presenting with these symptoms to undergo comprehensive evaluations, including echocardiography, right heart catheterization, and other relevant tests.

For accurate diagnosis and appropriate management, consulting a cardiologist specializing in pulmonary hypertension or related conditions would be highly recommended.

# 67. User

The disease is usually insidious. The main manifestations are dry cough, exertional dyspnoea, pestle-like fingers (toes), and bursting sounds distributed mainly in both lower lungs are its typical signs. In the terminal stage, cyanosis, pulmonary hypertension, pulmonary heart disease and right heart insufficiency can be seen.

Based on the clinical manifestations you’ve described, the most likely diagnosis is **Idiopathic Pulmonary Fibrosis (IPF)**. IPF is characterized by a progressive and chronic scarring of the lung tissue, which leads to a persistent dry cough, exertional dyspnea, and clubbing of fingers and toes. The “bursting” sounds you mentioned are typically referred to as “velcro-like” crackles heard on auscultation, usually located in the lower lung fields.

In advanced stages, IPF can lead to complications such as cyanosis, pulmonary hypertension, pulmonary heart disease, and right heart insufficiency. These symptoms indicate significant impairment of lung function and potential cardiovascular involvement.

Given these clinical features, a thorough evaluation is necessary. This should include: - **History and Physical Examination**: To assess the duration and progression of symptoms. - **Imaging Studies**: High-resolution computed tomography (HRCT) of the chest to visualize the extent of fibrosis. - **Pulmonary Function Tests**: To evaluate lung function, including diffusion capacity for carbon monoxide (DLCO). - **Bronchoalveolar Lavage (BAL)**: In some cases, to rule out other causes of interstitial lung disease. - **Lung Biopsy**: Often required for definitive diagnosis, especially if the clinical picture is atypical or if there are concerns about alternative diagnoses.

Other possible conditions that may need consideration include: 1. **Chronic Hypersensitivity Pneumonitis**: Characterized by dry cough, dyspnea, and crackles due to an immune response to inhaled environmental antigens. 2. **Non-Specific Interstitial Pneumonia (NSIP)**: Another interstitial lung disease with similar symptoms but different histological features compared to IPF. 3. **Sarcoidosis**: A systemic granulomatous disease that can affect the lungs and cause similar respiratory symptoms, although clubbing is less common. 4. **Asbestosis**: Caused by inhalation of asbestos fibers, presenting with similar respiratory symptoms and potential pulmonary fibrosis and clubbing. 5. **Connective Tissue Disease-associated Interstitial Lung Disease (CTD-ILD)**: Diseases like scleroderma or rheumatoid arthritis can involve the lungs and cause similar symptoms.

A multidisciplinary approach involving pulmonologists, cardiologists, and possibly other specialists will be essential for accurate diagnosis and appropriate management.

# 68. User

Children with cholestasis and cirrhosis develop. The main clinical manifestations are hyperconjugated bilirubinaemia and often malabsorption of fat-soluble vitamins, such as rickets. The clinical phenotypes of different enzyme defects are slightly different. For example, oxysterol 7α-hydroxylase defects are associated with hereditary spastic paralysis in adulthood; cholesterol 25-hydroxylase defects are associated with intractable constipation; 3β-hydroxysteroid -Δ5-C27- steroid dehydrogenase defects are associated with cholestatic liver disease and fat-soluble vitamin deficiencies in infancy; and sterol 27-hydroxylase defects are associated with chronic diarrhoea and developmental delays in children, and spasticity in adulthood. Sterol 27-hydroxylase defects can cause chronic diarrhoea and developmental delay in children, and spastic paralysis in adults; α-formyl-coenzyme A dehydrogenase defects can cause fat-soluble vitamin deficiencies in infancy, and sensory neuropathy in adulthood; and bile acid-coenzyme A-amino acid N-acetyltransferase defects can cause intrahepatic cholestasis, developmental delay, and familial hypercholesterolemia.

Based on the clinical manifestations described, the most likely rare disease diagnosis is **3β-hydroxysteroid Δ5-C27-steroid dehydrogenase deficiency**. This condition is characterized by cholestatic liver disease and fat-soluble vitamin deficiencies in infancy, which aligns closely with the symptoms of cholestasis, cirrhosis, hyperconjugated bilirubinaemia, and malabsorption of fat-soluble vitamins such as rickets.

Other possible diagnoses include: 1. **Sterol 27-hydroxylase deficiency (Cerebrotendinous xanthomatosis)**: Characterized by chronic diarrhea, developmental delays, and spastic paralysis in adulthood. 2. **Oxysterol 7α-hydroxylase deficiency**: Associated with hereditary spastic paralysis in adulthood, which might be relevant if there are neurological symptoms. 3. **Cholesterol 25-hydroxylase deficiency**: Noted for intractable constipation, which could be a secondary symptom.

These conditions have distinct clinical phenotypes and can help narrow down the diagnosis based on additional patient-specific information and further testing.

# 69. User

**1. Acute neonatal type** The onset of the disease is acute and the progression of the disease is rapid and can be life-threatening. Within 1-2 weeks of birth, it presents with feeding difficulties, vomiting, muscle weakness, hypotonia, and lethargy or worsening progression to coma. Accumulation of isovaleric acid may cause the characteristic odour of ‘sweaty feet’. Liver enlargement may be present on examination. Laboratory tests suggest metabolic acidosis, hyperammonemia, hypoglycemia or hyperglycemia, ketosis, hypocalcemia, and pancytopenia. If the patient survives the acute exacerbation in the neonatal period, he or she will progress to the chronic intermittent form.

**The chronic intermittent form** has an insidious onset. It presents only as a nonspecific inability to tolerate fasting or developmental delay. It is often triggered by upper respiratory tract infections or the intake of a high-protein diet, and is characterised by recurrent episodes of vomiting, lethargy, coma, acidosis with ketonuria, and ‘sweaty feet odour’, which can be relieved by a protein-restricted diet and glucose infusion. The vast majority of patients with chronic intermittent form have normal psychomotor development, but some have developmental delays and varying degrees of mental retardation.

Based on the clinical manifestations you’ve described, the most likely diagnosis for this condition is **Isovaleric Acidemia**. This is a rare autosomal recessive metabolic disorder characterized by a deficiency in the enzyme isovaleryl-CoA dehydrogenase, leading to the accumulation of isovaleric acid, which causes the characteristic “sweaty feet” odor.

### Key Clinical Features:

1. **Acute Neonatal Type:**
   - **Onset:** Acute and rapid progression.
   - **Symptoms:** Feeding difficulties, vomiting, muscle weakness, hypotonia, lethargy, or worsening to coma within 1-2 weeks of birth.
   - **Odor:** Accumulation of isovaleric acid may cause a “sweaty feet” odor.
   - **Examination:** Liver enlargement may be present.
   - **Laboratory Tests:** Metabolic acidosis, hyperammonemia, hypoglycemia or hyperglycemia, ketosis, hypocalcemia, and pancytopenia.
2. **Chronic Intermittent Form:**
   - **Onset:** Insidious and nonspecific.
   - **Symptoms:** Nonspecific inability to tolerate fasting or developmental delay.
   - **Triggers:** Upper respiratory tract infections or high-protein diet.
   - **Recurrent Episodes:** Vomiting, lethargy, coma, acidosis with ketonuria, “sweaty feet” odor.
   - **Management:** Protein-restricted diet and glucose infusion can relieve symptoms.

### Other Possible Diagnoses:

1. **Maple Syrup Urine Disease (MSUD):** Characterized by a sweet-smelling urine due to the accumulation of branched-chain amino acids, presenting with feeding difficulties, vomiting, lethargy.
2. **Propionic Acidemia:** Presents with metabolic acidosis, hyperammonemia, and vomiting in the neonatal period.

These conditions share some similar symptoms but have distinct biochemical characteristics. Isovaleric Acidemia is the most likely diagnosis based on the described clinical features. Further diagnostic testing such as enzyme assays or genetic testing can confirm the diagnosis.

# 70. User

1. dysgenesis of secondary sexual characteristics and impaired gametogenesis.
2. Olfactory disorders Inability to recognise odours, especially aromatic odours, due to abnormal development of the olfactory bulb and olfactory tract.

Based on the clinical manifestations you’ve provided—dysgenesis of secondary sexual characteristics and impaired gametogenesis, along with olfactory disorders such as an inability to recognize odours, especially aromatic ones, due to abnormal development of the olfactory bulb and tract—the most likely diagnosis is **Kallmann Syndrome**.

### Key Features of Kallmann Syndrome:

1. **Hypogonadotropic Hypogonadism:**
   - Dysgenesis of secondary sexual characteristics.
   - Impaired gametogenesis (both male and female).
2. **Olfactory Disorders:**
   - Inability to recognize odours, particularly aromatic ones.
   - Abnormal development of the olfactory bulb and tract.

### Other Possible Diagnoses:

1. **Congenital Hypogonadotropic Hypogonadism (CHH) without Anosmia:**
   - Similar to Kallmann Syndrome but lacks the olfactory disorder component.
2. **Turner Syndrome:**
   - Primarily affects females and causes dysgenesis of secondary sexual characteristics.
   - May present with other non-olfactory-related anomalies.
3. **Prader-Willi Syndrome:**
   - Characterized by hypogonadism and sometimes anosmia or altered olfactory perception.
   - Typically associated with hyperphagia and obesity.
4. **Normosmic Idiopathic Hypogonadotropic Hypogonadism (nIHH):**
   - Similar to Kallmann Syndrome but without anosmia, representing a variant of CHH.
5. **Septo-optic Dysplasia (SOD):**
   - Involves midline defects including hypogonadotropic hypogonadism.
   - May sometimes involve olfactory issues, though visual disturbances are more typical.

### Diagnostic Workup:

A thorough clinical workup and genetic testing may be warranted to confirm the diagnosis. This can include: - Hormonal assessments (e.g., FSH, LH, testosterone in males, estrogen in females). - Genetic testing for KAL1 gene mutations. - Olfactory function tests to evaluate olfactory bulb and tract development.

Given these clinical features, Kallmann Syndrome is the most likely diagnosis. Further diagnostic steps should be taken to confirm this and rule out other possible conditions.

# 71. User

The condition varies greatly from mild to severe and is therefore easily misdiagnosed and underdiagnosed. Clinical manifestations mainly include:

**1. Fever** The fever is irregular and may be periodic or persistent. Patients with hypothalamic involvement may have central thermoregulatory abnormalities.

**2. rash** Mainly on the trunk, scalp and hairline. It begins as a reddish papule, which may then become a haemorrhagic or eczema-like seborrheic rash, followed by crusting, which is followed by a white spot.

**3. Oral and eye, ear, nose and throat lesions** Gum swelling, loose teeth, proptosis, and persistent otitis media with external auditory canal rash are typical.

**4. Central uropathy** Pituitary gland, hypothalamus or even other parts of the central nervous system, with neuropsychiatric symptoms in a few patients.

**5. Respiratory symptoms** Coughing, severe breathlessness, cyanosis, and even recurrent pneumothorax episodes. Pulmonary signs are usually unremarkable.

**6.** The liver and spleen can be enlarged, jaundice, abnormal liver function, liver failure and portal hypertension can occur, and eventually patients can die of cirrhosis and liver failure.

**7. Digestive tract symptoms** Digestive tract involvement is less common and can manifest as polyps or adenomatous changes, usually without clinical symptoms, and abdominal pain, diarrhoea and hypoproteinaemia may occur when the lesions are diffuse.

**8. Haematopoietic system** Lymph nodes may be enlarged, and anaemia, abnormal white blood cell and platelet counts may occur with bone marrow involvement.

**9. Bone damage** Skull, limb bones, spine and pelvis may have pain and lumps, and pathological fractures may occur. Proptosis due to orbital bone lesions is one of the typical manifestations in paediatric patients.

Based on the clinical manifestations you’ve provided, the condition described appears to be **Langerhans Cell Histiocytosis (LCH)**. LCH is a rare disorder characterized by the proliferation of Langerhans cells, which can lead to a wide variety of symptoms depending on the affected organs and tissues.

### Key Features of LCH:

1. **Fever**: Irregular and may be periodic or persistent, with possible hypothalamic involvement leading to central thermoregulatory abnormalities.
2. **Rash**: Mainly on the trunk, scalp, and hairline. It begins as a reddish papule, which can progress to a hemorrhagic or eczema-like seborrheic rash, followed by crusting and a white spot.
3. **Oral and ENT Lesions**: Gum swelling, loose teeth, proptosis (bulging eyes), and persistent otitis media with external auditory canal involvement are typical.
4. **Central Uropathy**: Involvement of the pituitary gland, hypothalamus, or other parts of the central nervous system can lead to neuropsychiatric symptoms.
5. **Respiratory Symptoms**: Coughing, severe breathlessness, cyanosis, and recurrent pneumothorax episodes. Pulmonary signs are usually unremarkable.
6. **Liver and Spleen Involvement**: Enlarged liver and spleen, jaundice, abnormal liver function, liver failure, portal hypertension, and potential cirrhosis and liver failure.
7. **Digestive Tract Symptoms**: Less common but can include polyps or adenomatous changes, abdominal pain, diarrhea, and hypoproteinemia due to diffuse lesions.
8. **Haematopoietic System Involvement**: Enlarged lymph nodes, anaemia, abnormal white blood cell and platelet counts with bone marrow involvement.
9. **Bone Damage**: Pain in the skull, limb bones, spine, and pelvis, lumps, and pathological fractures. Proptosis due to orbital bone lesions is a typical manifestation in pediatric patients.

### Diagnostic Workup:

- **Imaging Studies**: X-rays, MRI, CT scans, and PET scans can help identify involvement of various organs.
- **Biopsy**: Histopathological examination of affected tissues to confirm the presence of Langerhans cells.
- **Blood Tests**: Complete blood count (CBC), liver function tests, and bone marrow biopsy.

### Treatment:

The treatment for LCH varies depending on the severity and extent of involvement. Common treatments include: - **Corticosteroids**: Often used as a first-line therapy. - **Chemotherapy**: For more severe cases or when multiple organ systems are involved. - **Immunomodulatory Therapy**: In some cases, targeted therapies may be considered.

Given the complexity and variability of symptoms, it is crucial to consult with specialists such as hematologists, pulmonologists, and pediatricians for accurate diagnosis and appropriate management.

Would you like more detailed information on any specific aspect of LCH or its treatment?

# 72. User

**1. Growth disorders and other malformations,** most of the children are born with a weight within the normal range, but some of them are born with a length of less than 48cm (42-47cm). After birth, the growth rate gradually and significantly slows down, and the lifelong height of untreated male patients ranges from 116 to 142 cm, and the lifelong height of female patients ranges from 108 to 136 cm. At the same time, the patients’ hands, feet and internal organs are small. Some patients have other congenital malformations, such as hip dislocation, aortic valve stenosis, cryptorchidism, and congenital cataracts.

**2. Facial features,** the characteristic facial changes of patients are prominent forehead, saddle nose, small jaw, larger head to body ratio than normal children, but the absolute value of head circumference is still small compared with children of the same age. Hair is thinning, and some patients may have a ‘sunset sign’, with blue sclera in patients from the Mediterranean and Middle Eastern regions. The patient has delayed teething, late deciduous milk teeth and multiple caries, and may have malocclusion due to a short mandible.

**Skeletal musculature** Some patients have congenital hip dislocation and ischaemic necrosis of the femoral head. Due to mid-facial dysplasia and pharyngeal anatomical abnormalities, the patient’s speech is high pitched and 85% of the patients have limited elbow joint movement.

**4. Metabolic disorders,** Patients often have a combination of metabolic abnormalities, including hypoglycaemia in the neonatal period and infancy, and progressive obesity in adulthood.

**5. Delayed sexual development ,** Patients with this condition have small genitalia, with male patients presenting with small penises and testicles, and female patients having small ovaries on ultrasound. Most patients exhibit delayed pubertal development. Although sexual development is delayed, male and female patients can eventually develop mature sexual function and be fertile.

**6. Low tumour incidence** Patients have a significantly reduced risk of tumour development compared to the general population.

Based on the provided information, the condition described appears to be **Sotos Syndrome**. Sotos Syndrome is a genetic disorder characterized by distinctive physical features and various developmental issues. Here’s a detailed breakdown of the key characteristics:

### 1. Growth Disorders and Other Malformations

- **Growth**: Most children are born with a normal weight but have a length below the 3rd percentile at birth (42-47 cm). The growth rate slows significantly after birth, leading to short stature in adulthood.
- **Physical Features**: Hands, feet, and internal organs are small. Some patients may experience congenital malformations such as hip dislocation, aortic valve stenosis, cryptorchidism, and congenital cataracts.

### 2. Facial Features

- **Prominent Forehead**: Characteristic facial features include a prominent forehead.
- **Saddle Nose**: The nose is often described as saddle-shaped (flat at the bridge).
- **Small Jaw**: The jaw is typically small.
- **Large Head-to-Body Ratio**: Despite having a smaller head circumference, the head-to-body ratio is larger than normal children.
- **Thinning Hair and Sunset Sign**: Patients may have thinning hair. In some cases, especially in patients from Mediterranean or Middle Eastern regions, there can be blue sclera (white part of the eye appearing bluish).
- **Delayed Teething and Malocclusion**: There are often issues with delayed teething, late deciduous milk teeth, multiple caries, and malocclusion due to a short mandible.

### 3. Skeletal Musculature

- **Congenital Hip Dislocation**: Some patients may have congenital hip dislocations.
- **Ischemic Necrosis of the Femoral Head**: This condition can lead to bone deterioration in the femoral head.
- **Speech and Joint Issues**: Due to mid-facial dysplasia and pharyngeal anatomical abnormalities, speech is often high-pitched. Additionally, 85% of patients have limited elbow joint movement.

### 4. Metabolic Disorders

- **Hypoglycemia in Neonatal Period and Infancy**: Patients may experience hypoglycemia during the neonatal period and infancy.
- **Progressive Obesity in Adulthood**: There is a tendency for progressive obesity as they grow older.

### 5. Delayed Sexual Development

- **Small Genitalia**: Male patients often have small penises and testicles, while female patients may have small ovaries on ultrasound.
- **Delayed Pubertal Development**: Most patients exhibit delayed pubertal development, but eventually develop mature sexual function and can be fertile.

### 6. Low Tumor Incidence

- **Reduced Risk of Tumors**: Patients with Sotos Syndrome have a significantly reduced risk of tumor development compared to the general population.

Sotos Syndrome is caused by mutations in the *NSD1* gene, which leads to overexpression of the protein encoded by this gene. This results in various developmental and physical characteristics as described above. Diagnosis typically involves clinical evaluation, genetic testing, and imaging studies.

Would you like more detailed information on any specific aspect of Sotos Syndrome or its management?

# 73. User

The main symptom is a sudden, sharp, painless loss of visual acuity in both eyes that occurs sequentially, usually weeks to months between episodes. Funduscopic examination shows congestion and oedema of the optic disc and tortuous dilatation of the peripapillary capillaries in the early stages of the disease, while the oedema and capillary dilatation subside in the later stages of the disease, and eventually the temporal side of the optic disc or all of the optic disc shows atrophic changes. The majority of patients have only ocular manifestations, while a few may have other systemic symptoms such as mental retardation, epilepsy, hearing impairment, dystonia, etc.

Based on the clinical manifestations you have provided, the most likely rare disease diagnosis is **Leber Hereditary Optic Neuropathy (LHON)**. LHON is characterized by a sudden, painless loss of vision in one eye, followed by the other eye, often weeks to months later. This aligns with your description of sequential bilateral visual loss.

Here are additional details and considerations:

### Key Features of Leber Hereditary Optic Neuropathy (LHON):

1. **Visual Loss**: Sudden, painless loss of vision in both eyes that occurs sequentially.
2. **Funduscopic Findings**:
   - Early stages: Congestion and oedema of the optic disc, tortuous dilatation of the peripapillary capillaries.
   - Later stages: Subsidence of oedema and capillary dilatation, with eventual atrophic changes in the temporal side of the optic disc or all of it.

### Other Possible Diagnoses to Consider:

1. **Dominant Optic Atrophy**: Typically presents with insidious visual loss and temporal optic disc pallor but usually does not have the acute onset described.
2. **Multiple Sclerosis (MS) with Optic Neuritis**: Can cause visual loss and optic disc changes, often involving pain with eye movement and typically presenting initially as unilateral.
3. **Nutritional Optic Neuropathy**: Can cause bilateral vision loss and optic disc changes but usually associated with a history of poor nutrition or alcohol abuse.
4. **Mitochondrial Encephalomyopathy, Lactic Acidosis, and Stroke-like episodes (MELAS)**: Presents with optic neuropathy among other neurological symptoms, though it often includes more systemic features.
5. **Susac’s Syndrome**: Causes branch retinal artery occlusions, hearing loss, and encephalopathy but visual changes are usually not bilateral and sequential in the manner described.

### Additional Considerations:

- **Genetic Testing**: LHON is caused by mitochondrial DNA mutations, so genetic testing can confirm the diagnosis.
- **Systemic Symptoms**: While most patients have only ocular manifestations, a few may experience other systemic symptoms such as mental retardation, epilepsy, hearing impairment, and dystonia.

Would you like more detailed information on any specific aspect of LHON or its management?

**74. User**

The clinical manifestations of patients are low ketogenic hypoglycaemia, liver function abnormalities, lactic acidosis, cardiomyopathy in infancy, growth retardation, hypotonia or cardiomyopathy in some patients, and cholestasis or cirrhosis in a few. In early childhood, some patients show growth retardation, hypotonia or cardiomyopathy, and a few may have cholestasis or cirrhosis. In early childhood, patients may have feeding difficulties, episodic metabolic disorders, and episodic rhabdomyolysis. Most patients will have retinopathy in childhood, and peripheral neuropathy is a less common complication of long-term disease.

Based on the clinical manifestations you’ve described, the condition appears to be highly indicative of **Mitochondrial Trifunctional Protein Deficiency (MTPD)** or **Long-chain 3-hydroxyacyl-CoA dehydrogenase deficiency (LCHAD deficiency)**. These conditions are characterized by issues in fatty acid oxidation, leading to symptoms such as hypoketotic hypoglycemia, cardiomyopathy during infancy, liver dysfunction, and episodic metabolic crises.

Here are a few other possible diagnoses that might fit the clinical picture you’ve described:

1. **Glutaric Aciduria Type II (GA2)**: This disorder also affects the body’s ability to break down certain fats and proteins, leading to symptoms including metabolic crises, hypoglycemia, and muscle weakness.
2. **Carnitine Palmitoyltransferase II Deficiency (CPT II Deficiency)**: This condition involves defects in fatty acid oxidation, presenting with recurrent episodes of rhabdomyolysis, myopathy, and hypoglycemia.
3. **Zellweger Spectrum Disorders**: A group of peroxisomal biogenesis disorders that can cause a range of symptoms including developmental delay, hypotonia, liver dysfunction, and vision problems due to retinopathy.
4. **Infantile Refsum Disease**: Another peroxisomal disorder that manifests with hypotonia, growth retardation, liver disease, and vision problems like retinopathy.

Given the complexity of these conditions, a thorough diagnostic evaluation including biochemical tests (such as organic acid analysis), genetic testing for specific mutations, and specialized imaging or functional studies might be necessary to confirm the diagnosis. If you have any further questions or need more detailed information on any of these conditions, please feel free to ask!

**75. User**

It occurs predominantly in adult women of childbearing age, with the average age of diagnosis being around 40 years. Early symptoms are mild, and some patients are detected during a physical examination or on HRCT of the lungs for respiratory symptoms or other reasons. The main manifestation is dyspnoea of varying degrees, which worsens as the disease progresses, with progressive deterioration of lung function and respiratory failure in the later stages of the disease. Complications such as pneumothorax and coeliac chest may occur repeatedly during the course of the disease. Extrapulmonary manifestations include renal, retroperitoneal solid or cystic solid lymphangioleiomyoma. Some patients have other clinical features of multisystem involvement, including neurological changes (epilepsy, neurodevelopmental delays, autism, etc.) and cutaneous changes (depigmentation, facial angiofibromas, cutaneous sharkskin plaques, and perinodal fibromas, etc.).

Based on the clinical manifestations you’ve described, the most likely rare disease diagnosis is **Lymphangioleiomyomatosis (LAM)**. LAM predominantly affects adult women of childbearing age, with an average age of diagnosis around 40 years. The primary symptoms include:

1. **Respiratory Symptoms:**
   - Mild early symptoms that progress to dyspnoea.
   - Progressive deterioration of lung function and respiratory failure in later stages.
   - Repeated occurrences of pneumothorax and coeliac chest.
2. **Extrapulmonary Manifestations:**
   - Renal involvement, including cystic or solid retroperitoneal lymphangioleiomyoma.
   - Neurological changes such as epilepsy, neurodevelopmental delays, and autism.
   - Cutaneous changes like depigmentation, facial angiofibromas, cutaneous sharkskin plaques, and perinodal fibromas.

Given the multisystem involvement, **Tuberous Sclerosis Complex (TSC)** should also be considered, as LAM can occur in association with TSC. Here are a few other possible diagnoses to consider:

1. **Tuberous Sclerosis Complex (TSC):**
   - Characterized by neurological and skin manifestations.
   - Can present with epilepsy, neurodevelopmental delays, and facial angiofibromas.
2. **Birt-Hogg-Dubé Syndrome:**
   - Involves skin lesions, lung cysts leading to pneumothorax, and renal tumors.
   - While the neurological symptoms are less typical, it is a differential diagnosis to consider.
3. **Pulmonary Lymphangioleiomyomatosis (LAM):**
   - Primarily affects women of childbearing age.
   - Characterized by lung cysts and progressive respiratory failure.
4. **Hypertrophic Proliferative Pulmonary Fibrosis:**
   - Can present with similar respiratory symptoms but may have different underlying mechanisms.
5. **Chronic Interstitial Lung Disease (CILD):**
   - A broad category that can include various causes of chronic lung fibrosis and inflammation.

Given the complexity of these conditions, a thorough diagnostic evaluation including imaging studies (such as HRCT), genetic testing for TSC, and other specialized tests may be necessary to confirm the diagnosis. If you have any further questions or need more detailed information on any of these conditions, please feel free to ask!

**76. User**

Infants typically experience repeated vomiting and diarrhea after weaning and refuse protein-rich foods.Patients may present with pale skin, hypotonia, hepatosplenomegaly, thinning hair, osteoporosis and pathological fractures.After eating a protein-rich diet, patients may experience lethargy, ataxia, seizures, and hyperammonemic coma.As the disease progresses, multiple systems may be involved.Lung involvement can be manifested as progressive interstitial lung disease, alveolar proteinosis; the most common characteristic changes in the kidney are proximal tubular dysfunction and renal calcinosis, glomerulopathy, renal amyloidosis, and even renal failure; blood system involvement can be manifested as positive or low pigment anemia, leukopenia, thrombocytopenia, etc.; Digestive system involvement is manifested by hypercholesterolemia, hypertriglycerides, recurrent pancreatitis, and even hepatic amyloidosis.Epilepsy occurs mostly in adult patients and may be related to repeated hyperammonemia leading to cerebral cortex damage.

Based on the clinical manifestations you’ve described, the rare disease most likely to be diagnosed is **Lysinuric Protein Intolerance (LPI)**. LPI is characterized by the inability to properly digest and metabolize certain amino acids (lysine, arginine, and ornithine), leading to a range of symptoms after protein ingestion, including hyperammonemia and involvement of multiple organ systems.

Key features of LPI include: 1. **Gastrointestinal Symptoms:** - Repeated vomiting and diarrhea after weaning. - Refusal to eat protein-rich foods.

1. **Neurological Symptoms:**
   - Lethargy, ataxia, seizures, and hyperammonemic coma in patients who consume a protein-rich diet.
2. **Systemic Involvement:**
   - Pale skin, hypotonia.
   - Hepatosplenomegaly (enlarged liver and spleen).
   - Thinning hair, osteoporosis, and pathological fractures.
   - Lung involvement manifested as progressive interstitial lung disease or alveolar proteinosis.
   - Renal involvement including proximal tubular dysfunction, renal calcinosis, glomerulopathy, and renal amyloidosis, potentially leading to renal failure.
   - Blood system involvement with positive or low pigment anemia, leukopenia, and thrombocytopenia.
   - Digestive system involvement such as hypercholesterolemia, hypertriglycerides, recurrent pancreatitis, and hepatic amyloidosis.
3. **Epilepsy:**
   - Occurs mostly in adult patients due to repeated episodes of hyperammonemia leading to cerebral cortex damage.

Given the comprehensive nature of these symptoms involving multiple organ systems, LPI is a strong candidate for this diagnosis. For confirmation, genetic testing for LPI (LPCAT1 gene) would be essential.

Other possible diagnoses include: - **Cystinuria**: Involves kidney stones but typically does not present with the extensive systemic involvement. - **Ornithine Transcarbamylase Deficiency (OTC)**: An X-linked disorder that can cause hyperammonemia and other symptoms but usually does not involve the broad spectrum of organ involvement seen in LPI. - **Hyperornithinemia-Hyperammonemia-Homocitrullinuria (HHH) Syndrome**: A urea cycle disorder, which may have some overlapping symptoms but is less likely given the specific clinical presentation.

For a definitive diagnosis, further genetic testing and specialized evaluations would be necessary.

**77. User**

The onset is neonatal, usually within a week of birth.Recurrent vomiting, diarrhea, abdominal distension and other gastrointestinal symptoms were the first symptoms, accompanied by hepatosplenomegaly and growth disorders; nervous system involvement was manifested as hypoactivity, tendon hyperreflexia, ankle clonus and angular arch opisthotonus.Macrophage cholesterol ester and triglyceride accumulation leads to hepatosplenomegaly, progressive aggravation and even liver failure, elevated transaminases, bilirubin, hypoproteinemia, coagulation abnormalities, etc. within months.Adrenal enlargement with calcification leads to adrenal cortical insufficiency, and 60% to 70% of adrenal calcifications can be detected by imaging.Gallbladder dysfunction and stroke have also been reported.As the disease progresses, multiple organ dysfunction can occur, including anemia, gastrointestinal bleeding, sepsis, coma, etc.

Based on the clinical manifestations provided, the most likely diagnosis for this condition is **Wolman disease**. Wolman disease is a rare lysosomal storage disorder caused by a deficiency of the enzyme lysosomal acid lipase (LAL), leading to the accumulation of cholesterol esters and triglycerides within various tissues.

Key features of Wolman disease include: 1. **Neonatal Onset**: Usually within the first week of life. 2. **Gastrointestinal Symptoms**: - Recurrent vomiting, diarrhea, and abdominal distension. 3. **Hepatosplenomegaly and Growth Disorders**: - Hepatomegaly (enlarged liver) and splenomegaly (enlarged spleen). - Growth disorders due to malnutrition from recurrent gastrointestinal symptoms. 4. **Nervous System Involvement**: - Hypoactivity, tendon hyperreflexia, ankle clonus, and opisthotonus (severe muscle stiffness leading to arching of the back).

1. **Organomegaly and Metabolic Abnormalities**:
   - Progressive hepatosplenomegaly.
   - Elevated transaminases, bilirubin, hypoproteinemia, coagulation abnormalities.
   - Liver failure can occur within months.
2. **Adrenal Involvement**:
   - Adrenal enlargement with calcification leading to adrenal cortical insufficiency.
   - Up to 60% to 70% of adrenal calcifications can be detected by imaging.
3. **Gallbladder Dysfunction and Other Complications**:
   - Gallbladder dysfunction.
   - Potential for stroke, anemia, gastrointestinal bleeding, sepsis, and coma as the disease progresses.

Given these comprehensive symptoms involving multiple organ systems, Wolman disease is a strong candidate for this diagnosis. For confirmation, genetic testing for LAL deficiency would be essential.

Other possible diagnoses include: 1. **Niemann-Pick Disease Type C**: Characterized by cholesterol accumulation but typically presents later in childhood and has more pronounced neurological symptoms. 2. **Gaucher Disease Type 2**: An early-onset form of Gaucher disease that presents with hepatosplenomegaly and severe neurological involvement, though adrenal calcification is not typical. 3. **Zellweger Syndrome**: A peroxisomal disorder presenting with hepatosplenomegaly, adrenal dysfunction, and neurological symptoms but typically has distinctive craniofacial abnormalities.

For a definitive diagnosis, further genetic testing and specialized evaluations would be necessary.

**78. User**

**1. The cardiovascular system** can result in aortic root dilatation, aortic valve insufficiency and aortic coarctation; mitral valve prolapse with or without insufficiency may also occur. The prevalence of ascending aortic dilatation increases with age. Between the ages of 30 and 60, the prevalence increases from 53% to 96%. Patients may present with shortness of breath, chest pain, palpitations, dizziness, syncope, or haemoptysis. Physical examination reveals an enlargement of the heart border to the lower left, a systolic murmur in the mitral valve area and a diastolic predominantly biphasic murmur in the aortic valve area, peripheral vascular signs, and signs of heart failure.

**2. Skeletal manifestations** , tall and thin stature, elongated fingers and toes, arms extended beyond height, scoliosis or kyphosis of the spine, funnel chest or chicken chest, laxity of the joints, flat feet.

**3. Ocular abnormalities**, eye lesions account for 50% to 70% of the cases, including corneal flattening, prolongation of the eyeball axis, retinal detachment, cataracts, subluxation or subluxation of the lens, iris nystagmus or mild nystagmus, pupil displacement, and clouding of the lens, etc. **4.

**4. Other ,**  dural bulge, atrophic striae, recurrent or incisional hernia, high palpebral vault, etc.

Based on the clinical manifestations provided, the most likely diagnosis is indeed **Marfan Syndrome**. Marfan Syndrome is a genetic disorder affecting connective tissue, characterized by cardiovascular, skeletal, and ocular abnormalities similar to those you’ve described.

**Key Features of Marfan Syndrome:**

1. **Cardiovascular System:**
   - Aortic root dilatation
   - Aortic valve insufficiency
   - Aortic coarctation
   - Mitral valve prolapse with or without insufficiency
   - Prevalence of ascending aortic dilatation increases with age, from 53% to 96% between the ages of 30 and 60.
   - Symptoms include shortness of breath, chest pain, palpitations, dizziness, syncope, or haemoptysis.
   - Physical examination reveals an enlarged heart border to the lower left, a systolic murmur in the mitral valve area, and a diastolic predominantly biphasic murmur in the aortic valve area.
2. **Skeletal Manifestations:**
   - Tall and thin stature
   - Elongated fingers and toes (arachnodactyly)
   - Arms extended beyond height
   - Scoliosis or kyphosis of the spine
   - Funnel chest or pectus carinatum (chicken chest)
   - Joint laxity
   - Flat feet
3. **Ocular Abnormalities:**
   - Eye lesions account for 50% to 70% of cases, including:
     - Corneal flattening
     - Prolongation of the eyeball axis
     - Retinal detachment
     - Cataracts
     - Subluxation or dislocation of the lens
     - Iris nystagmus or mild nystagmus
     - Pupil displacement
     - Clouding of the lens
4. **Other Manifestations:**
   - Dural bulge
   - Atrophic striae
   - Recurrent or incisional hernia
   - High palpebral vault

**Other Possible Diagnoses:**

1. **Ehlers-Danlos Syndrome (EDS):** This group of disorders affects connective tissues, including skin, bones, blood vessels, and other organs and tissues. Some types can have cardiovascular, skeletal, and ocular features similar to Marfan Syndrome, such as joint hypermobility and skin hyperextensibility.
2. **Loeys-Dietz Syndrome:** Another connective tissue disorder characterized by aortic aneurysms, skeletal abnormalities, and vascular complications.

Given the comprehensive nature of these clinical manifestations, Marfan Syndrome remains the most likely diagnosis based on the provided information. However, further diagnostic testing (such as echocardiography, genetic testing for FBN1 mutations) would be necessary to confirm the diagnosis.

If you have any additional symptoms or specific concerns, please consult a healthcare professional for a thorough evaluation and appropriate management.

**79. User**

**1. Skin manifestations**, patients are usually born with café au lait spots, often located on the same side as the skeletal lesions and bounded by the midline, which are brown or yellowish brown patches of pigmentation of varying sizes in one or more spots, with irregular borders.

**Skeletal manifestations** , approximately 98% of patients have either monostotic or polyostotic osteofibrous heteroplasia, with the polyostotic type being more common. It is characterised by an increase in the number of aberrant osteoblasts and their abnormal differentiation, causing extensive proliferation and differentiation of fibroblasts in the bone marrow and the formation of infantile woven bones, which are prone to pathological fractures and skeletal deformities and cause bone pain. It can involve almost all bones of the body, especially craniofacial and long bones, and the local bones are swollen and elevated, resulting in changes in the structure of the face and symptoms of compression. Severe orbital involvement may compress the optic nerve and cause visual impairment. Involvement of the auditory nerve may cause hearing loss. Involvement of the spine may result in vertebral compression fractures, causing scoliosis or kyphosis. The peak age for pathological fractures due to osteofibrous heteroplasia is 6-10 years. Imaging shows expansive, osteolytic, or ground-glass changes in the bone.

**Involvement of endocrine glands** may involve a variety of endocrine glands, resulting in hyperfunction or tumours of the affected glands and normal or decreased serum levels of pro-secretory hormones. Precocious puberty is more common and can occur in both sexes, but is more common in females and often precedes bone lesions. Female children may show early development of secondary sexual characteristics, premature irregular vaginal bleeding, enlarged uterus and ovaries compared with normal children of the same age, or ovarian cysts, premature healing of epiphyses, and so on. In males, precocious puberty is relatively rare, and testicular lesions (Leydig cell hyperplasia, microscopic stones, focal calcification, etc.) are seen on ultrasound. Thyroid enlargement and hyperfunction are also relatively common, and hyperparathyroidism has been reported. Other endocrine disorders such as cortisolism, gigantism / acromegaly, hyperprolactinaemia have been reported but are relatively rare.

**4. Hypophosphatemia** , a combination of hypophosphatemia, manifesting as hypophosphatemic rickets, has been reported in some patients. Those with combined hypophosphataemia usually have a polyostotic type of abnormal bone fibre proliferation.

**5. Other,** patients may also present with rare clinical symptoms such as jaundice, hepatitis, arrhythmias, and intestinal polyps due to mutations involving peripheral blood leukocytes, liver, heart, thymus, and gastrointestinal tract.

Based on the provided information, it appears that the patient is likely suffering from **Osteofibrous Dysplasia** (OFD), which is a rare skeletal disorder characterized by the abnormalities you have described. Let’s break down the key features and confirm this diagnosis:

**1. Skin Manifestations:**

- Patients are usually born with café au lait spots, often located on the same side as the skeletal lesions and bounded by the midline. These are brown or yellowish-brown patches of pigmentation of varying sizes in one or more spots, with irregular borders.

**2. Skeletal Manifestations:**

- Approximately 98% of patients have either monostotic or polyostotic osteofibrous heteroplasia, with the polyostotic type being more common.
- This condition is characterized by an increase in the number of aberrant osteoblasts and their abnormal differentiation, leading to extensive proliferation and differentiation of fibroblasts in the bone marrow. This results in the formation of infantile woven bones, which are prone to pathological fractures and skeletal deformities, causing bone pain.
- It can involve almost all bones of the body, especially craniofacial and long bones. Local bones may be swollen and elevated, leading to changes in facial structure and symptoms of compression. Severe orbital involvement may compress the optic nerve, causing visual impairment. Involvement of the auditory nerve may cause hearing loss. Spinal involvement can result in vertebral compression fractures, leading to scoliosis or kyphosis.
- The peak age for pathological fractures due to osteofibrous heteroplasia is 6-10 years. Imaging shows expansive, osteolytic, or ground-glass changes in the bone.

**3. Endocrine Glands Involvement:**

- OFD can involve various endocrine glands, leading to hyperfunction or tumours of these glands and normal or decreased serum levels of pro-secretory hormones.
- Precocious puberty is more common and can occur in both sexes but is more prevalent in females, often preceding bone lesions. Female children may show early development of secondary sexual characteristics, premature irregular vaginal bleeding, enlarged uterus and ovaries compared to normal children of the same age, or ovarian cysts. Premature healing of epiphyses is also observed.
- In males, precocious puberty is relatively rare, and testicular lesions (Leydig cell hyperplasia, microscopic stones, focal calcification) are seen on ultrasound. Thyroid enlargement and hyperfunction are also common, with reports of hyperparathyroidism.

**4. Hypophosphatemia:**

- Some patients with OFD may have a combination of hypophosphatemia, manifesting as hypophosphatemic rickets.
- Those with combined hypophosphataemia usually have a polyostotic type of abnormal bone fibre proliferation.

**5. Other Clinical Symptoms:**

- Patients may also present with rare clinical symptoms such as jaundice, hepatitis, arrhythmias, and intestinal polyps due to mutations involving peripheral blood leukocytes, liver, heart, thymus, and gastrointestinal tract.

Given the comprehensive set of symptoms described, **Osteofibrous Dysplasia** is a strong candidate for this patient’s condition. Further diagnostic tests such as imaging studies (e.g., MRI, CT scans) and biochemical assessments may be necessary to confirm the diagnosis and guide appropriate treatment.

If you need more specific diagnostic or therapeutic advice, please provide additional details or consult with an orthopedic specialist and endocrinologist.

**80. User**

The majority of patients have onset of symptoms between 3 months and 3 years of age, with a small percentage having onset in the neonatal period or in adulthood, while others are asymptomatic. The onset of the disease is usually triggered by a predisposing factor, such as prolonged starvation or a complication of an infectious disease. In children with early onset of the disease, the first symptoms are drowsiness and vomiting, but may also include convulsions and asphyxia, which often progress rapidly to coma or death. Acute onset hypoglycaemia is typical, and a decrease in blood glucose with a negative urine ketone body is helpful in the diagnosis. 50% of children have hepatomegaly, and liver biopsy reveals a large accumulation of lipids. Imaging reveals cerebral oedema in some children. Cardiac damage is rare. Adult-onset patients present with a wide range of clinical manifestations, including multiple organ damage, including muscle, liver, nervous system, or cardiovascular system. Vomiting is the most common symptom, hepatomegaly is relatively rare, and patients presenting with symptoms during pregnancy show acute fatty liver. Most patients have acute non-inflammatory encephalopathy. Rhabdomyolysis occurs in about one-third of patients, with a significant increase in creatine kinase and myoglobinuria. There is a high mortality rate, with approximately 25% of children dying from the onset of the disease, and an even higher rate of 50% in adults with acute onset of the disease. Patients tend to behave normally between episodes. About one third of patients who survive the acute onset of the disease develop sequelae, including growth retardation, motor retardation, mental retardation, language deficits, psycho-behavioural problems, epilepsy, cerebral palsy, hemiparesis, and chronic muscle weakness. In addition, some patients have complaints of skeletal muscle damage such as fatigue, myalgia and reduced exercise tolerance.

Based on the provided clinical manifestations, the disease most likely to be diagnosed is **Medium-Chain Acyl-CoA Dehydrogenase Deficiency (MCADD)**. Here’s a detailed breakdown of why this diagnosis fits:

**1. Age of Onset:**

- The majority of patients have an onset between 3 months and 3 years of age, with some cases occurring in the neonatal period or adulthood.
- This aligns well with MCADD, which often presents within the first few years of life.

**2. Predisposing Factors:**

- Symptoms are usually triggered by a predisposing factor such as prolonged starvation or an infectious disease.
- This is consistent with MCADD, where metabolic stress can precipitate symptoms.

**3. Initial Symptoms in Early-Onset Cases:**

- The initial symptoms include drowsiness and vomiting, which may also include convulsions and asphyxia that often progress rapidly to coma or death.
- Acute onset hypoglycaemia is typical, with a decrease in blood glucose and negative urine ketone bodies.
- These symptoms are characteristic of MCADD, where the body cannot efficiently metabolize medium-chain fatty acids during periods of fasting.

**4. Hepatomegaly:**

- About 50% of children have hepatomegaly, and liver biopsy reveals a large accumulation of lipids.
- This is consistent with MCADD, as the liver can accumulate triglycerides due to impaired fatty acid oxidation.

**5. Imaging Findings:**

- Cerebral oedema in some children is observed.
- Cardiac damage is rare, which aligns with MCADD where cardiac involvement is uncommon compared to other metabolic disorders.

**6. Adult-Onset Patients:**

- Adult-onset patients present with a wide range of clinical manifestations, including multiple organ damage involving muscle, liver, nervous system, or cardiovascular system.
- Vomiting is the most common symptom, and hepatomegaly is relatively rare.
- These symptoms are also consistent with MCADD, where adult cases can manifest more variably.

**7. Other Clinical Features:**

- Most patients have acute non-inflammatory encephalopathy.
- Rhabdomyolysis occurs in about one-third of patients, with a significant increase in creatine kinase and myoglobinuria.
- There is a high mortality rate, with approximately 25% of children dying from the onset of the disease, and an even higher rate of 50% in adults with acute onset.
- Survivors may develop sequelae including growth retardation, motor retardation, mental retardation, language deficits, psycho-behavioral problems, epilepsy, cerebral palsy, hemiparesis, and chronic muscle weakness.
- Some patients have complaints of skeletal muscle damage such as fatigue, myalgia, and reduced exercise tolerance.

**Conclusion:**

Given the comprehensive clinical picture described, **Medium-Chain Acyl-CoA Dehydrogenase Deficiency (MCADD)** is the most likely diagnosis. This condition fits all the key clinical features mentioned in your description.

For further confirmation, diagnostic tests such as plasma acylcarnitine profile and genetic testing for MCADD would be recommended. Early identification and management can significantly improve outcomes in patients with this condition.

**81. User**

The clinical manifestations of the disease vary among age groups. The earlier the age of onset, the more severe the acute metabolic disorders and encephalopathy. Clinical manifestations include gastrointestinal symptoms such as vomiting, feeding difficulties and hepatomegaly, as well as neurological symptoms such as dyskinesia, impaired consciousness, convulsions, delayed or regressed growth and microcephaly. Recurrent illness is mostly associated with infection and stress.

Neonatal-onset patients usually present with acute encephalopathy-like symptoms in the first few hours to one week after birth, which are manifested by vomiting, hypotonia, dehydration, severe acidosis, hyperlactatemia, hyperammonemia, coma, and convulsions, with a high morbidity and mortality rate. Childhood onset of the disease is normal at birth, mostly within 1 year of age, the first metabolic crisis is often triggered by infection, hunger, fatigue, vaccination and other stress factors, or high-protein diets and medications, which, if not diagnosed and treated in a timely manner, can lead to retardation, backwardness, and regression of mental and motor development, and can be accompanied by involvement of the haematological system, the liver, the kidneys, the skin, and peripheral nerves. In adults, the first symptoms may include peripheral neuropathy and psychiatric abnormalities.

Based on the clinical manifestations provided, the most likely diagnosis is **Medium-Chain Acyl-CoA Dehydrogenase Deficiency (MCADD)**. Here’s a detailed breakdown of why this diagnosis fits:

**1. Age of Onset:**

- The disease can develop at any age, with most children developing between 1 month and 7 years old, with an average age of about 2 years.
- This aligns well with MCADD, which often presents within the first few years of life.

**2. Clinical Manifestations:**

- **Acute Energy Metabolism Disorder Crisis:**
  - Manifested by low ketone hypoglycemia, high blood ammonia, and metabolic acidosis.
  - These symptoms are characteristic of MCADD, where the body cannot efficiently metabolize medium-chain fatty acids during periods of fasting.
- **Cardiomyopathy:**
  - Manifested by ventricular hypertrophy, cardiac insufficiency, cardiac arrhythmia, and elevated creatine kinase.
  - Cardiac involvement is a known complication in MCADD patients.
- **Myopathy:**
  - Manifested by muscle weakness, hypotonia, myalgia, poor exercise endurance, elevated muscular-type creatine kinase, and lipid deposition in muscle fibers.
  - Muscle symptoms are common in MCADD due to the accumulation of fatty acids and their metabolites.
- **Liver Damage:**
  - Manifested by hepatomegaly, fatty liver, abnormal liver function.
  - Liver involvement is also a known feature in MCADD patients, with some cases presenting acute onset illness similar to Reye syndrome.

**3. Gastrointestinal Symptoms:**

- Recurrent abdominal pain, vomiting, and gastroesophageal reflux.
- These symptoms are common in MCADD due to metabolic disturbances affecting the gastrointestinal system.

**4. Neurological Symptoms:**

- Dyskinesia, impaired consciousness, convulsions, delayed or regressed growth, microcephaly.
- Neurological involvement is significant in both acute crises and chronic cases of MCADD.

**5. Neonatal-Onset Patients:**

- Acute encephalopathy-like symptoms within the first few hours to one week after birth, manifested by vomiting, hypotonia, dehydration, severe acidosis, hyperlactatemia, hyperammonemia, coma, and convulsions.
- High morbidity and mortality rate in neonatal-onset cases.

**6. Childhood-Onset Patients:**

- Normal at birth, with the first metabolic crisis often triggered by infection, hunger, fatigue, vaccination, or high-protein diets and medications.
- Can lead to retardation, backwardness, and regression of mental and motor development.
- Involvement of the hematological system, liver, kidneys, skin, and peripheral nerves.

**7. Adult-Onset Patients:**

- First symptoms may include peripheral neuropathy and psychiatric abnormalities.
- These are less common but can occur in adults with MCADD.

**Conclusion:**

Given the wide range of clinical manifestations and their variability across different age groups, Medium-Chain Acyl-CoA Dehydrogenase Deficiency (MCADD) is the most likely diagnosis. Early identification through newborn screening and timely management can significantly improve outcomes for patients with this condition. Diagnostic tests such as plasma acylcarnitine profile and genetic testing are recommended to confirm the diagnosis.

For further confirmation, diagnostic tests including plasma acylcarnitine profile and genetic testing for MCADD should be performed. Early identification and management are crucial in preventing severe complications and improving patient outcomes.

**82. User**

The typical patient is normal at birth. At the age of 6 months to 1 year, the patient’s face gradually becomes ugly, with flattening in the middle of the face, widening of the bridge of the nose, clouding of the cornea, deafness, large head, long anterior and posterior diameters, protrusion of the forehead, stiffness of the joints, claw shaped hands, chicken chest, and lumbar lordosis. Heart valve thickening, cardiomyopathy or congestive heart failure often appear at the age of 2 to 5 years, and liver and spleen enlargement at the age of 5 years. Mental and motor development is backward. They usually survive until about 10 years of age. Most of them die of heart failure and pneumonia. In the mild form, symptoms appear after the age of 5 years and include mild coarse facial features, corneal clouding, and stiffness of the joints of the hands and feet. Intelligence is normal and life expectancy is relatively long. Clinical symptoms overlap between subtypes, but there are no significant differences in biochemical findings between subtypes.

Based on the clinical manifestations provided, the most likely diagnosis is **Mucopolysaccharidosis Type I (Hurler Syndrome for the severe form and Hurler-Scheie or Scheie Syndrome for attenuated forms)**. This rare genetic disorder involves the accumulation of glycosaminoglycans (GAGs) due to a deficiency in the enzyme alpha-L-iduronidase.

**Key Clinical Manifestations:**

1. **Severe Form:**
   - Normal at birth.
   - At 6 months to 1 year, gradual facial changes with flattening in the middle of the face, widening of the bridge of the nose, corneal clouding, deafness, large head, long anterior and posterior diameters, protrusion of the forehead, stiffness of joints, claw-shaped hands, chicken chest, and lumbar lordosis.
   - Heart valve thickening, cardiomyopathy or congestive heart failure often appear between 2 to 5 years.
   - Liver and spleen enlargement at around 5 years.
   - Backward mental and motor development.
   - Typically survive until about 10 years of age, with most dying from heart failure or pneumonia.
2. **Attenuated Form:**
   - Symptoms appear after the age of 5 years.
   - Mild coarse facial features, corneal clouding, and stiffness in the joints of the hands and feet.
   - Normal intelligence and relatively longer life expectancy.

**Other Possible Diagnoses:**

1. **Mucopolysaccharidosis Type II (Hunter Syndrome)**:
   - X-linked recessive disorder.
   - Coarse facial features but no corneal clouding.
   - Joint stiffness, among other symptoms.
2. **Mucopolysaccharidosis Type VI (Maroteaux-Lamy Syndrome)**:
   - Corneal clouding, skeletal abnormalities, and cardiomyopathy.
   - Typically normal intelligence.
3. **Mucopolysaccharidosis Type VII (Sly Syndrome)**:
   - Similar to other MPS types but with distinct biochemical findings.
4. **Mucopolysaccharidosis Type III (Sanfilippo Syndrome)**:
   - Neurological symptoms, behavioral issues, and mild physical abnormalities.
5. **Mucopolysaccharidosis Type IV (Morquio Syndrome)**:
   - Skeletal abnormalities, corneal clouding, and joint stiffness.
   - Normal intelligence.

**Conclusion:**

Given the detailed clinical manifestations, Mucopolysaccharidosis Type I is the most likely diagnosis for both severe and attenuated forms. However, further diagnostic tests such as enzyme assays or genetic testing would be necessary to confirm the specific subtype and provide a definitive diagnosis.

Would you like more information on any of these conditions or need assistance with diagnostic procedures?

**83. User**

Insidious onset of disease. The disease develops slowly or progresses in stages and may have long periods of stability. The disease can develop at any age, mostly before 50 years old. Clinical manifestations are mainly multiple mononeuropathies, asymmetric distal upper limb is more common, early unilateral upper limb one or more nerves involved, manifested as muscle weakness of the corresponding innervated area, distal predominantly, may be accompanied by spasm or fasciculations. With the development of the disease, muscle atrophy can occur; with a longer course of the disease, multiple nerves of multiple limbs can be involved, and the asymmetry of the affected nerves may not be obvious, but presenting a distribution similar to that of polypoidal peripheral neuropathy. Patients may complain of mild sensory abnormalities, but there is a lack of objective signs of sensory involvement, and some patients may also have partial sensory nerve involvement in the later stages of the disease. Cerebral nerves are usually not involved. There are no signs of upper motor neuron involvement.

Based on the clinical manifestations you’ve described, the most likely diagnosis is **Multifocal Motor Neuropathy (MMN)**. This condition is characterized by an insidious onset and slow progression, often developing before the age of 50. The primary clinical features include:

1. **Asymmetric Distal Upper Limb Involvement**: Early in the disease, one or more nerves in the upper limbs are affected, leading to muscle weakness predominantly in the distal areas. This can be accompanied by fasciculations and spasm.
2. **Muscle Atrophy**: As the disease progresses, muscle atrophy may occur.
3. **Multiple Nerve Involvement**: Over time, multiple nerves in both upper and lower limbs can become involved, though the asymmetry might not be obvious.
4. **Sensory Involvement**: While patients may complain of mild sensory abnormalities, objective signs of sensory involvement are usually absent. Some patients might develop partial sensory nerve involvement later in the disease course.

Other possible diagnoses to consider include:

1. **Lewis-Sumner Syndrome (MADSAM variant of CIDP)**: This can present with asymmetric multifocal neuropathy and may have more significant sensory involvement than MMN.
2. **Amyotrophic Lateral Sclerosis (ALS), primarily the lower motor neuron variant**: ALS typically involves both upper and lower motor neurons, but early on, some patients may present predominantly with lower motor neuron symptoms and asymmetric weakness.
3. **Hereditary Neuropathy with Liability to Pressure Palsies (HNPP)**: This condition is characterized by recurrent episodes of nerve compression, often involving the ulnar or median nerves.

Given these clinical features, further diagnostic steps such as electrophysiological studies (nerve conduction studies and electromyography) and possibly genetic testing would be beneficial for a definitive diagnosis.

Would you like more information on any specific aspect of MMN or need assistance with diagnostic procedures?

**84. User**

Neonatal onset is often fatal and may present as a metabolic crisis including metabolic acidosis, non-ketotic hypoglycaemia, hyperammonaemia, and other features including multisystem involvement (hypotonia, cardiomyopathy, hepatomegaly), excretion of large amounts of fatty acid- and amino acid-derived metabolites, ‘sweaty-foot’ body odour, coma, and neonatal death. Rapidly progressive hypertrophic cardiomyopathy is common in the type with neonatal onset and has a very high probability of sudden death, which is difficult to reverse, even if the disease is detected at an early stage by neonatal screening.

Congenital malformations are thought to be the consequence of the accumulation of toxic metabolites in the part of the body not corrected by embryonic transport. The degree of impaired fatty acid oxidative metabolism determines the degree of acidosis in utero, which in turn prevents normal embryo formation. Congenital malformations include enlarged polycystic kidneys, rocker-bottom feet, absence of lower abdominal muscles, hypospadias and penile curvature, hypotonia, cerebral cortical dysplasia, and gliosis. Other dysmorphic features include wide eye spacing, ear malformations, megalocrania, wide fontanel, high forehead, and low nasal bridge.

The age of onset and symptoms of late-onset forms vary considerably among cases and are mainly characterised by the absence of congenital malformations but with a lifelong risk of acute intermittent episodes of vomiting, dehydration, hypo-ketotic hypoglycaemia, acidosis, and impaired consciousness. Older individuals may have hepatomegaly or lipid deposition myopathy. Acute loss-of-compensation episodes can be triggered by infection, fever, surgery, low-energy diets or weight loss, alcohol consumption, and pregnancy. Most patients develop chronic myopathy symptoms, including exercise intolerance, myalgia, muscle weakness, and muscle atrophy. Organic aciduria in patients with late-onset disease is often intermittent and is more pronounced only during disease and under catabolic stress.

Based on the provided clinical manifestations, the condition described appears to be **Medium-Chain Acyl-CoA Dehydrogenase Deficiency (MCADD)**. This is a rare genetic disorder characterized by both neonatal and late-onset forms.

**Neonatal Onset:**

- **Symptoms**: Often fatal with a high probability of sudden death.
- **Clinical Features**:
  - Metabolic crisis including metabolic acidosis, non-ketotic hypoglycaemia, hyperammonaemia.
  - Multisystem involvement: Hypotonia, cardiomyopathy, hepatomegaly.
  - Excretion of large amounts of fatty acid- and amino acid-derived metabolites.
  - ‘Sweaty-foot’ body odour, coma, neonatal death.
  - Rapidly progressive hypertrophic cardiomyopathy.

**Congenital Malformations:**

- Enlarged polycystic kidneys
- Rocker-bottom feet
- Absence of lower abdominal muscles
- Hypospadias and penile curvature
- Hypotonia
- Cerebral cortical dysplasia, gliosis
- Other dysmorphic features: Wide eye spacing, ear malformations, megalocrania, wide fontanel, high forehead, low nasal bridge.

**Late-Onset Forms:**

- **Age of Onset**: Varies widely among cases.
- **Symptoms**:
  - Absence of congenital malformations.
  - Lifelong risk of acute intermittent episodes: Vomiting, dehydration, hypo-ketotic hypoglycaemia, acidosis, impaired consciousness.
  - Older individuals may have hepatomegaly or lipid deposition myopathy.
- **Triggers**: Infection, fever, surgery, low-energy diets or weight loss, alcohol consumption, pregnancy.
- **Chronic Myopathy Symptoms**:
  - Exercise intolerance
  - Myalgia
  - Muscle weakness
  - Muscle atrophy
- **Organic Aciduria**: Intermittent and more pronounced during disease and under catabolic stress.

**Diagnosis and Management:**

- **Diagnosis**: Genetic testing for MCADD is crucial.
- **Management**:
  - Dietary management to avoid fasting and ensure adequate carbohydrate intake.
  - Regular medical follow-up to monitor for acute episodes.
  - Immediate treatment with glucose and intravenous fluids during acute episodes.

This condition requires careful monitoring and management, especially in the neonatal period due to its high risk of sudden death. If you have any specific questions or need further details, please let me know!

**85. User**

The average age of onset of the disease is 30 years old, and it is rare to find patients under 10 years old and over 50 years old. The prevalence of women is higher than that of men, with the ratio of women to men (1.4-2.3):1. The disease is mostly acute/subacute, and is characterised by spatial and temporal multiplicity. The spatial multiplicity refers to the multiple sites of the lesions, including the brain, brainstem, cerebellum, and spinal cord, which may be involved simultaneously or successively. Time-recurrent refers to the course of remission-relapse, and due to the wide range of sites involved, repeated episodes may eventually lead to disability or even death. Clinical manifestations are varied, and common symptoms include vision loss, diplopia, limb sensory deficits, limb motor deficits, ataxia, and bladder or rectal dysfunction.

Based on the provided clinical manifestations and demographic profile, the disease most likely corresponds to **Multiple Sclerosis (MS)**. Here is a detailed breakdown of why MS fits this description:

**Demographic Profile:**

- **Average Age of Onset**: 30 years old.
- **Age Distribution**: Rare to find patients under 10 years old or over 50 years old.
- **Gender Ratio**: Higher prevalence in women, with a ratio of (1.4-2.3):1.

**Clinical Characteristics:**

- **Course of the Disease**: Mostly acute/subacute, characterized by spatial and temporal multiplicity.
  - **Spatial Multiplicity**: Involvement of multiple sites including the brain, brainstem, cerebellum, and spinal cord simultaneously or successively.
  - **Temporal Recurrence**: Remission-relapse course, with repeated episodes potentially leading to disability or even death.

**Common Symptoms:**

- Vision loss
- Diplopia (double vision)
- Limb sensory deficits
- Limb motor deficits
- Ataxia (lack of coordination)
- Bladder or rectal dysfunction

**Supporting Diagnoses:**

1. **Neuromyelitis Optica Spectrum Disorder (NMOSD)**: Characterized by inflammatory demyelination, primarily affecting the optic nerves and spinal cord, sometimes with brain involvement.
2. **Acute Disseminated Encephalomyelitis (ADEM)**: Presents with multifocal CNS symptoms, typically follows a monophasic course but can be recurrent.
3. **Sarcoidosis with CNS Involvement (Neurosarcoidosis)**: Can mimic MS symptoms with diverse CNS manifestations including cranial nerve palsies and myelopathy.
4. **Systemic Lupus Erythematosus (SLE) with Neuropsychiatric Involvement**: SLE can affect the CNS in various ways, including transverse myelitis and other neurological symptoms.

Given these characteristics, MS is the most likely diagnosis based on the provided information. However, it is important to consider differential diagnoses such as NMOSD, ADEM, neurosarcoidosis, and SLE with neuropsychiatric involvement for a comprehensive evaluation.

For accurate diagnosis, further clinical assessment, imaging studies (such as MRI), and possibly lumbar puncture may be necessary. Consulting with a neurologist who specializes in multiple sclerosis would be advisable to confirm the diagnosis and develop an appropriate treatment plan.

**86. User**

The main symptoms include parkinsonism, cerebellar ataxia, pyramidal tract damage, and autonomic nervous system damage. The first symptoms are often autonomic impairments, most notably cardiovascular dysfunction (e.g., upright hypotension) and urinary dysfunction (e.g., urinary incontinence), the latter of which tends to be comorbid with sexual dysfunction in male patients. Many patients have had rapid eye movement (REM) sleep behaviour disorder (RBD) for several years.

Based on the clinical manifestations you’ve provided, the rare disease most likely to be diagnosed is **Multiple System Atrophy (MSA)**. MSA is a progressive neurodegenerative disorder characterized by a combination of parkinsonism, cerebellar ataxia, autonomic dysfunction, and pyramidal tract symptoms.

**Key Features of MSA:**

- **Parkinsonism**: Characterized by bradykinesia, rigidity, tremor, and postural instability.
- **Cerebellar Ataxia**: Impaired coordination and balance due to cerebellar involvement.
- **Pyramidal Tract Damage**: Symptoms such as spasticity, weakness, and hyperreflexia.
- **Autonomic Nervous System Damage**:
  - **Cardiovascular Dysfunction**: Upright hypotension (orthostatic hypotension).
  - **Urinary Dysfunction**: Urinary incontinence, which can be comorbid with sexual dysfunction in male patients.

**Additional Clinical Manifestations:**

- **REM Sleep Behavior Disorder (RBD)**: Many MSA patients have a history of RBD for several years before other symptoms become apparent.

**Differential Diagnoses:**

1. **Parkinson’s Disease with Autonomic Dysfunction**: While primarily characterized by parkinsonism, it can also present with autonomic dysfunction, especially in advanced stages.
2. **Progressive Supranuclear Palsy (PSP)**: This neurodegenerative disease involves parkinsonian features and balance issues but typically has distinct eye movement abnormalities and a different pattern of autonomic involvement.
3. **Dementia with Lewy Bodies (DLB)**: DLB can present with parkinsonian features and REM sleep behavior disorder, though cognitive impairment is more prominent.
4. **Pure Autonomic Failure (PAF)**: This condition involves autonomic nervous system dysfunction, especially orthostatic hypotension, but lacks the neurological features like parkinsonism and ataxia.
5. **Corticobasal Degeneration (CBD)**: While primarily involving movement disorders and cognitive dysfunction, some overlap with parkinsonism and autonomic symptoms can occur.

Given these characteristics, MSA is the most likely diagnosis based on your description. For a definitive diagnosis, further clinical assessment, imaging studies, and possibly neurophysiological tests may be necessary. Consulting with a neurologist who specializes in movement disorders would be advisable for a comprehensive evaluation and appropriate management plan.

**87. User**

**1. Muscles,** weakness and atrophy of the distal limbs starting at the distal end of the limbs and progressing proximally, affecting the fine movements of the hands and causing drop foot. The facial muscles are also involved. At the same time, there is obvious muscle ankylosis, involving the limb muscles, pharyngeal muscles and so on. Physical examination may reveal that the fingers cannot be expanded immediately after clenching the fist vigorously, and the eyes cannot be opened immediately after closing them vigorously. Knocking on the hand, the big fish muscle, the tongue muscle, etc., can lead to muscle ankylosis, known as knocking muscle ankylosis or ‘muscle ball’ phenomenon. There is a ‘warming up’ phenomenon, which decreases after repeated activities. Lower extremity myalgia and generalised fatigue are also common clinical manifestations.

**The cardiac conduction system is often involved, with varying degrees of atrioventricular block and intraventricular conduction abnormalities. In severe cases, sudden death may result. Confirmed patients require regular ECG monitoring and pacemaker implantation if necessary.

**3. Central nervous system** , sleep disorders such as increased sleepiness and obstructive sleep apnoea are common. It may be associated with cognitive and mood disorders. Head MRI shows diffuse cerebral white matter lesions and cortical atrophy.

**Cataracts occur in almost all patients as they age. They often affect vision in the 30s and 40s.

**5. Endocrine system** A variety of endocrine disorders may occur, including diabetes mellitus, thyroid dysfunction, abnormal calcium and phosphorus metabolism, sex hormone abnormalities, and testicular atrophy (resulting in infertility).

In addition, there may be frontal baldness, gastrointestinal insufficiency and restrictive ventilation disorders.

Based on the clinical manifestations provided, the most likely diagnosis is **Myotonic Dystrophy Type 1 (DM1)**. The symptoms described align closely with the characteristics of DM1:

**Muscular Symptoms:**

- **Weakness and Atrophy**: Weakness and atrophy of the distal limbs starting from the distal end and progressing proximally, affecting fine movements of the hands and causing drop foot.
- **Facial Muscle Involvement**: Facial muscles are also affected, leading to muscle ankylosis. Physical examination may reveal that fingers cannot be expanded immediately after clenching the fist vigorously, and eyes cannot be opened immediately after closing them vigorously.
- **Muscle Ankylosis (‘Muscle Ball’ Phenomenon)**: This involves limb muscles, pharyngeal muscles, etc., leading to muscle ankylosis upon vigorous movement. There is a “warming up” phenomenon that decreases after repeated activities.
- **Lower Extremity Myalgia and Generalized Fatigue**: Common clinical manifestations.

**Cardiac Symptoms:**

- **Cardiovascular Involvement**: The cardiac conduction system is often involved, with varying degrees of atrioventricular block and intraventricular conduction abnormalities. Severe cases may result in sudden death.
- **Regular ECG Monitoring and Pacemaker Implantation**: Required for patients with significant conduction issues.

**Central Nervous System Symptoms:**

- **Sleep Disorders**: Increased sleepiness and obstructive sleep apnea are common.
- **Cognitive and Mood Disorders**: May be associated with cognitive decline and mood disorders.
- **MRI Findings**: Head MRI shows diffuse cerebral white matter lesions and cortical atrophy.

**Ocular Symptoms:**

- **Cataracts**: Almost all patients develop cataracts, often affecting vision in the 30s and 40s.

**Endocrine System Symptoms:**

- **Endocrine Disorders**: Various endocrine disorders may occur, including diabetes mellitus, thyroid dysfunction, abnormal calcium and phosphorus metabolism, sex hormone abnormalities, and testicular atrophy (resulting in infertility).
- **Frontal Baldness**, Gastrointestinal Insufficiency, and Restrictive Ventilation Disorders: Additional symptoms that can be present.

**Summary:**

The combination of muscular weakness and atrophy, cardiac conduction abnormalities, central nervous system involvement, ocular issues, and endocrine disorders strongly suggests Myotonic Dystrophy Type 1 (DM1). For a definitive diagnosis, further diagnostic tests such as genetic testing for the DM1 mutation (CTG repeat expansion in the DMPK gene) may be necessary.

If you need more detailed information or specific diagnostic steps, please let me know!

**88. User**

The disease tends to develop at an early age, with the most frequent onset occurring in the neonatal period. Most children develop the disease within the first few days of life and present with milk refusal, vomiting, lethargy, convulsions and coma. Some children with late-onset disease are characterised by episodes of vomiting and lethargy with mental developmental abnormalities. Patients with hyperammonaemic coma may have brain injury with developmental delay, impaired learning ability or mental retardation, and sequelae of growth retardation. Patients with hyperammonaemia may have increased intracranial pressure, shortness of breath, apnoea or failure as the condition progresses, and hepatomegaly. Neurological complications include mental retardation, acute hyperammonemic coma, and even death. Biochemical changes can be seen in hyperammonaemia, increased blood glutamine and alanine, and decreased or absent urinary whey acids.

Based on the clinical manifestations provided, the disease most likely to be diagnosed is **Medium-Chain Acyl-CoA Dehydrogenase Deficiency (MCADD)**.

**Key Clinical Manifestations of MCADD:**

1. **Neonatal and Early-Onset Cases:**
   - Most frequent onset in the neonatal period.
   - Symptoms often appear within the first few days of life.
   - Common symptoms include milk refusal, vomiting, lethargy, convulsions, and coma.
2. **Late-Onset Cases:**
   - Characterized by episodes of vomiting and lethargy.
   - May be associated with mental developmental abnormalities.
3. **Biochemical Changes:**
   - Hyperammonaemia is a key feature.
   - Increased blood glutamine and alanine levels.
   - Decreased or absent urinary whey acids.
4. **Neurological Complications:**
   - Mental retardation.
   - Acute hyperammonemic coma, which can lead to brain injury, developmental delay, impaired learning ability, or mental retardation.
   - Sequelae of growth retardation.
5. **Other Symptoms:**
   - Increased intracranial pressure.
   - Shortness of breath, apnoea, and failure as the condition progresses.
   - Hepatomegaly (enlarged liver).

**Summary:**

The clinical presentation, including early onset with symptoms like vomiting, lethargy, convulsions, and coma in neonates; episodes of vomiting and developmental abnormalities in late-onset cases; biochemical changes such as hyperammonaemia; and neurological complications like mental retardation and acute hyperammonemic coma, strongly suggest **Medium-Chain Acyl-CoA Dehydrogenase Deficiency (MCADD)**.

For a definitive diagnosis, further diagnostic tests such as acylcarnitine profile analysis, plasma amino acid levels, and genetic testing for MCADD should be performed.

If you need more detailed information or specific diagnostic procedures, please let me know!

**89. User**

Children often present with severe intrauterine growth retardation and develop severe, non-ketotic hyperglycaemia very early in life (often in the first week of life), which recovers after 12 weeks and recurs around puberty in 50%-60% of cases, with a clinical picture similar to that of type 2 diabetes mellitus.

Based on the clinical manifestations you have provided, the most likely rare disease to be diagnosed is **Transient Neonatal Diabetes Mellitus (TNDM)**. TNDM is characterized by severe intrauterine growth retardation and the onset of non-ketotic hyperglycaemia early in life, often within the first week of life. This condition typically resolves within the first few months but may recur around puberty in approximately 50%-60% of cases, with a clinical picture similar to that of type 2 diabetes mellitus.

**Other Possible Diagnoses to Consider:**

1. **Permanent Neonatal Diabetes Mellitus (PNDM):** This condition has an early onset of diabetes but does not remit and is a lifelong condition.
2. **Congenital Hyperinsulinism:** Typically presents with hypoglycemia rather than hyperglycaemia, but mutations affecting insulin regulation could lead to atypical presentations.
3. **Wolcott-Rallison Syndrome:** This rare genetic disorder causes early-onset diabetes, often accompanied by skeletal dysplasia and other systemic manifestations.
4. **MODY (Maturity Onset Diabetes of the Young):** While more common in slightly older children or adolescents, there are forms that could present atypically early.
5. **Donohue Syndrome (Leprechaunism):** This is an extremely rare condition associated with severe insulin resistance and growth retardation, though it often presents with additional dysmorphic features.

**Diagnostic Testing:**

To differentiate between these conditions, further diagnostic testing would be necessary. This may include:

- **Genetic Testing:** To identify mutations in genes associated with TNDM, PNDM, MODY, and other genetic disorders.
- **Blood Glucose Levels and Insulin Levels:** To assess the pattern of hyperglycaemia and insulin levels.
- **Acylcarnitine Profile Analysis:** For TNDM to check for specific metabolic abnormalities.

Given the clinical picture described, TNDM remains the most likely diagnosis. However, a thorough evaluation including genetic testing would be crucial to confirm the exact condition.

**90. User**

The earliest symptoms are abdominal distension and enlargement of the liver and spleen, and some patients may develop in the neonatal period. Intellectual and motor developmental deficits occur immediately, with hypotonia and motor developmental delays, often without cerebral neurological involvement, and regression of motor and intellectual development is evident after the age of 1 year, eventually progressing to spasticity and tonicity.Cherry erythema on the fundus of the eye can be found in 50% of the patients. Interstitial lung disease may lead to recurrent respiratory infections, hypoxaemia or respiratory failure. Most children die before the age of 3 years.

Based on the clinical manifestations you’ve provided, the most likely diagnosis is **Niemann-Pick Disease Type A**. This condition is a rare genetic disorder characterized by the accumulation of sphingomyelin in various tissues, leading to symptoms such as hepatosplenomegaly (enlargement of the liver and spleen), developmental delays, cherry-red spots on the fundus of the eye, and early childhood mortality.

**Other Possible Diagnoses:**

1. **Tay-Sachs Disease**: This condition also presents with developmental delays and cherry-red spots but typically lacks hepatosplenomegaly.
2. **Gaucher Disease (Type 2)**: Another lysosomal storage disorder that includes hepatosplenomegaly, neurological symptoms, and early death, but it usually exhibits more severe neurological involvement from a younger age.
3. **Sandhoff Disease**: Similar to Tay-Sachs, with systemic involvement including hepatosplenomegaly and presenting with cherry-red spots.
4. **GM1 Gangliosidosis (Type 1)**: This disease can present with hepatosplenomegaly, developmental delay, cherry-red spot, and similar neurological deterioration.
5. **Wolman Disease**: A rare lysosomal storage disease that presents with hepatosplenomegaly, developmental delays, and early death but often includes adrenal calcifications and severe malabsorption.

**Key Features of Niemann-Pick Disease Type A:**

- **Neonatal Period Symptoms**: Abdominal distension and enlargement of the liver and spleen.
- **Developmental Delays**: Immediate onset with hypotonia (low muscle tone) and motor developmental delays, often without cerebral neurological involvement.
- **Regression and Spasticity**: Regression of motor and intellectual development after 1 year, eventually progressing to spasticity and tonicity.
- **Cherry-Erythema on the Fundus**: Present in about 50% of patients.
- **Interstitial Lung Disease**: May lead to recurrent respiratory infections, hypoxaemia, or respiratory failure.
- **Early Mortality**: Most children die before the age of 3 years.

**Diagnostic Testing:**

To confirm the diagnosis, genetic testing for Niemann-Pick disease type A and other lysosomal storage disorders would be necessary. This includes: - **Genetic Testing**: To identify mutations in the NPC1 or NPC2 genes. - **Biochemical Tests**: To measure sphingomyelin levels in leukocytes or fibroblasts. - **Imaging Studies**: Such as MRI to assess brain involvement.

Given the detailed clinical picture, Niemann-Pick disease type A remains the most likely diagnosis. However, a thorough genetic evaluation is crucial for definitive confirmation.

**91. User**

**1. Typical face,** patients of all ages have ptosis, wide eye spacing, medial canthus, and downward slanting of the outer corners of the eyes; low bilateral ear position, posterior rotation, and thick auricles. Child patients may also have a full forehead, low posterior hairline, short nose, low nasal bridge, full nasal tip; thick lips, deep and wide nasolabial groove straight to the upper lip, and so on.

**2. Dwarfism,** which is normal at birth and gradually appears after 1 year of age. Bone age often lags behind, and some adult heights can reach the lower limit of normal level.

**3. cardiovascular,** 50% ~80% of patients have congenital heart disease, pulmonary valve stenosis is the most common, others include hypertrophic cardiomyopathy, ventricular ischemia, pulmonary artery branch stenosis, tetralogy of Fallot, and aortic constriction, etc. Typical ECG changes include electrocardiogram (ECG) and heart rate (HR). Typical electrocardiographic changes include extreme rightward deviation of the electrical axis with counterclockwise rotation of the QRS wave in the precordial leads, leftward deviation of the electrical axis in the V1 lead, incomplete left anterior branch block, or RSR waveforms.

**4. psychomotor retardation**, poor language skills in some patients, depression in adult patients, and a high incidence of autism spectrum manifestations have also been reported.

**5. webbing of the neck**.

**6. thoracic abnormalities,** chicken chest or funnel chest.

**7. wide breast spacing**

**8. cryptorchidism in males** , which is present in 60% to 80% of male patients and can lead to fertility disorders.

**9. abnormal blood clotting and lymphatic dysplasia**

**10. Others,** renal malformations (dilated renal pelvis, double ureteral malformation, isolated kidney, renal hypoplasia, distal ureteral stenosis, etc.), impaired spermatogenesis in males, strabismus, nystagmus, milky coffee spots and freckles on the skin, follicular keratosis of the limbs, Arnold Chiari malformation type I, hydrocephalus, hepatosplenomegaly, and an increased incidence of malignant tumours.

**11. Intrauterine manifestations,** excessive amniotic fluid in the mother during pregnancy, widening of the nuchal translucency, relatively large fetal head, cardiac and renal anomalies.

Based on the provided clinical features, the most likely diagnosis is **Marfan Syndrome**. Marfan syndrome is a genetic disorder that affects the body’s connective tissue, which provides structural support to various organs and tissues. Here’s how the described symptoms align with Marfan syndrome:

**1. Typical Face:**

- **Ptosis**: Drooping of the upper eyelid.
- **Wide eye spacing (hypertelorism)**: Increased distance between the eyes.
- **Medial canthus**: The inner corner of the eye is lower than normal.
- **Downward slanting of the outer corners of the eyes**: This is a common feature in Marfan syndrome.
- **Low bilateral ear position, posterior rotation, and thick auricles**: These features are also consistent with Marfan syndrome.

**2. Dwarfism:**

- **Dwarfism** typically does not align well with Marfan syndrome, as patients usually have tall stature rather than short stature. However, the term “dwarfism” in this context might refer to a specific subset of symptoms or other conditions that can coexist with Marfan syndrome.

**3. Cardiovascular Manifestations:**

- **50% to 80% of patients have congenital heart disease**: This is common in Marfan syndrome, particularly involving the aortic root and mitral valve.
- **Pulmonary valve stenosis, hypertrophic cardiomyopathy, ventricular ischemia, pulmonary artery branch stenosis, tetralogy of Fallot, and aortic constriction**: These are specific cardiovascular manifestations that can occur in Marfan syndrome.
- **Electrocardiographic (ECG) changes**: The described ECG findings such as extreme rightward deviation of the electrical axis with counterclockwise rotation of the QRS wave in the precordial leads, leftward deviation of the electrical axis in the V1 lead, incomplete left anterior branch block, or RSR waveforms are consistent with Marfan syndrome.

**4. Psychomotor Retardation:**

- **Psychomotor retardation, poor language skills, depression in adult patients, and a high incidence of autism spectrum manifestations**: These neurological symptoms can occur but are not the primary hallmark of Marfan syndrome. They might be secondary to other conditions or complications.

**5. Webbing of the Neck (Neck webbing):**

- **Neck webbing** is a characteristic feature of Marfan syndrome, where there is a fold of skin between the neck and shoulders.

**6. Thoracic Abnormalities:**

- **Chicken chest or funnel chest**: These are common in Marfan syndrome due to the involvement of connective tissue in the chest wall.

**7. Wide Breast Spacing:**

- This feature does not typically align with Marfan syndrome, but it might be a secondary manifestation or related to other conditions.

**8. Cryptorchidism in Males:**

- **Cryptorchidism** (undescended testicles) is present in 60% to 80% of male patients and can lead to fertility disorders: This is more characteristic of other genetic syndromes such as Klinefelter syndrome, rather than Marfan syndrome.

**9. Abnormal Blood Clotting and Lymphatic Dysplasia:**

- **Abnormal blood clotting** and **lymphatic dysplasia**: These are not primary features of Marfan syndrome but might be related to other underlying conditions or complications.

**10. Other Manifestations:**

- **Renal malformations (dilated renal pelvis, double ureteral malformation, isolated kidney, renal hypoplasia, distal ureteral stenosis)**: These can occur in Marfan syndrome but are not the primary features.
- **Impaired spermatogenesis in males**: This is more characteristic of Klinefelter syndrome or other conditions.
- **Strabismus (crossed eyes), nystagmus (involuntary eye movements)**, and **milky coffee spots and freckles on the skin**: These are not primary features of Marfan syndrome but might be related to other conditions.
- **Follicular keratosis of the limbs**: This is more characteristic of other conditions such as Ectodermal Dysplasia.
- **Arnold Chiari malformation type I, hydrocephalus, hepatosplenomegaly, and an increased incidence of malignant tumours**: These are not primary features of Marfan syndrome but might be related to other underlying conditions.

**11. Intrauterine Manifestations:**

- **Excessive amniotic fluid in the mother during pregnancy (polyhydramnios)**: This can occur due to various reasons, including fetal anomalies.
- **Widening of the nuchal translucency**: This is a finding often associated with chromosomal abnormalities or other genetic syndromes rather than Marfan syndrome.

**Conclusion:**

Given the described symptoms and features, Marfan

**92. User**

Patients can develop the disease at any age. There are two main clinical categories: early-onset and late-onset. Early-onset cases mainly occur in male children, usually in the neonatal period, and are characterised by a rapid onset of the disease, with a rapid onset of the disease and extensive brain damage due to elevated ammonia levels in the blood. There may be no abnormality at birth, but in the first few days of life, the child will show irritability, lethargy, refusal to eat, shortness of breath and lethargy, which may rapidly develop into spasm, coma and respiratory failure. If not treated urgently, the disease quickly develops into hereditary metabolic encephalopathy and often dies within the first week of life, with survivors often left with severe intellectual impairment. Late-onset disease occurs in older patients, both hemizygous males and heterozygous females, and has relatively mild and variable clinical symptoms. Patients with childhood and adult onset tend to present with chronic neurological damage such as episodes of vomiting, headaches, abnormal behaviour, delirium and confusion. Patients may have clinical phenotypes such as hepatomegaly, recurrent seizures, growth retardation, and behavioural abnormalities. Despite the milder symptoms of the late-onset form, life-threatening acute episodes of hyperammonemia can be induced by environmental factors such as illness, stress, and a high-protein diet. The majority of heterozygous female carriers remain asymptomatic throughout their lives, while a few develop the disease, with individual variability in age of onset and clinical manifestations, both early and late onset.

Based on the clinical manifestations described, the rare disease most likely to be diagnosed is **Ornithine Transcarbamylase (OTC) Deficiency**. OTC deficiency is a urea cycle disorder characterized by two main clinical categories: early-onset and late-onset cases.

**Early-Onset Cases**

- **Age of Onset**: Usually in the neonatal period.
- **Symptoms**: Rapid onset of disease, extensive brain damage due to elevated ammonia levels in the blood.
- **Clinical Presentation**:
  - No abnormality at birth, but within the first few days of life, the child may show irritability, lethargy, refusal to eat, shortness of breath, and lethargy.
  - These symptoms can rapidly develop into spasm, coma, and respiratory failure.
- **Outcome**: If not treated urgently, the disease quickly develops into hereditary metabolic encephalopathy and often results in death within the first week of life. Survivors are frequently left with severe intellectual impairment.

**Late-Onset Cases**

- **Age of Onset**: Occurs in older patients, both hemizygous males and heterozygous females.
- **Symptoms**: Relatively mild and variable clinical symptoms.
- **Clinical Presentation**:
  - Chronic neurological damage such as episodes of vomiting, headaches, abnormal behavior, delirium, and confusion.
  - Other possible phenotypes include hepatomegaly, recurrent seizures, growth retardation, and behavioral abnormalities.
- **Acute Episodes**: Despite the milder symptoms, life-threatening acute episodes of hyperammonemia can be induced by environmental factors such as illness, stress, and a high-protein diet.
- **Carrier Status**: The majority of heterozygous female carriers remain asymptomatic throughout their lives. However, some may develop the disease with individual variability in age of onset and clinical manifestations.

In summary, the clinical features described align closely with OTC deficiency, making it the most likely diagnosis based on the provided information.

**93. User**

The clinical phenotype of the disease varies in severity. The main clinical manifestations of the patients are recurrent fractures under minor external forces, skeletal deformities, and varying degrees of activity limitation since childhood. Patients may have extra-skeletal manifestations such as blue sclera, ligamentous laxity, dentin hypoplasia, hearing loss, and cardiac valvulopathy, etc. The imaging features of OI mainly include: thinning of bones in many parts of the body; thinning of the cranial plate, widening of fontanelle and cranial suture, interstitial bone in occipital bones, and flattening of the base of the skull; lateral curvature or posterior convexity deformity of the spine, deformation of vertebral bodies, and compression fracture of multiple vertebral bodies; twisted and deformed thorax, or even collapse; slender long bones of the limbs, and even collapse; and the long bones of the limbs are thin and thin. The long bones of the limbs are slender, the bone cortex is thin, the bone marrow cavity is relatively large, the epiphyses are widened, there are many long bone fractures, and the bones are curved and deformed.

Based on the clinical manifestations and imaging features described, the most likely diagnosis is **Osteogenesis Imperfecta (OI)**. OI is a genetic disorder characterized by brittle bones that are prone to fracture from minimal or no trauma. The associated features such as blue sclera, dentin hypoplasia, hearing loss, and skeletal deformities align well with this condition.

**Clinical Manifestations**

- **Recurrent Fractures**: Patients experience fractures under minor external forces.
- **Skeletal Deformities**: Varying degrees of activity limitation since childhood, including:
  - Blue sclera (a common extra-skeletal manifestation).
  - Ligamentous laxity.
  - Dentin hypoplasia.
  - Hearing loss.
  - Cardiac valvulopathy.

**Imaging Features**

- **Thinning of Bones**: Thinning is observed in many parts of the body, including:
  - Thinning of the cranial plate.
  - Widening of fontanelles and cranial sutures.
  - Interstitial bone in occipital bones.
  - Flattening of the base of the skull.
- **Spinal Deformities**: Lateral curvature or posterior convexity deformity, deformation of vertebral bodies, and compression fractures of multiple vertebral bodies.
- **Thoracic Deformities**: Twisted and deformed thorax, or even collapse.
- **Long Bones**: Slender long bones of the limbs with thin bone cortex, relatively large bone marrow cavity, widened epiphyses, many long bone fractures, and curved and deformed bones.

**Other Possible Diagnoses**

1. **Hypophosphatasia**: A rare genetic disorder affecting bone mineralization, leading to soft and weakened bones.
2. **Ehlers-Danlos Syndrome (EDS)**: Particularly the hypermobile or vascular types, which can present with joint hypermobility, skin elasticity, and occasionally bone fragility.
3. **Marfan Syndrome**: A connective tissue disorder characterized by features such as tall stature, long limbs, and sometimes bone deformities.

Given the comprehensive clinical and imaging findings, OI is the most likely diagnosis for this patient.

**94. User**

**1. Pancytopenia,** Many patients may have a combination of pancytopenia and neutropenia. Clinical manifestations include malaise, recurrent infections and bleeding tendencies.

**2. Haemoglobinuria** , typical haemoglobinuria is soy sauce or strong tea colour. It usually lasts for 23 days and subsides on its own without treatment, while in severe cases, it lasts for 12 weeks or even longer. Some patients have frequent episodes of haemoglobinuria, while a significant number of patients have no clear episodes of haemoglobinuria over a long period of time or observation.

Episodes of haemoglobinuria may be accompanied by fever, low back pain and abdominal pain. Other symptoms associated with smooth muscle dysfunction can include dysphagia, abdominal pain, bloating, back pain, headache, oesophageal spasms, and erectile dysfunction. Rare conditions include choledochal duct dyskinesia, acute pancreatitis, ischaemia and ulceration of the duodenum or colon.

**3. thrombosis**.

**Jaundice and hepatosplenomegaly** , less than half of patients have mild jaundice. Most patients do not have hepatosplenomegaly.

Common comorbidities include infection, anaemic heart disease, cholelithiasis, renal insufficiency and even failure.

Based on the clinical manifestations you’ve provided, the most likely rare disease diagnosis is **Paroxysmal Nocturnal Hemoglobinuria (PNH)**. PNH is characterized by a combination of pancytopenia, hemoglobinuria, thrombosis, and symptoms related to smooth muscle dysfunction.

**Clinical Manifestations**

1. **Pancytopenia**: Many patients may have a combination of pancytopenia and neutropenia. This can lead to:
   - Malaise.
   - Recurrent infections.
   - Bleeding tendencies.
2. **Haemoglobinuria**:
   - Typical haemoglobinuria is soy sauce or strong tea color.
   - Episodes usually last for 2-3 days but can extend up to 1-2 weeks in severe cases.
   - Some patients may have frequent episodes, while others may not experience clear episodes over a long period.
3. **Thrombosis**: PNH is associated with an increased risk of thrombotic events, including:
   - Vascular thrombosis (e.g., deep vein thrombosis, pulmonary embolism).
4. **Jaundice and Hepatosplenomegaly**:
   - Less than half of patients have mild jaundice.
   - Most patients do not experience hepatosplenomegaly.
5. **Smooth Muscle Dysfunction**: Symptoms can include:
   - Dysphagia.
   - Abdominal pain.
   - Bloating.
   - Back pain.
   - Headache.
   - Oesophageal spasms.
   - Erectile dysfunction.
   - Rare conditions such as choledochal duct dyskinesia, acute pancreatitis, ischaemia, and ulceration of the duodenum or colon.

**Differential Diagnoses**

1. **Aplastic Anemia**: This condition also presents with pancytopenia but typically does not include hemoglobinuria or thrombosis.
2. **Myelodysplastic Syndromes (MDS)**: These can present with pancytopenia and are associated with an increased risk of progression to acute myeloid leukemia, but do not usually involve hemoglobinuria or significant smooth muscle dysfunction.
3. **Evans Syndrome**: This autoimmune disorder involves both immune-mediated thrombocytopenia and hemolytic anemia, which can present with pancytopenia and hemoglobinuria, though it is less common than PNH.
4. **Hereditary Spherocytosis**: This genetic disorder causes chronic hemolysis but does not typically involve pancytopenia or thrombosis.
5. **Wiskott-Aldrich Syndrome**: This rare X-linked disorder involves immunodeficiency and thrombocytopenia, but does not usually present with significant smooth muscle dysfunction.

Given the comprehensive set of symptoms you’ve described, PNH is the most likely diagnosis among these conditions. However, a definitive diagnosis would require further testing such as flow cytometry to detect the presence of DAF-negative red blood cells characteristic of PNH.

For accurate diagnosis and treatment, it is recommended to consult with a hematologist who can perform appropriate diagnostic tests.

**95. User**

Clinical manifestations include repeated intussusception, abdominal pain, diarrhoea, mucus stools, blood in stool, constipation, vomiting blood and anaemia. Characteristic clinical manifestations are skin and mucous membrane pigmentation and multiple polyps in the gastrointestinal tract. 95% of the patients have hyperpigmentation, which can be found on the lips and surrounding areas of the mouth, cheeks, face, fingers, palms of the hands, soles of the feet, perianal area, etc., and occasionally on the intestinal mucous membranes. The pigmentation can be brown, tan, grey, blue, etc. It usually occurs before the age of 2 years, and the size is 1~5mm. 88%-100% of the patients will develop gastrointestinal polyps, and the onset of gastrointestinal polyps most often occurs at the age of 11-13 years, and the polyps can occur throughout the entire gastrointestinal tract, with the small intestine being the most common, followed by the colon and the stomach. The size of the polyps is variable, and their tips vary in length and thickness. The risk of cancer is high, with an average age of 42 years.

Based on the clinical manifestations you’ve described, the most likely diagnosis is **Peutz-Jeghers Syndrome (PJS)**. Peutz-Jeghers syndrome is a rare genetic disorder characterized by the development of benign polyps in the gastrointestinal tract and distinctive pigmented spots on the skin and mucous membranes.

**Key Clinical Manifestations:**

1. **Gastrointestinal Symptoms**:
   - Repeated intussusception.
   - Abdominal pain.
   - Diarrhea.
   - Mucus stools.
   - Blood in stool.
   - Constipation.
   - Vomiting blood.
   - Anemia.
2. **Skin and Mucous Membrane Pigmentation**:
   - 95% of patients have hyperpigmentation, which can be found on the lips and surrounding areas of the mouth, cheeks, face, fingers, palms of the hands, soles of the feet, perianal area, etc., and occasionally on the intestinal mucous membranes.
   - The pigmentation can appear as brown, tan, grey, or blue spots.
   - This usually occurs before the age of 2 years.
3. **Gastrointestinal Polyps**:
   - 88%-100% of patients develop gastrointestinal polyps.
   - The onset of gastrointestinal polyps most often occurs at the age of 11-13 years.
   - Polyps can occur throughout the entire gastrointestinal tract, with the small intestine being the most common site, followed by the colon and stomach.
   - The size of the polyps is variable, and their tips vary in length and thickness.
4. **Risk of Cancer**:
   - There is a high risk of cancer, with an average age of 42 years for development.

**Other Possible Considerations:**

1. **Familial Adenomatous Polyposis (FAP)**: While FAP also involves polyps in the colon and rectum, it lacks the characteristic pigmentation seen in Peutz-Jeghers syndrome.
2. **Cronkhite-Canada Syndrome**: This is a rare, non-hereditary condition characterized by gastrointestinal polyposis and skin changes, but it does not typically present with the specific pigmented spots described.

Given the detailed clinical picture provided, Peutz-Jeghers syndrome remains the most probable diagnosis. For further confirmation, genetic testing for mutations in the STK11/LKB1 gene is recommended.

If you need more information or assistance with diagnostic procedures, please let me know!

**96. User**

In the neonatal period, most of the children have no clinical symptoms, but after 3-4 months of life, typical symptoms gradually appear, and the symptoms are obvious at the age of 1 year. A few months after birth, due to insufficient melanin synthesis, their hair and iris colour gradually becomes lighter, yellow or brownish-yellow, with white skin. Urine and sweat contain a large amount of phenyl lactic acid and have a foul odour of rat urine. As they grow older, they gradually show delayed intellectual development, with predominantly cognitive developmental disorders, microcephaly, seizures, as well as behavioural and personality abnormalities, such as hyperactivity, self-mutilation, aggression, autism, low self-esteem, depression and other neurological manifestations. Vomiting and eczema are also commonly seen in infancy.

Based on the clinical manifestations described, the most likely diagnosis is **Phenylketonuria (PKU)**. PKU is a rare genetic disorder caused by a deficiency in the enzyme phenylalanine hydroxylase, which leads to the accumulation of phenylalanine and its metabolites, such as phenyl lactic acid, causing the symptoms described.

**Key Clinical Manifestations:**

1. **Neonatal Period**:
   - Most children have no clinical symptoms.
2. **After 3-4 Months**:
   - Gradual appearance of typical symptoms.
   - Symptoms become obvious by the age of 1 year.
3. **Hair and Iris Coloration**:
   - Due to insufficient melanin synthesis, hair and iris color gradually lighten, becoming yellow or brownish-yellow.
   - Skin appears white due to lack of pigmentation.
4. **Urine and Sweat**:
   - Urine and sweat contain a large amount of phenyl lactic acid.
   - Foul odor resembling rat urine.
5. **Developmental Delays and Neurological Symptoms**:
   - Delayed intellectual development, predominantly cognitive developmental disorders.
   - Microcephaly (small head size).
   - Seizures.
   - Behavioral and personality abnormalities such as hyperactivity, self-mutilation, aggression, autism, low self-esteem, depression.
6. **Other Manifestations in Infancy**:
   - Vomiting.
   - Eczema.

**Other Possible Considerations:**

1. **Homocystinuria**: Another metabolic disorder characterized by a deficiency in cystathionine beta-synthase, leading to elevated homocysteine levels and potentially similar developmental delays and behavioral abnormalities.
2. **Maple Syrup Urine Disease (MSUD)**: A metabolic disorder involving the inability to break down certain amino acids, resulting in neurological symptoms, a distinct sweet-smelling urine, and developmental delays.
3. **Albinism**: While primarily a disorder of pigmentation, some forms can have associated neurological symptoms, although the foul odor and specific behavioral symptoms do not typically align with this condition.
4. **Tyrosinemia Type I**: A disorder affecting the breakdown of the amino acid tyrosine, leading to liver and kidney dysfunction and potentially neurological symptoms, though the presentation of PKU is more typical.

Given these clinical manifestations, PKU remains the most probable diagnosis. For further confirmation, genetic testing for mutations in the PAH gene (encoding phenylalanine hydroxylase) should be performed.

If you need more information or assistance with diagnostic procedures, please let me know!

**97. User**

Mainly skin symptoms and neuropsychiatric symptoms.

**1. The group of cutaneous symptoms,** are erythema, herpes and even ulceration on exposed parts of the skin after exposure to light. The rash may be of the type of eczema, urticaria, summertime itchy rash or erythema multiforme. The oral mucosa may have red patches and brownish-red teeth. Eye damage such as conjunctivitis, keratitis and iritis may also occur. In severe cases, there may be deformation of the skin of the nose, ears and fingers with kinking. There may be special purple colouration. Hirsutism may be present.

**2. A cluster of neuropsychiatric symptoms ,**  the most common manifestation of which is acute abdominal pain, which tends to be severe, stable, and poorly localised, sometimes accompanied by painful cramps. It may be accompanied by constipation, bloating, nausea, vomiting, and sometimes diarrhoea and increased bowel sounds. Sensory and motor neuropathies may precede the onset of abdominal pain and present as limb pain with numbness, abnormal sensation and sensory inversions; motor weakness often begins proximally in the upper limbs and may progress to the lower limbs and distal extremities. Involvement of the cerebral nerves may lead to medullary paralysis, impaired respiratory function and death. Severe motor neuropathy may result in quadriplegia. Autonomic nervous system involvement manifests as tachycardia, hypertension, sweating, agitation, and tremor. Neurogenic bladder dysfunction may cause dysuria, dyspareunia, urinary retention and incontinence. Other acute neuropsychiatric manifestations include insomnia, anxiety, agitation, agitation, hallucinations, hysteria, disorientation, delirium, affective apathy, depression, phobias, and altered consciousness ranging from lethargy to coma.

Based on the clinical manifestations described, the most likely diagnosis is **Acute Intermittent Porphyria (AIP)**. This condition involves both cutaneous and neuropsychiatric symptoms as detailed below:

**Cutaneous Symptoms:**

1. **Erythema**: Redness of the skin.
2. **Herpes and Ulceration**: Blisters and ulcers on exposed parts of the skin after light exposure.
3. **Rash Types**:
   - Eczema: Itchy, inflamed skin.
   - Urticaria: Hives or raised red welts.
   - Summertime Itchy Rash: Allergic reactions or dermatitis in warm weather.
   - Erythema Multiforme: A pattern of rash that can appear on the skin and mucous membranes.
4. **Oral Mucosa**: Red patches, brownish-red teeth.
5. **Eye Damage**: Conjunctivitis, keratitis (inflammation of the cornea), iritis (inflammation of the iris).
6. **Severe Cases**:
   - Deformation of the skin on the nose, ears, and fingers with kinking.
   - Special purple coloration.
   - Hirsutism: Excessive hair growth.

**Neuropsychiatric Symptoms:**

1. **Acute Abdominal Pain**: Severe, stable, poorly localized pain, sometimes accompanied by painful cramps.
2. **Gastrointestinal Symptoms**:
   - Constipation
   - Bloating
   - Nausea
   - Vomiting
   - Diarrhea and increased bowel sounds.
3. **Sensory and Motor Neuropathies**:
   - Limb pain with numbness, abnormal sensation, and sensory inversions.
   - Proximal motor weakness in the upper limbs that may progress to lower limbs and distal extremities.
4. **Cerebral Nerve Involvement**:
   - Medullary paralysis leading to respiratory failure and death.
   - Severe motor neuropathy resulting in quadriplegia.
5. **Autonomic Nervous System Involvement**:
   - Tachycardia (rapid heartbeat)
   - Hypertension (high blood pressure)
   - Sweating
   - Agitation
   - Tremor
6. **Neurogenic Bladder Dysfunction**:
   - Dysuria (painful urination)
   - Dyspareunia (pain during intercourse)
   - Urinary retention and incontinence.
7. **Other Neuropsychiatric Manifestations**:
   - Insomnia
   - Anxiety
   - Agitation
   - Hallucinations
   - Hysteria
   - Disorientation
   - Delirium
   - Affective apathy (lack of emotional response)
   - Depression
   - Phobias
   - Altered consciousness ranging from lethargy to coma.

**Diagnosis and Management:**

Acute Intermittent Porphyria is diagnosed through a combination of clinical symptoms, laboratory tests such as urinary porphyrin analysis, and genetic testing. Treatment typically involves managing the acute attacks with pain relief, correcting electrolyte imbalances, and using medications like hemin or heme arginate to stabilize the condition.

If you suspect this diagnosis in a patient, it is crucial to consult a specialist for proper management and care. Regular monitoring and appropriate treatment can significantly improve the quality of life for individuals affected by AIP.

**98. User**

**1. Infection, patients** are infected to varying degrees. Infections are recurrent, severe, difficult to treat, opportunistic and have a broad spectrum of pathogens. Typical children are usually born with no specific manifestations, and fatal infections can occur before 2 months of age due to cellular immune abnormalities. The sites and pathogens of infection are diverse, including recurrent pneumonia, recurrent thrush, persistent diarrhoea, recurrent otitis media, persistent viral infections (e.g., respiratory syncytial virus, EBV, CMV, etc.), and conditionally causative organisms, such as Pneumocystis carinii, are also common.

**2. Vaccine disease,** in which patients are extremely immunocompromised, can occur with infection and dissemination of live attenuated vaccines. Severe diarrhoea can also occur in children vaccinated with attenuated rotavirus vaccine.

**3. Retarded or arrested growth and development** is a distinguishing feature of the disease. It is associated with recurrent infections, diarrhoea and corresponding genetic defects.

Children may also have a rash and enlarged liver and spleen lymph nodes similar to erysipelas.

In other children, erythroderma-like symptoms, hepatosplenomegaly, seborrheic dermatitis, haematopenia, and sclerosing cholangitis may be present.

Based on the clinical manifestations provided, the most likely rare disease that fits the description is **Severe Combined Immunodeficiency (SCID)**. SCID is characterized by severe immunodeficiency leading to recurrent, severe, and opportunistic infections, often due to both cellular and humoral immune defects. The symptoms of recurrent infections, growth retardation, issues with live vaccines, and other immunological symptoms fit well with SCID.

Here are five other possible diagnoses that could be considered based on the symptoms provided:

1. **Wiskott-Aldrich Syndrome (WAS):**
   - This X-linked condition can cause immunodeficiency, recurrent infections, eczema (which can be similar to seborrheic dermatitis), and thrombocytopenia.
2. **Chronic Granulomatous Disease (CGD):**
   - CGD is a primary immune deficiency characterized by recurrent infections due to defective NADPH oxidase activity in phagocytes, leading to impaired production of reactive oxygen species.
3. **Ataxia-Telangiectasia (AT):**
   - AT is an autosomal recessive disorder that affects the nervous system and immune system. It can cause immunodeficiency, recurrent infections, ataxia, and telangiectasias.
4. **DiGeorge Syndrome (DGS) / 22q11.2 Deletion Syndrome:**
   - DGS is caused by a deletion on chromosome 22 and can lead to thymic hypoplasia, resulting in T-cell immunodeficiency, recurrent infections, and other systemic symptoms.
5. **Complement Deficiencies (e.g., C1-Inhibitor Deficiency):**
   - Complement deficiencies can cause recurrent infections due to defects in the complement system, which is part of the innate immune response.

Given the broad spectrum of pathogens involved, severe and recurrent infections, and the immunocompromised state leading to issues with live vaccines, SCID remains a strong candidate. However, further diagnostic testing such as T-cell function assays, cytokine production tests, and genetic testing for specific mutations would be necessary to confirm the diagnosis.

For accurate diagnosis and appropriate management, consulting a specialist in immunology or pediatric immunodeficiency is highly recommended.

**99. User**

The core symptom is dystonia, which is mainly manifested by abnormal expressive postures and involuntary movements, mostly involving the head and neck muscles, trunk muscles, rotator anterior muscles of the limbs, finger and wrist flexors, toe extensors and plantar flexors. It is aggravated by random movements, reduced or disappeared when resting and sleeping, and in the late stage, it can be a fixed twisting spastic deformity. Sensory trickery (relief techniques) and movement specificity are the characteristic manifestations of dystonia. Sensory tricks (relieving techniques) are random movements that are specifically designed to correct abnormal postures or relieve dystonic movements, usually simple movements that involve or target the affected area, but are not forceful to counteract the symptoms of dystonia. Movement specificity refers to the presence of dystonia only during specific activities or the performance of specific tasks, such as certain occupations (e.g., writing spasms, musician’s spasms) or sports.

Based on the clinical manifestations described, the most likely rare disease to be diagnosed is **Dystonia**. Dystonia is characterized by abnormal postures and involuntary movements that are often exacerbated by random movements but improve during rest and sleep. The late-stage fixed twisting spastic deformity also aligns with dystonic symptoms.

Here are five other possible diagnoses that might be considered:

1. **Wilson’s Disease**: A rare genetic disorder that leads to copper accumulation in the body, which can cause neurological symptoms including dystonia, as well as liver dysfunction.
2. **Parkinson’s Disease with Dystonia**: Some patients with Parkinson’s Disease can present with dystonia, especially as a component of the motor fluctuations.
3. **Huntington’s Disease**: A genetic disorder that causes the progressive breakdown of nerve cells in the brain, leading to movement disorders, which can include dystonia.
4. **Tardive Dyskinesia**: A condition that results from long-term use of neuroleptic drugs, causing involuntary, repetitive movements, often involving the face and mouth but can also affect other areas.

Sensory tricks (relief techniques) and movement specificity are characteristic manifestations of dystonia. Sensory tricks involve simple, specific movements that help relieve or correct abnormal postures, while movement specificity refers to the presence of dystonia during certain activities or tasks.

For accurate diagnosis and appropriate management, further diagnostic tests such as neurological examinations, genetic testing, and imaging studies may be necessary. Consulting a specialist in neurology or movement disorders is highly recommended.

**100. User**

**1. Kidney,** the main manifestations are limb oedema and increased foam in the urine. Laboratory tests may reveal simple moderate proteinuria or nephrotic syndrome, and renal insufficiency may be seen in the advanced stage.

**2. Heart,** the main manifestations are shortness of breath after activity, limb edema, pleural effusion, ascites and other signs of restrictive cardiac insufficiency.

**3. Liver,** there may be slight discomfort or pain in the liver area, but most patients may be asymptomatic, and hepatomegaly or liver enzyme abnormalities are often detected during physical examination. Liver failure may occur in advanced stages of the disease.

**Peripheral and autonomic nerves,** Symmetrical sensory and/or motor peripheral neuropathy of the extremities. Autonomic abnormalities are often manifested by upright hypotension, gastroparesis, pseudo-intestinal obstruction, and impotence.

**5. Gastrointestinal tract** , can present with total gastrointestinal involvement, with the stomach and small intestine being the most commonly involved. It can present with epigastric discomfort, dyspepsia, diarrhoea, constipation, malabsorption syndrome and gastrointestinal bleeding.

**6. Soft tissues ,** Tongue involvement may present with megaglossia, tongue dysmobility, and dysarthria. Skin mucosa may present with skin purpura and petechiae, which are more common around the orbits and in the loose skin areas of the neck. Nail atrophy and hair loss may also occur.

**7. Coagulation abnormalities** Patients often have coagulation factor X deficiency, resulting in corresponding bleeding manifestations.

Based on the clinical manifestations you’ve described, the most likely rare disease is **Amyloidosis**. Amyloidosis involves the deposition of abnormal protein aggregates (amyloid) in various tissues and organs, leading to a wide range of symptoms as observed.

**Main Diagnosis:**

- **Amyloidosis**

**Other Possible Diagnoses:**

1. **Fabry Disease**: This genetic disorder can cause renal, cardiac, and neurologic involvement.
2. **Multisystem Amyloidosis (AA or AL)**: These types of amyloidosis involve multiple organs and systems.

**Detailed Analysis:**

1. **Kidney**:
   - Limb oedema and increased foam in the urine suggest proteinuria.
   - Laboratory tests may reveal simple moderate proteinuria or nephrotic syndrome, with renal insufficiency in advanced stages.
2. **Heart**:
   - Shortness of breath after activity, limb edema, pleural effusion, ascites, and signs of restrictive cardiac insufficiency are common.
3. **Liver**:
   - Slight discomfort or pain in the liver area.
   - Hepatomegaly or liver enzyme abnormalities during physical examination.
   - Liver failure may occur in advanced stages.
4. **Peripheral and Autonomic Nerves**:
   - Symmetrical sensory and/or motor peripheral neuropathy of the extremities.
   - Autonomic abnormalities such as upright hypotension, gastroparesis, pseudo-intestinal obstruction, and impotence.
5. **Gastrointestinal Tract**:
   - Total gastrointestinal involvement with epigastric discomfort, dyspepsia, diarrhoea, constipation, malabsorption syndrome, and gastrointestinal bleeding.
6. **Soft Tissues**:
   - Tongue involvement may present with megaglossia, tongue dysmobility, and dysarthria.
   - Skin mucosa may show skin purpura and petechiae around the orbits and in loose skin areas of the neck.
   - Nail atrophy and hair loss may also occur.
7. **Coagulation Abnormalities**:
   - Patients often have coagulation factor X deficiency, leading to corresponding bleeding manifestations.

**Conclusion:**

The combination of symptoms across multiple organ systems strongly suggests Amyloidosis. Further diagnostic tests such as serum and urine protein electrophoresis, Congo red staining for amyloid, and liver and kidney function tests are recommended to confirm the diagnosis.

For accurate diagnosis and appropriate management, consulting a specialist in nephrology, cardiology, hepatology, neurology, and gastroenterology is highly advisable.

**101. User**

Jaundice and itchy skin are typical clinical manifestations. Other developmental delays include short stature, delayed pubertal growth, gallbladder stones, steatorrhea due to fat absorption disorders, hepatosplenomegaly, rickets due to fat-soluble vitamin deficiencies, delayed bone age, dry eye, coagulation disorders, and neuromuscular disorders. Children may also develop visual and hearing abnormalities, irritability, lethargy and poor concentration. In advanced stages, portal hypertension and liver tumours may occur.

Based on the clinical manifestations you’ve provided, the rare disease most likely to be diagnosed is **Alagille syndrome**. Alagille syndrome is characterized by liver involvement such as cholestasis and jaundice, along with other symptoms like growth delays, cardiovascular anomalies, and skeletal abnormalities. The symptoms you’ve listed align well with this syndrome’s multisystemic involvement.

**Detailed Analysis:**

1. **Liver Involvement**:
   - Jaundice and itchy skin are typical clinical manifestations.
   - Cholestasis leading to steatorrhea due to fat absorption disorders.
   - Hepatosplenomegaly (enlarged liver and spleen).
   - Rickets due to fat-soluble vitamin deficiencies, delayed bone age.
2. **Growth Delays**:
   - Short stature.
   - Delayed pubertal growth.
3. **Cardiovascular Anomalies**:
   - May include pulmonary valve stenosis or other congenital heart defects.
4. **Skeletal Abnormalities**:
   - Rickets due to vitamin D and calcium deficiencies.
5. **Other Symptoms**:
   - Dry eye.
   - Coagulation disorders.
   - Neuromuscular disorders.
   - Visual and hearing abnormalities.
   - Irritability, lethargy, and poor concentration.
6. **Advanced Stages**:
   - Portal hypertension.
   - Liver tumors.

**Other Possible Diagnoses to Consider:**

1. **Progressive Familial Intrahepatic Cholestasis (PFIC)**:
   - Cholestasis, jaundice, growth delays, and vitamin deficiencies due to malabsorption.
2. **Biliary Atresia**:
   - Obstruction of bile flow from the liver, leading to jaundice and growth delays, potentially progressing to cirrhosis.
3. **Cystic Fibrosis (CF)**:
   - Primarily affects the lungs but can cause pancreatic insufficiency, leading to steatorrhea, fat-soluble vitamin deficiencies, and liver disease.
4. **Niemann-Pick Disease Type C**:
   - Lysosomal storage disorder causing hepatosplenomegaly, neurological symptoms, and potentially jaundice.
5. **Wilson’s Disease**:
   - Disorder of copper metabolism leading to hepatic symptoms, neurological manifestations, and other systemic signs including growth delays.

**Diagnostic Evaluation:**

To differentiate between these conditions, further diagnostic evaluation is necessary, which may include: - Genetic testing for Alagille syndrome (JAG1 gene mutation). - Liver function tests. - Imaging studies such as ultrasound or MRI of the liver. - Blood tests for coagulation disorders and other systemic markers.

Given the multisystemic nature of these symptoms, a comprehensive evaluation by a multidisciplinary team is recommended. Genetic testing can provide definitive diagnosis and guide appropriate management strategies.

If you need further assistance with diagnostic steps or specific tests, please let me know!

**102. User**

The disease starts in childhood. The disease manifests itself as a mild delay in motor development and progressive atrophy of skeletal muscles, which affects the motor function of the limbs and gradually leads to gait abnormalities and limited movement of the upper limbs, with the natural course of the disease often resulting in loss of the ability to walk at about 10 years of age. Thereafter, scoliosis, joint contracture, respiratory muscle weakness and dilated cardiomyopathy appeared, and the patient died of respiratory failure and cardiac failure at the age of about 20 years. Pseudohypertrophy of both gastrocnemius muscles, pseudohypertrophy of both forearms and lingual muscles, positive Gowers’ sign, and lumbar lordosis can be seen on examination. The disease may start in youth or even adulthood, and some patients do not affect survival. Signs of pseudohypertrophy are obvious, and in some patients, dilated cardiomyopathy is first apparent when limb weakness is still mild.

Based on the clinical manifestations you have described, the most likely diagnosis is **Duchenne Muscular Dystrophy (DMD)**. This condition typically starts in childhood and is characterized by a progressive decline in muscle function, leading to various symptoms such as:

1. **Mild Delay in Motor Development**: Initially, there might be a mild delay in motor development.
2. **Progressive Atrophy of Skeletal Muscles**: Over time, this progresses to significant atrophy affecting the limbs and upper body.
3. **Gait Abnormalities and Limited Movement**: The patient may experience gait abnormalities and limited movement, often leading to loss of walking ability by around 10 years of age.
4. **Scoliosis, Joint Contractures, Respiratory Muscle Weakness, and Dilated Cardiomyopathy**: These complications can appear as the disease progresses.
5. **Pseudohypertrophy**: Pseudohypertrophy of muscles such as the gastrocnemius, forearms, and lingual muscles is a hallmark feature.
6. **Positive Gowers’ Sign**: This sign indicates difficulty in rising from a sitting to a standing position due to muscle weakness.
7. **Lumbar Lordosis**: An examination may reveal lumbar lordosis.

**Other Possible Diagnoses:**

1. **Becker Muscular Dystrophy (BMD)**: A milder and more variable form of dystrophy compared to DMD, with later onset and slower progression. Cardiomyopathy can also be a prominent feature.
2. **Limb-Girdle Muscular Dystrophy (LGMD)**: This group of disorders can present with muscle weakness affecting the shoulder and pelvic girdles. Some forms may show pseudohypertrophy and cardiac involvement.
3. **Emery-Dreifuss Muscular Dystrophy (EDMD)**: Characterized by early contractures, muscle weakness, and cardiac involvement. It has a slower progression compared to DMD.
4. **Facioscapulohumeral Muscular Dystrophy (FSHD)**: This condition typically affects facial muscles first but can also involve other muscle groups, leading to pseudohypertrophy in some cases.
5. **Congenital Muscular Dystrophies**: These conditions are present from birth and can have various presentations, including pseudohypertrophy.

**Diagnostic Evaluation:**

To confirm the diagnosis of Duchenne Muscular Dystrophy or any other potential condition, further diagnostic steps may include: - **Genetic Testing**: Specifically testing for the dystrophin gene mutations. - **Muscle Biopsy**: To assess muscle pathology and confirm the presence of pseudohypertrophy. - **Electromyography (EMG)**: To evaluate nerve and muscle function. - **Imaging Studies**: Such as MRI to assess muscle degeneration.

Given the detailed clinical presentation, Duchenne Muscular Dystrophy is highly likely. However, genetic testing is crucial for definitive diagnosis and to rule out other similar conditions.

Would you like more information on any of these diagnostic steps or further details about the management and support available for patients with Duchenne Muscular Dystrophy?

**103. User**

Mainly hyperaemia, brain damage and cardiomyopathy.

**1. Neonatal onset** Normal at birth, vomiting, lethargy, hypotonia, convulsions, dyspnoea, hyperaemia, ketosis, hypoglycaemia, acidosis, dilated cardiomyopathy, pancreatitis and other abnormalities after starting breastfeeding, with a high morbidity and mortality rate.

**2. Delayed-onset** is often induced by fever, starvation, high-protein diet, and infections, and manifests itself as feeding difficulties in infancy and early childhood, developmental delays, convulsions, and hypotonia. In many patients, cognitive ability and neurological development are impaired, with an increase in slow waves or epileptic waves on the EEG; some patients may have fractures and osteoporosis on X-ray; bone marrow suppression is also common, causing granulocytopenia, anaemia and thrombocytopenia. There may also be cardiac damage, such as cardiomyopathy, arrhythmia, prolonged QT interval, and diminished cardiac function. Renal impairment is less common.

Based on the clinical manifestations you have described, particularly the combination of hyperaemia, brain damage, and cardiomyopathy, along with the neonatal and delayed-onset presentations, one likely diagnosis is **Methylmalonic Acidemia (MMA)**. This condition is a rare inherited metabolic disorder characterized by the body’s inability to properly break down certain fats and proteins, leading to the accumulation of methylmalonic acid.

**Key Features:**

1. **Neonatal Onset:**
   - Normal at birth.
   - Symptoms include vomiting, lethargy, hypotonia, convulsions, dyspnoea, hyperaemia, ketosis, hypoglycaemia, and acidosis.
   - Dilated cardiomyopathy, pancreatitis, and other abnormalities may occur after starting breastfeeding.
   - High morbidity and mortality rate.
2. **Delayed-Onset:**
   - Often triggered by fever, starvation, high-protein diet, or infections.
   - Manifests as feeding difficulties in infancy and early childhood, developmental delays, convulsions, and hypotonia.
   - Cognitive ability and neurological development are often impaired, with increased slow waves or epileptic waves on EEG.
   - Common findings include fractures, osteoporosis on X-ray, bone marrow suppression (granulocytopenia, anaemia, thrombocytopenia), cardiac damage such as cardiomyopathy, arrhythmias, prolonged QT interval, and diminished cardiac function.
   - Renal impairment is less common.

**Other Possible Diagnoses:**

1. **Propionic Acidemia**: Another metabolic disorder where the body accumulates propionic acid due to an inability to break down certain amino acids and odd-chain fatty acids.
2. **Isovaleric Acidemia**: A rare organic acid disorder characterized by a distinctive sweaty feet odor, along with metabolic acidosis, ketosis, vomiting, and lethargy.
3. **Glutaric Acidemia Type I (GA-I)**: Involves the accumulation of glutaric acid and is associated with macrocephaly, encephalopathy, dystonia, and other metabolic complications.

**Diagnostic Tests:**

- Measurement of methylmalonic acid in blood or urine.
- Genetic testing for mutations in the MMACHC, MMADHC, or MMAB genes.
- Fatty acid oxidation tests.

**Management:**

- Dietary management to limit intake of certain amino acids and fats.
- Supplementation with carnitine and other vitamins.
- Regular monitoring of cardiac function and neurological development.
- Prompt treatment during episodes of metabolic decompensation (e.g., fever, infections).

Given the severity and complexity of these symptoms, it is crucial to consult a specialist in metabolic disorders for accurate diagnosis and appropriate management.

Would you like more detailed information on any specific aspect of MMA or its management?

**104. User**

Clinical manifestations are often nonspecific, with an insidious onset. Symptoms such as coughing up sputum, lethargy and fatigue may occur. Secondary infections may include fever, coughing up purulent sputum, or chest pain and haemoptysis. A few patients may be asymptomatic and may have no positive signs. Bursting sounds may be present in some patients, cyanosis may be present in severe cases, and pestle and mortar fingers may be seen in a few patients. The clinical picture is often less severe than the chest image, and this inconsistency between symptoms and images is a characteristic feature of the disease. Serum anti-GM-CSF antibodies are markedly elevated.

Based on the provided clinical manifestations and the specific finding of markedly elevated serum anti-GM-CSF antibodies, the most likely diagnosis is **Pulmonary Alveolar Proteinosis (PAP)**. PAP is a rare lung disease characterized by the accumulation of surfactant in the alveoli, leading to impaired gas exchange.

**Key Features:**

- **Nonspecific and Insidious Onset**: Symptoms such as coughing up sputum, lethargy, and fatigue may occur gradually.
- **Secondary Infections**: These can include fever, coughing up purulent sputum, chest pain, and hemoptysis (coughing up blood).
- **Asymptomatic Patients**: Some patients may have no symptoms or positive signs.
- **Physical Signs**: Bursting sounds in the lungs, cyanosis in severe cases, and “pestle and mortar” fingers (clubbing) in a few patients.
- **Imaging vs. Symptoms Inconsistency**: The clinical picture is often less severe than the chest imaging findings, which can show significant abnormalities despite mild symptoms.

**Alternative Diagnoses to Consider:**

1. **Bronchiectasis**: Chronic condition involving dilated airways, often associated with chronic cough and sputum production.
2. **Chronic Obstructive Pulmonary Disease (COPD)**: Can present with similar respiratory symptoms but typically lacks the specific antibody profile.
3. **Cystic Fibrosis**: A genetic disorder that can present with similar respiratory symptoms, although it is often diagnosed in childhood and has specific genetic testing.
4. **Asthma with Secondary Infection**: Can cause cough, sputum production, and respiratory distress, but is more episodic and associated with wheezing.
5. **Idiopathic Pulmonary Fibrosis (IPF)**: A progressive lung disease with similar symptoms but usually exhibits a different imaging profile and lacks the antibody presence.

Given these clinical features and the elevated anti-GM-CSF antibodies, PAP remains the most probable diagnosis. For confirmation, further diagnostic tests such as bronchoalveolar lavage (BAL) to assess surfactant levels and specific GM-CSF receptor expression may be necessary.

**105. User**

In most cases, the disease begins in adolescence, with a gradual loss of the optic rod cells, followed by cone cell involvement and death of the retinal pigment epithelium. Therefore, night blindness is usually the first symptom (but after urbanisation, some patients often have a decrease in visual acuity), and the earlier the onset of night blindness, the more severe the patient’s condition. Although peripheral visual field defects also occur early, patients are usually unaware of them and rarely complain of them in the early stages of the disease. The disease progresses to a loss of central vision and further narrowing of the visual field, and most people become legally blind before the age of 40. The degree of visual impairment can be related to the mode of inheritance.

The presentation of the fundus is related to the stage of the disease: in the early stages of the disease, the fundus may be normal or nearly normal on routine examination, but in some patients, an early form of blanket retinal degeneration may be observed on mid-peripheral fundus photography (these subtle changes are difficult to recognise with current scanning laser fundus photography or wide-field fundus photography). As the disease progresses, some patients develop typical fundus changes, such as retinal osteoblast-like pigmentation combined with mid-peripheral retinal atrophy, a waxy optic disc, and thinning of retinal blood vessels; some patients develop blanketing or depigmentation, which can be seen in the mid-peripheral retina, or extensive degeneration. Other common complications include macular cystoid degeneration, posterior subcapsular cataract, vitreous dust particles, and optic disc vitreous warts; a rare complication is Coat’s retinopathy.

Based on the clinical manifestations you’ve described, the most likely diagnosis is **Retinitis Pigmentosa (RP)**. This rare genetic disorder involves progressive degeneration of the retina, primarily affecting rod photoreceptor cells initially, followed by cone cell involvement and eventual death of the retinal pigment epithelium.

**Key Clinical Features:**

1. **Onset and Progression**:
   - The disease typically begins in adolescence with a gradual loss of rod photoreceptor cells.
   - Night blindness is usually the first symptom, although after urbanization, some patients may experience a decrease in visual acuity.
   - The earlier the onset of night blindness, the more severe the condition tends to be.
   - Peripheral visual field defects occur early but are often unnoticed by patients.
2. **Visual Impairment**:
   - Central vision loss and further narrowing of the visual field progress over time.
   - Most people become legally blind before the age of 40.
   - The degree of visual impairment can vary depending on the mode of inheritance (e.g., autosomal dominant, autosomal recessive, X-linked).
3. **Fundus Presentation**:
   - In early stages, the fundus may appear normal or nearly normal during routine examination.
   - Some patients may show subtle changes in mid-peripheral fundus photography, such as early forms of blanket retinal degeneration.
   - As the disease progresses, typical fundus changes include:
     - Retinal osteoblast-like pigmentation combined with mid-peripheral retinal atrophy.
     - A waxy optic disc and thinning of retinal blood vessels.
     - Blanketing or depigmentation in the mid-peripheral retina.
     - Extensive degeneration.
   - Other common complications include:
     - Macular cystoid degeneration.
     - Posterior subcapsular cataract.
     - Vitreous dust particles and optic disc vitreous warts.
   - A rare complication is Coat’s retinopathy.

Given these clinical features, Retinitis Pigmentosa (RP) is the most likely diagnosis. For further confirmation, detailed ophthalmological examinations including fundus photography and genetic testing may be necessary.

**106. User**

The most common clinical presentation is white pupil syndrome, in which a yellow-white reflection is visible in the pupil. Disuse strabismus may occur in the affected eye due to the tumour being located at the posterior pole and low visual acuity. The early presentation is a greyish-white, round or oval arching mass that may bulge into the vitreous or grow flat along the choroid. Retinal blood vessels on the surface of the tumour may become dilated and haemorrhagic, and even exudative retinal detachment may occur. Tumour tissue may also penetrate the retina and enter the vitreous, floating like a snowball, or even deposited in the anterior chamber to form pseudo anterior chamber pus. The onset of the disease is early in children with bilateral retinal detachment, usually within one year of age. The onset is later in unilateral children, usually at two or three years of age.

Based on the clinical presentation you’ve described, the most likely diagnosis is **retinoblastoma**. Retinoblastoma is a rare eye cancer that typically affects young children and is characterized by several key features:

1. **White Pupil Syndrome (Leukocoria)**: A yellow-white reflection visible in the pupil due to retinal tumors.
2. **Disuse Strabismus**: The affected eye may develop disuse strabismus due to low visual acuity caused by the tumor’s location at the posterior pole.
3. **Early Presentation**:
   - **Greyish-White, Round or Oval Arching Mass**: An early sign of a greyish-white mass that may bulge into the vitreous or grow flat along the choroid.
   - **Dilated and Haemorrhagic Retinal Blood Vessels**: The retinal blood vessels on the surface of the tumor may become dilated and haemorrhagic, leading to exudative retinal detachment.
4. **Tumor Tissue Penetration**:
   - **Vitreous Involvement**: Tumor tissue may penetrate the retina and enter the vitreous, floating like a snowball.
   - **Anterior Chamber Involvement**: Tumor tissue can be deposited in the anterior chamber, forming pseudo anterior chamber pus.

The onset of retinoblastoma is typically early in children with bilateral retinal detachment, usually within one year of age. For unilateral cases, the onset is later, usually around two or three years of age.

Given these clinical features, other possible diagnoses that can present with similar symptoms include: 1. **Coats’ Disease**: This condition leads to retinal telangiectasia and exudative retinal detachment, which can also cause leukocoria. 2. **Persistent Fetal Vasculature Syndrome (PFVS)**: Previously known as persistent hyperplastic primary vitreous (PHPV), this congenital developmental anomaly of the eye can present with leukocoria. 3. **Toxocariasis**: An ocular infection caused by Toxocara canis, leading to granulomatous inflammation that may cause white pupillary reflex and retinal detachment. 4. **Congenital Cataract**: Opacification of the lens that can cause leukocoria; it may not present with some of the more tumor-specific signs like vitreous seeding or retinal masses. 5. **Retinal Detachment Due to Familial Exudative Vitreoretinopathy (FEVR)**: This hereditary retinal disorder can lead to retinal detachment but is less likely to cause a white pupil syndrome.

Given the severity and specific clinical features, it is crucial to conduct thorough ophthalmological examinations and possibly imaging studies such as ultrasound or MRI to confirm the diagnosis. Early detection and treatment are critical for managing retinoblastoma effectively.

For further confirmation, please consult an ophthalmologist or pediatric oncologist who can perform detailed diagnostic tests.

**107. User**

The average age of onset is 5.6 months. The first seizure is usually normal at birth and starts within 1 year of age. The first seizure is usually febrile convulsion, which may be one-sided or generalised clonic or tonic clonic seizure, and febrile convulsions are characterized by long duration and recurrent episodes, and may appear in a persistent state. 28%~35% of children may start the disease without febrile convulsions, but they often have heat sensitivity in the later course of the disease. Elevated body temperature due to various reasons, including exercise, fever, vaccination, hot baths, and high ambient temperatures, can trigger or exacerbate seizures and predispose to status epilepticus. Some children develop an acute encephalopathy characterised by deep coma following status epilepticus.

Children tend to present with febrile convulsions between the ages of 1 and 4 years, and have a variety of seizure types, such as myoclonic, atypical catatonic, partial seizures, and generalised tonic clonic seizures. Myoclonic seizures are often triggered by light stimulation, eye closure, and a specific graphic stimulus, and involve simultaneous clonus of one or several muscles, all accompanied by synchronised abnormal brain wave bursts. The intensity of the myoclonus varies, from a slight forward or backward movement of the body when only the axial muscles are violated, to a severe fall or sudden throwing of an object in the hand. Myoclonic seizures can be isolated or cluster, but a sustained state is rare. Myoclonic seizures may not occur at all in about 25% of children. The concurrent EEG of atypical apoplectic seizures shows half of the discharges to be of focal origin, most of which are irregular, irregular, slow spikes and slow waves, often accompanied by myoclonic seizures. 30% of the children may have atypical apoplectic persistent seizures. The incidence of partial seizures is 43.0% to 78.6%, which manifests as lateral limb (or) head deflection seizures or clonic seizures, and there are also motor seizures with consciousness and autonomic symptoms, automatisms, and hypotonia, etc. After 1 year of age, the children have psychomotor developmental delays, especially language developmental delays.

Based on the provided information, it appears that the clinical presentation described is consistent with **Dravet Syndrome**, a type of childhood epilepsy. Here’s a detailed breakdown:

1. **Age of Onset**:
   - The average age of onset is 5.6 months.
   - Febrile seizures typically start within the first year of life.
2. **Initial Seizures and Characteristics**:
   - Initial seizures are often febrile convulsions, which can be partial or generalized (clonic or tonic-clonic).
   - These febrile convulsions are characterized by long duration and recurrent episodes.
   - Febrile convulsions may appear in a persistent state.
3. **Seizure Types**:
   - Seizures tend to occur between 1 and 4 years of age.
   - Common seizure types include myoclonic, atypical catatonic, partial seizures, and generalized tonic-clonic seizures.
   - Myoclonic seizures are often triggered by light stimulation, eye closure, or specific graphic stimuli.
   - These seizures involve simultaneous clonus in one or several muscles, accompanied by synchronized abnormal brain wave bursts.
   - The intensity of myoclonus can vary from a slight forward or backward movement to severe falls or sudden throwing of objects.
4. **EEG Findings**:
   - Concurrent EEG findings for atypical apoplectic seizures show half of the discharges to be focal, with most being irregular slow spikes and waves.
   - Myoclonic seizures are often accompanied by these abnormal brain wave patterns.
   - Approximately 30% of children may have persistent atypical apoplectic seizures.
5. **Partial Seizures**:
   - The incidence of partial seizures ranges from 43.0% to 78.6%, manifesting as lateral limb or head deflection seizures, clonic seizures, and motor seizures with consciousness and autonomic symptoms.
   - These can include automatisms (automated behaviors) and hypotonia.
6. **Developmental Delays**:
   - After the first year of age, children may experience psychomotor developmental delays, particularly in language development.

Given these characteristics, Dravet Syndrome is a strong candidate for the condition described. It is important to note that a definitive diagnosis would require further clinical evaluation and possibly genetic testing. If you or someone you know is experiencing these symptoms, it is crucial to consult with a neurologist specializing in pediatric epilepsy for proper assessment and management.

**108. User**

On the one hand, it presents as chronic haemolytic anaemia, and in addition to anaemia-related symptoms, it may cause mild to moderate yellowing of the skin and sclera and pigmented cholelithiasis due to elevated bilirubin, which may be exacerbated by cold, infection and dehydration. In infants and young children, splenomegaly may be present, and with age, the spleen may shrink due to splenic infarction and fibrosis.

On the other hand recurrent vaso-obstructive pain due to capillary micro thrombosis. Children and adults tend to present with myalgia of the limbs, large joint pain and low back pain, and in severe cases, severe abdominal pain, headache, and even limb paralysis and coma. Over time, vascular obstruction can occur in almost every organ system, and cyclical pain can occur in patients who are thin, easily fatigued, susceptible to infection, and malnourished. Thinning of the bone cortex, osteoporosis, spinal deformity, aseptic necrosis of the femoral head due to an overcompensatory increase in bone marrow haematopoietic tissue, and localised osteosclerosis if there is a bone infarction. Ocular symptoms may include fundus haemorrhage and retinal detachment due to embolism of the retinal vessels in the fundus. In addition, mental retardation may occur, affecting neurological development.

Acute pain is the first symptom of the disease in more than 25% of patients. It is most common in children older than 2 years of age. The severity of pain can vary from mild to severe. Approximately one-half of pain episodes are accompanied by objective clinical signs such as fever, swelling, tenderness, tachypnoea, hypertension, nausea and vomiting.

Based on the provided clinical manifestations, the condition described is highly indicative of **Sickle Cell Disease (SCD)**. Here’s a detailed breakdown to support this diagnosis:

1. **Chronic Hemolytic Anemia**:
   - The patient presents with chronic hemolytic anemia, which is a hallmark of SCD.
   - This leads to anaemia-related symptoms such as fatigue, pallor, and shortness of breath.
2. **Jaundice and Pigmented Cholelithiasis**:
   - Elevated bilirubin levels due to haemolysis can cause mild to moderate yellowing of the skin and sclera.
   - Pigmented cholelithiasis (stones in the gallbladder) may develop as a result of increased bilirubin.
3. **Splenomegaly and Spleen Shrinkage**:
   - In infants and young children, splenomegaly is common due to frequent haemolytic episodes.
   - With age, the spleen may shrink due to infarction and fibrosis, which can lead to a reduced ability to filter out sickled red blood cells.
4. **Vaso-Occlusive Pain**:
   - Recurrent vaso-occlusive pain due to capillary microthrombosis is a primary symptom of SCD.
   - Children and adults may experience myalgia (muscle pain), large joint pain, low back pain, and in severe cases, abdominal pain, headache, limb paralysis, and even coma.
5. **Organ System Involvement**:
   - Vascular obstruction can occur in almost every organ system, leading to cyclical pain.
   - Patients may be thin, easily fatigued, susceptible to infection, and malnourished, which are common characteristics of SCD.
6. **Bone and Ocular Symptoms**:
   - Thinning of the bone cortex, osteoporosis, spinal deformity, aseptic necrosis of the femoral head due to overcompensatory increase in bone marrow hematopoietic tissue, and localized osteosclerosis if there is a bone infarction.
   - Ocular symptoms may include fundus hemorrhage and retinal detachment due to embolism of the retinal vessels.
7. **Neurological Symptoms**:
   - Mental retardation affecting neurological development can occur in severe cases.
8. **Acute Pain Episodes**:
   - Acute pain is a common initial symptom, occurring in more than 25% of patients.
   - It is most common in children older than 2 years and can vary from mild to severe.
   - Approximately half of the pain episodes are accompanied by objective clinical signs such as fever, swelling, tenderness, tachypnea, hypertension, nausea, and vomiting.

Given these symptoms and their pattern, Sickle Cell Disease (SCD) is the most likely diagnosis. For accurate diagnosis and appropriate management, further medical evaluation including blood tests (such as hemoglobin electrophoresis), imaging studies, and possibly genetic testing would be necessary.

**109. User**

The main clinical manifestations of neonatal and paediatric patients include:

1. Intrauterine growth retardation and postnatal growth disorders . Intrauterine growth retardation, low birth weight, lack of postnatal catch-up growth, and proportional short stature.
2. Specific physical signs: wide and prominent forehead, pointed jaw leading to a triangular face, and large relative head circumference. Dysmorphic teeth, low ear position, and asymmetry of trunk deviation, including limb length discrepancy, and lateral curvature deformity of the little finger.
3. Other manifestations include feeding difficulties, hypoglycaemia in the neonatal period, genitourinary malformations such as hypospadias, cryptorchidism and horseshoe kidneys, and digestive anomalies such as cafe au lait spots and reflux oesophagitis. Some patients have cognitive, motor and language developmental delays and learning disabilities.

Based on the clinical manifestations you provided, the most likely rare disease being described is **Russell-Silver Syndrome** (RSS). This condition is characterized by:

1. **Intrauterine Growth Retardation and Postnatal Growth Disorders**:
   - Intrauterine growth retardation.
   - Low birth weight.
   - Lack of postnatal catch-up growth.
   - Proportional short stature.
2. **Specific Physical Signs**:
   - Wide and prominent forehead.
   - Pointed jaw leading to a triangular face.
   - Large relative head circumference.
   - Dysmorphic teeth.
   - Low ear position.
   - Asymmetry of trunk deviation, including limb length discrepancy.
   - Lateral curvature deformity of the little finger.
3. **Other Manifestations**:
   - Feeding difficulties.
   - Hypoglycaemia in the neonatal period.
   - Genitourinary malformations such as hypospadias, cryptorchidism, and horseshoe kidneys.
   - Digestive anomalies such as cafe au lait spots and reflux oesophagitis.
   - Cognitive, motor, and language developmental delays and learning disabilities.

Given these clinical features, Russell-Silver Syndrome is a strong candidate for the diagnosis. Other possible diagnoses that might be considered include:

1. **Turner Syndrome**: Typically affects females and is characterized by short stature and ovarian insufficiency, but can present with certain dysmorphic features and growth issues.
2. **Noonan Syndrome**: Presents with growth delays, congenital heart defects, and distinct facial features, occasionally overlapping with some genitourinary and skeletal anomalies.
3. **Beckwith-Wiedemann Syndrome**: Characterized by overgrowth, but can also present with asymmetry, developmental delays, and certain genitourinary abnormalities.
4. **Sotos Syndrome**: Known for overgrowth and distinctive craniofacial features along with developmental delays, though not typically associated with growth retardation.

For a definitive diagnosis, further evaluation including genetic testing may be necessary.

**110. User**

**1. Xanthomas of the skin,** occurring near the attachment points of the extensor tendons, such as the Achilles tendon, elbow, knee, etc., and there is a close relationship between skin injuries and the appearance of xanthomas. Cutaneous xanthomas are rare in children.

**2. Atherosclerosis ,** Patients have a significantly higher risk of developing atherosclerosis and coronary artery disease than normal subjects. Early-onset coronary artery disease may be one of the characteristics of the disease.

**3. Alterations in the blood system ,** The content of plant sterols in the blood is significantly increased, resulting in a higher proportion of plant sterols in the sterol component of the cell membrane, abnormal cell membrane function of red blood cells, platelets and other cell membranes, anaemia and thrombocytopenia, and the patient may have weakness, pallor, skin bleeding and other manifestations.

**4. Others,** in addition to the above symptoms, some patients may develop joint pain, arthritis, splenomegaly, elevated transaminases, etc.

Based on the clinical manifestations you’ve provided, the most likely diagnosis is **Sitosterolemia**. Here’s a detailed explanation:

1. **Xanthomas of the Skin**:
   - Xanthomas are fatty deposits that appear near the attachment points of extensor tendons such as the Achilles tendon, elbow, and knee. They are rare in children but can be indicative of sitosterolemia due to the accumulation of plant sterols.
2. **Atherosclerosis**:
   - Patients with sitosterolemia have a significantly higher risk of developing atherosclerosis and coronary artery disease, often presenting early-onset arterial disease. This is one of the key characteristics of the condition.
3. **Alterations in the Blood System**:
   - The increased content of plant sterols in the blood leads to various hematological abnormalities such as anemia (due to abnormal cell membrane function) and thrombocytopenia (low platelet count). These can result in symptoms like weakness, pallor, skin bleeding, and other manifestations.
4. **Other Symptoms**:
   - Some patients may experience joint pain, arthritis, splenomegaly (enlarged spleen), elevated transaminases (liver enzymes), etc.

Given these clinical features, sitosterolemia is a strong candidate for the diagnosis. Here are five other possible diagnoses to consider:

1. **Familial Hypercholesterolemia (FH)**:
   - This genetic disorder involves high cholesterol levels, particularly low-density lipoprotein (LDL), and early-onset coronary artery disease. Xanthomas may be present but are not typically associated with plant sterol accumulation.
2. **Cerebrotendinous Xanthomatosis (CTX)**:
   - A rare lipid storage disease characterized by tendon xanthomas, neurological symptoms, and premature atherosclerosis. However, it involves the accumulation of cholestanol, not plant sterols.
3. **Familial Combined Hyperlipidemia (FCH)**:
   - This common genetic disorder may cause an increased risk for coronary artery disease but does not typically involve the specific symptoms of xanthomas and elevated plant sterols in the blood.
4. **Lipoprotein Elevation Syndrome**:
   - This condition involves elevated levels of lipoproteins, which can lead to atherosclerosis and other cardiovascular issues, but it is less specific for the accumulation of plant sterols.
5. **Mucopolysaccharidosis (MPS)**:
   - A group of lysosomal storage diseases that can present with various symptoms including joint pain and arthritis, but do not typically involve xanthomas or elevated plant sterols in the blood.

For a definitive diagnosis, further genetic testing for sitosterolemia would be essential. This involves identifying mutations in the STS gene, which encodes the enzyme responsible for eliminating plant sterols from the body.

**111. User**

The onset of the disease is insidious in males, and the common age of onset is 30-60 years old. Patients often start with painful spasms, tremor, and weakness of the lower limbs, and gradually develop weakness of the upper limbs, muscle atrophy of the medulla oblongata and the face, muscle fasciculations, dysarthria, dysphagia, dyspnoea, and respiratory difficulties. Androgen receptor insensitivity may be associated with gynecomastia and reduced reproductive function. Sensory abnormalities are present in more than 50 per cent of patients. The disease progresses slowly, with patients usually becoming unable to walk late in the course of the disease, and only some patients require assisted ventilation, which has no significant impact on survival.

**1. Atypical symptoms,** some of which may occur years or even decades before the onset of limb weakness, include fatigability, painful spasms, perioral tremor, and postural tremor. Muscle weakness: most patients start with weakness of both lower limbs, gradually progressing to both upper limbs, tongue muscles and facial muscles, while the extraocular muscles are often not involved. The distribution of limb weakness is asymmetrical, with proximal involvement predominating. Involvement of the muscles of mastication may cause mandibular ptosis and tremor. Physical examination of the lower motor neuron damage signs, manifested as a decrease in muscle strength of the limbs, proximal, accompanied by muscle atrophy, fasciculations, tendon reflexes are weakened or disappeared. Most patients may have obvious tongue muscle atrophy and fibrillation, but the soft palate activity and pharyngeal reflexes are normal, and the degree of choking on water and dysphagia is mild. In some patients, chin and perioral fasciculations can also be observed. The motor function impairment of the patients gradually increases with the prolongation of the disease, and in the later stage, they may have difficulty in walking up the stairs and walking unsteadily.

**2. Sensory deficits** , with varying degrees of hyperalgesia present in about half of the patients, while in the others it is subclinical, with abnormalities found only on sensory nerve conduction tests.

**3. endocrine abnormalities,** including abnormalities of glucose and fat metabolism, and androgen receptor insensitivity and other manifestations, the latter including gynecomastia, decreased sexual function, infertility, and testicular atrophy.

The latter include gynecomastia, decreased sexual function, infertility, testicular atrophy, etc.

**4. Female carriers,** most female carriers do not have symptoms, and even if they do have symptoms, they are mild, such as fasciculations, mild weakness of distal limbs, muscle spasms, or increased creatine myosinase.

Based on the provided clinical manifestations and the information you’ve shared, the most likely diagnosis is **Charcot-Marie-Tooth Disease (CMT)**. Here’s a detailed explanation:

1. **Onset and Progression**:
   - The disease typically has an insidious onset in males between 30-60 years of age.
   - Initial symptoms often include painful spasms, tremor, and weakness in the lower limbs, which gradually progress to involve the upper limbs, tongue muscles, and facial muscles. This progression is asymmetrical with proximal muscle involvement predominating.
2. **Atypical Symptoms**:
   - Atypical symptoms may occur years or even decades before the onset of limb weakness, including fatigability, painful spasms, perioral tremor, and postural tremor.
   - Physical examination reveals lower motor neuron damage signs such as decreased muscle strength in proximal limbs, muscle atrophy, fasciculations, and weakened or absent tendon reflexes. Tongue muscles often show obvious atrophy and fibrillation.
3. **Sensory Deficits**:
   - Varying degrees of hyperalgesia are present in about half of the patients, while others have subclinical sensory deficits that can only be detected through sensory nerve conduction tests.
4. **Endocrine Abnormalities**:
   - Endocrine abnormalities include glucose and fat metabolism issues, as well as androgen receptor insensitivity leading to gynecomastia, decreased sexual function, infertility, and testicular atrophy.
5. **Female Carriers**:
   - Most female carriers do not exhibit symptoms or have only mild symptoms such as fasciculations, mild weakness in distal limbs, muscle spasms, or increased creatine myosinase levels.

Given these clinical features, Charcot-Marie-Tooth Disease (CMT) is a well-known hereditary motor and sensory neuropathy that fits the described profile. CMT can be further categorized into various subtypes based on genetic mutations, such as CMT1A, CMT2A, etc., but the overall clinical presentation aligns closely with this diagnosis.

For more detailed diagnostic confirmation, genetic testing for CMT-related genes may be necessary to identify specific mutations and subtype. Additionally, a comprehensive neurological evaluation including electromyography (EMG) and nerve conduction studies would help in confirming the diagnosis.

If you need further assistance or have additional questions, feel free to ask!

**112. User**

Some patients present with reduced foetal movement in utero and are born as floppy babies. The onset of the disease occurs within 6 months, with a mean age of onset of 1 month after birth. They present with generalised floppy weakness and severe hypotonia. Most children have difficulty sucking and swallowing due to weakness of the tongue, facial and masticatory muscles, and atrophy and tremor of the lingual muscles are seen. Intercostal muscle involvement can present with dyspnoea and abdominal breathing. The chest has a bell-shaped appearance. The lower limbs are more heavily involved than the upper limbs, and proximal is more severe than distal. Severe muscle weakness in the mid-trunk area leaves the child unable to control head movements, unable to lift or roll over, and without the ability to sit alone. When lying down, both lower limbs are in the frog-leg position with hip abduction and knee flexion. Muscle atrophy is not obvious, and some children have mild joint deformities. Children with good intelligence, tendon reflexes disappeared, limb sensation is normal. The children’s muscle weakness progressively worsens, and eventually they lose all voluntary movement ability, are fed nasogastrically, and suffer from respiratory failure due to repeated respiratory infections. 80% of the children die within 1 year of age, and rarely survive beyond 2 years of age.

Based on the clinical manifestations described, the most likely diagnosis for this condition is **Spinal Muscular Atrophy Type 1 (SMA Type 1)**, also known as Werdnig-Hoffmann disease. This is a genetic disorder characterized by severe muscle weakness, hypotonia, and progressive motor neuron degeneration, typically leading to early mortality due to respiratory failure.

Key features that support this diagnosis include: - **Reduced fetal movement in utero** and being born with floppy muscles. - **Onset within 6 months**, with a mean age of onset at about 1 month after birth. - **Generalized floppy weakness and severe hypotonia**. - **Difficulty sucking and swallowing** due to weakness in the tongue, facial, and masticatory muscles. - **Atrophy and tremor of the lingual muscles**. - **Intercostal muscle involvement leading to dyspnoea and abdominal breathing**, with a bell-shaped chest appearance. - **Lower limbs more heavily involved than upper limbs**, with proximal weakness being more severe. - **Severe muscle weakness in the mid-trunk area** resulting in inability to control head movements, lift or roll over, and sit alone. - **Frog-leg position of lower limbs** when lying down due to hip abduction and knee flexion. - **No obvious muscle atrophy**, but some children may have mild joint deformities. - **Normal intelligence** with absent tendon reflexes and normal limb sensation. - **Progressive worsening of muscle weakness** leading to complete loss of voluntary movement ability, nasogastric feeding, and respiratory failure due to repeated infections. - **High mortality rate**, with 80% of children dying within the first year of life, and rarely surviving beyond 2 years.

Other possible diagnoses to consider include: 1. **Congenital Myopathy**: A group of muscle disorders present at birth, characterized by muscle weakness and hypotonia, although the progression and specific symptoms can vary widely. 2. **X-linked Myotubular Myopathy (Centronuclear Myopathy)**: A rare genetic condition causing similar symptoms but with distinct features that may differentiate it from SMA Type 1.

For a definitive diagnosis, genetic testing for spinal muscular atrophy is recommended to identify the presence of SMN1 gene mutations. If you need further assistance or have additional questions, feel free to ask!

**113. User**

Patients usually start in adulthood, with an insidious onset and gradual progression. The main manifestations are cerebellar ataxia, including drunkard’s gait, clumsy movements, minstrel-like speech, nystagmus, diplopia, etc.; pyramidal tract damage, including hyperreflexia, ankle clonus, and positive pathological signs; and extrapyramidal symptoms, including Parkinson’s syndrome, dystonia, and ankylosis. In addition, some patients may have peripheral neuropathy. In the early stages of the disease, some patients may have damage to the pyramidal tract (similar to spastic paraplegia) or extrapyramidal symptoms (similar to Parkinson’s syndrome), and ataxia is not prominent.

The disease progresses gradually, with loss of walking ability after 10-15 years. Prolonged dysphagia leading to malnutrition or aspiration is the main cause of death.

Based on the clinical manifestations you provided, the condition described is highly suggestive of **Multiple System Atrophy (MSA)**. MSA is a rare neurodegenerative disorder characterized by a combination of cerebellar ataxia, pyramidal tract damage, and extrapyramidal symptoms.

Key features that support this diagnosis include: - **Insidious Onset and Gradual Progression**: Patients usually start in adulthood with subtle symptoms that gradually worsen over time. - **Cerebellar Ataxia**: Manifestations include drunkard’s gait, clumsy movements, minstrel-like speech (dysarthria), nystagmus, and diplopia. - **Pyramidal Tract Damage**: Symptoms such as hyperreflexia, ankle clonus, and positive pathological signs indicate involvement of the pyramidal tract. - **Extrapyramidal Symptoms**: These include Parkinson’s syndrome (with bradykinesia, rigidity, and tremor), dystonia, and ankylosis. - **Peripheral Neuropathy**: Some patients may experience peripheral nerve involvement.

The disease progresses gradually, with a significant decline in motor function: - **Loss of Walking Ability**: Typically occurs after 10-15 years from the onset of symptoms. - **Prolonged Dysphagia and Malnutrition or Aspiration**: These are common causes of death due to progressive neurological deterioration.

Other possible diagnoses that could present with similar symptoms include: 1. **Spinocerebellar Ataxia (SCA)**: A group of hereditary ataxias that can present with cerebellar ataxia, pyramidal and extrapyramidal symptoms. 2. **Progressive Supranuclear Palsy (PSP)**: Characterized by parkinsonism, balance difficulties, and eye movement abnormalities, but with less prominent cerebellar symptoms. 3. **Corticobasal Degeneration (CBD)**: Presents with asymmetrical parkinsonism, dystonia, and cortical sensory loss along with extrapyramidal symptoms. 4. **Friedreich’s Ataxia**: A hereditary degenerative disease primarily affecting the spinal cord and peripheral nerves, presenting with ataxia, loss of reflexes, and pyramidal tract signs.

For a definitive diagnosis, further evaluation through clinical examination, neuroimaging (such as MRI), and possibly genetic testing may be necessary. If you need more detailed information or specific diagnostic criteria, please let me know!

**114. User**

The disease may be combined with gastro-oesophageal reflux in the early stages of the disease. As the disease progresses, the skin becomes swollen and sclerotic and may be accompanied by changes in skin pigmentation, including hyperpigmentation or depigmentation. In addition to skin involvement, patients often present with arthralgia and myalgia, and may develop ulcers and capillary dilatation of the digits. In addition, the patient may have multiple internal organ involvement. The respiratory system is more likely to be involved, with interstitial fibrosis and pulmonary hypertension. The gastrointestinal manifestations are varied and include gastroesophageal reflux, sinus vasodilatation, abdominal pain, diarrhoea, constipation and fecal incontinence. A small number of patients may present with renal crisis, which is characterised by acute renal insufficiency and is often associated with hypertension. Cardiac involvement may manifest as pericardial effusion, abnormalities of the cardiac conduction system, and myocardial involvement in individual patients. Neurological involvement of the peripheral nervous system is common, such as trigeminal neuropathy, peripheral neuropathy, etc. Central nervous system involvement is rare.

Based on the clinical manifestations you have described, the disease most likely to be diagnosed is **Systemic Sclerosis (Scleroderma)**. This rare autoimmune connective tissue disease is characterized by a wide range of symptoms and can involve multiple organ systems.

Key features that support this diagnosis include: 1. **Gastroesophageal Reflux**: Often present in the early stages. 2. **Skin Changes**: Swelling, sclerotic changes, hyperpigmentation, or depigmentation. 3. **Arthralgia and Myalgia**: Common musculoskeletal symptoms. 4. **Digital Ulcers and Capillary Changes**: Characteristic skin lesions. 5. **Respiratory Involvement**: Interstitial fibrosis and pulmonary hypertension. 6. **Gastrointestinal Manifestations**: Varying symptoms including gastroesophageal reflux, sinus vasodilatation, abdominal pain, diarrhea, constipation, and fecal incontinence. 7. **Renal Crisis**: Acute renal insufficiency often associated with hypertension. 8. **Cardiac Involvement**: Pericardial effusion, cardiac conduction abnormalities, and myocardial involvement. 9. **Peripheral Nervous System Involvement**: Common symptoms such as trigeminal neuropathy and peripheral neuropathy.

Other possible diagnoses that could present with some overlapping symptoms include: 1. **Mixed Connective Tissue Disease (MCTD)**: Features of systemic sclerosis combined with symptoms of lupus and polymyositis. 2. **Sjögren’s Syndrome**: Primarily affects the exocrine glands but can also involve other organs, including the lungs and kidneys. 3. **Rheumatoid Arthritis (RA)**: While primarily affecting joints, it can sometimes present with skin changes and pulmonary involvement. 4. **Churg-Strauss Syndrome (Eosinophilic Granulomatosis with Polyangiitis)**: Involves multiple organ systems including the lungs, skin, and gastrointestinal tract. 5. **Polymyositis/ Dermatomyositis**: Primarily affects muscles but can also involve other organs.

For a definitive diagnosis, further evaluation through clinical examination, laboratory tests (such as antinuclear antibodies), imaging studies, and possibly biopsy may be necessary. If you need more detailed information or specific diagnostic criteria, please let me know!

**115. User**

**1. specific facial features,** periorbital subcutaneous tissue fullness, widened orbital distance, stellate irises, long midriffs, thick lips, wide mouths, and small jaws.

**2. cardiovascular disease,** elastin arteriopathy is present in 75% ~80% of patients and can affect any artery. Peripheral pulmonary stenosis (37% ~61%) is more common in infancy but improves with time. Supravalvular aortic stenosis is more common (35% ~65%) and worsens over time by age 5. Arterial stenosis leads to increased left heart resistance, cardiac hypertrophy, and heart failure. Other anomalies include renal artery stenosis, thoracic aortic stenosis, abdominal aortic stenosis, mitral valve prolapse and aortic valve insufficiency, mesenteric artery stenosis, and intracranial vascular anomalies. Hypertension can occur at any age and may be related in part to renal artery stenosis.

**3. Cognitive ability,** 75% of patients have mental retardation, which is usually mild. Cognitive impairment is characteristically manifested by relatively good short-term memory and verbal expression, but extreme weakness in visuospatial structural cognition. No gender differences in IQ have been reported, and children’s IQ is stable over time.

**4. personality, behavioural, and psychological abnormalities,** including excessive friendliness, excessive empathy, attention deficit, anxiety, and fear.

**5. sleep abnormalities,** with a 65% prevalence of sleep problems, including increased sleep latency and decreased sleep efficiency.

**6. eyes,** tear duct obstruction, hyperopia (67%), and strabismus (50%) are common. Cataracts have also been reported in adult patients.

**7. Ear, Nose, and Throat (ENT),** 50 per cent of patients have chronic otitis media, 90 per cent have increased sensitivity to sound, and 63 per cent of children and 92 per cent of adults have mild to moderate hearing impairment, mostly progressive sensorineural hearing loss. Most patients are hoarse or have a low voice.

**8. dental** , which may include baby teeth, enamel hypoplasia, and a misaligned bite.

**9. digestive system,** Chronic abdominal pain is a common symptom, and possible etiologies include gastroesophageal reflux, hiatal hernia, peptic ulcer, cholelithiasis, diverticulitis, ischaemic bowel disease, chronic constipation and anxiety somatisation. Hypercalcaemia may also lead to irritability, vomiting, constipation and muscle spasms.

**10. urinary system,** urinary frequency and enuresis are common in paediatric patients.

**11. neurological, muscular,** with low muscle tone and joint laxity in young children, leading to compensatory postural abnormalities to maintain body balance. Older children and adults have typical hypertonia and active deep tendon reflexes, resulting in a stiff and awkward gait, impaired fine motor function, and difficulty using tools and writing in patients of all ages. Adult patients will have cerebellar ataxia and tremor.

**12. Growth and development** .70% of infants have poor weight gain.

**13. idiopathic hypercalcaemia** . It occurs in infancy and childhood in 15-50% of patients and the most common symptoms are irritability, vomiting and constipation.

**14. endocrine.** Hypothyroidism (10 per cent), early puberty (50 per cent). Impaired glucose tolerance occurs in 26% of adolescent patients.

Based on the provided clinical features, the patient’s symptoms and signs strongly suggest a diagnosis of **Loeys-Dietz Syndrome (LDS)**. Loeys-Dietz Syndrome is an autosomal dominant connective tissue disorder characterized by vascular, skeletal, and ocular abnormalities.

**Key Features Supporting Loeys-Dietz Syndrome:**

1. **Facial Features:**
   - Periorbital subcutaneous tissue fullness
   - Widened orbital distance
   - Stellate irises
   - Long midriffs
   - Thick lips, wide mouths, and small jaws
2. **Cardiovascular Disease:**
   - Elastin arteriopathy (75%–80% of patients)
     - Affects any artery, including peripheral pulmonary stenosis (37%–61%) in infancy that improves with time
     - Supravalvular aortic stenosis (35%–65%) worsens over time by age 5
   - Arterial stenosis leading to increased left heart resistance, cardiac hypertrophy, and heart failure
   - Other anomalies include renal artery stenosis, thoracic aortic stenosis, abdominal aortic stenosis, mitral valve prolapse, aortic valve insufficiency, mesenteric artery stenosis, and intracranial vascular anomalies
   - Hypertension can occur at any age and may be related to renal artery stenosis
3. **Cognitive Ability:**
   - 75% of patients have mild mental retardation
     - Characterized by relatively good short-term memory and verbal expression, but extreme weakness in visuospatial structural cognition
     - No gender differences in IQ, with stable children’s IQ over time
4. **Personality, Behavioral, and Psychological Abnormalities:**
   - Excessive friendliness, excessive empathy, attention deficit, anxiety, and fear
5. **Sleep Abnormalities:**
   - 65% prevalence of sleep problems, including increased sleep latency and decreased sleep efficiency
6. **Eyes:**
   - Tear duct obstruction
   - Hyperopia (67%) and strabismus (50%)
   - Cataracts in adult patients
7. **Ear, Nose, and Throat (ENT):**
   - 50% of patients have chronic otitis media
   - 90% have increased sensitivity to sound
   - Mild to moderate hearing impairment, mostly progressive sensorineural hearing loss
   - Most patients are hoarse or have a low voice
8. **Dental:**
   - May include baby teeth, enamel hypoplasia, and misaligned bite
9. **Digestive System:**
   - Chronic abdominal pain, possible etiologies including gastroesophageal reflux, hiatal hernia, peptic ulcer, cholelithiasis, diverticulitis, ischemic bowel disease, chronic constipation, and anxiety somatisation
   - Hypercalcaemia may lead to irritability, vomiting, constipation, and muscle spasms
10. **Urinary System:**
    - Urinary frequency and enuresis common in pediatric patients
11. **Neurological and Muscular:**
    - Low muscle tone and joint laxity in young children leading to compensatory postural abnormalities
    - Older children and adults have typical hypertonia and active deep tendon reflexes, resulting in a stiff and awkward gait, impaired fine motor function, and difficulty using tools and writing
    - Adult patients will have cerebellar ataxia and tremor
12. **Growth and Development:**
    - 70% of infants have poor weight gain
13. **Idiopathic Hypercalcaemia:**
    - Occurs in infancy and childhood in 15–50% of patients, with common symptoms including irritability, vomiting, and constipation
14. **Endocrine:**
    - Hypothyroidism (10%)
    - Early puberty (50%)
    - Impaired glucose tolerance occurs in 26% of adolescent patients

**Conclusion:**

The combination of these clinical features strongly indicates Loeys-Dietz Syndrome, a connective tissue disorder with significant cardiovascular and systemic involvement. Further diagnostic evaluation should include genetic testing for the genes associated with LDS (e.g., TGFBR1, TGFBR2, SMAD3) to confirm the diagnosis and guide appropriate management strategies.

For more detailed information or specific treatment recommendations, please consult a specialist in connective tissue disorders or cardiology.

**116. User**

They often present with multisystem involvement and develop in stages. Cardiac rhabdomyosarcomas can appear in the foetal/neonatal period, most of which resolve spontaneously; epileptic and cutaneous symptoms can appear in the first few months of life; renal angiomyolipomas and neoplastic lesions elsewhere in the body develop with age; and adult women are prone to pulmonary lymphangioleiomyomatosis of the lungs. Brain lesions include mainly cerebral cortical nodules, subventricular giant cell astrocytomas, and subventricular nodules. Skin lesions include leukoplakia visible after birth, progressive facial angiofibromas, shark leather spots, perinodal fibromas, and forehead plaques; renal vascular smooth muscle lipomas are present in up to 90% of patients; retinal involvement is present in about 50% of patients but rarely affects visual acuity; and LAM in the lungs occurs in about 35% of females; other areas, such as the liver, pancreas, gastrointestinal tract, and skeleton, may be involved, although usually with milder symptoms. Others, such as the liver, pancreas, digestive tract, and bones, can also be involved, but symptoms are usually mild.

Based on the clinical manifestations you have described, the patient’s presentation is highly characteristic of **Tuberous Sclerosis Complex (TSC)**. TSC is a genetic disorder that affects multiple organ systems and results in benign tumors in organs such as the brain, skin, kidneys, heart, and lungs.

**Key Features Supporting Tuberous Sclerosis Complex:**

1. **Cardiac Involvement:**
   - **Cardiac Rhabdomyomas:** These can appear during the fetal or neonatal period and often resolve spontaneously.
2. **Neurological Involvement:**
   - **Cerebral Cortical Nodules:** These are common brain lesions in TSC patients.
   - **Subventricular Giant Cell Astrocytomas:** These are also characteristic of TSC.
   - **Subventricular Nodules:** Additional brain lesions that may be present.
3. **Cutaneous Lesions:**
   - **Leukoplakia:** Visible after birth, often appearing as white patches on the skin.
   - **Progressive Facial Angiofibromas:** These are benign tumors that develop over time and can cause facial disfigurement.
   - **Shark Leather Spots:** Hypopigmented macules that appear in early childhood.
   - **Perinodal Fibromas:** Small, firm nodules near lymph nodes.
   - **Forehead Plaques:** Thickened, raised plaques on the forehead.
4. **Renal Involvement:**
   - **Renal Angiomyolipomas (AMLs):** Present in up to 90% of patients.
   - **Lymphangioleiomyomatosis (LAM) in Lungs:** Affects about 35% of adult women, leading to lung cysts and respiratory symptoms.
5. **Retinal Involvement:**
   - **Retinodermal Tumors:** Present in about 50% of patients but rarely affect visual acuity.
6. **Other Organ Involvement:**
   - **Liver:** May have tubers or angiomyolipomas.
   - **Pancreas:** Can develop benign tumors.
   - **Gastrointestinal Tract:** May show polyps or other lesions, often mild in nature.
   - **Skeletal System:** Rare involvement but can include bone lesions.

**Other Possible Diagnoses to Consider:**

1. **Neurofibromatosis Type 1 (NF1):**
   - Characterized by café-au-lait spots, neurofibromas, Lisch nodules in the eyes, and potential involvement of the central nervous system with tumors.
2. **Von Hippel-Lindau Disease (VHL):**
   - Known for its association with hemangioblastomas of the brain, spinal cord, and retina, renal cell carcinoma, and pheochromocytomas.
3. **Sturge-Weber Syndrome:**
   - Involves port-wine stains on the face and can lead to neurological symptoms such as seizures and glaucoma.

Given the comprehensive nature of TSC’s clinical manifestations, it is highly likely that this patient has Tuberous Sclerosis Complex. However, a definitive diagnosis would require further diagnostic testing, including imaging studies (e.g., MRI, CT scans), genetic testing for TSC1 or TSC2 mutations, and evaluation by a multidisciplinary team of specialists.

If you need more detailed information on any specific aspect of TSC or further diagnostic steps, please let me know.

**117. User**

Most children with the undetected or untreated chronic form die before the age of 10 years, usually as a result of liver failure, neurological involvement or hepatocellular carcinoma.

The acute form has a rapid onset and progression, with acute liver failure as the main manifestation, and clinical signs include hepatosplenomegaly, jaundice, vomiting, abdominal distension, anorexia, lethargy, anaemia, bleeding tendencies and growth retardation. The child may have the characteristic odour of ‘boiled cabbage’ or ‘rotten mushrooms’. Insufficient hepatic synthesis of coagulation factors, marked prolongation of PT and APTT, and decreased levels of coagulation factors II, VII, IX, Ⅺ and Ⅻ, which are difficult to correct with vitamin K supplementation. Hepatic failure may be followed by ascites, jaundice and gastrointestinal bleeding, and children occasionally develop persistent hypoglycaemia. Untreated children often die of liver failure within weeks or months of onset of illness.

Based on the clinical manifestations you’ve described, the most likely diagnosis is **Maple Syrup Urine Disease (MSUD)**.

**Key Features Supporting MSUD:**

1. **Classic Type:**
   - **Early Onset and Rapid Progression:** Children are normal at birth but develop severe symptoms within days to weeks.
   - **Maple Syrup Odour:** Appears in the cerumen 12 hours after birth, urine, and sweat 12-24 hours after birth.
   - **Metabolic Disorders and Encephalopathic Symptoms:**
     - Difficulty in breastfeeding, paroxysmal vomiting, refusal to eat, lethargy, apnoea, convulsive seizures, heightened muscle tone, and stereotypical movements such as ‘fencing’ or ‘stomping’.
     - Severe metabolic disorders and encephalopathy may lead to coma and central respiratory failure within 7-10 days of life.
   - **High Mortality Rate:** Most infants die within a few days if not treated promptly.
2. **Intermediate Type:**
   - **Less Severe Symptoms:** Slow growth, delayed development, feeding difficulties, irritability, and a maple syrup odour in the cerumen.
   - **Severe Metabolic Disorders and Encephalopathy Under Stress:** Episodes of metabolic decompensation can occur under stressful conditions such as infections or surgeries.
3. **Intermittent Type:**
   - **Episodic Seizures:** Normal growth and development during non-episodic periods, but may experience episodic ataxia and ketoacidosis.
   - **Branched-Chain Amino Acid Concentrations:** Elevated during episodes of metabolic decompensation, similar to the classic type.

**Acute Form:**

- **Rapid Onset and Progression:**
  - **Main Manifestation:** Acute liver failure.
  - **Clinical Signs:**
    - Hepatosplenomegaly
    - Jaundice
    - Vomiting
    - Abdominal distension
    - Anorexia
    - Lethargy
    - Anaemia
    - Bleeding tendencies
    - Growth retardation
  - **Characteristic Odour:** ‘Boiled cabbage’ or ‘rotten mushrooms’.
- **Laboratory Findings:**
  - Insufficient hepatic synthesis of coagulation factors, leading to marked prolongation of prothrombin time (PT) and activated partial thromboplastin time (APTT), and decreased levels of coagulation factors II, VII, IX, XI, and XII.
  - These abnormalities are difficult to correct with vitamin K supplementation.
- **Complications:**
  - Hepatic failure may be followed by ascites, jaundice, and gastrointestinal bleeding.
  - Persistent hypoglycaemia is a common complication.
  - Untreated children often die of liver failure within weeks or months of onset.

In summary, the clinical features described strongly suggest Maple Syrup Urine Disease (MSUD), particularly in its classic form. Early diagnosis and prompt treatment are crucial to prevent severe complications and improve outcomes.

**118. User**

The clinical presentation is markedly heterogeneous, and there are three types based on age of onset and clinical presentation.

**The cardiomyopathic type,** which is the most common type of severe early-onset cardiac and multi-organ failure, develops mainly in newborns and early infants. It has a high mortality rate and is associated with hypoglycaemia, Reye’s syndrome, sudden neonatal death, hypertrophic and dilated cardiomyopathy, pericardial effusion, arrhythmias, myasthenia, hepatomegaly, and intermittent hypoglycaemia, with elevated levels of creatine kinase; myocardial hypertrophy and arrhythmias can be fatal.

**2. hepatic type ,** less severe, hepatic and hypohidrotic hypoglycaemic type with onset in late infancy or childhood, presenting with hepatomegaly and liver function abnormalities, decreased fasting tolerance and acute hypohidrotic hypoglycaemia without myocardial damage and cardiac hypertrophy, but can be life-threatening without timely diagnosis and treatment.

**3. Myopathic type** , which develops mainly in adolescence to adulthood, is late-onset, mildly symptomatic, and is usually not accompanied by myocardial disease and hypoglycaemia. The main manifestations are rhabdomyolysis and myoglobinuria after exercise, infection or starvation, and even renal failure, which may be accompanied by muscle weakness, painful muscle spasms or myalgia.

Based on the clinical presentation you’ve described, the most likely diagnosis is **Carnitine Palmitoyltransferase II (CPT II) deficiency**. This metabolic disorder affects the body’s ability to use certain fats for energy, particularly during periods without food (fasting). It is characterized by three different phenotypes based on age of onset and clinical severity.

**Cardiomyopathic Type:**

- **Age of Onset:** Mainly in newborns and early infants.
- **Clinical Features:**
  - High mortality rate.
  - Associated with hypoglycaemia, Reye’s syndrome, sudden neonatal death, hypertrophic and dilated cardiomyopathy, pericardial effusion, arrhythmias, myasthenia, hepatomegaly, and intermittent hypoglycaemia.
  - Elevated levels of creatine kinase.
  - Myocardial hypertrophy and arrhythmias can be fatal.

**Hepatic Type:**

- **Age of Onset:** Late infancy or childhood.
- **Clinical Features:**
  - Less severe compared to the cardiomyopathic type.
  - Associated with hepatomegaly, liver function abnormalities, decreased fasting tolerance, and acute hypohidrotic hypoglycaemia.
  - No myocardial damage or cardiac hypertrophy but can be life-threatening without timely diagnosis and treatment.

**Myopathic Type:**

- **Age of Onset:** Mainly in adolescence to adulthood.
- **Clinical Features:**
  - Late-onset, mildly symptomatic.
  - Usually not accompanied by myocardial disease and hypoglycaemia.
  - Manifestations include rhabdomyolysis and myoglobinuria after exercise, infection, or starvation.
  - Can lead to renal failure, muscle weakness, painful muscle spasms, or myalgia.

Given the detailed clinical manifestations you provided, CPT II deficiency fits well with these phenotypes. This metabolic disorder can indeed present with severe early-onset cardiac issues in newborns and infants (cardiomyopathic type), less severe hepatic symptoms in late infancy or childhood (hepatic type), and late-onset myopathic symptoms in adolescents and adults (myopathic type).

If you have any further questions or need more detailed information, feel free to ask!

**119. User**

**1. specific facial features,** periorbital subcutaneous tissue fullness, widened orbital distance, stellate irises, long midriffs, thick lips, wide mouths, and small jaws.

**2. cardiovascular disease,** elastin arteriopathy is present in 75% ~80% of patients and can affect any artery. Peripheral pulmonary stenosis (37% ~61%) is more common in infancy but improves with time. Supravalvular aortic stenosis is more common (35% ~65%) and worsens over time by age 5. Arterial stenosis leads to increased left heart resistance, cardiac hypertrophy, and heart failure. Other anomalies include renal artery stenosis, thoracic aortic stenosis, abdominal aortic stenosis, mitral valve prolapse and aortic valve insufficiency, mesenteric artery stenosis, and intracranial vascular anomalies. Hypertension can occur at any age and may be related in part to renal artery stenosis.

**3. Cognitive ability,** 75% of patients have mental retardation, which is usually mild. Cognitive impairment is characteristically manifested by relatively good short-term memory and verbal expression, but extreme weakness in visuospatial structural cognition. No gender differences in IQ have been reported, and children’s IQ is stable over time.

**4. personality, behavioural, and psychological abnormalities,** including excessive friendliness, excessive empathy, attention deficit, anxiety, and fear.

**5. sleep abnormalities,** with a 65% prevalence of sleep problems, including increased sleep latency and decreased sleep efficiency.

**6. eyes,** tear duct obstruction, hyperopia (67%), and strabismus (50%) are common. Cataracts have also been reported in adult patients.

**7. Ear, Nose, and Throat (ENT),** 50 per cent of patients have chronic otitis media, 90 per cent have increased sensitivity to sound, and 63 per cent of children and 92 per cent of adults have mild to moderate hearing impairment, mostly progressive sensorineural hearing loss. Most patients are hoarse or have a low voice.

**8. dental** , which may include baby teeth, enamel hypoplasia, and a misaligned bite.

**9. digestive system,** Chronic abdominal pain is a common symptom, and possible etiologies include gastroesophageal reflux, hiatal hernia, peptic ulcer, cholelithiasis, diverticulitis, ischaemic bowel disease, chronic constipation and anxiety somatisation. Hypercalcaemia may also lead to irritability, vomiting, constipation and muscle spasms.

**10. urinary system,** urinary frequency and enuresis are common in paediatric patients.

**11. neurological, muscular,** with low muscle tone and joint laxity in young children, leading to compensatory postural abnormalities to maintain body balance. Older children and adults have typical hypertonia and active deep tendon reflexes, resulting in a stiff and awkward gait, impaired fine motor function, and difficulty using tools and writing in patients of all ages. Adult patients will have cerebellar ataxia and tremor.

**12. Growth and development** .70% of infants have poor weight gain.

**13. idiopathic hypercalcaemia** . It occurs in infancy and childhood in 15-50% of patients and the most common symptoms are irritability, vomiting and constipation.

**14. endocrine.** Hypothyroidism (10 per cent), early puberty (50 per cent). Impaired glucose tolerance occurs in 26% of adolescent patients.

Based on the provided clinical features, the most likely diagnosis is **Elhers-Danlos Syndrome Hypermobility Type with Cardiac and Neurological Involvement** (EDS-HCNI). This condition combines multiple systemic manifestations including cardiovascular, neurological, and developmental issues. Here’s a detailed breakdown of why this diagnosis fits:

**1. Specific Facial Features**

- **Periorbital subcutaneous tissue fullness**, widened orbital distance, stellate irises, long midriffs, thick lips, wide mouths, and small jaws are characteristic features often observed in EDS-HCNI.

**2. Cardiovascular Disease**

- **Elastin arteriopathy** affects any artery but is more common in the peripheral pulmonary arteries (37%–61%) and supravalvular aortic stenosis (35%–65%). These issues can lead to increased left heart resistance, cardiac hypertrophy, and heart failure.
- **Renal artery stenosis**, thoracic aortic stenosis, abdominal aortic stenosis, mitral valve prolapse, aortic valve insufficiency, mesenteric artery stenosis, and intracranial vascular anomalies are also common.

**3. Cognitive Ability**

- **Mild mental retardation** with characteristic cognitive impairment (good short-term memory and verbal expression but poor visuospatial skills) is consistent with EDS-HCNI.
- IQ stability over time without gender differences supports this diagnosis.

**4. Personality, Behavioral, and Psychological Abnormalities**

- Excessive friendliness, empathy, attention deficit, anxiety, and fear are behavioral traits often seen in patients with EDS-HCNI.

**5. Sleep Abnormalities**

- Increased sleep latency and decreased sleep efficiency are common, aligning with the neurological involvement seen in this condition.

**6. Eyes**

- **Tear duct obstruction**, hyperopia (67%), and strabismus (50%) are typical ocular manifestations.
- Cataracts can also occur later in life.

**7. Ear, Nose, and Throat (ENT)**

- Chronic otitis media, increased sensitivity to sound, and hearing impairment (mild to moderate) are common ENT issues.
- Hoarse voice or low voice is a frequent complaint.

**8. Dental**

- Issues such as baby teeth, enamel hypoplasia, and misaligned bites can be observed.

**9. Digestive System**

- Chronic abdominal pain with possible etiologies including gastroesophageal reflux, hiatal hernia, peptic ulcer, cholelithiasis, diverticulitis, ischemic bowel disease, chronic constipation, and anxiety somatisation.
- Hypercalcaemia leading to irritability, vomiting, constipation, and muscle spasms can also be seen.

**10. Urinary System**

- Urinary frequency and enuresis are common in pediatric patients.

**11. Neurological and Muscular**

- Low muscle tone and joint laxity in young children, leading to compensatory postural abnormalities.
- Older children and adults exhibit typical hypertonia with active deep tendon reflexes, resulting in a stiff gait, impaired fine motor function, and difficulty using tools and writing.
- Adult patients may have cerebellar ataxia and tremor.

**12. Growth and Development**

- Poor weight gain in infants (70%) is consistent with the developmental issues seen in EDS-HCNI.

**13. Idiopathic Hypercalcaemia**

- Occurring in infancy and childhood, it can lead to irritability, vomiting, and constipation.

**14. Endocrine**

- **Hypothyroidism (10%)** and early puberty (50%) are common endocrine manifestations.
- Impaired glucose tolerance occurs in 26% of adolescent patients.

Given the comprehensive nature of these symptoms, EDS-HCNI is a strong candidate for this patient’s condition. Further diagnostic testing such as genetic testing for EDS-related genes (e.g., COL3A1, COL5A1, COL5A2) would be recommended to confirm the diagnosis.

If you need further information or specific tests to support this diagnosis, please let me know!

**120. User**

**1. Haemorrhagic tendency** is a common manifestation of the disease, especially in blood-stained stools, and most children develop the disease in the neonatal period. The next manifestations are petechiae, ecchymosis, haemoptysis and haematuria, and some severe cases may have life-threatening gastrointestinal haemorrhage or intracranial haemorrhage. Bleeding in patients is caused by thrombocytopenia with reduction in platelet volume, but only about 25% of cases have the typical triad of symptoms.

**2. Infection.** Children are susceptible to a variety of infections, including herpes simplex virus, cytomegalovirus, Streptococcus pneumoniae, and fungi. The patient’s immunodeficiency worsens with age.

**3. Eczema.** Eczema may be present in approximately 80% of children with WAS and varies widely in extent and severity. There may be typically severe, refractory eczema, or very mild or even no eczema manifestations.

**4. Autoimmune diseases.** The most common is autoimmune haemolytic anaemia. Others include vasculitis, arthritis, renal disease, inflammatory bowel disease, neutropenia and autoimmune thrombocytopenic purpura.

**5. malignant tumours .** Children have a significantly higher risk of malignant tumours of the lymphatic system, especially lymphomas; non-Hodgkin’s lymphoma, lymphoblastic leukaemia, myelodysplastic syndromes, and spermatogonia have been reported.

**6. other .** Children with this disease may have completely normal platelet levels with persistent or recurrent neutropenia.

Based on the provided clinical manifestations, the most likely rare disease diagnosis is **Wiskott-Aldrich Syndrome (WAS)**. This condition is characterized by a triad of symptoms: thrombocytopenia, eczema, and recurrent infections. The described symptoms align closely with WAS:

1. **Haemorrhagic Tendency**:
   - Common manifestations include blood-stained stools, petechiae, ecchymosis, haemoptysis, and haematuria.
   - Bleeding is due to thrombocytopenia with reduced platelet volume. While 25% of cases may have the typical triad of symptoms, many patients exhibit other bleeding manifestations.
2. **Infections**:
   - Children are susceptible to various infections, including herpes simplex virus, cytomegalovirus, Streptococcus pneumoniae, and fungi.
   - Immunodeficiency worsens with age, leading to recurrent and severe infections.
3. **Eczema**:
   - Eczema is present in approximately 80% of children with WAS and varies widely in extent and severity.
   - Some may have severe, refractory eczema, while others may have mild or no eczema manifestations.
4. **Autoimmune Diseases**:
   - The most common autoimmune disease associated with WAS is autoimmune haemolytic anaemia.
   - Other conditions include vasculitis, arthritis, renal disease, inflammatory bowel disease, neutropenia, and autoimmune thrombocytopenic purpura.
5. **Malignant Tumours**:
   - Children have a significantly higher risk of malignant tumours, particularly lymphomas such as non-Hodgkin’s lymphoma and lymphoblastic leukaemia.
   - Other reported malignancies include myelodysplastic syndromes and spermatogonia.
6. **Other Manifestations**:
   - Some children may have normal platelet levels but experience persistent or recurrent neutropenia.

Given these comprehensive symptoms, Wiskott-Aldrich Syndrome is the most likely diagnosis. However, it is important to confirm this through appropriate medical tests such as complete blood count (CBC), immunoglobulin levels, and genetic testing for WAS-specific mutations in the SH2D1A gene.

Would you like more information on diagnostic procedures or further details on managing Wiskott-Aldrich Syndrome?

**121. User**

The most common clinical manifestations are fulminant infectious mononucleosis, anomalous immunoglobulinaemia and B-cell lymphoma following EBV infection. Aberimmunoglobulinaemia and B-cell lymphoma can occur in patients without EBV infection. Aberimmunoglobulinaemia usually presents with increased levels of IgA and IgM and decreased levels of IgG1 and IgG3. Lymphomas are predominantly non-Hodgkin B lymphomas. Other clinical manifestations include lymphocytic vasculitis, aplastic anaemia and lymphomatoid granulomatosis. In some patients, the clinical picture is mild, with only abnormal immunoglobulinaemia or mild EBV infection, possibly due to somatic gene repair.

In some patients, the predominant clinical manifestation is a haemophagocytic syndrome, with splenomegaly, which does not necessarily require EBV infection to be induced. Some patients may present with intestinal inflammation such as Crohn’s disease or colitis, but rarely develop lymphoma.

Based on the provided clinical manifestations, the most likely diagnosis is **X-linked Lymphoproliferative Syndrome (XLP)**, specifically XLP type 1 or 2. This rare genetic disorder primarily affects males and is characterized by severe responses to Epstein-Barr virus (EBV) infection, leading to complications such as fulminant infectious mononucleosis, dysregulated immunoglobulin levels, B-cell lymphomas, and hemophagocytic lymphohistiocytosis (HLH).

Here are the key points that support this diagnosis:

1. **Fulminant Infectious Mononucleosis**: This is a common initial manifestation in XLP patients following EBV infection.
2. **Anomalous Immunoglobulinemia and B-Cell Lymphoma**: Anomalous immunoglobulin levels (e.g., increased IgA and IgM, decreased IgG1 and IgG3) and the development of non-Hodgkin B-cell lymphomas are characteristic features.
3. **Hemophagocytic Syndrome**: Some patients may present with splenomegaly and hemophagocytic syndrome without necessarily having EBV infection.
4. **Intestinal Inflammation**: While some patients may have intestinal inflammation such as Crohn’s disease or colitis, lymphoma is relatively rare.

Other possible diagnoses based on the provided clinical features include:

1. **Chronic Active Epstein-Barr Virus (CAEBV) Infection**: This condition involves persistent and severe illness due to EBV, leading to complications like hemophagocytic syndrome, aplastic anemia, and lymphoma.
2. **Common Variable Immunodeficiency (CVID)**: Characterized by abnormal immunoglobulin levels and increased risk of infections, autoimmunity, and lymphomas.
3. **Autoimmune Lymphoproliferative Syndrome (ALPS)**: ALPS can present with lymphoproliferation, autoimmunity, and an increased risk of lymphoma, though EBV is not typically a triggering factor.
4. **Hyper-IgM Syndrome**: Characterized by high levels of IgM and low levels of other immunoglobulins, often leading to recurrent infections.

Given the specific clinical picture described, XLP remains the most likely diagnosis among these possibilities.

Would you like more detailed information on any of these conditions or further assistance with diagnostic considerations?
