# Supplementary material for: Performance of ChatGPT-4o and Four Open-Source Large Language Models in Generating Diagnoses Based on China’s Rare Disease Catalog: Comparative Study
Source: J Med Internet Res. 2025 Jun 18;27:e69929. doi: 10.2196/69929 (PMC12192912; doi:10.2196/69929)
Supplement: Multimedia Appendix 1 [file jmir-v27-e69929-s001.zip › numbered_qwen2.5-72b-cn.docx]

# numbered_qwen2.5_72b_cn

**1. user 2024-11-30T23:33:48Z:**

该疾病可于儿童期早期起病。典型的临床表现包括肾脏、眼部改变及听力受损。肾脏表现为血尿、蛋白尿和肾功能进行性恶化，其中血尿常为持续性镜下血尿，可在运动后或发热时出现肉眼血尿，常见于青少年型。听力改变为感音神经性听力损失， 并随病程逐渐加重。眼部异常表现为近视、青年环和白内障，但缺乏特异性。三种具有诊断意义的眼部改变包括：前圆锥形晶状体、后多形性角膜萎缩和视网膜斑（视网膜中心凹区域周围的白色或黄色颗粒）。我国男性患者听力受损发生率高于国外报道（68％比 55％），女性则低于国外报道（7％比 45％），眼异常发生率与国外报道相近。少数患者还可出现平滑肌瘤，可累及呼吸道、胃肠道及女性生殖道；偶有动脉瘤病变、面中部发育异常和精神发育迟滞的报道。

患者常有家族史。常染色体隐性遗传患者和 X 连锁遗传的男性患者病情进展较快，常于 16~35 岁进入终末期肾病；而常染色体显性遗传和 X 连锁遗传的女性患者多病程较为缓慢，肾功能衰竭出现较晚。在同一个患病家系中，所有男性成员发生肾功能衰竭的年龄相似，但不同家系中的男性成员发生肾功能衰竭的年龄可以不同。因此，可根据患病家系发生肾功能衰竭的年龄分为：青少年型（肾衰竭发生在青少年时期） 与成人型（肾衰竭发生在成年以后）。青少年型肾外表现更为突出，男孩通常在繁衍后代前即死亡，致病基因常为新发突变；而成人型家系中的患者较年长，少为新发突变。

**qwen2.5_72b** 2024-11-30T23:34:41Z:

根据您提供的临床表现和疾病特征，最有可能的诊断是 **Alport综合征**。

### 1. Alport综合征

- **遗传方式**：X连锁隐性、常染色体显性和常染色体隐性。
- **主要特征**：
  - 肾脏损害（血尿、蛋白尿、肾功能进行性恶化）
  - 感音神经性听力损失
  - 眼部异常（前圆锥形晶状体、后多形性角膜萎缩和视网膜斑）

### 其他五种可能的诊断

#### 2. Fabry病

- **遗传方式**：X连锁隐性。
- **主要特征**：
  - 肾脏损害（蛋白尿、血尿）
  - 眼部异常（角膜涡旋状混浊）
  - 心血管系统受累（心肌肥厚、冠状动脉病变）

#### 3. Bardet-Biedl综合征

- **遗传方式**：常染色体隐性。
- **主要特征**：
  - 肥胖
  - 多指（趾）畸形
  - 视网膜色素变性
  - 性腺发育不良
  - 智力障碍

#### 4. Marfan综合征

- **遗传方式**：常染色体显性。
- **主要特征**：
  - 心血管系统受累（主动脉瘤、瓣膜异常）
  - 眼部异常（晶状体脱位）
  - 骨骼系统异常（长指趾、脊柱侧弯）

#### 5. Lowe综合征

- **遗传方式**：X连锁隐性。
- **主要特征**：
  - 肾小管酸中毒
  - 白内障
  - 精神发育迟滞

#### 6. Cockayne综合征

- **遗传方式**：常染色体隐性。
- **主要特征**：
  - 生长发育迟缓
  - 感觉神经性听力损失
  - 眼部异常（视网膜色素变性、角膜混浊）
  - 脑萎缩

### 总结

Alport综合征是最符合您描述的临床表现和遗传特征的罕见病。其他几种疾病虽然也表现出一些相似的症状，但它们的典型特征与Alport综合征有所不同。建议进一步进行基因检测以确诊。

**2. user 2024-11-30T23:36:01Z:**

患者在新生儿期常无异常表现。首发症状是 6 月龄左右出现发育迟滞表现，但典型临床表现多在 1 岁后出现，临床确定诊断往往需要数年时间。按照临床表现出现频率不同分为：均出现的表现（100％患者）、经常性表现（80％以上患者）、相关性表现（20％ ~80％ 患者）3 类。

**1. 均出现的表现** 正常孕产史和出生头围；无出生缺陷和生化指标异常；颅脑 MRI/CT 除轻微皮质萎缩、髓鞘发育不良外，多无结构异常；运动里程碑落后而无倒退；6~12 个月出现严重发育迟缓；语言障碍显示为无或极少量词汇，重复性语言和非语言交往能力强于表达性语言能力；运动或平衡障碍常表现为共济失调以及四肢震颤；异常的行为特征表现为频繁大笑或微笑、明显的兴奋动作或快乐举止、常伴拍手或多动。

**2. 经常性表现** 头围增长落后，随访至 2 岁仍表现为小头畸形；3 岁前常出现癫痫；异常脑电图：特征性高波幅棘 - 慢波。

**3. 相关性表现** 枕部扁平；喜吐舌；吸吮或吞咽障碍；婴儿期喂养困难；肌张力低；巨大下颌；牙间隙宽；频繁流涎；过度咀嚼动作；斜视；皮肤色素减退，与家人相比头发和眼睛颜色浅（仅见于缺失型）；下肢腱反射亢进；上举或弯曲上肢，尤其是行走时；步基宽；对热敏感性高；异常睡眠 - 觉醒周期、睡眠少；迷恋水、纸、塑料；进食相关行为异常；肥胖（多见于年长、非缺失型患者）；脊柱侧凸；便秘。

**qwen2.5_72b** 2024-11-30T23:36:45Z:

根据您提供的详细临床表现，最有可能的诊断是**安格曼综合症 (Angelman syndrome)**。这是一种由15号染色体上的UBE3A基因突变或缺失引起的罕见遗传病。以下是其他五种可能的诊断：

- **雷特综合症（Rett Syndrome）**: 这是一种主要影响女性的神经发育障碍，特征为手部刻板动作、语言和社交技能丧失等。
- **普拉德-威利综合症 (Prader-Willi syndrome)**: 该病同样与15号染色体异常有关，但症状侧重于过度饥饿导致肥胖、肌张力低下等问题。尽管此病症的一些特征如喂养困难、发育迟缓和小头畸形等也出现在安格曼综合征患者中，但它们的临床表现还是有显著区别的。
- **自闭症谱系障碍 (Autism Spectrum Disorder, ASD)**: 某些ASD患者的症状可能与安格曼综合症相似，如社交沟通缺陷、重复行为模式及兴趣狭窄等。不过，ASD没有特定的遗传学标记，而安格曼综合症则有明确的基因突变或缺失作为诊断依据。
- **脑性瘫痪 (Cerebral Palsy, CP)**: 脑损伤导致的一组永久性的运动障碍和姿势异常。虽然CP患者可能会表现出肌肉控制问题、语言困难等症状，但其病因与安格曼综合症不同，且通常不会出现频繁大笑等行为特征。
- **唐氏综合征 (Down Syndrome)**: 一种因21号染色体三体现象引起的遗传性疾病，表现为智力障碍、特殊面容、心脏缺陷等多个系统的异常。尽管部分症状如发育迟缓和小头畸形可能与安格曼综合症有重叠之处，但两者间存在明显的区别。

上述五种疾病虽然在某些临床表现上有所交集，但是每一种都有其独特的病因及特异性标志，在诊断时需要通过详细的家族史、实验室检测和影像学检查来加以区分。

**3. user 2024-11-30T23:36:57Z:**

- 骨骼异常表现：疾病严重程度和相关并发症的类型存在差异。所有 病例均存在胸腔狭窄，胸廓可呈钟形或长而窄，呼吸窘迫通常由小而僵硬的胸腔引起。根据胸廓类型和潜在的肺发育不良的程度，呼吸功能不全严重程度不同，发病年龄不同。呼吸功能不全严重程度与 该疾病诊断年龄呈负相关。存在严重的限制性胸廓和钟形胸廓的婴儿更多见急性呼吸系统疾病，在新生儿早期可出现严重和致命的呼吸衰竭。胸腔长而窄的婴儿，更多表现为轻度呼吸系统受累，最终可逐渐发展为慢性限制性肺病伴反复呼吸道感染。肋骨短小、畸形引起肋间肌无力、呼吸受限，进而出现呼吸衰竭。多数患儿于 2 岁内死亡，其中约有半数患儿于生后 6 个月内死亡。患者其他骨骼异常包括肋骨短、四肢短小、骨盆形状异常、短指、多指（趾） 畸形等。约有 60％患者伴有近端颈椎狭窄。
- 其他脏器受累表现： 约 40％的患者会出现肾脏病变。患者肾脏病变的最早表现是尿液浓缩功能受损，其他表现包括多尿、蛋白尿、血尿素氮和肌酐升高以及高血压。肾功能衰竭是 该患者在 3~10 岁死亡的主要原因。约 30％的病例出现肝脏受累，表现为肝肿大、转氨酶升高、胆汁淤积、肝硬化和门静脉高压。该疾病偶与眼睛发育异常相关，包括视神经发育不全、视网膜营养不良、视网膜色素异常、眼球震颤、近视和进行性视力障碍。亦有少数报道提示这些患者也可出现神经系统异常如发育落后、肌张力低下、中枢神经系统MRI 异常等。

**qwen2.5_72b** 2024-11-30T23:37:43Z:

根据您提供的临床表现，最有可能的诊断是**成骨不全症（Osteogenesis Imperfecta, OI）**中的一个亚型，但更具体地讲，患者的表型和病程描述与**短肋多指综合征（Short-Rib Polydactyly Syndrome, SRPS）**，尤其是Jeune 综合征或称作“肋骨-胸廓发育不良”更为吻合。

除了SRPS外，以下五种可能的诊断也应该考虑：

- **Ellis-van Creveld 综合征（EVC综合征）**：这是一种非常罕见的遗传性骨骼发育障碍，表现为四肢短小、多指畸形及心脏缺陷等。患者通常会有指甲和牙齿异常，并且可能伴有呼吸困难等症状。
- **成骨不全症（Osteogenesis Imperfecta, OI）**：虽然OI主要特征为易骨折，但某些亚型也可能会出现胸廓狭窄以及相关的呼吸问题。此外，还可能出现骨骼外的表现如蓝巩膜、听力损失等。
- **Pierre Robin 序列征（Pierre Robin Sequence, PRS）**：此病以小下颌、腭裂和舌后坠为主要特点，可导致新生儿期严重的喂养困难及呼吸问题。虽然PRS主要影响面部结构，但也有文献报告过类似胸廓异常的情况。
- **肾性骨营养不良症（Renal Osteodystrophy）**：这是慢性肾脏疾病中常见的一种并发症，可能会影响骨骼的正常发育和代谢，导致骨骼变形、软化等问题。若伴有显著的呼吸系统受累，则需要进一步评估是否为先天性疾病的一部分。
- **Marfan 综合征**：尽管Marfan综合征主要表现为心血管系统异常及大血管扩张，但患者也可能出现胸廓形态改变（如漏斗胸或鸡胸），并且可能存在多指畸形。不过，它与您描述的症状并不完全吻合，但仍应考虑作为鉴别诊断之一。

综上所述，鉴于患者的临床表现特征，Jeune 综合征可能是最合适的初步诊断选择，同时上述其他几种疾病也应在临床实践中进行综合评估和排除。

**4. user 2024-11-30T23:37:45Z:**

主要症状包括精神行为异常、认知障碍、记忆力下降、癫痫发作、言语障碍、运动障碍、不自主运动，意识水平下降、自主神经功能障碍等。其他伴随症状包括睡眠障碍、CNS 局灶性损害、脑干、小脑症状以及周围神经和神经肌肉接头受累等。

临床可分为 3 种主要类型：①抗 NMDAR 脑炎，是 最主要类型，其特征性临床表现符合弥漫性脑炎。②边缘性脑炎，以精神行为异常、颞叶癫痫和近记忆力障碍为主要症状，常见的边缘性脑炎有抗 LGI1 抗体、抗 GABABR 抗体与抗 AMPAR 抗体相关的脑炎。③其他类型，包括莫旺综合征、抗 DPPX 抗体相关脑炎、抗 IgLON5 抗体相关脑病、自身免疫性小脑性共济失调等。

**qwen2.5_72b** 2024-11-30T23:38:16Z:

根据您提供的临床表现和分类信息，最有可能的诊断是**抗 NMDAR 脑炎**。这种疾病是一种由针对 N-甲基-D-天冬氨酸受体（NMDA 受体）的抗体引起的自身免疫性脑炎，其特征确实包括精神行为异常、认知障碍、记忆力下降和癫痫发作等症状。

除了抗 NMDAR 脑炎外，还有以下五种可能的诊断：

- **边缘性脑炎**：特别是与抗 LGI1 抗体或抗 GABABR 抗体相关的类型。这类疾病通常表现为精神行为异常、颞叶癫痫和记忆力障碍。
- **莫旺综合征（Morvan’s syndrome）**：这是一种罕见的自身免疫性疾病，可引起不自主运动、肌阵挛、睡眠障碍等症状。
- **抗 DPPX 抗体相关脑炎**：这种类型的脑炎可以导致严重的神经症状，包括认知障碍和自主神经系统功能障碍。
- **抗 IgLON5 抗体相关脑病**：这是一种罕见的自身免疫性疾病，主要影响脑干和小脑，可引起睡眠障碍、运动障碍等症状。
- **自身免疫性小脑共济失调**：虽然这是一类相对较广的疾病，但它们通常会导致小脑症状，如步态不稳和协调障碍。

诊断这些疾病的准确性和治疗方案的选择需要依赖于详细的临床评估、实验室检查（包括血液中的特定抗体检测）及影像学检查等。

**5. user 2024-11-30T23:38:31Z:**

疾病临床表现复杂多样，缺乏特异性。多累及神经、皮肤、呼吸、消化和免疫等多系统，单从临床表现难以确诊。可以分为完全性及部分性两种类型。

- 完全性： 又可分为早发型及晚发型两种。①早发型患者多在新生儿或婴儿早期起病，通常症状出现在生后早期起病，通常症状出现在生后 1 周至 10 岁期间，平均发病年龄为 3 个月，最常见的表现为神经系统症状，如肌张力低下、癫痫、发育迟缓、感音性耳聋、共济失调、进行性意识障碍等，大约一半的患者出现共济失调，发育迟缓，结膜炎和视觉问题，包括视神经萎缩。常合并代谢性酸中毒、低血糖、酮症等代谢紊乱，皮肤表现包括各种形态的难治性皮疹如湿疹、全身性红斑、尿布疹，患者也可出现过度通气、喉部喘鸣和呼吸暂停等呼吸系统症状，此外还有消化系统的症状如喂养困难、呕吐、腹泻等。感染、发热、疲劳、高蛋白饮食等可诱发急性发作。若治疗不及时，常可导致不可逆损害。②晚发型患者常于青春期发病，之前可无任何症状，临床表型较早发型不同，通常表现为周围神经病，肌无力、痉挛性轻瘫，皮疹和眼部问题，如视力丧失和视力下降等，如不能及时治疗，常导致不可逆性损害。
- 部分性 ： 可在各个年龄段发病，甚至不发病。患儿整体症状及体征相对较轻，仅在感染、饥饿等应急状态下起病。患者可能有肌张力减退、皮疹、脱发，可出现急性神经系统症状癫痫、意识障碍等。

**qwen2.5_72b** 2024-11-30T23:39:14Z:

根据您提供的复杂且多系统受累的临床表现，考虑以下几种罕见病可能性：

- **线粒体脑肌病**：患者的临床特征与完全性和部分性线粒体脑肌病非常吻合，尤其是早发型患者在新生儿或婴儿早期出现的神经系统症状、代谢紊乱以及皮肤和呼吸系统的异常。晚发型则表现为周围神经病变和其他系统功能障碍。
- **遗传性共济失调症（如弗里德赖希共济失调）**：这种疾病主要影响小脑及其连接，导致步态不稳、手部协调能力下降等共济失调症状，并伴有言语困难、心脏问题和脊柱侧弯等症状。但与描述中的代谢障碍不符。
- **尼曼-皮克病（Niemann-Pick disease）**：这是一种脂质沉积病，可引起肝脾肿大、肺功能受损、神经系统退行性变等广泛的身体损害，符合多系统受累特点，但通常不以肌张力低下和发育迟缓为主要表现。
- **威尔森氏症（Wilson’s Disease）**：此病症由于铜代谢异常导致肝脏损伤及神经系统症状如震颤、吞咽困难、言语障碍等。然而它较少出现早发性神经精神症状和皮肤问题，且发病年龄一般较晚。
- **代谢性脑病**：某些先天性代谢缺陷可能导致类似的表现，例如有机酸血症、脂肪酸氧化障碍或糖原累积病等，这些疾病可以导致严重的多系统损害，并表现出特定的代谢异常。具体类型需要通过详细检查确定。
- **自身免疫性疾病（如系统性红斑狼疮）**：这类疾病同样可以影响多个器官和组织，产生广泛的临床症状，但通常伴有较为明显的自身抗体阳性结果。

综上所述，根据提供的信息，最有可能诊断为线粒体脑肌病。当然，最终确诊还需结合详细的家族史、实验室检查（如血液学检测、遗传学分析）及影像学评估等多方面资料进行综合判断。

**6. user 2024-11-30T23:39:32Z:**

高发年龄段为婴儿期，表现为以失盐为主的肾上腺皮质功能不全，常见症状包括高血钾、低血钠与脱水、代谢性酸中毒、低血糖、呕吐、休克、皮肤色素沉着等。多数患者在青春期表现为不发育或发育不完全，或生育能力受损，少精和无精症等。少数患者可以表现为儿童早期高雄激素血症、单纯性盐皮质激素缺乏，青春期可出现假性性早熟或自发性青春发育等。轻者可成年期起病。

患者皮肤有色素沉着，尤其是在暴露处、摩擦处、瘢痕及乳晕、腋窝、阴囊处。

患者对感染、外伤等各种应激的抵抗力有不同程度的减弱。在发生应激情况时，可出现肾上腺危象，表现为恶心、呕吐、腹痛或腹泻、严重脱水、血压降低、心率快、脉细弱、精神失常，常有高热、低血糖症、低钠血症，甚至危及生命。

**qwen2.5_72b** 2024-11-30T23:40:14Z:

根据您提供的临床表现描述，最可能的诊断是先天性肾上腺皮质增生（CAH），这是一种由于肾上腺皮质激素合成过程中所需酶的遗传缺陷导致的罕见病。

**其他五种可能的诊断包括：**

- **艾迪生病（Addison’s disease）**：一种慢性肾上腺功能减退症，可由自身免疫性破坏、感染或肿瘤等原因引起。其症状与先天性肾上腺皮质增生类似，但通常在成年后发病。
- **假性盐皮质激素缺乏症（Pseudohypoaldosteronism）**：这是一种非常罕见的遗传性疾病，患者的肾脏无法正常响应盐皮质激素的作用，导致钠丢失增加和钾水平升高。症状可能与先天性肾上腺皮质增生相似。
- **低钠血症伴有高血钾性周期性麻痹（Hypokalemic Periodic Paralysis with Hyponatremia）**：这种罕见病的特点是反复发作的肌肉无力或瘫痪，伴随有电解质紊乱如低钠和高钾。但是，它通常不会导致严重的肾上腺功能障碍症状。
- **家族性醛固酮缺乏症（Familial Hypoaldosteronism）**：这是一种遗传性的内分泌疾病，表现为盐皮质激素分泌不足，引起类似先天性肾上腺皮质增生的临床表现如低血钠、高血钾等。然而，该病通常不伴随皮肤色素沉着。
- **肾上腺脑白质营养不良（Adrenoleukodystrophy, ALD）**：这是一种X连锁隐性遗传病，主要影响男性患者。除了肾上腺功能障碍外，还伴有中枢神经系统症状如视力减退、听力下降和认知障碍等。然而，并非所有患者都会出现典型的皮肤色素沉着现象。

在实际临床工作中，医生会结合详细的家族史、个人生长发育情况以及实验室检查结果来综合判断并确诊上述疾病。

**7. user 2024-11-30T23:40:27Z:**

从出生到成年早期均可发病。其中新生儿起病最为常见，常表现为巨大儿、摄食不良、不耐受饥饿和持续性低血糖等。低血糖发作时可表现为嗜睡、肌张力低下等，也可表现为神经系统后遗症甚至死亡，如呼吸暂停、癫痫发作和昏迷等。在迟发性类型中，患者常表现为低血糖反应，包括面色苍白、出汗和心悸等症状。

**qwen2.5_72b** 2024-11-30T23:40:54Z:

根据您提供的临床表现，最有可能的诊断是先天性高胰岛素血症（Congenital Hyperinsulinism, CHI）。这是一种由于胰腺β细胞功能异常导致的持续性或反复性低血糖疾病。

除此之外，还有以下五种可能的诊断：

- **糖原贮积病**：这是一组遗传性疾病，因体内特定酶缺陷而导致糖原代谢障碍。其中某些类型可表现为新生儿期巨大儿、肌张力低下和低血糖等症状。
- **先天性肾上腺皮质增生症（CAH）**：尤其是11β-羟化酶缺乏型，在婴儿早期可能会因为电解质失衡而出现类似症状，但通常还伴有其他内分泌异常的表现如女性假两性畸形等。
- **线粒体疾病**：某些线粒体病会影响能量代谢，导致新生儿或婴幼儿期出现低血糖、喂养困难和发育迟缓等问题。
- **脂肪酸氧化障碍**：这类遗传性代谢缺陷影响到长链、中链或者短链脂肪酸的正常β-氧化过程，在饥饿状态下容易引发严重的低血糖症状。
- **生长激素缺乏症或其他垂体功能减退症候群**：这些条件可能导致儿童生长期出现持续性的低血糖现象，尤其是夜间或空腹时更为明显。

**8. user 2024-11-30T23:41:06Z:**

- Ⅰ型 ，腹部膨隆、生长迟缓、低血糖抽搐、反复鼻出血、腹泻和呕吐为儿童患者主要就诊原因，极少数以肉眼血尿、便血、反复骨折、贫血或痛风等为首发表现。从未确诊及治疗的成年患者可以多发肝腺瘤、慢性肾衰、严重痛风伴多发痛风石、骨质疏松等就诊。其他少见表现包括肺动脉高压、糖尿病、脑血管病和肝腺瘤癌变等。查体可见身材矮小和肝脏明显增大。
- Ⅰb 型 ， 患者除以上表现外，还可有反复感染伴中性粒细胞减少、口腔溃疡、炎症性肠病、肛周溃疡、关节炎和脾大等。
- Ⅱ型婴儿型， 根据预后分为经典婴儿型和非经典婴儿型。经典婴儿型大部分在生后第 1 个月即出现全身性肌肉无力，运动发育迟缓，胸部 X 线片示心脏增大，心电图见高 QRS 波和短 PR 间期，心脏彩超见肥厚型心肌病改变，血肌酸激酶不同程度升高等， 多于生后 1 年之内死于左心衰竭或肺部感染后心肺功能衰竭。非经典婴儿型在生后 1 年内出现肌肉无力，运动发育落后，多于幼儿期死于呼吸衰竭。
- Ⅱ型晚发型 ， 患者于 1 岁后起病，可晚至 60 岁发病。多表现为慢性进行性近端肌力下降和呼吸功能不全，心脏受累少见，呼吸功能衰竭是主要的致死原因。临床表现为易疲劳、仰卧起坐、上下楼梯、蹲起困难和行走无力，少数以突发呼吸衰竭起病。

**qwen2.5_72b** 2024-11-30T23:41:47Z:

根据您提供的信息，最可能诊断的是糖原贮积症（Glycogen Storage Disease, GSD）。这是由于体内分解或合成糖原的酶缺陷导致糖原代谢障碍而引起的一组遗传性代谢性疾病。不同类型的糖原贮积症有不同的临床表现和预后。

具体来说： - **Ⅰ型**：这可能是糖原贮积症I型（Von Gierke病），其特征是由于葡萄糖6磷酸酶缺乏导致的低血糖、肝脏肿大和高脂血症等。 - **Ⅰb 型**：除了上述症状外，还伴有免疫功能低下，如反复感染、中性粒细胞减少等问题，这提示可能是GSD I型b亚型。 - **Ⅱ型婴儿型（经典与非经典）**：这是糖原贮积症II型（Pompe病），主要由于酸性α-葡萄糖苷酶缺陷引起的心肌和骨骼肌损伤。经典婴儿型表现为心脏肥大、呼吸衰竭；而非经典型则发展较慢，但同样影响肌肉功能。 - **Ⅱ型晚发型**：与经典的婴儿期起病形式不同，该类型患者可能在成年后才出现症状，主要表现为缓慢进展的肌肉无力和呼吸困难。

其他五种可能的诊断包括： 1. 糖尿病酮症酸中毒：虽然不是罕见病，但低血糖、腹部不适等症状也可见于糖尿病患者。 2. 肝豆状核变性（Wilson病）：该疾病可能导致肝脏损害及神经系统症状。 3. 先天性代谢障碍综合症：例如有机酸血症等，可以引起类似的多系统受累表现。 4. 线粒体肌病/脑肌病：这类遗传性疾病常表现为肌肉无力、生长发育迟缓等症状。 5. 某些类型的溶酶体贮积病（如法布雷病）：这些疾病也可能出现心脏、肾脏等器官功能障碍。

**9. user 2024-11-30T23:42:09Z:**

- 神经 - 肌肉系统， 引起神经系统的异常，如惊厥、昏迷、共济失调、甚至精神症状；也可出现肌肉症状，包括腱反射亢进、震颤、手足搐搦、肌无力等。
- 心血管系统， 引起心电图的改变，可见 PR 间期延长、QRS 波增宽等，严重者可引起室性心律失常。
- 肾脏 ，可引起肾结石、肾钙质沉着、肾功能不全。
- 骨关节系统， 可引起骨质疏松、骨软化。
- 消化系统， 可引起肠梗阻表现。
- 内分泌， 患者可见糖尿病或者糖耐量减低。
- 眼部症状， 部分类型可合并眼部病变，包括眼球震颤、视野缺损、近视等。

该组疾病通常为慢性病程，其中新生儿期、婴儿期起病的类型往往病情重、预后差。部分患者在青春期就发展为慢性肾衰竭，部分患者若未能及时治疗，会导致患儿严重精神发育迟滞甚至死亡。

**qwen2.5_72b** 2024-11-30T23:42:41Z:

根据您提供的临床表现，最有可能的诊断是**低钙血症（Hypocalcemia）**。这是一种由于血液中钙水平过低导致的一系列症状，可以影响多个系统。

此外，还有其他五种可能的罕见病诊断： 1. **高草酸尿症（Hyperoxaluria）**：这种代谢性疾病会导致肾脏和其它器官中的草酸盐沉积，引起肾结石、肾功能衰竭等问题。 2. **线粒体脑肌病伴乳酸血症及中风样发作（MELAS Syndrome）**：这是一种遗传性线粒体疾病，影响神经系统和肌肉系统，可导致癫痫发作、认知障碍、肌肉无力等症状。 3. **维生素D依赖型佝偻病（Vitamin D-Dependent Rickets）**：因维生素D代谢异常引起的一类罕见骨骼疾病，表现为生长迟缓、骨软化等。 4. **原发性甲状旁腺功能减退症（Primary Hypoparathyroidism）**：由于甲状旁腺素分泌不足造成的钙磷代谢失常，常见症状有低血钙引起的神经肌肉兴奋性增高。 5. **Fanconi综合征**：一种以近端肾小管重吸收障碍为特征的综合症，可导致多尿、脱水、电解质紊乱（包括低钾血症和低钙血症）、酸中毒等表现。

以上这些疾病均可能表现出您提到的症状组合。不过，最终的确诊需要结合详细的病史采集、体格检查及实验室检测结果来确定。

**10. user 2024-11-30T23:42:55Z:**

可分为 5 种类型，其中以经典型和中间型最常见。

**1. 经典型** 最常见，占 75％。患儿出生时正常，发病早、症状重并发展迅速。通常患儿出生 12 小时后，在耵聍中即出现枫糖浆气味；生后 12~24 小时在尿液和汗液中有特殊的枫糖味；2~3 天出现酮尿、易激惹和喂养困难；4~5 天开始出现严重的代谢紊乱和脑病症状，表现为哺乳困难、阵发性呕吐、拒食、嗜睡、呼吸暂停、惊厥发作、肌张力增高以及“击剑”样和“蹬车”样刻板动作等；生后 7~10 天可出现昏迷和中枢性呼吸衰竭，若不及时治疗， 大多数的患儿在出生后数天死于严重的代谢紊乱。

**2. 中间型** 在任何年龄阶段均可发病。患者症状较经典型轻，表现为生长缓慢、发育落后、喂养困难、易激惹、在耵聍中有枫糖浆气味，应激情况下可以出现严重的代谢紊乱和脑病。

**3. 间歇型** 呈间歇发作，早期生长发育均正常，多在感染和手术等应激情况下发作，表现为发作性共济失调和酮症酸中毒，极少数的严重者可引起死亡。间歇期支链氨基酸浓度正常，发作期与经典型类似有支链氨基酸浓度升高。

**qwen2.5_72b** 2024-11-30T23:43:31Z:

根据您提供的信息，最有可能诊断为枫糖尿病（Maple Syrup Urine Disease, MSUD）。这是一种罕见的遗传性代谢病，由于支链酮酸脱氢酶复合体缺乏所导致。该病主要影响身体处理特定氨基酸的能力，导致这些物质在体内积累，并产生类似于枫糖浆的特殊气味。

**其他五种可能的诊断：**

- **高苯丙氨酸血症（Phenylketonuria, PKU）**：这是一种由于苯丙氨酸羟化酶缺乏引起的代谢障碍疾病。患者不能正常代谢苯丙氨酸，导致其在血液中积累，进而影响大脑发育。
- **同型半胱氨酸尿症（Homocystinuria）**：这种遗传性代谢病是由体内同型半胱氨酸代谢途径中的多种酶缺陷造成的。症状包括生长迟缓、智力障碍等。
- **有机酸血症（Organic Acidemias）**：这是一组由于特定氨基酸或脂肪酸分解过程中所需的酶缺失而导致的代谢疾病，可能导致严重的神经系统问题和生命危险。
- **尿黑酸尿症（Alkaptonuria）**：这是一种罕见遗传性代谢障碍，因体内无法正常分解酪氨酸而产生过多的尿黑酸。主要症状为深色尿液、关节炎症及皮肤色素沉着等。
- **丙酮酸脱氢酶缺乏症（Pyruvate Dehydrogenase Deficiency, PDD）**：这是一种由于丙酮酸不能被有效转化为乙酰辅酶A而引发的代谢障碍，可能引起多种神经系统症状如发育迟缓、肌张力异常和癫痫发作等。

不过，请注意最终的确诊需要通过详细的临床检查和实验室检测来确定。

**11. user 2024-11-30T23:43:45Z:**

出生后至老年均可起病，可急性或隐袭起病。几乎任何系统或器官均可受累，以能量需求旺盛的器官，如脑、心、肾、眼、耳、肌肉、胃肠道等多见。患者可以表现为某种临床综合征，也可仅表现单个症状或者多种症状组合。

**qwen2.5_72b** 2024-11-30T23:44:20Z:

根据您提供的疾病临床表现，这类疾病的特征是几乎任何系统或器官均可受累，并且在能量需求旺盛的器官中更为常见，这使得线粒体病成为最可能的诊断。

- **线粒体病**：是一组由线粒体功能障碍引起的遗传性代谢病。线粒体作为细胞的能量工厂，如果其功能受损，则会导致全身多系统受累，尤其是对能量需求高的器官和组织如脑、心脏、肌肉等影响更为明显。
- **自身免疫性疾病（例如系统性红斑狼疮）**：这类疾病也可导致多个器官系统的损害。它们通常表现为多发性的症状，包括但不限于关节痛、皮疹、疲劳、肾炎等。
- **多发性硬化症**：这是一种中枢神经系统慢性炎症脱髓鞘疾病，可引起视力障碍、肌肉无力、感觉异常等症状，影响大脑和脊髓功能。
- **帕金森病**：虽然主要表现为运动障碍（如震颤、僵硬、动作迟缓），但也有非运动症状，比如认知功能下降、睡眠障碍等。随着病情进展，可能累及多个系统。
- **慢性疲劳综合症/肌痛性脑脊髓炎**：这种疾病以持续性的严重疲劳为主要特征，并伴有多种身体和精神健康问题，如肌肉疼痛、记忆力减退、注意力难以集中等。
- **线粒体脑肌病伴高乳酸血症及卒中样发作（MELAS综合征）**：作为特定类型的线粒体病之一，该病除了具备上述线粒体疾病特点外，还有典型的临床表现如反复出现的偏头痛、癫痫发作以及卒中样事件。

**12. user 2024-11-30T23:44:46Z:**

好发年龄 5~50 岁，中位发病年龄 39 岁。女性患病率明显高于男性，女：男比例为(9~11) ：1。多急性 / 亚急性起病，临床表现包括 6 组核心症候群：①视神经炎；②急性脊髓炎；③极后区综合征；④急性脑干综合征；⑤症状性睡眠发作或急性间脑临床综合征伴典型的间脑 MRI 病灶；⑥症状性大脑综合征伴典型的脑部病变。 为高复发、高致残性疾病，90％以上的患者为复发性病程，多数患者遗留较为严重的神经功能残疾。

**qwen2.5_72b** 2024-11-30T23:45:25Z:

根据您提供的信息，最可能的诊断是视神经脊髓炎谱系疾病（Neuromyelitis Optica Spectrum Disorders, NMOSD）。这是一种主要影响视神经和脊髓的自身免疫性疾病，符合您描述的特点，包括好发年龄、性别比例以及临床核心症状群。

其他五种可能的诊断还包括：

- **多发性硬化症 (Multiple Sclerosis, MS)**：尽管MS与NMOSD有一些相似的症状，如视神经炎和急性脊髓炎，但它们是不同的疾病。MS是一种中枢神经系统（CNS）的慢性炎症性疾病，可影响大脑、脊髓和视神经。
- **系统性红斑狼疮 (Systemic Lupus Erythematosus, SLE)**：一种自身免疫性疾病，可以引起多个器官系统的广泛损害，包括神经系统受累。SLE患者的神经系统表现有时会与NMOSD相似。
- **急性播散性脑脊髓炎 (Acute Disseminated Encephalomyelitis, ADEM)**：这是一种罕见的、通常发生在感染或疫苗接种后的自身免疫反应引起的中枢神经系统炎症性疾病，特点为迅速出现的症状和多灶性的神经损伤。
- **副肿瘤综合征 (Paraneoplastic Syndromes)**：某些癌症可以引发远程效应，导致神经系统异常。这类症状可能与NMOSD的部分表现相似，尤其是在无明显肿瘤迹象的情况下诊断较为困难。
- **自身免疫性脑炎 (Autoimmune Encephalitis)**：由身体对大脑的异常免疫反应引起的一组疾病，可表现为认知障碍、精神症状、癫痫发作等。在某些情况下，这些症状可能与NMOSD中观察到的症状重叠。

每种诊断都有其独特的特点和诊断标准，在临床实践中需要通过详细的病史询问、体格检查以及辅助检查（如MRI、血液检测、脊液分析等）来最终确定。

**13. user 2024-11-30T23:45:42Z:**

听力损伤。以往多认为该耳聋为听力筛查即可明确的先天性， 初生即外显，多累及双侧，对称的重度语前聋。近年来逐渐发现个别病例可表现为不对称或单侧听力受损，听力损失程度变异较大，不同病人的表现可从轻度至极重度不等；渐进性、出生后才发生的耳聋也并不少见。

**qwen2.5_72b** 2024-11-30T23:46:21Z:

根据您描述的症状，首先考虑的罕见疾病是：

**1. Usher 综合征**

这是一种与遗传相关的综合症，主要影响听力和视力，患者常表现为先天性的双侧重度或极重度听力损失，并伴有视网膜色素变性导致的夜盲症和视野缩小。但也有部分病例表现出不对称或者单侧听力受损。

其他可能的诊断包括：

**2. 大前庭水管综合征（LARGE Vestibular Aqueduct Syndrome）**

该病特征为内耳结构异常，特别是大前庭水管扩大，通常导致儿童期出现渐进性、波动性的双侧感音神经性聋。但也有部分患者表现为单侧或不对称听力下降。

**3. Pendred 综合征**

这是一种常染色体隐性遗传病，除了双侧重度感音神经性聋外，还可能伴有甲状腺肿大（即单纯性甲状腺肿）。听力损害多在婴儿期至学龄前发生，且呈进行性发展。

**4. Alport 综合征**

该病为X连锁或常染色体遗传的疾病，主要影响肾脏、耳朵和眼睛。听力损失通常始于青少年晚期或成年早期，表现为双侧对称性高频下降型感音神经性聋，但也有报告指出少数患者可能出现单侧或不对称听力受损。

**5. 线粒体基因突变相关耳聋**

线粒体DNA中的某些特定位点突变（如A1555G、C1494T）可导致遗传性耳聋。这类耳聋通常表现为双侧对称性，但也有报道指出存在个体间差异较大的情况，包括单侧或不对称听力损失。

这些疾病都是相对罕见的，并且临床表现可能存在重叠，因此最终的确诊需要依赖详细的家族史、体格检查及相应的辅助检查（如基因检测等）。

**14. user 2024-11-30T23:47:31Z:**

Bradykinesia, tremor, and myotonia. Levodopa-induced motor complications are more likely to occur after treatment. In terms of non-motor symptoms, mood disorders (e.g., depression, anxiety, irritability, etc.) and behavioural disorders (e.g., compulsive dosage increases, impulse control disorders, stereotyped behaviour, etc.) are likely to occur.

**qwen2.5_72b** 2024-11-30T23:48:11Z:

根据您提供的临床表现，最有可能诊断的罕见病是**多系统萎缩症（Multiple System Atrophy, MSA）**。然而，这些症状也可能是其他几种疾病的特征。以下是另外五种可能的诊断：

- **帕金森病（Parkinson’s Disease, PD）**：虽然PD是一种较为常见的神经退行性疾病，但其核心症状如运动迟缓、震颤和肌阵挛与您描述的症状相似。此外，左旋多巴诱导的运动并发症也是PD患者中常见的一种现象。
- **进行性核上性麻痹（Progressive Supranuclear Palsy, PSP）**：这是一种罕见的神经退行性疾病，主要表现为眼球运动障碍、步态不稳以及认知功能减退等症状。但PSP患者也可能出现类似帕金森病的症状，如震颤和肌张力增高。
- **皮质基底节变性（Corticobasal Degeneration, CBD）**：这是一种以异侧肢体僵硬无力、失用症等为特点的罕见神经系统退行性疾病。部分患者可能会表现出类似帕金森病的症状，如震颤和运动迟缓。
- **亨廷顿舞蹈病（Huntington’s Disease, HD）**：尽管该疾病的主要特征是舞蹈样动作异常，但其早期阶段也可能出现运动缓慢、肌肉僵硬等症状，并且伴有抑郁等精神症状。
- **Lewy体痴呆（Dementia with Lewy Bodies, DLB）**：DLB是一种以波动性认知障碍、视幻觉和帕金森病样运动障碍为特点的神经退行性疾病。部分患者会经历左旋多巴治疗后出现的副作用，如异动症等。

请注意，正确诊断需要综合考虑患者的详细病史、体格检查结果及辅助检查（如影像学检查、实验室检测）等多种信息。建议您与专业的医疗团队合作以获得准确的诊断和有效的治疗方案。

**15. user 2024-11-30T23:48:18Z:**

**1. 多发神经病** ，往往表现为对称性的四肢感觉和(或) 运动性周围神经病，逐步由远端向近端进展。

**2. 器官肿大，** 包括肝大、脾大或淋巴结肿大；淋巴结活检常提示为 Castleman 病。

**3. 内分泌异常 ，**常见的包括性功能减退(如男性阳痿、乳房发育)、甲状腺功能减退、糖代谢异常(如糖尿病)、肾上腺功能不全。

**4. 皮肤改变，** 可表现为皮肤颜色加深、皮肤肾小球样血管瘤、白甲、多血质、多毛症和手足发绀等。

**5. 循环外水负荷增加，** 包括肢体水肿、腹水、胸腔积液、心包积液、视乳头水肿等。

**6. 硬化性骨病 ，**是重要临床表现。可表现为骨痛，亦可无临床症状。骨骼 CT 检查可以显著提高硬化性骨病的检出率，影像学上可以表现为单纯骨骼硬化、或硬化和溶骨混合病灶、或者单纯溶骨性改变。

**7. 红细胞增多和(或) 血小板增多**

**8. 肺动脉高压** ，患者可出现肺动脉高压，表现为活动耐量减低、低氧血症等。

**9. 脑梗死**

**qwen2.5_72b** 2024-11-30T23:48:54Z:

根据您提供的临床表现，这些症状非常符合一种罕见病——POEMS综合征（多发性周围神经病变、器官肿大、内分泌病变、M蛋白和皮肤改变综合症）。这种疾病是一种与浆细胞增生有关的复杂多系统性疾病。主要特征为：

- **多发性周围神经病变**：对称性的四肢感觉或运动障碍。
- **器官肿大**：如肝脾淋巴结肿大等。
- **内分泌异常**：包括但不限于性腺功能减退、甲状腺问题和糖尿病等。
- **皮肤改变**：色素沉着、血管瘤形成、甲床苍白等症状。
- **其他症状**：骨硬化或溶骨病变、血液系统异常（如红细胞增多）、循环外水负荷增加及肺动脉高压等。

除此之外，还有以下五种可能的诊断：

- **Castleman病**：尽管它通常是POEMS综合征的一部分，但有时也可能独立存在。特别是如果患者的主要问题是淋巴结肿大和相关的炎症反应。
- **多发性骨髓瘤**：这是一种浆细胞恶性肿瘤，可导致骨骼破坏、贫血、高钙血症等症状，部分临床表现与POEMS重叠。
- **慢性炎症性脱髓鞘性多发神经根神经病(CIDP)**：此病会导致四肢无力和感觉异常，但通常没有其他系统受累的表现。
- **淀粉样变性**：这是一种蛋白质沉积疾病，可以影响心脏、肾脏等多个器官，并可能导致类似POEMS的多系统损害。
- **副蛋白血症相关性疾病**：包括由单克隆免疫球蛋白引起的一系列病症，如轻链沉积病等，可能表现出与POEMS相似的临床特征。

**16. user 2024-11-30T23:49:12Z:**

胎儿期的常见表现包括胎儿活动减少、羊水过多以及臀位。在新生儿期肌张力过低是这一疾病的标志性特征之一，严重的肌张力降低可导致窒息、喂养困难、吮吸无力、哭声无力。婴幼儿期主要表现为生长迟滞，认知、运动及语言发育落后。随着年龄的增长，食欲逐渐旺盛，如果不限制进食会出现肥胖。学龄期通常存在身材矮小，逐渐出现脾气暴躁、固执、强迫症等性格特点。异常行为包括强烈的觅食、搔抓皮肤、孤独症样行为等。约 25％的患者会出现癫痫。青春期男性和女性患者均可出现第二性征出现延迟或出现不完全。由于肥胖可导致相关并发症，如呼吸睡眠暂停、肺源性心脏病、胰岛素抵抗甚至糖尿病等。脊柱侧突、骨质疏松、胃食管反流也较为常见。成人期主要表现为肥胖及其并发症，包括心血管问题、糖尿病和睡眠呼吸暂停。其他表现为精神异常，不孕不育。

致命性危险：由口部运动不协调、肌张力过低、多食、狼吞虎咽的进食习惯和咀嚼不充分引起窒息以及由于暴饮暴食导致胃破裂。

体格检查可以发现特征性面容逐渐典型，包括杏仁眼、小嘴、上唇薄、嘴角向下、小手小脚、身材矮小、皮肤白皙、肌张力低、生殖器发育不良(如隐睾、阴囊发育不全或阴蒂发育不全)。

**qwen2.5_72b** 2024-11-30T23:49:52Z:

根据您提供的临床表现，最有可能诊断的是普拉德-威利综合症（Prader-Willi Syndrome, PWS）。这是一种罕见的遗传性疾病，由15号染色体上的特定区域缺失或功能异常引起。

另外五种可能的诊断包括：

- **天使人综合症 (Angelman Syndrome)**：与PWS一样，也是一种与15号染色体相关的神经发育障碍。但其症状如语言发展迟缓、共济失调和频繁笑等，与普拉德-威利综合症有显著区别。
- **唐氏综合症（Down Syndrome）**：一种因21号染色体多出一条而导致的遗传性智力障碍疾病。患者通常具有独特的面部特征，如扁平的脸、眼睛上斜等，并伴有生长发育迟缓等问题。
- **努南综合症 (Noonan Syndrome)**：是一种影响多个身体系统的遗传病，常见症状包括心脏缺陷、身材矮小和特殊面容（如宽鼻梁、下垂的眼睑）等。不过，它通常不会导致严重的肌张力低下或过度饥饿感。
- **苯丙酮尿症（Phenylketonuria, PKU）**：这是一种代谢障碍疾病，如果未经治疗，则会导致智力发育迟缓和其他神经系统问题。然而，PKU并不会引起肌肉无力、肥胖或者性腺功能减退等症状。
- **鲁宾斯坦-泰比综合症 (Rubinstein-Taybi Syndrome)**：此病特点是生长发育迟滞、特殊面部特征（如短鼻梁、外翻的拇指）和智力障碍等。尽管它可以导致肌张力下降，但通常不表现为过度饥饿或性腺功能减退。

总之，在面对如此复杂的临床表现时，普拉德-威利综合症是首选考虑的诊断。然而，最终的确诊需要通过遗传学检测来确定。

**17. user 2024-11-30T23:50:05Z:**

患者常在婴幼儿或青少年期起病，男女比例大致相等。呼吸道、胰腺和胆道等上皮细胞的分泌物含水量减低，分泌物变黏稠而难以清除。肺脏是最常见的受累器官，患者呼吸道出现慢性细菌感染及病原体的定植，最初常为流感嗜血杆菌和金黄色葡萄球菌，最终出现铜绿假单胞菌或洋葱克雷伯杆菌的慢性定植和(或) 感染。一旦出现感染，中性粒细胞被大量募集到肺组织中，并释放弹性蛋白酶，从而造成肺组织的破坏，导致支气管扩张形成。患者的支气管扩张常在幼年起病，且常从上叶起病，与多数感染后形成的支气管扩张症主要位于中、下肺不同。

患者常表现为深色服装上遗留的汗渍，或是父母发现患儿皮肤发咸。胆汁和胰液的流动异常可以导致消化不良和吸收不良，也可出现肝脏或胰腺疾病，严重时可以出现相关的糖尿病。黏稠的肠液还可能使 患者更容易出现肠梗阻和直肠脱垂。此外，男性患者可以出现输精管内精子移动障碍以及先天性输精管缺失(CBAVD)。

**qwen2.5_72b** 2024-11-30T23:50:41Z:

根据您提供的临床表现，最有可能诊断为囊性纤维化（Cystic Fibrosis, CF）。这是一种常见的常染色体隐性遗传病，主要影响肺部、胰腺、肝脏和肠道等部位的外分泌腺功能，导致黏液变得异常浓稠，从而引起反复感染、炎症以及器官功能障碍。

另外五种可能的诊断： 1. **原发性纤毛运动障碍 (Primary Ciliary Dyskinesia, PCD)**：这是一种罕见的遗传性疾病，特征为呼吸道上皮细胞纤毛结构或功能异常，导致黏液清除能力下降，易发生反复感染。 2. **α1-抗胰蛋白酶缺乏症 (Alpha-1 Antitrypsin Deficiency)**：这种遗传缺陷可引起肺部及肝脏损伤，特别是儿童期即出现的呼吸系统症状需考虑此病可能性。 3. **支气管扩张症 (Bronchiectasis)**：虽然您提到患者早期发病与感染后支气管扩张不同，但鉴于其临床表现中存在长期呼吸道疾病的特点，仍值得排除该诊断。 4. **慢性肉芽肿性疾病 (Chronic Granulomatous Disease, CGD)**：这是一种免疫缺陷病，由于吞噬细胞功能障碍导致反复严重细菌和真菌感染，尤其是肺部。 5. **先天性氯离子通道异常 (Congenital Chloride Channel Disorders)**：这类疾病同样会导致体内某些部位（如肺、胰腺）分泌物变得粘稠，进而引发一系列症状。例如，汗腺氯化物转运缺陷可表现为多汗症或低血钠等症状。

以上各病种虽有重叠之处，但通过详细的家族史询问、体格检查及实验室检测可以进一步明确诊断。

**18. user 2024-11-30T23:51:20Z:**

患者临床表现为口咽炎、中耳炎、呼吸系统感染、蜂窝织炎及皮肤感染，常由葡萄球菌及链球菌感染所致。在出现骨髓储备下降的重度中性粒细胞减少症患者中，2 岁之前大部分患儿都发生过口腔溃疡及疼痛性牙龈炎。如果伴弥漫性胃肠道病变，可引起类似克罗恩病的腹痛和腹泻表现。中性粒细胞减少症可导致各个部位遭受反复细菌或真菌感染，其中主要受累部位为皮肤黏膜、耳鼻、咽喉和肺。感染通常非常严重甚至是致命性的。约 15％的患者进展为急性白血病或骨髓增生异常综合征。患儿通常不伴有特征性畸形。

**qwen2.5_72b** 2024-11-30T23:51:59Z:

根据您描述的临床表现，最有可能诊断的是**周期性中性粒细胞减少症（Cyclic Neutropenia）**。这是一种少见的血液系统疾病，其特点是患者中性粒细胞数量周期性下降，导致易感染。

除了周期性中性粒细胞减少症外，还有其他五种可能的诊断：

- **严重先天性中性粒细胞减少症（Severe Congenital Neutropenia, SCN）**：这是一种从婴儿期或儿童早期开始的罕见遗传性疾病，特点是持续性的中性粒细胞缺乏，导致患者容易发生严重的细菌和真菌感染。
- **慢性肉芽肿病（Chronic Granulomatous Disease, CGD）**：虽然这主要是免疫缺陷疾病，但也可以出现反复感染的症状，尤其是由特定类型的细菌或真菌引起。
- **白细胞黏附缺陷I型（Leukocyte Adhesion Deficiency Type I, LAD-I）**：这是一种罕见的遗传性免疫缺陷病，由于白细胞无法正常移动到感染部位，导致患者容易遭受严重的细菌和真菌感染。
- **X-连锁无丙种球蛋白血症（X-linked Agammaglobulinemia, XLA）**：尽管主要表现为低免疫球蛋白水平导致的反复呼吸道和其他系统感染，但在一些病例中也可见到中性粒细胞减少的现象。
- **家族性地中海热（Familial Mediterranean Fever, FMF）**：虽然这是一种与炎症反应异常有关的遗传性疾病，但部分患者在急性发作期间可能会出现白细胞计数变化，包括中性粒细胞减少。

以上这些疾病都需要通过详细的病史采集、体格检查以及特定实验室检测来确诊。对于疑似周期性中性粒细胞减少症等疾病的患者，建议尽早进行骨髓穿刺或活检、遗传学分析等相关检查以明确诊断，并制定合适的治疗方案。

**19. user 2024-11-30T23:52:20Z:**

典型 患者出现中枢神经系统损害和肾上腺皮质功能低下两类症状。神经系统症状和肾上腺皮质功能减退的症状可同时出现或相继出现。少数患者可单独出现。肾上腺皮质功能不全的表现轻重不一。儿童患病最为常见，占所有患者 35％。多在 4~8 岁发病。初期表现为注意力不集中、记忆力减退、学习困难、步态不稳、行为异常，后逐渐出现视觉和听觉下降、构音障碍、癫痫发作、共济失调、瘫痪、痴呆等症状。临床表现进行性加重， 最终失明或耳聋，完全瘫痪。有的表现为去大脑强直状态，最终死于中枢性呼吸衰竭、脑疝、感染。多数患者在首次出现神经系统症状时，已有肾上腺皮质功能受损的表现。

**qwen2.5_72b** 2024-11-30T23:53:01Z:

根据您提供的临床表现，最可能的诊断是**肾上腺脑白质营养不良（Adrenoleukodystrophy, ALD）**。这是一种X连锁隐性遗传病，主要影响男性儿童。其特征为中枢神经系统脱髓鞘和肾上腺皮质功能不全。

### 其他五种可能的诊断：

- **肾上腺脑白质营养不良成人型（AMN, Adrenomyeloneuropathy）**：是ALD的一种较为温和的形式，主要影响成年人。患者表现出进行性脊髓症状、周围神经病变和轻度认知障碍，肾上腺功能不全也是常见的表现。
- **异染性脑白质营养不良（Metachromatic Leukodystrophy, MLD）**：一种溶酶体贮积症，由芳基硫酸酯酶A缺乏导致。临床表现为进行性的神经系统退化，包括运动障碍、语言困难、认知衰退等。
- **Canavan病**：是一种遗传性脑白质营养不良，由于天冬氨酰胺合成酶缺陷引起，主要影响婴儿和幼儿，特征为严重的神经发育迟缓、肌张力低下及头部异常增大。
- **Pelizaeus-Merzbacher disease (PMD)**：这是一种与X染色体相关的遗传性白质脑病，由髓鞘形成障碍导致。患者通常表现为眼球震颤、运动技能发展延迟以及肌肉僵硬。
- **Alexander病**：是一种罕见的神经退行性疾病，主要影响婴幼儿。其病理特征是在大脑和脊髓中出现异常的蛋白质沉积（Rosenthal纤维），临床表现多样，但常伴有发育迟缓、癫痫发作等症状。

以上几种疾病虽然都涉及中枢神经系统损害及可能伴有的内分泌功能障碍，但具体的遗传机制、生化缺陷以及治疗方案各不相同。因此，在诊断时需要结合详细的家族史、实验室检查结果（如基因检测）和其他辅助诊断手段来确定最终的诊断。

**20. user 2024-11-30T23:53:06Z:**

患儿多于生后 4~12 个月开始出现感染症状。最突出的临床表现是反复严重的细菌感染，感染部位主要包括呼吸系统、消化系统及中枢神经系统，其中以呼吸道感染最为常见，常见病原菌包括流感嗜血杆菌、肺炎链球菌、金黄色葡萄球菌和假单胞菌属细菌等。长期、反复的呼吸道感染可导致支气管扩张、杵状指等不可逆转的器官功能损害。消化道感染常见的症状有腹泻、腹痛、胃食管反流和胃肠炎。引起感染性腹泻的病原包括蓝氏贾第鞭毛虫、沙门菌、空肠弯曲菌、隐孢子虫等。

另外，患儿容易出现中耳炎、慢性鼻窦炎、营养不良、贫血、粒细胞减少、血小板减少、生长激素缺乏症及甲状腺激素紊乱等并发症状。患儿还可发生肿瘤、自身免疫和炎症性疾病，包括类风湿性关节炎、皮肌炎等。据报道，患儿的感音神经性听力损失和湿疹的发生率也较高。

**qwen2.5_72b** 2024-11-30T23:53:49Z:

根据您提供的临床表现，最有可能诊断的是**严重联合免疫缺陷病（Severe Combined Immunodeficiency, SCID）**，这是一种以T细胞功能缺失为主要特征的原发性免疫缺陷病，患者通常在出生后4-12个月内出现症状，表现为反复、严重的细菌、病毒和真菌感染。

另外五种可能的诊断包括：

- **慢性肉芽肿病（Chronic Granulomatous Disease, CGD）**：这是一种由吞噬细胞功能障碍引起的原发性免疫缺陷病。患者易患革兰氏阴性和某些分枝杆菌导致的反复严重感染，如假单胞菌属和诺卡氏菌等。
- **X-连锁无丙种球蛋白血症（X-linked Agammaglobulinemia, XLA）**：也称Bruton型无丙种球蛋白血症。该病主要表现为低免疫球蛋白G水平，易发细菌感染，特别是化脓性链球菌、金黄色葡萄球菌和流感嗜血杆菌等。
- **慢性活动性EB病毒感染（Chronic Active Epstein-Barr Virus Infection, CAEBV）**：表现为持续或反复的EB病毒感染相关症状，如发热、肝脾肿大、淋巴结病以及多系统受累，包括免疫介导性疾病。
- **周期性中性粒细胞减少症（Cyclic Neutropenia）**：一种罕见的骨髓衰竭疾病，以中性粒细胞数量周期性下降为特点。典型表现为每3周左右出现一次持续3-6天的中性粒细胞缺乏期，期间易患感染。
- **Wiskott-Aldrich综合征（Wiskott-Aldrich Syndrome, WAS）**：这是一种影响血液和免疫系统的遗传性疾病，其特征是血小板减少、湿疹以及反复细菌和病毒性感染。患者常伴有自身免疫性疾病。

以上六种疾病均属于罕见病范畴，临床表现具有一定的相似性和重叠性，在具体诊断时需要结合详细的家族史、体格检查及实验室检测结果综合判断。

### 21. User

患者的临床表现包括不同程度的失盐和高雄激素血症两大类。新生儿起病的患儿表现为不同程度肾上腺皮质功能不足的表现，如软弱无力、恶心、呕吐、喂养困难、腹泻、慢性脱水、皮肤色素沉着和生长迟缓等。肾上腺危象常是该疾病在新生儿期的首发表现，表现为严重低血钠、高血钾、低血容量性休克，可伴有低血糖，由应激诱发。严重的低钠血症可导致抽搐等中枢神经系统表现，严重的高钾血症则可引起致命的心律失常。

高雄激素血症在不同年龄表现不一。经典型疾病的女性患儿出生时外生殖器有不同程度男性化。轻者表现为孤立性阴蒂肥大，重者外生殖器可接近男性，大阴唇呈阴囊样，阴蒂呈尿道下裂型阴茎样，并具共通的尿道阴道口。但大阴唇内不能触及性腺，有完全正常的女性内生殖器结构（卵巢和子宫）。男性新生儿期和婴儿期时可能无阴茎增大等外生殖器异常，是延误诊断的常见原因。至幼儿期，两性均会呈现外周性性早熟。男孩患儿呈现阴茎增大，伴或不伴阴毛早现；女性患儿呈现异性性早熟。长期高水平性激素刺激下丘脑促性腺激素释放激素（GnRH）神经元，发展为中枢性性早熟。女性还可有第二性征发育不良和原发性闭经或月经稀发。两性均在幼年期开始发生线性生长伴骨龄增长加速，使终身高受损。

其他表现包括皮肤和黏膜色素增深，以乳晕和外阴为显，部分患儿可无此改变。

非经典型的该类疾病儿童期和青春期甚至成年期临床呈不同程度的高雄激素血症表现，也可仅表现为生长加速和骨龄快速进展。

### Qwen2.5_72b

根据您提供的临床表现，最可能的诊断是先天性肾上腺增生症（Congenital Adrenal Hyperplasia, CAH），特别是21-羟化酶缺乏症，这是CAH中最常见的类型。这种疾病是由于肾上腺皮质激素合成过程中某些酶的缺陷导致的，主要影响皮质醇和/或醛固酮的合成，导致盐皮质激素缺乏和雄激素过多。

### 其他可能的诊断包括：

- **肾上腺皮质功能不全（Addison病）**：
  - 临床表现包括乏力、恶心、呕吐、腹泻、慢性脱水、皮肤色素沉着和生长迟缓等，与CAH相似，但通常没有高雄激素血症的表现。
- **先天性肾上腺发育不全（Adrenal Hypoplasia Congenita）**：
  - 由于肾上腺发育不全导致皮质醇和醛固酮缺乏，表现为肾上腺皮质功能不足的症状，但通常没有高雄激素血症的表现。
- **11β-羟化酶缺乏症**：
  - 这是另一种类型的CAH，表现为盐皮质激素缺乏和雄激素过多，但临床表现可能与21-羟化酶缺乏症有所不同，如高血压和低血钾。
- **3β-羟类固醇脱氢酶缺乏症**：
  - 这种类型的CAH表现为皮质醇和性激素合成障碍，导致雄激素过多和盐皮质激素缺乏，但临床表现可能更为复杂，包括性发育异常和生殖器畸形。
- **先天性肾上腺皮质增生症（非21-羟化酶缺乏症）**：
  - 其他类型的CAH，如17α-羟化酶缺乏症，也可能导致类似的临床表现，但具体症状和生化异常有所不同，如高血压和低血钾。

### 总结：

- **最可能的诊断**：先天性肾上腺增生症（21-羟化酶缺乏症）
- **其他可能的诊断**：
  1. 肾上腺皮质功能不全（Addison病）
  2. 先天性肾上腺发育不全
  3. 11β-羟化酶缺乏症
  4. 3β-羟类固醇脱氢酶缺乏症
  5. 先天性肾上腺皮质增生症（非21-羟化酶缺乏症）

这些诊断需要通过详细的病史、体格检查和实验室检查（如血电解质、皮质醇、17-羟孕酮等）来进一步确认。

### 22. User

患者出生即有临床表现，因全身皮肤色素缺乏，皮肤毛细血管显露，皮肤薄而柔软，呈白色及红色，并伴有不同程度的血管扩张。患者对紫外线高度敏感，常出现日光性皮炎、光化性唇炎、基底细胞癌及鳞状细胞癌等疾病。毛发呈纯白、银白、黄色、金色或茶色。眼睫毛及眉毛呈白色或淡黄色。视网膜、虹膜颜色减退，瞳孔颜色减退。白天畏光明显，夜视正常，同时还可以伴有其他视觉改变，如视野敏锐度下降、视网膜中央凹发育不良、视交叉处神经路径改变、眼球震颤等。

### Qwen2.5_72b

根据您提供的临床表现，最可能的诊断是**白化病（Oculocutaneous Albinism, OCA）**。这是一种遗传性罕见病，主要特征是皮肤、毛发和眼睛的色素缺乏，导致对紫外线高度敏感，以及视力问题。

### 1. 白化病（Oculocutaneous Albinism, OCA）

- **遗传方式**：常染色体隐性遗传
- **主要特征**：
  - 皮肤色素缺乏，皮肤薄而柔软，呈白色及红色
  - 毛发呈纯白、银白、黄色、金色或茶色
  - 眼睫毛及眉毛呈白色或淡黄色
  - 视网膜、虹膜颜色减退，瞳孔颜色减退
  - 白天畏光明显，夜视正常
  - 视力问题，如视野敏锐度下降、视网膜中央凹发育不良、视交叉处神经路径改变、眼球震颤等
  - 对紫外线高度敏感，常出现日光性皮炎、光化性唇炎、基底细胞癌及鳞状细胞癌等

### 其他可能的诊断

- **着色性干皮病（Xeroderma Pigmentosum, XP）**
  - **遗传方式**：常染色体隐性遗传
  - **主要特征**：
    - 对紫外线高度敏感，易发生日光性皮炎
    - 皮肤色素沉着不均，出现斑点
    - 皮肤癌风险增加
    - 眼部症状，如畏光、结膜炎
    - 神经系统症状，如智力障碍、听力下降
- **先天性无色素痣（Congenital Achromic Nevus）**
  - **遗传方式**：常染色体显性遗传或散发
  - **主要特征**：
    - 局部皮肤色素缺乏，形成白色斑块
    - 皮肤薄而柔软，呈白色
    - 无全身性色素缺乏
    - 无眼部症状
- **瓦登伯格综合征（Waardenburg Syndrome）**
  - **遗传方式**：常染色体显性遗传
  - **主要特征**：
    - 皮肤色素缺乏，尤其是面部和头发
    - 眼睛颜色异色或浅色
    - 听力损失
    - 面部特征异常，如宽鼻梁、内眦赘皮
- **特纳综合征（Turner Syndrome）**
  - **遗传方式**：X染色体单体性
  - **主要特征**：
    - 矮小身材
    - 颈部皮肤松弛
    - 心血管异常
    - 卵巢发育不全，导致性腺功能减退
    - 皮肤色素沉着不均
- **赫尔勒综合征（Hurler Syndrome）**
  - **遗传方式**：常染色体隐性遗传
  - **主要特征**：
    - 皮肤粗糙，多毛
    - 骨骼异常，如短肢、脊柱侧弯
    - 肝脾肿大
    - 心血管异常
    - 智力障碍

根据您提供的临床表现，白化病是最可能的诊断，但其他几种疾病也需考虑，特别是着色性干皮病，因为它们也有类似的皮肤和眼部症状。进一步的基因检测和专科评估将有助于确诊。

### 23. User

一种上、下运动神经元同时受累的神经系统变性疾病。临床主要表现为球部、四肢、胸腹部肌肉进行性无力和萎缩。而眼球运动神经和括约肌功能一般并不受累，但在有些患者晚期也可能会被累及。20％ ~50％的患者可以表现有认知功能障碍，5％ ~15％的患者甚至会发展为额颞叶痴呆。发病后平均 3~5 年因呼吸衰竭死亡，但 5％ ~10％的患者可以存活 10 年以上。

通过详细的病史和体格检查，在脑干、颈、胸、腰骶 4 个区域中寻找上、下运动神经元共同受累的证据，是诊断该疾病的基础。查体发现在同一区域，同时存在上、下运动神经元受累的体征是诊断的要点。其中下运动神经元受累体征主要包括肌肉无力、萎缩和肌束颤动。通常检查舌肌、面肌、咽喉肌、颈肌、四肢不同肌群、背肌和胸腹肌。上运动神经元受累体征主要包括病理征阳性、腱反射亢进、肌张力增高、阵挛等。通常检查吸吮反射、咽反射、下颌反射、掌颌反射，有无强哭、强笑等假性延髓麻痹表现，四肢腱反射、肌张力、霍夫曼征、下肢病理征、腹壁反射等。临床查体是发现上运动神经元受累的主要方法，在出现萎缩、无力的区域，如果腱反射不低或活跃，即使没有病理征，也可以提示锥体束受损。

### Qwen2.5_72b

根据您提供的临床表现，最可能的诊断是肌萎缩侧索硬化症（Amyotrophic Lateral Sclerosis, ALS）。ALS是一种进行性的神经退行性疾病，主要影响大脑和脊髓中的运动神经元，导致肌肉无力和萎缩，最终影响呼吸功能。ALS的特征是上、下运动神经元同时受累，这与您描述的临床表现相符。

以下是其他五种可能的诊断：

- **进行性肌萎缩（Progressive Muscular Atrophy, PMA）**：
  - 与ALS类似，PMA主要影响下运动神经元，导致肌肉无力和萎缩。但与ALS不同的是，PMA通常不涉及上运动神经元，因此腱反射和病理征可能正常。
- **原发性侧索硬化症（Primary Lateral Sclerosis, PLS）**：
  - PLS主要影响上运动神经元，导致肌肉僵硬、腱反射亢进和病理征阳性。与ALS不同的是，PLS通常不引起明显的肌肉萎缩和无力。
- **肯尼迪病（Kennedy’s Disease）**：
  - 一种X连锁隐性遗传病，主要影响下运动神经元，导致肌肉无力和萎缩，尤其是面部、咽喉和四肢肌肉。患者可能伴有乳房发育和睾丸萎缩。
- **多灶性运动神经病（Multifocal Motor Neuropathy, MMN）**：
  - 一种免疫介导的周围神经病，主要影响下运动神经元，导致不对称的肌肉无力和萎缩。患者可能伴有传导阻滞，但通常不涉及上运动神经元。
- **脊髓性肌萎缩症（Spinal Muscular Atrophy, SMA）**：
  - 一种遗传性神经肌肉病，主要影响下运动神经元，导致肌肉无力和萎缩。SMA有多种类型，根据发病年龄和严重程度不同，可分为婴儿型、青少年型和成人型。

这些疾病在临床表现上可能有重叠，但通过详细的病史、体格检查和辅助检查（如电生理检查、基因检测等）可以进行鉴别诊断。

### 24. User

一种影响尿素循环的常染色体隐性疾病，可导致高氨血症、脑病和呼吸道阻塞。 其特征是精氨酸和氨在血液中积聚，导致发育迟缓、癫痫发作、智力残疾、痉挛和共济失调等症状。 这种情况通常在3岁左右出现，症状包括僵硬、生长缓慢和认知能力下降。 如果不进行治疗，它可能会发展为严重的痉挛状态，失去肌肉和智力残疾。

### Qwen2.5_72b

根据您提供的临床表现，最可能的诊断是**精氨酸血症**（Argininemia），这是一种尿素循环障碍，属于常染色体隐性遗传病。精氨酸血症是由于精氨酸酶缺乏导致精氨酸和氨在血液中积聚，进而引起高氨血症、脑病和一系列神经系统症状。

以下是五种其他可能的诊断：

- **鸟氨酸转氨甲酰酶缺乏症**（OTC Deficiency）：这是一种X连锁隐性遗传病，也是尿素循环障碍的一种。患者会出现高氨血症、脑病、发育迟缓和癫痫发作等症状。
- **高氨血症I型**（Citrullinemia Type I）：这是一种常染色体隐性遗传病，由于精氨琥珀酸合成酶缺乏导致高氨血症和代谢紊乱，症状包括发育迟缓、癫痫发作和共济失调。
- **高氨血症II型**（Citrullinemia Type II）：这是一种常染色体隐性遗传病，由于天冬氨酸-谷氨酸载体1（ASL）基因突变导致高氨血症和代谢紊乱，症状与I型类似。
- **甲基丙二酸血症**（Methylmalonic Acidemia）：这是一种有机酸代谢障碍，由于甲基丙二酸辅酶A变位酶缺乏导致代谢产物在体内积聚，引起高氨血症、脑病和神经系统症状。
- **丙酸血症**（Propionic Acidemia）：这是一种有机酸代谢障碍，由于丙酸辅酶A羧化酶缺乏导致代谢产物在体内积聚，引起高氨血症、脑病和神经系统症状。

这些疾病都可能导致类似的临床表现，因此需要通过详细的病史、体格检查和实验室检测（如血液氨水平、尿有机酸分析、基因检测等）来确诊。

### 25. User

典型的临床表现包括微血管病性溶血性贫血、血小板减少及急性肾功能衰竭三联征。微血栓形成所导致的非免疫性红细胞破坏，血红蛋白水平常低于 80g/L，Coombs 试验阴性， 外周血涂片可见红细胞碎片；血小板计数下降通常低于 50×109/L，但皮肤紫癜及活动性出血少见；急性肾功能损害的严重程度因人而异，部分患者需要透析支持，常伴血压升高。

### Qwen2.5_72b

根据您提供的临床表现，最可能的诊断是血栓性血小板减少性紫癜（Thrombotic Thrombocytopenic Purpura, TTP）。TTP 是一种罕见的血液疾病，其特征是微血管病性溶血性贫血、血小板减少和急性肾功能衰竭。以下是其他五种可能的诊断：

- **溶血性尿毒症综合征（Hemolytic Uremic Syndrome, HUS）**：
  - 与 TTP 类似，HUS 也表现为微血管病性溶血性贫血、血小板减少和急性肾功能衰竭。但 HUS 常见于儿童，且常与产志贺毒素的大肠杆菌感染有关。
- **弥散性血管内凝血（Disseminated Intravascular Coagulation, DIC）**：
  - DIC 是一种严重的凝血功能障碍，可导致微血管病性溶血性贫血和血小板减少。DIC 常见于严重感染、恶性肿瘤、产科并发症等情况下。
- **自身免疫性溶血性贫血（Autoimmune Hemolytic Anemia, AIHA）**：
  - AIHA 以免疫介导的红细胞破坏为特征，Coombs 试验阳性。虽然 AIHA 通常不伴有血小板减少和急性肾功能衰竭，但在某些情况下也可能出现这些症状。
- **恶性高血压（Malignant Hypertension）**：
  - 恶性高血压可导致急性肾功能衰竭和微血管病性溶血性贫血，但通常伴有严重的高血压和视网膜病变。
- **系统性红斑狼疮（Systemic Lupus Erythematosus, SLE）**：
  - SLE 是一种自身免疫性疾病，可累及多个器官系统，包括肾脏、血液系统等。SLE 患者可能出现溶血性贫血、血小板减少和肾功能损害，但通常伴有其他自身免疫性症状，如皮疹、关节炎等。

这些疾病在临床表现上有一定的重叠，但通过详细的病史、体格检查和实验室检查可以进行鉴别诊断。

### 26. User

患者临床表现多样。取决于病变的部位、范围和进展速度。临床症状主要包含：

- 增大垂体的占位效应，大多数患者会因增大的垂体对鞍区及周围结构（如视交叉、海绵窦等） 造成压迫而产生相应症状或由于炎症浸润产生的作用。最常见的临床表现为持续的剧烈头痛和进行性加重的视功能障碍，包括视力下降和视野缺损。
- 腺垂体功能减退，根据受累的程度不同，垂体的各个轴系会出现部分甚至全部的功能减低的临床表现，如闭经、性功能减低、纳差、乏力、怕冷、性毛脱落、体重下降等。
- 中枢性尿崩症 ， 约 70％以上患者会因为垂体后叶、垂体柄甚至下丘脑区受累出现多尿、烦渴和多饮等中枢性尿崩症的症状。部分患者因垂体柄阻断效应导致血清中泌乳素水平轻度升高。
- 下丘脑综合征， 当病变累及区域发展至下丘脑时，会出现嗜睡、缺乏饱腹感、体重严重增加、渴感缺乏、体温波动、出汗障碍和情绪神志障碍等临床表现。
- 系统性疾病的相关表现 ， 继发性的患者常合并有系统性疾病特征性的临床表现，如 LCH 患者常合并甲状腺、肺、骨、肝脏和皮肤病变。GPA 患者常为中老年男性， 呼吸道症状常早于下丘脑垂体受累。IgG4 相关疾病垂体炎患者常合并有泪腺、颌下腺和胰腺等其他脏器受累的临床表现等。

### Qwen2.5_72b

根据您提供的临床表现，最可能的诊断是**垂体炎**，特别是**IgG4相关性垂体炎**。这种疾病可以导致垂体增大，产生占位效应，引起头痛、视功能障碍、腺垂体功能减退、中枢性尿崩症和下丘脑综合征等症状。此外，IgG4相关性垂体炎患者常合并有其他器官受累的表现，如泪腺、颌下腺和胰腺等。

以下是其他五种可能的诊断：

- **朗格汉斯细胞组织细胞增生症（LCH）**：LCH是一种罕见的炎症性疾病，可以累及多个器官，包括垂体、甲状腺、肺、骨、肝脏和皮肤。LCH患者可能会出现垂体功能减退、中枢性尿崩症和下丘脑综合征等症状。
- **肉芽肿性多血管炎（GPA）**：GPA是一种自身免疫性疾病，主要影响小血管，尤其是呼吸道和肾脏。中老年男性患者常有呼吸道症状，随后可能出现下丘脑垂体受累的表现，如腺垂体功能减退和中枢性尿崩症。
- **垂体腺瘤**：垂体腺瘤是一种良性肿瘤，可以导致垂体增大，产生占位效应，引起头痛、视功能障碍和腺垂体功能减退。如果肿瘤侵犯垂体后叶或垂体柄，也可能导致中枢性尿崩症。
- **垂体结核**：垂体结核是一种罕见的感染性疾病，可以导致垂体增大，产生占位效应，引起头痛、视功能障碍和腺垂体功能减退。结核感染还可能导致全身症状，如发热、体重下降和乏力。
- **淋巴细胞性垂体炎**：淋巴细胞性垂体炎是一种自身免疫性疾病，可以导致垂体增大，产生占位效应，引起头痛、视功能障碍和腺垂体功能减退。这种疾病在产后女性中较为常见，但也可能发生在其他人群中。

这些疾病都有类似的临床表现，需要通过详细的病史、体格检查、实验室检查和影像学检查来进一步明确诊断。

### 27. User

高血糖是该疾病的最常见的临床表现。但在没有干预的情况下患者可以自发缓解而维持血糖水平正常。该疾病是自身免疫性低血糖的主要原因之一，约 25％的患者在病程中会出现自发性低血糖。约 50％的患者存在黑棘皮征。此外，多囊卵巢、高雄激素血症在该类患者中也不少见。 患者常伴发其他的自身免疫性疾病，其中以系统性红斑狼疮最为常见，约占 50％。此外，还有原发性胆汁性肝硬化、皮肌炎、硬皮病等。该疾病还可以是霍奇金淋巴瘤、多发性骨髓瘤等的副肿瘤综合征表现。

### Qwen2.5_72b

根据您提供的临床表现，最可能的诊断是**胰岛素自身免疫综合征（IAS，Insulin Autoimmune Syndrome）**，也称为Hirata病。这是一种罕见的自身免疫性疾病，其特点是患者体内产生针对胰岛素的自身抗体，导致血糖水平的波动，包括高血糖和低血糖。以下是其他五种可能的诊断：

- **多发性内分泌腺瘤病1型（MEN1）**：这是一种遗传性综合症，患者可能同时或先后出现多个内分泌腺体的肿瘤，包括胰岛细胞瘤，可能导致血糖水平的波动。
- **2型糖尿病伴自身免疫现象**：虽然2型糖尿病通常与胰岛素抵抗有关，但部分患者可能伴有自身免疫现象，如胰岛细胞抗体阳性，导致血糖控制不稳定。
- **自身免疫性多内分泌腺病综合征（APS）**：这是一种以多个内分泌腺体受累为特征的自身免疫性疾病，患者可能同时或先后出现多种内分泌功能障碍，包括胰岛素自身免疫综合征。
- **系统性红斑狼疮（SLE）伴胰岛素抵抗**：SLE患者可能因自身免疫反应导致胰岛素抵抗，进而出现血糖水平的波动。
- **副肿瘤性低血糖**：某些恶性肿瘤，如霍奇金淋巴瘤和多发性骨髓瘤，可能通过分泌胰岛素样生长因子（IGF）或其他激素导致低血糖。

这些疾病都可能表现出类似的临床特征，因此需要通过详细的病史、体格检查和实验室检查来进一步明确诊断。

### 28. User

婴儿及儿童期起病。首次发作时年龄在 5 个月至 7 岁。临床表现为反复发作的难治性呕吐及重症酮症酸中毒，伴有萎靡、脱水、呼吸急促、昏迷等临床表现。通常有明确诱因，可在禁食、胃肠道及上呼吸道感染、发热、应激或过量摄入蛋白质后急性起病。常规的实验室检查可见血糖升高或正常，部分出现低血糖，血气显示代谢性酸中毒，血氨可以正常或轻度升高，肝功能、肾功能及正常，尿酮体阳性。酮症及酸中毒可反复出现，发作间期临床及实验室检查可以表现为正常。

约 1/5 的病例出现神经系统的改变，表现为认知障碍、智力发育落后及锥体外系异常及脑卒中的症状，头颅 MRI 可发现包括白质广泛的脱髓鞘，基底节区在 T2 可见对称性高信号改变。

患者早期可无症状，在新生儿期或高危人群通过血、尿代谢筛查时被诊断。起病年龄、发作频率与预后无明确关联。发作随年龄增长有减少或停止趋势，多数病例随访其生长发育未受影响。反复发作及严重者可致多器官功能障碍甚至死亡。

### Qwen2.5_72b

根据您提供的临床表现，最可能的诊断是**丙酸血症（Propionic Acidemia）**。这是一种罕见的代谢性疾病，属于有机酸血症的一种，由于丙酸代谢途径中的酶缺陷导致丙酸及其代谢产物在体内积累，引起一系列临床症状。

### 丙酸血症（Propionic Acidemia）

- **临床表现**：反复发作的难治性呕吐、重症酮症酸中毒，伴有萎靡、脱水、呼吸急促、昏迷等。
- **实验室检查**：血糖升高或正常，部分出现低血糖，血气显示代谢性酸中毒，血氨可以正常或轻度升高，尿酮体阳性。
- **神经系统表现**：约 1/5 的病例出现认知障碍、智力发育落后及锥体外系异常及脑卒中的症状，头颅 MRI 可发现包括白质广泛的脱髓鞘，基底节区在 T2 可见对称性高信号改变。
- **诱因**：禁食、胃肠道及上呼吸道感染、发热、应激或过量摄入蛋白质后急性起病。
- **预后**：发作随年龄增长有减少或停止趋势，多数病例随访其生长发育未受影响，但反复发作及严重者可致多器官功能障碍甚至死亡。

### 其他可能的诊断

- **甲基丙二酸血症（Methylmalonic Acidemia）**
  - **临床表现**：与丙酸血症类似，反复发作的难治性呕吐、重症酮症酸中毒，伴有萎靡、脱水、呼吸急促、昏迷等。
  - **实验室检查**：血气显示代谢性酸中毒，血氨可以正常或轻度升高，尿酮体阳性。
  - **神经系统表现**：可有认知障碍、智力发育落后及锥体外系异常。
  - **诱因**：禁食、感染、应激等。
- **异戊酸血症（Isovaleric Acidemia）**
  - **临床表现**：反复发作的难治性呕吐、重症酮症酸中毒，伴有萎靡、脱水、呼吸急促、昏迷等。
  - **实验室检查**：血气显示代谢性酸中毒，血氨可以正常或轻度升高，尿酮体阳性。
  - **神经系统表现**：可有认知障碍、智力发育落后。
  - **诱因**：禁食、感染、应激等。
- **戊二酸血症 I 型（Glutaric Acidemia Type I）**
  - **临床表现**：反复发作的难治性呕吐、重症酮症酸中毒，伴有萎靡、脱水、呼吸急促、昏迷等。
  - **实验室检查**：血气显示代谢性酸中毒，血氨可以正常或轻度升高，尿酮体阳性。
  - **神经系统表现**：可有认知障碍、智力发育落后及锥体外系异常。
  - **诱因**：禁食、感染、应激等。
- **尿素循环障碍（Urea Cycle Disorders）**
  - **临床表现**：反复发作的难治性呕吐、重症酮症酸中毒，伴有萎靡、脱水、呼吸急促、昏迷等。
  - **实验室检查**：血氨显著升高，血气显示代谢性酸中毒。
  - **神经系统表现**：可有认知障碍、智力发育落后。
  - **诱因**：禁食、感染、应激等。
- **糖原贮积病（Glycogen Storage Disease）**
  - **临床表现**：反复发作的难治性呕吐、低血糖，伴有萎靡、脱水、呼吸急促、昏迷等。
  - **实验室检查**：血糖降低，血气显示代谢性酸中毒。
  - **神经系统表现**：可有认知障碍、智力发育落后。
  - **诱因**：禁食、感染、应激等。

这些疾病都需要通过详细的病史、体格检查、实验室检查及代谢筛查来确诊。建议进一步进行基因检测和代谢产物分析以明确诊断。

### 29. User

- 发病年龄小，女性多见。
- 常有晕厥或猝死家族史。
- 不同基因型和表现型，可累及钾、钠、钙通道。常有特征性尖端扭转型室速（TdP），导致晕厥或猝死。
- ECG 表现为 QT 间期延长。
- 心电图一般有如下特征：①表现为 QT 间期延长，短 - 长 - 短周期现象诱发；②心室率 160-240 次 / 分，QRS 波振幅与形态围绕等电位线扭转，5-20 个心动周期主波围绕基线扭转一次，多数能自行终止；③ ATP 较难终止的 TdP。

### Qwen2.5_72b

根据您提供的临床表现，最可能的诊断是**长QT综合征（Long QT Syndrome, LQTS）**。这是一种心脏离子通道病，主要特征是心电图上的QT间期延长，容易引发尖端扭转型室速（TdP），导致晕厥或猝死。LQTS可以由多种基因突变引起，影响钾、钠、钙通道的功能。

以下是五种其他可能的诊断：

- **布鲁加达综合征（Brugada Syndrome）**：
  - 也属于心脏离子通道病，心电图表现为右束支阻滞和ST段抬高，容易引发室性心动过速和室颤，导致晕厥或猝死。
  - 通常在中青年男性中多见，但也有女性病例。
- **儿茶酚胺敏感性多形性室性心动过速（Catecholaminergic Polymorphic Ventricular Tachycardia, CPVT）**：
  - 通常在运动或情绪激动时发作，表现为双向性或多形性室性心动过速，心电图在静息状态下可能正常。
  - 有晕厥或猝死家族史，多见于儿童和青少年。
- **短QT综合征（Short QT Syndrome）**：
  - 心电图表现为QT间期缩短，容易引发房颤、室性心动过速和室颤，导致晕厥或猝死。
  - 通常在中青年中多见，但也有儿童病例。
- **先天性心脏病**：
  - 如先天性长QT综合征的某些类型，可能伴有其他心脏结构异常。
  - 心电图表现和临床症状与LQTS相似，但可能有其他心脏结构异常的证据。
- **药物引起的QT间期延长**：
  - 某些药物（如抗心律失常药、抗精神病药、抗生素等）可以引起QT间期延长，导致尖端扭转型室速。
  - 有药物使用史，停药后QT间期可能恢复正常。

这些诊断都需要通过详细的病史、体格检查、心电图、基因检测等手段进行进一步确认。

### 30. User

可于任何年龄发病，多数患儿于 1 个月至 7 岁发病，平均年龄在 2 岁左右。不同患者临床表现有较大差异，主要有：①急性能量代谢障碍危象，表现为低酮型低血糖、高血氨及代谢性酸中毒等；②心肌病，表现为心室肥厚、心功能不全、心律失常及肌酸激酶升高等；③肌病，表现为肌无力、肌张力减退、肌痛、运动耐力差、肌肉型肌酸激酶升高、肌纤维内脂质沉积等；④肝脏损害，表现为肝肿大、脂肪肝、肝功能异常等，一些肝损患儿急性起病，表现为抽搐、进行性意识障碍等，常被误诊为 Reye 综合征。此外，反复腹痛、呕吐、胃食管反流等消化道症状，反复感染、喘息等呼吸道表现以及贫血等也有报道。

新生儿筛查：确诊的患儿可无临床表现。 是一种潜在的致死性疾病，患儿可因急性能量代谢障碍危象或急性心衰而猝死。临床表现多样，与发病年龄、发病时间和病情轻重有关。最常见的症状是婴幼儿期和儿童期心肌和骨骼肌受损。成年期症状较轻或无症状，多为耐力降低或易疲劳。妊娠期，由于能量消耗和血浆生理性的肉碱水平降低，孕妇可有疲劳和心律失常等不典型表现。

### Qwen2.5_72b

根据您提供的临床表现，最可能的诊断是**原发性肉碱缺乏症（Primary Carnitine Deficiency, PCD）**。这是一种罕见的代谢性疾病，由于肉碱转运蛋白缺陷导致肉碱吸收障碍，进而影响脂肪酸的氧化和能量代谢。以下是其他五种可能的诊断：

- **Reye 综合征**：这是一种急性脑病和肝功能障碍的综合征，多见于儿童，常在病毒感染后出现，表现为急性代谢性脑病、肝肿大和肝功能异常。临床表现与PCD相似，但Reye综合征通常有明确的病毒感染史。
- **线粒体肌病**：这是一种线粒体功能障碍导致的疾病，表现为肌无力、肌张力减退、心肌病、肝功能异常等。线粒体肌病的临床表现多样，与PCD有部分重叠。
- **脂肪酸氧化障碍（如中链酰基辅酶A脱氢酶缺乏症，MCADD）**：这是一种脂肪酸代谢障碍，表现为低酮型低血糖、高血氨、代谢性酸中毒等。MCADD的临床表现与PCD非常相似，需要通过代谢筛查和基因检测来鉴别。
- **糖原贮积病（如Pompe病）**：这是一种糖原代谢障碍，表现为心肌病、肌无力、肝肿大等。Pompe病的临床表现与PCD有部分重叠，但通常有更明显的肌病和心肌病表现。
- **有机酸血症（如丙酸血症）**：这是一种有机酸代谢障碍，表现为代谢性酸中毒、高血氨、低酮型低血糖等。有机酸血症的临床表现与PCD相似，但通常有更明显的代谢紊乱和神经系统症状。

确诊这些疾病通常需要进行详细的代谢筛查、基因检测和酶活性测定。希望这些信息对您有所帮助。

### 31. User

单中心型该疾病往往仅表现为淋巴结肿大，而无全身症状。肿大淋巴结的中位直径约 5.5cm， 常见于胸部（24 ％）、颈部（20 ％）、腹部（18 ％）、腹膜后（14 ％）。部分单中心型疾病的患者容易合并副肿瘤性天疱疮、闭塞性细支气管炎以及生长发育迟缓。多中心型 除了多发淋巴结肿大外，往往还伴有发热、盗汗、乏力、体重下降、贫血、肝功能不全、肾功能不全和容量负荷过多（全身水肿、胸腔积液、腹水等） 等全身表现。多中心型 还有一种特殊亚型，患者可以表现为血小板减少（thrombocytopenia）、重度水肿（anasarca）、骨髓纤维化（myelofibrosis）、肾功能不全（renal dysfunction）、肝脾肿大（organomegaly）。

### Qwen2.5_72b

根据您提供的临床表现，最可能的诊断是 **Castleman病**，特别是多中心型Castleman病（MCD）。Castleman病是一种罕见的淋巴组织增生性疾病，分为单中心型（UCD）和多中心型（MCD）。单中心型通常表现为孤立的淋巴结肿大，而多中心型则表现为多发淋巴结肿大，并伴有全身症状，如发热、盗汗、乏力、体重下降、贫血、肝功能不全、肾功能不全和容量负荷过多等。

以下是五种其他可能的诊断：

- **淋巴瘤**：特别是霍奇金淋巴瘤和非霍奇金淋巴瘤，可以表现为淋巴结肿大和全身症状，如发热、盗汗和体重下降。
- **系统性红斑狼疮（SLE）**：一种自身免疫性疾病，可以表现为多发淋巴结肿大、发热、乏力、贫血和多器官受累。
- **结节病**：一种多系统受累的肉芽肿性疾病，可以表现为淋巴结肿大、发热、乏力和肝功能异常。
- **Kaposi肉瘤**：一种与人类疱疹病毒8型（HHV-8）感染相关的血管肿瘤，可以表现为淋巴结肿大和多器官受累，尤其是在免疫抑制的患者中。
- **慢性活动性EB病毒感染**：可以表现为持续的淋巴结肿大、发热、乏力、肝功能异常和多器官受累。

这些疾病在临床表现上有一定的重叠，因此需要通过详细的病史、体格检查、实验室检查和影像学检查来进一步明确诊断。

### 32. User

主要临床表现为下肢远端为主，并逐渐向近端发展的肢体肌肉萎缩、无力及感觉丧失。常见临床表现为运动能力不如同龄人，跑步困难，易扭脚，足下垂，小腿腓肠肌萎缩形似“鹤腿”；查体可发现弓形足、锤状趾，远端肢体为主的无力萎缩，深感觉减退。患者通常 20 岁前起病，缓慢进展，疾病后期可能严重影响活动，但很少导致完全残疾， 也不影响正常寿命。但有些特殊类型可能起病早且严重。

### Qwen2.5_72b

根据您提供的临床表现，最可能的诊断是**遗传性运动感觉神经病（Hereditary Motor and Sensory Neuropathy, HMSN）**，也称为**Charcot-Marie-Tooth病（CMT病）**。这是一种常见的遗传性周围神经病，主要表现为下肢远端肌肉萎缩、无力及感觉丧失，逐渐向近端发展。CMT病通常在20岁前起病，进展缓慢，但很少导致完全残疾，也不影响正常寿命。

以下是其他五种可能的诊断：

- **脊髓性肌萎缩症（Spinal Muscular Atrophy, SMA）**：
  - 一种遗传性神经肌肉病，主要影响脊髓前角细胞，导致肌肉无力和萎缩。根据起病年龄和严重程度不同，可分为多种类型。SMA通常在儿童期起病，进展较快，严重者可导致呼吸肌受累。
- **远端肌病（Distal Myopathy）**：
  - 一组以远端肌肉无力和萎缩为主要表现的肌病。起病年龄和进展速度因具体类型而异，但通常进展较慢，影响下肢远端肌肉。
- **进行性肌营养不良症（Progressive Muscular Dystrophy）**：
  - 一组遗传性肌肉病，主要表现为肌肉无力和萎缩。根据受累肌肉和遗传方式不同，可分为多种类型，如Duchenne型肌营养不良症和Becker型肌营养不良症。这些疾病通常在儿童期或青少年期起病，进展较快，严重者可导致完全残疾。
- **多发性神经病（Polyneuropathy）**：
  - 一种影响周围神经的疾病，可由多种原因引起，如糖尿病、自身免疫性疾病、感染、中毒等。主要表现为远端肢体的无力、萎缩和感觉丧失，但通常伴有其他系统的表现。
- **脊髓小脑共济失调（Spinocerebellar Ataxia, SCA）**：
  - 一组遗传性神经退行性疾病，主要影响小脑和脊髓，导致共济失调、步态不稳、肌肉无力和萎缩。起病年龄和进展速度因具体类型而异，但通常进展较慢。

这些疾病的临床表现有部分重叠，但通过详细的病史、体格检查、电生理检查、基因检测等手段可以进一步明确诊断。

### 33. User

患儿于出生后至数月内发病。发病年龄多在 2 月龄内，很少晚于 5 月龄。最常表现为出生后黄疸持续不退伴或不伴大便颜色浅淡，肝脏轻度肿大或无明显肿大。 还有其他多种非特异临床表现，包括喂养困难、生长发育迟缓、腹泻、低血糖、凝血时间延长等。大多数患者上述症状及体征在 6 个月至 1 岁左右可自然缓解或经过饮食结构调整和药物治疗缓解，其中少部分的患者在青春期以后疾病进一步发展。发展后的疾病大部分是在成人后发病。通常在饮酒、摄入甜食、服用某些药物或感染后突然出现高氨血症所致的谵妄，意识混乱等精神异常表现。严重的病情发展急剧，得不到正确治疗可危及生命。患者多为消瘦体形，大部分患者有明显的饮食偏好，嗜食豆类、高蛋白、高脂食物，而厌食高糖类。

### Qwen2.5_72b

根据您提供的临床表现，最可能的诊断是**糖原贮积病I型（Glycogen Storage Disease Type I, GSD I）**，也称为冯·吉尔克病（von Gierke disease）。这是一种由于葡萄糖-6-磷酸酶缺乏导致的代谢障碍，主要影响肝脏和肾脏。以下是详细的诊断依据和五种其他可能的诊断：

### 最可能的诊断：糖原贮积病I型（GSD I）

- **临床表现**：
  - 出生后黄疸持续不退，伴或不伴大便颜色浅淡。
  - 肝脏轻度肿大或无明显肿大。
  - 喂养困难、生长发育迟缓、腹泻、低血糖、凝血时间延长。
  - 6 个月至 1 岁左右症状可自然缓解或通过饮食结构调整和药物治疗缓解。
  - 青春期后部分患者病情进一步发展，表现为高氨血症所致的谵妄、意识混乱等精神异常。
  - 患者多为消瘦体形，有明显的饮食偏好，嗜食豆类、高蛋白、高脂食物，而厌食高糖类。

### 其他可能的诊断：

- **先天性胆道闭锁（Biliary Atresia）**：
  - 出生后黄疸持续不退，大便颜色浅淡。
  - 肝脏肿大。
  - 喂养困难、生长发育迟缓。
  - 通常需要手术治疗。
- **半乳糖血症（Galactosemia）**：
  - 出生后黄疸持续不退。
  - 喂养困难、生长发育迟缓、腹泻、低血糖。
  - 肝脏肿大。
  - 通过限制乳糖摄入可以缓解症状。
- **先天性甲状腺功能减退症（Congenital Hypothyroidism）**：
  - 喂养困难、生长发育迟缓。
  - 低血糖、便秘。
  - 通过甲状腺激素替代治疗可以缓解症状。
- **先天性肾上腺皮质增生症（Congenital Adrenal Hyperplasia, CAH）**：
  - 喂养困难、生长发育迟缓。
  - 低血糖、电解质紊乱。
  - 通过皮质激素替代治疗可以缓解症状。
- **威尔森病（Wilson’s Disease）**：
  - 肝脏肿大、黄疸。
  - 喂养困难、生长发育迟缓。
- 低血糖、凝血时间延长。
- 青春期后部分患者出现神经系统症状，如震颤、肌张力障碍等。
- 通过铜代谢调节治疗可以缓解症状。

### 诊断建议：

- **实验室检查**：
  - 血糖、肝功能、凝血功能、血氨水平。
  - 尿常规、尿糖、尿酮体。
  - 基因检测（G6PC基因突变）。
- **影像学检查**：
  - 腹部超声检查肝脏和胆道系统。
- **代谢筛查**：
  - 血液和尿液代谢产物分析。

通过上述检查，可以进一步明确诊断并制定相应的治疗方案。

### 34. User

可于出生后出现症状，也可于婴幼儿期至成年早期起病。主要表现为波动性肌肉无力，不耐疲劳，多分布于眼外肌、面肌、延髓、四肢和呼吸肌，婴儿期甚至可能出现危及生命的呼吸暂停发作。单纯的综合征主要表现为骨骼肌无力，不伴有先天畸形。该疾病的糖基化缺陷和肌病重叠综合征往往伴有先天发育异常。不同类型的临床表现也有差异，部分患者，一般为出生后近端肌肉无力，胆碱酯酶抑制剂有效，严重者可出现突发的呼吸窘迫和延髓麻痹引起的呼吸暂停，可由应激、寒冷、感染等诱发。部分患者，出生后或婴儿期起病，可能出现比较严重、轴性分布的肌肉无力，但眼外肌可不受累。

### Qwen2.5_72b

根据您提供的临床表现，最可能的诊断是**先天性肌无力综合征（Congenital Myasthenic Syndromes, CMS）**。这是一种罕见的神经肌肉接头传递障碍疾病，主要表现为波动性肌肉无力和易疲劳，尤其是在眼外肌、面肌、延髓、四肢和呼吸肌。这些症状与您描述的临床表现非常吻合。

### 其他可能的诊断包括：

- **低钾性周期性麻痹（Hypokalemic Periodic Paralysis）**：
  - 特点是反复发作的肌肉无力，通常与血钾水平降低有关。发作常由应激、寒冷、高碳水化合物饮食等诱发。
- **线粒体肌病（Mitochondrial Myopathy）**：
  - 一种由线粒体功能障碍引起的肌肉疾病，表现为慢性进行性肌肉无力和易疲劳，可累及眼外肌、四肢和呼吸肌。
- **重症肌无力（Myasthenia Gravis, MG）**：
  - 一种自身免疫性疾病，主要表现为波动性肌肉无力和易疲劳，尤其是眼外肌和面肌。胆碱酯酶抑制剂治疗有效。
- **糖基化缺陷和肌病重叠综合征（Congenital Disorders of Glycosylation, CDG）**：
  - 一种遗传性代谢病，表现为多系统受累，包括肌肉无力、发育异常等。部分患者可能伴有先天畸形。
- **脊髓性肌萎缩症（Spinal Muscular Atrophy, SMA）**：
  - 一种遗传性神经肌肉疾病，表现为进行性肌肉无力和萎缩，主要累及近端肌肉。婴儿期起病的患者病情较重，可出现呼吸肌受累。

这些疾病在临床表现上有一些相似之处，但通过详细的病史、体格检查和辅助检查（如血液检查、肌电图、基因检测等）可以进一步明确诊断。

### 35. User

- 通常婴幼儿期至儿童期起病，少数患者成年后起病。
- 全身横纹肌，包括肢体骨骼肌、面肌、眼外肌、舌肌均可出现肌强直现象。
- 肌强直在休息后突然运动时明显，反复动作后减轻，称之为“热身（warm-up）”现象。可在突然启动时出现短暂肌肉无力。病程较长的的患者，可有持续性肢体远端无力，甚至累及肢体近端。
- 查体可见患者常肌肉发达，呈运动员体型。大力握拳后，手指不能及时展开；大力闭目后，不能立即睁开。叩击手部大鱼际肌、舌肌等部位，可引起肌肉持续性收缩（叩击性肌强直、“肌球”现象）。

### Qwen2.5_72b

根据您提供的临床表现，最可能的诊断是**先天性肌强直（Myotonia Congenita）**。这是一种罕见的遗传性肌肉疾病，主要特征是肌肉在收缩后不能迅速放松，特别是在休息后突然运动时更为明显。这种疾病通常在婴幼儿期至儿童期起病，但也有少数患者成年后起病。患者常表现为肌肉发达，呈运动员体型，且有典型的“热身”现象，即反复动作后肌强直减轻。

### 其他可能的诊断：

- **强直性肌营养不良（Myotonic Dystrophy）**：
  - 也是一种肌强直性疾病，但通常伴有肌无力和肌萎缩，且多在成年后起病。
  - 可能伴有白内障、心脏传导阻滞、内分泌异常等多系统受累。
- **先天性肌无力综合征（Congenital Myasthenic Syndromes, CMS）**：
  - 以肌无力为主要表现，尤其是在活动后加重，休息后可部分缓解。
  - 可能伴有眼外肌受累，导致眼睑下垂和复视。
- **多发性肌炎/皮肌炎（Polymyositis/Dermatomyositis）**：
  - 以对称性近端肌无力为主要表现，可伴有皮肤病变。
  - 肌肉活检和血清肌酶水平升高有助于诊断。
- **周期性麻痹（Periodic Paralysis）**：
  - 以反复发作的肌肉无力和麻痹为特征，通常与血钾水平异常有关。
  - 可分为低钾型、高钾型和正常钾型。
- **线粒体肌病（Mitochondrial Myopathy）**：
  - 以慢性进行性肌无力和易疲劳为特征，可伴有其他系统受累，如视网膜病变、心脏病变等。
  - 肌肉活检可见“破碎红纤维”（Ragged Red Fibers）。

这些疾病在临床表现上可能有部分重叠，但通过详细的病史、体格检查、实验室检查和特殊检查（如肌电图、肌肉活检等）可以进一步明确诊断。

### 36. User

由于脊柱侧凸所引起的继发性胸廓畸形可引起患儿心肺发育不全，心肺储备差，另可能出现包括心脏移位、心律失常、肺活量减少、肺炎等；腹腔畸形可引起胃肠功能紊乱， 导致消化不良，食欲缺乏。此外，由于椎体畸形和椎间盘退变引起的神经根牵拉可产生相应躯体部位的刺激性疼痛，严重者可引起脊髓功能障碍甚至瘫痪。

体格检查：大体表现为直立时两肩不等高、一侧腰部皱褶、腰前屈出现背部不对称（“剃刀背”），有时可见骨盆两侧不对称、步态异常等。此外，还需进行神经系统检查，观察患者有无肌张力增高或低下，有无四肢感觉异常。

临床上根据影像学特点通常分为以下 3 型：

Ⅰ型——椎体形成障碍型，即一个或多个椎体部分或完全形成障碍，如楔形椎、半椎体或蝴蝶椎等；

Ⅱ型——椎体分节不良型，即两个或多个椎体部分或完全分节障碍，如阻滞椎、骨桥形成等；

Ⅲ型——混合型，既有椎体形成障碍，又有椎体分节不良。

### Qwen2.5_72b

根据您提供的临床表现，最可能的诊断是先天性脊柱侧凸（Congenital Scoliosis）。先天性脊柱侧凸是一种罕见的脊柱发育异常，通常在出生时或婴儿期即存在，由于脊椎在胚胎期发育过程中出现椎体形成障碍或分节不良，导致脊柱侧弯。这种疾病可以引起一系列的并发症，包括胸廓畸形、心肺功能障碍、腹腔畸形、神经根牵拉症状等，与您描述的临床表现相符。

### 其他可能的诊断包括：

- **特发性脊柱侧凸（Idiopathic Scoliosis）**：
  - 特发性脊柱侧凸是最常见的脊柱侧凸类型，通常在青少年期发现，病因不明。虽然通常不伴有明显的胸廓畸形或心肺功能障碍，但在严重病例中也可能出现类似症状。
- **神经肌肉型脊柱侧凸（Neuromuscular Scoliosis）**：
  - 由神经或肌肉系统疾病引起的脊柱侧凸，如脑瘫、脊髓灰质炎、肌营养不良等。这些疾病可能导致脊柱侧弯和胸廓畸形，进而影响心肺功能。
- **综合征型脊柱侧凸（Syndromic Scoliosis）**：
  - 与某些遗传综合征相关，如马凡综合征（Marfan Syndrome）、埃勒斯-丹洛斯综合征（Ehlers-Danlos Syndrome）等。这些综合征可能伴有多种系统性异常，包括脊柱侧凸、胸廓畸形和心肺功能障碍。
- **脊柱裂（Spina Bifida）**：
  - 脊柱裂是一种神经管缺陷，可能导致脊柱和脊髓发育异常，引起脊柱侧弯、神经功能障碍和胸廓畸形。严重病例可能伴有心肺功能障碍。
- **脊柱肿瘤或感染（Spinal Tumor or Infection）**：
  - 脊柱肿瘤或感染（如脊柱结核）可能导致脊柱结构异常和侧弯，进而引起胸廓畸形和心肺功能障碍。这些疾病还可能伴有局部疼痛、发热等症状。

### 总结：

- **最可能的诊断**：先天性脊柱侧凸（Congenital Scoliosis）
- **其他可能的诊断**：
  1. 特发性脊柱侧凸（Idiopathic Scoliosis）
  2. 神经肌肉型脊柱侧凸（Neuromuscular Scoliosis）
  3. 综合征型脊柱侧凸（Syndromic Scoliosis）
  4. 脊柱裂（Spina Bifida）
  5. 脊柱肿瘤或感染（Spinal Tumor or Infection）

为了进一步确诊，建议进行详细的影像学检查（如X线、MRI等）和必要的实验室检查，以排除其他可能的病因。

### 37. User

男性多于女性。部分患者胸痛症状不典型，在行冠脉造影或冠脉 CT 时发现。当存在以下情况时，患者往往有临床症状：①合并阻塞性动脉粥样硬化性疾病、血栓、血流缓慢、夹层、破裂时可有劳力性心绞痛或急性冠脉综合征表现；②压迫邻近组织；③少见的有动脉瘤破裂，可引起急性心脏压塞；④虽然无显著的冠状动脉狭窄，也可发生因微血管功能障碍导致的心肌缺血。

最多见的临床表现是心绞痛，少数 患者会并发心肌梗死、严重心律失常甚至猝死。疾病可破入右房、右室或冠状窦，产生左向右分流，临床上可闻及连续性杂音，严重者会影响心脏功能。最严重的并发症是瘤体破入心包，可导致出血、心脏压塞甚至死亡。

### Qwen2.5_72b

根据您提供的临床表现，最可能的诊断是**冠状动脉瘤**（Coronary Artery Aneurysm, CAA）。冠状动脉瘤是一种罕见的冠状动脉疾病，其特点是冠状动脉局部扩张，直径超过正常冠状动脉的1.5倍。冠状动脉瘤可以是先天性的，也可以是后天性的，后者常见于动脉粥样硬化、感染（如川崎病）、炎症性疾病、创伤或医源性因素等。

### 最可能的诊断：

- **冠状动脉瘤**（Coronary Artery Aneurysm, CAA）

### 其他可能的诊断：

- **冠状动脉夹层**（Coronary Artery Dissection）：冠状动脉内膜撕裂，导致血流受阻，可引起急性冠脉综合征。
- **冠状动脉血栓形成**（Coronary Artery Thrombosis）：血栓形成导致冠状动脉阻塞，可引起急性心肌梗死。
- **冠状动脉痉挛**（Coronary Artery Spasm）：冠状动脉的暂时性收缩，可导致心绞痛或急性冠脉综合征。
- **冠状动脉瘘**（Coronary Artery Fistula）：冠状动脉与心脏腔室或其他血管之间的异常通道，可引起左向右分流和心脏杂音。
- **冠状动脉微血管疾病**（Coronary Microvascular Disease, CMD）：冠状动脉微血管功能障碍，导致心肌缺血，但无显著的冠状动脉狭窄。

这些疾病都可能导致类似的临床表现，如心绞痛、心肌梗死、心律失常等。确诊需要结合详细的病史、体格检查、心电图、心脏超声、冠脉造影或冠脉CT等检查结果。

### 38. User

- 骨髓衰竭 35％患儿出生时即表现为贫血，常于生后 2 周至 2 年确诊。贫血为大细胞或正细胞正色素性、网织红细胞减少、白细胞正常或轻度降低、血小板正常或轻度增高、骨髓红系增生低下而粒系和巨核细胞系增生活跃。
- 先天发育异常 30％ ~50％ 患者可能存在先天发育异常，主要涉及头部、上肢、心脏和泌尿生殖系统。如身材矮小，颅面部畸形包括颅面部过宽、宽平鼻梁、小头、先天性白内障、青光眼、斜视、硬腭高拱及唇腭裂甚至特纳综合征外貌，拇指畸形，先天性心血管发育异常，泌尿生殖器官畸形。存在身体的异常并不能预测患者血液系统疾病的严重程度。30％受累患者可出现生后的生长迟缓，通常伴有其他先天畸形。
- 癌症易感性增加的患者肿瘤发生率约为 4％，高于同年龄正常人群。且肿瘤发生年龄早。包括血液系统急性白血病、骨髓增生异常综合征、淋巴瘤及实体瘤如骨肉瘤、胸腺癌、肝细胞癌、黑色素瘤、纤维组织细胞瘤、胃癌和肠癌等。患肿瘤后，较患同样肿瘤的一般人群预后差，病死率高。

### Qwen2.5_72b

根据您提供的临床表现，最可能的诊断是**范可尼贫血（Fanconi Anemia, FA）**。范可尼贫血是一种罕见的遗传性骨髓衰竭综合征，其主要特征包括骨髓衰竭、先天发育异常和癌症易感性增加。以下是详细的诊断依据：

- **骨髓衰竭**：35%的患儿出生时即表现为贫血，常于生后2周至2年确诊。贫血为大细胞或正细胞正色素性，网织红细胞减少，白细胞正常或轻度降低，血小板正常或轻度增高，骨髓红系增生低下而粒系和巨核细胞系增生活跃。这些表现与范可尼贫血的骨髓衰竭特征相符。
- **先天发育异常**：30%~50%的患者可能存在先天发育异常，主要涉及头部、上肢、心脏和泌尿生殖系统。这些异常包括身材矮小、颅面部畸形、拇指畸形、先天性心血管发育异常和泌尿生殖器官畸形。这些先天性异常在范可尼贫血中较为常见。
- **癌症易感性增加**：患者肿瘤发生率约为4%，高于同年龄正常人群。肿瘤类型包括急性白血病、骨髓增生异常综合征、淋巴瘤及实体瘤如骨肉瘤、胸腺癌、肝细胞癌、黑色素瘤、纤维组织细胞瘤、胃癌和肠癌等。患肿瘤后，预后较差，病死率高。

### 其他可能的诊断

- **先天性角化不良症（Dyskeratosis Congenita, DC）**：这是一种以骨髓衰竭、皮肤色素沉着、甲营养不良和黏膜白斑为特征的遗传性疾病。患者也可能有先天性发育异常和癌症易感性增加。
- **Shwachman-Diamond 综合征（Shwachman-Diamond Syndrome, SDS）**：这是一种以胰腺外分泌功能不全、骨髓衰竭和骨骼异常为特征的遗传性疾病。患者可能有生长迟缓和癌症易感性增加。
- **先天性无巨核细胞性血小板减少症（Congenital Amegakaryocytic Thrombocytopenia, CAT）**：这是一种以血小板减少和骨髓巨核细胞缺乏为特征的遗传性疾病。患者可能有骨髓衰竭和生长迟缓。
- **先天性纯红细胞再生障碍性贫血（Congenital Pure Red Cell Aplasia, CPRCA）**：这是一种以骨髓红系增生低下为特征的遗传性疾病。患者可能有贫血和生长迟缓。
- **Seckel 综合征**：这是一种以生长迟缓、小头畸形和多发性先天性异常为特征的遗传性疾病。患者可能有骨髓衰竭和癌症易感性增加。

这些疾病在临床表现上与范可尼贫血有部分重叠，但通过详细的家族史、基因检测和特定的实验室检查可以进行鉴别诊断。

### 39. User

病变可累及全身各系统，因此其临床表现因受累部位不同而表现各异。一般症状包括发热、乏力、盗汗、体重下降等。其余受累系统主要包括：

- 骨骼系统， 绝大多数患者在诊断时候都有骨骼受累，以下肢骨最为常见，但仅有 50％患者有骨痛表现。
- 心血管系统 ，病变累及心血管系统，多数患者并无明显临床表现，但如心包受累，造成大量心包积液可导致胸闷、憋气甚至心脏压塞表现；病变累及冠状动脉则可造成心肌缺血样表现，如胸闷、胸痛等；弥漫性心肌受累亦有报道，可导致心力衰竭样表现；病变包绕肾动脉则可造成肾性高血压；病变包绕肠系膜血管可有肠道缺血表现，如腹痛等。
- 呼吸系统 呼吸系统受累多数患者无症状，少数患者有干咳、呼吸困难等表现。
- 中枢神经系统 中枢神经系统受累可因不同部位脑实质受累而出现相应的临床表现，如肢体活动障碍、步态异常、意识改变等；脊髓受累可因不同受累节段而出现不同临床表现；垂体病灶则可表现为多饮、多尿以及其他激素分泌减少相关表现。
- 眶后病变 眶后受累为， 常见临床表现之一，表现为单侧或双侧突眼，甚至造成失明。
- 腹膜后空间 / 肾脏受累 ， 腹膜后病变可类似腹膜后纤维化，若病灶包绕输尿管可造成单侧或双侧肾后性梗阻。
- 皮肤受累， 最为常见的皮肤病变为黄瘤样改变，约 1/3 患者发生在眼睑，面部、颈部、腋窝、躯干、腹股沟亦可出现。

### Qwen2.5_72b

根据您提供的临床表现，最可能的诊断是**IgG4相关性疾病（IgG4-related disease, IgG4-RD）**。这是一种全身性自身免疫性疾病，可以影响多个器官系统，包括但不限于骨骼、心血管系统、呼吸系统、中枢神经系统、眶后区域、腹膜后空间和皮肤。其特征是血清IgG4水平升高和受累组织中IgG4阳性浆细胞的浸润。

### 五种其他可能的诊断：

- **Castleman病**：这是一种淋巴组织增生性疾病，可以表现为多系统受累，包括淋巴结肿大、发热、乏力等。根据病变的分布，可以分为单中心型和多中心型。
- **系统性红斑狼疮（SLE）**：这是一种多系统受累的自身免疫性疾病，可以影响皮肤、关节、肾脏、心脏、肺、神经系统等，临床表现多样，包括发热、乏力、皮疹、关节痛、肾炎等。
- **多发性骨髓瘤**：这是一种浆细胞恶性肿瘤，主要影响骨骼系统，可以导致骨痛、骨折、高钙血症、贫血等。多发性骨髓瘤也可以累及肾脏，导致肾功能不全。
- **结节病（Sarcoidosis）**：这是一种多系统肉芽肿性疾病，可以影响肺、淋巴结、皮肤、眼睛、心脏、神经系统等。临床表现包括发热、乏力、干咳、呼吸困难、皮疹等。
- **淋巴瘤**：特别是霍奇金淋巴瘤和非霍奇金淋巴瘤，可以表现为多系统受累，包括淋巴结肿大、发热、盗汗、体重下降、皮肤病变等。

### 诊断思路：

- **详细病史和体格检查**：了解患者的症状、病程、家族史等。
- **实验室检查**：包括血常规、血沉、C反应蛋白、血清IgG4水平、自身抗体谱等。
- **影像学检查**：如X线、CT、MRI、PET-CT等，评估受累器官的范围和程度。
- **组织活检**：对受累组织进行病理学检查，特别是IgG4阳性浆细胞的浸润情况。
- **排除其他疾病**：通过上述检查排除其他可能的多系统受累疾病。

希望这些信息对您有所帮助。如果您有更多具体的临床资料，可以进一步讨论。

### 40. User

病常表现为多器官、多系统受累，男性重于女性患者。临床表现随着年龄增长而常常累及不同的器官。

- 面容， 男性患者多在 12~14 岁出现特征性面容，表现为眶上嵴外凸，额部隆起和嘴唇增厚。
- 皮肤血管角质瘤， 常见于经典型患者，多见于“坐浴区”即脐膝之间的外生殖器、阴囊、臀部和大腿内侧，凸出皮肤表面的红色斑点。
- 神经系统 ，多数患者会出现周围神经疼痛，表现为足底和手掌难以忍受的烧灼感，并放射到四肢近端，甚至出现痛性痉挛；自主神经受累时表现为少汗或无汗；中枢神经系统受累时多表现为早发的短暂性脑缺血发作或缺血性卒中。
- 眼， 特征性的表现包括结膜血管迂曲、角膜涡状混浊、晶状体后囊混浊和视网膜血管迂曲，严重者可导致视力降低甚至丧失。常为女性患者就诊的主要原因之一。
- 消化道 ，多在进食后出现恶心、呕吐、腹胀、痉挛性腹痛和腹泻等症状，也可表现为吸收不良或便秘。
- 肾脏 ， 早期表现为尿浓缩功能障碍如夜尿增多、多尿和遗尿，随病程进展可逐渐出现蛋白尿，甚至达肾病综合征水平，伴随肾功能损害，多在 30 岁左右进入终末期肾病。
- 心血管系统， 可表现为高血压、冠状动脉受累导致的心肌缺血，心脏瓣膜病变和肥厚型心肌病，严重者可表现为心绞痛、心肌梗死和心力衰竭。多为疾病的晚期表现和主要的死亡原因。

### Qwen2.5_72b

根据您提供的临床表现，最可能的诊断是法布里病（Fabry disease）。这是一种X连锁遗传的溶酶体贮积症，由于α-半乳糖苷酶A（α-Gal A）活性缺乏，导致其底物三己糖酰基鞘脂醇（Gb3）在多种组织中积累，引起多系统受累。男性患者通常症状较重，且发病年龄较早，而女性患者症状较轻，且发病年龄较晚。

以下是其他五种可能的诊断：

- **戈谢病（Gaucher disease）**：一种溶酶体贮积症，由于葡萄糖脑苷脂酶缺乏导致葡萄糖脑苷脂在多种组织中积累，引起肝脾肿大、骨痛、贫血等症状。
- **尼曼-皮克病（Niemann-Pick disease）**：一种溶酶体贮积症，由于鞘磷脂酶缺乏导致鞘磷脂在多种组织中积累，引起肝脾肿大、神经系统受累等症状。
- **黏多糖贮积症（Mucopolysaccharidoses, MPS）**：一组溶酶体贮积症，由于多种酶缺乏导致黏多糖在多种组织中积累，引起多系统受累，包括骨骼异常、心血管病变、神经系统受累等。
- **安德森-法布里病（Anderson-Fabry disease）**：虽然名称相似，但与法布里病不同，这是一种罕见的代谢性疾病，表现为多系统受累，但具体机制和临床表现有所不同。
- **线粒体脑肌病（Mitochondrial encephalomyopathy）**：一组由线粒体功能障碍引起的疾病，表现为多系统受累，包括神经系统、肌肉、心脏、肾脏等，常见症状包括肌无力、运动障碍、视力和听力下降等。

这些疾病都有多系统受累的表现，但具体的临床特征和实验室检查结果可以帮助进一步鉴别诊断。

### 41. User

疾病分为 1 型和 2 型。

- 1 型 主要表现为反复发作的炎性反应和浆膜炎，包括反复发热、腹痛及关节炎。发热反复发作，可有寒冷、剧烈运动、手术、感染等诱因，持续 13 天自行缓解。腹痛为最常见临床表现，见于 95％的患者。表现为突然发作的全腹痛，常伴发热，腹部查体可有腹胀，腹部压痛、反跳痛、肌紧张，听诊肠鸣音消失。立位腹平片可见小肠气液平。腹痛常在 24-48 小时后自行缓解。关节炎亦为常见临床表现，见于 75％的患者。常累及下肢大关节，如髋关节、膝关节。可有关节肿胀、疼痛。皮疹见于 7％ ~40％的患者，主要累及下肢伸侧，典型皮疹为丹毒样红斑。胸膜炎见于 45％的患者，表现为突然发作的单侧胸痛，常伴发热。心包炎较少见。
- 2 型 以淀粉样变起病。表现为持续大量的蛋白尿，常致终末期肾病。淀粉样物质亦可在心脏、胃肠道、肝脏等器官沉积，并导致相应临床表现。

### Qwen2.5_72b

根据您提供的临床表现，1 型和 2 型疾病的描述，最可能的诊断是家族性地中海热（Familial Mediterranean Fever, FMF）。这是一种遗传性自身炎症性疾病，主要影响地中海沿岸地区的人群，如土耳其、亚美尼亚、阿拉伯和犹太人。1 型的临床表现与 FMF 的典型症状非常吻合，包括反复发作的发热、腹痛、关节炎、胸膜炎和心包炎等。2 型的描述则符合 FMF 患者中较少见的淀粉样变性并发症，这通常发生在未经治疗或治疗不当的 FMF 患者中，导致肾功能衰竭。

### 其他可能的诊断：

- **周期性发热综合征（Periodic Fever Syndromes）**：如 TNF 受体相关周期性综合征（TRAPS）、高 IgD 综合征（HIDS）等，这些疾病也表现为反复发作的发热和炎症反应，但具体症状和触发因素可能有所不同。
- **系统性红斑狼疮（Systemic Lupus Erythematosus, SLE）**：SLE 也可以表现为反复发热、关节炎、胸膜炎和腹痛，但通常伴有其他自身免疫性疾病的特征，如皮疹、口腔溃疡、光敏感等。
- **炎症性肠病（Inflammatory Bowel Disease, IBD）**：如克罗恩病和溃疡性结肠炎，这些疾病可以引起腹痛、发热和关节炎，但通常伴有消化道症状，如腹泻、便血等。
- **感染性疾病**：如结核病、布鲁氏菌病等，这些疾病也可以引起反复发热、腹痛和关节炎，但通常有明确的感染证据，如病原体培养阳性。
- **肿瘤性疾病**：如淋巴瘤，特别是霍奇金淋巴瘤，可以表现为反复发热、腹痛和淋巴结肿大，但通常伴有其他肿瘤相关的症状，如体重下降、夜间盗汗等。

这些诊断需要通过详细的病史采集、体格检查和实验室检查来进一步确认。

### 42. User

主要表现为先天性发育异常，进行性骨髓衰竭和肿瘤发生率增高，也有患者无发育异常或骨髓衰竭表现。

- 发育异常 ，患者可以表现为多种形体和智力发育异常。生长缺陷、异常皮肤色素沉着、上肢单侧或双侧骨骼畸形、体格矮小、小头畸形、眼部异常、泌尿生殖道畸形、内分泌异常、听力损失、外耳异常、先天性心脏缺陷、消化道异常、中枢神经系统发育异常及面部畸形、脊柱畸形、颈部畸形及发育迟缓和（或） 智力残疾等。这些表现多为非特异性，拇指及上肢畸形较为特异。
- 进行性骨髓衰竭 ，血液系统的异常为范科尼贫血最严重的临床特征。可表现为单一或全血细胞减少。血细胞减少起病年龄差异较大，即使同胞发病年龄也不一。中位发病年龄约为 7.6 岁，罕见于婴儿和幼童，90％患者的血液学异常在 40 岁前发病。血小板减少或白细胞减少通常先于贫血出现，常伴随大红细胞，胎儿血红蛋白（HbF 1 ） 增加，全血细胞减少逐渐加重。
- 肿瘤发生风险增高， 包括血液系统恶性疾病及非血液系统恶性实体瘤。急性髓系白血病（AML） 发生危险增加约 500 倍，50 岁时 AML 累计发生率约 13％，多发生于15-35 岁。-7，del（7q） 与骨髓增生异常综合征（MDS）/AML 发生率增高相关。急性淋巴细胞白血病和淋巴瘤也有报道。实体肿瘤可以是该疾病的首发表现。头颈部鳞状细胞癌（HNSCCs） 是 患者最常见的实体瘤，而且发病年龄更早（20~40 岁），大多数发生于口腔（如舌癌），多处于进展期，对治疗反应差。皮肤、食管癌、肝肿瘤和泌尿生殖道肿瘤发生率也增加。少数患者可见到多种肿瘤并发，或者血液系统及非血液系统肿瘤并发。而且对化疗或放疗明显不耐受。

### Qwen2.5_72b

根据您提供的临床表现，最可能的诊断是范科尼贫血（Fanconi Anemia, FA）。这是一种罕见的遗传性血液病，主要特征包括先天性发育异常、进行性骨髓衰竭和肿瘤发生风险增高。以下是其他五种可能的诊断：

- **先天性角化不良症（Dyskeratosis Congenita, DC）**：
  - 也表现为先天性发育异常、进行性骨髓衰竭和肿瘤发生风险增高。特征性表现包括网状皮肤色素沉着、甲营养不良和口腔黏膜白斑。
- **Shwachman-Diamond 综合征（Shwachman-Diamond Syndrome, SDS）**：
  - 主要表现为胰腺功能不全、生长发育迟缓和中性粒细胞减少。部分患者可出现骨髓衰竭和肿瘤发生风险增高。
- **先天性无巨核细胞性血小板减少症（Congenital Amegakaryocytic Thrombocytopenia, CAMT）**：
  - 主要表现为血小板减少和进行性骨髓衰竭，但通常不伴有明显的发育异常或肿瘤发生风险增高。
- **Seckel 综合征**：
  - 以严重的生长发育迟缓、小头畸形和智力残疾为特征，部分患者可出现骨髓衰竭，但肿瘤发生风险增高不常见。
- **Werner 综合征**：
  - 一种早老症，表现为早发性白内障、皮肤硬化、糖尿病、动脉硬化和肿瘤发生风险增高，但通常不伴有明显的先天性发育异常或骨髓衰竭。

范科尼贫血是最符合您描述的临床表现的疾病，但其他几种疾病也应考虑在鉴别诊断中。建议进行进一步的基因检测和血液学检查以明确诊断。

### 43. User

经典型患儿常在围生期发病，在摄取母乳或含乳糖配方奶粉数天内，患儿会出现危及生命的并发症：喂养问题、腹泻、呕吐、低血糖、肝功能损伤、出血、黄疸、 白内障。如果未及时治疗，可能会发生败血症、休克和死亡。存活至婴儿期的患儿，如果继续摄取乳糖，可能会出现严重的脑损伤。一些患儿有智力落后、生长发育延迟、共济失调、失明以及女性患者的卵巢功能障碍等远期并发症。

### Qwen2.5_72b

根据您提供的临床表现，最可能的诊断是**半乳糖血症**（Galactosemia）。这是一种罕见的遗传性代谢病，由于半乳糖代谢途径中的酶缺陷导致半乳糖及其代谢产物在体内积累，引起一系列严重的临床症状。

### 最可能的诊断：

- **半乳糖血症**（Galactosemia）

### 其他可能的诊断：

- **先天性代谢异常**（如枫糖尿症、苯丙酮尿症等）
- **新生儿败血症**（Neonatal Sepsis）
- **新生儿肝炎**（Neonatal Hepatitis）
- **先天性甲状腺功能减退症**（Congenital Hypothyroidism）
- **先天性乳糖不耐受**（Congenital Lactose Intolerance）

### 详细解释：

- **半乳糖血症**：
  - **病因**：由于半乳糖-1-磷酸尿苷转移酶（GALT）缺乏，导致半乳糖代谢障碍。
  - **临床表现**：包括喂养问题、腹泻、呕吐、低血糖、肝功能损伤、出血、黄疸、白内障等。如果不及时治疗，可能会导致败血症、休克和死亡。长期摄取乳糖会导致严重的脑损伤、智力落后、生长发育延迟、共济失调、失明以及女性患者的卵巢功能障碍。
- **先天性代谢异常**：
  - **病因**：多种遗传性代谢病，如枫糖尿症、苯丙酮尿症等。
  - **临床表现**：类似半乳糖血症，包括喂养问题、呕吐、腹泻、低血糖、肝功能损伤等。
- **新生儿败血症**：
  - **病因**：细菌感染。
  - **临床表现**：包括喂养问题、呕吐、腹泻、低血糖、肝功能损伤、黄疸、败血症、休克等。
- **新生儿肝炎**：
  - **病因**：病毒感染或代谢异常。
  - **临床表现**：包括黄疸、肝功能损伤、喂养问题、呕吐、腹泻等。
- **先天性甲状腺功能减退症**：
  - **病因**：甲状腺激素合成或分泌不足。
  - **临床表现**：包括喂养问题、生长发育延迟、黄疸、低血糖等。
- **先天性乳糖不耐受**：
  - **病因**：乳糖酶缺乏。
  - **临床表现**：包括喂养问题、腹泻、呕吐等。

### 诊断建议：

- **新生儿筛查**：早期进行新生儿筛查，特别是半乳糖血症的筛查。
- **血液检查**：检测半乳糖、半乳糖-1-磷酸、GALT酶活性等。
- **基因检测**：确认GALT基因突变。
- **肝功能检查**：评估肝功能损伤程度。
- **代谢产物检测**：检测尿液中的代谢产物。

希望这些信息对您有所帮助。如果有更多详细信息或需要进一步的诊断建议，请随时告知。

### 44. User

- Ⅰ型最常见亚型。无原发性中枢神经系统受累表现，一些患者随着疾病进展可能出现继发神经系统临床表现（如脊髓受压等）。各年龄段均可发病，约 2/3 患者在儿童期发病。脏器表现主要为肝脾肿大，尤以脾肿大显著，常伴脾功能亢进，甚至出现脾梗死、脾破裂等。血液学主要表现为血小板减少和贫血，部分患者白细胞减少，可伴有凝血功能异常。患者表现为面色苍白、疲乏无力、皮肤及牙龈出血、月经增多，甚至出现危及生命的出血现象。多数患者有骨骼受侵，但轻重不一。早期受侵犯部位主要包括腰椎、长骨干骺端和骨干，中后期可累及骨骺和骨突。患者常有急性或慢性弥漫性骨痛，严重者可出现骨坏死（缺血性坏死），随后可出现关节塌陷累及股骨近端和远端、胫骨近端和肱骨近端。还可发生溶骨性病变、病理性骨折、椎体压缩骨折及其他因骨密度降低导致的脆性骨折。骨骼病变可影响日常活动，并可致残。儿童患者常见的表现依次是骨质疏松、长骨干骺端烧瓶样畸形、长骨干骺端密度不同程度的减低、骨皮质变薄等，可有生长发育迟缓。部分患者可有肺部受累，主要表现为间质性肺病、肺实变、肺动脉高压等。此外，患者还会出现糖和脂类代谢异常、多发性骨髓瘤等恶性肿瘤发病风险增高、胆石症、免疫系统异常等表现。
- Ⅱ型，Ⅱ型患者除有与Ⅰ型相似的肝脾肿大、贫血、血小板减少等表现外，主要为急性神经系统受累表现。常发病于新生儿期至婴儿期，进展较快，病死率高。有迅速进展的延髓麻痹、动眼障碍、癫痫发作、角弓反张及认知障碍等急性神经系统受损表现，生长发育落后，2~4 岁前死亡。一些重度患者会出现关节挛缩。
- Ⅲ型早期表现与Ⅰ型相似，逐渐出现神经系统受累表现，常发病于儿童期，病情进展缓慢，寿命可较长。患者常有动眼神经受侵、眼球运动障碍，并有共济失调、角弓反张、癫痫、肌阵挛，伴发育迟缓、智力落后。Ⅲ型可分为 3 种亚型，即以较快进展的神经系统症状（眼球运动障碍、小脑共济失调、痉挛、肌阵挛及痴呆） 及肝脾肿大为主要表现的Ⅲa 型；以肝脾肿大及骨骼症状为主要表现而中枢神经系统症状较少的Ⅲb 型；其他症状较轻，以心脏瓣膜钙化及角膜混浊为特殊表现， 主要出现在德鲁兹人群的Ⅲc型。

### Qwen2.5_72b

根据您提供的临床表现，最可能的诊断是戈谢病（Gaucher Disease, GD），这是一种罕见的遗传性代谢病，主要由于葡萄糖脑苷脂酶（glucocerebrosidase）缺乏导致葡萄糖脑苷脂在体内蓄积，引起多系统受累。根据您的描述，可以进一步分为以下亚型：

- **戈谢病Ⅰ型**：这是最常见的亚型，无原发性中枢神经系统受累表现，但随着疾病进展可能出现继发神经系统临床表现。主要表现为肝脾肿大、血小板减少、贫血、骨骼受侵、生长发育迟缓等。部分患者可有肺部受累、糖和脂类代谢异常、多发性骨髓瘤等恶性肿瘤发病风险增高、胆石症、免疫系统异常等表现。
- **戈谢病Ⅱ型**：主要表现为急性神经系统受累，常发病于新生儿期至婴儿期，进展较快，病死率高。有迅速进展的延髓麻痹、动眼障碍、癫痫发作、角弓反张及认知障碍等急性神经系统受损表现，生长发育落后，2~4 岁前死亡。一些重度患者会出现关节挛缩。
- **戈谢病Ⅲ型**：早期表现与Ⅰ型相似，逐渐出现神经系统受累表现，常发病于儿童期，病情进展缓慢，寿命可较长。患者常有动眼神经受侵、眼球运动障碍，并有共济失调、角弓反张、癫痫、肌阵挛，伴发育迟缓、智力落后。Ⅲ型可分为 3 种亚型，即Ⅲa 型、Ⅲb 型和Ⅲc 型。

### 其他可能的诊断：

- **尼曼-皮克病（Niemann-Pick Disease）**：这是一种溶酶体贮积症，主要由于鞘磷脂酶缺乏导致鞘磷脂在体内蓄积。临床表现包括肝脾肿大、神经系统受累、肺部受累等，与戈谢病有部分相似之处。
- **法布里病（Fabry Disease）**：这是一种X连锁遗传性溶酶体贮积症，主要由于α-半乳糖苷酶A缺乏导致鞘糖脂在体内蓄积。临床表现包括疼痛、皮肤病变、肾功能不全、心脏病变等，部分患者可有神经系统受累。
- **黏多糖贮积症（Mucopolysaccharidoses, MPS）**：这是一组溶酶体贮积症，主要由于溶酶体酶缺乏导致黏多糖在体内蓄积。临床表现包括肝脾肿大、骨骼异常、心脏病变、神经系统受累等。
- **克罗恩病（Krabbe Disease）**：这是一种溶酶体贮积症，主要由于半乳糖脑苷脂酶缺乏导致半乳糖脑苷脂在体内蓄积。临床表现包括神经系统受累、肌张力异常、发育迟缓等，常在婴儿期发病，进展迅速。
- **异染性脑白质营养不良（Metachromatic Leukodystrophy, MLD）**：这是一种溶酶体贮积症，主要由于芳基硫酸酯酶A缺乏导致硫酸脑苷脂在体内蓄积。临床表现包括神经系统受累、运动障碍、认知障碍等，常在儿童期发病，进展缓慢。

这些疾病在临床表现上有一定的重叠，但通过详细的病史、体格检查、实验室检查（如酶活性测定、基因检测等）可以进行鉴别诊断。

### 45. User

经常从一组肌群开始，逐渐累及其他肌群，以上睑下垂和复视为最常见的发病症状， 占 80％。骨骼肌无力表现为波动性和易疲劳性，晨轻暮重。根据临床表现，分成五型。Ⅰ型：眼肌型，表现为上睑下垂、复视等。ⅡA 型：轻度全身型，四肢肌群轻度受累。ⅡB 型：中度全身型，四肢肌群中度受累，通常有咀嚼、吞咽和构音障碍。Ⅲ型： 重度激进型，进展快，数周或数月内累及咽喉肌，半年内累及呼吸肌。Ⅳ型：迟发重度型，缓慢进展，2 年内累及呼吸肌。Ⅴ型：肌萎缩型，起病半年内出现骨骼肌萎缩。神经科查体可见四肢近端、躯干、颈部、颅面部肌肉无力，不耐疲劳，Jolly 试验阳性，腱反射正常，无感觉障碍，无病理征。部分病人可进展至呼吸衰竭，出现重症肌无力危象。

### Qwen2.5_72b

根据您提供的临床表现，最可能的诊断是重症肌无力（Myasthenia Gravis, MG）。这是一种自身免疫性疾病，主要影响神经肌肉接头，导致骨骼肌易疲劳和波动性无力。MG 的典型症状包括上睑下垂、复视、四肢肌群的无力和疲劳，且症状常表现为晨轻暮重。根据您的描述，可以进一步分为以下五型：

- **眼肌型（Ⅰ型）**：主要表现为上睑下垂和复视。
- **轻度全身型（ⅡA型）**：四肢肌群轻度受累。
- **中度全身型（ⅡB型）**：四肢肌群中度受累，常伴有咀嚼、吞咽和构音障碍。
- **重度激进型（Ⅲ型）**：进展迅速，数周或数月内累及咽喉肌，半年内累及呼吸肌。
- **迟发重度型（Ⅳ型）**：缓慢进展，2年内累及呼吸肌。
- **肌萎缩型（Ⅴ型）**：起病半年内出现骨骼肌萎缩。

### 其他可能的诊断

- **Lambert-Eaton 肌无力综合征（LEMS）**：
  - 也是一种神经肌肉接头疾病，但与 MG 不同，LEMS 通常表现为肌无力和肌疲劳，但晨轻暮重不明显。LEMS 常见于伴有小细胞肺癌的患者。
- **多发性肌炎（Polymyositis, PM）**：
  - 一种自身免疫性炎症性肌病，主要表现为对称性近端肌群无力，常伴有肌肉疼痛和压痛。肌电图和肌肉活检有助于诊断。
- **皮肌炎（Dermatomyositis, DM）**：
  - 与多发性肌炎类似，但伴有特征性的皮肤病变，如紫红色皮疹、Gottron丘疹等。
- **线粒体肌病**：
  - 一种遗传性代谢性疾病，表现为慢性进行性肌无力和易疲劳，常伴有其他系统受累，如心脏、神经系统等。
- **慢性炎症性脱髓鞘性多发性神经病（CIDP）**：
  - 一种慢性自身免疫性周围神经病，表现为对称性四肢无力和感觉障碍，进展缓慢，电生理检查有助于诊断。

这些疾病在临床表现上可能有重叠，但通过详细的病史、体格检查、实验室检查和电生理检查可以进行鉴别诊断。

### 46. User

常于青少年或成年早期起病。临床表现主要与低血钾和低血镁相关，轻型患者可无症状或表现为轻度乏力和纳差；严重患者会出现四肢抽搐、软瘫、痛性痉挛、晕厥和横纹肌溶解继发急性肾损伤，甚至因为严重室性心律失常导致心搏骤停。常见临床表现如下：

- 全身症状 疲乏、口渴、多饮、嗜盐。
- 神经 - 肌肉系统 肌无力、痛性痉挛、抽搐、惊厥发作、肢体麻木、感觉异常、横纹肌溶解、瘫痪、头晕、眩晕、共济失调和假性脑瘤等。
- 心血管系统 心悸、晕厥、血压正常或偏低和室性心律失常等。
- 消化系统 便秘、呕吐等。
- 泌尿系统 多尿、夜尿增多、遗尿、蛋白尿和肾功能不全。
- 骨关节系统 关节痛、软骨钙质沉着症。
- 内分泌和生长发育 生长迟缓、青春期延迟；长期低钾和低镁的患者糖尿病或者糖耐量减低的比例并不少见。
- 眼部症状 少数患者会出现视物模糊和巩膜脉络膜钙化。

### Qwen2.5_72b

根据您提供的临床表现，最可能的诊断是**Gitelman综合征**。这是一种罕见的遗传性肾小管疾病，主要特征是肾小管对钠、钾、镁和氯的重吸收障碍，导致低血钾、低血镁和代谢性碱中毒。

### 1. Gitelman综合征

- **遗传方式**：常染色体隐性遗传。
- **主要特征**：
  - 低血钾（<3.5 mmol/L）
  - 低血镁（<0.7 mmol/L）
  - 代谢性碱中毒
  - 低尿钙
  - 高血压不常见
- **临床表现**：
  - 疲乏、乏力
  - 肌无力、痛性痉挛、抽搐
  - 心悸、晕厥、室性心律失常
  - 便秘、呕吐
  - 多尿、夜尿增多
  - 关节痛、软骨钙质沉着症
  - 生长迟缓、青春期延迟
  - 糖尿病或糖耐量减低
  - 视物模糊和巩膜脉络膜钙化

### 2. 其他可能的诊断

#### 2.1 Bartter综合征

- **遗传方式**：常染色体隐性遗传。
- **主要特征**：
  - 低血钾
  - 低血氯
  - 代谢性碱中毒
  - 高尿钙
  - 高血压不常见
- **临床表现**：
  - 疲乏、乏力
  - 肌无力、痛性痉挛、抽搐
  - 心悸、晕厥、室性心律失常
  - 便秘、呕吐
  - 多尿、夜尿增多
  - 生长迟缓、青春期延迟

#### 2.2 Liddle综合征

- **遗传方式**：常染色体显性遗传。
- **主要特征**：
  - 高血压
  - 低血钾
  - 代谢性碱中毒
  - 低尿钾
- **临床表现**：
  - 高血压
  - 疲乏、乏力
  - 肌无力、痛性痉挛、抽搐
  - 心悸、晕厥、室性心律失常
  - 便秘、呕吐
  - 多尿、夜尿增多

#### 2.3 原发性醛固酮增多症

- **主要特征**：
  - 高血压
  - 低血钾
  - 代谢性碱中毒
- **临床表现**：
  - 高血压
  - 疲乏、乏力
  - 肌无力、痛性痉挛、抽搐
  - 心悸、晕厥、室性心律失常
  - 便秘、呕吐
  - 多尿、夜尿增多

#### 2.4 肾小管酸中毒

- **主要特征**：
  - 代谢性酸中毒
  - 低血钾
  - 低血镁
  - 高尿钙
- **临床表现**：
  - 疲乏、乏力
  - 肌无力、痛性痉挛、抽搐
  - 心悸、晕厥、室性心律失常
  - 便秘、呕吐
  - 多尿、夜尿增多
  - 肾结石、肾钙化

#### 2.5 艾迪生病（Addison病）

- **主要特征**：
  - 低血钾
  - 低血压
  - 皮肤色素沉着
- **临床表现**：
  - 疲乏、乏力
  - 肌无力、痛性痉挛、抽搐
  - 心悸、晕厥、室性心律失常
  - 便秘、呕吐
  - 皮肤色素沉着
  - 低血压

这些疾病都有类似的临床表现，但通过详细的病史、体格检查和实验室检查可以进行鉴别诊断。特别是电解质水平、尿液分析、肾功能检查和基因检测等，可以帮助确定最终诊断。

### 47. User

大多数患儿于婴幼儿期发病，表现多样。发病越早，症状越重，预后越差。大部分患者生后 1 年内表现相对正常，约 75％的患者最早出现的体征是头大，多数患儿出生时头围较同龄儿大或生后不久头围迅速增大，可伴轻微的非特异性症状，包括易激惹、喂养困难和呕吐等。患儿通常生后 3~36 月龄间，在发热、感染、手术或预防接种等诱因后出现酮症、呕吐、肝大和急性脑病危象表现，包括肌张力低下、意识丧失和惊厥发作等，对症治疗后症状可缓解，但不能完全恢复。如果急性脑病危象反复发生，神经系统损伤将进行性加重，可有发育倒退现象，最终可出现认知功能障碍。部分患儿在生后数年逐渐出现运动延缓、肌张力异常和随意运动障碍，但智力发育基本正常。患者常在 10 岁内死于伴发疾病或 Reye 样发作，随年龄增长发作减少。极少数患者于青春期甚至成年时期发病，首次发病之前可无症状。

### Qwen2.5_72b

根据您提供的临床表现，最可能的诊断是**Canavan病**，这是一种罕见的遗传性神经退行性疾病，主要影响大脑的白质。然而，由于症状的多样性和复杂性，还有其他几种可能的诊断需要考虑：

- **Reye综合征**：这是一种急性脑病和肝功能障碍的综合征，通常发生在病毒感染后，特别是在使用水杨酸盐（如阿司匹林）治疗后。症状包括呕吐、意识丧失、肝大和酮症。
- **线粒体脑肌病**：这是一组由线粒体功能障碍引起的疾病，可以表现为肌张力低下、发育迟缓、肝大和酮症。症状通常在婴幼儿期出现，且反复发作。
- **糖原贮积病（如Pompe病）**：这是一种由于糖原代谢酶缺陷导致的疾病，可以表现为肌张力低下、肝大、发育迟缓和酮症。症状通常在婴幼儿期出现，且病情进展迅速。
- **先天性代谢异常（如有机酸血症）**：这是一组由于代谢途径缺陷导致的疾病，可以表现为酮症、呕吐、肝大和急性脑病危象。症状通常在婴幼儿期出现，且反复发作。
- **Alexander病**：这是一种罕见的神经退行性疾病，主要影响大脑的白质。症状包括头大、肌张力低下、发育迟缓和反复发作的急性脑病危象。

这些疾病都需要通过详细的病史、体格检查和实验室检查（如基因检测、代谢筛查、脑影像学检查等）来进一步确诊。如果您需要更详细的诊断建议或治疗方案，请提供更多的临床信息。

### 48. User

主要表现为关节、肌肉和深部组织出血，也可表现为胃肠道、中枢神经系统等内部脏器出血等。若反复出血，不及时治疗可导致关节畸形和(或) 假肿瘤形成，严重者可危及生命。外伤或手术后延迟性出血是本病的特点。

### Qwen2.5_72b

根据您提供的临床表现，最可能的诊断是血友病（Hemophilia）。血友病是一种遗传性凝血功能障碍的出血性疾病，主要表现为关节、肌肉和深部组织出血，以及胃肠道、中枢神经系统等内部脏器出血。反复出血可导致关节畸形和假肿瘤形成，严重者可危及生命。外伤或手术后延迟性出血是血友病的典型特点。

以下是其他五种可能的诊断：

- **血管性血友病（Von Willebrand Disease, VWD）**：这是一种常见的遗传性出血性疾病，由于血管性血友病因子（vWF）的缺乏或功能异常导致。临床表现与血友病类似，包括关节、肌肉和深部组织出血，以及鼻出血、牙龈出血等。
- **获得性血友病（Acquired Hemophilia）**：这是一种罕见的自身免疫性疾病，由于体内产生了针对凝血因子的自身抗体，导致凝血功能障碍。临床表现与遗传性血友病相似，但通常发生在没有家族史的患者中。
- **血小板功能障碍（Platelet Function Disorders）**：包括血小板无力症（Glanzmann’s thrombasthenia）和储存池病（storage pool disease）等，这些疾病导致血小板功能异常，从而引起出血倾向。临床表现包括皮肤瘀斑、鼻出血、牙龈出血等。
- **弥散性血管内凝血（Disseminated Intravascular Coagulation, DIC）**：这是一种严重的凝血功能障碍，通常继发于其他疾病，如感染、肿瘤、产科并发症等。临床表现包括多部位出血和血栓形成，严重者可导致多器官功能衰竭。
- **纤维蛋白原缺乏症（Fibrinogen Deficiency）**：这是一种罕见的遗传性凝血障碍，由于纤维蛋白原的缺乏或功能异常导致。临床表现包括出血倾向，如关节、肌肉和深部组织出血，以及手术后出血等。

这些疾病都需要通过详细的病史、体格检查和实验室检查来确诊。如果您怀疑患者有上述任何一种疾病，建议进行进一步的检查和评估。

### 49. User

可累及全身多个脏器，临床表现多样。发病年龄多为 3~60 岁，也有8 个月及 70 多岁诊断的患者报道。儿童患者多以肝脏受累为首发表现，青少年及成人以神经系统受累为首发症状的比例增高。

可表现为无症状的转氨酶持续升高、慢性肝炎、肝硬化和急性肝功能衰竭。神经系统受累可表现为运动功能障碍、震颤、共济失调、舞蹈症、自主运动障碍、肌张力障碍，一些患者表现为面具脸、四肢僵硬、步态异常等。一些患者合并精神行为异常，如淡漠、攻击行为、性格改变等。肾脏受累可表现为血尿、蛋白尿等。血液系统受累可表现为溶血性贫血、肝硬化、脾功能亢进致血液三系下降、凝血功能异常等。相对少见的受累系统还包括骨关节、心脏、内分泌和生殖系统等。

### Qwen2.5_72b

根据您提供的临床表现，最可能的诊断是**威尔森病（Wilson病）**。威尔森病是一种罕见的遗传性铜代谢障碍疾病，主要影响肝脏和神经系统，但也可累及其他多个器官系统。该病通常在3-60岁之间发病，但也有更年轻或更年老的病例报道。肝脏受累可表现为无症状的转氨酶升高、慢性肝炎、肝硬化甚至急性肝功能衰竭。神经系统受累可表现为运动障碍、震颤、共济失调、舞蹈症、肌张力障碍等，一些患者还可能出现面具脸、四肢僵硬、步态异常等帕金森病样症状。此外，威尔森病患者还可能出现精神行为异常、肾脏受累（如血尿、蛋白尿）、血液系统受累（如溶血性贫血、肝硬化、脾功能亢进致血液三系下降、凝血功能异常）等。

以下是其他五种可能的诊断：

- **线粒体病**：线粒体病是一组由于线粒体功能障碍导致的遗传性疾病，可累及多个器官系统，包括神经系统、肝脏、心脏、肌肉、肾脏等。临床表现多样，可包括运动障碍、肌张力异常、肝功能异常、肾功能异常等。
- **肝豆状核变性（Hepatolenticular degeneration）**：这是一种与威尔森病非常相似的疾病，也是由于铜代谢障碍导致的，主要影响肝脏和神经系统。临床表现与威尔森病类似，包括肝功能异常、运动障碍、震颤、共济失调等。
- **多系统萎缩（Multiple System Atrophy, MSA）**：MSA是一种进行性神经系统退行性疾病，主要影响自主神经系统、小脑和基底节。临床表现包括运动障碍、自主神经功能障碍、共济失调、帕金森病样症状等。
- **自身免疫性肝病**：自身免疫性肝病包括自身免疫性肝炎、原发性胆汁性胆管炎和原发性硬化性胆管炎等，可表现为慢性肝炎、肝硬化、肝功能异常等。部分患者可合并神经系统受累，表现为运动障碍、肌张力异常等。
- **溶酶体贮积症（Lysosomal Storage Disorders）**：溶酶体贮积症是一组由于溶酶体酶缺陷导致的遗传性疾病，可累及多个器官系统，包括神经系统、肝脏、心脏、骨骼等。临床表现多样，可包括肝脾肿大、肝功能异常、神经系统受累（如运动障碍、肌张力异常、共济失调等）。

这些疾病在临床表现上有一定的重叠，因此需要通过详细的病史、体格检查、实验室检查（如血清铜蓝蛋白、24小时尿铜、基因检测等）以及影像学检查（如MRI、CT等）来进一步明确诊断。

### 50. User

临床表现具有很大的异质性，甚至同一家系中的患者也有很大差异。 通常在 30 岁前起病，青春期加重，水肿常呈急性发作。临床上以反复发作、难以预测的皮肤和黏膜下水肿为特征。水肿的特点呈发作性，自限性，一般 3~5 天自然缓解，非对称性，非可凹性。水肿可累及身体任何部位，以四肢、颜面、生殖器、呼吸道和胃肠道黏膜较为常见。其中最致命的是呼吸道黏膜水肿，可因喉水肿迅速进展导致呼吸困难或窒息， 如抢救不及时可窒息死亡，致死率高达 11％ ~40％，是 主要死因之一。消化道黏膜水肿发作表现为剧烈腹痛，伴恶心、呕吐，因此常被误诊为急腹症，导致不必要的腹部手术。发生于肢体等部位的皮肤水肿则会影响其功能。

### Qwen2.5_72b

根据您提供的临床表现，最可能的诊断是**遗传性血管性水肿（Hereditary Angioedema, HAE）**。这是一种罕见的遗传性疾病，主要由于C1酯酶抑制剂（C1-INH）的功能或数量缺陷导致。这种疾病的特点是反复发作的皮肤和黏膜下水肿，尤其是四肢、颜面、生殖器、呼吸道和胃肠道黏膜，且水肿呈急性发作，自限性，非对称性，非可凹性。其中，呼吸道黏膜水肿是最致命的并发症，可迅速进展导致呼吸困难或窒息，致死率较高。消化道黏膜水肿则常表现为剧烈腹痛，伴恶心、呕吐，容易被误诊为急腹症。

以下是其他五种可能的诊断：

- **获得性血管性水肿（Acquired Angioedema, AAE）**：这是一种与C1酯酶抑制剂功能障碍相关的获得性疾病，可能与自身免疫性疾病、淋巴增生性疾病或某些药物（如ACE抑制剂）有关。临床表现与HAE相似，但通常在成年后发病。
- **特发性血管性水肿（Idiopathic Angioedema）**：这是一种原因不明的血管性水肿，可能与过敏反应、食物或药物过敏有关。临床表现与HAE相似，但通常没有家族史。
- **荨麻疹性血管性水肿（Urticarial Angioedema）**：这是一种与荨麻疹同时发生的血管性水肿，可能与过敏反应、感染、自身免疫性疾病或某些药物有关。临床表现包括皮肤红斑、风团和水肿，但通常不累及呼吸道和消化道黏膜。
- **药物诱发的血管性水肿（Drug-Induced Angioedema）**：某些药物，如ACE抑制剂、非甾体抗炎药（NSAIDs）等，可引起血管性水肿。临床表现与HAE相似，但通常有明确的用药史。
- **过敏性血管性水肿（Allergic Angioedema）**：这是一种由过敏反应引起的血管性水肿，可能与食物、药物、昆虫叮咬等过敏原有关。临床表现包括皮肤红斑、风团和水肿，但通常不累及呼吸道和消化道黏膜。

这些诊断需要通过详细的病史采集、体格检查和实验室检查来进一步明确。特别是对于HAE，可以通过检测C1酯酶抑制剂的水平和功能来确诊。

### 51. User

出生即起病，临床表现的轻重与疾病亚型相关。临床表现为手部、肘部、膝及足部和其他容易摩擦的部位或周身出现水疱或大疱。可以出现口腔黏膜水疱或溃疡，导致吞咽困难和声音嘶哑。部分患者伴有斑驳性色素异常、秃发、掌跖角化过度、牙釉质缺陷、甲增厚和甲营养不良。其他损害包括反复瘢痕形成导致的指端残毁、贫血、骨质疏松、肌肉萎缩、心肌病、耳聋。部分亚型晚期可伴发基底细胞癌、鳞状细胞癌及恶性黑色素瘤。

### Qwen2.5_72b

根据您提供的临床表现，最可能的诊断是**大疱性表皮松解症（Epidermolysis Bullosa, EB）**。这是一种罕见的遗传性皮肤病，主要特征是皮肤和黏膜在轻微摩擦或创伤后容易形成水疱和大疱。根据临床表现的严重程度和遗传方式，EB可以分为多个亚型，包括单纯型EB（EBS）、交界型EB（JEB）、营养不良型EB（DEB）和Kindler综合征。

### 其他可能的诊断：

- **遗传性大疱性表皮松解症（Dystrophic Epidermolysis Bullosa, DEB）**：
  - 与您描述的临床表现非常相似，但通常伴有更严重的瘢痕形成和甲营养不良。
- **交界性大疱性表皮松解症（Junctional Epidermolysis Bullosa, JEB）**：
  - 通常在新生儿期起病，表现为广泛的水疱和大疱，伴有严重的黏膜受累和高死亡率。
- **单纯性大疱性表皮松解症（Epidermolysis Bullosa Simplex, EBS）**：
  - 通常在手部和足部受累较重，但黏膜受累较轻，预后相对较好。
- **Kindler综合征**：
  - 一种罕见的EB亚型，表现为皮肤光敏感、反复水疱和大疱、掌跖角化过度和甲营养不良。
- **线状IgA大疱性皮肤病（Linear IgA Bullous Dermatosis）**：
  - 一种自身免疫性疾病，表现为水疱和大疱，但通常不伴有严重的瘢痕形成和甲营养不良，且多在儿童期起病。

### 诊断建议：

- **皮肤活检**：通过皮肤活检可以观察到表皮和真皮的分离情况，有助于区分不同亚型的EB。
- **基因检测**：通过基因检测可以明确具体的遗传突变，有助于确诊和分型。
- **免疫荧光**：用于检测皮肤中的自身抗体，有助于排除自身免疫性疾病。

希望这些信息对您有所帮助。如果您需要进一步的诊断或治疗建议，请咨询专业的皮肤科医生。

### 52. User

患者的临床表现随年龄、接触果糖的量和时间而存在差异。一般而言，发病年龄越小，症状越重。经典型患儿于 6 个月左右起病，通常在首次食用了含有果糖的食物，如水果、蜂蜜和某些蔬菜时发病。常见临床症状包括进食含果糖食物后出现低血糖发作，表现为恶心、呕吐、腹痛、腹胀、出汗、震颤、抽搐甚至昏迷。

如果诊断未明，患者长期慢性摄入果糖食物，除了低血糖表现外，会出现生长发育迟缓，肝肿大、腹水、黄疸及代谢紊乱，如低血糖、代谢性酸中毒、低磷酸盐血症、高尿酸血症、高镁血症、高丙酮酸血症。病情严重者出现多脏器损害，肝、肾功能衰竭，抽搐、昏迷，甚至死亡。

在急性发作时，实验室检查会发现低血糖、低血磷和低血钾，同时伴有尿酸、乳酸、丙酮酸、游离脂肪酸和甘油三酯升高。慢性患者出现肝功能损害及肝纤维化的指征，包括血清胆红素，转氨酶升高，凝血障碍。尿液检查可见蛋白尿及果糖阳性。

### Qwen2.5_72b

根据您提供的临床表现，最可能的诊断是**遗传性果糖不耐受（Hereditary Fructose Intolerance, HFI）**。这是一种罕见的遗传性代谢病，由于果糖-1-磷酸醛缩酶（Aldolase B）的缺陷导致果糖代谢障碍，从而引起一系列临床症状。

### 1. 遗传性果糖不耐受（Hereditary Fructose Intolerance, HFI）

- **临床表现**：进食含果糖食物后出现低血糖、恶心、呕吐、腹痛、腹胀、出汗、震颤、抽搐甚至昏迷。长期慢性摄入果糖食物可导致生长发育迟缓、肝肿大、腹水、黄疸及代谢紊乱，如低血糖、代谢性酸中毒、低磷酸盐血症、高尿酸血症、高镁血症、高丙酮酸血症。严重者可出现多脏器损害，肝、肾功能衰竭，抽搐、昏迷，甚至死亡。
- **实验室检查**：急性发作时可见低血糖、低血磷和低血钾，尿酸、乳酸、丙酮酸、游离脂肪酸和甘油三酯升高。慢性患者出现肝功能损害及肝纤维化的指征，包括血清胆红素、转氨酶升高，凝血障碍。尿液检查可见蛋白尿及果糖阳性。

### 其他可能的诊断

- **糖原贮积病（Glycogen Storage Disease, GSD）**
  - **临床表现**：低血糖、肝肿大、生长发育迟缓、代谢紊乱。
  - **实验室检查**：低血糖、高乳酸、高尿酸、高甘油三酯。
- **半乳糖血症（Galactosemia）**
  - **临床表现**：进食含半乳糖食物后出现呕吐、黄疸、肝肿大、生长发育迟缓、代谢紊乱。
  - **实验室检查**：高半乳糖血症、低血糖、肝功能异常。
- **丙酸血症（Propionic Acidemia）**
  - **临床表现**：呕吐、腹痛、低血糖、代谢性酸中毒、肝肿大、生长发育迟缓。
  - **实验室检查**：高丙酸、高乳酸、高氨血症。
- **酪氨酸血症（Tyrosinemia）**
  - **临床表现**：呕吐、腹痛、肝肿大、生长发育迟缓、代谢紊乱。
  - **实验室检查**：高酪氨酸、高延胡索酸、肝功能异常。
- **先天性高胰岛素血症（Congenital Hyperinsulinism）**
  - **临床表现**：低血糖、抽搐、昏迷、生长发育迟缓。
  - **实验室检查**：低血糖、高胰岛素血症。

这些疾病在临床表现和实验室检查上有一些相似之处，但通过详细的病史、家族史、实验室检查和基因检测可以进行鉴别诊断。

### 53. User

**1. 偏头痛** 先兆偏头痛常为该疾病的首发症状，见于 20％ ~40％的患者，是普通人群的 5倍；也可表现为无先兆偏头痛，发病率与普通人群无差异。亚裔患者偏头痛发生率较欧美患者低。

**2. 皮层下缺血性事件** 短暂性脑缺血发作（TIA） 和缺血性卒中是该疾病最常见的临床表现，见于 60％ ~85％的症状性患者，且大多数患者缺乏常见的脑血管病危险因素。2/3 的患者表现为经典的腔隙综合征。

**3. 认知缺陷** 60％的患者逐渐出现认知功能障碍与痴呆。

**4. 情感障碍与淡漠** 20％的患者具有情感障碍，抑郁状态最常见，极少数可表现为抑郁与躁狂状态交替出现；淡漠见于 40％的患者。

此外，5％ ~10％患者有癫痫发作，也有听力下降、意识障碍、脊髓梗死、颅内出血以及合并流域性梗死的报道。

### Qwen2.5_72b

根据您提供的临床表现，最可能的诊断是 **CADASIL（脑动脉病伴皮质下梗死和白质脑病）**。CADASIL 是一种遗传性脑血管病，主要影响小血管，导致反复的皮层下缺血性事件、认知功能障碍和情感障碍。以下是五个其他可能的诊断：

- **Fabry 病**：一种罕见的遗传性代谢病，由于α-半乳糖苷酶 A 缺乏导致脂质在血管内皮细胞中积累，可引起脑血管病变、偏头痛、认知障碍和情感障碍。
- **MELAS 综合征**：线粒体脑肌病伴乳酸酸中毒和卒中样发作，可表现为反复的偏头痛、皮层下缺血性事件、认知障碍和情感障碍。
- **遗传性出血性毛细血管扩张症 (HHT)**：一种遗传性血管发育异常病，可导致脑血管畸形，引起反复的脑缺血事件和认知障碍。
- **脑淀粉样血管病 (CAA)**：一种与淀粉样蛋白在脑血管壁沉积有关的疾病，可导致反复的皮层下缺血性事件、认知障碍和情感障碍。
- **多发性硬化症 (MS)**：一种中枢神经系统的自身免疫性疾病，可引起反复的皮层下缺血性事件、认知障碍和情感障碍。

这些疾病都有类似的临床表现，但具体的诊断需要结合详细的病史、家族史、实验室检查和影像学检查结果。

### 54. User

通常在青年早期（但 1 岁至 76 岁起病均有报道） 隐匿起病。主要表现为缓慢进展的双下肢痉挛性瘫痪，查体可见锥体束征（腱反射亢进，踝阵挛阳性、病理征阳性）。双上肢反射也可亢进，但一般不影响上肢活动。超过一半的患者会有下肢近端力弱表现。约 50％的患者查体中可发现双踝音叉震动觉减低（一般不会消失），另约 1/3 的患者有排尿障碍。

随着年龄增长，疾病逐渐进展，约 20％的患者最终会丧失行走能力。在同一家系中， 携带同一突变的不同患者，临床症状轻重可有较大差异。

### Qwen2.5_72b

根据您提供的临床表现，最可能的诊断是**遗传性痉挛性截瘫（Hereditary Spastic Paraplegia, HSP）**。HSP 是一组以双下肢痉挛性瘫痪为主要特征的遗传性疾病，通常在青年早期起病，但起病年龄可以从婴儿期到老年期不等。主要临床表现为双下肢痉挛、腱反射亢进、踝阵挛和病理征阳性，部分患者可有上肢反射亢进，但上肢活动一般不受影响。此外，约 50% 的患者有下肢近端肌力减退，约 50% 的患者有双踝音叉震动觉减低，约 1/3 的患者有排尿障碍。随着疾病的进展，部分患者最终会丧失行走能力。同一家系中，携带同一突变的不同患者临床症状轻重可有较大差异。

以下是五种其他可能的诊断：

- **多发性硬化症（Multiple Sclerosis, MS）**：MS 是一种中枢神经系统的自身免疫性疾病，可表现为双下肢痉挛性瘫痪、腱反射亢进、病理征阳性等。但 MS 的病程通常为复发-缓解型或进展型，且常伴有视力障碍、感觉异常、共济失调等症状。
- **脊髓型肌萎缩症（Spinal Muscular Atrophy, SMA）**：SMA 是一种遗传性神经肌肉疾病，主要表现为进行性肌无力和肌萎缩。虽然 SMA 通常影响四肢，但某些亚型（如 SMA 4）可能在成年期起病，表现为下肢痉挛和肌力减退。
- **进行性脊髓性肌萎缩症（Primary Lateral Sclerosis, PLS）**：PLS 是一种罕见的运动神经元疾病，主要表现为缓慢进展的双下肢痉挛性瘫痪和腱反射亢进。与 HSP 不同的是，PLS 患者通常没有感觉障碍，且上肢受累更为常见。
- **脊髓小脑共济失调（Spinocerebellar Ataxia, SCA）**：SCA 是一组遗传性共济失调疾病，可表现为步态不稳、共济失调、腱反射亢进等。部分 SCA 亚型（如 SCA 3）可伴有下肢痉挛和肌力减退。
- **进行性多灶性白质脑病（Progressive Multifocal Leukoencephalopathy, PML）**：PML 是一种由JC病毒引起的中枢神经系统脱髓鞘疾病，多见于免疫抑制患者。临床表现可包括进行性神经功能障碍、痉挛性瘫痪、感觉异常等，但通常伴有认知功能下降和视力障碍。

这些疾病在临床表现上可能有重叠，但通过详细的病史、体格检查、辅助检查（如基因检测、MRI、电生理检查等）可以进一步明确诊断。

### 55. User

患儿多在新生儿、婴儿早期发病。临床表现与生物素酶缺乏相似，无特异性，主要有喂养困难、呕吐、腹泻、肌张力低下、惊厥、精神萎靡、嗜睡、呼吸困难、发育落后、顽固皮疹、脱皮，可合并酮症、代谢性酸中毒、高乳酸血症等。皮肤损害中大部分患者在头面部、颈部、躯干、臀部等部位皮肤红疹或红斑、溃烂或水疱、糠状或片状鳞屑，或皮肤干糙、脱皮等，少数仅在口周、眼周、肛周局部出现皮疹。患者不伴听力或视力障碍，不同于生物素酶缺乏症。

### Qwen2.5_72b

根据您提供的临床表现，最可能的诊断是**生物素反应性基底细胞营养不良（Biotin-responsive basal ganglia disease, BRBGD）**。这是一种罕见的遗传性代谢病，主要由于SLC19A3基因突变导致生物素代谢障碍。BRBGD的临床表现与生物素酶缺乏症相似，但不伴有听力或视力障碍，这与您描述的患者情况相符。

以下是其他五种可能的诊断：

- **生物素酶缺乏症（Biotinidase deficiency）**：
  - 临床表现包括喂养困难、呕吐、腹泻、肌张力低下、惊厥、精神萎靡、嗜睡、呼吸困难、发育落后、顽固皮疹、脱皮等。但通常伴有听力或视力障碍。
- **有机酸血症（Organic acidemias）**：
  - 一组代谢障碍，包括多种亚型，如甲基丙二酸血症（Methylmalonic acidemia）和丙酸血症（Propionic acidemia）。临床表现包括喂养困难、呕吐、腹泻、肌张力低下、惊厥、代谢性酸中毒、高乳酸血症等。
- **线粒体疾病（Mitochondrial disorders）**：
  - 一组由线粒体功能障碍引起的代谢病，临床表现多样，包括喂养困难、呕吐、腹泻、肌张力低下、惊厥、发育落后、代谢性酸中毒、高乳酸血症等。
- **先天性代谢异常（Inborn errors of metabolism, IEMs）**：
  - 一组遗传性代谢病，包括多种亚型，如尿素循环障碍、氨基酸代谢障碍等。临床表现包括喂养困难、呕吐、腹泻、肌张力低下、惊厥、代谢性酸中毒、高乳酸血症等。
- **维生素B12缺乏症（Vitamin B12 deficiency）**：
  - 临床表现包括喂养困难、呕吐、腹泻、肌张力低下、惊厥、发育落后、代谢性酸中毒等。但通常伴有贫血和神经系统症状。

建议进行进一步的实验室检查，如血液和尿液代谢筛查、基因检测等，以明确诊断。

### 56. User

该病患儿出生时多无异常，婴儿期临床表现并无特异性，仅表现为生长和发育迟缓。常于 3 岁有眼部症状和体征后得以诊断。

- 视力受损， 异位晶状体所致的近视和虹膜震颤等症状可在 3 岁后出现，晚期可合并散光、青光眼、葡萄肿、白内障、视网膜剥离及视神经萎缩。
- 智力障碍较常见，主要表现为进展性智力障碍。维生素 B6 治疗敏感型患者智力障碍较轻。
- 精神或行为障碍， 大约 50％患儿可出现，20％患儿合并惊厥。
- 骨骼受累， 类似马方综合征表现，或合并脊柱侧凸，漏斗胸，鸡胸，膝外翻及弓形足，X 线可见全身性骨质疏松。
- 血栓性疾病， 发生于各个年龄段，可累及各类血管，尤其是脑血管。高水平同型半胱氨酸血症可改变血管壁及增加血小板黏附性，出现视神经萎缩、瘫痪、肺心病及肾性高血压。
- 自发性气胸和急性胰腺炎 较少见。

### Qwen2.5_72b

根据您提供的临床表现，最可能的诊断是**马凡综合征（Marfan syndrome）**。然而，考虑到一些特定的特征，如维生素 B6 治疗敏感型患者智力障碍较轻，以及高水平同型半胱氨酸血症，**高胱氨酸尿症（Homocystinuria）** 也应被考虑。以下是详细的诊断列表：

### 最可能的诊断：

- **高胱氨酸尿症（Homocystinuria）**：
  - **临床表现**：视力受损（异位晶状体、近视、虹膜震颤）、智力障碍（进展性，维生素 B6 治疗敏感型患者较轻）、精神或行为障碍（50% 患儿出现，20% 合并惊厥）、骨骼受累（类似马方综合征表现，如脊柱侧凸、漏斗胸、鸡胸、膝外翻及弓形足）、血栓性疾病（高水平同型半胱氨酸血症导致血管壁改变及血小板黏附性增加）、自发性气胸和急性胰腺炎（少见）。

### 其他可能的诊断：

1. **马凡综合征（Marfan syndrome）**：
   - **临床表现**：视力受损（异位晶状体、近视、虹膜震颤）、骨骼受累（脊柱侧凸、漏斗胸、鸡胸、膝外翻及弓形足）、心血管受累（主动脉瘤、主动脉夹层）。
2. **埃勒斯-当洛斯综合征（Ehlers-Danlos syndrome）**：
   - **临床表现**：皮肤弹性增加、关节过度活动、易瘀伤、心血管受累（主动脉瘤、主动脉夹层）、自发性气胸。
3. **普拉德-威利综合征（Prader-Willi syndrome）**：
   - **临床表现**：生长和发育迟缓、智力障碍、行为问题、肥胖、肌肉张力低下、骨骼异常（如脊柱侧凸）。
4. **特纳综合征（Turner syndrome）**：
   - **临床表现**：生长迟缓、智力障碍（轻度）、心血管受累（如主动脉缩窄）、骨骼异常（如脊柱侧凸）、视力问题（如近视）。
5. **成骨不全症（Osteogenesis imperfecta）**：
   - **临床表现**：骨骼脆弱、易骨折、生长迟缓、蓝巩膜、听力损失、关节松弛、脊柱侧凸。

这些疾病都有类似的临床表现，但具体的诊断需要通过详细的病史、体格检查以及实验室检查（如基因检测、血液检查等）来确定。

### 57. User

患者的主要表现有：出生后即发现 LDL-C 水平明显升高，胆固醇沉积在皮肤、眼睛以及肌腱形成黄色瘤和脂性角膜弓。由于机体暴露于高水平的 LDL-C 中， 患者多数会在 20 岁前就出现冠状动脉粥样硬化性心脏病并可能因此死亡。

需要详细询问患者的家族史，部分患者父母均为杂合子并因此出现 LDL-C 水平升高（通常大于本国特定年龄和性别标准的 95百分位数） 以及早发动脉粥样硬化性心血管疾病强阳性家族史。

### Qwen2.5_72b

根据您提供的临床表现，最可能的诊断是**家族性高胆固醇血症（Familial Hypercholesterolemia, FH）**。这是一种常染色体显性遗传病，由于低密度脂蛋白受体（LDL receptor）基因突变导致LDL-C水平显著升高，进而引起皮肤、眼睛和肌腱的黄色瘤以及脂性角膜弓。患者通常在20岁前就会出现冠状动脉粥样硬化性心脏病。

以下是其他五种可能的诊断：

- **混合型高脂血症（Mixed Hyperlipidemia）**：这是一种代谢紊乱，表现为高胆固醇和高甘油三酯水平。虽然患者的LDL-C水平也会升高，但通常不会像FH那样显著，且黄色瘤和脂性角膜弓的出现较少见。
- **甲状腺功能减退症（Hypothyroidism）**：甲状腺功能减退可以导致血脂水平升高，包括LDL-C水平。患者可能有其他甲状腺功能减退的症状，如疲劳、体重增加、便秘等。
- **糖尿病（Diabetes Mellitus）**：糖尿病患者常伴有血脂异常，包括高LDL-C水平。但糖尿病患者通常有高血糖、多饮、多尿、体重下降等症状。
- **肾病综合征（Nephrotic Syndrome）**：肾病综合征患者由于大量蛋白尿和低蛋白血症，常伴有血脂异常，包括高LDL-C水平。患者可能有水肿、低蛋白血症、高脂血症等症状。
- **药物引起的高胆固醇血症（Drug-Induced Hypercholesterolemia）**：某些药物，如糖皮质激素、抗精神病药、β-阻滞剂等，可以导致血脂水平升高。患者可能有使用这些药物的病史。

为了进一步确诊，建议进行以下检查： - **基因检测**：检测LDLR、APOB、PCSK9等基因突变。 - **血脂谱**：包括总胆固醇、LDL-C、HDL-C和甘油三酯水平。 - **家族史**：详细询问家族中是否有类似症状的患者。 - **心血管评估**：如心电图、冠状动脉造影等，评估冠状动脉粥样硬化情况。

希望这些信息对您有所帮助。

### 58. User

临床特征表现为运动障碍、精神症状和认知障碍三联征，通常隐匿起病，缓慢进展。发病年龄从儿童期至 79 岁不等，最常见于 30~50 岁。

疾病累及面部、躯干和肢体的快速，不自主，无节律运动；早期异常动作轻微，患者可能难以意识到舞蹈症状的存在，或将舞蹈样动作整合到随意运动中，使得正常动作的控制出现困难或偏差；随着疾病的进展，舞蹈症的范围和程度常逐渐加重，甚至影响膈肌、咽和喉部肌肉，从而产生构音障碍、吞咽困难和不自主发声；疾病晚期舞蹈症常消失，代之以僵直、少动为主的帕金森症样表现，可伴有局灶性肌张力障碍；最后常导致卧床。运动保持困难也是该病常见表现，即不能保持某些简单的自主动作，如保持伸舌动作困难等。

精神障碍可先于舞蹈症出现，常见症状包括情绪低落、抑郁、易激惹、淡漠和焦虑， 也可出现偏执、妄想和幻觉；少见症状包括强迫行为和精神病表现，以上症状多呈进行性加重。

认知障碍最主要的特征是执行功能障碍，表现为做决定、执行多重任务和转换认知目标的能力下降。患者通常对其认知缺陷缺乏自知力。记忆减退或丧失多出现在病程晚期， 随疾病进展，最终可发展为痴呆。

除以上三联征外，眼球运动异常也是一个突出表现。早期表现为眼球扫视速度减慢，但眼球追随运动保留；晚期则眼球追随运动、自主扫视和再固定全部受损。此外，体重减轻和恶病质也是该病的常见特征，患者的不自主运动大量消耗能量可使体重明显下降，而通常无食欲减退。睡眠和(或) 性功能障碍也比较常见。

运动和认知功能的逐渐恶化会导致显著的并发症，如咽喉部肌肉受累导致吞咽困难， 运动障碍、活动减少甚至卧床可导致吸入性肺炎和其他感染、褥疮等。

### Qwen2.5_72b

根据您提供的临床表现，最可能的诊断是亨廷顿病（Huntington’s disease, HD）。亨廷顿病是一种遗传性神经退行性疾病，主要影响运动、认知和精神功能，通常在30-50岁之间发病，但也有儿童期和老年期发病的病例。以下是亨廷顿病的典型临床表现：

- **运动障碍**：包括快速、不自主、无节律的舞蹈样动作，逐渐加重，最终可能导致帕金森症样表现，如僵直和少动。
- **精神症状**：情绪低落、抑郁、易激惹、淡漠和焦虑，以及偏执、妄想和幻觉等。
- **认知障碍**：执行功能障碍，表现为做决定、执行多重任务和转换认知目标的能力下降，晚期可发展为痴呆。
- **眼球运动异常**：早期眼球扫视速度减慢，晚期眼球追随运动、自主扫视和再固定受损。
- **体重减轻和恶病质**：不自主运动大量消耗能量，导致体重下降，但通常无食欲减退。
- **睡眠和性功能障碍**：常见并发症。

### 其他可能的诊断

- **Wilson病**：一种铜代谢障碍的遗传性疾病，可导致肝功能异常、神经精神症状和运动障碍，如震颤、肌张力障碍和舞蹈样动作。
- **多系统萎缩（MSA）**：一种进行性神经退行性疾病，主要影响自主神经系统、小脑和基底节，表现为帕金森症样症状、小脑共济失调和自主神经功能障碍。
- **进行性核上性麻痹（PSP）**：一种罕见的神经退行性疾病，主要影响运动功能，表现为垂直眼球运动障碍、步态不稳、跌倒和帕金森症样症状。
- **皮质基底节变性（CBD）**：一种罕见的神经退行性疾病，表现为不对称的肌张力障碍、失用症、异己手综合征和帕金森症样症状。
- **遗传性共济失调**：如弗里德赖希共济失调（Friedreich’s ataxia），表现为进行性的小脑共济失调、脊髓病变和心脏问题，但通常不伴有明显的舞蹈样动作。

这些疾病虽然有相似的临床表现，但通过详细的病史、体格检查、实验室检查和影像学检查可以进行鉴别诊断。

### 59. User

临床症状、体征主要以神经系统症状为主，临床表现严重程度亦不同。发病年龄可以从新生儿期到成年期（晚发型），并且具有广泛的表型谱。

- 新生儿期, 约24~48 小时内无明显症状，随后出现与高氨血症相关的症状［包括嗜睡、困倦、拒食、呕吐，伴呼吸性碱中毒和（或） 癫痫发作］。与新生儿发病的其他尿素循环障碍疾病难以区分。
- 晚发型 , 该型患者占 88％，其中 40％发生在 3 岁前，29％在儿童期，19％在成人期。主要表现为：①慢性神经认知缺陷，包括发育迟缓、共济失调、痉挛、学习障碍、认知缺陷和（或） 无法解释的癫痫发作；②由多种因素（如感染，禁食或损伤） 诱发的继发于高氨血症危象的急性脑病；③慢性肝功能障碍，肝转氨酶不明原因升高伴有或不伴有轻度凝血功能障碍，伴或不伴轻度高氨血症和蛋白质不耐受。

### Qwen2.5_72b

根据您提供的临床表现，最可能的诊断是**尿素循环障碍**，特别是**鸟氨酸转氨甲酰酶缺乏症（OTC 缺乏症）**。这是一种X连锁遗传病，主要影响男性，但也可能在女性中出现症状。该病的主要特点是高氨血症，导致新生儿期或晚发型的神经系统症状。

### 其他可能的诊断包括：

- **丙酸血症**：这是一种有机酸血症，由于丙酸代谢途径中的酶缺陷导致。临床表现包括高氨血症、代谢性酸中毒、呕吐、嗜睡和发育迟缓。
- **甲基丙二酸血症**：这也是一种有机酸血症，由于甲基丙二酸代谢途径中的酶缺陷导致。临床表现包括高氨血症、代谢性酸中毒、呕吐、嗜睡和发育迟缓。
- **肉碱棕榈酰转移酶 II 缺乏症（CPT II 缺乏症）**：这是一种脂肪酸氧化障碍，可能导致高氨血症、低血糖、肌肉无力和急性脑病。
- **线粒体脑肌病伴乳酸酸中毒和中风样发作（MELAS 综合征）**：这是一种线粒体疾病，表现为慢性神经认知缺陷、癫痫发作、乳酸酸中毒和肝功能障碍。
- **威尔森病（Wilson 病）**：这是一种铜代谢障碍，可能导致慢性肝功能障碍、神经认知缺陷和高氨血症。

这些疾病都可能表现出类似的神经系统症状和高氨血症，因此需要通过详细的病史、体格检查和实验室检查（如血氨水平、尿有机酸分析、基因检测等）来进一步确诊。

### 60. User

部分患儿在新生儿期多无临床症状，出生 3-4 个月后逐渐出现典型症状：头发由黑变黄，皮肤颜色浅淡，尿液、汗液鼠臭味。随着年龄增长，逐渐表现出智力发育落后、小头畸形、癫痫发作，也可出现行为、性格、神经认知等异常。如多动、自残、攻击、自闭症、自卑、忧郁等，婴儿期还常出现呕吐、湿疹等。 部分 患者在新生儿期也多无临床症状，出生 1-3 个月后除表现 PKU 症状外，主要表现为运动障碍、嗜睡、躯干肌张力低下、四肢肌张力增高或低下、眼震颤、吞咽困难、口水增多、松软、角弓反张，反应迟钝、失眠、智力发育严重障碍等。

### Qwen2.5_72b

根据您提供的临床表现，最可能的诊断是苯丙酮尿症（Phenylketonuria, PKU）。这是一种由于苯丙氨酸羟化酶（PAH）基因突变导致的遗传性代谢病，患者体内无法正常代谢苯丙氨酸，导致苯丙氨酸及其代谢产物在体内积累，进而影响神经系统发育，引起一系列临床症状。

以下是其他五种可能的诊断：

- **枫糖尿病（Maple Syrup Urine Disease, MSUD）**：
  - 临床表现包括新生儿期无症状，随后出现喂养困难、呕吐、肌张力异常、嗜睡、发育迟缓、癫痫发作等。尿液有枫糖浆样气味。
- **高胱氨酸尿症（Homocystinuria）**：
  - 临床表现包括智力发育迟缓、小头畸形、骨骼异常、眼底异常、血栓形成等。尿液中高胱氨酸水平升高。
- **酪氨酸血症 I 型（Tyrosinemia Type I）**：
  - 临床表现包括新生儿期无症状，随后出现肝功能异常、肾功能异常、生长发育迟缓、呕吐、腹痛、肝肿大等。尿液中酪氨酸及其代谢产物水平升高。
- **半乳糖血症（Galactosemia）**：
  - 临床表现包括新生儿期出现黄疸、肝脾肿大、呕吐、腹泻、生长发育迟缓、白内障等。尿液中半乳糖水平升高。
- **甲基丙二酸血症（Methylmalonic Acidemia）**：
  - 临床表现包括新生儿期无症状，随后出现喂养困难、呕吐、肌张力异常、发育迟缓、代谢性酸中毒等。尿液中甲基丙二酸水平升高。

这些疾病都是遗传性代谢病，需要通过血液和尿液的生化检测以及基因检测来确诊。如果您怀疑患者有这些疾病，建议进行进一步的实验室检查和遗传咨询。

### 61. User

临床表现的严重程度具有很大的差异。患者可以表现为骨骼矿化异常、牙齿或关节病变、肌肉乏力等。目前，疾病主要分为 7 种类型。

- 单纯牙型， 是最轻、也是最常见的类型，仅有牙齿受累表现，无佝偻病或骨软化症的表现。
- 成人型，常中年发病，表现为牙齿异常或骨软化症。由于反复骨折、骨骼和关节疼痛或肌肉无力，可引起活动障碍。
- 儿童型，常在 6 月龄后发病，可轻度到重度，患儿可有不同程度的牙齿脱落、佝偻病的表现。
- 婴儿型 ，于 6 月龄前发病，常表现为纳差、难以存活、衰弱，可有囟门宽阔或头颅畸形。50％的婴儿型 HPP 会死于各种并发症。
- 围生期型，是极端类型，在宫内即发病，出生时症状明显，几乎均是致死型。患儿四肢短小、严重骨骼矿化不足，可表现为高调哭喊、癫痫发作、周期性呼吸暂停、伴有发绀和心动过缓、不明原因发热、易激惹、骨髓抑制性贫血和颅内出血、肺不张。
- 假性低磷酸酯酶症， 类似于婴儿型，患者具有婴儿型低磷酸酯酶症的表现，但血清 ALP 正常或升高。
- 良性型， 有时在宫内出现骨骼畸形，有围生期低磷酸酶活性，但出生后病情缓解，没有婴儿型低磷酸酯酶症或单纯牙型低磷酸酯酶症的相关表现。

### Qwen2.5_72b

根据您提供的临床表现和疾病类型描述，最可能的诊断是低磷酸酯酶症（Hypophosphatasia, HPP）。这是一种罕见的代谢性疾病，主要由于碱性磷酸酶（ALP）活性降低导致骨骼矿化障碍，从而引起一系列临床表现。

### 最可能的诊断：

- **低磷酸酯酶症（Hypophosphatasia, HPP）**

### 其他可能的诊断：

- **佝偻病（Rickets）**：表现为骨骼矿化不足，常见于儿童，可引起骨骼畸形、肌肉乏力等症状。
- **骨软化症（Osteomalacia）**：成人中较为常见，表现为骨骼疼痛、肌肉无力、骨折等。
- **成骨不全（Osteogenesis Imperfecta）**：一种遗传性骨骼疾病，表现为骨骼脆弱、易骨折、蓝巩膜等。
- **维生素D缺乏症（Vitamin D Deficiency）**：可引起骨骼矿化不足，表现为佝偻病或骨软化症。
- **氟骨症（Fluorosis）**：长期摄入过量氟化物导致的骨骼和牙齿病变，表现为牙齿斑点、骨骼硬化和疼痛。

这些疾病在临床表现上可能有重叠，但通过详细的病史、体格检查和实验室检查（如血清ALP水平、骨密度检查、基因检测等）可以进行鉴别诊断。

### 62. User

患儿主要表现为方颅、鸡胸、串珠肋、手镯征、足镯征、亨利氏沟，多在将近周岁开始负重时出现下肢畸形，可表现为膝内翻（O 形腿） 或膝外翻（X 形腿），并伴有生长迟缓、身材矮小、步态摇摆、进行性加重的骨畸形、多发性骨折、骨骼疼痛以及牙齿发育异常（牙质差、牙痛、脱落后不易再生） 等。影像学表现为骨骼畸形、长骨干骺端增宽和模糊，呈杯口样，杯口内可见许多细条状钙化影如毛刷状。在成人期主要表现为肢体乏力、活动受限、骨痛、多发病理性骨折（四肢长骨、肋骨、骨盆和椎体均可发生）、身高变矮。影像学表现为骨密度普遍减低，骨小梁模糊，呈毛玻璃状，骨盆畸形、假骨折线、椎体呈双凹变形等。

### Qwen2.5_72b

根据您提供的临床表现，最可能的诊断是**维生素D缺乏性佝偻病**（Rickets）。这种疾病主要影响儿童，尤其是在骨骼生长发育期，由于维生素D缺乏导致钙磷代谢障碍，进而影响骨骼的正常矿化。

### 其他五种可能的诊断：

- **成骨不全症**（Osteogenesis Imperfecta）：这是一种遗传性结缔组织病，主要表现为骨骼脆弱，易发生骨折，伴有蓝色巩膜、听力下降等症状。影像学可见多发性骨折、骨皮质变薄等。
- **低磷血症性佝偻病**（Hypophosphatemic Rickets）：这是一种由于肾脏对磷的重吸收障碍导致的疾病，表现为低磷血症、骨骼畸形、生长迟缓等。影像学表现与维生素D缺乏性佝偻病类似，但血磷水平显著降低。
- **甲状腺功能减退症**（Hypothyroidism）：甲状腺激素缺乏可导致生长发育迟缓、骨骼发育不良、智力障碍等。影像学可见骨骼发育迟缓、骨龄落后等。
- **骨软化症**（Osteomalacia）：这是成人维生素D缺乏或钙磷代谢障碍导致的疾病，表现为骨痛、肌肉无力、骨密度降低等。影像学可见骨密度减低、骨小梁模糊等。
- **成骨细胞功能障碍**（Osteoblast Dysfunction）：这是一种罕见的遗传性疾病，影响成骨细胞的功能，导致骨骼矿化不良、骨质疏松、骨折等。影像学可见骨密度减低、骨小梁模糊等。

### 总结：

- **最可能的诊断**：维生素D缺乏性佝偻病
- **其他可能的诊断**：
  1. 成骨不全症
  2. 低磷血症性佝偻病
  3. 甲状腺功能减退症
  4. 骨软化症
  5. 成骨细胞功能障碍

建议进一步进行血液检查（如血钙、血磷、碱性磷酸酶、25-羟基维生素D等）和基因检测，以明确诊断并制定相应的治疗方案。

### 63. User

临床表现有心力衰竭、胸痛、低血压、晕厥、心源性猝死、心律失常和血栓栓塞等。部分患者还会有心脏外的表现，如周围神经病、自主神经病和胃肠道症状等。

### Qwen2.5_72b

根据您提供的临床表现，这些症状可能指向一种罕见的心脏疾病，特别是当患者除了心脏症状外还表现出周围神经病、自主神经病和胃肠道症状时。最可能的诊断是**法布里病（Fabry Disease）**。这是一种X连锁遗传性溶酶体贮积症，由于α-半乳糖苷酶A（α-Gal A）活性缺乏，导致其底物（主要是GL-3）在多种细胞中蓄积，影响心脏、肾脏、神经系统等多个器官系统。

以下是其他五种可能的诊断：

- **心肌淀粉样变性（Cardiac Amyloidosis）**：这是一种由于异常蛋白质（淀粉样蛋白）在心肌中沉积导致的心肌病，可引起心力衰竭、心律失常等症状。根据淀粉样蛋白的类型，可分为原发性（AL型）和继发性（AA型）等。
- **肥厚型心肌病（Hypertrophic Cardiomyopathy, HCM）**：这是一种以心肌非对称性肥厚为特征的心肌病，可导致心力衰竭、心律失常、晕厥甚至猝死。部分患者可能有家族史。
- **心肌炎（Myocarditis）**：这是一种心肌炎症性疾病，可由病毒感染、自身免疫反应等多种原因引起。症状包括心力衰竭、胸痛、低血压等。
- **长QT综合征（Long QT Syndrome, LQTS）**：这是一种心电图异常导致的心律失常综合征，可引起晕厥、心源性猝死等症状。部分患者可能有家族史。
- **多发性硬化症（Multiple Sclerosis, MS）**：虽然主要影响中枢神经系统，但部分患者可能伴有自主神经功能障碍，导致低血压、晕厥等症状。此外，周围神经病和胃肠道症状也可能出现。

这些诊断需要通过详细的病史采集、体格检查、实验室检查（如血液检查、心电图、心脏超声、基因检测等）来进一步明确。如果您怀疑患者可能患有法布里病，建议进行α-半乳糖苷酶A活性检测和基因检测以确诊。

### 64. User

**1. 第二性征不发育和配子生成障碍** 男性表现为童声、小阴茎、无阴毛生长、小睾丸或隐睾、无精子生成；女性表现为乳腺不发育、幼稚外阴和原发闭经。

**2. 骨骺闭合延迟** 上部量 / 下部量<1，指尖距 > 身高，易患骨质疏松症。

**3. 嗅觉障碍** 因嗅球和嗅束发育异常，40％ ~60％ IHH 患者合并嗅觉丧失，不能识别气味。

**4. 其他表现** 面中线缺陷，如唇腭裂；孤立肾；短指(趾)、并指(趾)；骨骼畸形或牙齿发育不良；超重和肥胖；镜像运动等。

### Qwen2.5_72b

根据您提供的临床表现，最可能的诊断是**先天性低促性腺激素性性腺功能减退症（Congenital Hypogonadotropic Hypogonadism, IHH）**，特别是伴有嗅觉障碍的Kallmann综合征。以下是详细的诊断分析和五个其他可能的诊断：

### 1. 先天性低促性腺激素性性腺功能减退症（Congenital Hypogonadotropic Hypogonadism, IHH）

- **临床表现**：
  - **第二性征不发育和配子生成障碍**：男性表现为童声、小阴茎、无阴毛生长、小睾丸或隐睾、无精子生成；女性表现为乳腺不发育、幼稚外阴和原发闭经。
  - **骨骺闭合延迟**：上部量 / 下部量 < 1，指尖距 > 身高，易患骨质疏松症。
  - **嗅觉障碍**：因嗅球和嗅束发育异常，40％ ~ 60％ IHH 患者合并嗅觉丧失，不能识别气味。
  - **其他表现**：面中线缺陷，如唇腭裂；孤立肾；短指(趾)、并指(趾)；骨骼畸形或牙齿发育不良；超重和肥胖；镜像运动等。

### 2. 其他可能的诊断

- **Turner 综合征**：
  - **临床表现**：女性患者，身材矮小，第二性征不发育，原发闭经，颈部蹼状皮肤，盾形胸，肘外翻等。
  - **鉴别点**：Turner 综合征患者通常有染色体异常（45,X 或其他嵌合体），而 IHH 患者染色体正常。
- **Prader-Willi 综合征**：
  - **临床表现**：婴儿期肌张力低下，喂养困难，随后出现过度食欲和肥胖，智力障碍，第二性征不发育。
  - **鉴别点**：Prader-Willi 综合征患者有特定的基因缺失或印记异常，而 IHH 患者无此基因异常。
- **Klinefelter 综合征**：
  - **临床表现**：男性患者，身材高大，小睾丸，无精子生成，第二性征不发育，可能有智力障碍。
  - **鉴别点**：Klinefelter 综合征患者有染色体异常（47,XXY 或其他嵌合体），而 IHH 患者染色体正常。
- **垂体功能减退症**：
  - **临床表现**：多种垂体激素缺乏，包括促性腺激素、生长激素、促甲状腺激素等，导致第二性征不发育、生长迟缓、骨骺闭合延迟等。
  - **鉴别点**：垂体功能减退症患者通常有其他垂体激素缺乏的表现，如甲状腺功能减退、肾上腺功能减退等，而 IHH 患者主要表现为性腺功能减退。
- **Müllerian 管发育不全**：
  - **临床表现**：女性患者，第二性征发育正常，但子宫和阴道发育不全，原发闭经。
  - **鉴别点**：Müllerian 管发育不全患者第二性征发育正常，而 IHH 患者第二性征不发育。

### 总结

根据您提供的临床表现，最可能的诊断是**先天性低促性腺激素性性腺功能减退症（Congenital Hypogonadotropic Hypogonadism, IHH）**，特别是伴有嗅觉障碍的Kallmann综合征。其他可能的诊断包括Turner综合征、Prader-Willi综合征、Klinefelter综合征、垂体功能减退症和Müllerian管发育不全。建议进一步进行基因检测、染色体分析和内分泌功能检查以明确诊断。

### 65. User

最常见的症状是活动后气短，其他症状包括乏力、头晕、胸痛、胸闷、心悸、黑矇、晕厥等，一旦出现晕厥或眩晕，提示患者心搏量可能已明显下降。由于症状、体征不典型，绝大多数患者就诊时间明显延误，至少 1/5 患者从症状出现至确诊时间超过 2 年；患者可出现肺动脉瓣第二心音亢进，三尖瓣关闭不全引起三尖瓣区收缩期杂音； 右心衰竭体征包括颈静脉充盈或怒张、肝脏肿大、下肢水肿、多浆膜腔积液、黄疸和发绀等。

### Qwen2.5_72b

根据您提供的临床表现，最可能的诊断是**肺动脉高压**（Pulmonary Arterial Hypertension, PAH）。这是一种罕见的疾病，主要特征是肺动脉压力升高，导致右心室负荷增加，最终可能导致右心衰竭。以下是其他五种可能的诊断：

- **慢性血栓栓塞性肺高压**（Chronic Thromboembolic Pulmonary Hypertension, CTEPH）：这是一种由慢性血栓栓塞引起的肺高压，临床表现与PAH相似，但病史中可能有深静脉血栓或肺栓塞的证据。
- **特发性肺纤维化**（Idiopathic Pulmonary Fibrosis, IPF）：这是一种慢性、进行性的间质性肺病，主要表现为进行性呼吸困难、干咳、乏力等，但通常不会出现明显的右心衰竭体征。
- **心肌病**（Cardiomyopathy）：特别是右心室心肌病，可以导致右心功能不全，出现类似PAH的症状，如气短、胸痛、晕厥等。
- **先天性心脏病**（Congenital Heart Disease, CHD）：某些类型的先天性心脏病，如房间隔缺损或室间隔缺损，可能导致肺血流量增加，进而引起肺高压和右心衰竭。
- **结缔组织病相关肺高压**（Connective Tissue Disease-Associated Pulmonary Hypertension）：如系统性红斑狼疮、硬皮病等，这些疾病可以引起肺血管病变，导致肺高压。

为了进一步确诊，建议进行以下检查： - **超声心动图**：评估肺动脉压力和右心功能。 - **肺功能测试**：评估肺通气和换气功能。 - **CT肺动脉造影**：排除慢性血栓栓塞性肺高压。 - **右心导管检查**：直接测量肺动脉压力，是诊断肺高压的金标准。 - **血液检查**：排除结缔组织病等其他可能的病因。

希望这些信息对您有所帮助。如果有更多详细信息或需要进一步的诊断建议，请随时告知。

### 66. User

一般起病隐匿。主要表现为干咳、劳力性呼吸困难，杵状指(趾)、双下肺分布为主的爆裂音是其典型体征。终末期可以出现发绀、肺动脉高压、肺心病、右心功能不全的相关临床表现。

### Qwen2.5_72b

根据您提供的临床表现，最可能的诊断是特发性肺纤维化（Idiopathic Pulmonary Fibrosis, IPF）。这是一种慢性、进行性的间质性肺疾病，主要影响中老年人，其特征是肺泡壁的炎症和纤维化，导致肺功能逐渐下降。IPF的典型症状包括干咳、劳力性呼吸困难、杵状指（趾）以及双下肺分布为主的爆裂音（Velcro啰音）。

以下是五种其他可能的诊断：

- **结节病（Sarcoidosis）**：这是一种多系统受累的肉芽肿性疾病，可以影响肺部，导致类似的症状，如干咳、呼吸困难和肺部啰音。但结节病患者通常有其他器官受累的表现，如皮肤、眼睛或淋巴结肿大。
- **慢性过敏性肺炎（Chronic Hypersensitivity Pneumonitis, CHP）**：这是一种由于长期暴露于某些有机粉尘或化学物质引起的肺部炎症反应，症状包括干咳、呼吸困难和肺部啰音。CHP患者通常有明确的职业或环境暴露史。
- **系统性硬化症（Systemic Sclerosis, Scleroderma）**：这是一种自身免疫性疾病，可以导致皮肤和内脏器官的纤维化，包括肺部。患者可能有皮肤硬化、雷诺现象、关节疼痛等症状，肺部受累时可出现干咳、呼吸困难和肺部啰音。
- **药物性肺病（Drug-Induced Lung Disease）**：某些药物可以引起肺部炎症和纤维化，导致类似的症状。常见的致病药物包括胺碘酮、博来霉素、甲氨蝶呤等。患者通常有明确的用药史。
- **肺泡蛋白沉积症（Pulmonary Alveolar Proteinosis, PAP）**：这是一种罕见的肺部疾病，由于肺泡内积聚大量脂蛋白样物质，导致肺功能受损。患者可出现干咳、呼吸困难和肺部啰音，但通常没有杵状指（趾）。

这些疾病的诊断需要结合详细的病史、体格检查、实验室检查（如血液检查、肺功能测试、高分辨率CT扫描等）以及必要时的肺活检。

### 67. User

一种多器官、多系统受累的疾病，可累及几乎全身所有器官和组织。临床表现多种多样，患者因不同器官受累就诊于不同专科，容易漏诊。该病多数患者起病缓， 慢性进展。可以单器官起病，逐渐累及多个器官，也可以多种器官同时发病。少数患者病程呈自限性。

泪腺、唾液腺为最常见受累器官。主要表现为对称性、无痛性泪腺，颌下腺或腮腺肿大，硬结，伴或不伴有鼻窦炎或眼外肌增厚。消化系统受累可表现为自身免疫性胰腺炎、硬化性胆管炎、硬化性肠系膜炎等，临床可出现腹部隐痛、腹胀、消化不良、黄疸等。肺部受累时可表现为支气管壁增厚、肺间质病变、肺部结节 / 肿块以及胸膜增厚等，患者可无症状或咳嗽、气短及喘憋等。腹膜后纤维化或腹主动脉周围炎也是较常见的临床表现之一，主要为腹主动脉周围软组织增厚，可包绕输尿管造成输尿管狭窄和肾盂积水，少数患者发生腹主动脉瘤。临床表现为腹痛、腰痛或下肢水肿等。不到 10% 的患者发生 IgG4 相关性肾脏疾病，多表现为小管间质性肾病、肾盂占位性病变、输尿管管壁增厚或肾脏肿块样病变。其他受累器官包括甲状腺（硬化性甲状腺炎）、前列腺等。中枢神经系统病变少见，如自身免疫性垂体炎、肥厚性硬脑膜炎、颅内炎性假瘤等，患者可出现头痛、尿崩症、垂体功能减低等。除上述器官受累外，多数患者合并浅表或深部淋巴结肿大。另外，约半数患者有过敏相关病史，可以伴发哮喘。

### Qwen2.5_72b

根据您提供的临床表现，最可能的诊断是IgG4相关性疾病（IgG4-related disease, IgG4-RD）。这是一种以IgG4阳性浆细胞浸润为特征的多器官、多系统受累的疾病，可累及泪腺、唾液腺、胰腺、胆管、肺、腹膜后、肾脏、甲状腺、前列腺、中枢神经系统等多个器官和组织。该病的临床表现多样，起病缓慢，慢性进展，且容易漏诊。

以下是五种可能的其他诊断：

- **系统性红斑狼疮（Systemic Lupus Erythematosus, SLE）**：SLE是一种多系统受累的自身免疫性疾病，可累及皮肤、关节、肾脏、肺、心脏、神经系统等多个器官。患者可出现多种症状，如皮疹、关节痛、肾炎、胸膜炎、神经系统症状等。
- **干燥综合征（Sjögren’s Syndrome）**：干燥综合征是一种以泪腺和唾液腺受累为主的自身免疫性疾病，主要表现为口干、眼干。此外，还可累及肺、肾、肝、神经系统等多器官，出现相应的临床表现。
- **多发性肌炎/皮肌炎（Polymyositis/Dermatomyositis）**：这是一种以肌肉炎症和皮肤损害为特征的自身免疫性疾病，可累及多个器官，如肺、心脏、食管等。患者可出现肌无力、肌痛、皮疹、间质性肺病等症状。
- **Castleman病（Castleman Disease）**：Castleman病是一种罕见的淋巴组织增生性疾病，可分为单中心型和多中心型。多中心型Castleman病可累及多个淋巴结，出现全身症状，如发热、体重下降、疲劳等，还可累及肺、肝、肾等器官。
- **韦格纳肉芽肿（Wegener’s Granulomatosis）**：韦格纳肉芽肿是一种以坏死性肉芽肿性炎症和血管炎为特征的自身免疫性疾病，主要累及上呼吸道、肺和肾脏。患者可出现鼻窦炎、肺部结节、肾小球肾炎等症状。

这些疾病在临床表现上与IgG4相关性疾病有部分重叠，但通过详细的病史、体格检查、实验室检查和影像学检查，可以进行鉴别诊断。

### 68. User

患儿有胆汁淤积和肝硬化的发生。其主要临床表现为高结合胆红素血症，常出现脂溶性维生素吸收不良，如佝偻病等。而不同酶缺陷的临床表型略有不同，如氧固醇 7α- 羟化酶缺陷可出现成人期遗传性痉挛瘫痪；胆固醇 25 羟化酶缺陷可发生顽固性便秘；3β- 羟基类固醇 -Δ5-C27- 类固醇脱氢酶缺陷可发生婴儿期胆汁淤积性肝病和脂溶性维生素缺乏；固醇27- 羟化酶缺陷可发生儿童慢性腹泻及发育迟缓、成人期痉挛性瘫痪；α- 甲酰辅酶 A 消旋酶缺陷可发生婴儿期脂溶性维生素缺乏、成人期感觉神经病变；胆汁酸辅酶 A 氨基酸N- 乙酰转移酶缺陷可发生肝内胆汁淤积、发育迟缓、家族性高胆烷血症等。

### Qwen2.5_72b

根据您提供的临床表现，最可能的诊断是**胆汁酸合成障碍**，特别是**3β-羟基类固醇-Δ5-C27-类固醇脱氢酶缺陷**。这种缺陷会导致婴儿期胆汁淤积性肝病和脂溶性维生素缺乏，与您描述的高结合胆红素血症和脂溶性维生素吸收不良（如佝偻病）相符。

以下是其他五种可能的诊断：

- **进行性家族性肝内胆汁淤积症（PFIC）**：
  - PFIC 是一种遗传性肝病，主要表现为胆汁淤积和肝硬化。根据不同的基因突变，可分为 PFIC1、PFIC2 和 PFIC3。这些类型都可能导致高结合胆红素血症和脂溶性维生素吸收不良。
- **Alagille 综合征**：
  - Alagille 综合征是一种多系统受累的遗传病，主要表现为胆汁淤积、心脏缺陷、面部特征异常和蝶形椎骨。患者常有高结合胆红素血症和脂溶性维生素吸收不良。
- **Byler 病**：
  - Byler 病是一种罕见的遗传性肝病，主要表现为进行性肝内胆汁淤积和肝硬化。患者常有高结合胆红素血症和脂溶性维生素吸收不良。
- **Crigler-Najjar 综合征**：
  - Crigler-Najjar 综合征是一种遗传性高胆红素血症，分为 I 型和 II 型。I 型患者缺乏尿苷二磷酸葡萄糖醛酸转移酶（UGT1A1），导致严重的高未结合胆红素血症。虽然主要表现为高未结合胆红素血症，但某些患者也可能出现脂溶性维生素吸收不良。
- **Zellweger 综合征**：
  - Zellweger 综合征是一种过氧化物酶体生物发生障碍的遗传病，表现为多系统受累，包括肝功能异常、胆汁淤积和脂溶性维生素吸收不良。患者常有高结合胆红素血症和发育迟缓。

这些疾病都需要通过详细的临床评估、实验室检查和基因检测来确诊。希望这些信息对您有所帮助。

### 69. User

**1. 急性新生儿型** 起病急骤，病情进展迅速，可威胁生命。出生后 1~2 周内表现为喂养困难、呕吐、肌无力、肌张力减退，嗜睡或加重进展至昏迷。异戊酸蓄积可造成“汗脚”的特征性气味。查体可有肝大。化验提示代谢性酸中毒、高氨血症、低或高血糖、酮症、低钙血症及全血细胞减少。如果患者能度过新生儿期的急性发作，将会进展为慢性间歇型。

**2. 慢性间歇型** 起病隐匿。仅表现为非特异性不能耐受空腹或发育落后。常因上呼道感染或摄入高蛋白质饮食诱发，发作呈反复发生呕吐、嗜睡、昏迷、酸中毒伴酮尿， 会出现“汗脚气味”，限制蛋白质饮食并输注葡萄糖时可以缓解发作。绝大部分慢性间歇型患者精神运动发育正常，但是一些患者会表现为发育延迟和不同程度的智力低下。

### Qwen2.5_72b

根据您提供的临床表现，最可能的诊断是**异戊酸血症**（Isovaleric Acidemia）。这是一种罕见的有机酸代谢障碍，由于异戊酰辅酶A脱氢酶（IVD）缺乏导致异戊酸蓄积，引起一系列临床症状。

### 1. 最可能的诊断

- **异戊酸血症**（Isovaleric Acidemia）

### 2. 其他可能的诊断

- **甲基丙二酸血症**（Methylmalonic Acidemia）：这是一种由于甲基丙二酸辅酶A变位酶缺乏或其辅因子钴胺素代谢障碍导致的代谢病，临床表现包括代谢性酸中毒、高氨血症、喂养困难、肌张力减退等。
- **丙酸血症**（Propionic Acidemia）：这是一种由于丙酰辅酶A羧化酶缺乏导致的代谢病，临床表现与异戊酸血症类似，包括代谢性酸中毒、高氨血症、喂养困难、肌张力减退等。
- **尿素循环障碍**（Urea Cycle Disorders）：如鸟氨酸转氨甲酰酶缺乏症（OTC 缺乏症），临床表现包括高氨血症、代谢性酸中毒、喂养困难、肌张力减退等。
- **糖原贮积病**（Glycogen Storage Disease）：如庞贝病（Pompe Disease），临床表现包括肌无力、肝大、低血糖等。
- **线粒体疾病**（Mitochondrial Disorders）：如 Leigh 综合征，临床表现包括代谢性酸中毒、肌张力减退、发育迟缓等。

### 诊断依据

- **异戊酸血症**：特征性的“汗脚”气味、代谢性酸中毒、高氨血症、低或高血糖、酮症、低钙血症及全血细胞减少。
- **甲基丙二酸血症**：代谢性酸中毒、高氨血症、喂养困难、肌张力减退。
- **丙酸血症**：代谢性酸中毒、高氨血症、喂养困难、肌张力减退。
- **尿素循环障碍**：高氨血症、代谢性酸中毒、喂养困难、肌张力减退。
- **糖原贮积病**：肌无力、肝大、低血糖。
- **线粒体疾病**：代谢性酸中毒、肌张力减退、发育迟缓。

### 进一步检查建议

- **血液和尿液代谢筛查**：检测有机酸、氨基酸、酰基肉碱谱等。
- **基因检测**：确认特定的基因突变。
- **酶活性测定**：如异戊酰辅酶A脱氢酶活性测定。
- **影像学检查**：如头颅MRI，评估脑部病变。

希望这些信息对您有所帮助。如果您需要进一步的详细信息或有其他问题，请随时告知。

### 70. User

- 第二性征不发育和配子生成障碍。
- 嗅觉障碍， 因嗅球和嗅束发育异常，导致不能识别气味，尤其不能识别芳香气味。

### Qwen2.5_72b

根据您提供的临床表现，最可能的诊断是卡特尔-特纳综合征（Kallmann syndrome）。这是一种罕见的遗传性疾病，主要特征包括第二性征不发育（性腺功能减退）和嗅觉障碍（嗅觉缺失或减退）。以下是其他五种可能的诊断：

- **先天性低促性腺激素性性腺功能减退症（CHH）**：这是一种与卡特尔-特纳综合征相似的疾病，主要表现为性腺功能减退，但不伴有嗅觉障碍。
- **普拉德-威利综合征（Prader-Willi syndrome）**：这是一种遗传性疾病，主要特征包括肌肉张力低下、生长迟缓、性腺功能减退和肥胖。虽然不常见，但某些患者可能伴有嗅觉障碍。
- **卡尔曼-德朗热综合征（Kallmann-De Lange syndrome）**：这是一种罕见的遗传性疾病，主要特征包括智力障碍、生长迟缓、面部畸形和性腺功能减退。部分患者可能伴有嗅觉障碍。
- **先天性嗅觉缺失**：这是一种孤立的嗅觉障碍，可能与嗅球和嗅束的发育异常有关，但不伴有性腺功能减退。
- **特发性低促性腺激素性性腺功能减退症**：这是一种原因不明的性腺功能减退，可能与下丘脑-垂体轴的功能障碍有关，但不伴有嗅觉障碍。

如果您需要进一步的诊断或治疗建议，请咨询专业的内分泌科医生或遗传学专家。

### 71. User

病情从轻至重差异很大，因此容易被误诊和漏诊。临床表现主要包括：

**1. 发热** 热型不规则，可呈周期性或者持续性高热。下丘脑受累患者可以出现中枢性体温调节异常。

**2. 皮疹** 主要分布于躯干、头皮和发际。初起为淡红色丘疹，然后可以为出血性或湿疹样皮脂溢出样皮疹，继而结痂，结痂脱落后留有白斑。

**3. 口腔及眼耳鼻喉病变** 牙龈肿胀、牙齿松动，突眼，顽固性中耳炎伴外耳道皮疹为典型表现。

**4. 中枢性尿崩症** 垂体、下丘脑甚至其他部位中枢神经系统病变，少数患者出现神经精神症状。

**5. 呼吸道症状** 咳嗽，重者憋喘、发绀，甚至反复气胸发作。肺部体征通常不明显。

**6.** 肝脾可以肿大，可以出现黄疸、肝功能异常、肝功能衰竭和门脉高压，最终患者可死于肝硬化和肝功能衰竭。

**7. 消化道症状** 消化道累及较少见，可以表现为息肉或腺瘤样改变，通常不出现临床症状，病变弥漫时可能出现腹痛、腹泻和低蛋白血症等。

**8. 造血系统** 淋巴结可见肿大，骨髓受累时可以出现贫血、白细胞和血小板计数异常。

**9. 骨质损害** 颅骨、四肢骨、脊柱、骨盆等可有疼痛及肿块，可以发生病理性骨折。眼眶骨质病变所致突眼为儿童患者典型表现之一。

### Qwen2.5_72b

根据您提供的临床表现，这些症状可能指向一种罕见的疾病——**Langerhans细胞组织细胞增生症（Langerhans Cell Histiocytosis, LCH）**。LCH是一种罕见的疾病，其特征是Langerhans细胞的异常增生，这些细胞通常在皮肤、骨骼、肺、肝、脾、淋巴结和垂体等部位形成病灶。LCH的临床表现多样，从轻微的皮肤损害到严重的多系统受累，均可发生。您描述的症状，如发热、皮疹、口腔及眼耳鼻喉病变、中枢性尿崩症、呼吸道症状、肝脾肿大、消化道症状、造血系统异常以及骨质损害，均与LCH的临床表现相符。

**其他可能的诊断包括：**

- **Rosai-Dorfman病**：这是一种罕见的组织细胞增生性疾病，主要表现为淋巴结肿大，但也可累及皮肤、眼眶、中枢神经系统等，临床表现与LCH有部分重叠。
- **Erdheim-Chester病**：这是一种罕见的非Langerhans细胞组织细胞增生症，主要累及骨骼、皮肤、眼眶、中枢神经系统等，临床表现与LCH相似，但通常不累及淋巴结。
- **恶性组织细胞增生症**：这是一种罕见的恶性疾病，以组织细胞的异常增生为特征，可累及多个器官，临床表现与LCH有部分相似之处，但病情进展更快，预后较差。
- **Castleman病**：这是一种罕见的淋巴组织增生性疾病，主要表现为淋巴结肿大，但也可累及多个器官，临床表现与LCH有部分重叠。
- **系统性红斑狼疮**：这是一种自身免疫性疾病，可累及多个器官，包括皮肤、关节、肾脏、心脏、肺、神经系统等，临床表现与LCH有部分相似之处，但通常伴有抗核抗体等自身免疫指标的异常。

为了确诊，需要进行详细的病史采集、体格检查、实验室检查（如血液学检查、生化检查、免疫学检查等）、影像学检查（如X线、CT、MRI等）以及组织病理学检查（如皮肤、骨髓、淋巴结等的活检）。

### 72. User

**1. 生长障碍及其他畸形，**患儿出生时体重大多在正常范围内，但部分患儿的出生身长低于 48cm（42~47cm）。出生后逐渐显著地生长速度减慢，未治疗的男性患者终身高在 116_142cm，女性患者终身高在108_136cm。同时患者手、足和内脏器官均小。部分患者伴有其他的先天性畸形，如髋关节脱位、主动脉瓣狭窄、隐睾、先天性白内障等。

**2. 面容特点，**患者特征性的面容改变为前额突出、鞍鼻、小下颌，头部与身体比例较正常儿童大，但与同龄儿童相比头围绝对值仍偏小。患者头发稀疏，部分患者可出现“落日征”，地中海地区和中东地区的患者可表现出蓝巩膜。患者出牙延迟，乳牙脱落较晚并且易合并多发龋齿，由于下颌骨较短小，可有牙列不齐。

**3. 骨骼肌肉系统** 部分患者具有先天性髋关节脱位、股骨头缺血性坏死等表现。由于中面部骨骼发育不良和咽部解剖结构异常，患者说话音调高尖。85％患者肘关节活动受限。

**4. 代谢紊乱 ，**患者常合并代谢异常，包括新生儿期和婴儿期的低血糖，而成年后出现逐渐加重的肥胖。

**5. 性发育延迟 ，**患者生殖器较小，男性患者表现为小阴茎和小睾丸，女性患者超声检查发现卵巢体积较小。大多数患者表现为青春发育延迟。虽然性发育落后，但是男性和女性患者的性功能最终可发育成熟，具有生育能力。

**6. 肿瘤发生率低，**患者的肿瘤发生的风险较一般人群显著降低。

### Qwen2.5_72b

根据您提供的临床表现，最可能的诊断是**努南综合征（Noonan Syndrome）**。这是一种相对常见的遗传性综合症，但仍然属于罕见病范畴，其特征包括生长障碍、特殊的面容、骨骼异常、心脏缺陷、生殖系统发育不良等。努南综合征是由基因突变引起的，最常见的突变基因包括PTPN11、SOS1、RAF1、KRAS、NRAS等。

以下是其他五种可能的诊断：

- **特纳综合征（Turner Syndrome）**：这是一种影响女性的遗传病，通常由X染色体的部分或全部缺失引起。患者常表现为身材矮小、生殖器发育不良、心脏缺陷等。
- **普拉德-威利综合征（Prader-Willi Syndrome）**：这是一种由15号染色体上的基因缺陷引起的遗传病，患者常表现为新生儿期肌张力低下、喂养困难、生长迟缓、肥胖、性发育延迟等。
- **科斯特洛综合征（Costello Syndrome）**：这是一种由HRAS基因突变引起的罕见遗传病，患者常表现为身材矮小、特殊的面容、心脏缺陷、皮肤松弛、发育迟缓等。
- **卡特赖特-特纳综合征（Carter-Turner Syndrome）**：这是一种罕见的遗传病，患者常表现为身材矮小、特殊的面容、骨骼异常、生殖器发育不良等。
- **拉塞尔-西尔弗综合征（Russell-Silver Syndrome）**：这是一种影响生长和发育的遗传病，患者常表现为身材矮小、生长迟缓、特殊的面容、肢体不对称等。

努南综合征的临床表现与您提供的信息最为吻合，但最终诊断需要通过基因检测和详细的临床评估来确定。

### 73. User

主要症状为双眼先后发生的无痛性视力突然急剧下降，双眼发作间隔一般为数周到数月。眼底检查在疾病早期表现为视盘充血水肿、视盘旁毛细血管迂曲扩张，疾病后期视盘水肿和毛细血管扩张消退，最终视盘颞侧或全部呈萎缩性改变。多数病人只存在眼部表现，少数可合并全身其他系统症状如智力障碍、癫痫、听力障碍、肌张力障碍等。

### Qwen2.5_72b

根据您提供的临床表现，最可能的诊断是Leber遗传性视神经病变（Leber’s Hereditary Optic Neuropathy, LHON）。LHON是一种主要影响视神经的线粒体遗传病，通常表现为双眼先后发生的无痛性视力急剧下降，眼底检查早期可见视盘充血水肿、视盘旁毛细血管迂曲扩张，后期视盘水肿和毛细血管扩张消退，最终视盘颞侧或全部呈萎缩性改变。多数患者仅表现为眼部症状，少数患者可合并全身其他系统症状，如智力障碍、癫痫、听力障碍、肌张力障碍等。

以下是其他五种可能的诊断：

- **视神经炎（Optic Neuritis）**：
  - 视神经炎通常表现为单眼或双眼的视力下降，伴有眼痛，尤其是眼球运动时。眼底检查早期可见视盘充血水肿，但通常不会出现视盘旁毛细血管迂曲扩张。后期视盘可出现萎缩。
- **视网膜静脉阻塞（Retinal Vein Occlusion, RVO）**：
  - RVO表现为视力下降，眼底检查可见视网膜静脉扩张、迂曲，视盘充血水肿，视网膜出血和棉絮斑。但RVO通常不会导致双眼先后发病，且视盘萎缩不常见。
- **多发性硬化（Multiple Sclerosis, MS）**：
  - MS是一种中枢神经系统脱髓鞘疾病，可表现为视神经炎，导致视力下降和眼痛。眼底检查可见视盘充血水肿，但通常不会出现视盘旁毛细血管迂曲扩张。MS患者常有其他神经系统症状，如肢体无力、感觉异常等。
- **视神经脊髓炎谱系疾病（Neuromyelitis Optica Spectrum Disorders, NMOSD）**：
  - NMOSD是一种自身免疫性疾病，可表现为视神经炎和脊髓炎。视神经炎导致视力下降和眼痛，眼底检查可见视盘充血水肿。但NMOSD患者常有脊髓受累的症状，如肢体无力、感觉异常、膀胱功能障碍等。
- **视神经萎缩（Optic Atrophy）**：
  - 视神经萎缩是一种视神经损伤后的最终表现，可由多种原因引起，如缺血、炎症、遗传性疾病等。眼底检查可见视盘苍白萎缩，但通常不会出现视盘充血水肿和视盘旁毛细血管迂曲扩张。

综上所述，LHON是最可能的诊断，但其他几种疾病也需要考虑，特别是当患者有其他系统症状时。进一步的基因检测和辅助检查（如MRI、视觉诱发电位等）有助于确诊。

### 74. User

患者临床表现为婴儿期低酮性低血糖、肝功能异常、乳酸酸中毒、心肌病，部分患者表现为生长迟缓，肌张力低下或心肌病，少数可有胆汁淤积或肝硬化。幼儿期患分患者表现为生长迟缓，肌张力低下或心肌病，少数可有胆汁淤积或肝硬化。幼儿期患者可有喂养困难，发作性代谢紊乱，发作性横纹肌溶解。多数患者儿童期会有视网膜病变，周围神经病是一种较少见的长期病并发症。

### Qwen2.5_72b

根据您提供的临床表现，最可能的诊断是**糖原贮积病I型（Glycogen Storage Disease Type I, GSD I）**，特别是GSD Ia型。这种疾病是由于葡萄糖-6-磷酸酶（Glucose-6-Phosphatase, G6Pase）缺乏导致的，主要影响肝脏和肾脏，导致低血糖、乳酸酸中毒、肝功能异常、生长迟缓、肌张力低下和心肌病等症状。

以下是其他五种可能的诊断：

- **糖原贮积病III型（Glycogen Storage Disease Type III, GSD III）**：
  - 由于脱支酶（Amylo-1,6-glucosidase）缺乏导致，主要影响肝脏和肌肉，表现为低血糖、肝肿大、生长迟缓、肌张力低下和心肌病。
- **糖原贮积病VI型（Glycogen Storage Disease Type VI, GSD VI）**：
  - 由于肝磷酸化酶（Liver Phosphorylase）缺乏导致，主要影响肝脏，表现为低血糖、肝肿大、生长迟缓和乳酸酸中毒。
- **线粒体病（Mitochondrial Disease）**：
  - 由于线粒体功能障碍导致，表现为低血糖、乳酸酸中毒、心肌病、肌张力低下、生长迟缓和视网膜病变。
- **脂肪酸氧化障碍（Fatty Acid Oxidation Disorders, FAOD）**：
  - 由于脂肪酸代谢途径中的酶缺陷导致，表现为低血糖、肝功能异常、心肌病、肌张力低下和代谢性酸中毒。
- **糖原贮积病IX型（Glycogen Storage Disease Type IX, GSD IX）**：
  - 由于肝磷酸化酶激酶（Phosphorylase Kinase）缺乏导致，主要影响肝脏，表现为低血糖、肝肿大、生长迟缓和乳酸酸中毒。

这些疾病都有一些相似的临床表现，但具体的酶缺陷和病理机制不同，因此需要通过详细的生化检测和基因检测来确诊。

### 75. User

主要发生在成年育龄女性，平均诊断年龄在 40 岁左右。早期症状轻微，部分患者在查体时被发现，或因为呼吸症状或其他原因检查肺部 HRCT 时发现。主要表现为不同程度的呼吸困难，随疾病进展，呼吸困难症状也逐渐加重，肺功能进行性恶化，晚期出现呼吸衰竭。在病程中可反复出现气胸、乳糜胸等并发症。肺外表现包括肾， 腹膜后实性或囊实性淋巴管肌瘤。部分患者具有其他多系统受累临床特征，主要包括神经系统改变(癫痫、神经发育迟缓和自闭症等) 和皮肤改变(色素脱色斑、面部血管纤维瘤、皮肤鲨革斑和甲周纤维瘤等) 等临床表现。

### Qwen2.5_72b

根据您提供的临床表现，最可能的诊断是淋巴管肌瘤病（Lymphangioleiomyomatosis, LAM）。LAM 是一种罕见的、主要影响育龄女性的疾病，其特征是肺部、肾脏和腹膜后的平滑肌细胞异常增生，导致肺功能逐渐恶化，最终可能导致呼吸衰竭。LAM 患者常伴有气胸、乳糜胸等并发症，且部分患者可有多系统受累，包括神经系统和皮肤改变。

以下是其他五种可能的诊断：

- **结节性硬化症（Tuberous Sclerosis Complex, TSC）**：TSC 是一种遗传性疾病，可导致多个器官（包括肺、肾、脑和皮肤）的良性肿瘤。LAM 有时与 TSC 相关，特别是在女性患者中。
- **肺朗格汉斯细胞组织细胞增生症（Pulmonary Langerhans Cell Histiocytosis, PLCH）**：PLCH 是一种罕见的肺部疾病，主要影响吸烟者，表现为肺部多发囊性病变，可导致呼吸困难和气胸。
- **特发性肺纤维化（Idiopathic Pulmonary Fibrosis, IPF）**：IPF 是一种慢性、进行性的肺部疾病，主要表现为进行性呼吸困难和肺功能下降，但通常不伴有气胸和乳糜胸等并发症。
- **肺淋巴瘤**：某些类型的淋巴瘤可以累及肺部，导致呼吸困难和肺功能下降，但通常伴有其他全身症状，如发热、体重下降等。
- **肺囊性纤维化（Pulmonary Lymphangiomatosis）**：这是一种罕见的淋巴管异常增生性疾病，可导致肺部多发囊性病变，类似于 LAM，但通常在儿童和青少年中更为常见。

这些诊断需要通过详细的病史、体格检查、影像学检查（如 HRCT）和实验室检查（如基因检测、肺功能测试等）来进一步确认。

### 76. User

婴儿的典型表现是在断奶后出现反复呕吐和腹泻，拒食富含蛋白质的食物。患者可出现皮肤苍白、肌张力减低、肝脾大、头发稀疏、骨质疏松伴病理性骨折。进食富含蛋白质的膳食后患者可出现嗜睡、共济失调、癫痫发作和高氨血症昏迷等。随着病程的发展，可出现多系统受累表现。肺部受累可表现为进行性肺间质病变，肺泡蛋白沉积症；肾脏最常见的特征性改变是近端肾小管功能障碍和肾钙质沉积，也可出现肾小球病变，肾脏淀粉样变，甚至出现肾功能衰竭；血液系统受累表现为正色素或低色素性贫血，白细胞减少，血小板减少等；消化系统受累的表现为高胆固醇血症，高甘油三酯血症，反复发作的胰腺炎，甚至肝脏淀粉样变。癫痫多发生于成年患者，可能与反复发生高氨血症导致大脑皮质受损有关。

### Qwen2.5_72b

根据您提供的临床表现，最可能的诊断是**高氨血症**，特别是由于**尿素循环障碍**（如鸟氨酸转氨甲酰酶缺乏症，OTC缺乏症）引起的。尿素循环障碍是一组罕见的遗传性代谢病，主要影响肝脏中尿素的合成，导致血液中氨的水平升高。氨的积累会对神经系统造成损害，导致嗜睡、共济失调、癫痫发作和高氨血症昏迷等症状。此外，患者还可能出现肝脾大、骨质疏松、多系统受累等表现。

以下是其他五种可能的诊断：

- **枫糖尿症（Maple Syrup Urine Disease, MSUD）**：
  - 一种罕见的遗传性代谢病，由于支链氨基酸（亮氨酸、异亮氨酸和缬氨酸）代谢障碍导致。患者会出现反复呕吐、腹泻、拒食富含蛋白质的食物、皮肤苍白、肌张力减低、肝脾大、头发稀疏、骨质疏松等症状。高氨血症也是其常见表现之一。
- **苯丙酮尿症（Phenylketonuria, PKU）**：
  - 一种遗传性代谢病，由于苯丙氨酸羟化酶缺乏导致苯丙氨酸代谢障碍。患者会出现皮肤苍白、头发稀疏、智力障碍、癫痫发作等症状。高氨血症在严重病例中也可能出现。
- **酪氨酸血症I型（Tyrosinemia Type I）**：
  - 一种罕见的遗传性代谢病，由于酪氨酸代谢障碍导致。患者会出现肝脾大、肝功能异常、骨质疏松、肾小管功能障碍等症状。高氨血症也是其常见表现之一。
- **半乳糖血症（Galactosemia）**：
  - 一种遗传性代谢病，由于半乳糖代谢障碍导致。患者会出现反复呕吐、腹泻、拒食富含蛋白质的食物、肝脾大、黄疸、白细胞减少、血小板减少等症状。高氨血症在严重病例中也可能出现。
- **有机酸血症（Organic Acidemias）**：
  - 一组罕见的遗传性代谢病，由于有机酸代谢障碍导致。患者会出现反复呕吐、腹泻、拒食富含蛋白质的食物、皮肤苍白、肌张力减低、肝脾大、头发稀疏、骨质疏松等症状。高氨血症也是其常见表现之一。

这些疾病都具有类似的代谢障碍和多系统受累的表现，因此在临床诊断时需要通过详细的病史、体格检查和实验室检查（如血液氨水平、代谢筛查、基因检测等）来进一步明确诊断。

### 77. User

新生儿期起病，通常在生后 1 周内起病。以反复呕吐、腹泻、腹胀等胃肠道症状为首发症状，伴肝脾大和生长发育障碍；神经系统受累表现为活动少、腱反射亢进、踝阵挛和角弓反张。巨噬细胞内胆固醇酯和三酰甘油贮积导致肝脾肿大，进行性加重甚至肝衰竭，数月内出现转氨酶、胆红素升高，低蛋白血症，凝血异常等。肾上腺增大伴钙化导致肾上腺皮质功能不全，60％ ~70％通过影像学检查可发现肾上腺钙化。也有胆囊功能障碍、脑卒中报道。随着病情进展，可出现多器官功能障碍，包括贫血、消化道出血、败血症、昏迷等。

### Qwen2.5_72b

根据您提供的临床表现，最可能的诊断是尼曼-皮克病（Niemann-Pick disease，NPD），特别是NPD C型。这是一种罕见的遗传性代谢病，由于鞘磷脂酶活性降低或缺失导致鞘磷脂在细胞内蓄积，特别是巨噬细胞和神经细胞，引起一系列临床症状，包括肝脾肿大、神经系统症状、肾上腺钙化等。

以下是其他五种可能的诊断：

- **戈谢病（Gaucher disease）**：这是一种溶酶体贮积症，由于葡萄糖脑苷脂酶缺乏导致葡萄糖脑苷脂在巨噬细胞内蓄积，引起肝脾肿大、骨痛、贫血等症状。戈谢病的患者也可能有肾上腺钙化，但神经系统症状相对较少。
- **法布里病（Fabry disease）**：这是一种X连锁遗传的溶酶体贮积症，由于α-半乳糖苷酶A缺乏导致鞘糖脂在多种细胞内蓄积，引起肾功能不全、心脏病变、神经系统症状等。法布里病的患者通常有典型的皮肤病变和角膜混浊。
- **泰-萨克斯病（Tay-Sachs disease）**：这是一种罕见的遗传性神经退行性疾病，由于己糖胺酶A缺乏导致GM2神经节苷脂在神经细胞内蓄积，引起进行性神经系统退化，表现为肌张力异常、反射亢进、角弓反张等。泰-萨克斯病通常在婴儿期起病，进展迅速。
- **黏多糖贮积症（Mucopolysaccharidoses, MPS）**：这是一组溶酶体贮积症，由于溶酶体酶缺乏导致黏多糖在多种组织中蓄积，引起肝脾肿大、骨骼异常、心脏病变、神经系统症状等。MPS的患者通常有典型的面容和生长发育障碍。
- **威尔森病（Wilson disease）**：这是一种遗传性铜代谢障碍疾病，由于铜转运蛋白ATP7B基因突变导致铜在肝脏、脑、肾等器官中蓄积，引起肝功能异常、神经系统症状、肾功能不全等。威尔森病的患者通常有肝硬化、震颤、肌张力障碍等症状，但肾上腺钙化较少见。

这些疾病都有类似的临床表现，但具体的诊断需要通过基因检测、酶活性测定、影像学检查等进一步确认。

### 78. User

**1. 心血管系统** ，可以导致主动脉根部扩张、主动脉瓣关闭不全和主动脉夹层；也可能会有二尖瓣脱垂伴或不伴关闭不全。升主动脉扩张患病率随着患者年龄的增长而增加。从 30 岁至 60 岁，患病率从 53％上升至 96％。患者可以表现为气促、胸痛、心悸、头晕、晕厥或者咯血。查体表现为心界向左下扩大，听诊在二尖瓣区可以闻及收缩期杂音，在主动脉瓣听诊区可闻及舒张期为主的双期杂音，可有周围血管征以及心力衰竭的体征。

**2. 骨骼表现** ，瘦高身材、手指和脚趾细长、两臂平伸的距离超过身高、脊柱侧凸或后凸、漏斗胸或者鸡胸、关节松弛、扁平足等。

**3. 眼部异常** ，眼部病变占 50％ ~70％，包括角膜扁平、眼球轴延长、视网膜剥离、白内障、晶状体脱位或半脱位、虹膜震颤或轻度震颤、瞳孔移位、晶状体混浊等。

**4. 其他 ，**硬脊膜膨出、萎缩纹、复发性疝或切口疝、高腭穹等。

### Qwen2.5_72b

根据您提供的临床表现，最可能的诊断是**马凡综合征（Marfan syndrome）**。这是一种遗传性结缔组织疾病，主要影响心血管系统、骨骼系统和眼睛。以下是五个其他可能的诊断：

- **埃勒斯-当洛斯综合征（Ehlers-Danlos syndrome, EDS）**：这是一种以皮肤弹性过度、关节松弛和易受伤为特征的遗传性结缔组织病。心血管系统受累可表现为血管型EDS，导致主动脉根部扩张和主动脉夹层。
- **洛伊-迪茨综合征（Loeys-Dietz syndrome）**：这是一种以主动脉根部扩张、主动脉夹层和多发性动脉瘤为特征的遗传性结缔组织病。患者也可能有骨骼和面部异常。
- **努南综合征（Noonan syndrome）**：这是一种以心脏缺陷、生长迟缓和特殊面容为特征的遗传性疾病。心脏缺陷可能包括主动脉瓣关闭不全和二尖瓣脱垂。
- **成骨不全症（Osteogenesis imperfecta）**：这是一种以骨骼脆弱和易骨折为特征的遗传性结缔组织病。患者可能有蓝巩膜、关节松弛和听力损失。
- **普拉德-威利综合征（Prader-Willi syndrome）**：这是一种以肥胖、肌肉张力减低和智力障碍为特征的遗传性疾病。虽然心血管系统受累较少见，但患者可能有心脏缺陷和骨骼异常。

这些疾病都有部分与马凡综合征相似的临床表现，但具体的诊断需要结合详细的家族史、基因检测和影像学检查来确定。

### 79. User

**1. 皮肤表现** ，患者一般出生时即有牛奶咖啡斑，常位于骨骼病变同侧且以中线为界，为一处或多处点片状大小不等的褐色或黄棕色斑片状色素沉着，边界不规则。

**2. 骨骼系统表现** ，大约 98％的 患者有单骨型或多骨型骨纤维异样增殖症，以多骨型更为常见。其特征为畸变的骨母细胞增多并异常分化，引起骨髓内纤维细胞的广泛增殖、分化，形成幼稚编织骨，易发生病理性骨折和骨骼畸形，并引发骨痛，几乎可累及全身骨骼，尤以颅面骨和长骨受累多见，局部骨骼膨胀、隆起，引起面部结构改变和压迫症状。眼眶受累严重可压迫视神经，造成视力障碍。听神经受累可致听力下降。脊柱受累可致椎体压缩性骨折，引起脊柱侧弯或后凸畸形。骨纤维异样增殖症导致病理性骨折的高峰年龄多在 6~10 岁。影像学示骨骼呈膨胀性、溶骨性改变或磨玻璃样改变。

**3. 内分泌腺体受累表现** ，可累及多种内分泌腺，导致受累腺体功能亢进或发生肿瘤，血清促分泌激素的水平正常或降低。其中性早熟较多见，男女均可发病，但女性患者多见，且常早于骨病变。女性患儿可表现为第二性征早发育、过早阴道不规则出血、子宫及卵巢较正常同龄儿童增大，或发生卵巢囊肿，骨骺提前愈合等。男性患儿性早熟相对少见，超声见睾丸病变(Leydig 细胞增生、微小结石、局灶钙化等)。甲状腺肿大及功能亢进也较常见，也有报道发生甲状旁腺功能亢进者。其他内分泌疾病也有报道，如皮质醇增多症、巨人症 / 肢端肥大症、高泌乳素血症等，但相对较罕见。

**4. 低磷血症** ，部分患者合并低磷血症，表现为低血磷性佝偻病。合并低磷血症者通常为多骨型骨纤维异常增殖症。

**5. 其他，**患者还可因突变累及外周血白细胞、肝脏、心脏、胸腺和胃肠道，而出现相应的罕见临床症状，如黄疸、肝炎、心律失常、肠息肉等。

### Qwen2.5_72b

根据您提供的临床表现，最可能的诊断是**McCune-Albright综合征**（MAS）。这是一种罕见的遗传性疾病，主要特征包括皮肤的牛奶咖啡斑、多骨型骨纤维异常增殖症和内分泌腺体功能亢进。以下是详细的诊断依据和五种其他可能的诊断：

### 最可能的诊断

- **McCune-Albright综合征（MAS）**
  - **皮肤表现**：牛奶咖啡斑，边界不规则，常位于骨骼病变同侧。
  - **骨骼系统表现**：多骨型骨纤维异常增殖症，骨骼膨胀、隆起，易发生病理性骨折和骨骼畸形。
  - **内分泌腺体受累表现**：性早熟（尤其是女性），甲状腺功能亢进，其他内分泌疾病如皮质醇增多症、巨人症/肢端肥大症等。
  - **低磷血症**：部分患者合并低磷血症，表现为低血磷性佝偻病。
  - **其他**：可能累及外周血白细胞、肝脏、心脏、胸腺和胃肠道，出现相应的罕见临床症状。

### 其他可能的诊断

- **神经纤维瘤病1型（NF1）**
  - **皮肤表现**：牛奶咖啡斑，但数量更多，常超过6个。
  - **骨骼系统表现**：骨发育不良，但通常不表现为骨纤维异常增殖症。
  - **内分泌腺体受累表现**：较少见。
  - **其他**：神经纤维瘤，视神经胶质瘤等。
- **骨纤维异常增殖症（孤立型）**
  - **骨骼系统表现**：单骨型或多骨型骨纤维异常增殖症，但不伴有皮肤和内分泌系统的典型表现。
  - **其他**：无其他系统受累。
- **性早熟**
  - **内分泌腺体受累表现**：性早熟，但无骨纤维异常增殖症和牛奶咖啡斑。
  - **其他**：可能伴有其他内分泌疾病。
- **甲状腺功能亢进**
  - **内分泌腺体受累表现**：甲状腺功能亢进，但无骨纤维异常增殖症和牛奶咖啡斑。
  - **其他**：可能伴有其他甲状腺疾病。
- **低磷血症性佝偻病**
  - **低磷血症**：低血磷性佝偻病，但无骨纤维异常增殖症和牛奶咖啡斑。
  - **其他**：可能伴有其他代谢性疾病。

### 总结

根据您提供的临床表现，McCune-Albright综合征是最可能的诊断。然而，为了确诊，建议进行进一步的实验室检查和影像学检查，如基因检测、骨密度检查、内分泌功能检测等。

### 80. User

患者大多在出生后 3 个月至 3 岁之间发病，少部分在新生儿期或成人期发病， 也有患者无症状。患者发病通常有诱发因素，如多见于长时间饥饿或并发感染性疾病等。早期发病的患儿，其首发症状以嗜睡和呕吐常见，也可表现为抽搐、窒息等，常迅速进展为昏迷或死亡。急性发病期常有典型的低血糖表现，血糖降低伴尿酮体阴性有助于诊断。50％的患儿伴有肝脏肿大，肝活检可见大量脂质堆积。影像学检查发现部分患儿发生脑水肿。心脏损害表现较为罕见。成人期发病的患者临床表现多样，可有多器官受损，包括肌肉、肝脏、神经系统或心血管系统等。其中呕吐是最常见的症状，肝脏肿大相对少见，妊娠期出现症状的患者表现为急性脂肪肝。多数患者有急性非炎症性脑病。约 1/3 的患者出现横纹肌溶解，肌酸激酶显著增高，并伴有肌红蛋白尿。患者病死率较高，发病的患儿中约25％死亡，成人期急性发病的患者病死率更高，可达到 50％。疾病发作间歇期患者往往表现正常。约 1/3 的急性发病后存活的患者出现后遗症，包括生长发育迟滞、运动发育迟缓、智力障碍、语言发育缺陷、心理行为问题、癫痫、脑瘫、偏瘫、慢性肌无力等。另外，部分患者有疲劳、肌痛和运动耐力减退等骨骼肌损害的主诉症状。

### Qwen2.5_72b

根据您提供的临床表现，最可能的诊断是**肉碱棕榈酰转移酶 II 缺乏症 (Carnitine Palmitoyltransferase II Deficiency, CPT II Deficiency)**。这是一种罕见的代谢性疾病，主要影响脂肪酸的氧化过程，导致能量代谢障碍。以下是详细的诊断依据和五种其他可能的诊断：

### 最可能的诊断

- **肉碱棕榈酰转移酶 II 缺乏症 (CPT II Deficiency)**
  - **临床表现**：早期发病的患儿常有嗜睡、呕吐、抽搐、昏迷等症状，急性期常有低血糖表现，尿酮体阴性。肝脏肿大、脑水肿、横纹肌溶解、肌酸激酶显著增高、肌红蛋白尿等也是常见表现。成人期发病的患者症状多样，包括多器官受损，如肌肉、肝脏、神经系统或心血管系统等。妊娠期出现症状的患者表现为急性脂肪肝。病死率较高，急性发病后存活的患者可能出现多种后遗症，如生长发育迟滞、运动发育迟缓、智力障碍等。

### 其他可能的诊断

1. **中链酰基辅酶 A 脱氢酶缺乏症 (MCADD, Medium-Chain Acyl-CoA Dehydrogenase Deficiency)**
   - **临床表现**：多在婴儿期发病，常见症状包括呕吐、嗜睡、低血糖、肝肿大、脑病等。急性期常有低血糖和尿酮体阴性。
2. **长链 3-羟基酰基辅酶 A 脱氢酶缺乏症 (LCHAD, Long-Chain 3-Hydroxyacyl-CoA Dehydrogenase Deficiency)**
   - **临床表现**：多在婴儿期或儿童期发病，常见症状包括低血糖、肝肿大、肌病、脑病、急性脂肪肝等。成人期发病的患者症状多样，包括多器官受损。
3. **肉碱转运缺陷 (Carnitine Transport Defect)**
   - **临床表现**：多在婴儿期或儿童期发病，常见症状包括低血糖、肝肿大、肌病、脑病等。急性期常有低血糖和尿酮体阴性。
4. **线粒体脂肪酸氧化障碍 (Mitochondrial Fatty Acid Oxidation Disorders)**
   - **临床表现**：多在婴儿期或儿童期发病，常见症状包括低血糖、肝肿大、肌病、脑病等。急性期常有低血糖和尿酮体阴性。不同类型的线粒体脂肪酸氧化障碍可能有不同的临床表现。
5. **糖原贮积病 (Glycogen Storage Disease)**
   - **临床表现**：多在婴儿期或儿童期发病，常见症状包括低血糖、肝肿大、肌病、生长发育迟滞等。不同类型的糖原贮积病可能有不同的临床表现。

这些疾病都属于代谢性疾病，需要通过详细的代谢筛查、基因检测和生化检测来确诊。希望这些信息对您有所帮助。

### 81. User

疾病在各年龄段中的临床表现不尽相同。通常发病年龄越早，急性代谢紊乱和脑病表现越严重。临床表现主要包括消化系统症状如呕吐、喂养困难、肝大等以及神经系统症状如运动障碍、意识障碍、抽搐、发育迟缓或倒退、小头畸形等。病情反复多与感染和应激相关。

新生儿期发病者多在生后数小时至 1 周内出现急性脑病样症状，表现为呕吐、肌张力低下、脱水、严重酸中毒、高乳酸血症、高氨血症、昏迷和惊厥，病死率高。儿童期发病的出生时正常，多在 1 岁以内发病，首次代谢危象的诱因常为感染、饥饿、疲劳、疫苗注射等应激因素刺激或高蛋白饮食和药物，如果不及时诊治，可导致智力发育和运动发育迟缓、落后和倒退，可伴发血液系统、肝脏、肾脏、皮肤和周围神经受累。成人患者首发症状可为周围神经病变和精神心理异常等。

### Qwen2.5_72b

根据您提供的临床表现，最可能的诊断是**尿素循环障碍（Urea Cycle Disorders, UCDs）**，特别是**鸟氨酸转氨甲酰酶缺乏症（Ornithine Transcarbamylase Deficiency, OTC 缺乏症）**。这是一种罕见的遗传性代谢病，主要影响尿素循环，导致氨在体内积累，引起急性代谢紊乱和脑病。

### 1. 尿素循环障碍（Urea Cycle Disorders, UCDs）

- **主要表现**：新生儿期急性脑病样症状，如呕吐、肌张力低下、脱水、严重酸中毒、高乳酸血症、高氨血症、昏迷和惊厥。儿童期发病者可有反复的代谢危象，导致智力和运动发育迟缓或倒退，伴有肝大、喂养困难等消化系统症状。
- **遗传方式**：常染色体隐性遗传或X连锁隐性遗传（如OTC缺乏症）。

### 其他可能的诊断：

### 2. 有机酸血症（Organic Acidemias）

- **主要表现**：新生儿期急性脑病样症状，如呕吐、肌张力低下、脱水、严重酸中毒、高乳酸血症、昏迷和惊厥。儿童期发病者可有反复的代谢危象，导致智力和运动发育迟缓或倒退，伴有肝大、喂养困难等消化系统症状。
- **遗传方式**：常染色体隐性遗传。

### 3. 糖原贮积病（Glycogen Storage Diseases）

- **主要表现**：肝大、低血糖、生长迟缓、肌无力、代谢性酸中毒。儿童期发病者可有反复的代谢危象，导致智力和运动发育迟缓或倒退。
- **遗传方式**：常染色体隐性遗传。

### 4. 肝豆状核变性（Wilson Disease）

- **主要表现**：肝功能异常、肝大、神经系统症状如运动障碍、意识障碍、抽搐、发育迟缓或倒退。成人患者可有周围神经病变和精神心理异常。
- **遗传方式**：常染色体隐性遗传。

### 5. 线粒体疾病（Mitochondrial Disorders）

- **主要表现**：多系统受累，包括神经系统症状如运动障碍、意识障碍、抽搐、发育迟缓或倒退，消化系统症状如呕吐、喂养困难、肝大，以及代谢性酸中毒、高乳酸血症。
- **遗传方式**：线粒体遗传或常染色体遗传。

### 6. 黏多糖贮积病（Mucopolysaccharidoses）

- **主要表现**：多系统受累，包括神经系统症状如运动障碍、意识障碍、抽搐、发育迟缓或倒退，消化系统症状如肝大、喂养困难，以及骨骼异常、心脏病变。
- **遗传方式**：常染色体隐性遗传。

这些疾病都需要通过详细的病史、体格检查、实验室检查（如血氨、乳酸、有机酸、尿素循环酶活性测定等）以及基因检测来确诊。

### 82. User

典型患者出生时正常。于 6 个月 ~1 岁面容逐渐变丑，面中部变扁、鼻梁增宽、角膜混浊、耳聋、头大、前后径长，前额突出，关节僵硬、爪形手、鸡胸和腰椎后突等。常于 2~5 岁出现心脏瓣膜增厚、心肌病或充血性心力衰竭。5 岁出现肝脾大。精神运动发育落后。一般存活至 10 岁左右。多死于心衰和肺炎。轻型患者 5 岁以后出现症状，轻度面容粗陋、角膜混浊和手足关节僵硬。智力正常，寿命相对较长。临床症状在各亚型之间有互相重叠，但生化检查结果在各型之间无显著差异。

### Qwen2.5_72b

根据您提供的临床表现，最可能的诊断是**黏多糖贮积症（Mucopolysaccharidosis, MPS）**，特别是**MPS I型（Hurler综合征）**。MPS I型是一种罕见的遗传性代谢病，由于α-L-艾杜糖苷酸酶（IDUA）缺乏导致黏多糖（主要是硫酸皮肤素和硫酸肝素）在体内积累，引起多系统受累。

### 最可能的诊断：

- **MPS I型（Hurler综合征）**

### 其他可能的诊断：

- **MPS II型（Hunter综合征）**：X连锁隐性遗传，由于艾杜糖醛酸-2-硫酸酯酶缺乏导致类似症状。
- **MPS VI型（Maroteaux-Lamy综合征）**：由于N-乙酰半乳糖胺-4-硫酸酯酶缺乏导致类似症状。
- **MPS IV型（Morquio综合征）**：由于N-乙酰半乳糖胺-6-硫酸酯酶或半乳糖-6-硫酸酯酶缺乏导致类似症状。
- **MPS VII型（Sly综合征）**：由于β-葡萄糖醛酸酶缺乏导致类似症状。
- **黏脂贮积症（Mucolipidosis）**：由于溶酶体酶缺陷导致类似症状，但通常伴有更明显的神经系统受累。

### 诊断依据：

- **面容逐渐变丑**：MPS患者常有典型的面容改变，如面中部扁平、鼻梁增宽、角膜混浊等。
- **心脏瓣膜增厚、心肌病或充血性心力衰竭**：MPS患者常有心脏受累，尤其是瓣膜病变。
- **肝脾大**：MPS患者常有肝脾肿大。
- **关节僵硬、爪形手、鸡胸和腰椎后突**：MPS患者常有关节和骨骼系统受累。
- **精神运动发育落后**：MPS I型患者常有智力障碍。
- **生化检查**：酶活性测定和尿液中黏多糖的检测有助于确诊。

### 进一步检查建议：

- **酶活性测定**：检测α-L-艾杜糖苷酸酶活性。
- **尿液黏多糖检测**：检测尿液中硫酸皮肤素和硫酸肝素的水平。
- **基因检测**：确认IDUA基因突变。

这些检查有助于确诊MPS I型并与其他类型的MPS和其他类似疾病进行鉴别诊断。

### 83. User

隐袭起病。缓慢发展或阶段性进展，可有长时间的稳定期。任何年龄均可发病，多于50 岁前起病。临床主要表现为多发性单神经病，不对称性上肢远端起病较常见，早期以单侧上肢某一根或多根神经受累多见，表现为相应神经支配区域的肌肉无力， 远端为主，可伴有痉挛或束颤。随着病情发展，可以出现肌肉萎缩；病程较长者，可有多个肢体的多根神经受累，受累神经的不对称性可不明显，而呈现为类似多发性周围神经病的分布。患者可有轻微感觉异常的主诉，但缺乏客观感觉受累的体征，病程后期部分患者也可出现部分感觉神经受累。脑神经通常不受累。无上运动神经元受累体征。

### Qwen2.5_72b

根据您提供的临床表现，最可能的诊断是**遗传性压迫易感性神经病变（Hereditary Neuropathy with Liability to Pressure Palsies, HNPP）**。HNPP 是一种遗传性周围神经病，主要表现为多发性单神经病，常不对称，早期以单侧上肢远端神经受累为主，可伴有肌肉无力、痉挛或束颤，随着病情进展，可出现肌肉萎缩和多根神经受累。

### 其他五种可能的诊断：

- **多灶性运动神经病（Multifocal Motor Neuropathy, MMN）**：
  - 临床表现与 HNPP 有相似之处，但 MMN 通常表现为不对称的多灶性运动神经病，无感觉异常，且常伴有抗 GM1 抗体阳性。
- **慢性炎症性脱髓鞘性多发性神经病（Chronic Inflammatory Demyelinating Polyneuropathy, CIDP）**：
  - CIDP 通常表现为对称性的多发性周围神经病，可有感觉和运动受累，病程呈慢性进展或阶段性进展，电生理检查常有脱髓鞘改变。
- **Charcot-Marie-Tooth 病（CMT）**：
  - CMT 是一种常见的遗传性周围神经病，表现为对称性的远端肌肉无力和萎缩，常伴有感觉异常，病程呈慢性进展。
- **糖尿病性多发性神经病**：
  - 糖尿病患者可出现多发性周围神经病，表现为对称性的远端感觉和运动受累，常伴有糖尿病的其他表现，如高血糖、多饮、多尿等。
- **免疫介导性神经病（如 POEMS 综合征）**：
  - POEMS 综合征是一种与浆细胞增生相关的多系统疾病，表现为多发性周围神经病，常伴有感觉和运动受累，还可有其他系统的表现，如皮肤改变、内分泌异常、器官肿大等。

这些诊断需要通过详细的病史、体格检查、电生理检查、血液检查、基因检测等进一步明确。

### 84. User

新生儿期起病常致命，可表现为代谢危象，包括代谢性酸中毒、非酮症性低血糖、高氨血症，其他特征包括多系统受累(肌张力低下、心肌病、肝肿大)、排泄大量脂肪酸和氨基酸衍生的代谢产物、“汗脚”味体臭、昏迷、新生儿期死亡。快速进展的肥厚型心肌病常见于新生儿期起病的类型，猝死概率非常大，即使经过新生儿筛查在早期即检测出有此病，也难以逆转。

先天畸形被认为是未被胚胎转运纠正的那部分有毒代谢物累积的后果。脂肪酸氧化代谢受阻的程度决定了子宫内的酸中毒程度，进而阻碍正常的胚胎形成。先天畸形包括增大的多囊肾、摇篮底足(rocker-bottom feet)、下腹部肌肉缺失、尿道下裂和阴茎下弯、肌张力低下、脑皮质发育不良和胶质增生。其他畸形特征包括眼距过宽、耳畸形、巨颅、前囟过宽、高前额、鼻梁低平。

晚发型的发病年龄和症状在不同病例中差异很大，主要表现为不伴有先天畸形，但终身伴有急性间歇发作的呕吐、脱水、低酮性低血糖、酸中毒及意识障碍的风险。年龄大者可有肝脏肿大或脂质沉积性肌病。急性失代偿发作可由感染、发热、手术、低能量饮食或减肥、饮酒、妊娠等诱发。大多数患者发展为慢性肌病症状，包括运动不耐受、肌痛、肌无力、肌肉萎缩。晚发型病人的有机酸尿症常为间歇性，仅在疾病期间和处于分解代谢压力下较为显著。

### Qwen2.5_72b

根据您提供的临床表现，最可能的诊断是**中链酰基辅酶A脱氢酶缺乏症（MCADD, Medium-Chain Acyl-CoA Dehydrogenase Deficiency）**。这是一种脂肪酸氧化障碍，通常在新生儿期或婴儿早期出现症状，表现为代谢危象，包括代谢性酸中毒、非酮症性低血糖、高氨血症等。此外，患者常有多系统受累，如肌张力低下、心肌病、肝肿大等。MCADD患者还可能有“汗脚”味体臭，这是由于代谢产物的排泄所致。快速进展的肥厚型心肌病和新生儿期猝死也是该病的特征。

### 其他可能的诊断：

- **丙酸血症（Propionic Acidemia）**：这是一种有机酸血症，由于丙酸代谢途径中的酶缺陷导致。临床表现包括代谢性酸中毒、高氨血症、低血糖、肌张力低下、心肌病、肝肿大等。新生儿期起病，症状严重，可导致死亡。
- **甲基丙二酸血症（Methylmalonic Acidemia）**：这是一种有机酸血症，由于甲基丙二酸代谢途径中的酶缺陷导致。临床表现包括代谢性酸中毒、高氨血症、低血糖、肌张力低下、心肌病、肝肿大等。新生儿期起病，症状严重，可导致死亡。
- **肉碱棕榈酰转移酶II缺乏症（CPT II Deficiency）**：这是一种脂肪酸氧化障碍，影响肉碱转运。临床表现包括代谢性酸中毒、低血糖、心肌病、肝肿大、肌张力低下等。新生儿期或婴儿早期起病，症状严重，可导致死亡。
- **线粒体脂肪酸氧化障碍（Mitochondrial Fatty Acid Oxidation Disorders）**：这是一组影响线粒体脂肪酸氧化的代谢障碍，包括多种酶缺陷。临床表现包括代谢性酸中毒、低血糖、心肌病、肝肿大、肌张力低下等。新生儿期或婴儿早期起病，症状严重，可导致死亡。
- **尿素循环障碍（Urea Cycle Disorders）**：这是一组影响尿素循环的代谢障碍，导致高氨血症。临床表现包括代谢性酸中毒、高氨血症、低血糖、肌张力低下、心肌病、肝肿大等。新生儿期起病，症状严重，可导致死亡。

这些疾病都需要通过详细的代谢筛查和基因检测来确诊。如果您怀疑患者有这些疾病，建议尽快进行相关检查和咨询遗传代谢病专家。

### 85. User

平均发病年龄为 30 岁，10 岁以下和 50 岁以上患者少见。女性患病率高于男性，女：男比例为（1.4~2.3） ：1。多急性 / 亚急性起病，具有空间多发和时间多发特点。空间多发指病变部位多发，大脑、脑干、小脑、脊髓可同时或相继受累。时间多发指缓解—复发的病程，由于累及部位广泛，反复发作后可最终致残，甚至致死。临床表现多样，常见症状包括视力下降、复视、肢体感觉障碍、肢体运动障碍、共济失调、膀胱或直肠功能障碍等。

### Qwen2.5_72b

根据您提供的临床表现，最可能的诊断是多发性硬化症（Multiple Sclerosis, MS）。这是一种中枢神经系统的慢性炎症性疾病，其特点与您描述的空间多发和时间多发相符，且女性发病率高于男性，发病年龄多在20-40岁之间，与您提供的信息一致。

以下是五种其他可能的诊断：

- **视神经脊髓炎谱系疾病（Neuromyelitis Optica Spectrum Disorders, NMOSD）**：这是一种主要影响视神经和脊髓的自身免疫性疾病，临床表现与多发性硬化症有重叠，但通常病情更为严重，且女性患者更多。
- **急性播散性脑脊髓炎（Acute Disseminated Encephalomyelitis, ADEM）**：这是一种急性炎症性脱髓鞘疾病，通常发生在感染或疫苗接种后，临床表现与多发性硬化症相似，但通常为单相病程。
- **自身免疫性脑炎（Autoimmune Encephalitis）**：这是一种由自身免疫反应引起的脑炎，可导致多种神经系统症状，包括认知障碍、精神症状、运动障碍等。
- **遗传性共济失调（Hereditary Ataxias）**：这是一组遗传性疾病，主要表现为进行性共济失调，可伴有其他神经系统症状，如视力下降、感觉障碍等。
- **慢性炎症性脱髓鞘性多发性神经病（Chronic Inflammatory Demyelinating Polyneuropathy, CIDP）**：这是一种慢性进行性或复发性脱髓鞘性周围神经病，临床表现包括对称性肢体无力和感觉障碍，但主要影响周围神经系统，而非中枢神经系统。

这些疾病在临床表现上可能有重叠，但通过详细的病史、体格检查、实验室检查和影像学检查可以进行鉴别诊断。

### 86. User

主要包括帕金森症、小脑性共济失调、锥体束损害以及自主神经系统损害。首发症状常为自主神经损害，最主要的是心血管功能障碍(如直立性低血压) 和排尿障碍(如尿失禁)，后者在男性患者还容易合并性功能障碍。很多患者病前有长达数年的快速眼动期睡眠行为障碍(RBD)。

### Qwen2.5_72b

根据您提供的临床表现，最可能的诊断是多系统萎缩（Multiple System Atrophy, MSA）。这是一种罕见的神经退行性疾病，主要影响自主神经系统、小脑和锥体束，导致多种症状，包括自主神经功能障碍（如直立性低血压和尿失禁）、小脑性共济失调、帕金森症样症状和锥体束损害。此外，许多患者在疾病早期会出现快速眼动期睡眠行为障碍（RBD）。

以下是五种其他可能的诊断：

- **帕金森病（Parkinson’s Disease, PD）**：
  - 虽然帕金森病也会出现帕金森症样症状，但自主神经功能障碍和小脑性共济失调在PD中相对少见。PD的主要症状是震颤、僵硬、运动迟缓和平衡障碍。
- **进行性核上性麻痹（Progressive Supranuclear Palsy, PSP）**：
  - PSP是一种罕见的神经退行性疾病，主要表现为进行性平衡障碍、垂直凝视麻痹、帕金森症样症状和认知功能障碍。自主神经功能障碍在PSP中不如MSA常见。
- **皮质基底节变性（Corticobasal Degeneration, CBD）**：
  - CBD是一种罕见的神经退行性疾病，主要表现为不对称的运动障碍、肌张力障碍、失用症和认知功能障碍。自主神经功能障碍在CBD中相对少见。
- **路易体痴呆（Dementia with Lewy Bodies, DLB）**：
  - DLB是一种以认知功能障碍、波动性认知障碍、视幻觉和帕金森症样症状为特征的神经退行性疾病。自主神经功能障碍在DLB中可以出现，但不如MSA明显。
- **脊髓小脑性共济失调（Spinocerebellar Ataxia, SCA）**：
  - SCA是一组遗传性神经退行性疾病，主要表现为小脑性共济失调、步态不稳和协调障碍。自主神经功能障碍在SCA中相对少见，但某些亚型（如SCA3）可能会出现类似症状。

综上所述，多系统萎缩（MSA）是最可能的诊断，但需要进一步的临床评估和辅助检查来确诊。

### 87. User

**1. 肌肉，** 四肢远端起始并逐渐向近端进展的无力萎缩，影响手部精细动作，造成垂足。面肌亦可受累。同时有明显肌强直现象，累及肢体肌、咽喉肌等。查体可发现大力握拳后手指不能立即展开，用力闭目后不能立即睁开。叩击手部大鱼际肌、舌肌等，可引出肌强直，称为叩击性肌强直或“肌球”现象。肌强直存在“热身”现象，反复活动后，肌强直减轻。下肢肌痛和全身疲乏感也是常见临床表现。

**2. 心脏，** 心脏传导系统常有受累，出现不同程度的房室传导阻滞和室内传导异常。严重时可造成猝死。确诊患者需定期监测心电图，必要时植入起搏器。

**3. 中枢神经系统** ，睡眠增多、阻塞性睡眠呼吸暂停等睡眠障碍多见。可伴有认知及情绪障碍。头 MRI 可见弥漫性脑白质病变及皮层萎缩。

**4. 眼** ，随着年龄增长，几乎所有患者均会出现白内障。常在 30~40 岁影响视力。

**5. 内分泌系统** ，可出现多种内分泌疾病，包括糖尿病、甲状腺功能异常、钙磷代谢异常、性激素异常、睾丸萎缩(造成不育) 等。

此外，还可有额秃、胃肠动力不足、限制性通气障碍等。

### Qwen2.5_72b

根据您提供的临床表现，最可能的诊断是**强直性肌营养不良症1型（Myotonic Dystrophy Type 1, DM1）**。DM1是一种多系统受累的遗传性神经肌肉疾病，其特征包括肌肉无力和萎缩、肌强直、心脏传导异常、睡眠障碍、白内障、内分泌异常等，与您描述的症状高度吻合。

### 其他可能的诊断：

- **强直性肌营养不良症2型（Myotonic Dystrophy Type 2, DM2）**：
  - 与DM1类似，但通常症状较轻，进展较慢。DM2患者也常有肌肉无力、肌强直、心脏传导异常等症状，但通常不出现明显的白内障和内分泌异常。
- **多发性肌炎（Polymyositis, PM）**：
  - 以对称性近端肌肉无力和炎症为主要特征，可伴有肌痛和肌酶升高。但通常不出现肌强直和心脏传导异常。
- **肢带型肌营养不良症（Limb-Girdle Muscular Dystrophy, LGMD）**：
  - 以肩胛带和骨盆带肌肉无力和萎缩为主要特征，但通常不伴有肌强直和心脏传导异常。
- **进行性肌阵挛性癫痫（Progressive Myoclonic Epilepsy, PME）**：
  - 以肌阵挛、癫痫发作和进行性神经功能衰退为特征，但通常不伴有明显的肌肉无力和萎缩。
- **线粒体肌病（Mitochondrial Myopathy）**：
  - 以肌肉无力、运动不耐受和乳酸酸中毒为特征，可伴有心脏传导异常和内分泌异常，但通常不出现肌强直和白内障。

### 总结：

- **最可能的诊断**：强直性肌营养不良症1型（DM1）
- **其他可能的诊断**：
  1. 强直性肌营养不良症2型（DM2）
  2. 多发性肌炎（PM）
  3. 肢带型肌营养不良症（LGMD）
  4. 进行性肌阵挛性癫痫（PME）
  5. 线粒体肌病

建议进一步进行基因检测、肌电图、心电图、肌肉活检等检查以明确诊断。

### 88. User

该病发病年龄往往较早，最常发病的年龄为新生儿时期。大多患儿生后数日内发病，表现为拒奶、呕吐、嗜睡、惊厥、昏迷。部分晚发型患儿以发作性呕吐、嗜睡，伴精神发育异常为特点。高氨血症昏迷的患者可能有脑部伤害与发展迟缓、学习能力受损或智力障碍以及生长发育落后的后遗症。高氨血症患者可能造成颅内压增高、呼吸急促，病情进展会出现呼吸暂停或衰竭，肝肿大。神经系统并发症包括智力障碍、急性高血氨昏迷，甚至死亡。生化改变可见高氨血症、血谷氨酰胺和丙氨酸增高，尿中乳清酸降低或缺如。

### Qwen2.5_72b

根据您提供的临床表现，最可能的诊断是**尿素循环障碍（Urea Cycle Disorders, UCDs）**，特别是**鸟氨酸转氨甲酰酶缺乏症（Ornithine Transcarbamylase Deficiency, OTC 缺乏症）**。这是一种罕见的遗传性代谢病，主要影响尿素循环，导致体内氨的清除障碍，从而引起高氨血症。

### 1. 尿素循环障碍（Urea Cycle Disorders, UCDs）

- **最可能的诊断**：鸟氨酸转氨甲酰酶缺乏症（OTC 缺乏症）
- **特点**：新生儿期发病，表现为拒奶、呕吐、嗜睡、惊厥、昏迷。高氨血症导致的神经系统并发症包括智力障碍、急性高血氨昏迷，甚至死亡。生化改变可见高氨血症、血谷氨酰胺和丙氨酸增高，尿中乳清酸降低或缺如。

### 2. 其他可能的诊断

#### 2.1 丙酸血症（Propionic Acidemia）

- **特点**：新生儿期或婴儿期发病，表现为呕吐、嗜睡、惊厥、昏迷。高氨血症和代谢性酸中毒是常见表现。生化改变可见血中丙酸和甲基枸橼酸增高。

#### 2.2 异戊酸血症（Isovaleric Acidemia）

- **特点**：新生儿期或婴儿期发病，表现为呕吐、嗜睡、惊厥、昏迷。高氨血症和代谢性酸中毒是常见表现。生化改变可见血中异戊酸增高。

#### 2.3 高胱氨酸尿症（Homocystinuria）

- **特点**：新生儿期或婴儿期发病，表现为生长发育迟缓、智力障碍、惊厥。高氨血症和高同型半胱氨酸血症是常见表现。生化改变可见血中同型半胱氨酸增高。

#### 2.4 肝豆状核变性（Wilson Disease）

- **特点**：多在儿童或青少年期发病，表现为肝功能异常、神经系统症状（如震颤、肌张力障碍）、精神症状。高氨血症和肝肿大是常见表现。生化改变可见血清铜蓝蛋白降低、尿铜增高。

#### 2.5 肝性脑病（Hepatic Encephalopathy）

- **特点**：多见于肝功能衰竭的患者，表现为意识障碍、嗜睡、昏迷。高氨血症是主要生化改变。肝肿大和肝功能异常是常见表现。

这些疾病都有类似的临床表现，但具体的生化改变和遗传背景不同，需要通过详细的实验室检查和基因检测来确诊。

### 89. User

患儿常表现为严重的宫内发育迟缓，出生后很早(常在出生后 1 周) 即出现严重的、非酮症性高血糖，12 周后可恢复，50％ ~60％在青春期前后复发，复发后临床表现类似于2 型糖尿病。

### Qwen2.5_72b

根据您提供的临床表现，最可能的诊断是**暂时性新生儿糖尿病 (Transient Neonatal Diabetes Mellitus, TNDM)**。TNDM 是一种罕见的新生儿代谢性疾病，通常在出生后 1 周内出现严重的高血糖，但大多数患儿在 12 周后可以自行缓解，部分患儿在青春期前后会复发，复发后的临床表现类似于 2 型糖尿病。

以下是其他五种可能的诊断：

- **永久性新生儿糖尿病 (Permanent Neonatal Diabetes Mellitus, PNDM)**：这是一种与 TNDM 类似的疾病，但高血糖不会自行缓解，需要长期治疗。PNDM 通常由基因突变引起，如 KCNJ11 或 ABCC8 基因突变。
- **1 型糖尿病 (Type 1 Diabetes Mellitus)**：虽然 1 型糖尿病通常在儿童或青少年期发病，但极少数情况下，新生儿也可能出现 1 型糖尿病。这种情况下，患儿的高血糖不会自行缓解，需要终身胰岛素治疗。
- **先天性高胰岛素血症 (Congenital Hyperinsulinism)**：这是一种罕见的代谢性疾病，表现为新生儿期严重的低血糖，但有时也可能出现高血糖。这种疾病通常需要药物或手术治疗。
- **胰腺发育不全 (Pancreatic Agenesis or Hypoplasia)**：这是一种罕见的先天性疾病，表现为胰腺发育不全或完全缺失，导致胰岛素分泌不足，从而引起高血糖。这种情况下，患儿需要长期胰岛素治疗。
- **线粒体糖尿病 (Mitochondrial Diabetes)**：这是一种由线粒体基因突变引起的糖尿病，通常表现为早发性糖尿病和听力损失。虽然这种疾病通常在儿童或青少年期发病，但极少数情况下，新生儿也可能受到影响。

这些诊断都需要通过详细的病史、体格检查和实验室检查来进一步确认。特别是基因检测，对于明确诊断和制定治疗方案非常重要。

### 90. User

患者最早出现的症状是腹部膨隆，肝脾增大，部分患者可在新生儿期发病。智力和运动发育落后随即出现，肌张力低下，运动发育迟缓，而脑神经功能常不受累，1 岁后运动智力发育倒退明显，最后进展为痉挛强直状态。50％患者可以发现眼底樱桃红斑。间质性肺部病变可导致反复呼吸道感染、低氧血症或呼吸功能衰竭。多数患儿 3 岁前死亡。

### Qwen2.5_72b

根据您提供的临床表现，最可能的诊断是：

- **戈谢病（Gaucher Disease）**：这是一种溶酶体贮积症，由于葡萄糖脑苷脂酶（glucocerebrosidase）缺乏导致葡萄糖脑苷脂在单核-巨噬细胞系统中积累。临床表现包括肝脾肿大、骨骼病变、贫血、血小板减少等。部分患者可出现神经系统症状，但通常不涉及脑神经功能。眼底樱桃红斑是戈谢病的一个特征性表现，但并非所有患者都会出现。

其他可能的诊断包括：

1. **尼曼-皮克病（Niemann-Pick Disease）**：这是一种溶酶体贮积症，由于鞘磷脂酶（sphingomyelinase）缺乏导致鞘磷脂在细胞内积累。临床表现包括肝脾肿大、神经系统退行性病变、肌张力低下、运动发育迟缓、眼底樱桃红斑等。尼曼-皮克病的类型A和B通常在婴儿期或儿童早期发病，多数患儿在3岁前死亡。
2. **法布里病（Fabry Disease）**：这是一种X连锁遗传的溶酶体贮积症，由于α-半乳糖苷酶A缺乏导致鞘糖脂在多种细胞中积累。临床表现包括疼痛、皮肤血管角质瘤、角膜混浊、肾功能不全、心脏和脑血管病变等。虽然法布里病通常不以肝脾肿大为主要表现，但部分患者可出现类似症状。
3. **黏多糖贮积症（Mucopolysaccharidoses, MPS）**：这是一组溶酶体贮积症，由于特定的溶酶体酶缺乏导致黏多糖在组织中积累。临床表现包括肝脾肿大、骨骼异常、关节僵硬、心脏病变、智力障碍等。不同类型MPS的临床表现有所不同，但多数患者在儿童期或青少年期发病。
4. **泰-萨克斯病（Tay-Sachs Disease）**：这是一种溶酶体贮积症，由于己糖胺酶A缺乏导致GM2神经节苷脂在神经细胞中积累。临床表现包括进行性神经系统退行性病变、肌张力低下、运动发育迟缓、智力障碍等。眼底樱桃红斑是泰-萨克斯病的一个特征性表现。多数患儿在3岁前死亡。

这些疾病都属于罕见病，且有相似的临床表现，因此需要通过详细的病史、体格检查和实验室检查（如酶活性测定、基因检测等）来确诊。

### 91. User

**1. 典型面容，** 所有年龄患者均有上睑下垂，眼距宽，内眦赘皮，双眼外角下斜；双耳位低，后旋，耳廓厚。儿童患者还可有前额饱满，后发际低，鼻短，鼻梁低，鼻尖饱满；唇厚，鼻唇沟深而宽直达上唇等。

**2. 矮小，，** 出生时正常，1 岁后渐出现矮小。骨龄常落后，部分成年身高可达正常下限水平。

**3. 心血管 ，**50％ ~80％患者有先心病，肺动脉瓣狭窄最常见，其他包括肥厚型心肌病，室缺，肺动脉分支狭窄，法洛四联症和主动脉缩窄等。典型心电图改变包括心电轴极度右偏伴胸前导联 QRS 波逆时针旋转，V1 导联心电轴左偏、左前支不全传导阻滞或 RSR 波形。

**4. 精神运动发育落后** ，部分患者语言能力差，成年患者易有抑郁情绪，也有报道此病患者自闭症谱系表现发生率较高。

**5. 颈蹼，**

**6. 胸廓异常 ，**鸡胸或漏斗胸。

**7. 乳距宽**

**8. 男性隐睾** ，60％ ~80％男性患者合并隐睾，可致生育障碍。

**9. 凝血功能异常和淋巴管发育不良**

**10. 其他，** 肾脏畸形(肾盂扩张、双输尿管畸形、孤立肾、肾发育不良、远端输尿管狭窄等)，男性精子生成障碍，斜视，眼颤，皮肤牛奶咖啡斑和雀斑，四肢毛囊角化症， Ⅰ型阿诺德 - 基亚里畸形，脑积水，肝脾大，恶性肿瘤发生率增加等。

**11. 宫内表现，** 母亲孕期羊水过多，胎儿颈部透明带增宽，胎头相对较大，心脏和肾脏异常。

### Qwen2.5_72b

根据您提供的临床表现，最可能的诊断是**努南综合征（Noonan Syndrome）**。努南综合征是一种遗传性综合症，主要由基因突变引起，特别是与RAS/MAPK信号通路相关的基因，如PTPN11、SOS1、RAF1、KRAS、NRAS等。该病的典型特征包括特殊面容、心脏缺陷、生长迟缓、发育迟缓、生殖系统异常等，与您描述的症状高度吻合。

**其他可能的诊断包括：**

- **特纳综合征（Turner Syndrome）**：这是一种影响女性的遗传病，通常由X染色体部分或全部缺失引起。患者常有矮小、颈蹼、心脏缺陷、生殖系统发育不良等特征，但特纳综合征患者通常没有努南综合征中的特殊面容和精神运动发育迟缓。
- **威廉姆斯综合征（Williams Syndrome）**：这是一种由7号染色体微缺失引起的遗传病，患者常有特殊面容（如“精灵脸”）、心血管异常、发育迟缓、社交行为异常等，但威廉姆斯综合征患者通常没有努南综合征中的矮小和生殖系统异常。
- **科斯特洛综合征（Costello Syndrome）**：这是一种由HRAS基因突变引起的遗传病，患者常有特殊面容、生长迟缓、心脏缺陷、皮肤异常、发育迟缓等，但科斯特洛综合征患者通常有更明显的皮肤松弛和多汗症。
- **卡波西样血管内皮瘤病（Klippel-Trenaunay Syndrome）**：这是一种血管和淋巴管发育异常的疾病，患者常有肢体不对称、血管瘤、淋巴管瘤等，但通常没有努南综合征中的特殊面容和心脏缺陷。
- **艾尔-卡菲综合征（Alagille Syndrome）**：这是一种由JAG1或NOTCH2基因突变引起的遗传病，患者常有特殊面容、心脏缺陷、肝胆系统异常、眼异常等，但艾尔-卡菲综合征患者通常有更明显的肝功能异常和眼异常。

努南综合征的诊断通常需要结合临床表现、家族史和基因检测。如果您怀疑患者有努南综合征，建议进行基因检测以确诊。

### 92. User

患者可以在任何年龄阶段发病。临床主要分为早发型和晚发型两种。早发型主要发生在男性患儿，一般在新生儿期发病，临床表现为起病急，病情凶险，因血氨升高，使得大脑广泛性损害。出生时可无异常，生后数天即表现出易激惹、嗜睡、拒食、呼吸急促和昏睡等，可迅速发展为痉挛、昏迷和呼吸衰竭。如果不给予紧急处理，很快发展成遗传代谢性脑病，并常在刚出生的 1 周内死亡，幸存者多遗留严重的智力损害。晚发型多发生在较大年龄的患者中，可以是半合子的男性和杂合子的女性，临床症状相对轻，且表现多样。儿童期和成人期发病的患者多表现为慢性神经系统损伤，如发作性呕吐、头痛、行为异常、谵妄、精神错乱等。患者可有肝大、反复癫痫发作、生长发育迟缓、行为异常等临床表型。尽管晚发型症状较轻，但是在疾病、应激、高蛋白饮食等环境因素应激下会诱发高氨血症的急性发作而威胁生命。杂合子女性携带者多数终身无症状，少数发病，发病年龄及临床表现有个体差异性，既有早发型，也可表现为晚发型。

### Qwen2.5_72b

根据您提供的临床表现，最可能的诊断是**鸟氨酸氨甲酰转移酶缺乏症（OTC 缺乏症）**。这是一种X连锁遗传的尿素循环障碍，主要影响男性，但女性携带者也可能表现出症状。该病的临床表现与您描述的早发型和晚发型症状非常吻合，特别是血氨升高导致的神经系统损害、肝大、生长发育迟缓等。

以下是其他五种可能的诊断：

- **高苯丙氨酸血症（苯丙酮尿症，PKU）**：这是一种常染色体隐性遗传病，由于苯丙氨酸羟化酶缺乏导致苯丙氨酸代谢障碍。患者可表现为智力发育迟缓、癫痫发作、皮肤湿疹等。新生儿筛查可以早期发现。
- **甲基丙二酸血症**：这是一种有机酸代谢障碍，可导致代谢性酸中毒、高氨血症、神经系统损害、肝大、生长发育迟缓等。症状可以是急性的，也可以是慢性的。
- **丙酸血症**：这是一种有机酸代谢障碍，由于丙酸代谢途径中的酶缺陷导致丙酸积累。患者可表现为代谢性酸中毒、高氨血症、神经系统损害、肝大、生长发育迟缓等。
- **肉碱棕榈酰转移酶 II 缺乏症（CPT II 缺乏症）**：这是一种脂肪酸氧化障碍，可导致低血糖、代谢性酸中毒、高氨血症、肌肉无力、肝大等。症状可以是急性的，也可以是慢性的。
- **线粒体脑肌病伴乳酸中毒和中风样发作（MELAS 综合征）**：这是一种线粒体遗传病，可导致代谢性脑病、乳酸中毒、癫痫发作、头痛、呕吐、生长发育迟缓等。症状多在儿童期或青少年期出现，但也可在成年期发病。

这些疾病都可能表现出类似的临床症状，因此需要通过详细的病史、体格检查和实验室检查（如血氨、代谢筛查、基因检测等）来进一步确诊。

### 93. User

疾病的临床表型轻重不一。患者的主要临床表现是自幼起病的轻微外力下反复骨折、骨骼畸形，不同程度活动受限。患者可有蓝巩膜、韧带松弛、牙本质发育不全、听力下降、心脏瓣膜病变等骨骼外表现。OI 的影像学特征主要包括：全身多部位骨质稀疏；颅板变薄，囟门和颅缝增宽，枕骨可有缝间骨， 颅底扁平；脊柱可有侧弯或后凸畸形，椎体呈变形，可有多椎体压缩性骨折；胸廓可扭曲、变形，甚至塌陷；四肢长骨纤细、骨皮质菲薄，骨髓腔相对较大，干骺端增宽，多发长骨骨折，骨骼弯曲畸形等。

### Qwen2.5_72b

根据您提供的临床表现和影像学特征，最可能的诊断是成骨不全症（Osteogenesis Imperfecta, OI），这是一种以骨骼脆弱和反复骨折为特征的遗传性罕见病。此外，还有五种可能的诊断需要考虑：

- **成骨不全症（Osteogenesis Imperfecta, OI）**：
  - **临床表现**：自幼起病的轻微外力下反复骨折、骨骼畸形，不同程度活动受限，蓝巩膜、韧带松弛、牙本质发育不全、听力下降、心脏瓣膜病变等。
  - **影像学特征**：全身多部位骨质稀疏；颅板变薄，囟门和颅缝增宽，枕骨可有缝间骨，颅底扁平；脊柱可有侧弯或后凸畸形，椎体呈变形，可有多椎体压缩性骨折；胸廓可扭曲、变形，甚至塌陷；四肢长骨纤细、骨皮质菲薄，骨髓腔相对较大，干骺端增宽，多发长骨骨折，骨骼弯曲畸形等。
- **骨质疏松症（Osteoporosis）**：
  - **临床表现**：骨质疏松导致的反复骨折，尤其是脊柱和髋部骨折。
  - **影像学特征**：骨密度降低，骨质稀疏，椎体压缩性骨折。
- **马凡综合征（Marfan Syndrome）**：
  - **临床表现**：骨骼异常（如长骨细长、脊柱侧弯）、心血管异常（如主动脉根部扩张）、眼部异常（如晶状体脱位）。
  - **影像学特征**：长骨细长，脊柱侧弯，主动脉根部扩张。
- **埃勒斯-当洛斯综合征（Ehlers-Danlos Syndrome）**：
  - **临床表现**：皮肤弹性增加、关节过度活动、易受伤和骨折。
  - **影像学特征**：关节过度活动，可能有脊柱侧弯和骨质稀疏。
- **软骨发育不全（Achondroplasia）**：
  - **临床表现**：短肢侏儒症，脊柱侧弯，胸廓畸形。
  - **影像学特征**：短肢，脊柱侧弯，胸廓畸形，长骨短而粗。
- **成骨发育不良（Hypophosphatasia）**：
  - **临床表现**：骨骼矿化障碍，反复骨折，骨骼畸形，牙本质发育不全。
  - **影像学特征**：骨质稀疏，骨骼矿化不良，长骨骨折，骨骼畸形。

这些疾病都有类似的临床表现和影像学特征，但具体的诊断需要结合详细的病史、家族史、实验室检查和基因检测来确定。

### 94. User

**1. 全血细胞减少，** 许多患者可以合并全血细胞减少、中性粒细胞降低。临床上表现为乏力、反复感染和出血倾向。

**2. 血红蛋白尿** ，典型的血红蛋白尿呈酱油或浓茶色。一般持续 2~3 天，不加处理自行消退，重者 1~2 周，甚至持续更长时间。有些患者的血红蛋白尿频繁发作，也有相当多的患者在很长的病程或观察期内始终无明确血红蛋白尿发作。

血红蛋白尿发作时可伴有发热、腰痛、腹痛等症状。其他与平滑肌功能障碍相关的症状可以有吞咽困难、腹痛、胃胀、背痛、头痛、食管痉挛、勃起障碍等。少见的情况包括：胆囊管运动障碍、急性胰腺炎、十二指肠或结肠的缺血和溃疡。

**3. 血栓形成**

**4. 黄疸与肝脾肿大** ，不到一半 患者有轻度黄疸。多数患者没有肝脾肿大。

常见的合并症包括感染、贫血性心脏病、胆石症、肾功能不全甚至衰竭等。

### Qwen2.5_72b

根据您提供的临床表现，最可能的诊断是**阵发性睡眠性血红蛋白尿症（PNH）**。这是一种罕见的血液疾病，其特征是红细胞对补体介导的溶血异常敏感，导致血红蛋白尿、贫血、血栓形成和骨髓功能障碍。

### 1. 阵发性睡眠性血红蛋白尿症（PNH）

- **全血细胞减少**：PNH患者常伴有骨髓功能障碍，导致全血细胞减少，表现为乏力、反复感染和出血倾向。
- **血红蛋白尿**：典型的血红蛋白尿呈酱油或浓茶色，发作时可伴有发热、腰痛、腹痛等症状。
- **血栓形成**：PNH患者有较高的血栓形成风险，尤其是静脉血栓。
- **黄疸与肝脾肿大**：部分患者可出现轻度黄疸，但肝脾肿大不常见。

### 2. 其他可能的诊断

#### 2.1 骨髓增生异常综合征（MDS）

- **全血细胞减少**：MDS患者常有全血细胞减少，表现为贫血、感染和出血。
- **血红蛋白尿**：不常见，但溶血性贫血可能导致血红蛋白尿。
- **血栓形成**：不常见，但有血栓风险。
- **黄疸与肝脾肿大**：不常见，但肝脾肿大可能与肝功能异常有关。

#### 2.2 再生障碍性贫血（AA）

- **全血细胞减少**：AA患者有严重的全血细胞减少，表现为贫血、感染和出血。
- **血红蛋白尿**：不常见，但溶血性贫血可能导致血红蛋白尿。
- **血栓形成**：不常见。
- **黄疸与肝脾肿大**：不常见。

#### 2.3 自身免疫性溶血性贫血（AIHA）

- **全血细胞减少**：AIHA患者常有贫血，但全血细胞减少不常见。
- **血红蛋白尿**：溶血性贫血可能导致血红蛋白尿。
- **血栓形成**：不常见。
- **黄疸与肝脾肿大**：部分患者可有轻度黄疸和肝脾肿大。

#### 2.4 遗传性球形红细胞增多症（HS）

- **全血细胞减少**：不常见，但溶血性贫血可能导致贫血。
- **血红蛋白尿**：溶血性贫血可能导致血红蛋白尿。
- **血栓形成**：不常见。
- **黄疸与肝脾肿大**：部分患者可有轻度黄疸和肝脾肿大。

#### 2.5 药物诱导的溶血性贫血

- **全血细胞减少**：不常见，但溶血性贫血可能导致贫血。
- **血红蛋白尿**：溶血性贫血可能导致血红蛋白尿。
- **血栓形成**：不常见。
- **黄疸与肝脾肿大**：部分患者可有轻度黄疸和肝脾肿大。

这些诊断中，PNH是最符合您提供的临床表现的，但其他疾病也需要考虑，特别是当某些症状不典型或有其他合并症时。建议进一步进行相关实验室检查和影像学检查以明确诊断。

### 95. User

临床表现为反复肠套叠、腹痛、腹泻、黏液便、便血、便秘、呕血、贫血等。特征性临床表现为皮肤黏膜色素斑和胃肠道多发息肉。95% 以上患者出现色素沉着，色素沉着多见于口唇及其四周、颊部、面部、手指、手掌、足底、肛周等，偶见于肠黏膜。色素可呈褐色、棕褐色、灰色和蓝色等，通常发生 2 岁前，大小 1_5mm。约88%_100% 患者会发生胃肠道息肉，胃肠道息肉发病多发生于 11~13 岁，息肉可发生在整个胃肠道，以小肠多见，其次是结肠和胃。息肉大小不定，蒂长短粗细不一。该类疾病患癌风险较高，平均患癌年龄为 42 岁。

### Qwen2.5_72b

根据您提供的临床表现，最可能的诊断是**Peutz-Jeghers 综合征**（PJS）。这是一种罕见的遗传性综合症，主要特征是皮肤和黏膜的色素沉着以及胃肠道多发性息肉。PJS 患者有较高的癌症风险，尤其是胃肠道癌症。

以下是其他五种可能的诊断：

- **家族性腺瘤性息肉病（FAP）**：这是一种遗传性综合症，主要表现为结肠和直肠内多发性腺瘤性息肉，患者也有较高的结直肠癌风险。虽然 FAP 通常不伴有皮肤和黏膜的色素沉着，但其临床表现中的多发性息肉与 PJS 有相似之处。
- **黑斑息肉病（Peutz-Jeghers 综合征的同义词）**：虽然这是同一个疾病，但有时可能会被误诊为其他类似的综合症，如 Cowden 综合征。
- **Cowden 综合征**：这是一种以多发性错构瘤为特征的遗传性综合症，常伴有皮肤、黏膜、乳腺、甲状腺和胃肠道的病变。虽然 Cowden 综合征的皮肤和黏膜病变与 PJS 有相似之处，但其胃肠道息肉的特征不同。
- **幼年性息肉病（Juvenile Polyposis Syndrome, JPS）**：这是一种以胃肠道多发性幼年性息肉为特征的遗传性综合症。虽然 JPS 也表现为胃肠道多发性息肉，但通常不伴有皮肤和黏膜的色素沉着。
- **神经纤维瘤病 1 型（Neurofibromatosis Type 1, NF1）**：这是一种以皮肤咖啡牛奶斑、神经纤维瘤和多发性错构瘤为特征的遗传性综合症。虽然 NF1 也表现为皮肤色素沉着，但其胃肠道息肉的特征与 PJS 不同。

综上所述，根据您提供的临床表现，最可能的诊断是 Peutz-Jeghers 综合征（PJS），但其他几种综合症也需要考虑和排除。

### 96. User

患儿在新生儿期多无临床症状，出生 3~4 个月后逐渐出现典型症状，1 岁时症状明显。出生数月后因黑色素合成不足，其毛发和虹膜色泽逐渐变浅，为黄色或棕黄色，皮肤白。由于尿液、汗液含有大量苯乳酸而有鼠尿臭味。随着年龄增长，逐渐表现出智能发育迟缓，以认知发育障碍为主、小头畸形、癫痫发作，也可出现行为、性格等异常，如多动、自残、攻击、自闭症、自卑、忧郁等神经系统表现。婴儿期还常出现呕吐、湿疹等。

### Qwen2.5_72b

根据您提供的临床表现，最可能的诊断是苯丙酮尿症（Phenylketonuria, PKU）。这是一种由于苯丙氨酸羟化酶（PAH）基因突变导致苯丙氨酸代谢障碍的遗传性代谢病。以下是其他五种可能的诊断：

- **高胱氨酸尿症（Homocystinuria）**：
  - 临床表现包括智力发育迟缓、小头畸形、癫痫发作、行为异常等。但高胱氨酸尿症患者通常有高胱氨酸尿和血中高胱氨酸水平升高，且常有视力问题和骨骼异常。
- **枫糖尿病（Maple Syrup Urine Disease, MSUD）**：
  - 临床表现包括智力发育迟缓、癫痫发作、行为异常等。但枫糖尿病患者尿液有枫糖浆样气味，且常有代谢性酸中毒和酮症。
- **酪氨酸血症（Tyrosinemia）**：
  - 临床表现包括智力发育迟缓、癫痫发作、行为异常等。但酪氨酸血症患者常有肝功能异常、肝肿大、肾功能异常等症状。
- **先天性甲状腺功能减退症（Congenital Hypothyroidism）**：
  - 临床表现包括智力发育迟缓、生长发育迟缓、小头畸形等。但先天性甲状腺功能减退症患者常有黄疸持续不退、便秘、腹胀等症状，且血中甲状腺激素水平降低。
- **先天性肾上腺皮质增生症（Congenital Adrenal Hyperplasia, CAH）**：
  - 临床表现包括智力发育迟缓、生长发育迟缓、行为异常等。但先天性肾上腺皮质增生症患者常有电解质紊乱、高血压、性征异常等症状。

综合以上信息，苯丙酮尿症是最符合您描述的临床表现的诊断。建议进行苯丙氨酸水平检测和基因检测以确诊。

### 97. User

主要为皮肤症状和神经精神症状。

**1. 皮肤症状群，** 为光照后在皮肤暴露部位出现红斑、疱疹甚至溃烂。皮疹可为湿疹、荨麻疹、夏令痒疹或多形性红斑等类型。口腔黏膜可有红色斑点，牙呈棕红色。同时可并发眼损害如结膜炎、角膜炎及虹膜炎等。严重者可有鼻、耳、手指皮肤结瘢变形。可有特殊紫色面容。可有多毛症。

**2. 神经精神症状群 ，**最常见的表现为急性腹痛，腹痛往往为重度、稳定、定位不明，有时会伴有痛性痉挛。可以伴随便秘、腹胀、恶心、呕吐，有时出现腹泻和肠鸣音增加。感觉和运动神经病可能先于腹痛出现，表现为肢体痛，伴麻木感、感觉异常和感觉倒错； 运动无力往往始于上肢近端，可向下肢和远端进展。累及脑神经可能导致延髓麻痹、呼吸功能受损和死亡。严重运动神经病可导致四肢瘫痪。自主神经系统受累表现为心动过速、高血压、出汗、躁动和震颤等。神经源性膀胱功能障碍可能引起尿痛、排尿困难、尿潴留和尿失禁。其他急性神经精神表现包括失眠、焦虑、躁动、激越、幻觉、癔症、定向障碍、谵妄、情感淡漠、抑郁、恐惧症和意识改变，从嗜睡到昏迷程度不一。

### Qwen2.5_72b

根据您提供的临床表现，最可能的诊断是**卟啉病（Porphyria）**。这是一种由于血红素生物合成途径中酶缺陷导致的代谢障碍性疾病，主要分为急性神经型卟啉病和皮肤型卟啉病两大类。您描述的皮肤症状和神经精神症状都与卟啉病的典型表现相符。

### 1. 卟啉病（Porphyria）

- **皮肤症状**：光照后出现红斑、疱疹、溃烂，多形性红斑，口腔黏膜红色斑点，牙呈棕红色，眼损害如结膜炎、角膜炎及虹膜炎，鼻、耳、手指皮肤结瘢变形，特殊紫色面容，多毛症。
- **神经精神症状**：急性腹痛，便秘、腹胀、恶心、呕吐，感觉和运动神经病，肢体痛，麻木感、感觉异常和感觉倒错，运动无力，延髓麻痹，呼吸功能受损，心动过速、高血压、出汗、躁动和震颤，神经源性膀胱功能障碍，失眠、焦虑、躁动、激越、幻觉、癔症、定向障碍、谵妄、情感淡漠、抑郁、恐惧症和意识改变。

### 2. 其他可能的诊断

- **系统性红斑狼疮（Systemic Lupus Erythematosus, SLE）**
  - **皮肤症状**：光照后出现红斑、蝶形红斑、口腔溃疡、多形性红斑。
  - **神经精神症状**：头痛、认知障碍、抑郁、焦虑、癫痫发作、精神症状。
- **硬皮病（Systemic Sclerosis）**
  - **皮肤症状**：皮肤硬化、色素沉着、雷诺现象、多毛症。
  - **神经精神症状**：肌肉无力、关节痛、吞咽困难、消化道症状。
- **多发性硬化症（Multiple Sclerosis, MS）**
  - **皮肤症状**：较少见，但可有感觉异常。
  - **神经精神症状**：视力障碍、肌肉无力、感觉异常、平衡失调、认知障碍、抑郁、焦虑。
- **维生素B12缺乏症（Vitamin B12 Deficiency）**
  - **皮肤症状**：皮肤苍白、黄疸。
  - **神经精神症状**：感觉异常、肌肉无力、步态不稳、认知障碍、抑郁、焦虑。
- **自身免疫性脑炎（Autoimmune Encephalitis）**
  - **皮肤症状**：较少见，但可有感觉异常。
  - **神经精神症状**：认知障碍、精神症状、癫痫发作、运动障碍、自主神经功能障碍。

这些疾病虽然也有类似的症状，但根据您提供的详细临床表现，卟啉病是最可能的诊断。建议进一步进行实验室检查，如尿卟啉、血卟啉、粪卟啉等，以确诊。

### 98. User

**1. 感染，患者**均有不同程度感染。 感染具有反复、严重、难治、机会致病、病原谱广等特点。典型患儿出生时一般没有特别表现，由于患儿存在细胞免疫异常，2 月龄前也可发生致命感染。感染部位及病原多种多样，包括反复肺炎，反复鹅口疮，持续腹泻，反复中耳炎，持续病毒感染(如呼吸道合胞病毒、EBV、CMV 等)，卡氏肺囊虫等条件致病菌感染也较常见。

**2. 疫苗病，** 患者免疫功能极度低下，可发生减毒活疫苗感染和播散。 患儿接种减毒轮状病毒疫苗也可发生严重腹泻。

**3. 生长发育落后或停滞** 是 疾病的显著特征。与患儿反复感染、腹泻和相应基因缺陷均有关。

患儿还可伴有皮疹、肝脾淋巴结肿大类似红皮病表现

另一部分患儿可出现类似红皮病、肝脾肿大、脂溢性皮炎、血细胞减少症，硬化性胆管炎。

### Qwen2.5_72b

根据您提供的临床表现，最可能的诊断是**严重联合免疫缺陷病（Severe Combined Immunodeficiency, SCID）**。SCID 是一种罕见的原发性免疫缺陷病，主要由于 T 细胞和 B 细胞功能缺陷导致严重的免疫功能低下，患者常在婴儿期出现反复、严重的感染，生长发育落后，以及接种减毒活疫苗后发生播散性感染。

### 1. 严重联合免疫缺陷病（Severe Combined Immunodeficiency, SCID）

- **临床表现**：反复、严重的感染，包括肺炎、鹅口疮、腹泻、中耳炎、病毒感染（如呼吸道合胞病毒、EBV、CMV）、卡氏肺囊虫感染等。生长发育落后或停滞，皮疹，肝脾淋巴结肿大，类似红皮病表现。

### 2. 其他可能的诊断

- **X-连锁无丙种球蛋白血症（X-linked Agammaglobulinemia, XLA）**
  - **临床表现**：反复细菌感染，尤其是呼吸道和消化道感染，生长发育落后，但通常不涉及 T 细胞缺陷，因此对减毒活疫苗的反应可能不同。
- **慢性肉芽肿病（Chronic Granulomatous Disease, CGD）**
  - **临床表现**：反复细菌和真菌感染，尤其是卡氏肺囊虫感染，皮疹，肝脾肿大，生长发育落后。
- **Wiskott-Aldrich 综合征（Wiskott-Aldrich Syndrome, WAS）**
  - **临床表现**：反复感染，血小板减少，湿疹，生长发育落后，肝脾肿大。
- **高 IgE 综合征（Hyper-IgE Syndrome, HIES）**
  - **临床表现**：反复皮肤和肺部感染，高 IgE 水平，湿疹，生长发育落后，皮疹，肝脾肿大。
- **DiGeorge 综合征（DiGeorge Syndrome, DGS）**
  - **临床表现**：T 细胞缺陷导致的反复感染，心脏畸形，低钙血症，生长发育落后，皮疹，肝脾肿大。

这些疾病都可能导致类似的临床表现，但具体的诊断需要通过详细的病史、体格检查、实验室检查（如免疫功能检测、基因检测等）来进一步确认。

### 99. User

核心症状是肌张力不全，主要表现为异常的表情姿势和不自主的动作，多累及头颈部肌肉、躯干肌、肢体的旋前肌、指腕屈肌、趾伸肌和跖屈肌等。随意运动时加重，休息睡眠时减轻或消失，晚期可呈固定扭曲痉挛畸形。感觉诡计(缓解技巧) 和动作特异性是肌张力不全的特征性表现。感觉诡计(缓解技巧) 是指专门用于纠正异常姿势或缓解肌张力不全性运动的随意动作，通常是涉及或针对受累部位的简单运动，但不是用力对抗肌张力不全症状。动作特异性是指仅在特定活动或执行特定任务时出现肌张力不全，例如某些职业(如书写痉挛、音乐家痉挛) 或运动。

### Qwen2.5_72b

根据您提供的临床表现，最可能的诊断是**原发性肌张力障碍（Primary Dystonia）**。这是一种神经系统疾病，主要特征是持续的肌肉收缩导致异常的姿势和不自主的动作。原发性肌张力障碍通常没有其他神经系统疾病的证据，如脑部病变或代谢异常。

以下是其他五种可能的诊断：

- **继发性肌张力障碍（Secondary Dystonia）**：
  - 继发性肌张力障碍是由其他疾病或因素引起的，如脑部损伤、代谢异常、药物副作用等。临床表现与原发性肌张力障碍相似，但通常伴有其他神经系统症状。
- **帕金森病（Parkinson’s Disease）**：
  - 帕金森病是一种慢性进行性神经系统疾病，主要表现为震颤、僵硬、运动迟缓和平衡障碍。部分患者可能伴有肌张力障碍，尤其是在疾病晚期。
- **亨廷顿病（Huntington’s Disease）**：
  - 亨廷顿病是一种遗传性神经退行性疾病，主要表现为不自主的舞蹈样动作、认知障碍和精神症状。部分患者可能伴有肌张力障碍。
- **多巴反应性肌张力障碍（Dopa-Responsive Dystonia, DRD）**：
  - DRD是一种遗传性肌张力障碍，通常在儿童或青少年期发病。患者对多巴胺前体药物（如左旋多巴）反应良好，症状可以显著改善。
- **肌阵挛性癫痫（Myoclonic Epilepsy）**：
  - 肌阵挛性癫痫是一种以短暂、快速的肌肉抽搐为特征的癫痫类型。虽然主要表现为肌阵挛，但部分患者可能伴有肌张力障碍。

为了进一步确诊，建议进行详细的神经系统检查、基因检测、影像学检查（如MRI）和实验室检查（如血液和尿液检查），以排除其他可能的病因。

### 100. User

**1. 肾脏 ，**主要表现为肢体水肿和尿中泡沫增多，实验室检查可以发现单纯的中等量蛋白尿或肾病综合征，晚期可出现肾功能不全。

**2. 心脏 ，**主要表现为活动后气短、肢体水肿、胸腔积液、腹水等限制性心功能不全表现。

**3. 肝脏 ，**可有轻微肝区不适或疼痛，但多数患者可无症状，往往为体检时发现肝大或者肝酶异常。疾病晚期可出现肝功能衰竭。

**4. 周围神经和自主神经 ，**为对称性的四肢感觉和(或) 运动性周围神经病。自主神经异常多表现为直立性低血压、胃轻瘫、假性肠梗阻和阳痿等。

**5. 胃肠道** ，可以出现全胃肠道受累，以胃部和小肠受累多见。可以表现为上腹不适、消化不良、腹泻、便秘、吸收不良综合征和消化道出血等。

**6. 软组织 ，**舌体受累可出现巨舌、舌体活动障碍和构音异常等。皮肤黏膜可以出现皮肤紫癜和瘀斑，以眼眶周围和颈部皮肤松弛部位较为常见。也可以出现指甲萎缩脱落和毛发脱落等。

**7. 凝血功能异常** 患者常会伴发凝血因子Ⅹ缺乏，造成相应的出血表现。

### Qwen2.5_72b

根据您提供的临床表现，这些症状高度提示一种罕见疾病：**淀粉样变性**（Amyloidosis）。淀粉样变性是一种由于异常蛋白质（淀粉样蛋白）在组织中沉积导致器官功能障碍的疾病。淀粉样蛋白可以沉积在多个器官，包括肾脏、心脏、肝脏、周围神经、胃肠道、软组织等，导致相应的临床表现。

### 1. 淀粉样变性（Amyloidosis）

- **肾脏**：蛋白尿、肾病综合征、肾功能不全。
- **心脏**：限制性心功能不全、气短、水肿、胸腔积液、腹水。
- **肝脏**：肝大、肝酶异常、肝功能衰竭。
- **周围神经和自主神经**：对称性周围神经病、直立性低血压、胃轻瘫、假性肠梗阻、阳痿。
- **胃肠道**：全胃肠道受累、上腹不适、消化不良、腹泻、便秘、吸收不良综合征、消化道出血。
- **软组织**：巨舌、舌体活动障碍、构音异常、皮肤紫癜和瘀斑、指甲萎缩脱落、毛发脱落。
- **凝血功能异常**：凝血因子Ⅹ缺乏、出血表现。

### 2. 其他可能的诊断

- **多发性骨髓瘤（Multiple Myeloma）**
  - **肾脏**：蛋白尿、肾功能不全。
  - **心脏**：心功能不全。
  - **肝脏**：肝功能异常。
  - **周围神经**：周围神经病。
  - **软组织**：骨痛、骨折、高钙血症。
- **系统性硬化症（Systemic Sclerosis）**
  - **肾脏**：肾功能不全。
  - **心脏**：心功能不全。
  - **肝脏**：肝功能异常。
  - **周围神经**：周围神经病。
  - **胃肠道**：胃轻瘫、假性肠梗阻。
  - **软组织**：皮肤硬化、雷诺现象。
- **系统性红斑狼疮（Systemic Lupus Erythematosus, SLE）**
  - **肾脏**：肾炎、蛋白尿、肾功能不全。
  - **心脏**：心包炎、心肌炎。
  - **肝脏**：肝功能异常。
  - **周围神经**：周围神经病。
  - **胃肠道**：腹痛、腹泻。
  - **软组织**：皮疹、关节痛。
- **慢性淋巴细胞性甲状腺炎（Hashimoto’s Thyroiditis）**
  - **肾脏**：蛋白尿。
  - **心脏**：心功能不全。
  - **肝脏**：肝功能异常。
  - **周围神经**：周围神经病。
  - **胃肠道**：消化不良、便秘。
  - **软组织**：皮肤干燥、毛发脱落。
- **遗传性出血性毛细血管扩张症（Hereditary Hemorrhagic Telangiectasia, HHT）**
  - **肾脏**：蛋白尿。
  - **心脏**：心功能不全。
  - **肝脏**：肝功能异常。
  - **周围神经**：周围神经病。
  - **胃肠道**：消化道出血。
  - **软组织**：皮肤和黏膜出血点。

这些疾病虽然也有类似的临床表现，但淀粉样变性的多器官受累和特定的病理特征使其成为最可能的诊断。建议进一步进行淀粉样蛋白的特异性检查，如组织活检和免疫组化，以确诊。

### 101. User

黄疸和皮肤瘙痒是典型临床表现。其他有身材矮小、青春期发育落后等发育迟缓表现，胆囊结石，脂肪吸收障碍所致的脂肪泻，肝脾肿大以及脂溶性维生素缺乏所致佝偻病、骨龄延迟、干眼症、凝血障碍和神经肌肉病变等症状。患儿亦可以出现视觉及听力异常，出现烦躁、嗜睡及注意力不集中等改变。晚期可出现门静脉高压症和肝脏肿瘤等。

### Qwen2.5_72b

根据您提供的临床表现，最可能的诊断是**Alagille综合征**（Alagille syndrome, ALGS）。这是一种罕见的遗传性疾病，主要影响肝脏、心脏、骨骼、眼睛和面部特征。Alagille综合征的典型临床表现包括黄疸、皮肤瘙痒、胆汁淤积、肝脾肿大、身材矮小、青春期发育迟缓、胆囊结石、脂肪吸收障碍、脂溶性维生素缺乏（如维生素A、D、E、K缺乏）所致的佝偻病、骨龄延迟、干眼症、凝血障碍和神经肌肉病变等。此外，患者还可能出现视觉和听力异常，以及烦躁、嗜睡和注意力不集中等神经系统症状。晚期可出现门静脉高压症和肝脏肿瘤等严重并发症。

以下是其他五种可能的诊断：

- **进行性家族性肝内胆汁淤积症**（Progressive Familial Intrahepatic Cholestasis, PFIC）：这是一种遗传性肝病，主要表现为肝内胆汁淤积，导致黄疸、皮肤瘙痒、肝脾肿大、脂肪吸收障碍和脂溶性维生素缺乏等症状。
- **胆道闭锁**（Biliary Atresia）：这是一种婴儿期常见的胆道发育异常，表现为黄疸、皮肤瘙痒、肝脾肿大、脂肪吸收障碍和脂溶性维生素缺乏。如果不及时治疗，可导致肝硬化和肝功能衰竭。
- **囊性纤维化**（Cystic Fibrosis）：这是一种遗传性疾病，主要影响肺部和消化系统，表现为脂肪吸收障碍、脂肪泻、肝脾肿大、生长发育迟缓和脂溶性维生素缺乏。
- **α1-抗胰蛋白酶缺乏症**（Alpha-1 Antitrypsin Deficiency）：这是一种遗传性疾病，主要影响肺部和肝脏，表现为肝功能异常、黄疸、肝脾肿大、脂肪吸收障碍和脂溶性维生素缺乏。
- **威尔森病**（Wilson’s Disease）：这是一种遗传性铜代谢障碍疾病，主要影响肝脏和神经系统，表现为肝功能异常、黄疸、肝脾肿大、神经肌肉病变、视觉和听力异常等。

这些疾病都有类似的临床表现，但具体的诊断需要通过详细的病史、体格检查、实验室检查和影像学检查来确定。

### 102. User

在儿童期起病。表现为运动发育轻度迟滞，骨骼肌进行性无力萎缩，影响肢体运动功能，逐渐出现步态异常、上肢活动受限，自然病程常在 10 岁左右丧失行走能力。此后出现脊柱侧弯、关节挛缩、呼吸肌无力、扩张型心肌病，20 岁左右因呼吸衰竭、心功能衰竭死亡。查体可见双腓肠肌假肥大，同时可有双前臂及舌肌假肥大，Gowers 征阳性，腰椎前凸等。可青年甚至成年起病， 部分患者不影响生存期。假肥大体征明显，部分患者在肢体无力尚轻时，先出现明显的扩张型心肌病。

### Qwen2.5_72b

根据您提供的临床表现，最可能的诊断是**杜氏肌营养不良（Duchenne Muscular Dystrophy, DMD）**。DMD 是一种 X 连锁隐性遗传病，主要影响男性，其特征是进行性骨骼肌无力和萎缩，通常在儿童期起病，最终导致行走能力丧失、脊柱侧弯、关节挛缩、呼吸肌无力和扩张型心肌病。以下是其他五种可能的诊断：

- **贝克尔型肌营养不良（Becker Muscular Dystrophy, BMD）**：
  - 与 DMD 类似，但症状较轻，起病年龄较晚，进展较慢，部分患者不影响生存期。
  - 也表现为进行性肌肉无力和萎缩，但通常在青少年或成年起病。
- **肢带型肌营养不良（Limb-Girdle Muscular Dystrophy, LGMD）**：
  - 一种遗传性肌病，影响肩带和骨盆带肌肉。
  - 表现为进行性肌肉无力和萎缩，步态异常，Gowers 征阳性，但通常不伴有明显的假肥大。
- **脊髓性肌萎缩症（Spinal Muscular Atrophy, SMA）**：
  - 一种常染色体隐性遗传病，影响脊髓前角细胞，导致肌肉无力和萎缩。
  - 根据起病年龄和严重程度分为不同类型，部分类型在儿童期起病，进展较快。
- **先天性肌营养不良（Congenital Muscular Dystrophy, CMD）**：
  - 一组遗传性肌病，通常在出生时或婴儿期起病。
  - 表现为肌无力、肌张力低下、关节挛缩，部分类型伴有智力障碍。
- **糖原贮积病（Glycogen Storage Disease, GSD）**：
  - 一组代谢性疾病，影响糖原代谢，导致肌肉无力和萎缩。
  - 不同类型有不同的临床表现，部分类型可表现为进行性肌肉无力和心肌病。

这些疾病在临床表现上有一定的相似性，但通过详细的病史、体格检查、实验室检查（如基因检测、肌电图、肌肉活检等）可以进行鉴别诊断。

### 103. User

主要为高血氨、脑损伤和心肌病等。

**1. 新生儿起病型** 出生时正常，开始哺乳后出现呕吐、嗜睡、肌张力低下、惊厥、呼吸困难、高血氨、酮症、低血糖、酸中毒、扩张型心肌病、胰腺炎等异常，病死率高。

**2. 迟发型** 常因发热、饥饿、高蛋白饮食和感染等诱发，表现为婴幼儿期喂养困难、发育落后、惊厥、肌张力低下等。许多患者的认知能力及神经系统发育受到损害，脑电图慢波增多或见癫痫波；一些患者可有骨折，X 线见骨质疏松；还常造成骨髓抑制，引起粒细胞减少、贫血、血小板减少。也可有心脏损害，如心肌病、心律失常、QT 间期延长、心功能减弱等。肾功能损害较为少见。

### Qwen2.5_72b

根据您提供的临床表现，最可能的诊断是**尿素循环障碍（Urea Cycle Disorders, UCDs）**，特别是**鸟氨酸转氨甲酰酶缺乏症（OTC 缺乏症）**。尿素循环障碍是一组罕见的遗传性代谢病，主要影响肝脏中尿素的合成，导致血氨水平升高，进而引起一系列神经系统和心脏症状。

### 1. 尿素循环障碍（Urea Cycle Disorders, UCDs）

- **新生儿起病型**：出生时正常，开始哺乳后出现呕吐、嗜睡、肌张力低下、惊厥、呼吸困难、高血氨、酮症、低血糖、酸中毒、扩张型心肌病、胰腺炎等异常，病死率高。
- **迟发型**：常因发热、饥饿、高蛋白饮食和感染等诱发，表现为婴幼儿期喂养困难、发育落后、惊厥、肌张力低下等。许多患者的认知能力及神经系统发育受到损害，脑电图慢波增多或见癫痫波；一些患者可有骨折，X 线见骨质疏松；还常造成骨髓抑制，引起粒细胞减少、贫血、血小板减少。也可有心脏损害，如心肌病、心律失常、QT 间期延长、心功能减弱等。肾功能损害较为少见。

### 其他可能的诊断：

- **有机酸血症（Organic Acidemias）**：
  - 例如：丙酸血症、甲基丙二酸血症等。这些疾病也常表现为高血氨、代谢性酸中毒、酮症、低血糖、神经系统症状等。
- **线粒体疾病（Mitochondrial Disorders）**：
  - 例如：MELAS 综合征（线粒体脑肌病伴乳酸酸中毒和中风样发作）。这些疾病可导致多系统受累，包括脑损伤、心肌病、代谢异常等。
- **糖原贮积病（Glycogen Storage Diseases）**：
  - 例如：Pompe 病（II 型糖原贮积病）。这些疾病可导致心肌病、肝肿大、低血糖、代谢性酸中毒等。
- **氨基酸代谢障碍（Amino Acid Metabolism Disorders）**：
  - 例如：枫糖尿病（Maple Syrup Urine Disease, MSUD）。这些疾病可导致高血氨、代谢性酸中毒、神经系统症状等。
- **脂肪酸氧化障碍（Fatty Acid Oxidation Disorders）**：
  - 例如：中链酰基辅酶 A 脱氢酶缺乏症（MCADD）。这些疾病可导致代谢性酸中毒、低血糖、高血氨、神经系统症状等。

这些疾病都需要通过详细的病史、体格检查、实验室检查（如血氨、氨基酸谱、有机酸谱、基因检测等）来进一步确诊。

### 104. User

临床表现常没有特异性，隐匿起病。可出现咳痰、消瘦、乏力等症状。继发感染时可有发热、咳脓性痰，也可出现胸痛、咯血。少数患者可无症状，可以没有阳性体征。部分患者可以出现爆裂音，重症患者可有发绀，少数患者可见杵状指。临床表现常比胸部影像受累的程度轻，这种症状和影像不一致的表现也是该病 的一个特征。血清抗 GM-CSF 抗体测定明显增高。

### Qwen2.5_72b

根据您提供的临床表现，最可能的诊断是**肺泡蛋白沉积症（Pulmonary Alveolar Proteinosis, PAP）**。这是一种罕见的肺部疾病，其特征是肺泡内积聚了大量的脂蛋白物质，导致呼吸功能障碍。血清抗GM-CSF抗体测定明显增高是PAP的一个重要诊断指标。

以下是其他五种可能的诊断：

- **肺结核**：肺结核可以表现为慢性咳嗽、咳痰、发热、消瘦和乏力等症状。影像学上可能显示肺部浸润或空洞形成，但血清抗GM-CSF抗体通常不会增高。
- **特发性肺纤维化（Idiopathic Pulmonary Fibrosis, IPF）**：IPF是一种慢性、进行性的间质性肺病，表现为干咳、进行性呼吸困难、爆裂音和杵状指。影像学上可见蜂窝肺改变，但血清抗GM-CSF抗体通常不会增高。
- **慢性阻塞性肺疾病（Chronic Obstructive Pulmonary Disease, COPD）**：COPD表现为慢性咳嗽、咳痰、呼吸困难和乏力。影像学上可见肺气肿改变，但血清抗GM-CSF抗体通常不会增高。
- **肺部感染（如肺炎）**：肺部感染可以表现为发热、咳脓性痰、胸痛和咯血。影像学上可见肺部浸润或实变，但血清抗GM-CSF抗体通常不会增高。
- **肺癌**：肺癌可以表现为慢性咳嗽、咳血、胸痛、消瘦和乏力。影像学上可见肺部肿块或结节，但血清抗GM-CSF抗体通常不会增高。

综合考虑，PAP是最符合您提供的临床表现和实验室检查结果的诊断。建议进一步进行高分辨率CT（HRCT）和支气管肺泡灌洗（BAL）等检查以确诊。

### 105. User

大多患者青少年时期起病，首先是视杆细胞逐渐丧失，随后出现视锥细胞受累， 视网膜色素上皮细胞死亡。因此通常以夜盲为首发症状(但城市化后部分患者常以视力下降为首发症状)，而且夜盲出现得越早，患者的病情往往越严重。虽然周边视野缺损也发生较早，但患者通常意识不到，很少以此作为疾病早期的主诉。病情逐渐发展为中心视力下降，视野进一步缩窄，多数在 40 岁之前就成为法定盲人。患者的视功能损伤程度可与遗传方式相关。

患者的眼底表现与疾病所处的阶段相关：疾病早期患者眼底在常规检查下可表现正常或接近正常，但部分患者在中周部眼底照相下可以观察到早期毯层样视网膜变性(这类细微改变在目前的扫描激光眼底照相或广域眼底照相下很难识别)。随着病情的进展，部分 患者会出现典型的眼底改变，表现为视网膜骨细胞样色素沉积合并中周部视网膜萎缩，视盘蜡黄，视网膜血管变细等；部分病人会出现毯层样视网膜变性或脱色素改变，见于中周部视网膜或广泛变性。其他常见的并发症还包括黄斑囊样变性、后囊下白内障、玻璃体尘样颗粒、视盘玻璃膜疣等并发症；少见的并发症有 Coat’s 样视网膜病变。

### Qwen2.5_72b

根据您提供的临床表现，最可能的诊断是视网膜色素变性（Retinitis Pigmentosa, RP）。这是一种遗传性视网膜疾病，主要影响视杆细胞和视锥细胞，导致夜盲、视野逐渐缩小，最终可能导致中心视力丧失。视网膜色素变性的患者眼底表现多样，早期可能无明显异常，但随着病情进展，可出现骨细胞样色素沉着、视网膜萎缩、视盘蜡黄、视网膜血管变细等特征性改变。

以下是五种其他可能的诊断：

- **先天性静止性夜盲症（Congenital Stationary Night Blindness, CSNB）**：
  - 这是一种遗传性视网膜疾病，主要表现为夜盲，但与视网膜色素变性不同，CSNB的视力和视野通常保持稳定，不会进行性恶化。
- **Usher 综合征**：
  - 这是一种遗传性综合征，包括视网膜色素变性和听力损失。患者通常在青少年时期出现夜盲和听力下降，随后逐渐发展为视野缩小和中心视力丧失。
- **Stargardt 病**：
  - 这是一种遗传性黄斑变性，主要影响中心视力，通常在儿童或青少年时期起病。眼底检查可见黄斑区有黄色或白色斑点，称为“鱼子酱”样改变。
- **Best 病**：
  - 这是一种遗传性黄斑变性，主要影响中心视力，通常在儿童或青少年时期起病。眼底检查可见黄斑区有黄色或橙色的脂质沉积，称为“蛋黄”样改变。
- **Coat’s 病**：
  - 这是一种非遗传性视网膜血管异常，主要影响儿童和青少年，表现为视网膜血管异常扩张和渗漏，导致视网膜脱离和视力下降。眼底检查可见视网膜血管异常和白色渗出物。

这些疾病在临床表现上可能有部分重叠，但通过详细的病史、家族史、眼底检查和辅助检查（如视野检查、电生理检查、基因检测等）可以进行鉴别诊断。

### 106. User

最常见的临床表现是白瞳症，即瞳孔可见黄白色反光。患眼可因肿瘤位于后极部，视力低下，而发生失用性斜视。早期表现为灰白色，圆形或椭圆形拱状肿物，可向玻璃体隆起，或沿脉络膜扁平生长。肿瘤表面的视网膜血管可出现扩张、出血，甚至发生渗出性视网膜脱离。瘤组织也可穿破视网膜进入玻璃体，如雪球状漂浮，甚至沉积于前房形成假性前房积脓。双侧性患儿发病较早，通常一岁以内发病。单侧性患儿发病较迟，通常在两岁或三岁时发生。

### Qwen2.5_72b

根据您提供的临床表现，最可能的诊断是视网膜母细胞瘤（Retinoblastoma）。这是一种主要发生在儿童中的罕见眼内恶性肿瘤，其特征性表现包括白瞳症（Leukocoria）、视力下降、斜视、视网膜下肿物、视网膜血管异常、视网膜脱离等。视网膜母细胞瘤可以是单侧或双侧，双侧病例通常发病较早，而单侧病例发病较晚。

另外，根据您描述的症状，还有以下五种可能的诊断：

- **永存原始玻璃体增生症（Persistent Hyperplastic Primary Vitreous, PHPV）**：这是一种先天性眼病，表现为白瞳症、斜视、视力下降等，但通常没有恶性肿瘤的特征，如视网膜下肿物或视网膜血管异常。
- **Coats病**：这是一种以视网膜血管异常为特征的疾病，表现为视网膜血管扩张、渗出性视网膜脱离和白瞳症，但通常不伴有肿瘤。
- **早产儿视网膜病变（Retinopathy of Prematurity, ROP）**：这是一种主要发生在早产儿中的疾病，表现为视网膜血管异常、视网膜脱离和白瞳症，但通常没有肿瘤的特征。
- **眼内炎（Endophthalmitis）**：这是一种严重的眼内感染，可以导致白瞳症、视力下降、眼红、眼痛等症状，但通常伴有明显的炎症体征，如前房积脓。
- **视网膜血管瘤（Retinal Hemangioma）**：这是一种良性肿瘤，表现为视网膜血管异常、视网膜脱离和白瞳症，但通常生长缓慢，不具有恶性肿瘤的侵袭性特征。

综上所述，视网膜母细胞瘤是最可能的诊断，但需要进一步的检查和评估来确诊，如眼底检查、B超、CT或MRI等影像学检查，以及必要时的遗传学检测。

### 107. User

患儿出生时多正常，在 1 岁以内起病，平均发病年龄为 5.6 个月。首次发作常为热性惊厥，可为一侧性或全面性阵挛或强直阵挛发作，热性惊厥具有发作持续时间长和反复发作特点，可出现持续状态。28％ ~35％患儿可能以无热惊厥起病，但在以后病程中常有热敏感特点。各种原因引起的体温升高，包括运动、发热、疫苗接种、洗热水澡和环境温度高等，均容易诱发或使发作加重，并易发生癫痫持续状态。有些患儿会出现以癫痫持续状态之后深昏迷为特征的急性脑病。

患儿多于 1~4 岁期间出现无热惊厥，具有多种发作类型，如肌阵挛、不典型失神、部分性发作、全面强直阵挛发作等。肌阵挛发作常由光刺激、闭眼和某种特定的图形刺激诱发，一块或数块肌肉同时阵挛，均伴有同步异常脑电波阵发性发放。肌阵挛强度不一，仅侵犯轴性肌肉时，出现身体轻微向前或向后的动作，严重者跌倒或突然抛掷手中物体。肌阵挛发作可以是孤立的，也可群发，但持续状态比较罕见。约 25％的 儿可以始终不出现肌阵挛发作。不典型失神发作同期脑电图显示半数为局灶起源放电，多数呈不规则不节律的慢棘慢波发放，经常伴随肌阵挛发作。30％的患儿可发生不典型失神持续状态。部分性发作发生率为 43.0％ ~78.6％，表现为偏侧肢体(或) 头部偏转性发作或阵挛性发作，亦有运动性发作伴有意识障碍和自主神经症状、自动症、肌张力降低等。1 岁以后患儿出现精神运动发育迟滞，尤其是语言发育迟缓。

### Qwen2.5_72b

根据您提供的临床表现，最可能的诊断是**Dravet 综合征**（Dravet Syndrome）。这是一种罕见的、严重的儿童癫痫性脑病，通常在婴儿期起病，以热性惊厥为首发症状，随后出现多种类型的癫痫发作，包括肌阵挛、不典型失神、部分性发作和全面性强直阵挛发作。此外，Dravet 综合征患儿常有热敏感性，体温升高容易诱发或加重癫痫发作，并且在 1 岁以后出现精神运动发育迟滞，尤其是语言发育迟缓。

以下是其他五种可能的诊断：

- **Lennox-Gastaut 综合征**（Lennox-Gastaut Syndrome, LGS）：
  - 通常在 2-8 岁之间起病。
  - 多种类型的癫痫发作，包括不典型失神、肌阵挛、强直性发作和部分性发作。
  - 脑电图显示慢棘慢波发放。
  - 精神运动发育迟滞。
- **Doose 综合征**（Myoclonic-Astatic Epilepsy, MAE）：
  - 通常在 1-4 岁之间起病。
  - 主要表现为肌阵挛-失张力发作和不典型失神发作。
  - 脑电图显示广泛性棘慢波发放。
  - 精神运动发育迟滞。
- **大田原综合征**（Ohtahara Syndrome）：
  - 通常在出生后几周内起病。
  - 以强直性发作和部分性发作为主。
  - 脑电图显示爆发-抑制模式。
  - 严重的发育迟滞。
- **West 综合征**（West Syndrome）：
  - 通常在 3-8 个月之间起病。
  - 以婴儿痉挛为主要表现。
  - 脑电图显示高峰失律。
  - 精神运动发育迟滞。
- **进行性肌阵挛癫痫**（Progressive Myoclonus Epilepsy, PME）：
  - 通常在儿童期或青少年期起病。
  - 以肌阵挛和全面性强直阵挛发作为主。
  - 逐渐加重的肌阵挛和进行性神经功能退化。
  - 可能伴有共济失调和认知功能下降。

这些诊断都需要通过详细的病史、体格检查、脑电图、影像学检查和基因检测等进一步确诊。

### 108. User

一方面表现为慢性溶血性贫血，除贫血相关症状，因胆红素升高可引起皮肤、巩膜轻中度黄染、色素性胆石症，当寒冷、感染、脱水时症状可能加重。婴幼儿可出现脾大，随年龄增长，脾脏可因脾梗死、纤维化而缩小。

另一方面由于毛细血管微血栓而引起反复发作的血管阻塞性疼痛。儿童和成人多表现为四肢肌痛、大关节疼痛和腰背疼痛，严重者可出现剧烈腹痛、头痛，甚至肢体瘫痪、昏迷。随着时间的推移，血管阻塞几乎可发生于每个器官系统，可以出现周期性疼痛，患者瘦弱、易疲劳、易感染、营养不良。因骨髓造血组织过度代偿性增加使骨皮质变薄、骨质疏松、脊柱变形、股骨头无菌坏死，如有骨梗死则可导致局部骨质硬化。眼部症状可能出现眼底视网膜血管栓塞引起眼底出血、视网膜脱离。另外，尚可影响神经系统的发育而出现智力低下。

在超过 25％的患者中急性疼痛为疾病的首发症状。在大于 2 岁的患儿中是最常见的症状。疼痛的严重程度可从轻微到剧烈不等。大约 1/2 的疼痛发作伴随客观的临床体征，如发热、肿胀、压痛、呼吸过速、高血压、恶心和呕吐。

### Qwen2.5_72b

根据您提供的临床表现，最可能的诊断是**镰状细胞病（Sickle Cell Disease, SCD）**。这是一种遗传性血液病，主要影响红细胞，导致红细胞变形为镰刀状，从而引发一系列临床症状，包括慢性溶血性贫血、黄疸、脾大、血管阻塞性疼痛、骨髓病变、眼部并发症等。

### 其他可能的诊断：

- **遗传性球形红细胞增多症（Hereditary Spherocytosis, HS）**：
  - 慢性溶血性贫血，黄疸，脾大，但通常没有血管阻塞性疼痛和骨髓病变。
  - 实验室检查可见球形红细胞增多，红细胞渗透脆性增加。
- **地中海贫血（Thalassemia）**：
  - 慢性溶血性贫血，黄疸，脾大，但通常没有血管阻塞性疼痛和骨髓病变。
  - 实验室检查可见小细胞低色素性贫血，血红蛋白电泳异常。
- **阵发性睡眠性血红蛋白尿（Paroxysmal Nocturnal Hemoglobinuria, PNH）**：
  - 慢性溶血性贫血，黄疸，但通常没有血管阻塞性疼痛和骨髓病变。
  - 实验室检查可见尿液中血红蛋白尿，Ham试验阳性。
- **冷凝集素病（Cold Agglutinin Disease, CAD）**：
  - 慢性溶血性贫血，黄疸，但通常没有血管阻塞性疼痛和骨髓病变。
  - 实验室检查可见冷凝集素抗体阳性，冷热溶血试验阳性。
- **慢性粒细胞白血病（Chronic Myeloid Leukemia, CML）**：
  - 慢性贫血，脾大，但通常没有血管阻塞性疼痛和骨髓病变。
  - 实验室检查可见白细胞增多，Ph染色体阳性。

### 诊断依据：

- **镰状细胞病**的典型临床表现包括慢性溶血性贫血、黄疸、脾大、血管阻塞性疼痛、骨髓病变、眼部并发症等。
- **实验室检查**：血常规可见镰状红细胞，血红蛋白电泳可见HbS，基因检测可确诊。

### 进一步检查建议：

- **血常规**：检查红细胞形态，血红蛋白水平。
- **血红蛋白电泳**：检测HbS。
- **基因检测**：确认镰状细胞病的基因突变。
- **骨髓穿刺**：评估骨髓造血功能。
- **影像学检查**：如X线、CT、MRI，评估骨髓病变和器官受累情况。

希望这些信息对您有帮助。如有其他问题，请随时告知。

### 109. User

新生儿和儿童患者的主要临床表现包括：

**1. 宫内发育迟缓及出生后生长障碍** 宫内发育迟缓，低出生体重，出生后缺乏追赶生长，成比例的身材矮小。

**2. 特殊体征** 前额宽而突出、尖下颌导致三角形脸，相对头围偏大。齿列不齐、耳位低，躯体偏身不对称，包括四肢长度差异，小指侧弯畸形等。

**3. 其他表现** 喂养困难、新生儿期可出现低血糖，尿道下裂、隐睾和马蹄肾等泌尿生殖系统畸形，牛奶咖啡斑，反流性食管炎等消化系统异常。部分患者出现认知、运动和语言发育迟缓，学习障碍等。

### Qwen2.5_72b

根据您提供的临床表现，最可能的诊断是**Silver-Russell 综合征 (Silver-Russell Syndrome, SRS)**。这是一种罕见的生长发育障碍性疾病，主要特征包括宫内发育迟缓、出生后生长障碍、特殊体征（如前额宽而突出、尖下颌、三角形脸、相对头围偏大、齿列不齐、耳位低、躯体偏身不对称、小指侧弯畸形等）、喂养困难、新生儿期低血糖、泌尿生殖系统畸形（如尿道下裂、隐睾、马蹄肾）、牛奶咖啡斑、反流性食管炎等消化系统异常，以及认知、运动和语言发育迟缓等。

### 其他可能的诊断包括：

- **Prader-Willi 综合征 (Prader-Willi Syndrome, PWS)**：
  - 宫内发育迟缓，出生后生长障碍。
  - 特殊面容，如小嘴、窄额、下颌小。
  - 喂养困难，新生儿期肌张力低下。
  - 儿童期出现过度食欲和肥胖。
  - 认知和行为问题。
- **Beckwith-Wiedemann 综合征 (Beckwith-Wiedemann Syndrome, BWS)**：
  - 宫内和出生后过度生长。
  - 特殊面容，如大舌、耳垂裂。
  - 腹壁缺陷，如脐疝。
  - 低血糖，泌尿生殖系统畸形。
  - 增加肿瘤风险，如肾母细胞瘤。
- **Noonan 综合征 (Noonan Syndrome)**：
  - 宫内发育迟缓，出生后生长障碍。
  - 特殊面容，如宽鼻梁、下颌小、耳位低。
  - 心脏缺陷，如肺动脉瓣狭窄。
  - 出血倾向，淋巴水肿。
  - 认知和发育迟缓。
- **Russell-Silver 综合征 (Russell-Silver Syndrome, RSS)**：
  - 与 Silver-Russell 综合征非常相似，但可能有更明显的躯体不对称。
  - 宫内发育迟缓，出生后生长障碍。
  - 特殊面容，如前额宽而突出、尖下颌、三角形脸。
  - 喂养困难，新生儿期低血糖。
  - 泌尿生殖系统畸形，如尿道下裂、隐睾、马蹄肾。
- **Turner 综合征 (Turner Syndrome)**：
  - 宫内发育迟缓，出生后生长障碍。
  - 特殊面容，如宽颈、低耳位、下颌小。
  - 心血管畸形，如主动脉缩窄。
  - 泌尿生殖系统畸形，如卵巢发育不全。
  - 认知和发育迟缓，尤其是空间和数学能力。

这些诊断需要通过详细的临床评估、家族史、实验室检查和基因检测来进一步确认。

### 110. User

**1. 皮肤黄瘤，** 好发于伸肌肌腱附着点附近，例如跟腱、肘关节、膝关节等，皮肤损伤与黄瘤出现关系密切。皮肤黄瘤少见于儿童患者。

**2. 动脉粥样硬化 ，**患者出现动脉粥样硬化、冠心病的风险显著高于正常人。早发性冠心病可以作为本病的特点之一。

**3. 血液系统改变 ，**血液中植物固醇含量明显增加，导致细胞膜中固醇成分中植物固醇比例增高，红细胞、血小板等细胞膜功能异常，出现贫血和血小板减少，患者可出现乏力、面色苍白、皮肤出血等表现。

**4. 其他，**除上述症状，部分患者会出现关节痛、关节炎、脾大、转氨酶升高等。

### Qwen2.5_72b

根据您提供的临床表现，最可能的诊断是**植物固醇血症（Sitosterolemia）**。这是一种罕见的遗传性代谢病，由于患者体内无法正常代谢植物固醇，导致植物固醇在血液中积累，进而引发一系列临床症状，包括皮肤黄瘤、动脉粥样硬化、血液系统改变等。

以下是其他五种可能的诊断：

- **家族性高胆固醇血症（Familial Hypercholesterolemia, FH）**：这是一种常见的遗传性高脂血症，患者血中低密度脂蛋白胆固醇（LDL-C）水平显著升高，可导致皮肤黄瘤和早发性冠心病。
- **混合型高脂血症（Mixed Hyperlipidemia）**：这是一种代谢综合征，患者血中胆固醇和甘油三酯水平均升高，可导致皮肤黄瘤和动脉粥样硬化。
- **Wolman病（Wolman Disease）**：这是一种罕见的脂质代谢障碍疾病，患者体内胆固醇和甘油三酯代谢异常，可导致皮肤黄瘤、肝脾肿大、贫血和血小板减少。
- **Niemann-Pick病（Niemann-Pick Disease）**：这是一种溶酶体贮积症，患者体内胆固醇和鞘磷脂代谢异常，可导致肝脾肿大、贫血、血小板减少和神经系统症状。
- **Gilbert综合征（Gilbert Syndrome）**：这是一种常见的遗传性高胆红素血症，患者血中未结合胆红素水平升高，可导致黄疸、乏力等症状，但通常不伴有皮肤黄瘤和动脉粥样硬化。

这些疾病虽然有部分相似的临床表现，但通过详细的病史、体格检查和实验室检查，可以进一步明确诊断。

### 111. User

男性发病，起病隐匿，常见发病年龄为 30~60 岁。患者常以痛性痉挛、震颤、双下肢无力等症状起病，逐渐出现双上肢无力、延髓部和面部的肌肉萎缩、肌束颤动、构音障碍、吞咽困难、呼吸困难等。可伴有男性乳房发育、生殖功能降低等雄激素受体不敏感表现。超过 50％的患者存在感觉异常。该病进展缓慢，患者通常在病程晚期才出现行走不能，仅有部分患者需要辅助通气，对生存期无显著影响。

**1. 不典型症状 ，**某些不典型症状可在出现肢体力弱前数年甚至数十年出现，包括易疲劳、痛性痉挛、口周震颤及姿势性震颤。肌无力：多数患者以双下肢无力起病，逐渐进展至双上肢、舌肌以及面肌，而眼外肌往往不受累。肢体无力的分布多不对称，以近端受累为主。咀嚼肌受累时可引起下颌下垂及震颤。体检以下运动神经元损害体征为主，表现为肢体肌力下降，近端为著，伴肌肉萎缩、束颤，腱反射多减弱或消失。多数患者可出现明显舌肌萎缩、纤颤，但软腭活动、咽反射多正常，患者饮水呛咳、吞咽困难的程度也较轻。某些患者还可观察到颏部以及口周束颤。患者运动功能损害随病程延长逐渐加重，后期可出现上楼费力、行走不稳等。

**2. 感觉障碍** ，约半数患者存在不同程度的感觉减退，而在其他患者为亚临床性，仅在感觉神经传导检查中发现异常。

**3. 内分泌异常，** 包括葡萄糖及脂肪代谢的异常，雄激素受体不敏感等表现，后者包括男性乳房发育、性功能下降、不孕不育、睾丸萎缩等。

**4. 女性携带者，** 多数女性携带者并不出现症状，即使出现症状程度也较轻，可仅表现为束颤、轻度远端肢体无力、肌肉痉挛或肌酸肌酶增高等。

### Qwen2.5_72b

根据您提供的临床表现，最可能的诊断是**脊髓延髓肌萎缩症（Kennedy病）**。这是一种X连锁隐性遗传病，主要影响男性，其特征为进行性肌无力和萎缩，特别是四肢近端肌肉，伴有肌束颤动、痛性痉挛、震颤等。此外，患者常有雄激素受体不敏感的表现，如男性乳房发育、生殖功能降低等。感觉障碍在部分患者中也较为常见。

### 其他可能的诊断：

- **肌萎缩侧索硬化症（ALS）**：虽然ALS通常表现为上下运动神经元损害，但某些亚型可能以下运动神经元损害为主，且进展速度较慢。ALS患者也可能出现感觉障碍和内分泌异常，但雄激素受体不敏感的表现较少见。
- **进行性肌阵挛性癫痫（PME）**：这是一种罕见的遗传性神经退行性疾病，表现为肌阵挛、癫痫发作、进行性肌无力和萎缩。但PME患者通常有明显的癫痫发作，且感觉障碍和内分泌异常较少见。
- **多灶性运动神经病（MMN）**：这是一种慢性炎症性脱髓鞘性多神经病，表现为不对称的肌无力和萎缩，但通常不伴有感觉障碍或内分泌异常。
- **脊髓小脑共济失调（SCA）**：某些类型的SCA可能表现为肌无力、肌萎缩和震颤，但通常伴有小脑共济失调的表现，如步态不稳、眼球运动异常等。
- **慢性炎症性脱髓鞘性多发性神经病（CIDP）**：这是一种慢性进行性的周围神经病，表现为对称性肌无力和感觉障碍，但通常不伴有肌束颤动或内分泌异常。

### 总结：

- **最可能的诊断**：脊髓延髓肌萎缩症（Kennedy病）
- **其他可能的诊断**：
  1. 肌萎缩侧索硬化症（ALS）
  2. 进行性肌阵挛性癫痫（PME）
  3. 多灶性运动神经病（MMN）
  4. 脊髓小脑共济失调（SCA）
  5. 慢性炎症性脱髓鞘性多发性神经病（CIDP）

这些诊断需要通过详细的病史、体格检查、神经电生理检查、基因检测等进一步确认。

### 112. User

部分患者在宫内表现为胎动减少，出生时为松软儿。患者在 6 个月内发病，平均发病年龄在生后 1 个月。表现为全身松软无力，严重肌张力低下。由于舌、面和咀嚼肌无力，大多数患儿出现吸吮和吞咽困难，可见舌肌萎缩和震颤。肋间肌受累可以出现呼吸困难，腹式呼吸。胸部呈钟型外观。下肢较上肢受累重，近端较远端严重。严重躯体中轴部位肌无力使患儿不能控制头部运动，不会抬头或翻身，没有独坐能力。卧位时，双下肢呈髋外展、膝屈曲的蛙腿体位。肌肉萎缩多不明显， 部分患儿轻度关节畸形。患儿智力好，腱反射消失，四肢感觉正常。患儿肌无力进行性加重，最终失去所有自主运动能力，鼻饲喂养，反复呼吸道感染而致呼吸衰竭。80％患儿 1岁内死亡，很少活过 2 岁。

### Qwen2.5_72b

根据您提供的临床表现，最可能的诊断是**婴儿型脊髓性肌萎缩症（SMA, Spinal Muscular Atrophy）**，特别是**SMA 1型**（Werdnig-Hoffmann病）。这是一种由运动神经元存活基因1（SMN1）突变引起的常染色体隐性遗传病，主要影响脊髓前角细胞，导致进行性肌无力和肌萎缩。

### 诊断依据：

- **宫内胎动减少**：SMA 1型患者在宫内可能表现为胎动减少。
- **出生时为松软儿**：新生儿期即表现为全身松软无力。
- **肌张力低下**：严重肌张力低下是SMA 1型的典型表现。
- **吸吮和吞咽困难**：由于舌、面和咀嚼肌无力，患儿常出现吸吮和吞咽困难。
- **呼吸困难**：肋间肌受累导致呼吸困难，腹式呼吸。
- **下肢较上肢受累重**：近端肌肉受累较远端严重。
- **不能控制头部运动**：严重躯体中轴部位肌无力使患儿不能控制头部运动，不会抬头或翻身。
- **蛙腿体位**：卧位时，双下肢呈髋外展、膝屈曲的蛙腿体位。
- **进行性加重**：肌无力进行性加重，最终失去所有自主运动能力。
- **高死亡率**：80%的患儿1岁内死亡，很少活过2岁。

### 其他可能的诊断：

- **先天性肌营养不良症（CMD, Congenital Muscular Dystrophy）**：表现为肌无力、肌张力低下，但通常伴有明显的肌肉萎缩和关节畸形。
- **先天性肌病（Congenital Myopathy）**：如中央轴空病、多发性肌纤维发育不良等，表现为肌无力和肌张力低下，但通常不伴有进行性加重。
- **先天性代谢性肌病**：如糖原贮积病、脂质贮积病等，表现为肌无力、肌张力低下，但通常伴有代谢异常。
- **先天性神经肌肉接头疾病**：如先天性重症肌无力，表现为肌无力、肌张力低下，但通常伴有波动性症状。
- **先天性脊柱裂**：表现为肌无力、肌张力低下，但通常伴有脊柱和神经系统的异常。

### 进一步检查建议：

- **基因检测**：特别是SMN1基因检测，以确诊SMA。
- **肌电图（EMG）**：评估神经肌肉功能。
- **肌肉活检**：排除其他肌病。
- **代谢筛查**：排除代谢性肌病。
- **影像学检查**：如MRI，评估脊髓和神经系统的结构。

希望这些信息对您有所帮助。如果您有更多详细信息或需要进一步的诊断建议，请随时告知。

### 113. User

患者通常成年起病，隐匿起始，逐渐进展。主要表现为小脑性共济失调，包括醉汉步态、动作笨拙、吟诗样语言、眼震、复视等；锥体束损害，包括腱反射亢进、踝阵挛、病理征阳性；锥体外系症状，包括帕金森综合征、肌张力障碍、强直等。此外，部分患者可有周围神经病。部分患者在疾病早期可以锥体束损害(类似痉挛性截瘫) 或锥体外系症状(类似帕金森综合征) 为主，共济失调症状不突出。

疾病逐渐进展，起病 10~15 年后丧失行走能力。长期吞咽困难致营养不良或误吸是导致死亡的主要原因。

### Qwen2.5_72b

根据您提供的临床表现，最可能的诊断是**脊髓小脑性共济失调（Spinocerebellar Ataxia, SCA）**。SCA 是一组遗传性神经退行性疾病，主要影响小脑、脊髓和周围神经，导致共济失调、锥体束损害和锥体外系症状。SCA 有多种亚型，其中 SCA1、SCA2、SCA3（Machado-Joseph 病）、SCA6 和 SCA7 是较为常见的类型。

以下是五种其他可能的诊断：

- **多系统萎缩（Multiple System Atrophy, MSA）**：
  - MSA 是一种进行性神经退行性疾病，主要影响自主神经系统、小脑和基底节。临床表现包括小脑性共济失调、帕金森综合征、自主神经功能障碍等。
- **进行性核上性麻痹（Progressive Supranuclear Palsy, PSP）**：
  - PSP 是一种罕见的神经退行性疾病，主要影响脑干和基底节。临床表现包括垂直凝视障碍、步态不稳、帕金森综合征、认知障碍等。
- **弗里德赖希共济失调（Friedreich’s Ataxia）**：
  - 弗里德赖希共济失调是一种常染色体隐性遗传的神经退行性疾病，主要影响脊髓后索和周围神经。临床表现包括小脑性共济失调、腱反射消失、脊柱侧弯、心脏病变等。
- **遗传性痉挛性截瘫（Hereditary Spastic Paraplegia, HSP）**：
  - HSP 是一组以进行性下肢痉挛和无力为主要特征的遗传性神经退行性疾病。部分患者可伴有共济失调、周围神经病等。
- **亨廷顿病（Huntington’s Disease）**：
  - 亨廷顿病是一种常染色体显性遗传的神经退行性疾病，主要影响基底节。临床表现包括舞蹈样动作、认知障碍、精神症状、共济失调等。

这些疾病在临床表现上有一定的重叠，但通过详细的病史、体格检查、基因检测和影像学检查可以进行鉴别诊断。

### 114. User

疾病早期可能会合并胃食管反流。随着病程的进展，患者逐渐出现皮肤肿胀及硬化，并可伴皮肤色素改变，包括色素沉着或色素脱失。除皮肤受累之外，患者常会伴有关节痛及肌痛等症状，并可出现指端溃疡和毛细血管扩张。此外，患者可能会出现多个内脏器官受累。其中呼吸系统较易受累，主要表现为肺间质纤维化和肺动脉高压。患者的消化系统表现多样， 包括胃食管反流、胃窦血管扩张症、腹痛、腹泻、便秘、大便失禁等。少数患者可能会出现肾危象，主要表现为急性肾功能不全，并且常合并高血压。心脏受累可表现为心包积液、心脏传导系统异常，个别患者可出现心肌受累。神经系统方面，外周神经系统受累多见，例如三叉神经病变、周围神经病变等，罕见中枢神经受累。

### Qwen2.5_72b

根据您提供的临床表现，最可能的诊断是**系统性硬化症（Systemic Sclerosis, SS）**，也称为硬皮病。这是一种以皮肤和内脏器官纤维化为特征的自身免疫性疾病。以下是其他五种可能的诊断：

- **混合性结缔组织病（Mixed Connective Tissue Disease, MCTD）**：
  - 临床表现与系统性硬化症相似，但通常病情较轻，患者常有高滴度的抗U1-RNP抗体。
- **皮肌炎（Dermatomyositis, DM）**：
  - 皮肤表现包括紫红色皮疹、Gottron丘疹等，肌肉受累表现为肌无力和肌痛，也可累及肺、心脏等器官。
- **系统性红斑狼疮（Systemic Lupus Erythematosus, SLE）**：
  - 多系统受累，包括皮肤、关节、肾脏、心脏、肺、神经系统等，常有蝶形红斑、光敏感、口腔溃疡等表现。
- **干燥综合征（Sjögren’s Syndrome, SS）**：
  - 主要表现为口干、眼干，也可累及皮肤、关节、肺、肾等器官，部分患者可出现雷诺现象和皮肤硬化。
- **硬肿病（Eosinophilic Fasciitis, EF）**：
  - 以皮肤和筋膜的硬化为特征，常伴有嗜酸性粒细胞增多，关节痛和肌痛也较常见，但内脏受累较少见。

这些疾病在临床表现上有一定的重叠，确诊需要结合详细的病史、体格检查、实验室检查（如自身抗体检测）和影像学检查等。

### 115. User

患儿在出生 1~3 个月后除了出现类似苯丙酮尿症的临床症状外，还表现为多巴胺缺乏症状，如运动障碍、肌张力低下、嗜睡、眼震颤、吞咽困难等。5- 羟色胺缺乏相关症状，如反应迟钝、抑郁、失眠等。去甲肾上腺素缺乏相关症状，如躯干肌张力低下、眼睑下垂、小脑发育障碍等。还会有顽固性抽搐、反复发热，运动里程碑发育迟滞， 全身瘫痪，智能发育严重障碍等。

### Qwen2.5_72b

根据您提供的临床表现，最可能的诊断是**高苯丙氨酸血症伴多巴胺和5-羟色胺缺乏症**，也称为**BH4缺乏症**（Tetrahydrobiopterin deficiency）。BH4是一种重要的辅因子，参与苯丙氨酸、酪氨酸和色氨酸的代谢，这些代谢途径的异常会导致多巴胺、5-羟色胺和去甲肾上腺素的缺乏，从而引起您描述的多种症状。

### 最可能的诊断：

- **BH4缺乏症（Tetrahydrobiopterin deficiency）**

### 其他可能的诊断：

- **经典苯丙酮尿症（Classic Phenylketonuria, PKU）**：虽然PKU也会导致高苯丙氨酸血症，但通常不会出现多巴胺和5-羟色胺缺乏的症状。
- **多巴反应性肌张力障碍（Dopa-Responsive Dystonia, DRD）**：这种疾病主要表现为运动障碍，对多巴胺治疗反应良好。
- **线粒体脑肌病（Mitochondrial Encephalomyopathy）**：这类疾病可以导致多系统受累，包括运动障碍、肌张力低下和智能发育障碍。
- **先天性甲状腺功能减退症（Congenital Hypothyroidism）**：甲状腺激素缺乏可以导致生长发育迟缓、智能发育障碍和肌张力低下。
- **遗传性痉挛性截瘫（Hereditary Spastic Paraplegia, HSP）**：虽然主要表现为下肢痉挛，但某些亚型也可能伴有智能发育障碍和运动障碍。

### 诊断建议：

- **血液检查**：检测苯丙氨酸、酪氨酸、多巴胺、5-羟色胺和去甲肾上腺素的水平。
- **尿液检查**：检测尿蝶呤谱型，以确定BH4代谢途径的异常。
- **基因检测**：检测与BH4代谢途径相关的基因突变，如GTP-CH、6-PTS、DHPR等。
- **脑电图（EEG）**：评估是否存在癫痫活动。
- **影像学检查**：如MRI，评估脑部结构异常。

这些检查可以帮助进一步明确诊断，并指导治疗方案的选择。

### 116. User

常表现为多系统受累，其发生发展有一定的阶段性。在胎儿 / 新生儿期可以出现心脏横纹肌瘤，大部分可自发消退；出生后数月就可出现癫痫症状和皮肤症状；随着年龄的增大，出现肾血管平滑肌脂肪瘤和全身其他部位的肿瘤性病变；成年女性肺部容易出现肺部淋巴管肌瘤病。脑部病变主要包括脑皮质结节、室管膜下巨细胞星形细胞瘤和室管膜下结节等。皮肤病变包括出生后可见的白斑，逐渐生长的面部血管纤维瘤、鲨革斑、甲周纤维瘤及前额斑块等；高达 90％的患者有肾脏血管平滑肌脂肪瘤；约 50％患者病变累及视网膜，但很少影响视力；约 35％女性患者肺部出现 LAM；其他如肝脏、胰腺、消化道、骨骼也可以受累，但一般症状较轻。

### Qwen2.5_72b

根据您提供的临床表现，最可能的诊断是结节性硬化症（Tuberous Sclerosis Complex, TSC）。这是一种遗传性多系统受累的疾病，其特征包括心脏横纹肌瘤、癫痫、皮肤病变、肾血管平滑肌脂肪瘤、脑部病变（如脑皮质结节、室管膜下巨细胞星形细胞瘤和室管膜下结节）、肺淋巴管肌瘤病（LAM）等。

以下是五种其他可能的诊断：

- **神经纤维瘤病 1 型（Neurofibromatosis Type 1, NF1）**：
  - 皮肤表现：咖啡牛奶斑、神经纤维瘤、腋窝和腹股沟雀斑。
  - 神经系统：多发性神经纤维瘤、视神经胶质瘤、脑白质异常。
  - 骨骼：骨缺损、脊柱侧弯。
  - 其他：虹膜 Lisch 结节、心血管系统受累。
- **von Hippel-Lindau 病（VHL）**：
  - 肾脏：肾细胞癌、肾囊肿。
  - 中枢神经系统：脑和脊髓血管母细胞瘤。
  - 眼部：视网膜血管母细胞瘤。
  - 耳部：内耳血管母细胞瘤。
  - 胰腺：囊肿和胰岛细胞瘤。
  - 肾上腺：嗜铬细胞瘤。
- **Sturge-Weber 综合征**：
  - 皮肤：面部葡萄酒色斑。
  - 神经系统：脑皮质血管瘤、癫痫、偏瘫。
  - 眼部：青光眼、脉络膜血管瘤。
- **Birt-Hogg-Dubé 综合征**：
  - 皮肤：面部和躯干的纤维毛囊瘤。
  - 肾脏：肾细胞癌、肾囊肿。
  - 肺部：自发性气胸、肺囊肿。
  - 其他：皮肤纤维瘤、毛囊瘤。
- **Cowden 综合征（PTEN 错构瘤综合征）**：
  - 皮肤：面部和口腔黏膜的乳头状瘤、皮肤错构瘤。
  - 乳腺：乳腺癌。
  - 甲状腺：甲状腺癌。
  - 子宫：子宫内膜癌。
  - 其他：胃肠道息肉、肾囊肿。

这些疾病都有多系统受累的表现，但具体的临床特征和遗传背景有所不同。确诊需要结合详细的病史、体格检查、影像学检查和基因检测。

### 117. User

未被发现或未治疗的慢性型儿童大多在 10 岁以前死亡，死因通常是肝功能衰竭、神经系统受累或肝细胞癌。

急性型起病急骤、进展迅速，以急性肝功能衰竭为主要表现，临床表现为肝脾肿大、黄疸、呕吐、腹胀、厌食、嗜睡、贫血、出血倾向及生长迟缓。患儿可能具有“煮白菜” 或“烂蘑菇”的特征性气味。肝脏合成凝血因子不足，PT、APTT 明显延长，凝血因子Ⅱ、Ⅶ、Ⅸ、Ⅺ和Ⅻ水平降低，补充维生素 K 后难以纠正。肝功能衰竭可继发腹水、黄疸和消化道出血，患儿偶尔会出现持续低血糖。未治疗的患儿多于起病数周或数月内因肝功能衰竭死亡。

### Qwen2.5_72b

根据您提供的临床表现，最可能的诊断是**急性肝豆状核变性（Wilson病）**。这是一种罕见的遗传性铜代谢障碍疾病，会导致铜在肝脏、脑、肾和角膜等部位沉积，引起肝功能衰竭、神经系统症状等。

### 五种其他可能的诊断：

- **急性病毒性肝炎**：尤其是甲型、乙型或戊型肝炎，可以引起急性肝功能衰竭，表现为黄疸、肝脾肿大、呕吐等症状。
- **先天性代谢缺陷**：如糖原贮积病（例如 von Gierke 病）、尿素循环障碍等，这些疾病可以导致急性肝功能衰竭和代谢紊乱。
- **自身免疫性肝炎**：这是一种免疫介导的肝脏炎症，可以导致急性或慢性肝功能衰竭，表现为肝脾肿大、黄疸、贫血等症状。
- **药物或毒素引起的急性肝损伤**：某些药物或毒素（如对乙酰氨基酚过量、毒蕈中毒等）可以引起急性肝功能衰竭，表现为肝脾肿大、黄疸、凝血功能障碍等。
- **胆道闭锁**：这是一种婴儿期常见的胆道发育异常，可以导致进行性黄疸、肝脾肿大、肝功能衰竭等症状。

### 诊断依据：

- **急性肝豆状核变性**：特征性的“煮白菜”或“烂蘑菇”气味，铜代谢障碍导致的肝功能衰竭，凝血功能障碍，以及神经系统受累。
- **急性病毒性肝炎**：病毒学检测（如HBsAg、HAV IgM等）可以确诊。
- **先天性代谢缺陷**：特定的代谢产物检测（如血糖、乳酸、尿素等）和基因检测。
- **自身免疫性肝炎**：自身抗体检测（如ANA、SMA、LKM-1等）和肝活检。
- **药物或毒素引起的急性肝损伤**：详细的用药史和毒素暴露史，以及肝功能检测。
- **胆道闭锁**：腹部超声、肝胆核素扫描和肝活检。

希望这些信息对您有帮助。如果您需要进一步的详细信息或有其他问题，请随时告知。

### 118. User

临床表现具有明显异质性，根据起病年龄和临床表现分为 3 个类型。

**1. 心肌病型，** 是最常见的一种严重早发心脏和多脏器衰竭型，主要在新生儿和婴儿早期发病。此型发病凶险，患儿病死率高，表现为低血糖、瑞氏综合征、新生儿猝死、肥厚型和扩张型心肌病、心包积液、心律失常、肌无力、肝肿大和间歇性低血糖，肌酸激酶水平升高；心肌肥厚和心律失常可致死。

**2. 肝型 ，**较轻，为肝脏和低酮性低血糖型，婴儿后期或儿童期发病，表现为肝大和肝功能异常，空腹耐力下降和急性低酮性低血糖，不伴心肌损害和心肌肥厚，但未经及时诊断和治疗也会有生命危险。

**3. 肌病型** ，主要在青少年至成年期发病，为迟发型，症状轻，一般不伴有心肌疾病和低血糖。主要表现为运动、感染或饥饿后的横纹肌溶解和肌红蛋白尿，甚至可发生肾功能衰竭，可伴有肌无力、肌肉痛性痉挛或肌痛。

### Qwen2.5_72b

根据您提供的临床表现，最可能的诊断是**肉碱棕榈酰转移酶 II 缺乏症 (CPT II 缺乏症)**。这是一种罕见的代谢性疾病，属于脂肪酸氧化障碍的一种，主要影响心脏、肝脏和肌肉。CPT II 缺乏症的临床表现与您描述的三种类型非常吻合，特别是心肌病型、肝型和肌病型。

### 1. 最可能的诊断：

- **肉碱棕榈酰转移酶 II 缺乏症 (CPT II 缺乏症)**

### 2. 其他可能的诊断：

- **长链酰基辅酶 A 脱氢酶缺乏症 (LC-ADH 缺乏症)**：这是一种类似的脂肪酸氧化障碍，也可表现为心肌病、肝大和低血糖。
- **肉碱转运缺陷**：这是一种影响肉碱转运的代谢障碍，可导致心肌病、肝功能异常和低血糖。
- **中链酰基辅酶 A 脱氢酶缺乏症 (MC-ADH 缺乏症)**：这种代谢障碍也可表现为低血糖、肝大和肌肉症状。
- **线粒体心肌病**：这是一种线粒体功能障碍导致的心肌病，可伴有肝功能异常和低血糖。
- **糖原贮积病**：特别是糖原贮积病 II 型（Pompe 病），可表现为心肌病、肝大和低血糖。

这些疾病都需要通过详细的代谢筛查、基因检测和酶活性测定来确诊。如果您怀疑患者有这些症状，建议进行进一步的实验室检查和专科会诊。

### 119. User

**1. 特殊面容，** 眶周皮下组织饱满、眶距增宽、星状虹膜、人中长、嘴唇厚、宽嘴以及小下颌。

**2. 心血管疾病，** 75％ ~80％的患者存在弹性蛋白动脉病，可影响任何动脉。周围型肺动脉狭窄(37％ ~61％) 在婴儿期较常见，但随着时间的推移可有改善。瓣上型主动脉狭窄较常见(35％ ~65％)，并且随着时间的推移在 5 岁前恶化。动脉狭窄导致左心阻力增高、心肌肥厚和心力衰竭。其他异常包括肾动脉狭窄、胸主动脉狭窄、腹主动脉狭窄、二尖瓣脱垂和主动脉瓣关闭不全、肠系膜动脉狭窄、颅内血管异常等。高血压可出现在任何年龄，部分可能和肾动脉狭窄有关。

**3. 认知能力，** 75％的患者有智力障碍，通常较为轻微。认知障碍具有特征性，表现为短期记忆和语言表达能力相对较好，但在视觉空间结构性认知方面却极其薄弱。在智商方面没有性别差异的报道，且随着时间的推移，儿童的智商是稳定的。

**4. 性格、行为、心理异常，** 包括过度友好、过度移情、注意力缺陷、焦虑、恐惧。

**5. 睡眠异常 ，**睡眠问题的发生率为 65％，包括睡眠潜伏期增加和睡眠效率降低。

**6. 眼睛，** 泪道阻塞、远视(67％) 和斜视(50％) 常见。成人患者也有白内障的报道。

**7. 耳鼻喉，** 50％的患者有慢性中耳炎，90％对声音的敏感性增加，63％的儿童和92％的成人有轻度至中度听力损害，多为进行性感音神经性听力损失。大多数患者声音嘶哑或声音低沉。

**8. 牙齿** ，可有小牙、牙釉质发育不良和错位咬合。

**9. 消化系统，** 慢性腹痛是常见症状，可能的病因包括胃食管反流、裂孔疝、消化性溃疡、胆石症、憩室炎、缺血性肠病、慢性便秘和焦虑躯体化。高钙血症也可能导致易怒、呕吐、便秘和肌肉痉挛。

**10. 泌尿系统，** 尿频和遗尿症在儿童患者中很常见。

**11. 神经、肌肉** ，幼童的肌张力偏低，关节松弛，导致代偿性姿势异常，以保持身体平衡。年龄较大的儿童和成人有典型的肌张力增高和深肌腱反射活跃，导致步态僵硬而笨拙，精细运动功能受损，所有年龄段患者都有使用工具和书写困难。成人患者会有小脑共济失调和震颤。

**12. 生长发育** 。70％的婴儿体重增长不佳。

**13. 特发性高钙血症** 。多在婴幼儿期出现，见于 15％ ~50％的患者，最常见的症状为易激惹、呕吐和便秘。

**14. 内分泌。**甲状腺功能减退(10％)、青春期提前(50％)。糖耐量受损在青少年患者中的发生率为 26％。

### Qwen2.5_72b

根据您提供的临床表现，最可能的诊断是**威廉姆斯综合征（Williams Syndrome）**。这是一种由7号染色体长臂上的基因缺失引起的罕见遗传病，其特征包括特殊面容、心血管疾病、认知障碍、性格和行为异常、睡眠问题、眼部异常、耳鼻喉问题、牙齿异常、消化系统症状、泌尿系统症状、神经肌肉症状、生长发育问题、特发性高钙血症和内分泌异常等。

### 其他可能的诊断：

- **特纳综合征（Turner Syndrome）**：女性患者，常有特殊面容、心血管异常、生长发育迟缓、内分泌异常等。
- **努南综合征（Noonan Syndrome）**：特殊面容、心血管异常、生长发育迟缓、认知障碍等。
- **普拉德-威利综合征（Prader-Willi Syndrome）**：特殊面容、生长发育迟缓、内分泌异常、肌张力低下等。
- **天使人综合征（Angelman Syndrome）**：特殊面容、认知障碍、肌张力低下、睡眠问题等。
- **22q11.2缺失综合征（DiGeorge Syndrome）**：特殊面容、心血管异常、免疫缺陷、内分泌异常等。

这些疾病也有类似的临床表现，但通过详细的病史、体格检查和基因检测可以进一步明确诊断。

### 120. User

**1. 出血倾向** ，为患者常见表现，尤其血丝便多见，大部分患儿在新生儿期即可发病。其次表现为瘀斑、瘀点、咯血和血尿等出血倾向，部分严重者可出现威胁生命的消化道大出血或颅内出血等。患者出血由血小板减少引起，同时伴有血小板体积减小，但仅约 25％的病例同时具有典型三联征表现。

**2. 感染 。**患儿易患各种感染，包括单纯疱疹病毒、巨细胞病毒、肺炎链球菌、真菌等。患者免疫缺陷随年龄增长而加重。

**3. 湿疹。** 约 80％的 WAS 患儿可出现湿疹，范围和严重程度差异很大。可有典型严重、难治性湿疹，也可非常轻微，甚至无湿疹表现。

**4. 自身免疫性疾病。** 最常见为自身免疫性溶血性贫血。其他包括血管炎、关节炎、肾脏疾病、炎症性肠病、中性粒细胞减少症和自身免疫性血小板减少性紫癜等。

**5. 恶性肿瘤 。**患儿发生淋巴系统恶性肿瘤的风险明显增高，尤其是淋巴瘤，非霍奇金淋巴瘤、淋巴细胞白血病、骨髓增生异常综合征、精原细胞瘤等均有报道。

**6. 其他 。**该病患儿可具有完全正常的血小板水平，但中性粒细胞持续或反复减少。

### Qwen2.5_72b

根据您提供的临床表现，最可能的诊断是Wiskott-Aldrich综合征（WAS）。WAS是一种罕见的X连锁遗传性免疫缺陷病，主要影响男性，其特征包括血小板减少（伴血小板体积减小）、易感染、湿疹、自身免疫性疾病和恶性肿瘤的风险增加。这些症状与您描述的临床表现高度吻合。

### 1. Wiskott-Aldrich综合征（WAS）

- **出血倾向**：血小板减少，血小板体积减小，常见血丝便、瘀斑、瘀点、咯血和血尿等。
- **感染**：易患各种感染，包括单纯疱疹病毒、巨细胞病毒、肺炎链球菌、真菌等。
- **湿疹**：约80%的WAS患儿可出现湿疹，范围和严重程度差异很大。
- **自身免疫性疾病**：最常见为自身免疫性溶血性贫血，其他包括血管炎、关节炎、肾脏疾病、炎症性肠病等。
- **恶性肿瘤**：淋巴系统恶性肿瘤风险增加，尤其是淋巴瘤。
- **其他**：中性粒细胞持续或反复减少。

### 其他可能的诊断

- **先天性血小板减少症（如Bernard-Soulier综合征）**
  - **出血倾向**：血小板减少，血小板体积增大。
  - **感染**：不常见。
  - **湿疹**：不常见。
  - **自身免疫性疾病**：不常见。
  - **恶性肿瘤**：不常见。
- **免疫性血小板减少性紫癜（ITP）**
  - **出血倾向**：血小板减少，血小板体积正常。
  - **感染**：不常见。
  - **湿疹**：不常见。
  - **自身免疫性疾病**：不常见。
  - **恶性肿瘤**：不常见。
- **慢性肉芽肿病（CGD）**
  - **出血倾向**：不常见。
  - **感染**：易患细菌和真菌感染，尤其是卡氏肺囊虫和曲霉菌。
  - **湿疹**：不常见。
  - **自身免疫性疾病**：不常见。
  - **恶性肿瘤**：不常见。
- **X连锁无丙种球蛋白血症（XLA）**
  - **出血倾向**：不常见。
  - **感染**：易患细菌感染，尤其是呼吸道和消化道感染。
  - **湿疹**：不常见。
  - **自身免疫性疾病**：不常见。
  - **恶性肿瘤**：不常见。
- **自身免疫性淋巴细胞增生综合征（ALPS）**
  - **出血倾向**：不常见。
  - **感染**：不常见。
  - **湿疹**：不常见。
  - **自身免疫性疾病**：常见，包括自身免疫性溶血性贫血、血小板减少性紫癜等。
  - **恶性肿瘤**：淋巴瘤风险增加。

这些诊断中，Wiskott-Aldrich综合征（WAS）是最符合您描述的临床表现的。建议进一步进行基因检测和免疫学检查以确诊。

### 121. User

最常见的临床表现是 EB 病毒感染后的暴发性传染性单核细胞增多症、异常免疫球蛋白血症及 B 细胞淋巴瘤。异常免疫球蛋白血症及 B 细胞来源的淋巴瘤可发生于没有 EB 病毒感染的患者。异常免疫球蛋白血症通常表现为 IgA 及 IgM水平升高，而 IgG1 和 IgG3 减少。淋巴瘤以非霍奇金 B 淋巴细胞瘤为主。其他临床表现包括淋巴细胞性脉管炎、再生障碍性贫血和淋巴瘤样肉芽肿病等。某些患者临床表现轻微， 仅有异常免疫球蛋白血症或轻度 EB 病毒感染，可能是由于发生了体细胞基因修复。

部分患者主要临床表现为噬血细胞综合征，以脾肿大多见，且不一定需要 EBV 感染诱发。部分患者可出现肠道炎症如克罗恩病或结肠炎，但很少发展为淋巴瘤。

### Qwen2.5_72b

根据您提供的临床表现，最可能的诊断是与EB病毒感染相关的**噬血细胞性淋巴组织细胞增生症（Hemophagocytic Lymphohistiocytosis, HLH）**。这种疾病是一种严重的免疫反应失调，通常由病毒感染（如EB病毒）触发，导致过度的炎症反应和多器官功能障碍。主要临床表现包括发热、脾肿大、肝功能异常、血细胞减少、异常免疫球蛋白血症等。

以下是其他五种可能的诊断：

- **EB病毒相关性传染性单核细胞增多症（Infectious Mononucleosis）**：这是EB病毒感染最常见的临床表现，通常表现为发热、咽痛、淋巴结肿大、脾肿大等。部分患者可能伴有异常免疫球蛋白血症。
- **非霍奇金淋巴瘤（Non-Hodgkin Lymphoma, NHL）**：特别是B细胞来源的淋巴瘤，可能与EB病毒感染有关。临床表现包括淋巴结肿大、发热、体重下降、夜间盗汗等。
- **再生障碍性贫血（Aplastic Anemia）**：这是一种骨髓功能衰竭性疾病，可能导致全血细胞减少。虽然不一定是EB病毒感染的直接结果，但EB病毒感染可能是一个触发因素。
- **淋巴细胞性脉管炎（Lymphocytic Vasculitis）**：这是一种血管炎症性疾病，可能与EB病毒感染有关。临床表现包括皮肤病变、关节痛、发热等。
- **克罗恩病（Crohn’s Disease）**：这是一种慢性炎症性肠病，虽然不一定是EB病毒感染的直接结果，但部分患者可能在EB病毒感染后出现肠道炎症。临床表现包括腹痛、腹泻、体重下降等。

这些诊断需要通过详细的病史、体格检查、实验室检查（如血液学检查、免疫学检查、病毒学检查）和影像学检查（如超声、CT、MRI）来进一步确认。
