# Supplementary material for: Performance of ChatGPT-4o and Four Open-Source Large Language Models in Generating Diagnoses Based on China’s Rare Disease Catalog: Comparative Study
Source: J Med Internet Res. 2025 Jun 18;27:e69929. doi: 10.2196/69929 (PMC12192912; doi:10.2196/69929)
Supplement: Multimedia Appendix 1 [file jmir-v27-e69929-s001.zip › numbered_qwen2.5-72b-en.docx]

numbered_qwen2.5_72b_en

**1. user 2024-12-01T00:07:26Z:**

The disease can begin in early childhood. Typical clinical manifestations include kidney and eye changes and hearing impairment. Renal manifestations are hematuria, proteinuria and progressive deterioration of renal function, in which hematuria is often persistent microscopic hematuria, which can occur after exercise or fever, and is common in adolescents. Hearing changes were sensorineural hearing loss, which gradually aggravated with the course of disease. Eye abnormalities manifest as myopia, youth ring and cataract, but lack specificity. Three ocular changes of diagnostic significance include anterior conical lens, posterior pleomorphic corneal atrophy, and retinal macula (white or yellow particles around the fovea). The incidence of hearing impairment in male patients in China was higher than that reported abroad (68% vs 55%), while that in female patients was lower than that reported abroad (7% vs 45%). The incidence of ocular abnormalities was similar to that reported abroad. Leiomyomas may also occur in a few patients, involving the respiratory tract, gastrointestinal tract, and female reproductive tract; occasionally aneurysmal lesions, midfacial dysplasia, and mental retardation have been reported.

Patients often have a family history. Male patients with autosomal recessive inheritance and X-linked inheritance progressed faster, often entering end-stage renal disease at the age of 16~35, while female patients with autosomal dominant inheritance and X-linked inheritance had a slower course of disease and later onset of renal failure. The age at which renal failure occurs is similar for all male members of the same affected family, but can vary among male members of different families. Therefore, renal failure can be divided into juvenile type (renal failure occurs in adolescence) and adult type (renal failure occurs in adulthood) according to the age of the affected family. Adolescent type is more prominent in extrarenal manifestations, boys usually die before reproduction, disease genes are often new mutations, while adult type families have older patients and fewer new mutations.

**qwen2.5_72b** 2024-12-01T00:08:27Z:

Based on the clinical manifestations and genetic information provided, the most likely diagnosis is **Alport Syndrome**.

Alport Syndrome:

- **Genetics**: Inherited in an X-linked recessive pattern (most common), autosomal recessive, or autosomal dominant.
- **Clinical Features**:
  - **Renal Manifestations**: Persistent microscopic hematuria, proteinuria, and progressive renal failure. Hematuria can be exacerbated by exercise or fever.
  - **Hearing Impairment**: Sensorineural hearing loss that worsens over time.
  - **Ocular Abnormalities**: Myopia, anterior lenticonus (conical shape of the lens), posterior polymorphous corneal dystrophy, and retinal macular changes.
- **Gender Differences**: Higher incidence of hearing impairment in male patients in China compared to international reports; lower incidence in female patients. Female patients have a slower disease progression and later onset of renal failure.

Other Possible Diagnoses:

- **Fabry Disease**:
  - **Genetics**: X-linked recessive.
  - **Clinical Features**: Renal dysfunction, angiokeratomas (vascular lesions), hypohidrosis (reduced sweating), and peripheral neuropathy. Can also present with hearing loss and corneal dystrophy.
- **Thin Basement Membrane Nephropathy (TBMN)**:
  - **Genetics**: Autosomal dominant or sporadic.
  - **Clinical Features**: Persistent microscopic hematuria, often benign course without significant renal impairment. No associated hearing loss or ocular abnormalities.
- **Collagen IV-Related Nephropathies**:
  - **Genetics**: Mutations in collagen IV genes (COL4A3, COL4A4, COL4A5).
  - **Clinical Features**: Similar to Alport Syndrome but can present with a broader range of phenotypes, including benign familial hematuria and more severe forms.
- **Nail-Patella Syndrome**:
  - **Genetics**: Autosomal dominant.
  - **Clinical Features**: Renal involvement (hematuria, proteinuria), nail abnormalities, patellar hypoplasia or aplasia, elbow dysplasia, and iliac horns. Can also present with ocular abnormalities but not typically sensorineural hearing loss.
- **Mitochondrial Disorders**:
  - **Genetics**: Maternally inherited (mitochondrial DNA) or autosomal recessive.
  - **Clinical Features**: Multi-organ involvement including renal dysfunction, sensorineural hearing loss, and ophthalmological abnormalities. Can also present with myopathy, neuropathy, and other systemic symptoms.

Summary:

Alport Syndrome is the most likely diagnosis given the combination of renal manifestations (hematuria, proteinuria), sensorineural hearing loss, specific ocular changes, and family history consistent with X-linked or autosomal inheritance patterns. The other diagnoses listed are also possible but less likely based on the provided clinical information.

**2. user 2024-12-01T00:08:32Z:**

Patients often have no abnormal performance in the neonatal period. The first symptom is developmental retardation at about 6 months of age, but the typical clinical manifestations appear after 1 year of age, and it often takes several years for clinical diagnosis to be confirmed. According to the frequency of clinical manifestations, they were divided into three categories: common manifestations (100% patients), frequent manifestations (more than 80% patients) and correlated manifestations (20% ~80% patients).

- All of them showed normal pregnancy history and head circumference at birth; no birth defects and abnormal biochemical indexes; brain MRI/CT showed no structural abnormalities except mild cortical atrophy and myelin dysplasia; motor milestones lagged behind without regression; severe developmental retardation occurred at 6~12 months; language disorders showed no or very little vocabulary, repetitive language and non-verbal communication ability were stronger than expressive language ability; motor or balance disorders often showed ataxia and tremor of limbs; Abnormal behavioral characteristics are frequent laughter or smiling, obvious excited movements or happy behavior, often accompanied by clapping or hyperactivity.
- The head circumference often showed backward growth, and microcephaly was still present at the age of 2. Epilepsy often occurred before the age of 3. Abnormal EEG: characteristic high amplitude spike-slow wave.
- Correlation: flat occipital region; tongue preference; sucking or swallowing disorders; infantile feeding difficulties; low muscle tone; large jaw; wide tooth space; frequent salivation; excessive chewing movements; strabismus; hypopigmentation of the skin; light hair and eye color compared to family (absent type only); hyperreflexia of lower limb tendons; lifting or bending of upper limbs, especially when walking; wide gait base; high sensitivity to heat; abnormal sleep-wake cycle, less sleep; obsession with water, paper, and plastic; Eating related behavioral abnormalities; Obesity (more common in older, non-deficit patients); scoliosis; constipation.

**qwen2.5_72b** 2024-12-01T00:09:29Z:

Based on the clinical manifestations you’ve provided, the rare disease most likely to be diagnosed is **Angelman Syndrome**. Here’s a breakdown of how the symptoms align with Angelman Syndrome:

Common Manifestations (100% of Patients)

- **Normal pregnancy and birth history**: No abnormalities at birth.
- **Developmental delay starting around 6 months**: Severe developmental retardation becomes apparent by 6-12 months.
- **Severe language disorders**: Minimal or no vocabulary, with non-verbal communication being more prominent.
- **Motor and balance disorders**: Ataxia and tremor of limbs.
- **Abnormal behavioral characteristics**: Frequent laughter or smiling, hyperactivity, and excited movements.

Frequent Manifestations (More than 80% of Patients)

- **Microcephaly by age 2**.
- **Epilepsy often before age 3**.
- **Characteristic EEG findings**: High amplitude spike-slow wave complexes.

Correlated Manifestations (20%-80% of Patients)

- Flat occipital region.
- Tongue preference and sucking or swallowing disorders.
- Feeding difficulties in infancy.
- Low muscle tone.
- Large jaw and wide tooth spacing.
- Frequent salivation and excessive chewing movements.
- Strabismus.
- Hypopigmentation of skin, light hair, and eye color (in the deletion type).
- Hyperreflexia of lower limb tendons.
- Abnormal arm movements when walking.
- Wide gait base.
- Heat sensitivity.
- Abnormal sleep-wake cycle with less sleep.
- Obsession with water, paper, and plastic.
- Eating-related behavioral abnormalities.
- Obesity in older, non-deletion patients.
- Scoliosis.
- Constipation.

Differential Diagnoses

- **Rett Syndrome**: Primarily affects girls and involves regression of skills after a period of normal development, hand wringing, and breathing difficulties.
- **Prader-Willi Syndrome**: Characterized by severe hypotonia in infancy, hyperphagia leading to obesity, intellectual disability, and behavioral issues.
- **Phelan-McDermid Syndrome**: Associated with deletions on chromosome 22q13, causing developmental delay, language impairment, and autistic features.
- **Cri-du-Chat Syndrome**: Caused by a deletion on the short arm of chromosome 5, characterized by a high-pitched cry in infancy, intellectual disability, and distinctive facial features.
- **Williams Syndrome**: Characterized by cardiovascular problems, distinctive facial features, hyperacusis (sensitivity to sound), and a “cocktail party” personality.

Given the specific combination of developmental delay, severe language impairment, characteristic behavioral features, and EEG findings, Angelman Syndrome is the most likely diagnosis among rare diseases with these clinical manifestations.

**3. user 2024-12-01T00:09:33Z:**

- Skeletal abnormalities: There is variation in the severity of the disease and the type of associated complications. Thoracic stenosis is present in all cases, and the thorax may be bell-shaped or long and narrow, with respiratory distress usually caused by a small, rigid thorax. The severity of respiratory insufficiency varies according to the type of thorax and the degree of underlying pulmonary dysplasia, and the age of onset varies. There is a negative correlation between the severity of respiratory insufficiency and the age at which the disease is diagnosed. Acute respiratory disease is more common in infants with severe restrictive and bell-shaped chests, and severe and fatal respiratory failure can occur in the early neonatal period. Infants with a long, narrow chest are more likely to present with mild respiratory involvement, which can eventually progress to chronic restrictive lung disease with recurrent respiratory infections. Short, deformed ribs cause intercostal muscle weakness and restricted breathing, leading to respiratory failure. Most children die within 2 years of age, with about half dying within 6 months of birth. Other skeletal anomalies include short ribs, short limbs, abnormally shaped pelvis, short fingers, and polydactyly. About 60% of patients have proximal cervical spinal stenosis.
- Involvement of other organs: Renal lesions are present in about 40% of patients. The earliest sign of renal disease is impaired urinary concentration. Other signs include polyuria, proteinuria, elevated blood urea nitrogen and creatinine, and hypertension. Renal failure is the leading cause of death in this patient between the ages of 3 and 10 years. Hepatic involvement occurs in about 30% of cases and is manifested by hepatomegaly, elevated aminotransferases, cholestasis, cirrhosis and portal hypertension. The disease is occasionally associated with ocular developmental abnormalities, including optic nerve hypoplasia, retinal dystrophy, retinal pigmentation abnormalities, nystagmus, myopia and progressive visual impairment. There are also a few reports suggesting that these patients may also present with neurological abnormalities such as growth retardation, hypotonia, and central nervous system MRI abnormalities.

**qwen2.5_72b** 2024-12-01T00:10:29Z:

Based on the clinical manifestations you have provided, the most likely rare disease is **Jeune Syndrome (Asphyxiating Thoracic Dystrophy)**.

Jeune Syndrome (Asphyxiating Thoracic Dystrophy)

- **Skeletal Abnormalities**: Characterized by a small, bell-shaped or long, narrow thorax leading to respiratory distress due to a rigid thorax and pulmonary dysplasia.
- **Other Organ Involvement**:
  - Renal: Impaired urinary concentration, polyuria, proteinuria, elevated blood urea nitrogen and creatinine, hypertension, and renal failure.
  - Hepatic: Hepatomegaly, elevated aminotransferases, cholestasis, cirrhosis, and portal hypertension.
  - Ocular: Optic nerve hypoplasia, retinal dystrophy, retinal pigmentation abnormalities, nystagmus, myopia, and progressive visual impairment.
  - Neurological: Growth retardation, hypotonia, and central nervous system MRI abnormalities.

Other Possible Diagnoses

- **Ellis-van Creveld Syndrome**
  - **Skeletal Abnormalities**: Short ribs, short limbs, polydactyly.
  - **Other Organ Involvement**: Cardiac defects (e.g., atrial septal defect), nail dysplasia, and dental abnormalities.
- **Short Rib-Polydactyly Syndrome (SRPS)**
  - **Skeletal Abnormalities**: Short ribs, short limbs, polydactyly.
  - **Other Organ Involvement**: Pulmonary hypoplasia, renal anomalies, cardiac defects, and liver fibrosis.
- **Metatropic Dysplasia**
  - **Skeletal Abnormalities**: Short limbs, thoracic deformity, kyphoscoliosis.
  - **Other Organ Involvement**: Spinal cord compression, respiratory issues, and joint contractures.
- **Campomelic Dysplasia**
  - **Skeletal Abnormalities**: Bowing of long bones, short ribs, small chest.
  - **Other Organ Involvement**: Respiratory distress, genital anomalies, and hearing loss.
- **Osteogenesis Imperfecta Type II**
  - **Skeletal Abnormalities**: Severe bone fragility, short limbs, small thorax.
  - **Other Organ Involvement**: Blue sclerae, respiratory issues, and multiple fractures in utero or at birth.

Each of these conditions has overlapping features with Jeune Syndrome, but the combination of skeletal abnormalities, renal involvement, hepatic complications, ocular anomalies, and neurological symptoms is most consistent with Jeune Syndrome.

**4. user 2024-12-01T00:10:34Z:**

The main symptoms include mental behavioural abnormalities, cognitive impairment, memory loss, seizures, speech disorders, movement disorders, involuntary movements, decreased level of consciousness, and autonomic dysfunction. Other accompanying symptoms include sleep disorders, focal damage to the CNS, brainstem and cerebellar symptoms, and involvement of peripheral nerves and neuromuscular junctions.

There are three main clinical types: (1) anti-NMDAR encephalitis, which is the most predominant type and is characterised by diffuse encephalitis. (ii) Borderline encephalitis, with mental behavioural abnormalities, temporal lobe epilepsy and near-memory deficits as the main symptoms. The common types of borderline encephalitis are anti-LGI1 antibody, anti-GABABR antibody and anti-AMPAR antibody-related encephalitis. (iii) Other types, including Morvant syndrome, anti-DPPX antibody-associated encephalitis, anti-IgLON5 antibody-associated encephalopathy, and autoimmune cerebellar ataxia.

**qwen2.5_72b** 2024-12-01T00:11:24Z:

Based on the clinical manifestations you have provided, **anti-NMDAR encephalitis** is the most likely diagnosis among the rare diseases. This condition often presents with a wide range of neurological and psychiatric symptoms, including mental behavioral abnormalities, cognitive impairment, memory loss, seizures, speech disorders, movement disorders, involuntary movements, decreased level of consciousness, and autonomic dysfunction.

Differential Diagnoses:

- **Anti-LGI1 Antibody-Related Encephalitis**:
  - Characterized by prominent faciobrachial dystonic seizures (FBDS), memory deficits, and psychiatric symptoms.
  - Often involves the medial temporal lobe and can present with focal damage to the CNS.
- **Anti-GABABR Antibody-Related Encephalitis**:
  - Commonly presents with severe encephalopathy, memory impairment, and seizures.
  - Can also involve autonomic dysfunction and behavioral changes.
- **Anti-AMPAR Antibody-Related Encephalitis**:
  - Typically characterized by subacute cognitive decline, memory loss, and psychiatric symptoms.
  - May present with focal neurological deficits and cerebellar ataxia.
- **Morvan Syndrome**:
  - Associated with peripheral nerve hyperexcitability, autonomic dysfunction, and encephalopathy.
  - Patients often have insomnia, muscle cramps, and significant neuromuscular involvement.
- **Anti-IgLON5 Antibody-Associated Encephalopathy**:
  - Presents with a unique combination of sleep disorders (e.g., REM sleep behavior disorder), cerebellar ataxia, and brainstem signs.
  - Often includes prominent autonomic dysfunction and movement disorders.

Summary:

- **Most Likely Diagnosis**: Anti-NMDAR Encephalitis
- **Differential Diagnoses**:
  1. Anti-LGI1 Antibody-Related Encephalitis
  2. Anti-GABABR Antibody-Related Encephalitis
  3. Anti-AMPAR Antibody-Related Encephalitis
  4. Morvan Syndrome
  5. Anti-IgLON5 Antibody-Associated Encephalopathy

These conditions share overlapping symptoms but have distinct clinical and immunological features that can help in making a precise diagnosis. Further diagnostic testing, such as cerebrospinal fluid (CSF) analysis for specific antibodies and neuroimaging, will be essential to confirm the diagnosis.

**5. user 2024-12-01T00:11:27Z:**

The clinical manifestations of the disease are complex and varied, and lack specificity. Most of them involve multiple systems such as nerve, skin, respiratory, digestive and immune systems, and it is difficult to confirm the diagnosis from the clinical manifestations alone. It can be divided into two types: complete and partial.

- Complete: It can be divided into two types: early-onset and late-onset. The most common manifestations are neurological symptoms, such as hypotonia, epilepsy, developmental delay, sensorineural deafness, ataxia, and progressive disorders of consciousness, etc. About half of the patients have ataxia, developmental delay, conjunctivitis, and visual problems, including visual problems. conjunctivitis, and visual problems, including optic atrophy. Metabolic disorders such as metabolic acidosis, hypoglycaemia and ketosis are often comorbid, and cutaneous manifestations include various forms of refractory rashes such as eczema, generalised erythema, and nappy rash, and patients may also present with respiratory symptoms such as hyperventilation, laryngeal wheezing, and apnoea, as well as digestive symptoms such as feeding difficulties, vomiting, and diarrhoea. Acute attacks can be triggered by infection, fever, fatigue, and a high protein diet. If treatment is not timely, it can often lead to irreversible damage. Patients with late-onset disease often develop in adolescence and may have no symptoms beforehand. The clinical phenotype is different from that of early-onset disease, which usually presents with peripheral neuropathy, muscle weakness, spastic paraparesis, skin rashes, and ocular problems, such as loss of vision and visual acuity, etc. Failure to treat the disease in a timely manner often leads to irreversible damage.
- Partial : May develop at all ages or even without onset. The overall signs and symptoms of the child are relatively mild, starting only in emergency situations such as infections and starvation. Patients may have hypotonia, skin rashes, hair loss, acute neurological symptoms such as epilepsy and impaired consciousness.

**qwen2.5_72b** 2024-12-01T00:12:15Z:

Given the complex and varied clinical manifestations involving multiple systems (neurological, skin, respiratory, digestive, and immune), along with metabolic disturbances, it is likely that you are dealing with a rare disease known as **MELAS Syndrome** (Mitochondrial Encephalomyopathy, Lactic Acidosis, and Stroke-like Episodes).

Most Likely Diagnosis:

- **MELAS Syndrome**: This mitochondrial disorder presents with a wide range of symptoms, including neurological manifestations like hypotonia, epilepsy, developmental delay, sensorineural deafness, ataxia, and progressive disorders of consciousness. It also includes metabolic acidosis, hypoglycemia, and ketosis, as well as skin rashes and respiratory and digestive symptoms.

Other Possible Diagnoses:

1. ** Leigh Syndrome**: A mitochondrial disease that often presents with developmental delay, hypotonia, ataxia, and respiratory issues. Metabolic disturbances such as lactic acidosis are common.
2. **Kearns-Sayre Syndrome (KSS)**: Characterized by progressive external ophthalmoplegia, pigmentary retinopathy, cardiac conduction defects, and cerebellar ataxia. It can also present with hypoglycemia and metabolic acidosis.
3. **Alpers-Huttenlocher Syndrome**: A severe mitochondrial DNA depletion syndrome that typically presents in infancy with developmental delay, epilepsy, liver failure, and hypotonia.
4. **Zellweger Spectrum Disorders (Peroxisomal Biogenesis Disorders)**: These disorders can present with neurological symptoms, hypotonia, liver dysfunction, and characteristic facial features. They also involve metabolic disturbances and skin rashes.

Diagnostic Approach:

- **Genetic Testing**: Sequencing of mitochondrial DNA (mtDNA) for MELAS syndrome or other relevant genes.
- **Metabolic Panels**: Blood tests to check for lactic acidosis, ketone bodies, and amino acid levels.
- **MRI and MRS**: Brain imaging can show characteristic findings like leukoencephalopathy and stroke-like lesions in MELAS.
- **Muscle Biopsy**: Can help identify mitochondrial abnormalities and mtDNA mutations.
- **Ophthalmologic Evaluation**: To assess for optic atrophy, retinopathy, or other ocular manifestations.

These steps will help narrow down the diagnosis and guide appropriate treatment and management strategies.

**6. user 2024-12-01T00:13:25Z:**

The high prevalence age group is infancy, which manifests as adrenocortical insufficiency with a predominant salt loss, and common symptoms include hyperkalemia, hyponatremia with dehydration, metabolic acidosis, hypoglycaemia, vomiting, shock, and skin pigmentation. Most patients present with failure or incomplete development during puberty, or impaired fertility, oligospermia and azoospermia. A few patients may present with early childhood hyperandrogenaemia, simple saline corticosteroid deficiency, and pseudo-precocious puberty or spontaneous pubertal development during puberty. In mild cases, the disease may start in adulthood.

Patients have hyperpigmentation of the skin, especially in exposed areas, friction areas, scarring and areola, axilla, scrotum.

Patients have varying degrees of reduced resistance to various stresses such as infection and trauma. In the event of a stressful situation, adrenal crisis can occur, manifested by nausea, vomiting, abdominal pain or diarrhoea, severe dehydration, lowered blood pressure, rapid heart rate, weak pulse, mental disorders, and often high fever, hypoglycaemia, hyponatraemia and even life-threatening.

**qwen2.5_72b** 2024-12-01T00:14:31Z:

Based on the clinical manifestations you’ve provided, the most likely diagnosis is **Congenital Adrenal Hyperplasia (CAH) due to 21-hydroxylase deficiency**. This is a rare genetic disorder that affects the adrenal glands, leading to deficiencies in cortisol and aldosterone production, as well as excess androgen production.

Differential Diagnoses:

- **Addison’s Disease**: An autoimmune condition where the adrenal cortex is damaged, leading to adrenocortical insufficiency with similar symptoms such as hyperkalemia, hyponatremia, metabolic acidosis, and skin pigmentation.
- **Salt-Wasting Nephropathy**: A renal disorder that can cause significant salt loss and electrolyte imbalances, leading to dehydration, hypotension, and metabolic disturbances.
- **Primary Aldosteronism with Renin Deficiency**: A condition where aldosterone production is impaired, leading to similar symptoms of hyponatremia, hyperkalemia, and metabolic acidosis.
- **Familial Glucocorticoid Deficiency (FGD)**: A rare autosomal recessive disorder characterized by isolated adrenal insufficiency with cortisol deficiency but normal aldosterone levels.
- **X-linked Adrenoleukodystrophy (ALD)**: A genetic disorder that primarily affects males and can present with adrenal insufficiency, along with neurological symptoms.

Key Features of Congenital Adrenal Hyperplasia (CAH):

- **High Prevalence in Infancy**: CAH often presents in early infancy.
- **Adrenocortical Insufficiency**: Deficiency in cortisol and aldosterone production.
- **Salt Loss**: Prominent feature due to aldosterone deficiency, leading to hyponatremia and hyperkalemia.
- **Metabolic Disturbances**: Metabolic acidosis, hypoglycemia, and dehydration.
- **Skin Pigmentation**: Hyperpigmentation of the skin, especially in areas subject to friction or exposure.
- **Pubertal Development Issues**: Delayed or incomplete puberty, impaired fertility, oligospermia, and azoospermia.
- **Hyperandrogenism**: In some cases, early childhood hyperandrogenaemia leading to pseudo-precocious puberty.
- **Stress Sensitivity**: Increased vulnerability to adrenal crisis during stress, infection, or trauma.

Diagnostic Approach:

- **Biochemical Tests**:
  - Serum electrolytes (sodium, potassium)
  - Cortisol levels
  - 17-hydroxyprogesterone (17-OHP) levels (elevated in CAH due to 21-hydroxylase deficiency)
  - ACTH stimulation test
- **Genetic Testing**:
  - Sequencing of the CYP21A2 gene for confirmation of 21-hydroxylase deficiency.
- **Imaging Studies**:
  - Ultrasound or MRI to assess adrenal gland size and structure.
- **Endocrine Function Tests**:
  - Adrenal function tests to evaluate cortisol and aldosterone production.

Early diagnosis and treatment are crucial in managing CAH to prevent life-threatening complications such as adrenal crisis and to ensure proper development and quality of life for the patient.

**7. user 2024-12-01T00:14:37Z:**

The disease can develop from birth to early adulthood. Neonatal onset is the most common, often presenting as macrosomia, poor feeding, hunger intolerance and persistent hypoglycaemia. Hypoglycaemic episodes may present with lethargy and hypotonia, as well as neurological sequelae or even death, such as apnoea, seizures and coma. In the delayed type, patients often show a hypoglycaemic reaction, including symptoms such as pallor, sweating and palpitations.

**qwen2.5_72b** 2024-12-01T00:15:34Z:

Based on the clinical manifestations you have provided, the most likely rare disease is **Congenital Hyperinsulinism (CHI)**.

Congenital Hyperinsulinism (CHI)

- **Onset**: Neonatal to early childhood.
- **Clinical Features**:
  - **Macrosomia**: Large body size at birth.
  - **Poor Feeding and Hunger Intolerance**: Difficulty in feeding, excessive hunger.
  - **Persistent Hypoglycaemia**: Low blood sugar levels that can be severe and recurrent.
  - **Hypoglycaemic Episodes**:
    - **Lethargy and Hypotonia**: Decreased muscle tone and activity.
    - **Neurological Sequelae**: Severe hypoglycaemia can lead to apnoea, seizures, and coma.
  - **Delayed Type Symptoms**: Pallor, sweating, palpitations.

Other Possible Diagnoses

- **Glycogen Storage Diseases (GSDs)**:
  - **Type I (von Gierke Disease)**: Impaired glucose production leading to hypoglycaemia, hepatomegaly, and lactic acidosis.
  - **Type III (Cori or Forbes Disease)**: Enzyme deficiency affecting glycogen breakdown.
- **Ketotic Hypoglycaemia**:
  - Characterized by low blood sugar levels with ketone production during fasting or illness.
  - Symptoms include pallor, sweating, and irritability.
- **Glucagon Deficiency**:
  - Reduced levels of glucagon can lead to hypoglycaemia, especially in infants.
  - Symptoms are similar to those seen in CHI, including lethargy and seizures.
- **Mitochondrial Disorders**:
  - Affect energy production in cells, leading to hypoglycaemia and other systemic symptoms.
  - Can present with poor feeding, developmental delay, and muscle weakness.
- **Isolated Growth Hormone Deficiency (IGHD)**:
  - Lack of growth hormone can lead to short stature and potential issues with glucose metabolism.
  - Hypoglycaemia is a possible symptom, especially in neonates.

Diagnostic Approach

- **Blood Glucose Monitoring**: Frequent monitoring to detect hypoglycaemic episodes.
- **Insulin Levels**: Elevated insulin levels during hypoglycaemia support the diagnosis of CHI.
- **Genetic Testing**: Specific gene mutations (e.g., *ABCC8*, *KCNJ11*) can confirm CHI.
- **Imaging and Scanning**: Ultrasound or MRI to assess for focal lesions in the pancreas.

If you suspect Congenital Hyperinsulinism, it is crucial to consult with a pediatric endocrinologist for further evaluation and management.

**8. user 2024-12-01T00:15:53Z:**

- Type I, abdominal distension, growth retardation, hypoglycemic convulsions, recurrent nosebleeds, diarrhoea and vomiting are the main reasons for children’s patients to visit the clinic, and very rarely, the first manifestation is hematuria, blood in the stool, recurrent bone fracture, anaemia or gout. Adult patients who are never diagnosed and treated may present with multiple hepatic adenomas, chronic renal failure, severe gout with multiple gout stones, and osteoporosis. Other rare presentations include pulmonary hypertension, diabetes mellitus, cerebrovascular disease, and hepatic adenoma carcinoma. On physical examination, short stature and markedly enlarged liver can be seen.
- Type Ib, in addition to the above manifestations, patients may also have recurrent infections with neutropenia, oral ulcers, inflammatory bowel disease, perianal ulcers, arthritis and splenomegaly.
- Infantile type II, which is divided into classical infantile type and non-classical infantile type according to its prognosis. Most of the classical infants present with generalised muscle weakness in the first month of life, delayed motor development, enlarged heart on chest X-ray, high QRS waves and short PR intervals on ECG, hypertrophic cardiomyopathy on cardiac ultrasound, and varying degrees of elevation of creatinine kinase, etc. Most of the infants die of left heart failure or cardiorespiratory failure after pulmonary infections in the first year of life. The non-classical infantile form presents with muscle weakness within the first year of life, lags in motor development, and most often dies of respiratory failure in early childhood.
- Type II late-onset, with onset of disease after 1 year of age and as late as 60 years of age. Most of the patients present with chronic progressive proximal muscle weakness and respiratory insufficiency, cardiac involvement is rare, and respiratory failure is the main cause of death. Clinical manifestations include easy fatigue, difficulty in sitting up, walking up and down stairs, squatting and walking, and in a few cases, sudden onset of respiratory failure.

**qwen2.5_72b** 2024-12-01T00:17:07Z:

Based on the clinical manifestations provided, the most likely diagnosis is **Hereditary Fructose Intolerance (HFI)**, specifically Type I. HFI is a rare metabolic disorder caused by a deficiency of aldolase B, an enzyme involved in fructose metabolism.

Main Diagnosis:

- **Hereditary Fructose Intolerance (HFI)**
  - **Clinical Manifestations:**
    - Abdominal distension
    - Growth retardation
    - Hypoglycemic convulsions
    - Recurrent nosebleeds
    - Diarrhoea and vomiting
    - Hematuria, blood in the stool (rare)
    - Recurrent bone fractures
    - Anemia or gout
    - Multiple hepatic adenomas (in adults)
    - Chronic renal failure
    - Severe gout with multiple tophi
    - Osteoporosis
    - Pulmonary hypertension
    - Diabetes mellitus
    - Cerebrovascular disease
    - Hepatic adenoma carcinoma

Other Possible Diagnoses:

1. **Glycogen Storage Disease Type I (von Gierke’s Disease)**
   - **Clinical Manifestations:**
     - Hypoglycemia
     - Lactic acidosis
     - Hyperlipidemia
     - Hepatomegaly
     - Growth retardation
     - Uric acid nephrolithiasis
2. **Galactosemia**
   - **Clinical Manifestations:**
     - Jaundice
     - Hepatomegaly
     - Hypoglycemia
     - Cataracts
     - Liver failure
     - Renal tubular dysfunction
     - Intellectual disability
3. **Maple Syrup Urine Disease (MSUD)**
   - **Clinical Manifestations:**
     - Vomiting
     - Lethargy
     - Coma
     - Seizures
     - Hypotonia
     - Characteristic maple syrup odor in urine
4. **Mucopolysaccharidosis Type I (Hurler Syndrome)**
   - **Clinical Manifestations:**
     - Coarse facial features
     - Short stature
     - Hepatosplenomegaly
     - Cardiac involvement
     - Respiratory problems
     - Developmental delay
5. **Pompe Disease (Glycogen Storage Disease Type II)**
   - **Clinical Manifestations:**
     - Generalized muscle weakness (infantile form)
     - Hypertrophic cardiomyopathy (infantile form)
     - Chronic progressive proximal muscle weakness (late-onset form)
     - Respiratory insufficiency
     - Enlarged liver and heart

Additional Considerations:

- **Type Ib** of HFI can present with additional features such as recurrent infections, neutropenia, oral ulcers, inflammatory bowel disease, perianal ulcers, arthritis, and splenomegaly.
- **Infantile Type II** (both classical and non-classical) is characterized by muscle weakness, delayed motor development, hypertrophic cardiomyopathy, and respiratory failure.
- **Type II Late-Onset** presents with chronic progressive proximal muscle weakness, respiratory insufficiency, and rare cardiac involvement.

Given the wide range of symptoms and the specific mention of hypoglycemic convulsions, hepatomegaly, and growth retardation, HFI remains the most likely diagnosis. However, further laboratory tests such as fructose tolerance testing, genetic analysis, and metabolic screening are essential to confirm the diagnosis.

**9. user 2024-12-01T00:17:35Z:**

- Neuro-muscular system, causing neurological abnormalities, such as convulsions, coma, ataxia, and even psychiatric symptoms; muscle symptoms can also occur, including hyperreflexia, tremor, tetany, and muscle weakness. 2. Cardiovascular system, causing ECG changes, such as PR interval prolongation, QRS wave widening, etc. In severe cases, it can cause ventricular arrhythmia.
- cardiovascular system, causing changes in electrocardiogram, such as prolongation of PR interval, widening of QRS wave, and so on, and in severe cases, it may cause ventricular arrhythmias.
- Kidney, which may cause kidney stones, renal calcium deposits, renal insufficiency.
- bone and joint system, can cause osteoporosis and osteochondrosis.
- digestive system, may cause intestinal obstruction.
- endocrine, patients can see diabetes mellitus or impaired glucose tolerance.
- ocular symptoms, some types may be combined with ocular lesions, including nystagmus, visual field defects, myopia, etc. This group of diseases usually has a chronic course.

This group of diseases usually has a chronic course, with the neonatal and infantile onset types often being more severe and having a poorer prognosis. Some patients develop chronic renal failure during adolescence, and in some cases, failure to treat the disease in time can lead to severe mental retardation or even death.

**qwen2.5_72b** 2024-12-01T00:19:12Z:

Based on the clinical manifestations you’ve provided, the most likely rare disease is **Bartter Syndrome**. However, several other conditions share similar symptoms and should be considered as differential diagnoses. Here’s a detailed analysis:

Most Likely Diagnosis:

- **Bartter Syndrome**
  - **Neuro-muscular system:** Neurological abnormalities like convulsions, ataxia, and psychiatric symptoms can occur due to electrolyte imbalances.
  - **Cardiovascular system:** ECG changes such as PR interval prolongation and QRS wave widening are common due to hypokalemia and metabolic alkalosis.
  - **Kidney:** Kidney stones, renal calcium deposits, and renal insufficiency are frequent complications.
  - **Bone and joint system:** Osteoporosis and osteochondrosis can occur due to chronic electrolyte imbalances.
  - **Digestive system:** Intestinal obstruction may be a complication of severe dehydration or hypokalemia.
  - **Endocrine:** Diabetes mellitus or impaired glucose tolerance can occur secondary to metabolic disturbances.
  - **Ocular symptoms:** Ocular lesions, including nystagmus and visual field defects, are less common but can occur due to systemic electrolyte imbalances.

Other Possible Diagnoses:

1. **Gitelman Syndrome**
   - **Neuro-muscular system:** Similar neurological manifestations due to hypokalemia.
   - **Cardiovascular system:** ECG changes such as prolonged QT interval, PR interval prolongation, and QRS wave widening.
   - **Kidney:** Hypocalciuria and metabolic alkalosis.
   - **Bone and joint system:** Osteoporosis can occur.
   - **Digestive system:** Intestinal issues are less common but can occur due to dehydration.
   - **Endocrine:** Diabetes insipidus or diabetes mellitus can be seen.
   - **Ocular symptoms:** Less commonly reported.
2. **Hyperparathyroidism**
   - **Neuro-muscular system:** Neuromuscular irritability, including hyperreflexia and tetany.
   - **Cardiovascular system:** ECG changes due to hypercalcemia.
   - **Kidney:** Kidney stones, renal calcium deposits, and renal insufficiency.
   - **Bone and joint system:** Osteoporosis and bone pain.
   - **Digestive system:** Peptic ulcers and constipation.
   - **Endocrine:** Hypercalcemia can affect glucose metabolism.
   - **Ocular symptoms:** Cataracts may occur.
3. **Hypokalemic Periodic Paralysis**
   - **Neuro-muscular system:** Muscle weakness, hyperreflexia, and tetany.
   - **Cardiovascular system:** ECG changes such as U waves, ST-segment depression, and QT interval prolongation.
   - **Kidney:** Less commonly associated with renal issues.
   - **Bone and joint system:** Osteoporosis is rare.
   - **Digestive system:** Intestinal obstruction can occur due to severe hypokalemia.
   - **Endocrine:** Can be associated with thyrotoxicosis in some cases.
   - **Ocular symptoms:** Less commonly reported.
4. **Wilson Disease**
   - **Neuro-muscular system:** Neurological manifestations like tremor, ataxia, and psychiatric symptoms.
   - **Cardiovascular system:** Cardiomyopathy can occur due to copper toxicity.
   - **Kidney:** Renal tubular acidosis and kidney stones.
   - **Bone and joint system:** Arthritis and bone pain.
   - **Digestive system:** Hepatic cirrhosis leading to portal hypertension and esophageal varices.
   - **Endocrine:** Diabetes mellitus can occur due to liver damage.
   - **Ocular symptoms:** Kayser-Fleischer rings in the cornea.
5. **Multiple Endocrine Neoplasia Type 1 (MEN1)**
   - **Neuro-muscular system:** Neuromuscular symptoms can occur due to hypocalcemia from parathyroid disease.
   - **Cardiovascular system:** Hypertension and ECG changes.
   - **Kidney:** Kidney stones from hyperparathyroidism.
   - **Bone and joint system:** Osteoporosis.
   - **Digestive system:** Peptic ulcers from gastrinomas.
   - **Endocrine:** Diabetes mellitus or impaired glucose tolerance from pancreatic islet cell tumors.
   - **Ocular symptoms:** Less commonly reported.

Conclusion:

While Bartter Syndrome fits the clinical picture most closely, it’s essential to consider and rule out other differential diagnoses through appropriate laboratory tests, imaging, and genetic testing.

**10. user 2024-12-01T00:20:03Z:**

There are five types, of which the classic and intermediate types are the most common.

- Classic type is the most common, accounting for 75 per cent of cases. Children are normal at birth, with early onset, severe symptoms and rapid progression. Usually, maple syrup odour appears in the cerumen 12 hours after birth; a special maple sugar odour is found in urine and sweat 12-24 hours after birth; ketonuria, irritability and feeding difficulties appear in 2-3 days; severe metabolic disorders and encephalopathic symptoms begin to appear in 4-5 days, which are manifested as difficulty in breastfeeding, paroxysmal vomiting, refusal to eat, lethargy, apnoea, convulsive seizures, heightened muscle tone and ‘fencing’. The symptoms of severe metabolic disorders and encephalopathy may include difficulty in breastfeeding, refusal to eat, lethargy, apnoea, convulsive seizures, increased muscle tone, and stereotypical movements such as ‘fencing’-like and ‘stomping’-like movements, etc. Coma and central respiratory failure may appear in the first 7-10 days of life, and most of the infants will die of severe metabolic disorders a few days after birth if they are not treated promptly.
- Intermediate type can develop at any age. The symptoms are less severe than those of the classic type and include slow growth, delayed development, feeding difficulties, irritability, a maple syrup odour in the cerumen, and severe metabolic disorders and encephalopathy under stress. 3. Intermittent Type The intermittent type is characterised by episodic seizures.
- Intermittent type: intermittent seizures, normal growth and development in the early stage, mostly under stressful situations such as infections and surgeries, manifested by episodic ataxia and ketoacidosis, and a very small number of severe cases may cause death. Branched-chain amino acid concentrations are normal during the intermittent period, and are elevated during the episodic period, similar to the classic type.

**qwen2.5_72b** 2024-12-01T00:21:08Z:

Based on the clinical manifestations you’ve described, it is highly likely that the patient has **Maple Syrup Urine Disease (MSUD)**, a rare metabolic disorder caused by a deficiency in the branched-chain alpha-keto acid dehydrogenase complex. Here’s a breakdown of the most likely diagnosis and five other possible diagnoses:

Most Likely Diagnosis:

- **Maple Syrup Urine Disease (MSUD) - Classic Type**:
  - **Clinical Features**:
    - Maple syrup odor in cerumen, urine, and sweat within 24 hours of birth.
    - Early onset with severe symptoms including ketonuria, irritability, feeding difficulties.
    - Rapid progression to metabolic disorders and encephalopathic symptoms (difficulty feeding, vomiting, lethargy, apnoea, convulsions, increased muscle tone).
    - Potential for coma and central respiratory failure within the first 10 days of life.
  - **Genetics**: Caused by mutations in the BCKDHA, BCKDHB, or DBT genes.

Other Possible Diagnoses:

1. **Medium-Chain Acyl-CoA Dehydrogenase Deficiency (MCADD)**:
   - **Clinical Features**:
     - Hypoglycemia, vomiting, lethargy, and coma during fasting or illness.
     - Lack of characteristic odor but may present with metabolic acidosis and ketonuria.
   - **Genetics**: Caused by mutations in the ACADM gene.
2. **Glycogen Storage Disease Type I (von Gierke Disease)**:
   - **Clinical Features**:
     - Hypoglycemia, lactic acidosis, hepatomegaly, and growth retardation.
     - Feeding difficulties and vomiting may be present.
   - **Genetics**: Caused by mutations in the G6PC gene.
3. **Phenylketonuria (PKU)**:
   - **Clinical Features**:
     - Musty or mousy odor to urine, developmental delay, seizures, and hypopigmentation.
     - Early symptoms may include irritability and feeding difficulties.
   - **Genetics**: Caused by mutations in the PAH gene.
4. **Organic Acidemias (e.g., Propionic Acidemia, Methylmalonic Acidemia)**:
   - **Clinical Features**:
     - Metabolic acidosis, ketonuria, vomiting, lethargy, and developmental delay.
     - May present with a characteristic odor in the urine.
   - **Genetics**: Caused by mutations in various genes involved in organic acid metabolism (e.g., PCCB for Propionic Acidemia).
5. **Tyrosinemia Type I**:
   - **Clinical Features**:
     - Hepatic and renal dysfunction, failure to thrive, vomiting, and lethargy.
     - May present with a cabbage-like odor in the urine.
   - **Genetics**: Caused by mutations in the FAH gene.

Each of these conditions has overlapping symptoms but can be distinguished through specific metabolic testing, genetic analysis, and clinical evaluation. Early diagnosis and treatment are crucial for managing these disorders effectively.

**11. user 2024-12-01T00:21:27Z:**

The disease can start from birth to old age and can be acute or insidious. Almost any system or organ can be involved, with a predominance of energy-demanding organs such as the brain, heart, kidneys, eyes, ears, muscles, and gastrointestinal tract. Patients may present with a clinical syndrome of some kind, a single symptom or a combination of symptoms.

**qwen2.5_72b** 2024-12-01T00:22:15Z:

Based on the provided clinical manifestations, one rare disease that fits this description is **Mitochondrial Disease**. Mitochondrial diseases are a group of disorders caused by dysfunctional mitochondria, which can affect multiple organ systems due to their role in energy production.

Primary Diagnosis:

- **Mitochondrial Disease**: This includes a wide range of conditions such as MELAS (Mitochondrial Encephalomyopathy, Lactic Acidosis, and Stroke-like episodes), Leigh Syndrome, and Kearns-Sayre Syndrome. These conditions can present with a variety of symptoms affecting the brain, heart, kidneys, eyes, ears, muscles, and gastrointestinal tract.

Other Possible Diagnoses:

- **Lysosomal Storage Disorders**: Conditions like Gaucher Disease, Fabry Disease, and Niemann-Pick Disease can affect multiple organ systems and present with a wide range of symptoms.
- **Metabolic Disorders**: Such as Maple Syrup Urine Disease, Phenylketonuria (PKU), and Homocystinuria, which can lead to systemic involvement due to the accumulation of toxic metabolites.
- **Autoimmune Diseases**: Conditions like Systemic Lupus Erythematosus (SLE) or Sjögren’s Syndrome can affect multiple organs and present with a variety of symptoms.
- **Inherited Neurometabolic Disorders**: Such as Wilson Disease, which primarily affects the liver and brain but can also involve other systems.
- **Chronic Inflammatory Demyelinating Polyneuropathy (CIDP)**: This condition can affect the peripheral nerves and may present with muscle weakness, fatigue, and sensory symptoms, potentially affecting multiple organ systems indirectly.

Diagnostic Approach:

- **Detailed Medical History and Physical Examination**: To identify patterns of symptoms and affected organs.
- **Laboratory Tests**: Including blood tests, urine analysis, and metabolic screening to look for markers of mitochondrial dysfunction or other specific disorders.
- **Imaging Studies**: Such as MRI or CT scans to evaluate organ involvement.
- **Genetic Testing**: To identify specific genetic mutations associated with mitochondrial diseases or other inherited conditions.
- **Biopsy**: Muscle biopsy can be particularly useful in diagnosing mitochondrial diseases.

Given the complexity and variability of symptoms, a multidisciplinary approach involving specialists such as neurologists, cardiologists, nephrologists, and geneticists may be necessary to reach a definitive diagnosis.

**12. user 2024-12-01T00:22:22Z:**

The prevalence of the disease is between 5 and 50 years old, with a median age of onset of 39 years. The prevalence of women is significantly higher than that of men, with a female:male ratio of (9~11):1. Most of the cases have an acute/subacute onset, and the clinical manifestations include six core syndromes: (1) optic neuritis; (2) acute myelitis; (3) posterior pole syndrome; (4) acute brainstem syndrome; (5) symptomatic sleep attacks or acute mesencephalic clinical syndrome with typical MRI foci of the mesencephalon; and (6) symptomatic cerebral syndrome with typical brain lesions. It is a highly recurrent and disabling disease, with more than 90 per cent of patients having a recurrent course, and the majority of patients are left with more severe neurological disability.

**qwen2.5_72b** 2024-12-01T00:23:43Z:

Based on the clinical manifestations and epidemiological features you have provided, the most likely rare disease diagnosis is **Neuromyelitis Optica Spectrum Disorders (NMOSD)**.

Neuromyelitis Optica Spectrum Disorders (NMOSD)

- **Prevalence**: Affects individuals between 5 and 50 years old, with a median age of onset around 39 years.
- **Sex Ratio**: Predominantly affects women, with a female-to-male ratio of approximately 9:1 to 11:1.
- **Clinical Manifestations**:
  - **Optic Neuritis**: Inflammation of the optic nerve leading to vision loss.
  - **Acute Myelitis**: Inflammation of the spinal cord causing weakness, numbness, and sometimes paralysis.
  - **Posterior Pole Syndrome**: Involvement of the posterior part of the brain affecting visual pathways.
  - **Acute Brainstem Syndrome**: Symptoms related to the brainstem, such as cranial nerve palsies, ataxia, and vertigo.
  - **Symptomatic Sleep Attacks or Acute Mesencephalic Clinical Syndrome with Typical MRI Foci of the Mesencephalon**: Sleep disorders and symptoms related to mesencephalic involvement.
  - **Symptomatic Cerebral Syndrome with Typical Brain Lesions**: Involvement of cerebral regions leading to various neurological deficits.
- **Course**: Highly recurrent and disabling, with more than 90% of patients experiencing a relapsing course.

Other Possible Diagnoses

- **Multiple Sclerosis (MS)**:
  - **Prevalence**: Affects individuals between 20 and 50 years old.
  - **Sex Ratio**: More common in women, with a female-to-male ratio of about 3:1.
  - **Clinical Manifestations**: Similar to NMOSD but typically involves more diffuse white matter lesions and a broader range of symptoms.
- **Systemic Lupus Erythematosus (SLE) with Central Nervous System Involvement**:
  - **Prevalence**: Affects individuals between 15 and 45 years old.
  - **Sex Ratio**: Predominantly affects women, with a female-to-male ratio of about 9:1.
  - **Clinical Manifestations**: Can present with optic neuritis, myelitis, and brainstem syndromes, but also includes systemic symptoms like rash, joint pain, and renal involvement.
- **Sarcoidosis**:
  - **Prevalence**: Affects individuals between 20 and 40 years old.
  - **Sex Ratio**: More common in women.
  - **Clinical Manifestations**: Can present with optic neuritis, myelitis, and brainstem syndromes, but also includes systemic granulomatous inflammation affecting multiple organs.
- **Behçet’s Disease**:
  - **Prevalence**: Affects individuals between 20 and 30 years old.
  - **Sex Ratio**: More common in men in some populations, but can affect women as well.
  - **Clinical Manifestations**: Can present with optic neuritis, myelitis, and brainstem syndromes, but also includes oral and genital ulcers, skin lesions, and uveitis.
- **Anti-NMDA Receptor Encephalitis**:
  - **Prevalence**: Affects individuals of any age, but more common in young adults.
  - **Sex Ratio**: More common in women, particularly those with ovarian teratomas.
  - **Clinical Manifestations**: Can present with psychiatric symptoms, movement disorders, and encephalopathy, but can also involve optic neuritis and myelitis.

Each of these conditions has overlapping clinical features with NMOSD, but specific diagnostic tests such as serological markers (e.g., anti-AQP4 antibodies for NMOSD) and imaging studies will help differentiate between them.

**13. user 2024-12-01T00:23:46Z:**

Hearing impairment. In the past, it was thought that the deafness was congenital, which could be identified by hearing screening, and that it would be apparent from birth, mostly involving both sides, and symmetrically severe prelingual deafness. In recent years, it has been found that individual cases may present with asymmetrical or unilateral hearing loss, and the degree of hearing loss is highly variable, ranging from mild to very severe in different patients; progressive deafness that occurs after birth is also not uncommon.

**qwen2.5_72b** 2024-12-01T00:24:41Z:

Based on the clinical manifestations you’ve provided—congenital or early-onset bilateral symmetrical prelingual deafness, with some cases presenting asymmetrical or unilateral hearing loss and a wide range of severity from mild to very severe, including progressive deafness—there are several rare diseases that could be considered. Here is the most likely diagnosis followed by five other possible diagnoses:

Most Likely Diagnosis:

- **Pendred Syndrome (OMIM #274600)**
  - **Description:** Pendred syndrome is a genetic disorder characterized by congenital bilateral sensorineural hearing loss and goiter (enlarged thyroid). The hearing loss is often severe and can be progressive. It is caused by mutations in the SLC26A4 gene.

Other Possible Diagnoses:

1. **Usher Syndrome Type I (OMIM #276900)**
   - **Description:** Usher syndrome type I is a genetic disorder characterized by congenital bilateral profound sensorineural hearing loss and retinitis pigmentosa, leading to progressive vision loss. Some cases can present with variable severity of hearing loss.
2. **DFNB1 (Connexin 26-Related Deafness) (OMIM #220290)**
   - **Description:** DFNB1 is the most common form of congenital non-syndromic sensorineural deafness, caused by mutations in the GJB2 gene. It typically presents as severe to profound bilateral hearing loss from birth but can also present with milder forms and variable severity.
3. **Alport Syndrome (OMIM #301050)**
   - **Description:** Alport syndrome is a genetic disorder characterized by progressive sensorineural hearing loss, kidney disease, and ocular abnormalities. The hearing loss usually begins in childhood or adolescence but can be present at birth in some cases.
4. **Jervell and Lange-Nielsen Syndrome (OMIM #220400)**
   - **Description:** Jervell and Lange-Nielsen syndrome is a rare genetic disorder characterized by congenital bilateral sensorineural hearing loss and long QT syndrome, which can lead to cardiac arrhythmias. The hearing loss is typically severe and symmetrical.
5. **Branchio-Oto-Renal Syndrome (OMIM #113650)**
   - **Description:** Branchio-oto-renal syndrome is a genetic disorder characterized by hearing loss, branchial anomalies, and renal abnormalities. The hearing loss can be congenital or progressive, and its severity can vary from mild to severe.

These conditions are all rare and have specific genetic causes that would need to be confirmed through genetic testing and further clinical evaluation.

**14. user 2024-12-01T00:25:35Z:**

Bradykinesia, tremor, and myotonia. Levodopa-induced motor complications are more likely to occur after treatment. In terms of non-motor symptoms, mood disorders (e.g., depression, anxiety, irritability, etc.) and behavioural disorders (e.g., compulsive dosage increases, impulse control disorders, stereotyped behaviour, etc.) are likely to occur.

**qwen2.5_72b** 2024-12-01T00:26:14Z:

Based on the clinical manifestations you’ve provided—bradykinesia, tremor, myotonia, levodopa-induced motor complications, and non-motor symptoms such as mood and behavioral disorders—the most likely diagnosis is **Dystonic Parkinsonism**. However, it’s important to consider other differential diagnoses that could present similarly. Here are five additional possible diagnoses:

- **Parkinson’s Disease (PD)**: While classic PD typically does not include myotonia, the combination of bradykinesia, tremor, and mood/behavioral symptoms is highly characteristic. Levodopa-induced motor complications are common in advanced PD.
- **Multiple System Atrophy (MSA)**: MSA can present with parkinsonian features, including bradykinesia and tremor, but often includes additional signs such as autonomic dysfunction, cerebellar ataxia, and myoclonus. Myotonia is less common but can occur.
- **Progressive Supranuclear Palsy (PSP)**: PSP can cause parkinsonian symptoms like bradykinesia and tremor, but it often includes prominent postural instability, vertical gaze palsy, and cognitive decline. Mood and behavioral changes are also common.
- **Corticobasal Syndrome (CBS)**: CBS can present with parkinsonism, including bradykinesia and tremor, as well as myoclonus. It is often associated with apraxia, alien limb phenomenon, and cognitive/behavioral changes.
- **Lewy Body Dementia (LBD)**: LBD can cause parkinsonian features along with significant cognitive fluctuations, visual hallucinations, and mood disorders such as depression and anxiety. Levodopa-induced motor complications are also possible.

Each of these conditions has overlapping symptoms, making a thorough clinical evaluation, including detailed history, physical examination, and possibly neuroimaging or other diagnostic tests, essential for accurate diagnosis.

**15. user 2024-12-01T00:26:44Z:**

**1. polyneuropathy**, often presenting as a symmetrical sensory and/or motor peripheral neuropathy of the extremities, progressing gradually from distal to proximal.

**2. organomegaly,** including hepatomegaly, splenomegaly, or lymph node enlargement; lymph node biopsy is often suggestive of Castleman disease.

**Endocrine abnormalities,** including hypogonadism (e.g., male impotence, breast development), hypothyroidism, abnormal glucose metabolism (e.g., diabetes mellitus), and adrenal insufficiency.

**4. skin changes,** which may be manifested by deepening of skin colour, cutaneous glomeruloid haemangiomas, white nails, polycythemia, hirsutism, and cyanosis of the hands and feet.

**5. increased extracirculatory water load,** including limb oedema, ascites, pleural effusion, pericardial effusion, and optic papilla oedema.

**Sclerosing bone disease,** is an important clinical manifestation. It may be manifested as bone pain or without clinical symptoms. Sclerosing bone disease can be detected significantly more frequently with CT scanning of the bones, and can be visualised as simple sclerosis of the bones, mixed sclerotic and osteolytic lesions, or simple osteolytic changes.

**7. erythrocytosis and/or thrombocytosis** **8.

**8. pulmonary hypertension** , patients may present with pulmonary hypertension, as evidenced by reduced activity tolerance, hypoxaemia, etc.

**9. cerebral infarction**.

**qwen2.5_72b** 2024-12-01T00:27:43Z:

Based on the clinical manifestations you have provided, the most likely rare disease is **POEMS syndrome (Polyneuropathy, Organomegaly, Endocrinopathy, M-protein, and Skin changes)**. POEMS syndrome is a rare multisystemic disorder that involves multiple organ systems and is characterized by the presence of an underlying plasma cell dyscrasia.

Other Possible Diagnoses:

- **Castleman Disease (Unicentric or Multicentric):**
  - Similar to POEMS, Castleman disease can present with lymphadenopathy, hepatosplenomegaly, and constitutional symptoms. However, it typically lacks the neuropathy and endocrine abnormalities seen in POEMS.
- **Amyloidosis:**
  - Amyloidosis can present with similar systemic involvement, including neuropathy, organomegaly, and skin changes. However, amyloid deposits are a key feature, which can be detected through biopsy and Congo red staining.
- **Chronic Inflammatory Demyelinating Polyneuropathy (CIDP):**
  - CIDP primarily presents with progressive symmetric peripheral neuropathy but lacks the systemic manifestations such as organomegaly and endocrine abnormalities seen in POEMS.
- **Systemic Lupus Erythematosus (SLE):**
  - SLE can present with a wide range of multisystem involvement, including skin changes, endocrine abnormalities, and serositis (e.g., pleural and pericardial effusions). However, it typically lacks the specific combination of neuropathy and bone lesions seen in POEMS.
- **Multiple Myeloma:**
  - Multiple myeloma can present with bone pain, hypercalcemia, renal insufficiency, and anemia. It may also cause endocrine abnormalities and extramedullary plasmacytomas. However, it typically does not have the neuropathy and skin changes characteristic of POEMS.

Key Features of POEMS Syndrome:

- **Polyneuropathy:** Symmetrical sensory and motor peripheral neuropathy.
- **Organomegaly:** Hepatomegaly, splenomegaly, or lymphadenopathy.
- **Endocrinopathy:** Hypogonadism, hypothyroidism, diabetes mellitus, adrenal insufficiency.
- **Monoclonal gammopathy (M-protein):** Presence of an M-spike on serum protein electrophoresis.
- **Skin changes:** Hyperpigmentation, cutaneous hemangiomas, white nails, polycythemia, hirsutism, and cyanosis.

Given the combination of neuropathy, organomegaly, endocrine abnormalities, skin changes, sclerosing bone disease, and other systemic manifestations, POEMS syndrome is the most likely diagnosis. Further diagnostic workup should include serum protein electrophoresis, bone marrow biopsy, and imaging studies to confirm the presence of an M-spike and bone lesions.

**16. user 2024-12-01T00:27:46Z:**

Common manifestations in the foetal period include reduced foetal activity, excess amniotic fluid and breech presentation. In the neonatal period hypotonia is one of the hallmark features of this disorder, and severe hypotonia can lead to asphyxia, feeding difficulties, weak sucking, and weak crying. In infancy and early childhood, the main manifestations are growth retardation and lagging cognitive, motor and language development. Appetite increases with age and obesity can occur if eating is not restricted. Short stature is usually present in school age, and personality traits such as temper tantrums, stubbornness, and obsessive-compulsive disorder gradually appear. Abnormal behaviours include intense foraging, skin scratching, and autistic-like behaviour. Seizures occur in about 25 per cent of patients. Delayed or incomplete appearance of secondary sexual characteristics may occur in adolescent males and females. Obesity can lead to complications such as sleep apnoea, pulmonary heart disease, insulin resistance and even diabetes. Scoliosis, osteoporosis, and gastroesophageal reflux are also more common. The main manifestations in adulthood are obesity and its complications, including cardiovascular problems, diabetes and sleep apnoea. Other manifestations are mental abnormalities and infertility.

Fatal risks: choking due to uncoordinated mouth movements, hypotonia, polyphagia, gulping habits and inadequate chewing, and gastric rupture due to overeating.

Physical examination reveals characteristic facial features that become progressively more typical, including almond eyes, small mouth, thin upper lip, downward-facing corners of the mouth, small hands and feet, short stature, fair skin, low muscle tone, and genital hypoplasia (e.g., cryptorchidism, scrotal hypoplasia, or clitoral hypoplasia).

**qwen2.5_72b** 2024-12-01T00:28:44Z:

Based on the clinical manifestations you have provided, the most likely rare disease is **Prader-Willi Syndrome**. Here are some key points that align with this diagnosis:

- **Fetal Period**: Reduced fetal activity, excess amniotic fluid (polyhydramnios), and breech presentation.
- **Neonatal Period**: Hypotonia (floppy infant syndrome) leading to feeding difficulties, weak sucking, and weak crying.
- **Infancy and Early Childhood**: Growth retardation, lagging cognitive, motor, and language development.
- **Childhood and Adolescence**: Increased appetite with age, obesity if eating is not restricted, short stature, behavioral issues (temper tantrums, stubbornness, obsessive-compulsive disorder), abnormal behaviors (intense foraging, skin scratching, autistic-like behavior), seizures in about 25% of patients, delayed or incomplete appearance of secondary sexual characteristics.
- **Adulthood**: Obesity and its complications (cardiovascular problems, diabetes, sleep apnoea), mental abnormalities, infertility.
- **Physical Examination**: Characteristic facial features (almond eyes, small mouth, thin upper lip, downward-facing corners of the mouth), small hands and feet, short stature, fair skin, low muscle tone, genital hypoplasia (cryptorchidism, scrotal hypoplasia, or clitoral hypoplasia).

Five Other Possible Diagnoses:

- **Angelman Syndrome**: Characterized by severe intellectual disability, developmental delay, speech impairment, ataxia, and a happy demeanor with frequent laughing and smiling.
- **Williams Syndrome**: Features include developmental delays, cardiovascular problems (e.g., supravalvular aortic stenosis), distinctive facial features (elfin facies), hypercalcemia, and an outgoing personality.
- **Russell-Silver Syndrome**: Characterized by intrauterine growth restriction, postnatal growth deficiency, body asymmetry, and characteristic facial features (triangular face, prominent forehead).
- **Sotos Syndrome**: Features include overgrowth, macrocephaly, advanced bone age, developmental delay, and distinctive facial features (broad and prominent forehead, small chin).
- **22q11.2 Deletion Syndrome (DiGeorge Syndrome)**: Characterized by a wide range of symptoms including heart defects, immune deficiencies, hypoparathyroidism (leading to low calcium levels), cleft palate or other abnormalities of the palate, and developmental delays.

Each of these conditions has overlapping features with Prader-Willi Syndrome, but the combination of specific clinical manifestations, particularly the unique behavioral and physical characteristics, strongly suggests Prader-Willi Syndrome. Genetic testing for a deletion or mutation in the 15q11-q13 region on chromosome 15 would be definitive for confirming this diagnosis.

**17. user 2024-12-01T00:29:18Z:**

Patients often start in infancy or adolescence, with roughly equal male and female ratios. The water content of secretions from the epithelial cells of the respiratory tract, pancreas and biliary tract is reduced, and the secretions become viscous and difficult to clear. The lungs are the most commonly affected organ, and patients present with chronic bacterial infections and colonisation of the respiratory tract with pathogens, often initially Haemophilus influenzae and Staphylococcus aureus, and ultimately with chronic colonisation and/or infection with Pseudomonas aeruginosa or Klebsiella onionis. Once infection occurs, neutrophils are recruited in large numbers into the lung tissue and release elastase, which causes destruction of the lung tissue, leading to the formation of bronchiectasis. The patient’s bronchiectasis often starts at an early age and often begins in the upper lobes, in contrast to the majority of infections that result in the formation of bronchiectasis that is primarily located in the middle and lower lungs.

Patients often present with sweat stains left on dark-coloured clothing, or parents notice salty skin in their children. Abnormalities in the flow of bile and pancreatic fluid can lead to indigestion and malabsorption, as well as liver or pancreatic disease, and in severe cases, associated diabetes. The viscous intestinal fluid may also make the patient more susceptible to intestinal obstruction and rectal prolapse. In addition, male patients may develop impaired sperm movement in the vas deferens and congenital absence of the vas deferens (CBAVD).

**qwen2.5_72b** 2024-12-01T00:30:16Z:

Based on the clinical manifestations you’ve described, the most likely rare disease is **Cystic Fibrosis (CF)**.

Key Features of Cystic Fibrosis:

- **Age of Onset:** Often starts in infancy or adolescence.
- **Gender Ratio:** Equal male and female ratios.
- **Respiratory Tract:** Reduced water content in secretions, leading to viscous mucus. Chronic bacterial infections with pathogens like *Haemophilus influenzae*, *Staphylococcus aureus*, and later *Pseudomonas aeruginosa* or *Klebsiella pneumoniae*. Early onset bronchiectasis, often starting in the upper lobes.
- **Sweat:** High salt content leading to salty skin and sweat stains on clothing.
- **Digestive System:** Abnormalities in bile and pancreatic fluid flow causing indigestion, malabsorption, liver or pancreatic disease. Increased risk of intestinal obstruction and rectal prolapse.
- **Reproductive System:** Male patients may have impaired sperm movement and congenital absence of the vas deferens (CBAVD).

Other Possible Diagnoses:

- **Primary Ciliary Dyskinesia (PCD):**
  - **Features:** Chronic respiratory infections, bronchiectasis, situs inversus (reversed internal organs), and infertility due to immotile cilia.
- **Alpha-1 Antitrypsin Deficiency:**
  - **Features:** Chronic obstructive pulmonary disease (COPD), liver disease, and panniculitis. Can present with bronchiectasis but less commonly in the upper lobes.
- **Chronic Granulomatous Disease (CGD):**
  - **Features:** Recurrent bacterial and fungal infections, granulomas in various organs including the lungs, liver, and skin. Not typically associated with viscous secretions or high salt content in sweat.
- **Shwachman-Diamond Syndrome:**
  - **Features:** Chronic pancreatitis leading to malabsorption, bone marrow failure, and increased susceptibility to infections. Can present with respiratory issues but not typically with the specific pattern of bronchiectasis seen in CF.
- **Young’s Syndrome:**
  - **Features:** Bronchiectasis, sinusitis, and male infertility due to obstructive azoospermia. Often presents in adulthood rather than infancy or adolescence and does not have the high salt content in sweat characteristic of CF.

Given the combination of chronic respiratory infections, bronchiectasis starting in the upper lobes, high salt content in sweat, and digestive system involvement, Cystic Fibrosis is the most likely diagnosis. However, a definitive diagnosis would require genetic testing for mutations in the *CFTR* gene and possibly other specific tests like a sweat chloride test.

**18. user 2024-12-01T00:30:19Z:**

Patients present with oropharyngitis, otitis media, respiratory infections, cellulitis and skin infections, often due to staphylococcal and streptococcal infections. Oral ulcers and painful gingivitis occur in most children before the age of 2 years in severe neutropenia with decreased bone marrow reserve. If associated with diffuse gastrointestinal lesions, it may cause abdominal pain and diarrhoea similar to Crohn’s disease. Neutropenia can lead to recurrent bacterial or fungal infections in various areas, with the main sites of involvement being the skin and mucous membranes, ears, nose, throat and lungs. The infections are often severe or even fatal. About 15% of patients progress to acute leukaemia or myelodysplastic syndrome. The children usually do not have characteristic malformations.

**qwen2.5_72b** 2024-12-01T00:31:33Z:

Based on the clinical manifestations you’ve described, the most likely diagnosis is **Chronic Granulomatous Disease (CGD)**. However, given that neutropenia and recurrent infections are key features, another primary consideration could be **Severe Congenital Neutropenia (Kostmann Syndrome)**. Here’s a detailed breakdown:

Most Likely Diagnosis:

- **Severe Congenital Neutropenia (Kostmann Syndrome)**
  - **Clinical Features**: Severe neutropenia with decreased bone marrow reserve, recurrent infections (often staphylococcal and streptococcal), oropharyngitis, otitis media, respiratory infections, cellulitis, and skin infections. Oral ulcers and painful gingivitis are common in early childhood. Gastrointestinal involvement can cause abdominal pain and diarrhea.
  - **Pathophysiology**: Deficiency in the neutrophil elastase (ELA2) gene or other genes involved in granulopoiesis leads to a significant reduction in circulating neutrophils.

Other Possible Diagnoses:

- **Chronic Granulomatous Disease (CGD)**
  - **Clinical Features**: Recurrent bacterial and fungal infections, especially involving the skin, lungs, and lymph nodes. Neutropenia is not a primary feature but can occur secondary to chronic inflammation.
  - **Pathophysiology**: Deficiency in NADPH oxidase, leading to impaired bactericidal activity of phagocytes.
- **Shwachman-Diamond Syndrome**
  - **Clinical Features**: Pancreatic insufficiency, neutropenia, skeletal abnormalities, and a predisposition to myelodysplastic syndrome and acute leukemia.
  - **Pathophysiology**: Mutations in the SBDS gene affect hematopoiesis and exocrine pancreatic function.
- **Cyclic Neutropenia**
  - **Clinical Features**: Periodic episodes of neutropenia lasting 3-6 days, occurring every 21 days on average. Symptoms include recurrent infections, fever, and oral ulcers during periods of low neutrophil counts.
  - **Pathophysiology**: Autosomal dominant mutations in the ELA2 gene cause cyclic fluctuations in neutrophil production.
- **Myelodysplastic Syndrome (MDS)**
  - **Clinical Features**: Progressive bone marrow failure, leading to cytopenias including neutropenia. Recurrent infections are common due to inadequate neutrophil function.
  - **Pathophysiology**: Clonal hematopoietic stem cell disorders with dysplasia and ineffective hematopoiesis.
- **Leukocyte Adhesion Deficiency Type 1 (LAD-1)**
  - **Clinical Features**: Recurrent severe infections, delayed separation of the umbilical cord, and poor wound healing due to impaired neutrophil migration.
  - **Pathophysiology**: Deficiency in CD18 integrin subunit, which is essential for leukocyte adhesion and extravasation.

Summary:

- **Most Likely Diagnosis**: Severe Congenital Neutropenia (Kostmann Syndrome)
- **Other Possible Diagnoses**:
  1. Chronic Granulomatous Disease (CGD)
  2. Shwachman-Diamond Syndrome
  3. Cyclic Neutropenia
  4. Myelodysplastic Syndrome (MDS)
  5. Leukocyte Adhesion Deficiency Type 1 (LAD-1)

Further diagnostic testing, such as bone marrow examination, genetic analysis, and functional assays of neutrophil function, will be necessary to confirm the diagnosis.

**19. user 2024-12-01T00:31:43Z:**

Typical Patients present with both CNS damage and hypoadrenocorticism. Neurological symptoms and symptoms of hypoadrenocorticism may occur simultaneously or sequentially. In a minority of patients, they may occur alone. The manifestations of adrenocortical insufficiency vary in severity. It is most common in children, accounting for 35% of all patients. The onset of symptoms occurs between the ages of 4 and 8 years. The initial symptoms include poor concentration, memory loss, learning difficulties, unsteady gait and abnormal behaviour, followed by visual and auditory loss, dysarthria, seizures, ataxia, paralysis and dementia. Clinical manifestations progressively worsen, culminating in blindness or deafness and complete paralysis. Some exhibit deafferentation and eventually die of central respiratory failure, brain herniation, and infection. Most patients have adrenocortical impairment when they first develop neurological symptoms.

**qwen2.5_72b** 2024-12-01T00:32:34Z:

Based on the clinical manifestations you’ve provided, **Adrenoleukodystrophy (ALD)** is the most likely diagnosis. ALD is a rare genetic disorder that affects the adrenal glands and the white matter of the brain, leading to both central nervous system (CNS) damage and hypoadrenocorticism.

Other Possible Diagnoses:

- **Adrenomyeloneuropathy (AMN)**: This is a milder form of ALD that primarily affects adults and can present with similar neurological symptoms and adrenal insufficiency, but typically progresses more slowly.
- **Multiple Sclerosis (MS)**: A chronic inflammatory condition affecting the CNS, which can cause similar neurological symptoms such as cognitive impairment, ataxia, and visual loss.
- **Wilson’s Disease**: This is a genetic disorder that leads to copper accumulation in various organs, including the brain and liver. It can present with neurological symptoms and adrenal insufficiency, though adrenal involvement is less common.
- **Mitochondrial Encephalomyopathies (e.g., MELAS syndrome)**: These are a group of genetic disorders affecting mitochondria, which can cause a wide range of neurological symptoms including cognitive decline, seizures, and ataxia.
- **Autoimmune Addison’s Disease**: This is an autoimmune condition that primarily affects the adrenal glands but can also present with neurological symptoms due to chronic cortisol deficiency.

Rationale for ALD:

- **Genetic Basis**: ALD is caused by mutations in the ABCD1 gene, which leads to the accumulation of very long-chain fatty acids (VLCFAs) in the brain and adrenal cortex.
- **CNS Involvement**: The white matter damage in ALD can lead to a wide range of neurological symptoms, including cognitive decline, ataxia, seizures, and paralysis.
- **Adrenal Insufficiency**: Hypoadrenocorticism is a hallmark of ALD and often occurs alongside neurological symptoms.
- **Age of Onset**: ALD typically affects children between the ages of 4 and 8 years, which matches the age range you provided.

Diagnostic Confirmation:

- **Blood Tests**: Elevated levels of VLCFAs in the blood are diagnostic for ALD.
- **MRI**: Brain MRI can show characteristic white matter lesions.
- **Genetic Testing**: Confirmatory genetic testing for mutations in the ABCD1 gene.

If ALD is suspected, early diagnosis and treatment with Lorenzo’s oil and hematopoietic stem cell transplantation (HSCT) can significantly improve outcomes.

**20. user 2024-12-01T00:33:05Z:**

Most of the children start to show symptoms of infection from 4 to 12 months after birth. The most prominent clinical manifestation is recurrent severe bacterial infections in the respiratory system, digestive system and central nervous system, with respiratory tract infections being the most common, and common pathogens include Haemophilus influenzae, Streptococcus pneumoniae, Staphylococcus aureus, and Pseudomonas aeruginosa, etc. The most common infections are respiratory tract infections. Prolonged and repeated respiratory infections can lead to irreversible organ function damage such as bronchiectasis and pestle finger. Common symptoms of GI infections include diarrhoea, abdominal pain, gastro-oesophageal reflux and gastroenteritis. Pathogens causing infectious diarrhoea include Giardia lamblia, Salmonella, Campylobacter jejuni and Cryptosporidium.

In addition, children are prone to otitis media, chronic sinusitis, malnutrition, anaemia, granulocytopenia, thrombocytopenia, growth hormone deficiency and thyroid hormone disorders. Children may also develop tumours, autoimmune and inflammatory diseases, including rheumatoid arthritis and dermatomyositis. A higher incidence of sensorineural hearing loss and eczema has also been reported in children.

**qwen2.5_72b** 2024-12-01T00:33:46Z:

Based on the clinical manifestations you’ve provided, the most likely rare disease is **Chronic Granulomatous Disease (CGD)**. CGD is a primary immunodeficiency disorder characterized by recurrent and severe bacterial and fungal infections due to an inability of phagocytes to produce reactive oxygen species necessary for microbial killing.

Most Likely Diagnosis:

- **Chronic Granulomatous Disease (CGD)**

Other Possible Diagnoses:

1. **Severe Combined Immunodeficiency (SCID)**: This is another primary immunodeficiency characterized by a lack of functional T and B cells, leading to severe and recurrent infections.
2. **Hyper-IgM Syndrome**: A primary immunodeficiency where patients have low levels of IgG, IgA, and IgE but elevated or normal levels of IgM, leading to recurrent infections and increased susceptibility to opportunistic pathogens.
3. **Chronic Mucocutaneous Candidiasis (CMC)**: This condition is characterized by persistent and recurrent candida infections of the skin, nails, and mucous membranes.
4. **Common Variable Immunodeficiency (CVID)**: A primary immunodeficiency characterized by low levels of antibodies, leading to recurrent respiratory and gastrointestinal infections.

Additional Considerations:

- **Shwachman-Diamond Syndrome**: This is a rare genetic disorder affecting multiple systems, including the bone marrow, pancreas, and skeletal system. It can present with recurrent infections due to neutropenia.
- **Wiskott-Aldrich Syndrome**: A primary immunodeficiency characterized by eczema, thrombocytopenia, and recurrent infections.

Each of these conditions has overlapping symptoms, but the specific combination of recurrent bacterial infections, particularly those involving the respiratory tract, along with other systemic manifestations such as gastrointestinal issues, otitis media, and autoimmune disorders, strongly suggests Chronic Granulomatous Disease. Further diagnostic testing, including functional assays for phagocyte function and genetic testing, would be necessary to confirm the diagnosis.

### **21. User**

The clinical manifestations of the patients included salt loss and hyperandrogenism. Neonatal onset was characterized by varying degrees of adrenal insufficiency, such as weakness, nausea, vomiting, feeding difficulties, diarrhea, chronic dehydration, skin pigmentation, and growth retardation. Adrenal crisis is often the first manifestation of the disease in the neonatal period, manifested as severe hyponatremia, hyperkalemia, hypovolemic shock, may be accompanied by hypoglycemia, induced by stress. Severe hyponatremia can lead to central nervous system manifestations such as convulsions, and severe hyperkalemia can cause fatal arrhythmias.

Hyperandrogenism manifests differently at different ages. Female children with classical diseases have different degrees of masculinity at birth. Light performance for isolated clitoris hypertrophy, heavy external genitalia can approach men, labia majora scrotal shape, clitoris hypospadia-like penis, and with a common urethral vaginal orifice. However, the labia majora cannot touch the gonads and has completely normal female internal genital structures (ovary and uterus). Males may not have external genital abnormalities such as enlarged penis during neonatal and infancy, which is a common cause of delayed diagnosis. In early childhood, both sexes will present peripheral precocious puberty. Boys presented with enlarged penis with or without pubic hair; females presented with heterosexual precocious puberty. Chronic high levels of sex hormones stimulate hypothalamic gonadotropin-releasing hormone (GnRH) neurons and develop into central precocious puberty. Women may also have secondary sexual dysplasia and primary amenorrhea or oligomenorrhea. Linear growth with accelerated bone age occurs in both sexes from infancy, resulting in impaired terminal height.

Other findings include skin and mucous membrane pigmentation, especially areola and vulva, some children may not have this change.

Non-classical hyperandrogenism in childhood, adolescence and even adulthood may be characterized by accelerated growth and rapid bone age progression.

### Qwen2.5_72b

Based on the clinical manifestations you’ve provided, the most likely diagnosis is **Congenital Adrenal Hyperplasia (CAH)**, specifically the most common form, **21-Hydroxylase Deficiency (21-OHD)**. This condition is a rare genetic disorder that affects the adrenal glands, leading to deficiencies in cortisol and aldosterone production and excess androgen production.

### Most Likely Diagnosis:

- **Congenital Adrenal Hyperplasia (21-Hydroxylase Deficiency)**

### Other Possible Diagnoses:

- **11β-Hydroxylase Deficiency**: This is another form of CAH that can also cause salt loss and hyperandrogenism, but it is less common than 21-OHD. It can lead to hypertension and virilization in both males and females.
- **3β-Hydroxysteroid Dehydrogenase Deficiency**: This form of CAH can also cause salt loss and hyperandrogenism, but it is rarer and can lead to ambiguous genitalia in both sexes.
- **Classic Galactosemia**: This is a rare genetic disorder that can cause feeding difficulties, vomiting, and liver problems in neonates, but it does not typically cause hyperandrogenism or adrenal insufficiency.
- **Androgen Insensitivity Syndrome (AIS)**: This is a condition where the body is resistant to androgens, leading to ambiguous genitalia in genetic males. However, it does not cause adrenal insufficiency or salt loss.
- **Non-Classical Congenital Adrenal Hyperplasia (NCAH)**: This is a milder form of CAH that can present later in childhood or adulthood with symptoms such as hirsutism, acne, and menstrual irregularities, but it does not typically cause severe adrenal insufficiency or salt loss in the neonatal period.

### Summary:

- **Primary Diagnosis**: Congenital Adrenal Hyperplasia (21-Hydroxylase Deficiency)
- **Other Considerations**: 11β-Hydroxylase Deficiency, 3β-Hydroxysteroid Dehydrogenase Deficiency, Classic Galactosemia, Androgen Insensitivity Syndrome, Non-Classical Congenital Adrenal Hyperplasia

These conditions should be considered and ruled out through appropriate diagnostic testing, including genetic testing, hormone level measurements, and imaging studies if necessary.

### **22. User**

The patient has clinical manifestations at birth, due to lack of pigment in the whole body, skin capillaries are exposed, skin is thin and soft, white and red, and accompanied by varying degrees of vasodilation. Patients are highly sensitive to ultraviolet light, often solar dermatitis, actinic cheilitis, basal cell carcinoma and squamous cell carcinoma and other diseases. Hair is white, silver, yellow, gold or brown. Eyelashes and eyebrows are white or pale yellow. Retina, iris color decline, pupil color decline. Photophobia is obvious during the day, night vision is normal, and other visual changes can also be accompanied, such as decreased visual acuity, hypoplasia of the fovea, changes in neural pathways at the optic chiasm, and nystagmus.

### Qwen2.5_72b

Based on the clinical manifestations you’ve described, the most likely diagnosis is **Oculocutaneous Albinism (OCA)**. This is a group of genetic disorders characterized by a lack of pigmentation in the skin, hair, and eyes, leading to the symptoms you’ve outlined.

### Most Likely Diagnosis:

- **Oculocutaneous Albinism (OCA)**

### Other Possible Diagnoses:

1. **Ocular Albinism (OA)**: This condition primarily affects the eyes and is less severe in terms of skin and hair pigmentation compared to OCA.
2. **Chediak-Higashi Syndrome**: This is a rare genetic disorder that affects pigmentation, the immune system, and blood clotting. Patients may have light-colored skin and hair, and are prone to infections and bleeding.
3. **Hermansky-Pudlak Syndrome (HPS)**: This is a rare genetic disorder that affects pigmentation, vision, and can cause bleeding disorders and lung problems.
4. **Griscelli Syndrome**: This is a rare genetic disorder characterized by hypopigmentation, immunodeficiency, and neurological abnormalities. It can present with silvery-gray hair and skin.
5. **Waardenburg Syndrome**: This is a genetic disorder that can cause hearing loss and changes in pigmentation of the hair, skin, and eyes, but it typically does not cause the severe hypopigmentation seen in OCA.

### Key Features of Oculocutaneous Albinism (OCA):

- **Hypopigmentation**: Lack of melanin in the skin, hair, and eyes.
- **Photophobia**: Increased sensitivity to light.
- **Visual Impairments**: Including decreased visual acuity, nystagmus, and foveal hypoplasia.
- **Increased Risk of Skin Cancer**: Due to lack of protective pigmentation, patients are more susceptible to sun damage and skin cancers.

If you suspect OCA, genetic testing can confirm the diagnosis by identifying mutations in specific genes associated with the condition, such as OCA1 (tyrosinase gene), OCA2, OCA3, and OCA4.

### **23. User**

A degenerative disease of the nervous system in which both upper and lower motor neurons are involved. The main clinical manifestations are progressive weakness and atrophy of the muscles of the bulbar, limbs, chest and abdomen. Eye motor nerve and sphincter function are generally not affected, but may be affected later in some patients. Between 20% and 50% of patients may exhibit cognitive impairment, and between 5% and 15% may even develop frontotemporal dementia. Death from respiratory failure occurs an average of 3 to 5 years after onset, but 5% to 10% of patients survive for more than 10 years.

Detailed history and physical examination to find evidence of common involvement of upper and lower motor neurons in brain stem, neck, chest and lumbosacral regions is the basis for diagnosis of the disease. Physical examination showed that both upper and lower motor neurons were involved in the same area, which was the key point of diagnosis. The main signs of lower motor neuron involvement included muscle weakness, atrophy and fascicular fibrillation. The tongue muscles, facial muscles, throat muscles, neck muscles, different muscle groups of the limbs, back muscles and pectoralis muscles are usually examined. The signs of upper motor neuron involvement mainly included positive pathological signs, tendon hyperreflexia, hypertonia, clonus, etc. Usually check sucking reflex, pharyngeal reflex, mandibular reflex, palmar jaw reflex, whether there is strong crying, strong laughter and other pseudobulbar paralysis manifestations, limb tendon reflex, muscle tension, Hoffman sign, lower limb pathology, abdominal wall reflex, etc. Clinical examination is the primary method of detecting upper motor neuron involvement. In areas of atrophy and weakness, if tendon reflexes are not low or active, it may indicate pyramidal tract damage even if there are no pathological signs.

### Qwen2.5_72b

Based on the clinical manifestations and detailed history and physical examination, the most likely diagnosis is **Amyotrophic Lateral Sclerosis (ALS)**. Here are the five other possible diagnoses to consider:

- **Primary Lateral Sclerosis (PLS)**: This is a rare motor neuron disease that affects only the upper motor neurons. It causes progressive weakness and stiffness in the muscles, but unlike ALS, it does not involve the lower motor neurons, and the disease progression is generally slower.
- **Progressive Muscular Atrophy (PMA)**: This is a motor neuron disease that primarily affects the lower motor neurons, leading to muscle weakness and atrophy. Unlike ALS, PMA does not typically involve the upper motor neurons, and the disease progression is generally slower.
- **Multifocal Motor Neuropathy (MMN)**: This is a chronic inflammatory disorder of the peripheral nerves that causes progressive weakness and atrophy in the muscles, particularly in the hands and arms. It can mimic ALS, but it typically does not involve the upper motor neurons, and electrophysiological studies can help differentiate it from ALS.
- **Kennedy’s Disease (Spinal and Bulbar Muscular Atrophy)**: This is an X-linked recessive motor neuron disease that primarily affects males. It causes muscle weakness and atrophy, particularly in the bulbar and limb muscles, and can also cause endocrine and reproductive system issues. Unlike ALS, it does not typically involve the upper motor neurons, and the disease progression is generally slower.
- **Hereditary Spastic Paraplegia (HSP)**: This is a group of inherited disorders that primarily affect the upper motor neurons, leading to progressive weakness and spasticity in the legs. It can sometimes be confused with ALS, but it does not typically involve the lower motor neurons, and the disease progression is generally slower.

### Key Points for Diagnosis:

- **ALS**: Involves both upper and lower motor neurons, with progressive muscle weakness, atrophy, and fasciculations. Cognitive impairment and frontotemporal dementia can occur.
- **PLS**: Involves only upper motor neurons, with slower progression and no lower motor neuron involvement.
- **PMA**: Involves only lower motor neurons, with slower progression and no upper motor neuron involvement.
- **MMN**: Involves peripheral nerves, with no upper motor neuron involvement, and can be differentiated by electrophysiological studies.
- **Kennedy’s Disease**: X-linked, affects lower motor neurons, with slower progression and additional endocrine/reproductive issues.
- **HSP**: Involves upper motor neurons, with progressive spasticity and weakness in the legs, and no lower motor neuron involvement.

Given the clinical manifestations and the involvement of both upper and lower motor neurons, ALS is the most likely diagnosis. However, a thorough evaluation, including electrophysiological studies, imaging, and genetic testing, may be necessary to confirm the diagnosis and rule out other conditions.

### **24. User**

An autosomal recessive disorder affecting the urea cycle, leading to hyperammonemia, encephalopathy, and respiratory alkalosis. It is characterized by a buildup of arginine and ammonia in the blood, causing symptoms such as developmental delays, seizures, intellectual disability, spasticity, and ataxia. The condition typically manifests around age 3, with symptoms including stiffness, slow growth, and cognitive decline. Without treatment, it can progress to severe spasticity, loss of ambulation, and intellectual disability.

### Qwen2.5_72b

Based on the clinical manifestations and the description provided, the most likely diagnosis is **Argininosuccinic Aciduria (ASA)**, a rare urea cycle disorder. Here are five other possible diagnoses to consider:

- **Citrullinemia Type I (CTLN1)**: Another urea cycle disorder that can present with hyperammonemia, encephalopathy, and respiratory alkalosis. Symptoms can include developmental delays, seizures, and intellectual disability.
- **Ornithine Transcarbamylase Deficiency (OTC Deficiency)**: This is the most common urea cycle disorder, also characterized by hyperammonemia and encephalopathy. It can present with similar symptoms, including developmental delays, seizures, and intellectual disability.
- **Carbamoyl Phosphate Synthetase I Deficiency (CPS1 Deficiency)**: Another urea cycle disorder that can cause hyperammonemia and encephalopathy. Symptoms can include developmental delays, seizures, and intellectual disability.
- **N-Acetylglutamate Synthase Deficiency (NAGS Deficiency)**: A rare urea cycle disorder that can present with hyperammonemia and encephalopathy. Symptoms can include developmental delays, seizures, and intellectual disability.
- **Propionic Acidemia**: A metabolic disorder that can present with symptoms similar to urea cycle disorders, including developmental delays, seizures, and intellectual disability. It is caused by a deficiency in the enzyme propionyl-CoA carboxylase.

Each of these conditions can present with overlapping symptoms, making a detailed clinical evaluation, laboratory tests, and genetic testing essential for accurate diagnosis and appropriate management.

### **25. User**

The typical clinical picture includes the triad of microangiopathic haemolytic anaemia, thrombocytopenia and acute renal failure. Non-immune erythrocyte destruction due to microthrombosis often results in a haemoglobin level below 80 g/L, a negative Coombs test, and erythrocyte fragments seen on peripheral blood smears; platelet counts usually fall below 50×10^9 /L, but skin purpura and active bleeding are rare; the severity of the acute renal impairment varies from person to person, with some patients requiring dialysis support, often accompanied by elevated blood pressure.

### Qwen2.5_72b

Based on the clinical manifestations you’ve described, the most likely diagnosis is **Thrombotic Thrombocytopenic Purpura (TTP)**. TTP is a rare blood disorder characterized by the triad of microangiopathic hemolytic anemia (MAHA), thrombocytopenia, and acute renal failure. The presence of schistocytes (erythrocyte fragments) on peripheral blood smears, a negative Coombs test, and the need for dialysis in severe cases are highly suggestive of TTP.

### Differential Diagnoses:

- **Hemolytic Uremic Syndrome (HUS)**: Similar to TTP, HUS also presents with MAHA, thrombocytopenia, and acute renal failure. However, HUS is often associated with a history of diarrhea, particularly due to Shiga toxin-producing Escherichia coli (STEC) infection.
- **Disseminated Intravascular Coagulation (DIC)**: DIC can present with similar findings, including MAHA, thrombocytopenia, and organ dysfunction. However, DIC is typically associated with a more widespread coagulopathy, including elevated D-dimers and fibrin degradation products, and is often secondary to a known underlying condition such as sepsis, trauma, or malignancy.
- **Postpartum Hemolytic Uremic Syndrome (Postpartum HUS)**: This condition can occur in the postpartum period and presents similarly to TTP and HUS, with MAHA, thrombocytopenia, and renal failure. It is often associated with pregnancy-related complications.
- **Malignant Hypertension**: Severe hypertension can lead to renal failure, MAHA, and thrombocytopenia. However, the presence of retinal changes and a history of poorly controlled hypertension can help differentiate this condition.
- **Systemic Lupus Erythematosus (SLE) with Renal Involvement**: SLE can present with MAHA, thrombocytopenia, and renal failure. However, other systemic symptoms and positive autoantibodies (e.g., anti-dsDNA, ANA) are typically present in SLE.

### Summary:

- **Primary Diagnosis**: Thrombotic Thrombocytopenic Purpura (TTP)
- **Differential Diagnoses**:
  1. Hemolytic Uremic Syndrome (HUS)
  2. Disseminated Intravascular Coagulation (DIC)
  3. Postpartum Hemolytic Uremic Syndrome (Postpartum HUS)
  4. Malignant Hypertension
  5. Systemic Lupus Erythematosus (SLE) with Renal Involvement

Further diagnostic testing, such as ADAMTS13 activity and inhibitor assays, can help confirm the diagnosis of TTP. Prompt treatment with plasma exchange is critical in managing TTP and improving outcomes.

### **26. User**

Patients have a variety of clinical presentations. It depends on the location, extent and rate of progression of the lesion. Clinical symptoms mainly include:

- The occupying effect of an enlarged pituitary gland, which in most patients results from compression of the enlarged pituitary gland on the anterior region and surrounding structures (e.g., optic crossings, cavernous sinus, etc.), or from an inflammatory infiltrate. The most common clinical manifestations are persistent severe headaches and progressively worsening visual dysfunction, including decreased visual acuity and visual field defects.
- hypopituitarism. Depending on the degree of involvement, the various axes of the pituitary gland may show partial or even total hypopituitarism, such as amenorrhoea, hypogonadism, nausea, malaise, chills, loss of sexual hair and weight loss.
- Central dysuria, more than 70% of the patients will have symptoms of central dysuria such as polyuria, irritable thirst and polydipsia due to the involvement of the posterior pituitary, pituitary stalk and even hypothalamus region. In some patients, the blocking effect of the pituitary stalk leads to a mild increase in serum prolactin levels.
- Hypothalamic syndrome, when the lesion involves the region of the hypothalamus, there will be drowsiness, lack of satiety, severe weight gain, lack of thirst, temperature fluctuation, sweating disorders, and emotional and psychiatric disorders.
- Systemic disease-related manifestations. Patients with secondary disease often have a combination of clinical manifestations characteristic of systemic disease, such as LCH patients often have a combination of thyroid, lung, bone, liver, and skin lesions. patients with GPA are often middle-aged or older men, and respiratory symptoms often precede hypothalamic pituitary involvement. patients with IgG4-related pituitary inflammation often have a combination of clinical manifestations with the involvement of other organs such as lacrimal glands, submandibular glands, and pancreas. The disease is often associated with clinical signs of involvement of other organs such as the lacrimal gland and submaxillary gland and pancreas.

### Qwen2.5_72b

Based on the clinical manifestations you have provided, the most likely diagnosis is **Langerhans Cell Histiocytosis (LCH)**, particularly when considering the systemic involvement and the specific involvement of the pituitary gland and hypothalamus. Here are the five other possible diagnoses:

- **Langerhans Cell Histiocytosis (LCH)**:
  - **Rationale**: LCH can present with a variety of systemic symptoms, including pituitary involvement leading to hypopituitarism, central diabetes insipidus, and hypothalamic syndrome. The involvement of other organs such as the thyroid, lung, bone, liver, and skin is also characteristic.
- **Granulomatosis with Polyangiitis (GPA)**:
  - **Rationale**: GPA is a systemic vasculitis that can involve the respiratory system and can lead to pituitary and hypothalamic involvement, causing hypopituitarism and central diabetes insipidus. Middle-aged or older men are more commonly affected, and respiratory symptoms often precede hypothalamic-pituitary involvement.
- **IgG4-Related Disease**:
  - **Rationale**: IgG4-related disease can affect multiple organs, including the pituitary gland, leading to hypopituitarism and central diabetes insipidus. It often involves other organs such as the lacrimal glands, submandibular glands, and pancreas, which can present with characteristic clinical manifestations.
- **Pituitary Adenoma**:
  - **Rationale**: A pituitary adenoma can cause symptoms due to its mass effect, including headaches, visual dysfunction, and hypopituitarism. However, it is less likely to cause central diabetes insipidus unless it involves the posterior pituitary or pituitary stalk.
- **Hypothalamic-Pituitary Tumor**:
  - **Rationale**: Tumors in the hypothalamic-pituitary region can cause a combination of symptoms including headaches, visual dysfunction, hypopituitarism, and hypothalamic syndrome. These tumors can also lead to central diabetes insipidus if they involve the posterior pituitary or pituitary stalk.
- **Inflammatory Conditions (e.g., Sarcoidosis, Tuberculosis)**:
  - **Rationale**: Inflammatory conditions such as sarcoidosis or tuberculosis can involve the hypothalamic-pituitary region, leading to hypopituitarism, central diabetes insipidus, and other systemic symptoms. These conditions can also cause systemic involvement, including lung, liver, and bone lesions.

Each of these conditions can present with overlapping symptoms, and a thorough evaluation including imaging studies, laboratory tests, and sometimes biopsy may be necessary to confirm the diagnosis.

### **27. User**

Hyperglycaemia is the most common clinical manifestation of the disease. However, patients can spontaneously remit without intervention and maintain normal blood glucose levels. The disease is one of the leading causes of autoimmune hypoglycaemia, with about 25% of patients experiencing spontaneous hypoglycaemia during the course of the disease. Acanthosis nigricans is present in about 50 per cent of patients. In addition, polycystic ovaries and hyperandrogenism are not uncommon in this group of patients. Other autoimmune diseases are common, with systemic lupus erythematosus (SLE) being the most common, accounting for about 50 per cent of cases. Primary biliary cirrhosis, dermatomyositis, and scleroderma are also present. The disease can also be a paraneoplastic syndrome manifestation of Hodgkin’s lymphoma, multiple myeloma, and others.

### Qwen2.5_72b

Based on the clinical manifestations you’ve provided, the most likely rare disease is **Insulin Autoimmune Syndrome (IAS), also known as Hirata’s disease**. Here’s a detailed explanation and five other possible diagnoses:

### Most Likely Diagnosis:

- **Insulin Autoimmune Syndrome (IAS) or Hirata’s Disease:**
  - **Hyperglycaemia:** Common due to the presence of autoantibodies against insulin, which can lead to fluctuating blood glucose levels.
  - **Spontaneous Remission:** Some patients can experience spontaneous remission of hyperglycaemia.
  - **Autoimmune Hypoglycaemia:** About 25% of patients experience hypoglycaemia due to the binding of autoantibodies to insulin, leading to its release and subsequent hypoglycaemia.
  - **Acanthosis Nigricans:** Present in about 50% of patients, often associated with insulin resistance.
  - **Polycystic Ovaries and Hyperandrogenism:** Not uncommon, possibly due to underlying insulin resistance.
  - **Other Autoimmune Diseases:** Common, with SLE being the most frequent, followed by primary biliary cirrhosis, dermatomyositis, and scleroderma.
  - **Paraneoplastic Syndrome:** Can be associated with Hodgkin’s lymphoma, multiple myeloma, and other malignancies.

### Other Possible Diagnoses:

1. **Type B Insulin Resistance Syndrome:**
   - **Hyperglycaemia:** Due to autoantibodies against the insulin receptor.
   - **Acanthosis Nigricans:** Common.
   - **Polycystic Ovaries and Hyperandrogenism:** Frequent.
   - **Autoimmune Diseases:** Common, including SLE and others.
2. **Latent Autoimmune Diabetes in Adults (LADA):**
   - **Hyperglycaemia:** Gradual onset of insulin deficiency due to autoimmune destruction of beta cells.
   - **Spontaneous Remission:** Less common but possible in early stages.
   - **Autoimmune Diseases:** Common, including SLE and others.
3. **Autoimmune Polyendocrine Syndrome Type 2 (APS-2):**
   - **Hyperglycaemia:** Can occur due to autoimmune destruction of beta cells.
   - **Acanthosis Nigricans:** Possible.
   - **Polycystic Ovaries and Hyperandrogenism:** Possible.
   - **Other Autoimmune Diseases:** Common, including SLE, primary biliary cirrhosis, and others.
4. **Paraneoplastic Endocrinopathy:**
   - **Hyperglycaemia:** Can be due to paraneoplastic production of hormones or cytokines.
   - **Spontaneous Remission:** Possible if the underlying tumor is treated.
   - **Acanthosis Nigricans:** Possible.
   - **Polycystic Ovaries and Hyperandrogenism:** Possible.
   - **Other Autoimmune Diseases:** Less common but can occur.
5. **Metabolic Syndrome with Autoimmune Features:**
   - **Hyperglycaemia:** Due to insulin resistance.
   - **Acanthosis Nigricans:** Common.
   - **Polycystic Ovaries and Hyperandrogenism:** Common.
   - **Autoimmune Diseases:** Possible, but not as frequently associated as in IAS.

### Conclusion:

The clinical presentation strongly suggests **Insulin Autoimmune Syndrome (IAS) or Hirata’s disease** due to the combination of hyperglycaemia, spontaneous remission, autoimmune hypoglycaemia, acanthosis nigricans, polycystic ovaries, hyperandrogenism, and the presence of other autoimmune diseases. However, the other listed conditions should also be considered and ruled out through appropriate diagnostic testing.

### **28. User**

The disease starts in infancy and childhood. The first attack occurs between the ages of 5 months and 7 years. The clinical picture is one of recurrent episodes of intractable vomiting and severe ketoacidosis with depression, dehydration, shortness of breath, and coma. There is usually a clear trigger, and the disease may begin acutely after fasting, gastrointestinal and upper respiratory tract infections, fever, stress, or excessive protein intake. Routine laboratory tests show elevated or normal blood glucose, some hypoglycaemia, blood gases show metabolic acidosis, blood ammonia can be normal or mildly elevated, liver function, renal function and normal, and urine ketone bodies are positive. Ketosis and acidosis may recur, and clinical and laboratory tests may be normal between attacks.

Neurological changes occur in about 1/5 of the cases, manifested by cognitive impairment, mental retardation, extrapyramidal abnormalities and symptoms of stroke. Cranial MRI reveals extensive demyelination, including white matter, and symmetric high signal changes in the basal ganglia at T2.

Patients may be asymptomatic in the early stages and are diagnosed in the neonatal period or in high-risk groups by blood and urine metabolic screening. There is no clear association between age of onset, frequency of attacks and prognosis. There is a tendency for the attacks to decrease or stop with age, and in most cases growth is unaffected on follow-up. Recurrent and severe attacks can lead to multi-organ dysfunction or even death.

### Qwen2.5_72b

Based on the clinical manifestations and laboratory findings you’ve provided, the most likely diagnosis is **MCADD (Medium-Chain Acyl-CoA Dehydrogenase Deficiency)**. This is a rare genetic disorder that affects the body’s ability to break down certain fats, leading to recurrent episodes of metabolic crisis, particularly during periods of fasting or illness.

### Most Likely Diagnosis:

- **MCADD (Medium-Chain Acyl-CoA Dehydrogenase Deficiency)**

### Other Possible Diagnoses:

1. **Ketotic Hypoglycemia**: This condition can also present with recurrent episodes of hypoglycemia and ketosis, often triggered by fasting or illness.
2. **Mitochondrial Disorders**: Conditions such as MELAS (Mitochondrial Encephalomyopathy, Lactic Acidosis, and Stroke-like episodes) can present with recurrent metabolic crises, neurological symptoms, and MRI findings of white matter changes and basal ganglia involvement.
3. **Glutaric Aciduria Type I (GA1)**: This disorder can present with episodes of metabolic acidosis, particularly after protein intake, and can also cause neurological symptoms and MRI changes.
4. **Propionic Acidemia**: This is another metabolic disorder that can cause recurrent metabolic crises, including vomiting, acidosis, and neurological symptoms, often triggered by illness or dietary factors.
5. **Maple Syrup Urine Disease (MSUD)**: This condition can present with metabolic crises, including vomiting, acidosis, and neurological symptoms, particularly after protein intake.

### Key Points for MCADD:

- **Genetics**: MCADD is caused by mutations in the ACADM gene.
- **Metabolic Crisis**: Triggered by fasting, illness, or stress.
- **Laboratory Findings**: Elevated or normal blood glucose, metabolic acidosis, positive urine ketones.
- **Neurological Symptoms**: Cognitive impairment, mental retardation, extrapyramidal abnormalities, and stroke-like symptoms in some cases.
- **MRI Findings**: Extensive demyelination and symmetric high signal changes in the basal ganglia.
- **Management**: Avoidance of fasting, dietary management, and prompt treatment of metabolic crises.

If you have any further details or specific tests that have been performed, please provide them for a more refined differential diagnosis.

### **29. User**

- the onset is at a young age and is more common in women.
- there is often a family history of syncope or sudden death.
- different genotypes and phenotypes, can involve potassium, sodium and calcium channels. Characteristic tip-distortion ventricular tachycardia (TdP) is often present, leading to syncope or sudden death. 4.
- The ECG shows a prolonged QT interval.
- ECG generally has the following characteristics: (1) QT interval prolongation, short - long - short cycle phenomenon induced; (2) ventricular rate of 160-240 beats / minute, QRS wave amplitude and morphology around the equipotential line of reversal, 5-20 cardiac cycles of the main wave around the baseline of the reversal of the majority of the time can be terminated; (3) ATP is more difficult to terminate the TdP.

### Qwen2.5_72b

Based on the clinical manifestations and ECG findings you have provided, the most likely diagnosis is **Long QT Syndrome (LQTS)**. This is a rare genetic disorder that affects the heart’s electrical system, leading to prolonged QT intervals on the ECG and an increased risk of torsades de pointes (TdP), a type of ventricular tachycardia that can cause syncope or sudden death.

### Additional Possible Diagnoses:

- **Brugada Syndrome**:
  - **Onset**: Can occur at any age but is more common in adults.
  - **Family History**: Often positive for sudden unexplained death.
  - **ECG Characteristics**: ST-segment elevation in leads V1-V3, right bundle branch block pattern, and increased risk of ventricular fibrillation.
  - **Clinical Manifestations**: Syncope, sudden death, often triggered by fever or certain medications.
- **Catecholaminergic Polymorphic Ventricular Tachycardia (CPVT)**:
  - **Onset**: Usually in childhood or adolescence.
  - **Family History**: Often positive for syncope or sudden death.
  - **ECG Characteristics**: Normal at rest, but exercise or stress can induce bidirectional or polymorphic ventricular tachycardia.
  - **Clinical Manifestations**: Syncope, especially during physical activity or emotional stress, and sudden death.
- **Short QT Syndrome**:
  - **Onset**: Can occur at any age.
  - **Family History**: Often positive for sudden death.
  - **ECG Characteristics**: Shortened QT interval, often less than 300 ms.
  - **Clinical Manifestations**: Syncope, atrial fibrillation, and sudden death.
- **Hypertrophic Cardiomyopathy (HCM)**:
  - **Onset**: Can occur at any age but often diagnosed in young adults.
  - **Family History**: Often positive for HCM or sudden death.
  - **ECG Characteristics**: Abnormal Q waves, left ventricular hypertrophy, and ST-T wave changes.
  - **Clinical Manifestations**: Dyspnea, chest pain, syncope, and sudden death.
- **Arrhythmogenic Right Ventricular Cardiomyopathy (ARVC)**:
  - **Onset**: Usually in young adults.
  - **Family History**: Often positive for ARVC or sudden death.
  - **ECG Characteristics**: Epsilon waves, T-wave inversions in leads V1-V3, and ventricular tachycardia with a left bundle branch block pattern.
  - **Clinical Manifestations**: Palpitations, syncope, and sudden death, often during physical activity.

### Summary:

- **Primary Diagnosis**: Long QT Syndrome (LQTS)
- **Additional Diagnoses**:
  1. Brugada Syndrome
  2. Catecholaminergic Polymorphic Ventricular Tachycardia (CPVT)
  3. Short QT Syndrome
  4. Hypertrophic Cardiomyopathy (HCM)
  5. Arrhythmogenic Right Ventricular Cardiomyopathy (ARVC)

Each of these conditions has distinct ECG and clinical features, and a thorough evaluation, including genetic testing, may be necessary to confirm the diagnosis.

### **30. User**

The disease can develop at any age, and most of the children develop between 1 month and 7 years old, with an average age of about 2 years old. Clinical manifestations vary greatly among patients, mainly including: (1) acute energy metabolism disorder crisis, manifested by low ketone hypoglycemia, high blood ammonia and metabolic acidosis; (2) cardiomyopathy, manifested by ventricular hypertrophy, cardiac insufficiency, cardiac arrhythmia, and elevated creatine kinase, etc.; (3) myopathy, manifested by muscle weakness, hypotonia, myalgia, poor exercise endurance, elevated muscular-type creatine kinase, and lipid deposition in muscle fibres, etc. (4) liver damage, manifested by hepatomegaly, fatty liver, liver function abnormalities, etc. Some liver-damaged patients have liver enlargement, fatty liver, and liver function abnormalities; Liver damage, manifested by hepatomegaly, fatty liver, abnormal liver function, etc. Some children with liver damage have acute onset of illness, manifested by convulsions, progressive consciousness disorder, etc., which is often misdiagnosed as Reye syndrome. In addition, gastrointestinal symptoms such as recurrent abdominal pain, vomiting and gastroesophageal reflux, respiratory symptoms such as recurrent infections and wheezing, and anaemia have also been reported.

Newborn screening: diagnosed children may have no clinical manifestations. It is a potentially fatal disease, and children can die suddenly from acute energy metabolism disorder crisis or acute heart failure. Clinical manifestations are varied and related to age of onset, time of onset and severity of illness. The most common symptoms are cardiac and skeletal muscle damage in infancy and childhood. Symptoms in adulthood are mild or asymptomatic, mostly due to reduced endurance or easy fatigue. During pregnancy, pregnant women may have atypical manifestations such as fatigue and arrhythmias due to energy expenditure and reduced physiological plasma carnitine levels.

### Qwen2.5_72b

Based on the clinical manifestations and the age of onset, the most likely diagnosis is **Carnitine Palmitoyltransferase II Deficiency (CPT II Deficiency)**. This is a rare metabolic disorder that affects the body’s ability to convert certain fats into energy, particularly during periods of fasting or increased energy demand.

### Differential Diagnoses:

- **Medium-Chain Acyl-CoA Dehydrogenase Deficiency (MCADD)**: This is another fatty acid oxidation disorder that can present with hypoglycemia, metabolic acidosis, and liver dysfunction, particularly in young children.
- **Very Long-Chain Acyl-CoA Dehydrogenase Deficiency (VLCADD)**: This disorder can cause similar symptoms, including cardiomyopathy, hypotonia, and liver dysfunction, and can also present in early childhood.
- **Reye Syndrome**: This is a rare but serious condition that can cause liver and brain damage, often following a viral infection. It can present with symptoms like hypoglycemia, metabolic acidosis, and liver dysfunction, which can be mistaken for CPT II deficiency.
- **Kearns-Sayre Syndrome (KSS)**: This is a mitochondrial disorder that can present with muscle weakness, cardiomyopathy, and liver dysfunction, although it typically has additional features like progressive external ophthalmoplegia and pigmentary retinopathy.
- **Pompe Disease (Glycogen Storage Disease Type II)**: This is a lysosomal storage disorder that can cause cardiomyopathy, muscle weakness, and liver enlargement, particularly in infants and young children.

### Key Points:

- **CPT II Deficiency** is characterized by acute energy metabolism disorder crises, cardiomyopathy, myopathy, and liver damage, which aligns well with the clinical manifestations you described.
- **Newborn screening** can help in early diagnosis, even before symptoms appear, which is crucial for managing this potentially fatal condition.
- **Differential diagnoses** should be considered to ensure accurate diagnosis and appropriate management.

If you have any further details or specific test results, they can help narrow down the diagnosis even further.

### **31. User**

In the unicentric form, the disease often presents only with enlarged lymph nodes without systemic symptoms. The median diameter of the enlarged lymph nodes is about 5.5 cm, and they are commonly found in the chest (24%), neck (20%), abdomen (18%), and retroperitoneum (14%). Some patients with unicentric disease are prone to paraneoplastic aspergillosis, occlusive bronchiolitis, and growth retardation. In addition to multiple lymph node enlargements, multicentric disease is often associated with systemic manifestations such as fever, night sweats, malaise, weight loss, anaemia, hepatic insufficiency, renal insufficiency, and volume overload (generalised oedema, pleural effusion, ascites, etc.). There is also a specific subtype in which patients may present with thrombocytopenia, severe oedema (anasarca), myelofibrosis, renal dysfunction, and hepatosplenomegaly (organomegaly).

### Qwen2.5_72b

Based on the clinical manifestations you’ve provided, the most likely diagnosis is **Castleman’s Disease (CD)**, specifically the unicentric and multicentric forms. Here’s a detailed breakdown:

### Most Likely Diagnosis:

- **Castleman’s Disease (CD)**
  - **Unicentric CD**: Characterized by isolated, enlarged lymph nodes, often in the chest, neck, abdomen, or retroperitoneum. The median diameter of the enlarged lymph nodes is about 5.5 cm. Unicentric CD is typically asymptomatic or presents with localized symptoms.
  - **Multicentric CD**: Involves multiple lymph nodes and is often associated with systemic symptoms such as fever, night sweats, malaise, weight loss, anemia, hepatic and renal insufficiency, and volume overload (generalized edema, pleural effusion, ascites). A specific subtype, known as **Plasma Cell Variant of Multicentric CD**, can present with thrombocytopenia, severe edema (anasarca), myelofibrosis, renal dysfunction, and hepatosplenomegaly.

### Other Possible Diagnoses:

1. **Lymphoma (Hodgkin’s or Non-Hodgkin’s)**
   - **Hodgkin’s Lymphoma**: Often presents with enlarged lymph nodes, particularly in the neck, underarm, or groin. Systemic symptoms such as fever, night sweats, and weight loss are common.
   - **Non-Hodgkin’s Lymphoma**: Can involve multiple lymph nodes and can present with systemic symptoms similar to multicentric CD.
2. **Kikuchi-Fujimoto Disease (KFD)**
   - A self-limiting condition characterized by cervical lymphadenopathy, fever, and systemic symptoms. It is more common in young women and can mimic CD.
3. **Sarcoidosis**
   - A systemic granulomatous disease that can present with enlarged lymph nodes, particularly in the chest, and systemic symptoms such as fever, weight loss, and fatigue. It can also involve the liver, spleen, and other organs.
4. **Tuberculosis (TB)**
   - Can present with enlarged lymph nodes, particularly in the neck, and systemic symptoms such as fever, night sweats, and weight loss. TB can also involve the lungs, liver, and other organs.
5. **Systemic Lupus Erythematosus (SLE)**
   - An autoimmune disorder that can present with lymphadenopathy, fever, weight loss, and systemic symptoms affecting multiple organs, including the kidneys, liver, and lungs.

### Summary:

- **Primary Diagnosis**: Castleman’s Disease (Unicentric and Multicentric)
- **Other Possible Diagnoses**:
  1. Lymphoma (Hodgkin’s or Non-Hodgkin’s)
  2. Kikuchi-Fujimoto Disease
  3. Sarcoidosis
  4. Tuberculosis
  5. Systemic Lupus Erythematosus

Further diagnostic tests, such as biopsy, imaging studies, and laboratory tests, will be necessary to confirm the diagnosis and rule out other conditions.

### **32. User**

The main clinical manifestations are muscle atrophy, weakness and sensory loss of the limbs, mainly in the distal part of the lower limbs and gradually developing towards the proximal part of the limbs. Common clinical manifestations include poorer locomotor ability than people of the same age, difficulty in running, easy to twist the foot, foot drop, calf gastrocnemius muscle atrophy resembling a ‘crane leg’; examination can find bowed feet, hammertoes, distal limb weakness and atrophy, and loss of deep sensation. The disease usually starts before the age of 20 and progresses slowly. Later stages of the disease may severely affect activity, but rarely result in total disability and do not affect normal life expectancy. However, there are specific types that may have an early and severe onset.

### Qwen2.5_72b

Based on the clinical manifestations you’ve described, the most likely diagnosis is **Charcot-Marie-Tooth disease (CMT)**, a hereditary motor and sensory neuropathy. CMT is characterized by progressive muscle atrophy and weakness, particularly in the distal parts of the limbs, and sensory loss. The disease often begins in childhood or adolescence and progresses slowly, leading to symptoms such as foot drop, calf muscle atrophy, and difficulty with running and walking.

### Differential Diagnoses:

- **Spinal Muscular Atrophy (SMA)**:
  - **Clinical Features**: Progressive muscle weakness and atrophy, primarily affecting the proximal muscles. SMA can present in childhood and can vary in severity from mild to severe.
  - **Key Differences**: SMA typically affects the proximal muscles more than the distal muscles, and there is often a more rapid progression and more severe impact on motor function.
- **Hereditary Neuropathy with Liability to Pressure Palsies (HNPP)**:
  - **Clinical Features**: Recurrent, painless episodes of weakness and sensory loss, often triggered by minor trauma or pressure. The symptoms can be focal and may resolve spontaneously.
  - **Key Differences**: HNPP is characterized by episodic symptoms rather than a progressive, chronic course.
- **Amyotrophic Lateral Sclerosis (ALS)**:
  - **Clinical Features**: Progressive muscle weakness and atrophy, often with both upper and lower motor neuron signs. ALS can affect both proximal and distal muscles and progresses more rapidly than CMT.
  - **Key Differences**: ALS typically has a more rapid progression and a poorer prognosis, often leading to significant disability and shortened life expectancy.
- **Diabetic Neuropathy**:
  - **Clinical Features**: Symmetrical distal sensory loss and weakness, often accompanied by autonomic symptoms. Diabetic neuropathy is more common in older adults with a history of diabetes.
  - **Key Differences**: Diabetic neuropathy is associated with a history of diabetes and may have additional symptoms such as autonomic dysfunction.
- **Chronic Inflammatory Demyelinating Polyneuropathy (CIDP)**:
  - **Clinical Features**: Progressive or relapsing-remitting symmetric weakness and sensory loss, often with areflexia. CIDP can affect both proximal and distal muscles.
  - **Key Differences**: CIDP can be distinguished by the presence of areflexia and the potential for improvement with immunotherapy.

### Summary:

- **Most Likely Diagnosis**: Charcot-Marie-Tooth Disease (CMT)
- **Differential Diagnoses**:
  1. Spinal Muscular Atrophy (SMA)
  2. Hereditary Neuropathy with Liability to Pressure Palsies (HNPP)
  3. Amyotrophic Lateral Sclerosis (ALS)
  4. Diabetic Neuropathy
  5. Chronic Inflammatory Demyelinating Polyneuropathy (CIDP)

Further diagnostic testing, such as nerve conduction studies, electromyography (EMG), and genetic testing, would be necessary to confirm the diagnosis and rule out other conditions.

### **33. User**

The onset of the disease occurs from birth to several months. The onset of jaundice is usually within 2 months of age and rarely later than 5 months of age. The most common presentation is persistent jaundice after birth with or without light coloured stools and mild or no liver enlargement. There are many other non-specific clinical signs, including feeding difficulties, growth retardation, diarrhoea, hypoglycaemia and prolonged clotting time. Most of these symptoms and signs resolve spontaneously or with dietary modification and medication around 6 months to 1 year of age, with a small proportion of patients developing further disease after puberty. The majority of developed disease develops in adulthood. Delirium, confusion, and other mental abnormalities caused by hyperammonemia usually appear suddenly after drinking alcohol, consuming sweets, taking certain medications, or having an infection. In severe cases, the disease progresses rapidly and can be life-threatening without proper treatment. Most patients are slim, and most of them have obvious dietary preference, addicted to legumes, high protein, high fat food, and anorexic to high sugar.

### Qwen2.5_72b

Based on the clinical manifestations you’ve provided, the most likely rare disease is **Citrullinemia Type I (CTLN1)**, a urea cycle disorder. Here’s a detailed explanation and five other possible diagnoses:

### Most Likely Diagnosis: Citrullinemia Type I (CTLN1)

- **Onset**: From birth to several months.
- **Jaundice**: Usually within 2 months of age, rarely later than 5 months.
- **Liver Enlargement**: Mild or no liver enlargement.
- **Other Symptoms**: Feeding difficulties, growth retardation, diarrhea, hypoglycaemia, prolonged clotting time.
- **Resolution**: Symptoms often resolve spontaneously or with dietary modification and medication around 6 months to 1 year of age.
- **Adult Onset**: A small proportion of patients may develop further disease after puberty.
- **Hyperammonemia**: Delirium, confusion, and other mental abnormalities can occur after triggers such as alcohol, sweets, certain medications, or infections.
- **Physical Characteristics**: Patients are often slim and have dietary preferences for high protein, high fat foods, and an aversion to high sugar.

### Other Possible Diagnoses:

- **Neonatal Hemochromatosis**:
  - **Onset**: From birth to several months.
  - **Jaundice**: Early onset.
  - **Liver Enlargement**: Significant liver involvement.
  - **Other Symptoms**: Feeding difficulties, growth retardation, hypoglycaemia.
  - **Resolution**: Poor prognosis without liver transplantation.
  - **Physical Characteristics**: No specific dietary preferences.
- **Alpha-1 Antitrypsin Deficiency**:
  - **Onset**: From birth to several months.
  - **Jaundice**: Early onset.
  - **Liver Enlargement**: Mild to moderate.
  - **Other Symptoms**: Feeding difficulties, growth retardation, respiratory issues.
  - **Resolution**: Chronic liver disease in some cases.
  - **Physical Characteristics**: No specific dietary preferences.
- **Galactosemia**:
  - **Onset**: From birth to several months.
  - **Jaundice**: Early onset.
  - **Liver Enlargement**: Significant.
  - **Other Symptoms**: Feeding difficulties, growth retardation, hypoglycaemia, vomiting, diarrhea.
  - **Resolution**: Lifelong dietary restriction of galactose.
  - **Physical Characteristics**: No specific dietary preferences.
- **Tyrosinemia Type I**:
  - **Onset**: From birth to several months.
  - **Jaundice**: Early onset.
  - **Liver Enlargement**: Significant.
  - **Other Symptoms**: Feeding difficulties, growth retardation, hypoglycaemia, vomiting, diarrhea, renal tubular acidosis.
  - **Resolution**: Lifelong dietary restriction of tyrosine and phenylalanine, and treatment with nitisinone.
  - **Physical Characteristics**: No specific dietary preferences.
- **Hereditary Fructose Intolerance (HFI)**:
  - **Onset**: From birth to several months.
  - **Jaundice**: Early onset.
  - **Liver Enlargement**: Mild to moderate.
  - **Other Symptoms**: Feeding difficulties, growth retardation, hypoglycaemia, vomiting, diarrhea.
  - **Resolution**: Lifelong dietary restriction of fructose and sucrose.
  - **Physical Characteristics**: Aversion to sweet foods.

### Conclusion

Citrullinemia Type I (CTLN1) is the most likely diagnosis based on the provided clinical manifestations, especially the combination of early onset jaundice, feeding difficulties, growth retardation, and the specific dietary preferences and aversions. However, the other listed conditions should also be considered and ruled out through appropriate diagnostic testing.

### **34. User**

Symptoms may appear after birth or may start in infancy to early adulthood. It is characterised by fluctuating muscle weakness and intolerance to fatigue, mostly in the extraocular muscles, facial muscles, medulla oblongata, limbs and respiratory muscles, and even life-threatening apnoeic episodes may occur in infancy. The simplex syndrome is characterised mainly by skeletal muscle weakness without congenital malformations. The overlapping syndromes of glycosylation defects and myopathies of the disease are often accompanied by congenital developmental abnormalities. Clinical manifestations vary between the different types, with some patients, usually born with proximal muscle weakness, cholinesterase inhibitors effective, and in severe cases, sudden respiratory distress and apnoea due to medullary palsy, which can be induced by stress, cold, and infection. Some patients, starting at birth or in infancy, may present with more severe, axially distributed muscle weakness, but the extraocular muscles may not be involved.

### Qwen2.5_72b

Based on the clinical manifestations you have provided, the most likely rare disease is **Congenital Myasthenic Syndrome (CMS)**. CMS is a group of inherited neuromuscular disorders characterized by fatigable muscle weakness, often affecting the extraocular muscles, facial muscles, and limbs. The symptoms can vary widely, and some patients may experience life-threatening apnoeic episodes, especially in infancy.

### Other Possible Diagnoses:

- **Mitochondrial Myopathy**: This is a group of neuromuscular diseases caused by damage to the mitochondria, which are responsible for producing energy in cells. Symptoms can include muscle weakness, fatigue, and respiratory issues, similar to CMS.
- **Lambert-Eaton Myasthenic Syndrome (LEMS)**: This is an autoimmune disorder that affects the neuromuscular junction, leading to muscle weakness and fatigue. It is often associated with cancer, particularly small cell lung cancer.
- **Duchenne Muscular Dystrophy (DMD)**: This is a genetic disorder characterized by progressive muscle degeneration and weakness. While it typically presents in early childhood with proximal muscle weakness, it can sometimes be confused with CMS, especially in the early stages.
- **Glycogen Storage Disease Type II (Pompe Disease)**: This is a rare genetic disorder caused by the buildup of glycogen in the body’s cells. It can lead to muscle weakness, particularly in the proximal muscles and respiratory muscles, and can present in infancy or later in life.
- **Spinal Muscular Atrophy (SMA)**: This is a genetic disorder that affects the motor neurons in the spinal cord, leading to muscle weakness and atrophy. It can present in infancy or early childhood and can vary in severity, from mild to severe, with some patients experiencing respiratory issues.

### Key Points for Differentiation:

- **CMS**: Fluctuating muscle weakness, particularly in extraocular and facial muscles, with fatigue and potential for life-threatening apnoeic episodes.
- **Mitochondrial Myopathy**: Muscle weakness, fatigue, and often multisystem involvement.
- **LEMS**: Muscle weakness and fatigue, often associated with cancer.
- **DMD**: Progressive muscle weakness, typically starting in the proximal muscles, with a male predominance.
- **Pompe Disease**: Muscle weakness, particularly in the proximal and respiratory muscles, with glycogen buildup.
- **SMA**: Muscle weakness and atrophy, often with a rapid progression in severe forms.

Further diagnostic testing, such as genetic testing, electromyography (EMG), and specific enzyme assays, would be necessary to confirm the diagnosis.

### **35. User**

- The disease usually starts in infancy or childhood, and in a few cases it starts in adulthood.
- Tonicity can occur throughout the entire body, including the skeletal muscles of the limbs, facial muscles, extraocular muscles, and lingual muscles.
- Tonicity is apparent during sudden movement after rest and is reduced by repeated movements, which is called the ‘warm-up’ phenomenon. Transient muscle weakness may occur at the start of a sudden movement. In patients with a longer course of the disease, there may be persistent weakness of the distal limbs, even involving the proximal limbs.
- Physical examination reveals that the patient is often muscular and has an athlete’s body shape. After clenching the fist vigorously, the fingers cannot be unfolded in time; after closing the eyes vigorously, they cannot be opened immediately. Knocking on the hand interosseous muscle, lingual muscle and other parts of the hand can cause persistent muscle contraction (percussive muscle ankylosis, ‘muscle ball’ phenomenon).

### Qwen2.5_72b

Based on the clinical manifestations you have provided, the most likely diagnosis is **Myotonia Congenita (Thomsen Disease or Becker Myotonia)**. Myotonia congenita is a rare genetic disorder characterized by muscle stiffness and difficulty in relaxing muscles after contraction. The key features you described, such as the onset in infancy or childhood, the presence of tonicity throughout the body, the ‘warm-up’ phenomenon, and the muscle ball phenomenon, are highly characteristic of this condition.

### Other Possible Diagnoses:

- **Paramyotonia Congenita (Eulenburg Disease)**: This is another form of myotonia that is often confused with myotonia congenita. It is characterized by muscle stiffness that worsens with cold and repeated activity, unlike the ‘warm-up’ phenomenon seen in myotonia congenita.
- **Congenital Myasthenic Syndromes (CMS)**: These are a group of genetic disorders that affect the neuromuscular junction, leading to muscle weakness and fatigability. However, the ‘warm-up’ phenomenon and muscle stiffness are not typical features.
- **Lambert-Eaton Myasthenic Syndrome (LEMS)**: This is an autoimmune disorder that affects the neuromuscular junction, causing muscle weakness and fatigability. It typically presents in adulthood and is associated with other autoimmune conditions or malignancies.
- **Dystrophic Myotonia (Myotonic Dystrophy Type 1 and Type 2)**: These are genetic disorders characterized by muscle stiffness, weakness, and atrophy. They often present with additional systemic features such as cataracts, cardiac conduction defects, and endocrine abnormalities.
- **Hypokalemic Periodic Paralysis**: This is a rare genetic disorder characterized by episodes of muscle weakness or paralysis, often triggered by factors such as high carbohydrate meals, exercise, or stress. It is not typically associated with the ‘warm-up’ phenomenon or muscle stiffness.

### Summary:

- **Most Likely Diagnosis**: Myotonia Congenita (Thomsen Disease or Becker Myotonia)
- **Other Possible Diagnoses**:
  1. Paramyotonia Congenita (Eulenburg Disease)
  2. Congenital Myasthenic Syndromes (CMS)
  3. Lambert-Eaton Myasthenic Syndrome (LEMS)
  4. Dystrophic Myotonia (Myotonic Dystrophy Type 1 and Type 2)
  5. Hypokalemic Periodic Paralysis

These differential diagnoses should be considered and further evaluated with appropriate diagnostic tests, such as genetic testing, electromyography (EMG), and muscle biopsy, to confirm the specific condition.

### **36. User**

Secondary thoracic deformities caused by scoliosis can lead to cardiopulmonary hypoplasia, poor cardiopulmonary reserve, and may include cardiac displacement, arrhythmia, reduced lung capacity, pneumonia, etc. Abdominal deformities can cause gastrointestinal disorders, leading to dyspepsia and lack of appetite. In addition, the nerve root pulling caused by vertebral malformation and disc degeneration can produce irritating pain in the corresponding body parts, and in severe cases, it can cause spinal cord dysfunction or even paralysis.

Physical examination: the general manifestations are unequal shoulders when standing upright, one side of the lumbar folds, lumbar flexion with back asymmetry (‘razor back’), sometimes asymmetry on both sides of the pelvis, and gait abnormalities. In addition, neurological examination is also needed to observe whether the patient has increased or low muscle tone and whether there are sensory abnormalities in the limbs.

Clinically, it is usually classified into the following 3 types according to the imaging characteristics:

Type I - Vertebral formation disorder type, i.e., one or more vertebrae are partially or completely formed with disorder, such as wedge vertebrae, hemivertebrae or butterfly vertebrae;

Type II - poor vertebral segmentation type, i.e., two or more vertebrae are partially or completely segmented and impaired, e.g., blocked vertebrae, bone bridge formation, etc;

Type III - mixed type, where there is both vertebral body formation disorder and vertebral body mal-segmentation.

### Qwen2.5_72b

Based on the clinical manifestations and imaging characteristics you’ve provided, the most likely diagnosis is **Congenital Scoliosis**. This is a rare condition that results from abnormal vertebral development during fetal development, leading to the types of deformities and complications you’ve described.

### Most Likely Diagnosis:

- **Congenital Scoliosis** - This condition is characterized by vertebral formation and segmentation abnormalities, leading to thoracic and abdominal deformities, cardiopulmonary complications, and neurological symptoms.

### Other Possible Diagnoses:

1. **Idiopathic Scoliosis** - While not congenital, idiopathic scoliosis can also present with similar thoracic and abdominal deformities, and can lead to cardiopulmonary and neurological complications.
2. **Neuromuscular Scoliosis** - This type of scoliosis is associated with neuromuscular conditions such as cerebral palsy, muscular dystrophy, or spinal cord injuries, which can cause similar deformities and complications.
3. **Syndromic Scoliosis** - Scoliosis that is part of a broader genetic syndrome, such as Marfan syndrome, Ehlers-Danlos syndrome, or neurofibromatosis, can present with similar clinical features.
4. **Scheuermann’s Disease (Juvenile Kyphosis)** - This condition involves vertebral wedging and can lead to thoracic deformities, though it is more commonly associated with kyphosis rather than scoliosis.

### Summary:

- **Primary Diagnosis**: Congenital Scoliosis
- **Other Possible Diagnoses**:
  - Idiopathic Scoliosis
  - Neuromuscular Scoliosis
  - Syndromic Scoliosis
  - Scheuermann’s Disease

Each of these conditions can present with similar clinical manifestations, but the specific imaging characteristics and additional clinical findings will help in making the correct diagnosis.

### **37. User**

There are more men than women. Some patients have atypical symptoms of chest pain, which are detected during coronary angiography or coronary CT. Patients are often clinically symptomatic when there is (1) a combination of obstructive atherosclerotic disease, thrombosis, slow flow, entrapment, or rupture with exertional angina or acute coronary syndromes; (2) compression of adjacent tissues; (3) rupture of an aneurysm, which is rare and can cause acute cardiac tamponade; and (4) myocardial ischemia due to microvascular dysfunction, which can occur even in the absence of significant coronary stenosis. The most common clinical manifestation is cardiac ischaemia.

The most common clinical manifestation is angina pectoris, and in a few patients, myocardial infarction, severe arrhythmia, or even sudden death may occur. The disease may break into the right atrium, right ventricle, or coronary sinus, creating a left-to-right shunt, which can be heard as a continuous murmur and, in severe cases, affect cardiac function. The most serious complication is rupture of the tumour into the pericardium, which can lead to bleeding, cardiac tamponade or even death.

### Qwen2.5_72b

Based on the clinical manifestations and the provided details, the most likely rare disease is **Coronary Artery Fistula (CAF)**. Here’s a detailed explanation and five other possible diagnoses:

### Most Likely Diagnosis:

- **Coronary Artery Fistula (CAF)**
  - **Description**: A coronary artery fistula is an abnormal connection between a coronary artery and a heart chamber or another blood vessel. This can lead to various clinical manifestations, including angina pectoris, myocardial infarction, arrhythmias, and even sudden death.
  - **Symptoms**: Atypical chest pain, exertional angina, acute coronary syndromes, myocardial ischemia, and in severe cases, rupture leading to cardiac tamponade or death.
  - **Diagnostic Tests**: Coronary angiography, coronary CT, echocardiography, and cardiac MRI.

### Other Possible Diagnoses:

1. **Coronary Artery Aneurysm (CAA)**
   - **Description**: An abnormal dilation of a segment of a coronary artery. It can lead to thrombosis, embolization, or rupture.
   - **Symptoms**: Chest pain, myocardial infarction, arrhythmias, and sudden death.
   - **Diagnostic Tests**: Coronary angiography, coronary CT, echocardiography.
2. **Coronary Artery Dissection (CAD)**
   - **Description**: A tear in the wall of a coronary artery, which can lead to blood accumulation and blockage of blood flow.
   - **Symptoms**: Severe chest pain, myocardial infarction, arrhythmias, and sudden death.
   - **Diagnostic Tests**: Coronary angiography, coronary CT, echocardiography.
3. **Coronary Artery Spasm (Prinzmetal’s Angina)**
   - **Description**: A temporary spasm of the coronary arteries that can reduce blood flow to the heart muscle.
   - **Symptoms**: Chest pain, often at rest, myocardial ischemia, and arrhythmias.
   - **Diagnostic Tests**: Coronary angiography, provocative testing with ergonovine or acetylcholine.
4. **Coronary Artery Ectasia (CAE)**
   - **Description**: A condition where the coronary arteries are abnormally dilated, which can lead to reduced blood flow and ischemia.
   - **Symptoms**: Chest pain, myocardial infarction, arrhythmias, and sudden death.
   - **Diagnostic Tests**: Coronary angiography, coronary CT, echocardiography.
5. **Coronary Artery Embolism**
   - **Description**: A blockage of a coronary artery by an embolus, which can be a blood clot, air bubble, or other debris.
   - **Symptoms**: Sudden onset of chest pain, myocardial infarction, arrhythmias, and sudden death.
   - **Diagnostic Tests**: Coronary angiography, coronary CT, echocardiography.

### Summary:

- **Most Likely Diagnosis**: Coronary Artery Fistula (CAF)
- **Other Possible Diagnoses**: Coronary Artery Aneurysm (CAA), Coronary Artery Dissection (CAD), Coronary Artery Spasm (Prinzmetal’s Angina), Coronary Artery Ectasia (CAE), Coronary Artery Embolism

These conditions share similar clinical manifestations and can be differentiated through detailed imaging and diagnostic tests.

### **38. User**

- Bone marrow failure Anaemia is present at birth in 35 per cent of children and is often diagnosed between 2 weeks and 2 years after birth. The anaemia is macrocytic or normocytic orthochromatic, with decreased reticulocytes, normal or mildly decreased white blood cells, normal or mildly increased platelets, and hypoplasia of the erythroid lineage of the bone marrow and hyperproliferation of the granulocyte and megakaryocyte lineages.
- Congenital developmental anomalies 30% ~50% of patients may have congenital developmental anomalies, mainly involving the head, upper limbs, heart and genitourinary system. Examples include short stature, craniofacial anomalies including craniofacial hypertelorism, wide flat nose, small head, congenital cataracts, glaucoma, strabismus, high arched hard palate and cleft lip and palate or even Turner’s syndrome appearance, bunions, congenital cardiovascular anomalies, and genitourinary anomalies. The presence of physical abnormalities does not predict the severity of the patient’s haematological disorders. 30% of affected patients may present with postnatal growth retardation, often associated with other congenital malformations.
- The incidence of tumours in patients with increased susceptibility to cancer is about 4%, which is higher than in the normal population of the same age. And the tumour occurs at an early age. These include acute leukaemia of the haematological system, myelodysplastic syndromes, lymphomas and solid tumours such as osteosarcoma, thymic carcinoma, hepatocellular carcinoma, melanoma, fibrous histiocytoma, gastric cancer and intestinal cancer. The prognosis of the tumours is poorer than that of the general population suffering from the same tumour, with a higher mortality rate.

### Qwen2.5_72b

Based on the clinical manifestations you have provided, the most likely rare disease is **Diamond-Blackfan Anemia (DBA)**. Here are the key features that align with DBA:

- **Bone Marrow Failure and Anemia**:
  - **Macrocytic or normocytic anemia** present at birth or diagnosed in early infancy.
  - **Decreased reticulocytes**.
  - **Hypoplasia of the erythroid lineage** of the bone marrow.
  - **Normal or mildly decreased white blood cells**.
  - **Normal or mildly increased platelets**.
  - **Hyperproliferation of the granulocyte and megakaryocyte lineages**.
- **Congenital Developmental Anomalies**:
  - **Short stature**.
  - **Craniofacial anomalies** (hypertelorism, wide flat nose, small head, congenital cataracts, glaucoma, strabismus, high arched palate, cleft lip and palate).
  - **Congenital cardiovascular anomalies**.
  - **Genitourinary anomalies**.
  - **Postnatal growth retardation** often associated with other congenital malformations.
- **Increased Susceptibility to Cancer**:
  - **Higher incidence of tumors** (acute leukemia, myelodysplastic syndromes, lymphomas, solid tumors such as osteosarcoma, thymic carcinoma, hepatocellular carcinoma, melanoma, fibrous histiocytoma, gastric cancer, and intestinal cancer).
  - **Poorer prognosis** and higher mortality rate for these tumors compared to the general population.

### Other Possible Diagnoses

- **Fanconi Anemia (FA)**:
  - **Bone marrow failure** with pancytopenia.
  - **Congenital anomalies** (skeletal, renal, cardiac, and genitourinary).
  - **Increased risk of cancer** (leukemia, myelodysplastic syndromes, and solid tumors).
- **Shwachman-Diamond Syndrome (SDS)**:
  - **Bone marrow failure** with neutropenia.
  - **Pancreatic insufficiency**.
  - **Skeletal abnormalities**.
  - **Increased risk of myelodysplastic syndromes and leukemia**.
- **Severe Congenital Neutropenia (Kostmann Syndrome)**:
  - **Severe neutropenia**.
  - **Recurrent infections**.
  - **Increased risk of myelodysplastic syndromes and acute myeloid leukemia**.
- **Thrombocytopenia with Absent Radii (TAR Syndrome)**:
  - **Thrombocytopenia**.
  - **Absent radii**.
  - **Bilateral thumb hypoplasia or absence**.
  - **Short stature**.
  - **Increased risk of bleeding**.
- **Congenital Dyserythropoietic Anemia (CDA)**:
  - **Macrocytic anemia**.
  - **Hypoplastic bone marrow**.
  - **Abnormal red blood cell morphology**.
  - **Increased risk of iron overload**.

Each of these conditions has overlapping features with Diamond-Blackfan Anemia, but the specific combination of bone marrow failure, congenital anomalies, and increased cancer risk is most characteristic of DBA. Further diagnostic testing, such as genetic analysis, would be necessary to confirm the diagnosis.

### **39. User**

The lesions may involve all systems of the body, so their clinical manifestations vary depending on the site of involvement. General symptoms include fever, malaise, night sweats, and weight loss. The rest of the affected systems mainly include:

- Skeletal system, most patients have skeletal involvement at the time of diagnosis, with lower limb bones being the most common, but only 50% of patients have bone pain.
- cardiovascular system, lesions involving the cardiovascular system, most patients do not have obvious clinical manifestations, but such as pericardial involvement, resulting in a large amount of pericardial effusion can lead to chest tightness, breathlessness, or even cardiac tamponade; lesions involving coronary arteries can cause myocardial ischemia-like manifestations, such as chest tightness, chest pain, etc.; diffuse myocardial involvement has also been reported, which can lead to cardiac failure-like manifestations; lesions encircling the renal arteries can cause renal hypertension; lesions encircling the mesenteric artery can cause renal hypertension; lesions encircling the mesenteric artery can cause renal hypertension. Diffuse myocardial involvement has also been reported, leading to heart failure-like symptoms; lesions encircling the renal arteries may cause renal hypertension; lesions encircling the mesenteric vessels may cause intestinal ischaemia, such as abdominal pain.
- respiratory system Most patients with respiratory system involvement are asymptomatic, but a few patients have dry cough and dyspnoea.
- central nervous system central nervous system involvement may appear corresponding clinical manifestations due to different parts of brain parenchyma involvement, such as limb movement disorder, gait abnormality, alteration of consciousness, etc.; spinal cord involvement may appear different clinical manifestations due to different affected segments; pituitary gland lesions may manifest polydipsia, polyuria, and other hormone secretion related manifestations.
- Retro-orbital lesions Retro-orbital involvement is one of the most common clinical manifestations, which may manifest as unilateral or bilateral proptosis, or even cause blindness.
- Retroperitoneal space/renal involvement. Retroperitoneal lesions may resemble retroperitoneal fibrosis and may cause unilateral or bilateral retrorenal obstruction if the lesion encircles the ureter.
- cutaneous involvement, the most common cutaneous lesion is a xanthomatous change, which occurs on the eyelids in about 1/3 of patients, and may also occur on the face, neck, axillae, trunk, and groin.

### Qwen2.5_72b

Based on the clinical manifestations you have provided, the most likely rare disease is **IgG4-related disease (IgG4-RD)**. This is a systemic fibroinflammatory condition that can affect multiple organs and systems, leading to a wide range of clinical manifestations, including those you have described.

### Most Likely Diagnosis:

- **IgG4-related disease (IgG4-RD)**

### Other Possible Diagnoses:

- **Sarcoidosis**: A systemic granulomatous disorder that can affect multiple organs, including the lungs, heart, skin, and eyes. It can present with similar symptoms such as fever, malaise, and involvement of multiple organ systems.
- **Lymphoma (e.g., Hodgkin’s or Non-Hodgkin’s)**: Lymphomas can present with systemic symptoms like fever, night sweats, and weight loss, and can involve multiple organs, including the bones, heart, lungs, and central nervous system.
- **Systemic Sclerosis (Scleroderma)**: A connective tissue disease that can cause fibrosis and inflammation in multiple organs, including the skin, lungs, heart, and gastrointestinal tract.
- **Chronic Inflammatory Demyelinating Polyneuropathy (CIDP)**: While primarily affecting the peripheral nervous system, CIDP can sometimes present with central nervous system involvement and systemic symptoms.
- **Amyloidosis**: A group of diseases characterized by the deposition of amyloid proteins in various organs, leading to organ dysfunction. It can affect the heart, kidneys, liver, and other organs, causing symptoms similar to those described.

### Key Features of IgG4-RD:

- **Multisystem Involvement**: IgG4-RD can affect multiple organs, including the salivary glands, pancreas, kidneys, lungs, and retroperitoneum.
- **Characteristic Lesions**: Lesions often present as masses or nodules and can cause organ dysfunction.
- **Elevated Serum IgG4 Levels**: Elevated levels of serum IgG4 are a hallmark of the disease.
- **Histopathology**: Histological examination typically shows lymphoplasmacytic infiltration, storiform fibrosis, and obliterative phlebitis.
- **Response to Corticosteroids**: IgG4-RD often responds well to corticosteroid therapy.

Given the wide range of symptoms and organ involvement, a thorough evaluation, including imaging studies, serological tests, and biopsy, is essential for a definitive diagnosis.

### **40. User**

The disease often presents with multi-organ and multi-system involvement, with males being more affected than female patients. Clinical manifestations often involve different organs as the patient grows older.

- Facial appearance, male patients usually appear at the age of 12-14 years with characteristic facial features, such as convexity of the supraorbital ridge, frontal bulge and thickening of the lips. 2.
- cutaneous angiokeratomas, which are common in patients with the classic form, are red patches of raised skin in the ‘sitting area’, i.e., between the umbilicus and knees, the scrotum, the buttocks, and the inner thighs.
- Neurological system, most patients will have peripheral nerve pain, which manifests as intolerable burning sensation on the soles of the feet and palms of the hands and radiates to the proximal limbs, and even painful spasms; autonomic nerve involvement manifests as little or no sweating; and central nervous system involvement manifests as early-onset transient ischemic attacks or ischemic strokes.
- Eye: Characteristic manifestations include conjunctival vascular tortuosity, corneal turbidity, posterior lens capsule clouding, and retinal vascular tortuosity, which in severe cases may lead to reduced or even loss of vision. It is often one of the main reasons for female patients to visit the clinic.
- Digestive tract, most often after eating nausea, vomiting, abdominal distension, cramping abdominal pain and diarrhoea, may also manifest as malabsorption or constipation.
- Kidney, early manifestation of urinary concentration dysfunction such as increased nocturia, polyuria and enuresis, with the progress of the disease can gradually appear proteinuria, and even up to the level of nephrotic syndrome, accompanied by renal function damage, mostly around 30 years old into the end-stage renal disease.
- Cardiovascular system, can be manifested as hypertension, myocardial ischemia caused by coronary artery involvement, heart valve lesions and hypertrophic cardiomyopathy, and in severe cases, it can be manifested as angina pectoris, myocardial infarction and heart failure. It is mostly a late manifestation of the disease and a major cause of death.

### Qwen2.5_72b

Based on the clinical manifestations you have provided, the most likely diagnosis is **Fabry Disease**. Fabry Disease is an X-linked lysosomal storage disorder caused by a deficiency of the enzyme alpha-galactosidase A (α-Gal A). This leads to the accumulation of globotriaosylceramide (Gb3) in various tissues, resulting in multi-organ and multi-system involvement.

### Differential Diagnoses:

- **Gaucher Disease**:
  - **Clinical Features**: Enlarged liver and spleen, bone pain, and anemia. Neurological involvement can occur in the more severe forms.
  - **Pathophysiology**: Deficiency of glucocerebrosidase leading to accumulation of glucocerebroside.
- **Niemann-Pick Disease**:
  - **Clinical Features**: Hepatosplenomegaly, neurological deficits, and pulmonary involvement. Type A is often fatal in early childhood.
  - **Pathophysiology**: Deficiency of sphingomyelinase leading to accumulation of sphingomyelin.
- **Hurler Syndrome (MPS I)**:
  - **Clinical Features**: Coarse facial features, corneal clouding, hepatosplenomegaly, and skeletal abnormalities. Early-onset and rapid progression.
  - **Pathophysiology**: Deficiency of alpha-L-iduronidase leading to accumulation of dermatan sulfate and heparan sulfate.
- **Tay-Sachs Disease**:
  - **Clinical Features**: Progressive neurological deterioration, cherry-red spot on the retina, and developmental delay. Often fatal in early childhood.
  - **Pathophysiology**: Deficiency of hexosaminidase A leading to accumulation of GM2 ganglioside.
- **Anderson-Fabry Disease (Variant)**:
  - **Clinical Features**: Similar to classic Fabry Disease but with a later onset and less severe symptoms. May present with isolated cardiac or renal involvement.
  - **Pathophysiology**: Partial deficiency of α-Gal A leading to less severe accumulation of Gb3.

### Key Points for Fabry Disease:

- **X-linked inheritance**: Males are more severely affected.
- **Multi-organ involvement**: Skin, nervous system, eyes, gastrointestinal tract, kidneys, and cardiovascular system.
- **Characteristic findings**:
  - **Facial features**: Convex supraorbital ridge, frontal bulge, thickened lips.
  - **Cutaneous angiokeratomas**: Red, raised patches in the “sitting area.”
  - **Neurological symptoms**: Peripheral neuropathy with burning pain, autonomic dysfunction, and central nervous system involvement.
  - **Ocular findings**: Conjunctival vascular tortuosity, corneal turbidity, and retinal vascular tortuosity.
  - **Gastrointestinal symptoms**: Nausea, vomiting, abdominal pain, and diarrhea.
  - **Renal involvement**: Early urinary concentration dysfunction, proteinuria, and progression to end-stage renal disease.
  - **Cardiovascular involvement**: Hypertension, myocardial ischemia, heart valve lesions, and hypertrophic cardiomyopathy.

Given the detailed and specific clinical manifestations, Fabry Disease is the most likely diagnosis. However, further diagnostic testing, such as enzyme activity assays and genetic testing, would be necessary to confirm the diagnosis.

### **41. User**

The disease is classified as Type 1 and Type 2.

- Type 1 is mainly characterised by recurrent inflammatory reactions and plasma membrane inflammation, including recurrent fever, abdominal pain and arthritis. Fever is recurrent and may be triggered by cold, strenuous exercise, surgery, infection, etc. and resolves spontaneously within 13 days. Abdominal pain is the most common clinical manifestation, seen in 95% of patients. It is a sudden onset of abdominal pain, often accompanied by fever, abdominal examination may have abdominal distension, abdominal pressure, rebound pain, muscle tension, and the loss of bowel sounds on auscultation. Stereotactic abdominal radiographs show flat air and fluid in the small intestine. Abdominal pain often resolves spontaneously after 24-48 hours. Arthritis is also a common clinical manifestation, seen in 75% of patients. It often involves the large joints of the lower limbs, such as the hip and knee joints. There may be joint swelling and pain. Rash is seen in 7% ~40% of patients, mainly involving the extensor side of the lower limbs, and the typical rash is erysipelas of tansy-like erythema. Pleurisy is seen in 45% of patients and presents as a sudden onset of unilateral chest pain, often accompanied by fever. Pericarditis is less common.
- Type 2 starts with amyloidosis. It presents with persistent proteinuria and often leads to end-stage renal disease. Amyloid can also be deposited in the heart, gastrointestinal tract, liver and other organs, leading to corresponding clinical manifestations.

### Qwen2.5_72b

Based on the clinical manifestations you’ve provided, the most likely diagnosis is **Familial Mediterranean Fever (FMF)**, which is a hereditary autoinflammatory disorder. Here’s a detailed breakdown:

### Most Likely Diagnosis:

- **Familial Mediterranean Fever (FMF)**
  - **Type 1**: Characterized by recurrent inflammatory attacks, including fever, abdominal pain, and arthritis. The symptoms you described, such as recurrent fever, abdominal pain (often with signs of peritonitis), arthritis affecting large joints, and occasional rash (erysipelas-like erythema), are classic for FMF.
  - **Type 2**: Involves amyloidosis, which can lead to persistent proteinuria and end-stage renal disease. Amyloid deposition can also affect other organs, leading to various clinical manifestations.

### Other Possible Diagnoses:

- **Tumor Necrosis Factor Receptor-Associated Periodic Syndrome (TRAPS)**
  - **Type 1**: Recurrent fever, abdominal pain, and arthritis. TRAPS can also present with prolonged fever (lasting more than 14 days) and a characteristic rash.
  - **Type 2**: Amyloidosis can occur, leading to renal involvement and proteinuria.
- **Hyperimmunoglobulin D Syndrome (HIDS)**
  - **Type 1**: Recurrent fever, abdominal pain, and arthritis. HIDS can also present with rash, particularly a tender, erythematous rash.
  - **Type 2**: Amyloidosis is less common in HIDS but can occur.
- **Mevalonate Kinase Deficiency (MKD)**
  - **Type 1**: Similar to HIDS, MKD presents with recurrent fever, abdominal pain, and arthritis. The rash is often tender and erythematous.
  - **Type 2**: Amyloidosis can occur, leading to renal involvement.
- **Cryopyrin-Associated Periodic Syndromes (CAPS)**
  - **Type 1**: Recurrent fever, abdominal pain, and arthritis. CAPS can also present with rash (urticaria-like) and other systemic symptoms.
  - **Type 2**: Amyloidosis can occur, leading to renal involvement and proteinuria.
- **Periodic Fever, Aphthous Stomatitis, Pharyngitis, and Cervical Adenitis (PFAPA) Syndrome**
  - **Type 1**: Recurrent fever, but typically without the severe abdominal pain and arthritis seen in FMF. PFAPA is more common in children and often resolves spontaneously.
  - **Type 2**: Amyloidosis is not a feature of PFAPA.

### Summary:

- **Most Likely Diagnosis**: Familial Mediterranean Fever (FMF)
- **Other Possible Diagnoses**:
  1. Tumor Necrosis Factor Receptor-Associated Periodic Syndrome (TRAPS)
  2. Hyperimmunoglobulin D Syndrome (HIDS)
  3. Mevalonate Kinase Deficiency (MKD)
  4. Cryopyrin-Associated Periodic Syndromes (CAPS)
  5. Periodic Fever, Aphthous Stomatitis, Pharyngitis, and Cervical Adenitis (PFAPA) Syndrome

Each of these conditions has overlapping symptoms, but the combination of recurrent fever, abdominal pain, arthritis, and the potential for amyloidosis in Type 2 strongly points to FMF. Further genetic testing and specific inflammatory markers can help confirm the diagnosis.

### **42. User**

The main manifestations are congenital developmental anomalies, progressive bone marrow failure and increased incidence of tumours, while some patients may show no developmental anomalies or bone marrow failure.

- Developmental anomalies, patients may show a variety of physical and mental developmental abnormalities. Growth defects, abnormal skin pigmentation, unilateral or bilateral skeletal malformations of the upper limbs, short stature, microcephaly, ocular anomalies, genitourinary malformations, endocrine anomalies, hearing loss, outer ear anomalies, congenital heart defects, digestive anomalies, abnormalities of the central nervous system, facial deformities, spinal deformities, neck deformities, and developmental delays and/or intellectual disabilities. These manifestations are mostly non-specific, with thumb and upper limb deformities being more specific.
- Progressive bone marrow failure with haematological abnormalities is the most serious clinical feature of Fanconi anaemia. It may present as a single or total haematopenia. The age of onset of haemopenia varies widely, even among siblings. The median age of onset is about 7.6 years, and it is rare in infants and young children; 90% of patients with haematological abnormalities develop before the age of 40 years. Thrombocytopenia or leukopenia usually precedes anaemia and is often accompanied by an increase in macrocytes, foetal haemoglobin (HbF 1), and a gradual increase in total haematopoiesis.
- There is an increased risk of neoplasms, including haematological malignancies and non-haematological malignant solid tumours. The risk of acute myeloid leukaemia (AML) is increased about 500-fold, and the cumulative incidence of AML is about 13% at the age of 50 years, with most cases occurring between the ages of 15 and 35 years. -7, del (7q) is associated with an increased incidence of myelodysplastic syndromes (MDS)/AML. Acute lymphoblastic leukaemia and lymphoma have also been reported. Solid tumours can be the first manifestation of the disease. Head and neck squamous cell carcinomas (HNSCCs) are the most common solid tumours in patients and have an earlier age of onset (20-40 years), mostly occurring in the oral cavity (e.g., tongue cancers), are mostly in the progressive stage, and respond poorly to treatment. There is also an increased incidence of skin, oesophageal, liver and genitourinary tract tumours. In a few patients, multiple tumours may be seen concurrently, or haematological and non-haematological tumours may be seen concurrently. And there is a marked intolerance to chemotherapy or radiotherapy.

### Qwen2.5_72b

Based on the clinical manifestations you have provided, the most likely diagnosis is **Fanconi Anemia (FA)**. Fanconi Anemia is a rare genetic disorder characterized by congenital developmental anomalies, progressive bone marrow failure, and an increased risk of malignancies, which aligns well with the symptoms you have described.

### Differential Diagnoses:

- **Dyskeratosis Congenita (DC)**:
  - **Clinical Features**: Developmental anomalies, bone marrow failure, and increased risk of malignancies. Patients may also have characteristic nail changes, reticulated skin pigmentation, and mucosal leukoplakia.
  - **Key Differences**: DC often presents with characteristic nail changes and mucosal leukoplakia, which are not typically seen in FA.
- **Shwachman-Diamond Syndrome (SDS)**:
  - **Clinical Features**: Congenital developmental anomalies, bone marrow failure, and increased risk of malignancies. Patients may also have exocrine pancreatic insufficiency, short stature, and skeletal abnormalities.
  - **Key Differences**: SDS is often associated with exocrine pancreatic insufficiency, which is not a feature of FA.
- **Severe Congenital Neutropenia (SCN)**:
  - **Clinical Features**: Recurrent infections due to neutropenia, bone marrow failure, and increased risk of malignancies. Patients may also have developmental delays and skeletal abnormalities.
  - **Key Differences**: SCN is primarily characterized by severe neutropenia and recurrent infections, which are not the primary features of FA.
- **Diamond-Blackfan Anemia (DBA)**:
  - **Clinical Features**: Congenital developmental anomalies, bone marrow failure (primarily red cell aplasia), and increased risk of malignancies. Patients may also have short stature, craniofacial abnormalities, and thumb abnormalities.
  - **Key Differences**: DBA is primarily characterized by red cell aplasia, whereas FA involves pancytopenia and a broader range of developmental anomalies.
- **Ataxia-Telangiectasia (AT)**:
  - **Clinical Features**: Progressive cerebellar ataxia, oculocutaneous telangiectasias, immunodeficiency, and increased risk of malignancies. Patients may also have developmental delays and increased sensitivity to ionizing radiation.
  - **Key Differences**: AT is characterized by cerebellar ataxia and oculocutaneous telangiectasias, which are not typical features of FA.

### Summary:

- **Primary Diagnosis**: Fanconi Anemia (FA)
- **Differential Diagnoses**:
  1. Dyskeratosis Congenita (DC)
  2. Shwachman-Diamond Syndrome (SDS)
  3. Severe Congenital Neutropenia (SCN)
  4. Diamond-Blackfan Anemia (DBA)
  5. Ataxia-Telangiectasia (AT)

Each of these conditions has overlapping features, but the combination of congenital developmental anomalies, progressive bone marrow failure, and increased risk of malignancies is most consistent with Fanconi Anemia. Further genetic testing and specific diagnostic criteria can help confirm the diagnosis.

### **43. User**

The perinatal onset of the disease is often typical, and within a few days of ingestion of breast milk or lactose-containing formula, the child develops life-threatening complications: feeding problems, diarrhoea, vomiting, hypoglycaemia, liver impairment, haemorrhage, jaundice, and cataracts. If left untreated, sepsis, shock and death may occur. Children who survive to infancy may develop severe brain damage if they continue to ingest lactose. Some children have long-term complications such as mental retardation, delayed growth, ataxia, blindness, and ovarian dysfunction in female patients.

### Qwen2.5_72b

Based on the clinical manifestations you’ve described, the most likely diagnosis is **Galactosemia**. This is a rare genetic disorder that affects the body’s ability to metabolize the sugar galactose, which is a component of lactose found in breast milk and formula. The symptoms you’ve listed, including feeding problems, diarrhea, vomiting, hypoglycemia, liver impairment, hemorrhage, jaundice, and cataracts, are characteristic of this condition. If left untreated, galactosemia can lead to severe complications such as sepsis, shock, and death. Surviving infants may develop long-term complications like mental retardation, delayed growth, ataxia, blindness, and ovarian dysfunction in females.

### Other Possible Diagnoses:

- **Congenital Adrenal Hyperplasia (CAH)**: This is a group of genetic disorders affecting the adrenal glands, leading to an imbalance in hormone production. Symptoms can include feeding problems, vomiting, dehydration, and electrolyte imbalances, which can be life-threatening if not treated.
- **Maple Syrup Urine Disease (MSUD)**: This is a rare metabolic disorder that affects the body’s ability to break down certain amino acids. Symptoms can include poor feeding, vomiting, lethargy, and a characteristic sweet-smelling urine. If untreated, it can lead to brain damage and death.
- **Phenylketonuria (PKU)**: This is a genetic disorder that results in the inability to break down the amino acid phenylalanine. High levels of phenylalanine can cause brain damage, leading to intellectual disability, seizures, and other neurological problems.
- **Tyrosinemia Type I**: This is a rare genetic disorder that affects the body’s ability to break down the amino acid tyrosine. Symptoms can include liver and kidney problems, vomiting, diarrhea, and developmental delays. If untreated, it can lead to liver failure, kidney failure, and neurological damage.
- **Biotinidase Deficiency**: This is a rare genetic disorder that affects the body’s ability to recycle the vitamin biotin. Symptoms can include skin rashes, hair loss, seizures, and developmental delays. If untreated, it can lead to severe neurological damage and other complications.

Each of these conditions can present with similar symptoms in the neonatal period, making a thorough evaluation and specific diagnostic tests essential for accurate diagnosis and timely treatment.

### **44. User**

- Type I is the most common subtype. There is no primary central nervous system involvement, but some patients may develop secondary neurological manifestations (e.g., spinal cord compression) as the disease progresses. The disease can develop in all age groups, with about 2/3 of patients developing in childhood. Organ manifestations are mainly hepatosplenomegaly, especially splenomegaly, often accompanied by hypersplenism, and even splenic infarction and splenic rupture. The haematological manifestations are mainly thrombocytopenia and anaemia, and some patients have leucopenia, which may be accompanied by coagulation abnormalities. Patients present with pallor, fatigue, skin and gum bleeding, increased menstruation, and even life-threatening bleeding. Most patients have skeletal invasion, but the severity varies. Early invasion sites mainly include lumbar vertebrae, long bone epiphyses and diaphyses, and in the middle and late stages, epiphyses and bony prominences may be involved. Patients often experience acute or chronic diffuse bone pain, and in severe cases, osteonecrosis (ischaemic necrosis), followed by joint collapse involving the proximal and distal femur, proximal tibia and proximal humerus. Osteolytic lesions, pathological fractures, vertebral compression fractures and other fragility fractures due to reduced bone density may also occur. Skeletal lesions can interfere with daily activities and can be disabling. In children, the most common manifestations are osteoporosis, flask-like deformity of the metaphysis of the long bones, varying degrees of hypodensity of the metaphysis of the long bones, thinning of the bone cortex, etc. There may be growth retardation. Some patients may have lung involvement, which is mainly manifested as interstitial lung disease, pulmonary solid changes, and pulmonary hypertension. In addition, patients may have abnormalities of sugar and lipid metabolism, increased risk of malignant tumours such as multiple myeloma, cholelithiasis, and abnormalities of the immune system.
- Type II, in addition to hepatosplenomegaly, anaemia, thrombocytopenia and other manifestations similar to those of type I, patients with type II mainly have acute neurological involvement. It often develops in the neonatal period to infancy, progresses rapidly and has a high morbidity and mortality rate. There are rapidly progressive medullary palsy, dyskinesia, seizures, angular reflexes and cognitive deficits and other signs of acute neurological damage, and growth is retarded, with death occurring before the age of 2 to 4 years. Some patients with severe disease may develop joint contractures.
- The early manifestations of type III are similar to those of type I, with gradual onset of neurological involvement, which often develops in childhood and has a slow progression of disease and a longer life expectancy. The patients often have the invasion of the motor nerve, eye movement disorders, ataxia, anisocoria, epilepsy, myoclonus, accompanied by developmental delays and intellectual backwardness. Type III can be divided into three subtypes, namely, type IIIa, which is characterised by more rapidly progressive neurological symptoms (ocular motility disorders, cerebellar ataxia, spasticity, myoclonus, and dementia) and hepatosplenomegaly; type IIIb, which is characterised by hepatosplenomegaly and skeletal symptoms with fewer CNS symptoms; and type IIIc, which is characterised by milder symptoms, including calcification of the cardiac valves and corneal clouding, and is predominantly found in the Druze population. Type IIIc.

### Qwen2.5_72b

Based on the clinical manifestations provided, the most likely diagnosis is **Gaucher Disease**. Gaucher Disease is a rare genetic disorder caused by a deficiency of the enzyme glucocerebrosidase, leading to the accumulation of glucocerebroside in various organs, particularly the liver, spleen, and bones.

### Most Likely Diagnosis:

- **Gaucher Disease** (Type I, II, or III based on the specific clinical manifestations)

### Other Possible Diagnoses:

- **Niemann-Pick Disease** (Type A, B, or C):
  - **Type A**: Rapidly progressive neurological involvement, hepatosplenomegaly, and early death in infancy.
  - **Type B**: Hepatosplenomegaly, pulmonary involvement, and no primary neurological involvement.
  - **Type C**: Progressive neurological involvement, hepatosplenomegaly, and variable age of onset.
- **Fabry Disease**:
  - X-linked lysosomal storage disorder with multisystem involvement, including pain, skin lesions, renal failure, and cardiovascular and cerebrovascular complications.
- **Mucopolysaccharidosis (MPS) Type I (Hurler Syndrome, Scheie Syndrome, and Hurler-Scheie Syndrome)**:
  - Characterized by skeletal abnormalities, hepatosplenomegaly, and progressive neurological involvement, with varying degrees of severity.
- **Pompe Disease** (Glycogen Storage Disease Type II):
  - Characterized by muscle weakness, cardiomyopathy, and respiratory insufficiency, with infantile and late-onset forms.
- **Tay-Sachs Disease**:
  - A lysosomal storage disorder with progressive neurological deterioration, leading to early death, often in infancy.

### Summary:

- **Most Likely Diagnosis**: Gaucher Disease (Type I, II, or III)
- **Other Possible Diagnoses**:
  1. Niemann-Pick Disease (Type A, B, or C)
  2. Fabry Disease
  3. Mucopolysaccharidosis Type I
  4. Pompe Disease
  5. Tay-Sachs Disease

Each of these conditions has overlapping clinical features, and a definitive diagnosis would require specific enzymatic and genetic testing.

### **45. User**

It often starts in one muscle group and progresses to others, with ptosis and diplopia being the most common onset, accounting for 80 per cent of cases. Skeletal muscle weakness is fluctuating and easily fatigued, with a light morning and heavy evening. According to the clinical manifestations, it is divided into five types. Type I: ocular muscle type, manifested by ptosis and diplopia. Type ⅡA: mild generalised type, with mild involvement of the limb muscle groups. Type IIB: Moderate generalised type with moderate involvement of the limb muscle groups, usually with mastication, swallowing and dysarthria. Type III: Severe aggressive type with rapid progression, involving the pharyngeal muscles within weeks or months and the respiratory muscles within six months. Type IV: Late-onset severe type, slowly progressive, involving the respiratory muscles within 2 years. Type V: myasthenia gravis, skeletal muscle atrophy within six months of disease onset. Neurological examination can be seen in the proximal limbs, trunk, neck, craniofacial muscle weakness, intolerance to fatigue, Jolly test is positive, tendon reflexes are normal, no sensory deficits, no pathological signs. Some patients may progress to respiratory failure and develop myasthenia gravis crisis.

### Qwen2.5_72b

Based on the clinical manifestations you have provided, the most likely diagnosis is **Myasthenia Gravis (MG)**. Myasthenia Gravis is an autoimmune neuromuscular disorder characterized by fluctuating weakness and fatigability of skeletal muscles. The symptoms you described, including ptosis, diplopia, fluctuating muscle weakness, and the progression of symptoms from morning to evening, are classic for MG.

### Differential Diagnoses:

- **Lambert-Eaton Myasthenic Syndrome (LEMS)**:
  - Characterized by muscle weakness and fatigability, but often with a more gradual onset and improvement with repeated use of the affected muscles.
  - Associated with small cell lung cancer in about 50% of cases.
  - Electromyography (EMG) shows a characteristic increment in muscle action potentials after brief exercise.
- **Chronic Inflammatory Demyelinating Polyneuropathy (CIDP)**:
  - A progressive or relapsing-remitting disorder of the peripheral nerves.
  - Symptoms include symmetric weakness and sensory loss, often with areflexia.
  - Nerve conduction studies show slowed conduction velocities and conduction block.
- **Botulism**:
  - Caused by toxins produced by the bacterium *Clostridium botulinum*.
  - Symptoms include descending flaccid paralysis, ptosis, and diplopia.
  - Often associated with a history of food poisoning or wound infection.
- **Polymyositis**:
  - An inflammatory myopathy characterized by proximal muscle weakness and fatigue.
  - Often associated with elevated muscle enzymes (e.g., creatine kinase) and muscle inflammation on biopsy.
  - May present with dysphagia and respiratory muscle weakness.
- **Guillain-Barré Syndrome (GBS)**:
  - An acute inflammatory demyelinating polyneuropathy.
  - Symptoms include rapidly progressive muscle weakness, areflexia, and sometimes respiratory muscle involvement.
  - Cerebrospinal fluid (CSF) analysis may show albuminocytological dissociation (elevated protein with normal cell count).

### Key Points for Myasthenia Gravis:

- **Clinical Features**: Ptosis, diplopia, fluctuating muscle weakness, fatigue, and improvement with rest.
- **Types**: Ocular (Type I), mild generalized (Type IIa), moderate generalized (Type IIb), severe aggressive (Type III), late severe (Type IV), and myasthenic crisis (Type V).
- **Diagnostic Tests**:
  - **Edrophonium Test**: A positive test (improvement in muscle strength) is highly suggestive of MG.
  - **Repetitive Nerve Stimulation (RNS)**: Shows a decremental response in muscle action potentials.
  - **Single-Fiber EMG**: Abnormal jitter is a sensitive test for MG.
  - **Serum Antibodies**: Anti-acetylcholine receptor (AChR) antibodies are present in about 80-90% of generalized MG cases.

If you have any further questions or need more detailed information, feel free to ask!

### **46. User**

The disease often starts in adolescence or early adulthood. Clinical manifestations are mainly related to hypokalemia and hypomagnesemia. Mild patients may be asymptomatic or show mild fatigue and poor appetite; severe patients may have twitching of limbs, flaccid paralysis, painful spasms, fainting, and transverse myelinolysis secondary to acute renal injury, and may even suffer from cardiac arrest due to severe ventricular arrhythmias. The common clinical manifestations are as follows:

- systemic symptoms fatigue, thirst, polydipsia, salt craving.
- Neuro-muscular system Muscle weakness, painful spasms, convulsions, convulsive seizures, numbness of limbs, sensory abnormalities, rhabdomyolysis, paralysis, dizziness, vertigo, ataxia, and pseudotumor cerebri.
- Cardiovascular system Palpitation, syncope, normal or low blood pressure and ventricular arrhythmia, etc.
- Digestive system Constipation, vomiting, etc.
- Urinary system Polyuria, nocturia, enuresis, proteinuria and renal insufficiency.
- Bone and joint system Arthralgia, cartilage calcium sedimentation.
- endocrine and growth retardation, delayed puberty; diabetes mellitus or impaired glucose tolerance is not uncommon in patients with chronically low potassium and magnesium. 8. ocular symptoms.
- Ocular symptoms Blurred vision and scleral choroidal calcification may occur in a small number of patients.

### Qwen2.5_72b

Based on the clinical manifestations you’ve provided, the most likely rare disease is **Bartter Syndrome**. Bartter Syndrome is a group of rare, inherited disorders that affect the kidneys, leading to the loss of potassium, sodium, chloride, and water into the urine. This results in hypokalemia and hypomagnesemia, which can cause a wide range of symptoms, including those you’ve described.

### Differential Diagnoses:

- **Gitelman Syndrome**: Another rare genetic disorder affecting the kidneys, leading to hypokalemia and hypomagnesemia. However, Gitelman Syndrome typically presents later in life and is less severe than Bartter Syndrome. Symptoms include muscle weakness, cramps, and fatigue.
- **Liddle Syndrome**: A rare genetic disorder characterized by hypertension and hypokalemia due to a defect in the renal tubules. However, unlike Bartter Syndrome, Liddle Syndrome typically presents with high blood pressure rather than low or normal blood pressure.
- **Primary Aldosteronism (Conn’s Syndrome)**: A condition where the adrenal glands produce too much aldosterone, leading to hypertension and hypokalemia. However, it typically does not cause hypomagnesemia and is more commonly associated with high blood pressure.
- **Diabetes Insipidus**: A condition characterized by excessive thirst and urination. While it can cause dehydration and electrolyte imbalances, it does not typically cause hypokalemia and hypomagnesemia.
- **Renal Tubular Acidosis (RTA)**: A group of kidney disorders that affect the kidney’s ability to excrete acids into the urine, leading to a buildup of acid in the blood. RTA can cause hypokalemia and hypomagnesemia, but it is often associated with metabolic acidosis, which is not a prominent feature in the clinical manifestations you’ve described.

### Summary:

- **Most Likely Diagnosis**: Bartter Syndrome
- **Differential Diagnoses**:
  1. Gitelman Syndrome
  2. Liddle Syndrome
  3. Primary Aldosteronism (Conn’s Syndrome)
  4. Diabetes Insipidus
  5. Renal Tubular Acidosis (RTA)

Further diagnostic testing, such as genetic testing, renal function tests, and electrolyte panels, would be necessary to confirm the diagnosis and rule out other conditions.

### **47. User**

The majority of children develop the disease in infancy or early childhood, with a variety of presentations. The earlier the onset, the more severe the symptoms and the worse the prognosis. The majority of patients have a relatively normal presentation in the first year of life. The earliest sign that appears in about 75% of patients is a large head size, and most children are born with a larger head circumference than their peers or have a rapid increase in head circumference soon after birth, which may be accompanied by mild non-specific symptoms, including irritability, difficulty in feeding, and vomiting. Children usually develop ketosis, vomiting, hepatomegaly and acute encephalopathic crisis, including hypotonia, loss of consciousness and convulsive seizures, between 3 and 36 months of age after fever, infection, surgery, or vaccination, which may resolve with symptomatic treatment, but not fully recover. If acute encephalopathic crisis occurs repeatedly, the neurological damage will be progressively aggravated, and there may be developmental regression, and eventually cognitive dysfunction may occur. In some children, delayed movement, dystonia, and random movement disorders occur gradually over the first few years of life, but mental development is almost normal. Patients often die within 10 years of age from concomitant diseases or Reye-like seizures, which decrease with age. Rarely, the onset of the disease occurs during adolescence or even adulthood, and may be asymptomatic until the first episode.

### Qwen2.5_72b

Based on the clinical manifestations you have provided, the most likely rare disease is **Canavan Disease**. However, there are several other conditions that share similar features and should be considered in the differential diagnosis. Here are the top five possible diagnoses:

- **Canavan Disease**:
  - **Description**: A rare inherited leukodystrophy that affects the brain’s white matter. It is caused by a deficiency of the enzyme aspartoacylase (ASPA), which leads to the accumulation of N-acetylaspartic acid (NAA) in the brain.
  - **Key Features**: Large head size (macrocephaly), developmental delay, hypotonia, and progressive neurological deterioration.
- **Zellweger Syndrome (Peroxisomal Biogenesis Disorders)**:
  - **Description**: A group of rare, inherited disorders that affect the development and function of peroxisomes.
  - **Key Features**: Macrocephaly, hepatomegaly, hypotonia, developmental delay, and recurrent infections.
- **Methylmalonic Acidemia**:
  - **Description**: A rare inherited metabolic disorder characterized by the inability to metabolize certain amino acids and fats.
  - **Key Features**: Ketosis, vomiting, hepatomegaly, and acute encephalopathic crises triggered by infections or other stressors.
- **Maple Syrup Urine Disease (MSUD)**:
  - **Description**: A rare inherited metabolic disorder characterized by the inability to break down certain amino acids.
  - **Key Features**: Ketosis, vomiting, hepatomegaly, and acute encephalopathic crises, often triggered by infections or metabolic stress.
- **Reye Syndrome**:
  - **Description**: A rare but serious condition that causes swelling in the liver and brain, often following a viral infection.
  - **Key Features**: Rapid onset of vomiting, confusion, and seizures, often following a viral illness and aspirin use.
- **Alexander Disease**:
  - **Description**: A rare, progressive leukodystrophy that primarily affects the brain and spinal cord.
  - **Key Features**: Macrocephaly, developmental delay, spasticity, and seizures.

Each of these conditions has overlapping symptoms, but the specific clinical features and laboratory findings can help in making a definitive diagnosis. Genetic testing and metabolic screening are often crucial in confirming the diagnosis.

### **48. User**

It mainly manifests as bleeding from joints, muscles and deep tissues, but also internal organs such as gastrointestinal tract and central nervous system. If repeated bleeding occurs, untreated bleeding can lead to joint deformity and/or pseudotumour formation, which can be life-threatening in severe cases. Delayed haemorrhage after trauma or surgery is characteristic of this disease.

### Qwen2.5_72b

Based on the clinical manifestations you’ve described, the most likely diagnosis is **Hemophilia A (Factor VIII deficiency)** or **Hemophilia B (Factor IX deficiency)**. Both are rare bleeding disorders characterized by spontaneous and trauma-induced bleeding, particularly into joints, muscles, and deep tissues. Repeated bleeding can lead to joint deformities and pseudotumors, and delayed hemorrhage after trauma or surgery is a hallmark of these conditions.

### Most Likely Diagnosis:

- **Hemophilia A (Factor VIII deficiency)**

### Other Possible Diagnoses:

1. **Hemophilia B (Factor IX deficiency)**
2. **Von Willebrand Disease (VWD)** - A common inherited bleeding disorder, but can present with similar symptoms, especially if severe.
3. **Disseminated Intravascular Coagulation (DIC)** - A condition where small blood clots form throughout the body, leading to bleeding and organ dysfunction.
4. **Acquired Hemophilia** - A rare autoimmune disorder where the body produces antibodies against its own clotting factors, leading to spontaneous bleeding.
5. **Factor XI Deficiency (Rosenthal Syndrome)** - A rare bleeding disorder that can present with similar symptoms, though typically less severe than Hemophilia A or B.

Each of these conditions can present with significant bleeding episodes, but the specific clinical features and laboratory findings will help in making the correct diagnosis. For example, factor VIII and IX levels will be low in Hemophilia A and B, respectively, while von Willebrand factor levels and activity will be reduced in VWD. DIC will show evidence of both clotting and bleeding, and acquired hemophilia will show the presence of inhibitors against clotting factors.

### **49. User**

It can involve multiple organs of the body and has a variety of clinical manifestations. The age of onset is mostly 3-60 years old, and there are also reports of patients diagnosed at 8 months and over 70 years old. In children, hepatic involvement is the first manifestation of the disease, while in adolescents and adults, neurological involvement is the first manifestation of the disease.

It may present with asymptomatic persistent elevation of aminotransferases, chronic hepatitis, cirrhosis, and acute liver failure. Neurological involvement may manifest as motor dysfunction, tremor, ataxia, chorea, dysautonomia, dystonia, and in some patients masked face, stiff limbs, and gait abnormalities. Some patients combine with mental behavioural abnormalities such as apathy, aggressive behaviour, and personality changes. Kidney involvement may manifest as haematuria, proteinuria, etc. Involvement of the blood system may be manifested as haemolytic anaemia, liver cirrhosis, splenic hyperfunction leading to a decrease in the blood tertiary system, and coagulation abnormalities. Relatively rare systems involved include bone and joint, heart, endocrine and reproductive systems.

### Qwen2.5_72b

Based on the clinical manifestations and the age of onset you’ve described, the most likely rare disease is **Wilson’s Disease**. Wilson’s Disease is a genetic disorder characterized by the accumulation of copper in the liver, brain, and other vital organs. Here are the key features that align with the clinical manifestations you’ve provided:

- **Hepatic Involvement**:
  - Asymptomatic persistent elevation of aminotransferases
  - Chronic hepatitis
  - Cirrhosis
  - Acute liver failure
- **Neurological Involvement**:
  - Motor dysfunction
  - Tremor
  - Ataxia
  - Chorea
  - Dysautonomia
  - Dystonia
  - Masked face
  - Stiff limbs
  - Gait abnormalities
  - Mental and behavioral abnormalities (e.g., apathy, aggressive behavior, personality changes)
- **Renal Involvement**:
  - Hematuria
  - Proteinuria
- **Hematological Involvement**:
  - Hemolytic anemia
  - Liver cirrhosis and splenic hyperfunction leading to a decrease in the blood tertiary system
  - Coagulation abnormalities
- **Other Systems**:
  - Bone and joint involvement
  - Cardiac involvement
  - Endocrine and reproductive system involvement

### Other Possible Diagnoses

- **Hepatolenticular Degeneration (Wilson’s Disease)**: As already mentioned, this is the most likely diagnosis.
- **Autoimmune Hepatitis**: This can present with similar hepatic symptoms and can also involve neurological manifestations, though it is not typically associated with copper accumulation.
- **Chronic Viral Hepatitis (e.g., Hepatitis B or C)**: These can cause chronic liver disease and may have some neurological manifestations, but they are not typically associated with the specific neurological and renal symptoms seen in Wilson’s Disease.
- **Hereditary Hemochromatosis**: This is a genetic disorder characterized by iron overload, which can lead to liver disease and some neurological symptoms, but it does not typically present with the specific neurological and renal symptoms seen in Wilson’s Disease.
- **Mitochondrial Disorders**: These can present with a wide range of symptoms, including liver disease, neurological involvement, and renal issues, but they are typically associated with more generalized metabolic abnormalities and often have a different age of onset.

Given the specific combination of hepatic, neurological, renal, and hematological symptoms, Wilson’s Disease is the most likely diagnosis. However, further diagnostic testing, such as serum ceruloplasmin levels, 24-hour urinary copper, and liver biopsy, would be necessary to confirm the diagnosis.

### **50. User**

The clinical picture is very heterogeneous, even within the same family. The disease usually begins before the age of 30 years, worsens during adolescence, and the oedema often has an acute onset. The clinical picture is characterised by recurrent, unpredictable cutaneous and submucosal oedema. The oedema is episodic, self-limited, usually resolves spontaneously in 3-5 days, asymmetric and non-concave. Oedema can involve any part of the body, with the extremities, face, genitals, respiratory tract and gastrointestinal tract mucosa being the most common. The most fatal is respiratory mucosal oedema, which can rapidly progress to respiratory distress or asphyxiation due to laryngeal oedema, and can lead to death by asphyxiation if not resuscitated in time, with a lethality rate as high as 11%-40%, which is one of the main causes of death. Episodes of mucosal oedema of the digestive tract manifest as severe abdominal pain, accompanied by nausea and vomiting, and are therefore often misdiagnosed as acute abdomen, leading to unnecessary abdominal surgery. Skin oedema occurring in the limbs and other parts of the body can affect its function.

### Qwen2.5_72b

Based on the clinical manifestations you have provided, the most likely diagnosis is **Hereditary Angioedema (HAE)**. HAE is a rare genetic disorder characterized by recurrent episodes of swelling (angioedema) that can affect various parts of the body, including the skin, gastrointestinal tract, and airways. The condition is typically caused by a deficiency or dysfunction of the C1 esterase inhibitor (C1-INH) protein.

### Most Likely Diagnosis:

- **Hereditary Angioedema (HAE)**

### Other Possible Diagnoses:

- **Acquired Angioedema (AAE)**: This condition can occur in individuals without a family history and is often associated with other underlying conditions, such as lymphoproliferative disorders or autoimmune diseases.
- **Allergic Angioedema**: This can be triggered by allergens such as foods, medications, or environmental factors. It often occurs with urticaria (hives) and can be managed with antihistamines and corticosteroids.
- **Idiopathic Angioedema**: This is angioedema that occurs without a known cause. It can be recurrent and may require a thorough evaluation to rule out other conditions.
- **Angioedema Due to ACE Inhibitors**: This is a common side effect of angiotensin-converting enzyme (ACE) inhibitors, a class of medications used to treat hypertension and heart failure. The swelling can be severe and life-threatening, especially if it affects the airways.
- **Hereditary Angioedema with Normal C1 Inhibitor (HAE type III)**: This is a rare form of HAE that is not associated with C1-INH deficiency but is linked to mutations in the F12 gene, which encodes factor XII.

### Key Points for HAE:

- **Genetic Basis**: HAE is usually inherited in an autosomal dominant pattern.
- **C1 Esterase Inhibitor Deficiency**: Most cases are due to a deficiency or dysfunction of C1-INH.
- **Symptoms**: Recurrent, unpredictable, and self-limited episodes of swelling, often involving the skin, gastrointestinal tract, and airways.
- **Severity**: Respiratory involvement can be life-threatening, and gastrointestinal involvement can mimic acute abdomen.
- **Management**: Treatment includes prophylactic and acute management with C1-INH concentrates, bradykinin receptor antagonists, and kallikrein inhibitors.

If you suspect HAE, it is important to confirm the diagnosis with laboratory tests, including C1-INH levels and function, and genetic testing if necessary. Early diagnosis and appropriate management are crucial to prevent severe complications.

### **51. User**

The disease starts at birth and the severity of clinical manifestations is related to the subtype of the disease. Clinical manifestations include blisters or maculopapular blisters on the hands, elbows, knees and feet and other areas prone to friction, or around the body. Blisters or ulcers of the oral mucosa may occur, leading to dysphagia and hoarseness. Some patients have mottled pigmentation abnormalities, baldness, palmoplantar hyperkeratosis, enamel defects, nail thickening and nail dystrophy. Other impairments include disfigurement of the digits due to repeated scar formation, anaemia, osteoporosis, muscle atrophy, cardiomyopathy, and deafness. Some subtypes may be associated with basal cell carcinoma, squamous cell carcinoma, and malignant melanoma in advanced stages.

### Qwen2.5_72b

Based on the clinical manifestations you’ve described, the most likely diagnosis is **Epidermolysis Bullosa (EB)**. EB is a group of rare genetic disorders characterized by extremely fragile skin and recurrent blister formation, resulting from minor mechanical friction or trauma. The severity and specific clinical features can vary widely depending on the subtype of EB.

### Most Likely Diagnosis:

- **Epidermolysis Bullosa (EB)**

### Other Possible Diagnoses:

- **Dystrophic Epidermolysis Bullosa (DEB)**: This subtype of EB is characterized by the presence of blisters, scarring, and nail abnormalities, which aligns with the clinical manifestations you described.
- **Junctional Epidermolysis Bullosa (JEB)**: This subtype often presents with severe blistering, mucosal involvement, and can lead to significant complications such as dysphagia and hoarseness.
- **Kindler Syndrome**: This is a rare form of EB characterized by skin fragility, blistering, and progressive poikiloderma (mottled pigmentation, atrophy, and telangiectasia).
- **Bullous Pemphigoid**: While not a genetic disorder, this autoimmune condition can present with blisters, especially in areas prone to friction, and can sometimes be confused with EB.
- **Dermatitis Herpetiformis**: This is another autoimmune condition that can cause blistering, particularly on the elbows, knees, and buttocks, but it is typically associated with gluten sensitivity and celiac disease.

### Additional Considerations:

- **Anemia** and **osteoporosis** can be secondary complications in EB due to chronic inflammation and nutritional deficiencies.
- **Cardiomyopathy** and **muscle atrophy** are less common but can occur in severe cases of EB.
- **Malignant transformations** such as basal cell carcinoma, squamous cell carcinoma, and malignant melanoma are known complications in certain subtypes of EB, particularly in the dystrophic forms.

Given the complexity and severity of the symptoms, a multidisciplinary approach involving dermatologists, geneticists, and other specialists is crucial for accurate diagnosis and management. Genetic testing and skin biopsy can help confirm the diagnosis and identify the specific subtype of EB.

### **52. User**

The clinical presentation of patients varies with age, amount and duration of fructose exposure. In general, the younger the age of onset, the more severe the symptoms. The onset of the disease is typically around 6 months of age and usually occurs when fructose-containing foods, such as fruits, honey and certain vegetables, are first consumed. Common clinical symptoms include hypoglycaemic episodes after eating fructose-containing foods, with nausea, vomiting, abdominal pain, bloating, sweating, tremors, convulsions and even coma.

If the diagnosis is not clear and the patient chronically ingests fructose foods for a long period of time, in addition to hypoglycemic manifestations, growth retardation, hepatomegaly, ascites, jaundice and metabolic disorders such as hypoglycemia, metabolic acidosis, hypophosphatemia, hyperuricemia, hypermagnesemia, and hyperpyruvatemia may occur. In severe cases, multiple organ damage, liver and renal failure, convulsions, coma, and even death occur.

In acute attacks, laboratory tests reveal hypoglycaemia, hypophosphatemia and hypokalemia, accompanied by elevated uric acid, lactate, pyruvate, free fatty acids and triglycerides. Chronic patients present with hepatic impairment and indications of hepatic fibrosis, including serum bilirubin, elevated transaminases, and coagulation disorders. Urinalysis reveals proteinuria and fructose positivity.

### Qwen2.5_72b

Based on the clinical manifestations and laboratory findings you’ve provided, the most likely diagnosis is **Hereditary Fructose Intolerance (HFI)**. HFI is a rare genetic disorder caused by a deficiency in the enzyme aldolase B, which is essential for the metabolism of fructose. This leads to the accumulation of toxic metabolites, causing the symptoms you described.

### Most Likely Diagnosis:

- **Hereditary Fructose Intolerance (HFI)**

### Other Possible Diagnoses:

- **Galactosemia**: A genetic disorder affecting the metabolism of galactose, which can present with similar symptoms such as vomiting, jaundice, and liver dysfunction.
- **Glycogen Storage Disease Type I (von Gierke Disease)**: A disorder of glycogen metabolism that can cause hypoglycemia, hepatomegaly, and metabolic acidosis.
- **Wilson’s Disease**: A genetic disorder of copper metabolism that can lead to liver dysfunction, neurological symptoms, and metabolic abnormalities.
- **Reye Syndrome**: A rare but serious condition that can cause liver and brain dysfunction, often following a viral illness and aspirin use.
- **Non-Alcoholic Fatty Liver Disease (NAFLD)**: While not typically associated with fructose intolerance, chronic exposure to fructose can exacerbate liver damage and lead to similar symptoms.

### Key Points for HFI:

- **Age of Onset**: Typically around 6 months when fructose-containing foods are introduced.
- **Symptoms**: Hypoglycemia, nausea, vomiting, abdominal pain, bloating, sweating, tremors, convulsions, and coma.
- **Chronic Symptoms**: Growth retardation, hepatomegaly, ascites, jaundice, metabolic disorders (hypoglycemia, metabolic acidosis, hypophosphatemia, hyperuricemia, hypermagnesemia, hyperpyruvatemia).
- **Laboratory Findings**: Hypoglycemia, hypophosphatemia, hypokalemia, elevated uric acid, lactate, pyruvate, free fatty acids, and triglycerides. Chronic patients may show hepatic impairment, elevated serum bilirubin, transaminases, and coagulation disorders. Urinalysis may reveal proteinuria and fructose positivity.

If HFI is suspected, genetic testing for mutations in the *ALDOB* gene and enzyme activity assays can confirm the diagnosis. Early diagnosis and strict avoidance of fructose and sucrose are crucial for managing the condition and preventing severe complications.

### **53. User**

**1. Migraine** Migraine with aura is often the first symptom of the disease and is seen in 20-40% of patients, which is five times higher than in the general population; it can also manifest as migraine without aura, with an incidence not different from that of the general population. The incidence of migraine is lower in Asian patients than in European and American patients.

**2. Subcortical ischemic events** Transient ischemic attacks (TIAs) and ischemic strokes are the most common clinical manifestations of the disease, occurring in 60% to 85% of symptomatic patients, most of whom lack common risk factors for cerebrovascular disease. 2/3 of patients present with the classic lacunar syndrome.

**3. Cognitive deficits** Cognitive dysfunction with dementia develops progressively in 60% of patients.

**Affective disorders and apathy** 20% of patients have affective disorders, with depressive states being the most common, and rarely alternating between depressive and manic states; apathy is seen in 40% of patients.

In addition, seizures occur in 5-10% of patients, and hearing loss, impaired consciousness, spinal cord infarction, intracranial haemorrhage, and combined basin infarction have also been reported.

### Qwen2.5_72b

Based on the clinical manifestations you have provided, the most likely rare disease is **CADASIL (Cerebral Autosomal Dominant Arteriopathy with Subcortical Infarcts and Leukoencephalopathy)**. CADASIL is a genetic disorder that primarily affects the small blood vessels in the brain, leading to a variety of neurological symptoms.

### Most Likely Diagnosis:

- **CADASIL (Cerebral Autosomal Dominant Arteriopathy with Subcortical Infarcts and Leukoencephalopathy)**

### Other Possible Diagnoses:

- **Fabry Disease** - A lysosomal storage disorder that can cause cerebrovascular events, cognitive decline, and affective disorders.
- **MELAS Syndrome (Mitochondrial Encephalomyopathy, Lactic Acidosis, and Stroke-like Episodes)** - A mitochondrial disorder that can present with stroke-like episodes, cognitive decline, and migraine.
- **Sickle Cell Disease** - Can cause cerebrovascular events, cognitive deficits, and affective disorders, particularly in individuals with sickle cell anemia.
- **Cerebral Amyloid Angiopathy (CAA)** - A condition characterized by the deposition of amyloid in the walls of cerebral blood vessels, leading to recurrent cerebral hemorrhages and cognitive decline.
- **Primary Progressive Aphasia (PPA)** - A type of frontotemporal dementia that can present with cognitive deficits, particularly in language, and may be associated with affective disorders.

### Rationale:

- **CADASIL** is the most likely diagnosis because it fits the pattern of migraine with aura, subcortical ischemic events, cognitive deficits, and affective disorders. The high incidence of these symptoms, particularly the subcortical ischemic events and cognitive decline, is characteristic of CADASIL.
- **Fabry Disease** and **MELAS Syndrome** can also present with similar neurological symptoms but are less likely due to the specific pattern of subcortical ischemic events and the high incidence of migraine with aura.
- **Sickle Cell Disease** can cause cerebrovascular events but is less likely to present with the specific combination of symptoms seen in CADASIL.
- **Cerebral Amyloid Angiopathy (CAA)** can cause recurrent cerebral hemorrhages and cognitive decline but is less likely to present with the high incidence of migraine with aura.
- **Primary Progressive Aphasia (PPA)** is a type of frontotemporal dementia that can present with cognitive deficits but is less likely to present with the specific combination of subcortical ischemic events and migraine with aura.

If you have any further clinical details or genetic testing results, they would be helpful in confirming the diagnosis.

### **54. User**

The onset of the disease is usually insidious in early youth (but has been reported from 1 to 76 years of age). The main manifestation is a slowly progressive spastic paralysis of both lower limbs, with pyramidal signs (hyperreflexia, positive ankle clonus, positive pathology). Bilateral upper limb reflexes may also be hyperreflexic, but generally do not affect upper limb activity. More than half of the patients will have proximal weakness of the lower limbs. Decreased vibratory sensation of the ankle tuning forks (which usually does not disappear) is detected on examination in about 50% of patients, and urinary dysfunction is present in about one third of patients.

The disease progresses with age, with about 20% of patients eventually losing the ability to walk. The severity of clinical symptoms varies considerably between patients carrying the same mutation in the same family.

### Qwen2.5_72b

Based on the clinical manifestations you have provided, the most likely diagnosis is **Hereditary Spastic Paraplegia (HSP)**. HSP is a group of inherited neurological disorders characterized by progressive weakness and spasticity of the lower limbs. The insidious onset in early youth, progressive spastic paralysis, pyramidal signs, and variable involvement of upper limb reflexes and urinary dysfunction are all consistent with HSP.

### Other Possible Diagnoses:

- **Multiple Sclerosis (MS)**: MS can present with progressive spasticity and weakness, particularly in the lower limbs. However, MS typically has a more variable course with relapses and remissions, and may involve other neurological symptoms such as visual disturbances and cognitive impairment.
- **Amyotrophic Lateral Sclerosis (ALS)**: ALS can present with progressive muscle weakness and spasticity, but it typically involves both upper and lower motor neurons and affects both upper and lower limbs more symmetrically. Additionally, ALS often progresses more rapidly and is associated with muscle atrophy and fasciculations.
- **Primary Lateral Sclerosis (PLS)**: PLS is a rare motor neuron disease that primarily affects the upper motor neurons, leading to spasticity and weakness. It can present similarly to HSP but typically involves more extensive upper motor neuron signs and may progress more rapidly.
- **Spinal Muscular Atrophy (SMA)**: SMA is a genetic disorder that affects the motor neurons in the spinal cord, leading to muscle weakness and atrophy. While it can present with lower limb weakness, it typically involves more severe muscle atrophy and is less likely to present with spasticity.
- **Cerebral Palsy (CP)**: CP is a group of disorders that affect movement and muscle tone or posture. It can present with spasticity and weakness, particularly in the lower limbs, but it is usually associated with a history of perinatal or early childhood brain injury and may involve other neurological deficits such as cognitive impairment and sensory issues.

### Conclusion:

Given the insidious onset, progressive spastic paralysis, and pyramidal signs, **Hereditary Spastic Paraplegia (HSP)** is the most likely diagnosis. However, the other conditions listed should be considered and ruled out through appropriate clinical and diagnostic evaluations.

### **55. User**

Most of the affected children develop the disease in newborns and early infants. Clinical manifestations are similar to biotinidase deficiency, without specificity, mainly feeding difficulties, vomiting, diarrhoea, hypotonia, convulsions, depression, lethargy, respiratory difficulties, developmental delays, intractable rashes, desquamation, which can be combined with ketosis, metabolic acidosis, hyperlactic acidemia and so on. Skin damage in most patients in the head and face, neck, trunk, buttocks and other parts of the skin rash or erythema, ulceration or blisters, furfuraceous or flaky scales, or dry and rough skin, peeling skin, etc., and a few patients only in the perioral, periocular, perianal local appearance of the rash. Patients are not associated with hearing or visual impairment, unlike biotinidase deficiency.

### Qwen2.5_72b

Based on the clinical manifestations you’ve provided, the most likely diagnosis is **Multiple Carboxylase Deficiency (MCD)**, also known as **Holocarboxylase Synthetase Deficiency**. This condition is a rare metabolic disorder that results from a deficiency in the enzyme holocarboxylase synthetase, which is essential for the activation of biotin-dependent carboxylases. The symptoms you described, including feeding difficulties, vomiting, diarrhea, hypotonia, convulsions, lethargy, respiratory difficulties, developmental delays, and skin manifestations, are characteristic of MCD.

### Differential Diagnoses:

- **Biotinidase Deficiency**: While the symptoms are similar, biotinidase deficiency is distinguished by the presence of hearing and visual impairments, which are not mentioned in your case.
- **Maple Syrup Urine Disease (MSUD)**: This is a metabolic disorder characterized by a sweet, maple syrup-like odor in the urine, along with vomiting, lethargy, and developmental delays. However, the skin manifestations are not typically seen in MSUD.
- **Methylmalonic Acidemia**: This is a disorder of vitamin B12 metabolism that can present with vomiting, lethargy, hypotonia, and metabolic acidosis. Skin manifestations are less common.
- **Propionic Acidemia**: Similar to methylmalonic acidemia, this condition can present with vomiting, lethargy, hypotonia, and metabolic acidosis. Skin manifestations are not a typical feature.
- **Zellweger Syndrome**: This is a peroxisomal disorder that can present with hypotonia, feeding difficulties, and developmental delays. However, the skin manifestations are not a prominent feature, and there are often additional neurological and liver abnormalities.

### Key Points for Diagnosis:

- **Multiple Carboxylase Deficiency (MCD)**: The most likely diagnosis given the clinical manifestations and the absence of hearing or visual impairment.
- **Biotinidase Deficiency**: A close differential, but ruled out by the lack of hearing and visual impairments.
- **Maple Syrup Urine Disease (MSUD)**: Considered due to the metabolic disturbances but less likely due to the absence of the characteristic urine odor and skin manifestations.
- **Methylmalonic Acidemia**: Considered due to the metabolic acidosis but less likely due to the absence of skin manifestations.
- **Propionic Acidemia**: Considered due to the metabolic disturbances but less likely due to the absence of skin manifestations.
- **Zellweger Syndrome**: Considered due to the hypotonia and developmental delays but less likely due to the absence of additional neurological and liver abnormalities.

### Next Steps:

- **Biotin Challenge Test**: Administering biotin and monitoring for improvement in symptoms can help confirm the diagnosis of MCD.
- **Genetic Testing**: Confirming the diagnosis through genetic testing for mutations in the *HLCS* gene (holocarboxylase synthetase gene).
- **Metabolic Screening**: Comprehensive metabolic screening to evaluate for other potential metabolic disorders.

Early diagnosis and treatment with biotin supplementation are crucial for improving outcomes in MCD.

### **56. User**

Children are born without abnormalities, and the clinical presentation in infancy is not specific and is characterised only by growth and developmental delays. It is often diagnosed at 3 years of age after the onset of ocular signs and symptoms.

- visual impairment, myopia and iris tremor due to ectopic lens may appear after 3 years of age, and may be combined with astigmatism, glaucoma, staphylococcus, cataract, retinal detachment and optic nerve atrophy in later stages. 2. intellectual disability is common.
- Intellectual disability is more common, mainly manifested as progressive mental retardation. Vitamin B6 treatment-sensitive patients are less mentally impaired. 3.
- mental or behavioural disorders, which may occur in about 50% of children and are associated with convulsions in 20%.
- Skeletal involvement, similar to the manifestations of Marfontein syndrome, or in combination with scoliosis, funnel chest, chicken chest, knee valgus, and bowed feet, with generalised osteoporosis seen on X-ray.
- Thrombophilia, which occurs at all ages and may involve all types of blood vessels, especially the cerebral vessels. High levels of homocysteinemia may alter the vessel wall and increase platelet adhesion, resulting in optic nerve atrophy, paralysis, pulmonary heart disease, and renal hypertension.
- Spontaneous pneumothorax and acute pancreatitis are less common.

### Qwen2.5_72b

Based on the clinical manifestations you have provided, the most likely rare disease is **Homocystinuria** (specifically, the classical form caused by cystathionine beta-synthase deficiency). Here are the key points that support this diagnosis:

- **Delayed Diagnosis**: Homocystinuria is often diagnosed in early childhood, around 3 years of age, after the onset of ocular signs and symptoms.
- **Ocular Signs**: Visual impairment, myopia, and iris tremor due to ectopic lens (ectopia lentis) are hallmark features of homocystinuria. These can be accompanied by astigmatism, glaucoma, cataracts, retinal detachment, and optic nerve atrophy.
- **Intellectual Disability**: Progressive mental retardation is common in homocystinuria, and some patients may show less severe intellectual impairment if treated with vitamin B6 (pyridoxine).
- **Mental or Behavioral Disorders**: About 50% of children with homocystinuria may have mental or behavioral disorders, and 20% may experience convulsions.
- **Skeletal Involvement**: Skeletal abnormalities similar to those seen in Marfan syndrome, such as scoliosis, funnel chest, chicken chest, knee valgus, and bowed feet, are common. X-rays may show generalized osteoporosis.
- **Thrombophilia**: High levels of homocysteine can lead to thrombophilia, affecting all types of blood vessels, including cerebral vessels. This can result in optic nerve atrophy, paralysis, pulmonary heart disease, and renal hypertension.
- **Other Rare Manifestations**: Spontaneous pneumothorax and acute pancreatitis are less common but can occur.

### Other Possible Diagnoses

- **Marfan Syndrome**: While it shares some skeletal and ocular features, Marfan syndrome typically does not present with intellectual disability or thrombophilia.
- **Ehlers-Danlos Syndrome (EDS)**: EDS can present with joint hypermobility, skin hyperextensibility, and vascular fragility, but it does not typically cause intellectual disability or ocular lens dislocation.
- **Zellweger Spectrum Disorders (Peroxisomal Biogenesis Disorders)**: These disorders can cause intellectual disability, skeletal abnormalities, and visual impairment, but they usually present with more severe and early-onset symptoms.
- **Mucopolysaccharidoses (MPS)**: MPS can cause skeletal abnormalities, intellectual disability, and visual impairment, but it typically presents with additional features such as coarse facial features, hepatosplenomegaly, and cardiac issues.
- **Neurofibromatosis Type 1 (NF1)**: NF1 can cause intellectual disability, skeletal abnormalities, and visual impairment, but it is characterized by café-au-lait spots, neurofibromas, and Lisch nodules in the iris.

Given the specific combination of ocular, intellectual, skeletal, and thrombotic features, **Homocystinuria** is the most likely diagnosis. However, further diagnostic testing, such as plasma homocysteine levels and genetic testing, would be necessary to confirm the diagnosis.

### **57. User**

The main manifestations of the disease are markedly elevated levels of LDL-C from birth, cholesterol deposits in the skin, eyes and tendons, and the formation of xanthomas and lipomatous corneal arches. As a result of exposure to high levels of LDL-C, most patients develop coronary atherosclerotic heart disease before the age of 20 and may die as a result.

A detailed family history is needed, as some patients have heterozygous parents and therefore have elevated LDL-C levels (usually greater than the 95th percentile of their country’s age- and sex-specific norms) and a strong positive family history of premature atherosclerotic cardiovascular disease.

### Qwen2.5_72b

Based on the clinical manifestations you’ve provided, the most likely diagnosis is **Familial Hypercholesterolemia (FH)**. This is a genetic disorder characterized by markedly elevated levels of low-density lipoprotein cholesterol (LDL-C) from birth, leading to the formation of xanthomas, corneal arcus, and premature atherosclerotic cardiovascular disease.

### Differential Diagnoses:

- **Polygenic Hypercholesterolemia**: This condition is not caused by a single gene mutation but rather by multiple genetic variants, each contributing a small effect. It can also present with elevated LDL-C levels and a family history of hypercholesterolemia, but the severity and early onset are typically less pronounced than in FH.
- **Sitosterolemia**: This is a rare autosomal recessive disorder characterized by the accumulation of plant sterols (such as sitosterol) in the body. Patients can present with xanthomas, corneal arcus, and premature atherosclerosis, but the cholesterol levels are not as high as in FH.
- **Lecithin-Cholesterol Acyltransferase (LCAT) Deficiency**: This is a rare genetic disorder that affects the metabolism of lipoproteins, leading to low levels of high-density lipoprotein (HDL) and high levels of unesterified cholesterol. Patients can have corneal opacities and renal disease, but the LDL-C levels are not typically as elevated as in FH.
- **Cholesteryl Ester Transfer Protein (CETP) Deficiency**: This condition is characterized by high levels of HDL and low levels of LDL. While it can present with xanthomas, the LDL-C levels are usually not as markedly elevated as in FH.
- **Homozygous Apolipoprotein E Deficiency**: This is a rare genetic disorder that can lead to severe hypercholesterolemia and premature atherosclerosis. However, it is less common than FH and often presents with more variable clinical features.

### Summary:

- **Primary Diagnosis**: Familial Hypercholesterolemia (FH)
- **Differential Diagnoses**:
  1. Polygenic Hypercholesterolemia
  2. Sitosterolemia
  3. Lecithin-Cholesterol Acyltransferase (LCAT) Deficiency
  4. Cholesteryl Ester Transfer Protein (CETP) Deficiency
  5. Homozygous Apolipoprotein E Deficiency

A detailed family history, genetic testing, and lipid profile analysis will be crucial in confirming the diagnosis and ruling out other differential diagnoses.

### **58. User**

It is characterised by a triad of movement disorders, psychiatric symptoms and cognitive deficits, usually with an insidious onset and slow progression. The age of onset ranges from childhood to 79 years, with the most common being 30-50 years of age.

The disease involves rapid, involuntary, non-rhythmic movements of the face, trunk, and limbs. Early abnormal movements are mild, and the patient may have difficulty recognising the presence of chorea or integrating chorea-like movements into random movements, which makes the control of normal movements difficult or deviant; as the disease progresses, the extent and degree of chorea often increases, and even affects the diaphragm, pharynx, and laryngeal muscles, resulting in dysarthria, dysphagia, and involuntary movement. As the disease progresses, the extent and degree of chorea often increases, even affecting the pharyngeal and laryngeal muscles, resulting in dysarthria, dysphagia, and involuntary vocalisations. In the later stages of the disease, the chorea often disappears, and is replaced by a Parkinson’s-like pattern of stiffness and hyperkinesis, which may be accompanied by focal dystonia. Difficulty in maintaining movement is also a common manifestation of the disease, i.e., the inability to maintain certain simple voluntary movements, such as difficulty in maintaining tongue extension.

Mental disorders may precede chorea, and common symptoms include depressed mood, depression, irritability, apathy, and anxiety, as well as paranoia, delusions, and hallucinations; rare symptoms include obsessive-compulsive behaviours and psychotic manifestations, which tend to worsen progressively.

Cognitive impairment is most often characterised by executive dysfunction, which is manifested by a reduced ability to make decisions, multi-task and shift cognitive goals. Patients usually lack self-awareness of their cognitive deficits. Memory loss or impairment occurs late in the course of the disease and progresses to dementia as the disease progresses.

In addition to the triad of symptoms, eye movement abnormalities are also prominent. Early in the disease, there is a slowing down of eye-sweeping speed, but eye-following movements are preserved; in later stages, eye-following movements, voluntary sweeping and refixation are all impaired. In addition, weight loss and cachexia are common features of the disease, with patients experiencing significant weight loss as a result of the high energy expenditure of involuntary movements, usually without loss of appetite. Sleep and/or sexual dysfunction are also common.

Progressive deterioration of motor and cognitive function can lead to significant complications, such as dysphagia due to involvement of the pharyngeal muscles, dyskinesia, reduced activity and even bed rest, which can lead to aspiration pneumonia and other infections, and decubitus ulcers.

### Qwen2.5_72b

Based on the clinical manifestations you have provided, the most likely diagnosis is **Huntington’s Disease (HD)**. Huntington’s Disease is a progressive neurodegenerative disorder characterized by a triad of movement disorders, psychiatric symptoms, and cognitive deficits. The disease is caused by a mutation in the HTT gene, which leads to the production of an abnormal huntingtin protein that gradually damages neurons, particularly in the basal ganglia and cerebral cortex.

### Differential Diagnoses:

- **Wilson’s Disease**:
  - **Clinical Features**: Hepatic dysfunction, Kayser-Fleischer rings, tremor, dystonia, psychiatric symptoms, and cognitive decline.
  - **Key Differentiation**: Wilson’s Disease is caused by a mutation in the ATP7B gene, leading to copper accumulation in the liver and brain. It can present with similar movement disorders and psychiatric symptoms but is often associated with liver disease and characteristic eye findings.
- **Sydenham’s Chorea**:
  - **Clinical Features**: Involuntary, rapid, jerky movements, particularly in the face, hands, and feet, often following a streptococcal infection. Psychiatric symptoms can include emotional lability and obsessive-compulsive behaviors.
  - **Key Differentiation**: Sydenham’s Chorea is typically seen in children and adolescents and is associated with a recent streptococcal infection. It is usually self-limiting and does not progress to dementia.
- **Neuroacanthocytosis**:
  - **Clinical Features**: Chorea, dystonia, parkinsonism, psychiatric symptoms, and cognitive decline. Additional features include acanthocytes on blood smear, muscle weakness, and peripheral neuropathy.
  - **Key Differentiation**: Neuroacanthocytosis is a group of rare disorders characterized by the presence of acanthocytes (spiked red blood cells) and can be associated with specific genetic mutations. The presence of acanthocytes on a blood smear is a key diagnostic feature.
- **Dentatorubral-Pallidoluysian Atrophy (DRPLA)**:
  - **Clinical Features**: Chorea, ataxia, myoclonus, epilepsy, and cognitive decline. Psychiatric symptoms can include depression, anxiety, and psychosis.
  - **Key Differentiation**: DRPLA is caused by a CAG repeat expansion in the ATN1 gene. It often presents with ataxia and myoclonus, which are less prominent in Huntington’s Disease.
- **Progressive Supranuclear Palsy (PSP)**:
  - **Clinical Features**: Supranuclear gaze palsy, parkinsonism, postural instability, and cognitive decline. Psychiatric symptoms can include depression, apathy, and irritability.
  - **Key Differentiation**: PSP is characterized by a supranuclear gaze palsy, which is a specific type of eye movement disorder where patients have difficulty moving their eyes vertically. It also typically presents with a more rapid progression and a higher risk of falls.

### Summary:

- **Most Likely Diagnosis**: Huntington’s Disease
- **Differential Diagnoses**:
  1. Wilson’s Disease
  2. Sydenham’s Chorea
  3. Neuroacanthocytosis
  4. Dentatorubral-Pallidoluysian Atrophy (DRPLA)
  5. Progressive Supranuclear Palsy (PSP)

Each of these conditions has distinct clinical features that can help differentiate them from Huntington’s Disease, but a thorough clinical evaluation, including genetic testing, is essential for a definitive diagnosis.

### **59. User**

Clinical signs and symptoms are mainly neurological and vary in severity. The age of onset can range from neonatal to adulthood (late-onset), and there is a broad phenotypic spectrum.

- In the neonatal period, symptoms associated with hyperammonemia [including lethargy, drowsiness, refusal to eat, vomiting with respiratory alkalosis and/or seizures] appear after about 24-48 hours of asymptomatic onset. It is indistinguishable from other urea cycle disorders of neonatal onset.
- Late-onset, which accounts for 88 per cent of patients, 40 per cent of whom occur before the age of 3 years, 29 per cent in childhood and 19 per cent in adulthood. The main manifestations are: (i) chronic neurocognitive deficits, including developmental delay, ataxia, spasticity, learning disabilities, cognitive deficits, and/or unexplained seizures; (ii) acute encephalopathy secondary to hyperammonaemic crisis induced by a variety of factors (e.g., infections, fasting, or injuries); and (iii) chronic hepatic dysfunction, with unexplained elevation of hepatic aminotransferases with or without mild coagulation disorders, with or without mild hyperammonaemia, and protein intolerance. Ammonemia and protein intolerance.

### Qwen2.5_72b

Based on the clinical manifestations and the age of onset, the most likely diagnosis is **Citrullinemia Type I (CTLN1)**, a urea cycle disorder caused by a deficiency in the enzyme argininosuccinate synthetase (ASS1). Here are the key points that support this diagnosis:

- **Neonatal Onset**: Symptoms of hyperammonemia, including lethargy, drowsiness, refusal to eat, vomiting, respiratory alkalosis, and seizures, are characteristic of urea cycle disorders, including Citrullinemia Type I.
- **Late-Onset**: The majority of patients (88%) present with late-onset symptoms, which can include chronic neurocognitive deficits, developmental delay, ataxia, spasticity, learning disabilities, cognitive deficits, and unexplained seizures. Acute encephalopathy secondary to hyperammonemic crises and chronic hepatic dysfunction with elevated hepatic aminotransferases and protein intolerance are also common.

### Other Possible Diagnoses

- **Argininosuccinic Aciduria (ASA)**: Another urea cycle disorder caused by a deficiency in the enzyme argininosuccinate lyase (ASL). Symptoms are similar to those of Citrullinemia Type I, including hyperammonemia, neurological symptoms, and hepatic dysfunction.
- **Carbamoyl Phosphate Synthetase I Deficiency (CPS1)**: A urea cycle disorder characterized by hyperammonemia, neurological symptoms, and hepatic dysfunction. Symptoms can range from neonatal to late-onset.
- **Ornithine Transcarbamylase Deficiency (OTC)**: The most common urea cycle disorder, particularly in males. Symptoms include hyperammonemia, neurological symptoms, and hepatic dysfunction, with a broad phenotypic spectrum.
- **Glutaric Aciduria Type I (GA1)**: A metabolic disorder caused by a deficiency in the enzyme glutaryl-CoA dehydrogenase. Symptoms can include developmental delay, ataxia, and acute encephalopathy, often triggered by metabolic stress.
- **Wilson’s Disease**: A genetic disorder of copper metabolism that can present with neurological symptoms, including tremors, ataxia, and cognitive deficits, as well as hepatic dysfunction and elevated aminotransferases.

### Diagnostic Approach

To confirm the diagnosis, the following tests should be considered:

- **Plasma Ammonia Levels**: Elevated ammonia levels are a key indicator of urea cycle disorders.
- **Plasma Amino Acid Profile**: Abnormal levels of citrulline and other amino acids can help differentiate between different urea cycle disorders.
- **Urine Organic Acids**: Can help identify specific metabolic disorders, such as Glutaric Aciduria Type I.
- **Genetic Testing**: Sequencing of the ASS1 gene for Citrullinemia Type I or other relevant genes for other urea cycle disorders.
- **Liver Function Tests**: To assess hepatic dysfunction and elevated aminotransferases.

These tests will help narrow down the diagnosis and guide appropriate management and treatment.

### **60. User**

Some children have no clinical symptoms in the neonatal period, but after 3-4 months of life, the typical symptoms appear gradually: the hair turns from black to yellow, the skin is light-coloured, and the urine and sweat are foul-smelling. As the child grows older, he/she gradually shows backward intellectual development, microcephaly, epileptic seizures, and may also show abnormalities in behaviour, personality, and neurocognition. For example, hyperactivity, self-mutilation, aggression, autism, low self-esteem, depression, etc. Vomiting and eczema are also common in infancy.

In some patients, there are no clinical symptoms in the neonatal period, and after 1-3 months of life, in addition to the symptoms of PKU, the main manifestations are dyskinesia, lethargy, hypotonia of the trunk, increased or decreased muscle tone of the limbs, nystagmus, dysphagia, increased drooling, flaccidity, torticollis, unresponsiveness, insomnia, and severe intellectual developmental disorders, and so on.

### Qwen2.5_72b

Based on the clinical manifestations you’ve described, the most likely diagnosis is **Phenylketonuria (PKU)**. PKU is a rare inherited metabolic disorder characterized by the body’s inability to break down the amino acid phenylalanine, leading to its accumulation in the blood and brain, which can cause severe intellectual disability and other neurological issues if left untreated.

### Differential Diagnoses:

- **Homocystinuria**: This is another inherited metabolic disorder that can present with intellectual disability, seizures, and developmental delay. However, it also typically includes features such as tall stature, long limbs, and a high arched palate.
- **Maple Syrup Urine Disease (MSUD)**: This is a rare metabolic disorder characterized by a sweet, maple syrup-like odor in the urine. It can present with vomiting, lethargy, and developmental delay, but the odor is a distinctive feature.
- **Tyrosinemia Type I**: This is a rare genetic disorder that can cause liver and kidney damage, as well as neurological symptoms. It can present with vomiting, diarrhea, and developmental delay, but liver and kidney issues are more prominent.
- **Galactosemia**: This is a rare genetic disorder that affects the body’s ability to process the sugar galactose. It can present with jaundice, vomiting, and liver problems in infancy, and if untreated, can lead to intellectual disability and other neurological issues.
- **Zellweger Syndrome**: This is a rare genetic disorder that affects the development of peroxisomes, leading to a wide range of symptoms including developmental delay, seizures, and liver and kidney problems. It can present with hypotonia, nystagmus, and other neurological symptoms.

### Key Features of PKU:

- **Hair and Skin Changes**: Hair turning from black to yellow and light-colored skin.
- **Foul-Smelling Urine and Sweat**: Due to the accumulation of phenylalanine.
- **Intellectual Disability**: Gradual backward intellectual development and microcephaly.
- **Neurological Symptoms**: Epileptic seizures, hyperactivity, self-mutilation, aggression, autism, low self-esteem, and depression.
- **Gastrointestinal Symptoms**: Vomiting and eczema in infancy.
- **Motor Symptoms**: In some cases, dyskinesia, lethargy, hypotonia, nystagmus, dysphagia, increased drooling, flaccidity, torticollis, unresponsiveness, insomnia, and severe intellectual developmental disorders.

### Management:

- **Dietary Restriction**: A low-phenylalanine diet is the primary treatment for PKU. This involves avoiding foods high in phenylalanine, such as meat, fish, eggs, and dairy products.
- **Regular Monitoring**: Blood phenylalanine levels need to be monitored regularly to ensure they remain within a safe range.
- **Supplements**: Special formulas and supplements may be necessary to ensure adequate nutrition.

If PKU is suspected, early diagnosis and treatment are crucial to prevent severe intellectual disability and other complications.

### **61. User**

The severity of clinical manifestations is highly variable. Patients may present with abnormalities in bone mineralisation, dental or joint pathology, muscle weakness, etc. Currently, there are seven main types of the disease.

- simple dental type, which is the mildest and most common type, with only dental involvement and no rickets or osteomalacia. 2. adult type, which often develops in middle age.
- Adult type, which often develops in middle age and presents with dental abnormalities or osteochondrosis. It may cause impaired mobility due to repeated fractures, bone and joint pain, or muscle weakness. 3.
- the childhood type, which usually develops after 6 months of age, can be mild to severe, and the children may have different degrees of tooth loss and rickets.
- the infantile form, with onset before 6 months of age, is often poorly ventilated, difficult to survive, debilitating, and may have a wide fontanelle or cranial deformity. 50 per cent of infantile HPP die of complications. 5. the perinatal form, with onset before 6 months of age, is the most severe form of HPP, with onset before 6 months of age.
- The perinatal form, which is the extreme form, develops in utero, is evident at birth, and is almost always fatal. Children with short limbs and severe skeletal undermineralisation may present with high-pitched crying, seizures, periodic apnoea with cyanosis and bradycardia, unexplained fever, irritability, myelosuppressive anaemia and intracranial haemorrhage, and pulmonary atelectasis.
- Pseudohypophosphatasia, similar to the infantile form, in which the patient has the manifestations of infantile hypophosphatasia with normal or elevated serum ALP. 7.
- Benign type, sometimes presenting with skeletal malformations in utero, with perinatal hypophosphatase activity, but resolving after birth without the associated manifestations of infantile hypophosphatasia or simple dental hypophosphatasia.

### Qwen2.5_72b

Based on the clinical manifestations and the types of disease you have described, the most likely diagnosis is **Hypophosphatasia (HPP)**. HPP is a rare genetic disorder characterized by defective bone mineralization, which can lead to a wide range of clinical manifestations, including dental abnormalities, bone and joint pathology, muscle weakness, and more severe complications depending on the type and severity of the disease.

### Most Likely Diagnosis:

- **Hypophosphatasia (HPP)**

### Other Possible Diagnoses:

- **Osteogenesis Imperfecta (OI)**: This is a genetic disorder characterized by brittle bones that fracture easily. It can present with dental abnormalities, bone and joint pain, and muscle weakness, similar to some forms of HPP.
- **Rickets**: A condition caused by a deficiency in vitamin D, calcium, or phosphorus, leading to soft and weak bones. It can present with bone deformities, dental issues, and muscle weakness.
- **Osteomalacia**: A condition characterized by softening of the bones due to a deficiency in vitamin D, calcium, or phosphorus. It can cause bone pain, muscle weakness, and fractures.
- **Paget’s Disease of Bone**: A chronic disorder that can result in enlarged and misshapen bones. It can cause bone pain, deformities, and increased risk of fractures.
- **Chondrodysplasia Punctata**: A group of rare genetic disorders characterized by abnormal bone development, often with calcifications in the cartilage. It can present with skeletal abnormalities, joint issues, and sometimes dental problems.

Each of these conditions can present with overlapping symptoms, making a detailed clinical evaluation, including imaging studies, laboratory tests, and genetic testing, essential for a definitive diagnosis.

### **62. User**

Children with square skulls, chicken breasts, beaded ribs, bracelet sign, foot bracelet sign, Henry’s groove, and lower limb deformities, which may be inversion of the knee (O-shaped leg) or eversion of the knee (X-shaped leg), appearing when they start weight-bearing near the age of one week, are accompanied by growth retardation, short stature, wobbly gait, progressively aggravating bone deformities, multiple bone fractures, bone pain, and abnormalities of tooth development (poor tooth quality, toothache, difficulty in regeneration after loss). Imaging is characterised by skeletal deformities, such as Imaging manifestations include skeletal deformities, widening and blurring of the epiphysis of the long bones, a cupped shape, and many fine strips of calcification like brushes in the cupped shape. In adults, the main manifestations are limb weakness, activity limitation, bone pain, multiple pathological fractures (long bones of the limbs, ribs, pelvis and vertebrae can occur), and shortening of height. Imaging manifestations include a generalised decrease in bone density, blurred bone trabeculae with a hairy glass appearance, pelvic deformity, false fracture lines, and biconcave deformity of the vertebral body.

### Qwen2.5_72b

Based on the clinical manifestations and imaging findings you have provided, the most likely diagnosis is **Rickets** in children and **Osteomalacia** in adults. These conditions are characterized by impaired mineralization of the bone matrix, leading to the described skeletal deformities and bone pain.

### Most Likely Diagnosis:

- **Rickets (in children) / Osteomalacia (in adults)**

### Other Possible Diagnoses:

1. **Hypophosphatasia**: This is a rare genetic disorder characterized by low levels of the enzyme alkaline phosphatase, leading to poor mineralization of bones and teeth. Clinical features include skeletal deformities, bone pain, and increased risk of fractures.
2. **Vitamin D-Dependent Rickets Type I (VDDR I)**: This is a rare genetic disorder characterized by a deficiency in the renal 1α-hydroxylase enzyme, which is essential for converting 25-hydroxyvitamin D to the active form, 1,25-dihydroxyvitamin D. Symptoms are similar to those of rickets.
3. **X-Linked Hypophosphatemia (XLH)**: This is a genetic disorder characterized by low levels of phosphate in the blood, leading to rickets in children and osteomalacia in adults. Clinical features include skeletal deformities, bone pain, and dental abnormalities.
4. **Scurvy**: This is a nutritional deficiency caused by a lack of vitamin C. It can lead to bone pain, joint swelling, and skeletal deformities, particularly in children. However, it is less likely given the specific imaging findings.

### Summary:

- **Primary Diagnosis**: Rickets (in children) / Osteomalacia (in adults)
- **Other Possible Diagnoses**:
  1. Hypophosphatasia
  2. Vitamin D-Dependent Rickets Type I (VDDR I)
  3. X-Linked Hypophosphatemia (XLH)
  4. Scurvy

These conditions should be considered and further evaluated through specific laboratory tests, such as serum calcium, phosphate, alkaline phosphatase, and vitamin D levels, as well as genetic testing if necessary.

### **63. User**

Based on the clinical manifestations you’ve described, the rare disease most likely to be diagnosed is **Cardiac Amyloidosis**. This condition is characterized by the deposition of amyloid proteins in the heart tissue, leading to heart failure, arrhythmias, and other cardiac symptoms. The extracardiac manifestations like peripheral neuropathy and autonomic neuropathy further support this diagnosis.

Here are five other possible diagnoses:

- **Hypertrophic Cardiomyopathy (HCM):** This genetic condition can cause heart failure, arrhythmias, syncope, and sudden cardiac death. While primarily cardiac, it can have systemic implications.
- **Arrhythmogenic Right Ventricular Cardiomyopathy (ARVC):** This condition causes arrhythmias and can lead to heart failure and sudden cardiac death. Although mainly cardiac, it can have some systemic effects.
- **Fabry Disease:** This is a lysosomal storage disorder that can cause cardiac involvement, including hypertrophic cardiomyopathy, arrhythmias, and conduction blocks. It often presents with peripheral neuropathy and gastrointestinal symptoms.
- **Sarcoidosis:** Cardiac involvement in sarcoidosis can cause heart failure, arrhythmias, and sudden cardiac death. It also frequently leads to extracardiac symptoms, including neuropathy and other systemic manifestations.
- **Chagas Disease:** A parasitic disease that can lead to chronic heart issues like heart failure and arrhythmias. It can also cause peripheral and autonomic neuropathy and gastrointestinal symptoms.

Each of these conditions has distinct features and requires specific diagnostic tests for confirmation. A thorough clinical evaluation, including imaging and possibly a biopsy, would be necessary for accurate diagnosis.

### Qwen2.5_72b

Thank you for the detailed clinical manifestations. Based on the information provided, **Cardiac Amyloidosis** is indeed a strong candidate for the diagnosis. Here are the five other possible diagnoses, along with a brief explanation of each:

- **Hypertrophic Cardiomyopathy (HCM):**
  - **Description:** A genetic disorder characterized by thickening of the heart muscle, particularly the left ventricle, which can lead to heart failure, arrhythmias, and sudden cardiac death.
  - **Clinical Features:** Dyspnea, chest pain, syncope, and palpitations. Extracardiac manifestations are less common but can include peripheral neuropathy in some cases.
- **Fabry Disease:**
  - **Description:** A rare X-linked lysosomal storage disorder caused by a deficiency of the enzyme alpha-galactosidase A, leading to the accumulation of glycosphingolipids in various tissues.
  - **Clinical Features:** Cardiac involvement (hypertrophic cardiomyopathy, arrhythmias), peripheral neuropathy, gastrointestinal symptoms, and characteristic skin lesions (angiokeratomas).
- **Sarcoidosis:**
  - **Description:** A systemic inflammatory disorder characterized by the formation of non-caseating granulomas in multiple organs, including the heart.
  - **Clinical Features:** Cardiac involvement (heart failure, arrhythmias, conduction blocks), extracardiac manifestations (pulmonary, skin, eye, and nervous system involvement).
- **Chagas Disease:**
  - **Description:** A parasitic infection caused by Trypanosoma cruzi, transmitted by triatomine bugs. Chronic infection can lead to cardiac, digestive, and neurological complications.
  - **Clinical Features:** Cardiac involvement (heart failure, arrhythmias, conduction blocks), peripheral neuropathy, gastrointestinal symptoms (megaesophagus, megacolon).
- **Multiple Myeloma:**
  - **Description:** A cancer of plasma cells that can produce monoclonal proteins (M proteins) which can deposit in various tissues, including the heart, leading to amyloidosis.
  - **Clinical Features:** Bone pain, anemia, renal failure, hypercalcemia, and cardiac involvement (heart failure, arrhythmias). Extracardiac manifestations can include neuropathy and other systemic symptoms.

### Diagnostic Approach:

- **Cardiac Amyloidosis:**
  - **Imaging:** Echocardiography, cardiac MRI, and nuclear imaging (e.g., technetium-99m pyrophosphate scan).
  - **Biopsy:** Endomyocardial biopsy for definitive diagnosis.
  - **Blood Tests:** Serum and urine protein electrophoresis, free light chain assay.
- **Hypertrophic Cardiomyopathy:**
  - **Imaging:** Echocardiography, cardiac MRI.
  - **Genetic Testing:** For familial HCM.
- **Fabry Disease:**
  - **Enzyme Assay:** Alpha-galactosidase A activity.
  - **Genetic Testing:** Mutation analysis of the GLA gene.
- **Sarcoidosis:**
  - **Imaging:** Chest X-ray, CT scan, PET scan.
  - **Biopsy:** Tissue biopsy from affected organs (e.g., skin, lung, lymph nodes).
- **Chagas Disease:**
  - **Serology:** Anti-T. cruzi antibodies.
  - **PCR:** For detecting parasite DNA in blood.
  - **Imaging:** Echocardiography for cardiac involvement.
- **Multiple Myeloma:**
  - **Blood Tests:** Complete blood count, serum protein electrophoresis, free light chain assay.
  - **Imaging:** Bone marrow biopsy, skeletal survey.

Each of these conditions has distinct diagnostic criteria and treatment approaches, so a comprehensive evaluation is essential for accurate diagnosis and management.

### **64. User**

- secondary sexual characteristics and impaired gametogenesis in males: childish voice, small penis, no pubic hair growth, small testes or cryptorchid testes, no spermatogenesis; in females: mammary gland dysplasia, infantile vulva, primary amenorrhoea.
- delayed epiphyseal closure upper volume/lower volume <1, fingertip distance > height, susceptible to osteoporosis.
- Olfactory disorders 40% ~60% of IHH patients suffer from loss of sense of smell due to abnormal development of the olfactory bulb and olfactory tract, and are unable to identify odours.
- Other manifestations Facial midline defects, such as cleft lip and palate; isolated kidneys; short fingers (toes), syndactyly; skeletal deformities or dental hypoplasia; overweight and obesity; mirror image movement.

### Qwen2.5_72b

Based on the clinical manifestations you have provided, the most likely diagnosis is **Kallmann Syndrome**. Kallmann Syndrome is a form of hypogonadotropic hypogonadism (IHH) characterized by the failure of the hypothalamus to release gonadotropin-releasing hormone (GnRH), leading to impaired development of secondary sexual characteristics and gametogenesis. It is often associated with anosmia or hyposmia due to the abnormal development of the olfactory bulb and tract.

### Other Possible Diagnoses:

- **Isolated Hypogonadotropic Hypogonadism (IHH) without Kallmann Syndrome**: This condition is similar to Kallmann Syndrome but does not include the olfactory disorders. It is characterized by delayed or absent puberty and impaired gametogenesis.
- **Prader-Willi Syndrome**: This is a genetic disorder characterized by severe hypotonia and feeding difficulties in infancy, followed by hyperphagia and obesity in childhood. It can also include hypogonadism, short stature, and cognitive impairment.
- **Turner Syndrome**: This is a chromosomal disorder affecting females, characterized by the partial or complete absence of one of the X chromosomes. It can lead to short stature, primary amenorrhea, and other physical features such as webbed neck and low-set ears.
- **Androgen Insensitivity Syndrome (AIS)**: This is a condition where the body is unable to respond to androgens (male hormones), leading to underdeveloped male genitalia and secondary sexual characteristics. It can result in a range of phenotypes from complete to partial androgen insensitivity.
- **Congenital Adrenal Hyperplasia (CAH)**: This is a group of genetic disorders affecting the adrenal glands, leading to the overproduction or underproduction of certain hormones. It can cause ambiguous genitalia, delayed puberty, and other endocrine issues.

### Summary:

- **Most Likely Diagnosis**: Kallmann Syndrome
- **Other Possible Diagnoses**:
  1. Isolated Hypogonadotropic Hypogonadism (IHH) without Kallmann Syndrome
  2. Prader-Willi Syndrome
  3. Turner Syndrome
  4. Androgen Insensitivity Syndrome (AIS)
  5. Congenital Adrenal Hyperplasia (CAH)

Each of these conditions has overlapping symptoms, but the combination of hypogonadism, olfactory disorders, and specific physical features strongly suggests Kallmann Syndrome. Further genetic testing and hormonal evaluations would be necessary to confirm the diagnosis.

### **65. User**

The most common symptom is shortness of breath after activity, and other symptoms include fatigue, dizziness, chest pain, chest tightness, palpitations, blackouts, and syncope, etc. Once syncope or vertigo occurs, it suggests that the patient’s cardiac output may have decreased significantly. Due to atypical symptoms and signs, the majority of patients have a significant delay in consultation, with at least 1/5 of patients taking more than 2 years from the onset of symptoms to diagnosis; patients may have a hyperactive second heart sound of the pulmonary valve, and tricuspid insufficiency may cause a systolic murmur in the tricuspid region; signs of right heart failure include jugular venous congestion or rupture, enlarged liver, oedema of the lower extremities, multiple plasma cavities, jaundice, and cyanosis, among other signs.

### Qwen2.5_72b

Based on the clinical manifestations you’ve provided, the most likely rare disease is **Pulmonary Arterial Hypertension (PAH)**. PAH is a progressive disorder characterized by elevated blood pressure in the pulmonary arteries, which can lead to right heart failure and is often associated with the symptoms you’ve described.

### Differential Diagnoses:

- **Chronic Thromboembolic Pulmonary Hypertension (CTEPH)**: This condition results from chronic blood clots in the pulmonary arteries, leading to similar symptoms as PAH. However, CTEPH can sometimes be treated with surgical thromboendarterectomy.
- **Left Heart Failure**: Conditions such as mitral valve disease or dilated cardiomyopathy can cause symptoms similar to PAH, including shortness of breath, fatigue, and right heart failure.
- **Eisenmenger Syndrome**: This is a condition where a congenital heart defect leads to pulmonary hypertension and right-to-left shunting of blood, resulting in cyanosis and other symptoms.
- **Sarcoidosis**: This is a systemic inflammatory disorder that can affect the lungs and heart, leading to pulmonary hypertension and right heart failure.
- **Connective Tissue Disease-Associated Pulmonary Hypertension**: Conditions such as systemic sclerosis, lupus, and rheumatoid arthritis can cause pulmonary hypertension as a complication, leading to similar symptoms.

### Key Points for PAH:

- **Shortness of Breath**: Especially on exertion, due to increased pulmonary vascular resistance.
- **Fatigue and Dizziness**: Common due to reduced cardiac output.
- **Chest Pain and Tightness**: May be due to ischemia in the right ventricle.
- **Palpitations and Syncope**: Indicative of significant hemodynamic compromise.
- **Right Heart Failure Signs**: Jugular venous congestion, hepatomegaly, edema, and cyanosis.

### Diagnostic Workup:

- **Echocardiogram**: To assess right ventricular function and estimate pulmonary artery pressure.
- **Right Heart Catheterization**: Gold standard for confirming PAH and measuring pulmonary artery pressure.
- **Pulmonary Function Tests**: To rule out other lung diseases.
- **CT Pulmonary Angiography**: To rule out CTEPH.
- **Blood Tests**: To check for markers of inflammation, connective tissue diseases, and other systemic conditions.

Early diagnosis and treatment are crucial in PAH to improve outcomes and quality of life.

### **66. User**

The disease is usually insidious. The main manifestations are dry cough, exertional dyspnoea, pestle-like fingers (toes), and bursting sounds distributed mainly in both lower lungs are its typical signs. In the terminal stage, cyanosis, pulmonary hypertension, pulmonary heart disease and right heart insufficiency can be seen.

### Qwen2.5_72b

Based on the clinical manifestations you’ve provided, the most likely rare disease is **Idiopathic Pulmonary Fibrosis (IPF)**. IPF is a chronic, progressive fibrosing interstitial pneumonia of unknown cause, primarily affecting the lungs. The key features you’ve described, such as dry cough, exertional dyspnea, clubbing of the fingers (and toes), and bibasilar crackles (bursting sounds), are characteristic of IPF. In the terminal stages, cyanosis, pulmonary hypertension, cor pulmonale (pulmonary heart disease), and right heart failure are common complications.

### Other Possible Diagnoses:

- **Sarcoidosis**: A systemic inflammatory disease that can affect multiple organs, but most commonly the lungs. It can present with similar symptoms, including dry cough, dyspnea, and bibasilar crackles. However, sarcoidosis often has additional features such as skin lesions, eye involvement, and lymphadenopathy.
- **Hypersensitivity Pneumonitis (HP)**: A form of interstitial lung disease caused by inhalation of organic dusts or chemicals. It can present with chronic cough, dyspnea, and bibasilar crackles. Clubbing is less common in HP compared to IPF.
- **Connective Tissue Disease-Associated Interstitial Lung Disease (CTD-ILD)**: Conditions such as systemic sclerosis, rheumatoid arthritis, and systemic lupus erythematosus can cause interstitial lung disease. These conditions can present with similar respiratory symptoms and may also have extrapulmonary manifestations.
- **Pulmonary Langerhans Cell Histiocytosis (PLCH)**: A rare condition characterized by the proliferation of Langerhans cells in the lungs. It can present with dry cough, dyspnea, and clubbing. However, PLCH often has a more variable clinical course and may be associated with smoking.
- **Lymphangioleiomyomatosis (LAM)**: A rare lung disease that primarily affects women of childbearing age. It is characterized by the proliferation of smooth muscle cells in the lungs, leading to cystic lung destruction. Symptoms include dyspnea, cough, and recurrent pneumothoraces. Clubbing is less common in LAM compared to IPF.

Each of these conditions has overlapping symptoms with IPF, but the clinical history, physical examination, and additional diagnostic tests (such as high-resolution CT scans, pulmonary function tests, and sometimes lung biopsy) are crucial for making an accurate diagnosis.

### **67. User**

It is a multi-organ and multi-system disease that can involve almost all organs and tissues of the body. Clinical manifestations are varied, and it is easy to miss the diagnosis when patients consult different specialties for different organ involvement. Most of the patients have a slow onset of the disease, with a chronic progression. The disease may start in a single organ and gradually involve multiple organs, or it may develop in multiple organs at the same time. In a minority of patients, the disease is self-limiting.

Lacrimal and salivary glands are the most commonly affected organs. The main manifestations are symmetrical, painless enlargement of the lacrimal gland, submandibular gland or parotid gland, hard nodules, with or without sinusitis or thickening of extraocular muscles. Involvement of the digestive system may manifest as autoimmune pancreatitis, sclerosing cholangitis, sclerosing mesenteritis, etc. Clinical manifestations may include vague abdominal pain, abdominal distension, dyspepsia, and jaundice. Lung involvement may be manifested as bronchial wall thickening, interstitial lung lesions, lung nodules/lumps and pleural thickening, etc. Patients may be asymptomatic or have cough, shortness of breath and wheezing. Retroperitoneal fibrosis or peri-abdominal aortitis is also one of the more common clinical manifestations, which is mainly a thickening of the soft tissue around the abdominal aorta, which can encircle the ureter and cause ureteral stenosis and pyelonephrosis, and in a small number of patients, abdominal aortic aneurysm occurs. Clinical manifestations include abdominal pain, low back pain, or oedema of the lower extremities. Less than 10% of patients develop IgG4-associated renal disease, most often manifesting as tubulointerstitial nephropathy, renal pelvic space-occupying lesions, ureteral wall thickening, or renal mass-like lesions. Other organs involved include the thyroid (sclerosing thyroiditis) and prostate. CNS lesions are rare, such as autoimmune pituitary inflammation, hypertrophic dural meningitis, and intracranial inflammatory pseudotumours, and patients may present with headaches, urolithiasis, and hypopituitarism. In addition to the above organ involvement, most patients combine with superficial or deep lymph node enlargement. In addition, about half of the patients have a history of allergy-related disease, which can be accompanied by asthma.

### Qwen2.5_72b

Based on the clinical manifestations you have provided, the most likely diagnosis is **IgG4-related disease (IgG4-RD)**. This is a systemic fibroinflammatory condition characterized by the infiltration of IgG4-positive plasma cells and fibrosis in multiple organs, leading to a wide range of clinical manifestations.

### Differential Diagnoses:

- **Sarcoidosis**: A systemic granulomatous disease that can affect multiple organs, including the lungs, lymph nodes, skin, and eyes. It often presents with non-caseating granulomas and can have similar multi-organ involvement.
- **Systemic Lupus Erythematosus (SLE)**: A chronic autoimmune disease that can affect multiple organs, including the skin, joints, kidneys, and central nervous system. It often presents with a variety of symptoms, including fatigue, joint pain, and rash.
- **Sjögren’s Syndrome**: An autoimmune disease primarily affecting the exocrine glands, leading to dry eyes and dry mouth. It can also involve other organs, including the lungs, kidneys, and liver.
- **Chronic Graft-versus-Host Disease (cGVHD)**: A complication of allogeneic hematopoietic stem cell transplantation, characterized by chronic inflammation and fibrosis in multiple organs, including the skin, eyes, and mouth.
- **Amyloidosis**: A group of diseases characterized by the deposition of amyloid proteins in various organs, leading to organ dysfunction. It can affect the heart, kidneys, liver, and other organs, and can present with a wide range of symptoms.

### Key Features of IgG4-RD:

- **Multi-organ involvement**: Commonly affects the lacrimal and salivary glands, pancreas, biliary tree, lungs, retroperitoneum, and kidneys.
- **Symmetrical, painless enlargement of lacrimal and salivary glands**.
- **Autoimmune pancreatitis**: A characteristic feature, often presenting with jaundice and abdominal pain.
- **Lung involvement**: Bronchial wall thickening, interstitial lung disease, and lung nodules.
- **Retroperitoneal fibrosis**: Thickening of the soft tissue around the abdominal aorta, potentially leading to ureteral stenosis and pyelonephrosis.
- **Renal involvement**: Tubulointerstitial nephritis, renal pelvic space-occupying lesions, and ureteral wall thickening.
- **Lymphadenopathy**: Enlargement of superficial or deep lymph nodes.
- **Allergic history**: Many patients have a history of allergic diseases, including asthma.

### Diagnostic Criteria:

- **Clinical manifestations** consistent with IgG4-RD.
- **Elevated serum IgG4 levels**.
- **Histopathological findings**: Lymphoplasmacytic infiltration with numerous IgG4-positive plasma cells and storiform fibrosis.
- **Response to corticosteroid therapy**.

If you suspect IgG4-RD, further diagnostic workup, including serological tests, imaging studies, and biopsy, should be performed to confirm the diagnosis and rule out other differential diagnoses.

### **68. User**

Children with cholestasis and cirrhosis develop. The main clinical manifestations are hyperconjugated bilirubinaemia and often malabsorption of fat-soluble vitamins, such as rickets. The clinical phenotypes of different enzyme defects are slightly different. For example, oxysterol 7α-hydroxylase defects are associated with hereditary spastic paralysis in adulthood; cholesterol 25-hydroxylase defects are associated with intractable constipation; 3β-hydroxysteroid -Δ5-C27- steroid dehydrogenase defects are associated with cholestatic liver disease and fat-soluble vitamin deficiencies in infancy; and sterol 27-hydroxylase defects are associated with chronic diarrhoea and developmental delays in children, and spasticity in adulthood. Sterol 27-hydroxylase defects can cause chronic diarrhoea and developmental delay in children, and spastic paralysis in adults; α-formyl-coenzyme A dehydrogenase defects can cause fat-soluble vitamin deficiencies in infancy, and sensory neuropathy in adulthood; and bile acid-coenzyme A-amino acid N-acetyltransferase defects can cause intrahepatic cholestasis, developmental delay, and familial hypercholesterolemia.

### Qwen2.5_72b

Based on the clinical manifestations you’ve provided, the most likely diagnosis is a **bile acid synthesis defect**, specifically a **sterol 27-hydroxylase deficiency**. This condition is associated with cholestasis, cirrhosis, malabsorption of fat-soluble vitamins, and developmental delays in children, which aligns well with the symptoms described.

### Differential Diagnoses:

- **Oxysterol 7α-hydroxylase deficiency**: This condition can also cause cholestasis and cirrhosis, but it is more commonly associated with hereditary spastic paralysis in adulthood.
- **Cholesterol 25-hydroxylase deficiency**: This defect is associated with intractable constipation and may also present with cholestasis and cirrhosis.
- **3β-hydroxysteroid-Δ5-C27-steroid dehydrogenase deficiency**: This condition is characterized by cholestatic liver disease and fat-soluble vitamin deficiencies in infancy, which overlaps with the symptoms described.
- **α-formyl-coenzyme A dehydrogenase deficiency**: This can cause fat-soluble vitamin deficiencies in infancy and sensory neuropathy in adulthood, but it is less commonly associated with the severe liver disease described.
- **Bile acid-coenzyme A-amino acid N-acetyltransferase deficiency**: This condition can cause intrahepatic cholestasis, developmental delay, and familial hypercholesterolemia, but it is less specific to the combination of symptoms you’ve described.

### Summary:

- **Most Likely Diagnosis**: Sterol 27-hydroxylase deficiency
- **Differential Diagnoses**:
  1. Oxysterol 7α-hydroxylase deficiency
  2. Cholesterol 25-hydroxylase deficiency
  3. 3β-hydroxysteroid-Δ5-C27-steroid dehydrogenase deficiency
  4. α-formyl-coenzyme A dehydrogenase deficiency
  5. Bile acid-coenzyme A-amino acid N-acetyltransferase deficiency

These conditions should be considered and further evaluated through specific biochemical and genetic testing to confirm the diagnosis.

### **69. User**

**1. Acute neonatal type** The onset of the disease is acute and the progression of the disease is rapid and can be life-threatening. Within 1-2 weeks of birth, it presents with feeding difficulties, vomiting, muscle weakness, hypotonia, and lethargy or worsening progression to coma. Accumulation of isovaleric acid may cause the characteristic odour of ‘sweaty feet’. Liver enlargement may be present on examination. Laboratory tests suggest metabolic acidosis, hyperammonemia, hypoglycemia or hyperglycemia, ketosis, hypocalcemia, and pancytopenia. If the patient survives the acute exacerbation in the neonatal period, he or she will progress to the chronic intermittent form.

**The chronic intermittent form** has an insidious onset. It presents only as a nonspecific inability to tolerate fasting or developmental delay. It is often triggered by upper respiratory tract infections or the intake of a high-protein diet, and is characterised by recurrent episodes of vomiting, lethargy, coma, acidosis with ketonuria, and ‘sweaty feet odour’, which can be relieved by a protein-restricted diet and glucose infusion. The vast majority of patients with chronic intermittent form have normal psychomotor development, but some have developmental delays and varying degrees of mental retardation.

### Qwen2.5_72b

Based on the clinical manifestations and laboratory findings you’ve provided, the most likely diagnosis is **Isovaleric Acidemia (IVA)**, a rare inherited metabolic disorder characterized by a deficiency in the enzyme isovaleryl-CoA dehydrogenase, which leads to the accumulation of isovaleric acid and its metabolites.

### Most Likely Diagnosis:

- **Isovaleric Acidemia (IVA)**

### Other Possible Diagnoses:

1. **Maple Syrup Urine Disease (MSUD)**: This is another inherited metabolic disorder characterized by a deficiency in the branched-chain alpha-keto acid dehydrogenase complex, leading to the accumulation of branched-chain amino acids and their corresponding ketoacids. Symptoms can include feeding difficulties, vomiting, lethargy, and a characteristic maple syrup odor in the urine.
2. **Methylmalonic Acidemia (MMA)**: This is a disorder of vitamin B12 metabolism that results in the accumulation of methylmalonic acid. Symptoms can include vomiting, lethargy, hypotonia, and metabolic acidosis.
3. **Propionic Acidemia (PA)**: This is a disorder of propionate metabolism that leads to the accumulation of propionic acid and its metabolites. Symptoms can include vomiting, lethargy, hypotonia, and metabolic acidosis.
4. **Glutaric Acidemia Type I (GA1)**: This is a disorder of lysine and tryptophan metabolism that results in the accumulation of glutaric acid. Symptoms can include feeding difficulties, vomiting, hypotonia, and developmental delay, particularly after a catabolic stressor like an infection.

### Key Points for Differentiation:

- **Isovaleric Acidemia (IVA)**: Characterized by a “sweaty feet” odor, metabolic acidosis, hyperammonemia, and hypoglycemia.
- **Maple Syrup Urine Disease (MSUD)**: Characterized by a “maple syrup” odor in the urine, metabolic acidosis, and neurological symptoms.
- **Methylmalonic Acidemia (MMA)**: Characterized by metabolic acidosis, hyperammonemia, and ketosis.
- **Propionic Acidemia (PA)**: Characterized by metabolic acidosis, hyperammonemia, and ketosis.
- **Glutaric Acidemia Type I (GA1)**: Characterized by developmental delay, hypotonia, and a risk of dystonia after catabolic stress.

Each of these conditions can present with similar symptoms, but the specific metabolic abnormalities and characteristic odors can help in making a differential diagnosis. Confirmatory testing, such as tandem mass spectrometry and genetic testing, is essential for definitive diagnosis.

### **70. User**

- dysgenesis of secondary sexual characteristics and impaired gametogenesis.
- Olfactory disorders Inability to recognise odours, especially aromatic odours, due to abnormal development of the olfactory bulb and olfactory tract.

### Qwen2.5_72b

Based on the clinical manifestations you’ve provided—dysgenesis of secondary sexual characteristics and impaired gametogenesis, along with olfactory disorders—Kallmann syndrome is the most likely diagnosis. Kallmann syndrome is a rare genetic disorder characterized by hypogonadotropic hypogonadism (HH) and anosmia or hyposmia due to the abnormal development or function of the olfactory bulbs and tracts.

### Most Likely Diagnosis:

- **Kallmann Syndrome**

### Other Possible Diagnoses:

- **Congenital Hypogonadotropic Hypogonadism (CHH) without Anosmia**: This condition is similar to Kallmann syndrome but does not include the olfactory dysfunction.
- **Prader-Willi Syndrome**: This is a genetic disorder that can cause hypogonadism and delayed puberty, but it is typically associated with other features such as obesity, intellectual disability, and hypotonia.
- **Turner Syndrome**: This is a chromosomal disorder affecting females, characterized by the partial or complete absence of one X chromosome. It can lead to hypogonadism and delayed puberty, but it does not typically include olfactory disorders.
- **Androgen Insensitivity Syndrome (AIS)**: This is a condition where the body is resistant to androgens, leading to underdeveloped secondary sexual characteristics. However, it does not typically involve olfactory disorders.
- **McCune-Albright Syndrome**: This is a rare genetic disorder characterized by polyostotic fibrous dysplasia, café-au-lait spots, and endocrine disorders, including precocious puberty. It does not typically include olfactory dysfunction.

### Summary:

- **Primary Diagnosis**: Kallmann Syndrome
- **Other Possible Diagnoses**:
  1. Congenital Hypogonadotropic Hypogonadism (CHH) without Anosmia
  2. Prader-Willi Syndrome
  3. Turner Syndrome
  4. Androgen Insensitivity Syndrome (AIS)
  5. McCune-Albright Syndrome

These diagnoses should be considered and further evaluated through appropriate clinical and genetic testing to confirm the specific condition.

### **71. User**

The condition varies greatly from mild to severe and is therefore easily misdiagnosed and underdiagnosed. Clinical manifestations mainly include:

**1. Fever** The fever is irregular and may be periodic or persistent. Patients with hypothalamic involvement may have central thermoregulatory abnormalities.

**2. rash** Mainly on the trunk, scalp and hairline. It begins as a reddish papule, which may then become a haemorrhagic or eczema-like seborrheic rash, followed by crusting, which is followed by a white spot.

**3. Oral and eye, ear, nose and throat lesions** Gum swelling, loose teeth, proptosis, and persistent otitis media with external auditory canal rash are typical.

**4. Central uropathy** Pituitary gland, hypothalamus or even other parts of the central nervous system, with neuropsychiatric symptoms in a few patients.

**5. Respiratory symptoms** Coughing, severe breathlessness, cyanosis, and even recurrent pneumothorax episodes. Pulmonary signs are usually unremarkable.

**6.** The liver and spleen can be enlarged, jaundice, abnormal liver function, liver failure and portal hypertension can occur, and eventually patients can die of cirrhosis and liver failure.

**7. Digestive tract symptoms** Digestive tract involvement is less common and can manifest as polyps or adenomatous changes, usually without clinical symptoms, and abdominal pain, diarrhoea and hypoproteinaemia may occur when the lesions are diffuse.

**8. Haematopoietic system** Lymph nodes may be enlarged, and anaemia, abnormal white blood cell and platelet counts may occur with bone marrow involvement.

**9. Bone damage** Skull, limb bones, spine and pelvis may have pain and lumps, and pathological fractures may occur. Proptosis due to orbital bone lesions is one of the typical manifestations in paediatric patients.

### Qwen2.5_72b

Based on the clinical manifestations you’ve provided, the most likely rare disease is **Langerhans Cell Histiocytosis (LCH)**. LCH is a rare disorder characterized by the abnormal proliferation of Langerhans cells, which can affect multiple organs and systems, leading to a wide range of symptoms.

### Most Likely Diagnosis:

- **Langerhans Cell Histiocytosis (LCH)**

### Other Possible Diagnoses:

1. **Juvenile Xanthogranuloma (JXG)**: This is a benign histiocytic disorder that can present with skin lesions, eye involvement, and rarely, systemic involvement.
2. **Rosai-Dorfman Disease (RDD)**: Also known as sinus histiocytosis with massive lymphadenopathy, it can present with lymphadenopathy, skin lesions, and systemic symptoms.
3. **Erdheim-Chester Disease (ECD)**: A rare form of non-Langerhans cell histiocytosis that can affect multiple organs, including the bones, skin, and central nervous system.
4. **Hemophagocytic Lymphohistiocytosis (HLH)**: A severe and often fatal syndrome characterized by excessive activation of the immune system, leading to fever, hepatosplenomegaly, cytopenias, and multi-organ dysfunction.

### Rationale:

- **Langerhans Cell Histiocytosis (LCH)**: The clinical manifestations you described, including fever, rash, oral and eye lesions, central nervous system involvement, respiratory symptoms, hepatosplenomegaly, digestive tract involvement, hematopoietic system involvement, and bone damage, are highly characteristic of LCH. The disease can vary in severity and can affect multiple organ systems, making it a likely diagnosis.
- **Juvenile Xanthogranuloma (JXG)**: While JXG is typically a benign condition, it can present with skin lesions and, in rare cases, systemic involvement, which might overlap with some of the symptoms you described.
- **Rosai-Dorfman Disease (RDD)**: RDD can present with lymphadenopathy, skin lesions, and systemic symptoms, but it is less likely to cause the extensive multi-organ involvement seen in LCH.
- **Erdheim-Chester Disease (ECD)**: ECD can affect multiple organs, including the bones, skin, and central nervous system, but it is less likely to present with the specific combination of symptoms you described.
- **Hemophagocytic Lymphohistiocytosis (HLH)**: HLH is a severe syndrome that can present with fever, hepatosplenomegaly, cytopenias, and multi-organ dysfunction, but it is typically more acute and severe than the chronic course often seen in LCH.

Given the wide range of symptoms and the chronic, multi-organ involvement, LCH is the most likely diagnosis, but further diagnostic testing, including biopsy and imaging, would be necessary to confirm the diagnosis.

### **72. User**

**1. Growth disorders and other malformations,** most of the children are born with a weight within the normal range, but some of them are born with a length of less than 48cm (42-47cm). After birth, the growth rate gradually and significantly slows down, and the lifelong height of untreated male patients ranges from 116 to 142 cm, and the lifelong height of female patients ranges from 108 to 136 cm. At the same time, the patients’ hands, feet and internal organs are small. Some patients have other congenital malformations, such as hip dislocation, aortic valve stenosis, cryptorchidism, and congenital cataracts.

**2. Facial features,** the characteristic facial changes of patients are prominent forehead, saddle nose, small jaw, larger head to body ratio than normal children, but the absolute value of head circumference is still small compared with children of the same age. Hair is thinning, and some patients may have a ‘sunset sign’, with blue sclera in patients from the Mediterranean and Middle Eastern regions. The patient has delayed teething, late deciduous milk teeth and multiple caries, and may have malocclusion due to a short mandible.

**Skeletal musculature** Some patients have congenital hip dislocation and ischaemic necrosis of the femoral head. Due to mid-facial dysplasia and pharyngeal anatomical abnormalities, the patient’s speech is high pitched and 85% of the patients have limited elbow joint movement.

**4. Metabolic disorders,** Patients often have a combination of metabolic abnormalities, including hypoglycaemia in the neonatal period and infancy, and progressive obesity in adulthood.

**5. Delayed sexual development ,** Patients with this condition have small genitalia, with male patients presenting with small penises and testicles, and female patients having small ovaries on ultrasound. Most patients exhibit delayed pubertal development. Although sexual development is delayed, male and female patients can eventually develop mature sexual function and be fertile.

**6. Low tumour incidence** Patients have a significantly reduced risk of tumour development compared to the general population.

### Qwen2.5_72b

Based on the clinical manifestations you have provided, the most likely diagnosis is **Russell-Silver Syndrome (RSS)**. This is a rare genetic disorder characterized by growth retardation, characteristic facial features, and other congenital malformations.

### Russell-Silver Syndrome (RSS)

- **Growth Disorders and Malformations:** Intrauterine growth restriction (IUGR) and postnatal growth failure are hallmark features. Patients are often born with a length of less than 48 cm and have a lifelong short stature.
- **Facial Features:** Prominent forehead, saddle nose, small jaw, and a larger head-to-body ratio.
- **Skeletal Musculature:** Congenital hip dislocation, ischaemic necrosis of the femoral head, and limited elbow joint movement.
- **Metabolic Disorders:** Hypoglycemia in the neonatal period and progressive obesity in adulthood.
- **Delayed Sexual Development:** Small genitalia, delayed pubertal development, but eventual development of mature sexual function.
- **Low Tumor Incidence:** Reduced risk of tumor development compared to the general population.

### Other Possible Diagnoses

- **Prader-Willi Syndrome (PWS)**
  - **Growth Disorders and Malformations:** Hypotonia, feeding difficulties in infancy, and obesity in later childhood.
  - **Facial Features:** Narrow bifrontal diameter, almond-shaped eyes, and a thin upper lip.
  - **Skeletal Musculature:** Hypotonia and joint laxity.
  - **Metabolic Disorders:** Hypoglycemia in infancy and hyperphagia leading to obesity.
  - **Delayed Sexual Development:** Hypogonadism and delayed or incomplete pubertal development.
  - **Low Tumor Incidence:** Not a specific feature.
- **Turner Syndrome (TS)**
  - **Growth Disorders and Malformations:** Short stature, webbed neck, and low posterior hairline.
  - **Facial Features:** Broad chest, low-set ears, and a shield-shaped chest.
  - **Skeletal Musculature:** Cubitus valgus and short fourth metacarpals.
  - **Metabolic Disorders:** Hypothyroidism and insulin resistance.
  - **Delayed Sexual Development:** Gonadal dysgenesis and primary amenorrhea.
  - **Low Tumor Incidence:** Not a specific feature.
- **Noonan Syndrome (NS)**
  - **Growth Disorders and Malformations:** Short stature and webbed neck.
  - **Facial Features:** Wide-set eyes, ptosis, and low-set ears.
  - **Skeletal Musculature:** Pectus excavatum or pectus carinatum.
  - **Metabolic Disorders:** Hypothyroidism and bleeding disorders.
  - **Delayed Sexual Development:** Delayed puberty and hypogonadism.
  - **Low Tumor Incidence:** Not a specific feature.
- **Costello Syndrome (CS)**
  - **Growth Disorders and Malformations:** Short stature and loose, redundant skin.
  - **Facial Features:** Coarse facial features, thick lips, and a wide mouth.
  - **Skeletal Musculature:** Joint hypermobility and contractures.
  - **Metabolic Disorders:** Hypoglycemia and feeding difficulties.
  - **Delayed Sexual Development:** Delayed pubertal development.
  - **Low Tumor Incidence:** Increased risk of certain tumors, particularly rhabdomyosarcoma.
- **Beckwith-Wiedemann Syndrome (BWS)**
  - **Growth Disorders and Malformations:** Macrosomia, macroglossia, and omphalocele.
  - **Facial Features:** Ear creases or pits.
  - **Skeletal Musculature:** Hemihyperplasia.
  - **Metabolic Disorders:** Hypoglycemia in the neonatal period.
  - **Delayed Sexual Development:** Not a specific feature.
  - **Low Tumor Incidence:** Increased risk of embryonal tumors, particularly Wilms tumor.

Each of these conditions has overlapping features, but the combination of growth retardation, characteristic facial features, and other specific clinical manifestations in Russell-Silver Syndrome makes it the most likely diagnosis in this case.

### **73. User**

The main symptom is a sudden, sharp, painless loss of visual acuity in both eyes that occurs sequentially, usually weeks to months between episodes. Funduscopic examination shows congestion and oedema of the optic disc and tortuous dilatation of the peripapillary capillaries in the early stages of the disease, while the oedema and capillary dilatation subside in the later stages of the disease, and eventually the temporal side of the optic disc or all of the optic disc shows atrophic changes. The majority of patients have only ocular manifestations, while a few may have other systemic symptoms such as mental retardation, epilepsy, hearing impairment, dystonia, etc.

### Qwen2.5_72b

Based on the clinical manifestations you’ve described, the most likely rare disease is **Leber Hereditary Optic Neuropathy (LHON)**. However, the presence of systemic symptoms such as mental retardation, epilepsy, hearing impairment, and dystonia suggests a broader differential diagnosis. Here are the top five possible diagnoses:

- **Leber Hereditary Optic Neuropathy (LHON)**:
  - **Description**: LHON is a mitochondrial disorder characterized by bilateral, painless, subacute loss of central vision due to optic nerve dysfunction. The funduscopic findings you described, including optic disc congestion and edema followed by atrophy, are typical of LHON.
- **Leber’s Optic Atrophy with Neurological Features (LHON Plus)**:
  - **Description**: This is a variant of LHON where patients exhibit additional neurological symptoms such as mental retardation, epilepsy, hearing impairment, and dystonia. The presence of these systemic symptoms in a patient with optic neuropathy strongly suggests LHON Plus.
- **Mitochondrial Encephalomyopathy, Lactic Acidosis, and Stroke-like Episodes (MELAS)**:
  - **Description**: MELAS is a mitochondrial disorder that can present with optic neuropathy, along with other neurological symptoms such as seizures, stroke-like episodes, and cognitive decline. The funduscopic findings and systemic symptoms are consistent with MELAS.
- **Kearns-Sayre Syndrome (KSS)**:
  - **Description**: KSS is a mitochondrial disorder characterized by chronic progressive external ophthalmoplegia, pigmentary retinopathy, and cardiac conduction defects. It can also present with optic neuropathy and other systemic symptoms like hearing loss and ataxia.
- **Neuropathy, Ataxia, and Retinitis Pigmentosa (NARP)**:
  - **Description**: NARP is another mitochondrial disorder that can cause optic neuropathy, ataxia, and retinitis pigmentosa. It can also present with cognitive impairment and other neurological symptoms.
- **Multiple Sclerosis (MS)**:
  - **Description**: While not a rare disease, MS can present with optic neuritis, which can cause sudden, painless loss of vision. However, the presence of systemic symptoms and the specific funduscopic findings make it less likely compared to the mitochondrial disorders listed above.

Given the specific clinical manifestations and the presence of systemic symptoms, **Leber Hereditary Optic Neuropathy (LHON) Plus** is the most likely diagnosis. However, further genetic testing and a detailed family history would be essential to confirm the diagnosis and rule out other mitochondrial disorders.

### **74. User**

The clinical manifestations of patients are low ketogenic hypoglycaemia, liver function abnormalities, lactic acidosis, cardiomyopathy in infancy, growth retardation, hypotonia or cardiomyopathy in some patients, and cholestasis or cirrhosis in a few. In early childhood, some patients show growth retardation, hypotonia or cardiomyopathy, and a few may have cholestasis or cirrhosis. In early childhood, patients may have feeding difficulties, episodic metabolic disorders, and episodic rhabdomyolysis. Most patients will have retinopathy in childhood, and peripheral neuropathy is a less common complication of long-term disease.

### Qwen2.5_72b

Based on the clinical manifestations you’ve provided, the most likely rare disease is **Glycogen Storage Disease Type IV (GSD IV) or Andersen Disease**. This condition is characterized by the inability to properly break down glycogen, leading to the accumulation of abnormal glycogen in various organs, particularly the liver and muscles. The symptoms you’ve described, such as low ketogenic hypoglycemia, liver function abnormalities, lactic acidosis, cardiomyopathy, growth retardation, hypotonia, and cholestasis or cirrhosis, are consistent with GSD IV.

However, there are several other rare diseases that could present with similar clinical manifestations. Here are five other possible diagnoses:

- **Pompe Disease (Glycogen Storage Disease Type II)**:
  - **Symptoms**: Hypotonia, cardiomyopathy, liver enlargement, feeding difficulties, and respiratory issues.
  - **Pathophysiology**: Deficiency of acid alpha-glucosidase (GAA) enzyme, leading to glycogen accumulation in lysosomes.
- **Mitochondrial Disorders (e.g., MELAS Syndrome)**:
  - **Symptoms**: Lactic acidosis, cardiomyopathy, growth retardation, hypotonia, and episodic metabolic decompensation.
  - **Pathophysiology**: Mutations in mitochondrial DNA affecting energy production in cells.
- **Galactosemia**:
  - **Symptoms**: Liver dysfunction, hypoglycemia, jaundice, and feeding difficulties.
  - **Pathophysiology**: Deficiency of galactose-1-phosphate uridyltransferase (GALT) enzyme, leading to toxic accumulation of galactose metabolites.
- **Tyrosinemia Type I**:
  - **Symptoms**: Liver dysfunction, hypoglycemia, lactic acidosis, and cardiomyopathy.
  - **Pathophysiology**: Deficiency of fumarylacetoacetate hydrolase (FAH) enzyme, leading to accumulation of toxic metabolites.
- **Zellweger Spectrum Disorders (Peroxisomal Biogenesis Disorders)**:
  - **Symptoms**: Hepatomegaly, hypotonia, growth retardation, and metabolic abnormalities.
  - **Pathophysiology**: Defects in peroxisome biogenesis, leading to impaired fatty acid metabolism and other metabolic functions.

Each of these conditions has overlapping symptoms, and a definitive diagnosis would require specific biochemical and genetic testing.

### **75. User**

It occurs predominantly in adult women of childbearing age, with the average age of diagnosis being around 40 years. Early symptoms are mild, and some patients are detected during a physical examination or on HRCT of the lungs for respiratory symptoms or other reasons. The main manifestation is dyspnoea of varying degrees, which worsens as the disease progresses, with progressive deterioration of lung function and respiratory failure in the later stages of the disease. Complications such as pneumothorax and coeliac chest may occur repeatedly during the course of the disease. Extrapulmonary manifestations include renal, retroperitoneal solid or cystic solid lymphangioleiomyoma. Some patients have other clinical features of multisystem involvement, including neurological changes (epilepsy, neurodevelopmental delays, autism, etc.) and cutaneous changes (depigmentation, facial angiofibromas, cutaneous sharkskin plaques, and perinodal fibromas, etc.).

### Qwen2.5_72b

Based on the clinical manifestations you’ve provided, the most likely rare disease is **Lymphangioleiomyomatosis (LAM)**. LAM is a rare lung disease that predominantly affects women of childbearing age, and it is characterized by the abnormal growth of smooth muscle-like cells (lymphangioleiomyoma) in the lungs, lymph nodes, and other organs. The disease can lead to progressive lung destruction, respiratory failure, and extrapulmonary manifestations, as you have described.

### Other Possible Diagnoses:

- **Tuberous Sclerosis Complex (TSC)**: TSC is a genetic disorder that can cause tumors to form in various organs, including the lungs, kidneys, brain, and skin. It often presents with neurological symptoms, skin changes, and lung involvement similar to LAM, especially in women.
- **Pulmonary Lymphoma**: This can be a rare form of non-Hodgkin lymphoma that primarily affects the lungs. It can present with respiratory symptoms, including dyspnea, and may involve other organs.
- **Sarcoidosis**: A systemic inflammatory disease that can affect multiple organs, including the lungs, skin, and lymph nodes. It can present with respiratory symptoms and extrapulmonary manifestations, but it is more common in a younger age group and has a different histopathological appearance.
- **Birt-Hogg-Dubé Syndrome (BHD)**: A rare genetic disorder that can cause multiple benign skin tumors, lung cysts, and an increased risk of spontaneous pneumothorax. It can also present with renal tumors.
- **Neurofibromatosis Type 1 (NF1)**: A genetic disorder that can cause tumors to grow along nerves and can affect various organs, including the skin, brain, and lungs. It can present with neurological symptoms and skin changes, but lung involvement is less common.

### Summary:

- **Most Likely Diagnosis**: Lymphangioleiomyomatosis (LAM)
- **Other Possible Diagnoses**:
  1. Tuberous Sclerosis Complex (TSC)
  2. Pulmonary Lymphoma
  3. Sarcoidosis
  4. Birt-Hogg-Dubé Syndrome (BHD)
  5. Neurofibromatosis Type 1 (NF1)

These conditions share some overlapping features, but LAM is the most consistent with the clinical presentation you have described. Further diagnostic testing, such as genetic testing, imaging, and lung function tests, would be necessary to confirm the diagnosis.

### **76. User**

Infants typically experience repeated vomiting and diarrhea after weaning and refuse protein-rich foods.Patients may present with pale skin, hypotonia, hepatosplenomegaly, thinning hair, osteoporosis and pathological fractures.After eating a protein-rich diet, patients may experience lethargy, ataxia, seizures, and hyperammonemic coma.As the disease progresses, multiple systems may be involved.Lung involvement can be manifested as progressive interstitial lung disease, alveolar proteinosis; the most common characteristic changes in the kidney are proximal tubular dysfunction and renal calcinosis, glomerulopathy, renal amyloidosis, and even renal failure; blood system involvement can be manifested as positive or low pigment anemia, leukopenia, thrombocytopenia, etc.; Digestive system involvement is manifested by hypercholesterolemia, hypertriglycerides, recurrent pancreatitis, and even hepatic amyloidosis.Epilepsy occurs mostly in adult patients and may be related to repeated hyperammonemia leading to cerebral cortex damage.

### Qwen2.5_72b

Based on the clinical manifestations you’ve provided, the most likely rare disease is **Cystinosis**. Cystinosis is a lysosomal storage disorder caused by a mutation in the CTNS gene, leading to the accumulation of the amino acid cystine in various tissues and organs. The symptoms you described, such as repeated vomiting and diarrhea, protein aversion, hypotonia, hepatosplenomegaly, thinning hair, osteoporosis, and pathological fractures, are consistent with the early stages of cystinosis. As the disease progresses, the involvement of multiple systems, including the lungs, kidneys, blood, and digestive system, as well as the neurological symptoms like lethargy, ataxia, seizures, and hyperammonemic coma, are also characteristic of cystinosis.

### Other Possible Diagnoses:

- **Wilson’s Disease**: A genetic disorder that results in copper accumulation in the liver, brain, and other vital organs. Symptoms can include liver dysfunction, neurological symptoms (ataxia, seizures), and hematological abnormalities (anemia, thrombocytopenia).
- **Gaucher’s Disease**: A lysosomal storage disorder caused by a deficiency in the enzyme glucocerebrosidase. Symptoms can include hepatosplenomegaly, bone pain, and fractures, anemia, and thrombocytopenia.
- **Niemann-Pick Disease**: A group of lysosomal storage disorders caused by deficiencies in enzymes that break down lipids. Symptoms can include hepatosplenomegaly, neurological symptoms (ataxia, seizures), and hematological abnormalities (anemia, thrombocytopenia).
- **Fanconi Anemia**: A rare genetic disorder that affects the bone marrow, leading to decreased production of all types of blood cells. Symptoms can include anemia, thrombocytopenia, and increased risk of cancer. Some patients may also have renal and skeletal abnormalities.
- **Tyrosinemia Type I**: A metabolic disorder caused by a deficiency in the enzyme fumarylacetoacetate hydrolase. Symptoms can include liver and kidney dysfunction, hypotonia, and neurological symptoms (seizures, ataxia).

Each of these conditions can present with overlapping symptoms, but the specific combination and progression of symptoms you’ve described are most consistent with cystinosis. Further diagnostic testing, such as cystine level measurement in white blood cells, genetic testing, and renal function tests, would be necessary to confirm the diagnosis.

### **77. User**

The onset is neonatal, usually within a week of birth.Recurrent vomiting, diarrhea, abdominal distension and other gastrointestinal symptoms were the first symptoms, accompanied by hepatosplenomegaly and growth disorders; nervous system involvement was manifested as hypoactivity, tendon hyperreflexia, ankle clonus and angular arch opisthotonus.Macrophage cholesterol ester and triglyceride accumulation leads to hepatosplenomegaly, progressive aggravation and even liver failure, elevated transaminases, bilirubin, hypoproteinemia, coagulation abnormalities, etc. within months.Adrenal enlargement with calcification leads to adrenal cortical insufficiency, and 60% to 70% of adrenal calcifications can be detected by imaging.Gallbladder dysfunction and stroke have also been reported.As the disease progresses, multiple organ dysfunction can occur, including anemia, gastrointestinal bleeding, sepsis, coma, etc.

### Qwen2.5_72b

Based on the clinical manifestations you have provided, the most likely diagnosis is **Niemann-Pick Disease Type C (NPC)**. This is a rare, inherited lysosomal storage disorder characterized by the accumulation of cholesterol and other lipids in various organs, particularly the liver, spleen, and brain.

### Most Likely Diagnosis:

- **Niemann-Pick Disease Type C (NPC)**

### Other Possible Diagnoses:

1. **Gaucher Disease Type 2 (Acute Neuronopathic Gaucher Disease)**
   - **Clinical Features:** Neonatal onset, hepatosplenomegaly, neurological involvement (hypotonia, seizures, brainstem dysfunction), and often rapid progression leading to early death.
2. **Wolman Disease (Cholesterol Ester Storage Disease)**
   - **Clinical Features:** Neonatal onset, severe hepatosplenomegaly, adrenal calcification, failure to thrive, and often death within the first few months of life.
3. **Zellweger Syndrome (Peroxisomal Biogenesis Disorders)**
   - **Clinical Features:** Neonatal onset, hepatomegaly, hypotonia, seizures, characteristic facial features, and progressive neurological deterioration.
4. **Mucopolysaccharidosis Type I (Hurler Syndrome)**
   - **Clinical Features:** Neonatal or early infantile onset, hepatosplenomegaly, skeletal abnormalities, coarse facial features, and progressive neurological decline.
5. **Adrenoleukodystrophy (ALD)**
   - **Clinical Features:** While typically presenting later in childhood or adulthood, neonatal forms can occur with hepatosplenomegaly, adrenal insufficiency, and neurological symptoms.

### Key Points for NPC:

- **Neonatal Onset:** Symptoms often begin within the first week of life.
- **Gastrointestinal Symptoms:** Recurrent vomiting, diarrhea, and abdominal distension.
- **Hepatosplenomegaly:** Enlarged liver and spleen due to lipid accumulation.
- **Neurological Involvement:** Hypoactivity, tendon hyperreflexia, ankle clonus, and opisthotonus.
- **Adrenal Calcification:** Commonly detected by imaging.
- **Progressive Multisystem Dysfunction:** Anemia, gastrointestinal bleeding, sepsis, and coma.

If NPC is suspected, further diagnostic tests such as filipin staining, cholesterol esterification assays, and genetic testing for NPC1 and NPC2 mutations should be considered.

### **78. User**

**1. The cardiovascular system** can result in aortic root dilatation, aortic valve insufficiency and aortic coarctation; mitral valve prolapse with or without insufficiency may also occur. The prevalence of ascending aortic dilatation increases with age. Between the ages of 30 and 60, the prevalence increases from 53% to 96%. Patients may present with shortness of breath, chest pain, palpitations, dizziness, syncope, or haemoptysis. Physical examination reveals an enlargement of the heart border to the lower left, a systolic murmur in the mitral valve area and a diastolic predominantly biphasic murmur in the aortic valve area, peripheral vascular signs, and signs of heart failure.

**2. Skeletal manifestations** , tall and thin stature, elongated fingers and toes, arms extended beyond height, scoliosis or kyphosis of the spine, funnel chest or chicken chest, laxity of the joints, flat feet.

**3. Ocular abnormalities**, eye lesions account for 50% to 70% of the cases, including corneal flattening, prolongation of the eyeball axis, retinal detachment, cataracts, subluxation or subluxation of the lens, iris nystagmus or mild nystagmus, pupil displacement, and clouding of the lens, etc. **4.

**4. Other ,**  dural bulge, atrophic striae, recurrent or incisional hernia, high palpebral vault, etc.

### Qwen2.5_72b

Based on the clinical manifestations you have provided, the most likely diagnosis is **Marfan Syndrome**. This is a genetic disorder that affects the body’s connective tissue and is characterized by a range of cardiovascular, skeletal, and ocular abnormalities, as well as other systemic features.

### Most Likely Diagnosis:

- **Marfan Syndrome**

### Other Possible Diagnoses:

1. **Ehlers-Danlos Syndrome (EDS)**: This is a group of connective tissue disorders characterized by joint hypermobility, skin hyperextensibility, and tissue fragility. Some subtypes can present with cardiovascular issues, such as aortic aneurysms and mitral valve prolapse.
2. **Loeys-Dietz Syndrome**: This is a genetic disorder that affects the body’s connective tissue, similar to Marfan Syndrome. It is characterized by aortic aneurysms, arterial tortuosity, and skeletal abnormalities.
3. **Beals Syndrome (Congenital Contractural Arachnodactyly)**: This is a genetic disorder that shares some features with Marfan Syndrome, including long limbs, joint contractures, and cardiovascular issues.
4. **Homocystinuria**: This is a rare genetic disorder that results in the buildup of homocysteine in the body. It can cause a range of symptoms, including tall stature, lens dislocation, and cardiovascular issues.

### Summary:

- **Marfan Syndrome** is the most likely diagnosis given the combination of cardiovascular, skeletal, and ocular abnormalities.
- **Ehlers-Danlos Syndrome**, **Loeys-Dietz Syndrome**, **Beals Syndrome**, and **Homocystinuria** are other possible diagnoses that share some of the clinical features you have described.

### **79. User**

**1. Skin manifestations**, patients are usually born with café au lait spots, often located on the same side as the skeletal lesions and bounded by the midline, which are brown or yellowish brown patches of pigmentation of varying sizes in one or more spots, with irregular borders.

**Skeletal manifestations** , approximately 98% of patients have either monostotic or polyostotic osteofibrous heteroplasia, with the polyostotic type being more common. It is characterised by an increase in the number of aberrant osteoblasts and their abnormal differentiation, causing extensive proliferation and differentiation of fibroblasts in the bone marrow and the formation of infantile woven bones, which are prone to pathological fractures and skeletal deformities and cause bone pain. It can involve almost all bones of the body, especially craniofacial and long bones, and the local bones are swollen and elevated, resulting in changes in the structure of the face and symptoms of compression. Severe orbital involvement may compress the optic nerve and cause visual impairment. Involvement of the auditory nerve may cause hearing loss. Involvement of the spine may result in vertebral compression fractures, causing scoliosis or kyphosis. The peak age for pathological fractures due to osteofibrous heteroplasia is 6-10 years. Imaging shows expansive, osteolytic, or ground-glass changes in the bone.

**Involvement of endocrine glands** may involve a variety of endocrine glands, resulting in hyperfunction or tumours of the affected glands and normal or decreased serum levels of pro-secretory hormones. Precocious puberty is more common and can occur in both sexes, but is more common in females and often precedes bone lesions. Female children may show early development of secondary sexual characteristics, premature irregular vaginal bleeding, enlarged uterus and ovaries compared with normal children of the same age, or ovarian cysts, premature healing of epiphyses, and so on. In males, precocious puberty is relatively rare, and testicular lesions (Leydig cell hyperplasia, microscopic stones, focal calcification, etc.) are seen on ultrasound. Thyroid enlargement and hyperfunction are also relatively common, and hyperparathyroidism has been reported. Other endocrine disorders such as cortisolism, gigantism / acromegaly, hyperprolactinaemia have been reported but are relatively rare.

**4. Hypophosphatemia** , a combination of hypophosphatemia, manifesting as hypophosphatemic rickets, has been reported in some patients. Those with combined hypophosphataemia usually have a polyostotic type of abnormal bone fibre proliferation.

**5. Other,** patients may also present with rare clinical symptoms such as jaundice, hepatitis, arrhythmias, and intestinal polyps due to mutations involving peripheral blood leukocytes, liver, heart, thymus, and gastrointestinal tract.

### Qwen2.5_72b

Based on the clinical manifestations you have provided, the most likely diagnosis is **McCune-Albright Syndrome (MAS)**. This is a rare genetic disorder characterized by the triad of polyostotic fibrous dysplasia, café au lait spots, and endocrine abnormalities, which are all present in your patient’s clinical presentation.

### Most Likely Diagnosis:

- **McCune-Albright Syndrome (MAS)**

### Other Possible Diagnoses:

1. **Neurofibromatosis Type 1 (NF1)**: This condition also presents with café au lait spots, but it is typically associated with neurofibromas, Lisch nodules (pigmented iris hamartomas), and other neurological manifestations rather than the skeletal and endocrine abnormalities seen in MAS.
2. **Ollier Disease**: This is a non-hereditary condition characterized by multiple enchondromas, which can cause skeletal deformities and pathological fractures. However, it does not typically present with café au lait spots or endocrine abnormalities.
3. **Maffucci Syndrome**: This is a rare disorder characterized by multiple enchondromas and hemangiomas. While it can cause skeletal deformities, it does not typically present with café au lait spots or endocrine abnormalities.
4. **Fibrous Dysplasia of Bone**: This can be monostotic or polyostotic and can cause skeletal deformities and pathological fractures. However, it does not typically present with café au lait spots or endocrine abnormalities unless it is part of MAS.
5. **Hypophosphatemic Rickets**: This condition can present with hypophosphatemia and rickets, but it does not typically present with café au lait spots or the specific endocrine abnormalities seen in MAS.

### Summary:

- **McCune-Albright Syndrome (MAS)** is the most likely diagnosis given the combination of café au lait spots, polyostotic fibrous dysplasia, endocrine abnormalities, and hypophosphatemia.
- Other conditions like Neurofibromatosis Type 1, Ollier Disease, Maffucci Syndrome, and isolated Fibrous Dysplasia of Bone can present with some similar features but lack the full triad of symptoms seen in MAS.

### **80. User**

The majority of patients have onset of symptoms between 3 months and 3 years of age, with a small percentage having onset in the neonatal period or in adulthood, while others are asymptomatic. The onset of the disease is usually triggered by a predisposing factor, such as prolonged starvation or a complication of an infectious disease. In children with early onset of the disease, the first symptoms are drowsiness and vomiting, but may also include convulsions and asphyxia, which often progress rapidly to coma or death. Acute onset hypoglycaemia is typical, and a decrease in blood glucose with a negative urine ketone body is helpful in the diagnosis. 50% of children have hepatomegaly, and liver biopsy reveals a large accumulation of lipids. Imaging reveals cerebral oedema in some children. Cardiac damage is rare. Adult-onset patients present with a wide range of clinical manifestations, including multiple organ damage, including muscle, liver, nervous system, or cardiovascular system. Vomiting is the most common symptom, hepatomegaly is relatively rare, and patients presenting with symptoms during pregnancy show acute fatty liver. Most patients have acute non-inflammatory encephalopathy. Rhabdomyolysis occurs in about one-third of patients, with a significant increase in creatine kinase and myoglobinuria. There is a high mortality rate, with approximately 25% of children dying from the onset of the disease, and an even higher rate of 50% in adults with acute onset of the disease. Patients tend to behave normally between episodes. About one third of patients who survive the acute onset of the disease develop sequelae, including growth retardation, motor retardation, mental retardation, language deficits, psycho-behavioural problems, epilepsy, cerebral palsy, hemiparesis, and chronic muscle weakness. In addition, some patients have complaints of skeletal muscle damage such as fatigue, myalgia and reduced exercise tolerance.

### Qwen2.5_72b

Based on the clinical manifestations you have provided, the most likely rare disease is **Medium-Chain Acyl-CoA Dehydrogenase Deficiency (MCADD)**. This is a metabolic disorder that affects the body’s ability to break down certain fats, leading to the symptoms you described, particularly the acute onset of hypoglycemia, hepatomegaly, and the risk of coma or death, especially in young children.

### Other Possible Diagnoses:

- **Kearns-Sayre Syndrome (KSS)**: This is a mitochondrial disorder that can present with muscle weakness, ptosis, and ophthalmoplegia. It can also cause cardiac conduction defects and cerebellar ataxia. However, the acute onset of hypoglycemia and hepatomegaly are less typical.
- **Reye Syndrome**: This is a rare but serious condition that can cause swelling in the liver and brain. It often occurs in children recovering from a viral infection and can present with vomiting, confusion, and rapid onset of coma. However, it is not typically associated with a genetic predisposition.
- **Fatty Acid Oxidation Disorders (FAODs) other than MCADD**: Other FAODs, such as Long-Chain 3-Hydroxyacyl-CoA Dehydrogenase Deficiency (LCHADD) or Very Long-Chain Acyl-CoA Dehydrogenase Deficiency (VLCADD), can present with similar symptoms, including hypoglycemia, hepatomegaly, and muscle weakness. These disorders can also be triggered by fasting or illness.
- **Glycogen Storage Disease Type I (von Gierke Disease)**: This is a genetic disorder that affects the body’s ability to break down glycogen into glucose, leading to hypoglycemia, hepatomegaly, and lactic acidosis. However, it typically presents with more chronic symptoms and is less likely to cause acute, life-threatening episodes.
- **Acute Intermittent Porphyria (AIP)**: This is a rare genetic disorder that can cause abdominal pain, vomiting, and neurological symptoms, including seizures and mental status changes. However, it is less likely to present with hypoglycemia and hepatomegaly, and the symptoms are more often triggered by certain medications or hormonal changes.

### Summary:

- **Most Likely Diagnosis**: Medium-Chain Acyl-CoA Dehydrogenase Deficiency (MCADD)
- **Other Possible Diagnoses**:
  1. Kearns-Sayre Syndrome (KSS)
  2. Reye Syndrome
  3. Fatty Acid Oxidation Disorders (FAODs) other than MCADD
  4. Glycogen Storage Disease Type I (von Gierke Disease)
  5. Acute Intermittent Porphyria (AIP)

Given the specific clinical features, particularly the acute onset of hypoglycemia, hepatomegaly, and the risk of rapid progression to coma or death, MCADD is the most likely diagnosis. However, further diagnostic testing, such as genetic testing and metabolic screening, would be necessary to confirm the diagnosis.

### **81. User**

The clinical manifestations of the disease vary among age groups. The earlier the age of onset, the more severe the acute metabolic disorders and encephalopathy. Clinical manifestations include gastrointestinal symptoms such as vomiting, feeding difficulties and hepatomegaly, as well as neurological symptoms such as dyskinesia, impaired consciousness, convulsions, delayed or regressed growth and microcephaly. Recurrent illness is mostly associated with infection and stress.

Neonatal-onset patients usually present with acute encephalopathy-like symptoms in the first few hours to one week after birth, which are manifested by vomiting, hypotonia, dehydration, severe acidosis, hyperlactatemia, hyperammonemia, coma, and convulsions, with a high morbidity and mortality rate. Childhood onset of the disease is normal at birth, mostly within 1 year of age, the first metabolic crisis is often triggered by infection, hunger, fatigue, vaccination and other stress factors, or high-protein diets and medications, which, if not diagnosed and treated in a timely manner, can lead to retardation, backwardness, and regression of mental and motor development, and can be accompanied by involvement of the haematological system, the liver, the kidneys, the skin, and peripheral nerves. In adults, the first symptoms may include peripheral neuropathy and psychiatric abnormalities.

### Qwen2.5_72b

Based on the clinical manifestations you’ve provided, the most likely rare disease is **Maple Syrup Urine Disease (MSUD)**. MSUD is a rare inherited metabolic disorder characterized by a deficiency in the branched-chain alpha-keto acid dehydrogenase complex, leading to the accumulation of branched-chain amino acids (leucine, isoleucine, and valine) and their corresponding ketoacids. The disease can present with a wide range of symptoms, including those you’ve described, such as vomiting, feeding difficulties, hepatomegaly, neurological symptoms, and metabolic crises.

### Differential Diagnoses:

- **Methylmalonic Acidemia (MMA)**: This is another inherited metabolic disorder characterized by the accumulation of methylmalonic acid. Symptoms can include vomiting, feeding difficulties, lethargy, and metabolic acidosis, similar to MSUD.
- **Propionic Acidemia (PA)**: This is a rare metabolic disorder caused by a deficiency in the enzyme propionyl-CoA carboxylase. Symptoms can include vomiting, lethargy, hypotonia, and metabolic acidosis, which are also seen in MSUD.
- **Urea Cycle Disorders (UCDs)**: These are a group of rare genetic disorders affecting the urea cycle, leading to hyperammonemia. Symptoms can include vomiting, lethargy, coma, and seizures, which are similar to those seen in MSUD.
- **Glutaric Aciduria Type I (GA1)**: This is a rare metabolic disorder caused by a deficiency in the enzyme glutaryl-CoA dehydrogenase. Symptoms can include vomiting, hypotonia, and dystonia, particularly after a metabolic crisis.
- **Tyrosinemia Type I (HT-1)**: This is a rare metabolic disorder caused by a deficiency in the enzyme fumarylacetoacetate hydrolase. Symptoms can include vomiting, hepatomegaly, and neurological symptoms, which can be similar to those seen in MSUD.

### Summary:

- **Primary Diagnosis**: Maple Syrup Urine Disease (MSUD)
- **Differential Diagnoses**:
  1. Methylmalonic Acidemia (MMA)
  2. Propionic Acidemia (PA)
  3. Urea Cycle Disorders (UCDs)
  4. Glutaric Aciduria Type I (GA1)
  5. Tyrosinemia Type I (HT-1)

Each of these conditions requires prompt and accurate diagnosis to initiate appropriate treatment and management, as they can be life-threatening if left untreated.

### **82. User**

The typical patient is normal at birth. At the age of 6 months to 1 year, the patient’s face gradually becomes ugly, with flattening in the middle of the face, widening of the bridge of the nose, clouding of the cornea, deafness, large head, long anterior and posterior diameters, protrusion of the forehead, stiffness of the joints, claw shaped hands, chicken chest, and lumbar lordosis. Heart valve thickening, cardiomyopathy or congestive heart failure often appear at the age of 2 to 5 years, and liver and spleen enlargement at the age of 5 years. Mental and motor development is backward. They usually survive until about 10 years of age. Most of them die of heart failure and pneumonia. In the mild form, symptoms appear after the age of 5 years and include mild coarse facial features, corneal clouding, and stiffness of the joints of the hands and feet. Intelligence is normal and life expectancy is relatively long. Clinical symptoms overlap between subtypes, but there are no significant differences in biochemical findings between subtypes.

### Qwen2.5_72b

Based on the clinical manifestations you have provided, the most likely diagnosis is **Mucopolysaccharidosis Type I (MPS I)**, also known as Hurler syndrome (severe form) and Scheie syndrome (mild form). This is a rare genetic disorder caused by a deficiency in the enzyme alpha-L-iduronidase, which leads to the accumulation of glycosaminoglycans (GAGs) in various tissues and organs.

### Most Likely Diagnosis:

- **Mucopolysaccharidosis Type I (MPS I)**
  - **Severe Form (Hurler Syndrome):** Early onset, severe symptoms, and shortened life expectancy.
  - **Mild Form (Scheie Syndrome):** Later onset, milder symptoms, and normal or near-normal life expectancy.

### Other Possible Diagnoses:

1. **Mucopolysaccharidosis Type II (MPS II, Hunter Syndrome):**
   - X-linked recessive disorder with similar symptoms but typically milder in females.
   - Enzyme deficiency: Iduronate-2-sulfatase.
2. **Mucopolysaccharidosis Type VI (MPS VI, Maroteaux-Lamy Syndrome):**
   - Autosomal recessive disorder with symptoms similar to MPS I but often with less severe intellectual disability.
   - Enzyme deficiency: Arylsulfatase B.
3. **Mucopolysaccharidosis Type IV (MPS IV, Morquio Syndrome):**
   - Autosomal recessive disorder with skeletal abnormalities, corneal clouding, and mild to no intellectual disability.
   - Enzyme deficiencies: N-acetylgalactosamine-6-sulfatase (MPS IVA) or beta-galactosidase (MPS IVB).
4. **Mucolipidosis Type II (ML II, I-Cell Disease):**
   - Autosomal recessive disorder with similar clinical features to MPS I but with a different underlying mechanism.
   - Deficiency in the enzyme N-acetylglucosamine-1-phosphotransferase.
5. **Mucolipidosis Type III (ML III, Pseudo-Hurler Polydystrophy):**
   - Autosomal recessive disorder with milder symptoms compared to ML II, including coarse facial features, joint stiffness, and mild intellectual disability.
   - Deficiency in the enzyme N-acetylglucosamine-1-phosphodiester alpha-N-acetylglucosaminidase.

### Key Features to Consider:

- **Facial Dysmorphism:** Coarse facial features, flattening of the midface, and widening of the nasal bridge.
- **Skeletal Abnormalities:** Joint stiffness, claw hands, chicken chest, and lumbar lordosis.
- **Ocular and Auditory Issues:** Corneal clouding and deafness.
- **Cardiac Involvement:** Heart valve thickening, cardiomyopathy, and congestive heart failure.
- **Hepatosplenomegaly:** Enlargement of the liver and spleen.
- **Developmental Delay:** Intellectual and motor developmental delays.
- **Life Expectancy:** Shortened in severe forms, longer in mild forms.

### Diagnostic Confirmation:

- **Enzyme Assay:** Measurement of alpha-L-iduronidase activity in leukocytes or fibroblasts.
- **Genetic Testing:** Identification of mutations in the IDUA gene.
- **Urine GAGs:** Elevated levels of dermatan sulfate and heparan sulfate in urine.

Early diagnosis and treatment, including enzyme replacement therapy (ERT) and hematopoietic stem cell transplantation (HSCT), can significantly improve the quality of life and prognosis for patients with MPS I.

### **83. User**

Insidious onset of disease. The disease develops slowly or progresses in stages and may have long periods of stability. The disease can develop at any age, mostly before 50 years old. Clinical manifestations are mainly multiple mononeuropathies, asymmetric distal upper limb is more common, early unilateral upper limb one or more nerves involved, manifested as muscle weakness of the corresponding innervated area, distal predominantly, may be accompanied by spasm or fasciculations. With the development of the disease, muscle atrophy can occur; with a longer course of the disease, multiple nerves of multiple limbs can be involved, and the asymmetry of the affected nerves may not be obvious, but presenting a distribution similar to that of polypoidal peripheral neuropathy. Patients may complain of mild sensory abnormalities, but there is a lack of objective signs of sensory involvement, and some patients may also have partial sensory nerve involvement in the later stages of the disease. Cerebral nerves are usually not involved. There are no signs of upper motor neuron involvement.

### Qwen2.5_72b

Based on the clinical manifestations you’ve provided, the most likely diagnosis is **Multifocal Motor Neuropathy (MMN)**. This is a rare disorder characterized by slowly progressive, asymmetric weakness and atrophy of the distal muscles, particularly in the upper limbs. The involvement of multiple mononeuropathies, the absence of sensory loss (or minimal sensory involvement), and the absence of upper motor neuron signs are key features of MMN.

### Other Possible Diagnoses:

- **Amyotrophic Lateral Sclerosis (ALS)**:
  - **Rationale**: ALS can present with asymmetric muscle weakness and atrophy, but it typically involves both upper and lower motor neurons, leading to signs of upper motor neuron involvement (e.g., spasticity, hyperreflexia, and Babinski sign). MMN, in contrast, lacks these signs.
- **Chronic Inflammatory Demyelinating Polyneuropathy (CIDP)**:
  - **Rationale**: CIDP can present with symmetric or asymmetric weakness and sensory involvement. However, CIDP often involves both motor and sensory nerves and can have more prominent sensory symptoms and signs.
- **Hereditary Neuropathy with Liability to Pressure Palsies (HNPP)**:
  - **Rationale**: HNPP can cause recurrent, focal neuropathies that can be asymmetric and involve the upper limbs. However, HNPP is typically associated with a family history and specific genetic mutations (e.g., PMP22 deletion).
- **Parsonage-Turner Syndrome (Brachial Neuritis)**:
  - **Rationale**: This condition can cause acute or subacute onset of pain followed by weakness and atrophy in the shoulder and upper limb. However, it is usually more acute in onset and can be associated with pain, which is not a prominent feature in MMN.
- **Lyme Neuropathy**:
  - **Rationale**: Lyme neuropathy can present with multifocal neuropathies, including cranial nerve involvement and peripheral nerve involvement. However, it is often associated with a history of Lyme disease exposure and other systemic symptoms.

### Summary:

- **Most Likely Diagnosis**: Multifocal Motor Neuropathy (MMN)
- **Other Possible Diagnoses**:
  1. Amyotrophic Lateral Sclerosis (ALS)
  2. Chronic Inflammatory Demyelinating Polyneuropathy (CIDP)
  3. Hereditary Neuropathy with Liability to Pressure Palsies (HNPP)
  4. Parsonage-Turner Syndrome (Brachial Neuritis)
  5. Lyme Neuropathy

Further diagnostic testing, such as nerve conduction studies (NCS), electromyography (EMG), and serum anti-GM1 antibodies, can help confirm the diagnosis of MMN and rule out other conditions.

### **84. User**

Neonatal onset is often fatal and may present as a metabolic crisis including metabolic acidosis, non-ketotic hypoglycaemia, hyperammonaemia, and other features including multisystem involvement (hypotonia, cardiomyopathy, hepatomegaly), excretion of large amounts of fatty acid- and amino acid-derived metabolites, ‘sweaty-foot’ body odour, coma, and neonatal death. Rapidly progressive hypertrophic cardiomyopathy is common in the type with neonatal onset and has a very high probability of sudden death, which is difficult to reverse, even if the disease is detected at an early stage by neonatal screening.

Congenital malformations are thought to be the consequence of the accumulation of toxic metabolites in the part of the body not corrected by embryonic transport. The degree of impaired fatty acid oxidative metabolism determines the degree of acidosis in utero, which in turn prevents normal embryo formation. Congenital malformations include enlarged polycystic kidneys, rocker-bottom feet, absence of lower abdominal muscles, hypospadias and penile curvature, hypotonia, cerebral cortical dysplasia, and gliosis. Other dysmorphic features include wide eye spacing, ear malformations, megalocrania, wide fontanel, high forehead, and low nasal bridge.

The age of onset and symptoms of late-onset forms vary considerably among cases and are mainly characterised by the absence of congenital malformations but with a lifelong risk of acute intermittent episodes of vomiting, dehydration, hypo-ketotic hypoglycaemia, acidosis, and impaired consciousness. Older individuals may have hepatomegaly or lipid deposition myopathy. Acute loss-of-compensation episodes can be triggered by infection, fever, surgery, low-energy diets or weight loss, alcohol consumption, and pregnancy. Most patients develop chronic myopathy symptoms, including exercise intolerance, myalgia, muscle weakness, and muscle atrophy. Organic aciduria in patients with late-onset disease is often intermittent and is more pronounced only during disease and under catabolic stress.

### Qwen2.5_72b

Based on the clinical manifestations you have provided, the most likely diagnosis is **Methylmalonic Acidemia (MMA)**, specifically the neonatal form. This is a rare inherited metabolic disorder characterized by the body’s inability to process certain proteins and fats (lipids) properly, leading to the accumulation of toxic substances in the body.

### Most Likely Diagnosis:

- **Methylmalonic Acidemia (MMA)**

### Other Possible Diagnoses:

- **Maple Syrup Urine Disease (MSUD)**
  - **Clinical Features:** Neonatal onset, metabolic crisis, metabolic acidosis, hypoglycemia, hyperammonemia, hypotonia, hepatomegaly, characteristic maple syrup odor of urine, and coma.
- **Propionic Acidemia (PA)**
  - **Clinical Features:** Neonatal onset, metabolic crisis, metabolic acidosis, hypoglycemia, hyperammonemia, hypotonia, cardiomyopathy, hepatomegaly, and a distinctive odor (sweaty feet or musty smell).
- **Glutaric Aciduria Type I (GA1)**
  - **Clinical Features:** Neonatal or early infantile onset, hypotonia, dystonia, macrocephaly, and characteristic facial features. Later onset can present with episodic metabolic decompensation.
- **Isobutyryl-CoA Dehydrogenase Deficiency (IBD)**
  - **Clinical Features:** Neonatal or early infantile onset, metabolic acidosis, hypoglycemia, hyperammonemia, hypotonia, and developmental delay.
- **3-Methylcrotonyl-CoA Carboxylase Deficiency (3-MCC)**
  - **Clinical Features:** Neonatal or early infantile onset, metabolic acidosis, hypoglycemia, hyperammonemia, hypotonia, and developmental delay. Some patients may have a milder phenotype with intermittent symptoms.

### Key Points:

- **Methylmalonic Acidemia (MMA)** is characterized by the accumulation of methylmalonic acid and other organic acids, leading to metabolic acidosis, hypoglycemia, and hyperammonemia. The neonatal form is often severe and can be rapidly fatal.
- **Maple Syrup Urine Disease (MSUD)** involves the accumulation of branched-chain amino acids and their corresponding ketoacids, leading to a characteristic odor and severe metabolic decompensation.
- **Propionic Acidemia (PA)** is similar to MMA but involves the accumulation of propionic acid and its metabolites, leading to similar clinical features.
- **Glutaric Aciduria Type I (GA1)** is associated with the accumulation of glutaric acid and its metabolites, leading to neurological symptoms and characteristic facial features.
- **Isobutyryl-CoA Dehydrogenase Deficiency (IBD)** and **3-Methylcrotonyl-CoA Carboxylase Deficiency (3-MCC)** are less common but can present with similar metabolic crises and developmental issues.

Given the specific clinical features and the rapid progression of symptoms, **Methylmalonic Acidemia (MMA)** is the most likely diagnosis, but the other conditions should be considered and ruled out through appropriate diagnostic testing, including metabolic screening, genetic testing, and enzyme assays.

### **85. User**

The average age of onset of the disease is 30 years old, and it is rare to find patients under 10 years old and over 50 years old. The prevalence of women is higher than that of men, with the ratio of women to men (1.4-2.3):1. The disease is mostly acute/subacute, and is characterised by spatial and temporal multiplicity. The spatial multiplicity refers to the multiple sites of the lesions, including the brain, brainstem, cerebellum, and spinal cord, which may be involved simultaneously or successively. Time-recurrent refers to the course of remission-relapse, and due to the wide range of sites involved, repeated episodes may eventually lead to disability or even death. Clinical manifestations are varied, and common symptoms include vision loss, diplopia, limb sensory deficits, limb motor deficits, ataxia, and bladder or rectal dysfunction.

### Qwen2.5_72b

Based on the clinical manifestations and epidemiological characteristics you’ve provided, the most likely diagnosis is **Multiple Sclerosis (MS)**. However, there are several other rare diseases that can present with similar symptoms and should be considered in the differential diagnosis. Here are the top five other possible diagnoses:

- **Neuromyelitis Optica Spectrum Disorders (NMOSD)**:
  - **Epidemiology**: Higher prevalence in women, often presenting in the third to fourth decade of life.
  - **Clinical Features**: Characterized by severe optic neuritis and transverse myelitis. Unlike MS, NMOSD often affects the optic nerves and spinal cord more severely and can lead to more rapid disability.
- **Acute Disseminated Encephalomyelitis (ADEM)**:
  - **Epidemiology**: Can occur at any age but is more common in children and young adults.
  - **Clinical Features**: Typically presents as a monophasic illness following an infection or vaccination. Symptoms include encephalopathy, fever, and multifocal neurological deficits.
- **Systemic Lupus Erythematosus (SLE) with Central Nervous System (CNS) Involvement**:
  - **Epidemiology**: Higher prevalence in women, often presenting in the second to fourth decade of life.
  - **Clinical Features**: Can present with a wide range of neurological symptoms, including cognitive dysfunction, seizures, and focal neurological deficits. Other systemic symptoms such as rash, joint pain, and renal involvement are common.
- **Sarcoidosis with CNS Involvement**:
  - **Epidemiology**: Can occur at any age but is more common in young to middle-aged adults. No significant gender predilection.
  - **Clinical Features**: Can present with a variety of neurological symptoms, including cranial neuropathies, meningitis, and spinal cord involvement. Pulmonary and systemic manifestations are also common.
- **Lyme Neuroborreliosis**:
  - **Epidemiology**: Can occur at any age but is more common in young to middle-aged adults. No significant gender predilection.
  - **Clinical Features**: Can present with a variety of neurological symptoms, including cranial neuropathies (especially facial nerve palsy), radiculoneuritis, and meningitis. A history of tick exposure and erythema migrans rash is often present.

### Summary

- **Most Likely Diagnosis**: Multiple Sclerosis (MS)
- **Other Possible Diagnoses**:
  1. Neuromyelitis Optica Spectrum Disorders (NMOSD)
  2. Acute Disseminated Encephalomyelitis (ADEM)
  3. Systemic Lupus Erythematosus (SLE) with CNS Involvement
  4. Sarcoidosis with CNS Involvement
  5. Lyme Neuroborreliosis

Each of these conditions has distinct clinical and diagnostic features that can help in making a definitive diagnosis. Further diagnostic testing, such as MRI, cerebrospinal fluid analysis, and specific serological tests, will be essential to confirm the diagnosis.

### **86. User**

The main symptoms include parkinsonism, cerebellar ataxia, pyramidal tract damage, and autonomic nervous system damage. The first symptoms are often autonomic impairments, most notably cardiovascular dysfunction (e.g., upright hypotension) and urinary dysfunction (e.g., urinary incontinence), the latter of which tends to be comorbid with sexual dysfunction in male patients. Many patients have had rapid eye movement (REM) sleep behaviour disorder (RBD) for several years.

### Qwen2.5_72b

Based on the clinical manifestations you’ve provided, the most likely diagnosis is **Multiple System Atrophy (MSA)**. MSA is a rare neurodegenerative disorder characterized by a combination of parkinsonism, cerebellar ataxia, and pyramidal tract signs, along with significant autonomic dysfunction. The early onset of autonomic symptoms, such as orthostatic hypotension and urinary incontinence, is a hallmark of MSA. Additionally, the presence of REM sleep behavior disorder (RBD) is a common feature and often precedes the motor symptoms.

Here are five other possible diagnoses to consider:

- **Parkinson’s Disease (PD)**: While PD can present with parkinsonism, it typically does not have the prominent autonomic dysfunction or cerebellar ataxia seen in MSA. RBD can also occur in PD but is less common.
- **Progressive Supranuclear Palsy (PSP)**: PSP can present with parkinsonism and pyramidal tract signs, but it is less likely to have cerebellar ataxia or significant autonomic dysfunction. PSP is also characterized by vertical gaze palsy, which is not mentioned in your case.
- **Corticobasal Syndrome (CBS)**: CBS can present with parkinsonism and pyramidal tract signs, but it typically includes asymmetric limb apraxia, alien limb phenomenon, and cognitive decline. Autonomic dysfunction is not a prominent feature.
- **Lewy Body Dementia (LBD)**: LBD can present with parkinsonism and autonomic dysfunction, including RBD. However, it is more likely to have significant cognitive impairment and visual hallucinations, which are not mentioned in your case.
- **Spinocerebellar Ataxias (SCAs)**: SCAs can present with cerebellar ataxia and pyramidal tract signs, but they typically do not have the prominent autonomic dysfunction or parkinsonism seen in MSA. SCAs are also usually inherited in an autosomal dominant pattern.

Given the combination of symptoms, MSA is the most likely diagnosis, but a thorough evaluation, including imaging and possibly genetic testing, would be necessary to confirm the diagnosis and rule out other conditions.

### **87. User**

**1. Muscles,** weakness and atrophy of the distal limbs starting at the distal end of the limbs and progressing proximally, affecting the fine movements of the hands and causing drop foot. The facial muscles are also involved. At the same time, there is obvious muscle ankylosis, involving the limb muscles, pharyngeal muscles and so on. Physical examination may reveal that the fingers cannot be expanded immediately after clenching the fist vigorously, and the eyes cannot be opened immediately after closing them vigorously. Knocking on the hand, the big fish muscle, the tongue muscle, etc., can lead to muscle ankylosis, known as knocking muscle ankylosis or ‘muscle ball’ phenomenon. There is a ‘warming up’ phenomenon, which decreases after repeated activities. Lower extremity myalgia and generalised fatigue are also common clinical manifestations.

**The cardiac conduction system is often involved, with varying degrees of atrioventricular block and intraventricular conduction abnormalities. In severe cases, sudden death may result. Confirmed patients require regular ECG monitoring and pacemaker implantation if necessary.

**3. Central nervous system** , sleep disorders such as increased sleepiness and obstructive sleep apnoea are common. It may be associated with cognitive and mood disorders. Head MRI shows diffuse cerebral white matter lesions and cortical atrophy.

**Cataracts occur in almost all patients as they age. They often affect vision in the 30s and 40s.

**5. Endocrine system** A variety of endocrine disorders may occur, including diabetes mellitus, thyroid dysfunction, abnormal calcium and phosphorus metabolism, sex hormone abnormalities, and testicular atrophy (resulting in infertility).

In addition, there may be frontal baldness, gastrointestinal insufficiency and restrictive ventilation disorders.

### Qwen2.5_72b

Based on the clinical manifestations you have provided, the most likely diagnosis is **Myotonic Dystrophy Type 1 (DM1)**. This is a rare genetic disorder characterized by muscle weakness and atrophy, myotonia (muscle stiffness), and involvement of multiple organ systems, including the heart, central nervous system, eyes, and endocrine system.

### Most Likely Diagnosis:

- **Myotonic Dystrophy Type 1 (DM1)**

### Other Possible Diagnoses:

- **Myotonic Dystrophy Type 2 (DM2)**: Similar to DM1 but typically presents with milder symptoms and less involvement of the distal muscles.
- **Facioscapulohumeral Muscular Dystrophy (FSHD)**: Characterized by progressive weakness and atrophy of the facial, scapular, and upper arm muscles.
- **Limb-Girdle Muscular Dystrophy (LGMD)**: A group of disorders that primarily affect the muscles of the shoulders and hips, leading to progressive weakness and atrophy.
- **Charcot-Marie-Tooth Disease (CMT)**: A hereditary disorder affecting the peripheral nerves, leading to muscle weakness and atrophy, particularly in the distal limbs.
- **Duchenne Muscular Dystrophy (DMD)**: A severe form of muscular dystrophy that primarily affects males, causing progressive muscle weakness and atrophy, often leading to early loss of ambulation and respiratory failure.

### Key Features of Myotonic Dystrophy Type 1 (DM1):

- **Muscle Weakness and Atrophy**: Distal muscle involvement, particularly in the hands and feet, leading to drop foot and difficulty with fine movements.
- **Myotonia**: Muscle stiffness and difficulty relaxing muscles after contraction, evident in the “muscle ball” phenomenon.
- **Cardiac Involvement**: Atrioventricular block and intraventricular conduction abnormalities, potentially leading to sudden death.
- **Central Nervous System**: Sleep disorders, cognitive and mood disorders, and diffuse cerebral white matter lesions.
- **Ocular Involvement**: Cataracts, often developing in the 30s and 40s.
- **Endocrine Disorders**: Diabetes mellitus, thyroid dysfunction, abnormal calcium and phosphorus metabolism, and sex hormone abnormalities.
- **Other Features**: Frontal baldness, gastrointestinal insufficiency, and restrictive ventilation disorders.

Given the multifaceted nature of the symptoms and the specific involvement of the distal muscles, myotonia, and multiple organ systems, Myotonic Dystrophy Type 1 (DM1) is the most likely diagnosis. However, further diagnostic testing, including genetic testing, electromyography (EMG), and muscle biopsy, would be necessary to confirm the diagnosis.

### **88. User**

The disease tends to develop at an early age, with the most frequent onset occurring in the neonatal period. Most children develop the disease within the first few days of life and present with milk refusal, vomiting, lethargy, convulsions and coma. Some children with late-onset disease are characterised by episodes of vomiting and lethargy with mental developmental abnormalities. Patients with hyperammonaemic coma may have brain injury with developmental delay, impaired learning ability or mental retardation, and sequelae of growth retardation. Patients with hyperammonaemia may have increased intracranial pressure, shortness of breath, apnoea or failure as the condition progresses, and hepatomegaly. Neurological complications include mental retardation, acute hyperammonemic coma, and even death. Biochemical changes can be seen in hyperammonaemia, increased blood glutamine and alanine, and decreased or absent urinary whey acids.

### Qwen2.5_72b

Based on the clinical manifestations and biochemical changes you’ve described, the most likely diagnosis is **Ornithine Transcarbamylase Deficiency (OTC Deficiency)**, a urea cycle disorder. Here are the key points that support this diagnosis:

- **Early Onset**: OTC deficiency often presents in the neonatal period, with symptoms such as milk refusal, vomiting, lethargy, convulsions, and coma.
- **Hyperammonemia**: Elevated blood ammonia levels are a hallmark of OTC deficiency.
- **Neurological Complications**: Hyperammonemic coma, mental retardation, and neurological sequelae are common.
- **Biochemical Changes**: Increased blood glutamine and alanine, and decreased or absent urinary organic acids are typical findings.

### Other Possible Diagnoses

- **Citrullinemia Type I (CTLN1)**:
  - **Symptoms**: Neonatal onset with vomiting, lethargy, and hyperammonemia.
  - **Biochemical Changes**: Elevated citrulline in blood and urine.
- **Argininosuccinic Aciduria (ASA)**:
  - **Symptoms**: Neonatal onset with vomiting, lethargy, and hyperammonemia.
  - **Biochemical Changes**: Elevated argininosuccinic acid in blood and urine.
- **Carbamoyl Phosphate Synthetase I Deficiency (CPS1 Deficiency)**:
  - **Symptoms**: Neonatal onset with vomiting, lethargy, and hyperammonemia.
  - **Biochemical Changes**: Elevated orotic acid in urine.
- **Glutaric Aciduria Type I (GA1)**:
  - **Symptoms**: Neonatal or early childhood onset with vomiting, lethargy, and developmental delay.
  - **Biochemical Changes**: Elevated glutaric acid and 3-hydroxyglutaric acid in urine.
- **Methylmalonic Acidemia (MMA)**:
  - **Symptoms**: Neonatal or early childhood onset with vomiting, lethargy, and developmental delay.
  - **Biochemical Changes**: Elevated methylmalonic acid in urine and blood.

### Summary

- **Most Likely Diagnosis**: Ornithine Transcarbamylase Deficiency (OTC Deficiency)
- **Other Possible Diagnoses**:
  1. Citrullinemia Type I (CTLN1)
  2. Argininosuccinic Aciduria (ASA)
  3. Carbamoyl Phosphate Synthetase I Deficiency (CPS1 Deficiency)
  4. Glutaric Aciduria Type I (GA1)
  5. Methylmalonic Acidemia (MMA)

These conditions are all rare metabolic disorders that can present with similar clinical and biochemical features, making a thorough evaluation and specific diagnostic tests essential for accurate diagnosis and appropriate management.

### **89. User**

Children often present with severe intrauterine growth retardation and develop severe, non-ketotic hyperglycaemia very early in life (often in the first week of life), which recovers after 12 weeks and recurs around puberty in 50%-60% of cases, with a clinical picture similar to that of type 2 diabetes mellitus.

### Qwen2.5_72b

Based on the clinical manifestations you’ve provided, the most likely rare disease is **MODY 13 (Maturity-Onset Diabetes of the Young 13)**, also known as **KCNJ11-Neonatal Diabetes Mellitus (NDM)**. This condition is caused by mutations in the KCNJ11 gene, which encodes a subunit of the ATP-sensitive potassium channel in pancreatic beta cells. These mutations lead to impaired insulin secretion, resulting in severe hyperglycemia early in life.

### Most Likely Diagnosis:

- **MODY 13 (KCNJ11-Neonatal Diabetes Mellitus)**

### Other Possible Diagnoses:

1. **Transient Neonatal Diabetes Mellitus (TNDM)**: This condition is often caused by genetic abnormalities, such as imprinting disorders or mutations in the ZFP57 gene. It typically presents with severe hyperglycemia in the first few weeks of life and can resolve spontaneously within a few months, but may recur later in life.
2. **Permanent Neonatal Diabetes Mellitus (PNDM)**: This is a rare form of diabetes that appears within the first six months of life and persists throughout life. It can be caused by mutations in various genes, including KCNJ11, ABCC8, and INS.
3. **Wolfram Syndrome**: This is a rare genetic disorder characterized by diabetes insipidus, diabetes mellitus, optic atrophy, and deafness. While it typically presents later in childhood, some cases can present with early-onset diabetes.
4. **Fanconi-Bickel Syndrome**: This is a rare genetic disorder characterized by proximal renal tubular acidosis, hepatic glycogen accumulation, and severe growth retardation. It can present with hyperglycemia and other metabolic abnormalities in early life.

### Additional Considerations:

- **Congenital Hyperinsulinism**: While this condition typically presents with hypoglycemia rather than hyperglycemia, it is important to rule out in cases of severe metabolic disturbances in neonates.
- **Congenital Adrenal Hyperplasia (CAH)**: This can present with hyperglycemia and other endocrine abnormalities, but it is less likely given the specific pattern of hyperglycemia and growth retardation described.

### Diagnostic Approach:

- **Genetic Testing**: Sequencing of the KCNJ11 gene and other relevant genes (e.g., ABCC8, INS, ZFP57) is crucial for confirming the diagnosis.
- **Metabolic Panel**: To assess for other metabolic abnormalities that might be associated with the condition.
- **Imaging and Other Tests**: Depending on the clinical presentation, additional tests such as MRI, renal function tests, and hearing assessments may be necessary.

Early and accurate diagnosis is essential for appropriate management and long-term outcomes in these patients.

### **90. User**

The earliest symptoms are abdominal distension and enlargement of the liver and spleen, and some patients may develop in the neonatal period. Intellectual and motor developmental deficits occur immediately, with hypotonia and motor developmental delays, often without cerebral neurological involvement, and regression of motor and intellectual development is evident after the age of 1 year, eventually progressing to spasticity and tonicity.Cherry erythema on the fundus of the eye can be found in 50% of the patients. Interstitial lung disease may lead to recurrent respiratory infections, hypoxaemia or respiratory failure. Most children die before the age of 3 years.

### Qwen2.5_72b

Based on the clinical manifestations you’ve provided, the most likely diagnosis is **Niemann-Pick Disease Type A (NPA)**. This is a rare lysosomal storage disorder characterized by the accumulation of sphingomyelin in cells, leading to the symptoms you’ve described.

### Most Likely Diagnosis:

- **Niemann-Pick Disease Type A (NPA)**

### Other Possible Diagnoses:

1. **Gaucher Disease Type 2 (Acute Neuronopathic Gaucher Disease)**: This is another lysosomal storage disorder that can present with hepatosplenomegaly, neurological involvement, and early mortality.
2. **Mucopolysaccharidosis Type I (Hurler Syndrome)**: This condition can present with hepatosplenomegaly, developmental delays, and respiratory issues, but it typically also includes skeletal abnormalities and coarse facial features.
3. **Tay-Sachs Disease (Infantile Onset)**: This is a lysosomal storage disorder that can present with hepatosplenomegaly, developmental delays, and cherry-red spot on the retina, but it typically does not include interstitial lung disease.
4. **Krabbe Disease (Infantile Onset)**: This is a lysosomal storage disorder that can present with developmental delays, hypotonia, and spasticity, but it typically does not include hepatosplenomegaly or interstitial lung disease.
5. **Zellweger Syndrome (Peroxisomal Biogenesis Disorder)**: This condition can present with hepatomegaly, developmental delays, and respiratory issues, but it typically also includes distinctive facial features and liver dysfunction.

Each of these conditions has overlapping symptoms, but the combination of hepatosplenomegaly, developmental delays, cherry-red spot on the retina, and early mortality is most characteristic of Niemann-Pick Disease Type A.

### **91. User**

**1. Typical face,** patients of all ages have ptosis, wide eye spacing, medial canthus, and downward slanting of the outer corners of the eyes; low bilateral ear position, posterior rotation, and thick auricles. Child patients may also have a full forehead, low posterior hairline, short nose, low nasal bridge, full nasal tip; thick lips, deep and wide nasolabial groove straight to the upper lip, and so on.

**2. Dwarfism,** which is normal at birth and gradually appears after 1 year of age. Bone age often lags behind, and some adult heights can reach the lower limit of normal level.

**3. cardiovascular,** 50% ~80% of patients have congenital heart disease, pulmonary valve stenosis is the most common, others include hypertrophic cardiomyopathy, ventricular ischemia, pulmonary artery branch stenosis, tetralogy of Fallot, and aortic constriction, etc. Typical ECG changes include electrocardiogram (ECG) and heart rate (HR). Typical electrocardiographic changes include extreme rightward deviation of the electrical axis with counterclockwise rotation of the QRS wave in the precordial leads, leftward deviation of the electrical axis in the V1 lead, incomplete left anterior branch block, or RSR waveforms.

**4. psychomotor retardation**, poor language skills in some patients, depression in adult patients, and a high incidence of autism spectrum manifestations have also been reported.

**5. webbing of the neck**.

**6. thoracic abnormalities,** chicken chest or funnel chest.

**7. wide breast spacing**

**8. cryptorchidism in males** , which is present in 60% to 80% of male patients and can lead to fertility disorders.

**9. abnormal blood clotting and lymphatic dysplasia**

**10. Others,** renal malformations (dilated renal pelvis, double ureteral malformation, isolated kidney, renal hypoplasia, distal ureteral stenosis, etc.), impaired spermatogenesis in males, strabismus, nystagmus, milky coffee spots and freckles on the skin, follicular keratosis of the limbs, Arnold Chiari malformation type I, hydrocephalus, hepatosplenomegaly, and an increased incidence of malignant tumours.

**11. Intrauterine manifestations,** excessive amniotic fluid in the mother during pregnancy, widening of the nuchal translucency, relatively large fetal head, cardiac and renal anomalies.

### Qwen2.5_72b

Based on the clinical manifestations you have provided, the most likely diagnosis is **Noonan Syndrome**. Noonan Syndrome is a genetic disorder that can cause a wide range of features, including those you have described. Here is a detailed breakdown:

### Most Likely Diagnosis:

- **Noonan Syndrome**
  - **Typical Face:** Ptosis, wide eye spacing, downward slanting of the outer corners of the eyes, low-set and posteriorly rotated ears, thick auricles, full forehead, low posterior hairline, short nose, low nasal bridge, thick lips, deep and wide nasolabial groove.
  - **Dwarfism:** Normal at birth, gradual onset after 1 year of age, bone age lagging behind.
  - **Cardiovascular:** 50-80% of patients have congenital heart disease, most commonly pulmonary valve stenosis, hypertrophic cardiomyopathy, ventricular ischemia, pulmonary artery branch stenosis, tetralogy of Fallot, and aortic constriction. ECG changes include extreme rightward deviation of the electrical axis, counterclockwise rotation of the QRS wave, leftward deviation of the electrical axis in V1 lead, incomplete left anterior branch block, or RSR waveforms.
  - **Psychomotor Retardation:** Poor language skills, depression in adults, high incidence of autism spectrum manifestations.
  - **Webbing of the Neck:** Common in Noonan Syndrome.
  - **Thoracic Abnormalities:** Chicken chest or funnel chest.
  - **Wide Breast Spacing:** Characteristic feature.
  - **Cryptorchidism in Males:** Present in 60-80% of male patients, leading to fertility disorders.
  - **Abnormal Blood Clotting and Lymphatic Dysplasia:** Common.
  - **Other:** Renal malformations, impaired spermatogenesis, strabismus, nystagmus, milky coffee spots and freckles on the skin, follicular keratosis of the limbs, Arnold Chiari malformation type I, hydrocephalus, hepatosplenomegaly, increased incidence of malignant tumors.
  - **Intrauterine Manifestations:** Excessive amniotic fluid, widening of the nuchal translucency, relatively large fetal head, cardiac and renal anomalies.

### Other Possible Diagnoses:

- **Turner Syndrome**
  - **Typical Face:** Webbed neck, low-set ears, ptosis, wide eye spacing.
  - **Dwarfism:** Short stature, normal at birth, gradual onset.
  - **Cardiovascular:** Congenital heart defects, including coarctation of the aorta, bicuspid aortic valve.
  - **Psychomotor Retardation:** Normal intelligence, but some may have learning disabilities.
  - **Webbing of the Neck:** Common.
  - **Thoracic Abnormalities:** Shield chest.
  - **Wide Breast Spacing:** Characteristic feature.
  - **Cryptorchidism in Males:** Not applicable (females only).
  - **Abnormal Blood Clotting and Lymphatic Dysplasia:** Lymphedema of the hands and feet.
  - **Other:** Renal malformations, infertility, ovarian dysgenesis.
  - **Intrauterine Manifestations:** Low birth weight, lymphedema.
- **Williams Syndrome**
  - **Typical Face:** Elfin facies (wide mouth, full cheeks, small upturned nose, full lips, widely spaced teeth).
  - **Dwarfism:** Short stature.
  - **Cardiovascular:** Supravalvular aortic stenosis, peripheral pulmonary artery stenosis.
  - **Psychomotor Retardation:** Intellectual disability, hyperacusis, attention deficit hyperactivity disorder (ADHD).
  - **Webbing of the Neck:** Not common.
  - **Thoracic Abnormalities:** Pectus excavatum or pectus carinatum.
  - **Wide Breast Spacing:** Not a characteristic feature.
  - **Cryptorchidism in Males:** Not common.
  - **Abnormal Blood Clotting and Lymphatic Dysplasia:** Not common.
  - **Other:** Hypercalcemia, renal abnormalities, cardiovascular issues, distinctive personality (overly friendly, outgoing).
  - **Intrauterine Manifestations:** Low birth weight, feeding difficulties.
- **Alagille Syndrome**
  - **Typical Face:** Prominent forehead, deep-set eyes, broad nasal bridge, pointed chin.
  - **Dwarfism:** Short stature.
  - **Cardiovascular:** Peripheral pulmonary artery stenosis, tetralogy of Fallot.
  - **Psychomotor Retardation:** Normal intelligence, but some may have learning disabilities.
  - **Webbing of the Neck:** Not common.
  - **Thoracic Abnormalities:** Rib anomalies, scoliosis.
  - **Wide Breast Spacing:** Not a characteristic feature.
  - **Cryptorchidism in Males:** Not common.
  - **Abnormal Blood Clotting and Lymphatic Dysplasia:** Not common.
  - **Other:** Liver disease (cholestasis), renal anomalies, characteristic butterfly vertebrae on X-ray.
  - **Intrauterine Manifestations:** Low birth weight, liver issues.
- **DiGeorge Syndrome (22q11.2 Deletion Syndrome)**
  - **Typical Face:** Micrognathia, cleft palate, low-set ears, wide eye spacing.
  - **Dwarfism:** Short stature.
  - **Cardiovascular:** Tetralogy of Fallot, interrupted aortic arch, ventricular septal defect.
  - **Psychomotor Retardation:** Intellectual disability, speech and language delays, autism spectrum disorder.
  - **Webbing of the Neck:** Not common.
  - **Thoracic Abnormalities:** Rib anomalies, scoliosis.
  - **Wide Breast Spacing:** Not a characteristic feature.
  - **Cryptorchidism in Males:** Not common.
  - **Abnormal Blood Clotting and Lymphatic Dysplasia:** Immune deficiencies, hypocalcemia.
  - **Other:** Renal anomalies, hypoparathyroidism, psychiatric disorders.
  - **Intrauterine Manifestations:** Low birth weight, feeding difficulties, immune deficiencies.
- **Prader-Willi Syndrome**
  - **Typical Face:** Narrow bifrontal diameter, almond-shaped eyes, thin upper lip, downturned mouth.
  - **Dwarfism:** Short stature.
  - **Cardiovascular:** Not a primary feature.
  - **Psychomotor Retardation:** Intellectual disability, developmental delays, behavioral issues.
  - **Webbing of the Neck:** Not common.
  - **Thoracic Abnormalities:** Not a primary feature.
  - **Wide Breast Spacing:** Not a characteristic feature.
  - **Cryptorchidism in Males:** Common.
  - **Abnormal Blood Clotting and Lymphatic Dysplasia:** Not common.
  - **Other:** Hypotonia, obesity, hypogonadism, hyperphagia.
  - **Intrauterine Manifestations:** Hypotonia, feeding difficulties, low birth weight.

These differential diagnoses share some overlapping features with Noonan Syndrome, but the combination of facial features, cardiovascular anomalies, and other systemic manifestations strongly points to Noonan Syndrome as the most likely diagnosis.

### **92. User**

Patients can develop the disease at any age. There are two main clinical categories: early-onset and late-onset. Early-onset cases mainly occur in male children, usually in the neonatal period, and are characterised by a rapid onset of the disease, with a rapid onset of the disease and extensive brain damage due to elevated ammonia levels in the blood. There may be no abnormality at birth, but in the first few days of life, the child will show irritability, lethargy, refusal to eat, shortness of breath and lethargy, which may rapidly develop into spasm, coma and respiratory failure. If not treated urgently, the disease quickly develops into hereditary metabolic encephalopathy and often dies within the first week of life, with survivors often left with severe intellectual impairment. Late-onset disease occurs in older patients, both hemizygous males and heterozygous females, and has relatively mild and variable clinical symptoms. Patients with childhood and adult onset tend to present with chronic neurological damage such as episodes of vomiting, headaches, abnormal behaviour, delirium and confusion. Patients may have clinical phenotypes such as hepatomegaly, recurrent seizures, growth retardation, and behavioural abnormalities. Despite the milder symptoms of the late-onset form, life-threatening acute episodes of hyperammonemia can be induced by environmental factors such as illness, stress, and a high-protein diet. The majority of heterozygous female carriers remain asymptomatic throughout their lives, while a few develop the disease, with individual variability in age of onset and clinical manifestations, both early and late onset.

### Qwen2.5_72b

Based on the clinical manifestations and the pattern of inheritance described, the most likely diagnosis is **Ornithine Transcarbamylase Deficiency (OTC Deficiency)**. OTC deficiency is an X-linked disorder of the urea cycle, which leads to hyperammonemia and can present with the symptoms you have described.

### Most Likely Diagnosis:

- **Ornithine Transcarbamylase Deficiency (OTC Deficiency)**

### Other Possible Diagnoses:

1. **Citrullinemia Type I (ASS1 Deficiency)**: Another urea cycle disorder that can present with hyperammonemia, particularly in the neonatal period, with symptoms similar to OTC deficiency.
2. **Argininosuccinic Aciduria (ASL Deficiency)**: Another urea cycle disorder that can cause hyperammonemia, with symptoms ranging from neonatal onset to later onset in childhood or adulthood.
3. **Glutaric Aciduria Type II (GA II)**: A metabolic disorder that can present with hyperammonemia, particularly in the neonatal period, and can cause neurological damage and other systemic symptoms.
4. **Maple Syrup Urine Disease (MSUD)**: A metabolic disorder that can present with hyperammonemia, particularly in the neonatal period, and can cause neurological damage, lethargy, and other systemic symptoms.

### Key Points:

- **OTC Deficiency** is the most likely diagnosis given the X-linked inheritance pattern, the rapid onset in male neonates, and the variability in late-onset cases.
- **Citrullinemia Type I** and **Argininosuccinic Aciduria** are also urea cycle disorders that can present similarly, but they are less likely to be X-linked.
- **Glutaric Aciduria Type II** and **Maple Syrup Urine Disease** are other metabolic disorders that can cause hyperammonemia and similar clinical presentations, but they have different genetic and biochemical bases.

If you have any specific test results or additional clinical details, they can further refine the differential diagnosis.

### **93. User**

The clinical phenotype of the disease varies in severity. The main clinical manifestations of the patients are recurrent fractures under minor external forces, skeletal deformities, and varying degrees of activity limitation since childhood. Patients may have extra-skeletal manifestations such as blue sclera, ligamentous laxity, dentin hypoplasia, hearing loss, and cardiac valvulopathy, etc. The imaging features of OI mainly include: thinning of bones in many parts of the body; thinning of the cranial plate, widening of fontanelle and cranial suture, interstitial bone in occipital bones, and flattening of the base of the skull; lateral curvature or posterior convexity deformity of the spine, deformation of vertebral bodies, and compression fracture of multiple vertebral bodies; twisted and deformed thorax, or even collapse; slender long bones of the limbs, and even collapse; and the long bones of the limbs are thin and thin. The long bones of the limbs are slender, the bone cortex is thin, the bone marrow cavity is relatively large, the epiphyses are widened, there are many long bone fractures, and the bones are curved and deformed.

### Qwen2.5_72b

Based on the clinical manifestations and imaging features you have provided, the most likely diagnosis is **Osteogenesis Imperfecta (OI)**, also known as brittle bone disease. OI is a genetic disorder characterized by bones that break easily, often from minor trauma or even without any apparent cause. The condition can vary widely in severity, from mild forms with few fractures to severe forms that can be life-threatening.

### Other Possible Diagnoses:

- **Ehlers-Danlos Syndrome (EDS)**:
  - **Clinical Features**: Hypermobile joints, skin that bruises easily, and frequent dislocations. Some types of EDS can also present with skeletal deformities and dental issues.
  - **Imaging Features**: May show joint hypermobility and ligamentous laxity, but not the characteristic bone thinning and fractures seen in OI.
- **Marfan Syndrome**:
  - **Clinical Features**: Tall stature, long limbs, arachnodactyly, lens dislocation, and cardiovascular issues such as aortic aneurysm.
  - **Imaging Features**: May show skeletal abnormalities like pectus excavatum or carinatum, but not the bone fragility and fractures characteristic of OI.
- **Rickets**:
  - **Clinical Features**: Bowing of the legs, rachitic rosary, and delayed closure of fontanelles. Often associated with vitamin D deficiency or other metabolic bone disorders.
  - **Imaging Features**: Characterized by widened growth plates, cupping and fraying of the metaphyses, and undermineralization of the bones, but not the thinning and fractures seen in OI.
- **Osteoporosis**:
  - **Clinical Features**: Increased risk of fractures, especially in the spine, hip, and wrist. Often seen in older adults but can occur in younger individuals with certain risk factors.
  - **Imaging Features**: Generalized bone loss and thinning, but not the specific skeletal deformities and blue sclera seen in OI.
- **Skeletal Dysplasias**:
  - **Clinical Features**: Short stature, disproportionate limb lengths, and various skeletal deformities. There are many types of skeletal dysplasias, each with its own specific features.
  - **Imaging Features**: May show a variety of bone abnormalities, but the specific combination of thin bones, fractures, and extra-skeletal manifestations seen in OI is unique.

### Conclusion:

While the clinical and imaging features strongly suggest Osteogenesis Imperfecta, it is important to consider and rule out other differential diagnoses, especially if the clinical presentation is atypical or if there are additional findings that do not fit the classic OI profile. Genetic testing and a detailed family history can be helpful in confirming the diagnosis.

### **94. User**

**1. Pancytopenia,** Many patients may have a combination of pancytopenia and neutropenia. Clinical manifestations include malaise, recurrent infections and bleeding tendencies.

**2. Haemoglobinuria** , typical haemoglobinuria is soy sauce or strong tea colour. It usually lasts for 23 days and subsides on its own without treatment, while in severe cases, it lasts for 12 weeks or even longer. Some patients have frequent episodes of haemoglobinuria, while a significant number of patients have no clear episodes of haemoglobinuria over a long period of time or observation.

Episodes of haemoglobinuria may be accompanied by fever, low back pain and abdominal pain. Other symptoms associated with smooth muscle dysfunction can include dysphagia, abdominal pain, bloating, back pain, headache, oesophageal spasms, and erectile dysfunction. Rare conditions include choledochal duct dyskinesia, acute pancreatitis, ischaemia and ulceration of the duodenum or colon.

**3. thrombosis**.

**Jaundice and hepatosplenomegaly** , less than half of patients have mild jaundice. Most patients do not have hepatosplenomegaly.

Common comorbidities include infection, anaemic heart disease, cholelithiasis, renal insufficiency and even failure.

### Qwen2.5_72b

Based on the clinical manifestations you’ve provided, the most likely rare disease is **Paroxysmal Nocturnal Hemoglobinuria (PNH)**. Here’s a detailed explanation and five other possible diagnoses:

### 1. **Paroxysmal Nocturnal Hemoglobinuria (PNH)**

- **Pancytopenia**: Common in PNH due to bone marrow failure.
- **Hemoglobinuria**: Characteristic of PNH, often described as soy sauce or strong tea color.
- **Thrombosis**: PNH patients are at increased risk of thrombosis, particularly in unusual sites like the hepatic, portal, and cerebral veins.
- **Jaundice and hepatosplenomegaly**: Mild jaundice can occur due to hemolysis, and hepatosplenomegaly is less common but can be present.
- **Smooth muscle dysfunction**: Symptoms like dysphagia, abdominal pain, and erectile dysfunction can occur due to smooth muscle involvement.

### 2. **Aplastic Anemia**

- **Pancytopenia**: Severe reduction in all blood cell lines.
- **Thrombosis**: Less common but can occur due to reduced platelet count.
- **Jaundice and hepatosplenomegaly**: Not typically associated with aplastic anemia.
- **Smooth muscle dysfunction**: Not a typical feature.

### 3. **Hemolytic Uremic Syndrome (HUS)**

- **Pancytopenia**: Can occur due to hemolysis and bone marrow suppression.
- **Hemoglobinuria**: Hematuria is common, but hemoglobinuria is less typical.
- **Thrombosis**: Microangiopathic thrombosis is a hallmark.
- **Jaundice and hepatosplenomegaly**: Jaundice can occur due to hemolysis, but hepatosplenomegaly is less common.
- **Smooth muscle dysfunction**: Not a typical feature.

### 4. **Thrombotic Thrombocytopenic Purpura (TTP)**

- **Pancytopenia**: Thrombocytopenia and microangiopathic hemolytic anemia are common.
- **Hemoglobinuria**: Hematuria can occur but hemoglobinuria is less typical.
- **Thrombosis**: Microangiopathic thrombosis is a hallmark.
- **Jaundice and hepatosplenomegaly**: Jaundice can occur due to hemolysis, but hepatosplenomegaly is less common.
- **Smooth muscle dysfunction**: Not a typical feature.

### 5. **Systemic Lupus Erythematosus (SLE)**

- **Pancytopenia**: Can occur due to autoimmune destruction of blood cells.
- **Hemoglobinuria**: Hematuria can occur but hemoglobinuria is less typical.
- **Thrombosis**: Increased risk of thrombosis, particularly in antiphospholipid syndrome.
- **Jaundice and hepatosplenomegaly**: Hepatitis and hepatosplenomegaly can occur.
- **Smooth muscle dysfunction**: Can occur due to vasculitis and other systemic effects.

### 6. **Evan’s Syndrome**

- **Pancytopenia**: Autoimmune destruction of red blood cells and platelets.
- **Hemoglobinuria**: Hematuria can occur but hemoglobinuria is less typical.
- **Thrombosis**: Increased risk of thrombosis due to autoimmune mechanisms.
- **Jaundice and hepatosplenomegaly**: Jaundice can occur due to hemolysis, and hepatosplenomegaly can be present.
- **Smooth muscle dysfunction**: Not a typical feature.

### Conclusion

The most likely diagnosis based on the provided clinical manifestations is **Paroxysmal Nocturnal Hemoglobinuria (PNH)**. However, the other conditions listed should also be considered and ruled out through appropriate diagnostic testing.

### **95. User**

Clinical manifestations include repeated intussusception, abdominal pain, diarrhoea, mucus stools, blood in stool, constipation, vomiting blood and anaemia. Characteristic clinical manifestations are skin and mucous membrane pigmentation and multiple polyps in the gastrointestinal tract. 95% of the patients have hyperpigmentation, which can be found on the lips and surrounding areas of the mouth, cheeks, face, fingers, palms of the hands, soles of the feet, perianal area, etc., and occasionally on the intestinal mucous membranes. The pigmentation can be brown, tan, grey, blue, etc. It usually occurs before the age of 2 years, and the size is 1~5mm. 88%-100% of the patients will develop gastrointestinal polyps, and the onset of gastrointestinal polyps most often occurs at the age of 11-13 years, and the polyps can occur throughout the entire gastrointestinal tract, with the small intestine being the most common, followed by the colon and the stomach. The size of the polyps is variable, and their tips vary in length and thickness. The risk of cancer is high, with an average age of 42 years.

### Qwen2.5_72b

Based on the clinical manifestations you’ve provided, the most likely diagnosis is **Peutz-Jeghers Syndrome (PJS)**. This is a rare genetic disorder characterized by the presence of multiple hamartomatous polyps in the gastrointestinal tract and characteristic mucocutaneous pigmentation.

### Differential Diagnoses:

- **Familial Adenomatous Polyposis (FAP)**:
  - **Clinical Features**: Multiple adenomatous polyps in the colon and rectum, often leading to colorectal cancer. Extracolonic manifestations include desmoid tumors, osteomas, and epidermoid cysts.
  - **Distinguishing Features**: No characteristic mucocutaneous pigmentation, polyps are adenomatous rather than hamartomatous.
- **Juvenile Polyposis Syndrome (JPS)**:
  - **Clinical Features**: Multiple juvenile polyps in the gastrointestinal tract, particularly in the colon and rectum. Increased risk of colorectal cancer.
  - **Distinguishing Features**: No characteristic mucocutaneous pigmentation, polyps are juvenile rather than hamartomatous.
- **Cowden Syndrome**:
  - **Clinical Features**: Multiple hamartomas in various tissues, including the skin, mucous membranes, and gastrointestinal tract. Increased risk of breast, thyroid, and endometrial cancers.
  - **Distinguishing Features**: Characteristic facial trichilemmomas, acral keratoses, and macrocephaly. Mucocutaneous pigmentation is not a hallmark.
- **Neurofibromatosis Type 1 (NF1)**:
  - **Clinical Features**: Multiple café-au-lait spots, neurofibromas, and Lisch nodules (iris hamartomas). Gastrointestinal involvement can include polyps, but it is less common.
  - **Distinguishing Features**: Characteristic café-au-lait spots, axillary and inguinal freckling, and neurofibromas.
- **Hereditary Nonpolyposis Colorectal Cancer (Lynch Syndrome)**:
  - **Clinical Features**: Increased risk of colorectal cancer and other cancers, particularly endometrial, ovarian, and gastric cancers. No characteristic mucocutaneous pigmentation.
  - **Distinguishing Features**: No multiple polyps or characteristic mucocutaneous pigmentation, but a strong family history of cancer.

### Summary:

- **Primary Diagnosis**: Peutz-Jeghers Syndrome (PJS)
- **Differential Diagnoses**:
  1. Familial Adenomatous Polyposis (FAP)
  2. Juvenile Polyposis Syndrome (JPS)
  3. Cowden Syndrome
  4. Neurofibromatosis Type 1 (NF1)
  5. Hereditary Nonpolyposis Colorectal Cancer (Lynch Syndrome)

Each of these conditions has distinct clinical features that can help differentiate them from PJS. Genetic testing and further clinical evaluation are essential for a definitive diagnosis.

### **96. User**

In the neonatal period, most of the children have no clinical symptoms, but after 3-4 months of life, typical symptoms gradually appear, and the symptoms are obvious at the age of 1 year. A few months after birth, due to insufficient melanin synthesis, their hair and iris colour gradually becomes lighter, yellow or brownish-yellow, with white skin. Urine and sweat contain a large amount of phenyl lactic acid and have a foul odour of rat urine. As they grow older, they gradually show delayed intellectual development, with predominantly cognitive developmental disorders, microcephaly, seizures, as well as behavioural and personality abnormalities, such as hyperactivity, self-mutilation, aggression, autism, low self-esteem, depression and other neurological manifestations. Vomiting and eczema are also commonly seen in infancy.

### Qwen2.5_72b

Based on the clinical manifestations you’ve provided, the most likely diagnosis is **Phenylketonuria (PKU)**. PKU is a rare inherited metabolic disorder characterized by a deficiency in the enzyme phenylalanine hydroxylase, which leads to the accumulation of phenylalanine in the blood and brain, causing a range of neurological and developmental issues.

### Most Likely Diagnosis:

- **Phenylketonuria (PKU)**

### Other Possible Diagnoses:

1. **Tyrosinemia Type I**: This is another metabolic disorder that can cause liver and kidney damage, as well as neurological symptoms. However, it typically presents with more severe liver symptoms and a different metabolic profile.
2. **Homocystinuria**: This is a disorder of methionine metabolism that can cause intellectual disability, seizures, and other neurological symptoms, but it is often associated with tall stature, lens dislocation, and thromboembolic events.
3. **Maple Syrup Urine Disease (MSUD)**: This is a disorder of branched-chain amino acid metabolism that can cause neurological symptoms and a characteristic sweet odor in the urine, but it typically presents more acutely in the neonatal period.
4. **Zellweger Syndrome**: This is a peroxisomal disorder that can cause developmental delay, seizures, and other neurological symptoms, but it is usually associated with more severe and early-onset symptoms, including facial dysmorphism and liver dysfunction.
5. **Congenital Hypothyroidism**: This can cause developmental delay and neurological symptoms, but it is typically associated with other signs such as jaundice, poor feeding, and hypotonia in the neonatal period.

### Key Features of PKU:

- **Hair and Iris Lightening**: Due to reduced melanin synthesis.
- **Foul Odor**: Characteristic “mousy” or “rat-like” odor in urine and sweat due to the accumulation of phenyl lactic acid.
- **Neurological Symptoms**: Delayed intellectual development, microcephaly, seizures, and behavioral abnormalities.
- **Eczema and Vomiting**: Common in infancy.

If PKU is suspected, early diagnosis and treatment with a low-phenylalanine diet are crucial to prevent severe neurological damage and developmental delays.

### **97. User**

Mainly skin symptoms and neuropsychiatric symptoms.

**1. The group of cutaneous symptoms,** are erythema, herpes and even ulceration on exposed parts of the skin after exposure to light. The rash may be of the type of eczema, urticaria, summertime itchy rash or erythema multiforme. The oral mucosa may have red patches and brownish-red teeth. Eye damage such as conjunctivitis, keratitis and iritis may also occur. In severe cases, there may be deformation of the skin of the nose, ears and fingers with kinking. There may be special purple colouration. Hirsutism may be present.

**2. A cluster of neuropsychiatric symptoms ,**  the most common manifestation of which is acute abdominal pain, which tends to be severe, stable, and poorly localised, sometimes accompanied by painful cramps. It may be accompanied by constipation, bloating, nausea, vomiting, and sometimes diarrhoea and increased bowel sounds. Sensory and motor neuropathies may precede the onset of abdominal pain and present as limb pain with numbness, abnormal sensation and sensory inversions; motor weakness often begins proximally in the upper limbs and may progress to the lower limbs and distal extremities. Involvement of the cerebral nerves may lead to medullary paralysis, impaired respiratory function and death. Severe motor neuropathy may result in quadriplegia. Autonomic nervous system involvement manifests as tachycardia, hypertension, sweating, agitation, and tremor. Neurogenic bladder dysfunction may cause dysuria, dyspareunia, urinary retention and incontinence. Other acute neuropsychiatric manifestations include insomnia, anxiety, agitation, agitation, hallucinations, hysteria, disorientation, delirium, affective apathy, depression, phobias, and altered consciousness ranging from lethargy to coma.

### Qwen2.5_72b

Based on the clinical manifestations you’ve provided, the most likely rare disease is **Porphyria Cutanea Tarda (PCT)**, a type of porphyria. However, the neuropsychiatric symptoms are more characteristic of **Acute Intermittent Porphyria (AIP)**, another type of porphyria. Given the combination of cutaneous and neuropsychiatric symptoms, **AIP** is the most likely diagnosis.

### Most Likely Diagnosis:

- **Acute Intermittent Porphyria (AIP)**

### Other Possible Diagnoses:

1. **Porphyria Cutanea Tarda (PCT)**
2. **Hereditary Coproporphyria (HCP)**
3. **Variegate Porphyria (VP)**
4. **Erythropoietic Protoporphyria (EPP)**
5. **Systemic Lupus Erythematosus (SLE)**

### Explanation:

- **Acute Intermittent Porphyria (AIP)**: This is a genetic disorder characterized by acute attacks of abdominal pain, neuropathy, and neuropsychiatric symptoms. The cutaneous symptoms are less common in AIP but can occur in some cases, especially if there is a mixed porphyria.
- **Porphyria Cutanea Tarda (PCT)**: This is the most common form of porphyria and primarily affects the skin, causing photosensitivity, blistering, and scarring. However, it does not typically present with the severe neuropsychiatric symptoms described.
- **Hereditary Coproporphyria (HCP)**: This condition can present with both cutaneous and neuropsychiatric symptoms, similar to AIP, but the cutaneous symptoms are more prominent.
- **Variegate Porphyria (VP)**: This condition also presents with both cutaneous and neuropsychiatric symptoms, making it a possible differential diagnosis.
- **Erythropoietic Protoporphyria (EPP)**: This condition primarily affects the skin, causing photosensitivity and pain, but does not typically present with the severe neuropsychiatric symptoms described.
- **Systemic Lupus Erythematosus (SLE)**: This autoimmune disorder can present with a wide range of symptoms, including skin rashes, photosensitivity, and neuropsychiatric manifestations, but the specific combination of symptoms is more characteristic of porphyria.

Given the specific combination of cutaneous and neuropsychiatric symptoms, **Acute Intermittent Porphyria (AIP)** is the most likely diagnosis. However, further diagnostic testing, such as urinary porphobilinogen (PBG) and porphyrin levels, is essential to confirm the diagnosis.

### **98. User**

**1. Infection, patients** are infected to varying degrees. Infections are recurrent, severe, difficult to treat, opportunistic and have a broad spectrum of pathogens. Typical children are usually born with no specific manifestations, and fatal infections can occur before 2 months of age due to cellular immune abnormalities. The sites and pathogens of infection are diverse, including recurrent pneumonia, recurrent thrush, persistent diarrhoea, recurrent otitis media, persistent viral infections (e.g., respiratory syncytial virus, EBV, CMV, etc.), and conditionally causative organisms, such as Pneumocystis carinii, are also common.

**2. Vaccine disease,** in which patients are extremely immunocompromised, can occur with infection and dissemination of live attenuated vaccines. Severe diarrhoea can also occur in children vaccinated with attenuated rotavirus vaccine.

**3. Retarded or arrested growth and development** is a distinguishing feature of the disease. It is associated with recurrent infections, diarrhoea and corresponding genetic defects.

Children may also have a rash and enlarged liver and spleen lymph nodes similar to erysipelas.

In other children, erythroderma-like symptoms, hepatosplenomegaly, seborrheic dermatitis, haematopenia, and sclerosing cholangitis may be present.

### Qwen2.5_72b

Based on the clinical manifestations you have provided, the most likely rare disease is **Severe Combined Immunodeficiency (SCID)**. SCID is a group of rare, life-threatening genetic disorders characterized by a lack of functional T cells and B cells, leading to severe immunodeficiency. The clinical features you described, such as recurrent and severe infections, opportunistic infections, failure to thrive, and vaccine-related complications, are highly indicative of SCID.

### Differential Diagnoses:

- **Chronic Granulomatous Disease (CGD):** This is an inherited primary immunodeficiency characterized by recurrent and severe bacterial and fungal infections. Patients with CGD often have granulomas and can present with recurrent pneumonia, skin infections, and lymphadenopathy.
- **Hyper-IgM Syndrome:** This is a primary immunodeficiency characterized by low levels of IgG, IgA, and IgE, and normal or elevated levels of IgM. Patients often present with recurrent infections, particularly Pneumocystis jirovecii pneumonia, and can have hepatosplenomegaly and lymphadenopathy.
- **Wiskott-Aldrich Syndrome (WAS):** This is an X-linked recessive disorder characterized by eczema, thrombocytopenia, and immunodeficiency. Patients can have recurrent infections, autoimmune disorders, and an increased risk of malignancies.
- **X-linked Agammaglobulinemia (Bruton’s Agammaglobulinemia):** This is a primary immunodeficiency characterized by a lack of mature B cells and low levels of immunoglobulins. Patients often present with recurrent bacterial infections, particularly respiratory and gastrointestinal infections.
- **Common Variable Immunodeficiency (CVID):** This is a heterogeneous group of disorders characterized by low levels of immunoglobulins and an increased susceptibility to infections. Patients can have recurrent respiratory infections, gastrointestinal symptoms, and autoimmune manifestations.

### Summary:

- **Most Likely Diagnosis:** Severe Combined Immunodeficiency (SCID)
- **Differential Diagnoses:**
  1. Chronic Granulomatous Disease (CGD)
  2. Hyper-IgM Syndrome
  3. Wiskott-Aldrich Syndrome (WAS)
  4. X-linked Agammaglobulinemia (Bruton’s Agammaglobulinemia)
  5. Common Variable Immunodeficiency (CVID)

These differential diagnoses should be considered and ruled out through appropriate laboratory tests and clinical evaluation.

### **99. User**

The core symptom is dystonia, which is mainly manifested by abnormal expressive postures and involuntary movements, mostly involving the head and neck muscles, trunk muscles, rotator anterior muscles of the limbs, finger and wrist flexors, toe extensors and plantar flexors. It is aggravated by random movements, reduced or disappeared when resting and sleeping, and in the late stage, it can be a fixed twisting spastic deformity. Sensory trickery (relief techniques) and movement specificity are the characteristic manifestations of dystonia. Sensory tricks (relieving techniques) are random movements that are specifically designed to correct abnormal postures or relieve dystonic movements, usually simple movements that involve or target the affected area, but are not forceful to counteract the symptoms of dystonia. Movement specificity refers to the presence of dystonia only during specific activities or the performance of specific tasks, such as certain occupations (e.g., writing spasms, musician’s spasms) or sports.

### Qwen2.5_72b

Based on the clinical manifestations you’ve provided, the most likely diagnosis is **Primary (Idiopathic) Dystonia**. This is a neurological disorder characterized by sustained or intermittent muscle contractions causing abnormal, often repetitive, movements, postures, or both. The symptoms you described, such as abnormal postures, involuntary movements, and the characteristic sensory tricks and movement specificity, are hallmark features of dystonia.

### Other Possible Diagnoses:

- **Secondary Dystonia**: This can be caused by various underlying conditions such as brain injuries, infections, toxins, or genetic disorders. The clinical presentation can be similar to primary dystonia, but there is usually a known cause.
- **Wilson’s Disease**: A genetic disorder that results in copper accumulation in the liver, brain, and other organs. It can present with dystonia, particularly in the head and neck, along with other neurological and hepatic symptoms.
- **Huntington’s Disease**: A progressive neurodegenerative disorder that can present with dystonia, chorea, and cognitive decline. The dystonic movements are often part of a broader movement disorder.
- **Parkinson’s Disease**: While primarily characterized by tremor, bradykinesia, and rigidity, some patients can develop dystonic symptoms, especially in the early stages or as a side effect of medication.
- **Tardive Dystonia**: A form of tardive dyskinesia that can occur as a side effect of long-term use of certain medications, particularly antipsychotics. It can present with dystonic movements, often involving the face, neck, and limbs.

### Summary:

- **Primary (Idiopathic) Dystonia**: Most likely diagnosis based on the provided symptoms.
- **Secondary Dystonia**: Consider if there is a known underlying cause.
- **Wilson’s Disease**: Consider if there are additional hepatic or other neurological symptoms.
- **Huntington’s Disease**: Consider if there is a family history or other neurological symptoms.
- **Parkinson’s Disease**: Consider if there are other parkinsonian features.
- **Tardive Dystonia**: Consider if there is a history of medication use.

Further diagnostic testing, such as genetic testing, imaging studies, and a detailed patient history, would be necessary to confirm the diagnosis and rule out other conditions.

### **100. User**

**1. Kidney,** the main manifestations are limb oedema and increased foam in the urine. Laboratory tests may reveal simple moderate proteinuria or nephrotic syndrome, and renal insufficiency may be seen in the advanced stage.

**2. Heart,** the main manifestations are shortness of breath after activity, limb edema, pleural effusion, ascites and other signs of restrictive cardiac insufficiency.

**3. Liver,** there may be slight discomfort or pain in the liver area, but most patients may be asymptomatic, and hepatomegaly or liver enzyme abnormalities are often detected during physical examination. Liver failure may occur in advanced stages of the disease.

**Peripheral and autonomic nerves,** Symmetrical sensory and/or motor peripheral neuropathy of the extremities. Autonomic abnormalities are often manifested by upright hypotension, gastroparesis, pseudo-intestinal obstruction, and impotence.

**5. Gastrointestinal tract** , can present with total gastrointestinal involvement, with the stomach and small intestine being the most commonly involved. It can present with epigastric discomfort, dyspepsia, diarrhoea, constipation, malabsorption syndrome and gastrointestinal bleeding.

**6. Soft tissues ,** Tongue involvement may present with megaglossia, tongue dysmobility, and dysarthria. Skin mucosa may present with skin purpura and petechiae, which are more common around the orbits and in the loose skin areas of the neck. Nail atrophy and hair loss may also occur.

**7. Coagulation abnormalities** Patients often have coagulation factor X deficiency, resulting in corresponding bleeding manifestations.

### Qwen2.5_72b

Based on the clinical manifestations you have provided, the most likely rare disease is **Amyloidosis**. Amyloidosis is a group of diseases characterized by the deposition of amyloid proteins in various organs, leading to organ dysfunction. The symptoms you described, including kidney involvement (proteinuria, nephrotic syndrome), heart involvement (restrictive cardiomyopathy), liver involvement (hepatomegaly, liver enzyme abnormalities), peripheral and autonomic neuropathy, gastrointestinal involvement, soft tissue involvement, and coagulation abnormalities, are all consistent with systemic amyloidosis.

### Other Possible Diagnoses:

- **Multiple Myeloma**: This is a cancer of plasma cells that can cause similar symptoms, including kidney damage, anemia, bone pain, and increased protein in the urine. It can also lead to amyloid-like deposits in various organs.
- **Systemic Sclerosis (Scleroderma)**: This is an autoimmune disease that can affect multiple organs, including the kidneys, heart, and gastrointestinal tract. It can cause proteinuria, restrictive cardiomyopathy, and gastrointestinal dysmotility.
- **Hepatic Amyloidosis**: This is a specific form of amyloidosis that primarily affects the liver, leading to hepatomegaly and liver enzyme abnormalities. It can also involve other organs, but the liver is the primary site of amyloid deposition.
- **Familial Mediterranean Fever (FMF)**: This is an inherited autoinflammatory disorder that can cause recurrent fever, serositis (pleuritis, peritonitis), and can lead to amyloidosis, particularly in the kidneys.
- **Light Chain Deposition Disease (LCDD)**: This is a rare condition where monoclonal light chains deposit in the kidneys, leading to proteinuria and nephrotic syndrome. It can also affect other organs, including the heart and liver.

### Conclusion:

While amyloidosis is the most likely diagnosis given the wide range of organ involvement and the specific symptoms described, it is important to consider and rule out other conditions that can present with similar clinical manifestations. Further diagnostic testing, such as tissue biopsy, serum and urine protein electrophoresis, and genetic testing, may be necessary to confirm the diagnosis.

### **101. User**

Jaundice and itchy skin are typical clinical manifestations. Other developmental delays include short stature, delayed pubertal growth, gallbladder stones, steatorrhea due to fat absorption disorders, hepatosplenomegaly, rickets due to fat-soluble vitamin deficiencies, delayed bone age, dry eye, coagulation disorders, and neuromuscular disorders. Children may also develop visual and hearing abnormalities, irritability, lethargy and poor concentration. In advanced stages, portal hypertension and liver tumours may occur.

### Qwen2.5_72b

Based on the clinical manifestations you’ve provided, the most likely rare disease is **Alagille Syndrome (ALGS)**. Alagille Syndrome is a genetic disorder that can affect the liver, heart, kidneys, eyes, face, and skeleton. The liver manifestations, including jaundice, pruritus (itchy skin), and hepatosplenomegaly, are particularly characteristic. Other features such as short stature, delayed pubertal growth, gallbladder stones, steatorrhea, rickets, and coagulation disorders are also consistent with ALGS.

### Other Possible Diagnoses:

- **Biliary Atresia**: This is a rare condition in newborns where the bile ducts outside the liver are blocked or absent, leading to jaundice, hepatosplenomegaly, and steatorrhea. However, it typically presents in the first few weeks of life and does not usually include the other systemic features you’ve described.
- **Cystic Fibrosis**: This genetic disorder affects the lungs, pancreas, liver, and intestines. It can cause steatorrhea, hepatosplenomegaly, and growth delays, but it is less likely to present with the specific liver and eye abnormalities seen in Alagille Syndrome.
- **Wilson’s Disease**: This is a genetic disorder that results in copper accumulation in the liver, brain, and other organs. It can cause jaundice, hepatosplenomegaly, and neuromuscular disorders, but it typically presents later in childhood or adolescence and includes Kayser-Fleischer rings in the eyes.
- **Primary Sclerosing Cholangitis (PSC)**: This is a chronic liver disease characterized by inflammation and fibrosis of the bile ducts. It can cause jaundice, pruritus, and hepatosplenomegaly, but it is more common in adults and is often associated with inflammatory bowel disease.
- **Zellweger Spectrum Disorders (ZSD)**: These are a group of rare genetic disorders affecting the peroxisomes, which can lead to liver dysfunction, growth delays, and neurological abnormalities. However, ZSD typically presents in infancy with more severe and early-onset symptoms.

### Summary:

- **Most Likely Diagnosis**: Alagille Syndrome (ALGS)
- **Other Possible Diagnoses**:
  1. Biliary Atresia
  2. Cystic Fibrosis
  3. Wilson’s Disease
  4. Primary Sclerosing Cholangitis (PSC)
  5. Zellweger Spectrum Disorders (ZSD)

Further diagnostic testing, including genetic testing, liver function tests, and imaging studies, would be necessary to confirm the diagnosis.

### **102. User**

The disease starts in childhood. The disease manifests itself as a mild delay in motor development and progressive atrophy of skeletal muscles, which affects the motor function of the limbs and gradually leads to gait abnormalities and limited movement of the upper limbs, with the natural course of the disease often resulting in loss of the ability to walk at about 10 years of age. Thereafter, scoliosis, joint contracture, respiratory muscle weakness and dilated cardiomyopathy appeared, and the patient died of respiratory failure and cardiac failure at the age of about 20 years. Pseudohypertrophy of both gastrocnemius muscles, pseudohypertrophy of both forearms and lingual muscles, positive Gowers’ sign, and lumbar lordosis can be seen on examination. The disease may start in youth or even adulthood, and some patients do not affect survival. Signs of pseudohypertrophy are obvious, and in some patients, dilated cardiomyopathy is first apparent when limb weakness is still mild.

### Qwen2.5_72b

Based on the clinical manifestations you have provided, the most likely diagnosis is **Duchenne Muscular Dystrophy (DMD)**. DMD is a rare, progressive muscle-wasting disease that primarily affects boys and is caused by mutations in the dystrophin gene, leading to a lack of the dystrophin protein, which is essential for muscle function.

### Differential Diagnoses:

- **Becker Muscular Dystrophy (BMD)**: Similar to DMD but generally less severe and with a later onset. BMD is also caused by mutations in the dystrophin gene but results in a partially functional dystrophin protein.
- **Limb-Girdle Muscular Dystrophy (LGMD)**: A group of disorders that primarily affect the muscles of the shoulders and hips. LGMD can be inherited in various ways and can have a wide range of severities.
- **Spinal Muscular Atrophy (SMA)**: A group of genetic disorders that affect the motor neurons in the spinal cord, leading to muscle weakness and atrophy. SMA can present in infancy, childhood, or adulthood.
- **Congenital Muscular Dystrophy (CMD)**: A group of muscular dystrophies that are present at birth or in early infancy. CMD can be caused by various genetic mutations and can have a wide range of severities.
- **Myotonic Dystrophy (DM)**: A genetic disorder characterized by progressive muscle weakness and myotonia (difficulty relaxing muscles after contraction). DM can affect multiple systems, including the heart, eyes, and endocrine system.

### Key Features of Duchenne Muscular Dystrophy (DMD):

- **Onset in childhood**: Typically between 3 and 5 years of age.
- **Progressive muscle weakness**: Begins in the pelvic and shoulder girdle muscles, leading to difficulty walking, running, and jumping.
- **Gait abnormalities**: Waddling gait and frequent falls.
- **Pseudohypertrophy**: Enlargement of the calf muscles due to fat and connective tissue replacing muscle fibers.
- **Positive Gowers’ sign**: Difficulty rising from a sitting or lying position, often using the hands to “climb” up the legs.
- **Scoliosis and joint contractures**: Common as the disease progresses.
- **Respiratory muscle weakness**: Leading to respiratory failure.
- **Dilated cardiomyopathy**: Often develops in the teenage years.
- **Prognosis**: Most patients lose the ability to walk by age 10-12 and have a life expectancy of around 20-30 years, although advances in care have improved outcomes.

If you have any further questions or need more detailed information, please let me know!

### **103. User**

Mainly hyperaemia, brain damage and cardiomyopathy.

**1. Neonatal onset** Normal at birth, vomiting, lethargy, hypotonia, convulsions, dyspnoea, hyperaemia, ketosis, hypoglycaemia, acidosis, dilated cardiomyopathy, pancreatitis and other abnormalities after starting breastfeeding, with a high morbidity and mortality rate.

**2. Delayed-onset** is often induced by fever, starvation, high-protein diet, and infections, and manifests itself as feeding difficulties in infancy and early childhood, developmental delays, convulsions, and hypotonia. In many patients, cognitive ability and neurological development are impaired, with an increase in slow waves or epileptic waves on the EEG; some patients may have fractures and osteoporosis on X-ray; bone marrow suppression is also common, causing granulocytopenia, anaemia and thrombocytopenia. There may also be cardiac damage, such as cardiomyopathy, arrhythmia, prolonged QT interval, and diminished cardiac function. Renal impairment is less common.

### Qwen2.5_72b

Based on the clinical manifestations you’ve provided, the most likely rare disease is **Maple Syrup Urine Disease (MSUD)**. MSUD is a metabolic disorder caused by a deficiency in the branched-chain alpha-keto acid dehydrogenase complex, leading to the accumulation of branched-chain amino acids (leucine, isoleucine, and valine) and their corresponding ketoacids.

### Main Clinical Manifestations:

- **Neonatal Onset:**
  - Normal at birth
  - Vomiting, lethargy, hypotonia, convulsions, dyspnoea
  - Hyperaemia (often due to metabolic acidosis)
  - Ketosis, hypoglycaemia, acidosis
  - Dilated cardiomyopathy
  - Pancreatitis
  - High morbidity and mortality rate
- **Delayed-Onset:**
  - Induced by fever, starvation, high-protein diet, and infections
  - Feeding difficulties in infancy and early childhood
  - Developmental delays, convulsions, hypotonia
  - Cognitive impairment and neurological development issues
  - Slow waves or epileptic waves on EEG
  - Fractures and osteoporosis on X-ray
  - Bone marrow suppression (granulocytopenia, anaemia, thrombocytopenia)
  - Cardiac damage (cardiomyopathy, arrhythmia, prolonged QT interval, diminished cardiac function)
  - Less common renal impairment

### Other Possible Diagnoses:

- **Methylmalonic Acidemia (MMA):**
  - Metabolic disorder due to a deficiency in methylmalonyl-CoA mutase or cobalamin metabolism
  - Neonatal onset with vomiting, lethargy, hypotonia, convulsions, and metabolic acidosis
  - Cardiomyopathy and renal impairment
- **Propionic Acidemia:**
  - Metabolic disorder due to a deficiency in propionyl-CoA carboxylase
  - Neonatal onset with vomiting, lethargy, hypotonia, convulsions, and metabolic acidosis
  - Cardiomyopathy and renal impairment
- **Glutaric Aciduria Type I (GA1):**
  - Metabolic disorder due to a deficiency in glutaryl-CoA dehydrogenase
  - Delayed onset with developmental delays, hypotonia, and dystonia
  - Cardiomyopathy and renal impairment
- **Organic Acidemias:**
  - A group of metabolic disorders characterized by the accumulation of organic acids
  - Neonatal or delayed onset with vomiting, lethargy, hypotonia, convulsions, and metabolic acidosis
  - Cardiomyopathy and renal impairment
- **Mitochondrial Disorders:**
  - A group of genetic disorders affecting mitochondrial function
  - Neonatal or delayed onset with vomiting, lethargy, hypotonia, convulsions, and metabolic acidosis
  - Cardiomyopathy, neurological impairment, and renal impairment

### Conclusion:

While Maple Syrup Urine Disease (MSUD) is the most likely diagnosis based on the provided clinical manifestations, other metabolic disorders such as Methylmalonic Acidemia, Propionic Acidemia, Glutaric Aciduria Type I, and mitochondrial disorders should also be considered and ruled out through appropriate diagnostic testing.

### **104. User**

Clinical manifestations are often nonspecific, with an insidious onset. Symptoms such as coughing up sputum, lethargy and fatigue may occur. Secondary infections may include fever, coughing up purulent sputum, or chest pain and haemoptysis. A few patients may be asymptomatic and may have no positive signs. Bursting sounds may be present in some patients, cyanosis may be present in severe cases, and pestle and mortar fingers may be seen in a few patients. The clinical picture is often less severe than the chest image, and this inconsistency between symptoms and images is a characteristic feature of the disease. Serum anti-GM-CSF antibodies are markedly elevated.

### Qwen2.5_72b

Based on the clinical manifestations and the presence of markedly elevated serum anti-GM-CSF antibodies, the most likely diagnosis is **Pulmonary Alveolar Proteinosis (PAP)**. PAP is a rare lung disease characterized by the accumulation of surfactant proteins and lipids in the alveoli, leading to impaired gas exchange and respiratory symptoms.

### Differential Diagnoses:

- **Idiopathic Pulmonary Fibrosis (IPF)**: This is a chronic, progressive fibrosing interstitial pneumonia of unknown cause. Patients may present with similar symptoms such as cough, fatigue, and dyspnea, but the chest imaging typically shows honeycombing and reticular patterns.
- **Chronic Eosinophilic Pneumonia (CEP)**: This condition is characterized by the accumulation of eosinophils in the lungs. Patients may present with cough, fever, and fatigue. Chest imaging often shows peripheral and peribronchial infiltrates.
- **Hypersensitivity Pneumonitis (HP)**: This is an immune-mediated lung disease caused by inhalation of organic antigens. Symptoms include cough, dyspnea, and fatigue. Chest imaging may show ground-glass opacities and nodular infiltrates.
- **Sarcoidosis**: This is a systemic inflammatory disease characterized by the formation of non-caseating granulomas. Patients may present with cough, fatigue, and chest pain. Chest imaging often shows bilateral hilar lymphadenopathy and parenchymal infiltrates.
- **Lymphangioleiomyomatosis (LAM)**: This is a rare lung disease that primarily affects women of childbearing age. It is characterized by the proliferation of abnormal smooth muscle cells in the lungs, leading to cystic lung destruction. Patients may present with dyspnea, cough, and chest pain. Chest imaging shows multiple thin-walled cysts.

### Summary:

- **Most Likely Diagnosis**: Pulmonary Alveolar Proteinosis (PAP)
- **Differential Diagnoses**:
  1. Idiopathic Pulmonary Fibrosis (IPF)
  2. Chronic Eosinophilic Pneumonia (CEP)
  3. Hypersensitivity Pneumonitis (HP)
  4. Sarcoidosis
  5. Lymphangioleiomyomatosis (LAM)

Further diagnostic testing, such as bronchoalveolar lavage (BAL) and lung biopsy, may be necessary to confirm the diagnosis of PAP and rule out other conditions.

### **105. User**

In most cases, the disease begins in adolescence, with a gradual loss of the optic rod cells, followed by cone cell involvement and death of the retinal pigment epithelium. Therefore, night blindness is usually the first symptom (but after urbanisation, some patients often have a decrease in visual acuity), and the earlier the onset of night blindness, the more severe the patient’s condition. Although peripheral visual field defects also occur early, patients are usually unaware of them and rarely complain of them in the early stages of the disease. The disease progresses to a loss of central vision and further narrowing of the visual field, and most people become legally blind before the age of 40. The degree of visual impairment can be related to the mode of inheritance.

The presentation of the fundus is related to the stage of the disease: in the early stages of the disease, the fundus may be normal or nearly normal on routine examination, but in some patients, an early form of blanket retinal degeneration may be observed on mid-peripheral fundus photography (these subtle changes are difficult to recognise with current scanning laser fundus photography or wide-field fundus photography). As the disease progresses, some patients develop typical fundus changes, such as retinal osteoblast-like pigmentation combined with mid-peripheral retinal atrophy, a waxy optic disc, and thinning of retinal blood vessels; some patients develop blanketing or depigmentation, which can be seen in the mid-peripheral retina, or extensive degeneration. Other common complications include macular cystoid degeneration, posterior subcapsular cataract, vitreous dust particles, and optic disc vitreous warts; a rare complication is Coat’s retinopathy.

### Qwen2.5_72b

Based on the clinical manifestations you have provided, the most likely diagnosis is **Retinitis Pigmentosa (RP)**. This is a group of rare, genetic disorders that involve a progressive degeneration of the retina, leading to significant visual impairment and often legal blindness by middle age.

### Most Likely Diagnosis:

- **Retinitis Pigmentosa (RP)**

### Other Possible Diagnoses:

2. **Usher Syndrome**: This is a genetic disorder that affects both hearing and vision. It is characterized by RP and sensorineural hearing loss. The progression of visual symptoms is similar to RP, but the presence of hearing loss is a distinguishing feature.
3. **Choroideremia**: This is an X-linked recessive disorder characterized by progressive degeneration of the choroid, retinal pigment epithelium, and retina. It typically presents with night blindness and progressive loss of peripheral vision, similar to RP.
4. **Best Disease (Vitelliform Macular Dystrophy)**: This is a rare genetic disorder that primarily affects the macula, leading to a decline in central vision. While it does not typically present with night blindness or peripheral vision loss, it can cause significant visual impairment.
5. **Stargardt Disease**: This is a form of juvenile macular degeneration that causes progressive vision loss, primarily affecting the macula. It can present with decreased visual acuity and central vision loss, but night blindness and peripheral vision loss are not typical features.

### Summary:

- **Most Likely Diagnosis**: Retinitis Pigmentosa (RP)
- **Other Possible Diagnoses**:
  - Usher Syndrome
  - Choroideremia
  - Best Disease (Vitelliform Macular Dystrophy)
  - Stargardt Disease

Each of these conditions has distinct clinical features and genetic bases, and further diagnostic testing, such as genetic testing, electroretinography (ERG), and fundus imaging, would be necessary to confirm the specific diagnosis.

### **106. User**

The most common clinical presentation is white pupil syndrome, in which a yellow-white reflection is visible in the pupil. Disuse strabismus may occur in the affected eye due to the tumour being located at the posterior pole and low visual acuity. The early presentation is a greyish-white, round or oval arching mass that may bulge into the vitreous or grow flat along the choroid. Retinal blood vessels on the surface of the tumour may become dilated and haemorrhagic, and even exudative retinal detachment may occur. Tumour tissue may also penetrate the retina and enter the vitreous, floating like a snowball, or even deposited in the anterior chamber to form pseudo anterior chamber pus. The onset of the disease is early in children with bilateral retinal detachment, usually within one year of age. The onset is later in unilateral children, usually at two or three years of age.

### Qwen2.5_72b

Based on the clinical manifestations you have provided, the most likely diagnosis is **Retinoblastoma**. This is a rare pediatric cancer of the retina that typically presents with leukocoria (white pupil), strabismus, and a greyish-white mass in the eye. The other symptoms you described, such as dilated and hemorrhagic retinal blood vessels, exudative retinal detachment, and the presence of tumor tissue in the vitreous or anterior chamber, are also characteristic of retinoblastoma.

Here are five other possible diagnoses to consider:

- **Coats’ Disease**: This is a rare, non-neoplastic condition characterized by abnormal development of retinal blood vessels, leading to exudative retinal detachment and leukocoria. It is more common in males and typically affects one eye.
- **Persistent Hyperplastic Primary Vitreous (PHPV)**: This is a congenital anomaly where the primary vitreous and hyaloid vasculature fail to regress, leading to leukocoria, strabismus, and cataract. It can cause retinal detachment and is usually unilateral.
- **Toxocariasis**: This is an infection caused by the larvae of the roundworm Toxocara canis or Toxocara cati. It can cause leukocoria, uveitis, and retinal detachment, particularly in young children.
- **Congenital Cataract**: This is a clouding of the lens present at birth or in early childhood. It can cause leukocoria and may be associated with other ocular or systemic abnormalities.
- **Retinal Astrocytoma**: This is a benign hamartoma of the retina, often associated with von Hippel-Lindau disease. It can present with a white or greyish-white mass in the retina and may cause retinal detachment or exudation.

Each of these conditions has distinct clinical features and diagnostic criteria, but retinoblastoma remains the most likely diagnosis given the specific combination of symptoms you have described.

### **107. User**

The average age of onset is 5.6 months. The first seizure is usually normal at birth and starts within 1 year of age. The first seizure is usually febrile convulsion, which may be one-sided or generalised clonic or tonic clonic seizure, and febrile convulsions are characterized by long duration and recurrent episodes, and may appear in a persistent state. 28%~35% of children may start the disease without febrile convulsions, but they often have heat sensitivity in the later course of the disease. Elevated body temperature due to various reasons, including exercise, fever, vaccination, hot baths, and high ambient temperatures, can trigger or exacerbate seizures and predispose to status epilepticus. Some children develop an acute encephalopathy characterised by deep coma following status epilepticus.

Children tend to present with febrile convulsions between the ages of 1 and 4 years, and have a variety of seizure types, such as myoclonic, atypical catatonic, partial seizures, and generalised tonic clonic seizures. Myoclonic seizures are often triggered by light stimulation, eye closure, and a specific graphic stimulus, and involve simultaneous clonus of one or several muscles, all accompanied by synchronised abnormal brain wave bursts. The intensity of the myoclonus varies, from a slight forward or backward movement of the body when only the axial muscles are violated, to a severe fall or sudden throwing of an object in the hand. Myoclonic seizures can be isolated or cluster, but a sustained state is rare. Myoclonic seizures may not occur at all in about 25% of children. The concurrent EEG of atypical apoplectic seizures shows half of the discharges to be of focal origin, most of which are irregular, irregular, slow spikes and slow waves, often accompanied by myoclonic seizures. 30% of the children may have atypical apoplectic persistent seizures. The incidence of partial seizures is 43.0% to 78.6%, which manifests as lateral limb (or) head deflection seizures or clonic seizures, and there are also motor seizures with consciousness and autonomic symptoms, automatisms, and hypotonia, etc. After 1 year of age, the children have psychomotor developmental delays, especially language developmental delays.

### Qwen2.5_72b

Based on the clinical manifestations you have provided, the most likely diagnosis is **Dravet Syndrome (Severe Myoclonic Epilepsy of Infancy, SMEI)**. Dravet Syndrome is a rare and severe form of epilepsy that typically begins in the first year of life, often with febrile seizures. The condition is characterized by a wide range of seizure types, including febrile convulsions, myoclonic seizures, atypical absence seizures, and focal seizures. Developmental delays, particularly in language, are common, and the seizures are often triggered by fever, heat, or other environmental factors.

### Other Possible Diagnoses:

- **Epilepsy with Myoclonic-Atonic Seizures (Doose Syndrome)**:
  - This is a generalized epilepsy syndrome that typically begins in early childhood, often between 6 months and 6 years of age. It is characterized by myoclonic-atonic seizures, which can cause sudden drops or falls. Febrile seizures are less common, and developmental delays can occur.
- **Lennox-Gastaut Syndrome (LGS)**:
  - LGS is a severe form of epilepsy that usually begins in early childhood, often between 2 and 8 years of age. It is characterized by multiple seizure types, including tonic, atonic, and atypical absence seizures. Developmental delays and cognitive impairment are common.
- **Febrile Seizure Plus (FS+)**:
  - FS+ is a genetic condition that includes a spectrum of epilepsy syndromes characterized by febrile seizures that persist beyond the age of 6 years. Children with FS+ may also have afebrile seizures, and some may develop other types of epilepsy, such as generalized epilepsy with febrile seizures plus (GEFS+).
- **Epilepsy with Generalized Tonic-Clonic Seizures on Awakening (GTCSA)**:
  - This is a form of generalized epilepsy where seizures, particularly generalized tonic-clonic seizures, occur shortly after waking up. It can begin in childhood or adolescence and is often associated with a family history of epilepsy.
- **Rett Syndrome**:
  - Rett Syndrome is a rare genetic disorder that primarily affects girls and is characterized by developmental regression, particularly in motor and language skills. Seizures are common in Rett Syndrome, and they can be of various types, including myoclonic and generalized tonic-clonic seizures. However, the onset of seizures in Rett Syndrome is typically later than in Dravet Syndrome.

### Summary:

- **Most Likely Diagnosis**: Dravet Syndrome
- **Other Possible Diagnoses**:
  1. Epilepsy with Myoclonic-Atonic Seizures (Doose Syndrome)
  2. Lennox-Gastaut Syndrome (LGS)
  3. Febrile Seizure Plus (FS+)
  4. Epilepsy with Generalized Tonic-Clonic Seizures on Awakening (GTCSA)
  5. Rett Syndrome

Each of these conditions has overlapping features, but the specific combination of early onset, febrile seizures, multiple seizure types, and developmental delays strongly suggests Dravet Syndrome. Genetic testing, particularly for mutations in the SCN1A gene, can help confirm the diagnosis.

### **108. User**

On the one hand, it presents as chronic haemolytic anaemia, and in addition to anaemia-related symptoms, it may cause mild to moderate yellowing of the skin and sclera and pigmented cholelithiasis due to elevated bilirubin, which may be exacerbated by cold, infection and dehydration. In infants and young children, splenomegaly may be present, and with age, the spleen may shrink due to splenic infarction and fibrosis.

On the other hand recurrent vaso-obstructive pain due to capillary micro thrombosis. Children and adults tend to present with myalgia of the limbs, large joint pain and low back pain, and in severe cases, severe abdominal pain, headache, and even limb paralysis and coma. Over time, vascular obstruction can occur in almost every organ system, and cyclical pain can occur in patients who are thin, easily fatigued, susceptible to infection, and malnourished. Thinning of the bone cortex, osteoporosis, spinal deformity, aseptic necrosis of the femoral head due to an overcompensatory increase in bone marrow haematopoietic tissue, and localised osteosclerosis if there is a bone infarction. Ocular symptoms may include fundus haemorrhage and retinal detachment due to embolism of the retinal vessels in the fundus. In addition, mental retardation may occur, affecting neurological development.

Acute pain is the first symptom of the disease in more than 25% of patients. It is most common in children older than 2 years of age. The severity of pain can vary from mild to severe. Approximately one-half of pain episodes are accompanied by objective clinical signs such as fever, swelling, tenderness, tachypnoea, hypertension, nausea and vomiting.

### Qwen2.5_72b

Based on the clinical manifestations you have provided, the most likely diagnosis is **Sickle Cell Disease (SCD)**. Sickle Cell Disease is a group of inherited red blood cell disorders characterized by abnormal hemoglobin, which causes the red blood cells to become crescent-shaped (sickle-shaped) under certain conditions. This leads to chronic hemolytic anemia, vaso-occlusive pain crises, and a range of other complications.

### Most Likely Diagnosis:

- **Sickle Cell Disease (SCD)**

### Other Possible Diagnoses:

- **Hereditary Spherocytosis**: This is another inherited hemolytic anemia characterized by the production of spherical red blood cells that are more fragile and prone to hemolysis. It can cause chronic hemolytic anemia, jaundice, and gallstones, but it does not typically present with vaso-occlusive pain crises.
- **Thalassemia**: This is a group of inherited blood disorders characterized by abnormal hemoglobin production. Beta-thalassemia major can cause severe anemia, jaundice, and splenomegaly, but it does not typically present with the vaso-occlusive pain crises seen in SCD.
- **Glucose-6-Phosphate Dehydrogenase (G6PD) Deficiency**: This is an X-linked genetic disorder that leads to red blood cell breakdown (hemolysis) when exposed to certain triggers such as infections, certain foods, or medications. It can cause hemolytic anemia and jaundice but does not typically present with vaso-occlusive pain crises.
- **Paroxysmal Nocturnal Hemoglobinuria (PNH)**: This is an acquired hematopoietic stem cell disorder characterized by intravascular hemolysis, thrombosis, and bone marrow failure. It can cause hemolytic anemia, jaundice, and thrombosis, but it does not typically present with the vaso-occlusive pain crises seen in SCD.
- **Erythropoietic Protoporphyria (EPP)**: This is a rare genetic disorder that affects the production of heme, leading to the accumulation of protoporphyrin in the red blood cells. It can cause photosensitivity, abdominal pain, and neurological symptoms, but it does not typically present with chronic hemolytic anemia or vaso-occlusive pain crises.

### Summary:

- **Most Likely Diagnosis**: Sickle Cell Disease (SCD)
- **Other Possible Diagnoses**:
  1. Hereditary Spherocytosis
  2. Thalassemia
  3. Glucose-6-Phosphate Dehydrogenase (G6PD) Deficiency
  4. Paroxysmal Nocturnal Hemoglobinuria (PNH)
  5. Erythropoietic Protoporphyria (EPP)

Given the specific combination of chronic hemolytic anemia, vaso-occlusive pain crises, and other associated symptoms, Sickle Cell Disease is the most likely diagnosis. However, further diagnostic testing, such as hemoglobin electrophoresis and genetic testing, would be necessary to confirm the diagnosis.

### **109. User**

The main clinical manifestations of neonatal and paediatric patients include:

- Intrauterine growth retardation and postnatal growth disorders . Intrauterine growth retardation, low birth weight, lack of postnatal catch-up growth, and proportional short stature.
- Specific physical signs: wide and prominent forehead, pointed jaw leading to a triangular face, and large relative head circumference. Dysmorphic teeth, low ear position, and asymmetry of trunk deviation, including limb length discrepancy, and lateral curvature deformity of the little finger.
- Other manifestations include feeding difficulties, hypoglycaemia in the neonatal period, genitourinary malformations such as hypospadias, cryptorchidism and horseshoe kidneys, and digestive anomalies such as cafe au lait spots and reflux oesophagitis. Some patients have cognitive, motor and language developmental delays and learning disabilities.

### Qwen2.5_72b

Based on the clinical manifestations you have provided, the most likely diagnosis is **Silver-Russell Syndrome (SRS)**. SRS is a rare genetic disorder characterized by intrauterine growth retardation, postnatal growth disorders, and specific physical features such as a wide and prominent forehead, pointed jaw, and asymmetry of the trunk and limbs.

### Differential Diagnoses:

- **Prader-Willi Syndrome (PWS)**:
  - **Features**: Intrauterine growth retardation, postnatal obesity, hypotonia, hypogonadism, cognitive and behavioral issues.
  - **Distinguishing Features**: Unlike SRS, PWS patients often develop obesity in childhood and have hypotonia, which is not a prominent feature in SRS.
- **Russell-Silver Syndrome (SRS) with Uniparental Disomy (UPD) or Epimutations**:
  - **Features**: Similar to SRS, but with specific genetic mechanisms such as UPD or epimutations.
  - **Distinguishing Features**: Genetic testing can help differentiate between SRS and other genetic causes of growth retardation.
- **Noonan Syndrome**:
  - **Features**: Short stature, webbed neck, low-set ears, heart defects, and bleeding disorders.
  - **Distinguishing Features**: Noonan syndrome often includes heart defects and a webbed neck, which are not typically seen in SRS.
- **Turner Syndrome**:
  - **Features**: Short stature, webbed neck, low-set ears, and ovarian dysfunction.
  - **Distinguishing Features**: Turner syndrome is associated with ovarian dysfunction and a 45,X karyotype, which is not seen in SRS.
- **Fanconi Anemia**:
  - **Features**: Short stature, bone marrow failure, and various congenital anomalies including radial ray defects, kidney anomalies, and skin pigmentation.
  - **Distinguishing Features**: Fanconi anemia is associated with bone marrow failure and a higher risk of leukemia, which are not typical in SRS.

### Summary:

- **Primary Diagnosis**: Silver-Russell Syndrome (SRS)
- **Differential Diagnoses**:
  1. Prader-Willi Syndrome (PWS)
  2. Russell-Silver Syndrome (SRS) with Uniparental Disomy (UPD) or Epimutations
  3. Noonan Syndrome
  4. Turner Syndrome
  5. Fanconi Anemia

Genetic testing and a detailed clinical evaluation are essential to confirm the diagnosis and rule out other differential diagnoses.

### **110. User**

**1. Xanthomas of the skin,** occurring near the attachment points of the extensor tendons, such as the Achilles tendon, elbow, knee, etc., and there is a close relationship between skin injuries and the appearance of xanthomas. Cutaneous xanthomas are rare in children.

**2. Atherosclerosis ,** Patients have a significantly higher risk of developing atherosclerosis and coronary artery disease than normal subjects. Early-onset coronary artery disease may be one of the characteristics of the disease.

**3. Alterations in the blood system ,** The content of plant sterols in the blood is significantly increased, resulting in a higher proportion of plant sterols in the sterol component of the cell membrane, abnormal cell membrane function of red blood cells, platelets and other cell membranes, anaemia and thrombocytopenia, and the patient may have weakness, pallor, skin bleeding and other manifestations.

**4. Others,** in addition to the above symptoms, some patients may develop joint pain, arthritis, splenomegaly, elevated transaminases, etc.

### Qwen2.5_72b

Based on the clinical manifestations you have provided, the most likely rare disease is **Sitosterolemia (Phytosterolemia)**. This is a rare autosomal recessive disorder characterized by the excessive absorption and decreased excretion of plant sterols, leading to their accumulation in tissues and blood.

### Primary Diagnosis:

- **Sitosterolemia (Phytosterolemia)**

### Other Possible Diagnoses:

- **Familial Hypercholesterolemia (FH):** This is a genetic disorder characterized by high levels of low-density lipoprotein (LDL) cholesterol, leading to early atherosclerosis and coronary artery disease. However, it does not typically involve the accumulation of plant sterols.
- **Tangier Disease:** This is a rare genetic disorder characterized by very low levels of high-density lipoprotein (HDL) cholesterol and the accumulation of cholesterol in tissues, particularly the tonsils and peripheral nerves. It can cause xanthomas and atherosclerosis but does not involve plant sterols.
- **Wolman Disease:** This is a rare lysosomal storage disorder characterized by the accumulation of lipids in various tissues, leading to hepatosplenomegaly, adrenal calcification, and early death. It does not typically present with xanthomas or plant sterol accumulation.
- **Niemann-Pick Disease Type C (NPC):** This is a rare genetic disorder characterized by the accumulation of cholesterol and other lipids in various organs, leading to neurological symptoms, hepatosplenomegaly, and xanthomas. However, it does not involve plant sterols.
- **Cholesteryl Ester Storage Disease (CESD):** This is a rare lysosomal storage disorder characterized by the accumulation of cholesteryl esters in various tissues, leading to hepatosplenomegaly, liver dysfunction, and atherosclerosis. It does not typically involve plant sterols.

### Summary:

- **Primary Diagnosis:** Sitosterolemia (Phytosterolemia)
- **Other Possible Diagnoses:**
  1. Familial Hypercholesterolemia (FH)
  2. Tangier Disease
  3. Wolman Disease
  4. Niemann-Pick Disease Type C (NPC)
  5. Cholesteryl Ester Storage Disease (CESD)

These differential diagnoses should be considered and further evaluated through specific laboratory tests and genetic analysis to confirm the diagnosis.

### **111. User**

The onset of the disease is insidious in males, and the common age of onset is 30-60 years old. Patients often start with painful spasms, tremor, and weakness of the lower limbs, and gradually develop weakness of the upper limbs, muscle atrophy of the medulla oblongata and the face, muscle fasciculations, dysarthria, dysphagia, dyspnoea, and respiratory difficulties. Androgen receptor insensitivity may be associated with gynecomastia and reduced reproductive function. Sensory abnormalities are present in more than 50 per cent of patients. The disease progresses slowly, with patients usually becoming unable to walk late in the course of the disease, and only some patients require assisted ventilation, which has no significant impact on survival.

**1. Atypical symptoms,** some of which may occur years or even decades before the onset of limb weakness, include fatigability, painful spasms, perioral tremor, and postural tremor. Muscle weakness: most patients start with weakness of both lower limbs, gradually progressing to both upper limbs, tongue muscles and facial muscles, while the extraocular muscles are often not involved. The distribution of limb weakness is asymmetrical, with proximal involvement predominating. Involvement of the muscles of mastication may cause mandibular ptosis and tremor. Physical examination of the lower motor neuron damage signs, manifested as a decrease in muscle strength of the limbs, proximal, accompanied by muscle atrophy, fasciculations, tendon reflexes are weakened or disappeared. Most patients may have obvious tongue muscle atrophy and fibrillation, but the soft palate activity and pharyngeal reflexes are normal, and the degree of choking on water and dysphagia is mild. In some patients, chin and perioral fasciculations can also be observed. The motor function impairment of the patients gradually increases with the prolongation of the disease, and in the later stage, they may have difficulty in walking up the stairs and walking unsteadily.

**2. Sensory deficits** , with varying degrees of hyperalgesia present in about half of the patients, while in the others it is subclinical, with abnormalities found only on sensory nerve conduction tests.

**3. endocrine abnormalities,** including abnormalities of glucose and fat metabolism, and androgen receptor insensitivity and other manifestations, the latter including gynecomastia, decreased sexual function, infertility, and testicular atrophy.

The latter include gynecomastia, decreased sexual function, infertility, testicular atrophy, etc.

**4. Female carriers,** most female carriers do not have symptoms, and even if they do have symptoms, they are mild, such as fasciculations, mild weakness of distal limbs, muscle spasms, or increased creatine myosinase.

### Qwen2.5_72b

Based on the clinical manifestations you’ve provided, the most likely diagnosis is **Kennedy’s Disease (Spinal and Bulbar Muscular Atrophy, SBMA)**. Kennedy’s Disease is a rare X-linked recessive neuromuscular disorder characterized by progressive muscle weakness and atrophy, particularly affecting the lower motor neurons. The disease is caused by a CAG trinucleotide repeat expansion in the androgen receptor gene, leading to androgen receptor insensitivity.

### Most Likely Diagnosis:

- **Kennedy’s Disease (Spinal and Bulbar Muscular Atrophy, SBMA)**

### Other Possible Diagnoses:

- **Amyotrophic Lateral Sclerosis (ALS)**: ALS is a progressive neurodegenerative disease that affects both upper and lower motor neurons. However, ALS typically progresses more rapidly and involves both upper and lower motor neuron signs, which can help differentiate it from Kennedy’s Disease.
- **Progressive Muscular Atrophy (PMA)**: PMA is a motor neuron disease that primarily affects the lower motor neurons, similar to Kennedy’s Disease. However, PMA does not typically involve androgen receptor insensitivity or endocrine abnormalities.
- **Multifocal Motor Neuropathy (MMN)**: MMN is a chronic immune-mediated neuropathy characterized by progressive asymmetrical weakness and muscle atrophy, often with conduction block on nerve conduction studies. It does not typically involve endocrine abnormalities or androgen receptor insensitivity.
- **Hereditary Spastic Paraplegia (HSP)**: HSP is a group of inherited disorders characterized by progressive weakness and spasticity of the lower limbs. However, HSP does not typically involve muscle atrophy, fasciculations, or endocrine abnormalities.
- **Charcot-Marie-Tooth Disease (CMT)**: CMT is a group of hereditary motor and sensory neuropathies. While it can cause muscle weakness and atrophy, it typically involves both sensory and motor symptoms and does not include androgen receptor insensitivity or endocrine abnormalities.

### Key Differentiating Features:

- **Kennedy’s Disease**: Slow progression, lower motor neuron signs, androgen receptor insensitivity, gynecomastia, and reduced reproductive function.
- **ALS**: Rapid progression, both upper and lower motor neuron signs, no androgen receptor insensitivity.
- **PMA**: Lower motor neuron signs, no androgen receptor insensitivity.
- **MMN**: Asymmetrical weakness, conduction block on nerve conduction studies, no androgen receptor insensitivity.
- **HSP**: Spasticity and weakness of the lower limbs, no muscle atrophy or androgen receptor insensitivity.
- **CMT**: Sensory and motor symptoms, no androgen receptor insensitivity.

Given the specific combination of lower motor neuron signs, androgen receptor insensitivity, and endocrine abnormalities, Kennedy’s Disease is the most likely diagnosis.

### **112. User**

Some patients present with reduced foetal movement in utero and are born as floppy babies. The onset of the disease occurs within 6 months, with a mean age of onset of 1 month after birth. They present with generalised floppy weakness and severe hypotonia. Most children have difficulty sucking and swallowing due to weakness of the tongue, facial and masticatory muscles, and atrophy and tremor of the lingual muscles are seen. Intercostal muscle involvement can present with dyspnoea and abdominal breathing. The chest has a bell-shaped appearance. The lower limbs are more heavily involved than the upper limbs, and proximal is more severe than distal. Severe muscle weakness in the mid-trunk area leaves the child unable to control head movements, unable to lift or roll over, and without the ability to sit alone. When lying down, both lower limbs are in the frog-leg position with hip abduction and knee flexion. Muscle atrophy is not obvious, and some children have mild joint deformities. Children with good intelligence, tendon reflexes disappeared, limb sensation is normal. The children’s muscle weakness progressively worsens, and eventually they lose all voluntary movement ability, are fed nasogastrically, and suffer from respiratory failure due to repeated respiratory infections. 80% of the children die within 1 year of age, and rarely survive beyond 2 years of age.

### Qwen2.5_72b

Based on the clinical manifestations you’ve described, the most likely diagnosis is **Spinal Muscular Atrophy Type 1 (SMA Type 1)**, also known as Werdnig-Hoffmann disease. This is a severe form of spinal muscular atrophy characterized by the onset of symptoms within the first 6 months of life, severe hypotonia, and progressive muscle weakness leading to respiratory failure and early death.

### Differential Diagnoses:

- **Congenital Myopathies**:
  - These are a group of muscle disorders that are present at birth or in early infancy. They can present with hypotonia, muscle weakness, and feeding difficulties. However, the progression and severity may vary, and muscle atrophy is often more pronounced.
- **Congenital Myasthenic Syndromes (CMS)**:
  - These are a group of genetic disorders that affect the neuromuscular junction, leading to muscle weakness and fatigability. Symptoms can include hypotonia, feeding difficulties, and respiratory issues. However, the weakness often improves with rest and can be exacerbated by activity.
- **Mitochondrial Myopathies**:
  - These are a group of disorders affecting the mitochondria, the energy-producing structures within cells. Symptoms can include hypotonia, muscle weakness, and respiratory issues. However, other systemic symptoms such as developmental delay, seizures, and lactic acidosis may also be present.
- **Metabolic Myopathies**:
  - These are disorders of muscle metabolism that can present with hypotonia, muscle weakness, and feeding difficulties. However, they often have additional features such as metabolic acidosis, hepatomegaly, and specific biochemical abnormalities.
- **Central Hypotonia**:
  - This can be caused by various central nervous system disorders, such as cerebral palsy or brain malformations. Symptoms can include hypotonia, muscle weakness, and feeding difficulties. However, the presence of other neurological signs such as spasticity, seizures, and developmental delay can help differentiate it from SMA.

### Key Features of SMA Type 1:

- **Onset**: Within the first 6 months of life.
- **Hypotonia**: Severe and generalized.
- **Muscle Weakness**: Progressive and severe, affecting the proximal muscles more than the distal muscles.
- **Respiratory Issues**: Intercostal muscle involvement leading to dyspnea and abdominal breathing.
- **Feeding Difficulties**: Due to weakness of the tongue, facial, and masticatory muscles.
- **Bell-Shaped Chest**: Due to intercostal muscle weakness.
- **Frog-Leg Position**: Lower limbs in hip abduction and knee flexion.
- **Head Control**: Inability to control head movements.
- **Tendon Reflexes**: Absent.
- **Sensation**: Normal.
- **Prognosis**: Most children die within 1 year of age, and rarely survive beyond 2 years.

Given the clinical presentation, SMA Type 1 is the most likely diagnosis, but further diagnostic testing, such as genetic testing for the SMN1 gene, would be necessary to confirm the diagnosis.

### **113. User**

Patients usually start in adulthood, with an insidious onset and gradual progression. The main manifestations are cerebellar ataxia, including drunkard’s gait, clumsy movements, minstrel-like speech, nystagmus, diplopia, etc.; pyramidal tract damage, including hyperreflexia, ankle clonus, and positive pathological signs; and extrapyramidal symptoms, including Parkinson’s syndrome, dystonia, and ankylosis. In addition, some patients may have peripheral neuropathy. In the early stages of the disease, some patients may have damage to the pyramidal tract (similar to spastic paraplegia) or extrapyramidal symptoms (similar to Parkinson’s syndrome), and ataxia is not prominent.

The disease progresses gradually, with loss of walking ability after 10-15 years. Prolonged dysphagia leading to malnutrition or aspiration is the main cause of death.

### Qwen2.5_72b

Based on the clinical manifestations you’ve provided, the most likely rare disease is **Multiple System Atrophy (MSA)**. MSA is a progressive neurodegenerative disorder that affects multiple systems in the body, including the cerebellar, pyramidal, and extrapyramidal systems, leading to a combination of ataxia, parkinsonism, and autonomic dysfunction.

### Most Likely Diagnosis:

- **Multiple System Atrophy (MSA)**

### Other Possible Diagnoses:

2. **Progressive Supranuclear Palsy (PSP)**: This is another neurodegenerative disorder that can present with ataxia, parkinsonism, and extrapyramidal symptoms. However, PSP often has more prominent vertical gaze palsy and falls early in the disease course.
3. **Spinocerebellar Ataxia (SCA)**: This is a group of hereditary ataxias that can present with cerebellar ataxia, pyramidal signs, and sometimes peripheral neuropathy. However, the genetic basis and family history are key differentiators.
4. **Corticobasal Degeneration (CBD)**: This is a rare neurodegenerative disorder that can present with parkinsonism, dystonia, and ataxia. However, it often has more prominent apraxia and alien limb phenomenon.
5. **Friedreich’s Ataxia**: This is a hereditary ataxia that can present with cerebellar ataxia, pyramidal signs, and peripheral neuropathy. However, it typically starts in childhood or adolescence and is associated with a specific genetic mutation (GAA repeat expansion in the FXN gene).

### Key Points for Differentiation:

- **MSA**: Characterized by a combination of cerebellar ataxia, parkinsonism, and autonomic dysfunction. The disease progresses rapidly, and patients often lose the ability to walk within 10-15 years.
- **PSP**: Prominent vertical gaze palsy and early falls.
- **SCA**: Hereditary, with a family history and specific genetic mutations.
- **CBD**: Prominent apraxia and alien limb phenomenon.
- **Friedreich’s Ataxia**: Early onset, typically in childhood or adolescence, and associated with a specific genetic mutation.

These differential diagnoses should be considered and further evaluated with appropriate clinical, genetic, and imaging studies.

### **114. User**

The disease may be combined with gastro-oesophageal reflux in the early stages of the disease. As the disease progresses, the skin becomes swollen and sclerotic and may be accompanied by changes in skin pigmentation, including hyperpigmentation or depigmentation. In addition to skin involvement, patients often present with arthralgia and myalgia, and may develop ulcers and capillary dilatation of the digits. In addition, the patient may have multiple internal organ involvement. The respiratory system is more likely to be involved, with interstitial fibrosis and pulmonary hypertension. The gastrointestinal manifestations are varied and include gastroesophageal reflux, sinus vasodilatation, abdominal pain, diarrhoea, constipation and fecal incontinence. A small number of patients may present with renal crisis, which is characterised by acute renal insufficiency and is often associated with hypertension. Cardiac involvement may manifest as pericardial effusion, abnormalities of the cardiac conduction system, and myocardial involvement in individual patients. Neurological involvement of the peripheral nervous system is common, such as trigeminal neuropathy, peripheral neuropathy, etc. Central nervous system involvement is rare.

### Qwen2.5_72b

Based on the clinical manifestations you have provided, the most likely diagnosis is **Systemic Sclerosis (Scleroderma)**, specifically the diffuse cutaneous form. This condition is characterized by skin thickening and sclerosis, which can progress to involve multiple internal organs, including the lungs, gastrointestinal tract, kidneys, and heart. The presence of gastro-oesophageal reflux, skin changes (swelling, sclerosis, hyperpigmentation, and depigmentation), arthralgia, myalgia, digital ulcers, capillary dilatation, interstitial lung disease, pulmonary hypertension, gastrointestinal symptoms, renal crisis, and neurological involvement are all consistent with systemic sclerosis.

### Other Possible Diagnoses:

- **Mixed Connective Tissue Disease (MCTD)**: This condition shares many features with systemic sclerosis, including skin changes, arthralgia, myalgia, and internal organ involvement. However, MCTD is often associated with high titers of anti-U1 RNP antibodies.
- **Sjögren’s Syndrome**: While primarily characterized by dry eyes and mouth, Sjögren’s syndrome can also present with skin changes, arthralgia, myalgia, and gastrointestinal symptoms. However, the presence of renal crisis and significant skin sclerosis is less common.
- **Ehlers-Danlos Syndrome (EDS), Hypermobile Type**: This condition can present with joint hypermobility, arthralgia, and myalgia, but the skin changes are typically hyperelastic rather than sclerotic. Gastrointestinal symptoms and internal organ involvement are less common in EDS.
- **Systemic Lupus Erythematosus (SLE)**: SLE can present with a wide range of symptoms, including skin changes (such as a malar rash), arthralgia, myalgia, and internal organ involvement. However, the specific skin changes and the presence of renal crisis are more characteristic of systemic sclerosis.
- **Chronic Graft-versus-Host Disease (cGVHD)**: This condition can occur after allogeneic hematopoietic stem cell transplantation and can present with skin sclerosis, arthralgia, myalgia, and gastrointestinal symptoms. However, the presence of renal crisis and the specific pattern of skin changes are less common in cGVHD.

Each of these conditions has overlapping symptoms, but the combination and progression of symptoms you described are most consistent with systemic sclerosis. Further diagnostic testing, including serological tests (e.g., anti-Scl-70, anti-centromere antibodies), imaging studies, and possibly a skin biopsy, would be necessary to confirm the diagnosis.

### **115. User**

In addition to clinical symptoms similar to phenylketonuria, children 1-3 months after birth also show symptoms of dopamine deficiency, such as dyskinesia, hypotonia, lethargy, nystagmus, dysphagia, etc. Symptoms related to serotonin deficiency, such as unresponsiveness, depression, insomnia, etc. Norepinephrine deficiency, such as low muscle tone in the trunk, ptosis, cerebellar dysgenesis, etc., may be associated with dopamine deficiency. Symptoms associated with norepinephrine deficiency, such as hypotonia of the trunk, ptosis, and cerebellar hypoplasia. There may also be persistent convulsions, recurrent fever, delayed development of motor milestones, total paralysis, and severe impairment of intellectual development.

### Qwen2.5_72b

Based on the clinical manifestations you’ve provided, the most likely rare disease is **Tetrahydrobiopterin (BH4) Deficiency**. This condition is a rare inherited disorder that affects the metabolism of amino acids and neurotransmitters, leading to a deficiency in BH4, which is essential for the production of neurotransmitters such as dopamine, serotonin, and norepinephrine.

### Most Likely Diagnosis:

- **Tetrahydrobiopterin (BH4) Deficiency**

### Other Possible Diagnoses:

2. **Phenylketonuria (PKU) with Atypical Presentation**: While PKU typically presents with high phenylalanine levels, atypical presentations can include neurological symptoms similar to those described.
3. **Dopa-Responsive Dystonia (DRD)**: This condition is characterized by dystonia and parkinsonism, which can improve with dopa therapy. It can also present with hypotonia and developmental delays.
4. **Neurodegeneration with Brain Iron Accumulation (NBIA)**: This group of disorders can present with dystonia, hypotonia, and intellectual disability, along with other neurological symptoms.
5. **Mitochondrial Disorders**: These can present with a wide range of symptoms, including muscle weakness, developmental delay, and neurological deficits.
6. **Congenital Disorders of Glycosylation (CDG)**: These are a group of rare genetic disorders that affect the glycosylation process, leading to a variety of symptoms, including developmental delay, hypotonia, and neurological issues.

### Key Points:

- **Tetrahydrobiopterin (BH4) Deficiency** is the most likely diagnosis given the combination of symptoms related to neurotransmitter deficiencies and developmental delays.
- **Phenylketonuria (PKU)** should be considered, especially if there are high phenylalanine levels.
- **Dopa-Responsive Dystonia (DRD)** and **Neurodegeneration with Brain Iron Accumulation (NBIA)** are other neurological conditions that can present with similar symptoms.
- **Mitochondrial Disorders** and **Congenital Disorders of Glycosylation (CDG)** are broader categories that can also explain the diverse symptoms.

Further diagnostic testing, including genetic testing, metabolic screening, and imaging studies, will be necessary to confirm the diagnosis.

### **116. User**

They often present with multisystem involvement and develop in stages. Cardiac rhabdomyosarcomas can appear in the foetal/neonatal period, most of which resolve spontaneously; epileptic and cutaneous symptoms can appear in the first few months of life; renal angiomyolipomas and neoplastic lesions elsewhere in the body develop with age; and adult women are prone to pulmonary lymphangioleiomyomatosis of the lungs. Brain lesions include mainly cerebral cortical nodules, subventricular giant cell astrocytomas, and subventricular nodules. Skin lesions include leukoplakia visible after birth, progressive facial angiofibromas, shark leather spots, perinodal fibromas, and forehead plaques; renal vascular smooth muscle lipomas are present in up to 90% of patients; retinal involvement is present in about 50% of patients but rarely affects visual acuity; and LAM in the lungs occurs in about 35% of females; other areas, such as the liver, pancreas, gastrointestinal tract, and skeleton, may be involved, although usually with milder symptoms. Others, such as the liver, pancreas, digestive tract, and bones, can also be involved, but symptoms are usually mild.

### Qwen2.5_72b

Based on the clinical manifestations you’ve provided, the most likely diagnosis is **Tuberous Sclerosis Complex (TSC)**. TSC is a rare genetic disorder that causes non-cancerous (benign) tumors to grow in many parts of the body, including the brain, skin, kidneys, heart, eyes, and lungs. The multisystem involvement and the specific clinical features you’ve described are highly characteristic of TSC.

### Other Possible Diagnoses:

- **Neurofibromatosis Type 1 (NF1)**:
  - **Clinical Features**: Multiple café-au-lait spots, neurofibromas, Lisch nodules (pigmented iris hamartomas), axillary and inguinal freckling, optic gliomas, and skeletal abnormalities.
  - **Differential**: While NF1 also involves multiple systems, the specific skin lesions and brain tumors in TSC are more characteristic.
- **von Hippel-Lindau Disease (VHL)**:
  - **Clinical Features**: Hemangioblastomas of the brain, spinal cord, and retina; renal cell carcinomas; pheochromocytomas; and pancreatic cysts or tumors.
  - **Differential**: VHL does not typically present with the skin lesions or the specific brain and lung manifestations seen in TSC.
- **Sturge-Weber Syndrome**:
  - **Clinical Features**: Port-wine stain on the face, leptomeningeal angiomatosis, seizures, and glaucoma.
  - **Differential**: Sturge-Weber Syndrome primarily affects the skin and brain, but the renal and lung involvement is not typical.
- **Ataxia-Telangiectasia**:
  - **Clinical Features**: Progressive cerebellar ataxia, oculocutaneous telangiectasias, immunodeficiency, and increased risk of malignancies.
  - **Differential**: Ataxia-Telangiectasia does not typically present with the skin lesions, brain tumors, or renal and lung involvement seen in TSC.
- **Lymphangioleiomyomatosis (LAM)**:
  - **Clinical Features**: Progressive cystic lung disease, spontaneous pneumothorax, and chylous effusions.
  - **Differential**: LAM can occur in TSC, but it is a separate entity that can also occur sporadically. The other systemic manifestations in TSC are not typically seen in isolated LAM.

### Conclusion:

While the other conditions listed can present with some overlapping features, the combination of multisystem involvement, specific skin lesions, brain tumors, renal angiomyolipomas, and pulmonary lymphangioleiomyomatosis in females is highly suggestive of Tuberous Sclerosis Complex (TSC). Further genetic testing and imaging studies would be necessary to confirm the diagnosis.

### **117. User**

Most children with the undetected or untreated chronic form die before the age of 10 years, usually as a result of liver failure, neurological involvement or hepatocellular carcinoma.

The acute form has a rapid onset and progression, with acute liver failure as the main manifestation, and clinical signs include hepatosplenomegaly, jaundice, vomiting, abdominal distension, anorexia, lethargy, anaemia, bleeding tendencies and growth retardation. The child may have the characteristic odour of ‘boiled cabbage’ or ‘rotten mushrooms’. Insufficient hepatic synthesis of coagulation factors, marked prolongation of PT and APTT, and decreased levels of coagulation factors II, VII, IX, Ⅺ and Ⅻ, which are difficult to correct with vitamin K supplementation. Hepatic failure may be followed by ascites, jaundice and gastrointestinal bleeding, and children occasionally develop persistent hypoglycaemia. Untreated children often die of liver failure within weeks or months of onset of illness.

### Qwen2.5_72b

Based on the clinical manifestations and laboratory findings you’ve provided, the most likely diagnosis is **Tyrosinemia Type I (HT-1)**, a rare genetic disorder caused by a deficiency in the enzyme fumarylacetoacetate hydrolase (FAH). This enzyme is essential for the breakdown of tyrosine, and its deficiency leads to the accumulation of toxic metabolites, particularly succinylacetone, which can cause severe liver and kidney damage, as well as neurological complications.

### Most Likely Diagnosis:

- **Tyrosinemia Type I (HT-1)**

### Other Possible Diagnoses:

2. **Wilson’s Disease**: A genetic disorder that results in copper accumulation in the liver, brain, and other organs. It can present with liver failure, neurological symptoms, and Kayser-Fleischer rings in the eyes.
3. **Acute Fatty Liver of Pregnancy (AFLP)**: Although more common in pregnant women, it can present similarly with acute liver failure, jaundice, and coagulopathy.
4. **Hemophagocytic Lymphohistiocytosis (HLH)**: A rare, life-threatening disorder characterized by excessive activation of the immune system, leading to fever, hepatosplenomegaly, cytopenias, and coagulopathy.
5. **Acute Viral Hepatitis**: Viral infections such as hepatitis A, B, or E can cause acute liver failure, jaundice, and coagulopathy, especially in children.
6. **Reye’s Syndrome**: A rare but serious condition that can cause swelling in the liver and brain, often following a viral infection and aspirin use in children.

### Key Features of Tyrosinemia Type I:

- **Rapid onset and progression** in the acute form.
- **Acute liver failure** with hepatosplenomegaly, jaundice, vomiting, abdominal distension, anorexia, lethargy, and anemia.
- **Characteristic odor** of “boiled cabbage” or “rotten mushrooms.”
- **Coagulopathy** with prolonged PT and APTT, and decreased levels of coagulation factors II, VII, IX, XI, and XII.
- **Hepatic failure** leading to ascites, jaundice, and gastrointestinal bleeding.
- **Persistent hypoglycemia** in some cases.
- **High risk of hepatocellular carcinoma** in the chronic form.

Early diagnosis and treatment with a low-tyrosine and low-phenylalanine diet, and the use of nitisinone (an inhibitor of 4-hydroxyphenylpyruvate dioxygenase), can significantly improve outcomes and prevent liver failure and other complications.

### **118. User**

The clinical presentation is markedly heterogeneous, and there are three types based on age of onset and clinical presentation.

**The cardiomyopathic type,** which is the most common type of severe early-onset cardiac and multi-organ failure, develops mainly in newborns and early infants. It has a high mortality rate and is associated with hypoglycaemia, Reye’s syndrome, sudden neonatal death, hypertrophic and dilated cardiomyopathy, pericardial effusion, arrhythmias, myasthenia, hepatomegaly, and intermittent hypoglycaemia, with elevated levels of creatine kinase; myocardial hypertrophy and arrhythmias can be fatal.

**2. hepatic type ,** less severe, hepatic and hypohidrotic hypoglycaemic type with onset in late infancy or childhood, presenting with hepatomegaly and liver function abnormalities, decreased fasting tolerance and acute hypohidrotic hypoglycaemia without myocardial damage and cardiac hypertrophy, but can be life-threatening without timely diagnosis and treatment.

**3. Myopathic type** , which develops mainly in adolescence to adulthood, is late-onset, mildly symptomatic, and is usually not accompanied by myocardial disease and hypoglycaemia. The main manifestations are rhabdomyolysis and myoglobinuria after exercise, infection or starvation, and even renal failure, which may be accompanied by muscle weakness, painful muscle spasms or myalgia.

### Qwen2.5_72b

Based on the clinical manifestations you’ve provided, the most likely rare disease is **Carnitine Palmitoyltransferase II Deficiency (CPT II Deficiency)**. This is a metabolic disorder that affects the body’s ability to convert certain fats into energy, particularly during periods of fasting or increased energy demand.

### Most Likely Diagnosis:

- **Carnitine Palmitoyltransferase II Deficiency (CPT II Deficiency)**

### Other Possible Diagnoses:

- **Medium-Chain Acyl-CoA Dehydrogenase Deficiency (MCADD)**
  - **Clinical Features:** Hypoglycemia, hepatomegaly, Reye-like syndrome, and sudden death in infants and young children. MCADD can also present with vomiting, lethargy, and seizures.
- **Very Long-Chain Acyl-CoA Dehydrogenase Deficiency (VLCADD)**
  - **Clinical Features:** Similar to CPT II deficiency, VLCADD can present with cardiomyopathy, hepatomegaly, hypoglycemia, and muscle weakness. It can also cause pericardial effusion and arrhythmias.
- **Long-Chain 3-Hydroxyacyl-CoA Dehydrogenase Deficiency (LCHADD)**
  - **Clinical Features:** Hypoglycemia, hepatomegaly, cardiomyopathy, and muscle weakness. LCHADD can also present with retinal pigmentary changes and hypotonia.
- **Carnitine-Acylcarnitine Translocase Deficiency (CACTD)**
  - **Clinical Features:** Severe hypoglycemia, hepatomegaly, cardiomyopathy, and muscle weakness. CACTD can be fatal in early infancy if not diagnosed and treated promptly.
- **Glutaric Aciduria Type I (GA I)**
  - **Clinical Features:** While primarily associated with neurological symptoms, GA I can also present with cardiomyopathy, hepatomegaly, and hypoglycemia. It is often triggered by infections or metabolic stress.

### Summary:

- **CPT II Deficiency** is the most likely diagnosis given the specific clinical presentations of cardiomyopathic, hepatic, and myopathic types.
- **MCADD, VLCADD, LCHADD, CACTD, and GA I** are other metabolic disorders that can present with similar clinical features and should be considered in the differential diagnosis.

### **119. User**

**1. specific facial features,** periorbital subcutaneous tissue fullness, widened orbital distance, stellate irises, long midriffs, thick lips, wide mouths, and small jaws.

**2. cardiovascular disease,** elastin arteriopathy is present in 75% ~80% of patients and can affect any artery. Peripheral pulmonary stenosis (37% ~61%) is more common in infancy but improves with time. Supravalvular aortic stenosis is more common (35% ~65%) and worsens over time by age 5. Arterial stenosis leads to increased left heart resistance, cardiac hypertrophy, and heart failure. Other anomalies include renal artery stenosis, thoracic aortic stenosis, abdominal aortic stenosis, mitral valve prolapse and aortic valve insufficiency, mesenteric artery stenosis, and intracranial vascular anomalies. Hypertension can occur at any age and may be related in part to renal artery stenosis.

**3. Cognitive ability,** 75% of patients have mental retardation, which is usually mild. Cognitive impairment is characteristically manifested by relatively good short-term memory and verbal expression, but extreme weakness in visuospatial structural cognition. No gender differences in IQ have been reported, and children’s IQ is stable over time.

**4. personality, behavioural, and psychological abnormalities,** including excessive friendliness, excessive empathy, attention deficit, anxiety, and fear.

**5. sleep abnormalities,** with a 65% prevalence of sleep problems, including increased sleep latency and decreased sleep efficiency.

**6. eyes,** tear duct obstruction, hyperopia (67%), and strabismus (50%) are common. Cataracts have also been reported in adult patients.

**7. Ear, Nose, and Throat (ENT),** 50 per cent of patients have chronic otitis media, 90 per cent have increased sensitivity to sound, and 63 per cent of children and 92 per cent of adults have mild to moderate hearing impairment, mostly progressive sensorineural hearing loss. Most patients are hoarse or have a low voice.

**8. dental** , which may include baby teeth, enamel hypoplasia, and a misaligned bite.

**9. digestive system,** Chronic abdominal pain is a common symptom, and possible etiologies include gastroesophageal reflux, hiatal hernia, peptic ulcer, cholelithiasis, diverticulitis, ischaemic bowel disease, chronic constipation and anxiety somatisation. Hypercalcaemia may also lead to irritability, vomiting, constipation and muscle spasms.

**10. urinary system,** urinary frequency and enuresis are common in paediatric patients.

**11. neurological, muscular,** with low muscle tone and joint laxity in young children, leading to compensatory postural abnormalities to maintain body balance. Older children and adults have typical hypertonia and active deep tendon reflexes, resulting in a stiff and awkward gait, impaired fine motor function, and difficulty using tools and writing in patients of all ages. Adult patients will have cerebellar ataxia and tremor.

**12. Growth and development** .70% of infants have poor weight gain.

**13. idiopathic hypercalcaemia** . It occurs in infancy and childhood in 15-50% of patients and the most common symptoms are irritability, vomiting and constipation.

**14. endocrine.** Hypothyroidism (10 per cent), early puberty (50 per cent). Impaired glucose tolerance occurs in 26% of adolescent patients.

### Qwen2.5_72b

Based on the clinical manifestations provided, the most likely diagnosis is **Williams Syndrome** (WS). Williams Syndrome is a rare genetic disorder caused by a deletion of about 26-28 genes on chromosome 7, including the elastin gene, which leads to a wide range of physical, cognitive, and behavioral features.

### Most Likely Diagnosis:

- **Williams Syndrome (WS)**

### Other Possible Diagnoses:

- **Noonan Syndrome** - Characterized by short stature, distinctive facial features, and heart defects, but typically lacks the specific cardiovascular and cognitive profile of Williams Syndrome.
- **DiGeorge Syndrome (22q11.2 Deletion Syndrome)** - Features include heart defects, immune deficiencies, and characteristic facial features, but the cognitive and behavioral profile is different from Williams Syndrome.
- **Prader-Willi Syndrome** - Known for obesity, hypotonia, and cognitive impairment, but lacks the specific cardiovascular and facial features of Williams Syndrome.
- **Angelman Syndrome** - Characterized by severe intellectual disability, ataxia, and a happy, excitable demeanor, but lacks the cardiovascular and specific facial features of Williams Syndrome.
- **Smith-Magenis Syndrome** - Features include intellectual disability, sleep disturbances, and characteristic facial features, but the cardiovascular and specific cognitive profile differ from Williams Syndrome.

### Summary:

- **Williams Syndrome** is the most likely diagnosis given the combination of specific facial features, cardiovascular issues, cognitive and behavioral abnormalities, and other systemic manifestations.
- **Noonan Syndrome, DiGeorge Syndrome, Prader-Willi Syndrome, Angelman Syndrome, and Smith-Magenis Syndrome** are other rare genetic disorders that share some features but can be distinguished by their unique clinical profiles.

### **120. User**

**1. Haemorrhagic tendency** is a common manifestation of the disease, especially in blood-stained stools, and most children develop the disease in the neonatal period. The next manifestations are petechiae, ecchymosis, haemoptysis and haematuria, and some severe cases may have life-threatening gastrointestinal haemorrhage or intracranial haemorrhage. Bleeding in patients is caused by thrombocytopenia with reduction in platelet volume, but only about 25% of cases have the typical triad of symptoms.

**2. Infection.** Children are susceptible to a variety of infections, including herpes simplex virus, cytomegalovirus, Streptococcus pneumoniae, and fungi. The patient’s immunodeficiency worsens with age.

**3. Eczema.**  Eczema may be present in approximately 80% of children with WAS and varies widely in extent and severity. There may be typically severe, refractory eczema, or very mild or even no eczema manifestations.

**4. Autoimmune diseases.**  The most common is autoimmune haemolytic anaemia. Others include vasculitis, arthritis, renal disease, inflammatory bowel disease, neutropenia and autoimmune thrombocytopenic purpura.

**5. malignant tumours .** Children have a significantly higher risk of malignant tumours of the lymphatic system, especially lymphomas; non-Hodgkin’s lymphoma, lymphoblastic leukaemia, myelodysplastic syndromes, and spermatogonia have been reported.

**6. other .** Children with this disease may have completely normal platelet levels with persistent or recurrent neutropenia.

### Qwen2.5_72b

Based on the clinical manifestations you have provided, the most likely diagnosis is **Wiskott-Aldrich Syndrome (WAS)**. This is a rare X-linked recessive disorder characterized by a combination of immune deficiency, eczema, and thrombocytopenia with small platelets. The symptoms you described, including hemorrhagic tendency, infections, eczema, autoimmune diseases, and an increased risk of malignant tumors, are highly characteristic of WAS.

### Differential Diagnoses:

- **Severe Combined Immunodeficiency (SCID)**: This is another X-linked recessive disorder characterized by severe immune deficiency, leading to recurrent and severe infections. However, SCID typically does not present with the triad of eczema, thrombocytopenia, and hemorrhagic tendency.
- **Chédiak-Higashi Syndrome**: This is a rare autosomal recessive disorder characterized by partial albinism, recurrent infections, and bleeding tendencies. Patients may also have neutropenia and platelet abnormalities, but the eczema and autoimmune manifestations are less common.
- **Hyper-IgE Syndrome (Job’s Syndrome)**: This is a primary immunodeficiency characterized by recurrent skin and lung infections, eczema, and elevated IgE levels. However, thrombocytopenia and hemorrhagic tendency are not typical features.
- **Chronic Granulomatous Disease (CGD)**: This is an X-linked or autosomal recessive disorder characterized by recurrent bacterial and fungal infections due to impaired neutrophil function. While patients may have eczema, thrombocytopenia and hemorrhagic tendency are not common.
- **Autoimmune Lymphoproliferative Syndrome (ALPS)**: This is a rare genetic disorder characterized by chronic lymphadenopathy, splenomegaly, and autoimmune cytopenias. While patients may have autoimmune manifestations and increased risk of lymphoma, the combination of eczema and thrombocytopenia with small platelets is not typical.

### Summary:

- **Primary Diagnosis**: Wiskott-Aldrich Syndrome (WAS)
- **Differential Diagnoses**:
  1. Severe Combined Immunodeficiency (SCID)
  2. Chédiak-Higashi Syndrome
  3. Hyper-IgE Syndrome (Job’s Syndrome)
  4. Chronic Granulomatous Disease (CGD)
  5. Autoimmune Lymphoproliferative Syndrome (ALPS)

Further genetic testing, including sequencing of the *WAS* gene, would be essential to confirm the diagnosis of Wiskott-Aldrich Syndrome.

### **121. User**

The most common clinical manifestations are fulminant infectious mononucleosis, anomalous immunoglobulinaemia and B-cell lymphoma following EBV infection. Aberimmunoglobulinaemia and B-cell lymphoma can occur in patients without EBV infection. Aberimmunoglobulinaemia usually presents with increased levels of IgA and IgM and decreased levels of IgG1 and IgG3. Lymphomas are predominantly non-Hodgkin B lymphomas. Other clinical manifestations include lymphocytic vasculitis, aplastic anaemia and lymphomatoid granulomatosis. In some patients, the clinical picture is mild, with only abnormal immunoglobulinaemia or mild EBV infection, possibly due to somatic gene repair.

In some patients, the predominant clinical manifestation is a haemophagocytic syndrome, with splenomegaly, which does not necessarily require EBV infection to be induced. Some patients may present with intestinal inflammation such as Crohn’s disease or colitis, but rarely develop lymphoma.

### Qwen2.5_72b

Based on the clinical manifestations you’ve provided, the most likely rare disease is **X-linked Lymphoproliferative Syndrome (XLP)**, specifically **XLP-1 (caused by mutations in the SH2D1A gene)**. XLP is a primary immunodeficiency disorder characterized by an inability to control Epstein-Barr virus (EBV) infection, leading to severe and often fatal complications such as fulminant infectious mononucleosis, B-cell lymphomas, and hemophagocytic lymphohistiocytosis (HLH).

### Most Likely Diagnosis:

- **X-linked Lymphoproliferative Syndrome (XLP-1)**

### Other Possible Diagnoses:

- **Common Variable Immunodeficiency (CVID)**: CVID is a primary immunodeficiency characterized by hypogammaglobulinemia, recurrent infections, and an increased risk of autoimmune disorders and lymphomas. It can present with similar immunoglobulin abnormalities and lymphoproliferative disorders.
- **Hemophagocytic Lymphohistiocytosis (HLH)**: HLH is a life-threatening syndrome of excessive immune activation and cytokine storm, which can be triggered by infections, including EBV. It can present with symptoms such as fever, splenomegaly, and hemophagocytosis.
- **Chronic Active EBV Infection (CAEBV)**: CAEBV is a condition where EBV infection persists and causes chronic symptoms, including lymphadenopathy, hepatosplenomegaly, and hematological abnormalities. It can lead to lymphoproliferative disorders and lymphomas.
- **Wiskott-Aldrich Syndrome (WAS)**: WAS is an X-linked immunodeficiency characterized by eczema, thrombocytopenia, and recurrent infections. It can also present with an increased risk of lymphomas and autoimmune disorders.
- **Immunodeficiency with Hyper-IgM Syndrome (HIGM)**: HIGM is a group of primary immunodeficiencies characterized by low levels of IgG, IgA, and IgE, and normal or elevated levels of IgM. It can lead to recurrent infections and an increased risk of lymphomas and other malignancies.

These conditions share overlapping clinical features and should be considered in the differential diagnosis, especially when evaluating a patient with the described clinical manifestations.
