# Supplementary material for: Performance of ChatGPT-4o and Four Open-Source Large Language Models in Generating Diagnoses Based on China’s Rare Disease Catalog: Comparative Study
Source: J Med Internet Res. 2025 Jun 18;27:e69929. doi: 10.2196/69929 (PMC12192912; doi:10.2196/69929)
Supplement: Multimedia Appendix 1 [file jmir-v27-e69929-s001.zip › ChatGPT-4o_en.docx]

# ChatGPT-4o_en

**1. user - 2024-10-14 15:15:52**

The clinical manifestations of the patients included salt loss and hyperandrogenism. Neonatal onset was characterized by varying degrees of adrenal insufficiency, such as weakness, nausea, vomiting, feeding difficulties, diarrhea, chronic dehydration, skin pigmentation, and growth retardation. Adrenal crisis is often the first manifestation of the disease in the neonatal period, manifested as severe hyponatremia, hyperkalemia, hypovolemic shock, may be accompanied by hypoglycemia, induced by stress. Severe hyponatremia can lead to central nervous system manifestations such as convulsions, and severe hyperkalemia can cause fatal arrhythmias.

Hyperandrogenism manifests differently at different ages. Female children with classical diseases have different degrees of masculinity at birth. Light performance for isolated clitoris hypertrophy, heavy external genitalia can approach men, labia majora scrotal shape, clitoris hypospadia-like penis, and with a common urethral vaginal orifice. However, the labia majora cannot touch the gonads and has completely normal female internal genital structures (ovary and uterus). Males may not have external genital abnormalities such as enlarged penis during neonatal and infancy, which is a common cause of delayed diagnosis. In early childhood, both sexes will present peripheral precocious puberty. Boys presented with enlarged penis with or without pubic hair; females presented with heterosexual precocious puberty. Chronic high levels of sex hormones stimulate hypothalamic gonadotropin-releasing hormone (GnRH) neurons and develop into central precocious puberty. Women may also have secondary sexual dysplasia and primary amenorrhea or oligomenorrhea. Linear growth with accelerated bone age occurs in both sexes from infancy, resulting in impaired terminal height.

Other findings include skin and mucous membrane pigmentation, especially areola and vulva, some children may not have this change.

Non-classical hyperandrogenism in childhood, adolescence and even adulthood may be characterized by accelerated growth and rapid bone age progression.

**ChatGPT-4o** - 2024-10-14 15:15:52

Based on the clinical manifestations provided, the most likely diagnosis is **Congenital Adrenal Hyperplasia (CAH)** due to 21-hydroxylase deficiency. This condition commonly leads to salt loss (due to aldosterone deficiency) and hyperandrogenism (due to excess androgen production). The described symptoms, such as adrenal insufficiency in neonates, hyperandrogenism with varying presentations in females and males, and associated metabolic crises, are all characteristic of CAH.

Here are five other possible differential diagnoses that should be considered:

1. **11β-Hydroxylase Deficiency**: Another form of CAH that can also present with hyperandrogenism and adrenal insufficiency, although it typically includes hypertension due to excess deoxycorticosterone.

2. **17α-Hydroxylase Deficiency**: A different form of CAH that can lead to ambiguous genitalia and adrenal insufficiency but is usually associated with hypertension and hypokalemia rather than the electrolyte disturbances described.

3. **Addison's Disease**: While typically not associated with hyperandrogenism, primary adrenal insufficiency can present with many of the same symptoms like hyperpigmentation, salt loss, and adrenal crisis.

4. **Androgen Insensitivity Syndrome (AIS)**: This condition can cause ambiguous genitalia in genetic males and may present with features of hyperandrogenism, though it would not explain the adrenal insufficiency.

5. **Familial Glucocorticoid Deficiency (FGD)**: This rare condition primarily results in adrenal insufficiency symptoms such as hypoglycemia and hyperpigmentation, but without hyperandrogenism.

Each of these conditions has unique features that can help differentiate them from CAH due to 21-hydroxylase deficiency, but they share some overlapping symptoms necessitating careful diagnostic evaluation.

**2. user - 2024-10-14 15:28:54**

The patient has clinical manifestations at birth, due to lack of pigment in the whole body, skin capillaries are exposed, skin is thin and soft, white and red, and accompanied by varying degrees of vasodilation. Patients are highly sensitive to ultraviolet light, often solar dermatitis, actinic cheilitis, basal cell carcinoma and squamous cell carcinoma and other diseases. Hair is white, silver, yellow, gold or brown. Eyelashes and eyebrows are white or pale yellow. Retina, iris color decline, pupil color decline. Photophobia is obvious during the day, night vision is normal, and other visual changes can also be accompanied, such as decreased visual acuity, hypoplasia of the fovea, changes in neural pathways at the optic chiasm, and nystagmus.

**ChatGPT-4o** - 2024-10-14 15:28:54

Based on the clinical manifestations described, the most likely diagnosis is **Oculocutaneous albinism (OCA)**. This rare genetic condition results in a significant reduction or complete absence of melanin pigment in the skin, hair, and eyes, leading to many of the symptoms you've described.

Here are five other possible diagnoses that could be considered based on the provided symptoms:

1. **Hermansky-Pudlak Syndrome (HPS)**: This is a rare form of albinism that also involves bleeding disorders and can include lung, kidney, or bowel disease. It presents with similar pigmentation issues but also includes systemic complications.

2. **Chediak-Higashi Syndrome**: Another rare autosomal recessive disorder that involves albinism, it is associated with immunodeficiency, recurrent infections, and neurological issues, along with characteristic large granules in white blood cells.

3. **Griscelli Syndrome**: This rare condition presents with pigmentary dilution of the skin and hair, similar to albinism, and it can also involve immunodeficiency and neurological issues.

4. **Waardenburg Syndrome**: Though primarily known for causing hearing loss, this genetic condition can also cause pigmentary abnormalities of the skin, hair, and eyes. It can present with heterochromia (different colored eyes) and patches of depigmented skin and hair.

5. **Piebaldism**: A rare autosomal dominant disorder characterized by the absence of melanocytes in certain areas of the skin and hair, leading to patches of depigmentation. Unlike albinism, piebaldism usually doesn't affect the eyes.

Each of these conditions has distinctive features that can help differentiate them from Oculocutaneous albinism and from each other, and a definitive diagnosis would typically involve genetic testing and a thorough clinical evaluation.

**3. user - 2024-10-14 15:38:01**

The disease can begin in early childhood. Typical clinical manifestations include kidney and eye changes and hearing impairment. Renal manifestations are hematuria, proteinuria and progressive deterioration of renal function, in which hematuria is often persistent microscopic hematuria, which can occur after exercise or fever, and is common in adolescents. Hearing changes were sensorineural hearing loss, which gradually aggravated with the course of disease. Eye abnormalities manifest as myopia, youth ring and cataract, but lack specificity. Three ocular changes of diagnostic significance include anterior conical lens, posterior pleomorphic corneal atrophy, and retinal macula (white or yellow particles around the fovea). The incidence of hearing impairment in male patients in China was higher than that reported abroad (68% vs 55%), while that in female patients was lower than that reported abroad (7% vs 45%). The incidence of ocular abnormalities was similar to that reported abroad. Leiomyomas may also occur in a few patients, involving the respiratory tract, gastrointestinal tract, and female reproductive tract; occasionally aneurysmal lesions, midfacial dysplasia, and mental retardation have been reported.

 Patients often have a family history. Male patients with autosomal recessive inheritance and X-linked inheritance progressed faster, often entering end-stage renal disease at the age of 16~35, while female patients with autosomal dominant inheritance and X-linked inheritance had a slower course of disease and later onset of renal failure. The age at which renal failure occurs is similar for all male members of the same affected family, but can vary among male members of different families. Therefore, renal failure can be divided into juvenile type (renal failure occurs in adolescence) and adult type (renal failure occurs in adulthood) according to the age of the affected family. Adolescent type is more prominent in extrarenal manifestations, boys usually die before reproduction, disease genes are often new mutations, while adult type families have older patients and fewer new mutations.

**ChatGPT-4o** - 2024-10-14 15:38:01

Based on the clinical manifestations you've described, Alport Syndrome is the most likely diagnosis. The combination of kidney changes, hearing impairment, and specific eye abnormalities align with this condition.

Five other possible diagnoses could include:

1. Fabry Disease
2. Nail-Patella Syndrome
3. Thin Basement Membrane Nephropathy
4. Branchio-Oto-Renal Syndrome
5. Hereditary Nephritis

Each of these conditions can share some overlapping symptoms but differ in specific clinical features and genetic patterns.

**4. user - 2024-10-14 16:34:54**

A degenerative disease of the nervous system in which both upper and lower motor neurons are involved. The main clinical manifestations are progressive weakness and atrophy of the muscles of the bulbar, limbs, chest and abdomen. Eye motor nerve and sphincter function are generally not affected, but may be affected later in some patients. Between 20% and 50% of patients may exhibit cognitive impairment, and between 5% and 15% may even develop frontotemporal dementia. Death from respiratory failure occurs an average of 3 to 5 years after onset, but 5% to 10% of patients survive for more than 10 years.

Detailed history and physical examination to find evidence of common involvement of upper and lower motor neurons in brain stem, neck, chest and lumbosacral regions is the basis for diagnosis of the disease. Physical examination showed that both upper and lower motor neurons were involved in the same area, which was the key point of diagnosis. The main signs of lower motor neuron involvement included muscle weakness, atrophy and fascicular fibrillation. The tongue muscles, facial muscles, throat muscles, neck muscles, different muscle groups of the limbs, back muscles and pectoralis muscles are usually examined. The signs of upper motor neuron involvement mainly included positive pathological signs, tendon hyperreflexia, hypertonia, clonus, etc. Usually check sucking reflex, pharyngeal reflex, mandibular reflex, palmar jaw reflex, whether there is strong crying, strong laughter and other pseudobulbar paralysis manifestations, limb tendon reflex, muscle tension, Hoffman sign, lower limb pathology, abdominal wall reflex, etc. Clinical examination is the primary method of detecting upper motor neuron involvement. In areas of atrophy and weakness, if tendon reflexes are not low or active, it may indicate pyramidal tract damage even if there are no pathological signs.

**ChatGPT-4o** - 2024-10-14 16:34:54

Based on the clinical manifestations provided, the most likely diagnosis is Amyotrophic Lateral Sclerosis (ALS).

Five other possible diagnoses could include:

1. **Primary Lateral Sclerosis (PLS):** Affects only upper motor neurons.
2. **Progressive Muscular Atrophy (PMA):** Primarily affects lower motor neurons.
3. **Spinal Muscular Atrophy (SMA):** Genetic disorder affecting lower motor neurons.
4. **Multifocal Motor Neuropathy (MMN):** Affects peripheral nerves, leading to muscle weakness.
5. **Kennedy's Disease (Spinal and Bulbar Muscular Atrophy):** X-linked disorder affecting motor neurons, leading to muscle atrophy and weakness.

**5. user - 2024-10-14 16:46:12**

Patients often have no abnormal performance in the neonatal period. The first symptom is developmental retardation at about 6 months of age, but the typical clinical manifestations appear after 1 year of age, and it often takes several years for clinical diagnosis to be confirmed. According to the frequency of clinical manifestations, they were divided into three categories: common manifestations (100% patients), frequent manifestations (more than 80% patients) and correlated manifestations (20% ~80% patients).

 1. All of them showed normal pregnancy history and head circumference at birth; no birth defects and abnormal biochemical indexes; brain MRI/CT showed no structural abnormalities except mild cortical atrophy and myelin dysplasia; motor milestones lagged behind without regression; severe developmental retardation occurred at 6~12 months; language disorders showed no or very little vocabulary, repetitive language and non-verbal communication ability were stronger than expressive language ability; motor or balance disorders often showed ataxia and tremor of limbs; Abnormal behavioral characteristics are frequent laughter or smiling, obvious excited movements or happy behavior, often accompanied by clapping or hyperactivity.

 2. The head circumference often showed backward growth, and microcephaly was still present at the age of 2. Epilepsy often occurred before the age of 3. Abnormal EEG: characteristic high amplitude spike-slow wave.

 3. Correlation: flat occipital region; tongue preference; sucking or swallowing disorders; infantile feeding difficulties; low muscle tone; large jaw; wide tooth space; frequent salivation; excessive chewing movements; strabismus; hypopigmentation of the skin; light hair and eye color compared to family (absent type only); hyperreflexia of lower limb tendons; lifting or bending of upper limbs, especially when walking; wide gait base; high sensitivity to heat; abnormal sleep-wake cycle, less sleep; obsession with water, paper, and plastic; Eating related behavioral abnormalities; Obesity (more common in older, non-deficit patients); scoliosis; constipation.

**ChatGPT-4o** - 2024-10-14 16:46:12

Based on the clinical manifestations you described, the most likely diagnosis is **Angelman Syndrome**. This rare neurogenetic disorder is characterized by developmental delays, lack of speech, seizures, and a happy demeanor.

Here are five other possible diagnoses that could be considered based on the symptoms:

1. **Rett Syndrome**: This condition often presents with normal development followed by loss of purposeful hand skills, stereotypic movements, and developmental regression.

2. **Prader-Willi Syndrome**: Characterized by hypotonia, feeding difficulties in infancy, followed by hyperphagia, obesity, and developmental delays.

3. **Smith-Magenis Syndrome**: Includes developmental delay, distinctive facial features, sleep disturbances, and behavior problems.

4. **Williams Syndrome**: Known for unique cognitive profile, distinctive facial features, cardiovascular issues, and an overly sociable personality.

5. **Sotos Syndrome**: Characterized by overgrowth, developmental delay, and distinctive facial features.

Each of these conditions shares some overlapping symptoms with Angelman Syndrome but has distinct features that differentiate them.

**6. user - 2024-10-14 22:57:05**

An autosomal recessive disorder affecting the urea cycle, leading to hyperammonemia, encephalopathy, and respiratory alkalosis. It is characterized by a buildup of arginine and ammonia in the blood, causing symptoms such as developmental delays, seizures, intellectual disability, spasticity, and ataxia. The condition typically manifests around age 3, with symptoms including stiffness, slow growth, and cognitive decline. Without treatment, it can progress to severe spasticity, loss of ambulation, and intellectual disability.

**ChatGPT-4o** - 2024-10-14 22:57:05

Based on the clinical manifestations you've described, the most likely diagnosis is **Arginase Deficiency**, a rare urea cycle disorder.

Here are five other possible diagnoses to consider:

1. **Ornithine Transcarbamylase Deficiency (OTC Deficiency)**
2. **Carbamoyl Phosphate Synthetase I Deficiency**
3. **Citrullinemia Type I**
4. **Argininosuccinic Aciduria**
5. **Hyperornithinemia-Hyperammonemia-Homocitrullinuria Syndrome (HHH Syndrome)**

Each of these disorders involves issues with the urea cycle and can present with similar symptoms but may have distinct biochemical markers.

**7. user - 2024-11-23 09:35:17**

1. Skeletal abnormalities: There is variation in the severity of the disease and the type of associated complications. Thoracic stenosis is present in all cases, and the thorax may be bell-shaped or long and narrow, with respiratory distress usually caused by a small, rigid thorax. The severity of respiratory insufficiency varies according to the type of thorax and the degree of underlying pulmonary dysplasia, and the age of onset varies. There is a negative correlation between the severity of respiratory insufficiency and the age at which the disease is diagnosed. Acute respiratory disease is more common in infants with severe restrictive and bell-shaped chests, and severe and fatal respiratory failure can occur in the early neonatal period. Infants with a long, narrow chest are more likely to present with mild respiratory involvement, which can eventually progress to chronic restrictive lung disease with recurrent respiratory infections. Short, deformed ribs cause intercostal muscle weakness and restricted breathing, leading to respiratory failure. Most children die within 2 years of age, with about half dying within 6 months of birth. Other skeletal anomalies include short ribs, short limbs, abnormally shaped pelvis, short fingers, and polydactyly. About 60% of patients have proximal cervical spinal stenosis.

2. Involvement of other organs: Renal lesions are present in about 40% of patients. The earliest sign of renal disease is impaired urinary concentration. Other signs include polyuria, proteinuria, elevated blood urea nitrogen and creatinine, and hypertension. Renal failure is the leading cause of death in this patient between the ages of 3 and 10 years. Hepatic involvement occurs in about 30% of cases and is manifested by hepatomegaly, elevated aminotransferases, cholestasis, cirrhosis and portal hypertension. The disease is occasionally associated with ocular developmental abnormalities, including optic nerve hypoplasia, retinal dystrophy, retinal pigmentation abnormalities, nystagmus, myopia and progressive visual impairment. There are also a few reports suggesting that these patients may also present with neurological abnormalities such as growth retardation, hypotonia, and central nervous system MRI abnormalities.

**ChatGPT-4o** - 2024-11-23 09:35:17

Based on the clinical manifestations you've described, the disease most likely to be diagnosed is **Jeune Syndrome** (also known as Asphyxiating Thoracic Dystrophy). This rare genetic disorder is characterized by the skeletal and organ involvement you've detailed.

Five other possible diagnoses that could present with some overlapping features include:

1. **Ellis-van Creveld Syndrome**: This condition shares features like short ribs, short limbs, and polydactyly. However, it typically includes additional features like dental abnormalities and ectodermal dysplasia.

2. **Short Rib Polydactyly Syndromes**: This group of disorders includes several types such as Majewski, Saldino-Noonan, and Verma-Naumoff types, which share features like short ribs, polydactyly, and thoracic insufficiency.

3. **Bardet-Biedl Syndrome (BBS)**: It shares some features such as renal and hepatic involvement, polydactyly, and retinal dystrophy. However, BBS typically includes obesity and intellectual disability.

4. **Saldino-Mainzer Syndrome**: A form of short rib polydactyly syndrome with features like renal disease and retinal dystrophy, which can overlap with the findings in Jeune Syndrome.

5. **Meckel-Gruber Syndrome**: Known for features like polydactyly, renal cysts, and liver fibrosis, although it is often more severe and presents prenatally or shortly after birth with additional central nervous system abnormalities.

Each of these conditions would require a careful evaluation, including genetic testing, to differentiate them accurately.

**8. user - 2024-11-24 22:59:14**

The typical clinical picture includes the triad of microangiopathic haemolytic anaemia, thrombocytopenia and acute renal failure. Non-immune erythrocyte destruction due to microthrombosis often results in a haemoglobin level below 80 g/L, a negative Coombs test, and erythrocyte fragments seen on peripheral blood smears; platelet counts usually fall below 50×10^9 /L, but skin purpura and active bleeding are rare; the severity of the acute renal impairment varies from person to person, with some patients requiring dialysis support, often accompanied by elevated blood pressure.

**ChatGPT-4o** - 2024-11-24 22:59:14

Based on the clinical manifestations you provided, the most likely diagnosis is Hemolytic Uremic Syndrome (HUS). HUS is characterized by the triad of microangiopathic hemolytic anemia, thrombocytopenia, and acute renal failure.

Here are five other possible diagnoses that could be considered:

1. **Thrombotic Thrombocytopenic Purpura (TTP):** Shares similarities with HUS, including microangiopathic hemolytic anemia and thrombocytopenia. However, TTP often presents with neurological symptoms and fever, distinguishing it from typical HUS.

2. **Atypical Hemolytic Uremic Syndrome (aHUS):** A variant of HUS that is not associated with Shiga toxin-producing E. coli infection and often involves genetic or acquired complement dysregulation.

3. **Systemic Lupus Erythematosus (SLE):** With renal involvement leading to lupus nephritis, it can present with anemia, thrombocytopenia, and renal failure. Autoimmune features would be present.

4. **Disseminated Intravascular Coagulation (DIC):** Can present with thrombocytopenia, microangiopathic hemolytic anemia, and renal impairment, although typically associated with systemic bleeding and clotting issues.

5. **Malignant Hypertension:** Severe hypertension can lead to microangiopathic hemolytic anemia and renal failure. Elevated blood pressure is a key component here, consistent with the provided symptoms.

It’s important to perform specific diagnostic tests and consider the patient’s complete clinical history to differentiate between these conditions accurately.

**9. user - 2024-11-24 23:04:53**

The main symptoms include mental behavioural abnormalities, cognitive impairment, memory loss, seizures, speech disorders, movement disorders, involuntary movements, decreased level of consciousness, and autonomic dysfunction. Other accompanying symptoms include sleep disorders, focal damage to the CNS, brainstem and cerebellar symptoms, and involvement of peripheral nerves and neuromuscular junctions.

There are three main clinical types: (1) anti-NMDAR encephalitis, which is the most predominant type and is characterised by diffuse encephalitis. (ii) Borderline encephalitis, with mental behavioural abnormalities, temporal lobe epilepsy and near-memory deficits as the main symptoms. The common types of borderline encephalitis are anti-LGI1 antibody, anti-GABABR antibody and anti-AMPAR antibody-related encephalitis. (iii) Other types, including Morvant syndrome, anti-DPPX antibody-associated encephalitis, anti-IgLON5 antibody-associated encephalopathy, and autoimmune cerebellar ataxia.

**ChatGPT-4o** - 2024-11-24 23:04:53

Based on the clinical manifestations you've provided, the most likely diagnosis is **autoimmune encephalitis**, specifically **anti-NMDAR (N-methyl-D-aspartate receptor) encephalitis**. This condition is characterized by a range of neuropsychiatric symptoms including cognitive impairment, memory loss, seizures, and movement disorders, all of which align with the symptoms you have described.

Here are five other possible diagnoses that could fit the presentation:

1. **Anti-LGI1 (Leucine-rich Glioma Inactivated 1) antibody encephalitis**: This is a form of autoimmune encephalitis that often presents with seizures, cognitive impairment, and faciobrachial dystonic seizures.

2. **Anti-GABABR (gamma-aminobutyric acid B receptor) antibody encephalitis**: This type can present with seizures, cognitive decline, and behavioral changes, often associated with small cell lung cancer.

3. **Anti-AMPAR (alpha-amino-3-hydroxy-5-methyl-4-isoxazolepropionic acid receptor) antibody encephalitis**: Patients usually present with psychiatric symptoms, memory impairment, and seizures.

4. **Morvan syndrome**: Characterized by peripheral nerve hyperexcitability, autonomic instability, and central nervous system symptoms. It often involves antibodies against voltage-gated potassium channels.

5. **Anti-IgLON5 antibody-associated encephalopathy**: This is associated with a mix of sleep disorders, movement disorders, and cognitive impairment, with some overlap with neurodegenerative diseases.

These conditions share overlapping symptoms and often require specific antibody testing, clinical context, and neuroimaging to make a definitive diagnosis.

**10. user - 2024-11-24 23:07:48**

Patients have a variety of clinical presentations. It depends on the location, extent and rate of progression of the lesion. Clinical symptoms mainly include:

1. The occupying effect of an enlarged pituitary gland, which in most patients results from compression of the enlarged pituitary gland on the anterior region and surrounding structures (e.g., optic crossings, cavernous sinus, etc.), or from an inflammatory infiltrate. The most common clinical manifestations are persistent severe headaches and progressively worsening visual dysfunction, including decreased visual acuity and visual field defects.

2. hypopituitarism. Depending on the degree of involvement, the various axes of the pituitary gland may show partial or even total hypopituitarism, such as amenorrhoea, hypogonadism, nausea, malaise, chills, loss of sexual hair and weight loss.

3. Central dysuria, more than 70% of the patients will have symptoms of central dysuria such as polyuria, irritable thirst and polydipsia due to the involvement of the posterior pituitary, pituitary stalk and even hypothalamus region. In some patients, the blocking effect of the pituitary stalk leads to a mild increase in serum prolactin levels.

4. Hypothalamic syndrome, when the lesion involves the region of the hypothalamus, there will be drowsiness, lack of satiety, severe weight gain, lack of thirst, temperature fluctuation, sweating disorders, and emotional and psychiatric disorders.

5. Systemic disease-related manifestations. Patients with secondary disease often have a combination of clinical manifestations characteristic of systemic disease, such as LCH patients often have a combination of thyroid, lung, bone, liver, and skin lesions. patients with GPA are often middle-aged or older men, and respiratory symptoms often precede hypothalamic pituitary involvement. patients with IgG4-related pituitary inflammation often have a combination of clinical manifestations with the involvement of other organs such as lacrimal glands, submandibular glands, and pancreas. The disease is often associated with clinical signs of involvement of other organs such as the lacrimal gland and submaxillary gland and pancreas.

**ChatGPT-4o** - 2024-11-24 23:07:48

Based on the clinical manifestations provided, the most likely diagnosis is **Langerhans Cell Histiocytosis (LCH)** affecting the hypothalamic-pituitary axis. This is suggested by the combination of symptoms, including the occupying effect of an enlarged pituitary, hypopituitarism, central dysuria, and systemic disease-related manifestations that often include involvement of other organs such as the thyroid, lung, bone, liver, and skin.

Here are five other possible diagnoses:

1. **Pituitary Adenoma**: While more common than rare, large adenomas can cause similar symptoms due to compression effects and involvement of the hypothalamic-pituitary axis.

2. **Granulomatosis with Polyangiitis (GPA)**: This condition, formerly known as Wegener's granulomatosis, can involve the pituitary gland and present with systemic manifestations, particularly respiratory symptoms, preceding pituitary involvement.

3. **IgG4-related Disease**: This autoimmune condition can cause pituitary inflammation and is often associated with involvement of other organs, including the lacrimal and submandibular glands and pancreas.

4. **Primary Hypophysitis**: This is an inflammation of the pituitary gland that can mimic the symptoms of an expanding pituitary lesion and often presents with hypopituitarism and diabetes insipidus.

5. **Craniopharyngioma**: These benign tumors can cause similar symptoms due to their location near the pituitary gland and hypothalamus, leading to compression effects, hypopituitarism, and hypothalamic disturbances.

Each of these conditions can have overlapping symptoms and may require imaging, endocrine workup, and possibly biopsy for definitive diagnosis.

**11. user - 2024-11-24 23:14:10**

Hyperglycaemia is the most common clinical manifestation of the disease. However, patients can spontaneously remit without intervention and maintain normal blood glucose levels. The disease is one of the leading causes of autoimmune hypoglycaemia, with about 25% of patients experiencing spontaneous hypoglycaemia during the course of the disease. Acanthosis nigricans is present in about 50 per cent of patients. In addition, polycystic ovaries and hyperandrogenism are not uncommon in this group of patients. Other autoimmune diseases are common, with systemic lupus erythematosus (SLE) being the most common, accounting for about 50 per cent of cases. Primary biliary cirrhosis, dermatomyositis, and scleroderma are also present. The disease can also be a paraneoplastic syndrome manifestation of Hodgkin's lymphoma, multiple myeloma, and others.

**ChatGPT-4o** - 2024-11-24 23:14:10

Based on the clinical manifestations you've described, the most likely diagnosis is Type B Insulin Resistance Syndrome. This condition is characterized by severe insulin resistance often caused by autoantibodies against the insulin receptor, leading to hyperglycemia. Patients can experience spontaneous remissions and episodes of hypoglycemia due to fluctuating autoantibody levels. Acanthosis nigricans is commonly observed, and it is associated with other autoimmune conditions like systemic lupus erythematosus (SLE). It can also be associated with certain malignancies, aligning with your description of paraneoplastic syndrome features.

Other possible diagnoses to consider include:

1. **Polyendocrine Autoimmune Syndrome**: This encompasses a cluster of autoimmune diseases that might include diabetes, thyroiditis, and others, often with accompanying skin manifestations and polycystic ovarian disease.

2. **Polycystic Ovary Syndrome (PCOS) with Insulin Resistance and Autoimmunity**: Although primarily a reproductive disorder, PCOS can present with insulin resistance and has been associated with autoimmune conditions.

3. **Autoimmune Polyglandular Syndrome Type 1 or 2**: These involve multiple autoimmune conditions, often affecting endocrine glands, and can manifest with hypoglycemia due to adrenal insufficiency or other factors.

4. **Paraneoplastic Autoimmune Syndrome**: Some paraneoplastic syndromes might lead to autoimmune manifestations affecting glucose metabolism, especially in the presence of underlying malignancies.

5. **Insulin Autoimmune Syndrome (Hirata's Syndrome)**: Although more commonly associated with hypoglycemia due to insulin autoantibodies, it could potentially explain some of the autoimmune aspects and glycemic variability described.

While Type B Insulin Resistance Syndrome fits most closely with your description, further clinical investigation, including serological and immunological testing, would be necessary to confirm the diagnosis.

**12. user - 2024-11-24 23:17:51**

The disease starts in infancy and childhood. The first attack occurs between the ages of 5 months and 7 years. The clinical picture is one of recurrent episodes of intractable vomiting and severe ketoacidosis with depression, dehydration, shortness of breath, and coma. There is usually a clear trigger, and the disease may begin acutely after fasting, gastrointestinal and upper respiratory tract infections, fever, stress, or excessive protein intake. Routine laboratory tests show elevated or normal blood glucose, some hypoglycaemia, blood gases show metabolic acidosis, blood ammonia can be normal or mildly elevated, liver function, renal function and normal, and urine ketone bodies are positive. Ketosis and acidosis may recur, and clinical and laboratory tests may be normal between attacks.
Neurological changes occur in about 1/5 of the cases, manifested by cognitive impairment, mental retardation, extrapyramidal abnormalities and symptoms of stroke. Cranial MRI reveals extensive demyelination, including white matter, and symmetric high signal changes in the basal ganglia at T2.
Patients may be asymptomatic in the early stages and are diagnosed in the neonatal period or in high-risk groups by blood and urine metabolic screening. There is no clear association between age of onset, frequency of attacks and prognosis. There is a tendency for the attacks to decrease or stop with age, and in most cases growth is unaffected on follow-up. Recurrent and severe attacks can lead to multi-organ dysfunction or even death.

**ChatGPT-4o** - 2024-11-24 23:17:51

Based on the clinical manifestations you've described, the most likely diagnosis is **Glutaric Aciduria Type I (GA-I)**. This is a rare metabolic disorder that can present with the symptoms and clinical findings you've noted: recurrent ketoacidosis, neurological changes, and MRI findings of white matter abnormalities and basal ganglia changes. The episodes are often triggered by stressors such as fasting or infections, and the metabolic profile can include ketosis and metabolic acidosis.

Here are five other possible diagnoses:

1. **Maple Syrup Urine Disease (MSUD):** This disorder involves episodes of metabolic decompensation with ketoacidosis and neurological symptoms, often precipitated by infections or protein intake. However, it typically presents with a distinctive sweet-smelling urine, which wasn't noted in your description.

2. **Propionic Acidemia:** This is another organic acidemia that can cause recurrent episodes of metabolic acidosis, vomiting, and neurological issues. It can present with high blood ammonia levels, which might overlap with your case.

3. **Methylmalonic Acidemia (MMA):** Similar to propionic acidemia, MMA is characterized by episodes of ketoacidosis and neurological manifestations. Elevated ammonia can occur, and symptoms are often precipitated by stress or illness.

4. **Isovaleric Acidemia:** This condition can present with metabolic acidosis and neurological symptoms, often triggered by fasting or infections. It typically has a characteristic "sweaty feet" odor, which may not always be noted.

5. **Medium-Chain Acyl-CoA Dehydrogenase (MCAD) Deficiency:** Although it primarily presents with hypoglycemia and metabolic acidosis, the episodic nature and triggers (fasting, illness) could mimic the described condition. However, it usually presents with lower ketone levels compared to other disorders listed.

It's important for a healthcare professional to consider comprehensive metabolic testing, genetic analysis, and collaboration with a metabolic specialist for an accurate diagnosis.

**13. user - 2024-11-24 23:24:28**

The clinical manifestations of the disease are complex and varied, and lack specificity. Most of them involve multiple systems such as nerve, skin, respiratory, digestive and immune systems, and it is difficult to confirm the diagnosis from the clinical manifestations alone. It can be divided into two types: complete and partial.

1. Complete: It can be divided into two types: early-onset and late-onset. The most common manifestations are neurological symptoms, such as hypotonia, epilepsy, developmental delay, sensorineural deafness, ataxia, and progressive disorders of consciousness, etc. About half of the patients have ataxia, developmental delay, conjunctivitis, and visual problems, including visual problems. conjunctivitis, and visual problems, including optic atrophy. Metabolic disorders such as metabolic acidosis, hypoglycaemia and ketosis are often comorbid, and cutaneous manifestations include various forms of refractory rashes such as eczema, generalised erythema, and nappy rash, and patients may also present with respiratory symptoms such as hyperventilation, laryngeal wheezing, and apnoea, as well as digestive symptoms such as feeding difficulties, vomiting, and diarrhoea. Acute attacks can be triggered by infection, fever, fatigue, and a high protein diet. If treatment is not timely, it can often lead to irreversible damage. Patients with late-onset disease often develop in adolescence and may have no symptoms beforehand. The clinical phenotype is different from that of early-onset disease, which usually presents with peripheral neuropathy, muscle weakness, spastic paraparesis, skin rashes, and ocular problems, such as loss of vision and visual acuity, etc. Failure to treat the disease in a timely manner often leads to irreversible damage.

2. Partial : May develop at all ages or even without onset. The overall signs and symptoms of the child are relatively mild, starting only in emergency situations such as infections and starvation. Patients may have hypotonia, skin rashes, hair loss, acute neurological symptoms such as epilepsy and impaired consciousness.

**ChatGPT-4o** - 2024-11-24 23:24:28

Based on the clinical manifestations you've provided, the disease you are describing is suggestive of a rare metabolic disorder. Given the complex, multi-system involvement, the most likely diagnosis could be **Mitochondrial Disease**, specifically a disorder within the spectrum of mitochondrial encephalomyopathies such as MELAS (Mitochondrial Encephalomyopathy, Lactic Acidosis, and Stroke-like episodes) or Leigh syndrome. These conditions often present with neurological, muscular, and metabolic symptoms and can involve various other systems, including the skin and eyes.

Here are five other possible diagnoses that could fit the described symptoms:

1. **Metabolic Disorders (e.g., Maple Syrup Urine Disease, Propionic Acidemia, or Urea Cycle Disorders):** These can present with acute metabolic crises, neurological symptoms, and multisystem involvement.

2. **Lysosomal Storage Diseases (e.g., Niemann-Pick Disease, Gaucher Disease):** These diseases may present with neurological decline, skin changes, and visceral involvement.

3. **Peroxisomal Disorders (e.g., Zellweger Spectrum Disorders):** This group of disorders can present with developmental delay, neurological symptoms, and multiple organ involvement.

4. **Congenital Disorders of Glycosylation (CDG):** These conditions can cause a wide range of symptoms, including neurological impairment, skin issues, and metabolic abnormalities.

5. **Biotinidase Deficiency:** This is a metabolic disorder that can present with neurological symptoms, skin rashes, and metabolic crises, especially during stress situations like infections or fasting.

Diagnosis of these conditions often requires a combination of clinical evaluation, biochemical testing, genetic testing, and sometimes a muscle biopsy or other specialized investigations to confirm the specific disorder.

**14. user - 2024-11-24 23:29:53**

1. the onset is at a young age and is more common in women.

2. there is often a family history of syncope or sudden death.

3. different genotypes and phenotypes, can involve potassium, sodium and calcium channels. Characteristic tip-distortion ventricular tachycardia (TdP) is often present, leading to syncope or sudden death. 4.

4. The ECG shows a prolonged QT interval.

5. ECG generally has the following characteristics: (1) QT interval prolongation, short - long - short cycle phenomenon induced; (2) ventricular rate of 160-240 beats / minute, QRS wave amplitude and morphology around the equipotential line of reversal, 5-20 cardiac cycles of the main wave around the baseline of the reversal of the majority of the time can be terminated; (3) ATP is more difficult to terminate the TdP.

**ChatGPT-4o** - 2024-11-24 23:29:53

Based on the clinical manifestations provided, the most likely diagnosis is Long QT Syndrome (LQTS). This is a genetic condition characterized by prolonged QT intervals on an ECG and can lead to torsades de pointes (TdP), syncope, or sudden death. The condition often presents in young individuals and is more common in females. It involves mutations that affect cardiac ion channels (potassium, sodium, and calcium channels).

Five other possible diagnoses to consider could include:

1. **Brugada Syndrome**: Another genetic disorder affecting cardiac sodium channels that can lead to sudden cardiac death, though it more commonly presents with a distinct ECG pattern and not prolonged QT intervals.

2. **Catecholaminergic Polymorphic Ventricular Tachycardia (CPVT)**: This is triggered by physical activity or stress, leading to syncope or sudden death, but usually involves normal resting ECGs.

3. **Short QT Syndrome**: Characterized by a short QT interval and increased risk of arrhythmias, though it presents with a shortened rather than prolonged QT interval.

4. **Arrhythmogenic Right Ventricular Cardiomyopathy (ARVC)**: A genetic condition that can lead to ventricular arrhythmias and syncope, often with a specific ECG pattern different from Long QT Syndrome.

5. **Familial Hyperkalemic Periodic Paralysis**: This is a channelopathy-related condition involving potassium channels, leading to muscle weakness and potentially cardiac issues, but it’s primarily a neuromuscular disorder.

Each of these conditions involves genetic factors affecting cardiac function, though they differ in clinical presentation and ECG characteristics.

**15. user - 2024-11-24 23:33:23**

The disease can develop at any age, and most of the children develop between 1 month and 7 years old, with an average age of about 2 years old. Clinical manifestations vary greatly among patients, mainly including: (1) acute energy metabolism disorder crisis, manifested by low ketone hypoglycemia, high blood ammonia and metabolic acidosis; (2) cardiomyopathy, manifested by ventricular hypertrophy, cardiac insufficiency, cardiac arrhythmia, and elevated creatine kinase, etc.; (3) myopathy, manifested by muscle weakness, hypotonia, myalgia, poor exercise endurance, elevated muscular-type creatine kinase, and lipid deposition in muscle fibres, etc. (4) liver damage, manifested by hepatomegaly, fatty liver, liver function abnormalities, etc. Some liver-damaged patients have liver enlargement, fatty liver, and liver function abnormalities; Liver damage, manifested by hepatomegaly, fatty liver, abnormal liver function, etc. Some children with liver damage have acute onset of illness, manifested by convulsions, progressive consciousness disorder, etc., which is often misdiagnosed as Reye syndrome. In addition, gastrointestinal symptoms such as recurrent abdominal pain, vomiting and gastroesophageal reflux, respiratory symptoms such as recurrent infections and wheezing, and anaemia have also been reported.

Newborn screening: diagnosed children may have no clinical manifestations. It is a potentially fatal disease, and children can die suddenly from acute energy metabolism disorder crisis or acute heart failure. Clinical manifestations are varied and related to age of onset, time of onset and severity of illness. The most common symptoms are cardiac and skeletal muscle damage in infancy and childhood. Symptoms in adulthood are mild or asymptomatic, mostly due to reduced endurance or easy fatigue. During pregnancy, pregnant women may have atypical manifestations such as fatigue and arrhythmias due to energy expenditure and reduced physiological plasma carnitine levels.

**ChatGPT-4o** - 2024-11-24 23:33:23

Based on the clinical manifestations provided, the most likely diagnosis is a fatty acid oxidation disorder, specifically Medium-Chain Acyl-CoA Dehydrogenase Deficiency (MCADD). This disorder is characterized by the inability to break down medium-chain fatty acids, leading to symptoms such as hypoketotic hypoglycemia, metabolic acidosis, cardiomyopathy, myopathy, liver dysfunction, and potentially sudden death during metabolic stress or fasting.

Other possible diagnoses to consider with similar presentations include:

1. **Carnitine Palmitoyltransferase II Deficiency (CPT II Deficiency)**: This disorder also affects fatty acid oxidation and can present with muscle weakness, rhabdomyolysis, hypoglycemia, and liver dysfunction.

2. **Very Long-Chain Acyl-CoA Dehydrogenase Deficiency (VLCADD)**: Another fatty acid oxidation disorder that presents with hypoglycemia, cardiomyopathy, and muscle weakness.

3. **Multiple Acyl-CoA Dehydrogenase Deficiency (MADD)**: Also known as Glutaric Aciduria Type II, this can present with metabolic acidosis, hypoglycemia, cardiomyopathy, and muscle weakness.

4. **Pompe Disease**: A glycogen storage disorder that can present with cardiomyopathy, muscle weakness, and respiratory distress, although it typically involves lysosomal storage dysfunction.

5. **Reye Syndrome**: Though not a direct match, the acute liver dysfunction and encephalopathy can mimic some presentations, particularly in the context of metabolic crises in children.

These conditions are all rare metabolic disorders that can have overlapping symptoms, particularly involving energy metabolism, muscle, and liver dysfunction. A thorough metabolic workup, including enzyme activity assays and genetic testing, is crucial for accurate diagnosis.

**16. user - 2024-11-25 09:12:18**

In the unicentric form, the disease often presents only with enlarged lymph nodes without systemic symptoms. The median diameter of the enlarged lymph nodes is about 5.5 cm, and they are commonly found in the chest (24%), neck (20%), abdomen (18%), and retroperitoneum (14%). Some patients with unicentric disease are prone to paraneoplastic aspergillosis, occlusive bronchiolitis, and growth retardation. In addition to multiple lymph node enlargements, multicentric disease is often associated with systemic manifestations such as fever, night sweats, malaise, weight loss, anaemia, hepatic insufficiency, renal insufficiency, and volume overload (generalised oedema, pleural effusion, ascites, etc.). There is also a specific subtype in which patients may present with thrombocytopenia, severe oedema (anasarca), myelofibrosis, renal dysfunction, and hepatosplenomegaly (organomegaly).

**ChatGPT-4o** - 2024-11-25 09:12:18

Based on the clinical manifestations described, the most likely rare disease to be diagnosed is **Castleman disease**. Castleman disease can be classified into unicentric and multicentric forms. The unicentric form typically involves the enlargement of a single lymph node region without systemic symptoms, whereas the multicentric form involves multiple regions and is associated with systemic inflammatory symptoms. The specific subtype mentioned, with symptoms like thrombocytopenia, severe edema, myelofibrosis, renal dysfunction, and hepatosplenomegaly, aligns with TAFRO syndrome, a subtype of multicentric Castleman disease.

Here are five other possible diagnoses that might present with similar symptoms:

1. **Lymphoma**: Both Hodgkin and non-Hodgkin lymphomas can present with lymphadenopathy and systemic symptoms such as fever, night sweats, and weight loss.

2. **Sarcoidosis**: This can present with lymph node enlargement and systemic symptoms, although it typically has specific pulmonary involvement and non-caseating granulomas on biopsy.

3. **Systemic Lupus Erythematosus (SLE)**: SLE can present with systemic symptoms and lymphadenopathy, along with organ involvement, such as renal and hepatic dysfunction.

4. **Adult-onset Still's Disease**: Characterized by fever, rash, arthralgia, lymphadenopathy, and systemic inflammation, it could be considered in the differential diagnosis.

5. **Multicentric Kaposi Sarcoma associated with Human Herpesvirus 8 (HHV-8)**: Particularly in immunocompromised individuals, this can present with lymphadenopathy and systemic symptoms, though typically with cutaneous findings as well.

Each of these conditions has distinguishing features and would require specific diagnostic investigations to differentiate them accurately.

**17. user - 2024-11-25 09:16:30**

The main clinical manifestations are muscle atrophy, weakness and sensory loss of the limbs, mainly in the distal part of the lower limbs and gradually developing towards the proximal part of the limbs. Common clinical manifestations include poorer locomotor ability than people of the same age, difficulty in running, easy to twist the foot, foot drop, calf gastrocnemius muscle atrophy resembling a ‘crane leg’; examination can find bowed feet, hammertoes, distal limb weakness and atrophy, and loss of deep sensation. The disease usually starts before the age of 20 and progresses slowly. Later stages of the disease may severely affect activity, but rarely result in total disability and do not affect normal life expectancy. However, there are specific types that may have an early and severe onset.

**ChatGPT-4o** - 2024-11-25 09:16:30

Based on the clinical manifestations you provided, the most likely diagnosis is Charcot-Marie-Tooth disease (CMT), which is a group of inherited disorders that affect the peripheral nerves.

Here are five other possible diagnoses that could present with similar symptoms:

1. **Hereditary Neuropathy with Liability to Pressure Palsies (HNPP):** This is another inherited neuropathy that can cause similar symptoms, though it often involves episodic nerve compression.

2. **Friedreich's Ataxia:** While it primarily affects coordination and balance, it can also present with distal muscle weakness and atrophy, particularly in the lower limbs.

3. **Spinal Muscular Atrophy (SMA):** Some forms can present with gradual muscle weakness and atrophy, although sensory loss is not a typical feature.

4. **Distal Hereditary Motor Neuropathy (dHMN):** This condition primarily affects motor nerves, leading to muscle weakness and atrophy without significant sensory involvement.

5. **Refsum Disease:** A rare metabolic disorder that can present with peripheral neuropathy among other systemic symptoms, though it often includes additional features like retinitis pigmentosa and ichthyosis.

Each of these conditions has its own distinct features, but overlapping symptoms with Charcot-Marie-Tooth disease can sometimes make differential diagnosis challenging. Genetic testing and nerve conduction studies are often used to distinguish between these conditions.

**18. user - 2024-11-25 09:20:22**

The onset of the disease occurs from birth to several months. The onset of jaundice is usually within 2 months of age and rarely later than 5 months of age. The most common presentation is persistent jaundice after birth with or without light coloured stools and mild or no liver enlargement. There are many other non-specific clinical signs, including feeding difficulties, growth retardation, diarrhoea, hypoglycaemia and prolonged clotting time. Most of these symptoms and signs resolve spontaneously or with dietary modification and medication around 6 months to 1 year of age, with a small proportion of patients developing further disease after puberty. The majority of developed disease develops in adulthood. Delirium, confusion, and other mental abnormalities caused by hyperammonemia usually appear suddenly after drinking alcohol, consuming sweets, taking certain medications, or having an infection. In severe cases, the disease progresses rapidly and can be life-threatening without proper treatment. Most patients are slim, and most of them have obvious dietary preference, addicted to legumes, high protein, high fat food, and anorexic to high sugar.

**ChatGPT-4o** - 2024-11-25 09:20:22

Based on the clinical manifestations you've described, the most likely diagnosis is **Hereditary Fructose Intolerance (HFI)**. This is a metabolic disorder that can present with jaundice, feeding difficulties, and growth retardation in infancy, and can lead to severe symptoms like hypoglycemia and liver dysfunction after ingestion of fructose, sucrose, or sorbitol-containing foods. Symptoms might improve with dietary modifications as patients learn to avoid these substances. The mental abnormalities related to hyperammonemia after consuming certain foods or alcohol also align with metabolic issues related to fructose metabolism.

Other possible diagnoses include:

1. **Galactosemia**: A genetic disorder affecting the body's ability to process the sugar galactose properly. It also presents with jaundice, feeding difficulties, and liver issues in infancy.

2. **Tyrosinemia Type I**: A rare metabolic condition that can lead to liver failure, renal tubular dysfunction, and neurologic crises, often presenting in infancy with jaundice and liver enlargement.

3. **Glycogen Storage Disease Type I (Von Gierke Disease)**: It can present early in life with hypoglycemia, hepatomegaly, and lactic acidosis, and can lead to growth retardation.

4. **Urea Cycle Disorder**: A group of genetic conditions that result in hyperammonemia, especially after protein intake, and can lead to neurological symptoms.

5. **Progressive Familial Intrahepatic Cholestasis (PFIC)**: A group of disorders causing cholestasis, jaundice, and liver dysfunction starting in infancy.

Each of these conditions has distinct biochemical markers and genetic tests that can help confirm the diagnosis. A thorough metabolic workup and genetic testing are often necessary for definitive diagnosis.

**19. user - 2024-11-25 09:32:55**

The high prevalence age group is infancy, which manifests as adrenocortical insufficiency with a predominant salt loss, and common symptoms include hyperkalemia, hyponatremia with dehydration, metabolic acidosis, hypoglycaemia, vomiting, shock, and skin pigmentation. Most patients present with failure or incomplete development during puberty, or impaired fertility, oligospermia and azoospermia. A few patients may present with early childhood hyperandrogenaemia, simple saline corticosteroid deficiency, and pseudo-precocious puberty or spontaneous pubertal development during puberty. In mild cases, the disease may start in adulthood.

Patients have hyperpigmentation of the skin, especially in exposed areas, friction areas, scarring and areola, axilla, scrotum.

Patients have varying degrees of reduced resistance to various stresses such as infection and trauma. In the event of a stressful situation, adrenal crisis can occur, manifested by nausea, vomiting, abdominal pain or diarrhoea, severe dehydration, lowered blood pressure, rapid heart rate, weak pulse, mental disorders, and often high fever, hypoglycaemia, hyponatraemia and even life-threatening.

**ChatGPT-4o** - 2024-11-25 09:32:55

Based on the clinical manifestations you've provided, the most likely rare disease diagnosis is **Congenital Adrenal Hyperplasia (CAH)**, specifically due to 21-hydroxylase deficiency. This condition is characterized by adrenocortical insufficiency, salt-wasting symptoms, hyperkalemia, hyponatremia, and hyperpigmentation due to excess ACTH. The symptoms of precocious puberty, impaired fertility, and adrenal crisis align with CAH.

Five other possible diagnoses to consider include:

1. **Addison's Disease (Primary Adrenal Insufficiency)**: This condition also features adrenal insufficiency, hyperpigmentation, and electrolyte imbalances, although it is less commonly associated with congenital or early onset symptoms as seen in CAH.

2. **X-linked Adrenal Hypoplasia Congenita**: This can present with adrenal insufficiency and salt-wasting and often involves issues with puberty and fertility.

3. **Familial Glucocorticoid Deficiency**: Primarily presents with glucocorticoid deficiency, hyperpigmentation, and can sometimes be associated with mineralocorticoid insufficiency.

4. **21-Hydroxylase Deficiency (Non-classic)**: A milder form of CAH, where symptoms could present later in life with milder degrees of virilization and adrenal insufficiency.

5. **11-beta-Hydroxylase Deficiency**: Another form of CAH, typically with hypertension due to excess deoxycorticosterone, but can sometimes present with signs of adrenal insufficiency and hyperpigmentation.

These differential diagnoses would require further diagnostic tests such as hormone assays, genetic testing, and possibly imaging to confirm the specific disorder.

**20. user - 2024-11-25 09:34:47**

The disease can develop from birth to early adulthood. Neonatal onset is the most common, often presenting as macrosomia, poor feeding, hunger intolerance and persistent hypoglycaemia. Hypoglycaemic episodes may present with lethargy and hypotonia, as well as neurological sequelae or even death, such as apnoea, seizures and coma. In the delayed type, patients often show a hypoglycaemic reaction, including symptoms such as pallor, sweating and palpitations.

**ChatGPT-4o** - 2024-11-25 09:34:47

Based on the clinical manifestations you've provided, the most likely diagnosis is Congenital Hyperinsulinism (CHI). This condition is characterized by excessive insulin secretion and resultant hypoglycemia, with symptoms often presenting from birth or early childhood and manifesting as poor feeding, persistent hypoglycemia, hypotonia, and neurological concerns.

Here are five other possible diagnoses that could present with similar symptoms:

1. **Glycogen Storage Disease Type 1 (Von Gierke Disease):** This condition can lead to hypoglycemia, particularly during fasting, along with hepatomegaly, hyperlipidemia, and lactic acidosis.

2. **Congenital Adrenal Hyperplasia (CAH):** This condition can cause hypoglycemia due to adrenal insufficiency, along with electrolyte imbalances and poor feeding.

3. **Beckwith-Wiedemann Syndrome:** Characterized by neonatal hypoglycemia, macrosomia, and other physical anomalies.

4. **Mitochondrial Disorders:** These can cause energy production issues leading to hypoglycemia, hypotonia, and neurological symptoms.

5. **Insulinoma (in cases of very young onset):** Although extremely rare in neonates and young children, an insulin-producing tumor can cause persistent hypoglycemia.

Each of these conditions requires specific diagnostic tests and assessments to confirm the diagnosis and guide treatment.

**21. user - 2024-11-25 09:38:51**

Symptoms may appear after birth or may start in infancy to early adulthood. It is characterised by fluctuating muscle weakness and intolerance to fatigue, mostly in the extraocular muscles, facial muscles, medulla oblongata, limbs and respiratory muscles, and even life-threatening apnoeic episodes may occur in infancy. The simplex syndrome is characterised mainly by skeletal muscle weakness without congenital malformations. The overlapping syndromes of glycosylation defects and myopathies of the disease are often accompanied by congenital developmental abnormalities. Clinical manifestations vary between the different types, with some patients, usually born with proximal muscle weakness, cholinesterase inhibitors effective, and in severe cases, sudden respiratory distress and apnoea due to medullary palsy, which can be induced by stress, cold, and infection. Some patients, starting at birth or in infancy, may present with more severe, axially distributed muscle weakness, but the extraocular muscles may not be involved.

**ChatGPT-4o** - 2024-11-25 09:38:51

Based on the clinical symptoms you've provided, the most likely diagnosis could be Congenital Myasthenic Syndromes (CMS). This group of disorders is characterized by defects at the neuromuscular junction, leading to the fluctuating muscle weakness and fatigue you described. The symptoms, such as involvement of extraocular muscles, facial muscles, and potentially life-threatening respiratory issues, align with CMS presentations. The effectiveness of cholinesterase inhibitors in some cases is particularly indicative of CMS, as these medications can help improve neuromuscular transmission.

Here are five other possible diagnoses to consider:

1. **Myasthenia Gravis (Juvenile or Congenital):** While typically presenting with similar muscle weakness, it may not start as early as infancy and is more often acquired.

2. **Congenital Muscular Dystrophy (CMD):** This includes a variety of disorders that could present with muscle weakness and developmental abnormalities, though they might not exhibit the specific responsiveness to cholinesterase inhibitors.

3. **Pompe Disease (Glycogen Storage Disease Type II):** This lysosomal storage disorder can cause muscle weakness and respiratory distress, especially in infancy, though it has distinct metabolic and histological findings.

4. **Mitochondrial Myopathies:** These can cause muscle weakness and respiratory difficulties, with potential involvement of extraocular muscles and developmental issues, often associated with other systemic symptoms.

5. **Nemaline Myopathy:** Characterized by muscle weakness that can vary from mild to severe, this condition might include facial and respiratory muscle involvement but would typically show characteristic rod-like structures on muscle biopsy.

Each of these differential diagnoses would need to be evaluated further through clinical examination, family history, genetic testing, and possibly muscle biopsy or other specific tests to ascertain the correct diagnosis.

**22. user - 2024-11-25 09:41:16**

1. The disease usually starts in infancy or childhood, and in a few cases it starts in adulthood.

2. Tonicity can occur throughout the entire body, including the skeletal muscles of the limbs, facial muscles, extraocular muscles, and lingual muscles.

3. Tonicity is apparent during sudden movement after rest and is reduced by repeated movements, which is called the ‘warm-up’ phenomenon. Transient muscle weakness may occur at the start of a sudden movement. In patients with a longer course of the disease, there may be persistent weakness of the distal limbs, even involving the proximal limbs.

4. Physical examination reveals that the patient is often muscular and has an athlete's body shape. After clenching the fist vigorously, the fingers cannot be unfolded in time; after closing the eyes vigorously, they cannot be opened immediately. Knocking on the hand interosseous muscle, lingual muscle and other parts of the hand can cause persistent muscle contraction (percussive muscle ankylosis, ‘muscle ball’ phenomenon).

**ChatGPT-4o** - 2024-11-25 09:41:16

Based on the clinical manifestations you've provided, the most likely rare disease diagnosis is Myotonia Congenita. This condition is characterized by muscle stiffness (myotonia) that typically improves with repeated activity (the "warm-up" phenomenon), often starting in childhood or infancy. The description of the patient having a muscular build and experiencing difficulty relaxing muscles after contraction (such as not being able to quickly open the hand or eyes) aligns with this condition.

Here are five other possible diagnoses that share some overlapping features:

1. **Paramyotonia Congenita:** Similar to Myotonia Congenita, but worsens with cold and exercise, and does not exhibit the "warm-up" phenomenon.

2. **Myotonic Dystrophy (Type 1 or Type 2):** A multi-system disorder that includes progressive muscle wasting and weakness, myotonia, cataracts, and other systemic features. Unlike Myotonia Congenita, it often involves progressive weakness and atrophy.

3. **Hyperkalemic Periodic Paralysis:** Episodes of muscle weakness that can be accompanied by myotonia, often triggered by potassium intake or rest after exercise.

4. **Brody Myopathy:** Characterized by exercise-induced muscle stiffness due to impaired muscle relaxation, but lacks the prominent myotonia seen in Myotonia Congenita.

5. **Isaac's Syndrome (Neuromyotonia):** Characterized by continuous muscle fiber activity, stiffness, and muscle cramping, which can persist during sleep and involves peripheral nerve hyperexcitability.

Each of these conditions presents with unique features that may differentiate them from Myotonia Congenita, and a thorough clinical examination and genetic testing can help establish the correct diagnosis.

**23. user - 2024-11-25 09:47:18**

Secondary thoracic deformities caused by scoliosis can lead to cardiopulmonary hypoplasia, poor cardiopulmonary reserve, and may include cardiac displacement, arrhythmia, reduced lung capacity, pneumonia, etc. Abdominal deformities can cause gastrointestinal disorders, leading to dyspepsia and lack of appetite. In addition, the nerve root pulling caused by vertebral malformation and disc degeneration can produce irritating pain in the corresponding body parts, and in severe cases, it can cause spinal cord dysfunction or even paralysis.

Physical examination: the general manifestations are unequal shoulders when standing upright, one side of the lumbar folds, lumbar flexion with back asymmetry (‘razor back’), sometimes asymmetry on both sides of the pelvis, and gait abnormalities. In addition, neurological examination is also needed to observe whether the patient has increased or low muscle tone and whether there are sensory abnormalities in the limbs.

Clinically, it is usually classified into the following 3 types according to the imaging characteristics:

Type I - Vertebral formation disorder type, i.e., one or more vertebrae are partially or completely formed with disorder, such as wedge vertebrae, hemivertebrae or butterfly vertebrae;

Type II - poor vertebral segmentation type, i.e., two or more vertebrae are partially or completely segmented and impaired, e.g., blocked vertebrae, bone bridge formation, etc;

Type III - mixed type, where there is both vertebral body formation disorder and vertebral body mal-segmentation.

**ChatGPT-4o** - 2024-11-25 09:47:18

Based on the clinical manifestations and imaging characteristics you have described, the most likely rare disease diagnosis is **Congenital Scoliosis**. Congenital scoliosis is characterized by spinal deformities due to malformations of the vertebrae, which can lead to thoracic and abdominal deformities, impacting cardiopulmonary and gastrointestinal functions. The classification into vertebral formation disorder, poor vertebral segmentation, and mixed type aligns with the description of congenital scoliosis.

Here are five other possible diagnoses that could present with similar features:

1. **Klippel-Feil Syndrome**: This condition involves congenital fusion of cervical vertebrae, leading to a short neck, restricted neck movement, and sometimes scoliosis. It can also be associated with other spinal and systemic abnormalities.

2. **Kyphoscoliosis**: A condition characterized by a combination of kyphosis and scoliosis, potentially leading to thoracic deformities and impacting respiratory function. It can be congenital or acquired.

3. **Marfan Syndrome**: While primarily affecting connective tissue, Marfan syndrome can lead to skeletal abnormalities, including scoliosis, pectus excavatum, and other deformities impacting cardiopulmonary function.

4. **Neurofibromatosis Type 1 (NF1)**: This genetic disorder can lead to skeletal abnormalities, including scoliosis, due to the presence of neurofibromas and other bone dysplasias.

5. **Spinal Muscular Atrophy (SMA)**: Although primarily a neuromuscular disorder, severe forms can lead to scoliosis and thoracic deformities due to muscle weakness and imbalance.

Each of these conditions can present with some overlapping features, and detailed clinical and genetic evaluations would be necessary to narrow down the diagnosis accurately.

**24. user - 2024-11-25 09:55:49**

There are more men than women. Some patients have atypical symptoms of chest pain, which are detected during coronary angiography or coronary CT. Patients are often clinically symptomatic when there is (1) a combination of obstructive atherosclerotic disease, thrombosis, slow flow, entrapment, or rupture with exertional angina or acute coronary syndromes; (2) compression of adjacent tissues; (3) rupture of an aneurysm, which is rare and can cause acute cardiac tamponade; and (4) myocardial ischemia due to microvascular dysfunction, which can occur even in the absence of significant coronary stenosis. The most common clinical manifestation is cardiac ischaemia.

The most common clinical manifestation is angina pectoris, and in a few patients, myocardial infarction, severe arrhythmia, or even sudden death may occur. The disease may break into the right atrium, right ventricle, or coronary sinus, creating a left-to-right shunt, which can be heard as a continuous murmur and, in severe cases, affect cardiac function. The most serious complication is rupture of the tumour into the pericardium, which can lead to bleeding, cardiac tamponade or even death.

**ChatGPT-4o** - 2024-11-25 09:55:49

Based on the clinical manifestations you've described, the rare disease you are likely describing is a **coronary artery fistula**. This condition involves an abnormal connection between a coronary artery and a heart chamber or another blood vessel. The symptoms and complications you mentioned, including atypical chest pain, myocardial ischemia, coronary anomalies, flow issues, potential aneurysm rupture, and left-to-right shunting with continuous murmurs, align with the presentation of coronary artery fistulas.

Here are five other possible diagnoses for consideration:

1. **Coronary Artery Anomalies (CAAs)** - These can lead to atypical coronary circulation issues, including ischemia, especially under exertion.

2. **Coronary Artery Aneurysm** - While less common, they can present with ischemic symptoms and risk of rupture, possibly leading to tamponade.

3. **Kawasaki Disease (later stage)** - Known for coronary artery aneurysms and ischemic heart disease risk in younger patients.

4. **Myocardial Bridging** - A congenital coronary anomaly where a coronary artery tunnel under the myocardium can lead to ischemia due to compression during systole.

5. **Takayasu Arteritis** - A rare vasculitis that can affect the aorta and its branches, including coronary arteries, leading to ischemia and atypical presentations.

These diagnoses reflect conditions that can involve coronary artery abnormalities and atypical presentations of ischemia or angina, corresponding to the symptoms you described.

**25. user - 2024-11-25 10:03:31**

1. Bone marrow failure Anaemia is present at birth in 35 per cent of children and is often diagnosed between 2 weeks and 2 years after birth. The anaemia is macrocytic or normocytic orthochromatic, with decreased reticulocytes, normal or mildly decreased white blood cells, normal or mildly increased platelets, and hypoplasia of the erythroid lineage of the bone marrow and hyperproliferation of the granulocyte and megakaryocyte lineages.

2. Congenital developmental anomalies 30% ~50% of patients may have congenital developmental anomalies, mainly involving the head, upper limbs, heart and genitourinary system. Examples include short stature, craniofacial anomalies including craniofacial hypertelorism, wide flat nose, small head, congenital cataracts, glaucoma, strabismus, high arched hard palate and cleft lip and palate or even Turner's syndrome appearance, bunions, congenital cardiovascular anomalies, and genitourinary anomalies. The presence of physical abnormalities does not predict the severity of the patient's haematological disorders. 30% of affected patients may present with postnatal growth retardation, often associated with other congenital malformations.

3. The incidence of tumours in patients with increased susceptibility to cancer is about 4%, which is higher than in the normal population of the same age. And the tumour occurs at an early age. These include acute leukaemia of the haematological system, myelodysplastic syndromes, lymphomas and solid tumours such as osteosarcoma, thymic carcinoma, hepatocellular carcinoma, melanoma, fibrous histiocytoma, gastric cancer and intestinal cancer. The prognosis of the tumours is poorer than that of the general population suffering from the same tumour, with a higher mortality rate.

**ChatGPT-4o** - 2024-11-25 10:03:31

Based on the clinical manifestations you have provided, the most likely diagnosis is Diamond-Blackfan Anemia (DBA). This rare condition is characterized by:

1. Bone marrow failure typically presenting as macrocytic anemia in infancy or early childhood, with erythroid hypoplasia and normal or slightly decreased white blood cells and platelets.
2. Congenital anomalies, often affecting the face, head, limbs, heart, and genitourinary system.
3. An increased risk of malignancies, especially hematologic cancers, in affected individuals.

Other possible diagnoses to consider based on these clinical features include:

1. **Fanconi Anemia**: This is another bone marrow failure syndrome that can present with congenital abnormalities and a predisposition to cancer, although it often has more pronounced hematologic findings and a broader range of congenital anomalies.

2. **Shwachman-Diamond Syndrome**: Characterized by bone marrow dysfunction, skeletal abnormalities, and an increased risk of myelodysplastic syndrome and leukemia, although it more frequently involves exocrine pancreatic insufficiency.

3. **Congenital Dyserythropoietic Anemia (CDA)**: This group of rare hereditary anemias presents with ineffective erythropoiesis and congenital anomalies, but usually has distinct hematologic findings on blood smear and bone marrow examination.

4. **VACTERL Association with hematologic abnormalities**: Although not a single disease, VACTERL association involves a combination of congenital anomalies (vertebral defects, anal atresia, cardiac defects, tracheoesophageal fistula, renal anomalies, and limb abnormalities) and may sometimes overlap with hematologic disorders.

5. **Thrombocytopenia with Absent Radius (TAR) Syndrome**: This condition is characterized by skeletal abnormalities, especially involving the upper limbs, and bone marrow abnormalities leading to thrombocytopenia although typically not the macrocytic anemia seen in DBA.

A thorough evaluation including genetic testing and a detailed examination of clinical features can help in differentiating these conditions.

**26. user - 2024-11-25 10:06:37**

The lesions may involve all systems of the body, so their clinical manifestations vary depending on the site of involvement. General symptoms include fever, malaise, night sweats, and weight loss. The rest of the affected systems mainly include:

1. Skeletal system, most patients have skeletal involvement at the time of diagnosis, with lower limb bones being the most common, but only 50% of patients have bone pain.

2. cardiovascular system, lesions involving the cardiovascular system, most patients do not have obvious clinical manifestations, but such as pericardial involvement, resulting in a large amount of pericardial effusion can lead to chest tightness, breathlessness, or even cardiac tamponade; lesions involving coronary arteries can cause myocardial ischemia-like manifestations, such as chest tightness, chest pain, etc.; diffuse myocardial involvement has also been reported, which can lead to cardiac failure-like manifestations; lesions encircling the renal arteries can cause renal hypertension; lesions encircling the mesenteric artery can cause renal hypertension; lesions encircling the mesenteric artery can cause renal hypertension. Diffuse myocardial involvement has also been reported, leading to heart failure-like symptoms; lesions encircling the renal arteries may cause renal hypertension; lesions encircling the mesenteric vessels may cause intestinal ischaemia, such as abdominal pain.

3. respiratory system Most patients with respiratory system involvement are asymptomatic, but a few patients have dry cough and dyspnoea.

4. central nervous system central nervous system involvement may appear corresponding clinical manifestations due to different parts of brain parenchyma involvement, such as limb movement disorder, gait abnormality, alteration of consciousness, etc.; spinal cord involvement may appear different clinical manifestations due to different affected segments; pituitary gland lesions may manifest polydipsia, polyuria, and other hormone secretion related manifestations.

5. Retro-orbital lesions Retro-orbital involvement is one of the most common clinical manifestations, which may manifest as unilateral or bilateral proptosis, or even cause blindness.

6. Retroperitoneal space/renal involvement. Retroperitoneal lesions may resemble retroperitoneal fibrosis and may cause unilateral or bilateral retrorenal obstruction if the lesion encircles the ureter.

7. cutaneous involvement, the most common cutaneous lesion is a xanthomatous change, which occurs on the eyelids in about 1/3 of patients, and may also occur on the face, neck, axillae, trunk, and groin.

**ChatGPT-4o** - 2024-11-25 10:06:37

Based on the clinical manifestations provided, the most likely rare disease diagnosis is Erdheim-Chester Disease (ECD). ECD is a rare form of non-Langerhans cell histiocytosis characterized by the abnormal multiplication of histiocytes and can involve multiple systems throughout the body. The description of xanthomatous skin lesions, skeletal involvement, cardiovascular, central nervous system, and retro-orbital lesions align with the typical presentation of ECD.

Five other possible diagnoses that could present with some overlapping symptoms include:

1. **Sarcoidosis**: This systemic disease involves abnormal collections of inflammatory cells (granulomas) and can affect multiple organ systems, including the skin, lungs, and central nervous system.

2. **Wegener's Granulomatosis (Granulomatosis with Polyangiitis)**: This is a form of vasculitis that can affect the respiratory tract, kidneys, and other organs, presenting with systemic symptoms such as fever, malaise, and weight loss.

3. **Langerhans Cell Histiocytosis (LCH)**: Another histiocytic disorder that can involve multiple systems, including bone lesions, skin rashes, and potentially central nervous system involvement.

4. **Multicentric Castleman Disease**: This rare lymphoproliferative disorder can present with systemic inflammatory symptoms, lymphadenopathy, and may involve various organs leading to systemic symptoms.

5. **IgG4-Related Disease**: A systemic condition characterized by tissue infiltration with IgG4-positive plasma cells, which can affect multiple organs including the pancreas, salivary glands, kidneys, and retroperitoneum, among others.

Each of these conditions has distinct features that may help differentiate them from Erdheim-Chester Disease, but their overlapping symptoms warrant consideration in the differential diagnosis.

**27. user - 2024-11-25 10:11:10**

The disease often presents with multi-organ and multi-system involvement, with males being more affected than female patients. Clinical manifestations often involve different organs as the patient grows older.

1. Facial appearance, male patients usually appear at the age of 12-14 years with characteristic facial features, such as convexity of the supraorbital ridge, frontal bulge and thickening of the lips. 2.

2. cutaneous angiokeratomas, which are common in patients with the classic form, are red patches of raised skin in the ‘sitting area’, i.e., between the umbilicus and knees, the scrotum, the buttocks, and the inner thighs.

3. Neurological system, most patients will have peripheral nerve pain, which manifests as intolerable burning sensation on the soles of the feet and palms of the hands and radiates to the proximal limbs, and even painful spasms; autonomic nerve involvement manifests as little or no sweating; and central nervous system involvement manifests as early-onset transient ischemic attacks or ischemic strokes.

4. Eye: Characteristic manifestations include conjunctival vascular tortuosity, corneal turbidity, posterior lens capsule clouding, and retinal vascular tortuosity, which in severe cases may lead to reduced or even loss of vision. It is often one of the main reasons for female patients to visit the clinic.

5. Digestive tract, most often after eating nausea, vomiting, abdominal distension, cramping abdominal pain and diarrhoea, may also manifest as malabsorption or constipation.

6. Kidney, early manifestation of urinary concentration dysfunction such as increased nocturia, polyuria and enuresis, with the progress of the disease can gradually appear proteinuria, and even up to the level of nephrotic syndrome, accompanied by renal function damage, mostly around 30 years old into the end-stage renal disease.

7. Cardiovascular system, can be manifested as hypertension, myocardial ischemia caused by coronary artery involvement, heart valve lesions and hypertrophic cardiomyopathy, and in severe cases, it can be manifested as angina pectoris, myocardial infarction and heart failure. It is mostly a late manifestation of the disease and a major cause of death.

**ChatGPT-4o** - 2024-11-25 10:11:10

Based on the clinical manifestations you've described, the disease most likely being characterized is **Fabry disease**. Fabry disease is a rare X-linked lysosomal storage disorder caused by deficient activity of the enzyme alpha-galactosidase A. It results in the accumulation of globotriaosylceramide in various tissues, leading to the multi-systemic involvement you noted, with a predilection for affecting males more severely.

Here are five other possible differential diagnoses that could present with some overlapping symptoms:

1. **Niemann-Pick disease**: Another lysosomal storage disorder that can present with neurological symptoms, hepatosplenomegaly, and progressive organ involvement, although the specific symptom profile differs from Fabry disease.

2. **Gaucher disease**: This is a genetic disorder due to deficient glucocerebrosidase enzyme, leading to multi-organ involvement including spleen and liver enlargement, bone pains, and neurological symptoms in some types. Cutaneous symptoms and kidney involvement are less common.

3. **Alport syndrome**: A genetic condition characterized by kidney disease, hearing loss, and eye abnormalities, but without the angiokeratomas or classical nervous system involvement seen in Fabry disease.

4. **Chronic Inflammatory Demyelinating Polyneuropathy (CIDP)**: A neurological disorder that can cause peripheral nerve pain and motor dysfunction, but lacks the systemic organ involvement typical in Fabry disease.

5. **Hereditary Amyloidosis**: This can present with multi-system involvement including neuropathy, renal impairment, and cardiac issues which might mimic the systemic involvement in Fabry disease, but typically involves amyloid deposits rather than glycolipid accumulation.

Each of these possibilities would have distinguishing features and would require specific diagnostic testing to confirm, such as enzyme assays, genetic testing, or tissue biopsies.

**28. user - 2024-11-25 10:17:24**

The disease is classified as Type 1 and Type 2.

1. Type 1 is mainly characterised by recurrent inflammatory reactions and plasma membrane inflammation, including recurrent fever, abdominal pain and arthritis. Fever is recurrent and may be triggered by cold, strenuous exercise, surgery, infection, etc. and resolves spontaneously within 13 days. Abdominal pain is the most common clinical manifestation, seen in 95% of patients. It is a sudden onset of abdominal pain, often accompanied by fever, abdominal examination may have abdominal distension, abdominal pressure, rebound pain, muscle tension, and the loss of bowel sounds on auscultation. Stereotactic abdominal radiographs show flat air and fluid in the small intestine. Abdominal pain often resolves spontaneously after 24-48 hours. Arthritis is also a common clinical manifestation, seen in 75% of patients. It often involves the large joints of the lower limbs, such as the hip and knee joints. There may be joint swelling and pain. Rash is seen in 7% ~40% of patients, mainly involving the extensor side of the lower limbs, and the typical rash is erysipelas of tansy-like erythema. Pleurisy is seen in 45% of patients and presents as a sudden onset of unilateral chest pain, often accompanied by fever. Pericarditis is less common.

2. Type 2 starts with amyloidosis. It presents with persistent proteinuria and often leads to end-stage renal disease. Amyloid can also be deposited in the heart, gastrointestinal tract, liver and other organs, leading to corresponding clinical manifestations.

**ChatGPT-4o** - 2024-11-25 10:17:24

Based on the clinical manifestations you have provided, the most likely diagnosis is Familial Mediterranean Fever (FMF). FMF is a hereditary inflammatory disorder characterized by recurrent episodes of fever and inflammation affecting the abdomen, joints, and lungs. It is typical for FMF to have two distinct phenotypes as you described (Type 1 and Type 2), with Type 1 involving acute inflammatory episodes and Type 2 involving secondary amyloidosis, particularly affecting the kidneys.

Other possible diagnoses based on the symptoms presented could include:

1. **Hyper-IgD Syndrome (HIDS):** This is another autoinflammatory disorder characterized by recurrent fevers and can include abdominal pain, arthritis, and skin rash. However, HIDS typically presents with elevated IgD levels and may have different triggers and response patterns compared to FMF.

2. **Cryopyrin-Associated Periodic Syndromes (CAPS):** These include conditions like Muckle-Wells syndrome which involve recurrent fevers, rash, joint pain, and can lead to amyloidosis. The frequency and type of symptoms can sometimes overlap with those seen in FMF.

3. **TNF Receptor-Associated Periodic Syndrome (TRAPS):** TRAPS can present with prolonged episodes of fever, abdominal pain, and arthritis. Skin rashes and chest pain can also occur, with symptoms potentially leading to amyloidosis over time.

4. **Mevalonate Kinase Deficiency (MKD):** This condition can present with recurrent fever, abdominal pain, and arthritis. It is closely related to HIDS and may involve other systemic symptoms.

5. **Adult-Onset Still's Disease (AOSD):** Mainly affecting adults, this disorder can present with recurrent fevers, arthritis, and rash. The presentation can be similar, but AOSD often involves more systemic symptoms and different lab markers.

Each of these conditions shares some overlapping features with FMF but has distinct characteristics and diagnostic markers that help differentiate them.

**29. user - 2024-11-25 10:23:13**

The main manifestations are congenital developmental anomalies, progressive bone marrow failure and increased incidence of tumours, while some patients may show no developmental anomalies or bone marrow failure.

1. Developmental anomalies, patients may show a variety of physical and mental developmental abnormalities. Growth defects, abnormal skin pigmentation, unilateral or bilateral skeletal malformations of the upper limbs, short stature, microcephaly, ocular anomalies, genitourinary malformations, endocrine anomalies, hearing loss, outer ear anomalies, congenital heart defects, digestive anomalies, abnormalities of the central nervous system, facial deformities, spinal deformities, neck deformities, and developmental delays and/or intellectual disabilities. These manifestations are mostly non-specific, with thumb and upper limb deformities being more specific.

2. Progressive bone marrow failure with haematological abnormalities is the most serious clinical feature of Fanconi anaemia. It may present as a single or total haematopenia. The age of onset of haemopenia varies widely, even among siblings. The median age of onset is about 7.6 years, and it is rare in infants and young children; 90% of patients with haematological abnormalities develop before the age of 40 years. Thrombocytopenia or leukopenia usually precedes anaemia and is often accompanied by an increase in macrocytes, foetal haemoglobin (HbF 1), and a gradual increase in total haematopoiesis.

3. There is an increased risk of neoplasms, including haematological malignancies and non-haematological malignant solid tumours. The risk of acute myeloid leukaemia (AML) is increased about 500-fold, and the cumulative incidence of AML is about 13% at the age of 50 years, with most cases occurring between the ages of 15 and 35 years. -7, del (7q) is associated with an increased incidence of myelodysplastic syndromes (MDS)/AML. Acute lymphoblastic leukaemia and lymphoma have also been reported. Solid tumours can be the first manifestation of the disease. Head and neck squamous cell carcinomas (HNSCCs) are the most common solid tumours in patients and have an earlier age of onset (20-40 years), mostly occurring in the oral cavity (e.g., tongue cancers), are mostly in the progressive stage, and respond poorly to treatment. There is also an increased incidence of skin, oesophageal, liver and genitourinary tract tumours. In a few patients, multiple tumours may be seen concurrently, or haematological and non-haematological tumours may be seen concurrently. And there is a marked intolerance to chemotherapy or radiotherapy.

**ChatGPT-4o** - 2024-11-25 10:23:13

Based on the clinical manifestations provided, the rare disease most likely to be diagnosed is **Fanconi Anemia** (FA). This diagnosis is supported by the combination of congenital developmental anomalies, progressive bone marrow failure, and an increased incidence of both hematological malignancies and non-hematological solid tumors. The specific mention of thumb and upper limb deformities, along with the hematological features and cancer predisposition, aligns closely with Fanconi Anemia.

Here are five other possible differential diagnoses:

1. **Dyskeratosis Congenita (DC):** This is another rare inherited bone marrow failure syndrome that can present with mucocutaneous features, progressive bone marrow failure, and an increased cancer risk. It can also involve developmental anomalies.

2. **Shwachman-Diamond Syndrome (SDS):** Characterized by bone marrow failure, exocrine pancreatic insufficiency, and skeletal abnormalities. There is also an increased risk of myelodysplastic syndrome and leukemia.

3. **Diamond-Blackfan Anemia (DBA):** This is a rare blood disorder characterized by a failure to produce red blood cells, congenital anomalies, and an increased risk for cancer.

4. **Bloom Syndrome:** Features include growth deficiencies, sun-sensitive skin changes, cancer predisposition, and, occasionally, hematological abnormalities. It involves chromosomal instability, much like Fanconi Anemia.

5. **Noonan Syndrome with Multiple Lentigines (formerly LEOPARD syndrome):** Although primarily associated with developmental anomalies and certain distinct features, there can be hematological issues and increased cancer risk.

These alternate diagnoses share overlapping features with Fanconi Anemia, such as bone marrow failure, congenital anomalies, and increased malignancy risk, but vary in specific clinical manifestations and genetic causes.

**30. user - 2024-11-25 10:25:05**

The perinatal onset of the disease is often typical, and within a few days of ingestion of breast milk or lactose-containing formula, the child develops life-threatening complications: feeding problems, diarrhoea, vomiting, hypoglycaemia, liver impairment, haemorrhage, jaundice, and cataracts. If left untreated, sepsis, shock and death may occur. Children who survive to infancy may develop severe brain damage if they continue to ingest lactose. Some children have long-term complications such as mental retardation, delayed growth, ataxia, blindness, and ovarian dysfunction in female patients.

**ChatGPT-4o** - 2024-11-25 10:25:05

Based on the clinical manifestations you've described, the most likely diagnosis is **Galactosemia**. This is a rare genetic metabolic disorder that affects an individual's ability to metabolize the sugar galactose properly. In classic Galactosemia, infants present with symptoms soon after ingesting breast milk or lactose-containing formula due to the inability to break down galactose, leading to the severe complications mentioned.

Here are five other possible differential diagnoses:

1. **Hereditary Fructose Intolerance (HFI):** A condition where the body is unable to break down fructose properly, leading to liver and kidney damage after ingestion of fructose-containing foods, though this typically presents later when fructose is introduced into the diet.

2. **Tyrosinemia Type I:** A metabolic disorder resulting from a deficiency of the enzyme fumarylacetoacetate hydrolase, leading to liver dysfunction, renal tubular acidosis, and a risk of bleeding, although cataracts are not typically a feature.

3. **Glycogen Storage Disease Type I (Von Gierke Disease):** Characterized by hypoglycemia, lactic acidosis, hyperuricemia, and hepatomegaly, but typically manifests with different symptoms related to glycogen metabolism rather than immediate post-ingestion reactions.

4. **Neonatal Hemochromatosis:** A disorder resulting in congenital liver disease with iron overload, presenting with liver failure, but does not typically involve cataracts or direct dietary triggers like lactose.

5. **Congenital Lactase Deficiency:** While rare and causing symptoms after lactose ingestion, it primarily causes gastrointestinal symptoms without systemic complications like liver damage or cataracts.

Each of these conditions has distinct features, so further diagnostic tests, including specific enzyme assays and genetic testing, would be necessary to confirm the diagnosis.

**31. user - 2024-11-25 10:29:00**

1. Type I is the most common subtype. There is no primary central nervous system involvement, but some patients may develop secondary neurological manifestations (e.g., spinal cord compression) as the disease progresses. The disease can develop in all age groups, with about 2/3 of patients developing in childhood. Organ manifestations are mainly hepatosplenomegaly, especially splenomegaly, often accompanied by hypersplenism, and even splenic infarction and splenic rupture. The haematological manifestations are mainly thrombocytopenia and anaemia, and some patients have leucopenia, which may be accompanied by coagulation abnormalities. Patients present with pallor, fatigue, skin and gum bleeding, increased menstruation, and even life-threatening bleeding. Most patients have skeletal invasion, but the severity varies. Early invasion sites mainly include lumbar vertebrae, long bone epiphyses and diaphyses, and in the middle and late stages, epiphyses and bony prominences may be involved. Patients often experience acute or chronic diffuse bone pain, and in severe cases, osteonecrosis (ischaemic necrosis), followed by joint collapse involving the proximal and distal femur, proximal tibia and proximal humerus. Osteolytic lesions, pathological fractures, vertebral compression fractures and other fragility fractures due to reduced bone density may also occur. Skeletal lesions can interfere with daily activities and can be disabling. In children, the most common manifestations are osteoporosis, flask-like deformity of the metaphysis of the long bones, varying degrees of hypodensity of the metaphysis of the long bones, thinning of the bone cortex, etc. There may be growth retardation. Some patients may have lung involvement, which is mainly manifested as interstitial lung disease, pulmonary solid changes, and pulmonary hypertension. In addition, patients may have abnormalities of sugar and lipid metabolism, increased risk of malignant tumours such as multiple myeloma, cholelithiasis, and abnormalities of the immune system.

2. Type II, in addition to hepatosplenomegaly, anaemia, thrombocytopenia and other manifestations similar to those of type I, patients with type II mainly have acute neurological involvement. It often develops in the neonatal period to infancy, progresses rapidly and has a high morbidity and mortality rate. There are rapidly progressive medullary palsy, dyskinesia, seizures, angular reflexes and cognitive deficits and other signs of acute neurological damage, and growth is retarded, with death occurring before the age of 2 to 4 years. Some patients with severe disease may develop joint contractures.

3. The early manifestations of type III are similar to those of type I, with gradual onset of neurological involvement, which often develops in childhood and has a slow progression of disease and a longer life expectancy. The patients often have the invasion of the motor nerve, eye movement disorders, ataxia, anisocoria, epilepsy, myoclonus, accompanied by developmental delays and intellectual backwardness. Type III can be divided into three subtypes, namely, type IIIa, which is characterised by more rapidly progressive neurological symptoms (ocular motility disorders, cerebellar ataxia, spasticity, myoclonus, and dementia) and hepatosplenomegaly; type IIIb, which is characterised by hepatosplenomegaly and skeletal symptoms with fewer CNS symptoms; and type IIIc, which is characterised by milder symptoms, including calcification of the cardiac valves and corneal clouding, and is predominantly found in the Druze population. Type IIIc.

**ChatGPT-4o** - 2024-11-25 10:29:00

Based on the clinical manifestations and subtypes described, the most likely diagnosis is **Gaucher's disease**. This is a lysosomal storage disorder caused by a deficiency in the enzyme glucocerebrosidase, leading to the accumulation of glucocerebroside in cells. The descriptions provided match with the three types of Gaucher's disease:

1. **Type I (Non-neuronopathic Gaucher's Disease):** Characterized by hepatosplenomegaly, bone involvement, and hematological issues such as anemia and thrombocytopenia, but without primary CNS involvement.

2. **Type II (Acute Neuronopathic Gaucher's Disease):** Presents with severe neurological involvement in infants, with rapid progression and high mortality.

3. **Type III (Chronic Neuronopathic Gaucher's Disease):** Features neurological involvement with a slower progression compared to Type II, and includes motor nerve issues and ocular movement disorders.

Other possible diagnoses that could present with similar overlapping symptoms include:

1. **Niemann-Pick Disease:** Another lysosomal storage disorder that can present with hepatosplenomegaly, neurological symptoms, and bone involvement.

2. **Fabry Disease:** Though primarily characterized by skin, renal, and cardiac involvement, it can occasionally present with bone pain and organomegaly.

3. **Mucopolysaccharidoses (e.g., Hurler Syndrome):** These disorders involve storage of glycosaminoglycans, leading to organomegaly, skeletal abnormalities, and often CNS involvement.

4. **Chronic Myelogenous Leukemia (CML):** This hematological malignancy can cause splenomegaly, anemia, and bone pain, but typically lacks the storage disorder component.

5. **Sickle Cell Disease:** This can present with anemia, bone pain, and splenic complications, but it is a hemoglobinopathy rather than a storage disorder.

A thorough clinical assessment, including enzyme assays and genetic testing, would be required for a definitive diagnosis.

**32. user - 2024-11-25 10:31:08**

It often starts in one muscle group and progresses to others, with ptosis and diplopia being the most common onset, accounting for 80 per cent of cases. Skeletal muscle weakness is fluctuating and easily fatigued, with a light morning and heavy evening. According to the clinical manifestations, it is divided into five types. Type I: ocular muscle type, manifested by ptosis and diplopia. Type ⅡA: mild generalised type, with mild involvement of the limb muscle groups. Type IIB: Moderate generalised type with moderate involvement of the limb muscle groups, usually with mastication, swallowing and dysarthria. Type III: Severe aggressive type with rapid progression, involving the pharyngeal muscles within weeks or months and the respiratory muscles within six months. Type IV: Late-onset severe type, slowly progressive, involving the respiratory muscles within 2 years. Type V: myasthenia gravis, skeletal muscle atrophy within six months of disease onset. Neurological examination can be seen in the proximal limbs, trunk, neck, craniofacial muscle weakness, intolerance to fatigue, Jolly test is positive, tendon reflexes are normal, no sensory deficits, no pathological signs. Some patients may progress to respiratory failure and develop myasthenia gravis crisis.

**ChatGPT-4o** - 2024-11-25 10:31:08

Based on the clinical manifestations provided, the most likely diagnosis is **Myasthenia Gravis**. This condition is characterized by fluctuating muscle weakness and fatigue, often starting with ocular symptoms like ptosis and diplopia. The progression and types described align with the classification of Myasthenia Gravis, which can range from ocular to generalized forms, and severe cases can lead to respiratory involvement and crisis.

Here are five other possible diagnoses to consider:

1. **Lambert-Eaton Myasthenic Syndrome (LEMS):** Although less common, LEMS also involves muscle weakness and fatigue, but it typically starts with weakness in the proximal leg muscles rather than ocular symptoms, and can improve with exertion.

2. **Mitochondrial Myopathy:** These disorders can cause progressive muscle weakness, ptosis, and external ophthalmoplegia, but they often have additional systemic features like hearing loss or diabetes.

3. **Congenital Myasthenic Syndromes:** These are inherited disorders with symptoms similar to Myasthenia Gravis, but often present from birth or early childhood and have a genetic basis.

4. **Chronic Progressive External Ophthalmoplegia (CPEO):** This mitochondrial disorder leads to ptosis and ophthalmoplegia, but generally lacks the fluctuating weakness seen in Myasthenia Gravis.

5. **Botulism:** Caused by toxin exposure, this condition can present with muscle weakness and cranial nerve involvement, but usually has an acute onset and includes autonomic symptoms.

These alternative diagnoses consider similar symptoms but often have distinguishing features in their onset, progression, and associated systemic involvement.

**33. user - 2024-11-25 10:33:40**

The disease often starts in adolescence or early adulthood. Clinical manifestations are mainly related to hypokalemia and hypomagnesemia. Mild patients may be asymptomatic or show mild fatigue and poor appetite; severe patients may have twitching of limbs, flaccid paralysis, painful spasms, fainting, and transverse myelinolysis secondary to acute renal injury, and may even suffer from cardiac arrest due to severe ventricular arrhythmias. The common clinical manifestations are as follows:

1. systemic symptoms fatigue, thirst, polydipsia, salt craving.

2. Neuro-muscular system Muscle weakness, painful spasms, convulsions, convulsive seizures, numbness of limbs, sensory abnormalities, rhabdomyolysis, paralysis, dizziness, vertigo, ataxia, and pseudotumor cerebri.

3. Cardiovascular system Palpitation, syncope, normal or low blood pressure and ventricular arrhythmia, etc.

4. Digestive system Constipation, vomiting, etc.

5. Urinary system Polyuria, nocturia, enuresis, proteinuria and renal insufficiency.

6. Bone and joint system Arthralgia, cartilage calcium sedimentation.

7. endocrine and growth retardation, delayed puberty; diabetes mellitus or impaired glucose tolerance is not uncommon in patients with chronically low potassium and magnesium. 8. ocular symptoms.

8. Ocular symptoms Blurred vision and scleral choroidal calcification may occur in a small number of patients.

**ChatGPT-4o** - 2024-11-25 10:33:40

Based on the clinical manifestations you provided, the disease most likely to be diagnosed is Gitelman syndrome. Gitelman syndrome is a rare inherited disorder characterized by a specific defect in the distal convoluted tubule of the kidney, leading to hypokalemia (low potassium), hypomagnesemia (low magnesium), metabolic alkalosis, and low calcium excretion. The symptoms align with those described, such as muscle weakness, cramps, fatigue, and cardiac issues due to the electrolyte imbalances.

Other possible diagnoses to consider include:

1. **Bartter syndrome**: This is another rare inherited disorder affecting kidney function, leading to hypokalemia and metabolic alkalosis. However, hypomagnesemia is more characteristic of Gitelman syndrome.

2. **Familial hypokalemic periodic paralysis**: This condition is characterized by episodes of muscle weakness associated with low potassium levels, though it typically does not have hypomagnesemia.

3. **Primary hyperaldosteronism (Conn's syndrome)**: This involves excessive production of aldosterone, leading to hypokalemia and hypertension, though it usually doesn't include hypomagnesemia.

4. **Diuretic abuse**: Use of diuretics can lead to similar electrolyte imbalances, including hypokalemia and hypomagnesemia, often seen in individuals with an eating disorder or those attempting weight control.

5. **Renal tubular acidosis (RTA)**: Specifically, type 1 (distal) RTA can lead to hypokalemia and muscle weakness, but it usually presents with acidosis rather than alkalosis.

These possibilities should be considered in the context of comprehensive clinical evaluation and laboratory testing to narrow down the differential diagnosis.

**34. user - 2024-11-25 10:35:28**

The majority of children develop the disease in infancy or early childhood, with a variety of presentations. The earlier the onset, the more severe the symptoms and the worse the prognosis. The majority of patients have a relatively normal presentation in the first year of life. The earliest sign that appears in about 75% of patients is a large head size, and most children are born with a larger head circumference than their peers or have a rapid increase in head circumference soon after birth, which may be accompanied by mild non-specific symptoms, including irritability, difficulty in feeding, and vomiting. Children usually develop ketosis, vomiting, hepatomegaly and acute encephalopathic crisis, including hypotonia, loss of consciousness and convulsive seizures, between 3 and 36 months of age after fever, infection, surgery, or vaccination, which may resolve with symptomatic treatment, but not fully recover. If acute encephalopathic crisis occurs repeatedly, the neurological damage will be progressively aggravated, and there may be developmental regression, and eventually cognitive dysfunction may occur. In some children, delayed movement, dystonia, and random movement disorders occur gradually over the first few years of life, but mental development is almost normal. Patients often die within 10 years of age from concomitant diseases or Reye-like seizures, which decrease with age. Rarely, the onset of the disease occurs during adolescence or even adulthood, and may be asymptomatic until the first episode.

**ChatGPT-4o** - 2024-11-25 10:35:28

Based on the clinical manifestations provided, the most likely diagnosis for this condition is Medium-Chain Acyl-CoA Dehydrogenase Deficiency (MCAD deficiency). This inherited disorder affects the body's ability to break down medium-chain fatty acids into energy, especially during periods of fasting or illness, leading to the symptoms described.

Five other possible diagnoses to consider include:

1. **Ornithine Transcarbamylase Deficiency (OTC Deficiency):** An X-linked genetic disorder affecting the urea cycle, leading to ammonia accumulation with onset typically in infancy, presenting with vomiting, lethargy, seizures, and coma during metabolic stress.

2. **Maple Syrup Urine Disease (MSUD):** A disorder affecting the breakdown of certain amino acids, leading to symptoms such as poor feeding, vomiting, lethargy, hypotonia or hypertonia, and sweet-smelling urine, especially during illness or fasting.

3. **Glutaric Aciduria Type 1 (GA1):** An organic acidemia characterized by macrocephaly, metabolic crises with encephalopathy, and movement disorders, often triggered by intercurrent illnesses.

4. **Propionic Acidemia:** A metabolic disorder that can result in episodes of metabolic acidosis, vomiting, lethargy, seizures, developmental delay, and hyperammonemia, often precipitated by infection or fasting.

5. **Carnitine Palmitoyltransferase II Deficiency (CPT II Deficiency):** A disorder of fatty acid oxidation that can present with recurrent episodes of muscle pain, weakness, and myoglobinuria, typically triggered by prolonged exercise, fasting, or illness.

Each of these conditions shares some overlapping features with the described symptoms, but they have distinct biochemical and genetic underpinnings that necessitate specific diagnostic testing for confirmation.

**35. user - 2024-11-25 10:38:27**

1. Type I, abdominal distension, growth retardation, hypoglycemic convulsions, recurrent nosebleeds, diarrhoea and vomiting are the main reasons for children's patients to visit the clinic, and very rarely, the first manifestation is hematuria, blood in the stool, recurrent bone fracture, anaemia or gout. Adult patients who are never diagnosed and treated may present with multiple hepatic adenomas, chronic renal failure, severe gout with multiple gout stones, and osteoporosis. Other rare presentations include pulmonary hypertension, diabetes mellitus, cerebrovascular disease, and hepatic adenoma carcinoma. On physical examination, short stature and markedly enlarged liver can be seen.

2. Type Ib, in addition to the above manifestations, patients may also have recurrent infections with neutropenia, oral ulcers, inflammatory bowel disease, perianal ulcers, arthritis and splenomegaly.

3. Infantile type II, which is divided into classical infantile type and non-classical infantile type according to its prognosis. Most of the classical infants present with generalised muscle weakness in the first month of life, delayed motor development, enlarged heart on chest X-ray, high QRS waves and short PR intervals on ECG, hypertrophic cardiomyopathy on cardiac ultrasound, and varying degrees of elevation of creatinine kinase, etc. Most of the infants die of left heart failure or cardiorespiratory failure after pulmonary infections in the first year of life. The non-classical infantile form presents with muscle weakness within the first year of life, lags in motor development, and most often dies of respiratory failure in early childhood.

4. Type II late-onset, with onset of disease after 1 year of age and as late as 60 years of age. Most of the patients present with chronic progressive proximal muscle weakness and respiratory insufficiency, cardiac involvement is rare, and respiratory failure is the main cause of death. Clinical manifestations include easy fatigue, difficulty in sitting up, walking up and down stairs, squatting and walking, and in a few cases, sudden onset of respiratory failure.

**ChatGPT-4o** - 2024-11-25 10:38:27

Based on the clinical manifestations you provided, the most likely rare disease being described is **Glycogen Storage Disease Type I (GSD I)**, also known as **Von Gierke's Disease**, specifically Type I and Type Ib. This condition is characterized by the features you mentioned: abdominal distension, hypoglycemia, growth retardation, enlarged liver, and in some cases, features like hepatic adenomas and renal problems.

### Main Diagnosis:
- **Glycogen Storage Disease Type I (Von Gierke's Disease)**

### Differential Diagnoses with Similar Presentations:

1. **Glycogen Storage Disease Type II (Pompe Disease)**:
 - Detailed in your description as Type II infantile and late-onset with muscle weakness, cardiomyopathy, and respiratory failure.

2. **Fructose-1,6-bisphosphatase Deficiency**:
 - Presents with hypoglycemia, lactic acidosis, and hepatomegaly, similar metabolic disturbances as seen in GSD I.

3. **Niemann-Pick Disease**:
 - Presents with hepatomegaly, splenomegaly, and growth retardation. However, it also includes neurodegenerative symptoms not prominently described here.

4. **Tyrosinemia Type I**:
 - Presents with liver dysfunction, renal tubular dysfunction, and risk of hepatocellular carcinoma. It shares liver and renal symptoms similar to late-stage GSD I.

5. **Fanconi-Bickel Syndrome**:
 - Characterized by glycogen storage issues, hepatomegaly, growth retardation, and renal tubular dysfunction. It shares overlapping metabolic and renal symptoms.

These conditions represent a range of metabolic and storage disorders that could present with overlapping symptoms of hepatomegaly, growth retardation, and metabolic disturbances, but each has distinctive features or additional symptoms that guide diagnoses.

**36. user - 2024-11-25 10:40:08**

It mainly manifests as bleeding from joints, muscles and deep tissues, but also internal organs such as gastrointestinal tract and central nervous system. If repeated bleeding occurs, untreated bleeding can lead to joint deformity and/or pseudotumour formation, which can be life-threatening in severe cases. Delayed haemorrhage after trauma or surgery is characteristic of this disease.

**ChatGPT-4o** - 2024-11-25 10:40:08

Based on the clinical manifestations you have provided, the most likely diagnosis is Hemophilia. Hemophilia is a rare genetic disorder characterized by a deficiency in specific clotting factors, leading to prolonged bleeding episodes. The bleeding into joints, muscles, and deep tissues, as well as delayed hemorrhage after trauma or surgery, aligns with the typical presentation of hemophilia.

Five other possible diagnoses to consider include:

1. **Von Willebrand Disease**: Although more common than hemophilia, severe forms can present with joint and tissue bleeding, particularly when Von Willebrand factor is significantly decreased.

2. **Acquired Hemophilia**: This is an autoimmune disorder where the body produces antibodies against its own clotting factors, leading to similar bleeding symptoms as congenital hemophilia.

3. **Factor XIII Deficiency**: This is a rare bleeding disorder that can cause delayed bleeding, poor wound healing, and spontaneous bleeding similar to hemophilia.

4. **Factor VII Deficiency**: Another rare bleeding disorder, where deficiency of factor VII leads to bleeding symptoms, though joint bleeding is less common than in hemophilia.

5. **Platelet Function Disorders**: While classically these disorders result in mucocutaneous bleeding, severe cases can sometimes present with bleeding symptoms that mimic those of hemophilia, especially after surgery or trauma.

**37. user - 2024-11-25 10:42:18**

It can involve multiple organs of the body and has a variety of clinical manifestations. The age of onset is mostly 3-60 years old, and there are also reports of patients diagnosed at 8 months and over 70 years old. In children, hepatic involvement is the first manifestation of the disease, while in adolescents and adults, neurological involvement is the first manifestation of the disease.

It may present with asymptomatic persistent elevation of aminotransferases, chronic hepatitis, cirrhosis, and acute liver failure. Neurological involvement may manifest as motor dysfunction, tremor, ataxia, chorea, dysautonomia, dystonia, and in some patients masked face, stiff limbs, and gait abnormalities. Some patients combine with mental behavioural abnormalities such as apathy, aggressive behaviour, and personality changes. Kidney involvement may manifest as haematuria, proteinuria, etc. Involvement of the blood system may be manifested as haemolytic anaemia, liver cirrhosis, splenic hyperfunction leading to a decrease in the blood tertiary system, and coagulation abnormalities. Relatively rare systems involved include bone and joint, heart, endocrine and reproductive systems.

**ChatGPT-4o** - 2024-11-25 10:42:18

Based on the clinical manifestations provided, the disease most likely to be diagnosed is Wilson's Disease. Wilson's Disease is a rare genetic disorder characterized by excessive copper accumulation in the body, affecting primarily the liver and brain, but potentially involving other organs.

Other possible diagnoses could include:

1. **Hemochromatosis**: A condition characterized by excessive iron accumulation, affecting the liver, heart, and other organs, with symptoms of liver disease and neurological involvement.

2. **Autoimmune Hepatitis**: An autoimmune condition that can cause liver inflammation and may present with neurological symptoms and other systemic manifestations.

3. **Alpha-1 Antitrypsin Deficiency**: A genetic disorder that can lead to lung and liver disease, and may present with liver dysfunction and other systemic symptoms.

4. **Porphyria**: A group of disorders caused by abnormalities in the synthesis of heme, leading to neurological and skin manifestations, and potentially liver involvement.

5. **Lysosomal Storage Disorders (e.g., Niemann-Pick Disease)**: A group of metabolic disorders that can involve multiple systems, including the liver and neurological system, and present with a wide range of symptoms.

Each of these conditions has unique features and requires specific diagnostic testing for confirmation.

**38. user - 2024-11-25 10:46:46**

The clinical picture is very heterogeneous, even within the same family. The disease usually begins before the age of 30 years, worsens during adolescence, and the oedema often has an acute onset. The clinical picture is characterised by recurrent, unpredictable cutaneous and submucosal oedema. The oedema is episodic, self-limited, usually resolves spontaneously in 3-5 days, asymmetric and non-concave. Oedema can involve any part of the body, with the extremities, face, genitals, respiratory tract and gastrointestinal tract mucosa being the most common. The most fatal is respiratory mucosal oedema, which can rapidly progress to respiratory distress or asphyxiation due to laryngeal oedema, and can lead to death by asphyxiation if not resuscitated in time, with a lethality rate as high as 11%-40%, which is one of the main causes of death. Episodes of mucosal oedema of the digestive tract manifest as severe abdominal pain, accompanied by nausea and vomiting, and are therefore often misdiagnosed as acute abdomen, leading to unnecessary abdominal surgery. Skin oedema occurring in the limbs and other parts of the body can affect its function.

**ChatGPT-4o** - 2024-11-25 10:46:46

Based on the clinical manifestations you've described, the most likely diagnosis is **Hereditary Angioedema (HAE)**. This condition is characterized by recurrent episodes of severe swelling (angioedema) in various parts of the body, including the extremities, face, airway, and gastrointestinal tract, which matches the symptoms you've outlined. HAE is typically hereditary and can be life-threatening, especially if the swelling affects the respiratory system.

Five other possible diagnoses to consider include:

1. **Acquired Angioedema (AAE)**: Similar clinical features to hereditary angioedema but occurs later in life and is often associated with another condition that affects the immune system.

2. **Idiopathic Angioedema**: Angioedema without a known cause, which can present with episodic swelling similar to HAE but typically without a hereditary component.

3. **Angioedema due to ACE Inhibitors**: This is drug-induced angioedema related to the use of angiotensin-converting enzyme inhibitors, commonly used to treat hypertension.

4. **Hypereosinophilic Syndrome**: A rare, heterogeneous group of disorders characterized by high levels of eosinophils which can cause tissue damage and edema.

5. **Systemic Mastocytosis**: A condition where mast cells accumulate in the body, leading to symptoms such as skin lesions, gastrointestinal issues, and, in some cases, angioedema.

Each of these conditions has unique aspects that might be confirmed or ruled out through further diagnostic tests, family history examination, and consideration of additional symptoms.

**39. user - 2024-11-25 10:48:32**

The disease starts at birth and the severity of clinical manifestations is related to the subtype of the disease. Clinical manifestations include blisters or maculopapular blisters on the hands, elbows, knees and feet and other areas prone to friction, or around the body. Blisters or ulcers of the oral mucosa may occur, leading to dysphagia and hoarseness. Some patients have mottled pigmentation abnormalities, baldness, palmoplantar hyperkeratosis, enamel defects, nail thickening and nail dystrophy. Other impairments include disfigurement of the digits due to repeated scar formation, anaemia, osteoporosis, muscle atrophy, cardiomyopathy, and deafness. Some subtypes may be associated with basal cell carcinoma, squamous cell carcinoma, and malignant melanoma in advanced stages.

**ChatGPT-4o** - 2024-11-25 10:48:32

The clinical manifestations you've described are highly indicative of a group of disorders known as Epidermolysis Bullosa (EB). This is a rare genetic condition characterized by skin fragility resulting in blister formation in response to minor mechanical trauma.

### Most Likely Diagnosis:
**Epidermolysis Bullosa** (specifically, the Dystrophic form or other severe subtypes such as Junctional EB)

### Other Possible Diagnoses:
1. **Kindler Syndrome**: A rare genetic condition characterized by skin blistering, photosensitivity, and progressive poikiloderma.

2. **Porphyria**: A group of disorders affecting the skin and/or nervous system, some types can cause blistering on sun-exposed areas.

3. **Pemphigus Vulgaris**: An autoimmune disorder causing painful blistering of the skin and mucous membranes.

4. **Bullous Pemphigoid**: Another autoimmune blistering disorder, although it's more common in older adults, not at birth.

5. **Dyskeratosis Congenita**: A rare genetic disorder with features of nail dystrophy, skin pigmentation changes, and oral leukoplakia, which can also lead to blistering and other multi-systemic impacts.

These differential diagnoses consider the various aspects of blistering and systemic involvement you described. However, Epidermolysis Bullosa, particularly the severe subtypes, is the most fitting match based on the comprehensive clinical presentation provided. Genetic testing would be essential to confirm the diagnosis and determine the specific subtype.

**40. user - 2024-11-25 10:52:30**

The clinical presentation of patients varies with age, amount and duration of fructose exposure. In general, the younger the age of onset, the more severe the symptoms. The onset of the disease is typically around 6 months of age and usually occurs when fructose-containing foods, such as fruits, honey and certain vegetables, are first consumed. Common clinical symptoms include hypoglycaemic episodes after eating fructose-containing foods, with nausea, vomiting, abdominal pain, bloating, sweating, tremors, convulsions and even coma.

If the diagnosis is not clear and the patient chronically ingests fructose foods for a long period of time, in addition to hypoglycemic manifestations, growth retardation, hepatomegaly, ascites, jaundice and metabolic disorders such as hypoglycemia, metabolic acidosis, hypophosphatemia, hyperuricemia, hypermagnesemia, and hyperpyruvatemia may occur. In severe cases, multiple organ damage, liver and renal failure, convulsions, coma, and even death occur.

In acute attacks, laboratory tests reveal hypoglycaemia, hypophosphatemia and hypokalemia, accompanied by elevated uric acid, lactate, pyruvate, free fatty acids and triglycerides. Chronic patients present with hepatic impairment and indications of hepatic fibrosis, including serum bilirubin, elevated transaminases, and coagulation disorders. Urinalysis reveals proteinuria and fructose positivity.

**ChatGPT-4o** - 2024-11-25 10:52:30

Based on the clinical manifestations you've described, the most likely diagnosis is Hereditary Fructose Intolerance (HFI). This is a rare metabolic disorder caused by a deficiency of the enzyme aldolase B, which results in an inability to properly metabolize fructose, leading to the symptoms you've outlined.

Here are five other possible diagnoses that might be considered based on the symptoms:

1. **Fructose-1,6-bisphosphatase deficiency**: This is a disorder that affects gluconeogenesis and can present with hypoglycemia, metabolic acidosis, and liver dysfunction, particularly after fructose ingestion.

2. **Glycogen storage disease type I (Von Gierke disease)**: This condition is characterized by hypoglycemia, lactic acidosis, hyperuricemia, and hepatomegaly. While it doesn't specifically relate to fructose ingestion, the metabolic disturbances can overlap.

3. **Galactosemia**: An inability to metabolize galactose can cause liver dysfunction, hypoglycemia, and metabolic derangements. While it typically relates to galactose, the hepatic and metabolic symptoms may be similar.

4. **Tyrosinemia type I**: This is a disorder that can cause liver and renal dysfunction, hypoglycemia, and metabolic acidosis. It can present with some overlapping features, despite being unrelated to fructose metabolism.

5. **Wilson's disease**: Though primarily a copper metabolism disorder, Wilson's disease can present with liver dysfunction and metabolic abnormalities, including hypoglycemia in severe cases.

Each of these conditions has distinct biochemical and genetic profiles, so specific tests would be needed to confirm the diagnosis.

**41. user - 2024-11-25 10:57:34**

1. Neuro-muscular system, causing neurological abnormalities, such as convulsions, coma, ataxia, and even psychiatric symptoms; muscle symptoms can also occur, including hyperreflexia, tremor, tetany, and muscle weakness. 2. Cardiovascular system, causing ECG changes, such as PR interval prolongation, QRS wave widening, etc. In severe cases, it can cause ventricular arrhythmia.

2. cardiovascular system, causing changes in electrocardiogram, such as prolongation of PR interval, widening of QRS wave, and so on, and in severe cases, it may cause ventricular arrhythmias.

3. Kidney, which may cause kidney stones, renal calcium deposits, renal insufficiency.

4. bone and joint system, can cause osteoporosis and osteochondrosis. 5. digestive system, can cause intestinal tract infection.

5. digestive system, may cause intestinal obstruction.

6. endocrine, patients can see diabetes mellitus or impaired glucose tolerance.

7. ocular symptoms, some types may be combined with ocular lesions, including nystagmus, visual field defects, myopia, etc. This group of diseases usually has a chronic course.

This group of diseases usually has a chronic course, with the neonatal and infantile onset types often being more severe and having a poorer prognosis. Some patients develop chronic renal failure during adolescence, and in some cases, failure to treat the disease in time can lead to severe mental retardation or even death.

**ChatGPT-4o** - 2024-11-25 10:57:34

Based on the provided clinical manifestations, the most likely rare disease diagnosis is **Bartter syndrome** or **Gitelman syndrome**. These are renal tubular disorders characterized by abnormalities in electrolyte handling that can lead to various systemic symptoms, including those affecting the neuromuscular and cardiovascular systems, as well as potential effects on the kidneys, bones, and more.

### Five Other Possible Diagnoses:

1. **Hypoparathyroidism**:
 - Can cause neuromuscular symptoms such as tetany and seizures due to hypocalcemia, as well as cardiovascular changes like prolonged QT interval.

2. **Familial Hypocalciuric Hypercalcemia (FHH)**:
 - Though typically benign, it can cause mild hypercalcemia that might lead to neuromuscular symptoms, and rarely, renal and skeletal changes.

3. **Primary Hyperparathyroidism**:
 - Causes hypercalcemia, which can affect neuromuscular and cardiovascular systems, as well as lead to kidney stones and bone demineralization.

4. **Wilson's Disease**:
 - A disorder of copper metabolism that can cause neurological, psychiatric, hepatic, renal, and musculoskeletal symptoms.

5. **X-linked Hypophosphatemia**:
 - Causes rickets or osteomalacia due to phosphate wasting, leading to bone pain and muscle weakness, and sometimes associated with dental and renal effects.

These diagnoses consider the various systemic effects described, including neurological, cardiovascular, renal, skeletal, and endocrine manifestations.

**42. user - 2024-11-25 12:13:04**

**1. Migraine** Migraine with aura is often the first symptom of the disease and is seen in 20-40% of patients, which is five times higher than in the general population; it can also manifest as migraine without aura, with an incidence not different from that of the general population. The incidence of migraine is lower in Asian patients than in European and American patients.

**2. Subcortical ischemic events** Transient ischemic attacks (TIAs) and ischemic strokes are the most common clinical manifestations of the disease, occurring in 60% to 85% of symptomatic patients, most of whom lack common risk factors for cerebrovascular disease. 2/3 of patients present with the classic lacunar syndrome.

**3. Cognitive deficits** Cognitive dysfunction with dementia develops progressively in 60% of patients.

**Affective disorders and apathy** 20% of patients have affective disorders, with depressive states being the most common, and rarely alternating between depressive and manic states; apathy is seen in 40% of patients.

In addition, seizures occur in 5-10% of patients, and hearing loss, impaired consciousness, spinal cord infarction, intracranial haemorrhage, and combined basin infarction have also been reported.

**ChatGPT-4o** - 2024-11-25 12:13:04

Based on the clinical manifestations you've provided, the disease most likely to be diagnosed is **CADASIL (Cerebral Autosomal Dominant Arteriopathy with Subcortical Infarcts and Leukoencephalopathy)**. CADASIL is a genetic disorder that often presents with migraines, especially with aura, and subcortical ischemic events, such as transient ischemic attacks (TIAs) and strokes. Cognitive deficits, affective disorders, and apathy are common in CADASIL patients, aligning with the symptoms you've described.

Here are five other possible diagnoses that could present with similar symptoms:

1. **Fabry Disease**: A genetic disorder that can lead to strokes, cognitive impairment, and sometimes migraines. It also includes symptoms such as neuropathic pain and angiokeratomas, which are not mentioned but can overlap in presentation.

2. **MELAS Syndrome (Mitochondrial Encephalomyopathy, Lactic Acidosis, and Stroke-like episodes)**: A mitochondrial disorder that presents with stroke-like episodes, migraines, seizures, and progressive cognitive decline.

3. **Multiple Sclerosis (MS)**: An autoimmune disease characterized by demyelination in the brain and spinal cord, leading to symptoms such as migraines, cognitive impairment, affective disorders, and occasionally stroke-like symptoms.

4. **Binswanger's Disease**: A form of small vessel vascular dementia that presents with subcortical ischemic events, cognitive decline, mood disorders, and gait disturbances.

5. **Susac's Syndrome**: A rare autoimmune disorder that involves the microvasculature of the brain, retina, and inner ear, leading to migraines, cognitive dysfunction, stroke-like episodes, and hearing loss.

Each of these conditions has its own distinct set of additional symptoms and diagnostic criteria, so clinical evaluation, family history, and specific diagnostic tests would be necessary to differentiate them accurately.

**43. user - 2024-11-25 12:15:04**

The onset of the disease is usually insidious in early youth (but has been reported from 1 to 76 years of age). The main manifestation is a slowly progressive spastic paralysis of both lower limbs, with pyramidal signs (hyperreflexia, positive ankle clonus, positive pathology). Bilateral upper limb reflexes may also be hyperreflexic, but generally do not affect upper limb activity. More than half of the patients will have proximal weakness of the lower limbs. Decreased vibratory sensation of the ankle tuning forks (which usually does not disappear) is detected on examination in about 50% of patients, and urinary dysfunction is present in about one third of patients.

The disease progresses with age, with about 20% of patients eventually losing the ability to walk. The severity of clinical symptoms varies considerably between patients carrying the same mutation in the same family.

**ChatGPT-4o** - 2024-11-25 12:15:04

Based on the clinical manifestations you've described, the most likely diagnosis is Hereditary Spastic Paraplegia (HSP), specifically the pure form. HSP is a group of inherited disorders characterized by progressive weakness and spasticity of the legs.

Here are five other possible diagnoses that could be considered:

1. **Primary Lateral Sclerosis (PLS):** A rare motor neuron disease that causes progressive weakness and stiffness of the muscles, especially in the legs.

2. **Multiple Sclerosis (MS):** An autoimmune disease that affects the central nervous system, potentially causing similar spasticity and gait disturbances.

3. **Adrenomyeloneuropathy (AMN):** A form of adrenoleukodystrophy that mainly affects the spinal cord and peripheral nerves, leading to spastic paraparesis.

4. **Spinal Cord Tumor:** Though less likely given the genetic implications, a tumor could cause similar progressive spastic paraplegia.

5. **Tropical Spastic Paraparesis (TSP)/HTLV-1 associated myelopathy (HAM):** A disease caused by the human T-lymphotropic virus type 1, leading to spasticity in the legs.

Each of these conditions has distinguishing features and would be differentiated through further clinical evaluation, genetic testing, and imaging studies.

**44. user - 2024-11-25 12:17:11**

Most of the affected children develop the disease in newborns and early infants. Clinical manifestations are similar to biotinidase deficiency, without specificity, mainly feeding difficulties, vomiting, diarrhoea, hypotonia, convulsions, depression, lethargy, respiratory difficulties, developmental delays, intractable rashes, desquamation, which can be combined with ketosis, metabolic acidosis, hyperlactic acidemia and so on. Skin damage in most patients in the head and face, neck, trunk, buttocks and other parts of the skin rash or erythema, ulceration or blisters, furfuraceous or flaky scales, or dry and rough skin, peeling skin, etc., and a few patients only in the perioral, periocular, perianal local appearance of the rash. Patients are not associated with hearing or visual impairment, unlike biotinidase deficiency.

**ChatGPT-4o** - 2024-11-25 12:17:11

Based on the clinical manifestations you've described, the most likely diagnosis is **holocarboxylase synthetase deficiency**. This condition is part of the biotin-thiamine-responsive basal ganglia disease spectrum and presents similarly to biotinidase deficiency but with some differences in symptoms and severity.

Other possible diagnoses to consider include:

1. **Multiple Carboxylase Deficiency**: This includes both biotinidase deficiency and holocarboxylase synthetase deficiency. Symptoms overlap, and genetic testing is required to differentiate them.

2. **Propionic Acidemia**: A metabolic disorder that can cause similar symptoms, including metabolic acidosis, vomiting, hypotonia, and developmental delays.

3. **Methylmalonic Acidemia**: Another metabolic disorder presenting with similar metabolic derangements, such as metabolic acidosis and lethargy.

4. **Organic Acidemias**: A broader category that includes disorders like isovaleric acidemia and glutaric acidemia, which can present with metabolic crises and dermatologic manifestations.

5. **Maple Syrup Urine Disease (MSUD)**: Although not typically characterized by skin manifestations, it can cause metabolic acidosis and neurological symptoms, including lethargy and hypotonia.

These conditions should be differentiated through biochemical testing, enzyme assays, and genetic testing to confirm the exact diagnosis and guide treatment.

**45. user - 2024-11-25 12:19:10**

Children are born without abnormalities, and the clinical presentation in infancy is not specific and is characterised only by growth and developmental delays. It is often diagnosed at 3 years of age after the onset of ocular signs and symptoms.

1. visual impairment, myopia and iris tremor due to ectopic lens may appear after 3 years of age, and may be combined with astigmatism, glaucoma, staphylococcus, cataract, retinal detachment and optic nerve atrophy in later stages. 2. intellectual disability is common.

2. Intellectual disability is more common, mainly manifested as progressive mental retardation. Vitamin B6 treatment-sensitive patients are less mentally impaired. 3.

3. mental or behavioural disorders, which may occur in about 50% of children and are associated with convulsions in 20%.

4. Skeletal involvement, similar to the manifestations of Marfontein syndrome, or in combination with scoliosis, funnel chest, chicken chest, knee valgus, and bowed feet, with generalised osteoporosis seen on X-ray.

5. Thrombophilia, which occurs at all ages and may involve all types of blood vessels, especially the cerebral vessels. High levels of homocysteinemia may alter the vessel wall and increase platelet adhesion, resulting in optic nerve atrophy, paralysis, pulmonary heart disease, and renal hypertension.

6. Spontaneous pneumothorax and acute pancreatitis are less common.

**ChatGPT-4o** - 2024-11-25 12:19:10

The clinical manifestations you described are most suggestive of **Homocystinuria**. Homocystinuria is a rare autosomal recessive metabolic disorder characterized by a deficiency of the enzyme cystathionine beta-synthase, leading to high levels of homocysteine in the blood and urine. This condition fits well with the ocular findings, skeletal abnormalities similar to Marfan syndrome, intellectual disability, and thrombotic events.

Here are five other possible diagnoses to consider based on the symptoms provided:

1. **Marfan Syndrome**: This is a connective tissue disorder that can also present with ocular, skeletal, and cardiovascular abnormalities. However, it typically does not feature intellectual disabilities or thrombotic events without high homocysteine levels.

2. **Ehlers-Danlos Syndrome**: This is a group of connective tissue disorders that might share some skeletal features with your description, though intellectual disability and thrombotic complications are not typical.

3. **Lens Dislocation due to Sulfite Oxidase Deficiency**: This metabolic disorder can cause ectopia lentis (lens dislocation), intellectual disability, and neurological symptoms, but it is quite rare and usually presents with additional symptoms like epilepsy.

4. **Mucopolysaccharidoses (e.g., Maroteaux-Lamy Syndrome)**: These are lysosomal storage diseases that can present with developmental delay, skeletal abnormalities, and sometimes ocular issues, but the specific combination of symptoms might not match perfectly.

5. **Zellweger Spectrum Disorders**: These are a group of peroxisomal biogenesis disorders that can present with intellectual disability, ocular problems, and skeletal abnormalities, among other symptoms, but they typically present much earlier and with more severe neurological deficits.

While these conditions could share some overlapping symptoms, Homocystinuria remains the most likely diagnosis given your description.

**46. user - 2024-11-25 12:38:38**

The main manifestations of the disease are markedly elevated levels of LDL-C from birth, cholesterol deposits in the skin, eyes and tendons, and the formation of xanthomas and lipomatous corneal arches. As a result of exposure to high levels of LDL-C, most patients develop coronary atherosclerotic heart disease before the age of 20 and may die as a result.

A detailed family history is needed, as some patients have heterozygous parents and therefore have elevated LDL-C levels (usually greater than the 95th percentile of their country's age- and sex-specific norms) and a strong positive family history of premature atherosclerotic cardiovascular disease.

**ChatGPT-4o** - 2024-11-25 12:38:38

Based on the clinical manifestations you provided, the most likely diagnosis is Homozygous Familial Hypercholesterolemia (HoFH). This rare genetic disorder is characterized by extremely high levels of low-density lipoprotein cholesterol (LDL-C) from birth, leading to early-onset atherosclerotic cardiovascular disease, xanthomas, and other cholesterol deposits.

Here are five other possible diagnoses to consider, although they are less likely given the specific findings you described:

1. **Heterozygous Familial Hypercholesterolemia (HeFH):** Although usually less severe than HoFH, it can still lead to elevated LDL-C levels and an increased risk of cardiovascular disease, especially if both parents have the condition.

2. **Sitosterolemia:** A rare metabolic disorder leading to increased absorption and decreased excretion of plant sterols and cholesterol, causing xanthomas and early atherosclerosis.

3. **Polygenic Hypercholesterolemia:** A condition where multiple genetic factors contribute to high cholesterol levels, though it's typically less severe than familial hypercholesterolemia.

4. **Type III Hyperlipoproteinemia (Dysbetalipoproteinemia):** Characterized by elevated cholesterol and triglycerides, along with xanthomas, although the lipid profile is different from that of HoFH.

5. **Lecithin-Cholesterol Acyltransferase (LCAT) Deficiency:** A rare disorder affecting cholesterol metabolism that can lead to corneal opacities and kidney disease, but typically not as elevated LDL-C or such early heart disease as HoFH.

It is crucial to obtain a detailed family history and possibly perform genetic testing to confirm the diagnosis and differentiate between these conditions.

**47. user - 2024-11-25 12:41:23**

It is characterised by a triad of movement disorders, psychiatric symptoms and cognitive deficits, usually with an insidious onset and slow progression. The age of onset ranges from childhood to 79 years, with the most common being 30-50 years of age.

The disease involves rapid, involuntary, non-rhythmic movements of the face, trunk, and limbs. Early abnormal movements are mild, and the patient may have difficulty recognising the presence of chorea or integrating chorea-like movements into random movements, which makes the control of normal movements difficult or deviant; as the disease progresses, the extent and degree of chorea often increases, and even affects the diaphragm, pharynx, and laryngeal muscles, resulting in dysarthria, dysphagia, and involuntary movement. As the disease progresses, the extent and degree of chorea often increases, even affecting the pharyngeal and laryngeal muscles, resulting in dysarthria, dysphagia, and involuntary vocalisations. In the later stages of the disease, the chorea often disappears, and is replaced by a Parkinson's-like pattern of stiffness and hyperkinesis, which may be accompanied by focal dystonia. Difficulty in maintaining movement is also a common manifestation of the disease, i.e., the inability to maintain certain simple voluntary movements, such as difficulty in maintaining tongue extension.

Mental disorders may precede chorea, and common symptoms include depressed mood, depression, irritability, apathy, and anxiety, as well as paranoia, delusions, and hallucinations; rare symptoms include obsessive-compulsive behaviours and psychotic manifestations, which tend to worsen progressively.

Cognitive impairment is most often characterised by executive dysfunction, which is manifested by a reduced ability to make decisions, multi-task and shift cognitive goals. Patients usually lack self-awareness of their cognitive deficits. Memory loss or impairment occurs late in the course of the disease and progresses to dementia as the disease progresses.

In addition to the triad of symptoms, eye movement abnormalities are also prominent. Early in the disease, there is a slowing down of eye-sweeping speed, but eye-following movements are preserved; in later stages, eye-following movements, voluntary sweeping and refixation are all impaired. In addition, weight loss and cachexia are common features of the disease, with patients experiencing significant weight loss as a result of the high energy expenditure of involuntary movements, usually without loss of appetite. Sleep and/or sexual dysfunction are also common.

Progressive deterioration of motor and cognitive function can lead to significant complications, such as dysphagia due to involvement of the pharyngeal muscles, dyskinesia, reduced activity and even bed rest, which can lead to aspiration pneumonia and other infections, and decubitus ulcers.

**ChatGPT-4o** - 2024-11-25 12:41:23

Based on the clinical manifestations you've described, the most likely diagnosis is Huntington's disease. This rare, inherited neurodegenerative disorder is characterized by a triad of movement disorders (notably chorea), psychiatric symptoms, and cognitive deficits, with an insidious onset and slow progression. The age of onset and specific symptoms you've described align well with Huntington's disease.

Other possible diagnoses to consider include:

1. **Wilson's Disease**: This is a genetic disorder that leads to excessive copper accumulation in the body, affecting the liver and brain, and can cause movement disorders, psychiatric symptoms, and cognitive impairment.

2. **Neuroacanthocytosis**: This is a group of rare disorders characterized by the presence of acanthocytes in the blood and neurological symptoms, including chorea, cognitive decline, and psychiatric disorders.

3. **Spinocerebellar Ataxias (SCAs)**: SCAs are a group of genetic disorders marked by progressive ataxia, which can be accompanied by movement disorders and cognitive decline, although the specific presentation can vary.

4. **Sydenham's Chorea**: This is a disorder associated with rheumatic fever, leading to involuntary movements and potentially accompanied by psychiatric symptoms. However, it usually affects children or adolescents and is not progressive.

5. **Lesch-Nyhan Syndrome**: This is a rare genetic disorder caused by HPRT1 gene mutations, leading to neurological and behavioral abnormalities, including chorea, cognitive impairments, and self-mutilating behaviors. However, it's typically diagnosed in early childhood.

While these are possibilities, the combination of symptoms you provided most closely aligns with Huntington's disease.

**48. user - 2024-11-25 12:44:05**

Clinical signs and symptoms are mainly neurological and vary in severity. The age of onset can range from neonatal to adulthood (late-onset), and there is a broad phenotypic spectrum.

1. In the neonatal period, symptoms associated with hyperammonemia [including lethargy, drowsiness, refusal to eat, vomiting with respiratory alkalosis and/or seizures] appear after about 24-48 hours of asymptomatic onset. It is indistinguishable from other urea cycle disorders of neonatal onset.

2. Late-onset, which accounts for 88 per cent of patients, 40 per cent of whom occur before the age of 3 years, 29 per cent in childhood and 19 per cent in adulthood. The main manifestations are: (i) chronic neurocognitive deficits, including developmental delay, ataxia, spasticity, learning disabilities, cognitive deficits, and/or unexplained seizures; (ii) acute encephalopathy secondary to hyperammonaemic crisis induced by a variety of factors (e.g., infections, fasting, or injuries); and (iii) chronic hepatic dysfunction, with unexplained elevation of hepatic aminotransferases with or without mild coagulation disorders, with or without mild hyperammonaemia, and protein intolerance. Ammonemia and protein intolerance.

**ChatGPT-4o** - 2024-11-25 12:44:05

The clinical manifestations you've described are characteristic of Ornithine Transcarbamylase (OTC) Deficiency, a rare X-linked urea cycle disorder. The condition is known for causing hyperammonemia and has a broad phenotypic spectrum, ranging from severe neonatal onset to milder late-onset forms. OTC deficiency is the most common urea cycle disorder and fits the described symptoms well.

Five other possible diagnoses considering the symptoms and presentation include:

1. **Carbamoyl Phosphate Synthetase I (CPS1) Deficiency**: Another urea cycle disorder that can present with severe neonatal hyperammonemia and similar late-onset symptoms.

2. **Argininosuccinate Synthetase Deficiency (Citrullinemia Type I)**: This urea cycle disorder also leads to hyperammonemia, with neonatal and late-onset forms that can cause similar neurological and hepatic symptoms.

3. **Argininosuccinate Lyase Deficiency (Argininosuccinic Aciduria)**: Presents with hyperammonemia and neurologic deficits. Hepatic dysfunction is also common in this disorder.

4. **N-acetylglutamate Synthase (NAGS) Deficiency**: Results in hyperammonemic episodes due to the lack of cofactor required for CPS1 activity, causing similar presentations.

5. **Liver Mitochondrial DNA Depletion Syndrome**: A rare metabolic disorder that can cause similar symptoms, including hepatic dysfunction and neurological symptoms due to energy metabolism issues.

These conditions should be differentiated based on specific enzymatic assays, genetic testing, and metabolic profiles.

**49. user - 2024-11-25 12:48:10**

Some children have no clinical symptoms in the neonatal period, but after 3-4 months of life, the typical symptoms appear gradually: the hair turns from black to yellow, the skin is light-coloured, and the urine and sweat are foul-smelling. As the child grows older, he/she gradually shows backward intellectual development, microcephaly, epileptic seizures, and may also show abnormalities in behaviour, personality, and neurocognition. For example, hyperactivity, self-mutilation, aggression, autism, low self-esteem, depression, etc. Vomiting and eczema are also common in infancy.
In some patients, there are no clinical symptoms in the neonatal period, and after 1-3 months of life, in addition to the symptoms of PKU, the main manifestations are dyskinesia, lethargy, hypotonia of the trunk, increased or decreased muscle tone of the limbs, nystagmus, dysphagia, increased drooling, flaccidity, torticollis, unresponsiveness, insomnia, and severe intellectual developmental disorders, and so on.

**ChatGPT-4o** - 2024-11-25 12:48:10

Based on the clinical manifestations described, the most likely diagnosis is **Phenylketonuria (PKU)**. PKU is a rare genetic disorder caused by a deficiency of the enzyme phenylalanine hydroxylase, leading to the accumulation of phenylalanine in the body. The symptoms you described, such as changes in hair and skin pigmentation, intellectual disability, neurological issues, and a musty odor in the urine and sweat, are classic signs of untreated PKU.

Here are five other possible diagnoses that could be considered based on the symptoms:

1. **Hyperphenylalaninemia**: This refers to a group of conditions with elevated levels of phenylalanine, including variants of PKU with milder symptoms or other enzyme deficiencies affecting phenylalanine metabolism.

2. **Tyrosinemia Type I**: This is another metabolic disorder that can present with neurological symptoms, failure to thrive, and liver problems, although it typically has more severe hepatic manifestations.

3. **Maple Syrup Urine Disease (MSUD)**: This is a disorder of branched-chain amino acid metabolism. Symptoms can include poor feeding, lethargy, developmental delay, and a distinctive sweet-smelling urine, but it usually presents earlier in life.

4. **Nonketotic Hyperglycinemia (NKH)**: This condition is characterized by a build-up of glycine and can result in severe neurological impairment, hypotonia, seizures, and developmental delay.

5. **Biopterin Cofactor Deficiency**: This involves issues with the metabolism of tetrahydrobiopterin, a cofactor for phenylalanine metabolism, and can mimic PKU symptoms but may also involve neurotransmitter deficiencies causing more complex neurological symptoms.

Prompt medical evaluation, including metabolic screening and genetic testing, is crucial for accurate diagnosis and management of these conditions.

**50. user - 2024-11-25 12:52:51**

The severity of clinical manifestations is highly variable. Patients may present with abnormalities in bone mineralisation, dental or joint pathology, muscle weakness, etc. Currently, there are seven main types of the disease.

1. simple dental type, which is the mildest and most common type, with only dental involvement and no rickets or osteomalacia. 2. adult type, which often develops in middle age.

2. Adult type, which often develops in middle age and presents with dental abnormalities or osteochondrosis. It may cause impaired mobility due to repeated fractures, bone and joint pain, or muscle weakness. 3.

3. the childhood type, which usually develops after 6 months of age, can be mild to severe, and the children may have different degrees of tooth loss and rickets.

4. the infantile form, with onset before 6 months of age, is often poorly ventilated, difficult to survive, debilitating, and may have a wide fontanelle or cranial deformity. 50 per cent of infantile HPP die of complications. 5. the perinatal form, with onset before 6 months of age, is the most severe form of HPP, with onset before 6 months of age.

5. The perinatal form, which is the extreme form, develops in utero, is evident at birth, and is almost always fatal. Children with short limbs and severe skeletal undermineralisation may present with high-pitched crying, seizures, periodic apnoea with cyanosis and bradycardia, unexplained fever, irritability, myelosuppressive anaemia and intracranial haemorrhage, and pulmonary atelectasis.

6. Pseudohypophosphatasia, similar to the infantile form, in which the patient has the manifestations of infantile hypophosphatasia with normal or elevated serum ALP. 7.

7. Benign type, sometimes presenting with skeletal malformations in utero, with perinatal hypophosphatase activity, but resolving after birth without the associated manifestations of infantile hypophosphatasia or simple dental hypophosphatasia.

**ChatGPT-4o** - 2024-11-25 12:52:51

Based on the clinical manifestations described, the disease you are referring to is most likely **Hypophosphatasia (HPP)**. This is a rare, inherited disorder characterized by defective bone mineralization and deficiency of the enzyme alkaline phosphatase. The disease has several forms, ranging from mild to severe, with variable clinical presentations as you described.

Five other possible diagnoses that could present with similar symptoms include:

1. **Osteogenesis Imperfecta**: A genetic disorder characterized by fragile bones that break easily, often with other features such as blue sclerae, dental issues, and hearing loss.

2. **Rickets**: Typically caused by vitamin D deficiency, leading to soft and weak bones in children, which can result in skeletal deformities.

3. **Metaphyseal Dysplasia**: A disorder that affects the growth and development of bone, leading to abnormalities in the metaphysis of long bones and potentially causing joint issues and fractures.

4. **Cleidocranial Dysplasia**: A genetic condition that primarily affects the development of bones and teeth, leading to clavicular hypoplasia, delayed closure of fontanels, and dental abnormalities.

5. **X-linked Hypophosphatemia**: A genetic disorder that leads to low levels of phosphate in the blood, resulting in weak and soft bones, often with bone pain and dental abnormalities.

Each of these conditions has its unique pathophysiology and clinical spectrum, so careful clinical evaluation, genetic testing, and biochemical analysis are crucial for accurate diagnosis.

**51. user - 2024-11-27 12:15:47**

Children with square skulls, chicken breasts, beaded ribs, bracelet sign, foot bracelet sign, Henry's groove, and lower limb deformities, which may be inversion of the knee (O-shaped leg) or eversion of the knee (X-shaped leg), appearing when they start weight-bearing near the age of one week, are accompanied by growth retardation, short stature, wobbly gait, progressively aggravating bone deformities, multiple bone fractures, bone pain, and abnormalities of tooth development (poor tooth quality, toothache, difficulty in regeneration after loss). Imaging is characterised by skeletal deformities, such as Imaging manifestations include skeletal deformities, widening and blurring of the epiphysis of the long bones, a cupped shape, and many fine strips of calcification like brushes in the cupped shape. In adults, the main manifestations are limb weakness, activity limitation, bone pain, multiple pathological fractures (long bones of the limbs, ribs, pelvis and vertebrae can occur), and shortening of height. Imaging manifestations include a generalised decrease in bone density, blurred bone trabeculae with a hairy glass appearance, pelvic deformity, false fracture lines, and biconcave deformity of the vertebral body.

**ChatGPT-4o** - 2024-11-27 12:15:47

The clinical manifestations you have described are highly suggestive of **Rickets**, particularly Vitamin D-resistant rickets or X-linked hypophosphatemic rickets (XLH). This condition is characterized by skeletal deformities due to impaired mineralization of bone, which is consistent with the symptoms and imaging findings you've mentioned.

Here are five other possible diagnoses to consider:

1. **Osteogenesis Imperfecta**: This genetic disorder is characterized by brittle bones that fracture easily, bone deformities, and growth issues, which can overlap with some of the symptoms described.

2. **Hypophosphatasia**: A rare metabolic bone disease that affects mineralization of bones and teeth, leading to rickets-like symptoms, bone pain, and fractures.

3. **Metaphyseal Dysplasia (Pyle's Disease)**: A rare bone disorder that can cause widened metaphyses and resemble rickets on imaging, leading to bone pain and deformities.

4. **Fanconi Syndrome**: A disorder affecting the kidneys, leading to phosphate wasting and rickets-like symptoms due to subsequent bone demineralization.

5. **Renal Tubular Acidosis**: This condition can lead to metabolic acidosis and bone demineralization, resulting in rickets-like presentations, bone pain, and fractures.

These conditions share some overlapping features with the described clinical picture, but the presence of specific signs and the imaging findings make Rickets, particularly the vitamin D-resistant form, the most likely diagnosis.

**52. user - 2024-11-27 12:20:29**

Clinical manifestations include heart failure, chest pain, hypotension, syncope, sudden cardiac death, arrhythmias and thromboembolism. Some patients also have extracardiac manifestations such as peripheral neuropathy, autonomic neuropathy and gastrointestinal symptoms.

**ChatGPT-4o** - 2024-11-27 12:20:29

Based on the clinical manifestations you've described, the rare disease most likely to be diagnosed is **Cardiac Amyloidosis**. This condition is characterized by the deposition of amyloid proteins in the heart tissue, leading to heart failure, arrhythmias, and other cardiac symptoms. The extracardiac manifestations like peripheral neuropathy and autonomic neuropathy further support this diagnosis.

Here are five other possible diagnoses:

1. **Hypertrophic Cardiomyopathy (HCM):** This genetic condition can cause heart failure, arrhythmias, syncope, and sudden cardiac death. While primarily cardiac, it can have systemic implications.

2. **Arrhythmogenic Right Ventricular Cardiomyopathy (ARVC):** This condition causes arrhythmias and can lead to heart failure and sudden cardiac death. Although mainly cardiac, it can have some systemic effects.

3. **Fabry Disease:** This is a lysosomal storage disorder that can cause cardiac involvement, including hypertrophic cardiomyopathy, arrhythmias, and conduction blocks. It often presents with peripheral neuropathy and gastrointestinal symptoms.

4. **Sarcoidosis:** Cardiac involvement in sarcoidosis can cause heart failure, arrhythmias, and sudden cardiac death. It also frequently leads to extracardiac symptoms, including neuropathy and other systemic manifestations.

5. **Chagas Disease:** A parasitic disease that can lead to chronic heart issues like heart failure and arrhythmias. It can also cause peripheral and autonomic neuropathy and gastrointestinal symptoms.

Each of these conditions has distinct features and requires specific diagnostic tests for confirmation. A thorough clinical evaluation, including imaging and possibly a biopsy, would be necessary for accurate diagnosis.

**53. user - 2024-11-27 12:24:32**

1. secondary sexual characteristics and impaired gametogenesis in males: childish voice, small penis, no pubic hair growth, small testes or cryptorchid testes, no spermatogenesis; in females: mammary gland dysplasia, infantile vulva, primary amenorrhoea.

2. delayed epiphyseal closure upper volume/lower volume <1, fingertip distance > height, susceptible to osteoporosis.

3. Olfactory disorders 40% ~60% of IHH patients suffer from loss of sense of smell due to abnormal development of the olfactory bulb and olfactory tract, and are unable to identify odours.

4. Other manifestations Facial midline defects, such as cleft lip and palate; isolated kidneys; short fingers (toes), syndactyly; skeletal deformities or dental hypoplasia; overweight and obesity; mirror image movement.

**ChatGPT-4o** - 2024-11-27 12:24:32

Based on the clinical manifestations you've provided, the most likely rare disease to be diagnosed is **Kallmann Syndrome**. This condition is characterized by a combination of hypogonadotropic hypogonadism and anosmia (loss of sense of smell), along with other potential associated features such as midline defects and skeletal abnormalities.

### Five Other Possible Diagnoses:

1. **Congenital Hypogonadotropic Hypogonadism (CHH) without anosmia**: While similar to Kallmann Syndrome, this condition presents with hypogonadotropic hypogonadism but typically without the loss of smell.

2. **Prader-Willi Syndrome**: This genetic disorder can present with hypogonadism, obesity, and characteristic behavioral features, though typically not associated with anosmia.

3. **CHARGE Syndrome**: A genetic condition that includes coloboma, heart defects, atresia of the choanae, growth retardation, genital abnormalities, and ear abnormalities, which could overlap with some of the features described.

4. **Idiopathic Hypogonadotropic Hypogonadism (IHH)**: This condition involves a similar hormonal deficiency without the associated anosmia, possibly linked to other genetic factors.

5. **Turner Syndrome (in females)**: Although primarily a chromosomal condition, it can present with some overlapping features such as delayed sexual development and short stature, though it lacks the anosmia component.

Each of these diseases shares some overlapping symptoms, but Kallmann Syndrome is the most fitting primary diagnosis given the specific combination of hypogonadism and olfactory disorders.

**54. user - 2024-11-27 12:26:40**

The most common symptom is shortness of breath after activity, and other symptoms include fatigue, dizziness, chest pain, chest tightness, palpitations, blackouts, and syncope, etc. Once syncope or vertigo occurs, it suggests that the patient's cardiac output may have decreased significantly. Due to atypical symptoms and signs, the majority of patients have a significant delay in consultation, with at least 1/5 of patients taking more than 2 years from the onset of symptoms to diagnosis; patients may have a hyperactive second heart sound of the pulmonary valve, and tricuspid insufficiency may cause a systolic murmur in the tricuspid region; signs of right heart failure include jugular venous congestion or rupture, enlarged liver, oedema of the lower extremities, multiple plasma cavities, jaundice, and cyanosis, among other signs.

**ChatGPT-4o** - 2024-11-27 12:26:40

Based on the clinical manifestations provided, the most likely rare disease that fits the described symptomatology is **Pulmonary Arterial Hypertension (PAH)**. This condition is characterized by the increased pressure within the pulmonary arteries, leading to right heart strain and failure, which aligns with the symptoms of shortness of breath, fatigue, syncope, and signs of right heart failure described.

Other possible diagnoses that could explain these symptoms include:

1. **Chronic Thromboembolic Pulmonary Hypertension (CTEPH)**: This is a form of pulmonary hypertension caused by unresolved blood clots in the lungs, leading to similar symptoms of PAH.

2. **Eisenmenger Syndrome**: This is a type of congenital heart defect that has resulted in pulmonary hypertension, causing symptoms such as cyanosis, fatigue, and syncope.

3. **Constrictive Pericarditis**: This condition can lead to reduced cardiac output and mimic right heart failure due to the heart being constricted by a thickened pericardium.

4. **Cardiomyopathy**: Various forms of cardiomyopathy, particularly those affecting the right side of the heart, can cause these symptoms.

5. **Cor Pulmonale**: This is right heart failure caused by chronic lung disease, where increased resistance in the pulmonary circulation leads to right ventricular hypertrophy and failure, showing similar clinical manifestations.

A detailed clinical evaluation, imaging studies, and hemodynamic assessments are crucial for differentiating these conditions and confirming the diagnosis.

**55. user - 2024-11-27 12:47:50**

The disease is usually insidious. The main manifestations are dry cough, exertional dyspnoea, pestle-like fingers (toes), and bursting sounds distributed mainly in both lower lungs are its typical signs. In the terminal stage, cyanosis, pulmonary hypertension, pulmonary heart disease and right heart insufficiency can be seen.

**ChatGPT-4o** - 2024-11-27 12:47:50

Based on the clinical manifestations you've described, the most likely diagnosis is **Idiopathic Pulmonary Fibrosis (IPF)**. IPF is characterized by a progressive and chronic scarring of the lung tissue, which leads to a persistent dry cough, exertional dyspnea, and clubbing of fingers and toes. The "bursting" sounds you mentioned likely refer to "velcro-like" crackles heard on auscultation, typically at the lung bases. In advanced stages, IPF can lead to complications like cyanosis, pulmonary hypertension, and cor pulmonale (pulmonary heart disease).

Five other possible diagnoses include:

1. **Chronic Hypersensitivity Pneumonitis**: This condition is also characterized by dry cough, dyspnea, and crackles, resulting from an immune response to inhaled environmental antigens.

2. **Non-Specific Interstitial Pneumonia (NSIP)**: Another interstitial lung disease with similar symptoms but with different histological features compared to IPF. It can also present with clubbing and crackles.

3. **Sarcoidosis**: A systemic granulomatous disease that can affect the lungs and cause similar respiratory symptoms, although clubbing is less common.

4. **Asbestosis**: Caused by inhalation of asbestos fibers, it presents with similar respiratory symptoms and can lead to pulmonary fibrosis and clubbing.

5. **Connective Tissue Disease-associated Interstitial Lung Disease (CTD-ILD)**: Diseases like scleroderma or rheumatoid arthritis can involve the lungs and cause similar symptoms.

Each of these conditions warrants a thorough clinical evaluation, including history, physical examination, imaging studies, and possibly lung biopsy, to confirm the diagnosis.

**56. user - 2024-11-27 12:51:37**

It is a multi-organ and multi-system disease that can involve almost all organs and tissues of the body. Clinical manifestations are varied, and it is easy to miss the diagnosis when patients consult different specialties for different organ involvement. Most of the patients have a slow onset of the disease, with a chronic progression. The disease may start in a single organ and gradually involve multiple organs, or it may develop in multiple organs at the same time. In a minority of patients, the disease is self-limiting.

Lacrimal and salivary glands are the most commonly affected organs. The main manifestations are symmetrical, painless enlargement of the lacrimal gland, submandibular gland or parotid gland, hard nodules, with or without sinusitis or thickening of extraocular muscles. Involvement of the digestive system may manifest as autoimmune pancreatitis, sclerosing cholangitis, sclerosing mesenteritis, etc. Clinical manifestations may include vague abdominal pain, abdominal distension, dyspepsia, and jaundice. Lung involvement may be manifested as bronchial wall thickening, interstitial lung lesions, lung nodules/lumps and pleural thickening, etc. Patients may be asymptomatic or have cough, shortness of breath and wheezing. Retroperitoneal fibrosis or peri-abdominal aortitis is also one of the more common clinical manifestations, which is mainly a thickening of the soft tissue around the abdominal aorta, which can encircle the ureter and cause ureteral stenosis and pyelonephrosis, and in a small number of patients, abdominal aortic aneurysm occurs. Clinical manifestations include abdominal pain, low back pain, or oedema of the lower extremities. Less than 10% of patients develop IgG4-associated renal disease, most often manifesting as tubulointerstitial nephropathy, renal pelvic space-occupying lesions, ureteral wall thickening, or renal mass-like lesions. Other organs involved include the thyroid (sclerosing thyroiditis) and prostate. CNS lesions are rare, such as autoimmune pituitary inflammation, hypertrophic dural meningitis, and intracranial inflammatory pseudotumours, and patients may present with headaches, urolithiasis, and hypopituitarism. In addition to the above organ involvement, most patients combine with superficial or deep lymph node enlargement. In addition, about half of the patients have a history of allergy-related disease, which can be accompanied by asthma.

**ChatGPT-4o** - 2024-11-27 12:51:37

Based on the clinical manifestations you've provided, the most likely diagnosis is **IgG4-Related Disease (IgG4-RD)**. This condition is characterized by a fibro-inflammatory process that can affect multiple organs, including the lacrimal and salivary glands, pancreas, lungs, kidneys, and others, consistent with the symptoms you've described.

Here are five other possible diagnoses that could present with some of the symptoms you've described:

1. **Sarcoidosis**: A multi-system inflammatory disease that often affects the lungs and lymph nodes but can also involve the eyes, salivary glands, and other organs. It can present with pulmonary infiltrates, enlarged lymph nodes, and salivary gland involvement.

2. **Sjogren's Syndrome**: An autoimmune disorder primarily affecting the lacrimal and salivary glands, leading to dry eyes and mouth. It can also involve other organs, and patients may present with glandular enlargement.

3. **Granulomatosis with Polyangiitis (Wegener's Granulomatosis)**: A vasculitis that can present with upper and lower respiratory tract symptoms, renal involvement, and sometimes parotid gland swelling.

4. **Chronic Lymphocytic Inflammation with Pontine Perivascular Enhancement Responsive to Steroids (CLIPPERS)**: A rare CNS inflammatory disorder that could account for some of the neurological symptoms and meningeal enhancement.

5. **Castleman Disease**: A rare disorder that involves lymph node enlargement and can occasionally present with a variety of systemic symptoms, sometimes affecting multiple organs.

These conditions highlight the complexity of assessing multi-organ diseases, and further diagnostic testing, including laboratory work and histopathological examination, would be essential for an accurate diagnosis.

**57. user - 2024-11-27 12:56:30**

Children with cholestasis and cirrhosis develop. The main clinical manifestations are hyperconjugated bilirubinaemia and often malabsorption of fat-soluble vitamins, such as rickets. The clinical phenotypes of different enzyme defects are slightly different. For example, oxysterol 7α-hydroxylase defects are associated with hereditary spastic paralysis in adulthood; cholesterol 25-hydroxylase defects are associated with intractable constipation; 3β-hydroxysteroid -Δ5-C27- steroid dehydrogenase defects are associated with cholestatic liver disease and fat-soluble vitamin deficiencies in infancy; and sterol 27-hydroxylase defects are associated with chronic diarrhoea and developmental delays in children, and spasticity in adulthood. Sterol 27-hydroxylase defects can cause chronic diarrhoea and developmental delay in children, and spastic paralysis in adults; α-formyl-coenzyme A dehydrogenase defects can cause fat-soluble vitamin deficiencies in infancy, and sensory neuropathy in adulthood; and bile acid-coenzyme A-amino acid N-acetyltransferase defects can cause intrahepatic cholestasis, developmental delay, and familial hypercholesterolemia.

**ChatGPT-4o** - 2024-11-27 12:56:30

Based on the clinical manifestations described, the most likely rare disease diagnosis is **3β-hydroxysteroid Δ5-C27-steroid dehydrogenase deficiency**. This condition is characterized by cholestatic liver disease and fat-soluble vitamin deficiencies in infancy, which aligns closely with the symptoms of cholestasis, cirrhosis, hyperconjugated bilirubinaemia, and fat-soluble vitamin malabsorption leading to conditions such as rickets.

Five other possible diagnoses could include:

1. **Sterol 27-hydroxylase deficiency (Cerebrotendinous xanthomatosis)**: Characterized by chronic diarrhea, developmental delays, and spastic paralysis in adulthood.

2. **Oxysterol 7α-hydroxylase deficiency**: Associated with hereditary spastic paralysis in adulthood, which might be relevant if there are neurological symptoms.

3. **Cholesterol 25-hydroxylase deficiency**: Noted for intractable constipation, which could be a secondary symptom or in cases where constipation is present.

4. **α-Formyl-coenzyme A dehydrogenase deficiency**: Involves fat-soluble vitamin deficiencies and can present with sensory neuropathy in adulthood.

5. **Bile acid-coenzyme A-amino acid N-acetyltransferase deficiency**: Causes intrahepatic cholestasis, developmental delay, and familial hypercholesterolemia.

Each of these conditions involves different enzyme defects and has overlapping symptoms, but they can be distinguished by the specific combination of clinical features present in the patient.

**58. user - 2024-11-27 13:07:10**

**1. Acute neonatal type** The onset of the disease is acute and the progression of the disease is rapid and can be life-threatening. Within 1-2 weeks of birth, it presents with feeding difficulties, vomiting, muscle weakness, hypotonia, and lethargy or worsening progression to coma. Accumulation of isovaleric acid may cause the characteristic odour of ‘sweaty feet’. Liver enlargement may be present on examination. Laboratory tests suggest metabolic acidosis, hyperammonemia, hypoglycemia or hyperglycemia, ketosis, hypocalcemia, and pancytopenia. If the patient survives the acute exacerbation in the neonatal period, he or she will progress to the chronic intermittent form.

**The chronic intermittent form** has an insidious onset. It presents only as a nonspecific inability to tolerate fasting or developmental delay. It is often triggered by upper respiratory tract infections or the intake of a high-protein diet, and is characterised by recurrent episodes of vomiting, lethargy, coma, acidosis with ketonuria, and ‘sweaty feet odour’, which can be relieved by a protein-restricted diet and glucose infusion. The vast majority of patients with chronic intermittent form have normal psychomotor development, but some have developmental delays and varying degrees of mental retardation.

**ChatGPT-4o** - 2024-11-27 13:07:10

Based on the clinical manifestations you've described, the most likely diagnosis for the disease is **Isovaleric Acidemia**. This is a rare autosomal recessive metabolic disorder characterized by a deficiency in the enzyme isovaleryl-CoA dehydrogenase, leading to the accumulation of isovaleric acid, which causes the characteristic "sweaty feet" odor.

Five other possible diagnoses that can present with similar symptoms include:

1. **Maple Syrup Urine Disease (MSUD)**: Another metabolic disorder that can present with feeding difficulties, vomiting, lethargy, and a distinctive sweet-smelling urine due to the accumulation of branched-chain amino acids.

2. **Propionic Acidemia**: This condition can also present with metabolic acidosis, hyperammonemia, and vomiting in the neonatal period, and is due to a deficiency in propionyl-CoA carboxylase.

3. **Methylmalonic Acidemia**: Similar to propionic acidemia, it presents with metabolic acidosis and hyperammonemia. It results from defects in methylmalonyl-CoA mutase or related pathways.

4. **Urea Cycle Disorders**: These disorders can cause hyperammonemia, lethargy, and vomiting shortly after birth due to defects in the enzymes involved in the urea cycle.

5. **Glutaric Acidemia Type I**: This condition involves a deficiency in glutaryl-CoA dehydrogenase and can present with irritability, vomiting, and ketosis, along with hypotonia and metabolic acidosis.

These differential diagnoses are based on the overlapping symptoms in the neonatal period, especially those involving metabolic disturbances and characteristic odors. Further biochemical and genetic testing would be essential to confirm the diagnosis.

**59. user - 2024-11-27 13:09:27**

1. dysgenesis of secondary sexual characteristics and impaired gametogenesis. 2.

2. Olfactory disorders Inability to recognise odours, especially aromatic odours, due to abnormal development of the olfactory bulb and olfactory tract.

**ChatGPT-4o** - 2024-11-27 13:09:27

Based on the clinical manifestations you've provided, the most likely rare disease to be diagnosed is Kallmann Syndrome. Kallmann Syndrome is characterized by hypogonadotropic hypogonadism (leading to dysgenesis of secondary sexual characteristics and impaired gametogenesis) and anosmia or hyposmia (olfactory disorders due to abnormal development of the olfactory bulb and tract).

Five other possible diagnoses to consider include:

1. **Congenital Hypogonadotropic Hypogonadism (CHH) without Anosmia**: This is similar to Kallmann Syndrome but without the olfactory disorder component.

2. **Turner Syndrome**: While primarily affecting females and causing dysgenesis of secondary sexual characteristics, it can sometimes present with non-olfactory-related anomalies.

3. **Prader-Willi Syndrome**: Characterized by hypogonadism and sometimes anosmia or altered olfactory perception, though it has a distinct set of other features like hyperphagia and obesity.

4. **Normosmic Idiopathic Hypogonadotropic Hypogonadism (nIHH)**: Similar to Kallmann Syndrome but without anosmia, representing a variant of CHH.

5. **Septo-optic Dysplasia (SOD)**: This can involve midline defects including hypogonadotropic hypogonadism and may sometimes involve olfactory issues, though visual disturbances are more typical.

Each of these conditions has its own specific set of diagnostic criteria and associated features, so a thorough clinical workup and genetic testing may be warranted for a definitive diagnosis.

**60. user - 2024-11-27 13:10:55**

The condition varies greatly from mild to severe and is therefore easily misdiagnosed and underdiagnosed. Clinical manifestations mainly include:

**1. Fever** The fever is irregular and may be periodic or persistent. Patients with hypothalamic involvement may have central thermoregulatory abnormalities.

**2. rash** Mainly on the trunk, scalp and hairline. It begins as a reddish papule, which may then become a haemorrhagic or eczema-like seborrheic rash, followed by crusting, which is followed by a white spot.

**3. Oral and eye, ear, nose and throat lesions** Gum swelling, loose teeth, proptosis, and persistent otitis media with external auditory canal rash are typical.

**4. Central uropathy** Pituitary gland, hypothalamus or even other parts of the central nervous system, with neuropsychiatric symptoms in a few patients.

**5. Respiratory symptoms** Coughing, severe breathlessness, cyanosis, and even recurrent pneumothorax episodes. Pulmonary signs are usually unremarkable.

**6.** The liver and spleen can be enlarged, jaundice, abnormal liver function, liver failure and portal hypertension can occur, and eventually patients can die of cirrhosis and liver failure.

**7. Digestive tract symptoms** Digestive tract involvement is less common and can manifest as polyps or adenomatous changes, usually without clinical symptoms, and abdominal pain, diarrhoea and hypoproteinaemia may occur when the lesions are diffuse.

**8. Haematopoietic system** Lymph nodes may be enlarged, and anaemia, abnormal white blood cell and platelet counts may occur with bone marrow involvement.

**9. Bone damage** Skull, limb bones, spine and pelvis may have pain and lumps, and pathological fractures may occur. Proptosis due to orbital bone lesions is one of the typical manifestations in paediatric patients.

**ChatGPT-4o** - 2024-11-27 13:10:55

Based on the clinical manifestations you've provided, the most likely rare disease diagnosis is **Langerhans Cell Histiocytosis (LCH)**. LCH is a rare disorder characterized by the proliferation of Langerhans cells, which can lead to a wide variety of symptoms depending on which organs are involved.

Here are five other possible diagnoses that could be considered based on your description:

1. **Systemic Lupus Erythematosus (SLE)**: This autoimmune disease can present with a wide range of symptoms, including skin rashes, oral ulcers, fever, hematological abnormalities, and organ involvement.

2. **Wegener's Granulomatosis (Granulomatosis with Polyangiitis)**: This small to medium vessel vasculitis can cause respiratory symptoms, renal involvement, and otolaryngeal manifestations.

3. **Sarcoidosis**: A condition characterized by the growth of tiny collections of inflammatory cells in different parts of the body, which can lead to skin rashes, pulmonary symptoms, and other systemic manifestations.

4. **Hemophagocytic Lymphohistiocytosis (HLH)**: A severe systemic inflammatory syndrome that includes symptoms such as fever, hepatosplenomegaly, cytopenias, and sometimes skin rashes.

5. **Chronic Granulomatous Disease**: A genetic disorder that affects the immune system's ability to fight off certain infections, which can lead to recurrent infections, skin and soft tissue abscesses, and granuloma formation in various organs.

Each of these conditions can present with symptoms that might overlap with those described, and a detailed clinical evaluation, patient history, and appropriate diagnostic tests would be necessary to reach a definitive diagnosis.

**61. user - 2024-11-27 15:19:38**

**1. Growth disorders and other malformations,** most of the children are born with a weight within the normal range, but some of them are born with a length of less than 48cm (42-47cm). After birth, the growth rate gradually and significantly slows down, and the lifelong height of untreated male patients ranges from 116 to 142 cm, and the lifelong height of female patients ranges from 108 to 136 cm. At the same time, the patients' hands, feet and internal organs are small. Some patients have other congenital malformations, such as hip dislocation, aortic valve stenosis, cryptorchidism, and congenital cataracts.

**2. Facial features,** the characteristic facial changes of patients are prominent forehead, saddle nose, small jaw, larger head to body ratio than normal children, but the absolute value of head circumference is still small compared with children of the same age. Hair is thinning, and some patients may have a ‘sunset sign’, with blue sclera in patients from the Mediterranean and Middle Eastern regions. The patient has delayed teething, late deciduous milk teeth and multiple caries, and may have malocclusion due to a short mandible.

**Skeletal musculature** Some patients have congenital hip dislocation and ischaemic necrosis of the femoral head. Due to mid-facial dysplasia and pharyngeal anatomical abnormalities, the patient's speech is high pitched and 85% of the patients have limited elbow joint movement.

**4. Metabolic disorders, **Patients often have a combination of metabolic abnormalities, including hypoglycaemia in the neonatal period and infancy, and progressive obesity in adulthood.

**5. Delayed sexual development , **Patients with this condition have small genitalia, with male patients presenting with small penises and testicles, and female patients having small ovaries on ultrasound. Most patients exhibit delayed pubertal development. Although sexual development is delayed, male and female patients can eventually develop mature sexual function and be fertile.

**6. Low tumour incidence** Patients have a significantly reduced risk of tumour development compared to the general population.

**ChatGPT-4o** - 2024-11-27 15:19:38

Based on the clinical manifestations provided, the most likely diagnosis is **Mulibrey Nanism (Muscle-Liver-Brain-Eye syndrome)**. Mulibrey Nanism is a rare genetic disorder characterized by growth retardation, distinctive facial features, metabolic abnormalities, and delayed sexual development, which align closely with the symptoms you described.

Here are five other possible diagnoses that could also be considered based on the symptoms detailed:

1. **Russell-Silver Syndrome**: A growth disorder characterized by intrauterine growth retardation, postnatal growth deficiency, typical facial features, and limb asymmetry. Some patients may have hypoglycemia as well.

2. **Seckel Syndrome**: Known as "bird-headed dwarfism," this condition involves growth retardation, microcephaly, distinctive facial features, and delayed development.

3. **Noonan Syndrome**: A genetic disorder that can cause short stature, congenital heart defects, distinctive facial features, and a variety of other physical problems, including delayed puberty and cryptorchidism.

4. **Laron Syndrome (Growth Hormone Insensitivity)**: Characterized by short stature, reduced growth rate, some facial dysmorphisms, and often features like small genitals and delayed puberty.

5. **Williams Syndrome**: Though primarily associated with cardiovascular problems, distinctive facial features, and developmental delay, it can sometimes present with growth abnormalities and other endocrine issues.

Each of these conditions shares overlapping features with the symptoms described, but Mulibrey Nanism best fits the detailed complex of signs and symptoms in this case.

**62. user - 2024-11-27 15:22:24**

The main symptom is a sudden, sharp, painless loss of visual acuity in both eyes that occurs sequentially, usually weeks to months between episodes. Funduscopic examination shows congestion and oedema of the optic disc and tortuous dilatation of the peripapillary capillaries in the early stages of the disease, while the oedema and capillary dilatation subside in the later stages of the disease, and eventually the temporal side of the optic disc or all of the optic disc shows atrophic changes. The majority of patients have only ocular manifestations, while a few may have other systemic symptoms such as mental retardation, epilepsy, hearing impairment, dystonia, etc.

**ChatGPT-4o** - 2024-11-27 15:22:24

Based on the clinical manifestations you have provided, the most likely rare disease diagnosis is **Leber Hereditary Optic Neuropathy (LHON)**. LHON is characterized by a sudden, painless loss of vision in one eye, followed by the other eye, often weeks to months later. Fundoscopic findings can include optic disc edema and vascular changes, aligning with your description.

Here are five other possible diagnoses to consider:

1. **Dominant Optic Atrophy**: Typically presents with insidious visual loss and temporal optic disc pallor, though it usually doesn't have the acute onset described.

2. **Multiple Sclerosis (MS) with Optic Neuritis**: Can cause visual loss and optic disc changes, but usually involves pain with eye movement and is often unilateral initially.

3. **Nutritional Optic Neuropathy**: Can cause bilateral vision loss and optic disc changes, but usually with a history of poor nutrition or alcohol abuse.

4. **Mitochondrial Encephalomyopathy, Lactic Acidosis, and Stroke-like episodes (MELAS)**: Can present with optic neuropathy among other neurological symptoms, though it usually has more systemic features.

5. **Susac's Syndrome**: Causes branch retinal artery occlusions, hearing loss, and encephalopathy, but visual changes are usually not bilateral and sequential in the manner described.

Each of these conditions has distinct features that may overlap with the symptoms provided, but LHON remains the most likely based on the key clinical manifestations.

**63. user - 2024-11-27 15:29:24**

The clinical manifestations of patients are low ketogenic hypoglycaemia, liver function abnormalities, lactic acidosis, cardiomyopathy in infancy, growth retardation, hypotonia or cardiomyopathy in some patients, and cholestasis or cirrhosis in a few. In early childhood, some patients show growth retardation, hypotonia or cardiomyopathy, and a few may have cholestasis or cirrhosis. In early childhood, patients may have feeding difficulties, episodic metabolic disorders, and episodic rhabdomyolysis. Most patients will have retinopathy in childhood, and peripheral neuropathy is a less common complication of long-term disease.

**ChatGPT-4o** - 2024-11-27 15:29:24

Based on the clinical manifestations you have provided, the condition is highly suggestive of a mitochondrial disorder, particularly *Mitochondrial Trifunctional Protein Deficiency (MTPD)*, or its related condition, *Long-chain 3-hydroxyacyl-CoA dehydrogenase deficiency (LCHAD deficiency)*. These disorders are characterized by problems in fatty acid oxidation, which can lead to symptoms like hypoketotic hypoglycemia, cardiomyopathy, liver dysfunction, and episodic metabolic crises.

Here are five other possible diagnoses that might fit the provided clinical picture:

1. **Glutaric Aciduria Type II (GA2)**, also known as Multiple Acyl-CoA Dehydrogenase Deficiency: This disorder also affects the body's ability to break down certain fats and proteins, leading to a variety of symptoms including metabolic crises, hypoglycemia, and muscle weakness.

2. **Carnitine Palmitoyltransferase II Deficiency (CPT II Deficiency)**: This disorder also involves fatty acid oxidation defects, presenting with recurrent episodes of rhabdomyolysis, myopathy, and hypoglycemia.

3. **Zellweger Spectrum Disorders**: A group of peroxisomal biogenesis disorders that can cause a range of symptoms including developmental delay, hypotonia, liver dysfunction, and vision problems due to retinopathy.

4. **Infantile Refsum Disease**: Another peroxisomal disorder that can manifest with hypotonia, growth retardation, liver disease, and vision problems like retinopathy.

5. **Glycogen Storage Disease Type IV (Andersen’s Disease)**: This disease can lead to liver cirrhosis, hypotonia, cardiomyopathy, and growth retardation, though it primarily affects glycogen metabolism rather than fatty acid oxidation.

Each of these conditions has overlapping symptoms, but the presence of hypoketotic hypoglycemia, liver dysfunction, cardiomyopathy, and retinopathy points strongly toward a mitochondrial fatty acid oxidation defect, such as MTPD or LCHAD deficiency. Accurate diagnosis would require biochemical testing, genetic analysis, and possibly a muscle or liver biopsy to confirm the specific disorder.

**64. user - 2024-11-27 15:33:17**

It occurs predominantly in adult women of childbearing age, with the average age of diagnosis being around 40 years. Early symptoms are mild, and some patients are detected during a physical examination or on HRCT of the lungs for respiratory symptoms or other reasons. The main manifestation is dyspnoea of varying degrees, which worsens as the disease progresses, with progressive deterioration of lung function and respiratory failure in the later stages of the disease. Complications such as pneumothorax and coeliac chest may occur repeatedly during the course of the disease. Extrapulmonary manifestations include renal, retroperitoneal solid or cystic solid lymphangioleiomyoma. Some patients have other clinical features of multisystem involvement, including neurological changes (epilepsy, neurodevelopmental delays, autism, etc.) and cutaneous changes (depigmentation, facial angiofibromas, cutaneous sharkskin plaques, and perinodal fibromas, etc.).

**ChatGPT-4o** - 2024-11-27 15:33:17

Based on the clinical manifestations you've described, the most likely rare disease diagnosis is Lymphangioleiomyomatosis (LAM). LAM is a rare, progressive lung disease that primarily affects women, often those of childbearing age. It is characterized by abnormal smooth muscle cell proliferation, leading to cystic lung destruction and respiratory issues such as dyspnea and pneumothorax. The extrapulmonary manifestations involving kidneys and retroperitoneal areas, along with the neurological and cutaneous changes, suggest a potential association with Tuberous Sclerosis Complex (TSC), as LAM can occur sporadically or in association with TSC.

Here are five other possible diagnoses based on the symptoms provided:

1. **Tuberous Sclerosis Complex (TSC):** Given the neurological and skin manifestations, this genetic disorder may be considered, especially since LAM can be a part of TSC.

2. **Birt-Hogg-Dubé Syndrome:** This syndrome involves skin lesions, lung cysts leading to pneumothorax, and renal tumors, making it a differential diagnosis, although the neurological symptoms are less typical.

3. **Pulmonary Langerhans Cell Histiocytosis (PLCH):** A rare lung disease that might present with dyspnea and pneumothorax, primarily affecting young adults, although it typically lacks the extrapulmonary manifestations mentioned.

4. **Neurofibromatosis Type 1 (NF1):** This genetic disorder involves skin manifestations and potential lung involvement, but the specific combination of symptoms might not fully align without other characteristic signs like café-au-lait spots and neurofibromas.

5. **Churg-Strauss Syndrome (Eosinophilic Granulomatosis with Polyangiitis):** Although less likely due to the lack of prominent asthma and allergic features, it can involve lung issues and neurological symptoms.

Each of these alternative diagnoses has its own characteristic features and would require further investigation through clinical history, imaging, and genetic testing to confirm.

**65. user - 2024-11-27 15:37:25**

Infants typically experience repeated vomiting and diarrhea after weaning and refuse protein-rich foods.Patients may present with pale skin, hypotonia, hepatosplenomegaly, thinning hair, osteoporosis and pathological fractures.After eating a protein-rich diet, patients may experience lethargy, ataxia, seizures, and hyperammonemic coma.As the disease progresses, multiple systems may be involved.Lung involvement can be manifested as progressive interstitial lung disease, alveolar proteinosis; the most common characteristic changes in the kidney are proximal tubular dysfunction and renal calcinosis, glomerulopathy, renal amyloidosis, and even renal failure; blood system involvement can be manifested as positive or low pigment anemia, leukopenia, thrombocytopenia, etc.; Digestive system involvement is manifested by hypercholesterolemia, hypertriglycerides, recurrent pancreatitis, and even hepatic amyloidosis.Epilepsy occurs mostly in adult patients and may be related to repeated hyperammonemia leading to cerebral cortex damage.

**ChatGPT-4o** - 2024-11-27 15:37:25

Based on these clinical manifestations, the rare disease most likely to be diagnosed is **Lysinuric Protein Intolerance (LPI)**. LPI is characterized by the inability to properly digest and metabolize certain amino acids (lysine, arginine, and ornithine), leading to a range of symptoms after protein ingestion, including hyperammonemia and the involvement of multiple organ systems.

Five other possible diagnoses to consider include:

1. **Cystinuria**: A disorder that affects renal handling of certain amino acids leading to kidney stones, though it typically doesn't involve the broader systemic manifestations described here.

2. **Ornithine Transcarbamylase Deficiency (OTC)**: An X-linked disorder that can cause hyperammonemia, vomiting, and lethargy after protein intake, but typically does not have the extensive organ involvement like bone, hair, or interstitial lung disease.

3. **Hyperornithinemia-Hyperammonemia-Homocitrullinuria (HHH) Syndrome**: A urea cycle disorder that leads to a buildup of ornithine and ammonia, causing neurological issues and possibly hepatomegaly, but usually not the full multi-system involvement described.

4. **Hunter Syndrome (MPS II)**: A mucopolysaccharidosis that can cause multi-system involvement including hepatosplenomegaly, though it often presents with dysmorphic features not described here, and usually doesn't involve hyperammonemia.

5. **Gaucher Disease**: A lysosomal storage disorder that can mimic some of the systemic manifestations including bone issues and hematological abnormalities, though it typically doesn't cause hyperammonemia or issues specifically tied to protein intake.

Each of these conditions has distinct differences and would require further diagnostic testing to confirm or exclude.

**66. user - 2024-11-27 15:39:40**

The onset is neonatal, usually within a week of birth.Recurrent vomiting, diarrhea, abdominal distension and other gastrointestinal symptoms were the first symptoms, accompanied by hepatosplenomegaly and growth disorders; nervous system involvement was manifested as hypoactivity, tendon hyperreflexia, ankle clonus and angular arch opisthotonus.Macrophage cholesterol ester and triglyceride accumulation leads to hepatosplenomegaly, progressive aggravation and even liver failure, elevated transaminases, bilirubin, hypoproteinemia, coagulation abnormalities, etc. within months.Adrenal enlargement with calcification leads to adrenal cortical insufficiency, and 60% to 70% of adrenal calcifications can be detected by imaging.Gallbladder dysfunction and stroke have also been reported.As the disease progresses, multiple organ dysfunction can occur, including anemia, gastrointestinal bleeding, sepsis, coma, etc.

**ChatGPT-4o** - 2024-11-27 15:39:40

Based on the clinical manifestations provided, the most likely diagnosis for this condition is Wolman disease. Wolman disease is a rare lysosomal storage disorder caused by a deficiency of the enzyme lysosomal acid lipase (LAL), leading to the accumulation of cholesterol esters and triglycerides within various tissues. The signs and symptoms you described align well with this condition, including early onset, gastrointestinal symptoms, hepatosplenomegaly, adrenal calcification, and severe multisystem involvement.

Five other possible diagnoses could include:

1. **Niemann-Pick Disease Type C**: Another lysosomal storage disorder characterized by cholesterol accumulation, though it typically presents later in childhood and has more pronounced neurological symptoms.

2. **Gaucher Disease Type 2**: An early-onset form of Gaucher disease that presents with hepatosplenomegaly and severe neurological involvement, though adrenal calcification is not typical.

3. **Zellweger Syndrome**: A peroxisomal disorder that can present with hepatosplenomegaly, adrenal dysfunction, and neurological symptoms, but typically has distinctive craniofacial abnormalities.

4. **Lysosomal Acid Lipase Deficiency (LAL-D)**: While Wolman disease is a severe form of LAL-D, the condition has a spectrum, and milder forms might present differently.

5. **Congenital Adrenal Hyperplasia (CAH)**: While primarily an endocrine disorder, certain forms can present early with adrenal insufficiency and possibly hepatomegaly, but the lipid accumulation pattern is not typical.

These conditions share overlapping features but differ in the specific enzyme deficiencies, metabolic pathways affected, and consequent symptomatology.

**67. user - 2024-11-27 15:42:35**

There are five types, of which the classic and intermediate types are the most common.

1. Classic type is the most common, accounting for 75 per cent of cases. Children are normal at birth, with early onset, severe symptoms and rapid progression. Usually, maple syrup odour appears in the cerumen 12 hours after birth; a special maple sugar odour is found in urine and sweat 12-24 hours after birth; ketonuria, irritability and feeding difficulties appear in 2-3 days; severe metabolic disorders and encephalopathic symptoms begin to appear in 4-5 days, which are manifested as difficulty in breastfeeding, paroxysmal vomiting, refusal to eat, lethargy, apnoea, convulsive seizures, heightened muscle tone and ‘fencing’. The symptoms of severe metabolic disorders and encephalopathy may include difficulty in breastfeeding, refusal to eat, lethargy, apnoea, convulsive seizures, increased muscle tone, and stereotypical movements such as ‘fencing’-like and ‘stomping’-like movements, etc. Coma and central respiratory failure may appear in the first 7-10 days of life, and most of the infants will die of severe metabolic disorders a few days after birth if they are not treated promptly.

2. Intermediate type can develop at any age. The symptoms are less severe than those of the classic type and include slow growth, delayed development, feeding difficulties, irritability, a maple syrup odour in the cerumen, and severe metabolic disorders and encephalopathy under stress. 3. Intermittent Type The intermittent type is characterised by episodic seizures.

3. Intermittent type: intermittent seizures, normal growth and development in the early stage, mostly under stressful situations such as infections and surgeries, manifested by episodic ataxia and ketoacidosis, and a very small number of severe cases may cause death. Branched-chain amino acid concentrations are normal during the intermittent period, and are elevated during the episodic period, similar to the classic type.

**ChatGPT-4o** - 2024-11-27 15:42:35

Based on the clinical manifestations you've described, the most likely diagnosis is **Maple Syrup Urine Disease (MSUD)**. This rare metabolic disorder is characterized by the inability to break down certain amino acids, which leads to the symptoms you've detailed, such as the distinctive maple syrup odor in cerumen, urine, and sweat, along with severe metabolic disturbances.

Here are five other possible diagnoses, although they are less likely given the specific symptoms described:

1. **Phenylketonuria (PKU)**: A metabolic disorder characterized by the inability to break down phenylalanine, leading to intellectual disability and other symptoms without dietary management. However, it lacks the classic maple syrup odor.

2. **Isovaleric Acidemia**: A disorder of leucine metabolism that may present with a "sweaty feet" odor due to isovaleric acid accumulation and can cause episodes of metabolic crisis.

3. **Propionic Acidemia**: Another disorder of amino acid metabolism, causing vomiting, lethargy, and metabolic acidosis, but without the specific maple syrup odor.

4. **Methylmalonic Acidemia**: Similar to propionic acidemia, it involves defects in propionate metabolism and results in metabolic acidosis and neurological symptoms.

5. **Glutaric Acidemia Type I**: A metabolic disorder involving the breakdown of lysine, hydroxylysine, and tryptophan, leading to neurological symptoms and metabolic crises.

While these conditions share some overlapping symptoms with MSUD, particularly in terms of metabolic crises, the specific combination of symptoms and the characteristic odor make MSUD the most fitting diagnosis for your description.

**68. user - 2024-11-27 15:44:27**

**1. The cardiovascular system** can result in aortic root dilatation, aortic valve insufficiency and aortic coarctation; mitral valve prolapse with or without insufficiency may also occur. The prevalence of ascending aortic dilatation increases with age. Between the ages of 30 and 60, the prevalence increases from 53% to 96%. Patients may present with shortness of breath, chest pain, palpitations, dizziness, syncope, or haemoptysis. Physical examination reveals an enlargement of the heart border to the lower left, a systolic murmur in the mitral valve area and a diastolic predominantly biphasic murmur in the aortic valve area, peripheral vascular signs, and signs of heart failure.

**2. Skeletal manifestations** , tall and thin stature, elongated fingers and toes, arms extended beyond height, scoliosis or kyphosis of the spine, funnel chest or chicken chest, laxity of the joints, flat feet.

**3. Ocular abnormalities**, eye lesions account for 50% to 70% of the cases, including corneal flattening, prolongation of the eyeball axis, retinal detachment, cataracts, subluxation or subluxation of the lens, iris nystagmus or mild nystagmus, pupil displacement, and clouding of the lens, etc. **4.

**4. Other , ** dural bulge, atrophic striae, recurrent or incisional hernia, high palpebral vault, etc.

**ChatGPT-4o** - 2024-11-27 15:44:27

Based on the clinical manifestations provided, the most likely diagnosis is **Marfan Syndrome**. Marfan Syndrome is a genetic disorder affecting connective tissue, characterized by cardiovascular, skeletal, and ocular manifestations similar to those you've described.

Here are five other possible diagnoses that could present with some overlapping features:

1. **Ehlers-Danlos Syndrome**: This group of disorders affects connective tissues supporting the skin, bones, blood vessels, and other organs and tissues. Some types can have cardiovascular, skeletal, and ocular features similar to Marfan Syndrome, such as joint hypermobility and skin hyperextensibility.

2. **Loeys-Dietz Syndrome**: Another connective tissue disorder characterized by aortic aneurysms, skeletal abnormalities (such as scoliosis), and other features that overlap with those of Marfan Syndrome, including ocular issues.

3. **Homocystinuria**: This metabolic disorder can lead to symptoms similar to Marfan Syndrome, including tall stature, lens dislocation, and skeletal abnormalities, but is also associated with intellectual disability and thromboembolic events.

4. **Stickler Syndrome**: This condition primarily affects the eyes, ears, and joints. It can lead to joint problems and ocular issues like retinal detachment, though it typically does not have the cardiovascular issues seen in Marfan Syndrome.

5. **Beals Syndrome (Congenital Contractural Arachnodactyly)**: Similar to Marfan Syndrome, this condition features tall stature, joint contractures, and similar skeletal features, but typically lacks the significant cardiovascular manifestations.

It's important to conduct genetic testing and a thorough clinical evaluation to differentiate between these conditions, as they may require different management strategies.

**69. user - 2024-11-27 15:46:39**

**1. Skin manifestations**, patients are usually born with café au lait spots, often located on the same side as the skeletal lesions and bounded by the midline, which are brown or yellowish brown patches of pigmentation of varying sizes in one or more spots, with irregular borders.

**Skeletal manifestations** , approximately 98% of patients have either monostotic or polyostotic osteofibrous heteroplasia, with the polyostotic type being more common. It is characterised by an increase in the number of aberrant osteoblasts and their abnormal differentiation, causing extensive proliferation and differentiation of fibroblasts in the bone marrow and the formation of infantile woven bones, which are prone to pathological fractures and skeletal deformities and cause bone pain. It can involve almost all bones of the body, especially craniofacial and long bones, and the local bones are swollen and elevated, resulting in changes in the structure of the face and symptoms of compression. Severe orbital involvement may compress the optic nerve and cause visual impairment. Involvement of the auditory nerve may cause hearing loss. Involvement of the spine may result in vertebral compression fractures, causing scoliosis or kyphosis. The peak age for pathological fractures due to osteofibrous heteroplasia is 6-10 years. Imaging shows expansive, osteolytic, or ground-glass changes in the bone.

**Involvement of endocrine glands** may involve a variety of endocrine glands, resulting in hyperfunction or tumours of the affected glands and normal or decreased serum levels of pro-secretory hormones. Precocious puberty is more common and can occur in both sexes, but is more common in females and often precedes bone lesions. Female children may show early development of secondary sexual characteristics, premature irregular vaginal bleeding, enlarged uterus and ovaries compared with normal children of the same age, or ovarian cysts, premature healing of epiphyses, and so on. In males, precocious puberty is relatively rare, and testicular lesions (Leydig cell hyperplasia, microscopic stones, focal calcification, etc.) are seen on ultrasound. Thyroid enlargement and hyperfunction are also relatively common, and hyperparathyroidism has been reported. Other endocrine disorders such as cortisolism, gigantism / acromegaly, hyperprolactinaemia have been reported but are relatively rare.

**4. Hypophosphatemia** , a combination of hypophosphatemia, manifesting as hypophosphatemic rickets, has been reported in some patients. Those with combined hypophosphataemia usually have a polyostotic type of abnormal bone fibre proliferation.

**5. Other,** patients may also present with rare clinical symptoms such as jaundice, hepatitis, arrhythmias, and intestinal polyps due to mutations involving peripheral blood leukocytes, liver, heart, thymus, and gastrointestinal tract.

**ChatGPT-4o** - 2024-11-27 15:46:39

Based on the clinical manifestations you have provided, the most likely rare disease diagnosis is **McCune-Albright Syndrome**. This condition is characterized by the triad of café-au-lait spots, polyostotic fibrous dysplasia, and various endocrine abnormalities, including precocious puberty. The combination of skin, skeletal, and endocrine manifestations aligns closely with this syndrome.

Five other possible diagnoses to consider could include:

1. **Neurofibromatosis Type 1 (NF1)**: This condition can also present with café-au-lait spots and skeletal abnormalities; however, the fibrous dysplasia and endocrine issues are not typical features.

2. **Fibrous Dysplasia (Without McCune-Albright)**: This condition involves similar skeletal manifestations but lacks the endocrine and café-au-lait features unless it's part of the McCune-Albright syndrome.

3. **Proteus Syndrome**: A rare condition that might present with asymmetric overgrowth and bone abnormalities, though it typically does not include endocrine abnormalities or café-au-lait spots.

4. **Paget’s Disease of Bone**: This involves abnormal bone remodeling and can lead to bone pain, deformities, and increased fractures but usually occurs later in life and lacks the café-au-lait spots and endocrinopathies.

5. **Cherubism**: This genetic disorder involves fibrous dysplasia of the jaw, which can lead to facial disfigurement and other skeletal changes, but it does not include endocrine issues or café-au-lait spots.

Each of these conditions has overlapping features with McCune-Albright Syndrome but lacks the full complement of symptoms described in your case.

**70. user - 2024-11-27 15:48:53**

The majority of patients have onset of symptoms between 3 months and 3 years of age, with a small percentage having onset in the neonatal period or in adulthood, while others are asymptomatic. The onset of the disease is usually triggered by a predisposing factor, such as prolonged starvation or a complication of an infectious disease. In children with early onset of the disease, the first symptoms are drowsiness and vomiting, but may also include convulsions and asphyxia, which often progress rapidly to coma or death. Acute onset hypoglycaemia is typical, and a decrease in blood glucose with a negative urine ketone body is helpful in the diagnosis. 50% of children have hepatomegaly, and liver biopsy reveals a large accumulation of lipids. Imaging reveals cerebral oedema in some children. Cardiac damage is rare. Adult-onset patients present with a wide range of clinical manifestations, including multiple organ damage, including muscle, liver, nervous system, or cardiovascular system. Vomiting is the most common symptom, hepatomegaly is relatively rare, and patients presenting with symptoms during pregnancy show acute fatty liver. Most patients have acute non-inflammatory encephalopathy. Rhabdomyolysis occurs in about one-third of patients, with a significant increase in creatine kinase and myoglobinuria. There is a high mortality rate, with approximately 25% of children dying from the onset of the disease, and an even higher rate of 50% in adults with acute onset of the disease. Patients tend to behave normally between episodes. About one third of patients who survive the acute onset of the disease develop sequelae, including growth retardation, motor retardation, mental retardation, language deficits, psycho-behavioural problems, epilepsy, cerebral palsy, hemiparesis, and chronic muscle weakness. In addition, some patients have complaints of skeletal muscle damage such as fatigue, myalgia and reduced exercise tolerance.

**ChatGPT-4o** - 2024-11-27 15:48:53

Based on the provided clinical manifestations, the disease most likely to be diagnosed is Medium-Chain Acyl-CoA Dehydrogenase Deficiency (MCADD). This rare metabolic disorder is known for causing hypoglycemia without ketonuria, often triggered by fasting or illness. The symptoms, including drowsiness, vomiting, convulsions, and coma, as well as liver involvement and acute non-inflammatory encephalopathy, align well with MCADD.

Here are five other possible diagnoses:

1. **Reye Syndrome**: This condition involves acute non-inflammatory encephalopathy and liver dysfunction, often following viral infections, with symptoms like vomiting, confusion, seizures, and potential progression to coma.

2. **Carnitine Palmitoyltransferase II (CPT II) Deficiency**: This disorder affects fatty acid oxidation and can lead to hypoglycemia, muscle pain, rhabdomyolysis, and myoglobinuria, especially after prolonged fasting or exercise.

3. **Glutaric Acidemia Type II**: Also known as Multiple Acyl-CoA Dehydrogenase Deficiency, this disorder can cause hypoglycemia, metabolic acidosis, hepatomegaly, and muscle weakness, with symptoms triggered by fasting or illness.

4. **Fatty Acid Oxidation Disorders (general)**: Other disorders in this category, such as Very-Long-Chain Acyl-CoA Dehydrogenase Deficiency (VLCADD) or Long-Chain 3-Hydroxyacyl-CoA Dehydrogenase Deficiency (LCHADD), share similar symptoms, including hypoglycemia, rhabdomyolysis, and liver dysfunction.

5. **Mitochondrial Trifunctional Protein Deficiency**: This disorder also affects fatty acid oxidation, leading to episodes of hypoglycemia, liver dysfunction, rhabdomyolysis, and potential cardiac involvement.

The clinical presentation is crucial for narrowing down the diagnosis, and confirmatory genetic or enzymatic testing is typically required to distinguish between these conditions.

**71. user - 2024-11-27 16:05:51**

The clinical manifestations of the disease vary among age groups. The earlier the age of onset, the more severe the acute metabolic disorders and encephalopathy. Clinical manifestations include gastrointestinal symptoms such as vomiting, feeding difficulties and hepatomegaly, as well as neurological symptoms such as dyskinesia, impaired consciousness, convulsions, delayed or regressed growth and microcephaly. Recurrent illness is mostly associated with infection and stress.

Neonatal-onset patients usually present with acute encephalopathy-like symptoms in the first few hours to one week after birth, which are manifested by vomiting, hypotonia, dehydration, severe acidosis, hyperlactatemia, hyperammonemia, coma, and convulsions, with a high morbidity and mortality rate. Childhood onset of the disease is normal at birth, mostly within 1 year of age, the first metabolic crisis is often triggered by infection, hunger, fatigue, vaccination and other stress factors, or high-protein diets and medications, which, if not diagnosed and treated in a timely manner, can lead to retardation, backwardness, and regression of mental and motor development, and can be accompanied by involvement of the haematological system, the liver, the kidneys, the skin, and peripheral nerves. In adults, the first symptoms may include peripheral neuropathy and psychiatric abnormalities.

**ChatGPT-4o** - 2024-11-27 16:05:51

Based on the clinical manifestations you've described, the disease most likely to be diagnosed is **Maple Syrup Urine Disease (MSUD)**. This rare metabolic disorder is characterized by the body's inability to properly break down certain amino acids, leading to the acute metabolic crises and neurological symptoms you've outlined, particularly in the neonatal period. The description fits with the severe metabolic derangements such as acidosis, hyperammonemia, and the involvement of neurological symptoms and other organ systems you mentioned.

Here are five other possible diagnoses:

1. **Urea Cycle Disorders**: These disorders, such as ornithine transcarbamylase deficiency, can cause hyperammonemia and neurological symptoms, often presenting acutely in newborns.

2. **Methylmalonic Acidemia**: This is a type of organic acidemia which can present with similar acute metabolic crises, neurological symptoms, and multi-system involvement.

3. **Propionic Acidemia**: Another organic acidemia, it shares overlapping features with methylmalonic acidemia, including acute metabolic disturbances and developmental delays.

4. **Isovaleric Acidemia**: This condition can cause acute encephalopathic crises, often triggered by protein intake or stress, and presents with metabolic acidosis and hyperammonemia.

5. **Glutaric Aciduria Type I**: This rare metabolic disorder may present with macrocephaly initially but can progress to encephalopathic crises, often triggered by stress or infection, and affects neurological development.

These disorders often require a detailed metabolic and genetic workup for accurate diagnosis due to their overlapping clinical features.

**72. user - 2024-11-27 16:08:24**

The disease can start from birth to old age and can be acute or insidious. Almost any system or organ can be involved, with a predominance of energy-demanding organs such as the brain, heart, kidneys, eyes, ears, muscles, and gastrointestinal tract. Patients may present with a clinical syndrome of some kind, a single symptom or a combination of symptoms.

**ChatGPT-4o** - 2024-11-27 16:08:24

Based on the clinical manifestations provided, the symptoms suggest a possible mitochondrial disease. Mitochondrial diseases are a group of disorders caused by dysfunctional mitochondria, which are the energy-producing structures within cells. These disorders can affect multiple systems, especially those with high-energy demands, such as the brain, heart, muscles, and other organs you mentioned.

Here are five other possible diagnoses that could also fit the broad and variable symptom presentation:

1. **Lysosomal Storage Disorders** - These are a group of metabolic disorders resulting from defects in lysosomal function, which can similarly affect multiple organ systems and have a wide range of symptoms.

2. **Peroxisomal Disorders** - These involve dysfunctions in peroxisomes, another type of cell organelle, leading to symptoms that can affect various organs, especially the nervous system and liver.

3. **Congenital Disorders of Glycosylation (CDG)** - These are a group of inherited metabolic disorders that lead to problems in glycosylation, affecting numerous systems including the nervous system, liver, and muscles.

4. **Metabolic Myopathies** - This group includes conditions like glycogen storage diseases and fatty acid oxidation disorders, primarily affecting muscle and sometimes leading to multi-systemic involvement.

5. **Multi-systemic Atrophy Disorders** - Disorders like multi-system atrophy can present with various neurological symptoms that affect multiple systems and can have a variable onset.

These conditions can be challenging to diagnose due to their broad and overlapping symptoms across multiple organ systems.

**73. user - 2024-11-27 16:11:17**

The typical patient is normal at birth. At the age of 6 months to 1 year, the patient's face gradually becomes ugly, with flattening in the middle of the face, widening of the bridge of the nose, clouding of the cornea, deafness, large head, long anterior and posterior diameters, protrusion of the forehead, stiffness of the joints, claw shaped hands, chicken chest, and lumbar lordosis. Heart valve thickening, cardiomyopathy or congestive heart failure often appear at the age of 2 to 5 years, and liver and spleen enlargement at the age of 5 years. Mental and motor development is backward. They usually survive until about 10 years of age. Most of them die of heart failure and pneumonia. In the mild form, symptoms appear after the age of 5 years and include mild coarse facial features, corneal clouding, and stiffness of the joints of the hands and feet. Intelligence is normal and life expectancy is relatively long. Clinical symptoms overlap between subtypes, but there are no significant differences in biochemical findings between subtypes.

**ChatGPT-4o** - 2024-11-27 16:11:17

Based on the clinical manifestations provided, the most likely diagnosis is **Mucopolysaccharidosis Type I (Hurler Syndrome for the severe form and Hurler-Scheie or Scheie Syndrome for attenuated forms)**. This rare genetic disorder is characterized by the accumulation of glycosaminoglycans (GAGs) due to the deficiency of the enzyme alpha-L-iduronidase. The symptoms and progression you described align closely with the severe and attenuated forms of MPS I.

Here are five other possible diagnoses that could be considered based on the symptoms presented:

1. **Mucopolysaccharidosis Type II (Hunter Syndrome)**: Similar to MPS I but X-linked recessive and typically lacks corneal clouding. It presents with coarse facial features and joint stiffness, among other symptoms.

2. **Mucopolysaccharidosis Type VI (Maroteaux-Lamy Syndrome)**: This condition also involves corneal clouding, skeletal abnormalities, and cardiomyopathy, but typically normal intelligence.

3. **Mucopolysaccharidosis Type VII (Sly Syndrome)**: Similar to other MPS disorders, it includes developmental delay, skeletal changes, and organomegaly, but tends to be rarer and with variable presentation.

4. **Gaucher Disease Type 3**: This lysosomal storage disorder can present with some overlapping symptoms, such as organomegaly, joint stiffness, and neurological involvement, though typically there are different biochemical findings and a broader range of symptoms.

5. **I-cell Disease (Mucolipidosis II)**: Presents with severe developmental delay, skeletal abnormalities, coarse facial features, and cardiomyopathy similar to MPS, but with distinct biochemical profiles due to defects in the mannose-6-phosphate pathway.

In clinical practice, specific biochemical tests and genetic analysis would be critical to confirm the diagnosis and differentiate between these conditions.

**74. user - 2024-11-27 16:14:06**

Insidious onset of disease. The disease develops slowly or progresses in stages and may have long periods of stability. The disease can develop at any age, mostly before 50 years old. Clinical manifestations are mainly multiple mononeuropathies, asymmetric distal upper limb is more common, early unilateral upper limb one or more nerves involved, manifested as muscle weakness of the corresponding innervated area, distal predominantly, may be accompanied by spasm or fasciculations. With the development of the disease, muscle atrophy can occur; with a longer course of the disease, multiple nerves of multiple limbs can be involved, and the asymmetry of the affected nerves may not be obvious, but presenting a distribution similar to that of polypoidal peripheral neuropathy. Patients may complain of mild sensory abnormalities, but there is a lack of objective signs of sensory involvement, and some patients may also have partial sensory nerve involvement in the later stages of the disease. Cerebral nerves are usually not involved. There are no signs of upper motor neuron involvement.

**ChatGPT-4o** - 2024-11-27 16:14:06

Based on the clinical manifestations you've described, the most likely diagnosis is **Multifocal Motor Neuropathy (MMN)**. This rare disease is characterized by asymmetric muscle weakness, primarily affecting the distal upper limbs, with fasciculations and atrophy over time, and typically without sensory involvement or upper motor neuron signs.

Five other possible diagnoses to consider might include:

1. **Lewis-Sumner Syndrome (MADSAM variant of CIDP, or Multifocal Acquired Demyelinating Sensory and Motor Neuropathy)**: While it often has more sensory involvement than MMN, it can present with asymmetric multifocal neuropathy.

2. **Amyotrophic Lateral Sclerosis (ALS), primarily the lower motor neuron variant**: ALS typically involves both upper and lower motor neurons, but early on, some patients may present predominantly with lower motor neuron symptoms and asymmetric weakness.

3. **Hereditary Neuropathy with Liability to Pressure Palsies (HNPP)**: Although classically involves recurrent pressure palsies, it can present with asymmetric neuropathic symptoms.

4. **Isolated mononeuritis multiplex**: This is a pattern of nerve damage that can be due to various causes (e.g., vasculitis, diabetes), presenting with asymmetric motor and sensory deficits.

5. **Paraneoplastic Neuropathy**: Though usually more sensory, some paraneoplastic syndromes can present predominantly with motor neuropathy and mimic MMN.

Each of these conditions would require distinct diagnostic approaches, including clinical evaluation, electrophysiological studies, and possibly laboratory and genetic testing, to distinguish them from MMN.

**75. user - 2024-11-27 16:16:09**

Neonatal onset is often fatal and may present as a metabolic crisis including metabolic acidosis, non-ketotic hypoglycaemia, hyperammonaemia, and other features including multisystem involvement (hypotonia, cardiomyopathy, hepatomegaly), excretion of large amounts of fatty acid- and amino acid-derived metabolites, ‘sweaty-foot’ body odour, coma, and neonatal death. Rapidly progressive hypertrophic cardiomyopathy is common in the type with neonatal onset and has a very high probability of sudden death, which is difficult to reverse, even if the disease is detected at an early stage by neonatal screening.

Congenital malformations are thought to be the consequence of the accumulation of toxic metabolites in the part of the body not corrected by embryonic transport. The degree of impaired fatty acid oxidative metabolism determines the degree of acidosis in utero, which in turn prevents normal embryo formation. Congenital malformations include enlarged polycystic kidneys, rocker-bottom feet, absence of lower abdominal muscles, hypospadias and penile curvature, hypotonia, cerebral cortical dysplasia, and gliosis. Other dysmorphic features include wide eye spacing, ear malformations, megalocrania, wide fontanel, high forehead, and low nasal bridge.

The age of onset and symptoms of late-onset forms vary considerably among cases and are mainly characterised by the absence of congenital malformations but with a lifelong risk of acute intermittent episodes of vomiting, dehydration, hypo-ketotic hypoglycaemia, acidosis, and impaired consciousness. Older individuals may have hepatomegaly or lipid deposition myopathy. Acute loss-of-compensation episodes can be triggered by infection, fever, surgery, low-energy diets or weight loss, alcohol consumption, and pregnancy. Most patients develop chronic myopathy symptoms, including exercise intolerance, myalgia, muscle weakness, and muscle atrophy. Organic aciduria in patients with late-onset disease is often intermittent and is more pronounced only during disease and under catabolic stress.

**ChatGPT-4o** - 2024-11-27 16:16:09

Based on the clinical manifestations provided, the most likely diagnosis is a mitochondrial fatty acid oxidation disorder, specifically Glutaric Acidemia Type II (also known as Multiple Acyl-CoA Dehydrogenase Deficiency, or MADD). This disorder is known for its neonatal-onset form that presents with severe metabolic crises, non-ketotic hypoglycemia, hyperammonemia, and characteristic multisystem involvement such as cardiomyopathy and hepatomegaly. The description of a "sweaty-foot" odor is particularly indicative of MADD due to the accumulation of specific organic acids.

Here are five other possible diagnoses:

1. **Medium-Chain Acyl-CoA Dehydrogenase Deficiency (MCAD Deficiency):** While typically presenting with hypoketotic hypoglycemia and a risk of sudden death, it usually lacks the severe congenital malformations described.

2. **Propionic Acidemia:** Another metabolic disorder that presents with metabolic acidosis and can cause neonatal metabolic crises. It may also include hypotonia and cardiac issues, though the specific malformations and odor described are less characteristic.

3. **Methylmalonic Acidemia:** This disorder can also present with similar metabolic disturbances and cardiomyopathy but usually involves elevated methylmalonic acid levels.

4. **Carnitine Palmitoyltransferase II Deficiency (Neonatal form):** Known for causing severe neonatal metabolic crises, with hypoketotic hypoglycemia and cardiomyopathy, but generally lacking the specific malformations and odor.

5. **Holocarboxylase Synthetase Deficiency:** A form of multiple carboxylase deficiency that can present with metabolic crises, skin rash, and developmental delay, but usually has other distinct biochemical findings.

Each of these disorders has overlapping features with the described condition but lacks the full constellation of findings presented in Glutaric Acidemia Type II, especially the combination of clinical and biochemical features such as the "sweaty-foot" odor and specific congenital malformations.

**76. user - 2024-11-27 16:18:44**

The average age of onset of the disease is 30 years old, and it is rare to find patients under 10 years old and over 50 years old. The prevalence of women is higher than that of men, with the ratio of women to men (1.4-2.3):1. The disease is mostly acute/subacute, and is characterised by spatial and temporal multiplicity. The spatial multiplicity refers to the multiple sites of the lesions, including the brain, brainstem, cerebellum, and spinal cord, which may be involved simultaneously or successively. Time-recurrent refers to the course of remission-relapse, and due to the wide range of sites involved, repeated episodes may eventually lead to disability or even death. Clinical manifestations are varied, and common symptoms include vision loss, diplopia, limb sensory deficits, limb motor deficits, ataxia, and bladder or rectal dysfunction.

**ChatGPT-4o** - 2024-11-27 16:18:44

Based on the clinical manifestations and demographic profile you provided, the most likely rare disease could be Multiple Sclerosis (MS). The description fits MS well, as it commonly presents with varied symptoms due to lesions in different parts of the central nervous system (CNS), follows a relapsing-remitting course, and primarily affects adults with a higher prevalence in women.

Five other possible diagnoses to consider could include:

1. **Neuromyelitis Optica Spectrum Disorder (NMOSD):** Also characterized by inflammatory demyelination, primarily affecting the optic nerves and spinal cord, sometimes with brain involvement, and can present with relapses and remissions.

2. **Acute Disseminated Encephalomyelitis (ADEM):** Although more common in children, it can occur in adults and presents with multifocal CNS symptoms. It typically follows a monophasic course but can be recurrent.

3. **Sarcoidosis with CNS Involvement (Neurosarcoidosis):** Can mimic MS symptoms with diverse CNS manifestations including cranial nerve palsies and myelopathy, and can have a relapsing course.

4. **Systemic Lupus Erythematosus (SLE) with Neuropsychiatric Involvement:** SLE can affect the CNS in varied ways, including transverse myelitis and optic neuritis, and is more common in women.

5. **Chronic Inflammatory Demyelinating Polyneuropathy (CIDP):** While primarily a peripheral nervous system disorder, symptoms can overlap with MS, including sensory and motor deficits, and it can have a relapsing course.

These conditions share some overlapping clinical features with Multiple Sclerosis, and additional diagnostic work-up, including imaging, laboratory tests, and possibly a lumbar puncture, would be necessary to differentiate them.

**77. user - 2024-11-27 16:20:37**

The main symptoms include parkinsonism, cerebellar ataxia, pyramidal tract damage, and autonomic nervous system damage. The first symptoms are often autonomic impairments, most notably cardiovascular dysfunction (e.g., upright hypotension) and urinary dysfunction (e.g., urinary incontinence), the latter of which tends to be comorbid with sexual dysfunction in male patients. Many patients have had rapid eye movement (REM) sleep behaviour disorder (RBD) for several years.

**ChatGPT-4o** - 2024-11-27 16:20:37

Based on the clinical manifestations you've provided, the rare disease most likely to be diagnosed is Multiple System Atrophy (MSA). MSA is a progressive neurodegenerative disorder characterized by a combination of parkinsonism, cerebellar ataxia, autonomic dysfunction, and pyramidal tract symptoms. The presence of autonomic symptoms such as cardiovascular dysfunction and urinary incontinence, along with REM sleep behavior disorder, are particularly indicative of MSA.

Five other possible diagnoses that could present with similar symptoms include:

1. **Parkinson’s Disease with Autonomic Dysfunction**: Although primarily characterized by parkinsonism, it can also present with autonomic dysfunction, especially in advanced stages.

2. **Progressive Supranuclear Palsy (PSP)**: This neurodegenerative disease can involve parkinsonism and balance issues, although it typically has distinct eye movement abnormalities and differs in the pattern of autonomic involvement.

3. **Dementia with Lewy Bodies (DLB)**: This disorder can present with parkinsonian features and REM sleep behavior disorder, though cognitive impairment is more prominent.

4. **Pure Autonomic Failure (PAF)**: This involves autonomic nervous system dysfunction, especially orthostatic hypotension, but lacks the neurological features like parkinsonism and ataxia.

5. **Corticobasal Degeneration (CBD)**: While it primarily involves movement disorders and cognitive dysfunction, some overlap with parkinsonism and autonomic symptoms can occur.

Each of these conditions has a different pattern of onset, progression, and additional symptoms that help in distinguishing them from MSA. A thorough clinical evaluation, including history, examination, and possibly further testing such as imaging or autonomic function tests, would be essential for an accurate diagnosis.

**78. user - 2024-11-27 16:23:02**

**1. Muscles,** weakness and atrophy of the distal limbs starting at the distal end of the limbs and progressing proximally, affecting the fine movements of the hands and causing drop foot. The facial muscles are also involved. At the same time, there is obvious muscle ankylosis, involving the limb muscles, pharyngeal muscles and so on. Physical examination may reveal that the fingers cannot be expanded immediately after clenching the fist vigorously, and the eyes cannot be opened immediately after closing them vigorously. Knocking on the hand, the big fish muscle, the tongue muscle, etc., can lead to muscle ankylosis, known as knocking muscle ankylosis or ‘muscle ball’ phenomenon. There is a ‘warming up’ phenomenon, which decreases after repeated activities. Lower extremity myalgia and generalised fatigue are also common clinical manifestations.

**The cardiac conduction system is often involved, with varying degrees of atrioventricular block and intraventricular conduction abnormalities. In severe cases, sudden death may result. Confirmed patients require regular ECG monitoring and pacemaker implantation if necessary.

**3. Central nervous system** , sleep disorders such as increased sleepiness and obstructive sleep apnoea are common. It may be associated with cognitive and mood disorders. Head MRI shows diffuse cerebral white matter lesions and cortical atrophy.

**Cataracts occur in almost all patients as they age. They often affect vision in the 30s and 40s.

**5. Endocrine system** A variety of endocrine disorders may occur, including diabetes mellitus, thyroid dysfunction, abnormal calcium and phosphorus metabolism, sex hormone abnormalities, and testicular atrophy (resulting in infertility).

In addition, there may be frontal baldness, gastrointestinal insufficiency and restrictive ventilation disorders.

**ChatGPT-4o** - 2024-11-27 16:23:02

Based on the clinical manifestations provided, the most likely diagnosis is **Myotonic Dystrophy**. The description matches the typical presentation of Myotonic Dystrophy, particularly Myotonic Dystrophy Type 1 (DM1), also known as Steinert's Disease, which is characterized by:

1. **Muscle Symptoms**: Distal muscle weakness and atrophy, facial muscle involvement, myotonia (difficulty relaxing muscles after contraction), and "warming up" phenomenon.
2. **Cardiac Involvement**: Conduction abnormalities, which can lead to arrhythmias and sudden death.
3. **Central Nervous System**: Sleep disturbances, cognitive and mood disorders, and MRI findings.
4. **Ocular Symptoms**: Cataracts, often developing in early adulthood.
5. **Endocrine Dysfunction**: Including diabetes, thyroid issues, and testicular atrophy.
6. **Additional Features**: Frontal baldness, gastrointestinal issues, and respiratory problems.

Here are five other possible differential diagnoses that could partly match some of the symptoms described:

1. **Facioscapulohumeral Muscular Dystrophy (FSHD)**:
 - A muscular dystrophy primarily affecting the face, shoulders, and upper arms, but less likely to present with myotonia or cardiac involvement.

2. **Limb-Girdle Muscular Dystrophy (LGMD)**:
 - Characterized by weakness and wasting of the limb-girdle muscles, though it typically doesn't involve myotonia or the extensive multi-systemic involvement seen in your description.

3. **Amyotrophic Lateral Sclerosis (ALS)**:
 - Could present with muscle weakness and atrophy but typically lacks the myotonia, cataracts, and specific endocrine features.

4. **Pompe Disease**:
 - A glycogen storage disorder that can cause muscle weakness and respiratory issues, but it lacks the cataracts and myotonia.

5. **Mitochondrial Myopathy**:
 - Presents with muscle weakness, possible central nervous involvement, and multi-systemic features, but lacks the specific myotonia and classic cardiac conduction issues.

Each of these conditions has distinct features that differentiate them from Myotonic Dystrophy, which is the most comprehensive match based on the extensive multi-systemic involvement described.

**79. user - 2024-11-27 16:28:23**

The disease tends to develop at an early age, with the most frequent onset occurring in the neonatal period. Most children develop the disease within the first few days of life and present with milk refusal, vomiting, lethargy, convulsions and coma. Some children with late-onset disease are characterised by episodes of vomiting and lethargy with mental developmental abnormalities. Patients with hyperammonaemic coma may have brain injury with developmental delay, impaired learning ability or mental retardation, and sequelae of growth retardation. Patients with hyperammonaemia may have increased intracranial pressure, shortness of breath, apnoea or failure as the condition progresses, and hepatomegaly. Neurological complications include mental retardation, acute hyperammonemic coma, and even death. Biochemical changes can be seen in hyperammonaemia, increased blood glutamine and alanine, and decreased or absent urinary whey acids.

**ChatGPT-4o** - 2024-11-27 16:28:23

Based on the clinical manifestations you've provided, the most likely diagnosis is a Urea Cycle Disorder (UCD), specifically Ornithine Transcarbamylase (OTC) Deficiency. This condition is characterized by hyperammonemia and the symptoms you've described, such as vomiting, lethargy, and neurological complications, are common in UCDs. OTC deficiency often presents in neonates with severe symptoms due to the accumulation of ammonia, which is toxic to the brain.

Five other possible diagnoses include:

1. **Carbamoyl Phosphate Synthetase I Deficiency (CPS1 Deficiency):** Another urea cycle disorder that can also present with hyperammonemia and similar symptoms to OTC deficiency.

2. **Argininosuccinate Synthetase Deficiency (Citrullinemia Type I):** This is another urea cycle disorder with neonatal onset, hyperammonemia, and associated neurological symptoms.

3. **Argininosuccinate Lyase Deficiency (Argininosuccinic Aciduria):** A urea cycle disorder with similar presentations, including hyperammonemia and developmental delays.

4. **Methylmalonic Acidemia (MMA):** Although primarily an organic acidemia, MMA can also present with hyperammonemia and neurological symptoms. It involves metabolic crises with vomiting and lethargy.

5. **Propionic Acidemia:** Another organic acidemia that can cause hyperammonemia and presents with similar symptoms like vomiting, lethargy, and metabolic disturbances.

These conditions can often be differentiated by specific biochemical tests and genetic analyses.

**80. user - 2024-11-27 16:32:18**

Children often present with severe intrauterine growth retardation and develop severe, non-ketotic hyperglycaemia very early in life (often in the first week of life), which recovers after 12 weeks and recurs around puberty in 50%-60% of cases, with a clinical picture similar to that of type 2 diabetes mellitus.

**ChatGPT-4o** - 2024-11-27 16:32:18

Based on the clinical manifestations you have provided, the most likely rare disease to be diagnosed is Transient Neonatal Diabetes Mellitus (TNDM). TNDM is characterized by intrauterine growth retardation, the onset of diabetes early in life (typically within the first week), which usually remits within the first few months, and may recur later, often around puberty.

Five other possible diagnoses to consider include:

1. **Permanent Neonatal Diabetes Mellitus (PNDM):** Similar early onset of diabetes, but unlike TNDM, it does not remit. It is a lifelong condition.

2. **Congenital Hyperinsulinism:** Although it typically presents with hypoglycemia rather than hyperglycemia, mutations affecting insulin regulation could lead to an atypical presentation.

3. **Wolcott-Rallison Syndrome:** This rare genetic disorder causes early-onset diabetes, but it is usually accompanied by skeletal dysplasia and other systemic manifestations.

4. **MODY (Maturity Onset Diabetes of the Young):** While more common in slightly older children or adolescents and not typically associated with early neonatal onset, there are forms that could present atypically early.

5. **Donohue Syndrome (Leprechaunism):** This is an extremely rare condition associated with severe insulin resistance that can present with growth retardation and diabetes-like symptoms, though it is often more severe and with additional dysmorphic features.

Each of these conditions has distinct genetic and clinical features that would need to be differentiated through further diagnostic testing.

**81. user - 2024-11-27 17:09:29**

The prevalence of the disease is between 5 and 50 years old, with a median age of onset of 39 years. The prevalence of women is significantly higher than that of men, with a female:male ratio of (9~11):1. Most of the cases have an acute/subacute onset, and the clinical manifestations include six core syndromes: (1) optic neuritis; (2) acute myelitis; (3) posterior pole syndrome; (4) acute brainstem syndrome; (5) symptomatic sleep attacks or acute mesencephalic clinical syndrome with typical MRI foci of the mesencephalon; and (6) symptomatic cerebral syndrome with typical brain lesions. It is a highly recurrent and disabling disease, with more than 90 per cent of patients having a recurrent course, and the majority of patients are left with more severe neurological disability.

**ChatGPT-4o** - 2024-11-27 17:09:29

Based on the clinical manifestations and the demographic information provided, the most likely diagnosis is Neuromyelitis Optica Spectrum Disorder (NMOSD). NMOSD is characterized by optic neuritis, acute myelitis, and various brainstem syndromes, often showing a higher prevalence in women and usually with a relapsing course leading to significant neurological disability.

Here are five other possible diagnoses that could also be considered:

1. **Multiple Sclerosis (MS):** Similar to NMOSD, it presents with optic neuritis and myelitis but tends to have a different demographic profile and MRI findings.

2. **Sarcoidosis (neurosarcoidosis):** Can present with neurological symptoms including optic neuritis and myelitis, and can also affect the brainstem.

3. **Systemic Lupus Erythematosus (SLE) with CNS involvement:** Can cause a variety of neurological symptoms including optic neuritis and myelitis, and is more common in females.

4. **Antiphospholipid Syndrome (APS) with central nervous system manifestations:** May cause recurrent neurological symptoms, including those affecting vision and spinal cord.

5. **Behçet's Disease with neurological involvement (Neuro-Behçet's):** Can lead to recurrent neurological symptoms, including brainstem and cerebral involvement, and has a higher prevalence among young adults.

Each of these conditions has overlapping symptoms with NMOSD but would be differentiated based on specific clinical, laboratory, and imaging findings.

**82. user - 2024-11-27 17:11:03**

The earliest symptoms are abdominal distension and enlargement of the liver and spleen, and some patients may develop in the neonatal period. Intellectual and motor developmental deficits occur immediately, with hypotonia and motor developmental delays, often without cerebral neurological involvement, and regression of motor and intellectual development is evident after the age of 1 year, eventually progressing to spasticity and tonicity.Cherry erythema on the fundus of the eye can be found in 50% of the patients. Interstitial lung disease may lead to recurrent respiratory infections, hypoxaemia or respiratory failure. Most children die before the age of 3 years.

**ChatGPT-4o** - 2024-11-27 17:11:03

Based on the clinical manifestations you've provided, the most likely diagnosis is Niemann-Pick disease type A. This is a rare genetic disorder characterized by the accumulation of sphingomyelin in various tissues, leading to the symptoms you've described, including hepatosplenomegaly, developmental delays, cherry-red spot on the fundus, and early childhood mortality.

Here are five other possible diagnoses:

1. **Tay-Sachs Disease**: This condition also presents with developmental delays, cherry-red spots, and early mortality, but typically lacks the hepatosplenomegaly seen in Niemann-Pick disease.

2. **Gaucher Disease (Type 2)**: Another lysosomal storage disorder with hepatosplenomegaly, neurological symptoms, and early death, but it usually exhibits more severe neurological involvement from a younger age.

3. **Sandhoff Disease**: Similar to Tay-Sachs, but with systemic involvement including hepatosplenomegaly, and also presenting with a cherry-red spot.

4. **GM1 Gangliosidosis (Type 1)**: This disease can present with hepatosplenomegaly, developmental delay, cherry-red spot, and similar neurological deterioration.

5. **Wolman Disease**: A rare lysosomal storage disease that presents with hepatosplenomegaly, developmental delays, and early death, but more often includes adrenal calcifications and severe malabsorption.

In each of these conditions, the presence of specific genetic mutations and enzyme deficiencies would be crucial for accurate diagnosis.

**83. user - 2024-11-27 17:15:09**

Hearing impairment. In the past, it was thought that the deafness was congenital, which could be identified by hearing screening, and that it would be apparent from birth, mostly involving both sides, and symmetrically severe prelingual deafness. In recent years, it has been found that individual cases may present with asymmetrical or unilateral hearing loss, and the degree of hearing loss is highly variable, ranging from mild to very severe in different patients; progressive deafness that occurs after birth is also not uncommon.

**ChatGPT-4o** - 2024-11-27 17:15:09

Based on the clinical manifestations you have described, the most likely rare disease to consider is **Pendred syndrome**. It is a genetic disorder that often causes early hearing loss in children. It can also affect the thyroid gland and sometimes cause balance issues.

Five other possible diagnoses to consider are:

1. **Usher Syndrome**: A rare genetic disorder characterized by hearing loss and a progressive vision disorder called retinitis pigmentosa.

2. **Alport Syndrome**: A genetic condition that can cause progressive hearing loss, kidney disease, and eye abnormalities.

3. **Jervell and Lange-Nielsen Syndrome**: A rare condition characterized by congenital deafness and long QT syndrome, which affects the heart's electrical system.

4. **Wolfram Syndrome**: A rare genetic disorder that causes diabetes insipidus, diabetes mellitus, optic atrophy, and deafness.

5. **Branchio-Oto-Renal (BOR) Syndrome**: A genetic disorder that can lead to hearing loss, as well as kidney issues and branchial cysts or fistulas.

These conditions involve genetic factors that can lead to variable manifestations of hearing loss and should be considered based on the specific clinical context and genetic testing availability.

**84. user - 2024-11-27 17:17:57**

**1. Typical face,** patients of all ages have ptosis, wide eye spacing, medial canthus, and downward slanting of the outer corners of the eyes; low bilateral ear position, posterior rotation, and thick auricles. Child patients may also have a full forehead, low posterior hairline, short nose, low nasal bridge, full nasal tip; thick lips, deep and wide nasolabial groove straight to the upper lip, and so on.

**2. Dwarfism,** which is normal at birth and gradually appears after 1 year of age. Bone age often lags behind, and some adult heights can reach the lower limit of normal level.

**3. cardiovascular,** 50% ~80% of patients have congenital heart disease, pulmonary valve stenosis is the most common, others include hypertrophic cardiomyopathy, ventricular ischemia, pulmonary artery branch stenosis, tetralogy of Fallot, and aortic constriction, etc. Typical ECG changes include electrocardiogram (ECG) and heart rate (HR). Typical electrocardiographic changes include extreme rightward deviation of the electrical axis with counterclockwise rotation of the QRS wave in the precordial leads, leftward deviation of the electrical axis in the V1 lead, incomplete left anterior branch block, or RSR waveforms.

**4. psychomotor retardation**, poor language skills in some patients, depression in adult patients, and a high incidence of autism spectrum manifestations have also been reported.

**5. webbing of the neck**.

**6. thoracic abnormalities,** chicken chest or funnel chest.

**7. wide breast spacing**

**8. cryptorchidism in males** , which is present in 60% to 80% of male patients and can lead to fertility disorders.

**9. abnormal blood clotting and lymphatic dysplasia**

**10. Others,** renal malformations (dilated renal pelvis, double ureteral malformation, isolated kidney, renal hypoplasia, distal ureteral stenosis, etc.), impaired spermatogenesis in males, strabismus, nystagmus, milky coffee spots and freckles on the skin, follicular keratosis of the limbs, Arnold Chiari malformation type I, hydrocephalus, hepatosplenomegaly, and an increased incidence of malignant tumours.

**11. Intrauterine manifestations,** excessive amniotic fluid in the mother during pregnancy, widening of the nuchal translucency, relatively large fetal head, cardiac and renal anomalies.

**ChatGPT-4o** - 2024-11-27 17:17:57

Based on the clinical manifestations provided, the condition most likely to be diagnosed is **Noonan Syndrome**. Noonan Syndrome is a genetic disorder that causes a wide range of features, many of which you have described:

1. **Typical facial features** - including ptosis, wide-set eyes, low-set ears, and a wide nasal bridge are characteristic.
2. **Short stature/dwarfism** - often becomes apparent after the first year.
3. **Congenital heart defects** - such as pulmonary valve stenosis and hypertrophic cardiomyopathy.
4. **Developmental delays/psychomotor retardation** and potential issues related to language skills.
5. **Webbed neck** and thoracic deformities like pectus excavatum (funnel chest) or pectus carinatum (chicken chest).
6. **Cryptorchidism in males** is common and can lead to fertility issues.
7. **Other features** - such as bleeding disorders, lymphatic dysplasia, renal anomalies, and skin findings.

Five other possible diagnoses that could be considered based on the combination of the clinical features provided are:

1. **Turner Syndrome** - While it mostly affects females, it shares features like short stature, webbed neck, and cardiac issues.
2. **Williams Syndrome** - Characterized by specific facial features and congenital heart defects, especially supravalvular aortic stenosis.
3. **DiGeorge Syndrome (22q11.2 deletion syndrome)** - Involves cardiac anomalies, distinctive facial features, developmental delays, and immune deficiencies.
4. **Cardiofaciocutaneous (CFC) Syndrome** - Similar to Noonan Syndrome with overlapping features such as heart defects, distinctive facial appearance, and developmental delays.
5. **Costello Syndrome** - Involves similar growth delays, cardiac issues, and characteristic craniofacial features.

Each of these conditions has overlapping clinical features with Noonan Syndrome, but specific differences and genetic testing can help in establishing a definitive diagnosis.

**85. user - 2024-11-27 17:19:51**

Patients can develop the disease at any age. There are two main clinical categories: early-onset and late-onset. Early-onset cases mainly occur in male children, usually in the neonatal period, and are characterised by a rapid onset of the disease, with a rapid onset of the disease and extensive brain damage due to elevated ammonia levels in the blood. There may be no abnormality at birth, but in the first few days of life, the child will show irritability, lethargy, refusal to eat, shortness of breath and lethargy, which may rapidly develop into spasm, coma and respiratory failure. If not treated urgently, the disease quickly develops into hereditary metabolic encephalopathy and often dies within the first week of life, with survivors often left with severe intellectual impairment. Late-onset disease occurs in older patients, both hemizygous males and heterozygous females, and has relatively mild and variable clinical symptoms. Patients with childhood and adult onset tend to present with chronic neurological damage such as episodes of vomiting, headaches, abnormal behaviour, delirium and confusion. Patients may have clinical phenotypes such as hepatomegaly, recurrent seizures, growth retardation, and behavioural abnormalities. Despite the milder symptoms of the late-onset form, life-threatening acute episodes of hyperammonemia can be induced by environmental factors such as illness, stress, and a high-protein diet. The majority of heterozygous female carriers remain asymptomatic throughout their lives, while a few develop the disease, with individual variability in age of onset and clinical manifestations, both early and late onset.

**ChatGPT-4o** - 2024-11-27 17:19:51

Based on the clinical manifestations described, the rare disease most likely to be diagnosed is Ornithine Transcarbamylase (OTC) Deficiency. OTC deficiency is a urea cycle disorder and presents with the described symptoms: early-onset cases with severe hyperammonemia in male infants, leading to rapid neurological decline, and later-onset cases in both males and females with milder, variable symptoms but potential acute crises.

Here are five other possible diagnoses that could present with similar symptoms:

1. **Carbamoyl Phosphate Synthetase 1 Deficiency (CPS1 Deficiency)**: Another urea cycle disorder leading to hyperammonemia, which can cause similar early-onset and late-onset symptoms as described.

2. **Argininosuccinate Synthetase Deficiency (Citrullinemia Type I)**: Also a urea cycle disorder that can present with hyperammonemia and neurological symptoms. It can have neonatal and later-onset forms.

3. **Arginase Deficiency (Argininemia)**: A urea cycle disorder that might present with hyperammonemia, growth retardation, and neurological symptoms, although it generally presents with more progressive spasticity.

4. **Propionic Acidemia**: An organic acidemia that can cause episodic metabolic crises leading to neurological symptoms, hyperammonemia, and developmental delays.

5. **Methylmalonic Acidemia**: Another organic acidemia that presents with recurrent vomiting, lethargy, and potentially acute hyperammonemia, along with neurological symptoms and growth delays.

These conditions all involve metabolic pathways that can lead to hyperammonemia and associated neurological complications, with varying degrees of severity and age of onset.

**86. user - 2024-11-27 17:21:27**

The clinical phenotype of the disease varies in severity. The main clinical manifestations of the patients are recurrent fractures under minor external forces, skeletal deformities, and varying degrees of activity limitation since childhood. Patients may have extra-skeletal manifestations such as blue sclera, ligamentous laxity, dentin hypoplasia, hearing loss, and cardiac valvulopathy, etc. The imaging features of OI mainly include: thinning of bones in many parts of the body; thinning of the cranial plate, widening of fontanelle and cranial suture, interstitial bone in occipital bones, and flattening of the base of the skull; lateral curvature or posterior convexity deformity of the spine, deformation of vertebral bodies, and compression fracture of multiple vertebral bodies; twisted and deformed thorax, or even collapse; slender long bones of the limbs, and even collapse; and the long bones of the limbs are thin and thin. The long bones of the limbs are slender, the bone cortex is thin, the bone marrow cavity is relatively large, the epiphyses are widened, there are many long bone fractures, and the bones are curved and deformed.

**ChatGPT-4o** - 2024-11-27 17:21:27

Based on the clinical manifestations described, the most likely diagnosis is Osteogenesis Imperfecta (OI). OI is a genetic disorder characterized by brittle bones that are prone to fracture, often from minimal or no trauma. The associated features such as blue sclera, dentinogenesis imperfecta, hearing loss, and skeletal deformities align well with this condition.

Five other possible diagnoses that could present with similar symptoms include:

1. **Hypophosphatasia**: A rare genetic disorder that affects bone mineralization, leading to soft and weakened bones, and can present with fractures and deformities.

2. **Ehlers-Danlos Syndrome (EDS)**: Particularly the hypermobile or vascular types, which can present with joint hypermobility, skin elasticity, and occasionally bone fragility.

3. **Marfan Syndrome**: A connective tissue disorder characterized by features such as tall stature, long limbs, and sometimes bone deformities, though fractures are less common.

4. **Paget's Disease of Bone**: Although more common in adults, juvenile forms can cause bone deformities and pain due to abnormal bone remodeling.

5. **Idiopathic Juvenile Osteoporosis**: A condition that can cause bone pain, fractures, and deformities, typically presenting in children before puberty without a clear underlying cause.

Each of these conditions has distinct features and pathophysiology, and a comprehensive clinical evaluation including genetic testing may be necessary to confirm the diagnosis.

**87. user - 2024-11-27 17:24:26**

Bradykinesia, tremor, and myotonia. Levodopa-induced motor complications are more likely to occur after treatment. In terms of non-motor symptoms, mood disorders (e.g., depression, anxiety, irritability, etc.) and behavioural disorders (e.g., compulsive dosage increases, impulse control disorders, stereotyped behaviour, etc.) are likely to occur.

**ChatGPT-4o** - 2024-11-27 17:24:26

Based on the clinical manifestations you've described, it seems most likely that the rare disease is **Dopa-Responsive Dystonia (DRD)**, also known as Segawa Syndrome. This condition is characterized by a combination of motor symptoms such as bradykinesia, tremor, and myotonia, and can also involve non-motor symptoms including mood and behavioral disorders. DRD often responds well initially to levodopa treatment, but there can be complications associated with long-term use.

Here are five other possible diagnoses based on the symptoms provided:

1. **Juvenile Parkinsonism**: This condition shares several similarities with Parkinson's disease but occurs at a younger age. It can present with bradykinesia, tremor, and mood disorders. Levodopa treatment might also lead to complications.

2. **Myotonic Dystrophy**: While primarily a muscular disorder characterized by myotonia, it can have neurological complications, including mood and behavioral disorders.

3. **Wilson's Disease**: This genetic disorder affects copper metabolism and can cause neurological symptoms like tremor and bradykinesia, as well as mood disturbances.

4. **Parkinson's Plus Syndromes**: Conditions such as Multiple System Atrophy (MSA) or Progressive Supranuclear Palsy (PSP) can present with Parkinson-like symptoms and mood/behavioral disorders, though they're generally less responsive to levodopa.

5. **Essential Tremor with Co-occurring Mood Disorders**: Primarily a tremor disorder, essential tremor can sometimes be accompanied by mood disorders, though the levodopa effect isn't typical for this condition.

For accurate diagnosis, it would be crucial to consider detailed clinical history, genetic testing, and possibly neuroimaging, depending on the suspected condition.

**88. user - 2024-11-27 18:18:04**

**1. Pancytopenia,** Many patients may have a combination of pancytopenia and neutropenia. Clinical manifestations include malaise, recurrent infections and bleeding tendencies.

**2. Haemoglobinuria** , typical haemoglobinuria is soy sauce or strong tea colour. It usually lasts for 2~3 days and subsides on its own without treatment, while in severe cases, it lasts for 1~2 weeks or even longer. Some patients have frequent episodes of haemoglobinuria, while a significant number of patients have no clear episodes of haemoglobinuria over a long period of time or observation.

Episodes of haemoglobinuria may be accompanied by fever, low back pain and abdominal pain. Other symptoms associated with smooth muscle dysfunction can include dysphagia, abdominal pain, bloating, back pain, headache, oesophageal spasms, and erectile dysfunction. Rare conditions include choledochal duct dyskinesia, acute pancreatitis, ischaemia and ulceration of the duodenum or colon.

**3. thrombosis**.

**Jaundice and hepatosplenomegaly** , less than half of patients have mild jaundice. Most patients do not have hepatosplenomegaly.

Common comorbidities include infection, anaemic heart disease, cholelithiasis, renal insufficiency and even failure.

**ChatGPT-4o** - 2024-11-27 18:18:04

Based on the clinical manifestations you've provided, the most likely rare disease diagnosis is **Paroxysmal Nocturnal Hemoglobinuria (PNH)**. PNH is characterized by the combination of pancytopenia, hemoglobinuria, thrombosis, and symptoms related to smooth muscle dysfunction. The episodic hemoglobinuria, recurrent infections, bleeding tendencies, and the potential complications such as renal insufficiency and thrombosis further support this diagnosis.

Here are five other possible differential diagnoses:

1. **Aplastic Anemia**: This condition also presents with pancytopenia and could lead to recurrent infections and bleeding tendencies. However, it does not typically present with hemoglobinuria or thrombosis.

2. **Myelodysplastic Syndromes (MDS)**: These can present with pancytopenia and are associated with an increased risk of progression to leukemia, but hemoglobinuria is not a common feature.

3. **Hemolytic Uremic Syndrome (HUS)**: This could present with thrombosis, renal impairment, and hemolysis, but the pancytopenia and classic hemoglobinuria pattern are not typical features.

4. **Thrombotic Thrombocytopenic Purpura (TTP)**: This condition presents with thrombocytopenia, hemolytic anemia, renal dysfunction, and neurological symptoms. Hemoglobinuria and pancytopenia are not typical.

5. **Autoimmune Hemolytic Anemia (AIHA)**: This can cause hemolysis and hemoglobinuria, but typically doesn't cause pancytopenia or the diverse symptoms related to smooth muscle dysfunction seen in PNH.

These conditions should be differentiated based on specific laboratory tests, clinical history, and sometimes bone marrow examination.

**89. user - 2024-11-27 18:20:41**

Clinical manifestations include repeated intussusception, abdominal pain, diarrhoea, mucus stools, blood in stool, constipation, vomiting blood and anaemia. Characteristic clinical manifestations are skin and mucous membrane pigmentation and multiple polyps in the gastrointestinal tract. 95% of the patients have hyperpigmentation, which can be found on the lips and surrounding areas of the mouth, cheeks, face, fingers, palms of the hands, soles of the feet, perianal area, etc., and occasionally on the intestinal mucous membranes. The pigmentation can be brown, tan, grey, blue, etc. It usually occurs before the age of 2 years, and the size is 1~5mm. 88%-100% of the patients will develop gastrointestinal polyps, and the onset of gastrointestinal polyps most often occurs at the age of 11-13 years, and the polyps can occur throughout the entire gastrointestinal tract, with the small intestine being the most common, followed by the colon and the stomach. The size of the polyps is variable, and their tips vary in length and thickness. The risk of cancer is high, with an average age of 42 years.

**ChatGPT-4o** - 2024-11-27 18:20:41

Based on the clinical manifestations you've described, the most likely diagnosis is **Peutz-Jeghers syndrome**.

This is a rare genetic disorder characterized by the development of benign polyps in the gastrointestinal tract and distinctive pigmented spots on the skin and mucous membranes, particularly around the mouth, lips, and fingers. Individuals with Peutz-Jeghers syndrome are at an increased risk for developing certain types of cancer.

Five other possible diagnoses that might be considered based on overlapping symptoms, though less likely given the specific presentation, include:

1. **Familial Adenomatous Polyposis (FAP)**: A hereditary condition that leads to the development of numerous polyps in the colon and rectum, and an increased risk of colorectal cancer. However, it lacks the characteristic pigmentation seen in Peutz-Jeghers syndrome.

2. **Cronkhite-Canada Syndrome**: A rare, non-hereditary condition characterized by gastrointestinal polyposis, skin pigmentation, alopecia, and onychodystrophy. The polyps in this syndrome tend to be hamartomatous, and there is no genetic inheritance pattern.

3. **Juvenile Polyposis Syndrome**: An inherited condition characterized by the appearance of multiple benign polyps in the gastrointestinal tract during childhood. Unlike Peutz-Jeghers, it does not typically involve mucocutaneous pigmentation.

4. **Cowden Syndrome**: A genetic disorder characterized by multiple noncancerous, tumor-like growths called hamartomas and an increased risk of certain cancers. Skin pigmentation may occur but is not as distinctive as in Peutz-Jeghers syndrome.

5. **Neurofibromatosis Type 1 (NF1)**: Although it primarily involves skin manifestations like café-au-lait spots and neurofibromas, it can occasionally present with gastrointestinal involvement, albeit not typically with polyps or the specific pigmentation pattern of Peutz-Jeghers syndrome.

Each of these conditions has distinct features, but given the combination of gastrointestinal polyps and characteristic pigmentation, Peutz-Jeghers syndrome is the most fitting diagnosis.

**90. user - 2024-11-27 18:22:16**

In the neonatal period, most of the children have no clinical symptoms, but after 3-4 months of life, typical symptoms gradually appear, and the symptoms are obvious at the age of 1 year. A few months after birth, due to insufficient melanin synthesis, their hair and iris colour gradually becomes lighter, yellow or brownish-yellow, with white skin. Urine and sweat contain a large amount of phenyl lactic acid and have a foul odour of rat urine. As they grow older, they gradually show delayed intellectual development, with predominantly cognitive developmental disorders, microcephaly, seizures, as well as behavioural and personality abnormalities, such as hyperactivity, self-mutilation, aggression, autism, low self-esteem, depression and other neurological manifestations. Vomiting and eczema are also commonly seen in infancy.

**ChatGPT-4o** - 2024-11-27 18:22:16

Based on the clinical manifestations described, the most likely diagnosis is **Phenylketonuria (PKU)**. PKU is a rare genetic disorder caused by a deficiency in the enzyme phenylalanine hydroxylase, which leads to the accumulation of phenylalanine and its metabolites, such as phenyl lactic acid, causing the symptoms described.

Five other possible diagnoses to consider could include:

1. **Homocystinuria**: Another metabolic disorder that can lead to developmental delays and behavioral abnormalities. It is characterized by a deficiency in cystathionine beta-synthase, leading to elevated homocysteine levels.

2. **Maple Syrup Urine Disease (MSUD)**: A metabolic disorder involving the inability to break down certain amino acids, which can result in neurological symptoms, a distinct sweet-smelling urine, and developmental delays.

3. **Albinism**: While primarily a disorder of pigmentation, some forms can have associated neurological symptoms, although the foul odor and specific behavioral symptoms don't typically align.

4. **Tyrosinemia Type I**: A disorder affecting the breakdown of the amino acid tyrosine, leading to liver and kidney dysfunction, and potentially neurological symptoms, though the presentation of PKU is more typical for the described symptoms.

5. **Lesch-Nyhan Syndrome**: Though not a perfect fit, it's a rare X-linked disorder characterized by behavioral and neurological symptoms, including self-mutilation, but lacks the pigmentation changes and urine odor typical of PKU.

Given the described symptoms, particularly the presence of phenyl lactic acid and a mousey odor in urine, PKU remains the most consistent diagnosis.

**91. user - 2024-11-27 18:24:46**

**1. polyneuropathy**, often presenting as a symmetrical sensory and/or motor peripheral neuropathy of the extremities, progressing gradually from distal to proximal.

**2. organomegaly,** including hepatomegaly, splenomegaly, or lymph node enlargement; lymph node biopsy is often suggestive of Castleman disease.

**Endocrine abnormalities,** including hypogonadism (e.g., male impotence, breast development), hypothyroidism, abnormal glucose metabolism (e.g., diabetes mellitus), and adrenal insufficiency.

**4. skin changes,** which may be manifested by deepening of skin colour, cutaneous glomeruloid haemangiomas, white nails, polycythemia, hirsutism, and cyanosis of the hands and feet.

**5. increased extracirculatory water load,** including limb oedema, ascites, pleural effusion, pericardial effusion, and optic papilla oedema.

**Sclerosing bone disease,** is an important clinical manifestation. It may be manifested as bone pain or without clinical symptoms. Sclerosing bone disease can be detected significantly more frequently with CT scanning of the bones, and can be visualised as simple sclerosis of the bones, mixed sclerotic and osteolytic lesions, or simple osteolytic changes.

**7. erythrocytosis and/or thrombocytosis** **8.

**8. pulmonary hypertension** , patients may present with pulmonary hypertension, as evidenced by reduced activity tolerance, hypoxaemia, etc.

**9. cerebral infarction**.

**ChatGPT-4o** - 2024-11-27 18:24:46

Based on the clinical manifestations you have described, the most likely rare disease diagnosis is **POEMS syndrome**. POEMS syndrome is a rare multisystem disorder characterized by the constellation of features you listed: Polyneuropathy, Organomegaly, Endocrinopathy, Monoclonal gammopathy, and Skin changes. Here are five other possible diagnoses that could present with some of these features:

1. **Castleman Disease**: This condition can be associated with organomegaly, endocrinopathy, and polyneuropathy, as well as inflammatory symptoms. It sometimes overlaps with POEMS syndrome.

2. **Amyloidosis**: This condition can cause peripheral neuropathy, organomegaly, skin changes, and cardiac symptoms like pulmonary hypertension.

3. **Sclerosing Bone Dysplasias**: Conditions like osteopetrosis could explain the sclerosing bone disease, although they typically lack the systemic involvements like polyneuropathy and endocrinopathy.

4. **Chronic Myeloproliferative Disorders**: Such as polycythemia vera or essential thrombocythemia, which may cause erythrocytosis or thrombocytosis and could overlap with some features like pulmonary hypertension or organomegaly.

5. **Systemic Mastocytosis**: This can cause multi-organ involvement, including skin changes, bone pain, and possible organomegaly, but it usually presents with additional symptoms like anaphylaxis and flushing.

Each of these conditions can present with varying combinations of the symptoms you've described, but POEMS syndrome most comprehensively aligns with the full spectrum of manifestations.

**92. user - 2024-11-27 18:27:05**

Mainly skin symptoms and neuropsychiatric symptoms.

**1. The group of cutaneous symptoms,** are erythema, herpes and even ulceration on exposed parts of the skin after exposure to light. The rash may be of the type of eczema, urticaria, summertime itchy rash or erythema multiforme. The oral mucosa may have red patches and brownish-red teeth. Eye damage such as conjunctivitis, keratitis and iritis may also occur. In severe cases, there may be deformation of the skin of the nose, ears and fingers with kinking. There may be special purple colouration. Hirsutism may be present.

**2. A cluster of neuropsychiatric symptoms , ** the most common manifestation of which is acute abdominal pain, which tends to be severe, stable, and poorly localised, sometimes accompanied by painful cramps. It may be accompanied by constipation, bloating, nausea, vomiting, and sometimes diarrhoea and increased bowel sounds. Sensory and motor neuropathies may precede the onset of abdominal pain and present as limb pain with numbness, abnormal sensation and sensory inversions; motor weakness often begins proximally in the upper limbs and may progress to the lower limbs and distal extremities. Involvement of the cerebral nerves may lead to medullary paralysis, impaired respiratory function and death. Severe motor neuropathy may result in quadriplegia. Autonomic nervous system involvement manifests as tachycardia, hypertension, sweating, agitation, and tremor. Neurogenic bladder dysfunction may cause dysuria, dyspareunia, urinary retention and incontinence. Other acute neuropsychiatric manifestations include insomnia, anxiety, agitation, agitation, hallucinations, hysteria, disorientation, delirium, affective apathy, depression, phobias, and altered consciousness ranging from lethargy to coma.

**ChatGPT-4o** - 2024-11-27 18:27:05

Based on the provided clinical manifestations, the most likely diagnosis is **Porphyria**, specifically Acute Intermittent Porphyria (AIP) with potential cutaneous involvement similar to Porphyria Cutanea Tarda (PCT) or Variegate Porphyria. Porphyria is a group of rare disorders characterized by problems with the production of heme, a component of hemoglobin. The symptoms you've described align well with porphyria, especially the combination of skin photosensitivity and a wide array of neuropsychiatric symptoms.

Here are five other possible diagnoses that could be considered given the symptomatology:

1. **Systemic Lupus Erythematosus (SLE):** This autoimmune disease can cause a wide range of symptoms, including skin rashes, photosensitivity, and neuropsychiatric symptoms due to central nervous system involvement.

2. **Erythropoietic Protoporphyria (EPP):** Like porphyria, EPP involves photosensitivity leading to skin damage, but typically has less severe neuropsychiatric involvement.

3. **Multiple Sclerosis (MS):** While primarily a neurological disorder, it can present a variety of neurological symptoms, and in rare cases, there may be associated skin manifestations due to secondary complications.

4. **Sjögren's Syndrome:** This autoimmune condition can cause skin and neuropsychiatric symptoms, including neuropathy and cognitive difficulties, although it is better known for causing dry eyes and mouth.

5. **Vitamin B12 Deficiency:** Can cause neuropsychiatric symptoms, including neuropathies and mood disturbances, and in some cases, skin changes such as hyperpigmentation.

Each of these conditions has its own distinct set of diagnostic criteria and clinical features, so further specific testing and clinical evaluation would be required to differentiate between them.

**93. user - 2024-11-27 18:36:50**

Common manifestations in the foetal period include reduced foetal activity, excess amniotic fluid and breech presentation. In the neonatal period hypotonia is one of the hallmark features of this disorder, and severe hypotonia can lead to asphyxia, feeding difficulties, weak sucking, and weak crying. In infancy and early childhood, the main manifestations are growth retardation and lagging cognitive, motor and language development. Appetite increases with age and obesity can occur if eating is not restricted. Short stature is usually present in school age, and personality traits such as temper tantrums, stubbornness, and obsessive-compulsive disorder gradually appear. Abnormal behaviours include intense foraging, skin scratching, and autistic-like behaviour. Seizures occur in about 25 per cent of patients. Delayed or incomplete appearance of secondary sexual characteristics may occur in adolescent males and females. Obesity can lead to complications such as sleep apnoea, pulmonary heart disease, insulin resistance and even diabetes. Scoliosis, osteoporosis, and gastroesophageal reflux are also more common. The main manifestations in adulthood are obesity and its complications, including cardiovascular problems, diabetes and sleep apnoea. Other manifestations are mental abnormalities and infertility.

Fatal risks: choking due to uncoordinated mouth movements, hypotonia, polyphagia, gulping habits and inadequate chewing, and gastric rupture due to overeating.

Physical examination reveals characteristic facial features that become progressively more typical, including almond eyes, small mouth, thin upper lip, downward-facing corners of the mouth, small hands and feet, short stature, fair skin, low muscle tone, and genital hypoplasia (e.g., cryptorchidism, scrotal hypoplasia, or clitoral hypoplasia).

**ChatGPT-4o** - 2024-11-27 18:36:50

Based on the clinical manifestations and features provided, the most likely diagnosis is Prader-Willi syndrome (PWS). This genetic disorder is characterized by the symptoms you've described, including hypotonia, developmental delays, hyperphagia leading to obesity, endocrine problems, characteristic facial features, and behavioral issues.

Here are five other possible diagnoses that could be considered based on some overlapping features, although they are less likely given the specific combination of symptoms:

1. **Angelman Syndrome**: Although it shares genetic proximity with Prader-Willi syndrome (both can involve abnormalities on chromosome 15), Angelman syndrome typically presents with severe intellectual disability, frequent smiling/laughter, and ataxia rather than hypotonia and obesity.

2. **Bardet-Biedl Syndrome**: This genetic disorder can also present with obesity, intellectual disability, and hypogonadism, but it is often associated with additional features such as retinal dystrophy, renal abnormalities, and polydactyly.

3. **Beckwith-Wiedemann Syndrome**: Known for neonatal hypoglycemia and macrosomia, this syndrome can involve developmental delays, but typically presents with overgrowth rather than short stature.

4. **Cohen Syndrome**: This condition can include hypotonia, obesity, intellectual disability, and characteristic facial features, but often involves neutropenia and myopia, and lacks the hyperphagia seen in Prader-Willi syndrome.

5. **Smith-Magenis Syndrome**: This syndrome involves developmental delay, sleep disturbances, and behavioral problems, but these patients typically have distinct craniofacial and behavioral features (e.g., self-hugging, self-injurious behavior) different from those seen in Prader-Willi syndrome.

Each of these syndromes has unique features that differentiate them from Prader-Willi syndrome, but they share enough symptoms to be considered in a differential diagnosis. Genetic testing remains crucial for confirming the diagnosis of Prader-Willi syndrome.

**94. user - 2024-11-27 18:41:14**

**1. Infection, patients** are infected to varying degrees. Infections are recurrent, severe, difficult to treat, opportunistic and have a broad spectrum of pathogens. Typical children are usually born with no specific manifestations, and fatal infections can occur before 2 months of age due to cellular immune abnormalities. The sites and pathogens of infection are diverse, including recurrent pneumonia, recurrent thrush, persistent diarrhoea, recurrent otitis media, persistent viral infections (e.g., respiratory syncytial virus, EBV, CMV, etc.), and conditionally causative organisms, such as Pneumocystis carinii, are also common.

**2. Vaccine disease,** in which patients are extremely immunocompromised, can occur with infection and dissemination of live attenuated vaccines. Severe diarrhoea can also occur in children vaccinated with attenuated rotavirus vaccine.

**3. Retarded or arrested growth and development** is a distinguishing feature of the disease. It is associated with recurrent infections, diarrhoea and corresponding genetic defects.

Children may also have a rash and enlarged liver and spleen lymph nodes similar to erysipelas.

In other children, erythroderma-like symptoms, hepatosplenomegaly, seborrheic dermatitis, haematopenia, and sclerosing cholangitis may be present.

**ChatGPT-4o** - 2024-11-27 18:41:14

Based on the clinical manifestations provided, the most likely rare disease that fits the description is **Severe Combined Immunodeficiency (SCID)**. SCID is characterized by severe immunodeficiency leading to recurrent, severe, and opportunistic infections, often due to both cellular and humoral immune defects. The symptoms of recurrent infections, growth retardation, issues with live vaccines, and other immunological symptoms fit well with SCID.

Here are five other possible diagnoses that could be considered based on the symptoms provided:

1. **Wiskott-Aldrich Syndrome:** This X-linked condition can cause immunodeficiency, recurrent infections, eczema (which can be similar to seborrheic dermatitis), and thrombocytopenia.

2. **Chronic Granulomatous Disease (CGD):** This is a disorder where phagocytes have difficulty killing certain bacteria and fungi, leading to recurrent and severe infections.

3. **Leukocyte Adhesion Deficiency (LAD):** This is an immunodeficiency where the inability of white blood cells to adhere and migrate properly leads to recurrent bacterial infections and poor wound healing.

4. **X-linked Agammaglobulinemia (XLA):** This condition is marked by the absence of mature B cells and results in a lack of antibody production, leading to recurrent bacterial infections.

5. **Hyper IgM Syndrome:** This immunodeficiency involves defects in the CD40 ligand, resulting in normal or elevated levels of IgM antibodies but low levels of other immunoglobulins, leading to infections and possibly growth retardation.

**95. user - 2024-11-27 18:46:47**

The core symptom is dystonia, which is mainly manifested by abnormal expressive postures and involuntary movements, mostly involving the head and neck muscles, trunk muscles, rotator anterior muscles of the limbs, finger and wrist flexors, toe extensors and plantar flexors. It is aggravated by random movements, reduced or disappeared when resting and sleeping, and in the late stage, it can be a fixed twisting spastic deformity. Sensory trickery (relief techniques) and movement specificity are the characteristic manifestations of dystonia. Sensory tricks (relieving techniques) are random movements that are specifically designed to correct abnormal postures or relieve dystonic movements, usually simple movements that involve or target the affected area, but are not forceful to counteract the symptoms of dystonia. Movement specificity refers to the presence of dystonia only during specific activities or the performance of specific tasks, such as certain occupations (e.g., writing spasms, musician's spasms) or sports.

**ChatGPT-4o** - 2024-11-27 18:46:47

Based on the clinical manifestations described, the most likely rare disease to be diagnosed is **Dystonia**. Dystonia is characterized by the features you've mentioned: abnormal postures, involuntary movements that worsen with action, and improvement during rest and sleep. Sensory tricks and task-specific dystonia are also hallmark features.

Here are five other possible diagnoses that might be considered:

1. **Wilson's Disease**: A rare genetic disorder that leads to copper accumulation in the body, which can cause neurological symptoms including dystonia, as well as liver dysfunction.

2. **Parkinson's Disease with Dystonia**: Some patients with Parkinson's Disease can present with dystonia, especially as a component of the motor fluctuations.

3. **Huntington's Disease**: A genetic disorder that causes the progressive breakdown of nerve cells in the brain, leading to movement disorders, which can include dystonia.

4. **Tardive Dyskinesia**: A condition that results from long-term use of neuroleptic drugs, causing involuntary, repetitive body movements, which can include dystonic postures.

5. **Dopa-Responsive Dystonia (Segawa's Syndrome)**: A condition characterized by dystonia and parkinsonism, with symptoms that improve with levodopa treatment, often showing diurnal variation.

Each of these conditions has unique features and diagnostic criteria, so careful assessment and testing would be needed to differentiate them from primary dystonia and among themselves.

**96. user - 2024-11-27 19:42:47**

**1. Kidney,** the main manifestations are limb oedema and increased foam in the urine. Laboratory tests may reveal simple moderate proteinuria or nephrotic syndrome, and renal insufficiency may be seen in the advanced stage.

**2. Heart,** the main manifestations are shortness of breath after activity, limb edema, pleural effusion, ascites and other signs of restrictive cardiac insufficiency.

**3. Liver,** there may be slight discomfort or pain in the liver area, but most patients may be asymptomatic, and hepatomegaly or liver enzyme abnormalities are often detected during physical examination. Liver failure may occur in advanced stages of the disease.

**Peripheral and autonomic nerves, **Symmetrical sensory and/or motor peripheral neuropathy of the extremities. Autonomic abnormalities are often manifested by upright hypotension, gastroparesis, pseudo-intestinal obstruction, and impotence.

**5. Gastrointestinal tract** , can present with total gastrointestinal involvement, with the stomach and small intestine being the most commonly involved. It can present with epigastric discomfort, dyspepsia, diarrhoea, constipation, malabsorption syndrome and gastrointestinal bleeding.

**6. Soft tissues , **Tongue involvement may present with megaglossia, tongue dysmobility, and dysarthria. Skin mucosa may present with skin purpura and petechiae, which are more common around the orbits and in the loose skin areas of the neck. Nail atrophy and hair loss may also occur.

**7. Coagulation abnormalities** Patients often have coagulation factor X deficiency, resulting in corresponding bleeding manifestations.

**ChatGPT-4o** - 2024-11-27 19:42:47

The clinical manifestations you've described suggest a multisystem involvement. A rare disease that can cause this combination of symptoms is **Amyloidosis**. Amyloidosis is a condition characterized by the deposition of abnormal protein aggregates (amyloid) in various tissues, leading to organ dysfunction.

### Main Diagnosis:
- **Amyloidosis**

### Other Possible Diagnoses:
1. **Fabry Disease**: This is a genetic disorder that can cause renal, cardiac, and neurological manifestations, along with gastrointestinal symptoms.

2. **Systemic Lupus Erythematosus (SLE)**: Although more common than some rare diseases, SLE can cause multiorgan involvement, including kidney, heart, liver, nervous system, skin, and gastrointestinal tract.

3. **Sarcoidosis**: This is a systemic inflammatory disease that can affect multiple organs, including the heart, liver, peripheral nerves, and skin.

4. **Systemic Sclerosis (Scleroderma)**: This condition can cause skin changes, gastrointestinal involvement, renal and cardiac issues, and peripheral neuropathy.

5. **Mixed Connective Tissue Disease (MCTD)**: This is an autoimmune condition that can present with features of several connective tissue disorders, leading to multiorgan involvement similar to your description.

Each of these conditions has a different pathophysiological mechanism and requires specific tests for accurate diagnosis. Clinical correlation and further diagnostic workup, including biopsy and genetic testing, might be necessary to confirm the diagnosis.

**97. user - 2024-11-27 19:45:34**

Jaundice and itchy skin are typical clinical manifestations. Other developmental delays include short stature, delayed pubertal growth, gallbladder stones, steatorrhea due to fat absorption disorders, hepatosplenomegaly, rickets due to fat-soluble vitamin deficiencies, delayed bone age, dry eye, coagulation disorders, and neuromuscular disorders. Children may also develop visual and hearing abnormalities, irritability, lethargy and poor concentration. In advanced stages, portal hypertension and liver tumours may occur.

**ChatGPT-4o** - 2024-11-27 19:45:34

Based on the clinical manifestations you've provided, the rare disease most likely to be diagnosed is **Alagille syndrome**. Alagille syndrome is characterized by liver involvement such as cholestasis and jaundice, along with other symptoms like growth delays, cardiovascular anomalies, and skeletal abnormalities. The symptoms you've listed align well with this syndrome's multisystemic involvement.

Five other possible diagnoses to consider include:

1. **Progressive Familial Intrahepatic Cholestasis (PFIC)** - A group of disorders with cholestasis, jaundice, and liver disease, leading to growth delays and vitamin deficiencies due to malabsorption.

2. **Biliary Atresia** - A condition in infants that involves obstruction of bile flow from the liver, leading to jaundice, growth delays, and can progress to cirrhosis.

3. **Cystic Fibrosis (CF)** - While primarily affecting the lungs, it can also cause pancreatic insufficiency, leading to steatorrhea, fat-soluble vitamin deficiencies, and liver disease.

4. **Niemann-Pick Disease Type C** - A lysosomal storage disorder leading to hepatosplenomegaly, neurological symptoms, and potentially jaundice.

5. **Wilson's Disease** - A disorder of copper metabolism that can cause hepatic symptoms, neurological manifestations, and other systemic signs including growth delays.

Each of these conditions presents with overlapping symptoms, and further diagnostic evaluation, including genetic testing and liver function tests, would be necessary to differentiate between them.

**98. user - 2024-11-27 19:47:32**

The disease starts in childhood. The disease manifests itself as a mild delay in motor development and progressive atrophy of skeletal muscles, which affects the motor function of the limbs and gradually leads to gait abnormalities and limited movement of the upper limbs, with the natural course of the disease often resulting in loss of the ability to walk at about 10 years of age. Thereafter, scoliosis, joint contracture, respiratory muscle weakness and dilated cardiomyopathy appeared, and the patient died of respiratory failure and cardiac failure at the age of about 20 years. Pseudohypertrophy of both gastrocnemius muscles, pseudohypertrophy of both forearms and lingual muscles, positive Gowers' sign, and lumbar lordosis can be seen on examination. The disease may start in youth or even adulthood, and some patients do not affect survival. Signs of pseudohypertrophy are obvious, and in some patients, dilated cardiomyopathy is first apparent when limb weakness is still mild.

**ChatGPT-4o** - 2024-11-27 19:47:32

Based on the clinical manifestations you have described, the most likely diagnosis is **Duchenne Muscular Dystrophy (DMD)**. This condition is characterized by childhood onset, progressive muscle weakness, pseudohypertrophy of muscles, positive Gowers' sign, and complications such as scoliosis, joint contractures, respiratory muscle weakness, and cardiomyopathy, often leading to respiratory and cardiac failure.

Here are five other possible diagnoses that could be considered:

1. **Becker Muscular Dystrophy (BMD):** A milder and more variable form of dystrophy compared to Duchenne, with later onset and slower progression. Cardiomyopathy can also be a prominent feature, and pseudohypertrophy can be present.

2. **Limb-Girdle Muscular Dystrophy (LGMD):** This group of disorders can present with muscle weakness affecting the shoulder and pelvic girdles. Some forms may show pseudohypertrophy and cardiac involvement.

3. **Emery-Dreifuss Muscular Dystrophy (EDMD):** Characterized by early contractures, muscle weakness, and cardiac involvement. It has a slower progression compared to DMD.

4. **Congenital Muscular Dystrophy (CMD):** Although typically presenting earlier, some forms can have overlapping symptoms with juvenile-onset dystrophies, including muscle weakness, joint contractures, and possible cardiac involvement.

5. **Pompe Disease (Glycogen Storage Disease Type II):** This lysosomal storage disorder can present with proximal muscle weakness, respiratory involvement, and cardiomyopathy. The juvenile or late-onset forms may resemble dystrophies.

Proper genetic testing and muscle biopsy can help differentiate between these conditions and confirm the diagnosis.

**99. user - 2024-11-27 19:50:10**

Mainly hyperaemia, brain damage and cardiomyopathy.

**1. Neonatal onset** Normal at birth, vomiting, lethargy, hypotonia, convulsions, dyspnoea, hyperaemia, ketosis, hypoglycaemia, acidosis, dilated cardiomyopathy, pancreatitis and other abnormalities after starting breastfeeding, with a high morbidity and mortality rate.

**2. Delayed-onset** is often induced by fever, starvation, high-protein diet, and infections, and manifests itself as feeding difficulties in infancy and early childhood, developmental delays, convulsions, and hypotonia. In many patients, cognitive ability and neurological development are impaired, with an increase in slow waves or epileptic waves on the EEG; some patients may have fractures and osteoporosis on X-ray; bone marrow suppression is also common, causing granulocytopenia, anaemia and thrombocytopenia. There may also be cardiac damage, such as cardiomyopathy, arrhythmia, prolonged QT interval, and diminished cardiac function. Renal impairment is less common.

**ChatGPT-4o** - 2024-11-27 19:50:10

The clinical manifestations you've provided, particularly the combination of hyperaemia, brain damage, cardiomyopathy, and the progression from neonatal to delayed onset with the symptoms described, are suggestive of a metabolic disorder. Based on the description, one likely diagnosis is **Methylmalonic Acidemia (MMA)**. This is a rare inherited metabolic disorder characterized by the body's inability to break down certain fats and proteins, leading to the accumulation of methylmalonic acid.

### Other Possible Diagnoses:

1. **Propionic Acidemia**: Another metabolic disorder similar to MMA, where the body accumulates propionic acid due to an inability to break down certain amino acids and odd-chain fatty acids.

2. **Isovaleric Acidemia**: A rare organic acid disorder characterized by a distinctive sweaty feet odor, along with metabolic acidosis, ketosis, vomiting, and lethargy.

3. **Glutaric Acidemia Type I (GA-I)**: A disorder that involves the accumulation of glutaric acid and is associated with macrocephaly, encephalopathy, and dystonia, along with other metabolic crisis symptoms.

4. **Long-chain 3-hydroxyacyl-CoA dehydrogenase deficiency (LCHAD)**: This is a fatty acid oxidation disorder, which can also present with cardiomyopathy, muscle weakness, hypoglycemia, and other metabolic disturbances.

5. **Urea Cycle Disorders (e.g., Ornithine Transcarbamylase Deficiency)**: Although not typically associated with ketosis, these disorders can present with encephalopathy, hyperammonemia, seizures, and developmental delays, especially in situations that trigger catabolism.

These conditions require careful biochemical and genetic testing to confirm the diagnosis and differentiate between them, given their overlapping clinical spectrums.

**100. user - 2024-11-27 19:53:09**

Clinical manifestations are often nonspecific, with an insidious onset. Symptoms such as coughing up sputum, lethargy and fatigue may occur. Secondary infections may include fever, coughing up purulent sputum, or chest pain and haemoptysis. A few patients may be asymptomatic and may have no positive signs. Bursting sounds may be present in some patients, cyanosis may be present in severe cases, and pestle and mortar fingers may be seen in a few patients. The clinical picture is often less severe than the chest image, and this inconsistency between symptoms and images is a characteristic feature of the disease. Serum anti-GM-CSF antibodies are markedly elevated.

**ChatGPT-4o** - 2024-11-27 19:53:09

Based on the clinical manifestations and the specific finding of markedly elevated serum anti-GM-CSF antibodies, the most likely diagnosis is **Pulmonary Alveolar Proteinosis (PAP)**. PAP is a rare lung disease characterized by the accumulation of surfactant in the alveoli, leading to impaired gas exchange.

Five other possible diagnoses to consider, based on the symptoms provided, might include:

1. **Bronchiectasis**: Chronic condition involving dilated airways, often associated with chronic cough, sputum production, and recurrent infections.

2. **Chronic Obstructive Pulmonary Disease (COPD)**: Though more common, it can present with cough, sputum production, and fatigue. However, it typically lacks the specific antibody profile.

3. **Cystic Fibrosis**: A genetic disorder that can present with similar respiratory symptoms, although it is often diagnosed in childhood and has specific genetic testing.

4. **Asthma with Secondary Infection**: Can cause cough, sputum, and respiratory distress, but is more episodic and associated with wheezing.

5. **Idiopathic Pulmonary Fibrosis (IPF)**: A progressive lung disease with cough and fatigue, but usually exhibits a different imaging profile and lacks the antibody presence.

These alternative diagnoses consider the overlap in presenting symptoms and the rarity of certain features, with the primary differentiator being the elevated anti-GM-CSF antibodies specific to PAP.

**101. user - 2024-11-28 13:04:27**

Patients often start in infancy or adolescence, with roughly equal male and female ratios. The water content of secretions from the epithelial cells of the respiratory tract, pancreas and biliary tract is reduced, and the secretions become viscous and difficult to clear. The lungs are the most commonly affected organ, and patients present with chronic bacterial infections and colonisation of the respiratory tract with pathogens, often initially Haemophilus influenzae and Staphylococcus aureus, and ultimately with chronic colonisation and/or infection with Pseudomonas aeruginosa or Klebsiella onionis. Once infection occurs, neutrophils are recruited in large numbers into the lung tissue and release elastase, which causes destruction of the lung tissue, leading to the formation of bronchiectasis. The patient's bronchiectasis often starts at an early age and often begins in the upper lobes, in contrast to the majority of infections that result in the formation of bronchiectasis that is primarily located in the middle and lower lungs.

Patients often present with sweat stains left on dark-coloured clothing, or parents notice salty skin in their children. Abnormalities in the flow of bile and pancreatic fluid can lead to indigestion and malabsorption, as well as liver or pancreatic disease, and in severe cases, associated diabetes. The viscous intestinal fluid may also make the patient more susceptible to intestinal obstruction and rectal prolapse. In addition, male patients may develop impaired sperm movement in the vas deferens and congenital absence of the vas deferens (CBAVD).

**ChatGPT-4o** - 2024-11-28 13:04:27

Based on the clinical manifestations you have described, the most likely diagnosis is Cystic Fibrosis (CF). This condition is characterized by the production of thick, viscous secretions in various organs, primarily affecting the lungs, pancreas, and biliary system. The symptoms described fit well with CF, including chronic respiratory infections, bronchiectasis, salty sweat, malabsorption, and male infertility due to congenital bilateral absence of the vas deferens (CBAVD).

Here are five other possible diagnoses that may present with some similar features:

1. **Primary Ciliary Dyskinesia (PCD):** This genetic disorder affects the cilia lining the respiratory tract, leading to chronic respiratory infections and similar respiratory symptoms, though it generally does not present with salty sweat or pancreatic insufficiency.

2. **Alpha-1 Antitrypsin Deficiency:** This can lead to liver disease and early onset emphysema due to protease imbalance, potentially causing bronchiectasis, but typically would not lead to pancreatic insufficiency or salty sweat.

3. **Shwachman-Diamond Syndrome:** A rare inherited disorder that can cause pancreatic insufficiency, neutropenia, and skeletal abnormalities; however, it does not usually present with the lung symptoms typical of CF or salty sweat.

4. **Kartagener Syndrome:** A subset of PCD which includes situs inversus, chronic sinusitis, and bronchiectasis. While respiratory symptoms are similar, other systemic manifestations and genetic markers differ from CF.

5. **Young's Syndrome:** Characterized by a triad of bronchiectasis, rhinosinusitis, and obstructive azoospermia. It shares some respiratory manifestations with CF but lacks the pancreatic and sweat gland involvement.

Each of these conditions has distinct clinical and genetic markers that differentiate them from cystic fibrosis, which should be considered in the differential diagnosis process.

**102. user - 2024-11-28 13:08:44**

In most cases, the disease begins in adolescence, with a gradual loss of the optic rod cells, followed by cone cell involvement and death of the retinal pigment epithelium. Therefore, night blindness is usually the first symptom (but after urbanisation, some patients often have a decrease in visual acuity), and the earlier the onset of night blindness, the more severe the patient's condition. Although peripheral visual field defects also occur early, patients are usually unaware of them and rarely complain of them in the early stages of the disease. The disease progresses to a loss of central vision and further narrowing of the visual field, and most people become legally blind before the age of 40. The degree of visual impairment can be related to the mode of inheritance.

The presentation of the fundus is related to the stage of the disease: in the early stages of the disease, the fundus may be normal or nearly normal on routine examination, but in some patients, an early form of blanket retinal degeneration may be observed on mid-peripheral fundus photography (these subtle changes are difficult to recognise with current scanning laser fundus photography or wide-field fundus photography). As the disease progresses, some patients develop typical fundus changes, such as retinal osteoblast-like pigmentation combined with mid-peripheral retinal atrophy, a waxy optic disc, and thinning of retinal blood vessels; some patients develop blanketing or depigmentation, which can be seen in the mid-peripheral retina, or extensive degeneration. Other common complications include macular cystoid degeneration, posterior subcapsular cataract, vitreous dust particles, and optic disc vitreous warts; a rare complication is Coat's retinopathy.

**ChatGPT-4o** - 2024-11-28 13:08:44

Based on the clinical manifestations you've described, the most likely diagnosis is **Retinitis Pigmentosa (RP)**. This rare genetic disorder is characterized by progressive degeneration of the retina, affecting the rod photoreceptor cells first, leading to night blindness, and later involving the cone cells, resulting in a constricted visual field and eventual loss of central vision. The fundoscopic findings you described, including retinal pigmentation changes and retinal atrophy, are classic for RP.

Here are five other possible diagnoses that might be considered, based on the symptoms described:

1. **Leber Congenital Amaurosis (LCA):** Although it typically presents in infancy or early childhood, some forms can have later onset and share retinal degeneration characteristics similar to RP.

2. **Cone-Rod Dystrophy:** This condition also involves both cone and rod cells but typically starts with loss of central vision (cones) and later night blindness and peripheral vision loss (rods), unlike the sequence in classic RP.

3. **Choroideremia:** An X-linked condition characterized by progressive vision loss due to degeneration of the choroid, retinal pigment epithelium, and photoreceptors. It can present with night blindness and peripheral vision loss.

4. **Usher Syndrome:** A condition involving both hearing loss and retinitis pigmentosa. It's an important differential if the patient also has hearing abnormalities.

5. **Bardet-Biedl Syndrome:** A syndromic retinal dystrophy that includes features of retinitis pigmentosa along with polydactyly, obesity, renal anomalies, and sometimes cognitive impairments.

Further diagnostic tests, such as genetic testing and detailed retinal imaging, can help differentiate these conditions and confirm the diagnosis of retinitis pigmentosa or another similar retinal dystrophy.

**103. user - 2024-11-28 13:11:16**

The most common clinical presentation is white pupil syndrome, in which a yellow-white reflection is visible in the pupil. Disuse strabismus may occur in the affected eye due to the tumour being located at the posterior pole and low visual acuity. The early presentation is a greyish-white, round or oval arching mass that may bulge into the vitreous or grow flat along the choroid. Retinal blood vessels on the surface of the tumour may become dilated and haemorrhagic, and even exudative retinal detachment may occur. Tumour tissue may also penetrate the retina and enter the vitreous, floating like a snowball, or even deposited in the anterior chamber to form pseudo anterior chamber pus. The onset of the disease is early in children with bilateral retinal detachment, usually within one year of age. The onset is later in unilateral children, usually at two or three years of age.

**ChatGPT-4o** - 2024-11-28 13:11:16

Based on the clinical manifestations you've described, the most likely diagnosis is **retinoblastoma**. Retinoblastoma is a rare eye cancer that typically affects young children and is characterized by a white pupillary reflex (leukocoria), strabismus, and other features mentioned.

Five other possible diagnoses that can present with similar symptoms include:

1. **Coats' disease**: This is a condition that leads to retinal telangiectasia and exudative retinal detachment, which can also present with leukocoria and strabismus.

2. **Persistent Fetal Vasculature Syndrome (PFVS)**: Previously known as persistent hyperplastic primary vitreous (PHPV), this congenital developmental anomaly of the eye can present with leukocoria.

3. **Toxocariasis**: An ocular infection caused by Toxocara canis, leading to granulomatous inflammation that may cause white pupillary reflex and retinal detachment.

4. **Congenital cataract**: Opacification of the lens that can cause leukocoria; it may not present with some of the more tumor-specific signs like vitreous seeding or retinal masses.

5. **Retinal detachment due to familial exudative vitreoretinopathy (FEVR)**: This hereditary retinal disorder can mimic some features of retinoblastoma, including retinal detachment and possibly leukocoria.

Each of these conditions has distinct clinical and diagnostic features, so careful evaluation including imaging and possibly genetic testing is crucial for an accurate diagnosis.

**104. user - 2024-11-28 13:12:38**

Patients present with oropharyngitis, otitis media, respiratory infections, cellulitis and skin infections, often due to staphylococcal and streptococcal infections. Oral ulcers and painful gingivitis occur in most children before the age of 2 years in severe neutropenia with decreased bone marrow reserve. If associated with diffuse gastrointestinal lesions, it may cause abdominal pain and diarrhoea similar to Crohn's disease. Neutropenia can lead to recurrent bacterial or fungal infections in various areas, with the main sites of involvement being the skin and mucous membranes, ears, nose, throat and lungs. The infections are often severe or even fatal. About 15% of patients progress to acute leukaemia or myelodysplastic syndrome. The children usually do not have characteristic malformations.

**ChatGPT-4o** - 2024-11-28 13:12:38

Based on the clinical manifestations you described, the most likely diagnosis is Severe Congenital Neutropenia (SCN), also known as Kostmann syndrome. This condition is characterized by severe neutropenia leading to recurrent bacterial infections, especially involving the skin, mucous membranes, and respiratory tract, as well as an increased risk of progression to acute leukemia or myelodysplastic syndrome.

Five other possible diagnoses to consider are:

1. **Cyclic Neutropenia**: This is characterized by periodic drops in neutrophil count, leading to cyclic episodes of infection. Although not constant like SCN, it can present similarly during the neutropenic phases.

2. **Chronic Granulomatous Disease (CGD)**: This is an immunodeficiency that affects the ability of phagocytes to kill certain bacteria and fungi, resulting in recurrent infections and granuloma formation, but typically does not present with neutropenia.

3. **Shwachman-Diamond Syndrome**: This is a rare congenital disorder that can present with neutropenia, exocrine pancreatic insufficiency, and skeletal abnormalities, with increased risk for hematological malignancies.

4. **Dysgammaglobulinemia**: Although primarily an immunoglobulin disorder, it can lead to recurrent infections similar to those seen in neutropenia.

5. **Hyper-IgE Syndrome (Job's Syndrome)**: This immunodeficiency syndrome presents with recurrent staphylococcal infections, eczema, and elevated serum IgE levels, and can sometimes be confused with neutropenic disorders due to recurrent infections.

Each of these conditions can mimic aspects of the clinical presentation you described, but Severe Congenital Neutropenia remains the most fitting primary diagnosis based on the information provided.

**105. user - 2024-11-28 13:14:48**

The average age of onset is 5.6 months. The first seizure is usually normal at birth and starts within 1 year of age. The first seizure is usually febrile convulsion, which may be one-sided or generalised clonic or tonic clonic seizure, and febrile convulsions are characterized by long duration and recurrent episodes, and may appear in a persistent state. 28%~35% of children may start the disease without febrile convulsions, but they often have heat sensitivity in the later course of the disease. Elevated body temperature due to various reasons, including exercise, fever, vaccination, hot baths, and high ambient temperatures, can trigger or exacerbate seizures and predispose to status epilepticus. Some children develop an acute encephalopathy characterised by deep coma following status epilepticus.

Children tend to present with febrile convulsions between the ages of 1 and 4 years, and have a variety of seizure types, such as myoclonic, atypical catatonic, partial seizures, and generalised tonic clonic seizures. Myoclonic seizures are often triggered by light stimulation, eye closure, and a specific graphic stimulus, and involve simultaneous clonus of one or several muscles, all accompanied by synchronised abnormal brain wave bursts. The intensity of the myoclonus varies, from a slight forward or backward movement of the body when only the axial muscles are violated, to a severe fall or sudden throwing of an object in the hand. Myoclonic seizures can be isolated or cluster, but a sustained state is rare. Myoclonic seizures may not occur at all in about 25% of children. The concurrent EEG of atypical apoplectic seizures shows half of the discharges to be of focal origin, most of which are irregular, irregular, slow spikes and slow waves, often accompanied by myoclonic seizures. 30% of the children may have atypical apoplectic persistent seizures. The incidence of partial seizures is 43.0% to 78.6%, which manifests as lateral limb (or) head deflection seizures or clonic seizures, and there are also motor seizures with consciousness and autonomic symptoms, automatisms, and hypotonia, etc. After 1 year of age, the children have psychomotor developmental delays, especially language developmental delays.

**ChatGPT-4o** - 2024-11-28 13:14:48

Based on the clinical manifestations you've described, the rare disease that is most likely to be diagnosed is Dravet Syndrome. Dravet Syndrome is a severe form of epilepsy that begins in the first year of life with frequent, prolonged seizures that are often triggered by fever. The description of a child with normal development in early infancy, followed by recurrent seizures, including febrile seizures and various other types of seizures, fits well with Dravet Syndrome. The seizures you mentioned, such as febrile convulsions, myoclonic, and partial seizures, as well as the developmental delays, are characteristic of this condition.

Five other possible diagnoses that could present with similar features might include:

1. **Genetic Epilepsy with Febrile Seizures Plus (GEFS+)**: This condition can present with febrile seizures and other seizure types, but generally has a broader age range and less severe developmental impact.

2. **Lennox-Gastaut Syndrome**: This syndrome is characterized by multiple types of seizures, including tonic and atonic seizures, and can present with cognitive impairment, but typically starts between ages 3 and 5.

3. **Doose Syndrome (Myoclonic-Astatic Epilepsy)**: This is another epilepsy syndrome that starts in childhood and is characterized by myoclonic-atonic seizures, but it may start a bit later than Dravet Syndrome.

4. **Infantile Spasms (West Syndrome)**: This is a more distinct form of epilepsy with spasms and hypsarrhythmia on EEG, typically beginning in the first year of life, but with a different seizure pattern.

5. **Complex Febrile Seizures**: Although not a specific syndrome, complex febrile seizures can involve prolonged and recurrent febrile seizures and might precede other epilepsy syndromes, but generally do not involve the spectrum of other seizure types you described.

Each of these conditions has specific clinical, genetic, and often EEG features that can help differentiate them from Dravet Syndrome.

**106. user - 2024-11-28 15:44:42**

On the one hand, it presents as chronic haemolytic anaemia, and in addition to anaemia-related symptoms, it may cause mild to moderate yellowing of the skin and sclera and pigmented cholelithiasis due to elevated bilirubin, which may be exacerbated by cold, infection and dehydration. In infants and young children, splenomegaly may be present, and with age, the spleen may shrink due to splenic infarction and fibrosis.

On the other hand recurrent vaso-obstructive pain due to capillary micro thrombosis. Children and adults tend to present with myalgia of the limbs, large joint pain and low back pain, and in severe cases, severe abdominal pain, headache, and even limb paralysis and coma. Over time, vascular obstruction can occur in almost every organ system, and cyclical pain can occur in patients who are thin, easily fatigued, susceptible to infection, and malnourished. Thinning of the bone cortex, osteoporosis, spinal deformity, aseptic necrosis of the femoral head due to an overcompensatory increase in bone marrow haematopoietic tissue, and localised osteosclerosis if there is a bone infarction. Ocular symptoms may include fundus haemorrhage and retinal detachment due to embolism of the retinal vessels in the fundus. In addition, mental retardation may occur, affecting neurological development.

Acute pain is the first symptom of the disease in more than 25% of patients. It is most common in children older than 2 years of age. The severity of pain can vary from mild to severe. Approximately one-half of pain episodes are accompanied by objective clinical signs such as fever, swelling, tenderness, tachypnoea, hypertension, nausea and vomiting.

**ChatGPT-4o** - 2024-11-28 15:44:42

Based on the clinical manifestations provided, the most likely rare disease is **Sickle Cell Disease (SCD)**. This condition is characterized by chronic hemolytic anemia, jaundice due to elevated bilirubin, and vaso-occlusive crises which lead to recurrent pain episodes and potential organ damage. The pain crises are often triggered by factors such as cold, infection, and dehydration, aligning with the presented symptoms. The skeletal, ocular, and neurological complications further support this diagnosis, particularly the mention of aseptic necrosis of the femoral head and the described ocular symptoms, which are common in SCD.

Five other possible diagnoses that could present with similar features include:

1. **Thalassemia Intermedia**: Although less likely due to the description of vaso-occlusive crises, it can present with chronic hemolytic anemia, splenomegaly, and possible bone changes due to marrow expansion.

2. **Hereditary Spherocytosis**: This condition also causes chronic hemolytic anemia and splenomegaly, but lacks the vaso-occlusive crises typical of sickle cell disease.

3. **Paroxysmal Nocturnal Hemoglobinuria (PNH)**: PNH can cause hemolytic anemia and jaundice, with potential episodes of thrombosis, but it usually presents with hemoglobinuria and lacks the specific pain crisis pattern.

4. **G6PD Deficiency**: This can cause acute hemolysis and jaundice, especially after oxidative stress, but it's not associated with chronic pain crises or the extensive organ involvement described.

5. **Pyruvate Kinase Deficiency**: Another hemolytic anemia that can cause splenomegaly and jaundice, but typically does not have the vaso-occlusive complications seen in sickle cell disease.

Each of these conditions shares some overlapping features with the described case, but sickle cell disease is the most consistent with the complete spectrum of symptoms provided.

**107. user - 2024-11-28 15:48:14**

The main clinical manifestations of neonatal and paediatric patients include:

1. Intrauterine growth retardation and postnatal growth disorders . Intrauterine growth retardation, low birth weight, lack of postnatal catch-up growth, and proportional short stature.

2. Specific physical signs: wide and prominent forehead, pointed jaw leading to a triangular face, and large relative head circumference. Dysmorphic teeth, low ear position, and asymmetry of trunk deviation, including limb length discrepancy, and lateral curvature deformity of the little finger.

3. Other manifestations include feeding difficulties, hypoglycaemia in the neonatal period, genitourinary malformations such as hypospadias, cryptorchidism and horseshoe kidneys, and digestive anomalies such as cafe au lait spots and reflux oesophagitis. Some patients have cognitive, motor and language developmental delays and learning disabilities.

**ChatGPT-4o** - 2024-11-28 15:48:14

Based on the clinical manifestations you have provided, the most likely rare disease being described is Russell-Silver Syndrome. This is a congenital condition characterized by intrauterine growth retardation, postnatal growth disorders, and distinct facial features as well as potential asymmetry of limbs.

Here are five other possible diagnoses that might be considered based on the clinical manifestations:

1. **Turner Syndrome**: While typically affecting females and characterized by short stature and ovarian insufficiency, Turner syndrome can also present with certain dysmorphic features and growth issues.

2. **Noonan Syndrome**: This can present with growth delays, congenital heart defects, and distinct facial features. It also occasionally overlaps with some genitourinary and skeletal anomalies.

3. **Beckwith-Wiedemann Syndrome**: Although it typically involves overgrowth, some features such as asymmetry, developmental delays, and some genitourinary abnormalities can overlap.

4. **Sotos Syndrome**: Characterized by overgrowth and distinctive craniofacial features, along with developmental delays. While not typically associated with growth retardation, the facial features and developmental concerns can be similar.

5. **Prader-Willi Syndrome**: Presents with hypotonia, feeding difficulties in infancy, and developmental delays. While the growth pattern differs, there can be overlapping cognitive and physical manifestations.

Each of these syndromes has distinct features, so clinical genetic testing and expert consultation would be necessary to confirm the diagnosis.

**108. user - 2024-11-28 15:51:57**

**1. Xanthomas of the skin,** occurring near the attachment points of the extensor tendons, such as the Achilles tendon, elbow, knee, etc., and there is a close relationship between skin injuries and the appearance of xanthomas. Cutaneous xanthomas are rare in children.

**2. Atherosclerosis , **Patients have a significantly higher risk of developing atherosclerosis and coronary artery disease than normal subjects. Early-onset coronary artery disease may be one of the characteristics of the disease.

**3. Alterations in the blood system , **The content of plant sterols in the blood is significantly increased, resulting in a higher proportion of plant sterols in the sterol component of the cell membrane, abnormal cell membrane function of red blood cells, platelets and other cell membranes, anaemia and thrombocytopenia, and the patient may have weakness, pallor, skin bleeding and other manifestations.

**4. Others,** in addition to the above symptoms, some patients may develop joint pain, arthritis, splenomegaly, elevated transaminases, etc.

**ChatGPT-4o** - 2024-11-28 15:51:57

Based on the clinical manifestations you've provided, the most likely diagnosis is **sitosterolemia**. This rare genetic disorder is characterized by the accumulation of plant sterols in the blood, leading to symptoms such as xanthomas, premature atherosclerosis, and hematological abnormalities like anemia and thrombocytopenia.

Here are five other possible diagnoses to consider:

1. **Familial Hypercholesterolemia (FH):** This genetic disorder is characterized by high cholesterol levels, specifically low-density lipoprotein (LDL), and early-onset coronary artery disease. Xanthomas may also be present, although plant sterol accumulation is not typical.

2. **Cerebrotendinous Xanthomatosis (CTX):** A rare lipid storage disease that presents with tendon xanthomas, neurological symptoms, and premature atherosclerosis. However, CTX involves the accumulation of cholestanol, not plant sterols.

3. **Familial Combined Hyperlipidemia (FCH):** This common genetic disorder may cause increased risk for coronary artery disease and sometimes xanthomas, though it does not typically involve elevated plant sterols or hematological changes.

4. **Hypobetalipoproteinemia:** A disorder characterized by low levels of LDL cholesterol, which can sometimes have features like xanthomas and liver abnormalities without the typical plant sterol increase.

5. **Niemann-Pick Disease Type C:** This condition can cause hepatosplenomegaly, neurological symptoms, and sometimes lipid accumulation that might mimic xanthomas, though it differs in terms of sterol profile and atherosclerosis risk.

Each of these conditions has distinct differences in pathophysiology and clinical presentation that would need to be considered when making a differential diagnosis.

**109. user - 2024-11-28 16:52:10**

The onset of the disease is insidious in males, and the common age of onset is 30-60 years old. Patients often start with painful spasms, tremor, and weakness of the lower limbs, and gradually develop weakness of the upper limbs, muscle atrophy of the medulla oblongata and the face, muscle fasciculations, dysarthria, dysphagia, dyspnoea, and respiratory difficulties. Androgen receptor insensitivity may be associated with gynecomastia and reduced reproductive function. Sensory abnormalities are present in more than 50 per cent of patients. The disease progresses slowly, with patients usually becoming unable to walk late in the course of the disease, and only some patients require assisted ventilation, which has no significant impact on survival.

**1. Atypical symptoms,** some of which may occur years or even decades before the onset of limb weakness, include fatigability, painful spasms, perioral tremor, and postural tremor. Muscle weakness: most patients start with weakness of both lower limbs, gradually progressing to both upper limbs, tongue muscles and facial muscles, while the extraocular muscles are often not involved. The distribution of limb weakness is asymmetrical, with proximal involvement predominating. Involvement of the muscles of mastication may cause mandibular ptosis and tremor. Physical examination of the lower motor neuron damage signs, manifested as a decrease in muscle strength of the limbs, proximal, accompanied by muscle atrophy, fasciculations, tendon reflexes are weakened or disappeared. Most patients may have obvious tongue muscle atrophy and fibrillation, but the soft palate activity and pharyngeal reflexes are normal, and the degree of choking on water and dysphagia is mild. In some patients, chin and perioral fasciculations can also be observed. The motor function impairment of the patients gradually increases with the prolongation of the disease, and in the later stage, they may have difficulty in walking up the stairs and walking unsteadily.

**2. Sensory deficits** , with varying degrees of hyperalgesia present in about half of the patients, while in the others it is subclinical, with abnormalities found only on sensory nerve conduction tests.

**3. endocrine abnormalities,** including abnormalities of glucose and fat metabolism, and androgen receptor insensitivity and other manifestations, the latter including gynecomastia, decreased sexual function, infertility, and testicular atrophy.

The latter include gynecomastia, decreased sexual function, infertility, testicular atrophy, etc.

**4. Female carriers,** most female carriers do not have symptoms, and even if they do have symptoms, they are mild, such as fasciculations, mild weakness of distal limbs, muscle spasms, or increased creatine myosinase.

**ChatGPT-4o** - 2024-11-28 16:52:10

Based on the clinical manifestations described, the most likely diagnosis is **Kennedy's Disease**, also known as Spinal and Bulbar Muscular Atrophy (SBMA). This condition is characterized by insidious onset of muscle weakness, atrophy, and fasciculations, particularly affecting males due to its X-linked recessive inheritance. The involvement of androgen receptor insensitivity leading to symptoms like gynecomastia and infertility further supports this diagnosis.

Here are five other possible diagnoses to consider:

1. **Amyotrophic Lateral Sclerosis (ALS)**: While ALS typically progresses more rapidly than Kennedy's Disease and doesn't usually include endocrine symptoms, the muscle weakness, atrophy, and fasciculations might initially suggest ALS.

2. **Myotonic Dystrophy**: This can present with muscle weakness, endocrine abnormalities, and facial muscle involvement, but it usually includes myotonia (difficulty relaxing muscles) as a key feature.

3. **Friedreich's Ataxia**: This condition involves progressive weakness and sensory deficits, but it typically starts in childhood or adolescence and is often associated with ataxia and other systemic findings.

4. **Facioscapulohumeral Muscular Dystrophy (FSHD)**: This condition involves facial and shoulder girdle muscle weakness, but it doesn't typically involve endocrine abnormalities or the specific pattern of sensory and motor deficits described.

5. **Charcot-Marie-Tooth Disease (CMT)**: This group of inherited neuropathies can cause muscle weakness and sensory deficits, but the endocrine features and pattern of progression described here are not typical for CMT.

Each of these alternatives would require careful differentiation based on clinical, genetic, and sometimes electrophysiological or laboratory findings.

**110. user - 2024-11-28 16:54:08**

Some patients present with reduced foetal movement in utero and are born as floppy babies. The onset of the disease occurs within 6 months, with a mean age of onset of 1 month after birth. They present with generalised floppy weakness and severe hypotonia. Most children have difficulty sucking and swallowing due to weakness of the tongue, facial and masticatory muscles, and atrophy and tremor of the lingual muscles are seen. Intercostal muscle involvement can present with dyspnoea and abdominal breathing. The chest has a bell-shaped appearance. The lower limbs are more heavily involved than the upper limbs, and proximal is more severe than distal. Severe muscle weakness in the mid-trunk area leaves the child unable to control head movements, unable to lift or roll over, and without the ability to sit alone. When lying down, both lower limbs are in the frog-leg position with hip abduction and knee flexion. Muscle atrophy is not obvious, and some children have mild joint deformities. Children with good intelligence, tendon reflexes disappeared, limb sensation is normal. The children's muscle weakness progressively worsens, and eventually they lose all voluntary movement ability, are fed nasogastrically, and suffer from respiratory failure due to repeated respiratory infections. 80% of the children die within 1 year of age, and rarely survive beyond 2 years of age.

**ChatGPT-4o** - 2024-11-28 16:54:08

Based on the clinical manifestations described, the most likely diagnosis for this condition is **Spinal Muscular Atrophy Type 1 (SMA Type 1)**, also known as Werdnig-Hoffmann disease. This is a genetic disorder characterized by severe muscle weakness, hypotonia, and progressive motor neuron degeneration, typically leading to early mortality due to respiratory failure.

Other possible diagnoses to consider include:

1. **Congenital Myopathy**: A group of muscle disorders present at birth, characterized by muscle weakness and hypotonia, although the progression and specific symptoms can vary widely.

2. **X-linked Myotubular Myopathy (Centronuclear Myopathy)**: A rare genetic condition causing severe muscle weakness and hypotonia, particularly affecting males, with similar early onset symptoms.

3. **Pompe Disease (Infantile-Onset Glycogen Storage Disease Type II)**: A metabolic disorder caused by the buildup of glycogen in the body's cells, leading to muscle weakness and respiratory problems, though often accompanied by cardiomyopathy as well.

4. **Congenital Muscular Dystrophy**: A group of genetic conditions causing muscle weakness and hypotonia from birth, although typically with more variability in symptoms and progression.

5. **Nemaline Myopathy**: A congenital myopathy characterized by muscle weakness and hypotonia, often presenting with similar early life symptoms, though usually with distinct histological features seen on muscle biopsy.

These alternative diagnoses should be considered based on additional clinical evaluations, genetic testing, and possibly muscle biopsy findings.

**111. user - 2024-11-28 17:17:22**

Patients usually start in adulthood, with an insidious onset and gradual progression. The main manifestations are cerebellar ataxia, including drunkard's gait, clumsy movements, minstrel-like speech, nystagmus, diplopia, etc.; pyramidal tract damage, including hyperreflexia, ankle clonus, and positive pathological signs; and extrapyramidal symptoms, including Parkinson's syndrome, dystonia, and ankylosis. In addition, some patients may have peripheral neuropathy. In the early stages of the disease, some patients may have damage to the pyramidal tract (similar to spastic paraplegia) or extrapyramidal symptoms (similar to Parkinson's syndrome), and ataxia is not prominent.

The disease progresses gradually, with loss of walking ability after 10-15 years. Prolonged dysphagia leading to malnutrition or aspiration is the main cause of death.

**ChatGPT-4o** - 2024-11-28 17:17:22

Based on the clinical manifestations you provided, the condition described is suggestive of Multiple System Atrophy (MSA). MSA is a rare neurodegenerative disorder characterized by a combination of cerebellar ataxia, autonomic dysfunction, parkinsonism, and, in some cases, pyramidal tract signs.

Other possible diagnoses that could present with similar symptoms include:

1. **Spinocerebellar Ataxia (SCA):** A group of hereditary ataxias that present with cerebellar ataxia and may include pyramidal and extrapyramidal symptoms.

2. **Progressive Supranuclear Palsy (PSP):** A condition that can present with parkinsonism, difficulties with balance, and eye movement abnormalities, though cerebellar symptoms are less prominent.

3. **Corticobasal Degeneration (CBD):** A rare condition that can present with asymmetrical parkinsonism, dystonia, and cortical sensory loss, along with extrapyramidal symptoms.

4. **Friedreich's Ataxia:** A hereditary degenerative disease that primarily affects the spinal cord and peripheral nerves, presenting with ataxia, loss of reflexes, and pyramidal tract signs.

5. **Wilson's Disease:** A genetic disorder that typically presents with hepatic, neurological, and psychiatric symptoms, including tremors, dystonia, ataxia, and parkinsonism, due to copper accumulation.

It is important to consider the entire clinical picture, including any genetic, biochemical, and imaging studies, to arrive at a definitive diagnosis.

**112. user - 2024-11-28 17:20:36**

The disease may be combined with gastro-oesophageal reflux in the early stages of the disease. As the disease progresses, the skin becomes swollen and sclerotic and may be accompanied by changes in skin pigmentation, including hyperpigmentation or depigmentation. In addition to skin involvement, patients often present with arthralgia and myalgia, and may develop ulcers and capillary dilatation of the digits. In addition, the patient may have multiple internal organ involvement. The respiratory system is more likely to be involved, with interstitial fibrosis and pulmonary hypertension. The gastrointestinal manifestations are varied and include gastroesophageal reflux, sinus vasodilatation, abdominal pain, diarrhoea, constipation and fecal incontinence. A small number of patients may present with renal crisis, which is characterised by acute renal insufficiency and is often associated with hypertension. Cardiac involvement may manifest as pericardial effusion, abnormalities of the cardiac conduction system, and myocardial involvement in individual patients. Neurological involvement of the peripheral nervous system is common, such as trigeminal neuropathy, peripheral neuropathy, etc. Central nervous system involvement is rare.

**ChatGPT-4o** - 2024-11-28 17:20:36

Based on the clinical manifestations you have described, the disease most likely to be diagnosed is **Systemic Sclerosis (Scleroderma)**. This rare autoimmune connective tissue disease is characterized by skin involvement, arthralgia, myalgia, and potential internal organ involvement, including the gastrointestinal tract, lungs, kidneys, and heart. The presence of gastroesophageal reflux, skin changes (such as hyperpigmentation and sclerotic changes), digital ulcers, and capillary changes are hallmark features. Pulmonary involvement with interstitial lung disease and pulmonary hypertension further supports this diagnosis.

Here are five other possible diagnoses that could present with some overlapping symptoms:

1. **Mixed Connective Tissue Disease (MCTD)**: Features of systemic sclerosis, along with symptoms of lupus and polymyositis. It can present with arthralgia, myalgia, and internal organ involvement.

2. **Dermatomyositis**: An inflammatory condition affecting skin and muscles, which may have overlapping systemic features, involving the lung and gastrointestinal tract.

3. **Eosinophilic Fasciitis**: Characterized by skin thickening and sometimes scleroderma-like features, with muscular pain and possible systemic involvement, although typically less severe than systemic sclerosis.

4. **Sarcoidosis**: A multi-system inflammatory disease that can cause skin changes, joint pain, and lung involvement, albeit with different histopathological findings.

5. **Lupus Erythematosus (Systemic Lupus Erythematosus - SLE)**: An autoimmune disease that can present with skin involvement, organ manifestations, arthralgia, and potential renal crisis, although with distinct immunological markers and systemic features.

These alternative diagnoses would require further investigation to rule out, often involving serological tests, biopsy, imaging, and clinical correlation to confirm the primary diagnosis.

**113. user - 2024-11-28 17:39:16**

In addition to clinical symptoms similar to phenylketonuria, children 1-3 months after birth also show symptoms of dopamine deficiency, such as dyskinesia, hypotonia, lethargy, nystagmus, dysphagia, etc. Symptoms related to serotonin deficiency, such as unresponsiveness, depression, insomnia, etc. Norepinephrine deficiency, such as low muscle tone in the trunk, ptosis, cerebellar dysgenesis, etc., may be associated with dopamine deficiency. Symptoms associated with norepinephrine deficiency, such as hypotonia of the trunk, ptosis, and cerebellar hypoplasia. There may also be persistent convulsions, recurrent fever, delayed development of motor milestones, total paralysis, and severe impairment of intellectual development.

**ChatGPT-4o** - 2024-11-28 17:39:16

Based on the clinical manifestations you provided, the symptoms suggest a rare neurotransmitter disorder, with a primary suspicion of Aromatic L-amino acid decarboxylase (AADC) deficiency. This condition is characterized by a deficiency in the enzyme responsible for the synthesis of neurotransmitters such as dopamine, norepinephrine, and serotonin, leading to the constellation of symptoms you've described.

Here are five other possible diagnoses to consider:

1. **Tyrosine Hydroxylase Deficiency**: This condition also affects dopamine production and can present with similar symptoms, including movement disorders, developmental delays, and autonomic dysfunction.

2. **Segawa Syndrome (Dopa-Responsive Dystonia)**: Although typically with later onset, in some cases, symptoms may appear early and include dystonia, hypotonia, and developmental delay, potentially overlapping with your clinical picture.

3. **Succinic Semialdehyde Dehydrogenase Deficiency (SSADH deficiency)**: While this disorder primarily affects GABA metabolism rather than catecholamines, it may present with hypotonia, developmental delay, and seizures, overlapping with some of the symptoms described.

4. **Cerebral Creatine Deficiency Syndromes**: These disorders (e.g., GAMT deficiency, AGAT deficiency) can also present with hypotonia, developmental delays, and seizures, although the specific neurotransmitter profile would differ.

5. **Pyridoxine-dependent Epilepsy**: This rare condition can present with early-onset seizures, developmental delays, and may include hypotonia. It is treatable with vitamin B6 supplementation.

Each of these conditions has distinct biochemical and genetic markers that can be investigated to reach a definitive diagnosis.

**114. user - 2024-11-28 17:48:33**

They often present with multisystem involvement and develop in stages. Cardiac rhabdomyosarcomas can appear in the foetal/neonatal period, most of which resolve spontaneously; epileptic and cutaneous symptoms can appear in the first few months of life; renal angiomyolipomas and neoplastic lesions elsewhere in the body develop with age; and adult women are prone to pulmonary lymphangioleiomyomatosis of the lungs. Brain lesions include mainly cerebral cortical nodules, subventricular giant cell astrocytomas, and subventricular nodules. Skin lesions include leukoplakia visible after birth, progressive facial angiofibromas, shark leather spots, perinodal fibromas, and forehead plaques; renal vascular smooth muscle lipomas are present in up to 90% of patients; retinal involvement is present in about 50% of patients but rarely affects visual acuity; and LAM in the lungs occurs in about 35% of females; other areas, such as the liver, pancreas, gastrointestinal tract, and skeleton, may be involved, although usually with milder symptoms. Others, such as the liver, pancreas, digestive tract, and bones, can also be involved, but symptoms are usually mild.

**ChatGPT-4o** - 2024-11-28 17:48:33

The clinical manifestations you describe are characteristic of Tuberous Sclerosis Complex (TSC). TSC is a genetic disorder that affects multiple organ systems and results in benign tumors in organs such as the brain, skin, kidneys, heart, and lungs. The presence of cardiac rhabdomyomas, renal angiomyolipomas, pulmonary lymphangioleiomyomatosis (LAM), and various skin lesions are hallmark features of TSC.

Five other possible diagnoses to consider based on similar multisystem involvement include:

1. **Neurofibromatosis Type 1 (NF1)**: Characterized by café-au-lait spots, neurofibromas, Lisch nodules in the eyes, and potential involvement of the central nervous system with tumors.

2. **Von Hippel-Lindau Disease (VHL)**: Known for its association with hemangioblastomas of the brain, spinal cord, and retina, renal cell carcinoma, and pheochromocytomas.

3. **Sturge-Weber Syndrome**: Involves port-wine stains on the face, neurological abnormalities (like seizures), and leptomeningeal angiomas.

4. **Birt-Hogg-Dubé Syndrome**: Characterized by skin lesions, pulmonary cysts, and an increased risk of renal tumors.

5. **Hereditary Leiomyomatosis and Renal Cell Cancer (HLRCC)**: Somewhat similar in the multiorgan involvement, includes cutaneous and uterine leiomyomas and renal cell cancer.

Each of these conditions has distinct features that differentiate them from TSC, but they share some overlapping symptoms, particularly in the dermatological and renal manifestations.

**115. user - 2024-11-28 17:51:17**

Most children with the undetected or untreated chronic form die before the age of 10 years, usually as a result of liver failure, neurological involvement or hepatocellular carcinoma.

The acute form has a rapid onset and progression, with acute liver failure as the main manifestation, and clinical signs include hepatosplenomegaly, jaundice, vomiting, abdominal distension, anorexia, lethargy, anaemia, bleeding tendencies and growth retardation. The child may have the characteristic odour of ‘boiled cabbage’ or ‘rotten mushrooms’. Insufficient hepatic synthesis of coagulation factors, marked prolongation of PT and APTT, and decreased levels of coagulation factors II, VII, IX, Ⅺ and Ⅻ, which are difficult to correct with vitamin K supplementation. Hepatic failure may be followed by ascites, jaundice and gastrointestinal bleeding, and children occasionally develop persistent hypoglycaemia. Untreated children often die of liver failure within weeks or months of onset of illness.

**ChatGPT-4o** - 2024-11-28 17:51:17

Based on the clinical manifestations provided, the most likely diagnosis is Tyrosinemia Type I (Hepatorenal Tyrosinemia). This rare metabolic disorder is characterized by a deficiency of the enzyme fumarylacetoacetate hydrolase, leading to accumulation of toxic metabolites that cause severe liver and renal dysfunction.

### Five Other Possible Diagnoses:

1. **Galactosemia**: A hereditary disorder affecting the metabolism of galactose, leading to liver damage, renal dysfunction, and cataracts. It can cause symptoms like jaundice, hepatomegaly, and failure to thrive in infants.

2. **Glycogen Storage Disease Type IV (Andersen's Disease)**: A disorder characterized by the accumulation of abnormal glycogen in the liver, muscle, and other tissues, leading to liver failure, cirrhosis, and potentially cardiomyopathy.

3. **Neonatal Hemochromatosis**: A condition causing iron overload in the liver and other organs of newborns, leading to liver failure and systemic involvement.

4. **Alpha-1 Antitrypsin Deficiency**: A genetic condition that can cause liver disease in children due to the accumulation of abnormal alpha-1 antitrypsin protein in the liver.

5. **Wilson's Disease**: A genetic disorder leading to copper accumulation in the liver and brain, causing hepatic and neurological symptoms. It can manifest early in life with liver disease symptoms such as jaundice and hepatomegaly.

These conditions share clinical features such as liver failure and metabolic disturbances, making them important considerations in the differential diagnosis given your described symptoms.

**116. user - 2024-11-28 17:55:00**

The clinical presentation is markedly heterogeneous, and there are three types based on age of onset and clinical presentation.

**The cardiomyopathic type,** which is the most common type of severe early-onset cardiac and multi-organ failure, develops mainly in newborns and early infants. It has a high mortality rate and is associated with hypoglycaemia, Reye's syndrome, sudden neonatal death, hypertrophic and dilated cardiomyopathy, pericardial effusion, arrhythmias, myasthenia, hepatomegaly, and intermittent hypoglycaemia, with elevated levels of creatine kinase; myocardial hypertrophy and arrhythmias can be fatal.

**2. hepatic type , **less severe, hepatic and hypohidrotic hypoglycaemic type with onset in late infancy or childhood, presenting with hepatomegaly and liver function abnormalities, decreased fasting tolerance and acute hypohidrotic hypoglycaemia without myocardial damage and cardiac hypertrophy, but can be life-threatening without timely diagnosis and treatment.

**3. Myopathic type** , which develops mainly in adolescence to adulthood, is late-onset, mildly symptomatic, and is usually not accompanied by myocardial disease and hypoglycaemia. The main manifestations are rhabdomyolysis and myoglobinuria after exercise, infection or starvation, and even renal failure, which may be accompanied by muscle weakness, painful muscle spasms or myalgia.

**ChatGPT-4o** - 2024-11-28 17:55:00

Based on the clinical manifestations you provided, the most likely diagnosis is Carnitine Palmitoyltransferase II (CPT II) deficiency. CPT II deficiency is a metabolic disorder that affects the body's ability to use certain fats for energy, particularly during periods without food (fasting). It is characterized by three different phenotypes based on age of onset and clinical severity.

**Reasons for this diagnosis:**
- The cardiomyopathic type in infants, characterized by hypoketotic hypoglycemia, cardiomyopathy, and liver dysfunction, aligns with the severe early-onset manifestations typically seen in CPT II deficiency.
- The hepatic type with late infancy or childhood onset and symptoms predominantly involving the liver without cardiac involvement also matches the milder phenotype of CPT II deficiency.
- The myopathic type presenting in adolescents and adults with exercise-induced rhabdomyolysis and myoglobinuria is classic for the adult form of CPT II deficiency.

**Five other possible diagnoses could include:**

1. **Glycogen Storage Disease Type II (Pompe Disease):** Can present with cardiomyopathy in infants, muscle weakness, and progressive myopathy in late-onset forms.

2. **Very Long-Chain Acyl-CoA Dehydrogenase (VLCAD) Deficiency:** Similar spectrum with cardiomyopathy in early-onset cases, hypoglycemia, and rhabdomyolysis in later-onset forms.

3. **Medium-Chain Acyl-CoA Dehydrogenase (MCAD) Deficiency:** Typically presents with hypoketotic hypoglycemia, but cardiac involvement is less common.

4. **Mitochondrial Trifunctional Protein (TFP) Deficiency:** Can present with cardiomyopathy, hypoglycemia, and rhabdomyolysis.

5. **Multiple Acyl-CoA Dehydrogenase Deficiency (MADD, also known as Glutaric Aciduria Type II):** Can present with metabolic crises, hypoglycemia, cardiomyopathy, and muscle weakness.

These conditions all involve fatty acid oxidation or mitochondrial energy metabolism abnormalities and can present with overlapping clinical features.

**117. user - 2024-11-28 18:14:46**

**1. specific facial features,** periorbital subcutaneous tissue fullness, widened orbital distance, stellate irises, long midriffs, thick lips, wide mouths, and small jaws.

**2. cardiovascular disease,** elastin arteriopathy is present in 75% ~80% of patients and can affect any artery. Peripheral pulmonary stenosis (37% ~61%) is more common in infancy but improves with time. Supravalvular aortic stenosis is more common (35% ~65%) and worsens over time by age 5. Arterial stenosis leads to increased left heart resistance, cardiac hypertrophy, and heart failure. Other anomalies include renal artery stenosis, thoracic aortic stenosis, abdominal aortic stenosis, mitral valve prolapse and aortic valve insufficiency, mesenteric artery stenosis, and intracranial vascular anomalies. Hypertension can occur at any age and may be related in part to renal artery stenosis.

**3. Cognitive ability,** 75% of patients have mental retardation, which is usually mild. Cognitive impairment is characteristically manifested by relatively good short-term memory and verbal expression, but extreme weakness in visuospatial structural cognition. No gender differences in IQ have been reported, and children's IQ is stable over time.

**4. personality, behavioural, and psychological abnormalities,** including excessive friendliness, excessive empathy, attention deficit, anxiety, and fear.

**5. sleep abnormalities,** with a 65% prevalence of sleep problems, including increased sleep latency and decreased sleep efficiency.

**6. eyes,** tear duct obstruction, hyperopia (67%), and strabismus (50%) are common. Cataracts have also been reported in adult patients.

**7. Ear, Nose, and Throat (ENT),** 50 per cent of patients have chronic otitis media, 90 per cent have increased sensitivity to sound, and 63 per cent of children and 92 per cent of adults have mild to moderate hearing impairment, mostly progressive sensorineural hearing loss. Most patients are hoarse or have a low voice.

**8. dental** , which may include baby teeth, enamel hypoplasia, and a misaligned bite.

**9. digestive system,** Chronic abdominal pain is a common symptom, and possible etiologies include gastroesophageal reflux, hiatal hernia, peptic ulcer, cholelithiasis, diverticulitis, ischaemic bowel disease, chronic constipation and anxiety somatisation. Hypercalcaemia may also lead to irritability, vomiting, constipation and muscle spasms.

**10. urinary system,** urinary frequency and enuresis are common in paediatric patients.

**11. neurological, muscular,** with low muscle tone and joint laxity in young children, leading to compensatory postural abnormalities to maintain body balance. Older children and adults have typical hypertonia and active deep tendon reflexes, resulting in a stiff and awkward gait, impaired fine motor function, and difficulty using tools and writing in patients of all ages. Adult patients will have cerebellar ataxia and tremor.

**12. Growth and development** .70% of infants have poor weight gain.

**13. idiopathic hypercalcaemia** . It occurs in infancy and childhood in 15-50% of patients and the most common symptoms are irritability, vomiting and constipation.

**14. endocrine. **Hypothyroidism (10 per cent), early puberty (50 per cent). Impaired glucose tolerance occurs in 26% of adolescent patients.

**ChatGPT-4o** - 2024-11-28 18:14:46

Based on the clinical manifestations you've provided, the most likely diagnosis is **Williams Syndrome**. Williams Syndrome is characterized by distinctive facial features, cardiovascular problems (such as supravalvular aortic stenosis), intellectual disabilities, unique personality traits, and various other systemic manifestations, many of which align with the symptoms you've outlined.

Here are five other possible differential diagnoses to consider:

1. **Noonan Syndrome**: This condition shares some cardiovascular manifestations with Williams Syndrome, such as pulmonary stenosis, and may also present with facial dysmorphisms and learning disabilities.

2. **DiGeorge Syndrome (22q11.2 deletion syndrome)**: This disorder can present with cardiovascular anomalies, immune deficiencies, distinctive facial features, and developmental delays.

3. **Alagille Syndrome**: This syndrome involves congenital heart defects, distinct facial features, and may also include liver problems and skeletal abnormalities.

4. **Rubinstein-Taybi Syndrome**: Characterized by facial abnormalities, intellectual disabilities, and broad thumbs and toes, this syndrome can sometimes present features similar to those seen in Williams Syndrome.

5. **Kabuki Syndrome**: This syndrome includes distinctive facial features, developmental delay, and congenital heart defects, which may overlap with some aspects of the clinical picture presented.

Each of these conditions can present with overlapping symptoms but differ in specific genetic and clinical features. Further genetic testing and clinical evaluation would help narrow down these options to confirm the diagnosis.

**118. user - 2024-11-28 18:18:38**

**1. Haemorrhagic tendency** is a common manifestation of the disease, especially in blood-stained stools, and most children develop the disease in the neonatal period. The next manifestations are petechiae, ecchymosis, haemoptysis and haematuria, and some severe cases may have life-threatening gastrointestinal haemorrhage or intracranial haemorrhage. Bleeding in patients is caused by thrombocytopenia with reduction in platelet volume, but only about 25% of cases have the typical triad of symptoms.

**2. Infection. **Children are susceptible to a variety of infections, including herpes simplex virus, cytomegalovirus, Streptococcus pneumoniae, and fungi. The patient's immunodeficiency worsens with age.

**3. Eczema. ** Eczema may be present in approximately 80% of children with WAS and varies widely in extent and severity. There may be typically severe, refractory eczema, or very mild or even no eczema manifestations.

**4. Autoimmune diseases. ** The most common is autoimmune haemolytic anaemia. Others include vasculitis, arthritis, renal disease, inflammatory bowel disease, neutropenia and autoimmune thrombocytopenic purpura.

**5. malignant tumours . **Children have a significantly higher risk of malignant tumours of the lymphatic system, especially lymphomas; non-Hodgkin's lymphoma, lymphoblastic leukaemia, myelodysplastic syndromes, and spermatogonia have been reported.

**6. other . **Children with this disease may have completely normal platelet levels with persistent or recurrent neutropenia.

**ChatGPT-4o** - 2024-11-28 18:18:38

Based on the provided clinical manifestations, the most likely rare disease diagnosis is Wiskott-Aldrich Syndrome (WAS). This disease is characterized by the triad of thrombocytopenia (low platelet count), eczema, and recurrent infections. The described symptoms, such as haemorrhagic tendencies due to thrombocytopenia, susceptibility to various infections, eczema, autoimmune diseases, and an increased risk for malignancies, align closely with WAS.

Here are five other possible diagnoses that could present with somewhat overlapping symptoms:

1. **X-Linked Thrombocytopenia (XLT)**: A milder form of WAS, characterized primarily by low platelet counts and bleeding issues but typically with less severe eczema and infections.

2. **Chédiak-Higashi Syndrome**: This rare disorder includes immune deficiencies, bleeding problems, and increased risk of infections, but it is also characterized by albinism, which is not mentioned here.

3. **Hyper-IgM Syndrome**: This immunodeficiency can lead to recurrent infections and autoimmune issues. However, it doesn't typically present with thrombocytopenia or eczema.

4. **Severe Combined Immunodeficiency (SCID)**: Children with SCID have severe immune deficiencies, leading to recurrent infections, but SCID does not typically feature eczema or thrombocytopenia.

5. **Immune Thrombocytopenic Purpura (ITP)**: Characterized by low platelet counts and bleeding tendencies. It does not typically present with eczema, frequent infections, or the other autoimmune features described here.

While these alternatives could explain some of the manifestations, Wiskott-Aldrich Syndrome remains the most fitting diagnosis given the full spectrum of symptoms described.

**119. user - 2024-11-28 18:21:10**

Typical Patients present with both CNS damage and hypoadrenocorticism. Neurological symptoms and symptoms of hypoadrenocorticism may occur simultaneously or sequentially. In a minority of patients, they may occur alone. The manifestations of adrenocortical insufficiency vary in severity. It is most common in children, accounting for 35% of all patients. The onset of symptoms occurs between the ages of 4 and 8 years. The initial symptoms include poor concentration, memory loss, learning difficulties, unsteady gait and abnormal behaviour, followed by visual and auditory loss, dysarthria, seizures, ataxia, paralysis and dementia. Clinical manifestations progressively worsen, culminating in blindness or deafness and complete paralysis. Some exhibit deafferentation and eventually die of central respiratory failure, brain herniation, and infection. Most patients have adrenocortical impairment when they first develop neurological symptoms.

**ChatGPT-4o** - 2024-11-28 18:21:10

Based on the clinical manifestations provided, the rare disease most likely to be diagnosed is **Adrenoleukodystrophy (ALD)**. ALD is a genetic disorder that affects the nervous system and adrenal glands, often leading to central nervous system damage and adrenal insufficiency (hypoadrenocorticism). It usually presents in childhood, with the most common form being the cerebral form which begins between ages 4-8, and it aligns with the symptoms described.

Here are five other possible diagnoses that could present with similar symptoms:

1. **Refsum Disease**: This is a peroxisomal disorder that can lead to neurological symptoms, ataxia, and peripheral neuropathy, but typically includes retinitis pigmentosa and anosmia, which weren't specifically mentioned.

2. **Zellweger Spectrum Disorders**: These are a group of peroxisomal biogenesis disorders that can lead to neurological symptoms, including hypotonia, seizures, and hearing loss, as well as adrenal insufficiency.

3. **Metachromatic Leukodystrophy**: Another leukodystrophy that affects the central nervous system and can lead to progressive neurodegeneration and adrenal gland involvement.

4. **Neurodegeneration with Brain Iron Accumulation (NBIA)**: A group of disorders characterized by abnormal iron deposition in the brain, which can cause progressive neurological symptoms, though adrenal insufficiency is less common.

5. **Wolfram Syndrome (DIDMOAD)**: A rare genetic disorder that includes diabetes insipidus, diabetes mellitus, optic atrophy, and deafness, which can also involve neurological decline and adrenal insufficiency.

Each of these conditions has unique features, but they can present with overlapping symptoms, making differential diagnosis crucial.

**120. user - 2024-11-28 18:23:43**

Most of the children start to show symptoms of infection from 4 to 12 months after birth. The most prominent clinical manifestation is recurrent severe bacterial infections in the respiratory system, digestive system and central nervous system, with respiratory tract infections being the most common, and common pathogens include Haemophilus influenzae, Streptococcus pneumoniae, Staphylococcus aureus, and Pseudomonas aeruginosa, etc. The most common infections are respiratory tract infections. Prolonged and repeated respiratory infections can lead to irreversible organ function damage such as bronchiectasis and pestle finger. Common symptoms of GI infections include diarrhoea, abdominal pain, gastro-oesophageal reflux and gastroenteritis. Pathogens causing infectious diarrhoea include Giardia lamblia, Salmonella, Campylobacter jejuni and Cryptosporidium.

In addition, children are prone to otitis media, chronic sinusitis, malnutrition, anaemia, granulocytopenia, thrombocytopenia, growth hormone deficiency and thyroid hormone disorders. Children may also develop tumours, autoimmune and inflammatory diseases, including rheumatoid arthritis and dermatomyositis. A higher incidence of sensorineural hearing loss and eczema has also been reported in children.

**ChatGPT-4o** - 2024-11-28 18:23:43

Based on the provided clinical manifestations, the most likely rare disease diagnosis would be Chronic Granulomatous Disease (CGD). CGD is a primary immunodeficiency disorder characterized by recurrent bacterial and fungal infections due to the inability of phagocytes (a type of white blood cell) to produce reactive oxygen species to kill certain bacteria and fungi.

Here are five other possible diagnoses that could present with similar symptoms:

1. **Severe Combined Immunodeficiency (SCID):** This is a group of rare, potentially fatal disorders characterized by the absence of functional T-lymphocytes and B-lymphocytes, leading to severe recurrent infections.

2. **Common Variable Immunodeficiency (CVID):** A disorder characterized by low levels of serum immunoglobulins and an impaired ability to produce specific antibodies, leading to frequent infections, especially of the respiratory and gastrointestinal tracts.

3. **Hyper-IgM Syndrome:** A group of genetic disorders characterized by normal or increased levels of IgM antibodies but low levels of other immunoglobulins, resulting in increased susceptibility to infections.

4. **Leukocyte Adhesion Deficiency (LAD):** A rare immunodeficiency that leads to recurrent bacterial infections, impaired pus formation, and delayed wound healing due to defects in neutrophil adhesion.

5. **Wiskott-Aldrich Syndrome:** A rare X-linked immunodeficiency characterized by a triad of eczema, recurrent infections, and thrombocytopenia (low platelet count).

Each of these conditions involves some form of immune dysfunction leading to recurrent infections and may overlap with some of the described symptoms. A definitive diagnosis would require specific laboratory tests, genetic testing, and clinical evaluation by an immunologist.

**121. user - 2024-11-28 18:37:20**

The most common clinical manifestations are fulminant infectious mononucleosis, anomalous immunoglobulinaemia and B-cell lymphoma following EBV infection. Aberimmunoglobulinaemia and B-cell lymphoma can occur in patients without EBV infection. Aberimmunoglobulinaemia usually presents with increased levels of IgA and IgM and decreased levels of IgG1 and IgG3. Lymphomas are predominantly non-Hodgkin B lymphomas. Other clinical manifestations include lymphocytic vasculitis, aplastic anaemia and lymphomatoid granulomatosis. In some patients, the clinical picture is mild, with only abnormal immunoglobulinaemia or mild EBV infection, possibly due to somatic gene repair.

In some patients, the predominant clinical manifestation is a haemophagocytic syndrome, with splenomegaly, which does not necessarily require EBV infection to be induced. Some patients may present with intestinal inflammation such as Crohn's disease or colitis, but rarely develop lymphoma.

**ChatGPT-4o** - 2024-11-28 18:37:20

The clinical manifestations you describe are suggestive of X-linked lymphoproliferative syndrome (XLP), specifically XLP type 1 or 2. This rare genetic disorder primarily affects males and is often characterized by a severe response to Epstein-Barr virus (EBV) infection, leading to complications such as fulminant infectious mononucleosis, dysregulated immunoglobulin levels, B-cell lymphomas, and hemophagocytic lymphohistiocytosis (HLH).

Five other possible diagnoses based on the provided clinical features could include:

1. **Chronic Active EBV Infection (CAEBV):** This condition involves persistent and severe illness due to EBV, leading to complications like hemophagocytic syndrome, aplastic anemia, and lymphoma.

2. **Common Variable Immunodeficiency (CVID):** This immunodeficiency is characterized by abnormal immunoglobulin levels and an increased risk of infections, autoimmunity, and lymphomas.

3. **Autoimmune Lymphoproliferative Syndrome (ALPS):** ALPS can present with lymphoproliferation, autoimmunity, and an increased risk of lymphoma, though EBV is not typically a triggering factor.

4. **Hyper-IgM Syndrome:** This immunodeficiency involves elevated IgM levels with low levels of other immunoglobulins, leading to infection susceptibility and potential lymphoma development.

5. **Wiskott-Aldrich Syndrome:** Although less common, this syndrome can present with immunodeficiency, increased lymphoma risk, and autoimmunity, though it is typically associated with thrombocytopenia and eczema.

Each of these conditions has overlapping features with the described symptoms and would require specific genetic and clinical evaluations to differentiate and confirm the diagnosis.
